# Supplementary figures and images for: Tunnel and underground engineering rock mass water inrush damage and acoustic emission characteristics
Source: PLoS One. 2024 Sep 20;19(9):e0307700. doi: 10.1371/journal.pone.0307700 (PMC11414891; doi:10.1371/journal.pone.0307700)

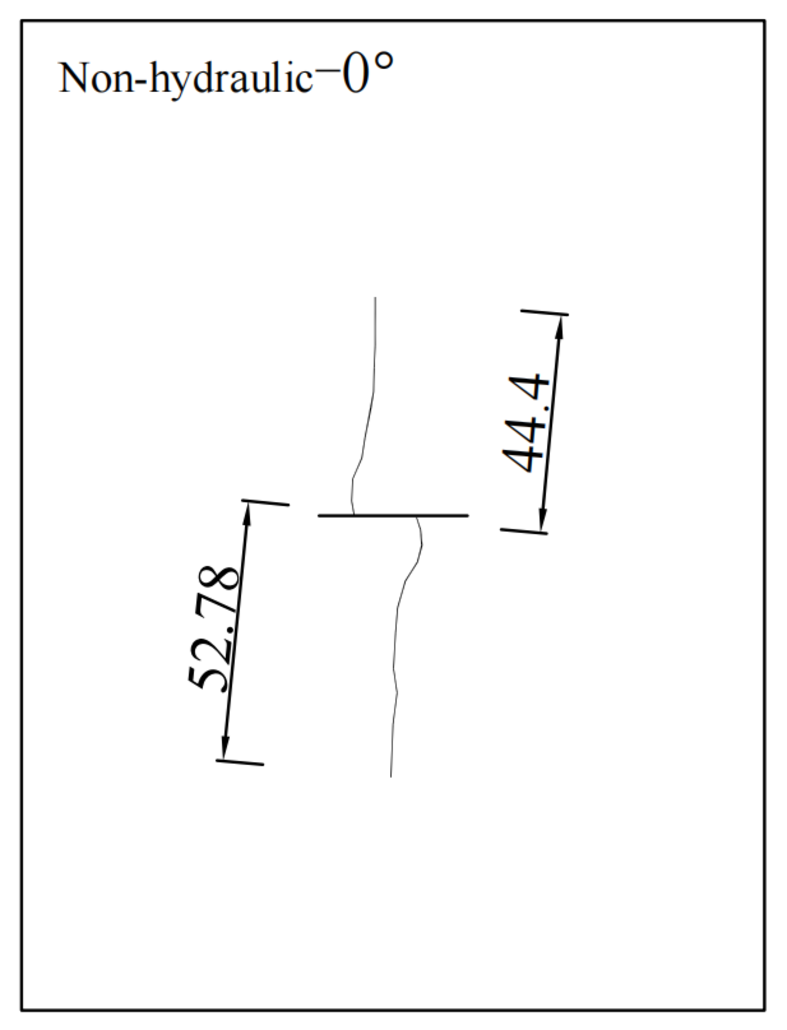

Supplement: S1 Table — (ZIP) [file pone.0307700.s003.zip › Table 1/Non-hydraulic-0íπ-Diagrammatic sketch.tif]

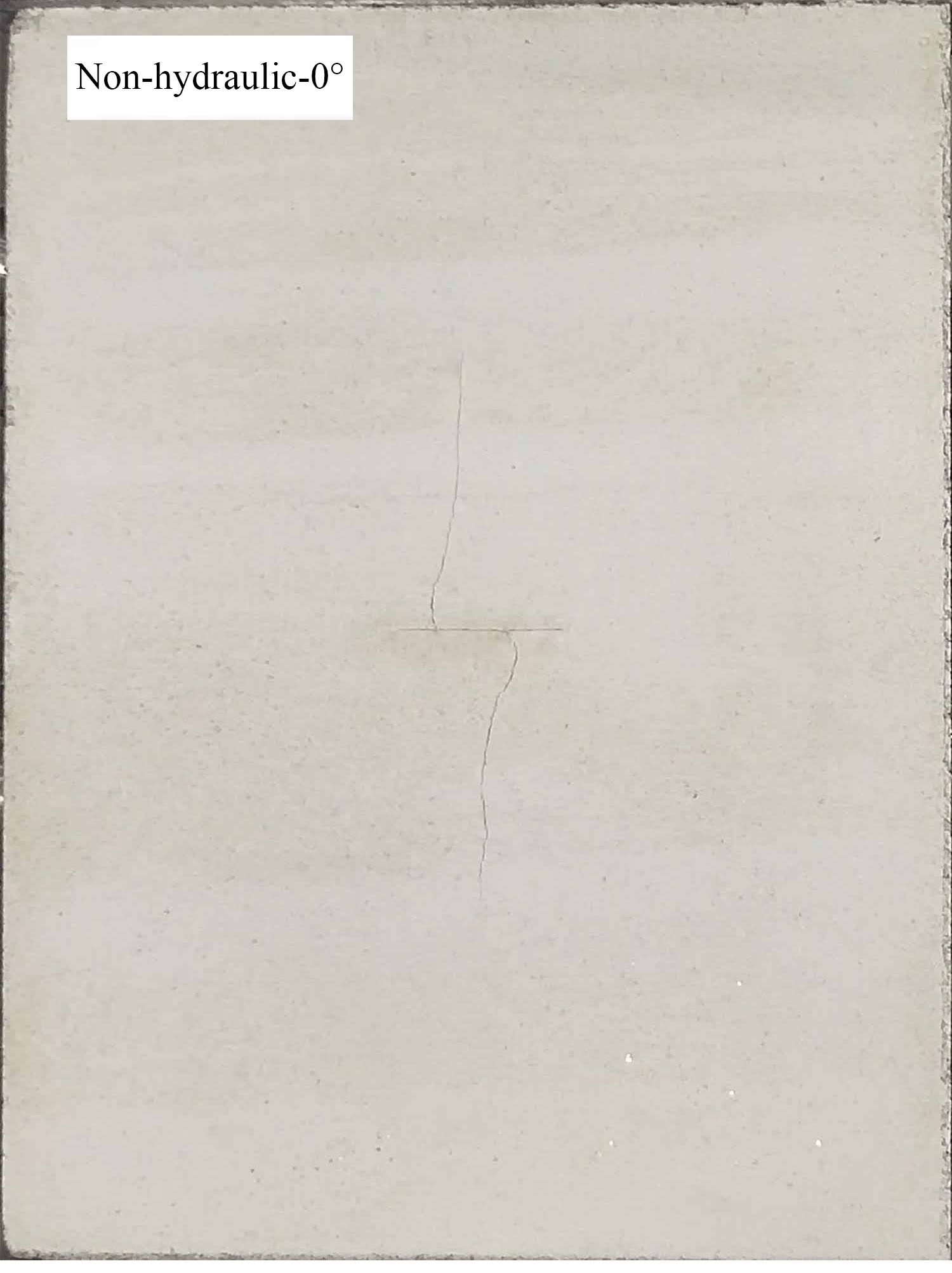

Supplement: S1 Table — (ZIP) [file pone.0307700.s003.zip › Table 1/Non-hydraulic-0íπ-Test result.tif]

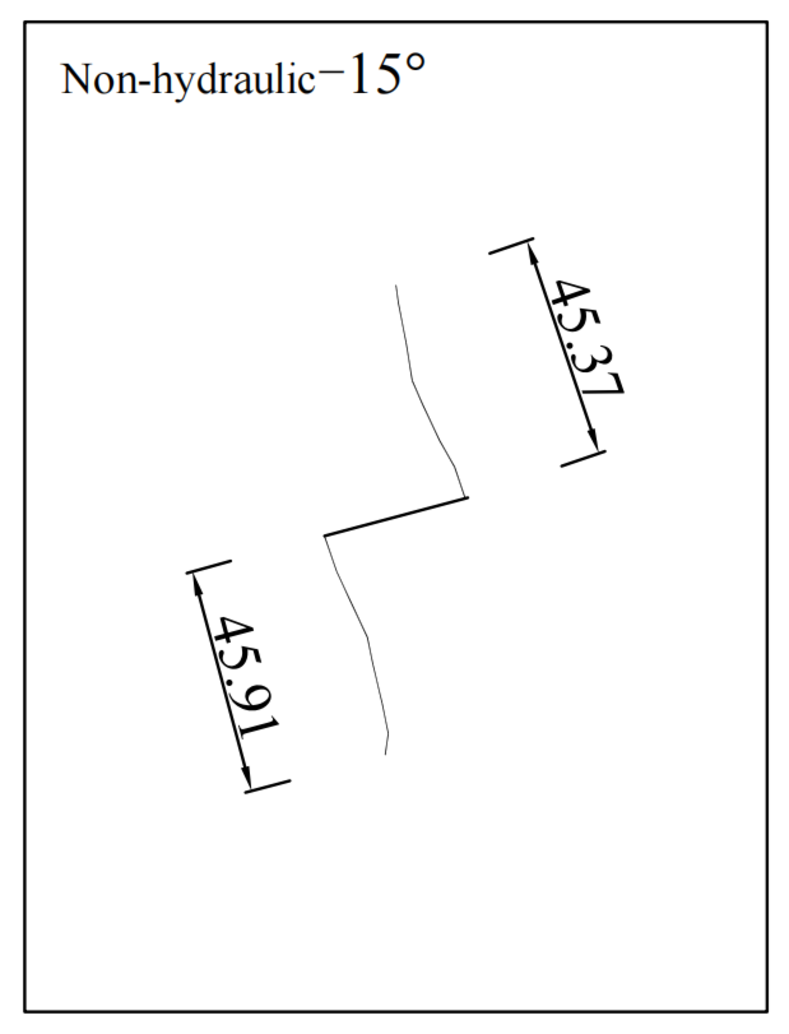

Supplement: S1 Table — (ZIP) [file pone.0307700.s003.zip › Table 1/Non-hydraulic-15íπ-Diagrammatic sketch.tif]

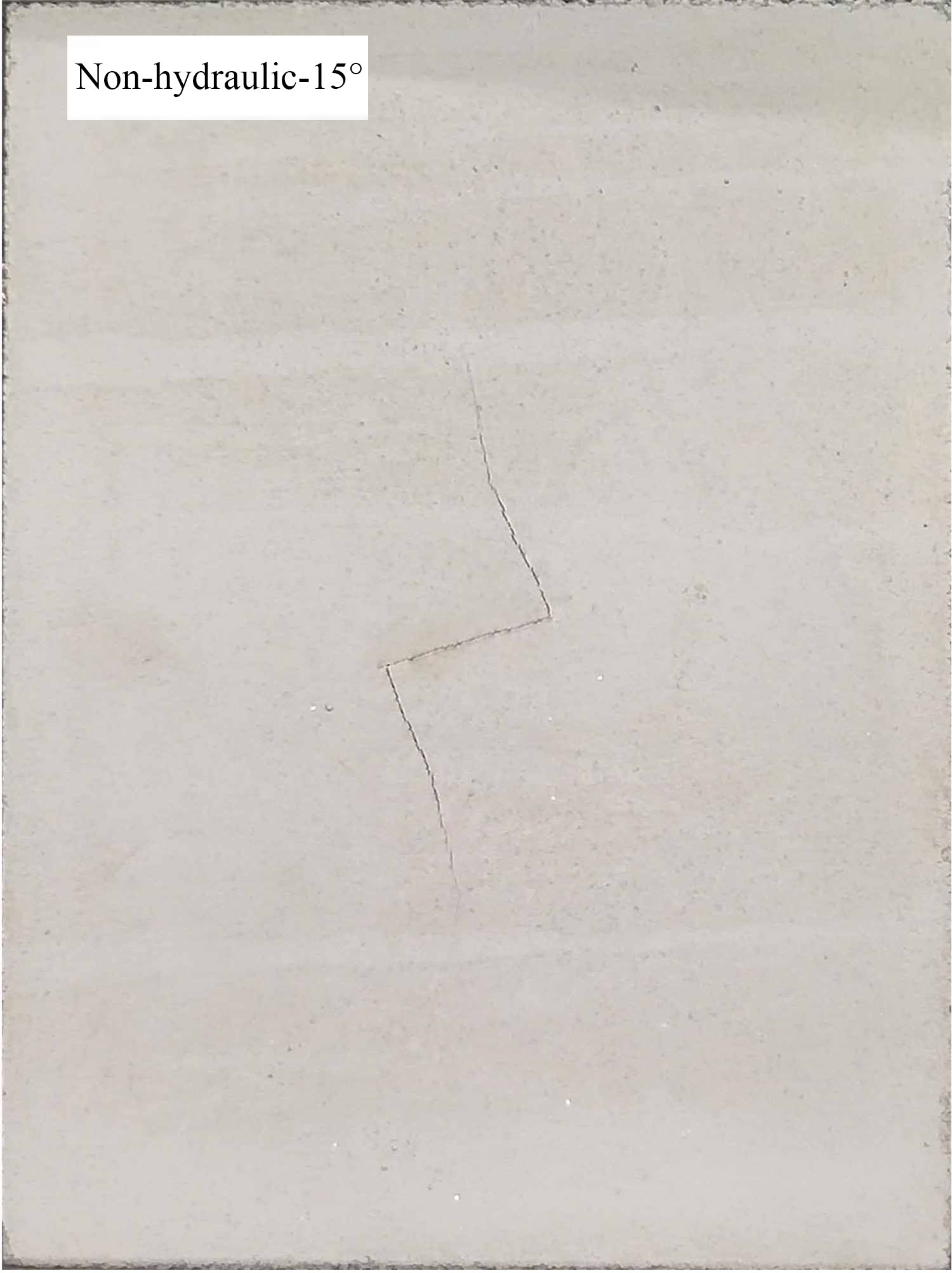

Supplement: S1 Table — (ZIP) [file pone.0307700.s003.zip › Table 1/Non-hydraulic-15íπ-Test result.tif]

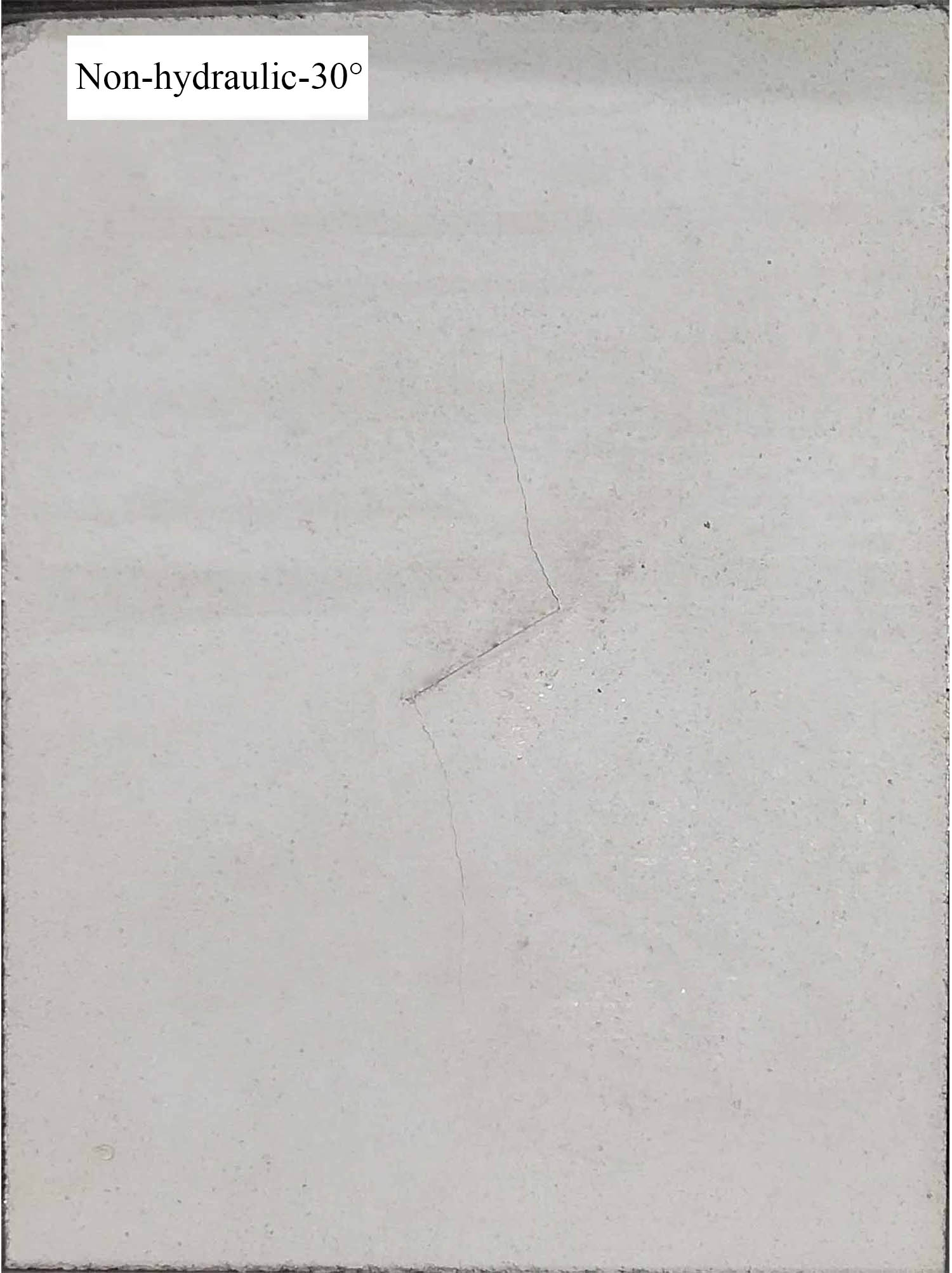

Supplement: S1 Table — (ZIP) [file pone.0307700.s003.zip › Table 1/Non-hydraulic-30íπ-Test result.tif]

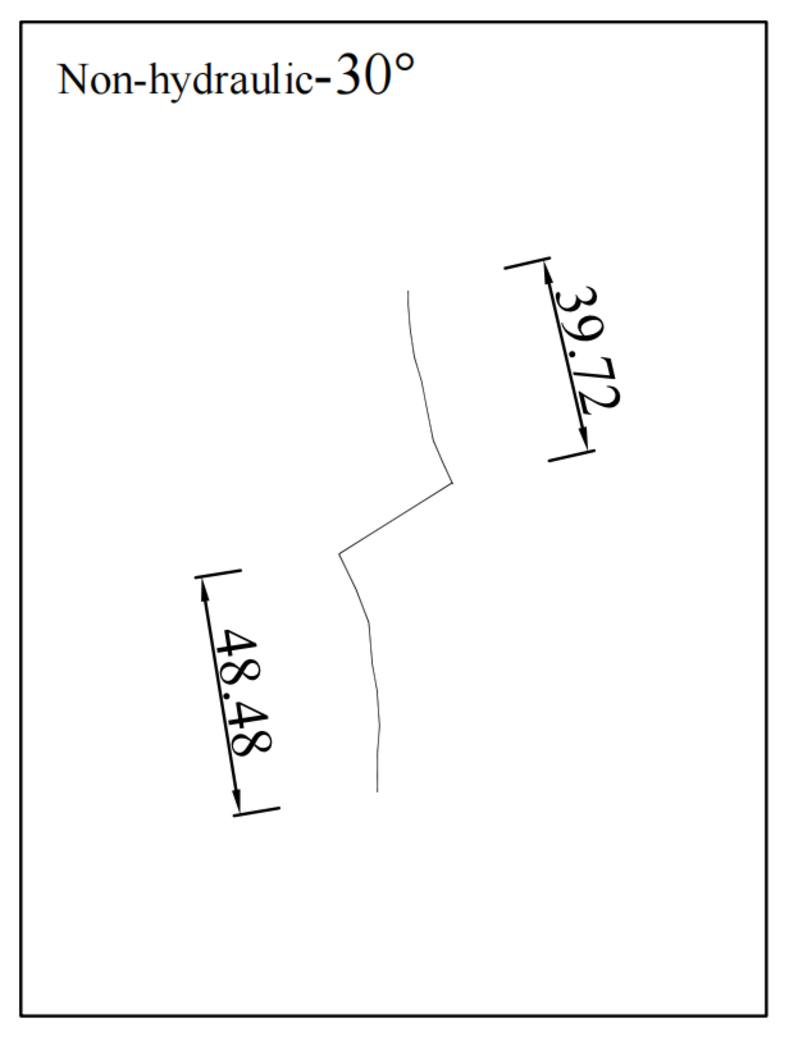

Supplement: S1 Table — (ZIP) [file pone.0307700.s003.zip › Table 1/Non-hydraulic-45íπ-Diagrammatic sketch.tif]

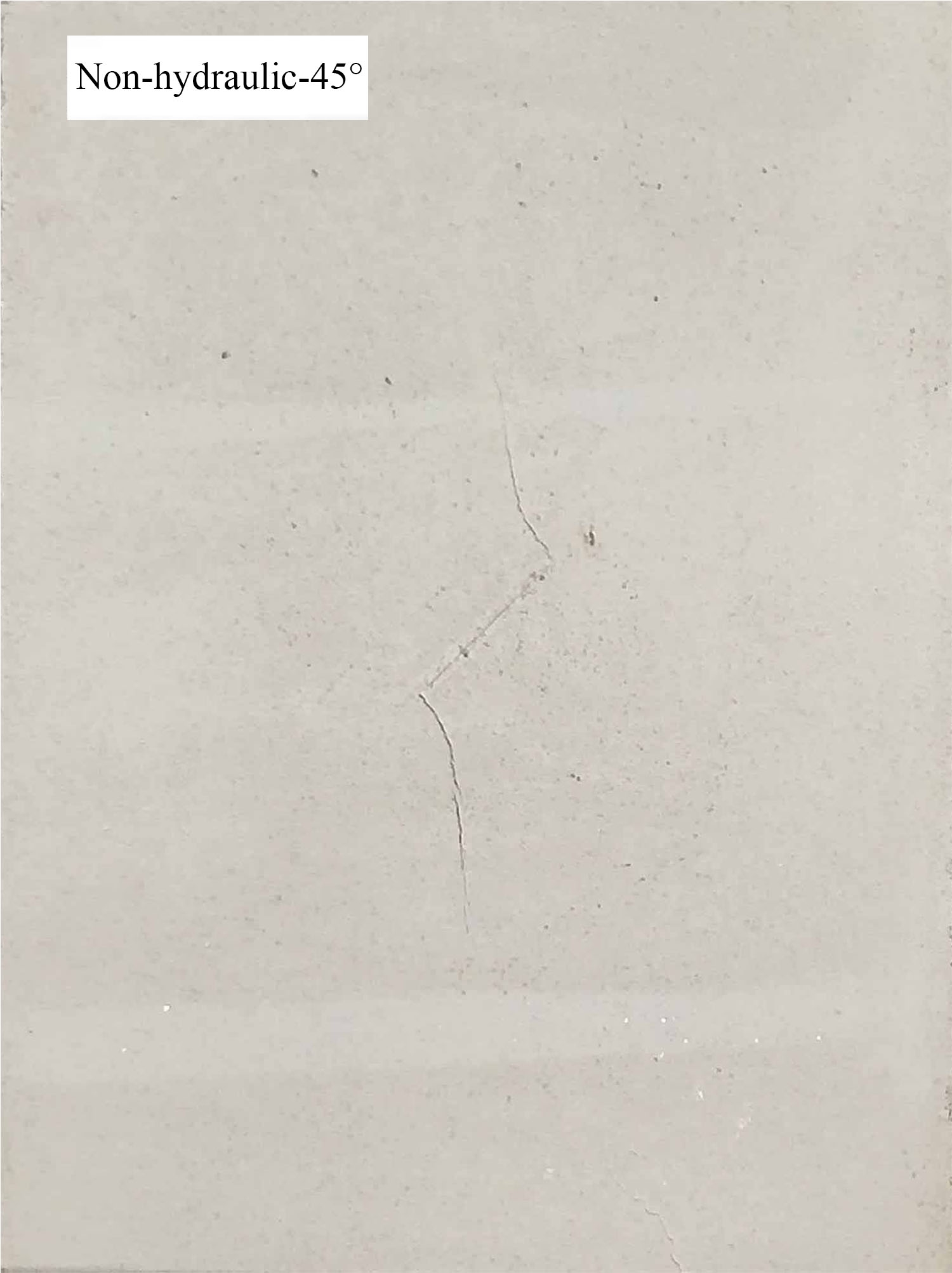

Supplement: S1 Table — (ZIP) [file pone.0307700.s003.zip › Table 1/Non-hydraulic-45íπ-Test result.tif]

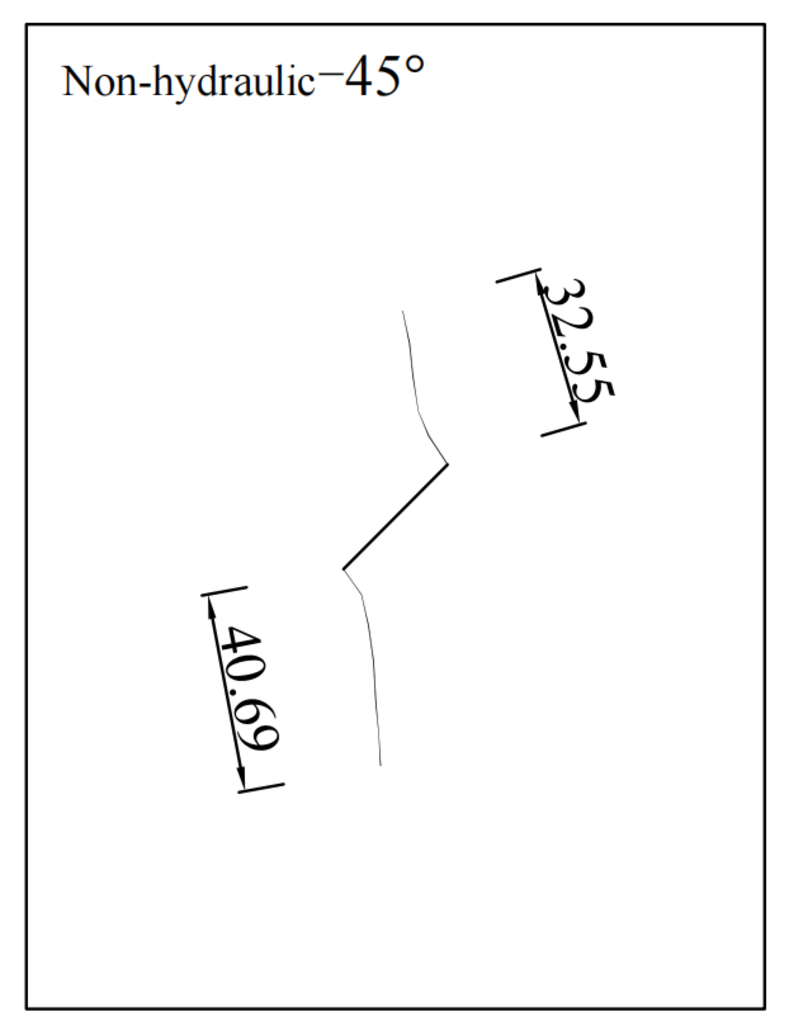

Supplement: S1 Table — (ZIP) [file pone.0307700.s003.zip › Table 1/Non-hydraulic-60íπ-Diagrammatic sketch.tif]

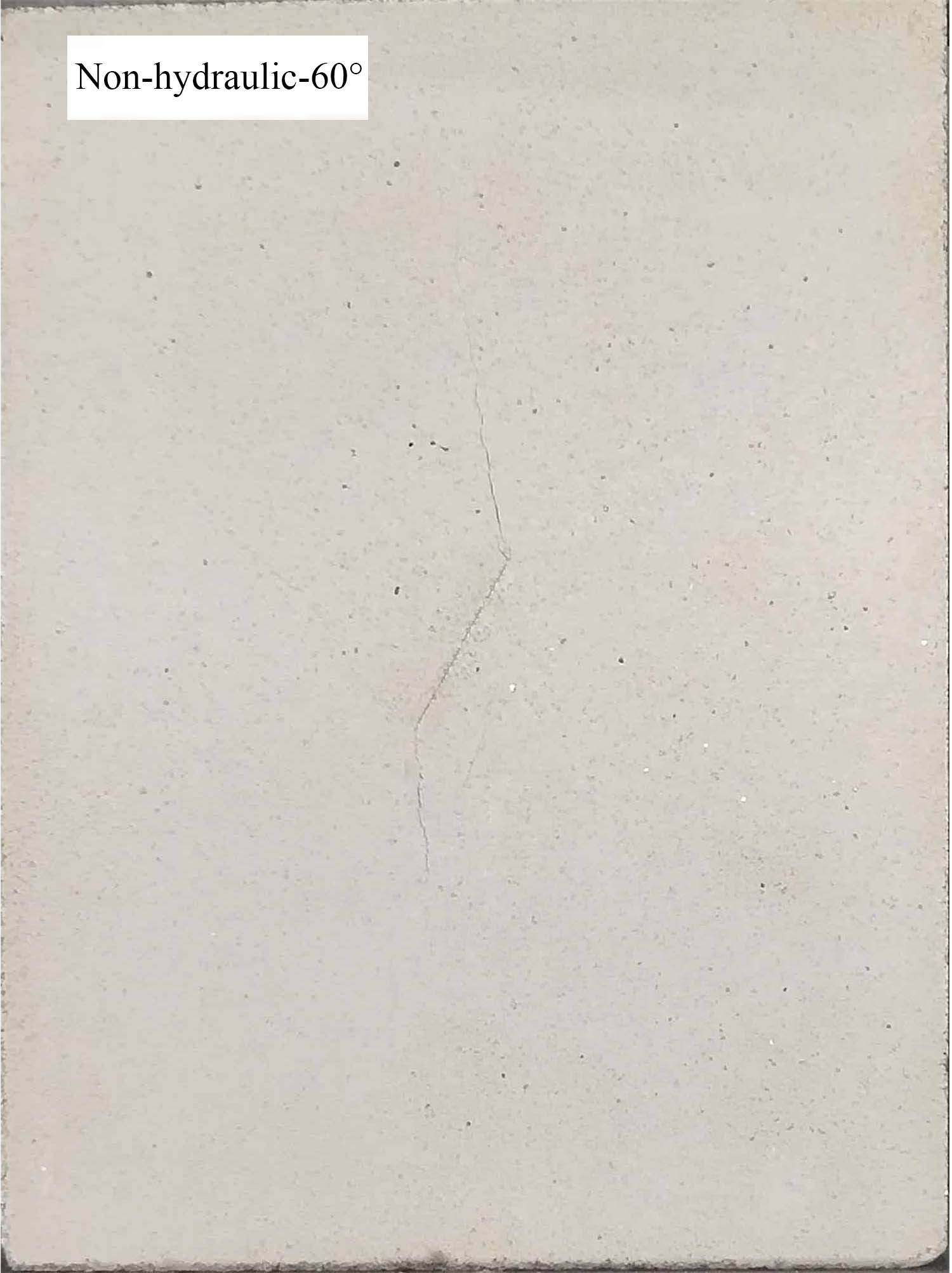

Supplement: S1 Table — (ZIP) [file pone.0307700.s003.zip › Table 1/Non-hydraulic-60íπ-Test result.tif]

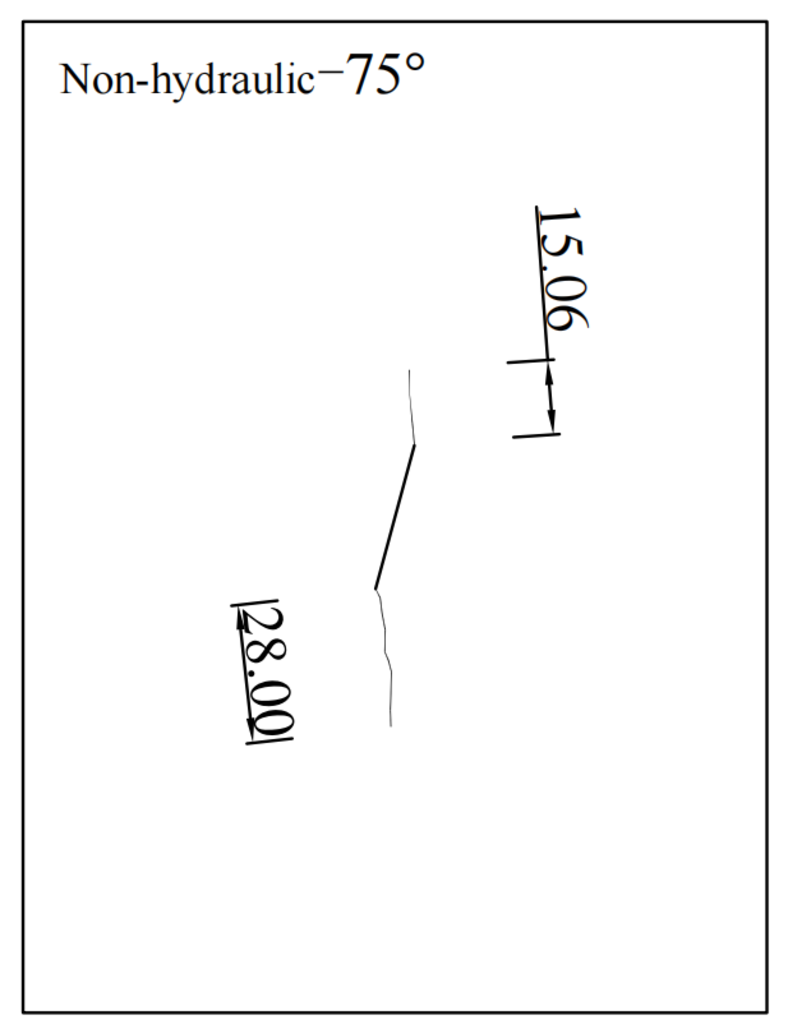

Supplement: S1 Table — (ZIP) [file pone.0307700.s003.zip › Table 1/Non-hydraulic-75íπ-Diagrammatic sketch.tif]

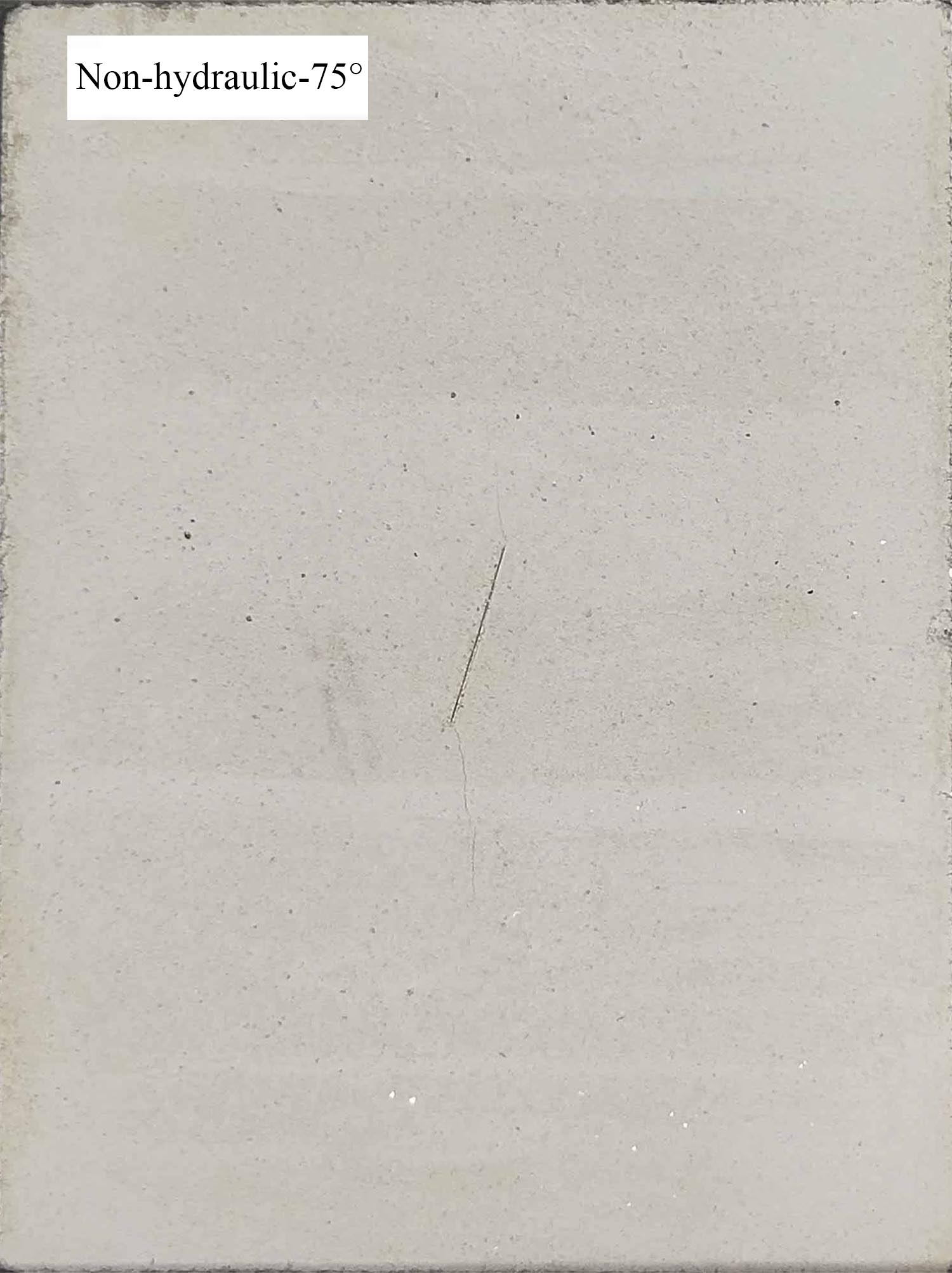

Supplement: S1 Table — (ZIP) [file pone.0307700.s003.zip › Table 1/Non-hydraulic-75íπ-Test result.tif]

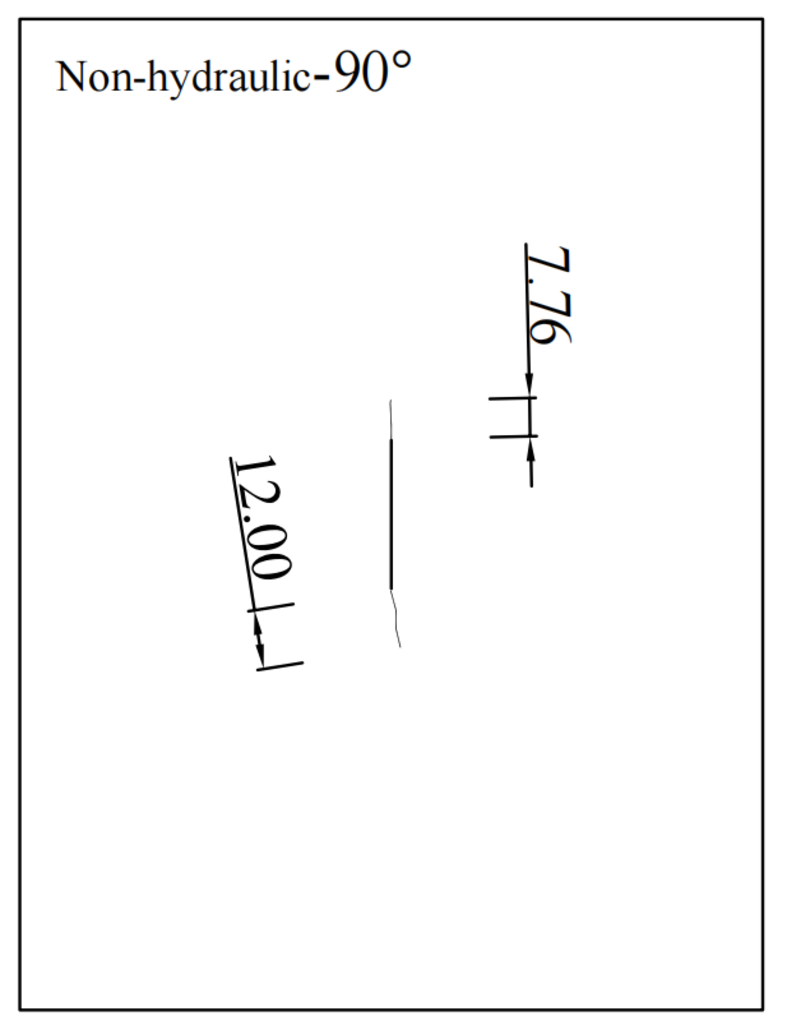

Supplement: S1 Table — (ZIP) [file pone.0307700.s003.zip › Table 1/Non-hydraulic-90íπ-Diagrammatic sketch.tif]

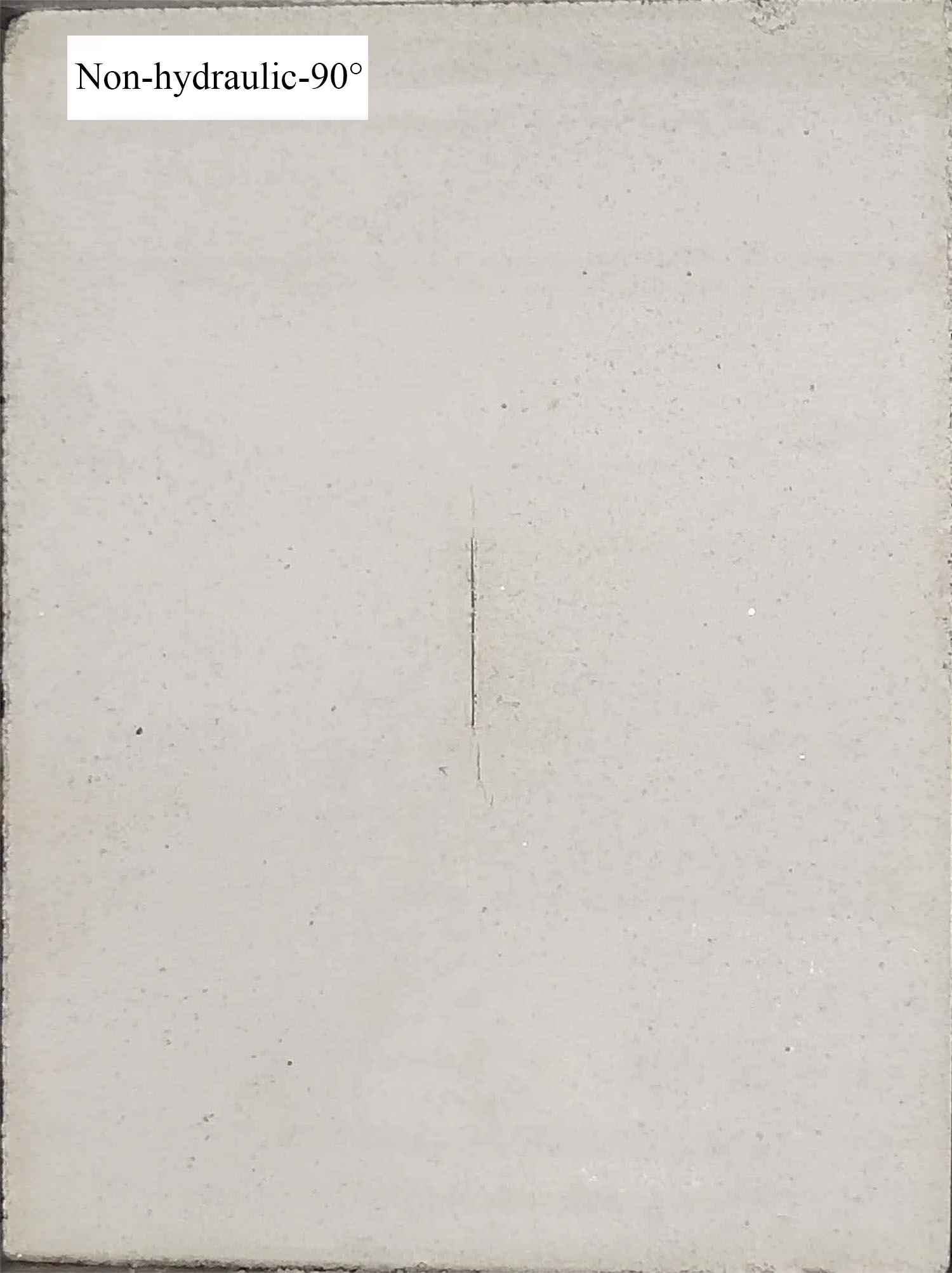

Supplement: S1 Table — (ZIP) [file pone.0307700.s003.zip › Table 1/Non-hydraulic-90íπ-Test result.tif]

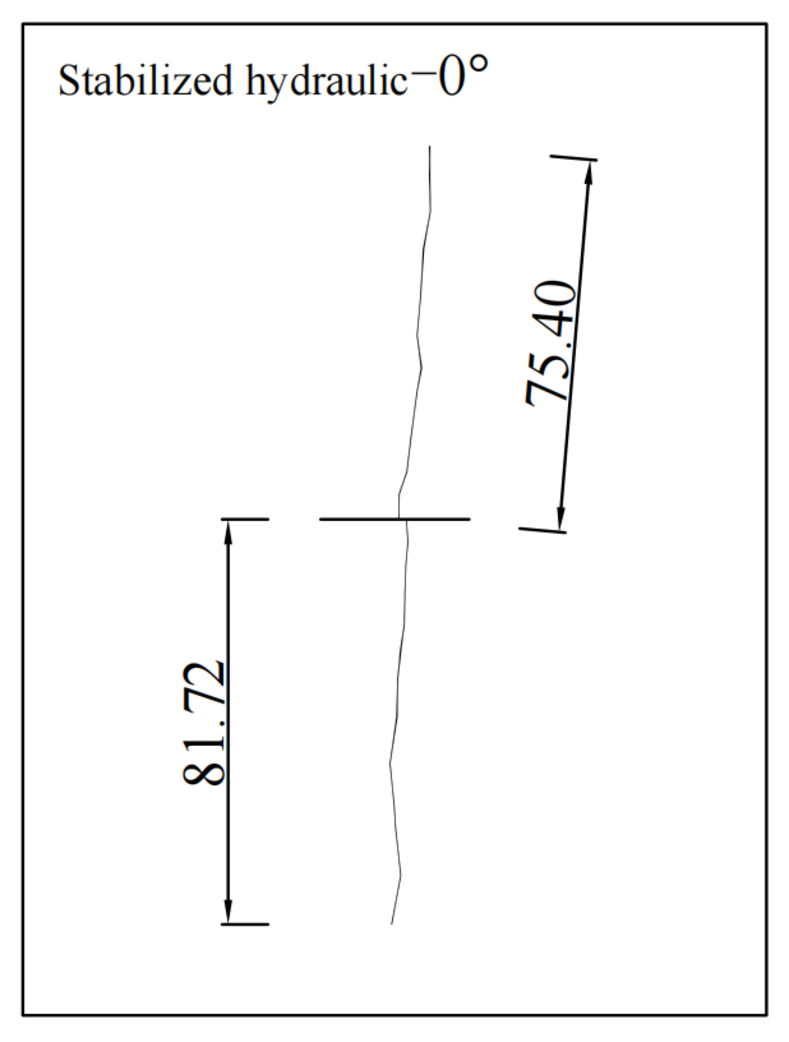

Supplement: S1 Table — (ZIP) [file pone.0307700.s003.zip › Table 1/Stabilized hydraulic-0íπ-Diagrammatic sketch.tif]

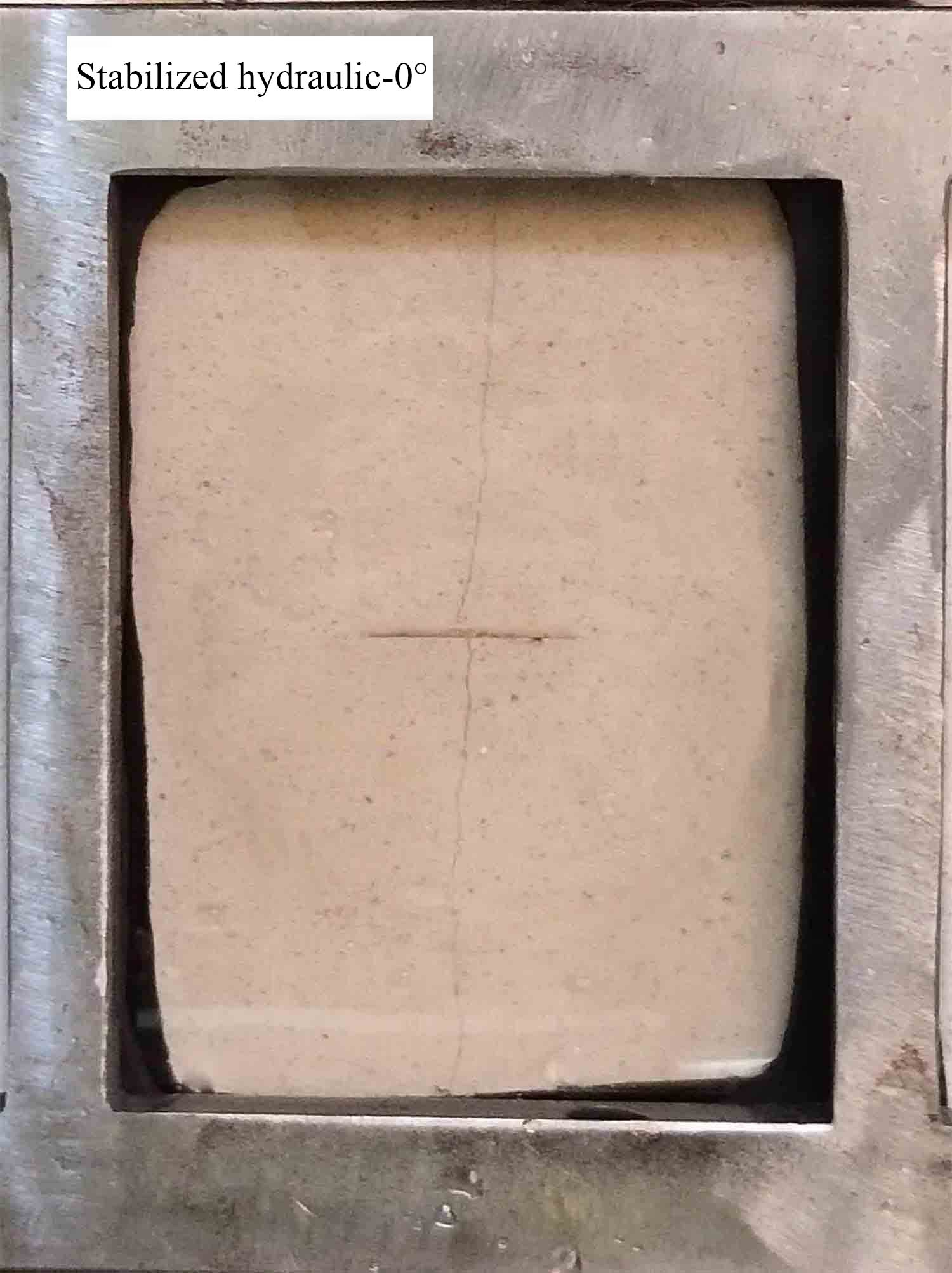

Supplement: S1 Table — (ZIP) [file pone.0307700.s003.zip › Table 1/Stabilized hydraulic-0íπ-Test result.tif]

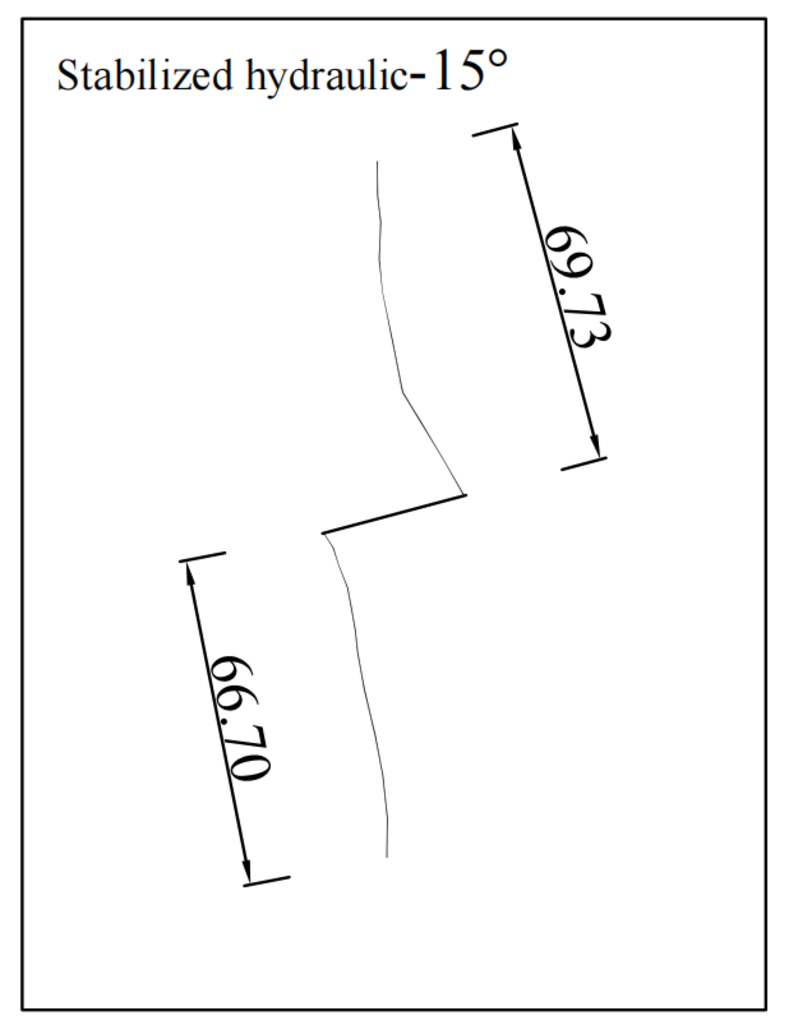

Supplement: S1 Table — (ZIP) [file pone.0307700.s003.zip › Table 1/Stabilized hydraulic-15íπ-Diagrammatic sketch.tif]

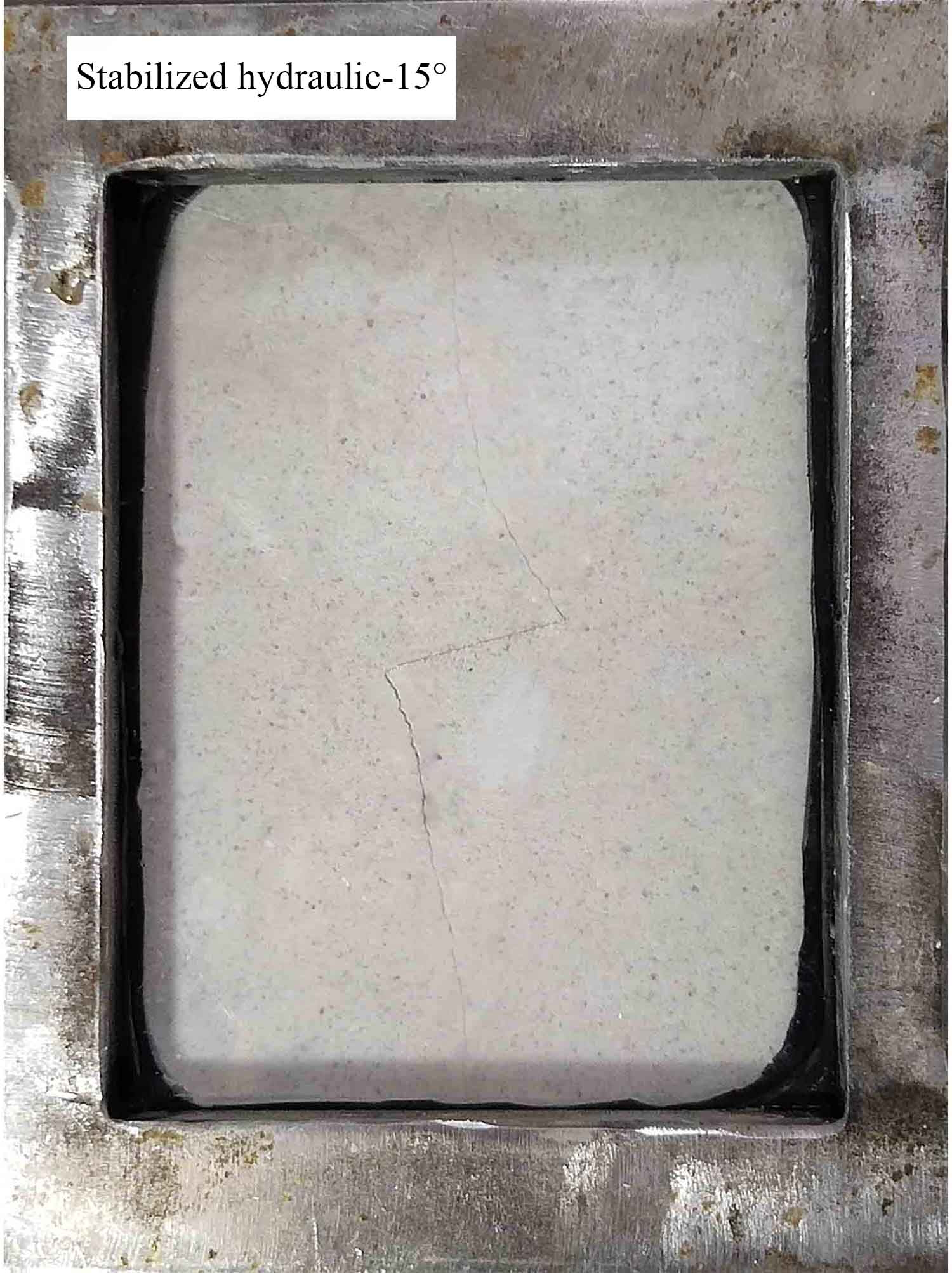

Supplement: S1 Table — (ZIP) [file pone.0307700.s003.zip › Table 1/Stabilized hydraulic-15íπ-Test result.tif]

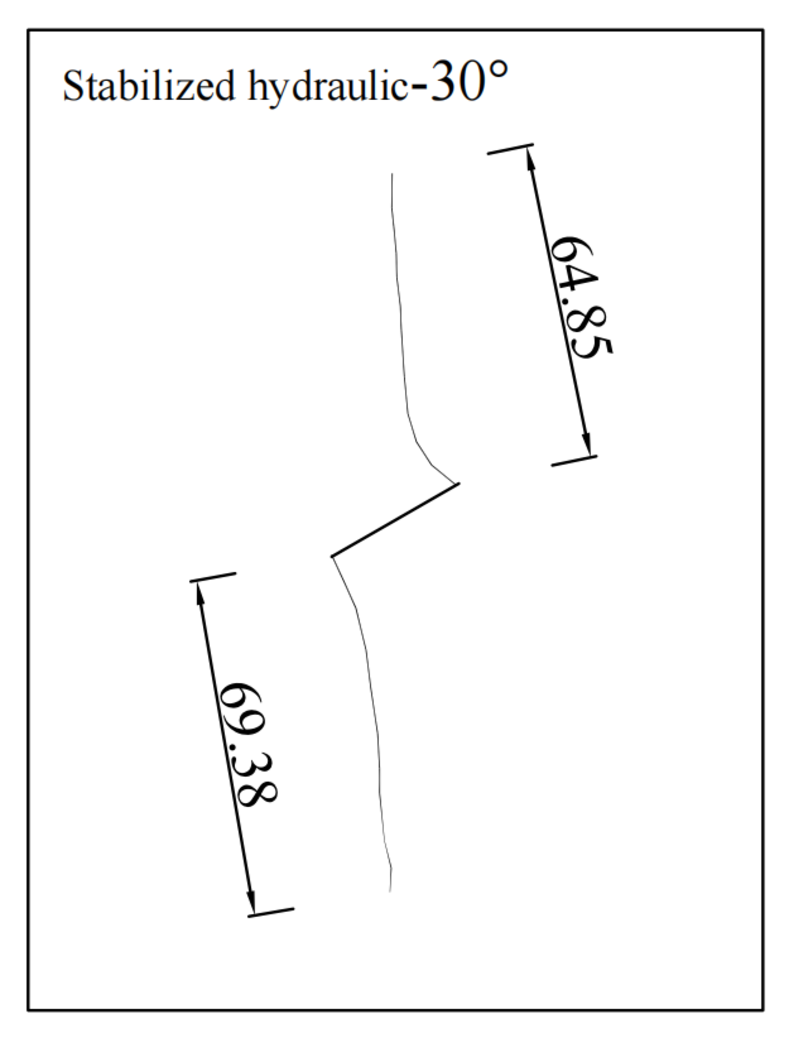

Supplement: S1 Table — (ZIP) [file pone.0307700.s003.zip › Table 1/Stabilized hydraulic-30íπ-Diagrammatic sketch.tif]

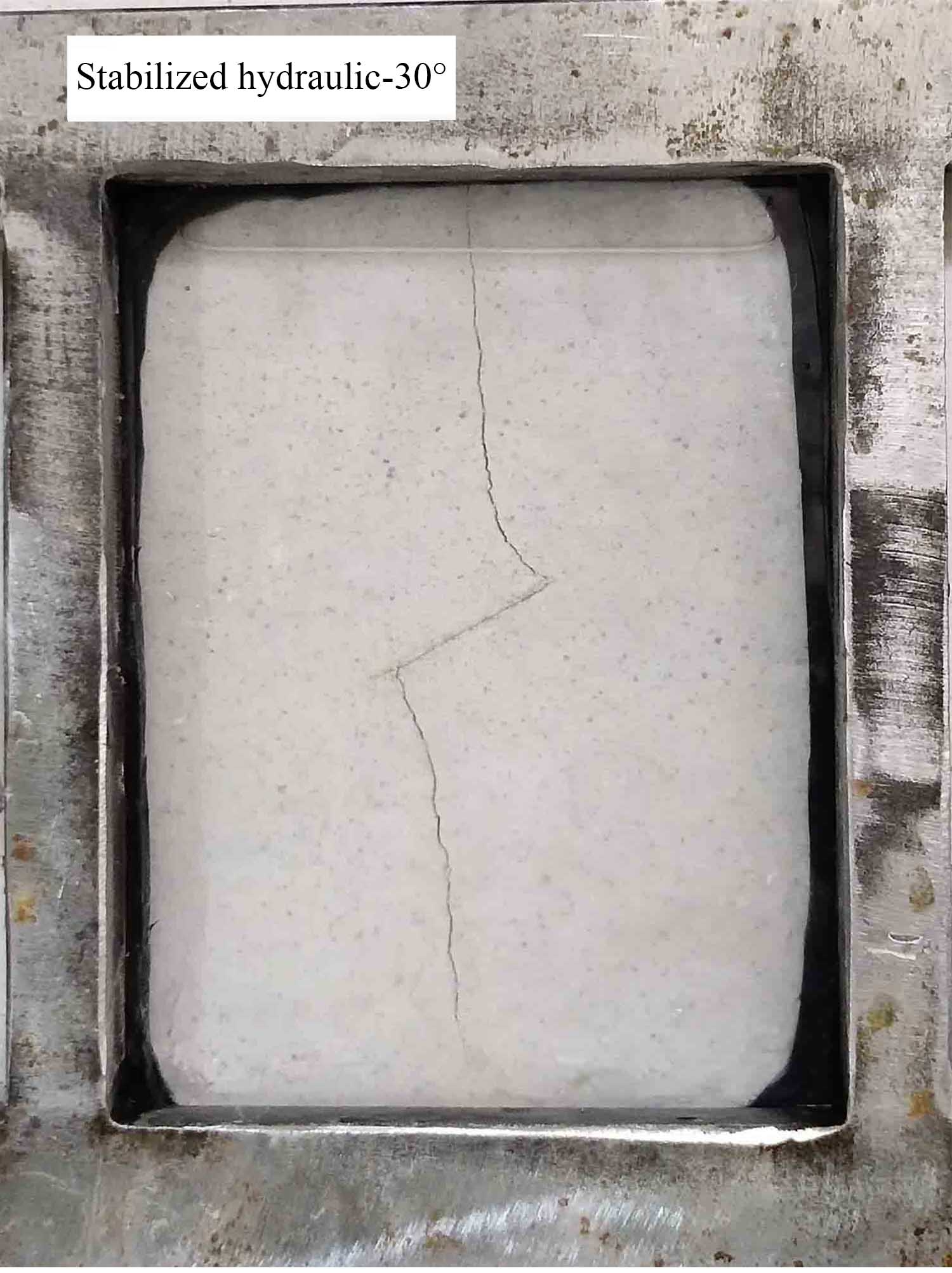

Supplement: S1 Table — (ZIP) [file pone.0307700.s003.zip › Table 1/Stabilized hydraulic-30íπ-Test result.tif]

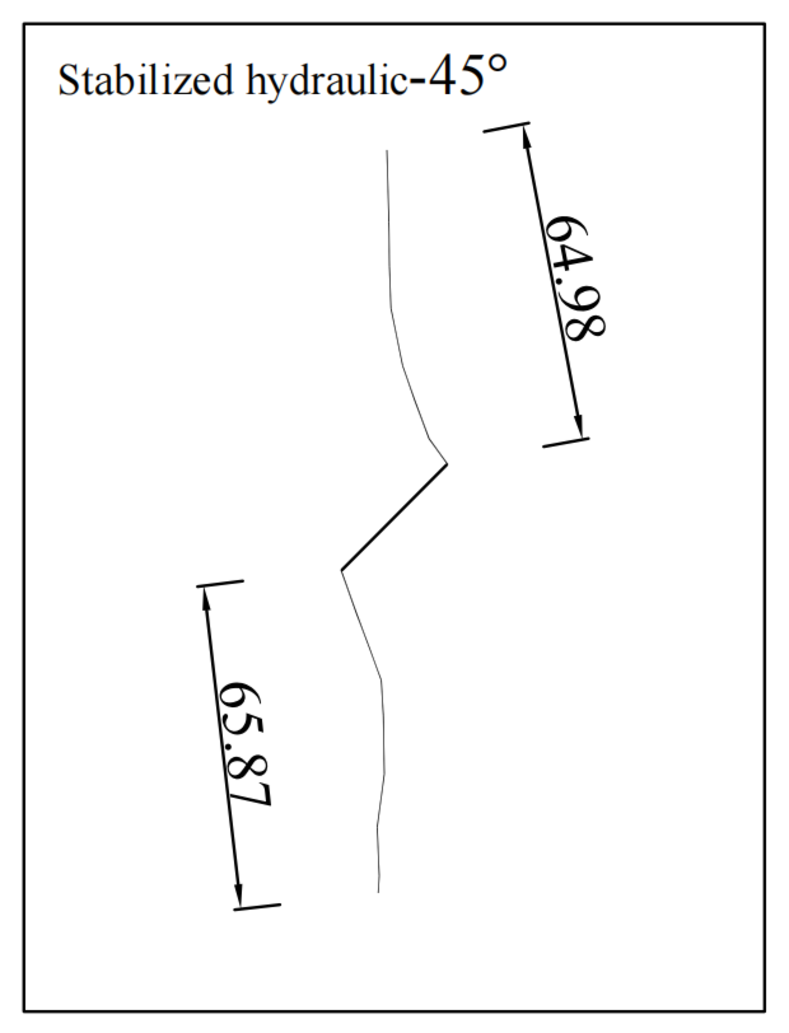

Supplement: S1 Table — (ZIP) [file pone.0307700.s003.zip › Table 1/Stabilized hydraulic-45íπ-Diagrammatic sketch.tif]

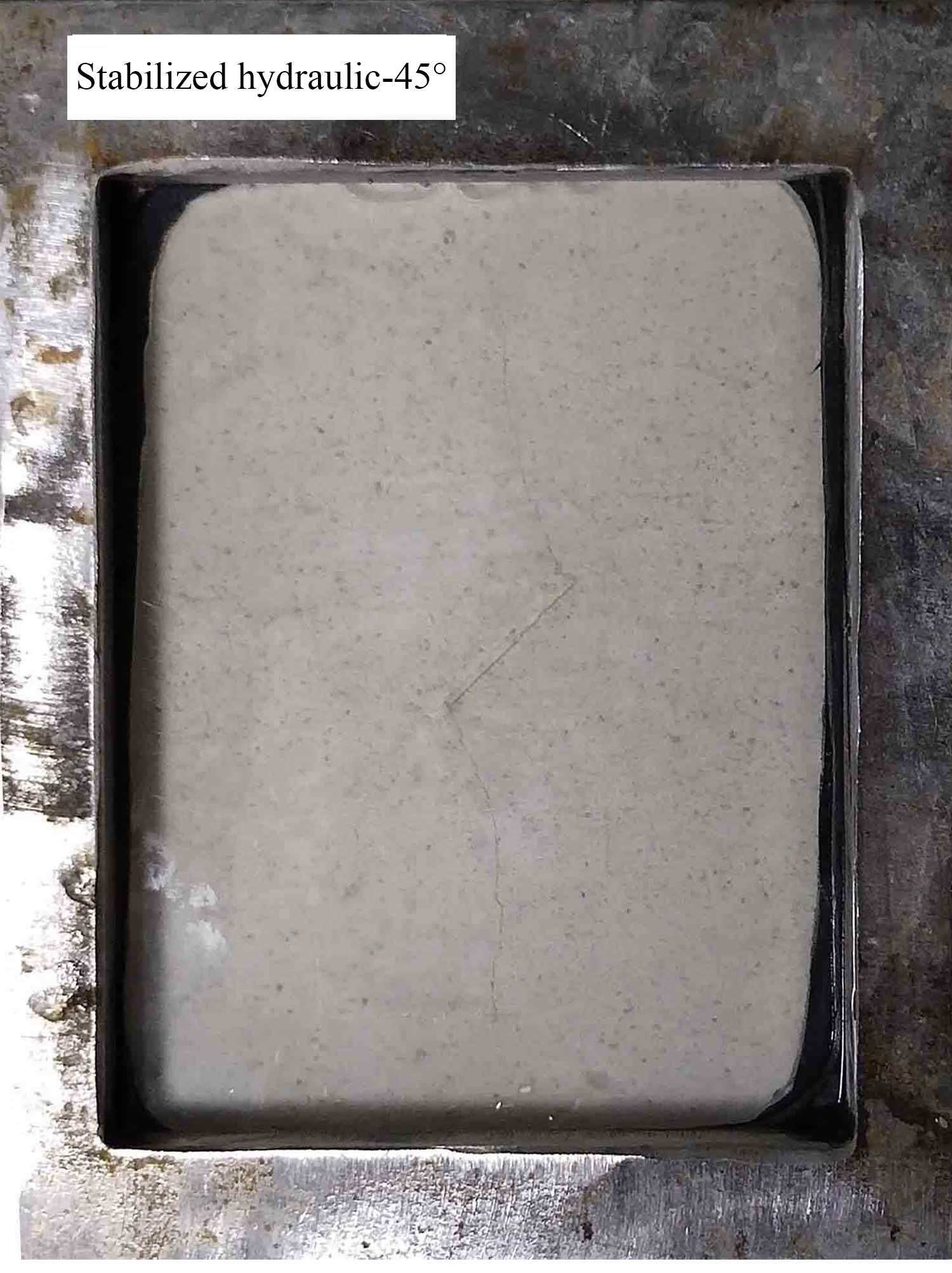

Supplement: S1 Table — (ZIP) [file pone.0307700.s003.zip › Table 1/Stabilized hydraulic-45íπ-Test result.tif]

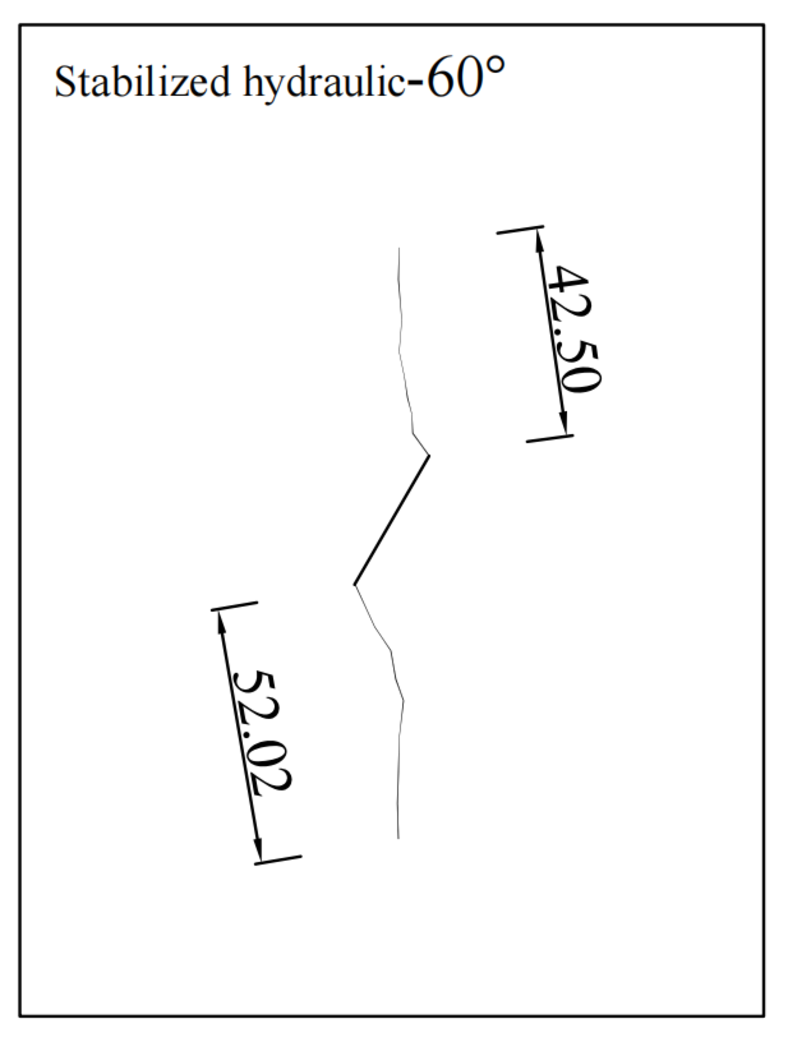

Supplement: S1 Table — (ZIP) [file pone.0307700.s003.zip › Table 1/Stabilized hydraulic-60íπ-Diagrammatic sketch.tif]

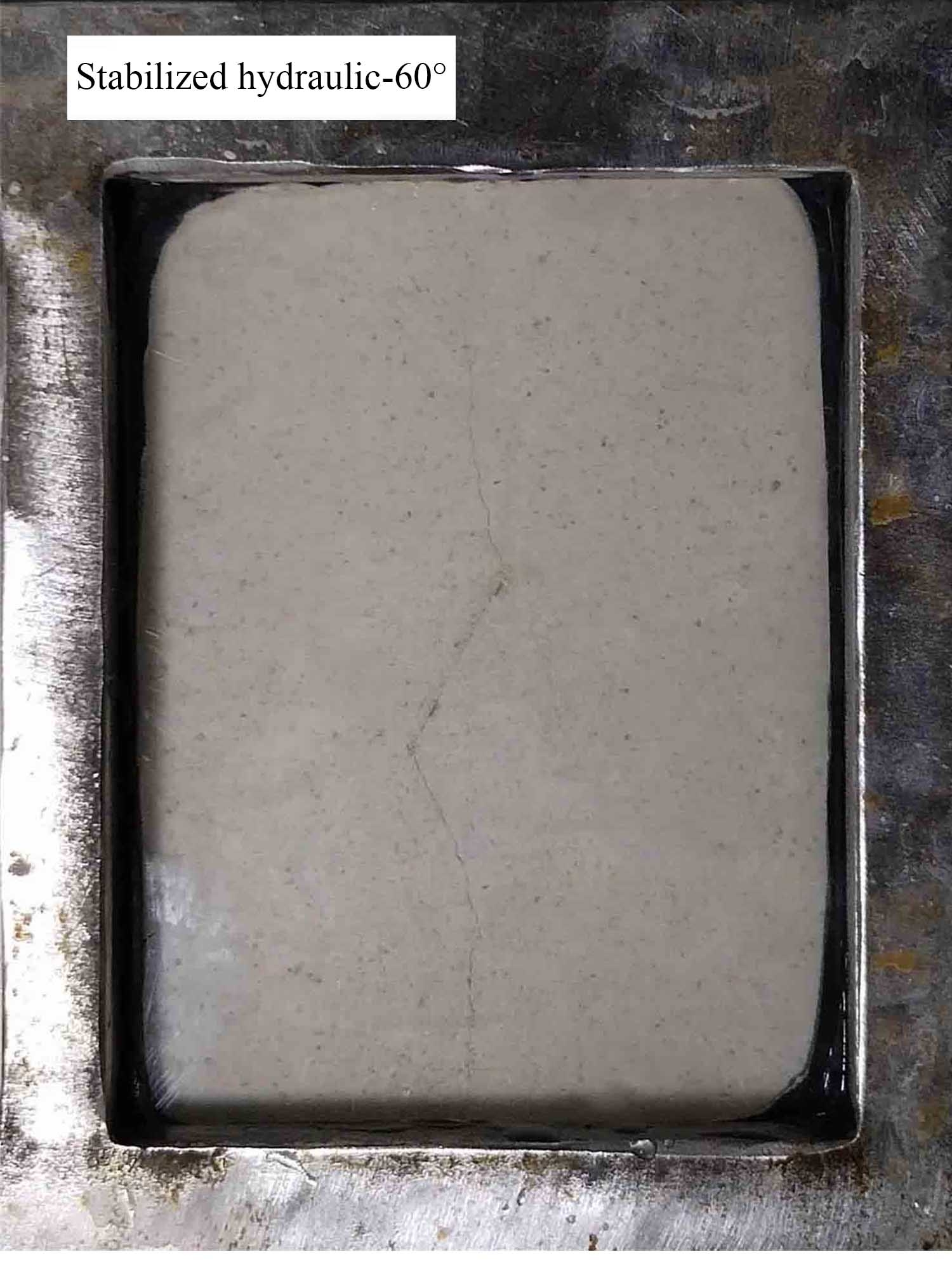

Supplement: S1 Table — (ZIP) [file pone.0307700.s003.zip › Table 1/Stabilized hydraulic-60íπ-Test result.tif]

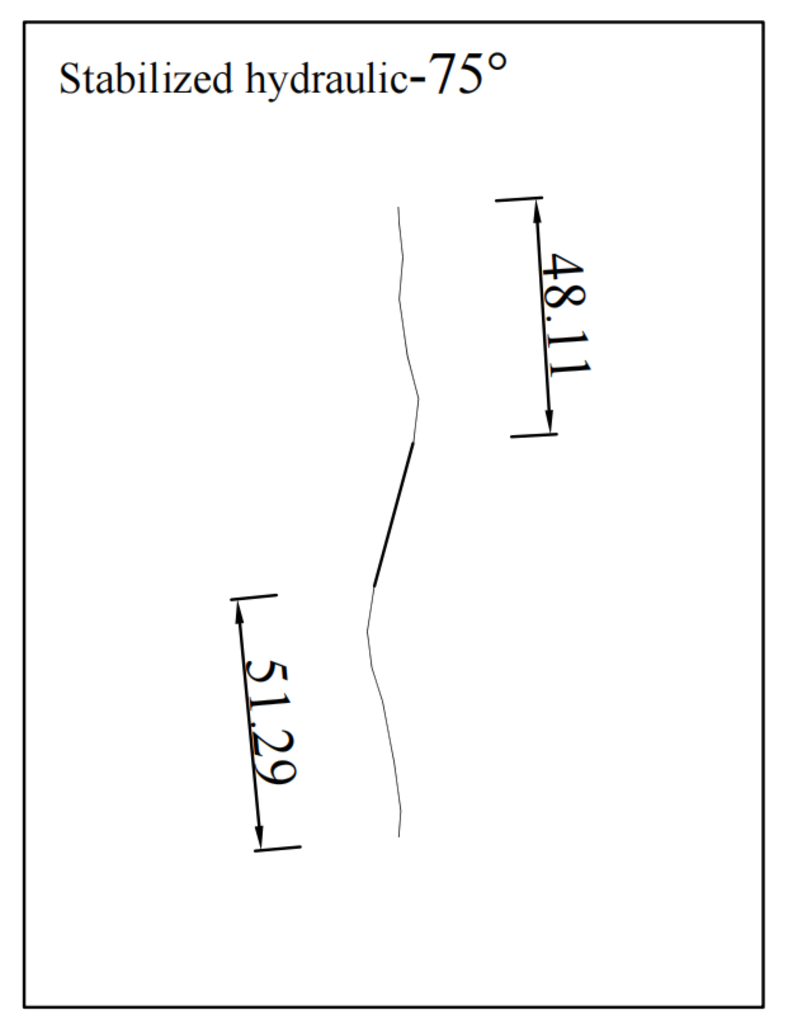

Supplement: S1 Table — (ZIP) [file pone.0307700.s003.zip › Table 1/Stabilized hydraulic-75íπ-Diagrammatic sketch.tif]

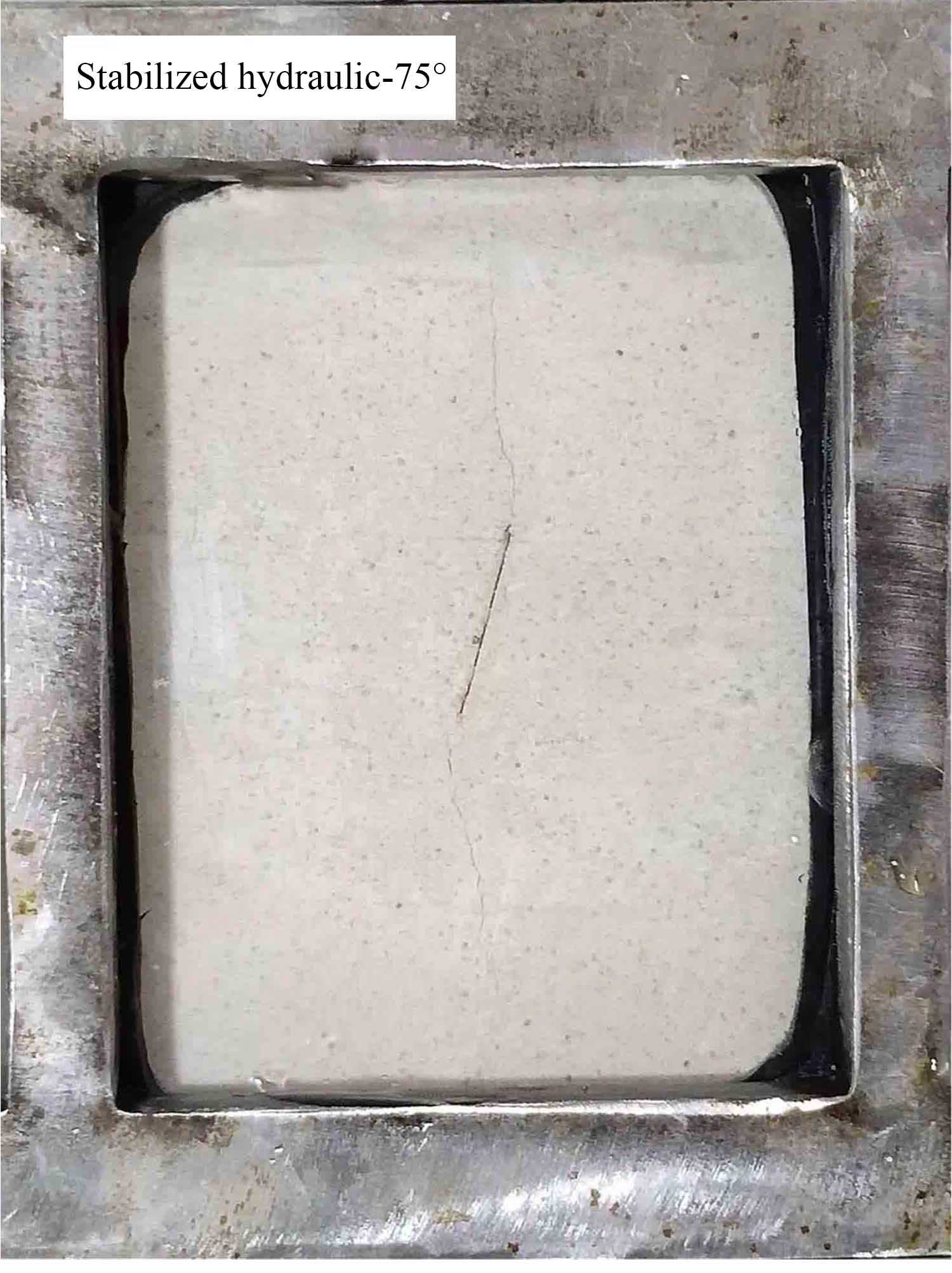

Supplement: S1 Table — (ZIP) [file pone.0307700.s003.zip › Table 1/Stabilized hydraulic-75íπ-Test result.tif]

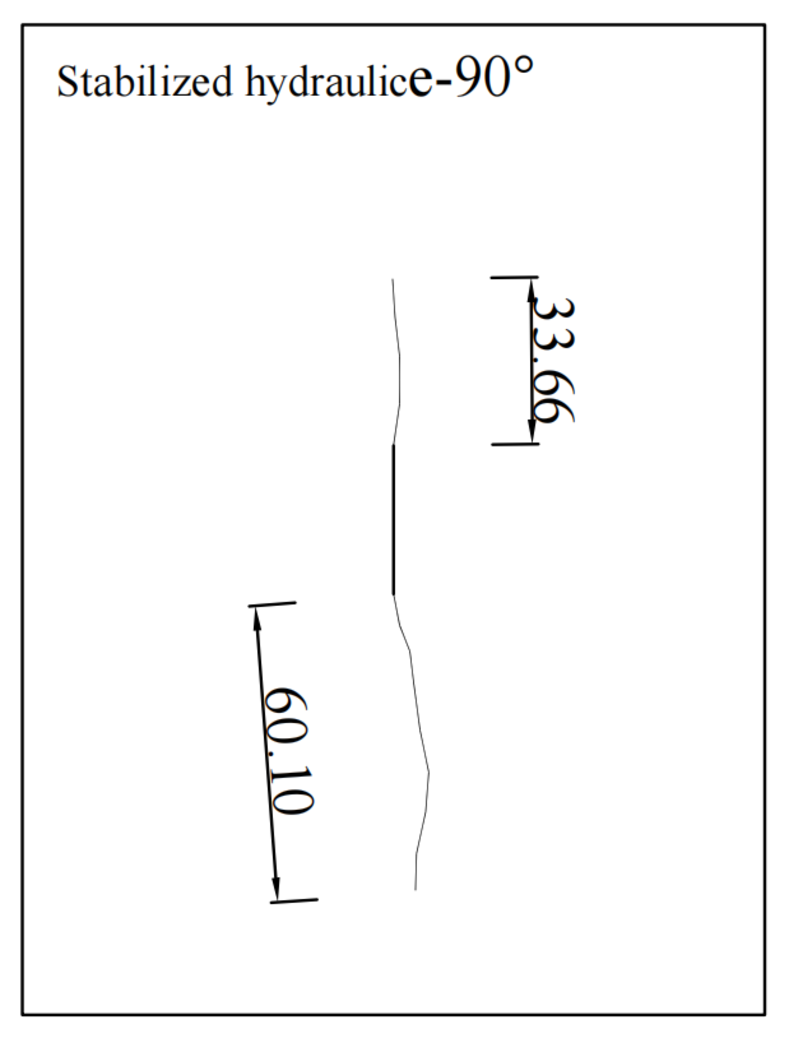

Supplement: S1 Table — (ZIP) [file pone.0307700.s003.zip › Table 1/Stabilized hydraulic-90íπ-Diagrammatic sketch.tif]

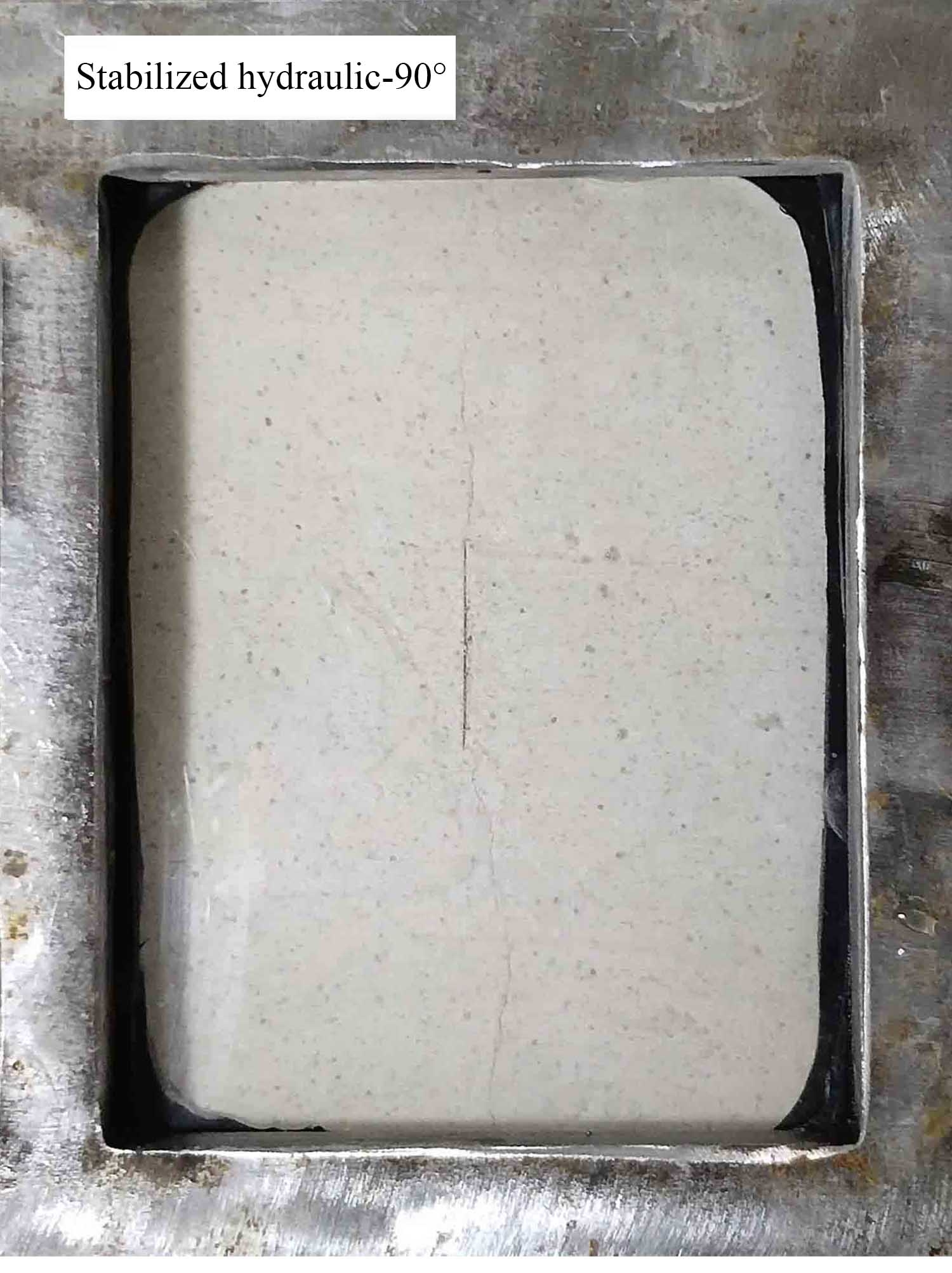

Supplement: S1 Table — (ZIP) [file pone.0307700.s003.zip › Table 1/Stabilized hydraulic-90íπ-Test result.tif]
