# Supplementary material for: Tunnel and underground engineering rock mass water inrush damage and acoustic emission characteristics
Source: PLoS One. 2024 Sep 20;19(9):e0307700. doi: 10.1371/journal.pone.0307700 (PMC11414891; doi:10.1371/journal.pone.0307700)
Supplement: S1 Dataset — (DOCX) [file pone.0307700.s001.docx]

**Fig 5. Stress-strain and hydraulic pressure curves**

9.00E-05	0.00147	1	1001

1.90E-04	0.00147	2	1.00E+03

2.90E-04	0.0056	3	1001.2

3.85E-04	0.0084	4	1001.2

4.55E-04	0.0084	5	1.00E+03

5.50E-04	0.0084	6	1001

6.65E-04	0.01387	7	1001

7.90E-04	0.01253	8	1.00E+03

8.85E-04	0.01947	9	1.00E+03

9.75E-04	0.0208	10	1.00E+03

0.00104	0.0208	11	1.00E+03

0.00113	0.0236	12	1001.2

0.0012	0.02787	13	1001.2

0.00127	0.0292	14	1001.2

0.0013	0.0292	15	1001.2

0.00137	0.0292	16	1001

0.00145	0.0292	17	1001

0.00156	0.03333	18	1001

0.00165	0.03613	19	1001

0.00176	0.03747	20	1001.2

0.00186	0.04027	21	1001.2

0.00198	0.03893	22	1001.2

0.00212	0.04587	23	1001

0.00221	0.05	24	1001

0.00231	0.04867	25	1001

0.00238	0.05	26	1001

0.00246	0.04867	27	1001

0.00253	0.0528	28	1001

0.00262	0.05413	29	1001

0.0027	0.0556	30	1001

0.00279	0.0584	31	1001

0.00293	0.0612	32	1001

0.00303	0.06253	33	1001

0.00313	0.06533	34	1001

0.00324	0.06813	35	1001

0.00331	0.06947	36	1001

0.00337	0.06947	37	1001

0.00346	0.0736	38	1001

0.00354	0.07227	39	1001

0.00364	0.07507	40	1001.2

0.00375	0.07787	41	1001

0.00385	0.07787	42	1001

0.00395	0.08053	43	1001

0.00404	0.0848	44	1001.2

0.00413	0.0848	45	1001.2

0.00424	0.0848	46	1001

0.00433	0.08893	47	1001

0.0044	0.09307	48	1001

0.00448	0.09453	49	1001

0.00454	0.09307	50	1001

0.00461	0.0972	51	1001

0.00469	0.09867	52	1001

0.00478	0.1	53	1001

0.00488	0.1028	54	1001.2

0.00496	0.10427	55	1001

0.00505	0.10693	56	1001

0.00513	0.1084	57	1001

0.00521	0.10973	58	1001

0.0053	0.1112	59	1001

0.00537	0.11387	60	1001

0.00544	0.11533	61	1001

0.0055	0.11667	62	1001

0.00556	0.11813	63	1001

0.00566	0.12093	64	1001

0.00576	0.1236	65	1001

0.00584	0.12507	66	1001

0.00588	0.12787	67	1001

0.00597	0.1264	68	1001.2

0.00605	0.1292	69	1001.2

0.0061	0.132	70	1001

0.0062	0.1348	71	1001

0.00629	0.13613	72	1001

0.00637	0.14027	73	1001

0.00645	0.14027	74	1001

0.00653	0.14173	75	1001

0.0066	0.14453	76	1001

0.00664	0.14587	77	1001

0.00667	0.1472	78	1001

0.00674	0.14867	79	1001

0.0068	0.15147	80	1001.2

0.00687	0.15	81	1001.2

0.00697	0.1528	82	1001.2

0.00705	0.15693	83	1001

0.00714	0.1612	84	1001

0.0072	0.16387	85	1001.2

0.00729	0.16253	86	1001

0.00735	0.16533	87	1001

0.00739	0.16667	88	1001.2

0.00747	0.16813	89	1001

0.0075	0.16947	90	1001.2

0.0076	0.17227	91	1001.2

0.00767	0.17093	92	1001

0.00776	0.17787	93	1001

0.00782	0.17787	94	1001

0.00788	0.17787	95	1001

0.00795	0.1792	96	1001

0.00803	0.1848	97	1001.2

0.0081	0.1848	98	1001

0.00816	0.1876	99	1001

0.00821	0.18893	100	1001

0.00826	0.19307	101	1001

0.00832	0.19173	102	1001

0.00836	0.19307	103	1001

0.00842	0.19453	104	1001

0.00847	0.19733	105	1001

0.00855	0.20147	106	1001

0.00861	0.20147	107	1001

0.00866	0.2028	108	1001

0.00872	0.2056	109	1001

0.00877	0.20693	110	1001

0.00882	0.20973	111	1001.2

0.00888	0.2112	112	1001

0.00895	0.20973	113	1001

0.00901	0.214	114	1001

0.00907	0.21667	115	1001

0.00912	0.21813	116	1001

0.00919	0.21947	117	1001

0.00923	0.22227	118	1001

0.00932	0.22507	119	1001

0.00936	0.22507	120	1001

0.00942	0.2292	121	1001.2

0.00947	0.2292	122	1001

0.00951	0.232	123	1001

0.00957	0.2348	124	1001

0.00961	0.23333	125	1001.2

0.00967	0.23613	126	1001

0.00972	0.24027	127	1001.2

0.00978	0.24307	128	1001.2

0.00984	0.24307	129	1001.2

0.00989	0.24587	130	1001.2

0.00992	0.24453	131	1001

0.00998	0.24867	132	1001

0.01003	0.25	133	1001

0.01008	0.2528	134	1001.2

0.01011	0.2528	135	1001.2

0.01016	0.25427	136	1001

0.01022	0.25693	137	1001

0.01028	0.25693	138	1001.2

0.01033	0.264	139	1001.2

0.01035	0.26253	140	1001.2

0.01042	0.26667	141	1001

0.01048	0.26947	142	1001.2

0.01053	0.26813	143	1001.2

0.01056	0.27093	144	1001.2

0.01059	0.27507	145	1001.2

0.01064	0.27507	146	1001.2

0.01069	0.2764	147	1001

0.01075	0.27787	148	1001

0.01078	0.2764	149	1001

0.01084	0.282	150	1001

0.0109	0.2848	151	1001

0.01095	0.2876	152	1001

0.01098	0.2876	153	1001.2

0.01101	0.29027	154	1001

0.01105	0.29027	155	1001

0.01112	0.29173	156	1001

0.01117	0.29307	157	1001

0.0112	0.29733	158	1001.2

0.01125	0.29733	159	1001.2

0.01127	0.29867	160	1001

0.01132	0.3028	161	1001

0.01137	0.3028	162	1001

0.01142	0.3084	163	1001

0.01149	0.3084	164	1001.2

0.01152	0.3112	165	1001.2

0.01155	0.3112	166	1001

0.01158	0.31533	167	1001.2

0.01162	0.31533	168	1001

0.01167	0.31947	169	1001.2

0.01172	0.31947	170	1001

0.01173	0.32227	171	1001

0.01178	0.32373	172	1001

0.01184	0.32507	173	1001

0.01186	0.32787	174	1001

0.0119	0.32787	175	1001.2

0.01196	0.33067	176	1001

0.012	0.33333	177	1001

0.01205	0.3348	178	1001

0.01211	0.33613	179	1001

0.01213	0.3376	180	1001

0.01216	0.3404	181	1001

0.01219	0.3404	182	1001

0.01223	0.34453	183	1001

0.01225	0.34733	184	1001

0.0123	0.34867	185	1001

0.01231	0.35	186	1001

0.01236	0.35	187	1001

0.0124	0.3528	188	1001

0.01245	0.35427	189	1001

0.0125	0.35707	190	1001.2

0.01254	0.35973	191	1001

0.01258	0.36253	192	1001

0.01262	0.364	193	1001.2

0.01262	0.36533	194	1001

0.01265	0.36667	195	1001

0.0127	0.364	196	1001

0.01272	0.37093	197	1001

0.01277	0.36947	198	1001

0.01282	0.37373	199	1001

0.01286	0.3764	200	1001.2

0.0129	0.3792	201	1001.2

0.01293	0.382	202	1001

0.01297	0.382	203	1001

0.01299	0.38333	204	1001

0.01302	0.38613	205	1001

0.01304	0.38613	206	1001

0.01308	0.3876	207	1001

0.01313	0.3904	208	1001

0.01317	0.3904	209	1001

0.01321	0.39453	210	1001

0.01325	0.39733	211	1001

0.01327	0.4	212	1001

0.01331	0.40147	213	1001

0.01331	0.40147	214	1001

0.01334	0.4028	215	1001

0.01336	0.4056	216	1001

0.01341	0.40707	217	1001.2

0.01346	0.40973	218	1001.2

0.01351	0.40973	219	1001

0.01357	0.41253	220	1001

0.01362	0.41533	221	1001

0.01366	0.41947	222	1001

0.01371	0.42093	223	1001

0.01374	0.42227	224	1001

0.01376	0.42507	225	1001

0.01377	0.42373	226	1001

0.01379	0.42787	227	1001

0.01381	0.4292	228	1001

0.01384	0.4292	229	1001

0.01388	0.432	230	1001

0.01393	0.432	231	1001

0.01399	0.43347	232	1001

0.01403	0.4376	233	1001

0.01407	0.4404	234	1001

0.01411	0.44307	235	1001

0.01413	0.44587	236	1001

0.01416	0.44307	237	1001

0.01417	0.44453	238	1001

0.0142	0.44867	239	1001

0.01424	0.44867	240	1001

0.01428	0.44867	241	1001

0.01434	0.4528	242	1001

0.01438	0.45973	243	1001

0.01442	0.45707	244	1001

0.01446	0.4612	245	1001.2

0.01451	0.46253	246	1001

0.01451	0.46253	247	1001

0.01453	0.46533	248	1001

0.01455	0.46533	249	1001

0.01459	0.4668	250	1001

0.01462	0.47093	251	1001

0.01467	0.47093	252	1001

0.01472	0.47507	253	1001

0.01474	0.47373	254	1001

0.01478	0.4792	255	1001

0.01481	0.48067	256	1001

0.01486	0.482	257	1001

0.01488	0.48347	258	1001

0.01491	0.4848	259	1001

0.01492	0.4876	260	1001

0.01496	0.4904	261	1001

0.01498	0.49173	262	1001

0.01502	0.49307	263	1001

0.01502	0.49733	264	1001

0.01504	0.49587	265	1001

0.01508	0.50013	266	1001

0.01513	0.50013	267	1001

0.01517	0.5028	268	1001

0.01519	0.50427	269	1001

0.01522	0.5084	270	1001

0.01523	0.5084	271	1001

0.01528	0.51253	272	1001

0.01529	0.51253	273	1001

0.01534	0.514	274	1001

0.01537	0.51533	275	1001

0.0154	0.51813	276	1001

0.01541	0.51947	277	1001

0.01545	0.52373	278	1001

0.01548	0.5264	279	1001

0.01551	0.5264	280	1001

0.01553	0.52507	281	1001

0.01558	0.52787	282	1001

0.01561	0.5292	283	1001

0.01567	0.532	284	1001

0.01573	0.53613	285	1001

0.01577	0.53893	286	1001

0.01579	0.53893	287	1001

0.01582	0.54173	288	1001

0.01583	0.54453	289	1001

0.01586	0.54173	290	1001

0.01587	0.54453	291	1001

0.0159	0.54733	292	1001

0.01592	0.54867	293	1001

0.01596	0.55147	294	1001

0.01599	0.5528	295	1001

0.01604	0.55427	296	1001

0.01607	0.55707	297	1001

0.01612	0.56253	298	1001

0.01613	0.55987	299	1001

0.01616	0.56253	300	1001

0.0162	0.56813	301	1001

0.0162	0.56533	302	1001

0.01623	0.56813	303	1001

0.01625	0.56813	304	1001

0.01627	0.57227	305	1001

0.01629	0.57227	306	1001

0.01632	0.57653	307	1001

0.0163	0.5792	308	1001

0.01633	0.58347	309	1001

0.01636	0.58067	310	1001

0.0164	0.58347	311	1001

0.01642	0.58613	312	1001

0.01646	0.5848	313	1001

0.01648	0.58893	314	1001

0.01653	0.59173	315	1001

0.01656	0.59587	316	1001

0.01657	0.59587	317	1001

0.01661	0.59867	318	1001

0.01664	0.60013	319	1001

0.01667	0.59867	320	1001

0.01668	0.60147	321	1001

0.01669	0.6056	322	1001

0.01674	0.6084	323	1001

0.01678	0.60707	324	1001

0.01682	0.60987	325	1001

0.01684	0.6112	326	1001

0.01686	0.614	327	1001

0.01689	0.61533	328	1001

0.01691	0.61813	329	1001

0.01694	0.6168	330	1001

0.01698	0.61813	331	1001

0.01699	0.62227	332	1001

0.01703	0.62373	333	1001

0.01707	0.62653	334	1001

0.01711	0.6292	335	1001

0.01713	0.63067	336	1001

0.01715	0.63067	337	1001

0.01716	0.632	338	1001

0.01718	0.6348	339	1001

0.01722	0.6404	340	1001

0.01723	0.63893	341	1001

0.01727	0.6432	342	1001

0.01729	0.6404	343	1001

0.01731	0.64587	344	1001

0.01734	0.64733	345	1001

0.01736	0.64867	346	1001

0.01739	0.65147	347	1001

0.01745	0.65147	348	1001

0.0175	0.65427	349	1001

0.01754	0.65707	350	1001

0.01758	0.6584	351	1001

0.01757	0.6612	352	1001

0.01762	0.664	353	1001

0.01764	0.664	354	1001

0.01769	0.66533	355	1001

0.01773	0.6668	356	1001

0.01775	0.6696	357	1001

0.01778	0.67093	358	1001

0.01779	0.67507	359	1001

0.01779	0.67653	360	1001

0.01783	0.67787	361	1001

0.01784	0.67787	362	1001

0.01787	0.68347	363	1001

0.0179	0.682	364	1001

0.01793	0.6848	365	1001

0.01796	0.68627	366	1001

0.01798	0.68893	367	1001

0.018	0.69173	368	1001

0.01801	0.6904	369	1001

0.01802	0.69453	370	1001

0.01804	0.69453	371	1001

0.01806	0.69453	372	1001

0.0181	0.69867	373	1001

0.01814	0.70013	374	1001

0.01817	0.7056	375	1001

0.0182	0.70427	376	1001

0.01823	0.7084	377	1001

0.01824	0.70987	378	1001

0.01826	0.7112	379	1001

0.01827	0.7112	380	1000.4

0.01831	0.71533	381	1001

0.01834	0.71533	382	1000.4

0.01836	0.71813	383	1001

0.0184	0.7196	384	1001

0.01843	0.72373	385	1001

0.01846	0.72653	386	1000.4

0.01845	0.72653	387	1001

0.01847	0.7292	388	1001

0.01847	0.72787	389	1001

0.0185	0.7292	390	1001

0.01851	0.732	391	1001

0.01856	0.73347	392	1001

0.01858	0.73627	393	1001

0.01862	0.73893	394	1001

0.01864	0.7432	395	1001

0.01865	0.74453	396	1001

0.01866	0.74587	397	1001

0.01866	0.74867	398	1001

0.01866	0.74733	399	1001

0.01867	0.74867	400	1001

0.0187	0.75013	401	1001

0.01874	0.75147	402	1001

0.01879	0.75707	403	1000.4

0.0188	0.7556	404	1001

0.01884	0.75987	405	1000.4

0.01888	0.764	406	1001

0.0189	0.76533	407	1000.4

0.0189	0.76533	408	1001

0.01891	0.7668	409	1001

0.01892	0.7668	410	1001

0.01896	0.7696	411	1001

0.019	0.77093	412	1001

0.01903	0.77507	413	1001

0.01905	0.77653	414	1000.4

0.01906	0.77653	415	1001

0.0191	0.7792	416	1001

0.01912	0.782	417	1000.4

0.01913	0.78347	418	1001

0.01913	0.78347	419	1001

0.01917	0.7876	420	1001

0.01919	0.78893	421	1001

0.01922	0.7904	422	1001

0.01923	0.796	423	1001

0.01926	0.796	424	1001

0.01928	0.79867	425	1001

0.01931	0.79867	426	1000.4

0.01934	0.80013	427	1000.4

0.01935	0.80293	428	1000.4

0.01939	0.8056	429	1000.4

0.01941	0.80707	430	1000.4

0.01942	0.8084	431	1001

0.01944	0.8112	432	1001

0.01947	0.80987	433	1001

0.01949	0.814	434	1001

0.01952	0.81533	435	1001

0.01955	0.8168	436	1001

0.01956	0.82093	437	1001

0.01958	0.82227	438	1001

0.01959	0.82373	439	1001

0.01962	0.82653	440	1001

0.01963	0.82787	441	1000.4

0.01966	0.82933	442	1000.4

0.01969	0.82933	443	1001

0.01971	0.832	444	1001

0.01972	0.8348	445	1001

0.01973	0.83627	446	1001

0.01975	0.83627	447	1001

0.01977	0.83893	448	1001

0.01979	0.84173	449	1001

0.01979	0.84453	450	1001

0.01983	0.8432	451	1000.4

0.01986	0.84733	452	1001

0.01991	0.84867	453	1001

0.01992	0.85147	454	1001

0.01992	0.85293	455	1001

0.01993	0.85293	456	1000.4

0.01996	0.8556	457	1000.4

0.01999	0.85707	458	1000.4

0.02002	0.8612	459	1001

0.02003	0.864	460	1000.4

0.02003	0.86533	461	1000.4

0.02005	0.864	462	1000.4

0.02003	0.86813	463	1000.4

0.02005	0.86813	464	1001

0.02007	0.87227	465	1000.4

0.02008	0.87227	466	1000.4

0.0201	0.87507	467	1000.4

0.0201	0.87653	468	1000.4

0.02014	0.87933	469	1000.4

0.02016	0.88067	470	1000.4

0.02018	0.8848	471	1000.4

0.02019	0.8848	472	1000.4

0.02021	0.88627	473	1001

0.02023	0.88893	474	1001

0.02025	0.8904	475	1001

0.02029	0.89173	476	1000.4

0.02029	0.89453	477	1000.4

0.02032	0.896	478	1000.4

0.02034	0.90013	479	1001

0.02037	0.90013	480	1000.4

0.02038	0.90293	481	1000.4

0.02039	0.9056	482	1000.4

0.0204	0.9056	483	1000.4

0.02043	0.90707	484	1000.4

0.02047	0.9112	485	1000.4

0.02049	0.91267	486	1000.4

0.02049	0.91267	487	1000.4

0.02048	0.91533	488	1000.4

0.02049	0.914	489	1000.4

0.02051	0.91813	490	1000.4

0.02053	0.91813	491	1000.4

0.02055	0.9224	492	1000.4

0.02057	0.92507	493	1000.4

0.02061	0.92653	494	1000.4

0.02063	0.92653	495	1000.4

0.02066	0.93067	496	1000.4

0.02068	0.93347	497	1000.4

0.0207	0.93347	498	1000.4

0.02074	0.93347	499	1000.4

0.02075	0.93627	500	1001

0.02075	0.9376	501	1000.4

0.02077	0.9432	502	1000.4

0.02078	0.9432	503	1000.4

0.02081	0.9432	504	1001

0.02081	0.94733	505	1001

0.02082	0.94867	506	1000.4

0.02086	0.95147	507	1000.4

0.0209	0.95147	508	1000.4

0.02092	0.95293	509	1000.4

0.02093	0.95573	510	1000.4

0.02096	0.95707	511	1001

0.02098	0.9584	512	1001

0.02101	0.9612	513	1000.4

0.02101	0.9612	514	1000.4

0.02105	0.964	515	1001

0.02109	0.9668	516	1001

0.02109	0.9724	517	1001

0.0211	0.97093	518	1001

0.02113	0.97093	519	1001

0.02118	0.97653	520	1000.4

0.0212	0.97787	521	1001

0.02121	0.97933	522	1000.4

0.02122	0.97933	523	1000.4

0.02125	0.982	524	1000.4

0.02127	0.982	525	1000.4

0.02128	0.98627	526	1000.4

0.02131	0.98907	527	1001

0.02137	0.9876	528	1000.4

0.02138	0.98907	529	1000.4

0.0214	0.9932	530	1000.4

0.02142	0.99453	531	1000.4

0.02143	1.00013	532	1000.4

0.02144	1.00013	533	1001

0.02146	1.00013	534	1001

0.02148	1.00293	535	1001

0.02151	1.00427	536	1000.4

0.02156	1.00427	537	1000.4

0.02157	1.00987	538	1000.4

0.0216	1.0112	539	1001

0.02159	1.014	540	1000.4

0.02159	1.014	541	1000.4

0.02162	1.01533	542	1000.4

0.02166	1.01813	543	1000.4

0.02167	1.0196	544	1000.4

0.02168	1.02093	545	1000.4

0.02173	1.0224	546	1000.4

0.02173	1.02653	547	1001

0.02175	1.02653	548	1000.4

0.02179	1.02933	549	1000.4

0.02181	1.02933	550	1001

0.02182	1.03213	551	1000.4

0.02186	1.03627	552	1001

0.02189	1.0376	553	1001

0.02189	1.0376	554	1000.4

0.02189	1.03907	555	1000.4

0.02194	1.04173	556	1000.4

0.02193	1.04173	557	1001

0.02194	1.05013	558	1001

0.02198	1.0488	559	1000.4

0.022	1.05013	560	1000.4

0.02201	1.05013	561	1000.4

0.02206	1.05573	562	1000.4

0.02209	1.05573	563	1000.4

0.02212	1.05707	564	1000.4

0.02213	1.0584	565	1000.4

0.02215	1.05987	566	1000.4

0.02217	1.0612	567	1000.4

0.02219	1.064	568	1000.4

0.02223	1.0668	569	1000.4

0.02225	1.0696	570	1000.4

0.02227	1.0696	571	1000.4

0.02228	1.07373	572	1000.4

0.02232	1.07653	573	1000.4

0.02231	1.07653	574	1000.4

0.02236	1.07507	575	1000.4

0.02237	1.07933	576	1000.4

0.02241	1.08347	577	1000.4

0.02243	1.07933	578	1000.4

0.02244	1.08347	579	1000.4

0.02244	1.0876	580	1000.4

0.02249	1.0876	581	1000.4

0.02251	1.09173	582	1000.4

0.02253	1.09173	583	1000.4

0.02256	1.09453	584	1000.4

0.02257	1.09733	585	1000.4

0.02258	1.0988	586	1000.4

0.02261	1.0988	587	1000.4

0.02263	1.10147	588	1000.4

0.02264	1.10147	589	1000.4

0.02267	1.10573	590	1000.4

0.02269	1.1084	591	1000.4

0.02274	1.1084	592	1000.4

0.02277	1.1112	593	1000.4

0.02277	1.11267	594	1000.4

0.02281	1.1168	595	1000.4

0.02283	1.11813	596	1000.4

0.02286	1.1196	597	1000.4

0.02288	1.12093	598	1000.4

0.0229	1.12093	599	1000.4

0.02293	1.12653	600	1000.4

0.02295	1.12507	601	1000.4

0.02298	1.12933	602	1000.4

0.02299	1.13067	603	1000.4

0.02302	1.13213	604	1000.4

0.02303	1.13347	605	1000.4

0.02308	1.1348	606	1000.4

0.02311	1.1376	607	1000.4

0.02313	1.1404	608	1000.4

0.02315	1.1404	609	1000.4

0.02316	1.14173	610	1000.4

0.02318	1.146	611	1000.4

0.02319	1.1488	612	1000.4

0.02321	1.15013	613	1000.4

0.02323	1.15147	614	1000.4

0.02325	1.15147	615	1000.4

0.02328	1.15293	616	1000.4

0.02331	1.15427	617	1000.4

0.02332	1.15853	618	1000.4

0.02336	1.1612	619	1000.4

0.02338	1.1612	620	1000.4

0.0234	1.16267	621	1000.4

0.0234	1.1668	622	1000.4

0.02342	1.16813	623	1000.4

0.02343	1.1696	624	1000.4

0.02346	1.17093	625	1000.4

0.02349	1.17373	626	1000.4

0.02353	1.1752	627	1000.4

0.02355	1.17933	628	1000.4

0.02356	1.18067	629	1000.4

0.02356	1.17933	630	1000.4

0.02357	1.18347	631	1000.4

0.0236	1.18347	632	1000.4

0.02362	1.18627	633	1000.4

0.02365	1.18907	634	1000.4

0.02366	1.18907	635	1000.4

0.02368	1.1932	636	1000.4

0.0237	1.19187	637	1000.4

0.02373	1.19733	638	1000.4

0.02374	1.196	639	1000.4

0.02377	1.196	640	1000.4

0.02379	1.20293	641	1000.4

0.02381	1.20293	642	1000.4

0.02381	1.20573	643	1000.4

0.02383	1.20293	644	1000.4

0.02385	1.20853	645	1000.4

0.02388	1.20853	646	1000.4

0.0239	1.21267	647	1000.4

0.02394	1.214	648	1000.4

0.02394	1.21547	649	1000.4

0.02398	1.21813	650	1000.4

0.02398	1.2196	651	1000.4

0.02399	1.22093	652	1000.4

0.02401	1.2224	653	1000.4

0.02404	1.2252	654	1000.4

0.02406	1.22653	655	1000.4

0.02408	1.22933	656	1000.4

0.0241	1.23213	657	1000.4

0.02412	1.23627	658	1000.4

0.02415	1.23627	659	1000.4

0.02417	1.23627	660	1000.4

0.02418	1.2376	661	1000.4

0.02421	1.24187	662	1000.4

0.02422	1.2404	663	1000.4

0.02423	1.246	664	1000.4

0.02423	1.24733	665	1000.4

0.02423	1.25013	666	1000.4

0.02424	1.25013	667	1000.4

0.02427	1.25147	668	1000.4

0.02429	1.25293	669	1000.4

0.02431	1.25427	670	1000.4

0.02431	1.25853	671	1000.4

0.02434	1.25853	672	1000.4

0.02436	1.26267	673	1000.4

0.02439	1.264	674	1000.4

0.02442	1.26267	675	1000.4

0.02444	1.26547	676	1000.4

0.02446	1.2696	677	1000.4

0.02448	1.27093	678	1000.4

0.0245	1.2724	679	1000.4

0.0245	1.2724	680	1000.4

0.02451	1.2752	681	1000.4

0.02453	1.27787	682	1000.4

0.02453	1.28067	683	1000.4

0.02455	1.28213	684	1000.4

0.02458	1.28067	685	1000.4

0.02461	1.2876	686	1000.4

0.02464	1.2876	687	1000.4

0.02465	1.28907	688	1000.4

0.02466	1.29187	689	1000.4

0.02468	1.29453	690	1000.4

0.02468	1.2932	691	1000.4

0.0247	1.296	692	1000.4

0.02473	1.29733	693	1000.4

0.02477	1.30293	694	1000.4

0.02477	1.30013	695	1000.4

0.02479	1.30427	696	1000.4

0.02481	1.30707	697	1000.4

0.02482	1.30707	698	1000.4

0.02482	1.30987	699	1000.4

0.02486	1.3112	700	1000.4

0.02489	1.31267	701	1000.4

0.02492	1.314	702	1000.4

0.02495	1.3168	703	1000.4

0.02497	1.3196	704	1000.4

0.02496	1.3196	705	1000.4

0.02499	1.32373	706	1000.4

0.02501	1.32373	707	1000.4

0.02506	1.3252	708	1000.4

0.02507	1.32653	709	1000.4

0.02508	1.33213	710	1000.4

0.02508	1.33213	711	1000.4

0.02511	1.33493	712	1000.4

0.02514	1.33493	713	1000.4

0.02518	1.33627	714	1000.4

0.0252	1.33907	715	1000.4

0.02524	1.34187	716	1000.4

0.02526	1.3432	717	1000.4

0.02528	1.34453	718	1000.4

0.02528	1.34733	719	1000.4

0.0253	1.35013	720	1000.4

0.02531	1.35013	721	1000.4

0.02534	1.35293	722	1000.4

0.02536	1.35427	723	1000.4

0.02541	1.35707	724	1000.4

0.0254	1.36267	725	1000.4

0.02544	1.36267	726	1000.4

0.02546	1.36267	727	1000.4

0.02549	1.364	728	1000.4

0.02548	1.36547	729	1000.4

0.02553	1.3668	730	1000.4

0.02553	1.3696	731	1000.4

0.02555	1.3752	732	1000.4

0.02558	1.3724	733	1000.4

0.02559	1.37653	734	1000.4

0.02562	1.3752	735	1000.4

0.02565	1.37787	736	1000.4

0.02566	1.37933	737	1000.4

0.02569	1.38493	738	1000.4

0.02572	1.38627	739	1000.4

0.02571	1.3876	740	1000.4

0.02575	1.38907	741	1000.4

0.02577	1.39187	742	1000.4

0.02577	1.3904	743	1000.4

0.02579	1.3904	744	1000.4

0.02583	1.39733	745	1000.4

0.02585	1.3988	746	1000.4

0.0259	1.4016	747	1000.4

0.02594	1.40013	748	1000.4

0.02594	1.40293	749	1000.4

0.02597	1.40573	750	1000.4

0.02597	1.40573	751	1000.4

0.02601	1.40853	752	1000.4

0.02604	1.41267	753	1000.4

0.02607	1.41133	754	1000.4

0.02607	1.414	755	1000.4

0.0261	1.4168	756	1000.4

0.02612	1.41827	757	1000.4

0.02614	1.4196	758	1000.4

0.02618	1.4224	759	1000.4

0.02619	1.42373	760	1000.4

0.02623	1.4252	761	1000.4

0.02624	1.428	762	1000.4

0.02625	1.42933	763	1000.4

0.02628	1.42933	764	1000.4

0.02628	1.43347	765	1000.4

0.02632	1.43493	766	1000.4

0.02634	1.4376	767	1000.4

0.02636	1.43627	768	1000.4

0.02638	1.44187	769	1000.4

0.02639	1.44187	770	1000.4

0.02642	1.4432	771	1000.4

0.02644	1.44467	772	1000.4

0.02647	1.44733	773	1000.4

0.02647	1.4516	774	1000.4

0.02651	1.45013	775	1000.4

0.02654	1.45573	776	1000.4

0.02659	1.45707	777	1000.4

0.02662	1.45707	778	1000.4

0.02661	1.45707	779	1000.4

0.02663	1.464	780	1000.4

0.02665	1.464	781	1000.4

0.02668	1.464	782	1000.4

0.02671	1.4668	783	1000.4

0.02672	1.4724	784	1000.4

0.02675	1.47093	785	1000.4

0.02676	1.47093	786	1000.4

0.02677	1.47653	787	1000.4

0.0268	1.47933	788	1000.4

0.02682	1.47933	789	1000.4

0.02683	1.47933	790	1000.4

0.02686	1.48213	791	1000.4

0.0269	1.48627	792	1000.4

0.02693	1.48627	793	1000.4

0.02694	1.4876	794	1000.4

0.02696	1.48907	795	1000.4

0.02698	1.4904	796	1000.4

0.027	1.49467	797	1000.4

0.02701	1.496	798	1000.4

0.02704	1.4988	799	1000.4

0.02704	1.49733	800	1000.4

0.02708	1.50013	801	1000.4

0.0271	1.5016	802	1000.4

0.02711	1.50293	803	1000.4

0.02715	1.5044	804	1000.4

0.02714	1.50573	805	1000.4

0.02719	1.51267	806	1000.4

0.02721	1.51133	807	1000.4

0.02722	1.51547	808	1000.4

0.02725	1.51133	809	1000.4

0.02727	1.5168	810	1000.4

0.02728	1.5196	811	1000.4

0.02728	1.5196	812	1000.4

0.02731	1.52373	813	1000.4

0.02735	1.5224	814	1000.4

0.02737	1.528	815	1000.4

0.02738	1.528	816	1000.4

0.02741	1.53213	817	1000.4

0.02745	1.53347	818	1000.4

0.02745	1.53493	819	1000.4

0.02749	1.53627	820	1000.4

0.02751	1.53627	821	1000.4

0.02755	1.5432	822	1000.4

0.02757	1.54187	823	1000.4

0.0276	1.54467	824	1000.4

0.02762	1.54467	825	1000.4

0.02765	1.54733	826	1000.4

0.02766	1.5516	827	1000.4

0.02768	1.55013	828	1000.4

0.0277	1.55293	829	1000.4

0.02774	1.55293	830	1000.4

0.02776	1.55573	831	1000.4

0.02779	1.55707	832	1000.4

0.02782	1.56267	833	1000.4

0.02783	1.56267	834	1000.4

0.02784	1.56547	835	1000.4

0.02786	1.5696	836	1000.4

0.02788	1.56547	837	1000.4

0.0279	1.56827	838	1000.4

0.02791	1.57373	839	1000.4

0.02792	1.57653	840	1000.4

0.02795	1.5752	841	1000.4

0.02798	1.57373	842	1000.4

0.02799	1.57933	843	1000.4

0.028	1.58067	844	1000.4

0.02803	1.58213	845	1000.4

0.02804	1.58627	846	1000.4

0.02808	1.58773	847	1000.4

0.0281	1.58627	848	1000.4

0.02812	1.58907	849	1000.4

0.02813	1.59187	850	1000.4

0.02816	1.59467	851	1000.4

0.02818	1.59467	852	1000.4

0.02821	1.5988	853	1000.4

0.02823	1.6016	854	1000.4

0.02825	1.60293	855	1000.4

0.02826	1.60293	856	1000.4

0.02827	1.60573	857	1000.4

0.0283	1.6044	858	1000.4

0.02832	1.61133	859	1000.4

0.02834	1.61133	860	1000.4

0.02837	1.61267	861	1000.4

0.02838	1.614	862	1000.4

0.02841	1.61547	863	1000.4

0.02844	1.6168	864	1000.4

0.02846	1.62107	865	1000.4

0.02849	1.6224	866	1000.4

0.0285	1.62373	867	1000.4

0.02852	1.6252	868	1000.4

0.02854	1.62653	869	1000.4

0.02855	1.62933	870	1000.4

0.02858	1.6308	871	1000.4

0.0286	1.63347	872	1000.4

0.02864	1.63627	873	1000.4

0.02865	1.63627	874	1000.4

0.02869	1.63907	875	1000.4

0.02872	1.64187	876	1000.4

0.02874	1.64187	877	1000.4

0.02874	1.64467	878	1000.4

0.02875	1.6488	879	1000.4

0.02877	1.64747	880	1000.4

0.0288	1.65013	881	1000.4

0.02881	1.6516	882	1000.4

0.02882	1.65293	883	1000.4

0.02883	1.65573	884	1000.4

0.02886	1.65853	885	1000.4

0.02888	1.65853	886	1000.4

0.02889	1.66267	887	1000.4

0.02892	1.66267	888	1000.4

0.02894	1.66267	889	1000.4

0.02896	1.66547	890	1000.4

0.02898	1.67107	891	1000.4

0.029	1.6696	892	1000.4

0.02902	1.6724	893	1000.4

0.02903	1.6752	894	1000.4

0.02905	1.67653	895	1000.4

0.02906	1.678	896	1000.4

0.02908	1.67933	897	1000.4

0.0291	1.68347	898	1000.4

0.02911	1.68493	899	1000.4

0.02915	1.68627	900	1000.4

0.02917	1.68773	901	1000.4

0.02919	1.68907	902	1000.4

0.02921	1.6904	903	1000.4

0.02923	1.696	904	1000.4

0.02923	1.696	905	1000.4

0.02926	1.6932	906	1000.4

0.02929	1.7016	907	1000.4

0.02932	1.70293	908	1000.4

0.02935	1.7044	909	1000.4

0.02936	1.70573	910	1000.4

0.02936	1.7044	911	1000.4

0.02939	1.70987	912	1000.4

0.02942	1.71267	913	1000.4

0.02942	1.71413	914	1000.4

0.02944	1.7168	915	1000.4

0.02946	1.71547	916	1000.4

0.02948	1.71827	917	1000.4

0.02952	1.7168	918	1000.4

0.02954	1.7224	919	1000.4

0.02954	1.7224	920	1000.4

0.02956	1.7252	921	1000.4

0.02959	1.7252	922	1000.4

0.02962	1.7308	923	1000.4

0.02964	1.72933	924	1000.4

0.02964	1.73213	925	1000.4

0.02967	1.73347	926	1000.4

0.02971	1.73493	927	1000.4

0.02974	1.73627	928	1000.4

0.02976	1.74187	929	1000.4

0.02977	1.74187	930	1000.4

0.0298	1.7432	931	1000.4

0.0298	1.746	932	1000.4

0.02985	1.74747	933	1000.4

0.02984	1.7516	934	1000.4

0.02985	1.7488	935	1000.4

0.02987	1.75293	936	1000.4

0.02988	1.75573	937	1000.4

0.02991	1.7572	938	1000.4

0.02991	1.75853	939	1000.4

0.02994	1.76267	940	1000.4

0.02996	1.76413	941	1000.4

0.02998	1.76267	942	1000.4

0.03001	1.7668	943	1000.4

0.03003	1.76827	944	1000.4

0.03003	1.77387	945	1000.4

0.03006	1.77107	946	1000.4

0.03008	1.7752	947	1000.4

0.03012	1.7752	948	1000.4

0.03014	1.77933	949	1000.4

0.03013	1.77933	950	1000.4

0.03015	1.7808	951	1000.4

0.03018	1.78493	952	1000.4

0.03022	1.78347	953	1000.4

0.03023	1.78627	954	1000.4

0.03024	1.79053	955	1000.4

0.03025	1.79053	956	1000.4

0.0303	1.79187	957	1000.4

0.03031	1.79467	958	1000.4

0.03034	1.79747	959	1000.4

0.03035	1.79747	960	1000.4

0.03035	1.80013	961	1000.4

0.03038	1.8016	962	1000.4

0.03037	1.80573	963	1000.4

0.03041	1.80293	964	1000.4

0.03044	1.80573	965	1000.4

0.03045	1.80987	966	1000.4

0.0305	1.81267	967	1000.4

0.0305	1.81267	968	1000.4

0.03052	1.81413	969	1000.4

0.03055	1.8168	970	1000.4

0.03058	1.8196	971	1000.4

0.03057	1.8224	972	1000.4

0.0306	1.82387	973	1000.4

0.03062	1.8252	974	1000.4

0.03066	1.8252	975	1000

0.0307	1.828	976	1000.4

0.03071	1.8308	977	1000.4

0.03074	1.83347	978	1000

0.03075	1.83493	979	1000

0.03076	1.83347	980	999.8

0.03077	1.83773	981	999.8

0.03082	1.83907	982	999.8

0.03083	1.84053	983	999.8

0.03087	1.84187	984	999.8

0.0309	1.846	985	999.8

0.03092	1.8488	986	999.8

0.03093	1.84747	987	999.8

0.03093	1.84747	988	999.8

0.03094	1.85293	989	999.8

0.03096	1.8544	990	999.8

0.03099	1.85573	991	999.8

0.03101	1.8572	992	999.8

0.03107	1.86133	993	999.8

0.03108	1.86133	994	999.8

0.0311	1.86547	995	999.8

0.03111	1.86413	996	999.8

0.03117	1.86547	997	999.8

0.03116	1.86827	998	999.8

0.03119	1.8696	999	999.8

0.03123	1.87387	1000	999.8

0.03125	1.87387	1001	1000

0.03126	1.87933	1002	1000

0.03132	1.878	1003	1000

0.03132	1.88213	1004	999.8

0.03134	1.88493	1005	999.8

0.03135	1.88493	1006	999.8

0.03139	1.88493	1007	999.8

0.0314	1.88773	1008	999.8

0.03141	1.88907	1009	999.8

0.03144	1.89187	1010	1000

0.03145	1.8932	1011	1000

0.03146	1.8932	1012	999.8

0.03152	1.8988	1013	999.8

0.03153	1.90027	1014	999.8

0.03155	1.90293	1015	999.8

0.03157	1.8988	1016	1000

0.03162	1.90573	1017	999.8

0.03164	1.90573	1018	999.8

0.03166	1.9072	1019	999.8

0.03168	1.90853	1020	999.8

0.03171	1.91133	1021	999.8

0.03171	1.91267	1022	1000

0.03176	1.91547	1023	999.8

0.03177	1.9224	1024	999.8

0.03177	1.9196	1025	999.8

0.03177	1.91827	1026	999.8

0.03178	1.91547	1027	999.8

0.03177	1.91693	1028	1000

0.03179	1.91693	1029	999.8

0.03179	1.91827	1030	999.8

0.0318	1.91547	1031	1000

0.03182	1.91827	1032	999.8

0.03184	1.91413	1033	1000

0.03186	1.91547	1034	999.8

0.03186	1.91693	1035	1000

0.03184	1.91693	1036	1000

0.03185	1.91547	1037	999.8

0.03187	1.91827	1038	999.8

0.03187	1.91827	1039	999.8

0.03188	1.91693	1040	999.8

0.03187	1.91827	1041	999.8

0.0319	1.91693	1042	999.8

0.03189	1.91827	1043	999.8

0.03189	1.91827	1044	999.8

0.03189	1.91827	1045	999.8

0.0319	1.91413	1046	999.8

0.03188	1.91693	1047	999.8

0.03191	1.91547	1048	999.8

0.03194	1.91693	1049	999.8

0.03195	1.91547	1050	999.8

0.03195	1.91827	1051	999.8

0.03194	1.91827	1052	999.8

0.03195	1.91693	1053	999.8

0.03194	1.91547	1054	999.8

0.03193	1.9196	1055	999.8

0.03195	1.91547	1056	999.8

0.03196	1.91547	1057	1000

0.03195	1.91827	1058	999.8

0.03195	1.91413	1059	999.8

0.03196	1.91693	1060	1000

0.03198	1.91547	1061	999.8

0.03199	1.91693	1062	1000

0.03201	1.91693	1063	1000

0.03201	1.91827	1064	1000

0.03202	1.91693	1065	1000

0.032	1.91547	1066	999.8

0.032	1.91547	1067	999.8

0.03203	1.91693	1068	999.8

0.03204	1.91693	1069	1000

0.03206	1.91547	1070	999.8

0.03209	1.91827	1071	999.8

0.03209	1.91413	1072	1000

0.03208	1.91827	1073	1000

0.03209	1.91693	1074	1000

0.0321	1.91693	1075	1000

0.03211	1.9196	1076	1000

0.03212	1.91693	1077	1000

0.03213	1.91693	1078	999.8

0.03213	1.91827	1079	999.8

0.03212	1.91413	1080	999.8

0.03211	1.9196	1081	1000

0.03211	1.91693	1082	1000

0.03213	1.91827	1083	1000

0.03215	1.91827	1084	1000

0.03214	1.91693	1085	999.8

0.03213	1.91827	1086	1000

0.03215	1.91827	1087	999.8

0.03216	1.91413	1088	999.8

0.03215	1.91547	1089	1000

0.03215	1.91413	1090	1000

0.03218	1.91693	1091	999.8

0.03219	1.91547	1092	1000

0.03218	1.91827	1093	999.8

0.03218	1.91547	1094	1000

0.03218	1.91693	1095	1000

0.03218	1.91827	1096	1000

0.03217	1.91693	1097	1000

0.03218	1.9196	1098	1000

0.03218	1.91547	1099	1000

0.03216	1.91693	1100	1000

0.03215	1.91693	1101	1000

0.03214	1.91547	1102	999.8

0.03216	1.91827	1103	999.8

0.03217	1.91827	1104	999.8

0.03216	1.91693	1105	1000

0.03219	1.91547	1106	999.8

0.03219	1.91827	1107	1000

0.03218	1.91827	1108	1000

0.0322	1.91547	1109	1000

0.03219	1.91693	1110	1000

0.03222	1.9196	1111	1000

0.03224	1.91413	1112	1000

0.03224	1.91547	1113	1000

0.03226	1.91693	1114	1000

0.03227	1.91827	1115	1000

0.03226	1.91547	1116	999.8

0.03226	1.9196	1117	999.8

0.03228	1.91413	1118	999.8

0.03227	1.91693	1119	1000

0.03229	1.91827	1120	1000

0.03227	1.91547	1121	1000

0.03226	1.91413	1122	999.8

0.03228	1.91693	1123	999.8

0.03228	1.91827	1124	1000

0.03228	1.91547	1125	1000

0.03228	1.91827	1126	1000

0.03229	1.91827	1127	1000

0.03229	1.91827	1128	1000

0.0323	1.91827	1129	1000

0.0323	1.91827	1130	1000

0.03229	1.91693	1131	1000

0.03231	1.91693	1132	1000

0.03231	1.91693	1133	1000

0.03232	1.91693	1134	1000

0.03232	1.91547	1135	1000

0.0323	1.91693	1136	1000

0.0323	1.91693	1137	1000

0.03232	1.91547	1138	1000

0.03233	1.91827	1139	1000

0.03234	1.91693	1140	1000

0.03232	1.91827	1141	1000

0.03232	1.91547	1142	1000

0.03235	1.91693	1143	1000

0.03236	1.91693	1144	1000

0.03237	1.91827	1145	1000

0.03236	1.91547	1146	1000

0.03237	1.91827	1147	1000

0.03236	1.91547	1148	1000

0.03237	1.91827	1149	1000

0.03237	1.91693	1150	1000

0.03238	1.91827	1151	1000

0.03238	1.91693	1152	1000

0.03237	1.91693	1153	1000

0.03237	1.91413	1154	1000

0.03237	1.91693	1155	1000

0.03237	1.91693	1156	1000

0.03238	1.91827	1157	1000

0.0324	1.91693	1158	1000

0.0324	1.91827	1159	1000

0.03239	1.91547	1160	1000

0.03239	1.91547	1161	1000

0.03239	1.91827	1162	1000

0.03241	1.91693	1163	1000

0.03242	1.91693	1164	1000

0.03242	1.91693	1165	1000

0.0324	1.91693	1166	1000

0.03239	1.91547	1167	1000

0.03239	1.91693	1168	1000

0.03239	1.92107	1169	1000

0.03239	1.91413	1170	1000

0.0324	1.91827	1171	1000

0.0324	1.9196	1172	1000

0.03241	1.91133	1173	1000

0.03242	1.91827	1174	1000

0.03241	1.91547	1175	1000

0.03241	1.91693	1176	1000

0.03241	1.9196	1177	1000

0.0324	1.91827	1178	1000

0.0324	1.91827	1179	1000

0.0324	1.91547	1180	1000

0.03241	1.91827	1181	1000

0.03243	1.91693	1182	1000

0.03241	1.91693	1183	999.8

0.03243	1.91693	1184	1000

0.03243	1.91693	1185	1000

0.03244	1.91693	1186	1000

0.03243	1.91547	1187	1000

0.03245	1.9196	1188	1000

0.03247	1.91547	1189	1000

0.03248	1.91413	1190	1000

0.03247	1.91693	1191	1000

0.03246	1.91827	1192	1000

0.03248	1.91693	1193	1000

0.03249	1.91827

0.03249	1.91693

0.03248	1.91547

0.03247	1.91547

0.03249	1.9196

0.03247	1.91547

0.03247	1.91547

0.03247	1.91547

0.03248	1.91827

0.03249	1.91413

0.03248	1.91827

0.0325	1.91827

0.03251	1.91413

0.03253	1.91693

0.03252	1.91693

0.03251	1.91827

0.0325	1.91547

0.03251	1.9196

0.03251	1.91693

0.03252	1.91547

0.03253	1.91827

0.03252	1.91547

0.03252	1.91413

0.03251	1.9196

0.03251	1.91693

0.03252	1.91547

0.03252	1.91827

0.03252	1.91693

0.03252	1.91413

0.03254	1.91827

0.03255	1.91693

0.03255	1.91827

0.03253	1.91547

0.03254	1.91693

0.03256	1.91547

0.03257	1.91413

0.03256	1.91827

0.03254	1.91693

0.03255	1.91827

0.03256	1.91827

0.03256	1.91827

0.03257	1.91693

0.03257	1.91413

0.0326	1.91827

0.0326	1.91547

0.0326	1.91547

0.03259	1.91827

0.03259	1.91827

0.0326	1.91547

0.03259	1.91693

0.0326	1.9196

0.03259	1.91827

0.03261	1.91413

0.03261	1.91693

0.0326	1.91827

0.0326	1.91413

0.0326	1.91693

0.03262	1.9196

0.03262	1.91693

0.03263	1.91547

0.03263	1.9196

0.03264	1.91693

0.03262	1.91547

0.03261	1.91547

0.03261	1.91547

0.03262	1.92107

0.03263	1.91693

0.03262	1.91693

0.03262	1.91547

0.03264	1.9196

0.03264	1.91547

0.03264	1.91413

0.03265	1.91827

0.03265	1.91827

0.03265	1.91547

0.03264	1.91827

0.03264	1.91547

0.03262	1.91413

0.03263	1.91693

0.03263	1.9196

0.03263	1.91693

0.03264	1.91827

0.03265	1.91693

0.03267	1.91547

0.03265	1.91547

0.03266	1.91693

0.03265	1.91827

0.03266	1.91693

0.03265	1.91547

0.03265	1.91547

0.03264	1.92107

0.03266	1.91547

0.03268	1.91547

0.03269	1.91827

0.03267	1.91693

0.03268	1.91827

0.03266	1.91693

0.03267	1.91693

0.03267	1.91547

0.03269	1.91547

0.0327	1.91827

0.03271	1.91547

0.03273	1.91827

0.03271	1.91827

0.03272	1.91547

0.03272	1.91693

0.03272	1.92107

0.03271	1.91547

0.03271	1.91827

0.03272	1.91827

0.03273	1.91827

0.03272	1.91547

0.03271	1.91827

0.03271	1.91693

0.03272	1.91693

0.03272	1.91693

0.03271	1.9196

0.03271	1.91693

0.0327	1.91827

0.03274	1.9196

0.03274	1.91693

0.03274	1.91413

0.03273	1.91693

0.03274	1.92107

0.03274	1.91547

0.03274	1.91547

0.03273	1.91827

0.03274	1.9196

0.03274	1.91267

0.03274	1.92107

0.03275	1.91693

0.03275	1.91413

0.03276	1.91693

0.03277	1.91827

0.03276	1.91827

0.03276	1.91413

0.03276	1.91827

0.03277	1.91693

0.03277	1.91547

0.03276	1.91827

0.03276	1.91547

0.03275	1.91693

0.03278	1.91827

0.03279	1.91413

0.03278	1.91547

0.03278	1.92107

0.03278	1.91547

0.03279	1.91827

0.03278	1.91693

0.03277	1.91693

0.03279	1.91413

0.03278	1.91693

0.03278	1.9196

0.03278	1.91547

0.03278	1.91693

0.0328	1.9196

0.03279	1.9196

0.0328	1.91693

0.03278	1.91413

0.03278	1.91693

0.03278	1.91547

0.03278	1.91547

0.03279	1.91827

0.03279	1.91693

0.03279	1.9196

0.03279	1.91547

0.03279	1.91693

0.03277	1.91827

0.03278	1.91827

0.03279	1.91693

0.0328	1.91693

0.0328	1.91693

0.03279	1.91547

0.03278	1.91693

0.03278	1.91547

0.03279	1.91827

0.03281	1.91827

0.0328	1.91693

0.0328	1.91693

0.03281	1.91827

0.0328	1.91413

0.0328	1.91693

0.03281	1.91827

0.03281	1.91547

0.03282	1.91827

0.03282	1.9196

0.03283	1.91413

0.03282	1.91827

0.03282	1.91827

0.03284	1.91413

0.03284	1.91547

0.03284	1.9196

0.03284	1.91827

0.03285	1.91267

0.03285	1.92107

0.03284	1.91547

0.03284	1.91693

0.03284	1.91827

0.03284	1.91827

0.03284	1.91547

0.03283	1.91267

0.03284	1.91693

0.03282	1.91693

0.03283	1.91547

0.03282	1.91693

0.03283	1.9196

0.03284	1.91693

0.03284	1.91547

0.03285	1.91693

0.03285	1.91693

0.03286	1.91827

0.03286	1.91547

0.03286	1.91827

0.03286	1.91827

0.03285	1.91547

0.03284	1.91827

0.03283	1.91827

0.03285	1.91547

0.03285	1.91547

0.03287	1.9196

0.03286	1.91693

0.03286	1.91693

0.03287	1.91827

0.03288	1.91547

0.03287	1.91693

0.03288	1.91413

0.03288	1.91693

0.03289	1.91827

0.03289	1.91547

0.03288	1.91827

0.03289	1.91693

0.03289	1.91693

0.03292	1.91693

0.03291	1.91693

0.03291	1.91693

0.03291	1.91693

0.03291	1.91693

0.03292	1.91693

0.03292	1.91693

0.0329	1.91693

0.03291	1.91827

0.03291	1.91693

0.03291	1.91547

0.0329	1.9196

0.03289	1.91693

0.0329	1.91693

0.03289	1.91413

0.03291	1.91693

0.03291	1.91827

0.03289	1.91693

0.0329	1.91827

0.03291	1.91693

0.03293	1.91827

0.03292	1.91547

0.03292	1.91693

0.03292	1.91827

0.03292	1.91547

0.03294	1.91827

0.03295	1.91693

0.03293	1.91693

0.03293	1.91693

0.03291	1.91827

0.03293	1.91413

0.03293	1.91547

0.03291	1.91547

0.0329	1.91547

0.03291	1.91413

0.03293	1.91693

0.03294	1.91827

0.03294	1.91547

0.03295	1.91693

0.03293	1.91827

0.03293	1.91547

0.03293	1.91413

0.03293	1.91693

0.03295	1.91827

0.03296	1.91693

0.03297	1.91827

0.03297	1.91693

0.03296	1.91827

0.03295	1.91693

0.03295	1.91693

0.03294	1.9196

0.03295	1.91827

0.03295	1.91413

0.03295	1.91827

0.03295	1.91827

0.03293	1.91827

0.03294	1.91267

0.03294	1.91827

0.03295	1.9196

0.03295	1.91267

0.03294	1.91827

0.03295	1.91827

0.03295	1.91693

0.03295	1.91547

0.03296	1.91693

0.03296	1.92107

0.03296	1.91413

0.03297	1.91413

0.03296	1.91827

0.03296	1.91693

0.03296	1.91693

0.03296	1.91547

0.03297	1.91693

0.03295	1.91693

0.03294	1.91413

0.03294	1.91693

0.03294	1.9196

0.03297	1.91693

0.03295	1.92107

0.03294	1.91827

0.03293	1.91413

0.03293	1.91827

0.03295	1.9196

0.03295	1.91413

0.03296	1.91547

0.03295	1.9196

0.03295	1.91693

0.03295	1.91267

0.03295	1.9196

0.03295	1.91827

0.03296	1.91547

0.03296	1.91693

0.03299	1.9196

0.03299	1.91547

0.03299	1.91413

0.03296	1.91693

0.03295	1.91547

0.03297	1.91827

0.03299	1.91693

0.033	1.9196

0.033	1.91413

0.033	1.91693

0.033	1.9196

0.03299	1.91693

0.03298	1.91547

0.03297	1.91827

0.03297	1.91693

0.03299	1.91413

0.03299	1.91693

0.03298	1.91827

0.03298	1.91693

0.03297	1.91827

0.03299	1.91693

0.03299	1.9196

0.03299	1.91693

0.03299	1.91827

0.03298	1.91413

0.03299	1.91827

0.03299	1.91547

0.03299	1.91693

0.03299	1.91827

0.03299	1.91693

0.033	1.91827

0.03299	1.91547

0.03298	1.91827

0.03296	1.91827

0.03297	1.91547

0.03299	1.91827

0.03298	1.91827

0.03299	1.91547

0.03299	1.9196

0.03299	1.91413

0.033	1.91547

0.033	1.91693

0.03301	1.91693

0.033	1.91827

0.033	1.91547

0.033	1.91693

0.033	1.91827

0.03301	1.91693

0.033	1.91547

0.03301	1.91827

0.03302	1.91413

0.03302	1.91827

0.03303	1.91693

0.03301	1.91693

0.03301	1.91693

0.03302	1.91827

0.03301	1.91693

0.03302	1.91827

0.03301	1.91693

0.03302	1.91693

0.03302	1.91827

0.033	1.91547

0.03301	1.9196

0.03298	1.91827

0.03298	1.91547

0.03298	1.91827

0.03299	1.91827

0.03301	1.91547

0.033	1.91693

0.03301	1.9196

0.03301	1.91413

0.03301	1.91827

0.03302	1.9196

0.033	1.91693

0.033	1.91267

0.03301	1.91693

0.03302	1.91827

0.03302	1.91547

0.03301	1.91827

0.033	1.91693

0.03301	1.91547

0.03301	1.91547

0.03301	1.91827

0.03301	1.91547

0.033	1.91827

0.03299	1.91413

0.03298	1.91827

0.03299	1.92107

0.03298	1.91413

0.03301	1.9196

0.03301	1.91827

0.03301	1.91547

0.033	1.91267

0.033	1.9196

0.03302	1.91547

0.03304	1.91693

0.03303	1.91827

0.03303	1.91413

0.03299	1.91827

0.033	1.91693

0.03301	1.91547

0.03302	1.91693

0.03302	1.91827

0.033	1.91827

0.033	1.91547

0.03302	1.91827

0.03303	1.91547

0.03304	1.91693

0.03303	1.91693

0.03303	1.91547

0.03303	1.91693

0.03304	1.91693

0.03303	1.91693

0.03303	1.91693

0.03303	1.91547

0.03304	1.91693

0.03304	1.9196

0.03304	1.91547

0.03303	1.91547

0.03303	1.91693

0.03304	1.91547

0.03302	1.91827

0.03303	1.91827

0.03302	1.91547

0.03303	1.91547

0.03304	1.9196

0.03304	1.91693

0.03304	1.91827

0.03303	1.91547

0.03302	1.91693

0.03303	1.91413

0.03303	1.91827

0.03303	1.91693

0.03304	1.91547

0.03304	1.91827

0.03304	1.91693

0.03305	1.91693

0.03305	1.91693

0.03304	1.91547

0.03305	1.91693

0.03305	1.91413

0.03306	1.91827

0.03306	1.9196

0.03307	1.91413

0.03307	1.91693

0.03308	1.9196

0.03308	1.91547

0.03309	1.91547

0.03308	1.9196

0.03307	1.92107

0.03307	1.91547

0.03306	1.91413

0.03306	1.91693

0.03305	1.91693

0.03308	1.91693

0.03309	1.91693

0.03308	1.92107

0.03307	1.91547

0.03305	1.91413

0.03305	1.9196

0.03306	1.91547

0.03307	1.91413

0.03308	1.91827

0.03307	1.91693

0.03307	1.91547

0.03307	1.91413

0.03307	1.91827

0.03308	1.91547

0.03307	1.9196

0.03307	1.9196

0.03306	1.91267

0.03308	1.91693

0.03309	1.91827

0.03309	1.91827

0.0331	1.91547

0.03308	1.92107

0.03309	1.91693

0.03309	1.91267

0.03308	1.91827

0.03307	1.91827

0.03306	1.91413

0.03309	1.91693

0.03308	1.91693

0.03307	1.91547

0.03306	1.91693

0.03306	1.91693

0.03306	1.91693

0.03305	1.91547

0.03306	1.91547

0.03306	1.9196

0.03308	1.91827

0.03308	1.91267

0.03309	1.91693

0.03307	1.92107

0.03306	1.91267

0.03305	1.91693

0.03306	1.91827

0.03305	1.91413

0.03307	1.91547

0.03308	1.91827

0.03309	1.91827

0.0331	1.91547

0.0331	1.9196

0.0331	1.91827

0.03309	1.91413

0.03308	1.91693

0.03309	1.9196

0.03311	1.91267

0.03311	1.91827

0.03309	1.9196

0.03309	1.91413

0.03309	1.91827

0.0331	1.9196

0.03311	1.91413

0.03311	1.91413

0.03311	1.9196

0.0331	1.91693

0.0331	1.91413

0.03309	1.91827

0.03311	1.91693

0.03309	1.91693

0.0331	1.9196

0.0331	1.91413

0.03311	1.91827

0.0331	1.91827

0.03308	1.91693

0.03311	1.91693

0.03311	1.91693

0.03311	1.91693

0.0331	1.91547

0.0331	1.91693

0.03308	1.92107

0.03311	1.91693

0.0331	1.91413

0.0331	1.91827

0.03309	1.9196

0.03309	1.91267

0.03308	1.91547

0.03308	1.91693

0.03307	1.91413

0.03307	1.91547

0.03307	1.9196

0.03308	1.91693

0.03309	1.91547

0.03309	1.91827

0.03309	1.9196

0.03309	1.91413

0.03309	1.91547

0.0331	1.9196

0.03309	1.9196

0.03309	1.91413

0.03309	1.9196

0.0331	1.9224

0.0331	1.91267

0.0331	1.91693

0.03309	1.9196

0.03308	1.91547

0.03308	1.91547

0.03309	1.91827

0.03309	1.91693

0.03308	1.91547

0.0331	1.91547

0.03309	1.9196

0.03309	1.91413

0.03308	1.91693

0.03308	1.9196

0.03309	1.91693

0.03309	1.91547

0.0331	1.91827

0.0331	1.91267

0.0331	1.91547

0.03311	1.91827

0.03311	1.91547

0.0331	1.91693

0.03309	1.91693

0.0331	1.91693

0.0331	1.91693

0.0331	1.91413

0.03312	1.9196

0.03312	1.91827

0.0331	1.91547

0.0331	1.91413

0.03311	1.9196

0.03312	1.91827

0.03313	1.91693

0.03313	1.91693

0.03313	1.91827

0.03314	1.91827

0.03313	1.91693

0.03313	1.91827

0.03311	1.91693

0.03312	1.91547

0.03313	1.91547

0.03314	1.91827

0.03315	1.91693

0.03315	1.91693

0.03314	1.91827

0.03314	1.91693

0.03314	1.91693

0.03313	1.9196

0.03313	1.91693

0.03314	1.91547

0.03315	1.91693

0.03314	1.91827

0.03314	1.91547

0.03316	1.91413

0.03315	1.91827

0.03315	1.91693

0.03315	1.91827

0.03317	1.91693

0.03318	1.91827

0.03318	1.91693

0.03316	1.91413

0.03314	1.91827

0.03314	1.91547

0.03316	1.91693

0.03317	1.91547

0.03316	1.91827

0.03314	1.91693

0.03314	1.91693

0.03314	1.9196

0.03314	1.91693

0.03314	1.91267

0.03315	1.91827

0.03315	1.9196

0.03316	1.91133

0.03314	1.9196

0.03316	1.91827

0.03315	1.91547

0.03316	1.91693

0.03316	1.91693

0.03316	1.91693

0.03316	1.91693

0.03316	1.91547

0.03316	1.91547

0.03315	1.91827

0.03315	1.91827

0.03314	1.91693

0.03314	1.91547

0.03313	1.91827

0.03315	1.91547

0.03315	1.91413

0.03317	1.91827

0.03315	1.91827

0.03314	1.91547

0.03314	1.91827

0.03314	1.9196

0.03316	1.91413

0.03317	1.91547

0.03317	1.9196

0.03315	1.91547

0.03314	1.91547

0.03315	1.91693

0.03316	1.92107

0.03316	1.91547

0.03316	1.91547

0.03315	1.9196

0.03315	1.91827

0.03316	1.91547

0.03316	1.91693

0.03316	1.91827

0.03317	1.91547

0.03318	1.91413

0.03319	1.9196

0.0332	1.91547

0.03319	1.91547

0.03318	1.91827

0.03318	1.91547

0.03319	1.91827

0.03318	1.91827

0.03316	1.91413

0.03317	1.91267

0.03317	1.91827

0.03318	1.91827

0.03319	1.91413

0.03319	1.91827

0.03319	1.91827

0.03319	1.91693

0.03321	1.91413

0.03321	1.9196

0.03319	1.91547

0.03318	1.91547

0.03318	1.91693

0.03319	1.91693

0.03319	1.91693

0.03318	1.92107

0.03319	1.91693

0.03319	1.91267

0.0332	1.9196

0.0332	1.91827

0.03318	1.91693

0.03319	1.91693

0.03318	1.91827

0.03319	1.91547

0.03319	1.91547

0.03321	1.91693

0.03323	1.91827

0.03323	1.91827

0.03321	1.91693

0.0332	1.91827

0.0332	1.91693

0.0332	1.91827

0.03321	1.91413

0.03319	1.91693

0.0332	1.91693

0.03321	1.91827

0.03321	1.91547

0.0332	1.91547

0.0332	1.91827

0.03321	1.91413

0.03322	1.9196

0.03322	1.91693

0.03322	1.91547

0.03321	1.91547

0.03321	1.91693

0.03321	1.91693

0.0332	1.91693

0.03323	1.91693

0.03322	1.91693

0.03322	1.91693

0.03321	1.91693

0.03322	1.91547

0.03322	1.91827

0.03321	1.91267

0.03321	1.9196

0.03323	1.91827

0.03323	1.91547

0.03322	1.91693

0.03321	1.9196

0.03321	1.91693

0.03321	1.91693

0.0332	1.91547

0.03321	1.9196

0.0332	1.91547

0.03321	1.92107

0.03319	1.91693

0.03318	1.91827

0.03319	1.91693

0.03319	1.91827

0.0332	1.91547

0.0332	1.91547

0.03318	1.91827

0.03318	1.91693

0.03319	1.9196

0.0332	1.91547

0.03321	1.91827

0.03322	1.9196

0.03322	1.91267

0.03323	1.91827

0.03324	1.92107

0.03323	1.91693

0.03323	1.91547

0.03323	1.91693

0.03322	1.91547

0.03322	1.91693

0.03322	1.91827

0.03322	1.91693

0.0332	1.91827

0.03321	1.91693

0.03322	1.91693

0.03323	1.91693

0.03323	1.91693

0.03322	1.91547

0.03324	1.91547

0.03325	1.91827

0.03328	1.91693

0.03327	1.91267

0.03328	1.91827

0.03327	1.91827

0.03327	1.9196

0.03325	1.91547

0.03326	1.91693

0.03325	1.91693

0.03326	1.91547

0.03325	1.91827

0.03326	1.91547

0.03326	1.9196

0.03326	1.91413

0.03325	1.91827

0.03326	1.91827

0.03327	1.91693

0.03327	1.91693

0.03327	1.9196

0.03325	1.91693

0.03325	1.91413

0.03326	1.91693

0.03327	1.9196

0.03326	1.91267

0.03326	1.91693

0.03327	1.91827

0.03327	1.91547

0.03327	1.91693

0.03325	1.9196

0.03325	1.91413

0.03325	1.91693

0.03326	1.9196

0.03326	1.91693

0.03326	1.91413

0.03328	1.9196

0.03328	1.91693

0.03329	1.91693

0.03328	1.91693

0.03327	1.91827

0.03326	1.91267

0.03326	1.9196

0.03325	1.9196

0.03323	1.91413

0.03325	1.91547

0.03327	1.9196

0.03328	1.91267

0.03327	1.91267

0.03327	1.92107

0.03327	1.91693

0.03328	1.91547

0.03327	1.91547

0.03326	1.91827

0.03326	1.91413

0.03326	1.91693

0.03327	1.91547

0.03326	1.91693

0.03325	1.91827

0.03325	1.91827

0.03326	1.91693

0.03328	1.91693

0.03328	1.91827

0.03328	1.91693

0.03328	1.91413

0.03328	1.91547

0.03329	1.91827

0.0333	1.91547

0.03331	1.91547

0.03329	1.91827

0.03328	1.91547

0.03328	1.91413

0.03328	1.9196

0.03328	1.91547

0.03329	1.91827

0.03329	1.91827

0.03329	1.91693

0.03329	1.91693

0.03328	1.91693

0.03328	1.91413

0.0333	1.91827

0.03329	1.91413

0.0333	1.9196

0.0333	1.91693

0.0333	1.91547

0.03331	1.9196

0.0333	1.91547

0.03331	1.91547

0.03331	1.91827

0.03332	1.9196

0.03332	1.91547

0.0333	1.91547

0.03331	1.91693

0.03331	1.9196

0.03333	1.91693

0.03333	1.91547

0.03332	1.9196

0.03333	1.91827

0.03331	1.91133

0.03333	1.91827

0.03332	1.91827

0.03331	1.91267

0.0333	1.9196

0.03329	1.91547

0.0333	1.90987

0.03331	1.91827

0.03333	1.92107

0.03332	1.91547

0.03333	1.91693

0.03332	1.92107

0.03332	1.91547

0.03331	1.91267

0.03332	1.9196

0.03333	1.91827

0.03332	1.91547

0.03333	1.91547

0.03333	1.91827

0.03334	1.92107

0.03332	1.91413

0.03331	1.91827

0.03333	1.91827

0.03334	1.91547

0.03335	1.91413

0.03335	1.9196

0.03335	1.91827

0.03333	1.91693

0.03332	1.91827

0.03331	1.91827

0.03332	1.91547

0.03333	1.91547

0.03334	1.91693

0.03334	1.91827

0.03333	1.91413

0.03334	1.91693

0.03335	1.9196

0.03334	1.91413

0.03334	1.9196

0.03334	1.91693

0.03335	1.91547

0.03334	1.91827

0.03334	1.91827

0.03333	1.91547

0.03333	1.91413

0.03333	1.91827

0.03334	1.91547

0.03335	1.9196

0.03335	1.91547

0.03336	1.91413

0.03335	1.9196

0.03336	1.91547

0.03336	1.91547

0.03335	1.9196

0.03336	1.91827

0.03335	1.91693

0.03335	1.91693

0.03336	1.91547

0.03336	1.92107

0.03336	1.91547

0.03335	1.91547

0.03336	1.91827

0.03337	1.91827

0.03337	1.91547

0.03336	1.91827

0.03338	1.91547

0.03338	1.91693

0.03336	1.91693

0.03337	1.91827

0.03336	1.91693

0.03338	1.91547

0.03336	1.91693

0.03337	1.91547

0.03337	1.91693

0.03338	1.91547

0.03338	1.91547

0.03339	1.9196

0.03338	1.91827

0.03337	1.91547

0.03336	1.91547

0.03336	1.91693

0.03336	1.91827

0.03337	1.91267

0.03336	1.9196

0.03335	1.91547

0.03334	1.91547

0.03334	1.91413

0.03335	1.9196

0.03336	1.91267

0.03336	1.91693

0.03337	1.9196

0.03336	1.91693

0.03336	1.91413

0.03336	1.91827

0.03335	1.91693

0.03335	1.91267

0.03336	1.9196

0.03336	1.91693

0.03335	1.91413

0.03335	1.9196

0.03335	1.91693

0.03334	1.91413

0.03333	1.91827

0.03334	1.91827

0.03335	1.91693

0.03335	1.91693

0.03335	1.91547

0.03335	1.91827

0.03336	1.91693

0.03336	1.91547

0.03336	1.92107

0.03336	1.91827

0.03337	1.91693

0.03338	1.91693

0.03337	1.9196

0.03337	1.91693

0.03336	1.91547

0.03337	1.91827

0.03337	1.91693

0.03338	1.91693

0.03338	1.91547

0.03338	1.91413

0.03338	1.9196

0.03337	1.91827

0.03337	1.91413

0.03337	1.91693

0.03338	1.9196

0.03339	1.91693

0.03339	1.91413

0.03338	1.9224

0.03338	1.91413

0.03337	1.91267

0.03338	1.92107

0.03338	1.91693

0.03337	1.91267

0.03338	1.91827

0.03338	1.9196

0.03338	1.91693

0.03337	1.91413

0.03337	1.9196

0.03338	1.91547

0.03338	1.91133

0.03338	1.9196

0.03337	1.9196

0.03337	1.91267

0.03338	1.92107

0.03339	1.91827

0.03341	1.91267

0.03341	1.91547

0.03341	1.9196

0.03342	1.91693

0.0334	1.91547

0.0334	1.91827

0.03339	1.91413

0.0334	1.91547

0.0334	1.91827

0.0334	1.91547

0.03342	1.91693

0.03341	1.91827

0.03342	1.91827

0.03342	1.91547

0.03343	1.91547

0.03343	1.91693

0.03343	1.91547

0.03344	1.91413

0.03344	1.91413

0.03344	1.92107

0.03343	1.91547

0.03343	1.91693

0.03343	1.9196

0.03344	1.91693

0.03343	1.91413

0.03343	1.92107

0.03341	1.91547

0.03342	1.91413

0.03342	1.91827

0.03343	1.91693

0.03343	1.91413

0.03343	1.91827

0.03343	1.9196

0.03342	1.91547

0.03343	1.91413

0.03343	1.9196

0.03344	1.91547

0.03345	1.91413

0.03345	1.92107

0.03345	1.91547

0.03344	1.91547

0.03344	1.91827

0.03344	1.91693

0.03343	1.91547

0.03343	1.91413

0.03343	1.91827

0.03344	1.91693

0.03343	1.91413

0.03343	1.91827

0.03343	1.9196

0.03343	1.91547

0.03344	1.91693

0.03343	1.9196

0.03343	1.91413

0.03344	1.91693

0.03345	1.9196

0.03345	1.91413

0.03345	1.91827

0.03346	1.9196

0.03346	1.91693

0.03345	1.91413

0.03346	1.92107

0.03344	1.91827

0.03344	1.91267

0.03343	1.91827

0.03344	1.9196

0.03346	1.91547

0.03347	1.91547

0.03347	1.9196

0.03347	1.91413

0.03346	1.91413

0.03346	1.9196

0.03346	1.91547

0.03345	1.91693

0.03345	1.9196

0.03345	1.91693

0.03346	1.91413

0.03347	1.9196

0.03348	1.91413

0.03347	1.91693

0.03347	1.91827

0.03347	1.9196

0.03347	1.91827

0.03346	1.91413

0.03346	1.91827

0.03346	1.9196

0.03347	1.91547

0.03347	1.91693

0.03346	1.91827

0.03345	1.91693

0.03346	1.91413

0.03346	1.9196

0.03347	1.9196

0.03346	1.91547

0.03345	1.9196

0.03345	1.91547

0.03345	1.91267

0.03346	1.9196

0.03346	1.91693

0.03346	1.91413

0.03346	1.91693

0.03347	1.91827

0.03349	1.91827

0.0335	1.91693

0.03349	1.91827

0.03349	1.91693

0.03348	1.91693

0.03348	1.91693

0.03348	1.91547

0.03349	1.91547

0.03349	1.91693

0.03348	1.91827

0.03348	1.91547

0.0335	1.91547

0.0335	1.92107

0.0335	1.91827

0.0335	1.91547

0.03349	1.91827

0.03349	1.91693

0.0335	1.91413

0.0335	1.91827

0.0335	1.91827

0.03349	1.91413

0.03348	1.91547

0.03347	1.9224

0.03348	1.91693

0.03349	1.91413

0.0335	1.92107

0.0335	1.91547

0.0335	1.91693

0.03349	1.91547

0.03349	1.91827

0.03348	1.91693

0.03349	1.91547

0.03349	1.91693

0.0335	1.91693

0.03349	1.91413

0.03349	1.92107

0.03349	1.91827

0.03349	1.91413

0.03349	1.91827

0.03349	1.91693

0.0335	1.91413

0.0335	1.9196

0.03351	1.91827

0.03351	1.91413

0.0335	1.91827

0.0335	1.91547

0.03349	1.91693

0.0335	1.91547

0.03351	1.91693

0.03352	1.91547

0.03351	1.91693

0.03351	1.91827

0.03351	1.91413

0.03351	1.91827

0.03352	1.91693

0.03352	1.91413

0.03352	1.91693

0.03352	1.91827

0.03351	1.91693

0.0335	1.91413

0.0335	1.91827

0.0335	1.91827

0.03352	1.91547

0.03353	1.9224

0.03352	1.91693

0.03354	1.91413

0.03354	1.92107

0.03354	1.91693

0.03355	1.91547

0.03354	1.91827

0.03354	1.91693

0.03354	1.91547

0.03354	1.91547

0.03353	1.9196

0.03354	1.91547

0.03354	1.91693

0.03355	1.91693

0.03354	1.91693

0.03355	1.91547

0.03355	1.91413

0.03356	1.91827

0.03355	1.91413

0.03355	1.91413

0.03357	1.91693

0.03358	1.91827

0.03357	1.91693

0.03357	1.91827

0.03356	1.91413

0.03356	1.91827

0.03356	1.91693

0.03356	1.91547

0.03355	1.91413

0.03356	1.9196

0.03357	1.91693

0.03357	1.91413

0.03355	1.91693

0.03356	1.9196

0.03356	1.91547

0.03356	1.91693

0.03356	1.9196

0.03357	1.91827

0.03356	1.91547

0.03356	1.9196

0.03355	1.91693

0.03354	1.91547

0.03355	1.91693

0.03356	1.9196

0.03356	1.91547

0.03356	1.91413

0.03355	1.91827

0.03355	1.91547

0.03356	1.91693

0.03355	1.91827

0.03354	1.91413

0.03354	1.91827

0.03356	1.91693

0.03356	1.91547

0.03355	1.91827

0.03356	1.9196

0.03355	1.91693

0.03357	1.91693

0.03355	1.91547

0.03356	1.91413

0.03354	1.91693

0.03354	1.91693

0.03355	1.91693

0.03355	1.9196

0.03355	1.9196

0.03356	1.91267

0.03355	1.91547

0.03353	1.9196

0.03351	1.91413

0.03353	1.91693

0.03354	1.92107

0.03355	1.91413

0.03355	1.91693

0.03354	1.91827

0.03355	1.92107

0.03355	1.91413

0.03356	1.91827

0.03357	1.9196

0.03357	1.91547

0.03354	1.91693

0.03354	1.91547

0.03354	1.91413

0.03358	1.91547

0.03357	1.9224

0.03356	1.91413

0.03357	1.91547

0.03356	1.91827

0.03355	1.9196

0.03353	1.91133

0.03354	1.91827

0.03356	1.91827

0.03356	1.91413

0.03357	1.91693

0.03355	1.9196

0.03356	1.91547

0.03355	1.91693

0.03355	1.9196

0.03356	1.91693

0.03356	1.91413

0.03355	1.9196

0.03355	1.91827

0.03355	1.91133

0.03354	1.91547

0.03354	1.9196

0.03355	1.91547

0.03356	1.91547

0.03358	1.9196

0.03358	1.91693

0.03358	1.91547

0.03357	1.91827

0.03358	1.91693

0.03358	1.91547

0.03358	1.91547

0.03358	1.91693

0.03358	1.91693

0.03357	1.91547

0.03356	1.92107

0.03357	1.91693

0.03357	1.91547

0.03356	1.91827

0.03354	1.91413

0.03354	1.91547

0.03355	1.91693

0.03353	1.9196

0.03354	1.91547

0.03354	1.91693

0.03355	1.91827

0.03356	1.91693

0.03356	1.91693

0.03357	1.91693

0.03357	1.91693

0.03357	1.9196

0.03358	1.91693

0.03357	1.91693

0.03358	1.91413

0.03358	1.91827

0.03358	1.91413

0.03359	1.91827

0.03358	1.92107

0.03359	1.91133

0.03358	1.91547

0.0336	1.92107

0.03359	1.91413

0.03359	1.91547

0.03358	1.91827

0.03358	1.91693

0.03358	1.91547

0.03358	1.91827

0.03359	1.91827

0.03358	1.91267

0.03358	1.91827

0.03358	1.9196

0.03357	1.91693

0.03357	1.91413

0.03357	1.91827

0.03358	1.9196

0.03359	1.91133

0.03359	1.9196

0.03359	1.92107

0.03359	1.91547

0.0336	1.91547

0.03361	1.9196

0.03361	1.91547

0.0336	1.91267

0.03358	1.92387

0.03358	1.91693

0.03359	1.91547

0.0336	1.92107

0.03362	1.91547

0.03361	1.91267

0.03361	1.92107

0.0336	1.91693

0.03362	1.91547

0.03361	1.91827

0.03362	1.92107

0.03361	1.91267

0.03362	1.91413

0.03361	1.91693

0.03361	1.91827

0.03361	1.91693

0.03363	1.91547

0.03363	1.9196

0.03361	1.91547

0.0336	1.91547

0.0336	1.91693

0.03359	1.91693

0.03359	1.91413

0.0336	1.92107

0.0336	1.91693

0.0336	1.91547

0.03362	1.9196

0.03362	1.91827

0.03361	1.91827

0.03359	1.91693

0.03359	1.91827

0.03361	1.9196

0.03362	1.91413

0.03363	1.91693

0.03365	1.91693

0.03364	1.91827

0.03364	1.91693

0.03364	1.91547

0.03364	1.92107

0.03364	1.91547

0.03363	1.91693

0.03365	1.91547

0.03364	1.91693

0.03364	1.91547

0.03363	1.91693

0.03361	1.91693

0.03363	1.9196

0.03364	1.91413

0.03364	1.91413

0.03365	1.91827

0.03365	1.91693

0.03365	1.91267

0.03365	1.92107

0.03362	1.91827

0.03362	1.91693

0.03362	1.92107

0.03362	1.91693

0.03363	1.91547

0.03363	1.9196

0.03364	1.91547

0.03364	1.91827

0.03365	1.91827

0.03364	1.91413

0.03363	1.91547

0.03365	1.9196

0.03364	1.91693

0.03363	1.91547

0.0336	1.91827

0.0336	1.91827

0.03361	1.91267

0.03361	1.91547

0.03362	1.92107

0.03362	1.9196

0.03361	1.91413

0.03361	1.91693

0.03362	1.9196

0.03362	1.91413

0.03362	1.91693

0.03361	1.9196

0.03359	1.91693

0.0336	1.91547

0.03361	1.91693

0.03362	1.9196

0.03363	1.91693

0.03363	1.91547

0.03364	1.91827

0.03364	1.91547

0.03365	1.91547

0.03363	1.91827

0.03363	1.91827

0.03361	1.91827

0.03363	1.91413

0.03361	1.91693

0.03361	1.91827

0.03361	1.91693

0.03362	1.91413

0.03364	1.9196

0.03366	1.91693

0.03366	1.91413

0.03365	1.91693

0.03364	1.91693

0.03363	1.91693

0.03363	1.91547

0.03363	1.91827

0.03363	1.91267

0.03363	1.92107

0.03364	1.91413

0.03364	1.91827

0.03364	1.91547

0.03365	1.91413

0.03365	1.91827

0.03366	1.91827

0.03364	1.91693

0.03364	1.91693

0.03364	1.91827

0.03364	1.91413

0.03364	1.91547

0.03363	1.91693

0.03363	1.91693

0.03364	1.91547

0.03364	1.91827

0.03364	1.9196

0.03364	1.91267

0.03365	1.91547

0.03365	1.91827

0.03364	1.91267

0.03363	1.91547

0.03364	1.92107

0.03364	1.91547

0.03365	1.91413

0.03365	1.92107

0.03365	1.91693

0.03365	1.91267

0.03364	1.9196

0.03365	1.92107

0.03365	1.91267

0.03365	1.91413

0.03363	1.92107

0.03365	1.91413

0.03365	1.91267

0.03365	1.92107

0.03366	1.9224

0.03366	1.91133

0.03364	1.91693

0.03364	1.92107

0.03364	1.91547

0.03365	1.91547

0.03365	1.92107

0.03365	1.91413

0.03366	1.91547

0.03366	1.92107

0.03367	1.91693

0.03367	1.90987

0.03366	1.91827

0.03366	1.91827

0.03366	1.91267

0.03367	1.91547

0.03366	1.91827

0.03366	1.91267

0.03367	1.91693

0.03366	1.91827

0.03366	1.91827

0.03366	1.91547

0.03367	1.91827

0.03366	1.91547

0.03366	1.91413

0.03367	1.92107

0.03367	1.91827

0.03367	1.91413

0.03369	1.91827

0.03368	1.91827

0.03368	1.91547

0.03365	1.91693

0.03364	1.91693

0.03365	1.91267

0.03366	1.9196

0.03368	1.91827

0.03368	1.91267

0.03369	1.91827

0.03368	1.92107

0.03368	1.91267

0.03367	1.91693

0.03368	1.91827

0.03368	1.91547

0.03369	1.91547

0.03369	1.92107

0.03369	1.91693

0.0337	1.91547

0.03369	1.91693

0.03369	1.91693

0.03369	1.91547

0.03368	1.91547

0.03367	1.91827

0.03369	1.91693

0.0337	1.91547

0.03371	1.91827

0.0337	1.91547

0.03367	1.91547

0.03368	1.92107

0.03368	1.91693

0.03369	1.91267

0.0337	1.92107

0.0337	1.9196

0.0337	1.91267

0.03371	1.91827

0.0337	1.92107

0.03371	1.91267

0.03371	1.91413

0.03371	1.92387

0.03369	1.91133

0.03368	1.91267

0.03368	1.9224

0.03367	1.91547

0.03368	1.91267

0.03368	1.9196

0.03368	1.91827

0.03369	1.91267

0.03369	1.91693

0.03369	1.92107

0.03369	1.91413

0.03368	1.91413

0.03369	1.91547

0.03368	1.91827

0.03368	1.91413

0.03367	1.91267

0.03368	1.91827

0.03369	1.92107

0.03369	1.91413

0.0337	1.91827

0.0337	1.9196

0.0337	1.91413

0.03371	1.91413

0.0337	1.9196

0.0337	1.91413

0.03371	1.91547

0.03372	1.91827

0.03373	1.91827

0.03374	1.91547

0.03373	1.91693

0.03372	1.91827

0.03371	1.91693

0.03371	1.91693

0.0337	1.9196

0.03371	1.91547

0.03372	1.91693

0.03373	1.91693

0.03373	1.91693

0.03373	1.91693

0.03372	1.91413

0.03372	1.9196

0.03372	1.91693

0.03372	1.91413

0.03372	1.9196

0.03373	1.91827

0.03372	1.91267

0.03371	1.91693

0.03371	1.9196

0.03371	1.91413

0.0337	1.91547

0.03372	1.92107

0.03372	1.91547

0.03372	1.91693

0.03372	1.91827

0.03372	1.91547

0.03372	1.91827

0.03372	1.91827

0.03372	1.91827

0.0337	1.91693

0.03371	1.91693

0.0337	1.91413

0.03371	1.91693

0.0337	1.91547

0.0337	1.91693

0.0337	1.91827

0.0337	1.91827

0.03369	1.91547

0.03369	1.91413

0.03369	1.9196

0.03369	1.91693

0.03369	1.91413

0.03369	1.91547

0.03369	1.92107

0.0337	1.91413

0.0337	1.91547

0.03371	1.9196

0.0337	1.91413

0.03371	1.91693

0.0337	1.91827

0.03371	1.91693

0.0337	1.91413

0.0337	1.91827

0.0337	1.9196

0.03369	1.91267

0.03371	1.91693

0.03371	1.92107

0.03371	1.91693

0.0337	1.91267

0.0337	1.92107

0.0337	1.91413

0.03372	1.91413

0.03372	1.9196

0.03371	1.91827

0.0337	1.91413

0.03369	1.91547

0.03369	1.92107

0.03368	1.91693

0.03368	1.91267

0.03369	1.91827

0.03369	1.91693

0.0337	1.91693

0.03369	1.91693

0.0337	1.91827

0.03371	1.91693

0.0337	1.91547

0.03372	1.9196

0.03372	1.91547

0.03372	1.91413

0.03372	1.91827

0.03372	1.91693

0.03374	1.91413

0.03374	1.9196

0.03374	1.9196

0.03374	1.91413

0.03373	1.91547

0.03373	1.91827

0.03373	1.91413

0.03373	1.91547

0.03372	1.9196

0.03372	1.91133

0.03372	1.9196

0.03372	1.92107

0.03372	1.91547

0.03372	1.91547

0.03372	1.9196

0.03372	1.91693

0.03371	1.91827

0.03371	1.91413

0.03372	1.9196

0.03372	1.91693

0.03373	1.91413

0.03372	1.92107

0.03372	1.91693

0.03372	1.91547

0.03372	1.91827

0.03372	1.91547

0.03371	1.91413

0.0337	1.91547

0.03371	1.91693

0.03371	1.91413

0.03372	1.92107

0.03372	1.91827

0.03372	1.91693

0.03371	1.91413

0.03372	1.91827

0.03374	1.9196

0.03374	1.91547

0.03373	1.91693

0.03372	1.9196

0.03373	1.91267

0.03372	1.91547

0.03373	1.91827

0.03373	1.91547

0.03374	1.91413

0.03373	1.91827

0.03373	1.91547

0.03373	1.91547

0.03374	1.91827

0.03374	1.91693

0.03374	1.91693

0.03373	1.91827

0.03374	1.91827

0.03373	1.90987

0.03375	1.9224

0.03376	1.9196

0.03375	1.91267

0.03374	1.91693

0.03374	1.9224

0.03372	1.91547

0.03372	1.91693

0.03372	1.9196

0.03373	1.91827

0.03373	1.91413

0.03373	1.91827

0.03372	1.92107

0.03373	1.90987

0.03373	1.91693

0.03373	1.9196

0.03374	1.91693

0.03374	1.91547

0.03376	1.91693

0.03375	1.91267

0.03375	1.91693

0.03375	1.91827

0.03376	1.91267

0.03376	1.91547

0.03375	1.9196

0.03376	1.9196

0.03376	1.91267

0.03374	1.91693

0.03373	1.9196

0.03373	1.91267

0.03374	1.91827

0.03375	1.91827

0.03375	1.91827

0.03375	1.91547

0.03375	1.91827

0.03375	1.91827

0.03375	1.91267

0.03375	1.91827

0.03375	1.91693

0.03374	1.91413

0.03372	1.91693

0.03373	1.91547

0.03373	1.91693

0.03372	1.91693

0.03372	1.9196

0.03372	1.91827

0.03373	1.90987

0.03373	1.91827

0.03375	1.91827

0.03373	1.91267

0.03373	1.91693

0.03373	1.9196

0.03373	1.91267

0.03374	1.91693

0.03375	1.92387

0.03373	1.91413

0.03374	1.91693

0.03372	1.92107

0.03374	1.91547

0.03373	1.91413

0.03374	1.91827

0.03374	1.91827

0.03374	1.90987

0.03373	1.91827

0.03374	1.9196

0.03375	1.91133

0.03375	1.91827

0.03375	1.92107

0.03374	1.91693

0.03375	1.91267

0.03374	1.9196

0.03375	1.9196

0.03375	1.90987

0.03375	1.9196

0.03375	1.92107

0.03376	1.91413

0.03377	1.91547

0.03376	1.92107

0.03376	1.91547

0.03376	1.91693

0.03376	1.91827

0.03375	1.92107

0.03375	1.91413

0.03374	1.91827

0.03375	1.9196

0.03374	1.91693

0.03374	1.91267

0.03373	1.92107

0.03374	1.91693

0.03374	1.91547

0.03375	1.91693

0.03375	1.9196

0.03376	1.91413

0.03375	1.91693

0.03375	1.91827

0.03375	1.91693

0.03375	1.91413

0.03375	1.91693

0.03374	1.9224

0.03373	1.91413

0.03373	1.91413

0.03374	1.9196

0.03373	1.91547

0.03375	1.91547

0.03375	1.91693

0.03376	1.91827

0.03377	1.91413

0.03377	1.91693

0.03376	1.92107

0.03376	1.91547

0.03375	1.9196

0.03375	1.92107

0.03375	1.91267

0.03376	1.91827

0.03376	1.91693

0.03376	1.91267

0.03377	1.91827

0.03378	1.9196

0.03378	1.91547

0.03377	1.91547

0.03376	1.9196

0.03376	1.91413

0.03375	1.91413

0.03376	1.91827

0.03376	1.91827

0.03377	1.91267

0.03378	1.91827

0.03377	1.9196

0.03377	1.91413

0.03376	1.91547

0.03375	1.9224

0.03375	1.91267

0.03375	1.91267

0.03375	1.9196

0.03375	1.9196

0.03375	1.91133

0.03376	1.92107

0.03377	1.92107

0.03377	1.91267

0.03378	1.91827

0.03378	1.9196

0.03377	1.91547

0.03375	1.91693

0.03375	1.91827

0.03375	1.91547

0.03377	1.91547

0.03376	1.91827

0.03377	1.9196

0.03375	1.91413

0.03375	1.91547

0.03376	1.92107

0.03377	1.91547

0.03376	1.91413

0.03375	1.91827

0.03375	1.91413

0.03377	1.91413

0.03377	1.91827

0.03376	1.91547

0.03376	1.91267

0.03376	1.9196

0.03376	1.91827

0.03377	1.91413

0.03376	1.91827

0.03377	1.91827

0.03376	1.91413

0.03377	1.91547

0.03378	1.91547

0.03378	1.91547

0.03377	1.9196

0.03377	1.91547

0.03377	1.91547

0.03378	1.91827

0.03378	1.91413

0.03379	1.91547

0.03379	1.91693

0.03379	1.91827

0.03379	1.91413

0.03379	1.91693

0.03378	1.91827

0.03379	1.91413

0.03379	1.91827

0.03379	1.91827

0.0338	1.91547

0.0338	1.91693

0.03379	1.9196

0.03378	1.91413

0.03379	1.91827

0.03379	1.91827

0.03379	1.91267

0.03381	1.91547

0.0338	1.9196

0.03381	1.91547

0.03381	1.91547

0.03382	1.9224

0.03382	1.9196

0.03381	1.91133

0.03381	1.92107

0.03382	1.91827

0.03382	1.91133

0.03382	1.92107

0.03381	1.9196

0.03381	1.91827

0.0338	1.91693

0.03381	1.91827

0.03379	1.91413

0.03379	1.91693

0.0338	1.9224

0.0338	1.91693

0.0338	1.91267

0.03379	1.92107

0.03381	1.91693

0.03382	1.91267

0.03384	1.91827

0.03384	1.9196

0.03383	1.91413

0.03381	1.91693

0.03381	1.91693

0.0338	1.91547

0.03379	1.91827

0.0338	1.91827

0.03381	1.91547

0.03382	1.91693

0.03382	1.91827

0.03383	1.9196

0.03381	1.91547

0.03381	1.91547

0.03382	1.9196

0.03382	1.91827

0.03382	1.91693

0.03382	1.91827

0.03381	1.9196

0.03382	1.91267

0.03381	1.91547

0.03381	1.92107

0.03382	1.91827

0.03382	1.91267

0.03383	1.9196

0.03382	1.91827

0.03381	1.91267

0.03381	1.91693

0.0338	1.91827

0.0338	1.91133

0.03379	1.91267

0.03379	1.9224

0.03378	1.91827

0.03376	1.91133

0.03376	1.9224

0.03378	1.9196

0.03379	1.90987

0.03379	1.91827

0.03378	1.9196

0.03378	1.91547

0.0338	1.91267

0.0338	1.91827

0.0338	1.91827

0.03378	1.91267

0.03377	1.9196

0.03378	1.91827

0.03379	1.91133

0.0338	1.91827

0.03382	1.9196

0.03381	1.91413

0.03382	1.91827

0.0338	1.91827

0.0338	1.91267

0.0338	1.91827

0.03379	1.91693

0.0338	1.91693

0.0338	1.91693

0.03381	1.91693

0.0338	1.91547

0.03378	1.91413

0.03379	1.91693

0.03378	1.9224

0.03379	1.91693

0.03379	1.91413

0.03379	1.92107

0.03378	1.91413

0.03378	1.91547

0.03379	1.92107

0.03379	1.91547

0.03379	1.91413

0.03379	1.91693

0.03379	1.91827

0.03379	1.91547

0.0338	1.91693

0.0338	1.92107

0.03382	1.91413

0.03381	1.91827

0.0338	1.91693

0.03382	1.91693

0.03381	1.91267

0.03383	1.9196

0.03382	1.91693

0.03381	1.91413

0.03382	1.91693

0.03381	1.91827

0.03381	1.91693

0.0338	1.91693

0.03381	1.9224

0.03381	1.91827

0.03381	1.91413

0.03381	1.9196

0.0338	1.91693

0.03379	1.91547

0.03378	1.91693

0.03379	1.9196

0.03379	1.91693

0.03378	1.91547

0.03378	1.9196

0.03379	1.91547

0.0338	1.91547

0.0338	1.91827

0.03379	1.91547

0.0338	1.91267

0.0338	1.91827

0.03379	1.9196

0.03379	1.91413

0.03379	1.91827

0.03379	1.9196

0.0338	1.91547

0.03379	1.91547

0.0338	1.92107

0.03381	1.91267

0.0338	1.91547

0.03381	1.92107

0.03381	1.91693

0.03381	1.91413

0.03382	1.91827

0.03382	1.91827

0.03383	1.91413

0.03382	1.9196

0.03383	1.91827

0.03382	1.91693

0.03383	1.91827

0.03385	1.91693

0.03386	1.91547

0.03386	1.91547

0.03386	1.91693

0.03385	1.91827

0.03384	1.91413

0.03383	1.91547

0.03383	1.9196

0.03385	1.91827

0.03385	1.91267

0.03386	1.91693

0.03386	1.9224

0.03386	1.91267

0.03386	1.91413

0.03386	1.92387

0.03385	1.91267

0.03385	1.91267

0.03385	1.92107

0.03384	1.91547

0.03385	1.91413

0.03385	1.91827

0.03385	1.92107

0.03385	1.91267

0.03384	1.9196

0.03385	1.9196

0.03385	1.91133

0.03385	1.9196

0.03385	1.91827

0.03384	1.91413

0.03384	1.91547

0.03386	1.92387

0.03387	1.91267

0.03387	1.91693

0.03385	1.9196

0.03385	1.91413

0.03385	1.91547

0.03384	1.9224

0.03384	1.91133

0.03384	1.91547

0.03386	1.9224

0.03386	1.91413

0.03385	1.91413

0.03384	1.92107

0.03384	1.91547

0.03384	1.91547

0.03384	1.92107

0.03384	1.91693

0.03385	1.91267

0.03384	1.91827

0.03386	1.92107

0.03386	1.91547

0.03385	1.91267

0.03385	1.92107

0.03385	1.91413

0.03386	1.91413

0.03386	1.91827

0.03387	1.91693

0.03388	1.91547

0.03388	1.91547

0.03387	1.9196

0.03387	1.9196

0.03385	1.91547

0.03386	1.91413

0.03386	1.92107

0.03387	1.91267

0.03387	1.91693

0.03387	1.91827

0.03387	1.91827

0.03386	1.91413

0.03387	1.91827

0.03387	1.91827

0.03386	1.91547

0.03386	1.91693

0.03385	1.91547

0.03385	1.91827

0.03385	1.91547

0.03385	1.91693

0.03385	1.91693

0.03385	1.92107

0.03385	1.91413

0.03384	1.91547

0.03383	1.9224

0.03384	1.91547

0.03384	1.91693

0.03385	1.91827

0.03385	1.9196

0.03385	1.91413

0.03385	1.9196

0.03384	1.92107

0.03385	1.91547

0.03386	1.91547

0.03385	1.91827

0.03385	1.91413

0.03383	1.91693

0.03383	1.92107

0.03383	1.91693

0.03384	1.91547

0.03384	1.92107

0.03384	1.91547

0.03385	1.91693

0.03386	1.92107

0.03384	1.91267

0.03384	1.91413

0.03385	1.91827

0.03385	1.91413

0.03386	1.91413

0.03385	1.92107

0.03385	1.91547

0.03384	1.91267

0.03385	1.92107

0.03384	1.91693

0.03385	1.91547

0.03385	1.91693

0.03383	1.91547

0.03384	1.91693

0.03385	1.91827

0.03384	1.91413

0.03384	1.92107

0.03385	1.9196

0.03385	1.91693

0.03385	1.91547

0.03386	1.91827

0.03385	1.91693

0.03385	1.91267

0.03386	1.91827

0.03386	1.91413

0.03387	1.91547

0.03386	1.91693

0.03386	1.9196

0.03385	1.91547

0.03385	1.91547

0.03385	1.92107

0.03385	1.91827

0.03384	1.91547

0.03385	1.9196

0.03385	1.91827

0.03386	1.91413

0.03388	1.91693

0.03387	1.9196

0.03387	1.91547

0.03386	1.91547

0.03384	1.9196

0.03383	1.91547

0.03384	1.91413

0.03383	1.92387

0.03384	1.91693

0.03385	1.91267

0.03385	1.92107

0.03386	1.9196

0.03385	1.91267

0.03386	1.91827

0.03385	1.92107

0.03383	1.91413

0.03383	1.91547

0.03383	1.9224

0.03384	1.91547

0.03384	1.91267

0.03386	1.91693

0.03385	1.91827

0.03384	1.91547

0.03383	1.91827

0.03383	1.9196

0.03386	1.91413

0.03387	1.9196

0.03388	1.91693

0.03387	1.91693

0.03387	1.91547

0.03387	1.91693

0.03386	1.92107

0.03386	1.91133

0.03386	1.91693

0.03386	1.9196

0.03385	1.91547

0.03386	1.91413

0.03387	1.91827

0.03387	1.91693

0.03386	1.91413

0.03387	1.92107

0.03386	1.9196

0.03387	1.91267

0.03387	1.91547

0.03388	1.91827

0.03388	1.91547

0.03388	1.91693

0.03389	1.91827

0.03388	1.9196

0.03388	1.91413

0.03388	1.91827

0.03388	1.91693

0.03388	1.91413

0.03389	1.91693

0.03388	1.91827

0.0339	1.91413

0.03389	1.91827

0.03389	1.9196

0.03388	1.91827

0.03387	1.91133

0.03385	1.91827

0.03387	1.91693

0.03387	1.91547

0.03388	1.91547

0.03387	1.91693

0.03386	1.91827

0.03387	1.91547

0.03387	1.91827

0.03388	1.91693

0.03387	1.9196

0.03388	1.91413

0.03387	1.91827

0.03388	1.9196

0.03387	1.91413

0.03387	1.91547

0.03388	1.91693

0.03389	1.91693

0.03389	1.91827

0.03388	1.91547

0.03388	1.91827

0.03387	1.91547

0.03388	1.91267

0.03387	1.91693

0.03388	1.91827

0.03388	1.91413

0.03388	1.91827

0.03388	1.9196

0.03387	1.91547

0.03387	1.91413

0.03388	1.9196

0.03388	1.91547

0.03387	1.91267

0.03387	1.91827

0.03388	1.91827

0.03387	1.91547

0.03388	1.9196

0.03388	1.91693

0.03387	1.91693

0.03388	1.91693

0.0339	1.91827

0.03389	1.91693

0.03387	1.91693

0.03387	1.91413

0.03388	1.9196

0.03387	1.91827

0.03387	1.91413

0.03387	1.9196

0.03388	1.91693

0.03388	1.91693

0.03387	1.9196

0.03387	1.91413

0.03385	1.91413

0.03387	1.91693

0.03386	1.91693

0.03387	1.91693

0.03387	1.91547

0.03388	1.91693

0.03388	1.91827

0.03387	1.9196

0.03386	1.91547

0.03386	1.91547

0.03386	1.91693

0.03385	1.91693

0.03386	1.91547

0.03387	1.9224

0.03388	1.91693

0.03388	1.91547

0.03389	1.92107

0.03388	1.91693

0.03389	1.91267

0.03389	1.9224

0.03389	1.91413

0.03389	1.91413

0.03389	1.9196

0.0339	1.9196

0.0339	1.91267

0.0339	1.91827

0.03388	1.91827

0.03388	1.91413

0.03389	1.91693

0.0339	1.91827

0.0339	1.91827

0.0339	1.91267

0.0339	1.91827

0.03389	1.9224

0.03389	1.91413

0.03389	1.91547

0.03389	1.92387

0.03389	1.91267

0.03389	1.91827

0.03389	1.92107

0.03389	1.91547

0.0339	1.91693

0.03391	1.9196

0.0339	1.91693

0.0339	1.91267

0.0339	1.92107

0.03391	1.91547

0.03391	1.91413

0.03392	1.91693

0.03391	1.92107

0.03391	1.91267

0.0339	1.91133

0.0339	1.92107

0.0339	1.91693

0.03392	1.91413

0.03392	1.91827

0.03391	1.91827

0.03391	1.91547

0.0339	1.91547

0.03391	1.92107

0.03389	1.91693

0.0339	1.91267

0.03389	1.9196

0.03391	1.9196

0.0339	1.90987

0.0339	1.9196

0.0339	1.9196

0.0339	1.91267

0.03391	1.91547

0.03391	1.9196

0.03389	1.91547

0.0339	1.91547

0.03391	1.91827

0.03392	1.91693

0.03392	1.91267

0.0339	1.91827

0.03391	1.91827

0.03392	1.91133

0.03392	1.91547

0.03391	1.9196

0.03391	1.91827

0.03391	1.91413

0.03391	1.91693

0.03392	1.9224

0.03392	1.91547

0.03394	1.91693

0.03394	1.91827

0.03394	1.91547

0.03393	1.91547

0.03393	1.9196

0.03393	1.91693

0.03392	1.91693

0.03391	1.91693

0.0339	1.91547

0.03391	1.91827

0.0339	1.91693

0.03392	1.91413

0.03393	1.91827

0.03393	1.9196

0.03392	1.91413

0.03392	1.91693

0.03391	1.9196

0.03393	1.91547

0.03393	1.91827

0.03393	1.91827

0.03393	1.91413

0.03393	1.91693

0.03393	1.9224

0.03393	1.91413

0.03393	1.91547

0.03393	1.9224

0.03393	1.91827

0.03392	1.91267

0.03393	1.91693

0.03393	1.92107

0.03394	1.91267

0.03394	1.91693

0.03392	1.9196

0.03391	1.91133

0.0339	1.91547

0.03391	1.9196

0.03392	1.91693

0.03392	1.91547

0.03392	1.9196

0.03392	1.91693

0.03393	1.91547

0.03392	1.91547

0.03392	1.91547

0.03391	1.91827

0.03391	1.91267

0.03391	1.91547

0.03392	1.92107

0.03393	1.91693

0.03393	1.90853

0.03392	1.9224

0.03392	1.91693

0.03392	1.91133

0.03393	1.92107

0.03392	1.9196

0.03393	1.90987

0.03393	1.92107

0.03394	1.92107

0.03394	1.91267

0.03395	1.91413

0.03395	1.9224

0.03396	1.91693

0.03396	1.91267

0.03396	1.92107

0.03396	1.91693

0.03396	1.91133

0.03396	1.92107

0.03396	1.92107

0.03394	1.91133

0.03394	1.91827

0.03393	1.92107

0.03394	1.91267

0.03396	1.91267

0.03397	1.9252

0.03397	1.91413

0.03397	1.91267

0.03398	1.9224

0.03397	1.91413

0.03397	1.91267

0.03397	1.92107

0.03397	1.91693

0.03398	1.91133

0.03397	1.9196

0.03397	1.9196

0.03396	1.90987

0.03397	1.91693

0.03396	1.92107

0.03396	1.91267

0.03395	1.91547

0.03396	1.9224

0.03396	1.91133

0.03398	1.91547

0.03397	1.9224

0.03398	1.91693

0.03396	1.91547

0.03397	1.92107

0.03396	1.91693

0.03395	1.91267

0.03395	1.91547

0.03396	1.9196

0.03395	1.91413

0.03395	1.91413

0.03395	1.92107

0.03395	1.91413

0.03395	1.91267

0.03395	1.91827

0.03395	1.9196

0.03395	1.91693

0.03393	1.91693

0.03392	1.91827

0.03393	1.91547

0.03393	1.91827

0.03392	1.91827

0.03393	1.92107

0.03393	1.91547

0.03393	1.91693

0.03394	1.91693

0.03394	1.91693

0.03394	1.91547

0.03393	1.91827

0.03392	1.91827

0.03393	1.91547

0.03393	1.92107

0.03393	1.91693

0.03393	1.91547

0.03392	1.91693

0.03393	1.91827

0.03393	1.91547

0.03393	1.91693

0.03393	1.9196

0.03393	1.91267

0.03393	1.91693

0.03393	1.91693

0.03394	1.91827

0.03394	1.91267

0.03393	1.91827

0.03392	1.91547

0.03392	1.9196

0.03391	1.91827

0.03391	1.91693

0.03392	1.9196

0.03392	1.91547

0.03393	1.91547

0.03392	1.91827

0.03393	1.91693

0.03393	1.91693

0.03393	1.91827

0.03392	1.91693

0.03392	1.91547

0.03392	1.9196

0.03392	1.91827

0.03393	1.91547

0.03392	1.91827

0.03393	1.91693

0.03393	1.91547

0.03394	1.91693

0.03393	1.9224

0.03393	1.91413

0.03394	1.91693

0.03394	1.9196

0.03393	1.91547

0.03394	1.91267

0.03394	1.91827

0.03394	1.91827

0.03395	1.91133

0.03395	1.9224

0.03396	1.92107

0.03396	1.91547

0.03397	1.91547

0.03396	1.92107

0.03395	1.91547

0.03396	1.91693

0.03396	1.9196

0.03397	1.91547

0.03397	1.91693

0.03397	1.9196

0.03396	1.91693

0.03394	1.91267

0.03394	1.92387

0.03395	1.91547

0.03397	1.91267

0.03398	1.9196

0.03397	1.91693

0.03398	1.91547

0.03397	1.91693

0.03397	1.92107

0.03398	1.91547

0.034	1.91827

0.03401	1.91827

0.03402	1.91267

0.034	1.91547

0.03399	1.92107

0.03399	1.91547

0.03399	1.91547

0.03399	1.91827

0.03399	1.91827

0.03399	1.91547

0.03399	1.91693

0.034	1.91693

0.03399	1.91827

0.03398	1.91413

0.03399	1.91693

0.03397	1.91827

0.03397	1.91693

0.03398	1.91693

0.03399	1.92107

0.03401	1.91413

0.034	1.91693

0.03398	1.91693

0.03398	1.91827

0.03398	1.91693

0.034	1.91547

0.03401	1.91693

0.03401	1.91827

0.03401	1.91413

0.03401	1.91827

0.03402	1.9196

0.03402	1.91547

0.03402	1.91413

0.03401	1.92107

0.034	1.91693

0.034	1.91267

0.03399	1.91693

0.034	1.9196

0.034	1.91547

0.034	1.91827

0.03401	1.91693

0.03402	1.91547

0.03402	1.91693

0.03401	1.9224

0.03401	1.91413

0.03399	1.91693

0.03399	1.9196

0.03399	1.91267

0.034	1.91693

0.03399	1.9196

0.03401	1.91267

0.03398	1.91693

0.03401	1.92107

0.03401	1.91693

0.03402	1.91693

0.03401	1.91693

0.034	1.91827

0.034	1.91693

0.034	1.91827

0.03401	1.91693

0.03402	1.91413

0.03402	1.91827

0.03402	1.91693

0.03402	1.9196

0.03402	1.91547

0.034	1.91827

0.03402	1.9196

0.03401	1.91267

0.03401	1.9196

0.03403	1.91547

0.03402	1.91413

0.03402	1.91693

0.03401	1.91547

0.03399	1.91547

0.034	1.92107

0.034	1.9196

0.03401	1.91413

0.03402	1.91827

0.03401	1.9196

0.03401	1.91267

0.03401	1.91827

0.03401	1.91827

0.03402	1.91133

0.03403	1.91693

0.03403	1.92107

0.03403	1.91267

0.03403	1.91693

0.03402	1.92107

0.03402	1.91827

0.03402	1.91133

0.03404	1.91547

0.03405	1.9196

0.03406	1.91547

0.03402	1.91547

0.03403	1.92107

0.03402	1.91547

0.03403	1.91693

0.03405	1.9196

0.03404	1.91693

0.03405	1.91267

0.03404	1.9196

0.03405	1.9196

0.03403	1.91413

0.03403	1.91413

0.03404	1.9196

0.03405	1.91413

0.03405	1.91413

0.03404	1.91827

0.03403	1.91827

0.03403	1.91693

0.03405	1.91693

0.03405	1.91693

0.03405	1.91827

0.03405	1.91547

0.03405	1.91693

0.03404	1.91827

0.03404	1.91547

0.03404	1.91547

0.03403	1.9196

0.03404	1.91267

0.03404	1.9196

0.03404	1.92107

0.03403	1.91547

0.03404	1.91267

0.03404	1.92107

0.03404	1.91693

0.03405	1.91267

0.03405	1.9196

0.03405	1.9196

0.03406	1.91413

0.03405	1.9196

0.03407	1.9196

0.03406	1.91547

0.03405	1.91827

0.03405	1.91693

0.03406	1.91133

0.03407	1.9196

0.03407	1.91827

0.03404	1.91133

0.03404	1.9196

0.03402	1.91827

0.03402	1.91413

0.03402	1.9196

0.03402	1.91827

0.03403	1.91267

0.03403	1.91547

0.03403	1.9196

0.03401	1.91547

0.034	1.91693

0.03401	1.9196

0.03403	1.91827

0.03404	1.91267

0.03403	1.91827

0.03402	1.92107

0.03401	1.91413

0.03402	1.91693

0.03402	1.9196

0.03402	1.91693

0.03401	1.91413

0.03401	1.92107

0.03399	1.91693

0.03399	1.91693

0.03398	1.91827

0.034	1.91693

0.03402	1.91267

0.03402	1.91827

0.03401	1.9196

0.034	1.91133

0.03401	1.91693

0.03401	1.91827

0.034	1.91267

0.034	1.91547

0.03401	1.92107

0.03399	1.91693

0.03399	1.91547

0.034	1.91827

0.03399	1.91547

0.034	1.91413

0.034	1.91547

0.03401	1.9196

0.034	1.91827

0.03402	1.91547

0.03401	1.91693

0.03401	1.9196

0.03402	1.91267

0.03402	1.91413

0.03404	1.92107

0.03402	1.91547

0.03403	1.91133

0.03402	1.9224

0.03402	1.91693

0.03402	1.91693

0.03402	1.91827

0.03401	1.91827

0.03401	1.91693

0.03402	1.91693

0.03403	1.91547

0.03403	1.91827

0.03402	1.91547

0.03403	1.91413

0.03403	1.91827

0.03402	1.91827

0.03401	1.91413

0.03401	1.91693

0.03401	1.91693

0.03403	1.91267

0.03403	1.91693

0.03403	1.91693

0.03403	1.91547

0.03402	1.91827

0.03403	1.9196

0.03403	1.91693

0.03404	1.91547

0.03402	1.92107

0.03403	1.9196

0.03403	1.91133

0.03404	1.91693

0.03403	1.92387

0.03404	1.91413

0.03404	1.91413

0.03403	1.9196

0.03404	1.91827

0.03404	1.91267

0.03402	1.9196

0.034	1.91693

0.034	1.91267

0.034	1.91693

0.03399	1.91693

0.034	1.91547

0.03401	1.91547

0.03401	1.91827

0.034	1.9196

0.03402	1.91693

0.03402	1.91693

0.03402	1.9196

0.03402	1.91413

0.03403	1.91693

0.03403	1.9196

0.03403	1.91547

0.03402	1.91693

0.03403	1.91547

0.03402	1.92107

0.03402	1.91413

0.03402	1.91547

0.03402	1.9196

0.03401	1.91547

0.03403	1.91413

0.03403	1.9196

0.03403	1.91827

0.03402	1.91267

0.03402	1.9196

0.03402	1.91827

0.03402	1.91267

0.03402	1.9196

0.03403	1.9196

0.03403	1.91267

0.03403	1.91547

0.03404	1.92107

0.03403	1.91267

0.03403	1.91547

0.03402	1.9196

0.03403	1.91827

0.03403	1.91267

0.03403	1.91827

0.03402	1.9196

0.03401	1.91267

0.03402	1.9196

0.03402	1.92107

0.03403	1.91133

0.03404	1.9196

0.03404	1.92107

0.03403	1.91267

0.03402	1.91827

0.03402	1.92107

0.03403	1.91267

0.03404	1.91547

0.03405	1.92107

0.03405	1.91827

0.03405	1.91133

0.03404	1.92107

0.03403	1.91547

0.03402	1.91267

0.03403	1.92107

0.03402	1.9196

0.03402	1.91547

0.03402	1.91547

0.03401	1.92107

0.03402	1.91267

0.03401	1.91547

0.03402	1.92107

0.03402	1.91413

0.03401	1.91547

0.03401	1.9196

0.03402	1.91693

0.03403	1.91413

0.03402	1.91827

0.03402	1.9224

0.03401	1.91267

0.03402	1.91693

0.03402	1.92107

0.03401	1.91547

0.03399	1.91413

0.03399	1.9196

0.034	1.91547

0.03401	1.91267

0.034	1.92107

0.03399	1.91827

0.03399	1.90987

0.03398	1.92107

0.034	1.91827

0.03401	1.90987

0.03401	1.91827

0.03402	1.9196

0.03402	1.91133

0.03403	1.91693

0.03403	1.9224

0.03402	1.91547

0.03402	1.91413

0.03402	1.9196

0.03402	1.91827

0.03402	1.91547

0.03402	1.91827

0.03401	1.9196

0.034	1.91547

0.03401	1.91547

0.03402	1.9196

0.03402	1.91827

0.03401	1.90987

0.03401	1.91827

0.03402	1.91827

0.03402	1.91133

0.03402	1.91693

0.03402	1.92107

0.03403	1.91133

0.03403	1.91827

0.03404	1.9196

0.03404	1.91413

0.03405	1.91827

0.03404	1.92107

0.03402	1.91413

0.03402	1.91693

0.03401	1.91827

0.03402	1.91693

0.03401	1.91267

0.03402	1.92107

0.03401	1.91413

0.03401	1.91413

0.034	1.9196

0.03401	1.91547

0.03402	1.91413

0.03402	1.9196

0.03403	1.91693

0.03402	1.91693

0.03403	1.91827

0.03402	1.91547

0.03402	1.91547

0.03402	1.9196

0.03402	1.91827

0.03402	1.91413

0.03402	1.91827

0.03402	1.91827

0.03402	1.91267

0.03402	1.91547

0.03401	1.91827

0.03399	1.91693

0.03399	1.91133

0.034	1.91693

0.03401	1.9224

0.03402	1.91413

0.03403	1.91547

0.03403	1.9196

0.03404	1.91693

0.03403	1.91413

0.03403	1.9196

0.03403	1.9196

0.03403	1.91267

0.03403	1.91693

0.03403	1.9196

0.03402	1.91547

0.034	1.91693

0.03402	1.9224

0.03402	1.91827

0.03401	1.91267

0.03401	1.9196

0.03401	1.91827

0.03402	1.91413

0.03403	1.91413

0.03402	1.9196

0.03402	1.91827

0.03402	1.91693

0.034	1.92387

0.034	1.91827

0.03402	1.91267

0.03402	1.92107

0.03402	1.91827

0.03401	1.91133

0.03401	1.91693

0.03402	1.9224

0.03404	1.91267

0.03404	1.91693

0.03404	1.92107

0.03402	1.91827

0.03403	1.91267

0.03402	1.92107

0.03402	1.91547

0.03401	1.91413

0.03402	1.91693

0.03402	1.91693

0.03402	1.91827

0.03402	1.91693

0.03401	1.91827

0.034	1.91413

0.034	1.91547

0.034	1.9196

0.03399	1.9196

0.03399	1.91413

0.03401	1.91693

0.03402	1.9196

0.03401	1.91547

0.03401	1.91827

0.03402	1.91693

0.034	1.91693

0.03399	1.91547

0.03399	1.9196

0.03399	1.91547

0.03399	1.91267

0.03399	1.91693

0.03398	1.91827

0.03398	1.91413

0.03399	1.91693

0.03399	1.9196

0.034	1.91693

0.03401	1.91413

0.03401	1.92107

0.03402	1.91827

0.03401	1.91267

0.03401	1.91693

0.03401	1.92107

0.03401	1.91413

0.034	1.91547

0.03401	1.92107

0.034	1.91693

0.03401	1.91267

0.03402	1.92107

0.03401	1.91547

0.03401	1.91413

0.034	1.91827

0.034	1.92107

0.034	1.91693

0.03399	1.90987

0.03399	1.9196

0.034	1.91827

0.034	1.91267

0.03402	1.9196

0.03401	1.91693

0.03401	1.91547

0.034	1.91693

0.03399	1.91693

0.03401	1.91693

0.03401	1.91693

0.03401	1.91547

0.03401	1.91693

0.03399	1.91693

0.03397	1.91827

0.03397	1.91413

0.03395	1.91547

0.03396	1.91827

0.03398	1.91827

0.03398	1.91693

0.034	1.91413

0.034	1.91827

0.03399	1.92107

0.03397	1.91413

0.03397	1.91693

0.03397	1.9224

0.03397	1.91413

0.03397	1.91267

0.03397	1.92107

0.03399	1.91827

0.03399	1.91133

0.03399	1.92107

0.03399	1.92107

0.03399	1.90987

0.03398	1.91547

0.034	1.9224

0.03399	1.91547

0.03398	1.91267

0.03399	1.92107

0.034	1.91693

0.03401	1.91547

0.034	1.9196

0.03401	1.9196

0.03399	1.91413

0.03399	1.91693

0.034	1.91693

0.03398	1.91693

0.03399	1.91693

0.03399	1.91547

0.03397	1.9196

0.03398	1.91413

0.03398	1.91693

0.03398	1.91827

0.03398	1.91267

0.03398	1.91693

0.03398	1.9196

0.03397	1.91413

0.03396	1.91827

0.03397	1.91827

0.03398	1.91693

0.03399	1.91413

0.034	1.91693

0.03399	1.92107

0.03399	1.91267

0.03398	1.91827

0.03399	1.92107

0.03399	1.91133

0.03398	1.91267

0.03399	1.9224

0.03397	1.91693

0.03396	1.91267

0.03395	1.9224

0.03396	1.91547

0.03395	1.91133

0.03396	1.92107

0.03395	1.91827

0.03395	1.91267

0.03396	1.92107

0.03397	1.9224

0.03398	1.91133

0.03397	1.91827

0.03398	1.91827

0.03397	1.91547

0.03398	1.91547

0.03396	1.9224

0.03396	1.91547

0.03396	1.91413

0.03397	1.92107

0.03398	1.91547

0.03396	1.91413

0.03395	1.9196

0.03395	1.91547

0.03396	1.91547

0.03397	1.91693

0.03397	1.91693

0.03397	1.91267

0.03395	1.91693

0.03396	1.92107

0.03397	1.91413

0.03397	1.91547

0.03397	1.9196

0.03398	1.91827

0.03399	1.91413

0.03399	1.91547

0.03398	1.9196

0.03398	1.91827

0.03398	1.91547

0.03398	1.91547

0.03399	1.9196

0.03398	1.91547

0.03398	1.91547

0.03396	1.91693

0.03395	1.91827

0.03396	1.91413

0.03397	1.91267

0.03397	1.9196

0.03397	1.91547

0.034	1.91413

0.03399	1.92107

0.03399	1.91693

0.03397	1.91547

0.034	1.9196

0.034	1.91547

0.03399	1.91267

0.03397	1.91693

0.03396	1.9196

0.03396	1.91547

0.03397	1.91547

0.03397	1.91827

0.03398	1.91827

0.03396	1.91413

0.03397	1.92107

0.03398	1.9196

0.03399	1.91133

0.03396	1.91693

0.03396	1.91827

0.03396	1.91413

0.03395	1.91547

0.03395	1.91827

0.03395	1.91413

0.03394	1.91547

0.03394	1.91827

0.03395	1.91693

0.03395	1.91267

0.03396	1.92107

0.03396	1.91827

0.03397	1.91547

0.03397	1.91693

0.03397	1.9196

0.03395	1.91547

0.03396	1.91267

0.03396	1.9196

0.03396	1.91693

0.03395	1.91413

0.03395	1.91827

0.03394	1.91827

0.03395	1.91547

0.03396	1.91413

0.03394	1.9196

0.03395	1.91547

0.03394	1.91547

0.03395	1.9196

0.03396	1.91693

0.03395	1.91267

0.03395	1.92107

0.03394	1.91693

0.03394	1.91133

0.03394	1.91827

0.03396	1.9196

0.03394	1.91133

0.03397	1.91693

0.03397	1.92107

0.03397	1.91693

0.03396	1.91133

0.03397	1.92107

0.03397	1.91693

0.03397	1.91547

0.03396	1.91547

0.03396	1.92107

0.03397	1.91413

0.03397	1.91547

0.03397	1.92107

0.03397	1.91413

0.03395	1.91413

0.03396	1.9252

0.03396	1.91267

0.03398	1.91413

0.03396	1.9224

0.03396	1.9196

0.03394	1.91267

0.03393	1.92107

0.03394	1.9196

0.03393	1.90987

0.03394	1.91827

0.03394	1.9196

0.03394	1.91133

0.03393	1.9196

0.03393	1.92107

0.03392	1.91413

0.03394	1.91547

0.03395	1.9196

0.03395	1.91693

0.03394	1.91547

0.03393	1.91827

0.03393	1.9224

0.03393	1.91547

0.03395	1.91693

0.03395	1.9196

0.03395	1.91547

0.03396	1.91413

0.03395	1.9196

0.03396	1.91547

0.03396	1.91267

0.03395	1.91827

0.03395	1.91827

0.03396	1.91547

0.03395	1.91547

0.03396	1.9196

0.03397	1.91547

0.03397	1.91413

0.03398	1.92107

0.03398	1.91827

0.03397	1.91413

0.03398	1.91693

0.03399	1.92107

0.03399	1.91547

0.03398	1.91413

0.03399	1.9196

0.034	1.9196

0.034	1.91693

0.03399	1.91547

0.03397	1.9196

0.03397	1.91827

0.03397	1.91133

0.03396	1.91827

0.03398	1.91827

0.03398	1.91547

0.03399	1.91827

0.034	1.91827

0.034	1.91547

0.03401	1.91693

0.034	1.91827

0.034	1.91827

0.03399	1.91413

0.03399	1.91827

0.03399	1.91827

0.03399	1.91413

0.03397	1.91547

0.03398	1.91827

0.03397	1.92107

0.03399	1.91133

0.03398	1.91827

0.03397	1.9196

0.03397	1.91133

0.03396	1.91693

0.03398	1.9196

0.03398	1.91413

0.03399	1.91133

0.03397	1.9196

0.03397	1.91547

0.03396	1.91267

0.03396	1.92107

0.03395	1.91827

0.03396	1.91547

0.03396	1.91693

0.03397	1.92107

0.03398	1.91267

0.03398	1.91547

0.03397	1.9196

0.03396	1.91693

0.03395	1.91267

0.03396	1.9196

0.03397	1.91827

0.03397	1.91267

0.03397	1.92107

0.03398	1.91693

0.03398	1.91267

0.03397	1.91547

0.03397	1.91693

0.03397	1.91547

0.03399	1.91693

0.03398	1.9196

0.03398	1.91693

0.03398	1.91133

0.03397	1.91693

0.03399	1.9196

0.03398	1.91547

0.03399	1.91827

0.03399	1.91827

0.03399	1.91547

0.03399	1.91547

0.03399	1.91693

0.03399	1.9196

0.03397	1.91413

0.03398	1.91693

0.03397	1.92107

0.03399	1.91547

0.03399	1.91547

0.03398	1.91827

0.03399	1.91827

0.03399	1.91413

0.03399	1.91827

0.03398	1.91827

0.03397	1.91267

0.03396	1.91693

0.03398	1.92107

0.03397	1.91267

0.03399	1.91267

0.03399	1.92107

0.034	1.91693

0.034	1.91267

0.03401	1.9196

0.03401	1.91827

0.034	1.91413

0.03399	1.91827

0.03399	1.9196

0.03399	1.91267

0.03398	1.91547

0.03399	1.92107

0.03399	1.91693

0.03398	1.91267

0.03398	1.9224

0.03398	1.91547

0.03398	1.91267

0.03399	1.9196

0.034	1.91693

0.034	1.91267

0.03399	1.91693

0.03399	1.9196

0.03399	1.91267

0.03399	1.91547

0.03398	1.92107

0.03399	1.91413

0.034	1.91547

0.03401	1.91827

0.03401	1.91693

0.03401	1.91267

0.03401	1.91827

0.034	1.91827

0.03399	1.91267

0.03399	1.91693

0.03398	1.92107

0.03399	1.91413

0.034	1.91547

0.03402	1.9196

0.03402	1.91693

0.03402	1.91267

0.03401	1.9196

0.03401	1.91693

0.03401	1.91547

0.03401	1.91827

0.03402	1.91693

0.03401	1.91547

0.03402	1.9196

0.03401	1.91827

0.034	1.91693

0.03401	1.91827

0.034	1.91827

0.03399	1.91547

0.03398	1.91547

0.03399	1.9196

0.03399	1.91827

0.034	1.91547

0.03401	1.91413

0.03402	1.92107

0.03402	1.91413

0.03402	1.91827

0.03401	1.9196

0.03401	1.91693

0.03401	1.91413

0.03401	1.91827

0.034	1.91693

0.03402	1.91547

0.03401	1.91827

0.03402	1.91547

0.03402	1.91413

0.03402	1.91547

0.03401	1.91827

0.03402	1.91693

0.03402	1.91413

0.03402	1.9224

0.03402	1.91267

0.03402	1.91413

0.03402	1.92107

0.03402	1.91693

0.03403	1.91267

0.03403	1.92107

0.03405	1.91693

0.03405	1.91413

0.03403	1.91827

0.03402	1.9196

0.03402	1.91547

0.03401	1.91413

0.03401	1.91827

0.03401	1.91547

0.03401	1.91827

0.03402	1.91827

0.03402	1.91547

0.03401	1.91547

0.034	1.91827

0.034	1.91693

0.03399	1.91693

0.03399	1.91827

0.034	1.9196

0.034	1.91693

0.03401	1.91693

0.034	1.9196

0.034	1.91413

0.03401	1.91413

0.03402	1.91693

0.03402	1.91827

0.03402	1.91547

0.03402	1.91547

0.03402	1.9196

0.03402	1.91827

0.03402	1.91267

0.03401	1.91547

0.03401	1.92107

0.03402	1.91267

0.03401	1.91693

0.03401	1.9224

0.03399	1.91547

0.034	1.91693

0.03402	1.91827

0.03402	1.91827

0.03401	1.91693

0.034	1.91827

0.03399	1.91693

0.034	1.91547

0.034	1.91547

0.03401	1.92107

0.034	1.91693

0.034	1.91413

0.034	1.9196

0.034	1.91827

0.03398	1.91413

0.03398	1.9196

0.03398	1.9196

0.03399	1.91133

0.034	1.91693

0.03399	1.9252

0.03399	1.91267

0.03399	1.91133

0.03398	1.9224

0.03399	1.91827

0.03401	1.91133

0.03402	1.92107

0.03403	1.91827

0.03402	1.91413

0.03402	1.91827

0.03402	1.9196

0.03403	1.91547

0.03403	1.91413

0.03402	1.92107

0.03402	1.91693

0.03402	1.91693

0.03403	1.91693

0.03403	1.91827

0.03403	1.91827

0.03403	1.91547

0.03402	1.91827

0.03402	1.91827

0.03402	1.91547

0.03401	1.91693

0.03402	1.91827

0.03401	1.91693

0.03402	1.91547

0.03402	1.91693

0.03403	1.91827

0.03403	1.91547

0.03403	1.92107

0.03403	1.91693

0.03403	1.91413

0.03402	1.91693

0.03402	1.92107

0.03402	1.91547

0.03402	1.91693

0.03403	1.91827

0.03403	1.91693

0.03404	1.91267

0.03404	1.91693

0.03404	1.91827

0.03404	1.91267

0.03405	1.91413

0.03406	1.92107

0.03407	1.91547

0.03405	1.91693

0.03405	1.91827

0.03403	1.91827

0.03403	1.91547

0.03404	1.9196

0.03404	1.91693

0.03403	1.91413

0.03404	1.91827

0.03404	1.91827

0.03404	1.91693

0.03404	1.91413

0.03405	1.9196

0.03406	1.91827

0.03405	1.91693

0.03404	1.91827

0.03404	1.91827

0.03405	1.91547

0.03403	1.91693

0.03404	1.91827

0.03404	1.91547

0.03403	1.91827

0.03404	1.9196

0.03402	1.91413

0.03403	1.91267

0.03403	1.9196

0.03405	1.91267

0.03405	1.91693

0.03405	1.9196

0.03403	1.91547

0.03403	1.91413

0.03403	1.91827

0.03405	1.91693

0.03405	1.91547

0.03405	1.91547

0.03405	1.92107

0.03405	1.91413

0.03404	1.91827

0.03405	1.91693

0.03404	1.91693

0.03404	1.91413

0.03404	1.91827

0.03404	1.91693

0.03405	1.91827

0.03405	1.91547

0.03405	1.91693

0.03404	1.92107

0.03403	1.91693

0.03404	1.91267

0.03404	1.9196

0.03405	1.91827

0.03405	1.91413

0.03404	1.91693

0.03404	1.92107

0.03404	1.91267

0.03404	1.91547

0.03405	1.92107

0.03406	1.91413

0.03405	1.91547

0.03404	1.92107

0.03404	1.91547

0.03404	1.91267

0.03403	1.9196

0.03405	1.91827

0.03405	1.91413

0.03408	1.91827

0.03407	1.91827

0.03408	1.91413

0.03407	1.91827

0.03406	1.91547

0.03405	1.91413

0.03404	1.91547

0.03403	1.91827

0.03405	1.91547

0.03406	1.91547

0.03407	1.91547

0.03406	1.92107

0.03406	1.91413

0.03406	1.91547

0.03405	1.92107

0.03406	1.91413

0.03405	1.91413

0.03406	1.9224

0.03405	1.91693

0.03405	1.91267

0.03406	1.92107

0.03407	1.91547

0.03407	1.91267

0.03406	1.91693

0.03406	1.91693

0.03406	1.91693

0.03407	1.91693

0.03406	1.91827

0.03405	1.91413

0.03405	1.91827

0.03405	1.9196

0.03404	1.91827

0.03405	1.91413

0.03402	1.92107

0.03402	1.91547

0.03403	1.91547

0.03402	1.9196

0.03403	1.91693

0.03403	1.91133

0.03405	1.9196

0.03407	1.9196

0.03407	1.91547

0.03407	1.91547

0.03407	1.9196

0.03407	1.91413

0.03407	1.91693

0.03406	1.91693

0.03406	1.9196

0.03405	1.91413

0.03404	1.91413

0.03407	1.91827

0.03407	1.91693

0.03407	1.91547

0.03408	1.91827

0.03408	1.9196

0.03407	1.91827

0.03407	1.91693

0.03407	1.91413

0.03405	1.91827

0.03407	1.92107

0.03406	1.91413

0.03407	1.91547

0.03408	1.9224

0.03409	1.91413

0.03409	1.91547

0.03408	1.91827

0.03409	1.92107

0.03408	1.91413

0.03409	1.91693

0.03408	1.91693

0.03407	1.91693

0.03408	1.91693

0.03409	1.91547

0.03409	1.91693

0.03409	1.91827

0.03409	1.91827

0.0341	1.91693

0.0341	1.91827

0.0341	1.91693

0.03408	1.91267

0.03407	1.91693

0.03408	1.91827

0.03409	1.91547

0.03409	1.91827

0.03409	1.91547

0.03408	1.91693

0.03408	1.91413

0.03407	1.91827

0.03408	1.91693

0.03407	1.91413

0.03407	1.9196

0.03409	1.91693

0.0341	1.91413

0.0341	1.9196

0.0341	1.91827

0.0341	1.91267

0.0341	1.91827

0.03409	1.9196

0.03409	1.91413

0.03408	1.91693

0.03408	1.91827

0.03408	1.91827

0.03408	1.91413

0.03409	1.91693

0.03407	1.91827

0.03408	1.91547

0.03407	1.91547

0.03407	1.91827

0.03406	1.91827

0.03406	1.91827

0.03407	1.91413

0.03407	1.91693

0.03408	1.91827

0.03409	1.91413

0.03409	1.91413

0.0341	1.9196

0.03409	1.91693

0.0341	1.91693

0.0341	1.91827

0.0341	1.91547

0.0341	1.91547

0.03409	1.91693

0.0341	1.91827

0.0341	1.91413

0.03413	1.9196

0.03413	1.91693

0.03411	1.91827

0.03411	1.91413

0.03408	1.91693

0.03408	1.9196

0.03407	1.91413

0.03409	1.9196

0.03407	1.91693

0.03407	1.91413

0.03408	1.91547

0.03409	1.92107

0.0341	1.91413

0.03409	1.91693

0.03409	1.91827

0.03408	1.9196

0.03409	1.91267

0.03409	1.91827

0.03409	1.9196

0.03412	1.91413

0.03411	1.91547

0.0341	1.91827

0.0341	1.91827

0.0341	1.91547

0.03411	1.9196

0.03411	1.91827

0.03412	1.91693

0.0341	1.91547

0.03411	1.91827

0.0341	1.91413

0.0341	1.91827

0.03409	1.9196

0.03409	1.91413

0.03409	1.91693

0.03411	1.9196

0.03411	1.91547

0.03411	1.9196

0.0341	1.91827

0.03412	1.91547

0.0341	1.91413

0.0341	1.91827

0.0341	1.91547

0.03409	1.91693

0.0341	1.91827

0.03409	1.91827

0.03411	1.91693

0.0341	1.91547

0.0341	1.9196

0.0341	1.91547

0.03411	1.91547

0.03411	1.91827

0.0341	1.9196

0.03409	1.90987

0.0341	1.9196

0.03411	1.91547

0.03411	1.91547

0.03411	1.91693

0.03409	1.91827

0.03409	1.92107

0.03408	1.91413

0.03408	1.91693

0.03409	1.91827

0.03409	1.91547

0.03409	1.91693

0.0341	1.91693

0.03411	1.9196

0.03411	1.91413

0.03412	1.91827

0.03413	1.9196

0.03412	1.91547

0.03412	1.91547

0.03414	1.9196

0.03412	1.91547

0.03412	1.91413

0.0341	1.91827

0.0341	1.91827

0.0341	1.91547

0.0341	1.9196

0.03409	1.9196

0.03409	1.91413

0.03409	1.91827

0.03408	1.92107

0.03408	1.91413

0.03408	1.91413

0.03407	1.9196

0.03408	1.91413

0.03408	1.91547

0.03408	1.9196

0.0341	1.91547

0.03411	1.91693

0.03411	1.91827

0.03412	1.91827

0.03412	1.91547

0.03412	1.91693

0.03412	1.9196

0.03414	1.91547

0.03413	1.91693

0.03413	1.91693

0.03413	1.91827

0.03412	1.91267

0.03412	1.91547

0.03413	1.92107

0.03413	1.91547

0.03411	1.91693

0.03412	1.91693

0.03411	1.91693

0.03412	1.91693

0.03414	1.91133

0.03414	1.91693

0.03413	1.92107

0.03413	1.91267

0.03413	1.9224

0.03413	1.91827

0.03414	1.91267

0.03413	1.91827

0.03414	1.9196

0.03415	1.91267

0.03416	1.91827

0.03414	1.91827

0.03413	1.91693

0.03414	1.91693

0.03414	1.9196

0.03413	1.91827

0.03413	1.91547

0.03413	1.91413

0.03414	1.9196

0.03414	1.91267

0.03414	1.91693

0.03414	1.9224

0.03414	1.91547

0.03413	1.91547

0.03413	1.9224

0.03412	1.91693

0.03412	1.91413

0.03413	1.9196

0.03413	1.91827

0.03412	1.91413

0.03412	1.91547

0.03412	1.9196

0.03413	1.91547

0.03413	1.91827

0.03413	1.9196

0.03413	1.91693

0.03413	1.91693

0.03412	1.91693

0.03413	1.91693

0.03414	1.91413

0.03414	1.91693

0.03415	1.91693

0.03416	1.91693

0.03416	1.91547

0.03415	1.91693

0.03414	1.91827

0.03414	1.91693

0.03414	1.91693

0.03414	1.91693

0.03415	1.91547

0.03415	1.91827

0.03416	1.91693

0.03415	1.91547

0.03414	1.91693

0.03415	1.9224

0.03413	1.91547

0.03414	1.91547

0.03415	1.9196

0.03415	1.91693

0.03414	1.91267

0.03415	1.91827

0.03415	1.92107

0.03416	1.91413

0.03415	1.91693

0.03417	1.92107

0.03415	1.91413

0.03417	1.91693

0.03415	1.91827

0.03413	1.91693

0.03412	1.91133

0.03413	1.91827

0.03414	1.91827

0.03416	1.91413

0.03415	1.91827

0.03415	1.91827

0.03414	1.91413

0.03414	1.91693

0.03414	1.92107

0.03415	1.91413

0.03415	1.91413

0.03414	1.92107

0.03415	1.91827

0.03415	1.91133

0.03417	1.9196

0.03417	1.9196

0.03417	1.91133

0.03419	1.91827

0.03418	1.91827

0.03416	1.9196

0.03416	1.91413

0.03416	1.91693

0.03416	1.92107

0.03416	1.91413

0.03416	1.91133

0.03418	1.92107

0.03417	1.91547

0.03417	1.91413

0.03418	1.9224

0.03417	1.91693

0.03416	1.91547

0.03415	1.91413

0.03415	1.91827

0.03417	1.91547

0.03416	1.91413

0.03416	1.91827

0.03415	1.9196

0.03414	1.91547

0.03415	1.91693

0.03414	1.9196

0.03415	1.91413

0.03416	1.91547

0.03417	1.92107

0.03417	1.91693

0.03415	1.91413

0.03416	1.9196

0.03416	1.91693

0.03417	1.91547

0.03417	1.91827

0.03417	1.91827

0.03416	1.91547

0.03417	1.91693

0.03416	1.91827

0.03418	1.91413

0.03417	1.91413

0.03417	1.92107

0.03417	1.91827

0.03416	1.91693

0.03418	1.91693

0.03418	1.91827

0.03419	1.91693

0.03419	1.91413

0.03419	1.92107

0.03418	1.91827

0.03417	1.91547

0.03418	1.91693

0.03419	1.91827

0.0342	1.91547

0.03418	1.91693

0.03417	1.91827

0.03417	1.91547

0.03418	1.91547

0.03416	1.91827

0.03417	1.91827

0.03417	1.91827

0.03418	1.91547

0.03418	1.91547

0.03418	1.91827

0.03419	1.91547

0.03418	1.91693

0.03419	1.91693

0.03418	1.9196

0.03418	1.91547

0.03418	1.91693

0.03417	1.91693

0.03418	1.91547

0.03418	1.91413

0.03418	1.91827

0.03416	1.91693

0.03417	1.91693

0.03416	1.91547

0.03417	1.9196

0.03417	1.91827

0.03418	1.91693

0.03417	1.91693

0.03419	1.91693

0.03419	1.9196

0.03418	1.91413

0.03417	1.91547

0.03419	1.92107

0.03417	1.91693

0.03418	1.91413

0.03417	1.91693

0.03417	1.91693

0.03416	1.91693

0.03416	1.91547

0.03417	1.91827

0.03418	1.91413

0.03417	1.91547

0.03416	1.9196

0.03417	1.91547

0.03416	1.91413

0.03416	1.9196

0.03416	1.91413

0.03417	1.91547

0.03416	1.91547

0.03415	1.91827

0.03414	1.91827

0.03415	1.91413

0.03415	1.91827

0.03415	1.92107

0.03416	1.91267

0.03417	1.91693

0.03417	1.91693

0.03417	1.91693

0.03416	1.91413

0.03415	1.91693

0.03414	1.91827

0.03415	1.91547

0.03417	1.91693

0.03416	1.91693

0.03416	1.91693

0.03415	1.91267

0.03414	1.9196

0.03413	1.91547

0.03413	1.91547

0.03414	1.9196

0.03415	1.91547

0.03416	1.91547

0.03415	1.91413

0.03415	1.92107

0.03416	1.91827

0.03415	1.91547

0.03416	1.91693

0.03416	1.91693

0.03417	1.91693

0.03417	1.91693

0.03417	1.91827

0.03416	1.91547

0.03416	1.9196

0.03416	1.91693

0.03417	1.91547

0.03417	1.91827

0.03417	1.92107

0.03415	1.91547

0.03416	1.91547

0.03417	1.9196

0.03417	1.91413

0.03418	1.91547

0.03417	1.91827

0.03418	1.9196

0.03416	1.91413

0.03417	1.91693

0.03418	1.9196

0.03415	1.91547

0.03415	1.91133

0.03413	1.9224

0.03414	1.91547

0.03415	1.91693

0.03418	1.9224

0.03418	1.91693

0.03419	1.91413

0.03416	1.9196

0.03417	1.91693

0.03416	1.91413

0.03416	1.91693

0.03416	1.92107

0.03417	1.91413

0.03416	1.91547

0.03417	1.9224

0.03416	1.91267

0.03417	1.91267

0.03416	1.92107

0.03418	1.91547

0.03417	1.91547

0.03419	1.9196

0.03416	1.91693

0.03415	1.91827

0.03415	1.91693

0.03415	1.91693

0.03415	1.91693

0.03416	1.91547

0.03416	1.91693

0.03414	1.91827

0.03415	1.91413

0.03415	1.91413

0.03416	1.92107

0.03416	1.91693

0.03417	1.91547

0.03417	1.92107

0.03418	1.91693

0.03418	1.91267

0.03418	1.92107

0.03417	1.91827

0.03418	1.91267

0.03418	1.91413

0.03417	1.92107

0.03418	1.91693

0.03417	1.91413

0.03416	1.91827

0.03418	1.91827

0.03416	1.91413

0.03418	1.91693

0.03418	1.91693

0.03419	1.91693

0.03419	1.91267

0.03418	1.92107

0.03419	1.91133

0.03419	1.91693

0.03419	1.92107

0.0342	1.91413

0.03419	1.91693

0.03419	1.9196

0.03419	1.91693

0.03419	1.91413

0.0342	1.91693

0.0342	1.92107

0.03419	1.91413

0.03418	1.91547

0.03419	1.92107

0.03419	1.91413

0.03418	1.91133

0.03419	1.92387

0.03419	1.91413

0.03419	1.91133

0.03419	1.92107

0.0342	1.91827

0.0342	1.91267

0.0342	1.9196

0.0342	1.91827

0.0342	1.91413

0.0342	1.91693

0.03419	1.9196

0.0342	1.91413

0.03419	1.91413

0.03419	1.91693

0.0342	1.91547

0.0342	1.91693

0.03419	1.91827

0.0342	1.9196

0.03418	1.91693

0.0342	1.91693

0.03419	1.9196

0.03418	1.91693

0.0342	1.91547

0.03418	1.91827

0.03419	1.9196

0.03418	1.91413

0.03418	1.91827

0.03418	1.92107

0.03418	1.91413

0.03417	1.91827

0.03418	1.9196

0.03419	1.91267

0.03421	1.91693

0.03422	1.91827

0.03422	1.91693

0.03421	1.91413

0.03422	1.91827

0.03422	1.9224

0.03422	1.91133

0.03423	1.9196

0.03422	1.92387

0.0342	1.91267

0.0342	1.91827

0.03419	1.91827

0.0342	1.91267

0.0342	1.91693

0.03422	1.9196

0.03423	1.91827

0.03423	1.91693

0.03421	1.91827

0.0342	1.9196

0.03421	1.91827

0.03422	1.91547

0.03422	1.91547

0.03421	1.91827

0.03421	1.91693

0.03422	1.91133

0.03421	1.91827

0.03421	1.91693

0.03421	1.91413

0.03421	1.92107

0.03421	1.91547

0.03422	1.91267

0.03422	1.9196

0.03421	1.92107

0.03422	1.91413

0.03422	1.91693

0.03423	1.9196

0.03423	1.91693

0.03423	1.91547

0.03421	1.9224

0.0342	1.91547

0.0342	1.91413

0.0342	1.91827

0.0342	1.91693

0.03422	1.91547

0.03422	1.91827

0.03421	1.91547

0.03421	1.91547

0.03419	1.91827

0.03422	1.91827

0.03421	1.91547

0.03422	1.91827

0.03422	1.91693

0.0342	1.91547

0.03419	1.91827

0.0342	1.91827

0.03421	1.91413

0.03422	1.91547

0.03421	1.9196

0.0342	1.91267

0.03418	1.91693

0.03418	1.91827

0.03419	1.91267

0.0342	1.91547

0.0342	1.9224

0.03422	1.91547

0.03423	1.91133

0.03423	1.92107

0.03425	1.91827

0.03423	1.91267

0.03422	1.91547

0.03422	1.9224

0.03422	1.91547

0.03422	1.91267

0.03423	1.92387

0.03423	1.91267

0.03424	1.91827

0.03424	1.91547

0.03425	1.91827

0.03424	1.91547

0.03424	1.91693

0.03424	1.9196

0.03425	1.91693

0.03425	1.91413

0.03425	1.9196

0.03424	1.9196

0.03424	1.91267

0.03424	1.91693

0.03425	1.9224

0.03425	1.91133

0.03423	1.91693

0.03423	1.92107

0.03423	1.91413

0.03423	1.91267

0.03423	1.91827

0.03426	1.91827

0.03426	1.91413

0.03425	1.91693

0.03426	1.9196

0.03425	1.91267

0.03424	1.91827

0.03423	1.92107

0.03424	1.91413

0.03425	1.91547

0.03426	1.92107

0.03426	1.91693

0.03426	1.91267

0.03425	1.9196

0.03424	1.91827

0.03424	1.91133

0.03425	1.9196

0.03426	1.92107

0.03425	1.91547

0.03425	1.91413

0.03424	1.92107

0.03423	1.91547

0.03424	1.91413

0.03424	1.91827

0.03424	1.91827

0.03423	1.91547

0.03423	1.9196

0.03423	1.91827

0.03423	1.91827

0.03422	1.91133

0.03423	1.92107

0.03423	1.9196

0.03424	1.90987

0.03424	1.91827

0.03424	1.9196

0.03425	1.91413

0.03425	1.91827

0.03424	1.9196

0.03424	1.91693

0.03424	1.91267

0.03424	1.9196

0.03423	1.91827

0.03424	1.91547

0.03422	1.91547

0.03424	1.9196

0.03424	1.91547

0.03425	1.91267

0.03426	1.9196

0.03425	1.91827

0.03424	1.91267

0.03424	1.9196

0.03424	1.91827

0.03425	1.91267

0.03424	1.91693

0.03425	1.91827

0.03425	1.91267

0.03425	1.91693

0.03424	1.9196

0.03423	1.91413

0.03423	1.91827

0.03424	1.92107

0.03425	1.91133

0.03426	1.91547

0.03426	1.92107

0.03427	1.91693

0.03427	1.91133

0.03428	1.91827

0.03429	1.91693

0.03427	1.90987

0.03424	1.92107

0.03424	1.91693

0.03424	1.91413

0.03424	1.91693

0.03425	1.9196

0.03424	1.91547

0.03425	1.91413

0.03425	1.92107

0.03426	1.91547

0.03425	1.91547

0.03424	1.91693

0.03423	1.91693

0.03422	1.91413

0.03423	1.9196

0.03422	1.91827

0.03423	1.91693

0.03425	1.92107

0.03424	1.91547

0.03424	1.91413

0.03424	1.91827

0.03424	1.91827

0.03425	1.91693

0.03425	1.91827

0.03426	1.92107

0.03425	1.91413

0.03426	1.91547

0.03426	1.91693

0.03426	1.91693

0.03426	1.91413

0.03425	1.91827

0.03427	1.91693

0.03428	1.91693

0.03428	1.91413

0.03425	1.9196

0.03423	1.91827

0.03424	1.91547

0.03423	1.91693

0.03423	1.91693

0.03422	1.91547

0.03423	1.91413

0.03423	1.9196

0.03425	1.91547

0.03425	1.91413

0.03425	1.92107

0.03423	1.91827

0.03424	1.91267

0.03422	1.91827

0.03424	1.92107

0.03426	1.91413

0.03426	1.91547

0.03425	1.9196

0.03424	1.91827

0.03424	1.91413

0.03425	1.91827

0.03427	1.91827

0.03425	1.91413

0.03428	1.91547

0.03426	1.9196

0.03425	1.91413

0.03424	1.91547

0.03425	1.91693

0.03426	1.91827

0.03427	1.91693

0.03428	1.91547

0.0343	1.9196

0.0343	1.91693

0.03431	1.91267

0.03429	1.91693

0.03429	1.92107

0.03429	1.91413

0.03428	1.91693

0.03427	1.91827

0.03427	1.91827

0.03425	1.91267

0.03426	1.91693

0.03426	1.9196

0.03427	1.91693

0.03427	1.91413

0.03426	1.92107

0.03427	1.91827

0.03428	1.91267

0.03427	1.9196

0.03426	1.91827

0.03425	1.91413

0.03425	1.91413

0.03425	1.91827

0.03426	1.91827

0.03426	1.91133

0.03425	1.9196

0.03425	1.91827

0.03425	1.91267

0.03426	1.92107

0.03425	1.91827

0.03426	1.91413

0.03424	1.92107

0.03426	1.91827

0.03426	1.91267

0.03427	1.92107

0.03427	1.92107

0.03428	1.91267

0.03427	1.91827

0.03427	1.9196

0.03427	1.91267

0.03426	1.91267

0.03424	1.92107

0.03423	1.91547

0.03424	1.91693

0.03426	1.92107

0.03427	1.91693

0.03427	1.91267

0.03426	1.9196

0.03425	1.91547

0.03425	1.91133

0.03425	1.9196

0.03425	1.9196

0.03425	1.90987

0.03426	1.92107

0.03427	1.9196

0.03427	1.91413

0.03426	1.9196

0.03427	1.9224

0.03427	1.91693

0.03429	1.91267

0.03428	1.9224

0.0343	1.91693

0.03429	1.91133

0.03427	1.9196

0.03427	1.91547

0.03425	1.91413

0.03424	1.92107

0.03423	1.91693

0.03423	1.91413

0.03422	1.91827

0.03423	1.91693

0.03423	1.91413

0.03422	1.91693

0.03422	1.9196

0.03424	1.91547

0.03424	1.91693

0.03424	1.9196

0.03423	1.91827

0.03424	1.91547

0.03423	1.91827

0.03424	1.91547

0.03426	1.91547

0.03426	1.9224

0.03427	1.91547

0.03426	1.91547

0.03426	1.9196

0.03425	1.91693

0.03425	1.91547

0.03425	1.91693

0.03426	1.92107

0.03426	1.90987

0.03426	1.91693

0.03425	1.92107

0.03425	1.91413

0.03426	1.91413

0.03425	1.9224

0.03425	1.91547

0.03426	1.91267

0.03426	1.9224

0.03427	1.91547

0.03426	1.91133

0.03426	1.9224

0.03425	1.91827

0.03426	1.91133

0.03425	1.92387

0.03425	1.9196

0.03425	1.91133

0.03424	1.91693

0.03425	1.9196

0.03426	1.91547

0.03426	1.91693

0.03427	1.92107

0.03426	1.91693

0.03426	1.91547

0.03426	1.91693

0.03425	1.91693

0.03426	1.90987

0.03426	1.91693

0.03427	1.91827

0.03425	1.91413

0.03426	1.9196

0.03426	1.91827

0.03427	1.91547

0.03427	1.91547

0.03427	1.91827

0.03427	1.91693

0.03427	1.91413

0.03428	1.91547

0.03426	1.91827

0.03426	1.91547

0.03426	1.91827

0.03426	1.91693

0.03426	1.9196

0.03427	1.91693

0.03428	1.91547

0.03428	1.91693

0.03429	1.91827

0.03427	1.91693

0.03427	1.91413

0.03427	1.91827

0.03428	1.91827

0.03429	1.91413

0.03428	1.91547

0.03429	1.9196

0.03428	1.91547

0.03429	1.91267

0.03429	1.92107

0.0343	1.91547

0.03429	1.91133

0.0343	1.9196

0.0343	1.9196

0.03431	1.91267

0.03431	1.91413

0.03429	1.91827

0.03428	1.91547

0.03429	1.91267

0.0343	1.91693

0.0343	1.91827

0.03431	1.91267

0.03432	1.91827

0.03431	1.9224

0.03431	1.91267

0.03432	1.91413

0.03431	1.92387

0.03431	1.91267

0.03431	1.91413

0.03431	1.9196

0.03431	1.91827

0.03431	1.91413

0.0343	1.91827

0.0343	1.92107

0.0343	1.91267

0.0343	1.91827

0.03429	1.9196

0.03429	1.91413

0.03429	1.91547

0.03429	1.9196

0.03429	1.91413

0.0343	1.91693

0.03429	1.92107

0.0343	1.91267

0.03431	1.91267

0.03431	1.92107

0.03431	1.91547

0.03431	1.91267

0.0343	1.9196

0.03431	1.91547

0.03431	1.91413

0.03429	1.91693

0.03429	1.92107

0.03429	1.91547

0.0343	1.91547

0.0343	1.9224

0.03432	1.91547

0.03431	1.91133

0.03431	1.9224

0.0343	1.91547

0.03432	1.91133

0.03431	1.92107

0.03433	1.91827

0.03432	1.90853

0.03432	1.92107

0.03432	1.9196

0.03433	1.91267

0.03433	1.91547

0.03431	1.9196

0.03429	1.91267

0.03429	1.91267

0.03431	1.9224

0.03433	1.91267

0.03433	1.91413

0.03433	1.92107

0.03433	1.91547

0.03433	1.91413

0.03431	1.9196

0.03431	1.91693

0.03432	1.91267

0.03431	1.92107

0.03432	1.91693

0.03431	1.91267

0.03431	1.9196

0.03431	1.91693

0.03433	1.91413

0.03432	1.9224

0.03433	1.91547

0.03432	1.91133

0.03432	1.9196

0.03431	1.9224

0.03432	1.91267

0.03432	1.91827

0.03431	1.92107

0.03431	1.91267

0.03431	1.91693

0.03431	1.92107

0.03432	1.91413

0.03433	1.91547

0.03434	1.92107

0.03434	1.91693

0.03433	1.91413

0.03432	1.9196

0.03432	1.91547

0.03432	1.91547

0.03433	1.91693

0.03434	1.91827

0.03435	1.91267

0.03433	1.91827

0.03432	1.9196

0.03433	1.91693

0.03433	1.91693

0.03434	1.9196

0.03432	1.91413

0.03431	1.91413

0.03431	1.91827

0.03432	1.91827

0.03431	1.91413

0.03431	1.9196

0.03431	1.91827

0.03432	1.91827

0.03433	1.91267

0.03435	1.9196

0.03436	1.91827

0.03434	1.91693

0.03435	1.91413

0.03434	1.9224

0.03435	1.91547

0.03436	1.91413

0.03436	1.92387

0.03437	1.91693

0.03435	1.91267

0.03436	1.92107

0.03435	1.91693

0.03436	1.91413

0.03437	1.92107

0.03435	1.91547

0.03434	1.91267

0.03434	1.91827

0.03435	1.91547

0.03436	1.91547

0.03434	1.9196

0.03435	1.91547

0.03435	1.91547

0.03435	1.9196

0.03433	1.91827

0.03432	1.91413

0.03432	1.91693

0.03431	1.91693

0.03431	1.91267

0.03433	1.9196

0.03434	1.91693

0.03434	1.91547

0.03434	1.91547

0.03435	1.91547

0.03434	1.91547

0.03435	1.91693

0.03434	1.9196

0.03434	1.91267

0.03433	1.91827

0.03434	1.91827

0.03434	1.91547

0.03435	1.91693

0.03435	1.91693

0.03435	1.91547

0.03435	1.91693

0.03434	1.9196

0.03434	1.91693

0.03434	1.91547

0.03435	1.91547

0.03436	1.91547

0.03435	1.91693

0.03435	1.91827

0.03436	1.91693

0.03435	1.91693

0.03435	1.91693

0.03435	1.91827

0.03433	1.91693

0.03432	1.9196

0.03433	1.91547

0.03433	1.91693

0.03432	1.91693

0.03434	1.91547

0.03434	1.91547

0.03434	1.91827

0.03435	1.91547

0.03434	1.91413

0.03433	1.9196

0.03433	1.91693

0.03434	1.91133

0.03435	1.9196

0.03436	1.92107

0.03435	1.91133

0.03435	1.91693

0.03435	1.9196

0.03437	1.91547

0.03435	1.91547

0.03435	1.92107

0.03434	1.91693

0.03435	1.91413

0.03434	1.91827

0.03436	1.91413

0.03434	1.91693

0.03436	1.91827

0.03435	1.9196

0.03435	1.91413

0.03435	1.91547

0.03436	1.9224

0.03435	1.91413

0.03432	1.91413

0.03432	1.91827

0.03434	1.91693

0.03435	1.91133

0.03436	1.91827

0.03436	1.92107

0.03436	1.91413

0.03435	1.91547

0.03437	1.9196

0.03436	1.91693

0.03437	1.91693

0.03438	1.91693

0.03437	1.91827

0.03436	1.91547

0.03437	1.9196

0.03437	1.91547

0.03438	1.91547

0.03438	1.91827

0.03438	1.91693

0.03439	1.91267

0.0344	1.9196

0.03439	1.91693

0.03438	1.91413

0.03438	1.91827

0.03439	1.91827

0.03438	1.91693

0.03438	1.91693

0.0344	1.9196

0.03438	1.91547

0.03439	1.91413

0.03438	1.92107

0.03437	1.91693

0.03437	1.91413

0.03439	1.91827

0.03439	1.9196

0.03438	1.91827

0.03438	1.91267

0.03438	1.91827

0.03437	1.91547

0.03437	1.91267

0.03436	1.9196

0.03438	1.91693

0.03437	1.91267

0.03439	1.91827

0.03439	1.9196

0.0344	1.91547

0.03441	1.91827

0.03439	1.91827

0.03439	1.91547

0.03438	1.91827

0.03438	1.91827

0.03438	1.91693

0.03438	1.91827

0.0344	1.9196

0.03438	1.92107

0.03439	1.91693

0.03439	1.91547

0.03438	1.91827

0.03437	1.91693

0.03437	1.91267

0.03437	1.91693

0.03438	1.9196

0.03438	1.91413

0.03436	1.91693

0.03436	1.9196

0.03437	1.91827

0.03437	1.91267

0.03437	1.91693

0.03437	1.91827

0.03438	1.91267

0.03437	1.91693

0.03438	1.9196

0.03439	1.91133

0.03438	1.91547

0.03437	1.9196

0.03437	1.91547

0.03437	1.91413

0.03437	1.9224

0.03439	1.91413

0.03439	1.91267

0.03439	1.9196

0.03438	1.9224

0.03437	1.90987

0.03437	1.91693

0.03436	1.92107

0.03436	1.91547

0.03436	1.91547

0.03436	1.92107

0.03435	1.91693

0.03435	1.91413

0.03436	1.91827

0.03436	1.9196

0.03435	1.91413

0.03435	1.91693

0.03435	1.92107

0.03435	1.91547

0.03436	1.91267

0.03437	1.9196

0.03437	1.91693

0.03437	1.90987

0.03436	1.92107

0.03436	1.92107

0.03434	1.91267

0.03435	1.91693

0.03436	1.92107

0.03436	1.91413

0.03435	1.91267

0.03436	1.91827

0.03437	1.91693

0.03436	1.91267

0.03436	1.91693

0.03437	1.91827

0.03436	1.91413

0.03436	1.91693

0.03435	1.9196

0.03435	1.91827

0.03436	1.90987

0.03438	1.9224

0.03438	1.9196

0.03438	1.91267

0.03438	1.91827

0.03438	1.9196

0.03438	1.91413

0.03436	1.91547

0.03437	1.91693

0.03438	1.91693

0.03438	1.91133

0.03436	1.91827

0.03436	1.91693

0.03436	1.91547

0.03435	1.91827

0.03435	1.91827

0.03437	1.91547

0.03437	1.91413

0.03438	1.92107

0.03438	1.91547

0.0344	1.91133

0.03439	1.9224

0.0344	1.91827

0.03439	1.91133

0.03438	1.9196

0.03437	1.92107

0.03438	1.91547

0.03437	1.91547

0.03438	1.92107

0.03438	1.9196

0.03437	1.91267

0.03437	1.91693

0.03437	1.92107

0.03438	1.91267

0.03438	1.91413

0.03438	1.9224

0.03438	1.91413

0.03436	1.91693

0.03436	1.9196

0.03436	1.91547

0.03437	1.91267

0.03437	1.91827

0.03437	1.91827

0.03436	1.91413

0.03437	1.91547

0.03439	1.91827

0.0344	1.9196

0.03438	1.91267

0.03438	1.91693

0.03438	1.9196

0.03439	1.91267

0.0344	1.91827

0.03437	1.9196

0.03437	1.91693

0.03436	1.91827

0.03437	1.91693

0.03436	1.91693

0.03438	1.91827

0.03438	1.91827

0.03438	1.91547

0.03438	1.91547

0.03438	1.91693

0.03437	1.91693

0.03436	1.91827

0.03436	1.91693

0.03436	1.91547

0.03436	1.91827

0.03438	1.91693

0.03438	1.91547

0.0344	1.91827

0.0344	1.9196

0.0344	1.91413

0.03438	1.91547

0.03438	1.9196

0.03437	1.91693

0.03437	1.91267

0.03438	1.9196

0.03439	1.91547

0.0344	1.91413

0.0344	1.91413

0.03441	1.9224

0.03439	1.91413

0.03438	1.91413

0.03436	1.9196

0.03439	1.91693

0.03438	1.91133

0.03439	1.91827

0.03438	1.91827

0.03437	1.91547

0.03439	1.91693

0.03439	1.9196

0.03438	1.91133

0.03439	1.91693

0.03439	1.91827

0.03439	1.91827

0.03439	1.91413

0.03438	1.91693

0.03437	1.91693

0.03439	1.91693

0.03438	1.91693

0.03439	1.91827

0.03438	1.9196

0.03439	1.91267

0.0344	1.91547

0.03439	1.9196

0.03437	1.91413

0.03436	1.91413

0.03437	1.9196

0.03438	1.91547

0.03439	1.91693

0.03438	1.92107

0.03436	1.91547

0.03436	1.91413

0.03437	1.91827

0.03439	1.9196

0.03439	1.91133

0.03439	1.92107

0.03439	1.9196

0.03438	1.91267

0.03438	1.9196

0.03437	1.9196

0.03437	1.91547

0.03437	1.91827

0.03437	1.91827

0.03439	1.91693

0.03439	1.91693

0.03439	1.91547

0.0344	1.91693

0.03439	1.91827

0.0344	1.91547

0.03439	1.91693

0.03439	1.91827

0.03439	1.91693

0.03439	1.91693

0.03438	1.91827

0.03439	1.91547

0.03438	1.91693

0.03439	1.91693

0.03439	1.91693

0.03438	1.91547

0.03436	1.9196

0.03438	1.91693

0.03437	1.91267

0.03438	1.9224

0.03438	1.91693

0.03437	1.91133

0.03438	1.92107

0.03439	1.91827

0.03441	1.91267

0.03441	1.91693

0.0344	1.92107

0.03438	1.91267

0.03439	1.91547

0.03439	1.92107

0.0344	1.91413

0.0344	1.91413

0.03442	1.91827

0.0344	1.9196

0.0344	1.91267

0.0344	1.91693

0.03441	1.92107

0.03439	1.91547

0.03439	1.91413

0.03438	1.9224

0.03439	1.91827

0.03438	1.91267

0.0344	1.91827

0.03439	1.9224

0.03439	1.91267

0.03438	1.91827

0.03439	1.92107

0.03438	1.91267

0.0344	1.91693

0.0344	1.9196

0.0344	1.91413

0.0344	1.91547

0.0344	1.9196

0.03439	1.91693

0.0344	1.91267

0.03439	1.91547

0.0344	1.92107

0.0344	1.91693

0.0344	1.91267

0.0344	1.91827

0.03439	1.91827

0.03439	1.91413

0.0344	1.91827

0.03441	1.91827

0.03441	1.91413

0.03439	1.91547

0.0344	1.91827

0.03441	1.91547

0.03441	1.91413

0.03442	1.9196

0.03441	1.91827

0.03441	1.91693

0.0344	1.91547

0.03443	1.92107

0.03442	1.91547

0.03444	1.91133

0.03444	1.91693

0.03443	1.91827

0.03443	1.91413

0.03444	1.92107

0.03444	1.91827

0.03444	1.91267

0.03443	1.91547

0.03442	1.92107

0.03442	1.91267

0.0344	1.91693

0.0344	1.9196

0.03441	1.91547

0.03441	1.91827

0.03442	1.9196

0.03443	1.91693

0.03443	1.91693

0.03442	1.91547

0.03443	1.9196

0.03442	1.91547

0.03443	1.91547

0.03443	1.91827

0.03444	1.91827

0.03442	1.91413

0.03442	1.91693

0.03441	1.9196

0.0344	1.91693

0.03441	1.91413

0.03443	1.91827

0.03443	1.91413

0.03444	1.91827

0.03442	1.92107

0.03444	1.90987

0.03443	1.9224

0.03442	1.92107

0.03443	1.91133

0.03441	1.91827

0.03443	1.9196

0.03442	1.91413

0.03441	1.91413

0.03441	1.92107

0.03441	1.91693

0.03443	1.91413

0.03444	1.91693

0.03444	1.91827

0.03443	1.91413

0.03443	1.91693

0.03445	1.92107

0.03445	1.91547

0.03445	1.91267

0.03442	1.9196

0.03442	1.91693

0.03442	1.90987

0.03444	1.9224

0.03445	1.91827

0.03445	1.91267

0.03446	1.91827

0.03446	1.9224

0.03444	1.91413

0.03444	1.91693

0.03444	1.92107

0.03444	1.91267

0.03444	1.91413

0.03445	1.92107

0.03444	1.91693

0.03444	1.91547

0.03444	1.91693

0.03445	1.91827

0.03444	1.91413

0.03443	1.91693

0.03442	1.9196

0.03442	1.91547

0.03441	1.91413

0.03442	1.91413

0.03443	1.91827

0.03442	1.91693

0.03442	1.91827

0.03442	1.9196

0.03442	1.91547

0.03443	1.91693

0.03442	1.9196

0.03441	1.91693

0.03443	1.91547

0.03445	1.9196

0.03446	1.91827

0.03446	1.91547

0.03445	1.91547

0.03446	1.92107

0.03445	1.91547

0.03445	1.91413

0.03444	1.91827

0.03443	1.91827

0.03444	1.91693

0.03443	1.91827

0.03444	1.91827

0.03445	1.91693

0.03444	1.91827

0.03443	1.91827

0.03445	1.91547

0.03443	1.91267

0.03444	1.9196

0.03445	1.91693

0.03445	1.91547

0.03444	1.91693

0.03445	1.91547

0.03445	1.91413

0.03446	1.91547

0.03446	1.9196

0.03447	1.91413

0.03446	1.91547

0.03445	1.92107

0.03445	1.91827

0.03445	1.91693

0.03445	1.91827

0.03445	1.91693

0.03446	1.91827

0.03446	1.91827

0.03446	1.91547

0.03448	1.91693

0.03447	1.9196

0.03447	1.91827

0.03447	1.91267

0.03448	1.9196

0.03446	1.91547

0.03446	1.91547

0.03446	1.9196

0.03445	1.91547

0.03445	1.91267

0.03446	1.92107

0.03446	1.91827

0.03447	1.91413

0.03446	1.91827

0.03445	1.91827

0.03445	1.91133

0.03446	1.91547

0.03445	1.92107

0.03446	1.91413

0.03445	1.91413

0.03445	1.9224

0.03446	1.91547

0.03447	1.91267

0.03447	1.9196

0.03445	1.91693

0.03446	1.90987

0.03445	1.9224

0.03446	1.91827

0.03446	1.91547

0.03445	1.9196

0.03446	1.9196

0.03445	1.91267

0.03445	1.91547

0.03443	1.9224

0.03444	1.91413

0.03445	1.91413

0.03445	1.91693

0.03444	1.91693

0.03446	1.91413

0.03445	1.9196

0.03445	1.91827

0.03445	1.91413

0.03444	1.91547

0.03446	1.9224

0.03446	1.91547

0.03445	1.91547

0.03446	1.91693

0.03446	1.91547

0.03447	1.91547

0.03446	1.91827

0.03445	1.91693

0.03445	1.91693

0.03444	1.91693

0.03443	1.91693

0.03445	1.91413

0.03446	1.9196

0.03445	1.91547

0.03444	1.91547

0.03444	1.91827

0.03444	1.91547

0.03445	1.91413

0.03444	1.9196

0.03444	1.9196

0.03444	1.91267

0.03445	1.91547

0.03446	1.9196

0.03446	1.91547

0.03447	1.91413

0.03446	1.91827

0.03447	1.91827

0.03446	1.91267

0.03444	1.9196

0.03444	1.9196

0.03445	1.91413

0.03444	1.91547

0.03446	1.92107

0.03447	1.91547

0.03446	1.91547

0.03446	1.91827

0.03446	1.91693

0.03447	1.91413

0.03446	1.9196

0.03446	1.91693

0.03446	1.91693

0.03447	1.91693

0.03446	1.92107

0.03448	1.91413

0.03449	1.91693

0.03449	1.9196

0.03447	1.91267

0.03447	1.91267

0.03448	1.92107

0.03449	1.91827

0.03451	1.91267

0.03451	1.91827

0.0345	1.92107

0.03449	1.91267

0.0345	1.91693

0.03449	1.91827

0.03449	1.91693

0.03449	1.91267

0.03449	1.92107

0.03447	1.91547

0.03449	1.91413

0.03447	1.91827

0.03448	1.91827

0.03448	1.91133

0.03448	1.9196

0.03447	1.91827

0.03447	1.91547

0.03449	1.91413

0.03449	1.91827

0.0345	1.91693

0.03449	1.91267

0.03448	1.91827

0.03446	1.9196

0.03449	1.91267

0.03449	1.91827

0.0345	1.91827

0.0345	1.91827

0.03451	1.91133

0.03451	1.9224

0.0345	1.91827

0.03449	1.91133

0.03448	1.91827

0.03448	1.91827

0.03448	1.91693

0.03449	1.91827

0.03449	1.91547

0.03447	1.91827

0.03448	1.91693

0.03448	1.91547

0.03449	1.91827

0.03448	1.91693

0.03447	1.9196

0.03447	1.91547

0.03447	1.91547

0.03447	1.9196

0.03448	1.91827

0.03446	1.91547

0.03447	1.91827

0.03449	1.91693

0.0345	1.91267

0.03449	1.9196

0.03449	1.91547

0.03449	1.91547

0.03448	1.91693

0.03447	1.91693

0.0345	1.9196

0.03448	1.91547

0.03448	1.91827

0.03447	1.91693

0.03449	1.91413

0.03447	1.91693

0.03448	1.91693

0.03448	1.91693

0.03447	1.91693

0.03448	1.91547

0.03448	1.9196

0.03449	1.91827

0.03449	1.91827

0.03449	1.9196

0.03448	1.91827

0.03447	1.91827

0.03447	1.91827

0.03447	1.91413

0.03446	1.91547

0.03446	1.91827

0.03446	1.91693

0.03447	1.91413

0.03446	1.91827

0.03446	1.91827

0.03447	1.91413

0.03446	1.91413

0.03447	1.91827

0.03449	1.91547

0.03448	1.91413

0.03448	1.91547

0.03446	1.92107

0.03446	1.91547

0.03446	1.91547

0.03446	1.92107

0.03446	1.91827

0.03446	1.91267

0.03446	1.91827

0.03448	1.91827

0.03447	1.91267

0.03448	1.91547

0.03447	1.91827

0.03448	1.91547

0.03446	1.91547

0.03447	1.91827

0.03446	1.91827

0.03446	1.91547

0.03446	1.91693

0.03448	1.9196

0.03448	1.91413

0.03449	1.91267

0.03448	1.9196

0.03448	1.91133

0.03447	1.91693

0.03446	1.9224

0.03447	1.91693

0.03448	1.91547

0.03448	1.91827

0.03447	1.92107

0.03447	1.91267

0.03447	1.91693

0.03447	1.9224

0.03449	1.91133

0.03449	1.91693

0.03448	1.92107

0.03447	1.91413

0.03448	1.91413

0.03447	1.92107

0.03448	1.91413

0.03448	1.91267

0.03447	1.91827

0.03447	1.92107

0.03447	1.91133

0.03448	1.9224

0.03446	1.91827

0.03445	1.91547

0.03446	1.91693

0.03446	1.91827

0.03446	1.91267

0.03446	1.91693

0.03446	1.9224

0.03445	1.91267

0.03446	1.91413

0.03448	1.92107

0.03449	1.91693

0.03448	1.91413

0.03447	1.9196

0.03447	1.91693

0.03446	1.91413

0.03447	1.9196

0.03447	1.91827

0.03446	1.91267

0.03446	1.91827

0.03446	1.91827

0.03448	1.91547

0.03447	1.91693

0.03447	1.9196

0.03447	1.91693

0.03447	1.91693

0.03446	1.91827

0.03447	1.91413

0.03447	1.91827

0.03447	1.91827

0.03448	1.91547

0.03448	1.91693

0.03448	1.9196

0.03449	1.91133

0.0345	1.91547

0.0345	1.92107

0.0345	1.91693

0.03449	1.91413

0.0345	1.91693

0.03449	1.91693

0.03449	1.91693

0.03448	1.91827

0.03446	1.91827

0.03446	1.91693

0.03447	1.91267

0.03447	1.92107

0.03447	1.91413

0.03448	1.91547

0.03448	1.92107

0.03449	1.91547

0.03447	1.91413

0.03447	1.9196

0.03447	1.91827

0.03449	1.91133

0.03449	1.91693

0.03449	1.91827

0.03447	1.91693

0.03447	1.91413

0.03447	1.92107

0.03447	1.91413

0.03446	1.91413

0.03447	1.9196

0.03448	1.91827

0.03446	1.91547

0.03446	1.91413

0.03446	1.9196

0.03446	1.91693

0.03446	1.91693

0.03447	1.91693

0.03447	1.91413

0.03446	1.91547

0.03446	1.9196

0.03446	1.91693

0.03446	1.91413

0.03446	1.9196

0.03446	1.91827

0.03446	1.91547

0.03447	1.91827

0.03447	1.91693

0.03446	1.91547

0.03445	1.9196

0.03446	1.92107

0.03446	1.91413

0.03447	1.91693

0.03447	1.9196

0.03448	1.91693

0.03449	1.91547

0.03448	1.91693

0.03448	1.91827

0.03449	1.91267

0.03448	1.91693

0.03448	1.92107

0.03448	1.91547

0.03447	1.91413

0.03447	1.92107

0.03449	1.91413

0.0345	1.91547

0.03449	1.9224

0.03448	1.91547

0.03447	1.91693

0.03446	1.9196

0.03449	1.91693

0.03447	1.91413

0.03448	1.91547

0.03448	1.92107

0.03449	1.91693

0.03449	1.91267

0.03449	1.9196

0.03448	1.91693

0.03447	1.91267

0.03448	1.91827

0.03447	1.9196

0.03447	1.91267

0.03447	1.9196

0.03447	1.91693

0.03448	1.91547

0.03448	1.91693

0.03448	1.91827

0.03449	1.91827

0.03448	1.91413

0.03448	1.9196

0.03448	1.91827

0.03447	1.91547

0.0345	1.9196

0.0345	1.91827

0.03448	1.91413

0.03448	1.9196

0.03447	1.91693

0.03446	1.91413

0.03447	1.9196

0.03449	1.91547

0.03449	1.91267

0.03448	1.92107

0.0345	1.91693

0.03449	1.91413

0.03449	1.9196

0.03449	1.91827

0.03449	1.91133

0.03449	1.91413

0.0345	1.9196

0.03451	1.91693

0.03451	1.91267

0.0345	1.9196

0.03449	1.91827

0.03448	1.91267

0.03448	1.91693

0.03448	1.9196

0.03449	1.91413

0.03448	1.9196

0.03449	1.91827

0.0345	1.91547

0.0345	1.91547

0.03449	1.9196

0.0345	1.91693

0.03449	1.91413

0.03449	1.9196

0.0345	1.9196

0.03448	1.91267

0.03449	1.9196

0.03448	1.9196

0.0345	1.91413

0.0345	1.91827

0.03449	1.92107

0.03448	1.91413

0.03448	1.91547

0.03447	1.91827

0.03447	1.91547

0.03447	1.91693

0.03449	1.91693

0.03449	1.91547

0.0345	1.91547

0.03451	1.92107

0.0345	1.91693

0.0345	1.91547

0.0345	1.91827

0.03449	1.92107

0.03449	1.91413

0.03449	1.91267

0.0345	1.92107

0.03449	1.91693

0.03451	1.91413

0.0345	1.91827

0.0345	1.91547

0.03449	1.91827

0.03451	1.9196

0.0345	1.91547

0.03449	1.91547

0.03449	1.91413

0.03451	1.92107

0.0345	1.91827

0.0345	1.91133

0.0345	1.92387

0.03451	1.91547

0.03449	1.91267

0.0345	1.9224

0.03449	1.91827

0.03449	1.91413

0.03449	1.91547

0.03451	1.91693

0.03451	1.91413

0.03451	1.91693

0.0345	1.92107

0.0345	1.91413

0.0345	1.91547

0.0345	1.9196

0.0345	1.91693

0.03448	1.91413

0.03447	1.91693

0.03449	1.9196

0.03447	1.91413

0.03448	1.91693

0.03448	1.9196

0.03449	1.91547

0.0345	1.91547

0.03451	1.91827

0.0345	1.91693

0.0345	1.91547

0.03449	1.91827

0.03449	1.91413

0.03448	1.91547

0.03449	1.91827

0.03449	1.91547

0.0345	1.91547

0.03449	1.9196

0.03449	1.91827

0.0345	1.91267

0.0345	1.91827

0.03451	1.9196

0.0345	1.91267

0.03452	1.91693

0.03451	1.92107

0.03451	1.91547

0.0345	1.91413

0.03449	1.9196

0.0345	1.91827

0.03451	1.91413

0.03451	1.91547

0.0345	1.92107

0.0345	1.91827

0.03451	1.91413

0.03452	1.9224

0.03452	1.91547

0.03452	1.91547

0.03452	1.91827

0.03451	1.91693

0.0345	1.91413

0.0345	1.92107

0.0345	1.91693

0.0345	1.91413

0.03451	1.91693

0.0345	1.92107

0.03449	1.91133

0.0345	1.91693

0.03451	1.9196

0.03451	1.91413

0.0345	1.91693

0.03451	1.91827

0.03451	1.9196

0.0345	1.91267

0.03451	1.91827

0.03451	1.92107

0.03452	1.91267

0.0345	1.91827

0.03452	1.91827

0.0345	1.91413

0.03451	1.91547

0.0345	1.91827

0.03448	1.91827

0.03447	1.91693

0.03448	1.91693

0.03449	1.9196

0.03449	1.91413

0.03449	1.91547

0.03449	1.91827

0.03449	1.91547

0.0345	1.91693

0.03449	1.91413

0.0345	1.91693

0.03448	1.91547

0.03447	1.91547

0.03447	1.91827

0.03447	1.91827

0.03448	1.91547

0.0345	1.91827

0.03449	1.91547

0.03447	1.91547

0.03447	1.92107

0.03449	1.91693

0.03448	1.91547

0.03447	1.91693

0.03448	1.91827

0.03446	1.91413

0.03446	1.91547

0.03446	1.92107

0.03447	1.91693

0.03446	1.91413

0.03446	1.92107

0.03446	1.92107

0.03445	1.91133

0.03445	1.9196

0.03446	1.9196

0.03447	1.91133

0.03448	1.91413

0.03447	1.9196

0.03447	1.91693

0.03446	1.91133

0.03448	1.9196

0.03447	1.91827

0.03448	1.91547

0.03448	1.91547

0.03449	1.9224

0.0345	1.91133

0.03449	1.91827

0.03448	1.9196

0.03449	1.91133

0.03448	1.91693

0.03447	1.9224

0.03449	1.91693

0.03448	1.91413

0.03449	1.91827

0.03447	1.91693

0.03449	1.91267

0.03449	1.91547

0.03448	1.92107

0.03448	1.91267

0.03448	1.91693

0.03448	1.9196

0.03448	1.91693

0.03448	1.91547

0.03449	1.92107

0.03449	1.91827

0.0345	1.91267

0.03449	1.91827

0.0345	1.91827

0.03449	1.91547

0.0345	1.91693

0.03449	1.9196

0.0345	1.91693

0.03452	1.91413

0.03451	1.9196

0.0345	1.91827

0.0345	1.91267

0.0345	1.91827

0.03449	1.91827

0.0345	1.90987

0.03451	1.9196

0.0345	1.91827

0.03451	1.91547

0.03453	1.91693

0.03452	1.92107

0.03452	1.91693

0.03452	1.91547

0.03451	1.9224

0.0345	1.91693

0.03451	1.91267

0.03451	1.92107

0.03451	1.91547

0.0345	1.91547

0.03452	1.9196

0.03453	1.91827

0.03453	1.91547

0.03452	1.91693

0.03452	1.91547

0.03451	1.91413

0.03451	1.91827

0.03453	1.91693

0.03453	1.91413

0.03452	1.91547

0.03453	1.92107

0.03453	1.91413

0.03451	1.91827

0.03451	1.92107

0.03452	1.91693

0.03452	1.91133

0.03452	1.9196

0.03453	1.91693

0.03453	1.91267

0.03453	1.91827

0.03455	1.9196

0.03455	1.91547

0.03454	1.91413

0.03453	1.9196

0.03454	1.91827

0.03453	1.91547

0.03453	1.92107

0.03452	1.91547

0.03452	1.91267

0.03451	1.91827

0.03452	1.91827

0.03453	1.91413

0.03453	1.91693

0.03455	1.92107

0.03455	1.91547

0.03453	1.91693

0.03455	1.91693

0.03456	1.91547

0.03456	1.91547

0.03456	1.91827

0.03456	1.91827

0.03457	1.91267

0.03456	1.91827

0.03457	1.92107

0.03456	1.91133

0.03456	1.91547

0.03456	1.9196

0.03457	1.91413

0.03457	1.91413

0.03458	1.9196

0.03457	1.91413

0.03458	1.91413

0.03456	1.91827

0.03456	1.9196

0.03456	1.91413

0.03455	1.91827

0.03456	1.9196

0.03457	1.91133

0.03458	1.91693

0.03456	1.91693

0.03456	1.91693

0.03456	1.91413

0.03456	1.92107

0.03456	1.91547

0.03455	1.91267

0.03455	1.9196

0.03456	1.91827

0.03455	1.91693

0.03456	1.91693

0.03456	1.91693

0.03457	1.91413

0.03455	1.91547

0.03455	1.91827

0.03455	1.91693

0.03455	1.91827

0.03457	1.91547

0.03458	1.91413

0.03458	1.91693

0.03457	1.9196

0.03455	1.91547

0.03456	1.91547

0.03454	1.91693

0.03454	1.91693

0.03454	1.91413

0.03454	1.9196

0.03455	1.91547

0.03454	1.91547

0.03454	1.91827

0.03454	1.91693

0.03454	1.91413

0.03453	1.91827

0.03454	1.9196

0.03454	1.91413

0.03455	1.91413

0.03456	1.9196

0.03458	1.91547

0.03458	1.91547

0.03457	1.92107

0.03458	1.91547

0.03457	1.91693

0.03455	1.91693

0.03454	1.91693

0.03454	1.91827

0.03454	1.91413

0.03455	1.91827

0.03455	1.91547

0.03456	1.91827

0.03455	1.9196

0.03455	1.91547

0.03454	1.91413

0.03454	1.9196

0.03454	1.91827

0.03455	1.91413

0.03453	1.91547

0.03453	1.91693

0.03455	1.91413

0.03455	1.91693

0.03454	1.9196

0.03453	1.91693

0.03454	1.91547

0.03453	1.91827

0.03454	1.91827

0.03454	1.91547

0.03454	1.91413

0.03454	1.91827

0.03456	1.91693

0.03456	1.91547

0.03456	1.91827

0.03456	1.91547

0.03456	1.91693

0.03455	1.9196

0.03457	1.91827

0.03457	1.91267

0.03457	1.9196

0.03457	1.91547

0.03458	1.91547

0.03458	1.9196

0.03458	1.91693

0.03457	1.91547

0.03458	1.91693

0.03457	1.92107

0.03456	1.91267

0.03456	1.91547

0.03457	1.92107

0.03457	1.91413

0.03456	1.91547

0.03455	1.9196

0.03453	1.91267

0.03452	1.91693

0.03454	1.91693

0.03454	1.91693

0.03456	1.91693

0.03456	1.91267

0.03457	1.9196

0.03455	1.91547

0.03456	1.91547

0.03456	1.91827

0.03456	1.9196

0.03456	1.91547

0.03458	1.91547

0.03458	1.91827

0.03458	1.92107

0.03457	1.91547

0.03457	1.91827

0.03456	1.9196

0.03457	1.91693

0.03458	1.91693

0.03455	1.91693

0.03456	1.91827

0.03456	1.91413

0.03457	1.9196

0.03457	1.9196

0.03458	1.91413

0.03456	1.91413

0.03456	1.92107

0.03458	1.92107

0.03459	1.91133

0.03458	1.9224

0.03459	1.91827

0.03459	1.91133

0.03458	1.9224

0.03456	1.9224

0.03457	1.91267

0.03455	1.91827

0.03457	1.92107

0.03456	1.91693

0.03458	1.91693

0.03458	1.92107

0.03458	1.91693

0.03458	1.91693

0.03457	1.91693

0.03457	1.92107

0.03457	1.91547

0.03458	1.91547

0.03458	1.91827

0.03458	1.91693

0.03458	1.91267

0.03458	1.92107

0.0346	1.91827

0.03459	1.91267

0.0346	1.91693

0.03459	1.91827

0.03462	1.91547

0.03462	1.91133

0.0346	1.91827

0.0346	1.91547

0.0346	1.91267

0.03459	1.91827

0.03459	1.91693

0.03462	1.91413

0.03464	1.91693

0.03468	1.92107

0.03468	1.91547

0.03464	1.91547

0.0346	1.9196

0.03459	1.9196

0.03459	1.91267

0.03458	1.91827

0.0346	1.91693

0.03459	1.91267

0.03458	1.91827

0.03457	1.91827

0.03458	1.91413

0.03458	1.91827

0.0346	1.92107

0.0346	1.91693

0.03459	1.90987

0.03456	1.92107

0.03456	1.9196

0.03457	1.91267

0.03457	1.91693

0.03457	1.92107

0.03458	1.91547

0.03458	1.91413

0.03458	1.92107

0.03458	1.91827

0.03459	1.91413

0.03459	1.91547

0.0346	1.92107

0.0346	1.91547

0.03458	1.91267

0.03458	1.92107

0.03459	1.9196

0.0346	1.91413

0.03459	1.9196

0.0346	1.91693

0.0346	1.91547

0.03459	1.91413

0.03459	1.91827

0.03457	1.9196

0.03456	1.91267

0.03456	1.91827

0.03458	1.92107

0.03457	1.91413

0.03457	1.91547

0.03456	1.92387

0.03457	1.91413

0.03456	1.91693

0.03457	1.92387

0.03458	1.91267

0.03457	1.91267

0.03458	1.9252

0.03459	1.91693

0.03459	1.91133

0.03459	1.9196

0.03458	1.92107

0.03458	1.91133

0.03457	1.91693

0.03458	1.9196

0.03458	1.91133

0.0346	1.91693

0.03459	1.9196

0.0346	1.91693

0.0346	1.91693

0.0346	1.91547

0.03459	1.91693

0.03459	1.91827

0.03459	1.91827

0.03458	1.91413

0.03459	1.91693

0.03457	1.9196

0.03457	1.91547

0.03459	1.91413

0.03459	1.92387

0.0346	1.91547

0.03461	1.91547

0.03463	1.91693

0.03461	1.9196

0.0346	1.91267

0.0346	1.91827

0.03459	1.91547

0.03459	1.91267

0.03461	1.91693

0.03461	1.91827

0.03462	1.91547

0.03462	1.91547

0.03463	1.9196

0.03462	1.91547

0.03461	1.91693

0.0346	1.91693

0.03459	1.91827

0.03459	1.91547

0.0346	1.91547

0.03461	1.91693

0.03461	1.91827

0.0346	1.91547

0.0346	1.91827

0.03459	1.9196

0.03459	1.91413

0.03459	1.91693

0.0346	1.92107

0.0346	1.91267

0.03461	1.91547

0.0346	1.9196

0.0346	1.91693

0.0346	1.91267

0.0346	1.9224

0.0346	1.92107

0.0346	1.91413

0.03461	1.91547

0.03461	1.91827

0.0346	1.91693

0.0346	1.91547

0.03459	1.91827

0.03459	1.9196

0.03458	1.91547

0.0346	1.91547

0.0346	1.91827

0.0346	1.91827

0.03461	1.91413

0.03461	1.91827

0.0346	1.9196

0.03459	1.91547

0.03459	1.91547

0.03459	1.92107

0.0346	1.91547

0.0346	1.91413

0.03459	1.92107

0.0346	1.91547

0.0346	1.91547

0.0346	1.91827

0.03458	1.91693

0.03458	1.91413

0.03458	1.91693

0.03459	1.91827

0.0346	1.91693

0.03461	1.91547

0.03461	1.92107

0.0346	1.91693

0.0346	1.91133

0.03461	1.91693

0.03462	1.91827

0.03462	1.91133

0.03463	1.91827

0.03462	1.91547

0.03462	1.91267

0.03463	1.91827

0.03461	1.91827

0.03462	1.91827

0.03461	1.9196

0.03462	1.91547

0.03461	1.91693

0.03462	1.9196

0.03461	1.91827

0.0346	1.91133

0.03459	1.9196

0.0346	1.91693

0.0346	1.91547

0.0346	1.91693

0.03461	1.9196

0.03461	1.91413

0.0346	1.91827

0.03462	1.91827

0.03463	1.91547

0.03464	1.91547

0.03463	1.91693

0.03464	1.91413

0.03463	1.91693

0.03463	1.91547

0.03461	1.91693

0.0346	1.9196

0.0346	1.91413

0.03461	1.91693

0.03461	1.9196

0.03461	1.91413

0.03461	1.91827

0.03461	1.91693

0.03462	1.91267

0.03462	1.91827

0.03462	1.91693

0.03461	1.91413

0.0346	1.91693

0.03462	1.9196

0.03462	1.91547

0.03461	1.91693

0.03462	1.91827

0.03462	1.91547

0.03462	1.91413

0.03464	1.91827

0.03463	1.91693

0.03463	1.91693

0.03462	1.91827

0.03464	1.91827

0.03464	1.91827

0.03464	1.91827

0.03463	1.91693

0.03464	1.91413

0.0346	1.91267

0.0346	1.92107

0.03461	1.91693

0.03462	1.91267

0.03464	1.9196

0.03464	1.92107

0.03463	1.91413

0.03462	1.91827

0.03462	1.91827

0.03461	1.91547

0.0346	1.91547

0.0346	1.91693

0.03461	1.91693

0.0346	1.91693

0.0346	1.91547

0.0346	1.91827

0.03459	1.91693

0.03459	1.91693

0.03459	1.91547

0.0346	1.91693

0.0346	1.91693

0.03462	1.91547

0.03461	1.91413

0.0346	1.91827

0.0346	1.91547

0.03463	1.91693

0.03462	1.9196

0.03462	1.91827

0.03462	1.91413

0.03462	1.91267

0.03462	1.9196

0.0346	1.91827

0.03461	1.91413

0.0346	1.91547

0.03461	1.91827

0.03461	1.91827

0.03462	1.91547

0.03462	1.9196

0.03462	1.91693

0.03461	1.91267

0.03459	1.91827

0.03459	1.91693

0.0346	1.91413

0.03461	1.9196

0.0346	1.91693

0.03461	1.91413

0.03461	1.9196

0.03461	1.91547

0.03462	1.91547

0.03463	1.91827

0.03462	1.91827

0.03462	1.91413

0.03463	1.91693

0.03464	1.92107

0.03463	1.91133

0.03464	1.91413

0.03463	1.9196

0.03463	1.91693

0.03462	1.91547

0.03462	1.91827

0.03461	1.91547

0.03462	1.91547

0.03462	1.9196

0.0346	1.91693

0.03461	1.91413

0.03462	1.9196

0.03462	1.91547

0.03462	1.91547

0.03462	1.9196

0.03461	1.91693

0.03461	1.91267

0.03462	1.9196

0.03461	1.92107

0.03462	1.91133

0.0346	1.91827

0.03461	1.9196

0.03461	1.91413

0.03461	1.91413

0.03462	1.92107

0.03462	1.91547

0.0346	1.91413

0.03461	1.92107

0.0346	1.91693

0.0346	1.91413

0.0346	1.91827

0.03461	1.91693

0.0346	1.91267

0.03461	1.91547

0.0346	1.9196

0.03461	1.91413

0.03459	1.91413

0.0346	1.9224

0.0346	1.91413

0.03461	1.91267

0.03463	1.9252

0.03462	1.91547

0.03462	1.91133

0.03462	1.9224

0.03464	1.9196

0.03465	1.91133

0.03465	1.92107

0.03465	1.92107

0.03466	1.90987

0.03465	1.91413

0.03465	1.92107

0.03464	1.91413

0.03464	1.91267

0.03463	1.9196

0.03463	1.91693

0.03463	1.91413

0.03462	1.9224

0.03463	1.91693

0.03463	1.91267

0.03463	1.9224

0.03463	1.91693

0.03462	1.91133

0.03462	1.92107

0.03463	1.91413

0.03462	1.91693

0.03464	1.91827

0.03463	1.91693

0.03464	1.91133

0.03464	1.9196

0.03462	1.91827

0.03462	1.91133

0.03464	1.91827

0.03464	1.91827

0.03464	1.91413

0.03465	1.9196

0.03465	1.91827

0.03464	1.91827

0.03464	1.91547

0.03464	1.91547

0.03463	1.91827

0.03464	1.91547

0.03464	1.91413

0.03465	1.9196

0.03465	1.91547

0.03466	1.91413

0.03466	1.9224

0.03465	1.91547

0.03467	1.91267

0.03466	1.9196

0.03466	1.9196

0.03466	1.91133

0.03467	1.9196

0.03466	1.91827

0.03465	1.91413

0.03466	1.92107

0.03465	1.91827

0.03466	1.91693

0.03466	1.91413

0.03465	1.92107

0.03464	1.9196

0.03464	1.91267

0.03463	1.9224

0.03463	1.91693

0.03461	1.91267

0.03464	1.9196

0.03466	1.91693

0.03468	1.91547

0.03467	1.91693

0.03466	1.9224

0.03464	1.91413

0.03466	1.91693

0.03465	1.9224

0.03466	1.91413

0.03465	1.91413

0.03467	1.9196

0.03465	1.91693

0.03465	1.91413

0.03464	1.9196

0.03463	1.91827

0.03462	1.91693

0.03461	1.91693

0.0346	1.9196

0.03463	1.91413

0.03465	1.91133

0.03467	1.9224

0.03466	1.91413

0.03464	1.91413

0.03465	1.9196

0.03465	1.91827

0.03465	1.91547

0.03465	1.91827

0.03465	1.91693

0.03465	1.91413

0.03464	1.91547

0.03464	1.91547

0.03464	1.91693

0.03465	1.91547

0.03465	1.91267

0.03466	1.91827

0.03466	1.91547

0.03465	1.91413

0.03465	1.91693

0.03464	1.9196

0.03463	1.91547

0.03461	1.91693

0.03461	1.9224

0.03464	1.91267

0.03466	1.91693

0.03466	1.9224

0.03464	1.91547

0.03465	1.91693

0.03463	1.9224

0.03462	1.91413

0.03462	1.91547

0.03463	1.92107

0.03462	1.91693

0.03464	1.91547

0.03463	1.9224

0.03463	1.91827

0.0346	1.90987

0.03458	1.9196

0.03458	1.92107

0.03458	1.91267

0.03459	1.91693

0.0346	1.9196

0.03461	1.91267

0.03462	1.91827

0.03462	1.91693

0.03462	1.91413

0.03461	1.91827

0.03461	1.92107

0.03461	1.91267

0.03461	1.91693

0.03462	1.9224

0.03462	1.91267

0.03461	1.91413

0.03462	1.9196

0.03462	1.91827

0.0346	1.91547

0.03461	1.91827

0.0346	1.9196

0.03459	1.91267

0.03459	1.91693

0.0346	1.91827

0.0346	1.91547

0.03461	1.91693

0.03462	1.9196

0.03462	1.91693

0.03463	1.91413

0.03462	1.92107

0.03462	1.91827

0.03461	1.91133

0.03462	1.9196

0.03462	1.91827

0.03463	1.91413

0.03462	1.92107

0.03464	1.91413

0.03463	1.91413

0.03464	1.9196

0.03464	1.91267

0.03463	1.91693

0.03462	1.9196

0.03462	1.91827

0.03462	1.91413

0.03461	1.91547

0.03461	1.91827

0.03462	1.91547

0.03462	1.91693

0.03464	1.91693

0.03463	1.91413

0.03464	1.91547

0.03462	1.92107

0.03464	1.91693

0.03463	1.91547

0.03462	1.91693

0.03462	1.91547

0.03463	1.91547

0.03462	1.91693

0.03462	1.9196

0.03462	1.91693

0.03463	1.91547

0.03464	1.91693

0.03463	1.91547

0.03464	1.91413

0.03464	1.9224

0.03467	1.91413

0.03467	1.91267

0.03466	1.92107

0.03465	1.91547

0.03464	1.91413

0.03465	1.9196

0.03464	1.91827

0.03465	1.91693

0.03465	1.91547

0.03464	1.9196

0.03464	1.91693

0.03465	1.91827

0.03465	1.91827

0.03465	1.91547

0.03464	1.91413

0.03464	1.91827

0.03461	1.91693

0.03462	1.91133

0.03462	1.9224

0.03464	1.91693

0.03464	1.91547

0.03467	1.92107

0.03467	1.91827

0.03467	1.91413

0.03467	1.9196

0.03467	1.91693

0.03465	1.91267

0.03465	1.91693

0.03465	1.91693

0.03464	1.91413

0.03465	1.91693

0.03466	1.91693

0.03466	1.91693

0.03466	1.91693

0.03467	1.91827

0.03467	1.91827

0.03464	1.91693

0.03464	1.91693

0.03464	1.91827

0.03464	1.91693

0.03465	1.91827

0.03466	1.91827

0.03464	1.91827

0.03464	1.91413

0.03464	1.9196

0.03465	1.9196

0.03465	1.91267

0.03463	1.91827

0.03465	1.91827

0.03465	1.91413

0.03465	1.91827

0.03466	1.91693

0.03465	1.91827

0.03465	1.91547

0.03464	1.91547

0.03465	1.92107

0.03464	1.91547

0.03465	1.91693

0.03463	1.9224

0.03462	1.90987

0.03461	1.91133

0.03462	1.92387

0.03462	1.91413

0.03462	1.91267

0.03461	1.92387

0.03461	1.91693

0.0346	1.91133

0.0346	1.9224

0.03461	1.9224

0.0346	1.90853

0.03462	1.91693

0.03462	1.92387

0.03462	1.91133

0.03461	1.91547

0.03464	1.9224

0.03463	1.91547

0.03461	1.91693

0.03462	1.9196

0.03462	1.91693

0.03461	1.90987

0.0346	1.9224

0.0346	1.91827

0.0346	1.91267

0.03463	1.91827

0.03465	1.9196

0.03464	1.91413

0.03463	1.91413

0.03462	1.92107

0.03463	1.91267

0.03463	1.91547

0.03463	1.91827

0.03463	1.91547

0.03463	1.91547

0.03464	1.91827

0.03465	1.91693

0.03465	1.91547

0.03465	1.91693

0.03465	1.91693

0.03465	1.91827

0.03462	1.91547

0.03463	1.91693

0.03462	1.9196

0.03462	1.91827

0.03461	1.91413

0.03461	1.9196

0.03461	1.91693

0.0346	1.91547

0.0346	1.91547

0.03462	1.91827

0.03462	1.91693

0.03462	1.91547

0.03462	1.9196

0.03462	1.91693

0.03462	1.91413

0.03463	1.91693

0.03463	1.91693

0.03464	1.91693

0.03463	1.9196

0.03462	1.91827

0.03462	1.91547

0.03463	1.91413

0.03464	1.9196

0.03463	1.91547

0.03464	1.91547

0.03465	1.9224

0.03465	1.91413

0.03464	1.91133

0.03463	1.9252

0.03464	1.91547

0.03463	1.91413

0.03463	1.9196

0.03463	1.91547

0.03463	1.91413

0.03463	1.91827

0.03466	1.91827

0.03465	1.91413

0.03464	1.91693

0.03464	1.91827

0.03465	1.91693

0.03465	1.91547

0.03465	1.91693

0.03465	1.91827

0.03465	1.91267

0.03464	1.91827

0.03464	1.91827

0.03464	1.91267

0.03466	1.91827

0.03468	1.91827

0.03468	1.91547

0.03467	1.91693

0.03466	1.9196

0.03465	1.91693

0.03465	1.91413

0.03465	1.91827

0.03465	1.91693

0.03464	1.91827

0.03464	1.91547

0.03466	1.92107

0.03465	1.91547

0.03464	1.91547

0.03464	1.9224

0.03463	1.91413

0.03462	1.91547

0.03462	1.91827

0.03463	1.91827

0.03463	1.91547

0.03465	1.91827

0.03466	1.91693

0.03463	1.9196

0.03463	1.91547

0.03462	1.91413

0.03463	1.9224

0.03464	1.91413

0.03465	1.91413

0.03464	1.91827

0.03464	1.9196

0.03465	1.91547

0.03466	1.91547

0.03465	1.92107

0.03464	1.91547

0.03464	1.91547

0.03462	1.91827

0.03463	1.91693

0.03462	1.91693

0.03461	1.91693

0.03462	1.91693

0.03463	1.91827

0.03464	1.91413

0.03464	1.91827

0.03464	1.91693

0.03463	1.91267

0.03462	1.91693

0.03462	1.9224

0.03462	1.91413

0.03462	1.91413

0.03461	1.9224

0.03462	1.91693

0.0346	1.91133

0.03461	1.92107

0.03461	1.91827

0.03463	1.90853

0.03463	1.92387

0.03464	1.9196

0.03463	1.91267

0.03465	1.9196

0.03465	1.92107

0.03466	1.90987

0.03466	1.91693

0.03464	1.92107

0.03463	1.91547

0.03463	1.91547

0.03462	1.9224

0.03463	1.91267

0.03464	1.91413

0.03462	1.9224

0.03462	1.91547

0.03463	1.91133

0.03465	1.9224

0.03464	1.91827

0.03465	1.91267

0.03464	1.9196

0.03463	1.91547

0.03463	1.91413

0.03462	1.9196

0.03462	1.91827

0.03463	1.91413

0.03466	1.91413

0.03464	1.9196

0.03465	1.91693

0.03465	1.91547

0.03464	1.9196

0.03464	1.9196

0.03465	1.91693

0.03466	1.91413

0.03465	1.9196

0.03465	1.91547

0.03466	1.91413

0.03467	1.92107

0.03466	1.91413

0.03466	1.91547

0.03464	1.91827

0.03462	1.91693

0.03463	1.91413

0.03464	1.91827

0.03463	1.91827

0.03462	1.91413

0.03463	1.91547

0.03463	1.9196

0.03462	1.91413

0.03464	1.91547

0.03464	1.92107

0.03464	1.91547

0.03465	1.91267

0.03465	1.9224

0.03463	1.91547

0.03462	1.91547

0.03462	1.9196

0.03462	1.91827

0.03462	1.91547

0.03463	1.91547

0.03462	1.92107

0.0346	1.91547

0.03461	1.91693

0.03461	1.9224

0.03463	1.91547

0.03463	1.91413

0.03464	1.92107

0.03463	1.91693

0.03461	1.91267

0.03462	1.91827

0.03463	1.91693

0.03462	1.91547

0.03462	1.91827

0.03463	1.91413

0.03463	1.92107

0.03463	1.91413

0.03463	1.91827

0.03463	1.91827

0.03462	1.91547

0.03463	1.91547

0.03463	1.92107

0.03464	1.91547

0.03465	1.91547

0.03465	1.91693

0.03463	1.91827

0.03462	1.91547

0.03462	1.91693

0.03462	1.91547

0.03462	1.91413

0.03463	1.9196

0.03465	1.91693

0.03463	1.91267

0.03465	1.91827

0.03464	1.9224

0.03464	1.91133

0.03464	1.91827

0.03464	1.92107

0.03463	1.91413

0.03463	1.91547

0.03464	1.9196

0.03465	1.91413

0.03465	1.91547

0.03465	1.92107

0.03464	1.91827

0.03463	1.91267

0.03463	1.92107

0.03465	1.91547

0.03465	1.91267

0.03465	1.92107

0.03466	1.91693

0.03467	1.90987

0.03466	1.9196

0.03467	1.9196

0.03468	1.91413

0.03468	1.91827

0.03467	1.91827

0.03465	1.91827

0.03465	1.91267

0.03463	1.9196

0.03464	1.9196

0.03463	1.91133

0.03462	1.91827

0.03464	1.91693

0.03465	1.91413

0.03463	1.91827

0.03464	1.92107

0.03463	1.91133

0.03464	1.91547

0.03465	1.92387

0.03465	1.91267

0.03466	1.91693

0.03465	1.91827

0.03466	1.91547

0.03465	1.91413

0.03465	1.91693

0.03465	1.9196

0.03465	1.91547

0.03465	1.91827

0.03464	1.92107

0.03464	1.91267

0.03465	1.91693

0.03467	1.9224

0.03466	1.91133

0.03466	1.91413

0.03466	1.92107

0.03467	1.91693

0.03465	1.91133

0.03465	1.92107

0.03466	1.9196

0.03467	1.91413

0.03466	1.91693

0.03465	1.9196

0.03466	1.91547

0.03466	1.91413

0.03467	1.9224

0.03467	1.91547

0.03465	1.91267

0.03466	1.91827

0.03466	1.91693

0.03467	1.91413

0.03466	1.91827

0.03468	1.91693

0.03468	1.91547

0.03468	1.91547

0.03467	1.91827

0.03469	1.91413

0.03466	1.91693

0.03466	1.92387

0.03467	1.91413

0.03466	1.91547

0.03467	1.9196

0.03468	1.91547

0.03467	1.91413

0.03466	1.9224

0.03467	1.91827

0.03468	1.91413

0.03467	1.91547

0.03466	1.92387

0.03467	1.91827

0.03467	1.91133

0.03468	1.91827

0.03468	1.92107

0.03468	1.91133

0.03466	1.91547

0.03468	1.91827

0.03468	1.91827

0.03468	1.91413

0.03467	1.91693

0.03468	1.91693

0.03468	1.91413

0.03469	1.9196

0.03468	1.91547

0.03468	1.91693

0.03467	1.9196

0.03468	1.91547

0.03469	1.91693

0.03469	1.91827

0.03469	1.91693

0.03468	1.91547

0.03465	1.9224

0.03465	1.91693

0.03468	1.9196

0.0347	1.9252

0.03473	1.9252

0.03472	1.92387

0.03474	1.928

0.03474	1.93213

0.03478	1.9308

0.03482	1.9336

0.0349	1.9336

0.03495	1.93627

0.035	1.9432

0.03503	1.9432

0.03507	1.94467

0.03508	1.946

0.03509	1.946

0.03507	1.94747

0.03507	1.9516

0.0351	1.9516

0.0351	1.9544

0.03512	1.95573

0.03512	1.9572

0.03513	1.95987

0.03513	1.96133

0.03512	1.96547

0.03515	1.96827

0.03516	1.96827

0.03518	1.9696

0.03519	1.9724

0.03519	1.9724

0.03519	1.97667

0.03519	1.978

0.03522	1.978

0.03524	1.98213

0.03525	1.98213

0.03527	1.98627

0.03527	1.98627

0.03527	1.98907

0.03529	1.99053

0.03531	1.99333

0.0353	1.996

0.03532	1.99747

0.03532	1.99747

0.03534	2.0016

0.03535	2.00293

0.03537	2.0016

0.0354	2.00573

0.0354	2.0072

0.0354	2.01

0.03541	2.01267

0.03544	2.01267

0.03545	2.01547

0.03547	2.0196

0.03548	2.0196

0.03549	2.02107

0.03551	2.02387

0.03551	2.028

0.03552	2.02667

0.03553	2.02933

0.03557	2.03213

0.03557	2.0336

0.03557	2.03213

0.03558	2.03627

0.03558	2.04187

0.0356	2.04053

0.03559	2.04187

0.03558	2.04467

0.0356	2.04747

0.03562	2.0488

0.03564	2.05027

0.03565	2.0516

0.03564	2.05573

0.03566	2.05573

0.03568	2.05573

0.03571	2.05853

0.03573	2.06267

0.03575	2.06267

0.03575	2.06547

0.03576	2.06693

0.03578	2.0696

0.03578	2.07107

0.03579	2.0724

0.03582	2.07387

0.03582	2.07667

0.03585	2.078

0.03585	2.07933

0.03587	2.08213

0.0359	2.08493

0.03591	2.08493

0.03593	2.08627

0.03592	2.09187

0.03593	2.08907

0.03597	2.096

0.03599	2.096

0.03601	2.0988

0.03601	2.0988

0.03602	2.10027

0.03607	2.1044

0.03607	2.1072

0.03608	2.11

0.0361	2.10853

0.03612	2.11267

0.03613	2.11413

0.03614	2.11547

0.03616	2.11827

0.03619	2.11973

0.03622	2.11973

0.03623	2.12667

0.03624	2.1224

0.03627	2.128

0.03629	2.12933

0.03631	2.12933

0.03631	2.13213

0.03632	2.1336

0.03635	2.13773

0.03636	2.13773

0.03637	2.14053

0.0364	2.14187

0.03642	2.14333

0.03644	2.14747

0.03644	2.146

0.03646	2.146

0.03649	2.1516

0.03652	2.15307

0.03654	2.15573

0.03654	2.1572

0.03657	2.15853

0.03657	2.16133

0.03659	2.16133

0.03659	2.16413

0.0366	2.16693

0.03663	2.16973

0.03664	2.17107

0.03666	2.1724

0.03668	2.1752

0.0367	2.178

0.03672	2.178

0.03673	2.178

0.03674	2.18213

0.03677	2.1836

0.03678	2.1864

0.0368	2.1864

0.03682	2.18907

0.03683	2.19053

0.03687	2.19467

0.03688	2.19467

0.0369	2.196

0.03693	2.20027

0.03695	2.20027

0.03697	2.20027

0.03697	2.20307

0.037	2.20573

0.037	2.20853

0.03702	2.21

0.03703	2.20853

0.03705	2.21547

0.03707	2.21413

0.03706	2.21827

0.03707	2.21973

0.03709	2.21973

0.03713	2.2224

0.03715	2.22387

0.03718	2.22667

0.03719	2.228

0.03721	2.22947

0.03722	2.2308

0.03725	2.23213

0.03726	2.23773

0.0373	2.23773

0.0373	2.23773

0.03731	2.23907

0.03733	2.24187

0.03735	2.24613

0.03736	2.24613

0.03738	2.2488

0.0374	2.2488

0.03741	2.2544

0.03743	2.2572

0.03742	2.25573

0.03746	2.25853

0.03747	2.26

0.0375	2.26133

0.03751	2.26413

0.03754	2.26133

0.03755	2.26547

0.03755	2.27107

0.03757	2.2724

0.03758	2.27387

0.0376	2.2752

0.03765	2.278

0.03766	2.27947

0.03767	2.2808

0.03768	2.28213

0.0377	2.28493

0.03771	2.28493

0.03772	2.28773

0.03774	2.29333

0.03777	2.29187

0.03779	2.29333

0.03782	2.29467

0.03784	2.29747

0.03784	2.2988

0.03785	2.30027

0.03785	2.30307

0.03785	2.3044

0.03787	2.3072

0.03789	2.31133

0.0379	2.31

0.0379	2.3128

0.03792	2.31413

0.03794	2.31413

0.03797	2.31693

0.03799	2.31827

0.03803	2.3224

0.03804	2.32387

0.03806	2.32387

0.03808	2.328

0.0381	2.32947

0.03811	2.33213

0.03814	2.33213

0.03816	2.3336

0.0382	2.33493

0.03822	2.34053

0.03825	2.34053

0.03827	2.34187

0.03829	2.34187

0.0383	2.34467

0.03833	2.34747

0.03836	2.35027

0.03838	2.3516

0.03841	2.35587

0.03842	2.35587

0.03846	2.3572

0.03849	2.36133

0.0385	2.35853

0.0385	2.36547

0.0385	2.3628

0.03852	2.36547

0.03855	2.36973

0.03857	2.37253

0.03859	2.37253

0.03862	2.37387

0.03862	2.3752

0.03866	2.3752

0.03867	2.378

0.0387	2.3808

0.03871	2.38213

0.03872	2.3892

0.03874	2.38493

0.03875	2.3892

0.03878	2.39333

0.0388	2.39333

0.03881	2.39333

0.03883	2.39747

0.03885	2.3988

0.03888	2.40027

0.03889	2.40307

0.03891	2.40307

0.03893	2.40587

0.03894	2.4072

0.03895	2.41133

0.03897	2.4128

0.039	2.4128

0.03901	2.41413

0.03903	2.41693

0.03906	2.41973

0.0391	2.41827

0.03912	2.42253

0.03915	2.42387

0.03916	2.42387

0.03918	2.428

0.0392	2.4308

0.03922	2.4308

0.03923	2.4364

0.03925	2.4364

0.03926	2.43493

0.0393	2.44053

0.0393	2.44053

0.03933	2.44467

0.03935	2.44467

0.03938	2.44467

0.03939	2.44893

0.03943	2.45027

0.03945	2.4544

0.03946	2.45307

0.03947	2.45587

0.03947	2.46

0.03951	2.46

0.03955	2.4628

0.03957	2.4628

0.03959	2.4656

0.0396	2.46973

0.03964	2.46827

0.03967	2.47107

0.0397	2.47107

0.03974	2.4752

0.03975	2.47667

0.03978	2.478

0.0398	2.4808

0.0398	2.4836

0.03984	2.4836

0.03984	2.48493

0.03986	2.4864

0.03988	2.4892

0.03991	2.49333

0.03994	2.49613

0.03996	2.49613

0.03997	2.49893

0.03999	2.49893

0.04002	2.50027

0.04004	2.50307

0.04006	2.5044

0.04009	2.5072

0.0401	2.50853

0.04012	2.51133

0.04012	2.5128

0.04015	2.5128

0.04021	2.5156

0.04025	2.51827

0.04026	2.52107

0.04027	2.52387

0.0403	2.52387

0.04032	2.5252

0.04035	2.528

0.04036	2.52947

0.0404	2.5308

0.04044	2.5336

0.04042	2.5364

0.04046	2.5364

0.04048	2.53773

0.04051	2.54053

0.04052	2.54053

0.04057	2.54467

0.0406	2.54613

0.0406	2.55027

0.04064	2.54893

0.04067	2.55307

0.0407	2.55307

0.04073	2.55587

0.04074	2.55853

0.04077	2.55853

0.04079	2.56133

0.04081	2.56413

0.04084	2.5656

0.04087	2.56693

0.04087	2.56973

0.0409	2.57253

0.04092	2.57253

0.04093	2.57387

0.04094	2.57533

0.041	2.578

0.04104	2.5808

0.04105	2.5808

0.04109	2.58227

0.0411	2.5864

0.04111	2.58773

0.04113	2.59053

0.04116	2.592

0.04119	2.59333

0.04121	2.59333

0.04124	2.59613

0.04126	2.59893

0.0413	2.6016

0.04132	2.60307

0.04133	2.6044

0.04137	2.6072

0.04139	2.61133

0.0414	2.6072

0.04141	2.61133

0.04147	2.6128

0.04147	2.61693

0.0415	2.61827

0.04153	2.61827

0.04154	2.62387

0.04158	2.62387

0.04161	2.62387

0.04164	2.62533

0.04167	2.62947

0.04172	2.6308

0.04173	2.6336

0.04179	2.6364

0.0418	2.6364

0.0418	2.63773

0.04182	2.64053

0.04184	2.64053

0.04188	2.64333

0.04191	2.64613

0.04193	2.64893

0.04194	2.64893

0.04198	2.65027

0.042	2.6544

0.04205	2.65587

0.04208	2.6572

0.04209	2.65867

0.04212	2.6572

0.04214	2.6628

0.04217	2.6656

0.04221	2.66693

0.04223	2.66827

0.04223	2.67107

0.04225	2.66973

0.04227	2.67667

0.04232	2.67533

0.04235	2.678

0.04237	2.67667

0.04241	2.67947

0.04243	2.6864

0.04247	2.68493

0.04247	2.68773

0.04249	2.68773

0.04251	2.6892

0.04252	2.692

0.04255	2.692

0.04258	2.69613

0.04261	2.70027

0.04265	2.70027

0.04267	2.70173

0.04269	2.70027

0.04272	2.70587

0.04274	2.7072

0.04277	2.71

0.04278	2.71

0.04281	2.71413

0.04285	2.71413

0.04289	2.71693

0.04293	2.7184

0.04296	2.72107

0.04298	2.72387

0.04299	2.72387

0.04303	2.72533

0.04306	2.728

0.0431	2.72947

0.04311	2.72947

0.04313	2.73227

0.04317	2.7364

0.04319	2.7364

0.04322	2.7392

0.04325	2.742

0.04328	2.74467

0.04329	2.74333

0.04332	2.74467

0.04336	2.74893

0.0434	2.75027

0.04341	2.75027

0.04344	2.75587

0.04348	2.7544

0.04352	2.75587

0.04354	2.76

0.04355	2.76

0.04357	2.76413

0.04361	2.76413

0.04365	2.76693

0.04368	2.76973

0.04371	2.77107

0.04373	2.77667

0.04373	2.77253

0.04378	2.77533

0.0438	2.7808

0.04384	2.78227

0.04387	2.7836

0.04391	2.78227

0.04394	2.7864

0.04396	2.7892

0.04397	2.79053

0.04399	2.792

0.04402	2.79467

0.04406	2.79333

0.04409	2.79747

0.04413	2.79747

0.04415	2.80027

0.04417	2.80587

0.0442	2.80587

0.04423	2.80587

0.04426	2.80867

0.04428	2.80867

0.04432	2.81147

0.04434	2.8128

0.04438	2.81693

0.04439	2.81973

0.04443	2.82107

0.04446	2.82107

0.0445	2.82387

0.04453	2.82533

0.04456	2.82813

0.04459	2.8308

0.04462	2.8308

0.04466	2.8308

0.04467	2.83507

0.0447	2.8392

0.04473	2.8392

0.04477	2.842

0.0448	2.84333

0.04483	2.8448

0.04483	2.84333

0.04487	2.84613

0.0449	2.84893

0.04493	2.84747

0.04497	2.85027

0.045	2.8572

0.04504	2.85587

0.04504	2.85867

0.04508	2.8628

0.0451	2.86147

0.04513	2.8656

0.04516	2.86693

0.04517	2.86973

0.04522	2.87107

0.04524	2.87253

0.04528	2.87253

0.04531	2.87253

0.04532	2.87667

0.04537	2.88227

0.04541	2.8808

0.04545	2.88227

0.04547	2.88227

0.0455	2.8864

0.04552	2.89053

0.04555	2.892

0.04558	2.892

0.0456	2.892

0.04565	2.89893

0.04568	2.89747

0.0457	2.90027

0.04573	2.90027

0.04575	2.90587

0.0458	2.90587

0.04583	2.9072

0.04587	2.91

0.0459	2.91413

0.04591	2.91413

0.04595	2.91413

0.04598	2.91693

0.04601	2.91973

0.04607	2.91973

0.04611	2.92253

0.04613	2.92667

0.04615	2.92667

0.04616	2.92813

0.0462	2.92813

0.04622	2.93507

0.04623	2.93507

0.04627	2.9336

0.0463	2.93787

0.04635	2.9392

0.04641	2.94333

0.04642	2.94333

0.04645	2.9448

0.04648	2.94613

0.04649	2.94747

0.04654	2.95027

0.04657	2.95173

0.04658	2.95453

0.04663	2.95453

0.04665	2.95587

0.04668	2.9572

0.0467	2.9656

0.04673	2.96147

0.04676	2.96693

0.04682	2.9684

0.04686	2.9712

0.04687	2.97253

0.04691	2.97533

0.04693	2.97253

0.04696	2.97533

0.04698	2.97813

0.04702	2.97813

0.04703	2.98227

0.04707	2.9836

0.04712	2.9864

0.04714	2.9892

0.04717	2.99053

0.04721	2.992

0.04724	2.99333

0.04727	2.99747

0.0473	2.99747

0.04732	3.00027

0.04734	3.00307

0.0474	3.00307

0.04744	3.00307

0.04746	3.0072

0.0475	3.0072

0.04754	3.01147

0.04757	3.0128

0.04758	3.01413

0.04761	3.01413

0.04766	3.01693

0.0477	3.0212

0.04772	3.01973

0.04777	3.02533

0.04777	3.02253

0.04779	3.02667

0.04783	3.02947

0.04788	3.0336

0.04789	3.03507

0.04791	3.0336

0.04794	3.03787

0.04798	3.03507

0.04804	3.0392

0.04808	3.04333

0.0481	3.04333

0.04811	3.0476

0.04814	3.04613

0.04818	3.05027

0.04822	3.05173

0.04824	3.05173

0.04828	3.05453

0.04832	3.0572

0.04834	3.06

0.04835	3.06427

0.04839	3.06427

0.04842	3.06693

0.04846	3.06693

0.04849	3.06973

0.04852	3.06973

0.04855	3.0712

0.04858	3.07533

0.04862	3.07533

0.04865	3.07813

0.04871	3.08093

0.04874	3.08507

0.04877	3.08507

0.04879	3.08507

0.04883	3.08507

0.04888	3.09053

0.04892	3.09053

0.04896	3.0948

0.04898	3.0948

0.04901	3.09613

0.04903	3.0976

0.04907	3.10027

0.0491	3.10173

0.04912	3.10307

0.04914	3.10453

0.04918	3.10867

0.04921	3.10587

0.04925	3.11427

0.04928	3.1128

0.0493	3.1156

0.04934	3.11693

0.04937	3.1184

0.04941	3.12253

0.04943	3.12387

0.04947	3.12667

0.04948	3.12947

0.04951	3.12667

0.04954	3.13093

0.04958	3.13227

0.04961	3.13507

0.04965	3.13507

0.04969	3.1392

0.04972	3.1392

0.04975	3.142

0.04979	3.142

0.04983	3.1476

0.04985	3.1476

0.04988	3.14893

0.04992	3.15173

0.04996	3.15307

0.04998	3.15587

0.05002	3.15587

0.05005	3.15867

0.05009	3.16147

0.05011	3.16147

0.05013	3.1656

0.05015	3.1684

0.05018	3.1684

0.0502	3.16973

0.05023	3.174

0.05028	3.1712

0.05031	3.17533

0.05034	3.17813

0.0504	3.17813

0.05041	3.17947

0.05043	3.18227

0.05044	3.1836

0.05045	3.1864

0.05049	3.18787

0.05053	3.192

0.05059	3.19333

0.05062	3.192

0.05063	3.1948

0.05068	3.1976

0.05069	3.19893

0.05073	3.20173

0.05076	3.20173

0.05079	3.20587

0.05082	3.20733

0.05083	3.21147

0.05087	3.21

0.05088	3.21147

0.05091	3.21693

0.05095	3.21427

0.05099	3.2184

0.05101	3.2184

0.05105	3.22253

0.05108	3.22533

0.05109	3.22533

0.05112	3.22947

0.05112	3.22947

0.05115	3.23093

0.05119	3.23227

0.05122	3.23507

0.05124	3.2364

0.05129	3.24067

0.05132	3.242

0.05134	3.24067

0.05138	3.242

0.05141	3.2448

0.05145	3.24893

0.05148	3.24893

0.05149	3.25307

0.05154	3.25307

0.05156	3.25453

0.05159	3.25867

0.05161	3.26

0.05164	3.26147

0.05168	3.26427

0.0517	3.2628

0.05172	3.2684

0.05176	3.2684

0.0518	3.2712

0.05183	3.2712

0.05187	3.27533

0.05189	3.27667

0.05193	3.27947

0.05196	3.27947

0.05199	3.27813

0.05203	3.28227

0.05205	3.28787

0.05209	3.28787

0.05214	3.28787

0.05216	3.29067

0.0522	3.292

0.05222	3.29333

0.05226	3.2976

0.0523	3.3004

0.05233	3.30173

0.05234	3.30307

0.05235	3.30173

0.05239	3.30453

0.05243	3.30733

0.05246	3.30867

0.0525	3.31

0.05254	3.3128

0.05256	3.31427

0.05257	3.3156

0.0526	3.31973

0.05264	3.31973

0.05267	3.32253

0.05268	3.324

0.05273	3.324

0.05276	3.32947

0.05279	3.33093

0.05281	3.33093

0.05284	3.33507

0.05288	3.33507

0.05294	3.34067

0.05298	3.33787

0.05303	3.342

0.05305	3.34333

0.05307	3.3448

0.0531	3.34893

0.05312	3.34893

0.05316	3.35173

0.05318	3.35307

0.0532	3.35453

0.05324	3.35733

0.05327	3.36147

0.0533	3.36

0.05333	3.3656

0.05337	3.36427

0.0534	3.3656

0.05345	3.36427

0.05348	3.3712

0.05351	3.3712

0.05352	3.37253

0.05356	3.37533

0.0536	3.37947

0.05363	3.38227

0.05366	3.38093

0.05369	3.38227

0.05373	3.38373

0.05376	3.38373

0.0538	3.3892

0.05382	3.3864

0.05383	3.39333

0.05385	3.39333

0.05388	3.3976

0.05393	3.39613

0.05396	3.4004

0.054	3.40173

0.05404	3.40307

0.05406	3.40587

0.05409	3.40587

0.05412	3.41013

0.05417	3.40867

0.0542	3.4128

0.05425	3.4156

0.05426	3.4184

0.05428	3.41973

0.05432	3.4212

0.05436	3.42253

0.05441	3.42533

0.05444	3.42533

0.05446	3.42813

0.05449	3.43093

0.05452	3.43227

0.05453	3.43373

0.05456	3.43507

0.05461	3.4364

0.05464	3.44067

0.05468	3.442

0.0547	3.44347

0.05473	3.44347

0.05475	3.44613

0.0548	3.44893

0.05483	3.45307

0.05485	3.45307

0.05488	3.45307

0.05492	3.45587

0.05498	3.45587

0.05502	3.45733

0.05504	3.46147

0.05507	3.4628

0.05508	3.4656

0.05511	3.4656

0.05513	3.4684

0.05517	3.4712

0.05522	3.4712

0.05522	3.4768

0.05525	3.4768

0.05529	3.4768

0.05532	3.48093

0.05536	3.48093

0.05538	3.48373

0.05542	3.48507

0.05547	3.49067

0.0555	3.4892

0.05553	3.49347

0.05553	3.49613

0.05557	3.4976

0.05562	3.49613

0.05565	3.49893

0.05567	3.50307

0.0557	3.50307

0.05575	3.50587

0.05578	3.50733

0.05579	3.50587

0.05583	3.51147

0.05587	3.51147

0.0559	3.5156

0.05593	3.5156

0.05595	3.5156

0.056	3.5184

0.05602	3.52253

0.05604	3.524

0.05606	3.52533

0.05613	3.52533

0.05615	3.52947

0.05618	3.52947

0.05621	3.53507

0.05625	3.53507

0.05628	3.53653

0.0563	3.53653

0.05632	3.5392

0.05637	3.54347

0.0564	3.5448

0.05641	3.5476

0.05645	3.54613

0.05649	3.55173

0.05652	3.5504

0.05657	3.55173

0.05663	3.55453

0.05666	3.55587

0.05669	3.56147

0.05672	3.56013

0.05675	3.56013

0.05676	3.5684

0.0568	3.5656

0.05685	3.56707

0.05687	3.5712

0.05691	3.5712

0.05695	3.574

0.05698	3.57533

0.057	3.57947

0.05704	3.57947

0.05707	3.58653

0.05711	3.58373

0.05712	3.58653

0.05715	3.58507

0.05718	3.58787

0.05723	3.59347

0.05727	3.592

0.05729	3.59613

0.05731	3.59893

0.05735	3.59893

0.05738	3.59893

0.05743	3.6032

0.05746	3.6032

0.05748	3.60733

0.0575	3.60733

0.05755	3.60733

0.05758	3.61013

0.05761	3.61427

0.05765	3.6156

0.05766	3.61707

0.05772	3.61987

0.05775	3.61987

0.05777	3.624

0.05781	3.6212

0.05782	3.6268

0.05786	3.62947

0.0579	3.63093

0.05793	3.63227

0.05796	3.63787

0.05799	3.63373

0.05804	3.63653

0.05805	3.64067

0.0581	3.642

0.05812	3.6392

0.05815	3.64627

0.05818	3.64893

0.05821	3.64893

0.05825	3.65173

0.05828	3.6532

0.05833	3.6532

0.05836	3.65733

0.0584	3.65453

0.05842	3.66013

0.05845	3.66293

0.05848	3.66427

0.05852	3.6656

0.05855	3.66707

0.05859	3.6684

0.05862	3.6712

0.05865	3.6712

0.05869	3.67533

0.05872	3.6768

0.05875	3.6768

0.05878	3.6796

0.05881	3.68227

0.05885	3.68373

0.05888	3.68653

0.05894	3.68787

0.05895	3.6892

0.059	3.692

0.05902	3.6948

0.05904	3.69627

0.05911	3.69893

0.05914	3.69627

0.05918	3.7004

0.0592	3.70453

0.05923	3.70587

0.05926	3.70733

0.05929	3.71013

0.05931	3.71147

0.05935	3.71427

0.05939	3.71293

0.05943	3.7184

0.05948	3.7184

0.0595	3.7212

0.05954	3.72253

0.05956	3.72533

0.0596	3.72813

0.05962	3.72813

0.05966	3.73093

0.05969	3.7296

0.05971	3.73227

0.05974	3.73507

0.05978	3.73787

0.05981	3.74067

0.05984	3.7392

0.05988	3.7392

0.05988	3.74627

0.05992	3.74627

0.05996	3.7448

0.06	3.74893

0.06005	3.7532

0.06008	3.756

0.06011	3.756

0.06012	3.756

0.06015	3.76013

0.06019	3.76013

0.06023	3.76013

0.06026	3.7656

0.06028	3.76707

0.06031	3.7684

0.06036	3.7712

0.06037	3.774

0.06041	3.774

0.06042	3.7768

0.06047	3.77813

0.06049	3.78227

0.06056	3.78093

0.06056	3.78653

0.06059	3.78787

0.06064	3.78787

0.06069	3.78933

0.06071	3.792

0.06072	3.7948

0.06077	3.79347

0.06078	3.7976

0.06084	3.79893

0.06086	3.8004

0.06089	3.80173

0.06093	3.80453

0.06096	3.80733

0.061	3.806

0.06103	3.80867

0.06107	3.81293

0.06108	3.81147

0.06114	3.81427

0.06115	3.8156

0.0612	3.81987

0.06122	3.8212

0.06125	3.8212

0.06129	3.824

0.06132	3.82533

0.06136	3.82813

0.06138	3.8296

0.06143	3.83093

0.06148	3.83373

0.06148	3.83653

0.06153	3.842

0.06153	3.83653

0.06157	3.83787

0.06163	3.842

0.06166	3.8448

0.0617	3.84627

0.06172	3.84893

0.06172	3.84893

0.06176	3.8532

0.06177	3.8532

0.06183	3.85733

0.06185	3.856

0.06187	3.85733

0.06191	3.86293

0.06193	3.86293

0.06197	3.86707

0.06201	3.8684

0.06204	3.86707

0.0621	3.8684

0.06212	3.874

0.06216	3.87533

0.06221	3.8768

0.06223	3.88093

0.06227	3.8824

0.0623	3.88507

0.06231	3.88507

0.06233	3.88787

0.06237	3.88787

0.06239	3.89067

0.06245	3.89347

0.06247	3.89347

0.0625	3.89627

0.06253	3.89907

0.06255	3.9004

0.06257	3.90453

0.06261	3.9032

0.06265	3.9032

0.06268	3.906

0.06274	3.91013

0.06277	3.90867

0.06281	3.91427

0.06283	3.91573

0.06287	3.91707

0.06289	3.91987

0.06292	3.9212

0.06294	3.9212

0.06298	3.9212

0.06301	3.9268

0.06306	3.9296

0.06309	3.92813

0.06313	3.9324

0.06318	3.93373

0.06321	3.93507

0.06325	3.93507

0.06329	3.93787

0.06332	3.94067

0.06335	3.94347

0.06339	3.94347

0.06342	3.9476

0.06344	3.94907

0.06349	3.9504

0.06352	3.9532

0.06356	3.956

0.06358	3.95867

0.06361	3.95733

0.06364	3.96013

0.06368	3.96147

0.06371	3.96427

0.06375	3.96573

0.06378	3.96707

0.06383	3.96707

0.06387	3.9712

0.06389	3.9712

0.06393	3.974

0.06395	3.97533

0.064	3.97813

0.06402	3.97813

0.06405	3.9824

0.0641	3.98373

0.06412	3.98787

0.06414	3.98653

0.06416	3.99067

0.0642	3.99347

0.06426	3.99347

0.0643	3.9976

0.06433	3.9948

0.06436	3.99627

0.06439	4.0004

0.06443	4.0004

0.06446	4.0032

0.06448	4.006

0.06452	4.00733

0.06456	4.01013

0.06459	4.01147

0.06463	4.01427

0.06466	4.0184

0.0647	4.01987

0.06474	4.0184

0.06476	4.02267

0.0648	4.02267

0.06482	4.024

0.06486	4.02813

0.06489	4.03093

0.06493	4.03093

0.06495	4.0324

0.06498	4.03507

0.06501	4.03507

0.06504	4.03373

0.06509	4.04067

0.06511	4.04347

0.06513	4.04213

0.06514	4.04627

0.06518	4.0476

0.06523	4.0476

0.06526	4.05173

0.06529	4.0532

0.06531	4.056

0.06534	4.05733

0.06538	4.06147

0.06543	4.06147

0.06546	4.06427

0.0655	4.06573

0.06553	4.06707

0.06555	4.06987

0.06558	4.06987

0.06564	4.074

0.06567	4.074

0.06569	4.0768

0.06569	4.07813

0.06572	4.0768

0.06576	4.0796

0.06582	4.08507

0.06585	4.08787

0.0659	4.08933

0.06593	4.08787

0.06596	4.08933

0.06599	4.09347

0.06602	4.0948

0.06604	4.0976

0.06608	4.09907

0.0661	4.09907

0.06613	4.1004

0.06616	4.106

0.06619	4.106

0.06622	4.1088

0.06626	4.1088

0.06628	4.11573

0.06636	4.11573

0.06641	4.11573

0.06643	4.1212

0.06646	4.1212

0.06648	4.124

0.06651	4.12547

0.06655	4.12813

0.06656	4.12547

0.06658	4.1324

0.06661	4.13093

0.06666	4.1324

0.06672	4.13653

0.06674	4.13787

0.06676	4.13933

0.0668	4.14347

0.06685	4.14067

0.06688	4.14627

0.06693	4.1504

0.06697	4.14907

0.06701	4.1504

0.06705	4.15453

0.06706	4.15187

0.06708	4.1588

0.06711	4.15733

0.06715	4.16147

0.06717	4.16147

0.0672	4.16427

0.06723	4.16293

0.06727	4.16987

0.0673	4.16987

0.06734	4.1712

0.06737	4.17267

0.06738	4.174

0.06742	4.17813

0.06746	4.1796

0.06748	4.1796

0.06752	4.1852

0.06755	4.1852

0.06759	4.18653

0.06763	4.18933

0.06767	4.18933

0.0677	4.19213

0.06772	4.19213

0.06775	4.19347

0.06777	4.1976

0.0678	4.20187

0.06782	4.2032

0.06786	4.206

0.06789	4.206

0.06792	4.206

0.06795	4.2088

0.06798	4.21147

0.06801	4.21293

0.06802	4.21427

0.06804	4.21427

0.06807	4.21707

0.06811	4.224

0.06814	4.22267

0.06818	4.224

0.06821	4.22547

0.06824	4.2268

0.06828	4.2268

0.06831	4.23093

0.06834	4.2352

0.06836	4.2352

0.06838	4.23933

0.06842	4.23787

0.06846	4.24067

0.0685	4.24213

0.06855	4.24347

0.06861	4.2504

0.06862	4.24907

0.06864	4.25187

0.06868	4.25187

0.06872	4.25453

0.06876	4.25453

0.06877	4.2588

0.06879	4.26013

0.06882	4.2616

0.06887	4.2588

0.06891	4.26293

0.06894	4.26427

0.06895	4.26853

0.06899	4.27267

0.06903	4.26987

0.06906	4.27267

0.06907	4.2768

0.06911	4.27827

0.06914	4.28093

0.06917	4.2824

0.06919	4.2824

0.06922	4.28653

0.06925	4.2852

0.06928	4.28933

0.06931	4.28933

0.06934	4.29213

0.06938	4.29627

0.06939	4.29493

0.06943	4.3004

0.06945	4.3004

0.0695	4.3032

0.06953	4.30453

0.06955	4.306

0.06957	4.31013

0.06959	4.30733

0.06961	4.3116

0.06965	4.31293

0.06967	4.3116

0.06971	4.31573

0.06975	4.31853

0.06977	4.31987

0.06981	4.32267

0.06989	4.32547

0.06988	4.3268

0.06993	4.33093

0.06996	4.3324

0.07	4.3352

0.07003	4.33933

0.07004	4.33653

0.07007	4.34213

0.0701	4.34347

0.07013	4.34627

0.07017	4.34347

0.07021	4.3476

0.07027	4.34907

0.0703	4.35187

0.07032	4.35467

0.07034	4.3532

0.07036	4.356

0.07038	4.36013

0.07041	4.356

0.07045	4.36293

0.07048	4.36293

0.07048	4.36573

0.0705	4.36573

0.07053	4.36987

0.07058	4.37133

0.07063	4.374

0.07064	4.37547

0.07065	4.3768

0.07068	4.38093

0.07073	4.3824

0.07076	4.38373

0.07076	4.3852

0.07079	4.38653

0.07082	4.39213

0.07085	4.388

0.07089	4.39493

0.07093	4.39627

0.07096	4.3976

0.07097	4.39907

0.07103	4.40187

0.07103	4.406

0.07106	4.40733

0.07107	4.4088

0.0711	4.4088

0.07113	4.4088

0.07119	4.41427

0.07121	4.41427

0.07123	4.41573

0.07123	4.41707

0.07127	4.41853

0.07131	4.42267

0.07134	4.42267

0.07137	4.4268

0.0714	4.4268

0.07145	4.43093

0.07146	4.43093

0.07149	4.4324

0.07151	4.4324

0.07155	4.4352

0.07158	4.438

0.07159	4.44067

0.07163	4.44347

0.07167	4.44493

0.07171	4.4476

0.07171	4.4476

0.07174	4.45187

0.07177	4.4532

0.07181	4.45187

0.07185	4.45467

0.07186	4.4588

0.0719	4.4616

0.07194	4.46293

0.07196	4.46573

0.07197	4.46293

0.072	4.46707

0.07204	4.46853

0.07208	4.46987

0.0721	4.47267

0.07214	4.47133

0.07219	4.4768

0.0722	4.48107

0.07221	4.4824

0.07224	4.48107

0.07227	4.4852

0.07229	4.4824

0.07233	4.48933

0.07237	4.48933

0.07239	4.49067

0.07239	4.49493

0.07243	4.49347

0.07245	4.49907

0.07248	4.49773

0.0725	4.5032

0.07255	4.5004

0.07257	4.50733

0.07261	4.506

0.07263	4.5088

0.07267	4.5116

0.07269	4.51293

0.07272	4.5144

0.07274	4.51573

0.07276	4.51987

0.07281	4.51707

0.07285	4.52267

0.07287	4.52547

0.07288	4.52547

0.07289	4.5296

0.07293	4.5296

0.07296	4.53107

0.07299	4.5324

0.07303	4.5352

0.07304	4.5352

0.07307	4.5352

0.0731	4.53933

0.07313	4.54213

0.07317	4.54213

0.0732	4.54493

0.07323	4.54773

0.07324	4.55187

0.07326	4.5504

0.07328	4.5532

0.07332	4.5532

0.07334	4.55467

0.07337	4.55733

0.07338	4.5588

0.07342	4.56573

0.07346	4.5644

0.07348	4.56707

0.07351	4.56573

0.07354	4.57267

0.07356	4.56853

0.07361	4.5768

0.07362	4.5768

0.07366	4.5796

0.07369	4.57827

0.07373	4.58107

0.07376	4.5824

0.07379	4.5852

0.07382	4.588

0.07388	4.588

0.0739	4.59213

0.07393	4.59347

0.07395	4.59627

0.07398	4.59773

0.07399	4.59493

0.07402	4.6004

0.07405	4.60187

0.07407	4.6032

0.07412	4.60467

0.07413	4.60747

0.07417	4.6116

0.07418	4.61013

0.0742	4.61293

0.07424	4.61293

0.07427	4.61987

0.07431	4.61853

0.07434	4.62267

0.07435	4.62133

0.0744	4.6268

0.07441	4.62547

0.07446	4.62827

0.07448	4.63107

0.07449	4.6324

0.07454	4.6324

0.07454	4.63653

0.07457	4.63653

0.07459	4.63933

0.07463	4.64213

0.07466	4.64347

0.07468	4.64493

0.07472	4.64493

0.07472	4.65187

0.07478	4.6504

0.07479	4.65187

0.07482	4.656

0.07484	4.65747

0.07488	4.6588

0.07493	4.66293

0.07493	4.6644

0.07496	4.66707

0.07497	4.6644

0.075	4.66853

0.07502	4.66573

0.07507	4.67267

0.07508	4.67413

0.07512	4.67413

0.07515	4.67547

0.07517	4.6768

0.0752	4.68107

0.0752	4.68107

0.07525	4.68373

0.07525	4.68653

0.07529	4.688

0.07529	4.69493

0.07533	4.69213

0.07535	4.69347

0.07538	4.69627

0.07542	4.7004

0.07544	4.69773

0.07548	4.7004

0.0755	4.7032

0.07552	4.706

0.07552	4.70747

0.07556	4.706

0.07557	4.71293

0.07563	4.7116

0.07563	4.71573

0.07568	4.71573

0.07568	4.71853

0.07574	4.72267

0.07574	4.71987

0.07579	4.72413

0.07579	4.72547

0.07583	4.72827

0.07582	4.72827

0.07585	4.73107

0.07585	4.73107

0.0759	4.73387

0.07592	4.738

0.07597	4.738

0.07598	4.7408

0.07603	4.74213

0.07602	4.74493

0.07609	4.74773

0.07608	4.74907

0.07612	4.75053

0.07611	4.75187

0.07616	4.75467

0.07616	4.7532

0.07621	4.75747

0.07623	4.7588

0.07626	4.76013

0.07627	4.7644

0.07631	4.76293

0.07633	4.77133

0.07634	4.7672

0.07638	4.77133

0.0764	4.77133

0.07643	4.77547

0.07645	4.77547

0.0765	4.7768

0.07652	4.77827

0.07656	4.78107

0.07657	4.7852

0.07661	4.7852

0.07665	4.7852

0.07666	4.7908

0.07669	4.7908

0.07671	4.79627

0.07674	4.79213

0.07674	4.79773

0.07678	4.79907

0.07678	4.79907

0.07681	4.80187

0.07681	4.8032

0.07683	4.8088

0.07688	4.81013

0.0769	4.8116

0.07694	4.8116

0.07695	4.8172

0.07698	4.81573

0.07698	4.81573

0.07702	4.82413

0.07706	4.81987

0.07708	4.82133

0.07712	4.82413

0.07713	4.82547

0.07716	4.82827

0.07717	4.8296

0.07719	4.83107

0.07722	4.83387

0.07725	4.8324

0.07728	4.838

0.07729	4.8408

0.07732	4.8408

0.07736	4.84213

0.07739	4.84627

0.07743	4.84773

0.07745	4.84907

0.07748	4.85053

0.0775	4.8532

0.07753	4.85187

0.07756	4.85747

0.07757	4.856

0.0776	4.86027

0.07761	4.86293

0.07766	4.8616

0.07771	4.8644

0.07774	4.86853

0.07777	4.87133

0.0778	4.87133

0.07781	4.86853

0.07786	4.87267

0.07787	4.87827

0.07789	4.8796

0.07791	4.8824

0.07794	4.88387

0.07798	4.88653

0.07801	4.88387

0.07804	4.8908

0.07806	4.89213

0.07807	4.8936

0.07811	4.89493

0.07815	4.89773

0.07819	4.89773

0.07819	4.90053

0.07821	4.90187

0.07824	4.90467

0.07826	4.90467

0.07828	4.90747

0.0783	4.91027

0.07833	4.91293

0.07837	4.91027

0.07839	4.91293

0.07841	4.91573

0.07842	4.92133

0.07845	4.92413

0.07849	4.92267

0.07853	4.92267

0.07855	4.92693

0.07857	4.92827

0.07859	4.93107

0.07861	4.9324

0.07864	4.9324

0.07865	4.93653

0.07869	4.93653

0.07871	4.938

0.07875	4.9408

0.07879	4.94213

0.07883	4.94493

0.07884	4.94773

0.07888	4.94773

0.0789	4.94907

0.07893	4.94907

0.07893	4.95187

0.07895	4.95747

0.07898	4.9588

0.07899	4.96027

0.07903	4.9588

0.07907	4.96027

0.07909	4.96853

0.07913	4.9672

0.07916	4.96573

0.07917	4.96853

0.07918	4.97413

0.07921	4.97267

0.07923	4.97547

0.07926	4.97693

0.07929	4.98107

0.07933	4.98387

0.07935	4.98387

0.07937	4.98667

0.0794	4.9852

0.07941	4.98933

0.07943	4.9908

0.07945	4.99213

0.0795	4.99773

0.07952	4.9936

0.07952	5.00053

0.07954	5.00053

0.07957	5.00187

0.0796	5.006

0.07963	5.006

0.07966	5.0088

0.07969	5.0088

0.07969	5.0116

0.07973	5.01027

0.07977	5.01573

0.0798	5.01853

0.07982	5.0172

0.07983	5.02413

0.07987	5.02413

0.07989	5.02547

0.07991	5.02827

0.07993	5.02547

0.07997	5.0324

0.08	5.0352

0.08	5.03387

0.08002	5.03667

0.08006	5.0352

0.08009	5.03933

0.08011	5.0436

0.08014	5.04493

0.08017	5.04773

0.08021	5.04627

0.08023	5.04907

0.08025	5.05187

0.08029	5.05187

0.08032	5.05467

0.08034	5.05747

0.08033	5.06027

0.08035	5.0616

0.08038	5.0588

0.0804	5.06573

0.08042	5.0672

0.08043	5.06853

0.08046	5.07133

0.0805	5.07133

0.08053	5.07413

0.08055	5.07547

0.08057	5.07693

0.08059	5.07827

0.08063	5.08107

0.08066	5.08387

0.08068	5.08387

0.08069	5.08667

0.0807	5.08667

0.08074	5.08933

0.0808	5.0908

0.08082	5.0908

0.08084	5.09773

0.08085	5.09493

0.08088	5.09773

0.0809	5.10187

0.08093	5.10333

0.08094	5.106

0.08095	5.11027

0.08099	5.1088

0.08101	5.11027

0.08105	5.11307

0.08107	5.11573

0.08107	5.1144

0.08109	5.11853

0.08111	5.11853

0.08114	5.12267

0.08117	5.12133

0.08118	5.12827

0.0812	5.12693

0.08122	5.12827

0.08125	5.12973

0.08128	5.13387

0.0813	5.1352

0.08132	5.13387

0.08134	5.1352

0.08137	5.1408

0.08139	5.1436

0.08141	5.14907

0.08145	5.1436

0.08148	5.14907

0.08151	5.14773

0.08153	5.15187

0.08156	5.14907

0.08158	5.156

0.08162	5.15747

0.08163	5.1616

0.08166	5.1588

0.08168	5.1644

0.0817	5.16573

0.08173	5.16573

0.08175	5.17

0.08179	5.17

0.08182	5.17267

0.08184	5.17547

0.08186	5.17827

0.08189	5.17693

0.08189	5.1824

0.0819	5.18387

0.08193	5.18107

0.08195	5.18667

0.08198	5.18947

0.082	5.188

0.08203	5.1936

0.08205	5.19493

0.08208	5.1936

0.08211	5.19773

0.08215	5.20053

0.08217	5.19773

0.08218	5.20333

0.0822	5.20467

0.08223	5.2088

0.08225	5.20613

0.08228	5.2088

0.08231	5.21307

0.08233	5.2144

0.08236	5.2144

0.08236	5.21853

0.0824	5.21853

0.08241	5.2228

0.08243	5.22413

0.08244	5.2228

0.08247	5.22693

0.08252	5.22827

0.08253	5.23107

0.08257	5.22973

0.0826	5.23667

0.08261	5.238

0.08264	5.238

0.08267	5.2436

0.08269	5.24213

0.08271	5.2436

0.08275	5.24493

0.08277	5.24773

0.0828	5.25053

0.08282	5.25467

0.08285	5.25187

0.08288	5.25747

0.08291	5.2588

0.08294	5.26027

0.08296	5.25747

0.08298	5.26307

0.08299	5.2672

0.08299	5.2672

0.08301	5.26573

0.08305	5.27133

0.08308	5.27133

0.08311	5.27547

0.08311	5.27693

0.08313	5.27827

0.08316	5.27827

0.08319	5.28107

0.08322	5.28387

0.08325	5.288

0.08328	5.28667

0.08329	5.2908

0.08331	5.288

0.08331	5.29493

0.08334	5.29493

0.08334	5.2936

0.08337	5.2964

0.08338	5.30187

0.08341	5.30187

0.08343	5.30187

0.08346	5.30747

0.08348	5.30747

0.08349	5.3088

0.08351	5.3116

0.08354	5.31027

0.08357	5.31307

0.08358	5.3144

0.0836	5.31853

0.08362	5.32133

0.08366	5.3228

0.08367	5.32547

0.08369	5.32693

0.08372	5.32973

0.08375	5.33107

0.08377	5.33253

0.08379	5.33387

0.08381	5.338

0.08383	5.3352

0.08384	5.33667

0.08386	5.33947

0.08389	5.34493

0.08391	5.35053

0.08392	5.34493

0.08395	5.34213

0.08397	5.35053

0.08399	5.35467

0.08401	5.35467

0.08403	5.35747

0.08404	5.3616

0.08405	5.35747

0.08406	5.3588

0.08411	5.3616

0.08414	5.36587

0.08417	5.36853

0.08417	5.36853

0.08417	5.37133

0.0842	5.37413

0.08422	5.3728

0.08426	5.37547

0.08429	5.37973

0.08431	5.38107

0.08434	5.38253

0.08434	5.38253

0.08437	5.38667

0.08439	5.388

0.08441	5.3908

0.08443	5.3908

0.08446	5.39213

0.08449	5.40053

0.0845	5.3992

0.08451	5.39773

0.08453	5.40613

0.08454	5.40053

0.08456	5.40053

0.08458	5.40467

0.0846	5.41027

0.08462	5.4116

0.08464	5.41587

0.08465	5.4144

0.08468	5.41853

0.08471	5.42

0.08475	5.42133

0.08475	5.4228

0.08478	5.42133

0.08481	5.4256

0.08484	5.42693

0.08486	5.43253

0.08486	5.4256

0.08487	5.43387

0.0849	5.438

0.08492	5.438

0.08493	5.438

0.08496	5.4408

0.08498	5.4408

0.08501	5.44493

0.08504	5.44493

0.08506	5.45187

0.08509	5.4492

0.0851	5.4492

0.08514	5.45613

0.08516	5.45467

0.08519	5.45613

0.08519	5.45893

0.08518	5.4616

0.0852	5.4644

0.08522	5.4644

0.08525	5.46307

0.08527	5.46853

0.08529	5.47133

0.08531	5.47133

0.08532	5.4728

0.08534	5.4756

0.08535	5.47973

0.08538	5.48107

0.08538	5.47827

0.08541	5.48107

0.08543	5.48387

0.08547	5.48947

0.0855	5.48947

0.08554	5.4908

0.08556	5.49493

0.08558	5.4936

0.08558	5.49493

0.0856	5.4992

0.08562	5.49773

0.08563	5.50333

0.08565	5.50467

0.0857	5.50613

0.08573	5.50893

0.08575	5.51027

0.08577	5.5144

0.08578	5.51307

0.08578	5.51587

0.08582	5.51587

0.08583	5.52133

0.08586	5.52

0.08588	5.52413

0.0859	5.52693

0.08592	5.52693

0.08594	5.52973

0.08595	5.52827

0.08596	5.53107

0.08598	5.53253

0.086	5.53667

0.08601	5.53947

0.08603	5.5436

0.08605	5.54227

0.08607	5.53947

0.08609	5.54493

0.08613	5.5464

0.08616	5.55053

0.08618	5.55467

0.08619	5.55467

0.08621	5.55467

0.08622	5.55333

0.08626	5.55747

0.08629	5.56307

0.0863	5.5616

0.08631	5.5616

0.08631	5.56307

0.08634	5.56867

0.08635	5.57133

0.08639	5.57133

0.08641	5.57

0.08645	5.57693

0.08645	5.5756

0.08647	5.58107

0.08649	5.57693

0.08653	5.58107

0.08655	5.58387

0.08658	5.58533

0.08657	5.58667

0.08658	5.588

0.08662	5.5964

0.08665	5.5936

0.08668	5.59493

0.08669	5.59773

0.08671	5.5992

0.08673	5.602

0.08676	5.60467

0.08679	5.60747

0.08681	5.60893

0.08682	5.60747

0.08685	5.61027

0.08688	5.61027

0.08692	5.6116

0.08692	5.61867

0.08695	5.6172

0.08698	5.6172

0.08702	5.62133

0.08705	5.6256

0.08705	5.62413

0.08707	5.62827

0.08708	5.63107

0.08711	5.63107

0.08714	5.63253

0.08716	5.63107

0.08717	5.63947

0.08719	5.63947

0.08723	5.64493

0.08725	5.6436

0.08727	5.64227

0.08728	5.64773

0.08729	5.6492

0.08732	5.64773

0.08734	5.65467

0.08736	5.652

0.08737	5.65467

0.0874	5.65467

0.08742	5.65893

0.08744	5.66307

0.08745	5.66027

0.08747	5.66587

0.08749	5.66587

0.0875	5.66867

0.08752	5.67

0.08752	5.6728

0.08754	5.67693

0.08757	5.67693

0.0876	5.6784

0.08763	5.67693

0.08766	5.68253

0.08768	5.68387

0.08768	5.688

0.08769	5.68667

0.08773	5.68947

0.08776	5.6908

0.08778	5.69227

0.08779	5.69227

0.08781	5.69773

0.08783	5.69773

0.08786	5.70053

0.08788	5.70333

0.08791	5.70467

0.08793	5.70613

0.08795	5.71027

0.08798	5.71027

0.08799	5.71173

0.08802	5.71307

0.08802	5.71587

0.08805	5.72

0.08806	5.71867

0.08809	5.72133

0.08812	5.72133

0.08815	5.72133

0.08817	5.72693

0.08818	5.72973

0.08821	5.73107

0.08823	5.72973

0.08826	5.73387

0.08827	5.73667

0.08829	5.7408

0.08831	5.74227

0.08835	5.7408

0.08837	5.7408

0.08839	5.74773

0.08841	5.74507

0.08842	5.7492

0.08844	5.75053

0.08847	5.75333

0.0885	5.75467

0.08853	5.75613

0.08856	5.75747

0.08858	5.76027

0.08858	5.76307

0.08859	5.76587

0.08861	5.7672

0.08864	5.77

0.08865	5.77

0.08867	5.77413

0.0887	5.77413

0.08872	5.7756

0.08875	5.7756

0.08876	5.77693

0.08879	5.78107

0.0888	5.78387

0.08881	5.78387

0.08884	5.78813

0.08883	5.7908

0.08886	5.78813

0.08888	5.79227

0.08889	5.79507

0.0889	5.79773

0.08892	5.79773

0.08892	5.7992

0.08895	5.7992

0.08899	5.80747

0.08899	5.80747

0.08901	5.80053

0.08904	5.81307

0.08905	5.81173

0.08905	5.8144

0.08908	5.8144

0.0891	5.8172

0.08913	5.81587

0.08917	5.82147

0.08918	5.8228

0.08918	5.82413

0.0892	5.82693

0.08922	5.8284

0.08925	5.83387

0.08928	5.8256

0.08929	5.83387

0.08932	5.83533

0.08935	5.83667

0.08937	5.83947

0.08941	5.83947

0.08943	5.84227

0.08945	5.84773

0.08948	5.84507

0.0895	5.8464

0.0895	5.852

0.08952	5.85053

0.08954	5.85333

0.08958	5.852

0.0896	5.85893

0.08961	5.85613

0.08962	5.86307

0.08966	5.86307

0.08967	5.8672

0.0897	5.86867

0.08972	5.86587

0.08974	5.86867

0.08979	5.8728

0.08978	5.8784

0.08979	5.8756

0.08981	5.87693

0.08981	5.88387

0.08986	5.8784

0.08987	5.88533

0.08988	5.88667

0.08991	5.88667

0.08992	5.88813

0.08996	5.8936

0.08997	5.89507

0.08998	5.89787

0.09003	5.8964

0.09003	5.89787

0.09008	5.90053

0.09009	5.90613

0.0901	5.90333

0.09014	5.90613

0.09015	5.90747

0.09017	5.91027

0.09018	5.91453

0.09018	5.91173

0.09023	5.9172

0.09023	5.92

0.09027	5.92

0.0903	5.9228

0.09031	5.92693

0.09035	5.9284

0.09036	5.9256

0.0904	5.92693

0.09041	5.93387

0.09043	5.93667

0.09046	5.93533

0.09047	5.93533

0.09049	5.93667

0.09047	5.9408

0.09047	5.94227

0.0905	5.94227

0.09052	5.94507

0.09056	5.95053

0.09057	5.952

0.09062	5.95333

0.09062	5.9548

0.09064	5.95613

0.09067	5.95747

0.09069	5.9548

0.09071	5.96173

0.09072	5.96173

0.09076	5.96587

0.09074	5.96453

0.09077	5.9672

0.09078	5.9756

0.09081	5.9728

0.09084	5.97413

0.09084	5.9756

0.09089	5.9756

0.09088	5.9812

0.09091	5.98253

0.09091	5.9812

0.09094	5.9812

0.09096	5.98947

0.09099	5.9908

0.09102	5.98947

0.09102	5.9908

0.09106	5.9936

0.09107	5.9992

0.09109	5.9992

0.09108	5.9992

0.09111	6.00333

0.09112	6.00333

0.09116	6.0076

0.09119	6.00613

0.09122	6.01027

0.09122	6.00893

0.09123	6.01307

0.09125	6.0172

0.09126	6.0172

0.09129	6.02

0.09129	6.0228

0.09133	6.0228

0.09134	6.02147

0.09137	6.02427

0.09136	6.0284

0.09141	6.03253

0.09143	6.03253

0.09147	6.03533

0.09145	6.03813

0.09148	6.03667

0.0915	6.04227

0.09152	6.0436

0.09153	6.04093

0.09157	6.04507

0.09159	6.04227

0.09162	6.05053

0.0916	6.052

0.09164	6.05333

0.09165	6.0576

0.09167	6.0548

0.09169	6.0576

0.09171	6.0576

0.09171	6.06027

0.09174	6.06867

0.09175	6.06307

0.09178	6.06587

0.0918	6.07

0.09184	6.07

0.09185	6.07427

0.09187	6.07693

0.09189	6.0784

0.09191	6.0784

0.09192	6.07693

0.09193	6.0812

0.09194	6.08533

0.09197	6.08667

0.092	6.09093

0.09203	6.08947

0.09204	6.08813

0.09205	6.0936

0.09207	6.09227

0.0921	6.09787

0.09211	6.09787

0.09213	6.102

0.09214	6.102

0.09216	6.10613

0.09216	6.10333

0.09219	6.1048

0.0922	6.11453

0.09223	6.11307

0.09225	6.1172

0.09227	6.11307

0.09228	6.11453

0.0923	6.11867

0.09233	6.1228

0.09234	6.1228

0.09238	6.1284

0.09238	6.1284

0.09241	6.12427

0.09244	6.134

0.09247	6.134

0.09247	6.12973

0.09249	6.13667

0.09252	6.1436

0.09253	6.14227

0.09254	6.13947

0.09254	6.1464

0.09257	6.14787

0.09258	6.1492

0.09259	6.14787

0.0926	6.152

0.09263	6.152

0.09266	6.15613

0.09269	6.15893

0.0927	6.16027

0.0927	6.16027

0.09272	6.15893

0.09274	6.16453

0.09276	6.1728

0.09278	6.17

0.0928	6.17147

0.09282	6.17147

0.09285	6.17427

0.09288	6.1784

0.09288	6.1756

0.09292	6.184

0.09294	6.18253

0.09295	6.18253

0.09297	6.18253

0.093	6.18667

0.09303	6.18947

0.09305	6.19227

0.09306	6.19507

0.09307	6.1936

0.0931	6.19787

0.09314	6.1992

0.09317	6.1992

0.09318	6.2048

0.0932	6.20613

0.09321	6.20893

0.09323	6.20613

0.09325	6.2076

0.09326	6.21307

0.0933	6.21027

0.09332	6.21173

0.09334	6.22147

0.09335	6.2228

0.09335	6.22147

0.09339	6.21587

0.09341	6.2256

0.09342	6.22973

0.09344	6.2284

0.09344	6.2312

0.09346	6.2312

0.09349	6.23667

0.09349	6.23813

0.09351	6.23533

0.09353	6.23533

0.09357	6.23813

0.09358	6.24507

0.09359	6.24507

0.09363	6.24507

0.09366	6.2492

0.09366	6.252

0.09369	6.25333

0.0937	6.2576

0.09371	6.2604

0.09373	6.25613

0.09375	6.26453

0.09378	6.26587

0.09379	6.26453

0.0938	6.26587

0.09383	6.26587

0.09384	6.2728

0.09387	6.2728

0.09389	6.27427

0.0939	6.2728

0.09392	6.27707

0.09396	6.27707

0.09396	6.27973

0.09399	6.28667

0.094	6.28947

0.09399	6.284

0.09403	6.28667

0.09404	6.28947

0.09406	6.29227

0.09411	6.29507

0.09413	6.29787

0.09414	6.2992

0.09416	6.29787

0.09416	6.2992

0.09419	6.2992

0.09421	6.30333

0.09423	6.30893

0.09424	6.30893

0.09427	6.30893

0.09427	6.31307

0.0943	6.31587

0.09433	6.31867

0.09434	6.31453

0.09435	6.32147

0.09437	6.32707

0.09436	6.3256

0.09439	6.3256

0.09442	6.32707

0.09442	6.3312

0.09443	6.334

0.09447	6.32973

0.09448	6.33533

0.09449	6.33813

0.09453	6.33947

0.09453	6.33947

0.09455	6.34373

0.09458	6.34373

0.09458	6.3464

0.0946	6.34507

0.09463	6.3492

0.09464	6.3464

0.09466	6.35067

0.09469	6.3548

0.09469	6.35893

0.09469	6.35893

0.09472	6.3576

0.09472	6.36173

0.09473	6.36733

0.09475	6.36733

0.09478	6.36867

0.0948	6.36453

0.09483	6.37013

0.09486	6.3756

0.09486	6.37707

0.09488	6.38253

0.09492	6.37973

0.09492	6.3812

0.09492	6.38813

0.09495	6.38813

0.09496	6.384

0.09497	6.39093

0.095	6.39787

0.09503	6.39227

0.09503	6.39373

0.09503	6.3964

0.09506	6.39787

0.09509	6.39787

0.09509	6.402

0.09513	6.40067

0.09516	6.40613

0.09517	6.40893

0.09517	6.4076

0.0952	6.4104

0.09522	6.41733

0.09522	6.41453

0.09526	6.41587

0.0953	6.42147

0.0953	6.42013

0.09531	6.4228

0.09534	6.4256

0.09535	6.42707

0.09535	6.42707

0.09538	6.4312

0.09541	6.42973

0.09543	6.43533

0.09543	6.43533

0.09547	6.43947

0.09549	6.43947

0.0955	6.43947

0.0955	6.4464

0.09553	6.44507

0.09556	6.4464

0.09556	6.4492

0.09557	6.4492

0.09561	6.45613

0.09564	6.45347

0.09565	6.45893

0.09567	6.4604

0.09569	6.45893

0.0957	6.46307

0.0957	6.46867

0.09571	6.47013

0.09575	6.46587

0.09576	6.46867

0.09576	6.47147

0.09576	6.4756

0.0958	6.47147

0.09583	6.4756

0.09584	6.4812

0.09585	6.47973

0.09588	6.47973

0.09591	6.4868

0.09591	6.48947

0.09592	6.4868

0.09594	6.49093

0.09598	6.49227

0.09601	6.49787

0.09602	6.49507

0.09603	6.49787

0.09606	6.49507

0.09607	6.4992

0.09609	6.50613

0.09609	6.5076

0.09612	6.5076

0.09615	6.5104

0.09616	6.5104

0.09615	6.50893

0.09616	6.51733

0.09619	6.52147

0.0962	6.51453

0.09623	6.52147

0.09625	6.5228

0.09629	6.52707

0.09631	6.52427

0.09633	6.5256

0.09633	6.52987

0.09635	6.534

0.09638	6.534

0.0964	6.53533

0.09639	6.534

0.09638	6.53813

0.09637	6.54227

0.09641	6.54093

0.09643	6.54653

0.09645	6.54507

0.09644	6.54787

0.09647	6.55347

0.09649	6.55613

0.0965	6.5548

0.09651	6.55613

0.09651	6.55613

0.09654	6.5576

0.09657	6.55893

0.09661	6.56587

0.09663	6.56587

0.09663	6.56867

0.09665	6.56733

0.09667	6.57147

0.0967	6.57147

0.09672	6.57427

0.09674	6.5784

0.09674	6.5784

0.09677	6.5756

0.09679	6.58533

0.09682	6.59093

0.09682	6.5868

0.09682	6.5868

0.09683	6.59093

0.09688	6.59227

0.0969	6.59227

0.09693	6.59653

0.09693	6.59373

0.09695	6.59653

0.09697	6.60347

0.09697	6.60067

0.097	6.5992

0.09701	6.60893

0.09704	6.6104

0.09705	6.61173

0.09707	6.61173

0.0971	6.61453

0.09713	6.61733

0.09714	6.61733

0.09714	6.61867

0.09716	6.62013

0.09717	6.62293

0.09719	6.62427

0.0972	6.6312

0.09722	6.6312

0.09726	6.62987

0.09728	6.6284

0.09731	6.6368

0.09732	6.64093

0.09735	6.63813

0.09737	6.63533

0.09737	6.64507

0.09739	6.65067

0.0974	6.64373

0.09741	6.64373

0.09743	6.65347

0.09746	6.652

0.09746	6.65347

0.09749	6.652

0.0975	6.6604

0.09751	6.66173

0.09754	6.66173

0.09754	6.66733

0.09756	6.6632

0.09757	6.66587

0.0976	6.67013

0.09762	6.67427

0.09764	6.67293

0.09764	6.66867

0.09763	6.67707

0.09764	6.6784

0.09766	6.68533

0.09768	6.6784

0.09771	6.67987

0.09773	6.68253

0.09775	6.69093

0.09776	6.6868

0.09778	6.6868

0.09781	6.6896

0.09782	6.69507

0.09784	6.69653

0.09783	6.70067

0.09785	6.6992

0.09787	6.69787

0.09788	6.7076

0.0979	6.71173

0.09791	6.7048

0.09793	6.70347

0.09793	6.71173

0.09794	6.71733

0.09795	6.71587

0.09797	6.71587

0.09799	6.72293

0.09799	6.72293

0.09801	6.72293

0.09805	6.7284

0.09806	6.7256

0.09808	6.7284

0.09809	6.7312

0.0981	6.7312

0.09811	6.7312

0.09811	6.7396

0.09813	6.74373

0.09815	6.7368

0.09817	6.73533

0.09819	6.74093

0.09819	6.74933

0.09821	6.75347

0.09823	6.74227

0.09825	6.74653

0.09826	6.752

0.09826	6.752

0.09828	6.75347

0.09831	6.7604

0.09833	6.7632

0.09835	6.7632

0.09836	6.76733

0.09837	6.77293

0.09838	6.76733

0.09838	6.766

0.09839	6.76867

0.0984	6.77147

0.09842	6.77427

0.09843	6.77427

0.09844	6.77707

0.09846	6.784

0.09847	6.7868

0.09849	6.7896

0.0985	6.784

0.09851	6.7868

0.09851	6.79227

0.09853	6.79373

0.09854	6.79507

0.09856	6.79227

0.09857	6.79227

0.09858	6.8048

0.0986	6.80347

0.0986	6.80067

0.09861	6.80347

0.09862	6.8104

0.09862	6.8104

0.09864	6.8132

0.09865	6.81453

0.09867	6.8132

0.0987	6.816

0.0987	6.81733

0.09871	6.82147

0.09874	6.8256

0.09876	6.82013

0.09877	6.82987

0.09878	6.8312

0.0988	6.82987

0.09881	6.83267

0.09882	6.83533

0.09883	6.834

0.09884	6.83533

0.09885	6.8396

0.09886	6.8396

0.09888	6.8424

0.0989	6.84507

0.09892	6.8424

0.09893	6.85067

0.09895	6.85067

0.09897	6.84787

0.09899	6.85907

0.099	6.8604

0.099	6.85627

0.09901	6.85627

0.09901	6.85907

0.09902	6.8632

0.09903	6.866

0.09904	6.86173

0.09907	6.866

0.09908	6.87147

0.09908	6.87293

0.0991	6.87013

0.0991	6.87707

0.09913	6.8784

0.09914	6.87987

0.09914	6.88267

0.09917	6.88267

0.09918	6.88533

0.09922	6.88533

0.09924	6.88813

0.09926	6.89507

0.09927	6.89653

0.09927	6.8924

0.09928	6.89507

0.09928	6.90067

0.09929	6.90067

0.09931	6.90347

0.09932	6.90627

0.09936	6.9104

0.09936	6.9076

0.09937	6.9104

0.09939	6.916

0.09941	6.91733

0.09942	6.9132

0.09945	6.92013

0.09948	6.92427

0.0995	6.92147

0.0995	6.92427

0.09949	6.92427

0.09951	6.92427

0.09952	6.92987

0.09953	6.9312

0.09955	6.9396

0.09957	6.934

0.09959	6.9312

0.09962	6.94373

0.09963	6.94093

0.09965	6.94093

0.09965	6.9424

0.09966	6.94507

0.09968	6.94787

0.09971	6.95067

0.09973	6.95347

0.09973	6.95627

0.09974	6.9548

0.09973	6.9548

0.09974	6.952

0.09975	6.9632

0.09978	6.966

0.09981	6.96453

0.09982	6.966

0.09982	6.9688

0.09984	6.9688

0.09985	6.97293

0.09987	6.97573

0.09988	6.97707

0.09989	6.97013

0.09991	6.97707

0.09991	6.984

0.09992	6.9896

0.09992	6.9812

0.09995	6.97987

0.09994	6.9924

0.09996	6.99653

0.09999	6.99507

0.10001	6.99093

0.10001	6.9924

0.10002	6.99933

0.10004	7.00067

0.10006	6.99933

0.10007	7.00627

0.1001	7.0104

0.10012	7.00213

0.10014	7.0104

0.10014	7.0132

0.10015	7.00907

0.10015	7.01173

0.10016	7.01733

0.10015	7.0188

0.10017	7.02293

0.10018	7.02427

0.10019	7.02293

0.1002	7.02147

0.10019	7.02573

0.10022	7.0312

0.10025	7.0312

0.10027	7.03547

0.10027	7.03813

0.10028	7.034

0.10031	7.03813

0.10033	7.0424

0.10034	7.04093

0.10035	7.04093

0.10037	7.04653

0.10038	7.04787

0.10039	7.05347

0.10042	7.0548

0.10043	7.05213

0.10044	7.05213

0.10045	7.0576

0.10045	7.06453

0.10047	7.066

0.10049	7.0632

0.1005	7.0604

0.10052	7.07293

0.10054	7.07147

0.10056	7.06733

0.10059	7.07293

0.1006	7.07427

0.10061	7.07293

0.1006	7.07987

0.10063	7.084

0.10064	7.0868

0.10066	7.08813

0.10066	7.0896

0.10067	7.08813

0.10067	7.0812

0.10068	7.09093

0.10069	7.09653

0.1007	7.0924

0.10071	7.09093

0.10073	7.10067

0.10074	7.1076

0.10075	7.10627

0.10077	7.09933

0.10077	7.10907

0.10078	7.11187

0.10081	7.1104

0.10083	7.11187

0.10083	7.1104

0.10084	7.116

0.10084	7.12013

0.10086	7.12147

0.10087	7.11733

0.10088	7.12293

0.10089	7.13267

0.1009	7.13267

0.10092	7.12707

0.10094	7.12427

0.10096	7.1312

0.10099	7.1396

0.101	7.13547

0.10101	7.1312

0.10102	7.134

0.10102	7.1452

0.10103	7.1424

0.10103	7.14653

0.10104	7.15067

0.10105	7.15213

0.10108	7.15067

0.1011	7.15213

0.10113	7.1604

0.10114	7.16187

0.10114	7.15907

0.10115	7.1548

0.10117	7.1632

0.10117	7.17013

0.10117	7.17013

0.10117	7.17013

0.1012	7.16187

0.10122	7.17573

0.10126	7.17987

0.10125	7.17573

0.10126	7.17427

0.10125	7.17987

0.10127	7.184

0.1013	7.1812

0.10131	7.184

0.10132	7.1896

0.10133	7.1924

0.10136	7.184

0.10138	7.18547

0.1014	7.19653

0.10141	7.20213

0.10142	7.19933

0.10144	7.1952

0.10145	7.20067

0.10147	7.20493

0.10149	7.20213

0.10152	7.20907

0.10152	7.20627

0.10154	7.216

0.10154	7.2188

0.10155	7.2132

0.10157	7.21187

0.10157	7.22427

0.10159	7.22293

0.10161	7.216

0.10163	7.22293

0.10164	7.22987

0.10166	7.22987

0.10165	7.22987

0.10166	7.23267

0.10168	7.234

0.10171	7.23547

0.10174	7.234

0.10174	7.2396

0.10175	7.24373

0.10177	7.24653

0.10178	7.24653

0.10178	7.2452

0.10179	7.24787

0.1018	7.25907

0.10182	7.25493

0.10183	7.24787

0.10184	7.25213

0.10184	7.2576

0.10184	7.26453

0.10186	7.26187

0.10188	7.25907

0.10191	7.266

0.10193	7.2716

0.10193	7.2688

0.10192	7.26187

0.10195	7.27013

0.10196	7.27987

0.102	7.28267

0.10201	7.27707

0.10203	7.27573

0.10204	7.27987

0.10205	7.28267

0.10206	7.284

0.10207	7.2896

0.10209	7.29093

0.1021	7.29093

0.10211	7.29093

0.10211	7.29373

0.10212	7.30213

0.10214	7.30213

0.10216	7.29933

0.10218	7.29933

0.10219	7.30493

0.1022	7.30907

0.10222	7.3104

0.10222	7.30493

0.10224	7.30907

0.10228	7.32013

0.10229	7.3188

0.10231	7.316

0.10231	7.31467

0.10231	7.32987

0.10234	7.32987

0.10234	7.32013

0.10236	7.32013

0.10237	7.33133

0.10239	7.33547

0.1024	7.32707

0.10241	7.32987

0.10245	7.3396

0.10246	7.34373

0.10249	7.3396

0.1025	7.3424

0.10252	7.3424

0.10253	7.34653

0.10254	7.34933

0.10254	7.35213

0.10257	7.35213

0.10257	7.35347

0.10258	7.35213

0.1026	7.3576

0.10262	7.36733

0.10264	7.35907

0.10264	7.3576

0.10265	7.36467

0.10266	7.37573

0.10268	7.3716

0.1027	7.3688

0.10272	7.36733

0.10273	7.37427

0.10275	7.38267

0.10275	7.38133

0.10275	7.37573

0.10278	7.37987

0.1028	7.39093

0.10282	7.38827

0.10284	7.37987

0.10284	7.38547

0.10285	7.39093

0.10286	7.39093

0.10288	7.3868

0.10289	7.3952

0.1029	7.40347

0.10291	7.40627

0.10293	7.40347

0.10295	7.40067

0.10296	7.40627

0.10299	7.41187

0.103	7.4076

0.10301	7.40627

0.10302	7.41187

0.10302	7.41733

0.10305	7.41733

0.10307	7.41733

0.10309	7.4216

0.10311	7.42707

0.10312	7.42427

0.10314	7.42573

0.10315	7.43133

0.10315	7.43267

0.10317	7.42987

0.10318	7.43267

0.10321	7.434

0.10322	7.4368

0.10325	7.4424

0.10326	7.4424

0.10326	7.4396

0.10326	7.44653

0.10327	7.45213

0.10329	7.45627

0.10331	7.448

0.10333	7.44653

0.10334	7.45627

0.10336	7.4604

0.10337	7.45907

0.10338	7.46187

0.10338	7.4632

0.10339	7.46187

0.10342	7.46733

0.10344	7.4688

0.10345	7.4688

0.10345	7.47573

0.10347	7.47853

0.10348	7.4716

0.10351	7.47293

0.10353	7.48133

0.10354	7.4896

0.10356	7.48547

0.10357	7.48133

0.10358	7.4868

0.10359	7.4896

0.1036	7.4896

0.10362	7.4896

0.10364	7.4952

0.10366	7.50067

0.10367	7.49933

0.10368	7.498

0.1037	7.50347

0.10371	7.50907

0.10373	7.5132

0.10375	7.50627

0.10374	7.50493

0.10377	7.51187

0.10378	7.516

0.1038	7.52013

0.10379	7.5104

0.10381	7.5104

0.10384	7.52013

0.10386	7.52573

0.10385	7.52987

0.10388	7.52573

0.10391	7.52013

0.10392	7.52573

0.10391	7.53267

0.10393	7.534

0.10394	7.53827

0.10394	7.53547

0.10394	7.5368

0.10396	7.5396

0.10398	7.5368

0.10398	7.5452

0.10399	7.5508

0.104	7.548

0.10402	7.5452

0.10401	7.55347

0.10403	7.55907

0.10406	7.55907

0.10406	7.55907

0.10406	7.55627

0.10408	7.55627

0.1041	7.5632

0.10411	7.56747

0.10412	7.56187

0.10414	7.56747

0.10414	7.5688

0.10414	7.57987

0.10416	7.57573

0.10418	7.5744

0.10418	7.57573

0.1042	7.58133

0.10423	7.5868

0.10423	7.58547

0.10422	7.58413

0.10424	7.5868

0.10426	7.5924

0.10425	7.5924

0.10424	7.58547

0.10426	7.58547

0.10428	7.59933

0.10429	7.6008

0.10431	7.59933

0.10433	7.598

0.10432	7.60213

0.10434	7.6104

0.10437	7.61187

0.10436	7.60773

0.10436	7.60627

0.10439	7.6132

0.1044	7.61467

0.1044	7.62013

0.10442	7.6216

0.10444	7.62013

0.10444	7.6244

0.10446	7.6188

0.10447	7.62013

0.10448	7.63133

0.10449	7.63547

0.10449	7.63547

0.10449	7.62853

0.10448	7.62987

0.10451	7.64107

0.10453	7.64933

0.10454	7.64653

0.10456	7.64107

0.10459	7.64653

0.10458	7.6452

0.10459	7.64653

0.10462	7.65347

0.10462	7.6604

0.10462	7.6604

0.10464	7.65347

0.10465	7.65907

0.10465	7.6632

0.10466	7.65627

0.10469	7.65493

0.10468	7.666

0.10469	7.6772

0.1047	7.67293

0.1047	7.66747

0.10472	7.67573

0.10475	7.6772

0.10476	7.67293

0.10478	7.6772

0.10479	7.68547

0.1048	7.68827

0.10481	7.68413

0.10484	7.68267

0.10484	7.68547

0.10484	7.6952

0.10487	7.7008

0.10487	7.69653

0.10488	7.6924

0.1049	7.69107

0.10489	7.7008

0.10491	7.70627

0.10494	7.70347

0.10494	7.7008

0.10494	7.70493

0.10496	7.716

0.10496	7.716

0.10498	7.716

0.105	7.70907

0.10501	7.70907

0.10502	7.7216

0.10507	7.72293

0.10506	7.72293

0.10507	7.72013

0.10508	7.7216

0.10507	7.73133

0.10508	7.73547

0.10511	7.73413

0.10511	7.7272

0.10513	7.72853

0.10515	7.7368

0.10515	7.74653

0.10517	7.748

0.10518	7.73413

0.10517	7.7368

0.10518	7.7452

0.1052	7.7508

0.10518	7.7508

0.10522	7.748

0.10523	7.75213

0.10523	7.75627

0.10525	7.75907

0.10526	7.76053

0.10526	7.75773

0.10529	7.76187

0.1053	7.7716

0.10531	7.7744

0.10534	7.76467

0.10535	7.76467

0.10536	7.7688

0.10539	7.7744

0.10538	7.77987

0.10542	7.77853

0.10543	7.7744

0.10543	7.77853

0.10545	7.78547

0.10546	7.7896

0.10545	7.7896

0.10549	7.78547

0.10548	7.78827

0.10549	7.7952

0.10552	7.79933

0.10552	7.7896

0.10554	7.7952

0.10557	7.80493

0.10557	7.80907

0.10561	7.81053

0.10561	7.8036

0.10561	7.8036

0.10564	7.80773

0.10562	7.81747

0.10565	7.816

0.10567	7.80907

0.10568	7.816

0.10571	7.8216

0.10572	7.8272

0.10574	7.8244

0.10575	7.816

0.10574	7.82293

0.10576	7.83133

0.10577	7.83547

0.10577	7.83413

0.1058	7.8272

0.10579	7.83413

0.10583	7.8396

0.10585	7.84653

0.10585	7.84933

0.10588	7.8424

0.10587	7.84107

0.1059	7.84387

0.10591	7.8536

0.1059	7.85627

0.10593	7.85627

0.10594	7.8508

0.10596	7.84933

0.10599	7.85773

0.10598	7.866

0.106	7.866

0.106	7.8632

0.10601	7.8688

0.10602	7.86467

0.10602	7.86467

0.10604	7.87293

0.10605	7.87987

0.10605	7.87987

0.10606	7.87573

0.10605	7.87853

0.1061	7.88133

0.10608	7.88827

0.10612	7.88547

0.10615	7.88133

0.10616	7.88827

0.10618	7.8952

0.10616	7.8952

0.10618	7.8924

0.10621	7.8896

0.10622	7.9008

0.10623	7.90773

0.10623	7.89933

0.10625	7.89387

0.10626	7.90627

0.10628	7.91187

0.10628	7.90907

0.10628	7.916

0.10631	7.91467

0.10631	7.90773

0.10633	7.91333

0.10635	7.9244

0.10634	7.9272

0.10637	7.91747

0.10635	7.91467

0.10638	7.92293

0.10638	7.93267

0.1064	7.93133

0.10642	7.92573

0.10643	7.93267

0.10646	7.9396

0.10645	7.94387

0.10646	7.9396

0.10646	7.93693

0.10647	7.9424

0.10649	7.9536

0.10648	7.9508

0.10651	7.9396

0.10651	7.948

0.10654	7.9536

0.10655	7.95773

0.10656	7.95213

0.10658	7.95213

0.10657	7.95627

0.10659	7.96747

0.10658	7.96187

0.10662	7.966

0.10663	7.966

0.10664	7.9716

0.10663	7.97853

0.10662	7.97573

0.10665	7.96747

0.10666	7.97293

0.10669	7.98133

0.10669	7.9896

0.10673	7.9896

0.10673	7.98267

0.10675	7.9772

0.10676	7.98267

0.10677	7.99387

0.10679	7.99933

0.1068	7.99387

0.10681	7.98827

0.10682	7.9924

0.10684	8.0036

0.10686	8.00907

0.10687	8.00907

0.10688	8.0008

0.1069	8.0008

0.10691	8.01053

0.10691	8.01747

0.10692	8.01467

0.10691	8.01187

0.10694	8.01747

0.10694	8.016

0.10697	8.02027

0.10697	8.0244

0.107	8.02307

0.10701	8.0272

0.10702	8.02853

0.10704	8.02853

0.10704	8.03413

0.10705	8.03693

0.10705	8.03267

0.10706	8.03

0.10706	8.03547

0.10707	8.0452

0.10707	8.04933

0.10711	8.04667

0.10711	8.04107

0.10715	8.04387

0.10715	8.05213

0.10717	8.0564

0.10716	8.05907

0.10718	8.04933

0.10719	8.048

0.1072	8.05907

0.10722	8.06747

0.10723	8.0688

0.10724	8.05907

0.10725	8.05907

0.10727	8.066

0.10727	8.0772

0.10729	8.08

0.10729	8.07307

0.10732	8.0716

0.10732	8.07307

0.10735	8.08133

0.10735	8.08693

0.10738	8.09107

0.10738	8.08973

0.1074	8.08

0.10741	8.08547

0.10741	8.0952

0.10742	8.09933

0.10743	8.09933

0.10743	8.09107

0.10745	8.0924

0.10745	8.09667

0.10748	8.10213

0.10748	8.10907

0.1075	8.1064

0.10752	8.1008

0.10753	8.1036

0.10752	8.11467

0.10753	8.1216

0.10753	8.1188

0.10754	8.11467

0.10757	8.11187

0.10759	8.12027

0.1076	8.12573

0.10761	8.12853

0.10762	8.1272

0.10764	8.1272

0.10765	8.13133

0.10767	8.13133

0.10768	8.13267

0.10769	8.13693

0.10768	8.14107

0.1077	8.1452

0.10771	8.14107

0.10774	8.13547

0.10774	8.1424

0.10777	8.1508

0.10778	8.15773

0.10779	8.15493

0.1078	8.148

0.1078	8.148

0.10781	8.15773

0.10783	8.16747

0.10784	8.1688

0.10785	8.16053

0.10786	8.16187

0.10785	8.16747

0.10786	8.1744

0.10789	8.1688

0.10791	8.16467

0.10793	8.17027

0.10793	8.17573

0.10794	8.1828

0.10794	8.1828

0.10796	8.17573

0.10798	8.1772

0.10799	8.18133

0.108	8.18693

0.108	8.19387

0.10799	8.18827

0.10801	8.18547

0.10804	8.19107

0.10806	8.20213

0.10806	8.2064

0.10806	8.19667

0.10807	8.19387

0.10808	8.2008

0.1081	8.2064

0.10811	8.2008

0.10812	8.21333

0.10813	8.22027

0.10813	8.21747

0.10815	8.20907

0.10816	8.20773

0.10817	8.21613

0.10819	8.2272

0.1082	8.23

0.1082	8.22027

0.10822	8.2188

0.10822	8.2244

0.10824	8.2328

0.10824	8.2272

0.10827	8.2272

0.10827	8.2328

0.10828	8.2452

0.10829	8.2452

0.1083	8.23973

0.10831	8.23547

0.10831	8.24107

0.10832	8.2508

0.10833	8.25213

0.10834	8.2508

0.10834	8.2508

0.10836	8.24947

0.10837	8.2508

0.10838	8.25493

0.10839	8.26333

0.10841	8.26053

0.10842	8.26187

0.10844	8.2564

0.10846	8.2688

0.10849	8.2716

0.1085	8.27587

0.1085	8.2772

0.1085	8.2688

0.10851	8.26747

0.10853	8.2716

0.10852	8.2828

0.10854	8.29107

0.10856	8.28827

0.10857	8.28133

0.10856	8.2828

0.10858	8.28693

0.10859	8.29253

0.1086	8.29667

0.1086	8.29667

0.10861	8.29667

0.10862	8.29947

0.10863	8.30213

0.10864	8.30213

0.10866	8.3008

0.10866	8.30493

0.10868	8.3092

0.10866	8.3188

0.1087	8.31333

0.10869	8.30493

0.1087	8.30773

0.1087	8.32027

0.10872	8.3216

0.10873	8.3188

0.10875	8.3216

0.10875	8.3244

0.10875	8.33

0.10878	8.3272

0.1088	8.3272

0.10881	8.3328

0.10883	8.34253

0.10883	8.33827

0.10885	8.34107

0.10884	8.33413

0.10886	8.33693

0.10886	8.34387

0.10887	8.35213

0.10889	8.35493

0.1089	8.34947

0.10893	8.3452

0.10893	8.34667

0.10894	8.35773

0.10894	8.36333

0.10895	8.36467

0.10897	8.35773

0.10898	8.35493

0.109	8.36187

0.109	8.36613

0.109	8.37587

0.109	8.3716

0.109	8.3744

0.10902	8.3744

0.10903	8.38133

0.10904	8.38413

0.10906	8.37853

0.10906	8.38

0.10909	8.3772

0.1091	8.3772

0.10911	8.38973

0.10913	8.398

0.10915	8.3952

0.10918	8.3856

0.10918	8.38413

0.10919	8.3952

0.1092	8.4064

0.1092	8.40493

0.10923	8.39667

0.10923	8.39253

0.10925	8.4064

0.10928	8.41187

0.10929	8.41613

0.1093	8.41467

0.10928	8.40773

0.10929	8.4092

0.10931	8.41613

0.10931	8.4216

0.10934	8.41613

0.10932	8.41187

0.10933	8.42587

0.10935	8.4356

0.10936	8.4356

0.10938	8.4272

0.10939	8.4216

0.10939	8.42853

0.10941	8.43693

0.1094	8.43973

0.1094	8.44387

0.10942	8.44387

0.10941	8.44253

0.10945	8.43413

0.10945	8.44253

0.10948	8.4536

0.1095	8.4592

0.10949	8.45227

0.10951	8.44387

0.10951	8.45493

0.10951	8.46333

0.10954	8.46613

0.10954	8.46333

0.10955	8.46053

0.10957	8.46053

0.10958	8.4716

0.1096	8.47307

0.10961	8.4716

0.10962	8.4716

0.10964	8.47853

0.10964	8.47587

0.10965	8.46747

0.10967	8.4772

0.10968	8.48827

0.1097	8.4952

0.10971	8.4856

0.10971	8.48413

0.10973	8.48

0.10971	8.49107

0.10973	8.4952

0.10976	8.5008

0.10976	8.5092

0.10978	8.50227

0.1098	8.4952

0.10978	8.49253

0.10982	8.50493

0.10982	8.512

0.10983	8.51613

0.10985	8.5092

0.10984	8.5036

0.10986	8.50493

0.10988	8.51333

0.10989	8.5244

0.10989	8.5216

0.10989	8.51747

0.10989	8.51613

0.10991	8.5244

0.10994	8.53413

0.10993	8.5356

0.10997	8.53827

0.10997	8.53413

0.10995	8.53

0.10998	8.52867

0.10998	8.54107

0.10999	8.54533

0.11002	8.54667

0.11003	8.548

0.11003	8.53827

0.11005	8.53973

0.11006	8.548

0.11008	8.55493

0.11012	8.562

0.1101	8.562

0.11011	8.5536

0.11012	8.55227

0.11011	8.5592

0.11012	8.56467

0.11016	8.5716

0.11015	8.56747

0.11018	8.56613

0.1102	8.56333

0.1102	8.56747

0.1102	8.57027

0.11021	8.5828

0.1102	8.58413

0.11024	8.58413

0.11026	8.58

0.11025	8.5744

0.11025	8.58413

0.11028	8.58827

0.11027	8.598

0.11031	8.59533

0.11033	8.58693

0.11032	8.58693

0.11034	8.59667

0.11035	8.6064

0.11035	8.6092

0.11036	8.6064

0.11037	8.6008

0.11037	8.59947

0.11038	8.60773

0.11042	8.61467

0.11041	8.62027

0.11042	8.61747

0.11045	8.61333

0.11043	8.6092

0.11044	8.61747

0.11046	8.62307

0.11046	8.62867

0.11047	8.63

0.11051	8.6272

0.11051	8.62027

0.11052	8.6272

0.11054	8.63413

0.11055	8.63973

0.11054	8.6356

0.11057	8.62867

0.11058	8.63413

0.11058	8.64667

0.1106	8.65507

0.11061	8.64667

0.11061	8.63973

0.11062	8.64107

0.11066	8.6508

0.11067	8.65773

0.11067	8.662

0.11071	8.662

0.1107	8.65227

0.11069	8.6536

0.1107	8.65227

0.11071	8.66333

0.11071	8.66747

0.11075	8.67173

0.11077	8.67307

0.11077	8.6744

0.11077	8.66747

0.11079	8.66893

0.1108	8.68133

0.11079	8.68133

0.11083	8.6772

0.11084	8.67587

0.11084	8.6744

0.11085	8.68693

0.11088	8.69667

0.11088	8.69947

0.11089	8.69667

0.11091	8.6884

0.11093	8.69107

0.11093	8.7036

0.11096	8.7064

0.11099	8.70507

0.11099	8.7008

0.11098	8.69533

0.11099	8.70507

0.111	8.71467

0.11099	8.71747

0.111	8.712

0.11101	8.71053

0.11101	8.712

0.11102	8.71893

0.11105	8.7272

0.11107	8.7272

0.11107	8.7244

0.11108	8.72173

0.11111	8.72027

0.11109	8.73133

0.1111	8.7384

0.11112	8.73973

0.11115	8.73

0.11115	8.73

0.11115	8.7328

0.11116	8.73973

0.11115	8.74667

0.11116	8.74947

0.11118	8.74667

0.1112	8.73973

0.11122	8.74387

0.11123	8.75773

0.11125	8.7592

0.11126	8.75507

0.11125	8.7536

0.11126	8.75773

0.11128	8.762

0.11129	8.76893

0.11129	8.76747

0.11131	8.762

0.11132	8.7592

0.11132	8.76613

0.11131	8.77307

0.11135	8.78

0.11139	8.78413

0.11139	8.78693

0.11141	8.78147

0.11144	8.77307

0.11143	8.7772

0.11142	8.78693

0.11143	8.79387

0.11144	8.79667

0.11146	8.79107

0.11145	8.78413

0.11146	8.79107

0.11149	8.8008

0.11151	8.8036

0.1115	8.80227

0.11151	8.79813

0.11153	8.79387

0.11156	8.8008

0.11157	8.8092

0.11159	8.82027

0.1116	8.82173

0.11161	8.81893

0.1116	8.81333

0.1116	8.8092

0.11163	8.82027

0.11164	8.82587

0.11164	8.8328

0.11166	8.83

0.11168	8.82173

0.1117	8.81893

0.1117	8.82307

0.11172	8.8356

0.11174	8.84107

0.11175	8.84253

0.11174	8.83413

0.11174	8.8328

0.11174	8.83973

0.11174	8.84813

0.11175	8.85507

0.11176	8.84667

0.11179	8.84813

0.11182	8.84387

0.11182	8.84947

0.11181	8.862

0.11185	8.86333

0.11186	8.8592

0.11188	8.85227

0.11187	8.85227

0.11188	8.8648

0.11188	8.87453

0.1119	8.8772

0.1119	8.87173

0.11192	8.86893

0.11195	8.86613

0.11197	8.87173

0.11197	8.88147

0.11197	8.88693

0.11199	8.8828

0.11201	8.8772

0.11201	8.87587

0.11202	8.88

0.11203	8.89533

0.11204	8.89813

0.11206	8.89947

0.11206	8.89387

0.11206	8.88973

0.11208	8.88973

0.1121	8.89533

0.11212	8.91053

0.11212	8.912

0.11212	8.9092

0.11213	8.90227

0.11216	8.90227

0.11215	8.91333

0.11217	8.91747

0.11218	8.91747

0.11221	8.91333

0.11221	8.91333

0.11222	8.91333

0.11222	8.92587

0.11224	8.93

0.11226	8.93147

0.11226	8.92867

0.11227	8.92453

0.11226	8.92173

0.11228	8.92587

0.11231	8.93693

0.11231	8.94813

0.11231	8.94387

0.11231	8.93693

0.11232	8.9412

0.11233	8.9384

0.11233	8.94387

0.11232	8.9564

0.11234	8.95787

0.11235	8.95227

0.11238	8.94253

0.11239	8.94813

0.1124	8.95507

0.11239	8.96613

0.1124	8.96893

0.1124	8.96053

0.11241	8.9592

0.11242	8.9592

0.11242	8.9648

0.11244	8.9772

0.11244	8.9828

0.11247	8.98147

0.11248	8.97307

0.11249	8.97173

0.11249	8.97867

0.11251	8.98693

0.11251	8.99533

0.11252	8.99387

0.11252	8.98427

0.11253	8.9828

0.11255	8.9828

0.11256	8.99253

0.11259	9.00507

0.11261	9.0036

0.1126	9.00093

0.11261	8.99387

0.11262	8.99387

0.11263	9.00093

0.11265	9.0148

0.11265	9.01613

0.11265	9.0092

0.11266	9.00787

0.11267	9.0036

0.11269	9.01613

0.1127	9.02307

0.1127	9.0272

0.11271	9.02307

0.11271	9.02173

0.11272	9.0176

0.11273	9.02307

0.11275	9.02867

0.11276	9.03693

0.11278	9.03693

0.11278	9.0328

0.1128	9.0272

0.1128	9.0384

0.11282	9.04813

0.11282	9.04947

0.11281	9.03693

0.1128	9.03693

0.11281	9.04533

0.11283	9.05507

0.11286	9.0592

0.11288	9.05507

0.11288	9.04947

0.1129	9.04947

0.1129	9.05787

0.1129	9.06893

0.11291	9.0676

0.11291	9.05787

0.11292	9.05227

0.11294	9.0648

0.11295	9.07307

0.11296	9.07867

0.11297	9.08

0.11299	9.0772

0.11299	9.07453

0.113	9.06893

0.11301	9.0828

0.11303	9.09253

0.11304	9.0912

0.11306	9.0912

0.11307	9.08

0.11307	9.08

0.11308	9.0884

0.11308	9.09947

0.1131	9.10507

0.11311	9.1036

0.11313	9.0912

0.11314	9.09813

0.11316	9.09947

0.11315	9.09667

0.11318	9.1092

0.11318	9.12027

0.11319	9.11893

0.11318	9.11333

0.1132	9.1092

0.1132	9.1148

0.11322	9.12587

0.11324	9.13147

0.11325	9.12867

0.11325	9.12733

0.11326	9.1176

0.11327	9.12173

0.11329	9.13

0.11331	9.13973

0.11331	9.144

0.11333	9.13693

0.11334	9.12733

0.11335	9.12867

0.11336	9.14533

0.11337	9.15093

0.11337	9.15093

0.11338	9.14813

0.11339	9.144

0.11341	9.14253

0.11343	9.14947

0.11343	9.1592

0.11344	9.16613

0.11346	9.1676

0.11347	9.1592

0.11348	9.162

0.11348	9.1592

0.11349	9.15787

0.11349	9.162

0.11348	9.17027

0.11349	9.17453

0.1135	9.1828

0.11353	9.17867

0.11354	9.17453

0.11355	9.17173

0.11355	9.17307

0.11356	9.1856

0.11356	9.19253

0.11357	9.19667

0.11358	9.19253

0.11359	9.18693

0.11359	9.18147

0.1136	9.1856

0.11361	9.194

0.11362	9.20507

0.11363	9.20787

0.11364	9.20227

0.11366	9.19813

0.11367	9.19667

0.11369	9.19813

0.11372	9.19947

0.11375	9.21067

0.11376	9.2176

0.11377	9.22173

0.11378	9.21893

0.11378	9.21893

0.11377	9.2148

0.11378	9.21613

0.11379	9.22173

0.11381	9.2356

0.11382	9.2356

0.11383	9.22867

0.11385	9.2204

0.11387	9.22587

0.11388	9.22587

0.11388	9.2328

0.11389	9.24533

0.11391	9.25093

0.11391	9.24947

0.11392	9.244

0.11394	9.23973

0.11394	9.23707

0.11395	9.24947

0.11395	9.25787

0.11397	9.262

0.11398	9.26067

0.11398	9.25227

0.114	9.24813

0.11402	9.2564

0.11404	9.26067

0.11406	9.26893

0.11406	9.27587

0.11407	9.27173

0.11407	9.27173

0.11408	9.26613

0.11409	9.26893

0.1141	9.26893

0.1141	9.28427

0.11411	9.28427

0.11412	9.28973

0.11414	9.28973

0.11415	9.2856

0.11416	9.28147

0.11416	9.28

0.11417	9.2884

0.11418	9.29253

0.1142	9.30373

0.11421	9.30227

0.11423	9.29813

0.11424	9.29253

0.11424	9.28973

0.11424	9.30093

0.11426	9.31333

0.11426	9.31613

0.11428	9.31893

0.11429	9.3092

0.11431	9.30507

0.11433	9.312

0.11436	9.31893

0.11436	9.32453

0.11438	9.33013

0.11439	9.33013

0.1144	9.32867

0.11441	9.31613

0.11441	9.32173

0.11441	9.32733

0.11442	9.3384

0.11443	9.34253

0.11442	9.33973

0.11443	9.3356

0.11444	9.33707

0.11445	9.3468

0.11446	9.34813

0.11447	9.34533

0.11448	9.3384

0.11449	9.344

0.1145	9.3592

0.11452	9.36067

0.11453	9.36613

0.11455	9.35787

0.11457	9.3564

0.11459	9.34947

0.11461	9.3564

0.11462	9.36613

0.11464	9.37587

0.11463	9.37587

0.11465	9.3676

0.11465	9.36613

0.11467	9.36893

0.11467	9.37733

0.11468	9.3856

0.11467	9.38427

0.11468	9.37307

0.11469	9.37867

0.11471	9.38013

0.1147	9.3828

0.11472	9.38973

0.11471	9.394

0.11474	9.39813

0.11474	9.39253

0.11476	9.3884

0.11478	9.38973

0.11478	9.40373

0.1148	9.4064

0.11479	9.412

0.11482	9.41347

0.11483	9.40507

0.11485	9.40227

0.11486	9.40093

0.11486	9.40787

0.11487	9.41347

0.11488	9.42453

0.11488	9.42733

0.1149	9.42307

0.11491	9.42453

0.11492	9.4176

0.11494	9.4148

0.11494	9.42587

0.11495	9.43427

0.11496	9.43707

0.11497	9.4412

0.11498	9.43013

0.115	9.42733

0.115	9.43013

0.11501	9.4384

0.11503	9.44533

0.11504	9.45093

0.11505	9.45653

0.11507	9.45653

0.11506	9.45227

0.11507	9.44813

0.11508	9.44947

0.1151	9.44947

0.1151	9.45507

0.11511	9.46347

0.11511	9.4704

0.11512	9.4676

0.11513	9.46347

0.11514	9.4592

0.11514	9.46067

0.11517	9.4676

0.11516	9.4828

0.11518	9.48147

0.11518	9.48427

0.11519	9.4732

0.11519	9.4732

0.11519	9.4704

0.1152	9.48147

0.11521	9.48707

0.11524	9.49813

0.11524	9.494

0.11524	9.4828

0.11525	9.48427

0.11525	9.48987

0.11527	9.50507

0.11529	9.50227

0.1153	9.51067

0.11533	9.50653

0.11533	9.50227

0.11535	9.49813

0.11536	9.49813

0.11536	9.5092

0.11537	9.51613

0.11539	9.5204

0.11539	9.5204

0.11539	9.5148

0.1154	9.5092

0.11541	9.5148

0.11541	9.51893

0.11543	9.53013

0.11544	9.5328

0.11547	9.5356

0.11549	9.52587

0.11551	9.52173

0.11553	9.52867

0.11554	9.5356

0.11555	9.5468

0.11554	9.54813

0.11552	9.544

0.11553	9.53987

0.11555	9.53427

0.11555	9.54533

0.11556	9.5592

0.11557	9.562

0.11558	9.55507

0.1156	9.55507

0.11561	9.54533

0.11562	9.55227

0.11564	9.55653

0.11566	9.5732

0.11566	9.57587

0.11567	9.57173

0.11566	9.5676

0.11566	9.56627

0.11566	9.56067

0.11567	9.56627

0.1157	9.57453

0.1157	9.58427

0.11571	9.5884

0.11572	9.58147

0.11573	9.58147

0.11574	9.5704

0.11577	9.58013

0.11577	9.58707

0.11578	9.5968

0.11577	9.60653

0.11579	9.60093

0.11579	9.594

0.11579	9.5912

0.11579	9.5968

0.1158	9.60373

0.11581	9.612

0.11581	9.6092

0.11582	9.60653

0.11584	9.60227

0.11584	9.5996

0.11584	9.61067

0.11586	9.62453

0.11589	9.6232

0.1159	9.62453

0.1159	9.62587

0.1159	9.62173

0.11592	9.61893

0.11593	9.6176

0.11595	9.61893

0.11596	9.62453

0.11597	9.63427

0.11598	9.64253

0.11599	9.644

0.116	9.64253

0.11601	9.63293

0.11603	9.62867

0.11603	9.6412

0.11605	9.6468

0.11607	9.65507

0.11607	9.65507

0.11609	9.6468

0.11608	9.6384

0.11609	9.644

0.1161	9.65227

0.11611	9.6592

0.11612	9.66627

0.11613	9.6704

0.11614	9.6676

0.11614	9.66067

0.11615	9.65787

0.11616	9.66067

0.11617	9.67173

0.11618	9.67587

0.1162	9.6856

0.11621	9.6856

0.11623	9.68147

0.11622	9.67587

0.11626	9.67587

0.11627	9.6732

0.11628	9.68013

0.11628	9.68987

0.11628	9.6968

0.1163	9.6996

0.1163	9.70093

0.11631	9.69533

0.11632	9.68427

0.11632	9.6912

0.11632	9.69533

0.11632	9.69813

0.11634	9.712

0.11636	9.7176

0.11636	9.712

0.11638	9.70787

0.11637	9.70507

0.11639	9.7148

0.1164	9.72453

0.1164	9.726

0.11641	9.7232

0.11641	9.72453

0.11642	9.7176

0.11643	9.7148

0.11644	9.71893

0.11646	9.72453

0.11646	9.72733

0.11648	9.7412

0.11649	9.74533

0.11651	9.73707

0.11651	9.7356

0.11652	9.73147

0.11652	9.7356

0.11651	9.7412

0.11653	9.7496

0.11655	9.75373

0.11657	9.75787

0.11658	9.75653

0.11658	9.75227

0.11659	9.74533

0.11662	9.74533

0.11663	9.75227

0.11664	9.76627

0.11662	9.77173

0.11664	9.77173

0.11664	9.7704

0.11666	9.7704

0.11666	9.762

0.11668	9.762

0.11668	9.7732

0.11671	9.78293

0.11672	9.7856

0.11672	9.78427

0.11674	9.78013

0.11675	9.77453

0.11677	9.77733

0.11678	9.78293

0.11676	9.78987

0.11675	9.80093

0.11676	9.79813

0.11677	9.79533

0.11679	9.7884

0.11679	9.78427

0.11682	9.7912

0.11684	9.8024

0.11686	9.80933

0.11687	9.81627

0.11687	9.8176

0.11689	9.81067

0.1169	9.80093

0.1169	9.80373

0.11688	9.80653

0.11689	9.81907

0.11689	9.83013

0.11689	9.826

0.11692	9.82867

0.11693	9.82173

0.11694	9.8148

0.11695	9.81907

0.11695	9.83013

0.11696	9.83707

0.11698	9.84267

0.11701	9.844

0.117	9.83573

0.11702	9.82867

0.11703	9.83293

0.11705	9.83707

0.11706	9.8468

0.11706	9.85653

0.11709	9.85653

0.1171	9.85093

0.11713	9.84533

0.11713	9.844

0.11715	9.8468

0.11715	9.86067

0.11716	9.86347

0.11716	9.8732

0.11717	9.87453

0.11718	9.8676

0.11718	9.862

0.11718	9.862

0.11719	9.8648

0.11719	9.8704

0.1172	9.87867

0.11721	9.8884

0.11721	9.8884

0.11722	9.8912

0.11722	9.88147

0.11723	9.876

0.11724	9.87733

0.11725	9.88427

0.11726	9.8968

0.11726	9.9024

0.11725	9.90093

0.11728	9.89533

0.1173	9.8912

0.11731	9.88427

0.11732	9.89267

0.11733	9.89533

0.11734	9.90787

0.11736	9.9148

0.11737	9.9176

0.11738	9.91907

0.11739	9.91347

0.1174	9.91067

0.11741	9.90373

0.11742	9.90787

0.11743	9.91907

0.11743	9.92733

0.11743	9.93293

0.11745	9.9288

0.11746	9.9232

0.11747	9.92173

0.11748	9.9232

0.11748	9.93573

0.1175	9.93987

0.11751	9.94547

0.11752	9.94813

0.11753	9.94813

0.11754	9.9384

0.11756	9.93427

0.11756	9.93573

0.11758	9.94267

0.1176	9.95653

0.11761	9.96213

0.11762	9.95507

0.11763	9.94813

0.11764	9.94547

0.11766	9.95507

0.11766	9.9676

0.11767	9.97453

0.11767	9.97173

0.11767	9.96067

0.11768	9.95787

0.11768	9.96067

0.11771	9.9704

0.11775	9.97173

0.11775	9.98013

0.11775	9.9912

0.11774	9.98293

0.11774	9.9788

0.11774	9.98013

0.11775	9.98013

0.11775	9.98147

0.11777	9.99547

0.11779	9.99547

0.11781	10.0024

0.11781	10.00507

0.11783	10.00093

0.11785	9.99813

0.11785	9.99813

0.11787	9.994

0.11788	9.9968

0.11789	10.00653

0.11789	10.01627

0.1179	10.0204

0.11792	10.01067

0.11792	10.0024

0.11794	10.00507

0.11795	10.01067

0.11795	10.02453

0.11795	10.03013

0.11797	10.03013

0.11797	10.0288

0.11796	10.0232

0.11798	10.0204

0.11799	10.0232

0.118	10.02453

0.118	10.03987

0.11799	10.04547

0.118	10.04547

0.11802	10.0412

0.11802	10.03573

0.11804	10.03147

0.11805	10.04267

0.11808	10.05373

0.11809	10.05093

0.1181	10.06067

0.11812	10.05653

0.11813	10.04813

0.11813	10.04813

0.11815	10.0524

0.11816	10.05787

0.11816	10.0704

0.11817	10.07453

0.11819	10.07453

0.1182	10.07187

0.1182	10.0704

0.11822	10.06213

0.11823	10.06347

0.11825	10.0676

0.11826	10.08573

0.11827	10.08573

0.11827	10.08987

0.11827	10.08147

0.11829	10.08147

0.11831	10.0788

0.11832	10.0788

0.11831	10.08573

0.11832	10.0968

0.11831	10.1052

0.11833	10.10653

0.11834	10.0996

0.11835	10.09547

0.11837	10.0912

0.11838	10.0912

0.11839	10.094

0.11839	10.10373

0.11841	10.1176

0.11841	10.12187

0.11842	10.1204

0.11842	10.1176

0.11842	10.10787

0.11842	10.11213

0.11844	10.12733

0.11846	10.13013

0.11846	10.13427

0.11849	10.13427

0.1185	10.1288

0.11851	10.126

0.1185	10.126

0.11851	10.12453

0.11851	10.126

0.11852	10.13573

0.11854	10.1468

0.11857	10.15373

0.11857	10.1524

0.11857	10.1468

0.11856	10.1412

0.11859	10.14267

0.1186	10.144

0.11861	10.14813

0.1186	10.15787

0.11862	10.1676

0.11863	10.16067

0.11865	10.16213

0.11866	10.16067

0.11866	10.15787

0.11867	10.15373

0.11868	10.1552

0.1187	10.16493

0.1187	10.17187

0.11872	10.17733

0.11873	10.18707

0.11875	10.1788

0.11878	10.17733

0.11878	10.17453

0.11878	10.17453

0.11879	10.18427

0.11882	10.1968

0.11883	10.19827

0.11883	10.19267

0.11883	10.194

0.11884	10.18573

0.11887	10.18427

0.11889	10.18853

0.11888	10.19267

0.11888	10.20373

0.11889	10.21213

0.1189	10.20933

0.11892	10.2052

0.11891	10.20373

0.1189	10.20373

0.11892	10.2052

0.11894	10.21067

0.11895	10.22187

0.11895	10.22733

0.11895	10.2288

0.11897	10.2176

0.11899	10.2176

0.119	10.2176

0.11901	10.22733

0.11902	10.23573

0.11905	10.23853

0.11907	10.2412

0.11907	10.23707

0.11906	10.23293

0.11907	10.23427

0.11908	10.2316

0.11911	10.2468

0.1191	10.25653

0.11911	10.25373

0.11912	10.25933

0.11913	10.25093

0.11914	10.2412

0.11916	10.244

0.11917	10.24547

0.11919	10.2552

0.11917	10.26493

0.11918	10.2704

0.11921	10.2704

0.11923	10.26067

0.11923	10.2552

0.11923	10.25933

0.11924	10.2704

0.11925	10.27453

0.11926	10.2816

0.11924	10.28573

0.11926	10.28427

0.11927	10.276

0.11929	10.2732

0.11928	10.2732

0.11929	10.276

0.11931	10.2816

0.11934	10.294

0.11932	10.2996

0.11933	10.3024

0.11933	10.29827

0.11935	10.29267

0.11936	10.28987

0.11936	10.28853

0.11939	10.29547

0.11942	10.308

0.11942	10.31627

0.11941	10.31907

0.11942	10.3176

0.11944	10.31493

0.11942	10.3052

0.11942	10.30373

0.11942	10.30933

0.11945	10.31493

0.11945	10.3176

0.11947	10.32467

0.11948	10.33427

0.1195	10.33573

0.11952	10.33853

0.11951	10.33987

0.11953	10.33427

0.11955	10.32467

0.11956	10.326

0.11954	10.3316

0.11956	10.33987

0.11959	10.3496

0.1196	10.35373

0.11961	10.35373

0.11962	10.3552

0.11964	10.35093

0.11964	10.34547

0.11965	10.34267

0.11965	10.35373

0.11969	10.36493

0.11968	10.36493

0.11969	10.37187

0.11969	10.36213

0.11971	10.35653

0.1197	10.36213

0.11971	10.35933

0.11974	10.35933

0.11975	10.36907

0.11975	10.3816

0.11976	10.38427

0.11978	10.38427

0.1198	10.38853

0.11981	10.38707

0.1198	10.37733

0.11982	10.376

0.1198	10.3788

0.11982	10.376

0.11985	10.38707

0.11987	10.3968

0.11984	10.40107

0.11983	10.40373

0.11986	10.3996

0.11989	10.39133

0.11991	10.38853

0.11991	10.39267

0.11992	10.4024

0.11993	10.41347

0.11993	10.4204

0.11995	10.41493

0.11998	10.41213

0.11997	10.4052

0.11997	10.40653

0.11998	10.40933

0.11999	10.41627

0.11998	10.42467

0.12002	10.4288

0.12004	10.4344

0.12003	10.4344

0.12002	10.4316

0.12006	10.42733

0.12005	10.4288

0.12003	10.42467

0.12004	10.43707

0.12005	10.44267

0.12006	10.45107

0.12006	10.45373

0.12009	10.45107

0.12011	10.43853

0.12012	10.43707

0.12012	10.43853

0.12015	10.44133

0.12015	10.44827

0.12015	10.458

0.12016	10.46773

0.12016	10.4704

0.12016	10.4732

0.12018	10.4704

0.12019	10.46493

0.12018	10.45653

0.12017	10.4552

0.1202	10.45933

0.12022	10.46493

0.12023	10.4788

0.12026	10.48293

0.12028	10.4844

0.12027	10.476

0.12027	10.47467

0.12029	10.4704

0.1203	10.476

0.12031	10.4844

0.12033	10.48853

0.12034	10.50373

0.12034	10.5024

0.12037	10.49267

0.12038	10.48853

0.12037	10.48707

0.12039	10.49133

0.1204	10.49827

0.12039	10.5052

0.12039	10.508

0.12042	10.51213

0.12042	10.5204

0.12044	10.51213

0.12047	10.50373

0.12049	10.5024

0.12048	10.50373

0.1205	10.51213

0.12052	10.51907

0.12049	10.52747

0.1205	10.5344

0.12051	10.53707

0.12052	10.53573

0.12053	10.53293

0.12055	10.526

0.12054	10.52467

0.12055	10.5288

0.12057	10.53573

0.12058	10.54547

0.12058	10.55107

0.12062	10.5552

0.12062	10.5552

0.12061	10.55107

0.12063	10.54133

0.12064	10.53987

0.12062	10.53987

0.12067	10.5468

0.12067	10.558

0.12067	10.56347

0.1207	10.56627

0.1207	10.56907

0.1207	10.56493

0.12072	10.5608

0.12074	10.5552

0.12072	10.5608

0.12074	10.56493

0.12075	10.5732

0.12076	10.58293

0.12076	10.5844

0.12078	10.58573

0.12076	10.58013

0.1208	10.5816

0.1208	10.5788

0.12079	10.576

0.1208	10.57747

0.12083	10.58293

0.12083	10.58707

0.12085	10.59827

0.12086	10.6052

0.12087	10.60933

0.12088	10.6108

0.12089	10.6108

0.12089	10.6052

0.12093	10.59547

0.12094	10.5968

0.12093	10.60373

0.12096	10.61213

0.12097	10.62747

0.12097	10.62467

0.12098	10.6204

0.121	10.61907

0.12101	10.61773

0.12104	10.6108

0.12105	10.61627

0.12104	10.63013

0.12106	10.6344

0.12106	10.64267

0.12106	10.6372

0.12108	10.63013

0.12108	10.6288

0.12108	10.626

0.12111	10.626

0.12111	10.6372

0.12112	10.6524

0.12112	10.6524

0.12112	10.6524

0.12114	10.64827

0.12116	10.64267

0.12113	10.6372

0.12115	10.64547

0.12117	10.6552

0.12118	10.66213

0.1212	10.66907

0.1212	10.66907

0.1212	10.67053

0.12123	10.6608

0.12122	10.66213

0.12125	10.658

0.12127	10.66213

0.12126	10.67053

0.12127	10.68293

0.12127	10.69133

0.12128	10.6872

0.12131	10.6816

0.12132	10.67747

0.12131	10.67747

0.12134	10.67467

0.12133	10.6788

0.12134	10.68853

0.12135	10.7024

0.12136	10.7052

0.12139	10.7052

0.12139	10.70653

0.12139	10.69827

0.12141	10.68987

0.12141	10.68987

0.12142	10.69827

0.12145	10.70387

0.12144	10.7108

0.12144	10.72053

0.12145	10.71773

0.12144	10.71627

0.12145	10.7108

0.12145	10.7052

0.12147	10.708

0.12148	10.71773

0.12148	10.71773

0.1215	10.73573

0.12152	10.7372

0.12151	10.73573

0.12154	10.73573

0.12154	10.73293

0.12154	10.7232

0.12156	10.726

0.12155	10.73987

0.12154	10.74267

0.12155	10.7468

0.12156	10.75387

0.12158	10.7524

0.1216	10.74267

0.12159	10.74267

0.12161	10.74133

0.1216	10.73987

0.12162	10.74547

0.12162	10.75387

0.12162	10.7552

0.12164	10.76213

0.12165	10.77467

0.12166	10.7732

0.12167	10.76907

0.12167	10.76493

0.12171	10.76213

0.12171	10.7608

0.12173	10.76213

0.12175	10.77187

0.12174	10.7788

0.12175	10.78293

0.12176	10.79267

0.12177	10.79133

0.12179	10.78853

0.12177	10.7844

0.12179	10.7816

0.12179	10.776

0.1218	10.78293

0.12182	10.79547

0.12181	10.80107

0.12184	10.7996

0.12183	10.81493

0.12183	10.8108

0.12183	10.80653

0.12182	10.8052

0.12184	10.8024

0.12185	10.79827

0.12186	10.8024

0.12189	10.8136

0.12189	10.82187

0.1219	10.8288

0.12192	10.83027

0.12195	10.826

0.12197	10.82053

0.12196	10.82053

0.12196	10.82187

0.12197	10.81627

0.12198	10.82187

0.12199	10.83293

0.12199	10.8344

0.12201	10.8372

0.12201	10.84413

0.12201	10.8524

0.12204	10.84693

0.12202	10.8524

0.12205	10.84413

0.12205	10.84133

0.12206	10.8372

0.12207	10.84693

0.12206	10.8496

0.12208	10.8524

0.12209	10.858

0.1221	10.86907

0.12211	10.86907

0.12211	10.86773

0.12214	10.87053

0.12213	10.8636

0.12215	10.86213

0.12215	10.85933

0.12216	10.86213

0.12217	10.86907

0.12218	10.88027

0.12219	10.8816

0.12218	10.8872

0.12219	10.88293

0.12219	10.8816

0.12221	10.87333

0.12222	10.87333

0.12221	10.8844

0.12223	10.8872

0.12223	10.89547

0.12223	10.90933

0.12223	10.9052

0.12225	10.90107

0.12226	10.8996

0.12226	10.89693

0.12229	10.89267

0.12228	10.89693

0.12229	10.908

0.1223	10.91213

0.1223	10.91773

0.12231	10.92187

0.12231	10.926

0.12233	10.91493

0.12233	10.9136

0.12235	10.908

0.12236	10.9136

0.12238	10.91493

0.1224	10.92467

0.1224	10.92747

0.1224	10.93853

0.12241	10.94133

0.12241	10.93853

0.12241	10.9288

0.12242	10.926

0.12243	10.92747

0.12245	10.9288

0.12247	10.94267

0.12248	10.9552

0.12247	10.95933

0.12247	10.958

0.12248	10.95107

0.1225	10.94827

0.1225	10.9496

0.12251	10.94547

0.12251	10.95107

0.12253	10.96493

0.12254	10.976

0.12254	10.97053

0.12255	10.96773

0.12256	10.96493

0.12257	10.95667

0.12257	10.958

0.12258	10.96773

0.12259	10.97187

0.12259	10.9788

0.12261	10.98573

0.12261	10.9872

0.12263	10.97467

0.12264	10.97187

0.12265	10.97747

0.12265	10.97467

0.12266	10.98293

0.12266	10.99267

0.12268	10.99693

0.12269	10.99693

0.12271	11.0052

0.12272	11.008

0.12273	10.99973

0.12273	10.99827

0.12273	10.99827

0.12273	10.99547

0.12275	10.99973

0.12277	11.008

0.12279	11.0164

0.1228	11.02187

0.1228	11.026

0.12279	11.02467

0.12279	11.0288

0.1228	11.01773

0.1228	11.0164

0.12281	11.0108

0.12282	11.0164

0.12282	11.026

0.12283	11.0316

0.12284	11.03307

0.12285	11.04267

0.12285	11.04547

0.12285	11.03853

0.12286	11.03573

0.12287	11.0344

0.12289	11.03027

0.12291	11.0344

0.12292	11.04693

0.12292	11.05107

0.12293	11.05387

0.12294	11.0636

0.12294	11.0636

0.12295	11.0664

0.12295	11.058

0.12295	11.05387

0.12296	11.04827

0.12297	11.0552

0.12298	11.06213

0.12298	11.07053

0.12299	11.07053

0.12301	11.0816

0.12303	11.0844

0.12302	11.08027

0.12304	11.07747

0.12304	11.07333

0.12305	11.0664

0.12306	11.06907

0.12307	11.08027

0.12308	11.076

0.1231	11.0872

0.1231	11.09547

0.12312	11.10387

0.12314	11.1052

0.12315	11.10107

0.12316	11.1052

0.12316	11.09547

0.12318	11.08853

0.12319	11.08853

0.12318	11.09413

0.12319	11.10387

0.1232	11.1136

0.12322	11.11773

0.12322	11.12187

0.12322	11.12613

0.12323	11.12333

0.12324	11.11493

0.12325	11.10667

0.12325	11.11213

0.12327	11.11773

0.12328	11.12613

0.12331	11.13853

0.12329	11.13853

0.12329	11.13853

0.12328	11.14

0.12331	11.14

0.12331	11.1288

0.12331	11.1288

0.12331	11.1344

0.12333	11.14

0.12334	11.14693

0.12337	11.1524

0.12336	11.158

0.12337	11.1552

0.12337	11.1552

0.12338	11.158

0.12337	11.15667

0.12341	11.14827

0.1234	11.14827

0.12342	11.15107

0.12341	11.158

0.12344	11.16773

0.12344	11.1788

0.12345	11.18027

0.12343	11.18027

0.12342	11.1788

0.12343	11.17613

0.12345	11.1664

0.12345	11.16773

0.12349	11.16907

0.12349	11.17333

0.1235	11.1816

0.12351	11.18027

0.12351	11.19973

0.12352	11.19827

0.12354	11.20107

0.12355	11.19413

0.12358	11.18853

0.12357	11.18573

0.12357	11.1872

0.12358	11.18853

0.12359	11.19693

0.1236	11.208

0.12361	11.21493

0.12361	11.21493

0.12363	11.22333

0.12363	11.22333

0.12364	11.22187

0.12363	11.22467

0.12365	11.22053

0.12364	11.21773

0.12366	11.21213

0.12367	11.21213

0.12368	11.2164

0.12367	11.22613

0.12368	11.2288

0.12368	11.23853

0.12371	11.24693

0.12371	11.24

0.12371	11.24133

0.12371	11.23853

0.12371	11.23587

0.12373	11.2288

0.12374	11.2288

0.12375	11.2344

0.12376	11.24

0.12376	11.24827

0.12377	11.2636

0.12378	11.26213

0.12381	11.26493

0.12382	11.2636

0.12381	11.258

0.12383	11.25387

0.12383	11.24973

0.12384	11.25107

0.12384	11.25107

0.12386	11.25387

0.12386	11.2636

0.12386	11.27747

0.12386	11.2788

0.12388	11.28027

0.12389	11.29413

0.12391	11.2872

0.12391	11.2816

0.12393	11.2816

0.12393	11.27613

0.12392	11.27467

0.12394	11.27747

0.12396	11.28587

0.12398	11.29413

0.12399	11.3052

0.12401	11.3052

0.124	11.308

0.124	11.30667

0.12401	11.30387

0.12402	11.3052

0.12403	11.29827

0.12405	11.29413

0.12406	11.308

0.12408	11.31213

0.12409	11.31213

0.12408	11.32467

0.12408	11.32747

0.12408	11.33027

0.12409	11.33027

0.12409	11.33307

0.1241	11.32613

0.12409	11.3192

0.12411	11.31213

0.12411	11.31493

0.12411	11.31773

0.12412	11.32333

0.12414	11.33587

0.12417	11.3456

0.12417	11.34973

0.12418	11.35387

0.12418	11.34973

0.1242	11.34413

0.12419	11.3372

0.1242	11.34133

0.12419	11.33587

0.12421	11.3456

0.12421	11.35947

0.12424	11.36773

0.12425	11.3692

0.12425	11.3692

0.12426	11.37333

0.12425	11.3608

0.1243	11.35253

0.1243	11.35387

0.12432	11.358

0.12434	11.3636

0.12436	11.37747

0.12435	11.38027

0.12435	11.38853

0.12436	11.39

0.12434	11.3872

0.12437	11.3816

0.12438	11.37747

0.12441	11.37747

0.12441	11.37893

0.1244	11.38027

0.1244	11.38853

0.12441	11.40107

0.12442	11.40387

0.12443	11.40947

0.12445	11.4108

0.12444	11.4052

0.12447	11.39973

0.12449	11.39693

0.1245	11.40253

0.12452	11.3956

0.12451	11.39827

0.12452	11.40253

0.12452	11.408

0.12454	11.40667

0.12455	11.42187

0.12457	11.4316

0.12458	11.43587

0.12456	11.43027

0.12459	11.42467

0.12457	11.42187

0.12458	11.4164

0.12458	11.42467

0.1246	11.42747

0.12464	11.4316

0.12464	11.4428

0.12467	11.44973

0.12464	11.45107

0.12466	11.45253

0.12465	11.44973

0.12466	11.44413

0.12468	11.43853

0.12469	11.4372

0.12471	11.44

0.12469	11.4428

0.12471	11.4552

0.12472	11.46227

0.12472	11.4636

0.12473	11.47467

0.12471	11.47467

0.12476	11.47467

0.12475	11.47053

0.12479	11.4636

0.1248	11.46227

0.12478	11.4636

0.12481	11.4608

0.1248	11.46773

0.12483	11.47747

0.12484	11.4844

0.12484	11.48867

0.12486	11.49827

0.12485	11.49

0.12488	11.4872

0.12488	11.4816

0.12488	11.48587

0.12487	11.48027

0.12487	11.4844

0.12491	11.4956

0.1249	11.49973

0.12491	11.49973

0.12493	11.51227

0.12493	11.5164

0.12495	11.51493

0.12495	11.5136

0.12495	11.5164

0.12496	11.50947

0.12496	11.50667

0.12499	11.50253

0.12499	11.50533

0.12501	11.50387

0.12502	11.51227

0.12505	11.52613

0.12506	11.54133

0.12506	11.53307

0.12508	11.54

0.12507	11.5372

0.12508	11.53587

0.12509	11.53027

0.12509	11.52747

0.12513	11.52747

0.12512	11.53027

0.12513	11.54

0.12515	11.55387

0.12515	11.55533

0.12516	11.55947

0.12517	11.5608

0.12517	11.55253

0.1252	11.5456

0.12519	11.54827

0.12521	11.54827

0.12522	11.54693

0.1252	11.55107

0.12522	11.55387

0.12522	11.5608

0.12523	11.56493

0.12527	11.572

0.12524	11.57893

0.12527	11.5844

0.12526	11.58307

0.12526	11.59

0.12529	11.58307

0.12528	11.58307

0.12532	11.58027

0.12531	11.58027

0.1253	11.57613

0.12533	11.572

0.12532	11.57613

0.12535	11.58173

0.12537	11.5872

0.12538	11.59133

0.1254	11.59973

0.12538	11.6108

0.12538	11.60947

0.12539	11.60387

0.12539	11.60533

0.1254	11.59693

0.12541	11.5956

0.12542	11.59973

0.12542	11.59973

0.12543	11.60253

0.12542	11.61227

0.12546	11.62333

0.12545	11.62467

0.1255	11.63587

0.12552	11.63173

0.12552	11.63307

0.12552	11.62613

0.12551	11.62333

0.12551	11.62053

0.12553	11.62053

0.12552	11.62053

0.12555	11.622

0.12555	11.62747

0.12557	11.63307

0.12559	11.63587

0.1256	11.6428

0.1256	11.65533

0.12562	11.658

0.12562	11.6608

0.12562	11.66227

0.12565	11.65667

0.12562	11.65533

0.12564	11.64693

0.12565	11.65107

0.12565	11.64693

0.12567	11.6456

0.12568	11.65533

0.12568	11.65947

0.12572	11.6692

0.12572	11.672

0.12571	11.6844

0.12573	11.6844

0.12572	11.68307

0.12574	11.68587

0.12576	11.67893

0.12573	11.67333

0.12576	11.66773

0.12576	11.672

0.12578	11.67467

0.12581	11.67613

0.12581	11.6844

0.12579	11.6956

0.12581	11.70533

0.12581	11.70947

0.12582	11.70813

0.12583	11.70253

0.12584	11.6928

0.12585	11.6956

0.12589	11.69133

0.12587	11.69413

0.12588	11.6984

0.12589	11.7108

0.12589	11.71227

0.12591	11.7192

0.12594	11.72893

0.12593	11.72747

0.12596	11.72893

0.12596	11.72333

0.12596	11.72333

0.12596	11.72053

0.12596	11.72053

0.12596	11.72053

0.126	11.72053

0.12601	11.722

0.12601	11.72333

0.12604	11.73027

0.12605	11.74

0.12607	11.74973

0.12607	11.75533

0.12608	11.75253

0.12605	11.74973

0.12608	11.74147

0.12609	11.74

0.12607	11.73867

0.1261	11.74413

0.1261	11.74

0.1261	11.75107

0.12611	11.75813

0.12613	11.7664

0.12614	11.7692

0.12616	11.77333

0.12613	11.77613

0.12612	11.78027

0.12615	11.77613

0.12618	11.7748

0.12618	11.76507

0.12621	11.76227

0.12622	11.7636

0.12623	11.7692

0.12624	11.7748

0.12625	11.7844

0.12625	11.79413

0.12627	11.79973

0.12628	11.7956

0.12628	11.79693

0.12628	11.80533

0.12629	11.79973

0.12628	11.79693

0.12631	11.7956

0.12633	11.80107

0.12633	11.79147

0.12634	11.79147

0.12635	11.7928

0.12636	11.7956

0.12636	11.7984

0.12637	11.80533

0.12636	11.8108

0.12638	11.82053

0.12641	11.82613

0.12642	11.82613

0.12642	11.83027

0.12645	11.82893

0.12644	11.82893

0.12644	11.822

0.12646	11.82053

0.12647	11.81773

0.12646	11.8192

0.12648	11.82333

0.1265	11.82893

0.1265	11.83453

0.12651	11.84147

0.12653	11.84973

0.12655	11.85813

0.12655	11.85813

0.12657	11.85387

0.12658	11.8428

0.12659	11.83867

0.1266	11.8456

0.12662	11.84693

0.12662	11.84147

0.12662	11.8428

0.12662	11.85253

0.12662	11.85667

0.12664	11.86507

0.12665	11.86787

0.12667	11.88173

0.12668	11.88173

0.12669	11.88307

0.12671	11.88027

0.12669	11.88027

0.1267	11.87333

0.12672	11.87053

0.12672	11.8692

0.12673	11.872

0.12675	11.87053

0.12677	11.88453

0.12675	11.8956

0.12678	11.9012

0.12679	11.90387

0.1268	11.9012

0.12678	11.90387

0.12681	11.90667

0.12681	11.90387

0.12683	11.8956

0.12685	11.8984

0.12686	11.8928

0.12686	11.89413

0.12688	11.90667

0.12688	11.90667

0.12689	11.90947

0.12689	11.91787

0.12691	11.92747

0.12692	11.92747

0.12692	11.93027

0.12692	11.93173

0.12694	11.922

0.12693	11.9164

0.12694	11.9136

0.12696	11.91787

0.12698	11.92053

0.12699	11.922

0.127	11.93307

0.12701	11.93453

0.12701	11.94427

0.12702	11.9484

0.12703	11.95387

0.12705	11.9512

0.12705	11.95533

0.12705	11.94693

0.12705	11.94693

0.12708	11.9456

0.12709	11.94147

0.12709	11.94427

0.1271	11.95253

0.1271	11.95667

0.1271	11.9636

0.12713	11.97613

0.12715	11.97333

0.12717	11.98173

0.12716	11.97893

0.12716	11.972

0.12716	11.97053

0.12718	11.972

0.12719	11.9664

0.12719	11.96787

0.1272	11.9636

0.12721	11.97053

0.12724	11.9748

0.12726	11.98587

0.12727	11.99427

0.12728	11.9984

0.12727	12.00387

0.12729	11.99973

0.1273	12.00533

0.12732	12.00387

0.12732	11.99973

0.12733	11.9984

0.12731	11.9956

0.12732	11.99

0.12733	11.99427

0.12735	11.9956

0.12734	12.00387

0.12735	12.00813

0.12737	12.01787

0.12739	12.02053

0.12739	12.0276

0.12741	12.03173

0.12742	12.0276

0.12743	12.0276

0.12743	12.02613

0.12744	12.0248

0.12743	12.0164

0.12743	12.022

0.12744	12.0192

0.12746	12.022

0.12746	12.0248

0.12747	12.03587

0.12747	12.0372

0.12746	12.04693

0.12748	12.05667

0.12751	12.05947

0.12751	12.05667

0.12753	12.05533

0.12755	12.05667

0.12756	12.0456

0.12756	12.04427

0.12755	12.04147

0.12757	12.04427

0.12758	12.0456

0.12758	12.0512

0.12757	12.0636

0.12759	12.0748

0.12759	12.07333

0.12761	12.08027

0.12762	12.08173

0.12765	12.08453

0.12763	12.08453

0.12765	12.08587

0.12765	12.0776

0.12765	12.07893

0.12766	12.072

0.12766	12.0692

0.12769	12.07067

0.1277	12.0748

0.1277	12.07613

0.12769	12.08027

0.1277	12.08867

0.12771	12.09973

0.12771	12.09973

0.12773	12.104

0.12773	12.11227

0.12774	12.1164

0.12775	12.1192

0.12776	12.11507

0.12778	12.1192

0.12779	12.11093

0.12779	12.10813

0.12779	12.10947

0.12778	12.10533

0.1278	12.10667

0.1278	12.10667

0.12782	12.10813

0.12783	12.11093

0.12785	12.12613

0.12785	12.1276

0.12786	12.13173

0.12786	12.13867

0.12787	12.14693

0.12789	12.1456

0.1279	12.1512

0.1279	12.1484

0.1279	12.14427

0.1279	12.1428

0.12791	12.13867

0.12792	12.14427

0.12792	12.13733

0.12792	12.13733

0.12793	12.13867

0.12794	12.14147

0.12796	12.14427

0.12795	12.1512

0.12796	12.16093

0.12797	12.16093

0.12798	12.17333

0.12799	12.1804

0.12801	12.1804

0.12801	12.18173

0.12802	12.18173

0.12802	12.1748

0.12802	12.17613

0.12802	12.1664

0.12805	12.16507

0.12807	12.16507

0.12808	12.1692

0.12807	12.172

0.12807	12.1804

0.12808	12.18307

0.12808	12.18587

0.1281	12.1956

0.1281	12.19973

0.12811	12.204

0.12812	12.20813

0.12813	12.21227

0.12814	12.21373

0.12815	12.21507

0.12815	12.21373

0.12816	12.21093

0.12817	12.21373

0.12817	12.20813

0.12818	12.204

0.12818	12.20947

0.1282	12.2012

0.1282	12.20533

0.12821	12.20667

0.12822	12.21093

0.12822	12.222

0.12824	12.22613

0.12824	12.23867

0.12824	12.23867

0.12826	12.24147

0.12828	12.24707

0.12829	12.24707

0.1283	12.24973

0.12831	12.24

0.12831	12.24427

0.12832	12.23587

0.12833	12.23307

0.12833	12.23173

0.12834	12.23587

0.12835	12.23307

0.12836	12.23453

0.12836	12.23867

0.12839	12.2428

0.12838	12.2456

0.1284	12.2484

0.12838	12.25947

0.1284	12.26787

0.12842	12.27067

0.12843	12.27333

0.12844	12.2776

0.12844	12.27893

0.12845	12.28587

0.12846	12.28867

0.12846	12.28453

0.12848	12.29

0.12847	12.28307

0.12847	12.27613

0.12849	12.27893

0.12852	12.272

0.12853	12.27613

0.12854	12.272

0.12852	12.2776

0.12854	12.27893

0.12857	12.28173

0.12857	12.28587

0.12857	12.2984

0.12857	12.30813

0.12858	12.30813

0.12859	12.31093

0.12862	12.31507

0.12863	12.3164

0.12863	12.32067

0.12864	12.32613

0.12865	12.32067

0.12866	12.32613

0.12869	12.3192

0.12868	12.31373

0.12869	12.3192

0.12868	12.3164

0.12869	12.31787

0.12871	12.31507

0.12874	12.31227

0.12872	12.3164

0.12873	12.31787

0.12874	12.32067

0.12873	12.3248

0.12876	12.33173

0.12876	12.34013

0.12879	12.34427

0.12879	12.3512

0.12879	12.35813

0.12879	12.36093

0.1288	12.36373

0.12882	12.36093

0.12883	12.36507

0.12883	12.3664

0.12882	12.36787

0.12883	12.3664

0.12883	12.36093

0.12884	12.372

0.12884	12.36227

0.12886	12.36093

0.12886	12.36093

0.12886	12.35947

0.12887	12.35813

0.12889	12.36093

0.12891	12.3664

0.12893	12.37347

0.12893	12.37893

0.12893	12.3804

0.12895	12.38587

0.12897	12.3956

0.129	12.4012

0.129	12.39707

0.12902	12.3984

0.12901	12.40253

0.12901	12.4068

0.12901	12.40947

0.12902	12.40813

0.12903	12.41227

0.12904	12.41093

0.12905	12.40533

0.12905	12.41227

0.12909	12.40813

0.12911	12.41653

0.12913	12.41373

0.12912	12.40947

0.12912	12.41227

0.12912	12.4068

0.12912	12.40947

0.12913	12.40533

0.12915	12.40533

0.12915	12.40947

0.12918	12.40947

0.12917	12.41227

0.12917	12.41507

0.12917	12.42067

0.12918	12.422

0.1292	12.43173

0.12923	12.43587

0.12924	12.4456

0.12922	12.44987

0.12922	12.4568

0.12923	12.45813

0.12922	12.46093

0.12924	12.46373

0.12926	12.45947

0.12927	12.46653

0.12929	12.46093

0.12928	12.46507

0.1293	12.45947

0.12931	12.45533

0.12931	12.45533

0.12933	12.4484

0.12933	12.454

0.12935	12.45253

0.12935	12.45813

0.12934	12.46373

0.12935	12.45947

0.12935	12.45947

0.12936	12.46787

0.12937	12.4776

0.12938	12.47613

0.12939	12.48867

0.12939	12.48453

0.12939	12.4956

0.1294	12.4984

0.12942	12.4984

0.12943	12.50253

0.12945	12.5068

0.12948	12.50813

0.12948	12.5068

0.12948	12.5068

0.12947	12.50253

0.12947	12.50813

0.12949	12.5068

0.12951	12.50253

0.12951	12.504

0.12952	12.5012

0.12952	12.50253

0.12953	12.49707

0.12954	12.504

0.12954	12.51093

0.12955	12.52067

0.12957	12.5192

0.12959	12.5332

0.12961	12.53733

0.1296	12.53867

0.12962	12.54147

0.12961	12.54707

0.12962	12.54147

0.12962	12.5332

0.12962	12.53733

0.12963	12.53453

0.12964	12.53867

0.12964	12.53733

0.12965	12.53867

0.12964	12.53453

0.12966	12.5304

0.12967	12.53733

0.12967	12.53453

0.12968	12.53867

0.12968	12.54013

0.1297	12.54707

0.12972	12.5456

0.12974	12.55253

0.12973	12.55813

0.12975	12.5596

0.12976	12.56787

0.12976	12.57067

0.12975	12.5832

0.12975	12.58587

0.12976	12.58867

0.12977	12.58453

0.12977	12.58587

0.1298	12.57893

0.12981	12.572

0.12983	12.57627

0.12982	12.5692

0.12983	12.572

0.12984	12.5692

0.12984	12.5776

0.12985	12.57627

0.12986	12.5804

0.12987	12.58173

0.1299	12.59013

0.12991	12.6012

0.12992	12.6012

0.12992	12.61093

0.12992	12.61227

0.12993	12.61787

0.12992	12.61653

0.12993	12.62067

0.12995	12.62627

0.12995	12.62627

0.12997	12.6276

0.12998	12.62627

0.13	12.62627

0.13	12.6248

0.13001	12.62627

0.13001	12.6192

0.13001	12.61507

0.13002	12.61787

0.13002	12.61507

0.13004	12.6248

0.13007	12.61787

0.13008	12.6192

0.1301	12.6248

0.1301	12.62627

0.13009	12.6304

0.13009	12.6304

0.13008	12.6332

0.1301	12.6304

0.13011	12.63453

0.13012	12.63453

0.13014	12.64147

0.13015	12.65267

0.13016	12.65533

0.13015	12.64987

0.13017	12.66227

0.13021	12.66933

0.1302	12.67627

0.13021	12.6776

0.13021	12.6832

0.13022	12.6804

0.13021	12.68173

0.13022	12.68867

0.13023	12.69013

0.13023	12.69013

0.13023	12.69427

0.13024	12.6956

0.13026	12.69013

0.13026	12.68867

0.13029	12.6804

0.1303	12.68453

0.13029	12.6804

0.13029	12.6832

0.13029	12.68867

0.1303	12.69013

0.13031	12.68867

0.13031	12.68733

0.13033	12.68733

0.13035	12.68733

0.13036	12.69147

0.13036	12.69293

0.13037	12.70267

0.13038	12.7068

0.1304	12.70533

0.13041	12.71507

0.13041	12.71787

0.13043	12.72347

0.13042	12.72893

0.13043	12.7304

0.13043	12.736

0.13045	12.736

0.13047	12.7456

0.13048	12.74427

0.13047	12.74987

0.13048	12.7484

0.13049	12.7484

0.1305	12.7484

0.13051	12.75267

0.13051	12.74707

0.13052	12.74013

0.13053	12.7484

0.13055	12.7456

0.13055	12.7456

0.13056	12.7456

0.13057	12.74427

0.13058	12.74427

0.13058	12.74293

0.13058	12.74427

0.1306	12.74293

0.1306	12.74707

0.13062	12.7484

0.13062	12.74987

0.13062	12.75813

0.13063	12.7596

0.13063	12.76507

0.13065	12.76653

0.13066	12.772

0.13067	12.77907

0.13068	12.78173

0.1307	12.78867

0.13071	12.79293

0.13072	12.79573

0.13074	12.8012

0.13074	12.80267

0.13075	12.80267

0.13076	12.8068

0.13075	12.80533

0.13075	12.8068

0.13076	12.80533

0.13075	12.80267

0.13076	12.81093

0.13075	12.80267

0.13077	12.79987

0.13078	12.8012

0.13079	12.7984

0.1308	12.80533

0.13081	12.8012

0.13082	12.7984

0.13084	12.80267

0.13085	12.804

0.13085	12.80533

0.13085	12.80813

0.13088	12.8124

0.1309	12.81653

0.13092	12.82627

0.13091	12.8304

0.1309	12.83733

0.13091	12.84147

0.13092	12.84573

0.13093	12.8484

0.13095	12.84987

0.13096	12.85533

0.13096	12.85267

0.13096	12.85533

0.13097	12.85813

0.13099	12.85813

0.131	12.85813

0.13101	12.85533

0.13103	12.85813

0.13104	12.86093

0.13104	12.85813

0.13103	12.85533

0.13101	12.8512

0.13103	12.854

0.13103	12.8512

0.13104	12.8568

0.13102	12.8512

0.13103	12.86093

0.13104	12.86507

0.13107	12.86093

0.13109	12.86093

0.1311	12.86373

0.13112	12.86933

0.13113	12.86653

0.13113	12.872

0.13113	12.872

0.13113	12.8776

0.13114	12.886

0.13115	12.88173

0.13116	12.8888

0.13117	12.88453

0.13119	12.89147

0.1312	12.90267

0.1312	12.8984

0.13121	12.90813

0.13121	12.90267

0.13124	12.90547

0.13126	12.91507

0.13127	12.9124

0.13128	12.91653

0.13128	12.91653

0.13129	12.92067

0.1313	12.9332

0.13131	12.936

0.13132	12.936

0.13133	12.9388

0.13133	12.94013

0.13135	12.93453

0.13137	12.94147

0.13138	12.94013

0.13136	12.94573

0.13137	12.94573

0.13135	12.94707

0.13136	12.94573

0.13137	12.94013

0.13138	12.94707

0.13141	12.936

0.13142	12.936

0.13143	12.94293

0.13144	12.93733

0.13144	12.94147

0.13146	12.9388

0.13146	12.9388

0.13146	12.94147

0.13147	12.94147

0.13146	12.9484

0.13149	12.94573

0.13151	12.9484

0.13152	12.95267

0.13153	12.94987

0.13153	12.95547

0.13155	12.9568

0.13156	12.9568

0.13157	12.96093

0.13157	12.9624

0.13157	12.9624

0.13157	12.96933

0.13158	12.97627

0.13157	12.9776

0.1316	12.9832

0.13159	12.9832

0.13162	12.99013

0.13164	12.9984

0.13165	12.99293

0.13165	13.00267

0.13164	13.00267

0.13163	13.0068

0.13165	13.01093

0.13168	13.0152

0.13168	13.01787

0.13169	13.01787

0.13169	13.02347

0.1317	13.02347

0.13171	13.02907

0.13173	13.02907

0.13174	13.036

0.13173	13.03187

0.13175	13.03187

0.13176	13.036

0.13176	13.036

0.13178	13.04013

0.1318	13.04293

0.13181	13.03733

0.13182	13.0388

0.13183	13.0332

0.13183	13.03453

0.13183	13.0304

0.13184	13.02907

0.13185	13.0332

0.13186	13.0304

0.13185	13.04013

0.13186	13.03453

0.13187	13.0332

0.13186	13.036

0.13187	13.036

0.13186	13.036

0.13188	13.04147

0.13191	13.04427

0.13191	13.04427

0.13193	13.04987

0.13194	13.04987

0.13194	13.0568

0.13195	13.0624

0.13196	13.0568

0.13197	13.06787

0.13197	13.06653

0.13197	13.07627

0.13197	13.08187

0.132	13.086

0.13201	13.08733

0.13201	13.09147

0.13201	13.09573

0.13199	13.09707

0.13201	13.09987

0.13201	13.104

0.13203	13.1068

0.13205	13.11093

0.13206	13.11093

0.13208	13.11093

0.13208	13.1096

0.13209	13.11653

0.1321	13.11373

0.13209	13.11373

0.13211	13.11933

0.13211	13.11373

0.13212	13.11933

0.13213	13.11787

0.13213	13.11373

0.13214	13.11653

0.13215	13.11093

0.13217	13.11787

0.13217	13.11373

0.13218	13.11093

0.13218	13.12067

0.1322	13.11933

0.13221	13.12493

0.13224	13.11787

0.13225	13.11933

0.13225	13.12493

0.13225	13.12347

0.13225	13.1276

0.13226	13.12627

0.13227	13.12493

0.13227	13.1304

0.13228	13.1304

0.13229	13.1332

0.13229	13.1332

0.13231	13.136

0.1323	13.13733

0.13231	13.14013

0.13231	13.14293

0.13232	13.14987

0.13231	13.1568

0.13232	13.15827

0.13234	13.1624

0.13237	13.16653

0.13237	13.17067

0.13239	13.17627

0.13241	13.17493

0.13243	13.18453

0.13243	13.18187

0.13243	13.1916

0.13244	13.19427

0.13245	13.19013

0.13245	13.19853

0.13245	13.19573

0.13247	13.20547

0.13247	13.20827

0.13249	13.20547

0.13248	13.2096

0.1325	13.2096

0.13252	13.21093

0.13252	13.21093

0.13252	13.21373

0.13253	13.20827

0.13253	13.2124

0.13253	13.2124

0.13254	13.204

0.13255	13.21093

0.13256	13.20827

0.13256	13.2152

0.13255	13.21093

0.13256	13.2096

0.13258	13.21373

0.1326	13.2124

0.13261	13.21653

0.13262	13.21933

0.13263	13.2124

0.13263	13.2152

0.13264	13.21653

0.13264	13.2152

0.13266	13.21653

0.13268	13.21933

0.13268	13.22347

0.13268	13.22493

0.13271	13.23453

0.13271	13.22907

0.13272	13.22907

0.13273	13.2304

0.13273	13.236

0.13274	13.2332

0.13274	13.236

0.13276	13.23733

0.13277	13.24013

0.13276	13.24293

0.13278	13.24573

0.13278	13.24427

0.13279	13.24987

0.13279	13.25133

0.13279	13.24987

0.13281	13.25547

0.1328	13.25267

0.13281	13.25547

0.13281	13.2596

0.13282	13.2596

0.13285	13.26653

0.13285	13.26653

0.13285	13.26653

0.13287	13.27347

0.13288	13.26933

0.13288	13.27347

0.13288	13.27493

0.13289	13.27627

0.1329	13.27627

0.13292	13.2776

0.13294	13.27907

0.13295	13.2804

0.13295	13.28733

0.13295	13.28733

0.13295	13.29293

0.13295	13.29293

0.13297	13.29853

0.13297	13.30267

0.13298	13.304

0.133	13.31093

0.133	13.3096

0.133	13.318

0.133	13.31933

0.13301	13.31933

0.13303	13.32347

0.13304	13.31933

0.13304	13.32627

0.13306	13.32213

0.13305	13.32907

0.13308	13.33733

0.13308	13.3304

0.13309	13.3332

0.1331	13.336

0.13311	13.3332

0.13312	13.34013

0.13313	13.336

0.13313	13.3416

0.13314	13.34427

0.13316	13.34853

0.13316	13.3568

0.13318	13.35827

0.13319	13.36373

0.13319	13.37067

0.13321	13.368

0.1332	13.37493

0.13323	13.37627

0.13323	13.37907

0.13323	13.3832

0.13325	13.3804

0.13325	13.38187

0.13326	13.3916

0.13328	13.3888

0.13329	13.3888

0.13328	13.3916

0.13329	13.3916

0.13331	13.39293

0.13332	13.3888

0.13333	13.3888

0.13333	13.39293

0.13334	13.38733

0.13335	13.39573

0.13336	13.39573

0.13337	13.3888

0.13337	13.39707

0.13339	13.3916

0.13341	13.39573

0.13341	13.3944

0.13341	13.39987

0.13342	13.4068

0.13343	13.404

0.13344	13.41107

0.13345	13.40827

0.13344	13.404

0.13347	13.4096

0.13348	13.40547

0.13348	13.4152

0.13348	13.41107

0.13349	13.4096

0.13351	13.418

0.13351	13.4152

0.13352	13.42213

0.13354	13.42213

0.13354	13.41653

0.13354	13.42493

0.13355	13.42067

0.13356	13.42493

0.13357	13.42213

0.13358	13.42347

0.1336	13.42907

0.13361	13.4304

0.13359	13.436

0.13359	13.4332

0.1336	13.436

0.13363	13.4332

0.13364	13.44013

0.13365	13.4416

0.13365	13.44573

0.13365	13.44573

0.13365	13.44573

0.13366	13.44987

0.13369	13.44853

0.13369	13.45547

0.1337	13.4596

0.13372	13.4596

0.13373	13.46107

0.13373	13.46107

0.13374	13.468

0.13374	13.47493

0.13377	13.47627

0.13378	13.4832

0.1338	13.48467

0.1338	13.48747

0.13379	13.4944

0.13379	13.486

0.1338	13.48747

0.13381	13.4916

0.13383	13.49707

0.13384	13.50267

0.13384	13.50413

0.13384	13.5096

0.13385	13.49987

0.13388	13.5068

0.13388	13.51933

0.13388	13.518

0.13388	13.51653

0.13389	13.52213

0.1339	13.52907

0.13391	13.52773

0.13394	13.52907

0.13397	13.5388

0.13395	13.53467

0.13394	13.53747

0.13393	13.5388

0.13394	13.54013

0.13395	13.5444

0.13397	13.55133

0.13398	13.55133

0.13398	13.55413

0.13398	13.54987

0.13399	13.5568

0.13402	13.55827

0.13403	13.55827

0.13403	13.5568

0.13402	13.5652

0.13399	13.55827

0.134	13.56373

0.13402	13.56653

0.13403	13.56653

0.13404	13.57213

0.13405	13.56933

0.13405	13.57627

0.13407	13.57347

0.13409	13.568

0.13411	13.57347

0.13412	13.5708

0.13412	13.5804

0.13413	13.57907

0.13415	13.57907

0.13416	13.58187

0.13418	13.5832

0.13417	13.59293

0.13419	13.5888

0.13418	13.5888

0.1342	13.59293

0.13421	13.5888

0.13423	13.5944

0.13422	13.59707

0.13423	13.6068

0.13425	13.60547

0.13425	13.60547

0.13426	13.60827

0.13426	13.61107

0.13426	13.60547

0.13429	13.61107

0.13432	13.60547

0.13434	13.6124

0.13435	13.60413

0.13435	13.60267

0.13434	13.60413

0.13434	13.60267

0.13437	13.6068

0.13437	13.6068

0.13438	13.60267

0.13437	13.6124

0.13436	13.6124

0.13439	13.62213

0.13441	13.62347

0.13442	13.61933

0.13441	13.62907

0.13441	13.62213

0.13443	13.63053

0.13443	13.62907

0.13446	13.6208

0.13446	13.636

0.13447	13.62907

0.13448	13.636

0.1345	13.636

0.1345	13.636

0.13448	13.64013

0.13448	13.636

0.13451	13.64013

0.13453	13.64013

0.13454	13.6388

0.13453	13.64013

0.13453	13.64573

0.13455	13.64573

0.13456	13.64573

0.13457	13.64987

0.13456	13.65267

0.13456	13.65413

0.13456	13.65413

0.13457	13.6568

0.1346	13.668

0.13461	13.66387

0.13461	13.6652

0.13462	13.6652

0.13464	13.67213

0.13467	13.67627

0.13466	13.67493

0.13465	13.67627

0.13467	13.67493

0.13469	13.67907

0.1347	13.68187

0.13471	13.68187

0.13472	13.68467

0.13475	13.68467

0.13475	13.6888

0.13476	13.69573

0.13473	13.69293

0.13472	13.6972

0.13474	13.69293

0.13475	13.6972

0.13476	13.70133

0.13474	13.69987

0.13476	13.70413

0.1348	13.70547

0.1348	13.7068

0.1348	13.7068

0.13479	13.71107

0.13481	13.718

0.13484	13.7124

0.13485	13.71933

0.13483	13.7236

0.13482	13.7236

0.13484	13.72907

0.13487	13.72493

0.13489	13.72907

0.13488	13.73187

0.13491	13.72773

0.13491	13.72773

0.13492	13.72773

0.13491	13.7332

0.13493	13.7388

0.13495	13.73467

0.13497	13.73747

0.13496	13.7388

0.13496	13.74573

0.13497	13.7444

0.13501	13.74293

0.13502	13.74987

0.135	13.74853

0.135	13.74987

0.13501	13.75547

0.13503	13.75413

0.13503	13.7596

0.13503	13.75827

0.13505	13.76387

0.13507	13.7624

0.13508	13.7624

0.13507	13.76387

0.13508	13.76653

0.1351	13.7708

0.13511	13.7708

0.13509	13.7708

0.1351	13.77627

0.13513	13.77773

0.13514	13.78053

0.13514	13.7832

0.13514	13.78053

0.13516	13.786

0.13519	13.78467

0.1352	13.7888

0.1352	13.79293

0.1352	13.7944

0.13522	13.79853

0.13522	13.79573

0.13522	13.80693

0.13523	13.8152

0.13526	13.81387

0.13527	13.8152

0.13525	13.8124

0.13523	13.8208

0.13524	13.82493

0.13526	13.8236

0.13528	13.82907

0.13529	13.82493

0.13528	13.82773

0.13531	13.8332

0.1353	13.82627

0.13532	13.82493

0.13534	13.82773

0.13534	13.8332

0.13535	13.836

0.13535	13.8332

0.13536	13.83747

0.13537	13.83467

0.13536	13.84027

0.13536	13.83747

0.13539	13.84027

0.13541	13.8472

0.1354	13.84573

0.13541	13.85133

0.13543	13.85693

0.13546	13.85827

0.13544	13.86107

0.13544	13.86107

0.13546	13.86667

0.13548	13.8736

0.13547	13.868

0.13548	13.87213

0.1355	13.86933

0.13552	13.8736

0.13554	13.87907

0.13555	13.8736

0.13557	13.88187

0.13556	13.87627

0.13556	13.88187

0.13556	13.886

0.13558	13.88053

0.13559	13.88747

0.13559	13.88467

0.13562	13.89027

0.13562	13.89027

0.13561	13.89853

0.1356	13.90547

0.13561	13.9

0.13562	13.90413

0.13562	13.91107

0.13562	13.9096

0.13565	13.91387

0.13564	13.91387

0.13565	13.9208

0.13567	13.918

0.13569	13.9208

0.13569	13.92493

0.13567	13.91933

0.13568	13.92773

0.1357	13.93187

0.1357	13.92627

0.13571	13.93187

0.13573	13.92773

0.13574	13.936

0.13575	13.9444

0.13576	13.94027

0.13577	13.9416

0.13578	13.93467

0.13578	13.94027

0.13578	13.9444

0.1358	13.94027

0.13578	13.9444

0.13578	13.9416

0.13581	13.94573

0.13583	13.95413

0.13584	13.95133

0.13584	13.95267

0.13589	13.95

0.13587	13.95547

0.13587	13.9624

0.13587	13.95133

0.13589	13.95547

0.13588	13.95413

0.1359	13.95693

0.13593	13.96107

0.13593	13.96107

0.13593	13.95973

0.13593	13.96387

0.13594	13.96667

0.13594	13.9652

0.13596	13.96933

0.13597	13.97213

0.13598	13.97493

0.13598	13.98053

0.13599	13.986

0.13601	13.98053

0.136	13.98053

0.13602	13.98053

0.13605	13.98747

0.13606	13.99307

0.13606	13.9888

0.13608	13.9916

0.13609	13.99307

0.13609	13.9972

0.1361	14.00267

0.13614	14

0.13613	14.00693

0.13613	14.00547

0.13614	14.00827

0.13614	14.01107

0.13614	14.01107

0.13616	14.0152

0.13617	14.0152

0.13616	14.01667

0.13616	14.0208

0.13617	14.01667

0.13619	14.018

0.13621	14.01667

0.13623	14.02493

0.13624	14.02773

0.13623	14.02773

0.13626	14.02907

0.13627	14.02907

0.13626	14.03187

0.13629	14.03467

0.1363	14.04027

0.1363	14.04307

0.13633	14.0388

0.13634	14.04307

0.13635	14.0472

0.13634	14.04307

0.13637	14.04853

0.13637	14.0472

0.13637	14.05133

0.13639	14.05827

0.13639	14.05267

0.13638	14.05973

0.13638	14.05547

0.13638	14.05547

0.13637	14.05973

0.13639	14.0624

0.13639	14.0652

0.13639	14.0652

0.13641	14.06667

0.13643	14.06933

0.13642	14.07493

0.13643	14.0736

0.13646	14.08333

0.13645	14.08613

0.13646	14.08467

0.13649	14.09027

0.1365	14.08747

0.13651	14.1

0.13652	14.09853

0.13652	14.0944

0.13653	14.10693

0.13656	14.10133

0.13657	14.1152

0.13657	14.10973

0.13658	14.10693

0.1366	14.11667

0.13658	14.11667

0.13661	14.12773

0.13662	14.12213

0.13662	14.1208

0.13663	14.12773

0.13664	14.12213

0.13663	14.12773

0.13666	14.13187

0.13667	14.1264

0.13668	14.13613

0.13668	14.13333

0.13669	14.14573

0.13668	14.1444

0.13669	14.1388

0.1367	14.15413

0.13671	14.1444

0.13672	14.15693

0.13673	14.15547

0.13673	14.15133

0.13676	14.15827

0.13677	14.15827

0.13676	14.16947

0.13678	14.16947

0.1368	14.168

0.1368	14.1764

0.13682	14.17773

0.13681	14.18053

0.13681	14.18333

0.13681	14.18187

0.13683	14.1888

0.13685	14.1916

0.13687	14.1944

0.13687	14.1944

0.13688	14.19587

0.13689	14.19587

0.1369	14.19853

0.1369	14.20547

0.13691	14.2028

0.13692	14.20827

0.13692	14.2028

0.13694	14.20827

0.13695	14.20693

0.13696	14.21107

0.13698	14.21667

0.13698	14.20547

0.13699	14.21387

0.13699	14.20973

0.137	14.21667

0.13701	14.22493

0.13702	14.218

0.13702	14.21947

0.13703	14.21253

0.13703	14.21387

0.13704	14.2208

0.13705	14.2152

0.13706	14.2208

0.13706	14.21667

0.13708	14.218

0.13709	14.22773

0.1371	14.2236

0.13712	14.2236

0.13712	14.21947

0.13714	14.2208

0.13714	14.22493

0.13716	14.22213

0.13715	14.2264

0.13715	14.22773

0.13715	14.23187

0.13717	14.23613

0.13718	14.23467

0.13719	14.23613

0.1372	14.23467

0.13721	14.23747

0.13722	14.2416

0.13722	14.24027

0.13724	14.24587

0.13725	14.24307

0.13725	14.24307

0.13725	14.24853

0.13728	14.25

0.13728	14.2528

0.13729	14.25547

0.13728	14.25547

0.13728	14.25973

0.1373	14.25693

0.13731	14.25973

0.13732	14.26667

0.13734	14.26107

0.13733	14.268

0.13736	14.27213

0.13737	14.27213

0.13736	14.2764

0.13736	14.27773

0.13736	14.2764

0.13737	14.28467

0.13737	14.28613

0.13738	14.29307

0.13739	14.28747

0.13741	14.2916

0.13743	14.30413

0.13743	14.30133

0.13744	14.30827

0.13744	14.30827

0.13743	14.3208

0.13745	14.318

0.13747	14.318

0.13747	14.33053

0.13748	14.32773

0.13748	14.32773

0.13749	14.33613

0.13749	14.33053

0.13751	14.34027

0.13752	14.33893

0.13752	14.3416

0.13753	14.34853

0.13753	14.35

0.13754	14.35413

0.13755	14.3556

0.13757	14.35827

0.13758	14.3556

0.1376	14.35973

0.1376	14.35973

0.13761	14.36387

0.13762	14.36667

0.13763	14.36253

0.13765	14.37227

0.13767	14.37227

0.13768	14.3708

0.13768	14.37227

0.13769	14.3708

0.1377	14.37493

0.1377	14.3652

0.13771	14.36667

0.13771	14.3708

0.13773	14.37493

0.13772	14.36947

0.13771	14.36253

0.13772	14.36387

0.13773	14.36387

0.13773	14.36387

0.13775	14.36667

0.13775	14.36387

0.13776	14.36667

0.13777	14.37227

0.13777	14.36947

0.13778	14.37773

0.13779	14.37493

0.1378	14.37773

0.13781	14.3792

0.13782	14.38333

0.13783	14.38333

0.13784	14.38613

0.13784	14.3916

0.13785	14.38893

0.13786	14.39587

0.13787	14.3972

0.13786	14.4028

0.13787	14.3972

0.13788	14.4

0.1379	14.40973

0.13791	14.40827

0.13794	14.418

0.13796	14.41667

0.13796	14.41947

0.13796	14.4292

0.13797	14.42773

0.13799	14.43747

0.138	14.43613

0.138	14.43613

0.13801	14.4472

0.138	14.44587

0.13802	14.45693

0.13803	14.45413

0.13804	14.45973

0.13805	14.46253

0.13806	14.46253

0.13808	14.46533

0.13809	14.46947

0.1381	14.46667

0.13811	14.47227

0.1381	14.4736

0.13811	14.4736

0.1381	14.4736

0.13812	14.48053

0.13813	14.48053

0.13814	14.48467

0.13815	14.48467

0.13816	14.48333

0.13818	14.49027

0.13818	14.48053

0.13819	14.49307

0.13821	14.48333

0.13821	14.48893

0.13821	14.4916

0.13822	14.48613

0.13823	14.48747

0.13825	14.482

0.13825	14.48747

0.13828	14.4944

0.13827	14.4944

0.13828	14.4916

0.13828	14.48893

0.13828	14.4916

0.13829	14.49587

0.13829	14.49307

0.13831	14.4972

0.13833	14.4944

0.13834	14.49587

0.13831	14.50133

0.13831	14.4972

0.13834	14.49867

0.13835	14.49867

0.13837	14.50133

0.13837	14.5028

0.13838	14.50133

0.13838	14.5028

0.1384	14.5056

0.1384	14.50973

0.13842	14.50827

0.13843	14.51533

0.13844	14.51947

0.13844	14.53053

0.13845	14.52773

0.13846	14.5292

0.13846	14.54587

0.13847	14.5416

0.13846	14.55413

0.13848	14.55693

0.13848	14.55413

0.13849	14.56387

0.13851	14.56533

0.1385	14.56947

0.13851	14.56667

0.13851	14.56947

0.13854	14.5736

0.13855	14.57507

0.13857	14.57773

0.13859	14.58053

0.13859	14.582

0.13859	14.58613

0.13861	14.582

0.13859	14.58613

0.13861	14.58893

0.13862	14.58747

0.13863	14.58893

0.13864	14.59307

0.13864	14.59173

0.13865	14.59173

0.13866	14.6

0.13866	14.59587

0.13866	14.58893

0.13866	14.5944

0.13868	14.59867

0.13867	14.6056

0.1387	14.60413

0.13869	14.61533

0.13871	14.6056

0.13871	14.61107

0.13873	14.61387

0.13875	14.60973

0.13878	14.60133

0.13877	14.60693

0.13877	14.61667

0.13879	14.62227

0.13879	14.61533

0.13881	14.618

0.13882	14.61533

0.13883	14.61667

0.13883	14.6236

0.13885	14.61253

0.13886	14.61533

0.13889	14.61107

0.13887	14.618

0.13889	14.62227

0.13886	14.61947

0.13887	14.62773

0.13887	14.62227

0.13889	14.6264

0.13889	14.632

0.13888	14.62773

0.1389	14.63053

0.13891	14.62773

0.13893	14.6292

0.13892	14.632

0.13894	14.6292

0.13894	14.63467

0.13896	14.63747

0.13895	14.63747

0.13897	14.64027

0.13895	14.64587

0.13899	14.6472

0.13898	14.6584

0.139	14.65693

0.139	14.6584

0.13901	14.66947

0.13901	14.6736

0.13904	14.682

0.13902	14.6848

0.13904	14.6792

0.13903	14.69027

0.13905	14.68893

0.13905	14.69867

0.13907	14.69867

0.13909	14.70413

0.13909	14.70973

0.1391	14.70413

0.1391	14.7084

0.13912	14.70973

0.13912	14.71387

0.13913	14.71107

0.13913	14.71107

0.13916	14.7084

0.13916	14.70973

0.13917	14.70413

0.13917	14.7056

0.13918	14.71387

0.13919	14.7056

0.13922	14.71107

0.13921	14.71253

0.13924	14.70413

0.13923	14.70693

0.13925	14.70147

0.13925	14.7028

0.13926	14.7084

0.13925	14.70973

0.13927	14.70973

0.13926	14.71107

0.13931	14.71253

0.13928	14.71387

0.13931	14.7208

0.13929	14.7208

0.1393	14.73053

0.1393	14.7264

0.13931	14.72507

0.13931	14.732

0.13933	14.732

0.13932	14.74307

0.13936	14.74867

0.13935	14.7444

0.13937	14.75693

0.13935	14.7556

0.13938	14.76253

0.13938	14.76387

0.13941	14.76253

0.13939	14.7736

0.13941	14.77773

0.1394	14.782

0.13941	14.78333

0.13941	14.78613

0.13944	14.79027

0.13943	14.78893

0.13945	14.79453

0.13945	14.79453

0.13947	14.79867

0.13946	14.80147

0.1395	14.80413

0.13951	14.8

0.13953	14.8056

0.13951	14.8084

0.13952	14.8056

0.1395	14.81253

0.13952	14.8056

0.13953	14.80693

0.13955	14.80693

0.13954	14.80413

0.13954	14.79587

0.13957	14.80147

0.1396	14.80413

0.1396	14.81253

0.1396	14.80693

0.1396	14.8112

0.13961	14.8028

0.13963	14.80413

0.13964	14.8084

0.13965	14.8028

0.13965	14.80693

0.13968	14.8056

0.13968	14.80973

0.13969	14.80973

0.13969	14.80973

0.13971	14.81813

0.1397	14.81253

0.13973	14.81667

0.1397	14.81813

0.13975	14.8292

0.13973	14.8292

0.13974	14.84173

0.13973	14.84173

0.13976	14.84027

0.13978	14.85147

0.1398	14.85

0.1398	14.86253

0.13978	14.85973

0.13979	14.8612

0.13981	14.86947

0.13984	14.87227

0.13984	14.8792

0.13986	14.87507

0.13984	14.88053

0.13987	14.88053

0.13986	14.8848

0.13989	14.88613

0.13988	14.88893

0.13991	14.89027

0.13991	14.88747

0.13994	14.88747

0.13995	14.8848

0.13995	14.88747

0.13995	14.89027

0.13993	14.882

0.13996	14.882

0.13995	14.88053

0.14	14.882

0.13999	14.88893

0.14001	14.87787

0.14	14.88333

0.14003	14.88333

0.14003	14.88333

0.14004	14.8792

0.14007	14.8848

0.14008	14.88893

0.14009	14.88893

0.14007	14.89027

0.14009	14.89027

0.1401	14.89867

0.14012	14.89867

0.14012	14.9

0.14013	14.9028

0.14013	14.9028

0.14016	14.91387

0.14017	14.91253

0.14016	14.9264

0.14018	14.93053

0.14017	14.93333

0.1402	14.94867

0.14019	14.9472

0.14022	14.9556

0.14022	14.9556

0.14025	14.9556

0.14024	14.95427

0.14024	14.95693

0.14026	14.95147

0.14025	14.95427

0.14028	14.95973

0.14027	14.95427

0.1403	14.95973

0.14032	14.9556

0.14033	14.95973

0.14033	14.9584

0.14032	14.95693

0.14034	14.9472

0.14034	14.95147

0.14037	14.95427

0.14035	14.95973

0.14037	14.95427

0.14037	14.95693

0.14038	14.9528

0.14041	14.9556

0.1404	14.9612

0.14044	14.9584

0.14044	14.96253

0.14045	14.97093

0.14047	14.97227

0.14048	14.97227

0.14048	14.9848

0.14048	14.9972

0.1405	14.99587

0.14051	15

0.14054	15.00147

0.14055	15.00973

0.14054	15.00973

0.14056	15.01947

0.14054	15.02227

0.14057	15.02507

0.14057	15.02507

0.14057	15.01667

0.14059	15.02093

0.14059	15.02093

0.1406	15.0292

0.1406	15.01813

0.14062	15.0236

0.14062	15.01387

0.14065	15.01813

0.14065	15.01947

0.14065	15.01533

0.14065	15.0056

0.14064	15.00973

0.14067	15.0112

0.14067	15.01387

0.14069	15.01947

0.14071	15.01667

0.1407	15.0264

0.14071	15.03067

0.14067	15.02507

0.14068	15.03613

0.14071	15.03333

0.14073	15.04587

0.14075	15.04307

0.14074	15.04307

0.14077	15.0528

0.14077	15.05147

0.14077	15.06533

0.14079	15.06533

0.14078	15.06947

0.14081	15.07787

0.14082	15.08333

0.14082	15.08613

0.14084	15.08893

0.14083	15.0876

0.14085	15.0876

0.14086	15.0848

0.14086	15.0764

0.1409	15.0848

0.1409	15.08613

0.14093	15.08067

0.14092	15.0848

0.14092	15.08067

0.14094	15.0848

0.14093	15.09027

0.14094	15.08333

0.14096	15.082

0.14095	15.0848

0.14098	15.08613

0.14098	15.0792

0.14099	15.08333

0.14099	15.0876

0.14099	15.08613

0.14101	15.08613

0.14101	15.08613

0.14103	15.0876

0.14105	15.09027

0.14104	15.09307

0.14107	15.09867

0.14106	15.10147

0.14106	15.1112

0.14107	15.10973

0.14106	15.1236

0.1411	15.1264

0.1411	15.13067

0.14111	15.14173

0.14113	15.14733

0.14114	15.15

0.14116	15.15

0.14114	15.15427

0.14115	15.1528

0.14118	15.1584

0.14117	15.1556

0.14119	15.15427

0.14121	15.15707

0.14121	15.15

0.14125	15.15147

0.14123	15.14453

0.14124	15.14587

0.14126	15.15147

0.14127	15.14307

0.14129	15.14587

0.1413	15.14173

0.14128	15.14307

0.14129	15.14587

0.14128	15.14587

0.1413	15.14733

0.14134	15.1556

0.14134	15.15973

0.14136	15.16253

0.14135	15.17373

0.14134	15.17507

0.14136	15.1904

0.14138	15.19307

0.1414	15.19867

0.14142	15.2

0.1414	15.20707

0.14142	15.2

0.14142	15.20427

0.14142	15.20147

0.14144	15.19587

0.14143	15.19867

0.14146	15.19307

0.14148	15.19733

0.14148	15.19867

0.14151	15.19307

0.14153	15.19587

0.14151	15.19307

0.14152	15.19453

0.14154	15.19587

0.14154	15.19307

0.14155	15.19453

0.14156	15.19733

0.14156	15.19587

0.14158	15.20147

0.14157	15.21667

0.14158	15.21947

0.14159	15.232

0.14159	15.23613

0.14161	15.24453

0.14162	15.24867

0.14163	15.24733

0.14165	15.24867

0.14166	15.25707

0.14167	15.25707

0.14167	15.25707

0.14168	15.2556

0.14169	15.25

0.14171	15.24867

0.14171	15.25147

0.14172	15.24173

0.14173	15.24307

0.14173	15.23893

0.14171	15.24173

0.14173	15.24307

0.14174	15.24173

0.14176	15.24453

0.14177	15.24733

0.14178	15.24587

0.14179	15.25

0.1418	15.26533

0.14181	15.2668

0.14182	15.28067

0.14183	15.28067

0.14183	15.28893

0.14184	15.29307

0.14183	15.29453

0.14184	15.3028

0.14185	15.30013

0.14185	15.3056

0.14186	15.3028

0.14187	15.30013

0.14188	15.3028

0.14186	15.30427

0.14187	15.30707

0.14188	15.3028

0.14188	15.3084

0.14191	15.29733

0.14191	15.29867

0.14193	15.30147

0.14194	15.29587

0.14195	15.29733

0.14196	15.29173

0.14197	15.29173

0.14197	15.29733

0.14199	15.29453

0.14201	15.29733

0.14201	15.30147

0.14201	15.30707

0.14202	15.30973

0.14203	15.31947

0.14202	15.31947

0.14204	15.33347

0.14203	15.3348

0.14205	15.33347

0.14205	15.34587

0.14206	15.34587

0.14207	15.3556

0.14209	15.35973

0.1421	15.3556

0.14209	15.3612

0.1421	15.3584

0.1421	15.3528

0.14211	15.3556

0.14212	15.3556

0.14214	15.34307

0.14213	15.34453

0.14215	15.3376

0.14215	15.33893

0.14216	15.34173

0.14218	15.3404

0.14219	15.34587

0.14219	15.35147

0.1422	15.3556

0.14222	15.3584

0.14222	15.37093

0.14224	15.37373

0.14225	15.39173

0.14226	15.39173

0.14226	15.3932

0.14226	15.39867

0.14226	15.4028

0.14227	15.39867

0.1423	15.4056

0.14231	15.4028

0.14233	15.3904

0.14233	15.38347

0.14233	15.3848

0.14234	15.3848

0.14234	15.38893

0.14237	15.382

0.14236	15.3848

0.14238	15.3848

0.14238	15.38347

0.1424	15.38347

0.1424	15.3904

0.14239	15.39453

0.1424	15.4056

0.14238	15.4028

0.1424	15.4084

0.14241	15.42227

0.14241	15.43347

0.14242	15.43347

0.14243	15.4376

0.14246	15.44587

0.14248	15.44733

0.1425	15.45013

0.14249	15.44867

0.1425	15.4556

0.14252	15.45427

0.14253	15.4528

0.14254	15.4556

0.14254	15.44587

0.14257	15.44453

0.14256	15.45013

0.14257	15.43613

0.14258	15.4376

0.1426	15.43067

0.14259	15.432

0.14259	15.4348

0.1426	15.43893

0.14262	15.45147

0.14264	15.45707

0.14265	15.4584

0.14266	15.4584

0.14266	15.47093

0.14266	15.47373

0.14266	15.48347

0.14267	15.4876

0.14266	15.48893

0.14268	15.49453

0.1427	15.4932

0.14271	15.4904

0.14271	15.48613

0.14271	15.4876

0.14274	15.4792

0.14275	15.47227

0.14278	15.47227

0.14279	15.47227

0.14279	15.47373

0.14279	15.47507

0.14279	15.47507

0.14279	15.48067

0.14281	15.48613

0.14282	15.4848

0.14283	15.49867

0.14284	15.51813

0.14283	15.5168

0.14285	15.5168

0.14284	15.52093

0.14285	15.532

0.14284	15.5292

0.14284	15.5376

0.14285	15.53347

0.14286	15.5432

0.14288	15.52787

0.14291	15.5292

0.14291	15.51813

0.14292	15.52093

0.14292	15.52093

0.14293	15.5168

0.14293	15.5196

0.14293	15.51253

0.14295	15.51533

0.14297	15.51533

0.14299	15.52373

0.14299	15.52373

0.143	15.5348

0.143	15.53893

0.143	15.53627

0.143	15.55147

0.14304	15.55147

0.14303	15.5668

0.14303	15.57227

0.14305	15.57507

0.14306	15.5792

0.14309	15.58347

0.14309	15.57373

0.1431	15.57507

0.1431	15.58067

0.14312	15.5696

0.14312	15.5696

0.14313	15.55987

0.14316	15.5668

0.14316	15.56813

0.14317	15.56253

0.14317	15.56533

0.14316	15.56253

0.14316	15.564

0.14319	15.56533

0.14319	15.5696

0.1432	15.57093

0.14321	15.57787

0.14322	15.58067

0.14323	15.57787

0.14322	15.5932

0.14321	15.5932

0.14322	15.60987

0.14324	15.61253

0.14323	15.6112

0.14324	15.62093

0.14323	15.62227

0.14324	15.62933

0.14326	15.62653

0.14326	15.63067

0.14328	15.62653

0.14329	15.63067

0.14331	15.614

0.14331	15.61533

0.14331	15.60707

0.14334	15.60987

0.14334	15.614

0.14335	15.60707

0.14338	15.6084

0.14338	15.6112

0.14339	15.61253

0.14339	15.61253

0.14341	15.62653

0.14342	15.62653

0.14343	15.6376

0.14344	15.65147

0.14343	15.65427

0.14344	15.66533

0.14345	15.66813

0.14347	15.67373

0.14349	15.67093

0.1435	15.67093

0.14351	15.6668

0.14351	15.6668

0.14352	15.65707

0.14354	15.65707

0.14354	15.6556

0.14354	15.65293

0.14354	15.65147

0.14355	15.65293

0.14356	15.64867

0.14356	15.65147

0.14356	15.65707

0.14357	15.6584

0.14361	15.65987

0.14361	15.66533

0.14362	15.67653

0.14362	15.67507

0.14363	15.67787

0.14363	15.682

0.14363	15.69733

0.14363	15.69733

0.14364	15.7112

0.14364	15.71267

0.14363	15.714

0.14363	15.71533

0.14365	15.7168

0.14366	15.70707

0.14368	15.71533

0.14368	15.7196

0.14369	15.70147

0.1437	15.70707

0.14372	15.69867

0.14372	15.70147

0.14373	15.70707

0.14375	15.70013

0.14378	15.70293

0.14379	15.70293

0.14378	15.70707

0.14379	15.70293

0.1438	15.70987

0.14383	15.71267

0.14383	15.72507

0.14384	15.72653

0.14386	15.72787

0.14389	15.7432

0.1439	15.75707

0.1439	15.75427

0.14389	15.75987

0.14391	15.75987

0.14392	15.76813

0.14393	15.76813

0.14392	15.76267

0.14392	15.7584

0.14394	15.74867

0.14397	15.75013

0.14397	15.74453

0.14396	15.74173

0.14398	15.7432

0.14401	15.74453

0.14402	15.74173

0.14403	15.74453

0.14403	15.75013

0.14404	15.75293

0.14404	15.75573

0.14404	15.7668

0.14401	15.7696

0.14404	15.7848

0.14404	15.78627

0.14406	15.78627

0.14406	15.80013

0.14406	15.79867

0.14406	15.8084

0.14409	15.80707

0.14411	15.8084

0.14412	15.80427

0.1441	15.80707

0.14411	15.79453

0.14413	15.79867

0.14415	15.80147

0.14416	15.7932

0.14417	15.79733

0.1442	15.7904

0.14421	15.7932

0.14423	15.7932

0.14424	15.78907

0.14424	15.79173

0.14424	15.79867

0.14427	15.80427

0.14427	15.814

0.1443	15.81813

0.14429	15.8168

0.1443	15.832

0.14431	15.83347

0.14435	15.83347

0.14436	15.85147

0.14436	15.84867

0.14437	15.85293

0.14435	15.85013

0.14437	15.85427

0.14436	15.84733

0.14437	15.85293

0.14438	15.8376

0.14437	15.83907

0.1444	15.83907

0.14441	15.832

0.14442	15.83347

0.14444	15.83067

0.14443	15.8348

0.14445	15.8348

0.14445	15.8404

0.14447	15.84867

0.14449	15.85147

0.14448	15.85573

0.14448	15.8584

0.1445	15.87653

0.1445	15.8876

0.14453	15.8904

0.14454	15.89173

0.14454	15.896

0.14455	15.89453

0.14457	15.8904

0.14458	15.89173

0.1446	15.8904

0.1446	15.8932

0.1446	15.88067

0.14459	15.88627

0.14461	15.87933

0.14464	15.88347

0.14465	15.88627

0.14466	15.87787

0.14467	15.87787

0.14467	15.87787

0.14468	15.88067

0.1447	15.88067

0.14472	15.8876

0.14472	15.89173

0.14472	15.90293

0.14471	15.90293

0.14473	15.9084

0.14475	15.92093

0.14476	15.92653

0.14476	15.9376

0.14475	15.93347

0.14475	15.93907

0.14475	15.93907

0.14478	15.9432

0.14479	15.93347

0.14483	15.93627

0.14483	15.9348

0.14485	15.92507

0.14486	15.92653

0.14487	15.92093

0.14488	15.92093

0.14488	15.92507

0.14489	15.92093

0.1449	15.92507

0.14489	15.92933

0.1449	15.92933

0.14494	15.93347

0.14495	15.94733

0.14498	15.946

0.14499	15.964

0.14499	15.96547

0.14497	15.96547

0.14497	15.97787

0.14498	15.9848

0.145	15.9848

0.14502	15.98347

0.14501	15.98907

0.14503	15.99187

0.14501	15.98907

0.14502	15.98067

0.14502	15.9848

0.14505	15.9904

0.14506	15.97933

0.14507	15.97373

0.14508	15.96813

0.14509	15.9668

0.14509	15.97373

0.14509	15.9696

0.14511	15.97093

0.14511	15.97373

0.14513	15.97373

0.14514	15.97653

0.14515	15.98347

0.14515	16.00013

0.14515	15.9988

0.14516	16.00293

0.14519	16.00707

0.1452	16.02093

0.1452	16.02373

0.1452	16.02787

0.1452	16.0348

0.14521	16.03213

0.14521	16.0376

0.14521	16.02933

0.14524	16.02933

0.14526	16.02653

0.14525	16.02787

0.14528	16.01813

0.14528	16.0196

0.1453	16.0112

0.14531	16.0112

0.14532	16.014

0.14533	16.01547

0.14531	16.03067

0.14533	16.03347

0.14532	16.03907

0.14531	16.0348

0.14532	16.05013

0.14534	16.05573

0.14536	16.06267

0.14538	16.06813

0.14538	16.06267

0.14538	16.07373

0.14538	16.07373

0.14539	16.08067

0.1454	16.08213

0.14541	16.07653

0.14544	16.08347

0.14545	16.07093

0.14547	16.07373

0.14548	16.07373

0.14547	16.07787

0.14549	16.06267

0.14549	16.0668

0.14551	16.05987

0.14552	16.0612

0.14554	16.06267

0.14555	16.0612

0.14555	16.0696

0.14556	16.0724

0.14558	16.0752

0.14557	16.07093

0.14558	16.0848

0.14558	16.0848

0.14558	16.10293

0.14561	16.1112

0.14561	16.10987

0.14562	16.1252

0.14564	16.12373

0.14565	16.1224

0.14566	16.12373

0.14565	16.12933

0.14566	16.11827

0.14567	16.1252

0.14568	16.11827

0.14568	16.11827

0.1457	16.11827

0.14572	16.10853

0.14573	16.1016

0.14575	16.10573

0.14576	16.10573

0.14574	16.10573

0.14576	16.114

0.14576	16.12787

0.14576	16.12787

0.14577	16.13213

0.14579	16.13493

0.14579	16.1516

0.1458	16.1612

0.14582	16.164

0.14582	16.15987

0.14584	16.16547

0.14583	16.1668

0.14584	16.1696

0.14583	16.15573

0.14583	16.15987

0.14584	16.15853

0.14585	16.146

0.14588	16.15013

0.14589	16.14187

0.14591	16.14453

0.14593	16.14733

0.14595	16.14733

0.14595	16.1488

0.14596	16.15987

0.14595	16.16267

0.14595	16.1668

0.14596	16.18907

0.14598	16.18627

0.14599	16.2016

0.14601	16.20013

0.14603	16.20427

0.14603	16.20573

0.14602	16.20853

0.14603	16.19733

0.14605	16.20427

0.14605	16.2016

0.14606	16.18907

0.14607	16.18067

0.14608	16.18493

0.14608	16.1876

0.14609	16.18627

0.1461	16.18347

0.14612	16.18493

0.14615	16.19453

0.14617	16.19453

0.14616	16.19453

0.14619	16.20987

0.14618	16.2252

0.14618	16.228

0.14616	16.23067

0.14617	16.23347

0.14618	16.246

0.1462	16.24187

0.1462	16.25013

0.14623	16.24467

0.14623	16.25013

0.14623	16.24467

0.14625	16.24733

0.14626	16.2404

0.14628	16.24187

0.14628	16.2404

0.1463	16.23213

0.14632	16.22653

0.14633	16.22653

0.14632	16.22933

0.14634	16.23067

0.14635	16.23213

0.14635	16.23493

0.14635	16.2404

0.14636	16.24187

0.14638	16.24187

0.14639	16.25293

0.1464	16.2696

0.14641	16.27373

0.14641	16.27933

0.14643	16.28067

0.14643	16.28907

0.14642	16.29187

0.14643	16.2876

0.14644	16.296

0.14645	16.296

0.14647	16.28627

0.14647	16.2724

0.14647	16.2696

0.14647	16.2696

0.14649	16.27373

0.14651	16.26827

0.14651	16.2696

0.14651	16.2752

0.14653	16.2752

0.14654	16.27653

0.14655	16.2904

0.14657	16.29733

0.14656	16.31547

0.14659	16.32093

0.14659	16.32093

0.14662	16.33067

0.14663	16.32373

0.14664	16.3252

0.14664	16.32373

0.14664	16.3224

0.14664	16.31267

0.14665	16.31267

0.14668	16.30573

0.14669	16.30573

0.1467	16.30707

0.1467	16.30707

0.14673	16.31827

0.14674	16.3168

0.14676	16.3224

0.14678	16.3252

0.14678	16.33773

0.14677	16.33627

0.14677	16.3544

0.14678	16.35707

0.1468	16.35853

0.14681	16.3668

0.14681	16.36267

0.14681	16.364

0.14682	16.36133

0.14683	16.36547

0.14683	16.3544

0.14685	16.34467

0.14685	16.34187

0.14686	16.34733

0.14688	16.34733

0.14689	16.34467

0.1469	16.346

0.1469	16.35293

0.14691	16.35987

0.14693	16.364

0.14694	16.38627

0.14694	16.39733

0.14696	16.396

0.14697	16.39733

0.14699	16.39733

0.14699	16.40013

0.14701	16.3932

0.14702	16.396

0.14702	16.3932

0.14703	16.3988

0.14705	16.38493

0.14705	16.3752

0.14705	16.37653

0.14706	16.37653

0.14707	16.378

0.14708	16.38067

0.14709	16.39187

0.14708	16.3904

0.14709	16.39467

0.1471	16.40853

0.1471	16.41133

0.14712	16.42653

0.14713	16.43213

0.14716	16.43347

0.14717	16.43493

0.14717	16.4404

0.14719	16.43347

0.14718	16.43067

0.14719	16.42933

0.14719	16.428

0.14719	16.41827

0.1472	16.41827

0.14721	16.414

0.14722	16.414

0.14722	16.41547

0.14723	16.42107

0.14724	16.43627

0.14724	16.4404

0.14725	16.44747

0.14726	16.45573

0.14726	16.4668

0.14727	16.46827

0.14728	16.47373

0.14728	16.47373

0.1473	16.47653

0.14732	16.46413

0.14732	16.44747

0.14733	16.45013

0.14733	16.4516

0.14734	16.4516

0.14735	16.446

0.14736	16.4488

0.14737	16.4488

0.14737	16.45293

0.14738	16.4696

0.14739	16.4696

0.1474	16.47653

0.14742	16.49467

0.1474	16.496

0.14742	16.50013

0.14741	16.50707

0.14743	16.4988

0.14744	16.50013

0.14743	16.49747

0.14746	16.4988

0.14745	16.48627

0.14747	16.4752

0.14747	16.47653

0.14748	16.47933

0.14747	16.4808

0.14747	16.48627

0.14747	16.5044

0.14749	16.50707

0.1475	16.50853

0.14751	16.50987

0.14752	16.52933

0.14753	16.5404

0.14753	16.53627

0.14755	16.53907

0.14755	16.5404

0.14757	16.5404

0.14758	16.5224

0.1476	16.5252

0.14761	16.5252

0.14761	16.5252

0.14763	16.5168

0.14762	16.50987

0.14764	16.51413

0.14764	16.51827

0.14765	16.5196

0.14766	16.52653

0.14767	16.53907

0.14767	16.54467

0.14767	16.5516

0.14768	16.55987

0.14768	16.57107

0.1477	16.5696

0.14771	16.5668

0.14772	16.57107

0.14774	16.56827

0.14775	16.55573

0.14777	16.54467

0.14777	16.54467

0.14777	16.546

0.14776	16.55013

0.14777	16.5516

0.14779	16.55987

0.14779	16.56547

0.1478	16.5724

0.1478	16.578

0.14778	16.59187

0.14781	16.6016

0.14782	16.6044

0.14782	16.6016

0.14784	16.60573

0.14783	16.5988

0.14784	16.596

0.14785	16.5932

0.14785	16.5932

0.14785	16.58907

0.14785	16.58213

0.14786	16.57933

0.14787	16.57933

0.14788	16.57933

0.14789	16.58347

0.1479	16.58907

0.14791	16.6016

0.14793	16.60293

0.14794	16.6044

0.14794	16.60987

0.14796	16.62653

0.14796	16.64053

0.14797	16.64467

0.14799	16.64187

0.14801	16.6488

0.14799	16.64053

0.14799	16.6308

0.148	16.62653

0.148	16.63213

0.14802	16.62387

0.14805	16.61547

0.14805	16.61267

0.14806	16.61827

0.14804	16.62387

0.14806	16.628

0.14807	16.64467

0.14808	16.66133

0.14808	16.66267

0.14809	16.66413

0.1481	16.66267

0.14811	16.6752

0.14813	16.67107

0.14814	16.6752

0.14814	16.6696

0.14816	16.67933

0.14816	16.65987

0.14817	16.65013

0.14817	16.6488

0.14817	16.6516

0.14818	16.65293

0.14819	16.6516

0.14819	16.65853

0.1482	16.65987

0.14821	16.66133

0.14821	16.66547

0.14823	16.69053

0.14824	16.70573

0.14825	16.7072

0.14824	16.70987

0.14825	16.70987

0.14825	16.70293

0.14827	16.69187

0.14829	16.696

0.1483	16.69467

0.14831	16.696

0.14831	16.68213

0.14833	16.678

0.14833	16.68213

0.14833	16.6836

0.14834	16.68627

0.14836	16.6988

0.14838	16.71547

0.14838	16.71693

0.14837	16.7252

0.14837	16.728

0.14838	16.73907

0.14838	16.73627

0.14839	16.73627

0.14841	16.73773

0.14842	16.74053

0.14842	16.72387

0.14843	16.71267

0.14844	16.71413

0.14843	16.71413

0.14846	16.71267

0.14846	16.71547

0.14847	16.7224

0.14849	16.728

0.14849	16.72653

0.1485	16.73213

0.14849	16.75293

0.14849	16.7724

0.14849	16.7696

0.14849	16.77387

0.1485	16.77387

0.14851	16.778

0.14854	16.76547

0.14854	16.76693

0.14856	16.7696

0.14856	16.7724

0.14858	16.76267

0.14856	16.7488

0.14858	16.7544

0.14858	16.7572

0.14858	16.75987

0.1486	16.7516

0.14862	16.75987

0.1486	16.76267

0.1486	16.76267

0.14862	16.77653

0.14863	16.7808

0.14865	16.80027

0.14866	16.80293

0.14868	16.8016

0.14868	16.8072

0.14868	16.81827

0.14869	16.81693

0.14869	16.82107

0.1487	16.8224

0.14872	16.8072

0.14873	16.8072

0.14874	16.80573

0.14873	16.7932

0.14874	16.79467

0.14874	16.78907

0.14878	16.78907

0.14877	16.79467

0.14877	16.80027

0.14877	16.8044

0.14878	16.80853

0.14879	16.82667

0.1488	16.84187

0.14883	16.84333

0.14884	16.84467

0.14884	16.85293

0.14882	16.85293

0.14881	16.84467

0.1488	16.84053

0.14881	16.84053

0.14883	16.84053

0.14885	16.82667

0.14886	16.8224

0.14887	16.82387

0.14886	16.8224

0.14886	16.82933

0.14887	16.83213

0.14887	16.85027

0.14888	16.8544

0.14888	16.85853

0.1489	16.86413

0.14892	16.8752

0.14893	16.88493

0.14893	16.88213

0.14895	16.88213

0.14896	16.8752

0.14897	16.878

0.14896	16.86133

0.14897	16.86133

0.14898	16.86267

0.14898	16.86267

0.14901	16.85573

0.14903	16.86267

0.14905	16.86693

0.14905	16.86547

0.14906	16.87387

0.14904	16.89333

0.14906	16.90853

0.14904	16.91133

0.14905	16.91413

0.14904	16.91827

0.14906	16.91547

0.14907	16.90573

0.14908	16.90573

0.14908	16.90853

0.14907	16.9072

0.14907	16.90027

0.14908	16.88907

0.14908	16.89053

0.14911	16.89053

0.14912	16.89187

0.14915	16.9016

0.14913	16.91413

0.14913	16.91827

0.14914	16.9252

0.14915	16.928

0.14917	16.946

0.14919	16.946

0.1492	16.95573

0.14921	16.9488

0.14922	16.94053

0.14923	16.94187

0.14924	16.92933

0.14925	16.928

0.14927	16.928

0.14927	16.9252

0.14929	16.91827

0.14928	16.93213

0.14929	16.9336

0.14929	16.94187

0.1493	16.96

0.14931	16.96133

0.14933	16.97667

0.14933	16.97667

0.14933	16.98213

0.14933	16.9808

0.14933	16.9836

0.14932	16.96693

0.14933	16.97387

0.14935	16.96973

0.14935	16.9752

0.14939	16.96133

0.1494	16.95307

0.14942	16.9544

0.14943	16.95573

0.14944	16.96

0.14943	16.96133

0.14944	16.97667

0.14945	16.97667

0.14947	16.98213

0.14946	16.9864

0.14946	17.00307

0.14945	17.01267

0.14948	17.01693

0.14949	17.02107

0.14951	17.01693

0.14953	17.01693

0.14953	17.0016

0.14954	17.0016

0.14954	17.00027

0.14955	17.0016

0.14955	16.98907

0.14957	16.9864

0.14957	16.98907

0.14959	16.99187

0.1496	16.99467

0.14961	17.00573

0.14962	17.0252

0.14962	17.0308

0.14963	17.03907

0.14963	17.04187

0.14965	17.0516

0.14964	17.04467

0.14964	17.04467

0.14965	17.04333

0.14966	17.04187

0.14969	17.02387

0.14969	17.01827

0.14969	17.01827

0.1497	17.02107

0.14972	17.0252

0.14971	17.03493

0.14973	17.0572

0.14974	17.06547

0.14975	17.06693

0.14975	17.0724

0.14975	17.0752

0.14976	17.078

0.14977	17.0808

0.14978	17.0808

0.1498	17.07947

0.1498	17.07107

0.14978	17.05853

0.14979	17.0572

0.1498	17.05853

0.1498	17.05573

0.14986	17.05307

0.14986	17.06693

0.14986	17.06827

0.14986	17.07107

0.14984	17.078

0.14983	17.1016

0.14984	17.10573

0.14985	17.11133

0.14986	17.1128

0.14985	17.11693

0.14984	17.1072

0.14988	17.1016

0.14989	17.0988

0.1499	17.1016

0.14989	17.10307

0.14988	17.09333

0.14989	17.0836

0.14992	17.08493

0.14993	17.08907

0.14994	17.08773

0.14994	17.10027

0.14994	17.11827

0.14995	17.1252

0.14997	17.128

0.14998	17.13213

0.15	17.14333

0.14999	17.14187

0.14999	17.14467

0.15	17.14187

0.15	17.14747

0.15002	17.128

0.15002	17.11973

0.15003	17.11693

0.15003	17.11693

0.15002	17.11827

0.15004	17.11827

0.15005	17.12667

0.15006	17.12667

0.15006	17.1364

0.15008	17.14053

0.15009	17.15587

0.15013	17.16973

0.15014	17.17253

0.15015	17.1752

0.15016	17.178

0.15015	17.1808

0.15018	17.1628

0.15018	17.16827

0.15019	17.16413

0.1502	17.16547

0.15019	17.15587

0.15021	17.1488

0.15022	17.15027

0.15023	17.1488

0.15024	17.1516

0.15023	17.16413

0.15026	17.1864

0.15028	17.18773

0.15029	17.1892

0.15028	17.19333

0.15026	17.20853

0.15029	17.21413

0.1503	17.2128

0.15032	17.21

0.15031	17.19613

0.15032	17.19333

0.15033	17.18213

0.15034	17.18493

0.15035	17.18493

0.15034	17.1864

0.15036	17.18213

0.15036	17.19053

0.15039	17.19187

0.1504	17.1988

0.15041	17.2016

0.15041	17.21973

0.1504	17.2392

0.15042	17.2364

0.15044	17.24187

0.15045	17.24187

0.15045	17.2488

0.15045	17.2364

0.15047	17.23213

0.15049	17.228

0.15051	17.2308

0.1505	17.21827

0.15051	17.21693

0.15052	17.21547

0.15053	17.21547

0.15053	17.21827

0.15052	17.22947

0.15051	17.2516

0.15053	17.25853

0.15056	17.2628

0.15056	17.26413

0.15055	17.27387

0.15057	17.27667

0.15058	17.2752

0.1506	17.26547

0.15061	17.2752

0.15061	17.26547

0.15062	17.2516

0.15064	17.2544

0.15064	17.2516

0.15066	17.25307

0.15064	17.25027

0.15064	17.26133

0.15064	17.26693

0.15066	17.26973

0.15068	17.27667

0.15069	17.29333

0.15072	17.3044

0.15072	17.3072

0.15072	17.30853

0.15071	17.30853

0.15073	17.29613

0.15076	17.2864

0.15078	17.2808

0.15079	17.28227

0.15079	17.28227

0.15079	17.27667

0.15081	17.2836

0.15081	17.29053

0.15081	17.29333

0.15081	17.3016

0.15083	17.32387

0.15084	17.33227

0.15085	17.3364

0.15086	17.33773

0.15088	17.3364

0.15089	17.32107

0.15089	17.3044

0.15089	17.30587

0.15089	17.3044

0.15091	17.30853

0.15093	17.30307

0.15094	17.3072

0.15093	17.3156

0.15094	17.32387

0.15097	17.3364

0.15098	17.34613

0.15097	17.36133

0.15098	17.35853

0.15099	17.36693

0.15101	17.3656

0.15098	17.36693

0.15102	17.34747

0.15104	17.34747

0.15105	17.34747

0.15104	17.35027

0.15103	17.33773

0.15106	17.33493

0.15106	17.3364

0.15107	17.33773

0.15107	17.34467

0.15108	17.3516

0.15108	17.37107

0.15108	17.37947

0.15109	17.3836

0.15111	17.39613

0.15112	17.39893

0.15111	17.39613

0.15112	17.39187

0.15115	17.39333

0.15118	17.39333

0.1512	17.378

0.1512	17.36693

0.15122	17.36693

0.15121	17.3656

0.15123	17.36693

0.15122	17.37107

0.15127	17.38773

0.15128	17.39467

0.15128	17.39893

0.15128	17.4044

0.15127	17.42107

0.15128	17.42533

0.15128	17.42667

0.15128	17.42667

0.15129	17.42667

0.15131	17.4128

0.1513	17.4016

0.15132	17.39893

0.15132	17.39893

0.15134	17.40027

0.15135	17.39747

0.15137	17.4072

0.15137	17.4128

0.15139	17.41827

0.15139	17.41973

0.15138	17.44613

0.1514	17.4572

0.15141	17.46

0.15141	17.45867

0.15142	17.4516

0.15144	17.45027

0.15145	17.4392

0.15144	17.4364

0.15146	17.4308

0.15145	17.4308

0.15147	17.42667

0.15146	17.43227

0.15148	17.43493

0.1515	17.44333

0.1515	17.46973

0.1515	17.47533

0.15153	17.4864

0.15155	17.4836

0.15156	17.4808

0.15154	17.48773

0.15157	17.47387

0.15158	17.45867

0.1516	17.46133

0.15159	17.4572

0.15161	17.46

0.15161	17.45307

0.15162	17.45867

0.15165	17.4628

0.15166	17.46827

0.15168	17.47253

0.15169	17.49053

0.15172	17.51413

0.15173	17.5128

0.15171	17.51413

0.15171	17.51133

0.1517	17.5044

0.15169	17.4864

0.15171	17.48493

0.15173	17.4864

0.15175	17.49053

0.15175	17.4836

0.15176	17.4892

0.15176	17.49613

0.15177	17.492

0.15177	17.49613

0.15179	17.51133

0.15179	17.53227

0.15181	17.53507

0.15182	17.542

0.15184	17.54747

0.15184	17.54613

0.15186	17.5392

0.15186	17.53227

0.15187	17.53773

0.15188	17.53507

0.1519	17.52107

0.15192	17.5128

0.15193	17.5128

0.15193	17.5156

0.15193	17.51413

0.15194	17.528

0.15195	17.55027

0.15196	17.5544

0.15197	17.55587

0.15199	17.55867

0.15199	17.57533

0.152	17.578

0.15201	17.57533

0.15201	17.57533

0.15202	17.57667

0.15203	17.5628

0.15205	17.54613

0.15207	17.54467

0.15208	17.54613

0.15208	17.54893

0.15209	17.54747

0.15209	17.56413

0.15209	17.5684

0.15208	17.57533

0.15209	17.57667

0.1521	17.59893

0.15211	17.60867

0.15213	17.60587

0.15213	17.60307

0.15214	17.59747

0.15214	17.59747

0.15215	17.5892

0.15216	17.5864

0.15215	17.5836

0.15218	17.58507

0.15221	17.57387

0.15221	17.5808

0.15221	17.6072

0.1522	17.58773

0.15222	17.6072

0.15224	17.60867

0.15225	17.62667

0.15224	17.628

0.15226	17.63507

0.15229	17.6392

0.15231	17.6448

0.15231	17.63227

0.15232	17.63227

0.15231	17.62533

0.15231	17.62533

0.15234	17.61133

0.15234	17.6072

0.15235	17.61133

0.15235	17.61133

0.15236	17.6128

0.15238	17.628

0.1524	17.65027

0.15241	17.6572

0.15241	17.65867

0.15242	17.6628

0.15243	17.66693

0.15242	17.66413

0.15245	17.65867

0.15244	17.6572

0.15245	17.6544

0.15247	17.642

0.15248	17.6336

0.15248	17.6336

0.15249	17.63773

0.1525	17.6392

0.1525	17.65173

0.15251	17.67533

0.15251	17.67667

0.15252	17.67947

0.15252	17.6892

0.15253	17.69893

0.15254	17.68773

0.15256	17.6864

0.15256	17.6836

0.15255	17.68227

0.15255	17.6684

0.15258	17.66147

0.15261	17.66413

0.15262	17.66693

0.15262	17.67107

0.15261	17.68507

0.15264	17.70587

0.15265	17.71

0.15265	17.71693

0.15265	17.72253

0.15264	17.72813

0.15267	17.7184

0.15268	17.7156

0.15271	17.71

0.15272	17.70867

0.15274	17.6948

0.15275	17.6892

0.15276	17.69333

0.15277	17.6948

0.15278	17.7044

0.15278	17.7072

0.15279	17.7308

0.1528	17.73507

0.15281	17.73773

0.15282	17.74053

0.1528	17.75027

0.15283	17.75453

0.15283	17.75307

0.15285	17.75587

0.15286	17.75587

0.15287	17.7392

0.15287	17.72253

0.15285	17.72387

0.15285	17.7184

0.15285	17.71973

0.15288	17.7308

0.1529	17.74893

0.1529	17.75307

0.15292	17.75867

0.15293	17.7656

0.15295	17.77813

0.15296	17.78227

0.15297	17.78227

0.15298	17.7808

0.15298	17.7808

0.153	17.75173

0.15301	17.75587

0.15303	17.75453

0.15305	17.75173

0.15307	17.75307

0.15306	17.75587

0.15306	17.7684

0.15308	17.76973

0.15309	17.77387

0.15311	17.77667

0.15309	17.79747

0.15309	17.81

0.15309	17.81147

0.1531	17.8128

0.15311	17.81973

0.15312	17.80587

0.15313	17.79053

0.15313	17.78787

0.15313	17.78787

0.15314	17.7892

0.15316	17.78227

0.15318	17.78227

0.15322	17.7836

0.15322	17.7864

0.15324	17.792

0.15325	17.8128

0.15326	17.8336

0.15328	17.8364

0.15329	17.8392

0.15327	17.8392

0.15328	17.8448

0.15328	17.8308

0.15331	17.8392

0.15331	17.8308

0.15332	17.8184

0.15332	17.81693

0.1533	17.81147

0.15332	17.81413

0.15333	17.8128

0.15334	17.81693

0.15333	17.8308

0.15333	17.85307

0.15334	17.8572

0.15337	17.86413

0.15337	17.87387

0.15338	17.87667

0.15339	17.8712

0.15339	17.8712

0.1534	17.8712

0.15341	17.85307

0.15343	17.85173

0.15347	17.8392

0.15347	17.842

0.15348	17.842

0.15349	17.8448

0.15348	17.85453

0.15351	17.87387

0.15351	17.87813

0.15352	17.8864

0.15354	17.892

0.15354	17.90173

0.15357	17.90453

0.15357	17.89893

0.15359	17.90027

0.1536	17.8948

0.1536	17.8836

0.15362	17.87253

0.1536	17.8712

0.15364	17.86973

0.15364	17.88093

0.15364	17.88787

0.15366	17.9072

0.15365	17.91

0.15368	17.9184

0.15369	17.92533

0.15368	17.9336

0.15369	17.92667

0.15369	17.92947

0.15371	17.92667

0.15372	17.9156

0.15374	17.91

0.15374	17.89893

0.15375	17.89893

0.15376	17.90027

0.15379	17.90027

0.15377	17.90587

0.15378	17.9212

0.15379	17.92947

0.15381	17.94053

0.15382	17.94333

0.15382	17.96

0.15383	17.95587

0.15384	17.96427

0.15387	17.96

0.15388	17.96693

0.15389	17.94613

0.15389	17.92813

0.15388	17.92813

0.15391	17.92947

0.1539	17.92947

0.15392	17.93227

0.15392	17.95173

0.15394	17.9572

0.15397	17.9628

0.15395	17.98507

0.15394	17.9836

0.15391	17.992

0.15392	17.9836

0.15393	17.98507

0.15392	17.9864

0.15396	17.97387

0.15397	17.95587

0.154	17.95587

0.15399	17.95453

0.15402	17.95453

0.15403	17.96

0.15403	17.97813

0.15404	17.98227

0.15404	17.99067

0.15406	17.99893

0.15408	18.01147

0.1541	18.0184

0.1541	18.01147

0.1541	18.0156

0.15411	18.0128

0.15412	18.00173

0.15415	17.98787

0.15414	17.98787

0.15415	17.9892

0.15415	17.9836

0.15416	17.9892

0.15416	17.9976

0.15417	18.00307

0.15419	18.01147

0.15421	18.01973

0.1542	18.042

0.1542	18.04613

0.15422	18.0476

0.15424	18.05027

0.15426	18.0364

0.15427	18.02947

0.15426	18.01427

0.15427	18.0156

0.15427	18.01427

0.15427	18.0156

0.15429	18.01427

0.15431	18.0336

0.15433	18.03507

0.15431	18.0392

0.15432	18.04333

0.15433	18.06427

0.15435	18.0684

0.15434	18.0684

0.15435	18.0656

0.15438	18.0656

0.15439	18.04613

0.15441	18.03507

0.15441	18.03787

0.15443	18.03787

0.15444	18.04333

0.15445	18.05453

0.15444	18.07947

0.15447	18.0864

0.15449	18.08787

0.15448	18.09333

0.15451	18.0976

0.15452	18.09067

0.15452	18.0836

0.15453	18.0836

0.15454	18.08093

0.15454	18.06693

0.15455	18.06693

0.15454	18.06693

0.15456	18.0684

0.15454	18.08507

0.15456	18.0948

0.15458	18.11147

0.1546	18.1184

0.15461	18.124

0.1546	18.12667

0.15463	18.124

0.15462	18.10867

0.15463	18.11

0.15462	18.10733

0.15467	18.0948

0.15466	18.09067

0.1547	18.092

0.15469	18.09333

0.15469	18.10173

0.15472	18.10173

0.1547	18.1212

0.1547	18.142

0.15471	18.1448

0.15474	18.15173

0.15473	18.1504

0.15477	18.15867

0.15478	18.1392

0.15479	18.14067

0.15479	18.1364

0.15481	18.1364

0.1548	18.12253

0.15483	18.11973

0.15481	18.12253

0.15485	18.12253

0.15486	18.12947

0.15486	18.14613

0.15485	18.1684

0.15489	18.17533

0.15489	18.17947

0.15489	18.17667

0.1549	18.17813

0.15491	18.15867

0.15491	18.15173

0.15491	18.15587

0.15493	18.15307

0.15491	18.14613

0.15492	18.15307

0.15492	18.1628

0.15497	18.1684

0.15495	18.17533

0.15496	18.1948

0.15495	18.2004

0.15499	18.21

0.15498	18.20587

0.155	18.21

0.15501	18.18787

0.15504	18.17533

0.15503	18.174

0.15506	18.17253

0.15507	18.1712

0.15508	18.17667

0.15506	18.19613

0.1551	18.2004

0.1551	18.21

0.1551	18.21427

0.15509	18.23227

0.15511	18.22947

0.1551	18.22533

0.15512	18.22253

0.15512	18.20173

0.15514	18.19893

0.15515	18.1948

0.15515	18.1976

0.15517	18.2004

0.15516	18.2184

0.15516	18.22533

0.15519	18.24613

0.15518	18.25307

0.1552	18.2504

0.15521	18.25587

0.15522	18.25307

0.15521	18.242

0.15524	18.23507

0.15523	18.2364

0.15526	18.2212

0.15526	18.224

0.15529	18.224

0.15528	18.23373

0.15531	18.2364

0.15531	18.25733

0.15535	18.26973

0.15535	18.28227

0.15539	18.28507

0.15538	18.28373

0.15537	18.28787

0.15539	18.2712

0.15539	18.25733

0.15539	18.25453

0.15541	18.25453

0.15541	18.2476

0.15545	18.24893

0.15544	18.26973

0.15546	18.274

0.15544	18.28373

0.15545	18.2892

0.15544	18.30733

0.15547	18.30173

0.15547	18.29347

0.15549	18.2976

0.15549	18.3004

0.1555	18.2892

0.15554	18.274

0.15555	18.274

0.15556	18.274

0.15558	18.2768

0.15557	18.28227

0.15556	18.2948

0.15558	18.29893

0.15558	18.30307

0.1556	18.32947

0.1556	18.33093

0.15562	18.33507

0.1556	18.33227

0.15563	18.33093

0.15563	18.31973

0.15566	18.31707

0.15565	18.29893

0.15566	18.3004

0.15566	18.30453

0.15567	18.30453

0.15568	18.30867

0.1557	18.32533

0.15572	18.33507

0.15571	18.342

0.1557	18.3504

0.15572	18.3656

0.15574	18.35733

0.15573	18.36147

0.15575	18.3532

0.15575	18.34067

0.15578	18.33227

0.15578	18.324

0.15579	18.3268

0.15579	18.32947

0.15582	18.34893

0.15583	18.35173

0.15584	18.37533

0.15587	18.37947

0.15588	18.38653

0.15586	18.38653

0.15588	18.38787

0.15589	18.36427

0.15589	18.3628

0.15594	18.36147

0.15594	18.3504

0.15596	18.34893

0.15596	18.35587

0.15597	18.36707

0.15596	18.36987

0.15599	18.37813

0.15599	18.4032

0.15599	18.41427

0.15602	18.40867

0.15603	18.41147

0.15603	18.40453

0.15601	18.38787

0.15603	18.3768

0.15603	18.37533

0.15605	18.37813

0.15604	18.38093

0.15606	18.3948

0.15607	18.42253

0.15608	18.42253

0.15609	18.4268

0.15609	18.43787

0.1561	18.44347

0.15612	18.42533

0.15611	18.42947

0.15613	18.4212

0.15614	18.40867

0.15617	18.40587

0.15617	18.3976

0.15619	18.4032

0.15619	18.40587

0.1562	18.42253

0.15619	18.4268

0.15617	18.4504

0.15618	18.45453

0.15617	18.46147

0.15618	18.46293

0.15619	18.46013

0.15621	18.4448

0.15622	18.43787

0.15624	18.43093

0.15625	18.43093

0.15624	18.42813

0.15624	18.442

0.15622	18.44893

0.15626	18.4504

0.15626	18.45733

0.1563	18.47533

0.15631	18.48507

0.15632	18.49067

0.15632	18.48787

0.15632	18.49067

0.15633	18.4796

0.15634	18.4656

0.15635	18.45733

0.15634	18.45453

0.15636	18.4504

0.15637	18.45173

0.15639	18.46293

0.15641	18.4656

0.15641	18.4768

0.15643	18.48093

0.15643	18.51293

0.15642	18.51707

0.15645	18.51707

0.15643	18.51987

0.15644	18.50587

0.15644	18.4976

0.15645	18.48093

0.15647	18.47813

0.15647	18.48093

0.15652	18.48093

0.15651	18.48373

0.15653	18.5032

0.15652	18.51427

0.15652	18.5184

0.15652	18.5268

0.15654	18.5392

0.15655	18.53787

0.15655	18.53093

0.15659	18.5268

0.15659	18.50867

0.15658	18.50587

0.15659	18.5032

0.15659	18.50733

0.15661	18.51427

0.15662	18.5156

0.15662	18.53653

0.15664	18.55733

0.15663	18.55867

0.15665	18.56427

0.15666	18.56707

0.15666	18.55867

0.1567	18.53653

0.1567	18.53093

0.15673	18.53093

0.15673	18.5296

0.15674	18.5296

0.15674	18.5476

0.15674	18.55173

0.15674	18.55867

0.15674	18.5796

0.15674	18.58227

0.15674	18.58787

0.15674	18.592

0.15675	18.59067

0.15677	18.5768

0.15676	18.57253

0.15678	18.55587

0.15678	18.55867

0.15679	18.55587

0.15678	18.5532

0.15679	18.55867

0.15682	18.5796

0.15682	18.58933

0.15684	18.5976

0.15684	18.60173

0.15686	18.61293

0.15687	18.61013

0.15686	18.6004

0.15688	18.6032

0.15689	18.58787

0.1569	18.58653

0.15691	18.57813

0.15691	18.5796

0.15692	18.58373

0.15694	18.59627

0.15695	18.60173

0.15697	18.62533

0.15698	18.63227

0.157	18.64067

0.157	18.64627

0.15702	18.64067

0.15704	18.6268

0.15705	18.624

0.15705	18.62533

0.15705	18.61293

0.15706	18.61147

0.15706	18.606

0.15707	18.60867

0.15707	18.61293

0.15707	18.62813

0.15706	18.63507

0.15709	18.66147

0.1571	18.66293

0.15711	18.6712

0.15711	18.66147

0.15711	18.6656

0.15712	18.64347

0.15714	18.6448

0.15715	18.64067

0.15717	18.6296

0.15719	18.63093

0.1572	18.63787

0.15722	18.64627

0.15721	18.65453

0.15721	18.66147

0.15723	18.68373

0.15724	18.69347

0.15724	18.68933

0.15724	18.68507

0.15725	18.6712

0.15726	18.66427

0.15728	18.6532

0.15729	18.6532

0.1573	18.656

0.15732	18.6656

0.15732	18.674

0.15734	18.692

0.15734	18.7004

0.15735	18.70867

0.15736	18.71427

0.15737	18.71707

0.15739	18.7032

0.15739	18.706

0.15739	18.7004

0.15739	18.68227

0.1574	18.68093

0.1574	18.68227

0.15743	18.68653

0.15744	18.68933

0.15747	18.71427

0.15749	18.71987

0.15748	18.73653

0.15749	18.73653

0.1575	18.74347

0.15753	18.73507

0.15753	18.73653

0.15753	18.71573

0.15754	18.71147

0.15754	18.71147

0.15758	18.70173

0.15759	18.706

0.15759	18.7184

0.15761	18.72813

0.15762	18.73507

0.15762	18.75733

0.15762	18.76427

0.15762	18.76573

0.15762	18.76707

0.15763	18.76573

0.15764	18.742

0.15765	18.742

0.15768	18.7296

0.15769	18.7268

0.15769	18.72813

0.15769	18.7448

0.15769	18.74907

0.1577	18.76987

0.15771	18.7768

0.15771	18.7796

0.15772	18.78787

0.15772	18.79627

0.15774	18.7824

0.15774	18.78373

0.15774	18.77533

0.15773	18.7768

0.15775	18.75733

0.15778	18.756

0.15779	18.756

0.15781	18.76013

0.15782	18.76707

0.15785	18.79067

0.15786	18.81147

0.15787	18.81707

0.15786	18.8212

0.15787	18.8184

0.15788	18.80173

0.1579	18.78787

0.15791	18.78093

0.15791	18.77813

0.1579	18.7796

0.15793	18.78507

0.15794	18.8004

0.15796	18.80733

0.15797	18.80867

0.15795	18.83373

0.15795	18.83933

0.15794	18.84067

0.15796	18.84347

0.15798	18.83787

0.158	18.82267

0.15801	18.8212

0.15802	18.81013

0.15803	18.80453

0.15804	18.806

0.15804	18.8184

0.15803	18.8296

0.15805	18.85173

0.15804	18.856

0.15807	18.86293

0.15808	18.86707

0.15808	18.8684

0.15809	18.84907

0.15812	18.84347

0.15814	18.83653

0.15815	18.8268

0.15816	18.82813

0.15815	18.84067

0.15815	18.84627

0.15818	18.8504

0.15818	18.8796

0.15816	18.88507

0.15818	18.88653

0.1582	18.88933

0.15821	18.88933

0.15823	18.86987

0.15825	18.86427

0.15826	18.8504

0.15826	18.84907

0.15827	18.84907

0.15828	18.85453

0.15831	18.86013

0.15833	18.88653

0.15832	18.89213

0.15832	18.90173

0.15832	18.906

0.15834	18.91573

0.15833	18.906

0.15834	18.9032

0.15837	18.8948

0.15839	18.8796

0.15841	18.87813

0.15842	18.87813

0.15845	18.8796

0.15844	18.8796

0.15845	18.90453

0.15846	18.91427

0.15845	18.9352

0.15846	18.9352

0.15848	18.93933

0.15849	18.93093

0.15848	18.93653

0.1585	18.91573

0.15853	18.91427

0.15852	18.91147

0.15852	18.89907

0.15854	18.9032

0.15855	18.906

0.15856	18.91573

0.15859	18.92267

0.15859	18.93093

0.1586	18.95453

0.15861	18.96293

0.15862	18.96427

0.15863	18.956

0.15864	18.93933

0.15868	18.9352

0.15869	18.924

0.15868	18.924

0.1587	18.9268

0.15871	18.93933

0.15871	18.9476

0.15872	18.97267

0.15873	18.9796

0.15873	18.98787

0.15873	18.98787

0.15874	18.9824

0.15874	18.96427

0.15875	18.96147

0.15875	18.95453

0.15876	18.94627

0.15879	18.9504

0.15879	18.96293

0.1588	18.96987

0.15882	18.9796

0.15883	19.0032

0.15882	19.00453

0.15884	19.01013

0.15886	19.0088

0.15885	19.0088

0.15885	18.98933

0.15887	18.98787

0.15888	18.974

0.15888	18.97267

0.15889	18.97547

0.15889	18.98373

0.15894	18.99067

0.15894	19.01573

0.15893	19.02267

0.15895	19.0296

0.15895	19.0324

0.15893	19.03373

0.15894	19.01013

0.15898	19.01293

0.15897	19.00733

0.15899	18.9976

0.159	18.99627

0.15904	19.00453

0.15906	19.01293

0.15906	19.02267

0.15907	19.04347

0.15909	19.0476

0.15908	19.05733

0.15908	19.0616

0.1591	19.06293

0.15913	19.04347

0.15911	19.03933

0.15913	19.024

0.15915	19.02267

0.15916	19.02267

0.15917	19.02813

0.15917	19.0352

0.1592	19.06293

0.15922	19.06707

0.1592	19.07267

0.1592	19.0824

0.15923	19.07827

0.15924	19.06293

0.15925	19.05453

0.15925	19.0504

0.15927	19.04213

0.15927	19.04213

0.15928	19.05187

0.15928	19.05187

0.15932	19.06013

0.15934	19.0824

0.15933	19.08787

0.15934	19.10453

0.15935	19.1088

0.15935	19.10453

0.15936	19.1004

0.15935	19.09907

0.15936	19.08093

0.15939	19.0824

0.15939	19.0768

0.1594	19.06853

0.15942	19.074

0.15943	19.0824

0.15945	19.09213

0.15943	19.09627

0.15943	19.11707

0.15944	19.12547

0.15945	19.1296

0.15945	19.1324

0.15944	19.1268

0.15947	19.1212

0.15949	19.1116

0.15952	19.09627

0.15951	19.09493

0.15953	19.0976

0.15954	19.1032

0.15956	19.11293

0.15957	19.13653

0.15956	19.14347

0.15958	19.15187

0.1596	19.1532

0.1596	19.1532

0.15961	19.1352

0.15959	19.13373

0.15962	19.1296

0.15963	19.11987

0.15963	19.11853

0.15963	19.11853

0.15964	19.1268

0.15966	19.1324

0.15967	19.156

0.15967	19.16293

0.15968	19.18093

0.15969	19.1796

0.1597	19.188

0.15971	19.16987

0.15971	19.16987

0.1597	19.1504

0.15971	19.15187

0.15973	19.14907

0.15975	19.14493

0.15975	19.1476

0.15974	19.16427

0.15978	19.174

0.15976	19.18093

0.15977	19.2004

0.15977	19.20733

0.15978	19.19493

0.15982	19.19213

0.15982	19.188

0.15984	19.1712

0.15985	19.16707

0.15987	19.16573

0.15988	19.16987

0.15986	19.1768

0.15985	19.1976

0.15987	19.206

0.15989	19.22547

0.15991	19.2268

0.15993	19.23373

0.15993	19.21707

0.15995	19.21853

0.15996	19.1976

0.16	19.1976

0.15999	19.19347

0.16	19.19213

0.16001	19.19627

0.16002	19.2088

0.16004	19.2116

0.16003	19.21707

0.16004	19.24213

0.16005	19.24627

0.16005	19.25467

0.16006	19.2476

0.16004	19.2532

0.16007	19.224

0.16009	19.22547

0.1601	19.21427

0.16013	19.21707

0.16011	19.22267

0.16013	19.238

0.16013	19.24493

0.16015	19.26427

0.16018	19.27133

0.16018	19.27547

0.16016	19.28093

0.16017	19.26987

0.16019	19.2532

0.16021	19.2504

0.1602	19.24493

0.16022	19.238

0.16022	19.238

0.16023	19.2476

0.16025	19.2532

0.16024	19.25733

0.16023	19.26293

0.16024	19.288

0.16026	19.30467

0.16027	19.3004

0.16029	19.30187

0.16032	19.2852

0.16032	19.2796

0.16032	19.26293

0.16033	19.26427

0.16036	19.26427

0.1604	19.274

0.16041	19.2796

0.1604	19.306

0.16038	19.31573

0.16038	19.31853

0.16038	19.31853

0.1604	19.3144

0.16042	19.29773

0.16042	19.29773

0.16042	19.29627

0.16042	19.288

0.16044	19.28653

0.16045	19.3004

0.16046	19.306

0.16047	19.31293

0.16047	19.33933

0.16049	19.34213

0.1605	19.34627

0.16054	19.34907

0.16053	19.34347

0.16056	19.324

0.16055	19.32267

0.16056	19.30733

0.16058	19.31013

0.16059	19.31293

0.16061	19.32547

0.16058	19.32827

0.1606	19.35187

0.16059	19.36013

0.16063	19.36573

0.16063	19.37547

0.16064	19.36853

0.16063	19.3588

0.16061	19.356

0.16065	19.35187

0.16067	19.338

0.1607	19.33653

0.1607	19.33653

0.16071	19.34067

0.1607	19.34493

0.16072	19.36293

0.16074	19.37133

0.16074	19.39347

0.16077	19.39493

0.16078	19.39627

0.16078	19.39213

0.16078	19.38933

0.16077	19.37133

0.16079	19.36573

0.1608	19.3616

0.16082	19.36293

0.16084	19.3644

0.16086	19.388

0.16086	19.39067

0.16086	19.39493

0.16087	19.4144

0.16087	19.41573

0.16089	19.4268

0.1609	19.42133

0.16091	19.42827

0.16092	19.4088

0.16094	19.40733

0.16095	19.39213

0.16094	19.38933

0.16094	19.38933

0.16096	19.38933

0.16096	19.39213

0.16099	19.406

0.16099	19.41573

0.16102	19.42267

0.16101	19.44347

0.16103	19.44773

0.16104	19.44627

0.16104	19.4408

0.16104	19.4408

0.16104	19.42133

0.16107	19.41573

0.16106	19.41013

0.16109	19.41573

0.16109	19.41707

0.1611	19.43373

0.16109	19.43933

0.16111	19.4616

0.16111	19.46573

0.16112	19.46987

0.16111	19.47547

0.16112	19.47547

0.16115	19.45187

0.16115	19.44627

0.16115	19.44347

0.16115	19.44347

0.16116	19.43373

0.16117	19.44627

0.16118	19.456

0.1612	19.46293

0.16121	19.48933

0.16124	19.4908

0.16127	19.49627

0.16127	19.49213

0.16128	19.4908

0.16128	19.47413

0.1613	19.47267

0.16129	19.4616

0.16129	19.4588

0.16131	19.46013

0.16134	19.46987

0.16133	19.47413

0.16132	19.5004

0.16133	19.51013

0.16134	19.51853

0.16134	19.52133

0.16135	19.52133

0.16135	19.51013

0.16136	19.5004

0.16136	19.49907

0.16139	19.4852

0.16139	19.48373

0.1614	19.48653

0.1614	19.49213

0.16143	19.5004

0.16143	19.52267

0.16146	19.53107

0.16146	19.54493

0.16147	19.54213

0.16146	19.54213

0.16148	19.52827

0.16149	19.52413

0.16151	19.50747

0.16153	19.5088

0.16153	19.5088

0.16152	19.51707

0.16153	19.52133

0.16156	19.54347

0.16156	19.55467

0.16156	19.5588

0.16157	19.56853

0.16158	19.56853

0.16158	19.56013

0.1616	19.55467

0.16161	19.5532

0.16164	19.5324

0.16165	19.5324

0.16166	19.53387

0.16167	19.53653

0.16167	19.54493

0.16167	19.57267

0.16168	19.5796

0.16169	19.59213

0.16169	19.59493

0.1617	19.59347

0.16171	19.5768

0.16171	19.57133

0.16173	19.556

0.16174	19.5532

0.16175	19.55467

0.16177	19.5616

0.16177	19.5672

0.1618	19.59627

0.1618	19.60467

0.16181	19.6088

0.16181	19.61573

0.16182	19.6116

0.16182	19.59493

0.16183	19.58653

0.16185	19.58387

0.16185	19.57547

0.16186	19.5796

0.16187	19.59773

0.16187	19.60467

0.16188	19.60467

0.16189	19.6116

0.16189	19.62827

0.1619	19.63653

0.16191	19.638

0.16192	19.63387

0.16193	19.61853

0.16194	19.6088

0.16196	19.60053

0.16197	19.60053

0.162	19.59773

0.162	19.6116

0.16201	19.6172

0.16201	19.64347

0.16202	19.65053

0.162	19.656

0.162	19.66573

0.162	19.66573

0.16203	19.64627

0.16204	19.64907

0.16206	19.64213

0.16207	19.62827

0.16208	19.62827

0.16209	19.62413

0.1621	19.6296

0.16209	19.6296

0.1621	19.65747

0.1621	19.66293

0.16211	19.68387

0.16212	19.68933

0.16213	19.688

0.16215	19.6796

0.16215	19.67827

0.16216	19.66293

0.16217	19.656

0.16217	19.65747

0.16218	19.64627

0.16219	19.65187

0.1622	19.6672

0.16222	19.6644

0.16223	19.67413

0.16224	19.69907

0.16224	19.706

0.16224	19.71293

0.16223	19.7144

0.16223	19.7088

0.16225	19.69213

0.16226	19.68933

0.16227	19.67693

0.16228	19.67693

0.1623	19.67693

0.16233	19.6824

0.16236	19.68387

0.16236	19.7088

0.16236	19.7172

0.16235	19.72547

0.16235	19.7352

0.16235	19.7352

0.16236	19.73107

0.16236	19.72547

0.16236	19.72413

0.16236	19.706

0.16239	19.706

0.16242	19.70467

0.16242	19.71027

0.16244	19.71573

0.16245	19.7324

0.16247	19.73653

0.16248	19.7588

0.1625	19.76293

0.1625	19.7616

0.16251	19.7616

0.16251	19.756

0.16252	19.738

0.16252	19.73387

0.16253	19.738

0.16253	19.72547

0.16254	19.72693

0.16255	19.74213

0.16257	19.7436

0.16258	19.7588

0.16258	19.78107

0.1626	19.7852

0.16261	19.78933

0.16262	19.7908

0.16262	19.77827

0.16263	19.76293

0.16265	19.76027

0.16266	19.74773

0.16268	19.74907

0.16269	19.74907

0.16269	19.7672

0.16268	19.76573

0.1627	19.7908

0.16272	19.80333

0.16274	19.806

0.16274	19.8144

0.16273	19.81293

0.16275	19.7936

0.16276	19.7852

0.16277	19.78387

0.1628	19.77133

0.1628	19.77133

0.16281	19.77827

0.16279	19.7852

0.1628	19.7936

0.16281	19.82

0.1628	19.82827

0.1628	19.83387

0.1628	19.83667

0.16282	19.82827

0.16283	19.8116

0.16284	19.806

0.16284	19.79213

0.16285	19.7936

0.16287	19.79907

0.16288	19.82133

0.16289	19.82827

0.16291	19.85187

0.16292	19.85187

0.16292	19.8588

0.16293	19.83933

0.16293	19.83667

0.16294	19.8172

0.16297	19.81853

0.16297	19.81573

0.16301	19.82827

0.16303	19.83667

0.16305	19.86027

0.16304	19.86853

0.16303	19.87267

0.16301	19.86853

0.16302	19.87133

0.16302	19.84907

0.16301	19.84627

0.16302	19.8408

0.16304	19.83667

0.16306	19.83933

0.16308	19.8616

0.16307	19.86853

0.16309	19.88107

0.16309	19.89773

0.1631	19.8964

0.16311	19.8824

0.16314	19.88107

0.16315	19.87267

0.16316	19.856

0.16317	19.8588

0.16316	19.87

0.16319	19.87827

0.1632	19.8824

0.16319	19.9088

0.16321	19.9144

0.1632	19.9116

0.16323	19.91307

0.16325	19.91027

0.16324	19.8964

0.16325	19.8908

0.16326	19.88107

0.16328	19.87827

0.16327	19.88107

0.16327	19.8964

0.16328	19.89907

0.16329	19.92

0.16331	19.92693

0.16331	19.9352

0.16331	19.9408

0.16332	19.9464

0.16333	19.93107

0.16335	19.92693

0.16336	19.92547

0.16337	19.90747

0.16338	19.906

0.16339	19.90467

0.16341	19.9116

0.16341	19.91307

0.16342	19.938

0.16342	19.9464

0.16344	19.96573

0.16345	19.96853

0.16346	19.97

0.16347	19.95467

0.16345	19.95333

0.16345	19.938

0.16347	19.93667

0.16348	19.938

0.1635	19.93387

0.16351	19.938

0.16352	19.94907

0.16353	19.95467

0.16355	19.9672

0.16357	19.98667

0.16358	19.99213

0.16359	19.98933

0.1636	19.9936

0.16361	19.98667

0.16361	19.96853

0.1636	19.96307

0.1636	19.95467

0.16362	19.956

0.16364	19.95747

0.16364	19.97133

0.16363	19.98107

0.16362	20.0088

0.16363	20.01027

0.16364	20.01573

0.16365	20.0172

0.16365	20.01573

0.16365	19.9908

0.16368	19.98933

0.16369	19.9852

0.16372	19.97547

0.16372	19.9824

0.16374	19.9936

0.16375	20.00053

0.16376	20.01307

0.16377	20.0352

0.16378	20.038

0.1638	20.0352

0.16379	20.02973

0.16379	20.03107

0.16382	20.0172

0.16382	20.0144

0.16383	20.00333

0.16384	19.99907

0.16385	20.00467

0.16383	20.0172

0.16384	20.0228

0.16383	20.0464

0.16384	20.05187

0.16386	20.05613

0.16386	20.06307

0.16388	20.0672

0.1639	20.0436

0.16389	20.0408

0.1639	20.0352

0.16391	20.02413

0.16392	20.02413

0.16395	20.03387

0.16395	20.038

0.16396	20.04493

0.16398	20.07413

0.16398	20.07973

0.16401	20.08947

0.16402	20.08947

0.16403	20.08947

0.16402	20.0728

0.16404	20.07693

0.16403	20.05613

0.16406	20.05747

0.16408	20.05747

0.16407	20.05187

0.16407	20.05613

0.16409	20.07

0.16409	20.07693

0.16411	20.07973

0.16414	20.10467

0.16413	20.11027

0.16416	20.1088

0.16417	20.1172

0.16417	20.1088

0.16419	20.09773

0.16419	20.08947

0.16418	20.07547

0.16419	20.07547

0.1642	20.07827

0.1642	20.08947

0.16422	20.09493

0.16421	20.12133

0.16421	20.12827

0.16423	20.13387

0.16426	20.1352

0.16426	20.13947

0.16429	20.11573

0.16428	20.1172

0.16429	20.1116

0.16431	20.10053

0.16432	20.10467

0.16432	20.1088

0.16434	20.11573

0.16436	20.12

0.16435	20.1464

0.16435	20.1492

0.16437	20.16307

0.16437	20.16307

0.16437	20.16587

0.16439	20.1492

0.16439	20.14213

0.1644	20.12693

0.16443	20.1228

0.16444	20.12413

0.16443	20.13107

0.16444	20.13947

0.16444	20.16587

0.16445	20.1728

0.16447	20.18107

0.16448	20.18947

0.16448	20.188

0.16451	20.16587

0.16452	20.16587

0.16455	20.16027

0.16455	20.14773

0.16457	20.14773

0.16454	20.1588

0.16454	20.1728

0.16455	20.17973

0.16457	20.20333

0.16458	20.20333

0.16461	20.20187

0.16461	20.1964

0.16461	20.1936

0.16464	20.17547

0.16465	20.17413

0.16464	20.16853

0.16466	20.17133

0.16466	20.17413

0.16466	20.1908

0.16467	20.19773

0.16467	20.21853

0.16467	20.22547

0.16467	20.22973

0.1647	20.23107

0.16471	20.22827

0.16471	20.2116

0.16473	20.2088

0.16475	20.20333

0.16473	20.1964

0.16475	20.19213

0.16477	20.20333

0.16479	20.21027

0.16479	20.22413

0.16481	20.2464

0.16479	20.25333

0.16479	20.25613

0.16479	20.25187

0.16481	20.25467

0.16479	20.22973

0.16479	20.22547

0.16483	20.2144

0.16484	20.21587

0.16485	20.22

0.16488	20.24227

0.1649	20.2492

0.1649	20.26853

0.16491	20.27693

0.16492	20.2756

0.16493	20.27

0.16494	20.26027

0.16497	20.2436

0.16499	20.23947

0.16499	20.23947

0.16499	20.24493

0.16501	20.25333

0.16501	20.27693

0.16501	20.28107

0.165	20.288

0.16501	20.29773

0.165	20.30053

0.16504	20.28387

0.16506	20.28253

0.16507	20.27693

0.16504	20.26307

0.16504	20.2616

0.16504	20.2644

0.16505	20.27

0.16507	20.27

0.16511	20.2936

0.16513	20.30187

0.16511	20.31853

0.16511	20.32413

0.16511	20.32693

0.16513	20.31853

0.16513	20.3116

0.16518	20.29227

0.16519	20.28947

0.16521	20.28387

0.1652	20.2852

0.16521	20.29493

0.16522	20.3144

0.16524	20.3256

0.16524	20.33253

0.16527	20.3464

0.16529	20.35187

0.16528	20.33253

0.16528	20.32827

0.16528	20.32133

0.16529	20.30893

0.16532	20.30613

0.16534	20.3144

0.16534	20.32

0.16534	20.32973

0.16533	20.35333

0.16534	20.36027

0.16535	20.36867

0.16535	20.37

0.16537	20.37413

0.16537	20.35053

0.16541	20.35187

0.1654	20.33387

0.16541	20.33107

0.16543	20.33253

0.16543	20.338

0.16546	20.3408

0.16547	20.3644

0.16547	20.3728

0.16547	20.37973

0.16547	20.3908

0.16547	20.3964

0.16549	20.38533

0.16552	20.37973

0.16553	20.38107

0.16555	20.36307

0.16555	20.35893

0.16555	20.35613

0.16556	20.35613

0.16556	20.3616

0.16559	20.37973

0.16559	20.388

0.16557	20.40613

0.16558	20.4116

0.1656	20.42

0.16562	20.41867

0.16563	20.41867

0.16566	20.39493

0.16567	20.39227

0.16568	20.38667

0.16566	20.37827

0.16566	20.38253

0.1657	20.3964

0.16571	20.40613

0.16571	20.41867

0.1657	20.43947

0.1657	20.438

0.16572	20.43387

0.16573	20.42693

0.16574	20.42827

0.16578	20.40613

0.16579	20.40747

0.16577	20.40613

0.16577	20.40893

0.16579	20.4144

0.1658	20.42973

0.16582	20.4408

0.16583	20.4616

0.16585	20.46587

0.16585	20.46587

0.16588	20.45747

0.16585	20.45467

0.16587	20.43947

0.16588	20.43253

0.1659	20.43107

0.16593	20.4228

0.16594	20.42827

0.16596	20.4408

0.16595	20.4492

0.16595	20.45747

0.16595	20.48387

0.16597	20.48667

0.166	20.48533

0.16602	20.48387

0.16602	20.48387

0.16601	20.4616

0.16603	20.46307

0.16606	20.44773

0.16607	20.452

0.16608	20.45053

0.16609	20.46587

0.1661	20.4784

0.16611	20.50053

0.16612	20.50747

0.16612	20.51173

0.16612	20.51307

0.16611	20.50747

0.16612	20.49227

0.16612	20.48667

0.16614	20.48533

0.16615	20.47413

0.16617	20.47693

0.16618	20.49227

0.16619	20.4964

0.1662	20.50613

0.16621	20.52413

0.16623	20.52973

0.16623	20.53387

0.16622	20.53667

0.16623	20.53667

0.16624	20.51307

0.16626	20.50467

0.16627	20.4964

0.16629	20.4992

0.16631	20.49773

0.1663	20.51173

0.16629	20.52133

0.16629	20.54227

0.16631	20.5492

0.16632	20.55467

0.16633	20.56587

0.16633	20.56173

0.16634	20.5464

0.16637	20.53947

0.16639	20.5408

0.16641	20.52413

0.1664	20.5228

0.16641	20.53107

0.1664	20.52973

0.1664	20.53387

0.16641	20.55467

0.16642	20.5644

0.16643	20.58387

0.16646	20.588

0.16647	20.588

0.16649	20.57973

0.16648	20.57413

0.16649	20.552

0.1665	20.54773

0.16651	20.54507

0.16651	20.54773

0.16651	20.55333

0.16654	20.5784

0.16654	20.58667

0.16655	20.5964

0.16655	20.60747

0.16656	20.61027

0.16657	20.5992

0.16658	20.59227

0.16659	20.5936

0.1666	20.57693

0.1666	20.5756

0.16662	20.5728

0.16663	20.57693

0.16663	20.57973

0.16664	20.60053

0.16666	20.60893

0.16667	20.6256

0.16669	20.63667

0.16669	20.62973

0.1667	20.62413

0.16671	20.62147

0.16672	20.60333

0.16672	20.59773

0.16673	20.60053

0.16675	20.59773

0.16675	20.5992

0.16677	20.61587

0.16677	20.62147

0.16679	20.6284

0.16682	20.64773

0.16682	20.6548

0.16684	20.65613

0.16684	20.65893

0.16685	20.6492

0.16686	20.63253

0.16687	20.62973

0.16686	20.61867

0.16686	20.62

0.16688	20.6256

0.16689	20.63387

0.16691	20.63813

0.1669	20.65893

0.16691	20.6644

0.16693	20.67

0.16694	20.68533

0.16696	20.68667

0.16698	20.6756

0.16699	20.6672

0.167	20.66173

0.167	20.6464

0.16701	20.65053

0.16701	20.6464

0.16702	20.6436

0.16703	20.6492

0.16705	20.6672

0.16704	20.6784

0.16705	20.6992

0.16707	20.7048

0.16708	20.71027

0.1671	20.70613

0.1671	20.702

0.16711	20.67973

0.16712	20.67693

0.16712	20.67693

0.16714	20.67147

0.16713	20.67413

0.16715	20.69507

0.16716	20.70053

0.16717	20.71027

0.16718	20.7284

0.1672	20.7284

0.16721	20.72973

0.16722	20.7312

0.16723	20.72973

0.16725	20.70613

0.16726	20.702

0.16727	20.69227

0.16728	20.69507

0.16729	20.6964

0.1673	20.71173

0.1673	20.7172

0.16731	20.73533

0.16732	20.74507

0.16733	20.75053

0.16734	20.76027

0.16735	20.75613

0.16737	20.74507

0.16736	20.73947

0.16737	20.73813

0.16738	20.72

0.16742	20.71867

0.16744	20.72147

0.16744	20.72693

0.16744	20.73813

0.16743	20.76027

0.16744	20.7672

0.16746	20.7784

0.16746	20.78253

0.16747	20.78253

0.16749	20.7728

0.1675	20.77

0.16753	20.7492

0.16754	20.7464

0.16756	20.74507

0.16757	20.74787

0.16757	20.74787

0.16758	20.76453

0.16758	20.76867

0.16759	20.77693

0.16759	20.802

0.16761	20.8048

0.16761	20.80613

0.16761	20.7992

0.16761	20.80333

0.16763	20.78387

0.16764	20.78533

0.16767	20.77147

0.16767	20.7728

0.16768	20.77

0.16768	20.7756

0.16768	20.78253

0.16768	20.80053

0.16769	20.81027

0.16771	20.81587

0.16774	20.83533

0.16775	20.83533

0.16776	20.82693

0.16777	20.8256

0.16777	20.82

0.16776	20.80333

0.16778	20.80053

0.16779	20.7964

0.1678	20.79787

0.16781	20.80613

0.16784	20.82147

0.16784	20.83253

0.16784	20.85333

0.16786	20.85613

0.16785	20.85893

0.16786	20.84787

0.16788	20.852

0.16789	20.82973

0.1679	20.8228

0.16791	20.82

0.1679	20.82147

0.16791	20.8228

0.16791	20.83947

0.16793	20.85053

0.16793	20.85613

0.16795	20.8756

0.16794	20.87973

0.16796	20.88387

0.16798	20.87693

0.16798	20.87693

0.16801	20.85333

0.16801	20.85053

0.16802	20.8436

0.16805	20.84093

0.16805	20.84507

0.16805	20.86173

0.16805	20.87147

0.16806	20.8964

0.16807	20.90053

0.16808	20.9076

0.1681	20.90053

0.16809	20.89787

0.16811	20.8784

0.16811	20.8756

0.16813	20.87427

0.16814	20.86867

0.16816	20.87147

0.16814	20.88947

0.16815	20.89227

0.16816	20.902

0.16817	20.92

0.16819	20.9256

0.1682	20.93253

0.1682	20.92147

0.16821	20.92147

0.16823	20.9048

0.16824	20.90613

0.16823	20.89507

0.16825	20.89507

0.16826	20.89227

0.16827	20.902

0.16828	20.90893

0.16829	20.92693

0.16829	20.93533

0.16831	20.94227

0.16831	20.95893

0.16831	20.95613

0.16831	20.93947

0.16833	20.92973

0.16834	20.92693

0.16835	20.9172

0.16836	20.91307

0.16837	20.92973

0.16837	20.93387

0.1684	20.94093

0.1684	20.96453

0.16841	20.96733

0.16842	20.97693

0.16843	20.97427

0.16843	20.97693

0.16845	20.95893

0.16846	20.95893

0.16849	20.9436

0.16851	20.9464

0.1685	20.94093

0.16851	20.94093

0.16852	20.94507

0.16853	20.96733

0.16854	20.97693

0.16856	20.984

0.16856	21.002

0.16856	21.00067

0.16857	20.98813

0.16857	20.9784

0.16859	20.97973

0.16861	20.96587

0.16861	20.96587

0.16863	20.96453

0.16864	20.97

0.16863	20.97427

0.16867	20.99227

0.16869	20.9992

0.16869	21.01867

0.16871	21.0228

0.16872	21.02693

0.16871	21.02427

0.16873	21.02147

0.16872	21.002

0.16873	21.002

0.16875	21.002

0.16874	20.98813

0.16874	20.98813

0.16878	21.00333

0.16877	21.01027

0.16879	21.01867

0.16882	21.04227

0.16883	21.04787

0.16886	21.05333

0.16887	21.05067

0.16886	21.0492

0.1689	21.02427

0.16889	21.02147

0.1689	21.00893

0.16893	21.01587

0.16893	21.01587

0.16894	21.0312

0.16895	21.03947

0.16893	21.06453

0.16893	21.07147

0.16895	21.07427

0.16894	21.07973

0.16898	21.07147

0.16898	21.05613

0.16901	21.052

0.16903	21.05067

0.16902	21.03667

0.16903	21.03947

0.16904	21.052

0.16904	21.0548

0.16906	21.06307

0.16907	21.084

0.16907	21.09373

0.16912	21.09507

0.16911	21.0964

0.16912	21.09787

0.16913	21.084

0.16916	21.06307

0.16917	21.06027

0.16918	21.06307

0.16919	21.06173

0.16922	21.07147

0.16923	21.0756

0.16924	21.0964

0.16924	21.10613

0.16922	21.10893

0.16926	21.12427

0.16924	21.12427

0.16927	21.12147

0.16925	21.11733

0.16924	21.11587

0.16926	21.09507

0.16928	21.09507

0.1693	21.08667

0.16931	21.09093

0.16931	21.09227

0.16935	21.1048

0.16936	21.11307

0.16937	21.13533

0.16937	21.14373

0.16937	21.14787

0.16939	21.15333

0.1694	21.1464

0.1694	21.12707

0.16942	21.12707

0.16942	21.1256

0.16945	21.11453

0.16946	21.11307

0.16946	21.11867

0.16947	21.12427

0.16947	21.13533

0.16948	21.1576

0.16948	21.16173

0.16949	21.1756

0.16951	21.17

0.1695	21.1728

0.16953	21.1548

0.16954	21.15613

0.16954	21.13667

0.16955	21.13667

0.16954	21.13667

0.16956	21.14373

0.16957	21.152

0.16958	21.1756

0.16959	21.1812

0.16959	21.18533

0.16961	21.1992

0.1696	21.1964

0.1696	21.1812

0.1696	21.17707

0.1696	21.1784

0.16962	21.16173

0.16964	21.16173

0.16966	21.16587

0.16968	21.17

0.16966	21.17707

0.16968	21.19787

0.16968	21.21173

0.16968	21.2228

0.16969	21.22013

0.16969	21.21587

0.16973	21.1992

0.16972	21.19373

0.16977	21.1812

0.16976	21.18813

0.16976	21.18813

0.16977	21.2048

0.16977	21.20613

0.16981	21.23253

0.16981	21.2368

0.16981	21.24373

0.16982	21.24227

0.16983	21.23813

0.16985	21.2228

0.16985	21.21867

0.16985	21.21587

0.16985	21.20347

0.16986	21.2048

0.16987	21.22013

0.16989	21.2284

0.16993	21.23533

0.16992	21.2576

0.16993	21.26587

0.1699	21.2604

0.1699	21.2604

0.16992	21.2548

0.16992	21.2368

0.16997	21.234

0.16996	21.22707

0.16999	21.2284

0.17001	21.23253

0.17001	21.2464

0.17002	21.2492

0.17002	21.2728

0.17005	21.2756

0.17007	21.284

0.17009	21.28947

0.17009	21.29227

0.17009	21.27707

0.17009	21.26587

0.17007	21.26307

0.17012	21.2492

0.17012	21.252

0.17015	21.26173

0.17014	21.27013

0.17015	21.2756

0.17016	21.29787

0.17015	21.30613

0.17016	21.3048

0.17018	21.31173

0.1702	21.29227

0.17021	21.28813

0.17022	21.284

0.17022	21.2756

0.17024	21.27427

0.17027	21.2784

0.17026	21.29507

0.17027	21.3048

0.17029	21.32987

0.17032	21.33533

0.17032	21.33947

0.17034	21.32427

0.17034	21.31867

0.17036	21.29507

0.17035	21.29507

0.17035	21.29373

0.17036	21.302

0.17037	21.31173

0.17038	21.33533

0.17039	21.34653

0.17039	21.34787

0.1704	21.3576

0.17043	21.35893

0.17044	21.3492

0.17047	21.34093

0.17047	21.34093

0.17047	21.3256

0.17046	21.3228

0.17047	21.32147

0.17049	21.3256

0.17049	21.33533

0.17051	21.34787

0.1705	21.3576

0.17052	21.3756

0.17052	21.3784

0.17053	21.38253

0.17054	21.37147

0.17056	21.36867

0.17058	21.35347

0.1706	21.35067

0.17059	21.34787

0.17061	21.34653

0.17062	21.3492

0.17065	21.3632

0.17066	21.37013

0.17065	21.37427

0.17066	21.3992

0.17066	21.39653

0.17067	21.40893

0.17067	21.40613

0.17069	21.40893

0.1707	21.39227

0.17073	21.39227

0.17073	21.3728

0.17075	21.3728

0.17073	21.37427

0.17076	21.3784

0.17077	21.38533

0.17079	21.40067

0.17079	21.4076

0.17079	21.4104

0.17079	21.42707

0.17079	21.43253

0.1708	21.42707

0.17081	21.42707

0.17082	21.4312

0.17083	21.40613

0.17083	21.39227

0.17087	21.39653

0.17087	21.39653

0.17088	21.40347

0.1709	21.42427

0.17089	21.43253

0.1709	21.45347

0.1709	21.45347

0.17094	21.4576

0.17095	21.45067

0.17096	21.452

0.17096	21.4284

0.17098	21.4256

0.17098	21.42147

0.171	21.41867

0.17099	21.42147

0.171	21.44093

0.171	21.45067

0.17102	21.45627

0.17103	21.47427

0.17106	21.4784

0.17107	21.4812

0.17109	21.47427

0.17109	21.47427

0.17111	21.45627

0.1711	21.45347

0.17113	21.44227

0.17113	21.44507

0.17115	21.45347

0.17116	21.46867

0.17119	21.47293

0.17119	21.49373

0.1712	21.50067

0.17122	21.5048

0.17123	21.50347

0.17123	21.5048

0.17122	21.4868

0.17124	21.484

0.17127	21.4784

0.1713	21.46867

0.17131	21.46867

0.17133	21.4756

0.17133	21.47987

0.17133	21.4868

0.17134	21.51587

0.17134	21.51867

0.17135	21.534

0.17136	21.5256

0.17136	21.53253

0.17138	21.51587

0.1714	21.51453

0.17142	21.49507

0.17142	21.49373

0.17144	21.49227

0.17145	21.49373

0.17147	21.5048

0.17145	21.5256

0.17147	21.5368

0.17145	21.54227

0.17148	21.55067

0.1715	21.54787

0.17149	21.54653

0.1715	21.54093

0.17152	21.5368

0.17154	21.52293

0.17155	21.52293

0.17155	21.51867

0.17156	21.51733

0.17158	21.52293

0.1716	21.53813

0.1716	21.5576

0.1716	21.56453

0.17163	21.56453

0.17162	21.5756

0.17164	21.58267

0.17164	21.58267

0.17165	21.55893

0.17167	21.55893

0.17168	21.54653

0.17169	21.54093

0.1717	21.54653

0.17169	21.55347

0.1717	21.56173

0.17171	21.56173

0.17172	21.58533

0.17176	21.59227

0.17176	21.602

0.17177	21.602

0.17176	21.6076

0.17178	21.59227

0.17179	21.59093

0.1718	21.57427

0.17182	21.57427

0.17181	21.57427

0.17181	21.56733

0.17183	21.57427

0.17183	21.5868

0.17186	21.59227

0.17186	21.5896

0.17188	21.61453

0.17188	21.6256

0.17191	21.634

0.17191	21.634

0.17193	21.62707

0.17195	21.6076

0.17196	21.60067

0.17196	21.59227

0.17197	21.59507

0.17198	21.59787

0.172	21.61453

0.17201	21.61867

0.17201	21.64653

0.17203	21.64933

0.17205	21.6576

0.17205	21.6548

0.17207	21.652

0.17206	21.634

0.17207	21.6312

0.17207	21.6256

0.17208	21.6132

0.1721	21.62013

0.17212	21.6256

0.17213	21.63533

0.17214	21.63533

0.17216	21.6604

0.17217	21.66733

0.17219	21.67707

0.1722	21.67987

0.17221	21.6812

0.1722	21.6632

0.17221	21.65893

0.17222	21.64653

0.17224	21.64227

0.17226	21.64653

0.17228	21.64653

0.17228	21.65067

0.17228	21.666

0.1723	21.67013

0.17232	21.68267

0.17234	21.69933

0.17236	21.70627

0.17236	21.69653

0.17236	21.69787

0.17237	21.68813

0.17238	21.67147

0.17238	21.67013

0.17239	21.6632

0.17241	21.67013

0.17242	21.6868

0.17244	21.69933

0.17244	21.7076

0.17245	21.72427

0.17247	21.72427

0.17247	21.7284

0.17248	21.72147

0.17247	21.71867

0.17248	21.69933

0.17246	21.69653

0.17249	21.69507

0.17249	21.6896

0.1725	21.69653

0.17252	21.70627

0.17254	21.71733

0.17255	21.72147

0.17256	21.74093

0.17257	21.74653

0.17259	21.75067

0.17259	21.75067

0.17259	21.74507

0.17262	21.734

0.17264	21.7284

0.17265	21.71867

0.17266	21.71733

0.17267	21.71867

0.1727	21.72147

0.1727	21.7284

0.1727	21.74933

0.17271	21.75627

0.17271	21.76453

0.17271	21.77427

0.17273	21.7784

0.17274	21.76453

0.17274	21.76173

0.17275	21.75907

0.17276	21.74653

0.17279	21.74373

0.17279	21.74507

0.17281	21.74933

0.17281	21.75067

0.17283	21.77013

0.17287	21.77147

0.17289	21.79373

0.17289	21.79653

0.1729	21.79933

0.17289	21.80067

0.17291	21.79653

0.17293	21.784

0.17294	21.77707

0.17296	21.77293

0.17297	21.76453

0.17297	21.77573

0.173	21.784

0.17299	21.79373

0.17301	21.79933

0.17301	21.82013

0.17302	21.82427

0.17303	21.82147

0.17303	21.82573

0.17305	21.816

0.17307	21.80213

0.17306	21.79653

0.17306	21.78813

0.17307	21.7896

0.17308	21.79507

0.17309	21.81173

0.1731	21.8368

0.1731	21.84373

0.17313	21.8424

0.17313	21.85213

0.17315	21.8424

0.17315	21.84507

0.17316	21.82427

0.17318	21.82427

0.17319	21.82427

0.17321	21.81733

0.17324	21.8188

0.17323	21.82147

0.17324	21.8284

0.17325	21.8284

0.17328	21.84933

0.17331	21.85627

0.17331	21.87147

0.17331	21.87427

0.1733	21.87427

0.17331	21.87293

0.17333	21.86453

0.17333	21.85067

0.17333	21.84653

0.17336	21.84933

0.17337	21.8396

0.17338	21.84653

0.17339	21.85907

0.17341	21.8632

0.17341	21.86733

0.17342	21.8868

0.17343	21.8924

0.17345	21.9048

0.17348	21.90627

0.17347	21.9048

0.17348	21.89653

0.1735	21.8924

0.17349	21.87147

0.1735	21.87013

0.17351	21.87013

0.17353	21.87427

0.17354	21.8784

0.17356	21.90067

0.17354	21.90627

0.17358	21.91453

0.17359	21.92707

0.17358	21.92853

0.17358	21.92853

0.17361	21.92427

0.17362	21.92013

0.17362	21.89787

0.17362	21.89653

0.17363	21.89093

0.17366	21.89507

0.17366	21.90213

0.17367	21.9188

0.1737	21.92573

0.17371	21.94653

0.1737	21.95213

0.17372	21.95347

0.17372	21.95347

0.17373	21.9452

0.17374	21.92987

0.17377	21.9312

0.17377	21.92987

0.17377	21.916

0.17377	21.91733

0.17377	21.92987

0.17379	21.93267

0.17379	21.9396

0.17381	21.95907

0.17382	21.966

0.17384	21.97987

0.17383	21.97853

0.17386	21.98267

0.17388	21.95347

0.17387	21.95627

0.17388	21.94093

0.17391	21.94373

0.17391	21.9424

0.17392	21.94787

0.17393	21.9688

0.17395	21.97427

0.17395	21.98547

0.17397	21.99373

0.17398	22.0048

0.17397	21.99787

0.17401	21.984

0.174	21.97573

0.17401	21.97707

0.17402	21.96733

0.17402	21.96733

0.17404	21.97853

0.17407	21.97707

0.17407	21.984

0.17406	22.00627

0.17409	22.01453

0.17411	22.02573

0.1741	22.02707

0.17412	22.02427

0.17413	22.016

0.17413	22.0048

0.17412	22.00347

0.17416	22.0048

0.17415	22.00067

0.17418	21.9896

0.17421	21.99093

0.17422	22.00347

0.17422	22.0076

0.17424	22.01733

0.17426	22.0396

0.17423	22.0452

0.17423	22.04933

0.17426	22.05213

0.17427	22.05627

0.17428	22.0368

0.1743	22.02573

0.17434	22.02013

0.17431	22.0216

0.17435	22.02013

0.17436	22.0216

0.17435	22.02427

0.17437	22.0424

0.17439	22.05213

0.17438	22.05907

0.17438	22.07707

0.17442	22.07853

0.17442	22.07013

0.17441	22.06453

0.17442	22.06187

0.17443	22.04787

0.17443	22.04653

0.17444	22.0396

0.17447	22.04093

0.17449	22.04933

0.1745	22.06453

0.17451	22.07293

0.17452	22.09373

0.17453	22.09653

0.17454	22.10067

0.17454	22.10213

0.17455	22.1076

0.17455	22.09093

0.17457	22.09093

0.17457	22.07987

0.17458	22.07013

0.17458	22.07013

0.17459	22.07013

0.17463	22.07707

0.17461	22.08547

0.17461	22.10907

0.17464	22.11733

0.17464	22.12707

0.17464	22.12707

0.17466	22.134

0.17467	22.12013

0.17466	22.1188

0.17468	22.09933

0.17472	22.10067

0.17472	22.10067

0.17474	22.09373

0.17473	22.09653

0.17473	22.0952

0.17474	22.10627

0.17474	22.1104

0.17475	22.13547

0.17479	22.1452

0.17479	22.15493

0.17479	22.16187

0.17481	22.1576

0.17484	22.14933

0.17482	22.13827

0.17484	22.12293

0.17487	22.1216

0.17488	22.1216

0.17488	22.12573

0.17487	22.12707

0.17488	22.1424

0.17492	22.15493

0.17491	22.15907

0.17493	22.17707

0.17492	22.17987

0.17495	22.17427

0.17494	22.17707

0.17496	22.16467

0.17496	22.15493

0.17497	22.148

0.17498	22.14373

0.17498	22.14933

0.17501	22.148

0.17502	22.16733

0.17502	22.17013

0.17504	22.1924

0.17507	22.19933

0.17508	22.20213

0.17509	22.20627

0.17509	22.20347

0.17509	22.184

0.1751	22.17707

0.1751	22.17707

0.17512	22.16733

0.17515	22.17293

0.17516	22.184

0.17516	22.1868

0.17518	22.1924

0.17518	22.2104

0.1752	22.21733

0.17521	22.22707

0.17521	22.234

0.17525	22.23267

0.17526	22.22293

0.17524	22.21467

0.17524	22.20213

0.17525	22.198

0.17526	22.20213

0.17528	22.20213

0.17529	22.20213

0.1753	22.21467

0.17533	22.22293

0.17532	22.22987

0.17533	22.25773

0.17534	22.25493

0.17533	22.25773

0.17537	22.25493

0.17537	22.25773

0.17539	22.2396

0.1754	22.24373

0.1754	22.22853

0.17542	22.22707

0.17541	22.22427

0.17543	22.22987

0.17545	22.23133

0.17547	22.25493

0.17545	22.26187

0.17547	22.266

0.17547	22.28267

0.17549	22.28133

0.1755	22.2744

0.17549	22.2716

0.17553	22.27573

0.17555	22.25773

0.17554	22.26187

0.17555	22.248

0.17554	22.24933

0.17556	22.25213

0.17558	22.26467

0.17558	22.2716

0.1756	22.2924

0.17561	22.30067

0.17563	22.30347

0.17563	22.3104

0.17563	22.29653

0.17564	22.29107

0.17565	22.28827

0.17567	22.2868

0.1757	22.27573

0.1757	22.2716

0.1757	22.27987

0.1757	22.28827

0.17572	22.29653

0.17571	22.3132

0.17573	22.32853

0.17573	22.33267

0.17576	22.33267

0.17577	22.33133

0.17579	22.316

0.17578	22.3132

0.17581	22.298

0.17583	22.30067

0.17585	22.30067

0.17587	22.30627

0.17585	22.30347

0.17588	22.3188

0.17589	22.32573

0.1759	22.32987

0.17592	22.35493

0.17591	22.35347

0.17593	22.35907

0.17593	22.35773

0.17596	22.36467

0.17597	22.34933

0.17596	22.3452

0.17597	22.33133

0.17598	22.32853

0.176	22.32853

0.17602	22.32853

0.17605	22.3396

0.17606	22.34373

0.17606	22.34933

0.17609	22.35213

0.17611	22.3744

0.17611	22.3896

0.17612	22.38827

0.17611	22.3896

0.17612	22.38413

0.17613	22.3716

0.17614	22.366

0.17614	22.35773

0.17612	22.35493

0.17615	22.35493

0.17617	22.36467

0.17619	22.3632

0.17619	22.38827

0.17619	22.3924

0.1762	22.398

0.17622	22.40907

0.17624	22.416

0.17624	22.416

0.17624	22.41747

0.17625	22.41187

0.17626	22.3952

0.17628	22.37987

0.17628	22.37987

0.1763	22.38413

0.17629	22.38267

0.17632	22.39107

0.17634	22.39107

0.17633	22.41187

0.17636	22.41747

0.17637	22.4244

0.1764	22.43827

0.1764	22.4368

0.17641	22.448

0.17641	22.4396

0.17643	22.44653

0.17645	22.42573

0.17644	22.4244

0.17647	22.4104

0.17648	22.4132

0.1765	22.41187

0.17651	22.41747

0.17651	22.42293

0.17653	22.43267

0.17654	22.44107

0.17654	22.44653

0.17653	22.46747

0.17653	22.47573

0.17656	22.4688

0.17656	22.4716

0.17654	22.46747

0.17655	22.44933

0.17658	22.448

0.1766	22.4424

0.17663	22.43827

0.17661	22.44107

0.17663	22.448

0.17664	22.46187

0.17666	22.4716

0.17667	22.48133

0.17669	22.4924

0.1767	22.498

0.1767	22.5008

0.1767	22.48267

0.1767	22.4924

0.17673	22.48267

0.17674	22.4744

0.17675	22.45907

0.17676	22.45907

0.17678	22.466

0.17678	22.4688

0.17681	22.4868

0.17681	22.4868

0.17681	22.5132

0.17683	22.5132

0.17685	22.5216

0.17687	22.5216

0.17687	22.52293

0.17687	22.5132

0.17686	22.50907

0.17686	22.50627

0.17686	22.4896

0.17688	22.4896

0.17688	22.49387

0.17689	22.49933

0.1769	22.50627

0.17692	22.52987

0.17694	22.53547

0.17694	22.54653

0.17696	22.548

0.17697	22.54933

0.17699	22.54653

0.177	22.52013

0.17702	22.5216

0.17702	22.5188

0.17704	22.5216

0.17705	22.516

0.17708	22.5216

0.17709	22.52853

0.17709	22.52853

0.17709	22.5368

0.17711	22.55493

0.17711	22.5632

0.17713	22.5744

0.17715	22.5772

0.17716	22.57573

0.17716	22.56747

0.17717	22.56053

0.1772	22.54653

0.17722	22.548

0.17723	22.5452

0.17722	22.54387

0.17723	22.54653

0.17723	22.566

0.17725	22.5688

0.17727	22.57573

0.17727	22.59107

0.17727	22.60213

0.17728	22.60773

0.17729	22.598

0.1773	22.6008

0.17731	22.58413

0.17733	22.58547

0.17734	22.57013

0.17736	22.5716

0.17738	22.566

0.17739	22.5688

0.17739	22.57573

0.17741	22.5868

0.17743	22.5924

0.17745	22.59387

0.17743	22.6188

0.17743	22.6216

0.17745	22.62987

0.17747	22.62853

0.17748	22.62987

0.17749	22.61467

0.17749	22.61053

0.17752	22.5952

0.17754	22.59107

0.17757	22.59933

0.17757	22.59933

0.17757	22.60627

0.17757	22.62293

0.17758	22.63413

0.17758	22.63547

0.17761	22.6536

0.17762	22.65907

0.17763	22.65627

0.17765	22.66187

0.17766	22.64653

0.17767	22.63693

0.17767	22.6216

0.17768	22.6244

0.17769	22.62573

0.17769	22.62853

0.1777	22.63413

0.17772	22.63547

0.17773	22.65213

0.17774	22.6508

0.17773	22.65907

0.17773	22.67853

0.17774	22.68133

0.17775	22.68827

0.17777	22.68693

0.17778	22.6896

0.17779	22.68133

0.1778	22.66467

0.17781	22.66053

0.17781	22.6632

0.17782	22.66187

0.17785	22.65907

0.17785	22.66053

0.17787	22.66747

0.17787	22.67027

0.17788	22.68133

0.17789	22.69107

0.17792	22.69387

0.17793	22.71467

0.17795	22.716

0.17796	22.72293

0.17797	22.71187

0.17798	22.71187

0.17799	22.698

0.178	22.6952

0.17802	22.69387

0.17803	22.68413

0.17806	22.68827

0.17807	22.69107

0.17809	22.7008

0.1781	22.70213

0.1781	22.72293

0.17812	22.7272

0.17814	22.7396

0.17816	22.74933

0.17817	22.748

0.17817	22.7508

0.17817	22.72853

0.17817	22.72853

0.17817	22.7244

0.17819	22.72293

0.17822	22.71187

0.17825	22.716

0.17825	22.72293

0.17826	22.7272

0.17827	22.73133

0.17828	22.7452

0.1783	22.75213

0.17831	22.7688

0.17832	22.7716

0.17836	22.77293

0.17836	22.76747

0.17835	22.75493

0.17836	22.7536

0.17838	22.75213

0.17838	22.74933

0.17839	22.7424

0.1784	22.7452

0.17842	22.7508

0.17843	22.7508

0.17844	22.75213

0.17846	22.76467

0.17847	22.78133

0.17848	22.78827

0.17848	22.79107

0.17848	22.798

0.17849	22.8036

0.1785	22.8036

0.17852	22.80213

0.17854	22.8008

0.17855	22.79387

0.17855	22.78

0.17856	22.7716

0.17854	22.77573

0.17855	22.77573

0.17857	22.7744

0.17857	22.78133

0.17861	22.798

0.17861	22.8036

0.17862	22.80627

0.17864	22.8188

0.17864	22.83267

0.17867	22.83267

0.17866	22.83413

0.17867	22.83

0.17866	22.83547

0.17868	22.80907

0.1787	22.80907

0.17872	22.80627

0.17872	22.80773

0.17873	22.80493

0.17875	22.80773

0.17873	22.8272

0.17876	22.83413

0.17877	22.84107

0.17877	22.848

0.17879	22.86467

0.17878	22.86333

0.1788	22.85907

0.17881	22.85213

0.17881	22.8536

0.17883	22.8396

0.17886	22.82853

0.17888	22.82853

0.17889	22.82853

0.17888	22.83547

0.1789	22.8452

0.1789	22.86187

0.17891	22.87027

0.17892	22.87853

0.17893	22.88267

0.17894	22.88547

0.17896	22.88

0.17899	22.87307

0.179	22.87307

0.17899	22.8688

0.17901	22.85907

0.179	22.85493

0.17903	22.8564

0.17904	22.86187

0.17905	22.86467

0.17907	22.88267

0.17907	22.89933

0.17909	22.90773

0.1791	22.90907

0.17911	22.91467

0.17913	22.916

0.17914	22.91053

0.17916	22.90907

0.17916	22.91053

0.17917	22.9036

0.1792	22.88693

0.17919	22.88547

0.17921	22.88693

0.17922	22.88827

0.17923	22.88547

0.17925	22.89933

0.17924	22.92307

0.17926	22.92853

0.17925	22.93693

0.17925	22.93693

0.17929	22.948

0.1793	22.93827

0.17931	22.9452

0.17933	22.93973

0.17931	22.9244

0.17934	22.9272

0.17934	22.91467

0.17936	22.91747

0.17937	22.91747

0.17937	22.91747

0.17938	22.92573

0.17939	22.93973

0.1794	22.93973

0.17941	22.948

0.17942	22.94933

0.17942	22.96747

0.17945	22.9716

0.17946	22.9772

0.17949	22.98133

0.17951	22.97853

0.17952	22.9744

0.17954	22.95493

0.17954	22.95907

0.17954	22.9564

0.17954	22.9564

0.17956	22.9508

0.17958	22.9536

0.17958	22.9564

0.17958	22.96053

0.17958	22.9688

0.17962	22.97853

0.17962	23.00213

0.17963	23.00493

0.17963	23.01053

0.17963	23.01187

0.17966	23.00213

0.17966	22.98693

0.17968	22.9828

0.17968	22.98547

0.17968	22.98

0.17971	22.97573

0.17973	22.97573

0.17974	22.98827

0.17975	22.98693

0.17977	22.9952

0.17977	23.01333

0.17978	23.02853

0.17979	23.03547

0.17978	23.0328

0.1798	23.03547

0.17983	23.02853

0.17985	23.01613

0.17987	23.00773

0.17986	23.0064

0.17989	23.00213

0.1799	23.0036

0.1799	23.0188

0.17992	23.01747

0.17994	23.0244

0.17995	23.0272

0.17993	23.0508

0.17995	23.06053

0.17995	23.06187

0.17996	23.06053

0.17998	23.05213

0.17998	23.0536

0.18002	23.03973

0.18003	23.048

0.18004	23.03973

0.18006	23.03413

0.18006	23.03413

0.18006	23.03547

0.18009	23.04107

0.18011	23.0452

0.18011	23.0508

0.1801	23.07027

0.18011	23.08

0.18011	23.08693

0.18013	23.09107

0.18014	23.08973

0.18016	23.09107

0.18019	23.0772

0.1802	23.08

0.18021	23.0744

0.18021	23.07573

0.18022	23.06333

0.18022	23.06333

0.18024	23.0688

0.18026	23.0688

0.18027	23.0772

0.18027	23.08827

0.18028	23.0952

0.18028	23.11187

0.18028	23.1092

0.1803	23.1188

0.18033	23.1188

0.18034	23.11467

0.18036	23.1064

0.18037	23.11187

0.1804	23.11053

0.18041	23.1008

0.1804	23.09387

0.1804	23.09387

0.18041	23.0952

0.18045	23.09947

0.18046	23.1008

0.18048	23.1064

0.18047	23.11333

0.18048	23.11187

0.18048	23.1188

0.18049	23.13693

0.18047	23.15213

0.18048	23.1564

0.18049	23.1536

0.18051	23.1592

0.18053	23.14253

0.18057	23.13133

0.18058	23.12853

0.1806	23.1328

0.1806	23.1328

0.18063	23.1272

0.18065	23.12853

0.18066	23.13827

0.18068	23.14253

0.1807	23.14253

0.18071	23.16613

0.18071	23.16613

0.18074	23.18413

0.18073	23.18413

0.18072	23.18693

0.18074	23.18973

0.18076	23.17307

0.18078	23.17307

0.18078	23.1688

0.18078	23.16613

0.18079	23.15773

0.1808	23.15773

0.1808	23.1592

0.18083	23.16187

0.18084	23.16053

0.18086	23.1744

0.18087	23.17587

0.18088	23.1952

0.18087	23.2036

0.18087	23.20213

0.18089	23.21613

0.18089	23.21467

0.18091	23.22027

0.18092	23.21613

0.18094	23.22027

0.18095	23.2008

0.18097	23.18973

0.18097	23.19253

0.18097	23.1952

0.181	23.19253

0.18102	23.19107

0.18103	23.20773

0.18105	23.2064

0.18105	23.21053

0.18107	23.21053

0.18108	23.23

0.1811	23.24947

0.1811	23.248

0.18114	23.25227

0.18115	23.248

0.18115	23.2452

0.18117	23.2328

0.18117	23.23413

0.1812	23.22853

0.1812	23.23

0.18122	23.21893

0.18122	23.22027

0.18124	23.22587

0.18124	23.22587

0.18124	23.23413

0.18126	23.24387

0.18126	23.26613

0.18128	23.26893

0.1813	23.2744

0.18132	23.2772

0.18133	23.2772

0.18135	23.28133

0.18134	23.27027

0.18136	23.2744

0.18137	23.27027

0.18139	23.26053

0.18139	23.25227

0.18139	23.2536

0.1814	23.2536

0.18141	23.25493

0.18145	23.27027

0.18145	23.2828

0.18148	23.29253

0.18148	23.29107

0.1815	23.29947

0.18151	23.3092

0.18151	23.3064

0.18152	23.31187

0.18152	23.3064

0.18153	23.30227

0.18153	23.2856

0.18154	23.28

0.18154	23.28413

0.18156	23.2828

0.18159	23.28133

0.18163	23.28413

0.18165	23.29947

0.18165	23.29947

0.18164	23.30493

0.18165	23.32307

0.18168	23.33133

0.1817	23.33827

0.18172	23.34533

0.18173	23.33827

0.18174	23.34253

0.18175	23.32867

0.18176	23.31467

0.18177	23.31613

0.18178	23.32307

0.18178	23.31747

0.1818	23.31333

0.18181	23.3216

0.18181	23.3216

0.18183	23.3244

0.18184	23.3244

0.18185	23.34387

0.18187	23.35773

0.18187	23.3592

0.18188	23.37027

0.18189	23.37027

0.1819	23.3716

0.18191	23.35773

0.18194	23.362

0.18195	23.35773

0.18198	23.36613

0.18198	23.348

0.182	23.34253

0.182	23.34667

0.182	23.34533

0.182	23.35227

0.18201	23.3536

0.18203	23.36893

0.18205	23.36893

0.18207	23.37867

0.18208	23.3856

0.18211	23.39947

0.18212	23.40493

0.18212	23.4008

0.18212	23.40227

0.18212	23.398

0.18215	23.38827

0.18217	23.3744

0.1822	23.37867

0.18222	23.3744

0.18223	23.37867

0.18225	23.38133

0.18225	23.39253

0.18225	23.40227

0.18224	23.4036

0.18226	23.40773

0.18226	23.41893

0.18227	23.43133

0.18227	23.4356

0.18227	23.4356

0.18228	23.43693

0.18232	23.4328

0.18234	23.41613

0.18235	23.4092

0.18236	23.41333

0.18237	23.412

0.18239	23.40773

0.18241	23.41053

0.18242	23.412

0.18243	23.41613

0.18244	23.41747

0.18246	23.4328

0.18247	23.45227

0.18248	23.4564

0.18248	23.4564

0.18248	23.4592

0.1825	23.47173

0.18251	23.46893

0.18254	23.46053

0.18257	23.46333

0.18258	23.46333

0.18258	23.44947

0.18259	23.43827

0.1826	23.44253

0.18262	23.44667

0.18264	23.44387

0.18265	23.4508

0.18269	23.45507

0.18269	23.46053

0.18268	23.46747

0.18268	23.47307

0.1827	23.48693

0.18272	23.49387

0.18274	23.49947

0.18276	23.49947

0.18275	23.5036

0.18276	23.49533

0.18277	23.49253

0.18279	23.49107

0.18281	23.49667

0.18281	23.49533

0.18283	23.48413

0.18283	23.4772

0.18285	23.4744

0.18285	23.47867

0.18286	23.48133

0.18287	23.4828

0.18289	23.49667

0.1829	23.50227

0.18292	23.5092

0.18293	23.51467

0.18293	23.5272

0.18295	23.5328

0.18298	23.5384

0.18299	23.5384

0.18301	23.54253

0.18302	23.5328

0.18304	23.52173

0.18304	23.52587

0.18304	23.52587

0.18305	23.5244

0.18308	23.51053

0.18308	23.51333

0.1831	23.512

0.1831	23.52173

0.18312	23.52027

0.18312	23.5384

0.18314	23.5508

0.18315	23.5536

0.18317	23.5564

0.18318	23.55773

0.18318	23.57027

0.1832	23.5744

0.18321	23.5772

0.18323	23.57027

0.18325	23.5648

0.18325	23.56053

0.18326	23.548

0.18328	23.5536

0.1833	23.5536

0.1833	23.55773

0.1833	23.5508

0.18333	23.5536

0.18335	23.5508

0.18338	23.55773

0.18339	23.55773

0.1834	23.57173

0.18339	23.58973

0.18339	23.59253

0.18339	23.59533

0.1834	23.59667

0.18341	23.6092

0.1834	23.6036

0.18343	23.6092

0.18344	23.6008

0.18347	23.6008

0.18347	23.58413

0.18349	23.5772

0.1835	23.5828

0.1835	23.5828

0.18351	23.5856

0.18353	23.58413

0.18354	23.59813

0.18356	23.6008

0.18358	23.61333

0.18359	23.6092

0.18358	23.6272

0.18362	23.63973

0.18364	23.6384

0.18365	23.64533

0.18364	23.6356

0.18366	23.63147

0.18365	23.61613

0.18366	23.61893

0.18366	23.61747

0.18368	23.6244

0.1837	23.61747

0.18374	23.61333

0.18376	23.61747

0.18377	23.61747

0.18378	23.62867

0.18382	23.6356

0.18382	23.65773

0.18383	23.6564

0.18383	23.66053

0.18385	23.662

0.18385	23.66893

0.18388	23.67027

0.18389	23.66747

0.1839	23.68

0.18391	23.67173

0.18392	23.66893

0.18394	23.65227

0.18397	23.6536

0.18396	23.65227

0.184	23.65227

0.18399	23.6508

0.18403	23.65507

0.18405	23.66613

0.18406	23.662

0.18407	23.66613

0.18411	23.67587

0.18409	23.69387

0.18411	23.69533

0.18413	23.69947

0.18414	23.70507

0.18413	23.7064

0.18416	23.70507

0.18416	23.69947

0.18418	23.7036

0.18418	23.69813

0.1842	23.69813

0.18422	23.68973

0.18423	23.69253

0.18423	23.6912

0.18425	23.69253

0.18425	23.68973

0.18423	23.6856

0.18425	23.69387

0.18425	23.69813

0.18429	23.7008

0.18427	23.7092

0.1843	23.73

0.18431	23.73413

0.18431	23.73693

0.18432	23.74387

0.18435	23.74387

0.18438	23.74667

0.1844	23.7412

0.18442	23.74947

0.18441	23.74813

0.18442	23.72587

0.18444	23.72867

0.18447	23.73147

0.18447	23.7272

0.18449	23.7272

0.18449	23.7272

0.18451	23.7384

0.18452	23.75227

0.18454	23.74947

0.18456	23.7564

0.18457	23.77027

0.18458	23.77867

0.1846	23.78413

0.1846	23.78147

0.18463	23.78

0.18464	23.77027

0.18464	23.7648

0.18467	23.75787

0.18468	23.76333

0.18468	23.7592

0.18471	23.7536

0.1847	23.762

0.18473	23.7592

0.18476	23.76747

0.18478	23.7648

0.18477	23.78

0.18478	23.79387

0.18478	23.79947

0.18481	23.79947

0.18482	23.80507

0.18483	23.81333

0.18486	23.80787

0.18487	23.81613

0.18487	23.81053

0.18489	23.8148

0.18491	23.8036

0.18494	23.79813

0.18495	23.79667

0.18495	23.79813

0.18496	23.79667

0.18498	23.79387

0.185	23.79387

0.18502	23.7912

0.18501	23.79947

0.18503	23.8036

0.18506	23.81893

0.18507	23.8356

0.18509	23.83973

0.1851	23.83973

0.18511	23.84533

0.18511	23.84947

0.18511	23.84813

0.18514	23.8564

0.18516	23.85227

0.18516	23.8592

0.18519	23.8592

0.1852	23.8592

0.18522	23.85227

0.18523	23.8564

0.18524	23.8536

0.18526	23.84667

0.18526	23.83693

0.18528	23.8384

0.1853	23.83973

0.18529	23.8412

0.1853	23.83973

0.18528	23.84533

0.18531	23.85093

0.18533	23.8536

0.18532	23.85507

0.18533	23.87453

0.18531	23.8856

0.18534	23.8884

0.18535	23.88973

0.18538	23.89533

0.18542	23.89533

0.18541	23.89387

0.18543	23.8912

0.18542	23.89253

0.18544	23.89253

0.18546	23.8856

0.18549	23.87867

0.1855	23.8772

0.18552	23.88147

0.18552	23.87587

0.18556	23.8772

0.18555	23.88147

0.18558	23.88693

0.18558	23.88973

0.18557	23.89533

0.18561	23.912

0.18561	23.92453

0.18564	23.93

0.18566	23.93

0.18566	23.9328

0.1857	23.9356

0.18571	23.93

0.18571	23.9356

0.18569	23.93427

0.18568	23.9356

0.1857	23.92587

0.18572	23.91613

0.18574	23.9148

0.18574	23.91893

0.18575	23.9176

0.18578	23.91333

0.18581	23.92173

0.18583	23.91893

0.18582	23.92587

0.18584	23.92867

0.18585	23.93973

0.18587	23.95093

0.1859	23.95507

0.18591	23.9536

0.18593	23.9592

0.18592	23.97027

0.18593	23.97453

0.18594	23.97733

0.18596	23.97307

0.18599	23.98

0.18598	23.96613

0.186	23.9564

0.18603	23.9564

0.18607	23.96333

0.18607	23.96067

0.18608	23.95227

0.1861	23.9564

0.18611	23.9564

0.18611	23.95787

0.18614	23.96333

0.18614	23.96893

0.18614	23.98427

0.18614	23.98693

0.18615	23.9884

0.18619	23.994

0.18619	24.0064

0.18621	24.0064

0.18622	24.012

0.18623	24.0148

0.18624	24.02027

0.18624	24.0148

0.18623	24.0092

0.18625	23.99947

0.18626	24.0064

0.18626	24.00093

0.18629	23.994

0.18631	23.9912

0.18634	23.9912

0.18634	23.99667

0.18636	23.994

0.18637	24.0064

0.18638	24.0176

0.18637	24.02587

0.18639	24.02453

0.1864	24.03707

0.18645	24.0356

0.18646	24.044

0.18648	24.04667

0.18648	24.04947

0.18646	24.05507

0.18647	24.05373

0.1865	24.05227

0.18653	24.044

0.18656	24.04947

0.18656	24.04813

0.18657	24.0384

0.18657	24.03147

0.18659	24.0328

0.1866	24.03427

0.18662	24.03147

0.18662	24.04533

0.18663	24.05507

0.18666	24.06067

0.18669	24.062

0.1867	24.07733

0.1867	24.07867

0.18671	24.08427

0.18672	24.0912

0.18673	24.08707

0.18676	24.09533

0.18677	24.08427

0.18679	24.07587

0.1868	24.07867

0.1868	24.07733

0.1868	24.07867

0.1868	24.06613

0.18682	24.06893

0.18683	24.0676

0.18686	24.07307

0.18687	24.07307

0.18688	24.07453

0.1869	24.07867

0.18693	24.08147

0.18694	24.0828

0.18694	24.08427

0.18694	24.09253

0.18696	24.1064

0.18698	24.11613

0.18699	24.12173

0.187	24.12587

0.187	24.13

0.18702	24.13

0.18703	24.13707

0.18705	24.13147

0.18708	24.13707

0.18709	24.12307

0.18711	24.112

0.18712	24.1148

0.18713	24.12453

0.18713	24.12173

0.18715	24.1176

0.18717	24.112

0.18718	24.1148

0.18719	24.1204

0.18719	24.1176

0.18721	24.12733

0.18723	24.1412

0.18723	24.14533

0.18726	24.14253

0.18727	24.14667

0.18729	24.1648

0.1873	24.16893

0.18731	24.17453

0.18733	24.16893

0.18736	24.17587

0.18738	24.17307

0.18738	24.17173

0.18738	24.1704

0.18739	24.16613

0.1874	24.17307

0.18741	24.16613

0.18743	24.15373

0.18745	24.1564

0.18747	24.15507

0.18748	24.1592

0.18748	24.16067

0.18749	24.1592

0.18749	24.16893

0.18752	24.16893

0.18754	24.17867

0.18755	24.17587

0.18757	24.18973

0.18758	24.194

0.18761	24.19253

0.18763	24.19533

0.18763	24.2092

0.18764	24.2176

0.18766	24.2176

0.18768	24.21893

0.18769	24.2176

0.1877	24.21347

0.1877	24.2064

0.1877	24.2064

0.18774	24.212

0.18776	24.21067

0.18778	24.20373

0.1878	24.19947

0.1878	24.19947

0.18783	24.20093

0.18784	24.20373

0.18787	24.2064

0.18787	24.21347

0.18788	24.21893

0.18788	24.2204

0.18787	24.22587

0.18789	24.2384

0.18791	24.25093

0.18793	24.25227

0.18795	24.25507

0.18795	24.25227

0.18795	24.25787

0.18797	24.262

0.18799	24.26347

0.18801	24.26893

0.18801	24.262

0.18802	24.26893

0.18804	24.25093

0.18806	24.24947

0.18807	24.24947

0.18807	24.25507

0.18809	24.25093

0.18813	24.24533

0.18814	24.24947

0.18816	24.24947

0.18817	24.25373

0.18821	24.25227

0.18821	24.26613

0.18822	24.2732

0.18822	24.2704

0.18823	24.2704

0.18824	24.28427

0.18828	24.29813

0.1883	24.29813

0.18831	24.30093

0.18833	24.30227

0.18832	24.31067

0.18832	24.30227

0.18831	24.30507

0.18833	24.30507

0.18837	24.31067

0.18839	24.30227

0.18841	24.2912

0.18842	24.294

0.18842	24.2912

0.18844	24.2884

0.18847	24.28987

0.1885	24.294

0.18852	24.29533

0.18852	24.2968

0.18853	24.3092

0.18855	24.30227

0.18855	24.3176

0.18858	24.31893

0.18856	24.32173

0.18859	24.32453

0.1886	24.33987

0.1886	24.35093

0.18863	24.34253

0.18863	24.35093

0.18867	24.35093

0.18869	24.35653

0.1887	24.34947

0.18872	24.35093

0.18872	24.3468

0.18876	24.35227

0.18878	24.33707

0.18877	24.32733

0.18876	24.3328

0.18879	24.33427

0.18879	24.3356

0.18882	24.3384

0.18884	24.34253

0.18881	24.3412

0.18884	24.35093

0.18885	24.34813

0.18887	24.3648

0.18888	24.37733

0.18888	24.38013

0.18892	24.3828

0.18893	24.3828

0.18894	24.394

0.18895	24.3912

0.18896	24.394

0.18899	24.3968

0.189	24.40227

0.189	24.39533

0.18902	24.3828

0.18903	24.38707

0.18905	24.38987

0.18905	24.39533

0.18907	24.3856

0.18908	24.38013

0.18909	24.38147

0.18908	24.3856

0.18909	24.38147

0.18912	24.3828

0.18914	24.3912

0.18916	24.3856

0.18915	24.3912

0.18917	24.38987

0.18919	24.40507

0.18921	24.41067

0.18923	24.412

0.18925	24.40507

0.18928	24.41347

0.1893	24.4232

0.18931	24.4356

0.1893	24.43707

0.18932	24.43147

0.18934	24.44253

0.18932	24.4412

0.18935	24.4496

0.18938	24.4496

0.18939	24.45093

0.1894	24.45653

0.18941	24.46347

0.18943	24.45787

0.18946	24.4496

0.18943	24.45373

0.18948	24.45093

0.1895	24.44813

0.18952	24.43707

0.18951	24.4412

0.18953	24.43987

0.18956	24.44533

0.18957	24.4412

0.18958	24.43987

0.18958	24.44533

0.1896	24.444

0.18962	24.4496

0.18962	24.4468

0.18963	24.46067

0.18966	24.462

0.18967	24.46347

0.1897	24.4732

0.18973	24.4704

0.18974	24.4856

0.18973	24.4856

0.18976	24.4884

0.18977	24.49813

0.18975	24.4996

0.18977	24.50653

0.18979	24.50653

0.18982	24.51067

0.18983	24.51347

0.18984	24.5148

0.18985	24.50093

0.18986	24.50653

0.18986	24.50227

0.18989	24.4996

0.1899	24.4968

0.1899	24.4912

0.18989	24.4912

0.18992	24.4968

0.18991	24.49813

0.18994	24.49533

0.18995	24.50653

0.18999	24.50373

0.18997	24.50653

0.19	24.50653

0.19001	24.512

0.19003	24.52733

0.19004	24.52733

0.19006	24.53147

0.19005	24.5356

0.19008	24.5496

0.19009	24.5496

0.19014	24.55373

0.19011	24.55787

0.19013	24.55653

0.19016	24.55933

0.19018	24.55787

0.19018	24.56347

0.19023	24.55933

0.19024	24.55507

0.19022	24.55093

0.19023	24.5468

0.19027	24.53987

0.19027	24.54267

0.1903	24.5468

0.19031	24.54533

0.19032	24.5496

0.19032	24.54813

0.19034	24.55653

0.19034	24.55507

0.19035	24.5648

0.19037	24.5676

0.19041	24.5732

0.19043	24.56893

0.19044	24.5856

0.19043	24.58427

0.19044	24.59533

0.19046	24.59813

0.19048	24.59813

0.19049	24.5996

0.1905	24.60373

0.19053	24.60507

0.19053	24.60787

0.19057	24.61067

0.19055	24.60933

0.19055	24.60653

0.19056	24.59813

0.19059	24.5996

0.19058	24.60227

0.19062	24.5996

0.19063	24.5968

0.19065	24.5912

0.19067	24.59533

0.19068	24.59533

0.1907	24.59813

0.19071	24.60093

0.19075	24.60373

0.19075	24.61067

0.19078	24.61067

0.19077	24.61627

0.19078	24.61627

0.19079	24.63427

0.19081	24.63013

0.19081	24.63013

0.19084	24.6412

0.19087	24.644

0.19088	24.65373

0.1909	24.6524

0.1909	24.65933

0.19092	24.65653

0.19094	24.6648

0.19097	24.6648

0.19095	24.66067

0.19099	24.66347

0.19098	24.6676

0.19102	24.66347

0.19103	24.65093

0.19103	24.65933

0.19105	24.65933

0.19105	24.66347

0.19107	24.65507

0.19108	24.65093

0.19111	24.64813

0.19112	24.65093

0.19113	24.65507

0.19114	24.65787

0.19116	24.6676

0.19119	24.6648

0.19119	24.6732

0.1912	24.67867

0.19122	24.68147

0.19122	24.69267

0.19123	24.69533

0.19124	24.694

0.19126	24.70093

0.19129	24.7024

0.19129	24.70787

0.19131	24.71067

0.1913	24.70787

0.19134	24.71067

0.19136	24.7176

0.1914	24.71347

0.19139	24.72453

0.19141	24.7204

0.19141	24.72867

0.19142	24.7176

0.19143	24.7176

0.19142	24.712

0.19145	24.7232

0.19147	24.7204

0.1915	24.71627

0.19151	24.712

0.19151	24.712

0.19154	24.71347

0.19156	24.71067

0.19156	24.7176

0.19158	24.7148

0.19159	24.71907

0.19161	24.7204

0.19164	24.73293

0.19165	24.72733

0.19166	24.73987

0.19168	24.7412

0.1917	24.73573

0.1917	24.75507

0.19172	24.75373

0.19173	24.7648

0.19174	24.76213

0.19174	24.76627

0.19175	24.77453

0.19176	24.77173

0.19178	24.78147

0.19179	24.7788

0.19181	24.78427

0.19182	24.78013

0.19184	24.78573

0.19187	24.76907

0.19187	24.77733

0.19189	24.77453

0.19189	24.78013

0.19189	24.76213

0.19193	24.76067

0.19194	24.7648

0.19195	24.76347

0.19196	24.7704

0.19197	24.76907

0.192	24.7732

0.192	24.77453

0.19202	24.7732

0.19203	24.77453

0.19205	24.78987

0.19206	24.79813

0.19207	24.7912

0.19208	24.79813

0.19208	24.7968

0.19211	24.81627

0.19212	24.8232

0.19214	24.82173

0.19216	24.8232

0.19218	24.8288

0.1922	24.83427

0.1922	24.82733

0.1922	24.83707

0.19222	24.8288

0.19225	24.83573

0.19228	24.82733

0.19231	24.82453

0.19233	24.82453

0.19235	24.82733

0.19236	24.82733

0.19238	24.8204

0.19239	24.8204

0.19241	24.82173

0.19242	24.82453

0.19243	24.82733

0.19244	24.82733

0.19246	24.8288

0.19247	24.82733

0.19248	24.83013

0.19247	24.83293

0.19249	24.83293

0.19252	24.83573

0.19254	24.83987

0.19256	24.83573

0.19259	24.8412

0.1926	24.8468

0.19262	24.85933

0.19264	24.86067

0.19267	24.86067

0.19268	24.86213

0.1927	24.87187

0.19271	24.88427

0.19272	24.88573

0.19271	24.88987

0.19271	24.88707

0.19272	24.8968

0.19273	24.90373

0.19275	24.8968

0.19279	24.9052

0.19279	24.8968

0.1928	24.9052

0.1928	24.89267

0.19282	24.89547

0.19283	24.8912

0.19285	24.88853

0.19288	24.88987

0.19289	24.88707

0.19291	24.88853

0.19291	24.88987

0.19295	24.894

0.19296	24.8912

0.19298	24.8912

0.19298	24.89267

0.193	24.8968

0.19302	24.90093

0.19303	24.90093

0.19305	24.91347

0.19306	24.9148

0.19308	24.9204

0.1931	24.93293

0.19311	24.93293

0.19312	24.9412

0.19313	24.93987

0.19314	24.93987

0.19316	24.9496

0.19318	24.9524

0.19321	24.94547

0.19321	24.9552

0.19322	24.9524

0.19324	24.95933

0.19326	24.9524

0.19327	24.9552

0.1933	24.9524

0.1933	24.95787

0.1933	24.94813

0.19331	24.9496

0.19334	24.94547

0.19333	24.9412

0.19334	24.94547

0.19335	24.944

0.19338	24.9496

0.19339	24.9496

0.19342	24.9524

0.19344	24.95373

0.19344	24.95787

0.19347	24.96627

0.19349	24.9648

0.1935	24.96907

0.19348	24.96627

0.1935	24.98573

0.19353	24.9968

0.19354	24.99547

0.19358	24.9968

0.19362	24.98987

0.19363	25.00653

0.19365	25.00373

0.19366	25.01347

0.19367	25.00933

0.19366	25.01347

0.19368	25.01347

0.19369	25.0176

0.19371	25.01627

0.19371	25.0176

0.19373	25.02733

0.19372	25.01907

0.19375	25.02453

0.19377	25.01347

0.19382	25.0232

0.19385	25.0176

0.19387	25.02187

0.19389	25.01627

0.1939	25.01067

0.19391	25.01213

0.1939	25.01493

0.19395	25.01213

0.19394	25.01493

0.19398	25.0176

0.19396	25.0176

0.194	25.0204

0.194	25.02453

0.19401	25.02453

0.19403	25.03427

0.19407	25.0316

0.1941	25.03427

0.1941	25.0412

0.19412	25.0468

0.19413	25.05093

0.19415	25.0524

0.19415	25.0496

0.19417	25.06347

0.19417	25.06067

0.19419	25.0704

0.19421	25.07187

0.19422	25.07187

0.19424	25.0704

0.19427	25.08013

0.19431	25.08427

0.1943	25.08853

0.19432	25.08987

0.19436	25.08707

0.19436	25.08853

0.19438	25.0912

0.19438	25.0912

0.19441	25.10093

0.19443	25.08707

0.19446	25.09547

0.19446	25.08853

0.19447	25.1024

0.1945	25.10373

0.19451	25.10933

0.19452	25.10373

0.19452	25.0912

0.19454	25.0968

0.19457	25.09547

0.19456	25.08987

0.19459	25.0912

0.19459	25.08853

0.19463	25.09267

0.19466	25.0968

0.19468	25.09827

0.19471	25.0968

0.19471	25.10933

0.19474	25.0996

0.19474	25.108

0.19476	25.11067

0.19477	25.1176

0.19479	25.1288

0.19481	25.13013

0.19481	25.1288

0.19482	25.14133

0.19484	25.14547

0.19486	25.144

0.19489	25.1496

0.19489	25.1468

0.19492	25.15653

0.19493	25.15653

0.19495	25.158

0.19499	25.16067

0.195	25.16493

0.19502	25.15933

0.19503	25.1552

0.19505	25.15933

0.19508	25.15653

0.1951	25.1676

0.1951	25.15373

0.19514	25.16067

0.19513	25.1552

0.19515	25.16067

0.19517	25.16347

0.19518	25.1524

0.1952	25.1552

0.1952	25.1524

0.19523	25.16067

0.19528	25.15933

0.19528	25.16627

0.19532	25.16067

0.19532	25.16213

0.19534	25.16347

0.19536	25.16493

0.19536	25.17733

0.19539	25.18707

0.19539	25.19133

0.1954	25.17733

0.19542	25.18573

0.19544	25.18853

0.19544	25.19133

0.19548	25.1996

0.1955	25.1996

0.19552	25.1968

0.19555	25.1996

0.19555	25.20653

0.19557	25.21347

0.19559	25.21907

0.19558	25.21347

0.19562	25.2204

0.19564	25.21907

0.19565	25.2288

0.19567	25.23293

0.19569	25.23013

0.19571	25.23853

0.19574	25.2344

0.19576	25.24267

0.19576	25.244

0.19579	25.244

0.19579	25.24827

0.19581	25.2496

0.19583	25.25933

0.19584	25.2524

0.19585	25.258

0.19589	25.2524

0.19589	25.26627

0.19591	25.258

0.19594	25.26627

0.19595	25.26213

0.19597	25.25933

0.196	25.26347

0.19601	25.26213

0.19603	25.27467

0.19603	25.26347

0.19605	25.2704

0.19606	25.258

0.19609	25.258

0.19612	25.25653

0.19614	25.26627

0.19618	25.25933

0.1962	25.26213

0.19621	25.26067

0.19622	25.258

0.19625	25.26347

0.19625	25.26493

0.19628	25.26907

0.1963	25.2732

0.19631	25.2788

0.19632	25.2704

0.19633	25.2816

0.19635	25.28573

0.19636	25.29267

0.19637	25.294

0.1964	25.29133

0.19641	25.3024

0.19644	25.29827

0.19644	25.31213

0.19645	25.31627

0.19647	25.31627

0.1965	25.32187

0.19653	25.31773

0.19654	25.326

0.19656	25.3232

0.19657	25.32733

0.19661	25.3232

0.19663	25.3316

0.19664	25.33853

0.19664	25.33573

0.19667	25.33853

0.19668	25.33853

0.1967	25.34547

0.19673	25.34133

0.19675	25.35107

0.19675	25.3496

0.19678	25.35107

0.1968	25.3496

0.19682	25.3496

0.19684	25.3552

0.19686	25.35653

0.19687	25.35933

0.19689	25.358

0.19691	25.3608

0.19694	25.3496

0.19696	25.36347

0.19697	25.35373

0.19698	25.36347

0.197	25.3552

0.19702	25.36213

0.19705	25.36493

0.19707	25.3608

0.1971	25.36493

0.19711	25.35653

0.19713	25.36493

0.19714	25.36627

0.19715	25.35933

0.19716	25.36347

0.19719	25.358

0.19721	25.36627

0.19723	25.36347

0.19724	25.37187

0.19726	25.3704

0.19728	25.38707

0.19729	25.38013

0.19731	25.3844

0.19733	25.38853

0.19735	25.38573

0.19736	25.38707

0.19738	25.38707

0.19741	25.38987

0.19744	25.39267

0.19745	25.39827

0.19744	25.40653

0.19746	25.4108

0.19749	25.4052

0.19752	25.41213

0.19752	25.4108

0.19752	25.4232

0.19753	25.42747

0.19755	25.43013

0.19756	25.43707

0.19759	25.43013

0.19761	25.43707

0.19762	25.44267

0.19765	25.44267

0.19767	25.44133

0.19768	25.44413

0.19771	25.44413

0.19773	25.44413

0.19775	25.45107

0.19775	25.43853

0.19777	25.44827

0.19779	25.43293

0.19781	25.44133

0.19782	25.43707

0.19783	25.43573

0.19784	25.43987

0.19787	25.43707

0.1979	25.44413

0.19791	25.44413

0.19793	25.4468

0.19794	25.44827

0.19795	25.44827

0.19798	25.4496

0.19801	25.4524

0.19805	25.45653

0.19807	25.4524

0.19808	25.46213

0.1981	25.45933

0.19811	25.46493

0.19813	25.4608

0.19817	25.46493

0.19817	25.47467

0.19818	25.476

0.1982	25.47747

0.19822	25.48853

0.19826	25.48853

0.19827	25.4996

0.19829	25.4996

0.19829	25.4996

0.19831	25.50933

0.19833	25.50933

0.19836	25.51213

0.19836	25.51773

0.19837	25.51627

0.19838	25.52187

0.1984	25.51493

0.19843	25.51773

0.19846	25.51213

0.19848	25.52467

0.19848	25.52053

0.19851	25.5288

0.19855	25.52053

0.19857	25.52053

0.19859	25.52053

0.1986	25.51213

0.19862	25.51773

0.19865	25.51347

0.19866	25.51907

0.19866	25.52053

0.1987	25.52053

0.19871	25.52053

0.1987	25.51347

0.19871	25.5232

0.19874	25.526

0.19878	25.53293

0.19879	25.52747

0.1988	25.53987

0.19882	25.53573

0.19884	25.53987

0.19885	25.54413

0.19887	25.54547

0.1989	25.55387

0.19893	25.5552

0.19893	25.55653

0.19895	25.56213

0.19897	25.56213

0.19899	25.56907

0.19901	25.57187

0.19903	25.56907

0.19906	25.5788

0.19908	25.58027

0.19907	25.5816

0.19908	25.59133

0.19908	25.5844

0.19911	25.58293

0.19915	25.59133

0.19918	25.59827

0.19919	25.59827

0.1992	25.60107

0.19921	25.6052

0.19924	25.60387

0.19928	25.6108

0.19928	25.60653

0.19931	25.6136

0.19936	25.60387

0.19937	25.6136

0.19938	25.5996

0.19938	25.608

0.19939	25.60107

0.19938	25.5996

0.19941	25.6024

0.19945	25.59827

0.19948	25.60387

0.1995	25.6136

0.19952	25.61493

0.19954	25.6136

0.19954	25.61627

0.19956	25.61493

0.19958	25.62467

0.1996	25.61627

0.19961	25.61493

0.19964	25.61907

0.19965	25.62053

0.19968	25.62053

0.19969	25.61773

0.19972	25.62187

0.1997	25.6288

0.19972	25.6344

0.19975	25.6344

0.19976	25.63853

0.1998	25.64693

0.19983	25.64133

0.19984	25.64267

0.19986	25.6372

0.19987	25.6496

0.19989	25.64413

0.19992	25.65933

0.19996	25.65387

0.19998	25.65933

0.19999	25.66493

0.20001	25.6636

0.20003	25.67187

0.20006	25.67053

0.20008	25.6732

0.20011	25.676

0.20012	25.6816

0.20015	25.6872

0.20016	25.68573

0.20017	25.68853

0.20018	25.68853

0.20023	25.69693

0.20024	25.69827

0.20026	25.6996

0.20027	25.6996

0.20028	25.70387

0.2003	25.70387

0.20033	25.708

0.20034	25.7136

0.20036	25.71213

0.2004	25.71773

0.20041	25.71213

0.20043	25.71907

0.20043	25.71627

0.20045	25.72467

0.20047	25.7136

0.20049	25.72747

0.2005	25.7108

0.20054	25.71773

0.20055	25.71907

0.20056	25.7108

0.20058	25.72053

0.2006	25.71493

0.20061	25.71773

0.20065	25.71627

0.20067	25.71773

0.2007	25.72187

0.2007	25.72467

0.20074	25.726

0.20076	25.7344

0.20079	25.7316

0.20079	25.73573

0.20082	25.7316

0.20083	25.7316

0.20085	25.74

0.20088	25.7372

0.20089	25.74

0.20092	25.7372

0.20094	25.74

0.20097	25.74413

0.20101	25.74267

0.201	25.7496

0.20102	25.74827

0.20104	25.75387

0.20105	25.7496

0.20109	25.75667

0.2011	25.7636

0.20111	25.7636

0.20112	25.76773

0.20113	25.7636

0.20115	25.77467

0.20116	25.76213

0.20119	25.776

0.20121	25.77333

0.20123	25.7788

0.20125	25.776

0.20129	25.77747

0.20131	25.7844

0.20134	25.77747

0.20136	25.7844

0.20138	25.7844

0.20139	25.78853

0.2014	25.79133

0.20143	25.79413

0.20145	25.79133

0.20148	25.80107

0.20149	25.8024

0.20152	25.8108

0.20154	25.81213

0.20155	25.81493

0.20158	25.82467

0.2016	25.82333

0.20163	25.83027

0.20163	25.83027

0.20165	25.83307

0.20167	25.8372

0.20168	25.83307

0.20171	25.84413

0.20172	25.8372

0.20176	25.84693

0.20177	25.84267

0.20179	25.84693

0.20181	25.84827

0.20183	25.85107

0.20185	25.84973

0.20187	25.84133

0.20189	25.84267

0.20192	25.84267

0.20195	25.84827

0.20197	25.84973

0.202	25.8524

0.202	25.84973

0.20201	25.84693

0.20202	25.8524

0.20205	25.8608

0.20209	25.85107

0.20212	25.8608

0.20215	25.86213

0.20218	25.86213

0.2022	25.86907

0.20222	25.86213

0.20224	25.87467

0.20227	25.8664

0.2023	25.87053

0.20232	25.87053

0.20233	25.87053

0.20234	25.87747

0.20236	25.87333

0.20238	25.87747

0.20241	25.876

0.20242	25.87747

0.20246	25.8788

0.20248	25.88027

0.20251	25.88307

0.20252	25.88027

0.20255	25.89133

0.20256	25.88573

0.2026	25.89827

0.20261	25.89413

0.20263	25.89547

0.20265	25.89973

0.20268	25.90387

0.20267	25.90667

0.20271	25.90933

0.20273	25.90933

0.20277	25.91213

0.20279	25.9164

0.20282	25.92053

0.20283	25.92187

0.20284	25.9164

0.20286	25.9288

0.20288	25.92333

0.2029	25.93307

0.20292	25.93573

0.20295	25.94

0.20298	25.94413

0.20299	25.94133

0.20301	25.94827

0.20302	25.94133

0.20306	25.94827

0.20309	25.9552

0.20311	25.95667

0.20313	25.9636

0.20318	25.9636

0.20319	25.96493

0.20322	25.96493

0.20324	25.97333

0.20326	25.96493

0.20327	25.97333

0.2033	25.97333

0.20333	25.97053

0.20336	25.97613

0.20338	25.96907

0.20339	25.98307

0.2034	25.9788

0.20343	25.98307

0.20346	25.9844

0.20348	25.98853

0.20351	25.98853

0.20352	25.99547

0.20353	25.9872

0.20354	25.98853

0.20357	25.9844

0.20361	25.9872

0.20364	25.99693

0.20367	25.99

0.20368	26.008

0.20371	25.99827

0.20371	26.0108

0.20375	26.00947

0.20376	26.00947

0.2038	26.01773

0.20382	26.01213

0.20383	26.01907

0.20385	26.01773

0.20387	26.02613

0.20391	26.02467

0.20393	26.02467

0.20396	26.02187

0.20397	26.0288

0.204	26.03027

0.20403	26.0316

0.20405	26.03307

0.2041	26.03027

0.20411	26.03573

0.20413	26.02467

0.20414	26.03853

0.20416	26.02613

0.20421	26.0316

0.20423	26.03573

0.20426	26.0372

0.20427	26.03853

0.20428	26.0316

0.20432	26.03853

0.20434	26.04133

0.20437	26.03853

0.20438	26.04547

0.20441	26.04547

0.20446	26.04693

0.20448	26.05253

0.2045	26.04827

0.2045	26.05107

0.20454	26.05107

0.20459	26.05107

0.20462	26.0552

0.20465	26.0552

0.20467	26.05667

0.2047	26.05667

0.20473	26.05947

0.20474	26.0636

0.20476	26.0692

0.2048	26.07613

0.20481	26.0788

0.20485	26.0816

0.20487	26.0788

0.20491	26.0872

0.20494	26.08027

0.20495	26.09693

0.20497	26.0928

0.20498	26.09413

0.20502	26.10253

0.20505	26.09547

0.2051	26.10107

0.20511	26.09413

0.20517	26.09973

0.2052	26.10253

0.20523	26.108

0.20525	26.1108

0.20527	26.11213

0.20529	26.1108

0.20531	26.1164

0.20533	26.12187

0.20537	26.12613

0.20543	26.13027

0.20544	26.12747

0.20546	26.13307

0.2055	26.1316

0.20553	26.13307

0.20556	26.14

0.2056	26.1344

0.20563	26.14

0.20566	26.13853

0.2057	26.14827

0.20574	26.14827

0.20577	26.15107

0.2058	26.15253

0.20584	26.15253

0.2059	26.15107

0.20594	26.14827

0.20596	26.15947

0.20603	26.15107

0.20607	26.1608

0.20613	26.15107

0.20617	26.158

0.20624	26.1552

0.2063	26.158

0.20636	26.15667

0.20644	26.15667

0.20651	26.1636

0.2066	26.1608

0.20667	26.17187

0.20676	26.17053

0.20684	26.17333

0.20693	26.17053

0.20702	26.17893

0.20711	26.17747

0.2072	26.1816

0.20734	26.18307

0.20742	26.1816

0.20754	26.19133

0.20765	26.1844

0.20777	26.19133

0.20789	26.19413

0.20799	26.19413

0.20811	26.19133

0.20825	26.19693

0.20838	26.19973

0.20852	26.1956

0.20865	26.2052

0.20878	26.20253

0.20894	26.20947

0.2091	26.20667

0.20924	26.21227

0.2094	26.21493

0.20955	26.21227

0.20971	26.2192

0.20987	26.2164

0.21003	26.22187

0.21019	26.22053

0.21032	26.22747

0.21051	26.22613

0.21066	26.22747

0.21085	26.2316

0.21102	26.23027

0.21117	26.23307

0.21138	26.2372

0.21156	26.23587

0.21177	26.24

0.21198	26.24133

0.21217	26.24

0.21238	26.24693

0.21256	26.24413

0.21279	26.24693

0.21299	26.24973

0.21318	26.25387

0.21336	26.25387

0.21353	26.25387

0.21375	26.25947

0.21394	26.25387

0.21414	26.26493

0.21435	26.25947

0.21455	26.26773

0.21481	26.26493

0.21502	26.2664

0.21524	26.2692

0.21546	26.27187

0.21571	26.27747

0.21593	26.27187

0.21615	26.27613

0.2164	26.27893

0.21668	26.2816

0.21694	26.28027

0.21725	26.2844

0.21755	26.2872

0.21785	26.29133

0.21822	26.29

0.21855	26.2928

0.21895	26.29693

0.21932	26.29413

0.21973	26.29827

0.2202	26.29693

0.22067	26.30387

0.22121	26.30667

0.22178	26.29827

0.2224	26.308

0.22317	26.30253

0.22396	26.30947

0.22496	26.30387

0.22607	26.29827

0.22755	26.258

0.22998	25.93573

0.23316	25.51907

0.23842	24.9288

0.24663	23.33

0.25695	14.38333

0.2691	7.6132

0.27825	4.92827

0.28624	3.50453

0.2928	2.61133

0.29903	1.99333

0.30569	1.6808

0.31166	1.54733

0.31811	1.47653

0.32392	1.43067

0.32969	1.39733

0.33607	1.37093

0.34179	1.346

**Fig 6. Stress-strain curves with Non-hydraulic and Stabilized hydraulic**

**（*a*）Non-hydraulic**

5.6827E-4	0.10917	2.27701E-4	0.00806	1.55E-4	0	1.55E-4	0	1.55E-4	0	1.55E-4	0	2.25E-4	0.04026

6.10364E-4	0.12684	2.97762E-4	0.03063	2.48E-4	0.02207	2.48E-4	0.02197	2.48E-4	0.02162	2.48E-4	0.02102	2.79E-4	0.06476

6.59474E-4	0.1445	3.67824E-4	0.04997	3.34E-4	0.05886	3.34E-4	0.05859	3.34E-4	0.05767	3.34E-4	0.05607	3.37E-4	0.09101

6.87537E-4	0.15894	4.20371E-4	0.0677	3.74E-4	0.0699	3.74E-4	0.06957	3.74E-4	0.06848	3.74E-4	0.06658	3.88E-4	0.10852

7.01568E-4	0.17339	4.6124E-4	0.08382	4.19E-4	0.09197	4.19E-4	0.09154	4.19E-4	0.0901	4.19E-4	0.0876	4.3E-4	0.12602

7.50678E-4	0.18945	4.90432E-4	0.09832	4.6E-4	0.11404	4.6E-4	0.11351	4.6E-4	0.11172	4.6E-4	0.10863	4.77E-4	0.14353

7.85756E-4	0.20551	5.25463E-4	0.11283	4.84E-4	0.1306	4.84E-4	0.13	4.84E-4	0.12795	4.84E-4	0.1244	5.12E-4	0.16628

8.13819E-4	0.22477	5.7801E-4	0.12895	5.08E-4	0.14348	5.08E-4	0.14281	5.08E-4	0.14056	5.08E-4	0.13667	5.62E-4	0.18903

8.2785E-4	0.23922	6.01363E-4	0.14507	5.45E-4	0.16371	5.45E-4	0.16295	5.45E-4	0.16038	5.45E-4	0.15594	6.01E-4	0.20654

8.83976E-4	0.25848	6.24717E-4	0.15796	5.78E-4	0.18578	5.78E-4	0.18492	5.78E-4	0.182	5.78E-4	0.17696	6.24E-4	0.21879

9.05023E-4	0.26973	6.77264E-4	0.17892	5.98E-4	0.2005	5.98E-4	0.19957	5.98E-4	0.19642	5.98E-4	0.19098	6.43E-4	0.23805

9.19054E-4	0.28418	7.12295E-4	0.19665	6.26E-4	0.22073	6.26E-4	0.21971	6.26E-4	0.21624	6.26E-4	0.21025	6.86E-4	0.2573

9.47117E-4	0.29862	7.35648E-4	0.21277	6.63E-4	0.2428	6.63E-4	0.24168	6.63E-4	0.23787	6.63E-4	0.23127	7.09E-4	0.27305

9.82195E-4	0.3195	7.70679E-4	0.22888	6.79E-4	0.26119	6.79E-4	0.25998	6.79E-4	0.25588	6.79E-4	0.24879	7.36E-4	0.29056

0.001	0.33716	7.99872E-4	0.245	6.96E-4	0.27591	6.96E-4	0.27463	6.96E-4	0.2703	6.96E-4	0.26281	7.52E-4	0.3063

0.00102	0.34679	8.29064E-4	0.26112	7.16E-4	0.29246	7.16E-4	0.29111	7.16E-4	0.28652	7.16E-4	0.27858	7.83E-4	0.32555

0.00105	0.36445	8.4658E-4	0.2724	7.37E-4	0.31454	7.37E-4	0.31308	7.37E-4	0.30815	7.37E-4	0.29961	8.14E-4	0.34656

0.00108	0.38212	8.75772E-4	0.28852	7.59E-4	0.32925	7.59E-4	0.32773	7.59E-4	0.32257	7.59E-4	0.31363	8.37E-4	0.36407

0.00111	0.39816	9.10803E-4	0.30787	7.74E-4	0.34396	7.74E-4	0.34237	7.74E-4	0.33697	7.74E-4	0.32764	8.45E-4	0.37457

0.00112	0.41262	9.39995E-4	0.32076	7.96E-4	0.36603	7.96E-4	0.36434	7.96E-4	0.3586	7.96E-4	0.34866	8.68E-4	0.39907

0.00115	0.43188	9.51672E-4	0.33527	8.25E-4	0.38259	8.25E-4	0.38083	8.25E-4	0.37482	8.25E-4	0.36443	8.99E-4	0.42008

0.00118	0.44793	9.75026E-4	0.35139	8.32E-4	0.39547	8.32E-4	0.39364	8.32E-4	0.38743	8.32E-4	0.3767	9.22E-4	0.43408

0.00119	0.46078	0.00102	0.37073	8.61E-4	0.41754	8.61E-4	0.41561	8.61E-4	0.40906	8.61E-4	0.39772	9.3E-4	0.44808

0.0012	0.47684	0.00103	0.38524	8.91E-4	0.43961	8.91E-4	0.43758	8.91E-4	0.43068	8.91E-4	0.41875	9.53E-4	0.46733

0.00124	0.4945	0.00106	0.40297	9.05E-4	0.45617	9.05E-4	0.45406	9.05E-4	0.4469	9.05E-4	0.43452	9.69E-4	0.48659

0.00126	0.50894	0.00109	0.41908	9.2E-4	0.47272	9.2E-4	0.47054	9.2E-4	0.46312	9.2E-4	0.45028	9.92E-4	0.50584

0.00126	0.5218	0.00113	0.43681	9.49E-4	0.49663	9.49E-4	0.49434	9.49E-4	0.48654	9.49E-4	0.47306	0.001	0.52159

0.00128	0.54106	0.00113	0.4481	9.71E-4	0.51319	9.71E-4	0.51082	9.71E-4	0.50277	9.71E-4	0.48883	0.00103	0.5356

0.00133	0.56353	0.00115	0.46583	9.71E-4	0.52607	9.71E-4	0.52364	9.71E-4	0.51538	9.71E-4	0.5011	0.00105	0.5531

0.00133	0.57317	0.00119	0.48356	0.001	0.5463	0.001	0.54378	0.001	0.5352	0.001	0.52037	0.00107	0.57586

0.00134	0.58761	0.00121	0.50129	0.00103	0.56469	0.00103	0.56208	0.00103	0.55322	0.00103	0.53789	0.00109	0.59335

0.00138	0.61009	0.00121	0.51418	0.00105	0.5886	0.00105	0.58588	0.00105	0.57664	0.00105	0.56066	0.00109	0.60561

0.0014	0.62294	0.00123	0.5303	0.00106	0.59964	0.00106	0.59687	0.00106	0.58745	0.00106	0.57118	0.00111	0.61961

0.00142	0.63578	0.00127	0.55126	0.00109	0.61804	0.00109	0.61518	0.00109	0.60548	0.00109	0.5887	0.00113	0.64237

0.00142	0.65185	0.0013	0.56899	0.00112	0.64195	0.00112	0.63898	0.00112	0.62891	0.00112	0.61148	0.00116	0.65987

0.00145	0.67432	0.0013	0.57866	0.00112	0.65114	0.00112	0.64814	0.00112	0.63792	0.00112	0.62024	0.00117	0.67912

0.00146	0.68395	0.00131	0.59639	0.00115	0.66953	0.00115	0.66644	0.00115	0.65593	0.00115	0.63776	0.00118	0.69137

0.00147	0.70161	0.00134	0.61251	0.00117	0.69344	0.00117	0.69024	0.00117	0.67936	0.00117	0.66053	0.00119	0.71238

0.0015	0.71606	0.00135	0.62863	0.00119	0.70816	0.00119	0.70489	0.00119	0.69378	0.00119	0.67455	0.00122	0.73338

0.00152	0.73532	0.00137	0.64152	0.0012	0.72655	0.0012	0.7232	0.0012	0.71179	0.0012	0.69207	0.00122	0.74914

0.00154	0.74977	0.00139	0.65925	0.00123	0.75046	0.00123	0.747	0.00123	0.73522	0.00123	0.71485	0.00123	0.76314

0.00154	0.76583	0.0014	0.67859	0.00126	0.76887	0.00126	0.76532	0.00126	0.75325	0.00126	0.73237	0.00125	0.78064

0.00156	0.78028	0.00144	0.69632	0.00128	0.78174	0.00128	0.77813	0.00128	0.76586	0.00128	0.74464	0.00127	0.7999

0.00159	0.79954	0.00142	0.70922	0.00129	0.80197	0.00129	0.79827	0.00129	0.78568	0.00129	0.76391	0.00129	0.8174

0.00159	0.81079	0.00145	0.72695	0.00133	0.8222	0.00133	0.81841	0.00133	0.8055	0.00133	0.78318	0.0013	0.83665

0.00161	0.83005	0.00148	0.74468	0.00135	0.83876	0.00135	0.83489	0.00135	0.82172	0.00135	0.79895	0.00131	0.84715

0.00164	0.84771	0.00148	0.75596	0.00136	0.85531	0.00136	0.85136	0.00136	0.83794	0.00136	0.81472	0.00132	0.8699

0.00166	0.86216	0.00149	0.77047	0.00138	0.87371	0.00138	0.86968	0.00138	0.85596	0.00138	0.83224	0.00135	0.88916

0.00166	0.8734	0.00151	0.79142	0.00141	0.89762	0.00141	0.89348	0.00141	0.87939	0.00141	0.85502	0.00136	0.90491

0.00167	0.88785	0.00154	0.80754	0.00143	0.91601	0.00143	0.91178	0.00143	0.8974	0.00143	0.87254	0.00136	0.92241

0.0017	0.90872	0.00154	0.81883	0.00144	0.93257	0.00144	0.92827	0.00144	0.91363	0.00144	0.88831	0.00138	0.93642

0.0017	0.92317	0.00155	0.83495	0.00147	0.95464	0.00147	0.95024	0.00147	0.93525	0.00147	0.90933	0.0014	0.95742

0.0017	0.93441	0.00158	0.85751	0.00149	0.97303	0.00149	0.96854	0.00149	0.95327	0.00149	0.92685	0.00141	0.97667

0.00173	0.95849	0.00159	0.87202	0.0015	0.98959	0.0015	0.98502	0.0015	0.96949	0.0015	0.94262	0.00143	0.99243

0.00175	0.97133	0.00159	0.88169	0.0015	1.00614	0.0015	1.00149	0.0015	0.9857	0.0015	0.95839	0.00144	1.00643

0.00175	0.98418	0.0016	0.89942	0.00153	1.03005	0.00153	1.0253	0.00153	1.00913	0.00153	0.98117	0.00144	1.02569

0.00177	1.01628	0.00162	0.92037	0.00155	1.04845	0.00155	1.04361	0.00155	1.02715	0.00155	0.99869	0.00147	1.04494

0.0018	1.03074	0.00165	0.93327	0.00155	1.065	0.00155	1.06008	0.00155	1.04337	0.00155	1.01445	0.00148	1.06594

0.00181	1.05001	0.00165	0.94616	0.00158	1.0834	0.00158	1.0784	0.00158	1.06139	0.00158	1.03198	0.00149	1.07994

0.00181	1.05964	0.00166	0.96389	0.00161	1.10179	0.00161	1.0967	0.00161	1.07941	0.00161	1.0495	0.0015	1.09745

0.00182	1.0757	0.00169	0.98485	0.00161	1.12018	0.00161	1.11501	0.00161	1.09742	0.00161	1.06701	0.0015	1.11495

0.00185	1.09496	0.00169	0.99774	0.00161	1.13858	0.00161	1.13332	0.00161	1.11545	0.00161	1.08454	0.00153	1.1342

0.00187	1.10781	0.00169	1.01064	0.00164	1.16065	0.00164	1.15529	0.00164	1.13707	0.00164	1.10557	0.00153	1.15171

0.00187	1.11904	0.00172	1.02998	0.00166	1.1772	0.00166	1.17176	0.00166	1.15329	0.00166	1.12133	0.00154	1.16221

0.00188	1.14473	0.00173	1.04932	0.00166	1.19376	0.00166	1.18825	0.00166	1.16951	0.00166	1.1371	0.00155	1.18146

0.00189	1.15436	0.00174	1.05899	0.00168	1.21767	0.00168	1.21205	0.00168	1.19294	0.00168	1.15988	0.00157	1.20422

0.00191	1.17203	0.00174	1.07511	0.00169	1.23422	0.00169	1.22852	0.00169	1.20915	0.00169	1.17564	0.00159	1.22347

0.00191	1.18647	0.00176	1.09607	0.00171	1.2471	0.00171	1.24134	0.00171	1.22176	0.00171	1.18791	0.00159	1.23397

0.00194	1.20413	0.00179	1.10896	0.00172	1.26917	0.00172	1.26331	0.00172	1.24339	0.00172	1.20893	0.0016	1.25147

0.00194	1.2202	0.00177	1.12347	0.00174	1.28757	0.00174	1.28162	0.00174	1.26141	0.00174	1.22646	0.00162	1.27248

0.00195	1.23464	0.00179	1.1412	0.00175	1.30964	0.00175	1.30359	0.00175	1.28304	0.00175	1.24748	0.00163	1.28999

0.00196	1.2523	0.00181	1.16215	0.00176	1.32068	0.00176	1.31458	0.00176	1.29385	0.00176	1.25799	0.00164	1.30749

0.00198	1.26996	0.00182	1.17666	0.00177	1.34091	0.00177	1.33471	0.00177	1.31367	0.00177	1.27727	0.00164	1.32149

0.00201	1.28441	0.00182	1.18794	0.0018	1.36298	0.0018	1.35668	0.0018	1.33529	0.0018	1.29829	0.00166	1.34074

0.00199	1.29564	0.00184	1.20567	0.0018	1.38138	0.0018	1.375	0.0018	1.35332	0.0018	1.31582	0.00167	1.3635

0.00201	1.31652	0.00186	1.22663	0.0018	1.39425	0.0018	1.38782	0.0018	1.36593	0.0018	1.32808	0.00168	1.37925

0.00202	1.33258	0.00187	1.2363	0.00182	1.41817	0.00182	1.41162	0.00182	1.38936	0.00182	1.35086	0.00169	1.3915

0.00203	1.34863	0.00187	1.25081	0.00184	1.43104	0.00184	1.42444	0.00184	1.40197	0.00184	1.36313	0.0017	1.41075

0.00203	1.35988	0.00188	1.27015	0.00183	1.44391	0.00183	1.43724	0.00183	1.41458	0.00183	1.37538	0.00172	1.43175

0.00206	1.38555	0.0019	1.2911	0.00185	1.46966	0.00185	1.46288	0.00185	1.43981	0.00185	1.39991	0.00173	1.44926

0.00208	1.3984	0.00189	1.304	0.00187	1.49174	0.00187	1.48485	0.00187	1.46143	0.00187	1.42094	0.00174	1.46501

0.00205	1.40803	0.00192	1.32012	0.00187	1.50645	0.00187	1.4995	0.00187	1.47585	0.00187	1.43496	0.00174	1.47902

0.00208	1.42249	0.00193	1.33946	0.00188	1.52484	0.00188	1.5178	0.00188	1.49387	0.00188	1.45247	0.00174	1.50002

0.0021	1.44497	0.00194	1.35558	0.0019	1.55244	0.0019	1.54527	0.0019	1.5209	0.0019	1.47876	0.00177	1.51927

0.00212	1.46102	0.00194	1.36364	0.00191	1.56531	0.00191	1.55809	0.00191	1.53352	0.00191	1.49102	0.00178	1.53503

0.0021	1.47226	0.00195	1.38137	0.00191	1.57451	0.00191	1.56724	0.00191	1.54252	0.00191	1.49978	0.00178	1.54903

0.00212	1.49312	0.00197	1.40232	0.00192	1.59474	0.00192	1.58738	0.00192	1.56234	0.00192	1.51905	0.00178	1.56654

0.00215	1.50757	0.00197	1.41683	0.00194	1.61681	0.00194	1.60935	0.00194	1.58397	0.00194	1.54008	0.0018	1.58754

0.00215	1.52203	0.00197	1.42811	0.00195	1.63337	0.00195	1.62583	0.00195	1.60019	0.00195	1.55585	0.00181	1.60504

0.00215	1.53808	0.00199	1.44907	0.00195	1.64993	0.00195	1.64231	0.00195	1.61641	0.00195	1.57162	0.00181	1.6208

0.00216	1.55735	0.00202	1.47002	0.00197	1.67016	0.00197	1.66245	0.00197	1.63623	0.00197	1.59089	0.00181	1.63479

0.00217	1.57019	0.00202	1.48291	0.00198	1.69407	0.00198	1.68625	0.00198	1.65966	0.00198	1.61367	0.00184	1.6558

0.00217	1.58303	0.00201	1.49742	0.00199	1.70511	0.00199	1.69724	0.00199	1.67047	0.00199	1.62418	0.00185	1.6768

0.00219	1.6007	0.00203	1.51515	0.00199	1.7235	0.00199	1.71554	0.00199	1.68849	0.00199	1.6417	0.00186	1.69256

0.0022	1.61996	0.00204	1.53127	0.00201	1.74557	0.00201	1.73751	0.00201	1.71011	0.00201	1.66272	0.00186	1.70481

0.00222	1.63923	0.00204	1.54255	0.00201	1.76213	0.00201	1.75399	0.00201	1.72633	0.00201	1.6785	0.00187	1.72406

0.0022	1.64725	0.00206	1.55867	0.00201	1.77685	0.00201	1.76864	0.00201	1.74075	0.00201	1.69252	0.00188	1.74682

0.00222	1.66491	0.00208	1.57801	0.00204	1.79892	0.00204	1.79061	0.00204	1.76237	0.00204	1.71354	0.00191	1.76257

0.00225	1.68579	0.00208	1.59574	0.00205	1.82283	0.00205	1.81441	0.00205	1.7858	0.00205	1.73632	0.0019	1.77482

0.00225	1.69702	0.00208	1.61025	0.00205	1.83571	0.00205	1.82723	0.00205	1.79842	0.00205	1.74858	0.00191	1.79058

0.00225	1.7163	0.00208	1.62637	0.00205	1.85594	0.00205	1.84737	0.00205	1.81824	0.00205	1.76785	0.00191	1.81333

0.00226	1.73075	0.00211	1.64571	0.00207	1.87985	0.00207	1.87117	0.00207	1.84166	0.00207	1.79063	0.00193	1.83258

0.00227	1.75001	0.00211	1.65861	0.00209	1.89273	0.00209	1.88399	0.00209	1.85428	0.00209	1.8029	0.00194	1.85008

0.00227	1.76446	0.00211	1.66989	0.00208	1.90376	0.00208	1.89497	0.00208	1.86509	0.00208	1.81341	0.00194	1.86234

0.00227	1.77569	0.00213	1.68762	0.0021	1.93135	0.0021	1.92243	0.0021	1.89211	0.0021	1.83968	0.00195	1.88159

0.0023	1.79818	0.00215	1.71019	0.00211	1.94975	0.00211	1.94074	0.00211	1.91014	0.00211	1.85721	0.00197	1.90259

0.0023	1.81263	0.00215	1.72308	0.00211	1.96445	0.00211	1.95538	0.00211	1.92455	0.00211	1.87122	0.00198	1.9201

0.0023	1.82386	0.00214	1.73598	0.00211	1.97733	0.00211	1.9682	0.00211	1.93716	0.00211	1.88348	0.00198	1.93235

0.00232	1.84152	0.00216	1.75693	0.00213	2.00493	0.00213	1.99567	0.00213	1.9642	0.00213	1.90977	0.00198	1.94986

0.00234	1.86079	0.00217	1.77466	0.00214	2.01964	0.00214	2.01032	0.00214	1.97862	0.00214	1.92379	0.00198	1.96911

0.00234	1.88165	0.00218	1.78917	0.00214	2.03252	0.00214	2.02314	0.00214	1.99123	0.00214	1.93606	0.00201	1.99011

0.00234	1.88647	0.00217	1.79884	0.00215	2.05643	0.00215	2.04694	0.00215	2.01466	0.00215	1.95883	0.00202	2.00586

0.00234	1.90735	0.00218	1.81979	0.00217	2.07666	0.00217	2.06708	0.00217	2.03448	0.00217	1.9781	0.00202	2.01986

0.00236	1.92661	0.00221	1.83591	0.00216	2.09138	0.00216	2.08173	0.00216	2.0489	0.00216	1.99212	0.00202	2.03736

0.00237	1.93464	0.00221	1.8472	0.00218	2.11161	0.00218	2.10186	0.00218	2.06872	0.00218	2.01139	0.00204	2.06012

0.00237	1.94908	0.00221	1.86493	0.00219	2.13368	0.00219	2.12383	0.00219	2.09034	0.00219	2.03242	0.00205	2.07588

0.00239	1.97157	0.00223	1.88427	0.0022	2.14656	0.0022	2.13665	0.00236	2.13688	0.0022	2.04468	0.00205	2.09162

0.00239	1.98762	0.00224	1.90361	0.00219	2.16311	0.00219	2.15312	0.00236	2.14363	0.00222	2.06045	0.00202	2.09513

0.00239	2.00047	0.00223	1.91489	0.00219	2.17646	0.00219	2.16466	0.00237	2.16562	0.0022	2.07622	0.00205	2.06888

0.00239	2.01974	0.00225	1.93101	0.00219	2.1986	0.00219	2.18297	0.00237	2.1859	0.00225	2.09724	0.00204	2.07412

0.00243	2.039	0.00227	1.95358	0.00219	2.21393	0.00219	2.20296	0.00238	2.28565	0.00226	2.09024	0.00204	2.09863

0.00243	2.05346	0.00227	1.96486	0.00219	2.22926	0.00221	2.21961	0.0024	2.30425	0.00227	2.106	0.00204	2.11964

0.00243	2.06629	0.00227	1.97614	0.0022	2.24969	0.0022	2.23293	0.0024	2.31269	0.00228	2.12353	0.00205	2.13538

0.00244	2.07913	0.00227	1.99387	0.00221	2.26843	0.00221	2.23959	0.0024	2.33298	0.00227	2.13754	0.00204	2.14413

0.00246	2.10322	0.00229	2.01322	0.0022	2.27694	0.00222	2.26624	0.00241	2.35327	0.00227	2.15856	0.00204	2.15989

0.00246	2.11606	0.0023	2.02772	0.0022	2.29398	0.00223	2.28455	0.00243	2.37187	0.00228	2.17258	0.00205	2.1844

0.00246	2.12571	0.0023	2.04223	0.00222	2.31781	0.00222	2.29287	0.00243	2.38201	0.00228	2.1936	0.00206	2.20365

0.00247	2.14818	0.0023	2.06319	0.00221	2.33314	0.00222	2.31452	0.00241	2.40229	0.00228	2.21113	0.00206	2.21766

0.00248	2.16263	0.00232	2.08414	0.00221	2.33995	0.00224	2.33617	0.00243	2.42427	0.00228	2.23216	0.00206	2.23165

0.00248	2.1835	0.00232	2.09542	0.00225	2.36891	0.00224	2.34949	0.00243	2.43104	0.0023	2.25143	0.00207	2.2509

0.00248	2.19313	0.00232	2.10671	0.00225	2.38424	0.00223	2.35948	0.00243	2.44964	0.0023	2.26369	0.00208	2.27366

0.0025	2.214	0.00234	2.12605	0.00223	2.39786	0.00224	2.38113	0.00243	2.4733	0.0023	2.27945	0.00208	2.29117

0.0025	2.22685	0.00235	2.14378	0.00225	2.41318	0.00225	2.39944	0.00245	2.48514	0.00231	2.29873	0.00208	2.30341

0.00251	2.23969	0.00235	2.15184	0.00226	2.43192	0.00225	2.40943	0.00245	2.49697	0.00232	2.31801	0.00208	2.31917

0.00251	2.25574	0.00235	2.16957	0.00226	2.44554	0.00226	2.42608	0.00245	2.52064	0.00231	2.33202	0.00208	2.34368

0.00254	2.27983	0.00236	2.19213	0.00226	2.46257	0.00228	2.45272	0.00246	2.53754	0.00232	2.34954	0.0021	2.35593

0.00254	2.29106	0.00238	2.20986	0.00227	2.48472	0.00228	2.46771	0.00245	2.55444	0.00234	2.36881	0.00209	2.36993

0.00254	2.30231	0.00237	2.21954	0.00228	2.50344	0.00228	2.48103	0.00246	2.56459	0.00234	2.38984	0.00209	2.38744

0.00254	2.32157	0.00237	2.23888	0.00228	2.51878	0.00227	2.49602	0.00248	2.59164	0.00233	2.39685	0.0021	2.41018

0.00257	2.34404	0.00241	2.25822	0.0023	2.5341	0.0023	2.51767	0.00248	2.60178	0.00236	2.42138	0.00211	2.42594

0.00257	2.36011	0.00242	2.26789	0.00231	2.55283	0.0023	2.52932	0.00248	2.6153	0.00236	2.43539	0.00211	2.44694

0.00255	2.36974	0.00239	2.28401	0.00231	2.56475	0.0023	2.54098	0.00248	2.63729	0.00235	2.44941	0.00211	2.45569

0.00258	2.389	0.00242	2.30174	0.00231	2.58009	0.00231	2.56595	0.00249	2.65757	0.00236	2.46868	0.00211	2.4732

0.0026	2.40827	0.00243	2.31947	0.00232	2.60222	0.00232	2.58594	0.00248	2.66941	0.00237	2.49497	0.00212	2.4977

0.00258	2.4147	0.00243	2.33398	0.00233	2.61756	0.00232	2.59759	0.00248	2.6897	0.00238	2.50723	0.00212	2.5117

0.0026	2.43396	0.00243	2.34687	0.00233	2.63288	0.00232	2.61424	0.0025	2.70998	0.00237	2.52299	0.00212	2.52571

0.00261	2.45483	0.00244	2.36783	0.00234	2.65502	0.00233	2.63422	0.00251	2.7235	0.00239	2.54928	0.00213	2.54321

0.00261	2.46928	0.00245	2.38556	0.00236	2.67715	0.00234	2.65254	0.00251	2.73196	0.00239	2.55628	0.00214	2.56597

0.00261	2.48213	0.00245	2.40006	0.00236	2.68567	0.00234	2.66086	0.00251	2.759	0.00239	2.57206	0.00214	2.57996

0.00262	2.50299	0.00246	2.41618	0.00236	2.70099	0.00234	2.67585	0.00253	2.7776	0.0024	2.59484	0.00215	2.59922

0.00262	2.52066	0.00248	2.43069	0.00237	2.72144	0.00236	2.69583	0.00252	2.78775	0.00242	2.61761	0.00215	2.61322

0.00262	2.53189	0.00249	2.44681	0.00239	2.73846	0.00236	2.70915	0.00252	2.80635	0.00242	2.62988	0.00217	2.63422

0.00265	2.54313	0.00248	2.4597	0.00238	2.74868	0.00236	2.72414	0.00254	2.82325	0.00242	2.6474	0.00217	2.65173

0.00265	2.56401	0.00249	2.47582	0.0024	2.77251	0.00236	2.74746	0.00254	2.84522	0.00244	2.67368	0.00218	2.66923

0.00267	2.58167	0.0025	2.49839	0.00242	2.79126	0.00238	2.76577	0.00253	2.85368	0.00244	2.68419	0.00218	2.68324

0.00267	2.58968	0.00251	2.51451	0.00242	2.80148	0.00238	2.77908	0.00254	2.87396	0.00244	2.69821	0.00218	2.70424

0.00267	2.61056	0.0025	2.52579	0.00242	2.82022	0.00238	2.79741	0.00255	2.89087	0.00245	2.71573	0.00219	2.72174

0.00269	2.62982	0.00251	2.54674	0.00245	2.84406	0.0024	2.81405	0.00254	2.90102	0.00246	2.74026	0.0022	2.74099

0.00269	2.64428	0.00252	2.56609	0.00245	2.85598	0.00241	2.82904	0.00255	2.9213	0.00246	2.75602	0.0022	2.755

0.00268	2.65711	0.00253	2.57576	0.00244	2.86959	0.0024	2.84403	0.00257	2.94159	0.00246	2.76829	0.00221	2.77425

0.00268	2.67318	0.00252	2.58704	0.00247	2.89174	0.00241	2.86401	0.00257	2.95511	0.00247	2.78756	0.00221	2.79001

0.00272	2.69726	0.00255	2.60638	0.00248	2.91217	0.00242	2.88066	0.00257	2.97202	0.00248	2.81034	0.00223	2.80926

0.00271	2.7085	0.00255	2.62573	0.00249	2.92579	0.00242	2.89731	0.00257	2.99399	0.00248	2.82786	0.00223	2.82501

0.00271	2.72135	0.00253	2.63862	0.00248	2.94454	0.00242	2.9123	0.0026	3.0109	0.00248	2.84013	0.00222	2.83901

0.00272	2.74061	0.00255	2.65313	0.0025	2.96155	0.00243	2.93228	0.00259	3.02781	0.0025	2.85765	0.00223	2.86001

0.00272	2.75666	0.00257	2.67892	0.0025	2.97519	0.00244	2.95059	0.0026	3.03965	0.0025	2.87867	0.00225	2.88102

0.00272	2.7695	0.00258	2.69504	0.0025	2.98881	0.00243	2.9556	0.00262	3.06161	0.0025	2.88918	0.00225	2.89502

0.00272	2.78396	0.00257	2.70471	0.0025	3.01095	0.00244	2.97724	0.00262	3.07683	0.0025	2.91722	0.00225	2.91252

0.00275	2.80965	0.00257	2.7176	0.00253	3.02797	0.00247	2.99889	0.00262	3.09204	0.00252	2.93474	0.00225	2.92828

0.00276	2.81928	0.00259	2.73694	0.00253	3.04331	0.00247	3.01553	0.00262	3.11572	0.00253	2.95051	0.00227	2.95104

0.00275	2.8273	0.0026	2.75145	0.00253	3.05862	0.00246	3.02387	0.00263	3.12754	0.00252	2.96102	0.00228	2.97204

0.00275	2.853	0.00259	2.76273	0.00255	3.08417	0.00247	3.05217	0.00262	3.14783	0.00253	2.98555	0.00228	2.98254

0.00278	2.87066	0.0026	2.78208	0.00255	3.0995	0.00248	3.06549	0.00263	3.16305	0.00254	3.00132	0.00228	2.99829

0.00278	2.88189	0.00263	2.80303	0.00255	3.10802	0.00249	3.08214	0.00265	3.18334	0.00254	3.01007	0.00229	3.01754

0.00278	2.89634	0.00263	2.81915	0.00258	3.12675	0.00249	3.09547	0.00265	3.19348	0.00255	3.03636	0.00231	3.0368

0.00278	2.91721	0.00262	2.83366	0.00258	3.1489	0.0025	3.11378	0.00265	3.21208	0.00255	3.05563	0.00232	3.0543

0.00279	2.93327	0.00264	2.853	0.00259	3.16422	0.00251	3.12543	0.00266	3.2273	0.00255	3.06614	0.00231	3.07005

0.00279	2.94772	0.00265	2.86589	0.00259	3.18125	0.00251	3.14209	0.00266	3.24589	0.00255	3.08717	0.00231	3.08581

0.00279	2.96217	0.00265	2.8804	0.0026	3.19828	0.00251	3.16207	0.00266	3.25941	0.00257	3.1117	0.00233	3.10856

0.00282	2.98625	0.00265	2.89329	0.0026	3.21701	0.00252	3.18371	0.00266	3.28139	0.00258	3.13097	0.00233	3.12782

0.00282	2.99589	0.00266	2.90941	0.0026	3.23233	0.00252	3.19704	0.00267	3.30337	0.00257	3.13974	0.00233	3.13832

0.00281	3.00713	0.00267	2.93037	0.0026	3.24937	0.00251	3.21369	0.00267	3.31182	0.00258	3.1555	0.00233	3.15932

0.00281	3.02318	0.00267	2.94165	0.00263	3.2698	0.00252	3.23033	0.00267	3.32704	0.0026	3.17303	0.00235	3.17682

0.00283	3.04566	0.00267	2.95777	0.00263	3.27832	0.00254	3.25864	0.0027	3.3524	0.0026	3.1923	0.00235	3.19958

0.00283	3.06011	0.00269	2.98195	0.00263	3.29875	0.00254	3.25864	0.00271	3.36423	0.0026	3.20456	0.00235	3.21008

0.00283	3.07615	0.0027	2.99968	0.00264	3.32089	0.00254	3.28029	0.0027	3.37944	0.00261	3.23084	0.00235	3.22759

0.00285	3.09061	0.00269	3.00935	0.00265	3.33794	0.00256	3.29694	0.0027	3.39804	0.00261	3.25361	0.00236	3.24684

0.00286	3.10827	0.0027	3.02547	0.00265	3.34644	0.00256	3.31193	0.00271	3.42002	0.00261	3.26413	0.00237	3.26959

0.00286	3.11791	0.00271	3.04481	0.00265	3.3686	0.00256	3.32858	0.00271	3.43354	0.00261	3.27639	0.00238	3.28359

0.00286	3.13717	0.00271	3.0577	0.00266	3.39072	0.00257	3.35023	0.00271	3.44537	0.00263	3.29917	0.00237	3.2976

0.00286	3.15804	0.00271	3.06738	0.00266	3.39924	0.00258	3.37021	0.00274	3.46905	0.00264	3.32195	0.00239	3.31509

0.00289	3.1757	0.00272	3.08672	0.00267	3.41457	0.00259	3.38353	0.00274	3.48089	0.00263	3.3237	0.00239	3.3361

0.00289	3.18694	0.00273	3.10767	0.00269	3.44182	0.00258	3.39352	0.00274	3.4961	0.00264	3.35348	0.0024	3.35361

0.00289	3.20461	0.00273	3.12218	0.00269	3.45374	0.00259	3.4135	0.00274	3.52146	0.00265	3.37101	0.0024	3.36411

0.0029	3.22226	0.00273	3.1383	0.00269	3.46224	0.0026	3.43181	0.00274	3.53498	0.00265	3.38502	0.0024	3.38511

0.00292	3.23511	0.00274	3.15764	0.0027	3.48779	0.0026	3.44514	0.00274	3.54682	0.00265	3.4043	0.00242	3.40787

0.0029	3.25116	0.00276	3.17376	0.00271	3.50313	0.0026	3.45846	0.00274	3.56541	0.00266	3.42357	0.00242	3.42537

0.0029	3.26561	0.00276	3.18182	0.00272	3.51845	0.00261	3.48843	0.00274	3.59076	0.00267	3.43933	0.00242	3.44113

0.00293	3.2913	0.00276	3.19632	0.00273	3.53719	0.00262	3.50341	0.00276	3.6026	0.00267	3.4481	0.00242	3.45163

0.00293	3.30093	0.00278	3.21567	0.00274	3.55931	0.00262	3.51341	0.00276	3.61443	0.00267	3.47088	0.00244	3.47613

0.00293	3.31538	0.00278	3.23501	0.00275	3.57465	0.00262	3.53505	0.00277	3.63979	0.00269	3.49015	0.00245	3.49188

0.00295	3.33143	0.00277	3.2479	0.00275	3.58486	0.00264	3.55337	0.00278	3.65332	0.00269	3.50591	0.00245	3.50938

0.00296	3.3507	0.00277	3.26564	0.00275	3.607	0.00264	3.56502	0.00277	3.66346	0.00269	3.52519	0.00245	3.52164

0.00295	3.36837	0.0028	3.28659	0.00277	3.62573	0.00263	3.57835	0.00277	3.68205	0.0027	3.54797	0.00245	3.54614

0.00295	3.37639	0.0028	3.3011	0.00277	3.64107	0.00265	3.59833	0.00279	3.70741	0.0027	3.56373	0.00247	3.56364

0.00297	3.39887	0.00281	3.31077	0.00277	3.65128	0.00265	3.61831	0.00279	3.72601	0.0027	3.576	0.00246	3.58114

0.00297	3.41653	0.0028	3.33011	0.00279	3.68023	0.00265	3.62997	0.00279	3.72939	0.0027	3.59527	0.00246	3.59515

0.00297	3.42294	0.00281	3.34623	0.00279	3.68875	0.00265	3.64829	0.00279	3.75475	0.00272	3.6198	0.00247	3.6144

0.00297	3.44222	0.00283	3.36235	0.00279	3.70748	0.00268	3.67159	0.0028	3.77504	0.00272	3.62331	0.00249	3.63365

0.00299	3.46309	0.00281	3.37202	0.00279	3.72622	0.00268	3.68492	0.0028	3.78518	0.00272	3.64433	0.00249	3.65466

0.00299	3.47753	0.00284	3.39458	0.00281	3.74666	0.00267	3.69491	0.0028	3.80547	0.00273	3.67236	0.00249	3.66516

0.00299	3.48878	0.00285	3.41231	0.00281	3.75857	0.00267	3.71322	0.00281	3.82575	0.00274	3.68638	0.00249	3.67741

0.003	3.51286	0.00285	3.43005	0.00281	3.77732	0.00269	3.73487	0.0028	3.83928	0.00273	3.70215	0.00249	3.70192

0.00302	3.53213	0.00285	3.44133	0.00283	3.79775	0.00269	3.74986	0.00281	3.85787	0.00274	3.72318	0.00251	3.72467

0.003	3.53695	0.00286	3.45906	0.00283	3.81648	0.00269	3.75985	0.00284	3.87816	0.00275	3.73544	0.00251	3.73868

0.003	3.55299	0.00287	3.48001	0.00283	3.82669	0.0027	3.78315	0.00284	3.89168	0.00275	3.74596	0.00251	3.75268

0.00303	3.57066	0.00286	3.48646	0.00285	3.84373	0.00271	3.80147	0.00282	3.90521	0.00275	3.76873	0.00252	3.77193

0.00303	3.59313	0.00286	3.50097	0.00285	3.86417	0.00271	3.81979	0.00284	3.9255	0.00277	3.79151	0.00252	3.79293

0.00302	3.59956	0.00288	3.52353	0.00285	3.87949	0.00271	3.83145	0.00284	3.94578	0.00277	3.81078	0.00253	3.80869

0.00302	3.61883	0.0029	3.54126	0.00285	3.89651	0.00273	3.84643	0.00284	3.95255	0.00277	3.82129	0.00252	3.82094

0.00304	3.63969	0.00288	3.55255	0.00287	3.92206	0.00273	3.86309	0.00284	3.97114	0.00277	3.84407	0.00252	3.83669

0.00304	3.66057	0.0029	3.56867	0.00288	3.93399	0.00272	3.87474	0.00287	3.98805	0.00279	3.8651	0.00254	3.86295

0.00303	3.6702	0.00291	3.59446	0.00287	3.94931	0.00273	3.89472	0.00287	4.00495	0.00279	3.8686	0.00255	3.88045

0.00306	3.68465	0.00291	3.60574	0.00288	3.96465	0.00275	3.91969	0.00285	4.02693	0.00279	3.88963	0.00256	3.89445

0.00306	3.70071	0.0029	3.61541	0.00289	3.98507	0.00274	3.93136	0.00288	4.04214	0.0028	3.91065	0.00256	3.9102

0.00307	3.71355	0.00291	3.63153	0.00288	3.9987	0.00275	3.94967	0.00288	4.06582	0.0028	3.92992	0.00256	3.92946

0.00307	3.728	0.00293	3.65409	0.00289	4.01743	0.00276	3.96632	0.00288	4.07427	0.0028	3.93868	0.00258	3.95222

0.00307	3.75047	0.00293	3.6686	0.00292	4.03788	0.00276	3.99297	0.00288	4.08779	0.0028	3.9597	0.00258	3.96621

0.00309	3.76814	0.00293	3.67827	0.00292	4.0566	0.00276	3.99796	0.00288	4.10977	0.00282	3.98774	0.00258	3.98022

0.00307	3.78259	0.00293	3.69923	0.00292	4.06172	0.00277	4.00961	0.00288	4.12836	0.00282	4.00001	0.00258	3.99597

0.00307	3.79543	0.00295	3.72018	0.00293	4.08726	0.00278	4.03293	0.00288	4.14189	0.00282	4.01577	0.00259	4.01872

0.0031	3.81469	0.00294	3.73308	0.00293	4.10599	0.00278	4.05124	0.00288	4.16556	0.00282	4.03504	0.00259	4.03973

0.00311	3.83235	0.00294	3.74758	0.00293	4.11791	0.00278	4.0629	0.00292	4.18246	0.00283	4.05081	0.00259	4.05023

0.00309	3.83717	0.00295	3.76692	0.00293	4.13324	0.00278	4.08288	0.00291	4.19092	0.00283	4.06133	0.0026	4.06423

0.0031	3.85805	0.00298	3.77982	0.00296	4.15197	0.0028	4.10453	0.00292	4.20782	0.00283	4.0806	0.0026	4.08699

0.00311	3.88213	0.00295	3.7911	0.00297	4.17241	0.0028	4.12117	0.00294	4.2298	0.00285	4.10864	0.00261	4.10624

0.00311	3.89176	0.00297	3.80561	0.00296	4.18604	0.0028	4.12617	0.00293	4.24332	0.00285	4.12266	0.00261	4.12374

0.00311	3.903	0.00299	3.82979	0.00297	4.20137	0.00281	4.15115	0.00292	4.25854	0.00285	4.13666	0.00261	4.1395

0.00314	3.92869	0.00299	3.84752	0.00299	4.22009	0.00282	4.16947	0.00293	4.28052	0.00285	4.15244	0.00261	4.157

0.00316	3.94795	0.00299	3.86041	0.00297	4.23713	0.00281	4.17446	0.00294	4.29911	0.00286	4.17871	0.00263	4.17451

0.00313	3.95599	0.00299	3.87653	0.00297	4.25246	0.00281	4.19277	0.00294	4.31602	0.00286	4.18573	0.00263	4.19725

0.00314	3.97364	0.003	3.89749	0.00299	4.2763	0.00283	4.21609	0.00294	4.33292	0.00286	4.20149	0.00263	4.20251

0.00316	3.99452	0.00301	3.91038	0.003	4.28993	0.00283	4.23107	0.00295	4.35152	0.00288	4.22251	0.00263	4.22351

0.00316	4.00415	0.00301	3.92166	0.003	4.29844	0.00283	4.2444	0.00295	4.36674	0.00289	4.24705	0.00265	4.24977

0.00316	4.01699	0.00301	3.94262	0.003	4.32056	0.00284	4.26437	0.00294	4.37518	0.00288	4.25756	0.00265	4.26377

0.00316	4.03787	0.00304	3.95712	0.00302	4.34442	0.00285	4.28602	0.00295	4.40054	0.00288	4.28209	0.00265	4.27427

0.00319	4.05713	0.00304	3.97002	0.00302	4.35804	0.00285	4.29934	0.00296	4.41745	0.0029	4.29961	0.00265	4.29178

0.00319	4.06837	0.00302	3.98614	0.00302	4.37677	0.00285	4.311	0.00296	4.42929	0.00289	4.31363	0.00266	4.31278

0.00317	4.08603	0.00305	4.01193	0.00303	4.39551	0.00287	4.33264	0.00296	4.44788	0.00289	4.3294	0.00267	4.33379

0.00319	4.10368	0.00305	4.02643	0.00304	4.40913	0.00287	4.34597	0.00298	4.47494	0.00291	4.34867	0.00267	4.34953

0.0032	4.11653	0.00305	4.03611	0.00303	4.41424	0.00287	4.35929	0.00299	4.48676	0.00291	4.37145	0.00266	4.36354

0.0032	4.12777	0.00306	4.05384	0.00304	4.44318	0.00287	4.3826	0.00298	4.4986	0.00291	4.3767	0.00268	4.38279

0.0032	4.14383	0.00307	4.07318	0.00306	4.46703	0.00287	4.40592	0.00299	4.5172	0.00291	4.40123	0.00269	4.40379

0.00321	4.17112	0.00307	4.08769	0.00306	4.47215	0.00288	4.41757	0.00301	4.53241	0.00293	4.42576	0.0027	4.42129

0.00321	4.18236	0.00307	4.09897	0.00306	4.49428	0.00287	4.42256	0.00299	4.54594	0.00293	4.43978	0.00269	4.43705

0.00321	4.19359	0.00307	4.1167	0.00308	4.51982	0.00288	4.44588	0.00301	4.56115	0.00293	4.44678	0.0027	4.45106

0.00321	4.21447	0.00309	4.13926	0.00308	4.53175	0.0029	4.47251	0.00301	4.58989	0.00294	4.47657	0.0027	4.47556

0.00323	4.23373	0.00308	4.15377	0.00308	4.53857	0.00289	4.47419	0.00301	4.60173	0.00294	4.48883	0.00271	4.49482

0.00323	4.24336	0.00308	4.16667	0.00308	4.56412	0.0029	4.49583	0.00301	4.60849	0.00293	4.50461	0.0027	4.50357

0.00323	4.25782	0.00309	4.1844	0.00309	4.57772	0.00291	4.51581	0.00302	4.63891	0.00294	4.51512	0.00271	4.51931

0.00324	4.2819	0.00309	4.20535	0.00309	4.59817	0.00292	4.53412	0.00302	4.64737	0.00295	4.54665	0.00272	4.54382

0.00324	4.29474	0.00311	4.21825	0.00309	4.61179	0.00291	4.54745	0.00301	4.66259	0.00296	4.55892	0.00273	4.56658

0.00324	4.30598	0.00311	4.22792	0.0031	4.63734	0.00292	4.56243	0.00302	4.6778	0.00296	4.57819	0.00273	4.57533

0.00324	4.32524	0.00311	4.24565	0.00311	4.64926	0.00294	4.58408	0.00302	4.70485	0.00296	4.59921	0.00272	4.59107

0.00326	4.34452	0.00312	4.26338	0.0031	4.66288	0.00294	4.59407	0.00304	4.72176	0.00297	4.62024	0.00273	4.61383

0.00327	4.36218	0.00312	4.2795	0.00311	4.6782	0.00293	4.60572	0.00302	4.7302	0.00297	4.62374	0.00274	4.63309

0.00326	4.37341	0.00312	4.29239	0.00313	4.69864	0.00294	4.6307	0.00305	4.75726	0.00297	4.63952	0.00274	4.64884

0.00326	4.38466	0.00314	4.31657	0.00311	4.71227	0.00295	4.64736	0.00305	4.77585	0.00298	4.66404	0.00274	4.65759

0.00328	4.41515	0.00314	4.3343	0.00313	4.731	0.00295	4.65901	0.00305	4.77923	0.00299	4.68157	0.00274	4.67684

0.00328	4.41998	0.00313	4.34397	0.00315	4.75655	0.00296	4.67732	0.00306	4.79783	0.00298	4.69908	0.00276	4.69784

0.00327	4.43282	0.00314	4.3617	0.00314	4.77017	0.00296	4.70397	0.00306	4.82489	0.00299	4.71836	0.00276	4.7206

0.00328	4.45369	0.00315	4.38266	0.00315	4.78038	0.00296	4.71896	0.00306	4.83333	0.003	4.74113	0.00276	4.73285

0.0033	4.47136	0.00315	4.39555	0.00315	4.80252	0.00296	4.73228	0.00306	4.84855	0.003	4.7499	0.00276	4.74686

0.0033	4.4874	0.00315	4.40361	0.00316	4.82297	0.00297	4.74559	0.00307	4.86206	0.003	4.75866	0.00276	4.77137

0.00328	4.50025	0.00316	4.42134	0.00316	4.83489	0.00298	4.77057	0.00308	4.89419	0.00301	4.79019	0.00277	4.79237

0.00331	4.52434	0.00319	4.44874	0.00316	4.8468	0.00298	4.78389	0.00307	4.89926	0.00301	4.80071	0.00277	4.80461

0.00331	4.53717	0.00319	4.4568	0.00319	4.87065	0.00297	4.79389	0.00307	4.92632	0.00301	4.81472	0.00277	4.81862

0.0033	4.54681	0.00318	4.47453	0.00319	4.88428	0.00299	4.81386	0.00309	4.93815	0.00302	4.83926	0.00278	4.83962

0.00331	4.55965	0.00319	4.49387	0.00316	4.9013	0.00299	4.83718	0.00309	4.95675	0.00303	4.86028	0.0028	4.86063

0.00333	4.58534	0.0032	4.50999	0.0032	4.92004	0.00299	4.84717	0.00308	4.97027	0.00303	4.86904	0.0028	4.87463

0.00333	4.59818	0.0032	4.5245	0.00321	4.94047	0.003	4.86715	0.0031	4.99225	0.00303	4.89006	0.0028	4.88513

0.00333	4.61263	0.0032	4.53417	0.0032	4.9507	0.00302	4.88713	0.0031	5.00915	0.00304	4.91635	0.0028	4.90789

0.00333	4.62708	0.00322	4.55351	0.00321	4.96942	0.00302	4.90212	0.0031	5.01761	0.00305	4.92686	0.00281	4.93065

0.00335	4.64796	0.00322	4.57124	0.00321	4.98986	0.00301	4.90878	0.0031	5.03958	0.00304	4.93562	0.00283	4.95165

0.00335	4.6608	0.00321	4.58414	0.00321	5.00178	0.00302	4.93209	0.00313	5.06494	0.00305	4.96015	0.00282	4.96215

0.00334	4.68006	0.00322	4.60026	0.00321	5.01711	0.00304	4.94874	0.00312	5.07001	0.00306	4.98467	0.00282	4.97439

0.00337	4.68969	0.00323	4.62282	0.00322	5.03925	0.00303	4.9654	0.00312	5.09368	0.00305	4.99694	0.00283	4.9989

0.00338	4.70897	0.00323	4.64055	0.00324	5.05799	0.00303	4.97705	0.00313	5.11058	0.00305	5.01446	0.00283	5.0164

0.00337	4.72181	0.00323	4.65023	0.00324	5.0682	0.00304	5.00036	0.00313	5.13257	0.00307	5.03549	0.00283	5.03216

0.00337	4.73627	0.00323	4.66796	0.00324	5.08183	0.00304	5.02034	0.00312	5.13594	0.00307	5.053	0.00283	5.04441

0.00338	4.76035	0.00326	4.68246	0.00326	5.11079	0.00305	5.03034	0.00313	5.15792	0.00307	5.06352	0.00284	5.06366

0.00338	4.78443	0.00326	4.69858	0.00326	5.1227	0.00305	5.04532	0.00316	5.17821	0.00307	5.08805	0.00285	5.08817

0.00338	4.78924	0.00326	4.71148	0.00326	5.13291	0.00306	5.06198	0.00316	5.19004	0.00308	5.10382	0.00286	5.10567

0.00338	4.80208	0.00326	4.72437	0.00326	5.15165	0.00306	5.07529	0.00315	5.20694	0.00309	5.11958	0.00285	5.11968

0.00341	4.82136	0.00328	4.75016	0.00327	5.1789	0.00305	5.09027	0.00318	5.23061	0.00308	5.13185	0.00285	5.13367

0.00341	4.8358	0.00328	4.76628	0.00326	5.19252	0.00306	5.11359	0.00317	5.24244	0.00309	5.15638	0.00287	5.15818

0.0034	4.84865	0.00328	4.77917	0.00327	5.20444	0.00307	5.13191	0.00316	5.24921	0.00309	5.17214	0.00287	5.17394

0.00341	4.86309	0.00328	4.79691	0.0033	5.22488	0.00308	5.15022	0.00317	5.27794	0.00309	5.18967	0.00287	5.18619

0.00342	4.89199	0.00329	4.81464	0.0033	5.24703	0.00308	5.15855	0.00318	5.29486	0.00309	5.20369	0.00287	5.20369

0.00342	4.90003	0.00329	4.82753	0.0033	5.25724	0.00309	5.18186	0.00318	5.30837	0.00311	5.22996	0.00287	5.22294

0.00341	4.91929	0.00329	4.83881	0.0033	5.27427	0.0031	5.19684	0.00318	5.32529	0.00312	5.23698	0.00289	5.24219

0.00344	4.93695	0.0033	4.86138	0.00331	5.29811	0.00309	5.20684	0.0032	5.34388	0.00311	5.2545	0.00288	5.25795

0.00345	4.95942	0.00332	4.87911	0.00331	5.31004	0.0031	5.22682	0.0032	5.36248	0.00312	5.27201	0.00289	5.2772

0.00344	4.96264	0.0033	4.88395	0.00331	5.32536	0.00311	5.2468	0.00319	5.37263	0.00313	5.29304	0.00289	5.29646

0.00344	4.9803	0.0033	4.90651	0.00333	5.3441	0.00311	5.26345	0.00319	5.39122	0.00312	5.31056	0.0029	5.31396

0.00345	4.99475	0.00333	4.92908	0.00333	5.36623	0.00311	5.28343	0.0032	5.41489	0.00312	5.33159	0.0029	5.32971

0.00345	5.01401	0.00333	4.9452	0.00331	5.37645	0.00312	5.29841	0.0032	5.42842	0.00314	5.3491	0.0029	5.34021

0.00345	5.02364	0.00332	4.95648	0.00333	5.39348	0.00313	5.31674	0.0032	5.44532	0.00315	5.37013	0.0029	5.35947

0.00345	5.04934	0.00333	4.9726	0.00335	5.41391	0.00313	5.32672	0.00322	5.4656	0.00314	5.37188	0.00291	5.38397

0.00347	5.0686	0.00334	4.99033	0.00335	5.42073	0.00313	5.34338	0.00322	5.48251	0.00315	5.39466	0.00292	5.39972

0.00347	5.08144	0.00335	5.00484	0.00335	5.44117	0.00314	5.36169	0.00322	5.49435	0.00315	5.41394	0.00291	5.41548

0.00348	5.08947	0.00334	5.01612	0.00336	5.4633	0.00314	5.38001	0.00322	5.51632	0.00315	5.42795	0.00292	5.43298

0.00348	5.11836	0.00335	5.03224	0.00337	5.48375	0.00314	5.39	0.00323	5.53323	0.00315	5.44723	0.00294	5.45224

0.00348	5.13122	0.00337	5.0548	0.00336	5.49736	0.00314	5.41331	0.00323	5.54168	0.00317	5.4735	0.00294	5.47149

0.00348	5.14245	0.00337	5.06609	0.00337	5.5144	0.00316	5.43662	0.00323	5.55859	0.00317	5.49103	0.00294	5.48199

0.00348	5.15369	0.00336	5.08221	0.00338	5.53313	0.00316	5.45161	0.00324	5.59071	0.00317	5.50504	0.00294	5.49599

0.00349	5.17616	0.00337	5.10316	0.00338	5.54335	0.00315	5.45827	0.00326	5.59916	0.00318	5.52257	0.00294	5.5205

0.00351	5.19865	0.00339	5.12411	0.00338	5.56207	0.00316	5.47159	0.00324	5.61438	0.00318	5.54535	0.00296	5.53975

0.00349	5.20186	0.00339	5.13378	0.0034	5.59103	0.00318	5.4949	0.00326	5.63466	0.00318	5.5541	0.00296	5.55551

0.00349	5.22274	0.00339	5.14829	0.0034	5.60124	0.00317	5.50655	0.00326	5.64649	0.00318	5.56987	0.00296	5.5695

0.00351	5.242	0.0034	5.1628	0.0034	5.61317	0.00318	5.52488	0.00326	5.65832	0.00319	5.59089	0.00296	5.58876

0.00352	5.25003	0.00341	5.18214	0.00341	5.6336	0.00319	5.54986	0.00326	5.68538	0.0032	5.61542	0.00297	5.61501

0.00351	5.26769	0.00341	5.19181	0.00341	5.65575	0.00319	5.56817	0.00327	5.69722	0.00318	5.62068	0.00297	5.63252

0.00352	5.28374	0.0034	5.20632	0.00341	5.65915	0.00319	5.57483	0.00327	5.71581	0.0032	5.63645	0.00297	5.64127

0.00354	5.30462	0.00343	5.23533	0.00342	5.67959	0.00319	5.59481	0.00326	5.72425	0.00321	5.66624	0.00297	5.66052

0.00352	5.31264	0.00343	5.24984	0.00344	5.70344	0.00321	5.6148	0.00329	5.75468	0.00321	5.682	0.00298	5.68328

0.00352	5.33029	0.00342	5.26112	0.00344	5.71705	0.00322	5.63144	0.00329	5.77497	0.00321	5.69602	0.00299	5.70078

0.00354	5.35438	0.00343	5.27724	0.00344	5.73067	0.0032	5.63644	0.00329	5.78681	0.00322	5.7188	0.00299	5.71128

0.00355	5.37043	0.00344	5.29819	0.00344	5.74771	0.00321	5.66308	0.00329	5.79358	0.00322	5.73282	0.00299	5.72879

0.00354	5.37846	0.00344	5.3127	0.00346	5.76814	0.00323	5.67973	0.00329	5.82738	0.00321	5.74333	0.00301	5.75329

0.00354	5.39772	0.00344	5.32076	0.00345	5.78348	0.00323	5.69971	0.00329	5.83584	0.00322	5.77136	0.00301	5.77254

0.00358	5.42021	0.00346	5.34172	0.00345	5.8022	0.00323	5.7097	0.00329	5.8426	0.00323	5.79589	0.00301	5.78654

0.00356	5.43305	0.00347	5.35783	0.00346	5.82774	0.00323	5.73302	0.00329	5.87134	0.00323	5.8064	0.00301	5.79529

0.00355	5.44589	0.00347	5.37234	0.00347	5.83627	0.00323	5.748	0.00332	5.89163	0.00323	5.81341	0.00301	5.81455

0.00358	5.46838	0.00346	5.38685	0.00346	5.85159	0.00323	5.758	0.00331	5.90177	0.00324	5.84145	0.00302	5.83905

0.00359	5.48443	0.00347	5.41264	0.00347	5.87033	0.00323	5.77964	0.00331	5.92037	0.00325	5.85546	0.00303	5.85481

0.00358	5.49567	0.00348	5.42714	0.00349	5.88905	0.00325	5.79463	0.00334	5.94066	0.00325	5.86772	0.00302	5.86531

0.00358	5.51493	0.00348	5.43843	0.00348	5.90097	0.00325	5.8146	0.00332	5.95417	0.00324	5.88525	0.00302	5.88632

0.00359	5.52618	0.00348	5.45616	0.00349	5.92142	0.00325	5.82627	0.00333	5.9694	0.00326	5.91328	0.00304	5.90907

0.00359	5.54705	0.00349	5.47228	0.0035	5.94186	0.00325	5.84458	0.00333	5.98292	0.00326	5.9273	0.00304	5.92482

0.00359	5.55026	0.00349	5.48517	0.0035	5.95547	0.00327	5.86956	0.00334	6.00827	0.00326	5.94131	0.00304	5.93707

0.00359	5.56952	0.00349	5.49807	0.0035	5.97251	0.00327	5.87788	0.00334	6.01842	0.00326	5.95883	0.00304	5.95632

0.00361	5.59199	0.00349	5.51741	0.00351	5.99294	0.00327	5.89287	0.00334	6.03194	0.00328	5.98161	0.00305	5.98083

0.00362	5.61127	0.0035	5.53836	0.00353	6.00997	0.00327	5.90785	0.00336	6.06069	0.00327	5.98861	0.00306	5.99833

0.00361	5.61608	0.0035	5.54965	0.00353	6.0202	0.00329	5.92784	0.00336	6.0742	0.00328	6.01314	0.00306	6.00883

0.00362	5.64498	0.0035	5.56576	0.00352	6.03893	0.00329	5.94116	0.00335	6.07928	0.00328	6.03592	0.00306	6.02634

0.00362	5.66425	0.00351	5.58994	0.00354	6.05935	0.00329	5.95614	0.00335	6.10126	0.00329	6.05345	0.00306	6.04735

0.00363	5.66907	0.00353	5.60445	0.00355	6.07298	0.00331	5.97945	0.00337	6.11985	0.00328	6.0657	0.00308	6.06485

0.00363	5.68512	0.00353	5.61573	0.00353	6.0832	0.00331	5.99111	0.00335	6.13506	0.00328	6.08848	0.00308	6.08585

0.00365	5.70438	0.00351	5.62701	0.00355	6.11045	0.0033	6.00943	0.00337	6.15367	0.0033	6.1025	0.00307	6.0981

0.00366	5.72204	0.00354	5.64958	0.00355	6.12918	0.00331	6.03607	0.0034	6.17564	0.0033	6.11652	0.00308	6.11385

0.00363	5.72847	0.00354	5.6657	0.00355	6.14282	0.00332	6.04939	0.00338	6.19085	0.0033	6.12528	0.00309	6.13661

0.00363	5.75255	0.00354	5.67537	0.00357	6.15303	0.00332	6.05438	0.00338	6.20269	0.00331	6.15682	0.00309	6.15236

0.00365	5.7686	0.00354	5.69471	0.00357	6.17858	0.00332	6.06937	0.0034	6.22635	0.00332	6.17434	0.00308	6.16636

0.00366	5.79268	0.00357	5.71728	0.00357	6.1939	0.00333	6.09935	0.0034	6.23819	0.00331	6.18135	0.00309	6.18562

0.00365	5.7975	0.00356	5.7334	0.00357	6.20753	0.00333	6.11266	0.0034	6.25341	0.00331	6.20412	0.00309	6.20663

0.00366	5.81998	0.00356	5.7479	0.00358	6.22796	0.00333	6.11932	0.0034	6.27876	0.00332	6.23215	0.0031	6.22412

0.00368	5.83604	0.00356	5.76402	0.00359	6.245	0.00333	6.14264	0.0034	6.28891	0.00332	6.24442	0.00311	6.23987

0.00368	5.84567	0.00357	5.78014	0.00358	6.26201	0.00335	6.16762	0.0034	6.30582	0.00332	6.25668	0.00311	6.25563

0.00366	5.85852	0.00358	5.79304	0.00359	6.27735	0.00335	6.17927	0.00341	6.32441	0.00334	6.28122	0.00311	6.27663

0.00369	5.88742	0.00357	5.80432	0.00359	6.3012	0.00334	6.18926	0.00343	6.3447	0.00334	6.30049	0.00312	6.29413

0.0037	5.90186	0.00358	5.82527	0.0036	6.31481	0.00336	6.21257	0.00343	6.35822	0.00333	6.30575	0.00312	6.31164

0.00368	5.90989	0.0036	5.843	0.00359	6.32843	0.00336	6.23088	0.00343	6.36837	0.00333	6.32151	0.00311	6.32039

0.00369	5.92915	0.00358	5.85751	0.00362	6.35058	0.00336	6.24255	0.00343	6.39541	0.00336	6.35305	0.00312	6.3414

0.00372	5.94681	0.0036	5.87363	0.00362	6.37272	0.00336	6.2542	0.00345	6.41739	0.00335	6.36531	0.00314	6.36415

0.00372	5.95966	0.00361	5.8962	0.00362	6.38293	0.00338	6.28251	0.00345	6.42078	0.00334	6.37933	0.00314	6.38165

0.00372	5.97572	0.00361	5.91393	0.00362	6.39827	0.00338	6.29582	0.00343	6.43599	0.00335	6.40036	0.00314	6.3939

0.0037	5.99177	0.00361	5.91876	0.00364	6.4204	0.00338	6.31415	0.00346	6.46304	0.00336	6.42313	0.00314	6.41316

0.00372	6.00943	0.00361	5.93488	0.00364	6.43743	0.00339	6.32746	0.00346	6.48164	0.00336	6.42839	0.00315	6.43591

0.00372	6.02228	0.00362	5.95583	0.00364	6.44254	0.0034	6.35411	0.00346	6.49008	0.00336	6.44591	0.00315	6.45516

0.00372	6.03833	0.00363	5.97034	0.00364	6.46809	0.0034	6.36077	0.00346	6.51037	0.00337	6.46518	0.00314	6.46567

0.00372	6.05599	0.00362	5.98324	0.00365	6.48001	0.0034	6.37075	0.00346	6.53235	0.00337	6.48621	0.00315	6.48142

0.00373	6.07686	0.00362	6.00097	0.00365	6.49534	0.00341	6.39407	0.00347	6.53404	0.00337	6.49847	0.00315	6.50067

0.00373	6.08649	0.00364	6.02192	0.00365	6.51918	0.00342	6.41405	0.00347	6.55601	0.00338	6.523	0.00317	6.52518

0.00373	6.10255	0.00364	6.03643	0.00368	6.5345	0.00342	6.42737	0.00349	6.58307	0.00339	6.54227	0.00317	6.53918

0.00375	6.12182	0.00364	6.04932	0.00368	6.55154	0.00342	6.44235	0.00349	6.59659	0.00339	6.55629	0.00317	6.54794

0.00376	6.14108	0.00364	6.06705	0.00368	6.56176	0.00342	6.46567	0.00348	6.60843	0.00339	6.57207	0.00318	6.57244

0.00373	6.1459	0.00367	6.08801	0.00369	6.58899	0.00343	6.48066	0.00349	6.63379	0.0034	6.59134	0.00318	6.59169

0.00375	6.16999	0.00365	6.1009	0.00369	6.60433	0.00342	6.48898	0.00351	6.6473	0.0034	6.60536	0.00318	6.61095

0.00376	6.19727	0.00364	6.11057	0.00369	6.61285	0.00343	6.51396	0.00348	6.65914	0.00339	6.61761	0.00318	6.62145

0.00376	6.20531	0.00367	6.13153	0.00369	6.63669	0.00344	6.53061	0.00349	6.67943	0.0034	6.63514	0.00318	6.6407

0.00376	6.21333	0.00368	6.14765	0.0037	6.65883	0.00344	6.54226	0.00351	6.70141	0.00342	6.66843	0.00319	6.66346

0.00377	6.2326	0.00367	6.15893	0.0037	6.67074	0.00344	6.56058	0.00351	6.71324	0.00342	6.68419	0.00321	6.6792

0.00379	6.24865	0.00367	6.17666	0.0037	6.68266	0.00344	6.57889	0.00351	6.72507	0.00341	6.69121	0.00319	6.68971

0.00377	6.25989	0.00368	6.19923	0.00371	6.70651	0.00346	6.59388	0.00352	6.75213	0.00342	6.71223	0.0032	6.71071

0.00377	6.27915	0.00369	6.21373	0.00372	6.72525	0.00346	6.60887	0.00352	6.77072	0.00343	6.73851	0.00321	6.73697

0.00379	6.30003	0.00368	6.2234	0.00372	6.73206	0.00346	6.62719	0.00352	6.77917	0.00342	6.74026	0.00321	6.75097

0.0038	6.3193	0.00369	6.23952	0.00372	6.75419	0.00348	6.64883	0.00352	6.7927	0.00342	6.75778	0.00321	6.76322

0.00379	6.32411	0.0037	6.26048	0.00374	6.77463	0.00348	6.65883	0.00353	6.81975	0.00344	6.78581	0.00321	6.78073

0.00379	6.34177	0.0037	6.27176	0.00374	6.78997	0.00348	6.66715	0.00354	6.82989	0.00344	6.80159	0.00322	6.80173

0.00383	6.36585	0.00369	6.27982	0.00374	6.80189	0.00348	6.69046	0.00353	6.84511	0.00343	6.81561	0.00322	6.81923

0.00383	6.3803	0.0037	6.30077	0.00374	6.82232	0.0035	6.70877	0.00353	6.86539	0.00345	6.84714	0.00322	6.83324

0.0038	6.38673	0.00371	6.32495	0.00376	6.84105	0.00349	6.71876	0.00355	6.8823	0.00345	6.8629	0.00322	6.84723

0.00382	6.40599	0.00371	6.33946	0.00375	6.85297	0.00349	6.74208	0.00354	6.89752	0.00345	6.86641	0.00324	6.87174

0.00383	6.43169	0.00371	6.35074	0.00376	6.87341	0.00351	6.76539	0.00355	6.91273	0.00345	6.88219	0.00324	6.89099

0.00383	6.4381	0.00372	6.37008	0.00376	6.89384	0.00351	6.7837	0.00357	6.93809	0.00347	6.91197	0.00324	6.91024

0.00383	6.45736	0.00374	6.38781	0.00377	6.91428	0.0035	6.79037	0.00357	6.95668	0.00347	6.91897	0.00324	6.9225

0.00384	6.47342	0.00374	6.40071	0.00376	6.91938	0.00351	6.81035	0.00357	6.95668	0.00346	6.9365	0.00325	6.94001

0.00384	6.4959	0.00372	6.41199	0.00378	6.93983	0.00351	6.832	0.00357	6.98035	0.00347	6.96277	0.00325	6.96276

0.00384	6.50393	0.00374	6.42972	0.00378	6.96196	0.00352	6.84365	0.00357	7.00402	0.00347	6.9838	0.00325	6.97851

0.00384	6.51357	0.00375	6.44907	0.00378	6.97048	0.00351	6.85364	0.00357	7.01586	0.00347	6.98731	0.00325	6.98901

0.00386	6.54086	0.00375	6.46035	0.00378	6.99262	0.00352	6.87695	0.00357	7.03783	0.00347	7.01184	0.00326	7.01002

0.00387	6.55692	0.00375	6.47969	0.0038	7.01476	0.00354	6.8986	0.00359	7.05474	0.00348	7.03286	0.00327	7.03102

0.00386	6.56173	0.00377	6.50064	0.0038	7.02838	0.00354	6.90859	0.0036	7.06488	0.00349	7.05213	0.00327	7.04852

0.00386	6.58259	0.00377	6.51676	0.0038	7.04201	0.00354	6.9269	0.00357	7.0784	0.00349	7.06264	0.00327	7.06077

0.00389	6.60668	0.00376	6.52805	0.0038	7.06585	0.00354	6.95022	0.00359	7.10376	0.0035	7.08367	0.00327	7.07653

0.00389	6.62113	0.00377	6.54417	0.00382	7.08457	0.00355	6.96354	0.0036	7.11897	0.0035	7.09944	0.00328	7.09929

0.00387	6.63237	0.00378	6.56512	0.00382	7.08969	0.00354	6.9702	0.00359	7.12574	0.0035	7.11871	0.00328	7.12029

0.00389	6.65325	0.00378	6.5764	0.00383	7.11012	0.00355	6.99018	0.0036	7.15448	0.0035	7.13973	0.00328	7.13254

0.00389	6.66769	0.00378	6.58769	0.00384	7.13738	0.00357	7.0085	0.00362	7.17477	0.0035	7.15551	0.00328	7.14479

0.00389	6.67732	0.0038	6.60864	0.00384	7.14418	0.00358	7.02848	0.00362	7.1866	0.00351	7.18004	0.00328	7.16579

0.00389	6.69819	0.00381	6.62798	0.00383	7.16463	0.00357	7.0418	0.00362	7.20013	0.00351	7.18529	0.0033	7.18855

0.00391	6.71906	0.0038	6.6441	0.00384	7.19018	0.00357	7.06845	0.00362	7.22041	0.00351	7.20106	0.0033	7.20606

0.0039	6.73512	0.00378	6.657	0.00385	7.2038	0.00359	7.07677	0.00362	7.23563	0.00353	7.22209	0.0033	7.21831

0.0039	6.74957	0.00381	6.67795	0.00385	7.21401	0.00358	7.09175	0.00362	7.24577	0.00352	7.23785	0.00331	7.23581

0.0039	6.76402	0.00382	6.69568	0.00386	7.22764	0.00359	7.1134	0.00363	7.26606	0.00353	7.25187	0.00331	7.25681

0.00393	6.78649	0.00381	6.70213	0.00387	7.25659	0.0036	7.12838	0.00363	7.29987	0.00353	7.28165	0.00332	7.27782

0.00393	6.79774	0.00382	6.71825	0.00388	7.26681	0.0036	7.14005	0.00364	7.30326	0.00354	7.30268	0.00332	7.29007

0.00391	6.80737	0.00383	6.74081	0.00387	7.29065	0.0036	7.15003	0.00363	7.31677	0.00353	7.30794	0.00332	7.30232

0.00393	6.82664	0.00382	6.75532	0.00387	7.29916	0.0036	7.18001	0.00365	7.33876	0.00354	7.32371	0.00332	7.32507

0.00394	6.84911	0.00382	6.76338	0.00388	7.32471	0.00361	7.19832	0.00366	7.36242	0.00355	7.34298	0.00333	7.34608

0.00394	6.86357	0.00382	6.78433	0.00388	7.33492	0.00361	7.21664	0.00365	7.37087	0.00355	7.35699	0.00334	7.36008

0.00393	6.8732	0.00384	6.8069	0.00388	7.35196	0.0036	7.22663	0.00366	7.38777	0.00355	7.37452	0.00333	7.37058

0.00394	6.89888	0.00384	6.82141	0.00391	7.37068	0.00361	7.24661	0.00367	7.41313	0.00355	7.39554	0.00333	7.39509

0.00396	6.91494	0.00383	6.83591	0.00391	7.39113	0.00363	7.2616	0.00367	7.42328	0.00356	7.42183	0.00334	7.41784

0.00394	6.91975	0.00384	6.85203	0.00391	7.40305	0.00362	7.26659	0.00367	7.43512	0.00356	7.43409	0.00335	7.43359

0.00396	6.94705	0.00385	6.87137	0.00391	7.42008	0.00363	7.28824	0.00368	7.46047	0.00356	7.45161	0.00334	7.44409

0.00397	6.9615	0.00385	6.88266	0.00392	7.44052	0.00364	7.30823	0.00368	7.47569	0.00358	7.47789	0.00334	7.45985

0.00397	6.97113	0.00385	6.89394	0.00391	7.44563	0.00364	7.33153	0.00368	7.48413	0.00358	7.48665	0.00335	7.48436

0.00394	6.98718	0.00385	6.91006	0.00392	7.46946	0.00364	7.33819	0.00368	7.50105	0.00357	7.49892	0.00335	7.50186

0.00397	7.00325	0.00387	6.93585	0.00393	7.4933	0.00364	7.35984	0.00369	7.5281	0.00358	7.51819	0.00335	7.51236

0.00398	7.02251	0.00387	6.94874	0.00395	7.51033	0.00366	7.37982	0.00369	7.54499	0.00358	7.53921	0.00335	7.53511

0.00397	7.04177	0.00387	6.96164	0.00393	7.52226	0.00366	7.39647	0.00369	7.55176	0.00358	7.55148	0.00337	7.55612

0.00397	7.05784	0.00387	6.98582	0.00393	7.54099	0.00366	7.40979	0.00371	7.57542	0.00358	7.56724	0.00337	7.57537

0.004	7.06906	0.00388	7.00032	0.00395	7.55803	0.00367	7.42811	0.00373	7.59234	0.0036	7.59528	0.00337	7.58587

0.004	7.08673	0.00388	7.00999	0.00395	7.56824	0.00368	7.44477	0.00371	7.60586	0.0036	7.6128	0.00338	7.60337

0.00398	7.09797	0.00388	7.0245	0.00395	7.59038	0.00367	7.45142	0.00373	7.62784	0.00358	7.61981	0.00339	7.62437

0.004	7.11563	0.00389	7.04707	0.00397	7.60911	0.00368	7.47306	0.00374	7.64982	0.00361	7.64259	0.00339	7.64188

0.004	7.1381	0.00389	7.05674	0.00397	7.63125	0.00369	7.50137	0.00371	7.65151	0.00361	7.6601	0.00339	7.65588

0.00401	7.15094	0.00388	7.06963	0.00397	7.64146	0.00369	7.51637	0.00373	7.67518	0.00361	7.67062	0.00339	7.66988

0.004	7.15898	0.0039	7.08897	0.00397	7.66021	0.00369	7.52302	0.00374	7.70222	0.00361	7.69164	0.00339	7.69089

0.004	7.18627	0.00391	7.11315	0.00398	7.67894	0.00369	7.53967	0.00374	7.72082	0.00361	7.71793	0.0034	7.71189

0.00403	7.20233	0.00391	7.12766	0.00397	7.69086	0.0037	7.56465	0.00373	7.72251	0.00361	7.73195	0.0034	7.72939

0.00403	7.21356	0.0039	7.14055	0.00397	7.70618	0.00369	7.56798	0.00374	7.74957	0.00361	7.74946	0.0034	7.73815

0.00404	7.22801	0.0039	7.15828	0.00399	7.73343	0.00369	7.5813	0.00376	7.76815	0.00362	7.76348	0.0034	7.76091

0.00404	7.25691	0.00391	7.17763	0.00399	7.74194	0.00372	7.6146	0.00376	7.77323	0.00364	7.78976	0.00341	7.78365

0.00404	7.26495	0.00392	7.18407	0.00399	7.75898	0.00371	7.63292	0.00374	7.78507	0.00363	7.79677	0.00342	7.79766

0.00404	7.27618	0.00392	7.20019	0.00401	7.78112	0.0037	7.64124	0.00376	7.8155	0.00364	7.80904	0.00342	7.80991

0.00404	7.28903	0.00394	7.22115	0.00402	7.79474	0.00372	7.66456	0.00376	7.83408	0.00364	7.83531	0.00342	7.82741

0.00406	7.31632	0.00395	7.23727	0.00401	7.80666	0.00374	7.67788	0.00376	7.84085	0.00364	7.85634	0.00343	7.85192

0.00407	7.32595	0.00395	7.25016	0.00402	7.82199	0.00374	7.6962	0.00377	7.86114	0.00364	7.86685	0.00343	7.86767

0.00406	7.34361	0.00395	7.27112	0.00403	7.85095	0.00373	7.70119	0.00378	7.8848	0.00364	7.89313	0.00343	7.88342

0.00406	7.35805	0.00395	7.29207	0.00403	7.86457	0.00374	7.72451	0.00378	7.89664	0.00365	7.91766	0.00343	7.89743

0.00408	7.38054	0.00396	7.30496	0.00402	7.8748	0.00375	7.74781	0.00378	7.91355	0.00365	7.92993	0.00344	7.92718

0.00408	7.38696	0.00396	7.31786	0.00403	7.89692	0.00375	7.75447	0.00379	7.93044	0.00366	7.94219	0.00345	7.93944

0.00407	7.40141	0.00396	7.33075	0.00404	7.91566	0.00375	7.77778	0.00379	7.94566	0.00367	7.95971	0.00345	7.95343

0.00408	7.42389	0.00397	7.35171	0.00403	7.92928	0.00375	7.8011	0.00378	7.9558	0.00367	7.97898	0.00345	7.96744

0.0041	7.43993	0.00397	7.36783	0.00404	7.94461	0.00377	7.81608	0.00379	7.98623	0.00366	7.98949	0.00345	7.99019

0.00408	7.45279	0.00397	7.3775	0.00404	7.97015	0.00376	7.8244	0.0038	8.00314	0.00366	8.00702	0.00346	8.0112

0.00408	7.46723	0.00397	7.39362	0.00405	7.98378	0.00376	7.84106	0.00379	8.01498	0.00368	8.0333	0.00345	8.02345

0.00411	7.48651	0.00398	7.41941	0.00404	7.99059	0.00377	7.86438	0.0038	8.0285	0.00368	8.04732	0.00345	8.0357

0.00411	7.50577	0.00398	7.4323	0.00407	8.01613	0.00378	7.86937	0.00381	8.05049	0.00368	8.06309	0.00345	8.0602

0.0041	7.51701	0.00398	7.44681	0.00407	8.03998	0.00378	7.88435	0.00382	8.06739	0.00369	8.08936	0.00348	8.08471

0.00411	7.52664	0.00399	7.46776	0.00407	8.0502	0.00378	7.90767	0.0038	8.07415	0.00369	8.10338	0.00347	8.09696

0.00411	7.54912	0.00399	7.48388	0.00407	8.06383	0.00379	7.93265	0.00381	8.10289	0.00369	8.11565	0.00347	8.11097

0.00413	7.56839	0.00399	7.49033	0.00408	8.08936	0.00379	7.9443	0.00382	8.11135	0.00369	8.13317	0.00348	8.12847

0.00411	7.57641	0.00398	7.50806	0.00408	8.10128	0.00378	7.96094	0.00381	8.12656	0.00371	8.1577	0.00349	8.14947

0.00413	7.6021	0.00401	7.52418	0.00408	8.11491	0.0038	7.98259	0.00384	8.14685	0.00371	8.17347	0.00349	8.16872

0.00413	7.62297	0.00401	7.5403	0.0041	8.13193	0.0038	8.00092	0.00385	8.1722	0.0037	8.18748	0.00349	8.18098

0.00414	7.6326	0.00399	7.55158	0.0041	8.15579	0.0038	8.00757	0.00384	8.18573	0.00371	8.20501	0.00349	8.19848

0.00413	7.64063	0.00401	7.57415	0.00409	8.166	0.00381	8.02256	0.00384	8.19587	0.00372	8.23654	0.00349	8.21948

0.00415	7.66311	0.00403	7.59671	0.0041	8.18303	0.00381	8.04254	0.00385	8.21616	0.00371	8.2383	0.0035	8.24048

0.00415	7.6904	0.00403	7.61283	0.00412	8.21029	0.00382	8.05752	0.00384	8.23982	0.00372	8.25406	0.0035	8.25274

0.00415	7.6888	0.00402	7.62411	0.00412	8.21879	0.00381	8.07751	0.00384	8.24828	0.00372	8.27684	0.0035	8.26499

0.00415	7.71448	0.00402	7.64023	0.0041	8.23412	0.00383	8.09748	0.00385	8.26011	0.00372	8.28735	0.0035	8.28424

0.00417	7.73054	0.00403	7.66119	0.00412	8.25455	0.00384	8.11581	0.00387	8.28885	0.00373	8.29961	0.00352	8.30875

0.00417	7.7482	0.00404	7.67247	0.00413	8.2716	0.00383	8.1208	0.00388	8.30407	0.00373	8.32765	0.00352	8.32276

0.00415	7.75462	0.00403	7.68214	0.00413	8.28181	0.00384	8.13912	0.00387	8.3159	0.00374	8.34342	0.00352	8.33501

0.00417	7.7755	0.00404	7.70309	0.00414	8.30736	0.00385	8.16576	0.00388	8.33618	0.00374	8.36444	0.00352	8.35251

0.00418	7.79476	0.00405	7.72405	0.00415	8.32778	0.00385	8.17242	0.00388	8.35647	0.00374	8.37846	0.00353	8.37527

0.00418	7.80279	0.00405	7.74017	0.00415	8.34141	0.00385	8.18241	0.00388	8.36661	0.00374	8.40299	0.00353	8.39276

0.00417	7.81724	0.00404	7.75145	0.00414	8.35844	0.00385	8.20739	0.00388	8.38352	0.00375	8.417	0.00353	8.40502

0.0042	7.83971	0.00405	7.77079	0.00415	8.37549	0.00386	8.2257	0.00388	8.41226	0.00375	8.42401	0.00353	8.42427

0.0042	7.85897	0.00405	7.79175	0.00416	8.3925	0.00387	8.24402	0.00388	8.42072	0.00375	8.44328	0.00354	8.44703

0.00418	7.8654	0.00405	7.80303	0.00415	8.40272	0.00387	8.25401	0.00388	8.43424	0.00377	8.46606	0.00355	8.46278

0.00418	7.88788	0.00406	7.8127	0.00416	8.42316	0.00388	8.27733	0.00391	8.4596	0.00377	8.48184	0.00355	8.47678

0.00421	7.91036	0.00408	7.83043	0.00417	8.44359	0.00388	8.29897	0.00392	8.47651	0.00376	8.50111	0.00355	8.49429

0.0042	7.92159	0.00409	7.84816	0.00418	8.46233	0.00387	8.30562	0.00392	8.48665	0.00377	8.53264	0.00355	8.51354

0.0042	7.93284	0.00406	7.85945	0.00417	8.47425	0.00389	8.32894	0.00391	8.49848	0.00377	8.54315	0.00356	8.53279

0.0042	7.95371	0.00406	7.87879	0.00418	8.49639	0.00389	8.3456	0.00393	8.52384	0.00377	8.55367	0.00356	8.55029

0.00422	7.96976	0.00408	7.90458	0.00419	8.51682	0.0039	8.35392	0.00393	8.53398	0.00377	8.56944	0.00356	8.56605

0.00422	7.98743	0.00409	7.92231	0.00419	8.52534	0.00389	8.3739	0.00392	8.55258	0.00379	8.58871	0.00356	8.58355

0.00422	7.99224	0.00408	7.93198	0.00419	8.53727	0.0039	8.39222	0.00393	8.56948	0.00379	8.60447	0.00357	8.60455

0.00422	8.02435	0.00409	7.94971	0.00421	8.56621	0.00391	8.41553	0.00394	8.59653	0.00378	8.61499	0.00358	8.61681

0.00424	8.03559	0.00411	7.96583	0.00421	8.57984	0.00392	8.43051	0.00394	8.60499	0.0038	8.64478	0.00357	8.62906

0.00422	8.04201	0.0041	7.98034	0.00421	8.59005	0.00392	8.43551	0.00394	8.61344	0.0038	8.66405	0.00358	8.65182

0.00422	8.05807	0.00409	7.99162	0.00421	8.61389	0.00391	8.45882	0.00396	8.64049	0.00379	8.67631	0.00359	8.67631

0.00425	8.08215	0.00409	8.00935	0.00421	8.63434	0.00393	8.4788	0.00395	8.65233	0.0038	8.69383	0.00359	8.69032

0.00425	8.09499	0.00411	8.02869	0.00421	8.64626	0.00392	8.48713	0.00395	8.66247	0.00382	8.71485	0.00359	8.70432

0.00425	8.11425	0.00411	8.03997	0.00423	8.65817	0.00393	8.50711	0.00396	8.6912	0.00382	8.72538	0.00359	8.72358

0.00425	8.1287	0.00411	8.06093	0.00424	8.68543	0.00394	8.53375	0.00398	8.70642	0.00381	8.73939	0.0036	8.74458

0.00428	8.14958	0.00412	8.08188	0.00425	8.69905	0.00395	8.54207	0.00398	8.71995	0.00382	8.76041	0.0036	8.76034

0.00428	8.15761	0.00413	8.10123	0.00424	8.71437	0.00394	8.5554	0.00396	8.7453	0.00382	8.78143	0.0036	8.77084

0.00427	8.17366	0.00412	8.1109	0.00425	8.73141	0.00394	8.57538	0.00398	8.7622	0.00382	8.79545	0.0036	8.78834

0.00428	8.19775	0.00412	8.12218	0.00426	8.75013	0.00396	8.59869	0.00398	8.76897	0.00382	8.81298	0.00361	8.81109

0.00428	8.20576	0.00415	8.14475	0.00425	8.76377	0.00396	8.60535	0.00398	8.78589	0.00383	8.84101	0.00362	8.82859

0.00428	8.21701	0.00415	8.16086	0.00426	8.77739	0.00396	8.62201	0.00398	8.81124	0.00383	8.86028	0.0036	8.8426

0.00428	8.23146	0.00413	8.16892	0.00429	8.80293	0.00396	8.64531	0.00398	8.82983	0.00383	8.86905	0.00362	8.8601

0.00431	8.25875	0.00415	8.18665	0.00426	8.82337	0.00396	8.65864	0.00398	8.84167	0.00383	8.87605	0.00362	8.88285

0.00431	8.26679	0.00416	8.21244	0.00426	8.83019	0.00396	8.67861	0.00399	8.86196	0.00384	8.90934	0.00363	8.90386

0.00428	8.27963	0.00416	8.22534	0.00429	8.85402	0.00396	8.6886	0.00401	8.88899	0.00384	8.9181	0.00363	8.91786

0.00431	8.30371	0.00416	8.23662	0.00429	8.87106	0.00398	8.71192	0.00402	8.89407	0.00384	8.93212	0.00363	8.92311

0.00432	8.32297	0.00416	8.25758	0.00429	8.88128	0.00399	8.7219	0.00402	8.90929	0.00385	8.96015	0.00363	8.94762

0.00432	8.33742	0.00418	8.27047	0.00429	8.89831	0.00398	8.73523	0.00401	8.92282	0.00386	8.98293	0.00364	8.97212

0.00431	8.34706	0.00417	8.28498	0.00431	8.92554	0.00399	8.75687	0.00402	8.94479	0.00385	8.98819	0.00365	8.98787

0.00432	8.37114	0.00416	8.29787	0.00431	8.93747	0.004	8.77519	0.00401	8.95832	0.00385	9.0057	0.00364	8.99837

0.00434	8.38238	0.00418	8.32044	0.00431	8.94938	0.004	8.78352	0.00402	8.97691	0.00387	9.02497	0.00363	9.02113

0.00434	8.39522	0.00418	8.33172	0.00431	8.97154	0.004	8.81016	0.00404	8.9972	0.00387	9.04425	0.00365	9.04388

0.00432	8.41449	0.00418	8.34623	0.00432	8.98516	0.00401	8.83348	0.00402	9.01072	0.00387	9.04775	0.00366	9.06138

0.00434	8.43215	0.00418	8.37202	0.00431	8.99709	0.00402	8.84679	0.00402	9.01918	0.00388	9.07754	0.00366	9.07189

0.00435	8.45302	0.00419	8.39458	0.00431	9.01581	0.00401	8.86011	0.00405	9.04284	0.00388	9.09506	0.00365	9.08764

0.00434	8.46104	0.00419	8.40426	0.00434	9.04135	0.00402	8.87677	0.00405	9.06314	0.00387	9.11083	0.00366	9.11389

0.00435	8.47871	0.00418	8.41876	0.00435	9.05498	0.00404	8.89175	0.00404	9.07497	0.00388	9.13186	0.00366	9.1279

0.00436	8.50279	0.00419	8.43005	0.00434	9.06861	0.00403	8.91007	0.00404	9.09525	0.00389	9.15288	0.00366	9.1419

0.00436	8.50761	0.0042	8.44939	0.00434	9.09075	0.00403	8.92006	0.00406	9.11723	0.00389	9.1669	0.00367	9.1559

0.00436	8.52527	0.00422	8.46389	0.00436	9.10608	0.00404	8.94004	0.00405	9.1223	0.0039	9.17566	0.00367	9.18216

0.00436	8.53971	0.0042	8.47518	0.00435	9.11799	0.00405	8.96501	0.00405	9.14597	0.0039	9.20369	0.00368	9.20316

0.00438	8.57022	0.0042	8.4913	0.00436	9.13503	0.00404	8.97168	0.00407	9.16794	0.00391	9.21771	0.00368	9.21542

0.00436	8.57986	0.00423	8.51547	0.00437	9.15717	0.00405	8.99166	0.00407	9.17471	0.0039	9.22822	0.00368	9.22592

0.00438	8.58949	0.00423	8.52837	0.00437	9.17079	0.00405	9.01497	0.00407	9.19161	0.00391	9.25275	0.00368	9.24867

0.00439	8.61358	0.00422	8.54449	0.00437	9.18441	0.00405	9.02829	0.00406	9.2119	0.00391	9.27377	0.0037	9.26617

0.00439	8.62802	0.00423	8.56383	0.00437	9.20826	0.00405	9.02995	0.00408	9.22543	0.00392	9.29655	0.0037	9.28193

0.00438	8.63605	0.00424	8.58478	0.00437	9.23039	0.00406	9.05826	0.00407	9.24909	0.00392	9.29831	0.0037	9.29768

0.00439	8.6521	0.00423	8.59123	0.00438	9.23381	0.00407	9.07658	0.00407	9.26093	0.00392	9.32108	0.0037	9.31693

0.00441	8.67297	0.00423	8.60735	0.00438	9.25424	0.00407	9.08657	0.0041	9.28121	0.00393	9.34211	0.0037	9.34319

0.00441	8.68742	0.00425	8.62186	0.0044	9.27467	0.00407	9.10322	0.0041	9.30487	0.00393	9.35612	0.00371	9.35719

0.00441	8.69705	0.00425	8.63475	0.0044	9.29001	0.00408	9.12487	0.00409	9.31164	0.00393	9.37189	0.0037	9.36769

0.00441	8.71954	0.00424	8.65087	0.0044	9.29851	0.00408	9.14652	0.0041	9.337	0.00395	9.39467	0.00372	9.3887

0.00442	8.74201	0.00424	8.66699	0.00441	9.33088	0.00409	9.15318	0.00412	9.35391	0.00395	9.41745	0.00372	9.4097

0.00442	8.75967	0.00426	8.69439	0.00441	9.34109	0.00408	9.17315	0.00409	9.36574	0.00393	9.42796	0.00372	9.42545

0.00441	8.75967	0.00425	8.71212	0.00441	9.35643	0.0041	9.18981	0.0041	9.3742	0.00394	9.44547	0.00372	9.43595

0.00442	8.78215	0.00425	8.72502	0.00441	9.37004	0.0041	9.20813	0.00412	9.40293	0.00395	9.47001	0.00372	9.45696

0.00443	8.7982	0.00425	8.73952	0.00443	9.39559	0.0041	9.21812	0.00413	9.41646	0.00396	9.47876	0.00373	9.47796

0.00442	8.80623	0.00427	8.75725	0.00443	9.41091	0.00411	9.23643	0.00412	9.43506	0.00395	9.49103	0.00373	9.49372

0.00442	8.82389	0.00427	8.77176	0.00443	9.41774	0.00412	9.25808	0.00412	9.45535	0.00396	9.51556	0.00373	9.50596

0.00445	8.85279	0.00426	8.77982	0.00443	9.45007	0.00412	9.26807	0.00413	9.46886	0.00397	9.53834	0.00373	9.52522

0.00445	8.87045	0.00426	8.79594	0.00445	9.46541	0.00411	9.29638	0.00413	9.47732	0.00396	9.54534	0.00375	9.54798

0.00443	8.87687	0.00429	8.82012	0.00445	9.47562	0.00412	9.3147	0.00412	9.49761	0.00396	9.56637	0.00374	9.56723

0.00445	8.89294	0.00429	8.83785	0.00445	9.49607	0.00414	9.32635	0.00415	9.52803	0.00397	9.59616	0.00375	9.57948

0.00446	8.91702	0.00429	8.85719	0.00446	9.51821	0.00413	9.33467	0.00415	9.5348	0.00397	9.61192	0.00375	9.59523

0.00446	8.92665	0.00429	8.87331	0.00447	9.52842	0.00413	9.35133	0.00413	9.54663	0.00397	9.62419	0.00375	9.61799

0.00445	8.93949	0.0043	8.8862	0.00445	9.54204	0.00415	9.3763	0.00416	9.5703	0.00399	9.63996	0.00376	9.63549

0.00446	8.96196	0.00429	8.89426	0.00447	9.56248	0.00415	9.3863	0.00416	9.59397	0.00399	9.65047	0.00376	9.65125

0.00448	8.97803	0.00429	8.90877	0.00448	9.58461	0.00414	9.40794	0.00416	9.59904	0.00399	9.66448	0.00376	9.66699

0.00448	8.98766	0.0043	8.93295	0.00447	9.59314	0.00415	9.42626	0.00416	9.61425	0.00399	9.68552	0.00376	9.68625

0.00448	9.01013	0.00431	8.95068	0.00448	9.60846	0.00416	9.44457	0.00416	9.63792	0.004	9.71179	0.00378	9.71075

0.00449	9.0294	0.00431	8.95712	0.0045	9.63742	0.00415	9.45623	0.00416	9.65482	0.004	9.72932	0.00378	9.72476

0.00449	9.04384	0.00431	8.96841	0.0045	9.65275	0.00416	9.46622	0.00416	9.67004	0.004	9.74333	0.00376	9.73701

0.00448	9.05027	0.00433	9.00548	0.0045	9.66296	0.00418	9.49286	0.00418	9.68865	0.00401	9.76435	0.00378	9.75101

0.00448	9.06793	0.00433	9.01999	0.0045	9.6851	0.00418	9.50452	0.00419	9.70386	0.00401	9.77837	0.00378	9.77727

0.00452	9.09362	0.00431	9.03288	0.00452	9.70384	0.00417	9.52117	0.00418	9.71568	0.00401	9.79414	0.0038	9.79301

0.00452	9.10968	0.00433	9.04578	0.0045	9.71234	0.00418	9.53782	0.00419	9.73936	0.00401	9.80641	0.00379	9.80702

0.00449	9.11609	0.00433	9.0619	0.00452	9.72768	0.0042	9.55947	0.00421	9.75626	0.00402	9.82919	0.00378	9.82278

0.00452	9.14179	0.00434	9.07801	0.00452	9.75323	0.0042	9.57445	0.00421	9.7664	0.00402	9.85196	0.00379	9.84553

0.00453	9.16586	0.00433	9.08607	0.00453	9.77365	0.00419	9.59111	0.0042	9.77824	0.00402	9.86773	0.0038	9.87003

0.00452	9.17068	0.00434	9.10864	0.00452	9.78048	0.0042	9.60609	0.0042	9.8036	0.00403	9.88875	0.0038	9.88228

0.00452	9.18193	0.00436	9.12959	0.00453	9.81112	0.00421	9.62607	0.00422	9.82896	0.00404	9.90628	0.0038	9.89279

0.00453	9.20922	0.00434	9.13926	0.00453	9.82475	0.00421	9.63773	0.00422	9.83403	0.00403	9.91679	0.0038	9.91204

0.00455	9.22045	0.00434	9.16022	0.00453	9.83157	0.00421	9.65437	0.00422	9.85263	0.00403	9.93255	0.00382	9.9313

0.00453	9.23169	0.00436	9.17795	0.00453	9.852	0.00423	9.67602	0.00422	9.87798	0.00405	9.95358	0.00382	9.94705

0.00455	9.24936	0.00437	9.19729	0.00455	9.87413	0.00423	9.68601	0.00423	9.88644	0.00404	9.97111	0.00381	9.96105

0.00456	9.27344	0.00437	9.20535	0.00455	9.88435	0.00423	9.701	0.00424	9.89996	0.00404	9.97986	0.00382	9.97855

0.00456	9.28628	0.00437	9.21825	0.00455	9.89968	0.00423	9.72431	0.00423	9.92024	0.00405	10.00789	0.00383	10.00831

0.00455	9.29591	0.00438	9.23759	0.00457	9.92353	0.00424	9.74762	0.00426	9.93208	0.00406	10.03242	0.00383	10.02406

0.00456	9.32161	0.00438	9.25048	0.00457	9.95077	0.00424	9.75928	0.00424	9.9473	0.00406	10.04294	0.00382	10.03456

0.00457	9.33927	0.00437	9.26015	0.00457	9.95588	0.00424	9.77094	0.00423	9.96927	0.00406	10.0552	0.00383	10.05382

0.00456	9.33927	0.00437	9.28272	0.00457	9.97803	0.00425	9.79592	0.00426	9.99294	0.00407	10.08149	0.00383	10.07482

0.00457	9.36334	0.00439	9.31012	0.00459	9.99505	0.00426	9.80923	0.00426	10.01323	0.00407	10.09375	0.00384	10.09232

0.00459	9.38582	0.00439	9.32463	0.00459	10.00697	0.00426	9.81756	0.00426	10.02337	0.00407	10.10601	0.00383	10.10108

0.00459	9.40027	0.00438	9.3343	0.00459	10.0189	0.00425	9.83421	0.00427	10.04197	0.00408	10.13229	0.00383	10.12033

0.00457	9.4099	0.00439	9.35848	0.00459	10.04273	0.00427	9.86419	0.00427	10.05719	0.00408	10.14807	0.00385	10.14483

0.00459	9.42757	0.0044	9.3746	0.00461	10.05806	0.00427	9.87418	0.00426	10.06563	0.00408	10.16909	0.00385	10.16234

0.0046	9.45004	0.0044	9.37782	0.00461	10.0785	0.00427	9.88749	0.00426	10.0893	0.00408	10.1831	0.00385	10.17633

0.0046	9.45647	0.0044	9.39716	0.00461	10.09552	0.00428	9.90748	0.00429	10.11635	0.00409	10.20763	0.00385	10.19209

0.0046	9.47413	0.0044	9.41006	0.00462	10.11597	0.00429	9.92579	0.00427	10.12312	0.00409	10.21639	0.00385	10.21485

0.0046	9.48697	0.00441	9.42779	0.00463	10.12789	0.00429	9.93579	0.00429	10.1434	0.00409	10.23041	0.00387	10.23235

0.00462	9.50623	0.00441	9.44391	0.00462	10.13469	0.00429	9.9541	0.00429	10.16369	0.0041	10.24443	0.00387	10.2446

0.00462	9.51747	0.0044	9.4697	0.00463	10.15854	0.0043	9.97242	0.00429	10.18398	0.00411	10.26545	0.00387	10.25861

0.0046	9.53995	0.00444	9.49226	0.00464	10.18238	0.00431	9.99073	0.00429	10.1806	0.0041	10.28648	0.00387	10.27786

0.00463	9.56082	0.00443	9.51161	0.00464	10.19261	0.0043	10.00406	0.00429	10.20764	0.0041	10.29523	0.00388	10.30061

0.00463	9.57848	0.00443	9.50999	0.00464	10.20792	0.00431	10.02237	0.00429	10.23132	0.00411	10.32852	0.00388	10.31811

0.00463	9.58652	0.00441	9.53095	0.00465	10.23858	0.00431	10.05234	0.00429	10.24146	0.00411	10.34254	0.00388	10.33037

0.00463	9.60256	0.00444	9.54062	0.00465	10.2471	0.00432	10.05734	0.00429	10.25329	0.00411	10.35481	0.00388	10.34962

0.00466	9.62344	0.00444	9.55674	0.00464	10.25731	0.00432	10.06566	0.00432	10.28372	0.00411	10.36532	0.00388	10.37413

0.00464	9.63789	0.00444	9.55996	0.00465	10.28456	0.00432	10.08398	0.00432	10.29555	0.00413	10.3951	0.00389	10.38463

0.00464	9.64912	0.00444	9.58253	0.00468	10.29478	0.00433	10.10396	0.00431	10.30232	0.00413	10.40737	0.00389	10.39688

0.00464	9.66518	0.00445	9.60993	0.00465	10.31182	0.00433	10.11895	0.00432	10.32768	0.00413	10.4249	0.0039	10.41438

0.00466	9.68926	0.00445	9.62444	0.00467	10.33055	0.00433	10.1306	0.00434	10.35811	0.00414	10.44766	0.00391	10.43889

0.00464	9.69729	0.00444	9.63894	0.00468	10.35099	0.00434	10.15558	0.00432	10.36148	0.00414	10.46168	0.0039	10.45814

0.00464	9.71495	0.00446	9.66473	0.00468	10.3697	0.00435	10.17889	0.00433	10.37163	0.00414	10.48271	0.0039	10.47039

0.00466	9.73743	0.00446	9.68407	0.00468	10.37994	0.00435	10.19221	0.00433	10.40207	0.00414	10.49848	0.0039	10.48789

0.00467	9.75669	0.00446	9.67924	0.00469	10.40036	0.00435	10.19887	0.00434	10.41728	0.00415	10.52126	0.00391	10.5124

0.00466	9.76312	0.00446	9.69858	0.00469	10.42251	0.00436	10.22884	0.00433	10.42742	0.00415	10.53528	0.00391	10.52815

0.00466	9.77757	0.00447	9.71792	0.00469	10.42762	0.00436	10.2355	0.00435	10.44432	0.00415	10.54404	0.00392	10.54215

0.00469	9.80326	0.00448	9.73404	0.00469	10.44976	0.00436	10.2455	0.00437	10.46461	0.00415	10.5598	0.00391	10.55441

0.00469	9.81289	0.00447	9.75016	0.00471	10.4719	0.00437	10.27213	0.00435	10.47307	0.00417	10.58784	0.00393	10.57716

0.00467	9.82412	0.00447	9.76789	0.00471	10.48893	0.00438	10.29379	0.00435	10.48828	0.00416	10.6001	0.00393	10.59992

0.00469	9.84821	0.00448	9.79207	0.00471	10.49914	0.00438	10.29711	0.00437	10.50857	0.00416	10.61762	0.00393	10.61217

0.0047	9.86908	0.0045	9.81141	0.00471	10.52298	0.00438	10.3171	0.00437	10.52885	0.00418	10.6474	0.00393	10.62792

0.0047	9.88192	0.00448	9.81947	0.00474	10.53832	0.00439	10.34207	0.00436	10.54744	0.00418	10.66142	0.00394	10.64892

0.0047	9.88514	0.00448	9.8372	0.00471	10.54683	0.0044	10.36039	0.00438	10.56605	0.00418	10.66843	0.00395	10.66993

0.00471	9.90922	0.00451	9.84848	0.00474	10.56216	0.00439	10.36538	0.00438	10.58464	0.00417	10.68595	0.00394	10.68393

0.00473	9.93009	0.00451	9.86299	0.00474	10.5826	0.0044	10.38537	0.00438	10.59648	0.00418	10.71224	0.00394	10.69443

0.00471	9.93651	0.0045	9.87427	0.00474	10.59792	0.00441	10.40535	0.00438	10.61677	0.00418	10.71924	0.00394	10.71719

0.00471	9.95096	0.0045	9.89362	0.00474	10.61836	0.00442	10.41701	0.00438	10.64043	0.00418	10.73326	0.00395	10.73994

0.00474	9.97665	0.00452	9.91779	0.00475	10.6371	0.0044	10.43033	0.0044	10.6455	0.0042	10.75778	0.00396	10.75569

0.00474	9.99752	0.00452	9.9323	0.00475	10.66093	0.00442	10.46196	0.0044	10.66579	0.0042	10.78231	0.00395	10.76795

0.00474	10.00876	0.00451	9.93875	0.00475	10.66945	0.00442	10.47861	0.0044	10.68607	0.0042	10.79809	0.00396	10.78545

0.00473	10.02001	0.00451	9.96293	0.00478	10.68478	0.00442	10.4936	0.00441	10.70636	0.0042	10.80158	0.00397	10.80995

0.00474	10.03927	0.00454	9.98388	0.00478	10.70691	0.00442	10.50525	0.0044	10.71482	0.00421	10.82787	0.00397	10.83096

0.00476	10.04568	0.00454	9.99194	0.00478	10.72224	0.00443	10.5319	0.0044	10.73003	0.00421	10.84364	0.00397	10.83971

0.00474	10.06174	0.00453	9.99839	0.00478	10.73587	0.00444	10.53523	0.00443	10.7537	0.00421	10.85415	0.00397	10.85546

0.00476	10.08422	0.00454	10.02095	0.00478	10.7529	0.00444	10.55187	0.00443	10.76215	0.00422	10.88043	0.00397	10.87647

0.0048	10.1083	0.00455	10.03707	0.0048	10.77333	0.00444	10.57352	0.00441	10.78075	0.00422	10.89971	0.00398	10.89397

0.00478	10.12115	0.00454	10.04674	0.00479	10.79376	0.00445	10.58851	0.00443	10.8061	0.00422	10.91372	0.00398	10.90797

0.00477	10.13078	0.00454	10.07576	0.00479	10.8023	0.00445	10.61016	0.00443	10.81963	0.00422	10.933	0.00398	10.92197

0.00478	10.15325	0.00457	10.09026	0.00482	10.83295	0.00445	10.62014	0.00443	10.83316	0.00425	10.95927	0.00399	10.94648

0.0048	10.17734	0.00457	10.11767	0.0048	10.84316	0.00446	10.64512	0.00443	10.85682	0.00425	10.97329	0.004	10.96398

0.00478	10.17895	0.00455	10.12895	0.00481	10.85678	0.00447	10.65844	0.00446	10.87373	0.00423	10.98205	0.00399	10.98149

0.0048	10.19179	0.00455	10.13378	0.00482	10.87893	0.00446	10.6701	0.00445	10.8788	0.00425	11.00483	0.00399	10.99199

0.00481	10.21749	0.00458	10.15152	0.00483	10.89254	0.00447	10.68675	0.00445	10.89402	0.00425	11.01885	0.004	11.01648

0.00481	10.22712	0.00458	10.16925	0.00482	10.90447	0.00448	10.71339	0.00445	10.92106	0.00424	11.03812	0.00401	11.0375

0.0048	10.23996	0.00457	10.18536	0.00482	10.9232	0.00449	10.71339	0.00446	10.94135	0.00425	11.05739	0.004	11.048

0.0048	10.25761	0.00458	10.18536	0.00484	10.95386	0.00448	10.73172	0.00445	10.94981	0.00426	11.08543	0.004	11.06024

0.00483	10.2833	0.00458	10.22244	0.00485	10.96579	0.0045	10.75669	0.00445	10.97009	0.00426	11.09769	0.00401	11.083

0.00483	10.29293	0.00458	10.2305	0.00484	10.97089	0.0045	10.77834	0.00447	10.99208	0.00426	11.1117	0.00401	11.10751

0.00483	10.31221	0.00459	10.25629	0.00486	10.99984	0.0045	10.79498	0.00448	11.00559	0.00427	11.12572	0.00403	11.12151

0.00483	10.32344	0.00458	10.27724	0.00486	11.01686	0.00451	10.80831	0.00447	11.02081	0.00428	11.1485	0.00401	11.13201

0.00485	10.3411	0.0046	10.29014	0.00485	11.02198	0.00451	10.83161	0.00449	11.04109	0.00427	11.15376	0.00402	11.15302

0.00484	10.34913	0.00459	10.30142	0.00486	11.04582	0.00451	10.83995	0.00449	11.05968	0.00428	11.17303	0.00403	11.17577

0.00484	10.3668	0.00459	10.31109	0.00488	11.06796	0.00451	10.84827	0.00448	11.07322	0.00428	11.20632	0.00403	11.19152

0.00485	10.39409	0.0046	10.33366	0.00488	11.08328	0.00451	10.86658	0.00449	11.08167	0.00428	11.21859	0.00403	11.20027

0.00485	10.40853	0.00461	10.34172	0.00487	11.09691	0.00453	10.88823	0.0045	11.1104	0.00428	11.23085	0.00404	11.22303

0.00484	10.41977	0.0046	10.35622	0.00489	11.12416	0.00453	10.90988	0.00449	11.12731	0.00428	11.24837	0.00404	11.24753

0.00485	10.43743	0.0046	10.38201	0.0049	11.13267	0.00452	10.9232	0.0045	11.13745	0.0043	11.2764	0.00404	11.25804

0.00487	10.46312	0.00462	10.40297	0.00489	11.14629	0.00453	10.93819	0.00451	11.16113	0.00429	11.27815	0.00404	11.27378

0.00487	10.47115	0.00462	10.42392	0.0049	11.15993	0.00454	10.9615	0.00452	11.17634	0.0043	11.29568	0.00404	11.29305

0.00487	10.48239	0.00461	10.43843	0.0049	11.18376	0.00454	10.96983	0.00451	11.1848	0.00431	11.31846	0.00406	11.31405

0.00487	10.50486	0.00462	10.4481	0.00492	11.19569	0.00454	10.97815	0.00451	11.20339	0.00431	11.34124	0.00406	11.33505

0.00488	10.51932	0.00464	10.45938	0.00491	11.21952	0.00455	11.00646	0.00453	11.22537	0.00431	11.35349	0.00406	11.34905

0.00488	10.53056	0.00464	10.47066	0.00492	11.23315	0.00457	11.02644	0.00453	11.23551	0.00431	11.37277	0.00406	11.35955

0.00487	10.54982	0.00462	10.48195	0.00492	11.25699	0.00456	11.0331	0.00452	11.24734	0.00432	11.39905	0.00407	11.38406

0.0049	10.5755	0.00465	10.5029	0.00492	11.26211	0.00456	11.05641	0.00453	11.27609	0.00431	11.40781	0.00408	11.40331

0.0049	10.58514	0.00465	10.52386	0.00492	11.28083	0.00457	11.08305	0.00454	11.29468	0.00433	11.42533	0.00408	11.41731

0.0049	10.59157	0.00465	10.54965	0.00495	11.30297	0.00457	11.09304	0.00453	11.30144	0.00433	11.43409	0.00408	11.42957

0.0049	10.60921	0.00465	10.56576	0.00495	11.3183	0.00457	11.10303	0.00454	11.32511	0.00434	11.45687	0.00408	11.45232

0.00491	10.6333	0.00466	10.58833	0.00494	11.33022	0.00459	11.12635	0.00454	11.3437	0.00433	11.46213	0.00408	11.47333

0.00491	10.64775	0.00467	10.60123	0.00495	11.36088	0.00459	11.14133	0.00455	11.35216	0.00434	11.49191	0.00408	11.48907

0.0049	10.65579	0.00467	10.59639	0.00496	11.37451	0.0046	11.15132	0.00455	11.37076	0.00435	11.52169	0.00408	11.50133

0.00493	10.67665	0.00466	10.61896	0.00495	11.38982	0.00459	11.16131	0.00457	11.3978	0.00435	11.53045	0.00408	11.51883

0.00494	10.69592	0.00468	10.63024	0.00495	11.40516	0.0046	11.19129	0.00457	11.41302	0.00434	11.55148	0.0041	11.54334

0.00493	10.71037	0.00468	10.64797	0.00497	11.41877	0.00461	11.2146	0.00457	11.42655	0.00435	11.569	0.0041	11.55734

0.00493	10.72804	0.00467	10.65442	0.00497	11.43411	0.0046	11.21793	0.00457	11.44176	0.00436	11.58827	0.00409	11.56784

0.00494	10.74889	0.00468	10.67859	0.00497	11.45454	0.0046	11.23957	0.00457	11.46542	0.00436	11.59703	0.0041	11.58709

0.00495	10.75854	0.00468	10.70922	0.00497	11.47328	0.00461	11.25956	0.00457	11.48064	0.00436	11.6128	0.0041	11.60985

0.00494	10.76977	0.00469	10.72695	0.00498	11.49201	0.00462	11.27121	0.00455	11.49078	0.00437	11.64434	0.00411	11.63085

0.00494	10.78743	0.00469	10.74468	0.00498	11.50563	0.00462	11.27787	0.00459	11.51276	0.00438	11.65661	0.00411	11.64311

0.00497	10.80991	0.00469	10.74307	0.00498	11.52267	0.00462	11.30951	0.0046	11.53305	0.00437	11.67062	0.0041	11.66061

0.00498	10.82114	0.00471	10.77531	0.005	11.54821	0.00463	11.32117	0.00457	11.54149	0.00438	11.6934	0.00411	11.68161

0.00495	10.83881	0.00471	10.78337	0.005	11.55673	0.00463	11.33116	0.0046	11.5601	0.00439	11.71267	0.00412	11.69736

0.00497	10.85327	0.00469	10.78981	0.005	11.57035	0.00464	11.3578	0.00462	11.58208	0.00438	11.72494	0.00413	11.71487

0.005	10.87735	0.00472	10.79142	0.005	11.58739	0.00465	11.37611	0.0046	11.59053	0.00439	11.73019	0.00412	11.72362

0.00498	10.89019	0.00473	10.81238	0.00502	11.61122	0.00465	11.38611	0.00462	11.61251	0.00439	11.76523	0.00411	11.74462

0.00498	10.8934	0.00472	10.83978	0.005	11.61293	0.00465	11.40276	0.0046	11.62941	0.0044	11.77925	0.00413	11.77088

0.005	10.9207	0.00472	10.86557	0.005	11.63336	0.00465	11.42108	0.00462	11.64462	0.00439	11.78276	0.00413	11.78312

0.00501	10.93193	0.00473	10.89458	0.00502	11.66232	0.00467	11.44438	0.0046	11.65815	0.0044	11.81605	0.00413	11.79713

0.005	10.94959	0.00473	10.89942	0.00502	11.67763	0.00467	11.44771	0.0046	11.6852	0.00442	11.83357	0.00413	11.81989

0.00501	10.96886	0.00473	10.90587	0.00502	11.68956	0.00467	11.4627	0.00463	11.7038	0.00441	11.84583	0.00414	11.84264

0.00501	10.99295	0.00473	10.93005	0.00503	11.70829	0.00468	11.48601	0.00463	11.71225	0.0044	11.86159	0.00414	11.85489

0.00502	11.00258	0.00475	10.93166	0.00503	11.72703	0.00469	11.50766	0.00463	11.72915	0.00442	11.88437	0.00414	11.86364

0.00501	11.01221	0.00474	10.95261	0.00503	11.73894	0.00468	11.52264	0.00465	11.74944	0.00442	11.89839	0.00414	11.88465

0.00501	11.02505	0.00475	10.95583	0.00503	11.76109	0.00468	11.5393	0.00465	11.76297	0.00442	11.90715	0.00415	11.9074

0.00502	11.04913	0.00475	10.98807	0.00506	11.78152	0.00469	11.56428	0.00464	11.7765	0.00442	11.92468	0.00416	11.9249

0.00504	11.05876	0.00476	11.01064	0.00506	11.80196	0.00469	11.57426	0.00464	11.80185	0.00443	11.95446	0.00415	11.9354

0.00502	11.07321	0.00475	11.02353	0.00506	11.80877	0.00469	11.57926	0.00466	11.82383	0.00443	11.96848	0.00415	11.95816

0.00505	11.09248	0.00475	11.05577	0.00506	11.82921	0.0047	11.59758	0.00464	11.82551	0.00443	11.98074	0.00416	11.97916

0.00507	11.11496	0.00475	11.05577	0.00507	11.84623	0.00472	11.61922	0.00465	11.85087	0.00445	12.00527	0.00418	11.99666

0.00504	11.12459	0.00478	11.08317	0.00507	11.85476	0.00471	11.63421	0.00466	11.87286	0.00445	12.01929	0.00418	12.00892

0.00504	11.13904	0.00478	11.0735	0.00507	11.87688	0.0047	11.64753	0.00466	11.8813	0.00445	12.02629	0.00416	12.02467

0.00507	11.16312	0.00476	11.1009	0.00508	11.89393	0.00472	11.67584	0.00466	11.89144	0.00445	12.04382	0.00418	12.04743

0.00507	11.17436	0.00478	11.11057	0.00508	11.91096	0.00473	11.68915	0.00467	11.91513	0.00446	12.0701	0.00418	12.06318

0.00505	11.18079	0.00479	11.1283	0.00507	11.91947	0.00472	11.70082	0.00468	11.93709	0.00447	12.08586	0.00418	12.07894

0.00507	11.20326	0.00478	11.14281	0.00509	11.94501	0.00473	11.71746	0.00468	11.94216	0.00446	12.1104	0.00418	12.09644

0.00508	11.22414	0.00478	11.1686	0.00511	11.96885	0.00474	11.73911	0.00468	11.97091	0.00446	12.12442	0.00419	12.11744

0.00508	11.23377	0.0048	11.19439	0.00509	11.97396	0.00474	11.7491	0.00469	11.98613	0.00447	12.14894	0.00419	12.13669

0.00507	11.24982	0.0048	11.2089	0.00511	11.99611	0.00474	11.76408	0.00468	12.00641	0.00447	12.15069	0.00419	12.15245

0.00509	11.2739	0.0048	11.22179	0.00512	12.01995	0.00475	11.7824	0.00469	12.01824	0.00447	12.16822	0.00419	12.1612

0.00509	11.28995	0.0048	11.22824	0.00512	12.03358	0.00476	11.80738	0.00471	12.03177	0.00448	12.19099	0.00419	12.18395

0.00509	11.3012	0.00482	11.23791	0.00511	12.04549	0.00476	11.8207	0.00469	12.05543	0.00449	12.21202	0.00421	12.20496

0.00508	11.31404	0.00482	11.25564	0.00512	12.07103	0.00477	11.83403	0.00469	12.0605	0.00448	12.22603	0.00421	12.2207

0.00511	11.34133	0.0048	11.2766	0.00513	12.08977	0.00478	11.859	0.00471	12.08249	0.0045	12.25056	0.00421	12.23646

0.00512	11.34937	0.00482	11.28949	0.00512	12.09658	0.00478	11.87732	0.00471	12.10615	0.0045	12.27334	0.00421	12.25396

0.00511	11.3606	0.00482	11.31689	0.00513	12.11702	0.00478	11.88065	0.00471	12.12306	0.0045	12.28385	0.00421	12.27847

0.00511	11.38468	0.00482	11.33301	0.00514	12.13746	0.00478	11.89896	0.00471	12.13151	0.0045	12.29962	0.00421	12.29072

0.00512	11.40877	0.00482	11.34268	0.00515	12.15449	0.00478	11.91561	0.00474	12.15518	0.0045	12.31539	0.00421	12.30472

0.00512	11.4184	0.00485	11.3717	0.00514	12.1647	0.00479	11.93393	0.00474	12.17885	0.0045	12.3259	0.00422	12.32572

0.00512	11.42963	0.00485	11.38137	0.00516	12.18515	0.00479	11.95391	0.00474	12.18053	0.0045	12.34692	0.00423	12.35023

0.00514	11.44409	0.00483	11.38781	0.00516	12.20387	0.0048	11.97889	0.00473	12.19406	0.00452	12.36269	0.00423	12.36248

0.00514	11.46978	0.00483	11.41038	0.00516	12.21408	0.00481	11.9972	0.00474	12.23295	0.00453	12.38898	0.00423	12.37299

0.00514	11.4778	0.00487	11.40232	0.00516	12.23793	0.0048	12.00554	0.00474	12.23971	0.00452	12.40299	0.00423	12.39399

0.00514	11.49225	0.00486	11.43456	0.00518	12.25667	0.0048	12.01719	0.00474	12.25154	0.00453	12.42227	0.00425	12.41674

0.00515	11.51472	0.00485	11.45229	0.00518	12.27539	0.00482	12.03883	0.00477	12.28028	0.00453	12.44505	0.00425	12.43249

0.00516	11.53239	0.00485	11.47969	0.00517	12.28393	0.00482	12.05049	0.00477	12.29042	0.00453	12.45556	0.00424	12.44299

0.00515	11.54202	0.00487	11.49742	0.00519	12.30605	0.00482	12.06547	0.00476	12.30057	0.00453	12.47308	0.00425	12.464

0.00516	11.55648	0.00487	11.51838	0.0052	12.32649	0.00482	12.08712	0.00477	12.32086	0.00454	12.48359	0.00425	12.48326

0.00518	11.57896	0.00487	11.52643	0.00518	12.33671	0.00484	12.10876	0.00477	12.34959	0.00455	12.50987	0.00426	12.50251

0.00518	11.59982	0.00487	11.53611	0.00518	12.35715	0.00484	12.11543	0.00478	12.35297	0.00454	12.51688	0.00425	12.51652

0.00516	11.59822	0.00489	11.55706	0.0052	12.37929	0.00483	12.13374	0.00476	12.37495	0.00454	12.53966	0.00426	12.53051

0.00518	11.62391	0.00488	11.56834	0.0052	12.38611	0.00485	12.15872	0.00478	12.39354	0.00456	12.57294	0.00426	12.55327

0.00521	11.64318	0.00487	11.56995	0.0052	12.40312	0.00486	12.1737	0.0048	12.41215	0.00456	12.58521	0.00427	12.57077

0.00518	11.65762	0.00489	11.58769	0.00522	12.42526	0.00485	12.18037	0.00479	12.42397	0.00455	12.59222	0.00427	12.58652

0.00519	11.67207	0.0049	11.61992	0.00522	12.44399	0.00485	12.19868	0.00479	12.43751	0.00457	12.61499	0.00427	12.60227

0.00521	11.69455	0.00489	11.62798	0.00522	12.45252	0.00487	12.222	0.00481	12.4578	0.00457	12.63252	0.00428	12.62328

0.00522	11.71061	0.0049	11.65055	0.00522	12.46954	0.00487	12.23365	0.00481	12.46963	0.00457	12.64128	0.00428	12.64603

0.00522	11.71864	0.0049	11.67795	0.00524	12.48998	0.00487	12.24863	0.00479	12.48484	0.00457	12.66055	0.00428	12.66004

0.00522	11.72827	0.00493	11.69246	0.00524	12.5053	0.00487	12.27028	0.00481	12.51188	0.00458	12.69033	0.00428	12.66879

0.00522	11.75716	0.0049	11.69407	0.00524	12.52745	0.00488	12.2886	0.00481	12.52541	0.00459	12.70084	0.00428	12.68979

0.00523	11.77482	0.0049	11.70535	0.00525	12.54106	0.00488	12.30525	0.00481	12.5457	0.00458	12.72012	0.00429	12.70904

0.00522	11.78124	0.00493	11.72631	0.00526	12.56321	0.00488	12.31357	0.00481	12.56091	0.00459	12.73413	0.00429	12.7283

0.00523	11.7989	0.00493	11.7392	0.00525	12.57342	0.00489	12.33356	0.00481	12.57274	0.0046	12.76217	0.00429	12.7423

0.00525	11.81978	0.00493	11.74726	0.00525	12.58536	0.0049	12.35187	0.00481	12.59134	0.0046	12.76392	0.00429	12.75981

0.00525	11.83744	0.00493	11.78433	0.00527	12.6143	0.0049	12.3602	0.00481	12.60149	0.0046	12.77969	0.00431	12.78606

0.00525	11.84707	0.00495	11.80368	0.00527	12.62622	0.0049	12.38184	0.00482	12.63023	0.00461	12.80422	0.00431	12.80357

0.00526	11.86633	0.00495	11.82141	0.00527	12.64326	0.00491	12.41348	0.00482	12.64206	0.00461	12.82349	0.0043	12.81232

0.00526	11.8856	0.00494	11.82785	0.00528	12.66368	0.00492	12.42681	0.00482	12.65559	0.00461	12.83225	0.00431	12.82982

0.00526	11.88721	0.00494	11.84558	0.00529	12.67902	0.00492	12.43347	0.00483	12.68263	0.00461	12.86379	0.00432	12.85432

0.00526	11.91129	0.00495	11.86331	0.00527	12.69265	0.00493	12.45344	0.00484	12.70462	0.00462	12.8778	0.00432	12.87007

0.00528	11.93538	0.00495	11.85848	0.00529	12.70968	0.00494	12.47343	0.00484	12.71306	0.00462	12.89533	0.00431	12.87882

0.00529	11.94983	0.00495	11.87943	0.0053	12.72841	0.00493	12.48508	0.00484	12.72152	0.00462	12.90058	0.00432	12.89633

0.00526	11.95785	0.00497	11.91167	0.00531	12.74203	0.00494	12.49508	0.00487	12.7418	0.00464	12.91986	0.00432	12.92433

0.00528	11.97712	0.00497	11.92295	0.0053	12.75736	0.00495	12.52171	0.00487	12.75703	0.00464	12.93738	0.00432	12.93834

0.00529	11.9996	0.00497	11.94552	0.00531	12.7812	0.00495	12.53338	0.00484	12.77392	0.00464	12.9584	0.00432	12.95409

0.0053	12.01564	0.00497	11.9568	0.00531	12.80333	0.00496	12.54336	0.00485	12.79083	0.00464	12.98469	0.00434	12.9716

0.00529	12.02689	0.00497	11.97292	0.00531	12.81356	0.00496	12.57001	0.00488	12.81619	0.00465	13.00921	0.00435	12.99435

0.0053	12.03813	0.00499	12.00032	0.00531	12.82888	0.00496	12.58998	0.00488	12.82633	0.00465	13.01972	0.00435	13.0101

0.00532	12.059	0.00499	12.01322	0.00533	12.84932	0.00496	12.60165	0.00488	12.84325	0.00465	13.03024	0.00434	13.02235

0.00532	12.07344	0.00497	12.01483	0.00533	12.86635	0.00496	12.61163	0.00488	12.86353	0.00466	13.05476	0.00434	13.0381

0.00532	12.09272	0.005	12.0374	0.00533	12.87657	0.00497	12.64161	0.0049	12.87705	0.00466	13.06703	0.00435	13.05736

0.00533	12.10877	0.00501	12.04707	0.00535	12.90382	0.00498	12.64993	0.00488	12.88719	0.00465	13.07579	0.00435	13.07837

0.00533	12.12803	0.005	12.05674	0.00535	12.91745	0.00499	12.66158	0.00488	12.90748	0.00466	13.10383	0.00435	13.09236

0.00532	12.13767	0.005	12.08253	0.00535	12.93447	0.00498	12.67658	0.00488	12.93453	0.00468	13.12836	0.00435	13.10987

0.00533	12.15212	0.00502	12.10993	0.00535	12.9515	0.005	12.70821	0.00488	12.94298	0.00468	13.14763	0.00436	13.13087

0.00535	12.17299	0.00502	12.13411	0.00536	12.97024	0.005	12.72153	0.00488	12.96496	0.00467	13.15639	0.00436	13.15363

0.00535	12.18904	0.00501	12.14539	0.00536	12.98726	0.00499	12.72985	0.00491	12.98525	0.00468	13.18092	0.00436	13.16413

0.00535	12.19065	0.00502	12.15667	0.00536	12.99237	0.005	12.75317	0.00493	12.99877	0.00469	13.2037	0.00436	13.17463

0.00533	12.21794	0.00504	12.16634	0.00536	13.01281	0.00502	12.76816	0.00492	13.01229	0.00468	13.19843	0.00438	13.20088

0.00536	12.23882	0.00503	12.18407	0.00537	13.03836	0.00501	12.78314	0.00493	13.03427	0.00469	13.21946	0.00438	13.22013

0.00536	12.25166	0.00502	12.19213	0.00537	13.05028	0.00501	12.79313	0.00492	13.04611	0.0047	13.2475	0.00438	13.23414

0.00535	12.26771	0.00504	12.21309	0.00536	13.07073	0.00503	12.81312	0.00493	13.05794	0.00471	13.26326	0.00438	13.24639

0.00537	12.28699	0.00504	12.22598	0.0054	13.09625	0.00504	12.83477	0.00492	13.07823	0.0047	13.28079	0.00438	13.26915

0.00539	12.30143	0.00504	12.24371	0.0054	13.10818	0.00503	12.84642	0.00494	13.1019	0.00471	13.29481	0.00439	13.29365

0.00537	12.30946	0.00504	12.26467	0.00539	13.1201	0.00503	12.86972	0.00494	13.12218	0.00472	13.32634	0.00439	13.30415

0.00537	12.32391	0.00506	12.28885	0.0054	13.14565	0.00505	12.89137	0.00494	13.13571	0.00471	13.33861	0.00439	13.31815

0.0054	12.34799	0.00507	12.30335	0.00541	13.15757	0.00505	12.90469	0.00495	13.14754	0.00472	13.34561	0.00439	13.33741

0.00539	12.36725	0.00507	12.30496	0.0054	13.16097	0.00505	12.90803	0.00495	13.16614	0.00472	13.36839	0.0044	13.35841

0.00539	12.3785	0.00506	12.32431	0.00541	13.18652	0.00505	12.92634	0.00496	13.18473	0.00473	13.37715	0.00441	13.37592

0.0054	12.39455	0.00507	12.34043	0.00544	13.21718	0.00506	12.95465	0.00494	13.19487	0.00473	13.39993	0.0044	13.38642

0.0054	12.42023	0.00507	12.35654	0.00542	13.22739	0.00506	12.9663	0.00496	13.22193	0.00472	13.4227	0.0044	13.40217

0.00542	12.42827	0.00507	12.36299	0.00544	13.2359	0.00507	12.98462	0.00498	13.23884	0.00474	13.44724	0.00441	13.43192

0.00542	12.43468	0.00509	12.38878	0.00544	13.26144	0.00508	13.0096	0.00496	13.24898	0.00474	13.45775	0.00441	13.44593

0.00542	12.46198	0.00509	12.41618	0.00544	13.28189	0.00508	13.02625	0.00498	13.27434	0.00474	13.47702	0.00442	13.45993

0.00543	12.47482	0.0051	12.43714	0.00544	13.28359	0.00507	13.04123	0.00498	13.28786	0.00475	13.49629	0.00441	13.48094

0.00542	12.48607	0.00509	12.4452	0.00545	13.30574	0.00509	13.0479	0.00498	13.2963	0.00475	13.51206	0.00442	13.49494

0.00542	12.50693	0.0051	12.4597	0.00545	13.33468	0.00509	13.0762	0.00498	13.31491	0.00475	13.52082	0.00443	13.51595

0.00544	12.5262	0.00511	12.47582	0.00545	13.3415	0.0051	13.08953	0.00499	13.33182	0.00475	13.53133	0.00443	13.53345

0.00546	12.54387	0.0051	12.49033	0.00545	13.35683	0.0051	13.10118	0.00499	13.35041	0.00477	13.56287	0.00442	13.54044

0.00544	12.55189	0.0051	12.48872	0.00546	13.38067	0.0051	13.11451	0.00499	13.36393	0.00477	13.5804	0.00443	13.5667

0.00546	12.56955	0.00513	12.52095	0.00547	13.39598	0.00511	13.13948	0.00499	13.38422	0.00477	13.5944	0.00444	13.5912

0.00547	12.59042	0.00513	12.52579	0.00547	13.40791	0.00511	13.15946	0.00499	13.40789	0.00478	13.61894	0.00444	13.6017

0.00547	12.60648	0.00513	12.55158	0.00547	13.42324	0.00512	13.17278	0.00501	13.41803	0.00478	13.63471	0.00444	13.61571

0.00547	12.6145	0.00511	12.56931	0.00548	13.45048	0.00513	13.1911	0.00502	13.43832	0.00478	13.63471	0.00444	13.63671

0.00547	12.63698	0.00513	12.59349	0.00547	13.46582	0.00513	13.20275	0.00502	13.4586	0.00478	13.65047	0.00445	13.65596

0.00549	12.64983	0.00515	12.61444	0.00548	13.46923	0.00513	13.20608	0.00504	13.47043	0.0048	13.68552	0.00446	13.67172

0.00547	12.66428	0.00514	12.61767	0.00548	13.49817	0.00513	13.2294	0.00502	13.48396	0.00479	13.70304	0.00445	13.68573

0.00549	12.68194	0.00513	12.63056	0.0055	13.52031	0.00514	13.25438	0.00502	13.49918	0.00479	13.71005	0.00445	13.70147

0.0055	12.70281	0.00516	12.64507	0.0055	13.52031	0.00515	13.2627	0.00504	13.52623	0.0048	13.73983	0.00446	13.72598

0.0055	12.72047	0.00516	12.65152	0.0055	13.54416	0.00514	13.27602	0.00504	13.53638	0.00481	13.76085	0.00447	13.74173

0.00549	12.72689	0.00515	12.66602	0.00551	13.568	0.00515	13.30765	0.00502	13.55666	0.0048	13.77136	0.00447	13.75573

0.0055	12.74776	0.00517	12.69342	0.00552	13.5731	0.00516	13.32098	0.00505	13.58032	0.00481	13.78538	0.00447	13.77674

0.00553	12.76543	0.00517	12.71438	0.0055	13.59525	0.00516	13.33763	0.00505	13.58202	0.00482	13.80116	0.00448	13.79774

0.00553	12.77506	0.00516	12.73211	0.00552	13.62078	0.00516	13.35427	0.00505	13.59554	0.00482	13.82218	0.00448	13.81175

0.00551	12.7879	0.00517	12.74339	0.00554	13.63612	0.00518	13.36927	0.00506	13.61921	0.00482	13.82919	0.00448	13.8275

0.00553	12.81359	0.00517	12.77079	0.00552	13.64633	0.00518	13.38591	0.00507	13.64118	0.00482	13.85021	0.00448	13.845

0.00553	12.83606	0.0052	12.7853	0.00552	13.66507	0.00518	13.38758	0.00506	13.65302	0.00484	13.88	0.00449	13.86601

0.00553	12.84089	0.00517	12.78691	0.00554	13.6855	0.00518	13.41089	0.00506	13.66654	0.00483	13.89226	0.00449	13.88526

0.00553	12.85694	0.00518	12.79819	0.00554	13.70425	0.00519	13.4392	0.00507	13.69697	0.00484	13.91329	0.00449	13.89401

0.00554	12.8762	0.0052	12.82721	0.00554	13.71275	0.0052	13.45085	0.00507	13.70544	0.00485	13.93081	0.00449	13.91326

0.00556	12.88905	0.00521	12.83849	0.00556	13.7349	0.00519	13.46751	0.00507	13.71726	0.00485	13.95008	0.0045	13.93952

0.00556	12.90671	0.0052	12.84655	0.00556	13.75533	0.00521	13.49081	0.00508	13.73924	0.00485	13.95709	0.00451	13.95352

0.00556	12.91956	0.00521	12.87234	0.00555	13.76725	0.00522	13.50581	0.00509	13.75445	0.00485	13.97636	0.0045	13.96577

0.00557	12.94524	0.00522	12.89974	0.00556	13.78939	0.00521	13.51246	0.00507	13.7646	0.00486	14.00264	0.0045	13.97978

0.00557	12.95808	0.00522	12.91264	0.00557	13.80472	0.00522	13.52245	0.00509	13.78151	0.00486	14.0149	0.00451	14.00253

0.00556	12.97414	0.00522	12.92231	0.00557	13.81664	0.00523	13.55076	0.0051	13.81194	0.00486	14.02542	0.00452	14.02178

0.00558	12.99662	0.00522	12.93359	0.00557	13.83027	0.00523	13.56575	0.00509	13.82038	0.00488	14.05871	0.00452	14.03754

0.00558	13.00465	0.00524	12.95938	0.00559	13.86432	0.00523	13.58406	0.00509	13.84237	0.00488	14.07623	0.00452	14.05154

0.00558	13.02231	0.00524	12.96422	0.00559	13.87624	0.00523	13.60072	0.00512	13.86434	0.00488	14.08499	0.00452	14.07429

0.00558	13.03354	0.00522	12.97389	0.00559	13.88305	0.00524	13.62403	0.00512	13.86941	0.00488	14.10602	0.00453	14.0953

0.00561	13.05602	0.00524	13.00129	0.00559	13.90179	0.00525	13.64068	0.00512	13.88463	0.00488	14.12353	0.00452	14.10755

0.00561	13.07368	0.00525	13.02063	0.00561	13.92394	0.00525	13.64068	0.00512	13.9083	0.00488	14.12704	0.00452	14.12505

0.0056	13.0785	0.00524	13.03836	0.00561	13.94267	0.00526	13.66733	0.00512	13.9269	0.00489	14.14631	0.00454	14.1443

0.00561	13.09937	0.00524	13.05609	0.00561	13.95799	0.00526	13.67731	0.00512	13.94042	0.0049	14.17786	0.00454	14.16531

0.00561	13.12024	0.00527	13.07705	0.00561	13.97502	0.00526	13.69396	0.00512	13.95563	0.0049	14.19888	0.00454	14.17756

0.00561	13.1363	0.00527	13.08833	0.00562	13.99036	0.00526	13.70729	0.00512	13.98437	0.0049	14.20238	0.00454	14.19156

0.00561	13.14593	0.00527	13.09317	0.00563	13.99546	0.00528	13.73059	0.00515	13.9979	0.00491	14.23042	0.00454	14.21257

0.00564	13.17804	0.00527	13.10928	0.00563	14.02441	0.00528	13.75391	0.00513	14.00466	0.00492	14.24618	0.00456	14.23357

0.00564	13.17965	0.00529	13.13346	0.00563	14.04824	0.00528	13.76556	0.00515	14.02326	0.00492	14.26195	0.00456	14.24758

0.00564	13.1941	0.00529	13.14152	0.00564	14.06017	0.00529	13.78222	0.00515	14.04523	0.00491	14.2672	0.00456	14.25983

0.00564	13.21818	0.00529	13.15603	0.00563	14.06868	0.0053	13.80886	0.00515	14.05706	0.00493	14.29524	0.00456	14.28083

0.00567	13.23263	0.0053	13.17698	0.00564	14.09593	0.0053	13.81385	0.00516	14.08074	0.00493	14.30926	0.00457	14.30883

0.00567	13.24868	0.0053	13.206	0.00567	14.11297	0.0053	13.8305	0.00516	14.10103	0.00492	14.33378	0.00457	14.31934

0.00565	13.26474	0.00531	13.22373	0.00564	14.12489	0.00531	13.85881	0.00518	14.11792	0.00493	14.35306	0.00456	14.32809

0.00567	13.27758	0.0053	13.23662	0.00566	14.13681	0.00532	13.86547	0.00516	14.12806	0.00495	14.37233	0.00457	14.34909

0.00568	13.30006	0.0053	13.24307	0.00568	14.15554	0.00532	13.87713	0.00518	14.1416	0.00495	14.3916	0.00458	14.3771

0.00568	13.3113	0.00532	13.26241	0.00567	14.17257	0.00532	13.90376	0.00518	14.16696	0.00495	14.39687	0.00459	14.3911

0.00568	13.31772	0.00532	13.27369	0.00567	14.18961	0.00533	13.92542	0.00518	14.17541	0.00495	14.41614	0.00458	14.39985

0.00568	13.34019	0.00531	13.28337	0.00569	14.21173	0.00533	13.93707	0.00516	14.19232	0.00496	14.43191	0.00458	14.42086

0.0057	13.36107	0.00532	13.30432	0.00569	14.23047	0.00533	13.95039	0.00519	14.20921	0.00496	14.44767	0.00459	14.44711

0.00571	13.3707	0.00535	13.32689	0.00568	14.2407	0.00533	13.96038	0.0052	14.22443	0.00496	14.4652	0.0046	14.46287

0.00571	13.38997	0.00534	13.33817	0.00569	14.25772	0.00535	13.97704	0.00519	14.24641	0.00497	14.49147	0.00459	14.47162

0.00571	13.40603	0.00532	13.36074	0.0057	14.28496	0.00534	13.98536	0.0052	14.26839	0.00498	14.51425	0.00459	14.49087

0.00572	13.42207	0.00535	13.38814	0.0057	14.29348	0.00535	14.00534	0.00522	14.28698	0.00497	14.52301	0.00461	14.51362

0.00571	13.43653	0.00535	13.39458	0.00569	14.3054	0.00536	14.03531	0.00521	14.30051	0.00498	14.53352	0.00461	14.53112

0.00571	13.45579	0.00535	13.40426	0.00572	14.33777	0.00537	14.05197	0.00521	14.31572	0.00499	14.56506	0.0046	14.53987

0.00574	13.47667	0.00535	13.41715	0.00573	14.3565	0.00536	14.06362	0.00522	14.33601	0.00499	14.56681	0.0046	14.56263

0.00574	13.48469	0.00537	13.43649	0.00573	14.36332	0.00537	14.0886	0.00522	14.34954	0.00499	14.57908	0.00461	14.58363

0.00574	13.50075	0.00537	13.44939	0.00573	14.38375	0.00539	14.10192	0.00521	14.35968	0.00499	14.60887	0.00462	14.60464

0.00575	13.5152	0.00537	13.45745	0.00574	14.39737	0.00539	14.11523	0.00522	14.38504	0.005	14.62814	0.00461	14.61689

0.00577	13.53446	0.00537	13.48968	0.00574	14.41441	0.00539	14.1269	0.00523	14.40871	0.00499	14.63514	0.00463	14.63265

0.00577	13.54571	0.00538	13.50903	0.00574	14.43654	0.0054	14.14855	0.00524	14.41378	0.005	14.66493	0.00463	14.65539

0.00575	13.56818	0.00539	13.52837	0.00575	14.45527	0.0054	14.17019	0.00524	14.429	0.00501	14.6877	0.00463	14.6694

0.00577	13.58263	0.00539	13.54126	0.00575	14.46889	0.0054	14.18184	0.00526	14.45097	0.00501	14.69297	0.00463	14.68165

0.00578	13.59547	0.00539	13.55899	0.00575	14.48251	0.00541	14.20515	0.00524	14.46111	0.00501	14.70873	0.00463	14.7009

0.00578	13.60671	0.00542	13.57189	0.00575	14.49955	0.00542	14.21848	0.00524	14.47633	0.00503	14.72801	0.00463	14.72191

0.00578	13.62918	0.00539	13.57511	0.00578	14.52681	0.00543	14.23846	0.00526	14.4983	0.00503	14.74903	0.00464	14.74116

0.00579	13.64846	0.00541	13.59607	0.00578	14.53872	0.00542	14.24678	0.00527	14.51859	0.00503	14.75954	0.00464	14.75341

0.00581	13.66451	0.00542	13.6138	0.00578	14.55236	0.00542	14.25678	0.00526	14.53887	0.00503	14.77706	0.00464	14.77092

0.00579	13.67254	0.00542	13.6283	0.00578	14.57108	0.00543	14.27675	0.00526	14.55241	0.00505	14.81035	0.00466	14.79542

0.00579	13.69823	0.00542	13.64926	0.00579	14.59151	0.00543	14.29674	0.00529	14.57437	0.00504	14.82086	0.00466	14.81293

0.00582	13.71107	0.00542	13.66538	0.00578	14.60173	0.00543	14.31172	0.00529	14.57945	0.00504	14.83138	0.00466	14.82343

0.00582	13.72231	0.00544	13.69117	0.00579	14.62558	0.00544	14.3417	0.00529	14.59298	0.00505	14.85415	0.00466	14.83918

0.00582	13.73034	0.00544	13.70084	0.00581	14.6443	0.00545	14.35835	0.00529	14.61834	0.00506	14.86993	0.00466	14.86018

0.00585	13.76085	0.00544	13.70729	0.00581	14.65453	0.00545	14.36335	0.00529	14.63186	0.00506	14.87868	0.00467	14.87943

0.00585	13.78332	0.00544	13.72179	0.0058	14.66815	0.00546	14.37833	0.00529	14.64877	0.00506	14.91197	0.00467	14.89519

0.00582	13.78974	0.00546	13.74436	0.00583	14.68689	0.00547	14.40164	0.00529	14.66736	0.00507	14.93124	0.00467	14.9092

0.00585	13.80258	0.00546	13.75564	0.00583	14.70731	0.00548	14.41163	0.00532	14.6961	0.00507	14.94702	0.00467	14.9302

0.00585	13.82666	0.00545	13.76692	0.00583	14.71753	0.00548	14.42829	0.00532	14.70793	0.00507	14.95578	0.00469	14.9512

0.00585	13.84754	0.00546	13.7911	0.00583	14.73457	0.00548	14.44493	0.00532	14.7147	0.00507	14.98381	0.00469	14.9687

0.00585	13.84915	0.00548	13.81528	0.00584	14.76352	0.00549	14.46492	0.00532	14.74006	0.00509	14.99433	0.00469	14.97745

0.00585	13.86841	0.00549	13.82818	0.00585	14.77204	0.00549	14.48656	0.00533	14.75696	0.00509	15.00133	0.00469	15.00021

0.00588	13.88607	0.00546	13.84591	0.00584	14.79247	0.00549	14.50488	0.00533	14.75865	0.00509	15.0206	0.0047	15.02296

0.00588	13.89891	0.00549	13.86041	0.00585	14.81121	0.00551	14.52486	0.00533	14.78908	0.0051	15.04689	0.0047	15.03871

0.00588	13.91979	0.0055	13.87653	0.00585	14.82142	0.00551	14.53818	0.00534	14.80936	0.0051	15.06791	0.0047	15.04572

0.00588	13.93584	0.00549	13.88781	0.00585	14.83675	0.0055	14.54318	0.00533	14.83135	0.0051	15.07667	0.0047	15.06672

0.00591	13.95832	0.0055	13.8991	0.00585	14.8623	0.00551	14.55982	0.00533	14.83303	0.00511	15.09945	0.0047	15.09122

0.00589	13.96796	0.00551	13.92005	0.00589	14.88785	0.00552	14.58647	0.00535	14.85671	0.00512	15.12573	0.00471	15.11048

0.00589	13.98241	0.00552	13.93133	0.00588	14.89124	0.00553	14.59313	0.00535	14.87023	0.00511	15.13098	0.0047	15.12098

0.00592	14.00327	0.0055	13.94423	0.00588	14.90827	0.00552	14.61145	0.00534	14.88206	0.00512	15.14676	0.0047	15.13848

0.00592	14.01933	0.00552	13.96841	0.00589	14.93211	0.00553	14.63476	0.00534	14.89897	0.00513	15.16603	0.00472	15.16474

0.00592	14.02255	0.00552	13.99259	0.0059	14.94575	0.00554	14.65307	0.00537	14.91756	0.00513	15.1888	0.00473	15.17699

0.00592	14.05304	0.00555	14.0087	0.0059	14.95766	0.00554	14.66473	0.00537	14.93954	0.00513	15.20282	0.00472	15.18749

0.00594	14.07231	0.00552	14.0216	0.0059	14.97641	0.00554	14.68471	0.00536	14.94968	0.00513	15.22385	0.00473	15.20849

0.00595	14.08034	0.00553	14.03288	0.00592	14.99853	0.00556	14.69803	0.00538	14.98011	0.00514	15.24487	0.00473	15.2295

0.00595	14.098	0.00555	14.049	0.00592	15.01217	0.00557	14.71136	0.0054	15.0004	0.00515	15.26239	0.00474	15.24876

0.00595	14.11245	0.00555	14.06673	0.00592	15.0326	0.00555	14.72634	0.00538	15.00379	0.00515	15.27991	0.00473	15.26101

0.00596	14.13011	0.00555	14.07318	0.00592	15.04623	0.00556	14.74466	0.00538	15.02915	0.00516	15.29392	0.00473	15.27851

0.00598	14.14135	0.00556	14.09574	0.00594	15.07348	0.00558	14.76463	0.0054	15.04774	0.00516	15.3097	0.00475	15.30127

0.00595	14.15098	0.00557	14.11831	0.00593	15.08199	0.00559	14.78628	0.0054	15.05451	0.00516	15.31846	0.00475	15.31877

0.00598	14.17667	0.00557	14.13443	0.00592	15.09049	0.00558	14.80127	0.0054	15.06465	0.00516	15.33773	0.00475	15.33102

0.00598	14.19112	0.00557	14.15055	0.00594	15.11434	0.0056	14.81959	0.00541	15.09169	0.00518	15.35876	0.00475	15.34503

0.00598	14.20075	0.00558	14.16667	0.00595	15.12967	0.0056	14.83124	0.00541	15.11537	0.00518	15.37803	0.00476	15.36777

0.00598	14.22644	0.00559	14.18279	0.00595	15.1467	0.0056	14.84124	0.00541	15.1272	0.00518	15.39205	0.00476	15.38703

0.00601	14.24892	0.00558	14.18762	0.00595	15.16883	0.0056	14.85955	0.00541	15.1441	0.00519	15.42359	0.00476	15.40278

0.00601	14.25695	0.00559	14.20374	0.00597	15.18246	0.00561	14.8812	0.00543	15.16777	0.0052	15.44461	0.00476	15.41854

0.00601	14.26658	0.00562	14.22147	0.00595	15.19609	0.00562	14.89618	0.00543	15.17284	0.00518	15.44461	0.00476	15.43779

0.00601	14.28745	0.00562	14.2392	0.00596	15.21653	0.00561	14.91117	0.00543	15.19144	0.0052	15.46213	0.00477	15.46055

0.00602	14.30832	0.0056	14.24887	0.00597	15.23014	0.00562	14.93281	0.00545	15.2168	0.00521	15.49017	0.00478	15.4728

0.00603	14.31474	0.0056	14.27789	0.00597	15.24888	0.00563	14.95613	0.00545	15.22863	0.0052	15.50068	0.00477	15.4833

0.00603	14.3324	0.00562	14.30045	0.00597	15.2608	0.00563	14.96612	0.00543	15.23708	0.0052	15.51294	0.00478	15.5078

0.00603	14.3597	0.00563	14.31496	0.00599	15.28635	0.00563	14.97944	0.00545	15.26751	0.00522	15.54623	0.00479	15.53055

0.00606	14.37093	0.00562	14.32302	0.00599	15.29997	0.00564	14.99442	0.00547	15.2878	0.00522	15.56726	0.00479	15.54106

0.00603	14.38379	0.00564	14.34397	0.00599	15.317	0.00566	15.01441	0.00546	15.29794	0.00522	15.5655	0.00479	15.55506

0.00605	14.39823	0.00565	14.36009	0.006	15.33573	0.00565	15.02939	0.00547	15.3064	0.00523	15.59704	0.00479	15.57606

0.00606	14.4191	0.00564	14.36493	0.00601	15.35958	0.00566	15.04604	0.00548	15.33176	0.00523	15.61456	0.0048	15.59882

0.00608	14.43034	0.00564	14.38104	0.00601	15.36981	0.00566	15.07435	0.00548	15.34359	0.00524	15.62682	0.0048	15.61632

0.00606	14.43997	0.00566	14.40683	0.00601	15.38002	0.00567	15.091	0.00547	15.35542	0.00524	15.63384	0.0048	15.62857

0.00608	14.46567	0.00566	14.42134	0.00603	15.39875	0.00568	15.10099	0.00549	15.38585	0.00525	15.65836	0.0048	15.64782

0.00609	14.48332	0.00566	14.42779	0.00603	15.41918	0.00567	15.12098	0.0055	15.40445	0.00525	15.68464	0.00482	15.66708

0.00609	14.49617	0.00566	14.45197	0.00602	15.4328	0.00569	15.13429	0.00549	15.41628	0.00525	15.69866	0.00482	15.68634

0.00609	14.51061	0.00569	14.48098	0.00603	15.45665	0.00569	15.14262	0.00551	15.42812	0.00525	15.71442	0.00482	15.69334

0.0061	14.5331	0.0057	14.48582	0.00605	15.47879	0.00569	15.15428	0.00551	15.45517	0.00526	15.7372	0.00482	15.71784

0.00612	14.54594	0.00568	14.49549	0.00606	15.49071	0.00569	15.18258	0.00551	15.46192	0.00527	15.74596	0.00483	15.73885

0.0061	14.55718	0.00569	14.51483	0.00605	15.50264	0.0057	15.2009	0.0055	15.48053	0.00526	15.75998	0.00483	15.7581

0.0061	14.57162	0.0057	14.52934	0.00606	15.52818	0.0057	15.21422	0.00551	15.49575	0.00527	15.781	0.00483	15.7686

0.00613	14.59891	0.00571	14.54223	0.00607	15.5418	0.0057	15.24086	0.00552	15.51603	0.00528	15.80378	0.00483	15.7861

0.00615	14.61176	0.0057	14.55835	0.00607	15.54691	0.00573	15.25251	0.00553	15.52448	0.00528	15.81429	0.00483	15.8071

0.00613	14.61979	0.00571	14.57769	0.00607	15.57076	0.00573	15.26584	0.00552	15.54984	0.00528	15.82656	0.00484	15.82635

0.00615	14.64227	0.00572	14.60026	0.00607	15.5946	0.00572	15.27416	0.00554	15.57012	0.0053	15.86511	0.00483	15.83686

0.00616	14.65833	0.00572	14.6196	0.00608	15.60822	0.00574	15.30081	0.00557	15.58196	0.0053	15.87738	0.00484	15.85437

0.00616	14.67759	0.00572	14.6325	0.00607	15.61844	0.00575	15.31412	0.00554	15.59041	0.0053	15.88963	0.00485	15.87712

0.00616	14.68722	0.00573	14.65023	0.0061	15.64739	0.00575	15.32912	0.00555	15.61069	0.00531	15.9054	0.00486	15.89637

0.00617	14.69686	0.00575	14.66473	0.00612	15.65931	0.00575	15.34909	0.00555	15.63268	0.00532	15.92994	0.00485	15.90863

0.00619	14.72095	0.00575	14.6744	0.0061	15.67124	0.00577	15.37407	0.00555	15.63944	0.00533	15.93869	0.00486	15.92613

0.00619	14.73378	0.00575	14.68407	0.00612	15.69507	0.00577	15.39072	0.00557	15.65634	0.00532	15.94921	0.00487	15.94888

0.00619	14.74662	0.00577	14.70825	0.00613	15.71551	0.00576	15.40072	0.00557	15.68847	0.00534	15.97374	0.00487	15.96989

0.00622	14.77071	0.00577	14.72598	0.00613	15.72913	0.00578	15.41403	0.00559	15.70537	0.00534	16.00001	0.00487	15.97688

0.00622	14.79158	0.00577	14.74533	0.00613	15.74106	0.00578	15.43235	0.00557	15.71721	0.00534	16.01754	0.00487	15.99614

0.0062	14.80924	0.00577	14.76144	0.00613	15.7666	0.00578	15.44068	0.00559	15.73242	0.00534	16.02981	0.00487	16.01189

0.00622	14.81085	0.00579	14.7824	0.00616	15.78364	0.00578	15.45899	0.00559	15.75271	0.00535	16.05083	0.00489	16.0364

0.00622	14.84456	0.00579	14.79368	0.00616	15.78874	0.00579	15.48563	0.00559	15.76116	0.00536	16.0631	0.00488	16.0469

0.00624	14.85098	0.00579	14.80658	0.00616	15.80748	0.0058	15.50395	0.00559	15.77468	0.00535	16.0771	0.00487	16.06791

0.00622	14.85901	0.00579	14.81947	0.00617	15.83473	0.0058	15.51561	0.0056	15.80173	0.00536	16.09814	0.00489	16.08891

0.00624	14.87989	0.00582	14.8372	0.00617	15.84153	0.0058	15.52393	0.0056	15.82201	0.00537	16.11916	0.0049	16.11166

0.00626	14.89755	0.00582	14.85816	0.00616	15.85005	0.00581	15.55224	0.0056	15.82878	0.00536	16.12967	0.0049	16.12391

0.00624	14.91521	0.00582	14.85977	0.00617	15.88071	0.00582	15.56557	0.00562	15.85584	0.00537	16.15595	0.0049	16.13267

0.00624	14.93607	0.00582	14.88717	0.00618	15.90284	0.00582	15.57222	0.00563	15.87105	0.00539	16.18574	0.0049	16.15717

0.00627	14.95855	0.00583	14.91135	0.00618	15.91817	0.00583	15.5922	0.00563	15.88964	0.00539	16.18399	0.0049	16.17817

0.00627	14.96979	0.00584	14.92263	0.00619	15.92838	0.00585	15.61219	0.00562	15.90316	0.00538	16.19801	0.00492	16.18692

0.00627	14.96979	0.00583	14.93553	0.0062	15.95393	0.00585	15.6305	0.00564	15.92345	0.00539	16.21903	0.00491	16.20268

0.00629	14.99387	0.00585	14.95003	0.0062	15.96586	0.00585	15.64882	0.00564	15.93191	0.0054	16.2383	0.00491	16.22368

0.0063	15.01795	0.00586	14.9726	0.0062	15.97607	0.00586	15.6738	0.00564	15.9488	0.00539	16.24881	0.00493	16.24819

0.00631	15.0308	0.00586	14.98227	0.00622	16.00162	0.00587	15.68878	0.00565	15.96741	0.0054	16.27335	0.00493	16.26394

0.0063	15.04043	0.00586	14.99516	0.00622	16.01865	0.00586	15.69377	0.00565	15.99107	0.00541	16.29437	0.00492	16.27444

0.0063	15.0597	0.00587	15.01451	0.00622	16.03228	0.00587	15.71209	0.00566	15.99953	0.00542	16.31539	0.00493	16.29544

0.00633	15.08057	0.00587	15.0274	0.00622	16.0476	0.00588	15.72874	0.00566	16.02657	0.00542	16.32766	0.00494	16.31644

0.00633	15.08539	0.00589	15.0403	0.00624	16.07655	0.00589	15.74373	0.00567	16.04517	0.00542	16.34868	0.00494	16.3322

0.00633	15.10626	0.00589	15.06609	0.00624	16.09017	0.00588	15.75538	0.00567	16.04686	0.00543	16.3627	0.00494	16.3427

0.00636	15.12713	0.0059	15.08704	0.00624	16.09699	0.00589	15.78536	0.00567	16.06208	0.00543	16.37497	0.00494	16.36196

0.00636	15.13837	0.00591	15.10155	0.00626	16.11401	0.00589	15.80867	0.00569	16.08743	0.00543	16.39073	0.00495	16.38821

0.00634	15.14962	0.0059	15.11122	0.00627	16.13616	0.0059	15.81367	0.00569	16.10265	0.00545	16.42051	0.00496	16.40396

0.00636	15.16888	0.0059	15.12734	0.00626	16.14978	0.0059	15.82532	0.00569	16.11786	0.00545	16.43453	0.00495	16.41446

0.00637	15.19135	0.00592	15.14829	0.00627	16.1583	0.00591	15.84531	0.00569	16.13646	0.00545	16.45031	0.00495	16.43022

0.00638	15.2042	0.00592	15.15635	0.00628	16.18724	0.00592	15.86195	0.00571	16.16182	0.00545	16.47308	0.00497	16.45998

0.00638	15.21383	0.00592	15.16925	0.00629	16.20598	0.00593	15.87194	0.00571	16.17535	0.00546	16.48885	0.00497	16.47398

0.00638	15.23309	0.00594	15.19504	0.00628	16.21279	0.00593	15.89359	0.00571	16.1838	0.00546	16.49936	0.00497	16.48448

0.00641	15.25397	0.00594	15.2176	0.0063	16.23494	0.00594	15.92023	0.00573	16.20916	0.00546	16.50987	0.00497	16.49848

0.0064	15.27163	0.00594	15.23211	0.0063	16.26047	0.00595	15.93522	0.00574	16.22268	0.00548	16.5344	0.00499	16.52649

0.0064	15.28126	0.00594	15.24662	0.0063	16.26389	0.00594	15.94354	0.00573	16.23114	0.00549	16.55367	0.00499	16.54224

0.00643	15.29731	0.00597	15.26596	0.00631	16.28433	0.00595	15.96519	0.00574	16.25142	0.00548	16.5712	0.00498	16.55624

0.00643	15.31819	0.00598	15.28208	0.00632	16.30305	0.00596	15.98185	0.00576	16.28185	0.00549	16.58696	0.00499	16.57199

0.00643	15.31979	0.00597	15.29014	0.00633	16.32349	0.00596	15.98517	0.00576	16.29368	0.0055	16.6185	0.00499	16.60175

0.00643	15.34388	0.00597	15.30948	0.00633	16.33371	0.00596	16.00349	0.00574	16.30382	0.0055	16.62376	0.00501	16.6175

0.00645	15.37118	0.00599	15.33043	0.00634	16.35586	0.00598	16.03013	0.00576	16.32751	0.0055	16.63953	0.00499	16.62625

0.00645	15.3792	0.00599	15.33204	0.00636	16.37798	0.00598	16.04844	0.00577	16.34272	0.0055	16.66581	0.005	16.64201

0.00645	15.39044	0.00599	15.34655	0.00635	16.38309	0.00598	16.06177	0.00576	16.34779	0.00552	16.67632	0.00502	16.66301

0.00647	15.41291	0.006	15.3804	0.00636	16.40013	0.00599	16.08008	0.00578	16.37145	0.00551	16.68859	0.00501	16.67876

0.00648	15.42898	0.00601	15.39329	0.00636	16.42908	0.006	16.10839	0.00579	16.39681	0.00552	16.7026	0.00501	16.69452

0.00648	15.44342	0.00603	15.40458	0.00636	16.44271	0.00601	16.11005	0.00579	16.40358	0.00553	16.72888	0.00501	16.71202

0.00648	15.44983	0.00601	15.4207	0.00636	16.45633	0.00601	16.12671	0.00579	16.41879	0.00554	16.75341	0.00502	16.73477

0.00651	15.48034	0.00601	15.42876	0.00639	16.47847	0.00602	16.14003	0.00581	16.44922	0.00553	16.76743	0.00504	16.75578

0.00651	15.49319	0.00604	15.45455	0.00639	16.50231	0.00602	16.16334	0.0058	16.46444	0.00554	16.7867	0.00502	16.76628

0.00651	15.50924	0.00604	15.46422	0.00639	16.50742	0.00603	16.16501	0.0058	16.47627	0.00555	16.80423	0.00503	16.78204

0.00651	15.52049	0.00604	15.48034	0.0064	16.52105	0.00603	16.19498	0.0058	16.48473	0.00555	16.80948	0.00504	16.80304

0.00654	15.54617	0.00606	15.49645	0.0064	16.54318	0.00605	16.21995	0.00583	16.51008	0.00555	16.82525	0.00504	16.82054

0.00654	15.55901	0.00607	15.51902	0.00641	16.55681	0.00605	16.23494	0.00582	16.51853	0.00557	16.85153	0.00504	16.83454

0.00652	15.56384	0.00607	15.53191	0.00641	16.57212	0.00605	16.24493	0.00582	16.5422	0.00557	16.87255	0.00504	16.85204

0.00654	15.58631	0.00607	15.55126	0.00643	16.59087	0.00605	16.26159	0.00584	16.56587	0.00557	16.88306	0.00504	16.86954

0.00655	15.60558	0.0061	15.57221	0.00644	16.6113	0.00606	16.28323	0.00584	16.58447	0.00558	16.90935	0.00507	16.89405

0.00657	15.61842	0.0061	15.58188	0.00643	16.62833	0.00607	16.28988	0.00584	16.59123	0.00559	16.93037	0.00505	16.90806

0.00657	15.63447	0.00608	15.59478	0.00643	16.64536	0.00606	16.30321	0.00584	16.6132	0.00559	16.93913	0.00505	16.92205

0.00657	15.65695	0.0061	15.61573	0.00645	16.6624	0.00608	16.32986	0.00584	16.6318	0.00559	16.95666	0.00506	16.94306

0.00659	15.67139	0.00612	15.62863	0.00645	16.67942	0.00609	16.34484	0.00584	16.63687	0.0056	16.97417	0.00508	16.96581

0.00658	15.68104	0.00612	15.64152	0.00645	16.68964	0.00609	16.36316	0.00585	16.65377	0.00561	16.98819	0.00508	16.98157

0.00659	15.69548	0.00612	15.65442	0.00646	16.71348	0.00609	16.38313	0.00588	16.6842	0.00561	16.99344	0.00507	16.98857

0.00661	15.71315	0.00612	15.68021	0.00647	16.73392	0.0061	16.40645	0.00588	16.69097	0.00561	17.02324	0.00508	17.00957

0.00662	15.73081	0.00614	15.69794	0.00645	16.73732	0.00611	16.4181	0.00588	16.71295	0.00562	17.05126	0.00508	17.03408

0.00661	15.74364	0.00614	15.7205	0.00649	16.75947	0.00611	16.41977	0.00588	16.73999	0.00562	17.06002	0.00509	17.04808

0.00662	15.76773	0.00614	15.72211	0.0065	16.78501	0.00612	16.44475	0.00588	16.7586	0.00563	17.08455	0.00508	17.06033

0.00664	15.79021	0.00617	15.74307	0.0065	16.80034	0.00614	16.46307	0.0059	16.76367	0.00564	17.10558	0.00508	17.08133

0.00665	15.79663	0.00617	15.76241	0.0065	16.81225	0.00614	16.47638	0.00588	16.77719	0.00565	17.11259	0.0051	17.10584

0.00664	15.80144	0.00617	15.76725	0.0065	16.8327	0.00613	16.4897	0.00592	16.79917	0.00564	17.1196	0.0051	17.11984

0.00665	15.82874	0.00618	15.78659	0.00651	16.84462	0.00615	16.52134	0.00593	16.80425	0.00564	17.14588	0.0051	17.13035

0.00668	15.84961	0.00619	15.80432	0.00651	16.86165	0.00615	16.53133	0.00592	16.82114	0.00566	17.1634	0.00511	17.1496

0.00666	15.85603	0.00619	15.82689	0.00651	16.87867	0.00615	16.53966	0.00593	16.84819	0.00566	17.17917	0.00512	17.17235

0.00666	15.86566	0.00619	15.83333	0.00653	16.90593	0.00616	16.56463	0.00595	16.87525	0.00566	17.2002	0.00512	17.1881

0.00669	15.89136	0.0062	15.85268	0.00655	16.91955	0.00618	16.58295	0.00592	16.87862	0.00567	17.23173	0.00511	17.20035

0.00671	15.91062	0.00622	15.87685	0.00655	16.92977	0.00618	16.58961	0.00593	16.89553	0.00569	17.24224	0.00512	17.21961

0.00669	15.92026	0.00624	15.88975	0.00655	16.9502	0.00617	16.5996	0.00596	16.92427	0.00567	17.25451	0.00514	17.24236

0.00669	15.93309	0.00621	15.89942	0.00655	16.97234	0.00619	16.6329	0.00596	16.92934	0.00568	17.26853	0.00514	17.26161

0.00674	15.95557	0.00624	15.91231	0.00655	16.96894	0.0062	16.65621	0.00595	16.94455	0.0057	17.28429	0.00513	17.27211

0.00675	15.97484	0.00626	15.94133	0.00656	16.99959	0.0062	16.66287	0.00597	16.96991	0.0057	17.30007	0.00514	17.28962

0.00674	15.98287	0.00626	15.94939	0.00658	17.01662	0.00621	16.68119	0.00598	16.98851	0.00569	17.31758	0.00515	17.31238

0.00675	16.00214	0.00626	15.95906	0.00658	17.03365	0.00622	16.70616	0.00598	16.99697	0.00571	17.34211	0.00514	17.32638

0.00676	16.023	0.00626	15.9784	0.00658	17.05239	0.00623	16.7145	0.00598	17.01725	0.00572	17.35963	0.00514	17.33863

0.00675	16.03264	0.00628	16.00258	0.00659	17.07452	0.00623	16.72116	0.00599	17.0443	0.00571	17.37891	0.00515	17.35788

0.00675	16.04227	0.00628	16.02353	0.00662	17.08815	0.00623	16.7378	0.00599	17.04937	0.00572	17.39292	0.00516	17.38064

0.00678	16.07277	0.00628	16.02837	0.00661	17.10007	0.00624	16.76278	0.00599	17.06797	0.00573	17.41745	0.00517	17.39989

0.00681	16.08402	0.00629	16.04932	0.00662	17.1137	0.00625	16.77443	0.00602	17.0815	0.00574	17.43147	0.00517	17.41389

0.00678	16.09205	0.00631	16.07028	0.00663	17.14434	0.00625	16.79775	0.00602	17.10178	0.00574	17.43848	0.00517	17.4314

0.00681	16.11131	0.00631	16.07511	0.00664	17.15287	0.00626	16.8194	0.00602	17.12037	0.00574	17.47352	0.00518	17.45415

0.00681	16.13539	0.00632	16.09284	0.00663	17.16138	0.00628	16.83771	0.00602	17.13559	0.00575	17.48403	0.00518	17.47166

0.00683	16.14985	0.00632	16.11541	0.00665	17.18693	0.00627	16.84604	0.00605	17.15418	0.00575	17.49805	0.00518	17.48041

0.00683	16.15786	0.00634	16.1283	0.00667	17.20735	0.00627	16.8577	0.00604	17.1694	0.00575	17.51557	0.00518	17.49791

0.00683	16.17714	0.00634	16.14765	0.00666	17.22098	0.00629	16.87935	0.00604	17.1863	0.00577	17.54361	0.00518	17.52242

0.00686	16.19801	0.00634	16.15893	0.00667	17.23461	0.00629	16.88601	0.00606	17.20321	0.00578	17.55762	0.0052	17.53642

0.00686	16.21566	0.00638	16.18956	0.00669	17.25674	0.0063	16.89599	0.00606	17.2235	0.00577	17.56112	0.0052	17.55042

0.00686	16.2253	0.00636	16.19278	0.00668	17.26866	0.0063	16.92763	0.00606	17.22857	0.00578	17.58039	0.0052	17.56968

0.00688	16.24296	0.00636	16.20084	0.00669	17.27549	0.00632	16.95095	0.00608	17.24886	0.00579	17.60142	0.00521	17.59593

0.00688	16.26223	0.00638	16.22179	0.00669	17.30273	0.00632	16.96426	0.0061	17.27084	0.00579	17.61544	0.00521	17.61169

0.00689	16.27347	0.00639	16.24436	0.00671	17.32827	0.00632	16.98257	0.0061	17.28606	0.0058	17.63821	0.00521	17.62219

0.0069	16.2847	0.00641	16.25725	0.00671	17.33338	0.00633	16.99923	0.0061	17.30464	0.00581	17.6645	0.00521	17.63793

0.00692	16.31682	0.0064	16.27337	0.00671	17.35892	0.00635	17.01755	0.0061	17.32324	0.00581	17.67851	0.00522	17.66244

0.00693	16.32324	0.00641	16.2911	0.00674	17.38277	0.00634	17.01755	0.0061	17.34015	0.00581	17.69253	0.00523	17.67994

0.00692	16.3425	0.00643	16.30883	0.00674	17.38789	0.00634	17.03753	0.0061	17.34522	0.00582	17.7118	0.00523	17.69395

0.00693	16.36498	0.00643	16.32012	0.00674	17.4015	0.00636	17.06251	0.0061	17.36382	0.00583	17.73108	0.00523	17.7097

0.00696	16.37622	0.00643	16.3314	0.00676	17.42194	0.00637	17.07915	0.00612	17.38749	0.00584	17.74334	0.00524	17.7342

0.00696	16.38907	0.00645	16.35719	0.00678	17.44068	0.00636	17.09415	0.00613	17.40439	0.00583	17.75735	0.00525	17.75521

0.00695	16.40191	0.00646	16.37008	0.00678	17.4492	0.00638	17.11079	0.00613	17.42468	0.00585	17.78715	0.00524	17.76571

0.00697	16.42921	0.00646	16.38298	0.00678	17.47304	0.0064	17.1391	0.00616	17.44835	0.00585	17.80116	0.00525	17.77971

0.00699	16.43722	0.00647	16.39265	0.00678	17.49687	0.0064	17.14243	0.00616	17.46186	0.00585	17.81693	0.00525	17.79897

0.00697	16.44044	0.00649	16.41683	0.00678	17.51391	0.0064	17.15576	0.00615	17.47539	0.00586	17.83971	0.00526	17.81997

0.00699	16.46773	0.00649	16.43456	0.00678	17.52583	0.00642	17.17573	0.00616	17.48553	0.00587	17.85547	0.00526	17.83573

0.00702	16.49343	0.00649	16.44262	0.00682	17.54286	0.00642	17.19405	0.00618	17.50582	0.00588	17.86248	0.00526	17.84623

0.00702	16.50146	0.00649	16.46357	0.00683	17.5667	0.00642	17.19738	0.00619	17.51766	0.00588	17.873	0.00526	17.86897

0.00702	16.51429	0.00653	16.49259	0.00683	17.5718	0.00642	17.22569	0.00618	17.5278	0.00588	17.89927	0.00527	17.89173

0.00704	16.53517	0.00653	16.49903	0.00683	17.58884	0.00644	17.25399	0.00619	17.55823	0.00589	17.92205	0.00528	17.90574

0.00704	16.55443	0.00653	16.51515	0.00684	17.61098	0.00645	17.26565	0.00621	17.58189	0.00589	17.92906	0.00527	17.91974

0.00706	16.56568	0.00654	16.52805	0.00685	17.63141	0.00644	17.28063	0.00619	17.59374	0.00591	17.95534	0.00528	17.93724

0.00707	16.57852	0.00656	16.54739	0.00685	17.64845	0.00646	17.29396	0.0062	17.61232	0.00592	17.98162	0.00529	17.96175

0.00709	16.60099	0.00656	16.55706	0.00686	17.66718	0.00647	17.31394	0.00622	17.63431	0.00592	17.99038	0.0053	17.97574

0.00709	16.61866	0.00656	16.56834	0.00688	17.68421	0.00647	17.30728	0.00623	17.63601	0.00592	18.00791	0.00529	17.988

0.00709	16.63149	0.00657	16.59574	0.00688	17.69612	0.00649	17.34391	0.00624	17.65459	0.00593	18.02016	0.0053	18.00725

0.00709	16.64114	0.00659	16.61348	0.00688	17.70805	0.0065	17.3639	0.00624	17.67658	0.00594	18.0447	0.00532	18.03176

0.00711	16.66361	0.00659	16.62798	0.0069	17.73699	0.0065	17.36556	0.00626	17.69179	0.00594	18.05345	0.00531	18.04751

0.00714	16.68127	0.00661	16.6441	0.0069	17.75063	0.00651	17.38554	0.00626	17.70361	0.00593	18.07449	0.00532	18.05976

0.00713	16.68608	0.00663	16.66022	0.0069	17.76084	0.00651	17.41551	0.00626	17.73404	0.00596	18.09901	0.00532	18.07902

0.00714	16.71017	0.00663	16.6715	0.00693	17.78639	0.00652	17.43049	0.00627	17.75095	0.00596	18.11829	0.00532	18.10352

0.00714	16.72301	0.00663	16.67795	0.00694	17.80512	0.00652	17.44049	0.00627	17.75772	0.00596	18.13055	0.00533	18.11927

0.00717	16.74067	0.00664	16.70213	0.00694	17.81704	0.00654	17.46546	0.00627	17.76955	0.00596	18.14983	0.00532	18.12627

0.00717	16.75351	0.00666	16.72469	0.00694	17.83406	0.00655	17.48378	0.0063	17.79491	0.00598	18.1691	0.00533	18.14552

0.00717	16.78563	0.00667	16.73275	0.00697	17.85281	0.00655	17.48711	0.0063	17.81013	0.00599	18.18136	0.00535	18.17178

0.0072	16.79205	0.00667	16.74887	0.00696	17.86472	0.00655	17.49876	0.0063	17.82196	0.00599	18.19712	0.00535	18.18928

0.0072	16.80329	0.00667	16.77789	0.00698	17.88346	0.00657	17.52874	0.00633	17.84224	0.00601	18.2199	0.00534	18.19978

0.0072	16.82095	0.00669	16.80045	0.00699	17.9005	0.00658	17.54539	0.00633	17.86591	0.006	18.23217	0.00535	18.21554

0.00723	16.84182	0.0067	16.8069	0.00699	17.92263	0.00658	17.56037	0.00634	17.88282	0.006	18.24794	0.00536	18.24705

0.00724	16.85467	0.00671	16.81818	0.00701	17.93626	0.00659	17.58369	0.00633	17.89127	0.00601	18.27072	0.00537	18.26105

0.00723	16.86269	0.00671	16.84075	0.00701	17.95158	0.0066	17.607	0.00636	17.91494	0.00603	18.29699	0.00537	18.2698

0.00725	16.88517	0.00674	16.85364	0.00702	17.97373	0.0066	17.61699	0.00636	17.92509	0.00602	18.30576	0.00537	18.2838

0.00728	16.90765	0.00674	16.87299	0.00704	17.99244	0.00661	17.62864	0.00636	17.93523	0.00603	18.31277	0.00537	18.31006

0.00728	16.92531	0.00674	16.8746	0.00702	17.99586	0.00662	17.64364	0.00637	17.9589	0.00604	18.33379	0.00539	18.32931

0.00728	16.93013	0.00676	16.89877	0.00707	18.0197	0.00663	17.65196	0.00638	17.98256	0.00606	18.35306	0.00538	18.33806

0.0073	16.94778	0.00678	16.91651	0.00707	18.03844	0.00664	17.67027	0.00637	17.99102	0.00604	18.36357	0.00539	18.35731

0.00731	16.96865	0.00677	16.93262	0.00707	18.05207	0.00665	17.68859	0.0064	18.01637	0.00606	18.3881	0.00539	18.38357

0.00731	16.97828	0.00678	16.95035	0.00707	18.0708	0.00666	17.71191	0.0064	18.03666	0.00607	18.41614	0.0054	18.40282

0.00731	16.99594	0.00681	16.9697	0.00711	18.09122	0.00667	17.73355	0.0064	18.05356	0.00607	18.43191	0.00539	18.41332

0.00735	17.01843	0.00681	16.97776	0.00711	18.10485	0.00667	17.74354	0.0064	18.05526	0.00607	18.43892	0.0054	18.42908

0.00735	17.03127	0.00681	16.99226	0.00711	18.12188	0.00669	17.76685	0.00643	18.07724	0.00609	18.46169	0.00541	18.45184

0.00735	17.0409	0.00683	17.01161	0.00713	18.14572	0.00669	17.78184	0.00643	18.09921	0.00609	18.47746	0.00542	18.46758

0.00735	17.06337	0.00684	17.02934	0.00715	18.15764	0.00669	17.7885	0.00644	18.10935	0.00609	18.48973	0.00542	18.47809

0.00739	17.07944	0.00687	17.04545	0.00716	18.16957	0.0067	17.80015	0.00644	18.12456	0.0061	18.50549	0.00542	18.49909

0.00741	17.0971	0.00687	17.05513	0.00716	18.19001	0.00672	17.81847	0.00648	18.15669	0.00612	18.53002	0.00543	18.52009

0.00739	17.10191	0.00687	17.07608	0.00718	18.21555	0.00672	17.84178	0.00647	18.16853	0.00612	18.55105	0.00543	18.53935

0.00741	17.1244	0.0069	17.09542	0.00719	18.22406	0.00673	17.86343	0.00647	18.18375	0.00612	18.56331	0.00543	18.55335

0.00744	17.14205	0.0069	17.10832	0.00719	18.2411	0.00673	17.87841	0.00648	18.19726	0.00613	18.5931	0.00543	18.56911

0.00744	17.15971	0.00691	17.12605	0.00722	18.25642	0.00676	17.90339	0.00649	18.21418	0.00614	18.60711	0.00545	18.59185

0.00744	17.16934	0.00692	17.14217	0.00723	18.27856	0.00677	17.91338	0.00651	18.22093	0.00614	18.61413	0.00545	18.60936

0.00746	17.19503	0.00694	17.16796	0.00723	18.28708	0.00677	17.92338	0.00651	18.24798	0.00615	18.6334	0.00545	18.61811

0.00749	17.21109	0.00695	17.1744	0.00726	18.30752	0.00678	17.94169	0.00653	18.27334	0.00617	18.65267	0.00545	18.63561

0.00746	17.22072	0.00696	17.18569	0.00727	18.32454	0.00679	17.95501	0.00653	18.28348	0.00617	18.66669	0.00546	18.66012

0.00749	17.23678	0.00698	17.2147	0.00729	18.33818	0.00678	17.96667	0.00652	18.29532	0.00618	18.67895	0.00547	18.67588

0.00752	17.25926	0.00699	17.22921	0.0073	18.3586	0.00679	17.99165	0.00654	18.31898	0.00619	18.714	0.00547	18.69338

0.00753	17.2753	0.00698	17.23404	0.00732	18.37904	0.00682	18.01662	0.00657	18.33758	0.00619	18.73327	0.00548	18.70563

0.00753	17.28655	0.00701	17.25338	0.00735	18.40118	0.00683	18.02828	0.00657	18.34097	0.0062	18.74378	0.00549	18.73188

0.00755	17.301	0.00703	17.27434	0.00737	18.40459	0.00683	18.03827	0.00657	18.36125	0.0062	18.75954	0.00549	18.75113

0.00756	17.31865	0.00704	17.29207	0.0074	18.42332	0.00684	18.06158	0.00659	18.38492	0.00621	18.77882	0.00549	18.76339

0.00756	17.33792	0.00705	17.29368	0.0075	18.44375	0.00687	18.08156	0.0066	18.39844	0.00623	18.79283	0.00549	18.77739

0.00758	17.35237	0.00705	17.31302	0.00752	18.45737	0.00687	18.08823	0.0066	18.42211	0.00623	18.80335	0.0055	18.80015

0.00759	17.36682	0.00708	17.3372	0.00756	18.4659	0.00687	18.09655	0.00662	18.44578	0.00623	18.82963	0.00551	18.81765

0.00762	17.3893	0.00709	17.34687	0.00764	18.49145	0.00688	18.11985	0.00663	18.45761	0.00624	18.84716	0.00552	18.8334

0.00762	17.39572	0.00709	17.3646	0.00771	18.50847	0.0069	18.14483	0.00663	18.46776	0.00625	18.86467	0.0055	18.84566

0.00765	17.41498	0.00711	17.38072	0.00779	18.51868	0.0069	18.15982	0.00663	18.48297	0.00625	18.8857	0.00552	18.86666

0.00766	17.42943	0.00713	17.4049	0.00788	18.53913	0.00691	18.17647	0.00666	18.50327	0.00627	18.90672	0.00553	18.89116

0.00768	17.45031	0.00713	17.41779	0.00804	18.44716	0.00693	18.19646	0.00666	18.51509	0.00628	18.91198	0.00553	18.90517

0.00768	17.45673	0.00715	17.42747	0.00832	17.55478	0.00694	18.21144	0.00666	18.52862	0.00627	18.93125	0.00552	18.91917

0.00768	17.48082	0.00718	17.44842	0.0085	16.65388	0.00694	18.22309	0.00668	18.55398	0.00628	18.95578	0.00553	18.93492

0.00772	17.5049	0.00718	17.46454	0.00871	15.74276	0.00695	18.23309	0.00671	18.57764	0.0063	18.9663	0.00555	18.95943

0.00772	17.51132	0.00719	17.4726	0.00897	14.74479	0.00697	18.25639	0.00669	18.58609	0.00631	18.98381	0.00555	18.97168

0.00773	17.52095	0.0072	17.49194	0.00926	13.87284	0.00698	18.26806	0.00669	18.59623	0.00631	19.00308	0.00555	18.98218

0.00773	17.54663	0.00723	17.51773	0.0095	12.80846	0.00698	18.28803	0.00671	18.62329	0.00633	19.03288	0.00556	19.00494

0.00776	17.55788	0.00725	17.52579	0.00983	11.8803	0.007	18.31301	0.00674	18.63343	0.00634	19.04514	0.00556	19.02768

0.00777	17.57715	0.00724	17.54191	0.01026	10.99132	0.00702	18.33466	0.00674	18.64527	0.00634	19.05215	0.00557	19.04344

0.00777	17.58517	0.00726	17.55642	0.01064	10.0785	0.00702	18.34631	0.00677	18.66893	0.00634	19.06966	0.00556	19.05745

0.0078	17.60925	0.0073	17.57737	0.01104	9.18951	0.00703	18.35797	0.00677	18.68922	0.00636	19.0977	0.00557	19.07845

0.0078	17.63013	0.0073	17.59188	0.0117	8.28181	0.00705	18.37629	0.00677	18.70443	0.00636	19.10471	0.00559	19.0977

0.00783	17.63976	0.00731	17.60477	0.01238	7.3741	0.00705	18.39127	0.00678	18.72472	0.00636	19.12048	0.00559	19.11345

0.00783	17.6526	0.00733	17.61767	0.01297	6.4187	0.00706	18.39127	0.0068	18.7467	0.00637	19.1415	0.00559	19.12395

0.00786	17.67026	0.00734	17.64184	0.01389	5.45649	0.00708	18.41458	0.0068	18.75177	0.00639	19.16603	0.00559	19.14146

0.00787	17.68471	0.00738	17.65635	0.01503	4.55389	0.00709	18.44456	0.00682	18.7636	0.00639	19.18004	0.00561	19.16771

0.00787	17.69916	0.00738	17.66602	0.00711	18.45954	0.00683	18.79066	0.0064	19.19932	0.00562	19.18347

0.0079	17.72004	0.0074	17.68375	0.00711	18.47786	0.00684	18.79742	0.00642	19.22735	0.00562	19.19747

0.00791	17.73769	0.00743	17.70471	0.00713	18.49118	0.00687	18.81602	0.00642	19.22735	0.00562	19.21672

0.00793	17.75214	0.00743	17.7176	0.00715	18.50783	0.00687	18.84138	0.00642	19.23611	0.00563	19.24297

0.00794	17.76177	0.00744	17.73533	0.00715	18.51616	0.00688	18.86335	0.00644	19.26415	0.00563	19.25698

0.00797	17.78585	0.00747	17.75306	0.00717	18.53447	0.00688	18.87856	0.00645	19.28342	0.00563	19.26398

0.00797	17.80513	0.00748	17.77079	0.00719	18.55945	0.0069	18.88195	0.00645	19.29744	0.00563	19.28323

0.00797	17.80994	0.00751	17.78046	0.00721	18.56944	0.00692	18.91068	0.00646	19.31846	0.00565	19.30423

0.008	17.83402	0.00752	17.79497	0.00722	18.5811	0.00692	18.92421	0.00647	19.34649	0.00566	19.32525

0.00803	17.85008	0.00755	17.81109	0.00723	18.61107	0.00693	18.93774	0.00648	19.36227	0.00566	19.33575

0.00804	17.86293	0.00758	17.82882	0.00726	18.63271	0.00695	18.95802	0.00648	19.36577	0.00566	19.35325

0.00804	17.87577	0.00758	17.84333	0.00726	18.63604	0.00697	18.97493	0.0065	19.38329	0.00566	19.37775

0.00807	17.89504	0.0076	17.86589	0.00728	18.65603	0.00698	18.99352	0.00651	19.40607	0.00568	19.4005

0.0081	17.9159	0.00764	17.88685	0.00731	18.67601	0.00701	19.00874	0.00652	19.40782	0.00568	19.40926

0.0081	17.92875	0.00766	17.89652	0.00732	18.68267	0.00702	19.02902	0.00653	19.43585	0.00568	19.42676

0.00811	17.94159	0.00766	17.91103	0.00733	18.69266	0.00702	19.03579	0.00654	19.45687	0.0057	19.44776

0.00814	17.96247	0.00768	17.92714	0.00735	18.71265	0.00702	19.04761	0.00655	19.48667	0.0057	19.46526

0.00815	17.97692	0.00773	17.94004	0.00739	18.73262	0.00704	19.07467	0.00655	19.49192	0.0057	19.47227

0.00817	17.98976	0.00775	17.95132	0.00741	18.75261	0.00707	19.09327	0.00656	19.51119	0.0057	19.49152

0.00818	18.00742	0.00777	17.96744	0.00742	18.76925	0.00707	19.10341	0.00658	19.53221	0.00571	19.51428

0.00821	18.02829	0.0078	17.98839	0.00745	18.78591	0.00709	19.11863	0.00659	19.54098	0.00573	19.53353

0.00822	18.04113	0.00784	18.00613	0.00749	18.80089	0.00713	19.14737	0.0066	19.55325	0.00572	19.54753

0.00824	18.05558	0.00786	18.02386	0.00751	18.81088	0.00712	19.15751	0.00661	19.57777	0.00573	19.56679

0.00825	18.06683	0.00788	18.03514	0.00754	18.82421	0.00713	19.16765	0.00662	19.59179	0.00573	19.58779

0.00828	18.09251	0.00792	18.05609	0.00759	18.83752	0.00716	19.18963	0.00663	19.61281	0.00575	19.60529

0.00829	18.10697	0.00795	18.06899	0.00766	18.83419	0.00718	19.20823	0.00664	19.63208	0.00575	19.61754

0.00831	18.1182	0.00798	18.07705	0.00791	17.98832	0.00718	19.22174	0.00666	19.65661	0.00575	19.6298

0.00833	18.13586	0.00801	18.09478	0.00809	17.14077	0.0072	19.23697	0.00666	19.66888	0.00576	19.65605

0.00835	18.15192	0.00806	18.12218	0.00824	16.26159	0.00723	19.25556	0.00666	19.67939	0.00576	19.6718

0.00836	18.16315	0.0081	18.13185	0.00844	15.28582	0.00726	19.27923	0.00669	19.70567	0.00576	19.68581

0.00838	18.18242	0.00812	18.14313	0.00865	14.33004	0.00724	19.28937	0.0067	19.71968	0.00578	19.70331

0.0084	18.20489	0.00817	18.16248	0.00885	13.43088	0.00727	19.30459	0.0067	19.72845	0.00579	19.72606

0.00843	18.22256	0.00823	18.18182	0.00908	12.39684	0.0073	19.32995	0.00671	19.75123	0.0058	19.74356

0.00845	18.22738	0.00828	18.19632	0.00934	11.39943	0.00733	19.33502	0.00673	19.77926	0.0058	19.75932

0.00846	18.24022	0.00831	18.20438	0.0096	10.49693	0.00735	19.35699	0.00674	19.79853	0.0058	19.77332

0.00849	18.26591	0.0084	18.22211	0.01001	9.54115	0.00738	19.37728	0.00674	19.80554	0.00581	19.79607

0.00852	18.28036	0.00847	18.24146	0.01022	8.53209	0.0074	19.39419	0.00677	19.82832	0.00582	19.81008

0.00852	18.29802	0.00854	18.2479	0.01053	7.62793	0.0074	19.40771	0.00677	19.84059	0.00583	19.82408

0.00856	18.31728	0.00863	18.2608	0.01107	6.7604	0.00744	19.42969	0.00678	19.8546	0.00583	19.83983

0.00857	18.32531	0.00878	18.26725	0.01178	5.87954	0.00746	19.4449	0.00679	19.87212	0.00583	19.86259

0.00857	18.43382	0.00903	18.1109	0.01332	5.032	0.00748	19.45675	0.00682	19.89139	0.00584	19.88359

0.00862	18.46359	0.00939	17.29046	0.01469	4.1728	0.00752	19.47364	0.00683	19.91417	0.00584	19.89585

0.00866	18.49382	0.00961	16.43295	0.00757	19.49392	0.00684	19.92993	0.00584	19.9151

0.00866	18.50394	0.00983	15.50935	0.0076	19.50069	0.00685	19.95447	0.00586	19.9326

0.00869	18.53399	0.01007	14.69375	0.00763	19.52436	0.00687	19.97374	0.00587	19.9536

0.0087	18.55942	0.01032	13.87331	0.00767	19.53789	0.00688	19.97549	0.00587	19.96585

0.00873	18.57444	0.01052	13.04481	0.00774	19.54972	0.00688	19.99126	0.00587	19.97811

0.00876	18.58455	0.01074	12.00999	0.00782	19.51084	0.00691	20.01755	0.00588	20.00086

0.00878	18.60467	0.011	10.94294	0.00796	19.42799	0.00692	20.03682	0.0059	20.02187

0.00881	18.62489	0.01129	10.04352	0.00836	18.56073	0.00693	20.04382	0.0059	20.03587

0.00884	18.65067	0.01168	9.20213	0.00867	17.6174	0.00694	20.06835	0.0059	20.04987

0.00892	18.67485	0.01234	8.34784	0.00885	16.66562	0.00696	20.09289	0.00591	20.07262

0.00894	18.69775	0.01316	7.4726	0.00916	15.78314	0.00698	20.1034	0.00593	20.09363

0.00897	18.7179	0.014	6.59574	0.00937	14.89897	0.00699	20.11566	0.00594	20.10763

0.00898	18.74966	0.01444	5.76725	0.00958	13.87617	0.00701	20.14194	0.00593	20.11638

0.009	18.80384	0.01472	4.67279	0.00981	12.85676	0.00702	20.15245	0.00594	20.13914

0.00901	18.84593	0.01502	3.84913	0.01002	11.883	0.00703	20.16822	0.00595	20.16365

0.00902	18.87532	0.01551	3.00613	0.01016	10.85852	0.00705	20.18399	0.00596	20.17415

0.00904	18.89513	0.01646	2.17602	0.0104	9.68188	0.00707	20.21203	0.00596	20.18814

0.00908	18.92134	0.01065	8.8011	0.00709	20.22954	0.00597	20.2109

0.00916	18.96168	0.01102	7.93214	0.00709	20.24181	0.00598	20.23015

0.0092	19.02942	0.01141	6.97359	0.00713	20.26108	0.006	20.24766

0.00927	19.08035	0.01169	5.9322	0.00715	20.2786	0.00599	20.25991

0.00946	18.24665	0.01213	4.24332	0.00715	20.29087	0.006	20.28092

0.00961	17.19824	0.0124	3.20701	0.00718	20.30663	0.00601	20.30367

0.01001	16.18677	0.01257	2.06419	0.0072	20.32241	0.00602	20.31417

0.01052	14.31956	0.01302	1.1834	0.00722	20.34694	0.00602	20.32992

0.0109	10.80188	0.01437	0.32966	0.00723	20.35745	0.00603	20.34568

0.01188	7.50417	0.00726	20.38723	0.00605	20.37368

0.01224	5.75576	0.00728	20.39599	0.00606	20.39118

0.01246	4.84865	0.0073	20.41001	0.00606	20.40168

0.01261	3.97525	0.00733	20.42578	0.00606	20.42269

0.01333	3.14359	0.00737	20.4503	0.00608	20.44544

0.01564	2.34083	0.00739	20.46257	0.00609	20.4612

0.00741	20.47659	0.00609	20.4682

0.00745	20.49937	0.00609	20.4892

0.00747	20.51688	0.00611	20.5102

0.0075	20.5309	0.00611	20.52596

0.00754	20.55719	0.00611	20.5382

0.00759	20.5677	0.00613	20.55922

0.00764	20.57997	0.00614	20.58372

0.00768	20.59398	0.00616	20.59772

0.00775	20.60799	0.00616	20.60998

0.00783	20.61675	0.00617	20.62923

0.00802	20.38372	0.00618	20.65373

0.00818	19.48316	0.00619	20.66598

0.0083	18.58434	0.00619	20.67823

0.00845	17.61719	0.0062	20.69574

0.00864	16.69034	0.00622	20.72024

0.00885	15.79502	0.00622	20.736

0.00906	14.88044	0.00624	20.75

0.00923	13.69253	0.00625	20.77101

0.00943	12.7429	0.00626	20.79201

0.00969	11.59178	0.00627	20.80601

0.00996	10.60886	0.00628	20.82176

0.01035	9.67675	0.00628	20.83751

0.01067	8.64127	0.00631	20.86027

0.01086	7.63557	0.00632	20.87427

0.01106	6.47569	0.00632	20.89002

0.01126	5.55586	0.00634	20.90928

0.01185	4.63952	0.00636	20.93028

0.0125	3.76347	0.00637	20.94778

0.01326	2.68244	0.00638	20.95829

0.00639	20.97579

0.0064	20.99679

0.00642	21.01605

0.00643	21.02655

0.00645	21.0458

0.00647	21.06681

0.00649	21.08606

0.00652	21.09831

0.00653	21.10881

0.00659	21.12456

0.00666	21.12631

0.00697	19.86506

0.00721	18.27582

0.00751	17.07617

0.0079	15.04764

0.00851	13.09961

0.00899	10.81729

0.01001	7.7089

0.01213	6.02509

**（*b*）Stabilized hydraulic**

0	0	0	0	2.5E-4	0.03061	1.73E-4	0	3.07E-4	0.05056	2.55E-4	0.03828	3.08E-4	0.03576

2.4404E-5	0	2.4404E-5	0	3.14E-4	0.04664	2.35E-4	0.01045	3.53E-4	0.06894	3.27E-4	0.06158	3.94E-4	0.05187

7.72796E-5	0	7.72796E-5	0	3.79E-4	0.06269	3.01E-4	0.02985	4.04E-4	0.09039	3.99E-4	0.08321	5.1E-4	0.07511

1.54558E-4	0	1.54558E-4	0	4.48E-4	0.07872	3.88E-4	0.05075	4.55E-4	0.10724	4.71E-4	0.10485	6.11E-4	0.10015

2.48107E-4	0.02038	2.48107E-4	0.02038	5.22E-4	0.09912	4.39E-4	0.06716	4.75E-4	0.11797	5.29E-4	0.12815	7.12E-4	0.12519

3.33521E-4	0.05435	3.33521E-4	0.05435	5.78E-4	0.11662	4.85E-4	0.08358	5.06E-4	0.13483	5.77E-4	0.1398	7.92E-4	0.15201

3.74195E-4	0.06454	3.74195E-4	0.06454	6.28E-4	0.13119	5.41E-4	0.10448	5.57E-4	0.1532	6.25E-4	0.16143	8.73E-4	0.17347

4.18935E-4	0.08492	4.18935E-4	0.08492	6.84E-4	0.14869	5.97E-4	0.1209	5.77E-4	0.17007	6.88E-4	0.18141	9.34E-4	0.19315

4.59609E-4	0.1053	4.59609E-4	0.1053	7.49E-4	0.17055	6.17E-4	0.12985	5.93E-4	0.18079	7.16E-4	0.19473	9.74E-4	0.21103

4.84012E-4	0.12059	4.84012E-4	0.12059	7.9E-4	0.18367	6.58E-4	0.14776	6.28E-4	0.19611	7.45E-4	0.2097	0.00102	0.22713

5.08417E-4	0.13248	5.08417E-4	0.13248	8.27E-4	0.19679	7.14E-4	0.16418	6.69E-4	0.21603	7.79E-4	0.22801	0.00105	0.24501

5.45023E-4	0.15116	5.45023E-4	0.15116	8.64E-4	0.21282	7.45E-4	0.17911	6.95E-4	0.22828	8.22E-4	0.24632	0.0011	0.26826

5.77561E-4	0.17154	5.77561E-4	0.17154	9.06E-4	0.22886	7.75E-4	0.19404	7.1E-4	0.24054	8.51E-4	0.26463	0.00114	0.28793

5.97898E-4	0.18513	5.97898E-4	0.18513	9.33E-4	0.24052	8.06E-4	0.20597	7.41E-4	0.25893	8.7E-4	0.27794	0.00116	0.30045

6.26369E-4	0.20381	6.26369E-4	0.20381	9.61E-4	0.25218	8.52E-4	0.22538	7.66E-4	0.27731	8.94E-4	0.29126	0.0012	0.32013

6.62975E-4	0.22419	6.62975E-4	0.22419	9.98E-4	0.27113	8.77E-4	0.23881	7.87E-4	0.28957	9.23E-4	0.30956	0.00123	0.3398

6.79245E-4	0.24117	6.79245E-4	0.24117	0.00103	0.28425	8.93E-4	0.25075	8.07E-4	0.30336	9.62E-4	0.32953	0.00128	0.35589

6.95514E-4	0.25476	6.95514E-4	0.25476	0.00105	0.29883	9.23E-4	0.26418	8.38E-4	0.32481	9.9E-4	0.34617	0.0013	0.37378

7.1585E-4	0.27005	7.1585E-4	0.27005	0.00107	0.31195	9.59E-4	0.2821	8.58E-4	0.3386	0.001	0.35949	0.00132	0.38987

7.37226E-4	0.29043	7.37226E-4	0.29043	0.00111	0.32798	9.84E-4	0.29702	8.74E-4	0.35085	0.00103	0.37613	0.00134	0.40954

7.59124E-4	0.30402	7.59124E-4	0.30402	0.00114	0.34402	0.001	0.30747	8.99E-4	0.36464	0.00106	0.39609	0.00137	0.431

7.73723E-4	0.3176	7.73723E-4	0.3176	0.00116	0.35421	0.00103	0.32389	9.3E-4	0.38762	0.00109	0.41275	0.00141	0.44532

7.9562E-4	0.33798	7.9562E-4	0.33798	0.00118	0.36734	0.00106	0.34479	9.4E-4	0.39835	0.0011	0.42606	0.00142	0.46141

8.24818E-4	0.35327	8.24818E-4	0.35327	0.00122	0.38775	0.0011	0.36121	9.55E-4	0.40907	0.00113	0.44437	0.00144	0.48108

8.32117E-4	0.36516	8.32117E-4	0.36516	0.00124	0.40087	0.0011	0.36866	9.71E-4	0.42592	0.00115	0.46434	0.00147	0.50076

8.61314E-4	0.38554	8.61314E-4	0.38554	0.00126	0.41398	0.00112	0.38359	0.001	0.44584	0.00117	0.48265	0.00149	0.51685

8.90511E-4	0.40592	8.90511E-4	0.40592	0.00128	0.42856	0.00115	0.40598	0.00102	0.4627	0.00119	0.49763	0.0015	0.53116

9.05109E-4	0.42121	9.05109E-4	0.42121	0.00131	0.44605	0.00117	0.41494	0.00103	0.47342	0.0012	0.50761	0.00153	0.55262

9.19708E-4	0.43649	9.19708E-4	0.43649	0.00134	0.46209	0.00117	0.42837	0.00104	0.49027	0.00122	0.52758	0.00156	0.5705

9.48905E-4	0.45857	9.48905E-4	0.45857	0.00135	0.47375	0.00121	0.44777	0.00106	0.50713	0.00124	0.54755	0.00157	0.59017

9.70803E-4	0.47386	9.70803E-4	0.47386	0.00138	0.48833	0.00124	0.46718	0.00108	0.51938	0.00126	0.56252	0.00159	0.60627

9.70803E-4	0.48575	9.70803E-4	0.48575	0.00141	0.50436	0.00123	0.47763	0.0011	0.53318	0.00126	0.57418	0.0016	0.62415

0.001	0.50443	0.001	0.50443	0.00143	0.5204	0.00125	0.48956	0.00111	0.55156	0.00129	0.59249	0.00164	0.64204

0.00103	0.52141	0.00103	0.52141	0.00144	0.52914	0.00128	0.50748	0.00113	0.56841	0.00131	0.61412	0.00166	0.6635

0.00105	0.54349	0.00105	0.54349	0.00146	0.54664	0.0013	0.51942	0.00115	0.58066	0.00133	0.62744	0.00166	0.6778

0.00106	0.55368	0.00106	0.55368	0.00149	0.56413	0.00131	0.53135	0.00115	0.59598	0.00134	0.64075	0.00168	0.6939

0.00109	0.57067	0.00109	0.57067	0.00151	0.5787	0.00133	0.54777	0.00118	0.61436	0.00136	0.65574	0.00169	0.71178

0.00112	0.59275	0.00112	0.59275	0.00152	0.5889	0.00135	0.56569	0.00121	0.63123	0.00137	0.67737	0.00172	0.73324

0.00112	0.60124	0.00112	0.60124	0.00153	0.6064	0.00136	0.58061	0.00121	0.64195	0.00139	0.69568	0.00173	0.75113

0.00115	0.61822	0.00115	0.61822	0.00156	0.62244	0.00137	0.59404	0.00123	0.65727	0.0014	0.71065	0.00173	0.76365

0.00117	0.6403	0.00117	0.6403	0.00157	0.63701	0.00139	0.60748	0.00125	0.67719	0.00141	0.72563	0.00175	0.78332

0.00119	0.65389	0.00119	0.65389	0.00158	0.64721	0.00142	0.62539	0.00127	0.69251	0.00143	0.7456	0.00178	0.80299

0.0012	0.67087	0.0012	0.67087	0.00161	0.66324	0.00143	0.63733	0.00127	0.7017	0.00146	0.76391	0.0018	0.82267

0.00123	0.69295	0.00123	0.69295	0.00164	0.6822	0.00144	0.65076	0.00129	0.71855	0.00147	0.78221	0.00181	0.83876

0.00126	0.70994	0.00126	0.70994	0.00165	0.69532	0.00145	0.66419	0.00131	0.73847	0.00147	0.79221	0.00181	0.85307

0.00128	0.72183	0.00128	0.72183	0.00166	0.70698	0.00147	0.68509	0.00133	0.75533	0.00149	0.80884	0.00183	0.87453

0.00129	0.74051	0.00129	0.74051	0.00168	0.72155	0.00149	0.70002	0.00132	0.76451	0.00152	0.82715	0.00185	0.89062

0.00133	0.75919	0.00133	0.75919	0.00171	0.74197	0.0015	0.71644	0.00134	0.77984	0.00154	0.84546	0.00186	0.90851

0.00135	0.77448	0.00135	0.77448	0.00172	0.75216	0.00152	0.73286	0.00138	0.80129	0.00154	0.85711	0.00186	0.9246

0.00136	0.78976	0.00136	0.78976	0.00172	0.76675	0.00155	0.75226	0.00137	0.81201	0.00155	0.87542	0.00187	0.9407

0.00138	0.80675	0.00138	0.80675	0.00175	0.77986	0.00156	0.7657	0.00137	0.82581	0.00156	0.89372	0.00191	0.96216

0.00141	0.82883	0.00141	0.82883	0.00177	0.79882	0.00156	0.77315	0.0014	0.84419	0.00159	0.9137	0.00192	0.98004

0.00143	0.84581	0.00143	0.84581	0.00178	0.81193	0.00158	0.78808	0.00141	0.85797	0.0016	0.92867	0.00193	0.99614

0.00144	0.8611	0.00144	0.8611	0.00178	0.82505	0.0016	0.80898	0.00141	0.87024	0.00161	0.94033	0.00194	1.01403

0.00147	0.88148	0.00147	0.88148	0.0018	0.83671	0.00162	0.81793	0.00143	0.88709	0.00162	0.95864	0.00195	1.03191

0.00149	0.89846	0.00149	0.89846	0.00183	0.85858	0.00162	0.83136	0.00145	0.90547	0.00163	0.97861	0.00197	1.05158

0.0015	0.91375	0.0015	0.91375	0.00183	0.8717	0.00165	0.85077	0.00146	0.92079	0.00163	0.99691	0.00197	1.06947

0.0015	0.92903	0.0015	0.92903	0.00184	0.87753	0.00167	0.87018	0.00146	0.93304	0.00165	1.01023	0.00197	1.0802

0.00153	0.95111	0.00153	0.95111	0.00185	0.89939	0.00167	0.88062	0.00147	0.94684	0.00166	1.02854	0.00198	1.10166

0.00155	0.9681	0.00155	0.9681	0.00187	0.91398	0.00168	0.89405	0.00149	0.96369	0.00168	1.04351	0.00201	1.11954

0.00155	0.98338	0.00155	0.98338	0.00189	0.93001	0.0017	0.91346	0.0015	0.97901	0.0017	1.06348	0.00203	1.13921

0.00158	1.00037	0.00158	1.00037	0.0019	0.94021	0.0017	0.92689	0.00151	0.99126	0.00171	1.07513	0.00203	1.15352

0.00161	1.01735	0.00161	1.01735	0.00192	0.9577	0.00171	0.93734	0.00152	1.00811	0.00171	1.09178	0.00204	1.16783

0.00161	1.03433	0.00161	1.03433	0.00192	0.97519	0.00172	0.95226	0.00154	1.02957	0.00173	1.10842	0.00205	1.19465

0.00161	1.05132	0.00161	1.05132	0.00193	0.98394	0.00174	0.97167	0.00155	1.04336	0.00175	1.12838	0.00207	1.21254

0.00164	1.0717	0.00164	1.0717	0.00194	0.99852	0.00175	0.98361	0.00155	1.05715	0.00175	1.14337	0.00208	1.22864

0.00166	1.08698	0.00166	1.08698	0.00197	1.01601	0.00175	0.99853	0.00156	1.07401	0.00175	1.15668	0.00208	1.23937

0.00166	1.10227	0.00166	1.10227	0.00198	1.02912	0.00176	1.01645	0.00158	1.08932	0.00177	1.17332	0.00209	1.26083

0.00168	1.12435	0.00168	1.12435	0.00199	1.03934	0.00178	1.03287	0.00159	1.10158	0.00179	1.19662	0.00211	1.28408

0.00169	1.13963	0.00169	1.13963	0.00199	1.05682	0.0018	1.04779	0.00159	1.11537	0.0018	1.21327	0.00211	1.30017

0.00171	1.15152	0.00171	1.15152	0.00201	1.07432	0.0018	1.05525	0.00161	1.13375	0.0018	1.22491	0.00213	1.31448

0.00172	1.1719	0.00172	1.1719	0.00201	1.09035	0.0018	1.07316	0.00163	1.14908	0.00182	1.23989	0.00214	1.33236

0.00174	1.18889	0.00174	1.18889	0.00201	1.10055	0.00182	1.08958	0.00162	1.15827	0.00184	1.26154	0.00216	1.35382

0.00175	1.20927	0.00175	1.20927	0.00202	1.11658	0.00185	1.10301	0.00165	1.17819	0.00185	1.27984	0.00217	1.37349

0.00176	1.21946	0.00176	1.21946	0.00205	1.13117	0.00184	1.11645	0.00166	1.19657	0.00185	1.29149	0.00217	1.3878

0.00177	1.23814	0.00177	1.23814	0.00206	1.1472	0.00186	1.13287	0.00168	1.21036	0.00186	1.30647	0.00218	1.4039

0.0018	1.25852	0.0018	1.25852	0.00207	1.15595	0.00188	1.15227	0.00168	1.22108	0.00187	1.32644	0.00218	1.42536

0.0018	1.27551	0.0018	1.27551	0.00207	1.17343	0.00189	1.16421	0.00169	1.23794	0.00188	1.34475	0.0022	1.44145

0.0018	1.2874	0.0018	1.2874	0.0021	1.19093	0.00188	1.17615	0.00171	1.25631	0.00189	1.36139	0.00222	1.46291

0.00182	1.30948	0.00182	1.30948	0.00211	1.20259	0.00191	1.19406	0.00171	1.27011	0.00189	1.37137	0.00221	1.47544

0.00184	1.32137	0.00184	1.32137	0.00211	1.21717	0.00192	1.21198	0.00171	1.28084	0.0019	1.39134	0.00222	1.49331

0.00183	1.33325	0.00183	1.33325	0.00212	1.23175	0.00192	1.21943	0.00173	1.30228	0.00193	1.41132	0.00225	1.51656

0.00185	1.35703	0.00185	1.35703	0.00213	1.24633	0.00193	1.23436	0.00174	1.31913	0.00192	1.4263	0.00226	1.53445

0.00187	1.37741	0.00187	1.37741	0.00214	1.2609	0.00195	1.25675	0.00174	1.33139	0.00193	1.43795	0.00226	1.54697

0.00187	1.391	0.00187	1.391	0.00214	1.27403	0.00196	1.27019	0.00174	1.34671	0.00194	1.45791	0.00227	1.56664

0.00188	1.40798	0.00188	1.40798	0.00215	1.2886	0.00196	1.28063	0.00177	1.36357	0.00195	1.47789	0.00228	1.5881

0.0019	1.43346	0.0019	1.43346	0.00216	1.30609	0.00197	1.30153	0.00178	1.37735	0.00197	1.49453	0.00229	1.60599

0.00191	1.44535	0.00191	1.44535	0.00218	1.31775	0.00199	1.31496	0.00178	1.38808	0.00197	1.50785	0.0023	1.62029

0.00191	1.45384	0.00191	1.45384	0.00217	1.32942	0.002	1.3284	0.0018	1.4034	0.00198	1.5245	0.0023	1.63639

0.00192	1.47252	0.00192	1.47252	0.00219	1.34837	0.00201	1.33884	0.00181	1.42485	0.00199	1.5428	0.00231	1.65785

0.00194	1.4929	0.00194	1.4929	0.00221	1.36003	0.00201	1.35377	0.00182	1.43711	0.00201	1.55944	0.00232	1.67931

0.00195	1.50819	0.00195	1.50819	0.00221	1.3746	0.00203	1.37317	0.00182	1.4509	0.00202	1.57442	0.00233	1.69362

0.00195	1.52348	0.00195	1.52348	0.00222	1.38335	0.00205	1.38959	0.00182	1.46622	0.00201	1.58607	0.00233	1.70792

0.00197	1.54216	0.00197	1.54216	0.00224	1.40521	0.00205	1.40004	0.00183	1.48614	0.00204	1.60771	0.00234	1.72938

0.00198	1.56424	0.00198	1.56424	0.00225	1.41834	0.00205	1.41646	0.00186	1.50146	0.00206	1.62601	0.00237	1.75084

0.00199	1.57443	0.00199	1.57443	0.00225	1.43291	0.00207	1.43736	0.00185	1.51065	0.00206	1.64765	0.00237	1.77052

0.00199	1.59141	0.00199	1.59141	0.00225	1.44603	0.00207	1.45228	0.00186	1.5275	0.00207	1.65763	0.00237	1.77768

0.00201	1.61179	0.00201	1.61179	0.00226	1.46206	0.00207	1.45974	0.00189	1.54435	0.00207	1.67428	0.00238	1.79914

0.00201	1.62708	0.00201	1.62708	0.00228	1.47664	0.00208	1.47467	0.00189	1.56121	0.00209	1.69424	0.00239	1.81881

0.00201	1.64067	0.00201	1.64067	0.00227	1.48976	0.0021	1.49407	0.00189	1.57041	0.00209	1.71089	0.0024	1.84027

0.00204	1.66105	0.00204	1.66105	0.00229	1.50434	0.00211	1.50452	0.0019	1.59032	0.0021	1.72753	0.00241	1.85457

0.00205	1.68313	0.00205	1.68313	0.0023	1.52183	0.00211	1.51944	0.00191	1.6087	0.0021	1.73918	0.0024	1.86888

0.00205	1.69502	0.00205	1.69502	0.00231	1.5364	0.00213	1.53736	0.00191	1.62096	0.00212	1.75916	0.00241	1.89034

0.00205	1.7137	0.00205	1.7137	0.00231	1.54661	0.00214	1.55378	0.00192	1.63475	0.00213	1.77746	0.00243	1.91538

0.00207	1.73578	0.00207	1.73578	0.00232	1.56264	0.00214	1.56571	0.00194	1.65313	0.00213	1.79577	0.00243	1.92969

0.00209	1.74767	0.00209	1.74767	0.00234	1.58014	0.00214	1.58064	0.00195	1.66845	0.00213	1.80575	0.00243	1.9422

0.00208	1.75786	0.00208	1.75786	0.00234	1.59325	0.00217	1.60005	0.00195	1.67612	0.00214	1.82573	0.00244	1.96009

0.0021	1.78333	0.0021	1.78333	0.00234	1.60637	0.00217	1.61199	0.00196	1.69297	0.00216	1.84403	0.00245	1.97977

0.00211	1.80032	0.00211	1.80032	0.00236	1.62095	0.00217	1.62392	0.00198	1.71442	0.00217	1.86234	0.00246	1.99944

0.00211	1.8139	0.00211	1.8139	0.00237	1.63845	0.00218	1.63736	0.00198	1.7282	0.00217	1.87232	0.00253	2.01553

0.00211	1.82579	0.00211	1.82579	0.00238	1.65156	0.00219	1.65826	0.00198	1.74046	0.00217	1.89064	0.00253	2.03342

0.00213	1.85127	0.00213	1.85127	0.00238	1.66322	0.00221	1.67019	0.002	1.75731	0.00219	1.90895	0.00253	2.0513

0.00214	1.86486	0.00214	1.86486	0.00239	1.67925	0.0022	1.68512	0.00201	1.77724	0.0022	1.92724	0.00255	2.07097

0.00214	1.87675	0.00214	1.87675	0.0024	1.69529	0.00221	1.70154	0.00202	1.78949	0.0022	1.9389	0.00256	2.08886

0.00215	1.89883	0.00215	1.89883	0.00241	1.70987	0.00224	1.72095	0.00202	1.80022	0.0022	1.95222	0.00256	2.10316

0.00217	1.91751	0.00217	1.91751	0.00241	1.72299	0.00223	1.73288	0.00202	1.81861	0.00222	1.97551	0.00257	2.12284

0.00216	1.9311	0.00216	1.9311	0.00242	1.73756	0.00223	1.74333	0.00204	1.83546	0.00223	1.99548	0.00258	2.14251

0.00218	1.94978	0.00218	1.94978	0.00243	1.7536	0.00224	1.75826	0.00204	1.84771	0.00223	2.01046	0.00258	2.16218

0.00219	1.97016	0.00219	1.97016	0.00243	1.76671	0.00227	1.77617	0.00205	1.8615	0.00223	2.02212	0.00258	2.17827

0.0022	1.98205	0.0022	1.98205	0.00244	1.7813	0.00227	1.79259	0.00206	1.87989	0.00224	2.03876	0.00258	2.19259

0.00219	1.99733	0.00219	1.99733	0.00245	1.79879	0.00226	1.80154	0.00207	1.89674	0.00226	2.06039	0.0026	2.21225

0.00219	2.00317	0.00219	2.00617	0.00246	1.81191	0.00228	1.81796	0.00208	1.91205	0.00227	2.07704	0.00261	2.23371

0.00221	2.01652	0.00218	2.02263	0.00246	1.82502	0.0023	1.83886	0.00208	1.92584	0.00226	2.08702	0.00262	2.2516

0.0022	2.02541	0.00217	2.0346	0.00247	1.83669	0.0023	1.85378	0.0021	1.94423	0.00227	2.10699	0.00261	2.26233

0.00221	2.04469	0.00219	2.05105	0.00248	1.85418	0.0023	1.86423	0.00212	1.95649	0.00229	2.12697	0.00261	2.282

0.00222	2.06248	0.00221	2.072	0.0025	1.87168	0.00231	1.88214	0.00212	1.96568	0.0023	2.14361	0.00263	2.30525

0.00222	2.07138	0.00222	2.07798	0.00249	1.88333	0.00233	1.89856	0.00212	1.9856	0.0023	2.15525	0.00264	2.32135

0.00222	2.08917	0.00221	2.08995	0.0025	1.89645	0.00232	1.90751	0.00215	2.00399	0.0023	2.1719	0.00264	2.33387

0.00224	2.10548	0.00222	2.10641	0.00251	1.9154	0.00233	1.91945	0.00216	2.01778	0.00232	2.19187	0.00264	2.35354

0.00224	2.11734	0.00222	2.11987	0.00252	1.92997	0.00234	1.93737	0.00215	2.03003	0.00233	2.21018	0.00265	2.375

0.00224	2.12772	0.00222	2.13483	0.00252	1.93873	0.00236	1.95677	0.00216	2.04842	0.00233	2.22349	0.00267	2.39467

0.00225	2.14996	0.00222	2.15129	0.00253	1.95476	0.00236	1.96871	0.00218	2.06833	0.00233	2.23681	0.00267	2.41077

0.00227	2.16034	0.00226	2.17223	0.00254	1.97371	0.00235	1.98364	0.00219	2.08212	0.00234	2.25512	0.00268	2.4215

0.00225	2.1722	0.00226	2.1842	0.00256	1.98682	0.00237	2.00155	0.00218	2.09285	0.00236	2.27841	0.00267	2.44296

0.00227	2.19445	0.00225	2.20066	0.00255	1.99849	0.00238	2.01498	0.00219	2.1051	0.00237	2.29339	0.00269	2.46978

0.00229	2.21076	0.00226	2.21263	0.00256	2.01307	0.00238	2.02543	0.00221	2.12502	0.00236	2.30504	0.00269	2.4841

0.00228	2.22262	0.00228	2.22908	0.00257	2.03202	0.00238	2.04035	0.00221	2.13881	0.00237	2.32168	0.00269	2.49483

0.00229	2.23596	0.00228	2.24105	0.00258	2.04513	0.0024	2.05827	0.0022	2.15107	0.00237	2.34	0.00269	2.5145

0.0023	2.25524	0.00228	2.25751	0.00258	2.05535	0.00241	2.07468	0.00223	2.17099	0.00238	2.35997	0.00271	2.53775

0.0023	2.2671	0.0023	2.27695	0.00259	2.07283	0.0024	2.08364	0.00224	2.18784	0.00238	2.36995	0.00272	2.55205

0.0023	2.276	0.00231	2.29191	0.0026	2.09033	0.00241	2.10006	0.00224	2.20162	0.00238	2.38493	0.00272	2.56994

0.00232	2.29824	0.0023	2.2994	0.00261	2.10199	0.00243	2.12245	0.00224	2.21695	0.00239	2.40491	0.00271	2.58424

0.00232	2.31306	0.00231	2.31884	0.0026	2.11511	0.00243	2.13588	0.00225	2.23533	0.0024	2.42486	0.00273	2.6057

0.00232	2.32344	0.00233	2.33829	0.00262	2.13114	0.00243	2.14483	0.00227	2.24759	0.00241	2.43985	0.00273	2.62538

0.00233	2.33975	0.00233	2.34876	0.00262	2.15009	0.00243	2.15976	0.00226	2.25831	0.00241	2.45316	0.00274	2.64505

0.00235	2.35903	0.00233	2.35624	0.00262	2.15884	0.00246	2.17767	0.00227	2.27364	0.00241	2.4698	0.00273	2.654

0.00234	2.36644	0.00234	2.38168	0.00263	2.17342	0.00247	2.1926	0.0023	2.29354	0.00243	2.48978	0.00274	2.67724

0.00235	2.38424	0.00236	2.39215	0.00264	2.19236	0.00246	2.20304	0.0023	2.30888	0.00243	2.50975	0.00275	2.69692

0.00236	2.40203	0.00236	2.40711	0.00266	2.20694	0.00247	2.22096	0.00229	2.31959	0.00243	2.52139	0.00275	2.71479

0.00237	2.41537	0.00237	2.42207	0.00266	2.21715	0.00248	2.23737	0.00231	2.33951	0.00243	2.53804	0.00275	2.72731

0.00237	2.42872	0.00239	2.44301	0.00265	2.23027	0.00248	2.2523	0.00232	2.35636	0.00245	2.55802	0.00276	2.74699

0.00238	2.44651	0.00239	2.45498	0.00267	2.25067	0.00249	2.26424	0.00232	2.37016	0.00246	2.57466	0.00277	2.76666

0.00241	2.46282	0.00239	2.46695	0.00268	2.2638	0.00249	2.28215	0.00233	2.38241	0.00246	2.58798	0.00277	2.78455

0.00239	2.47468	0.00239	2.4864	0.00268	2.27399	0.0025	2.29708	0.00235	2.39926	0.00247	2.60296	0.00279	2.80242

0.00241	2.48654	0.00241	2.49986	0.00268	2.29004	0.0025	2.30752	0.00235	2.41458	0.00248	2.62126	0.00279	2.81853

0.00242	2.50878	0.00241	2.51482	0.00271	2.30898	0.0025	2.32096	0.00235	2.42684	0.00249	2.64123	0.00279	2.83641

0.00242	2.51916	0.00242	2.52679	0.0027	2.32211	0.00252	2.34036	0.00235	2.44217	0.00249	2.65788	0.0028	2.85608

0.00242	2.53399	0.00244	2.54624	0.0027	2.33084	0.00252	2.35529	0.00237	2.45902	0.00249	2.66952	0.0028	2.87575

0.00243	2.5503	0.00245	2.5627	0.00271	2.34688	0.00252	2.36573	0.00237	2.4774	0.0025	2.68782	0.0028	2.88827

0.00245	2.56513	0.00244	2.57317	0.00273	2.36583	0.00253	2.38365	0.00237	2.49119	0.00251	2.7078	0.00281	2.90794

0.00244	2.57699	0.00245	2.58962	0.00274	2.38041	0.00255	2.40455	0.00238	2.50499	0.00252	2.72278	0.00282	2.92762

0.00245	2.5933	0.00246	2.60608	0.00273	2.39207	0.00256	2.41798	0.0024	2.5249	0.00252	2.73443	0.00282	2.94729

0.00247	2.61109	0.00245	2.61655	0.00274	2.40665	0.00255	2.42693	0.0024	2.53869	0.00252	2.75108	0.00282	2.96159

0.00246	2.61999	0.00246	2.63002	0.00275	2.42414	0.00257	2.44335	0.0024	2.54634	0.00253	2.77437	0.00282	2.97948

0.00246	2.6363	0.00249	2.64647	0.00275	2.43871	0.00258	2.46126	0.00241	2.56473	0.00254	2.79268	0.00283	3.00273

0.00249	2.65558	0.00249	2.66443	0.00275	2.45038	0.00259	2.47021	0.00242	2.58312	0.00253	2.80267	0.00284	3.01883

0.00249	2.66595	0.00249	2.6734	0.00275	2.46641	0.00258	2.48514	0.00244	2.59844	0.00254	2.81931	0.00283	3.03492

0.00249	2.6793	0.0025	2.69435	0.00277	2.48245	0.00258	2.50156	0.00243	2.60917	0.00255	2.83928	0.00284	3.05102

0.0025	2.69857	0.00251	2.7123	0.00277	2.4941	0.00261	2.52096	0.00245	2.62755	0.00256	2.85759	0.00284	3.0689

0.00252	2.71192	0.00251	2.72427	0.00276	2.50868	0.00261	2.53589	0.00246	2.64746	0.00256	2.87257	0.00285	3.09036

0.00251	2.7223	0.00253	2.73773	0.00278	2.52618	0.00261	2.54783	0.00247	2.66278	0.00256	2.88588	0.00286	3.10646

0.00252	2.74306	0.00254	2.75269	0.00279	2.54222	0.00261	2.56126	0.00246	2.67198	0.00257	2.90252	0.00285	3.12255

0.00254	2.75937	0.00255	2.76915	0.00279	2.55387	0.00263	2.57917	0.00247	2.69036	0.00258	2.92582	0.00286	3.13865

0.00254	2.76678	0.00254	2.77663	0.00279	2.56845	0.00264	2.59111	0.00248	2.70875	0.00258	2.93913	0.00288	3.16011

0.00254	2.78457	0.00255	2.79308	0.00281	2.58448	0.00263	2.60305	0.00248	2.71641	0.00258	2.95079	0.00288	3.17979

0.00255	2.80237	0.00257	2.82151	0.00282	2.59907	0.00264	2.61947	0.00248	2.73326	0.00259	2.97242	0.00287	3.19052

0.00256	2.81275	0.00257	2.826	0.00282	2.61072	0.00265	2.63589	0.0025	2.75319	0.0026	2.99072	0.00287	3.21018

0.00257	2.82609	0.00257	2.84245	0.00282	2.6253	0.00266	2.65082	0.00251	2.7685	0.00261	3.00737	0.00288	3.22807

0.00258	2.84537	0.00258	2.8619	0.00284	2.64279	0.00266	2.66574	0.0025	2.77923	0.00261	3.02069	0.00289	3.25132

0.00258	2.86168	0.00259	2.87537	0.00284	2.65737	0.00267	2.68365	0.00251	2.79762	0.00261	3.03566	0.00289	3.26563

0.00258	2.87057	0.00259	2.88584	0.00284	2.66611	0.00268	2.69858	0.00253	2.81752	0.00263	3.05231	0.00289	3.27815

0.0026	2.89133	0.0026	2.90379	0.00284	2.68215	0.00267	2.70903	0.00253	2.83132	0.00263	3.07062	0.00289	3.29961

0.00262	2.91061	0.00262	2.92025	0.00286	2.69964	0.00267	2.71948	0.00252	2.84051	0.00263	3.08892	0.00292	3.32107

0.00261	2.9195	0.00262	2.93521	0.00287	2.71568	0.00269	2.74186	0.00253	2.8543	0.00263	3.10057	0.00292	3.33895

0.00262	2.93136	0.00263	2.94718	0.00286	2.72587	0.0027	2.75679	0.00254	2.87422	0.00263	3.12054	0.0029	3.35326

0.00263	2.95064	0.00263	2.96812	0.00286	2.74046	0.00269	2.76724	0.00255	2.88647	0.00265	3.14052	0.00292	3.37114

0.00264	2.96102	0.00264	2.98158	0.00288	2.76232	0.0027	2.78216	0.00254	2.9018	0.00265	3.15715	0.00293	3.39081

0.00264	2.97436	0.00263	2.99505	0.00288	2.77398	0.00271	2.80306	0.00255	2.92171	0.00264	3.16715	0.00293	3.41048

0.00266	2.99512	0.00264	3.00702	0.00288	2.78418	0.00272	2.81799	0.00256	2.94163	0.00266	3.18545	0.00293	3.423

0.00267	3.01143	0.00266	3.02796	0.00288	2.80022	0.00271	2.82993	0.00257	2.95542	0.00267	3.20709	0.00293	3.43732

0.00266	3.02181	0.00267	3.03694	0.0029	2.81771	0.00272	2.84336	0.00256	2.96461	0.00267	3.22207	0.00294	3.45878

0.00267	3.03812	0.00267	3.0519	0.0029	2.83083	0.00274	2.86127	0.00259	2.98146	0.00267	3.23538	0.00294	3.47844

0.00269	3.0574	0.00267	3.07135	0.0029	2.84395	0.00274	2.87321	0.00259	2.99526	0.00267	3.25203	0.00295	3.49812

0.00268	3.06778	0.00269	3.09079	0.0029	2.85998	0.00275	2.88366	0.00259	3.0075	0.00268	3.27199	0.00295	3.50885

0.00269	3.08112	0.00269	3.09528	0.00292	2.87602	0.00275	2.90157	0.0026	3.02283	0.00269	3.29031	0.00295	3.52852

0.00271	3.09891	0.00269	3.10875	0.00292	2.88913	0.00276	2.91799	0.00261	3.04122	0.00269	3.30196	0.00296	3.54998

0.00271	3.11226	0.00272	3.13418	0.00292	2.90226	0.00276	2.9359	0.00263	3.05959	0.00269	3.3186	0.00296	3.56787

0.00271	3.12708	0.00271	3.14016	0.00292	2.91537	0.00276	2.94784	0.00262	3.07033	0.0027	3.33689	0.00296	3.58217

0.00272	3.14339	0.00271	3.15213	0.00294	2.93433	0.00277	2.96426	0.00262	3.08871	0.00271	3.35521	0.00296	3.59648

0.00274	3.15674	0.00273	3.17158	0.00294	2.94598	0.00277	2.97918	0.00265	3.10862	0.00271	3.36852	0.00296	3.61794

0.00272	3.16712	0.00274	3.18953	0.00294	2.95911	0.00278	2.99112	0.00265	3.11935	0.00271	3.38183	0.00298	3.63761

0.00274	3.18491	0.00273	3.19851	0.00294	2.97223	0.00278	3.00455	0.00264	3.12855	0.00271	3.40015	0.00298	3.65371

0.00275	3.2027	0.00274	3.22095	0.00295	2.99118	0.00279	3.02097	0.00266	3.14693	0.00273	3.42178	0.00298	3.66801

0.00275	3.21605	0.00275	3.23292	0.00296	3.00429	0.00281	3.03889	0.00266	3.16532	0.00273	3.43509	0.00298	3.68769

0.00275	3.22643	0.00275	3.24189	0.00296	3.01596	0.00281	3.04785	0.00266	3.17757	0.00272	3.44841	0.00299	3.70736

0.00277	3.25015	0.00275	3.25835	0.00296	3.0349	0.00281	3.06724	0.00266	3.18983	0.00274	3.46672	0.003	3.72345

0.00278	3.26201	0.00276	3.27181	0.00298	3.05095	0.00282	3.08665	0.00267	3.21127	0.00274	3.48669	0.00299	3.73777

0.00278	3.27239	0.00278	3.29126	0.00298	3.06114	0.00283	3.10158	0.00269	3.22966	0.00276	3.50167	0.00299	3.75565

0.00279	3.29315	0.00277	3.30473	0.00298	3.07427	0.00282	3.11053	0.00268	3.24192	0.00275	3.51664	0.003	3.7789

0.0028	3.3065	0.00277	3.31969	0.00299	3.09175	0.00283	3.12248	0.00268	3.25265	0.00275	3.53162	0.00301	3.79678

0.0028	3.31391	0.0028	3.34063	0.00299	3.10779	0.00286	3.14187	0.0027	3.27256	0.00277	3.54993	0.003	3.8093

0.0028	3.33318	0.00281	3.35559	0.00299	3.118	0.00285	3.15531	0.0027	3.28942	0.00278	3.56824	0.003	3.82718

0.00283	3.35246	0.0028	3.36906	0.003	3.13112	0.00284	3.16725	0.0027	3.29707	0.00279	3.58488	0.00301	3.84507

0.00282	3.36136	0.00281	3.37953	0.003	3.15006	0.00285	3.18218	0.00271	3.31392	0.00278	3.59654	0.00302	3.86653

0.00282	3.37915	0.00282	3.39598	0.00302	3.16464	0.00287	3.20456	0.00272	3.33231	0.00278	3.6165	0.00302	3.88084

0.00284	3.39398	0.00284	3.41394	0.00301	3.17631	0.00287	3.218	0.00272	3.34763	0.0028	3.63814	0.00302	3.89514

0.00284	3.41029	0.00282	3.4259	0.00302	3.18943	0.00286	3.22696	0.00271	3.36142	0.0028	3.65145	0.00302	3.91481

0.00285	3.42067	0.00284	3.44386	0.00303	3.20546	0.00287	3.24486	0.00272	3.37674	0.0028	3.66144	0.00305	3.93806

0.00286	3.43846	0.00286	3.46031	0.00303	3.2215	0.00288	3.26128	0.00273	3.39666	0.0028	3.68141	0.00306	3.95774

0.00287	3.45477	0.00285	3.47228	0.00303	3.23315	0.00289	3.27322	0.00274	3.40739	0.00281	3.69805	0.00304	3.96847

0.00287	3.46515	0.00284	3.48724	0.00304	3.24628	0.00289	3.28367	0.00274	3.41964	0.00282	3.7147	0.00305	3.98278

0.00287	3.48146	0.00286	3.5037	0.00304	3.26523	0.0029	3.29711	0.00274	3.43496	0.00282	3.73134	0.00306	4.00424

0.00289	3.50073	0.00287	3.51567	0.00306	3.27981	0.0029	3.31949	0.00276	3.45488	0.00282	3.74632	0.00306	4.0257

0.00289	3.5126	0.00286	3.52763	0.00305	3.29	0.00291	3.33442	0.00276	3.46561	0.00283	3.76297	0.00306	4.03822

0.00289	3.52594	0.00287	3.55007	0.00306	3.30604	0.00291	3.34487	0.00276	3.47786	0.00284	3.78293	0.00306	4.05431

0.00292	3.54373	0.0029	3.56204	0.00306	3.32354	0.00292	3.36427	0.00276	3.50085	0.00284	3.79957	0.00301	4.08114

0.00292	3.55559	0.00289	3.577	0.00307	3.3352	0.00293	3.3777	0.00278	3.51924	0.00285	3.81289	0.00308	4.09723

0.00292	3.56894	0.00289	3.58897	0.00306	3.34831	0.00293	3.39114	0.00278	3.53149	0.00284	3.82954	0.00308	4.11512

0.00292	3.58821	0.00291	3.61141	0.00307	3.36143	0.00293	3.40009	0.00278	3.54221	0.00286	3.84784	0.00307	4.12764

0.00294	3.60304	0.00292	3.62338	0.00307	3.38038	0.00294	3.4195	0.00279	3.56213	0.00286	3.86282	0.00308	4.14552

0.00294	3.61342	0.00291	3.63086	0.00309	3.39496	0.00295	3.43592	0.0028	3.57438	0.00285	3.87779	0.00308	4.16877

0.00293	3.62825	0.00292	3.6533	0.00308	3.4037	0.00295	3.44636	0.00279	3.58664	0.00286	3.89111	0.00309	4.18486

0.00296	3.64752	0.00293	3.66976	0.0031	3.41974	0.00295	3.46428	0.0028	3.60043	0.00287	3.91441	0.00308	4.19918

0.00296	3.66087	0.00292	3.67873	0.0031	3.43723	0.00297	3.48666	0.00281	3.62189	0.00287	3.93272	0.00308	4.21348

0.00296	3.67273	0.00294	3.69669	0.00311	3.45034	0.00298	3.50009	0.00281	3.6372	0.00287	3.94603	0.0031	4.23851

0.00297	3.69497	0.00295	3.71613	0.0031	3.46347	0.00297	3.51203	0.00281	3.65099	0.00287	3.96101	0.0031	4.25819

0.00299	3.71128	0.00296	3.7296	0.00311	3.47804	0.00297	3.52249	0.00281	3.66631	0.00288	3.97932	0.0031	4.2707

0.00298	3.71721	0.00296	3.73857	0.00312	3.49554	0.00299	3.54039	0.00283	3.68623	0.00288	3.99597	0.0031	4.28502

0.00298	3.73204	0.00296	3.75653	0.00311	3.50574	0.00299	3.55383	0.00283	3.69849	0.00288	4.00928	0.00311	4.30648

0.003	3.75132	0.00298	3.77448	0.00311	3.52032	0.00299	3.56577	0.00283	3.70921	0.00288	4.02425	0.00312	4.32615

0.00301	3.76318	0.00298	3.78196	0.00312	3.53635	0.00299	3.58219	0.00285	3.72453	0.00289	4.04256	0.00312	4.34224

0.003	3.77652	0.00298	3.79542	0.00313	3.55239	0.00301	3.60308	0.00286	3.74291	0.0029	4.06253	0.00311	4.35655

0.00302	3.79728	0.00299	3.81487	0.00313	3.56259	0.00301	3.61502	0.00285	3.75364	0.00291	4.07751	0.00312	4.37265

0.00304	3.81062	0.003	3.83881	0.00313	3.57572	0.00301	3.62994	0.00285	3.76895	0.0029	4.0925	0.00312	4.3959

0.00302	3.821	0.00299	3.84629	0.00314	3.5932	0.00301	3.64339	0.00286	3.79194	0.0029	4.10747	0.00313	4.41557

0.00304	3.83731	0.003	3.86125	0.00315	3.6107	0.00302	3.66278	0.00286	3.80573	0.00292	4.1291	0.00312	4.43346

0.00304	3.85659	0.00302	3.8777	0.00314	3.61944	0.00304	3.67324	0.00286	3.81799	0.00293	4.14409	0.00312	4.45134

0.00305	3.86549	0.00302	3.88967	0.00315	3.63256	0.00303	3.68518	0.00286	3.83331	0.00292	4.15407	0.00314	4.47101

0.00304	3.88031	0.00301	3.89865	0.00317	3.65296	0.00303	3.69712	0.00288	3.8517	0.00292	4.17237	0.00316	4.49068

0.00308	3.89959	0.00303	3.92109	0.00317	3.66609	0.00305	3.72099	0.00289	3.86701	0.00294	4.19234	0.00316	4.50499

0.00308	3.91145	0.00305	3.94203	0.00316	3.67775	0.00305	3.72995	0.00287	3.87467	0.00295	4.21065	0.00314	4.52109

0.00307	3.92776	0.00304	3.95101	0.00316	3.69087	0.00304	3.74787	0.00289	3.89153	0.00294	4.22231	0.00316	4.54076

0.00309	3.94555	0.00304	3.96447	0.00319	3.70982	0.00305	3.76726	0.00289	3.90992	0.00295	4.23895	0.00316	4.55864

0.00309	3.9589	0.00306	3.98243	0.00318	3.7244	0.00307	3.78518	0.0029	3.92218	0.00295	4.25893	0.00317	4.57831

0.00309	3.97224	0.00306	3.99739	0.00318	3.73314	0.00306	3.78966	0.00289	3.93749	0.00296	4.27722	0.00316	4.58904

0.00309	3.98855	0.00305	4.00487	0.00319	3.74918	0.00305	3.80309	0.0029	3.95894	0.00297	4.29386	0.00317	4.60872

0.00312	4.00486	0.00306	4.0303	0.0032	3.76813	0.00308	3.82697	0.0029	3.9758	0.00296	4.30385	0.00318	4.63375

0.00311	4.01524	0.00309	4.04376	0.0032	3.78125	0.00309	3.84189	0.00291	3.98652	0.00297	4.32049	0.00318	4.64985

0.00312	4.03007	0.00308	4.05274	0.00319	3.79291	0.00308	3.84339	0.00291	3.99878	0.00298	4.34214	0.00318	4.66594

0.00313	4.04934	0.00309	4.0662	0.00321	3.80895	0.00309	3.87025	0.00292	4.01564	0.00299	4.35877	0.00318	4.67846

0.00314	4.06269	0.0031	4.08864	0.00321	3.82352	0.00311	3.88816	0.00293	4.03402	0.00297	4.3671	0.00319	4.69813

0.00314	4.07307	0.00311	4.10211	0.00321	3.8381	0.0031	3.90757	0.00294	4.0432	0.00298	4.38874	0.00319	4.7196

0.00316	4.09531	0.0031	4.11109	0.00321	3.84975	0.00309	3.91503	0.00293	4.05547	0.00299	4.40704	0.0032	4.7357

0.00316	4.11162	0.0031	4.13203	0.00322	3.86871	0.00311	3.92995	0.00294	4.07691	0.003	4.42535	0.00319	4.74821

0.00316	4.11607	0.00312	4.15148	0.00323	3.88475	0.00312	3.94488	0.00295	4.0953	0.003	4.437	0.0032	4.7643

0.00317	4.13979	0.00312	4.15597	0.00323	3.89641	0.00312	3.95683	0.00294	4.10756	0.003	4.4553	0.0032	4.78935

0.00317	4.1561	0.00312	4.17392	0.00323	3.91098	0.00312	3.97026	0.00296	4.12134	0.00301	4.47028	0.00321	4.81081

0.00317	4.15907	0.00313	4.19187	0.00323	3.93139	0.00312	3.97921	0.00297	4.14126	0.00301	4.49026	0.0032	4.82154

0.00317	4.17686	0.00314	4.20683	0.00324	3.94451	0.00314	4.00608	0.00297	4.15199	0.00302	4.50357	0.0032	4.83763

0.0032	4.19762	0.00314	4.2203	0.00323	3.95326	0.00314	4.01951	0.00296	4.16424	0.00301	4.51855	0.00322	4.85909

0.0032	4.20948	0.00315	4.23675	0.00323	3.96783	0.00314	4.03443	0.00296	4.17957	0.00302	4.53851	0.00322	4.88234

0.00321	4.21986	0.00316	4.2547	0.00325	3.98387	0.00314	4.04787	0.00298	4.20102	0.00303	4.55516	0.00322	4.89487

0.00321	4.24507	0.00316	4.26069	0.00326	4.00136	0.00316	4.06429	0.00299	4.21327	0.00303	4.57014	0.00321	4.90738

0.00323	4.25693	0.00316	4.27266	0.00326	4.01157	0.00316	4.07325	0.00297	4.22706	0.00302	4.58345	0.00322	4.92706

0.00322	4.26879	0.00317	4.29659	0.00326	4.02614	0.00315	4.08817	0.00299	4.2485	0.00302	4.60343	0.00323	4.9503

0.00322	4.28362	0.00318	4.31155	0.00327	4.04655	0.00316	4.10459	0.003	4.26536	0.00303	4.62173	0.00323	4.9664

0.00325	4.30141	0.00317	4.32502	0.00328	4.06113	0.00318	4.12101	0.003	4.27455	0.00304	4.64004	0.00323	4.97891

0.00324	4.30882	0.00318	4.33699	0.00327	4.07132	0.00317	4.13594	0.003	4.28681	0.00303	4.65003	0.00323	5.00037

0.00324	4.32662	0.0032	4.35494	0.00328	4.0859	0.00317	4.15235	0.00301	4.30673	0.00304	4.66501	0.00325	5.02183

0.00326	4.34737	0.00321	4.37289	0.00329	4.10339	0.00318	4.17474	0.00302	4.32358	0.00304	4.68664	0.00324	5.03793

0.00327	4.35924	0.00319	4.38187	0.00329	4.11652	0.00319	4.19116	0.00301	4.32971	0.00305	4.70494	0.00324	5.05224

0.00326	4.3711	0.00322	4.39982	0.00329	4.12526	0.00318	4.19712	0.00301	4.3481	0.00305	4.7166	0.00325	5.06834

0.00328	4.38889	0.00322	4.41927	0.0033	4.14567	0.00319	4.21354	0.00303	4.37107	0.00306	4.73324	0.00325	5.0898

0.00329	4.4052	0.00322	4.42824	0.00331	4.1617	0.00321	4.23444	0.00303	4.38487	0.00306	4.75654	0.00326	5.11126

0.00329	4.41706	0.00322	4.44769	0.00331	4.17482	0.00321	4.24489	0.00302	4.39713	0.00307	4.77318	0.00326	5.12556

0.00329	4.43189	0.00324	4.46265	0.0033	4.18648	0.0032	4.25683	0.00303	4.41398	0.00307	4.78317	0.00326	5.14167

0.00331	4.4482	0.00324	4.47911	0.00331	4.20544	0.00321	4.27474	0.00304	4.43236	0.00307	4.79981	0.00326	5.16133

0.0033	4.45858	0.00322	4.48958	0.00333	4.22001	0.00322	4.29564	0.00304	4.44309	0.00308	4.82145	0.00328	5.18279

0.0033	4.47489	0.00324	4.50454	0.00332	4.23167	0.00322	4.31057	0.00304	4.45533	0.0031	4.83476	0.00328	5.20068

0.00333	4.4912	0.00326	4.518	0.00333	4.24333	0.00322	4.31952	0.00306	4.47372	0.00309	4.85141	0.00326	5.21498

0.00333	4.50751	0.00326	4.53296	0.00334	4.26229	0.00323	4.33146	0.00307	4.49057	0.00308	4.86472	0.00328	5.23108

0.00333	4.51789	0.00326	4.54493	0.00334	4.27686	0.00324	4.34191	0.00307	4.5059	0.00309	4.88635	0.00329	5.25076

0.00333	4.53716	0.00328	4.56289	0.00333	4.28706	0.00324	4.35982	0.00306	4.51816	0.00311	4.90466	0.00329	5.274

0.00336	4.55347	0.00328	4.58084	0.00334	4.30309	0.00324	4.36878	0.00307	4.53654	0.00311	4.92131	0.00329	5.28473

0.00333	4.56089	0.00328	4.59281	0.00335	4.3235	0.00324	4.38221	0.00308	4.55493	0.00311	4.93463	0.00329	5.30262

0.00336	4.5772	0.00328	4.61076	0.00336	4.33663	0.00326	4.41803	0.00308	4.56872	0.00311	4.95293	0.0033	5.32408

0.00338	4.59647	0.00331	4.62871	0.00335	4.34975	0.00326	4.43296	0.00308	4.57944	0.00313	4.97124	0.00331	5.34912

0.00337	4.60389	0.0033	4.63918	0.00335	4.3614	0.00326	4.44639	0.00309	4.5963	0.00313	4.98622	0.00331	5.35985

0.00338	4.62168	0.00329	4.65414	0.00336	4.38035	0.00326	4.45833	0.0031	4.61315	0.00313	4.99953	0.00331	5.37415

0.00339	4.64096	0.00331	4.67359	0.00336	4.39347	0.00327	4.46878	0.0031	4.62387	0.00313	5.01617	0.00331	5.39204

0.00339	4.65282	0.00332	4.68556	0.00336	4.40514	0.00327	4.48221	0.0031	4.6392	0.00313	5.03947	0.00332	5.41707

0.00338	4.66468	0.00331	4.69753	0.00336	4.42117	0.00327	4.48519	0.00311	4.66218	0.00314	5.05445	0.00333	5.43317

0.0034	4.68247	0.00332	4.71698	0.00337	4.44012	0.00329	4.50161	0.00312	4.67749	0.00315	5.06943	0.00332	5.44569

0.00341	4.6973	0.00334	4.73493	0.00338	4.45178	0.00329	4.52102	0.00311	4.68668	0.00314	5.08607	0.00332	5.46715

0.00341	4.71213	0.00334	4.7469	0.00337	4.46344	0.00328	4.53445	0.00311	4.70507	0.00315	5.10106	0.00333	5.48861

0.00342	4.72844	0.00334	4.76036	0.00337	4.48094	0.00329	4.55982	0.00313	4.72346	0.00316	5.12102	0.00333	5.50829

0.00344	4.74326	0.00336	4.77532	0.00339	4.49843	0.0033	4.58371	0.00313	4.73418	0.00317	5.136	0.00333	5.5208

0.00343	4.75809	0.00335	4.78879	0.00339	4.51301	0.0033	4.59714	0.00313	4.74337	0.00316	5.14764	0.00333	5.53511

0.00344	4.76847	0.00334	4.80075	0.00339	4.5232	0.00329	4.59864	0.00314	4.76024	0.00316	5.16596	0.00334	5.55835

0.00346	4.78775	0.00336	4.81871	0.00339	4.53924	0.0033	4.61207	0.00315	4.78168	0.00317	5.18592	0.00334	5.57803

0.00346	4.80257	0.00338	4.84115	0.0034	4.55819	0.00332	4.6255	0.00315	4.7924	0.00318	5.20257	0.00334	5.59055

0.00344	4.81444	0.00338	4.85012	0.00341	4.56986	0.00333	4.6449	0.00315	4.80772	0.00317	5.21755	0.00334	5.60665

0.00346	4.83075	0.00337	4.8606	0.0034	4.58006	0.00331	4.64788	0.00315	4.82918	0.00318	5.23419	0.00334	5.62632

0.00347	4.84854	0.00339	4.87556	0.00341	4.59755	0.00332	4.67923	0.00316	4.84603	0.00319	5.25583	0.00336	5.64778

0.00347	4.85892	0.00339	4.89351	0.00343	4.6165	0.00334	4.70013	0.00316	4.85828	0.00319	5.2708	0.00336	5.66746

0.00348	4.87226	0.00339	4.90548	0.00341	4.62525	0.00334	4.71953	0.00316	4.86901	0.00319	5.28412	0.00335	5.68176

0.0035	4.89747	0.00339	4.91744	0.00342	4.63982	0.00333	4.71953	0.00318	4.88586	0.00319	5.29743	0.00335	5.69786

0.0035	4.9034	0.00341	4.94587	0.00344	4.65732	0.00334	4.73147	0.00318	4.90271	0.0032	5.31908	0.00337	5.72289

0.00349	4.9123	0.00341	4.95784	0.00345	4.67335	0.00335	4.75237	0.00318	4.9119	0.0032	5.33572	0.00337	5.73898

0.00351	4.93602	0.00341	4.96681	0.00344	4.68501	0.00335	4.75983	0.00318	4.92876	0.00321	5.34903	0.00337	5.75151

0.00351	4.95381	0.00342	4.98776	0.00344	4.69668	0.00335	4.76878	0.0032	4.95022	0.0032	5.36566	0.00338	5.76939

0.00351	4.95826	0.00343	4.99973	0.00346	4.71708	0.00335	4.78222	0.0032	4.964	0.00321	5.38398	0.00337	5.78906

0.00352	4.9805	0.00343	5.01469	0.00346	4.7302	0.00336	4.81506	0.00319	4.97932	0.00322	5.40562	0.0034	5.81052

0.00354	4.99533	0.00343	5.02516	0.00345	4.74187	0.00337	4.82699	0.00319	4.99311	0.00322	5.42392	0.0034	5.82483

0.00354	5.00571	0.00345	5.04461	0.00345	4.75789	0.00337	4.84341	0.00321	5.01149	0.00321	5.43391	0.00338	5.84093

0.00354	5.0235	0.00346	5.05807	0.00347	4.77538	0.00338	4.85983	0.00321	5.02529	0.00322	5.44889	0.0034	5.86239

0.00355	5.04278	0.00345	5.06854	0.00348	4.79142	0.00338	4.87177	0.00321	5.03601	0.00323	5.47219	0.0034	5.88206

0.00356	5.05612	0.00345	5.0865	0.00346	4.80018	0.00338	4.87327	0.00321	5.05439	0.00325	5.48717	0.00341	5.89994

0.00355	5.0665	0.00348	5.10894	0.00347	4.81912	0.00339	4.88968	0.00322	5.06972	0.00323	5.49548	0.0034	5.91247

0.00358	5.08726	0.00347	5.1209	0.00348	4.83515	0.0034	4.91655	0.00322	5.08351	0.00325	5.51546	0.0034	5.93035

0.00358	5.10505	0.00346	5.12988	0.00348	4.84827	0.0034	4.92699	0.00322	5.09882	0.00326	5.53876	0.00341	5.95181

0.00358	5.1095	0.00349	5.15232	0.00348	4.85703	0.00339	4.94192	0.00323	5.12027	0.00326	5.55541	0.00342	5.97148

0.00358	5.12877	0.00349	5.16578	0.00348	4.87452	0.00339	4.96581	0.00324	5.13866	0.00326	5.57039	0.00341	5.98578

0.00359	5.14657	0.00348	5.17177	0.00349	4.89346	0.00341	4.98073	0.00323	5.14938	0.00326	5.5837	0.00342	6.00188

0.0036	5.15695	0.00348	5.19271	0.0035	4.90804	0.00341	4.99118	0.00323	5.16164	0.00327	5.602	0.00342	6.02692

0.00359	5.17029	0.00351	5.21067	0.00349	4.91679	0.0034	5.00312	0.00325	5.18156	0.00327	5.62198	0.00343	6.04659

0.00363	5.18957	0.00351	5.22413	0.00349	4.93428	0.00341	5.01804	0.00325	5.19381	0.00328	5.63196	0.00343	6.0609

0.00363	5.20439	0.0035	5.2361	0.00351	4.95323	0.00343	5.02999	0.00325	5.20454	0.00326	5.64527	0.00342	6.077

0.00362	5.21477	0.00351	5.25405	0.00351	4.96781	0.00343	5.04789	0.00325	5.21986	0.00328	5.66692	0.00343	6.09489

0.00363	5.23405	0.00353	5.272	0.00351	4.97801	0.00342	5.05387	0.00327	5.24285	0.00328	5.68687	0.00344	6.11455

0.00364	5.24888	0.00353	5.27948	0.00351	4.99404	0.00343	5.0673	0.00328	5.25663	0.00328	5.70186	0.00344	6.13422

0.00363	5.25926	0.00353	5.29295	0.00352	5.01007	0.00344	5.09267	0.00326	5.27041	0.00328	5.71517	0.00344	6.14674

0.00364	5.27557	0.00355	5.31539	0.00352	5.02175	0.00343	5.11357	0.00327	5.28727	0.00328	5.73181	0.00344	6.16641

0.00366	5.29336	0.00355	5.33035	0.00352	5.0334	0.00343	5.12999	0.00329	5.30566	0.0033	5.75512	0.00346	6.18788

0.00367	5.3067	0.00354	5.34381	0.00352	5.05235	0.00344	5.13894	0.00329	5.31484	0.0033	5.76843	0.00345	6.20398

0.00367	5.31708	0.00355	5.35728	0.00354	5.06984	0.00345	5.15387	0.00328	5.32558	0.0033	5.7834	0.00345	6.21649

0.00368	5.34081	0.00356	5.37224	0.00353	5.08151	0.00345	5.16581	0.0033	5.34702	0.0033	5.79839	0.00345	6.23438

0.00368	5.35563	0.00356	5.3842	0.00353	5.09463	0.00345	5.17178	0.00331	5.36235	0.00331	5.81836	0.00346	6.25763

0.00368	5.36305	0.00356	5.39468	0.00354	5.11211	0.00346	5.19715	0.0033	5.37306	0.00331	5.83666	0.00347	6.27372

0.00369	5.38232	0.00357	5.41712	0.00355	5.12669	0.00346	5.21805	0.0033	5.39145	0.00331	5.85331	0.00347	6.29339

0.00371	5.40011	0.00358	5.42759	0.00355	5.1369	0.00346	5.22999	0.00331	5.41443	0.00332	5.86829	0.00347	6.30591

0.0037	5.40605	0.00358	5.44554	0.00355	5.15148	0.00347	5.24342	0.00332	5.42975	0.00332	5.88494	0.00347	6.32737

0.00371	5.4268	0.00358	5.462	0.00356	5.16897	0.00348	5.26731	0.00331	5.43588	0.00333	5.9049	0.00348	6.34883

0.00373	5.44608	0.0036	5.47995	0.00357	5.18646	0.00348	5.27776	0.00332	5.45273	0.00332	5.91988	0.00348	6.36493

0.00372	5.45498	0.0036	5.48743	0.00356	5.19666	0.00347	5.28074	0.00333	5.46959	0.00332	5.93152	0.00347	6.38102

0.00371	5.46832	0.0036	5.5009	0.00356	5.20978	0.00348	5.29417	0.00333	5.48337	0.00333	5.94651	0.00348	6.4007

0.00375	5.48908	0.00362	5.52034	0.00359	5.23018	0.00349	5.31209	0.00333	5.4941	0.00334	5.96815	0.00349	6.42216

0.00375	5.50242	0.00362	5.53381	0.00359	5.24331	0.00349	5.32403	0.00334	5.51095	0.00334	5.98479	0.00349	6.44005

0.00375	5.51132	0.00362	5.54877	0.00359	5.25789	0.00348	5.33746	0.00335	5.53241	0.00335	5.99644	0.00348	6.44899

0.00376	5.52911	0.00363	5.56522	0.00359	5.26955	0.00349	5.36731	0.00335	5.54619	0.00335	6.01641	0.00348	6.46865

0.00376	5.54542	0.00363	5.58318	0.0036	5.28849	0.00351	5.38373	0.00334	5.56304	0.00336	6.03805	0.00349	6.49191

0.00376	5.55432	0.00363	5.59963	0.0036	5.30307	0.0035	5.40015	0.00335	5.57837	0.00337	6.05469	0.00352	6.51157

0.00378	5.57211	0.00363	5.61011	0.0036	5.31182	0.0035	5.40164	0.00336	5.59369	0.00336	6.06468	0.00352	6.52768

0.00378	5.59139	0.00364	5.63105	0.0036	5.3264	0.00351	5.42403	0.00336	5.60441	0.00336	6.08299	0.0035	6.54198

0.00379	5.60177	0.00365	5.64302	0.00361	5.34681	0.00351	5.43299	0.00336	5.61821	0.00337	6.10296	0.00352	6.56165

0.00379	5.61659	0.00365	5.6505	0.00361	5.36139	0.00351	5.44791	0.00337	5.63506	0.00338	6.11793	0.00352	6.5849

0.00381	5.63439	0.00365	5.66396	0.00361	5.37304	0.00351	5.45238	0.00338	5.65497	0.00338	6.13458	0.00353	6.60279

0.00381	5.64625	0.00367	5.69089	0.0036	5.38617	0.00352	5.47776	0.00338	5.6657	0.00338	6.14789	0.00352	6.61531

0.0038	5.65811	0.00367	5.70286	0.00362	5.40802	0.00353	5.50314	0.00337	5.67949	0.00338	6.16619	0.00353	6.62961

0.00381	5.67739	0.00367	5.71483	0.00362	5.4197	0.00353	5.50762	0.00338	5.70401	0.00339	6.18617	0.00354	6.65465

0.00383	5.69666	0.00368	5.73128	0.00362	5.43135	0.00354	5.52552	0.00338	5.71932	0.00339	6.20615	0.00354	6.67074

0.00383	5.70408	0.00369	5.74924	0.00362	5.44447	0.00354	5.54194	0.00338	5.72852	0.00338	6.21779	0.00353	6.68506

0.00383	5.72187	0.00368	5.75672	0.00364	5.46488	0.00354	5.55686	0.00338	5.74077	0.00339	6.23277	0.00353	6.70294

0.00385	5.73818	0.00368	5.77317	0.00364	5.47509	0.00354	5.56284	0.00339	5.76222	0.00341	6.2544	0.00354	6.72082

0.00384	5.75301	0.00371	5.79561	0.00364	5.4882	0.00354	5.57328	0.00341	5.77449	0.00341	6.26772	0.00355	6.74586

0.00384	5.7619	0.00371	5.80608	0.00364	5.50569	0.00355	5.5897	0.00339	5.78367	0.0034	6.27936	0.00355	6.76017

0.00386	5.77969	0.0037	5.81506	0.00365	5.52464	0.00356	5.61657	0.0034	5.80052	0.00341	6.29601	0.00354	6.77447

0.00387	5.80045	0.0037	5.8375	0.00364	5.53484	0.00356	5.62254	0.00342	5.82504	0.00342	6.31765	0.00354	6.79236

0.00386	5.8049	0.00372	5.85545	0.00365	5.54797	0.00355	5.64195	0.00341	5.8373	0.00343	6.33595	0.00356	6.81382

0.00386	5.82418	0.00371	5.86144	0.00366	5.56546	0.00357	5.67031	0.00341	5.85261	0.00342	6.34926	0.00357	6.8317

0.00388	5.84049	0.00372	5.87789	0.00366	5.5815	0.00358	5.67627	0.00342	5.871	0.00342	6.36591	0.00356	6.8478

0.00388	5.8568	0.00374	5.89734	0.00366	5.59169	0.00357	5.68523	0.00342	5.8848	0.00343	6.38422	0.00356	6.8621

0.00389	5.86569	0.00374	5.90632	0.00366	5.60482	0.00357	5.69568	0.00342	5.89399	0.00344	6.40086	0.00357	6.88714

0.00391	5.88497	0.00374	5.91829	0.00367	5.62376	0.00359	5.71658	0.00342	5.90624	0.00344	6.4175	0.00358	6.90681

0.00391	5.90128	0.00374	5.93774	0.00368	5.63835	0.00359	5.72702	0.00344	5.92615	0.00343	6.42915	0.00357	6.91933

0.0039	5.91018	0.00376	5.95718	0.00368	5.65	0.00358	5.74344	0.00345	5.94301	0.00345	6.44746	0.00357	6.93364

0.00392	5.92649	0.00375	5.97065	0.00368	5.66458	0.00359	5.76583	0.00343	5.95221	0.00345	6.47075	0.00358	6.95689

0.00393	5.94873	0.00375	5.98411	0.00369	5.68499	0.0036	5.78673	0.00344	5.97365	0.00345	6.48407	0.00358	6.97657

0.00392	5.95317	0.00376	6.00506	0.0037	5.70102	0.0036	5.80016	0.00345	5.99664	0.00345	6.49739	0.00359	6.99266

0.00392	5.97097	0.00377	6.02002	0.0037	5.70977	0.00359	5.8121	0.00345	6.01195	0.00346	6.51736	0.00359	7.00697

0.00395	5.99024	0.00377	6.02301	0.0037	5.72435	0.00361	5.81956	0.00345	6.01961	0.00347	6.53734	0.00359	7.02843

0.00395	6.0021	0.00378	6.04395	0.0037	5.74184	0.00361	5.84045	0.00346	6.03187	0.00348	6.55397	0.0036	7.0481

0.00395	6.01693	0.0038	6.0634	0.00371	5.75642	0.00361	5.84792	0.00347	6.05025	0.00346	6.56397	0.0036	7.06598

0.00397	6.03621	0.0038	6.07387	0.0037	5.76662	0.00361	5.85687	0.00347	6.06404	0.00347	6.5806	0.0036	7.08029

0.00397	6.04955	0.0038	6.08883	0.00371	5.78266	0.00362	5.88374	0.00347	6.07477	0.00348	6.59891	0.0036	7.0946

0.00397	6.05993	0.00381	6.10828	0.00372	5.80161	0.00363	5.89866	0.00347	6.09315	0.00348	6.61722	0.0036	7.11963

0.00398	6.08069	0.00382	6.12324	0.00372	5.81472	0.00363	5.91508	0.00349	6.11614	0.00348	6.63054	0.00361	7.13931

0.00399	6.09552	0.00381	6.12923	0.00372	5.82638	0.00362	5.92404	0.00348	6.12839	0.00348	6.64718	0.0036	7.15183

0.00398	6.10293	0.00381	6.14419	0.00372	5.84242	0.00364	5.94643	0.00348	6.14065	0.00349	6.66715	0.00361	7.16793

0.004	6.11776	0.00382	6.16513	0.00373	5.85991	0.00364	5.96135	0.00349	6.15904	0.0035	6.68878	0.00362	7.19117

0.00401	6.14	0.00383	6.18009	0.00373	5.86866	0.00364	5.96583	0.0035	6.17589	0.0035	6.70044	0.00362	7.20905

0.00401	6.15186	0.00382	6.18907	0.00372	5.88323	0.00363	5.97777	0.00349	6.18202	0.0035	6.71375	0.00362	7.22337

0.00401	6.16224	0.00383	6.20702	0.00374	5.90072	0.00365	6.00016	0.0035	6.19581	0.0035	6.73039	0.00361	7.24304

0.00403	6.18003	0.00385	6.22647	0.00374	5.91676	0.00366	6.01658	0.00352	6.22032	0.00351	6.75369	0.00362	7.25913

0.00404	6.20227	0.00384	6.23395	0.00374	5.92842	0.00365	6.02853	0.00352	6.23411	0.00351	6.76534	0.00365	7.28416

0.00403	6.20672	0.00385	6.2534	0.00374	5.943	0.00365	6.04643	0.00352	6.24484	0.0035	6.77699	0.00365	7.29669

0.00404	6.22303	0.00386	6.27135	0.00375	5.96195	0.00366	6.07032	0.00352	6.26169	0.00352	6.79697	0.00364	7.30921

0.00405	6.24379	0.00387	6.2893	0.00376	5.97799	0.00367	6.08076	0.00354	6.28467	0.00352	6.81859	0.00365	7.32888

0.00405	6.25417	0.00386	6.29828	0.00376	5.98964	0.00367	6.08375	0.00354	6.29999	0.00352	6.83857	0.00365	7.35213

0.00405	6.269	0.00387	6.31623	0.00376	6.00277	0.00367	6.10613	0.00353	6.30612	0.00351	6.85022	0.00365	7.3718

0.00407	6.28679	0.00388	6.33568	0.00377	6.02172	0.00369	6.11808	0.00353	6.32298	0.00351	6.86687	0.00365	7.38432

0.00408	6.30013	0.00387	6.34166	0.00377	6.03484	0.00368	6.12853	0.00355	6.34442	0.00352	6.88517	0.00366	7.40041

0.00407	6.30903	0.00387	6.35513	0.00376	6.04503	0.00367	6.14196	0.00355	6.35208	0.00353	6.90181	0.00367	7.42546

0.00408	6.33127	0.0039	6.37607	0.00376	6.05961	0.00368	6.16136	0.00355	6.36281	0.00353	6.91345	0.00367	7.44512

0.0041	6.34758	0.0039	6.39103	0.00378	6.07856	0.00369	6.18524	0.00355	6.38425	0.00352	6.92844	0.00366	7.45943

0.00408	6.355	0.00389	6.39552	0.00378	6.09315	0.00369	6.19867	0.00356	6.40417	0.00353	6.9534	0.00367	7.47553

0.00409	6.37131	0.00389	6.41048	0.00378	6.10188	0.00369	6.20763	0.00355	6.41643	0.00354	6.96838	0.00367	7.49163

0.00412	6.39355	0.00391	6.43591	0.00378	6.12084	0.0037	6.23002	0.00355	6.43634	0.00354	6.98336	0.00368	7.51309

0.00412	6.40244	0.00392	6.44638	0.0038	6.13979	0.0037	6.24345	0.00356	6.45167	0.00354	7.00167	0.00368	7.53097

0.00413	6.4143	0.00392	6.45835	0.0038	6.1529	0.0037	6.2539	0.00357	6.46392	0.00355	7.01665	0.00367	7.54349

0.00413	6.43655	0.00393	6.4793	0.00379	6.16311	0.0037	6.26286	0.00356	6.47618	0.00357	7.03496	0.00368	7.56137

0.00414	6.44692	0.00394	6.49426	0.0038	6.1806	0.00372	6.27629	0.00357	6.48844	0.00357	7.05492	0.00369	7.58641

0.00413	6.45879	0.00393	6.50623	0.00381	6.19664	0.00372	6.30166	0.00358	6.51295	0.00357	7.06491	0.00369	7.6025

0.00415	6.47954	0.00394	6.51969	0.00381	6.20684	0.00372	6.3136	0.00359	6.52521	0.00357	7.08155	0.00368	7.61682

0.00416	6.49585	0.00396	6.53764	0.0038	6.21996	0.00372	6.33301	0.00358	6.53594	0.00358	7.10486	0.00369	7.63648

0.00415	6.50623	0.00396	6.55111	0.00382	6.23745	0.00373	6.34942	0.00358	6.55584	0.00358	7.11651	0.0037	7.65616

0.00416	6.51958	0.00396	6.56457	0.00382	6.25641	0.00373	6.36286	0.0036	6.58037	0.00358	7.13149	0.0037	7.67762

0.00418	6.53885	0.00397	6.5915	0.00382	6.2666	0.00372	6.36435	0.0036	6.59416	0.00358	7.14812	0.0037	7.68835

0.00417	6.54923	0.00398	6.60496	0.00382	6.27972	0.00372	6.38077	0.00359	6.60181	0.00359	7.17143	0.0037	7.70623

0.00418	6.56109	0.00398	6.62591	0.00383	6.29867	0.00374	6.39719	0.0036	6.61713	0.00359	7.18641	0.00371	7.72769

0.0042	6.58037	0.00396	6.61843	0.00384	6.31471	0.00374	6.41361	0.00361	6.63399	0.00361	7.20139	0.00372	7.74558

0.00421	6.5952	0.00398	6.65134	0.00384	6.32637	0.00373	6.43152	0.00361	6.64625	0.0036	7.21137	0.00372	7.76346

0.0042	6.60706	0.00399	6.66032	0.00384	6.33803	0.00375	6.44495	0.00361	6.65697	0.00362	7.23467	0.00371	7.77956

0.00421	6.62189	0.00398	6.6663	0.00385	6.35552	0.00376	6.4763	0.00362	6.67842	0.00362	7.24965	0.00372	7.79923

0.00421	6.64413	0.00399	6.68874	0.00385	6.3701	0.00376	6.48226	0.00363	6.69527	0.00362	7.2663	0.00373	7.8207

0.00421	6.65451	0.00401	6.71567	0.00384	6.38176	0.00375	6.49122	0.00362	6.70753	0.00362	7.28127	0.00373	7.83857

0.00421	6.66489	0.004	6.71268	0.00385	6.3978	0.00376	6.50466	0.00363	6.72592	0.00362	7.29957	0.00372	7.8493

0.00424	6.68713	0.00401	6.73961	0.00386	6.41675	0.00377	6.52108	0.00364	6.74582	0.00363	7.31955	0.00372	7.86719

0.00424	6.69602	0.00403	6.75008	0.00386	6.43133	0.00377	6.53301	0.00364	6.75655	0.00363	7.33786	0.00374	7.89222

0.00424	6.71233	0.00403	6.76803	0.00385	6.44006	0.00377	6.54495	0.00363	6.76575	0.00363	7.34784	0.00374	7.90833

0.00426	6.73161	0.00403	6.77551	0.00385	6.45611	0.00377	6.57032	0.00365	6.78107	0.00363	7.36449	0.00373	7.92442

0.00426	6.74495	0.00403	6.79646	0.00387	6.47505	0.00378	6.58525	0.00365	6.80252	0.00364	7.38779	0.00374	7.94409

0.00425	6.75237	0.00404	6.81142	0.00387	6.48818	0.00378	6.59868	0.00366	6.81784	0.00364	7.39944	0.00374	7.96198

0.00427	6.77313	0.00404	6.82189	0.00387	6.49837	0.00378	6.6151	0.00365	6.82703	0.00364	7.41275	0.00376	7.98165

0.00428	6.78795	0.00404	6.83984	0.00387	6.51295	0.00379	6.63301	0.00365	6.84847	0.00364	7.42939	0.00376	7.99953

0.00428	6.8013	0.00405	6.85779	0.00389	6.5319	0.0038	6.64794	0.00366	6.8684	0.00365	7.45269	0.00374	8.01205

0.00428	6.81613	0.00404	6.87425	0.00389	6.54648	0.0038	6.65242	0.00367	6.88371	0.00366	7.471	0.00376	8.02993

0.00429	6.83688	0.00405	6.87724	0.00388	6.55814	0.00379	6.66735	0.00366	6.89444	0.00365	7.48432	0.00377	8.05497

0.00431	6.84578	0.00406	6.89071	0.00389	6.57563	0.00381	6.68526	0.00367	6.90823	0.00365	7.49929	0.00377	8.06928

0.00429	6.85912	0.00408	6.92063	0.0039	6.59313	0.00381	6.70167	0.00368	6.92815	0.00366	7.51927	0.00376	8.08537

0.00432	6.87988	0.00407	6.92511	0.0039	6.60624	0.00381	6.71212	0.00368	6.93428	0.00366	7.53924	0.00376	8.09969

0.00432	6.89619	0.00407	6.94007	0.00389	6.61645	0.00381	6.736	0.00368	6.94807	0.00367	7.54756	0.00378	8.12472

0.00432	6.90509	0.0041	6.95952	0.00391	6.63394	0.00383	6.75242	0.00369	6.97105	0.00367	7.56253	0.00379	8.14439

0.00433	6.91695	0.0041	6.97448	0.00391	6.64998	0.00383	6.76585	0.00369	6.9879	0.00367	7.58417	0.00378	8.15691

0.00434	6.94067	0.00409	6.98346	0.00391	6.66018	0.00382	6.77332	0.00368	7.00169	0.00368	7.60081	0.00378	8.173

0.00433	6.94957	0.0041	7.01039	0.0039	6.67476	0.00382	6.78675	0.00369	7.01701	0.00368	7.61746	0.00379	8.19446

0.00435	6.95995	0.00411	7.02086	0.00392	6.6937	0.00384	6.81063	0.0037	7.03693	0.00368	7.63077	0.0038	8.21592

0.00437	6.98367	0.00411	7.03732	0.00392	6.7112	0.00384	6.8181	0.0037	7.05224	0.00368	7.6524	0.0038	8.22845

0.00437	6.9985	0.0041	7.0418	0.00392	6.72286	0.00383	6.82705	0.0037	7.05838	0.00369	7.67404	0.0038	8.24454

0.00437	7.00888	0.00413	7.06574	0.00392	6.73452	0.00384	6.85242	0.0037	7.07369	0.0037	7.68902	0.0038	8.26422

0.00438	7.02519	0.00413	7.0822	0.00394	6.75055	0.00385	6.87033	0.00372	7.09362	0.00369	7.69734	0.00381	8.28568

0.00439	7.04447	0.00413	7.09267	0.00394	6.76659	0.00385	6.88227	0.00372	7.10893	0.00369	7.71565	0.00381	8.30356

0.00438	7.05485	0.00413	7.10314	0.00394	6.77825	0.00385	6.89869	0.00371	7.12119	0.0037	7.73562	0.00381	8.31787

0.00438	7.06967	0.00415	7.13007	0.00394	6.79429	0.00386	6.91212	0.00371	7.14264	0.00371	7.75227	0.0038	8.33933

0.0044	7.08598	0.00416	7.13755	0.00396	6.81032	0.00386	6.93302	0.00373	7.16103	0.00371	7.76392	0.00381	8.35722

0.00441	7.09636	0.00414	7.14952	0.00396	6.82344	0.00386	6.939	0.00373	7.17328	0.00371	7.7789	0.00382	8.37688

0.0044	7.10822	0.00415	7.16448	0.00395	6.83801	0.00386	6.95392	0.00372	7.18401	0.00372	7.80219	0.00382	8.38941

0.00442	7.1275	0.00416	7.18841	0.00395	6.85114	0.00387	6.96586	0.00372	7.19933	0.00374	7.82383	0.00382	8.40907

0.00442	7.14233	0.00417	7.19889	0.00396	6.86863	0.00388	6.98675	0.00374	7.21771	0.00373	7.83714	0.00382	8.42696

0.00442	7.15419	0.00416	7.20786	0.00397	6.88466	0.00388	7.00317	0.00373	7.22997	0.00374	7.85047	0.00383	8.44842

0.00442	7.17346	0.00417	7.2333	0.00396	6.89633	0.00387	7.01511	0.00373	7.23917	0.00373	7.86877	0.00383	8.46272

0.00445	7.19126	0.00419	7.24377	0.00397	6.9109	0.00388	7.02855	0.00375	7.26368	0.00374	7.88208	0.00383	8.48061

0.00444	7.19867	0.00417	7.25274	0.00397	6.92694	0.00389	7.04944	0.00375	7.28206	0.00375	7.90206	0.00383	8.4985

0.00444	7.21646	0.00418	7.2677	0.00397	6.94297	0.00389	7.05691	0.00374	7.29278	0.00375	7.91204	0.00384	8.51996

0.00446	7.23574	0.00421	7.28566	0.00397	6.95318	0.00389	7.07184	0.00374	7.30964	0.00375	7.93201	0.00384	8.53784

0.00446	7.24464	0.00421	7.30062	0.00397	6.96776	0.00391	7.08974	0.00376	7.33109	0.00376	7.95531	0.00384	8.55036

0.00446	7.25798	0.0042	7.31259	0.00398	6.9867	0.00391	7.10467	0.00376	7.34028	0.00376	7.97029	0.00384	8.56824

0.00448	7.2817	0.00421	7.33503	0.00398	7.00273	0.0039	7.12109	0.00375	7.34641	0.00376	7.98194	0.00385	8.59328

0.00448	7.29505	0.00422	7.35148	0.00397	7.01295	0.00391	7.13452	0.00375	7.3648	0.00376	8.00191	0.00386	8.61116

0.00448	7.30246	0.00422	7.35896	0.00399	7.02752	0.00392	7.1584	0.00378	7.38471	0.00377	8.02021	0.00385	8.62189

0.00448	7.3247	0.00421	7.37691	0.00401	7.04793	0.00392	7.16586	0.00378	7.3985	0.00377	8.03686	0.00385	8.63978

0.00451	7.34101	0.00422	7.39935	0.004	7.05813	0.00391	7.1778	0.00377	7.41076	0.00377	8.04851	0.00386	8.66303

0.0045	7.34694	0.00422	7.40983	0.004	7.0698	0.00392	7.19571	0.00379	7.43068	0.00377	8.06515	0.00387	8.68449

0.0045	7.36325	0.00423	7.4203	0.004	7.08583	0.00394	7.21064	0.00379	7.45213	0.00378	8.08679	0.00386	8.69701

0.00454	7.38253	0.00424	7.43676	0.00401	7.10332	0.00393	7.22258	0.00379	7.46438	0.00379	8.10176	0.00386	8.71132

0.00452	7.39143	0.00426	7.44872	0.00401	7.11498	0.00393	7.23005	0.00378	7.47664	0.00379	8.11507	0.00387	8.73278

0.00453	7.40774	0.00424	7.46069	0.00401	7.12956	0.00393	7.25243	0.00379	7.49196	0.00378	8.13505	0.00387	8.75066

0.00454	7.42405	0.00426	7.48014	0.00403	7.1485	0.00395	7.27333	0.0038	7.50728	0.00379	8.15502	0.00387	8.76855

0.00455	7.44184	0.00428	7.50108	0.00403	7.16163	0.00394	7.28527	0.0038	7.518	0.0038	8.17333	0.00387	8.78285

0.00454	7.45073	0.00427	7.51006	0.00403	7.17475	0.00394	7.30318	0.0038	7.53485	0.00381	8.18497	0.00387	8.80431

0.00455	7.46705	0.00427	7.52053	0.00402	7.18641	0.00395	7.31512	0.00381	7.55324	0.0038	8.19829	0.0039	8.82756

0.00457	7.4878	0.00428	7.53849	0.00404	7.20681	0.00396	7.33005	0.00382	7.57317	0.00381	8.21327	0.0039	8.84544

0.00457	7.49522	0.00429	7.55943	0.00404	7.22139	0.00397	7.34348	0.00381	7.58848	0.00381	8.23492	0.00389	8.85618

0.00457	7.51301	0.00428	7.56841	0.00404	7.2316	0.00396	7.35393	0.00381	7.60074	0.00383	8.25321	0.00389	8.87585

0.00458	7.52784	0.00428	7.58486	0.00405	7.24472	0.00396	7.37333	0.00382	7.62066	0.00381	8.26653	0.0039	8.89731

0.00458	7.54563	0.00431	7.60581	0.00405	7.26512	0.00397	7.38676	0.00383	7.63445	0.00381	8.28484	0.00391	8.91698

0.00458	7.55304	0.00431	7.61628	0.00406	7.27825	0.00397	7.40468	0.00382	7.64057	0.00383	8.30482	0.0039	8.9295

0.00459	7.57677	0.00429	7.62675	0.00405	7.28991	0.00398	7.41811	0.00383	7.65283	0.00383	8.31979	0.0039	8.94559

0.00462	7.59011	0.00431	7.6462	0.00405	7.30449	0.00398	7.43751	0.00384	7.67735	0.00383	8.33643	0.00392	8.96705

0.0046	7.60049	0.00432	7.66415	0.00407	7.32489	0.00398	7.44796	0.00384	7.6896	0.00383	8.34974	0.00392	8.98851

0.00462	7.61532	0.00432	7.67313	0.00407	7.33801	0.00398	7.45841	0.00384	7.70033	0.00385	8.36805	0.00392	9.0064

0.00463	7.63608	0.00432	7.6851	0.00406	7.34821	0.00399	7.47185	0.00384	7.72331	0.00385	8.38304	0.00392	9.01892

0.00463	7.64794	0.00433	7.70903	0.00407	7.36424	0.004	7.49274	0.00386	7.74322	0.00385	8.39801	0.00393	9.03681

0.00463	7.6598	0.00434	7.7195	0.00408	7.38174	0.004	7.50916	0.00385	7.75548	0.00385	8.41299	0.00393	9.06005

0.00464	7.67908	0.00434	7.72998	0.00408	7.39194	0.00399	7.51812	0.00385	7.76774	0.00385	8.43628	0.00393	9.07794

0.00465	7.69094	0.00434	7.75242	0.00408	7.40652	0.004	7.53454	0.00386	7.78766	0.00386	8.45626	0.00393	9.09046

0.00464	7.70428	0.00436	7.76439	0.00408	7.42255	0.00401	7.55692	0.00386	7.79991	0.00386	8.4729	0.00393	9.10476

0.00466	7.72504	0.00436	7.77785	0.00409	7.44005	0.00402	7.57334	0.00385	7.8091	0.00386	8.48122	0.00394	9.13338

0.00467	7.73839	0.00435	7.79281	0.00409	7.45171	0.00401	7.58378	0.00386	7.82596	0.00387	8.49953	0.00394	9.14768

0.00467	7.74728	0.00436	7.80927	0.00408	7.46629	0.00402	7.59722	0.00388	7.84587	0.00387	8.52117	0.00394	9.1602

0.00467	7.76359	0.00437	7.82572	0.00409	7.48232	0.00403	7.61513	0.00388	7.8612	0.00388	8.53615	0.00394	9.18166

0.00468	7.7799	0.00436	7.83769	0.00411	7.49836	0.00403	7.62558	0.00387	7.86886	0.00387	8.5478	0.00395	9.20134

0.00469	7.79473	0.00438	7.85564	0.00409	7.51001	0.00403	7.63454	0.00388	7.89185	0.00388	8.5661	0.00396	9.2228

0.00469	7.80659	0.00439	7.87659	0.00409	7.52314	0.00403	7.65543	0.00388	7.91329	0.00388	8.58774	0.00395	9.23353

0.00471	7.82735	0.00439	7.88556	0.00411	7.54209	0.00404	7.67334	0.00388	7.92248	0.00389	8.60438	0.00395	9.24784

0.00471	7.83921	0.00439	7.89903	0.00413	7.55667	0.00404	7.68827	0.00388	7.9332	0.00388	8.61603	0.00396	9.27287

0.00471	7.85107	0.00439	7.91698	0.00411	7.56832	0.00404	7.70468	0.00389	7.94853	0.00388	8.636	0.00397	9.29255

0.00471	7.8659	0.00441	7.93194	0.00411	7.57998	0.00405	7.71961	0.0039	7.96691	0.00389	8.65764	0.00397	9.30864

0.00473	7.88666	0.0044	7.94241	0.00413	7.59893	0.00406	7.73603	0.0039	7.97916	0.00389	8.67262	0.00396	9.31937

0.00473	7.89555	0.0044	7.96037	0.00413	7.61498	0.00405	7.7435	0.00389	7.99142	0.00389	8.6826	0.00397	9.34083

0.00473	7.91187	0.00441	7.98131	0.00413	7.62809	0.00406	7.7614	0.00389	8.00981	0.00389	8.69592	0.00398	9.36229

0.00475	7.93262	0.00443	7.99029	0.00413	7.64121	0.00407	7.77633	0.0039	8.02973	0.00389	8.71589	0.00398	9.38197

0.00475	7.94152	0.00442	8.00525	0.00414	7.65578	0.00407	7.78976	0.0039	8.04198	0.00391	8.73586	0.00397	9.39628

0.00476	7.95338	0.00443	8.0217	0.00414	7.67473	0.00407	7.80021	0.0039	8.0573	0.0039	8.74917	0.00398	9.41237

0.00476	7.97266	0.00444	8.03666	0.00413	7.68348	0.00407	7.81961	0.00392	8.08029	0.00391	8.76748	0.00399	9.43562

0.00477	7.99045	0.00444	8.03965	0.00415	7.70098	0.00408	7.842	0.00392	8.09101	0.00391	8.78912	0.00401	9.4535

0.00476	8.00083	0.00445	8.0621	0.00415	7.71847	0.00409	7.85544	0.00392	8.1002	0.00391	8.80576	0.00399	9.46602

0.00479	8.0201	0.00445	8.08304	0.00415	7.73012	0.00407	7.85992	0.00391	8.11093	0.00391	8.81907	0.00399	9.48212

0.00479	8.03641	0.00445	8.09052	0.00415	7.74179	0.00408	7.87484	0.00393	8.13697	0.00391	8.83239	0.00401	9.50358

0.00479	8.04828	0.00445	8.10398	0.00416	7.75928	0.0041	7.89573	0.00393	8.14924	0.00392	8.8457	0.00401	9.52504

0.00479	8.05717	0.00446	8.12493	0.00417	7.77678	0.0041	7.91215	0.00392	8.15842	0.00393	8.86901	0.00401	9.54292

0.0048	8.07941	0.00447	8.14288	0.00417	7.78843	0.0041	7.91813	0.00393	8.17988	0.00393	8.88399	0.00401	9.55544

0.00482	8.09276	0.00447	8.15186	0.00417	7.80155	0.0041	7.93902	0.00395	8.20132	0.00392	8.89564	0.00402	9.5769

0.00481	8.10462	0.00448	8.16831	0.00417	7.81758	0.00412	7.95693	0.00394	8.21664	0.00393	8.9156	0.00403	9.59657

0.00482	8.12241	0.00448	8.18477	0.00419	7.83217	0.00411	7.97186	0.00394	8.22431	0.00395	8.94057	0.00403	9.61267

0.00483	8.14021	0.00449	8.20272	0.00418	7.84382	0.00411	7.98231	0.00394	8.23962	0.00395	8.95554	0.00402	9.62519

0.00483	8.1491	0.0045	8.20422	0.00417	7.85694	0.00412	8.0032	0.00396	8.25801	0.00395	8.9672	0.00402	9.64665

0.00483	8.1669	0.0045	8.22965	0.00419	7.87589	0.00413	8.01663	0.00396	8.26874	0.00395	8.98218	0.00404	9.6699

0.00485	8.18321	0.00451	8.25059	0.0042	7.89193	0.00412	8.0241	0.00395	8.27945	0.00397	9.00381	0.00404	9.6842

0.00485	8.19358	0.00451	8.25658	0.00419	7.90213	0.00412	8.04052	0.00397	8.30091	0.00396	9.02045	0.00403	9.7003

0.00485	8.20841	0.00451	8.27004	0.00419	7.91671	0.00413	8.05842	0.00397	8.31623	0.00395	9.03211	0.00403	9.71819

0.00487	8.22769	0.00452	8.29248	0.00421	7.93712	0.00414	8.07634	0.00396	8.33155	0.00395	9.04875	0.00405	9.73786

0.00488	8.23955	0.00453	8.30595	0.00421	7.94878	0.00413	8.08827	0.00396	8.35147	0.00397	9.06872	0.00405	9.75753

0.00487	8.24993	0.00451	8.31492	0.00421	7.95898	0.00414	8.10768	0.00399	8.36986	0.00397	9.08703	0.00404	9.77184

0.00488	8.27069	0.00453	8.33288	0.00421	7.97356	0.00415	8.11962	0.00399	8.38364	0.00397	9.10367	0.00405	9.78972

0.00489	8.28996	0.00455	8.35382	0.00421	7.99251	0.00416	8.13455	0.00397	8.38976	0.00397	9.11698	0.00406	9.80761

0.00489	8.29589	0.00454	8.3628	0.00422	8.00709	0.00415	8.14649	0.00399	8.40663	0.00399	9.13862	0.00406	9.82907

0.0049	8.3122	0.00455	8.37776	0.00421	8.01729	0.00416	8.15843	0.004	8.42654	0.00399	9.15526	0.00406	9.84516

0.00492	8.33148	0.00456	8.3987	0.00421	8.03478	0.00416	8.18082	0.004	8.44033	0.00399	9.16691	0.00406	9.86126

0.00492	8.34186	0.00457	8.41067	0.00423	8.05373	0.00417	8.19574	0.00399	8.44953	0.00399	9.18022	0.00406	9.87915

0.00492	8.35669	0.00455	8.41665	0.00423	8.06686	0.00416	8.20321	0.004	8.46943	0.00399	9.19854	0.00407	9.89881

0.00492	8.373	0.00457	8.43461	0.00421	8.07705	0.00416	8.22112	0.00401	8.48936	0.00401	9.21851	0.00407	9.91849

0.00493	8.38931	0.00458	8.45705	0.00423	8.09018	0.00417	8.24351	0.00401	8.50467	0.00401	9.23348	0.00407	9.931

0.00493	8.40413	0.00457	8.46303	0.00424	8.10766	0.00418	8.25694	0.00401	8.51694	0.00401	9.25013	0.00407	9.95068

0.00493	8.41599	0.00458	8.48098	0.00424	8.12225	0.00417	8.26441	0.00401	8.53225	0.00401	9.26844	0.00408	9.97035

0.00495	8.4412	0.00458	8.50193	0.00423	8.13245	0.00418	8.27933	0.00402	8.54758	0.00401	9.28675	0.00408	9.98645

0.00495	8.44713	0.0046	8.51539	0.00425	8.1514	0.00419	8.29724	0.00403	8.55984	0.00402	9.30172	0.00408	10.00076

0.00495	8.46048	0.00458	8.52586	0.00425	8.16889	0.00419	8.31515	0.00402	8.57362	0.00401	9.31836	0.00408	10.02043

0.00497	8.4842	0.00459	8.54232	0.00425	8.1791	0.00419	8.32559	0.00403	8.59201	0.00402	9.33167	0.00409	10.04189

0.00497	8.49161	0.00461	8.55728	0.00425	8.19076	0.0042	8.34351	0.00403	8.60732	0.00403	9.34998	0.0041	10.06156

0.00497	8.50199	0.00462	8.57374	0.00425	8.2097	0.00421	8.36739	0.00403	8.61959	0.00403	9.36662	0.00409	10.07587

0.00498	8.52572	0.00461	8.58421	0.00427	8.22574	0.00421	8.37784	0.00403	8.63491	0.00403	9.38493	0.00408	10.09196

0.005	8.54203	0.00462	8.60067	0.00426	8.23595	0.0042	8.3853	0.00405	8.65942	0.00403	9.40157	0.0041	10.11342

0.00499	8.54944	0.00463	8.62161	0.00426	8.25052	0.00421	8.4047	0.00405	8.67474	0.00405	9.42155	0.00411	10.13309

0.005	8.56723	0.00462	8.62909	0.00428	8.26801	0.00422	8.41963	0.00404	8.68547	0.00406	9.44152	0.0041	10.14919

0.00502	8.58354	0.00463	8.64405	0.00428	8.28259	0.00421	8.43306	0.00404	8.69619	0.00406	9.45649	0.0041	10.16171

0.00501	8.59689	0.00464	8.66799	0.00427	8.29425	0.00421	8.445	0.00405	8.71458	0.00406	9.46649	0.00411	10.1796

0.00502	8.60727	0.00465	8.67846	0.00428	8.30737	0.00423	8.46142	0.00407	8.72836	0.00406	9.48312	0.00413	10.20642

0.00503	8.62803	0.00463	8.68295	0.00429	8.32486	0.00423	8.47784	0.00405	8.73756	0.00406	9.50143	0.00413	10.21894

0.00504	8.6384	0.00465	8.70988	0.00429	8.3409	0.00423	8.49127	0.00405	8.75595	0.00407	9.51641	0.00411	10.23325

0.00504	8.64878	0.00467	8.72035	0.00429	8.3511	0.00424	8.5062	0.00407	8.77893	0.00406	9.52806	0.00413	10.2565

0.00504	8.67251	0.00466	8.73531	0.0043	8.3686	0.00425	8.5256	0.00407	8.7927	0.00408	9.54969	0.00414	10.27438

0.00507	8.68585	0.00466	8.74877	0.00431	8.38608	0.00425	8.54352	0.00407	8.8065	0.00408	9.57134	0.00414	10.29226

0.00505	8.69475	0.00468	8.7742	0.00431	8.39775	0.00424	8.55545	0.00408	8.82642	0.00408	9.58798	0.00414	10.30836

0.00506	8.71551	0.00469	8.78617	0.00431	8.40941	0.00425	8.5659	0.00409	8.84328	0.00408	9.59963	0.00414	10.32446

0.00507	8.73033	0.00467	8.79814	0.00431	8.42253	0.00426	8.58232	0.00407	8.84787	0.00408	9.61794	0.00415	10.34413

0.00507	8.7422	0.00469	8.81161	0.00431	8.44148	0.00426	8.60322	0.00407	8.86165	0.0041	9.63623	0.00415	10.36202

0.00507	8.75702	0.00469	8.82956	0.00432	8.45752	0.00426	8.61217	0.00409	8.88311	0.0041	9.65455	0.00415	10.37811

0.00509	8.7763	0.00469	8.84153	0.00431	8.46917	0.00426	8.63008	0.00409	8.89996	0.0041	9.6662	0.00415	10.39421

0.0051	8.78816	0.00469	8.84751	0.00432	8.48522	0.00427	8.65098	0.00409	8.90762	0.0041	9.68284	0.00415	10.41387

0.0051	8.79854	0.0047	8.87593	0.00433	8.50125	0.00428	8.66143	0.00409	8.92754	0.00411	9.70281	0.00416	10.43533

0.00511	8.82078	0.00472	8.8894	0.00433	8.51583	0.00427	8.67038	0.00411	8.94898	0.00411	9.72112	0.00416	10.45501

0.00513	8.83857	0.00471	8.90286	0.00433	8.52602	0.00427	8.68829	0.00411	8.96583	0.0041	9.7361	0.00415	10.46932

0.0051	8.84747	0.00472	8.91633	0.00433	8.53915	0.00429	8.70919	0.0041	8.9735	0.00412	9.75108	0.00416	10.48541

0.00512	8.86378	0.00474	8.93578	0.00434	8.55955	0.00428	8.71516	0.00411	8.99342	0.00412	9.76939	0.00417	10.50509

0.00513	8.88306	0.00474	8.94326	0.00435	8.57268	0.00428	8.72561	0.00411	9.0072	0.00413	9.78769	0.00417	10.52476

0.00513	8.89195	0.00473	8.95822	0.00434	8.58433	0.0043	8.74949	0.00411	9.01946	0.00412	9.80101	0.00417	10.53728

0.00514	8.9053	0.00474	8.97617	0.00434	8.60037	0.0043	8.76591	0.00411	9.03172	0.00412	9.81598	0.00417	10.55516

0.00515	8.92012	0.00475	8.99262	0.00435	8.61786	0.0043	8.77785	0.00412	9.04858	0.00413	9.8343	0.00418	10.57662

0.00517	8.9394	0.00474	9.0031	0.00435	8.62953	0.00429	8.79128	0.00413	9.07002	0.00413	9.85426	0.00418	10.5945

0.00515	8.94978	0.00475	9.01357	0.00435	8.64264	0.0043	8.80471	0.00413	9.08228	0.00413	9.87091	0.00418	10.61239

0.00517	8.96757	0.00476	9.04499	0.00437	8.66159	0.00432	8.82412	0.00413	9.0976	0.00413	9.88255	0.00418	10.62669

0.00518	8.98536	0.00477	9.05546	0.00436	8.67617	0.00431	8.83307	0.00414	9.12057	0.00413	9.90086	0.00418	10.64637

0.00517	8.99426	0.00477	9.06144	0.00436	8.68782	0.00431	8.84502	0.00413	9.13437	0.00414	9.92416	0.00419	10.66783

0.00517	9.0076	0.00476	9.0764	0.00437	8.70095	0.00432	8.8689	0.00414	9.14204	0.00415	9.93914	0.0042	10.68393

0.00519	9.02836	0.00478	9.10034	0.00437	8.71844	0.00432	8.88681	0.00414	9.15889	0.00415	9.94914	0.00419	10.69823

0.0052	9.03874	0.00478	9.10632	0.00438	8.73302	0.00432	8.89277	0.00414	9.17574	0.00415	9.96577	0.0042	10.71432

0.00519	9.05209	0.00478	9.12278	0.00437	8.74322	0.00433	8.91367	0.00415	9.18953	0.00416	9.98741	0.00421	10.73758

0.00521	9.07136	0.00481	9.14522	0.00437	8.75925	0.00434	8.92711	0.00414	9.19872	0.00417	10.00073	0.00421	10.75367

0.00521	9.08767	0.00481	9.15569	0.00439	8.7782	0.00435	8.94054	0.00414	9.21863	0.00416	10.0157	0.0042	10.77156

0.00521	9.09657	0.00479	9.16616	0.00439	8.79133	0.00434	8.95397	0.00416	9.24008	0.00416	10.03235	0.00421	10.78944

0.00522	9.11881	0.00481	9.18561	0.00439	8.80298	0.00434	8.9689	0.00416	9.25235	0.00418	10.05564	0.00421	10.8109

0.00524	9.13215	0.00481	9.20805	0.00439	8.81902	0.00436	8.9883	0.00416	9.26613	0.00418	10.07395	0.00422	10.83057

0.00524	9.14402	0.00481	9.21104	0.00441	8.83505	0.00436	9.00323	0.00416	9.28605	0.00417	10.0856	0.00421	10.84309

0.00523	9.15588	0.00482	9.2275	0.00441	8.84817	0.00436	9.01517	0.00417	9.30597	0.00417	10.10224	0.00421	10.85919

0.00525	9.18108	0.00484	9.24994	0.00441	8.85837	0.00435	9.03308	0.00418	9.30903	0.00419	10.11888	0.00422	10.88065

0.00525	9.1885	0.00484	9.25443	0.00441	8.87441	0.00437	9.05099	0.00416	9.31975	0.0042	10.13719	0.00423	10.90032

0.00525	9.19739	0.00482	9.27388	0.00443	8.89336	0.00437	9.06144	0.00418	9.34427	0.00419	10.14885	0.00423	10.91284

0.00527	9.2226	0.00484	9.29333	0.00442	8.90502	0.00436	9.07487	0.00419	9.35959	0.00419	10.16382	0.00422	10.92893

0.00527	9.23743	0.00486	9.30829	0.00443	8.91814	0.00438	9.08681	0.00418	9.37031	0.0042	10.18545	0.00423	10.95219

0.00527	9.24633	0.00486	9.31726	0.00442	8.93418	0.00439	9.10771	0.00418	9.39024	0.0042	10.20543	0.00425	10.97007

0.00529	9.2656	0.00484	9.33372	0.00444	8.95313	0.00438	9.11666	0.0042	9.41014	0.00421	10.21875	0.00423	10.98617

0.00529	9.28339	0.00486	9.34569	0.00443	8.96333	0.00438	9.1301	0.0042	9.42394	0.00421	10.23539	0.00425	11.00047

0.00529	9.29674	0.00487	9.36663	0.00443	8.9779	0.0044	9.15249	0.00419	9.43467	0.0042	10.25204	0.00425	11.01836

0.00529	9.30119	0.00486	9.37561	0.00445	8.99394	0.0044	9.16741	0.0042	9.44692	0.00422	10.27366	0.00426	11.03982

0.00531	9.32491	0.00487	9.39057	0.00445	9.01143	0.00439	9.17786	0.00421	9.46837	0.00422	10.28698	0.00426	11.0577

0.00532	9.33677	0.00488	9.41151	0.00445	9.02018	0.0044	9.18981	0.00421	9.47603	0.00422	10.2953	0.00426	11.07201

0.00531	9.35012	0.00489	9.42498	0.00445	9.03476	0.00441	9.21518	0.0042	9.48676	0.00422	10.31527	0.00426	11.08989

0.00533	9.36939	0.00488	9.43395	0.00445	9.0537	0.0044	9.22562	0.0042	9.5082	0.00423	10.3369	0.00427	11.11135

0.00534	9.3857	0.0049	9.4534	0.00445	9.06974	0.0044	9.24055	0.00422	9.52659	0.00424	10.35522	0.00428	11.13102

0.00533	9.39756	0.00491	9.46836	0.00445	9.07995	0.00441	9.25547	0.00422	9.5419	0.00422	10.36687	0.00428	11.14354

0.00533	9.41387	0.0049	9.48033	0.00445	9.09452	0.00442	9.27339	0.00421	9.55876	0.00424	10.38517	0.00427	11.16143

0.00535	9.43018	0.0049	9.49978	0.00446	9.11201	0.00442	9.28981	0.00422	9.57408	0.00425	10.40348	0.00429	11.18289

0.00535	9.44056	0.00492	9.51474	0.00447	9.12513	0.00442	9.29876	0.00424	9.59093	0.00424	10.42345	0.00429	11.19899

0.00535	9.45242	0.00492	9.52521	0.00446	9.13681	0.00442	9.31219	0.00423	9.5986	0.00424	10.4351	0.00429	11.21687

0.00538	9.47467	0.00492	9.53867	0.00446	9.15138	0.00444	9.33458	0.00423	9.61238	0.00425	10.44841	0.00429	11.23118

0.00538	9.48356	0.00493	9.56112	0.00448	9.16741	0.00444	9.34353	0.00424	9.63231	0.00426	10.46672	0.00429	11.25085

0.00538	9.49691	0.00494	9.57608	0.00448	9.18053	0.00443	9.36443	0.00425	9.64762	0.00426	10.48836	0.0043	11.27231

0.00539	9.5147	0.00494	9.58655	0.00448	9.19365	0.00444	9.37189	0.00424	9.65835	0.00426	10.50333	0.0043	11.29198

0.00539	9.53101	0.00494	9.603	0.00448	9.21114	0.00445	9.38981	0.00424	9.67368	0.00426	10.51664	0.0043	11.30271

0.0054	9.54139	0.00495	9.62096	0.0045	9.22862	0.00445	9.40473	0.00426	9.69512	0.00426	10.53662	0.0043	11.3206

0.0054	9.55622	0.00495	9.63442	0.0045	9.24175	0.00444	9.41816	0.00426	9.71503	0.00428	10.55826	0.00431	11.34384

0.00542	9.57994	0.00494	9.63891	0.0045	9.25049	0.00445	9.43608	0.00424	9.72577	0.00428	10.57157	0.00431	11.35995

0.00542	9.58735	0.00496	9.66434	0.0045	9.26653	0.00446	9.451	0.00425	9.73955	0.00427	10.58323	0.00431	11.37425

0.00542	9.60366	0.00498	9.68678	0.00451	9.28548	0.00446	9.46891	0.00426	9.75794	0.00428	10.60153	0.00431	11.39035

0.00544	9.62294	0.00497	9.69127	0.00451	9.29569	0.00445	9.47936	0.00426	9.77019	0.0043	10.61983	0.00432	11.41181

0.00544	9.63628	0.00497	9.71072	0.00451	9.3088	0.00447	9.49429	0.00426	9.77785	0.0043	10.63482	0.00433	11.43147

0.00543	9.64963	0.00499	9.72717	0.00452	9.3263	0.00448	9.50622	0.00427	9.79777	0.00429	10.64813	0.00433	11.444

0.00545	9.66594	0.00499	9.74064	0.00452	9.34233	0.00447	9.51519	0.00428	9.81769	0.0043	10.66477	0.00432	11.46188

0.00546	9.68225	0.00499	9.74961	0.00452	9.35254	0.00448	9.5316	0.00428	9.83147	0.0043	10.68973	0.00433	11.48692

0.00546	9.68966	0.00499	9.76607	0.00453	9.36857	0.00449	9.5525	0.00428	9.84527	0.00432	10.70472	0.00433	11.5048

0.00546	9.70746	0.00501	9.78702	0.00453	9.38753	0.00449	9.57041	0.00428	9.86671	0.0043	10.71803	0.00433	11.51911

0.00547	9.72821	0.005	9.79599	0.00453	9.40064	0.00448	9.58981	0.00429	9.88203	0.00431	10.73467	0.00434	11.53342

0.00548	9.73859	0.00501	9.80646	0.00453	9.41084	0.00448	9.59429	0.00428	9.89276	0.00432	10.75464	0.00435	11.55131

0.00548	9.74897	0.00502	9.8319	0.00453	9.42542	0.0045	9.61668	0.00428	9.90349	0.00432	10.77295	0.00435	11.57277

0.00549	9.7727	0.00501	9.84386	0.00455	9.44438	0.0045	9.62862	0.0043	9.92188	0.00432	10.78294	0.00435	11.59064

0.0055	9.78752	0.00501	9.85134	0.00455	9.45895	0.00449	9.63757	0.00431	9.93719	0.00432	10.79625	0.00434	11.60496

0.0055	9.79345	0.00503	9.87528	0.00455	9.46769	0.0045	9.65399	0.0043	9.94945	0.00434	10.81955	0.00435	11.62284

0.0055	9.81273	0.00504	9.88875	0.00455	9.48226	0.00451	9.67489	0.0043	9.96323	0.00434	10.8362	0.00437	11.64608

0.00552	9.83349	0.00504	9.90071	0.00457	9.50413	0.00451	9.69131	0.00431	9.98775	0.00433	10.85284	0.00438	11.66397

0.00551	9.83942	0.00504	9.91567	0.00457	9.5158	0.00451	9.70474	0.00431	10.00767	0.00434	10.86948	0.00437	11.67649

0.00552	9.85425	0.00504	9.93662	0.00456	9.52892	0.00451	9.72265	0.00431	10.01686	0.00435	10.88613	0.00437	11.69259

0.00554	9.87352	0.00506	9.94859	0.00457	9.54495	0.00453	9.74057	0.00431	10.03218	0.00436	10.90276	0.00438	11.71584

0.00554	9.88983	0.00504	9.95756	0.00458	9.56098	0.00453	9.74802	0.00432	10.0521	0.00436	10.91774	0.00438	11.73551

0.00554	9.90169	0.00506	9.97551	0.00457	9.5741	0.00452	9.76146	0.00433	10.06435	0.00435	10.93106	0.00437	11.74624

0.00554	9.918	0.00508	9.98898	0.00457	9.58431	0.00452	9.77937	0.00432	10.07201	0.00436	10.94937	0.00438	11.76412

0.00555	9.93283	0.00508	10.00095	0.00458	9.6018	0.00454	9.79878	0.00433	10.08887	0.00437	10.97101	0.00439	11.78737

0.00555	9.94173	0.00508	10.01441	0.00458	9.61783	0.00454	9.80773	0.00433	10.10878	0.00437	10.98597	0.0044	11.80704

0.00556	9.95804	0.00508	10.03536	0.00458	9.62949	0.00453	9.82265	0.00433	10.12257	0.00437	10.99763	0.0044	11.82493

0.00558	9.98028	0.00509	10.05331	0.00458	9.6397	0.00454	9.84206	0.00433	10.13943	0.00437	11.0176	0.00439	11.83744

0.00558	9.98621	0.0051	10.06677	0.0046	9.66302	0.00456	9.854	0.00434	10.15628	0.00438	11.0409	0.00439	11.85533

0.00558	10.00104	0.0051	10.07874	0.0046	9.6776	0.00455	9.86444	0.00435	10.17619	0.00438	11.05256	0.00441	11.87679

0.00561	10.02476	0.00511	10.09819	0.00459	9.6878	0.00455	9.88236	0.00435	10.18845	0.00438	11.06254	0.00441	11.8911

0.00561	10.02921	0.00511	10.11165	0.00459	9.70091	0.00457	9.89728	0.00434	10.19765	0.00438	11.0825	0.0044	11.90898

0.0056	10.04404	0.0051	10.11764	0.0046	9.71987	0.00457	9.91071	0.00435	10.21297	0.00438	11.10081	0.00441	11.92507

0.00561	10.06628	0.00511	10.13709	0.00462	9.73445	0.00456	9.91968	0.00437	10.22982	0.00438	11.11913	0.00442	11.94475

0.00563	10.0811	0.00513	10.15504	0.0046	9.74611	0.00457	9.94206	0.00435	10.24055	0.00439	11.13078	0.00442	11.96621

0.00561	10.09	0.00512	10.17	0.00461	9.75777	0.00458	9.96147	0.00435	10.25587	0.00439	11.14909	0.00442	11.98052

0.00563	10.10928	0.00512	10.18197	0.00462	9.77818	0.00459	9.9749	0.00437	10.27731	0.0044	11.17238	0.00442	11.9984

0.00564	10.13003	0.00514	10.19992	0.00462	9.79421	0.00457	9.98982	0.00437	10.29417	0.0044	11.18736	0.00442	12.01629

0.00564	10.13597	0.00516	10.21937	0.00462	9.80296	0.00458	9.99878	0.00436	10.30949	0.0044	11.19901	0.00443	12.03596

0.00564	10.14783	0.00514	10.22834	0.00462	9.81608	0.00459	10.00923	0.00437	10.32482	0.0044	11.21566	0.00443	12.05205

0.00566	10.16859	0.00516	10.2448	0.00464	9.83795	0.0046	10.03908	0.00439	10.3432	0.00441	11.2323	0.00443	12.06637

0.00567	10.1849	0.00516	10.26275	0.00464	9.85106	0.00459	10.05102	0.00439	10.35392	0.00442	11.25559	0.00443	12.08424

0.00565	10.19379	0.00516	10.27622	0.00464	9.86127	0.00459	10.05997	0.00438	10.36311	0.00442	11.26725	0.00444	12.10392

0.00567	10.21158	0.00516	10.28968	0.00464	9.87584	0.0046	10.07938	0.00439	10.38303	0.00441	11.2789	0.00444	12.12359

0.00568	10.23086	0.00517	10.30614	0.00466	9.8948	0.00461	10.0958	0.00439	10.39989	0.00442	11.30053	0.00444	12.13968

0.00567	10.23976	0.00518	10.32858	0.00466	9.90645	0.0046	10.10774	0.00439	10.41061	0.00444	11.32217	0.00445	12.15757

0.00567	10.2531	0.00516	10.33007	0.00466	9.91812	0.00461	10.12266	0.00439	10.42593	0.00443	11.33381	0.00445	12.17903

0.00569	10.27238	0.00518	10.34952	0.00466	9.93416	0.00462	10.1346	0.00441	10.45044	0.00444	11.35212	0.00446	12.19692

0.00571	10.28276	0.0052	10.36299	0.00467	9.95019	0.00463	10.1555	0.0044	10.4673	0.00444	11.3721	0.00445	12.20943

0.00569	10.2961	0.0052	10.37795	0.00467	9.96331	0.00462	10.16744	0.00439	10.47802	0.00445	11.38707	0.00445	12.22374

0.00572	10.31686	0.0052	10.39141	0.00467	9.97789	0.00463	10.18087	0.00441	10.49487	0.00445	11.40039	0.00446	12.24699

0.00572	10.33317	0.00521	10.41086	0.00467	9.99246	0.00463	10.1958	0.00442	10.5102	0.00444	11.41204	0.00447	12.26666

0.00571	10.34355	0.00522	10.41984	0.00467	10.00995	0.00463	10.20774	0.00443	10.52092	0.00446	11.43034	0.00447	12.27918

0.00572	10.35838	0.00521	10.4333	0.00468	10.02162	0.00463	10.22117	0.00442	10.53318	0.00446	11.45032	0.00446	12.29885

0.00574	10.37469	0.00522	10.45424	0.00468	10.03474	0.00465	10.24655	0.00441	10.55309	0.00446	11.47029	0.00449	12.32031

0.00574	10.3821	0.00523	10.4692	0.00469	10.04931	0.00465	10.25998	0.00443	10.56994	0.00446	11.48194	0.00449	12.3382

0.00574	10.39841	0.00523	10.48416	0.00469	10.06535	0.00464	10.26595	0.00443	10.58526	0.00446	11.50191	0.00447	12.35429

0.00576	10.42213	0.00522	10.49763	0.00469	10.07701	0.00464	10.27789	0.00443	10.59752	0.00448	11.52022	0.00449	12.37218

0.00576	10.43399	0.00524	10.51259	0.00469	10.09013	0.00466	10.30775	0.00443	10.61897	0.00448	11.54019	0.00449	12.39006

0.00575	10.44437	0.00525	10.52905	0.0047	10.11054	0.00466	10.31222	0.00446	10.63889	0.00448	11.54685	0.0045	12.40974

0.00577	10.46365	0.00525	10.54101	0.0047	10.12657	0.00465	10.31968	0.00446	10.64961	0.00448	11.56349	0.0045	12.42404

0.00577	10.48292	0.00525	10.55897	0.0047	10.13677	0.00466	10.34356	0.00443	10.65728	0.00448	11.58679	0.0045	12.44013

0.00577	10.48737	0.00527	10.57841	0.0047	10.15135	0.00468	10.35103	0.00446	10.67566	0.0045	11.60343	0.0045	12.45802

0.00577	10.50665	0.00528	10.58889	0.00472	10.16739	0.00468	10.37341	0.00447	10.69098	0.00448	11.61508	0.00452	12.48127

0.0058	10.52592	0.00526	10.60085	0.00472	10.18342	0.00467	10.38834	0.00446	10.70171	0.00449	11.63339	0.00451	12.49558

0.0058	10.53482	0.00527	10.61881	0.00472	10.19216	0.00468	10.39581	0.00446	10.71856	0.0045	11.65502	0.00451	12.51167

0.0058	10.55261	0.00529	10.63526	0.00472	10.20674	0.00469	10.41968	0.00447	10.74001	0.00451	11.67333	0.00452	12.52956

0.00583	10.57041	0.00528	10.64574	0.00473	10.22569	0.00469	10.43461	0.00448	10.75839	0.00451	11.68498	0.00453	12.55102

0.00582	10.57634	0.00528	10.6607	0.00474	10.24173	0.00468	10.4361	0.00448	10.76912	0.00451	11.70163	0.00453	12.5707

0.00582	10.59116	0.0053	10.67715	0.00472	10.25193	0.00469	10.45998	0.00448	10.78904	0.00452	11.71661	0.00453	12.58143

0.00583	10.61785	0.00531	10.69361	0.00473	10.26504	0.0047	10.48237	0.00449	10.80895	0.00452	11.73491	0.00452	12.59752

0.00584	10.62675	0.0053	10.70558	0.00474	10.28546	0.00469	10.4973	0.00449	10.81509	0.00453	11.74989	0.00453	12.61898

0.00583	10.63416	0.00532	10.72353	0.00475	10.29857	0.0047	10.50625	0.00448	10.82428	0.00453	11.76487	0.00455	12.64044

0.00586	10.6564	0.00533	10.74148	0.00474	10.30878	0.0047	10.51968	0.00449	10.83959	0.00453	11.78151	0.00454	12.65654

0.00587	10.67271	0.00532	10.75046	0.00475	10.32481	0.00471	10.53461	0.0045	10.86257	0.00455	11.80314	0.00455	12.67263

0.00586	10.68013	0.00532	10.76243	0.00476	10.34231	0.00471	10.55252	0.0045	10.87636	0.00454	11.81979	0.00454	12.69052

0.00586	10.69792	0.00534	10.78487	0.00476	10.35542	0.00471	10.56297	0.00449	10.88862	0.00453	11.83144	0.00456	12.71198

0.00588	10.72164	0.00534	10.80132	0.00475	10.36709	0.00473	10.58237	0.0045	10.9116	0.00455	11.85142	0.00456	12.72807

0.00589	10.73351	0.00534	10.80731	0.00476	10.38458	0.00473	10.60029	0.00452	10.92999	0.00455	11.87138	0.00455	12.74238

0.00588	10.74092	0.00534	10.82825	0.00477	10.40062	0.00472	10.60477	0.00451	10.93918	0.00456	11.88469	0.00455	12.75847

0.00589	10.75871	0.00536	10.8477	0.00478	10.41373	0.00473	10.61969	0.00451	10.94991	0.00455	11.89636	0.00457	12.78172

0.00591	10.77502	0.00536	10.85817	0.00478	10.42539	0.00474	10.65103	0.00452	10.96676	0.00455	11.91299	0.00456	12.79782

0.00591	10.78689	0.00535	10.87313	0.00479	10.44288	0.00474	10.65252	0.00453	10.98208	0.00457	11.93463	0.00456	12.81212

0.00591	10.80023	0.00537	10.88959	0.00478	10.45893	0.00473	10.66894	0.00452	10.99127	0.00456	11.95293	0.00456	12.8318

0.00592	10.81951	0.00538	10.90754	0.00478	10.47204	0.00474	10.68387	0.00453	11.00966	0.00457	11.96459	0.00457	12.85505

0.00592	10.83137	0.00537	10.91053	0.00478	10.4837	0.00475	10.70327	0.00453	11.02958	0.00457	11.98289	0.00458	12.87293

0.00592	10.84471	0.00538	10.93297	0.0048	10.50265	0.00475	10.71969	0.00453	11.04336	0.00458	12.00286	0.00458	12.88545

0.00594	10.8714	0.00539	10.95093	0.0048	10.51577	0.00474	10.72566	0.00453	11.05868	0.00459	12.02117	0.00458	12.90334

0.00596	10.88178	0.00539	10.96289	0.0048	10.52743	0.00475	10.74506	0.00453	11.07707	0.00458	12.03116	0.00459	12.923

0.00594	10.88771	0.00538	10.97486	0.0048	10.54347	0.00477	10.76895	0.00455	11.09239	0.00458	12.04281	0.00459	12.94268

0.00595	10.91143	0.00539	10.99581	0.00482	10.56242	0.00477	10.78089	0.00455	11.10771	0.00459	12.06777	0.00459	12.95699

0.00596	10.9233	0.00541	11.01376	0.00482	10.57553	0.00477	10.78984	0.00454	11.11538	0.00461	12.08441	0.00459	12.97308

0.00596	10.93071	0.00541	11.02573	0.00481	10.58719	0.00477	10.80029	0.00455	11.1307	0.00459	12.09606	0.00461	12.99275

0.00596	10.95147	0.0054	11.0362	0.00482	10.60177	0.00478	10.82567	0.00456	11.14909	0.00459	12.11603	0.00462	13.016

0.00598	10.96926	0.00543	11.05565	0.00482	10.61781	0.00478	10.83462	0.00455	11.16134	0.00461	12.13434	0.00462	13.03031

0.00599	10.98112	0.00543	11.06911	0.00482	10.63384	0.00478	10.85104	0.00455	11.17665	0.00461	12.15598	0.00461	13.04462

0.00597	10.99595	0.00543	11.08108	0.00482	10.64259	0.00479	10.86746	0.00457	11.19964	0.00462	12.16929	0.00462	13.06429

0.006	11.01523	0.00544	11.09754	0.00482	10.65717	0.00479	10.88686	0.00457	11.21956	0.00461	12.18094	0.00463	13.08754

0.006	11.02412	0.00545	11.11549	0.00484	10.67612	0.00479	10.89582	0.00457	11.22875	0.00462	12.19759	0.00463	13.10363

0.00599	11.03598	0.00545	11.12446	0.00484	10.69069	0.00479	10.91373	0.00457	11.24407	0.00462	12.21756	0.00463	13.11616

0.006	11.05971	0.00544	11.14242	0.00484	10.7009	0.0048	10.92567	0.00458	11.26092	0.00462	12.2342	0.00463	13.13225

0.00602	11.07454	0.00546	11.16486	0.00484	10.71984	0.00481	10.94955	0.00459	11.27471	0.00462	12.24585	0.00463	13.15371

0.00602	11.08343	0.00546	11.17683	0.00485	10.73442	0.0048	10.95253	0.00458	11.28238	0.00463	12.26415	0.00464	13.17339

0.00602	11.09974	0.00546	11.18281	0.00485	10.74608	0.00481	10.96596	0.00459	11.29769	0.00464	12.28579	0.00463	13.1859

0.00604	11.11753	0.00546	11.20226	0.00484	10.75921	0.00482	10.98836	0.00459	11.31914	0.00464	12.30243	0.00463	13.20736

0.00604	11.1294	0.00548	11.2247	0.00486	10.77524	0.00482	11.00179	0.0046	11.33447	0.00464	12.31242	0.00465	13.22704

0.00604	11.13978	0.00549	11.23218	0.00486	10.79273	0.00481	11.01672	0.00459	11.34825	0.00464	12.33405	0.00465	13.24671

0.00604	11.16202	0.00548	11.24714	0.00486	10.80584	0.00482	11.02716	0.0046	11.36664	0.00466	12.35071	0.00465	13.2628

0.00606	11.17536	0.0055	11.27257	0.00486	10.81606	0.00483	11.06149	0.00461	11.38962	0.00466	12.36568	0.00465	13.27712

0.00606	11.18574	0.0055	11.28005	0.00488	10.835	0.00483	11.05701	0.00461	11.39881	0.00464	12.38065	0.00466	13.29499

0.00607	11.20205	0.0055	11.29651	0.00488	10.85104	0.00482	11.07642	0.0046	11.40494	0.00466	12.39563	0.00467	13.31645

0.00608	11.21984	0.0055	11.30698	0.00488	10.86124	0.00483	11.08388	0.00461	11.42179	0.00466	12.41394	0.00466	13.33255

0.00608	11.22874	0.00551	11.32194	0.00488	10.87437	0.00485	11.10328	0.00462	11.43864	0.00466	12.43225	0.00466	13.34686

0.00608	11.24505	0.00553	11.33391	0.0049	10.89331	0.00485	11.1197	0.00462	11.45243	0.00466	12.44723	0.00466	13.36475

0.00609	11.26729	0.00552	11.35186	0.0049	10.90789	0.00485	11.13015	0.00461	11.46623	0.00466	12.46387	0.00468	13.38799

0.00611	11.27619	0.00553	11.37131	0.0049	10.91955	0.00485	11.15105	0.00463	11.48767	0.00468	12.48551	0.00468	13.40588

0.00609	11.28805	0.00555	11.38627	0.00489	10.93267	0.00487	11.16448	0.00464	11.50452	0.00468	12.49882	0.00467	13.42197

0.00612	11.31029	0.00553	11.39525	0.00491	10.95307	0.00486	11.1809	0.00463	11.51984	0.00469	12.51214	0.00469	13.43449

0.00612	11.32215	0.00555	11.40871	0.00492	10.96475	0.00486	11.18836	0.00463	11.5367	0.00469	12.52878	0.00469	13.45953

0.00611	11.3355	0.00555	11.42816	0.0049	10.9764	0.00487	11.20926	0.00464	11.54895	0.00469	12.54875	0.00469	13.47741

0.00612	11.35181	0.00557	11.44312	0.00491	10.99244	0.00488	11.22866	0.00465	11.56274	0.00471	12.56706	0.00469	13.49172

0.00614	11.37405	0.00555	11.45359	0.00492	11.01139	0.00487	11.23314	0.00465	11.575	0.00471	12.58204	0.00469	13.50603

0.00614	11.38146	0.00557	11.47304	0.00493	11.02596	0.00487	11.24806	0.00465	11.58878	0.00471	12.59535	0.0047	13.52749

0.00614	11.39184	0.00557	11.4895	0.00492	11.03325	0.00489	11.26448	0.00466	11.60871	0.00471	12.61698	0.0047	13.54716

0.00616	11.41556	0.00557	11.49997	0.00494	11.0522	0.00489	11.28687	0.00466	11.62097	0.00471	12.63696	0.00471	13.56325

0.00616	11.42743	0.00557	11.51343	0.00494	11.06824	0.00489	11.29433	0.00465	11.63629	0.00473	12.6486	0.0047	13.57935

0.00616	11.43336	0.00559	11.53737	0.00494	11.0799	0.00489	11.31673	0.00466	11.6562	0.00472	12.66192	0.00471	13.60081

0.00617	11.45856	0.0056	11.54634	0.00494	11.0901	0.0049	11.32717	0.00468	11.67765	0.00472	12.68023	0.00473	13.61869

0.00618	11.47191	0.00558	11.5613	0.00494	11.10905	0.00491	11.34956	0.00467	11.69144	0.00473	12.70019	0.00473	13.63479

0.00617	11.4808	0.00561	11.57926	0.00495	11.12655	0.0049	11.35702	0.00466	11.70063	0.00474	12.71684	0.00471	13.64731

0.00617	11.49415	0.00562	11.59272	0.00494	11.1382	0.0049	11.37195	0.00467	11.71443	0.00474	12.72682	0.00473	13.6652

0.0062	11.51936	0.00562	11.60319	0.00494	11.15132	0.00491	11.39285	0.00468	11.73128	0.00473	12.7468	0.00475	13.69202

0.0062	11.52825	0.00562	11.62115	0.00496	11.16735	0.00492	11.41076	0.00468	11.74353	0.00475	12.76844	0.00475	13.70454

0.00621	11.54456	0.00563	11.6376	0.00496	11.18194	0.00491	11.41673	0.00468	11.75732	0.00475	12.78508	0.00474	13.71885

0.00622	11.56384	0.00563	11.65555	0.00496	11.19505	0.00492	11.43016	0.0047	11.77571	0.00475	12.79506	0.00474	13.74031

0.00621	11.57125	0.00563	11.66154	0.00496	11.20817	0.00493	11.45255	0.0047	11.79409	0.00475	12.81171	0.00475	13.76177

0.00621	11.58608	0.00563	11.67949	0.00497	11.22712	0.00494	11.46897	0.00469	11.80635	0.00477	12.83335	0.00476	13.78145

0.00622	11.60535	0.00565	11.69744	0.00497	11.24024	0.00493	11.47494	0.0047	11.82627	0.00477	12.84832	0.00476	13.79396

0.00625	11.62166	0.00566	11.70941	0.00496	11.25044	0.00493	11.49135	0.00471	11.84466	0.00477	12.85831	0.00475	13.81184

0.00624	11.62759	0.00565	11.71988	0.00497	11.2694	0.00494	11.51375	0.00471	11.85691	0.00477	12.87662	0.00477	13.83151

0.00625	11.65132	0.00567	11.74083	0.00499	11.28543	0.00495	11.52121	0.0047	11.86457	0.00478	12.89991	0.00477	13.8494

0.00625	11.66615	0.00568	11.75728	0.00499	11.29855	0.00494	11.53314	0.00471	11.88449	0.00479	12.91822	0.00476	13.86371

0.00625	11.67801	0.00567	11.76776	0.00498	11.31021	0.00494	11.55554	0.00472	11.89981	0.00478	12.92988	0.00477	13.87802

0.00626	11.68839	0.00567	11.7872	0.005	11.32479	0.00496	11.56897	0.00472	11.91054	0.00478	12.94818	0.00477	13.90305

0.00626	11.70766	0.00569	11.80217	0.005	11.34082	0.00496	11.5824	0.00472	11.92585	0.0048	12.96649	0.00478	13.92273

0.00628	11.71804	0.00569	11.81413	0.005	11.35686	0.00495	11.59583	0.00472	11.94271	0.00481	12.98147	0.00478	13.93882

0.00628	11.73435	0.00568	11.8276	0.005	11.3656	0.00496	11.61375	0.00473	11.96722	0.00479	12.99478	0.00477	13.95313

0.00629	11.7477	0.0057	11.84256	0.005	11.3831	0.00497	11.62718	0.00472	11.97795	0.00479	13.00809	0.00478	13.97101

0.0063	11.76697	0.0057	11.85752	0.00502	11.40204	0.00497	11.63912	0.00474	11.99326	0.00481	13.02972	0.0048	13.99426

0.00629	11.77883	0.0057	11.87098	0.00502	11.41079	0.00497	11.65853	0.00474	12.00859	0.00481	13.05303	0.00479	14.01036

0.00629	11.79366	0.00571	11.88893	0.00502	11.42682	0.00499	11.67644	0.00474	12.02085	0.00481	13.06302	0.00479	14.02109

0.00632	11.82035	0.00573	11.90988	0.00502	11.44286	0.00499	11.69136	0.00474	12.03157	0.00482	13.07633	0.00479	14.04076

0.00632	11.82628	0.00574	11.92035	0.00504	11.45889	0.00498	11.70181	0.00474	12.04843	0.00483	13.09962	0.0048	14.06222

0.00632	11.83814	0.00572	11.93232	0.00503	11.47202	0.00499	11.70778	0.00476	12.06681	0.00484	13.11628	0.00482	14.08368

0.00633	11.85594	0.00574	11.94578	0.00503	11.48367	0.00501	11.73166	0.00476	12.08213	0.00484	13.13292	0.00481	14.0962

0.00634	11.86928	0.00575	11.96074	0.00504	11.50117	0.005	11.74509	0.00476	12.09592	0.00483	13.14289	0.00482	14.11409

0.00634	11.88114	0.00574	11.9772	0.00505	11.5172	0.00499	11.75554	0.00476	12.1143	0.00484	13.15955	0.00482	14.13375

0.00634	11.90338	0.00574	11.98917	0.00504	11.52886	0.005	11.77644	0.00477	12.13576	0.00485	13.17952	0.00483	14.15343

0.00636	11.91673	0.00577	12.0146	0.00505	11.54344	0.00501	11.79435	0.00478	12.15261	0.00486	13.1945	0.00483	14.16774

0.00635	11.93007	0.00576	12.02657	0.00506	11.55947	0.00501	11.81226	0.00476	12.15413	0.00486	13.20947	0.00482	14.18562

0.00636	11.94045	0.00576	12.03555	0.00506	11.57551	0.005	11.81226	0.00478	12.17099	0.00485	13.22778	0.00483	14.2035

0.00638	11.95973	0.00577	12.052	0.00506	11.59008	0.00502	11.84062	0.00478	12.1909	0.00486	13.24942	0.00485	14.22318

0.00638	11.97011	0.00579	12.07295	0.00506	11.6003	0.00503	11.85405	0.00478	12.2047	0.00487	13.26439	0.00485	14.24106

0.00638	11.98642	0.00579	12.07893	0.00507	11.61923	0.00503	11.86599	0.00478	12.21388	0.00486	13.27438	0.00483	14.25537

0.00639	12.00569	0.00579	12.0909	0.00507	11.63382	0.00503	11.87494	0.00478	12.23687	0.00488	13.29268	0.00483	14.27504

0.0064	12.01904	0.00581	12.11932	0.00507	11.64402	0.00503	11.89584	0.0048	12.25679	0.00488	13.31266	0.00486	14.29651

0.00639	12.0309	0.00581	12.13279	0.00507	11.6586	0.00504	11.91376	0.00478	12.27057	0.00488	13.33097	0.00486	14.3126

0.00641	12.04721	0.00581	12.14176	0.00508	11.67754	0.00505	11.9242	0.0048	12.28436	0.00488	13.34262	0.00486	14.3269

0.00642	12.065	0.00581	12.16271	0.00509	11.69212	0.00504	11.93913	0.00481	12.30428	0.00488	13.35593	0.00486	14.34657

0.00642	12.07538	0.00582	12.17767	0.00508	11.70379	0.00504	11.95405	0.00481	12.31348	0.00489	13.38256	0.00488	14.36803

0.00642	12.08576	0.00582	12.19263	0.00509	11.71837	0.00505	11.97495	0.0048	12.3196	0.00489	13.39921	0.00488	14.38592

0.00642	12.11097	0.00582	12.2031	0.00511	11.73731	0.00505	11.98241	0.00481	12.33492	0.00489	13.41086	0.00488	14.39844

0.00644	12.12431	0.00584	12.21956	0.0051	11.74898	0.00505	12.00032	0.00483	12.35943	0.00489	13.42583	0.00488	14.41633

0.00643	12.13617	0.00585	12.23601	0.0051	11.7621	0.00506	12.01824	0.00483	12.37322	0.00489	13.44746	0.00489	14.43957

0.00645	12.15248	0.00584	12.25397	0.00511	11.77668	0.00507	12.03316	0.00482	12.38548	0.00491	13.46245	0.00489	14.45746

0.00646	12.16583	0.00586	12.26444	0.00512	11.79416	0.00507	12.04361	0.00483	12.40846	0.00491	13.47742	0.00489	14.47355

0.00646	12.18065	0.00586	12.28089	0.00512	11.80583	0.00507	12.05405	0.00484	12.42838	0.00491	13.48908	0.00489	14.48786

0.00646	12.19548	0.00586	12.29735	0.00511	11.81749	0.00508	12.08093	0.00483	12.44064	0.00491	13.50904	0.0049	14.51111

0.00647	12.21327	0.00586	12.30782	0.00512	11.83499	0.00509	12.09734	0.00483	12.45137	0.00493	13.53068	0.0049	14.52899

0.00648	12.2281	0.00587	12.32877	0.00512	11.85393	0.00508	12.10779	0.00485	12.46516	0.00493	13.54566	0.0049	14.5433

0.00648	12.24589	0.00588	12.33924	0.00513	11.8656	0.00508	12.11674	0.00485	12.48353	0.00493	13.56063	0.00491	14.5594

0.0065	12.25776	0.00587	12.34822	0.00512	11.87871	0.00511	12.14063	0.00485	12.4912	0.00493	13.57729	0.00491	14.57907

0.0065	12.2711	0.00588	12.36467	0.00514	11.89475	0.0051	12.15257	0.00485	12.50651	0.00494	13.59725	0.00492	14.60053

0.0065	12.28148	0.00591	12.38711	0.00515	11.91223	0.00509	12.16003	0.00485	12.52644	0.00494	13.61057	0.00492	14.61842

0.00651	12.30224	0.00591	12.3931	0.00514	11.91953	0.00511	12.17943	0.00486	12.54788	0.00493	13.62056	0.00491	14.63093

0.00652	12.323	0.0059	12.41254	0.00514	11.9341	0.00512	12.19735	0.00485	12.56168	0.00494	13.64052	0.00492	14.65418

0.00652	12.32893	0.00591	12.43349	0.00516	11.95596	0.00512	12.21526	0.00485	12.57853	0.00496	13.66217	0.00493	14.67207

0.00652	12.33931	0.00592	12.44995	0.00516	11.96763	0.00511	12.22122	0.00487	12.59538	0.00496	13.67547	0.00494	14.68816

0.00654	12.366	0.00592	12.46042	0.00516	11.98076	0.00512	12.24212	0.00488	12.60764	0.00495	13.69046	0.00492	14.70426

0.00654	12.37489	0.00592	12.47837	0.00516	11.99533	0.00513	12.25854	0.00487	12.61377	0.00496	13.70875	0.00494	14.72035

0.00653	12.38527	0.00594	12.49483	0.00518	12.01864	0.00513	12.266	0.00487	12.62909	0.00497	13.7304	0.00495	14.7436

0.00656	12.409	0.00593	12.5038	0.00518	12.03031	0.00513	12.28391	0.00489	12.64901	0.00497	13.74538	0.00495	14.7597

0.00657	12.41789	0.00593	12.51577	0.00518	12.0376	0.00514	12.30183	0.00489	12.66586	0.00497	13.75536	0.00494	14.774

0.00656	12.42827	0.00595	12.53223	0.00518	12.05509	0.00515	12.31526	0.00488	12.67658	0.00497	13.772	0.00495	14.79368

0.00656	12.45051	0.00596	12.55168	0.00519	12.07113	0.00515	12.32869	0.00489	12.69497	0.00499	13.79031	0.00496	14.81335

0.00658	12.46386	0.00595	12.56514	0.00519	12.08425	0.00515	12.34362	0.00491	12.71947	0.00499	13.80695	0.00496	14.83303

0.00658	12.48165	0.00596	12.57711	0.00519	12.09444	0.00515	12.36302	0.0049	12.73173	0.00499	13.82193	0.00498	14.8509

0.00658	12.48906	0.00598	12.59955	0.00519	12.1134	0.00516	12.38242	0.0049	12.744	0.00499	13.83858	0.00496	14.867

0.00661	12.51427	0.00598	12.61152	0.0052	12.13089	0.00516	12.38989	0.00491	12.75625	0.00501	13.86021	0.00496	14.88309

0.00661	12.52317	0.00598	12.62049	0.00519	12.14256	0.00516	12.40033	0.00493	12.77617	0.00501	13.87852	0.00499	14.90634

0.00659	12.53206	0.00598	12.63844	0.00519	12.15567	0.00517	12.4272	0.00491	12.7823	0.00501	13.8935	0.00499	14.92066

0.00661	12.55134	0.006	12.6564	0.0052	12.17608	0.00518	12.43317	0.00491	12.79762	0.005	13.90847	0.00498	14.93496

0.00663	12.57061	0.006	12.66238	0.00521	12.18774	0.00517	12.4466	0.00494	12.81753	0.00502	13.92844	0.00499	14.95285

0.00661	12.57951	0.006	12.68033	0.00521	12.1994	0.00517	12.46452	0.00494	12.83746	0.00502	13.94176	0.005	14.97609

0.00663	12.60027	0.00601	12.70277	0.00521	12.21252	0.00519	12.48989	0.00493	12.84664	0.00502	13.95507	0.00501	14.9922

0.00664	12.6151	0.00602	12.71624	0.00523	12.23294	0.00519	12.49586	0.00494	12.86503	0.00502	13.97005	0.005	15.00829

0.00664	12.62399	0.00602	12.72073	0.00523	12.24605	0.00518	12.50332	0.00496	12.88495	0.00503	13.99003	0.00501	15.02259

0.00664	12.64178	0.00602	12.74765	0.00523	12.25625	0.00519	12.52721	0.00496	12.89874	0.00505	14.01166	0.00502	15.04585

0.00666	12.65661	0.00604	12.76112	0.00523	12.27228	0.00521	12.54363	0.00494	12.90486	0.00504	14.02497	0.00502	15.06194

0.00667	12.66996	0.00604	12.77159	0.00525	12.28978	0.00521	12.55258	0.00495	12.91865	0.00504	14.03995	0.00502	15.07983

0.00667	12.6833	0.00604	12.78655	0.00525	12.30582	0.0052	12.56302	0.00496	12.94317	0.00504	14.05659	0.00502	15.09234

0.00667	12.70406	0.00605	12.8075	0.00524	12.31456	0.00521	12.58094	0.00497	12.95389	0.00506	14.07657	0.00503	15.11559

0.00668	12.71444	0.00606	12.82246	0.00525	12.33059	0.00522	12.59736	0.00496	12.96768	0.00506	14.09155	0.00503	15.13348

0.00669	12.72185	0.00605	12.82545	0.00526	12.34954	0.00522	12.61676	0.00498	12.98453	0.00506	14.1032	0.00503	15.14957

0.00668	12.74113	0.00606	12.8449	0.00527	12.36266	0.00522	12.6287	0.00499	13.00905	0.00506	14.1215	0.00503	15.16388

0.0067	12.76633	0.00608	12.86285	0.00526	12.37287	0.00522	12.64512	0.00499	13.02437	0.00508	14.14148	0.00504	15.18534

0.0067	12.7693	0.00608	12.87631	0.00526	12.3889	0.00523	12.65706	0.00498	13.0351	0.00508	14.15646	0.00505	15.21038

0.00671	12.79006	0.00608	12.88828	0.00528	12.40785	0.00523	12.67199	0.00499	13.055	0.00507	14.16977	0.00505	15.22468

0.00672	12.81082	0.00609	12.90923	0.00528	12.42097	0.00523	12.68243	0.00499	13.06573	0.00507	14.18973	0.00504	15.23541

0.00673	12.81971	0.0061	12.92718	0.00527	12.43118	0.00524	12.69885	0.00499	13.07186	0.00509	14.21138	0.00506	15.25866

0.00673	12.82713	0.0061	12.93915	0.00529	12.45304	0.00525	12.71826	0.00499	13.09025	0.0051	14.22802	0.00506	15.28012

0.00674	12.8553	0.0061	12.9556	0.0053	12.46616	0.00524	12.73019	0.00501	13.1117	0.00509	14.23634	0.00507	15.29622

0.00675	12.86271	0.00611	12.97056	0.00528	12.47928	0.00525	12.75109	0.00501	13.12549	0.0051	14.25299	0.00506	15.30874

0.00673	12.87606	0.00611	12.97954	0.00529	12.49093	0.00527	12.76602	0.00501	13.13775	0.0051	14.27295	0.00507	15.32841

0.00675	12.89681	0.00611	12.993	0.0053	12.50843	0.00527	12.77647	0.00501	13.16073	0.00511	14.28793	0.00508	15.34809

0.00676	12.91461	0.00613	13.01245	0.00531	12.52155	0.00526	12.7899	0.00502	13.17758	0.00511	14.30124	0.00508	15.36776

0.00676	12.92499	0.00613	13.0319	0.00531	12.53467	0.00527	12.80781	0.00503	13.19443	0.00511	14.31788	0.00508	15.38028

0.00677	12.93537	0.00613	13.04088	0.00531	12.54924	0.00527	12.82423	0.00501	13.20056	0.00513	14.33953	0.00508	15.39815

0.00679	12.95909	0.00613	13.05284	0.00531	12.56674	0.00528	12.83617	0.00503	13.21435	0.00512	14.35783	0.0051	15.41962

0.00679	12.97095	0.00615	13.07977	0.00531	12.57985	0.00527	12.84512	0.00503	13.2358	0.00512	14.36947	0.0051	15.43929

0.00678	12.97836	0.00616	13.09473	0.00531	12.59152	0.00528	12.86602	0.00505	13.24499	0.00512	14.39112	0.0051	15.45181

0.00679	13.00061	0.00615	13.1067	0.00531	12.60755	0.0053	12.8899	0.00504	13.25571	0.00513	14.40943	0.0051	15.46791

0.00681	13.0184	0.00616	13.11867	0.00533	12.62651	0.00529	12.89438	0.00505	13.27716	0.00513	14.42274	0.00511	15.49115

0.00681	13.02581	0.00618	13.13363	0.00533	12.64108	0.00529	12.9093	0.00505	13.29555	0.00513	14.43439	0.00512	15.50904

0.00681	13.03916	0.00618	13.1456	0.00533	12.65128	0.0053	12.92572	0.00505	13.31088	0.00514	14.45269	0.00512	15.52513

0.00683	13.06436	0.00618	13.15906	0.00533	12.6644	0.00532	12.94513	0.00505	13.32466	0.00515	14.47101	0.00511	15.53945

0.00683	13.07029	0.0062	13.18001	0.00535	12.68482	0.00532	12.95408	0.00507	13.34611	0.00516	14.48932	0.00513	15.55911

0.00683	13.08809	0.0062	13.19946	0.00535	12.69793	0.00531	12.96602	0.00507	13.35683	0.00515	14.50263	0.00513	15.58415

0.00684	13.10143	0.0062	13.20394	0.00535	12.70668	0.00532	12.98543	0.00507	13.3645	0.00515	14.52093	0.00514	15.59667

0.00686	13.11922	0.00621	13.22339	0.00535	12.72416	0.00533	13.00632	0.00507	13.38135	0.00516	14.53924	0.00514	15.61276

0.00684	13.12812	0.00622	13.24583	0.00537	12.74166	0.00533	13.01827	0.00509	13.39973	0.00518	14.55755	0.00514	15.63065

0.00686	13.15036	0.00623	13.25481	0.00537	12.75624	0.00532	13.0317	0.00509	13.41199	0.00516	14.57086	0.00516	15.6539

0.00688	13.16519	0.00622	13.26528	0.00536	12.76645	0.00533	13.04364	0.00508	13.42424	0.00517	14.58251	0.00515	15.66821

0.00687	13.17557	0.00624	13.28623	0.00537	12.78102	0.00534	13.06454	0.00509	13.44723	0.00518	14.60082	0.00515	15.68252

0.00687	13.18891	0.00625	13.30119	0.00539	12.7985	0.00534	13.0705	0.00509	13.46714	0.00518	14.62079	0.00515	15.70219

0.00689	13.20522	0.00624	13.31016	0.00539	12.81309	0.00534	13.08692	0.0051	13.484	0.00518	14.63577	0.00517	15.72365

0.0069	13.22005	0.00625	13.32811	0.00539	12.82476	0.00536	13.10782	0.00509	13.49166	0.00518	14.64908	0.00517	15.74332

0.0069	13.23191	0.00626	13.34457	0.00539	12.83933	0.00537	13.12424	0.00511	13.50851	0.0052	14.66906	0.00517	15.75763

0.00691	13.25415	0.00627	13.35654	0.0054	12.85827	0.00536	13.1302	0.00511	13.52536	0.0052	14.68903	0.00517	15.77552

0.00692	13.26453	0.00627	13.36851	0.00541	12.87139	0.00536	13.1511	0.00511	13.53609	0.00522	14.709	0.00518	15.79698

0.00692	13.27788	0.00628	13.38646	0.00541	12.88452	0.00538	13.1705	0.00511	13.54528	0.0052	14.71566	0.00519	15.81485

0.00692	13.29864	0.00628	13.39992	0.00542	12.90055	0.00538	13.18245	0.00511	13.5652	0.00521	14.73562	0.00518	15.82917

0.00693	13.31198	0.00628	13.4089	0.00542	12.91658	0.00538	13.19588	0.00513	13.58359	0.00522	14.75394	0.00518	15.84704

0.00693	13.31939	0.00628	13.42685	0.00542	12.92825	0.00538	13.20483	0.00513	13.59583	0.00522	14.76892	0.00519	15.86493

0.00693	13.33422	0.00632	13.4478	0.00542	12.93845	0.0054	13.22723	0.00513	13.61576	0.00522	14.7789	0.0052	15.88282

0.00696	13.35943	0.00631	13.46276	0.00543	12.95741	0.0054	13.23767	0.00514	13.63874	0.00523	14.79887	0.0052	15.9007

0.00695	13.36388	0.00632	13.47622	0.00543	12.97489	0.00539	13.24961	0.00515	13.651	0.00524	14.82051	0.00519	15.9168

0.00695	13.3787	0.00632	13.49567	0.00543	12.9851	0.0054	13.272	0.00514	13.66019	0.00524	14.83881	0.0052	15.93826

0.00697	13.40094	0.00634	13.50913	0.00543	13.00113	0.00541	13.28991	0.00515	13.66939	0.00524	14.85047	0.00522	15.95793

0.00697	13.41132	0.00632	13.51811	0.00544	13.01862	0.00541	13.29588	0.00516	13.69543	0.00524	14.8671	0.00523	15.97402

0.00696	13.42318	0.00633	13.53307	0.00545	13.03319	0.00541	13.31379	0.00516	13.70615	0.00526	14.88541	0.00522	15.98654

0.00699	13.44543	0.00635	13.55252	0.00544	13.04487	0.00542	13.32872	0.00516	13.71534	0.00526	14.90206	0.00523	16.008

0.007	13.46025	0.00635	13.56598	0.00545	13.05652	0.00543	13.34663	0.00516	13.73833	0.00526	14.91537	0.00524	16.02946

0.007	13.46915	0.00635	13.58244	0.00545	13.07402	0.00543	13.35708	0.00517	13.76131	0.00526	14.93035	0.00524	16.04556

0.007	13.48843	0.00636	13.60189	0.00547	13.09005	0.00543	13.37051	0.00517	13.77357	0.00528	14.95032	0.00524	16.05987

0.00701	13.5077	0.00637	13.61685	0.00545	13.10026	0.00544	13.38992	0.00518	13.78429	0.00528	14.96863	0.00524	16.07954

0.00701	13.51956	0.00637	13.62283	0.00547	13.11483	0.00545	13.40634	0.00518	13.80114	0.00528	14.98194	0.00526	16.09921

0.00702	13.52549	0.00637	13.63779	0.00548	13.13524	0.00544	13.41827	0.0052	13.81799	0.00528	14.99858	0.00526	16.11709

0.00703	13.54477	0.00639	13.65275	0.00547	13.14836	0.00544	13.43171	0.00519	13.82872	0.0053	15.01855	0.00525	16.13141

0.00704	13.56404	0.0064	13.66622	0.00547	13.16002	0.00546	13.44514	0.00519	13.83791	0.0053	15.03687	0.00526	16.15108

0.00704	13.57442	0.00639	13.68267	0.00548	13.1746	0.00547	13.46604	0.0052	13.86242	0.0053	15.05017	0.00527	16.17254

0.00704	13.59222	0.00639	13.69614	0.0055	13.19209	0.00546	13.47499	0.0052	13.87622	0.0053	15.06016	0.00528	16.1922

0.00708	13.61001	0.00641	13.71858	0.0055	13.20667	0.00546	13.48544	0.0052	13.89	0.0053	15.0818	0.00527	16.20473

0.00706	13.62039	0.00642	13.73055	0.0055	13.21541	0.00548	13.50783	0.0052	13.90992	0.00532	15.09845	0.00528	16.22082

0.00706	13.63522	0.00642	13.74251	0.00551	13.2329	0.00548	13.52574	0.00522	13.92984	0.00532	15.11343	0.00528	16.2405

0.00708	13.64856	0.00643	13.76196	0.00553	13.25039	0.00547	13.53619	0.00522	13.94363	0.00532	15.13006	0.0053	16.26196

0.00708	13.66635	0.00644	13.76944	0.00551	13.2606	0.00548	13.5541	0.00522	13.95129	0.00533	15.15171	0.0053	16.27626

0.00708	13.6797	0.00643	13.78739	0.00551	13.27372	0.0055	13.5735	0.00522	13.96661	0.00534	15.17	0.00529	16.29236

0.0071	13.69601	0.00645	13.79787	0.00553	13.29559	0.00549	13.58246	0.00524	13.98192	0.00534	15.18333	0.0053	16.31204

0.00712	13.70935	0.00645	13.81731	0.00554	13.31016	0.00549	13.59141	0.00524	13.99418	0.00533	15.19664	0.00531	16.33528

0.0071	13.72121	0.00646	13.82928	0.00553	13.32037	0.0055	13.61082	0.00524	14.00797	0.00533	15.21494	0.00531	16.35137

0.00712	13.74049	0.00646	13.84873	0.00553	13.3364	0.00552	13.63022	0.00524	14.02329	0.00535	15.23325	0.0053	16.36389

0.00713	13.76125	0.00647	13.86668	0.00555	13.3539	0.00552	13.63917	0.00525	14.04781	0.00535	15.24823	0.00531	16.38357

0.00713	13.76421	0.00648	13.88164	0.00555	13.36701	0.00552	13.6541	0.00526	14.0616	0.00535	15.25988	0.00532	16.40503

0.00713	13.77904	0.00648	13.88613	0.00555	13.37867	0.00552	13.675	0.00526	14.07692	0.00535	15.27819	0.00534	16.42113

0.00715	13.80276	0.0065	13.90708	0.00555	13.39179	0.00553	13.69142	0.00526	14.09072	0.00537	15.30148	0.00532	16.43543

0.00716	13.81759	0.0065	13.92353	0.00556	13.41075	0.00553	13.70037	0.00528	14.10603	0.00537	15.31813	0.00534	16.45511

0.00715	13.82945	0.00651	13.93251	0.00556	13.42386	0.00553	13.7138	0.00527	14.11828	0.00537	15.32811	0.00535	16.47657

0.00717	13.8428	0.00651	13.95196	0.00556	13.43552	0.00554	13.72873	0.00526	14.12901	0.00537	15.34809	0.00536	16.49624

0.00717	13.86059	0.00652	13.97141	0.00557	13.45301	0.00555	13.74515	0.00528	14.14893	0.00538	15.36639	0.00535	16.50697

0.00717	13.87245	0.00653	13.99085	0.00559	13.47051	0.00555	13.75858	0.00529	14.16731	0.00538	15.38137	0.00535	16.52664

0.00718	13.88283	0.00652	13.99833	0.00558	13.48217	0.00555	13.77201	0.00528	14.17803	0.00538	15.39302	0.00537	16.54811

0.0072	13.90804	0.00653	14.0118	0.00558	13.49383	0.00556	13.78993	0.00528	14.19489	0.00539	15.40966	0.00537	16.56598

0.0072	13.9199	0.00655	14.02825	0.00559	13.51132	0.00557	13.80635	0.00531	14.22093	0.0054	15.43296	0.00538	16.57671

0.0072	13.92583	0.00655	14.03873	0.0056	13.52882	0.00557	13.82127	0.00531	14.23779	0.0054	15.44794	0.00538	16.59639

0.00721	13.94807	0.00655	14.05518	0.00559	13.53901	0.00557	13.8362	0.0053	14.23932	0.0054	15.4596	0.00538	16.61606

0.00723	13.9629	0.00655	14.07014	0.00559	13.5536	0.00558	13.85262	0.00531	14.25617	0.0054	15.47957	0.00539	16.63395

0.00721	13.97328	0.00657	14.08211	0.00561	13.57109	0.00559	13.86455	0.00532	14.27303	0.00542	15.49954	0.00539	16.65361

0.00723	13.99107	0.00657	14.09857	0.00561	13.58567	0.00558	13.875	0.00532	14.28682	0.00542	15.51452	0.00539	16.66614

0.00725	14.00886	0.00657	14.11802	0.00561	13.59732	0.00559	13.89291	0.00532	14.3006	0.00542	15.52617	0.00539	16.6858

0.00725	14.01776	0.00659	14.13746	0.00561	13.60899	0.00561	13.91083	0.00533	14.31746	0.00542	15.54115	0.00542	16.70726

0.00725	14.03259	0.0066	14.14943	0.00563	13.63085	0.00561	13.92575	0.00534	14.34197	0.00544	15.56444	0.00541	16.72515

0.00726	14.05483	0.00658	14.15841	0.00563	13.64397	0.0056	13.93769	0.00534	14.35882	0.00544	15.58275	0.00541	16.73946

0.00727	14.06669	0.0066	14.17636	0.00563	13.65271	0.0056	13.9556	0.00534	14.37262	0.00544	15.59274	0.00541	16.75734

0.00726	14.083	0.00662	14.19731	0.00563	13.66875	0.00563	13.97501	0.00535	14.3864	0.00544	15.61271	0.00543	16.7788

0.00728	14.09486	0.00662	14.20479	0.00565	13.6877	0.00563	13.98396	0.00536	14.40019	0.00546	15.63268	0.00543	16.7949

0.00729	14.11266	0.00662	14.22274	0.00565	13.70227	0.00562	13.9959	0.00535	14.41091	0.00546	15.65099	0.00543	16.80921

0.00729	14.12304	0.00664	14.2377	0.00565	13.71248	0.00564	14.01232	0.00535	14.42318	0.00546	15.66431	0.00543	16.82888

0.00729	14.13638	0.00665	14.25565	0.00565	13.72851	0.00565	14.03023	0.00537	14.44003	0.00546	15.67928	0.00544	16.85034

0.00731	14.15862	0.00663	14.26762	0.00567	13.74747	0.00564	14.04217	0.00537	14.45534	0.00548	15.69759	0.00544	16.86823

0.00732	14.16455	0.00664	14.27809	0.00567	13.75912	0.00565	14.05859	0.00537	14.46913	0.00548	15.71256	0.00544	16.88075

0.00732	14.18086	0.00667	14.30053	0.00567	13.76934	0.00566	14.078	0.00538	14.48752	0.00548	15.72754	0.00546	16.89863

0.00733	14.2031	0.00667	14.30652	0.00567	13.78682	0.00567	14.09292	0.0054	14.50744	0.00549	15.74253	0.00547	16.92188

0.00734	14.21645	0.00666	14.31848	0.00568	13.80432	0.00567	14.10187	0.0054	14.53041	0.00549	15.7625	0.00548	16.93797

0.00734	14.22238	0.00669	14.34392	0.00569	13.81597	0.00567	14.1168	0.0054	14.53961	0.0055	15.78246	0.00547	16.95407

0.00735	14.24165	0.00669	14.35738	0.00568	13.82619	0.00568	14.13322	0.0054	14.55647	0.00551	15.79911	0.00548	16.97016

0.00737	14.25796	0.00669	14.36785	0.00569	13.84513	0.00569	14.14964	0.00542	14.56566	0.0055	15.81076	0.00548	16.98805

0.00737	14.26686	0.00669	14.3888	0.0057	13.86263	0.00568	14.15859	0.00542	14.57638	0.00551	15.83074	0.00549	17.0113

0.00737	14.28465	0.0067	14.40525	0.0057	13.87428	0.00569	14.1765	0.00542	14.59017	0.00552	15.84737	0.00549	17.02739

0.00738	14.30541	0.00672	14.41423	0.0057	13.88886	0.0057	14.19292	0.00542	14.61009	0.00553	15.86402	0.0055	17.04349

0.00738	14.31282	0.00672	14.43069	0.00571	13.90781	0.00571	14.21233	0.00544	14.62695	0.00553	15.874	0.0055	17.05958

0.00738	14.32913	0.00673	14.44415	0.00572	13.91947	0.0057	14.22128	0.00544	14.64073	0.00553	15.8923	0.00551	17.08284

0.00742	14.34989	0.00674	14.45761	0.00572	13.93114	0.00571	14.24218	0.00544	14.66219	0.00555	15.91395	0.00551	17.09893

0.00742	14.36176	0.00674	14.47557	0.00573	13.94426	0.00572	14.25263	0.00544	14.6775	0.00555	15.92893	0.00551	17.11323

0.00741	14.37213	0.00674	14.49352	0.00574	13.9632	0.00572	14.26755	0.00546	14.69129	0.00554	15.94225	0.00551	17.13112

0.00742	14.38844	0.00676	14.51297	0.00575	13.97924	0.00572	14.27501	0.00544	14.70354	0.00555	15.9622	0.00553	17.15258

0.00744	14.41069	0.00676	14.52194	0.00574	13.98945	0.00573	14.2974	0.00545	14.71427	0.00557	15.98218	0.00554	17.17047

0.00743	14.41513	0.00676	14.52643	0.00575	14.00548	0.00574	14.31382	0.00547	14.73726	0.00557	15.99882	0.00553	17.18477

0.00743	14.43293	0.00677	14.54289	0.00577	14.02297	0.00574	14.32427	0.00548	14.74798	0.00557	16.01048	0.00554	17.20266

0.00746	14.45368	0.00679	14.56982	0.00577	14.03754	0.00574	14.34517	0.00547	14.76024	0.00557	16.02878	0.00555	17.22591

0.00746	14.45665	0.00679	14.5773	0.00577	14.04775	0.00576	14.36159	0.00548	14.77861	0.00558	16.04709	0.00556	17.24558

0.00746	14.47444	0.0068	14.58927	0.00577	14.0638	0.00577	14.37353	0.00549	14.80313	0.00559	16.06374	0.00555	17.2581

0.00748	14.49817	0.00681	14.61171	0.00579	14.07982	0.00576	14.38397	0.0055	14.82152	0.00559	16.07372	0.00556	17.27599

0.00748	14.51448	0.00681	14.62517	0.00579	14.09731	0.00577	14.40188	0.00549	14.83071	0.00559	16.09369	0.00558	17.29745

0.00748	14.52189	0.00681	14.63863	0.00579	14.10314	0.00578	14.41532	0.0055	14.84143	0.0056	16.11533	0.00558	17.31354

0.0075	14.53523	0.00682	14.65659	0.00579	14.11918	0.00579	14.43173	0.00551	14.85522	0.00561	16.1303	0.00558	17.32964

0.00752	14.55451	0.00684	14.66706	0.0058	14.13813	0.00578	14.44218	0.00551	14.86902	0.00561	16.14362	0.00558	17.34573

0.0075	14.56489	0.00684	14.68202	0.0058	14.15271	0.00578	14.4586	0.00551	14.8828	0.00561	16.16192	0.00559	17.36898

0.00752	14.58268	0.00683	14.69698	0.0058	14.16437	0.0058	14.47801	0.00553	14.89965	0.00562	16.18189	0.0056	17.39044

0.00754	14.59751	0.00686	14.71942	0.00581	14.17894	0.0058	14.49442	0.00553	14.91345	0.00563	16.19687	0.0056	17.40296

0.00754	14.61085	0.00686	14.72989	0.00582	14.19498	0.0058	14.50786	0.00553	14.93643	0.00562	16.20685	0.0056	17.41906

0.00754	14.62123	0.00686	14.74186	0.00582	14.21101	0.0058	14.5198	0.00553	14.95174	0.00562	16.2235	0.00562	17.43873

0.00755	14.64792	0.00686	14.76131	0.00582	14.22268	0.00582	14.53771	0.00555	14.97013	0.00564	16.24847	0.00563	17.46198

0.00757	14.6583	0.00689	14.78225	0.00583	14.23871	0.00583	14.55263	0.00555	14.98698	0.00565	16.26345	0.00563	17.47628

0.00757	14.66868	0.00689	14.79273	0.00584	14.25475	0.00582	14.56159	0.00554	14.99157	0.00564	16.27675	0.00562	17.4888

0.00758	14.68203	0.00689	14.8032	0.00584	14.26786	0.00583	14.57651	0.00555	15.00691	0.00564	16.29673	0.00563	17.50669

0.00758	14.70427	0.00692	14.82414	0.00584	14.27953	0.00585	14.59891	0.00557	15.0253	0.00566	16.31504	0.00564	17.52993

0.00758	14.70871	0.00692	14.83461	0.00586	14.29993	0.00585	14.61234	0.00557	15.03295	0.00567	16.33001	0.00565	17.54603

0.00758	14.72354	0.00691	14.83761	0.00586	14.31306	0.00585	14.62577	0.00557	15.04828	0.00566	16.34499	0.00565	17.56212

0.00762	14.74578	0.00692	14.86603	0.00586	14.32179	0.00585	14.6392	0.00557	15.06819	0.00567	16.35831	0.00565	17.58001

0.00763	14.75616	0.00694	14.88997	0.00586	14.33929	0.00587	14.65861	0.00559	15.08504	0.00567	16.37495	0.00566	17.59969

0.00762	14.77247	0.00694	14.89595	0.00588	14.35532	0.00587	14.66756	0.00559	15.10342	0.00569	16.3966	0.00568	17.61936

0.00763	14.79175	0.00694	14.91091	0.00589	14.37137	0.00587	14.68099	0.00558	15.11875	0.00569	16.41323	0.00566	17.63545

0.00764	14.80509	0.00696	14.93186	0.00589	14.37864	0.00588	14.69741	0.00559	15.13254	0.00568	16.42655	0.00567	17.64975

0.00764	14.81399	0.00697	14.94233	0.00589	14.3976	0.00589	14.71383	0.00561	15.14939	0.00568	16.44485	0.0057	17.66943

0.00765	14.83475	0.00696	14.95579	0.00591	14.41655	0.00588	14.72726	0.00561	15.16318	0.00569	16.46316	0.0057	17.6891

0.00767	14.84809	0.00698	14.96776	0.00591	14.42967	0.00589	14.74368	0.00561	15.17237	0.00569	16.47814	0.00571	17.7052

0.00767	14.86144	0.007	14.99319	0.0059	14.44133	0.00591	14.76309	0.00562	15.18769	0.00571	16.49146	0.0057	17.72129

0.00767	14.87478	0.007	15.00217	0.00591	14.45591	0.00592	14.77652	0.00563	15.21221	0.00571	16.50976	0.00572	17.73918

0.00769	14.89554	0.00699	15.01264	0.00592	14.47632	0.00591	14.78398	0.00563	15.22292	0.00573	16.52806	0.00572	17.76243

0.0077	14.9074	0.00701	15.03359	0.00592	14.48506	0.00592	14.8004	0.00563	15.24745	0.00573	16.54305	0.00573	17.78032

0.0077	14.91926	0.00703	15.04855	0.00592	14.49818	0.00594	14.81682	0.00565	15.26582	0.00572	16.55636	0.00572	17.79462

0.00771	14.93557	0.00702	15.05902	0.00592	14.51276	0.00594	14.83324	0.00566	15.28269	0.00573	16.57467	0.00573	17.81251

0.00772	14.9504	0.00701	15.07099	0.00594	14.53317	0.00594	14.84518	0.00565	15.28115	0.00575	16.59631	0.00574	17.83038

0.00771	14.96374	0.00704	15.09343	0.00594	14.54337	0.00593	14.8601	0.00566	15.29954	0.00575	16.61128	0.00574	17.85006

0.00771	14.97857	0.00704	15.1039	0.00594	14.55648	0.00595	14.8825	0.00567	15.31333	0.00575	16.62626	0.00574	17.86079

0.00775	14.99785	0.00704	15.11736	0.00596	14.57689	0.00596	14.89742	0.00568	15.32558	0.00575	16.6429	0.00576	17.88404

0.00775	15.00823	0.00707	15.13831	0.00597	14.59147	0.00596	14.90787	0.00567	15.33477	0.00577	16.66122	0.00577	17.9055

0.00775	15.02305	0.00708	15.15925	0.00598	14.60021	0.00596	14.9213	0.00567	15.35929	0.00578	16.67618	0.00578	17.92517

0.00777	15.04381	0.00706	15.16524	0.00597	14.61625	0.00598	14.93772	0.0057	15.3838	0.00577	16.68784	0.00577	17.93769

0.00777	15.05716	0.00708	15.1802	0.00598	14.63374	0.00599	14.95115	0.0057	15.39912	0.00577	16.70781	0.00577	17.95379

0.00779	15.06012	0.0071	15.19815	0.006	14.64832	0.00599	14.96309	0.00569	15.41139	0.00579	16.72612	0.00579	17.97704

0.00779	15.08829	0.0071	15.21161	0.00599	14.65998	0.00599	14.97951	0.0057	15.43129	0.0058	16.74443	0.0058	17.99492

0.00781	15.10312	0.00709	15.2176	0.006	14.67456	0.00601	15.00041	0.00572	15.44509	0.0058	16.75775	0.00579	18.00744

0.0078	15.1135	0.00713	15.24004	0.00601	14.69205	0.00601	15.01384	0.00572	15.45887	0.0058	16.77438	0.00579	18.02353

0.00781	15.12981	0.00713	15.2535	0.00602	14.70809	0.006	15.02429	0.00572	15.46041	0.00582	16.79603	0.00582	18.04678

0.00783	15.1476	0.00712	15.26397	0.00602	14.71682	0.00602	15.04369	0.00572	15.47879	0.00582	16.811	0.00582	18.06467

0.00783	15.15947	0.00713	15.28492	0.00603	14.73432	0.00603	15.06011	0.00574	15.49718	0.00582	16.82099	0.00583	18.07897

0.00783	15.16984	0.00715	15.30287	0.00604	14.75036	0.00603	15.06906	0.00574	15.50943	0.00582	16.8393	0.00583	18.09507

0.00786	15.19357	0.00716	15.31484	0.00604	14.76494	0.00603	15.081	0.00574	15.53394	0.00584	16.86259	0.00584	18.11475

0.00787	15.20691	0.00715	15.32382	0.00604	14.77513	0.00605	15.1019	0.00576	15.55233	0.00584	16.87924	0.00585	18.13978

0.00787	15.21729	0.00716	15.34326	0.00605	14.79263	0.00606	15.11832	0.00576	15.57531	0.00584	16.89089	0.00585	18.1523

0.00788	15.23509	0.00718	15.35822	0.00606	14.80866	0.00605	15.12727	0.00575	15.57531	0.00584	16.91251	0.00585	18.17018

0.0079	15.24991	0.00718	15.37169	0.00606	14.82033	0.00605	15.14369	0.00576	15.59064	0.00586	16.92916	0.00586	18.18807

0.0079	15.26326	0.0072	15.38515	0.00606	14.83344	0.00608	15.16609	0.00578	15.60442	0.00587	16.94747	0.00587	18.20774

0.0079	15.2766	0.00721	15.40909	0.00608	14.85531	0.00608	15.18101	0.00579	15.62281	0.00586	16.95745	0.00588	18.22562

0.00792	15.29143	0.00722	15.42255	0.00608	14.86843	0.00607	15.18698	0.00578	15.62434	0.00587	16.9741	0.00587	18.23993

0.00793	15.30626	0.00722	15.42854	0.00607	14.88009	0.00609	15.20489	0.00578	15.64425	0.00588	16.99407	0.00588	18.2596

0.00792	15.32108	0.00722	15.44499	0.00608	14.89175	0.00611	15.21981	0.0058	15.67336	0.00589	17.01238	0.00589	18.28106

0.00795	15.33739	0.00725	15.46444	0.0061	14.91362	0.00611	15.23623	0.0058	15.68256	0.00588	17.0207	0.00591	18.29895

0.00796	15.35519	0.00726	15.47641	0.0061	14.92528	0.0061	15.24519	0.0058	15.70094	0.00588	17.03734	0.0059	18.31147

0.00796	15.36557	0.00725	15.48688	0.0061	14.93548	0.00611	15.2631	0.00582	15.72546	0.00589	17.06064	0.00591	18.33114

0.00797	15.38039	0.00727	15.51082	0.00612	14.95297	0.00614	15.284	0.00583	15.73618	0.00591	17.07562	0.00592	18.3526

0.00798	15.39819	0.00727	15.52279	0.00613	14.97046	0.00614	15.29892	0.00582	15.74538	0.00591	17.09392	0.00594	18.36869

0.00799	15.41005	0.00727	15.53775	0.00614	14.98359	0.00614	15.31086	0.00583	15.74997	0.00591	17.10891	0.00592	18.38301

0.008	15.42784	0.00728	15.55869	0.00613	14.9938	0.00614	15.32429	0.00585	15.77142	0.00592	17.12888	0.00594	18.39731

0.00801	15.44415	0.0073	15.57216	0.00615	15.01274	0.00616	15.34221	0.00585	15.77909	0.00593	17.14053	0.00595	18.42234

0.00802	15.45305	0.0073	15.58113	0.00616	15.02877	0.00617	15.35415	0.00585	15.7944	0.00593	17.15551	0.00596	18.43844

0.00802	15.46787	0.00731	15.5946	0.00616	15.04044	0.00616	15.36609	0.00587	15.82198	0.00593	17.17215	0.00597	18.45633

0.00804	15.48715	0.00733	15.61105	0.00616	15.05064	0.00617	15.3825	0.00588	15.83423	0.00594	17.19045	0.00597	18.47064

0.00805	15.50939	0.00734	15.62452	0.00617	15.06959	0.00618	15.40042	0.00589	15.85262	0.00595	17.2121	0.00598	18.49031

0.00805	15.50939	0.00734	15.63649	0.00618	15.08562	0.00618	15.41236	0.00588	15.86641	0.00596	17.22707	0.00599	18.51356

0.00806	15.53015	0.00735	15.65444	0.00618	15.09729	0.00619	15.42728	0.0059	15.89092	0.00596	17.24039	0.00599	18.52607

0.00808	15.54794	0.00737	15.67688	0.00618	15.11041	0.0062	15.44968	0.00591	15.9093	0.00597	17.25869	0.00599	18.54218

0.00808	15.55536	0.00737	15.69034	0.0062	15.12936	0.00622	15.46012	0.00592	15.90779	0.00598	17.277	0.006	18.56364

0.00808	15.5687	0.00737	15.69782	0.0062	15.14539	0.00621	15.46758	0.00592	15.92003	0.00599	17.29364	0.00601	18.5851

0.00811	15.59094	0.00738	15.72026	0.0062	15.1556	0.00622	15.48549	0.00592	15.93842	0.00599	17.30863	0.00601	18.5994

0.00812	15.60132	0.0074	15.72924	0.00622	15.16872	0.00624	15.50639	0.00593	15.95834	0.006	17.32526	0.00601	18.61549

0.00811	15.61466	0.0074	15.7427	0.00623	15.18621	0.00625	15.51684	0.00594	15.97059	0.00601	17.34357	0.00603	18.63517

0.00813	15.63839	0.00741	15.75766	0.00624	15.20224	0.00625	15.52878	0.00594	15.98438	0.00602	17.35855	0.00604	18.65663

0.00815	15.65173	0.00743	15.77861	0.00624	15.21245	0.00625	15.54669	0.00596	16.01195	0.00602	17.37353	0.00606	18.67094

0.00815	15.6547	0.00744	15.79207	0.00624	15.22702	0.00627	15.56759	0.00596	16.02729	0.00602	17.39184	0.00604	18.68882

0.00817	15.67842	0.00743	15.80554	0.00626	15.24452	0.00627	15.57953	0.00596	16.03494	0.00604	17.41181	0.00606	18.70671

0.00818	15.69622	0.00745	15.819	0.00626	15.2591	0.00627	15.59296	0.00596	16.04567	0.00604	17.42678	0.00607	18.72638

0.00818	15.70659	0.00747	15.83845	0.00626	15.27076	0.00628	15.6034	0.00598	16.06404	0.00604	17.43844	0.00609	18.74247

0.0082	15.72587	0.00746	15.84593	0.00627	15.28679	0.0063	15.6243	0.006	16.08857	0.00605	17.45675	0.00608	18.755

0.00821	15.7407	0.00747	15.86089	0.00628	15.30573	0.0063	15.63624	0.00598	16.08703	0.00606	17.47506	0.00609	18.77646

0.00823	15.75256	0.0075	15.88333	0.00629	15.32031	0.0063	15.64818	0.00599	16.10389	0.00607	17.49169	0.0061	18.79612

0.00821	15.76294	0.00751	15.8938	0.00628	15.32907	0.00631	15.66311	0.00601	16.12993	0.00606	17.50334	0.00611	18.8158

0.00824	15.78666	0.0075	15.90427	0.00629	15.34364	0.00632	15.6855	0.00602	16.14065	0.00608	17.52331	0.00611	18.82831

0.00825	15.79556	0.00752	15.92522	0.00631	15.36259	0.00633	15.69744	0.00601	16.15904	0.00609	17.54495	0.00612	18.8462

0.00825	15.80149	0.00754	15.94317	0.00631	15.37425	0.00633	15.71087	0.00603	16.18203	0.0061	17.56159	0.00613	18.86945

0.00827	15.82076	0.00754	15.95514	0.00631	15.38446	0.00635	15.72729	0.00604	16.20041	0.0061	17.57324	0.00614	18.88555

0.00829	15.84745	0.00754	15.96561	0.00633	15.40195	0.00636	15.7452	0.00604	16.20654	0.0061	17.59321	0.00614	18.90164

0.00829	15.85042	0.00757	15.98506	0.00634	15.4209	0.00636	15.75565	0.00604	16.21113	0.00612	17.61319	0.00615	18.91953

0.0083	15.87118	0.00757	16.00002	0.00636	15.43402	0.00636	15.76908	0.00605	16.22798	0.00613	17.62484	0.00616	18.93741

0.00832	15.88452	0.00757	16.009	0.00636	15.44713	0.00639	15.78699	0.00607	16.25403	0.00613	17.63815	0.00617	18.96066

0.00833	15.90083	0.00758	16.02994	0.00636	15.46025	0.0064	15.80341	0.00607	16.25556	0.00613	17.6548	0.00619	18.97675

0.00833	15.91418	0.00761	16.05238	0.00638	15.47774	0.00639	15.81386	0.00607	16.27549	0.00614	17.67477	0.00619	18.98927

0.00835	15.929	0.00761	16.05537	0.00638	15.49087	0.0064	15.82878	0.00609	16.29539	0.00614	17.69141	0.00619	19.00716

0.00837	15.94383	0.00763	16.07632	0.00638	15.50544	0.00642	15.84968	0.00609	16.32144	0.00614	17.70639	0.00621	19.03219

0.00837	15.95569	0.00764	16.09427	0.0064	15.52002	0.00643	15.86312	0.00609	16.31685	0.00615	17.72469	0.00621	19.04651

0.00838	15.97349	0.00765	16.09876	0.00641	15.53605	0.00642	15.86908	0.0061	16.34136	0.00616	17.74467	0.00622	19.0626

0.0084	15.98831	0.00765	16.11073	0.0064	15.54772	0.00643	15.88849	0.00612	16.35975	0.00618	17.76131	0.00622	19.08048

0.00841	16.00314	0.00766	16.13466	0.00641	15.56229	0.00645	15.90789	0.00613	16.37353	0.00618	17.77295	0.00625	19.10194

0.00842	16.01204	0.00769	16.15112	0.00643	15.58125	0.00646	15.91983	0.00613	16.38272	0.0062	17.7896	0.00625	19.11803

0.00844	16.03576	0.0077	16.16458	0.00643	15.59436	0.00646	15.93177	0.00614	16.39499	0.0062	17.80624	0.00625	19.13235

0.00846	16.04762	0.00769	16.17954	0.00643	15.60456	0.00647	15.95118	0.00615	16.4195	0.0062	17.82289	0.00625	19.15202

0.00846	16.05652	0.00771	16.19899	0.00644	15.61914	0.00649	15.96909	0.00616	16.43789	0.00621	17.8362	0.00627	19.17169

0.00847	16.07431	0.00773	16.21395	0.00646	15.63664	0.0065	15.98252	0.00616	16.4532	0.00622	17.85617	0.00628	19.18779

0.00849	16.09359	0.00773	16.22442	0.00647	15.65121	0.0065	15.99297	0.00617	16.46547	0.00624	17.87449	0.00628	19.20209

0.00849	16.09952	0.00774	16.23639	0.00647	15.66433	0.00651	16.00939	0.00618	16.48078	0.00624	17.89279	0.00629	19.22177

0.0085	16.11435	0.00777	16.25285	0.00647	15.6789	0.00652	16.02431	0.00618	16.49303	0.00624	17.9061	0.00631	19.24323

0.00854	16.144	0.00777	16.26631	0.0065	15.69786	0.00653	16.03924	0.00618	16.49763	0.00624	17.92274	0.00632	19.2629

0.00854	16.14845	0.00777	16.27828	0.0065	15.71098	0.00653	16.05267	0.0062	16.52369	0.00626	17.94105	0.00633	19.2772

0.00854	16.16328	0.00781	16.30371	0.0065	15.72264	0.00654	16.0676	0.00622	16.53287	0.00628	17.95936	0.00633	19.29152

0.00857	16.18255	0.00781	16.31867	0.00652	15.73867	0.00656	16.08551	0.00621	16.54513	0.00628	17.97102	0.00635	19.31118

0.00858	16.1959	0.00782	16.32765	0.00653	15.7547	0.00657	16.09894	0.00622	16.56505	0.00629	17.98765	0.00635	19.33086

0.00858	16.19886	0.00783	16.34261	0.00653	15.76637	0.00657	16.11237	0.00624	16.59263	0.00629	18.00429	0.00636	19.34696

0.00861	16.22852	0.00786	16.36206	0.00653	15.77949	0.00658	16.13029	0.00625	16.60948	0.00631	18.02593	0.00637	19.36305

0.00863	16.24038	0.00786	16.37702	0.00655	15.79698	0.0066	16.1482	0.00624	16.62021	0.00631	18.03925	0.00638	19.38629

0.00863	16.25224	0.00786	16.37851	0.00657	15.81155	0.0066	16.15715	0.00625	16.63552	0.00631	18.05423	0.00639	19.40418

0.00864	16.26707	0.0079	16.40245	0.00656	15.82467	0.00661	16.1676	0.00627	16.64472	0.00633	18.07586	0.0064	19.42028

0.00867	16.28486	0.00791	16.4234	0.00657	15.83925	0.00664	16.18999	0.00628	16.65085	0.00633	18.09416	0.0064	19.43637

0.00868	16.29672	0.00791	16.42788	0.00659	15.85966	0.00665	16.20342	0.00628	16.67076	0.00635	18.10748	0.00641	19.45605

0.00869	16.3071	0.00792	16.45332	0.00661	15.87278	0.00664	16.21536	0.00628	16.68455	0.00635	18.11914	0.00644	19.47751

0.00871	16.33082	0.00796	16.46828	0.00661	15.88298	0.00665	16.22879	0.0063	16.70294	0.00635	18.13744	0.00645	19.49181

0.00872	16.34269	0.00797	16.48024	0.00661	15.89464	0.00667	16.2482	0.00631	16.71826	0.00637	18.15741	0.00645	19.50791

0.00873	16.35455	0.00798	16.48473	0.00664	15.91505	0.00669	16.26313	0.0063	16.73664	0.00637	18.17073	0.00646	19.52579

0.00875	16.36938	0.00799	16.50418	0.00665	15.92817	0.00668	16.27357	0.00632	16.76422	0.00637	18.18737	0.00647	19.55083

0.00877	16.38717	0.00801	16.52363	0.00664	15.93692	0.00669	16.29298	0.00636	16.77189	0.00638	18.20568	0.00648	19.56693

0.00879	16.40051	0.00801	16.5356	0.00665	15.95586	0.00671	16.3079	0.00635	16.77648	0.0064	18.22564	0.00648	19.57944

0.0088	16.41831	0.00803	16.54906	0.00667	15.97482	0.00673	16.32134	0.00636	16.79639	0.0064	18.23729	0.0065	19.59912

0.00883	16.43165	0.00806	16.57001	0.00668	15.98647	0.00672	16.3303	0.00638	16.80252	0.00641	18.25227	0.00651	19.62236

0.00884	16.445	0.00808	16.58347	0.00669	15.9996	0.00673	16.34969	0.00639	16.8301	0.00641	18.27058	0.00653	19.63668

0.00885	16.45241	0.00808	16.59095	0.00669	16.01563	0.00676	16.36761	0.00638	16.83317	0.00643	18.29056	0.00653	19.65098

0.00888	16.4791	0.0081	16.6089	0.00671	16.03167	0.00676	16.38253	0.0064	16.86074	0.00644	18.30553	0.00653	19.66708

0.00891	16.49244	0.00813	16.62686	0.00672	16.04478	0.00676	16.39447	0.00642	16.88066	0.00644	18.31884	0.00656	19.68675

0.00891	16.50282	0.00813	16.63882	0.00673	16.05645	0.00678	16.4094	0.00642	16.89292	0.00645	18.33715	0.00657	19.70463

0.00892	16.51913	0.00815	16.65528	0.00673	16.07102	0.0068	16.42582	0.00642	16.91436	0.00647	18.35879	0.00658	19.72252

0.00896	16.53841	0.00818	16.67323	0.00675	16.09144	0.0068	16.43925	0.00644	16.92355	0.00647	18.37376	0.00659	19.73861

0.00897	16.54879	0.0082	16.6867	0.00677	16.10163	0.00681	16.45119	0.00644	16.95114	0.00647	18.38709	0.0066	19.76007

0.00899	16.56065	0.00822	16.70016	0.00677	16.11476	0.00683	16.46611	0.00646	16.94653	0.00649	18.40539	0.00661	19.77975

0.00902	16.58141	0.00823	16.71662	0.00678	16.1337	0.00685	16.48552	0.00646	16.95727	0.00651	18.42203	0.00663	19.79763

0.00904	16.59475	0.00826	16.73607	0.0068	16.15119	0.00685	16.49447	0.00647	16.97565	0.00651	18.44035	0.00663	19.81015

0.00904	16.60661	0.00828	16.74504	0.0068	16.15994	0.00685	16.50791	0.00648	17.00016	0.00651	18.45199	0.00665	19.82804

0.00908	16.62441	0.00829	16.75103	0.00681	16.17307	0.00688	16.5303	0.0065	17.01242	0.00653	18.46863	0.00667	19.8477

0.00911	16.63775	0.00833	16.77197	0.00683	16.19201	0.00689	16.54522	0.0065	17.03694	0.00655	18.49027	0.00668	19.86738

0.00913	16.64813	0.00835	16.78543	0.00684	16.20658	0.0069	16.55417	0.00651	17.04919	0.00655	18.50858	0.00668	19.87811

0.00915	16.66592	0.00837	16.80039	0.00685	16.2168	0.00691	16.5691	0.00653	17.0691	0.00655	18.52022	0.0067	19.90135

0.00919	16.68372	0.0084	16.81087	0.00687	16.23138	0.00693	16.59149	0.00653	17.0829	0.00656	18.53687	0.00672	19.92103

0.00921	16.69706	0.00844	16.8348	0.00689	16.24886	0.00694	16.60343	0.00653	17.07677	0.00659	18.5585	0.00674	19.9407

0.00922	16.70744	0.00847	16.84677	0.00689	16.26343	0.00694	16.61537	0.00656	17.10281	0.0066	18.57348	0.00673	19.95322

0.00928	16.72968	0.00849	16.85276	0.00689	16.2751	0.00696	16.6288	0.00657	17.10588	0.00659	18.58346	0.00675	19.96932

0.0093	16.74302	0.00854	16.8722	0.0069	16.29259	0.00698	16.64821	0.00657	17.12427	0.0066	18.60177	0.00677	19.99435

0.00931	16.74896	0.00863	16.87819	0.00692	16.31008	0.00699	16.66314	0.00657	17.14418	0.00662	18.62175	0.00679	20.01224

0.00936	16.76971	0.00877	16.76599	0.00694	16.32029	0.007	16.67806	0.00659	17.16103	0.00663	18.63839	0.0068	20.02655

0.0094	16.78751	0.00896	16.01199	0.00694	16.33341	0.00701	16.69149	0.00661	17.18708	0.00663	18.6517	0.0068	20.04265

0.00943	16.79492	0.00939	15.22807	0.00695	16.34798	0.00704	16.70941	0.00661	17.1978	0.00663	18.67168	0.00683	20.06231

0.00947	16.80827	0.00968	14.33494	0.00699	16.36693	0.00705	16.72135	0.00662	17.21466	0.00665	18.69164	0.00685	20.08377

0.00953	16.82458	0.00988	13.57197	0.00699	16.3786	0.00706	16.73328	0.00665	17.23764	0.00667	18.70829	0.00685	20.0963

0.00958	16.8394	0.0101	12.54719	0.00701	16.39172	0.00707	16.7497	0.00666	17.23764	0.00667	18.71828	0.00687	20.11596

0.00962	16.84682	0.01028	11.49398	0.00702	16.40921	0.00709	16.76463	0.00665	17.2499	0.00667	18.73658	0.00688	20.13922

0.0097	16.86757	0.01051	10.68613	0.00703	16.42524	0.0071	16.77806	0.00668	17.27287	0.00669	18.75655	0.00689	20.1571

0.00978	16.87499	0.01078	9.87528	0.00704	16.43545	0.00711	16.79747	0.0067	17.2836	0.00671	18.77154	0.00692	20.16425

0.00999	16.12769	0.01097	8.9268	0.00705	16.45149	0.00713	16.81687	0.00669	17.29279	0.00671	18.78652	0.00693	20.18393

0.0101	15.22174	0.01106	7.3814	0.00707	16.46752	0.00715	16.82881	0.0067	17.3173	0.00672	18.80149	0.00695	20.20539

0.01017	14.39734	0.01116	5.10445	0.00709	16.48064	0.00717	16.83926	0.00672	17.34488	0.00675	18.82479	0.00697	20.22506

0.01026	13.49732	0.01126	3.64283	0.0071	16.49229	0.00717	16.85121	0.00674	17.35561	0.00675	18.84144	0.00698	20.23758

0.01039	12.52168	0.01199	2.84245	0.00711	16.50979	0.0072	16.8706	0.00673	17.36173	0.00676	18.85475	0.00699	20.25546

0.0105	11.63946	0.01402	2.07349	0.00714	16.52728	0.00722	16.88404	0.00675	17.37706	0.00677	18.87138	0.00701	20.27692

0.01062	10.69051	--	--	0.00715	16.53895	0.00723	16.89598	0.00678	17.40005	0.00679	18.89136	0.00705	20.29838

0.01076	9.94024	0.00715	16.54914	0.00724	16.91239	0.00678	17.4123	0.0068	18.90467	0.00706	20.31091

0.01102	9.02688	0.00717	16.56518	0.00727	16.93329	0.00678	17.42149	0.0068	18.91966	0.00708	20.327

0.01128	8.23065	0.0072	16.58267	0.00729	16.94673	0.00681	17.44294	0.00682	18.9363	0.00709	20.34489

0.01166	7.42553	0.00722	16.59872	0.00729	16.95717	0.00683	17.45979	0.00684	18.9546	0.00712	20.36635

0.01217	6.60854	0.00723	16.61037	0.00731	16.97359	0.00684	17.47817	0.00685	18.97125	0.00715	20.38244

0.0129	5.78563	0.00725	16.62495	0.00735	16.9915	0.00685	17.49197	0.00686	18.98623	0.00715	20.39675

0.01351	5.01757	0.00727	16.64244	0.00736	17.00344	0.00686	17.50882	0.00686	19.00287	0.00717	20.41284

0.01403	4.19614	0.00728	16.65701	0.00736	17.0139	0.00688	17.52107	0.00688	19.02284	0.00721	20.43609

0.01481	3.41918	0.00729	16.66576	0.00739	17.0318	0.00689	17.5318	0.00689	19.03782	0.00723	20.45398

--	--	0.00732	16.6818	0.00743	17.04971	0.0069	17.5364	0.0069	19.05112	0.00725	20.47007

--	--	0.00734	16.69929	0.00744	17.06165	0.00692	17.55479	0.00691	19.06778	0.00728	20.48617

--	--	0.00736	16.71386	0.00745	17.07509	0.00694	17.5793	0.00692	19.08608	0.00731	20.50763

--	--	0.00737	16.72553	0.00748	17.093	0.00695	17.58849	0.00694	19.10439	0.00734	20.52552

0.00739	16.74156	0.0075	17.10792	0.00696	17.60993	0.00695	19.11771	0.00736	20.53803

0.00742	16.7576	0.00752	17.12285	0.00698	17.63139	0.00696	19.13434	0.0074	20.55949

0.00744	16.76925	0.00754	17.13181	0.007	17.64976	0.00698	19.15765	0.00744	20.57738

0.00745	16.78091	0.00757	17.14972	0.007	17.6513	0.007	19.17263	0.00747	20.59704

0.00749	16.80132	0.0076	17.16613	0.00701	17.66815	0.00701	19.18761	0.0075	20.60957

0.00751	16.81445	0.00761	17.17808	0.00704	17.685	0.00702	19.20092	0.00755	20.62924

0.00752	16.82464	0.00764	17.19301	0.00705	17.69267	0.00704	19.21922	0.0076	20.64534

0.00755	16.84068	0.00768	17.20942	0.00706	17.71566	0.00706	19.2342	0.00764	20.66501

0.00759	16.85817	0.00771	17.22733	0.00708	17.73251	0.00707	19.25084	0.00769	20.6811

0.00761	16.87421	0.00773	17.24076	0.00711	17.74017	0.00708	19.26416	0.00774	20.69542

0.00763	16.88295	0.00776	17.25271	0.00711	17.75549	0.00709	19.28579	0.00783	20.71151

0.00765	16.89899	0.00781	17.26913	0.00712	17.77387	0.00712	19.30244	0.00792	20.72761

0.0077	16.91648	0.00783	17.28405	0.00715	17.79379	0.00713	19.31742	0.00803	20.73654

0.00773	16.93106	0.00786	17.29599	0.00716	17.80911	0.00714	19.33406	0.00821	20.67037

0.00775	16.94127	0.0079	17.31241	0.00718	17.82289	0.00716	19.35071	0.00875	19.77617

0.00778	16.95439	0.00795	17.33181	0.0072	17.83363	0.00718	19.36901	0.00893	18.87124

0.00782	16.97333	0.00798	17.34524	0.00722	17.85661	0.0072	19.38399	0.00905	17.71593

0.00786	16.98791	0.00801	17.35271	0.00724	17.86733	0.0072	19.39731	0.00919	16.51233

0.00788	16.99957	0.00806	17.36913	0.00724	17.87346	0.00723	19.41727	0.00934	15.56091

0.00793	17.01706	0.00812	17.38704	0.00727	17.89337	0.00725	19.43559	0.00951	14.49859

0.00799	17.02873	0.00818	17.39898	0.00731	17.91483	0.00727	19.45222	0.00968	13.48635

0.00802	17.04185	0.00822	17.41241	0.00733	17.9332	0.00727	19.46554	0.01009	12.45623

0.00807	17.05642	0.00829	17.42734	0.00733	17.94087	0.0073	19.48385	0.01089	11.49586

0.00813	17.071	0.00837	17.44376	0.00736	17.96079	0.00733	19.50549	0.01152	10.56768

0.00819	17.08558	0.00846	17.45271	0.00738	17.97611	0.00735	19.51881	0.01191	9.52862

0.00826	17.09432	0.00856	17.46018	0.0074	17.98683	0.00735	19.53045	0.01225	8.42517

0.00835	17.10453	0.00872	17.46316	0.00742	17.99143	0.00737	19.54875	0.01257	7.32351

0.00846	17.1191	0.009	17.36465	0.00744	18.01595	0.0074	19.56706	0.01306	6.41321

0.00861	17.09724	0.00941	16.57657	0.00748	18.03126	0.00742	19.58204	0.01364	5.40277

0.009	16.4748	0.00961	15.7258	0.00748	18.04811	0.00743	19.59536	0.01435	4.42987

0.00935	15.73721	0.00974	14.77951	0.0075	18.06038	0.00746	19.61698	0.01522	3.49454

0.0096	14.98796	0.00985	14.00933	0.00754	18.08336	0.0075	19.63696	0.01633	2.57888

0.00982	14.25475	0.01	12.98841	0.00756	18.09714	0.00752	19.65194

0.01003	13.43552	0.01012	12.21227	0.00758	18.10633	0.00754	19.66192

0.01024	12.48657	0.01024	11.46449	0.00761	18.12013	0.00755	19.6819

0.01043	11.56239	0.01044	10.53909	0.00765	18.13851	0.00759	19.7002

0.01063	10.79127	0.01075	9.60921	0.00768	18.15536	0.00762	19.71518

0.01092	9.9429	0.01109	8.84502	0.0077	18.15843	0.00762	19.72684

0.01138	9.19947	0.01175	8.04052	0.00772	18.17835	0.00766	19.7468

0.01195	8.37443	0.01255	7.27034	0.00779	18.20593	0.0077	19.76511

0.01247	7.55521	0.01336	6.44047	0.00781	18.21205	0.00773	19.78341

0.01297	6.73452	0.01415	5.68821	0.00785	18.22278	0.00775	19.79508

0.01362	5.93717	0.01525	4.93446	0.00788	18.24422	0.00779	19.81171

0.00794	18.25955	0.00783	19.83169

0.00798	18.2672	0.00787	19.84667

0.00801	18.27946	0.0079	19.86164

0.00806	18.29479	0.00794	19.87663

0.00812	18.3147	0.008	19.89659

0.00817	18.33003	0.00805	19.91157

0.00824	18.34381	0.00809	19.92655

0.00832	18.35453	0.00814	19.93986

0.00842	18.36986	0.00822	19.96151

0.00854	18.37599	0.00831	19.97482

0.00871	18.34688	0.00839	19.98147

0.00917	17.58082	0.00853	19.9848

0.00929	16.71979	0.00876	19.90991

0.00938	15.81738	0.00908	19.07277

0.00948	15.0253	0.00925	18.19569

0.00962	14.2332	0.00941	17.26701

0.00982	13.23733	0.00959	16.35665

0.01024	12.4054	0.0098	15.48955

0.01111	11.63016	0.01002	14.39112

0.01167	10.81968	0.01027	13.27438

0.01206	9.92493	0.01049	12.25251

0.01229	9.02559	0.01074	11.02426

0.01254	8.23043	0.01098	10.09726

0.01288	7.22691	0.01121	9.21018

0.01321	6.31991	0.01145	8.28317

0.01354	5.46806	0.01178	7.26962

0.01388	4.64379	0.01217	6.27769

0.01428	3.79194	0.01271	5.34571

0.01479	2.99831	0.01334	4.46696

**Fig 7. Curve of peak strength change of specimen with Non-hydraulic and Stabilized hydraulic**

0	19.12	16.85

15	18.83	16.9

30	18.27	17.13

45	18.53	17.43

60	19.43	18.48

75	20.61	19.96

90	21.08	20.72

**Fig 8. Elastic modulus change curve with Non-hydraulic and Stabilized hydraulic**

0	4.09	2.57

15	4.27	3.05

30	4.41	3.47

45	4.62	3.72

60	4.88	4.18

75	5.43	4.86

90	5.68	5.39

**Fig 9. Characteristic curve of acoustic emission ringing count and its total number change**

**（*a*）Non-hydraulic-0°**

1	0.20551	0.87104	25	74

2	0.22477	0.96782	1	75

3	0.23922	1.0646	3	78

4	0.25848	1.16138	2	80

5	0.26973	1.25816	5	85

6	0.28418	1.35495	19	103

7	0.29862	1.45173	1	104

8	0.3195	1.54851	3	107

9	0.33716	1.64529	7	115

10	0.34679	1.74207	2	117

11	0.36445	1.83885	10	127

12	0.38212	1.93564	19	146

13	0.39816	2.03242	1	147

14	0.41262	2.1292	11	158

15	0.43188	2.22598	4	162

16	0.44793	2.32276	1	163

17	0.46078	2.41955	2	165

18	0.47684	2.51633	1	166

19	0.4945	2.61311	7	173

20	0.50894	2.70989	1	173

21	0.5218	2.80667	1	175

22	0.54106	2.90345	6	181

23	0.56353	3.00024	1	181

24	0.57317	3.09702	3	185

25	0.58761	3.1938	1	185

26	0.61009	3.29058	2	187

27	0.62294	3.38736	10	197

28	0.63578	3.48415	1	198

29	0.65185	3.58093	11	209

30	0.67432	3.67771	2	211

31	0.68395	3.77449	1	212

32	0.70161	3.87127	4	216

33	0.71606	3.96805	3	219

34	0.73532	4.06484	1	221

35	0.74977	4.16162	5	225

36	0.76583	4.2584	3	229

37	0.78028	4.35518	7	235

38	0.79954	4.45196	1	236

39	0.81079	4.54875	1	237

40	0.83005	4.64553	9	247

41	0.84771	4.74231	23	269

42	0.86216	4.83909	7	276

43	0.8734	4.93587	1	277

44	0.88785	5.03265	5	282

45	0.90872	5.12944	1	283

46	0.92317	5.22622	13	296

47	0.93441	5.323	3	299

48	0.95849	5.41978	1	299

49	0.97133	5.51656	1	300

50	0.98418	5.61335	1	301

51	1.01628	5.71013	1	301

52	1.03074	5.80691	1	303

53	1.05001	5.90369	3	305

54	1.05964	6.00047	14	319

55	1.0757	6.09725	1	320

56	1.09496	6.19404	25	345

57	1.10781	6.29082	1	345

58	1.11904	6.3876	15	361

59	1.14473	6.48438	4	365

60	1.15436	6.58116	1	365

61	1.17203	6.67794	5	370

62	1.18647	6.77473	6	376

63	1.20413	6.87151	5	381

64	1.2202	6.96829	1	381

65	1.23464	7.06507	13	394

66	1.2523	7.16185	2	396

67	1.26996	7.25864	5	401

68	1.28441	7.35542	7	408

69	1.29564	7.4522	1	409

70	1.31652	7.54898	1	410

71	1.33258	7.64576	1	411

72	1.34863	7.74254	6	417

73	1.35988	7.83933	5	423

74	1.38555	7.93611	17	439

75	1.3984	8.03289	2	441

76	1.40803	8.12967	7	449

77	1.42249	8.22645	7	456

78	1.44497	8.32324	1	457

79	1.46102	8.42002	29	486

80	1.47226	8.5168	3	489

81	1.49312	8.61358	1	489

82	1.50757	8.71036	19	508

83	1.52203	8.80714	18	526

84	1.53808	8.90393	9	535

85	1.55735	9.00071	8	543

86	1.57019	9.09749	1	544

87	1.58303	9.19427	11	555

88	1.6007	9.29105	3	557

89	1.61996	9.38784	22	579

90	1.63923	9.48462	1	580

91	1.64725	9.5814	3	583

92	1.66491	9.67818	1	585

93	1.68579	9.77496	4	589

94	1.69702	9.87174	3	592

95	1.7163	9.96853	19	611

96	1.73075	10.06531	46	657

97	1.75001	10.16209	5	662

98	1.76446	10.25887	14	676

99	1.77569	10.35565	7	683

100	1.79818	10.45244	9	692

101	1.81263	10.54922	1	693

102	1.82386	10.646	1	693

103	1.84152	10.74278	1	694

104	1.86079	10.83956	9	703

105	1.88165	10.93634	1	703

106	1.88647	11.03313	3	706

107	1.90735	11.12991	1	707

108	1.92661	11.22669	1	708

109	1.93464	11.32347	16	724

110	1.94908	11.42025	1	725

111	1.97157	11.51704	6	731

112	1.98762	11.61382	1	733

113	2.00047	11.7106	3	736

114	2.01974	11.80738	1	737

115	2.039	11.90416	1	737

116	2.05346	12.00094	1	738

117	2.06629	12.09773	5	743

118	2.07913	12.19451	5	748

119	2.10322	12.29129	7	755

120	2.11606	12.38807	1	755

121	2.12571	12.48485	4	759

122	2.14818	12.58164	3	763

123	2.16263	12.67842	15	778

124	2.1835	12.7752	1	779

125	2.19313	12.87198	1	780

126	2.214	12.96876	10	790

127	2.22685	13.06554	5	795

128	2.23969	13.16233	9	803

129	2.25574	13.25911	3	807

130	2.27983	13.35589	11	817

131	2.29106	13.45267	3	821

132	2.30231	13.54945	1	822

133	2.32157	13.64624	3	825

134	2.34404	13.74302	1	827

135	2.36011	13.8398	16	843

136	2.36974	13.93658	5	847

137	2.389	14.03336	5	853

138	2.40827	14.13014	5	857

139	2.4147	14.22693	4	861

140	2.43396	14.32371	9	870

141	2.45483	14.42049	2	872

142	2.46928	14.51727	1	873

143	2.48213	14.61405	3	877

144	2.50299	14.71084	1	878

145	2.52066	14.80762	1	879

146	2.53189	14.9044	17	897

147	2.54313	15.00118	5	901

148	2.56401	15.09796	10	911

149	2.58167	15.19474	7	918

150	2.58968	15.29153	11	929

151	2.61056	15.38831	1	931

152	2.62982	15.48509	7	937

153	2.64428	15.58187	1	939

154	2.65711	15.67865	3	942

155	2.67318	15.77544	1	943

156	2.69726	15.87222	4	947

157	2.7085	15.969	15	962

158	2.72135	16.06578	3	965

159	2.74061	16.16256	3	967

160	2.75666	16.25934	7	975

161	2.7695	16.35613	21	996

162	2.78396	16.45291	1	997

163	2.80965	16.54969	3	999

164	2.81928	16.64647	5	1005

165	2.8273	16.74325	1	1005

166	2.853	16.84004	2	1007

167	2.87066	16.93682	15	1023

168	2.88189	17.0336	1	1023

169	2.89634	17.13038	1	1024

170	2.91721	17.22716	13	1037

171	2.93327	17.32394	2	1039

172	2.94772	17.42073	2	1041

173	2.96217	17.51751	6	1047

174	2.98625	17.61429	2	1049

175	2.99589	17.71107	2	1051

176	3.00713	17.80785	12	1063

177	3.02318	17.90464	12	1075

178	3.04566	18.00142	1	1076

179	3.06011	18.0982	7	1083

180	3.07615	18.19498	1	1084

181	3.09061	18.29176	26	1110

182	3.10827	18.38854	3	1113

183	3.11791	18.48533	7	1120

184	3.13717	18.58211	11	1131

185	3.15804	18.67889	5	1137

186	3.1757	18.77567	5	1141

187	3.18694	18.87245	13	1154

188	3.20461	18.96924	14	1168

189	3.22226	19.06602	1	1169

190	3.23511	19.1628	1	1170

191	3.25116	19.25958	3	1173

192	3.26561	19.35636	18	1191

193	3.2913	19.45314	1	1191

194	3.30093	19.54993	4	1195

195	3.31538	19.64671	1	1196

196	3.33143	19.74349	3	1199

197	3.3507	19.84027	1	1200

198	3.36837	19.93705	1	1201

199	3.37639	20.03383	29	1230

200	3.39887	20.13062	1	1231

201	3.41653	20.2274	11	1242

202	3.42294	20.32418	1	1243

203	3.44222	20.42096	1	1243

204	3.46309	20.51774	1	1245

205	3.47753	20.61453	4	1249

206	3.48878	20.71131	1	1249

207	3.51286	20.80809	2	1251

208	3.53213	20.90487	2	1253

209	3.53695	21.00165	1	1254

210	3.55299	21.09843	1	1255

211	3.57066	21.19522	11	1265

212	3.59313	21.292	1	1266

213	3.59956	21.38878	1	1267

214	3.61883	21.48556	3	1270

215	3.63969	21.58234	1	1271

216	3.66057	21.67913	1	1272

217	3.6702	21.77591	3	1275

218	3.68465	21.87269	1	1275

219	3.70071	21.96947	1	1276

220	3.71355	22.06625	1	1277

221	3.728	22.16303	1	1278

222	3.75047	22.25982	4	1282

223	3.76814	22.3566	1	1283

224	3.78259	22.45338	1	1284

225	3.79543	22.55016	10	1294

226	3.81469	22.64694	1	1295

227	3.83235	22.74373	25	1320

228	3.83717	22.84051	1	1321

229	3.85805	22.93729	8	1329

230	3.88213	23.03407	1	1330

231	3.89176	23.13085	1	1331

232	3.903	23.22763	13	1344

233	3.92869	23.32442	3	1347

234	3.94795	23.4212	8	1355

235	3.95599	23.51798	1	1356

236	3.97364	23.61476	3	1359

237	3.99452	23.71154	31	1390

238	4.00415	23.80833	1	1391

239	4.01699	23.90511	3	1393

240	4.03787	24.00189	3	1397

241	4.05713	24.09867	1	1397

242	4.06837	24.19545	5	1402

243	4.08603	24.29223	2	1404

244	4.10368	24.38902	5	1409

245	4.11653	24.4858	1	1410

246	4.12777	24.58258	1	1411

247	4.14383	24.67936	1	1412

248	4.17112	24.77614	4	1416

249	4.18236	24.87293	13	1429

250	4.19359	24.96971	10	1439

251	4.21447	25.06649	5	1444

252	4.23373	25.16327	2	1446

253	4.24336	25.26005	1	1447

254	4.25782	25.35683	1	1449

255	4.2819	25.45362	12	1461

256	4.29474	25.5504	4	1465

257	4.30598	25.64718	1	1465

258	4.32524	25.74396	2	1467

259	4.34452	25.84074	3	1471

260	4.36218	25.93753	7	1477

261	4.37341	26.03431	2	1479

262	4.38466	26.13109	10	1489

263	4.41515	26.22787	3	1492

264	4.41998	26.32465	7	1499

265	4.43282	26.42143	3	1501

266	4.45369	26.51822	1	1502

267	4.47136	26.615	1	1503

268	4.4874	26.71178	3	1506

269	4.50025	26.80856	5	1511

270	4.52434	26.90534	1	1511

271	4.53717	27.00213	11	1523

272	4.54681	27.09891	1	1523

273	4.55965	27.19569	3	1527

274	4.58534	27.29247	1	1528

275	4.59818	27.38925	1	1529

276	4.61263	27.48603	9	1537

277	4.62708	27.58282	3	1541

278	4.64796	27.6796	5	1546

279	4.6608	27.77638	1	1547

280	4.68006	27.87316	5	1552

281	4.68969	27.96994	3	1555

282	4.70897	28.06673	2	1557

283	4.72181	28.16351	7	1564

284	4.73627	28.26029	16	1580

285	4.76035	28.35707	1	1581

286	4.78443	28.45385	13	1595

287	4.78924	28.55063	17	1611

288	4.80208	28.64742	3	1614

289	4.82136	28.7442	1	1615

290	4.8358	28.84098	3	1617

291	4.84865	28.93776	1	1618

292	4.86309	29.03454	3	1621

293	4.89199	29.13133	1	1622

294	4.90003	29.22811	1	1623

295	4.91929	29.32489	15	1639

296	4.93695	29.42167	1	1640

297	4.95942	29.51845	3	1643

298	4.96264	29.61523	1	1644

299	4.9803	29.71202	3	1647

300	4.99475	29.8088	1	1647

301	5.01401	29.90558	25	1672

302	5.02364	30.00236	1	1673

303	5.04934	30.09914	1	1673

304	5.0686	30.19593	2	1675

305	5.08144	30.29271	15	1691

306	5.08947	30.38949	1	1691

307	5.11836	30.48627	1	1693

308	5.13122	30.58305	5	1698

309	5.14245	30.67983	22	1720

310	5.15369	30.77662	8	1728

311	5.17616	30.8734	12	1740

312	5.19865	30.97018	12	1752

313	5.20186	31.06696	3	1755

314	5.22274	31.16374	23	1777

315	5.242	31.26053	1	1779

316	5.25003	31.35731	15	1793

317	5.26769	31.45409	7	1800

318	5.28374	31.55087	12	1812

319	5.30462	31.64765	3	1815

320	5.31264	31.74443	7	1821

321	5.33029	31.84122	5	1827

322	5.35438	31.938	1	1827

323	5.37043	32.03478	1	1828

324	5.37846	32.13156	2	1830

325	5.39772	32.22834	7	1837

326	5.42021	32.32512	2	1839

327	5.43305	32.42191	4	1843

328	5.44589	32.51869	7	1851

329	5.46838	32.61547	8	1859

330	5.48443	32.71225	1	1860

331	5.49567	32.80903	5	1865

332	5.51493	32.90582	5	1869

333	5.52618	33.0026	1	1870

334	5.54705	33.09938	8	1878

335	5.55026	33.19616	1	1879

336	5.56952	33.29294	3	1881

337	5.59199	33.38972	2	1883

338	5.61127	33.48651	3	1886

339	5.61608	33.58329	1	1887

340	5.64498	33.68007	15	1903

341	5.66425	33.77685	1	1904

342	5.66907	33.87363	1	1905

343	5.68512	33.97042	1	1905

344	5.70438	34.0672	1	1906

345	5.72204	34.16398	36	1942

346	5.72847	34.26076	2	1944

347	5.75255	34.35754	1	1945

348	5.7686	34.45432	1	1945

349	5.79268	34.55111	1	1947

350	5.7975	34.64789	5	1951

351	5.81998	34.74467	1	1953

352	5.83604	34.84145	1	1953

353	5.84567	34.93823	23	1977

354	5.85852	35.03502	10	1987

355	5.88742	35.1318	1	1987

356	5.90186	35.22858	1	1988

357	5.90989	35.32536	2	1990

358	5.92915	35.42214	1	1991

359	5.94681	35.51892	6	1997

360	5.95966	35.61571	19	2015

361	5.97572	35.71249	2	2017

362	5.99177	35.80927	1	2018

363	6.00943	35.90605	1	2019

364	6.02228	36.00283	3	2022

365	6.03833	36.09962	11	2033

366	6.05599	36.1964	3	2036

367	6.07686	36.29318	3	2039

368	6.08649	36.38996	7	2045

369	6.10255	36.48674	1	2046

370	6.12182	36.58352	12	2058

371	6.14108	36.68031	1	2059

372	6.1459	36.77709	4	2063

373	6.16999	36.87387	5	2068

374	6.19727	36.97065	8	2076

375	6.20531	37.06743	1	2077

376	6.21333	37.16422	2	2079

377	6.2326	37.261	2	2081

378	6.24865	37.35778	11	2092

379	6.25989	37.45456	1	2093

380	6.27915	37.55134	7	2099

381	6.30003	37.64812	1	2101

382	6.3193	37.74491	3	2103

383	6.32411	37.84169	6	2109

384	6.34177	37.93847	19	2129

385	6.36585	38.03525	1	2130

386	6.3803	38.13203	21	2151

387	6.38673	38.22882	30	2181

388	6.40599	38.3256	29	2211

389	6.43169	38.42238	3	2213

390	6.4381	38.51916	7	2221

391	6.45736	38.61594	5	2225

392	6.47342	38.71272	3	2228

393	6.4959	38.80951	25	2253

394	6.50393	38.90629	7	2260

395	6.51357	39.00307	7	2267

396	6.54086	39.09985	3	2271

397	6.55692	39.19663	6	2277

398	6.56173	39.29342	3	2280

399	6.58259	39.3902	1	2281

400	6.60668	39.48698	3	2284

401	6.62113	39.58376	1	2285

402	6.63237	39.68054	4	2289

403	6.65325	39.77732	1	2290

404	6.66769	39.87411	1	2291

405	6.67732	39.97089	11	2301

406	6.69819	40.06767	3	2305

407	6.71906	40.16445	5	2309

408	6.73512	40.26123	1	2310

409	6.74957	40.35802	1	2311

410	6.76402	40.4548	2	2313

411	6.78649	40.55158	10	2323

412	6.79774	40.64836	17	2340

413	6.80737	40.74514	6	2346

414	6.82664	40.84192	8	2354

415	6.84911	40.93871	8	2362

416	6.86357	41.03549	2	2364

417	6.8732	41.13227	17	2381

418	6.89888	41.22905	3	2384

419	6.91494	41.32583	1	2385

420	6.91975	41.42262	1	2386

421	6.94705	41.5194	2	2388

422	6.9615	41.61618	21	2409

423	6.97113	41.71296	2	2411

424	6.98718	41.80974	1	2413

425	7.00325	41.90652	13	2425

426	7.02251	42.00331	7	2432

427	7.04177	42.10009	1	2433

428	7.05784	42.19687	1	2433

429	7.06906	42.29365	3	2437

430	7.08673	42.39043	39	2475

431	7.09797	42.48722	1	2476

432	7.11563	42.584	3	2479

433	7.1381	42.68078	4	2483

434	7.15094	42.77756	5	2488

435	7.15898	42.87434	1	2489

436	7.18627	42.97112	4	2493

437	7.20233	43.06791	1	2494

438	7.21356	43.16469	1	2495

439	7.22801	43.26147	10	2505

440	7.25691	43.35825	1	2506

441	7.26495	43.45503	4	2510

442	7.27618	43.55182	1	2511

443	7.28903	43.6486	1	2511

444	7.31632	43.74538	2	2513

445	7.32595	43.84216	1	2514

446	7.34361	43.93894	2	2516

447	7.35805	44.03572	1	2517

448	7.38054	44.13251	1	2517

449	7.38696	44.22929	3	2521

450	7.40141	44.32607	31	2552

451	7.42389	44.42285	4	2556

452	7.43993	44.51963	1	2557

453	7.45279	44.61641	12	2569

454	7.46723	44.7132	1	2569

455	7.48651	44.80998	3	2572

456	7.50577	44.90676	1	2573

457	7.51701	45.00354	3	2576

458	7.52664	45.10032	1	2577

459	7.54912	45.19711	1	2578

460	7.56839	45.29389	1	2579

461	7.57641	45.39067	5	2583

462	7.6021	45.48745	5	2588

463	7.62297	45.58423	2	2590

464	7.6326	45.68101	1	2591

465	7.64063	45.7778	3	2594

466	7.66311	45.87458	8	2602

467	7.6904	45.97136	1	2603

468	7.6888	46.06814	1	2604

469	7.71448	46.16492	12	2616

470	7.73054	46.26171	7	2623

471	7.7482	46.35849	1	2624

472	7.75462	46.45527	6	2630

473	7.7755	46.55205	7	2637

474	7.79476	46.64883	1	2638

475	7.80279	46.74561	13	2651

476	7.81724	46.8424	3	2653

477	7.83971	46.93918	12	2665

478	7.85897	47.03596	5	2670

479	7.8654	47.13274	4	2674

480	7.88788	47.22952	34	2708

481	7.91036	47.32631	3	2711

482	7.92159	47.42309	1	2711

483	7.93284	47.51987	1	2712

484	7.95371	47.61665	27	2739

485	7.96976	47.71343	1	2741

486	7.98743	47.81021	9	2749

487	7.99224	47.907	18	2767

488	8.02435	48.00378	7	2774

489	8.03559	48.10056	1	2775

490	8.04201	48.19734	1	2776

491	8.05807	48.29412	1	2777

492	8.08215	48.39091	2	2779

493	8.09499	48.48769	1	2779

494	8.11425	48.58447	3	2782

495	8.1287	48.68125	1	2783

496	8.14958	48.77803	31	2815

497	8.15761	48.87481	19	2834

498	8.17366	48.9716	4	2838

499	8.19775	49.06838	1	2839

500	8.20576	49.16516	3	2842

501	8.21701	49.26194	1	2843

502	8.23146	49.35872	16	2859

503	8.25875	49.45551	9	2869

504	8.26679	49.55229	26	2895

505	8.27963	49.64907	7	2901

506	8.30371	49.74585	48	2949

507	8.32297	49.84263	2	2951

508	8.33742	49.93941	1	2952

509	8.34706	50.0362	1	2953

510	8.37114	50.13298	6	2959

511	8.38238	50.22976	5	2963

512	8.39522	50.32654	1	2964

513	8.41449	50.42332	1	2965

514	8.43215	50.52011	1	2966

515	8.45302	50.61689	3	2969

516	8.46104	50.71367	1	2970

517	8.47871	50.81045	6	2976

518	8.50279	50.90723	35	3011

519	8.50761	51.00401	9	3019

520	8.52527	51.1008	1	3020

521	8.53971	51.19758	1	3021

522	8.57022	51.29436	9	3030

523	8.57986	51.39114	4	3034

524	8.58949	51.48792	7	3041

525	8.61358	51.58471	1	3042

526	8.62802	51.68149	5	3047

527	8.63605	51.77827	1	3049

528	8.6521	51.87505	1	3049

529	8.67297	51.97183	3	3052

530	8.68742	52.06861	23	3075

531	8.69705	52.1654	9	3083

532	8.71954	52.26218	1	3085

533	8.74201	52.35896	1	3085

534	8.75967	52.45574	3	3089

535	8.75967	52.55252	3	3092

536	8.78215	52.64931	5	3097

537	8.7982	52.74609	3	3100

538	8.80623	52.84287	1	3101

539	8.82389	52.93965	1	3102

540	8.85279	53.03643	12	3114

541	8.87045	53.13321	3	3117

542	8.87687	53.23	6	3123

543	8.89294	53.32678	1	3125

544	8.91702	53.42356	5	3130

545	8.92665	53.52034	8	3138

546	8.93949	53.61712	3	3141

547	8.96196	53.71391	1	3142

548	8.97803	53.81069	3	3145

549	8.98766	53.90747	1	3145

550	9.01013	54.00425	2	3147

551	9.0294	54.10103	7	3155

552	9.04384	54.19781	4	3159

553	9.05027	54.2946	3	3161

554	9.06793	54.39138	3	3164

555	9.09362	54.48816	1	3165

556	9.10968	54.58494	1	3166

557	9.11609	54.68172	1	3167

558	9.14179	54.77851	4	3171

559	9.16586	54.87529	27	3198

560	9.17068	54.97207	3	3201

561	9.18193	55.06885	1	3202

562	9.20922	55.16563	1	3203

563	9.22045	55.26241	7	3209

564	9.23169	55.3592	1	3210

565	9.24936	55.45598	1	3211

566	9.27344	55.55276	6	3217

567	9.28628	55.64954	1	3218

568	9.29591	55.74632	1	3219

569	9.32161	55.84311	3	3222

570	9.33927	55.93989	4	3226

571	9.33927	56.03667	1	3227

572	9.36334	56.13345	16	3243

573	9.38582	56.23023	3	3247

574	9.40027	56.32701	3	3249

575	9.4099	56.4238	8	3257

576	9.42757	56.52058	1	3258

577	9.45004	56.61736	8	3266

578	9.45647	56.71414	6	3272

579	9.47413	56.81092	3	3275

580	9.48697	56.9077	1	3275

581	9.50623	57.00449	1	3277

582	9.51747	57.10127	17	3293

583	9.53995	57.19805	9	3302

584	9.56082	57.29483	1	3303

585	9.57848	57.39161	5	3308

586	9.58652	57.4884	9	3317

587	9.60256	57.58518	3	3320

588	9.62344	57.68196	1	3321

589	9.63789	57.77874	1	3321

590	9.64912	57.87552	2	3323

591	9.66518	57.9723	2	3325

592	9.68926	58.06909	1	3326

593	9.69729	58.16587	1	3327

594	9.71495	58.26265	1	3328

595	9.73743	58.35943	1	3329

596	9.75669	58.45621	9	3338

597	9.76312	58.553	1	3339

598	9.77757	58.64978	2	3341

599	9.80326	58.74656	29	3369

600	9.81289	58.84334	7	3376

601	9.82412	58.94012	1	3377

602	9.84821	59.0369	3	3380

603	9.86908	59.13369	1	3381

604	9.88192	59.23047	2	3383

605	9.88514	59.32725	11	3394

606	9.90922	59.42403	5	3399

607	9.93009	59.52081	23	3421

608	9.93651	59.6176	1	3422

609	9.95096	59.71438	4	3426

610	9.97665	59.81116	5	3431

611	9.99752	59.90794	2	3433

612	10.00876	60.00472	6	3439

613	10.02001	60.1015	1	3440

614	10.03927	60.19829	2	3442

615	10.04568	60.29507	33	3475

616	10.06174	60.39185	20	3495

617	10.08422	60.48863	13	3508

618	10.1083	60.58541	5	3513

619	10.12115	60.6822	2	3515

620	10.13078	60.77898	1	3516

621	10.15325	60.87576	8	3524

622	10.17734	60.97254	1	3525

623	10.17895	61.06932	1	3526

624	10.19179	61.1661	1	3527

625	10.21749	61.26289	7	3533

626	10.22712	61.35967	2	3535

627	10.23996	61.45645	1	3537

628	10.25761	61.55323	5	3541

629	10.2833	61.65001	1	3542

630	10.29293	61.7468	4	3546

631	10.31221	61.84358	1	3547

632	10.32344	61.94036	1	3548

633	10.3411	62.03714	7	3555

634	10.34913	62.13392	1	3556

635	10.3668	62.2307	4	3560

636	10.39409	62.32749	1	3561

637	10.40853	62.42427	2	3563

638	10.41977	62.52105	1	3563

639	10.43743	62.61783	1	3564

640	10.46312	62.71461	5	3569

641	10.47115	62.8114	1	3569

642	10.48239	62.90818	1	3570

643	10.50486	63.00496	1	3571

644	10.51932	63.10174	1	3572

645	10.53056	63.19852	1	3573

646	10.54982	63.2953	2	3575

647	10.5755	63.39209	1	3575

648	10.58514	63.48887	1	3577

649	10.59157	63.58565	1	3577

650	10.60921	63.68243	8	3585

651	10.6333	63.77921	4	3589

652	10.64775	63.876	7	3597

653	10.65579	63.97278	1	3597

654	10.67665	64.06956	1	3598

655	10.69592	64.16634	7	3605

656	10.71037	64.26312	1	3606

657	10.72804	64.3599	1	3607

658	10.74889	64.45669	1	3607

659	10.75854	64.55347	1	3609

660	10.76977	64.65025	3	3611

661	10.78743	64.74703	5	3616

662	10.80991	64.84381	1	3617

663	10.82114	64.9406	1	3618

664	10.83881	65.03738	1	3619

665	10.85327	65.13416	1	3619

666	10.87735	65.23094	1	3620

667	10.89019	65.32772	1	3621

668	10.8934	65.4245	7	3629

669	10.9207	65.52129	1	3629

670	10.93193	65.61807	1	3630

671	10.94959	65.71485	2	3632

672	10.96886	65.81163	2	3634

673	10.99295	65.90841	1	3635

674	11.00258	66.0052	3	3637

675	11.01221	66.10198	1	3639

676	11.02505	66.19876	1	3640

677	11.04913	66.29554	1	3641

678	11.05876	66.39232	5	3646

679	11.07321	66.4891	1	3647

680	11.09248	66.58589	1	3647

681	11.11496	66.68267	6	3653

682	11.12459	66.77945	3	3656

683	11.13904	66.87623	5	3661

684	11.16312	66.97301	1	3662

685	11.17436	67.0698	1	3663

686	11.18079	67.16658	18	3681

687	11.20326	67.26336	1	3682

688	11.22414	67.36014	1	3683

689	11.23377	67.45692	8	3691

690	11.24982	67.5537	1	3692

691	11.2739	67.65049	2	3694

692	11.28995	67.74727	4	3698

693	11.3012	67.84405	1	3699

694	11.31404	67.94083	1	3700

695	11.34133	68.03761	1	3701

696	11.34937	68.1344	3	3704

697	11.3606	68.23118	27	3731

698	11.38468	68.32796	1	3732

699	11.40877	68.42474	3	3735

700	11.4184	68.52152	4	3739

701	11.42963	68.6183	5	3744

702	11.44409	68.71509	1	3745

703	11.46978	68.81187	5	3749

704	11.4778	68.90865	1	3751

705	11.49225	69.00543	1	3752

706	11.51472	69.10221	1	3753

707	11.53239	69.199	4	3757

708	11.54202	69.29578	23	3781

709	11.55648	69.39256	1	3781

710	11.57896	69.48934	1	3782

711	11.59982	69.58612	1	3783

712	11.59822	69.6829	5	3788

713	11.62391	69.77969	3	3791

714	11.64318	69.87647	1	3792

715	11.65762	69.97325	1	3793

716	11.67207	70.07003	1	3794

717	11.69455	70.16681	2	3796

718	11.71061	70.26359	1	3797

719	11.71864	70.36038	1	3798

720	11.72827	70.45716	1	3799

721	11.75716	70.55394	1	3800

722	11.77482	70.65072	1	3801

723	11.78124	70.7475	1	3802

724	11.7989	70.84429	2	3804

725	11.81978	70.94107	1	3805

726	11.83744	71.03785	1	3805

727	11.84707	71.13463	1	3806

728	11.86633	71.23141	3	3809

729	11.8856	71.32819	2	3811

730	11.88721	71.42498	15	3826

731	11.91129	71.52176	1	3827

732	11.93538	71.61854	7	3833

733	11.94983	71.71532	1	3834

734	11.95785	71.8121	1	3835

735	11.97712	71.90889	1	3835

736	11.9996	72.00567	2	3837

737	12.01564	72.10245	2	3839

738	12.02689	72.19923	1	3840

739	12.03813	72.29601	18	3858

740	12.059	72.39279	1	3859

741	12.07344	72.48958	1	3860

742	12.09272	72.58636	1	3861

743	12.10877	72.68314	5	3865

744	12.12803	72.77992	1	3867

745	12.13767	72.8767	9	3875

746	12.15212	72.97349	17	3893

747	12.17299	73.07027	3	3895

748	12.18904	73.16705	5	3900

749	12.19065	73.26383	2	3902

750	12.21794	73.36061	1	3903

751	12.23882	73.45739	1	3904

752	12.25166	73.55418	1	3905

753	12.26771	73.65096	14	3919

754	12.28699	73.74774	7	3926

755	12.30143	73.84452	11	3937

756	12.30946	73.9413	27	3963

757	12.32391	74.03809	3	3966

758	12.34799	74.13487	1	3967

759	12.36725	74.23165	5	3973

760	12.3785	74.32843	26	3999

761	12.39455	74.42521	21	4020

762	12.42023	74.52199	1	4021

763	12.42827	74.61878	1	4021

764	12.43468	74.71556	5	4026

765	12.46198	74.81234	2	4028

766	12.47482	74.90912	5	4033

767	12.48607	75.0059	1	4035

768	12.50693	75.10269	11	4046

769	12.5262	75.19947	1	4047

770	12.54387	75.29625	31	4078

771	12.55189	75.39303	20	4098

772	12.56955	75.48981	2	4100

773	12.59042	75.58659	11	4111

774	12.60648	75.68338	1	4111

775	12.6145	75.78016	2	4113

776	12.63698	75.87694	23	4136

777	12.64983	75.97372	1	4137

778	12.66428	76.0705	1	4139

779	12.68194	76.16729	3	4141

780	12.70281	76.26407	1	4142

781	12.72047	76.36085	3	4145

782	12.72689	76.45763	41	4187

783	12.74776	76.55441	5	4192

784	12.76543	76.65119	5	4197

785	12.77506	76.74798	1	4198

786	12.7879	76.84476	1	4199

787	12.81359	76.94154	1	4199

788	12.83606	77.03832	3	4202

789	12.84089	77.1351	7	4209

790	12.85694	77.23189	5	4213

791	12.8762	77.32867	16	4229

792	12.88905	77.42545	1	4231

793	12.90671	77.52223	5	4235

794	12.91956	77.61901	10	4245

795	12.94524	77.71579	45	4290

796	12.95808	77.81258	1	4291

797	12.97414	77.90936	10	4301

798	12.99662	78.00614	9	4311

799	13.00465	78.10292	1	4311

800	13.02231	78.1997	19	4331

801	13.03354	78.29649	3	4333

802	13.05602	78.39327	2	4335

803	13.07368	78.49005	5	4340

804	13.0785	78.58683	1	4341

805	13.09937	78.68361	1	4342

806	13.12024	78.78039	16	4358

807	13.1363	78.87718	1	4359

808	13.14593	78.97396	1	4360

809	13.17804	79.07074	3	4363

810	13.17965	79.16752	9	4371

811	13.1941	79.2643	1	4372

812	13.21818	79.36109	7	4379

813	13.23263	79.45787	1	4380

814	13.24868	79.55465	15	4395

815	13.26474	79.65143	1	4396

816	13.27758	79.74821	2	4398

817	13.30006	79.84499	5	4403

818	13.3113	79.94178	1	4403

819	13.31772	80.03856	5	4409

820	13.34019	80.13534	3	4411

821	13.36107	80.23212	1	4412

822	13.3707	80.3289	1	4413

823	13.38997	80.42569	2	4415

824	13.40603	80.52247	1	4417

825	13.42207	80.61925	1	4417

826	13.43653	80.71603	1	4418

827	13.45579	80.81281	1	4419

828	13.47667	80.90959	7	4427

829	13.48469	81.00638	3	4429

830	13.50075	81.10316	12	4441

831	13.5152	81.19994	1	4442

832	13.53446	81.29672	1	4443

833	13.54571	81.3935	9	4452

834	13.56818	81.49029	1	4453

835	13.58263	81.58707	1	4454

836	13.59547	81.68385	1	4455

837	13.60671	81.78063	1	4456

838	13.62918	81.87741	4	4460

839	13.64846	81.97419	5	4465

840	13.66451	82.07098	2	4467

841	13.67254	82.16776	8	4475

842	13.69823	82.26454	4	4479

843	13.71107	82.36132	29	4508

844	13.72231	82.4581	2	4510

845	13.73034	82.55488	3	4513

846	13.76085	82.65167	1	4514

847	13.78332	82.74845	3	4517

848	13.78974	82.84523	1	4517

849	13.80258	82.94201	5	4522

850	13.82666	83.03879	1	4523

851	13.84754	83.13558	18	4541

852	13.84915	83.23236	2	4543

853	13.86841	83.32914	13	4556

854	13.88607	83.42592	24	4580

855	13.89891	83.5227	1	4581

856	13.91979	83.61948	1	4581

857	13.93584	83.71627	2	4583

858	13.95832	83.81305	1	4585

859	13.96796	83.90983	3	4587

860	13.98241	84.00661	17	4605

861	14.00327	84.10339	19	4623

862	14.01933	84.20018	1	4624

863	14.02255	84.29696	1	4625

864	14.05304	84.39374	1	4626

865	14.07231	84.49052	1	4627

866	14.08034	84.5873	1	4627

867	14.098	84.68408	1	4628

868	14.11245	84.78087	1	4629

869	14.13011	84.87765	4	4633

870	14.14135	84.97443	6	4639

871	14.15098	85.07121	1	4639

872	14.17667	85.16799	5	4645

873	14.19112	85.26478	16	4661

874	14.20075	85.36156	5	4665

875	14.22644	85.45834	9	4674

876	14.24892	85.55512	1	4675

877	14.25695	85.6519	1	4675

878	14.26658	85.74868	1	4676

879	14.28745	85.84547	7	4683

880	14.30832	85.94225	1	4684

881	14.31474	86.03903	7	4691

882	14.3324	86.13581	1	4692

883	14.3597	86.23259	9	4701

884	14.37093	86.32938	5	4706

885	14.38379	86.42616	1	4707

886	14.39823	86.52294	5	4713

887	14.4191	86.61972	9	4721

888	14.43034	86.7165	1	4722

889	14.43997	86.81328	1	4723

890	14.46567	86.91007	3	4726

891	14.48332	87.00685	3	4729

892	14.49617	87.10363	1	4730

893	14.51061	87.20041	1	4731

894	14.5331	87.29719	7	4738

895	14.54594	87.39398	3	4741

896	14.55718	87.49076	4	4745

897	14.57162	87.58754	5	4750

898	14.59891	87.68432	1	4751

899	14.61176	87.7811	1	4751

900	14.61979	87.87788	17	4769

901	14.64227	87.97467	1	4769

902	14.65833	88.07145	14	4783

903	14.67759	88.16823	3	4786

904	14.68722	88.26501	1	4787

905	14.69686	88.36179	1	4787

906	14.72095	88.45858	1	4788

907	14.73378	88.55536	1	4789

908	14.74662	88.65214	1	4789

909	14.77071	88.74892	1	4790

910	14.79158	88.8457	1	4791

911	14.80924	88.94248	5	4796

912	14.81085	89.03927	1	4797

913	14.84456	89.13605	1	4798

914	14.85098	89.23283	1	4799

915	14.85901	89.32961	8	4807

916	14.87989	89.42639	3	4810

917	14.89755	89.52318	19	4829

918	14.91521	89.61996	7	4836

919	14.93607	89.71674	1	4837

920	14.95855	89.81352	4	4841

921	14.96979	89.9103	1	4841

922	14.96979	90.00708	2	4843

923	14.99387	90.10387	1	4844

924	15.01795	90.20065	3	4847

925	15.0308	90.29743	1	4849

926	15.04043	90.39421	3	4851

927	15.0597	90.49099	1	4853

928	15.08057	90.58778	2	4855

929	15.08539	90.68456	1	4855

930	15.10626	90.78134	3	4858

931	15.12713	90.87812	7	4865

932	15.13837	90.9749	19	4884

933	15.14962	91.07168	13	4897

934	15.16888	91.16847	1	4897

935	15.19135	91.26525	1	4898

936	15.2042	91.36203	1	4899

937	15.21383	91.45881	1	4899

938	15.23309	91.55559	1	4900

939	15.25397	91.65238	1	4901

940	15.27163	91.74916	5	4905

941	15.28126	91.84594	1	4906

942	15.29731	91.94272	3	4909

943	15.31819	92.0395	2	4911

944	15.31979	92.13628	1	4913

945	15.34388	92.23307	1	4914

946	15.37118	92.32985	2	4916

947	15.3792	92.42663	27	4943

948	15.39044	92.52341	2	4945

949	15.41291	92.62019	3	4948

950	15.42898	92.71698	1	4949

951	15.44342	92.81376	3	4951

952	15.44983	92.91054	1	4952

953	15.48034	93.00732	13	4965

954	15.49319	93.1041	1	4966

955	15.50924	93.20088	3	4969

956	15.52049	93.29767	6	4975

957	15.54617	93.39445	22	4997

958	15.55901	93.49123	1	4997

959	15.56384	93.58801	1	4998

960	15.58631	93.68479	1	4999

961	15.60558	93.78158	1	4999

962	15.61842	93.87836	2	5001

963	15.63447	93.97514	8	5009

964	15.65695	94.07192	1	5010

965	15.67139	94.1687	1	5011

966	15.68104	94.26548	1	5012

967	15.69548	94.36227	1	5013

968	15.71315	94.45905	2	5015

969	15.73081	94.55583	2	5017

970	15.74364	94.65261	1	5017

971	15.76773	94.74939	27	5045

972	15.79021	94.84617	1	5045

973	15.79663	94.94296	2	5047

974	15.80144	95.03974	1	5048

975	15.82874	95.13652	19	5067

976	15.84961	95.2333	2	5069

977	15.85603	95.33008	1	5071

978	15.86566	95.42687	1	5071

979	15.89136	95.52365	1	5072

980	15.91062	95.62043	3	5075

981	15.92026	95.71721	2	5077

982	15.93309	95.81399	1	5079

983	15.95557	95.91077	4	5083

984	15.97484	96.00756	1	5084

985	15.98287	96.10434	13	5097

986	16.00214	96.20112	27	5123

987	16.023	96.2979	1	5124

988	16.03264	96.39468	1	5125

989	16.04227	96.49147	5	5131

990	16.07277	96.58825	11	5142

991	16.08402	96.68503	1	5143

992	16.09205	96.78181	14	5157

993	16.11131	96.87859	1	5157

994	16.13539	96.97537	2	5159

995	16.14985	97.07216	1	5161

996	16.15786	97.16894	1	5161

997	16.17714	97.26572	1	5162

998	16.19801	97.3625	1	5163

999	16.21566	97.45928	1	5163

1000	16.2253	97.55607	3	5166

1001	16.24296	97.65285	1	5167

1002	16.26223	97.74963	43	5210

1003	16.27347	97.84641	1	5211

1004	16.2847	97.94319	20	5231

1005	16.31682	98.03997	2	5233

1006	16.32324	98.13676	1	5233

1007	16.3425	98.23354	3	5236

1008	16.36498	98.33032	2	5238

1009	16.37622	98.4271	1	5239

1010	16.38907	98.52388	35	5274

1011	16.40191	98.62067	2	5276

1012	16.42921	98.71745	9	5285

1013	16.43722	98.81423	1	5285

1014	16.44044	98.91101	1	5286

1015	16.46773	99.00779	1	5287

1016	16.49343	99.10457	11	5298

1017	16.50146	99.20136	2	5300

1018	16.51429	99.29814	8	5308

1019	16.53517	99.39492	2	5310

1020	16.55443	99.4917	31	5341

1021	16.56568	99.58848	3	5344

1022	16.57852	99.68527	2	5346

1023	16.60099	99.78205	5	5351

1024	16.61866	99.87883	3	5353

1025	16.63149	99.97561	2	5355

1026	16.64114	100.07239	9	5365

1027	16.66361	100.16917	1	5365

1028	16.68127	100.26596	6	5371

1029	16.68608	100.36274	3	5375

1030	16.71017	100.45952	1	5375

1031	16.72301	100.5563	3	5378

1032	16.74067	100.65308	1	5379

1033	16.75351	100.74987	1	5380

1034	16.78563	100.84665	8	5388

1035	16.79205	100.94343	1	5389

1036	16.80329	101.04021	1	5389

1037	16.82095	101.13699	7	5397

1038	16.84182	101.23377	1	5397

1039	16.85467	101.33056	3	5400

1040	16.86269	101.42734	1	5401

1041	16.88517	101.52412	2	5403

1042	16.90765	101.6209	2	5405

1043	16.92531	101.71768	2	5407

1044	16.93013	101.81447	3	5409

1045	16.94778	101.91125	11	5421

1046	16.96865	102.00803	2	5423

1047	16.97828	102.10481	1	5423

1048	16.99594	102.20159	1	5424

1049	17.01843	102.29837	30	5454

1050	17.03127	102.39516	2	5456

1051	17.0409	102.49194	1	5457

1052	17.06337	102.58872	5	5461

1053	17.07944	102.6855	8	5469

1054	17.0971	102.78228	11	5480

1055	17.10191	102.87907	3	5483

1056	17.1244	102.97585	1	5485

1057	17.14205	103.07263	3	5488

1058	17.15971	103.16941	1	5489

1059	17.16934	103.26619	2	5491

1060	17.19503	103.36297	1	5491

1061	17.21109	103.45976	4	5495

1062	17.22072	103.55654	1	5496

1063	17.23678	103.65332	10	5506

1064	17.25926	103.7501	2	5508

1065	17.2753	103.84688	1	5509

1066	17.28655	103.94367	1	5510

1067	17.301	104.04045	1	5511

1068	17.31865	104.13723	3	5514

1069	17.33792	104.23401	1	5515

1070	17.35237	104.33079	7	5522

1071	17.36682	104.42757	5	5527

1072	17.3893	104.52436	1	5528

1073	17.39572	104.62114	1	5529

1074	17.41498	104.71792	1	5530

1075	17.42943	104.8147	14	5544

1076	17.45031	104.91148	1	5545

1077	17.45673	105.00827	3	5548

1078	17.48082	105.10505	1	5549

1079	17.5049	105.20183	14	5563

1080	17.51132	105.29861	5	5568

1081	17.52095	105.39539	1	5569

1082	17.54663	105.49217	1	5570

1083	17.55788	105.58896	1	5571

1084	17.57715	105.68574	5	5576

1085	17.58517	105.78252	1	5577

1086	17.60925	105.8793	1	5579

1087	17.63013	105.97608	9	5587

1088	17.63976	106.07287	3	5591

1089	17.6526	106.16965	1	5592

1090	17.67026	106.26643	2	5594

1091	17.68471	106.36321	2	5596

1092	17.69916	106.45999	11	5607

1093	17.72004	106.55677	83	5689

1094	17.73769	106.65356	1	5690

1095	17.75214	106.75034	5	5695

1096	17.76177	106.84712	1	5695

1097	17.78585	106.9439	1	5696

1098	17.80513	107.04068	4	5700

1099	17.80994	107.13747	14	5714

1100	17.83402	107.23425	3	5717

1101	17.85008	107.33103	10	5727

1102	17.86293	107.42781	1	5727

1103	17.87577	107.52459	5	5733

1104	17.89504	107.62137	49	5782

1105	17.9159	107.71816	1	5783

1106	17.92875	107.81494	5	5788

1107	17.94159	107.91172	2	5790

1108	17.96247	108.0085	1	5791

1109	17.97692	108.10528	23	5813

1110	17.98976	108.20206	15	5828

1111	18.00742	108.29885	6	5834

1112	18.02829	108.39563	7	5841

1113	18.04113	108.49241	6	5847

1114	18.05558	108.58919	5	5851

1115	18.06683	108.68597	17	5869

1116	18.09251	108.78276	7	5875

1117	18.10697	108.87954	1	5877

1118	18.1182	108.97632	1	5877

1119	18.13586	109.0731	3	5881

1120	18.15192	109.16988	3	5884

1121	18.16315	109.26666	3	5887

1122	18.18242	109.36345	1	5887

1123	18.20489	109.46023	9	5897

1124	18.22256	109.55701	5	5902

1125	18.22738	109.65379	1	5903

1126	18.24022	109.75057	13	5915

1127	18.26591	109.84736	3	5918

1128	18.28036	109.94414	6	5924

1129	18.29802	110.04092	16	5940

1130	18.31728	110.1377	7	5947

1131	18.32531	110.23448	1	5949

1132	18.33816	110.33126	1	5949

1133	18.35903	110.42805	1	5950

1134	18.38151	110.52483	2	5952

1135	18.39434	110.62161	7	5959

1136	18.39916	110.71839	2	5961

1137	18.42486	110.81517	1	5961

1138	18.44412	110.91196	1	5962

1139	18.45536	111.00874	1	5963

1140	18.46659	111.10552	8	5971

1141	18.48907	111.2023	1	5972

1142	18.50673	111.29908	4	5976

1143	18.54848	111.39586	3	5979

1144	18.56775	111.49265	1	5980

1145	18.57898	111.58943	2	5982

1146	18.59664	111.68621	37	6019

1147	18.63839	111.78299	1	6020

1148	18.65926	111.87977	1	6021

1149	18.67532	111.97656	9	6029

1150	18.85513	112.07334	19	6048

1151	18.94343	112.17012	1	6049

1152	19.11683	112.2669	16	6065

1153	18.92417	112.36368	1	6065

1154	18.63518	112.46046	5	6070

1155	18.24665	112.55725	1	6071

1156	17.19824	112.65403	1	6072

1157	16.18677	112.75081	1	6073

1158	14.31956	112.84759	1	6074

1159	10.80188	112.94437	3	6077

1160	7.50417	113.04116	1	6077

1161	5.75576	113.13794	1	6079

1162	4.84865	113.23472	1	6079

1163	3.97525	113.3315	2	6081

1164	3.14359	113.42828	3	6084

1165	2.34083	113.52506	1	6085

113.62185	16	6101

113.71863	1	6102

113.81541	1	6103

113.91219	3	6106

114.00897	3	6109

114.10576	1	6110

114.20254	2	6112

114.29932	10	6122

114.3961	1	6123

114.49288	2	6125

114.58966	11	6136

114.68645	1	6137

114.78323	7	6144

114.88001	2	6146

114.97679	4	6150

115.07357	21	6171

115.17036	5	6175

115.26714	3	6178

115.36392	2	6180

115.4607	4	6184

115.55748	1	6185

115.65426	1	6187

115.75105	3	6190

115.84783	1	6191

115.94461	1	6192

116.04139	1	6193

116.13817	3	6195

116.23496	1	6197

116.33174	4	6201

116.42852	2	6203

116.5253	1	6203

116.62208	10	6213

116.71886	6	6219

116.81565	3	6223

116.91243	1	6224

117.00921	14	6238

117.10599	1	6239

117.20277	1	6241

117.29956	2	6243

117.39634	5	6247

117.49312	11	6258

117.5899	1	6259

117.68668	5	6264

117.78346	1	6265

117.88025	1	6267

117.97703	4	6271

118.07381	39	6309

118.17059	1	6310

118.26737	1	6311

118.36416	2	6313

118.46094	1	6314

118.55772	6	6320

118.6545	1	6321

118.75128	11	6331

118.84806	3	6335

118.94485	1	6335

119.04163	1	6336

119.13841	1	6337

119.23519	5	6343

119.33197	17	6359

119.42876	1	6360

119.52554	1	6361

119.62232	1	6361

119.7191	1	6362

119.81588	21	6383

119.91266	4	6387

120.00945	3	6390

120.10623	5	6395

120.20301	1	6395

120.29979	13	6408

120.39657	1	6409

120.49335	1	6410

120.59014	1	6411

120.68692	1	6412

120.7837	1	6413

120.88048	8	6421

120.97726	2	6423

121.07405	2	6425

121.17083	1	6427

121.26761	1	6427

121.36439	1	6428

121.46117	1	6429

121.55795	1	6431

121.65474	7	6438

121.75152	2	6440

121.8483	1	6441

121.94508	1	6441

122.04186	2	6443

122.13865	1	6444

122.23543	1	6445

122.33221	1	6446

122.42899	25	6471

122.52577	2	6473

122.62255	3	6475

122.71934	1	6476

122.81612	1	6477

122.9129	2	6479

123.00968	16	6495

123.10646	1	6496

123.20325	1	6497

123.30003	1	6498

123.39681	1	6499

123.49359	1	6500

123.59037	6	6506

123.68715	1	6507

123.78394	9	6515

123.88072	1	6516

123.9775	1	6517

124.07428	6	6523

124.17106	1	6523

124.26785	10	6533

124.36463	27	6561

124.46141	2	6563

124.55819	11	6574

124.65497	27	6601

124.75175	1	6602

124.84854	3	6605

124.94532	20	6625

125.0421	3	6628

125.13888	1	6629

125.23566	19	6648

125.33245	1	6649

125.42923	1	6650

125.52601	1	6651

125.62279	1	6653

125.71957	1	6653

125.81635	6	6659

125.91314	2	6661

126.00992	1	6662

126.1067	31	6693

126.20348	35	6728

126.30026	3	6731

126.39705	17	6748

126.49383	3	6751

126.59061	5	6756

126.68739	1	6757

126.78417	1	6759

126.88095	1	6759

126.97774	4	6763

127.07452	1	6765

127.1713	2	6767

127.26808	4	6771

127.36486	1	6771

127.46165	12	6783

127.55843	13	6797

127.65521	4	6801

127.75199	3	6804

127.84877	1	6805

127.94555	13	6817

128.04234	2	6819

128.13912	3	6822

128.2359	1	6823

128.33268	2	6825

128.42946	10	6835

128.52625	5	6839

128.62303	11	6851

128.71981	15	6865

128.81659	10	6875

128.91337	7	6882

129.01015	1	6883

129.10694	3	6886

129.20372	1	6887

129.3005	5	6892

129.39728	5	6897

129.49406	11	6908

129.59085	10	6918

129.68763	1	6919

129.78441	2	6921

129.88119	1	6921

129.97797	1	6922

130.07475	1	6923

130.17154	1	6925

130.26832	1	6925

130.3651	6	6931

130.46188	1	6933

130.55866	10	6943

130.65545	2	6945

130.75223	1	6946

130.84901	1	6947

130.94579	4	6951

131.04257	5	6957

131.13935	4	6961

131.23614	1	6962

131.33292	6	6968

131.4297	17	6985

131.52648	1	6986

131.62326	23	7009

131.72005	1	7009

131.81683	3	7013

131.91361	1	7013

132.01039	2	7015

132.10717	1	7017

132.20395	1	7017

132.30074	1	7018

132.39752	2	7020

132.4943	1	7021

132.59108	1	7022

132.68786	6	7028

132.78465	5	7033

132.88143	14	7047

132.97821	1	7048

133.07499	3	7051

133.17177	5	7056

133.26855	1	7057

133.36534	1	7059

133.46212	3	7061

133.5589	8	7069

133.65568	3	7072

133.75246	43	7115

133.84924	5	7120

133.94603	2	7122

134.04281	19	7141

134.13959	2	7143

134.23637	1	7143

134.33315	8	7151

134.42994	1	7153

134.52672	5	7157

134.6235	3	7160

134.72028	1	7161

134.81706	1	7161

134.91384	3	7164

135.01063	3	7167

135.10741	1	7167

135.20419	2	7169

135.30097	1	7170

135.39775	9	7179

135.49454	1	7180

135.59132	4	7184

135.6881	7	7191

135.78488	1	7192

135.88166	2	7194

135.97844	3	7197

136.07523	1	7198

136.17201	1	7199

136.26879	1	7199

136.36557	1	7200

136.46235	1	7201

136.55914	5	7206

136.65592	3	7209

136.7527	1	7209

136.84948	5	7214

136.94626	1	7215

137.04304	13	7228

137.13983	3	7231

137.23661	2	7233

137.33339	2	7235

137.43017	1	7237

137.52695	2	7239

137.62374	8	7247

137.72052	1	7247

137.8173	3	7250

137.91408	1	7251

138.01086	1	7251

138.10764	5	7257

138.20443	3	7260

138.30121	14	7274

138.39799	11	7285

138.49477	1	7285

138.59155	4	7289

138.68834	1	7290

138.78512	7	7297

138.8819	1	7299

138.97868	10	7309

139.07546	4	7313

139.17224	5	7318

139.26903	1	7319

139.36581	2	7321

139.46259	1	7322

139.55937	1	7323

139.65615	1	7324

139.75294	5	7329

139.84972	3	7331

139.9465	1	7332

140.04328	1	7333

140.14006	3	7336

140.23684	6	7342

140.33363	1	7343

140.43041	4	7347

140.52719	1	7348

140.62397	9	7357

140.72075	1	7358

140.81754	1	7359

140.91432	20	7379

141.0111	3	7382

141.10788	13	7395

141.20466	1	7396

141.30144	1	7397

141.39823	1	7397

141.49501	1	7398

141.59179	2	7400

141.68857	1	7401

141.78535	1	7401

141.88214	1	7402

141.97892	1	7403

142.0757	1	7404

142.17248	1	7405

142.26926	1	7405

142.36604	1	7406

142.46283	17	7423

142.55961	1	7423

142.65639	3	7427

142.75317	1	7427

142.84995	4	7431

142.94674	19	7451

143.04352	1	7451

143.1403	12	7463

143.23708	1	7465

143.33386	1	7466

143.43064	7	7473

143.52743	1	7475

143.62421	1	7475

143.72099	1	7476

143.81777	1	7477

143.91455	3	7479

144.01134	1	7481

144.10812	1	7481

144.2049	3	7484

144.30168	6	7490

144.39846	12	7502

144.49524	7	7509

144.59203	1	7510

144.68881	11	7521

144.78559	30	7551

144.88237	2	7553

144.97915	2	7555

145.07593	1	7556

145.17272	5	7561

145.2695	6	7567

145.36628	8	7575

145.46306	1	7577

145.55984	1	7578

145.65663	1	7579

145.75341	4	7583

145.85019	1	7583

145.94697	4	7587

146.04375	1	7588

146.14053	1	7589

146.23732	3	7591

146.3341	13	7605

146.43088	18	7623

146.52766	5	7628

146.62444	1	7629

146.72123	5	7633

146.81801	1	7634

146.91479	1	7635

147.01157	1	7637

147.10835	1	7638

147.20513	1	7639

147.30192	5	7645

147.3987	4	7649

147.49548	1	7650

147.59226	3	7653

147.68904	1	7653

147.78583	1	7654

147.88261	1	7655

147.97939	6	7661

148.07617	5	7666

148.17295	1	7667

148.26973	1	7669

148.36652	1	7669

148.4633	1	7670

148.56008	3	7673

148.65686	1	7674

148.75364	1	7675

148.85043	1	7676

148.94721	1	7677

149.04399	56	7733

149.14077	11	7744

149.23755	5	7749

149.33433	1	7750

149.43112	2	7752

149.5279	1	7753

149.62468	1	7755

149.72146	1	7755

149.81824	1	7756

149.91503	3	7759

150.01181	5	7765

150.10859	1	7766

150.20537	1	7767

150.30215	1	7767

150.39893	9	7776

150.49572	17	7793

150.5925	4	7797

150.68928	1	7798

150.78606	1	7799

150.88284	3	7802

150.97963	5	7807

151.07641	1	7807

151.17319	2	7809

151.26997	3	7813

151.36675	1	7814

151.46353	1	7815

151.56032	1	7815

151.6571	1	7817

151.75388	1	7817

151.85066	1	7818

151.94744	3	7821

152.04423	6	7827

152.14101	1	7828

152.23779	6	7834

152.33457	2	7836

152.43135	3	7839

152.52813	1	7840

152.62492	1	7841

152.7217	1	7842

152.81848	1	7843

152.91526	1	7844

153.01204	1	7845

153.10883	4	7849

153.20561	1	7849

153.30239	1	7851

153.39917	1	7851

153.49595	1	7852

153.59273	1	7853

153.68952	3	7855

153.7863	1	7856

153.88308	2	7858

153.97986	3	7861

154.07664	3	7864

154.17343	1	7865

154.27021	1	7866

154.36699	1	7867

154.46377	21	7887

154.56055	1	7888

154.65733	1	7889

154.75412	1	7889

154.8509	1	7891

154.94768	3	7894

155.04446	3	7897

155.14124	1	7898

155.23803	2	7900

155.33481	3	7903

155.43159	1	7904

155.52837	5	7909

155.62515	2	7911

155.72193	2	7913

155.81872	25	7938

155.9155	1	7939

156.01228	1	7940

156.10906	3	7943

156.20584	1	7943

156.30263	9	7952

156.39941	1	7953

156.49619	4	7957

156.59297	1	7957

156.68975	1	7958

156.78653	1	7959

156.88332	1	7959

156.9801	3	7962

157.07688	1	7963

157.17366	6	7969

157.27044	2	7971

157.36722	1	7972

157.46401	1	7973

157.56079	10	7983

157.65757	21	8004

157.75435	29	8033

157.85113	3	8036

157.94792	6	8042

158.0447	5	8047

158.14148	5	8052

158.23826	2	8054

158.33504	1	8055

158.43183	1	8055

158.52861	1	8056

158.62539	1	8057

158.72217	9	8066

158.81895	1	8067

158.91573	1	8068

159.01252	1	8069

159.1093	1	8071

159.20608	1	8071

159.30286	2	8073

159.39964	1	8075

159.49642	2	8077

159.59321	1	8077

159.68999	23	8101

159.78677	1	8101

159.88355	1	8103

159.98033	1	8104

160.07712	32	8136

160.1739	9	8145

160.27068	1	8145

160.36746	1	8146

160.46424	8	8154

160.56102	1	8155

160.65781	2	8157

160.75459	2	8159

160.85137	6	8165

160.94815	3	8168

161.04493	5	8173

161.14172	1	8173

161.2385	3	8177

161.33528	1	8178

161.43206	2	8180

161.52884	2	8182

161.62562	1	8183

161.72241	19	8202

161.81919	1	8203

161.91597	19	8221

162.01275	7	8229

162.10953	6	8235

162.20632	11	8246

162.3031	1	8247

162.39988	5	8252

162.49666	1	8253

162.59344	1	8254

162.69022	1	8255

162.78701	1	8256

162.88379	21	8277

162.98057	1	8278

163.07735	1	8279

163.17413	17	8296

163.27092	2	8298

163.3677	9	8307

163.46448	15	8322

163.56126	6	8328

163.65804	1	8329

163.75482	1	8330

163.85161	12	8342

163.94839	5	8346

164.04517	8	8354

164.14195	5	8358

164.23873	2	8360

164.33552	9	8369

164.4323	11	8379

164.52908	2	8381

164.62586	2	8382

164.72264	3	8385

164.81942	2	8387

164.91621	15	8402

165.01299	2	8403

165.10977	3	8406

165.20655	2	8408

165.30333	3	8411

165.40012	2	8412

165.4969	3	8415

165.59368	3	8418

165.69046	2	8420

165.78724	35	8454

165.88402	2	8456

165.98081	5	8460

166.07759	11	8471

166.17437	5	8475

166.27115	3	8478

166.36793	11	8489

166.46472	23	8511

166.5615	3	8514

166.65828	2	8516

166.75506	2	8517

166.85184	3	8520

166.94862	9	8529

167.04541	3	8532

167.14219	9	8541

167.23897	2	8543

167.33575	2	8544

167.43253	9	8553

167.52932	2	8555

167.6261	11	8565

167.72288	42	8607

167.81966	3	8610

167.91644	45	8655

168.01322	18	8673

168.11001	2	8675

168.20679	9	8684

168.30357	2	8685

168.40035	63	8748

168.49713	3	8751

168.59392	2	8753

168.6907	3	8756

168.78748	5	8760

168.88426	6	8766

168.98104	26	8792

169.07782	17	8808

169.17461	27	8835

169.27139	2	8837

169.36817	2	8838

169.46495	2	8840

169.56173	24	8864

169.65852	2	8865

169.7553	5	8870

169.85208	2	8871

169.94886	2	8873

170.04564	2	8874

170.14242	2	8876

170.23921	2	8877

170.33599	11	8888

170.43277	3	8891

170.52955	3	8894

170.62633	18	8912

170.72312	2	8913

170.8199	2	8915

170.91668	3	8918

171.01346	6	8924

171.11024	11	8934

171.20702	6	8940

171.30381	2	8942

171.40059	2	8943

171.49737	3	8946

171.59415	18	8964

171.69093	2	8966

171.78771	26	8991

171.8845	26	9017

171.98128	2	9018

172.07806	17	9035

172.17484	2	9036

172.27162	3	9039

172.36841	3	9042

172.46519	3	9045

172.56197	3	9048

172.65875	9	9057

172.75553	17	9074

172.85231	17	9090

172.9491	2	9092

173.04588	3	9095

173.14266	3	9098

173.23944	2	9099

173.33622	29	9128

173.43301	14	9141

173.52979	8	9149

173.62657	2	9150

173.72335	2	9152

173.82013	3	9155

173.91691	2	9156

174.0137	2	9158

174.11048	2	9159

174.20726	9	9168

174.30404	3	9171

174.40082	2	9173

174.49761	2	9174

174.59439	2	9176

174.69117	2	9177

174.78795	2	9179

174.88473	2	9180

174.98151	5	9185

175.0783	3	9188

175.17508	26	9213

175.27186	27	9240

175.36864	2	9242

175.46542	17	9258

175.56221	2	9260

175.65899	2	9261

175.75577	2	9263

175.85255	2	9264

175.94933	9	9273

176.04611	15	9288

176.1429	2	9290

176.23968	3	9293

176.33646	5	9297

176.43324	2	9299

176.53002	2	9300

176.62681	24	9324

176.72359	14	9338

176.82037	2	9339

176.91715	2	9341

177.01393	3	9344

177.11071	3	9347

177.2075	2	9348

177.30428	15	9363

177.40106	12	9375

177.49784	2	9377

177.59462	2	9378

177.69141	5	9383

177.78819	14	9396

177.88497	5	9401

177.98175	2	9402

178.07853	5	9407

178.17531	3	9410

178.2721	2	9411

178.36888	2	9413

178.46566	18	9431

178.56244	3	9434

178.65922	2	9435

178.75601	78	9513

178.85279	48	9561

178.94957	18	9579

179.04635	23	9602

179.14313	3	9605

179.23991	2	9606

179.3367	17	9623

179.43348	5	9627

179.53026	3	9630

179.62704	3	9633

179.72382	3	9636

179.82061	5	9641

179.91739	3	9644

180.01417	3	9647

180.11095	6	9653

180.20773	2	9654

180.30451	5	9659

180.4013	2	9660

180.49808	2	9662

180.59486	23	9684

180.69164	5	9689

180.78842	2	9690

180.88521	2	9692

180.98199	2	9693

181.07877	2	9695

181.17555	24	9719

181.27233	3	9722

181.36911	2	9723

181.4659	2	9725

181.56268	2	9726

181.65946	3	9729

181.75624	3	9732

181.85302	8	9740

181.94981	2	9741

182.04659	2	9743

182.14337	2	9744

182.24015	2	9746

182.33693	2	9747

182.43371	2	9749

182.5305	8	9756

182.62728	39	9795

182.72406	2	9797

182.82084	2	9798

182.91762	2	9800

183.01441	3	9803

183.11119	2	9804

183.20797	12	9816

183.30475	2	9818

183.40153	3	9821

183.49831	2	9822

183.5951	2	9824

183.69188	30	9854

183.78866	2	9855

183.88544	2	9857

183.98222	2	9858

184.079	2	9860

184.17579	5	9864

184.27257	3	9867

184.36935	2	9869

184.46613	2	9870

184.56291	2	9872

184.6597	3	9875

184.75648	2	9876

184.85326	2	9878

184.95004	2	9879

185.04682	2	9881

185.1436	2	9882

185.24039	2	9884

185.33717	2	9885

185.43395	3	9888

185.53073	3	9891

185.62751	5	9896

185.7243	2	9897

185.82108	15	9912

185.91786	3	9915

186.01464	3	9918

186.11142	29	9947

186.2082	3	9950

186.30499	2	9951

186.40177	3	9954

186.49855	17	9971

186.59533	2	9972

186.69211	2	9974

186.7889	12	9986

186.88568	2	9987

186.98246	3	9990

187.07924	12	10002

187.17602	2	10004

187.2728	2	10005

187.36959	2	10007

187.46637	2	10008

187.56315	24	10032

187.65993	2	10034

187.75671	3	10037

187.8535	2	10038

187.95028	3	10041

188.04706	2	10043

188.14384	6	10049

188.24062	2	10050

188.3374	2	10052

188.43419	3	10055

188.53097	3	10058

188.62775	2	10059

188.72453	2	10061

188.82131	18	10079

188.9181	5	10083

189.01488	2	10085

189.11166	8	10092

189.20844	3	10095

189.30522	2	10097

189.402	8	10104

189.49879	2	10106

189.59557	3	10109

189.69235	6	10115

189.78913	2	10116

189.88591	2	10118

189.9827	5	10122

190.07948	2	10124

190.17626	6	10130

190.27304	2	10131

190.36982	5	10136

190.4666	6	10142

190.56339	14	10155

190.66017	2	10157

190.75695	2	10158

190.85373	2	10160

190.95051	5	10164

191.0473	2	10166

191.14408	2	10167

191.24086	5	10172

191.33764	2	10173

191.43442	2	10175

191.5312	2	10176

191.62799	8	10184

191.72477	2	10185

191.82155	11	10196

191.91833	5	10200

192.01511	3	10203

192.1119	8	10211

192.20868	5	10215

192.30546	3	10218

192.40224	2	10220

192.49902	2	10221

192.5958	2	10223

192.69259	2	10224

192.78937	2	10226

192.88615	11	10236

192.98293	2	10238

193.07971	3	10241

193.1765	2	10242

193.27328	15	10257

193.37006	2	10259

193.46684	2	10260

193.56362	2	10262

193.6604	2	10263

193.75719	2	10265

193.85397	2	10266

193.95075	2	10268

194.04753	29	10296

194.14431	2	10298

194.2411	8	10305

194.33788	2	10307

194.43466	2	10308

194.53144	3	10311

194.62822	44	10355

194.725	2	10356

194.82179	3	10359

194.91857	2	10361

195.01535	3	10364

195.11213	2	10365

195.20891	3	10368

195.30569	2	10370

195.40248	2	10371

195.49926	2	10373

195.59604	3	10376

195.69282	2	10377

195.7896	3	10380

195.88639	3	10383

195.98317	3	10386

196.07995	3	10389

196.17673	2	10391

196.27351	2	10392

196.3703	2	10394

196.46708	12	10406

196.56386	3	10409

196.66064	2	10410

196.75742	11	10421

196.8542	8	10428

196.95099	2	10430

197.04777	3	10433

197.14455	2	10434

197.24133	2	10436

197.33811	2	10437

197.43489	9	10446

197.53168	2	10448

197.62846	3	10451

197.72524	2	10452

197.82202	2	10454

197.9188	2	10455

198.01559	2	10457

198.11237	2	10458

198.20915	3	10461

198.30593	3	10464

198.40271	3	10467

198.49949	2	10469

198.59628	2	10470

198.69306	2	10472

198.78984	8	10479

198.88662	2	10481

198.9834	2	10482

199.08019	2	10484

199.17697	2	10485

199.27375	2	10487

199.37053	2	10488

199.46731	2	10490

199.56409	6	10496

199.66088	2	10497

199.75766	2	10499

199.85444	3	10502

199.95122	3	10505

200.048	2	10506

200.14479	2	10508

200.24157	2	10509

200.33835	3	10512

200.43513	2	10514

200.53191	2	10515

200.62869	2	10517

200.72548	2	10518

200.82226	2	10520

200.91904	30	10550

201.01582	2	10551

201.1126	2	10553

201.20939	6	10559

201.30617	2	10560

201.40295	2	10562

201.49973	3	10565

201.59651	2	10566

201.69329	5	10571

201.79008	2	10572

201.88686	2	10574

201.98364	2	10575

202.08042	2	10577

202.1772	8	10584

202.27399	3	10587

202.37077	2	10589

202.46755	2	10590

202.56433	2	10592

202.66111	3	10595

202.75789	5	10599

202.85468	2	10601

202.95146	2	10602

203.04824	2	10604

203.14502	2	10605

203.2418	2	10607

203.33859	3	10610

203.43537	2	10611

203.53215	5	10616

203.62893	14	10629

203.72571	3	10632

203.82249	2	10634

203.91928	3	10637

204.01606	2	10638

204.11284	2	10640

204.20962	2	10641

204.3064	2	10643

204.40319	2	10644

204.49997	8	10652

204.59675	5	10656

204.69353	2	10658

204.79031	6	10664

204.88709	2	10665

204.98388	3	10668

205.08066	3	10671

205.17744	2	10673

205.27422	2	10674

205.371	2	10676

205.46779	3	10679

205.56457	3	10682

205.66135	2	10683

205.75813	5	10688

205.85491	17	10704

205.95169	2	10706

206.04848	20	10725

206.14526	2	10727

206.24204	2	10728

206.33882	2	10730

206.4356	2	10731

206.53239	5	10736

206.62917	15	10751

206.72595	2	10752

206.82273	5	10757

206.91951	2	10758

207.01629	2	10760

207.11308	3	10763

207.20986	2	10764

207.30664	6	10770

207.40342	3	10773

207.5002	5	10778

207.59699	2	10779

207.69377	3	10782

207.79055	2	10784

207.88733	2	10785

207.98411	2	10787

208.08089	8	10794

208.17768	8	10802

208.27446	2	10803

208.37124	2	10805

208.46802	2	10806

208.5648	5	10811

208.66159	6	10817

208.75837	2	10818

208.85515	3	10821

208.95193	2	10823

209.04871	2	10824

209.14549	5	10829

209.24228	2	10830

209.33906	3	10833

209.43584	3	10836

209.53262	2	10838

209.6294	6	10844

209.72618	2	10845

209.82297	2	10847

209.91975	3	10850

210.01653	2	10851

210.11331	11	10862

210.21009	2	10863

210.30688	3	10866

210.40366	2	10868

210.50044	5	10872

210.59722	2	10874

210.694	2	10875

210.79078	5	10880

210.88757	3	10883

210.98435	2	10884

211.08113	2	10886

211.17791	2	10887

211.27469	2	10889

211.37148	6	10895

211.46826	2	10896

211.56504	2	10898

211.66182	2	10899

211.7586	2	10901

211.85538	2	10902

211.95217	2	10904

212.04895	6	10910

212.14573	6	10916

212.24251	2	10917

212.33929	3	10920

212.43608	3	10923

212.53286	5	10928

212.62964	2	10929

212.72642	32	10961

212.8232	2	10962

212.91998	2	10964

213.01677	2	10965

213.11355	2	10967

213.21033	3	10970

213.30711	2	10971

213.40389	2	10973

213.50068	6	10979

213.59746	5	10983

213.69424	3	10986

213.79102	2	10988

213.8878	18	11006

213.98458	18	11024

214.08137	17	11040

214.17815	2	11042

214.27493	2	11043

214.37171	2	11045

214.46849	2	11046

214.56528	5	11051

214.66206	3	11054

214.75884	2	11055

214.85562	2	11057

214.9524	2	11058

215.04918	15	11073

215.14597	2	11075

215.24275	2	11076

215.33953	5	11081

215.43631	12	11093

215.53309	2	11094

215.62988	2	11096

215.72666	3	11099

215.82344	2	11100

215.92022	3	11103

216.017	3	11106

216.11378	2	11108

216.21057	2	11109

216.30735	2	11111

216.40413	2	11112

216.50091	23	11135

216.59769	2	11136

216.69448	2	11138

216.79126	2	11139

216.88804	2	11141

216.98482	2	11142

217.0816	2	11144

217.17838	2	11145

217.27517	3	11148

217.37195	2	11150

217.46873	5	11154

217.56551	2	11156

217.66229	2	11157

217.75908	3	11160

217.85586	3	11163

217.95264	2	11165

218.04942	3	11168

218.1462	2	11169

218.24298	17	11186

218.33977	2	11187

218.43655	3	11190

218.53333	2	11192

218.63011	2	11193

218.72689	2	11195

218.82368	2	11196

218.92046	3	11199

219.01724	2	11201

219.11402	2	11202

219.2108	2	11204

219.30758	2	11205

219.40437	3	11208

219.50115	2	11210

219.59793	2	11211

219.69471	2	11213

219.79149	2	11214

219.88828	11	11225

219.98506	3	11228

220.08184	3	11231

220.17862	2	11232

220.2754	3	11235

220.37218	2	11237

220.46897	14	11250

220.56575	2	11252

220.66253	2	11253

220.75931	3	11256

220.85609	2	11258

220.95288	3	11261

221.04966	2	11262

221.14644	5	11267

221.24322	6	11273

221.34	2	11274

221.43678	2	11276

221.53357	2	11277

221.63035	11	11288

221.72713	2	11289

221.82391	3	11292

221.92069	2	11294

222.01747	5	11298

222.11426	2	11300

222.21104	3	11303

222.30782	5	11307

222.4046	2	11309

222.50138	2	11310

222.59817	2	11312

222.69495	3	11315

222.79173	2	11316

222.88851	3	11319

222.98529	2	11321

223.08207	41	11361

223.17886	2	11363

223.27564	2	11364

223.37242	5	11369

223.4692	3	11372

223.56598	2	11373

223.66277	2	11375

223.75955	3	11378

223.85633	17	11394

223.95311	2	11396

224.04989	2	11397

224.14667	2	11399

224.24346	2	11400

224.34024	27	11427

224.43702	3	11430

224.5338	2	11432

224.63058	2	11433

224.72737	9	11442

224.82415	2	11444

224.92093	3	11447

225.01771	3	11450

225.11449	2	11451

225.21127	2	11453

225.30806	2	11454

225.40484	5	11459

225.50162	3	11462

225.5984	2	11463

225.69518	2	11465

225.79197	3	11468

225.88875	3	11471

225.98553	3	11474

226.08231	2	11475

226.17909	3	11478

226.27587	2	11480

226.37266	2	11481

226.46944	5	11486

226.56622	2	11487

226.663	2	11489

226.75978	2	11490

226.85657	2	11492

226.95335	2	11493

227.05013	3	11496

227.14691	2	11498

227.24369	2	11499

227.34047	2	11501

227.43726	2	11502

227.53404	2	11504

227.63082	2	11505

227.7276	6	11511

227.82438	5	11516

227.92117	2	11517

228.01795	2	11519

228.11473	2	11520

228.21151	3	11523

228.30829	2	11525

228.40507	2	11526

228.50186	3	11529

228.59864	2	11531

228.69542	2	11532

228.7922	2	11534

228.88898	5	11538

228.98577	3	11541

229.08255	2	11543

229.17933	5	11547

229.27611	2	11549

229.37289	2	11550

229.46967	2	11552

229.56646	3	11555

229.66324	6	11561

229.76002	8	11568

229.8568	2	11570

229.95358	2	11571

230.05037	2	11573

230.14715	6	11579

230.24393	2	11580

230.34071	2	11582

230.43749	3	11585

230.53427	6	11591

230.63106	21	11612

230.72784	2	11613

230.82462	2	11615

230.9214	2	11616

231.01818	2	11618

231.11497	2	11619

231.21175	2	11621

231.30853	3	11624

231.40531	3	11627

231.50209	2	11628

231.59887	3	11631

231.69566	2	11633

231.79244	6	11639

231.88922	2	11640

231.986	2	11642

232.08278	2	11643

232.17957	2	11645

232.27635	18	11663

232.37313	2	11664

232.46991	2	11666

232.56669	2	11667

232.66347	2	11669

232.76026	2	11670

232.85704	3	11673

232.95382	2	11675

233.0506	6	11681

233.14738	2	11682

233.24417	2	11684

233.34095	2	11685

233.43773	2	11687

233.53451	3	11690

233.63129	48	11738

233.72807	2	11739

233.82486	5	11744

233.92164	2	11745

234.01842	2	11747

234.1152	2	11748

234.21198	2	11750

234.30876	2	11751

234.40555	2	11753

234.50233	2	11754

234.59911	3	11757

234.69589	9	11766

234.79267	2	11768

234.88946	6	11774

234.98624	6	11780

235.08302	2	11781

235.1798	2	11783

235.27658	2	11784

235.37336	2	11786

235.47015	2	11787

235.56693	2	11789

235.66371	2	11790

235.76049	3	11793

235.85727	2	11795

235.95406	2	11796

236.05084	2	11798

236.14762	2	11799

236.2444	3	11802

236.34118	2	11804

236.43796	2	11805

236.53475	2	11807

236.63153	2	11808

236.72831	3	11811

236.82509	2	11813

236.92187	3	11816

237.01866	2	11817

237.11544	3	11820

237.21222	5	11825

237.309	2	11826

237.40578	2	11828

237.50256	2	11829

237.59935	2	11831

237.69613	2	11832

237.79291	11	11843

237.88969	2	11844

237.98647	2	11846

238.08326	2	11847

238.18004	2	11849

238.27682	2	11850

238.3736	3	11853

238.47038	5	11858

238.56716	2	11859

238.66395	2	11861

238.76073	2	11862

238.85751	3	11865

238.95429	2	11867

239.05107	2	11868

239.14786	12	11880

239.24464	9	11889

239.34142	6	11895

239.4382	2	11897

239.53498	2	11898

239.63176	2	11900

239.72855	2	11901

239.82533	2	11903

239.92211	2	11904

240.01889	2	11906

240.11567	2	11907

240.21246	2	11909

240.30924	2	11910

240.40602	2	11912

240.5028	5	11916

240.59958	3	11919

240.69636	2	11921

240.79315	3	11924

240.88993	2	11925

240.98671	5	11930

241.08349	2	11931

241.18027	2	11933

241.27706	3	11936

241.37384	2	11937

241.47062	2	11939

241.5674	38	11976

241.66418	6	11982

241.76096	9	11991

241.85775	2	11993

241.95453	2	11994

242.05131	2	11996

242.14809	2	11997

242.24487	3	12000

242.34166	2	12002

242.43844	2	12003

242.53522	5	12008

242.632	5	12012

242.72878	6	12018

242.82556	15	12033

242.92235	2	12035

243.01913	3	12038

243.11591	2	12039

243.21269	2	12041

243.30947	2	12042

243.40626	2	12044

243.50304	2	12045

243.59982	2	12047

243.6966	5	12051

243.79338	5	12056

243.89016	3	12059

243.98695	2	12060

244.08373	2	12062

244.18051	3	12065

244.27729	3	12068

244.37407	2	12069

244.47086	2	12071

244.56764	2	12072

244.66442	2	12074

244.7612	2	12075

244.85798	2	12077

244.95476	2	12078

245.05155	2	12080

245.14833	2	12081

245.24511	2	12083

245.34189	2	12084

245.43867	2	12086

245.53546	8	12093

245.63224	3	12096

245.72902	5	12101

245.8258	24	12125

245.92258	9	12134

246.01936	2	12135

246.11615	17	12152

246.21293	5	12156

246.30971	2	12158

246.40649	2	12159

246.50327	2	12161

246.60006	2	12162

246.69684	2	12164

246.79362	2	12165

246.8904	3	12168

246.98718	2	12170

247.08396	5	12174

247.18075	3	12177

247.27753	3	12180

247.37431	2	12182

247.47109	2	12183

247.56787	2	12185

247.66465	2	12186

247.76144	3	12189

247.85822	3	12192

247.955	6	12198

248.05178	2	12200

248.14856	2	12201

248.24535	3	12204

248.34213	2	12206

248.43891	2	12207

248.53569	2	12209

248.63247	57	12266

248.72925	2	12267

248.82604	6	12273

248.92282	2	12275

249.0196	2	12276

249.11638	3	12279

249.21316	3	12282

249.30995	3	12285

249.40673	2	12287

249.50351	2	12288

249.60029	3	12291

249.69707	2	12293

249.79385	3	12296

249.89064	2	12297

249.98742	2	12299

250.0842	2	12300

250.18098	2	12302

250.27776	3	12305

250.37455	2	12306

250.47133	2	12308

250.56811	2	12309

250.66489	2	12311

250.76167	2	12312

250.85845	2	12314

250.95524	3	12317

251.05202	2	12318

251.1488	2	12320

251.24558	6	12326

251.34236	3	12329

251.43915	3	12332

251.53593	2	12333

251.63271	3	12336

251.72949	5	12341

251.82627	2	12342

251.92305	3	12345

252.01984	2	12347

252.11662	5	12351

252.2134	2	12353

252.31018	3	12356

252.40696	2	12357

252.50375	2	12359

252.60053	2	12360

252.69731	3	12363

252.79409	2	12365

252.89087	2	12366

252.98765	3	12369

253.08444	2	12371

253.18122	2	12372

253.278	3	12375

253.37478	3	12378

253.47156	2	12380

253.56835	2	12381

253.66513	2	12383

253.76191	3	12386

253.85869	2	12387

253.95547	2	12389

254.05225	2	12390

254.14904	3	12393

254.24582	5	12398

254.3426	3	12401

254.43938	5	12405

254.53616	2	12407

254.63295	3	12410

254.72973	6	12416

254.82651	2	12417

254.92329	2	12419

255.02007	6	12425

255.11685	2	12426

255.21364	5	12431

255.31042	2	12432

255.4072	3	12435

255.50398	2	12437

255.60076	6	12443

255.69755	2	12444

255.79433	8	12452

255.89111	2	12453

255.98789	5	12458

256.08467	2	12459

256.18145	3	12462

256.27824	2	12464

256.37502	6	12470

256.4718	3	12473

256.56858	3	12476

256.66536	3	12479

256.76215	2	12480

256.85893	2	12482

256.95571	2	12483

257.05249	24	12507

257.14927	2	12509

257.24605	6	12515

257.34284	2	12516

257.43962	8	12524

257.5364	11	12534

257.63318	2	12536

257.72996	3	12539

257.82675	2	12540

257.92353	2	12542

258.02031	2	12543

258.11709	2	12545

258.21387	2	12546

258.31065	2	12548

258.40744	3	12551

258.50422	2	12552

258.601	2	12554

258.69778	2	12555

258.79456	2	12557

258.89135	2	12558

258.98813	2	12560

259.08491	2	12561

259.18169	2	12563

259.27847	15	12578

259.37525	2	12579

259.47204	5	12584

259.56882	2	12585

259.6656	2	12587

259.76238	2	12588

259.85916	2	12590

259.95594	6	12596

260.05273	2	12597

260.14951	6	12603

260.24629	2	12605

260.34307	2	12606

260.43985	2	12608

260.53664	2	12609

260.63342	3	12612

260.7302	2	12614

260.82698	6	12620

260.92376	2	12621

261.02054	2	12623

261.11733	2	12624

261.21411	2	12626

261.31089	3	12629

261.40767	2	12630

261.50445	8	12638

261.60124	3	12641

261.69802	2	12642

261.7948	8	12650

261.89158	2	12651

261.98836	2	12653

262.08514	3	12656

262.18193	14	12669

262.27871	3	12672

262.37549	3	12675

262.47227	2	12677

262.56905	2	12678

262.66584	5	12683

262.76262	3	12686

262.8594	3	12689

262.95618	2	12690

263.05296	2	12692

263.14974	2	12693

263.24653	6	12699

263.34331	2	12701

263.44009	2	12702

263.53687	2	12704

263.63365	2	12705

263.73044	5	12710

263.82722	2	12711

263.924	3	12714

264.02078	9	12723

264.11756	2	12725

264.21434	2	12726

264.31113	2	12728

264.40791	3	12731

264.50469	3	12734

264.60147	2	12735

264.69825	5	12740

264.79504	6	12746

264.89182	2	12747

264.9886	2	12749

265.08538	2	12750

265.18216	3	12753

265.27894	2	12755

265.37573	3	12758

265.47251	2	12759

265.56929	2	12761

265.66607	6	12767

265.76285	3	12770

265.85964	5	12774

265.95642	3	12777

266.0532	2	12779

266.14998	2	12780

266.24676	2	12782

266.34354	2	12783

266.44033	2	12785

266.53711	5	12789

266.63389	11	12800

266.73067	2	12801

266.82745	8	12809

266.92424	2	12810

267.02102	2	12812

267.1178	3	12815

267.21458	3	12818

267.31136	2	12819

267.40814	3	12822

267.50493	2	12824

267.60171	2	12825

267.69849	3	12828

267.79527	5	12833

267.89205	5	12837

267.98884	3	12840

268.08562	2	12842

268.1824	5	12846

268.27918	9	12855

268.37596	2	12857

268.47274	2	12858

268.56953	3	12861

268.66631	3	12864

268.76309	3	12867

268.85987	2	12869

268.95665	2	12870

269.05344	2	12872

269.15022	6	12878

269.247	3	12881

269.34378	2	12882

269.44056	5	12887

269.53734	33	12920

269.63413	3	12923

269.73091	5	12927

269.82769	3	12930

269.92447	2	12932

270.02125	3	12935

270.11804	6	12941

270.21482	2	12942

270.3116	3	12945

270.40838	2	12947

270.50516	3	12950

270.60194	2	12951

270.69873	3	12954

270.79551	5	12959

270.89229	2	12960

270.98907	5	12965

271.08585	2	12966

271.18264	2	12968

271.27942	2	12969

271.3762	3	12972

271.47298	2	12974

271.56976	2	12975

271.66654	2	12977

271.76333	2	12978

271.86011	2	12980

271.95689	2	12981

272.05367	2	12983

272.15045	3	12986

272.24723	2	12987

272.34402	2	12989

272.4408	2	12990

272.53758	6	12996

272.63436	2	12998

272.73114	2	12999

272.82793	3	13002

272.92471	2	13004

273.02149	3	13007

273.11827	2	13008

273.21505	2	13010

273.31183	2	13011

273.40862	3	13014

273.5054	2	13016

273.60218	2	13017

273.69896	2	13019

273.79574	2	13020

273.89253	5	13025

273.98931	5	13029

274.08609	2	13031

274.18287	2	13032

274.27965	5	13037

274.37643	2	13038

274.47322	2	13040

274.57	2	13041

274.66678	2	13043

274.76356	5	13047

274.86034	3	13050

274.95713	2	13052

275.05391	2	13053

275.15069	3	13056

275.24747	3	13059

275.34425	2	13061

275.44103	2	13062

275.53782	2	13064

275.6346	2	13065

275.73138	2	13067

275.82816	3	13070

275.92494	2	13071

276.02173	2	13073

276.11851	5	13077

276.21529	18	13095

276.31207	2	13097

276.40885	2	13098

276.50563	6	13104

276.60242	3	13107

276.6992	2	13109

276.79598	3	13112

276.89276	2	13113

276.98954	3	13116

277.08633	2	13118

277.18311	2	13119

277.27989	3	13122

277.37667	5	13127

277.47345	2	13128

277.57023	11	13139

277.66702	2	13140

277.7638	5	13145

277.86058	2	13146

277.95736	3	13149

278.05414	2	13151

278.15093	2	13152

278.24771	2	13154

278.34449	5	13158

278.44127	2	13160

278.53805	2	13161

278.63483	8	13169

278.73162	2	13170

278.8284	2	13172

278.92518	18	13190

279.02196	2	13191

279.11874	3	13194

279.21553	2	13196

279.31231	2	13197

279.40909	3	13200

279.50587	2	13202

279.60265	14	13215

279.69943	5	13220

279.79622	2	13221

279.893	2	13223

279.98978	9	13232

280.08656	5	13236

280.18334	2	13238

280.28013	12	13250

280.37691	2	13251

280.47369	5	13256

280.57047	2	13257

280.66725	3	13260

280.76403	5	13265

280.86082	2	13266

280.9576	5	13271

281.05438	3	13274

281.15116	2	13275

281.24794	3	13278

281.34473	2	13280

281.44151	3	13283

281.53829	3	13286

281.63507	11	13296

281.73185	2	13298

281.82863	2	13299

281.92542	2	13301

282.0222	3	13304

282.11898	3	13307

282.21576	3	13310

282.31254	2	13311

282.40933	8	13319

282.50611	5	13323

282.60289	2	13325

282.69967	2	13326

282.79645	2	13328

282.89323	3	13331

282.99002	2	13332

283.0868	2	13334

283.18358	2	13335

283.28036	8	13343

283.37714	2	13344

283.47393	2	13346

283.57071	8	13353

283.66749	2	13355

283.76427	2	13356

283.86105	2	13358

283.95783	6	13364

284.05462	2	13365

284.1514	2	13367

284.24818	5	13371

284.34496	3	13374

284.44174	5	13379

284.53852	6	13385

284.63531	3	13388

284.73209	2	13389

284.82887	2	13391

284.92565	2	13392

285.02243	2	13394

285.11922	2	13395

285.216	3	13398

285.31278	2	13400

285.40956	5	13404

285.50634	6	13410

285.60312	2	13412

285.69991	2	13413

285.79669	6	13419

285.89347	2	13421

285.99025	2	13422

286.08703	12	13434

286.18382	2	13436

286.2806	2	13437

286.37738	3	13440

286.47416	2	13442

286.57094	2	13443

286.66772	2	13445

286.76451	2	13446

286.86129	6	13452

286.95807	2	13454

287.05485	2	13455

287.15163	6	13461

287.24842	3	13464

287.3452	8	13472

287.44198	2	13473

287.53876	2	13475

287.63554	2	13476

287.73232	2	13478

287.82911	2	13479

287.92589	2	13481

288.02267	2	13482

288.11945	2	13484

288.21623	2	13485

288.31302	3	13488

288.4098	3	13491

288.50658	3	13494

288.60336	2	13496

288.70014	2	13497

288.79692	2	13499

288.89371	2	13500

288.99049	3	13503

289.08727	2	13505

289.18405	2	13506

289.28083	3	13509

289.37762	2	13511

289.4744	3	13514

289.57118	2	13515

289.66796	2	13517

289.76474	5	13521

289.86152	2	13523

289.95831	2	13524

290.05509	5	13529

290.15187	3	13532

290.24865	2	13533

290.34543	2	13535

290.44222	2	13536

290.539	2	13538

290.63578	2	13539

290.73256	2	13541

290.82934	2	13542

290.92612	3	13545

291.02291	8	13553

291.11969	2	13554

291.21647	3	13557

291.31325	5	13562

291.41003	2	13563

291.50682	3	13566

291.6036	5	13571

291.70038	2	13572

291.79716	2	13574

291.89394	2	13575

291.99072	2	13577

292.08751	11	13587

292.18429	3	13590

292.28107	2	13592

292.37785	2	13593

292.47463	5	13598

292.57142	3	13601

292.6682	2	13602

292.76498	2	13604

292.86176	2	13605

292.95854	21	13626

293.05532	2	13628

293.15211	2	13629

293.24889	3	13632

293.34567	3	13635

293.44245	2	13637

293.53923	2	13638

293.63602	2	13640

293.7328	5	13644

293.82958	2	13646

293.92636	2	13647

294.02314	2	13649

294.11992	3	13652

294.21671	2	13653

294.31349	2	13655

294.41027	5	13659

294.50705	2	13661

294.60383	3	13664

294.70062	2	13665

294.7974	2	13667

294.89418	2	13668

294.99096	2	13670

295.08774	2	13671

295.18452	2	13673

295.28131	6	13679

295.37809	2	13680

295.47487	2	13682

295.57165	2	13683

295.66843	2	13685

295.76522	3	13688

295.862	3	13691

295.95878	2	13692

296.05556	2	13694

296.15234	2	13695

296.24912	3	13698

296.34591	5	13703

296.44269	3	13706

296.53947	3	13709

296.63625	8	13716

296.73303	2	13718

296.82982	2	13719

296.9266	2	13721

297.02338	5	13725

297.12016	2	13727

297.21694	3	13730

297.31372	2	13731

297.41051	2	13733

297.50729	3	13736

297.60407	6	13742

297.70085	3	13745

297.79763	2	13746

297.89441	2	13748

297.9912	2	13749

298.08798	2	13751

298.18476	2	13752

298.28154	3	13755

298.37832	2	13757

298.47511	2	13758

298.57189	6	13764

298.66867	2	13766

298.76545	3	13769

298.86223	2	13770

298.95901	2	13772

299.0558	2	13773

299.15258	2	13775

299.24936	3	13778

299.34614	3	13781

299.44292	2	13782

299.53971	3	13785

299.63649	2	13787

299.73327	3	13790

299.83005	2	13791

299.92683	2	13793

300.02361	3	13796

300.1204	2	13797

300.21718	5	13802

300.31396	2	13803

300.41074	2	13805

300.50752	3	13808

300.60431	3	13811

300.70109	24	13835

300.79787	2	13836

300.89465	2	13838

300.99143	2	13839

301.08821	2	13841

301.185	2	13842

301.28178	2	13844

301.37856	2	13845

301.47534	2	13847

301.57212	2	13848

301.66891	2	13850

301.76569	2	13851

301.86247	2	13853

301.95925	5	13857

302.05603	2	13859

302.15281	2	13860

302.2496	2	13862

302.34638	2	13863

302.44316	2	13865

302.53994	3	13868

302.63672	2	13869

302.73351	2	13871

302.83029	2	13872

302.92707	2	13874

303.02385	3	13877

303.12063	6	13883

303.21741	2	13884

303.3142	2	13886

303.41098	3	13889

303.50776	3	13892

303.60454	2	13893

303.70132	2	13895

303.79811	2	13896

303.89489	9	13905

303.99167	3	13908

304.08845	3	13911

304.18523	5	13916

304.28201	2	13917

304.3788	2	13919

304.47558	3	13922

304.57236	2	13923

304.66914	2	13925

304.76592	2	13926

304.86271	5	13931

304.95949	3	13934

305.05627	2	13935

305.15305	2	13937

305.24983	5	13941

305.34661	5	13946

305.4434	2	13947

305.54018	12	13959

305.63696	2	13961

305.73374	56	14016

305.83052	2	14018

305.92731	5	14022

306.02409	15	14037

306.12087	6	14043

306.21765	2	14045

306.31443	2	14046

306.41121	8	14054

306.508	3	14057

306.60478	2	14058

306.70156	9	14067

306.79834	2	14069

306.89512	2	14070

306.99191	2	14072

307.08869	2	14073

307.18547	3	14076

307.28225	2	14078

307.37903	2	14079

307.47581	2	14081

307.5726	2	14082

307.66938	9	14091

307.76616	2	14093

307.86294	2	14094

307.95972	3	14097

308.05651	6	14103

308.15329	2	14105

308.25007	3	14108

308.34685	2	14109

308.44363	2	14111

308.54041	2	14112

308.6372	2	14114

308.73398	2	14115

308.83076	2	14117

308.92754	2	14118

309.02432	3	14121

309.12111	5	14126

309.21789	2	14127

309.31467	2	14129

309.41145	2	14130

309.50823	2	14132

309.60501	3	14135

309.7018	2	14136

309.79858	5	14141

309.89536	3	14144

309.99214	3	14147

310.08892	3	14150

310.1857	11	14160

310.28249	2	14162

310.37927	5	14166

310.47605	2	14168

310.57283	2	14169

310.66961	2	14171

310.7664	3	14174

310.86318	2	14175

310.95996	3	14178

311.05674	3	14181

311.15352	2	14183

311.2503	2	14184

311.34709	2	14186

311.44387	2	14187

311.54065	5	14192

311.63743	3	14195

311.73421	2	14196

311.831	2	14198

311.92778	2	14199

312.02456	2	14201

312.12134	2	14202

312.21812	5	14207

312.3149	2	14208

312.41169	5	14213

312.50847	2	14214

312.60525	2	14216

312.70203	3	14219

312.79881	2	14220

312.8956	2	14222

312.99238	2	14223

313.08916	6	14229

313.18594	5	14234

313.28272	2	14235

313.3795	2	14237

313.47629	2	14238

313.57307	2	14240

313.66985	2	14241

313.76663	5	14246

313.86341	2	14247

313.9602	2	14249

314.05698	2	14250

314.15376	2	14252

314.25054	3	14255

314.34732	2	14256

314.4441	2	14258

314.54089	3	14261

314.63767	3	14264

314.73445	2	14265

314.83123	5	14270

314.92801	3	14273

315.0248	6	14279

315.12158	5	14283

315.21836	2	14285

315.31514	2	14286

315.41192	3	14289

315.5087	3	14292

315.60549	2	14294

315.70227	2	14295

315.79905	2	14297

315.89583	5	14301

315.99261	2	14303

316.0894	2	14304

316.18618	3	14307

316.28296	2	14309

316.37974	2	14310

316.47652	2	14312

316.5733	2	14313

316.67009	3	14316

316.76687	5	14321

316.86365	2	14322

316.96043	2	14324

317.05721	2	14325

317.154	3	14328

317.25078	2	14330

317.34756	5	14334

317.44434	2	14336

317.54112	3	14339

317.6379	2	14340

317.73469	2	14342

317.83147	2	14343

317.92825	3	14346

318.02503	2	14348

318.12181	2	14349

318.2186	3	14352

318.31538	6	14358

318.41216	3	14361

318.50894	3	14364

318.60572	2	14366

318.7025	2	14367

318.79929	2	14369

318.89607	2	14370

318.99285	2	14372

319.08963	5	14376

319.18641	3	14379

319.2832	2	14381

319.37998	6	14387

319.47676	2	14388

319.57354	2	14390

319.67032	12	14402

319.7671	2	14403

319.86389	2	14405

319.96067	8	14412

320.05745	3	14415

320.15423	2	14417

320.25101	2	14418

320.3478	2	14420

320.44458	5	14424

320.54136	2	14426

320.63814	2	14427

320.73492	2	14429

320.8317	3	14432

320.92849	3	14435

321.02527	2	14436

321.12205	8	14444

321.21883	2	14445

321.31561	2	14447

321.4124	5	14451

321.50918	2	14453

321.60596	2	14454

321.70274	2	14456

321.79952	2	14457

321.8963	5	14462

321.99309	3	14465

322.08987	2	14466

322.18665	2	14468

322.28343	3	14471

322.38021	2	14472

322.47699	2	14474

322.57378	2	14475

322.67056	2	14477

322.76734	2	14478

322.86412	2	14480

322.9609	3	14483

323.05769	5	14487

323.15447	2	14489

323.25125	2	14490

323.34803	3	14493

323.44481	2	14495

323.54159	6	14501

323.63838	2	14502

323.73516	2	14504

323.83194	2	14505

323.92872	2	14507

324.0255	5	14511

324.12229	3	14514

324.21907	17	14531

324.31585	2	14532

324.41263	3	14535

324.50941	2	14537

324.60619	11	14547

324.70298	3	14550

324.79976	3	14553

324.89654	3	14556

324.99332	9	14565

325.0901	2	14567

325.18689	2	14568

325.28367	2	14570

325.38045	5	14574

325.47723	2	14576

325.57401	3	14579

325.67079	2	14580

325.76758	2	14582

325.86436	2	14583

325.96114	2	14585

326.05792	2	14586

326.1547	2	14588

326.25149	2	14589

326.34827	5	14594

326.44505	2	14595

326.54183	2	14597

326.63861	3	14600

326.73539	2	14601

326.83218	2	14603

326.92896	3	14606

327.02574	5	14610

327.12252	2	14612

327.2193	5	14616

327.31609	2	14618

327.41287	2	14619

327.50965	2	14621

327.60643	2	14622

327.70321	3	14625

327.79999	2	14627

327.89678	2	14628

327.99356	2	14630

328.09034	2	14631

328.18712	3	14634

328.2839	2	14636

328.38069	2	14637

328.47747	2	14639

328.57425	2	14640

328.67103	5	14645

328.76781	2	14646

328.86459	2	14648

328.96138	3	14651

329.05816	2	14652

329.15494	6	14658

329.25172	2	14660

329.3485	21	14681

329.44529	2	14682

329.54207	3	14685

329.63885	2	14687

329.73563	2	14688

329.83241	5	14693

329.92919	2	14694

330.02598	8	14702

330.12276	3	14705

330.21954	2	14706

330.31632	8	14714

330.4131	3	14717

330.50989	2	14718

330.60667	2	14720

330.70345	2	14721

330.80023	2	14723

330.89701	2	14724

330.99379	2	14726

331.09058	2	14727

331.18736	2	14729

331.28414	3	14732

331.38092	8	14739

331.4777	2	14741

331.57449	2	14742

331.67127	3	14745

331.76805	2	14747

331.86483	2	14748

331.96161	2	14750

332.05839	2	14751

332.15518	2	14753

332.25196	6	14759

332.34874	2	14760

332.44552	3	14763

332.5423	5	14768

332.63909	3	14771

332.73587	2	14772

332.83265	2	14774

332.92943	2	14775

333.02621	2	14777

333.12299	2	14778

333.21978	2	14780

333.31656	5	14784

333.41334	2	14786

333.51012	3	14789

333.6069	2	14790

333.70369	2	14792

333.80047	5	14796

333.89725	2	14798

333.99403	5	14802

334.09081	2	14804

334.18759	6	14810

334.28438	6	14816

334.38116	5	14820

334.47794	5	14825

334.57472	2	14826

334.6715	3	14829

334.76828	2	14831

334.86507	2	14832

334.96185	2	14834

335.05863	3	14837

335.15541	3	14840

335.25219	2	14841

335.34898	8	14849

335.44576	2	14850

335.54254	2	14852

335.63932	2	14853

335.7361	2	14855

335.83288	2	14856

335.92967	5	14861

336.02645	3	14864

336.12323	2	14865

336.22001	6	14871

336.31679	2	14873

336.41358	3	14876

336.51036	2	14877

336.60714	2	14879

336.70392	3	14882

336.8007	3	14885

336.89748	2	14886

336.99427	2	14888

337.09105	8	14895

337.18783	2	14897

337.28461	2	14898

337.38139	2	14900

337.47818	2	14901

337.57496	3	14904

337.67174	2	14906

337.76852	2	14907

337.8653	3	14910

337.96208	2	14912

338.05887	2	14913

338.15565	2	14915

338.25243	2	14916

338.34921	2	14918

338.44599	3	14921

338.54278	2	14922

338.63956	3	14925

338.73634	5	14930

338.83312	2	14931

338.9299	2	14933

339.02668	3	14936

339.12347	2	14937

339.22025	3	14940

339.31703	3	14943

339.41381	3	14946

339.51059	2	14948

339.60738	9	14957

339.70416	5	14961

339.80094	5	14966

339.89772	6	14972

339.9945	2	14973

340.09128	2	14975

340.18807	2	14976

340.28485	6	14982

340.38163	2	14984

340.47841	3	14987

340.57519	5	14991

340.67198	2	14993

340.76876	3	14996

340.86554	2	14997

340.96232	2	14999

341.0591	2	15000

341.15588	5	15005

341.25267	2	15006

341.34945	2	15008

341.44623	11	15018

341.54301	5	15023

341.63979	9	15032

341.73658	2	15033

341.83336	3	15036

341.93014	2	15038

342.02692	2	15039

342.1237	3	15042

342.22048	6	15048

342.31727	3	15051

342.41405	2	15053

342.51083	2	15054

342.60761	3	15057

342.70439	2	15059

342.80118	2	15060

342.89796	5	15065

342.99474	2	15066

343.09152	2	15068

343.1883	2	15069

343.28508	2	15071

343.38187	2	15072

343.47865	2	15074

343.57543	9	15083

343.67221	3	15086

343.76899	15	15101

343.86578	3	15104

343.96256	2	15105

344.05934	2	15107

344.15612	5	15111

344.2529	2	15113

344.34968	8	15120

344.44647	2	15122

344.54325	3	15125

344.64003	2	15126

344.73681	2	15128

344.83359	2	15129

344.93038	3	15132

345.02716	3	15135

345.12394	6	15141

345.22072	2	15143

345.3175	6	15149

345.41428	5	15153

345.51107	3	15156

345.60785	2	15158

345.70463	2	15159

345.80141	60	15219

345.89819	9	15228

345.99498	2	15230

346.09176	2	15231

346.18854	5	15236

346.28532	2	15237

346.3821	3	15240

346.47888	3	15243

346.57567	2	15245

346.67245	3	15248

346.76923	2	15249

346.86601	8	15257

346.96279	3	15260

347.05958	2	15261

347.15636	2	15263

347.25314	3	15266

347.34992	8	15273

347.4467	29	15302

347.54348	2	15303

347.64027	2	15305

347.73705	2	15306

347.83383	3	15309

347.93061	2	15311

348.02739	3	15314

348.12417	2	15315

348.22096	6	15321

348.31774	5	15326

348.41452	3	15329

348.5113	2	15330

348.60808	3	15333

348.70487	2	15335

348.80165	3	15338

348.89843	3	15341

348.99521	2	15342

349.09199	2	15344

349.18877	2	15345

349.28556	2	15347

349.38234	2	15348

349.47912	2	15350

349.5759	2	15351

349.67268	5	15356

349.76947	2	15357

349.86625	2	15359

349.96303	3	15362

350.05981	2	15363

350.15659	2	15365

350.25337	2	15366

350.35016	3	15369

350.44694	2	15371

350.54372	2	15372

350.6405	2	15374

350.73728	11	15384

350.83407	2	15386

350.93085	2	15387

351.02763	11	15398

351.12441	2	15399

351.22119	2	15401

351.31797	2	15402

351.41476	2	15404

351.51154	5	15408

351.60832	5	15413

351.7051	12	15425

351.80188	3	15428

351.89867	2	15429

351.99545	2	15431

352.09223	3	15434

352.18901	2	15435

352.28579	6	15441

352.38257	2	15443

352.47936	2	15444

352.57614	6	15450

352.67292	3	15453

352.7697	2	15455

352.86648	2	15456

352.96327	3	15459

353.06005	2	15461

353.15683	3	15464

353.25361	2	15465

353.35039	2	15467

353.44717	2	15468

353.54396	6	15474

353.64074	2	15476

353.73752	6	15482

353.8343	2	15483

353.93108	2	15485

354.02787	20	15504

354.12465	6	15510

354.22143	2	15512

354.31821	33	15545

354.41499	9	15554

354.51177	3	15557

354.60856	2	15558

354.70534	3	15561

354.80212	2	15563

354.8989	3	15566

354.99568	3	15569

355.09247	2	15570

355.18925	2	15572

355.28603	2	15573

355.38281	2	15575

355.47959	5	15579

355.57637	2	15581

355.67316	2	15582

355.76994	5	15587

355.86672	2	15588

355.9635	2	15590

356.06028	3	15593

356.15707	2	15594

356.25385	2	15596

356.35063	2	15597

356.44741	2	15599

356.54419	2	15600

356.64097	2	15602

356.73776	2	15603

356.83454	11	15614

356.93132	6	15620

357.0281	2	15621

357.12488	5	15626

357.22167	2	15627

357.31845	41	15668

357.41523	2	15669

357.51201	3	15672

357.60879	2	15674

357.70557	3	15677

357.80236	2	15678

357.89914	2	15680

357.99592	2	15681

358.0927	2	15683

358.18948	3	15686

358.28627	2	15687

358.38305	2	15689

358.47983	2	15690

358.57661	3	15693

358.67339	2	15695

358.77017	2	15696

358.86696	3	15699

358.96374	6	15705

359.06052	2	15707

359.1573	2	15708

359.25408	2	15710

359.35087	5	15714

359.44765	5	15719

359.54443	3	15722

359.64121	2	15723

359.73799	2	15725

359.83477	2	15726

359.93156	5	15731

360.02834	3	15734

360.12512	5	15738

360.2219	2	15740

360.31868	2	15741

360.41546	3	15744

360.51225	2	15746

360.60903	2	15747

360.70581	5	15752

360.80259	3	15755

360.89937	2	15756

360.99616	12	15768

361.09294	6	15774

361.18972	3	15777

361.2865	2	15779

361.38328	3	15782

361.48006	3	15785

361.57685	3	15788

361.67363	3	15791

361.77041	2	15792

361.86719	2	15794

361.96397	2	15795

362.06076	2	15797

362.15754	2	15798

362.25432	5	15803

362.3511	6	15809

362.44788	2	15810

362.54466	8	15818

362.64145	2	15819

362.73823	2	15821

362.83501	3	15824

362.93179	2	15825

363.02857	2	15827

363.12536	8	15834

363.22214	2	15836

363.31892	2	15837

363.4157	3	15840

363.51248	2	15842

363.60926	2	15843

363.70605	2	15845

363.80283	3	15848

363.89961	2	15849

363.99639	2	15851

364.09317	9	15860

364.18996	3	15863

364.28674	2	15864

364.38352	2	15866

364.4803	3	15869

364.57708	3	15872

364.67386	2	15873

364.77065	6	15879

364.86743	2	15881

364.96421	5	15885

365.06099	2	15887

365.15777	3	15890

365.25456	3	15893

365.35134	3	15896

365.44812	5	15900

365.5449	2	15902

365.64168	6	15908

365.73846	2	15909

365.83525	6	15915

365.93203	2	15917

366.02881	2	15918

366.12559	3	15921

366.22237	2	15923

366.31916	5	15927

366.41594	2	15929

366.51272	8	15936

366.6095	3	15939

366.70628	2	15941

366.80306	2	15942

366.89985	2	15944

366.99663	9	15953

367.09341	3	15956

367.19019	3	15959

367.28697	2	15960

367.38376	2	15962

367.48054	2	15963

367.57732	2	15965

367.6741	2	15966

367.77088	2	15968

367.86766	2	15969

367.96445	2	15971

368.06123	6	15977

368.15801	23	15999

368.25479	29	16028

368.35157	5	16032

368.44836	5	16037

368.54514	3	16040

368.64192	2	16041

368.7387	2	16043

368.83548	2	16044

368.93226	6	16050

369.02905	2	16052

369.12583	2	16053

369.22261	2	16055

369.31939	3	16058

369.41617	2	16059

369.51296	2	16061

369.60974	3	16064

369.70652	2	16065

369.8033	3	16068

369.90008	2	16070

369.99686	2	16071

370.09365	2	16073

370.19043	2	16074

370.28721	2	16076

370.38399	6	16082

370.48077	2	16083

370.57756	2	16085

370.67434	8	16092

370.77112	2	16094

370.8679	2	16095

370.96468	2	16097

371.06146	2	16098

371.15825	2	16100

371.25503	3	16103

371.35181	2	16104

371.44859	2	16106

371.54537	5	16110

371.64216	2	16112

371.73894	2	16113

371.83572	3	16116

371.9325	2	16118

372.02928	5	16122

372.12606	2	16124

372.22285	2	16125

372.31963	3	16128

372.41641	3	16131

372.51319	5	16136

372.60997	2	16137

372.70675	5	16142

372.80354	3	16145

372.90032	5	16149

372.9971	2	16151

373.09388	2	16152

373.19066	2	16154

373.28745	2	16155

373.38423	6	16161

373.48101	2	16163

373.57779	2	16164

373.67457	2	16166

373.77135	2	16167

373.86814	2	16169

373.96492	2	16170

374.0617	3	16173

374.15848	2	16175

374.25526	2	16176

374.35205	2	16178

374.44883	6	16184

374.54561	2	16185

374.64239	6	16191

374.73917	2	16193

374.83595	2	16194

374.93274	2	16196

375.02952	2	16197

375.1263	8	16205

375.22308	3	16208

375.31986	2	16209

375.41665	3	16212

375.51343	3	16215

375.61021	2	16217

375.70699	2	16218

375.80377	5	16223

375.90055	8	16230

375.99734	3	16233

376.09412	2	16235

376.1909	2	16236

376.28768	2	16238

376.38446	2	16239

376.48125	27	16266

376.57803	3	16269

376.67481	2	16271

376.77159	2	16272

376.86837	2	16274

376.96515	3	16277

377.06194	2	16278

377.15872	5	16283

377.2555	6	16289

377.35228	5	16293

377.44906	2	16295

377.54585	2	16296

377.64263	2	16298

377.73941	6	16304

377.83619	2	16305

377.93297	2	16307

378.02975	2	16308

378.12654	2	16310

378.22332	2	16311

378.3201	8	16319

378.41688	2	16320

378.51366	3	16323

378.61045	14	16337

378.70723	2	16338

378.80401	3	16341

378.90079	11	16352

378.99757	3	16355

379.09435	2	16356

379.19114	2	16358

379.28792	11	16368

379.3847	2	16370

379.48148	3	16373

379.57826	11	16383

379.67505	2	16385

379.77183	6	16391

379.86861	3	16394

379.96539	2	16395

380.06217	2	16397

380.15895	2	16398

380.25574	2	16400

380.35252	2	16401

380.4493	5	16406

380.54608	2	16407

380.64286	2	16409

380.73965	2	16410

380.83643	12	16422

380.93321	2	16424

381.02999	6	16430

381.12677	2	16431

381.22355	2	16433

381.32034	2	16434

381.41712	2	16436

381.5139	5	16440

381.61068	12	16452

381.70746	2	16454

381.80425	2	16455

381.90103	3	16458

381.99781	2	16460

382.09459	2	16461

382.19137	2	16463

382.28815	3	16466

382.38494	2	16467

382.48172	2	16469

382.5785	2	16470

382.67528	2	16472

382.77206	5	16476

382.86885	3	16479

382.96563	3	16482

383.06241	2	16484

383.15919	2	16485

383.25597	2	16487

383.35275	3	16490

383.44954	2	16491

383.54632	2	16493

383.6431	2	16494

383.73988	8	16502

383.83666	2	16503

383.93345	2	16505

384.03023	6	16511

384.12701	5	16515

384.22379	2	16517

384.32057	3	16520

384.41735	15	16535

384.51414	3	16538

384.61092	2	16539

384.7077	2	16541

384.80448	11	16551

384.90126	6	16557

384.99804	2	16559

385.09483	3	16562

385.19161	2	16563

385.28839	2	16565

385.38517	8	16572

385.48195	2	16574

385.57874	2	16575

385.67552	11	16586

385.7723	2	16587

385.86908	2	16589

385.96586	5	16593

386.06264	2	16595

386.15943	2	16596

386.25621	2	16598

386.35299	3	16601

386.44977	2	16602

386.54655	2	16604

386.64334	5	16608

386.74012	3	16611

386.8369	3	16614

386.93368	5	16619

387.03046	5	16623

387.12724	2	16625

387.22403	2	16626

387.32081	2	16628

387.41759	3	16631

387.51437	2	16632

387.61115	6	16638

387.70794	2	16640

387.80472	3	16643

387.9015	2	16644

387.99828	2	16646

388.09506	6	16652

388.19184	8	16659

388.28863	2	16661

388.38541	3	16664

388.48219	3	16667

388.57897	2	16668

388.67575	5	16673

388.77254	6	16679

388.86932	3	16682

388.9661	3	16685

389.06288	2	16686

389.15966	2	16688

389.25644	2	16689

389.35323	5	16694

389.45001	2	16695

389.54679	5	16700

389.64357	5	16704

389.74035	3	16707

389.83714	5	16712

389.93392	3	16715

390.0307	2	16716

390.12748	3	16719

390.22426	6	16725

390.32104	2	16727

390.41783	3	16730

390.51461	5	16734

390.61139	8	16742

390.70817	3	16745

390.80495	3	16748

390.90174	8	16755

390.99852	3	16758

391.0953	2	16760

391.19208	2	16761

391.28886	2	16763

391.38564	2	16764

391.48243	2	16766

391.57921	3	16769

391.67599	2	16770

391.77277	2	16772

391.86955	5	16776

391.96634	3	16779

392.06312	3	16782

392.1599	2	16784

392.25668	2	16785

392.35346	3	16788

392.45024	3	16791

392.54703	2	16793

392.64381	5	16797

392.74059	2	16799

392.83737	3	16802

392.93415	3	16805

393.03094	2	16806

393.12772	3	16809

393.2245	5	16814

393.32128	2	16815

393.41806	6	16821

393.51484	2	16823

393.61163	2	16824

393.70841	2	16826

393.80519	2	16827

393.90197	17	16844

393.99875	2	16845

394.09554	2	16847

394.19232	2	16848

394.2891	3	16851

394.38588	2	16853

394.48266	2	16854

394.57944	9	16863

394.67623	2	16865

394.77301	12	16877

394.86979	5	16881

394.96657	2	16883

395.06335	2	16884

395.16014	2	16886

395.25692	6	16892

395.3537	3	16895

395.45048	5	16899

395.54726	3	16902

395.64404	2	16904

395.74083	5	16908

395.83761	3	16911

395.93439	3	16914

396.03117	2	16916

396.12795	2	16917

396.22474	3	16920

396.32152	5	16925

396.4183	2	16926

396.51508	8	16934

396.61186	14	16947

396.70864	5	16952

396.80543	3	16955

396.90221	2	16956

396.99899	3	16959

397.09577	2	16961

397.19255	2	16962

397.28933	2	16964

397.38612	9	16973

397.4829	2	16974

397.57968	12	16986

397.67646	2	16988

397.77324	3	16991

397.87003	2	16992

397.96681	2	16994

398.06359	2	16995

398.16037	2	16997

398.25715	2	16998

398.35393	2	17000

398.45072	3	17003

398.5475	2	17004

398.64428	3	17007

398.74106	2	17009

398.83784	3	17012

398.93463	2	17013

399.03141	6	17019

399.12819	5	17024

399.22497	3	17027

399.32175	2	17028

399.41853	3	17031

399.51532	2	17033

399.6121	2	17034

399.70888	12	17046

399.80566	2	17048

399.90244	2	17049

399.99923	3	17052

400.09601	5	17057

400.19279	2	17058

400.28957	3	17061

400.38635	2	17063

400.48313	32	17094

400.57992	3	17097

400.6767	2	17099

400.77348	3	17102

400.87026	2	17103

400.96704	2	17105

401.06383	2	17106

401.16061	2	17108

401.25739	2	17109

401.35417	2	17111

401.45095	2	17112

401.54773	2	17114

401.64452	3	17117

401.7413	2	17118

401.83808	11	17129

401.93486	5	17133

402.03164	2	17135

402.12843	2	17136

402.22521	5	17141

402.32199	2	17142

402.41877	3	17145

402.51555	2	17147

402.61233	6	17153

402.70912	2	17154

402.8059	6	17160

402.90268	2	17162

402.99946	3	17165

403.09624	3	17168

403.19303	2	17169

403.28981	2	17171

403.38659	2	17172

403.48337	6	17178

403.58015	2	17180

403.67693	3	17183

403.77372	3	17186

403.8705	2	17187

403.96728	3	17190

404.06406	6	17196

404.16084	2	17198

404.25763	9	17207

404.35441	3	17210

404.45119	6	17216

404.54797	2	17217

404.64475	2	17219

404.74153	3	17222

404.83832	3	17225

404.9351	2	17226

405.03188	2	17228

405.12866	2	17229

405.22544	5	17234

405.32223	2	17235

405.41901	3	17238

405.51579	2	17240

405.61257	2	17241

405.70935	2	17243

405.80613	3	17246

405.90292	3	17249

405.9997	2	17250

406.09648	2	17252

406.19326	2	17253

406.29004	2	17255

406.38683	2	17256

406.48361	2	17258

406.58039	2	17259

406.67717	3	17262

406.77395	8	17270

406.87073	5	17274

406.96752	2	17276

407.0643	2	17277

407.16108	2	17279

407.25786	2	17280

407.35464	3	17283

407.45143	8	17291

407.54821	2	17292

407.64499	2	17294

407.74177	2	17295

407.83855	3	17298

407.93533	2	17300

408.03212	3	17303

408.1289	2	17304

408.22568	5	17309

408.32246	3	17312

408.41924	3	17315

408.51603	3	17318

408.61281	2	17319

408.70959	2	17321

408.80637	2	17322

408.90315	2	17324

408.99993	5	17328

409.09672	5	17333

409.1935	3	17336

409.29028	2	17337

409.38706	3	17340

409.48384	2	17342

409.58063	2	17343

409.67741	3	17346

409.77419	6	17352

409.87097	2	17354

409.96775	2	17355

410.06453	12	17367

410.16132	2	17369

410.2581	3	17372

410.35488	2	17373

410.45166	2	17375

410.54844	2	17376

410.64522	2	17378

410.74201	2	17379

410.83879	2	17381

410.93557	3	17384

411.03235	6	17390

411.12913	29	17418

411.22592	2	17420

411.3227	3	17423

411.41948	2	17424

411.51626	2	17426

411.61304	2	17427

411.70982	2	17429

411.80661	2	17430

411.90339	2	17432

412.00017	2	17433

412.09695	2	17435

412.19373	2	17436

412.29052	8	17444

412.3873	2	17445

412.48408	5	17450

412.58086	3	17453

412.67764	2	17454

412.77442	2	17456

412.87121	3	17459

412.96799	2	17460

413.06477	2	17462

413.16155	2	17463

413.25833	5	17468

413.35512	2	17469

413.4519	2	17471

413.54868	6	17477

413.64546	2	17478

413.74224	5	17483

413.83902	11	17493

413.93581	3	17496

414.03259	2	17498

414.12937	2	17499

414.22615	2	17501

414.32293	5	17505

414.41972	2	17507

414.5165	12	17519

414.61328	2	17520

414.71006	2	17522

414.80684	2	17523

414.90362	2	17525

415.00041	2	17526

415.09719	3	17529

415.19397	2	17531

415.29075	5	17535

415.38753	5	17540

415.48432	2	17541

415.5811	2	17543

415.67788	2	17544

415.77466	2	17546

415.87144	2	17547

415.96822	8	17555

416.06501	2	17556

416.16179	2	17558

416.25857	2	17559

416.35535	2	17561

416.45213	11	17571

416.54892	2	17573

416.6457	3	17576

416.74248	2	17577

416.83926	5	17582

416.93604	2	17583

417.03282	2	17585

417.12961	2	17586

417.22639	2	17588

417.32317	2	17589

417.41995	2	17591

417.51673	2	17592

417.61352	2	17594

417.7103	2	17595

417.80708	2	17597

417.90386	2	17598

418.00064	2	17600

418.09742	2	17601

418.19421	3	17604

418.29099	2	17606

418.38777	3	17609

418.48455	2	17610

418.58133	5	17615

418.67812	2	17616

418.7749	3	17619

418.87168	2	17621

418.96846	3	17624

419.06524	3	17627

419.16202	3	17630

419.25881	8	17637

419.35559	11	17648

419.45237	2	17649

419.54915	3	17652

419.64593	5	17657

419.74272	2	17658

419.8395	2	17660

419.93628	20	17679

420.03306	6	17685

420.12984	5	17690

420.22662	3	17693

420.32341	2	17694

420.42019	2	17696

420.51697	15	17711

420.61375	2	17712

420.71053	2	17714

420.80732	3	17717

420.9041	3	17720

421.00088	23	17742

421.09766	2	17744

421.19444	3	17747

421.29122	2	17748

421.38801	2	17750

421.48479	2	17751

421.58157	3	17754

421.67835	2	17756

421.77513	3	17759

421.87192	2	17760

421.9687	3	17763

422.06548	5	17768

422.16226	2	17769

422.25904	8	17777

422.35582	5	17781

422.45261	17	17798

422.54939	3	17801

422.64617	3	17804

422.74295	6	17810

422.83973	2	17811

422.93651	3	17814

423.0333	2	17816

423.13008	3	17819

423.22686	2	17820

423.32364	2	17822

423.42042	2	17823

423.51721	2	17825

423.61399	2	17826

423.71077	2	17828

423.80755	2	17829

423.90433	2	17831

424.00111	3	17834

424.0979	3	17837

424.19468	3	17840

424.29146	3	17843

424.38824	2	17844

424.48502	2	17846

424.58181	2	17847

424.67859	5	17852

424.77537	5	17856

424.87215	5	17861

424.96893	2	17862

425.06571	6	17868

425.1625	2	17870

425.25928	2	17871

425.35606	2	17873

425.45284	2	17874

425.54962	2	17876

425.64641	2	17877

425.74319	2	17879

425.83997	2	17880

425.93675	5	17885

426.03353	9	17894

426.13031	6	17900

426.2271	2	17901

426.32388	3	17904

426.42066	6	17910

426.51744	2	17912

426.61422	2	17913

426.71101	2	17915

426.80779	20	17934

426.90457	2	17936

427.00135	9	17945

427.09813	2	17946

427.19491	5	17951

427.2917	2	17952

427.38848	2	17954

427.48526	2	17955

427.58204	3	17958

427.67882	5	17963

427.77561	5	17967

427.87239	6	17973

427.96917	2	17975

428.06595	2	17976

428.16273	3	17979

428.25951	2	17981

428.3563	3	17984

428.45308	2	17985

428.54986	2	17987

428.64664	2	17988

428.74342	2	17990

428.84021	5	17994

428.93699	2	17996

429.03377	2	17997

429.13055	3	18000

429.22733	8	18008

429.32411	5	18012

429.4209	3	18015

429.51768	2	18017

429.61446	9	18026

429.71124	2	18027

429.80802	2	18029

429.90481	2	18030

430.00159	3	18033

430.09837	2	18035

430.19515	2	18036

430.29193	8	18044

430.38871	2	18045

430.4855	2	18047

430.58228	3	18050

430.67906	3	18053

430.77584	2	18054

430.87262	2	18056

430.96941	3	18059

431.06619	2	18060

431.16297	2	18062

431.25975	3	18065

431.35653	2	18066

431.45331	8	18074

431.5501	6	18080

431.64688	2	18081

431.74366	2	18083

431.84044	2	18084

431.93722	3	18087

432.03401	2	18089

432.13079	9	18098

432.22757	14	18111

432.32435	2	18113

432.42113	2	18114

432.51791	14	18128

432.6147	2	18129

432.71148	5	18134

432.80826	2	18135

432.90504	5	18140

433.00182	2	18141

433.09861	2	18143

433.19539	2	18144

433.29217	5	18149

433.38895	2	18150

433.48573	2	18152

433.58251	8	18159

433.6793	2	18161

433.77608	3	18164

433.87286	2	18165

433.96964	2	18167

434.06642	2	18168

434.16321	2	18170

434.25999	8	18177

434.35677	2	18179

434.45355	6	18185

434.55033	6	18191

434.64711	3	18194

434.7439	3	18197

434.84068	2	18198

434.93746	8	18206

435.03424	6	18212

435.13102	3	18215

435.2278	2	18216

435.32459	2	18218

435.42137	3	18221

435.51815	2	18222

435.61493	3	18225

435.71171	2	18227

435.8085	2	18228

435.90528	2	18230

436.00206	2	18231

436.09884	3	18234

436.19562	6	18240

436.2924	5	18245

436.38919	2	18246

436.48597	2	18248

436.58275	2	18249

436.67953	2	18251

436.77631	2	18252

436.8731	2	18254

436.96988	2	18255

437.06666	3	18258

437.16344	2	18260

437.26022	2	18261

437.357	2	18263

437.45379	2	18264

437.55057	2	18266

437.64735	2	18267

437.74413	2	18269

437.84091	5	18273

437.9377	3	18276

438.03448	2	18278

438.13126	2	18279

438.22804	2	18281

438.32482	2	18282

438.4216	5	18287

438.51839	3	18290

438.61517	2	18291

438.71195	2	18293

438.80873	2	18294

438.90551	2	18296

439.0023	5	18300

439.09908	2	18302

439.19586	2	18303

439.29264	3	18306

439.38942	6	18312

439.4862	12	18324

439.58299	2	18326

439.67977	2	18327

439.77655	2	18329

439.87333	9	18338

439.97011	3	18341

440.0669	3	18344

440.16368	6	18350

440.26046	2	18351

440.35724	3	18354

440.45402	2	18356

440.5508	2	18357

440.64759	2	18359

440.74437	2	18360

440.84115	2	18362

440.93793	12	18374

441.03471	3	18377

441.1315	2	18378

441.22828	2	18380

441.32506	3	18383

441.42184	2	18384

441.51862	2	18386

441.6154	3	18389

441.71219	6	18395

441.80897	2	18396

441.90575	3	18399

442.00253	3	18402

442.09931	3	18405

442.1961	3	18408

442.29288	3	18411

442.38966	3	18414

442.48644	8	18422

442.58322	3	18425

442.68	3	18428

442.77679	2	18429

442.87357	3	18432

442.97035	2	18434

443.06713	3	18437

443.16391	6	18443

443.2607	2	18444

443.35748	2	18446

443.45426	2	18447

443.55104	2	18449

443.64782	2	18450

443.7446	2	18452

443.84139	3	18455

443.93817	6	18461

444.03495	2	18462

444.13173	3	18465

444.22851	5	18470

444.3253	5	18474

444.42208	3	18477

444.51886	3	18480

444.61564	2	18482

444.71242	3	18485

444.8092	2	18486

444.90599	2	18488

445.00277	2	18489

445.09955	2	18491

445.19633	5	18495

445.29311	3	18498

445.3899	3	18501

445.48668	3	18504

445.58346	2	18506

445.68024	2	18507

445.77702	2	18509

445.8738	3	18512

445.97059	8	18519

446.06737	2	18521

446.16415	2	18522

446.26093	2	18524

446.35771	2	18525

446.4545	3	18528

446.55128	3	18531

446.64806	2	18533

446.74484	2	18534

446.84162	2	18536

446.9384	2	18537

447.03519	5	18542

447.13197	2	18543

447.22875	3	18546

447.32553	15	18561

447.42231	2	18563

447.5191	3	18566

447.61588	6	18572

447.71266	2	18573

447.80944	2	18575

447.90622	2	18576

448.003	2	18578

448.09979	2	18579

448.19657	2	18581

448.29335	5	18585

448.39013	2	18587

448.48691	5	18591

448.58369	3	18594

448.68048	2	18596

448.77726	3	18599

448.87404	3	18602

448.97082	5	18606

449.0676	2	18608

449.16439	2	18609

449.26117	5	18614

449.35795	3	18617

449.45473	2	18618

449.55151	2	18620

449.64829	12	18632

449.74508	2	18633

449.84186	9	18642

449.93864	2	18644

450.03542	3	18647

450.1322	2	18648

450.22899	2	18650

450.32577	9	18659

450.42255	2	18660

450.51933	2	18662

450.61611	2	18663

450.71289	6	18669

450.80968	2	18671

450.90646	2	18672

451.00324	2	18674

451.10002	2	18675

451.1968	2	18677

451.29359	9	18686

451.39037	9	18695

451.48715	2	18696

451.58393	2	18698

451.68071	2	18699

451.77749	2	18701

451.87428	2	18702

451.97106	2	18704

452.06784	2	18705

452.16462	2	18707

452.2614	2	18708

452.35819	2	18710

452.45497	2	18711

452.55175	2	18713

452.64853	2	18714

452.74531	2	18716

452.84209	5	18720

452.93888	2	18722

453.03566	3	18725

453.13244	5	18729

453.22922	5	18734

453.326	2	18735

453.42279	2	18737

453.51957	3	18740

453.61635	3	18743

453.71313	3	18746

453.80991	6	18752

453.90669	2	18753

454.00348	2	18755

454.10026	6	18761

454.19704	2	18762

454.29382	5	18767

454.3906	2	18768

454.48739	2	18770

454.58417	2	18771

454.68095	6	18777

454.77773	2	18779

454.87451	3	18782

454.97129	2	18783

455.06808	2	18785

455.16486	2	18786

455.26164	8	18794

455.35842	3	18797

455.4552	2	18798

455.55199	2	18800

455.64877	2	18801

455.74555	3	18804

455.84233	2	18806

455.93911	2	18807

456.03589	2	18809

456.13268	2	18810

456.22946	5	18815

456.32624	2	18816

456.42302	2	18818

456.5198	2	18819

456.61659	2	18821

456.71337	3	18824

456.81015	8	18831

456.90693	2	18833

457.00371	6	18839

457.10049	5	18843

457.19728	2	18845

457.29406	3	18848

457.39084	3	18851

457.48762	2	18852

457.5844	2	18854

457.68119	3	18857

457.77797	2	18858

457.87475	2	18860

457.97153	2	18861

458.06831	2	18863

458.16509	2	18864

458.26188	2	18866

458.35866	2	18867

458.45544	15	18882

458.55222	2	18884

458.649	3	18887

458.74579	2	18888

458.84257	3	18891

458.93935	2	18893

459.03613	2	18894

459.13291	2	18896

459.22969	2	18897

459.32648	3	18900

459.42326	2	18902

459.52004	2	18903

459.61682	2	18905

459.7136	2	18906

459.81039	5	18911

459.90717	9	18920

460.00395	2	18921

460.10073	2	18923

460.19751	2	18924

460.29429	5	18929

460.39108	2	18930

460.48786	2	18932

460.58464	2	18933

460.68142	5	18938

460.7782	2	18939

460.87498	3	18942

460.97177	8	18950

461.06855	2	18951

461.16533	3	18954

461.26211	2	18956

461.35889	2	18957

461.45568	2	18959

461.55246	5	18963

461.64924	6	18969

461.74602	5	18974

461.8428	3	18977

461.93958	3	18980

462.03637	2	18981

462.13315	2	18983

462.22993	2	18984

462.32671	5	18989

462.42349	8	18996

462.52028	3	18999

462.61706	3	19002

462.71384	8	19010

462.81062	3	19013

462.9074	3	19016

463.00418	9	19025

463.10097	2	19026

463.19775	2	19028

463.29453	2	19029

463.39131	2	19031

463.48809	2	19032

463.58488	2	19034

463.68166	5	19038

463.77844	3	19041

463.87522	2	19043

463.972	2	19044

464.06878	12	19056

464.16557	5	19061

464.26235	2	19062

464.35913	2	19064

464.45591	2	19065

464.55269	3	19068

464.64948	2	19070

464.74626	2	19071

464.84304	2	19073

464.93982	2	19074

465.0366	2	19076

465.13338	2	19077

465.23017	2	19079

465.32695	3	19082

465.42373	2	19083

465.52051	2	19085

465.61729	5	19089

465.71408	2	19091

465.81086	3	19094

465.90764	3	19097

466.00442	3	19100

466.1012	2	19101

466.19798	2	19103

466.29477	3	19106

466.39155	2	19107

466.48833	2	19109

466.58511	2	19110

466.68189	2	19112

466.77868	3	19115

466.87546	2	19116

466.97224	8	19124

467.06902	2	19125

467.1658	2	19127

467.26258	2	19128

467.35937	2	19130

467.45615	2	19131

467.55293	2	19133

467.64971	2	19134

467.74649	2	19136

467.84328	2	19137

467.94006	2	19139

468.03684	2	19140

468.13362	5	19145

468.2304	2	19146

468.32718	3	19149

468.42397	2	19151

468.52075	2	19152

468.61753	2	19154

468.71431	2	19155

468.81109	2	19157

468.90788	5	19161

469.00466	2	19163

469.10144	2	19164

469.19822	2	19166

469.295	2	19167

469.39178	2	19169

469.48857	2	19170

469.58535	3	19173

469.68213	2	19175

469.77891	2	19176

469.87569	2	19178

469.97248	2	19179

470.06926	2	19181

470.16604	3	19184

470.26282	3	19187

470.3596	2	19188

470.45638	2	19190

470.55317	2	19191

470.64995	2	19193

470.74673	2	19194

470.84351	2	19196

470.94029	5	19200

471.03708	3	19203

471.13386	2	19205

471.23064	2	19206

471.32742	3	19209

471.4242	3	19212

471.52098	3	19215

471.61777	2	19217

471.71455	5	19221

471.81133	3	19224

471.90811	2	19226

472.00489	3	19229

472.10168	2	19230

472.19846	2	19232

472.29524	2	19233

472.39202	2	19235

472.4888	3	19238

472.58558	3	19241

472.68237	8	19248

472.77915	5	19253

472.87593	2	19254

472.97271	2	19256

473.06949	2	19257

473.16627	3	19260

473.26306	2	19262

473.35984	2	19263

473.45662	3	19266

473.5534	6	19272

473.65018	2	19274

473.74697	2	19275

473.84375	2	19277

473.94053	2	19278

474.03731	2	19280

474.13409	2	19281

474.23087	8	19289

474.32766	2	19290

474.42444	15	19305

474.52122	6	19311

474.618	3	19314

474.71478	2	19316

474.81157	2	19317

474.90835	2	19319

475.00513	3	19322

475.10191	2	19323

475.19869	2	19325

475.29547	3	19328

475.39226	3	19331

475.48904	3	19334

475.58582	3	19337

475.6826	2	19338

475.77938	2	19340

475.87617	5	19344

475.97295	3	19347

476.06973	2	19349

476.16651	9	19358

476.26329	3	19361

476.36007	2	19362

476.45686	2	19364

476.55364	6	19370

476.65042	3	19373

476.7472	2	19374

476.84398	2	19376

476.94077	2	19377

477.03755	5	19382

477.13433	2	19383

477.23111	2	19385

477.32789	8	19392

477.42467	2	19394

477.52146	2	19395

477.61824	5	19400

477.71502	2	19401

477.8118	2	19403

477.90858	2	19404

478.00537	2	19406

478.10215	2	19407

478.19893	2	19409

478.29571	2	19410

478.39249	2	19412

478.48927	2	19413

478.58606	3	19416

478.68284	2	19418

478.77962	2	19419

478.8764	2	19421

478.97318	2	19422

479.06997	6	19428

479.16675	3	19431

479.26353	2	19433

479.36031	2	19434

479.45709	5	19439

479.55387	2	19440

479.65066	2	19442

479.74744	2	19443

479.84422	2	19445

479.941	2	19446

480.03778	2	19448

480.13457	3	19451

480.23135	2	19452

480.32813	6	19458

480.42491	2	19460

480.52169	3	19463

480.61847	2	19464

480.71526	8	19472

480.81204	3	19475

480.90882	2	19476

481.0056	2	19478

481.10238	5	19482

481.19917	2	19484

481.29595	2	19485

481.39273	3	19488

481.48951	2	19490

481.58629	2	19491

481.68307	5	19496

481.77986	2	19497

481.87664	2	19499

481.97342	5	19503

482.0702	8	19511

482.16698	5	19515

482.26377	2	19517

482.36055	2	19518

482.45733	2	19520

482.55411	9	19529

482.65089	2	19530

482.74767	3	19533

482.84446	2	19535

482.94124	2	19536

483.03802	2	19538

483.1348	2	19539

483.23158	2	19541

483.32837	5	19545

483.42515	2	19547

483.52193	2	19548

483.61871	2	19550

483.71549	3	19553

483.81227	5	19557

483.90906	2	19559

484.00584	2	19560

484.10262	2	19562

484.1994	3	19565

484.29618	2	19566

484.39297	5	19571

484.48975	3	19574

484.58653	2	19575

484.68331	5	19580

484.78009	6	19586

484.87687	2	19587

484.97366	5	19592

485.07044	2	19593

485.16722	2	19595

485.264	2	19596

485.36078	6	19602

485.45756	2	19604

485.55435	6	19610

485.65113	2	19611

485.74791	2	19613

485.84469	5	19617

485.94147	2	19619

486.03826	3	19622

486.13504	5	19626

486.23182	2	19628

486.3286	2	19629

486.42538	3	19632

486.52216	2	19634

486.61895	5	19638

486.71573	2	19640

486.81251	3	19643

486.90929	2	19644

487.00607	2	19646

487.10286	2	19647

487.19964	3	19650

487.29642	3	19653

487.3932	3	19656

487.48998	2	19658

487.58676	2	19659

487.68355	2	19661

487.78033	2	19662

487.87711	5	19667

487.97389	3	19670

488.07067	2	19671

488.16746	2	19673

488.26424	2	19674

488.36102	2	19676

488.4578	5	19680

488.55458	5	19685

488.65136	2	19686

488.74815	2	19688

488.84493	2	19689

488.94171	2	19691

489.03849	2	19692

489.13527	3	19695

489.23206	6	19701

489.32884	2	19703

489.42562	2	19704

489.5224	5	19709

489.61918	2	19710

489.71596	3	19713

489.81275	2	19715

489.90953	2	19716

490.00631	2	19718

490.10309	2	19719

490.19987	2	19721

490.29666	2	19722

490.39344	2	19724

490.49022	2	19725

490.587	3	19728

490.68378	2	19730

490.78056	2	19731

490.87735	3	19734

490.97413	2	19736

491.07091	2	19737

491.16769	5	19742

491.26447	2	19743

491.36126	2	19745

491.45804	2	19746

491.55482	2	19748

491.6516	2	19749

491.74838	2	19751

491.84516	2	19752

491.94195	8	19760

492.03873	5	19764

492.13551	48	19812

492.23229	2	19814

492.32907	8	19821

492.42586	6	19827

492.52264	2	19829

492.61942	2	19830

492.7162	9	19839

492.81298	2	19841

492.90976	2	19842

493.00655	3	19845

493.10333	2	19847

493.20011	2	19848

493.29689	3	19851

493.39367	3	19854

493.49046	2	19856

493.58724	2	19857

493.68402	2	19859

493.7808	2	19860

493.87758	5	19865

493.97436	3	19868

494.07115	3	19871

494.16793	8	19878

494.26471	2	19880

494.36149	2	19881

494.45827	6	19887

494.55506	3	19890

494.65184	2	19892

494.74862	2	19893

494.8454	17	19910

494.94218	2	19911

495.03896	3	19914

495.13575	3	19917

495.23253	8	19925

495.32931	2	19926

495.42609	2	19928

495.52287	2	19929

495.61966	2	19931

495.71644	2	19932

495.81322	6	19938

495.91	2	19940

496.00678	3	19943

496.10356	2	19944

496.20035	3	19947

496.29713	2	19949

496.39391	8	19956

496.49069	2	19958

496.58747	2	19959

496.68426	2	19961

496.78104	2	19962

496.87782	2	19964

496.9746	2	19965

497.07138	8	19973

497.16816	2	19974

497.26495	2	19976

497.36173	2	19977

497.45851	2	19979

497.55529	2	19980

497.65207	2	19982

497.74886	3	19985

497.84564	2	19986

497.94242	8	19994

498.0392	9	20003

498.13598	2	20004

498.23276	2	20006

498.32955	5	20010

498.42633	3	20013

498.52311	2	20015

498.61989	5	20019

498.71667	2	20021

498.81345	5	20025

498.91024	3	20028

499.00702	2	20030

499.1038	2	20031

499.20058	11	20042

499.29736	2	20043

499.39415	2	20045

499.49093	3	20048

499.58771	2	20049

499.68449	5	20054

499.78127	2	20055

499.87805	2	20057

499.97484	2	20058

500.07162	2	20060

500.1684	2	20061

500.26518	2	20063

500.36196	3	20066

500.45875	5	20070

500.55553	2	20072

500.65231	6	20078

500.74909	2	20079

500.84587	3	20082

500.94265	3	20085

501.03944	3	20088

501.13622	3	20091

501.233	2	20093

501.32978	2	20094

501.42656	3	20097

501.52335	6	20103

501.62013	2	20105

501.71691	2	20106

501.81369	3	20109

501.91047	2	20111

502.00725	3	20114

502.10404	5	20118

502.20082	3	20121

502.2976	2	20123

502.39438	2	20124

502.49116	2	20126

502.58795	3	20129

502.68473	2	20130

502.78151	2	20132

502.87829	2	20133

502.97507	2	20135

503.07185	2	20136

503.16864	2	20138

503.26542	2	20139

503.3622	3	20142

503.45898	5	20147

503.55576	2	20148

503.65255	2	20150

503.74933	2	20151

503.84611	3	20154

503.94289	2	20156

504.03967	3	20159

504.13645	3	20162

504.23324	5	20166

504.33002	3	20169

504.4268	2	20171

504.52358	3	20174

504.62036	5	20178

504.71715	6	20184

504.81393	3	20187

504.91071	2	20189

505.00749	2	20190

505.10427	2	20192

505.20105	2	20193

505.29784	8	20201

505.39462	2	20202

505.4914	5	20207

505.58818	2	20208

505.68496	2	20210

505.78175	2	20211

505.87853	2	20213

505.97531	2	20214

506.07209	3	20217

506.16887	6	20223

506.26565	2	20225

506.36244	3	20228

506.45922	2	20229

506.556	2	20231

506.65278	2	20232

506.74956	2	20234

506.84635	2	20235

506.94313	5	20240

507.03991	5	20244

507.13669	2	20246

507.23347	3	20249

507.33025	2	20250

507.42704	2	20252

507.52382	3	20255

507.6206	2	20256

507.71738	2	20258

507.81416	3	20261

507.91095	5	20265

508.00773	2	20267

508.10451	3	20270

508.20129	2	20271

508.29807	5	20276

508.39485	2	20277

508.49164	2	20279

508.58842	5	20283

508.6852	2	20285

508.78198	3	20288

508.87876	2	20289

508.97555	3	20292

509.07233	2	20294

509.16911	3	20297

509.26589	3	20300

509.36267	3	20303

509.45945	5	20307

509.55624	2	20309

509.65302	2	20310

509.7498	3	20313

509.84658	2	20315

509.94336	5	20319

510.04015	2	20321

510.13693	3	20324

510.23371	2	20325

510.33049	2	20327

510.42727	2	20328

510.52405	2	20330

510.62084	2	20331

510.71762	5	20336

510.8144	6	20342

510.91118	2	20343

511.00796	3	20346

511.10474	2	20348

511.20153	2	20349

511.29831	6	20355

511.39509	2	20357

511.49187	2	20358

511.58865	2	20360

511.68544	2	20361

511.78222	8	20369

511.879	9	20378

511.97578	5	20382

512.07256	2	20384

512.16934	3	20387

512.26613	2	20388

512.36291	2	20390

512.45969	2	20391

512.55647	2	20393

512.65325	2	20394

512.75004	2	20396

512.84682	2	20397

512.9436	3	20400

513.04038	3	20403

513.13716	2	20405

513.23394	5	20409

513.33073	3	20412

513.42751	3	20415

513.52429	5	20420

513.62107	5	20424

513.71785	2	20426

513.81464	2	20427

513.91142	2	20429

514.0082	2	20430

514.10498	3	20433

514.20176	3	20436

514.29854	2	20438

514.39533	2	20439

514.49211	2	20441

514.58889	3	20444

514.68567	2	20445

514.78245	2	20447

514.87924	2	20448

514.97602	2	20450

515.0728	3	20453

515.16958	9	20462

515.26636	2	20463

515.36314	3	20466

515.45993	2	20468

515.55671	2	20469

515.65349	3	20472

515.75027	2	20474

515.84705	2	20475

515.94384	6	20481

516.04062	2	20483

516.1374	2	20484

516.23418	2	20486

516.33096	2	20487

516.42774	3	20490

516.52453	3	20493

516.62131	2	20495

516.71809	2	20496

516.81487	5	20501

516.91165	2	20502

517.00844	2	20504

517.10522	2	20505

517.202	14	20519

517.29878	5	20523

517.39556	2	20525

517.49234	2	20526

517.58913	3	20529

517.68591	5	20534

517.78269	2	20535

517.87947	2	20537

517.97625	2	20538

518.07304	2	20540

518.16982	5	20544

518.2666	2	20546

518.36338	3	20549

518.46016	2	20550

518.55694	2	20552

518.65373	2	20553

518.75051	2	20555

518.84729	2	20556

518.94407	5	20561

519.04085	2	20562

519.13764	2	20564

519.23442	2	20565

519.3312	2	20567

519.42798	2	20568

519.52476	2	20570

519.62154	2	20571

519.71833	2	20573

519.81511	3	20576

519.91189	6	20582

520.00867	5	20586

520.10545	2	20588

520.20224	12	20600

520.29902	2	20601

520.3958	6	20607

520.49258	3	20610

520.58936	3	20613

520.68614	9	20622

520.78293	2	20624

520.87971	2	20625

520.97649	3	20628

521.07327	5	20633

521.17005	2	20634

521.26684	2	20636

521.36362	5	20640

521.4604	2	20642

521.55718	2	20643

521.65396	3	20646

521.75074	2	20648

521.84753	3	20651

521.94431	3	20654

522.04109	2	20655

522.13787	2	20657

522.23465	2	20658

522.33144	2	20660

522.42822	2	20661

522.525	2	20663

522.62178	5	20667

522.71856	3	20670

522.81534	2	20672

522.91213	3	20675

523.00891	2	20676

523.10569	3	20679

523.20247	3	20682

523.29925	2	20684

523.39603	3	20687

523.49282	2	20688

523.5896	2	20690

523.68638	2	20691

523.78316	2	20693

523.87994	2	20694

523.97673	2	20696

524.07351	2	20697

524.17029	5	20702

524.26707	2	20703

524.36385	5	20708

524.46064	2	20709

524.55742	2	20711

524.6542	2	20712

524.75098	2	20714

524.84776	2	20715

524.94454	2	20717

525.04133	5	20721

525.13811	9	20730

525.23489	2	20732

525.33167	3	20735

525.42845	32	20766

525.52523	6	20772

525.62202	6	20778

525.7188	3	20781

525.81558	2	20783

525.91236	3	20786

526.00914	3	20789

526.10593	15	20804

526.20271	3	20807

526.29949	3	20810

526.39627	2	20811

526.49305	6	20817

526.58983	3	20820

526.68662	2	20822

526.7834	2	20823

526.88018	3	20826

526.97696	3	20829

527.07374	8	20837

527.17053	2	20838

527.26731	2	20840

527.36409	3	20843

527.46087	2	20844

527.55765	2	20846

527.65443	11	20856

527.75122	2	20858

527.848	5	20862

527.94478	2	20864

528.04156	8	20871

528.13834	2	20873

528.23513	2	20874

528.33191	2	20876

528.42869	3	20879

528.52547	2	20880

528.62225	5	20885

528.71903	6	20891

528.81582	2	20892

528.9126	2	20894

529.00938	3	20897

529.10616	2	20898

529.20294	2	20900

529.29973	3	20903

529.39651	2	20904

529.49329	2	20906

529.59007	2	20907

529.68685	2	20909

529.78363	3	20912

529.88042	12	20924

529.9772	6	20930

530.07398	6	20936

530.17076	5	20940

530.26754	2	20942

530.36433	2	20943

530.46111	2	20945

530.55789	3	20948

530.65467	2	20949

530.75145	2	20951

530.84823	2	20952

530.94502	3	20955

531.0418	6	20961

531.13858	2	20963

531.23536	2	20964

531.33214	2	20966

531.42893	2	20967

531.52571	3	20970

531.62249	2	20972

531.71927	3	20975

531.81605	2	20976

531.91283	3	20979

532.00962	2	20981

532.1064	3	20984

532.20318	2	20985

532.29996	2	20987

532.39674	2	20988

532.49353	2	20990

532.59031	2	20991

532.68709	2	20993

532.78387	2	20994

532.88065	2	20996

532.97743	5	21000

533.07422	2	21002

533.171	2	21003

533.26778	2	21005

533.36456	3	21008

533.46134	3	21011

533.55813	3	21014

533.65491	3	21017

533.75169	2	21018

533.84847	6	21024

533.94525	3	21027

534.04203	3	21030

534.13882	9	21039

534.2356	2	21041

534.33238	3	21044

534.42916	2	21045

534.52594	2	21047

534.62273	2	21048

534.71951	2	21050

534.81629	2	21051

534.91307	3	21054

535.00985	2	21056

535.10663	2	21057

535.20342	2	21059

535.3002	5	21063

535.39698	5	21068

535.49376	2	21069

535.59054	6	21075

535.68733	3	21078

535.78411	2	21080

535.88089	2	21081

535.97767	2	21083

536.07445	3	21086

536.17123	3	21089

536.26802	2	21090

536.3648	2	21092

536.46158	3	21095

536.55836	3	21098

536.65514	5	21102

536.75193	2	21104

536.84871	2	21105

536.94549	2	21107

537.04227	3	21110

537.13905	2	21111

537.23583	2	21113

537.33262	2	21114

537.4294	2	21116

537.52618	9	21125

537.62296	2	21126

537.71974	2	21128

537.81652	2	21129

537.91331	2	21131

538.01009	3	21134

538.10687	2	21135

538.20365	2	21137

538.30043	3	21140

538.39722	3	21143

538.494	2	21144

538.59078	3	21147

538.68756	2	21149

538.78434	2	21150

538.88112	24	21174

538.97791	3	21177

539.07469	3	21180

539.17147	2	21182

539.26825	3	21185

539.36503	5	21189

539.46182	3	21192

539.5586	3	21195

539.65538	2	21197

539.75216	2	21198

539.84894	3	21201

539.94572	2	21203

540.04251	2	21204

540.13929	5	21209

540.23607	6	21215

540.33285	2	21216

540.42963	2	21218

540.52642	2	21219

540.6232	2	21221

540.71998	2	21222

540.81676	3	21225

540.91354	2	21227

541.01032	2	21228

541.10711	3	21231

541.20389	5	21236

541.30067	2	21237

541.39745	2	21239

541.49423	3	21242

541.59102	3	21245

541.6878	9	21254

541.78458	3	21257

541.88136	3	21260

541.97814	3	21263

542.07492	3	21266

542.17171	3	21269

542.26849	3	21272

542.36527	3	21275

542.46205	2	21276

542.55883	3	21279

542.65562	2	21281

542.7524	2	21282

542.84918	2	21284

542.94596	8	21291

543.04274	8	21299

543.13952	3	21302

543.23631	2	21303

543.33309	2	21305

543.42987	5	21309

543.52665	5	21314

543.62343	3	21317

543.72022	2	21318

543.817	3	21321

543.91378	2	21323

544.01056	2	21324

544.10734	2	21326

544.20412	3	21329

544.30091	2	21330

544.39769	2	21332

544.49447	2	21333

544.59125	5	21338

544.68803	2	21339

544.78482	6	21345

544.8816	2	21347

544.97838	2	21348

545.07516	2	21350

545.17194	3	21353

545.26872	2	21354

545.36551	3	21357

545.46229	2	21359

545.55907	5	21363

545.65585	6	21369

545.75263	2	21371

545.84942	3	21374

545.9462	3	21377

546.04298	2	21378

546.13976	6	21384

546.23654	3	21387

546.33332	2	21389

546.43011	5	21393

546.52689	2	21395

546.62367	2	21396

546.72045	6	21402

546.81723	2	21404

546.91402	2	21405

547.0108	2	21407

547.10758	9	21416

547.20436	2	21417

547.30114	2	21419

547.39792	3	21422

547.49471	5	21426

547.59149	3	21429

547.68827	2	21431

547.78505	2	21432

547.88183	2	21434

547.97862	2	21435

548.0754	3	21438

548.17218	3	21441

548.26896	2	21443

548.36574	2	21444

548.46252	3	21447

548.55931	5	21452

548.65609	2	21453

548.75287	2	21455

548.84965	5	21459

548.94643	2	21461

549.04322	3	21464

549.14	2	21465

549.23678	3	21468

549.33356	2	21470

549.43034	2	21471

549.52712	2	21473

549.62391	2	21474

549.72069	2	21476

549.81747	6	21482

549.91425	2	21483

550.01103	2	21485

550.10781	3	21488

550.2046	2	21489

550.30138	3	21492

550.39816	2	21494

550.49494	2	21495

550.59172	2	21497

550.68851	5	21501

550.78529	2	21503

550.88207	2	21504

550.97885	2	21506

551.07563	2	21507

551.17241	6	21513

551.2692	12	21525

551.36598	2	21527

551.46276	5	21531

551.55954	2	21533

551.65632	2	21534

551.75311	2	21536

551.84989	2	21537

551.94667	3	21540

552.04345	2	21542

552.14023	2	21543

552.23701	2	21545

552.3338	2	21546

552.43058	2	21548

552.52736	2	21549

552.62414	3	21552

552.72092	2	21554

552.81771	2	21555

552.91449	2	21557

553.01127	3	21560

553.10805	2	21561

553.20483	2	21563

553.30161	3	21566

553.3984	3	21569

553.49518	2	21570

553.59196	5	21575

553.68874	6	21581

553.78552	9	21590

553.88231	2	21591

553.97909	3	21594

554.07587	2	21596

554.17265	2	21597

554.26943	2	21599

554.36621	2	21600

554.463	2	21602

554.55978	2	21603

554.65656	2	21605

554.75334	2	21606

554.85012	9	21615

554.94691	5	21620

555.04369	2	21621

555.14047	3	21624

555.23725	2	21626

555.33403	2	21627

555.43081	3	21630

555.5276	2	21632

555.62438	2	21633

555.72116	2	21635

555.81794	2	21636

555.91472	3	21639

556.01151	3	21642

556.10829	2	21644

556.20507	2	21645

556.30185	2	21647

556.39863	2	21648

556.49541	2	21650

556.5922	2	21651

556.68898	2	21653

556.78576	2	21654

556.88254	2	21656

556.97932	3	21659

557.07611	2	21660

557.17289	2	21662

557.26967	2	21663

557.36645	2	21665

557.46323	2	21666

557.56001	3	21669

557.6568	2	21671

557.75358	2	21672

557.85036	2	21674

557.94714	2	21675

558.04392	5	21680

558.14071	2	21681

558.23749	2	21683

558.33427	5	21687

558.43105	3	21690

558.52783	2	21692

558.62461	5	21696

558.7214	2	21698

558.81818	5	21702

558.91496	6	21708

559.01174	3	21711

559.10852	3	21714

559.20531	6	21720

559.30209	2	21722

559.39887	2	21723

559.49565	2	21725

559.59243	2	21726

559.68921	5	21731

559.786	2	21732

559.88278	3	21735

559.97956	2	21737

560.07634	2	21738

560.17312	3	21741

560.26991	3	21744

560.36669	2	21746

560.46347	5	21750

560.56025	2	21752

560.65703	2	21753

560.75381	6	21759

560.8506	2	21761

560.94738	3	21764

561.04416	2	21765

561.14094	2	21767

561.23772	5	21771

561.33451	2	21773

561.43129	3	21776

561.52807	3	21779

561.62485	3	21782

561.72163	2	21783

561.81841	2	21785

561.9152	2	21786

562.01198	2	21788

562.10876	2	21789

562.20554	2	21791

562.30232	2	21792

562.3991	3	21795

562.49589	3	21798

562.59267	2	21800

562.68945	5	21804

562.78623	3	21807

562.88301	2	21809

562.9798	2	21810

563.07658	2	21812

563.17336	2	21813

563.27014	2	21815

563.36692	2	21816

563.4637	3	21819

563.56049	2	21821

563.65727	2	21822

563.75405	3	21825

563.85083	3	21828

563.94761	2	21830

564.0444	2	21831

564.14118	2	21833

564.23796	2	21834

564.33474	6	21840

564.43152	2	21842

564.5283	2	21843

564.62509	2	21845

564.72187	2	21846

564.81865	2	21848

564.91543	2	21849

565.01221	3	21852

565.109	2	21854

565.20578	2	21855

565.30256	2	21857

565.39934	5	21861

565.49612	3	21864

565.5929	3	21867

565.68969	5	21872

565.78647	2	21873

565.88325	3	21876

565.98003	2	21878

566.07681	5	21882

566.1736	5	21887

566.27038	2	21888

566.36716	3	21891

566.46394	6	21897

566.56072	2	21899

566.6575	6	21905

566.75429	2	21906

566.85107	2	21908

566.94785	3	21911

567.04463	2	21912

567.14141	2	21914

567.2382	3	21917

567.33498	2	21918

567.43176	2	21920

567.52854	2	21921

567.62532	2	21923

567.7221	3	21926

567.81889	2	21927

567.91567	54	21981

568.01245	2	21983

568.10923	2	21984

568.20601	3	21987

568.3028	2	21989

568.39958	2	21990

568.49636	2	21992

568.59314	2	21993

568.68992	3	21996

568.7867	2	21998

568.88349	8	22005

568.98027	2	22007

569.07705	2	22008

569.17383	3	22011

569.27061	2	22013

569.3674	2	22014

569.46418	2	22016

569.56096	2	22017

569.65774	3	22020

569.75452	2	22022

569.8513	2	22023

569.94809	5	22028

570.04487	2	22029

570.14165	2	22031

570.23843	3	22034

570.33521	2	22035

570.432	2	22037

570.52878	2	22038

570.62556	2	22040

570.72234	2	22041

570.81912	2	22043

570.9159	3	22046

571.01269	2	22047

571.10947	3	22050

571.20625	2	22052

571.30303	3	22055

571.39981	5	22059

571.4966	2	22061

571.59338	2	22062

571.69016	2	22064

571.78694	2	22065

571.88372	2	22067

571.9805	2	22068

572.07729	2	22070

572.17407	3	22073

572.27085	2	22074

572.36763	3	22077

572.46441	2	22079

572.5612	2	22080

572.65798	2	22082

572.75476	2	22083

572.85154	5	22088

572.94832	2	22089

573.0451	2	22091

573.14189	3	22094

573.23867	2	22095

573.33545	3	22098

573.43223	2	22100

573.52901	5	22104

573.6258	5	22109

573.72258	6	22115

573.81936	2	22116

573.91614	6	22122

574.01292	3	22125

574.1097	29	22154

574.20649	2	22155

574.30327	5	22160

574.40005	2	22161

574.49683	2	22163

574.59361	2	22164

574.69039	3	22167

574.78718	2	22169

574.88396	2	22170

574.98074	2	22172

575.07752	2	22173

575.1743	2	22175

575.27109	5	22179

575.36787	2	22181

575.46465	2	22182

575.56143	2	22184

575.65821	6	22190

575.75499	2	22191

575.85178	2	22193

575.94856	2	22194

576.04534	2	22196

576.14212	2	22197

576.2389	2	22199

576.33569	3	22202

576.43247	2	22203

576.52925	2	22205

576.62603	3	22208

576.72281	2	22209

576.81959	2	22211

576.91638	3	22214

577.01316	2	22215

577.10994	3	22218

577.20672	2	22220

577.3035	2	22221

577.40029	2	22223

577.49707	2	22224

577.59385	11	22235

577.69063	2	22236

577.78741	6	22242

577.88419	2	22244

577.98098	2	22245

578.07776	2	22247

578.17454	2	22248

578.27132	5	22253

578.3681	5	22257

578.46489	2	22259

578.56167	3	22262

578.65845	5	22266

578.75523	2	22268

578.85201	2	22269

578.94879	2	22271

579.04558	2	22272

579.14236	5	22277

579.23914	5	22281

579.33592	3	22284

579.4327	2	22286

579.52949	3	22289

579.62627	2	22290

579.72305	3	22293

579.81983	2	22295

579.91661	2	22296

580.01339	2	22298

580.11018	3	22301

580.20696	2	22302

580.30374	6	22308

580.40052	3	22311

580.4973	2	22313

580.59409	2	22314

580.69087	2	22316

580.78765	12	22328

580.88443	2	22329

580.98121	2	22331

581.07799	2	22332

581.17478	2	22334

581.27156	2	22335

581.36834	2	22337

581.46512	2	22338

581.5619	3	22341

581.65869	2	22343

581.75547	2	22344

581.85225	3	22347

581.94903	2	22349

582.04581	6	22355

582.14259	8	22362

582.23938	2	22364

582.33616	3	22367

582.43294	2	22368

582.52972	2	22370

582.6265	2	22371

582.72329	8	22379

582.82007	2	22380

582.91685	2	22382

583.01363	2	22383

583.11041	2	22385

583.20719	2	22386

583.30398	2	22388

583.40076	2	22389

583.49754	3	22392

583.59432	6	22398

583.6911	2	22400

583.78789	8	22407

583.88467	2	22409

583.98145	2	22410

584.07823	6	22416

584.17501	2	22418

584.27179	3	22421

584.36858	2	22422

584.46536	2	22424

584.56214	2	22425

584.65892	2	22427

584.7557	3	22430

584.85249	2	22431

584.94927	5	22436

585.04605	2	22437

585.14283	2	22439

585.23961	3	22442

585.33639	2	22443

585.43318	2	22445

585.52996	2	22446

585.62674	6	22452

585.72352	3	22455

585.8203	3	22458

585.91709	2	22460

586.01387	2	22461

586.11065	2	22463

586.20743	2	22464

586.30421	2	22466

586.40099	2	22467

586.49778	3	22470

586.59456	2	22472

586.69134	2	22473

586.78812	8	22481

586.8849	2	22482

586.98168	2	22484

587.07847	2	22485

587.17525	2	22487

587.27203	2	22488

587.36881	2	22490

587.46559	3	22493

587.56238	2	22494

587.65916	2	22496

587.75594	2	22497

587.85272	3	22500

587.9495	5	22505

588.04628	2	22506

588.14307	2	22508

588.23985	2	22509

588.33663	2	22511

588.43341	11	22521

588.53019	2	22523

588.62698	2	22524

588.72376	2	22526

588.82054	2	22527

588.91732	3	22530

589.0141	2	22532

589.11088	2	22533

589.20767	5	22538

589.30445	2	22539

589.40123	2	22541

589.49801	3	22544

589.59479	3	22547

589.69158	2	22548

589.78836	2	22550

589.88514	2	22551

589.98192	2	22553

590.0787	2	22554

590.17548	2	22556

590.27227	3	22559

590.36905	3	22562

590.46583	9	22571

590.56261	8	22578

590.65939	14	22592

590.75618	5	22596

590.85296	2	22598

590.94974	2	22599

591.04652	2	22601

591.1433	6	22607

591.24008	2	22608

591.33687	2	22610

591.43365	2	22611

591.53043	2	22613

591.62721	2	22614

591.72399	2	22616

591.82078	2	22617

591.91756	2	22619

592.01434	2	22620

592.11112	3	22623

592.2079	5	22628

592.30468	5	22632

592.40147	2	22634

592.49825	2	22635

592.59503	2	22637

592.69181	5	22641

592.78859	3	22644

592.88538	2	22646

592.98216	3	22649

593.07894	3	22652

593.17572	2	22653

593.2725	3	22656

593.36928	6	22662

593.46607	2	22664

593.56285	2	22665

593.65963	2	22667

593.75641	5	22671

593.85319	3	22674

593.94998	5	22679

594.04676	2	22680

594.14354	3	22683

594.24032	2	22685

594.3371	3	22688

594.43388	2	22689

594.53067	5	22694

594.62745	6	22700

594.72423	6	22706

594.82101	3	22709

594.91779	3	22712

595.01458	5	22716

595.11136	2	22718

595.20814	2	22719

595.30492	6	22725

595.4017	2	22727

595.49848	5	22731

595.59527	2	22733

595.69205	6	22739

595.78883	3	22742

595.88561	2	22743

595.98239	3	22746

596.07918	2	22748

596.17596	2	22749

596.27274	2	22751

596.36952	3	22754

596.4663	2	22755

596.56308	2	22757

596.65987	5	22761

596.75665	3	22764

596.85343	2	22766

596.95021	2	22767

597.04699	2	22769

597.14378	2	22770

597.24056	3	22773

597.33734	2	22775

597.43412	2	22776

597.5309	3	22779

597.62768	2	22781

597.72447	8	22788

597.82125	2	22790

597.91803	2	22791

598.01481	2	22793

598.11159	2	22794

598.20838	2	22796

598.30516	3	22799

598.40194	2	22800

598.49872	2	22802

598.5955	3	22805

598.69228	3	22808

598.78907	3	22811

598.88585	2	22812

598.98263	3	22815

599.07941	2	22817

599.17619	3	22820

599.27297	2	22821

599.36976	2	22823

599.46654	2	22824

599.56332	2	22826

599.6601	6	22832

599.75688	2	22833

599.85367	5	22838

599.95045	3	22841

600.04723	5	22845

600.14401	2	22847

600.24079	2	22848

600.33758	2	22850

600.43436	2	22851

600.53114	2	22853

600.62792	3	22856

600.7247	2	22857

600.82148	8	22865

600.91827	2	22866

601.01505	2	22868

601.11183	2	22869

601.20861	3	22872

601.30539	2	22874

601.40217	3	22877

601.49896	2	22878

601.59574	2	22880

601.69252	2	22881

601.7893	2	22883

601.88608	3	22886

601.98287	2	22887

602.07965	2	22889

602.17643	2	22890

602.27321	2	22892

602.36999	2	22893

602.46677	2	22895

602.56356	3	22898

602.66034	3	22901

602.75712	3	22904

602.8539	2	22905

602.95068	2	22907

603.04747	2	22908

603.14425	3	22911

603.24103	2	22913

603.33781	2	22914

603.43459	2	22916

603.53137	5	22920

603.62816	3	22923

603.72494	2	22925

603.82172	3	22928

603.9185	3	22931

604.01528	3	22934

604.11207	8	22941

604.20885	2	22943

604.30563	3	22946

604.40241	2	22947

604.49919	2	22949

604.59597	9	22958

604.69276	2	22959

604.78954	2	22961

604.88632	2	22962

604.9831	2	22964

605.07988	2	22965

605.17667	5	22970

605.27345	2	22971

605.37023	2	22973

605.46701	2	22974

605.56379	2	22976

605.66057	2	22977

605.75736	2	22979

605.85414	2	22980

605.95092	2	22982

606.0477	2	22983

606.14448	2	22985

606.24127	3	22988

606.33805	17	23004

606.43483	2	23006

606.53161	2	23007

606.62839	2	23009

606.72517	2	23010

606.82196	6	23016

606.91874	2	23018

607.01552	2	23019

607.1123	2	23021

607.20908	3	23024

607.30587	3	23027

607.40265	2	23028

607.49943	2	23030

607.59621	2	23031

607.69299	6	23037

607.78977	8	23045

607.88656	2	23046

607.98334	3	23049

608.08012	3	23052

608.1769	2	23054

608.27368	3	23057

608.37047	2	23058

608.46725	6	23064

608.56403	2	23066

608.66081	6	23072

608.75759	3	23075

608.85437	6	23081

608.95116	2	23082

609.04794	3	23085

609.14472	2	23087

609.2415	2	23088

609.33828	2	23090

609.43507	3	23093

609.53185	2	23094

609.62863	2	23096

609.72541	2	23097

609.82219	2	23099

609.91897	2	23100

610.01576	3	23103

610.11254	3	23106

610.20932	2	23108

610.3061	2	23109

610.40288	2	23111

610.49967	5	23115

610.59645	2	23117

610.69323	3	23120

610.79001	5	23124

610.88679	2	23126

610.98357	2	23127

611.08036	2	23129

611.17714	3	23132

611.27392	2	23133

611.3707	5	23138

611.46748	3	23141

611.56427	5	23145

611.66105	2	23147

611.75783	5	23151

611.85461	8	23159

611.95139	15	23174

612.04817	2	23175

612.14496	2	23177

612.24174	3	23180

612.33852	3	23183

612.4353	8	23190

612.53208	3	23193

612.62887	2	23195

612.72565	2	23196

612.82243	5	23201

612.91921	5	23205

613.01599	2	23207

613.11277	2	23208

613.20956	3	23211

613.30634	2	23213

613.40312	2	23214

613.4999	2	23216

613.59668	2	23217

613.69346	2	23219

613.79025	6	23225

613.88703	3	23228

613.98381	2	23229

614.08059	2	23231

614.17737	2	23232

614.27416	2	23234

614.37094	6	23240

614.46772	2	23241

614.5645	3	23244

614.66128	8	23252

614.75806	2	23253

614.85485	2	23255

614.95163	3	23258

615.04841	2	23259

615.14519	5	23264

615.24197	2	23265

615.33876	2	23267

615.43554	3	23270

615.53232	2	23271

615.6291	5	23276

615.72588	3	23279

615.82266	2	23280

615.91945	2	23282

616.01623	2	23283

616.11301	8	23291

616.20979	3	23294

616.30657	2	23295

616.40336	8	23303

616.50014	2	23304

616.59692	2	23306

616.6937	2	23307

616.79048	3	23310

616.88726	2	23312

616.98405	2	23313

617.08083	3	23316

617.17761	2	23318

617.27439	3	23321

617.37117	3	23324

617.46796	2	23325

617.56474	2	23327

617.66152	2	23328

617.7583	3	23331

617.85508	2	23333

617.95186	5	23337

618.04865	5	23342

618.14543	2	23343

618.24221	2	23345

618.33899	3	23348

618.43577	2	23349

618.53256	3	23352

618.62934	2	23354

618.72612	2	23355

618.8229	2	23357

618.91968	9	23366

619.01646	3	23369

619.11325	5	23373

619.21003	3	23376

619.30681	2	23378

619.40359	2	23379

619.50037	2	23381

619.59716	3	23384

619.69394	2	23385

619.79072	2	23387

619.8875	2	23388

619.98428	2	23390

620.08106	2	23391

620.17785	3	23394

620.27463	3	23397

620.37141	3	23400

620.46819	5	23405

620.56497	8	23412

620.66176	2	23414

620.75854	2	23415

620.85532	2	23417

620.9521	3	23420

621.04888	9	23429

621.14566	2	23430

621.24245	3	23433

621.33923	2	23435

621.43601	3	23438

621.53279	2	23439

621.62957	2	23441

621.72636	3	23444

621.82314	2	23445

621.91992	3	23448

622.0167	2	23450

622.11348	5	23454

622.21026	2	23456

622.30705	2	23457

622.40383	6	23463

622.50061	2	23465

622.59739	2	23466

622.69417	2	23468

622.79096	2	23469

622.88774	6	23475

622.98452	3	23478

623.0813	2	23480

623.17808	2	23481

623.27486	3	23484

623.37165	2	23486

623.46843	6	23492

623.56521	2	23493

623.66199	3	23496

623.75877	6	23502

623.85556	2	23504

623.95234	3	23507

624.04912	2	23508

624.1459	2	23510

624.24268	5	23514

624.33946	2	23516

624.43625	2	23517

624.53303	44	23561

624.62981	2	23562

624.72659	2	23564

624.82337	3	23567

624.92016	2	23568

625.01694	3	23571

625.11372	9	23580

625.2105	3	23583

625.30728	5	23588

625.40406	3	23591

625.50085	8	23598

625.59763	8	23606

625.69441	3	23609

625.79119	6	23615

625.88797	3	23618

625.98475	2	23619

626.08154	2	23621

626.17832	2	23622

626.2751	2	23624

626.37188	2	23625

626.46866	3	23628

626.56545	2	23630

626.66223	2	23631

626.75901	2	23633

626.85579	2	23634

626.95257	5	23639

627.04935	2	23640

627.14614	5	23645

627.24292	3	23648

627.3397	9	23657

627.43648	2	23658

627.53326	2	23660

627.63005	2	23661

627.72683	2	23663

627.82361	2	23664

627.92039	2	23666

628.01717	3	23669

628.11395	2	23670

628.21074	3	23673

628.30752	2	23675

628.4043	2	23676

628.50108	2	23678

628.59786	3	23681

628.69465	5	23685

628.79143	2	23687

628.88821	2	23688

628.98499	3	23691

629.08177	3	23694

629.17855	2	23696

629.27534	2	23697

629.37212	2	23699

629.4689	6	23705

629.56568	5	23709

629.66246	5	23714

629.75925	3	23717

629.85603	2	23718

629.95281	2	23720

630.04959	2	23721

630.14637	2	23723

630.24315	2	23724

630.33994	2	23726

630.43672	3	23729

630.5335	6	23735

630.63028	6	23741

630.72706	2	23742

630.82385	2	23744

630.92063	2	23745

631.01741	2	23747

631.11419	2	23748

631.21097	9	23757

631.30775	2	23759

631.40454	5	23763

631.50132	3	23766

631.5981	3	23769

631.69488	3	23772

631.79166	3	23775

631.88845	3	23778

631.98523	2	23780

632.08201	2	23781

632.17879	2	23783

632.27557	2	23784

632.37235	3	23787

632.46914	2	23789

632.56592	3	23792

632.6627	6	23798

632.75948	2	23799

632.85626	8	23807

632.95305	2	23808

633.04983	5	23813

633.14661	2	23814

633.24339	3	23817

633.34017	2	23819

633.43695	2	23820

633.53374	2	23822

633.63052	2	23823

633.7273	3	23826

633.82408	2	23828

633.92086	3	23831

634.01765	5	23835

634.11443	2	23837

634.21121	2	23838

634.30799	2	23840

634.40477	2	23841

634.50155	2	23843

634.59834	2	23844

634.69512	2	23846

634.7919	3	23849

634.88868	2	23850

634.98546	3	23853

635.08225	3	23856

635.17903	5	23861

635.27581	8	23868

635.37259	2	23870

635.46937	2	23871

635.56615	3	23874

635.66294	2	23876

635.75972	2	23877

635.8565	3	23880

635.95328	2	23882

636.05006	2	23883

636.14685	5	23888

636.24363	5	23892

636.34041	2	23894

636.43719	2	23895

636.53397	2	23897

636.63075	2	23898

636.72754	2	23900

636.82432	2	23901

636.9211	3	23904

637.01788	2	23906

637.11466	2	23907

637.21145	2	23909

637.30823	2	23910

637.40501	2	23912

637.50179	5	23916

637.59857	3	23919

637.69535	6	23925

637.79214	6	23931

637.88892	2	23933

637.9857	2	23934

638.08248	2	23936

638.17926	2	23937

638.27604	6	23943

638.37283	5	23948

638.46961	2	23949

638.56639	2	23951

638.66317	2	23952

638.75995	2	23954

638.85674	5	23958

638.95352	2	23960

639.0503	2	23961

639.14708	5	23966

639.24386	5	23970

639.34064	3	23973

639.43743	2	23975

639.53421	2	23976

639.63099	2	23978

639.72777	2	23979

639.82455	2	23981

639.92134	2	23982

640.01812	3	23985

640.1149	5	23990

640.21168	3	23993

640.30846	14	24006

640.40524	5	24011

640.50203	2	24012

640.59881	5	24017

640.69559	2	24018

640.79237	2	24020

640.88915	3	24023

640.98594	2	24024

641.08272	2	24026

641.1795	2	24027

641.27628	2	24029

641.37306	2	24030

641.46984	2	24032

641.56663	5	24036

641.66341	3	24039

641.76019	2	24041

641.85697	2	24042

641.95375	2	24044

642.05054	2	24045

642.14732	3	24048

642.2441	6	24054

642.34088	3	24057

642.43766	2	24059

642.53444	2	24060

642.63123	6	24066

642.72801	2	24068

642.82479	2	24069

642.92157	3	24072

643.01835	2	24074

643.11514	2	24075

643.21192	2	24077

643.3087	8	24084

643.40548	3	24087

643.50226	3	24090

643.59904	2	24092

643.69583	2	24093

643.79261	2	24095

643.88939	3	24098

643.98617	8	24105

644.08295	2	24107

644.17974	2	24108

644.27652	3	24111

644.3733	3	24114

644.47008	6	24120

644.56686	2	24122

644.66364	12	24134

644.76043	2	24135

644.85721	2	24137

644.95399	2	24138

645.05077	2	24140

645.14755	2	24141

645.24434	3	24144

645.34112	8	24152

645.4379	2	24153

645.53468	2	24155

645.63146	2	24156

645.72824	2	24158

645.82503	2	24159

645.92181	8	24167

646.01859	3	24170

646.11537	5	24174

646.21215	2	24176

646.30894	2	24177

646.40572	3	24180

646.5025	2	24182

646.59928	2	24183

646.69606	3	24186

646.79284	3	24189

646.88963	2	24191

646.98641	5	24195

647.08319	2	24197

647.17997	2	24198

647.27675	3	24201

647.37354	2	24203

647.47032	3	24206

647.5671	3	24209

647.66388	2	24210

647.76066	2	24212

647.85744	2	24213

647.95423	3	24216

648.05101	5	24221

648.14779	2	24222

648.24457	2	24224

648.34135	2	24225

648.43814	2	24227

648.53492	6	24233

648.6317	3	24236

648.72848	2	24237

648.82526	2	24239

648.92204	2	24240

649.01883	6	24246

649.11561	3	24249

649.21239	6	24255

649.30917	2	24257

649.40595	2	24258

649.50274	3	24261

649.59952	2	24263

649.6963	2	24264

649.79308	2	24266

649.88986	2	24267

649.98664	2	24269

650.08343	2	24270

650.18021	3	24273

650.27699	2	24275

650.37377	2	24276

650.47055	5	24281

650.56733	3	24284

650.66412	2	24285

650.7609	3	24288

650.85768	2	24290

650.95446	2	24291

651.05124	3	24294

651.14803	2	24296

651.24481	3	24299

651.34159	2	24300

651.43837	2	24302

651.53515	2	24303

651.63193	2	24305

651.72872	3	24308

651.8255	2	24309

651.92228	8	24317

652.01906	6	24323

652.11584	2	24324

652.21263	2	24326

652.30941	2	24327

652.40619	3	24330

652.50297	2	24332

652.59975	2	24333

652.69653	2	24335

652.79332	5	24339

652.8901	2	24341

652.98688	2	24342

653.08366	2	24344

653.18044	3	24347

653.27723	3	24350

653.37401	2	24351

653.47079	116	24467

653.56757	2	24469

653.66435	2	24470

653.76113	2	24472

653.85792	3	24475

653.9547	2	24476

654.05148	2	24478

654.14826	2	24479

654.24504	8	24487

654.34183	6	24493

654.43861	2	24494

654.53539	2	24496

654.63217	6	24502

654.72895	3	24505

654.82573	2	24506

654.92252	6	24512

655.0193	2	24514

655.11608	2	24515

655.21286	2	24517

655.30964	2	24518

655.40643	2	24520

655.50321	3	24523

655.59999	2	24524

655.69677	2	24526

655.79355	3	24529

655.89033	2	24530

655.98712	3	24533

656.0839	3	24536

656.18068	2	24538

656.27746	6	24544

656.37424	2	24545

656.47103	2	24547

656.56781	2	24548

656.66459	2	24550

656.76137	2	24551

656.85815	2	24553

656.95493	2	24554

657.05172	3	24557

657.1485	2	24559

657.24528	2	24560

657.34206	2	24562

657.43884	2	24563

657.53563	2	24565

657.63241	3	24568

657.72919	9	24577

657.82597	8	24584

657.92275	2	24586

658.01953	3	24589

658.11632	3	24592

658.2131	2	24593

658.30988	5	24598

658.40666	2	24599

658.50344	2	24601

658.60023	2	24602

658.69701	3	24605

658.79379	5	24610

658.89057	3	24613

658.98735	5	24617

659.08413	2	24619

659.18092	2	24620

659.2777	2	24622

659.37448	2	24623

659.47126	2	24625

659.56804	2	24626

659.66483	5	24631

659.76161	5	24635

659.85839	3	24638

659.95517	2	24640

660.05195	8	24647

660.14873	3	24650

660.24552	2	24652

660.3423	3	24655

660.43908	5	24659

660.53586	2	24661

660.63264	2	24662

660.72943	2	24664

660.82621	2	24665

660.92299	2	24667

661.01977	2	24668

661.11655	2	24670

661.21333	2	24671

661.31012	2	24673

661.4069	3	24676

661.50368	2	24677

661.60046	2	24679

661.69724	5	24683

661.79403	2	24685

661.89081	2	24686

661.98759	2	24688

662.08437	2	24689

662.18115	2	24691

662.27793	2	24692

662.37472	2	24694

662.4715	2	24695

662.56828	2	24697

662.66506	2	24698

662.76184	2	24700

662.85862	2	24701

662.95541	3	24704

663.05219	2	24706

663.14897	2	24707

663.24575	2	24709

663.34253	5	24713

663.43932	2	24715

663.5361	5	24719

663.63288	5	24724

663.72966	2	24725

663.82644	2	24727

663.92322	2	24728

664.02001	2	24730

664.11679	5	24734

664.21357	2	24736

664.31035	2	24737

664.40713	3	24740

664.50392	3	24743

664.6007	2	24745

664.69748	2	24746

664.79426	2	24748

664.89104	5	24752

664.98782	2	24754

665.08461	2	24755

665.18139	2	24757

665.27817	9	24766

665.37495	2	24767

665.47173	2	24769

665.56852	2	24770

665.6653	2	24772

665.76208	2	24773

665.85886	3	24776

665.95564	6	24782

666.05242	2	24784

666.14921	2	24785

666.24599	2	24787

666.34277	2	24788

666.43955	2	24790

666.53633	2	24791

666.63312	5	24796

666.7299	2	24797

666.82668	3	24800

666.92346	2	24802

667.02024	6	24808

667.11702	3	24811

667.21381	2	24812

667.31059	2	24814

667.40737	2	24815

667.50415	3	24818

667.60093	6	24824

667.69772	9	24833

667.7945	5	24838

667.89128	5	24842

667.98806	2	24844

668.08484	2	24845

668.18162	3	24848

668.27841	3	24851

668.37519	2	24853

668.47197	2	24854

668.56875	2	24856

668.66553	3	24859

668.76232	2	24860

668.8591	5	24865

668.95588	2	24866

669.05266	2	24868

669.14944	6	24874

669.24622	2	24875

669.34301	3	24878

669.43979	2	24880

669.53657	2	24881

669.63335	3	24884

669.73013	2	24886

669.82692	2	24887

669.9237	2	24889

670.02048	3	24892

670.11726	2	24893

670.21404	2	24895

670.31082	3	24898

670.40761	2	24899

670.50439	2	24901

670.60117	2	24902

670.69795	2	24904

670.79473	3	24907

670.89152	2	24908

670.9883	5	24913

671.08508	2	24914

671.18186	6	24920

671.27864	2	24922

671.37542	2	24923

671.47221	2	24925

671.56899	2	24926

671.66577	2	24928

671.76255	2	24929

671.85933	2	24931

671.95612	2	24932

672.0529	3	24935

672.14968	2	24937

672.24646	2	24938

672.34324	5	24943

672.44002	2	24944

672.53681	2	24946

672.63359	2	24947

672.73037	2	24949

672.82715	2	24950

672.92393	2	24952

673.02072	2	24953

673.1175	3	24956

673.21428	2	24958

673.31106	5	24962

673.40784	8	24970

673.50462	9	24979

673.60141	3	24982

673.69819	2	24983

673.79497	5	24988

673.89175	2	24989

673.98853	11	25000

674.08532	2	25001

674.1821	2	25003

674.27888	6	25009

674.37566	5	25013

674.47244	6	25019

674.56922	3	25022

674.66601	3	25025

674.76279	73	25098

674.85957	2	25100

674.95635	2	25101

675.05313	6	25107

675.14991	3	25110

675.2467	2	25112

675.34348	2	25113

675.44026	2	25115

675.53704	3	25118

675.63382	2	25119

675.73061	2	25121

675.82739	6	25127

675.92417	11	25137

676.02095	2	25139

676.11773	2	25140

676.21451	6	25146

676.3113	5	25151

676.40808	2	25152

676.50486	2	25154

676.60164	3	25157

676.69842	2	25158

676.79521	2	25160

676.89199	2	25161

676.98877	3	25164

677.08555	3	25167

677.18233	2	25169

677.27911	2	25170

677.3759	3	25173

677.47268	2	25175

677.56946	6	25181

677.66624	5	25185

677.76302	2	25187

677.85981	3	25190

677.95659	2	25191

678.05337	2	25193

678.15015	2	25194

678.24693	2	25196

678.34371	2	25197

678.4405	2	25199

678.53728	6	25205

678.63406	5	25209

678.73084	2	25211

678.82762	2	25212

678.92441	3	25215

679.02119	2	25217

679.11797	2	25218

679.21475	3	25221

679.31153	2	25223

679.40831	2	25224

679.5051	2	25226

679.60188	11	25236

679.69866	2	25238

679.79544	2	25239

679.89222	2	25241

679.98901	2	25242

680.08579	5	25247

680.18257	3	25250

680.27935	2	25251

680.37613	2	25253

680.47291	2	25254

680.5697	3	25257

680.66648	3	25260

680.76326	2	25262

680.86004	3	25265

680.95682	2	25266

681.05361	2	25268

681.15039	3	25271

681.24717	8	25278

681.34395	2	25280

681.44073	2	25281

681.53751	8	25289

681.6343	2	25290

681.73108	11	25301

681.82786	2	25302

681.92464	2	25304

682.02142	2	25305

682.11821	2	25307

682.21499	3	25310

682.31177	5	25314

682.40855	2	25316

682.50533	2	25317

682.60211	2	25319

682.6989	2	25320

682.79568	2	25322

682.89246	2	25323

682.98924	2	25325

683.08602	20	25344

683.18281	3	25347

683.27959	2	25349

683.37637	3	25352

683.47315	3	25355

683.56993	2	25356

683.66671	2	25358

683.7635	3	25361

683.86028	2	25362

683.95706	3	25365

684.05384	2	25367

684.15062	2	25368

684.24741	3	25371

684.34419	3	25374

684.44097	2	25376

684.53775	2	25377

684.63453	2	25379

684.73131	11	25389

684.8281	3	25392

684.92488	2	25394

685.02166	8	25401

685.11844	2	25403

685.21522	5	25407

685.31201	3	25410

685.40879	2	25412

685.50557	6	25418

685.60235	2	25419

685.69913	2	25421

685.79591	6	25427

685.8927	2	25428

685.98948	2	25430

686.08626	2	25431

686.18304	2	25433

686.27982	3	25436

686.37661	2	25437

686.47339	2	25439

686.57017	3	25442

686.66695	2	25443

686.76373	8	25451

686.86051	2	25452

686.9573	3	25455

687.05408	2	25457

687.15086	2	25458

687.24764	8	25466

687.34442	2	25467

687.4412	2	25469

687.53799	2	25470

687.63477	2	25472

687.73155	2	25473

687.82833	2	25475

687.92511	2	25476

688.0219	3	25479

688.11868	3	25482

688.21546	2	25484

688.31224	2	25485

688.40902	2	25487

688.50581	2	25488

688.60259	3	25491

688.69937	2	25493

688.79615	5	25497

688.89293	2	25499

688.98971	2	25500

689.0865	2	25502

689.18328	2	25503

689.28006	2	25505

689.37684	2	25506

689.47362	2	25508

689.5704	5	25512

689.66719	15	25527

689.76397	2	25529

689.86075	2	25530

689.95753	2	25532

690.05431	2	25533

690.1511	2	25535

690.24788	2	25536

690.34466	2	25538

690.44144	2	25539

690.53822	2	25541

690.635	2	25542

690.73179	3	25545

690.82857	2	25547

690.92535	2	25548

691.02213	2	25550

691.11891	2	25551

691.2157	6	25557

691.31248	6	25563

691.40926	2	25565

691.50604	2	25566

691.60282	2	25568

691.6996	2	25569

691.79639	2	25571

691.89317	2	25572

691.98995	2	25574

692.08673	2	25575

692.18351	3	25578

692.2803	3	25581

692.37708	3	25584

692.47386	2	25586

692.57064	2	25587

692.66742	5	25592

692.7642	2	25593

692.86099	2	25595

692.95777	3	25598

693.05455	3	25601

693.15133	3	25604

693.24811	5	25608

693.3449	2	25610

693.44168	3	25613

693.53846	3	25616

693.63524	2	25617

693.73202	2	25619

693.8288	3	25622

693.92559	2	25623

694.02237	2	25625

694.11915	3	25628

694.21593	2	25629

694.31271	2	25631

694.4095	3	25634

694.50628	2	25635

694.60306	2	25637

694.69984	2	25638

694.79662	2	25640

694.8934	2	25641

694.99019	2	25643

695.08697	3	25646

695.18375	9	25655

695.28053	3	25658

695.37731	2	25659

695.4741	2	25661

695.57088	5	25665

695.66766	5	25670

695.76444	5	25674

695.86122	2	25676

695.958	3	25679

696.05479	2	25680

696.15157	2	25682

696.24835	2	25683

696.34513	2	25685

696.44191	2	25686

696.5387	2	25688

696.63548	5	25692

696.73226	2	25694

696.82904	2	25695

696.92582	8	25703

697.0226	5	25707

697.11939	2	25709

697.21617	6	25715

697.31295	2	25716

697.40973	2	25718

697.50651	2	25719

697.6033	5	25724

697.70008	2	25725

697.79686	2	25727

697.89364	3	25730

697.99042	2	25731

698.0872	2	25733

698.18399	2	25734

698.28077	3	25737

698.37755	3	25740

698.47433	3	25743

698.57111	2	25745

698.6679	2	25746

698.76468	3	25749

698.86146	2	25751

698.95824	3	25754

699.05502	2	25755

699.1518	2	25757

699.24859	2	25758

699.34537	2	25760

699.44215	2	25761

699.53893	2	25763

699.63571	3	25766

699.7325	3	25769

699.82928	2	25770

699.92606	2	25772

700.02284	2	25773

700.11962	2	25775

700.2164	3	25778

700.31319	2	25779

700.40997	2	25781

700.50675	3	25784

700.60353	5	25788

700.70031	2	25790

700.7971	2	25791

700.89388	2	25793

700.99066	8	25800

701.08744	2	25802

701.18422	2	25803

701.281	2	25805

701.37779	3	25808

701.47457	2	25809

701.57135	2	25811

701.66813	2	25812

701.76491	3	25815

701.86169	3	25818

701.95848	2	25820

702.05526	3	25823

702.15204	3	25826

702.24882	2	25827

702.3456	5	25832

702.44239	3	25835

702.53917	2	25836

702.63595	3	25839

702.73273	2	25841

702.82951	2	25842

702.92629	2	25844

703.02308	2	25845

703.11986	2	25847

703.21664	5	25851

703.31342	3	25854

703.4102	2	25856

703.50699	5	25860

703.60377	2	25862

703.70055	2	25863

703.79733	3	25866

703.89411	6	25872

703.99089	3	25875

704.08768	5	25880

704.18446	2	25881

704.28124	2	25883

704.37802	2	25884

704.4748	2	25886

704.57159	2	25887

704.66837	3	25890

704.76515	2	25892

704.86193	2	25893

704.95871	2	25895

705.05549	2	25896

705.15228	2	25898

705.24906	2	25899

705.34584	2	25901

705.44262	2	25902

705.5394	2	25904

705.63619	2	25905

705.73297	2	25907

705.82975	2	25908

705.92653	2	25910

706.02331	2	25911

706.12009	6	25917

706.21688	2	25919

706.31366	2	25920

706.41044	3	25923

706.50722	2	25925

706.604	2	25926

706.70079	3	25929

706.79757	14	25943

706.89435	5	25947

706.99113	14	25961

707.08791	2	25962

707.18469	3	25965

707.28148	2	25967

707.37826	3	25970

707.47504	3	25973

707.57182	2	25974

707.6686	6	25980

707.76539	2	25982

707.86217	2	25983

707.95895	2	25985

708.05573	2	25986

708.15251	8	25994

708.24929	2	25995

708.34608	5	26000

708.44286	2	26001

708.53964	2	26003

708.63642	2	26004

708.7332	2	26006

708.82999	3	26009

708.92677	2	26010

709.02355	2	26012

709.12033	3	26015

709.21711	2	26016

709.31389	2	26018

709.41068	3	26021

709.50746	3	26024

709.60424	2	26025

709.70102	2	26027

709.7978	2	26028

709.89459	6	26034

709.99137	5	26039

710.08815	2	26040

710.18493	2	26042

710.28171	2	26043

710.37849	2	26045

710.47528	2	26046

710.57206	3	26049

710.66884	2	26051

710.76562	5	26055

710.8624	2	26057

710.95919	2	26058

711.05597	2	26060

711.15275	3	26063

711.24953	2	26064

711.34631	6	26070

711.44309	2	26072

711.53988	2	26073

711.63666	2	26075

711.73344	5	26079

711.83022	2	26081

711.927	3	26084

712.02379	2	26085

712.12057	3	26088

712.21735	2	26090

712.31413	2	26091

712.41091	2	26093

712.50769	2	26094

712.60448	2	26096

712.70126	5	26100

712.79804	3	26103

712.89482	2	26105

712.9916	5	26109

713.08839	2	26111

713.18517	2	26112

713.28195	2	26114

713.37873	5	26118

713.47551	2	26120

713.57229	3	26123

713.66908	5	26127

713.76586	2	26129

713.86264	3	26132

713.95942	2	26133

714.0562	2	26135

714.15298	3	26138

714.24977	2	26139

714.34655	8	26147

714.44333	2	26148

714.54011	3	26151

714.63689	2	26153

714.73368	2	26154

714.83046	2	26156

714.92724	2	26157

715.02402	2	26159

715.1208	59	26218

715.21758	2	26219

715.31437	2	26221

715.41115	5	26225

715.50793	5	26230

715.60471	2	26231

715.70149	2	26233

715.79828	2	26234

715.89506	2	26236

715.99184	2	26237

716.08862	2	26239

716.1854	2	26240

716.28218	2	26242

716.37897	2	26243

716.47575	3	26246

716.57253	5	26251

716.66931	3	26254

716.76609	2	26255

716.86288	2	26257

716.95966	5	26261

717.05644	2	26263

717.15322	2	26264

717.25	2	26266

717.34678	3	26269

717.44357	2	26270

717.54035	2	26272

717.63713	3	26275

717.73391	3	26278

717.83069	6	26284

717.92748	2	26285

718.02426	9	26294

718.12104	2	26296

718.21782	2	26297

718.3146	2	26299

718.41138	2	26300

718.50817	5	26305

718.60495	3	26308

718.70173	2	26309

718.79851	5	26314

718.89529	2	26315

718.99208	3	26318

719.08886	2	26320

719.18564	2	26321

719.28242	2	26323

719.3792	2	26324

719.47598	2	26326

719.57277	2	26327

719.66955	3	26330

719.76633	2	26332

719.86311	2	26333

719.95989	2	26335

720.05668	2	26336

720.15346	2	26338

720.25024	2	26339

720.34702	2	26341

720.4438	2	26342

720.54058	2	26344

720.63737	2	26345

720.73415	5	26350

720.83093	2	26351

720.92771	3	26354

721.02449	3	26357

721.12128	3	26360

721.21806	2	26362

721.31484	2	26363

721.41162	2	26365

721.5084	3	26368

721.60518	3	26371

721.70197	6	26377

721.79875	2	26378

721.89553	3	26381

721.99231	2	26383

722.08909	2	26384

722.18588	2	26386

722.28266	3	26389

722.37944	2	26390

722.47622	2	26392

722.573	3	26395

722.66978	2	26396

722.76657	2	26398

722.86335	2	26399

722.96013	2	26401

723.05691	2	26402

723.15369	3	26405

723.25048	2	26407

723.34726	3	26410

723.44404	2	26411

723.54082	2	26413

723.6376	2	26414

723.73438	2	26416

723.83117	5	26420

723.92795	2	26422

724.02473	2	26423

724.12151	5	26428

724.21829	2	26429

724.31508	2	26431

724.41186	2	26432

724.50864	2	26434

724.60542	3	26437

724.7022	2	26438

724.79898	5	26443

724.89577	2	26444

724.99255	2	26446

725.08933	2	26447

725.18611	3	26450

725.28289	2	26452

725.37968	2	26453

725.47646	2	26455

725.57324	2	26456

725.67002	2	26458

725.7668	2	26459

725.86358	3	26462

725.96037	2	26464

726.05715	3	26467

726.15393	2	26468

726.25071	3	26471

726.34749	3	26474

726.44427	3	26477

726.54106	2	26479

726.63784	2	26480

726.73462	2	26482

726.8314	3	26485

726.92818	3	26488

727.02497	2	26489

727.12175	2	26491

727.21853	2	26492

727.31531	2	26494

727.41209	2	26495

727.50887	2	26497

727.60566	2	26498

727.70244	2	26500

727.79922	2	26501

727.896	3	26504

727.99278	2	26506

728.08957	2	26507

728.18635	2	26509

728.28313	2	26510

728.37991	2	26512

728.47669	2	26513

728.57347	2	26515

728.67026	2	26516

728.76704	2	26518

728.86382	2	26519

728.9606	2	26521

729.05738	2	26522

729.15417	2	26524

729.25095	2	26525

729.34773	3	26528

729.44451	2	26530

729.54129	8	26537

729.63807	2	26539

729.73486	3	26542

729.83164	2	26543

729.92842	3	26546

730.0252	2	26548

730.12198	2	26549

730.21877	2	26551

730.31555	2	26552

730.41233	2	26554

730.50911	3	26557

730.60589	2	26558

730.70267	2	26560

730.79946	2	26561

730.89624	3	26564

730.99302	2	26566

731.0898	3	26569

731.18658	2	26570

731.28337	2	26572

731.38015	5	26576

731.47693	2	26578

731.57371	6	26584

731.67049	6	26590

731.76727	2	26591

731.86406	3	26594

731.96084	2	26596

732.05762	6	26602

732.1544	2	26603

732.25118	2	26605

732.34797	2	26606

732.44475	2	26608

732.54153	2	26609

732.63831	3	26612

732.73509	3	26615

732.83187	2	26617

732.92866	2	26618

733.02544	2	26620

733.12222	2	26621

733.219	2	26623

733.31578	3	26626

733.41257	2	26627

733.50935	2	26629

733.60613	3	26632

733.70291	2	26633

733.79969	2	26635

733.89647	2	26636

733.99326	3	26639

734.09004	3	26642

734.18682	3	26645

734.2836	2	26647

734.38038	6	26653

734.47717	2	26654

734.57395	2	26656

734.67073	2	26657

734.76751	5	26662

734.86429	2	26663

734.96107	3	26666

735.05786	2	26668

735.15464	2	26669

735.25142	2	26671

735.3482	2	26672

735.44498	2	26674

735.54177	3	26677

735.63855	2	26678

735.73533	2	26680

735.83211	5	26684

735.92889	3	26687

736.02567	2	26689

736.12246	3	26692

736.21924	2	26693

736.31602	2	26695

736.4128	3	26698

736.50958	2	26699

736.60637	2	26701

736.70315	3	26704

736.79993	2	26705

736.89671	3	26708

736.99349	5	26713

737.09027	3	26716

737.18706	6	26722

737.28384	3	26725

737.38062	3	26728

737.4774	2	26729

737.57418	6	26735

737.67097	3	26738

737.76775	5	26743

737.86453	2	26744

737.96131	2	26746

738.05809	2	26747

738.15487	2	26749

738.25166	2	26750

738.34844	2	26752

738.44522	2	26753

738.542	3	26756

738.63878	3	26759

738.73556	3	26762

738.83235	2	26764

738.92913	2	26765

739.02591	2	26767

739.12269	2	26768

739.21947	2	26770

739.31626	2	26771

739.41304	2	26773

739.50982	3	26776

739.6066	5	26780

739.70338	2	26782

739.80016	5	26786

739.89695	2	26788

739.99373	2	26789

740.09051	2	26791

740.18729	2	26792

740.28407	3	26795

740.38086	3	26798

740.47764	5	26803

740.57442	2	26804

740.6712	2	26806

740.76798	5	26810

740.86476	6	26816

740.96155	2	26818

741.05833	2	26819

741.15511	2	26821

741.25189	3	26824

741.34867	2	26825

741.44546	3	26828

741.54224	3	26831

741.63902	3	26834

741.7358	2	26836

741.83258	2	26837

741.92936	2	26839

742.02615	2	26840

742.12293	2	26842

742.21971	3	26845

742.31649	2	26846

742.41327	2	26848

742.51006	3	26851

742.60684	3	26854

742.70362	3	26857

742.8004	5	26861

742.89718	2	26863

742.99396	2	26864

743.09075	2	26866

743.18753	2	26867

743.28431	2	26869

743.38109	2	26870

743.47787	2	26872

743.57466	3	26875

743.67144	3	26878

743.76822	2	26879

743.865	8	26887

743.96178	2	26888

744.05856	2	26890

744.15535	5	26894

744.25213	2	26896

744.34891	2	26897

744.44569	2	26899

744.54247	2	26900

744.63926	2	26902

744.73604	2	26903

744.83282	3	26906

744.9296	2	26908

745.02638	2	26909

745.12316	3	26912

745.21995	2	26914

745.31673	3	26917

745.41351	2	26918

745.51029	3	26921

745.60707	3	26924

745.70386	5	26929

745.80064	3	26932

745.89742	2	26933

745.9942	2	26935

746.09098	3	26938

746.18776	2	26939

746.28455	8	26947

746.38133	2	26948

746.47811	2	26950

746.57489	2	26951

746.67167	2	26953

746.76846	5	26957

746.86524	2	26959

746.96202	6	26965

747.0588	2	26966

747.15558	3	26969

747.25236	2	26971

747.34915	3	26974

747.44593	8	26981

747.54271	2	26983

747.63949	2	26984

747.73627	2	26986

747.83306	2	26987

747.92984	2	26989

748.02662	2	26990

748.1234	2	26992

748.22018	2	26993

748.31696	2	26995

748.41375	2	26996

748.51053	2	26998

748.60731	2	26999

748.70409	2	27001

748.80087	5	27005

748.89766	2	27007

748.99444	2	27008

749.09122	2	27010

749.188	2	27011

749.28478	5	27016

749.38156	2	27017

749.47835	2	27019

749.57513	2	27020

749.67191	2	27022

749.76869	2	27023

749.86547	2	27025

749.96226	2	27026

750.05904	2	27028

750.15582	2	27029

750.2526	2	27031

750.34938	2	27032

750.44616	2	27034

750.54295	3	27037

750.63973	2	27038

750.73651	2	27040

750.83329	3	27043

750.93007	2	27044

751.02685	5	27049

751.12364	3	27052

751.22042	2	27053

751.3172	2	27055

751.41398	2	27056

751.51076	2	27058

751.60755	5	27062

751.70433	2	27064

751.80111	32	27095

751.89789	2	27097

751.99467	2	27098

752.09145	5	27103

752.18824	3	27106

752.28502	3	27109

752.3818	2	27110

752.47858	2	27112

752.57536	2	27113

752.67215	8	27121

752.76893	2	27122

752.86571	2	27124

752.96249	2	27125

753.05927	2	27127

753.15605	2	27128

753.25284	2	27130

753.34962	5	27134

753.4464	2	27136

753.54318	2	27137

753.63996	2	27139

753.73675	3	27142

753.83353	2	27143

753.93031	3	27146

754.02709	2	27148

754.12387	3	27151

754.22065	2	27152

754.31744	3	27155

754.41422	3	27158

754.511	2	27160

754.60778	2	27161

754.70456	5	27166

754.80135	2	27167

754.89813	2	27169

754.99491	2	27170

755.09169	2	27172

755.18847	2	27173

755.28525	2	27175

755.38204	2	27176

755.47882	2	27178

755.5756	3	27181

755.67238	3	27184

755.76916	3	27187

755.86595	2	27188

755.96273	2	27190

756.05951	5	27194

756.15629	2	27196

756.25307	2	27197

756.34985	2	27199

756.44664	2	27200

756.54342	2	27202

756.6402	2	27203

756.73698	2	27205

756.83376	2	27206

756.93055	2	27208

757.02733	2	27209

757.12411	2	27211

757.22089	3	27214

757.31767	3	27217

757.41445	2	27218

757.51124	3	27221

757.60802	2	27223

757.7048	3	27226

757.80158	2	27227

757.89836	3	27230

757.99515	2	27232

758.09193	2	27233

758.18871	2	27235

758.28549	2	27236

758.38227	2	27238

758.47905	2	27239

758.57584	3	27242

758.67262	2	27244

758.7694	43	27287

758.86618	2	27288

758.96296	2	27290

759.05975	2	27291

759.15653	5	27296

759.25331	2	27297

759.35009	5	27302

759.44687	2	27303

759.54365	3	27306

759.64044	2	27308

759.73722	2	27309

759.834	2	27311

759.93078	2	27312

760.02756	2	27314

760.12435	2	27315

760.22113	3	27318

760.31791	3	27321

760.41469	2	27323

760.51147	2	27324

760.60825	2	27326

760.70504	2	27327

760.80182	2	27329

760.8986	2	27330

760.99538	2	27332

761.09216	2	27333

761.18895	5	27338

761.28573	2	27339

761.38251	6	27345

761.47929	2	27347

761.57607	2	27348

761.67285	2	27350

761.76964	2	27351

761.86642	2	27353

761.9632	3	27356

762.05998	2	27357

762.15676	2	27359

762.25355	3	27362

762.35033	2	27363

762.44711	5	27368

762.54389	2	27369

762.64067	2	27371

762.73745	2	27372

762.83424	2	27374

762.93102	2	27375

763.0278	2	27377

763.12458	2	27378

763.22136	2	27380

763.31814	2	27381

763.41493	2	27383

763.51171	2	27384

763.60849	2	27386

763.70527	2	27387

763.80205	2	27389

763.89884	8	27396

763.99562	2	27398

764.0924	2	27399

764.18918	3	27402

764.28596	2	27404

764.38275	3	27407

764.47953	2	27408

764.57631	2	27410

764.67309	2	27411

764.76987	2	27413

764.86665	2	27414

764.96344	6	27420

765.06022	2	27422

765.157	2	27423

765.25378	2	27425

765.35056	3	27428

765.44734	3	27431

765.54413	2	27432

765.64091	2	27434

765.73769	2	27435

765.83447	33	27468

765.93125	2	27470

766.02804	2	27471

766.12482	2	27473

766.2216	2	27474

766.31838	6	27480

766.41516	2	27482

766.51194	2	27483

766.60873	2	27485

766.70551	2	27486

766.80229	2	27488

766.89907	2	27489

766.99585	2	27491

767.09264	6	27497

767.18942	3	27500

767.2862	2	27501

767.38298	3	27504

767.47976	2	27506

767.57654	3	27509

767.67333	23	27532

767.77011	5	27536

767.86689	2	27538

767.96367	2	27539

768.06045	8	27547

768.15724	2	27548

768.25402	2	27550

768.3508	2	27551

768.44758	2	27553

768.54436	3	27556

768.64114	3	27559

768.73793	2	27560

768.83471	2	27562

768.93149	3	27565

769.02827	6	27571

769.12505	3	27574

769.22184	3	27577

769.31862	2	27578

769.4154	3	27581

769.51218	2	27583

769.60896	2	27584

769.70574	3	27587

769.80253	2	27589

769.89931	2	27590

769.99609	3	27593

770.09287	2	27595

770.18965	3	27598

770.28644	2	27599

770.38322	2	27601

770.48	2	27602

770.57678	56	27658

770.67356	2	27660

770.77034	2	27661

770.86713	2	27663

770.96391	3	27666

771.06069	2	27667

771.15747	2	27669

771.25425	2	27670

771.35104	6	27676

771.44782	3	27679

771.5446	2	27681

771.64138	2	27682

771.73816	2	27684

771.83494	2	27685

771.93173	3	27688

772.02851	2	27690

772.12529	3	27693

772.22207	2	27694

772.31885	5	27699

772.41564	2	27700

772.51242	2	27702

772.6092	2	27703

772.70598	3	27706

772.80276	6	27712

772.89954	3	27715

772.99633	3	27718

773.09311	2	27720

773.18989	5	27724

773.28667	2	27726

773.38345	2	27727

773.48024	2	27729

773.57702	2	27730

773.6738	2	27732

773.77058	3	27735

773.86736	3	27738

773.96414	2	27739

774.06093	3	27742

774.15771	6	27748

774.25449	2	27750

774.35127	3	27753

774.44805	2	27754

774.54484	2	27756

774.64162	2	27757

774.7384	2	27759

774.83518	2	27760

774.93196	9	27769

775.02874	2	27771

775.12553	2	27772

775.22231	5	27777

775.31909	2	27778

775.41587	2	27780

775.51265	2	27781

775.60944	3	27784

775.70622	2	27786

775.803	2	27787

775.89978	2	27789

775.99656	2	27790

776.09334	3	27793

776.19013	2	27795

776.28691	2	27796

776.38369	5	27801

776.48047	2	27802

776.57725	5	27807

776.67404	2	27808

776.77082	2	27810

776.8676	2	27811

776.96438	3	27814

777.06116	2	27816

777.15794	2	27817

777.25473	3	27820

777.35151	2	27822

777.44829	2	27823

777.54507	3	27826

777.64185	2	27828

777.73863	2	27829

777.83542	2	27831

777.9322	2	27832

778.02898	2	27834

778.12576	2	27835

778.22254	2	27837

778.31933	2	27838

778.41611	2	27840

778.51289	2	27841

778.60967	2	27843

778.70645	2	27844

778.80323	2	27846

778.90002	2	27847

778.9968	6	27853

779.09358	2	27855

779.19036	3	27858

779.28714	2	27859

779.38393	2	27861

779.48071	5	27865

779.57749	3	27868

779.67427	2	27870

779.77105	3	27873

779.86783	2	27874

779.96462	3	27877

780.0614	2	27879

780.15818	2	27880

780.25496	2	27882

780.35174	2	27883

780.44853	2	27885

780.54531	2	27886

780.64209	2	27888

780.73887	3	27891

780.83565	2	27892

780.93243	2	27894

781.02922	2	27895

781.126	5	27900

781.22278	2	27901

781.31956	2	27903

781.41634	3	27906

781.51313	3	27909

781.60991	3	27912

781.70669	2	27913

781.80347	3	27916

781.90025	3	27919

781.99703	2	27921

782.09382	2	27922

782.1906	5	27927

782.28738	2	27928

782.38416	6	27934

782.48094	2	27936

782.57773	2	27937

782.67451	2	27939

782.77129	3	27942

782.86807	2	27943

782.96485	3	27946

783.06163	2	27948

783.15842	2	27949

783.2552	2	27951

783.35198	2	27952

783.44876	2	27954

783.54554	2	27955

783.64233	2	27957

783.73911	3	27960

783.83589	2	27961

783.93267	5	27966

784.02945	2	27967

784.12623	3	27970

784.22302	2	27972

784.3198	3	27975

784.41658	2	27976

784.51336	2	27978

784.61014	2	27979

784.70693	2	27981

784.80371	2	27982

784.90049	3	27985

784.99727	2	27987

785.09405	2	27988

785.19083	2	27990

785.28762	2	27991

785.3844	2	27993

785.48118	2	27994

785.57796	2	27996

785.67474	2	27997

785.77153	2	27999

785.86831	2	28000

785.96509	2	28002

786.06187	3	28005

786.15865	2	28006

786.25543	2	28008

786.35222	3	28011

786.449	2	28012

786.54578	2	28014

786.64256	3	28017

786.73934	6	28023

786.83613	3	28026

786.93291	3	28029

787.02969	2	28030

787.12647	2	28032

787.22325	2	28033

787.32003	15	28048

787.41682	2	28050

787.5136	5	28054

787.61038	2	28056

787.70716	2	28057

787.80394	2	28059

787.90073	3	28062

787.99751	2	28063

788.09429	3	28066

788.19107	5	28071

788.28785	2	28072

788.38463	3	28075

788.48142	2	28077

788.5782	2	28078

788.67498	2	28080

788.77176	6	28086

788.86854	2	28087

788.96533	5	28092

789.06211	2	28093

789.15889	2	28095

789.25567	5	28099

789.35245	2	28101

789.44923	5	28105

789.54602	2	28107

789.6428	2	28108

789.73958	2	28110

789.83636	2	28111

789.93314	3	28114

790.02992	2	28116

790.12671	2	28117

790.22349	3	28120

790.32027	2	28122

790.41705	5	28126

790.51383	2	28128

790.61062	2	28129

790.7074	2	28131

790.80418	2	28132

790.90096	5	28137

790.99774	3	28140

791.09452	3	28143

791.19131	2	28144

791.28809	2	28146

791.38487	2	28147

791.48165	5	28152

791.57843	2	28153

791.67522	2	28155

791.772	5	28159

791.86878	2	28161

791.96556	2	28162

792.06234	2	28164

792.15912	2	28165

792.25591	2	28167

792.35269	2	28168

792.44947	2	28170

792.54625	2	28171

792.64303	2	28173

792.73982	2	28174

792.8366	3	28177

792.93338	2	28179

793.03016	2	28180

793.12694	2	28182

793.22372	3	28185

793.32051	3	28188

793.41729	2	28189

793.51407	3	28192

793.61085	2	28194

793.70763	8	28201

793.80442	2	28203

793.9012	2	28204

793.99798	2	28206

794.09476	5	28210

794.19154	2	28212

794.28832	3	28215

794.38511	2	28216

794.48189	2	28218

794.57867	2	28219

794.67545	2	28221

794.77223	2	28222

794.86902	2	28224

794.9658	3	28227

795.06258	3	28230

795.15936	2	28231

795.25614	5	28236

795.35292	3	28239

795.44971	2	28240

795.54649	2	28242

795.64327	2	28243

795.74005	3	28246

795.83683	2	28248

795.93362	2	28249

796.0304	3	28252

796.12718	2	28254

796.22396	8	28261

796.32074	3	28264

796.41752	6	28270

796.51431	5	28275

796.61109	2	28276

796.70787	2	28278

796.80465	2	28279

796.90143	2	28281

796.99822	2	28282

797.095	5	28287

797.19178	3	28290

797.28856	2	28291

797.38534	2	28293

797.48212	3	28296

797.57891	3	28299

797.67569	2	28300

797.77247	2	28302

797.86925	2	28303

797.96603	3	28306

798.06282	2	28308

798.1596	2	28309

798.25638	2	28311

798.35316	2	28312

798.44994	2	28314

798.54672	3	28317

798.64351	2	28318

798.74029	2	28320

798.83707	2	28321

798.93385	3	28324

799.03063	3	28327

799.12742	2	28329

799.2242	3	28332

799.32098	5	28336

799.41776	3	28339

799.51454	3	28342

799.61132	3	28345

799.70811	2	28347

799.80489	2	28348

799.90167	2	28350

799.99845	2	28351

800.09523	2	28353

800.19202	2	28354

800.2888	2	28356

800.38558	2	28357

800.48236	2	28359

800.57914	5	28363

800.67592	2	28365

800.77271	2	28366

800.86949	2	28368

800.96627	2	28369

801.06305	2	28371

801.15983	2	28372

801.25662	2	28374

801.3534	2	28375

801.45018	3	28378

801.54696	2	28380

801.64374	2	28381

801.74052	2	28383

801.83731	2	28384

801.93409	2	28386

802.03087	2	28387

802.12765	3	28390

802.22443	3	28393

802.32122	2	28395

802.418	3	28398

802.51478	2	28399

802.61156	6	28405

802.70834	2	28407

802.80512	2	28408

802.90191	2	28410

802.99869	2	28411

803.09547	2	28413

803.19225	8	28420

803.28903	2	28422

803.38581	8	28429

803.4826	2	28431

803.57938	2	28432

803.67616	3	28435

803.77294	3	28438

803.86972	2	28440

803.96651	2	28441

804.06329	2	28443

804.16007	2	28444

804.25685	5	28449

804.35363	2	28450

804.45041	12	28462

804.5472	2	28464

804.64398	2	28465

804.74076	2	28467

804.83754	2	28468

804.93432	2	28470

805.03111	2	28471

805.12789	2	28473

805.22467	3	28476

805.32145	2	28477

805.41823	2	28479

805.51501	3	28482

805.6118	2	28483

805.70858	5	28488

805.80536	3	28491

805.90214	2	28492

805.99892	2	28494

806.09571	2	28495

806.19249	2	28497

806.28927	2	28498

806.38605	2	28500

806.48283	2	28501

806.57961	2	28503

806.6764	2	28504

806.77318	3	28507

806.86996	2	28509

806.96674	2	28510

807.06352	2	28512

807.16031	3	28515

807.25709	3	28518

807.35387	2	28519

807.45065	2	28521

807.54743	2	28522

807.64421	2	28524

807.741	2	28525

807.83778	2	28527

807.93456	2	28528

808.03134	2	28530

808.12812	3	28533

808.22491	5	28537

808.32169	3	28540

808.41847	5	28545

808.51525	2	28546

808.61203	2	28548

808.70881	2	28549

808.8056	2	28551

808.90238	5	28555

808.99916	2	28557

809.09594	3	28560

809.19272	2	28561

809.28951	15	28576

809.38629	9	28585

809.48307	3	28588

809.57985	3	28591

809.67663	2	28593

809.77341	2	28594

809.8702	2	28596

809.96698	5	28600

810.06376	3	28603

810.16054	5	28608

810.25732	3	28611

810.35411	2	28612

810.45089	2	28614

810.54767	5	28618

810.64445	2	28620

810.74123	3	28623

810.83801	3	28626

810.9348	2	28627

811.03158	2	28629

811.12836	14	28642

811.22514	2	28644

811.32192	3	28647

811.41871	2	28648

811.51549	2	28650

811.61227	2	28651

811.70905	5	28656

811.80583	2	28657

811.90261	2	28659

811.9994	3	28662

812.09618	2	28663

812.19296	2	28665

812.28974	2	28666

812.38652	3	28669

812.48331	6	28675

812.58009	5	28680

812.67687	2	28681

812.77365	6	28687

812.87043	3	28690

812.96721	2	28692

813.064	2	28693

813.16078	3	28696

813.25756	2	28698

813.35434	3	28701

813.45112	2	28702

813.54791	2	28704

813.64469	2	28705

813.74147	2	28707

813.83825	2	28708

813.93503	6	28714

814.03181	2	28716

814.1286	5	28720

814.22538	2	28722

814.32216	2	28723

814.41894	3	28726

814.51572	3	28729

814.61251	2	28731

814.70929	5	28735

814.80607	2	28737

814.90285	3	28740

814.99963	3	28743

815.09641	2	28744

815.1932	2	28746

815.28998	3	28749

815.38676	6	28755

815.48354	2	28756

815.58032	2	28758

815.6771	2	28759

815.77389	2	28761

815.87067	5	28765

815.96745	2	28767

816.06423	2	28768

816.16101	2	28770

816.2578	2	28771

816.35458	2	28773

816.45136	9	28782

816.54814	3	28785

816.64492	2	28786

816.7417	2	28788

816.83849	2	28789

816.93527	2	28791

817.03205	32	28822

817.12883	2	28824

817.22561	3	28827

817.3224	3	28830

817.41918	2	28831

817.51596	2	28833

817.61274	3	28836

817.70952	2	28837

817.8063	2	28839

817.90309	2	28840

817.99987	2	28842

818.09665	2	28843

818.19343	5	28848

818.29021	2	28849

818.387	3	28852

818.48378	5	28857

818.58056	3	28860

818.67734	2	28861

818.77412	3	28864

818.8709	5	28869

818.96769	5	28873

819.06447	2	28875

819.16125	3	28878

819.25803	2	28879

819.35481	2	28881

819.4516	2	28882

819.54838	2	28884

819.64516	2	28885

819.74194	2	28887

819.83872	6	28893

819.9355	2	28894

820.03229	3	28897

820.12907	2	28899

820.22585	2	28900

820.32263	6	28906

820.41941	2	28908

820.5162	2	28909

820.61298	2	28911

820.70976	3	28914

820.80654	5	28918

820.90332	3	28921

821.0001	5	28926

821.09689	2	28927

821.19367	3	28930

821.29045	2	28932

821.38723	3	28935

821.48401	2	28936

821.5808	2	28938

821.67758	2	28939

821.77436	2	28941

821.87114	3	28944

821.96792	2	28945

822.0647	5	28950

822.16149	2	28951

822.25827	2	28953

822.35505	5	28957

822.45183	2	28959

822.54861	2	28960

822.6454	3	28963

822.74218	2	28965

822.83896	2	28966

822.93574	3	28969

823.03252	2	28971

823.1293	2	28972

823.22609	2	28974

823.32287	2	28975

823.41965	2	28977

823.51643	3	28980

823.61321	2	28981

823.71	2	28983

823.80678	2	28984

823.90356	2	28986

824.00034	2	28987

824.09712	2	28989

824.1939	3	28992

824.29069	2	28993

824.38747	3	28996

824.48425	2	28998

824.58103	2	28999

824.67781	3	29002

824.7746	3	29005

824.87138	2	29007

824.96816	2	29008

825.06494	2	29010

825.16172	3	29013

825.2585	5	29017

825.35529	6	29023

825.45207	2	29025

825.54885	2	29026

825.64563	2	29028

825.74241	5	29032

825.8392	2	29034

825.93598	2	29035

826.03276	2	29037

826.12954	3	29040

826.22632	5	29044

826.3231	6	29050

826.41989	2	29052

826.51667	2	29053

826.61345	2	29055

826.71023	2	29056

826.80701	3	29059

826.90379	2	29061

827.00058	2	29062

827.09736	2	29064

827.19414	2	29065

827.29092	3	29068

827.3877	2	29070

827.48449	2	29071

827.58127	2	29073

827.67805	2	29074

827.77483	3	29077

827.87161	2	29079

827.96839	2	29080

828.06518	2	29082

828.16196	3	29085

828.25874	2	29086

828.35552	2	29088

828.4523	2	29089

828.54909	3	29092

828.64587	2	29094

828.74265	2	29095

828.83943	3	29098

828.93621	5	29103

829.03299	2	29104

829.12978	3	29107

829.22656	2	29109

829.32334	2	29110

829.42012	3	29113

829.5169	2	29115

829.61369	2	29116

829.71047	3	29119

829.80725	2	29121

829.90403	5	29125

830.00081	2	29127

830.09759	8	29134

830.19438	2	29136

830.29116	2	29137

830.38794	2	29139

830.48472	2	29140

830.5815	3	29143

830.67829	2	29145

830.77507	3	29148

830.87185	3	29151

830.96863	2	29152

831.06541	2	29154

831.16219	5	29158

831.25898	3	29161

831.35576	2	29163

831.45254	5	29167

831.54932	5	29172

831.6461	2	29173

831.74289	3	29176

831.83967	2	29178

831.93645	2	29179

832.03323	3	29182

832.13001	2	29184

832.22679	3	29187

832.32358	2	29188

832.42036	2	29190

832.51714	2	29191

832.61392	2	29193

832.7107	2	29194

832.80749	2	29196

832.90427	5	29200

833.00105	2	29202

833.09783	2	29203

833.19461	2	29205

833.29139	3	29208

833.38818	2	29209

833.48496	2	29211

833.58174	2	29212

833.67852	2	29214

833.7753	2	29215

833.87209	3	29218

833.96887	2	29220

834.06565	2	29221

834.16243	2	29223

834.25921	8	29230

834.35599	2	29232

834.45278	2	29233

834.54956	2	29235

834.64634	2	29236

834.74312	2	29238

834.8399	3	29241

834.93669	2	29242

835.03347	2	29244

835.13025	2	29245

835.22703	2	29247

835.32381	3	29250

835.42059	3	29253

835.51738	2	29254

835.61416	2	29256

835.71094	2	29257

835.80772	2	29259

835.9045	3	29262

836.00129	2	29263

836.09807	2	29265

836.19485	2	29266

836.29163	2	29268

836.38841	2	29269

836.48519	6	29275

836.58198	5	29280

836.67876	2	29281

836.77554	2	29283

836.87232	3	29286

836.9691	3	29289

837.06589	2	29290

837.16267	2	29292

837.25945	2	29293

837.35623	2	29295

837.45301	2	29296

837.54979	3	29299

837.64658	2	29301

837.74336	3	29304

837.84014	2	29305

837.93692	2	29307

838.0337	2	29308

838.13049	2	29310

838.22727	2	29311

838.32405	2	29313

838.42083	5	29317

838.51761	6	29323

838.61439	2	29325

838.71118	2	29326

838.80796	2	29328

838.90474	2	29329

839.00152	2	29331

839.0983	2	29332

839.19508	3	29335

839.29187	3	29338

839.38865	2	29340

839.48543	3	29343

839.58221	5	29347

839.67899	3	29350

839.77578	2	29352

839.87256	2	29353

839.96934	3	29356

840.06612	2	29358

840.1629	2	29359

840.25968	2	29361

840.35647	2	29362

840.45325	3	29365

840.55003	2	29367

840.64681	2	29368

840.74359	2	29370

840.84038	2	29371

840.93716	2	29373

841.03394	2	29374

841.13072	2	29376

841.2275	2	29377

841.32428	8	29385

841.42107	3	29388

841.51785	2	29389

841.61463	3	29392

841.71141	6	29398

841.80819	6	29404

841.90498	2	29406

842.00176	2	29407

842.09854	2	29409

842.19532	2	29410

842.2921	2	29412

842.38888	2	29413

842.48567	2	29415

842.58245	2	29416

842.67923	2	29418

842.77601	2	29419

842.87279	2	29421

842.96958	2	29422

843.06636	2	29424

843.16314	6	29430

843.25992	3	29433

843.3567	2	29434

843.45348	3	29437

843.55027	2	29439

843.64705	8	29446

843.74383	5	29451

843.84061	2	29452

843.93739	3	29455

844.03418	2	29457

844.13096	3	29460

844.22774	2	29461

844.32452	2	29463

844.4213	2	29464

844.51808	2	29466

844.61487	2	29467

844.71165	2	29469

844.80843	3	29472

844.90521	2	29473

845.00199	2	29475

845.09878	2	29476

845.19556	2	29478

845.29234	2	29479

845.38912	8	29487

845.4859	2	29488

845.58268	2	29490

845.67947	3	29493

845.77625	2	29494

845.87303	2	29496

845.96981	2	29497

846.06659	2	29499

846.16338	3	29502

846.26016	3	29505

846.35694	2	29506

846.45372	2	29508

846.5505	2	29509

846.64728	2	29511

846.74407	2	29512

846.84085	2	29514

846.93763	2	29515

847.03441	2	29517

847.13119	3	29520

847.22798	9	29529

847.32476	2	29530

847.42154	5	29535

847.51832	2	29536

847.6151	2	29538

847.71188	2	29539

847.80867	5	29544

847.90545	2	29545

848.00223	2	29547

848.09901	2	29548

848.19579	2	29550

848.29258	2	29551

848.38936	3	29554

848.48614	2	29556

848.58292	2	29557

848.6797	2	29559

848.77648	2	29560

848.87327	9	29569

848.97005	9	29578

849.06683	2	29580

849.16361	3	29583

849.26039	5	29587

849.35718	2	29589

849.45396	2	29590

849.55074	2	29592

849.64752	2	29593

849.7443	2	29595

849.84108	2	29596

849.93787	2	29598

850.03465	3	29601

850.13143	3	29604

850.22821	2	29605

850.32499	2	29607

850.42178	2	29609

850.51856	5	29614

850.61534	1	29615

850.71212	4	29619

850.8089	1	29620

850.90568	1	29621

851.00247	4	29625

851.09925	6	29631

851.19603	3	29634

851.29281	6	29640

851.38959	1	29641

851.48638	4	29645

851.58316	9	29654

851.67994	1	29655

851.77672	1	29656

851.8735	8	29664

851.97028	3	29667

852.06707	3	29670

852.16385	2	29672

852.26063	5	29677

852.35741	1	29678

852.45419	1	29679

852.55097	1	29680

852.64776	1	29681

852.74454	1	29682

852.84132	3	29685

852.9381	1	29686

853.03488	1	29687

853.13167	1	29688

853.22845	5	29693

853.32523	2	29695

853.42201	2	29697

853.51879	1	29698

853.61557	2	29700

853.71236	4	29704

853.80914	10	29714

853.90592	1	29715

854.0027	2	29717

854.09948	3	29720

854.19627	1	29721

854.29305	1	29722

854.38983	1	29723

854.48661	4	29727

854.58339	1	29728

854.68017	1	29729

854.77696	1	29730

854.87374	5	29735

854.97052	1	29736

855.0673	10	29746

855.16408	4	29750

855.26087	1	29751

855.35765	3	29754

855.45443	1	29755

855.55121	3	29758

855.64799	2	29760

855.74477	3	29763

855.84156	1	29764

855.93834	3	29767

856.03512	2	29769

856.1319	12	29781

856.22868	1	29782

856.32547	1	29783

856.42225	4	29787

856.51903	1	29788

856.61581	9	29797

856.71259	1	29798

856.80937	1	29799

856.90616	2	29801

857.00294	1	29802

857.09972	10	29812

857.1965	1	29813

857.29328	1	29814

857.39007	1	29815

857.48685	7	29822

857.58363	1	29823

857.68041	5	29828

857.77719	2	29830

857.87397	1	29831

857.97076	2	29833

858.06754	1	29834

858.16432	2	29836

858.2611	1	29837

858.35788	2	29839

858.45467	1	29840

858.55145	1	29841

858.64823	1	29842

858.74501	1	29843

858.84179	1	29844

858.93857	2	29846

859.03536	1	29847

859.13214	1	29848

859.22892	1	29849

859.3257	2	29851

859.42248	1	29852

859.51927	5	29857

859.61605	1	29858

859.71283	3	29861

859.80961	2	29863

859.90639	3	29866

860.00317	2	29868

860.09996	4	29872

860.19674	1	29873

860.29352	2	29875

860.3903	1	29876

860.48708	1	29877

860.58387	2	29879

860.68065	4	29883

860.77743	5	29888

860.87421	2	29890

860.97099	2	29892

861.06777	1	29893

861.16456	3	29896

861.26134	2	29898

861.35812	4	29902

861.4549	2	29904

861.55168	1	29905

861.64847	1	29906

861.74525	13	29919

861.84203	1	29920

861.93881	7	29927

862.03559	2	29929

862.13237	1	29930

862.22916	6	29936

862.32594	2	29938

862.42272	1	29939

862.5195	1	29940

862.61628	2	29942

862.71307	2	29944

862.80985	4	29948

862.90663	6	29954

863.00341	6	29960

863.10019	5	29965

863.19697	14	29979

863.29376	4	29983

863.39054	3	29986

863.48732	7	29993

863.5841	9	30002

863.68088	6	30008

863.77767	2	30010

863.87445	1	30011

863.97123	3	30014

864.06801	1	30015

864.16479	3	30018

864.26157	1	30019

864.35836	1	30020

864.45514	1	30021

864.55192	12	30033

864.6487	6	30039

864.74548	3	30042

864.84227	1	30043

864.93905	1	30044

865.03583	4	30048

865.13261	7	30055

865.22939	1	30056

865.32617	3	30059

865.42296	7	30066

865.51974	1	30067

865.61652	18	30085

865.7133	1	30086

865.81008	2	30088

865.90686	5	30093

866.00365	1	30094

866.10043	1	30095

866.19721	1	30096

866.29399	2	30098

866.39077	6	30104

866.48756	1	30105

866.58434	11	30116

866.68112	1	30117

866.7779	4	30121

866.87468	10	30131

866.97146	1	30132

867.06825	1	30133

867.16503	16	30149

867.26181	2	30151

867.35859	9	30160

867.45537	1	30161

867.55216	34	30195

867.64894	1	30196

867.74572	1	30197

867.8425	5	30202

867.93928	2	30204

868.03606	8	30212

868.13285	1	30213

868.22963	5	30218

868.32641	4	30222

868.42319	1	30223

868.51997	1	30224

868.61676	3	30227

868.71354	1	30228

868.81032	2	30230

868.9071	4	30234

869.00388	1	30235

869.10066	2	30237

869.19745	2	30239

869.29423	5	30244

869.39101	1	30245

869.48779	6	30251

869.58457	8	30259

869.68136	5	30264

869.77814	4	30268

869.87492	4	30272

869.9717	1	30273

870.06848	1	30274

870.16526	7	30281

870.26205	2	30283

870.35883	3	30286

870.45561	8	30294

870.55239	7	30301

870.64917	1	30302

870.74596	1	30303

870.84274	1	30304

870.93952	2	30306

871.0363	2	30308

871.13308	2	30310

871.22986	2	30312

871.32665	3	30315

871.42343	4	30319

871.52021	10	30329

871.61699	2	30331

871.71377	2	30333

871.81056	1	30334

871.90734	3	30337

872.00412	1	30338

872.1009	6	30344

872.19768	1	30345

872.29446	2	30347

872.39125	1	30348

872.48803	6	30354

872.58481	5	30359

872.68159	2	30361

872.77837	3	30364

872.87516	14	30378

872.97194	20	30398

873.06872	2	30400

873.1655	24	30424

873.26228	3	30427

873.35906	1	30428

873.45585	7	30435

873.55263	1	30436

873.64941	2	30438

873.74619	6	30444

873.84297	1	30445

873.93976	7	30452

874.03654	1	30453

874.13332	1	30454

874.2301	3	30457

874.32688	4	30461

874.42366	1	30462

874.52045	5	30467

874.61723	1	30468

874.71401	8	30476

874.81079	1	30477

874.90757	1	30478

875.00436	3	30481

875.10114	2	30483

875.19792	3	30486

875.2947	4	30490

875.39148	2	30492

875.48826	5	30497

875.58505	3	30500

875.68183	4	30504

875.77861	2	30506

875.87539	1	30507

875.97217	1	30508

876.06896	2	30510

876.16574	11	30521

876.26252	2	30523

876.3593	1	30524

876.45608	8	30532

876.55286	2	30534

876.64965	1	30535

876.74643	15	30550

876.84321	4	30554

876.93999	5	30559

877.03677	2	30561

877.13356	14	30575

877.23034	2	30577

877.32712	7	30584

877.4239	1	30585

877.52068	9	30594

877.61746	3	30597

877.71425	1	30598

877.81103	4	30602

877.90781	3	30605

878.00459	5	30610

878.10137	1	30611

878.19816	9	30620

878.29494	1	30621

878.39172	5	30626

878.4885	1	30627

878.58528	2	30629

878.68206	1	30630

878.77885	1	30631

878.87563	1	30632

878.97241	1	30633

879.06919	10	30643

879.16597	1	30644

879.26275	10	30654

879.35954	1	30655

879.45632	1	30656

879.5531	1	30657

879.64988	8	30665

879.74666	1	30666

879.84345	5	30671

879.94023	7	30678

880.03701	2	30680

880.13379	1	30681

880.23057	11	30692

880.32735	2	30694

880.42414	10	30704

880.52092	1	30705

880.6177	2	30707

880.71448	4	30711

880.81126	13	30724

880.90805	2	30726

881.00483	2	30728

881.10161	1	30729

881.19839	5	30734

881.29517	8	30742

881.39195	1	30743

881.48874	1	30744

881.58552	4	30748

881.6823	3	30751

881.77908	2	30753

881.87586	2	30755

881.97265	1	30756

882.06943	1	30757

882.16621	4	30761

882.26299	2	30763

882.35977	1	30764

882.45655	1	30765

882.55334	1	30766

882.65012	7	30773

882.7469	3	30776

882.84368	1	30777

882.94046	1	30778

883.03725	4	30782

883.13403	6	30788

883.23081	11	30799

883.32759	1	30800

883.42437	1	30801

883.52115	27	30828

883.61794	1	30829

883.71472	2	30831

883.8115	3	30834

883.90828	3	30837

884.00506	1	30838

884.10185	6	30844

884.19863	3	30847

884.29541	1	30848

884.39219	6	30854

884.48897	1	30855

884.58575	10	30865

884.68254	1	30866

884.77932	12	30878

884.8761	9	30887

884.97288	4	30891

885.06966	6	30897

885.16645	1	30898

885.26323	5	30903

885.36001	12	30915

885.45679	6	30921

885.55357	3	30924

885.65035	1	30925

885.74714	1	30926

885.84392	1	30927

885.9407	1	30928

886.03748	3	30931

886.13426	3	30934

886.23105	1	30935

886.32783	1	30936

886.42461	2	30938

886.52139	3	30941

886.61817	1	30942

886.71495	9	30951

886.81174	1	30952

886.90852	2	30954

887.0053	1	30955

887.10208	3	30958

887.19886	1	30959

887.29565	2	30961

887.39243	5	30966

887.48921	2	30968

887.58599	10	30978

887.68277	3	30981

887.77955	1	30982

887.87634	9	30991

887.97312	5	30996

888.0699	13	31009

888.16668	2	31011

888.26346	1	31012

888.36025	2	31014

888.45703	1	31015

888.55381	10	31025

888.65059	1	31026

888.74737	1	31027

888.84415	2	31029

888.94094	2	31031

889.03772	2	31033

889.1345	6	31039

889.23128	1	31040

889.32806	1	31041

889.42485	4	31045

889.52163	13	31058

889.61841	7	31065

889.71519	1	31066

889.81197	2	31068

889.90875	11	31079

890.00554	1	31080

890.10232	2	31082

890.1991	1	31083

890.29588	4	31087

890.39266	1	31088

890.48945	2	31090

890.58623	2	31092

890.68301	3	31095

890.77979	2	31097

890.87657	1	31098

890.97335	5	31103

891.07014	3	31106

891.16692	8	31114

891.2637	2	31116

891.36048	11	31127

891.45726	2	31129

891.55404	10	31139

891.65083	1	31140

891.74761	1	31141

891.84439	4	31145

891.94117	1	31146

892.03795	1	31147

892.13474	1	31148

892.23152	1	31149

892.3283	6	31155

892.42508	1	31156

892.52186	3	31159

892.61864	4	31163

892.71543	2	31165

892.81221	2	31167

892.90899	1	31168

893.00577	8	31176

893.10255	2	31178

893.19934	1	31179

893.29612	1	31180

893.3929	2	31182

893.48968	1	31183

893.58646	1	31184

893.68324	10	31194

893.78003	9	31203

893.87681	2	31205

893.97359	2	31207

894.07037	1	31208

894.16715	1	31209

894.26394	7	31216

894.36072	3	31219

894.4575	2	31221

894.55428	1	31222

894.65106	1	31223

894.74784	4	31227

894.84463	1	31228

894.94141	13	31241

895.03819	1	31242

895.13497	11	31253

895.23175	1	31254

895.32854	1	31255

895.42532	3	31258

895.5221	4	31262

895.61888	1	31263

895.71566	1	31264

895.81244	4	31268

895.90923	1	31269

896.00601	1	31270

896.10279	1	31271

896.19957	2	31273

896.29635	1	31274

896.39314	3	31277

896.48992	1	31278

896.5867	6	31284

896.68348	3	31287

896.78026	1	31288

896.87704	1	31289

896.97383	9	31298

897.07061	2	31300

897.16739	2	31302

897.26417	1	31303

897.36095	1	31304

897.45774	7	31311

897.55452	1	31312

897.6513	8	31320

897.74808	1	31321

897.84486	1	31322

897.94164	1	31323

898.03843	17	31340

898.13521	1	31341

898.23199	6	31347

898.32877	1	31348

898.42555	2	31350

898.52234	4	31354

898.61912	1	31355

898.7159	11	31366

898.81268	1	31367

898.90946	1	31368

899.00624	8	31376

899.10303	5	31381

899.19981	3	31384

899.29659	1	31385

899.39337	1	31386

899.49015	2	31388

899.58694	4	31392

899.68372	1	31393

899.7805	3	31396

899.87728	16	31412

899.97406	1	31413

900.07084	1	31414

900.16763	2	31416

900.26441	2	31418

900.36119	2	31420

900.45797	4	31424

900.55475	1	31425

900.65154	1	31426

900.74832	3	31429

900.8451	12	31441

900.94188	1	31442

901.03866	7	31449

901.13544	1	31450

901.23223	9	31459

901.32901	2	31461

901.42579	1	31462

901.52257	1	31463

901.61935	2	31465

901.71614	7	31472

901.81292	1	31473

901.9097	1	31474

902.00648	1	31475

902.10326	3	31478

902.20004	1	31479

902.29683	1	31480

902.39361	1	31481

902.49039	14	31495

902.58717	1	31496

902.68395	1	31497

902.78074	1	31498

902.87752	1	31499

902.9743	6	31505

903.07108	1	31506

903.16786	1	31507

903.26464	6	31513

903.36143	1	31514

903.45821	9	31523

903.55499	1	31524

903.65177	3	31527

903.74855	2	31529

903.84533	4	31533

903.94212	8	31541

904.0389	1	31542

904.13568	1	31543

904.23246	1	31544

904.32924	1	31545

904.42603	17	31562

904.52281	3	31565

904.61959	5	31570

904.71637	1	31571

904.81315	3	31574

904.90993	8	31582

905.00672	3	31585

905.1035	7	31592

905.20028	1	31593

905.29706	9	31602

905.39384	6	31608

905.49063	1	31609

905.58741	1	31610

905.68419	2	31612

905.78097	1	31613

905.87775	1	31614

905.97453	11	31625

906.07132	3	31628

906.1681	6	31634

906.26488	1	31635

906.36166	1	31636

906.45844	9	31645

906.55523	1	31646

906.65201	8	31654

906.74879	2	31656

906.84557	6	31662

906.94235	2	31664

907.03913	1	31665

907.13592	2	31667

907.2327	4	31671

907.32948	17	31688

907.42626	1	31689

907.52304	2	31691

907.61983	10	31701

907.71661	1	31702

907.81339	6	31708

907.91017	2	31710

908.00695	1	31711

908.10373	4	31715

908.20052	1	31716

908.2973	1	31717

908.39408	9	31726

908.49086	1	31727

908.58764	7	31734

908.68443	1	31735

908.78121	1	31736

908.87799	1	31737

908.97477	21	31758

909.07155	1	31759

909.16833	2	31761

909.26512	8	31769

909.3619	1	31770

909.45868	4	31774

909.55546	1	31775

909.65224	1	31776

909.74903	1	31777

909.84581	11	31788

909.94259	1	31789

910.03937	1	31790

910.13615	1	31791

910.23293	3	31794

910.32972	3	31797

910.4265	1	31798

910.52328	16	31814

910.62006	6	31820

910.71684	3	31823

910.81363	18	31841

910.91041	1	31842

911.00719	1	31843

911.10397	11	31854

911.20075	1	31855

911.29753	1	31856

911.39432	10	31866

911.4911	1	31867

911.58788	2	31869

911.68466	1	31870

911.78144	1	31871

911.87823	1	31872

911.97501	2	31874

912.07179	8	31882

912.16857	1	31883

912.26535	8	31891

912.36213	2	31893

912.45892	1	31894

912.5557	1	31895

912.65248	2	31897

912.74926	8	31905

912.84604	9	31914

912.94283	2	31916

913.03961	6	31922

913.13639	2	31924

913.23317	1	31925

913.32995	4	31929

913.42673	19	31948

913.52352	5	31953

913.6203	3	31956

913.71708	7	31963

913.81386	1	31964

913.91064	12	31976

914.00743	1	31977

914.10421	1	31978

914.20099	2	31980

914.29777	4	31984

914.39455	1	31985

914.49133	5	31990

914.58812	5	31995

914.6849	1	31996

914.78168	13	32009

914.87846	1	32010

914.97524	1	32011

915.07203	2	32013

915.16881	3	32016

915.26559	1	32017

915.36237	31	32048

915.45915	1	32049

915.55593	1	32050

915.65272	5	32055

915.7495	3	32058

915.84628	5	32063

915.94306	1	32064

916.03984	7	32071

916.13662	1	32072

916.23341	7	32079

916.33019	1	32080

916.42697	11	32091

916.52375	1	32092

916.62053	2	32094

916.71732	1	32095

916.8141	1	32096

916.91088	1	32097

917.00766	10	32107

917.10444	1	32108

917.20122	1	32109

917.29801	2	32111

917.39479	1	32112

917.49157	2	32114

917.58835	1	32115

917.68513	22	32137

917.78192	8	32145

917.8787	1	32146

917.97548	8	32154

918.07226	17	32171

918.16904	13	32184

918.26582	10	32194

918.36261	1	32195

918.45939	1	32196

918.55617	5	32201

918.65295	3	32204

918.74973	1	32205

918.84652	1	32206

918.9433	1	32207

919.04008	1	32208

919.13686	2	32210

919.23364	7	32217

919.33042	4	32221

919.42721	1	32222

919.52399	2	32224

919.62077	4	32228

919.71755	5	32233

919.81433	2	32235

919.91112	7	32242

920.0079	1	32243

920.10468	1	32244

920.20146	14	32258

920.29824	1	32259

920.39502	9	32268

920.49181	1	32269

920.58859	1	32270

920.68537	9	32279

920.78215	12	32291

920.87893	1	32292

920.97572	5	32297

921.0725	2	32299

921.16928	8	32307

921.26606	2	32309

921.36284	2	32311

921.45962	15	32326

921.55641	1	32327

921.65319	2	32329

921.74997	1	32330

921.84675	9	32339

921.94353	13	32352

922.04032	3	32355

922.1371	1	32356

922.23388	16	32372

922.33066	5	32377

922.42744	1	32378

922.52422	23	32401

922.62101	2	32403

922.71779	4	32407

922.81457	7	32414

922.91135	2	32416

923.00813	1	32417

923.10492	5	32422

923.2017	2	32424

923.29848	11	32435

923.39526	5	32440

923.49204	6	32446

923.58882	1	32447

923.68561	1	32448

923.78239	2	32450

923.87917	6	32456

923.97595	4	32460

924.07273	3	32463

924.16952	1	32464

924.2663	20	32484

924.36308	16	32500

924.45986	1	32501

924.55664	13	32514

924.65342	1	32515

924.75021	3	32518

924.84699	5	32523

924.94377	2	32525

925.04055	2	32527

925.13733	1	32528

925.23412	3	32531

925.3309	4	32535

925.42768	2	32537

925.52446	3	32540

925.62124	19	32559

925.71802	10	32569

925.81481	3	32572

925.91159	1	32573

926.00837	8	32581

926.10515	4	32585

926.20193	4	32589

926.29872	11	32600

926.3955	1	32601

926.49228	1	32602

926.58906	8	32610

926.68584	6	32616

926.78262	4	32620

926.87941	11	32631

926.97619	2	32633

927.07297	10	32643

927.16975	1	32644

927.26653	6	32650

927.36331	2	32652

927.4601	1	32653

927.55688	11	32664

927.65366	6	32670

927.75044	5	32675

927.84722	5	32680

927.94401	9	32689

928.04079	1	32690

928.13757	7	32697

928.23435	1	32698

928.33113	4	32702

928.42791	2	32704

928.5247	9	32713

928.62148	4	32717

928.71826	2	32719

928.81504	1	32720

928.91182	2	32722

929.00861	9	32731

929.10539	2	32733

929.20217	6	32739

929.29895	6	32745

929.39573	2	32747

929.49251	2	32749

929.5893	1	32750

929.68608	13	32763

929.78286	3	32766

929.87964	11	32777

929.97642	9	32786

930.07321	1	32787

930.16999	4	32791

930.26677	1	32792

930.36355	3	32795

930.46033	1	32796

930.55711	1	32797

930.6539	5	32802

930.75068	4	32806

930.84746	12	32818

930.94424	2	32820

931.04102	4	32824

931.13781	2	32826

931.23459	1	32827

931.33137	1	32828

931.42815	1	32829

931.52493	9	32838

931.62171	9	32847

931.7185	4	32851

931.81528	38	32889

931.91206	1	32890

932.00884	13	32903

932.10562	1	32904

932.20241	15	32919

932.29919	1	32920

932.39597	1	32921

932.49275	2	32923

932.58953	15	32938

932.68631	1	32939

932.7831	12	32951

932.87988	9	32960

932.97666	2	32962

933.07344	1	32963

933.17022	17	32980

933.26701	12	32992

933.36379	2	32994

933.46057	6	33000

933.55735	1	33001

933.65413	1	33002

933.75091	6	33008

933.8477	14	33022

933.94448	3	33025

934.04126	2	33027

934.13804	12	33039

934.23482	1	33040

934.33161	1	33041

934.42839	6	33047

934.52517	1	33048

934.62195	1	33049

934.71873	1	33050

934.81551	2	33052

934.9123	1	33053

935.00908	3	33056

935.10586	1	33057

935.20264	2	33059

935.29942	2	33061

935.39621	2	33063

935.49299	19	33082

935.58977	1	33083

935.68655	38	33121

935.78333	1	33122

935.88011	1	33123

935.9769	19	33142

936.07368	2	33144

936.17046	8	33152

936.26724	1	33153

936.36402	10	33163

936.46081	1	33164

936.55759	1	33165

936.65437	4	33169

936.75115	3	33172

936.84793	24	33196

936.94471	1	33197

937.0415	16	33213

937.13828	1	33214

937.23506	10	33224

937.33184	1	33225

937.42862	4	33229

937.52541	1	33230

937.62219	6	33236

937.71897	5	33241

937.81575	1	33242

937.91253	17	33259

938.00931	1	33260

938.1061	9	33269

938.20288	1	33270

938.29966	4	33274

938.39644	6	33280

938.49322	4	33284

938.59001	10	33294

938.68679	1	33295

938.78357	1	33296

938.88035	1	33297

938.97713	2	33299

939.07391	2	33301

939.1707	17	33318

939.26748	2	33320

939.36426	8	33328

939.46104	1	33329

939.55782	1	33330

939.65461	2	33332

939.75139	12	33344

939.84817	4	33348

939.94495	3	33351

940.04173	14	33365

940.13851	1	33366

940.2353	9	33375

940.33208	1	33376

940.42886	1	33377

940.52564	9	33386

940.62242	1	33387

940.7192	4	33391

940.81599	2	33393

940.91277	5	33398

941.00955	13	33411

941.10633	2	33413

941.20311	11	33424

941.2999	3	33427

941.39668	13	33440

941.49346	3	33443

941.59024	15	33458

941.68702	1	33459

941.7838	3	33462

941.88059	3	33465

941.97737	1	33466

942.07415	7	33473

942.17093	6	33479

942.26771	1	33480

942.3645	9	33489

942.46128	6	33495

942.55806	1	33496

942.65484	5	33501

942.75162	1	33502

942.8484	9	33511

942.94519	2	33513

943.04197	10	33523

943.13875	1	33524

943.23553	7	33531

943.33231	9	33540

943.4291	15	33555

943.52588	1	33556

943.62266	2	33558

943.71944	1	33559

943.81622	14	33573

943.913	13	33586

944.00979	11	33597

944.10657	2	33599

944.20335	1	33600

944.30013	1	33601

944.39691	8	33609

944.4937	2	33611

944.59048	1	33612

944.68726	5	33617

944.78404	2	33619

944.88082	1	33620

944.9776	2	33622

945.07439	16	33638

945.17117	1	33639

945.26795	9	33648

945.36473	1	33649

945.46151	1	33650

945.5583	1	33651

945.65508	5	33656

945.75186	2	33658

945.84864	1	33659

945.94542	7	33666

946.0422	2	33668

946.13899	2	33670

946.23577	2	33672

946.33255	7	33679

946.42933	1	33680

946.52611	17	33697

946.6229	1	33698

946.71968	1	33699

946.81646	4	33703

946.91324	2	33705

947.01002	2	33707

947.1068	16	33723

947.20359	2	33725

947.30037	5	33730

947.39715	1	33731

947.49393	4	33735

947.59071	8	33743

947.6875	1	33744

947.78428	1	33745

947.88106	4	33749

947.97784	1	33750

948.07462	1	33751

948.1714	3	33754

948.26819	2	33756

948.36497	26	33782

948.46175	1	33783

948.55853	11	33794

948.65531	1	33795

948.7521	1	33796

948.84888	9	33805

948.94566	4	33809

949.04244	3	33812

949.13922	10	33822

949.236	2	33824

949.33279	1	33825

949.42957	1	33826

949.52635	11	33837

949.62313	3	33840

949.71991	6	33846

949.8167	3	33849

949.91348	1	33850

950.01026	11	33861

950.10704	1	33862

950.20382	11	33873

950.3006	4	33877

950.39739	4	33881

950.49417	16	33897

950.59095	1	33898

950.68773	3	33901

950.78451	5	33906

950.8813	2	33908

950.97808	2	33910

951.07486	3	33913

951.17164	2	33915

951.26842	3	33918

951.3652	1	33919

951.46199	1	33920

951.55877	3	33923

951.65555	2	33925

951.75233	3	33928

951.84911	4	33932

951.9459	3	33935

952.04268	2	33937

952.13946	19	33956

952.23624	2	33958

952.33302	8	33966

952.4298	10	33976

952.52659	9	33985

952.62337	1	33986

952.72015	2	33988

952.81693	1	33989

952.91371	6	33995

953.01049	1	33996

953.10728	1	33997

953.20406	1	33998

953.30084	21	34019

953.39762	2	34021

953.4944	27	34048

953.59119	1	34049

953.68797	3	34052

953.78475	4	34056

953.88153	2	34058

953.97831	2	34060

954.07509	9	34069

954.17188	2	34071

954.26866	2	34073

954.36544	1	34074

954.46222	12	34086

954.559	2	34088

954.65579	1	34089

954.75257	3	34092

954.84935	9	34101

954.94613	1	34102

955.04291	1	34103

955.13969	5	34108

955.23648	2	34110

955.33326	1	34111

955.43004	2	34113

955.52682	1	34114

955.6236	3	34117

955.72039	11	34128

955.81717	3	34131

955.91395	1	34132

956.01073	1	34133

956.10751	9	34142

956.20429	1	34143

956.30108	1	34144

956.39786	22	34166

956.49464	1	34167

956.59142	13	34180

956.6882	1	34181

956.78499	17	34198

956.88177	6	34204

956.97855	2	34206

957.07533	1	34207

957.17211	11	34218

957.26889	1	34219

957.36568	11	34230

957.46246	4	34234

957.55924	4	34238

957.65602	16	34254

957.7528	1	34255

957.84959	3	34258

957.94637	5	34263

958.04315	2	34265

958.13993	2	34267

958.23671	3	34270

958.33349	2	34272

958.43028	3	34275

958.52706	1	34276

958.62384	1	34277

958.72062	3	34280

958.8174	2	34282

958.91419	3	34285

959.01097	4	34289

959.10775	3	34292

959.20453	2	34294

959.30131	19	34313

959.39809	2	34315

959.49488	8	34323

959.59166	10	34333

959.68844	9	34342

959.78522	1	34343

959.882	2	34345

959.97879	1	34346

960.07557	6	34352

960.17235	1	34353

960.26913	1	34354

960.36591	1	34355

960.46269	21	34376

960.55948	2	34378

960.65626	27	34405

960.75304	1	34406

960.84982	3	34409

960.9466	4	34413

961.04339	2	34415

961.14017	2	34417

961.23695	9	34426

961.33373	2	34428

961.43051	2	34430

961.52729	1	34431

961.62408	12	34443

961.72086	2	34445

961.81764	1	34446

961.91442	3	34449

962.0112	9	34458

962.10799	1	34459

962.20477	1	34460

962.30155	5	34465

962.39833	2	34467

962.49511	1	34468

962.59189	2	34470

962.68868	1	34471

962.78546	3	34474

962.88224	11	34485

962.97902	3	34488

963.0758	1	34489

963.17259	1	34490

963.26937	9	34499

963.36615	1	34500

963.46293	1	34501

963.55971	22	34523

963.65649	1	34524

963.75328	13	34537

963.85006	1	34538

963.94684	17	34555

964.04362	2	34557

964.1404	2	34559

964.23719	4	34563

964.33397	1	34564

964.43075	5	34569

964.52753	3	34572

964.62431	31	34603

964.72109	1	34604

964.81788	24	34628

964.91466	14	34642

965.01144	3	34645

965.10822	1	34646

965.205	1	34647

965.30179	3	34650

965.39857	14	34664

965.49535	26	34690

965.59213	1	34691

965.68891	18	34709

965.78569	8	34717

965.88248	1	34718

965.97926	1	34719

966.07604	1	34720

966.17282	19	34739

966.2696	1	34740

966.36639	1	34741

966.46317	10	34751

966.55995	3	34754

966.65673	1	34755

966.75351	4	34759

966.85029	3	34762

966.94708	1	34763

967.04386	1	34764

967.14064	1	34765

967.23742	2	34767

967.3342	16	34783

967.43098	1	34784

967.52777	1	34785

967.62455	2	34787

967.72133	25	34812

967.81811	1	34813

967.91489	13	34826

968.01168	1	34827

968.10846	12	34839

968.20524	2	34841

968.30202	1	34842

968.3988	1	34843

968.49558	20	34863

968.59237	2	34865

968.68915	3	34868

968.78593	1	34869

968.88271	1	34870

968.97949	1	34871

969.07628	33	34904

969.17306	1	34905

969.26984	9	34914

969.36662	15	34929

969.4634	8	34937

969.56018	2	34939

969.65697	1	34940

969.75375	15	34955

969.85053	4	34959

969.94731	1	34960

970.04409	22	34982

970.14088	1	34983

970.23766	12	34995

970.33444	22	35017

970.43122	36	35053

970.528	2	35055

970.62478	13	35068

970.72157	8	35076

970.81835	2	35078

970.91513	1	35079

971.01191	24	35103

971.10869	27	35130

971.20548	2	35132

971.30226	28	35160

971.39904	1	35161

971.49582	9	35170

971.5926	8	35178

971.68938	1	35179

971.78617	27	35206

971.88295	4	35210

971.97973	7	35217

972.07651	1	35218

972.17329	6	35224

972.27008	3	35227

972.36686	24	35251

972.46364	2	35253

972.56042	17	35270

972.6572	1	35271

972.75398	1	35272

972.85077	23	35295

972.94755	3	35298

973.04433	1	35299

973.14111	1	35300

973.23789	2	35302

973.33468	16	35318

973.43146	1	35319

973.52824	3	35322

973.62502	1	35323

973.7218	1	35324

973.81858	1	35325

973.91537	6	35331

974.01215	15	35346

974.10893	1	35347

974.20571	1	35348

974.30249	1	35349

974.39928	1	35350

974.49606	1	35351

974.59284	19	35370

974.68962	9	35379

974.7864	1	35380

974.88318	13	35393

974.97997	3	35396

975.07675	1	35397

975.17353	16	35413

975.27031	1	35414

975.36709	1	35415

975.46388	5	35420

975.56066	7	35427

975.65744	1	35428

975.75422	1	35429

975.851	15	35444

975.94778	3	35447

976.04457	25	35472

976.14135	1	35473

976.23813	1	35474

976.33491	3	35477

976.43169	12	35489

976.52848	4	35493

976.62526	23	35516

976.72204	1	35517

976.81882	1	35518

976.9156	20	35538

977.01238	30	35568

977.10917	7	35575

977.20595	1	35576

977.30273	2	35578

977.39951	1	35579

977.49629	10	35589

977.59308	1	35590

977.68986	1	35591

977.78664	30	35621

977.88342	1	35622

977.9802	20	35642

978.07698	1	35643

978.17377	1	35644

978.27055	6	35650

978.36733	10	35660

978.46411	4	35664

978.56089	1	35665

978.65768	12	35677

978.75446	1	35678

978.85124	17	35695

978.94802	2	35697

979.0448	1	35698

979.14158	16	35714

979.23837	2	35716

979.33515	1	35717

979.43193	11	35728

979.52871	1	35729

979.62549	1	35730

979.72227	17	35747

979.81906	2	35749

979.91584	2	35751

980.01262	36	35787

980.1094	1	35788

980.20618	1	35789

980.30297	22	35811

980.39975	1	35812

980.49653	1	35813

980.59331	2	35815

980.69009	24	35839

980.78687	2	35841

980.88366	9	35850

980.98044	2	35852

981.07722	1	35853

981.174	1	35854

981.27078	17	35871

981.36757	1	35872

981.46435	2	35874

981.56113	13	35887

981.65791	5	35892

981.75469	2	35894

981.85147	25	35919

981.94826	1	35920

982.04504	13	35933

982.14182	2	35935

982.2386	4	35939

982.33538	1	35940

982.43217	3	35943

982.52895	4	35947

982.62573	1	35948

982.72251	6	35954

982.81929	3	35957

982.91607	8	35965

983.01286	2	35967

983.10964	1	35968

983.20642	2	35970

983.3032	1	35971

983.39998	4	35975

983.49677	2	35977

983.59355	2	35979

983.69033	24	36003

983.78711	1	36004

983.88389	9	36013

983.98067	1	36014

984.07746	1	36015

984.17424	4	36019

984.27102	4	36023

984.3678	1	36024

984.46458	2	36026

984.56137	4	36030

984.65815	10	36040

984.75493	1	36041

984.85171	2	36043

984.94849	6	36049

985.04527	26	36075

985.14206	1	36076

985.23884	29	36105

985.33562	1	36106

985.4324	1	36107

985.52918	2	36109

985.62597	9	36118

985.72275	1	36119

985.81953	1	36120

985.91631	1	36121

986.01309	2	36123

986.10987	15	36138

986.20666	4	36142

986.30344	1	36143

986.40022	40	36183

986.497	3	36186

986.59378	1	36187

986.69057	16	36203

986.78735	1	36204

986.88413	1	36205

986.98091	1	36206

987.07769	9	36215

987.17447	1	36216

987.27126	1	36217

987.36804	1	36218

987.46482	1	36219

987.5616	14	36233

987.65838	21	36254

987.75517	3	36257

987.85195	10	36267

987.94873	2	36269

988.04551	1	36270

988.14229	1	36271

988.23907	8	36279

988.33586	2	36281

988.43264	2	36283

988.52942	1	36284

988.6262	17	36301

988.72298	1	36302

988.81977	2	36304

988.91655	14	36318

989.01333	1	36319

989.11011	4	36323

989.20689	2	36325

989.30367	1	36326

989.40046	112	36438

989.49724	1	36439

989.59402	1	36440

989.6908	1	36441

989.78758	16	36457

989.88437	23	36480

989.98115	4	36484

990.07793	1	36485

990.17471	15	36500

990.27149	2	36502

990.36827	4	36506

990.46506	21	36527

990.56184	1	36528

990.65862	11	36539

990.7554	1	36540

990.85218	2	36542

990.94897	11	36553

991.04575	3	36556

991.14253	18	36574

991.23931	1	36575

991.33609	1	36576

991.43287	26	36602

991.52966	3	36605

991.62644	10	36615

991.72322	6	36621

991.82	10	36631

991.91678	1	36632

992.01356	32	36664

992.11035	11	36675

992.20713	1	36676

992.30391	30	36706

992.40069	1	36707

992.49747	3	36710

992.59426	6	36716

992.69104	33	36749

992.78782	1	36750

992.8846	1	36751

992.98138	123	36874

993.07816	1	36875

993.17495	14	36889

993.27173	2	36891

993.36851	12	36903

993.46529	1	36904

993.56207	18	36922

993.65886	1	36923

993.75564	1	36924

993.85242	1	36925

993.9492	20	36945

994.04598	1	36946

994.14276	1	36947

994.23955	17	36964

994.33633	1	36965

994.43311	2	36967

994.52989	3	36970

994.62667	1	36971

994.72346	2	36973

994.82024	23	36996

994.91702	1	36997

995.0138	19	37016

995.11058	1	37017

995.20736	2	37019

995.30415	12	37031

995.40093	1	37032

995.49771	4	37036

995.59449	10	37046

995.69127	23	37069

995.78806	1	37070

995.88484	16	37086

995.98162	2	37088

996.0784	24	37112

996.17518	2	37114

996.27196	21	37135

996.36875	1	37136

996.46553	14	37150

996.56231	7	37157

996.65909	2	37159

996.75587	25	37184

996.85266	2	37186

996.94944	17	37203

997.04622	2	37205

997.143	1	37206

997.23978	26	37232

997.33656	2	37234

997.43335	12	37246

997.53013	1	37247

997.62691	8	37255

997.72369	2	37257

997.82047	72	37329

997.91726	4	37333

998.01404	1	37334

998.11082	7	37341

998.2076	7	37348

998.30438	4	37352

998.40116	9	37361

998.49795	2	37363

998.59473	3	37366

998.69151	1	37367

998.78829	4	37371

998.88507	18	37389

998.98186	14	37403

999.07864	5	37408

999.17542	7	37415

999.2722	5	37420

999.36898	14	37434

999.46576	12	37446

999.56255	1	37447

999.65933	1	37448

999.75611	12	37460

999.85289	1	37461

999.94967	1	37462

1000.04646	14	37476

1000.14324	1	37477

1000.24002	1	37478

1000.3368	1	37479

1000.43358	32	37511

1000.53036	1	37512

1000.62715	20	37532

1000.72393	1	37533

1000.82071	7	37540

1000.91749	5	37545

1001.01427	1	37546

1001.11106	2	37548

1001.20784	1	37549

1001.30462	10	37559

1001.4014	19	37578

1001.49818	1	37579

1001.59496	17	37596

1001.69175	1	37597

1001.78853	14	37611

1001.88531	1	37612

1001.98209	1	37613

1002.07887	2	37615

1002.17566	8	37623

1002.27244	5	37628

1002.36922	1	37629

1002.466	2	37631

1002.56278	2	37633

1002.65956	19	37652

1002.75635	5	37657

1002.85313	2	37659

1002.94991	1	37660

1003.04669	1	37661

1003.14347	2	37663

1003.24026	6	37669

1003.33704	2	37671

1003.43382	1	37672

1003.5306	1	37673

1003.62738	2	37675

1003.72416	7	37682

1003.82095	2	37684

1003.91773	5	37689

1004.01451	1	37690

1004.11129	1	37691

1004.20807	33	37724

1004.30486	8	37732

1004.40164	2	37734

1004.49842	20	37754

1004.5952	4	37758

1004.69198	1	37759

1004.78876	27	37786

1004.88555	1	37787

1004.98233	1	37788

1005.07911	25	37813

1005.17589	58	37871

1005.27267	1	37872

1005.36946	2	37874

1005.46624	6	37880

1005.56302	3	37883

1005.6598	18	37901

1005.75658	1	37902

1005.85336	22	37924

1005.95015	3	37927

1006.04693	1	37928

1006.14371	2	37930

1006.24049	4	37934

1006.33727	3	37937

1006.43405	18	37955

1006.53084	1	37956

1006.62762	1	37957

1006.7244	5	37962

1006.82118	5	37967

1006.91796	2	37969

1007.01475	7	37976

1007.11153	3	37979

1007.20831	2	37981

1007.30509	2	37983

1007.40187	20	38003

1007.49866	1	38004

1007.59544	5	38009

1007.69222	15	38024

1007.789	2	38026

1007.88578	1	38027

1007.98256	1	38028

1008.07935	31	38059

1008.17613	1	38060

1008.27291	5	38065

1008.36969	4	38069

1008.46647	1	38070

1008.56326	4	38074

1008.66004	12	38086

1008.75682	1	38087

1008.8536	27	38114

1008.95038	1	38115

1009.04716	7	38122

1009.14395	1	38123

1009.24073	1	38124

1009.33751	6	38130

1009.43429	9	38139

1009.53107	2	38141

1009.62786	2	38143

1009.72464	4	38147

1009.82142	11	38158

1009.9182	3	38161

1010.01498	2	38163

1010.11176	3	38166

1010.20855	7	38173

1010.30533	1	38174

1010.40211	24	38198

1010.49889	1	38199

1010.59567	2	38201

1010.69246	9	38210

1010.78924	1	38211

1010.88602	2	38213

1010.9828	16	38229

1011.07958	6	38235

1011.17636	1	38236

1011.27315	17	38253

1011.36993	5	38258

1011.46671	16	38274

1011.56349	2	38276

1011.66027	1	38277

1011.75705	37	38314

1011.85384	1	38315

1011.95062	1	38316

1012.0474	1	38317

1012.14418	20	38337

1012.24096	1	38338

1012.33775	11	38349

1012.43453	2	38351

1012.53131	23	38374

1012.62809	1	38375

1012.72487	1	38376

1012.82165	2	38378

1012.91844	26	38404

1013.01522	2	38406

1013.112	1	38407

1013.20878	1	38408

1013.30556	1	38409

1013.40235	6	38415

1013.49913	6	38421

1013.59591	2	38423

1013.69269	33	38456

1013.78947	2	38458

1013.88625	17	38475

1013.98304	1	38476

1014.07982	12	38488

1014.1766	4	38492

1014.27338	1	38493

1014.37016	20	38513

1014.46695	2	38515

1014.56373	1	38516

1014.66051	12	38528

1014.75729	3	38531

1014.85407	2	38533

1014.95085	13	38546

1015.04764	1	38547

1015.14442	2	38549

1015.2412	6	38555

1015.33798	7	38562

1015.43476	2	38564

1015.53155	3	38567

1015.62833	1	38568

1015.72511	16	38584

1015.82189	1	38585

1015.91867	5	38590

1016.01545	2	38592

1016.11224	1	38593

1016.20902	13	38606

1016.3058	1	38607

1016.40258	1	38608

1016.49936	1	38609

1016.59615	13	38622

1016.69293	1	38623

1016.78971	18	38641

1016.88649	1	38642

1016.98327	24	38666

1017.08005	2	38668

1017.17684	1	38669

1017.27362	2	38671

1017.3704	23	38694

1017.46718	5	38699

1017.56396	13	38712

1017.66075	19	38731

1017.75753	22	38753

1017.85431	5	38758

1017.95109	29	38787

1018.04787	1	38788

1018.14465	1	38789

1018.24144	1	38790

1018.33822	1	38791

1018.435	12	38803

1018.53178	5	38808

1018.62856	9	38817

1018.72534	16	38833

1018.82213	2	38835

1018.91891	15	38850

1019.01569	3	38853

1019.11247	9	38862

1019.20925	2	38864

1019.30604	2	38866

1019.40282	2	38868

1019.4996	1	38869

1019.59638	3	38872

1019.69316	4	38876

1019.78995	1	38877

1019.88673	14	38891

1019.98351	2	38893

1020.08029	16	38909

1020.17707	2	38911

1020.27385	8	38919

1020.37064	18	38937

1020.46742	2	38939

1020.5642	1	38940

1020.66098	1	38941

1020.75776	40	38981

1020.85455	1	38982

1020.95133	16	38998

1021.04811	1	38999

1021.14489	1	39000

1021.24167	1	39001

1021.33845	5	39006

1021.43524	4	39010

1021.53202	1	39011

1021.6288	4	39015

1021.72558	2	39017

1021.82236	21	39038

1021.91915	1	39039

1022.01593	2	39041

1022.11271	11	39052

1022.20949	9	39061

1022.30627	16	39077

1022.40305	1	39078

1022.49984	3	39081

1022.59662	6	39087

1022.6934	2	39089

1022.79018	1	39090

1022.88696	17	39107

1022.98375	1	39108

1023.08053	14	39122

1023.17731	2	39124

1023.27409	1	39125

1023.37087	9	39134

1023.46765	2	39136

1023.56444	1	39137

1023.66122	12	39149

1023.758	8	39157

1023.85478	18	39175

1023.95156	1	39176

1024.04834	15	39191

1024.14513	3	39194

1024.24191	1	39195

1024.33869	2	39197

1024.43547	2	39199

1024.53225	8	39207

1024.62903	1	39208

1024.72582	2	39210

1024.8226	23	39233

1024.91938	2	39235

1025.01616	1	39236

1025.11294	11	39247

1025.20973	10	39257

1025.30651	6	39263

1025.40329	1	39264

1025.50007	8	39272

1025.59685	2	39274

1025.69364	9	39283

1025.79042	13	39296

1025.8872	2	39298

1025.98398	2	39300

1026.08076	2	39302

1026.17754	19	39321

1026.27433	2	39323

1026.37111	9	39332

1026.46789	3	39335

1026.56467	1	39336

1026.66145	9	39345

1026.75824	7	39352

1026.85502	2	39354

1026.9518	5	39359

1027.04858	20	39379

1027.14536	1	39380

1027.24214	16	39396

1027.33893	12	39408

1027.43571	4	39412

1027.53249	7	39419

1027.62927	10	39429

1027.72605	1	39430

1027.82283	4	39434

1027.91962	6	39440

1028.0164	1	39441

1028.11318	7	39448

1028.20996	6	39454

1028.30674	7	39461

1028.40353	2	39463

1028.50031	23	39486

1028.59709	2	39488

1028.69387	2	39490

1028.79065	1	39491

1028.88744	1	39492

1028.98422	21	39513

1029.081	3	39516

1029.17778	1	39517

1029.27456	1	39518

1029.37134	13	39531

1029.46813	1	39532

1029.56491	1	39533

1029.66169	12	39545

1029.75847	1	39546

1029.85525	2	39548

1029.95204	12	39560

1030.04882	2	39562

1030.1456	20	39582

1030.24238	2	39584

1030.33916	1	39585

1030.43594	8	39593

1030.53273	1	39594

1030.62951	1	39595

1030.72629	1	39596

1030.82307	5	39601

1030.91985	2	39603

1031.01664	3	39606

1031.11342	1	39607

1031.2102	15	39622

1031.30698	3	39625

1031.40376	2	39627

1031.50054	11	39638

1031.59733	27	39665

1031.69411	24	39689

1031.79089	2	39691

1031.88767	27	39718

1031.98445	8	39726

1032.08124	18	39744

1032.17802	16	39760

1032.2748	1	39761

1032.37158	2	39763

1032.46836	2	39765

1032.56514	5	39770

1032.66193	3	39773

1032.75871	1	39774

1032.85549	10	39784

1032.95227	2	39786

1033.04905	2	39788

1033.14584	1	39789

1033.24262	22	39811

1033.3394	2	39813

1033.43618	1	39814

1033.53296	4	39818

1033.62974	2	39820

1033.72653	2	39822

1033.82331	3	39825

1033.92009	1	39826

1034.01687	18	39844

1034.11365	3	39847

1034.21044	1	39848

1034.30722	5	39853

1034.404	2	39855

1034.50078	22	39877

1034.59756	1	39878

1034.69434	1	39879

1034.79113	1	39880

1034.88791	5	39885

1034.98469	6	39891

1035.08147	6	39897

1035.17825	17	39914

1035.27503	2	39916

1035.37182	2	39918

1035.4686	12	39930

1035.56538	2	39932

1035.66216	1	39933

1035.75894	23	39956

1035.85572	5	39961

1035.95251	11	39972

1036.04929	1	39973

1036.14607	6	39979

1036.24285	4	39983

1036.33963	10	39993

1036.43642	1	39994

1036.5332	31	40025

1036.62998	15	40040

1036.72676	1	40041

1036.82354	1	40042

1036.92033	22	40064

1037.01711	1	40065

1037.11389	15	40080

1037.21067	1	40081

1037.30745	11	40092

1037.40423	20	40112

1037.50102	3	40115

1037.5978	1	40116

1037.69458	1	40117

1037.79136	3	40120

1037.88814	9	40129

1037.98493	21	40150

1038.08171	1	40151

1038.17849	19	40170

1038.27527	2	40172

1038.37205	16	40188

1038.46883	2	40190

1038.56562	7	40197

1038.6624	4	40201

1038.75918	1	40202

1038.85596	12	40214

1038.95274	1	40215

1039.04952	14	40229

1039.14631	1	40230

1039.24309	1	40231

1039.33987	1	40232

1039.43665	1	40233

1039.53343	14	40247

1039.63022	1	40248

1039.727	1	40249

1039.82378	15	40264

1039.92056	5	40269

1040.01734	2	40271

1040.11413	11	40282

1040.21091	13	40295

1040.30769	8	40303

1040.40447	29	40332

1040.50125	1	40333

1040.59803	1	40334

1040.69482	2	40336

1040.7916	14	40350

1040.88838	3	40353

1040.98516	20	40373

1041.08194	1	40374

1041.17873	19	40393

1041.27551	3	40396

1041.37229	2	40398

1041.46907	1	40399

1041.56585	3	40402

1041.66263	13	40415

1041.75942	1	40416

1041.8562	15	40431

1041.95298	1	40432

1042.04976	31	40463

1042.14654	1	40464

1042.24332	20	40484

1042.34011	26	40510

1042.43689	1	40511

1042.53367	1	40512

1042.63045	1	40513

1042.72723	11	40524

1042.82402	32	40556

1042.9208	2	40558

1043.01758	18	40576

1043.11436	23	40599

1043.21114	4	40603

1043.30793	8	40611

1043.40471	2	40613

1043.50149	15	40628

1043.59827	1	40629

1043.69505	1	40630

1043.79183	2	40632

1043.88862	20	40652

1043.9854	1	40653

1044.08218	33	40686

1044.17896	1	40687

1044.27574	1	40688

1044.37253	2	40690

1044.46931	4	40694

1044.56609	4	40698

1044.66287	1	40699

1044.75965	3	40702

1044.85643	26	40728

1044.95322	4	40732

1045.05	1	40733

1045.14678	13	40746

1045.24356	1	40747

1045.34034	21	40768

1045.43712	1	40769

1045.53391	17	40786

1045.63069	2	40788

1045.72747	1	40789

1045.82425	15	40804

1045.92103	23	40827

1046.01782	1	40828

1046.1146	2	40830

1046.21138	10	40840

1046.30816	34	40874

1046.40494	1	40875

1046.50173	12	40887

1046.59851	6	40893

1046.69529	57	40950

1046.79207	12	40962

1046.88885	12	40974

1046.98563	1	40975

1047.08242	4	40979

1047.1792	6	40985

1047.27598	1	40986

1047.37276	1	40987

1047.46954	11	40998

1047.56633	16	41014

1047.66311	1	41015

1047.75989	24	41039

1047.85667	2	41041

1047.95345	1	41042

1048.05023	29	41071

1048.14702	1	41072

1048.2438	12	41084

1048.34058	1	41085

1048.43736	3	41088

1048.53414	2	41090

1048.63092	1	41091

1048.72771	16	41107

1048.82449	3	41110

1048.92127	2	41112

1049.01805	21	41133

1049.11483	5	41138

1049.21161	1	41139

1049.3084	22	41161

1049.40518	1	41162

1049.50196	23	41185

1049.59874	1	41186

1049.69552	21	41207

1049.79231	1	41208

1049.88909	1	41209

1049.98587	23	41232

1050.08265	2	41234

1050.17943	32	41266

1050.27622	2	41268

1050.373	1	41269

1050.46978	13	41282

1050.56656	1	41283

1050.66334	1	41284

1050.76012	21	41305

1050.85691	1	41306

1050.95369	10	41316

1051.05047	8	41324

1051.14725	1	41325

1051.24403	6	41331

1051.34082	2	41333

1051.4376	1	41334

1051.53438	1	41335

1051.63116	20	41355

1051.72794	18	41373

1051.82472	2	41375

1051.92151	2	41377

1052.01829	6	41383

1052.11507	5	41388

1052.21185	1	41389

1052.30863	5	41394

1052.40541	6	41400

1052.5022	1	41401

1052.59898	2	41403

1052.69576	6	41409

1052.79254	3	41412

1052.88932	2	41414

1052.98611	26	41440

1053.08289	19	41459

1053.17967	1	41460

1053.27645	1	41461

1053.37323	3	41464

1053.47002	12	41476

1053.5668	1	41477

1053.66358	1	41478

1053.76036	9	41487

1053.85714	38	41525

1053.95392	3	41528

1054.05071	26	41554

1054.14749	3	41557

1054.24427	22	41579

1054.34105	1	41580

1054.43783	2	41582

1054.53462	3	41585

1054.6314	21	41606

1054.72818	2	41608

1054.82496	16	41624

1054.92174	2	41626

1055.01852	1	41627

1055.11531	3	41630

1055.21209	5	41635

1055.30887	21	41656

1055.40565	3	41659

1055.50243	4	41663

1055.59922	4	41667

1055.696	1	41668

1055.79278	16	41684

1055.88956	1	41685

1055.98634	1	41686

1056.08312	1	41687

1056.17991	38	41725

1056.27669	1	41726

1056.37347	1	41727

1056.47025	3	41730

1056.56703	5	41735

1056.66382	1	41736

1056.7606	1	41737

1056.85738	1	41738

1056.95416	20	41758

1057.05094	4	41762

1057.14772	15	41777

1057.24451	1	41778

1057.34129	2	41780

1057.43807	16	41796

1057.53485	1	41797

1057.63163	1	41798

1057.72842	1	41799

1057.8252	8	41807

1057.92198	2	41809

1058.01876	2	41811

1058.11554	1	41812

1058.21232	12	41824

1058.30911	2	41826

1058.40589	10	41836

1058.50267	6	41842

1058.59945	1	41843

1058.69623	2	41845

1058.79302	1	41846

1058.8898	8	41854

1058.98658	2	41856

1059.08336	1	41857

1059.18014	10	41867

1059.27692	3	41870

1059.37371	3	41873

1059.47049	12	41885

1059.56727	10	41895

1059.66405	20	41915

1059.76083	1	41916

1059.85761	1	41917

1059.9544	18	41935

1060.05118	2	41937

1060.14796	1	41938

1060.24474	9	41947

1060.34152	5	41952

1060.4383	1	41953

1060.53509	3	41956

1060.63187	2	41958

1060.72865	2	41960

1060.82543	8	41968

1060.92222	3	41971

1061.019	6	41977

1061.11578	1	41978

1061.21256	2	41980

1061.30934	1	41981

1061.40612	28	42009

1061.50291	1	42010

1061.59969	1	42011

1061.69647	14	42025

1061.79325	1	42026

1061.89003	1	42027

1061.98681	16	42043

1062.0836	1	42044

1062.18038	3	42047

1062.27716	4	42051

1062.37394	2	42053

1062.47072	1	42054

1062.56751	12	42066

1062.66429	9	42075

1062.76107	26	42101

1062.85785	1	42102

1062.95463	2	42104

1063.05141	9	42113

1063.1482	21	42134

1063.24498	5	42139

1063.34176	1	42140

1063.43854	31	42171

1063.53532	3	42174

1063.6321	2	42176

1063.72889	2	42178

1063.82567	1	42179

1063.92245	1	42180

1064.01923	12	42192

1064.11601	7	42199

1064.2128	33	42232

1064.30958	1	42233

1064.40636	1	42234

1064.50314	22	42256

1064.59992	1	42257

1064.69671	1	42258

1064.79349	1	42259

1064.89027	5	42264

1064.98705	3	42267

1065.08383	24	42291

1065.18061	3	42294

1065.2774	2	42296

1065.37418	27	42323

1065.47096	32	42355

1065.56774	1	42356

1065.66452	2	42358

1065.76131	10	42368

1065.85809	2	42370

1065.95487	1	42371

1066.05165	21	42392

1066.14843	5	42397

1066.24521	1	42398

1066.342	16	42414

1066.43878	2	42416

1066.53556	1	42417

1066.63234	10	42427

1066.72912	4	42431

1066.8259	2	42433

1066.92269	1	42434

1067.01947	1	42435

1067.11625	24	42459

1067.21303	1	42460

1067.30981	26	42486

1067.4066	2	42488

1067.50338	1	42489

1067.60016	26	42515

1067.69694	1	42516

1067.79372	1	42517

1067.89051	7	42524

1067.98729	3	42527

1068.08407	1	42528

1068.18085	2	42530

1068.27763	17	42547

1068.37441	2	42549

1068.4712	2	42551

1068.56798	2	42553

1068.66476	9	42562

1068.76154	7	42569

1068.85832	3	42572

1068.95511	25	42597

1069.05189	1	42598

1069.14867	2	42600

1069.24545	14	42614

1069.34223	2	42616

1069.43901	8	42624

1069.5358	1	42625

1069.63258	1	42626

1069.72936	38	42664

1069.82614	21	42685

1069.92292	1	42686

1070.0197	1	42687

1070.11649	1	42688

1070.21327	16	42704

1070.31005	10	42714

1070.40683	6	42720

1070.50361	4	42724

1070.6004	3	42727

1070.69718	1	42728

1070.79396	1	42729

1070.89074	28	42757

1070.98752	3	42760

1071.08431	12	42772

1071.18109	8	42780

1071.27787	1	42781

1071.37465	1	42782

1071.47143	13	42795

1071.56821	5	42800

1071.665	3	42803

1071.76178	12	42815

1071.85856	5	42820

1071.95534	1	42821

1072.05212	27	42848

1072.14891	13	42861

1072.24569	2	42863

1072.34247	2	42865

1072.43925	21	42886

1072.53603	2	42888

1072.63281	19	42907

1072.7296	1	42908

1072.82638	1	42909

1072.92316	2	42911

1073.01994	20	42931

1073.11672	1	42932

1073.2135	1	42933

1073.31029	1	42934

1073.40707	24	42958

1073.50385	2	42960

1073.60063	1	42961

1073.69741	16	42977

1073.79419	4	42981

1073.89098	1	42982

1073.98776	35	43017

1074.08454	23	43040

1074.18132	1	43041

1074.2781	13	43054

1074.37489	2	43056

1074.47167	24	43080

1074.56845	15	43095

1074.66523	2	43097

1074.76201	1	43098

1074.8588	10	43108

1074.95558	7	43115

1075.05236	1	43116

1075.14914	10	43126

1075.24592	1	43127

1075.3427	1	43128

1075.43949	8	43136

1075.53627	1	43137

1075.63305	1	43138

1075.72983	18	43156

1075.82661	4	43160

1075.9234	19	43179

1076.02018	2	43181

1076.11696	1	43182

1076.21374	1	43183

1076.31052	7	43190

1076.4073	11	43201

1076.50409	1	43202

1076.60087	11	43213

1076.69765	2	43215

1076.79443	10	43225

1076.89121	10	43235

1076.988	2	43237

1077.08478	1	43238

1077.18156	4	43242

1077.27834	1	43243

1077.37512	2	43245

1077.4719	3	43248

1077.56869	1	43249

1077.66547	5	43254

1077.76225	4	43258

1077.85903	1	43259

1077.95581	16	43275

1078.0526	1	43276

1078.14938	11	43287

1078.24616	2	43289

1078.34294	16	43305

1078.43972	2	43307

1078.5365	1	43308

1078.63329	13	43321

1078.73007	1	43322

1078.82685	1	43323

1078.92363	1	43324

1079.02041	14	43338

1079.1172	1	43339

1079.21398	2	43341

1079.31076	24	43365

1079.40754	2	43367

1079.50432	5	43372

1079.6011	17	43389

1079.69789	4	43393

1079.79467	2	43395

1079.89145	1	43396

1079.98823	13	43409

1080.08501	11	43420

1080.1818	2	43422

1080.27858	1	43423

1080.37536	21	43444

1080.47214	1	43445

1080.56892	1	43446

1080.6657	1	43447

1080.76249	2	43449

1080.85927	1	43450

1080.95605	1	43451

1081.05283	6	43457

1081.14961	9	43466

1081.24639	3	43469

1081.34318	1	43470

1081.43996	24	43494

1081.53674	1	43495

1081.63352	37	43532

1081.7303	6	43538

1081.82709	4	43542

1081.92387	3	43545

1082.02065	27	43572

1082.11743	1	43573

1082.21421	1	43574

1082.311	18	43592

1082.40778	1	43593

1082.50456	5	43598

1082.60134	7	43605

1082.69812	2	43607

1082.7949	4	43611

1082.89169	11	43622

1082.98847	6	43628

1083.08525	2	43630

1083.18203	1	43631

1083.27881	33	43664

1083.3756	18	43682

1083.47238	3	43685

1083.56916	3	43688

1083.66594	2	43690

1083.76272	4	43694

1083.8595	7	43701

1083.95629	1	43702

1084.05307	11	43713

1084.14985	2	43715

1084.24663	1	43716

1084.34341	2	43718

1084.44019	4	43722

1084.53698	2	43724

1084.63376	1	43725

1084.73054	2	43727

1084.82732	30	43757

1084.9241	27	43784

1085.02089	1	43785

1085.11767	36	43821

1085.21445	2	43823

1085.31123	9	43832

1085.40801	6	43838

1085.5048	1	43839

1085.60158	1	43840

1085.69836	15	43855

1085.79514	4	43859

1085.89192	3	43862

1085.9887	24	43886

1086.08549	4	43890

1086.18227	12	43902

1086.27905	1	43903

1086.37583	23	43926

1086.47261	2	43928

1086.56939	38	43966

1086.66618	2	43968

1086.76296	15	43983

1086.85974	1	43984

1086.95652	3	43987

1087.0533	7	43994

1087.15009	2	43996

1087.24687	21	44017

1087.34365	2	44019

1087.44043	33	44052

1087.53721	2	44054

1087.63399	1	44055

1087.73078	14	44069

1087.82756	1	44070

1087.92434	14	44084

1088.02112	4	44088

1088.1179	2	44090

1088.21468	23	44113

1088.31147	2	44115

1088.40825	1	44116

1088.50503	4	44120

1088.60181	6	44126

1088.69859	1	44127

1088.79538	1	44128

1088.89216	1	44129

1088.98894	8	44137

1089.08572	11	44148

1089.1825	1	44149

1089.27929	1	44150

1089.37607	33	44183

1089.47285	4	44187

1089.56963	19	44206

1089.66641	1	44207

1089.76319	4	44211

1089.85998	2	44213

1089.95676	9	44222

1090.05354	12	44234

1090.15032	1	44235

1090.2471	24	44259

1090.34389	2	44261

1090.44067	26	44287

1090.53745	1	44288

1090.63423	17	44305

1090.73101	1	44306

1090.82779	28	44334

1090.92458	1	44335

1091.02136	1	44336

1091.11814	25	44361

1091.21492	3	44364

1091.3117	1	44365

1091.40848	33	44398

1091.50527	2	44400

1091.60205	16	44416

1091.69883	4	44420

1091.79561	15	44435

1091.89239	17	44452

1091.98918	1	44453

1092.08596	1	44454

1092.18274	32	44486

1092.27952	1	44487

1092.3763	4	44491

1092.47309	13	44504

1092.56987	6	44510

1092.66665	2	44512

1092.76343	1	44513

1092.86021	1	44514

1092.95699	3	44517

1093.05378	2	44519

1093.15056	1	44520

1093.24734	4	44524

1093.34412	1	44525

1093.4409	10	44535

1093.53769	4	44539

1093.63447	1	44540

1093.73125	2	44542

1093.82803	39	44581

1093.92481	1	44582

1094.02159	1	44583

1094.11838	5	44588

1094.21516	7	44595

1094.31194	1	44596

1094.40872	1	44597

1094.5055	9	44606

1094.60228	4	44610

1094.69907	1	44611

1094.79585	2	44613

1094.89263	5	44618

1094.98941	4	44622

1095.08619	1	44623

1095.18298	1	44624

1095.27976	13	44637

1095.37654	3	44640

1095.47332	1	44641

1095.5701	10	44651

1095.66689	5	44656

1095.76367	1	44657

1095.86045	2	44659

1095.95723	14	44673

1096.05401	4	44677

1096.15079	22	44699

1096.24758	1	44700

1096.34436	31	44731

1096.44114	3	44734

1096.53792	12	44746

1096.6347	8	44754

1096.73149	1	44755

1096.82827	2	44757

1096.92505	9	44766

1097.02183	2	44768

1097.11861	10	44778

1097.21539	1	44779

1097.31218	1	44780

1097.40896	30	44810

1097.50574	2	44812

1097.60252	1	44813

1097.6993	20	44833

1097.79608	3	44836

1097.89287	1	44837

1097.98965	21	44858

1098.08643	1	44859

1098.18321	4	44863

1098.27999	4	44867

1098.37678	1	44868

1098.47356	1	44869

1098.57034	36	44905

1098.66712	4	44909

1098.7639	15	44924

1098.86069	1	44925

1098.95747	1	44926

1099.05425	1	44927

1099.15103	18	44945

1099.24781	3	44948

1099.34459	5	44953

1099.44138	2	44955

1099.53816	14	44969

1099.63494	2	44971

1099.73172	10	44981

1099.8285	3	44984

1099.92528	1	44985

1100.02207	10	44995

1100.11885	1	44996

1100.21563	22	45018

1100.31241	2	45020

1100.40919	1	45021

1100.50598	4	45025

1100.60276	5	45030

1100.69954	21	45051

1100.79632	1	45052

1100.8931	1	45053

1100.98988	20	45073

1101.08667	5	45078

1101.18345	6	45084

1101.28023	12	45096

1101.37701	1	45097

1101.47379	1	45098

1101.57058	25	45123

1101.66736	13	45136

1101.76414	27	45163

1101.86092	1	45164

1101.9577	20	45184

1102.05448	11	45195

1102.15127	1	45196

1102.24805	1	45197

1102.34483	3	45200

1102.44161	17	45217

1102.53839	1	45218

1102.63517	15	45233

1102.73196	1	45234

1102.82874	3	45237

1102.92552	11	45248

1103.0223	1	45249

1103.11908	3	45252

1103.21587	18	45270

1103.31265	11	45281

1103.40943	7	45288

1103.50621	1	45289

1103.60299	18	45307

1103.69978	1	45308

1103.79656	1	45309

1103.89334	1	45310

1103.99012	14	45324

1104.0869	2	45326

1104.18368	1	45327

1104.28047	24	45351

1104.37725	7	45358

1104.47403	2	45360

1104.57081	21	45381

1104.66759	3	45384

1104.76438	1	45385

1104.86116	3	45388

1104.95794	16	45404

1105.05472	1	45405

1105.1515	21	45426

1105.24828	1	45427

1105.34507	2	45429

1105.44185	11	45440

1105.53863	4	45444

1105.63541	18	45462

1105.73219	1	45463

1105.82897	18	45481

1105.92576	5	45486

1106.02254	1	45487

1106.11932	2	45489

1106.2161	1	45490

1106.31288	2	45492

1106.40967	1	45493

1106.50645	5	45498

1106.60323	6	45504

1106.70001	2	45506

1106.79679	1	45507

1106.89358	27	45534

1106.99036	1	45535

1107.08714	17	45552

1107.18392	1	45553

1107.2807	3	45556

1107.37748	1	45557

1107.47427	28	45585

1107.57105	7	45592

1107.66783	4	45596

1107.76461	10	45606

1107.86139	1	45607

1107.95818	1	45608

1108.05496	10	45618

1108.15174	3	45621

1108.24852	1	45622

1108.3453	3	45625

1108.44208	1	45626

1108.53887	7	45633

1108.63565	13	45646

1108.73243	2	45648

1108.82921	1	45649

1108.92599	22	45671

1109.02277	4	45675

1109.11956	3	45678

1109.21634	1	45679

1109.31312	20	45699

1109.4099	1	45700

1109.50668	2	45702

1109.60347	4	45706

1109.70025	16	45722

1109.79703	23	45745

1109.89381	2	45747

1109.99059	20	45767

1110.08738	2	45769

1110.18416	10	45779

1110.28094	24	45803

1110.37772	1	45804

1110.4745	1	45805

1110.57128	25	45830

1110.66807	3	45833

1110.76485	10	45843

1110.86163	3	45846

1110.95841	4	45850

1111.05519	2	45852

1111.15198	1	45853

1111.24876	1	45854

1111.34554	2	45856

1111.44232	7	45863

1111.5391	14	45877

1111.63588	2	45879

1111.73267	1	45880

1111.82945	5	45885

1111.92623	2	45887

1112.02301	1	45888

1112.11979	11	45899

1112.21657	1	45900

1112.31336	2	45902

1112.41014	13	45915

1112.50692	1	45916

1112.6037	1	45917

1112.70048	1	45918

1112.79726	2	45920

1112.89405	3	45923

1112.99083	7	45930

1113.08761	40	45970

1113.18439	1	45971

1113.28117	1	45972

1113.37796	13	45985

1113.47474	1	45986

1113.57152	1	45987

1113.6683	8	45995

1113.76508	2	45997

1113.86187	14	46011

1113.95865	4	46015

1114.05543	2	46017

1114.15221	1	46018

1114.24899	1	46019

1114.34577	1	46020

1114.44256	24	46044

1114.53934	4	46048

1114.63612	10	46058

1114.7329	16	46074

1114.82968	1	46075

1114.92647	1	46076

1115.02325	14	46090

1115.12003	2	46092

1115.21681	5	46097

1115.31359	1	46098

1115.41037	1	46099

1115.50716	13	46112

1115.60394	2	46114

1115.70072	1	46115

1115.7975	4	46119

1115.89428	6	46125

1115.99106	1	46126

1116.08785	14	46140

1116.18463	3	46143

1116.28141	3	46146

1116.37819	1	46147

1116.47497	22	46169

1116.57176	6	46175

1116.66854	1	46176

1116.76532	19	46195

1116.8621	1	46196

1116.95888	1	46197

1117.05567	1	46198

1117.15245	1	46199

1117.24923	1	46200

1117.34601	13	46213

1117.44279	5	46218

1117.53957	5	46223

1117.63636	3	46226

1117.73314	11	46237

1117.82992	2	46239

1117.9267	1	46240

1118.02348	1	46241

1118.12027	14	46255

1118.21705	1	46256

1118.31383	5	46261

1118.41061	5	46266

1118.50739	2	46268

1118.60417	1	46269

1118.70096	3	46272

1118.79774	3	46275

1118.89452	1	46276

1118.9913	1	46277

1119.08808	34	46311

1119.18486	1	46312

1119.28165	10	46322

1119.37843	1	46323

1119.47521	2	46325

1119.57199	27	46352

1119.66877	1	46353

1119.76556	1	46354

1119.86234	5	46359

1119.95912	2	46361

1120.0559	3	46364

1120.15268	7	46371

1120.24947	1	46372

1120.34625	26	46398

1120.44303	1	46399

1120.53981	3	46402

1120.63659	6	46408

1120.73337	1	46409

1120.83016	1	46410

1120.92694	1	46411

1121.02372	6	46417

1121.1205	2	46419

1121.21728	2	46421

1121.31407	3	46424

1121.41085	3	46427

1121.50763	1	46428

1121.60441	1	46429

1121.70119	6	46435

1121.79797	2	46437

1121.89476	30	46467

1121.99154	18	46485

1122.08832	20	46505

1122.1851	19	46524

1122.28188	1	46525

1122.37867	28	46553

1122.47545	1	46554

1122.57223	12	46566

1122.66901	3	46569

1122.76579	1	46570

1122.86257	48	46618

1122.95936	19	46637

1123.05614	21	46658

1123.15292	3	46661

1123.2497	6	46667

1123.34648	10	46677

1123.44327	1	46678

1123.54005	22	46700

1123.63683	1	46701

1123.73361	25	46726

1123.83039	1	46727

1123.92717	6	46733

1124.02395	3	46736

1124.12074	2	46738

1124.21752	15	46753

1124.3143	15	46768

1124.41108	2	46770

1124.50786	17	46787

1124.60465	16	46803

1124.70143	1	46804

1124.79821	1	46805

1124.89499	3	46808

1124.99177	7	46815

1125.08856	2	46817

1125.18534	2	46819

1125.28212	27	46846

1125.3789	1	46847

1125.47568	2	46849

1125.57246	13	46862

1125.66925	21	46883

1125.76603	1	46884

1125.86281	12	46896

1125.95959	1	46897

1126.05637	14	46911

1126.15316	1	46912

1126.24994	3	46915

1126.34672	2	46917

1126.4435	3	46920

1126.54028	2	46922

1126.63706	26	46948

1126.73385	1	46949

1126.83063	17	46966

1126.92741	3	46969

1127.02419	13	46982

1127.12097	4	46986

1127.21775	26	47012

1127.31454	9	47021

1127.41132	32	47053

1127.5081	1	47054

1127.60488	1	47055

1127.70166	4	47059

1127.79845	3	47062

1127.89523	19	47081

1127.99201	14	47095

1128.08879	2	47097

1128.18557	1	47098

1128.28236	1	47099

1128.37914	11	47110

1128.47592	2	47112

1128.5727	11	47123

1128.66948	2	47125

1128.76626	1	47126

1128.86305	8	47134

1128.95983	2	47136

1129.05661	1	47137

1129.15339	1	47138

1129.25017	17	47155

1129.34696	1	47156

1129.44374	18	47174

1129.54052	1	47175

1129.6373	2	47177

1129.73408	22	47199

1129.83086	1	47200

1129.92765	20	47220

1130.02443	8	47228

1130.12121	3	47231

1130.21799	11	47242

1130.31477	16	47258

1130.41155	1	47259

1130.50834	1	47260

1130.60512	1	47261

1130.7019	7	47268

1130.79868	1	47269

1130.89546	2	47271

1130.99225	15	47286

1131.08903	6	47292

1131.18581	10	47302

1131.28259	3	47305

1131.37937	1	47306

1131.47616	21	47327

1131.57294	1	47328

1131.66972	15	47343

1131.7665	1	47344

1131.86328	7	47351

1131.96006	2	47353

1132.05685	27	47380

1132.15363	13	47393

1132.25041	4	47397

1132.34719	1	47398

1132.44397	1	47399

1132.54076	4	47403

1132.63754	11	47414

1132.73432	1	47415

1132.8311	11	47426

1132.92788	2	47428

1133.02466	14	47442

1133.12145	1	47443

1133.21823	2	47445

1133.31501	11	47456

1133.41179	15	47471

1133.50857	1	47472

1133.60535	1	47473

1133.70214	23	47496

1133.79892	1	47497

1133.8957	3	47500

1133.99248	12	47512

1134.08926	6	47518

1134.18605	3	47521

1134.28283	9	47530

1134.37961	1	47531

1134.47639	1	47532

1134.57317	4	47536

1134.66996	3	47539

1134.76674	1	47540

1134.86352	19	47559

1134.9603	1	47560

1135.05708	19	47579

1135.15386	1	47580

1135.25065	20	47600

1135.34743	1	47601

1135.44421	33	47634

1135.54099	1	47635

1135.63777	1	47636

1135.73456	6	47642

1135.83134	4	47646

1135.92812	1	47647

1136.0249	6	47653

1136.12168	4	47657

1136.21846	1	47658

1136.31525	6	47664

1136.41203	1	47665

1136.50881	2	47667

1136.60559	8	47675

1136.70237	1	47676

1136.79915	9	47685

1136.89594	5	47690

1136.99272	5	47695

1137.0895	1	47696

1137.18628	23	47719

1137.28306	8	47727

1137.37984	19	47746

1137.47663	1	47747

1137.57341	11	47758

1137.67019	4	47762

1137.76697	1	47763

1137.86375	13	47776

1137.96054	27	47803

1138.05732	1	47804

1138.1541	1	47805

1138.25088	1	47806

1138.34766	17	47823

1138.44445	18	47841

1138.54123	3	47844

1138.63801	4	47848

1138.73479	3	47851

1138.83157	21	47872

1138.92835	1	47873

1139.02514	2	47875

1139.12192	11	47886

1139.2187	4	47890

1139.31548	3	47893

1139.41226	7	47900

1139.50905	2	47902

1139.60583	25	47927

1139.70261	1	47928

1139.79939	33	47961

1139.89617	2	47963

1139.99295	30	47993

1140.08974	1	47994

1140.18652	12	48006

1140.2833	9	48015

1140.38008	7	48022

1140.47686	2	48024

1140.57364	16	48040

1140.67043	2	48042

1140.76721	32	48074

1140.86399	3	48077

1140.96077	1	48078

1141.05755	9	48087

1141.15434	3	48090

1141.25112	4	48094

1141.3479	1	48095

1141.44468	14	48109

1141.54146	1	48110

1141.63825	17	48127

1141.73503	2	48129

1141.83181	29	48158

1141.92859	6	48164

1142.02537	23	48187

1142.12215	9	48196

1142.21894	4	48200

1142.31572	7	48207

1142.4125	3	48210

1142.50928	7	48217

1142.60606	3	48220

1142.70285	8	48228

1142.79963	4	48232

1142.89641	24	48256

1142.99319	4	48260

1143.08997	2	48262

1143.18675	4	48266

1143.28354	1	48267

1143.38032	12	48279

1143.4771	1	48280

1143.57388	21	48301

1143.67066	1	48302

1143.76745	1	48303

1143.86423	11	48314

1143.96101	14	48328

1144.05779	8	48336

1144.15457	1	48337

1144.25135	14	48351

1144.34814	1	48352

1144.44492	1	48353

1144.5417	17	48370

1144.63848	18	48388

1144.73526	2	48390

1144.83205	1	48391

1144.92883	24	48415

1145.02561	1	48416

1145.12239	18	48434

1145.21917	9	48443

1145.31595	2	48445

1145.41274	1	48446

1145.50952	1	48447

1145.6063	1	48448

1145.70308	16	48464

1145.79986	4	48468

1145.89665	2	48470

1145.99343	6	48476

1146.09021	5	48481

1146.18699	21	48502

1146.28377	9	48511

1146.38055	13	48524

1146.47734	5	48529

1146.57412	13	48542

1146.6709	1	48543

1146.76768	14	48557

1146.86446	2	48559

1146.96125	3	48562

1147.05803	1	48563

1147.15481	2	48565

1147.25159	1	48566

1147.34837	19	48585

1147.44515	15	48600

1147.54194	16	48616

1147.63872	4	48620

1147.7355	1	48621

1147.83228	13	48634

1147.92906	3	48637

1148.02584	1	48638

1148.12263	7	48645

1148.21941	3	48648

1148.31619	1	48649

1148.41297	5	48654

1148.50975	15	48669

1148.60653	1	48670

1148.70332	17	48687

1148.8001	1	48688

1148.89688	1	48689

1148.99366	33	48722

1149.09045	2	48724

1149.18723	10	48734

1149.28401	5	48739

1149.38079	90	48829

1149.47757	15	48844

1149.57435	1	48845

1149.67114	11	48856

1149.76792	1	48857

1149.8647	2	48859

1149.96148	10	48869

1150.05826	1	48870

1150.15504	11	48881

1150.25183	18	48899

1150.34861	3	48902

1150.44539	30	48932

1150.54217	1	48933

1150.63895	6	48939

1150.73574	3	48942

1150.83252	2	48944

1150.9293	1	48945

1151.02608	2	48947

1151.12286	1	48948

1151.21964	5	48953

1151.31643	4	48957

1151.41321	1	48958

1151.50999	12	48970

1151.60677	1	48971

1151.70355	23	48994

1151.80033	88	49082

1151.89712	49082

1151.9939	2	49083

1152.09068	6	49089

1152.18746	6	49095

1152.28424	2	49097

1152.38103	6	49103

1152.47781	2	49104

1152.57459	2	49106

1152.67137	3	49109

1152.76815	2	49110

1152.86494	12	49122

1152.96172	2	49124

1153.0585	3	49127

1153.15528	2	49128

1153.25206	2	49130

1153.34884	2	49131

1153.44563	2	49133

1153.54241	2	49134

1153.63919	2	49136

1153.73597	2	49137

1153.83275	6	49143

1153.92954	3	49146

1154.02632	2	49148

1154.1231	3	49151

1154.21988	8	49158

1154.31666	2	49160

1154.41344	2	49161

1154.51023	5	49166

1154.60701	2	49167

1154.70379	2	49169

1154.80057	2	49170

1154.89735	2	49172

1154.99413	2	49173

1155.09092	2	49175

1155.1877	2	49176

1155.28448	3	49179

1155.38126	2	49181

1155.47804	3	49184

1155.57483	2	49185

1155.67161	2	49187

1155.76839	3	49190

1155.86517	2	49191

1155.96195	2	49193

1156.05874	2	49194

1156.15552	12	49206

1156.2523	8	49214

1156.34908	2	49215

1156.44586	9	49224

1156.54264	3	49227

1156.63943	2	49229

1156.73621	2	49230

1156.83299	2	49232

1156.92977	3	49235

1157.02655	5	49239

1157.12334	2	49241

1157.22012	63	49304

1157.3169	2	49305

1157.41368	3	49308

1157.51046	5	49313

1157.60724	8	49320

1157.70403	2	49322

1157.80081	2	49323

1157.89759	2	49325

1157.99437	2	49326

1158.09115	9	49335

1158.18793	2	49337

1158.28472	5	49341

1158.3815	2	49343

1158.47828	2	49344

1158.57506	3	49347

1158.67184	2	49349

1158.76863	2	49350

1158.86541	8	49358

1158.96219	8	49365

1159.05897	9	49374

1159.15575	2	49376

1159.25254	2	49377

1159.34932	2	49379

1159.4461	2	49380

1159.54288	3	49383

1159.63966	8	49391

1159.73644	2	49392

1159.83323	5	49397

1159.93001	6	49403

1160.02679	6	49409

1160.12357	2	49410

1160.22035	2	49412

1160.31714	2	49413

1160.41392	8	49421

1160.5107	5	49425

1160.60748	2	49427

1160.70426	5	49431

1160.80104	5	49436

1160.89783	218	49653

1160.99461	5	49658

1161.09139	2	49659

1161.18817	8	49667

1161.28495	2	49668

1161.38173	6	49674

1161.47852	2	49676

1161.5753	6	49682

1161.67208	3	49685

1161.76886	2	49686

1161.86564	3	49689

1161.96242	2	49691

1162.05921	2	49692

1162.15599	2	49694

1162.25277	2	49695

1162.34955	2	49697

1162.44633	2	49698

1162.54312	2	49700

1162.6399	2	49701

1162.73668	6	49707

1162.83346	5	49712

1162.93024	9	49721

1163.02703	9	49730

1163.12381	2	49731

1163.22059	3	49734

1163.31737	2	49736

1163.41415	6	49742

1163.51093	5	49746

1163.60772	2	49748

1163.7045	2	49749

1163.80128	2	49751

1163.89806	5	49755

1163.99484	3	49758

1164.09163	2	49760

1164.18841	5	49764

1164.28519	5	49769

1164.38197	252	50021

1164.47875	2	50022

1164.57553	267	50289

1164.67232	2	50291

1164.7691	3	50294

1164.86588	2	50295

1164.96266	14	50309

1165.05944	6	50315

1165.15622	2	50316

1165.25301	3	50319

1165.34979	2	50321

1165.44657	2	50322

1165.54335	2	50324

1165.64013	2	50325

1165.73692	2	50327

1165.8337	8	50334

1165.93048	2	50336

1166.02726	23	50358

1166.12404	2	50360

1166.22083	3	50363

1166.31761	5	50367

1166.41439	6	50373

1166.51117	3	50376

1166.60795	1	50377

1166.70473	5	50382

1166.80152	1	50383

1166.8983	4	50387

1166.99508	4	50391

1167.09186	2	50393

1167.18864	17	50410

1167.28543	9	50419

1167.38221	1	50420

1167.47899	1	50421

1167.57577	1	50422

1167.67255	1	50423

1167.76933	1	50424

1167.86612	5	50429

1167.9629	1	50430

1168.05968	2	50432

1168.15646	5	50437

1168.25324	7	50444

1168.35003	52	50496

1168.44681	3	50499

1168.54359	1	50500

1168.64037	58	50558

1168.73715	1	50559

1168.83393	6	50565

1168.93072	13	50578

1169.0275	1	50579

1169.12428	2	50581

1169.22106	20	50601

1169.31784	2	50603

1169.41462	15213	65816

1169.51141	10	65826

1169.60819	54	65880

1169.70497	8	65888

1169.80175	146	66034

1169.89853	2	66036

1169.99532	6	66042

1170.0921	5	66047

1170.18888	48	66095

1170.28566	33	66128

1170.38244	14	66142

1170.47923	6	66148

1170.57601	60	66208

1170.67279	6	66214

1170.76957	14	66228

1170.86635	5	66233

1170.96313	1	66234

1171.05992	18	66252

1171.1567	3	66255

1171.25348	1	66256

**（*b*）Stabilized hydraulic-0°**

1	0.04596	2.41066	2	2

2	0.06524	2.57137	2	4

3	0.08303	2.73208	4	8

4	0.10676	2.89279	1	9

5	0.1201	3.0535	1	10

6	0.13048	3.21421	1	11

7	0.14827	3.37492	1	12

8	0.16607	3.53563	2	14

9	0.17645	3.69634	1	15

10	0.19127	3.85706	1	16

11	0.21203	4.01777	1	17

12	0.22093	4.17848	2	19

13	0.23575	4.33919	1	20

14	0.25503	4.4999	1	21

15	0.26986	4.66061	1	22

16	0.27875	4.82132	1	23

17	0.29951	4.98203	2	25

18	0.31286	5.14274	2	27

19	0.32472	5.30345	1	28

20	0.34103	5.46416	12	40

21	0.35882	5.62487	2	42

22	0.37217	5.78558	1	43

23	0.38551	5.94629	1	44

24	0.40182	6.107	2	46

25	0.41961	6.26772	11	57

26	0.42999	6.42843	1	58

27	0.4463	6.58914	1	59

28	0.46261	6.74985	2	61

29	0.47447	6.91056	1	62

30	0.48634	7.07127	1	63

31	0.50709	7.23198	3	66

32	0.51896	7.39269	1	67

33	0.5323	7.5534	1	68

34	0.55009	7.71411	1	69

35	0.5664	7.87482	3	72

36	0.58271	8.03553	2	74

37	0.59161	8.19624	1	75

38	0.61237	8.35695	3	78

39	0.61978	8.51766	1	79

40	0.63609	8.67838	1	80

41	0.65389	8.83909	2	82

42	0.66871	8.9998	6	88

43	0.67909	9.16051	13	101

44	0.6954	9.32122	3	104

45	0.71171	9.48193	2	106

46	0.72357	9.64264	3	109

47	0.74137	9.80335	3	112

48	0.75619	9.96406	1	113

49	0.76954	10.12477	1	114

50	0.78437	10.28548	1	115

51	0.80216	10.44619	2	117

52	0.81847	10.6069	1	118

53	0.82588	10.76761	10	128

54	0.84368	10.92832	1	129

55	0.85999	11.08903	1	130

56	0.87333	11.24975	12	142

57	0.88816	11.41046	2	144

58	0.90743	11.57117	3	147

59	0.91929	11.73188	1	148

60	0.93116	11.89259	1	149

61	0.94895	12.0533	2	151

62	0.96229	12.21401	1	152

63	0.97564	12.37472	1	153

64	0.99343	12.53543	1	154

65	1.00974	12.69614	1	155

66	1.02012	12.85685	8	163

67	1.03643	13.01756	1	164

68	1.05571	13.17827	54	218

69	1.06757	13.33898	1	219

70	1.07943	13.49969	1	220

71	1.10019	13.66041	2	222

72	1.11353	13.82112	1	223

73	1.12243	13.98183	1	224

74	1.1417	14.14254	1	225

75	1.15801	14.30325	4	229

76	1.16839	14.46396	1	230

77	1.18322	14.62467	6	236

78	1.20398	14.78538	3	239

79	1.21732	14.94609	1	240

80	1.22474	15.1068	1	241

81	1.24401	15.26751	1	242

82	1.26181	15.42822	1	243

83	1.26922	15.58893	1	244

84	1.28701	15.74964	1	245

85	1.30629	15.91035	1	246

86	1.31815	16.07107	1	247

87	1.33001	16.23178	1	248

88	1.34929	16.39249	2	250

89	1.36411	16.5532	2	252

90	1.37746	16.71391	4	256

91	1.39525	16.87462	1	257

92	1.4086	17.03533	3	260

93	1.41749	17.19604	4	264

94	1.4338	17.35675	1	265

95	1.45456	17.51746	19	284

96	1.46791	17.67817	1	285

97	1.47977	17.83888	6	291

98	1.50053	17.99959	5	296

99	1.51239	18.1603	1	297

100	1.52277	18.32101	2	299

101	1.54056	18.48172	1	300

102	1.55687	18.64244	1	301

103	1.56577	18.80315	1	302

104	1.58356	18.96386	1	303

105	1.60432	19.12457	1	304

106	1.6147	19.28528	1	305

107	1.62656	19.44599	2	307

108	1.64732	19.6067	3	310

109	1.65918	19.76741	3	313

110	1.66956	19.92812	1	314

111	1.68883	20.08883	1	315

112	1.70663	20.24954	1	316

113	1.71552	20.41025	1	317

114	1.73035	20.57096	4	321

115	1.74963	20.73167	1	322

116	1.75852	20.89238	2	324

117	1.78225	21.0531	1	325

118	1.75259	21.21381	1	326

119	1.77187	21.37452	2	328

120	1.78076	21.53523	1	329

121	1.79559	21.69594	1	330

122	1.8119	21.85665	5	335

123	1.81931	22.01736	1	336

124	1.83414	22.17807	1	337

125	1.8549	22.33878	6	343

126	1.86824	22.49949	1	344

127	1.87862	22.6602	1	345

128	1.89938	22.82091	10	355

129	1.91273	22.98162	1	356

130	1.92607	23.14233	2	358

131	1.94238	23.30304	4	362

132	1.96017	23.46375	1	363

133	1.96907	23.62447	2	365

134	1.98241	23.78518	1	366

135	2.00317	23.94589	2	368

136	2.01652	24.1066	1	369

137	2.02541	24.26731	1	370

138	2.04469	24.42802	1	371

139	2.06248	24.58873	1	372

140	2.07138	24.74944	2	374

141	2.08917	24.91015	6	380

142	2.10548	25.07086	1	381

143	2.11734	25.23157	1	382

144	2.12772	25.39228	1	383

145	2.14996	25.55299	3	386

146	2.16034	25.7137	1	387

147	2.1722	25.87441	1	388

148	2.19445	26.03513	2	390

149	2.21076	26.19584	2	392

150	2.22262	26.35655	3	395

151	2.23596	26.51726	1	396

152	2.25524	26.67797	2	398

153	2.2671	26.83868	1	399

154	2.276	26.99939	3	402

155	2.29824	27.1601	2	404

156	2.31306	27.32081	8	412

157	2.32344	27.48152	4	416

158	2.33975	27.64223	2	418

159	2.35903	27.80294	1	419

160	2.36644	27.96365	3	422

161	2.38424	28.12436	1	423

162	2.40203	28.28507	2	425

163	2.41537	28.44579	1	426

164	2.42872	28.6065	17	443

165	2.44651	28.76721	4	447

166	2.46282	28.92792	1	448

167	2.47468	29.08863	3	451

168	2.48654	29.24934	1	452

169	2.50878	29.41005	2	454

170	2.51916	29.57076	1	455

171	2.53399	29.73147	1	456

172	2.5503	29.89218	3	459

173	2.56513	30.05289	6	465

174	2.57699	30.2136	1	466

175	2.5933	30.37431	26	492

176	2.61109	30.53502	1	493

177	2.61999	30.69573	2	495

178	2.6363	30.85644	3	498

179	2.65558	31.01716	3	501

180	2.66595	31.17787	6	507

181	2.6793	31.33858	4	511

182	2.69857	31.49929	2	513

183	2.71192	31.66	1	514

184	2.7223	31.82071	6	520

185	2.74306	31.98142	1	521

186	2.75937	32.14213	2	523

187	2.76678	32.30284	1	524

188	2.78457	32.46355	1	525

189	2.80237	32.62426	7	532

190	2.81275	32.78497	2	534

191	2.82609	32.94568	1	535

192	2.84537	33.10639	1	536

193	2.86168	33.2671	1	537

194	2.87057	33.42782	1	538

195	2.89133	33.58853	4	542

196	2.91061	33.74924	2	544

197	2.9195	33.90995	2	546

198	2.93136	34.07066	2	548

199	2.95064	34.23137	1	549

200	2.96102	34.39208	1	550

201	2.97436	34.55279	1	551

202	2.99512	34.7135	2	553

203	3.01143	34.87421	1	554

204	3.02181	35.03492	1	555

205	3.03812	35.19563	5	560

206	3.0574	35.35634	5	565

207	3.06778	35.51705	11	576

208	3.08112	35.67776	1	577

209	3.09891	35.83848	4	581

210	3.11226	35.99919	1	582

211	3.12708	36.1599	1	583

212	3.14339	36.32061	3	586

213	3.15674	36.48132	3	589

214	3.16712	36.64203	1	590

215	3.18491	36.80274	4	594

216	3.2027	36.96345	1	595

217	3.21605	37.12416	1	596

218	3.22643	37.28487	1	597

219	3.25015	37.44558	1	598

220	3.26201	37.60629	2	600

221	3.27239	37.767	6	606

222	3.29315	37.92771	3	609

223	3.3065	38.08842	1	610

224	3.31391	38.24913	1	611

225	3.33318	38.40985	1	612

226	3.35246	38.57056	1	613

227	3.36136	38.73127	1	614

228	3.37915	38.89198	2	616

229	3.39398	39.05269	1	617

230	3.41029	39.2134	27	644

231	3.42067	39.37411	1	645

232	3.43846	39.53482	1	646

233	3.45477	39.69553	1	647

234	3.46515	39.85624	1	648

235	3.48146	40.01695	1	649

236	3.50073	40.17766	1	650

237	3.5126	40.33837	2	652

238	3.52594	40.49908	1	653

239	3.54373	40.65979	2	655

240	3.55559	40.82051	1	656

241	3.56894	40.98122	7	663

242	3.58821	41.14193	1	664

243	3.60304	41.30264	2	666

244	3.61342	41.46335	1	667

245	3.62825	41.62406	1	668

246	3.64752	41.78477	3	671

247	3.66087	41.94548	1	672

248	3.67273	42.10619	2	674

249	3.69497	42.2669	4	678

250	3.71128	42.42761	3	681

251	3.71721	42.58832	1	682

252	3.73204	42.74903	5	687

253	3.75132	42.90974	2	689

254	3.76318	43.07045	1	690

255	3.77652	43.23116	1	691

256	3.79728	43.39188	2	693

257	3.81062	43.55259	2	695

258	3.821	43.7133	1	696

259	3.83731	43.87401	50	746

260	3.85659	44.03472	1	747

261	3.86549	44.19543	1	748

262	3.88031	44.35614	8	756

263	3.89959	44.51685	1	757

264	3.91145	44.67756	2	759

265	3.92776	44.83827	2	761

266	3.94555	44.99898	1	762

267	3.9589	45.15969	1	763

268	3.97224	45.3204	1	764

269	3.98855	45.48111	23	787

270	4.00486	45.64182	1	788

271	4.01524	45.80254	6	794

272	4.03007	45.96325	2	796

273	4.04934	46.12396	2	798

274	4.06269	46.28467	1	799

275	4.07307	46.44538	3	802

276	4.09531	46.60609	2	804

277	4.11162	46.7668	5	809

278	4.11607	46.92751	2	811

279	4.13979	47.08822	1	812

280	4.1561	47.24893	1	813

281	4.15907	47.40964	1	814

282	4.17686	47.57035	3	817

283	4.19762	47.73106	1	818

284	4.20948	47.89177	2	820

285	4.21986	48.05248	1	821

286	4.24507	48.2132	2	823

287	4.25693	48.37391	5	828

288	4.26879	48.53462	1	829

289	4.28362	48.69533	7	836

290	4.30141	48.85604	4	840

291	4.30882	49.01675	2	842

292	4.32662	49.17746	3	845

293	4.34737	49.33817	1	846

294	4.35924	49.49888	43	889

295	4.3711	49.65959	1	890

296	4.38889	49.8203	1	891

297	4.4052	49.98101	11	902

298	4.41706	50.14172	2	904

299	4.43189	50.30243	1	905

300	4.4482	50.46314	1	906

301	4.45858	50.62385	2	908

302	4.47489	50.78457	1	909

303	4.4912	50.94528	6	915

304	4.50751	51.10599	2	917

305	4.51789	51.2667	1	918

306	4.53716	51.42741	1	919

307	4.55347	51.58812	1	920

308	4.56089	51.74883	1	921

309	4.5772	51.90954	1	922

310	4.59647	52.07025	4	926

311	4.60389	52.23096	2	928

312	4.62168	52.39167	1	929

313	4.64096	52.55238	4	933

314	4.65282	52.71309	5	938

315	4.66468	52.8738	1	939

316	4.68247	53.03451	2	941

317	4.6973	53.19523	2	943

318	4.71213	53.35594	1	944

319	4.72844	53.51665	4	948

320	4.74326	53.67736	3	951

321	4.75809	53.83807	1	952

322	4.76847	53.99878	1	953

323	4.78775	54.15949	1	954

324	4.80257	54.3202	1	955

325	4.81444	54.48091	3	958

326	4.83075	54.64162	1	959

327	4.84854	54.80233	1	960

328	4.85892	54.96304	2	962

329	4.87226	55.12375	3	965

330	4.89747	55.28446	11	976

331	4.9034	55.44517	1	977

332	4.9123	55.60589	1	978

333	4.93602	55.7666	4	982

334	4.95381	55.92731	3	985

335	4.95826	56.08802	2	987

336	4.9805	56.24873	1	988

337	4.99533	56.40944	1	989

338	5.00571	56.57015	2	991

339	5.0235	56.73086	1	992

340	5.04278	56.89157	3	995

341	5.05612	57.05228	1	996

342	5.0665	57.21299	3	999

343	5.08726	57.3737	7	1006

344	5.10505	57.53441	2	1008

345	5.1095	57.69512	5	1013

346	5.12877	57.85583	3	1016

347	5.14657	58.01654	1	1017

348	5.15695	58.17726	1	1018

349	5.17029	58.33797	1	1019

350	5.18957	58.49868	1	1020

351	5.20439	58.65939	1	1021

352	5.21477	58.8201	1	1022

353	5.23405	58.98081	5	1027

354	5.24888	59.14152	1	1028

355	5.25926	59.30223	12	1040

356	5.27557	59.46294	2	1042

357	5.29336	59.62365	1	1043

358	5.3067	59.78436	3	1046

359	5.31708	59.94507	1	1047

360	5.34081	60.10578	4	1051

361	5.35563	60.26649	1	1052

362	5.36305	60.4272	5	1057

363	5.38232	60.58792	1	1058

364	5.40011	60.74863	3	1061

365	5.40605	60.90934	1	1062

366	5.4268	61.07005	2	1064

367	5.44608	61.23076	1	1065

368	5.45498	61.39147	1	1066

369	5.46832	61.55218	2	1068

370	5.48908	61.71289	1	1069

371	5.50242	61.8736	1	1070

372	5.51132	62.03431	3	1073

373	5.52911	62.19502	2	1075

374	5.54542	62.35573	3	1078

375	5.55432	62.51644	3	1081

376	5.57211	62.67715	1	1082

377	5.59139	62.83786	1	1083

378	5.60177	62.99858	1	1084

379	5.61659	63.15929	3	1087

380	5.63439	63.32	1	1088

381	5.64625	63.48071	1	1089

382	5.65811	63.64142	2	1091

383	5.67739	63.80213	4	1095

384	5.69666	63.96284	2	1097

385	5.70408	64.12355	2	1099

386	5.72187	64.28426	4	1103

387	5.73818	64.44497	1	1104

388	5.75301	64.60568	2	1106

389	5.7619	64.76639	1	1107

390	5.77969	64.9271	1	1108

391	5.80045	65.08781	2	1110

392	5.8049	65.24852	3	1113

393	5.82418	65.40923	2	1115

394	5.84049	65.56995	1	1116

395	5.8568	65.73066	1	1117

396	5.86569	65.89137	1	1118

397	5.88497	66.05208	2	1120

398	5.90128	66.21279	2	1122

399	5.91018	66.3735	1	1123

400	5.92649	66.53421	1	1124

401	5.94873	66.69492	4	1128

402	5.95317	66.85563	1	1129

403	5.97097	67.01634	2	1131

404	5.99024	67.17705	1	1132

405	6.0021	67.33776	1	1133

406	6.01693	67.49847	1	1134

407	6.03621	67.65918	22	1156

408	6.04955	67.81989	2	1158

409	6.05993	67.98061	11	1169

410	6.08069	68.14132	2	1171

411	6.09552	68.30203	9	1180

412	6.10293	68.46274	2	1182

413	6.11776	68.62345	1	1183

414	6.14	68.78416	5	1188

415	6.15186	68.94487	1	1189

416	6.16224	69.10558	1	1190

417	6.18003	69.26629	3	1193

418	6.20227	69.427	1	1194

419	6.20672	69.58771	1	1195

420	6.22303	69.74842	2	1197

421	6.24379	69.90913	2	1199

422	6.25417	70.06984	1	1200

423	6.269	70.23055	1	1201

424	6.28679	70.39126	3	1204

425	6.30013	70.55198	1	1205

426	6.30903	70.71269	1	1206

427	6.33127	70.8734	1	1207

428	6.34758	71.03411	1	1208

429	6.355	71.19482	1	1209

430	6.37131	71.35553	2	1211

431	6.39355	71.51624	6	1217

432	6.40244	71.67695	11	1228

433	6.4143	71.83766	2	1230

434	6.43655	71.99837	5	1235

435	6.44692	72.15908	3	1238

436	6.45879	72.31979	3	1241

437	6.47954	72.4805	1	1242

438	6.49585	72.64121	1	1243

439	6.50623	72.80192	18	1261

440	6.51958	72.96264	1	1262

441	6.53885	73.12335	2	1264

442	6.54923	73.28406	1	1265

443	6.56109	73.44477	8	1273

444	6.58037	73.60548	6	1279

445	6.5952	73.76619	52	1331

446	6.60706	73.9269	2	1333

447	6.62189	74.08761	1	1334

448	6.64413	74.24832	2	1336

449	6.65451	74.40903	2	1338

450	6.66489	74.56974	2	1340

451	6.68713	74.73045	4	1344

452	6.69602	74.89116	3	1347

453	6.71233	75.05187	10	1357

454	6.73161	75.21258	2	1359

455	6.74495	75.3733	1	1360

456	6.75237	75.53401	1	1361

457	6.77313	75.69472	1	1362

458	6.78795	75.85543	2	1364

459	6.8013	76.01614	2	1366

460	6.81613	76.17685	2	1368

461	6.83688	76.33756	1	1369

462	6.84578	76.49827	4	1373

463	6.85912	76.65898	8	1381

464	6.87988	76.81969	1	1382

465	6.89619	76.9804	1	1383

466	6.90509	77.14111	3	1386

467	6.91695	77.30182	1	1387

468	6.94067	77.46253	1	1388

469	6.94957	77.62324	3	1391

470	6.95995	77.78395	3	1394

471	6.98367	77.94467	2	1396

472	6.9985	78.10538	12	1408

473	7.00888	78.26609	1	1409

474	7.02519	78.4268	1	1410

475	7.04447	78.58751	5	1415

476	7.05485	78.74822	5	1420

477	7.06967	78.90893	2	1422

478	7.08598	79.06964	1	1423

479	7.09636	79.23035	1	1424

480	7.10822	79.39106	1	1425

481	7.1275	79.55177	1	1426

482	7.14233	79.71248	3	1429

483	7.15419	79.87319	1	1430

484	7.17346	80.0339	1	1431

485	7.19126	80.19461	4	1435

486	7.19867	80.35533	2	1437

487	7.21646	80.51604	2	1439

488	7.23574	80.67675	3	1442

489	7.24464	80.83746	2	1444

490	7.25798	80.99817	2	1446

491	7.2817	81.15888	1	1447

492	7.29505	81.31959	1	1448

493	7.30246	81.4803	1	1449

494	7.3247	81.64101	1	1450

495	7.34101	81.80172	1	1451

496	7.34694	81.96243	7	1458

497	7.36325	82.12314	1	1459

498	7.38253	82.28385	1	1460

499	7.39143	82.44456	1	1461

500	7.40774	82.60527	1	1462

501	7.42405	82.76599	13	1475

502	7.44184	82.9267	1	1476

503	7.45073	83.08741	6	1482

504	7.46705	83.24812	1	1483

505	7.4878	83.40883	1	1484

506	7.49522	83.56954	2	1486

507	7.51301	83.73025	1	1487

508	7.52784	83.89096	2	1489

509	7.54563	84.05167	15	1504

510	7.55304	84.21238	2	1506

511	7.57677	84.37309	2	1508

512	7.59011	84.5338	1	1509

513	7.60049	84.69451	9	1518

514	7.61532	84.85522	6	1524

515	7.63608	85.01593	2	1526

516	7.64794	85.17664	1	1527

517	7.6598	85.33736	1	1528

518	7.67908	85.49807	4	1532

519	7.69094	85.65878	1	1533

520	7.70428	85.81949	1	1534

521	7.72504	85.9802	1	1535

522	7.73839	86.14091	1	1536

523	7.74728	86.30162	1	1537

524	7.76359	86.46233	3	1540

525	7.7799	86.62304	1	1541

526	7.79473	86.78375	1	1542

527	7.80659	86.94446	3	1545

528	7.82735	87.10517	2	1547

529	7.83921	87.26588	1	1548

530	7.85107	87.42659	1	1549

531	7.8659	87.5873	4	1553

532	7.88666	87.74802	1	1554

533	7.89555	87.90873	1	1555

534	7.91187	88.06944	3	1558

535	7.93262	88.23015	3	1561

536	7.94152	88.39086	1	1562

537	7.95338	88.55157	5	1567

538	7.97266	88.71228	2	1569

539	7.99045	88.87299	1	1570

540	8.00083	89.0337	1	1571

541	8.0201	89.19441	5	1576

542	8.03641	89.35512	1	1577

543	8.04828	89.51583	2	1579

544	8.05717	89.67654	1	1580

545	8.07941	89.83725	3	1583

546	8.09276	89.99796	1	1584

547	8.10462	90.15867	1	1585

548	8.12241	90.31939	1	1586

549	8.14021	90.4801	1	1587

550	8.1491	90.64081	19	1606

551	8.1669	90.80152	2	1608

552	8.18321	90.96223	1	1609

553	8.19358	91.12294	1	1610

554	8.20841	91.28365	2	1612

555	8.22769	91.44436	1	1613

556	8.23955	91.60507	4	1617

557	8.24993	91.76578	2	1619

558	8.27069	91.92649	4	1623

559	8.28996	92.0872	1	1624

560	8.29589	92.24791	4	1628

561	8.3122	92.40862	1	1629

562	8.33148	92.56933	3	1632

563	8.34186	92.73005	4	1636

564	8.35669	92.89076	3	1639

565	8.373	93.05147	12	1651

566	8.38931	93.21218	3	1654

567	8.40413	93.37289	2	1656

568	8.41599	93.5336	1	1657

569	8.4412	93.69431	5	1662

570	8.44713	93.85502	4	1666

571	8.46048	94.01573	2	1668

572	8.4842	94.17644	1	1669

573	8.49161	94.33715	4	1673

574	8.50199	94.49786	3	1676

575	8.52572	94.65857	1	1677

576	8.54203	94.81928	1	1678

577	8.54944	94.97999	4	1682

578	8.56723	95.14071	8	1690

579	8.58354	95.30142	8	1698

580	8.59689	95.46213	1	1699

581	8.60727	95.62284	3	1702

582	8.62803	95.78355	2	1704

583	8.6384	95.94426	1	1705

584	8.64878	96.10497	1	1706

585	8.67251	96.26568	5	1711

586	8.68585	96.42639	5	1716

587	8.69475	96.5871	1	1717

588	8.71551	96.74781	1	1718

589	8.73033	96.90852	3	1721

590	8.7422	97.06923	1	1722

591	8.75702	97.22994	1	1723

592	8.7763	97.39065	5	1728

593	8.78816	97.55136	2	1730

594	8.79854	97.71208	1	1731

595	8.82078	97.87279	3	1734

596	8.83857	98.0335	1	1735

597	8.84747	98.19421	6	1741

598	8.86378	98.35492	1	1742

599	8.88306	98.51563	19	1761

600	8.89195	98.67634	12	1773

601	8.9053	98.83705	1	1774

602	8.92012	98.99776	1	1775

603	8.9394	99.15847	3	1778

604	8.94978	99.31918	1	1779

605	8.96757	99.47989	6	1785

606	8.98536	99.6406	2	1787

607	8.99426	99.80131	1	1788

608	9.0076	99.96202	1	1789

609	9.02836	100.12274	1	1790

610	9.03874	100.28345	1	1791

611	9.05209	100.44416	1	1792

612	9.07136	100.60487	2	1794

613	9.08767	100.76558	5	1799

614	9.09657	100.92629	1	1800

615	9.11881	101.087	1	1801

616	9.13215	101.24771	4	1805

617	9.14402	101.40842	5	1810

618	9.15588	101.56913	2	1812

619	9.18108	101.72984	5	1817

620	9.1885	101.89055	1	1818

621	9.19739	102.05126	5	1823

622	9.2226	102.21197	2	1825

623	9.23743	102.37268	2	1827

624	9.24633	102.5334	6	1833

625	9.2656	102.69411	1	1834

626	9.28339	102.85482	3	1837

627	9.29674	103.01553	1	1838

628	9.30119	103.17624	1	1839

629	9.32491	103.33695	1	1840

630	9.33677	103.49766	1	1841

631	9.35012	103.65837	1	1842

632	9.36939	103.81908	1	1843

633	9.3857	103.97979	1	1844

634	9.39756	104.1405	1	1845

635	9.41387	104.30121	4	1849

636	9.43018	104.46192	1	1850

637	9.44056	104.62263	1	1851

638	9.45242	104.78334	1	1852

639	9.47467	104.94405	1	1853

640	9.48356	105.10477	2	1855

641	9.49691	105.26548	2	1857

642	9.5147	105.42619	2	1859

643	9.53101	105.5869	1	1860

644	9.54139	105.74761	2	1862

645	9.55622	105.90832	1	1863

646	9.57994	106.06903	1	1864

647	9.58735	106.22974	3	1867

648	9.60366	106.39045	8	1875

649	9.62294	106.55116	1	1876

650	9.63628	106.71187	1	1877

651	9.64963	106.87258	1	1878

652	9.66594	107.03329	3	1881

653	9.68225	107.194	1	1882

654	9.68966	107.35471	3	1885

655	9.70746	107.51543	2	1887

656	9.72821	107.67614	29	1916

657	9.73859	107.83685	1	1917

658	9.74897	107.99756	2	1919

659	9.7727	108.15827	1	1920

660	9.78752	108.31898	3	1923

661	9.79345	108.47969	1	1924

662	9.81273	108.6404	1	1925

663	9.83349	108.80111	20	1945

664	9.83942	108.96182	1	1946

665	9.85425	109.12253	2	1948

666	9.87352	109.28324	1	1949

667	9.88983	109.44395	1	1950

668	9.90169	109.60466	20	1970

669	9.918	109.76537	31	2001

670	9.93283	109.92609	2	2003

671	9.94173	110.0868	3	2006

672	9.95804	110.24751	2	2008

673	9.98028	110.40822	2	2010

674	9.98621	110.56893	2	2012

675	10.00104	110.72964	3	2015

676	10.02476	110.89035	1	2016

677	10.02921	111.05106	3	2019

678	10.04404	111.21177	2	2021

679	10.06628	111.37248	1	2022

680	10.0811	111.53319	1	2023

681	10.09	111.6939	5	2028

682	10.10928	111.85461	1	2029

683	10.13003	112.01532	3	2032

684	10.13597	112.17603	3	2035

685	10.14783	112.33674	2	2037

686	10.16859	112.49746	1	2038

687	10.1849	112.65817	2	2040

688	10.19379	112.81888	1	2041

689	10.21158	112.97959	1	2042

690	10.23086	113.1403	1	2043

691	10.23976	113.30101	3	2046

692	10.2531	113.46172	1	2047

693	10.27238	113.62243	1	2048

694	10.28276	113.78314	1	2049

695	10.2961	113.94385	3	2052

696	10.31686	114.10456	1	2053

697	10.33317	114.26527	5	2058

698	10.34355	114.42598	1	2059

699	10.35838	114.58669	1	2060

700	10.37469	114.7474	5	2065

701	10.3821	114.90812	1	2066

702	10.39841	115.06883	1	2067

703	10.42213	115.22954	1	2068

704	10.43399	115.39025	3	2071

705	10.44437	115.55096	1	2072

706	10.46365	115.71167	2	2074

707	10.48292	115.87238	1	2075

708	10.48737	116.03309	2	2077

709	10.50665	116.1938	1	2078

710	10.52592	116.35451	1	2079

711	10.53482	116.51522	12	2091

712	10.55261	116.67593	4	2095

713	10.57041	116.83664	2	2097

714	10.57634	116.99735	1	2098

715	10.59116	117.15806	17	2115

716	10.61785	117.31877	1	2116

717	10.62675	117.47949	1	2117

718	10.63416	117.6402	6	2123

719	10.6564	117.80091	1	2124

720	10.67271	117.96162	3	2127

721	10.68013	118.12233	2	2129

722	10.69792	118.28304	3	2132

723	10.72164	118.44375	3	2135

724	10.73351	118.60446	15	2150

725	10.74092	118.76517	2	2152

726	10.75871	118.92588	2	2154

727	10.77502	119.08659	1	2155

728	10.78689	119.2473	17	2172

729	10.80023	119.40801	1	2173

730	10.81951	119.56872	1	2174

731	10.83137	119.72943	4	2178

732	10.84471	119.89015	3	2181

733	10.8714	120.05086	1	2182

734	10.88178	120.21157	3	2185

735	10.88771	120.37228	1	2186

736	10.91143	120.53299	3	2189

737	10.9233	120.6937	1	2190

738	10.93071	120.85441	2	2192

739	10.95147	121.01512	7	2199

740	10.96926	121.17583	1	2200

741	10.98112	121.33654	2	2202

742	10.99595	121.49725	1	2203

743	11.01523	121.65796	5	2208

744	11.02412	121.81867	1	2209

745	11.03598	121.97938	1	2210

746	11.05971	122.14009	32	2242

747	11.07454	122.30081	1	2243

748	11.08343	122.46152	1	2244

749	11.09974	122.62223	2	2246

750	11.11753	122.78294	1	2247

751	11.1294	122.94365	5	2252

752	11.13978	123.10436	1	2253

753	11.16202	123.26507	2	2255

754	11.17536	123.42578	3	2258

755	11.18574	123.58649	2	2260

756	11.20205	123.7472	6	2266

757	11.21984	123.90791	2	2268

758	11.22874	124.06862	1	2269

759	11.24505	124.22933	1	2270

760	11.26729	124.39004	1	2271

761	11.27619	124.55075	1	2272

762	11.28805	124.71146	4	2276

763	11.31029	124.87218	1	2277

764	11.32215	125.03289	2	2279

765	11.3355	125.1936	4	2283

766	11.35181	125.35431	1	2284

767	11.37405	125.51502	1	2285

768	11.38146	125.67573	2	2287

769	11.39184	125.83644	2	2289

770	11.41556	125.99715	2	2291

771	11.42743	126.15786	3	2294

772	11.43336	126.31857	1	2295

773	11.45856	126.47928	2	2297

774	11.47191	126.63999	1	2298

775	11.4808	126.8007	3	2301

776	11.49415	126.96141	1	2302

777	11.51936	127.12212	29	2331

778	11.52825	127.28284	2	2333

779	11.54456	127.44355	3	2336

780	11.56384	127.60426	3	2339

781	11.57125	127.76497	1	2340

782	11.58608	127.92568	1	2341

783	11.60535	128.08639	1	2342

784	11.62166	128.2471	1	2343

785	11.62759	128.40781	3	2346

786	11.65132	128.56852	1	2347

787	11.66615	128.72923	11	2358

788	11.67801	128.88994	1	2359

789	11.68839	129.05065	1	2360

790	11.70766	129.21136	1	2361

791	11.71804	129.37207	3	2364

792	11.73435	129.53278	1	2365

793	11.7477	129.6935	1	2366

794	11.76697	129.85421	2	2368

795	11.77883	130.01492	8	2376

796	11.79366	130.17563	2	2378

797	11.82035	130.33634	1	2379

798	11.82628	130.49705	5	2384

799	11.83814	130.65776	1	2385

800	11.85594	130.81847	2	2387

801	11.86928	130.97918	2	2389

802	11.88114	131.13989	1	2390

803	11.90338	131.3006	26	2416

804	11.91673	131.46131	1	2417

805	11.93007	131.62202	1	2418

806	11.94045	131.78273	2	2420

807	11.95973	131.94344	1	2421

808	11.97011	132.10415	5	2426

809	11.98642	132.26487	7	2433

810	12.00569	132.42558	2	2435

811	12.01904	132.58629	4	2439

812	12.0309	132.747	1	2440

813	12.04721	132.90771	1	2441

814	12.065	133.06842	1	2442

815	12.07538	133.22913	1	2443

816	12.08576	133.38984	2	2445

817	12.11097	133.55055	11	2456

818	12.12431	133.71126	1	2457

819	12.13617	133.87197	20	2477

820	12.15248	134.03268	1	2478

821	12.16583	134.19339	2	2480

822	12.18065	134.3541	1	2481

823	12.19548	134.51481	2	2483

824	12.21327	134.67553	5	2488

825	12.2281	134.83624	1	2489

826	12.24589	134.99695	1	2490

827	12.25776	135.15766	1	2491

828	12.2711	135.31837	1	2492

829	12.28148	135.47908	1	2493

830	12.30224	135.63979	1	2494

831	12.323	135.8005	3	2497

832	12.32893	135.96121	1	2498

833	12.33931	136.12192	2	2500

834	12.366	136.28263	1	2501

835	12.37489	136.44334	2	2503

836	12.38527	136.60405	9	2512

837	12.409	136.76476	1	2513

838	12.41789	136.92547	8	2521

839	12.42827	137.08618	1	2522

840	12.45051	137.2469	2	2524

841	12.46386	137.40761	11	2535

842	12.48165	137.56832	1	2536

843	12.48906	137.72903	14	2550

844	12.51427	137.88974	4	2554

845	12.52317	138.05045	2	2556

846	12.53206	138.21116	2	2558

847	12.55134	138.37187	1	2559

848	12.57061	138.53258	1	2560

849	12.57951	138.69329	1	2561

850	12.60027	138.854	2	2563

851	12.6151	139.01471	1	2564

852	12.62399	139.17542	1	2565

853	12.64178	139.33613	1	2566

854	12.65661	139.49684	1	2567

855	12.66996	139.65756	3	2570

856	12.6833	139.81827	2	2572

857	12.70406	139.97898	1	2573

858	12.71444	140.13969	4	2577

859	12.72185	140.3004	2	2579

860	12.74113	140.46111	1	2580

861	12.76633	140.62182	1	2581

862	12.7693	140.78253	1	2582

863	12.79006	140.94324	1	2583

864	12.81082	141.10395	19	2602

865	12.81971	141.26466	6	2608

866	12.82713	141.42537	3	2611

867	12.8553	141.58608	1	2612

868	12.86271	141.74679	2	2614

869	12.87606	141.9075	5	2619

870	12.89681	142.06822	6	2625

871	12.91461	142.22893	2	2627

872	12.92499	142.38964	1	2628

873	12.93537	142.55035	2	2630

874	12.95909	142.71106	1	2631

875	12.97095	142.87177	1	2632

876	12.97836	143.03248	1	2633

877	13.00061	143.19319	1	2634

878	13.0184	143.3539	3	2637

879	13.02581	143.51461	11	2648

880	13.03916	143.67532	1	2649

881	13.06436	143.83603	1	2650

882	13.07029	143.99674	4	2654

883	13.08809	144.15745	2	2656

884	13.10143	144.31816	1	2657

885	13.11922	144.47887	2	2659

886	13.12812	144.63959	9	2668

887	13.15036	144.8003	2	2670

888	13.16519	144.96101	1	2671

889	13.17557	145.12172	1	2672

890	13.18891	145.28243	1	2673

891	13.20522	145.44314	1	2674

892	13.22005	145.60385	1	2675

893	13.23191	145.76456	1	2676

894	13.25415	145.92527	5	2681

895	13.26453	146.08598	2	2683

896	13.27788	146.24669	20	2703

897	13.29864	146.4074	1	2704

898	13.31198	146.56811	2	2706

899	13.31939	146.72882	1	2707

900	13.33422	146.88953	1	2708

901	13.35943	147.05025	9	2717

902	13.36388	147.21096	2	2719

903	13.3787	147.37167	5	2724

904	13.40094	147.53238	5	2729

905	13.41132	147.69309	2	2731

906	13.42318	147.8538	1	2732

907	13.44543	148.01451	1	2733

908	13.46025	148.17522	6	2739

909	13.46915	148.33593	2	2741

910	13.48843	148.49664	3	2744

911	13.5077	148.65735	5	2749

912	13.51956	148.81806	1	2750

913	13.52549	148.97877	2	2752

914	13.54477	149.13948	2	2754

915	13.56404	149.30019	8	2762

916	13.57442	149.46091	1	2763

917	13.59222	149.62162	1	2764

918	13.61001	149.78233	1	2765

919	13.62039	149.94304	14	2779

920	13.63522	150.10375	2	2781

921	13.64856	150.26446	1	2782

922	13.66635	150.42517	8	2790

923	13.6797	150.58588	1	2791

924	13.69601	150.74659	1	2792

925	13.70935	150.9073	1	2793

926	13.72121	151.06801	1	2794

927	13.74049	151.22872	1	2795

928	13.76125	151.38943	2	2797

929	13.76421	151.55014	1	2798

930	13.77904	151.71085	1	2799

931	13.80276	151.87156	1	2800

932	13.81759	152.03228	1	2801

933	13.82945	152.19299	4	2805

934	13.8428	152.3537	8	2813

935	13.86059	152.51441	2	2815

936	13.87245	152.67512	2	2817

937	13.88283	152.83583	4	2821

938	13.90804	152.99654	1	2822

939	13.9199	153.15725	3	2825

940	13.92583	153.31796	1	2826

941	13.94807	153.47867	2	2828

942	13.9629	153.63938	1	2829

943	13.97328	153.80009	3	2832

944	13.99107	153.9608	1	2833

945	14.00886	154.12151	1	2834

946	14.01776	154.28222	4	2838

947	14.03259	154.44294	4	2842

948	14.05483	154.60365	3	2845

949	14.06669	154.76436	1	2846

950	14.083	154.92507	5	2851

951	14.09486	155.08578	2	2853

952	14.11266	155.24649	1	2854

953	14.12304	155.4072	1	2855

954	14.13638	155.56791	1	2856

955	14.15862	155.72862	1	2857

956	14.16455	155.88933	7	2864

957	14.18086	156.05004	1	2865

958	14.2031	156.21075	11	2876

959	14.21645	156.37146	2	2878

960	14.22238	156.53217	1	2879

961	14.24165	156.69288	4	2883

962	14.25796	156.85359	6	2889

963	14.26686	157.01431	1	2890

964	14.28465	157.17502	1	2891

965	14.30541	157.33573	6	2897

966	14.31282	157.49644	1	2898

967	14.32913	157.65715	2	2900

968	14.34989	157.81786	5	2905

969	14.36176	157.97857	13	2918

970	14.37213	158.13928	4	2922

971	14.38844	158.29999	1	2923

972	14.41069	158.4607	1	2924

973	14.41513	158.62141	1	2925

974	14.43293	158.78212	5	2930

975	14.45368	158.94283	2	2932

976	14.45665	159.10354	52	2984

977	14.47444	159.26425	1	2985

978	14.49817	159.42497	3	2988

979	14.51448	159.58568	1	2989

980	14.52189	159.74639	3	2992

981	14.53523	159.9071	1	2993

982	14.55451	160.06781	1	2994

983	14.56489	160.22852	6	3000

984	14.58268	160.38923	1	3001

985	14.59751	160.54994	2	3003

986	14.61085	160.71065	1	3004

987	14.62123	160.87136	2	3006

988	14.64792	161.03207	2	3008

989	14.6583	161.19278	1	3009

990	14.66868	161.35349	1	3010

991	14.68203	161.5142	1	3011

992	14.70427	161.67491	4	3015

993	14.70871	161.83563	1	3016

994	14.72354	161.99634	4	3020

995	14.74578	162.15705	1	3021

996	14.75616	162.31776	2	3023

997	14.77247	162.47847	1	3024

998	14.79175	162.63918	2	3026

999	14.80509	162.79989	1	3027

1000	14.81399	162.9606	3	3030

1001	14.83475	163.12131	1	3031

1002	14.84809	163.28202	2	3033

1003	14.86144	163.44273	1	3034

1004	14.87478	163.60344	3	3037

1005	14.89554	163.76415	1	3038

1006	14.9074	163.92486	1	3039

1007	14.91926	164.08557	3	3042

1008	14.93557	164.24628	1	3043

1009	14.9504	164.407	1	3044

1010	14.96374	164.56771	1	3045

1011	14.97857	164.72842	5	3050

1012	14.99785	164.88913	3	3053

1013	15.00823	165.04984	1	3054

1014	15.02305	165.21055	2	3056

1015	15.04381	165.37126	1	3057

1016	15.05716	165.53197	1	3058

1017	15.06012	165.69268	1	3059

1018	15.08829	165.85339	1	3060

1019	15.10312	166.0141	1	3061

1020	15.1135	166.17481	1	3062

1021	15.12981	166.33552	3	3065

1022	15.1476	166.49623	2	3067

1023	15.15947	166.65694	2	3069

1024	15.16984	166.81766	1	3070

1025	15.19357	166.97837	1	3071

1026	15.20691	167.13908	1	3072

1027	15.21729	167.29979	2	3074

1028	15.23509	167.4605	11	3085

1029	15.24991	167.62121	2	3087

1030	15.26326	167.78192	1	3088

1031	15.2766	167.94263	1	3089

1032	15.29143	168.10334	3	3092

1033	15.30626	168.26405	3	3095

1034	15.32108	168.42476	25	3120

1035	15.33739	168.58547	2	3122

1036	15.35519	168.74618	2	3124

1037	15.36557	168.90689	52	3176

1038	15.38039	169.0676	2	3178

1039	15.39819	169.22832	1	3179

1040	15.41005	169.38903	1	3180

1041	15.42784	169.54974	1	3181

1042	15.44415	169.71045	1	3182

1043	15.45305	169.87116	1	3183

1044	15.46787	170.03187	2	3185

1045	15.48715	170.19258	1	3186

1046	15.50939	170.35329	1	3187

1047	15.50939	170.514	1	3188

1048	15.53015	170.67471	3	3191

1049	15.54794	170.83542	1	3192

1050	15.55536	170.99613	1	3193

1051	15.5687	171.15684	1	3194

1052	15.59094	171.31755	2	3196

1053	15.60132	171.47826	1	3197

1054	15.61466	171.63897	8	3205

1055	15.63839	171.79969	3	3208

1056	15.65173	171.9604	1	3209

1057	15.6547	172.12111	1	3210

1058	15.67842	172.28182	7	3217

1059	15.69622	172.44253	1	3218

1060	15.70659	172.60324	1	3219

1061	15.72587	172.76395	3	3222

1062	15.7407	172.92466	1	3223

1063	15.75256	173.08537	4	3227

1064	15.76294	173.24608	3	3230

1065	15.78666	173.40679	1	3231

1066	15.79556	173.5675	1	3232

1067	15.80149	173.72821	1	3233

1068	15.82076	173.88892	2	3235

1069	15.84745	174.04963	1	3236

1070	15.85042	174.21035	2	3238

1071	15.87118	174.37106	1	3239

1072	15.88452	174.53177	1	3240

1073	15.90083	174.69248	4	3244

1074	15.91418	174.85319	3	3247

1075	15.929	175.0139	4	3251

1076	15.94383	175.17461	1	3252

1077	15.95569	175.33532	1	3253

1078	15.97349	175.49603	2	3255

1079	15.98831	175.65674	3	3258

1080	16.00314	175.81745	3	3261

1081	16.01204	175.97816	3	3264

1082	16.03576	176.13887	1	3265

1083	16.04762	176.29958	2	3267

1084	16.05652	176.46029	1	3268

1085	16.07431	176.621	7	3275

1086	16.09359	176.78172	2	3277

1087	16.09952	176.94243	6	3283

1088	16.11435	177.10314	1	3284

1089	16.144	177.26385	2	3286

1090	16.14845	177.42456	4	3290

1091	16.16328	177.58527	3	3293

1092	16.18255	177.74598	3	3296

1093	16.1959	177.90669	2	3298

1094	16.19886	178.0674	1	3299

1095	16.22852	178.22811	1	3300

1096	16.24038	178.38882	1	3301

1097	16.25224	178.54953	6	3307

1098	16.26707	178.71024	1	3308

1099	16.28486	178.87095	2	3310

1100	16.29672	179.03166	2	3312

1101	16.3071	179.19238	1	3313

1102	16.33082	179.35309	1	3314

1103	16.34269	179.5138	3	3317

1104	16.35455	179.67451	1	3318

1105	16.36938	179.83522	1	3319

1106	16.38717	179.99593	1	3320

1107	16.40051	180.15664	2	3322

1108	16.41831	180.31735	6	3328

1109	16.43165	180.47806	2	3330

1110	16.445	180.63877	1	3331

1111	16.45241	180.79948	2	3333

1112	16.4791	180.96019	1	3334

1113	16.49244	181.1209	3	3337

1114	16.50282	181.28161	2	3339

1115	16.51913	181.44232	1	3340

1116	16.53841	181.60304	1	3341

1117	16.54879	181.76375	5	3346

1118	16.56065	181.92446	1	3347

1119	16.58141	182.08517	2	3349

1120	16.59475	182.24588	26	3375

1121	16.60661	182.40659	3	3378

1122	16.62441	182.5673	1	3379

1123	16.63775	182.72801	19	3398

1124	16.64813	182.88872	1	3399

1125	16.66592	183.04943	4	3403

1126	16.68372	183.21014	5	3408

1127	16.69706	183.37085	1	3409

1128	16.70744	183.53156	1	3410

1129	16.72968	183.69227	6	3416

1130	16.74302	183.85298	1	3417

1131	16.74896	184.01369	2	3419

1132	16.76971	184.17441	1	3420

1133	16.78751	184.33512	16	3436

1134	16.79492	184.49583	1	3437

1135	16.80827	184.65654	3	3440

1136	16.82458	184.81725	2	3442

1137	16.8394	184.97796	4	3446

1138	16.84682	185.13867	1	3447

1139	16.86757	185.29938	3	3450

1140	16.87499	185.46009	1	3451

1141	16.12769	185.6208	1	3452

1142	15.22174	185.78151	2	3454

1143	14.39734	185.94222	2	3456

1144	13.49732	186.10293	1	3457

1145	12.52168	186.26364	1	3458

1146	11.63946	186.42435	1	3459

1147	10.69051	186.58507	1	3460

1148	9.94024	186.74578	3	3463

1149	9.02688	186.90649	3	3466

1150	8.23065	187.0672	1	3467

1151	7.42553	187.22791	2	3469

1152	6.60854	187.38862	7	3476

1153	5.78563	187.54933	1	3477

1154	5.01757	187.71004	1	3478

1155	4.19614	187.87075	3	3481

1156	3.41918	188.03146	1	3482

1157	2.64964	188.19217	7	3489

1158	2.64964	188.35288	1	3490

1159	2.08027	188.51359	1	3491

1160	1.39377	188.6743	1	3492

188.83501	2	3494

188.99572	4	3498

189.15644	2	3500

189.31715	1	3501

189.47786	4	3505

189.63857	1	3506

189.79928	4	3510

189.95999	1	3511

190.1207	4	3515

190.28141	5	3520

190.44212	1	3521

190.60283	1	3522

190.76354	17	3539

190.92425	3	3542

191.08496	3	3545

191.24567	3	3548

191.40638	2	3550

191.5671	4	3554

191.72781	7	3561

191.88852	1	3562

192.04923	2	3564

192.20994	1	3565

192.37065	3	3568

192.53136	3	3571

192.69207	2	3573

192.85278	2	3575

193.01349	11	3586

193.1742	1	3587

193.33491	1	3588

193.49562	1	3589

193.65633	1	3590

193.81704	1	3591

193.97776	3	3594

194.13847	2	3596

194.29918	1	3597

194.45989	1	3598

194.6206	1	3599

194.78131	3	3602

194.94202	3	3605

195.10273	1	3606

195.26344	1	3607

195.42415	1	3608

195.58486	1	3609

195.74557	2	3611

195.90628	1	3612

196.06699	2	3614

196.2277	4	3618

196.38842	2	3620

196.54913	4	3624

196.70984	3	3627

196.87055	1	3628

197.03126	1	3629

197.19197	1	3630

197.35268	3	3633

197.51339	1	3634

197.6741	1	3635

197.83481	1	3636

197.99552	8	3644

198.15623	3	3647

198.31694	1	3648

198.47765	1	3649

198.63836	3	3652

198.79907	2	3654

198.95979	2	3656

199.1205	3	3659

199.28121	5	3664

199.44192	1	3665

199.60263	4	3669

199.76334	1	3670

199.92405	1	3671

200.08476	1	3672

200.24547	3	3675

200.40618	4	3679

200.56689	1	3680

200.7276	17	3697

200.88831	1	3698

201.04902	1	3699

201.20973	1	3700

201.37045	2	3702

201.53116	2	3704

201.69187	2	3706

201.85258	1	3707

202.01329	3	3710

202.174	2	3712

202.33471	1	3713

202.49542	4	3717

202.65613	3	3720

202.81684	18	3738

202.97755	6	3744

203.13826	2	3746

203.29897	1	3747

203.45968	1	3748

203.62039	39	3787

203.7811	1	3788

203.94182	1	3789

204.10253	3	3792

204.26324	1	3793

204.42395	1	3794

204.58466	5	3799

204.74537	30	3829

204.90608	1	3830

205.06679	4	3834

205.2275	7	3841

205.38821	1	3842

205.54892	4	3846

205.70963	2	3848

205.87034	1	3849

206.03105	1	3850

206.19176	2	3852

206.35248	1	3853

206.51319	2	3855

206.6739	1	3856

206.83461	1	3857

206.99532	1	3858

207.15603	8	3866

207.31674	3	3869

207.47745	1	3870

207.63816	1	3871

207.79887	1	3872

207.95958	1	3873

208.12029	4	3877

208.281	1	3878

208.44171	3	3881

208.60242	1	3882

208.76314	1	3883

208.92385	5	3888

209.08456	1	3889

209.24527	10	3899

209.40598	3	3902

209.56669	6	3908

209.7274	1	3909

209.88811	3	3912

210.04882	5	3917

210.20953	1	3918

210.37024	2	3920

210.53095	1	3921

210.69166	3	3924

210.85237	2	3926

211.01308	1	3927

211.17379	6	3933

211.33451	2	3935

211.49522	2	3937

211.65593	1	3938

211.81664	2	3940

211.97735	1	3941

212.13806	4	3945

212.29877	4	3949

212.45948	1	3950

212.62019	1	3951

212.7809	7	3958

212.94161	4	3962

213.10232	1	3963

213.26303	1	3964

213.42374	2	3966

213.58445	1	3967

213.74517	1	3968

213.90588	1	3969

214.06659	1	3970

214.2273	1	3971

214.38801	2	3973

214.54872	2	3975

214.70943	20	3995

214.87014	4	3999

215.03085	2	4001

215.19156	1	4002

215.35227	4	4006

215.51298	1	4007

215.67369	1	4008

215.8344	3	4011

215.99511	8	4019

216.15582	6	4025

216.31654	2	4027

216.47725	7	4034

216.63796	3	4037

216.79867	1	4038

216.95938	1	4039

217.12009	2	4041

217.2808	5	4046

217.44151	2	4048

217.60222	6	4054

217.76293	2	4056

217.92364	2	4058

218.08435	2	4060

218.24506	1	4061

218.40577	2	4063

218.56648	5	4068

218.7272	2	4070

218.88791	2	4072

219.04862	2	4074

219.20933	26	4100

219.37004	2	4102

219.53075	3	4105

219.69146	24	4129

219.85217	1	4130

220.01288	1	4131

220.17359	1	4132

220.3343	3	4135

220.49501	17	4152

220.65572	2	4154

220.81643	27	4181

220.97714	1	4182

221.13786	1	4183

221.29857	5	4188

221.45928	1	4189

221.61999	1	4190

221.7807	13	4203

221.94141	7	4210

222.10212	3	4213

222.26283	1	4214

222.42354	3	4217

222.58425	1	4218

222.74496	1	4219

222.90567	2	4221

223.06638	4	4225

223.22709	2	4227

223.3878	3	4230

223.54851	1	4231

223.70923	4	4235

223.86994	3	4238

224.03065	2	4240

224.19136	2	4242

224.35207	1	4243

224.51278	1	4244

224.67349	1	4245

224.8342	1	4246

224.99491	1	4247

225.15562	1	4248

225.31633	3	4251

225.47704	3	4254

225.63775	1	4255

225.79846	2	4257

225.95917	1	4258

226.11989	1	4259

226.2806	3	4262

226.44131	1	4263

226.60202	1	4264

226.76273	1	4265

226.92344	3	4268

227.08415	2	4270

227.24486	1	4271

227.40557	1	4272

227.56628	1	4273

227.72699	1	4274

227.8877	1	4275

228.04841	1	4276

228.20912	2	4278

228.36983	8	4286

228.53055	2	4288

228.69126	1	4289

228.85197	4	4293

229.01268	2	4295

229.17339	2	4297

229.3341	2	4299

229.49481	6	4305

229.65552	2	4307

229.81623	1	4308

229.97694	7	4315

230.13765	1	4316

230.29836	1	4317

230.45907	5	4322

230.61978	1	4323

230.78049	3	4326

230.9412	1	4327

231.10192	4	4331

231.26263	1	4332

231.42334	5	4337

231.58405	1	4338

231.74476	2	4340

231.90547	1	4341

232.06618	1	4342

232.22689	2	4344

232.3876	1	4345

232.54831	4	4349

232.70902	1	4350

232.86973	5	4355

233.03044	1	4356

233.19115	1	4357

233.35186	4	4361

233.51258	5	4366

233.67329	3	4369

233.834	2	4371

233.99471	1	4372

234.15542	1	4373

234.31613	4	4377

234.47684	16	4393

234.63755	1	4394

234.79826	3	4397

234.95897	2	4399

235.11968	1	4400

235.28039	2	4402

235.4411	1	4403

235.60181	3	4406

235.76252	1	4407

235.92324	1	4408

236.08395	4	4412

236.24466	1	4413

236.40537	1	4414

236.56608	1	4415

236.72679	1	4416

236.8875	1	4417

237.04821	1	4418

237.20892	6	4424

237.36963	1	4425

237.53034	1	4426

237.69105	4	4430

237.85176	1	4431

238.01247	1	4432

238.17318	2	4434

238.33389	1	4435

238.49461	4	4439

238.65532	4	4443

238.81603	1	4444

238.97674	1	4445

239.13745	1	4446

239.29816	1	4447

239.45887	1	4448

239.61958	1	4449

239.78029	3	4452

239.941	1	4453

240.10171	4	4457

240.26242	2	4459

240.42313	2	4461

240.58384	1	4462

240.74455	3	4465

240.90527	3	4468

241.06598	2	4470

241.22669	7	4477

241.3874	64	4541

241.54811	2	4543

241.70882	8	4551

241.86953	2	4553

242.03024	2	4555

242.19095	2	4557

242.35166	1	4558

242.51237	2	4560

242.67308	1	4561

242.83379	1	4562

242.9945	3	4565

243.15521	2	4567

243.31592	21	4588

243.47664	1	4589

243.63735	1	4590

243.79806	1	4591

243.95877	1	4592

244.11948	4	4596

244.28019	1	4597

244.4409	1	4598

244.60161	3	4601

244.76232	1	4602

244.92303	1	4603

245.08374	1	4604

245.24445	1	4605

245.40516	2	4607

245.56587	1	4608

245.72658	1	4609

245.8873	2	4611

246.04801	2	4613

246.20872	6	4619

246.36943	3	4622

246.53014	2	4624

246.69085	1	4625

246.85156	11	4636

247.01227	3	4639

247.17298	9	4648

247.33369	2	4650

247.4944	3	4653

247.65511	6	4659

247.81582	1	4660

247.97653	1	4661

248.13724	5	4666

248.29796	1	4667

248.45867	1	4668

248.61938	3	4671

248.78009	1	4672

248.9408	1	4673

249.10151	2	4675

249.26222	5	4680

249.42293	1	4681

249.58364	4	4685

249.74435	3	4688

249.90506	1	4689

250.06577	2	4691

250.22648	3	4694

250.38719	3	4697

250.5479	1	4698

250.70861	1	4699

250.86933	2	4701

251.03004	1	4702

251.19075	4	4706

251.35146	3	4709

251.51217	1	4710

251.67288	2	4712

251.83359	4	4716

251.9943	1	4717

252.15501	2	4719

252.31572	3	4722

252.47643	1	4723

252.63714	3	4726

252.79785	1	4727

252.95856	7	4734

253.11927	5	4739

253.27999	1	4740

253.4407	1	4741

253.60141	1	4742

253.76212	7	4749

253.92283	8	4757

254.08354	1	4758

254.24425	5	4763

254.40496	2	4765

254.56567	2	4767

254.72638	1	4768

254.88709	2	4770

255.0478	1	4771

255.20851	1	4772

255.36922	2	4774

255.52993	1	4775

255.69064	2	4777

255.85136	1	4778

256.01207	1	4779

256.17278	1	4780

256.33349	2	4782

256.4942	1	4783

256.65491	1	4784

256.81562	1	4785

256.97633	3	4788

257.13704	1	4789

257.29775	1	4790

257.45846	1	4791

257.61917	1	4792

257.77988	20	4812

257.94059	2	4814

258.1013	2	4816

258.26202	2	4818

258.42273	2	4820

258.58344	2	4822

258.74415	2	4824

258.90486	1	4825

259.06557	6	4831

259.22628	1	4832

259.38699	1	4833

259.5477	18	4851

259.70841	1	4852

259.86912	1	4853

260.02983	1	4854

260.19054	1	4855

260.35125	2	4857

260.51196	2	4859

260.67268	2	4861

260.83339	4	4865

260.9941	1	4866

261.15481	1	4867

261.31552	4	4871

261.47623	1	4872

261.63694	3	4875

261.79765	1	4876

261.95836	3	4879

262.11907	2	4881

262.27978	1	4882

262.44049	1	4883

262.6012	4	4887

262.76191	2	4889

262.92262	4	4893

263.08333	1	4894

263.24405	3	4897

263.40476	1	4898

263.56547	6	4904

263.72618	1	4905

263.88689	1	4906

264.0476	1	4907

264.20831	6	4913

264.36902	1	4914

264.52973	11	4925

264.69044	1	4926

264.85115	1	4927

265.01186	2	4929

265.17257	2	4931

265.33328	1	4932

265.49399	17	4949

265.65471	1	4950

265.81542	1	4951

265.97613	9	4960

266.13684	1	4961

266.29755	5	4966

266.45826	4	4970

266.61897	6	4976

266.77968	1	4977

266.94039	1	4978

267.1011	1	4979

267.26181	3	4982

267.42252	1	4983

267.58323	2	4985

267.74394	1	4986

267.90465	2	4988

268.06537	1	4989

268.22608	1	4990

268.38679	3	4993

268.5475	1	4994

268.70821	2	4996

268.86892	1	4997

269.02963	4	5001

269.19034	2	5003

269.35105	17	5020

269.51176	1	5021

269.67247	1	5022

269.83318	2	5024

269.99389	2	5026

270.1546	2	5028

270.31531	2	5030

270.47602	1	5031

270.63674	2	5033

270.79745	2	5035

270.95816	2	5037

271.11887	7	5044

271.27958	1	5045

271.44029	2	5047

271.601	2	5049

271.76171	2	5051

271.92242	1	5052

272.08313	2	5054

272.24384	6	5060

272.40455	23	5083

272.56526	4	5087

272.72597	1	5088

272.88668	3	5091

273.0474	3	5094

273.20811	1	5095

273.36882	1	5096

273.52953	1	5097

273.69024	1	5098

273.85095	3	5101

274.01166	1	5102

274.17237	1	5103

274.33308	1	5104

274.49379	4	5108

274.6545	1	5109

274.81521	2	5111

274.97592	1	5112

275.13663	2	5114

275.29734	1	5115

275.45806	1	5116

275.61877	2	5118

275.77948	1	5119

275.94019	1	5120

276.1009	24	5144

276.26161	1	5145

276.42232	1	5146

276.58303	5	5151

276.74374	1	5152

276.90445	2	5154

277.06516	1	5155

277.22587	1	5156

277.38658	3	5159

277.54729	1	5160

277.708	3	5163

277.86871	2	5165

278.02943	2	5167

278.19014	1	5168

278.35085	1	5169

278.51156	5	5174

278.67227	7	5181

278.83298	1	5182

278.99369	1	5183

279.1544	3	5186

279.31511	3	5189

279.47582	3	5192

279.63653	1	5193

279.79724	2	5195

279.95795	3	5198

280.11866	2	5200

280.27937	1	5201

280.44009	2	5203

280.6008	2	5205

280.76151	1	5206

280.92222	1	5207

281.08293	2	5209

281.24364	1	5210

281.40435	2	5212

281.56506	6	5218

281.72577	1	5219

281.88648	1	5220

282.04719	2	5222

282.2079	1	5223

282.36861	1	5224

282.52932	1	5225

282.69003	2	5227

282.85074	3	5230

283.01146	1	5231

283.17217	1	5232

283.33288	2	5234

283.49359	1	5235

283.6543	5	5240

283.81501	1	5241

283.97572	1	5242

284.13643	14	5256

284.29714	5	5261

284.45785	1	5262

284.61856	1	5263

284.77927	1	5264

284.93998	6	5270

285.10069	2	5272

285.2614	4	5276

285.42212	1	5277

285.58283	2	5279

285.74354	3	5282

285.90425	1	5283

286.06496	2	5285

286.22567	1	5286

286.38638	2	5288

286.54709	4	5292

286.7078	1	5293

286.86851	1	5294

287.02922	2	5296

287.18993	1	5297

287.35064	3	5300

287.51135	4	5304

287.67206	2	5306

287.83278	13	5319

287.99349	2	5321

288.1542	1	5322

288.31491	1	5323

288.47562	1	5324

288.63633	1	5325

288.79704	3	5328

288.95775	1	5329

289.11846	1	5330

289.27917	1	5331

289.43988	1	5332

289.60059	1	5333

289.7613	1	5334

289.92201	2	5336

290.08272	1	5337

290.24343	1	5338

290.40415	3	5341

290.56486	4	5345

290.72557	1	5346

290.88628	1	5347

291.04699	6	5353

291.2077	3	5356

291.36841	1	5357

291.52912	3	5360

291.68983	1	5361

291.85054	1	5362

292.01125	1	5363

292.17196	1	5364

292.33267	1	5365

292.49338	4	5369

292.65409	2	5371

292.81481	1	5372

292.97552	4	5376

293.13623	2	5378

293.29694	1	5379

293.45765	6	5385

293.61836	2	5387

293.77907	2	5389

293.93978	1	5390

294.10049	1	5391

294.2612	1	5392

294.42191	2	5394

294.58262	1	5395

294.74333	5	5400

294.90404	3	5403

295.06475	1	5404

295.22546	5	5409

295.38618	1	5410

295.54689	1	5411

295.7076	3	5414

295.86831	2	5416

296.02902	4	5420

296.18973	12	5432

296.35044	1	5433

296.51115	1	5434

296.67186	1	5435

296.83257	1	5436

296.99328	1	5437

297.15399	1	5438

297.3147	1	5439

297.47541	1	5440

297.63612	1	5441

297.79684	3	5444

297.95755	1	5445

298.11826	1	5446

298.27897	1	5447

298.43968	5	5452

298.60039	1	5453

298.7611	2	5455

298.92181	5	5460

299.08252	2	5462

299.24323	1	5463

299.40394	1	5464

299.56465	1	5465

299.72536	1	5466

299.88607	1	5467

300.04678	3	5470

300.2075	2	5472

300.36821	1	5473

300.52892	1	5474

300.68963	1	5475

300.85034	4	5479

301.01105	1	5480

301.17176	1	5481

301.33247	4	5485

301.49318	2	5487

301.65389	1	5488

301.8146	1	5489

301.97531	10	5499

302.13602	2	5501

302.29673	5	5506

302.45744	3	5509

302.61816	1	5510

302.77887	2	5512

302.93958	2	5514

303.10029	1	5515

303.261	1	5516

303.42171	1	5517

303.58242	1	5518

303.74313	1	5519

303.90384	1	5520

304.06455	6	5526

304.22526	2	5528

304.38597	1	5529

304.54668	1	5530

304.70739	1	5531

304.8681	1	5532

305.02881	4	5536

305.18953	1	5537

305.35024	2	5539

305.51095	1	5540

305.67166	1	5541

305.83237	2	5543

305.99308	1	5544

306.15379	1	5545

306.3145	1	5546

306.47521	1	5547

306.63592	1	5548

306.79663	2	5550

306.95734	1	5551

307.11805	1	5552

307.27876	4	5556

307.43947	4	5560

307.60019	1	5561

307.7609	1	5562

307.92161	1	5563

308.08232	3	5566

308.24303	2	5568

308.40374	10	5578

308.56445	2	5580

308.72516	3	5583

308.88587	2	5585

309.04658	8	5593

309.20729	1	5594

309.368	4	5598

309.52871	2	5600

309.68942	2	5602

309.85013	2	5604

310.01084	1	5605

310.17156	1	5606

310.33227	1	5607

310.49298	1	5608

310.65369	7	5615

310.8144	4	5619

310.97511	1	5620

311.13582	1	5621

311.29653	3	5624

311.45724	2	5626

311.61795	2	5628

311.77866	2	5630

311.93937	1	5631

312.10008	6	5637

312.26079	12	5649

312.4215	1	5650

312.58222	1	5651

312.74293	3	5654

312.90364	1	5655

313.06435	6	5661

313.22506	5	5666

313.38577	2	5668

313.54648	1	5669

313.70719	1	5670

313.8679	1	5671

314.02861	1	5672

314.18932	2	5674

314.35003	1	5675

314.51074	1	5676

314.67145	1	5677

314.83216	1	5678

314.99288	2	5680

315.15359	1	5681

315.3143	1	5682

315.47501	1	5683

315.63572	2	5685

315.79643	3	5688

315.95714	47	5735

316.11785	1	5736

316.27856	1	5737

316.43927	2	5739

316.59998	3	5742

316.76069	3	5745

316.9214	1	5746

317.08211	1	5747

317.24282	1	5748

317.40353	2	5750

317.56425	3	5753

317.72496	1	5754

317.88567	1	5755

318.04638	1	5756

318.20709	1	5757

318.3678	1	5758

318.52851	2	5760

318.68922	2	5762

318.84993	3	5765

319.01064	19	5784

319.17135	1	5785

319.33206	1	5786

319.49277	1	5787

319.65348	7	5794

319.81419	3	5797

319.97491	3	5800

320.13562	2	5802

320.29633	1	5803

320.45704	4	5807

320.61775	1	5808

320.77846	5	5813

320.93917	46	5859

321.09988	1	5860

321.26059	1	5861

321.4213	8	5869

321.58201	1	5870

321.74272	1	5871

321.90343	2	5873

322.06414	1	5874

322.22485	1	5875

322.38556	1	5876

322.54628	1	5877

322.70699	7	5884

322.8677	2	5886

323.02841	1	5887

323.18912	1	5888

323.34983	3	5891

323.51054	2	5893

323.67125	4	5897

323.83196	2	5899

323.99267	12	5911

324.15338	2	5913

324.31409	1	5914

324.4748	2	5916

324.63551	2	5918

324.79622	1	5919

324.95694	7	5926

325.11765	3	5929

325.27836	1	5930

325.43907	3	5933

325.59978	1	5934

325.76049	1	5935

325.9212	1	5936

326.08191	2	5938

326.24262	4	5942

326.40333	1	5943

326.56404	2	5945

326.72475	4	5949

326.88546	1	5950

327.04617	2	5952

327.20688	7	5959

327.3676	1	5960

327.52831	1	5961

327.68902	3	5964

327.84973	11	5975

328.01044	1	5976

328.17115	2	5978

328.33186	1	5979

328.49257	5	5984

328.65328	2	5986

328.81399	1	5987

328.9747	3	5990

329.13541	1	5991

329.29612	2	5993

329.45683	1	5994

329.61754	1	5995

329.77825	3	5998

329.93897	4	6002

330.09968	1	6003

330.26039	1	6004

330.4211	9	6013

330.58181	5	6018

330.74252	1	6019

330.90323	1	6020

331.06394	1	6021

331.22465	2	6023

331.38536	2	6025

331.54607	2	6027

331.70678	2	6029

331.86749	1	6030

332.0282	3	6033

332.18891	1	6034

332.34963	2	6036

332.51034	2	6038

332.67105	1	6039

332.83176	1	6040

332.99247	2	6042

333.15318	1	6043

333.31389	1	6044

333.4746	1	6045

333.63531	1	6046

333.79602	1	6047

333.95673	3	6050

334.11744	1	6051

334.27815	3	6054

334.43886	3	6057

334.59957	1	6058

334.76029	1	6059

334.921	1	6060

335.08171	1	6061

335.24242	1	6062

335.40313	7	6069

335.56384	1	6070

335.72455	7	6077

335.88526	1	6078

336.04597	1	6079

336.20668	1	6080

336.36739	5	6085

336.5281	1	6086

336.68881	3	6089

336.84952	1	6090

337.01023	6	6096

337.17094	1	6097

337.33166	2	6099

337.49237	8	6107

337.65308	3	6110

337.81379	1	6111

337.9745	5	6116

338.13521	1	6117

338.29592	1	6118

338.45663	1	6119

338.61734	1	6120

338.77805	6	6126

338.93876	5	6131

339.09947	1	6132

339.26018	1	6133

339.42089	4	6137

339.5816	1	6138

339.74232	6	6144

339.90303	1	6145

340.06374	3	6148

340.22445	1	6149

340.38516	2	6151

340.54587	2	6153

340.70658	1	6154

340.86729	7	6161

341.028	1	6162

341.18871	1	6163

341.34942	3	6166

341.51013	4	6170

341.67084	1	6171

341.83155	2	6173

341.99226	1	6174

342.15298	1	6175

342.31369	1	6176

342.4744	3	6179

342.63511	1	6180

342.79582	1	6181

342.95653	1	6182

343.11724	3	6185

343.27795	4	6189

343.43866	2	6191

343.59937	2	6193

343.76008	1	6194

343.92079	3	6197

344.0815	1	6198

344.24221	4	6202

344.40292	1	6203

344.56363	5	6208

344.72435	1	6209

344.88506	3	6212

345.04577	1	6213

345.20648	1	6214

345.36719	2	6216

345.5279	1	6217

345.68861	1	6218

345.84932	1	6219

346.01003	5	6224

346.17074	1	6225

346.33145	2	6227

346.49216	1	6228

346.65287	1	6229

346.81358	4	6233

346.97429	1	6234

347.13501	2	6236

347.29572	2	6238

347.45643	2	6240

347.61714	1	6241

347.77785	2	6243

347.93856	1	6244

348.09927	14	6258

348.25998	6	6264

348.42069	1	6265

348.5814	7	6272

348.74211	1	6273

348.90282	3	6276

349.06353	5	6281

349.22424	4	6285

349.38495	2	6287

349.54566	3	6290

349.70638	16	6306

349.86709	1	6307

350.0278	2	6309

350.18851	8	6317

350.34922	2	6319

350.50993	1	6320

350.67064	1	6321

350.83135	4	6325

350.99206	6	6331

351.15277	2	6333

351.31348	1	6334

351.47419	2	6336

351.6349	1	6337

351.79561	7	6344

351.95632	5	6349

352.11704	3	6352

352.27775	1	6353

352.43846	11	6364

352.59917	1	6365

352.75988	1	6366

352.92059	1	6367

353.0813	2	6369

353.24201	2	6371

353.40272	3	6374

353.56343	1	6375

353.72414	7	6382

353.88485	2	6384

354.04556	5	6389

354.20627	1	6390

354.36698	2	6392

354.5277	15	6407

354.68841	1	6408

354.84912	7	6415

355.00983	5	6420

355.17054	2	6422

355.33125	2	6424

355.49196	1	6425

355.65267	1	6426

355.81338	2	6428

355.97409	2	6430

356.1348	2	6432

356.29551	10	6442

356.45622	14	6456

356.61693	11	6467

356.77764	1	6468

356.93835	1	6469

357.09907	1	6470

357.25978	1	6471

357.42049	1	6472

357.5812	9	6481

357.74191	3	6484

357.90262	2	6486

358.06333	1	6487

358.22404	2	6489

358.38475	1	6490

358.54546	4	6494

358.70617	1	6495

358.86688	1	6496

359.02759	1	6497

359.1883	1	6498

359.34901	3	6501

359.50973	1	6502

359.67044	2	6504

359.83115	1	6505

359.99186	1	6506

360.15257	2	6508

360.31328	5	6513

360.47399	1	6514

360.6347	1	6515

360.79541	1	6516

360.95612	1	6517

361.11683	8	6525

361.27754	1	6526

361.43825	1	6527

361.59896	2	6529

361.75967	1	6530

361.92039	12	6542

362.0811	1	6543

362.24181	5	6548

362.40252	2	6550

362.56323	4	6554

362.72394	1	6555

362.88465	2	6557

363.04536	2	6559

363.20607	1	6560

363.36678	4	6564

363.52749	5	6569

363.6882	1	6570

363.84891	1	6571

364.00962	4	6575

364.17033	1	6576

364.33104	2	6578

364.49176	1	6579

364.65247	1	6580

364.81318	1	6581

364.97389	1	6582

365.1346	2	6584

365.29531	1	6585

365.45602	1	6586

365.61673	5	6591

365.77744	2	6593

365.93815	4	6597

366.09886	5	6602

366.25957	2	6604

366.42028	1	6605

366.58099	3	6608

366.7417	3	6611

366.90242	1	6612

367.06313	2	6614

367.22384	1	6615

367.38455	2	6617

367.54526	2	6619

367.70597	2	6621

367.86668	1	6622

368.02739	6	6628

368.1881	4	6632

368.34881	1	6633

368.50952	1	6634

368.67023	26	6660

368.83094	24	6684

368.99165	1	6685

369.15236	4	6689

369.31307	2	6691

369.47379	1	6692

369.6345	1	6693

369.79521	3	6696

369.95592	1	6697

370.11663	4	6701

370.27734	1	6702

370.43805	4	6706

370.59876	2	6708

370.75947	1	6709

370.92018	1	6710

371.08089	1	6711

371.2416	3	6714

371.40231	1	6715

371.56302	3	6718

371.72373	2	6720

371.88445	8	6728

372.04516	1	6729

372.20587	1	6730

372.36658	4	6734

372.52729	2	6736

372.688	3	6739

372.84871	2	6741

373.00942	1	6742

373.17013	1	6743

373.33084	1	6744

373.49155	1	6745

373.65226	1	6746

373.81297	1	6747

373.97368	1	6748

374.13439	1	6749

374.29511	3	6752

374.45582	2	6754

374.61653	1	6755

374.77724	2	6757

374.93795	2	6759

375.09866	1	6760

375.25937	2	6762

375.42008	1	6763

375.58079	1	6764

375.7415	1	6765

375.90221	2	6767

376.06292	2	6769

376.22363	2	6771

376.38434	1	6772

376.54505	20	6792

376.70576	1	6793

376.86648	1	6794

377.02719	2	6796

377.1879	4	6800

377.34861	1	6801

377.50932	1	6802

377.67003	7	6809

377.83074	5	6814

377.99145	2	6816

378.15216	2	6818

378.31287	1	6819

378.47358	1	6820

378.63429	1	6821

378.795	1	6822

378.95571	4	6826

379.11642	1	6827

379.27714	1	6828

379.43785	2	6830

379.59856	1	6831

379.75927	1	6832

379.91998	2	6834

380.08069	2	6836

380.2414	1	6837

380.40211	9	6846

380.56282	7	6853

380.72353	1	6854

380.88424	1	6855

381.04495	1	6856

381.20566	3	6859

381.36637	1	6860

381.52708	8	6868

381.68779	1	6869

381.84851	3	6872

382.00922	5	6877

382.16993	1	6878

382.33064	1	6879

382.49135	2	6881

382.65206	1	6882

382.81277	1	6883

382.97348	1	6884

383.13419	1	6885

383.2949	4	6889

383.45561	1	6890

383.61632	7	6897

383.77703	1	6898

383.93774	1	6899

384.09845	1	6900

384.25917	1	6901

384.41988	1	6902

384.58059	4	6906

384.7413	1	6907

384.90201	1	6908

385.06272	2	6910

385.22343	1	6911

385.38414	9	6920

385.54485	1	6921

385.70556	1	6922

385.86627	2	6924

386.02698	1	6925

386.18769	15	6940

386.3484	4	6944

386.50911	1	6945

386.66983	5	6950

386.83054	4	6954

386.99125	1	6955

387.15196	6	6961

387.31267	1	6962

387.47338	5	6967

387.63409	1	6968

387.7948	6	6974

387.95551	1	6975

388.11622	2	6977

388.27693	1	6978

388.43764	4	6982

388.59835	1	6983

388.75906	1	6984

388.91977	3	6987

389.08048	1	6988

389.2412	1	6989

389.40191	1	6990

389.56262	3	6993

389.72333	1	6994

389.88404	1	6995

390.04475	5	7000

390.20546	3	7003

390.36617	3	7006

390.52688	1	7007

390.68759	1	7008

390.8483	1	7009

391.00901	4	7013

391.16972	1	7014

391.33043	2	7016

391.49114	3	7019

391.65186	1	7020

391.81257	9	7029

391.97328	1	7030

392.13399	2	7032

392.2947	1	7033

392.45541	3	7036

392.61612	1	7037

392.77683	13	7050

392.93754	1	7051

393.09825	3	7054

393.25896	1	7055

393.41967	2	7057

393.58038	2	7059

393.74109	1	7060

393.9018	1	7061

394.06252	1	7062

394.22323	1	7063

394.38394	8	7071

394.54465	1	7072

394.70536	2	7074

394.86607	1	7075

395.02678	1	7076

395.18749	1	7077

395.3482	1	7078

395.50891	1	7079

395.66962	1	7080

395.83033	1	7081

395.99104	1	7082

396.15175	3	7085

396.31246	2	7087

396.47317	3	7090

396.63389	1	7091

396.7946	1	7092

396.95531	1	7093

397.11602	3	7096

397.27673	2	7098

397.43744	5	7103

397.59815	4	7107

397.75886	1	7108

397.91957	5	7113

398.08028	1	7114

398.24099	8	7122

398.4017	1	7123

398.56241	1	7124

398.72312	1	7125

398.88383	1	7126

399.04455	6	7132

399.20526	1	7133

399.36597	1	7134

399.52668	33	7167

399.68739	1	7168

399.8481	2	7170

400.00881	2	7172

400.16952	1	7173

400.33023	1	7174

400.49094	4	7178

400.65165	2	7180

400.81236	1	7181

400.97307	4	7185

401.13378	3	7188

401.29449	4	7192

401.4552	1	7193

401.61592	5	7198

401.77663	3	7201

401.93734	1	7202

402.09805	1	7203

402.25876	12	7215

402.41947	2	7217

402.58018	1	7218

402.74089	1	7219

402.9016	2	7221

403.06231	2	7223

403.22302	1	7224

403.38373	3	7227

403.54444	8	7235

403.70515	2	7237

403.86586	1	7238

404.02658	2	7240

404.18729	1	7241

404.348	9	7250

404.50871	1	7251

404.66942	1	7252

404.83013	3	7255

404.99084	1	7256

405.15155	1	7257

405.31226	1	7258

405.47297	2	7260

405.63368	3	7263

405.79439	1	7264

405.9551	1	7265

406.11581	15	7280

406.27652	4	7284

406.43724	1	7285

406.59795	7	7292

406.75866	2	7294

406.91937	1	7295

407.08008	1	7296

407.24079	1	7297

407.4015	1	7298

407.56221	3	7301

407.72292	3	7304

407.88363	1	7305

408.04434	1	7306

408.20505	3	7309

408.36576	4	7313

408.52647	1	7314

408.68718	1	7315

408.84789	6	7321

409.00861	1	7322

409.16932	1	7323

409.33003	2	7325

409.49074	1	7326

409.65145	1	7327

409.81216	3	7330

409.97287	2	7332

410.13358	4	7336

410.29429	9	7345

410.455	1	7346

410.61571	1	7347

410.77642	1	7348

410.93713	1	7349

411.09784	2	7351

411.25855	2	7353

411.41927	1	7354

411.57998	1	7355

411.74069	3	7358

411.9014	4	7362

412.06211	1	7363

412.22282	1	7364

412.38353	2	7366

412.54424	1	7367

412.70495	1	7368

412.86566	1	7369

413.02637	3	7372

413.18708	2	7374

413.34779	6	7380

413.5085	3	7383

413.66921	5	7388

413.82993	6	7394

413.99064	3	7397

414.15135	3	7400

414.31206	1	7401

414.47277	1	7402

414.63348	3	7405

414.79419	5	7410

414.9549	2	7412

415.11561	4	7416

415.27632	2	7418

415.43703	3	7421

415.59774	3	7424

415.75845	5	7429

415.91916	3	7432

416.07987	1	7433

416.24058	1	7434

416.4013	1	7435

416.56201	5	7440

416.72272	2	7442

416.88343	1	7443

417.04414	4	7447

417.20485	1	7448

417.36556	2	7450

417.52627	1	7451

417.68698	2	7453

417.84769	1	7454

418.0084	1	7455

418.16911	3	7458

418.32982	1	7459

418.49053	1	7460

418.65124	8	7468

418.81196	3	7471

418.97267	2	7473

419.13338	1	7474

419.29409	2	7476

419.4548	1	7477

419.61551	2	7479

419.77622	2	7481

419.93693	1	7482

420.09764	3	7485

420.25835	1	7486

420.41906	1	7487

420.57977	5	7492

420.74048	2	7494

420.90119	5	7499

421.0619	2	7501

421.22262	6	7507

421.38333	1	7508

421.54404	1	7509

421.70475	2	7511

421.86546	1	7512

422.02617	1	7513

422.18688	2	7515

422.34759	1	7516

422.5083	1	7517

422.66901	1	7518

422.82972	1	7519

422.99043	1	7520

423.15114	2	7522

423.31185	8	7530

423.47256	3	7533

423.63327	1	7534

423.79399	1	7535

423.9547	2	7537

424.11541	1	7538

424.27612	4	7542

424.43683	3	7545

424.59754	2	7547

424.75825	1	7548

424.91896	13	7561

425.07967	1	7562

425.24038	2	7564

425.40109	2	7566

425.5618	3	7569

425.72251	7	7576

425.88322	3	7579

426.04393	1	7580

426.20465	1	7581

426.36536	1	7582

426.52607	1	7583

426.68678	3	7586

426.84749	2	7588

427.0082	10	7598

427.16891	1	7599

427.32962	1	7600

427.49033	1	7601

427.65104	2	7603

427.81175	1	7604

427.97246	1	7605

428.13317	3	7608

428.29388	1	7609

428.45459	1	7610

428.6153	1	7611

428.77602	2	7613

428.93673	1	7614

429.09744	3	7617

429.25815	1	7618

429.41886	2	7620

429.57957	2	7622

429.74028	2	7624

429.90099	4	7628

430.0617	2	7630

430.22241	4	7634

430.38312	2	7636

430.54383	1	7637

430.70454	4	7641

430.86525	1	7642

431.02596	6	7648

431.18668	2	7650

431.34739	1	7651

431.5081	1	7652

431.66881	2	7654

431.82952	1	7655

431.99023	1	7656

432.15094	1	7657

432.31165	1	7658

432.47236	6	7664

432.63307	6	7670

432.79378	1	7671

432.95449	12	7683

433.1152	1	7684

433.27591	1	7685

433.43662	1	7686

433.59734	4	7690

433.75805	1	7691

433.91876	6	7697

434.07947	5	7702

434.24018	1	7703

434.40089	1	7704

434.5616	6	7710

434.72231	1	7711

434.88302	13	7724

435.04373	6	7730

435.20444	10	7740

435.36515	1	7741

435.52586	1	7742

435.68657	1	7743

435.84728	1	7744

436.00799	1	7745

436.16871	4	7749

436.32942	1	7750

436.49013	1	7751

436.65084	9	7760

436.81155	2	7762

436.97226	1	7763

437.13297	1	7764

437.29368	1	7765

437.45439	1	7766

437.6151	1	7767

437.77581	1	7768

437.93652	1	7769

438.09723	3	7772

438.25794	1	7773

438.41865	10	7783

438.57937	1	7784

438.74008	1	7785

438.90079	1	7786

439.0615	1	7787

439.22221	6	7793

439.38292	1	7794

439.54363	2	7796

439.70434	4	7800

439.86505	1	7801

440.02576	1	7802

440.18647	2	7804

440.34718	2	7806

440.50789	4	7810

440.6686	2	7812

440.82931	3	7815

440.99003	2	7817

441.15074	1	7818

441.31145	3	7821

441.47216	3	7824

441.63287	4	7828

441.79358	1	7829

441.95429	1	7830

442.115	1	7831

442.27571	1	7832

442.43642	2	7834

442.59713	4	7838

442.75784	3	7841

442.91855	1	7842

443.07926	5	7847

443.23997	3	7850

443.40068	3	7853

443.5614	1	7854

443.72211	2	7856

443.88282	2	7858

444.04353	1	7859

444.20424	1	7860

444.36495	1	7861

444.52566	1	7862

444.68637	1	7863

444.84708	1	7864

445.00779	2	7866

445.1685	4	7870

445.32921	5	7875

445.48992	18	7893

445.65063	5	7898

445.81134	1	7899

445.97206	4	7903

446.13277	1	7904

446.29348	1	7905

446.45419	1	7906

446.6149	1	7907

446.77561	3	7910

446.93632	6	7916

447.09703	3	7919

447.25774	1	7920

447.41845	1	7921

447.57916	3	7924

447.73987	2	7926

447.90058	1	7927

448.06129	15	7942

448.222	1	7943

448.38272	5	7948

448.54343	2	7950

448.70414	1	7951

448.86485	1	7952

449.02556	3	7955

449.18627	1	7956

449.34698	1	7957

449.50769	2	7959

449.6684	6	7965

449.82911	2	7967

449.98982	1	7968

450.15053	4	7972

450.31124	1	7973

450.47195	1	7974

450.63266	21	7995

450.79337	1	7996

450.95409	1	7997

451.1148	1	7998

451.27551	5	8003

451.43622	1	8004

451.59693	5	8009

451.75764	9	8018

451.91835	7	8025

452.07906	1	8026

452.23977	2	8028

452.40048	1	8029

452.56119	3	8032

452.7219	1	8033

452.88261	1	8034

453.04332	4	8038

453.20403	1	8039

453.36475	1	8040

453.52546	3	8043

453.68617	2	8045

453.84688	2	8047

454.00759	1	8048

454.1683	1	8049

454.32901	2	8051

454.48972	2	8053

454.65043	1	8054

454.81114	6	8060

454.97185	1	8061

455.13256	1	8062

455.29327	8	8070

455.45398	1	8071

455.61469	3	8074

455.7754	2	8076

455.93612	11	8087

456.09683	2	8089

456.25754	1	8090

456.41825	2	8092

456.57896	2	8094

456.73967	3	8097

456.90038	1	8098

457.06109	1	8099

457.2218	1	8100

457.38251	4	8104

457.54322	1	8105

457.70393	1	8106

457.86464	4	8110

458.02535	1	8111

458.18606	1	8112

458.34678	1	8113

458.50749	3	8116

458.6682	5	8121

458.82891	1	8122

458.98962	5	8127

459.15033	2	8129

459.31104	2	8131

459.47175	3	8134

459.63246	3	8137

459.79317	1	8138

459.95388	11	8149

460.11459	3	8152

460.2753	14	8166

460.43601	9	8175

460.59672	1	8176

460.75744	4	8180

460.91815	1	8181

461.07886	7	8188

461.23957	11	8199

461.40028	1	8200

461.56099	13	8213

461.7217	1	8214

461.88241	2	8216

462.04312	1	8217

462.20383	8	8225

462.36454	2	8227

462.52525	5	8232

462.68596	2	8234

462.84667	1	8235

463.00738	5	8240

463.16809	1	8241

463.32881	1	8242

463.48952	4	8246

463.65023	1	8247

463.81094	1	8248

463.97165	1	8249

464.13236	2	8251

464.29307	1	8252

464.45378	4	8256

464.61449	1	8257

464.7752	1	8258

464.93591	5	8263

465.09662	1	8264

465.25733	1	8265

465.41804	3	8268

465.57875	2	8270

465.73947	1	8271

465.90018	9	8280

466.06089	10	8290

466.2216	2	8292

466.38231	1	8293

466.54302	2	8295

466.70373	1	8296

466.86444	1	8297

467.02515	1	8298

467.18586	1	8299

467.34657	6	8305

467.50728	2	8307

467.66799	1	8308

467.8287	5	8313

467.98941	2	8315

468.15013	1	8316

468.31084	1	8317

468.47155	2	8319

468.63226	1	8320

468.79297	3	8323

468.95368	2	8325

469.11439	1	8326

469.2751	5	8331

469.43581	4	8335

469.59652	1	8336

469.75723	3	8339

469.91794	1	8340

470.07865	1	8341

470.23936	1	8342

470.40007	8	8350

470.56078	2	8352

470.7215	1	8353

470.88221	1	8354

471.04292	1	8355

471.20363	2	8357

471.36434	1	8358

471.52505	2	8360

471.68576	56	8416

471.84647	12	8428

472.00718	1	8429

472.16789	1	8430

472.3286	1	8431

472.48931	1	8432

472.65002	1	8433

472.81073	3	8436

472.97144	2	8438

473.13216	1	8439

473.29287	1	8440

473.45358	2	8442

473.61429	2	8444

473.775	1	8445

473.93571	1	8446

474.09642	2	8448

474.25713	1	8449

474.41784	5	8454

474.57855	1	8455

474.73926	4	8459

474.89997	2	8461

475.06068	1	8462

475.22139	2	8464

475.3821	2	8466

475.54281	1	8467

475.70353	1	8468

475.86424	1	8469

476.02495	2	8471

476.18566	1	8472

476.34637	1	8473

476.50708	4	8477

476.66779	2	8479

476.8285	1	8480

476.98921	1	8481

477.14992	1	8482

477.31063	1	8483

477.47134	1	8484

477.63205	3	8487

477.79276	1	8488

477.95347	7	8495

478.11419	10	8505

478.2749	4	8509

478.43561	1	8510

478.59632	2	8512

478.75703	1	8513

478.91774	2	8515

479.07845	1	8516

479.23916	2	8518

479.39987	2	8520

479.56058	5	8525

479.72129	3	8528

479.882	10	8538

480.04271	3	8541

480.20342	1	8542

480.36413	3	8545

480.52485	6	8551

480.68556	3	8554

480.84627	1	8555

481.00698	5	8560

481.16769	2	8562

481.3284	15	8577

481.48911	4	8581

481.64982	2	8583

481.81053	7	8590

481.97124	11	8601

482.13195	1	8602

482.29266	1	8603

482.45337	1	8604

482.61408	3	8607

482.77479	2	8609

482.9355	3	8612

483.09622	12	8624

483.25693	2	8626

483.41764	5	8631

483.57835	1	8632

483.73906	1	8633

483.89977	12	8645

484.06048	1	8646

484.22119	4	8650

484.3819	1	8651

484.54261	17	8668

484.70332	1	8669

484.86403	7	8676

485.02474	7	8683

485.18545	1	8684

485.34616	1	8685

485.50688	1	8686

485.66759	1	8687

485.8283	1	8688

485.98901	3	8691

486.14972	5	8696

486.31043	4	8700

486.47114	12	8712

486.63185	2	8714

486.79256	2	8716

486.95327	3	8719

487.11398	1	8720

487.27469	2	8722

487.4354	3	8725

487.59611	26	8751

487.75682	8	8759

487.91753	3	8762

488.07825	14	8776

488.23896	6	8782

488.39967	5	8787

488.56038	4	8791

488.72109	4	8795

488.8818	16	8811

489.04251	1	8812

489.20322	6	8818

489.36393	1	8819

489.52464	12	8831

489.68535	1	8832

489.84606	1	8833

490.00677	5	8838

490.16748	1	8839

490.32819	1	8840

490.48891	2	8842

490.64962	2	8844

490.81033	5	8849

490.97104	1	8850

491.13175	4	8854

491.29246	22	8876

491.45317	1	8877

491.61388	1	8878

491.77459	9	8887

491.9353	1	8888

492.09601	7	8895

492.25672	1	8896

492.41743	2	8898

492.57814	1	8899

492.73885	1	8900

492.89957	5	8905

493.06028	1	8906

493.22099	1	8907

493.3817	1	8908

493.54241	1	8909

493.70312	15	8924

493.86383	2	8926

494.02454	3	8929

494.18525	1	8930

494.34596	9	8939

494.50667	1	8940

494.66738	3	8943

494.82809	2	8945

494.9888	1	8946

495.14951	1	8947

495.31023	7	8954

495.47094	4	8958

495.63165	6	8964

495.79236	3	8967

495.95307	1	8968

496.11378	6	8974

496.27449	1	8975

496.4352	1	8976

496.59591	1	8977

496.75662	3	8980

496.91733	2	8982

497.07804	2	8984

497.23875	2	8986

497.39946	2	8988

497.56017	3	8991

497.72088	3	8994

497.8816	2	8996

498.04231	7	9003

498.20302	17	9020

498.36373	4	9024

498.52444	7	9031

498.68515	6	9037

498.84586	2	9039

499.00657	8	9047

499.16728	1	9048

499.32799	1	9049

499.4887	1	9050

499.64941	3	9053

499.81012	6	9059

499.97083	2	9061

500.13154	4	9065

500.29226	1	9066

500.45297	1	9067

500.61368	3	9070

500.77439	2	9072

500.9351	1	9073

501.09581	5	9078

501.25652	2	9080

501.41723	1	9081

501.57794	3	9084

501.73865	9	9093

501.89936	1	9094

502.06007	1	9095

502.22078	4	9099

502.38149	2	9101

502.5422	2	9103

502.70291	1	9104

502.86363	1	9105

503.02434	1	9106

503.18505	3	9109

503.34576	4	9113

503.50647	2	9115

503.66718	3	9118

503.82789	4	9122

503.9886	1	9123

504.14931	4	9127

504.31002	3	9130

504.47073	2	9132

504.63144	2	9134

504.79215	1	9135

504.95286	4	9139

505.11357	4	9143

505.27429	2	9145

505.435	6	9151

505.59571	1	9152

505.75642	1	9153

505.91713	7	9160

506.07784	4	9164

506.23855	1	9165

506.39926	8	9173

506.55997	5	9178

506.72068	4	9182

506.88139	1	9183

507.0421	1	9184

507.20281	3	9187

507.36352	15	9202

507.52423	9	9211

507.68495	5	9216

507.84566	8	9224

508.00637	1	9225

508.16708	1	9226

508.32779	2	9228

508.4885	3	9231

508.64921	3	9234

508.80992	15	9249

508.97063	1	9250

509.13134	11	9261

509.29205	1	9262

509.45276	10	9272

509.61347	1	9273

509.77418	7	9280

509.93489	3	9283

510.0956	1	9284

510.25632	2	9286

510.41703	5	9291

510.57774	1	9292

510.73845	2	9294

510.89916	1	9295

511.05987	15	9310

511.22058	1	9311

511.38129	4	9315

511.542	1	9316

511.70271	2	9318

511.86342	1	9319

512.02413	13	9332

512.18484	1	9333

512.34555	1	9334

512.50626	1	9335

512.66698	6	9341

512.82769	1	9342

512.9884	1	9343

513.14911	1	9344

513.30982	4	9348

513.47053	6	9354

513.63124	1	9355

513.79195	1	9356

513.95266	3	9359

514.11337	3	9362

514.27408	2	9364

514.43479	2	9366

514.5955	1	9367

514.75621	12	9379

514.91692	1	9380

515.07763	1	9381

515.23835	1	9382

515.39906	5	9387

515.55977	1	9388

515.72048	4	9392

515.88119	3	9395

516.0419	6	9401

516.20261	5	9406

516.36332	14	9420

516.52403	7	9427

516.68474	3	9430

516.84545	2	9432

517.00616	1	9433

517.16687	8	9441

517.32758	1	9442

517.48829	3	9445

517.64901	3	9448

517.80972	1	9449

517.97043	1	9450

518.13114	1	9451

518.29185	2	9453

518.45256	6	9459

518.61327	1	9460

518.77398	1	9461

518.93469	1	9462

519.0954	4	9466

519.25611	1	9467

519.41682	1	9468

519.57753	1	9469

519.73824	2	9471

519.89895	1	9472

520.05967	3	9475

520.22038	31	9506

520.38109	1	9507

520.5418	1	9508

520.70251	1	9509

520.86322	5	9514

521.02393	3	9517

521.18464	2	9519

521.34535	1	9520

521.50606	2	9522

521.66677	9	9531

521.82748	2	9533

521.98819	1	9534

522.1489	2	9536

522.30961	7	9543

522.47032	1	9544

522.63104	1	9545

522.79175	1	9546

522.95246	1	9547

523.11317	1	9548

523.27388	5	9553

523.43459	1	9554

523.5953	3	9557

523.75601	2	9559

523.91672	14	9573

524.07743	2	9575

524.23814	1	9576

524.39885	3	9579

524.55956	2	9581

524.72027	5	9586

524.88098	1	9587

525.0417	9	9596

525.20241	2	9598

525.36312	1	9599

525.52383	3	9602

525.68454	2	9604

525.84525	2	9606

526.00596	9	9615

526.16667	8	9623

526.32738	1	9624

526.48809	10	9634

526.6488	3	9637

526.80951	1	9638

526.97022	4	9642

527.13093	1	9643

527.29164	1	9644

527.45236	1	9645

527.61307	4	9649

527.77378	1	9650

527.93449	11	9661

528.0952	1	9662

528.25591	2	9664

528.41662	1	9665

528.57733	1	9666

528.73804	1	9667

528.89875	6	9673

529.05946	1	9674

529.22017	2	9676

529.38088	13	9689

529.54159	8	9697

529.7023	1	9698

529.86301	5	9703

530.02373	1	9704

530.18444	17	9721

530.34515	1	9722

530.50586	1	9723

530.66657	2	9725

530.82728	3	9728

530.98799	2	9730

531.1487	1	9731

531.30941	1	9732

531.47012	1	9733

531.63083	1	9734

531.79154	4	9738

531.95225	1	9739

532.11296	1	9740

532.27367	4	9744

532.43439	1	9745

532.5951	1	9746

532.75581	1	9747

532.91652	1	9748

533.07723	1	9749

533.23794	4	9753

533.39865	4	9757

533.55936	5	9762

533.72007	1	9763

533.88078	5	9768

534.04149	1	9769

534.2022	4	9773

534.36291	1	9774

534.52362	3	9777

534.68433	2	9779

534.84505	13	9792

535.00576	1	9793

535.16647	1	9794

535.32718	3	9797

535.48789	1	9798

535.6486	1	9799

535.80931	2	9801

535.97002	4	9805

536.13073	1	9806

536.29144	1	9807

536.45215	5	9812

536.61286	2	9814

536.77357	8	9822

536.93428	1	9823

537.09499	4	9827

537.2557	8	9835

537.41642	1	9836

537.57713	1	9837

537.73784	10	9847

537.89855	1	9848

538.05926	1	9849

538.21997	3	9852

538.38068	2	9854

538.54139	1	9855

538.7021	5	9860

538.86281	1	9861

539.02352	1	9862

539.18423	1	9863

539.34494	3	9866

539.50565	1	9867

539.66636	3	9870

539.82708	2	9872

539.98779	1	9873

540.1485	1	9874

540.30921	2	9876

540.46992	2	9878

540.63063	2	9880

540.79134	2	9882

540.95205	1	9883

541.11276	3	9886

541.27347	1	9887

541.43418	5	9892

541.59489	2	9894

541.7556	1	9895

541.91631	1	9896

542.07702	11	9907

542.23773	1	9908

542.39845	2	9910

542.55916	1	9911

542.71987	1	9912

542.88058	2	9914

543.04129	1	9915

543.202	1	9916

543.36271	1	9917

543.52342	2	9919

543.68413	6	9925

543.84484	1	9926

544.00555	4	9930

544.16626	1	9931

544.32697	1	9932

544.48768	1	9933

544.64839	3	9936

544.80911	1	9937

544.96982	2	9939

545.13053	1	9940

545.29124	2	9942

545.45195	1	9943

545.61266	6	9949

545.77337	1	9950

545.93408	1	9951

546.09479	1	9952

546.2555	3	9955

546.41621	1	9956

546.57692	1	9957

546.73763	2	9959

546.89834	8	9967

547.05905	5	9972

547.21977	1	9973

547.38048	1	9974

547.54119	1	9975

547.7019	2	9977

547.86261	2	9979

548.02332	7	9986

548.18403	8	9994

548.34474	1	9995

548.50545	1	9996

548.66616	3	9999

548.82687	2	10001

548.98758	5	10006

549.14829	2	10008

549.309	6	10014

549.46971	8	10022

549.63042	1	10023

549.79114	2	10025

549.95185	1	10026

550.11256	2	10028

550.27327	1	10029

550.43398	2	10031

550.59469	1	10032

550.7554	2	10034

550.91611	4	10038

551.07682	2	10040

551.23753	10	10050

551.39824	1	10051

551.55895	6	10057

551.71966	1	10058

551.88037	1	10059

552.04108	2	10061

552.2018	1	10062

552.36251	1	10063

552.52322	1	10064

552.68393	1	10065

552.84464	4	10069

553.00535	1	10070

553.16606	4	10074

553.32677	2	10076

553.48748	1	10077

553.64819	2	10079

553.8089	1	10080

553.96961	1	10081

554.13032	2	10083

554.29103	1	10084

554.45174	1	10085

554.61245	2	10087

554.77317	20	10107

554.93388	2	10109

555.09459	1	10110

555.2553	1	10111

555.41601	6	10117

555.57672	1	10118

555.73743	15	10133

555.89814	2	10135

556.05885	3	10138

556.21956	5	10143

556.38027	1	10144

556.54098	1	10145

556.70169	1	10146

556.8624	1	10147

557.02311	1	10148

557.18383	3	10151

557.34454	1	10152

557.50525	7	10159

557.66596	4	10163

557.82667	10	10173

557.98738	4	10177

558.14809	6	10183

558.3088	1	10184

558.46951	6	10190

558.63022	1	10191

558.79093	4	10195

558.95164	1	10196

559.11235	4	10200

559.27306	8	10208

559.43377	1	10209

559.59449	16	10225

559.7552	3	10228

559.91591	1	10229

560.07662	10	10239

560.23733	2	10241

560.39804	4	10245

560.55875	3	10248

560.71946	1	10249

560.88017	1	10250

561.04088	3	10253

561.20159	1	10254

561.3623	8	10262

561.52301	1	10263

561.68372	1	10264

561.84443	2	10266

562.00514	7	10273

562.16586	1	10274

562.32657	24	10298

562.48728	1	10299

562.64799	3	10302

562.8087	3	10305

562.96941	31	10336

563.13012	2	10338

563.29083	1	10339

563.45154	2	10341

563.61225	1	10342

563.77296	1	10343

563.93367	2	10345

564.09438	6	10351

564.25509	5	10356

564.4158	6	10362

564.57652	3	10365

564.73723	10	10375

564.89794	2	10377

565.05865	2	10379

565.21936	5	10384

565.38007	4	10388

565.54078	3	10391

565.70149	7	10398

565.8622	3	10401

566.02291	3	10404

566.18362	9	10413

566.34433	4	10417

566.50504	1	10418

566.66575	1	10419

566.82646	4	10423

566.98718	14	10437

567.14789	10	10447

567.3086	3	10450

567.46931	1	10451

567.63002	2	10453

567.79073	2	10455

567.95144	3	10458

568.11215	1	10459

568.27286	4	10463

568.43357	3	10466

568.59428	5	10471

568.75499	1	10472

568.9157	2	10474

569.07641	7	10481

569.23712	5	10486

569.39783	2	10488

569.55855	8	10496

569.71926	6	10502

569.87997	3	10505

570.04068	1	10506

570.20139	9	10515

570.3621	1	10516

570.52281	4	10520

570.68352	3	10523

570.84423	6	10529

571.00494	3	10532

571.16565	2	10534

571.32636	4	10538

571.48707	6	10544

571.64778	1	10545

571.80849	2	10547

571.96921	1	10548

572.12992	7	10555

572.29063	12	10567

572.45134	3	10570

572.61205	1	10571

572.77276	11	10582

572.93347	1	10583

573.09418	2	10585

573.25489	6	10591

573.4156	4	10595

573.57631	6	10601

573.73702	3	10604

573.89773	6	10610

574.05844	3	10613

574.21915	2	10615

574.37987	2	10617

574.54058	1	10618

574.70129	4	10622

574.862	2	10624

575.02271	6	10630

575.18342	1	10631

575.34413	1	10632

575.50484	2	10634

575.66555	41	10675

575.82626	1	10676

575.98697	1	10677

576.14768	1	10678

576.30839	2	10680

576.4691	2	10682

576.62981	18	10700

576.79052	5	10705

576.95124	1	10706

577.11195	1	10707

577.27266	1	10708

577.43337	1	10709

577.59408	6	10715

577.75479	5	10720

577.9155	8	10728

578.07621	1	10729

578.23692	1	10730

578.39763	20	10750

578.55834	3	10753

578.71905	4	10757

578.87976	1	10758

579.04047	11	10769

579.20118	1	10770

579.3619	7	10777

579.52261	1	10778

579.68332	3	10781

579.84403	3	10784

580.00474	27	10811

580.16545	1	10812

580.32616	6	10818

580.48687	1	10819

580.64758	1	10820

580.80829	1	10821

580.969	2	10823

581.12971	2	10825

581.29042	2	10827

581.45113	1	10828

581.61184	1	10829

581.77255	3	10832

581.93327	1	10833

582.09398	1	10834

582.25469	6	10840

582.4154	1	10841

582.57611	7	10848

582.73682	1	10849

582.89753	3	10852

583.05824	2	10854

583.21895	5	10859

583.37966	1	10860

583.54037	1	10861

583.70108	1	10862

583.86179	2	10864

584.0225	2	10866

584.18321	1	10867

584.34393	5	10872

584.50464	1	10873

584.66535	1	10874

584.82606	7	10881

584.98677	1	10882

585.14748	2	10884

585.30819	4	10888

585.4689	1	10889

585.62961	2	10891

585.79032	3	10894

585.95103	2	10896

586.11174	4	10900

586.27245	1	10901

586.43316	6	10907

586.59387	1	10908

586.75459	1	10909

586.9153	1	10910

587.07601	1	10911

587.23672	2	10913

587.39743	5	10918

587.55814	1	10919

587.71885	2	10921

587.87956	10	10931

588.04027	1	10932

588.20098	1	10933

588.36169	6	10939

588.5224	2	10941

588.68311	1	10942

588.84382	4	10946

589.00453	1	10947

589.16524	1	10948

589.32596	1	10949

589.48667	13	10962

589.64738	1	10963

589.80809	1	10964

589.9688	3	10967

590.12951	1	10968

590.29022	4	10972

590.45093	5	10977

590.61164	5	10982

590.77235	1	10983

590.93306	6	10989

591.09377	2	10991

591.25448	7	10998

591.41519	2	11000

591.5759	1	11001

591.73662	2	11003

591.89733	11	11014

592.05804	1	11015

592.21875	1	11016

592.37946	3	11019

592.54017	3	11022

592.70088	1	11023

592.86159	36	11059

593.0223	1	11060

593.18301	2	11062

593.34372	3	11065

593.50443	6	11071

593.66514	1	11072

593.82585	1	11073

593.98656	1	11074

594.14728	4	11078

594.30799	2	11080

594.4687	2	11082

594.62941	2	11084

594.79012	1	11085

594.95083	1	11086

595.11154	2	11088

595.27225	2	11090

595.43296	4	11094

595.59367	2	11096

595.75438	4	11100

595.91509	1	11101

596.0758	1	11102

596.23651	4	11106

596.39722	1	11107

596.55793	2	11109

596.71865	4	11113

596.87936	3	11116

597.04007	1	11117

597.20078	1	11118

597.36149	3	11121

597.5222	3	11124

597.68291	2	11126

597.84362	2	11128

598.00433	1	11129

598.16504	1	11130

598.32575	2	11132

598.48646	4	11136

598.64717	1	11137

598.80788	1	11138

598.96859	4	11142

599.12931	3	11145

599.29002	2	11147

599.45073	1	11148

599.61144	1	11149

599.77215	2	11151

599.93286	3	11154

600.09357	1	11155

600.25428	1	11156

600.41499	2	11158

600.5757	9	11167

600.73641	3	11170

600.89712	1	11171

601.05783	1	11172

601.21854	1	11173

601.37925	10	11183

601.53997	2	11185

601.70068	3	11188

601.86139	1	11189

602.0221	2	11191

602.18281	1	11192

602.34352	1	11193

602.50423	3	11196

602.66494	3	11199

602.82565	4	11203

602.98636	3	11206

603.14707	9	11215

603.30778	5	11220

603.46849	4	11224

603.6292	1	11225

603.78991	5	11230

603.95062	1	11231

604.11134	3	11234

604.27205	2	11236

604.43276	1	11237

604.59347	2	11239

604.75418	1	11240

604.91489	5	11245

605.0756	6	11251

605.23631	1	11252

605.39702	8	11260

605.55773	1	11261

605.71844	3	11264

605.87915	1	11265

606.03986	6	11271

606.20057	4	11275

606.36128	1	11276

606.522	3	11279

606.68271	2	11281

606.84342	10	11291

607.00413	2	11293

607.16484	1	11294

607.32555	1	11295

607.48626	2	11297

607.64697	4	11301

607.80768	1	11302

607.96839	5	11307

608.1291	3	11310

608.28981	3	11313

608.45052	3	11316

608.61123	5	11321

608.77194	3	11324

608.93265	7	11331

609.09337	2	11333

609.25408	11	11344

609.41479	3	11347

609.5755	2	11349

609.73621	5	11354

609.89692	1	11355

610.05763	8	11363

610.21834	7	11370

610.37905	3	11373

610.53976	2	11375

610.70047	1	11376

610.86118	1	11377

611.02189	8	11385

611.1826	11	11396

611.34331	1	11397

611.50403	11	11408

611.66474	2	11410

611.82545	2	11412

611.98616	1	11413

612.14687	4	11417

612.30758	1	11418

612.46829	11	11429

612.629	5	11434

612.78971	2	11436

612.95042	8	11444

613.11113	1	11445

613.27184	14	11459

613.43255	1	11460

613.59326	1	11461

613.75397	3	11464

613.91469	1	11465

614.0754	1	11466

614.23611	6	11472

614.39682	4	11476

614.55753	1	11477

614.71824	3	11480

614.87895	1	11481

615.03966	6	11487

615.20037	1	11488

615.36108	1	11489

615.52179	5	11494

615.6825	2	11496

615.84321	1	11497

616.00392	3	11500

616.16463	1	11501

616.32534	8	11509

616.48606	1	11510

616.64677	1	11511

616.80748	2	11513

616.96819	8	11521

617.1289	2	11523

617.28961	3	11526

617.45032	6	11532

617.61103	3	11535

617.77174	2	11537

617.93245	1	11538

618.09316	2	11540

618.25387	1	11541

618.41458	1	11542

618.57529	1	11543

618.736	1	11544

618.89672	7	11551

619.05743	1	11552

619.21814	1	11553

619.37885	3	11556

619.53956	1	11557

619.70027	1	11558

619.86098	16	11574

620.02169	1	11575

620.1824	3	11578

620.34311	4	11582

620.50382	1	11583

620.66453	8	11591

620.82524	8	11599

620.98595	1	11600

621.14666	6	11606

621.30737	3	11609

621.46809	1	11610

621.6288	2	11612

621.78951	4	11616

621.95022	3	11619

622.11093	1	11620

622.27164	9	11629

622.43235	2	11631

622.59306	1	11632

622.75377	3	11635

622.91448	1	11636

623.07519	5	11641

623.2359	19	11660

623.39661	1	11661

623.55732	8	11669

623.71803	2	11671

623.87875	5	11676

624.03946	2	11678

624.20017	4	11682

624.36088	1	11683

624.52159	1	11684

624.6823	1	11685

624.84301	2	11687

625.00372	1	11688

625.16443	1	11689

625.32514	4	11693

625.48585	1	11694

625.64656	1	11695

625.80727	5	11700

625.96798	14	11714

626.12869	11	11725

626.28941	2	11727

626.45012	4	11731

626.61083	4	11735

626.77154	8	11743

626.93225	1	11744

627.09296	1	11745

627.25367	1	11746

627.41438	3	11749

627.57509	1	11750

627.7358	3	11753

627.89651	8	11761

628.05722	1	11762

628.21793	17	11779

628.37864	2	11781

628.53935	2	11783

628.70006	3	11786

628.86078	2	11788

629.02149	2	11790

629.1822	2	11792

629.34291	4	11796

629.50362	12	11808

629.66433	2	11810

629.82504	7	11817

629.98575	1	11818

630.14646	1	11819

630.30717	11	11830

630.46788	5	11835

630.62859	1	11836

630.7893	2	11838

630.95001	10	11848

631.11072	4	11852

631.27144	2	11854

631.43215	1	11855

631.59286	2	11857

631.75357	5	11862

631.91428	6	11868

632.07499	1	11869

632.2357	2	11871

632.39641	10	11881

632.55712	10	11891

632.71783	1	11892

632.87854	5	11897

633.03925	1	11898

633.19996	3	11901

633.36067	3	11904

633.52138	10	11914

633.6821	1	11915

633.84281	1	11916

634.00352	4	11920

634.16423	1	11921

634.32494	9	11930

634.48565	1	11931

634.64636	12	11943

634.80707	3	11946

634.96778	1	11947

635.12849	3	11950

635.2892	2	11952

635.44991	2	11954

635.61062	2	11956

635.77133	4	11960

635.93204	1	11961

636.09275	1	11962

636.25347	6	11968

636.41418	2	11970

636.57489	5	11975

636.7356	3	11978

636.89631	5	11983

637.05702	3	11986

637.21773	2	11988

637.37844	1	11989

637.53915	2	11991

637.69986	1	11992

637.86057	2	11994

638.02128	1	11995

638.18199	1	11996

638.3427	1	11997

638.50341	1	11998

638.66413	11	12009

638.82484	1	12010

638.98555	4	12014

639.14626	1	12015

639.30697	2	12017

639.46768	2	12019

639.62839	11	12030

639.7891	10	12040

639.94981	9	12049

640.11052	20	12069

640.27123	2	12071

640.43194	1	12072

640.59265	5	12077

640.75336	10	12087

640.91407	1	12088

641.07479	1	12089

641.2355	3	12092

641.39621	4	12096

641.55692	7	12103

641.71763	24	12127

641.87834	1	12128

642.03905	5	12133

642.19976	2	12135

642.36047	2	12137

642.52118	1	12138

642.68189	1	12139

642.8426	6	12145

643.00331	4	12149

643.16402	1	12150

643.32473	1	12151

643.48544	4	12155

643.64616	1	12156

643.80687	13	12169

643.96758	8	12177

644.12829	2	12179

644.289	4	12183

644.44971	3	12186

644.61042	1	12187

644.77113	2	12189

644.93184	3	12192

645.09255	1	12193

645.25326	2	12195

645.41397	9	12204

645.57468	4	12208

645.73539	1	12209

645.8961	1	12210

646.05682	3	12213

646.21753	4.5	12217.5

646.37824	4.5	12222

646.53895	2.25	12224.25

646.69966	6.75	12231

646.86037	2.25	12233.25

647.02108	4.5	12237.75

647.18179	2.25	12240

647.3425	2.25	12242.25

647.50321	2.25	12244.5

647.66392	6.75	12251.25

647.82463	2.25	12253.5

647.98534	2.25	12255.75

648.14605	2.25	12258

648.30676	2.25	12260.25

648.46747	4.5	12264.75

648.62819	2.25	12267

648.7889	2.25	12269.25

648.94961	2.25	12271.5

649.11032	6.75	12278.25

649.27103	2.25	12280.5

649.43174	9	12289.5

649.59245	2.25	12291.75

649.75316	4.5	12296.25

649.91387	2.25	12298.5

650.07458	4.5	12303

650.23529	2.25	12305.25

650.396	2.25	12307.5

650.55671	2.25	12309.75

650.71742	2.25	12312

650.87813	69	12321

651.03885	6.75	12327.75

651.19956	2.25	12330

651.36027	6.75	12336.75

651.52098	2.25	12339

651.68169	2.25	12341.25

651.8424	4.5	12345.75

652.00311	2.25	12348

652.16382	2.25	12350.25

652.32453	2.25	12352.5

652.48524	2.25	12354.75

652.64595	2.25	12357

652.80666	2.25	12359.25

652.96737	4.5	12363.75

653.12808	2.25	12366

653.28879	2.25	12368.25

653.44951	2.25	12370.5

653.61022	2.25	12372.75

653.77093	2.25	12375

653.93164	2.25	12377.25

654.09235	2.25	12379.5

654.25306	2.25	12381.75

654.41377	2.25	12384

654.57448	2.25	12386.25

654.73519	2.25	12388.5

654.8959	2.25	12390.75

655.05661	4.5	12395.25

655.21732	2.25	12397.5

655.37803	2.25	12399.75

655.53874	2.25	12402

655.69945	2.25	12404.25

655.86016	4.5	12408.75

656.02088	4.5	12413.25

656.18159	2.25	12415.5

656.3423	2.25	12417.75

656.50301	4.5	12422.25

656.66372	2.25	12424.5

656.82443	9	12433.5

656.98514	4.5	12438

657.14585	2.25	12440.25

657.30656	6.75	12447

657.46727	4.5	12451.5

657.62798	4.5	12456

657.78869	2.25	12458.25

657.9494	2.25	12460.5

658.11011	13.5	12474

658.27082	6.75	12480.75

658.43154	4.5	12485.25

658.59225	2.25	12487.5

658.75296	11.25	12498.75

658.91367	4.5	12503.25

659.07438	2.25	12505.5

659.23509	2.25	12507.75

659.3958	2.25	12510

659.55651	6.75	12516.75

659.71722	2.25	12519

659.87793	4.5	12523.5

660.03864	6.75	12530.25

660.19935	4.5	12534.75

660.36006	4.5	12539.25

660.52077	2.25	12541.5

660.68148	2.25	12543.75

660.84219	6.75	12550.5

661.00291	4.5	12555

661.16362	2.25	12557.25

661.32433	2.25	12559.5

661.48504	2.25	12561.75

661.64575	2.25	12564

661.80646	4.5	12568.5

661.96717	2.25	12570.75

662.12788	4.5	12575.25

662.28859	2.25	12577.5

662.4493	4.5	12582

662.61001	2.25	12584.25

662.77072	2.25	12586.5

662.93143	2.25	12588.75

663.09214	2.25	12591

663.25285	2.25	12593.25

663.41357	4.5	12597.75

663.57428	2.25	12600

663.73499	2.25	12602.25

663.8957	2.25	12604.5

664.05641	2.25	12606.75

664.21712	2.25	12609

664.37783	2.25	12611.25

664.53854	2.25	12613.5

664.69925	2.25	12615.75

664.85996	2.25	12618

665.02067	2.25	12620.25

665.18138	2.25	12622.5

665.34209	6.75	12629.25

665.5028	2.25	12631.5

665.66351	2.25	12633.75

665.82423	4.5	12638.25

665.98494	2.25	12640.5

666.14565	2.25	12642.75

666.30636	2.25	12645

666.46707	2.25	12647.25

666.62778	9	12656.25

666.78849	2.25	12658.5

666.9492	4.5	12663

667.10991	2.25	12665.25

667.27062	2.25	12667.5

667.43133	2.25	12669.75

667.59204	2.25	12672

667.75275	11.25	12683.25

667.91346	2.25	12685.5

668.07417	2.25	12687.75

668.23488	4.5	12692.25

668.3956	11.25	12703.5

668.55631	2.25	12705.75

668.71702	4.5	12710.25

668.87773	2.25	12712.5

669.03844	2.25	12714.75

669.19915	2.25	12717

669.35986	6.75	12723.75

669.52057	2.25	12726

669.68128	9	12735

669.84199	2.25	12737.25

670.0027	2.25	12739.5

670.16341	11.25	12750.75

670.32412	2.25	12753

670.48483	2.25	12755.25

670.64554	2.25	12757.5

670.80626	2.25	12759.75

670.96697	2.25	12762

671.12768	2.25	12764.25

671.28839	2.25	12766.5

671.4491	2.25	12768.75

671.60981	2.25	12771

671.77052	2.25	12773.25

671.93123	4.5	12777.75

672.09194	2.25	12780

672.25265	2.25	12782.25

672.41336	2.25	12784.5

672.57407	2.25	12786.75

672.73478	4.5	12791.25

672.89549	2.25	12793.5

673.0562	2.25	12795.75

673.21692	2.25	12798

673.37763	2.25	12800.25

673.53834	4.5	12804.75

673.69905	6.75	12811.5

673.85976	2.25	12813.75

674.02047	4.5	12818.25

674.18118	4.5	12822.75

674.34189	2.25	12825

674.5026	2.25	12827.25

674.66331	2.25	12829.5

674.82402	2.25	12831.75

674.98473	2.25	12834

675.14544	2.25	12836.25

675.30615	2.25	12838.5

675.46686	2.25	12840.75

675.62757	2.25	12843

675.78829	2.25	12845.25

675.949	2.25	12847.5

676.10971	62.25	12849.75

676.27042	2.25	12852

676.43113	2.25	12854.25

676.59184	4.5	12858.75

676.75255	6.75	12865.5

676.91326	6.75	12872.25

677.07397	2.25	12874.5

677.23468	4.5	12879

677.39539	4.5	12883.5

677.5561	2.25	12885.75

677.71681	6.75	12892.5

677.87752	2.25	12894.75

678.03823	2.25	12897

678.19895	2.25	12899.25

678.35966	2.25	12901.5

678.52037	4.5	12906

678.68108	2.25	12908.25

678.84179	2.25	12910.5

679.0025	2.25	12912.75

679.16321	2.25	12915

679.32392	2.25	12917.25

679.48463	2.25	12919.5

679.64534	2.25	12921.75

679.80605	2.25	12924

679.96676	2.25	12926.25

680.12747	2.25	12928.5

680.28818	11.25	12939.75

680.44889	4.5	12944.25

680.6096	2.25	12946.5

680.77032	2.25	12948.75

680.93103	2.25	12951

681.09174	2.25	12953.25

681.25245	2.25	12955.5

681.41316	2.25	12957.75

681.57387	2.25	12960

681.73458	2.25	12962.25

681.89529	2.25	12964.5

682.056	4.5	12969

682.21671	4.5	12973.5

682.37742	2.25	12975.75

682.53813	4.5	12980.25

682.69884	2.25	12982.5

682.85955	4.5	12987

683.02026	2.25	12989.25

683.18098	2.25	12991.5

683.34169	6.75	12998.25

683.5024	2.25	13000.5

683.66311	2.25	13002.75

683.82382	2.25	13005

683.98453	4.5	13009.5

684.14524	22.5	13032

684.30595	4.5	13036.5

684.46666	4.5	13041

684.62737	4.5	13045.5

684.78808	2.25	13047.75

684.94879	4.5	13052.25

685.1095	2.25	13054.5

685.27021	6.75	13061.25

685.43092	2.25	13063.5

685.59164	2.25	13065.75

685.75235	2.25	13068

685.91306	2.25	13070.25

686.07377	2.25	13072.5

686.23448	2.25	13074.75

686.39519	2.25	13077

686.5559	2.25	13079.25

686.71661	2.25	13081.5

686.87732	2.25	13083.75

687.03803	2.25	13086

687.19874	2.25	13088.25

687.35945	2.25	13090.5

687.52016	2.25	13092.75

687.68087	6.75	13099.5

687.84158	4.5	13104

688.00229	2.25	13106.25

688.16301	4.5	13110.75

688.32372	2.25	13113

688.48443	2.25	13115.25

688.64514	2.25	13117.5

688.80585	2.25	13119.75

688.96656	52.25	13122

689.12727	4.5	13126.5

689.28798	4.5	13131

689.44869	2.25	13133.25

689.6094	13.5	13146.75

689.77011	6.75	13153.5

689.93082	4.5	13158

690.09153	6.75	13164.75

690.25224	2.25	13167

690.41295	2.25	13169.25

690.57367	2.25	13171.5

690.73438	2.25	13173.75

690.89509	2.25	13176

691.0558	2.25	13178.25

691.21651	2.25	13180.5

691.37722	2.25	13182.75

691.53793	4.5	13187.25

691.69864	2.25	13189.5

691.85935	2.25	13191.75

692.02006	4.5	13196.25

692.18077	2.25	13198.5

692.34148	2.25	13200.75

692.50219	2.25	13203

692.6629	6.75	13209.75

692.82361	2.25	13212

692.98433	2.25	13214.25

693.14504	2.25	13216.5

693.30575	2.25	13218.75

693.46646	6.75	13225.5

693.62717	2.25	13227.75

693.78788	2.25	13230

693.94859	2.25	13232.25

694.1093	2.25	13234.5

694.27001	2.25	13236.75

694.43072	6.75	13243.5

694.59143	4.5	13248

694.75214	4.5	13252.5

694.91285	2.25	13254.75

695.07356	2.25	13257

695.23427	6.75	13263.75

695.39498	2.25	13266

695.5557	6.75	13272.75

695.71641	2.25	13275

695.87712	2.25	13277.25

696.03783	2.25	13279.5

696.19854	2.25	13281.75

696.35925	2.25	13284

696.51996	2.25	13286.25

696.68067	4.5	13290.75

696.84138	2.25	13293

697.00209	2.25	13295.25

697.1628	4.5	13299.75

697.32351	2.25	13302

697.48422	4.5	13306.5

697.64493	4.5	13311

697.80564	2.25	13313.25

697.96636	11.25	13324.5

698.12707	2.25	13326.75

698.28778	2.25	13329

698.44849	2.25	13331.25

698.6092	2.25	13333.5

698.76991	4.5	13338

698.93062	2.25	13340.25

699.09133	2.25	13342.5

699.25204	2.25	13344.75

699.41275	2.25	13347

699.57346	4.5	13351.5

699.73417	2.25	13353.75

699.89488	2.25	13356

700.05559	2.25	13358.25

700.2163	4.5	13362.75

700.37702	9	13371.75

700.53773	4.5	13376.25

700.69844	2.25	13378.5

700.85915	9	13387.5

701.01986	2.25	13389.75

701.18057	2.25	13392

701.34128	9	13401

701.50199	4.5	13405.5

701.6627	2.25	13407.75

701.82341	4.5	13412.25

701.98412	2.25	13414.5

702.14483	4.5	13419

702.30554	2.25	13421.25

702.46625	2.25	13423.5

702.62696	6.75	13430.25

702.78767	2.25	13432.5

702.94839	2.25	13434.75

703.1091	4.5	13439.25

703.26981	4.5	13443.75

703.43052	4.5	13448.25

703.59123	2.25	13450.5

703.75194	2.25	13452.75

703.91265	2.25	13455

704.07336	2.25	13457.25

704.23407	6.75	13464

704.39478	2.25	13466.25

704.55549	2.25	13468.5

704.7162	2.25	13470.75

704.87691	2.25	13473

705.03762	2.25	13475.25

705.19833	2.25	13477.5

705.35905	2.25	13479.75

705.51976	2.25	13482

705.68047	2.25	13484.25

705.84118	2.25	13486.5

706.00189	2.25	13488.75

706.1626	2.25	13491

706.32331	4.5	13495.5

706.48402	2.25	13497.75

706.64473	9	13506.75

706.80544	4.5	13511.25

706.96615	2.25	13513.5

707.12686	2.25	13515.75

707.28757	6.75	13522.5

707.44828	2.25	13524.75

707.60899	6.75	13531.5

707.76971	4.5	13536

707.93042	4.5	13540.5

708.09113	2.25	13542.75

708.25184	24.75	13567.5

708.41255	2.25	13569.75

708.57326	2.25	13572

708.73397	11.25	13583.25

708.89468	2.25	13585.5

709.05539	2.25	13587.75

709.2161	4.5	13592.25

709.37681	2.25	13594.5

709.53752	2.25	13596.75

709.69823	9	13605.75

709.85894	2.25	13608

710.01965	4.5	13612.5

710.18036	2.25	13614.75

710.34108	4.5	13619.25

710.50179	2.25	13621.5

710.6625	2.25	13623.75

710.82321	2.25	13626

710.98392	2.25	13628.25

711.14463	2.25	13630.5

711.30534	2.25	13632.75

711.46605	2.25	13635

711.62676	2.25	13637.25

711.78747	2.25	13639.5

711.94818	2.25	13641.75

712.10889	4.5	13646.25

712.2696	13.5	13659.75

712.43031	11.25	13671

712.59102	6.75	13677.75

712.75174	2.25	13680

712.91245	2.25	13682.25

713.07316	2.25	13684.5

713.23387	2.25	13686.75

713.39458	4.5	13691.25

713.55529	2.25	13693.5

713.716	4.5	13698

713.87671	2.25	13700.25

714.03742	9	13709.25

714.19813	9	13718.25

714.35884	4.5	13722.75

714.51955	4.5	13727.25

714.68026	2.25	13729.5

714.84097	11.25	13740.75

715.00168	9	13749.75

715.16239	2.25	13752

715.32311	2.25	13754.25

715.48382	6.75	13761

715.64453	2.25	13763.25

715.80524	4.5	13767.75

715.96595	4.5	13772.25

716.12666	2.25	13774.5

716.28737	2.25	13776.75

716.44808	2.25	13779

716.60879	2.25	13781.25

716.7695	2.25	13783.5

716.93021	2.25	13785.75

717.09092	2.25	13788

717.25163	4.5	13792.5

717.41234	2.25	13794.75

717.57305	2.25	13797

717.73377	2.25	13799.25

717.89448	2.25	13801.5

718.05519	2.25	13803.75

718.2159	4.5	13808.25

718.37661	11.25	13819.5

718.53732	2.25	13821.75

718.69803	2.25	13824

718.85874	2.25	13826.25

719.01945	2.25	13828.5

719.18016	6.75	13835.25

719.34087	2.25	13837.5

719.50158	2.25	13839.75

719.66229	2.25	13842

719.823	2.25	13844.25

719.98371	2.25	13846.5

720.14443	2.25	13848.75

720.30514	2.25	13851

720.46585	4.5	13855.5

720.62656	2.25	13857.75

720.78727	2.25	13860

720.94798	2.25	13862.25

721.10869	13.5	13875.75

721.2694	4.5	13880.25

721.43011	2.25	13882.5

721.59082	2.25	13884.75

721.75153	2.25	13887

721.91224	2.25	13889.25

722.07295	2.25	13891.5

722.23366	11.25	13902.75

722.39437	2.25	13905

722.55508	4.5	13909.5

722.7158	9	13918.5

722.87651	2.25	13920.75

723.03722	2.25	13923

723.19793	2.25	13925.25

723.35864	2.25	13927.5

723.51935	2.25	13929.75

723.68006	2.25	13932

723.84077	2.25	13934.25

724.00148	2.25	13936.5

724.16219	4.5	13941

724.3229	2.25	13943.25

724.48361	4.5	13947.75

724.64432	4.5	13952.25

724.80503	2.25	13954.5

724.96574	2.25	13956.75

725.12646	2.25	13959

725.28717	4.5	13963.5

725.44788	6.75	13970.25

725.60859	2.25	13972.5

725.7693	2.25	13974.75

725.93001	4.5	13979.25

726.09072	2.25	13981.5

726.25143	2.25	13983.75

726.41214	4.5	13988.25

726.57285	2.25	13990.5

726.73356	6.75	13997.25

726.89427	4.5	14001.75

727.05498	2.25	14004

727.21569	4.5	14008.5

727.3764	2.25	14010.75

727.53711	2.25	14013

727.69783	2.25	14015.25

727.85854	4.5	14019.75

728.01925	11.25	14031

728.17996	4.5	14035.5

728.34067	2.25	14037.75

728.50138	2.25	14040

728.66209	2.25	14042.25

728.8228	4.5	14046.75

728.98351	2.25	14049

729.14422	2.25	14051.25

729.30493	2.25	14053.5

729.46564	2.25	14055.75

729.62635	2.25	14058

729.78706	4.5	14062.5

729.94777	2.25	14064.75

730.10849	2.25	14067

730.2692	2.25	14069.25

730.42991	4.5	14073.75

730.59062	6.75	14080.5

730.75133	2.25	14082.75

730.91204	2.25	14085

731.07275	2.25	14087.25

731.23346	2.25	14089.5

731.39417	6.75	14096.25

731.55488	2.25	14098.5

731.71559	4.5	14103

731.8763	4.5	14107.5

732.03701	4.5	14112

732.19772	2.25	14114.25

732.35843	2.25	14116.5

732.51915	6.75	14123.25

732.67986	2.25	14125.5

732.84057	2.25	14127.75

733.00128	2.25	14130

733.16199	2.25	14132.25

733.3227	2.25	14134.5

733.48341	6.75	14141.25

733.64412	2.25	14143.5

733.80483	2.25	14145.75

733.96554	2.25	14148

734.12625	4.5	14152.5

734.28696	11.25	14163.75

734.44767	2.25	14166

734.60838	2.25	14168.25

734.76909	6.75	14175

734.9298	4.5	14179.5

735.09052	9	14188.5

735.25123	2.25	14190.75

735.41194	2.25	14193

735.57265	2.25	14195.25

735.73336	4.5	14199.75

735.89407	2.25	14202

736.05478	2.25	14204.25

736.21549	2.25	14206.5

736.3762	6.75	14213.25

736.53691	2.25	14215.5

736.69762	2.25	14217.75

736.85833	2.25	14220

737.01904	2.25	14222.25

737.17975	2.25	14224.5

737.34046	2.25	14226.75

737.50118	2.25	14229

737.66189	4.5	14233.5

737.8226	2.25	14235.75

737.98331	4.5	14240.25

738.14402	2.25	14242.5

738.30473	2.25	14244.75

738.46544	4.5	14249.25

738.62615	2.25	14251.5

738.78686	4.5	14256

738.94757	2.25	14258.25

739.10828	2.25	14260.5

739.26899	2.25	14262.75

739.4297	2.25	14265

739.59041	9	14274

739.75112	2.25	14276.25

739.91184	2.25	14278.5

740.07255	2.25	14280.75

740.23326	2.25	14283

740.39397	2.25	14285.25

740.55468	2.25	14287.5

740.71539	4.5	14292

740.8761	6.75	14298.75

741.03681	2.25	14301

741.19752	2.25	14303.25

741.35823	2.25	14305.5

741.51894	2.25	14307.75

741.67965	2.25	14310

741.84036	4.5	14314.5

742.00107	4.5	14319

742.16178	2.25	14321.25

742.32249	2.25	14323.5

742.48321	2.25	14325.75

742.64392	4.5	14330.25

742.80463	2.25	14332.5

742.96534	6.75	14339.25

743.12605	11.25	14350.5

743.28676	4.5	14355

743.44747	2.25	14357.25

743.60818	2.25	14359.5

743.76889	15.75	14375.25

743.9296	2.25	14377.5

744.09031	2.25	14379.75

744.25102	9	14388.75

744.41173	2.25	14391

744.57244	2.25	14393.25

744.73315	6.75	14400

744.89387	2.25	14402.25

745.05458	6.75	14409

745.21529	2.25	14411.25

745.376	2.25	14413.5

745.53671	2.25	14415.75

745.69742	2.25	14418

745.85813	2.25	14420.25

746.01884	2.25	14422.5

746.17955	2.25	14424.75

746.34026	4.5	14429.25

746.50097	2.25	14431.5

746.66168	9	14440.5

746.82239	2.25	14442.75

746.9831	6.75	14449.5

747.14381	4.5	14454

747.30453	2.25	14456.25

747.46524	2.25	14458.5

747.62595	4.5	14463

747.78666	4.5	14467.5

747.94737	27	14494.5

748.10808	2.25	14496.75

748.26879	2.25	14499

748.4295	2.25	14501.25

748.59021	4.5	14505.75

748.75092	2.25	14508

748.91163	4.5	14512.5

749.07234	2.25	14514.75

749.23305	2.25	14517

749.39376	2.25	14519.25

749.55447	4.5	14523.75

749.71518	2.25	14526

749.8759	4.5	14530.5

750.03661	2.25	14532.75

750.19732	9	14541.75

750.35803	4.5	14546.25

750.51874	2.25	14548.5

750.67945	4.5	14553

750.84016	4.5	14557.5

751.00087	2.25	14559.75

751.16158	2.25	14562

751.32229	2.25	14564.25

751.483	2.25	14566.5

751.64371	2.25	14568.75

751.80442	2.25	14571

751.96513	4.5	14575.5

752.12584	2.25	14577.75

752.28656	4.5	14582.25

752.44727	6.75	14589

752.60798	6.75	14595.75

752.76869	4.5	14600.25

752.9294	6.75	14607

753.09011	4.5	14611.5

753.25082	2.25	14613.75

753.41153	2.25	14616

753.57224	2.25	14618.25

753.73295	4.5	14622.75

753.89366	2.25	14625

754.05437	2.25	14627.25

754.21508	4.5	14631.75

754.37579	6.75	14638.5

754.5365	4.5	14643

754.69721	2.25	14645.25

754.85793	2.25	14647.5

755.01864	2.25	14649.75

755.17935	4.5	14654.25

755.34006	2.25	14656.5

755.50077	2.25	14658.75

755.66148	2.25	14661

755.82219	2.25	14663.25

755.9829	2.25	14665.5

756.14361	2.25	14667.75

756.30432	2.25	14670

756.46503	4.5	14674.5

756.62574	4.5	14679

756.78645	2.25	14681.25

756.94716	2.25	14683.5

757.10787	4.5	14688

757.26859	4.5	14692.5

757.4293	2.25	14694.75

757.59001	4.5	14699.25

757.75072	2.25	14701.5

757.91143	2.25	14703.75

758.07214	4.5	14708.25

758.23285	2.25	14710.5

758.39356	2.25	14712.75

758.55427	2.25	14715

758.71498	2.25	14717.25

758.87569	2.25	14719.5

759.0364	2.25	14721.75

759.19711	6.75	14728.5

759.35782	2.25	14730.75

759.51853	2.25	14733

759.67925	2.25	14735.25

759.83996	4.5	14739.75

760.00067	4.5	14744.25

760.16138	2.25	14746.5

760.32209	4.5	14751

760.4828	4.5	14755.5

760.64351	4.5	14760

760.80422	2.25	14762.25

760.96493	6.75	14769

761.12564	9	14778

761.28635	2.25	14780.25

761.44706	4.5	14784.75

761.60777	9	14793.75

761.76848	4.5	14798.25

761.92919	2.25	14800.5

762.0899	6.75	14807.25

762.25062	2.25	14809.5

762.41133	9	14818.5

762.57204	2.25	14820.75

762.73275	2.25	14823

762.89346	2.25	14825.25

763.05417	2.25	14827.5

763.21488	2.25	14829.75

763.37559	4.5	14834.25

763.5363	2.25	14836.5

763.69701	6.75	14843.25

763.85772	2.25	14845.5

764.01843	4.5	14850

764.17914	6.75	14856.75

764.33985	4.5	14861.25

764.50056	2.25	14863.5

764.66128	2.25	14865.75

764.82199	9	14874.75

764.9827	2.25	14877

765.14341	4.5	14881.5

765.30412	4.5	14886

765.46483	4.5	14890.5

765.62554	2.25	14892.75

765.78625	2.25	14895

765.94696	2.25	14897.25

766.10767	2.25	14899.5

766.26838	2.25	14901.75

766.42909	9	14910.75

766.5898	4.5	14915.25

766.75051	2.25	14917.5

766.91122	4.5	14922

767.07194	2.25	14924.25

767.23265	2.25	14926.5

767.39336	2.25	14928.75

767.55407	2.25	14931

767.71478	2.25	14933.25

767.87549	4.5	14937.75

768.0362	4.5	14942.25

768.19691	2.25	14944.5

768.35762	2.25	14946.75

768.51833	2.25	14949

768.67904	2.25	14951.25

768.83975	2.25	14953.5

769.00046	2.25	14955.75

769.16117	2.25	14958

769.32188	2.25	14960.25

769.48259	4.5	14964.75

769.64331	2.25	14967

769.80402	2.25	14969.25

769.96473	2.25	14971.5

770.12544	2.25	14973.75

770.28615	2.25	14976

770.44686	2.25	14978.25

770.60757	2.25	14980.5

770.76828	2.25	14982.75

770.92899	2.25	14985

771.0897	9	14994

771.25041	6.75	15000.75

771.41112	4.5	15005.25

771.57183	2.25	15007.5

771.73254	9	15016.5

771.89325	9	15025.5

772.05397	4.5	15030

772.21468	2.25	15032.25

772.37539	6.75	15039

772.5361	2.25	15041.25

772.69681	2.25	15043.5

772.85752	2.25	15045.75

773.01823	6.75	15052.5

773.17894	2.25	15054.75

773.33965	2.25	15057

773.50036	2.25	15059.25

773.66107	2.25	15061.5

773.82178	2.25	15063.75

773.98249	2.25	15066

774.1432	4.5	15070.5

774.30391	6.75	15077.25

774.46462	6.75	15084

774.62534	2.25	15086.25

774.78605	2.25	15088.5

774.94676	2.25	15090.75

775.10747	2.25	15093

775.26818	2.25	15095.25

775.42889	4.5	15099.75

775.5896	2.25	15102

775.75031	2.25	15104.25

775.91102	2.25	15106.5

776.07173	4.5	15111

776.23244	13.5	15124.5

776.39315	6.75	15131.25

776.55386	2.25	15133.5

776.71457	2.25	15135.75

776.87528	4.5	15140.25

777.036	2.25	15142.5

777.19671	6.75	15149.25

777.35742	4.5	15153.75

777.51813	4.5	15158.25

777.67884	2.25	15160.5

777.83955	2.25	15162.75

778.00026	2.25	15165

778.16097	2.25	15167.25

778.32168	2.25	15169.5

778.48239	4.5	15174

778.6431	2.25	15176.25

778.80381	2.25	15178.5

778.96452	9	15187.5

779.12523	2.25	15189.75

779.28594	4.5	15194.25

779.44666	2.25	15196.5

779.60737	4.5	15201

779.76808	2.25	15203.25

779.92879	2.25	15205.5

780.0895	4.5	15210

780.25021	2.25	15212.25

780.41092	2.25	15214.5

780.57163	2.25	15216.75

780.73234	4.5	15221.25

780.89305	9	15230.25

781.05376	2.25	15232.5

781.21447	2.25	15234.75

781.37518	2.25	15237

781.53589	2.25	15239.25

781.6966	2.25	15241.5

781.85731	2.25	15243.75

782.01803	2.25	15246

782.17874	13.5	15259.5

782.33945	2.25	15261.75

782.50016	4.5	15266.25

782.66087	9	15275.25

782.82158	2.25	15277.5

782.98229	2.25	15279.75

783.143	6.75	15286.5

783.30371	2.25	15288.75

783.46442	6.75	15295.5

783.62513	6.75	15302.25

783.78584	6.75	15309

783.94655	13.5	15322.5

784.10726	4.5	15327

784.26797	2.25	15329.25

784.42869	6.75	15336

784.5894	6.75	15342.75

784.75011	2.25	15345

784.91082	2.25	15347.25

785.07153	2.25	15349.5

785.23224	52.25	15401.75

785.39295	2.25	15404

785.55366	2.25	15406.25

785.71437	4.5	15410.75

785.87508	2.25	15413

786.03579	6.75	15419.75

786.1965	2.25	15422

786.35721	9	15431

786.51792	2.25	15433.25

786.67863	32.25	15465.5

786.83934	2.25	15467.75

787.00006	2.25	15470

787.16077	2.25	15472.25

787.32148	2.25	15474.5

787.48219	2.25	15476.75

787.6429	2.25	15479

787.80361	13.5	15492.5

787.96432	2.25	15494.75

788.12503	2.25	15497

788.28574	2.25	15499.25

788.44645	2.25	15501.5

788.60716	4.5	15506

788.76787	2.25	15508.25

788.92858	2.25	15510.5

789.08929	4.5	15515

789.25	4.5	15519.5

789.41072	2.25	15521.75

789.57143	2.25	15524

789.73214	2.25	15526.25

789.89285	2.25	15528.5

790.05356	6.75	15535.25

790.21427	6.75	15542

790.37498	2.25	15544.25

790.53569	2.25	15546.5

790.6964	2.25	15548.75

790.85711	6.75	15555.5

791.01782	2.25	15557.75

791.17853	9	15566.75

791.33924	2.25	15569

791.49995	9	15578

791.66066	4.5	15582.5

791.82138	2.25	15584.75

791.98209	2.25	15587

792.1428	2.25	15589.25

792.30351	2.25	15591.5

792.46422	4.5	15596

792.62493	4.5	15600.5

792.78564	4.5	15605

792.94635	15.75	15620.75

793.10706	4.5	15625.25

793.26777	4.5	15629.75

793.42848	2.25	15632

793.58919	2.25	15634.25

793.7499	44.5	15678.75

793.91061	2.25	15681

794.07132	2.25	15683.25

794.23204	2.25	15685.5

794.39275	9	15694.5

794.55346	4.5	15699

794.71417	22.5	15721.5

794.87488	6.75	15728.25

795.03559	2.25	15730.5

795.1963	6.75	15737.25

795.35701	2.25	15739.5

795.51772	2.25	15741.75

795.67843	9	15750.75

795.83914	2.25	15753

795.99985	2.25	15755.25

796.16056	2.25	15757.5

796.32127	4.5	15762

796.48198	2.25	15764.25

796.64269	2.25	15766.5

796.80341	4.5	15771

796.96412	2.25	15773.25

797.12483	2.25	15775.5

797.28554	4.5	15780

797.44625	2.25	15782.25

797.60696	2.25	15784.5

797.76767	11.25	15795.75

797.92838	2.25	15798

798.08909	2.25	15800.25

798.2498	2.25	15802.5

798.41051	2.25	15804.75

798.57122	2.25	15807

798.73193	2.25	15809.25

798.89264	2.25	15811.5

799.05335	4.5	15816

799.21407	2.25	15818.25

799.37478	9	15827.25

799.53549	2.25	15829.5

799.6962	2.25	15831.75

799.85691	4.5	15836.25

800.01762	2.25	15838.5

800.17833	4.5	15843

800.33904	2.25	15845.25

800.49975	2.25	15847.5

800.66046	4.5	15852

800.82117	2.25	15854.25

800.98188	4.5	15858.75

801.14259	2.25	15861

801.3033	2.25	15863.25

801.46401	62.25	15925.5

801.62472	2.25	15927.75

801.78544	2.25	15930

801.94615	2.25	15932.25

802.10686	2.25	15934.5

802.26757	2.25	15936.75

802.42828	2.25	15939

802.58899	2.25	15941.25

802.7497	4.5	15945.75

802.91041	4.5	15950.25

803.07112	4.5	15954.75

803.23183	6.75	15961.5

803.39254	2.25	15963.75

803.55325	4.5	15968.25

803.71396	2.25	15970.5

803.87467	2.25	15972.75

804.03538	2.25	15975

804.1961	2.25	15977.25

804.35681	2.25	15979.5

804.51752	11.25	15990.75

804.67823	2.25	15993

804.83894	2.25	15995.25

804.99965	2.25	15997.5

805.16036	2.25	15999.75

805.32107	4.5	16004.25

805.48178	2.25	16006.5

805.64249	4.5	16011

805.8032	2.25	16013.25

805.96391	2.25	16015.5

806.12462	2.25	16017.75

806.28533	4.5	16022.25

806.44604	4.5	16026.75

806.60676	2.25	16029

806.76747	2.25	16031.25

806.92818	2.25	16033.5

807.08889	2.25	16035.75

807.2496	2.25	16038

807.41031	2.25	16040.25

807.57102	4.5	16044.75

807.73173	6.75	16051.5

807.89244	2.25	16053.75

808.05315	6.75	16060.5

808.21386	4.5	16065

808.37457	2.25	16067.25

808.53528	2.25	16069.5

808.69599	4.5	16074

808.8567	2.25	16076.25

809.01741	2.25	16078.5

809.17813	2.25	16080.75

809.33884	6.75	16087.5

809.49955	2.25	16089.75

809.66026	2.25	16092

809.82097	4.5	16096.5

809.98168	9	16105.5

810.14239	4.5	16110

810.3031	2.25	16112.25

810.46381	2.25	16114.5

810.62452	6.75	16121.25

810.78523	4.5	16125.75

810.94594	9	16134.75

811.10665	2.25	16137

811.26736	9	16146

811.42807	2.25	16148.25

811.58879	2.25	16150.5

811.7495	2.25	16152.75

811.91021	4.5	16157.25

812.07092	4.5	16161.75

812.23163	4.5	16166.25

812.39234	2.25	16168.5

812.55305	36	16204.5

812.71376	4.5	16209

812.87447	2.25	16211.25

813.03518	2.25	16213.5

813.19589	6.75	16220.25

813.3566	2.25	16222.5

813.51731	2.25	16224.75

813.67802	4.5	16229.25

813.83873	2.25	16231.5

813.99945	2.25	16233.75

814.16016	2.25	16236

814.32087	4.5	16240.5

814.48158	2.25	16242.75

814.64229	2.25	16245

814.803	6.75	16251.75

814.96371	2.25	16254

815.12442	2.25	16256.25

815.28513	4.5	16260.75

815.44584	2.25	16263

815.60655	6.75	16269.75

815.76726	4.5	16274.25

815.92797	2.25	16276.5

816.08868	4.5	16281

816.24939	2.25	16283.25

816.4101	6.75	16290

816.57082	4.5	16294.5

816.73153	4.5	16299

816.89224	2.25	16301.25

817.05295	2.25	16303.5

817.21366	2.25	16305.75

817.37437	2.25	16308

817.53508	2.25	16310.25

817.69579	2.25	16312.5

817.8565	2.25	16314.75

818.01721	11.25	16326

818.17792	6.75	16332.75

818.33863	2.25	16335

818.49934	2.25	16337.25

818.66005	15.75	16353

818.82076	2.25	16355.25

818.98148	2.25	16357.5

819.14219	6.75	16364.25

819.3029	2.25	16366.5

819.46361	2.25	16368.75

819.62432	2.25	16371

819.78503	2.25	16373.25

819.94574	2.25	16375.5

820.10645	2.25	16377.75

820.26716	2.25	16380

820.42787	2.25	16382.25

820.58858	4.5	16386.75

820.74929	2.25	16389

820.91	4.5	16393.5

821.07071	2.25	16395.75

821.23142	2.25	16398

821.39213	4.5	16402.5

821.55285	2.25	16404.75

821.71356	2.25	16407

821.87427	2.25	16409.25

822.03498	4.5	16413.75

822.19569	2.25	16416

822.3564	2.25	16418.25

822.51711	2.25	16420.5

822.67782	4.5	16425

822.83853	2.25	16427.25

822.99924	2.25	16429.5

823.15995	4.5	16434

823.32066	2.25	16436.25

823.48137	2.25	16438.5

823.64208	2.25	16440.75

823.80279	2.25	16443

823.96351	2.25	16445.25

824.12422	2.25	16447.5

824.28493	2.25	16449.75

824.44564	2.25	16452

824.60635	11.25	16463.25

824.76706	4.5	16467.75

824.92777	2.25	16470

825.08848	6.75	16476.75

825.24919	2.25	16479

825.4099	4.5	16483.5

825.57061	4.5	16488

825.73132	2.25	16490.25

825.89203	2.25	16492.5

826.05274	2.25	16494.75

826.21345	2.25	16497

826.37417	6.75	16503.75

826.53488	2.25	16506

826.69559	2.25	16508.25

826.8563	4.5	16512.75

827.01701	9	16521.75

827.17772	2.25	16524

827.33843	2.25	16526.25

827.49914	9	16535.25

827.65985	6.75	16542

827.82056	4.5	16546.5

827.98127	15.75	16562.25

828.14198	2.25	16564.5

828.30269	4.5	16569

828.4634	2.25	16571.25

828.62411	2.25	16573.5

828.78482	4.5	16578

828.94554	2.25	16580.25

829.10625	4.5	16584.75

829.26696	18	16602.75

829.42767	2.25	16605

829.58838	6.75	16611.75

829.74909	2.25	16614

829.9098	2.25	16616.25

830.07051	2.25	16618.5

830.23122	6.75	16625.25

830.39193	2.25	16627.5

830.55264	2.25	16629.75

830.71335	4.5	16634.25

830.87406	2.25	16636.5

831.03477	2.25	16638.75

831.19548	2.25	16641

831.3562	2.25	16643.25

831.51691	6.75	16650

831.67762	2.25	16652.25

831.83833	2.25	16654.5

831.99904	4.5	16659

832.15975	76.75	16735.75

832.32046	20.25	16756

832.48117	2.25	16758.25

832.64188	4.5	16762.75

832.80259	4.5	16767.25

832.9633	2.25	16769.5

833.12401	2.25	16771.75

833.28472	6.75	16778.5

833.44543	4.5	16783

833.60614	4.5	16787.5

833.76685	2.25	16789.75

833.92757	2.25	16792

834.08828	2.25	16794.25

834.24899	2.25	16796.5

834.4097	2.25	16798.75

834.57041	13.5	16812.25

834.73112	2.25	16814.5

834.89183	2.25	16816.75

835.05254	4.5	16821.25

835.21325	4.5	16825.75

835.37396	2.25	16828

835.53467	2.25	16830.25

835.69538	6.75	16837

835.85609	4.5	16841.5

836.0168	6.75	16848.25

836.17751	2.25	16850.5

836.33823	2.25	16852.75

836.49894	2.25	16855

836.65965	2.25	16857.25

836.82036	2.25	16859.5

836.98107	2.25	16861.75

837.14178	2.25	16864

837.30249	9	16873

837.4632	2.25	16875.25

837.62391	2.25	16877.5

837.78462	2.25	16879.75

837.94533	13.5	16893.25

838.10604	2.25	16895.5

838.26675	2.25	16897.75

838.42746	2.25	16900

838.58817	2.25	16902.25

838.74889	4.5	16906.75

838.9096	27	16933.75

839.07031	2.25	16936

839.23102	2.25	16938.25

839.39173	2.25	16940.5

839.55244	2.25	16942.75

839.71315	6.75	16949.5

839.87386	4.5	16954

840.03457	2.25	16956.25

840.19528	2.25	16958.5

840.35599	2.25	16960.75

840.5167	2.25	16963

840.67741	6.75	16969.75

840.83812	2.25	16972

840.99883	4.5	16976.5

841.15954	2.25	16978.75

841.32026	15.75	16994.5

841.48097	6.75	17001.25

841.64168	2.25	17003.5

841.80239	2.25	17005.75

841.9631	2.25	17008

842.12381	2.25	17010.25

842.28452	2.25	17012.5

842.44523	2.25	17014.75

842.60594	9	17023.75

842.76665	2.25	17026

842.92736	2.25	17028.25

843.08807	4.5	17032.75

843.24878	2.25	17035

843.40949	9	17044

843.5702	4.5	17048.5

843.73092	6.75	17055.25

843.89163	2.25	17057.5

844.05234	2.25	17059.75

844.21305	4.5	17064.25

844.37376	2.25	17066.5

844.53447	2.25	17068.75

844.69518	4.5	17073.25

844.85589	2.25	17075.5

845.0166	2.25	17077.75

845.17731	2.25	17080

845.33802	6.75	17086.75

845.49873	4.5	17091.25

845.65944	2.25	17093.5

845.82015	4.5	17098

845.98086	6.75	17104.75

846.14158	2.25	17107

846.30229	2.25	17109.25

846.463	2.25	17111.5

846.62371	2.25	17113.75

846.78442	4.5	17118.25

846.94513	2.25	17120.5

847.10584	2.25	17122.75

847.26655	2.25	17125

847.42726	2.25	17127.25

847.58797	2.25	17129.5

847.74868	13.5	17143

847.90939	6.75	17149.75

848.0701	6.75	17156.5

848.23081	2.25	17158.75

848.39152	4.5	17163.25

848.55223	2.25	17165.5

848.71295	9	17174.5

848.87366	4.5	17179

849.03437	6.75	17185.75

849.19508	4.5	17190.25

849.35579	4.5	17194.75

849.5165	4.5	17199.25

849.67721	2.25	17201.5

849.83792	2.25	17203.75

849.99863	2.25	17206

850.15934	4.5	17210.5

850.32005	6.75	17217.25

850.48076	2.25	17219.5

850.64147	4.5	17224

850.80218	2.25	17226.25

850.96289	2.25	17228.5

851.12361	4.5	17233

851.28432	6.75	17239.75

851.44503	2.25	17242

851.60574	2.25	17244.25

851.76645	2.25	17246.5

851.92716	2.25	17248.75

852.08787	2.25	17251

852.24858	2.25	17253.25

852.40929	2.25	17255.5

852.57	2.25	17257.75

852.73071	4.5	17262.25

852.89142	2.25	17264.5

853.05213	4.5	17269

853.21284	2.25	17271.25

853.37355	2.25	17273.5

853.53427	2.25	17275.75

853.69498	2.25	17278

853.85569	2.25	17280.25

854.0164	2.25	17282.5

854.17711	2.25	17284.75

854.33782	2.25	17287

854.49853	2.25	17289.25

854.65924	2.25	17291.5

854.81995	2.25	17293.75

854.98066	2.25	17296

855.14137	2.25	17298.25

855.30208	11.25	17309.5

855.46279	2.25	17311.75

855.6235	6.75	17318.5

855.78421	4.5	17323

855.94492	6.75	17329.75

856.10564	6.75	17336.5

856.26635	6.75	17343.25

856.42706	2.25	17345.5

856.58777	4.5	17350

856.74848	2.25	17352.25

856.90919	6.75	17359

857.0699	2.25	17361.25

857.23061	2.25	17363.5

857.39132	2.25	17365.75

857.55203	4.5	17370.25

857.71274	2.25	17372.5

857.87345	2.25	17374.75

858.03416	2.25	17377

858.19487	2.25	17379.25

858.35558	2.25	17381.5

858.5163	2.25	17383.75

858.67701	6.75	17390.5

858.83772	2.25	17392.75

858.99843	2.25	17395

859.15914	4.5	17399.5

859.31985	2.25	17401.75

859.48056	2.25	17404

859.64127	6.75	17410.75

859.80198	2.25	17413

859.96269	2.25	17415.25

860.1234	2.25	17417.5

860.28411	4.5	17422

860.44482	2.25	17424.25

860.60553	2.25	17426.5

860.76624	2.25	17428.75

860.92695	4.5	17433.25

861.08767	2.25	17435.5

861.24838	2.25	17437.75

861.40909	2.25	17440

861.5698	2.25	17442.25

861.73051	4.5	17446.75

861.89122	2.25	17449

862.05193	4.5	17453.5

862.21264	2.25	17455.75

862.37335	6.75	17462.5

862.53406	2.25	17464.75

862.69477	2.25	17467

862.85548	2.25	17469.25

863.01619	2.25	17471.5

863.1769	2.25	17473.75

863.33761	6.75	17480.5

863.49833	2.25	17482.75

863.65904	4.5	17487.25

863.81975	4.5	17491.75

863.98046	2.25	17494

864.14117	2.25	17496.25

864.30188	2.25	17498.5

864.46259	2.25	17500.75

864.6233	2.25	17503

864.78401	2.25	17505.25

864.94472	4.5	17509.75

865.10543	2.25	17512

865.26614	2.25	17514.25

865.42685	2.25	17516.5

865.58756	2.25	17518.75

865.74827	4.5	17523.25

865.90899	2.25	17525.5

866.0697	2.25	17527.75

866.23041	4.5	17532.25

866.39112	6.75	17539

866.55183	2.25	17541.25

866.71254	2.25	17543.5

866.87325	2.25	17545.75

867.03396	2.25	17548

867.19467	6.75	17554.75

867.35538	2.25	17557

867.51609	4.5	17561.5

867.6768	4.5	17566

867.83751	18	17584

867.99822	2.25	17586.25

868.15893	4.5	17590.75

868.31964	4.5	17595.25

868.48036	76.75	17672

868.64107	2.25	17674.25

868.80178	2.25	17676.5

868.96249	2.25	17678.75

869.1232	6.75	17685.5

869.28391	6.75	17692.25

869.44462	2.25	17694.5

869.60533	2.25	17696.75

869.76604	4.5	17701.25

869.92675	13.5	17714.75

870.08746	4.5	17719.25

870.24817	2.25	17721.5

870.40888	2.25	17723.75

870.56959	2.25	17726

870.7303	4.5	17730.5

870.89102	2.25	17732.75

871.05173	4.5	17737.25

871.21244	2.25	17739.5

871.37315	4.5	17744

871.53386	2.25	17746.25

871.69457	4.5	17750.75

871.85528	2.25	17753

872.01599	9	17762

872.1767	2.25	17764.25

872.33741	2.25	17766.5

872.49812	11.25	17777.75

872.65883	2.25	17780

872.81954	4.5	17784.5

872.98025	2.25	17786.75

873.14096	2.25	17789

873.30168	2.25	17791.25

873.46239	6.75	17798

873.6231	4.5	17802.5

873.78381	2.25	17804.75

873.94452	6.75	17811.5

874.10523	2.25	17813.75

874.26594	2.25	17816

874.42665	2.25	17818.25

874.58736	2.25	17820.5

874.74807	2.25	17822.75

874.90878	2.25	17825

875.06949	6.75	17831.75

875.2302	4.5	17836.25

875.39091	2.25	17838.5

875.55162	6.75	17845.25

875.71233	2.25	17847.5

875.87305	4.5	17852

876.03376	2.25	17854.25

876.19447	4.5	17858.75

876.35518	15.75	17874.5

876.51589	2.25	17876.75

876.6766	4.5	17881.25

876.83731	2.25	17883.5

876.99802	2.25	17885.75

877.15873	2.25	17888

877.31944	4.5	17892.5

877.48015	6.75	17899.25

877.64086	2.25	17901.5

877.80157	2.25	17903.75

877.96228	6.75	17910.5

878.12299	6.75	17917.25

878.28371	2.25	17919.5

878.44442	4.5	17924

878.60513	2.25	17926.25

878.76584	2.25	17928.5

878.92655	2.25	17930.75

879.08726	4.5	17935.25

879.24797	2.25	17937.5

879.40868	11.25	17948.75

879.56939	2.25	17951

879.7301	2.25	17953.25

879.89081	2.25	17955.5

880.05152	20.25	17975.75

880.21223	4.5	17980.25

880.37294	2.25	17982.5

880.53365	2.25	17984.75

880.69436	13.5	17998.25

880.85508	9	18007.25

881.01579	6.75	18014

881.1765	4.5	18018.5

881.33721	4.5	18023

881.49792	2.25	18025.25

881.65863	2.25	18027.5

881.81934	6.75	18034.25

881.98005	4.5	18038.75

882.14076	9	18047.75

882.30147	2.25	18050

882.46218	4.5	18054.5

882.62289	4.5	18059

882.7836	2.25	18061.25

882.94431	2.25	18063.5

883.10502	2.25	18065.75

883.26574	2.25	18068

883.42645	2.25	18070.25

883.58716	2.25	18072.5

883.74787	4.5	18077

883.90858	4.5	18081.5

884.06929	6.75	18088.25

884.23	2.25	18090.5

884.39071	2.25	18092.75

884.55142	4.5	18097.25

884.71213	2.25	18099.5

884.87284	2.25	18101.75

885.03355	9	18110.75

885.19426	4.5	18115.25

885.35497	15.75	18131

885.51568	2.25	18133.25

885.6764	2.25	18135.5

885.83711	9	18144.5

885.99782	2.25	18146.75

886.15853	4.5	18151.25

886.31924	6.75	18158

886.47995	2.25	18160.25

886.64066	2.25	18162.5

886.80137	4.5	18167

886.96208	6.75	18173.75

887.12279	2.25	18176

887.2835	2.25	18178.25

887.44421	2.25	18180.5

887.60492	2.25	18182.75

887.76563	4.5	18187.25

887.92634	2.25	18189.5

888.08705	4.5	18194

888.24777	9	18203

888.40848	4.5	18207.5

888.56919	4.5	18212

888.7299	2.25	18214.25

888.89061	6.75	18221

889.05132	2.25	18223.25

889.21203	2.25	18225.5

889.37274	2.25	18227.75

889.53345	4.5	18232.25

889.69416	2.25	18234.5

889.85487	4.5	18239

890.01558	2.25	18241.25

890.17629	2.25	18243.5

890.337	2.25	18245.75

890.49771	6.75	18252.5

890.65843	2.25	18254.75

890.81914	2.25	18257

890.97985	9	18266

891.14056	2.25	18268.25

891.30127	2.25	18270.5

891.46198	6.75	18277.25

891.62269	4.5	18281.75

891.7834	2.25	18284

891.94411	2.25	18286.25

892.10482	6.75	18293

892.26553	9	18302

892.42624	4.5	18306.5

892.58695	2.25	18308.75

892.74766	2.25	18311

892.90837	2.25	18313.25

893.06908	11.25	18324.5

893.2298	11.25	18335.75

893.39051	2.25	18338

893.55122	2.25	18340.25

893.71193	2.25	18342.5

893.87264	6.75	18349.25

894.03335	6.75	18356

894.19406	2.25	18358.25

894.35477	2.25	18360.5

894.51548	4.5	18365

894.67619	2.25	18367.25

894.8369	2.25	18369.5

894.99761	13.5	18383

895.15832	2.25	18385.25

895.31903	2.25	18387.5

895.47974	2.25	18389.75

895.64046	2.25	18392

895.80117	2.25	18394.25

895.96188	9	18403.25

896.12259	4.5	18407.75

896.2833	2.25	18410

896.44401	4.5	18414.5

896.60472	2.25	18416.75

896.76543	2.25	18419

896.92614	2.25	18421.25

897.08685	2.25	18423.5

897.24756	2.25	18425.75

897.40827	2.25	18428

897.56898	6.75	18434.75

897.72969	2.25	18437

897.8904	6.75	18443.75

898.05112	2.25	18446

898.21183	9	18455

898.37254	4.5	18459.5

898.53325	4.5	18464

898.69396	9	18473

898.85467	15.75	18488.75

899.01538	2.25	18491

899.17609	4.5	18495.5

899.3368	4.5	18500

899.49751	2.25	18502.25

899.65822	2.25	18504.5

899.81893	4.5	18509

899.97964	4.5	18513.5

900.14035	2.25	18515.75

900.30106	4.5	18520.25

900.46177	2.25	18522.5

900.62249	13.5	18536

900.7832	13.5	18549.5

900.94391	6.75	18556.25

901.10462	2.25	18558.5

901.26533	4.5	18563

901.42604	2.25	18565.25

901.58675	2.25	18567.5

901.74746	2.25	18569.75

901.90817	2.25	18572

902.06888	2.25	18574.25

902.22959	4.5	18578.75

902.3903	2.25	18581

902.55101	2.25	18583.25

902.71172	4.5	18587.75

902.87243	4.5	18592.25

903.03315	6.75	18599

903.19386	2.25	18601.25

903.35457	2.25	18603.5

903.51528	6.75	18610.25

903.67599	6.75	18617

903.8367	2.25	18619.25

903.99741	4.5	18623.75

904.15812	2.25	18626

904.31883	4.5	18630.5

904.47954	2.25	18632.75

904.64025	2.25	18635

904.80096	4.5	18639.5

904.96167	2.25	18641.75

905.12238	2.25	18644

905.28309	2.25	18646.25

905.44381	2.25	18648.5

905.60452	4.5	18653

905.76523	2.25	18655.25

905.92594	2.25	18657.5

906.08665	2.25	18659.75

906.24736	2.25	18662

906.40807	6.75	18668.75

906.56878	6.75	18675.5

906.72949	2.25	18677.75

906.8902	2.25	18680

907.05091	9	18689

907.21162	6.75	18695.75

907.37233	6.75	18702.5

907.53304	4.5	18707

907.69375	4.5	18711.5

907.85446	2.25	18713.75

908.01518	2.25	18716

908.17589	6.75	18722.75

908.3366	4.5	18727.25

908.49731	2.25	18729.5

908.65802	6.75	18736.25

908.81873	2.25	18738.5

908.97944	2.25	18740.75

909.14015	2.25	18743

909.30086	4.5	18747.5

909.46157	4.5	18752

909.62228	6.75	18758.75

909.78299	2.25	18761

909.9437	2.25	18763.25

910.10441	4.5	18767.75

910.26512	4.5	18772.25

910.42584	2.25	18774.5

910.58655	2.25	18776.75

910.74726	2.25	18779

910.90797	2.25	18781.25

911.06868	2.25	18783.5

911.22939	4.5	18788

911.3901	2.25	18790.25

911.55081	2.25	18792.5

911.71152	4.5	18797

911.87223	6.75	18803.75

912.03294	6.75	18810.5

912.19365	2.25	18812.75

912.35436	4.5	18817.25

912.51507	2.25	18819.5

912.67578	2.25	18821.75

912.8365	2.25	18824

912.99721	4.5	18828.5

913.15792	2.25	18830.75

913.31863	4.5	18835.25

913.47934	2.25	18837.5

913.64005	11.25	18848.75

913.80076	2.25	18851

913.96147	4.5	18855.5

914.12218	2.25	18857.75

914.28289	2.25	18860

914.4436	4.5	18864.5

914.60431	2.25	18866.75

914.76502	2.25	18869

914.92573	13.5	18882.5

915.08644	2.25	18884.75

915.24715	2.25	18887

915.40787	2.25	18889.25

915.56858	2.25	18891.5

915.72929	2.25	18893.75

915.89	2.25	18896

916.05071	2.25	18898.25

916.21142	6.75	18905

916.37213	2.25	18907.25

916.53284	2.25	18909.5

916.69355	2.25	18911.75

916.85426	2.25	18914

917.01497	2.25	18916.25

917.17568	4.5	18920.75

917.33639	2.25	18923

917.4971	4.5	18927.5

917.65781	2.25	18929.75

917.81853	2.25	18932

917.97924	2.25	18934.25

918.13995	4.5	18938.75

918.30066	9	18947.75

918.46137	2.25	18950

918.62208	6.75	18956.75

918.78279	6.75	18963.5

918.9435	6.75	18970.25

919.10421	2.25	18972.5

919.26492	13.5	18986

919.42563	2.25	18988.25

919.58634	2.25	18990.5

919.74705	2.25	18992.75

919.90776	2.25	18995

920.06847	2.25	18997.25

920.22919	2.25	18999.5

920.3899	9	19008.5

920.55061	11.25	19019.75

920.71132	2.25	19022

920.87203	2.25	19024.25

921.03274	9	19033.25

921.19345	2.25	19035.5

921.35416	2.25	19037.75

921.51487	2.25	19040

921.67558	2.25	19042.25

921.83629	2.25	19044.5

921.997	13.5	19058

922.15771	2.25	19060.25

922.31842	4.5	19064.75

922.47913	4.5	19069.25

922.63984	4.5	19073.75

922.80056	9	19082.75

922.96127	2.25	19085

923.12198	2.25	19087.25

923.28269	2.25	19089.5

923.4434	9	19098.5

923.60411	2.25	19100.75

923.76482	4.5	19105.25

923.92553	2.25	19107.5

924.08624	9	19116.5

924.24695	2.25	19118.75

924.40766	4.5	19123.25

924.56837	2.25	19125.5

924.72908	6.75	19132.25

924.88979	2.25	19134.5

925.0505	2.25	19136.75

925.21122	2.25	19139

925.37193	6.75	19145.75

925.53264	4.5	19150.25

925.69335	4.5	19154.75

925.85406	6.75	19161.5

926.01477	6.75	19168.25

926.17548	2.25	19170.5

926.33619	2.25	19172.75

926.4969	9	19181.75

926.65761	4.5	19186.25

926.81832	2.25	19188.5

926.97903	2.25	19190.75

927.13974	6.75	19197.5

927.30045	2.25	19199.75

927.46116	2.25	19202

927.62187	2.25	19204.25

927.78259	2.25	19206.5

927.9433	2.25	19208.75

928.10401	6.75	19215.5

928.26472	2.25	19217.75

928.42543	4.5	19222.25

928.58614	2.25	19224.5

928.74685	2.25	19226.75

928.90756	4.5	19231.25

929.06827	11.25	19242.5

929.22898	4.5	19247

929.38969	13.5	19260.5

929.5504	2.25	19262.75

929.71111	4.5	19267.25

929.87182	11.25	19278.5

930.03253	4.5	19283

930.19325	2.25	19285.25

930.35396	2.25	19287.5

930.51467	4.5	19292

930.67538	2.25	19294.25

930.83609	4.5	19298.75

930.9968	2.25	19301

931.15751	4.5	19305.5

931.31822	2.25	19307.75

931.47893	6.75	19314.5

931.63964	2.25	19316.75

931.80035	4.5	19321.25

931.96106	4.5	19325.75

932.12177	9	19334.75

932.28248	2.25	19337

932.44319	2.25	19339.25

932.60391	2.25	19341.5

932.76462	4.5	19346

932.92533	2.25	19348.25

933.08604	2.25	19350.5

933.24675	6.75	19357.25

933.40746	4.5	19361.75

933.56817	4.5	19366.25

933.72888	2.25	19368.5

933.88959	4.5	19373

934.0503	4.5	19377.5

934.21101	6.75	19384.25

934.37172	4.5	19388.75

934.53243	9	19397.75

934.69314	11.25	19409

934.85385	4.5	19413.5

935.01456	27	19440.5

935.17528	4.5	19445

935.33599	2.25	19447.25

935.4967	2.25	19449.5

935.65741	2.25	19451.75

935.81812	6.75	19458.5

935.97883	2.25	19460.75

936.13954	2.25	19463

936.30025	2.25	19465.25

936.46096	2.25	19467.5

936.62167	2.25	19469.75

936.78238	2.25	19472

936.94309	6.75	19478.75

937.1038	4.5	19483.25

937.26451	2.25	19485.5

937.42522	4.5	19490

937.58594	2.25	19492.25

937.74665	15.75	19508

937.90736	2.25	19510.25

938.06807	2.25	19512.5

938.22878	9	19521.5

938.38949	2.25	19523.75

938.5502	2.25	19526

938.71091	2.25	19528.25

938.87162	2.25	19530.5

939.03233	2.25	19532.75

939.19304	2.25	19535

939.35375	4.5	19539.5

939.51446	2.25	19541.75

939.67517	2.25	19544

939.83588	4.5	19548.5

939.99659	2.25	19550.75

940.15731	4.5	19555.25

940.31802	4.5	19559.75

940.47873	6.75	19566.5

940.63944	2.25	19568.75

940.80015	2.25	19571

940.96086	32.25	19603.25

941.12157	6.75	19610

941.28228	9	19619

941.44299	4.5	19623.5

941.6037	2.25	19625.75

941.76441	6.75	19632.5

941.92512	2.25	19634.75

942.08583	4.5	19639.25

942.24654	2.25	19641.5

942.40725	2.25	19643.75

942.56797	6.75	19650.5

942.72868	2.25	19652.75

942.88939	2.25	19655

943.0501	2.25	19657.25

943.21081	2.25	19659.5

943.37152	2.25	19661.75

943.53223	2.25	19664

943.69294	4.5	19668.5

943.85365	4.5	19673

944.01436	18	19691

944.17507	2.25	19693.25

944.33578	2.25	19695.5

944.49649	11.25	19706.75

944.6572	2.25	19709

944.81791	2.25	19711.25

944.97863	2.25	19713.5

945.13934	4.5	19718

945.30005	6.75	19724.75

945.46076	4.5	19729.25

945.62147	2.25	19731.5

945.78218	2.25	19733.75

945.94289	4.5	19738.25

946.1036	2.25	19740.5

946.26431	42.25	19782.75

946.42502	2.25	19785

946.58573	2.25	19787.25

946.74644	6.75	19794

946.90715	2.25	19796.25

947.06786	2.25	19798.5

947.22857	4.5	19803

947.38928	4.5	19807.5

947.55	2.25	19809.75

947.71071	2.25	19812

947.87142	6.75	19818.75

948.03213	2.25	19821

948.19284	6.75	19827.75

948.35355	4.5	19832.25

948.51426	2.25	19834.5

948.67497	2.25	19836.75

948.83568	4.5	19841.25

948.99639	2.25	19843.5

949.1571	4.5	19848

949.31781	2.25	19850.25

949.47852	11.25	19861.5

949.63923	2.25	19863.75

949.79994	2.25	19866

949.96066	2.25	19868.25

950.12137	2.25	19870.5

950.28208	4.5	19875

950.44279	2.25	19877.25

950.6035	6.75	19884

950.76421	9	19893

950.92492	2.25	19895.25

951.08563	2.25	19897.5

951.24634	4.5	19902

951.40705	13.5	19915.5

951.56776	2.25	19917.75

951.72847	2.25	19920

951.88918	15.75	19935.75

952.04989	2.25	19938

952.2106	2.25	19940.25

952.37132	2.25	19942.5

952.53203	2.25	19944.75

952.69274	4.5	19949.25

952.85345	2.25	19951.5

953.01416	2.25	19953.75

953.17487	20.25	19974

953.33558	4.5	19978.5

953.49629	2.25	19980.75

953.657	2.25	19983

953.81771	2.25	19985.25

953.97842	2.25	19987.5

954.13913	4.5	19992

954.29984	4.5	19996.5

954.46055	2.25	19998.75

954.62126	2.25	20001

954.78197	4.5	20005.5

954.94269	2.25	20007.75

955.1034	13.5	20021.25

955.26411	2.25	20023.5

955.42482	6.75	20030.25

955.58553	2.25	20032.5

955.74624	2.25	20034.75

955.90695	6.75	20041.5

956.06766	2.25	20043.75

956.22837	6.75	20050.5

956.38908	11.25	20061.75

956.54979	2.25	20064

956.7105	2.25	20066.25

956.87121	6.75	20073

957.03192	2.25	20075.25

957.19263	2.25	20077.5

957.35335	4.5	20082

957.51406	4.5	20086.5

957.67477	4.5	20091

957.83548	4.5	20095.5

957.99619	6.75	20102.25

958.1569	13.5	20115.75

958.31761	2.25	20118

958.47832	9	20127

958.63903	2.25	20129.25

958.79974	4.5	20133.75

958.96045	4.5	20138.25

959.12116	4.5	20142.75

959.28187	2.25	20145

959.44258	2.25	20147.25

959.60329	2.25	20149.5

959.76401	4.5	20154

959.92472	2.25	20156.25

960.08543	9	20165.25

960.24614	4.5	20169.75

960.40685	4.5	20174.25

960.56756	2.25	20176.5

960.72827	9	20185.5

960.88898	2.25	20187.75

961.04969	4.5	20192.25

961.2104	2.25	20194.5

961.37111	4.5	20199

961.53182	2.25	20201.25

961.69253	2.25	20203.5

961.85324	4.5	20208

962.01395	2.25	20210.25

962.17466	6.75	20217

962.33538	2.25	20219.25

962.49609	4.5	20223.75

962.6568	2.25	20226

962.81751	2.25	20228.25

962.97822	2.25	20230.5

963.13893	2.25	20232.75

963.29964	4.5	20237.25

963.46035	2.25	20239.5

963.62106	2.25	20241.75

963.78177	6.75	20248.5

963.94248	4.5	20253

964.10319	2.25	20255.25

964.2639	13.5	20268.75

964.42461	4.5	20273.25

964.58532	2.25	20275.5

964.74604	4.5	20280

964.90675	9	20289

965.06746	4.5	20293.5

965.22817	4.5	20298

965.38888	2.25	20300.25

965.54959	2.25	20302.5

965.7103	2.25	20304.75

965.87101	2.25	20307

966.03172	2.25	20309.25

966.19243	9	20318.25

966.35314	4.5	20322.75

966.51385	2.25	20325

966.67456	2.25	20327.25

966.83527	4.5	20331.75

966.99598	2.25	20334

967.15669	4.5	20338.5

967.31741	4.5	20343

967.47812	2.25	20345.25

967.63883	13.5	20358.75

967.79954	2.25	20361

967.96025	2.25	20363.25

968.12096	2.25	20365.5

968.28167	2.25	20367.75

968.44238	4.5	20372.25

968.60309	2.25	20374.5

968.7638	6.75	20381.25

968.92451	9	20390.25

969.08522	2.25	20392.5

969.24593	2.25	20394.75

969.40664	6.75	20401.5

969.56735	6.75	20408.25

969.72807	2.25	20410.5

969.88878	27	20437.5

970.04949	2.25	20439.75

970.2102	6.75	20446.5

970.37091	2.25	20448.75

970.53162	18	20466.75

970.69233	2.25	20469

970.85304	9	20478

971.01375	2.25	20480.25

971.17446	2.25	20482.5

971.33517	2.25	20484.75

971.49588	2.25	20487

971.65659	2.25	20489.25

971.8173	4.5	20493.75

971.97801	4.5	20498.25

972.13873	4.5	20502.75

972.29944	4.5	20507.25

972.46015	2.25	20509.5

972.62086	13.5	20523

972.78157	2.25	20525.25

972.94228	2.25	20527.5

973.10299	6.75	20534.25

973.2637	15.75	20550

973.42441	2.25	20552.25

973.58512	2.25	20554.5

973.74583	2.25	20556.75

973.90654	4.5	20561.25

974.06725	4.5	20565.75

974.22796	2.25	20568

974.38867	2.25	20570.25

974.54938	4.5	20574.75

974.7101	4.5	20579.25

974.87081	11.25	20590.5

975.03152	6.75	20597.25

975.19223	2.25	20599.5

975.35294	2.25	20601.75

975.51365	4.5	20606.25

975.67436	2.25	20608.5

975.83507	2.25	20610.75

975.99578	2.25	20613

976.15649	15.75	20628.75

976.3172	2.25	20631

976.47791	4.5	20635.5

976.63862	2.25	20637.75

976.79933	2.25	20640

976.96004	2.25	20642.25

977.12076	4.5	20646.75

977.28147	4.5	20651.25

977.44218	2.25	20653.5

977.60289	2.25	20655.75

977.7636	11.25	20667

977.92431	6.75	20673.75

978.08502	6.75	20680.5

978.24573	4.5	20685

978.40644	6.75	20691.75

978.56715	4.5	20696.25

978.72786	2.25	20698.5

978.88857	4.5	20703

979.04928	2.25	20705.25

979.20999	6.75	20712

979.3707	2.25	20714.25

979.53142	2.25	20716.5

979.69213	2.25	20718.75

979.85284	4.5	20723.25

980.01355	4.5	20727.75

980.17426	2.25	20730

980.33497	2.25	20732.25

980.49568	4.5	20736.75

980.65639	2.25	20739

980.8171	2.25	20741.25

980.97781	2.25	20743.5

981.13852	2.25	20745.75

981.29923	11.25	20757

981.45994	4.5	20761.5

981.62065	2.25	20763.75

981.78136	2.25	20766

981.94207	2.25	20768.25

982.10279	2.25	20770.5

982.2635	2.25	20772.75

982.42421	2.25	20775

982.58492	2.25	20777.25

982.74563	2.25	20779.5

982.90634	4.5	20784

983.06705	6.75	20790.75

983.22776	2.25	20793

983.38847	2.25	20795.25

983.54918	2.25	20797.5

983.70989	2.25	20799.75

983.8706	2.25	20802

984.03131	2.25	20804.25

984.19202	2.25	20806.5

984.35273	2.25	20808.75

984.51345	2.25	20811

984.67416	4.5	20815.5

984.83487	9	20824.5

984.99558	2.25	20826.75

985.15629	2.25	20829

985.317	2.25	20831.25

985.47771	20.25	20851.5

985.63842	2.25	20853.75

985.79913	2.25	20856

985.95984	4.5	20860.5

986.12055	27	20887.5

986.28126	2.25	20889.75

986.44197	20.25	20910

986.60268	2.25	20912.25

986.76339	2.25	20914.5

986.9241	9	20923.5

987.08482	2.25	20925.75

987.24553	2.25	20928

987.40624	2.25	20930.25

987.56695	2.25	20932.5

987.72766	2.25	20934.75

987.88837	2.25	20937

988.04908	2.25	20939.25

988.20979	20.25	20959.5

988.3705	2.25	20961.75

988.53121	2.25	20964

988.69192	6.75	20970.75

988.85263	2.25	20973

989.01334	4.5	20977.5

989.17405	2.25	20979.75

989.33476	2.25	20982

989.49548	2.25	20984.25

989.65619	6.75	20991

989.8169	4.5	20995.5

989.97761	4.5	21000

990.13832	2.25	21002.25

990.29903	27	21029.25

990.45974	6.75	21036

990.62045	2.25	21038.25

990.78116	79	21117.25

990.94187	2.25	21119.5

991.10258	9	21128.5

991.26329	4.5	21133

991.424	6.75	21139.75

991.58471	9	21148.75

991.74542	2.25	21151

991.90614	2.25	21153.25

992.06685	9	21162.25

992.22756	42.75	21205

992.38827	2.25	21207.25

992.54898	4.5	21211.75

992.70969	4.5	21216.25

992.8704	13.5	21229.75

993.03111	2.25	21232

993.19182	2.25	21234.25

993.35253	9	21243.25

993.51324	2.25	21245.5

993.67395	2.25	21247.75

993.83466	15.75	21263.5

993.99537	2.25	21265.75

994.15608	2.25	21268

994.31679	2.25	21270.25

994.47751	4.5	21274.75

994.63822	2.25	21277

994.79893	2.25	21279.25

994.95964	4.5	21283.75

995.12035	18	21301.75

995.28106	4.5	21306.25

995.44177	20.25	21326.5

995.60248	2.25	21328.75

995.76319	4.5	21333.25

995.9239	2.25	21335.5

996.08461	15.75	21351.25

996.24532	2.25	21353.5

996.40603	2.25	21355.75

996.56674	11.25	21367

996.72745	4.5	21371.5

996.88817	2.25	21373.75

997.04888	2.25	21376

997.20959	15.75	21391.75

997.3703	9	21400.75

997.53101	2.25	21403

997.69172	2.25	21405.25

997.85243	2.25	21407.5

998.01314	2.25	21409.75

998.17385	4.5	21414.25

998.33456	4.5	21418.75

998.49527	6.75	21425.5

998.65598	2.25	21427.75

998.81669	4.5	21432.25

998.9774	15.75	21448

999.13811	2.25	21450.25

999.29882	18	21468.25

999.45954	4.5	21472.75

999.62025	2.25	21475

999.78096	13.5	21488.5

999.94167	4.5	21493

1000.10238	2.25	21495.25

1000.26309	2.25	21497.5

1000.4238	6.75	21504.25

1000.58451	4.5	21508.75

1000.74522	2.25	21511

1000.90593	2.25	21513.25

1001.06664	2.25	21515.5

1001.22735	34.5	21550

1001.38806	2.25	21552.25

1001.54877	2.25	21554.5

1001.70949	2.25	21556.75

1001.8702	2.25	21559

1002.03091	4.5	21563.5

1002.19162	4.5	21568

1002.35233	2.25	21570.25

1002.51304	6.75	21577

1002.67375	2.25	21579.25

1002.83446	4.5	21583.75

1002.99517	4.5	21588.25

1003.15588	2.25	21590.5

1003.31659	6.75	21597.25

1003.4773	2.25	21599.5

1003.63801	2.25	21601.75

1003.79872	2.25	21604

1003.95943	18	21622

1004.12015	2.25	21624.25

1004.28086	2.25	21626.5

1004.44157	6.75	21633.25

1004.60228	2.25	21635.5

1004.76299	2.25	21637.75

1004.9237	2.25	21640

1005.08441	6.75	21646.75

1005.24512	6.75	21653.5

1005.40583	6.75	21660.25

1005.56654	2.25	21662.5

1005.72725	2.25	21664.75

1005.88796	2.25	21667

1006.04867	6.75	21673.75

1006.20938	2.25	21676

1006.37009	2.25	21678.25

1006.5308	2.25	21680.5

1006.69152	2.25	21682.75

1006.85223	4.5	21687.25

1007.01294	4.5	21691.75

1007.17365	4.5	21696.25

1007.33436	2.25	21698.5

1007.49507	2.25	21700.75

1007.65578	6.75	21707.5

1007.81649	2.25	21709.75

1007.9772	2.25	21712

1008.13791	2.25	21714.25

1008.29862	2.25	21716.5

1008.45933	2.25	21718.75

1008.62004	2.25	21721

1008.78075	4.5	21725.5

1008.94146	11.25	21736.75

1009.10218	2.25	21739

1009.26289	2.25	21741.25

1009.4236	32.25	21773.5

1009.58431	9	21782.5

1009.74502	2.25	21784.75

1009.90573	2.25	21787

1010.06644	2.25	21789.25

1010.22715	9	21798.25

1010.38786	4.5	21802.75

1010.54857	2.25	21805

1010.70928	2.25	21807.25

1010.86999	4.5	21811.75

1011.0307	4.5	21816.25

1011.19141	2.25	21818.5

1011.35212	2.25	21820.75

1011.51283	2.25	21823

1011.67355	2.25	21825.25

1011.83426	4.5	21829.75

1011.99497	4.5	21834.25

1012.15568	2.25	21836.5

1012.31639	2.25	21838.75

1012.4771	2.25	21841

1012.63781	2.25	21843.25

1012.79852	2.25	21845.5

1012.95923	2.25	21847.75

1013.11994	2.25	21850

1013.28065	18	21868

1013.44136	4.5	21872.5

1013.60207	2.25	21874.75

1013.76278	2.25	21877

1013.92349	45	21922

1014.08421	6.75	21928.75

1014.24492	4.5	21933.25

1014.40563	2.25	21935.5

1014.56634	2.25	21937.75

1014.72705	2.25	21940

1014.88776	2.25	21942.25

1015.04847	2.25	21944.5

1015.20918	2.25	21946.75

1015.36989	13.5	21960.25

1015.5306	4.5	21964.75

1015.69131	2.25	21967

1015.85202	27	21994

1016.01273	4.5	21998.5

1016.17344	2.25	22000.75

1016.33415	6.75	22007.5

1016.49487	4.5	22012

1016.65558	2.25	22014.25

1016.81629	4.5	22018.75

1016.977	6.75	22025.5

1017.13771	4.5	22030

1017.29842	4.5	22034.5

1017.45913	2.25	22036.75

1017.61984	2.25	22039

1017.78055	2.25	22041.25

1017.94126	2.25	22043.5

1018.10197	9	22052.5

1018.26268	4.5	22057

1018.42339	2.25	22059.25

1018.5841	2.25	22061.5

1018.74481	4.5	22066

1018.90552	29.25	22095.25

1019.06624	4.5	22099.75

1019.22695	2.25	22102

1019.38766	9	22111

1019.54837	2.25	22113.25

1019.70908	4.5	22117.75

1019.86979	4.5	22122.25

1020.0305	2.25	22124.5

1020.19121	4.5	22129

1020.35192	2.25	22131.25

1020.51263	2.25	22133.5

1020.67334	2.25	22135.75

1020.83405	9	22144.75

1020.99476	59	22203.75

1021.15547	19	22222.75

1021.31618	4.5	22227.25

1021.4769	2.25	22229.5

1021.63761	2.25	22231.75

1021.79832	2.25	22234

1021.95903	2.25	22236.25

1022.11974	2.25	22238.5

1022.28045	2.25	22240.75

1022.44116	2.25	22243

1022.60187	6.75	22249.75

1022.76258	2.25	22252

1022.92329	11.25	22263.25

1023.084	2.25	22265.5

1023.24471	6.75	22272.25

1023.40542	6.75	22279

1023.56613	4.5	22283.5

1023.72684	2.25	22285.75

1023.88756	2.25	22288

1024.04826	4.5	22292.5

1024.20898	6.75	22299.25

1024.36969	4.5	22303.75

1024.5304	6.75	22310.5

1024.69111	4.5	22315

1024.85182	2.25	22317.25

1025.01253	9	22326.25

1025.17324	4.5	22330.75

1025.33395	2.25	22333

1025.49466	2.25	22335.25

1025.65537	6.75	22342

1025.81608	4.5	22346.5

1025.97679	4.5	22351

1026.1375	9	22360

1026.29821	4.5	22364.5

1026.45892	6.75	22371.25

1026.61964	2.25	22373.5

1026.78035	4.5	22378

1026.94106	2.25	22380.25

1027.10177	4.5	22384.75

1027.26248	2.25	22387

1027.42319	9	22396

1027.5839	2.25	22398.25

1027.74461	2.25	22400.5

1027.90532	2.25	22402.75

1028.06603	9	22411.75

1028.22674	4.5	22416.25

1028.38745	11.25	22427.5

1028.54816	9	22436.5

1028.70887	4.5	22441

1028.86958	2.25	22443.25

1029.0303	11.25	22454.5

1029.19101	6.75	22461.25

1029.35172	4.5	22465.75

1029.51243	2.25	22468

1029.67314	6.75	22474.75

1029.83385	2.25	22477

1029.99456	4.5	22481.5

1030.15527	6.75	22488.25

1030.31598	2.25	22490.5

1030.47669	9	22499.5

1030.6374	2.25	22501.75

1030.79811	2.25	22504

1030.95882	2.25	22506.25

1031.11953	4.5	22510.75

1031.28024	15.75	22526.5

1031.44096	2.25	22528.75

1031.60167	18	22546.75

1031.76238	2.25	22549

1031.92309	2.25	22551.25

1032.0838	4.5	22555.75

1032.24451	6.75	22562.5

1032.40522	4.5	22567

1032.56593	4.5	22571.5

1032.72664	4.5	22576

1032.88735	2.25	22578.25

1033.04806	2.25	22580.5

1033.20877	2.25	22582.75

1033.36948	2.25	22585

1033.53019	2.25	22587.25

1033.6909	6.75	22594

1033.85162	11.25	22605.25

1034.01233	6.75	22612

1034.17304	22.25	22634.25

1034.33375	2.25	22636.5

1034.49446	2.25	22638.75

1034.65517	2.25	22641

1034.81588	4.5	22645.5

1034.97659	2.25	22647.75

1035.1373	9	22656.75

1035.29801	2.25	22659

1035.45872	2.25	22661.25

1035.61943	4.5	22665.75

1035.78014	2.25	22668

1035.94085	2.25	22670.25

1036.10156	2.25	22672.5

1036.26228	2.25	22674.75

1036.42298	6.75	22681.5

1036.5837	6.75	22688.25

1036.74441	4.5	22692.75

1036.90512	4.5	22697.25

1037.06583	4.5	22701.75

1037.22654	6.75	22708.5

1037.38725	9	22717.5

1037.54796	4.5	22722

1037.70867	2.25	22724.25

1037.86938	2.25	22726.5

1038.03009	9	22735.5

1038.1908	2.25	22737.75

1038.35151	2.25	22740

1038.51222	2.25	22742.25

1038.67293	9	22751.25

1038.83364	2.25	22753.5

1038.99436	2.25	22755.75

1039.15507	2.25	22758

1039.31578	2.25	22760.25

1039.47649	9	22769.25

1039.6372	2.25	22771.5

1039.79791	2.25	22773.75

1039.95862	42.25	22816

1040.11933	2.25	22818.25

1040.28004	4.5	22822.75

1040.44075	9	22831.75

1040.60146	4.5	22836.25

1040.76217	2.25	22838.5

1040.92288	20.25	22858.75

1041.08359	2.25	22861

1041.2443	2.25	22863.25

1041.40502	2.25	22865.5

1041.56573	2.25	22867.75

1041.72644	2.25	22870

1041.88715	2.25	22872.25

1042.04786	2.25	22874.5

1042.20857	4.5	22879

1042.36928	11.25	22890.25

1042.52999	2.25	22892.5

1042.6907	4.5	22897

1042.85141	6.75	22903.75

1043.01212	2.25	22906

1043.17283	4.5	22910.5

1043.33354	2.25	22912.75

1043.49425	2.25	22915

1043.65496	6.75	22921.75

1043.81568	2.25	22924

1043.97639	2.25	22926.25

1044.1371	2.25	22928.5

1044.29781	2.25	22930.75

1044.45852	2.25	22933

1044.61923	2.25	22935.25

1044.77994	2.25	22937.5

1044.94065	9	22946.5

1045.10136	4.5	22951

1045.26207	2.25	22953.25

1045.42278	2.25	22955.5

1045.58349	2.25	22957.75

1045.7442	9	22966.75

1045.90491	2.25	22969

1046.06562	6.75	22975.75

1046.22634	2.25	22978

1046.38705	6.75	22984.75

1046.54776	2.25	22987

1046.70847	4.5	22991.5

1046.86918	9	23000.5

1047.02989	15.75	23016.25

1047.1906	13.5	23029.75

1047.35131	4.5	23034.25

1047.51202	2.25	23036.5

1047.67273	4.5	23041

1047.83344	2.25	23043.25

1047.99415	2.25	23045.5

1048.15486	4.5	23050

1048.31557	4.5	23054.5

1048.47628	9	23063.5

1048.637	2.25	23065.75

1048.79771	9	23074.75

1048.95842	2.25	23077

1049.11913	2.25	23079.25

1049.27984	2.25	23081.5

1049.44055	4.5	23086

1049.60126	2.25	23088.25

1049.76197	2.25	23090.5

1049.92268	4.5	23095

1050.08339	2.25	23097.25

1050.2441	2.25	23099.5

1050.40481	2.25	23101.75

1050.56552	2.25	23104

1050.72623	4.5	23108.5

1050.88694	2.25	23110.75

1051.04766	4.5	23115.25

1051.20837	9	23124.25

1051.36908	2.25	23126.5

1051.52979	6.75	23133.25

1051.6905	6.75	23140

1051.85121	6.75	23146.75

1052.01192	4.5	23151.25

1052.17263	6.75	23158

1052.33334	9	23167

1052.49405	2.25	23169.25

1052.65476	2.25	23171.5

1052.81547	2.25	23173.75

1052.97618	11.25	23185

1053.13689	22.5	23207.5

1053.2976	4.5	23212

1053.45831	2.25	23214.25

1053.61903	2.25	23216.5

1053.77974	6.75	23223.25

1053.94045	2.25	23225.5

1054.10116	2.25	23227.75

1054.26187	4.5	23232.25

1054.42258	2.25	23234.5

1054.58329	2.25	23236.75

1054.744	11.25	23248

1054.90471	15.75	23263.75

1055.06542	13.5	23277.25

1055.22613	4.5	23281.75

1055.38684	2.25	23284

1055.54755	2.25	23286.25

1055.70826	4.5	23290.75

1055.86897	2.25	23293

1056.02969	6.75	23299.75

1056.1904	2.25	23302

1056.35111	2.25	23304.25

1056.51182	9	23313.25

1056.67253	6.75	23320

1056.83324	2.25	23322.25

1056.99395	13.5	23335.75

1057.15466	59	23394.75

1057.31537	2.25	23397

1057.47608	2.25	23399.25

1057.63679	9	23408.25

1057.7975	2.25	23410.5

1057.95821	2.25	23412.75

1058.11892	2.25	23415

1058.27963	6.75	23421.75

1058.44034	2.25	23424

1058.60106	2.25	23426.25

1058.76177	2.25	23428.5

1058.92248	2.25	23430.75

1059.08319	2.25	23433

1059.2439	2.25	23435.25

1059.40461	4.5	23439.75

1059.56532	4.5	23444.25

1059.72603	2.25	23446.5

1059.88674	4.5	23451

1060.04745	6.75	23457.75

1060.20816	2.25	23460

1060.36887	2.25	23462.25

1060.52958	4.5	23466.75

1060.69029	13.5	23480.25

1060.851	4.5	23484.75

1061.01172	2.25	23487

1061.17243	2.25	23489.25

1061.33314	2.25	23491.5

1061.49385	4.5	23496

1061.65456	4.5	23500.5

1061.81527	2.25	23502.75

1061.97598	4.5	23507.25

1062.13669	2.25	23509.5

1062.2974	2.25	23511.75

1062.45811	22.5	23534.25

1062.61882	2.25	23536.5

1062.77953	2.25	23538.75

1062.94024	2.25	23541

1063.10095	4.5	23545.5

1063.26166	2.25	23547.75

1063.42238	11.25	23559

1063.58309	2.25	23561.25

1063.7438	2.25	23563.5

1063.90451	2.25	23565.75

1064.06522	2.25	23568

1064.22593	2.25	23570.25

1064.38664	2.25	23572.5

1064.54735	2.25	23574.75

1064.70806	2.25	23577

1064.86877	2.25	23579.25

1065.02948	4.5	23583.75

1065.19019	4.5	23588.25

1065.3509	9	23597.25

1065.51161	2.25	23599.5

1065.67232	15.75	23615.25

1065.83303	6.75	23622

1065.99375	2.25	23624.25

1066.15446	2.25	23626.5

1066.31517	15.75	23642.25

1066.47588	2.25	23644.5

1066.63659	2.25	23646.75

1066.7973	6.75	23653.5

1066.95801	2.25	23655.75

1067.11872	11.25	23667

1067.27943	2.25	23669.25

1067.44014	2.25	23671.5

1067.60085	18	23689.5

1067.76156	9	23698.5

1067.92227	2.25	23700.75

1068.08298	2.25	23703

1068.24369	4.5	23707.5

1068.40441	13.5	23721

1068.56512	4.5	23725.5

1068.72583	4.5	23730

1068.88654	2.25	23732.25

1069.04725	6.75	23739

1069.20796	2.25	23741.25

1069.36867	2.25	23743.5

1069.52938	2.25	23745.75

1069.69009	6.75	23752.5

1069.8508	6.75	23759.25

1070.01151	6.75	23766

1070.17222	2.25	23768.25

1070.33293	4.5	23772.75

1070.49364	6.75	23779.5

1070.65435	4.5	23784

1070.81507	9	23793

1070.97578	4.5	23797.5

1071.13649	2.25	23799.75

1071.2972	11.25	23811

1071.45791	6.75	23817.75

1071.61862	9	23826.75

1071.77933	6.75	23833.5

1071.94004	6.75	23840.25

1072.10075	4.5	23844.75

1072.26146	2.25	23847

1072.42217	9	23856

1072.58288	2.25	23858.25

1072.74359	11.25	23869.5

1072.9043	2.25	23871.75

1073.06501	2.25	23874

1073.22572	4.5	23878.5

1073.38643	2.25	23880.75

1073.54715	2.25	23883

1073.70786	2.25	23885.25

1073.86857	2.25	23887.5

1074.02928	4.5	23892

1074.18999	4.5	23896.5

1074.3507	2.25	23898.75

1074.51141	2.25	23901

1074.67212	4.5	23905.5

1074.83283	2.25	23907.75

1074.99354	2.25	23910

1075.15425	4.5	23914.5

1075.31496	11.25	23925.75

1075.47567	2.25	23928

1075.63638	4.5	23932.5

1075.79709	4.5	23937

1075.95781	2.25	23939.25

1076.11852	2.25	23941.5

1076.27923	2.25	23943.75

1076.43994	2.25	23946

1076.60065	9	23955

1076.76136	2.25	23957.25

1076.92207	2.25	23959.5

1077.08278	2.25	23961.75

1077.24349	2.25	23964

1077.4042	2.25	23966.25

1077.56491	2.25	23968.5

1077.72562	4.5	23973

1077.88633	6.75	23979.75

1078.04704	2.25	23982

1078.20775	11.25	23993.25

1078.36847	6.75	24000

1078.52918	2.25	24002.25

1078.68989	2.25	24004.5

1078.8506	6.75	24011.25

1079.01131	2.25	24013.5

1079.17202	2.25	24015.75

1079.33273	6.75	24022.5

1079.49344	2.25	24024.75

1079.65415	13.5	24038.25

1079.81486	11.25	24049.5

1079.97557	9	24058.5

1080.13628	9	24067.5

1080.29699	2.25	24069.75

1080.4577	15.75	24085.5

1080.61841	4.5	24090

1080.77913	4.5	24094.5

1080.93984	18	24112.5

1081.10055	2.25	24114.75

1081.26126	2.25	24117

1081.42197	2.25	24119.25

1081.58268	2.25	24121.5

1081.74339	4.5	24126

1081.9041	18	24144

1082.06481	2.25	24146.25

1082.22552	4.5	24150.75

1082.38623	2.25	24153

1082.54694	2.25	24155.25

1082.70765	4.5	24159.75

1082.86836	2.25	24162

1083.02907	2.25	24164.25

1083.18979	4.5	24168.75

1083.35049	24.75	24193.5

1083.51121	2.25	24195.75

1083.67192	2.25	24198

1083.83263	4.5	24202.5

1083.99334	2.25	24204.75

1084.15405	2.25	24207

1084.31476	4.5	24211.5

1084.47547	6.75	24218.25

1084.63618	2.25	24220.5

1084.79689	2.25	24222.75

1084.9576	2.25	24225

1085.11831	4.5	24229.5

1085.27902	2.25	24231.75

1085.43973	4.5	24236.25

1085.60044	4.5	24240.75

1085.76115	2.25	24243

1085.92187	6.75	24249.75

1086.08258	2.25	24252

1086.24329	2.25	24254.25

1086.404	11.25	24265.5

1086.56471	4.5	24270

1086.72542	11.25	24281.25

1086.88613	6.75	24288

1087.04684	4.5	24292.5

1087.20755	2.25	24294.75

1087.36826	2.25	24297

1087.52897	13.5	24310.5

1087.68968	2.25	24312.75

1087.85039	2.25	24315

1088.0111	2.25	24317.25

1088.17181	2.25	24319.5

1088.33253	9	24328.5

1088.49324	2.25	24330.75

1088.65395	6.75	24337.5

1088.81466	2.25	24339.75

1088.97537	2.25	24342

1089.13608	4.5	24346.5

1089.29679	6.75	24353.25

1089.4575	2.25	24355.5

1089.61821	2.25	24357.75

1089.77892	11.25	24369

1089.93963	4.5	24373.5

1090.10034	2.25	24375.75

1090.26105	2.25	24378

1090.42176	4.5	24382.5

1090.58247	4.5	24387

1090.74319	2.25	24389.25

1090.9039	4.5	24393.75

1091.06461	4.5	24398.25

1091.22532	13.5	24411.75

1091.38603	2.25	24414

1091.54674	9	24423

1091.70745	4.5	24427.5

1091.86816	11.25	24438.75

1092.02887	2.25	24441

1092.18958	2.25	24443.25

1092.35029	4.5	24447.75

1092.511	6.75	24454.5

1092.67171	2.25	24456.75

1092.83242	2.25	24459

1092.99313	2.25	24461.25

1093.15385	9	24470.25

1093.31456	2.25	24472.5

1093.47527	2.25	24474.75

1093.63598	6.75	24481.5

1093.79669	2.25	24483.75

1093.9574	6.75	24490.5

1094.11811	2.25	24492.75

1094.27882	6.75	24499.5

1094.43953	2.25	24501.75

1094.60024	2.25	24504

1094.76095	4.5	24508.5

1094.92166	6.75	24515.25

1095.08237	2.25	24517.5

1095.24308	11.25	24528.75

1095.40379	2.25	24531

1095.56451	6.75	24537.75

1095.72521	2.25	24540

1095.88593	18	24558

1096.04664	2.25	24560.25

1096.20735	9	24569.25

1096.36806	2.25	24571.5

1096.52877	4.5	24576

1096.68948	9	24585

1096.85019	2.25	24587.25

1097.0109	2.25	24589.5

1097.17161	6.75	24596.25

1097.33232	2.25	24598.5

1097.49303	4.5	24603

1097.65374	2.25	24605.25

1097.81445	2.25	24607.5

1097.97516	9	24616.5

1098.13587	2.25	24618.75

1098.29659	4.5	24623.25

1098.4573	4.5	24627.75

1098.61801	2.25	24630

1098.77872	4.5	24634.5

1098.93943	2.25	24636.75

1099.10014	2.25	24639

1099.26085	6.75	24645.75

1099.42156	6.75	24652.5

1099.58227	2.25	24654.75

1099.74298	2.25	24657

1099.90369	2.25	24659.25

1100.0644	2.25	24661.5

1100.22511	2.25	24663.75

1100.38582	2.25	24666

1100.54653	18	24684

1100.70725	4.5	24688.5

1100.86796	2.25	24690.75

1101.02867	2.25	24693

1101.18938	13.5	24706.5

1101.35009	4.5	24711

1101.5108	2.25	24713.25

1101.67151	2.25	24715.5

1101.83222	6.75	24722.25

1101.99293	4.5	24726.75

1102.15364	2.25	24729

1102.31435	2.25	24731.25

1102.47506	2.25	24733.5

1102.63577	42.75	24776.25

1102.79648	2.25	24778.5

1102.9572	2.25	24780.75

1103.11791	2.25	24783

1103.27862	4.5	24787.5

1103.43933	2.25	24789.75

1103.60004	9	24798.75

1103.76075	2.25	24801

1103.92146	2.25	24803.25

1104.08217	2.25	24805.5

1104.24288	13.5	24819

1104.40359	4.5	24823.5

1104.5643	2.25	24825.75

1104.72501	9	24834.75

1104.88572	15.75	24850.5

1105.04643	9	24859.5

1105.20714	4.5	24864

1105.36785	4.5	24868.5

1105.52857	4.5	24873

1105.68928	2.25	24875.25

1105.84999	2.25	24877.5

1106.0107	6.75	24884.25

1106.17141	2.25	24886.5

1106.33212	24.75	24911.25

1106.49283	2.25	24913.5

1106.65354	6.75	24920.25

1106.81425	2.25	24922.5

1106.97496	2.25	24924.75

1107.13567	2.25	24927

1107.29638	2.25	24929.25

1107.45709	4.5	24933.75

1107.6178	4.5	24938.25

1107.77851	2.25	24940.5

1107.93923	2.25	24942.75

1108.09994	15.75	24958.5

1108.26065	4.5	24963

1108.42136	4.5	24967.5

1108.58207	4.5	24972

1108.74278	2.25	24974.25

1108.90349	4.5	24978.75

1109.0642	2.25	24981

1109.22491	4.5	24985.5

1109.38562	2.25	24987.75

1109.54633	4.5	24992.25

1109.70704	4.5	24996.75

1109.86775	2.25	24999

1110.02846	2.25	25001.25

1110.18917	4.5	25005.75

1110.34989	2.25	25008

1110.5106	11.25	25019.25

1110.67131	4.5	25023.75

1110.83202	2.25	25026

1110.99273	2.25	25028.25

1111.15344	4.5	25032.75

1111.31415	6.75	25039.5

1111.47486	4.5	25044

1111.63557	6.75	25050.75

1111.79628	9	25059.75

1111.95699	2.25	25062

1112.1177	2.25	25064.25

1112.27841	2.25	25066.5

1112.43912	11.25	25077.75

1112.59983	2.25	25080

1112.76054	6.75	25086.75

1112.92126	4.5	25091.25

1113.08197	15.75	25107

1113.24268	13.5	25120.5

1113.40339	2.25	25122.75

1113.5641	6.75	25129.5

1113.72481	2.25	25131.75

1113.88552	2.25	25134

1114.04623	2.25	25136.25

1114.20694	4.5	25140.75

1114.36765	2.25	25143

1114.52836	4.5	25147.5

1114.68907	4.5	25152

1114.84978	2.25	25154.25

1115.01049	6.75	25161

1115.1712	6.75	25167.75

1115.33192	18	25185.75

1115.49263	13.5	25199.25

1115.65334	9	25208.25

1115.81405	2.25	25210.5

1115.97476	2.25	25212.75

1116.13547	4.5	25217.25

1116.29618	2.25	25219.5

1116.45689	4.5	25224

1116.6176	2.25	25226.25

1116.77831	4.5	25230.75

1116.93902	4.5	25235.25

1117.09973	2.25	25237.5

1117.26044	6.75	25244.25

1117.42115	4.5	25248.75

1117.58186	2.25	25251

1117.74257	2.25	25253.25

1117.90329	4.5	25257.75

1118.064	72.25	25330

1118.22471	4.5	25334.5

1118.38542	4.5	25339

1118.54613	2.25	25341.25

1118.70684	2.25	25343.5

1118.86755	2.25	25345.75

1119.02826	2.25	25348

1119.18897	2.25	25350.25

1119.34968	2.25	25352.5

1119.51039	4.5	25357

1119.6711	2.25	25359.25

1119.83181	4.5	25363.75

1119.99252	2.25	25366

1120.15323	15.75	25381.75

1120.31395	2.25	25384

1120.47466	2.25	25386.25

1120.63537	2.25	25388.5

1120.79608	4.5	25393

1120.95679	13.5	25406.5

1121.1175	9	25415.5

1121.27821	6.75	25422.25

1121.43892	2.25	25424.5

1121.59963	2.25	25426.75

1121.76034	11.25	25438

1121.92105	2.25	25440.25

1122.08176	2.25	25442.5

1122.24247	4.5	25447

1122.40318	9	25456

1122.56389	6.75	25462.75

1122.72461	2.25	25465

1122.88532	20.25	25485.25

1123.04603	13.5	25498.75

1123.20674	2.25	25501

1123.36745	6.75	25507.75

1123.52816	4.5	25512.25

1123.68887	4.5	25516.75

1123.84958	4.5	25521.25

1124.01029	2.25	25523.5

1124.171	2.25	25525.75

1124.33171	4.5	25530.25

1124.49242	2.25	25532.5

1124.65313	2.25	25534.75

1124.81384	6.75	25541.5

1124.97455	46.75	25588.25

1125.13526	6.75	25595

1125.29598	9	25604

1125.45669	4.5	25608.5

1125.6174	2.25	25610.75

1125.77811	2.25	25613

1125.93882	6.75	25619.75

1126.09953	4.5	25624.25

1126.26024	4.5	25628.75

1126.42095	2.25	25631

1126.58166	2.25	25633.25

1126.74237	2.25	25635.5

1126.90308	2.25	25637.75

1127.06379	11.25	25649

1127.2245	6.75	25655.75

1127.38521	2.25	25658

1127.54592	4.5	25662.5

1127.70664	2.25	25664.75

1127.86734	6.75	25671.5

1128.02806	2.25	25673.75

1128.18877	2.25	25676

1128.34948	13.5	25689.5

1128.51019	2.25	25691.75

1128.6709	2.25	25694

1128.83161	2.25	25696.25

1128.99232	2.25	25698.5

1129.15303	2.25	25700.75

1129.31374	4.5	25705.25

1129.47445	2.25	25707.5

1129.63516	2.25	25709.75

1129.79587	9	25718.75

1129.95658	6.75	25725.5

1130.1173	6.75	25732.25

1130.278	2.25	25734.5

1130.43872	4.5	25739

1130.59943	2.25	25741.25

1130.76014	4.5	25745.75

1130.92085	4.5	25750.25

1131.08156	4.5	25754.75

1131.24227	2.25	25757

1131.40298	2.25	25759.25

1131.56369	4.5	25763.75

1131.7244	2.25	25766

1131.88511	2.25	25768.25

1132.04582	2.25	25770.5

1132.20653	2.25	25772.75

1132.36724	2.25	25775

1132.52795	2.25	25777.25

1132.68866	13.5	25790.75

1132.84938	2.25	25793

1133.01009	6.75	25799.75

1133.1708	2.25	25802

1133.33151	4.5	25806.5

1133.49222	9	25815.5

1133.65293	4.5	25820

1133.81364	2.25	25822.25

1133.97435	2.25	25824.5

1134.13506	2.25	25826.75

1134.29577	2.25	25829

1134.45648	31.5	25860.5

1134.61719	15.75	25876.25

1134.7779	4.5	25880.75

1134.93861	4.5	25885.25

1135.09932	2.25	25887.5

1135.26004	2.25	25889.75

1135.42075	6.75	25896.5

1135.58146	2.25	25898.75

1135.74217	4.5	25903.25

1135.90288	2.25	25905.5

1136.06359	2.25	25907.75

1136.2243	9	25916.75

1136.38501	2.25	25919

1136.54572	2.25	25921.25

1136.70643	2.25	25923.5

1136.86714	2.25	25925.75

1137.02785	4.5	25930.25

1137.18856	2.25	25932.5

1137.34927	2.25	25934.75

1137.50998	11.25	25946

1137.6707	4.5	25950.5

1137.83141	2.25	25952.75

1137.99212	4.5	25957.25

1138.15283	6.75	25964

1138.31354	4.5	25968.5

1138.47425	2.25	25970.75

1138.63496	2.25	25973

1138.79567	2.25	25975.25

1138.95638	2.25	25977.5

1139.11709	2.25	25979.75

1139.2778	4.5	25984.25

1139.43851	4.5	25988.75

1139.59922	4.5	25993.25

1139.75993	2.25	25995.5

1139.92064	2.25	25997.75

1140.08136	2.25	26000

1140.24207	2.25	26002.25

1140.40278	2.25	26004.5

1140.56349	2.25	26006.75

1140.7242	6.75	26013.5

1140.88491	2.25	26015.75

1141.04562	2.25	26018

1141.20633	2.25	26020.25

1141.36704	2.25	26022.5

1141.52775	4.5	26027

1141.68846	4.5	26031.5

1141.84917	2.25	26033.75

1142.00988	4.5	26038.25

1142.17059	2.25	26040.5

1142.3313	2.25	26042.75

1142.49202	4.5	26047.25

1142.65272	2.25	26049.5

1142.81344	6.75	26056.25

1142.97415	9	26065.25

1143.13486	4.5	26069.75

1143.29557	2.25	26072

1143.45628	2.25	26074.25

1143.61699	4.5	26078.75

1143.7777	2.25	26081

1143.93841	2.25	26083.25

1144.09912	9	26092.25

1144.25983	4.5	26096.75

1144.42054	2.25	26099

1144.58125	2.25	26101.25

1144.74196	2.25	26103.5

1144.90267	2.25	26105.75

1145.06338	2.25	26108

1145.2241	2.25	26110.25

1145.38481	4.5	26114.75

1145.54552	9	26123.75

1145.70623	2.25	26126

1145.86694	6.75	26132.75

1146.02765	6.75	26139.5

1146.18836	6.75	26146.25

1146.34907	13.5	26159.75

1146.50978	2.25	26162

1146.67049	40.5	26202.5

1146.8312	11.25	26213.75

1146.99191	4	26217.75

1147.15262	6	26223.75

1147.31333	1	26224.75

1147.47404	1	26225.75

1147.63476	13	26238.75

1147.79547	2	26240.75

1147.95618	1	26241.75

1148.11689	6	26247.75

1148.2776	13	26260.75

1148.43831	16	26276.75

1148.59902	1	26277.75

1148.75973	22	26299.75

1148.92044	1	26300.75

1149.08115	14	26314.75

1149.24186	1	26315.75

1149.40257	1	26316.75

1149.56328	14	26330.75

1149.72399	6	26336.75

1149.8847	1	26337.75

1150.04542	9	26346.75

1150.20613	1	26347.75

1150.36684	3	26350.75

1150.52755	1	26351.75

1150.68826	1	26352.75

1150.84897	7	26359.75

1151.00968	2	26361.75

1151.17039	13	26374.75

1151.3311	1	26375.75

1151.49181	1	26376.75

1151.65252	6	26382.75

1151.81323	1	26383.75

1151.97394	3	26386.75

1152.13465	2	26388.75

1152.29536	8	26396.75

1152.45608	2	26398.75

1152.61679	4	26402.75

1152.7775	7	26409.75

1152.93821	1	26410.75

1153.09892	18	26428.75

1153.25963	6	26434.75

1153.42034	23	26457.75

1153.58105	3	26460.75

1153.74176	1	26461.75

1153.90247	7	26468.75

1154.06318	1	26469.75

1154.22389	1	26470.75

1154.3846	2	26472.75

1154.54531	4	26476.75

1154.70602	4	26480.75

1154.86674	1	26481.75

1155.02744	3	26484.75

1155.18816	2	26486.75

1155.34887	10	26496.75

1155.50958	1	26497.75

1155.67029	25	26522.75

1155.831	1	26523.75

1155.99171	15	26538.75

1156.15242	5	26543.75

1156.31313	21	26564.75

1156.47384	3	26567.75

1156.63455	9	26576.75

1156.79526	4	26580.75

1156.95597	1	26581.75

1157.11668	30	26611.75

1157.2774	3	26614.75

1157.43811	9	26623.75

1157.59882	2	26625.75

1157.75953	303	26928.75

1157.92024	176	27104.75

1158.08095	52	27156.75

1158.24166	2	27158.75

1158.40237	34	27192.75

1158.56308	164	27356.75

1158.72379	550	27906.75

1158.8845	36	27942.75

1159.04521	14595	42537.75

1159.20592	5	42542.75

1159.36663	2	42544.75

1159.52734	1	42545.75

1159.68805	9	42554.75

1159.84877	3	42557.75

1160.00948	2	42559.75

1160.17019	50	42609.75

1160.3309	1	42609.75

1160.49161	3	42609.75

1160.65232	11	42609.75

1160.81303	1	42609.75

1160.97374	4	42609.75

1161.13445	6	42609.75

1161.29516	1	42609.75

1161.45587	2	42609.75

**（*c*）Non-hydraulic-45°**

1	0.01596	0.119699	1	1

2	0.03524	0.29599	2	3

3	0.04303	0.429499	3	6

4	0.05471	0.59398	1.00E+00	7

5	0.0724	0.69298	1	8

6	0.0901	0.79198	4	12

7	0.11263	0.89097	2.00E+00	13

8	0.13033	0.98997	11	15

9	0.13998	1.08897	2	19

10	0.15929	1.18796	4.00E+00	21

11	0.17699	1.28696	1.00E+00	32

12	0.19308	1.38596	1.00E+00	34

13	0.20917	1.48496	1.00E+00	38

14	0.22204	1.58395	15	39

15	0.24296	1.68295	1	40

16	0.25744	1.78195	2	41

17	0.27031	1.88094	1	56

18	0.28479	1.97994	1	57

19	0.3041	2.07894	1	59

20	0.32019	2.17793	2	60

21	0.33145	2.27693	2	61

22	0.34915	2.37593	1	62

23	0.37168	2.47493	8	64

24	0.38938	2.57392	5	66

25	0.39742	2.67292	10	67

26	0.41351	2.77192	1	75

27	0.43765	2.87091	13	80

28	0.4473	2.96991	6	90

29	0.46178	3.06891	11	91

30	0.4827	3.1679	1	104

31	0.50362	3.2669	9	110

32	0.51488	3.3659	2	121

33	0.52775	3.4649	2	122

34	0.54706	3.56389	11	131

35	0.55993	3.66289	2	133

36	0.5728	3.76189	1	135

37	0.5905	3.86088	12	146

38	0.60981	3.95988	1	148

39	0.6259	4.05888	2	149

40	0.64038	4.15788	5	161

41	0.65486	4.25687	1	162

42	0.67417	4.35587	11	164

43	0.68704	4.45487	50	169

44	0.70152	4.55386	12	170

45	0.716	4.65286	2	181

46	0.73853	4.75186	1	231

47	0.75462	4.85085	2	243

48	0.77232	4.94985	1	245

49	0.79002	5.04885	2	246

50	0.81094	5.14785	1	248

51	0.82542	5.24684	1	249

52	0.83346	5.34584	1	251

53	0.84955	5.44484	11	252

54	0.87208	5.54383	2	253

55	0.88173	5.64283	3	254

56	0.89621	5.74183	2	265

57	0.91713	5.84082	3	267

58	0.93805	5.93982	7	270

59	0.94931	6.03882	2	272

60	0.96379	6.13782	1	275

61	0.98471	6.23681	5	282

62	0.99919	6.33581	3	284

63	1.01045	6.43481	16	285

64	1.02654	6.5338	23	290

65	1.04746	6.6328	2	293

66	1.06033	6.7318	1	309

67	1.07642	6.83079	2	332

68	1.09573	6.92979	16	334

69	1.11343	7.02879	2	335

70	1.12952	7.12779	1	337

71	1.13756	7.22678	1	353

72	1.15687	7.32578	1	355

73	1.17457	7.42478	2	356

74	1.18905	7.52377	1	357

75	1.20353	7.62277	2	358

76	1.22123	7.72177	7	360

77	1.24215	7.82077	1	361

78	1.25502	7.91976	4	363

79	1.26789	8.01876	4	370

80	1.2872	8.11776	3	371

81	1.30651	8.21675	1	375

82	1.31455	8.31575	1	379

83	1.33064	8.41475	1	382

84	1.35478	8.51374	5	383

85	1.36926	8.61274	2	384

86	1.38052	8.71174	1	385

87	1.40305	8.81074	1	390

88	1.41753	8.90973	16	392

89	1.43201	9.00873	4	393

90	1.44327	9.10773	1	394

91	1.45936	9.20672	1	410

92	1.48028	9.30572	1	414

93	1.49798	9.40472	13	415

94	1.50924	9.50371	2	416

95	1.52694	9.60271	5	417

96	1.54947	9.70171	7	430

97	1.56556	9.80071	7	432

98	1.5736	9.8997	6	437

99	1.58969	9.9987	2	444

100	1.61061	10.0977	1	451

101	1.62187	10.19669	2	457

102	1.63796	10.29569	1	459

103	1.65727	10.39469	2	460

104	1.67497	10.49368	1	462

105	1.68784	10.59268	1	463

106	1.70393	10.69168	1	465

107	1.72485	10.79068	3	466

108	1.73772	10.88967	2	467

109	1.75059	10.98867	19	468

110	1.76507	11.08767	4	471

111	1.7876	11.18666	1	473

112	1.80047	11.28566	1	492

113	1.81656	11.38466	2	496

114	1.83426	11.48366	3	497

115	1.85518	11.58265	1	498

116	1.86805	11.68165	29	500

117	1.87931	11.78065	1	503

118	1.8954	11.87964	13	504

119	1.91471	11.97864	13	533

120	1.93241	12.07764	2	534

121	1.94206	12.17663	2	547

122	1.95976	12.27563	1	560

123	1.98229	12.37463	2	562

124	1.99838	12.47363	12	564

125	2.00964	12.57262	18	565

126	2.02895	12.67162	1	567

127	2.04665	12.77062	2	579

128	2.0563	12.86961	1	597

129	2.06917	12.96861	5	598

130	2.08848	13.06761	3	600

131	2.1094	13.1666	66	601

132	2.12227	13.2656	4	606

133	2.13836	13.3646	1	609

134	2.15767	13.4636	7	675

135	2.17215	13.56259	4	679

136	2.18341	13.66159	1	680

137	2.1995	13.76059	1	687

138	2.21881	13.85958	16	691

139	2.23651	13.95858	10	692

140	2.24616	14.05758	6	693

141	2.26386	14.15658	1	709

142	2.288	14.25557	4	719

143	2.30248	14.35457	1	725

144	2.31213	14.45357	5	726

145	2.32822	14.55256	22	730

146	2.34753	14.65156	3	731

147	2.36362	14.75056	3	736

148	2.37488	14.84955	2	758

149	2.39419	14.94855	1	761

150	2.41189	15.04755	1	764

151	2.42798	15.14655	2	766

152	2.44085	15.24554	24	767

153	2.46016	15.34454	1	768

154	2.47625	15.44354	1	770

155	2.48751	15.54253	1	794

156	2.50199	15.64153	13	795

157	2.52291	15.74053	17	796

158	2.539	15.83952	12	797

159	2.55026	15.93852	2	810

160	2.56957	16.03752	2	827

161	2.5921	16.13652	6	839

162	2.60658	16.23551	1	841

163	2.61623	16.33451	2	843

164	2.63393	16.43351	3	849

165	2.65324	16.5325	1	850

166	2.66289	16.6315	1	852

167	2.67898	16.7305	9	855

168	2.69668	16.82949	42	856

169	2.7176	16.92849	3	857

170	2.73369	17.02749	7	866

171	2.74656	17.12649	9	908

172	2.76104	17.22548	7	911

173	2.78035	17.32448	2	918

174	2.79322	17.42348	3	927

175	2.80609	17.52247	1	934

176	2.82379	17.62147	3	936

177	2.84149	17.72047	1	939

178	2.85758	17.81947	3	940

179	2.87367	17.91846	2	943

180	2.89298	18.01746	66	944

181	2.90907	18.11646	2	947

182	2.92033	18.21545	5	949

183	2.9316	18.31445	1	1015

184	2.95573	18.41345	4	1017

185	2.97182	18.51244	5	1022

186	2.98308	18.61144	13	1023

187	2.99917	18.71044	1	1027

188	3.0217	18.80944	9	1032

189	3.03779	18.90843	10	1045

190	3.05066	19.00743	1	1046

191	3.06514	19.10643	1	1055

192	3.08445	19.20542	7	1065

193	3.09732	19.30442	1	1066

194	3.10859	19.40342	1	1067

195	3.12789	19.50241	1	1074

196	3.14559	19.60141	5	1075

197	3.1649	19.70041	2	1076

198	3.17777	19.79941	1	1077

199	3.19547	19.8984	2	1082

200	3.21156	19.9974	39	1084

201	3.22443	20.0964	2	1085

202	3.23891	20.19539	1	1087

203	3.25661	20.29439	3	1126

204	3.27592	20.39339	3	1128

205	3.28558	20.49238	1	1129

206	3.30649	20.59138	1	1132

207	3.32741	20.69038	23	1135

208	3.3435	20.78938	1	1136

209	3.35315	20.88837	6	1137

210	3.36603	20.98737	7	1160

211	3.38694	21.08637	2	1161

212	3.40142	21.18536	1	1167

213	3.4143	21.28436	2	1174

214	3.43039	21.38336	2	1176

215	3.45452	21.48236	1	1177

216	3.469	21.58135	7	1179

217	3.47866	21.68035	1	1181

218	3.49796	21.77935	2	1182

219	3.51566	21.87834	3	1189

220	3.52853	21.97734	2	1190

221	3.5398	22.07634	3	1192

222	3.55428	22.17533	1	1195

223	3.57841	22.27433	1	1197

224	3.5945	22.37333	1	1200

225	3.60577	22.47233	3	1201

226	3.62668	22.57132	3	1202

227	3.64116	22.67032	1	1203

228	3.65565	22.76932	1	1206

229	3.6653	22.86831	18	1209

230	3.68622	22.96731	1	1210

231	3.70392	23.06631	2	1211

232	3.71518	23.1653	4	1229

233	3.73449	23.2643	5	1230

234	3.75862	23.3633	1	1232

235	3.7731	23.4623	4	1236

236	3.78597	23.56129	1	1241

237	3.79724	23.66029	2	1242

238	3.81654	23.75929	2	1246

239	3.83103	23.85828	5	1247

240	3.8439	23.95728	1	1249

241	3.8616	24.05628	1	1251

242	3.88412	24.15527	1	1256

243	3.89699	24.25427	1	1257

244	3.91308	24.35327	3	1258

245	3.92757	24.45227	1	1259

246	3.94848	24.55126	2	1260

247	3.95975	24.65026	5	1263

248	3.97262	24.74926	1	1264

249	3.98549	24.84825	2	1266

250	4.01123	24.94725	1	1271

251	4.02089	25.04625	1	1272

252	4.0402	25.14525	1	1274

253	4.06111	25.24424	1	1275

254	4.08042	25.34324	1	1276

255	4.08525	25.44224	1	1277

256	4.09973	25.54123	1	1278

257	4.12547	25.64023	4	1279

258	4.14156	25.73923	9	1280

259	4.14317	25.83822	1	1281

260	4.17213	25.93722	1	1285

261	4.19144	26.03622	44	1294

262	4.21236	26.13522	1	1295

263	4.2204	26.23421	17	1296

264	4.23649	26.33321	2	1340

265	4.25258	26.43221	1	1341

266	4.26546	26.5312	2	1358

267	4.27994	26.6302	1	1360

268	4.28959	26.7292	2	1361

269	4.31855	26.82819	1	1363

270	4.33303	26.92719	2	1364

271	4.34912	27.02619	1	1366

272	4.3636	27.12519	2	1367

273	4.3813	27.22418	2	1369

274	4.39096	27.32318	2	1370

275	4.40705	27.42218	4	1372

276	4.42475	27.52117	36	1374

277	4.44245	27.62017	1	1376

278	4.45854	27.71917	1	1380

279	4.47623	27.81816	1	1416

280	4.50037	27.91716	1	1417

281	4.51807	28.01616	2	1418

282	4.5245	28.11516	1	1419

283	4.5422	28.21415	1	1420

284	4.56473	28.31315	1	1422

285	4.57599	28.41215	1	1423

286	4.58886	28.51114	1	1424

287	4.60817	28.61014	2	1425

288	4.6307	28.70914	3	1426

289	4.64679	28.80814	1	1427

290	4.65644	28.90713	1	1429

291	4.66931	29.00613	2	1432

292	4.68058	29.10513	1	1433

293	4.69989	29.20412	1	1434

294	4.70954	29.30312	1	1436

295	4.72402	29.40212	1	1437

296	4.76264	29.50111	9	1438

297	4.77873	29.60011	2	1439

298	4.79321	29.69911	2	1440

299	4.80608	29.79811	1	1449

300	4.81734	29.8971	2	1451

301	4.83182	29.9961	1	1453

302	4.83504	30.0951	1	1454

303	4.85274	30.19409	3	1456

304	4.87366	30.29309	1	1457

305	4.88814	30.39209	1	1458

306	4.91549	30.49108	1	1461

307	4.94124	30.59008	1	1462

308	4.95572	30.68908	3	1463

309	4.95733	30.78808	1	1464

310	4.97181	30.88707	1	1465

311	4.98629	30.98607	1	1468

312	5.0072	31.08507	4	1469

313	5.01042	31.18406	1	1470

314	5.04421	31.28306	1	1471

315	5.06674	31.38206	1	1475

316	5.08765	31.48105	1	1476

317	5.08765	31.58005	10	1477

318	5.10053	31.67905	1	1478

319	5.12305	31.77805	1	1479

320	5.1311	31.87704	10	1489

321	5.14075	31.97604	1	1490

322	5.15523	32.07504	2	1491

323	5.19063	32.17403	1	1501

324	5.2035	32.27303	1	1502

325	5.2212	32.37203	1	1504

326	5.2389	32.47103	1	1505

327	5.25177	32.57002	10	1506

328	5.25338	32.66902	1	1507

329	5.27108	32.76802	1	1508

330	5.30004	32.86701	1	1518

331	5.3113	32.96601	36	1519

332	5.32739	33.06501	3	1520

333	5.35314	33.164	4	1521

334	5.36923	33.263	6	1557

335	5.38049	33.362	1	1560

336	5.39336	33.461	3	1564

337	5.40945	33.55999	6	1570

338	5.42233	33.65899	1	1571

339	5.44163	33.75799	1	1574

340	5.44807	33.85698	3	1580

341	5.46255	33.95598	3	1581

342	5.4899	34.05498	1	1582

343	5.51243	34.15397	2	1585

344	5.53013	34.25297	1	1588

345	5.53978	34.35197	1	1589

346	5.55587	34.45097	1	1591

347	5.56874	34.54996	1	1592

348	5.57518	34.64896	1	1593

349	5.60253	34.74796	6	1594

350	5.62506	34.84695	1	1595

351	5.63793	34.94595	1	1596

352	5.65241	35.04495	2	1602

353	5.67816	35.14395	1	1603

354	5.68942	35.24294	1	1604

355	5.69264	35.34194	1	1606

356	5.70712	35.44094	1	1607

357	5.72643	35.53993	7	1608

358	5.7393	35.63893	1	1609

359	5.75378	35.73793	1	1610

360	5.78596	35.83692	3	1617

361	5.80366	35.93592	1	1618

362	5.82136	36.03492	1	1619

363	5.82297	36.13392	2	1622

364	5.8471	36.23291	1	1623

365	5.85676	36.33191	2	1624

366	5.87285	36.43091	2	1626

367	5.87767	36.5299	1	1627

368	5.90503	36.6289	1	1629

369	5.93238	36.7279	1	1631

370	5.93721	36.82689	2	1632

371	5.95651	36.92589	2	1633

372	5.97421	37.02489	1	1634

373	5.9903	37.12389	1	1636

374	5.99674	37.22288	1	1638

375	6.008	37.32188	1	1639

376	6.0257	37.42088	4	1640

377	6.05466	37.51987	1	1641

378	6.0611	37.61887	1	1642

379	6.08202	37.71787	15	1646

380	6.11259	37.81686	3	1647

381	6.11902	37.91586	1	1648

382	6.12868	38.01486	17	1663

383	6.13994	38.11386	27	1666

384	6.16247	38.21285	1	1667

385	6.17373	38.31185	1	1684

386	6.19143	38.41085	4	1711

387	6.21556	38.50984	1	1712

388	6.23809	38.60884	1	1713

389	6.25257	38.70784	1	1717

390	6.26544	38.80684	4	1718

391	6.27349	38.90583	1	1719

392	6.29601	39.00483	1	1720

393	6.30406	39.10383	1	1724

394	6.31371	39.20282	1	1725

395	6.34267	39.30182	1	1726

396	6.35876	39.40082	2	1727

397	6.37646	39.49981	1	1728

398	6.38612	39.59881	3	1729

399	6.41025	39.69781	1	1731

400	6.42634	39.79681	1	1732

401	6.43117	39.8958	1	1735

402	6.44404	39.9948	36	1736

403	6.46817	40.0938	16	1737

404	6.48587	40.19279	30	1738

405	6.49875	40.29179	1	1774

406	6.51805	40.39079	2	1790

407	6.5438	40.48978	8	1820

408	6.55506	40.58878	1	1821

409	6.55828	40.68778	1	1823

410	6.58241	40.78678	1	1831

411	6.59529	40.88577	1	1832

412	6.60655	40.98477	1	1833

413	6.62103	41.08377	1	1834

414	6.64195	41.18276	1	1835

415	6.66769	41.28176	1	1836

416	6.68217	41.38076	1	1837

417	6.69183	41.47975	1	1838

418	6.71596	41.57875	1	1839

419	6.73044	41.67775	1	1840

420	6.7417	41.77675	1	1841

421	6.75136	41.87574	1	1842

422	6.76584	41.97474	3	1843

423	6.79319	42.07374	1	1844

424	6.80606	42.17273	1	1845

425	6.82698	42.27173	1	1848

426	6.84468	42.37073	2	1849

427	6.85916	42.46973	1	1850

428	6.86077	42.56872	1	1851

429	6.87847	42.66772	2	1853

430	6.89617	42.76672	1	1854

431	6.91387	42.86571	4	1855

432	6.93318	42.96471	4	1857

433	6.94766	43.06371	1	1858

434	6.98145	43.1627	2	1862

435	6.98788	43.2617	1	1866

436	6.99754	43.3607	3	1867

437	7.01202	43.4597	1	1869

438	7.02972	43.55869	1	1870

439	7.04259	43.65769	8	1873

440	7.05546	43.75669	1	1874

441	7.08281	43.85568	1	1875

442	7.0989	43.95468	1	1883

443	7.11338	44.05368	1	1884

444	7.13108	44.15267	2	1885

445	7.15039	44.25167	1	1886

446	7.16648	44.35067	1	1887

447	7.17131	44.44967	8	1889

448	7.1874	44.54866	3	1890

449	7.20671	44.64766	1	1891

450	7.2244	44.74666	1	1899

451	7.23567	44.84565	1	1902

452	7.26141	44.94465	1	1903

453	7.27911	45.04365	3	1904

454	7.29359	45.14264	2	1905

455	7.30164	45.24164	3	1906

456	7.31612	45.34064	1	1909

457	7.34186	45.43964	2	1911

458	7.34991	45.53863	1	1914

459	7.35956	45.63763	1	1915

460	7.38691	45.73663	1	1917

461	7.40622	45.83562	1	1918

462	7.41909	45.93462	2	1919

463	7.43679	46.03362	2	1920

464	7.45127	46.13262	1	1921

465	7.4738	46.23161	1	1923

466	7.48024	46.33061	1	1925

467	7.49633	46.42961	1	1926

468	7.5092	46.5286	1	1927

469	7.53172	46.6276	1	1928

470	7.54942	46.7266	1	1929

471	7.56229	46.82559	1	1930

472	7.57678	46.92459	1	1931

473	7.5993	47.02359	2	1932

474	7.60735	47.12259	1	1933

475	7.62344	47.22158	2	1934

476	7.64274	47.32058	2	1936

477	7.65883	47.41958	2	1937

478	7.67653	47.51857	2	1939

479	7.69101	47.61757	3	1941

480	7.71676	47.71657	2	1943

481	7.7248	47.81556	1	1945

482	7.73767	47.91456	1	1948

483	7.75698	48.01356	1	1950

484	7.77307	48.11256	1	1951

485	7.78594	48.21155	1	1952

486	7.79399	48.31055	2	1953

487	7.81812	48.40955	1	1954

488	7.84065	48.50854	2	1955

489	7.85352	48.60754	1	1957

490	7.87283	48.70654	2	1958

491	7.8857	48.80553	5	1960

492	7.90179	48.90453	2	1961

493	7.91627	49.00353	2	1963

494	7.92754	49.10253	1	1968

495	7.94845	49.20152	1	1970

496	7.96293	49.30052	2	1972

497	7.98224	49.39952	2	1973

498	7.99672	49.49851	1	1974

499	8.01764	49.59751	1	1976

500	8.0289	49.69651	1	1978

501	8.04017	49.79551	1	1979

502	8.05465	49.8945	2	1980

503	8.07717	49.9935	2	1981

504	8.09487	50.0925	3	1982

505	8.10453	50.19149	1	1984

506	8.12223	50.29049	1	1986

507	8.14636	50.38949	13	1989

508	8.16406	50.48848	1	1990

509	8.17532	50.58748	1	1991

510	8.1898	50.68648	1	2004

511	8.20911	50.78548	1	2005

512	8.22037	50.88447	2	2006

513	8.23003	50.98347	1	2007

514	8.25255	51.08247	2	2008

515	8.27186	51.18146	1	2010

516	8.28795	51.28046	2	2011

517	8.30565	51.37946	2	2013

518	8.32174	51.47845	1	2014

519	8.33944	51.57745	1	2016

520	8.34749	51.67645	3	2018

521	8.36679	51.77545	1	2019

522	8.38288	51.87444	2	2020

523	8.39736	51.97344	3	2023

524	8.40863	52.07244	2	2024

525	8.42954	52.17143	11	2026

526	8.45368	52.27043	1	2029

527	8.46816	52.36943	1	2031

528	8.47299	52.46842	1	2042

529	8.48908	52.56742	5	2043

530	8.5116	52.66642	1	2044

531	8.5293	52.76542	3	2045

532	8.53574	52.86441	1	2050

533	8.55826	52.96341	4	2051

534	8.57757	53.06241	2	2054

535	8.59366	53.1614	1	2055

536	8.60493	53.2604	1	2059

537	8.62745	53.3594	1	2061

538	8.64193	53.4584	1	2062

539	8.64998	53.55739	2	2063

540	8.66768	53.65639	3	2064

541	8.68698	53.75539	3	2065

542	8.70629	53.85438	1	2067

543	8.71916	53.95338	1	2070

544	8.74008	54.05238	5	2073

545	8.75295	54.15137	3	2074

546	8.76904	54.25037	3	2075

547	8.78192	54.34937	1	2080

548	8.79479	54.44837	2	2083

549	8.81892	54.54736	2	2086

550	8.83501	54.64636	1	2087

551	8.84306	54.74536	1	2089

552	8.86237	54.84435	1	2091

553	8.8865	54.94335	3	2092

554	8.90098	55.04235	3	2093

555	8.90903	55.14134	1	2094

556	8.92512	55.24034	2	2097

557	8.94442	55.33934	1	2100

558	8.96373	55.43834	1	2101

559	8.97499	55.53733	2	2103

560	8.9943	55.63633	1	2104

561	9.02005	55.73533	1	2105

562	9.03131	55.83432	2	2107

563	9.03935	55.93332	2	2108

564	9.06027	56.03232	1	2109

565	9.07636	56.13132	5	2111

566	9.09084	56.23031	3	2113

567	9.10371	56.32931	3	2114

568	9.12141	56.42831	1	2119

569	9.13911	56.5273	1	2122

570	9.15359	56.6263	3	2125

571	9.16968	56.7253	1	2126

572	9.1906	56.82429	3	2127

573	9.20991	56.92329	1	2130

574	9.22278	57.02229	1	2131

575	9.23404	57.12129	2	2134

576	9.25174	57.22028	3	2135

577	9.27427	57.31928	1	2136

578	9.28392	57.41828	1	2138

579	9.30323	57.51727	1	2141

580	9.32576	57.61627	2	2142

581	9.33702	57.71527	2	2143

582	9.34667	57.81426	1	2144

583	9.36598	57.91326	8	2146

584	9.38851	58.01226	2	2148

585	9.39494	58.11126	1	2149

586	9.40621	58.21025	2	2157

587	9.43195	58.30925	2	2159

588	9.44965	58.40825	2	2160

589	9.46252	58.50724	1	2162

590	9.477	58.60624	2	2164

591	9.49148	58.70524	1	2166

592	9.5124	58.80423	1	2167

593	9.52205	58.90323	3	2169

594	9.53493	59.00223	6	2170

595	9.56067	59.10123	8	2171

596	9.57998	59.20022	2	2174

597	9.58641	59.29922	1	2180

598	9.60894	59.39822	2	2188

599	9.62342	59.49721	1	2190

600	9.6379	59.59621	1	2191

601	9.65238	59.69521	1	2193

602	9.66847	59.79421	2	2194

603	9.68939	59.8932	1	2195

604	9.70548	59.9922	1	2196

605	9.71835	60.0912	2	2198

606	9.73766	60.19019	1	2199

607	9.75697	60.28919	1	2200

608	9.76823	60.38819	1	2202

609	9.78271	60.48718	3	2203

610	9.79558	60.58618	2	2204

611	9.81811	60.68518	4	2205

612	9.82776	60.78418	1	2208

613	9.84225	60.88317	2	2210

614	9.86638	60.98217	4	2214

615	9.88247	61.08117	1	2215

616	9.89373	61.18016	1	2217

617	9.90661	61.27916	2	2221

618	9.93396	61.37816	1	2222

619	9.94522	61.47715	2	2223

620	9.96131	61.57615	1	2225

621	9.9774	61.67515	4	2226

622	9.99671	61.77415	1	2228

623	10.01441	61.87314	1	2229

624	10.02406	61.97214	1	2233

625	10.03854	62.07114	10	2234

626	10.06268	62.17013	5	2235

627	10.07233	62.26913	2	2236

628	10.09486	62.36813	3	2246

629	10.1029	62.46712	3	2251

630	10.12221	62.56612	3	2253

631	10.1383	62.66512	4	2256

632	10.15278	62.76412	1	2259

633	10.17209	62.86311	4	2262

634	10.18818	62.96211	1	2266

635	10.20749	63.06111	2	2267

636	10.21875	63.1601	2	2271

637	10.23484	63.2591	2	2272

638	10.24771	63.3581	1	2274

639	10.25737	63.4571	4	2276

640	10.27507	63.55609	2	2278

641	10.29759	63.65509	1	2279

642	10.3169	63.75409	8	2283

643	10.33782	63.85308	1	2285

644	10.34264	63.95208	1	2286

645	10.36678	64.05108	1	2294

646	10.37965	64.15007	2	2295

647	10.3893	64.24907	4	2296

648	10.407	64.34807	3	2297

649	10.42953	64.44707	3	2299

650	10.44723	64.54606	2	2303

651	10.46171	64.64506	1	2306

652	10.48102	64.74406	1	2309

653	10.50033	64.84305	1	2311

654	10.50837	64.94205	1	2312

655	10.52285	65.04105	1	2313

656	10.54216	65.14004	1	2314

657	10.56308	65.23904	4	2315

658	10.57273	65.33804	1	2316

659	10.58882	65.43704	1	2317

660	10.60974	65.53603	1	2321

661	10.62261	65.63503	1	2322

662	10.63387	65.73403	4	2323

663	10.65318	65.83302	3	2324

664	10.66927	65.93202	1	2325

665	10.68375	66.03102	1	2329

666	10.69341	66.13001	1	2332

667	10.71754	66.22901	1	2333

668	10.73846	66.32801	2	2334

669	10.75294	66.42701	1	2335

670	10.76903	66.526	1	2336

671	10.77868	66.625	1	2338

672	10.78995	66.724	4	2339

673	10.82213	66.82299	15	2340

674	10.835	66.92199	1	2341

675	10.84465	67.02099	1	2345

676	10.86557	67.11999	3	2360

677	10.88327	67.21898	1	2361

678	10.89614	67.31798	3	2362

679	10.91223	67.41698	4	2365

680	10.9251	67.51597	1	2366

681	10.94763	67.61497	4	2369

682	10.9605	67.71397	1	2373

683	10.97498	67.81296	2	2374

684	10.99107	67.91196	1	2378

685	11.00394	68.01096	2	2379

686	11.01842	68.10996	1	2381

687	11.04578	68.20895	1	2382

688	11.06026	68.30795	4	2384

689	11.06669	68.40695	1	2385

690	11.07957	68.50594	1	2386

691	11.11175	68.60494	2	2390

692	11.11657	68.70394	3	2391

693	11.12462	68.80293	5	2392

694	11.15036	68.90193	2	2394

695	11.15841	69.00093	5	2397

696	11.18254	69.09993	1	2402

697	11.19863	69.19892	6	2404

698	11.20668	69.29792	1	2409

699	11.23242	69.39692	1	2410

700	11.24851	69.49591	1	2416

701	11.25012	69.59491	3	2417

702	11.27586	69.69391	1	2418

703	11.3	69.7929	6	2419

704	11.31609	69.8919	2	2422

705	11.32574	69.9909	1	2423

706	11.34022	70.0899	3	2429

707	11.35631	70.18889	1	2431

708	11.37562	70.28789	8	2432

709	11.38688	70.38689	1	2435

710	11.4078	70.48588	6	2436

711	11.42711	70.58488	1	2444

712	11.43194	70.68388	2	2445

713	11.44803	70.78288	2	2451

714	11.48181	70.88187	1	2452

715	11.48342	70.98087	1	2454

716	11.50112	71.07987	3	2456

717	11.51721	71.17886	1	2457

718	11.53813	71.27786	7	2458

719	11.55583	71.37686	3	2461

720	11.56226	71.47585	1	2462

721	11.58318	71.57485	4	2469

722	11.60893	71.67385	10	2472

723	11.6218	71.77285	2	2473

724	11.63145	71.87184	3	2477

725	11.64271	71.97084	1	2487

726	11.67007	72.06984	1	2489

727	11.67972	72.16883	2	2492

728	11.69742	72.26783	1	2493

729	11.71512	72.36683	1	2494

730	11.73604	72.46582	1	2496

731	11.74569	72.56482	1	2497

732	11.765	72.66382	1	2498

733	11.77787	72.76282	1	2499

734	11.80361	72.86181	1	2500

735	11.80683	72.96081	4	2501

736	11.82131	73.05981	1	2502

737	11.84545	73.1588	1	2503

738	11.85993	73.2578	1	2507

739	11.87602	73.3568	2	2508

740	11.88728	73.45579	1	2509

741	11.92429	73.55479	3	2510

742	11.91946	73.65379	2	2512

743	11.94038	73.75279	1	2513

744	11.94842	73.85178	1	2516

745	11.96934	73.95078	3	2518

746	11.98704	74.04978	2	2519

747	11.9983	74.14877	1	2520

748	12.02083	74.24777	1	2523

749	12.03531	74.34677	1	2525

750	12.05301	74.44577	1	2526

751	12.06105	74.54476	1	2527

752	12.08358	74.64376	1	2528

753	12.1045	74.74276	1	2529

754	12.10932	74.84175	2	2530

755	12.12541	74.94075	2	2531

756	12.14311	75.03975	1	2532

757	12.16725	75.13874	2	2534

758	12.17529	75.23774	4	2536

759	12.19943	75.33674	1	2537

760	12.21069	75.43574	1	2539

761	12.23483	75.53473	6	2543

762	12.24287	75.63373	2	2544

763	12.25896	75.73273	2	2545

764	12.28149	75.83172	2	2551

765	12.3008	75.93072	1	2553

766	12.30723	76.02972	1	2555

767	12.32171	76.12871	1	2557

768	12.34585	76.22771	1	2558

769	12.36355	76.32671	1	2559

770	12.36998	76.42571	1	2560

771	12.38768	76.5247	2	2561

772	12.41182	76.6237	2	2562

773	12.41986	76.7227	1	2563

774	12.43273	76.82169	1	2565

775	12.45687	76.92069	1	2567

776	12.47135	77.01969	1	2568

777	12.48583	77.11868	4	2569

778	12.50031	77.21768	2	2570

779	12.51962	77.31668	1	2571

780	12.5341	77.41568	1	2575

781	12.54697	77.51467	1	2577

782	12.56789	77.61367	1	2578

783	12.5872	77.71267	1	2579

784	12.60329	77.81166	1	2580

785	12.61455	77.91066	1	2581

786	12.62099	78.00966	4	2582

787	12.64673	78.10866	3	2583

788	12.66121	78.20765	1	2584

789	12.67247	78.30665	1	2588

790	12.695	78.40565	3	2591

791	12.71431	78.50464	2	2592

792	12.73362	78.60364	1	2593

793	12.73362	78.70264	1	2596

794	12.76419	78.80163	1	2598

795	12.77867	78.90063	1	2599

796	12.79154	78.99963	1	2600

797	12.80119	79.09863	1	2601

798	12.82372	79.19762	1	2602

799	12.84303	79.29662	1	2603

800	12.85429	79.39562	1	2604

801	12.87038	79.49461	2	2605

802	12.88647	79.59361	6	2606

803	12.909	79.69261	2	2607

804	12.91704	79.7916	2	2609

805	12.93635	79.8906	1	2615

806	12.95566	79.9896	3	2617

807	12.97175	80.0886	1	2619

808	12.98301	80.18759	5	2620

809	12.99427	80.28659	2	2623

810	13.02324	80.38559	5	2624

811	13.04093	80.48458	1	2629

812	13.0522	80.58358	5	2631

813	13.06185	80.68258	1	2636

814	13.0876	80.78158	1	2637

815	13.10047	80.88057	1	2642

816	13.10851	80.97957	1	2643

817	13.12943	81.07857	1	2644

818	13.14874	81.17756	1	2645

819	13.16805	81.27656	2	2646

820	13.17448	81.37556	4	2647

821	13.19701	81.47455	4	2648

822	13.21471	81.57355	1	2650

823	13.22275	81.67255	2	2654

824	13.24206	81.77155	1	2658

825	13.26137	81.87054	1	2659

826	13.27585	81.96954	1	2661

827	13.29033	82.06854	6	2662

828	13.30642	82.16753	5	2663

829	13.32734	82.26653	1	2664

830	13.34825	82.36553	3	2670

831	13.3563	82.46452	1	2675

832	13.36756	82.56352	1	2676

833	13.39652	82.66252	2	2679

834	13.40296	82.76152	2	2680

835	13.41744	82.86051	2	2681

836	13.43675	82.95951	1	2683

837	13.4641	83.05851	1	2685

838	13.47054	83.1575	4	2687

839	13.47858	83.2565	1	2688

840	13.50433	83.3555	1	2689

841	13.52203	83.45449	1	2693

842	13.53168	83.55349	1	2694

843	13.54294	83.65249	3	2695

844	13.56225	83.75149	1	2696

845	13.57995	83.85048	1	2697

846	13.60087	83.94948	5	2700

847	13.61374	84.04848	1	2701

848	13.63144	84.14747	1	2702

849	13.64431	84.24647	1	2707

850	13.6604	84.34547	1	2708

851	13.67166	84.44447	4	2709

852	13.68936	84.54346	2	2710

853	13.71028	84.64246	5	2711

854	13.72315	84.74146	8	2715

855	13.74568	84.84045	1	2717

856	13.76177	84.93945	1	2722

857	13.77303	85.03845	1	2730

858	13.78751	85.13744	1	2731

859	13.80682	85.23644	7	2732

860	13.82452	85.33544	4	2733

861	13.83739	85.43444	2	2734

862	13.84704	85.53343	1	2741

863	13.86957	85.63243	1	2745

864	13.89531	85.73143	1	2747

865	13.90014	85.83042	15	2748

866	13.91623	85.92942	4	2749

867	13.93393	86.02842	1	2750

868	13.95485	86.12741	1	2765

869	13.9645	86.22641	1	2769

870	13.97737	86.32541	1	2770

871	13.99829	86.42441	1	2771

872	14.02081	86.5234	2	2772

873	14.03369	86.6224	1	2773

874	14.04817	86.7214	1	2774

875	14.06104	86.82039	1	2776

876	14.08357	86.91939	1	2777

877	14.09	87.01839	3	2778

878	14.1077	87.11738	5	2779

879	14.13023	87.21638	10	2780

880	14.14793	87.31538	3	2783

881	14.15436	87.41438	1	2788

882	14.17689	87.51337	2	2798

883	14.1978	87.61237	1	2801

884	14.21068	87.71137	11	2802

885	14.22516	87.81036	1	2804

886	14.23481	87.90936	3	2805

887	14.25895	88.00836	1	2816

888	14.27021	88.10736	5	2817

889	14.28308	88.20635	3	2820

890	14.30722	88.30535	1	2821

891	14.32652	88.40435	1	2826

892	14.33296	88.50334	1	2829

893	14.35227	88.60234	2	2830

894	14.36836	88.70134	2	2831

895	14.38767	88.80033	1	2832

896	14.39893	88.89933	2	2834

897	14.41341	88.99833	1	2836

898	14.43433	89.09733	2	2837

899	14.45203	89.19632	1	2839

900	14.4649	89.29532	1	2840

901	14.47938	89.39432	5	2842

902	14.49386	89.49331	30	2843

903	14.51639	89.59231	4	2844

904	14.52604	89.69131	1	2849

905	14.5373	89.7903	1	2879

906	14.56144	89.8893	9	2883

907	14.58075	89.9883	1	2884

908	14.59201	90.0873	40	2885

909	14.61132	90.18629	1	2894

910	14.63223	90.28529	4	2895

911	14.64189	90.38429	2	2935

912	14.65154	90.48328	1	2936

913	14.67246	90.58228	1	2940

914	14.69338	90.68128	1	2942

915	14.70303	90.78027	3	2943

916	14.71912	90.87927	1	2944

917	14.74165	90.97827	1	2945

918	14.75935	91.07727	1	2948

919	14.769	91.17626	1	2949

920	14.78348	91.27526	1	2950

921	14.79957	91.37426	2	2951

922	14.81727	91.47325	1	2952

923	14.83175	91.57225	1	2953

924	14.84623	91.67125	2	2955

925	14.86554	91.77025	1	2956

926	14.88324	91.86924	1	2957

927	14.89933	91.96824	1	2959

928	14.91542	92.06724	1	2960

929	14.93312	92.16623	2	2961

930	14.94599	92.26523	15	2962

931	14.95725	92.36423	3	2963

932	14.97656	92.46322	2	2965

933	14.99587	92.56222	1	2980

934	15.01196	92.66122	3	2983

935	15.02483	92.76022	1	2985

936	15.04414	92.85921	1	2986

937	15.06506	92.95821	1	2989

938	15.07471	93.05721	4	2990

939	15.08758	93.1562	1	2991

940	15.10528	93.2552	2	2992

941	15.12459	93.3542	3	2996

942	15.13746	93.45319	3	2997

943	15.15516	93.55219	4	2999

944	15.17608	93.65119	1	3002

945	15.19217	93.75019	4	3005

946	15.20182	93.84918	2	3009

947	15.21791	93.94818	1	3010

948	15.23561	94.04718	3	3014

949	15.25331	94.14617	1	3016

950	15.26296	94.24517	1	3017

951	15.28227	94.34417	2	3020

952	15.29997	94.44316	4	3021

953	15.32089	94.54216	10	3022

954	15.33054	94.64116	1	3024

955	15.35307	94.74016	1	3028

956	15.36433	94.83915	1	3038

957	15.38042	94.93815	1	3039

958	15.38846	95.03715	2	3040

959	15.4126	95.13614	1	3041

960	15.4303	95.23514	1	3042

961	15.44156	95.33414	1	3044

962	15.46409	95.43314	1	3045

963	15.48179	95.53213	1	3046

964	15.49466	95.63113	3	3047

965	15.50592	95.73013	3	3048

966	15.52523	95.82912	1	3049

967	15.53971	95.92812	1	3052

968	15.55741	96.02712	1	3055

969	15.56867	96.12611	1	3056

970	15.58637	96.22511	1	3057

971	15.60729	96.32411	1	3058

972	15.62499	96.42311	2	3059

973	15.63947	96.5221	3	3060

974	15.65234	96.6211	1	3061

975	15.67165	96.7201	4	3063

976	15.68774	96.81909	1	3066

977	15.69739	96.91809	3	3067

978	15.71348	97.01709	1	3071

979	15.73762	97.11608	1	3072

980	15.7521	97.21508	1	3075

981	15.76658	97.31408	1	3076

982	15.78106	97.41308	4	3077

983	15.80198	97.51207	1	3078

984	15.81163	97.61107	1	3079

985	15.82611	97.71007	1	3083

986	15.84381	97.80906	7	3084

987	15.86151	97.90806	1	3085

988	15.87599	98.00706	2	3086

989	15.89369	98.10605	6	3093

990	15.91461	98.20505	4	3094

991	15.92909	98.30405	1	3096

992	15.93713	98.40305	4	3102

993	15.95483	98.50204	2	3106

994	15.97253	98.60104	6	3107

995	15.99023	98.70004	1	3111

996	16.0031	98.79903	1	3113

997	16.01919	98.89803	1	3119

998	16.04333	98.99703	1	3120

999	16.05942	99.09603	1	3121

1000	16.07068	99.19502	1	3122

1001	16.08516	99.29402	2	3123

1002	16.10286	99.39302	1	3124

1003	16.11734	99.49201	1	3125

1004	16.13021	99.59101	1	3127

1005	16.14791	99.69001	1	3128

1006	16.17044	99.789	2	3129

1007	16.18492	99.888	1	3130

1008	16.19618	99.987	1	3131

1009	16.2171	100.086	3	3133

1010	16.2348	100.18499	1	3134

1011	16.24445	100.28399	4	3135

1012	16.25732	100.38299	12	3138

1013	16.27985	100.48198	1	3139

1014	16.29755	100.58098	4	3143

1015	16.3072	100.67998	4	3155

1016	16.3249	100.77897	1	3156

1017	16.34904	100.87797	2	3160

1018	16.36513	100.97697	2	3164

1019	16.37156	101.07597	3	3165

1020	16.39087	101.17496	6	3167

1021	16.40696	101.27396	1	3169

1022	16.42466	101.37296	2	3172

1023	16.43431	101.47195	1	3178

1024	16.45362	101.57095	2	3179

1025	16.47615	101.66995	1	3181

1026	16.49224	101.76895	1	3182

1027	16.50511	101.86794	1	3184

1028	16.51959	101.96694	1	3185

1029	16.5389	102.06594	1	3186

1030	16.55177	102.16493	1	3187

1031	16.56464	102.26393	8	3188

1032	16.58234	102.36293	4	3189

1033	16.60165	102.46192	2	3190

1034	16.61452	102.56092	5	3198

1035	16.63061	102.65992	1	3202

1036	16.65475	102.75892	5	3204

1037	16.66601	102.85791	1	3209

1038	16.67405	102.95691	3	3210

1039	16.69336	103.05591	5	3215

1040	16.71589	103.1549	5	3216

1041	16.72715	103.2539	2	3219

1042	16.74002	103.3529	1	3224

1043	16.75933	103.45189	3	3229

1044	16.78186	103.55089	4	3231

1045	16.79473	103.64989	2	3232

1046	16.80921	103.74889	1	3235

1047	16.82047	103.84788	3	3239

1048	16.843	103.94688	1	3241

1049	16.85587	104.04588	3	3242

1050	16.86874	104.14487	2	3245

1051	16.88483	104.24387	4	3246

1052	16.90897	104.34287	1	3249

1053	16.92184	104.44186	1	3251

1054	16.93632	104.54086	2	3255

1055	16.95402	104.63986	1	3256

1056	16.97333	104.73886	1	3257

1057	16.98459	104.83785	1	3259

1058	16.99907	104.93685	4	3260

1059	17.01838	105.03585	1	3261

1060	17.03608	105.13484	3	3262

1061	17.04734	105.23384	1	3266

1062	17.06343	105.33284	2	3267

1063	17.08596	105.43184	2	3270

1064	17.10044	105.53083	5	3271

1065	17.10687	105.62983	1	3273

1066	17.12779	105.72883	1	3275

1067	17.14871	105.82782	1	3280

1068	17.16158	105.92682	1	3281

1069	17.17445	106.02582	2	3282

1070	17.19537	106.12481	5	3283

1071	17.21468	106.22381	1	3284

1072	17.22916	106.32281	6	3286

1073	17.24042	106.42181	3	3291

1074	17.25812	106.5208	6	3292

1075	17.27421	106.6198	3	3298

1076	17.2903	106.7188	3	3301

1077	17.30478	106.81779	1	3307

1078	17.32087	106.91679	3	3310

1079	17.34018	107.01579	2	3313

1080	17.35466	107.11478	1	3314

1081	17.36914	107.21378	1	3317

1082	17.38845	107.31278	1	3319

1083	17.40776	107.41178	2	3320

1084	17.41741	107.51077	1	3321

1085	17.42867	107.60977	1	3322

1086	17.45281	107.70877	1	3324

1087	17.46729	107.80776	5	3325

1088	17.48016	107.90676	2	3326

1089	17.49464	108.00576	2	3327

1090	17.51556	108.10475	3	3332

1091	17.53165	108.20375	1	3334

1092	17.54291	108.30275	1	3336

1093	17.56383	108.40175	1	3339

1094	17.57992	108.50074	1	3340

1095	17.5944	108.59974	1	3341

1096	17.60406	108.69874	1	3342

1097	17.62497	108.79773	4	3343

1098	17.64428	108.89673	1	3344

1099	17.66037	108.99573	4	3345

1100	17.67324	109.09473	3	3349

1101	17.68933	109.19372	2	3350

1102	17.70703	109.29272	4	3354

1103	17.72151	109.39172	9	3357

1104	17.73438	109.49071	5	3359

1105	17.75047	109.58971	3	3363

1106	17.77139	109.68871	1	3372

1107	17.78104	109.7877	1	3377

1108	17.79553	109.8867	1	3380

1109	17.81966	109.9857	2	3381

1110	17.83575	110.0847	1	3382

1111	17.8454	110.18369	2	3383

1112	17.86149	110.28269	3	3385

1113	17.88563	110.38169	6	3386

1114	17.8985	110.48068	2	3388

1115	17.91137	110.57968	4	3391

1116	17.92585	110.67868	6	3397

1117	17.94677	110.77767	1	3399

1118	17.96286	110.87667	1	3403

1119	17.97895	110.97567	3	3409

1120	17.99343	111.07467	2	3410

1121	18.01274	111.17366	1	3411

1122	18.02561	111.27266	4	3414

1123	18.03848	111.37166	2	3416

1124	18.05618	111.47065	2	3417

1125	18.07227	111.56965	16	3421

1126	18.08675	111.66865	3	3423

1127	18.10767	111.76764	2	3425

1128	18.12859	111.86664	4	3441

1129	18.14146	111.96564	7	3444

1130	18.15272	112.06464	3	3446

1131	18.1656	112.16363	5	3450

1132	18.18651	112.26263	1	3457

1133	18.20099	112.36163	1	3460

1134	18.21387	112.46062	4	3465

1135	18.23156	112.55962	1	3466

1136	18.25409	112.65862	1	3467

1137	18.26857	112.75762	3	3471

1138	18.27983	112.85661	2	3472

1139	18.29753	112.95561	1	3473

1140	18.31684	113.05461	1	3476

1141	18.32971	113.1536	3	3478

1142	18.34098	113.2526	1	3479

1143	18.36028	113.3516	3	3480

1144	18.37959	113.45059	9	3483

1145	18.39246	113.54959	1	3484

1146	18.40695	113.64859	1	3487

1147	18.42625	113.74759	5	3496

1148	18.44234	113.84658	1	3497

1149	18.45843	113.94558	1	3498

1150	18.46809	114.04458	4	3503

1151	18.4874	114.14357	6	3504

1152	18.50509	114.24257	1	3505

1153	18.51797	114.34157	2	3509

1154	18.53406	114.44056	1	3515

1155	18.55176	114.53956	6	3516

1156	18.57106	114.63856	1	3518

1157	18.58554	114.73756	5	3519

1158	18.59842	114.83655	5	3525

1159	18.61612	114.93555	1	3526

1160	18.63221	115.03455	3	3531

1161	18.64508	115.13354	1	3536

1162	18.66278	115.23254	1	3537

1163	18.68369	115.33154	1	3540

1164	18.69817	115.43053	1	3541

1165	18.70622	115.52953	2	3542

1166	18.72392	115.62853	2	3543

1167	18.74323	115.72753	1	3544

1168	18.7561	115.82652	2	3546

1169	18.77058	115.92552	2	3548

1170	18.78667	116.02452	1	3549

1171	18.80437	116.12351	1	3551

1172	18.81402	116.22251	6	3553

1173	18.82207	116.32151	3	3554

1174	18.82529	116.42051	1	3555

1175	18.71909	116.5195	1	3561

1176	17.86954	116.6185	1	3564

1177	16.95241	116.7175	1	3565

1178	15.93231	116.81649	2	3566

1179	15.10206	116.91549	1	3567

1180	14.00151	117.01449	1	3568

1181	13.16483	117.11348	2	3570

1182	12.35872	117.21248	1	3571

1183	11.36114	117.31148	1	3572

1184	10.35873	117.41048	1	3574

1185	9.53493	117.50947	3	3575

1186	8.66768	117.60847	1	3576

1187	7.83743	117.70747	1	3577

1188	6.94283	117.80646	2	3580

1189	6.13189	117.90546	2	3581

1190	5.31935	118.00446	6	3582

1191	4.48428	118.10345	2	3584

1192	3.65404	118.20245	2	3586

1193	2.80288	118.30145	3	3592

1194	1.96137	118.40045	4	3594

1195	1.15365	118.49944	1	3596

1196	0.38133	118.59844	1	3599

1197	0.20595	118.69744	2	3603

118.79643	1	3604

118.89543	1	3605

118.99443	1	3607

119.09343	1	3608

119.19242	5	3609

119.29142	1	3610

119.39042	6	3611

119.48941	1	3616

119.58841	9	3617

119.68741	2	3623

119.7864	4	3624

119.8854	3	3633

119.9844	1	3635

120.0834	8	3639

120.18239	1	3642

120.28139	4	3643

120.38039	4	3651

120.47938	1	3652

120.57838	1	3656

120.67738	3	3660

120.77637	1	3661

120.87537	1	3662

120.97437	9	3665

121.07337	1	3666

121.17236	4	3667

121.27136	3	3676

121.37036	2	3677

121.46935	2	3681

121.56835	7	3684

121.66735	2	3686

121.76634	1	3688

121.86534	1	3695

121.96434	1	3697

122.06334	6	3698

122.16233	1	3699

122.26133	1	3700

122.36033	2	3706

122.45932	1	3707

122.55832	1	3708

122.65732	1	3710

122.75632	3	3711

122.85531	3	3712

122.95431	2	3713

123.05331	1	3716

123.1523	1	3719

123.2513	1	3721

123.3503	5	3722

123.44929	3	3723

123.54829	3	3724

123.64729	2	3729

123.74629	5	3732

123.84528	4	3735

123.94428	1	3737

124.04328	4	3742

124.14227	5	3746

124.24127	10	3747

124.34027	2	3751

124.43926	2	3756

124.53826	2	3766

124.63726	6	3768

124.73626	4	3770

124.83525	1	3772

124.93425	3	3778

125.03325	1	3782

125.13224	1	3783

125.23124	1	3786

125.33024	1	3787

125.42923	2	3788

125.52823	1	3789

125.62723	4	3790

125.72623	1	3792

125.82522	2	3793

125.92422	5	3797

126.02322	1	3798

126.12221	4	3800

126.22121	1	3805

126.32021	3	3806

126.41921	4	3810

126.5182	12	3811

126.6172	2	3814

126.7162	1	3818

126.81519	2	3830

126.91419	3	3832

127.01319	6	3833

127.11218	1	3835

127.21118	3	3838

127.31018	1	3844

127.40918	1	3845

127.50817	3	3848

127.60717	1	3849

127.70617	2	3850

127.80516	5	3853

127.90416	2	3854

128.00316	1	3856

128.10215	1	3861

128.20115	13	3863

128.30015	1	3864

128.39915	5	3865

128.49814	1	3878

128.59714	10	3879

128.69614	2	3884

128.79513	1	3885

128.89413	2	3895

128.99313	5	3897

129.09212	2	3898

129.19112	4	3900

129.29012	2	3905

129.38912	1	3907

129.48811	1	3911

129.58711	2	3913

129.68611	13	3914

129.7851	1	3915

129.8841	2	3917

129.9831	1	3930

130.0821	2	3931

130.18109	3	3933

130.28009	1	3934

130.37909	2	3936

130.47808	2	3939

130.57708	1	3940

130.67608	3	3942

130.77507	2	3944

130.87407	5	3945

130.97307	1	3948

131.07207	2	3950

131.17106	5	3955

131.27006	5	3956

131.36906	1	3958

131.46805	6	3963

131.56705	2	3968

131.66605	1	3969

131.76504	3	3975

131.86404	1	3977

131.96304	2	3978

132.06204	4	3981

132.16103	1	3982

132.26003	1	3984

132.35903	19	3988

132.45802	7	3989

132.55702	3	3990

132.65602	1	4009

132.75501	7	4016

132.85401	1	4019

132.95301	1	4020

133.05201	10	4027

133.151	3	4028

133.25	2	4029

133.349	1	4039

133.44799	1	4042

133.54699	1	4044

133.64599	6	4045

133.74499	13	4046

133.84398	2	4047

133.94298	1	4053

134.04198	1	4066

134.14097	3	4068

134.23997	3	4069

134.33897	1	4070

134.43796	4	4073

134.53696	1	4076

134.63596	2	4077

134.73496	3	4081

134.83395	2	4082

134.93295	5	4084

135.03195	1	4087

135.13094	1	4089

135.22994	1	4094

135.32894	1	4095

135.42793	2	4096

135.52693	3	4097

135.62593	5	4098

135.72493	1	4100

135.82392	3	4103

135.92292	1	4108

136.02192	50	4109

136.12091	4	4112

136.21991	1	4113

136.31891	4	4163

136.4179	2	4167

136.5169	2	4168

136.6159	1	4172

136.7149	6	4174

136.81389	6	4176

136.91289	1	4177

137.01189	4	4183

137.11088	1	4189

137.20988	2	4190

137.30888	2	4194

137.40788	5	4195

137.50687	4	4197

137.60587	1	4199

137.70487	2	4204

137.80386	1	4208

137.90286	3	4209

138.00186	1	4211

138.10085	1	4212

138.19985	1	4215

138.29885	1	4216

138.39785	1	4217

138.49684	1	4218

138.59584	1	4219

138.69484	1	4220

138.79383	1	4221

138.89283	9	4222

138.99183	4	4223

139.09082	1	4224

139.18982	2	4233

139.28882	1	4237

139.38782	1	4238

139.48681	1	4240

139.58581	1	4241

139.68481	2	4242

139.7838	4	4243

139.8828	1	4244

139.9818	1	4246

140.08079	2	4250

140.17979	1	4251

140.27879	1	4252

140.37779	1	4254

140.47678	1	4255

140.57578	1	4256

140.67478	1	4257

140.77377	3	4258

140.87277	1	4259

140.97177	2	4260

141.07077	1	4263

141.16976	1	4264

141.26876	2	4266

141.36776	2	4267

141.46675	1	4268

141.56575	1	4270

141.66475	2	4272

141.76374	3	4273

141.86274	6	4274

141.96174	2	4276

142.06074	2	4279

142.15973	6	4285

142.25873	6	4287

142.35773	3	4289

142.45672	3	4295

142.55572	1	4301

142.65472	4	4304

142.75371	4	4307

142.85271	1	4308

142.95171	1	4312

143.05071	5	4316

143.1497	1	4317

143.2487	4	4318

143.3477	1	4323

143.44669	1	4324

143.54569	2	4328

143.64469	1	4329

143.74369	1	4330

143.84268	3	4332

143.94168	1	4333

144.04068	1	4334

144.13967	1	4337

144.23867	2	4338

144.33767	1	4339

144.43666	2	4340

144.53566	10	4342

144.63466	1	4343

144.73366	1	4345

144.83265	7	4355

144.93165	2	4356

145.03065	2	4357

145.12964	1	4364

145.22864	1	4366

145.32764	8	4368

145.42663	3	4369

145.52563	2	4370

145.62463	1	4378

145.72363	2	4381

145.82262	3	4383

145.92162	4	4384

146.02062	2	4386

146.11961	1	4389

146.21861	12	4393

146.31761	10	4395

146.4166	1	4396

146.5156	1	4408

146.6146	2	4418

146.7136	1	4419

146.81259	3	4420

146.91159	2	4422

147.01059	1	4423

147.10958	1	4426

147.20858	2	4428

147.30758	2	4429

147.40658	2	4430

147.50557	4	4432

147.60457	1	4434

147.70357	2	4436

147.80256	2	4440

147.90156	3	4441

148.00056	4	4443

148.09955	2	4445

148.19855	3	4448

148.29755	1	4452

148.39655	3	4454

148.49554	1	4457

148.59454	1	4458

148.69354	2	4461

148.79253	1	4462

148.89153	10	4463

148.99053	1	4465

149.08952	1	4466

149.18852	7	4476

149.28752	3	4477

149.38652	1	4478

149.48551	2	4485

149.58451	16	4488

149.68351	1	4489

149.7825	1	4491

149.8815	1	4507

149.9805	7	4508

150.07949	2	4509

150.17849	2	4510

150.27749	9	4517

150.37649	1	4519

150.47548	1	4521

150.57448	4	4530

150.67348	1	4531

150.77247	1	4532

150.87147	5	4536

150.97047	1	4537

151.06947	5	4538

151.16846	2	4543

151.26746	2	4544

151.36646	1	4549

151.46545	1	4551

151.56445	1	4553

151.66345	1	4554

151.76244	4	4555

151.86144	3	4556

151.96044	3	4557

152.05944	1	4561

152.15843	2	4564

152.25743	1	4567

152.35643	1	4568

152.45542	2	4570

152.55442	4	4571

152.65342	5	4572

152.75241	3	4574

152.85141	1	4578

152.95041	1	4583

153.04941	1	4586

153.1484	4	4587

153.2474	3	4588

153.3464	3	4589

153.44539	7	4593

153.54439	4	4596

153.64339	1	4599

153.74238	2	4606

153.84138	6	4610

153.94038	1	4611

154.03938	5	4613

154.13837	4	4619

154.23737	2	4620

154.33637	1	4625

154.43536	14	4629

154.53436	8	4631

154.63336	4	4632

154.73236	1	4646

154.83135	3	4654

154.93035	1	4658

155.02935	2	4659

155.12834	7	4662

155.22734	2	4663

155.32634	1	4665

155.42533	3	4672

155.52433	2	4674

155.62333	1	4675

155.72233	2	4678

155.82132	1	4680

155.92032	1	4681

156.01932	5	4683

156.11831	1	4684

156.21731	3	4685

156.31631	8	4690

156.4153	5	4691

156.5143	3	4694

156.6133	1	4702

156.7123	1	4707

156.81129	2	4710

156.91029	4	4711

157.00929	2	4712

157.10828	1	4714

157.20728	1	4718

157.30628	1	4720

157.40527	4	4721

157.50427	3	4722

157.60327	2	4723

157.70227	1	4727

157.80126	1	4730

157.90026	1	4732

157.99926	1	4733

158.09825	6	4734

158.19725	2	4735

158.29625	2	4736

158.39525	1	4742

158.49424	1	4744

158.59324	11	4746

158.69224	20	4747

158.79123	1	4748

158.89023	2	4759

158.98923	3	4779

159.08822	2	4780

159.18722	1	4782

159.28622	1	4785

159.38522	1	4787

159.48421	1	4788

159.58321	2	4789

159.68221	2	4790

159.7812	1	4791

159.8802	1	4793

159.9792	1	4795

160.07819	2	4796

160.17719	1	4797

160.27619	4	4798

160.37519	1	4800

160.47418	3	4801

160.57318	4	4805

160.67218	1	4806

160.77117	1	4809

160.87017	1	4813

160.96917	1	4814

161.06816	1	4815

161.16716	1	4816

161.26616	1	4817

161.36516	1	4818

161.46415	1	4819

161.56315	2	4820

161.66215	1	4821

161.76114	1	4822

161.86014	5	4824

161.95914	2	4825

162.05814	1	4826

162.15713	1	4831

162.25613	5	4833

162.35513	5	4834

162.45412	4	4835

162.55312	4	4840

162.65212	1	4845

162.75111	1	4849

162.85011	3	4853

162.94911	1	4854

163.04811	11	4855

163.1471	3	4858

163.2461	2	4859

163.3451	2	4870

163.44409	6	4873

163.54309	1	4875

163.64209	1	4877

163.74108	3	4883

163.84008	1	4884

163.93908	1	4885

164.03808	3	4888

164.13707	13	4889

164.23607	5	4890

164.33507	1	4893

164.43406	1	4906

164.53306	2	4911

164.63206	1	4912

164.73106	1	4913

164.83005	4	4915

164.92905	12	4916

165.02805	1	4917

165.12704	9	4921

165.22604	1	4933

165.32504	2	4934

165.42403	1	4943

165.52303	5	4944

165.62203	1	4946

165.72103	4	4947

165.82002	5	4952

165.91902	3	4953

166.01802	2	4957

166.11701	1	4962

166.21601	4	4965

166.31501	5	4967

166.414	2	4968

166.513	2	4972

166.612	2	4977

166.711	77	4979

166.80999	7	4981

166.90899	1	4983

167.00799	1	5060

167.10698	1	5067

167.20598	1	5068

167.30498	2	5069

167.40397	1	5070

167.50297	5	5071

167.60197	2	5073

167.70097	3	5074

167.79996	1	5079

167.89896	11	5081

167.99796	1	5084

168.09695	9	5085

168.19595	2	5096

168.29495	5	5097

168.39395	2	5106

168.49294	2	5108

168.59194	3	5113

168.69094	2	5115

168.78993	1	5117

168.88893	1	5120

168.98793	1	5122

169.08692	1	5123

169.18592	1	5124

169.28492	1	5125

169.38392	1	5126

169.48291	15	5127

169.58191	12	5128

169.68091	2	5129

169.7799	3	5144

169.8789	1	5156

169.9779	8	5158

170.07689	2	5161

170.17589	1	5162

170.27489	1	5170

170.37389	1	5172

170.47288	9	5173

170.57188	7	5174

170.67088	4	5175

170.76987	1	5184

170.86887	4	5191

170.96787	4	5195

171.06686	1	5196

171.16586	3	5200

171.26486	1	5204

171.36386	1	5205

171.46285	5	5208

171.56185	1	5209

171.66085	3	5210

171.75984	5	5215

171.85884	3	5216

171.95784	2	5219

172.05684	2	5224

172.15583	10	5227

172.25483	6	5229

172.35383	1	5231

172.45282	1	5241

172.55182	1	5247

172.65082	5	5248

172.74981	8	5249

172.84881	1	5250

172.94781	2	5255

173.04681	11	5263

173.1458	1	5264

173.2448	5	5266

173.3438	1	5277

173.44279	4	5278

173.54179	5	5283

173.64079	4	5284

173.73978	1	5288

173.83878	1	5293

173.93778	1	5297

174.03678	8	5298

174.13577	2	5299

174.23477	1	5300

174.33377	1	5308

174.43276	2	5310

174.53176	2	5311

174.63076	2	5312

174.72975	1	5314

174.82875	1	5316

174.92775	9	5318

175.02675	2	5319

175.12574	3	5320

175.22474	2	5329

175.32374	2	5331

175.42273	1	5334

175.52173	2	5336

175.62073	1	5338

175.71973	1	5339

175.81872	2	5341

175.91772	5	5342

176.01672	1	5343

176.11571	2	5345

176.21471	3	5350

176.31371	3	5351

176.4127	3	5353

176.5117	1	5356

176.6107	1	5359

176.7097	2	5362

176.80869	5	5363

176.90769	4	5364

177.00669	1	5366

177.10568	1	5371

177.20468	6	5375

177.30368	3	5376

177.40267	2	5377

177.50167	3	5383

177.60067	3	5386

177.69967	2	5388

177.79866	1	5391

177.89766	16	5394

177.99666	1	5396

178.09565	3	5397

178.19465	2	5413

178.29365	1	5414

178.39264	6	5417

178.49164	1	5419

178.59064	2	5420

178.68964	2	5426

178.78863	1	5427

178.88763	1	5429

178.98663	2	5431

179.08562	2	5432

179.18462	3	5433

179.28362	1	5435

179.38262	3	5437

179.48161	2	5440

179.58061	2	5441

179.67961	6	5444

179.7786	2	5446

179.8776	1	5448

179.9766	2	5454

180.07559	2	5456

180.17459	1	5457

180.27359	7	5459

180.37259	3	5461

180.47158	1	5462

180.57058	1	5469

180.66958	4	5472

180.76857	2	5473

180.86757	6	5474

180.96657	2	5478

181.06556	2	5480

181.16456	6	5486

181.26356	2	5488

181.36256	2	5490

181.46155	9	5496

181.56055	1	5498

181.65955	1	5500

181.75854	2	5509

181.85754	5	5510

181.95654	1	5511

182.05553	5	5513

182.15453	1	5518

182.25353	7	5519

182.35253	7	5524

182.45152	1	5525

182.55052	4	5532

182.64952	4	5539

182.74851	1	5540

182.84751	1	5544

182.94651	1	5548

183.04551	1	5549

183.1445	2	5550

183.2435	4	5551

183.3425	18	5552

183.44149	2	5554

183.54049	1	5558

183.63949	1	5576

183.73848	2	5578

183.83748	11	5579

183.93648	2	5580

184.03548	4	5582

184.13447	2	5593

184.23347	1	5595

184.33247	2	5599

184.43146	3	5601

184.53046	1	5602

184.62946	1	5604

184.72845	1	5607

184.82745	2	5608

184.92645	1	5609

185.02545	2	5610

185.12444	2	5612

185.22344	3	5613

185.32244	1	5615

185.42143	1	5617

185.52043	1	5620

185.61943	5	5621

185.71843	2	5622

185.81742	1	5623

185.91642	4	5628

186.01542	2	5630

186.11441	4	5631

186.21341	2	5635

186.31241	14	5637

186.4114	3	5641

186.5104	1	5643

186.6094	2	5657

186.7084	1	5660

186.80739	2	5661

186.90639	1	5663

187.00539	9	5664

187.10438	1	5666

187.20338	1	5667

187.30238	8	5676

187.40137	1	5677

187.50037	1	5678

187.59937	1	5686

187.69837	1	5687

187.79736	3	5688

187.89636	7	5689

187.99536	2	5690

188.09435	1	5693

188.19335	2	5700

188.29235	4	5702

188.39134	2	5703

188.49034	1	5705

188.58934	7	5709

188.68834	1	5711

188.78733	1	5712

188.88633	1	5719

188.98533	9	5720

189.08432	1	5721

189.18332	22	5722

189.28232	1	5731

189.38132	9	5732

189.48031	1	5754

189.57931	1	5755

189.67831	2	5764

189.7773	1	5765

189.8763	2	5766

189.9753	2	5768

190.07429	1	5769

190.17329	1	5771

190.27229	2	5773

190.37129	5	5774

190.47028	1	5775

190.56928	2	5777

190.66828	2	5782

190.76727	2	5783

190.86627	1	5785

190.96527	6	5787

191.06426	3	5789

191.16326	2	5790

191.26226	2	5796

191.36126	2	5799

191.46025	16	5801

191.55925	1	5803

191.65825	22	5805

191.75724	1	5821

191.85624	2	5822

191.95524	2	5844

192.05423	1	5845

192.15323	1	5847

192.25223	1	5849

192.35123	2	5850

192.45022	5	5851

192.54922	17	5852

192.64822	2	5854

192.74721	5	5859

192.84621	22	5876

192.94521	2	5878

193.04421	3	5883

193.1432	8	5905

193.2422	2	5907

193.3412	3	5910

193.44019	1	5918

193.53919	2	5920

193.63819	1	5923

193.73718	1	5924

193.83618	2	5926

193.93518	2	5927

194.03418	1	5928

194.13317	11	5930

194.23217	1	5932

194.33117	3	5933

194.43016	1	5944

194.52916	2	5945

194.62816	4	5948

194.72715	2	5949

194.82615	2	5951

194.92515	1	5955

195.02415	12	5957

195.12314	1	5959

195.22214	1	5960

195.32114	1	5972

195.42013	4	5973

195.51913	1	5974

195.61813	4	5975

195.71712	12	5979

195.81612	1	5980

195.91512	3	5984

196.01412	1	5996

196.11311	2	5997

196.21211	6	6000

196.31111	1	6001

196.4101	3	6003

196.5091	1	6009

196.6081	2	6010

196.7071	3	6013

196.80609	1	6014

196.90509	1	6016

197.00409	1	6019

197.10308	1	6020

197.20208	1	6021

197.30108	2	6022

197.40007	8	6023

197.49907	1	6024

197.59807	1	6026

197.69707	6	6034

197.79606	7	6035

197.89506	4	6036

197.99406	9	6042

198.09305	3	6049

198.19205	2	6053

198.29105	1	6062

198.39004	2	6065

198.48904	6	6067

198.58804	1	6068

198.68704	1	6070

198.78603	3	6076

198.88503	5	6077

198.98403	8	6078

199.08302	9	6081

199.18202	1	6086

199.28102	1	6094

199.38001	1	6103

199.47901	1	6104

199.57801	1	6105

199.67701	1	6106

199.776	1	6107

199.875	1	6108

199.974	1	6109

200.07299	1	6110

200.17199	1	6111

200.27099	9	6112

200.36999	7	6113

200.46898	3	6114

200.56798	2	6123

200.66698	3	6130

200.76597	6	6133

200.86497	2	6135

200.96397	1	6138

201.06296	1	6144

201.16196	1	6146

201.26096	1	6147

201.35996	2	6148

201.45895	9	6149

201.55795	1	6150

201.65695	2	6152

201.75594	1	6161

201.85494	3	6162

201.95394	2	6164

202.05293	3	6165

202.15193	2	6168

202.25093	2	6170

202.34993	1	6173

202.44892	4	6175

202.54792	2	6177

202.64692	5	6178

202.74591	1	6182

202.84491	1	6184

202.94391	1	6189

203.0429	2	6190

203.1419	8	6191

203.2409	5	6192

203.3399	1	6194

203.43889	3	6202

203.53789	1	6207

203.63689	1	6208

203.73588	4	6211

203.83488	15	6212

203.93388	2	6213

204.03288	2	6217

204.13187	4	6232

204.23087	1	6234

204.32987	13	6236

204.42886	1	6240

204.52786	1	6241

204.62686	1	6254

204.72585	2	6255

204.82485	2	6256

204.92385	3	6257

205.02285	1	6259

205.12184	1	6261

205.22084	3	6264

205.31984	1	6265

205.41883	7	6266

205.51783	2	6269

205.61683	1	6270

205.71582	1	6277

205.81482	1	6279

205.91382	1	6280

206.01282	1	6281

206.11181	2	6282

206.21081	2	6283

206.30981	1	6284

206.4088	1	6286

206.5078	5	6288

206.6068	3	6289

206.70579	2	6290

206.80479	1	6295

206.90379	2	6298

207.00279	1	6300

207.10178	1	6301

207.20078	1	6303

207.29978	10	6304

207.39877	6	6305

207.49777	4	6306

207.59677	2	6316

207.69577	1	6322

207.79476	4	6326

207.89376	4	6328

207.99276	2	6329

208.09175	3	6333

208.19075	1	6337

208.28975	4	6339

208.38874	1	6342

208.48774	8	6343

208.58674	2	6347

208.68574	1	6348

208.78473	3	6356

208.88373	3	6358

208.98273	1	6359

209.08172	2	6362

209.18072	2	6365

209.27972	1	6366

209.37871	1	6368

209.47771	1	6370

209.57671	1	6371

209.67571	29	6372

209.7747	4	6373

209.8737	2	6374

209.9727	6	6403

210.07169	2	6407

210.17069	1	6409

210.26969	1	6415

210.36869	3	6417

210.46768	2	6418

210.56668	15	6419

210.66568	1	6422

210.76467	10	6424

210.86367	3	6439

210.96267	2	6440

211.06166	1	6450

211.16066	1	6453

211.25966	1	6455

211.35866	1	6456

211.45765	4	6457

211.55665	9	6458

211.65565	5	6459

211.75464	2	6463

211.85364	1	6472

211.95264	0.5	6477

212.05163	0.5	6479

212.15063	0.5	6480

212.24963	0.5	6480.5

212.34863	3.5	6481

212.44762	2.5	6481.5

212.54662	0.5	6482

212.64562	1.5	6485.5

212.74461	3.5	6488

212.84361	0.5	6488.5

212.94261	0.5	6490

213.0416	4	6493.5

213.1406	1.5	6494

213.2396	1	6494.5

213.3386	2.5	6498.5

213.43759	2.5	6500

213.53659	6	6501

213.63559	1.5	6503.5

213.73458	5.5	6506

213.83358	1.5	6512

213.93258	0.5	6513.5

214.03158	1	6519

214.13057	2	6520.5

214.22957	0.5	6521

214.32857	0.5	6522

214.42756	0.5	6524

214.52656	1	6524.5

214.62556	2.5	6525

214.72455	2	6525.5

214.82355	1	6526.5

214.92255	3	6529

215.02155	0.5	6531

215.12054	2	6532

215.21954	4	6535

215.31854	0.5	6535.5

215.41753	1	6537.5

215.51653	0.5	6541.5

215.61553	2	6542

215.71452	5	6543

215.81352	0.5	6543.5

215.91252	2	6545.5

216.01152	1	6550.5

216.11051	0.5	6551

216.20951	0.5	6553

216.30851	8.5	6554

216.4075	1.5	6554.5

216.5065	0.5	6555

216.6055	0.5	6563.5

216.70449	3	6565

216.80349	0.5	6565.5

216.90249	2	6566

217.00149	1	6569

217.10048	1	6569.5

217.19948	1	6571.5

217.29848	0.5	6572.5

217.39747	1.5	6573.5

217.49647	1	6574.5

217.59547	1	6575

217.69447	1	6576.5

217.79346	0.5	6577.5

217.89246	0.5	6578.5

217.99146	1.5	6579.5

218.09045	4.5	6580

218.18945	0.5	6580.5

218.28845	0.5	6582

218.38744	3.5	6586.5

218.48644	2	6587

218.58544	0.5	6587.5

218.68444	0.5	6591

218.78343	0.5	6593

218.88243	0.5	6593.5

218.98143	0.5	6594

219.08042	0.5	6594.5

219.17942	3	6595

219.27842	0.5	6595.5

219.37741	1	6596

219.47641	1.5	6599

219.57541	0.5	6599.5

219.67441	2	6600.5

219.7734	3	6602

219.8724	1	6602.5

219.9714	7	6604.5

220.07039	1	6607.5

220.16939	1.5	6608.5

220.26839	0.5	6615.5

220.36738	0.5	6616.5

220.46638	2	6618

220.56538	3.5	6618.5

220.66438	0.5	6619

220.76337	1.5	6621

220.86237	4	6624.5

220.96137	2.5	6625

221.06036	1.5	6626.5

221.15936	2.5	6630.5

221.25836	2	6633

221.35736	1.5	6634.5

221.45635	1	6637

221.55535	1.5	6639

221.65435	1	6640.5

221.75334	0.5	6641.5

221.85234	1.5	6643

221.95134	5	6644

222.05033	1	6644.5

222.14933	1	6646

222.24833	1.5	6651

222.34733	1	6652

222.44632	0.5	6653

222.54532	5	6654.5

222.64432	0.5	6655.5

222.74331	0.5	6656

222.84231	0.5	6661

222.94131	0.5	6661.5

223.0403	0.5	6662

223.1393	0.5	6662.5

223.2383	0.5	6663

223.3373	0.5	6663.5

223.43629	1.5	6664

223.53529	0.5	6664.5

223.63429	1	6665

223.73328	1.5	6666.5

223.83228	4.5	6667

223.93128	0.5	6668

224.03027	0.5	6669.5

224.12927	3.5	6674

224.22827	0.5	6674.5

224.32727	0.5	6675

224.42626	1	6678.5

224.52526	0.5	6679

224.62426	0.5	6679.5

224.72325	3	6680.5

224.82225	0.5	6681

224.92125	3	6681.5

225.02025	0.5	6684.5

225.11924	7	6685

225.21824	1	6688

225.31724	2	6688.5

225.41623	0.5	6695.5

225.51523	0.5	6696.5

225.61423	0.5	6698.5

225.71322	1	6699

225.81222	0.5	6699.5

225.91122	0.5	6700

226.01022	0.5	6701

226.10921	1	6701.5

226.20821	0.5	6702

226.30721	0.5	6702.5

226.4062	3.5	6703.5

226.5052	0.5	6704

226.6042	5.5	6704.5

226.70319	0.5	6708

226.80219	5	6708.5

226.90119	0.5	6714

227.00019	2	6714.5

227.09918	1.5	6719.5

227.19818	0.5	6720

227.29718	1.5	6722

227.39617	1	6723.5

227.49517	0.5	6724

227.59417	0.5	6725.5

227.69316	0.5	6726.5

227.79216	2	6727

227.89116	1.5	6727.5

227.99016	0.5	6728

228.08915	0.5	6730

228.18815	0.5	6731.5

228.28715	0.5	6732

228.38614	0.5	6732.5

228.48514	0.5	6733

228.58414	0.5	6733.5

228.68314	0.5	6734

228.78213	1	6734.5

228.88113	1.5	6735

228.98013	4	6735.5

229.07912	2	6736.5

229.17812	4.5	6738

229.27712	1.5	6742

229.37611	0.5	6744

229.47511	0.5	6748.5

229.57411	0.5	6750

229.67311	0.5	6750.5

229.7721	0.5	6751

229.8711	11	6751.5

229.9701	3	6752

230.06909	0.5	6752.5

230.16809	1	6763.5

230.26709	1	6766.5

230.36608	0.5	6767

230.46508	0.5	6768

230.56408	0.5	6769

230.66308	3	6769.5

230.76207	0.5	6770

230.86107	0.5	6770.5

230.96007	0.5	6773.5

231.05906	0.5	6774

231.15806	2.5	6774.5

231.25706	2	6775

231.35606	3.5	6775.5

231.45505	2.5	6778

231.55405	0.5	6780

231.65305	0.5	6783.5

231.75204	0.5	6786

231.85104	4.5	6786.5

231.95004	1.5	6787

232.04903	1.5	6787.5

232.14803	0.5	6792

232.24703	0.5	6793.5

232.34603	0.5	6795

232.44502	0.5	6795.5

232.54402	2.5	6796

232.64302	0.5	6796.5

232.74201	9.5	6797

232.84101	0.5	6799.5

232.94001	3.5	6800

233.039	0.5	6809.5

233.138	0.5	6810

233.237	3.5	6813.5

233.336	0.5	6814

233.43499	0.5	6814.5

233.53399	1.5	6818

233.63299	9.5	6818.5

233.73198	0.5	6819

233.83098	0.5	6820.5

233.92998	2	6830

234.02897	1	6830.5

234.12797	8	6831

234.22697	1.5	6833

234.32597	0.5	6834

234.42496	0.5	6842

234.52396	2	6843.5

234.62296	0.5	6844

234.72195	2.5	6844.5

234.82095	3	6846.5

234.91995	4	6847

235.01895	0.5	6849.5

235.11794	1.5	6852.5

235.21694	1	6856.5

235.31594	1	6857

235.41493	1.5	6858.5

235.51393	1	6859.5

235.61293	1.5	6860.5

235.71192	1.5	6862

235.81092	2	6863

235.90992	4	6864.5

236.00892	1	6866

236.10791	5	6868

236.20691	1	6872

236.30591	0.5	6873

236.4049	0.5	6878

236.5039	3	6879

236.6029	2.5	6879.5

236.70189	1	6880

236.80089	5.5	6883

236.89989	1	6885.5

236.99889	0.5	6886.5

237.09788	1	6892

237.19688	2	6893

237.29588	0.5	6893.5

237.39487	4	6894.5

237.49387	3.5	6896.5

237.59287	1	6897

237.69186	1	6901

237.79086	4	6904.5

237.88986	0.5	6905.5

237.98886	1	6906.5

238.08785	1	6910.5

238.18685	3.5	6911

238.28585	0.5	6912

238.38484	0.5	6913

238.48384	0.5	6916.5

238.58284	4	6917

238.68184	1.5	6917.5

238.78083	0.5	6918

238.87983	1	6922

238.97883	0.5	6923.5

239.07782	1	6924

239.17682	0.5	6925

239.27582	8	6925.5

239.37481	0.5	6926.5

239.47381	2	6927

239.57281	0.5	6935

239.67181	2.5	6935.5

239.7708	2.5	6937.5

239.8698	0.5	6938

239.9688	1	6940.5

240.06779	0.5	6943

240.16679	1	6943.5

240.26579	1	6944.5

240.36478	0.5	6945

240.46378	5	6946

240.56278	1.5	6947

240.66178	5.5	6947.5

240.76077	1	6952.5

240.85977	4.5	6954

240.95877	1.5	6959.5

241.05776	2	6960.5

241.15676	0.5	6965

241.25576	2	6966.5

241.35475	0.5	6968.5

241.45375	1	6969

241.55275	0.5	6971

241.65175	0.5	6971.5

241.75074	0.5	6972.5

241.84974	0.5	6973

241.94874	3	6973.5

242.04773	1.5	6974

242.14673	5	6974.5

242.24573	1	6977.5

242.34473	1	6979

242.44372	8	6984

242.54272	0.5	6985

242.64172	0.5	6986

242.74071	4.5	6994

242.83971	1.5	6994.5

242.93871	5.5	6995

243.0377	1	6999.5

243.1367	1.5	7001

243.2357	0.5	7006.5

243.3347	2.5	7007.5

243.43369	0.5	7009

243.53269	0.5	7009.5

243.63169	1	7012

243.73068	2	7012.5

243.82968	2.5	7013

243.92868	0.5	7014

244.02767	1	7016

244.12667	3	7018.5

244.22567	0.5	7019

244.32467	2.5	7020

244.42366	1	7023

244.52266	6.5	7023.5

244.62166	0.5	7026

244.72065	0.5	7027

244.81965	0.5	7033.5

244.91865	1.5	7034

245.01764	0.5	7034.5

245.11664	4.5	7035

245.21564	0.5	7036.5

245.31464	1	7037

245.41363	2.5	7041.5

245.51263	1.5	7042

245.61163	0.5	7043

245.71062	10	7045.5

245.80962	1.5	7047

245.90862	4	7047.5

246.00762	1	7057.5

246.10661	0.5	7059

246.20561	2.5	7063

246.30461	0.5	7064

246.4036	0.5	7064.5

246.5026	0.5	7067

246.6016	0.5	7067.5

246.70059	1	7068

246.79959	2.5	7068.5

246.89859	1.5	7069

246.99759	1.5	7070

247.09658	1	7072.5

247.19558	1	7074

247.29458	0.5	7075.5

247.39357	1	7076.5

247.49257	7	7077.5

247.59157	0.5	7078

247.69056	0.5	7079

247.78956	2	7086

247.88856	2	7086.5

247.98756	1	7087

248.08655	0.5	7089

248.18555	6.5	7091

248.28455	0.5	7092

248.38354	1.5	7092.5

248.48254	1	7099

248.58154	0.5	7099.5

248.68053	0.5	7101

248.77953	2	7102

248.87853	0.5	7102.5

248.97753	3	7103

249.07652	1	7105

249.17552	3	7105.5

249.27452	0.5	7108.5

249.37351	0.5	7109.5

249.47251	1	7112.5

249.57151	0.5	7113

249.67051	1	7113.5

249.7695	0.5	7114.5

249.8685	5.5	7115

249.9675	1	7116

250.06649	6.5	7116.5

250.16549	3	7122

250.26449	1	7123

250.36348	7.5	7129.5

250.46248	2	7132.5

250.56148	3	7133.5

250.66048	0.5	7141

250.75947	2.5	7143

250.85847	1.5	7146

250.95747	1	7146.5

251.05646	0.5	7149

251.15546	0.5	7150.5

251.25446	1.5	7151.5

251.35345	0.5	7152

251.45245	0.5	7152.5

251.55145	2.5	7154

251.65045	3	7154.5

251.74944	1.5	7155

251.84844	0.5	7157.5

251.94744	0.5	7160.5

252.04643	0.5	7162

252.14543	0.5	7162.5

252.24443	2	7163

252.34343	0.5	7163.5

252.44242	1	7164

252.54142	1.5	7166

252.64042	0.5	7166.5

252.73941	1	7167.5

252.83841	1	7169

252.93741	3	7169.5

253.0364	1	7170.5

253.1354	2	7171.5

253.2344	0.5	7174.5

253.3334	0.5	7175.5

253.43239	2.5	7177.5

253.53139	0.5	7178

253.63039	7	7178.5

253.72938	0.5	7181

253.82838	1	7181.5

253.92738	0.5	7188.5

254.02637	2	7189

254.12537	2.5	7190

254.22437	0.5	7190.5

254.32337	1	7192.5

254.42236	5	7195

254.52136	6.5	7195.5

254.62036	1	7196.5

254.71935	2	7201.5

254.81835	1.5	7208

254.91735	7	7209

255.01634	1.5	7211

255.11534	1	7212.5

255.21434	1.5	7219.5

255.31334	1	7221

255.41233	0.5	7222

255.51133	2.5	7223.5

255.61033	4	7224.5

255.70932	2.5	7225

255.80832	0.5	7227.5

255.90732	5.5	7231.5

256.00631	1	7234

256.10531	0.5	7234.5

256.20431	0.5	7240

256.30331	1	7241

256.4023	1.5	7241.5

256.5013	0.5	7242

256.6003	1.5	7243

256.69929	3	7244.5

256.79829	1	7245

256.89729	0.5	7246.5

256.99629	2	7249.5

257.09528	1	7250.5

257.19428	1	7251

257.29328	0.5	7253

257.39227	2	7254

257.49127	6	7255

257.59027	5.5	7255.5

257.68926	1	7257.5

257.78826	1	7263.5

257.88726	2.5	7269

257.98626	0.5	7270

258.08525	2	7271

258.18425	0.5	7273.5

258.28325	0.5	7274

258.38224	0.5	7276

258.48124	4	7276.5

258.58024	1	7277

258.67923	1	7277.5

258.77823	0.5	7281.5

258.87723	0.5	7282.5

258.97623	0.5	7283.5

259.07522	4.5	7284

259.17422	0.5	7284.5

259.27322	0.5	7285

259.37221	0.5	7289.5

259.47121	1.5	7290

259.57021	1	7290.5

259.66921	0.5	7291

259.7682	1.5	7292.5

259.8672	0.5	7293.5

259.9662	6	7294

260.06519	0.5	7295.5

260.16419	1	7296

260.26319	0.5	7302

260.36218	0.5	7302.5

260.46118	0.5	7303.5

260.56018	5	7304

260.65918	0.5	7304.5

260.75817	1	7305

260.85717	1	7310

260.95617	2.5	7310.5

261.05516	0.5	7311.5

261.15416	1.5	7312.5

261.25316	5.5	7315

261.35215	1	7315.5

261.45115	0.5	7317

261.55015	8	7322.5

261.64915	0.5	7323.5

261.74814	0.5	7324

261.84714	0.5	7332

261.94614	0.5	7332.5

262.04513	5	7333

262.14413	0.5	7333.5

262.24313	2	7334

262.34212	1	7339

262.44112	0.5	7339.5

262.54012	0.5	7341.5

262.63912	2.5	7342.5

262.73811	2	7343

262.83711	0.5	7343.5

262.93611	1	7346

263.0351	0.5	7348

263.1341	3.5	7348.5

263.2331	0.5	7349.5

263.3321	2	7350

263.43109	0.5	7353.5

263.53009	0.5	7354

263.62909	0.5	7356

263.72808	1	7356.5

263.82708	5.5	7357

263.92608	1	7357.5

264.02507	0.5	7358.5

264.12407	0.5	7364

264.22307	0.5	7365

264.32207	5.5	7365.5

264.42106	1.5	7366

264.52006	0.5	7366.5

264.61906	0.5	7372

264.71805	1.5	7373.5

264.81705	2	7374

264.91605	3.5	7374.5

265.01504	1.5	7376

265.11404	1	7378

265.21304	0.5	7381.5

265.31204	2.5	7383

265.41103	3	7384

265.51003	3	7384.5

265.60903	3	7387

265.70802	0.5	7390

265.80702	1	7393

265.90602	1	7396

266.00501	6	7396.5

266.10401	0.5	7397.5

266.20301	1	7398.5

266.30201	0.5	7404.5

266.401	0.5	7405

266.5	3.5	7406

266.599	1.5	7406.5

266.69799	1	7407

266.79699	0.5	7410.5

266.89599	0.5	7412

266.99499	1.5	7413

267.09398	4	7413.5

267.19298	1	7414

267.29198	0.5	7415.5

267.39097	6	7419.5

267.48997	0.5	7420.5

267.58897	6	7421

267.68796	2	7427

267.78696	0.5	7427.5

267.88596	0.5	7433.5

267.98496	2	7435.5

268.08395	1.5	7436

268.18295	1.5	7436.5

268.28195	1.5	7438.5

268.38094	1	7440

268.47994	0.5	7441.5

268.57894	0.5	7443

268.67793	0.5	7444

268.77693	1	7444.5

268.87593	3	7445

268.97493	2	7445.5

269.07392	1	7446.5

269.17292	0.5	7449.5

269.27192	0.5	7451.5

269.37091	7	7452.5

269.46991	0.5	7453

269.56891	2.5	7453.5

269.6679	1.5	7460.5

269.7669	2	7461

269.8659	0.5	7463.5

269.9649	0.5	7465

270.06389	8.5	7467

270.16289	0.5	7467.5

270.26189	3	7468

270.36088	0.5	7476.5

270.45988	0.5	7477

270.55888	5	7480

270.65788	1	7480.5

270.75687	1	7481

270.85587	4.5	7486

270.95487	4	7487

271.05386	0.5	7488

271.15286	2.5	7492.5

271.25186	16	7496.5

271.35085	3	7497

271.44985	1	7499.5

271.54885	3.5	7515.5

271.64785	1	7518.5

271.74684	0.5	7519.5

271.84584	10.5	7523

271.94484	3	7524

272.04383	3.5	7524.5

272.14283	1	7535

272.24183	1	7538

272.34082	11	7541.5

272.43982	3	7542.5

272.53882	3	7543.5

272.63782	0.5	7554.5

272.73681	0.5	7557.5

272.83581	2	7560.5

272.93481	2	7561

273.0338	0.5	7561.5

273.1328	3	7563.5

273.2318	0.5	7565.5

273.33079	3	7566

273.42979	6.5	7569

273.52879	1.5	7569.5

273.62779	0.5	7572.5

273.72678	4.5	7579

273.82578	2	7580.5

273.92478	18	7581

274.02377	1	7585.5

274.12277	0.5	7587.5

274.22177	1	7605.5

274.32077	2.5	7606.5

274.41976	3	7607

274.51876	1	7608

274.61776	1.5	7610.5

274.71675	1	7613.5

274.81575	0.5	7614.5

274.91475	0.5	7616

275.01374	1	7617

275.11274	1	7617.5

275.21174	7	7618

275.31074	0.5	7619

275.40973	2.5	7620

275.50873	0.5	7627

275.60773	1.5	7627.5

275.70672	2	7630

275.80572	0.5	7630.5

275.90472	0.5	7632

276.00371	1.5	7634

276.10271	6	7634.5

276.20171	0.5	7635

276.30071	1	7636.5

276.3997	2.5	7642.5

276.4987	0.5	7643

276.5977	0.5	7644

276.69669	0.5	7646.5

276.79569	4.5	7647

276.89469	0.5	7647.5

276.99369	8	7648

277.09268	8	7652.5

277.19168	0.5	7653

277.29068	0.5	7661

277.38967	3	7669

277.48867	2.5	7669.5

277.58767	8.5	7670

277.68666	2.5	7673

277.78566	5	7675.5

277.88466	0.5	7684

277.98366	0.5	7686.5

278.08265	0.5	7691.5

278.18165	3.5	7692

278.28065	0.5	7692.5

278.37964	2	7693

278.47864	4.5	7696.5

278.57764	0.5	7697

278.67663	4.5	7699

278.77563	0.5	7703.5

278.87463	1	7704

278.97363	1	7708.5

279.07262	2	7709

279.17162	0.5	7710

279.27062	1	7711

279.36961	5.5	7713

279.46861	2.5	7713.5

279.56761	0.5	7714.5

279.6666	1.5	7720

279.7656	1	7722.5

279.8646	1	7723

279.9636	0.5	7724.5

280.06259	1.5	7725.5

280.16159	2.5	7726.5

280.26059	2.5	7727

280.35958	0.5	7728.5

280.45858	0.5	7731

280.55758	1	7733.5

280.65657	0.5	7734

280.75557	0.5	7734.5

280.85457	7	7735.5

280.95357	0.5	7736

281.05256	0.5	7736.5

281.15156	1.5	7743.5

281.25056	3	7744

281.34955	4	7744.5

281.44855	2	7746

281.54755	0.5	7749

281.64655	1	7753

281.74554	0.5	7755

281.84454	0.5	7755.5

281.94354	4	7756.5

282.04253	0.5	7757

282.14153	4	7757.5

282.24053	2	7761.5

282.33952	5	7762

282.43852	2	7766

282.53752	1	7768

282.63652	1	7773

282.73551	5.5	7775

282.83451	1	7776

282.93351	0.5	7777

283.0325	2.5	7782.5

283.1315	2	7783.5

283.2305	1	7784

283.32949	1.5	7786.5

283.42849	2	7788.5

283.52749	1	7789.5

283.62649	1	7791

283.72548	3.5	7793

283.82448	1	7794

283.92348	1	7795

284.02247	2	7798.5

284.12147	0.5	7799.5

284.22047	3.5	7800.5

284.31947	8	7802.5

284.41846	1	7803

284.51746	6	7806.5

284.61646	0.5	7814.5

284.71545	0.5	7815.5

284.81445	1	7821.5

284.91345	2	7822

285.01244	1.5	7822.5

285.11144	2	7823.5

285.21044	1	7825.5

285.30944	12	7827

285.40843	4	7829

285.50743	0.5	7830

285.60643	6.5	7842

285.70542	1.5	7846

285.80442	0.5	7846.5

285.90342	9.5	7853

286.00241	1	7854.5

286.10141	0.5	7855

286.20041	0.5	7864.5

286.29941	6	7865.5

286.3984	2	7866

286.4974	2.5	7866.5

286.5964	1.5	7872.5

286.69539	1	7874.5

286.79439	0.5	7877

286.89339	4.5	7878.5

286.99238	1	7879.5

287.09138	1.5	7880

287.19038	5	7884.5

287.28938	0.5	7885.5

287.38837	6	7887

287.48737	2	7892

287.58637	0.5	7892.5

287.68536	6	7898.5

287.78436	0.5	7900.5

287.88336	13.5	7901

287.98236	7	7907

288.08135	5	7907.5

288.18035	0.5	7921

288.27935	3.5	7928

288.37834	1	7933

288.47734	1	7933.5

288.57634	3.5	7937

288.67533	2.5	7938

288.77433	1	7939

288.87333	1	7942.5

288.97233	11.5	7945

289.07132	0.5	7946

289.17032	5.5	7947

289.26932	0.5	7958.5

289.36831	0.5	7959

289.46731	2	7964.5

289.56631	6.5	7965

289.6653	0.5	7965.5

289.7643	1	7967.5

289.8633	2	7974

289.9623	0.5	7974.5

290.06129	0.5	7975.5

290.16029	0.5	7977.5

290.25929	3.5	7978

290.35828	3	7978.5

290.45728	0.5	7979

290.55628	0.5	7982.5

290.65527	2.5	7985.5

290.75427	2.5	7986

290.85327	2	7986.5

290.95227	0.5	7989

291.05126	2.5	7991.5

291.15026	6.5	7993.5

291.24926	0.5	7994

291.34825	8.5	7996.5

291.44725	2.5	8003

291.54625	2.5	8003.5

291.64525	1	8012

291.74424	8	8014.5

291.84324	11	8017

291.94224	0.5	8018

292.04123	0.5	8026

292.14023	0.5	8037

292.23923	4.5	8037.5

292.33822	1	8038

292.43722	0.5	8038.5

292.53622	0.5	8043

292.63522	0.5	8044

292.73421	4.5	8044.5

292.83321	0.5	8045

292.93221	0.5	8045.5

293.0312	10.5	8050

293.1302	7	8050.5

293.2292	7.5	8051

293.32819	0.5	8061.5

293.42719	2.5	8068.5

293.52619	0.5	8076

293.62519	3	8076.5

293.72418	0.5	8079

293.82318	13	8079.5

293.92218	5.5	8082.5

294.02117	1	8083

294.12017	3	8096

294.21917	3	8101.5

294.31816	1.5	8102.5

294.41716	1.5	8105.5

294.51616	2.5	8108.5

294.61516	10	8110

294.71415	0.5	8111.5

294.81315	2.5	8114

294.91215	6	8124

295.01114	1	8124.5

295.11014	0.5	8127

295.20914	2	8133

295.30814	4.5	8134

295.40713	16.5	8134.5

295.50613	2	8136.5

295.60513	2	8141

295.70412	1	8157.5

295.80312	1.5	8159.5

295.90212	1	8161.5

296.00111	4	8162.5

296.10011	0.5	8164

296.19911	3.5	8165

296.29811	0.5	8169

296.3971	5	8169.5

296.4961	0.5	8173

296.5951	1	8173.5

296.69409	0.5	8178.5

296.79309	1	8179

296.89209	1.5	8180

296.99108	0.5	8180.5

297.09008	11.5	8181.5

297.18908	11.5	8183

297.28808	1	8183.5

297.38707	0.5	8195

297.48607	1.5	8206.5

297.58507	7	8207.5

297.68406	9	8208

297.78306	2.5	8209.5

297.88206	7	8216.5

297.98106	2	8225.5

298.08005	4	8228

298.17905	2	8235

298.27805	1	8237

298.37704	8.5	8241

298.47604	3.5	8243

298.57504	1	8244

298.67403	6.5	8252.5

298.77303	0.5	8256

298.87203	0.5	8257

298.97103	0.5	8263.5

299.07002	2	8264

299.16902	4.5	8264.5

299.26802	3	8265

299.36701	0.5	8267

299.46601	1	8271.5

299.56501	2	8274.5

299.664	3	8275

299.763	3.5	8276

299.862	1.5	8278

299.961	1	8281

300.05999	8	8284.5

300.15899	0.5	8286

300.25799	4.5	8287

300.35698	1	8295

300.45598	0.5	8295.5

300.55498	10.5	8300

300.65397	0.5	8301

300.75297	6	8301.5

300.85197	1.5	8312

300.95097	0.5	8312.5

301.04996	0.5	8318.5

301.14896	3	8320

301.24796	1	8320.5

301.34695	1	8321

301.44595	0.5	8324

301.54495	1	8325

301.64394	4.5	8326

301.74294	8	8326.5

301.84194	5.5	8327.5

301.94094	10.5	8332

302.03993	1.5	8340

302.13893	0.5	8345.5

302.23793	15.5	8356

302.33692	2	8357.5

302.43592	1	8358

302.53492	3.5	8373.5

302.63392	0.5	8375.5

302.73291	5	8376.5

302.83191	0.5	8380

302.93091	0.5	8380.5

303.0299	2.5	8385.5

303.1289	9.5	8386

303.2279	3	8386.5

303.32689	1	8389

303.42589	1	8398.5

303.52489	0.5	8401.5

303.62389	0.5	8402.5

303.72288	2	8403.5

303.82188	0.5	8404

303.92088	0.5	8404.5

304.01987	4	8406.5

304.11887	0.5	8407

304.21787	0.5	8407.5

304.31686	1	8411.5

304.41586	8	8412

304.51486	2.5	8412.5

304.61386	0.5	8413.5

304.71285	4.5	8421.5

304.81185	4.5	8424

304.91085	0.5	8424.5

305.00984	0.5	8429

305.10884	15.5	8433.5

305.20784	0.5	8434

305.30684	15	8434.5

305.40583	0.5	8450

305.50483	1.5	8450.5

305.60383	6.5	8465.5

305.70282	0.5	8466

305.80182	0.5	8467.5

305.90082	1	8474

305.99981	6.5	8474.5

306.09881	1	8475

306.19781	14	8476

306.29681	0.5	8482.5

306.3958	0.5	8483.5

306.4948	1	8497.5

306.5938	2.5	8498

306.69279	1	8498.5

306.79179	1	8499.5

306.89079	8	8502

306.98978	2	8503

307.08878	6	8504

307.18778	0.5	8512

307.28678	3	8514

307.38577	4.5	8520

307.48477	2	8520.5

307.58377	0.5	8523.5

307.68276	4.5	8528

307.78176	5	8530

307.88076	1	8530.5

307.97975	1.5	8535

308.07875	0.5	8540

308.17775	2	8541

308.27675	1.5	8542.5

308.37574	15.5	8543

308.47474	1.5	8545

308.57374	0.5	8546.5

308.67273	3	8562

308.77173	3.5	8563.5

308.87073	2.5	8564

308.96973	1.5	8567

309.06872	5.5	8570.5

309.16772	0.5	8573

309.26672	17.5	8574.5

309.36571	1.5	8580

309.46471	0.5	8580.5

309.56371	1	8598

309.6627	10	8599.5

309.7617	0.5	8600

309.8607	10	8601

309.9597	8	8611

310.05869	1	8611.5

310.15769	0.5	8621.5

310.25669	2.5	8629.5

310.35568	0.5	8630.5

310.45468	8.5	8631

310.55368	1	8633.5

310.65267	2	8634

310.75167	0.5	8642.5

310.85067	2.5	8643.5

310.94967	4.5	8645.5

311.04866	2.5	8646

311.14766	5.5	8648.5

311.24666	1	8653

311.34565	0.5	8655.5

311.44465	3	8661

311.54365	2	8662

311.64264	15.5	8662.5

311.74164	0.5	8665.5

311.84064	5.5	8667.5

311.93964	2	8683

312.03863	7.5	8683.5

312.13763	2	8689

312.23663	8.5	8691

312.33562	5	8698.5

312.43462	6	8700.5

312.53362	2.5	8709

312.63262	1.5	8714

312.73161	0.5	8720

312.83061	4	8722.5

312.92961	7	8724

313.0286	0.5	8724.5

313.1276	4	8728.5

313.2266	1	8735.5

313.32559	0.5	8736

313.42459	17	8740

313.52359	0.5	8741

313.62259	0.5	8741.5

313.72158	0.5	8758.5

313.82058	2	8759

313.91958	2	8759.5

314.01857	4.5	8760

314.11757	0.5	8762

314.21657	13	8764

314.31556	1.5	8768.5

314.41456	6	8769

314.51356	1	8782

314.61256	0.5	8783.5

314.71155	3	8789.5

314.81055	2	8790.5

314.90955	0.5	8791

315.00854	0.5	8794

315.10754	2.5	8796

315.20654	3.5	8796.5

315.30553	6.5	8797

315.40453	4	8799.5

315.50353	0.5	8803

315.60253	2	8809.5

315.70152	6.5	8813.5

315.80052	0.5	8814

315.89952	2	8816

315.99851	1	8822.5

316.09751	0.5	8823

316.19651	0.5	8825

316.29551	10	8826

316.3945	0.5	8826.5

316.4935	0.5	8827

316.5925	1	8837

316.69149	3	8837.5

316.79049	4	8838

316.88949	0.5	8839

316.98848	1	8842

317.08748	5	8846

317.18648	1	8846.5

317.28548	0.5	8847.5

317.38447	5.5	8852.5

317.48347	9	8853.5

317.58247	1.5	8854

317.68146	0.5	8859.5

317.78046	2.5	8868.5

317.87946	0.5	8870

317.97845	2	8870.5

318.07745	1.5	8873

318.17645	0.5	8873.5

318.27545	1.5	8875.5

318.37444	1	8877

318.47344	17.5	8877.5

318.57244	0.5	8879

318.67143	0.5	8880

318.77043	6.5	8897.5

318.86943	1.5	8898

318.96843	1	8898.5

319.06742	7	8905

319.16642	2.5	8906.5

319.26542	13	8907.5

319.36441	0.5	8914.5

319.46341	1	8917

319.56241	3.5	8930

319.6614	2	8930.5

319.7604	0.5	8931.5

319.8594	9.5	8935

319.9584	1	8937

320.05739	0.5	8937.5

320.15639	3.5	8947

320.25539	5.5	8948

320.35438	1	8948.5

320.45338	23.5	8952

320.55238	11.5	8957.5

320.65137	1.5	8958.5

320.75037	0.5	8982

320.84937	2	8993.5

320.94837	0.5	8995

321.04736	13.5	8995.5

321.14636	0.5	8997.5

321.24536	14.5	8998

321.34435	1.5	9011.5

321.44335	1	9012

321.54235	3.5	9026.5

321.64134	1	9028

321.74034	2	9029

321.83934	1	9032.5

321.93834	8	9033.5

322.03733	3.5	9035.5

322.13633	6.5	9036.5

322.23533	0.5	9044.5

322.33432	5	9048

322.43332	2	9054.5

322.53232	2	9055

322.63131	7.5	9060

322.73031	4.5	9062

322.82931	1.5	9064

322.92831	1.5	9071.5

323.0273	6.5	9076

323.1263	0.5	9077.5

323.2253	1	9079

323.32429	10	9085.5

323.42329	0.5	9086

323.52229	0.5	9087

323.62129	2	9097

323.72028	7.5	9097.5

323.81928	0.5	9098

323.91828	4.5	9100

324.01727	2.5	9107.5

324.11627	0.5	9108

324.21527	6.5	9112.5

324.31426	5	9115

324.41326	3.5	9115.5

324.51226	1.5	9122

324.61126	0.5	9127

324.71025	5	9130.5

324.80925	2.5	9132

324.90825	10.5	9132.5

325.00724	1	9137.5

325.10624	0.5	9140

325.20524	6.5	9150.5

325.30423	0.5	9151.5

325.40323	0.5	9152

325.50223	0.5	9158.5

325.60123	2	9159

325.70022	3	9159.5

325.79922	2.5	9160

325.89822	4.5	9162

325.99721	1.5	9165

326.09621	2	9167.5

326.19521	3	9172

326.29421	0.5	9173.5

326.3932	13	9175.5

326.4922	0.5	9178.5

326.5912	14.5	9179

326.69019	1	9192

326.78919	3.5	9192.5

326.88819	0.5	9207

326.98718	0.5	9208

327.08618	5	9211.5

327.18518	1.5	9212

327.28418	0.5	9212.5

327.38317	16	9217.5

327.48217	3	9219

327.58117	4.5	9219.5

327.68016	0.5	9235.5

327.77916	4	9238.5

327.87816	0.5	9243

327.97715	0.5	9243.5

328.07615	8	9247.5

328.17515	2.5	9248

328.27415	1.5	9248.5

328.37314	0.5	9256.5

328.47214	2	9259

328.57114	8.5	9260.5

328.67013	1	9261

328.76913	22.5	9263

328.86813	4	9271.5

328.96712	2	9272.5

329.06612	0.5	9295

329.16512	0.5	9299

329.26412	5	9301

329.36311	1	9301.5

329.46211	2	9302

329.56111	6	9307

329.6601	1	9308

329.7591	0.5	9310

329.8581	0.5	9316

329.9571	2	9317

330.05609	4	9317.5

330.15509	0.5	9318

330.25409	3	9320

330.35308	12.5	9324

330.45208	1.5	9324.5

330.55108	1	9327.5

330.65007	3.5	9340

330.74907	0.5	9341.5

330.84807	1.5	9342.5

330.94707	7.5	9346

331.04606	2	9346.5

331.14506	1	9348

331.24406	0.5	9355.5

331.34305	10.5	9357.5

331.44205	1	9358.5

331.54105	3.5	9359

331.64004	1	9369.5

331.73904	1	9370.5

331.83804	3.5	9374

331.93704	1.5	9375

332.03603	10	9376

332.13503	1	9379.5

332.23403	0.5	9381

332.33302	9.5	9391

332.43202	1	9392

332.53102	3.5	9392.5

332.63001	19	9402

332.72901	0.5	9403

332.82801	11	9406.5

332.92701	0.5	9425.5

333.026	0.5	9426

333.125	1	9437

333.224	11	9437.5

333.32299	0.5	9438

333.42199	1	9439

333.52099	8	9450

333.61999	3	9450.5

333.71898	0.5	9451.5

333.81798	3.5	9459.5

333.91698	9	9462.5

334.01597	0.5	9463

334.11497	0.5	9466.5

334.21397	10	9475.5

334.31296	1	9476

334.41196	1	9476.5

334.51096	4.5	9486.5

334.60996	3.5	9487.5

334.70895	1	9488.5

334.80795	1.5	9493

334.90695	7	9496.5

335.00594	2	9497.5

335.10494	0.5	9499

335.20394	4.5	9506

335.30293	1.5	9508

335.40193	1	9508.5

335.50093	1	9513

335.59993	0.5	9514.5

335.69892	15.5	9515.5

335.79792	6.5	9516.5

335.89692	2	9517

335.99591	2	9532.5

336.09491	1	9539

336.19391	2	9541

336.2929	0.5	9543

336.3919	1.5	9544

336.4909	13	9546

336.5899	4.5	9546.5

336.68889	10	9548

336.78789	1	9561

336.88689	1	9565.5

336.98588	3	9575.5

337.08488	1.5	9576.5

337.18388	2	9577.5

337.28288	3.5	9580.5

337.38187	2.5	9582

337.48087	1.5	9584

337.57987	4.5	9587.5

337.67886	10.5	9590

337.77786	1.5	9591.5

337.87686	1	9596

337.97585	7	9606.5

338.07485	5.5	9608

338.17385	1	9609

338.27285	2.5	9616

338.37184	0.5	9621.5

338.47084	0.5	9622.5

338.56984	2.5	9625

338.66883	5.5	9625.5

338.76783	1.5	9626

338.86683	0.5	9628.5

338.96582	16.5	9634

339.06482	1	9635.5

339.16382	1.5	9636

339.26282	10.5	9652.5

339.36181	0.5	9653.5

339.46081	13	9655

339.55981	0.5	9665.5

339.6588	0.5	9666

339.7578	0.5	9679

339.8568	10	9679.5

339.95579	13.5	9680

340.05479	2.5	9680.5

340.15379	1	9690.5

340.25279	1	9704

340.35178	4	9706.5

340.45078	3.5	9707.5

340.54978	0.5	9708.5

340.64877	16.5	9712.5

340.74777	0.5	9716

340.84677	1	9716.5

340.94577	2	9733

341.04476	4.5	9733.5

341.14376	1	9734.5

341.24276	14	9736.5

341.34175	1.5	9741

341.44075	14.5	9742

341.53975	0.5	9756

341.63874	0.5	9757.5

341.73774	3	9772

341.83674	0.5	9772.5

341.93574	2.5	9773

342.03473	0.5	9776

342.13373	4.5	9776.5

342.23273	7	9779

342.33172	0.5	9779.5

342.43072	0.5	9784

342.52972	2.5	9791

342.62871	0.5	9791.5

342.72771	3	9792

342.82671	7.5	9794.5

342.92571	0.5	9795

343.0247	0.5	9798

343.1237	4.5	9805.5

343.2227	1	9806

343.32169	0.5	9806.5

343.42069	0.5	9811

343.51969	25	9812

343.61869	2	9812.5

343.71768	1.5	9813

343.81668	8.5	9838

343.91568	0.5	9840

344.01467	11.5	9841.5

344.11367	2	9850

344.21267	1	9850.5

344.31166	0.5	9862

344.41066	5.5	9864

344.50966	1.5	9865

344.60866	8	9865.5

344.70765	0.5	9871

344.80665	1	9872.5

344.90565	13	9880.5

345.00464	1.5	9881

345.10364	0.5	9882

345.20264	0.5	9895

345.30163	12.5	9896.5

345.40063	1	9897

345.49963	8	9897.5

345.59863	3	9910

345.69762	1.5	9911

345.79662	1	9919

345.89562	7.5	9922

345.99461	0.5	9923.5

346.09361	17	9924.5

346.19261	0.5	9932

346.2916	2	9932.5

346.3906	0.5	9949.5

346.4896	3.5	9950

346.5886	0.5	9952

346.68759	0.5	9952.5

346.78659	7.5	9956

346.88559	1	9956.5

346.98458	0.5	9957

347.08358	17	9964.5

347.18258	2	9965.5

347.28157	4	9966

347.38057	6.5	9983

347.47957	0.5	9985

347.57857	0.5	9989

347.67756	1.5	9995.5

347.77656	1	9996

347.87556	2.5	9996.5

347.97455	3	9998

348.07355	11.5	9999

348.17255	0.5	10001.5

348.27155	1	10004.5

348.37054	14.5	10016

348.46954	0.5	10016.5

348.56854	3	10017.5

348.66753	8	10032

348.76653	2.5	10032.5

348.86553	1.5	10035.5

348.96452	12	10043.5

349.06352	1.5	10046

349.16252	3.5	10047.5

349.26152	0.5	10059.5

349.36051	9.5	10061

349.45951	8	10064.5

349.55851	1	10065

349.6575	0.5	10074.5

349.7565	1	10082.5

349.8555	1.5	10083.5

349.95449	0.5	10084

350.05349	1.5	10085

350.15249	3	10086.5

350.25149	1.5	10087

350.35048	0.5	10088.5

350.44948	1	10091.5

350.54848	17	10093

350.64747	1	10093.5

350.74647	3	10094.5

350.84547	10	10111.5

350.94447	0.5	10112.5

351.04346	1	10115.5

351.14246	14	10125.5

351.24146	1	10126

351.34045	2.5	10127

351.43945	12	10141

351.53845	1	10142

351.63744	2.5	10144.5

351.73644	13.5	10156.5

351.83544	0.5	10157.5

351.93444	2.5	10160

352.03343	8.5	10173.5

352.13243	1	10174

352.23143	1	10176.5

352.33042	1	10185

352.42942	4.5	10186

352.52842	3	10187

352.62741	24	10188

352.72641	1	10192.5

352.82541	1.5	10195.5

352.92441	6.5	10219.5

353.0234	1.5	10220.5

353.1224	20.5	10222

353.2214	1	10228.5

353.32039	0.5	10230

353.41939	0.5	10250.5

353.51839	6.5	10251.5

353.61738	1	10252

353.71638	1	10252.5

353.81538	1	10259

353.91438	1	10260

354.01337	5.5	10261

354.11237	18.5	10262

354.21137	5.5	10263

354.31036	0.5	10268.5

354.40936	0.5	10287

354.50836	0.5	10292.5

354.60736	10.5	10293

354.70635	0.5	10293.5

354.80535	0.5	10294

354.90435	0.5	10304.5

355.00334	10	10305

355.10234	0.5	10305.5

355.20134	0.5	10306

355.30033	1.5	10316

355.39933	1	10316.5

355.49833	0.5	10317

355.59733	1	10318.5

355.69632	1	10319.5

355.79532	14.5	10320

355.89432	0.5	10321

355.99331	5	10322

356.09231	1	10336.5

356.19131	0.5	10337

356.2903	6.5	10342

356.3893	5	10343

356.4883	0.5	10343.5

356.5873	1	10350

356.68629	9	10355

356.78529	1	10355.5

356.88429	6.5	10356.5

356.98328	3	10365.5

357.08228	2	10366.5

357.18128	1.5	10373

357.28027	6	10376

357.37927	2.5	10378

357.47827	11	10379.5

357.57727	0.5	10385.5

357.67626	1	10388

357.77526	4.5	10399

357.87426	4.5	10399.5

357.97325	5.5	10400.5

358.07225	10.5	10405

358.17125	1	10409.5

358.27025	3	10415

358.36924	5.5	10425.5

358.46824	2	10426.5

358.56724	0.5	10429.5

358.66623	6.5	10435

358.76523	2	10437

358.86423	1	10437.5

358.96322	1	10444

359.06222	0.5	10446

359.16122	3.5	10447

359.26022	2.5	10448

359.35921	1.5	10448.5

359.45821	6.5	10452

359.55721	0.5	10454.5

359.6562	0.5	10456

359.7552	0.5	10462.5

359.8542	3	10463

359.95319	13	10463.5

360.05219	2.5	10464

360.15119	1.5	10467

360.25019	12.5	10480

360.34918	0.5	10482.5

360.44818	0.5	10484

360.54718	21	10496.5

360.64617	0.5	10497

360.74517	22	10497.5

360.84417	1	10518.5

360.94316	0.5	10519

361.04216	11.5	10541

361.14116	0.5	10542

361.24016	5	10542.5

361.33915	6.5	10554

361.43815	0.5	10554.5

361.53715	5.5	10559.5

361.63614	5	10566

361.73514	2.5	10566.5

361.83414	0.5	10572

361.93314	6	10577

362.03213	1	10579.5

362.13113	4.5	10580

362.23013	7	10586

362.32912	1.5	10587

362.42812	16.5	10591.5

362.52712	0.5	10598.5

362.62611	0.5	10600

362.72511	0.5	10616.5

362.82411	6	10617

362.92311	1.5	10617.5

363.0221	0.5	10618

363.1211	10.5	10624

363.2201	7	10625.5

363.31909	4.5	10626

363.41809	0.5	10636.5

363.51709	23	10643.5

363.61608	1	10648

363.71508	4.5	10648.5

363.81408	3.5	10671.5

363.91308	0.5	10672.5

364.01207	7	10677

364.11107	11.5	10680.5

364.21007	8	10681

364.30906	2	10688

364.40806	2.5	10699.5

364.50706	6.5	10707.5

364.60606	1.5	10709.5

364.70505	14	10712

364.80405	0.5	10718.5

364.90305	1	10720

365.00204	1.5	10734

365.10104	13	10734.5

365.20004	1	10735.5

365.29903	1	10737

365.39803	1.5	10750

365.49703	0.5	10751

365.59603	17.5	10752

365.69502	1.5	10753.5

365.79402	0.5	10754

365.89302	19	10771.5

365.99201	0.5	10773

366.09101	1.5	10773.5

366.19001	3.5	10792.5

366.289	7.5	10793

366.388	0.5	10794.5

366.487	1	10798

366.586	11	10805.5

366.68499	1	10806

366.78399	3	10807

366.88299	1.5	10818

366.98198	0.5	10819

367.08098	1	10822

367.17998	5.5	10823.5

367.27897	0.5	10824

367.37797	1	10825

367.47697	0.5	10830.5

367.57597	9.5	10831

367.67496	0.5	10832

367.77396	0.5	10832.5

367.87296	4.5	10842

367.97195	1	10842.5

368.07095	5	10843

368.16995	9.5	10847.5

368.26894	2	10848.5

368.36794	18.5	10853.5

368.46694	1	10863

368.56594	0.5	10865

368.66493	9.5	10883.5

368.76393	0.5	10884.5

368.86293	1	10885

368.96192	14.5	10894.5

369.06092	2	10895

369.15992	5	10896

369.25892	9.5	10910.5

369.35791	0.5	10912.5

369.45691	1	10917.5

369.55591	2	10927

369.6549	1.5	10927.5

369.7539	1	10928.5

369.8529	13.5	10930.5

369.95189	0.5	10932

370.05089	8	10933

370.14989	7.5	10946.5

370.24889	9.5	10947

370.34788	3.5	10955

370.44688	11.5	10962.5

370.54588	6.5	10972

370.64487	1	10975.5

370.74387	11	10987

370.84287	0.5	10993.5

370.94186	0.5	10994.5

371.04086	2	11005.5

371.13986	5	11006

371.23886	2	11006.5

371.33785	16.5	11008.5

371.43685	3	11013.5

371.53585	1	11015.5

371.63484	18	11032

371.73384	1	11035

371.83284	7	11036

371.93184	0.5	11054

372.03083	1.5	11055

372.12983	1	11062

372.22883	5.5	11062.5

372.32782	2	11064

372.42682	1	11065

372.52582	10.5	11070.5

372.62481	2	11072.5

372.72381	6	11073.5

372.82281	1	11084

372.92181	0.5	11086

373.0208	2	11092

373.1198	3.5	11093

373.2188	1	11093.5

373.31779	0.5	11095.5

373.41679	1	11099

373.51579	1.5	11100

373.61478	0.5	11100.5

373.71378	0.5	11101.5

373.81278	1.5	11103

373.91178	3	11103.5

374.01077	0.5	11104

374.10977	1	11105.5

374.20877	5	11108.5

374.30776	9	11109

374.40676	2.5	11110

374.50576	23.5	11115

374.60475	1	11124

374.70375	13	11126.5

374.80275	11.5	11150

374.90175	1.5	11151

375.00074	1.5	11164

375.09974	0.5	11175.5

375.19874	5.5	11177

375.29773	4.5	11178.5

375.39673	0.5	11179

375.49573	2	11184.5

375.59473	12.5	11189

375.69372	3	11189.5

375.79272	19	11191.5

375.89172	1	11204

375.99071	3	11207

376.08971	2.5	11226

376.18871	0.5	11227

376.2877	1.5	11230

376.3867	4	11232.5

376.4857	1.5	11233

376.5847	1	11234.5

376.68369	3	11238.5

376.78269	2	11240

376.88169	0.5	11241

376.98068	0.5	11244

377.07968	4.5	11246

377.17868	8.5	11246.5

377.27767	0.5	11247

377.37667	15.5	11251.5

377.47567	1.5	11260

377.57467	0.5	11260.5

377.67366	0.5	11276

377.77266	22.5	11277.5

377.87166	2	11278

377.97065	0.5	11278.5

378.06965	8	11301

378.16865	0.5	11303

378.26764	10	11303.5

378.36664	0.5	11311.5

378.46564	3.5	11312

378.56464	6.5	11322

378.66363	0.5	11322.5

378.76263	17	11326

378.86163	0.5	11332.5

378.96062	0.5	11333

379.05962	11	11350

379.15862	4	11350.5

379.25762	0.5	11351

379.35661	3.5	11362

379.45561	1	11366

379.55461	0.5	11366.5

379.6536	6	11370

379.7526	1	11371

379.8516	3	11371.5

379.95059	8.5	11377.5

380.04959	1	11378.5

380.14859	0.5	11381.5

380.24759	6	11390

380.34658	2.5	11391

380.44558	0.5	11391.5

380.54458	0.5	11397.5

380.64357	5.5	11400

380.74257	3	11400.5

380.84157	16.5	11401

380.94056	7	11406.5

381.03956	6	11409.5

381.13856	0.5	11426

381.23756	15	11433

381.33655	1.5	11439

381.43555	1	11439.5

381.53455	4.5	11454.5

381.63354	4	11456

381.73254	0.5	11457

381.83154	6	11461.5

381.93053	3	11465.5

382.02953	5	11466

382.12853	1	11472

382.22753	21.5	11475

382.32652	0.5	11480

382.42552	3	11481

382.52452	4	11502.5

382.62351	0.5	11503

382.72251	5.5	11506

382.82151	2	11510

382.92051	1	11510.5

383.0195	5.5	11516

383.1185	4	11518

383.2175	0.5	11519

383.31649	1	11524.5

383.41549	7.5	11528.5

383.51449	6	11529

383.61348	0.5	11530

383.71248	8.5	11537.5

383.81148	1	11543.5

383.91048	3.5	11544

384.00947	4.5	11552.5

384.10847	0.5	11553.5

384.20747	6	11557

384.30646	6.5	11561.5

384.40546	0.5	11562

384.50446	22.5	11568

384.60345	1.5	11574.5

384.70245	0.5	11575

384.80145	4.5	11597.5

384.90045	6.5	11599

384.99944	0.5	11599.5

385.09844	1.5	11604

385.19744	5	11610.5

385.29643	0.5	11611

385.39543	2	11612.5

385.49443	5.5	11617.5

385.59343	3	11618

385.69242	4	11620

385.79142	0.5	11625.5

385.89042	1	11628.5

385.98941	1	11632.5

386.08841	16.5	11633

386.18741	0.5	11634

386.2864	2.5	11635

386.3854	2	11651.5

386.4844	15.5	11652

386.5834	1	11654.5

386.68239	2	11656.5

386.78139	1.5	11672

386.88039	1	11673

386.97938	1	11675

387.07838	2	11676.5

387.17738	1.5	11677.5

387.27637	1.5	11678.5

387.37537	0.5	11680.5

387.47437	3	11682

387.57337	5	11683.5

387.67236	0.5	11684

387.77136	0.5	11687

387.87036	1.5	11692

387.96935	2	11692.5

388.06835	0.5	11693

388.16735	0.5	11694.5

388.26634	12.5	11696.5

388.36534	0.5	11697

388.46434	1	11697.5

388.56334	1	11710

388.66233	1	11710.5

388.76133	1.5	11711.5

388.86033	2	11712.5

388.95932	0.5	11713.5

389.05832	3.5	11715

389.15732	0.5	11717

389.25631	6	11717.5

389.35531	9.5	11721

389.45431	0.5	11721.5

389.55331	1	11727.5

389.6523	5	11737

389.7513	0.5	11737.5

389.8503	0.5	11738.5

389.94929	0.5	11743.5

390.04829	2	11744

390.14729	2.5	11744.5

390.24629	1	11745

390.34528	0.5	11747

390.44428	7.5	11749.5

390.54328	1	11750.5

390.64227	1	11751

390.74127	0.5	11758.5

390.84027	11	11759.5

390.93926	14	11760.5

391.03826	2	11761

391.13726	3.5	11772

391.23626	0.5	11786

391.33525	18.5	11788

391.43425	18	11791.5

391.53325	0.5	11792

391.63224	3	11810.5

391.73124	4.5	11828.5

391.83024	2	11829

391.92923	0.5	11832

392.02823	1.5	11836.5

392.12723	1	11838.5

392.22623	0.5	11839

392.32522	3.5	11840.5

392.42422	1	11841.5

392.52322	10	11842

392.62221	0.5	11845.5

392.72121	2	11846.5

392.82021	2	11856.5

392.91921	0.5	11857

393.0182	1	11859

393.1172	7.5	11861

393.2162	0.5	11861.5

393.31519	1.5	11862.5

393.41419	0.5	11870

393.51319	4.5	11870.5

393.61218	5	11872

393.71118	3.5	11872.5

393.81018	1	11877

393.90918	1	11882

394.00817	5	11885.5

394.10717	6.5	11886.5

394.20617	1.5	11887.5

394.30516	1	11892.5

394.40416	4	11899

394.50316	4	11900.5

394.60215	5.5	11901.5

394.70115	10	11905.5

394.80015	16.5	11909.5

394.89915	0.5	11915

394.99814	12.5	11925

395.09714	1.5	11941.5

395.19614	1	11942

395.29513	10	11954.5

395.39413	0.5	11956

395.49313	1.5	11957

395.59212	1	11967

395.69112	16.5	11967.5

395.79012	0.5	11969

395.88912	7	11970

395.98811	1.5	11986.5

396.08711	7.5	11987

396.18611	1	11994

396.2851	0.5	11995.5

396.3841	0.5	12003

396.4831	10.5	12004

396.5821	3	12004.5

396.68109	1.5	12005

396.78009	1.5	12015.5

396.87909	1.5	12018.5

396.97808	2.5	12020

397.07708	1	12021.5

397.17608	1	12023

397.27507	0.5	12025.5

397.37407	1.5	12026.5

397.47307	5.5	12027.5

397.57207	1	12028

397.67106	3.5	12029.5

397.77006	8	12035

397.86906	1	12036

397.96805	14	12039.5

398.06705	0.5	12047.5

398.16605	17.5	12048.5

398.26504	1.5	12062.5

398.36404	15.5	12063

398.46304	1	12080.5

398.56204	10.5	12082

398.66103	7.5	12097.5

398.76003	8	12098.5

398.85903	6.5	12109

398.95802	13	12116.5

399.05702	1	12124.5

399.15602	0.5	12131

399.25501	2	12144

399.35401	0.5	12145

399.45301	1.5	12145.5

399.55201	0.5	12147.5

399.651	13.5	12148

399.75	4	12149.5

399.849	0.5	12150

399.94799	2	12163.5

400.04699	1.5	12167.5

400.14599	1.5	12168

400.24499	15.5	12170

400.34398	2.5	12171.5

400.44298	1	12173

400.54198	22	12188.5

400.64097	1	12191

400.73997	11.5	12192

400.83897	0.5	12214

400.93796	1.5	12215

401.03696	11.5	12226.5

401.13596	2	12227

401.23496	3	12228.5

401.33395	1.5	12240

401.43295	14.5	12242

401.53195	0.5	12245

401.63094	6	12246.5

401.72994	0.5	12261

401.82894	1.5	12261.5

401.92793	2.5	12267.5

402.02693	9	12268

402.12593	3.5	12269.5

402.22493	12.5	12272

402.32392	0.5	12281

402.42292	2	12284.5

402.52192	1	12297

402.62091	18.5	12297.5

402.71991	0.5	12299.5

402.81891	0.5	12300.5

402.9179	1	12319

403.0169	1	12319.5

403.1159	9	12320

403.2149	0.5	12321

403.31389	1	12322

403.41289	7.5	12331

403.51189	1	12331.5

403.61088	16	12332.5

403.70988	1	12340

403.80888	1	12341

403.90788	2.5	12357

404.00687	2	12358

404.10587	1	12359

404.20487	1.5	12361.5

404.30386	6	12363.5

404.40286	1.5	12364.5

404.50186	1.5	12366

404.60085	0.5	12372

404.69985	7.5	12373.5

404.79885	1.5	12375

404.89785	1	12375.5

404.99684	16.5	12383

405.09584	0.5	12384.5

405.19484	1.5	12385.5

405.29383	1.5	12402

405.39283	24	12402.5

405.49183	18	12404

405.59082	1	12405.5

405.68982	1.5	12429.5

405.78882	0.5	12447.5

405.88782	4	12448.5

405.98681	6.5	12450

406.08581	0.5	12450.5

406.18481	3	12454.5

406.2838	3.5	12461

406.3828	6	12461.5

406.4818	0.5	12464.5

406.58079	8.5	12468

406.67979	1.5	12474

406.77879	4.5	12474.5

406.87779	16	12483

406.97678	0.5	12484.5

407.07578	2	12489

407.17478	13.5	12505

407.27377	0.5	12505.5

407.37277	7.5	12507.5

407.47177	3.5	12521

407.57077	20	12521.5

407.66976	1	12529

407.76876	7.5	12532.5

407.86776	1	12552.5

407.96675	14	12553.5

408.06575	0.5	12561

408.16475	5.5	12562

408.26374	13	12576

408.36274	1.5	12576.5

408.46174	4	12582

408.56074	18	12595

408.65973	2.5	12596.5

408.75873	8	12600.5

408.85773	0.5	12618.5

408.95672	5	12621

409.05572	2.5	12629

409.15472	2	12629.5

409.25371	5	12634.5

409.35271	1	12637

409.45171	0.5	12639

409.55071	0.5	12644

409.6497	18	12645

409.7487	2	12645.5

409.8477	0.5	12646

409.94669	2	12664

410.04569	1	12666

410.14469	4	12666.5

410.24369	9	12668.5

410.34268	0.5	12669.5

410.44168	1.5	12673.5

410.54068	0.5	12682.5

410.63967	10	12683

410.73867	6	12684.5

410.83767	0.5	12685

410.93666	11.5	12695

411.03566	0.5	12701

411.13466	0.5	12701.5

411.23366	0.5	12713

411.33265	3	12713.5

411.43165	6.5	12714

411.53065	0.5	12714.5

411.62964	0.5	12717.5

411.72864	10.5	12724

411.82764	1.5	12724.5

411.92663	0.5	12725

412.02563	14.5	12735.5

412.12463	1.5	12737

412.22363	9.5	12737.5

412.32262	9	12752

412.42162	0.5	12753.5

412.52062	3.5	12763

412.61961	12.5	12772

412.71861	0.5	12772.5

412.81761	1	12776

412.9166	1.5	12788.5

413.0156	4	12789

413.1146	0.5	12790

413.2136	0.5	12791.5

413.31259	8.5	12795.5

413.41159	4.5	12796

413.51059	0.5	12796.5

413.60958	1	12805

413.70858	17.5	12809.5

413.80758	0.5	12810

413.90657	0.5	12811

414.00557	4.5	12828.5

414.10457	4	12829

414.20357	2	12829.5

414.30256	5.5	12834

414.40156	1	12838

414.50056	13.5	12840

414.59955	3.5	12845.5

414.69855	0.5	12846.5

414.79755	0.5	12860

414.89655	0.5	12863.5

414.99554	0.5	12864

415.09454	1	12864.5

415.19354	2.5	12865

415.29253	2	12865.5

415.39153	8.5	12866.5

415.49053	12.5	12869

415.58952	13.5	12871

415.68852	1	12879.5

415.78752	9	12892

415.88652	2	12905.5

415.98551	13	12906.5

416.08451	5.5	12915.5

416.18351	0.5	12917.5

416.2825	14	12930.5

416.3815	0.5	12936

416.4805	6.5	12936.5

416.57949	7.5	12950.5

416.67849	0.5	12951

416.77749	9.5	12957.5

416.87649	1.5	12965

416.97548	0.5	12965.5

417.07448	0.5	12975

417.17348	9.5	12976.5

417.27247	0.5	12977

417.37147	1	12977.5

417.47047	10	12987

417.56947	1.5	12987.5

417.66846	0.5	12988.5

417.76746	5.5	12998.5

417.86646	8	13000

417.96545	0.5	13000.5

418.06445	2	13006

418.16345	9	13014

418.26244	3.5	13014.5

418.36144	6.5	13016.5

418.46044	8	13025.5

418.55944	3	13029

418.65843	1	13035.5

418.75743	0.5	13043.5

418.85643	13	13046.5

418.95542	0.5	13047.5

419.05442	11	13048

419.15342	4	13061

419.25241	2	13061.5

419.35141	0.5	13072.5

419.45041	5	13076.5

419.54941	1	13078.5

419.6484	3.5	13079

419.7474	9.5	13084

419.8464	3	13085

419.94539	8	13088.5

420.04439	1	13098

420.14339	0.5	13101

420.24238	11.5	13109

420.34138	0.5	13110

420.44038	12	13110.5

420.53938	0.5	13122

420.63837	1.5	13122.5

420.73737	9.5	13134.5

420.83637	0.5	13135

420.93536	0.5	13136.5

421.03436	8.5	13146

421.13336	1	13146.5

421.23236	3.5	13147

421.33135	1.5	13155.5

421.43035	2	13156.5

421.52935	1	13160

421.62834	1	13161.5

421.72734	24	13163.5

421.82634	1.5	13164.5

421.92533	4	13165.5

422.02433	0.5	13189.5

422.12333	2	13191

422.22233	1	13195

422.32132	8	13195.5

422.42032	1	13197.5

422.51932	8	13198.5

422.61831	2	13206.5

422.71731	1	13207.5

422.81631	1	13215.5

422.9153	22.5	13217.5

423.0143	0.5	13218.5

423.1133	13.5	13219.5

423.2123	0.5	13242

423.31129	1.5	13242.5

423.41029	10.5	13256

423.50929	15	13256.5

423.60828	0.5	13258

423.70728	0.5	13268.5

423.80628	0.5	13283.5

423.90527	17	13284

424.00427	1	13284.5

424.10327	13.5	13285

424.20227	1	13302

424.30126	2	13303

424.40026	13.5	13316.5

424.49926	0.5	13317.5

424.59825	12.5	13319.5

424.69725	2.5	13333

424.79625	7	13333.5

424.89525	0.5	13346

424.99424	2	13348.5

425.09324	0.5	13355.5

425.19224	4	13356

425.29123	0.5	13358

425.39023	9	13358.5

425.48923	2.5	13362.5

425.58822	2.5	13363

425.68722	0.5	13372

425.78622	0.5	13374.5

425.88522	16	13377

425.98421	1.5	13377.5

426.08321	0.5	13378

426.18221	20.5	13394

426.2812	13	13395.5

426.3802	1	13396

426.4792	2	13416.5

426.57819	5.5	13429.5

426.67719	0.5	13430.5

426.77619	3	13432.5

426.87519	8.5	13438

426.97418	1	13438.5

427.07318	0.5	13441.5

427.17218	6.5	13450

427.27117	1	13451

427.37017	1.5	13451.5

427.46917	0.5	13458

427.56816	17.5	13459

427.66716	1.5	13460.5

427.76616	0.5	13461

427.86516	11	13478.5

427.96415	2.5	13480

428.06315	2	13480.5

428.16215	0.5	13491.5

428.26114	6	13494

428.36014	0.5	13496

428.45914	3	13496.5

428.55814	11	13502.5

428.65713	0.5	13503

428.75613	1	13506

428.85513	7.5	13517

428.95412	0.5	13517.5

429.05312	18	13518.5

429.15212	0.5	13526

429.25111	1.5	13526.5

429.35011	1	13544.5

429.44911	8.5	13545

429.54811	0.5	13546.5

429.6471	0.5	13547.5

429.7461	0.5	13556

429.8451	15	13556.5

429.94409	3.5	13557

430.04309	4.5	13557.5

430.14209	1.5	13572.5

430.24108	1	13576

430.34008	1.5	13580.5

430.43908	6	13582

430.53808	3	13583

430.63707	0.5	13584.5

430.73607	17	13590.5

430.83507	0.5	13593.5

430.93406	7.5	13594

431.03306	10	13611

431.13206	3	13611.5

431.23106	0.5	13619

431.33005	1	13629

431.42905	19.5	13632

431.52805	25	13632.5

431.62704	0.5	13633.5

431.72604	14.5	13653

431.82504	1	13678

431.92403	2	13678.5

432.02303	11	13693

432.12203	1	13694

432.22103	7	13696

432.32002	1.5	13707

432.41902	0.5	13708

432.51802	0.5	13715

432.61701	0.5	13716.5

432.71601	12	13717

432.81501	0.5	13717.5

432.914	13	13718

433.013	4	13730

433.112	0.5	13730.5

433.211	0.5	13743.5

433.30999	19	13747.5

433.40899	1	13748

433.50799	12.5	13748.5

433.60698	1	13767.5

433.70598	19	13768.5

433.80498	0.5	13781

433.90397	11	13782

434.00297	1	13801

434.10197	19	13801.5

434.20097	1	13812.5

434.29996	14.5	13813.5

434.39896	1.5	13832.5

434.49796	17	13833.5

434.59695	0.5	13848

434.69595	3.5	13849.5

434.79495	5.5	13866.5

434.89394	3	13867

434.99294	21	13870.5

435.09194	0.5	13876

435.19094	0.5	13879

435.28993	14	13900

435.38893	2	13900.5

435.48793	1	13901

435.58692	0.5	13915

435.68592	17	13917

435.78492	2	13918

435.88392	1	13918.5

435.98291	1	13935.5

436.08191	17	13937.5

436.18091	0.5	13938.5

436.2799	3.5	13939.5

436.3789	2.5	13956.5

436.4779	1.5	13957

436.57689	8.5	13960.5

436.67589	1	13963

436.77489	2	13964.5

436.87389	0.5	13973

436.97288	5.5	13974

437.07188	5	13976

437.17088	8.5	13976.5

437.26987	0.5	13982

437.36887	11.5	13987

437.46787	1.5	13995.5

437.56686	0.5	13996

437.66586	21	14007.5

437.76486	0.5	14009

437.86386	0.5	14009.5

437.96285	0.5	14030.5

438.06185	4.5	14031

438.16085	15	14031.5

438.25984	1	14032

438.35884	1	14036.5

438.45784	1.5	14051.5

438.55684	14.5	14052.5

438.65583	0.5	14053.5

438.75483	10.5	14055

438.85383	1.5	14069.5

438.95282	12	14070

439.05182	2	14080.5

439.15082	0.5	14082

439.24981	2	14094

439.34881	11.5	14096

439.44781	0.5	14096.5

439.54681	14.5	14098.5

439.6458	3.5	14110

439.7448	0.5	14110.5

439.8438	0.5	14125

439.94279	3	14128.5

440.04179	0.5	14129

440.14079	5	14129.5

440.23978	14.5	14132.5

440.33878	2.5	14133

440.43778	5.5	14138

440.53678	16.5	14152.5

440.63577	11	14155

440.73477	3	14160.5

440.83377	15.5	14177

440.93276	0.5	14188

441.03176	13.5	14191

441.13076	2	14206.5

441.22975	1	14207

441.32875	9.5	14220.5

441.42775	1.5	14222.5

441.52675	0.5	14223.5

441.62574	14	14233

441.72474	1.5	14234.5

441.82374	13	14235

441.92273	1	14249

442.02173	0.5	14250.5

442.12073	15	14263.5

442.21973	1	14264.5

442.31872	5.5	14265

442.41772	2	14280

442.51672	2.5	14281

442.61571	7	14286.5

442.71471	2.5	14288.5

442.81371	1.5	14291

442.9127	19	14298

443.0117	0.5	14300.5

443.1107	16.5	14302

443.2097	0.5	14321

443.30869	11.5	14321.5

443.40769	14	14338

443.50669	1	14338.5

443.60568	10.5	14350

443.70468	0.5	14364

443.80368	8	14365

443.90267	0.5	14375.5

444.00167	1	14376

444.10067	0.5	14384

444.19967	4.5	14384.5

444.29866	2.5	14385.5

444.39766	0.5	14386

444.49666	0.5	14390.5

444.59565	19	14393

444.69465	1.5	14393.5

444.79365	7	14394

444.89264	3.5	14413

444.99164	7.5	14414.5

445.09064	0.5	14421.5

445.18964	0.5	14425

445.28863	13	14432.5

445.38763	0.5	14433

445.48663	7	14433.5

445.58562	1.5	14446.5

445.68462	1	14447

445.78362	0.5	14454

445.88262	14	14455.5

445.98161	2	14456.5

446.08061	0.5	14457

446.17961	9	14471

446.2786	0.5	14473

446.3776	3.5	14473.5

446.4766	9.5	14482.5

446.57559	0.5	14483

446.67459	1.5	14486.5

446.77359	5	14496

446.87259	1.5	14496.5

446.97158	0.5	14498

447.07058	0.5	14503

447.16958	3	14504.5

447.26857	18.5	14505

447.36757	1	14505.5

447.46657	17.5	14508.5

447.56556	0.5	14527

447.66456	18	14528

447.76356	1.5	14545.5

447.86256	18.5	14546

447.96155	4	14564

448.06055	25.5	14565.5

448.15955	18.5	14584

448.25854	1	14588

448.35754	0.5	14613.5

448.45654	20	14632

448.55553	16.5	14633

448.65453	1.5	14633.5

448.75353	1	14653.5

448.85253	12.5	14670

448.95152	16.5	14671.5

449.05052	0.5	14672.5

449.14952	2	14685

449.24851	3.5	14701.5

449.34751	5.5	14702

449.44651	2	14704

449.54551	0.5	14707.5

449.6445	2.5	14713

449.7435	2.5	14715

449.8425	2	14715.5

449.94149	1	14718

450.04049	2.5	14720.5

450.13949	11.5	14722.5

450.23848	1.5	14723.5

450.33748	2	14726

450.43648	4.5	14737.5

450.53548	4.5	14739

450.63447	0.5	14741

450.73347	26	14745.5

450.83247	0.5	14750

450.93146	1	14750.5

451.03046	14	14776.5

451.12946	1.5	14777

451.22845	16.5	14778

451.32745	0.5	14792

451.42645	0.5	14793.5

451.52545	3	14810

451.62444	7.5	14810.5

451.72344	1.5	14811

451.82244	0.5	14814

451.92143	14.5	14821.5

452.02043	2	14823

452.11943	1	14823.5

452.21843	2	14838

452.31742	1	14840

452.41642	5	14841

452.51542	7	14843

452.61441	0.5	14844

452.71341	0.5	14849

452.81241	7	14856

452.9114	5	14856.5

453.0104	0.5	14857

453.1094	2	14864

453.2084	0.5	14869

453.30739	1.5	14869.5

453.40639	1	14871.5

453.50539	2.5	14872

453.60438	0.5	14873.5

453.70338	3	14874.5

453.80238	0.5	14877

453.90137	2.5	14877.5

454.00037	0.5	14880.5

454.09937	0.5	14881

454.19837	1	14883.5

454.29736	8	14884

454.39636	0.5	14884.5

454.49536	0.5	14885.5

454.59435	8	14893.5

454.69335	2.5	14894

454.79235	2	14894.5

454.89134	0.5	14902.5

454.99034	16	14905

455.08934	4	14907

455.18834	2	14907.5

455.28733	1	14923.5

455.38633	3.5	14927.5

455.48533	2	14929.5

455.58432	1.5	14930.5

455.68332	11.5	14934

455.78232	0.5	14936

455.88131	4	14937.5

455.98031	11.5	14949

456.07931	0.5	14949.5

456.17831	0.5	14953.5

456.2773	9.5	14965

456.3763	7.5	14965.5

456.4753	1.5	14966

456.57429	18	14975.5

456.67329	13.5	14983

456.77229	0.5	14984.5

456.87129	15.5	15002.5

456.97028	19	15016

457.06928	0.5	15016.5

457.16828	1.5	15032

457.26727	7	15051

457.36627	1	15051.5

457.46527	0.5	15053

457.56426	3.5	15060

457.66326	7	15061

457.76226	22.5	15061.5

457.86126	0.5	15065

457.96025	0.5	15072

458.05925	10	15094.5

458.15825	0.5	15095

458.25724	3.5	15095.5

458.35624	2.5	15105.5

458.45524	0.5	15106

458.55423	1	15109.5

458.65323	0.5	15112

458.75223	17.5	15112.5

458.85123	0.5	15113.5

458.95022	1	15114

459.04922	9.5	15131.5

459.14822	1	15132

459.24721	1	15133

459.34621	7	15142.5

459.44521	1.5	15143.5

459.54421	1	15144.5

459.6432	8.5	15151.5

459.7422	1	15153

459.8412	2.5	15154

459.94019	3	15162.5

460.03919	1	15163.5

460.13819	5	15166

460.23718	1	15169

460.33618	13.5	15170

460.43518	0.5	15175

460.53418	19	15176

460.63317	0.5	15189.5

460.73217	0.5	15190

460.83117	10	15209

460.93016	1.5	15209.5

461.02916	0.5	15210

461.12816	6.5	15220

461.22715	1	15221.5

461.32615	3.5	15222

461.42515	0.5	15228.5

461.52415	9.5	15229.5

461.62314	1.5	15233

461.72214	0.5	15233.5

461.82114	1	15243

461.92013	2.5	15244.5

462.01913	0.5	15245

462.11813	15.5	15246

462.21712	0.5	15248.5

462.31612	11	15249

462.41512	8.5	15264.5

462.51412	3	15265

462.61311	5	15276

462.71211	7	15284.5

462.81111	1	15287.5

462.9101	1	15292.5

463.0091	1	15299.5

463.1081	0.5	15300.5

463.2071	2	15301.5

463.30609	9	15302.5

463.40509	1	15303

463.50409	2	15305

463.60308	0.5	15314

463.70208	1.5	15315

463.80108	1.5	15317

463.90007	1	15317.5

463.99907	9.5	15319

464.09807	0.5	15320.5

464.19707	24.5	15321.5

464.29606	1.5	15331

464.39506	0.5	15331.5

464.49406	19	15356

464.59305	0.5	15357.5

464.69205	0.5	15358

464.79105	0.5	15377

464.89004	3	15377.5

464.98904	6	15378

465.08804	1	15378.5

465.18704	2.5	15381.5

465.28603	8.5	15387.5

465.38503	0.5	15388.5

465.48403	0.5	15391

465.58302	21.5	15399.5

465.68202	0.5	15400

465.78102	3	15400.5

465.88001	2.5	15422

465.97901	21	15422.5

466.07801	4	15425.5

466.17701	3.5	15428

466.276	0.5	15449

466.375	3	15453

466.474	1.5	15456.5

466.57299	2	15457

466.67199	0.5	15460

466.77099	3.5	15461.5

466.86999	1.5	15463.5

466.96898	0.5	15464

467.06798	2	15467.5

467.16698	13	15469

467.26597	0.5	15469.5

467.36497	0.5	15471.5

467.46397	4	15484.5

467.56296	2	15485

467.66196	0.5	15485.5

467.76096	2.5	15489.5

467.85996	7	15491.5

467.95895	14	15492

468.05795	2	15494.5

468.15695	7.5	15501.5

468.25594	2	15515.5

468.35494	4.5	15517.5

468.45394	2.5	15525

468.55293	1	15527

468.65193	0.5	15531.5

468.75093	0.5	15534

468.84993	12.5	15535

468.94892	3	15535.5

469.04792	6.5	15536

469.14692	5	15548.5

469.24591	6.5	15551.5

469.34491	0.5	15558

469.44391	4.5	15563

469.5429	0.5	15569.5

469.6419	13	15570

469.7409	1	15574.5

469.8399	21.5	15575

469.93889	0.5	15588

470.03789	13.5	15589

470.13689	1	15610.5

470.23588	1.5	15611

470.33488	6.5	15624.5

470.43388	13	15625.5

470.53288	0.5	15627

470.63187	10	15633.5

470.73087	1	15646.5

470.82987	19.5	15647

470.92886	1	15657

471.02786	0.5	15658

471.12686	1.5	15677.5

471.22585	2	15678.5

471.32485	0.5	15679

471.42385	5	15680.5

471.52285	1	15682.5

471.62184	1	15683

471.72084	1	15688

471.81984	4	15689

471.91883	3	15690

472.01783	0.5	15691

472.11683	0.5	15695

472.21582	4.5	15698

472.31482	1.5	15698.5

472.41382	1.5	15699

472.51282	0.5	15703.5

472.61181	5.5	15705

472.71081	0.5	15706.5

472.80981	2	15707

472.9088	9.5	15712.5

473.0078	5	15713

473.1068	0.5	15715

473.20579	1	15724.5

473.30479	1	15729.5

473.40379	4	15730

473.50279	1	15731

473.60178	0.5	15732

473.70078	6	15736

473.79978	9	15737

473.89877	2.5	15737.5

473.99777	22	15743.5

474.09677	14	15752.5

474.19577	0.5	15755

474.29476	5	15777

474.39376	6	15791

474.49276	2	15791.5

474.59175	1	15796.5

474.69075	0.5	15802.5

474.78975	8	15804.5

474.88874	13.5	15805.5

474.98774	0.5	15806

475.08674	1.5	15814

475.18574	5	15827.5

475.28473	0.5	15828

475.38373	4.5	15829.5

475.48273	1	15834.5

475.58172	0.5	15835

475.68072	1	15839.5

475.77972	1.5	15840.5

475.87871	3	15841

475.97771	1	15842

476.07671	1.5	15843.5

476.17571	1	15846.5

476.2747	2	15847.5

476.3737	2	15849

476.4727	2.5	15850

476.57169	12	15852

476.67069	0.5	15854

476.76969	3.5	15856.5

476.86869	3	15868.5

476.96768	0.5	15869

477.06668	9	15872.5

477.16568	2.5	15875.5

477.26467	11.5	15876

477.36367	0.5	15885

477.46267	2.5	15887.5

477.56166	1.5	15899

477.66066	20.5	15899.5

477.75966	3	15902

477.85866	6	15903.5

477.95765	0.5	15924

478.05665	0.5	15927

478.15565	0.5	15933

478.25464	1	15933.5

478.35364	2.5	15934

478.45264	0.5	15934.5

478.55163	0.5	15935.5

478.65063	0.5	15938

478.74963	1.5	15938.5

478.84863	5.5	15939

478.94762	9	15939.5

479.04662	2	15941

479.14562	6.5	15946.5

479.24461	4.5	15955.5

479.34361	1.5	15957.5

479.44261	5.5	15964

479.5416	8.5	15968.5

479.6406	1.5	15970

479.7396	0.5	15975.5

479.8386	1	15984

479.93759	14.5	15985.5

480.03659	0.5	15986

480.13559	0.5	15987

480.23458	5.5	16001.5

480.33358	5	16002

480.43258	1.5	16002.5

480.53157	9	16008

480.63057	1.5	16013

480.72957	2.5	16014.5

480.82857	1	16023.5

480.92756	3.5	16025

481.02656	0.5	16027.5

481.12556	0.5	16028.5

481.22455	8.5	16032

481.32355	0.5	16032.5

481.42255	14	16033

481.52155	0.5	16041.5

481.62054	25.5	16042

481.71954	2	16056

481.81854	5.5	16056.5

481.91753	0.5	16082

482.01653	5	16084

482.11553	5.5	16089.5

482.21452	1	16090

482.31352	18	16095

482.41252	1	16100.5

482.51152	26	16101.5

482.61051	0.5	16119.5

482.70951	4	16120.5

482.80851	5	16146.5

482.9075	1.5	16147

483.0065	5.5	16151

483.1055	3.5	16156

483.20449	2	16157.5

483.30349	0.5	16163

483.40249	8	16166.5

483.50149	0.5	16168.5

483.60048	4.5	16169

483.69948	2.5	16177

483.79848	4.5	16177.5

483.89747	2.5	16182

483.99647	0.5	16184.5

484.09547	0.5	16189

484.19447	8	16191.5

484.29346	0.5	16192

484.39246	3	16192.5

484.49146	3.5	16200.5

484.59045	0.5	16201

484.68945	0.5	16204

484.78845	21.5	16207.5

484.88744	0.5	16208

484.98644	1	16208.5

485.08544	3.5	16230

485.18444	8	16230.5

485.28343	21	16231.5

485.38243	1	16235

485.48143	0.5	16243

485.58042	10.5	16264

485.67942	0.5	16265

485.77842	7	16265.5

485.87741	18	16276

485.97641	15	16276.5

486.07541	2.5	16283.5

486.17441	6	16301.5

486.2734	0.5	16316.5

486.3724	1	16319

486.4714	1.5	16325

486.57039	11	16325.5

486.66939	1	16326.5

486.76839	1.5	16328

486.86738	5	16339

486.96638	12	16340

487.06538	0.5	16341.5

487.16438	19	16346.5

487.26337	1	16358.5

487.36237	0.5	16359

487.46137	16	16378

487.56036	1	16379

487.65936	11.5	16379.5

487.75836	1.5	16395.5

487.85736	17	16396.5

487.95635	0.5	16408

488.05535	1	16409.5

488.15435	0.5	16426.5

488.25334	12.5	16427

488.35234	1	16428

488.45134	18	16428.5

488.55033	0.5	16441

488.64933	0.5	16442

488.74833	8.5	16460

488.84733	21.5	16460.5

488.94632	0.5	16461

489.04532	5.5	16469.5

489.14432	0.5	16491

489.24331	0.5	16491.5

489.34231	12	16497

489.44131	1	16497.5

489.5403	11.5	16498

489.6393	0.5	16510

489.7383	3	16511

489.8373	19	16522.5

489.93629	11	16523

490.03529	6.5	16526

490.13429	3	16545

490.23328	1.5	16556

490.33228	14	16562.5

490.43128	2	16565.5

490.53027	9.5	16567

490.62927	16.5	16581

490.72827	7.5	16583

490.82727	4	16592.5

490.92626	1	16609

491.02526	0.5	16616.5

491.12426	0.5	16620.5

491.22325	0.5	16621.5

491.32225	13.5	16622

491.42125	8	16622.5

491.52025	0.5	16623

491.61924	4	16636.5

491.71824	2.5	16644.5

491.81724	1	16645

491.91623	5.5	16649

492.01523	3	16651.5

492.11423	0.5	16652.5

492.21322	0.5	16658

492.31222	0.5	16661

492.41122	22	16661.5

492.51022	9.5	16662

492.60921	17	16662.5

492.70821	0.5	16684.5

492.80721	11	16694

492.9062	0.5	16711

493.0052	2.5	16711.5

493.1042	4	16722.5

493.20319	2	16723

493.30219	0.5	16725.5

493.40119	18	16729.5

493.50019	8.5	16731.5

493.59918	0.5	16732

493.69818	8.5	16750

493.79718	2	16758.5

493.89617	0.5	16759

493.99517	18	16767.5

494.09417	0.5	16769.5

494.19316	1	16770

494.29216	11	16788

494.39116	2	16788.5

494.49016	1	16789.5

494.58915	0.5	16800.5

494.68815	8	16802.5

494.78715	3.5	16803.5

494.88614	0.5	16804

494.98514	1	16812

495.08414	15	16815.5

495.18314	2	16816

495.28213	9	16817

495.38113	6.5	16832

495.48013	1.5	16834

495.57912	0.5	16843

495.67812	0.5	16849.5

495.77712	10.5	16851

495.87611	1	16851.5

495.97511	17.5	16852

496.07411	2.5	16862.5

496.17311	13.5	16863.5

496.2721	1	16881

496.3711	6	16883.5

496.4701	2	16897

496.56909	0.5	16898

496.66809	12.5	16904

496.76709	24	16906

496.86608	2	16906.5

496.96508	2.5	16919

497.06408	13	16943

497.16308	1	16945

497.26207	5	16947.5

497.36107	2.5	16960.5

497.46007	0.5	16961.5

497.55906	1.5	16966.5

497.65806	3	16969

497.75706	10	16969.5

497.85606	1	16971

497.95505	19.5	16974

498.05405	1	16984

498.15305	5	16985

498.25204	1	17004.5

498.35104	2	17005.5

498.45004	9.5	17010.5

498.54903	0.5	17011.5

498.64803	0.5	17013.5

498.74703	11.5	17023

498.84603	0.5	17023.5

498.94502	4	17024

499.04402	2	17035.5

499.14302	4.5	17036

499.24201	13	17040

499.34101	0.5	17042

499.44001	0.5	17046.5

499.539	5	17059.5

499.638	1	17060

499.737	1	17060.5

499.836	13	17065.5

499.93499	12.5	17066.5

500.03399	1.5	17067.5

500.13299	8	17080.5

500.23198	0.5	17093

500.33098	1.5	17094.5

500.42998	0.5	17102.5

500.52897	1	17103

500.62797	0.5	17104.5

500.72697	1	17105

500.82597	2	17106

500.92496	6.5	17106.5

501.02396	12.5	17107.5

501.12296	0.5	17109.5

501.22195	13.5	17116

501.32095	0.5	17128.5

501.41995	3.5	17129

501.51894	5.5	17142.5

501.61794	2.5	17143

501.71694	0.5	17146.5

501.81594	0.5	17152

501.91493	13.5	17154.5

502.01393	11.5	17155

502.11293	1.5	17155.5

502.21192	0.5	17169

502.31092	12	17180.5

502.40992	1	17182

502.50892	1	17182.5

502.60791	0.5	17194.5

502.70691	0.5	17195.5

502.80591	0.5	17196.5

502.9049	7	17197

503.0039	1.5	17197.5

503.1029	4	17198

503.20189	1.5	17205

503.30089	4	17206.5

503.39989	0.5	17210.5

503.49889	22.5	17212

503.59788	0.5	17216

503.69688	1	17216.5

503.79588	3	17239

503.89487	23.5	17239.5

503.99387	0.5	17240.5

504.09287	0.5	17243.5

504.19186	3.5	17267

504.29086	3	17267.5

504.38986	0.5	17268

504.48886	3	17271.5

504.58785	3	17274.5

504.68685	1.5	17275

504.78585	0.5	17278

504.88484	2.5	17281

504.98384	10	17282.5

505.08284	8.5	17283

505.18184	5	17285.5

505.28083	0.5	17295.5

505.37983	0.5	17304

505.47883	6	17309

505.57782	3.5	17309.5

505.67682	0.5	17310

505.77582	1.5	17316

505.87481	10.5	17319.5

505.97381	13.5	17320

506.07281	0.5	17321.5

506.17181	0.5	17332

506.2708	2	17345.5

506.3698	7	17346

506.4688	1.5	17346.5

506.56779	0.5	17348.5

506.66679	0.5	17355.5

506.76579	0.5	17357

506.86478	0.5	17357.5

506.96378	7.5	17358

507.06278	0.5	17358.5

507.16178	0.5	17359

507.26077	1.5	17366.5

507.35977	8.5	17367

507.45877	0.5	17367.5

507.55776	5.5	17369

507.65676	1.5	17377.5

507.75576	1	17378

507.85475	0.5	17383.5

507.95375	1.5	17385

508.05275	8.5	17386

508.15175	2.5	17386.5

508.25074	0.5	17388

508.34974	1	17396.5

508.44874	10.5	17399

508.54773	0.5	17399.5

508.64673	11.5	17400.5

508.74573	1	17411

508.84473	0.5	17411.5

508.94372	8.5	17423

509.04272	6	17424

509.14172	0.5	17424.5

509.24071	5.5	17433

509.33971	4	17439

509.43871	0.5	17439.5

509.5377	9	17445

509.6367	0.5	17449

509.7357	0.5	17449.5

509.8347	7.5	17458.5

509.93369	0.5	17459

510.03269	1.5	17459.5

510.13169	12.5	17467

510.23068	9.5	17467.5

510.32968	1.5	17469

510.42868	1	17481.5

510.52767	3	17491

510.62667	2.5	17492.5

510.72567	7	17493.5

510.82467	0.5	17496.5

510.92366	14.5	17499

511.02266	1	17506

511.12166	0.5	17506.5

511.22065	4.5	17521

511.31965	5	17522

511.41865	4	17522.5

511.51764	3.5	17527

511.61664	0.5	17532

511.71564	0.5	17536

511.81464	4.5	17539.5

511.91363	0.5	17540

512.01263	9.5	17540.5

512.11163	9	17545

512.21062	0.5	17545.5

512.30962	2	17555

512.40862	1.5	17564

512.50762	0.5	17564.5

512.60661	13	17566.5

512.70561	0.5	17568

512.80461	1	17568.5

512.9036	6	17581.5

513.0026	2	17582

513.1016	3.5	17583

513.20059	1.5	17589

513.29959	0.5	17591

513.39859	0.5	17594.5

513.49759	11	17596

513.59658	4.5	17596.5

513.69558	0.5	17597

513.79458	17	17608

513.89357	1	17612.5

513.99257	0.5	17613

514.09157	7.5	17630

514.19056	1	17631

514.28956	15.5	17631.5

514.38856	0.5	17639

514.48756	1	17640

514.58655	7.5	17655.5

514.68555	1.5	17656

514.78455	0.5	17657

514.88354	15.5	17664.5

514.98254	16.5	17666

515.08154	0.5	17666.5

515.18053	2	17682

515.27953	0.5	17698.5

515.37853	15	17699

515.47753	4.5	17701

515.57652	1	17701.5

515.67552	3.5	17716.5

515.77452	14	17721

515.87351	0.5	17722

515.97251	19.5	17725.5

516.07151	0.5	17739.5

516.17051	1.5	17740

516.2695	10.5	17759.5

516.3685	0.5	17760

516.4675	0.5	17761.5

516.56649	1	17772

516.66549	4	17772.5

516.76449	0.5	17773

516.86348	4.5	17774

516.96248	4	17778

517.06148	2	17778.5

517.16048	5	17783

517.25947	0.5	17787

517.35847	7	17789

517.45747	2	17794

517.55646	1.5	17794.5

517.65546	5.5	17801.5

517.75446	8	17803.5

517.85345	0.5	17805

517.95245	10.5	17810.5

518.05145	1.5	17818.5

518.15045	2	17819

518.24944	8.5	17829.5

518.34844	14	17831

518.44744	1.5	17833

518.54643	19	17841.5

518.64543	4	17855.5

518.74443	1	17857

518.84343	12.5	17876

518.94242	6	17880

519.04142	2.5	17881

519.14042	1	17893.5

519.23941	12	17899.5

519.33841	0.5	17902

519.43741	0.5	17903

519.5364	0.5	17915

519.6354	4.5	17915.5

519.7344	1	17916

519.8334	12.5	17916.5

519.93239	0.5	17921

520.03139	9	17922

520.13039	0.5	17934.5

520.22938	1.5	17935

520.32838	10	17944

520.42738	0.5	17944.5

520.52637	0.5	17946

520.62537	8	17956

520.72437	0.5	17956.5

520.82337	0.5	17957

520.92236	6	17965

521.02136	2.5	17965.5

521.12036	22	17966

521.21935	0.5	17972

521.31835	19.5	17974.5

521.41735	14.5	17996.5

521.51634	0.5	17997

521.61534	0.5	18016.5

521.71434	4	18031

521.81334	1	18031.5

521.91233	2.5	18032

522.01133	3.5	18036

522.11033	0.5	18037

522.20932	6.5	18039.5

522.30832	0.5	18043

522.40732	21.5	18043.5

522.50631	0.5	18050

522.60531	5.5	18050.5

522.70431	0.5	18072

522.80331	0.5	18072.5

522.9023	12	18078

523.0013	1	18078.5

523.1003	13.5	18079

523.19929	2	18091

523.29829	8	18092

523.39729	0.5	18105.5

523.49629	6	18107.5

523.59528	1	18115.5

523.69428	2.5	18116

523.79328	7.5	18122

523.89227	1	18123

523.99127	9.5	18125.5

524.09027	0.5	18133

524.18926	3	18134

524.28826	6	18143.5

524.38726	0.5	18144

524.48626	2	18147

524.58525	5.5	18153

524.68425	0.5	18153.5

524.78325	17.5	18155.5

524.88224	15	18161

524.98124	4	18161.5

525.08024	0.5	18179

525.17923	0.5	18194

525.27823	1	18198

525.37723	3	18198.5

525.47623	6.5	18199

525.57522	1.5	18200

525.67422	2	18203

525.77322	2.5	18209.5

525.87221	0.5	18211

525.97121	0.5	18213

526.07021	13	18215.5

526.16921	2.5	18216

526.2682	0.5	18216.5

526.3672	1.5	18229.5

526.4662	6.5	18232

526.56519	3	18232.5

526.66419	1.5	18234

526.76319	0.5	18240.5

526.86218	0.5	18243.5

526.96118	12	18245

527.06018	2	18245.5

527.15918	1	18246

527.25817	1.5	18258

527.35717	0.5	18260

527.45617	1	18261

527.55516	7	18262.5

527.65416	5	18263

527.75316	21	18264

527.85215	0.5	18271

527.95115	0.5	18276

528.05015	3	18297

528.14915	9.5	18297.5

528.24814	0.5	18298

528.34714	0.5	18301

528.44614	1.5	18310.5

528.54513	0.5	18311

528.64413	2	18311.5

528.74313	4	18313

528.84212	0.5	18313.5

528.94112	13.5	18315.5

529.04012	1	18319.5

529.13912	11	18320

529.23811	0.5	18333.5

529.33711	5.5	18334.5

529.43611	2.5	18345.5

529.5351	6.5	18346

529.6341	0.5	18351.5

529.7331	8.5	18354

529.8321	1.5	18360.5

529.93109	12	18361

530.03009	1.5	18369.5

530.12909	3	18371

530.22808	0.5	18383

530.32708	1	18384.5

530.42608	0.5	18387.5

530.52507	1	18388

530.62407	2.5	18389

530.72307	1	18389.5

530.82207	1	18390.5

530.92106	6	18393

531.02006	0.5	18394

531.11906	0.5	18395

531.21805	0.5	18401

531.31705	2	18401.5

531.41605	0.5	18402

531.51504	0.5	18402.5

531.61404	5	18404.5

531.71304	1	18405

531.81204	0.5	18405.5

531.91103	15	18410.5

532.01003	0.5	18411.5

532.10903	0.5	18412

532.20802	9.5	18427

532.30702	4	18427.5

532.40602	0.5	18428

532.50501	1.5	18437.5

532.60401	0.5	18441.5

532.70301	6	18442

532.80201	1.5	18443.5

532.901	13	18444

533	3.5	18450

533.099	1	18451.5

533.19799	0.5	18464.5

533.29699	0.5	18468

533.39599	1	18469

533.49499	0.5	18469.5

533.59398	1	18470

533.69298	1	18471

533.79198	1	18471.5

533.89097	0.5	18472.5

533.98997	7	18473.5

534.08897	2	18474.5

534.18796	22	18475

534.28696	4	18482

534.38596	0.5	18484

534.48496	2	18506

534.58395	5.5	18510

534.68295	2	18510.5

534.78195	2.5	18512.5

534.88094	9	18518

534.97994	0.5	18520

535.07894	12.5	18522.5

535.17793	1.5	18531.5

535.27693	1	18532

535.37593	4	18544.5

535.47493	1	18546

535.57392	1.5	18547

535.67292	8.5	18551

535.77192	1.5	18552

535.87091	3	18553.5

535.96991	11	18562

536.06891	18	18563.5

536.1679	0.5	18566.5

536.2669	5	18577.5

536.3659	4.5	18595.5

536.4649	0.5	18596

536.56389	12	18601

536.66289	1	18605.5

536.76189	11.5	18606

536.86088	2	18618

536.95988	3.5	18619

537.05888	6	18630.5

537.15788	0.5	18632.5

537.25687	19.5	18636

537.35587	5	18642

537.45487	2.5	18642.5

537.55386	1	18662

537.65286	3	18667

537.75186	1	18669.5

537.85085	2	18670.5

537.94985	9	18673.5

538.04885	0.5	18674.5

538.14785	9	18676.5

538.24684	0.5	18685.5

538.34584	0.5	18686

538.44484	1	18695

538.54383	10	18695.5

538.64283	0.5	18696

538.74183	1	18697

538.84082	16.5	18707

538.93982	0.5	18707.5

539.03882	2	18708.5

539.13782	2	18725

539.23681	0.5	18725.5

539.33581	1.5	18727.5

539.43481	5.5	18729.5

539.5338	0.5	18730

539.6328	1.5	18731.5

539.7318	0.5	18737

539.83079	3.5	18737.5

539.92979	1.5	18739

540.02879	1.5	18739.5

540.12779	1	18743

540.22678	1	18744.5

540.32578	12	18746

540.42478	1	18747

540.52377	0.5	18748

540.62277	13.5	18760

540.72177	6.5	18761

540.82077	4	18761.5

540.91976	0.5	18775

541.01876	3	18781.5

541.11776	4	18785.5

541.21675	1	18786

541.31575	4.5	18789

541.41475	1	18793

541.51374	1	18794

541.61274	4.5	18798.5

541.71174	1.5	18799.5

541.81074	0.5	18800.5

541.90973	15.5	18805

542.00873	0.5	18806.5

542.10773	0.5	18807

542.20672	12.5	18822.5

542.30572	0.5	18823

542.40472	1	18823.5

542.50371	2	18836

542.60271	4	18836.5

542.70171	7.5	18837.5

542.80071	0.5	18839.5

542.8997	6	18843.5

542.9987	4	18851

543.0977	0.5	18851.5

543.19669	14.5	18857.5

543.29569	0.5	18861.5

543.39469	0.5	18862

543.49369	9.5	18876.5

543.59268	0.5	18877

543.69168	0.5	18877.5

543.79068	1	18887

543.88967	9.5	18887.5

543.98867	0.5	18888

544.08767	14	18889

544.18666	0.5	18898.5

544.28566	10	18899

544.38466	0.5	18913

544.48366	0.5	18913.5

544.58265	8.5	18923.5

544.68165	0.5	18924

544.78065	11.5	18924.5

544.87964	2	18933

544.97864	8.5	18933.5

545.07764	1	18945

545.17663	5	18947

545.27563	1	18955.5

545.37463	0.5	18956.5

545.47363	0.5	18961.5

545.57262	0.5	18962.5

545.67162	10	18963

545.77062	0.5	18963.5

545.86961	1	18964

545.96861	3	18974

546.06761	1	18974.5

546.1666	4	18975.5

546.2656	1	18978.5

546.3646	1.5	18979.5

546.4636	3	18983.5

546.56259	0.5	18984.5

546.66159	20	18986

546.76059	0.5	18989

546.85958	7	18989.5

546.95858	1.5	19009.5

547.05758	1	19010

547.15657	2	19017

547.25557	5	19018.5

547.35457	0.5	19019.5

547.45357	13.5	19021.5

547.55256	1	19026.5

547.65156	9.5	19027

547.75056	1.5	19040.5

547.84955	0.5	19041.5

547.94855	0.5	19051

548.04755	24	19052.5

548.14655	1	19053

548.24554	3.5	19053.5

548.34454	3.5	19077.5

548.44354	0.5	19078.5

548.54253	1.5	19082

548.64153	6.5	19085.5

548.74053	0.5	19086

548.83952	1.5	19087.5

548.93852	1.5	19094

549.03752	1	19094.5

549.13652	23.5	19096

549.23551	0.5	19097.5

549.33451	3.5	19098.5

549.43351	4.5	19122

549.5325	0.5	19122.5

549.6315	0.5	19126

549.7305	4	19130.5

549.82949	6	19131

549.92849	15.5	19131.5

550.02749	8.5	19135.5

550.12649	1	19141.5

550.22548	0.5	19157

550.32448	0.5	19165.5

550.42348	9.5	19166.5

550.52247	1	19167

550.62147	7.5	19167.5

550.72047	2.5	19177

550.81947	1.5	19178

550.91846	4	19185.5

551.01746	0.5	19188

551.11646	1	19189.5

551.21545	1.5	19193.5

551.31445	4	19194

551.41345	12.5	19195

551.51244	1	19196.5

551.61144	9.5	19200.5

551.71044	4.5	19213

551.80944	1	19214

551.90843	5	19223.5

552.00743	4	19228

552.10643	7.5	19229

552.20542	1	19234

552.30442	5	19238

552.40342	9.5	19245.5

552.50241	0.5	19246.5

552.60141	10.5	19251.5

552.70041	3.5	19261

552.79941	14.5	19261.5

552.8984	1.5	19272

552.9974	9	19275.5

553.0964	2	19290

553.19539	0.5	19291.5

553.29439	16.5	19300.5

553.39339	0.5	19302.5

553.49238	16	19303

553.59138	1.5	19319.5

553.69038	5	19320

553.78938	1.5	19336

553.88837	2.5	19337.5

553.98737	1	19342.5

554.08637	4	19344

554.18536	14.5	19346.5

554.28436	0.5	19347.5

554.38336	10.5	19351.5

554.48236	0.5	19366

554.58135	1	19366.5

554.68035	5	19377

554.77935	2.5	19377.5

554.87834	0.5	19378.5

554.97734	0.5	19383.5

555.07634	9.5	19386

555.17533	0.5	19386.5

555.27433	0.5	19387

555.37333	4.5	19396.5

555.47233	0.5	19397

555.57132	0.5	19397.5

555.67032	9	19402

555.76932	2	19402.5

555.86831	0.5	19403

555.96731	14.5	19412

556.06631	2	19414

556.1653	16	19414.5

556.2643	2	19429

556.3633	10	19431

556.4623	0.5	19447

556.56129	1	19449

556.66029	9.5	19459

556.75929	0.5	19459.5

556.85828	5	19460.5

556.95728	3	19470

557.05628	17	19470.5

557.15527	0.5	19475.5

557.25427	1.5	19478.5

557.35327	8.5	19495.5

557.45227	11	19496

557.55126	0.5	19497.5

557.65026	1.5	19506

557.74926	2.5	19517

557.84825	1	19517.5

557.94725	1.5	19519

558.04625	3.5	19521.5

558.14525	0.5	19522.5

558.24424	2	19524

558.34324	2	19527.5

558.44224	11.5	19528

558.54123	0.5	19530

558.64023	0.5	19532

558.73923	6	19543.5

558.83822	0.5	19544

558.93722	2	19544.5

559.03622	0.5	19550.5

559.13522	0.5	19551

559.23421	5	19553

559.33321	3	19553.5

559.43221	1.5	19554

559.5312	13.5	19559

559.6302	0.5	19562

559.7292	0.5	19563.5

559.82819	8	19577

559.92719	1	19577.5

560.02619	6	19578

560.12519	5.5	19586

560.22418	4	19587

560.32318	3.5	19593

560.42218	0.5	19598.5

560.52117	0.5	19602.5

560.62017	10	19606

560.71917	1	19606.5

560.81816	0.5	19607

560.91716	1.5	19617

561.01616	6.5	19618

561.11516	11	19618.5

561.21415	1	19620

561.31315	1	19626.5

561.41215	0.5	19637.5

561.51114	0.5	19638.5

561.61014	12	19639.5

561.70914	0.5	19640

561.80814	1	19640.5

561.90713	3.5	19652.5

562.00613	7.5	19653

562.10513	8	19654

562.20412	0.5	19657.5

562.30312	22	19665

562.40212	18	19673

562.50111	5.5	19673.5

562.60011	1.5	19695.5

562.69911	0.5	19713.5

562.79811	0.5	19719

562.8971	8.5	19720.5

562.9961	0.5	19721

563.0951	9.5	19721.5

563.19409	3.5	19730

563.29309	0.5	19730.5

563.39209	3	19740

563.49108	4.5	19743.5

563.59008	0.5	19744

563.68908	5	19747

563.78808	4	19751.5

563.88707	0.5	19752

563.98607	0.5	19757

564.08507	7.5	19761

564.18406	2.5	19761.5

564.28306	2	19762

564.38206	9.5	19769.5

564.48105	0.5	19772

564.58005	0.5	19774

564.67905	2.5	19783.5

564.77805	1.5	19784

564.87704	0.5	19784.5

564.97604	0.5	19787

565.07504	5.5	19788.5

565.17403	0.5	19789

565.27303	1.5	19789.5

565.37203	0.5	19795

565.47103	10	19795.5

565.57002	1	19797

565.66902	0.5	19797.5

565.76802	2.5	19807.5

565.86701	6.5	19808.5

565.96601	1.5	19809

566.06501	1	19811.5

566.164	9	19818

566.263	0.5	19819.5

566.362	16	19820.5

566.461	12	19829.5

566.55999	1	19830

566.65899	0.5	19846

566.75799	1.5	19858

566.85698	5.5	19859

566.95598	4	19859.5

567.05498	20	19861

567.15397	8.5	19866.5

567.25297	1	19870.5

567.35197	0.5	19890.5

567.45097	10.5	19899

567.54996	0.5	19900

567.64896	1	19900.5

567.74796	1.5	19911

567.84695	1.5	19911.5

567.94595	0.5	19912.5

568.04495	0.5	19914

568.14395	1	19915.5

568.24294	2.5	19916

568.34194	2	19916.5

568.44094	0.5	19917.5

568.53993	4	19920

568.63893	5	19922

568.73793	0.5	19922.5

568.83692	0.5	19926.5

568.93592	20.5	19931.5

569.03492	0.5	19932

569.13392	0.5	19932.5

569.23291	3.5	19953

569.33191	1	19953.5

569.43091	1.5	19954

569.5299	6.5	19957.5

569.6289	1	19958.5

569.7279	0.5	19960

569.82689	3	19966.5

569.92589	4.5	19967.5

570.02489	1.5	19968

570.12389	0.5	19971

570.22288	6	19975.5

570.32188	0.5	19977

570.42088	0.5	19977.5

570.51987	25	19983.5

570.61887	0.5	19984

570.71787	1	19984.5

570.81686	13.5	20009.5

570.91586	2.5	20010

571.01486	3.5	20011

571.11386	8	20024.5

571.21285	1	20027

571.31185	1	20030.5

571.41085	15.5	20038.5

571.50984	0.5	20039.5

571.60884	0.5	20040.5

571.70784	14.5	20056

571.80684	1.5	20056.5

571.90583	4.5	20057

572.00483	1.5	20071.5

572.10383	1	20073

572.20282	11	20077.5

572.30182	3.5	20079

572.40082	4	20080

572.49981	0.5	20091

572.59881	1	20094.5

572.69781	7	20098.5

572.79681	1.5	20099

572.8958	0.5	20100

572.9948	1	20107

573.0938	1	20108.5

573.19279	24	20109

573.29179	18	20110

573.39079	1.5	20111

573.48978	5	20135

573.58878	4.5	20153

573.68778	17.5	20154.5

573.78678	0.5	20159.5

573.88577	8.5	20164

573.98477	2.5	20181.5

574.08377	1	20182

574.18276	7.5	20190.5

574.28176	0.5	20193

574.38076	18	20194

574.47975	1	20201.5

574.57875	2	20202

574.67775	10	20220

574.77675	4.5	20221

574.87574	0.5	20223

574.97474	13.5	20233

575.07374	1.5	20237.5

575.17273	5.5	20238

575.27173	4	20251.5

575.37073	1	20253

575.46973	7	20258.5

575.56872	1.5	20262.5

575.66772	12.5	20263.5

575.76672	0.5	20270.5

575.86571	2	20272

575.96471	6	20284.5

576.06371	0.5	20285

576.1627	6.5	20287

576.2617	2	20293

576.3607	1	20293.5

576.4597	9	20300

576.55869	0.5	20302

576.65769	2	20303

576.75669	1.5	20312

576.85568	2	20312.5

576.95468	1.5	20314.5

577.05368	4	20316

577.15267	1	20318

577.25167	1	20319.5

577.35067	10	20323.5

577.44967	2	20324.5

577.54866	0.5	20325.5

577.64766	0.5	20335.5

577.74666	5.5	20337.5

577.84565	20	20338

577.94465	0.5	20338.5

578.04365	16	20344

578.14264	1	20364

578.24164	0.5	20364.5

578.34064	0.5	20380.5

578.43964	1	20381.5

578.53863	16.5	20382

578.63763	2	20382.5

578.73663	0.5	20383.5

578.83562	10.5	20400

578.93462	0.5	20402

579.03362	0.5	20402.5

579.13262	0.5	20413

579.23161	2	20413.5

579.33061	4	20414

579.42961	2	20414.5

579.5286	3	20416.5

579.6276	4	20420.5

579.7266	7	20422.5

579.82559	17.5	20425.5

579.92459	1.5	20429.5

580.02359	2.5	20436.5

580.12259	2	20454

580.22158	1	20455.5

580.32058	13.5	20458

580.41958	4	20460

580.51857	0.5	20461

580.61757	6	20474.5

580.71657	2.5	20478.5

580.81556	1	20479

580.91456	1	20485

581.01356	10	20487.5

581.11256	2	20488.5

581.21155	0.5	20489.5

581.31055	2.5	20499.5

581.40955	2.5	20501.5

581.50854	2.5	20502

581.60754	7	20504.5

581.70654	5.5	20507

581.80553	0.5	20509.5

581.90453	0.5	20516.5

582.00353	3	20522

582.10253	2.5	20522.5

582.20152	0.5	20523

582.30052	0.5	20526

582.39952	2	20528.5

582.49851	0.5	20529

582.59751	1	20529.5

582.69651	2	20531.5

582.79551	4	20532

582.8945	0.5	20533

582.9935	0.5	20535

583.0925	12	20539

583.19149	10	20539.5

583.29049	20.5	20540

583.38949	6	20552

583.48848	4	20562

583.58748	18	20582.5

583.68648	2	20588.5

583.78548	11.5	20592.5

583.88447	0.5	20610.5

583.98347	2	20612.5

584.08247	12	20624

584.18146	0.5	20624.5

584.28046	0.5	20626.5

584.37946	18	20638.5

584.47845	2	20639

584.57745	4.5	20639.5

584.67645	0.5	20657.5

584.77545	2	20659.5

584.87444	0.5	20664

584.97344	8	20664.5

585.07244	1	20666.5

585.17143	2	20667

585.27043	0.5	20675

585.36943	10	20676

585.46843	0.5	20678

585.56742	5	20678.5

585.66642	8	20688.5

585.76542	0.5	20689

585.86441	1.5	20694

585.96341	1.5	20702

586.06241	0.5	20702.5

586.1614	0.5	20704

586.2604	1	20705.5

586.3594	4	20706

586.4584	0.5	20706.5

586.55739	8.5	20707.5

586.65639	0.5	20711.5

586.75539	1	20712

586.85438	1	20720.5

586.95338	7	20721

587.05238	0.5	20722

587.15137	12.5	20723

587.25037	0.5	20730

587.34937	0.5	20730.5

587.44837	2.5	20743

587.54736	2	20743.5

587.64636	0.5	20744

587.74536	12.5	20746.5

587.84435	0.5	20748.5

587.94335	17.5	20749

588.04235	0.5	20761.5

588.14134	0.5	20762

588.24034	0.5	20779.5

588.33934	2	20780

588.43834	1.5	20780.5

588.53733	4.5	20781

588.63633	2	20783

588.73533	1	20784.5

588.83432	2.5	20789

588.93332	7	20791

589.03232	0.5	20792

589.13131	2.5	20794.5

589.23031	4	20801.5

589.32931	4.5	20802

589.42831	3.5	20804.5

589.5273	1.5	20808.5

589.6263	0.5	20813

589.7253	0.5	20816.5

589.82429	0.5	20818

589.92329	10.5	20818.5

590.02229	0.5	20819

590.12129	4	20819.5

590.22028	7	20830

590.31928	2	20830.5

590.41828	0.5	20834.5

590.51727	5.5	20841.5

590.61627	4	20843.5

590.71527	15	20844

590.81426	0.5	20849.5

590.91326	0.5	20853.5

591.01226	12	20868.5

591.11126	2.5	20869

591.21025	11.5	20869.5

591.30925	2	20881.5

591.40825	1	20884

591.50724	0.5	20895.5

591.60624	15	20897.5

591.70524	0.5	20898.5

591.80423	10.5	20899

591.90323	0.5	20914

592.00223	14.5	20914.5

592.10123	10	20925

592.20022	3	20925.5

592.29922	1	20940

592.39822	9	20950

592.49721	3	20953

592.59621	4	20954

592.69521	0.5	20963

592.79421	0.5	20966

592.8932	19	20970

592.9922	0.5	20970.5

593.0912	2	20971

593.19019	14	20990

593.28919	1	20990.5

593.38819	1.5	20992.5

593.48718	1	21006.5

593.58618	0.5	21007.5

593.68518	0.5	21009

593.78418	1	21010

593.88317	0.5	21010.5

593.98217	3	21011

594.08117	2.5	21012

594.18016	0.5	21012.5

594.27916	6.5	21015.5

594.37816	5.5	21018

594.47715	4	21018.5

594.57615	8.5	21025

594.67515	13	21030.5

594.77415	1	21034.5

594.87314	10	21043

594.97214	1.5	21056

595.07114	14	21057

595.17013	1.5	21067

595.26913	0.5	21068.5

595.36813	8	21082.5

595.46712	1	21084

595.56612	1	21084.5

595.66512	2	21092.5

595.76412	0.5	21093.5

595.86311	0.5	21094.5

595.96211	6.5	21096.5

596.06111	1	21097

596.1601	5	21097.5

596.2591	3	21104

596.3581	0.5	21105

596.4571	2.5	21110

596.55609	6.5	21113

596.65509	0.5	21113.5

596.75409	11	21116

596.85308	1	21122.5

596.95208	5	21123

597.05108	0.5	21134

597.15007	3	21135

597.24907	11	21140

597.34807	1	21140.5

597.44707	4	21143.5

597.54606	0.5	21154.5

597.64506	0.5	21155.5

597.74406	0.5	21159.5

597.84305	1.5	21160

597.94205	8	21160.5

598.04105	0.5	21161

598.14004	6	21162.5

598.23904	0.5	21170.5

598.33804	0.5	21171

598.43704	7.5	21177

598.53603	3	21177.5

598.63503	1	21178

598.73403	1	21185.5

598.83302	16	21188.5

598.93202	0.5	21189.5

599.03102	14.5	21190.5

599.13001	0.5	21206.5

599.22901	0.5	21207

599.32801	8	21221.5

599.42701	0.5	21222

599.526	17	21222.5

599.625	2	21230.5

599.724	2.5	21231

599.82299	0.5	21248

599.92199	13.5	21250

600.02099	3	21252.5

600.11999	12	21253

600.21898	0.5	21266.5

600.31798	2.5	21269.5

600.41698	4	21281.5

600.51597	1	21282

600.61497	1	21284.5

600.71397	0.5	21288.5

600.81296	11.5	21289.5

600.91196	0.5	21290.5

601.01096	0.5	21291

601.10996	0.5	21302.5

601.20895	17.5	21303

601.30795	23	21303.5

601.40695	0.5	21304

601.50594	15	21321.5

601.60494	0.5	21344.5

601.70394	0.5	21345

601.80293	3.5	21360

601.90193	1.5	21360.5

602.00093	0.5	21361

602.09993	1	21364.5

602.19892	7.5	21366

602.29792	0.5	21366.5

602.39692	3.5	21367.5

602.49591	19.5	21375

602.59491	1	21375.5

602.69391	4	21379

602.7929	0.5	21398.5

602.8919	0.5	21399.5

602.9909	15	21403.5

603.0899	12.5	21404

603.18889	0.5	21404.5

603.28789	0.5	21419.5

603.38689	0.5	21432

603.48588	2.5	21432.5

603.58488	2	21433

603.68388	1.5	21433.5

603.78288	8	21436

603.88187	1.5	21438

603.98087	1	21439.5

604.07987	0.5	21447.5

604.17886	4.5	21449

604.27786	8	21450

604.37686	2	21450.5

604.47585	2	21455

604.57485	0.5	21463

604.67385	4.5	21465

604.77285	0.5	21467

604.87184	0.5	21467.5

604.97084	5	21472

605.06984	11.5	21472.5

605.16883	5	21473

605.26783	1.5	21478

605.36683	1.5	21489.5

605.46582	3.5	21494.5

605.56482	10.5	21496

605.66382	0.5	21497.5

605.76282	6.5	21501

605.86181	0.5	21511.5

605.96081	2.5	21512

606.05981	13.5	21518.5

606.1588	0.5	21519

606.2578	5.5	21521.5

606.3568	1	21535

606.45579	10.5	21535.5

606.55479	0.5	21541

606.65379	10	21542

606.75279	0.5	21552.5

606.85178	0.5	21553

606.95078	11	21563

607.04978	0.5	21563.5

607.14877	1	21564

607.24777	3	21575

607.34677	3	21575.5

607.44577	1.5	21576.5

607.54476	13	21579.5

607.64376	0.5	21582.5

607.74276	5	21584

607.84175	5	21597

607.94075	0.5	21597.5

608.03975	0.5	21602.5

608.13874	16	21607.5

608.23774	9.5	21608

608.33674	0.5	21608.5

608.43574	19.5	21624.5

608.53473	0.5	21634

608.63373	14	21634.5

608.73273	1.5	21654

608.83172	1.5	21654.5

608.93072	1	21668.5

609.02972	6.5	21670

609.12871	0.5	21671.5

609.22771	1.5	21672.5

609.32671	3	21679

609.42571	5.5	21679.5

609.5247	23.5	21681

609.6237	2	21684

609.7227	1	21689.5

609.82169	1.5	21713

609.92069	12.5	21715

610.01969	0.5	21716

610.11869	0.5	21717.5

610.21768	4	21730

610.31668	3.5	21730.5

610.41568	1.5	21731

610.51467	2	21735

610.61367	2.5	21738.5

610.71267	2	21740

610.81166	3.5	21742

610.91066	0.5	21744.5

611.00966	4	21746.5

611.10866	5	21750

611.20765	0.5	21750.5

611.30665	1	21754.5

611.40565	8.5	21759.5

611.50464	0.5	21760

611.60364	1.5	21761

611.70264	7	21769.5

611.80163	0.5	21770

611.90063	12.5	21771.5

611.99963	2	21778.5

612.09863	3	21779

612.19762	2	21791.5

612.29662	1	21793.5

612.39562	2.5	21796.5

612.49461	6.5	21798.5

612.59361	0.5	21799.5

612.69261	0.5	21802

612.7916	9	21808.5

612.8906	0.5	21809

612.9896	1	21809.5

613.0886	11.5	21818.5

613.18759	0.5	21819

613.28659	1	21820

613.38559	6.5	21831.5

613.48458	1.5	21832

613.58358	0.5	21833

613.68258	10.5	21839.5

613.78157	1	21841

613.88057	1	21841.5

613.97957	0.5	21852

614.07857	1	21853

614.17756	0.5	21854

614.27656	1	21854.5

614.37556	0.5	21855.5

614.47455	15.5	21856

614.57355	1	21857

614.67255	0.5	21857.5

614.77155	11.5	21873

614.87054	0.5	21874

614.96954	1	21874.5

615.06854	11.5	21886

615.16753	1	21886.5

615.26653	1	21887.5

615.36553	0.5	21899

615.46452	2.5	21900

615.56352	3.5	21901

615.66252	0.5	21901.5

615.76152	1	21904

615.86051	4.5	21907.5

615.95951	0.5	21908

616.05851	1.5	21909

616.1575	15.5	21913.5

616.2565	15	21914

616.3555	0.5	21915.5

616.45449	3.5	21931

616.55349	7.5	21946

616.65249	1	21946.5

616.75149	0.5	21950

616.85048	9	21957.5

616.94948	0.5	21958.5

617.04848	0.5	21959

617.14747	0.5	21968

617.24647	9.5	21968.5

617.34547	0.5	21969

617.44447	0.5	21969.5

617.54346	8	21979

617.64246	4	21979.5

617.74146	0.5	21980

617.84045	0.5	21988

617.93945	1.5	21992

618.03845	6.5	21992.5

618.13744	0.5	21993

618.23644	0.5	21994.5

618.33544	4.5	22001

618.43444	1	22001.5

618.53343	0.5	22002

618.63243	0.5	22006.5

618.73143	13	22007.5

618.83042	8.5	22008

618.92942	1.5	22008.5

619.02842	18.5	22021.5

619.12741	0.5	22030

619.22641	8	22031.5

619.32541	2.5	22050

619.42441	0.5	22050.5

619.5234	11	22058.5

619.6224	0.5	22061

619.7214	11.5	22061.5

619.82039	0.5	22072.5

619.91939	1	22073

620.01839	4	22084.5

620.11738	10	22085

620.21638	1.5	22086

620.31538	2.5	22090

620.41438	7	22100

620.51337	3	22101.5

620.61237	4.5	22104

620.71137	0.5	22111

620.81036	3	22114

620.90936	3.5	22118.5

621.00836	4	22119

621.10736	9.5	22122

621.20635	1	22125.5

621.30535	0.5	22129.5

621.40435	14	22139

621.50334	0.5	22140

621.60234	0.5	22140.5

621.70134	1.5	22154.5

621.80033	5.5	22155

621.89933	0.5	22155.5

621.99833	2.5	22157

622.09733	1.5	22162.5

622.19632	0.5	22163

622.29532	5	22165.5

622.39432	5.5	22167

622.49331	1.5	22167.5

622.59231	0.5	22172.5

622.69131	6.5	22178

622.7903	0.5	22179.5

622.8893	0.5	22180

622.9883	0.5	22186.5

623.0873	0.5	22187

623.18629	19.5	22187.5

623.28529	1	22188

623.38429	0.5	22188.5

623.48328	4.5	22208

623.58228	1	22209

623.68128	1	22209.5

623.78027	3	22214

623.87927	4	22215

623.97827	0.5	22216

624.07727	0.5	22219

624.17626	1	22223

624.27526	8.5	22223.5

624.37426	1.5	22224

624.47325	0.5	22225

624.57225	20	22233.5

624.67125	0.5	22235

624.77025	9	22235.5

624.86924	3	22255.5

624.96824	1	22256

625.06724	1.5	22265

625.16623	4.5	22268

625.26523	4.5	22269

625.36423	17.5	22270.5

625.46322	15.5	22275

625.56222	2	22279.5

625.66122	1	22297

625.76022	10	22312.5

625.85921	0.5	22314.5

625.95821	6.5	22315.5

626.05721	0.5	22325.5

626.1562	0.5	22326

626.2552	8.5	22332.5

626.3542	2	22333

626.45319	0.5	22333.5

626.55219	16.5	22342

626.65119	0.5	22344

626.75019	0.5	22344.5

626.84918	0.5	22361

626.94818	13	22361.5

627.04718	1	22362

627.14617	15	22362.5

627.24517	0.5	22375.5

627.34417	0.5	22376.5

627.44316	0.5	22391.5

627.54216	3	22392

627.64116	0.5	22392.5

627.74016	0.5	22393

627.83915	25	22396

627.93815	1	22396.5

628.03715	13.5	22397

628.13614	18.5	22422

628.23514	2.5	22423

628.33414	8	22436.5

628.43314	0.5	22455

628.53213	16.5	22457.5

628.63113	1	22465.5

628.73013	17.5	22466

628.82912	0.5	22482.5

628.92812	16.5	22483.5

629.02712	1	22501

629.12611	3	22501.5

629.22511	5	22518

629.32411	0.5	22519

629.42311	5	22522

629.5221	3	22527

629.6211	0.5	22527.5

629.7201	11	22532.5

629.81909	2.5	22535.5

629.91809	0.5	22536

630.01709	0.5	22547

630.11608	9	22549.5

630.21508	2	22550

630.31408	0.5	22550.5

630.41308	12	22559.5

630.51207	2	22561.5

630.61107	0.5	22562

630.71007	13.5	22574

630.80906	0.5	22576

630.90806	22	22576.5

631.00706	1	22590

631.10605	0.5	22590.5

631.20505	4	22612.5

631.30405	0.5	22613.5

631.40305	0.5	22614

631.50204	19.5	22618

631.60104	0.5	22618.5

631.70004	10.5	22619

631.79903	3	22638.5

631.89803	1.5	22639

631.99703	2	22649.5

632.09603	1.5	22652.5

632.19502	0.5	22654

632.29402	2	22656

632.39302	0.5	22657.5

632.49201	0.5	22658

632.59101	5.5	22660

632.69001	1.5	22660.5

632.789	0.5	22661

632.888	1	22666.5

632.987	3	22668

633.086	4.5	22668.5

633.18499	0.5	22669.5

633.28399	9.5	22672.5

633.38299	0.5	22677

633.48198	13	22677.5

633.58098	3.5	22687

633.67998	0.5	22687.5

633.77897	7	22700.5

633.87797	1	22704

633.97697	0.5	22704.5

634.07597	1	22711.5

634.17496	8	22712.5

634.27396	0.5	22713

634.37296	0.5	22714

634.47195	1	22722

634.57095	0.5	22722.5

634.66995	1.5	22723

634.76895	10	22724

634.86794	0.5	22724.5

634.96694	2.5	22726

635.06594	9.5	22736

635.16493	1	22736.5

635.26393	3.5	22739

635.36293	1.5	22748.5

635.46192	1	22749.5

635.56092	0.5	22753

635.65992	0.5	22754.5

635.75892	0.5	22755.5

635.85791	1.5	22756

635.95691	10	22756.5

636.05591	0.5	22757

636.1549	0.5	22758.5

636.2539	1.5	22768.5

636.3529	0.5	22769

636.45189	5	22769.5

636.55089	0.5	22771

636.64989	0.5	22771.5

636.74889	3	22776.5

636.84788	2.5	22777

636.94688	0.5	22777.5

637.04588	0.5	22780.5

637.14487	19	22783

637.24387	1.5	22783.5

637.34287	6	22784

637.44186	1	22803

637.54086	0.5	22804.5

637.63986	1.5	22810.5

637.73886	8	22811.5

637.83785	1	22812

637.93685	0.5	22813.5

638.03585	1	22821.5

638.13484	7	22822.5

638.23384	0.5	22823

638.33284	1.5	22824

638.43184	6.5	22831

638.53083	1	22831.5

638.62983	0.5	22833

638.72883	13	22839.5

638.82782	0.5	22840.5

638.92682	14	22841

639.02582	1	22854

639.12481	0.5	22854.5

639.22381	15.5	22868.5

639.32281	0.5	22869.5

639.42181	21	22870

639.5208	0.5	22885.5

639.6198	0.5	22886

639.7188	10.5	22907

639.81779	1	22907.5

639.91679	9	22908

640.01579	0.5	22918.5

640.11478	0.5	22919.5

640.21378	12.5	22928.5

640.31278	1	22929

640.41178	0.5	22929.5

640.51077	10.5	22942

640.60977	1.5	22943

640.70877	10.5	22943.5

640.80776	4.5	22954

640.90676	0.5	22955.5

641.00576	9.5	22966

641.10475	1.5	22970.5

641.20375	11.5	22971

641.30275	0.5	22980.5

641.40175	0.5	22982

641.50074	4	22993.5

641.59974	3.5	22994

641.69874	14	22994.5

641.79773	0.5	22998.5

641.89673	13.5	23002

641.99573	0.5	23016

642.09473	15.5	23016.5

642.19372	2	23030

642.29272	9.5	23030.5

642.39172	0.5	23046

642.49071	0.5	23048

642.58971	16.5	23057.5

642.68871	0.5	23058

642.7877	3	23058.5

642.8867	1.5	23075

642.9857	5	23075.5

643.0847	0.5	23078.5

643.18369	0.5	23080

643.28269	4.5	23085

643.38169	0.5	23085.5

643.48068	0.5	23086

643.57968	2	23090.5

643.67868	2.5	23091

643.77767	10	23091.5

643.87667	0.5	23093.5

643.97567	0.5	23096

644.07467	1.5	23106

644.17366	17.5	23106.5

644.27266	0.5	23107

644.37166	0.5	23108.5

644.47065	0.5	23126

644.56965	9	23126.5

644.66865	1	23127

644.76764	14.5	23127.5

644.86664	0.5	23136.5

644.96564	26.5	23137.5

645.06464	0.5	23152

645.16363	0.5	23152.5

645.26263	12	23179

645.36163	0.5	23179.5

645.46062	1	23180

645.55962	14.5	23192

645.65862	1	23192.5

645.75762	0.5	23193.5

645.85661	7	23208

645.95561	1	23209

646.05461	1	23209.5

646.1536	3.5	23216.5

646.2526	3	23217.5

646.3516	13	23218.5

646.45059	3.5	23222

646.54959	4	23225

646.64859	1	23238

646.74759	2	23241.5

646.84658	8	23245.5

646.94558	1.5	23246.5

647.04458	8	23248.5

647.14357	4.5	23256.5

647.24257	0.5	23258

647.34157	4.5	23266

647.44056	0.5	23270.5

647.53956	0.5	23271

647.63856	8	23275.5

647.73756	4	23276

647.83655	0.5	23276.5

647.93555	0.5	23284.5

648.03455	0.5	23288.5

648.13354	4.5	23289

648.23254	5	23289.5

648.33154	0.5	23290

648.43053	0.5	23294.5

648.52953	17.5	23299.5

648.62853	1	23300

648.72753	0.5	23300.5

648.82652	0.5	23318

648.92552	0.5	23319

649.02452	3.5	23319.5

649.12351	1.5	23320

649.22251	1	23320.5

649.32151	1	23324

649.42051	5	23325.5

649.5195	1.5	23326.5

649.6185	1	23327.5

649.7175	3.5	23332.5

649.81649	3.5	23334

649.91549	0.5	23335

650.01449	14	23338.5

650.11348	1	23342

650.21248	19	23342.5

650.31148	0.5	23356.5

650.41048	8.5	23357.5

650.50947	0.5	23376.5

650.60847	18	23377

650.70747	0.5	23385.5

650.80646	10.5	23386

650.90546	0.5	23404

651.00446	2	23404.5

651.10345	1	23415

651.20245	0.5	23415.5

651.30145	8.5	23417.5

651.40045	1	23418.5

651.49944	14.5	23419

651.59844	0.5	23427.5

651.69744	4.5	23428.5

651.79643	8.5	23443

651.89543	1	23443.5

651.99443	6	23448

652.09343	1	23456.5

652.19242	16	23457.5

652.29142	4	23463.5

652.39042	13	23464.5

652.48941	0.5	23480.5

652.58841	5	23484.5

652.68741	10.5	23497.5

652.7864	0.5	23498

652.8854	1.5	23503

652.9844	1	23513.5

653.0834	4	23514

653.18239	5.5	23515.5

653.28139	1	23516.5

653.38039	2.5	23520.5

653.47938	2.5	23526

653.57838	0.5	23527

653.67738	10.5	23529.5

653.77637	1	23532

653.87537	17	23532.5

653.97437	0.5	23543

654.07337	3	23544

654.17236	1.5	23561

654.27136	11.5	23561.5

654.37036	0.5	23564.5

654.46935	2	23566

654.56835	0.5	23577.5

654.66735	14	23578

654.76634	0.5	23580

654.86534	12.5	23580.5

654.96434	1	23594.5

655.06334	0.5	23595

655.16233	1	23607.5

655.26133	12.5	23608.5

655.36033	1	23609

655.45932	8	23610

655.55832	0.5	23622.5

655.65732	9.5	23623.5

655.75631	1	23631.5

655.85531	0.5	23632

655.95431	16	23641.5

656.05331	0.5	23642.5

656.1523	4	23643

656.2513	11	23659

656.3503	0.5	23659.5

656.44929	0.5	23663.5

656.54829	6	23674.5

656.64729	0.5	23675

656.74629	0.5	23675.5

656.84528	0.5	23681.5

656.94428	7	23682

657.04328	1	23682.5

657.14227	1.5	23683

657.24127	2	23690

657.34027	0.5	23691

657.43926	0.5	23692.5

657.53826	23.5	23694.5

657.63726	2.5	23695

657.73626	12	23695.5

657.83525	0.5	23719

657.93425	1	23721.5

658.03325	1.5	23733.5

658.13224	10	23734

658.23124	1	23735

658.33024	0.5	23736.5

658.42923	12.5	23746.5

658.52823	12	23747.5

658.62723	0.5	23748

658.72623	2	23760.5

658.82522	0.5	23772.5

658.92422	6.5	23773

659.02322	0.5	23775

659.12221	4.5	23775.5

659.22121	0.5	23782

659.32021	16.5	23782.5

659.41921	0.5	23787

659.5182	1	23787.5

659.6172	5.5	23804

659.7162	0.5	23804.5

659.81519	0.5	23805.5

659.91419	12.5	23811

660.01319	0.5	23811.5

660.11218	1.5	23812

660.21118	3	23824.5

660.31018	4	23825

660.40918	2.5	23826.5

660.50817	1	23829.5

660.60717	10	23833.5

660.70617	0.5	23836

660.80516	0.5	23837

660.90416	0.5	23847

661.00316	12.5	23847.5

661.10215	0.5	23848

661.20115	8.5	23848.5

661.30015	1	23861

661.39915	13.5	23861.5

661.49814	1	23870

661.59714	2.5	23871

661.69614	3.5	23884.5

661.79513	0.5	23885.5

661.89413	11	23888

661.99313	0.5	23891.5

662.09212	0.5	23892

662.19112	0.5	23903

662.29012	17.5	23903.5

662.38912	2	23904

662.48811	0.5	23904.5

662.58711	11.5	23922

662.68611	3	23924

662.7851	8	23924.5

662.8841	0.5	23936

662.9831	0.5	23939

663.0821	14.5	23947

663.18109	5.5	23947.5

663.28009	7	23948

663.37909	11	23962.5

663.47808	0.5	23968

663.57708	1.5	23975

663.67608	15.5	23986

663.77507	0.5	23986.5

663.87407	16	23988

663.97307	1.5	24003.5

664.07207	6.5	24004

664.17106	7	24020

664.27006	0.5	24021.5

664.36906	0.5	24028

664.46805	9	24035

664.56705	0.5	24035.5

664.66605	8.5	24036

664.76504	1	24045

664.86404	1.5	24045.5

664.96304	18.5	24054

665.06204	1	24055

665.16103	16	24056.5

665.26003	0.5	24075

665.35903	4	24076

665.45802	4	24092

665.55702	2.5	24092.5

665.65602	13	24096.5

665.75501	1	24100.5

665.85401	1	24103

665.95301	21	24116

666.05201	1	24117

666.151	2.5	24118

666.25	4	24139

666.349	7.5	24140

666.44799	0.5	24142.5

666.54699	2	24146.5

666.64599	1	24154

666.74499	13.5	24154.5

666.84398	0.5	24156.5

666.94298	8	24157.5

667.04198	1.5	24171

667.14097	0.5	24171.5

667.23997	16	24179.5

667.33897	0.5	24181

667.43796	1.5	24181.5

667.53696	8	24197.5

667.63596	2.5	24198

667.73496	0.5	24199.5

667.83395	20.5	24207.5

667.93295	1.5	24210

668.03195	2.5	24210.5

668.13094	2.5	24231

668.22994	0.5	24232.5

668.32894	0.5	24235

668.42793	5	24237.5

668.52693	1.5	24238

668.62593	1.5	24238.5

668.72493	8.5	24243.5

668.82392	1.5	24245

668.92292	1	24246.5

669.02192	1.5	24255

669.12091	16.5	24256.5

669.21991	1.5	24257.5

669.31891	3.5	24259

669.4179	13.5	24275.5

669.5169	14	24277

669.6159	1	24280.5

669.7149	0.5	24294

669.81389	4.5	24308

669.91289	2.5	24309

670.01189	0.5	24309.5

670.11088	10	24314

670.20988	0.5	24316.5

670.30888	9	24317

670.40788	1	24327

670.50687	14	24327.5

670.60587	2	24336.5

670.70487	0.5	24337.5

670.80386	0.5	24351.5

670.90286	9.5	24353.5

671.00186	0.5	24354

671.10085	0.5	24354.5

671.19985	17.5	24364

671.29885	0.5	24364.5

671.39785	9.5	24365

671.49684	1.5	24382.5

671.59584	1	24383

671.69484	9	24392.5

671.79383	2.5	24394

671.89283	0.5	24395

671.99183	13.5	24404

672.09082	17.5	24406.5

672.18982	11.5	24407

672.28882	1	24420.5

672.38782	14	24438

672.48681	1	24449.5

672.58581	1.5	24450.5

672.68481	13.5	24464.5

672.7838	0.5	24465.5

672.8828	1.5	24467

672.9818	0.5	24480.5

673.08079	2.5	24481

673.17979	2.5	24482.5

673.27879	0.5	24483

673.37779	1.5	24485.5

673.47678	14.5	24488

673.57578	1	24488.5

673.67478	0.5	24490

673.77377	3.5	24504.5

673.87277	0.5	24505.5

673.97177	1	24506

674.07077	0.5	24509.5

674.16976	1	24510

674.26876	0.5	24511

674.36776	3	24511.5

674.46675	0.5	24512.5

674.56575	0.5	24513

674.66475	15.5	24516

674.76374	1	24516.5

674.86274	8.5	24517

674.96174	0.5	24532.5

675.06074	5	24533.5

675.15973	8	24542

675.25873	1	24542.5

675.35773	0.5	24547.5

675.45672	0.5	24555.5

675.55572	15.5	24556.5

675.65472	0.5	24557

675.75371	0.5	24557.5

675.85271	0.5	24573

675.95171	1	24573.5

676.05071	6.5	24574

676.1497	12.5	24574.5

676.2487	1.5	24575.5

676.3477	1	24582

676.44669	10	24594.5

676.54569	1.5	24596

676.64469	0.5	24597

676.74369	0.5	24607

676.84268	13	24608.5

676.94168	4.5	24609

677.04068	1	24609.5

677.13967	10.5	24622.5

677.23867	5.5	24627

677.33767	0.5	24628

677.43666	1	24638.5

677.53566	0.5	24644

677.63466	5	24644.5

677.73366	12	24645.5

677.83265	0.5	24646

677.93165	20	24651

678.03065	2	24663

678.12964	9.5	24663.5

678.22864	4.5	24683.5

678.32764	0.5	24685.5

678.42663	25.5	24695

678.52563	0.5	24699.5

678.62463	0.5	24700

678.72363	18	24725.5

678.82262	0.5	24726

678.92162	18	24726.5

679.02062	0.5	24744.5

679.11961	16	24745

679.21861	0.5	24763

679.31761	14	24763.5

679.4166	1	24779.5

679.5156	20	24780

679.6146	3	24794

679.7136	0.5	24795

679.81259	11	24815

679.91159	2.5	24818

680.01059	1	24818.5

680.10958	14.5	24829.5

680.20858	0.5	24832

680.30758	3.5	24833

680.40657	2.5	24847.5

680.50557	0.5	24848

680.60457	7	24851.5

680.70357	2	24854

680.80256	1.5	24854.5

680.90156	1	24861.5

681.00056	8	24863.5

681.09955	0.5	24865

681.19855	0.5	24866

681.29755	15.5	24874

681.39655	1	24874.5

681.49554	7	24875

681.59454	0.5	24890.5

681.69354	1.5	24891.5

681.79253	7	24898.5

681.89153	1	24899

681.99053	3	24900.5

682.08952	7.5	24907.5

682.18852	0.5	24908.5

682.28752	0.5	24911.5

682.38652	14	24919

682.48551	0.5	24919.5

682.58451	13	24920

682.68351	0.5	24934

682.7825	0.5	24934.5

682.8815	0.5	24947.5

682.9805	0.5	24948

683.07949	1	24948.5

683.17849	6.5	24949

683.27749	0.5	24949.5

683.37649	9	24950.5

683.47548	0.5	24957

683.57448	0.5	24957.5

683.67348	18	24966.5

683.77247	2.5	24967

683.87147	3	24967.5

683.97047	1	24985.5

684.06947	13	24988

684.16846	0.5	24991

684.26746	0.5	24992

684.36646	12	25005

684.46545	0.5	25005.5

684.56445	1	25006

684.66345	14	25018

684.76244	0.5	25018.5

684.86144	0.5	25019.5

684.96044	14	25033.5

685.05944	2.5	25034

685.15843	0.5	25034.5

685.25743	8	25048.5

685.35643	1	25051

685.45542	0.5	25051.5

685.55442	2	25059.5

685.65342	3.5	25060.5

685.75241	0.5	25061

685.85141	1	25063

685.95041	1	25066.5

686.04941	1	25067

686.1484	3.5	25068

686.2474	4	25069

686.3464	2	25070

686.44539	25	25073.5

686.54439	0.5	25077.5

686.64339	4.5	25079.5

686.74238	4	25104.5

686.84138	0.5	25105

686.94038	26.5	25109.5

687.03938	1	25113.5

687.13837	14.5	25114

687.23737	0.5	25140.5

687.33637	0.5	25141.5

687.43536	0.5	25156

687.53436	9.5	25156.5

687.63336	5.5	25157

687.73236	0.5	25157.5

687.83135	13.5	25167

687.93035	6.5	25172.5

688.02935	1	25173

688.12834	11.5	25186.5

688.22734	4	25193

688.32634	0.5	25194

688.42533	18.5	25205.5

688.52433	0.5	25209.5

688.62333	0.5	25210

688.72233	1	25228.5

688.82132	4.5	25229

688.92032	2.5	25229.5

689.01932	11	25230.5

689.11831	0.5	25235

689.21731	0.5	25237.5

689.31631	1	25248.5

689.4153	0.5	25249

689.5143	25.5	25249.5

689.6133	0.5	25250.5

689.7123	12	25251

689.81129	0.5	25276.5

689.91029	20.5	25277

690.00929	0.5	25289

690.10828	3	25289.5

690.20728	11	25310

690.30628	0.5	25310.5

690.40527	0.5	25313.5

690.50427	10.5	25324.5

690.60327	1	25325

690.70227	0.5	25325.5

690.80126	0.5	25336

690.90026	10.5	25337

690.99926	1	25337.5

691.09825	2	25338

691.19725	6	25348.5

691.29625	2.5	25349.5

691.39525	11	25351.5

691.49424	0.5	25357.5

691.59324	5	25360

691.69224	3.5	25371

691.79123	4.5	25371.5

691.89023	1.5	25376.5

691.98923	0.5	25380

692.08822	1	25384.5

692.18722	0.5	25386

692.28622	14	25386.5

692.38522	1	25387.5

692.48421	3	25388

692.58321	1.5	25402

692.68221	1	25403

692.7812	1.5	25406

692.8802	13.5	25407.5

692.9792	0.5	25408.5

693.07819	1	25410

693.17719	1.5	25423.5

693.27619	3	25424

693.37519	3.5	25425

693.47418	0.5	25426.5

693.57318	1.5	25429.5

693.67218	11.5	25433

693.77117	0.5	25433.5

693.87017	2	25435

693.96917	2.5	25446.5

694.06816	6.5	25447

694.16716	0.5	25449

694.26616	14.5	25451.5

694.36516	0.5	25458

694.46415	19.5	25458.5

694.56315	2	25473

694.66215	6	25473.5

694.76114	1	25493

694.86014	1.5	25495

694.95914	0.5	25501

695.05814	13.5	25502

695.15713	1	25503.5

695.25613	0.5	25504

695.35513	0.5	25517.5

695.45412	0.5	25518.5

695.55312	18.5	25519

695.65212	1.5	25519.5

695.75111	0.5	25520

695.85011	17.5	25538.5

695.94911	2.5	25540

696.04811	4.5	25540.5

696.1471	0.5	25558

696.2461	0.5	25560.5

696.3451	0.5	25565

696.44409	1.5	25565.5

696.54309	7.5	25566

696.64209	0.5	25566.5

696.74108	1.5	25568

696.84008	10	25575.5

696.93908	4.5	25576

697.03808	1	25577.5

697.13707	9	25587.5

697.23607	2.5	25592

697.33507	2	25593

697.43406	19.5	25602

697.53306	8	25604.5

697.63206	2.5	25606.5

697.73105	0.5	25626

697.83005	8.5	25634

697.92905	3.5	25636.5

698.02805	0.5	25637

698.12704	1	25645.5

698.22604	2.5	25649

698.32504	8.5	25649.5

698.42403	1	25650.5

698.52303	0.5	25653

698.62203	13	25661.5

698.72103	2	25662.5

698.82002	2.5	25663

698.91902	2	25676

699.01802	0.5	25678

699.11701	24.5	25680.5

699.21601	0.5	25682.5

699.31501	0.5	25683

699.414	20.5	25707.5

699.513	2	25708

699.612	4.5	25708.5

699.711	1	25729

699.80999	1	25731

699.90899	0.5	25735.5

700.00799	0.5	25736.5

700.10698	6	25737.5

700.20598	4	25738

700.30498	0.5	25738.5

700.40397	9	25744.5

700.50297	0.5	25748.5

700.60197	0.5	25749

700.70097	1.5	25758

700.79996	8.5	25758.5

700.89896	0.5	25759

700.99796	0.5	25760.5

701.09695	6.5	25769

701.19595	5.5	25769.5

701.29495	2.5	25770

701.39395	12.5	25776.5

701.49294	4.5	25782

701.59194	0.5	25784.5

701.69094	2.5	25797

701.78993	2.5	25801.5

701.88893	0.5	25802

701.98793	0.5	25804.5

702.08692	4.5	25807

702.18592	0.5	25807.5

702.28492	0.5	25808

702.38392	2	25812.5

702.48291	8	25813

702.58191	17	25813.5

702.68091	0.5	25815.5

702.7799	15	25823.5

702.8789	15.5	25840.5

702.9779	0.5	25841

703.07689	1	25856

703.17589	20	25871.5

703.27489	0.5	25872

703.37389	17	25873

703.47288	1.5	25893

703.57188	3.5	25893.5

703.67088	2	25910.5

703.76987	0.5	25912

703.86887	11.5	25915.5

703.96787	0.5	25917.5

704.06686	14.5	25918

704.16586	0.5	25929.5

704.26486	1	25930

704.36386	10.5	25944.5

704.46285	1	25945

704.56185	15.5	25946

704.66085	2.5	25956.5

704.75984	2.5	25957.5

704.85884	0.5	25973

704.95784	6.5	25975.5

705.05684	2	25978

705.15583	4.5	25978.5

705.25483	13	25985

705.35383	2	25987

705.45282	13	25991.5

705.55182	0.5	26004.5

705.65082	19.5	26006.5

705.74981	0.5	26019.5

705.84881	2.5	26020

705.94781	7	26039.5

706.04681	0.5	26040

706.1458	3	26042.5

706.2448	6	26049.5

706.3438	0.5	26050

706.44279	0.5	26053

706.54179	8.5	26059

706.64079	1	26059.5

706.73978	0.5	26060

706.83878	16.5	26068.5

706.93778	1	26069.5

707.03678	0.5	26070

707.13577	9	26086.5

707.23477	1	26087.5

707.33377	0.5	26088

707.43276	0.5	26097

707.53176	1.5	26098

707.63076	0.5	26098.5

707.72975	1	26099

707.82875	19.5	26100.5

707.92775	1	26101

708.02675	1.5	26102

708.12574	10.5	26121.5

708.22474	0.5	26122.5

708.32374	6	26124

708.42273	0.5	26134.5

708.52173	0.5	26135

708.62073	1	26141

708.71973	0.5	26141.5

708.81872	4.5	26142

708.91772	5.5	26143

709.01672	3	26143.5

709.11571	2	26148

709.21471	1	26153.5

709.31371	18	26156.5

709.4127	7.5	26158.5

709.5117	0.5	26159.5

709.6107	1.5	26177.5

709.7097	0.5	26185

709.80869	1	26185.5

709.90769	1.5	26187

710.00669	4	26187.5

710.10568	3	26188.5

710.20468	16.5	26190

710.30368	1	26194

710.40267	0.5	26197

710.50167	16.5	26213.5

710.60067	3.5	26214.5

710.69967	0.5	26215

710.79866	8.5	26231.5

710.89766	1	26235

710.99666	1	26235.5

711.09565	3.5	26244

711.19465	8.5	26245

711.29365	1	26246

711.39264	0.5	26249.5

711.49164	19	26258

711.59064	4	26259

711.68964	3.5	26259.5

711.78863	0.5	26278.5

711.88763	0.5	26282.5

711.98663	7	26286

712.08562	1.5	26286.5

712.18462	0.5	26287

712.28362	12	26294

712.38262	19	26295.5

712.48161	0.5	26296

712.58061	1	26308

712.67961	11.5	26327

712.7786	1	26327.5

712.8776	2	26328.5

712.9766	3.5	26340

713.07559	0.5	26341

713.17459	0.5	26343

713.27359	0.5	26346.5

713.37259	0.5	26347

713.47158	0.5	26347.5

713.57058	0.5	26348

713.66958	8.5	26348.5

713.76857	0.5	26349

713.86757	0.5	26349.5

713.96657	0.5	26358

714.06556	0.5	26358.5

714.16456	5.5	26359

714.26356	8.5	26359.5

714.36256	1.5	26360

714.46155	23.5	26365.5

714.56055	16	26374

714.65955	1	26375.5

714.75854	0.5	26399

714.85754	16	26415

714.95654	2	26416

715.05553	1	26416.5

715.15453	9	26432.5

715.25353	1.5	26434.5

715.35253	2	26435.5

715.45152	0.5	26444.5

715.55052	0.5	26446

715.64952	10.5	26448

715.74851	0.5	26448.5

715.84751	4	26449

715.94651	2	26459.5

716.04551	0.5	26460

716.1445	0.5	26464

716.2435	13	26466

716.3425	0.5	26466.5

716.44149	22.5	26467

716.54049	9	26480

716.63949	3	26480.5

716.73848	1.5	26503

716.83748	16	26512

716.93648	1	26515

717.03548	0.5	26516.5

717.13447	13	26532.5

717.23347	0.5	26533.5

717.33247	0.5	26534

717.43146	1	26547

717.53046	19.5	26547.5

717.62946	5.5	26548

717.72845	4	26549

717.82745	0.5	26568.5

717.92645	23.5	26574

718.02545	1	26578

718.12444	0.5	26578.5

718.22344	12	26602

718.32244	0.5	26603

718.42143	13	26603.5

718.52043	0.5	26615.5

718.61943	1.5	26616

718.71843	0.5	26629

718.81742	11	26629.5

718.91642	0.5	26631

719.01542	0.5	26631.5

719.11441	1	26642.5

719.21341	11	26643

719.31241	8.5	26643.5

719.4114	1	26644.5

719.5104	1	26655.5

719.6094	13.5	26664

719.7084	0.5	26665

719.80739	1.5	26666

719.90639	9	26679.5

720.00539	0.5	26680

720.10438	0.5	26681.5

720.20338	23.5	26690.5

720.30238	1	26691

720.40137	9	26691.5

720.50037	0.5	26715

720.59937	0.5	26716

720.69837	0.5	26725

720.79736	26	26725.5

720.89636	0.5	26726

720.99536	2	26726.5

721.09435	9	26752.5

721.19335	0.5	26753

721.29235	2.5	26755

721.39134	5.5	26764

721.49034	3	26764.5

721.58934	0.5	26767

721.68834	19	26772.5

721.78733	3	26775.5

721.88633	0.5	26776

721.98533	2	26795

722.08432	7	26798

722.18332	0.5	26798.5

722.28232	2.5	26800.5

722.38131	2	26807.5

722.48031	1.5	26808

722.57931	0.5	26810.5

722.67831	1	26812.5

722.7773	7.5	26814

722.8763	4.5	26814.5

722.9753	1	26815.5

723.07429	0.5	26823

723.17329	26.5	26827.5

723.27229	0.5	26828.5

723.37129	17.5	26829

723.47028	0.5	26855.5

723.56928	1	26856

723.66828	0.5	26873.5

723.76727	9	26874

723.86627	2	26875

723.96527	0.5	26875.5

724.06426	22	26884.5

724.16326	1.5	26886.5

724.26226	0.5	26887

724.36126	0.5	26909

724.46025	11.5	26910.5

724.55925	1	26911

724.65825	10	26911.5

724.75724	5	26923

724.85624	10	26924

724.95524	1.5	26934

725.05423	0.5	26939

725.15323	10	26949

725.25223	2	26950.5

725.35123	13.5	26951

725.45022	1	26961

725.54922	0.5	26963

725.64822	15	26976.5

725.74721	0.5	26977.5

725.84621	11.5	26978

725.94521	0.5	26993

726.04421	5	26993.5

726.1432	0.5	27005

726.2422	11.5	27005.5

726.3412	1	27010.5

726.44019	0.5	27011

726.53919	0.5	27022.5

726.63819	16.5	27023.5

726.73718	1	27024

726.83618	1	27024.5

726.93518	17	27041

727.03418	0.5	27042

727.13317	11	27043

727.23217	0.5	27060

727.33117	0.5	27060.5

727.43016	2	27071.5

727.52916	1.5	27072

727.62816	14	27072.5

727.72715	1.5	27074.5

727.82615	1	27076

727.92515	4.5	27090

728.02415	1	27091.5

728.12314	17.5	27092.5

728.22214	0.5	27097

728.32114	1.5	27098

728.42013	15.5	27115.5

728.51913	0.5	27116

728.61813	9	27117.5

728.71712	2	27133

728.81612	1	27133.5

728.91512	1	27142.5

729.01412	13.5	27144.5

729.11311	0.5	27145.5

729.21211	0.5	27146.5

729.31111	14	27160

729.4101	0.5	27160.5

729.5091	20	27161

729.6081	0.5	27175

729.7071	2.5	27175.5

729.80609	5.5	27195.5

729.90509	1	27196

730.00409	0.5	27198.5

730.10308	14.5	27204

730.20208	1.5	27205

730.30108	18	27205.5

730.40007	2	27220

730.49907	7.5	27221.5

730.59807	1	27239.5

730.69707	14.5	27241.5

730.79606	1	27249

730.89506	1	27250

730.99406	0.5	27264.5

731.09305	9.5	27265.5

731.19205	0.5	27266.5

731.29105	1	27267

731.39004	14	27276.5

731.48904	0.5	27277

731.58804	1.5	27278

731.68704	11.5	27292

731.78603	1.5	27292.5

731.88503	1	27294

731.98403	1	27305.5

732.08302	17	27307

732.18202	1	27308

732.28102	0.5	27309

732.38001	9.5	27326

732.47901	0.5	27327

732.57801	0.5	27327.5

732.67701	0.5	27337

732.776	1	27337.5

732.875	15	27338

732.974	2	27338.5

733.07299	10	27339.5

733.17199	0.5	27354.5

733.27099	1.5	27356.5

733.36999	23	27366.5

733.46898	0.5	27367

733.56798	0.5	27368.5

733.66698	0.5	27391.5

733.76597	7	27392

733.86497	1	27392.5

733.96397	2	27393

734.06296	8.5	27400

734.16196	1.5	27401

734.26096	0.5	27403

734.35996	18	27411.5

734.45895	0.5	27413

734.55795	1.5	27413.5

734.65695	1	27431.5

734.75594	7.5	27432

734.85494	0.5	27433.5

734.95394	17.5	27434.5

735.05293	1	27442

735.15193	2.5	27442.5

735.25093	9	27460

735.34993	3.5	27461

735.44892	22	27463.5

735.54792	2.5	27472.5

735.64692	11.5	27476

735.74591	0.5	27498

735.84491	0.5	27500.5

735.94391	0.5	27512

736.0429	0.5	27512.5

736.1419	0.5	27513

736.2409	6	27513.5

736.3399	1	27514

736.43889	13.5	27514.5

736.53789	2	27520.5

736.63689	1	27521.5

736.73588	9.5	27535

736.83488	2	27537

736.93388	0.5	27538

737.03288	1	27547.5

737.13187	13.5	27549.5

737.23087	0.5	27550

737.32987	0.5	27551

737.42886	11.5	27564.5

737.52786	7	27565

737.62686	0.5	27565.5

737.72585	4.5	27577

737.82485	21	27584

737.92385	0.5	27584.5

738.02285	0.5	27589

738.12184	11.5	27610

738.22084	6.5	27610.5

738.31984	0.5	27611

738.41883	1.5	27622.5

738.51783	1	27629

738.61683	0.5	27629.5

738.71582	8.5	27631

738.81482	0.5	27632

738.91382	1	27632.5

739.01282	1	27641

739.11181	11	27641.5

739.21081	0.5	27642.5

739.30981	0.5	27643.5

739.4088	0.5	27654.5

739.5078	15	27655

739.6068	1	27655.5

739.70579	0.5	27656

739.80479	0.5	27671

739.90379	1	27672

740.00279	6.5	27672.5

740.10178	0.5	27673

740.20078	17	27674

740.29978	16	27680.5

740.39877	1.5	27681

740.49777	11.5	27698

740.59677	4	27714

740.69577	1	27715.5

740.79476	1.5	27727

740.89376	10	27731

740.99276	1	27732

741.09175	0.5	27733.5

741.19075	16	27743.5

741.28975	0.5	27744.5

741.38874	0.5	27745

741.48774	8.5	27761

741.58674	2	27761.5

741.68574	1.5	27762

741.78473	6	27770.5

741.88373	2.5	27772.5

741.98273	1.5	27774

742.08172	0.5	27780

742.18072	22.5	27782.5

742.27972	1	27784

742.37871	2	27784.5

742.47771	2.5	27807

742.57671	3	27808

742.67571	10	27810

742.7747	1	27812.5

742.8737	0.5	27815.5

742.9727	10	27825.5

743.07169	5.5	27826.5

743.17069	18.5	27827

743.26969	0.5	27837

743.36869	0.5	27842.5

743.46768	18.5	27861

743.56668	0.5	27861.5

743.66568	0.5	27862

743.76467	22.5	27880.5

743.86367	1	27881

743.96267	0.5	27881.5

744.06166	1	27904

744.16066	7.5	27905

744.25966	0.5	27905.5

744.35866	0.5	27906.5

744.45765	0.5	27914

744.55665	12	27914.5

744.65565	1	27915

744.75464	1	27915.5

744.85364	0.5	27927.5

744.95264	16.5	27928.5

745.05163	1	27929.5

745.15063	1.5	27930

745.24963	14	27946.5

745.34863	0.5	27947.5

745.44762	1.5	27949

745.54662	0.5	27963

745.64562	17.5	27963.5

745.74461	1.5	27965

745.84361	0.5	27965.5

745.94261	17.5	27983

746.0416	0.5	27984.5

746.1406	2	27985

746.2396	1	28002.5

746.3386	3.5	28003

746.43759	1	28005

746.53659	1	28006

746.63559	1	28009.5

746.73458	14.5	28010.5

746.83358	0.5	28011.5

746.93258	1	28012.5

747.03157	16	28027

747.13057	1.5	28027.5

747.22957	0.5	28028.5

747.32857	0.5	28044.5

747.42756	8	28046

747.52656	2.5	28046.5

747.62556	18.5	28047

747.72455	1	28055

747.82355	1	28057.5

747.92255	10	28076

748.02155	1.5	28077

748.12054	1	28078

748.21954	0.5	28088

748.31854	0.5	28089.5

748.41753	0.5	28090.5

748.51653	11	28091

748.61553	2.5	28091.5

748.71452	13.5	28092

748.81352	0.5	28103

748.91252	0.5	28105.5

749.01152	6.5	28119

749.11051	0.5	28119.5

749.20951	0.5	28120

749.30851	6	28126.5

749.4075	4	28127

749.5065	0.5	28127.5

749.6055	0.5	28133.5

749.70449	16.5	28137.5

749.80349	0.5	28138

749.90249	17	28138.5

750.00149	12	28155

750.10048	0.5	28155.5

750.19948	0.5	28172.5

750.29848	1.5	28184.5

750.39747	10	28185

750.49647	1	28185.5

750.59547	0.5	28187

750.69447	18	28197

750.79346	4	28198

750.89246	1	28198.5

750.99146	12.5	28216.5

751.09045	3.5	28220.5

751.18945	8.5	28221.5

751.28845	0.5	28234

751.38744	17	28237.5

751.48644	0.5	28246

751.58544	0.5	28246.5

751.68444	9.5	28263.5

751.78343	1.5	28264

751.88243	0.5	28264.5

751.98143	19.5	28274

752.08042	6.5	28275.5

752.17942	0.5	28276

752.27842	7	28295.5

752.37741	1	28302

752.47641	0.5	28302.5

752.57541	2	28309.5

752.67441	9	28310.5

752.7734	1.5	28311

752.8724	0.5	28313

752.9714	14	28322

753.07039	1	28323.5

753.16939	0.5	28324

753.26839	7.5	28338

753.36738	0.5	28339

753.46638	1	28339.5

753.56538	0.5	28347

753.66438	18.5	28347.5

753.76337	1	28348.5

753.86237	2	28349

753.96137	10.5	28367.5

754.06036	0.5	28368.5

754.15936	1.5	28370.5

754.25836	19.5	28381

754.35736	1	28381.5

754.45635	1	28383

754.55535	7.5	28402.5

754.65435	1	28403.5

754.75334	1	28404.5

754.85234	1.5	28412

754.95134	10.5	28413

755.05033	2.5	28414

755.14933	0.5	28415.5

755.24833	0.5	28426

755.34733	13.5	28428.5

755.44632	0.5	28429

755.54532	1.5	28429.5

755.64432	7	28443

755.74331	3	28443.5

755.84231	16	28445

755.94131	3.5	28452

756.0403	9	28455

756.1393	1	28471

756.2383	0.5	28474.5

756.3373	8.5	28483.5

756.43629	0.5	28484.5

756.53529	0.5	28485

756.63429	0.5	28493.5

756.73328	6	28494

756.83228	4	28494.5

756.93128	1.5	28495

757.03027	1.5	28501

757.12927	5.5	28505

757.22827	19.5	28506.5

757.32727	0.5	28508

757.42626	0.5	28513.5

757.52526	14	28533

757.62426	0.5	28533.5

757.72325	0.5	28534

757.82225	18.5	28548

757.92125	1.5	28548.5

758.02025	2	28549

758.11924	2.5	28567.5

758.21824	10.5	28569

758.31724	1.5	28571

758.41623	0.5	28573.5

758.51523	4.5	28584

758.61423	5	28585.5

758.71322	1	28586

758.81222	0.5	28590.5

758.91122	5	28595.5

759.01022	2	28596.5

759.10921	1.5	28597

759.20821	0.5	28602

759.30721	0.5	28604

759.4062	0.5	28605.5

759.5052	12.5	28606

759.6042	0.5	28606.5

759.70319	8	28607

759.80219	0.5	28619.5

759.90119	6	28620

760.00019	5.5	28628

760.09918	0.5	28628.5

760.19818	0.5	28634.5

760.29718	11	28640

760.39617	1.5	28640.5

760.49517	0.5	28641

760.59417	13	28652

760.69316	1	28653.5

760.79216	12	28654

760.89116	0.5	28667

760.99016	0.5	28668

761.08915	0.5	28680

761.18815	19	28680.5

761.28715	0.5	28681

761.38614	2	28681.5

761.48514	13.5	28700.5

761.58414	1.5	28701

761.68314	2.5	28703

761.78213	1	28716.5

761.88113	3	28718

761.98013	1.5	28720.5

762.07912	16	28721.5

762.17812	0.5	28724.5

762.27712	2	28726

762.37611	14	28742

762.47511	0.5	28742.5

762.57411	13	28744.5

762.67311	0.5	28758.5

762.7721	1.5	28759

762.8711	0.5	28772

762.9701	12.5	28772.5

763.06909	0.5	28774

763.16809	7	28774.5

763.26709	1.5	28787

763.36608	6	28787.5

763.46508	1.5	28794.5

763.56408	1	28796

763.66308	8	28802

763.76207	12.5	28803.5

763.86107	1	28804.5

763.96007	0.5	28812.5

764.05906	1.5	28825

764.15806	2.5	28826

764.25706	1	28826.5

764.35605	0.5	28828

764.45505	2.5	28830.5

764.55405	5.5	28831.5

764.65305	2	28832

764.75204	1	28834.5

764.85104	22.5	28840

764.95004	0.5	28842

765.04903	13	28843

765.14803	1.5	28865.5

765.24703	7.5	28866

765.34603	0.5	28879

765.44502	1	28880.5

765.54402	18.5	28888

765.64302	2.5	28888.5

765.74201	1	28889.5

765.84101	0.5	28908

765.94001	13.5	28910.5

766.039	0.5	28911.5

766.138	2	28912

766.237	9	28925.5

766.336	1	28926

766.43499	23.5	28928

766.53399	0.5	28937

766.63299	1	28938

766.73198	15.5	28961.5

766.83098	1.5	28962

766.92998	4.5	28963

767.02897	2	28978.5

767.12797	0.5	28980

767.22697	1	28984.5

767.32597	11	28986.5

767.42496	1	28987

767.52396	15	28988

767.62296	1.5	28999

767.72195	0.5	29000

767.82095	6.5	29015

767.91995	4.5	29016.5

768.01895	1	29017

768.11794	0.5	29023.5

768.21694	0.5	29028

768.31594	0.5	29029

768.41493	10.5	29029.5

768.51393	1	29030

768.61293	12.5	29030.5

768.71192	17.5	29041

768.81092	3	29042

768.90992	11.5	29054.5

769.00892	2	29072

769.10791	0.5	29075

769.20691	11.5	29086.5

769.30591	0.5	29088.5

769.4049	1.5	29089

769.5039	2	29100.5

769.6029	9	29101

769.70189	2	29102.5

769.80089	14	29104.5

769.89989	4.5	29113.5

769.99889	0.5	29115.5

770.09788	0.5	29129.5

770.19688	3	29134

770.29588	1	29134.5

770.39487	1.5	29135

770.49387	10.5	29138

770.59287	1	29139

770.69186	12.5	29140.5

770.79086	1	29151

770.88986	2.5	29152

770.98886	10.5	29164.5

771.08785	15.5	29165.5

771.18685	1	29168

771.28585	13	29178.5

771.38484	0.5	29194

771.48384	6.5	29195

771.58284	2	29208

771.68184	1	29208.5

771.78083	17.5	29215

771.87983	1.5	29217

771.97883	14.5	29218

772.07782	1	29235.5

772.17682	6.5	29237

772.27582	6.5	29251.5

772.37481	1.5	29252.5

772.47381	0.5	29259

772.57281	15	29265.5

772.67181	9	29267

772.7708	0.5	29267.5

772.8698	0.5	29282.5

772.9688	9.5	29291.5

773.06779	1	29292

773.16679	2	29292.5

773.26579	16	29302

773.36478	4.5	29303

773.46378	0.5	29305

773.56278	0.5	29321

773.66178	6.5	29325.5

773.76077	4.5	29326

773.85977	2.5	29326.5

773.95877	16.5	29333

774.05776	0.5	29337.5

774.15676	0.5	29340

774.25576	5	29356.5

774.35475	2	29357

774.45375	2	29357.5

774.55275	8	29362.5

774.65175	2.5	29364.5

774.75074	0.5	29366.5

774.84974	0.5	29374.5

774.94874	1.5	29377

775.04773	7	29377.5

775.14673	1	29378

775.24573	0.5	29379.5

775.34473	20.5	29386.5

775.44372	0.5	29387.5

775.54272	0.5	29388

775.64172	22.5	29408.5

775.74071	2	29409

775.83971	15.5	29409.5

775.93871	0.5	29432

776.0377	3.5	29434

776.1367	18	29449.5

776.2357	0.5	29450

776.3347	0.5	29453.5

776.43369	1	29471.5

776.53269	3	29472

776.63169	4	29472.5

776.73068	1	29473.5

776.82968	0.5	29476.5

776.92868	0.5	29480.5

777.02767	10	29481.5

777.12667	3	29482

777.22567	1	29482.5

777.32467	17	29492.5

777.42366	1	29495.5

777.52266	14.5	29496.5

777.62166	0.5	29513.5

777.72065	0.5	29514.5

777.81965	0.5	29529

777.91865	1	29529.5

778.01764	6	29530

778.11664	0.5	29530.5

778.21564	11	29531.5

778.31464	0.5	29537.5

778.41363	1	29538

778.51263	0.5	29549

778.61163	1.5	29549.5

778.71062	11.5	29550.5

778.80962	1	29551

778.90862	22.5	29552.5

779.00762	1	29564

779.10661	24	29565

779.20561	0.5	29587.5

779.30461	0.5	29588.5

779.4036	3.5	29612.5

779.5026	6.5	29613

779.6016	0.5	29613.5

779.70059	15.5	29617

779.79959	1.5	29623.5

779.89859	15.5	29624

779.99759	1.5	29639.5

780.09658	0.5	29641

780.19558	1.5	29656.5

780.29458	12.5	29658

780.39357	1	29658.5

780.49257	1.5	29660

780.59157	14.5	29672.5

780.69056	0.5	29673.5

780.78956	0.5	29675

780.88856	0.5	29689.5

780.98756	10.5	29690

781.08655	1	29690.5

781.18555	0.5	29691

781.28455	0.5	29701.5

781.38354	13.5	29702.5

781.48254	1.5	29703

781.58154	0.5	29703.5

781.68053	0.5	29717

781.77953	3.5	29718.5

781.87853	6.5	29719

781.97753	2	29719.5

782.07652	17.5	29723

782.17552	0.5	29729.5

782.27452	14.5	29731.5

782.37351	1	29749

782.47251	0.5	29749.5

782.57151	3.5	29764

782.67051	2	29765

782.7695	1.5	29765.5

782.8685	0.5	29769

782.9675	0.5	29771

783.06649	8.5	29772.5

783.16549	2	29773

783.26449	0.5	29773.5

783.36348	12.5	29782

783.46248	4.5	29784

783.56148	0.5	29784.5

783.66048	0.5	29797

783.75947	15.5	29801.5

783.85847	0.5	29802

783.95747	17	29802.5

784.05646	5.5	29818

784.15546	5	29818.5

784.25446	2	29835.5

784.35345	4	29841

784.45245	1	29846

784.55145	16	29848

784.65045	1	29852

784.74944	11	29853

784.84844	1	29869

784.94744	2.5	29870

785.04643	10	29881

785.14543	3	29882

785.24443	0.5	29884.5

785.34343	2	29894.5

785.44242	5.5	29897.5

785.54142	0.5	29898

785.64042	0.5	29900

785.73941	24	29905.5

785.83841	1.5	29906

785.93741	1	29906.5

786.0364	20	29930.5

786.1354	1.5	29932

786.2344	0.5	29933

786.3334	2.5	29953

786.43239	0.5	29954.5

786.53139	3.5	29955

786.63039	1	29957.5

786.72938	10.5	29958

786.82838	4.5	29961.5

786.92738	2.5	29962.5

787.02637	15	29973

787.12537	0.5	29977.5

787.22437	11	29980

787.32337	0.5	29995

787.42236	8	29995.5

787.52136	3	30006.5

787.62036	1.5	30007

787.71935	2	30015

787.81835	7.5	30018

787.91735	9	30019.5

788.01634	0.5	30021.5

788.11534	15	30029

788.21434	0.5	30038

788.31334	9.5	30038.5

788.41233	4	30053.5

788.51133	0.5	30054

788.61033	1.5	30063.5

788.70932	11	30067.5

788.80832	0.5	30068

788.90732	0.5	30069.5

789.00631	20.5	30080.5

789.10531	0.5	30081

789.20431	0.5	30081.5

789.30331	0.5	30102

789.4023	15.5	30102.5

789.5013	3	30103

789.6003	2	30103.5

789.69929	0.5	30119

789.79829	34.5	30122

789.89729	1.5	30124

789.99629	3.5	30124.5

790.09528	2.5	30159

790.19428	1.5	30160.5

790.29328	1.5	30164

790.39227	5	30166.5

790.49127	0.5	30168

790.59027	1	30169.5

790.68926	0.5	30174.5

790.78826	29	30175

790.88726	0.5	30176

790.98626	6.5	30176.5

791.08525	4.5	30205.5

791.18425	0.5	30206

791.28325	1	30212.5

791.38224	26.5	30217

791.48124	0.5	30217.5

791.58024	14	30218.5

791.67923	1	30245

791.77823	0.5	30245.5

791.87723	1	30259.5

791.97623	13	30260.5

792.07522	2.5	30261

792.17422	0.5	30262

792.27322	1	30275

792.37221	8.5	30277.5

792.47121	1.5	30278

792.57021	17	30279

792.66921	5	30287.5

792.7682	1	30289

792.8672	8	30306

792.9662	2	30311

793.06519	0.5	30312

793.16419	6.5	30320

793.26319	2	30322

793.36218	3	30322.5

793.46118	0.5	30329

793.56018	0.5	30331

793.65918	2	30334

793.75817	16	30334.5

793.85717	0.5	30335

793.95617	0.5	30337

794.05516	20.5	30353

794.15416	4	30353.5

794.25316	3	30354

794.35215	0.5	30374.5

794.45115	3	30378.5

794.55015	0.5	30381.5

794.64915	2	30382

794.74814	12.5	30385

794.84714	0.5	30385.5

794.94614	1	30387.5

795.04513	12	30400

795.14413	0.5	30400.5

795.24313	20.5	30401.5

795.34212	0.5	30413.5

795.44112	0.5	30414

795.54012	0.5	30434.5

795.63912	16	30435

795.73811	1.5	30435.5

795.83711	0.5	30436

795.93611	16	30452

796.0351	0.5	30453.5

796.1341	9	30454

796.2331	6.5	30470

796.3321	1	30470.5

796.43109	0.5	30479.5

796.53009	20.5	30486

796.62909	0.5	30487

796.72808	7.5	30487.5

796.82708	5	30508

796.92608	0.5	30508.5

797.02507	22.5	30516

797.12407	0.5	30521

797.22307	0.5	30521.5

797.32207	0.5	30544

797.42106	12.5	30544.5

797.52006	1	30545

797.61906	0.5	30545.5

797.71805	0.5	30558

797.81705	0.5	30559

797.91605	0.5	30559.5

798.01504	2	30560

798.11404	1	30560.5

798.21304	1	30561

798.31204	7.5	30563

798.41103	2.5	30564

798.51003	4	30565

798.60903	4	30572.5

798.70802	6	30575

798.80702	1.5	30579

798.90602	9	30583

799.00501	3	30589

799.10401	0.5	30590.5

799.20301	13.5	30599.5

799.30201	2	30602.5

799.401	0.5	30603

799.5	0.5	30616.5

799.599	0.5	30618.5

799.69799	1	30619

799.79699	3.5	30619.5

799.89599	2	30620

799.99499	0.5	30621

800.09398	3	30624.5

800.19298	1.5	30626.5

800.29198	4	30627

800.39097	5	30630

800.48997	1	30631.5

800.58897	0.5	30635.5

800.68796	1	30640.5

800.78696	10.5	30641.5

800.88596	0.5	30642

800.98496	0.5	30643

801.08395	0.5	30653.5

801.18295	20	30654

801.28195	1	30654.5

801.38094	2	30655

801.47994	13.5	30675

801.57894	2	30676

801.67793	0.5	30678

801.77693	10	30691.5

801.87593	1	30693.5

801.97493	0.5	30694

802.07392	14.5	30704

802.17292	0.5	30705

802.27192	2.5	30705.5

802.37091	0.5	30720

802.46991	11	30720.5

802.56891	22.5	30723

802.6679	1	30723.5

802.7669	0.5	30734.5

802.8659	0.5	30757

802.9649	16	30758

803.06389	8.5	30758.5

803.16289	3.5	30759

803.26189	2	30775

803.36088	8.5	30783.5

803.45988	0.5	30787

803.55888	0.5	30789

803.65788	0.5	30797.5

803.75687	2	30798

803.85587	4	30798.5

803.95487	0.5	30799

804.05386	1	30801

804.15286	14	30805

804.25186	1	30805.5

804.35085	1	30806.5

804.44985	11.5	30820.5

804.54885	2	30821.5

804.64785	15.5	30822.5

804.74684	0.5	30834

804.84584	1.5	30836

804.94484	16	30851.5

805.04383	0.5	30852

805.14283	0.5	30853.5

805.24183	0.5	30869.5

805.34082	16	30870

805.43982	0.5	30870.5

805.53882	1	30871

805.63782	10.5	30887

805.73681	0.5	30887.5

805.83581	13	30888.5

805.93481	0.5	30899

806.0338	0.5	30899.5

806.1328	10	30912.5

806.2318	0.5	30913

806.33079	2	30913.5

806.42979	15.5	30923.5

806.52879	0.5	30924

806.62779	3	30926

806.72678	2.5	30941.5

806.82578	0.5	30942

806.92478	1	30945

807.02377	0.5	30947.5

807.12277	20	30948

807.22177	1	30949

807.32077	2.5	30949.5

807.41976	0.5	30969.5

807.51876	4.5	30970.5

807.61776	0.5	30973

807.71675	13	30973.5

807.81575	0.5	30978

807.91475	2	30978.5

808.01374	13	30991.5

808.11274	2.5	30992

808.21174	8	30994

808.31074	1	31007

808.40973	1	31009.5

808.50873	5.5	31017.5

808.60773	1.5	31018.5

808.70672	1	31019.5

808.80572	9	31025

808.90472	0.5	31026.5

809.00371	2.5	31027.5

809.10271	0.5	31036.5

809.20171	3	31037

809.30071	5	31039.5

809.3997	0.5	31040

809.4987	0.5	31043

809.5977	0.5	31048

809.69669	16	31048.5

809.79569	1.5	31049

809.89469	9	31049.5

809.99369	2.5	31065.5

810.09268	1	31067

810.19168	1.5	31076

810.29068	9.5	31078.5

810.38967	1	31079.5

810.48867	0.5	31081

810.58767	18	31090.5

810.68666	2	31091.5

810.78566	18.5	31092

810.88466	0.5	31110

810.98366	0.5	31112

811.08265	20	31130.5

811.18165	0.5	31131

811.28065	0.5	31131.5

811.37964	0.5	31151.5

811.47864	11	31152

811.57764	3.5	31152.5

811.67663	0.5	31153

811.77563	12	31164

811.87463	0.5	31167.5

811.97363	0.5	31168

812.07262	0.5	31180

812.17162	0.5	31180.5

812.27062	15	31181

812.36961	1	31181.5

812.46861	0.5	31182

812.56761	21.5	31197

812.6666	1	31198

812.7656	1	31198.5

812.8646	17.5	31220

812.9636	1.5	31221

813.06259	0.5	31222

813.16159	0.5	31239.5

813.26059	11	31241

813.35958	1.5	31241.5

813.45858	0.5	31242

813.55758	20.5	31253

813.65657	2	31254.5

813.75557	0.5	31255

813.85457	5	31275.5

813.95357	0.5	31277.5

814.05256	1.5	31278

814.15156	0.5	31283

814.25056	8.5	31283.5

814.34955	0.5	31285

814.44855	1	31285.5

814.54755	0.5	31294

814.64655	1.5	31294.5

814.74554	10	31295.5

814.84454	1	31296

814.94354	11.5	31297.5

815.04253	1	31307.5

815.14153	1.5	31308.5

815.24053	1.5	31320

815.33952	0.5	31321

815.43852	0.5	31322.5

815.53752	0.5	31324

815.63652	1.5	31324.5

815.73551	11.5	31325

815.83451	2.5	31325.5

815.93351	0.5	31327

816.0325	0.5	31338.5

816.1315	16.5	31341

816.2305	2	31341.5

816.32949	0.5	31342

816.42849	2	31358.5

816.52749	3.5	31360.5

816.62649	5.5	31361

816.72548	3	31363

816.82448	0.5	31366.5

816.92348	16	31372

817.02247	8.5	31375

817.12147	1.5	31375.5

817.22047	2.5	31391.5

817.31947	4.5	31400

817.41846	9	31401.5

817.51746	0.5	31404

817.61646	0.5	31408.5

817.71545	17.5	31417.5

817.81445	1	31418

817.91345	14.5	31418.5

818.01244	4	31436

818.11144	0.5	31437

818.21044	18	31451.5

818.30944	1	31455.5

818.40843	0.5	31456

818.50743	0.5	31474

818.60643	9.5	31475

818.70542	1	31475.5

818.80442	1.5	31476

818.90342	1.5	31485.5

819.00241	11.5	31486.5

819.10141	0.5	31488

819.20041	11	31489.5

819.29941	3.5	31501

819.3984	1	31501.5

819.4974	12	31512.5

819.5964	0.5	31516

819.69539	0.5	31517

819.79439	2	31529

819.89339	13	31529.5

819.99238	2	31530

820.09138	0.5	31532

820.19038	0.5	31545

820.28938	13	31547

820.38837	0.5	31547.5

820.48737	0.5	31548

820.58637	0.5	31561

820.68536	10	31561.5

820.78436	1	31562

820.88336	14.5	31562.5

820.98236	1.5	31572.5

821.08135	14	31573.5

821.18035	1.5	31588

821.27935	4.5	31589.5

821.37834	5	31603.5

821.47734	0.5	31605

821.57634	8	31609.5

821.67533	3	31614.5

821.77433	0.5	31615

821.87333	12.5	31623

821.97233	1.5	31626

822.07132	1	31626.5

822.17032	0.5	31639

822.26932	3	31640.5

822.36831	4	31641.5

822.46731	3.5	31642

822.56631	0.5	31645

822.6653	10	31649

822.7643	0.5	31652.5

822.8633	1	31653

822.9623	0.5	31663

823.06129	0.5	31663.5

823.16029	0.5	31664.5

823.25929	9	31665

823.35828	0.5	31665.5

823.45728	0.5	31666

823.55628	9.5	31675

823.65527	1.5	31675.5

823.75427	2	31676

823.85327	1.5	31685.5

823.95227	0.5	31687

824.05126	7.5	31689

824.15026	0.5	31690.5

824.24926	0.5	31691

824.34825	1.5	31698.5

824.44725	16.5	31699

824.54625	1	31699.5

824.64525	2.5	31701

824.74424	11	31717.5

824.84324	3.5	31718.5

824.94224	0.5	31721

825.04123	9.5	31732

825.14023	2.5	31735.5

825.23923	23	31736

825.33822	0.5	31745.5

825.43722	1	31748

825.53622	22	31771

825.63522	0.5	31771.5

825.73421	1	31772.5

825.83321	17	31794.5

825.93221	0.5	31795

826.0312	0.5	31796

826.1302	1.5	31813

826.2292	16.5	31813.5

826.32819	1	31814

826.42719	1	31815.5

826.52619	0.5	31832

826.62519	8.5	31833

826.72418	2.5	31834

826.82318	2.5	31834.5

826.92218	0.5	31843

827.02117	1	31845.5

827.12017	18	31848

827.21917	0.5	31848.5

827.31816	20.5	31849.5

827.41716	3.5	31867.5

827.51616	24.5	31868

827.61516	0.5	31888.5

827.71415	0.5	31892

827.81315	0.5	31916.5

827.91215	0.5	31917

828.01114	4	31917.5

828.11014	27	31918

828.20914	1	31918.5

828.30814	0.5	31922.5

828.40713	22	31949.5

828.50613	15	31950.5

828.60513	2	31951

828.70412	2	31973

828.80312	7.5	31988

828.90212	1	31990

829.00111	0.5	31992

829.10011	16.5	31999.5

829.19911	0.5	32000.5

829.29811	21.5	32001

829.3971	0.5	32017.5

829.4961	17.5	32018

829.5951	1	32039.5

829.69409	0.5	32040

829.79309	16	32057.5

829.89209	1.5	32058.5

829.99108	11.5	32059

830.09008	0.5	32075

830.18908	1.5	32076.5

830.28808	32.5	32088

830.38707	0.5	32088.5

830.48607	19	32090

830.58507	2	32122.5

830.68406	0.5	32123

830.78306	1	32142

830.88206	22.5	32144

830.98105	3	32144.5

831.08005	1.5	32145.5

831.17905	16.5	32168

831.27805	4	32171

831.37704	1	32172.5

831.47604	0.5	32189

831.57504	6.5	32193

831.67403	1.5	32194

831.77303	1	32194.5

831.87203	20	32201

831.97103	0.5	32202.5

832.07002	6	32203.5

832.16902	13	32223.5

832.26802	0.5	32224

832.36701	1	32230

832.46601	14.5	32243

832.56501	1	32243.5

832.664	1	32244.5

832.763	20	32259

832.862	1	32260

832.961	24	32261

833.05999	0.5	32281

833.15899	1	32282

833.25799	10.5	32306

833.35698	2	32306.5

833.45598	0.5	32307.5

833.55498	16.5	32318

833.65397	1.5	32320

833.75297	0.5	32320.5

833.85197	0.5	32337

833.95097	1	32338.5

834.04996	3.5	32339

834.14896	5.5	32339.5

834.24796	0.5	32340.5

834.34695	0.5	32344

834.44595	11	32349.5

834.54495	1.5	32350

834.64395	8	32350.5

834.74294	1.5	32361.5

834.84194	0.5	32363

834.94094	2.5	32371

835.03993	2.5	32372.5

835.13893	18.5	32373

835.23793	0.5	32375.5

835.33692	15.5	32378

835.43592	0.5	32396.5

835.53492	0.5	32397

835.63392	17.5	32412.5

835.73291	20	32413

835.83191	1	32413.5

835.93091	0.5	32431

836.0299	1	32451

836.1289	8	32452

836.2279	2	32452.5

836.32689	11	32453.5

836.42589	0.5	32461.5

836.52489	0.5	32463.5

836.62389	1	32474.5

836.72288	2	32475

836.82188	10.5	32475.5

836.92088	1.5	32476.5

837.01987	15.5	32478.5

837.11887	0.5	32489

837.21787	0.5	32490.5

837.31686	0.5	32506

837.41586	21	32506.5

837.51486	17	32507

837.61386	2	32507.5

837.71285	2.5	32528.5

837.81185	13.5	32545.5

837.91085	0.5	32547.5

838.00984	1	32550

838.10884	4	32563.5

838.20784	1	32564

838.30684	4	32565

838.40583	0.5	32569

838.50483	1	32570

838.60383	11.5	32574

838.70282	0.5	32574.5

838.80182	3.5	32575.5

838.90082	24.5	32587

838.99981	1	32587.5

839.09881	9	32591

839.19781	1	32615.5

839.29681	0.5	32616.5

839.3958	0.5	32625.5

839.4948	0.5	32626.5

839.5938	19.5	32627

839.69279	5.5	32627.5

839.79179	12.5	32628

839.89079	1.5	32647.5

839.98978	8	32653

840.08878	0.5	32665.5

840.18778	0.5	32667

840.28678	1.5	32675

840.38577	16.5	32675.5

840.48477	4.5	32676

840.58377	0.5	32677.5

840.68276	3	32694

840.78176	3.5	32698.5

840.88076	1	32699

840.97975	0.5	32702

841.07875	3	32705.5

841.17775	4.5	32706.5

841.27675	17	32707

841.37574	0.5	32710

841.47474	2.5	32714.5

841.57374	0.5	32731.5

841.67273	0.5	32732

841.77173	22	32734.5

841.87073	0.5	32735

841.96973	1.5	32735.5

842.06872	25.5	32757.5

842.16772	0.5	32758

842.26672	1	32759.5

842.36571	0.5	32785

842.46471	14	32785.5

842.56371	0.5	32786.5

842.6627	16.5	32787

842.7617	1	32801

842.8607	1	32801.5

842.9597	9	32818

843.05869	1.5	32819

843.15769	8.5	32820

843.25669	2.5	32829

843.35568	1	32830.5

843.45468	0.5	32839

843.55368	0.5	32841.5

843.65267	17.5	32842.5

843.75167	1	32843

843.85067	9.5	32843.5

843.94967	0.5	32861

844.04866	0.5	32862

844.14766	0.5	32871.5

844.24666	0.5	32872

844.34565	11.5	32872.5

844.44465	1	32873

844.54365	0.5	32873.5

844.64264	1	32885

844.74164	16	32886

844.84064	0.5	32886.5

844.93964	1	32887.5

845.03863	18	32903.5

845.13763	0.5	32904

845.23663	0.5	32905

845.33562	10	32923

845.43462	1.5	32923.5

845.53362	0.5	32924

845.63262	3.5	32934

845.73161	1.5	32935.5

845.83061	0.5	32936

845.92961	1	32939.5

846.0286	1	32941

846.1276	0.5	32941.5

846.2266	19	32942.5

846.32559	0.5	32943.5

846.42459	6.5	32944

846.52359	4.5	32963

846.62259	2	32963.5

846.72158	0.5	32970

846.82058	1	32974.5

846.91958	11	32976.5

847.01857	1.5	32977

847.11757	0.5	32978

847.21657	1	32989

847.31556	3	32990.5

847.41456	1.5	32991

847.51356	0.5	32992

847.61256	0.5	32995

847.71155	1.5	32996.5

847.81055	11.5	32997

847.90955	0.5	32997.5

848.00854	0.5	32999

848.10754	3.5	33010.5

848.20654	11	33011

848.30553	0.5	33011.5

848.40453	22	33015

848.50353	1.5	33026

848.60253	5	33026.5

848.70152	5	33048.5

848.80052	0.5	33050

848.89952	0.5	33055

848.99851	6.5	33060

849.09751	0.5	33060.5

849.19651	2.5	33061

849.29551	4.5	33067.5

849.3945	6	33068

849.4935	1	33070.5

849.5925	2	33075

849.69149	0.5	33081

849.79049	3.5	33082

849.88949	8.5	33084

849.98848	0.5	33084.5

850.08748	2.5	33088

850.18648	0.5	33096.5

850.28548	4	33097

850.38447	4.5	33099.5

850.48347	5	33100

850.58247	6.5	33104

850.68146	0.5	33108.5

850.78046	20	33113.5

850.87946	1.5	33120

850.97845	0.5	33120.5

851.07745	2.5	33140.5

851.17645	7	33142

851.27545	0.5	33142.5

851.37444	7.5	33145

851.47344	1.5	33152

851.57244	0.5	33152.5

851.67143	0.5	33160

851.77043	0.5	33161.5

851.86943	17.5	33162

851.96843	3	33162.5

852.06742	1	33163

852.16642	1.5	33180.5

852.26542	0.5	33183.5

852.36441	0.5	33184.5

852.46341	1	33186

852.56241	0.5	33186.5

852.6614	14	33187

852.7604	3.5	33188

852.8594	0.5	33188.5

852.9584	18.5	33202.5

853.05739	3	33206

853.15639	0.5	33206.5

853.25539	21	33225

853.35438	1.5	33228

853.45338	6	33228.5

853.55238	1	33249.5

853.65137	1	33251

853.75037	7.5	33257

853.84937	5.5	33258

853.94837	0.5	33259

854.04736	14	33266.5

854.14636	0.5	33272

854.24536	0.5	33272.5

854.34435	22	33286.5

854.44335	2	33287

854.54235	0.5	33287.5

854.64134	3.5	33309.5

854.74034	9.5	33311.5

854.83934	1.5	33312

854.93834	21.5	33315.5

855.03733	1	33325

855.13633	2	33326.5

855.23533	7.5	33348

855.33432	5	33349

855.43332	0.5	33351

855.53232	25	33358.5

855.63131	20.5	33363.5

855.73031	1	33364

855.82931	0.5	33389

855.92831	0.5	33409.5

856.0273	13.5	33410.5

856.1263	2	33411

856.2253	15	33411.5

856.32429	1	33425

856.42329	0.5	33427

856.52229	16.5	33442

856.62129	0.5	33443

856.72028	1	33443.5

856.81928	0.5	33460

856.91828	9	33460.5

857.01727	1.5	33461.5

857.11627	0.5	33462

857.21527	1	33471

857.31426	2.5	33472.5

857.41326	4	33473

857.51226	1	33474

857.61126	0.5	33476.5

857.71025	19	33480.5

857.80925	1	33481.5

857.90825	24.5	33482

858.00724	12.5	33501

858.10624	0.5	33502

858.20524	1	33526.5

858.30423	2	33539

858.40323	12	33539.5

858.50223	1	33540.5

858.60123	10.5	33542.5

858.70022	6	33554.5

858.79922	2	33555.5

858.89822	12.5	33566

858.99721	0.5	33572

859.09621	1	33574

859.19521	14	33586.5

859.29421	0.5	33587

859.3932	0.5	33588

859.4922	7.5	33602

859.5912	0.5	33602.5

859.69019	0.5	33603

859.78919	1.5	33610.5

859.88819	17	33611

859.98718	4.5	33611.5

860.08618	1	33613

860.18518	1	33630

860.28418	0.5	33634.5

860.38317	10.5	33635.5

860.48217	3	33636.5

860.58117	1.5	33637

860.68016	1	33647.5

860.77916	0.5	33650.5

860.87816	1	33652

860.97715	7	33653

861.07615	2.5	33653.5

861.17515	0.5	33654.5

861.27415	14	33661.5

861.37314	0.5	33664

861.47214	0.5	33664.5

861.57114	22	33678.5

861.67013	3	33679

861.76913	1.5	33679.5

861.86813	1	33701.5

861.96712	1	33704.5

862.06612	7.5	33706

862.16512	0.5	33707

862.26412	5.5	33708

862.36311	8	33715.5

862.46211	0.5	33716

862.56111	0.5	33721.5

862.6601	9.5	33729.5

862.7591	5.5	33730

862.8581	1.5	33730.5

862.9571	0.5	33740

863.05609	1.5	33745.5

863.15509	13.5	33747

863.25409	2.5	33747.5

863.35308	0.5	33749

863.45208	0.5	33762.5

863.55108	0.5	33765

863.65007	17.5	33765.5

863.74907	1.5	33766

863.84807	3	33766.5

863.94707	17.5	33784

864.04606	1.5	33785.5

864.14506	1	33788.5

864.24406	9.5	33806

864.34305	0.5	33807.5

864.44205	0.5	33808.5

864.54105	8.5	33818

864.64004	1	33818.5

864.73904	0.5	33819

864.83804	0.5	33827.5

864.93704	1	33828.5

865.03603	15.5	33829

865.13503	1	33829.5

865.23403	1	33830.5

865.33302	1	33846

865.43202	1	33847

865.53102	24	33848

865.63001	2.5	33849

865.72901	14	33850

865.82801	0.5	33874

865.92701	1.5	33876.5

866.026	2	33890.5

866.125	11	33891

866.224	1.5	33892.5

866.32299	15	33894.5

866.42199	0.5	33905.5

866.52099	0.5	33907

866.61999	11.5	33922

866.71898	1	33922.5

866.81798	0.5	33923

866.91698	2	33934.5

867.01597	13	33935.5

867.11497	2	33936

867.21397	14	33938

867.31296	1.5	33951

867.41196	12	33953

867.51096	1.5	33967

867.60996	0.5	33968.5

867.70895	15.5	33980.5

867.80795	0.5	33982

867.90695	2.5	33982.5

868.00594	6	33998

868.10494	1.5	33998.5

868.20394	2	34001

868.30293	14.5	34007

868.40193	2	34008.5

868.50093	1	34010.5

868.59993	1.5	34025

868.69892	7	34027

868.79792	1	34028

868.89692	1	34029.5

868.99591	1.5	34036.5

869.09491	18	34037.5

869.19391	1.5	34038.5

869.2929	0.5	34040

869.3919	12.5	34058

869.4909	1.5	34059.5

869.5899	19	34060

869.68889	16	34072.5

869.78789	1.5	34074

869.88689	0.5	34093

869.98588	1	34109

870.08488	3.5	34110.5

870.18388	1	34111

870.28288	2.5	34112

870.38187	0.5	34115.5

870.48087	0.5	34116.5

870.57987	9	34119

870.67886	0.5	34119.5

870.77786	0.5	34120

870.87686	0.5	34129

870.97585	8	34129.5

871.07485	1.5	34130

871.17385	1.5	34130.5

871.27285	6	34138.5

871.37184	4.5	34140

871.47084	4	34141.5

871.56984	0.5	34147.5

871.66883	10.5	34152

871.76783	0.5	34156

871.86683	1.5	34156.5

871.96582	11.5	34167

872.06482	1	34167.5

872.16382	0.5	34169

872.26282	1	34180.5

872.36181	0.5	34181.5

872.46081	5.5	34182

872.55981	3.5	34183

872.6588	0.5	34183.5

872.7578	0.5	34189

872.8568	4	34192.5

872.95579	6	34193

873.05479	0.5	34193.5

873.15379	0.5	34197.5

873.25279	1	34203.5

873.35178	1	34204

873.45078	18	34204.5

873.54978	1	34205.5

873.64877	1	34206.5

873.74777	16.5	34224.5

873.84677	0.5	34225.5

873.94577	17	34226.5

874.04476	1	34243

874.14376	0.5	34243.5

874.24276	1	34260.5

874.34175	1	34261.5

874.44075	18.5	34262

874.53975	1	34263

874.63874	1	34264

874.73774	15.5	34282.5

874.83674	0.5	34283.5

874.93574	3	34284.5

875.03473	4.5	34300

875.13373	2	34300.5

875.23273	14	34303.5

875.33172	0.5	34308

875.43072	1	34310

875.52972	1.5	34324

875.62871	1	34324.5

875.72771	2.5	34325.5

875.82671	0.5	34327

875.92571	15	34328

876.0247	0.5	34330.5

876.1237	1.5	34331

876.2227	2.5	34346

876.32169	16	34346.5

876.42069	1	34348

876.51969	0.5	34350.5

876.61869	15.5	34366.5

876.71768	1	34367.5

876.81668	0.5	34368

876.91568	9	34383.5

877.01467	8.5	34384.5

877.11367	0.5	34385

877.21267	16	34394

877.31166	0.5	34402.5

877.41066	1	34403

877.50966	0.5	34419

877.60866	19	34419.5

877.70765	0.5	34420.5

877.80665	1	34421

877.90565	0.5	34440

878.00464	18.5	34440.5

878.10364	15	34441.5

878.20264	1.5	34442

878.30163	7	34460.5

878.40063	1.5	34475.5

878.49963	0.5	34477

878.59863	0.5	34484

878.69762	6	34485.5

878.79662	0.5	34486

878.89562	1	34486.5

878.99461	0.5	34492.5

879.09361	15	34493

879.19261	0.5	34494

879.2916	0.5	34494.5

879.3906	18	34509.5

879.4896	1	34510

879.5886	1	34510.5

879.68759	1	34528.5

879.78659	11.5	34529.5

879.88559	0.5	34530.5

879.98458	0.5	34531.5

880.08358	0.5	34543

880.18258	17	34543.5

880.28157	2	34544

880.38057	1	34544.5

880.47957	19.5	34561.5

880.57857	0.5	34563.5

880.67756	18	34564.5

880.77656	0.5	34584

880.87556	0.5	34584.5

880.97455	3	34602.5

881.07355	11	34603

881.17255	1	34603.5

881.27155	0.5	34606.5

881.37054	3	34617.5

881.46954	11	34618.5

881.56854	0.5	34619

881.66753	1	34622

881.76653	3	34633

881.86553	10.5	34633.5

881.96452	0.5	34634.5

882.06352	14	34637.5

882.16252	0.5	34648

882.26152	1	34648.5

882.36051	15	34662.5

882.45951	0.5	34663

882.55851	0.5	34664

882.6575	0.5	34679

882.7565	3	34679.5

882.8555	4	34680

882.95449	18.5	34680.5

883.05349	1.5	34683.5

883.15249	1	34687.5

883.25149	1	34706

883.35048	10.5	34707.5

883.44948	1.5	34708.5

883.54848	0.5	34709.5

883.64747	0.5	34720

883.74647	7.5	34721.5

883.84547	3.5	34722

883.94447	1	34722.5

884.04346	18.5	34730

884.14246	1.5	34733.5

884.24146	18.5	34734.5

884.34045	3.5	34753

884.43945	0.5	34754.5

884.53845	13.5	34773

884.63744	0.5	34776.5

884.73644	1	34777

884.83544	1	34790.5

884.93444	9	34791

885.03343	1.5	34792

885.13243	1	34793

885.23143	15	34802

885.33042	0.5	34803.5

885.42942	9.5	34804.5

885.52842	0.5	34819.5

885.62741	0.5	34820

885.72641	15.5	34829.5

885.82541	0.5	34830

885.92441	16.5	34830.5

886.0234	0.5	34846

886.1224	23.5	34846.5

886.2214	2	34863

886.32039	1.5	34863.5

886.41939	18	34887

886.51839	2.5	34889

886.61738	1	34890.5

886.71638	2	34908.5

886.81538	7.5	34911

886.91438	0.5	34912

887.01337	1	34914

887.11237	2	34921.5

887.21137	1	34922

887.31036	0.5	34923

887.40936	1	34925

887.50836	14	34926

887.60736	1	34926.5

887.70635	3.5	34927.5

887.80535	0.5	34941.5

887.90435	21	34942.5

888.00334	0.5	34946

888.10234	0.5	34946.5

888.20134	16.5	34967.5

888.30033	2.5	34968

888.39933	0.5	34968.5

888.49833	16	34985

888.59733	1	34987.5

888.69632	0.5	34988

888.79532	0.5	35004

888.89432	14	35005

888.99331	0.5	35005.5

889.09231	20	35006

889.19131	1.5	35020

889.2903	0.5	35020.5

889.3893	1	35040.5

889.4883	22.5	35042

889.5873	1	35042.5

889.68629	2	35043.5

889.78529	14.5	35066

889.88429	1	35067

889.98328	10.5	35069

890.08228	2.5	35083.5

890.18128	0.5	35084.5

890.28027	13	35095

890.37927	1	35097.5

890.47827	26.5	35098

890.57727	1.5	35111

890.67626	18	35112

890.77526	1	35138.5

890.87426	19	35140

890.97325	1	35158

891.07225	0.5	35159

891.17125	0.5	35178

891.27025	15	35179

891.36924	0.5	35179.5

891.46824	1	35180

891.56724	15.5	35195

891.66623	0.5	35195.5

891.76523	1	35196.5

891.86423	1.5	35212

891.96322	21.5	35212.5

892.06222	0.5	35213.5

892.16122	0.5	35215

892.26022	1	35236.5

892.35921	6.5	35237

892.45821	1	35237.5

892.55721	24	35238.5

892.6562	1.5	35245

892.7552	0.5	35246

892.8542	0.5	35270

892.95319	11	35271.5

893.05219	0.5	35272

893.15119	1	35272.5

893.25019	4.5	35283.5

893.34918	5.5	35284

893.44818	0.5	35285

893.54718	2	35289.5

893.64617	1	35295

893.74517	1	35295.5

893.84417	13.5	35297.5

893.94316	2.5	35298.5

894.04216	16.5	35299.5

894.14116	0.5	35313

894.24016	0.5	35315.5

894.33915	12.5	35332

894.43815	9	35332.5

894.53715	0.5	35333

894.63614	16.5	35345.5

894.73514	1	35354.5

894.83414	0.5	35355

894.93314	5.5	35371.5

895.03213	1.5	35372.5

895.13113	1.5	35373

895.23013	0.5	35378.5

895.32912	1.5	35380

895.42812	3	35381.5

895.52712	2.5	35382

895.62611	4.5	35383.5

895.72511	0.5	35386.5

895.82411	0.5	35389

895.92311	9	35393.5

896.0221	1	35394

896.1211	0.5	35394.5

896.2201	19.5	35403.5

896.31909	0.5	35404.5

896.41809	0.5	35405

896.51709	0.5	35424.5

896.61608	0.5	35425

896.71508	7	35425.5

896.81408	3	35426

896.91308	1	35426.5

897.01207	1.5	35433.5

897.11107	8	35436.5

897.21007	4	35437.5

897.30906	0.5	35439

897.40806	7	35447

897.50706	0.5	35451

897.60605	0.5	35451.5

897.70505	1.5	35458.5

897.80405	6	35459

897.90305	0.5	35459.5

898.00204	0.5	35461

898.10104	17.5	35467

898.20004	0.5	35467.5

898.29903	1	35468

898.39803	1	35485.5

898.49703	9	35486

898.59603	2	35487

898.69502	0.5	35488

898.79402	15.5	35497

898.89302	3	35499

898.99201	1	35499.5

899.09101	21.5	35515

899.19001	1	35518

899.289	2	35519

899.388	11	35540.5

899.487	1	35541.5

899.586	0.5	35543.5

899.68499	0.5	35554.5

899.78399	17	35555.5

899.88299	2	35556

899.98198	10	35556.5

900.08098	0.5	35573.5

900.17998	0.5	35575.5

900.27897	17	35585.5

900.37797	11.5	35586

900.47697	1.5	35586.5

900.57597	1.5	35603.5

900.67496	16	35615

900.77396	0.5	35616.5

900.87296	1.5	35618

900.97195	14	35634

901.07095	0.5	35634.5

901.16995	1	35636

901.26895	4	35650

901.36794	4.5	35650.5

901.46694	2	35651.5

901.56594	3	35655.5

901.66493	4	35660

901.76393	6.5	35662

901.86293	0.5	35665

901.96192	1.5	35669

902.06092	16	35675.5

902.15992	0.5	35676

902.25892	12	35677.5

902.35791	0.5	35693.5

902.45691	7	35694

902.55591	4	35706

902.6549	1	35706.5

902.7539	0.5	35713.5

902.8529	1	35717.5

902.95189	11	35718.5

903.05089	1	35719

903.14989	0.5	35720

903.24889	23	35731

903.34788	1.5	35732

903.44688	0.5	35732.5

903.54588	0.5	35755.5

903.64487	15.5	35757

903.74387	0.5	35757.5

903.84287	0.5	35758

903.94186	0.5	35773.5

904.04086	2.5	35774

904.13986	6.5	35774.5

904.23886	0.5	35775

904.33785	0.5	35777.5

904.43685	17	35784

904.53585	2	35784.5

904.63484	0.5	35785

904.73384	19	35802

904.83284	1	35804

904.93184	3	35804.5

905.03083	13.5	35823.5

905.12983	1	35824.5

905.22883	12.5	35827.5

905.32782	1.5	35841

905.42682	0.5	35842

905.52582	12.5	35854.5

905.62481	1	35856

905.72381	1	35856.5

905.82281	0.5	35869

905.92181	24.5	35870

906.0208	1.5	35871

906.1198	1	35871.5

906.2188	15	35896

906.31779	2	35897.5

906.41679	0.5	35898.5

906.51579	19.5	35913.5

906.61478	4.5	35915.5

906.71378	3.5	35916

906.81278	1.5	35935.5

906.91178	11	35940

907.01077	1.5	35943.5

907.10977	0.5	35945

907.20877	9.5	35956

907.30776	1	35957.5

907.40676	12.5	35958

907.50576	0.5	35967.5

907.60475	0.5	35968.5

907.70375	14.5	35981

907.80275	1.5	35981.5

907.90175	0.5	35982

908.00074	0.5	35996.5

908.09974	1	35998

908.19874	10	35998.5

908.29773	3.5	35999

908.39673	1.5	36000

908.49573	13	36010

908.59473	0.5	36013.5

908.69372	0.5	36015

908.79272	0.5	36028

908.89172	0.5	36028.5

908.99071	9.5	36029

909.08971	2	36029.5

909.18871	1	36030

909.2877	0.5	36039.5

909.3867	11.5	36041.5

909.4857	0.5	36042.5

909.5847	0.5	36043

909.68369	0.5	36054.5

909.78269	1	36055

909.88169	6.5	36055.5

909.98068	3.5	36056

910.07968	6	36057

910.17868	4.5	36063.5

910.27767	1.5	36067

910.37667	0.5	36073

910.47567	9	36077.5

910.57467	1	36079

910.67366	1.5	36079.5

910.77266	1	36088.5

910.87166	4	36089.5

910.97065	0.5	36091

911.06965	1.5	36092

911.16865	1	36096

911.26764	1	36096.5

911.36664	20.5	36098

911.46564	0.5	36099

911.56464	1	36100

911.66363	0.5	36120.5

911.76263	23.5	36121

911.86163	1	36122

911.96062	0.5	36122.5

912.05962	2	36146

912.15862	15	36147

912.25762	0.5	36147.5

912.35661	1	36149.5

912.45561	1.5	36164.5

912.55461	4	36165

912.6536	6.5	36166

912.7526	0.5	36167.5

912.8516	13	36171.5

912.95059	4	36178

913.04959	0.5	36178.5

913.14859	15.5	36191.5

913.24759	0.5	36195.5

913.34658	1	36196

913.44558	8	36211.5

913.54458	0.5	36212

913.64357	0.5	36213

913.74257	0.5	36221

913.84157	9	36221.5

913.94056	6.5	36222

914.03956	1.5	36222.5

914.13856	16.5	36231.5

914.23756	0.5	36238

914.33655	0.5	36239.5

914.43555	9.5	36256

914.53455	0.5	36256.5

914.63354	1	36257

914.73254	21.5	36266.5

914.83154	1.5	36267

914.93053	0.5	36268

915.02953	1	36289.5

915.12853	7	36291

915.22753	2	36291.5

915.32652	0.5	36292.5

915.42552	6.5	36299.5

915.52452	13	36301.5

915.62351	0.5	36302

915.72251	0.5	36308.5

915.82151	15	36321.5

915.92051	0.5	36322

916.0195	3	36322.5

916.1185	1	36337.5

916.2175	18.5	36338

916.31649	0.5	36341

916.41549	1	36342

916.51449	13.5	36360.5

916.61348	0.5	36361

916.71248	5	36362

916.81148	13	36375.5

916.91048	2	36376

917.00947	13	36381

917.10847	0.5	36394

917.20747	13.5	36396

917.30646	1	36409

917.40546	0.5	36409.5

917.50446	0.5	36423

917.60345	0.5	36424

917.70245	11.5	36424.5

917.80145	0.5	36425

917.90045	1	36425.5

917.99944	1	36437

918.09844	7	36437.5

918.19744	1	36438.5

918.29643	5	36439.5

918.39543	1	36446.5

918.49443	16	36447.5

918.59343	1.5	36452.5

918.69242	0.5	36453.5

918.79142	0.5	36469.5

918.89042	16	36471

918.98941	1.5	36471.5

919.08841	0.5	36472

919.18741	1	36488

919.2864	17.5	36489.5

919.3854	0.5	36490

919.4844	1	36491

919.5834	11.5	36508.5

919.68239	8.5	36509

919.78139	11.5	36510

919.88039	1.5	36521.5

919.97938	0.5	36530

920.07838	0.5	36541.5

920.17738	9.5	36543

920.27637	0.5	36543.5

920.37537	1	36544

920.47437	1.5	36553.5

920.57337	5.5	36554

920.67236	1.5	36555

920.77136	1	36556.5

920.87036	0.5	36562

920.96935	1.5	36563.5

921.06835	12.5	36564.5

921.16735	0.5	36565

921.26634	1.5	36566.5

921.36534	0.5	36579

921.46434	11.5	36579.5

921.56334	2.5	36581

921.66233	1.5	36581.5

921.76133	1	36593

921.86033	0.5	36595.5

921.95932	1.5	36597

922.05832	15	36598

922.15732	16	36598.5

922.25631	3	36600

922.35531	0.5	36615

922.45431	10	36631

922.55331	8	36634

922.6523	0.5	36634.5

922.7513	2.5	36644.5

922.8503	11	36652.5

922.94929	0.5	36653

923.04829	0.5	36655.5

923.14729	1.5	36666.5

923.24629	2.5	36667

923.34528	7	36667.5

923.44428	2	36669

923.54328	9	36671.5

923.64227	4.5	36678.5

923.74127	0.5	36680.5

923.84027	0.5	36689.5

923.93926	17.5	36694

924.03826	0.5	36694.5

924.13726	18	36695

924.23626	2	36712.5

924.33525	0.5	36713

924.43425	4.5	36731

924.53325	12	36733

924.63224	1	36733.5

924.73124	3	36738

924.83024	11	36750

924.92923	1.5	36751

925.02823	15	36754

925.12723	2	36765

925.22623	0.5	36766.5

925.32522	1	36781.5

925.42422	1.5	36783.5

925.52322	1.5	36784

925.62221	1	36785

925.72121	1	36786.5

925.82021	10.5	36788

925.91921	1.5	36789

926.0182	0.5	36790

926.1172	25	36800.5

926.2162	1	36802

926.31519	3.5	36802.5

926.41419	0.5	36827.5

926.51319	20.5	36828.5

926.61218	0.5	36832

926.71118	20	36832.5

926.81018	1	36853

926.90918	0.5	36853.5

927.00817	13.5	36873.5

927.10717	20.5	36874.5

927.20617	1	36875

927.30516	2.5	36888.5

927.40416	1	36909

927.50316	1	36910

927.60215	1.5	36912.5

927.70115	11.5	36913.5

927.80015	0.5	36914.5

927.89915	1	36916

927.99814	0.5	36927.5

928.09714	2	36928

928.19614	10	36929

928.29513	0.5	36929.5

928.39413	0.5	36931.5

928.49313	0.5	36941.5

928.59212	8	36942

928.69112	1.5	36942.5

928.79012	1	36943

928.88912	0.5	36951

928.98811	5.5	36952.5

929.08711	0.5	36953.5

929.18611	0.5	36954

929.2851	2	36959.5

929.3841	2	36960

929.4831	11.5	36960.5

929.5821	2.5	36962.5

929.68109	1	36964.5

929.78009	13.5	36976

929.87909	1.5	36978.5

929.97808	1.5	36979.5

930.07708	1.5	36993

930.17608	1	36994.5

930.27507	9	36996

930.37407	1.5	36997.5

930.47307	17.5	36998.5

930.57207	1	37007.5

930.67106	0.5	37009

930.77006	1.5	37026.5

930.86906	25	37027.5

930.96805	2.5	37028

931.06705	17	37029.5

931.16605	2	37054.5

931.26504	0.5	37057

931.36404	14	37074

931.46304	0.5	37076

931.56204	1.5	37076.5

931.66103	0.5	37090.5

931.76003	12.5	37091

931.85903	4.5	37092.5

931.95802	17.5	37093

932.05702	0.5	37105.5

932.15602	2.5	37110

932.25501	8	37127.5

932.35401	1	37128

932.45301	4	37130.5

932.55201	0.5	37138.5

932.651	17.5	37139.5

932.75	1.5	37143.5

932.849	0.5	37144

932.94799	13	37161.5

933.04699	1.5	37163

933.14599	1.5	37163.5

933.24499	1	37176.5

933.34398	0.5	37178

933.44298	3.5	37179.5

933.54198	5	37180.5

933.64097	1	37181

933.73997	1	37184.5

933.83897	0.5	37189.5

933.93796	7.5	37190.5

934.03696	0.5	37191.5

934.13596	0.5	37192

934.23496	16.5	37199.5

934.33395	0.5	37200

934.43295	2.5	37200.5

934.53195	1	37217

934.63094	0.5	37217.5

934.72994	6.5	37220

934.82894	3.5	37221

934.92793	1	37221.5

935.02693	1	37228

935.12593	11.5	37231.5

935.22493	1	37232.5

935.32392	0.5	37233.5

935.42292	8	37245

935.52192	1.5	37246

935.62091	17.5	37246.5

935.71991	2.5	37254.5

935.81891	0.5	37256

935.9179	1	37273.5

936.0169	0.5	37276

936.1159	16	37276.5

936.2149	3	37277.5

936.31389	21	37278

936.41289	1.5	37294

936.51189	0.5	37297

936.61088	0.5	37318

936.70988	10.5	37319.5

936.80888	1.5	37320

936.90788	0.5	37320.5

937.00687	13.5	37331

937.10587	2	37332.5

937.20487	4.5	37333

937.30386	10.5	37346.5

937.40286	1	37348.5

937.50186	0.5	37353

937.60085	23	37363.5

937.69985	1	37364.5

937.79885	0.5	37365

937.89785	3	37388

937.99684	5.5	37389

938.09584	1	37389.5

938.19484	1.5	37392.5

938.29383	18.5	37398

938.39283	1	37399

938.49183	0.5	37400.5

938.59082	0.5	37419

938.68982	13	37420

938.78882	1.5	37420.5

938.88782	1	37421

938.98681	2	37434

939.08581	5.5	37435.5

939.18481	5	37436.5

939.2838	13	37438.5

939.3828	1	37444

939.4818	10.5	37449

939.58079	17	37462

939.67979	0.5	37463

939.77879	0.5	37473.5

939.87779	1	37490.5

939.97678	12.5	37491

940.07578	2.5	37491.5

940.17478	10	37492.5

940.27377	1.5	37505

940.37277	0.5	37507.5

940.47177	10	37517.5

940.57077	0.5	37519

940.66976	0.5	37519.5

940.76876	0.5	37529.5

940.86776	12.5	37530

940.96675	2	37530.5

941.06575	12.5	37531

941.16475	0.5	37543.5

941.26374	2	37545.5

941.36274	0.5	37558

941.46174	17	37558.5

941.56074	0.5	37560.5

941.65973	1	37561

941.75873	0.5	37578

941.85773	1	37578.5

941.95672	14	37579.5

942.05572	0.5	37580

942.15472	3	37581

942.25371	16	37595

942.35271	3.5	37595.5

942.45171	1	37598.5

942.55071	0.5	37614.5

942.6497	18.5	37618

942.7487	1	37619

942.8477	0.5	37619.5

942.94669	9.5	37638

943.04569	3	37639

943.14469	0.5	37639.5

943.24369	1	37649

943.34268	2	37652

943.44168	5	37652.5

943.54068	1	37653.5

943.63967	1.5	37655.5

943.73867	1	37660.5

943.83767	1	37661.5

943.93666	14.5	37663

944.03566	1.5	37664

944.13466	1.5	37665

944.23366	6	37679.5

944.33265	14.5	37681

944.43165	2	37682.5

944.53065	1	37688.5

944.62964	15.5	37703

944.72864	0.5	37705

944.82764	0.5	37706

944.92663	2	37721.5

945.02563	7.5	37722

945.12463	1	37722.5

945.22363	1	37724.5

945.32262	9	37732

945.42162	1.5	37733

945.52062	2	37734

945.61961	10.5	37743

945.71861	1	37744.5

945.81761	1	37746.5

945.9166	7	37757

946.0156	1	37758

946.1146	1.5	37759

946.2136	1.5	37766

946.31259	18.5	37767

946.41159	11	37768.5

946.51059	0.5	37770

946.60958	0.5	37788.5

946.70858	0.5	37799.5

946.80758	0.5	37800

946.90657	18	37800.5

947.00557	0.5	37801

947.10457	15	37801.5

947.20357	0.5	37819.5

947.30256	1.5	37820

947.40156	13	37835

947.50056	1	37835.5

947.59955	15.5	37837

947.69855	3.5	37850

947.79755	15	37851

947.89655	1	37866.5

947.99554	0.5	37870

948.09454	0.5	37885

948.19354	21.5	37886

948.29253	1	37886.5

948.39153	7	37887

948.49053	3	37908.5

948.58952	9	37909.5

948.68852	0.5	37916.5

948.78752	3	37919.5

948.88652	11.5	37928.5

948.98551	1	37929

949.08451	1.5	37932

949.18351	1.5	37943.5

949.2825	11.5	37944.5

949.3815	1	37946

949.4805	0.5	37947.5

949.57949	2	37959

949.67849	1.5	37960

949.77749	1.5	37960.5

949.87649	10.5	37962.5

949.97548	2.5	37964

950.07448	2	37965.5

950.17348	3.5	37976

950.27247	6	37978.5

950.37147	0.5	37980.5

950.47047	1.5	37984

950.56947	0.5	37990

950.66846	10	37990.5

950.76746	1	37992

950.86646	1.5	37992.5

950.96545	1	38002.5

951.06445	15	38003.5

951.16345	4.5	38005

951.26244	0.5	38006

951.36144	0.5	38021

951.46044	10	38025.5

951.55944	0.5	38026

951.65843	0.5	38026.5

951.75743	0.5	38036.5

951.85643	15.5	38037

951.95542	0.5	38037.5

952.05442	0.5	38038

952.15342	1.5	38053.5

952.25241	10	38054

952.35141	0.5	38054.5

952.45041	0.5	38056

952.54941	0.5	38066

952.6484	4	38066.5

952.7474	9	38067

952.8464	1	38067.5

952.94539	0.5	38071.5

953.04439	0.5	38080.5

953.14339	3.5	38081.5

953.24238	1.5	38082

953.34138	0.5	38082.5

953.44038	4	38086

953.53938	1	38087.5

953.63837	13	38088

953.73737	0.5	38092

953.83637	0.5	38093

953.93536	12	38106

954.03436	0.5	38106.5

954.13336	0.5	38107

954.23236	17.5	38119

954.33135	1	38119.5

954.43035	0.5	38120

954.52935	1	38137.5

954.62834	13	38138.5

954.72734	2.5	38139

954.82634	1	38140

954.92533	10.5	38153

955.02433	1	38155.5

955.12333	0.5	38156.5

955.22233	1	38167

955.32132	22	38168

955.42032	3.5	38168.5

955.51932	23.5	38169.5

955.61831	2.5	38191.5

955.71731	0.5	38195

955.81631	12	38218.5

955.9153	10.5	38221

956.0143	1.5	38221.5

956.1133	1	38233.5

956.2123	3.5	38244

956.31129	5.5	38245.5

956.41029	4	38246.5

956.50929	0.5	38250

956.60828	0.5	38255.5

956.70728	10.5	38259.5

956.80628	0.5	38260

956.90527	2	38260.5

957.00427	0.5	38271

957.10327	0.5	38271.5

957.20227	15	38273.5

957.30126	6.5	38274

957.40026	2.5	38274.5

957.49926	2	38289.5

957.59825	13	38296

957.69725	0.5	38298.5

957.79625	0.5	38300.5

957.89525	22.5	38313.5

957.99424	0.5	38314

958.09324	19.5	38314.5

958.19224	7.5	38337

958.29123	1	38337.5

958.39023	0.5	38357

958.48923	1	38364.5

958.58822	0.5	38365.5

958.68722	0.5	38366

958.78622	21	38367

958.88522	1	38367.5

958.98421	1	38368

959.08321	13.5	38389

959.18221	2.5	38390

959.2812	1.5	38391

959.3802	0.5	38404.5

959.4792	2.5	38407

959.57819	12	38408.5

959.67719	0.5	38409

959.77619	14.5	38411.5

959.87519	2	38423.5

959.97418	0.5	38424

960.07318	1.5	38438.5

960.17218	0.5	38440.5

960.27117	17	38441

960.37017	1	38442.5

960.46917	0.5	38443

960.56816	17	38460

960.66716	18	38461

960.76616	2	38461.5

960.86516	1.5	38478.5

960.96415	0.5	38496.5

961.06315	11.5	38498.5

961.16215	1	38500

961.26114	0.5	38500.5

961.36014	3	38512

961.45914	4.5	38513

961.55814	5.5	38513.5

961.65713	2	38516.5

961.75613	0.5	38521

961.85513	0.5	38526.5

961.95412	5.5	38528.5

962.05312	0.5	38529

962.15212	1	38529.5

962.25111	0.5	38535

962.35011	2.5	38535.5

962.44911	1.5	38536.5

962.54811	3	38537

962.6471	0.5	38539.5

962.7461	1	38541

962.8451	0.5	38544

962.94409	14	38544.5

963.04309	3	38545.5

963.14209	0.5	38546

963.24108	9	38560

963.34008	0.5	38563

963.43908	0.5	38563.5

963.53808	0.5	38572.5

963.63707	8.5	38573

963.73607	2	38573.5

963.83507	3	38574

963.93406	2	38582.5

964.03306	18	38584.5

964.13206	0.5	38587.5

964.23105	0.5	38589.5

964.33005	1	38607.5

964.42905	10.5	38608

964.52805	0.5	38608.5

964.62704	1	38609.5

964.72604	1	38620

964.82504	9	38620.5

964.92403	0.5	38621.5

965.02303	14.5	38622.5

965.12203	0.5	38631.5

965.22103	1.5	38632

965.32002	2	38646.5

965.41902	8	38647

965.51802	0.5	38648.5

965.61701	19.5	38650.5

965.71601	4	38658.5

965.81501	2	38659

965.914	14.5	38678.5

966.013	0.5	38682.5

966.112	0.5	38684.5

966.211	17.5	38699

966.30999	0.5	38699.5

966.40899	1	38700

966.50799	13.5	38717.5

966.60698	4	38718

966.70598	13	38719

966.80498	1	38732.5

966.90397	14.5	38736.5

967.00297	5.5	38749.5

967.10197	1	38750.5

967.20097	9	38765

967.29996	1	38770.5

967.39896	2	38771.5

967.49796	0.5	38780.5

967.59695	11.5	38781.5

967.69595	4.5	38783.5

967.79495	15.5	38784

967.89395	0.5	38795.5

967.99294	16.5	38800

968.09194	2	38815.5

968.19094	1	38816

968.28993	1	38832.5

968.38893	19	38834.5

968.48793	0.5	38835.5

968.58692	0.5	38836.5

968.68592	2	38855.5

968.78492	2.5	38856

968.88392	1.5	38856.5

968.98291	0.5	38858.5

969.08191	0.5	38861

969.18091	13.5	38862.5

969.2799	0.5	38863

969.3789	10	38863.5

969.4779	0.5	38877

969.57689	0.5	38877.5

969.67589	14.5	38887.5

969.77489	2	38888

969.87389	1	38888.5

969.97288	0.5	38903

970.07188	0.5	38905

970.17088	19	38906

970.26987	3	38906.5

970.36887	0.5	38907

970.46787	0.5	38926

970.56686	16	38929

970.66586	1	38929.5

970.76486	1.5	38930

970.86386	18.5	38946

970.96285	1	38947

971.06185	0.5	38948.5

971.16085	0.5	38967

971.25984	17.5	38968

971.35884	2.5	38968.5

971.45784	8	38969

971.55684	1	38986.5

971.65583	0.5	38989

971.75483	1	38997

971.85383	15.5	38998

971.95282	18.5	38998.5

972.05182	0.5	38999.5

972.15082	0.5	39015

972.24981	14.5	39033.5

972.34881	1.5	39034

972.44781	0.5	39034.5

972.54681	0.5	39049

972.6458	9.5	39050.5

972.7448	0.5	39051

972.8438	0.5	39051.5

972.94279	10	39061

973.04179	2	39061.5

973.14079	0.5	39062

973.23978	14.5	39072

973.33878	3.5	39074

973.43778	3.5	39074.5

973.53678	17	39089

973.63577	0.5	39092.5

973.73477	1	39096

973.83377	11	39113

973.93276	0.5	39113.5

974.03176	0.5	39114.5

974.13076	2	39125.5

974.22975	3.5	39126

974.32875	0.5	39126.5

974.42775	22	39128.5

974.52675	0.5	39132

974.62574	0.5	39132.5

974.72474	14	39154.5

974.82374	0.5	39155

974.92273	1	39155.5

975.02173	17.5	39169.5

975.12073	0.5	39170

975.21973	1	39171

975.31872	0.5	39188.5

975.41772	1	39189

975.51672	8.5	39190

975.61571	2	39190.5

975.71471	0.5	39191.5

975.81371	20	39200

975.9127	0.5	39202

976.0117	1	39202.5

976.1107	0.5	39222.5

976.2097	3	39223

976.30869	2.5	39224

976.40769	12	39224.5

976.50669	0.5	39227.5

976.60568	1	39230

976.70468	0.5	39242

976.80368	23.5	39242.5

976.90267	1.5	39243.5

977.00167	3.5	39244

977.10067	15	39267.5

977.19967	0.5	39269

977.29866	0.5	39272.5

977.39766	1.5	39287.5

977.49666	17	39288

977.59565	0.5	39288.5

977.69465	1.5	39290

977.79365	1	39307

977.89264	8.5	39307.5

977.99164	2	39309

978.09064	1	39310

978.18964	0.5	39318.5

978.28863	19	39320.5

978.38763	1.5	39321.5

978.48663	0.5	39322

978.58562	9	39341

978.68462	0.5	39342.5

978.78362	16.5	39343

978.88262	0.5	39352

978.98161	1.5	39352.5

979.08061	1	39369

979.17961	1	39369.5

979.2786	7.5	39371

979.3776	0.5	39372

979.4766	1	39373

979.57559	2.5	39380.5

979.67459	0.5	39381

979.77359	13	39382

979.87259	0.5	39384.5

979.97158	0.5	39385

980.07058	9.5	39398

980.16958	1	39398.5

980.26857	0.5	39399

980.36757	6	39408.5

980.46657	1.5	39409.5

980.56556	1	39410

980.66456	6.5	39416

980.76356	1.5	39417.5

980.86256	2.5	39418.5

980.96155	16	39425

981.06055	0.5	39426.5

981.15955	1	39429

981.25854	17.5	39445

981.35754	2.5	39445.5

981.45654	1.5	39446.5

981.55553	0.5	39464

981.65453	9	39466.5

981.75353	1	39468

981.85253	2	39468.5

981.95152	19.5	39477.5

982.05052	1.5	39478.5

982.14952	0.5	39480.5

982.24851	3	39500

982.34751	12	39501.5

982.44651	0.5	39502

982.54551	0.5	39505

982.6445	1	39517

982.7435	4.5	39517.5

982.8425	7	39518

982.94149	2	39519

983.04049	0.5	39523.5

983.13949	14	39530.5

983.23848	1.5	39532.5

983.33748	3	39533

983.43648	7	39547

983.53548	0.5	39548.5

983.63447	7	39551.5

983.73347	2.5	39558.5

983.83247	3	39559

983.93146	1	39566

984.03046	0.5	39568.5

984.12946	9.5	39571.5

984.22845	0.5	39572.5

984.32745	2.5	39573

984.42645	5.5	39582.5

984.52545	1	39583

984.62444	1	39585.5

984.72344	1	39591

984.82244	18	39592

984.92143	1	39593

985.02043	13	39594

985.11943	1.5	39612

985.21843	0.5	39613

985.31742	11	39626

985.41642	0.5	39627.5

985.51542	0.5	39628

985.61441	0.5	39639

985.71341	17	39639.5

985.81241	0.5	39640

985.9114	0.5	39640.5

986.0104	0.5	39657.5

986.1094	7.5	39658

986.2084	7.5	39658.5

986.30739	3.5	39659

986.40639	0.5	39666.5

986.50539	9	39674

986.60438	0.5	39677.5

986.70338	1	39678

986.80238	17.5	39687

986.90137	1.5	39687.5

987.00037	0.5	39688.5

987.09937	19	39706

987.19837	0.5	39707.5

987.29736	1.5	39708

987.39636	15	39727

987.49536	1	39727.5

987.59435	0.5	39729

987.69335	0.5	39744

987.79235	16.5	39745

987.89134	2	39745.5

987.99034	0.5	39746

988.08934	2	39762.5

988.18834	15.5	39764.5

988.28733	1.5	39765

988.38633	0.5	39767

988.48533	7	39782.5

988.58432	1.5	39784

988.68332	1.5	39784.5

988.78232	12.5	39791.5

988.88131	1.5	39793

988.98031	1	39794.5

989.07931	0.5	39807

989.17831	8	39808.5

989.2773	2.5	39809.5

989.3763	1.5	39810

989.4753	3.5	39818

989.57429	11	39820.5

989.67329	1.5	39822

989.77229	0.5	39825.5

989.87129	0.5	39836.5

989.97028	21.5	39838

990.06928	1	39838.5

990.16828	0.5	39839

990.26727	0.5	39860.5

990.36627	0.5	39861.5

990.46527	23	39862

990.56426	0.5	39862.5

990.66326	13.5	39863

990.76226	0.5	39886

990.86126	0.5	39886.5

990.96025	0.5	39900

991.05925	12.5	39900.5

991.15825	0.5	39901

991.25724	0.5	39901.5

991.35624	0.5	39914

991.45524	12.5	39914.5

991.55423	22	39915

991.65323	1	39915.5

991.75223	1.5	39928

991.85123	3.5	39950

991.95022	1	39951

992.04922	2.5	39952.5

992.14822	1	39956

992.24721	13	39957

992.34621	1	39959.5

992.44521	0.5	39960.5

992.54421	16	39973.5

992.6432	2	39974.5

992.7422	10	39975

992.8412	0.5	39991

992.94019	1.5	39993

993.03919	2	40003

993.13819	0.5	40003.5

993.23718	0.5	40005

993.33618	4	40007

993.43518	3	40007.5

993.53418	0.5	40008

993.63317	0.5	40012

993.73217	1	40015

993.83117	15.5	40015.5

993.93016	0.5	40016

994.02916	17.5	40017

994.12816	1	40032.5

994.22715	0.5	40033

994.32615	0.5	40050.5

994.42515	0.5	40051.5

994.52415	6.5	40052

994.62314	2	40052.5

994.72214	0.5	40053

994.82114	3	40059.5

994.92013	1.5	40061.5

995.01913	2.5	40062

995.11813	0.5	40065

995.21712	13	40066.5

995.31612	0.5	40069

995.41512	0.5	40069.5

995.51412	0.5	40082.5

995.61311	14	40083

995.71211	1.5	40083.5

995.81111	1	40084

995.9101	9	40098

996.0091	6	40099.5

996.1081	1	40100.5

996.2071	1.5	40109.5

996.30609	1	40115.5

996.40509	21	40116.5

996.50409	2.5	40118

996.60308	0.5	40119

996.70208	25.5	40140

996.80108	16.5	40142.5

996.90007	0.5	40143

996.99907	0.5	40168.5

997.09807	1	40185

997.19707	8	40185.5

997.29606	0.5	40186

997.39506	1	40187

997.49406	0.5	40195

997.59305	14	40195.5

997.69205	2.5	40196.5

997.79105	1.5	40197

997.89004	1	40211

997.98904	2	40213.5

998.08804	7.5	40215

998.18704	0.5	40216

998.28603	1	40218

998.38503	7.5	40225.5

998.48403	0.5	40226

998.58302	0.5	40227

998.68202	0.5	40234.5

998.78102	15.5	40235

998.88001	2	40235.5

998.97901	1	40236

999.07801	0.5	40251.5

999.17701	19.5	40253.5

999.276	1	40254.5

999.375	0.5	40255

999.474	0.5	40274.5

999.57299	2	40275.5

999.67199	8	40276

999.77099	0.5	40276.5

999.86999	0.5	40278.5

999.96898	1.5	40286.5

1000.06798	0.5	40287

1000.16698	9	40287.5

1000.26597	1	40289

1000.36497	1	40289.5

1000.46397	1	40298.5

1000.56296	3.5	40299.5

1000.66196	2.5	40300.5

1000.76096	0.5	40301.5

1000.85996	1	40305

1000.95895	15	40307.5

1001.05795	1.5	40308

1001.15695	1	40309

1001.25594	1	40324

1001.35494	17	40325.5

1001.45394	1	40326.5

1001.55294	0.5	40327.5

1001.65193	12.5	40344.5

1001.75093	0.5	40345.5

1001.84993	1.5	40346

1001.94892	0.5	40358.5

1002.04792	26	40359

1002.14692	1	40360.5

1002.24591	16	40361

1002.34491	1	40387

1002.44391	5	40388

1002.54291	11	40404

1002.6419	0.5	40405

1002.7409	0.5	40410

1002.8399	1	40421

1002.93889	12.5	40421.5

1003.03789	0.5	40422

1003.13689	0.5	40423

1003.23588	0.5	40435.5

1003.33488	0.5	40436

1003.43388	7	40436.5

1003.53288	4	40437

1003.63187	1	40437.5

1003.73087	0.5	40444.5

1003.82987	0.5	40448.5

1003.92886	2.5	40449.5

1004.02786	10.5	40450

1004.12686	1	40450.5

1004.22585	1	40453

1004.32485	1.5	40463.5

1004.42385	0.5	40464.5

1004.52285	3	40465.5

1004.62184	13.5	40467

1004.72084	1	40467.5

1004.81984	0.5	40470.5

1004.91883	10.5	40484

1005.01783	2	40485

1005.11683	1	40485.5

1005.21583	20	40496

1005.31482	0.5	40498

1005.41382	1	40499

1005.51282	19	40519

1005.61181	0.5	40519.5

1005.71081	14.5	40520.5

1005.80981	0.5	40539.5

1005.9088	0.5	40540

1006.0078	1	40554.5

1006.1068	6	40555

1006.2058	11	40555.5

1006.30479	0.5	40556.5

1006.40379	9	40562.5

1006.50279	0.5	40573.5

1006.60178	0.5	40574

1006.70078	0.5	40583

1006.79978	9	40583.5

1006.89877	1	40584

1006.99777	1	40584.5

1007.09677	5	40593.5

1007.19577	14	40594.5

1007.29476	0.5	40595.5

1007.39376	1	40600.5

1007.49276	15.5	40614.5

1007.59175	2	40615

1007.69075	19.5	40616

1007.78975	1	40631.5

1007.88874	1	40633.5

1007.98774	1.5	40653

1008.08674	13.5	40654

1008.18574	1.5	40655

1008.28473	0.5	40656.5

1008.38373	0.5	40670

1008.48273	8.5	40671.5

1008.58172	0.5	40672

1008.68072	1	40672.5

1008.77972	15.5	40681

1008.87872	1.5	40681.5

1008.97771	0.5	40682.5

1009.07671	3	40698

1009.17571	6.5	40699.5

1009.2747	4	40700

1009.3737	25	40703

1009.4727	2	40709.5

1009.57169	1.5	40713.5

1009.67069	14.5	40738.5

1009.76969	3	40740.5

1009.86869	0.5	40742

1009.96768	19.5	40756.5

1010.06668	0.5	40759.5

1010.16568	0.5	40760

1010.26467	0.5	40779.5

1010.36367	17.5	40780

1010.46267	1	40780.5

1010.56166	17	40781

1010.66066	2.5	40798.5

1010.75966	0.5	40799.5

1010.85866	27.5	40816.5

1010.95765	0.5	40819

1011.05665	1	40819.5

1011.15565	14	40847

1011.25464	4	40847.5

1011.35364	0.5	40848.5

1011.45264	0.5	40862.5

1011.55163	13	40866.5

1011.65063	1	40867

1011.74963	0.5	40867.5

1011.84863	0.5	40880.5

1011.94762	14	40881.5

1012.04662	0.5	40882

1012.14562	0.5	40882.5

1012.24461	3	40896.5

1012.34361	9	40897

1012.44261	0.5	40897.5

1012.54161	22	40900.5

1012.6406	1	40909.5

1012.7396	0.5	40910

1012.8386	5	40932

1012.93759	11.5	40933

1013.03659	2	40933.5

1013.13559	0.5	40938.5

1013.23458	9	40950

1013.33358	1	40952

1013.43258	0.5	40952.5

1013.53158	1	40961.5

1013.63057	15	40962.5

1013.72957	1	40963

1013.82857	0.5	40964

1013.92756	12.5	40979

1014.02656	1.5	40980

1014.12556	3.5	40980.5

1014.22455	0.5	40993

1014.32355	1	40994.5

1014.42255	3	40998

1014.52154	1.5	40998.5

1014.62054	10.5	40999.5

1014.71954	1	41002.5

1014.81854	1.5	41004

1014.91753	1	41014.5

1015.01653	20	41015.5

1015.11553	0.5	41017

1015.21453	1	41018

1015.31352	0.5	41038

1015.41252	7.5	41038.5

1015.51152	0.5	41039.5

1015.61051	3	41040

1015.70951	11.5	41047.5

1015.80851	0.5	41048

1015.9075	25	41051

1016.0065	14	41062.5

1016.1055	3.5	41063

1016.2045	0.5	41088

1016.30349	0.5	41102

1016.40249	0.5	41105.5

1016.50149	19.5	41106

1016.60048	0.5	41106.5

1016.69948	1	41107

1016.79848	10.5	41126.5

1016.89747	1	41127

1016.99647	1.5	41128

1017.09547	0.5	41138.5

1017.19447	0.5	41139.5

1017.29346	0.5	41141

1017.39246	6.5	41141.5

1017.49146	2	41142

1017.59045	1	41142.5

1017.68945	0.5	41149

1017.78845	18.5	41151

1017.88744	0.5	41152

1017.98644	0.5	41152.5

1018.08544	3.5	41171

1018.18444	3	41171.5

1018.28343	0.5	41172

1018.38243	1.5	41175.5

1018.48143	6	41178.5

1018.58042	7	41179

1018.67942	0.5	41180.5

1018.77842	16	41186.5

1018.87742	1	41193.5

1018.97641	1	41194

1019.07541	0.5	41210

1019.17441	8	41211

1019.2734	2	41212

1019.3724	0.5	41212.5

1019.4714	1	41220.5

1019.57039	22	41222.5

1019.66939	0.5	41223

1019.76839	17	41224

1019.86739	0.5	41246

1019.96638	0.5	41246.5

1020.06538	13	41263.5

1020.16438	4	41264

1020.26337	0.5	41264.5

1020.36237	29	41277.5

1020.46137	1	41281.5

1020.56036	0.5	41282

1020.65936	2	41311

1020.75836	9.5	41312

1020.85736	1	41312.5

1020.95635	3	41314.5

1021.05535	0.5	41324

1021.15435	0.5	41325

1021.25334	0.5	41328

1021.35234	13	41328.5

1021.45134	1.5	41329

1021.55033	0.5	41329.5

1021.64933	18	41342.5

1021.74833	0.5	41344

1021.84733	0.5	41344.5

1021.94632	0.5	41362.5

1022.04532	2.5	41363

1022.14432	5	41363.5

1022.24331	3	41364

1022.34231	0.5	41366.5

1022.44131	0.5	41371.5

1022.54031	17.5	41374.5

1022.6393	1	41375

1022.7383	2	41375.5

1022.8373	15	41393

1022.93629	0.5	41394

1023.03529	0.5	41396

1023.13429	0.5	41411

1023.23328	0.5	41411.5

1023.33228	2.5	41412

1023.43128	6	41412.5

1023.53028	0.5	41413

1023.62927	3	41415.5

1023.72827	2.5	41421.5

1023.82727	10	41422

1023.92626	1	41425

1024.02526	0.5	41427.5

1024.12426	0.5	41437.5

1024.22325	0.5	41438.5

1024.32225	13	41439

1024.42125	0.5	41439.5

1024.52024	0.5	41440

1024.61924	15.5	41453

1024.71824	3	41453.5

1024.81724	14	41454

1024.91623	2	41469.5

1025.01523	0.5	41472.5

1025.11423	16	41486.5

1025.21322	0.5	41488.5

1025.31222	10.5	41489

1025.41122	1	41505

1025.51022	0.5	41505.5

1025.60921	23.5	41516

1025.70821	0.5	41517

1025.80721	3.5	41517.5

1025.9062	1	41541

1026.0052	10	41541.5

1026.1042	0.5	41545

1026.2032	1.5	41546

1026.30219	0.5	41556

1026.40119	1	41556.5

1026.50019	0.5	41558

1026.59918	9	41558.5

1026.69818	0.5	41559.5

1026.79718	1	41560

1026.89617	2	41569

1026.99517	14	41569.5

1027.09417	2	41570.5

1027.19316	0.5	41572.5

1027.29216	1	41586.5

1027.39116	1.5	41588.5

1027.49016	11.5	41589

1027.58915	0.5	41590

1027.68815	1	41591.5

1027.78715	2	41603

1027.88614	15.5	41603.5

1027.98514	1.5	41604.5

1028.08414	0.5	41606.5

1028.18314	23	41622

1028.28213	0.5	41623.5

1028.38113	16.5	41624

1028.48013	2.5	41647

1028.57912	0.5	41647.5

1028.67812	1	41664

1028.77712	9.5	41666.5

1028.87611	0.5	41667

1028.97511	3	41668

1029.07411	11.5	41677.5

1029.17311	2	41678

1029.2721	2	41681

1029.3711	0.5	41692.5

1029.4701	23	41694.5

1029.56909	1.5	41696.5

1029.66809	1	41697

1029.76709	1	41720

1029.86609	13.5	41721.5

1029.96508	0.5	41722.5

1030.06408	1.5	41723.5

1030.16308	7.5	41737

1030.26207	1.5	41737.5

1030.36107	1	41739

1030.46007	0.5	41746.5

1030.55906	25	41748

1030.65806	1	41749

1030.75706	0.5	41749.5

1030.85606	0.5	41774.5

1030.95505	16	41775.5

1031.05405	4.5	41776

1031.15305	0.5	41776.5

1031.25204	2.5	41792.5

1031.35104	6	41797

1031.45004	4.5	41797.5

1031.54903	1	41800

1031.64803	0.5	41806

1031.74703	20	41810.5

1031.84603	1	41811.5

1031.94502	14.5	41812

1032.04402	1	41832

1032.14302	16	41833

1032.24201	0.5	41847.5

1032.34101	1	41848.5

1032.44001	20	41864.5

1032.539	0.5	41865

1032.638	0.5	41866

1032.737	2.5	41886

1032.836	0.5	41886.5

1032.93499	6	41887

1033.03399	1.5	41889.5

1033.13299	0.5	41890

1033.23198	18	41896

1033.33098	1	41897.5

1033.42998	0.5	41898

1033.52897	0.5	41916

1033.62797	17	41917

1033.72697	1.5	41917.5

1033.82597	2	41918

1033.92496	14	41935

1034.02396	1	41936.5

1034.12296	0.5	41938.5

1034.22195	0.5	41952.5

1034.32095	18.5	41953.5

1034.41995	2	41954

1034.51895	15	41954.5

1034.61794	1	41973

1034.71694	2.5	41975

1034.81594	19.5	41990

1034.91493	1.5	41991

1035.01393	1.5	41993.5

1035.11293	9	42013

1035.21192	3	42014.5

1035.31092	1	42016

1035.40992	14.5	42025

1035.50891	3	42028

1035.60791	3.5	42029

1035.70691	0.5	42043.5

1035.80591	0.5	42046.5

1035.9049	17	42050

1036.0039	1.5	42050.5

1036.1029	0.5	42051

1036.20189	14.5	42068

1036.30089	1	42069.5

1036.39989	0.5	42070

1036.49889	0.5	42084.5

1036.59788	0.5	42085.5

1036.69688	15.5	42086

1036.79588	1	42086.5

1036.89487	2	42087

1036.99387	24.5	42102.5

1037.09287	0.5	42103.5

1037.19187	1	42105.5

1037.29086	24.5	42130

1037.38986	0.5	42130.5

1037.48886	1.5	42131.5

1037.58785	10	42156

1037.68685	4.5	42156.5

1037.78585	1	42158

1037.88484	3	42168

1037.98384	2.5	42172.5

1038.08284	0.5	42173.5

1038.18184	8.5	42176.5

1038.28083	3	42179

1038.37983	1.5	42179.5

1038.47883	1.5	42188

1038.57782	5.5	42191

1038.67682	11	42192.5

1038.77582	0.5	42194

1038.87481	9.5	42199.5

1038.97381	4	42210.5

1039.07281	1	42211

1039.1718	0.5	42220.5

1039.2708	2.5	42224.5

1039.3698	4	42225.5

1039.4688	1	42226

1039.56779	1	42228.5

1039.66679	10	42232.5

1039.76579	0.5	42233.5

1039.86479	11.5	42234.5

1039.96378	3.5	42244.5

1040.06278	1	42245

1040.16178	5	42256.5

1040.26077	12	42260

1040.35977	3.5	42261

1040.45877	1.5	42266

1040.55776	0.5	42278

1040.65676	16	42281.5

1040.75576	2.5	42283

1040.85476	0.5	42283.5

1040.95375	5	42299.5

1041.05275	7.5	42302

1041.15175	3.5	42302.5

1041.25074	1.5	42307.5

1041.34974	15	42315

1041.44874	1	42318.5

1041.54773	0.5	42320

1041.64673	11	42335

1041.74573	3	42336

1041.84472	1	42336.5

1041.94372	17	42347.5

1042.04272	1.5	42350.5

1042.14172	3	42351.5

1042.24071	13.5	42368.5

1042.33971	0.5	42370

1042.43871	2	42373

1042.5377	14	42386.5

1042.6367	0.5	42387

1042.7357	1	42389

1042.8347	12	42403

1042.93369	0.5	42403.5

1043.03269	0.5	42404.5

1043.13169	0.5	42416.5

1043.23068	7	42417

1043.32968	4	42417.5

1043.42868	3	42418

1043.52768	0.5	42425

1043.62667	0.5	42429

1043.72567	13.5	42432

1043.82467	1	42432.5

1043.92366	0.5	42433

1044.02266	11.5	42446.5

1044.12166	1.5	42447.5

1044.22065	0.5	42448

1044.31965	6.5	42459.5

1044.41865	2.5	42461

1044.51764	4	42461.5

1044.61664	0.5	42468

1044.71564	18.5	42470.5

1044.81464	0.5	42474.5

1044.91363	1	42475

1045.01263	0.5	42493.5

1045.11163	12	42494

1045.21062	1	42495

1045.30962	2	42495.5

1045.40862	19	42507.5

1045.50762	1	42508.5

1045.60661	1	42510.5

1045.70561	1	42529.5

1045.80461	7	42530.5

1045.9036	0.5	42531.5

1046.0026	19.5	42532.5

1046.1016	0.5	42539.5

1046.20059	1	42540

1046.29959	20	42559.5

1046.39859	2.5	42560

1046.49759	14	42561

1046.59658	1	42581

1046.69558	0.5	42583.5

1046.79458	0.5	42597.5

1046.89357	0.5	42598.5

1046.99257	24	42599

1047.09157	1	42599.5

1047.19057	1	42600

1047.28956	2	42624

1047.38856	4	42625

1047.48756	4	42626

1047.58655	11.5	42628

1047.68555	3.5	42632

1047.78455	0.5	42636

1047.88354	1.5	42647.5

1047.98254	0.5	42651

1048.08154	12	42651.5

1048.18054	1	42653

1048.27953	0.5	42653.5

1048.37853	11.5	42665.5

1048.47753	0.5	42666.5

1048.57652	1	42667

1048.67552	1	42678.5

1048.77452	0.5	42679

1048.87351	0.5	42680

1048.97251	0.5	42681

1049.07151	6	42681.5

1049.17051	0.5	42682

1049.2695	2	42682.5

1049.3685	2	42688.5

1049.4675	0.5	42689

1049.56649	1.5	42691

1049.66549	6.5	42693

1049.76449	3	42693.5

1049.86348	2	42695

1049.96248	9	42701.5

1050.06148	2	42704.5

1050.16047	1	42706.5

1050.25947	0.5	42715.5

1050.35847	2.5	42717.5

1050.45747	4	42718.5

1050.55646	10	42719

1050.65546	0.5	42721.5

1050.75446	1	42725.5

1050.85345	10.5	42735.5

1050.95245	2	42736

1051.05145	0.5	42737

1051.15045	1	42747.5

1051.24944	9.5	42749.5

1051.34844	0.5	42750

1051.44744	3.5	42751

1051.54643	16.5	42760.5

1051.64543	3	42761

1051.74443	2	42764.5

1051.84343	1	42781

1051.94242	8	42784

1052.04142	3	42786

1052.14042	7.5	42787

1052.23941	2.5	42795

1052.33841	1	42798

1052.43741	0.5	42805.5

1052.5364	1	42808

1052.6354	15	42809

1052.7344	0.5	42809.5

1052.8334	0.5	42810.5

1052.93239	13.5	42825.5

1053.03139	1	42826

1053.13039	1.5	42826.5

1053.22938	12	42840

1053.32838	1.5	42841

1053.42738	23.5	42842.5

1053.52637	1	42854.5

1053.62537	0.5	42856

1053.72437	0.5	42879.5

1053.82337	14	42880.5

1053.92236	2	42881

1054.02136	0.5	42881.5

1054.12036	2.5	42895.5

1054.21935	4.5	42897.5

1054.31835	1	42898

1054.41735	0.5	42900.5

1054.51635	0.5	42905

1054.61534	0.5	42906

1054.71434	0.5	42906.5

1054.81334	15.5	42907

1054.91233	0.5	42907.5

1055.01133	26.5	42908

1055.11033	0.5	42923.5

1055.20932	19	42924

1055.30832	0.5	42950.5

1055.40732	14	42951

1055.50632	0.5	42970

1055.60531	11.5	42970.5

1055.70431	4.5	42984.5

1055.80331	1	42985

1055.9023	18	42996.5

1056.0013	0.5	43001

1056.1003	1.5	43002

1056.19929	4	43020

1056.29829	1	43020.5

1056.39729	0.5	43022

1056.49628	0.5	43026

1056.59528	0.5	43027

1056.69428	12.5	43027.5

1056.79328	1.5	43028

1056.89227	0.5	43028.5

1056.99127	13.5	43041

1057.09027	1.5	43042.5

1057.18926	0.5	43043

1057.28826	18	43056.5

1057.38726	0.5	43058

1057.48626	1	43058.5

1057.58525	14	43076.5

1057.68425	10	43077

1057.78325	1.5	43078

1057.88224	0.5	43092

1057.98124	0.5	43102

1058.08024	0.5	43103.5

1058.17924	4.5	43104

1058.27823	5.5	43104.5

1058.37723	2	43105

1058.47623	0.5	43109.5

1058.57522	3	43115

1058.67422	12.5	43117

1058.77322	0.5	43117.5

1058.87221	12.5	43120.5

1058.97121	1	43133

1059.07021	0.5	43133.5

1059.1692	0.5	43146

1059.2682	0.5	43147

1059.3672	5	43147.5

1059.4662	7.5	43148

1059.56519	0.5	43148.5

1059.66419	7	43153.5

1059.76319	8	43161

1059.86218	0.5	43161.5

1059.96118	0.5	43168.5

1060.06018	4.5	43176.5

1060.15918	6	43177

1060.25817	0.5	43177.5

1060.35717	14	43182

1060.45617	0.5	43188

1060.55516	0.5	43188.5

1060.65416	10	43202.5

1060.75316	0.5	43203

1060.85216	0.5	43203.5

1060.95115	0.5	43213.5

1061.05015	11	43214

1061.14915	1	43214.5

1061.24814	1	43215

1061.34714	11.5	43226

1061.44614	0.5	43227

1061.54513	0.5	43228

1061.64413	1	43239.5

1061.74313	3	43240

1061.84212	1	43240.5

1061.94112	1.5	43241.5

1062.04012	0.5	43244.5

1062.13912	15	43245.5

1062.23811	4	43247

1062.33711	1.5	43247.5

1062.43611	6	43262.5

1062.5351	1.5	43266.5

1062.6341	0.5	43268

1062.7331	1	43274

1062.8321	5.5	43275.5

1062.93109	0.5	43276

1063.03009	0.5	43277

1063.12909	0.5	43282.5

1063.22808	8.5	43283

1063.32708	0.5	43283.5

1063.42608	0.5	43284

1063.52507	2	43292.5

1063.62407	11	43293

1063.72307	3.5	43293.5

1063.82207	0.5	43295.5

1063.92106	1.5	43306.5

1064.02006	6	43310

1064.11906	1	43310.5

1064.21805	0.5	43312

1064.31705	2	43318

1064.41605	14	43319

1064.51505	0.5	43319.5

1064.61404	0.5	43321.5

1064.71304	0.5	43335.5

1064.81204	0.5	43336

1064.91103	21.5	43336.5

1065.01003	1	43337

1065.10903	0.5	43337.5

1065.20802	21	43359

1065.30702	3	43360

1065.40602	1	43360.5

1065.50502	9.5	43381.5

1065.60401	2	43384.5

1065.70301	1.5	43385.5

1065.80201	3	43395

1065.901	0.5	43397

1066	18	43398.5

1066.099	10	43401.5

1066.19799	6	43402

1066.29699	1	43420

1066.39599	15.5	43430

1066.49499	1.5	43436

1066.59398	0.5	43437

1066.69298	6.5	43452.5

1066.79198	1	43454

1066.89097	6	43454.5

1066.98997	2.5	43461

1067.08897	15.5	43462

1067.18796	0.5	43468

1067.28696	17	43470.5

1067.38596	2.5	43486

1067.48495	1	43486.5

1067.58395	17.5	43503.5

1067.68295	2.5	43506

1067.78195	9.5	43507

1067.88094	2	43524.5

1067.97994	13	43527

1068.07894	1	43536.5

1068.17793	1	43538.5

1068.27693	1.5	43551.5

1068.37593	19	43552.5

1068.47493	3	43553.5

1068.57392	16	43555

1068.67292	14	43574

1068.77192	0.5	43577

1068.87091	0.5	43593

1068.96991	0.5	43607

1069.06891	0.5	43607.5

1069.16791	1	43608

1069.2669	15.5	43608.5

1069.3659	0.5	43609

1069.4649	0.5	43610

1069.56389	14.5	43625.5

1069.66289	0.5	43626

1069.76189	0.5	43626.5

1069.86088	1.5	43641

1069.95988	13	43641.5

1070.05888	0.5	43642

1070.15788	0.5	43643.5

1070.25687	12.5	43656.5

1070.35587	0.5	43657

1070.45487	1	43657.5

1070.55386	0.5	43670

1070.65286	1	43670.5

1070.75186	16.5	43671.5

1070.85085	2	43672

1070.94985	1.5	43673

1071.04885	11.5	43689.5

1071.14785	0.5	43691.5

1071.24684	0.5	43693

1071.34584	6.5	43704.5

1071.44484	0.5	43705

1071.54383	1	43705.5

1071.64283	0.5	43712

1071.74183	0.5	43712.5

1071.84083	11	43713.5

1071.93982	0.5	43714

1072.03882	0.5	43714.5

1072.13782	14	43725.5

1072.23681	0.5	43726

1072.33581	2	43726.5

1072.43481	16	43740.5

1072.5338	1	43741

1072.6328	0.5	43743

1072.7318	14.5	43759

1072.8308	2	43760

1072.92979	1	43760.5

1073.02879	10	43775

1073.12779	1	43777

1073.22678	14.5	43778

1073.32578	0.5	43788

1073.42478	0.5	43789

1073.52377	18.5	43803.5

1073.62277	2	43804

1073.72177	8	43804.5

1073.82076	8	43823

1073.91976	0.5	43825

1074.01876	0.5	43833

1074.11776	1	43841

1074.21675	13	43841.5

1074.31575	1	43842

1074.41475	1	43843

1074.51374	0.5	43856

1074.61274	0.5	43857

1074.71174	1.5	43858

1074.81074	3.5	43858.5

1074.90973	0.5	43859

1075.00873	13	43860.5

1075.10773	1	43864

1075.20672	0.5	43864.5

1075.30572	4	43877.5

1075.40472	4	43878.5

1075.50372	3	43879

1075.60271	0.5	43883

1075.70171	8.5	43887

1075.80071	0.5	43890

1075.8997	0.5	43890.5

1075.9987	0.5	43899

1076.0977	20	43899.5

1076.19669	2	43900

1076.29569	0.5	43900.5

1076.39469	15	43920.5

1076.49368	2	43922.5

1076.59268	0.5	43923

1076.69168	8	43938

1076.79068	11	43940

1076.88967	0.5	43940.5

1076.98867	1	43948.5

1077.08767	0.5	43959.5

1077.18666	9.5	43960

1077.28566	0.5	43961

1077.38466	0.5	43961.5

1077.48366	0.5	43971

1077.58265	26	43971.5

1077.68165	0.5	43972

1077.78065	0.5	43972.5

1077.87964	12	43998.5

1077.97864	1.5	43999

1078.07764	1	43999.5

1078.17663	11.5	44011.5

1078.27563	0.5	44013

1078.37463	1	44014

1078.47363	14.5	44025.5

1078.57262	0.5	44026

1078.67162	0.5	44027

1078.77062	0.5	44041.5

1078.86961	0.5	44042

1078.96861	2	44042.5

1079.06761	9.5	44043

1079.1666	0.5	44043.5

1079.2656	1.5	44045.5

1079.3646	1	44055

1079.4636	11	44055.5

1079.56259	0.5	44057

1079.66159	0.5	44058

1079.76059	14	44069

1079.85958	1	44069.5

1079.95858	1.5	44070

1080.05758	1	44084

1080.15658	15.5	44085

1080.25557	0.5	44086.5

1080.35457	17	44087.5

1080.45357	2.5	44103

1080.55256	1	44103.5

1080.65156	2.5	44120.5

1080.75056	3	44123

1080.84955	1	44124

1080.94855	0.5	44126.5

1081.04755	1.5	44129.5

1081.14655	6.5	44130.5

1081.24554	2	44131

1081.34454	0.5	44132.5

1081.44354	17	44139

1081.54253	0.5	44141

1081.64153	0.5	44141.5

1081.74053	4	44158.5

1081.83953	0.5	44159

1081.93852	0.5	44159.5

1082.03752	13	44163.5

1082.13652	0.5	44164

1082.23551	0.5	44164.5

1082.33451	4	44177.5

1082.43351	2.5	44178

1082.5325	1	44178.5

1082.6315	0.5	44182.5

1082.7305	3	44185

1082.8295	5	44186

1082.92849	0.5	44186.5

1083.02749	11	44189.5

1083.12649	1	44194.5

1083.22548	0.5	44195

1083.32448	1	44206

1083.42348	12.5	44207

1083.52247	1.5	44207.5

1083.62147	0.5	44208.5

1083.72047	0.5	44221

1083.81947	10.5	44222.5

1083.91846	0.5	44223

1084.01746	2.5	44223.5

1084.11646	1	44234

1084.21545	10.5	44234.5

1084.31445	0.5	44237

1084.41345	0.5	44238

1084.51244	0.5	44248.5

1084.61144	7.5	44249

1084.71044	0.5	44249.5

1084.80943	1.5	44250

1084.90843	11	44257.5

1085.00743	4	44258

1085.10643	0.5	44259.5

1085.20542	0.5	44270.5

1085.30442	2.5	44274.5

1085.40342	2	44275

1085.50241	2.5	44275.5

1085.60141	0.5	44278

1085.70041	0.5	44280

1085.79941	5	44282.5

1085.8984	0.5	44283

1085.9974	1	44283.5

1086.0964	2.5	44288.5

1086.19539	6	44289

1086.29439	0.5	44290

1086.39339	0.5	44292.5

1086.49239	0.5	44298.5

1086.59138	0.5	44299

1086.69038	13	44299.5

1086.78938	5	44300

1086.88837	1.5	44300.5

1086.98737	2	44313.5

1087.08637	3	44318.5

1087.18536	3.5	44320

1087.28436	1	44322

1087.38336	1.5	44325

1087.48236	16	44328.5

1087.58135	0.5	44329.5

1087.68035	0.5	44331

1087.77935	1	44347

1087.87834	2.5	44347.5

1087.97734	11	44348

1088.07634	4	44349

1088.17533	0.5	44351.5

1088.27433	5.5	44362.5

1088.37333	0.5	44366.5

1088.47233	0.5	44367

1088.57132	0.5	44372.5

1088.67032	2	44373

1088.76932	1	44373.5

1088.86831	1.5	44374

1088.96731	0.5	44376

1089.06631	8	44377

1089.16531	1.5	44378.5

1089.2643	3	44379

1089.3633	16	44387

1089.4623	2	44388.5

1089.56129	0.5	44391.5

1089.66029	5.5	44407.5

1089.75929	5	44409.5

1089.85828	1	44410

1089.95728	0.5	44415.5

1090.05628	1.5	44420.5

1090.15528	11.5	44421.5

1090.25427	0.5	44422

1090.35327	0.5	44423.5

1090.45227	4	44435

1090.55126	6	44435.5

1090.65026	0.5	44436

1090.74926	4	44440

1090.84825	2.5	44446

1090.94725	1	44446.5

1091.04625	0.5	44450.5

1091.14524	3.5	44453

1091.24424	2	44454

1091.34324	2	44454.5

1091.44224	0.5	44458

1091.54123	5	44460

1091.64023	1.5	44462

1091.73923	2	44462.5

1091.83822	0.5	44467.5

1091.93722	15	44469

1092.03622	2	44471

1092.13522	3.5	44471.5

1092.23421	5.5	44486.5

1092.33321	1	44488.5

1092.43221	0.5	44492

1092.5312	8.5	44497.5

1092.6302	5.5	44498.5

1092.7292	0.5	44499

1092.8282	15.5	44507.5

1092.92719	0.5	44513

1093.02619	15	44513.5

1093.12519	0.5	44529

1093.22418	0.5	44529.5

1093.32318	22.5	44544.5

1093.42218	16	44545

1093.52117	0.5	44545.5

1093.62017	2	44568

1093.71917	1	44584

1093.81816	11	44584.5

1093.91716	2	44586.5

1094.01616	0.5	44587.5

1094.11516	3	44598.5

1094.21415	3.5	44600.5

1094.31315	0.5	44601

1094.41215	1.5	44604

1094.51114	6	44607.5

1094.61014	0.5	44608

1094.70914	13	44609.5

1094.80814	5	44615.5

1094.90713	0.5	44616

1095.00613	7.5	44629

1095.10513	0.5	44634

1095.20412	0.5	44634.5

1095.30312	0.5	44642

1095.40212	10	44642.5

1095.50111	1	44643

1095.60011	0.5	44643.5

1095.69911	3.5	44653.5

1095.79811	9	44654.5

1095.8971	1.5	44655

1095.9961	17.5	44658.5

1096.0951	1.5	44667.5

1096.19409	5.5	44669

1096.29309	0.5	44686.5

1096.39209	1	44688

1096.49109	0.5	44693.5

1096.59008	0.5	44694

1096.68908	15	44695

1096.78808	1.5	44695.5

1096.88707	2.5	44696

1096.98607	3	44711

1097.08507	0.5	44712.5

1097.18406	0.5	44715

1097.28306	1	44718

1097.38206	9	44718.5

1097.48106	1	44719

1097.58005	4.5	44720

1097.67905	2	44729

1097.77805	0.5	44730

1097.87704	0.5	44734.5

1097.97604	20	44736.5

1098.07504	0.5	44737

1098.17403	0.5	44737.5

1098.27303	0.5	44757.5

1098.37203	13	44758

1098.47103	0.5	44758.5

1098.57002	0.5	44759

1098.66902	0.5	44772

1098.76802	3	44772.5

1098.86701	5	44773

1098.96601	0.5	44773.5

1099.06501	10.5	44776.5

1099.164	0.5	44781.5

1099.263	0.5	44782

1099.362	1.5	44792.5

1099.461	8.5	44793

1099.55999	0.5	44793.5

1099.65899	0.5	44795

1099.75799	1	44803.5

1099.85698	7	44804

1099.95598	11	44804.5

1100.05498	0.5	44805.5

1100.15397	15	44812.5

1100.25297	1	44823.5

1100.35197	1	44824

1100.45097	1.5	44839

1100.54996	0.5	44840

1100.64896	0.5	44841

1100.74796	3.5	44842.5

1100.84695	0.5	44843

1100.94595	12.5	44843.5

1101.04495	3	44847

1101.14395	0.5	44847.5

1101.24294	1	44860

1101.34194	5	44863

1101.44094	9.5	44863.5

1101.53993	1	44864.5

1101.63893	11	44869.5

1101.73793	0.5	44879

1101.83692	2	44880

1101.93592	2.5	44891

1102.03492	0.5	44891.5

1102.13391	3	44893.5

1102.23291	0.5	44896

1102.33191	0.5	44896.5

1102.43091	6	44899.5

1102.5299	1.5	44900

1102.6289	1	44900.5

1102.7279	0.5	44906.5

1102.82689	9	44908

1102.92589	3	44909

1103.02489	1.5	44909.5

1103.12389	5	44918.5

1103.22288	3	44921.5

1103.32188	0.5	44923

1103.42088	13.5	44928

1103.51987	2.5	44931

1103.61887	3	44931.5

1103.71787	10.5	44945

1103.81687	0.5	44947.5

1103.91586	1	44950.5

1104.01486	6	44961

1104.11386	1.5	44961.5

1104.21285	0.5	44962.5

1104.31185	3	44968.5

1104.41085	1	44970

1104.50984	2.5	44970.5

1104.60884	3	44973.5

1104.70784	0.5	44974.5

1104.80684	0.5	44977

1104.90583	1.5	44980

1105.00483	10	44980.5

1105.10383	0.5	44981

1105.20282	2	44982.5

1105.30182	8	44992.5

1105.40082	3	44993

1105.49981	17.5	44995

1105.59881	0.5	45003

1105.69781	0.5	45006

1105.7968	1	45023.5

1105.8958	0.5	45024

1105.9948	5.5	45024.5

1106.0938	0.5	45025.5

1106.19279	1.5	45026

1106.29179	8	45031.5

1106.39079	0.5	45032

1106.48979	1	45033.5

1106.58878	7.5	45041.5

1106.68778	0.5	45042

1106.78678	17	45043

1106.88577	0.5	45050.5

1106.98477	1	45051

1107.08377	1	45068

1107.18276	12	45068.5

1107.28176	3	45069.5

1107.38076	0.5	45070.5

1107.47976	2.5	45082.5

1107.57875	6.5	45085.5

1107.67775	2	45086

1107.77675	13	45088.5

1107.87574	1	45095

1107.97474	0.5	45097

1108.07374	12.5	45110

1108.17273	3	45111

1108.27173	6.5	45111.5

1108.37073	5	45124

1108.46972	3.5	45127

1108.56872	5.5	45133.5

1108.66772	0.5	45138.5

1108.76672	1	45142

1108.86571	3.5	45147.5

1108.96471	8	45148

1109.06371	0.5	45149

1109.1627	14	45152.5

1109.2617	0.5	45160.5

1109.3607	4	45161

1109.4597	1.5	45175

1109.55869	0.5	45175.5

1109.65769	2	45179.5

1109.75669	2.5	45181

1109.85568	0.5	45181.5

1109.95468	3.5	45183.5

1110.05368	1	45186

1110.15268	1	45186.5

1110.25167	15.5	45190

1110.35067	0.5	45191

1110.44967	4	45192

1110.54866	1.5	45207.5

1110.64766	1.5	45208

1110.74666	0.5	45212

1110.84565	14.5	45213.5

1110.94465	0.5	45215

1111.04365	7	45215.5

1111.14264	1.5	45230

1111.24164	1	45230.5

1111.34064	3	45237.5

1111.43964	0.5	45239

1111.53863	0.5	45240

1111.63763	16.5	45243

1111.73663	4.5	45243.5

1111.83562	3	45244

1111.93462	0.5	45260.5

1112.03362	7.5	45265

1112.13262	0.5	45268

1112.23161	1	45268.5

1112.33061	4	45276

1112.42961	0.5	45276.5

1112.5286	0.5	45277.5

1112.6276	13	45281.5

1112.7266	1	45282

1112.82559	0.5	45282.5

1112.92459	7	45295.5

1113.02359	1	45296.5

1113.12259	0.5	45297

1113.22158	0.5	45304

1113.32058	1	45305

1113.41958	1	45305.5

1113.51857	7.5	45306

1113.61757	1	45307

1113.71657	0.5	45308

1113.81557	2.5	45315.5

1113.91456	0.5	45316.5

1114.01356	2.5	45317

1114.11256	0.5	45319.5

1114.21155	0.5	45320

1114.31055	0.5	45322.5

1114.40955	8.5	45323

1114.50854	0.5	45323.5

1114.60754	1.5	45324

1114.70654	6.5	45332.5

1114.80554	1	45333

1114.90453	0.5	45334.5

1115.00353	1	45341

1115.10253	14	45342

1115.20152	1.5	45342.5

1115.30052	0.5	45343.5

1115.39952	2.5	45357.5

1115.49851	6.5	45359

1115.59751	0.5	45359.5

1115.69651	2	45362

1115.79551	4.5	45368.5

1115.8945	0.5	45369

1115.9935	5.5	45371

1116.0925	1.5	45375.5

1116.19149	1.5	45376

1116.29049	9.5	45381.5

1116.38949	1	45383

1116.48848	0.5	45384.5

1116.58748	3.5	45394

1116.68648	1	45395

1116.78547	1.5	45395.5

1116.88447	1.5	45399

1116.98347	5.5	45400

1117.08247	2.5	45401.5

1117.18146	0.5	45403

1117.28046	11	45408.5

1117.37946	1.5	45411

1117.47845	2	45411.5

1117.57745	0.5	45422.5

1117.67645	0.5	45424

1117.77545	2	45426

1117.87444	0.5	45426.5

1117.97344	1	45427

1118.07244	0.5	45429

1118.17143	2	45429.5

1118.27043	1	45430.5

1118.36943	0.5	45431

1118.46843	0.5	45433

1118.56742	1	45434

1118.66642	2.5	45434.5

1118.76542	4.5	45435

1118.86441	0.5	45436

1118.96341	4.5	45438.5

1119.06241	4.5	45443

1119.1614	2	45443.5

1119.2604	2.5	45448

1119.3594	8	45452.5

1119.4584	1	45454.5

1119.55739	1.5	45457

1119.65639	0.5	45465

1119.75539	0.5	45466

1119.85438	1.5	45467.5

1119.95338	1.5	45468

1120.05238	0.5	45468.5

1120.15137	0.5	45470

1120.25037	1	45471.5

1120.34937	2	45472

1120.44837	17	45472.5

1120.54736	0.5	45473.5

1120.64636	1.5	45475.5

1120.74536	12.5	45492.5

1120.84435	1	45493

1120.94335	1.5	45494.5

1121.04235	16.5	45507

1121.14135	1.5	45508

1121.24034	0.5	45509.5

1121.33934	0.5	45526

1121.43834	8	45527.5

1121.53733	1	45528

1121.63633	2	45528.5

1121.73533	1	45536.5

1121.83432	0.5	45537.5

1121.93332	0.5	45539.5

1122.03232	9	45540.5

1122.13132	0.5	45541

1122.23031	0.5	45541.5

1122.32931	0.5	45550.5

1122.42831	8	45551

1122.5273	1	45551.5

1122.6263	1	45552

1122.7253	19	45560

1122.82429	0.5	45561

1122.92329	13	45562

1123.02229	0.5	45581

1123.12128	4	45581.5

1123.22028	3.5	45594.5

1123.31928	1	45595

1123.41828	0.5	45599

1123.51727	1.5	45602.5

1123.61627	16.5	45603.5

1123.71527	1	45604

1123.81426	9.5	45605.5

1123.91326	1.5	45622

1124.01226	1	45623

1124.11126	1	45632.5

1124.21025	15.5	45634

1124.30925	1.5	45635

1124.40825	1.5	45636

1124.50724	0.5	45651.5

1124.60624	0.5	45653

1124.70524	11.5	45654.5

1124.80424	3.5	45655

1124.90323	0.5	45655.5

1125.00223	0.5	45667

1125.10123	2.5	45670.5

1125.20022	1	45671

1125.29922	1	45671.5

1125.39822	7	45674

1125.49721	2	45675

1125.59621	2.5	45676

1125.69521	7.5	45683

1125.7942	9.5	45685

1125.8932	13	45687.5

1125.9922	0.5	45695

1126.0912	6.5	45704.5

1126.19019	1.5	45717.5

1126.28919	1	45718

1126.38819	0.5	45724.5

1126.48718	0.5	45726

1126.58618	6.5	45727

1126.68518	0.5	45727.5

1126.78418	2	45728

1126.88317	3	45734.5

1126.98217	0.5	45735

1127.08117	14.5	45737

1127.18016	0.5	45740

1127.27916	1	45740.5

1127.37816	1.5	45755

1127.47716	1.5	45755.5

1127.57615	4	45756.5

1127.67515	0.5	45758

1127.77415	1.5	45759.5

1127.87314	8.5	45763.5

1127.97214	1.5	45764

1128.07114	0.5	45765.5

1128.17013	7	45774

1128.26913	0.5	45775.5

1128.36813	1	45776

1128.46712	17	45783

1128.56612	0.5	45783.5

1128.66512	0.5	45784.5

1128.76412	10	45801.5

1128.86311	1	45802

1128.96211	1	45802.5

1129.06111	8	45812.5

1129.1601	0.5	45813.5

1129.2591	0.5	45814.5

1129.3581	3	45822.5

1129.4571	7	45823

1129.55609	2.5	45823.5

1129.65509	1	45826.5

1129.75409	3.5	45833.5

1129.85308	0.5	45836

1129.95208	0.5	45837

1130.05108	17	45840.5

1130.15007	1	45841

1130.24907	7.5	45841.5

1130.34807	2.5	45858.5

1130.44707	0.5	45859.5

1130.54606	0.5	45867

1130.64506	2.5	45869.5

1130.74406	2	45870

1130.84305	1	45870.5

1130.94205	0.5	45873

1131.04105	15	45875

1131.14005	1	45876

1131.23904	1	45876.5

1131.33804	1	45891.5

1131.43704	1.5	45892.5

1131.53603	0.5	45893.5

1131.63503	0.5	45894.5

1131.73403	5	45896

1131.83302	3	45896.5

1131.93202	1.5	45897

1132.03102	1.5	45902

1132.13002	1	45905

1132.22901	16	45906.5

1132.32801	0.5	45908

1132.42701	1	45909

1132.526	8.5	45925

1132.625	0.5	45925.5

1132.724	1	45926.5

1132.82299	14.5	45935

1132.92199	1	45935.5

1133.02099	1	45936.5

1133.11999	0.5	45951

1133.21898	3.5	45952

1133.31798	2	45953

1133.41698	1	45953.5

1133.51597	4	45957

1133.61497	2	45959

1133.71397	1.5	45960

1133.81296	1	45964

1133.91196	0.5	45966

1134.01096	8	45967.5

1134.10995	11.5	45968.5

1134.20895	0.5	45969

1134.30795	4	45977

1134.40695	1.5	45988.5

1134.50594	1	45989

1134.60494	10.5	45993

1134.70394	2.5	45994.5

1134.80293	2	45995.5

1134.90193	1	46006

1135.00093	6	46008.5

1135.09993	3	46010.5

1135.19892	8.5	46011.5

1135.29792	1	46017.5

1135.39692	1	46020.5

1135.49591	1.5	46029

1135.59491	1.5	46030

1135.69391	0.5	46031

1135.79291	0.5	46032.5

1135.8919	6.5	46034

1135.9909	0.5	46034.5

1136.0899	1.5	46035

1136.18889	0.5	46041.5

1136.28789	0.5	46042

1136.38689	0.5	46043.5

1136.48588	4	46044

1136.58488	3	46044.5

1136.68388	0.5	46045

1136.78288	6.5	46049

1136.88187	4	46052

1136.98087	0.5	46052.5

1137.07987	6	46059

1137.17886	0.5	46063

1137.27786	1.5	46063.5

1137.37686	3.5	46069.5

1137.47585	0.5	46070

1137.57485	0.5	46071.5

1137.67385	1	46075

1137.77285	0.5	46075.5

1137.87184	0.5	46076

1137.97084	10	46077

1138.06984	0.5	46077.5

1138.16883	0.5	46078

1138.26783	6.5	46088

1138.36683	0.5	46088.5

1138.46583	1	46089

1138.56482	1.5	46095.5

1138.66382	0.5	46096

1138.76282	0.5	46097

1138.86181	0.5	46098.5

1138.96081	6.5	46099

1139.05981	0.5	46099.5

1139.1588	0.5	46100

1139.2578	1	46106.5

1139.3568	2	46107

1139.4558	1	46107.5

1139.55479	0.5	46108.5

1139.65379	1	46110.5

1139.75279	1.5	46111.5

1139.85178	1	46112

1139.95078	0.5	46113

1140.04978	4.5	46114.5

1140.14877	6.5	46115.5

1140.24777	0.5	46116

1140.34677	6	46120.5

1140.44576	4.5	46127

1140.54476	6	46127.5

1140.64376	5	46133.5

1140.74276	0.5	46138

1140.84175	0.5	46144

1140.94075	4.5	46149

1141.03975	2	46149.5

1141.13874	3.5	46150

1141.23774	7	46154.5

1141.33674	1.5	46156.5

1141.43574	1	46160

1141.53473	2.5	46167

1141.63373	1	46168.5

1141.73273	1	46169.5

1141.83172	1	46172

1141.93072	0.5	46173

1142.02972	1.5	46174

1142.12872	9	46175

1142.22771	1	46175.5

1142.32671	0.5	46177

1142.42571	8	46186

1142.5247	1	46187

1142.6237	1.5	46187.5

1142.7227	2	46195.5

1142.82169	2.5	46196.5

1142.92069	1	46198

1143.01969	1	46200

1143.11868	0.5	46202.5

1143.21768	3.5	46203.5

1143.31668	3	46204.5

1143.41568	1	46205

1143.51467	0.5	46208.5

1143.61367	15.5	46211.5

1143.71267	0.5	46212.5

1143.81166	0.5	46213

1143.91066	0.5	46228.5

1144.00966	1.5	46229

1144.10866	0.5	46229.5

1144.20765	1	46230

1144.30665	1	46231.5

1144.40565	9.5	46232

1144.50464	9	46233

1144.60364	1	46234

1144.70264	1.5	46243.5

1144.80163	9	46252.5

1144.90063	1.5	46253.5

1144.99963	0.5	46255

1145.09863	1	46264

1145.19762	2.5	46265.5

1145.29662	4	46266

1145.39562	0.5	46267

1145.49461	1	46269.5

1145.59361	0.5	46273.5

1145.69261	11	46274

1145.7916	0.5	46275

1145.8906	1	46275.5

1145.9896	0.5	46286.5

1146.0886	0.5	46287

1146.18759	0.5	46288

1146.28659	0.5	46288.5

1146.38559	1	46289

1146.48458	0.5	46289.5

1146.58358	5.5	46290

1146.68258	4.5	46291

1146.78158	0.5	46291.5

1146.88057	2	46297

1146.97957	1	46301.5

1147.07857	1.5	46302

1147.17756	2.5	46304

1147.27656	0.5	46305

1147.37556	0.5	46306.5

1147.47455	0.5	46309

1147.57355	0.5	46309.5

1147.67255	2	46310

1147.77155	1.5	46310.5

1147.87054	3	46311

1147.96954	0.5	46313

1148.06854	0.5	46314.5

1148.16753	0.5	46317.5

1148.26653	4	46318

1148.36553	0.5	46318.5

1148.46453	0.5	46319

1148.56352	2	46323

1148.66252	5.5	46323.5

1148.76152	0.5	46324

1148.86051	8.5	46326

1148.95951	0.5	46331.5

1149.05851	0.5	46332

1149.1575	0.5	46340.5

1149.2565	1.5	46341

1149.3555	2.5	46341.5

1149.4545	0.5	46342

1149.55349	0.5	46343.5

1149.65249	1	46346

1149.75149	0.5	46346.5

1149.85048	5	46347

1149.94948	0.5	46348

1150.04848	1	46348.5

1150.14747	3.5	46353.5

1150.24647	2	46354

1150.34547	0.5	46355

1150.44447	2	46358.5

1150.54346	5	46360.5

1150.64246	1	46361

1150.74146	1	46363

1150.84045	5	46368

1150.93945	1	46369

1151.03845	1.5	46370

1151.13744	1.5	46375

1151.23644	3	46376

1151.33544	1	46377.5

1151.43443	0.5	46379

1151.53343	1	46382

1151.63243	7	46383

1151.73143	0.5	46383.5

1151.83042	0.5	46384.5

1151.92942	0.5	46391.5

1152.02842	0.5	46392

1152.12741	1.5	46392.5

1152.22641	11	46393

1152.32541	1	46393.5

1152.42441	4	46395

1152.5234	0.5	46406

1152.6224	2	46407

1152.7214	1.5	46411

1152.82039	0.5	46411.5

1152.91939	0.5	46413.5

1153.01839	6	46415

1153.11739	2.5	46415.5

1153.21638	1.5	46416

1153.31538	0.5	46422

1153.41438	0.5	46424.5

1153.51337	0.5	46426

1153.61237	0.5	46426.5

1153.71137	6	46427

1153.81036	0.5	46427.5

1153.90936	1	46428

1154.00836	8	46434

1154.10736	2.5	46434.5

1154.20635	0.5	46435.5

1154.30535	3	46443.5

1154.40435	1.5	46446

1154.50334	2	46446.5

1154.60234	1.5	46449.5

1154.70134	3.5	46451

1154.80033	0.5	46453

1154.89933	3.5	46454.5

1154.99833	1	46458

1155.09733	1	46458.5

1155.19632	0.5	46462

1155.29532	0.5	46463

1155.39432	2	46464

1155.49331	0.5	46464.5

1155.59231	2.5	46465

1155.69131	0.5	46467

1155.79031	9	46467.5

1155.8893	0.5	46470

1155.9883	1	46470.5

1156.0873	1.5	46479.5

1156.18629	5.5	46480

1156.28529	4.5	46481

1156.38429	1	46482.5

1156.48328	1	46488

1156.58228	9.5	46492.5

1156.68128	6.5	46493.5

1156.78028	1	46494.5

1156.87927	1	46504

1156.97827	1	46510.5

1157.07727	3.5	46511.5

1157.17626	0.5	46512.5

1157.27526	0.5	46513.5

1157.37426	4.5	46517

1157.47325	1	46517.5

1157.57225	0.5	46518

1157.67125	4	46522.5

1157.77024	5	46523.5

1157.86924	0.5	46524

1157.96824	0.5	46528

1158.06724	1	46533

1158.16623	1.5	46533.5

1158.26523	0.5	46534

1158.36423	7.5	46535

1158.46322	1.5	46536.5

1158.56222	0.5	46537

1158.66122	2.5	46544.5

1158.76022	2.5	46546

1158.85921	0.5	46546.5

1158.95821	4.5	46549

1159.05721	1	46551.5

1159.1562	1	46552

1159.2552	12	46556.5

1159.3542	0.5	46557.5

1159.4532	1.5	46558.5

1159.55219	3.5	46570.5

1159.65119	1	46571

1159.75019	0.5	46572.5

1159.84918	2.5	46576

1159.94818	1	46577

1160.04718	3	46577.5

1160.14617	1.5	46580

1160.24517	1	46581

1160.34417	7	46584

1160.44316	2	46585.5

1160.54216	6.5	46586.5

1160.64116	4.5	46593.5

1160.74016	1.5	46595.5

1160.83915	1.5	46602

1160.93815	1	46606.5

1161.03715	1.5	46608

1161.13614	0.5	46609.5

1161.23514	2.5	46610.5

1161.33414	1	46612

1161.43314	0.5	46612.5

1161.53213	1.5	46615

1161.63113	0.5	46616

1161.73013	2	46616.5

1161.82912	2.5	46618

1161.92812	2.5	46618.5

1162.02712	0.5	46620.5

1162.12611	2	46623

1162.22511	7	46625.5

1162.32411	0.5	46626

1162.42311	2.5	46628

1162.5221	2	46635

1162.6211	9	46635.5

1162.7201	0.5	46638

1162.81909	1	46640

1162.91809	4	46649

1163.01709	8	46649.5

1163.11609	2.5	46650.5

1163.21508	0.5	46654.5

1163.31408	1	46662.5

1163.41308	2	46665

1163.51207	1.5	46665.5

1163.61107	1	46666.5

1163.71007	1	46668.5

1163.80906	0.5	46670

1163.90806	0.5	46671

1164.00706	5.5	46672

1164.10606	1.5	46672.5

1164.20505	0.5	46673

1164.30405	2.5	46678.5

1164.40305	1.5	46680

1164.50204	0.5	46680.5

1164.60104	0.5	46683

1164.70004	1.5	46684.5

1164.79903	1.5	46685

1164.89803	2.5	46685.5

1164.99703	4	46687

1165.09603	0.5	46688.5

1165.19502	3	46691

1165.29402	5.5	46695

1165.39302	3	46695.5

1165.49201	12	46698.5

1165.59101	0.5	46704

1165.69001	0.5	46707

1165.789	3	46719

1165.888	1	46719.5

1165.987	1.5	46720

1166.086	9	46723

1166.18499	1.5	46724

1166.28399	6	46725.5

1166.38299	1	46734.5

1166.48198	0.5	46736

1166.58098	1.5	46742

1166.67998	0.5	46743

1166.77897	1	46743.5

1166.87797	0.5	46745

1166.97697	1	46745.5

1167.07597	0.5	46746.5

1167.17496	1.5	46747

1167.27396	3.5	46748

1167.37296	2	46748.5

1167.47195	3.5	46750

1167.57095	0.5	46753.5

1167.66995	11.5	46755.5

1167.76895	0.5	46759

1167.86794	7.5	46759.5

1167.96694	4.5	46771

1168.06594	2	46771.5

1168.16493	1	46779

1168.26393	0.5	46783.5

1168.36293	8	46785.5

1168.46192	0.5	46786.5

1168.56092	0.5	46787

1168.65992	4	46795

1168.75891	1	46795.5

1168.85791	20	46796

1168.95691	3.5	46800

1169.05591	0.5	46801

1169.1549	11	46821

1169.2539	0.5	46824.5

1169.3529	0.5	46825

1169.45189	2	46836

1169.55089	2	46836.5

1169.64989	1.5	46837

1169.74889	0.5	46839

1169.84788	0.5	46841

1169.94688	1.5	46842.5

1170.04588	1	46843

1170.14487	0.5	46843.5

1170.24387	0.5	46845

1170.34287	1	46846

1170.44187	0.5	46846.5

1170.54086	4.5	46847

1170.63986	2	46848

1170.73886	17	46848.5

1170.83785	3	46853

1170.93685	0.5	46855

1171.03585	0.5	46872

1171.13484	0.5	46875

1171.23384	1	46875.5

1171.33284	5.5	46876

1171.43184	1.5	46876.5

1171.53083	0.5	46877.5

1171.62983	2.5	46883

1171.72883	0.5	46884.5

1171.82782	8	46885

1171.92682	0.5	46887.5

1172.02582	3.5	46888

1172.12481	3.5	46896

1172.22381	0.5	46896.5

1172.32281	0.5	46900

1172.4218	1	46903.5

1172.5208	3	46904

1172.6198	0.5	46904.5

1172.7188	0.5	46905.5

1172.81779	11	46908.5

1172.91679	2	46909

1173.01579	0.5	46909.5

1173.11479	1.5	46920.5

1173.21378	1.5	46922.5

1173.31278	2.5	46923

1173.41178	1	46924.5

1173.51077	0.5	46926

1173.60977	0.5	46928.5

1173.70877	1	46929.5

1173.80776	4.5	46930

1173.90676	1.5	46930.5

1174.00576	0.5	46931.5

1174.10476	8	46936

1174.20375	2	46937.5

1174.30275	2.5	46938

1174.40175	2.5	46946

1174.50074	2.5	46948

1174.59974	4	46950.5

1174.69874	2	46953

1174.79773	1.5	46955.5

1174.89673	0.5	46959.5

1174.99573	3.5	46961.5

1175.09472	1	46963

1175.19372	0.5	46963.5

1175.29272	0.5	46967

1175.39172	2.5	46968

1175.49071	6.5	46968.5

1175.58971	1	46969

1175.68871	12	46971.5

1175.7877	0.5	46978

1175.8867	2.5	46979

1175.9857	3.5	46991

1176.0847	0.5	46991.5

1176.18369	0.5	46994

1176.28269	2.5	46997.5

1176.38169	1.5	46998

1176.48068	1	46998.5

1176.57968	1.5	47001

1176.67868	0.5	47002.5

1176.77768	1.5	47003.5

1176.87667	0.5	47005

1176.97567	1.5	47005.5

1177.07467	34	47007

1177.17366	0.5	47007.5

1177.27266	0.5	47009

1177.37166	1	47043

1177.47065	6.5	47043.5

1177.56965	0.5	47044

1177.66865	0.5	47045

1177.76764	0.5	47051.5

1177.86664	0.5	47052

1177.96564	42	47052.5

1178.06464	1	47053

1178.16363	0.5	47053.5

1178.26263	1	47095.5

1178.36163	1	47096.5

1178.46062	1	47097

1178.55962	0.5	47098

1178.65862	0.5	47099

1178.75762	0.5	47100

1178.85661	0.5	47100.5

1178.95561	15	47101

1179.05461	0.5	47101.5

1179.1536	1.5	47102

1179.2526	1	47117

1179.3516	0.5	47117.5

1179.45059	0.5	47119

1179.54959	5.5	47120

1179.64859	0.5	47120.5

1179.74759	3	47121

1179.84658	24	47126.5

1179.94558	1.5	47127

1180.04458	2	47130

1180.14357	0.5	47154

1180.24257	1.5	47155.5

1180.34157	2	47157.5

1180.44057	3	47158

1180.53956	30	47159.5

1180.63856	0.5	47161.5

1180.73756	2	47164.5

1180.83655	0.5	47194.5

1180.93555	0.5	47195

1181.03455	24	47197

1181.13354	2	47197.5

1181.23254	0.5	47198

1181.33154	52	47222

1181.43054	0.5	47224

1181.52953	1.5	47224.5

1181.62853	2.5	47276.5

1181.72753	1.5	47277

1181.82652	0.5	47278.5

1181.92552	39	47281

1182.02452	5	47282.5

1182.12351	0.5	47283

1182.22251	3	47322

1182.32151	1	47327

1182.42051	69	47327.5

1182.5195	0.5	47330.5

1182.6185	0.5	47331.5

1182.7175	0.5	47400.5

1182.81649	1	47401

1182.91549	0.5	47401.5

1183.01449	1	47402

1183.11348	1	47403

1183.21248	1	47403.5

1183.31148	3.5	47404.5

1183.41047	3	47405.5

1183.50947	1	47406.5

1183.60847	0.5	47410

1183.70747	3.5	47413

1183.80646	1	47414

1183.90546	0.5	47414.5

1184.00446	47	47418

1184.10345	4.5	47419

1184.20245	1	47419.5

1184.30145	1.5	47466.5

1184.40045	7.5	47471

1184.49944	0.5	47472

1184.59844	0.5	47473.5

1184.69744	1	47481

1184.79643	1.5	47481.5

1184.89543	3	47482

1184.99443	0.5	47483

1185.09343	0.5	47484.5

1185.19242	35	47487.5

1185.29142	0.5	47488

1185.39042	1.5	47488.5

1185.48941	0.5	47523.5

1185.58841	0.5	47524

1185.68741	0.5	47525.5

1185.7864	0.5	47526

1185.8854	1	47526.5

1185.9844	1	47527

1186.0834	0.5	47527.5

1186.18239	44	47528.5

1186.28139	1.5	47529.5

1186.38039	1.5	47530

1186.47938	0.5	47574

1186.57838	1.5	47575.5

1186.67738	1.5	47577

1186.77637	0.5	47577.5

1186.87537	11	47579

1186.97437	0.5	47580.5

1187.07337	2	47581

1187.17236	0.5	47592

1187.27136	3	47592.5

1187.37036	1.5	47594.5

1187.46935	3.5	47595

1187.56835	2	47598

1187.66735	0.5	47599.5

1187.76635	50	47603

1187.86534	3	47605

1187.96434	0.5	47605.5

1188.06334	0.5	47655.5

1188.16233	1	47658.5

1188.26133	0.5	47659

1188.36033	5	47659.5

1188.45932	0.5	47660.5

1188.55832	1	47661

1188.65732	4.5	47666

1188.75632	5	47666.5

1188.85531	1	47667.5

1188.95431	2.5	47672

1189.05331	1.5	47677

1189.1523	2	47678

1189.2513	20	47680.5

1189.3503	1	47682

1189.44929	6	47684

1189.54829	7.5	47704

1189.64729	2	47705

1189.74628	0.5	47711

1189.84528	0.5	47718.5

1189.94428	2	47720.5

1190.04328	0.5	47721

1190.14227	60	47721.5

1190.24127	5.5	47723.5

1190.34027	2	47724

1190.43926	0.5	47784

1190.53826	50	47789.5

1190.63726	2.5	47791.5

1190.73626	2.5	47792

1190.83525	0.5	47842

1190.93425	1	47844.5

1191.03325	0.5	47847

1191.13224	8	47847.5

1191.23124	7	47848.5

1191.33024	20	47849

1191.42924	2.5	47857

1191.52823	2.5	47864

1191.62723	2	47884

1191.72623	44	47886.5

1191.82522	1	47889

1191.92422	3	47891

1192.02322	7.5	47935

1192.12221	1.5	47936

1192.22121	1	47939

1192.32021	3.5	47946.5

1192.4192	3.5	47948

1192.5182	2	47949

1192.6172	6	47952.5

1192.7162	7	47956

1192.81519	7	47958

1192.91419	68	47964

1193.01319	4	47971

1193.11218	3	47978

1193.21118	3	48046

1193.31018	20	48050

1193.40918	5	48053

1193.50817	3	48056

1193.60717	47	48076

1193.70617	8	48081

1193.80516	55	48084

1193.90416	6	48131

1194.00316	16	48139

1194.10216	4	48194

1194.20115	2	48200

1194.30015	8	48216

1194.39915	58	48220

1194.49814	59	48222

1194.59714	11	48230

1194.69614	199	48288

1194.79513	13081	48347

1194.89413	97	48358

1194.99313	258	48557

1195.09212	15	61638

1195.19112	3	61735

1195.29012	4	61993

1195.38912	1	62008

1195.48811	6	62011

1195.58711	9	62015

1195.68611	5	62016

1195.7851	3	62022

1195.8841	1	62031

1195.9831	33	62036

1196.0821	21	62039

1196.18109	6	62040

1196.28009	2	62073

1196.37909	18	62094

1196.47808	17	62100

1196.57708	2	62102

1196.67608	9	62120

1196.77507	6	62137

1196.87407	2	62139

1196.97307	11	62148

1197.07207	1	62154

1197.17106	3	62156

1197.27006	4	62167

1197.36906	9	62168

1197.46805	1	62171

1197.56705	26	62175

1197.66605	15	62184

1197.76505	1	62185

1197.86404	1	62211

1197.96304	10	62226

1198.06204	1	62227

1198.16103	8	62228

1198.26003	2	62238

1198.35903	2	62239

1198.45802	5	62247

1198.55702	12	62249

1198.65602	5	62251

1198.75502	4	62256

1198.85401	3	62268

1198.95301	4	62273

1199.05201	4	62277

1199.151	1	62280

1199.25	19	62296

**（*d*）Stabilized hydraulic-45°**

1	0.10349	1.94625	12	12

2	0.12048	2.076	1	13

3	0.13593	2.20575	2	15

4	0.1452	2.3355	8	23

5	0.16064	2.46525	1	24

6	0.17918	2.59501	4	28

7	0.18999	2.72476	1	29

8	0.20544	2.85451	2	31

9	0.22397	2.98426	3	34

10	0.24251	3.11401	1	35

11	0.25795	3.24376	1	36

12	0.27186	3.37351	1	37

13	0.29039	3.50326	1	38

14	0.30584	3.63301	9	47

15	0.31511	3.76276	3	50

16	0.3321	3.89251	1	51

17	0.35372	4.02226	1	52

18	0.36608	4.15201	1	53

19	0.37998	4.28176	14	67

20	0.39852	4.41151	1	68

21	0.41551	4.54126	1	69

22	0.42787	4.67101	2	71

23	0.44486	4.80076	1	72

24	0.46339	4.93051	2	74

25	0.47729	5.06026	7	81

26	0.48811	5.19001	4	85

27	0.50664	5.31976	1	86

28	0.52363	5.44951	4	90

29	0.53753	5.57926	2	92

30	0.55144	5.70901	2	94

31	0.56997	5.83876	3	97

32	0.58851	5.96851	10	107

33	0.59932	6.09826	1	108

34	0.61013	6.22801	2	110

35	0.62867	6.35776	1	111

36	0.64411	6.48751	1	112

37	0.65647	6.61726	1	113

38	0.67501	6.74701	2	115

39	0.69045	6.87676	14	129

40	0.7059	7.00651	1	130

41	0.71826	7.13626	1	131

42	0.73525	7.26601	1	132

43	0.75224	7.39576	1	133

44	0.7646	7.52552	1	134

45	0.78159	7.65527	4	138

46	0.80012	7.78502	6	144

47	0.81557	7.91477	1	145

48	0.82638	8.04452	1	146

49	0.84183	8.17427	1	147

50	0.86345	8.30402	1	148

51	0.87118	8.43377	1	149

52	0.88508	8.56352	2	151

53	0.9067	8.69327	1	152

54	0.92524	8.82302	1	153

55	0.93451	8.95277	3	156

56	0.94995	9.08252	1	157

57	0.97003	9.21227	1	158

58	0.98548	9.34202	2	160

59	0.9932	9.47177	4	164

60	1.01174	9.60152	1	165

61	1.03027	9.73127	2	167

62	1.04109	9.86102	2	169

63	1.05653	9.99077	1	170

64	1.07816	10.12052	2	172

65	1.09206	10.25027	1	173

66	1.09978	10.38002	1	174

67	1.11986	10.50977	2	176

68	1.15539	10.63952	8	184

69	1.15694	10.76927	13	197

70	1.1662	10.89902	3	200

71	1.18937	11.02877	2	202

72	1.20636	11.15852	8	210

73	1.21563	11.28827	1	211

74	1.23108	11.41802	2	213

75	1.24961	11.54777	1	214

76	1.2666	11.67752	2	216

77	1.27587	11.80727	8	224

78	1.28977	11.93702	1	225

79	1.31294	12.06677	29	254

80	1.3253	12.19652	1	255

81	1.3392	12.32627	2	257

82	1.35774	12.45602	10	267

83	1.37319	12.58578	1	268

84	1.38709	12.71553	1	269

85	1.40099	12.84528	1	270

86	1.41798	12.97503	22	292

87	1.43188	13.10478	6	298

88	1.44578	13.23453	1	299

89	1.46432	13.36428	1	300

90	1.48285	13.49403	5	305

91	1.49676	13.62378	3	308

92	1.50602	13.75353	1	309

93	1.52456	13.88328	11	320

94	1.54464	14.01303	5	325

95	1.55236	14.14278	1	326

96	1.5709	14.27253	3	329

97	1.59252	14.40228	1	330

98	1.60488	14.53203	1	331

99	1.61724	14.66178	2	333

100	1.63268	14.79153	1	334

101	1.64968	14.92128	1	335

102	1.66512	15.05103	4	339

103	1.67902	15.18078	1	340

104	1.69447	15.31053	5	345

105	1.70992	15.44028	29	374

106	1.72227	15.57003	1	375

107	1.74235	15.69978	1	376

108	1.76089	15.82953	1	377

109	1.77479	15.95928	1	378

110	1.78406	16.08903	1	379

111	1.80105	16.21878	1	380

112	1.81959	16.34853	2	382

113	1.83349	16.47828	1	383

114	1.85048	16.60803	2	385

115	1.82576	16.73778	3	388

116	1.83812	16.86753	1	389

117	1.85202	16.99728	1	390

118	1.86747	17.12703	1	391

119	1.89064	17.25678	1	392

120	1.90145	17.38653	1	393

121	1.91535	17.51629	4	397

122	1.93543	17.64604	3	400

123	1.94625	17.77579	1	401

124	1.95706	17.90554	7	408

125	1.97405	18.03529	6	414

126	1.99722	18.16504	16	430

127	2.00803	18.29479	1	431

128	2.02502	18.42454	4	435

129	2.04356	18.55429	3	438

130	2.05901	18.68404	1	439

131	2.07136	18.81379	2	441

132	2.07754	18.94354	1	442

133	2.10226	19.07329	11	453

134	2.11925	19.20304	19	472

135	2.12697	19.33279	1	473

136	2.14705	19.46254	1	474

137	2.16713	19.59229	6	480

138	2.17949	19.72204	1	481

139	2.18876	19.85179	3	484

140	2.20884	19.98154	1	485

141	2.22583	20.11129	1	486

142	2.23509	20.24104	1	487

143	2.25054	20.37079	4	491

144	2.27525	20.50054	1	492

145	2.28916	20.63029	2	494

146	2.30151	20.76004	2	496

147	2.31542	20.88979	1	497

148	2.3355	21.01954	4	501

149	2.34631	21.14929	1	502

150	2.35712	21.27904	2	504

151	2.38029	21.40879	1	505

152	2.39883	21.53854	1	506

153	2.40964	21.66829	1	507

154	2.42508	21.79804	1	508

155	2.44362	21.92779	1	509

156	2.46061	22.05754	1	510

157	2.46833	22.18729	1	511

158	2.48224	22.31704	1	512

159	2.50077	22.44679	4	516

160	2.51313	22.57655	1	517

161	2.52703	22.7063	1	518

162	2.54866	22.83605	4	522

163	2.56565	22.9658	1	523

164	2.578	23.09555	5	528

165	2.595	23.2253	1	529

166	2.61044	23.35505	1	530

167	2.62434	23.4848	1	531

168	2.63825	23.61455	1	532

169	2.65678	23.7443	1	533

170	2.67223	23.87405	1	534

171	2.68767	24.0038	1	535

172	2.70158	24.13355	2	537

173	2.72011	24.2633	1	538

174	2.7371	24.39305	1	539

175	2.74174	24.5228	12	551

176	2.76182	24.65255	1	552

177	2.7819	24.7823	2	554

178	2.79734	24.91205	6	560

179	2.80507	25.0418	5	565

180	2.83133	25.17155	5	570

181	2.84368	25.3013	4	574

182	2.85913	25.43105	2	576

183	2.87149	25.5608	1	577

184	2.88848	25.69055	1	578

185	2.89929	25.8203	1	579

186	2.91474	25.95005	3	582

187	2.93327	26.0798	1	583

188	2.95335	26.20955	1	584

189	2.96571	26.3393	1	585

190	2.98116	26.46905	1	586

191	2.9966	26.5988	1	587

192	3.02286	26.72855	1	588

193	3.02286	26.8583	2	590

194	3.04294	26.98805	1	591

195	3.05839	27.1178	1	592

196	3.07229	27.24755	1	593

197	3.08774	27.3773	1	594

198	3.10782	27.50706	2	596

199	3.12635	27.63681	2	598

200	3.13871	27.76656	2	600

201	3.14798	27.89631	3	603

202	3.16651	28.02606	1	604

203	3.1835	28.15581	2	606

204	3.19586	28.28556	2	608

205	3.20822	28.41531	1	609

206	3.23602	28.54506	3	612

207	3.24992	28.67481	2	614

208	3.25919	28.80456	11	625

209	3.27927	28.93431	1	626

210	3.29626	29.06406	1	627

211	3.30707	29.19381	4	631

212	3.31943	29.32356	1	632

213	3.33797	29.45331	5	637

214	3.3565	29.58306	1	638

215	3.36732	29.71281	1	639

216	3.38431	29.84256	2	641

217	3.40593	29.97231	11	652

218	3.41829	30.10206	4	656

219	3.42756	30.23181	1	657

220	3.44455	30.36156	1	658

221	3.46463	30.49131	1	659

222	3.47853	30.62106	1	660

223	3.4878	30.75081	1	661

224	3.50942	30.88056	2	663

225	3.52641	31.01031	3	666

226	3.5434	31.14006	1	667

227	3.55422	31.26981	1	668

228	3.56812	31.39956	2	670

229	3.58357	31.52931	1	671

230	3.59438	31.65906	3	674

231	3.61291	31.78881	1	675

232	3.63608	31.91856	2	677

233	3.6469	32.04831	1	678

234	3.66389	32.17806	1	679

235	3.67933	32.30781	1	680

236	3.70405	32.43756	1	681

237	3.70868	32.56732	5	686

238	3.71949	32.69707	1	687

239	3.74112	32.82682	1	688

240	3.75811	32.95657	1	689

241	3.76892	33.08632	1	690

242	3.78746	33.21607	1	691

243	3.80754	33.34582	2	693

244	3.82298	33.47557	2	695

245	3.82762	33.60532	1	696

246	3.85079	33.73507	7	703

247	3.86778	33.86482	1	704

248	3.87241	33.99457	2	706

249	3.8894	34.12432	1	707

250	3.91103	34.25407	1	708

251	3.92493	34.38382	1	709

252	3.93729	34.51357	2	711

253	3.95582	34.64332	1	712

254	3.9759	34.77307	1	713

255	3.98826	34.90282	1	714

256	3.99907	35.03257	2	716

257	4.01915	35.16232	2	718

258	4.03151	35.29207	5	723

259	4.04387	35.42182	1	724

260	4.06549	35.55157	1	725

261	4.08712	35.68132	1	726

262	4.09793	35.81107	2	728

263	4.10256	35.94082	2	730

264	4.12419	36.07057	5	735

265	4.1489	36.20032	1	736

266	4.15045	36.33007	1	737

267	4.17053	36.45982	15	752

268	4.18906	36.58957	1	753

269	4.20605	36.71932	1	754

270	4.21841	36.84907	17	771

271	4.23231	36.97882	2	773

272	4.25239	37.10857	2	775

273	4.26166	37.23832	2	777

274	4.27247	37.36807	3	780

275	4.29564	37.49783	9	789

276	4.31109	37.62758	2	791

277	4.3219	37.75733	3	794

278	4.33889	37.88708	4	798

279	4.36361	38.01683	1	799

280	4.37751	38.14658	2	801

281	4.38987	38.27633	3	804

282	4.40222	38.40608	6	810

283	4.42539	38.53583	1	811

284	4.43775	38.66558	1	812

285	4.44702	38.79533	1	813

286	4.46555	38.92508	3	816

287	4.48718	39.05483	2	818

288	4.49645	39.18458	5	823

289	4.51498	39.31433	1	824

290	4.53352	39.44408	5	829

291	4.54742	39.57383	1	830

292	4.5536	39.70358	1	831

293	4.57522	39.83333	1	832

294	4.59067	39.96308	1	833

295	4.60612	40.09283	1	834

296	4.61693	40.22258	1	835

297	4.63855	40.35233	1	836

298	4.65709	40.48208	1	837

299	4.66636	40.61183	1	838

300	4.68026	40.74158	1	839

301	4.69571	40.87133	2	841

302	4.70806	41.00108	1	842

303	4.72196	41.13083	34	876

304	4.74359	41.26058	1	877

305	4.76058	41.39033	1	878

306	4.77757	41.52008	1	879

307	4.7853	41.64983	2	881

308	4.80692	41.77958	4	885

309	4.82082	41.90933	2	887

310	4.83009	42.03908	3	890

311	4.84863	42.16883	3	893

312	4.86716	42.29858	2	895

313	4.88261	42.42833	1	896

314	4.90114	42.55809	1	897

315	4.91504	42.68784	2	899

316	4.93204	42.81759	7	906

317	4.9413	42.94734	1	907

318	4.95675	43.07709	1	908

319	4.97374	43.20684	1	909

320	4.99073	43.33659	1	910

321	5	43.46634	1	911

322	5.02162	43.59609	3	914

323	5.04325	43.72584	6	920

324	5.05715	43.85559	3	923

325	5.06333	43.98534	2	925

326	5.07569	44.11509	1	926

327	5.09731	44.24484	1	927

328	5.10812	44.37459	1	928

329	5.12512	44.50434	1	929

330	5.14829	44.63409	1	930

331	5.16528	44.76384	2	932

332	5.17146	44.89359	2	934

333	5.18999	45.02334	2	936

334	5.20853	45.15309	3	939

335	5.22397	45.28284	2	941

336	5.22861	45.41259	2	943

337	5.25332	45.54234	1	944

338	5.26877	45.67209	1	945

339	5.2873	45.80184	5	950

340	5.29657	45.93159	2	952

341	5.3182	46.06134	2	954

342	5.3321	46.19109	2	956

343	5.34137	46.32084	1	957

344	5.36145	46.45059	1	958

345	5.37535	46.58034	4	962

346	5.39388	46.71009	2	964

347	5.4047	46.83984	1	965

348	5.42169	46.96959	3	968

349	5.44486	47.09934	1	969

350	5.45258	47.22909	3	972

351	5.46648	47.35884	1	973

352	5.48038	47.4886	1	974

353	5.49892	47.61835	1	975

354	5.51128	47.7481	1	976

355	5.52518	47.87785	4	980

356	5.5468	48.0076	8	988

357	5.55762	48.13735	2	990

358	5.57461	48.2671	2	992

359	5.59932	48.39685	1	993

360	5.61168	48.5266	1	994

361	5.61631	48.65635	1	995

362	5.63021	48.7861	1	996

363	5.65802	48.91585	1	997

364	5.67037	49.0456	1	998

365	5.67655	49.17535	2	1000

366	5.69818	49.3051	1	1001

367	5.72135	49.43485	1	1002

368	5.73216	49.5646	2	1004

369	5.74143	49.69435	1	1005

370	5.76305	49.8241	1	1006

371	5.78004	49.95385	3	1009

372	5.79086	50.0836	1	1010

373	5.80167	50.21335	1	1011

374	5.82793	50.3431	1	1012

375	5.84028	50.47285	1	1013

376	5.85728	50.6026	1	1014

377	5.86963	50.73235	4	1018

378	5.89435	50.8621	1	1019

379	5.90053	50.99185	5	1024

380	5.90979	51.1216	15	1039

381	5.93142	51.25135	1	1040

382	5.94995	51.3811	1	1041

383	5.96231	51.51085	1	1042

384	5.97621	51.6406	1	1043

385	5.99784	51.77035	1	1044

386	6.01174	51.9001	2	1046

387	6.01946	52.02985	1	1047

388	6.04263	52.1596	2	1049

389	6.05808	52.28935	6	1055

390	6.06889	52.4191	1	1056

391	6.08588	52.54886	1	1057

392	6.10287	52.67861	38	1095

393	6.11677	52.80836	1	1096

394	6.13068	52.93811	4	1100

395	6.14767	53.06786	2	1102

396	6.16775	53.19761	1	1103

397	6.17702	53.32736	16	1119

398	6.18474	53.45711	1	1120

399	6.20636	53.58686	1	1121

400	6.22335	53.71661	1	1122

401	6.23262	53.84636	3	1125

402	6.25425	53.97611	1	1126

403	6.27587	54.10586	15	1141

404	6.29286	54.23561	1	1142

405	6.29904	54.36536	4	1146

406	6.31758	54.49511	1	1147

407	6.33766	54.62486	1	1148

408	6.34847	54.75461	2	1150

409	6.35774	54.88436	2	1152

410	6.37936	55.01411	2	1154

411	6.39944	55.14386	1	1155

412	6.40871	55.27361	1	1156

413	6.4257	55.40336	1	1157

414	6.44733	55.53311	1	1158

415	6.45968	55.66286	1	1159

416	6.46586	55.79261	3	1162

417	6.4844	55.92236	1	1163

418	6.50139	56.05211	2	1165

419	6.51993	56.18186	4	1169

420	6.53228	56.31161	2	1171

421	6.557	56.44136	1	1172

422	6.56472	56.57111	3	1175

423	6.57862	56.70086	1	1176

424	6.5987	56.83061	2	1178

425	6.6126	56.96036	1	1179

426	6.62342	57.09011	2	1181

427	6.63268	57.21986	1	1182

428	6.66049	57.34961	11	1193

429	6.67748	57.47937	1	1194

430	6.69447	57.60912	1	1195

431	6.70374	57.73887	2	1197

432	6.72227	57.86862	3	1200

433	6.73618	57.99837	51	1251

434	6.74081	58.12812	3	1254

435	6.76089	58.25787	2	1256

436	6.77943	58.38762	1	1257

437	6.80105	58.51737	2	1259

438	6.80723	58.64712	1	1260

439	6.82731	58.77687	1	1261

440	6.84584	58.90662	1	1262

441	6.86129	59.03637	3	1265

442	6.87365	59.16612	1	1266

443	6.89064	59.29587	8	1274

444	6.90609	59.42562	1	1275

445	6.91226	59.55537	2	1277

446	6.93234	59.68512	1	1278

447	6.9586	59.81487	1	1279

448	6.97251	59.94462	5	1284

449	6.97868	60.07437	1	1285

450	6.99413	60.20412	1	1286

451	7.0173	60.33387	1	1287

452	7.02039	60.46362	1	1288

453	7.03275	60.59337	4	1292

454	7.06364	60.72312	1	1293

455	7.08063	60.85287	3	1296

456	7.08835	60.98262	2	1298

457	7.10998	61.11237	2	1300

458	7.12234	61.24212	1	1301

459	7.13933	61.37187	1	1302

460	7.14396	61.50162	1	1303

461	7.16559	61.63137	1	1304

462	7.18721	61.76112	1	1305

463	7.19339	61.89087	1	1306

464	7.21501	62.02062	1	1307

465	7.23664	62.15037	1	1308

466	7.25054	62.28012	3	1311

467	7.25826	62.40987	1	1312

468	7.27371	62.53963	1	1313

469	7.29534	62.66938	1	1314

470	7.29997	62.79913	2	1316

471	7.31387	62.92888	2	1318

472	7.3355	63.05863	1	1319

473	7.35867	63.18838	1	1320

474	7.36948	63.31813	1	1321

475	7.38492	63.44788	2	1323

476	7.405	63.57763	1	1324

477	7.422	63.70738	3	1327

478	7.42817	63.83713	2	1329

479	7.44208	63.96688	2	1331

480	7.46061	64.09663	2	1333

481	7.47451	64.22638	1	1334

482	7.49305	64.35613	1	1335

483	7.51158	64.48588	1	1336

484	7.52858	64.61563	1	1337

485	7.53321	64.74538	1	1338

486	7.5502	64.87513	1	1339

487	7.57492	65.00488	12	1351

488	7.58109	65.13463	1	1352

489	7.59036	65.26438	2	1354

490	7.61353	65.39413	1	1355

491	7.63052	65.52388	3	1358

492	7.64751	65.65363	2	1360

493	7.65678	65.78338	1	1361

494	7.67841	65.91313	2	1363

495	7.69849	66.04288	5	1368

496	7.70466	66.17263	1	1369

497	7.72629	66.30238	1	1370

498	7.74174	66.43213	1	1371

499	7.74946	66.56188	3	1374

500	7.768	66.69163	1	1375

501	7.78499	66.82138	1	1376

502	7.80661	66.95113	4	1380

503	7.82051	67.08088	4	1384

504	7.82515	67.21063	2	1386

505	7.84677	67.34038	1	1387

506	7.86531	67.47014	4	1391

507	7.87303	67.59989	1	1392

508	7.89157	67.72964	2	1394

509	7.91628	67.85939	1	1395

510	7.924	67.98914	1	1396

511	7.93636	68.11889	3	1399

512	7.9549	68.24864	1	1400

513	7.97652	68.37839	1	1401

514	7.9827	68.50814	3	1404

515	7.99815	68.63789	1	1405

516	8.01977	68.76764	1	1406

517	8.03213	68.89739	1	1407

518	8.05066	69.02714	2	1409

519	8.05993	69.15689	1	1410

520	8.08156	69.28664	1	1411

521	8.09082	69.41639	1	1412

522	8.10318	69.54614	1	1413

523	8.12326	69.67589	1	1414

524	8.14025	69.80564	2	1416

525	8.14798	69.93539	1	1417

526	8.17269	70.06514	3	1420

527	8.19432	70.19489	4	1424

528	8.20667	70.32464	1	1425

529	8.21903	70.45439	1	1426

530	8.23448	70.58414	3	1429

531	8.24838	70.71389	7	1436

532	8.26537	70.84364	1	1437

533	8.27464	70.97339	1	1438

534	8.29317	71.10314	2	1440

535	8.31634	71.23289	1	1441

536	8.32252	71.36264	1	1442

537	8.34106	71.49239	1	1443

538	8.36268	71.62214	8	1451

539	8.37504	71.75189	3	1454

540	8.37658	71.88164	2	1456

541	8.40284	72.01139	1	1457

542	8.41983	72.14114	1	1458

543	8.4291	72.27089	2	1460

544	8.44455	72.40064	4	1464

545	8.46463	72.5304	1	1465

546	8.48471	72.66015	2	1467

547	8.49089	72.7899	1	1468

548	8.50942	72.91965	2	1470

549	8.52487	73.0494	2	1472

550	8.54186	73.17915	1	1473

551	8.55113	73.3089	4	1477

552	8.56812	73.43865	3	1480

553	8.5882	73.5684	1	1481

554	8.59747	73.69815	5	1486

555	8.62373	73.8279	5	1491

556	8.64072	73.95765	2	1493

557	8.65153	74.0874	1	1494

558	8.65925	74.21715	5	1499

559	8.6747	74.3469	35	1534

560	8.69787	74.47665	3	1537

561	8.70714	74.6064	1	1538

562	8.72722	74.73615	1	1539

563	8.74421	74.8659	4	1543

564	8.7612	74.99565	1	1544

565	8.77201	75.1254	1	1545

566	8.78128	75.25515	1	1546

567	8.80599	75.3849	2	1548

568	8.81681	75.51465	1	1549

569	8.83225	75.6444	3	1552

570	8.8477	75.77415	1	1553

571	8.86778	75.9039	2	1555

572	8.88168	76.03365	1	1556

573	8.89713	76.1634	8	1564

574	8.91103	76.29315	3	1567

575	8.92956	76.4229	7	1574

576	8.94038	76.55265	1	1575

577	8.95582	76.6824	1	1576

578	8.9759	76.81215	2	1578

579	8.98517	76.9419	4	1582

580	8.99907	77.07165	4	1586

581	9.0207	77.2014	1	1587

582	9.04232	77.33115	1	1588

583	9.05314	77.46091	1	1589

584	9.06395	77.59066	5	1594

585	9.08712	77.72041	3	1597

586	9.09947	77.85016	1	1598

587	9.1072	77.97991	3	1601

588	9.12264	78.10966	5	1606

589	9.15045	78.23941	1	1607

590	9.15972	78.36916	2	1609

591	9.17207	78.49891	1	1610

592	9.19061	78.62866	1	1611

593	9.2076	78.75841	1	1612

594	9.21687	78.88816	1	1613

595	9.23386	79.01791	1	1614

596	9.25085	79.14766	1	1615

597	9.26784	79.27741	2	1617

598	9.2802	79.40716	2	1619

599	9.29719	79.53691	1	1620

600	9.32499	79.66666	11	1631

601	9.32963	79.79641	1	1632

602	9.33735	79.92616	1	1633

603	9.35434	80.05591	1	1634

604	9.37288	80.18566	7	1641

605	9.38678	80.31541	1	1642

606	9.39759	80.44516	2	1644

607	9.42076	80.57491	1	1645

608	9.44238	80.70466	1	1646

609	9.45474	80.83441	1	1647

610	9.46092	80.96416	2	1649

611	9.48872	81.09391	2	1651

612	9.4949	81.22366	3	1654

613	9.50417	81.35341	2	1656

614	9.52888	81.48316	3	1659

615	9.54897	81.61291	1	1660

616	9.55205	81.74266	4	1664

617	9.57059	81.87241	1	1665

618	9.59376	82.00216	3	1668

619	9.61075	82.13191	1	1669

620	9.61538	82.26166	1	1670

621	9.63392	82.39141	2	1672

622	9.65246	82.52117	1	1673

623	9.66327	82.65092	2	1675

624	9.67563	82.78067	1	1676

625	9.70497	82.91042	1	1677

626	9.72042	83.04017	1	1678

627	9.73432	83.16992	1	1679

628	9.74513	83.29967	1	1680

629	9.76985	83.42942	1	1681

630	9.77294	83.55917	1	1682

631	9.78838	83.68892	3	1685

632	9.80846	83.81867	1	1686

633	9.82237	83.94842	1	1687

634	9.84245	84.07817	1	1688

635	9.85171	84.20792	1	1689

636	9.87488	84.33767	1	1690

637	9.88724	84.46742	2	1692

638	9.89805	84.59717	1	1693

639	9.9135	84.72692	1	1694

640	9.93821	84.85667	2	1696

641	9.93821	84.98642	10	1706

642	9.95521	85.11617	3	1709

643	9.97838	85.24592	2	1711

644	9.99846	85.37567	4	1715

645	10.0139	85.50542	2	1717

646	10.02626	85.63517	1	1718

647	10.04788	85.76492	1	1719

648	10.05561	85.89467	1	1720

649	10.06333	86.02442	2	1722

650	10.08032	86.15417	1	1723

651	10.1004	86.28392	2	1725

652	10.12048	86.41367	1	1726

653	10.13284	86.54342	9	1735

654	10.14674	86.67317	1	1736

655	10.16837	86.80292	1	1737

656	10.17609	86.93267	1	1738

657	10.18381	87.06242	1	1739

658	10.21007	87.19217	1	1740

659	10.22861	87.32192	21	1761

660	10.23479	87.45167	1	1762

661	10.25641	87.58143	10	1772

662	10.28112	87.71118	5	1777

663	10.29039	87.84093	7	1784

664	10.29966	87.97068	28	1812

665	10.32129	88.10043	1	1813

666	10.33519	88.23018	7	1820

667	10.34445	88.35993	1	1821

668	10.35372	88.48968	1	1822

669	10.38153	88.61943	5	1827

670	10.40315	88.74918	1	1828

671	10.40624	88.87893	1	1829

672	10.42632	89.00868	3	1832

673	10.44486	89.13843	1	1833

674	10.45567	89.26818	1	1834

675	10.46185	89.39793	1	1835

676	10.4912	89.52768	1	1836

677	10.50201	89.65743	1	1837

678	10.51128	89.78718	2	1839

679	10.53599	89.91693	1	1840

680	10.55298	90.04668	1	1841

681	10.56225	90.17643	3	1844

682	10.5777	90.30618	1	1845

683	10.59469	90.43593	1	1846

684	10.61631	90.56568	3	1849

685	10.6194	90.69543	1	1850

686	10.6333	90.82518	4	1854

687	10.65493	90.95493	1	1855

688	10.67501	91.08468	2	1857

689	10.68891	91.21443	2	1859

690	10.70436	91.34418	2	1861

691	10.72753	91.47393	1	1862

692	10.73679	91.60368	1	1863

693	10.74143	91.73343	1	1864

694	10.75842	91.86318	2	1866

695	10.7785	91.99293	6	1872

696	10.7924	92.12268	2	1874

697	10.80476	92.25243	1	1875

698	10.83102	92.38218	3	1878

699	10.84337	92.51194	1	1879

700	10.85419	92.64169	1	1880

701	10.86963	92.77144	7	1887

702	10.88971	92.90119	1	1888

703	10.89898	93.03094	1	1889

704	10.91288	93.16069	4	1893

705	10.92987	93.29044	1	1894

706	10.95304	93.42019	1	1895

707	10.9654	93.54994	2	1897

708	10.97776	93.67969	11	1908

709	11.00093	93.80944	1	1909

710	11.01792	93.93919	3	1912

711	11.02101	94.06894	1	1913

712	11.038	94.19869	1	1914

713	11.05344	94.32844	1	1915

714	11.07044	94.45819	3	1918

715	11.08897	94.58794	1	1919

716	11.11214	94.71769	17	1936

717	11.12913	94.84744	10	1946

718	11.13686	94.97719	4	1950

719	11.14767	95.10694	7	1957

720	11.16775	95.23669	3	1960

721	11.17856	95.36644	14	1974

722	11.19246	95.49619	1	1975

723	11.21254	95.62594	1	1976

724	11.23262	95.75569	2	1978

725	11.2388	95.88544	6	1984

726	11.25579	96.01519	5	1989

727	11.27896	96.14494	1	1990

728	11.29286	96.27469	1	1991

729	11.29904	96.40444	8	1999

730	11.31603	96.53419	2	2001

731	11.33766	96.66394	3	2004

732	11.34847	96.79369	2	2006

733	11.36237	96.92344	16	2022

734	11.38245	97.05319	2	2024

735	11.39944	97.18294	30	2054

736	11.41489	97.31269	2	2056

737	11.42261	97.44244	2	2058

738	11.44115	97.5722	1	2059

739	11.45814	97.70195	7	2066

740	11.46586	97.8317	1	2067

741	11.48594	97.96145	1	2068

742	11.51529	98.0912	1	2069

743	11.52765	98.22095	3	2072

744	11.53383	98.3507	1	2073

745	11.55236	98.48045	6	2079

746	11.5709	98.6102	1	2080

747	11.58171	98.73995	1	2081

748	11.59098	98.8697	1	2082

749	11.61569	98.99945	1	2083

750	11.62651	99.1292	2	2085

751	11.63577	99.25895	3	2088

752	11.66049	99.3887	7	2095

753	11.67902	99.51845	16	2111

754	11.68984	99.6482	1	2112

755	11.6991	99.77795	1	2113

756	11.72691	99.9077	1	2114

757	11.73463	100.03745	4	2118

758	11.74544	100.1672	1	2119

759	11.75935	100.29695	5	2124

760	11.78869	100.4267	2	2126

761	11.80105	100.55645	1	2127

762	11.80877	100.6862	4	2131

763	11.8304	100.81595	1	2132

764	11.8443	100.9457	1	2133

765	11.8582	101.07545	1	2134

766	11.86747	101.2052	3	2137

767	11.88601	101.33495	2	2139

768	11.90609	101.4647	2	2141

769	11.9169	101.59445	2	2143

770	11.93852	101.7242	2	2145

771	11.9586	101.85395	2	2147

772	11.97096	101.9837	1	2148

773	11.97405	102.11345	1	2149

774	11.99104	102.2432	1	2150

775	12.0173	102.37295	1	2151

776	12.02811	102.50271	3	2154

777	12.0451	102.63246	2	2156

778	12.06827	102.76221	1	2157

779	12.08372	102.89196	3	2160

780	12.09762	103.02171	1	2161

781	12.1038	103.15146	1	2162

782	12.13006	103.28121	5	2167

783	12.14242	103.41096	1	2168

784	12.15323	103.54071	2	2170

785	12.16559	103.67046	1	2171

786	12.18876	103.80021	2	2173

787	12.20729	103.92996	2	2175

788	12.21965	104.05971	1	2176

789	12.23664	104.18946	1	2177

790	12.24745	104.31921	1	2178

791	12.25054	104.44896	1	2179

792	12.27217	104.57871	1	2180

793	12.29534	104.70846	1	2181

794	12.30306	104.83821	4	2185

795	12.31542	104.96796	4	2189

796	12.34476	105.09771	1	2190

797	12.35712	105.22746	1	2191

798	12.37257	105.35721	7	2198

799	12.38801	105.48696	1	2199

800	12.40192	105.61671	1	2200

801	12.41736	105.74646	3	2203

802	12.41891	105.87621	6	2209

803	12.44053	106.00596	1	2210

804	12.46679	106.13571	3	2213

805	12.4776	106.26546	1	2214

806	12.49305	106.39521	1	2215

807	12.51467	106.52496	1	2216

808	12.52858	106.65471	1	2217

809	12.53475	106.78446	5	2222

810	12.54402	106.91421	1	2223

811	12.57028	107.04396	3	2226

812	12.58418	107.17371	3	2229

813	12.60117	107.30346	1	2230

814	12.61662	107.43322	1	2231

815	12.63825	107.56297	2	2233

816	12.65369	107.69272	9	2242

817	12.65369	107.82247	2	2244

818	12.67841	107.95222	5	2249

819	12.68767	108.08197	1	2250

820	12.70312	108.21172	2	2252

821	12.71548	108.34147	1	2253

822	12.7371	108.47122	6	2259

823	12.75873	108.60097	1	2260

824	12.76954	108.73072	1	2261

825	12.78499	108.86047	2	2263

826	12.8097	108.99022	2	2265

827	12.81433	109.11997	2	2267

828	12.82978	109.24972	1	2268

829	12.85604	109.37947	1	2269

830	12.86222	109.50922	2	2271

831	12.87303	109.63897	1	2272

832	12.89774	109.76872	1	2273

833	12.91783	109.89847	3	2276

834	12.92864	110.02822	8	2284

835	12.94099	110.15797	1	2285

836	12.95026	110.28772	7	2292

837	12.96571	110.41747	3	2295

838	12.97343	110.54722	1	2296

839	12.99197	110.67697	3	2299

840	13.01977	110.80672	2	2301

841	13.03522	110.93647	1	2302

842	13.04603	111.06622	1	2303

843	13.0692	111.19597	2	2305

844	13.08156	111.32572	2	2307

845	13.09391	111.45547	1	2308

846	13.10473	111.58522	1	2309

847	13.12481	111.71497	6	2315

848	13.14489	111.84472	1	2316

849	13.1557	111.97447	3	2319

850	13.17732	112.10422	1	2320

851	13.18968	112.23397	1	2321

852	13.20822	112.36372	4	2325

853	13.21594	112.49348	1	2326

854	13.22521	112.62323	2	2328

855	13.24374	112.75298	1	2329

856	13.26228	112.88273	2	2331

857	13.27618	113.01248	1	2332

858	13.30399	113.14223	1	2333

859	13.31943	113.27198	5	2338

860	13.32407	113.40173	1	2339

861	13.33797	113.53148	3	2342

862	13.35959	113.66123	1	2343

863	13.36886	113.79098	1	2344

864	13.38431	113.92073	1	2345

865	13.39975	114.05048	1	2346

866	13.41829	114.18023	1	2347

867	13.43837	114.30998	2	2349

868	13.45536	114.43973	2	2351

869	13.4739	114.56948	1	2352

870	13.48625	114.69923	1	2353

871	13.49089	114.82898	2	2355

872	13.50633	114.95873	2	2357

873	13.53105	115.08848	6	2363

874	13.53723	115.21823	1	2364

875	13.55422	115.34798	2	2366

876	13.57584	115.47773	2	2368

877	13.59283	115.60748	1	2369

878	13.60365	115.73723	3	2372

879	13.62218	115.86698	1	2373

880	13.63454	115.99673	6	2379

881	13.6469	116.12648	1	2380

882	13.6608	116.25623	4	2384

883	13.67779	116.38598	2	2386

884	13.69632	116.51573	2	2388

885	13.7164	116.64548	1	2389

886	13.73031	116.77523	4	2393

887	13.7473	116.90498	2	2395

888	13.75811	117.03473	1	2396

889	13.76738	117.16448	1	2397

890	13.78437	117.29423	1	2398

891	13.80445	117.42399	1	2399

892	13.81835	117.55374	1	2400

893	13.83225	117.68349	1	2401

894	13.85233	117.81324	1	2402

895	13.87396	117.94299	2	2404

896	13.88323	118.07274	1	2405

897	13.89558	118.20249	1	2406

898	13.90948	118.33224	5	2411

899	13.92802	118.46199	1	2412

900	13.94192	118.59174	3	2415

901	13.95737	118.72149	1	2416

902	13.98363	118.85124	1	2417

903	13.99907	118.98099	1	2418

904	14.00834	119.11074	1	2419

905	14.02688	119.24049	5	2424

906	14.03923	119.37024	1	2425

907	14.04696	119.49999	26	2451

908	14.05777	119.62974	1	2452

909	14.08403	119.75949	6	2458

910	14.10102	119.88924	3	2461

911	14.11338	120.01899	2	2463

912	14.13809	120.14874	3	2466

913	14.1489	120.27849	4	2470

914	14.16126	120.40824	2	2472

915	14.16898	120.53799	2	2474

916	14.1937	120.66774	2	2476

917	14.20605	120.79749	3	2479

918	14.21996	120.92724	20	2499

919	14.23849	121.05699	29	2528

920	14.26166	121.18674	47	2575

921	14.27711	121.31649	11	2586

922	14.28638	121.44624	1	2587

923	14.29873	121.57599	8	2595

924	14.31572	121.70574	3	2598

925	14.32345	121.83549	1	2599

926	14.34044	121.96524	1	2600

927	14.36515	122.09499	1	2601

928	14.38214	122.22474	1	2602

929	14.39296	122.35449	1	2603

930	14.40068	122.48425	2	2605

931	14.42694	122.614	3	2608

932	14.4393	122.74375	3	2611

933	14.44547	122.8735	5	2616

934	14.46401	123.00325	13	2629

935	14.48255	123.133	7	2636

936	14.49954	123.26275	2	2638

937	14.51653	123.3925	1	2639

938	14.5397	123.52225	1	2640

939	14.5536	123.652	2	2642

940	14.55823	123.78175	1	2643

941	14.57522	123.9115	1	2644

942	14.59067	124.04125	1	2645

943	14.60457	124.171	1	2646

944	14.61538	124.30075	2	2648

945	14.64319	124.4305	1	2649

946	14.66481	124.56025	2	2651

947	14.66945	124.69	1	2652

948	14.68026	124.81975	10	2662

949	14.6988	124.9495	1	2663

950	14.71424	125.07925	1	2664

951	14.72351	125.209	2	2666

952	14.74359	125.33875	1	2667

953	14.7683	125.4685	2	2669

954	14.78221	125.59825	1	2670

955	14.78993	125.728	1	2671

956	14.81001	125.85775	1	2672

957	14.82546	125.9875	1	2673

958	14.82854	126.11725	18	2691

959	14.84554	126.247	1	2692

960	14.87025	126.37675	1	2693

961	14.88724	126.5065	1	2694

962	14.8996	126.63625	2	2696

963	14.91659	126.766	3	2699

964	14.94285	126.89575	1	2700

965	14.94439	127.0255	3	2703

966	14.95984	127.15525	5	2708

967	14.9722	127.285	2	2710

968	14.99382	127.41476	1	2711

969	14.99537	127.54451	2	2713

970	15.02317	127.67426	1	2714

971	15.04634	127.80401	1	2715

972	15.06024	127.93376	1	2716

973	15.06951	128.06351	1	2717

974	15.08496	128.19326	5	2722

975	15.10504	128.32301	7	2729

976	15.11121	128.45276	1	2730

977	15.12357	128.58251	1	2731

978	15.14829	128.71226	4	2735

979	15.16219	128.84201	1	2736

980	15.17918	128.97176	1	2737

981	15.19771	129.10151	2	2739

982	15.21934	129.23126	1	2740

983	15.23015	129.36101	1	2741

984	15.2317	129.49076	2	2743

985	15.25487	129.62051	1	2744

986	15.27186	129.75026	1	2745

987	15.28421	129.88001	8	2753

988	15.29657	130.00976	2	2755

989	15.32592	130.13951	1	2756

990	15.33519	130.26926	1	2757

991	15.34291	130.39901	4	2761

992	15.36608	130.52876	2	2763

993	15.38307	130.65851	1	2764

994	15.38925	130.78826	1	2765

995	15.39852	130.91801	1	2766

996	15.42941	131.04776	1	2767

997	15.45103	131.17751	2	2769

998	15.45721	131.30726	3	2772

999	15.4742	131.43701	1	2773

1000	15.49737	131.56676	1	2774

1001	15.5051	131.69651	30	2804

1002	15.51128	131.82626	3	2807

1003	15.52672	131.95601	2	2809

1004	15.54989	132.08576	1	2810

1005	15.5607	132.21551	9	2819

1006	15.58233	132.34526	2	2821

1007	15.60241	132.47502	2	2823

1008	15.6194	132.60477	1	2824

1009	15.62712	132.73452	3	2827

1010	15.63794	132.86427	1	2828

1011	15.65802	132.99402	1	2829

1012	15.6642	133.12377	1	2830

1013	15.67346	133.25352	1	2831

1014	15.70281	133.38327	1	2832

1015	15.72444	133.51302	3	2835

1016	15.73679	133.64277	1	2836

1017	15.75378	133.77252	1	2837

1018	15.76923	133.90227	1	2838

1019	15.78622	134.03202	2	2840

1020	15.78622	134.16177	1	2841

1021	15.80476	134.29152	4	2845

1022	15.82793	134.42127	1	2846

1023	15.84337	134.55102	2	2848

1024	15.85728	134.68077	3	2851

1025	15.87272	134.81052	5	2856

1026	15.89898	134.94027	2	2858

1027	15.90207	135.07002	2	2860

1028	15.91443	135.19977	3	2863

1029	15.93296	135.32952	1	2864

1030	15.94995	135.45927	2	2866

1031	15.95304	135.58902	1	2867

1032	15.9793	135.71877	1	2868

1033	16.00556	135.84852	1	2869

1034	16.01637	135.97827	1	2870

1035	16.03027	136.10802	1	2871

1036	16.04263	136.23777	3	2874

1037	16.06117	136.36752	1	2875

1038	16.05499	136.49727	8	2883

1039	16.08897	136.62702	9	2892

1040	16.10751	136.75677	2	2894

1041	16.10905	136.88652	2	2896

1042	16.12759	137.01627	6	2902

1043	16.15539	137.14602	1	2903

1044	16.16929	137.27577	3	2906

1045	16.17856	137.40553	1	2907

1046	16.20173	137.53528	5	2912

1047	16.21872	137.66503	8	2920

1048	16.22181	137.79478	2	2922

1049	16.23262	137.92453	3	2925

1050	16.26043	138.05428	1	2926

1051	16.27587	138.18403	3	2929

1052	16.28977	138.31378	11	2940

1053	16.3114	138.44353	5	2945

1054	16.33302	138.57328	2	2947

1055	16.34229	138.70303	1	2948

1056	16.3531	138.83278	4	2952

1057	16.36701	138.96253	5	2957

1058	16.37473	139.09228	1	2958

1059	16.39172	139.22203	23	2981

1060	16.40871	139.35178	1	2982

1061	16.43034	139.48153	1	2983

1062	16.45042	139.61128	6	2989

1063	16.45968	139.74103	1	2990

1064	16.48131	139.87078	2	2992

1065	16.49521	140.00053	1	2993

1066	16.50139	140.13028	2	2995

1067	16.5122	140.26003	3	2998

1068	16.52919	140.38978	1	2999

1069	16.55082	140.51953	6	3005

1070	16.5709	140.64928	4	3009

1071	16.5848	140.77903	1	3010

1072	16.60797	140.90878	2	3012

1073	16.61724	141.03853	1	3013

1074	16.62651	141.16828	1	3014

1075	16.6435	141.29803	1	3015

1076	16.65585	141.42778	1	3016

1077	16.66667	141.55753	4	3020

1078	16.68984	141.68728	1	3021

1079	16.71301	141.81703	3	3024

1080	16.72382	141.94678	3	3027

1081	16.73309	142.07653	1	3028

1082	16.75471	142.20628	2	3030

1083	16.77325	142.33603	1	3031

1084	16.77943	142.46579	1	3032

1085	16.78715	142.59554	1	3033

1086	16.80877	142.72529	2	3035

1087	16.83194	142.85504	9	3044

1088	16.84584	142.98479	3	3047

1089	16.86129	143.11454	3	3050

1090	16.87983	143.24429	1	3051

1091	16.89373	143.37404	2	3053

1092	16.90454	143.50379	2	3055

1093	16.91381	143.63354	1	3056

1094	16.93543	143.76329	2	3058

1095	16.94625	143.89304	5	3063

1096	16.96478	144.02279	1	3064

1097	16.98795	144.15254	1	3065

1098	17.00803	144.28229	1	3066

1099	17.01884	144.41204	13	3079

1100	17.02966	144.54179	1	3080

1101	17.04665	144.67154	13	3093

1102	17.06055	144.80129	1	3094

1103	17.06055	144.93104	4	3098

1104	17.08217	145.06079	2	3100

1105	17.10998	145.19054	2	3102

1106	17.12388	145.32029	1	3103

1107	17.14087	145.45004	1	3104

1108	17.15323	145.57979	2	3106

1109	17.16867	145.70954	2	3108

1110	17.1764	145.83929	1	3109

1111	17.19339	145.96904	1	3110

1112	17.21656	146.09879	3	3113

1113	17.22583	146.22854	1	3114

1114	17.23664	146.35829	1	3115

1115	17.26444	146.48804	1	3116

1116	17.28452	146.61779	3	3119

1117	17.28761	146.74754	1	3120

1118	17.30615	146.87729	1	3121

1119	17.32468	147.00704	3	3124

1120	17.33086	147.13679	1	3125

1121	17.34013	147.26654	1	3126

1122	17.35867	147.3963	2	3128

1123	17.3772	147.52605	1	3129

1124	17.39574	147.6558	5	3134

1125	17.41118	147.78555	2	3136

1126	17.42663	147.9153	9	3145

1127	17.44053	148.04505	1	3146

1128	17.4498	148.1748	2	3148

1129	17.46216	148.30455	2	3150

1130	17.47451	148.4343	3	3153

1131	17.47142	148.56405	1	3154

1132	16.68675	148.6938	1	3155

1133	15.90053	148.82355	2	3157

1134	15.08496	148.9533	1	3158

1135	14.1798	149.08305	1	3159

1136	13.29317	149.2128	1	3160

1137	12.45907	149.34255	2	3162

1138	11.49985	149.4723	3	3165

1139	10.57461	149.60205	2	3167

1140	9.73741	149.7318	3	3170

149.86155	1	3171

149.9913	5	3176

150.12105	3	3179

150.2508	4	3183

150.38055	1	3184

150.5103	1	3185

150.64005	3	3188

150.7698	1	3189

150.89955	2	3191

151.0293	1	3192

151.15905	9	3201

151.2888	2	3203

151.41855	1	3204

151.5483	5	3209

151.67805	2	3211

151.8078	5	3216

151.93755	3	3219

152.0673	2	3221

152.19705	1	3222

152.3268	2	3224

152.45656	1	3225

152.58631	4	3229

152.71606	3	3232

152.84581	3	3235

152.97556	1	3236

153.10531	1	3237

153.23506	4	3241

153.36481	3	3244

153.49456	1	3245

153.62431	20	3265

153.75406	2	3267

153.88381	2	3269

154.01356	1	3270

154.14331	1	3271

154.27306	1	3272

154.40281	5	3277

154.53256	4	3281

154.66231	1	3282

154.79206	2	3284

154.92181	1	3285

155.05156	4	3289

155.18131	2	3291

155.31106	1	3292

155.44081	2	3294

155.57056	1	3295

155.70031	1	3296

155.83006	1	3297

155.95981	1	3298

156.08956	2	3300

156.21931	2	3302

156.34906	3	3305

156.47881	1	3306

156.60856	3	3309

156.73831	1	3310

156.86806	1	3311

156.99781	1	3312

157.12756	1	3313

157.25731	5	3318

157.38707	6	3324

157.51682	3	3327

157.64657	1	3328

157.77632	2	3330

157.90607	1	3331

158.03582	1	3332

158.16557	12	3344

158.29532	1	3345

158.42507	1	3346

158.55482	1	3347

158.68457	7	3354

158.81432	1	3355

158.94407	2	3357

159.07382	3	3360

159.20357	1	3361

159.33332	1	3362

159.46307	1	3363

159.59282	1	3364

159.72257	1	3365

159.85232	1	3366

159.98207	1	3367

160.11182	1	3368

160.24157	1	3369

160.37132	3	3372

160.50107	2	3374

160.63082	1	3375

160.76057	1	3376

160.89032	1	3377

161.02007	5	3382

161.14982	4	3386

161.27957	2	3388

161.40932	1	3389

161.53907	3	3392

161.66882	1	3393

161.79857	1	3394

161.92832	1	3395

162.05807	1	3396

162.18782	2	3398

162.31757	1	3399

162.44733	1	3400

162.57708	1	3401

162.70683	1	3402

162.83658	1	3403

162.96633	2	3405

163.09608	3	3408

163.22583	4	3412

163.35558	2	3414

163.48533	1	3415

163.61508	1	3416

163.74483	1	3417

163.87458	1	3418

164.00433	3	3421

164.13408	1	3422

164.26383	1	3423

164.39358	3	3426

164.52333	1	3427

164.65308	1	3428

164.78283	1	3429

164.91258	37	3466

165.04233	2	3468

165.17208	1	3469

165.30183	6	3475

165.43158	1	3476

165.56133	4	3480

165.69108	1	3481

165.82083	1	3482

165.95058	1	3483

166.08033	1	3484

166.21008	3	3487

166.33983	6	3493

166.46958	5	3498

166.59933	1	3499

166.72908	3	3502

166.85883	1	3503

166.98858	5	3508

167.11833	1	3509

167.24808	1	3510

167.37783	5	3515

167.50759	2	3517

167.63734	1	3518

167.76709	1	3519

167.89684	2	3521

168.02659	1	3522

168.15634	1	3523

168.28609	7	3530

168.41584	2	3532

168.54559	1	3533

168.67534	3	3536

168.80509	1	3537

168.93484	13	3550

169.06459	1	3551

169.19434	1	3552

169.32409	3	3555

169.45384	3	3558

169.58359	1	3559

169.71334	3	3562

169.84309	1	3563

169.97284	2	3565

170.10259	1	3566

170.23234	2	3568

170.36209	1	3569

170.49184	1	3570

170.62159	1	3571

170.75134	15	3586

170.88109	1	3587

171.01084	2	3589

171.14059	3	3592

171.27034	1	3593

171.40009	1	3594

171.52984	1	3595

171.65959	1	3596

171.78934	2	3598

171.91909	2	3600

172.04884	1	3601

172.17859	2	3603

172.30834	1	3604

172.4381	2	3606

172.56785	1	3607

172.6976	1	3608

172.82735	3	3611

172.9571	2	3613

173.08685	5	3618

173.2166	1	3619

173.34635	1	3620

173.4761	6	3626

173.60585	1	3627

173.7356	1	3628

173.86535	1	3629

173.9951	3	3632

174.12485	1	3633

174.2546	1	3634

174.38435	3	3637

174.5141	2	3639

174.64385	19	3658

174.7736	6	3664

174.90335	2	3666

175.0331	1	3667

175.16285	3	3670

175.2926	1	3671

175.42235	1	3672

175.5521	6	3678

175.68185	2	3680

175.8116	2	3682

175.94135	6	3688

176.0711	1	3689

176.20085	2	3691

176.3306	1	3692

176.46035	14	3706

176.5901	25	3731

176.71985	1	3732

176.8496	1	3733

176.97935	1	3734

177.1091	2	3736

177.23885	1	3737

177.3686	7	3744

177.49836	1	3745

177.62811	1	3746

177.75786	8	3754

177.88761	2	3756

178.01736	4	3760

178.14711	1	3761

178.27686	4	3765

178.40661	1	3766

178.53636	2	3768

178.66611	1	3769

178.79586	1	3770

178.92561	4	3774

179.05536	1	3775

179.18511	1	3776

179.31486	1	3777

179.44461	2	3779

179.57436	3	3782

179.70411	12	3794

179.83386	3	3797

179.96361	1	3798

180.09336	1	3799

180.22311	2	3801

180.35286	5	3806

180.48261	4	3810

180.61236	1	3811

180.74211	1	3812

180.87186	1	3813

181.00161	2	3815

181.13136	1	3816

181.26111	1	3817

181.39086	1	3818

181.52061	1	3819

181.65036	1	3820

181.78011	1	3821

181.90986	2	3823

182.03961	2	3825

182.16936	3	3828

182.29911	9	3837

182.42887	2	3839

182.55862	1	3840

182.68837	2	3842

182.81812	2	3844

182.94787	3	3847

183.07762	1	3848

183.20737	5	3853

183.33712	5	3858

183.46687	6	3864

183.59662	2	3866

183.72637	1	3867

183.85612	2	3869

183.98587	1	3870

184.11562	2	3872

184.24537	1	3873

184.37512	4	3877

184.50487	1	3878

184.63462	7	3885

184.76437	1	3886

184.89412	2	3888

185.02387	3	3891

185.15362	4	3895

185.28337	2	3897

185.41312	4	3901

185.54287	2	3903

185.67262	1	3904

185.80237	4	3908

185.93212	1	3909

186.06187	1	3910

186.19162	1	3911

186.32137	1	3912

186.45112	1	3913

186.58087	6	3919

186.71062	1	3920

186.84037	1	3921

186.97012	3	3924

187.09987	1	3925

187.22962	1	3926

187.35937	1	3927

187.48913	1	3928

187.61888	2	3930

187.74863	1	3931

187.87838	1	3932

188.00813	1	3933

188.13788	1	3934

188.26763	1	3935

188.39738	1	3936

188.52713	2	3938

188.65688	1	3939

188.78663	1	3940

188.91638	1	3941

189.04613	1	3942

189.17588	2	3944

189.30563	1	3945

189.43538	2	3947

189.56513	3	3950

189.69488	2	3952

189.82463	2	3954

189.95438	1	3955

190.08413	1	3956

190.21388	1	3957

190.34363	1	3958

190.47338	1	3959

190.60313	1	3960

190.73288	4	3964

190.86263	2	3966

190.99238	1	3967

191.12213	2	3969

191.25188	6	3975

191.38163	1	3976

191.51138	4	3980

191.64113	2	3982

191.77088	1	3983

191.90063	1	3984

192.03038	1	3985

192.16013	1	3986

192.28988	2	3988

192.41964	3	3991

192.54939	2	3993

192.67914	2	3995

192.80889	1	3996

192.93864	1	3997

193.06839	1	3998

193.19814	2	4000

193.32789	1	4001

193.45764	2	4003

193.58739	4	4007

193.71714	1	4008

193.84689	4	4012

193.97664	1	4013

194.10639	1	4014

194.23614	3	4017

194.36589	1	4018

194.49564	2	4020

194.62539	2	4022

194.75514	2	4024

194.88489	1	4025

195.01464	1	4026

195.14439	1	4027

195.27414	1	4028

195.40389	3	4031

195.53364	7	4038

195.66339	1	4039

195.79314	1	4040

195.92289	1	4041

196.05264	4	4045

196.18239	2	4047

196.31214	1	4048

196.44189	1	4049

196.57164	2	4051

196.70139	1	4052

196.83114	2	4054

196.96089	1	4055

197.09064	4	4059

197.22039	1	4060

197.35014	5	4065

197.4799	2	4067

197.60965	1	4068

197.7394	1	4069

197.86915	3	4072

197.9989	3	4075

198.12865	2	4077

198.2584	3	4080

198.38815	1	4081

198.5179	10	4091

198.64765	2	4093

198.7774	2	4095

198.90715	2	4097

199.0369	1	4098

199.16665	1	4099

199.2964	1	4100

199.42615	2	4102

199.5559	3	4105

199.68565	2	4107

199.8154	8	4115

199.94515	3	4118

200.0749	1	4119

200.20465	4	4123

200.3344	1	4124

200.46415	2	4126

200.5939	1	4127

200.72365	2	4129

200.8534	1	4130

200.98315	2	4132

201.1129	7	4139

201.24265	2	4141

201.3724	1	4142

201.50215	1	4143

201.6319	1	4144

201.76165	1	4145

201.8914	1	4146

202.02115	1	4147

202.1509	1	4148

202.28065	1	4149

202.41041	2	4151

202.54016	1	4152

202.66991	1	4153

202.79966	1	4154

202.92941	5	4159

203.05916	1	4160

203.18891	1	4161

203.31866	3	4164

203.44841	1	4165

203.57816	6	4171

203.70791	3	4174

203.83766	1	4175

203.96741	2	4177

204.09716	1	4178

204.22691	1	4179

204.35666	3	4182

204.48641	3	4185

204.61616	1	4186

204.74591	1	4187

204.87566	2	4189

205.00541	2	4191

205.13516	2	4193

205.26491	1	4194

205.39466	1	4195

205.52441	1	4196

205.65416	4	4200

205.78391	2	4202

205.91366	1	4203

206.04341	1	4204

206.17316	1	4205

206.30291	2	4207

206.43266	2	4209

206.56241	2	4211

206.69216	2	4213

206.82191	1	4214

206.95166	1	4215

207.08141	1	4216

207.21116	3	4219

207.34091	1	4220

207.47067	1	4221

207.60042	4	4225

207.73017	4	4229

207.85992	2	4231

207.98967	3	4234

208.11942	27	4261

208.24917	4	4265

208.37892	2	4267

208.50867	8	4275

208.63842	2	4277

208.76817	1	4278

208.89792	5	4283

209.02767	1	4284

209.15742	3	4287

209.28717	1	4288

209.41692	6	4294

209.54667	3	4297

209.67642	6	4303

209.80617	1	4304

209.93592	1	4305

210.06567	1	4306

210.19542	11	4317

210.32517	1	4318

210.45492	1	4319

210.58467	1	4320

210.71442	2	4322

210.84417	1	4323

210.97392	4	4327

211.10367	1	4328

211.23342	2	4330

211.36317	1	4331

211.49292	1	4332

211.62267	2	4334

211.75242	1	4335

211.88217	1	4336

212.01192	1	4337

212.14167	1	4338

212.27142	1	4339

212.40117	1	4340

212.53093	3	4343

212.66068	1	4344

212.79043	3	4347

212.92018	1	4348

213.04993	1	4349

213.17968	1	4350

213.30943	4	4354

213.43918	4	4358

213.56893	2	4360

213.69868	4	4364

213.82843	3	4367

213.95818	2	4369

214.08793	2	4371

214.21768	2	4373

214.34743	2	4375

214.47718	5	4380

214.60693	1	4381

214.73668	2	4383

214.86643	2	4385

214.99618	1	4386

215.12593	3	4389

215.25568	2	4391

215.38543	1	4392

215.51518	3	4395

215.64493	2	4397

215.77468	1	4398

215.90443	22	4420

216.03418	1	4421

216.16393	2	4423

216.29368	2	4425

216.42343	1	4426

216.55318	1	4427

216.68293	2	4429

216.81268	2	4431

216.94243	1	4432

217.07218	2	4434

217.20193	1	4435

217.33168	3	4438

217.46144	1	4439

217.59119	2	4441

217.72094	1	4442

217.85069	1	4443

217.98044	1	4444

218.11019	2	4446

218.23994	1	4447

218.36969	1	4448

218.49944	3	4451

218.62919	4	4455

218.75894	5	4460

218.88869	1	4461

219.01844	1	4462

219.14819	9	4471

219.27794	1	4472

219.40769	3	4475

219.53744	1	4476

219.66719	1	4477

219.79694	1	4478

219.92669	2	4480

220.05644	6	4486

220.18619	2	4488

220.31594	1	4489

220.44569	5	4494

220.57544	6	4499

220.70519	1	4500

220.83494	2	4502

220.96469	1	4503

221.09444	1	4504

221.22419	14	4517

221.35394	3	4520

221.48369	17	4536

221.61344	1	4537

221.74319	1	4537

221.87294	13	4550

222.00269	9	4559

222.13244	1	4559

222.26219	17	4576

222.39194	1	4577

222.5217	1	4577

222.65145	6	4583

222.7812	2	4584

222.91095	2	4586

223.0407	1	4586

223.17045	2	4588

223.3002	3	4591

223.42995	3	4593

223.5597	5	4598

223.68945	1	4598

223.8192	1	4599

223.94895	9	4608

224.0787	1	4609

224.20845	1	4609

224.3382	20	4629

224.46795	1	4629

224.5977	1	4630

224.72745	1	4630

224.8572	1	4631

224.98695	7	4638

225.1167	3	4641

225.24645	1	4642

225.3762	2	4643

225.50595	8	4651

225.6357	4	4655

225.76545	1	4656

225.8952	7	4663

226.02495	1	4663

226.1547	1	4664

226.28445	2	4665

226.4142	6	4671

226.54395	1	4672

226.6737	1	4672

226.80345	18	4690

226.9332	1	4690

227.06295	1	4691

227.1927	1	4692

227.32245	9	4701

227.45221	2	4703

227.58196	1	4704

227.71171	1	4705

227.84146	3	4708

227.97121	1	4709

228.10096	22	4730

228.23071	1	4731

228.36046	2	4733

228.49021	11	4744

228.61996	1	4745

228.74971	1	4746

228.87946	1	4746

229.00921	17	4763

229.13896	2	4765

229.26871	10	4775

229.39846	1	4776

229.52821	1	4776

229.65796	1	4777

229.78771	12	4789

229.91746	2	4790

230.04721	2	4792

230.17696	16	4808

230.30671	1	4808

230.43646	2	4810

230.56621	14	4824

230.69596	1	4824

230.82571	1	4825

230.95546	4	4829

231.08521	1	4830

231.21496	2	4832

231.34471	3	4835

231.47446	4	4839

231.60421	7	4846

231.73396	1	4846

231.86371	2	4848

231.99346	16	4864

232.12321	1	4864

232.25296	12	4876

232.38271	1	4877

232.51247	7	4884

232.64222	4	4888

232.77197	1	4889

232.90172	1	4889

233.03147	1	4890

233.16122	11	4901

233.29097	1	4902

233.42072	1	4903

233.55047	23	4926

233.68022	1	4927

233.80997	1	4927

233.93972	1	4928

234.06947	1	4929

234.19922	1	4929

234.32897	1	4930

234.45872	1	4930

234.58847	3	4933

234.71822	7	4939

234.84797	1	4940

234.97772	1	4940

235.10747	17	4957

235.23722	2	4959

235.36697	1	4960

235.49672	19	4979

235.62647	1	4980

235.75622	3	4983

235.88597	14	4996

236.01572	1	4997

236.14547	13	5010

236.27522	2	5011

236.40497	1	5012

236.53472	1	5013

236.66447	1	5014

236.79422	1	5015

236.92397	1	5015

237.05372	25	5040

237.18347	2	5041

237.31322	1	5042

237.44298	15	5057

237.57273	2	5059

237.70248	1	5060

237.83223	20	5079

237.96198	1	5080

238.09173	4	5084

238.22148	1	5085

238.35123	11	5096

238.48098	2	5097

238.61073	1	5098

238.74048	1	5099

238.87023	1	5100

238.99998	13	5112

239.12973	1	5113

239.25948	1	5113

239.38923	1	5114

239.51898	2	5116

239.64873	1	5116

239.77848	1	5117

239.90823	1	5118

240.03798	10	5128

240.16773	4	5131

240.29748	2	5133

240.42723	13	5146

240.55698	1	5146

240.68673	1	5147

240.81648	1	5147

240.94623	1	5148

241.07598	10	5157

241.20573	2	5159

241.33548	1	5160

241.46523	1	5161

241.59498	12	5172

241.72473	1	5173

241.85448	1	5173

241.98423	1	5174

242.11398	1	5175

242.24373	7	5181

242.37348	4	5185

242.50324	6	5191

242.63299	5	5195

242.76274	2	5197

242.89249	1	5197

243.02224	9	5206

243.15199	1	5207

243.28174	2	5209

243.41149	1	5210

243.54124	4	5214

243.67099	1	5214

243.80074	2	5216

243.93049	1	5217

244.06024	1	5218

244.18999	1	5219

244.31974	1	5219

244.44949	1	5220

244.57924	1	5221

244.70899	24	5244

244.83874	1	5245

244.96849	1	5246

245.09824	2	5248

245.22799	15	5263

245.35774	1	5263

245.48749	1	5264

245.61724	2	5266

245.74699	4	5270

245.87674	7	5276

246.00649	1	5277

246.13624	13	5290

246.26599	4	5294

246.39574	1	5294

246.52549	1	5295

246.65524	1	5296

246.78499	1	5297

246.91474	8	5305

247.04449	1	5305

247.17424	1	5306

247.30399	1	5306

247.43375	9	5315

247.5635	7	5322

247.69325	2	5323

247.823	17	5340

247.95275	1	5340

248.0825	1	5341

248.21225	1	5342

248.342	1	5342

248.47175	1	5343

248.6015	1	5344

248.73125	2	5346

248.861	1	5346

248.99075	1	5347

249.1205	7	5354

249.25025	2	5356

249.38	1	5357

249.50975	7	5363

249.6395	13	5376

249.76925	1	5377

249.899	1	5377

250.02875	15	5392

250.1585	1	5393

250.28825	3	5396

250.418	1	5397

250.54775	19	5415

250.6775	1	5416

250.80725	1	5417

250.937	1	5418

251.06675	1	5418

251.1965	5	5423

251.32625	13	5436

251.456	2	5438

251.58575	13	5451

251.7155	1	5452

251.84525	1	5453

251.975	1	5454

252.10475	1	5454

252.2345	1	5455

252.36426	1	5455

252.49401	12	5467

252.62376	1	5467

252.75351	1	5468

252.88326	1	5469

253.01301	7	5476

253.14276	1	5477

253.27251	5	5482

253.40226	1	5483

253.53201	16	5499

253.66176	2	5501

253.79151	1	5501

253.92126	1	5502

254.05101	16	5518

254.18076	2	5519

254.31051	1	5520

254.44026	1	5521

254.57001	1	5522

254.69976	1	5523

254.82951	1	5524

254.95926	1	5525

255.08901	9	5533

255.21876	12	5545

255.34851	2	5546

255.47826	1	5547

255.60801	1	5547

255.73776	10	5557

255.86751	1	5557

255.99726	1	5558

256.12701	2	5560

256.25676	6	5565

256.38651	2	5567

256.51626	1	5568

256.64601	1	5568

256.77576	2	5570

256.90551	1	5571

257.03526	1	5571

257.16501	2	5573

257.29476	1	5573

257.42452	12	5585

257.55427	3	5587

257.68402	2	5589

257.81377	1	5590

257.94352	1	5590

258.07327	2	5592

258.20302	15	5607

258.33277	16	5623

258.46252	3	5626

258.59227	1	5626

258.72202	1	5627

258.85177	8	5635

258.98152	1	5636

259.11127	3	5638

259.24102	11	5649

259.37077	1	5650

259.50052	1	5650

259.63027	2	5652

259.76002	1	5653

259.88977	7	5660

260.01952	2	5662

260.14927	9	5671

260.27902	1	5672

260.40877	1	5672

260.53852	1	5673

260.66827	18	5690

260.79802	1	5691

260.92777	1	5692

261.05752	2	5694

261.18727	1	5694

261.31702	1	5695

261.44677	12	5707

261.57652	1	5708

261.70627	3	5711

261.83602	1	5712

261.96577	2	5714

262.09552	15	5729

262.22527	2	5731

262.35503	1	5731

262.48478	1	5732

262.61453	2	5734

262.74428	2	5735

262.87403	1	5736

263.00378	1	5737

263.13353	11	5748

263.26328	2	5749

263.39303	1	5750

263.52278	25	5775

263.65253	1	5776

263.78228	4	5779

263.91203	1	5780

264.04178	1	5781

264.17153	1	5782

264.30128	20	5802

264.43103	1	5803

264.56078	1	5803

264.69053	14	5817

264.82028	1	5818

264.95003	1	5819

265.07978	3	5821

265.20953	1	5822

265.33928	1	5823

265.46903	2	5825

265.59878	12	5836

265.72853	1	5837

265.85828	1	5838

265.98803	1	5838

266.11778	2	5840

266.24753	10	5850

266.37728	1	5851

266.50703	1	5851

266.63678	1	5852

266.76653	8	5860

266.89628	2	5861

267.02603	1	5862

267.15578	1	5863

267.28553	1	5864

267.41529	1	5864

267.54504	1	5865

267.67479	2	5867

267.80454	2	5869

267.93429	12	5880

268.06404	3	5883

268.19379	1	5884

268.32354	1	5885

268.45329	2	5886

268.58304	2	5888

268.71279	2	5889

268.84254	1	5890

268.97229	9	5899

269.10204	2	5901

269.23179	1	5902

269.36154	1	5903

269.49129	1	5903

269.62104	2	5905

269.75079	25	5930

269.88054	3	5932

270.01029	17	5949

270.14004	2	5951

270.26979	1	5952

270.39954	14	5966

270.52929	1	5966

270.65904	2	5968

270.78879	1	5968

270.91854	1	5969

271.04829	5	5974

271.17804	1	5975

271.30779	1	5975

271.43754	3	5978

271.56729	8	5986

271.69704	1	5987

271.82679	4	5991

271.95654	1	5991

272.08629	1	5992

272.21604	2	5994

272.3458	1	5994

272.47555	13	6007

272.6053	2	6009

272.73505	2	6010

272.8648	1	6011

272.99455	1	6012

273.1243	4	6015

273.25405	5	6020

273.3838	1	6021

273.51355	1	6022

273.6433	1	6023

273.77305	8	6030

273.9028	1	6031

274.03255	1	6031

274.1623	17	6048

274.29205	1	6048

274.4218	3	6051

274.55155	1	6052

274.6813	1	6052

274.81105	1	6053

274.9408	4	6057

275.07055	1	6058

275.2003	1	6059

275.33005	1	6060

275.4598	1	6061

275.58955	1	6061

275.7193	8	6069

275.84905	2	6071

275.9788	1	6072

276.10855	3	6074

276.2383	1	6075

276.36805	1	6076

276.4978	1	6076

276.62755	16	6092

276.7573	3	6095

276.88705	21	6116

277.0168	2	6118

277.14655	1	6118

277.2763	1	6119

277.40606	1	6120

277.53581	2	6121

277.66556	1	6122

277.79531	1	6123

277.92506	2	6125

278.05481	5	6129

278.18456	1	6130

278.31431	1	6131

278.44406	1	6132

278.57381	23	6155

278.70356	1	6156

278.83331	1	6156

278.96306	3	6159

279.09281	6	6165

279.22256	1	6166

279.35231	2	6167

279.48206	19	6186

279.61181	1	6187

279.74156	1	6187

279.87131	1	6188

280.00106	13	6201

280.13081	2	6202

280.26056	1	6203

280.39031	2	6205

280.52006	1	6206

280.64981	5	6211

280.77956	1	6212

280.90931	1	6213

281.03906	11	6224

281.16881	17	6241

281.29856	1	6241

281.42831	1	6242

281.55806	1	6243

281.68781	13	6255

281.81756	1	6256

281.94731	10	6266

282.07706	2	6268

282.20681	1	6268

282.33657	10	6278

282.46632	1	6279

282.59607	1	6279

282.72582	1	6280

282.85557	13	6292

282.98532	2	6294

283.11507	13	6307

283.24482	1	6307

283.37457	2	6309

283.50432	1	6310

283.63407	1	6311

283.76382	1	6311

283.89357	1	6312

284.02332	1	6313

284.15307	1	6314

284.28282	14	6328

284.41257	1	6328

284.54232	3	6331

284.67207	16	6347

284.80182	4	6351

284.93157	1	6352

285.06132	1	6352

285.19107	1	6353

285.32082	1	6354

285.45057	1	6355

285.58032	1	6356

285.71007	3	6359

285.83982	1	6359

285.96957	1	6360

286.09932	2	6362

286.22907	5	6367

286.35882	1	6368

286.48857	2	6370

286.61832	1	6371

286.74807	1	6372

286.87782	15	6386

287.00757	2	6388

287.13732	2	6389

287.26707	6	6395

287.39683	15	6410

287.52658	2	6412

287.65633	1	6413

287.78608	1	6414

287.91583	1	6414

288.04558	1	6415

288.17533	2	6417

288.30508	8	6424

288.43483	1	6425

288.56458	1	6426

288.69433	9	6435

288.82408	2	6437

288.95383	2	6439

289.08358	1	6440

289.21333	1	6441

289.34308	1	6442

289.47283	7	6449

289.60258	1	6450

289.73233	2	6451

289.86208	2	6453

289.99183	1	6454

290.12158	11	6465

290.25133	1	6465

290.38108	1	6466

290.51083	1	6466

290.64058	1	6467

290.77033	5	6472

290.90008	1	6472

291.02983	15	6487

291.15958	1	6488

291.28933	2	6489

291.41908	1	6490

291.54883	1	6491

291.67858	16	6507

291.80833	1	6508

291.93808	1	6509

292.06783	1	6510

292.19758	1	6510

292.32734	1	6511

292.45709	1	6512

292.58684	1	6513

292.71659	7	6520

292.84634	3	6523

292.97609	9	6532

293.10584	1	6532

293.23559	3	6535

293.36534	1	6536

293.49509	1	6537

293.62484	2	6539

293.75459	2	6540

293.88434	1	6541

294.01409	1	6542

294.14384	1	6543

294.27359	2	6545

294.40334	2	6546

294.53309	2	6548

294.66284	11	6558

294.79259	3	6561

294.92234	2	6563

295.05209	1	6564

295.18184	6	6570

295.31159	1	6570

295.44134	2	6572

295.57109	1	6572

295.70084	10	6582

295.83059	1	6583

295.96034	2	6585

296.09009	1	6586

296.21984	15	6601

296.34959	1	6602

296.47934	1	6602

296.60909	1	6603

296.73884	1	6604

296.86859	1	6604

296.99834	1	6605

297.12809	1	6605

297.25784	16	6621

297.3876	1	6621

297.51735	1	6622

297.6471	2	6623

297.77685	10	6633

297.9066	1	6634

298.03635	1	6634

298.1661	1	6635

298.29585	4	6639

298.4256	9	6648

298.55535	1	6649

298.6851	1	6649

298.81485	1	6650

298.9446	1	6651

299.07435	2	6652

299.2041	1	6653

299.33385	4	6657

299.4636	1	6658

299.59335	13	6671

299.7231	1	6671

299.85285	1	6672

299.9826	12	6684

300.11235	1	6684

300.2421	1	6685

300.37185	1	6686

300.5016	1	6687

300.63135	1	6687

300.7611	1	6688

300.89085	13	6701

301.0206	3	6704

301.15035	1	6705

301.2801	11	6715

301.40985	1	6716

301.5396	1	6717

301.66935	1	6718

301.7991	1	6719

301.92885	1	6720

302.0586	5	6725

302.18835	3	6727

302.31811	1	6728

302.44786	12	6740

302.57761	1	6741

302.70736	2	6742

302.83711	1	6743

302.96686	4	6747

303.09661	1	6748

303.22636	4	6752

303.35611	1	6752

303.48586	1	6753

303.61561	11	6763

303.74536	1	6764

303.87511	2	6766

304.00486	1	6766

304.13461	1	6767

304.26436	15	6782

304.39411	1	6783

304.52386	3	6785

304.65361	2	6787

304.78336	13	6800

304.91311	1	6801

305.04286	1	6801

305.17261	23	6824

305.30236	1	6824

305.43211	1	6825

305.56186	1	6826

305.69161	1	6827

305.82136	1	6828

305.95111	1	6829

306.08086	1	6829

306.21061	1	6830

306.34036	21	6851

306.47011	1	6852

306.59986	1	6853

306.72961	1	6854

306.85936	3	6856

306.98911	2	6858

307.11886	1	6858

307.24861	3	6861

307.37837	12	6873

307.50812	1	6873

307.63787	1	6874

307.76762	2	6876

307.89737	1	6877

308.02712	2	6878

308.15687	1	6879

308.28662	1	6880

308.41637	1	6881

308.54612	1	6881

308.67587	2	6883

308.80562	18	6901

308.93537	2	6903

309.06512	2	6905

309.19487	1	6905

309.32462	1	6906

309.45437	1	6907

309.58412	1	6908

309.71387	3	6911

309.84362	5	6915

309.97337	1	6916

310.10312	2	6918

310.23287	1	6919

310.36262	1	6919

310.49237	6	6925

310.62212	1	6925

310.75187	1	6926

310.88162	1	6927

311.01137	3	6929

311.14112	2	6931

311.27087	3	6934

311.40062	1	6934

311.53037	1	6935

311.66012	1	6936

311.78987	14	6950

311.91962	3	6953

312.04937	1	6953

312.17912	9	6962

312.30888	1	6963

312.43863	1	6963

312.56838	1	6964

312.69813	9	6972

312.82788	2	6974

312.95763	3	6977

313.08738	2	6979

313.21713	18	6997

313.34688	1	6998

313.47663	1	6998

313.60638	1	6999

313.73613	11	7010

313.86588	1	7010

313.99563	1	7011

314.12538	1	7012

314.25513	9	7021

314.38488	1	7022

314.51463	15	7036

314.64438	1	7037

314.77413	2	7038

314.90388	2	7040

315.03363	8	7048

315.16338	1	7049

315.29313	1	7050

315.42288	4	7054

315.55263	2	7056

315.68238	1	7057

315.81213	1	7057

315.94188	1	7058

316.07163	1	7059

316.20138	1	7059

316.33113	1	7060

316.46088	14	7074

316.59063	4	7078

316.72038	13	7091

316.85013	1	7092

316.97988	15	7106

317.10963	1	7107

317.23938	1	7108

317.36914	9	7117

317.49889	1	7118

317.62864	2	7120

317.75839	1	7121

317.88814	1	7122

318.01789	5	7126

318.14764	16	7142

318.27739	1	7142

318.40714	1	7143

318.53689	1	7144

318.66664	1	7145

318.79639	1	7146

318.92614	1	7147

319.05589	1	7148

319.18564	1	7148

319.31539	2	7150

319.44514	3	7153

319.57489	2	7154

319.70464	1	7155

319.83439	1	7155

319.96414	14	7169

320.09389	1	7169

320.22364	10	7179

320.35339	1	7180

320.48314	1	7180

320.61289	1	7181

320.74264	2	7183

320.87239	1	7184

321.00214	1	7185

321.13189	1	7185

321.26164	19	7204

321.39139	3	7207

321.52114	1	7208

321.65089	1	7208

321.78064	16	7224

321.91039	1	7225

322.04014	2	7227

322.16989	1	7228

322.29965	1	7229

322.4294	1	7229

322.55915	1	7230

322.6889	1	7231

322.81865	3	7233

322.9484	8	7241

323.07815	1	7242

323.2079	1	7243

323.33765	1	7244

323.4674	1	7245

323.59715	19	7263

323.7269	1	7264

323.85665	1	7264

323.9864	1	7265

324.11615	1	7266

324.2459	1	7267

324.37565	1	7267

324.5054	10	7277

324.63515	1	7277

324.7649	1	7278

324.89465	10	7288

325.0244	2	7290

325.15415	1	7290

325.2839	1	7291

325.41365	4	7295

325.5434	4	7298

325.67315	17	7315

325.8029	1	7316

325.93265	1	7317

326.0624	1	7318

326.19215	1	7318

326.3219	1	7319

326.45165	2	7321

326.5814	1	7322

326.71115	1	7322

326.8409	1	7323

326.97065	1	7324

327.1004	1	7324

327.23015	1	7325

327.35991	1	7326

327.48966	1	7327

327.61941	18	7344

327.74916	1	7345

327.87891	1	7346

328.00866	1	7346

328.13841	1	7347

328.26816	1	7348

328.39791	2	7350

328.52766	1	7351

328.65741	1	7352

328.78716	1	7352

328.91691	1	7353

329.04666	1	7354

329.17641	3	7357

329.30616	3	7359

329.43591	12	7371

329.56566	1	7372

329.69541	1	7373

329.82516	1	7373

329.95491	5	7378

330.08466	2	7380

330.21441	4	7383

330.34416	1	7384

330.47391	1	7385

330.60366	1	7385

330.73341	2	7387

330.86316	17	7404

330.99291	1	7404

331.12266	2	7406

331.25241	1	7407

331.38216	1	7408

331.51191	2	7410

331.64166	1	7411

331.77141	1	7411

331.90116	19	7430

332.03091	2	7432

332.16066	1	7432

332.29042	9	7441

332.42017	1	7442

332.54992	1	7443

332.67967	1	7443

332.80942	2	7445

332.93917	1	7446

333.06892	1	7447

333.19867	8	7454

333.32842	1	7455

333.45817	1	7456

333.58792	3	7458

333.71767	1	7459

333.84742	13	7472

333.97717	1	7472

334.10692	1	7473

334.23667	1	7474

334.36642	1	7475

334.49617	1	7475

334.62592	6	7481

334.75567	2	7483

334.88542	1	7484

335.01517	7	7490

335.14492	2	7492

335.27467	3	7494

335.40442	16	7510

335.53417	1	7511

335.66392	1	7512

335.79367	18	7529

335.92342	3	7532

336.05317	2	7533

336.18292	1	7534

336.31267	9	7543

336.44242	1	7544

336.57217	2	7546

336.70192	1	7547

336.83167	2	7548

336.96142	1	7549

337.09117	3	7552

337.22092	12	7564

337.35068	1	7564

337.48043	1	7565

337.61018	1	7566

337.73993	1	7567

337.86968	7	7574

337.99943	2	7576

338.12918	1	7576

338.25893	14	7590

338.38868	2	7592

338.51843	3	7595

338.64818	7	7602

338.77793	1	7602

338.90768	7	7609

339.03743	3	7612

339.16718	3	7615

339.29693	1	7616

339.42668	1	7616

339.55643	10	7626

339.68618	1	7626

339.81593	3	7629

339.94568	6	7634

340.07543	1	7635

340.20518	1	7636

340.33493	1	7637

340.46468	18	7655

340.59443	1	7656

340.72418	5	7661

340.85393	2	7663

340.98368	1	7663

341.11343	1	7664

341.24318	1	7665

341.37293	1	7665

341.50268	1	7666

341.63243	17	7683

341.76218	1	7683

341.89193	1	7684

342.02168	1	7684

342.15143	1	7685

342.28118	1	7686

342.41094	4	7690

342.54069	1	7690

342.67044	9	7699

342.80019	1	7700

342.92994	1	7701

343.05969	18	7718

343.18944	2	7720

343.31919	1	7720

343.44894	5	7725

343.57869	1	7726

343.70844	2	7727

343.83819	15	7742

343.96794	1	7743

344.09769	1	7744

344.22744	1	7744

344.35719	17	7761

344.48694	2	7763

344.61669	1	7763

344.74644	2	7765

344.87619	3	7768

345.00594	2	7770

345.13569	1	7770

345.26544	7	7777

345.39519	2	7779

345.52494	2	7780

345.65469	1	7781

345.78444	2	7783

345.91419	1	7784

346.04394	1	7784

346.17369	8	7792

346.30344	3	7795

346.43319	2	7796

346.56294	1	7797

346.69269	11	7808

346.82244	2	7810

346.95219	1	7810

347.08194	1	7811

347.21169	1	7812

347.34145	1	7813

347.4712	1	7813

347.60095	1	7814

347.7307	1	7814

347.86045	1	7815

347.9902	1	7816

348.11995	2	7818

348.2497	1	7818

348.37945	1	7819

348.5092	1	7819

348.63895	1	7820

348.7687	1	7821

348.89845	1	7821

349.0282	1	7822

349.15795	1	7823

349.2877	5	7828

349.41745	1	7829

349.5472	2	7830

349.67695	1	7831

349.8067	1	7832

349.93645	3	7835

350.0662	1	7836

350.19595	13	7849

350.3257	1	7850

350.45545	1	7850

350.5852	1	7851

350.71495	2	7853

350.8447	10	7863

350.97445	1	7864

351.1042	2	7865

351.23395	2	7867

351.3637	1	7868

351.49345	1	7868

351.6232	4	7872

351.75295	3	7875

351.8827	1	7876

352.01245	1	7876

352.1422	1	7877

352.27195	1	7878

352.40171	1	7879

352.53146	1	7880

352.66121	1	7881

352.79096	1	7881

352.92071	1	7882

353.05046	1	7882

353.18021	1	7883

353.30996	2	7885

353.43971	1	7886

353.56946	3	7889

353.69921	2	7890

353.82896	3	7893

353.95871	1	7893

354.08846	13	7906

354.21821	1	7907

354.34796	1	7907

354.47771	1	7908

354.60746	14	7922

354.73721	2	7923

354.86696	1	7924

354.99671	9	7933

355.12646	6	7939

355.25621	1	7940

355.38596	2	7942

355.51571	1	7943

355.64546	21	7964

355.77521	3	7966

355.90496	1	7967

356.03471	3	7970

356.16446	1	7971

356.29421	1	7971

356.42396	1	7972

356.55371	1	7973

356.68346	8	7981

356.81321	1	7981

356.94296	1	7982

357.07271	1	7983

357.20246	14	7997

357.33222	3	7999

357.46197	2	8001

357.59172	1	8002

357.72147	2	8004

357.85122	1	8005

357.98097	1	8005

358.11072	1	8006

358.24047	8	8014

358.37022	1	8014

358.49997	1	8015

358.62972	1	8015

358.75947	1	8016

358.88922	2	8018

359.01897	1	8019

359.14872	1	8020

359.27847	5	8025

359.40822	1	8026

359.53797	1	8026

359.66772	1	8027

359.79747	2	8029

359.92722	8	8037

360.05697	1	8037

360.18672	1	8038

360.31647	2	8039

360.44622	1	8040

360.57597	9	8049

360.70572	1	8050

360.83547	1	8051

360.96522	1	8052

361.09497	1	8053

361.22472	3	8055

361.35447	1	8056

361.48422	1	8057

361.61397	15	8072

361.74372	2	8073

361.87347	1	8074

362.00322	1	8075

362.13297	1	8076

362.26272	1	8077

362.39248	1	8078

362.52223	13	8090

362.65198	1	8091

362.78173	2	8092

362.91148	1	8093

363.04123	26	8119

363.17098	1	8120

363.30073	16	8136

363.43048	1	8137

363.56023	5	8142

363.68998	11	8153

363.81973	1	8153

363.94948	1	8154

364.07923	1	8155

364.20898	1	8156

364.33873	1	8156

364.46848	1	8157

364.59823	1	8157

364.72798	1	8158

364.85773	7	8165

364.98748	4	8169

365.11723	1	8170

365.24698	1	8170

365.37673	1	8171

365.50648	3	8173

365.63623	11	8184

365.76598	1	8185

365.89573	1	8186

366.02548	2	8187

366.15523	1	8188

366.28498	3	8191

366.41473	1	8192

366.54448	1	8193

366.67423	1	8193

366.80398	1	8194

366.93373	2	8196

367.06348	1	8197

367.19323	20	8217

367.32299	1	8218

367.45274	1	8219

367.58249	19	8238

367.71224	1	8238

367.84199	15	8253

367.97174	1	8253

368.10149	1	8254

368.23124	1	8255

368.36099	6	8261

368.49074	6	8267

368.62049	1	8267

368.75024	9	8276

368.87999	1	8277

369.00974	1	8277

369.13949	1	8278

369.26924	9	8287

369.39899	1	8288

369.52874	1	8289

369.65849	1	8290

369.78824	1	8291

369.91799	1	8291

370.04774	1	8292

370.17749	1	8293

370.30724	2	8295

370.43699	1	8296

370.56674	1	8297

370.69649	1	8298

370.82624	2	8300

370.95599	1	8301

371.08574	2	8302

371.21549	1	8303

371.34524	1	8303

371.47499	1	8304

371.60474	1	8305

371.73449	1	8306

371.86424	1	8307

371.99399	2	8308

372.12374	1	8309

372.25349	3	8312

372.38325	1	8313

372.513	4	8317

372.64275	25	8342

372.7725	2	8344

372.90225	2	8345

373.032	15	8360

373.16175	3	8363

373.2915	1	8363

373.42125	1	8364

373.551	1	8365

373.68075	1	8365

373.8105	1	8366

373.94025	2	8368

374.07	1	8369

374.19975	17	8386

374.3295	3	8388

374.45925	1	8389

374.589	1	8390

374.71875	1	8390

374.8485	1	8391

374.97825	2	8393

375.108	4	8397

375.23775	1	8398

375.3675	1	8398

375.49725	13	8411

375.627	1	8412

375.75675	1	8413

375.8865	1	8413

376.01625	14	8427

376.146	1	8428

376.27575	1	8428

376.4055	3	8431

376.53525	9	8440

376.665	1	8441

376.79475	22	8463

376.9245	1	8464

377.05425	1	8464

377.184	5	8469

377.31376	1	8470

377.44351	2	8472

377.57326	1	8473

377.70301	1	8474

377.83276	1	8475

377.96251	1	8475

378.09226	1	8476

378.22201	1	8477

378.35176	1	8478

378.48151	1	8479

378.61126	13	8491

378.74101	2	8493

378.87076	4	8496

379.00051	1	8497

379.13026	1	8498

379.26001	3	8501

379.38976	2	8502

379.51951	1	8503

379.64926	1	8504

379.77901	2	8506

379.90876	1	8507

380.03851	1	8508

380.16826	1	8508

380.29801	1	8509

380.42776	1	8510

380.55751	8	8517

380.68726	1	8518

380.81701	3	8521

380.94676	1	8522

381.07651	1	8522

381.20626	1	8523

381.33601	2	8525

381.46576	4	8529

381.59551	1	8529

381.72526	1	8530

381.85501	1	8530

381.98476	20	8550

382.11451	1	8550

382.24426	1	8551

382.37402	1	8552

382.50377	1	8553

382.63352	2	8555

382.76327	1	8555

382.89302	1	8556

383.02277	1	8556

383.15252	7	8563

383.28227	2	8565

383.41202	1	8566

383.54177	1	8566

383.67152	1	8567

383.80127	1	8568

383.93102	1	8568

384.06077	4	8572

384.19052	3	8575

384.32027	1	8575

384.45002	2	8577

384.57977	6	8583

384.70952	7	8590

384.83927	1	8590

384.96902	1	8591

385.09877	1	8592

385.22852	1	8593

385.35827	1	8594

385.48802	8	8602

385.61777	2	8604

385.74752	1	8604

385.87727	1	8605

386.00702	22	8627

386.13677	1	8628

386.26652	17	8645

386.39627	1	8645

386.52602	1	8646

386.65577	1	8647

386.78552	4	8651

386.91527	1	8651

387.04502	2	8653

387.17477	1	8654

387.30453	1	8655

387.43428	2	8657

387.56403	1	8658

387.69378	1	8659

387.82353	3	8662

387.95328	1	8662

388.08303	1	8663

388.21278	1	8663

388.34253	13	8676

388.47228	2	8678

388.60203	1	8678

388.73178	2	8680

388.86153	1	8681

388.99128	1	8681

389.12103	1	8682

389.25078	3	8684

389.38053	5	8689

389.51028	3	8692

389.64003	1	8693

389.76978	1	8693

389.89953	1	8694

390.02928	1	8695

390.15903	2	8697

390.28878	15	8712

390.41853	1	8713

390.54828	1	8713

390.67803	1	8714

390.80778	1	8714

390.93753	3	8717

391.06728	6	8723

391.19703	1	8723

391.32678	3	8726

391.45653	3	8729

391.58628	10	8739

391.71603	1	8740

391.84578	1	8740

391.97553	1	8741

392.10528	1	8741

392.23503	1	8742

392.36479	1	8743

392.49454	1	8743

392.62429	16	8759

392.75404	3	8762

392.88379	1	8763

393.01354	2	8765

393.14329	1	8765

393.27304	16	8781

393.40279	1	8782

393.53254	5	8787

393.66229	1	8788

393.79204	1	8788

393.92179	1	8789

394.05154	1	8790

394.18129	4	8793

394.31104	1	8794

394.44079	1	8795

394.57054	1	8796

394.70029	2	8797

394.83004	1	8798

394.95979	1	8799

395.08954	1	8799

395.21929	9	8808

395.34904	1	8809

395.47879	1	8810

395.60854	2	8812

395.73829	1	8813

395.86804	2	8815

395.99779	1	8815

396.12754	1	8816

396.25729	2	8818

396.38704	1	8819

396.51679	1	8819

396.64654	1	8820

396.77629	2	8822

396.90604	16	8838

397.03579	2	8839

397.16554	1	8840

397.2953	23	8863

397.42505	1	8863

397.5548	2	8865

397.68455	3	8868

397.8143	1	8868

397.94405	1	8869

398.0738	10	8879

398.20355	1	8879

398.3333	3	8882

398.46305	1	8883

398.5928	2	8885

398.72255	2	8887

398.8523	1	8888

398.98205	23	8911

399.1118	2	8912

399.24155	1	8913

399.3713	1	8914

399.50105	1	8915

399.6308	1	8916

399.76055	1	8917

399.8903	1	8918

400.02005	2	8919

400.1498	1	8920

400.27955	1	8921

400.4093	25	8946

400.53905	1	8947

400.6688	1	8947

400.79855	1	8948

400.9283	1	8949

401.05805	5	8953

401.1878	1	8954

401.31755	3	8956

401.4473	6	8962

401.57705	2	8964

401.7068	1	8965

401.83655	1	8966

401.9663	1	8967

402.09605	1	8968

402.2258	2	8970

402.35556	1	8971

402.48531	16	8987

402.61506	1	8987

402.74481	1	8988

402.87456	20	9008

403.00431	1	9009

403.13406	1	9009

403.26381	3	9012

403.39356	1	9012

403.52331	6	9018

403.65306	2	9020

403.78281	1	9020

403.91256	18	9038

404.04231	1	9039

404.17206	1	9040

404.30181	1	9040

404.43156	17	9057

404.56131	2	9059

404.69106	2	9061

404.82081	14	9075

404.95056	1	9076

405.08031	1	9076

405.21006	1	9077

405.33981	1	9078

405.46956	2	9080

405.59931	5	9085

405.72906	1	9086

405.85881	3	9088

405.98856	2	9090

406.11831	2	9092

406.24806	2	9093

406.37781	1	9094

406.50756	3	9097

406.63731	1	9098

406.76706	15	9113

406.89681	3	9116

407.02656	4	9119

407.15631	1	9120

407.28607	1	9120

407.41582	17	9137

407.54557	2	9139

407.67532	1	9139

407.80507	2	9141

407.93482	1	9142

408.06457	1	9143

408.19432	1	9143

408.32407	1	9144

408.45382	1	9145

408.58357	1	9146

408.71332	2	9148

408.84307	8	9156

408.97282	1	9156

409.10257	1	9157

409.23232	2	9159

409.36207	1	9160

409.49182	2	9161

409.62157	10	9171

409.75132	5	9176

409.88107	1	9177

410.01082	3	9180

410.14057	3	9182

410.27032	1	9183

410.40007	2	9185

410.52982	3	9188

410.65957	2	9189

410.78932	2	9191

410.91907	6	9196

411.04882	11	9207

411.17857	1	9208

411.30832	2	9210

411.43807	4	9214

411.56782	1	9215

411.69757	1	9215

411.82732	3	9218

411.95707	4	9222

412.08682	1	9223

412.21657	1	9224

412.34633	10	9234

412.47608	1	9234

412.60583	1	9235

412.73558	4	9239

412.86533	1	9240

412.99508	5	9245

413.12483	5	9250

413.25458	4	9253

413.38433	2	9255

413.51408	1	9255

413.64383	16	9271

413.77358	3	9274

413.90333	1	9274

414.03308	5	9279

414.16283	2	9281

414.29258	4	9285

414.42233	2	9286

414.55208	2	9288

414.68183	1	9289

414.81158	1	9290

414.94133	11	9301

415.07108	3	9304

415.20083	1	9305

415.33058	17	9322

415.46033	2	9323

415.59008	3	9326

415.71983	1	9327

415.84958	1	9328

415.97933	2	9330

416.10908	14	9344

416.23883	1	9344

416.36858	1	9345

416.49833	1	9346

416.62808	1	9347

416.75783	1	9347

416.88758	1	9348

417.01733	7	9355

417.14708	4	9359

417.27684	3	9362

417.40659	1	9362

417.53634	1	9363

417.66609	1	9364

417.79584	1	9365

417.92559	1	9365

418.05534	1	9366

418.18509	2	9368

418.31484	1	9368

418.44459	1	9369

418.57434	3	9372

418.70409	4	9376

418.83384	1	9376

418.96359	19	9395

419.09334	1	9395

419.22309	1	9396

419.35284	1	9397

419.48259	12	9409

419.61234	1	9410

419.74209	2	9412

419.87184	1	9413

420.00159	1	9414

420.13134	1	9415

420.26109	1	9416

420.39084	7	9423

420.52059	1	9423

420.65034	1	9424

420.78009	1	9425

420.90984	1	9426

421.03959	20	9446

421.16934	3	9448

421.29909	14	9462

421.42884	1	9463

421.55859	1	9464

421.68834	1	9464

421.81809	1	9465

421.94784	24	9489

422.07759	1	9490

422.20734	1	9491

422.3371	2	9493

422.46685	4	9497

422.5966	4	9501

422.72635	1	9502

422.8561	4	9505

422.98585	1	9506

423.1156	2	9507

423.24535	1	9508

423.3751	12	9520

423.50485	1	9521

423.6346	1	9521

423.76435	1	9522

423.8941	1	9523

424.02385	1	9524

424.1536	1	9525

424.28335	1	9525

424.4131	1	9526

424.54285	1	9526

424.6726	6	9532

424.80235	1	9533

424.9321	2	9535

425.06185	2	9537

425.1916	1	9537

425.32135	2	9539

425.4511	7	9545

425.58085	3	9548

425.7106	2	9550

425.84035	1	9551

425.9701	2	9553

426.09985	1	9554

426.2296	1	9555

426.35935	3	9557

426.4891	4	9561

426.61885	1	9562

426.7486	1	9563

426.87835	1	9564

427.0081	11	9574

427.13785	2	9576

427.26761	1	9577

427.39736	1	9578

427.52711	1	9579

427.65686	1	9579

427.78661	4	9583

427.91636	17	9599

428.04611	3	9602

428.17586	2	9604

428.30561	1	9605

428.43536	8	9613

428.56511	3	9616

428.69486	1	9617

428.82461	3	9620

428.95436	1	9621

429.08411	1	9621

429.21386	1	9622

429.34361	15	9637

429.47336	1	9638

429.60311	1	9638

429.73286	1	9639

429.86261	1	9640

429.99236	2	9642

430.12211	12	9654

430.25186	2	9655

430.38161	1	9656

430.51136	1	9657

430.64111	1	9658

430.77086	1	9658

430.90061	14	9672

431.03036	2	9674

431.16011	1	9675

431.28986	3	9677

431.41961	5	9682

431.54936	1	9683

431.67911	1	9683

431.80886	1	9684

431.93861	1	9684

432.06836	1	9685

432.19811	1	9686

432.32787	1	9686

432.45762	1	9687

432.58737	1	9688

432.71712	19	9707

432.84687	1	9707

432.97662	14	9721

433.10637	1	9722

433.23612	1	9723

433.36587	5	9727

433.49562	1	9728

433.62537	18	9746

433.75512	1	9747

433.88487	2	9748

434.01462	4	9752

434.14437	1	9753

434.27412	1	9754

434.40387	1	9754

434.53362	1	9755

434.66337	13	9767

434.79312	2	9769

434.92287	1	9769

435.05262	1	9770

435.18237	2	9772

435.31212	1	9772

435.44187	18	9790

435.57162	1	9791

435.70137	1	9792

435.83112	14	9806

435.96087	1	9807

436.09062	2	9808

436.22037	1	9809

436.35012	1	9809

436.47987	1	9810

436.60962	1	9811

436.73937	1	9812

436.86912	2	9814

436.99887	1	9814

437.12862	3	9817

437.25838	2	9819

437.38813	1	9820

437.51788	13	9832

437.64763	1	9833

437.77738	1	9834

437.90713	1	9834

438.03688	1	9835

438.16663	5	9840

438.29638	8	9847

438.42613	1	9848

438.55588	7	9855

438.68563	8	9863

438.81538	1	9863

438.94513	1	9864

439.07488	5	9868

439.20463	6	9874

439.33438	1	9875

439.46413	14	9889

439.59388	1	9889

439.72363	1	9890

439.85338	10	9900

439.98313	1	9900

440.11288	1	9901

440.24263	1	9901

440.37238	11	9912

440.50213	1	9913

440.63188	1	9914

440.76163	2	9916

440.89138	1	9917

441.02113	1	9917

441.15088	1	9918

441.28063	3	9921

441.41038	1	9922

441.54013	2	9924

441.66988	1	9924

441.79963	1	9925

441.92938	4	9929

442.05913	2	9931

442.18888	6	9937

442.31864	2	9938

442.44839	1	9939

442.57814	1	9940

442.70789	1	9941

442.83764	1	9941

442.96739	1	9942

443.09714	1	9942

443.22689	2	9944

443.35664	1	9945

443.48639	1	9945

443.61614	2	9947

443.74589	11	9958

443.87564	2	9960

444.00539	1	9961

444.13514	2	9962

444.26489	6	9968

444.39464	1	9969

444.52439	1	9970

444.65414	2	9972

444.78389	2	9974

444.91364	1	9974

445.04339	1	9975

445.17314	1	9975

445.30289	1	9976

445.43264	22	9997

445.56239	1	9998

445.69214	1	9999

445.82189	21	10020

445.95164	3	10023

446.08139	1	10024

446.21114	2	10026

446.34089	2	10028

446.47064	2	10029

446.60039	3	10032

446.73014	1	10033

446.85989	18	10051

446.98964	10	10061

447.11939	6	10067

447.24915	1	10068

447.3789	2	10070

447.50865	2	10071

447.6384	1	10072

447.76815	1	10073

447.8979	1	10074

448.02765	6	10080

448.1574	3	10082

448.28715	1	10083

448.4169	1	10084

448.54665	17	10101

448.6764	3	10103

448.80615	1	10104

448.9359	2	10106

449.06565	3	10109

449.1954	10	10118

449.32515	2	10120

449.4549	13	10133

449.58465	1	10134

449.7144	1	10135

449.84415	2	10137

449.9739	2	10139

450.10365	3	10142

450.2334	2	10144

450.36315	2	10146

450.4929	1	10146

450.62265	1	10147

450.7524	1	10147

450.88215	1	10148

451.0119	1	10149

451.14165	16	10164

451.2714	1	10165

451.40115	1	10165

451.5309	1	10166

451.66065	1	10167

451.7904	1	10167

451.92015	2	10169

452.0499	13	10182

452.17965	1	10182

452.30941	1	10183

452.43916	1	10184

452.56891	1	10184

452.69866	1	10185

452.82841	1	10186

452.95816	1	10187

453.08791	1	10188

453.21766	2	10190

453.34741	2	10191

453.47716	12	10203

453.60691	1	10203

453.73666	1	10204

453.86641	2	10206

453.99616	1	10206

454.12591	1	10207

454.25566	1	10208

454.38541	1	10208

454.51516	11	10219

454.64491	1	10220

454.77466	1	10220

454.90441	14	10234

455.03416	1	10235

455.16391	2	10237

455.29366	16	10253

455.42341	1	10254

455.55316	1	10254

455.68291	15	10269

455.81266	2	10271

455.94241	1	10272

456.07216	10	10282

456.20191	1	10283

456.33166	1	10284

456.46141	1	10284

456.59116	1	10285

456.72091	2	10287

456.85066	2	10289

456.98041	8	10297

457.11016	8	10305

457.23992	1	10305

457.36967	1	10306

457.49942	1	10307

457.62917	13	10320

457.75892	1	10321

457.88867	1	10322

458.01842	1	10322

458.14817	1	10323

458.27792	2	10324

458.40767	4	10328

458.53742	1	10328

458.66717	13	10341

458.79692	1	10342

458.92667	1	10343

459.05642	4	10347

459.18617	4	10351

459.31592	3	10354

459.44567	1	10354

459.57542	9	10363

459.70517	1	10363

459.83492	1	10364

459.96467	1	10364

460.09442	20	10384

460.22417	2	10386

460.35392	1	10387

460.48367	2	10389

460.61342	2	10391

460.74317	1	10391

460.87292	8	10399

461.00267	11	10410

461.13242	1	10411

461.26217	1	10412

461.39192	1	10412

461.52167	10	10422

461.65142	1	10422

461.78117	1	10423

461.91092	1	10423

462.04067	26	10449

462.17042	1	10450

462.30018	1	10450

462.42993	2	10452

462.55968	2	10454

462.68943	1	10455

462.81918	1	10456

462.94893	1	10456

463.07868	1	10457

463.20843	1	10458

463.33818	1	10459

463.46793	1	10459

463.59768	1	10460

463.72743	1	10460

463.85718	2	10462

463.98693	2	10464

464.11668	1	10465

464.24643	2	10466

464.37618	1	10467

464.50593	11	10478

464.63568	1	10479

464.76543	1	10479

464.89518	2	10481

465.02493	1	10482

465.15468	2	10484

465.28443	1	10485

465.41418	16	10500

465.54393	1	10501

465.67368	2	10503

465.80343	3	10505

465.93318	1	10506

466.06293	3	10509

466.19268	3	10512

466.32243	1	10513

466.45218	1	10513

466.58193	2	10515

466.71168	7	10521

466.84143	2	10523

466.97118	1	10524

467.10093	17	10541

467.23068	1	10541

467.36044	1	10542

467.49019	1	10543

467.61994	1	10543

467.74969	1	10544

467.87944	13	10557

468.00919	1	10557

468.13894	1	10558

468.26869	4	10562

468.39844	3	10564

468.52819	1	10565

468.65794	1	10566

468.78769	3	10569

468.91744	5	10574

469.04719	1	10574

469.17694	1	10575

469.30669	1	10576

469.43644	1	10577

469.56619	1	10578

469.69594	13	10590

469.82569	2	10592

469.95544	1	10592

470.08519	1	10593

470.21494	2	10595

470.34469	1	10595

470.47444	3	10598

470.60419	1	10599

470.73394	1	10600

470.86369	1	10600

470.99344	1	10601

471.12319	1	10601

471.25294	1	10602

471.38269	1	10603

471.51244	2	10604

471.64219	11	10615

471.77194	4	10619

471.90169	1	10620

472.03144	1	10620

472.16119	3	10623

472.29095	2	10625

472.4207	3	10627

472.55045	1	10628

472.6802	1	10628

472.80995	5	10633

472.9397	1	10634

473.06945	1	10635

473.1992	3	10637

473.32895	6	10643

473.4587	1	10644

473.58845	1	10644

473.7182	1	10645

473.84795	1	10645

473.9777	13	10658

474.10745	5	10663

474.2372	2	10665

474.36695	2	10667

474.4967	3	10670

474.62645	4	10673

474.7562	1	10674

474.88595	2	10676

475.0157	16	10692

475.14545	1	10692

475.2752	1	10693

475.40495	1	10694

475.5347	3	10696

475.66445	2	10698

475.7942	4	10702

475.92395	1	10703

476.0537	1	10704

476.18345	1	10704

476.3132	1	10705

476.44295	1	10705

476.5727	2	10707

476.70245	1	10708

476.8322	2	10710

476.96195	1	10710

477.0917	8	10718

477.22145	2	10720

477.35121	1	10721

477.48096	1	10722

477.61071	2	10724

477.74046	1	10724

477.87021	1	10725

477.99996	5	10730

478.12971	1	10731

478.25946	1	10732

478.38921	2	10733

478.51896	12	10745

478.64871	1	10745

478.77846	1	10746

478.90821	4	10750

479.03796	6	10756

479.16771	1	10756

479.29746	4	10760

479.42721	3	10763

479.55696	1	10764

479.68671	1	10764

479.81646	4	10768

479.94621	2	10770

480.07596	2	10772

480.20571	1	10772

480.33546	5	10777

480.46521	2	10779

480.59496	2	10781

480.72471	1	10781

480.85446	15	10796

480.98421	2	10798

481.11396	4	10802

481.24371	6	10807

481.37346	1	10808

481.50321	1	10809

481.63296	9	10817

481.76271	6	10823

481.89246	1	10823

482.02221	1	10824

482.15196	1	10825

482.28172	15	10840

482.41147	1	10840

482.54122	1	10841

482.67097	2	10843

482.80072	16	10859

482.93047	1	10859

483.06022	2	10861

483.18997	1	10862

483.31972	11	10873

483.44947	2	10875

483.57922	1	10876

483.70897	3	10879

483.83872	4	10882

483.96847	1	10883

484.09822	2	10884

484.22797	6	10890

484.35772	1	10891

484.48747	13	10904

484.61722	5	10909

484.74697	1	10909

484.87672	2	10911

485.00647	1	10912

485.13622	1	10912

485.26597	1	10913

485.39572	10	10923

485.52547	1	10924

485.65522	1	10924

485.78497	4	10928

485.91472	9	10937

486.04447	2	10938

486.17422	18	10956

486.30397	2	10957

486.43372	1	10958

486.56347	1	10959

486.69322	1	10960

486.82297	1	10960

486.95272	1	10961

487.08247	15	10976

487.21222	2	10977

487.34198	3	10980

487.47173	3	10983

487.60148	1	10983

487.73123	1	10984

487.86098	1	10985

487.99073	9	10994

488.12048	1	10995

488.25023	5	10999

488.37998	2	11001

488.50973	1	11002

488.63948	1	11002

488.76923	2	11004

488.89898	1	11005

489.02873	1	11005

489.15848	1	11006

489.28823	13	11019

489.41798	1	11019

489.54773	1	11020

489.67748	1	11020

489.80723	3	11023

489.93698	5	11028

490.06673	1	11029

490.19648	11	11039

490.32623	1	11040

490.45598	1	11040

490.58573	2	11042

490.71548	9	11050

490.84523	1	11051

490.97498	1	11051

491.10473	1	11052

491.23448	7	11059

491.36423	11	11070

491.49398	1	11071

491.62373	15	11086

491.75348	1	11087

491.88323	1	11088

492.01298	2	11089

492.14273	1	11090

492.27249	1	11090

492.40224	4	11094

492.53199	1	11094

492.66174	13	11107

492.79149	3	11110

492.92124	1	11110

493.05099	1	11111

493.18074	5	11116

493.31049	1	11117

493.44024	1	11118

493.56999	11	11129

493.69974	1	11130

493.82949	2	11132

493.95924	3	11134

494.08899	1	11135

494.21874	3	11138

494.34849	1	11138

494.47824	1	11139

494.60799	6	11145

494.73774	2	11146

494.86749	1	11147

494.99724	1	11148

495.12699	9	11157

495.25674	3	11160

495.38649	2	11161

495.51624	5	11166

495.64599	3	11169

495.77574	1	11170

495.90549	14	11183

496.03524	3	11186

496.16499	3	11189

496.29474	2	11191

496.42449	1	11191

496.55424	1	11192

496.68399	6	11198

496.81374	2	11200

496.94349	1	11200

497.07324	3	11203

497.20299	1	11204

497.33275	3	11207

497.4625	3	11210

497.59225	1	11210

497.722	1	11211

497.85175	2	11212

497.9815	10	11222

498.11125	1	11223

498.241	2	11225

498.37075	8	11233

498.5005	3	11236

498.63025	18	11253

498.76	1	11254

498.88975	1	11254

499.0195	1	11255

499.14925	1	11256

499.279	1	11257

499.40875	1	11257

499.5385	2	11259

499.66825	8	11267

499.798	1	11267

499.92775	1	11268

500.0575	1	11269

500.18725	1	11270

500.317	17	11287

500.44675	1	11287

500.5765	1	11288

500.70625	1	11289

500.836	12	11301

500.96575	3	11304

501.0955	1	11305

501.22525	3	11307

501.355	1	11308

501.48475	2	11310

501.6145	13	11323

501.74425	1	11324

501.874	1	11325

502.00375	1	11326

502.1335	3	11329

502.26326	1	11330

502.39301	5	11335

502.52276	4	11338

502.65251	1	11339

502.78226	1	11340

502.91201	1	11341

503.04176	1	11342

503.17151	8	11350

503.30126	1	11350

503.43101	14	11364

503.56076	1	11365

503.69051	4	11369

503.82026	2	11370

503.95001	1	11371

504.07976	2	11373

504.20951	3	11375

504.33926	1	11376

504.46901	4	11379

504.59876	1	11380

504.72851	1	11381

504.85826	1	11382

504.98801	1	11383

505.11776	4	11387

505.24751	2	11388

505.37726	2	11390

505.50701	1	11390

505.63676	15	11405

505.76651	1	11405

505.89626	7	11412

506.02601	2	11414

506.15576	1	11415

506.28551	3	11418

506.41526	1	11418

506.54501	1	11419

506.67476	17	11435

506.80451	1	11436

506.93426	3	11439

507.06401	1	11440

507.19376	2	11442

507.32352	1	11442

507.45327	1	11443

507.58302	4	11447

507.71277	1	11448

507.84252	1	11448

507.97227	13	11461

508.10202	1	11462

508.23177	1	11463

508.36152	7	11470

508.49127	1	11471

508.62102	1	11471

508.75077	1	11472

508.88052	1	11473

509.01027	1	11474

509.14002	2	11476

509.26977	1	11477

509.39952	1	11477

509.52927	3	11480

509.65902	1	11480

509.78877	3	11483

509.91852	1	11483

510.04827	1	11484

510.17802	1	11484

510.30777	1	11485

510.43752	1	11486

510.56727	2	11487

510.69702	7	11494

510.82677	1	11495

510.95652	1	11495

511.08627	1	11496

511.21602	14	11510

511.34577	2	11512

511.47552	1	11512

511.60527	3	11515

511.73502	1	11516

511.86477	1	11516

511.99452	2	11518

512.12427	5	11523

512.25403	1	11523

512.38378	6	11529

512.51353	2	11530

512.64328	2	11532

512.77303	10	11541

512.90278	1	11542

513.03253	1	11543

513.16228	4	11546

513.29203	1	11547

513.42178	2	11549

513.55153	2	11550

513.68128	1	11551

513.81103	3	11554

513.94078	1	11554

514.07053	11	11565

514.20028	2	11567

514.33003	2	11569

514.45978	1	11569

514.58953	1	11570

514.71928	2	11572

514.84903	1	11572

514.97878	1	11573

515.10853	1	11574

515.23828	2	11576

515.36803	1	11577

515.49778	1	11577

515.62753	1	11578

515.75728	1	11579

515.88703	3	11581

516.01678	5	11586

516.14653	1	11586

516.27628	1	11587

516.40603	5	11592

516.53578	2	11594

516.66553	3	11596

516.79528	8	11604

516.92503	1	11605

517.05478	2	11607

517.18453	1	11607

517.31429	1	11608

517.44404	2	11609

517.57379	2	11611

517.70354	1	11611

517.83329	1	11612

517.96304	1	11613

518.09279	2	11615

518.22254	1	11616

518.35229	1	11616

518.48204	2	11618

518.61179	1	11619

518.74154	1	11620

518.87129	2	11621

519.00104	17	11638

519.13079	2	11639

519.26054	1	11640

519.39029	1	11640

519.52004	8	11648

519.64979	1	11649

519.77954	2	11651

519.90929	1	11652

520.03904	1	11653

520.16879	1	11653

520.29854	9	11662

520.42829	1	11663

520.55804	1	11663

520.68779	1	11664

520.81754	8	11672

520.94729	1	11673

521.07704	1	11674

521.20679	19	11693

521.33654	1	11693

521.46629	13	11706

521.59604	1	11707

521.72579	4	11711

521.85554	4	11714

521.98529	1	11715

522.11504	1	11716

522.2448	2	11717

522.37455	2	11719

522.5043	1	11720

522.63405	2	11722

522.7638	2	11724

522.89355	1	11725

523.0233	1	11726

523.15305	16	11741

523.2828	2	11743

523.41255	2	11744

523.5423	1	11745

523.67205	1	11745

523.8018	1	11746

523.93155	4	11750

524.0613	1	11750

524.19105	1	11751

524.3208	3	11753

524.45055	1	11754

524.5803	1	11755

524.71005	7	11762

524.8398	2	11764

524.96955	3	11767

525.0993	1	11768

525.22905	2	11770

525.3588	13	11783

525.48855	1	11783

525.6183	7	11790

525.74805	2	11791

525.8778	1	11792

526.00755	1	11793

526.1373	1	11793

526.26705	7	11800

526.3968	1	11800

526.52655	2	11802

526.6563	3	11805

526.78605	1	11806

526.9158	1	11807

527.04555	1	11807

527.1753	1	11808

527.30506	2	11810

527.43481	2	11811

527.56456	4	11815

527.69431	1	11816

527.82406	2	11817

527.95381	1	11818

528.08356	2	11820

528.21331	1	11820

528.34306	7	11827

528.47281	1	11828

528.60256	1	11829

528.73231	17	11846

528.86206	1	11846

528.99181	1	11847

529.12156	10	11857

529.25131	1	11858

529.38106	1	11859

529.51081	8	11867

529.64056	1	11867

529.77031	1	11868

529.90006	3	11871

530.02981	7	11878

530.15956	3	11880

530.28931	1	11881

530.41906	4	11885

530.54881	1	11885

530.67856	1	11886

530.80831	17	11903

530.93806	1	11904

531.06781	1	11905

531.19756	3	11907

531.32731	1	11908

531.45706	1	11908

531.58681	3	11911

531.71656	2	11913

531.84631	1	11914

531.97606	1	11914

532.10581	15	11929

532.23557	1	11930

532.36532	1	11931

532.49507	1	11932

532.62482	2	11934

532.75457	1	11934

532.88432	1	11935

533.01407	5	11940

533.14382	3	11943

533.27357	2	11944

533.40332	2	11946

533.53307	1	11947

533.66282	16	11963

533.79257	1	11963

533.92232	1	11964

534.05207	1	11965

534.18182	1	11966

534.31157	1	11967

534.44132	1	11968

534.57107	1	11969

534.70082	1	11970

534.83057	1	11971

534.96032	4	11974

535.09007	2	11976

535.21982	1	11977

535.34957	4	11981

535.47932	2	11983

535.60907	2	11985

535.73882	1	11986

535.86857	1	11986

535.99832	8	11994

536.12807	12	12006

536.25782	1	12006

536.38757	4	12010

536.51732	2	12012

536.64707	1	12013

536.77682	1	12014

536.90657	3	12016

537.03632	2	12018

537.16607	1	12019

537.29583	6	12025

537.42558	3	12028

537.55533	9	12037

537.68508	1	12038

537.81483	1	12039

537.94458	2	12040

538.07433	2	12042

538.20408	1	12042

538.33383	1	12043

538.46358	1	12044

538.59333	1	12044

538.72308	2	12046

538.85283	1	12046

538.98258	1	12047

539.11233	1	12047

539.24208	4	12051

539.37183	3	12054

539.50158	1	12055

539.63133	1	12056

539.76108	4	12060

539.89083	1	12060

540.02058	6	12066

540.15033	1	12067

540.28008	2	12068

540.40983	4	12072

540.53958	1	12072

540.66933	1	12073

540.79908	1	12074

540.92883	1	12074

541.05858	1	12075

541.18833	10	12085

541.31808	1	12085

541.44783	1	12086

541.57758	7	12092

541.70733	1	12093

541.83708	1	12094

541.96683	2	12095

542.09658	1	12096

542.22634	1	12096

542.35609	1	12097

542.48584	7	12103

542.61559	1	12104

542.74534	1	12104

542.87509	1	12105

543.00484	2	12107

543.13459	1	12108

543.26434	1	12109

543.39409	1	12110

543.52384	2	12111

543.65359	1	12112

543.78334	1	12113

543.91309	5	12117

544.04284	7	12124

544.17259	1	12124

544.30234	6	12130

544.43209	5	12135

544.56184	6	12141

544.69159	5	12146

544.82134	1	12146

544.95109	1	12147

545.08084	1	12148

545.21059	2	12150

545.34034	1	12151

545.47009	7	12158

545.59984	1	12159

545.72959	1	12160

545.85934	3	12162

545.98909	1	12163

546.11884	1	12164

546.24859	1	12165

546.37834	1	12166

546.50809	2	12167

546.63784	9	12176

546.76759	1	12177

546.89734	1	12178

547.02709	8	12186

547.15684	1	12187

547.2866	2	12188

547.41635	2	12190

547.5461	3	12193

547.67585	1	12194

547.8056	1	12195

547.93535	1	12195

548.0651	4	12199

548.19485	3	12202

548.3246	1	12203

548.45435	1	12203

548.5841	1	12204

548.71385	1	12205

548.8436	1	12205

548.97335	1	12206

549.1031	2	12207

549.23285	1	12208

549.3626	1	12209

549.49235	1	12210

549.6221	10	12219

549.75185	9	12228

549.8816	1	12229

550.01135	2	12231

550.1411	9	12240

550.27085	2	12241

550.4006	1	12242

550.53035	1	12243

550.6601	3	12245

550.78985	4	12249

550.9196	1	12250

551.04935	1	12251

551.1791	1	12251

551.30885	11	12262

551.4386	1	12263

551.56835	1	12264

551.6981	1	12264

551.82785	1	12265

551.9576	1	12265

552.08735	1	12266

552.21711	1	12267

552.34686	1	12267

552.47661	6	12273

552.60636	1	12274

552.73611	1	12274

552.86586	2	12276

552.99561	1	12277

553.12536	2	12279

553.25511	3	12281

553.38486	1	12282

553.51461	1	12282

553.64436	1	12283

553.77411	1	12283

553.90386	2	12285

554.03361	2	12287

554.16336	3	12290

554.29311	1	12290

554.42286	1	12291

554.55261	1	12291

554.68236	4	12295

554.81211	1	12296

554.94186	1	12296

555.07161	2	12298

555.20136	6	12304

555.33111	1	12304

555.46086	9	12313

555.59061	1	12313

555.72036	1	12314

555.85011	1	12314

555.97986	2	12316

556.10961	3	12318

556.23936	1	12319

556.36911	1	12319

556.49886	1	12320

556.62861	1	12321

556.75836	5	12326

556.88811	1	12326

557.01786	1	12327

557.14761	4	12331

557.27737	2	12333

557.40712	1	12333

557.53687	2	12335

557.66662	5	12340

557.79637	1	12341

557.92612	1	12342

558.05587	5	12347

558.18562	1	12348

558.31537	2	12350

558.44512	2	12351

558.57487	3	12354

558.70462	1	12355

558.83437	1	12356

558.96412	1	12357

559.09387	7	12364

559.22362	1	12364

559.35337	1	12365

559.48312	1	12365

559.61287	1	12366

559.74262	2	12367

559.87237	11	12378

560.00212	1	12379

560.13187	4	12383

560.26162	1	12384

560.39137	2	12386

560.52112	2	12387

560.65087	1	12388

560.78062	1	12388

560.91037	6	12394

561.04012	3	12397

561.16987	2	12398

561.29962	1	12399

561.42937	1	12399

561.55912	1	12400

561.68887	1	12400

561.81862	6	12406

561.94837	1	12407

562.07812	1	12408

562.20787	8	12416

562.33763	3	12418

562.46738	1	12419

562.59713	3	12422

562.72688	2	12423

562.85663	2	12425

562.98638	2	12427

563.11613	4	12430

563.24588	1	12431

563.37563	4	12434

563.50538	1	12435

563.63513	1	12436

563.76488	1	12437

563.89463	1	12437

564.02438	2	12439

564.15413	1	12440

564.28388	3	12442

564.41363	1	12443

564.54338	9	12452

564.67313	1	12452

564.80288	1	12453

564.93263	2	12455

565.06238	6	12460

565.19213	5	12465

565.32188	1	12466

565.45163	1	12467

565.58138	10	12476

565.71113	7	12483

565.84088	1	12484

565.97063	1	12485

566.10038	1	12486

566.23013	4	12489

566.35988	1	12490

566.48963	1	12490

566.61938	5	12495

566.74913	1	12496

566.87888	1	12496

567.00863	4	12500

567.13838	1	12501

567.26814	1	12502

567.39789	1	12502

567.52764	1	12503

567.65739	2	12505

567.78714	1	12505

567.91689	1	12506

568.04664	2	12508

568.17639	1	12508

568.30614	3	12511

568.43589	3	12513

568.56564	1	12514

568.69539	5	12518

568.82514	1	12519

568.95489	1	12520

569.08464	12	12532

569.21439	1	12533

569.34414	2	12534

569.47389	4	12538

569.60364	1	12539

569.73339	1	12539

569.86314	3	12542

569.99289	1	12543

570.12264	3	12546

570.25239	2	12547

570.38214	1	12548

570.51189	7	12555

570.64164	2	12557

570.77139	7	12564

570.90114	5	12568

571.03089	2	12570

571.16064	2	12571

571.29039	9	12580

571.42014	5	12585

571.54989	1	12586

571.67964	1	12587

571.80939	1	12588

571.93914	1	12589

572.06889	1	12590

572.19865	1	12591

572.3284	1	12592

572.45815	1	12593

572.5879	1	12594

572.71765	1	12595

572.8474	10	12605

572.97715	1	12606

573.1069	8	12614

573.23665	2	12616

573.3664	3	12619

573.49615	1	12620

573.6259	3	12623

573.75565	2	12625

573.8854	1	12626

574.01515	1	12627

574.1449	1	12628

574.27465	1	12629

574.4044	4	12633

574.53415	3	12636

574.6639	1	12637

574.79365	2	12639

574.9234	2	12641

575.05315	1	12642

575.1829	1	12643

575.31265	2	12645

575.4424	3	12648

575.57215	1	12649

575.7019	6	12655

575.83165	1	12656

575.9614	1	12657

576.09115	15	12672

576.2209	9	12681

576.35065	1	12682

576.4804	3	12685

576.61015	3	12688

576.7399	6	12694

576.86965	1	12695

576.9994	2	12697

577.12915	3	12700

577.25891	1	12701

577.38866	2	12703

577.51841	1	12704

577.64816	5	12709

577.77791	1	12710

577.90766	30	12740

578.03741	1	12741

578.16716	1	12742

578.29691	5	12747

578.42666	2	12749

578.55641	1	12750

578.68616	3	12753

578.81591	1	12754

578.94566	1	12755

579.07541	1	12756

579.20516	1	12757

579.33491	3	12760

579.46466	1	12761

579.59441	4	12765

579.72416	1	12766

579.85391	1	12767

579.98366	1	12768

580.11341	6	12774

580.24316	1	12775

580.37291	1	12776

580.50266	1	12777

580.63241	1	12778

580.76216	3	12781

580.89191	1	12782

581.02166	1	12783

581.15141	3	12786

581.28116	5	12791

581.41091	2	12793

581.54066	1	12794

581.67041	1	12795

581.80016	1	12796

581.92991	1	12797

582.05966	7	12804

582.18942	1	12805

582.31917	1	12806

582.44892	1	12807

582.57867	2	12809

582.70842	1	12810

582.83817	1	12811

582.96792	1	12812

583.09767	3	12815

583.22742	1	12816

583.35717	5	12821

583.48692	1	12822

583.61667	2	12824

583.74642	1	12825

583.87617	1	12826

584.00592	3	12829

584.13567	2	12831

584.26542	1	12832

584.39517	7	12839

584.52492	1	12840

584.65467	3	12843

584.78442	1	12844

584.91417	2	12846

585.04392	1	12847

585.17367	1	12848

585.30342	2	12850

585.43317	2	12852

585.56292	1	12853

585.69267	2	12855

585.82242	1	12856

585.95217	1	12857

586.08192	1	12858

586.21167	3	12861

586.34142	2	12863

586.47117	1	12864

586.60092	1	12865

586.73067	4	12869

586.86042	3	12872

586.99017	2	12874

587.11992	1	12875

587.24968	1	12876

587.37943	1	12877

587.50918	2	12879

587.63893	11	12890

587.76868	4	12894

587.89843	1	12895

588.02818	1	12896

588.15793	2	12898

588.28768	4	12902

588.41743	1	12903

588.54718	2	12905

588.67693	7	12912

588.80668	2	12914

588.93643	1	12915

589.06618	1	12916

589.19593	1	12917

589.32568	1	12918

589.45543	1	12919

589.58518	1	12920

589.71493	2	12922

589.84468	1	12923

589.97443	3	12926

590.10418	2	12928

590.23393	1	12929

590.36368	1	12930

590.49343	1	12931

590.62318	1	12932

590.75293	1	12933

590.88268	1	12934

591.01243	1	12935

591.14218	1	12936

591.27193	2	12938

591.40168	1	12939

591.53143	1	12940

591.66118	8	12948

591.79093	2	12950

591.92068	1	12951

592.05043	1	12952

592.18019	10	12962

592.30994	1	12963

592.43969	1	12964

592.56944	14	12978

592.69919	1	12979

592.82894	2	12981

592.95869	17	12998

593.08844	1	12999

593.21819	1	13000

593.34794	1	13001

593.47769	1	13002

593.60744	1	13003

593.73719	7	13010

593.86694	1	13011

593.99669	1	13012

594.12644	3	13015

594.25619	12	13027

594.38594	7	13034

594.51569	27	13061

594.64544	1	13062

594.77519	2	13064

594.90494	1	13065

595.03469	1	13066

595.16444	6	13072

595.29419	1	13073

595.42394	4	13077

595.55369	2	13079

595.68344	1	13080

595.81319	1	13081

595.94294	1	13082

596.07269	5	13087

596.20244	1	13088

596.33219	3	13091

596.46194	1	13092

596.59169	2	13094

596.72144	1	13095

596.85119	1	13096

596.98094	1	13097

597.11069	5	13102

597.24045	12	13114

597.3702	4	13118

597.49995	1	13119

597.6297	3	13122

597.75945	1	13123

597.8892	1	13124

598.01895	3	13127

598.1487	1	13128

598.27845	2	13130

598.4082	1	13131

598.53795	1	13132

598.6677	1	13133

598.79745	4	13137

598.9272	1	13138

599.05695	2	13140

599.1867	2	13142

599.31645	5	13147

599.4462	2	13149

599.57595	1	13150

599.7057	1	13151

599.83545	7	13158

599.9652	6	13164

600.09495	1	13165

600.2247	1	13166

600.35445	2	13168

600.4842	1	13169

600.61395	1	13170

600.7437	1	13171

600.87345	2	13173

601.0032	3	13176

601.13295	1	13177

601.2627	1	13178

601.39245	2	13180

601.5222	1	13181

601.65195	2	13183

601.7817	1	13184

601.91145	1	13185

602.0412	1	13186

602.17096	2	13188

602.30071	1	13189

602.43046	64	13253

602.56021	3	13256

602.68996	8	13264

602.81971	2	13266

602.94946	1	13267

603.07921	2	13269

603.20896	14	13283

603.33871	1	13284

603.46846	1	13285

603.59821	2	13287

603.72796	1	13288

603.85771	1	13289

603.98746	1	13290

604.11721	1	13291

604.24696	1	13292

604.37671	1	13293

604.50646	1	13294

604.63621	1	13295

604.76596	1	13296

604.89571	1	13297

605.02546	1	13298

605.15521	1	13299

605.28496	2	13301

605.41471	1	13302

605.54446	1	13303

605.67421	4	13307

605.80396	1	13308

605.93371	1	13309

606.06346	1	13310

606.19321	1	13311

606.32296	1	13312

606.45271	1	13313

606.58246	1	13314

606.71221	2	13316

606.84196	1	13317

606.97171	2	13319

607.10146	1	13320

607.23122	14	13334

607.36097	41	13375

607.49072	1	13376

607.62047	1	13377

607.75022	1	13378

607.87997	4	13382

608.00972	1	13383

608.13947	1	13384

608.26922	1	13385

608.39897	1	13386

608.52872	1	13387

608.65847	1	13388

608.78822	9	13397

608.91797	2	13399

609.04772	3	13402

609.17747	1	13403

609.30722	3	13406

609.43697	7	13413

609.56672	2	13415

609.69647	2	13417

609.82622	3	13420

609.95597	1	13421

610.08572	1	13422

610.21547	1	13423

610.34522	1	13424

610.47497	1	13425

610.60472	1	13426

610.73447	2	13428

610.86422	1	13429

610.99397	1	13430

611.12372	1	13431

611.25347	1	13432

611.38322	1	13433

611.51297	3	13436

611.64272	1	13437

611.77247	2	13439

611.90222	2	13441

612.03197	1	13442

612.16173	4	13446

612.29148	35	13481

612.42123	4	13485

612.55098	1	13486

612.68073	1	13487

612.81048	1	13488

612.94023	1	13489

613.06998	1	13490

613.19973	1	13491

613.32948	3	13494

613.45923	1	13495

613.58898	1	13496

613.71873	1	13497

613.84848	1	13498

613.97823	1	13499

614.10798	1	13500

614.23773	1	13501

614.36748	1	13502

614.49723	1	13503

614.62698	1	13504

614.75673	1	13505

614.88648	6	13511

615.01623	1	13512

615.14598	1	13513

615.27573	1	13514

615.40548	3	13517

615.53523	1	13518

615.66498	2	13520

615.79473	1	13521

615.92448	1	13522

616.05423	1	13523

616.18398	3	13526

616.31373	1	13527

616.44348	2	13529

616.57323	7	13536

616.70298	1	13537

616.83273	1	13538

616.96248	4	13542

617.09223	1	13543

617.22199	3	13546

617.35174	2	13548

617.48149	1	13549

617.61124	1	13550

617.74099	1	13551

617.87074	1	13552

618.00049	4	13556

618.13024	3	13559

618.25999	1	13560

618.38974	1	13561

618.51949	2	13563

618.64924	2	13565

618.77899	1	13566

618.90874	3	13569

619.03849	2	13571

619.16824	3	13574

619.29799	1	13575

619.42774	1	13576

619.55749	6	13582

619.68724	2	13584

619.81699	1	13585

619.94674	1	13586

620.07649	1	13587

620.20624	1	13588

620.33599	1	13589

620.46574	1	13590

620.59549	4	13594

620.72524	1	13595

620.85499	2	13597

620.98474	1	13598

621.11449	1	13599

621.24424	1	13600

621.37399	1	13601

621.50374	1	13602

621.63349	1	13603

621.76324	1	13604

621.89299	2	13606

622.02274	1	13607

622.1525	1	13608

622.28225	2	13610

622.412	3	13613

622.54175	2	13615

622.6715	3	13618

622.80125	1	13619

622.931	2	13621

623.06075	38	13659

623.1905	4	13663

623.32025	3	13666

623.45	1	13667

623.57975	1	13668

623.7095	1	13669

623.83925	1	13670

623.969	2	13672

624.09875	8	13680

624.2285	4	13684

624.35825	2	13686

624.488	1	13687

624.61775	1	13688

624.7475	1	13689

624.87725	1	13690

625.007	1	13691

625.13675	3	13694

625.2665	2	13696

625.39625	1	13697

625.526	1	13698

625.65575	1	13699

625.7855	2	13701

625.91525	1	13702

626.045	3	13705

626.17475	2	13707

626.3045	1	13708

626.43425	2	13710

626.564	1	13711

626.69375	3	13714

626.8235	2	13716

626.95325	5	13721

627.083	1	13722

627.21276	1	13723

627.34251	2	13725

627.47226	1	13726

627.60201	1	13727

627.73176	1	13728

627.86151	1	13729

627.99126	18	13747

628.12101	2	13749

628.25076	1	13750

628.38051	2	13752

628.51026	2	13754

628.64001	1	13755

628.76976	3	13758

628.89951	4	13762

629.02926	9	13771

629.15901	2	13773

629.28876	1	13774

629.41851	1	13775

629.54826	1	13776

629.67801	1	13777

629.80776	7	13784

629.93751	1	13785

630.06726	1	13786

630.19701	3	13789

630.32676	1	13790

630.45651	1	13791

630.58626	1	13792

630.71601	4	13796

630.84576	7	13803

630.97551	2	13805

631.10526	1	13806

631.23501	2	13808

631.36476	3	13811

631.49451	13	13824

631.62426	1	13825

631.75401	4	13829

631.88376	5	13834

632.01351	2	13836

632.14327	2	13838

632.27302	5	13843

632.40277	2	13845

632.53252	4	13849

632.66227	1	13850

632.79202	2	13852

632.92177	1	13853

633.05152	2	13855

633.18127	2	13857

633.31102	1	13858

633.44077	6	13864

633.57052	6	13870

633.70027	1	13871

633.83002	1	13872

633.95977	3	13875

634.08952	1	13876

634.21927	1	13877

634.34902	1	13878

634.47877	1	13879

634.60852	1	13880

634.73827	1	13881

634.86802	2	13883

634.99777	3	13886

635.12752	1	13887

635.25727	2	13889

635.38702	5	13894

635.51677	4	13898

635.64652	1	13899

635.77627	2	13901

635.90602	4	13905

636.03577	1	13906

636.16552	1	13907

636.29527	1	13908

636.42502	13	13921

636.55477	1	13922

636.68452	1	13923

636.81427	1	13924

636.94402	2	13926

637.07377	4	13930

637.20353	1	13931

637.33328	2	13933

637.46303	1	13934

637.59278	1	13935

637.72253	2	13937

637.85228	3	13940

637.98203	1	13941

638.11178	2	13943

638.24153	1	13944

638.37128	1	13945

638.50103	1	13946

638.63078	2	13948

638.76053	5	13953

638.89028	1	13954

639.02003	1	13955

639.14978	1	13956

639.27953	1	13957

639.40928	6	13963

639.53903	1	13964

639.66878	3	13967

639.79853	4	13971

639.92828	2	13973

640.05803	1	13974

640.18778	15	13989

640.31753	2	13991

640.44728	1	13992

640.57703	1	13993

640.70678	1	13994

640.83653	6	14000

640.96628	1	14001

641.09603	2	14003

641.22578	1	14004

641.35553	5	14009

641.48528	1	14010

641.61503	1	14011

641.74478	2	14013

641.87453	1	14014

642.00428	4	14018

642.13404	1	14019

642.26379	6	14025

642.39354	4	14029

642.52329	2	14031

642.65304	5	14036

642.78279	3	14039

642.91254	1	14040

643.04229	2	14042

643.17204	1	14043

643.30179	5	14048

643.43154	3	14051

643.56129	3	14054

643.69104	3	14057

643.82079	1	14058

643.95054	2	14060

644.08029	1	14061

644.21004	1	14062

644.33979	4	14066

644.46954	2	14068

644.59929	2	14070

644.72904	1	14071

644.85879	1	14072

644.98854	6	14078

645.11829	2	14080

645.24804	1	14081

645.37779	2	14083

645.50754	2	14086

645.63729	1	14087

645.76704	1	14087

645.89679	1	14088

646.02654	1	14089

646.15629	5	14094

646.28604	1	14095

646.41579	1	14095

646.54554	1	14096

646.67529	2	14098

646.80504	1	14099

646.93479	1	14099

647.06454	2	14101

647.1943	1	14102

647.32405	5	14107

647.4538	2	14108

647.58355	3	14111

647.7133	10	14122

647.84305	2	14123

647.9728	2	14125

648.10255	5	14130

648.2323	2	14131

648.36205	1	14132

648.4918	1	14133

648.62155	1	14134

648.7513	3	14137

648.88105	1	14138

649.0108	1	14139

649.14055	1	14139

649.2703	2	14142

649.40005	3	14145

649.5298	2	14147

649.65955	1	14147

649.7893	2	14149

649.91905	46	14195

650.0488	1	14196

650.17855	2	14198

650.3083	1	14199

650.43805	1	14199

650.5678	1	14200

650.69755	1	14201

650.8273	1	14202

650.95705	2	14203

651.0868	2	14205

651.21655	1	14206

651.3463	3	14209

651.47605	2	14211

651.6058	1	14212

651.73555	4	14216

651.8653	1	14217

651.99505	3	14220

652.12481	1	14221

652.25456	1	14222

652.38431	1	14223

652.51406	1	14223

652.64381	1	14224

652.77356	1	14225

652.90331	1	14226

653.03306	10	14236

653.16281	2	14238

653.29256	1	14239

653.42231	1	14239

653.55206	1	14240

653.68181	2	14242

653.81156	2	14244

653.94131	1	14245

654.07106	4	14249

654.20081	2	14251

654.33056	1	14252

654.46031	1	14253

654.59006	1	14254

654.71981	1	14255

654.84956	4	14259

654.97931	1	14259

655.10906	1	14260

655.23881	1	14261

655.36856	1	14262

655.49831	1	14263

655.62806	1	14263

655.75781	2	14265

655.88756	6	14271

656.01731	1	14271

656.14706	1	14272

656.27681	1	14273

656.40656	8	14281

656.53631	2	14283

656.66606	1	14283

656.79581	2	14286

656.92556	2	14287

657.05531	1	14288

657.18507	1	14289

657.31482	2	14291

657.44457	1	14291

657.57432	2	14294

657.70407	1	14295

657.83382	1	14295

657.96357	1	14296

658.09332	1	14297

658.22307	1	14298

658.35282	2	14300

658.48257	2	14303

658.61232	1	14303

658.74207	6	14309

658.87182	1	14310

659.00157	1	14311

659.13132	2	14313

659.26107	1	14314

659.39082	1	14315

659.52057	2	14316

659.65032	1	14317

659.78007	13	14330

659.90982	2	14331

660.03957	2	14333

660.16932	2	14335

660.29907	4	14339

660.42882	2	14340

660.55857	1	14341

660.68832	2	14343

660.81807	1	14344

660.94782	1	14345

661.07757	1	14346

661.20732	2	14347

661.33707	1	14348

661.46682	1	14349

661.59657	1	14350

661.72632	2	14351

661.85607	1	14352

661.98582	11	14363

662.11558	1	14364

662.24533	1	14365

662.37508	1	14366

662.50483	1	14367

662.63458	1	14367

662.76433	1	14368

662.89408	1	14369

663.02383	1	14370

663.15358	2	14371

663.28333	1	14372

663.41308	1	14373

663.54283	2	14375

663.67258	1	14375

663.80233	1	14376

663.93208	6	14382

664.06183	1	14383

664.19158	2	14384

664.32133	1	14385

664.45108	1	14386

664.58083	1	14387

664.71058	1	14387

664.84033	1	14388

664.97008	2	14391

665.09983	1	14391

665.22958	1	14392

665.35933	2	14394

665.48908	14	14407

665.61883	1	14408

665.74858	1	14409

665.87833	1	14410

666.00808	1	14411

666.13783	1	14411

666.26758	4	14415

666.39733	1	14416

666.52708	1	14417

666.65683	1	14418

666.78658	2	14419

666.91633	1	14420

667.04608	1	14421

667.17584	1	14422

667.30559	2	14424

667.43534	3	14427

667.56509	1	14428

667.69484	1	14429

667.82459	1	14430

667.95434	1	14431

668.08409	1	14431

668.21384	2	14433

668.34359	4	14437

668.47334	1	14438

668.60309	10	14447

668.73284	4	14451

668.86259	1	14452

668.99234	1	14453

669.12209	2	14455

669.25184	1	14456

669.38159	1	14457

669.51134	4	14461

669.64109	1	14462

669.77084	1	14463

669.90059	2	14465

670.03034	1	14466

670.16009	1	14467

670.28984	2	14468

670.41959	1	14469

670.54934	1	14470

670.67909	2	14471

670.80884	2	14474

670.93859	2	14476

671.06834	1	14477

671.19809	2	14479

671.32784	4	14483

671.45759	3	14486

671.58734	1	14487

671.71709	2	14488

671.84684	8	14496

671.97659	2	14499

672.10635	18	14517

672.2361	1	14518

672.36585	1	14519

672.4956	2	14520

672.62535	2	14522

672.7551	1	14523

672.88485	2	14524

673.0146	2	14527

673.14435	1	14527

673.2741	1	14528

673.40385	3	14531

673.5336	2	14533

673.66335	10	14543

673.7931	1	14544

673.92285	1	14545

674.0526	1	14546

674.18235	2	14547

674.3121	1	14548

674.44185	1	14549

674.5716	2	14551

674.70135	2	14554

674.8311	1	14555

674.96085	3	14558

675.0906	11	14569

675.22035	14	14583

675.3501	2	14584

675.47985	1	14585

675.6096	2	14587

675.73935	1	14587

675.8691	1	14588

675.99885	1	14589

676.1286	1	14590

676.25835	1	14591

676.3881	2	14592

676.51785	2	14595

676.6476	1	14595

676.77735	2	14597

676.9071	1	14598

677.03685	12	14610

677.16661	1	14611

677.29636	1	14611

677.42611	1	14612

677.55586	5	14617

677.68561	10	14627

677.81536	3	14631

677.94511	1	14631

678.07486	17	14648

678.20461	1	14649

678.33436	1	14650

678.46411	2	14651

678.59386	2	14653

678.72361	4	14657

678.85336	2	14659

678.98311	2	14661

679.11286	3	14664

679.24261	1	14665

679.37236	6	14671

679.50211	2	14672

679.63186	1	14673

679.76161	1	14674

679.89136	1	14675

680.02111	1	14675

680.15086	9	14684

680.28061	1	14685

680.41036	1	14686

680.54011	1	14687

680.66986	1	14687

680.79961	1	14688

680.92936	2	14690

681.05911	4	14694

681.18886	4	14698

681.31861	17	14715

681.44836	1	14715

681.57811	1	14716

681.70786	2	14719

681.83761	3	14722

681.96736	1	14723

682.09712	2	14724

682.22687	1	14725

682.35662	1	14726

682.48637	1	14727

682.61612	2	14729

682.74587	2	14731

682.87562	1	14732

683.00537	2	14734

683.13512	4	14738

683.26487	2	14739

683.39462	1	14740

683.52437	1	14741

683.65412	1	14742

683.78387	2	14744

683.91362	1	14745

684.04337	8	14753

684.17312	1	14754

684.30287	1	14755

684.43262	2	14756

684.56237	2	14758

684.69212	1	14759

684.82187	1	14759

684.95162	2	14761

685.08137	2	14763

685.21112	3	14766

685.34087	2	14767

685.47062	1	14768

685.60037	6	14775

685.73012	1	14775

685.85987	2	14777

685.98962	2	14779

686.11937	5	14784

686.24912	1	14785

686.37887	2	14787

686.50862	2	14788

686.63837	7	14795

686.76812	4	14799

686.89787	3	14803

687.02762	1	14803

687.15738	10	14814

687.28713	6	14820

687.41688	1	14821

687.54663	1	14822

687.67638	1	14823

687.80613	4	14827

687.93588	7	14834

688.06563	1	14835

688.19538	1	14835

688.32513	2	14837

688.45488	1	14838

688.58463	2	14839

688.71438	1	14840

688.84413	1	14841

688.97388	1	14842

689.10363	2	14844

689.23338	10	14854

689.36313	1	14855

689.49288	3	14858

689.62263	1	14859

689.75238	2	14861

689.88213	2	14863

690.01188	1	14863

690.14163	1	14864

690.27138	10	14875

690.40113	38	14913

690.53088	1	14914

690.66063	2	14916

690.79038	3	14919

690.92013	1	14920

691.04988	2	14922

691.17963	6	14928

691.30938	5	14933

691.43913	1	14934

691.56888	3	14937

691.69863	1	14938

691.82838	1	14939

691.95813	1	14939

692.08788	2	14942

692.21764	2	14944

692.34739	1	14945

692.47714	2	14947

692.60689	4	14951

692.73664	2	14953

692.86639	1	14954

692.99614	1	14955

693.12589	2	14956

693.25564	1	14957

693.38539	1	14958

693.51514	3	14961

693.64489	1	14962

693.77464	1	14963

693.90439	1	14963

694.03414	1	14964

694.16389	2	14966

694.29364	1	14967

694.42339	2	14969

694.55314	1	14970

694.68289	1	14971

694.81264	1	14971

694.94239	2	14973

695.07214	1	14974

695.20189	1	14975

695.33164	3	14978

695.46139	2	14979

695.59114	3	14983

695.72089	3	14986

695.85064	1	14987

695.98039	1	14987

696.11014	2	14990

696.23989	2	14991

696.36964	1	14992

696.49939	2	14994

696.62914	2	14995

696.75889	1	14996

696.88864	1	14997

697.01839	1	14998

697.14815	1	14999

697.2779	4	15003

697.40765	1	15003

697.5374	2	15006

697.66715	1	15007

697.7969	1	15007

697.92665	3	15011

698.0564	1	15011

698.18615	3	15015

698.3159	2	15017

698.44565	2	15019

698.5754	2	15020

698.70515	1	15021

698.8349	1	15022

698.96465	2	15023

699.0944	1	15024

699.22415	2	15026

699.3539	2	15028

699.48365	1	15029

699.6134	4	15033

699.74315	2	15035

699.8729	1	15035

700.00265	1	15036

700.1324	2	15038

700.26215	2	15040

700.3919	2	15042

700.52165	9	15051

700.6514	1	15051

700.78115	1	15052

700.9109	1	15053

701.04065	1	15054

701.1704	1	15055

701.30015	2	15056

701.4299	1	15057

701.55965	1	15058

701.6894	2	15059

701.81915	2	15062

701.9489	1	15063

702.07865	4	15067

702.20841	1	15067

702.33816	2	15069

702.46791	2	15071

702.59766	8	15079

702.72741	1	15079

702.85716	1	15080

702.98691	2	15082

703.11666	1	15083

703.24641	1	15083

703.37616	2	15085

703.50591	3	15088

703.63566	1	15089

703.76541	1	15090

703.89516	1	15091

704.02491	6	15096

704.15466	2	15098

704.28441	2	15099

704.41416	14	15113

704.54391	1	15114

704.67366	1	15115

704.80341	1	15115

704.93316	2	15118

705.06291	1	15119

705.19266	1	15119

705.32241	2	15122

705.45216	1	15123

705.58191	1	15123

705.71166	1	15124

705.84141	2	15127

705.97116	1	15127

706.10091	3	15131

706.23066	1	15131

706.36041	2	15134

706.49016	3	15137

706.61991	2	15139

706.74966	4	15143

706.87941	4	15147

707.00916	1	15147

707.13892	2	15150

707.26867	2	15151

707.39842	1	15152

707.52817	1	15153

707.65792	1	15154

707.78767	1	15155

707.91742	7	15162

708.04717	1	15163

708.17692	2	15164

708.30667	2	15166

708.43642	2	15168

708.56617	2	15171

708.69592	9	15179

708.82567	10	15189

708.95542	2	15191

709.08517	2	15192

709.21492	2	15194

709.34467	1	15195

709.47442	1	15195

709.60417	2	15197

709.73392	2	15199

709.86367	10	15209

709.99342	2	15211

710.12317	1	15211

710.25292	1	15212

710.38267	1	15213

710.51242	2	15215

710.64217	7	15222

710.77192	1	15223

710.90167	1	15223

711.03142	2	15226

711.16117	1	15227

711.29092	1	15227

711.42067	2	15229

711.55042	1	15230

711.68017	1	15231

711.80992	3	15234

711.93967	2	15236

712.06942	1	15237

712.19918	2	15239

712.32893	2	15240

712.45868	2	15243

712.58843	3	15246

712.71818	1	15247

712.84793	1	15247

712.97768	1	15248

713.10743	1	15249

713.23718	2	15251

713.36693	2	15253

713.49668	2	15255

713.62643	1	15255

713.75618	1	15256

713.88593	1	15257

714.01568	1	15258

714.14543	3	15261

714.27518	1	15262

714.40493	3	15265

714.53468	3	15268

714.66443	1	15269

714.79418	2	15271

714.92393	1	15271

715.05368	1	15272

715.18343	2	15274

715.31318	2	15275

715.44293	1	15276

715.57268	1	15277

715.70243	2	15279

715.83218	1	15279

715.96193	2	15281

716.09168	1	15282

716.22143	1	15283

716.35118	4	15287

716.48093	6	15293

716.61068	1	15294

716.74043	1	15295

716.87018	2	15296

716.99993	1	15297

717.12969	2	15299

717.25944	3	15302

717.38919	1	15303

717.51894	1	15303

717.64869	1	15304

717.77844	3	15307

717.90819	2	15309

718.03794	1	15310

718.16769	1	15311

718.29744	1	15311

718.42719	1	15312

718.55694	1	15313

718.68669	2	15315

718.81644	2	15317

718.94619	1	15318

719.07594	1	15319

719.20569	5	15323

719.33544	1	15324

719.46519	1	15325

719.59494	1	15326

719.72469	3	15329

719.85444	2	15331

719.98419	2	15333

720.11394	4	15337

720.24369	2	15339

720.37344	1	15340

720.50319	5	15345

720.63294	1	15346

720.76269	4	15350

720.89244	1	15351

721.02219	2	15352

721.15194	1	15353

721.28169	1	15354

721.41144	1	15355

721.54119	3	15358

721.67094	6	15364

721.80069	2	15366

721.93044	2	15367

722.06019	1	15368

722.18995	1	15369

722.3197	1	15370

722.44945	1	15371

722.5792	1	15371

722.70895	6	15377

722.8387	1	15378

722.96845	6	15384

723.0982	4	15388

723.22795	2	15391

723.3577	3	15394

723.48745	3	15397

723.6172	4	15401

723.74695	1	15402

723.8767	2	15403

724.00645	1	15404

724.1362	2	15407

724.26595	7	15414

724.3957	2	15416

724.52545	1	15417

724.6552	1	15418

724.78495	1	15419

724.9147	2	15420

725.04445	2	15422

725.1742	17	15439

725.30395	2	15440

725.4337	2	15443

725.56345	6	15448

725.6932	3	15451

725.82295	1	15452

725.9527	1	15453

726.08245	1	15454

726.2122	1	15455

726.34195	2	15456

726.4717	2	15458

726.60145	2	15460

726.7312	1	15461

726.86095	2	15463

726.9907	1	15463

727.12046	1	15464

727.25021	1	15465

727.37996	1	15466

727.50971	2	15467

727.63946	1	15468

727.76921	2	15470

727.89896	2	15472

728.02871	7	15479

728.15846	1	15480

728.28821	6	15486

728.41796	3	15489

728.54771	6	15495

728.67746	3	15498

728.80721	6	15503

728.93696	5	15508

729.06671	4	15512

729.19646	6	15518

729.32621	7	15525

729.45596	1	15526

729.58571	1	15527

729.71546	22	15549

729.84521	2	15551

729.97496	1	15552

730.10471	2	15555

730.23446	2	15557

730.36421	3	15560

730.49396	2	15562

730.62371	4	15566

730.75346	10	15575

730.88321	1	15576

731.01296	2	15578

731.14271	2	15580

731.27246	1	15581

731.40221	1	15582

731.53196	16	15598

731.66171	3	15601

731.79146	1	15602

731.92121	2	15604

732.05096	1	15605

732.18072	2	15607

732.31047	1	15607

732.44022	1	15608

732.56997	2	15611

732.69972	1	15611

732.82947	2	15614

732.95922	4	15618

733.08897	2	15619

733.21872	2	15621

733.34847	2	15623

733.47822	2	15625

733.60797	2	15627

733.73772	2	15629

733.86747	2	15631

733.99722	1	15631

734.12697	3	15635

734.25672	2	15636

734.38647	2	15639

734.51622	2	15640

734.64597	2	15643

734.77572	1	15643

734.90547	2	15645

735.03522	5	15650

735.16497	2	15652

735.29472	2	15654

735.42447	2	15655

735.55422	3	15659

735.68397	2	15660

735.81372	1	15661

735.94347	1	15662

736.07322	6	15668

736.20297	1	15669

736.33272	1	15670

736.46247	2	15672

736.59222	3	15675

736.72197	6	15682

736.85172	1	15683

736.98147	1	15683

737.11122	1	15684

737.24098	5	15689

737.37073	6	15695

737.50048	6	15701

737.63023	2	15703

737.75998	2	15704

737.88973	14	15718

738.01948	1	15719

738.14923	2	15720

738.27898	2	15722

738.40873	2	15723

738.53848	2	15726

738.66823	1	15727

738.79798	1	15727

738.92773	1	15728

739.05748	1	15729

739.18723	2	15731

739.31698	7	15739

739.44673	6	15744

739.57648	3	15747

739.70623	17	15764

739.83598	25	15789

739.96573	1	15790

740.09548	1	15791

740.22523	1	15791

740.35498	36	15827

740.48473	6	15834

740.61448	2	15835

740.74423	1	15836

740.87398	1	15837

741.00373	3	15840

741.13348	2	15843

741.26323	1	15843

741.39298	19	15863

741.52273	4	15867

741.65248	2	15868

741.78223	8	15876

741.91198	9	15885

742.04173	2	15887

742.17149	2	15889

742.30124	2	15891

742.43099	4	15895

742.56074	1	15896

742.69049	6	15902

742.82024	2	15903

742.94999	1	15904

743.07974	6	15911

743.20949	1	15911

743.33924	1	15912

743.46899	1	15913

743.59874	12	15925

743.72849	2	15927

743.85824	7	15934

743.98799	3	15937

744.11774	1	15938

744.24749	2	15939

744.37724	1	15940

744.50699	1	15941

744.63674	2	15943

744.76649	2	15945

744.89624	3	15948

745.02599	1	15949

745.15574	6	15955

745.28549	1	15955

745.41524	2	15957

745.54499	9	15966

745.67474	2	15967

745.80449	1	15968

745.93424	6	15975

746.06399	2	15976

746.19374	15	15991

746.32349	3	15995

746.45324	7	16002

746.58299	2	16003

746.71274	1	16004

746.84249	1	16005

746.97224	6	16011

747.10199	6	16016

747.23175	11	16027

747.3615	2	16030

747.49125	1	16031

747.621	19	16050

747.75075	1	16051

747.8805	6	16057

748.01025	2	16059

748.14	6	16065

748.26975	14	16079

748.3995	10	16088

748.52925	3	16091

748.659	1	16092

748.78875	6	16098

748.9185	1	16099

749.04825	1	16099

749.178	17	16116

749.30775	6	16122

749.4375	2	16123

749.56725	1	16124

749.697	6	16130

749.82675	1	16131

749.9565	1	16131

750.08625	2	16133

750.216	1	16134

750.34575	3	16137

750.4755	2	16139

750.60525	2	16140

750.735	1	16141

750.86475	1	16142

750.9945	1	16143

751.12425	1	16143

751.254	1	16144

751.38375	1	16145

751.5135	6	16151

751.64325	3	16154

751.773	5	16159

751.90275	1	16159

752.0325	2	16161

752.16226	2	16163

752.29201	3	16166

752.42176	2	16167

752.55151	1	16168

752.68126	1	16169

752.81101	1	16170

752.94076	1	16171

753.07051	2	16173

753.20026	2	16175

753.33001	2	16178

753.45976	1	16179

753.58951	2	16181

753.71926	1	16182

753.84901	2	16183

753.97876	2	16185

754.10851	10	16195

754.23826	1	16195

754.36801	1	16196

754.49776	1	16197

754.62751	10	16207

754.75726	1	16208

754.88701	1	16209

755.01676	19	16228

755.14651	6	16235

755.27626	2	16237

755.40601	10	16247

755.53576	1	16248

755.66551	2	16250

755.79526	2	16251

755.92501	1	16252

756.05476	1	16253

756.18451	1	16254

756.31426	2	16255

756.44401	1	16256

756.57376	1	16257

756.70351	3	16260

756.83326	2	16262

756.96301	10	16271

757.09276	3	16275

757.22252	6	16281

757.35227	2	16283

757.48202	2	16284

757.61177	1	16285

757.74152	1	16286

757.87127	3	16289

758.00102	1	16290

758.13077	1	16291

758.26052	3	16294

758.39027	2	16295

758.52002	1	16296

758.64977	3	16299

758.77952	1	16300

758.90927	2	16303

759.03902	1	16303

759.16877	1	16304

759.29852	2	16306

759.42827	1	16307

759.55802	2	16308

759.68777	1	16309

759.81752	2	16311

759.94727	1	16311

760.07702	2	16313

760.20677	2	16315

760.33652	4	16319

760.46627	1	16319

760.59602	4	16323

760.72577	6	16329

760.85552	6	16335

760.98527	1	16335

761.11502	1	16336

761.24477	6	16342

761.37452	6	16347

761.50427	1	16348

761.63402	1	16349

761.76377	3	16352

761.89352	2	16354

762.02327	3	16357

762.15303	6	16363

762.28278	1	16364

762.41253	2	16367

762.54228	1	16367

762.67203	1	16368

762.80178	1	16369

762.93153	1	16370

763.06128	1	16371

763.19103	5	16375

763.32078	2	16378

763.45053	2	16379

763.58028	3	16383

763.71003	1	16383

763.83978	5	16388

763.96953	2	16390

764.09928	3	16393

764.22903	7	16400

764.35878	1	16401

764.48853	3	16404

764.61828	21	16425

764.74803	2	16427

764.87778	1	16427

765.00753	2	16430

765.13728	1	16431

765.26703	2	16432

765.39678	2	16434

765.52653	10	16444

765.65628	2	16447

765.78603	1	16447

765.91578	1	16448

766.04553	1	16449

766.17528	1	16450

766.30503	6	16456

766.43478	3	16459

766.56453	5	16464

766.69428	2	16467

766.82403	10	16476

766.95378	1	16477

767.08353	2	16479

767.21329	1	16480

767.34304	2	16482

767.47279	2	16483

767.60254	1	16484

767.73229	1	16485

767.86204	5	16490

767.99179	1	16491

768.12154	32	16523

768.25129	1	16523

768.38104	2	16525

768.51079	4	16529

768.64054	11	16540

768.77029	1	16541

768.90004	2	16543

769.02979	3	16547

769.15954	5	16551

769.28929	5	16556

769.41904	2	16559

769.54879	1	16559

769.67854	1	16560

769.80829	3	16563

769.93804	2	16565

770.06779	13	16578

770.19754	1	16579

770.32729	9	16587

770.45704	3	16591

770.58679	3	16594

770.71654	6	16599

770.84629	1	16600

770.97604	2	16602

771.10579	1	16603

771.23554	4	16607

771.36529	2	16608

771.49504	9	16617

771.62479	2	16619

771.75454	8	16627

771.88429	1	16627

772.01404	1	16628

772.1438	2	16630

772.27355	2	16631

772.4033	1	16632

772.53305	1	16633

772.6628	2	16635

772.79255	6	16641

772.9223	1	16642

773.05205	2	16644

773.1818	1	16645

773.31155	2	16647

773.4413	2	16649

773.57105	1	16650

773.7008	2	16651

773.83055	15	16667

773.9603	2	16668

774.09005	2	16670

774.2198	2	16671

774.34955	5	16676

774.4793	1	16677

774.60905	1	16678

774.7388	8	16686

774.86855	4	16690

774.9983	1	16691

775.12805	2	16692

775.2578	2	16695

775.38755	1	16695

775.5173	2	16698

775.64705	1	16699

775.7768	1	16699

775.90655	1	16700

776.0363	1	16701

776.16605	2	16703

776.2958	2	16705

776.42555	1	16706

776.5553	3	16709

776.68505	1	16710

776.8148	1	16711

776.94455	2	16712

777.0743	2	16714

777.20406	2	16716

777.33381	1	16717

777.46356	7	16724

777.59331	3	16727

777.72306	6	16733

777.85281	6	16739

777.98256	1	16740

778.11231	1	16741

778.24206	1	16742

778.37181	4	16746

778.50156	2	16748

778.63131	1	16749

778.76106	2	16751

778.89081	2	16753

779.02056	2	16755

779.15031	1	16755

779.28006	1	16756

779.40981	2	16758

779.53956	6	16764

779.66931	4	16768

779.79906	1	16769

779.92881	3	16772

780.05856	2	16775

780.18831	1	16775

780.31806	10	16786

780.44781	2	16787

780.57756	2	16790

780.70731	1	16791

780.83706	10	16801

780.96681	1	16802

781.09656	2	16803

781.22631	2	16805

781.35606	2	16807

781.48581	1	16808

781.61556	3	16811

781.74531	2	16814

781.87506	7	16821

782.00481	1	16822

782.13457	2	16824

782.26432	1	16825

782.39407	1	16826

782.52382	1	16827

782.65357	1	16827

782.78332	2	16829

782.91307	5	16834

783.04282	2	16836

783.17257	6	16843

783.30232	14	16856

783.43207	1	16857

783.56182	3	16860

783.69157	2	16862

783.82132	6	16868

783.95107	1	16869

784.08082	2	16871

784.21057	2	16873

784.34032	2	16875

784.47007	2	16877

784.59982	5	16882

784.72957	1	16883

784.85932	6	16889

784.98907	2	16891

785.11882	1	16891

785.24857	13	16904

785.37832	9	16913

785.50807	3	16916

785.63782	1	16917

785.76757	7	16924

785.89732	2	16926

786.02707	1	16927

786.15682	2	16929

786.28657	1	16930

786.41632	28	16958

786.54607	7	16965

786.67582	13	16978

786.80557	3	16981

786.93532	1	16982

787.06507	1	16983

787.19483	4	16987

787.32458	1	16987

787.45433	5	16992

787.58408	5	16997

787.71383	3	17000

787.84358	2	17002

787.97333	2	17003

788.10308	3	17007

788.23283	2	17008

788.36258	1	17009

788.49233	1	17010

788.62208	4	17014

788.75183	1	17015

788.88158	1	17015

789.01133	3	17019

789.14108	3	17022

789.27083	1	17023

789.40058	3	17026

789.53033	2	17027

789.66008	5	17032

789.78983	1	17033

789.91958	2	17035

790.04933	1	17035

790.17908	3	17039

790.30883	1	17039

790.43858	3	17043

790.56833	6	17049

790.69808	2	17051

790.82783	3	17055

790.95758	9	17063

791.08733	16	17079

791.21708	1	17080

791.34683	1	17081

791.47658	2	17083

791.60633	12	17095

791.73608	1	17095

791.86583	2	17097

791.99558	2	17099

792.12534	10	17109

792.25509	26	17135

792.38484	4	17139

792.51459	2	17141

792.64434	3	17144

792.77409	2	17147

792.90384	1	17147

793.03359	1	17148

793.16334	1	17149

793.29309	3	17152

793.42284	5	17157

793.55259	30	17187

793.68234	1	17188

793.81209	1	17189

793.94184	1	17190

794.07159	1	17191

794.20134	6	17196

794.33109	2	17198

794.46084	2	17199

794.59059	2	17202

794.72034	1	17203

794.85009	3	17206

794.97984	1	17207

795.10959	5	17211

795.23934	13	17224

795.36909	5	17229

795.49884	1	17230

795.62859	2	17231

795.75834	1	17232

795.88809	1	17233

796.01784	1	17234

796.14759	1	17235

796.27734	1	17235

796.40709	7	17243

796.53684	1	17243

796.66659	2	17246

796.79634	3	17249

796.92609	2	17251

797.05584	11	17262

797.1856	2	17263

797.31535	1	17264

797.4451	3	17267

797.57485	28	17295

797.7046	1	17296

797.83435	2	17298

797.9641	2	17300

798.09385	1	17301

798.2236	1	17302

798.35335	1	17303

798.4831	4	17307

798.61285	1	17307

798.7426	6	17313

798.87235	4	17317

799.0021	1	17318

799.13185	2	17319

799.2616	2	17322

799.39135	5	17327

799.5211	3	17330

799.65085	3	17333

799.7806	1	17334

799.91035	4	17338

800.0401	2	17340

800.16985	4	17344

800.2996	1	17345

800.42935	1	17346

800.5591	4	17350

800.68885	4	17354

800.8186	2	17355

800.94835	2	17358

801.0781	2	17360

801.20785	1	17361

801.3376	2	17363

801.46735	3	17366

801.5971	10	17375

801.72685	19	17395

801.8566	2	17396

801.98635	15	17411

802.11611	3	17415

802.24586	1	17415

802.37561	1	17416

802.50536	3	17419

802.63511	1	17420

802.76486	2	17422

802.89461	1	17423

803.02436	12	17435

803.15411	1	17435

803.28386	1	17436

803.41361	14	17450

803.54336	10	17460

803.67311	1	17461

803.80286	3	17464

803.93261	1	17465

804.06236	1	17466

804.19211	1	17467

804.32186	2	17469

804.45161	1	17470

804.58136	3	17473

804.71111	2	17475

804.84086	12	17487

804.97061	1	17488

805.10036	2	17491

805.23011	7	17498

805.35986	5	17503

805.48961	1	17503

805.61936	7	17511

805.74911	49	17559

805.87886	1	17560

806.00861	4	17564

806.13836	2	17566

806.26811	6	17571

806.39786	1	17572

806.52761	6	17579

806.65736	3	17582

806.78711	1	17583

806.91686	1	17583

807.04661	3	17587

807.17637	6	17593

807.30612	12	17605

807.43587	1	17606

807.56562	2	17607

807.69537	8	17615

807.82512	5	17620

807.95487	2	17622

808.08462	2	17624

808.21437	3	17627

808.34412	3	17631

808.47387	2	17632

808.60362	2	17635

808.73337	2	17636

808.86312	2	17638

808.99287	6	17644

809.12262	6	17650

809.25237	2	17651

809.38212	8	17659

809.51187	13	17672

809.64162	2	17675

809.77137	5	17679

809.90112	2	17681

810.03087	2	17683

810.16062	6	17689

810.29037	5	17694

810.42012	3	17697

810.54987	3	17700

810.67962	4	17704

810.80937	1	17705

810.93912	2	17707

811.06887	2	17709

811.19862	1	17710

811.32837	4	17714

811.45812	18	17732

811.58787	26	17758

811.71762	12	17770

811.84737	2	17771

811.97712	9	17780

812.10688	4	17784

812.23663	2	17786

812.36638	1	17787

812.49613	2	17788

812.62588	3	17791

812.75563	18	17810

812.88538	14	17824

813.01513	1	17825

813.14488	8	17833

813.27463	2	17835

813.40438	2	17836

813.53413	2	17838

813.66388	1	17839

813.79363	6	17844

813.92338	1	17845

814.05313	14	17859

814.18288	2	17860

814.31263	2	17862

814.44238	3	17865

814.57213	47	17912

814.70188	15	17927

814.83163	1	17928

814.96138	11	17939

815.09113	2	17941

815.22088	2	17943

815.35063	6	17948

815.48038	4	17952

815.61013	1	17953

815.73988	14	17967

815.86963	1	17968

815.99938	4	17972

816.12913	10	17983

816.25888	6	17988

816.38863	2	17990

816.51838	2	17991

816.64813	1	17992

816.77788	2	17994

816.90763	2	17995

817.03738	1	17996

817.16714	5	18001

817.29689	2	18003

817.42664	2	18005

817.55639	16	18021

817.68614	2	18023

817.81589	22	18045

817.94564	1	18046

818.07539	2	18048

818.20514	8	18056

818.33489	4	18060

818.46464	22	18083

818.59439	4	18087

818.72414	4	18091

818.85389	1	18091

818.98364	1	18092

819.11339	9	18101

819.24314	5	18106

819.37289	2	18108

819.50264	4	18112

819.63239	1	18113

819.76214	9	18122

819.89189	3	18125

820.02164	5	18130

820.15139	15	18145

820.28114	24	18169

820.41089	2	18171

820.54064	14	18184

820.67039	6	18191

820.80014	2	18192

820.92989	12	18204

821.05964	3	18207

821.18939	1	18208

821.31914	4	18212

821.44889	2	18214

821.57864	1	18215

821.70839	5	18219

821.83814	6	18225

821.96789	1	18226

822.09765	4	18230

822.2274	2	18231

822.35715	2	18234

822.4869	5	18239

822.61665	2	18241

822.7464	1	18242

822.87615	1	18243

823.0059	2	18244

823.13565	2	18246

823.2654	14	18260

823.39515	1	18261

823.5249	11	18272

823.65465	9	18281

823.7844	1	18282

823.91415	3	18285

824.0439	25	18310

824.17365	1	18311

824.3034	2	18313

824.43315	6	18319

824.5629	6	18326

824.69265	6	18332

824.8224	6	18338

824.95215	2	18339

825.0819	1	18340

825.21165	9	18349

825.3414	59	18408

825.47115	4	18412

825.6009	1	18413

825.73065	10	18423

825.8604	1	18424

825.99015	1	18425

826.1199	1	18426

826.24965	10	18436

826.3794	2	18438

826.50915	1	18439

826.6389	2	18440

826.76865	2	18443

826.8984	12	18455

827.02815	2	18457

827.15791	1	18458

827.28766	2	18460

827.41741	2	18462

827.54716	2	18464

827.67691	2	18467

827.80666	1	18467

827.93641	4	18471

828.06616	1	18472

828.19591	1	18473

828.32566	5	18478

828.45541	1	18479

828.58516	2	18481

828.71491	2	18483

828.84466	2	18486

828.97441	1	18487

829.10416	15	18502

829.23391	1	18503

829.36366	24	18527

829.49341	2	18528

829.62316	8	18536

829.75291	8	18544

829.88266	21	18565

830.01241	8	18573

830.14216	2	18575

830.27191	6	18581

830.40166	10	18591

830.53141	25	18615

830.66116	1	18616

830.79091	10	18627

830.92066	18	18644

831.05041	9	18653

831.18016	4	18657

831.30991	5	18662

831.43966	21	18683

831.56941	3	18686

831.69916	11	18697

831.82891	6	18703

831.95866	2	18704

832.08842	14	18719

832.21817	7	18726

832.34792	2	18728

832.47767	8	18736

832.60742	19	18755

832.73717	4	18759

832.86692	1	18760

832.99667	2	18763

833.12642	9	18771

833.25617	18	18789

833.38592	1	18790

833.51567	2	18791

833.64542	3	18795

833.77517	1	18795

833.90492	1	18796

834.03467	11	18807

834.16442	17	18824

834.29417	6	18830

834.42392	3	18833

834.55367	2	18835

834.68342	6	18840

834.81317	2	18843

834.94292	5	18847

835.07267	1	18848

835.20242	1	18849

835.33217	6	18855

835.46192	2	18856

835.59167	1	18857

835.72142	4	18861

835.85117	66	18927

835.98092	3	18930

836.11067	2	18932

836.24042	10	18942

836.37017	6	18947

836.49992	13	18960

836.62967	19	18979

836.75942	13	18992

836.88917	7	18999

837.01892	10	19010

837.14868	37	19047

837.27843	6	19053

837.40818	14	19067

837.53793	1	19068

837.66768	13	19081

837.79743	2	19083

837.92718	7	19090

838.05693	32	19122

838.18668	2	19124

838.31643	15	19139

838.44618	3	19143

838.57593	1	19143

838.70568	2	19145

838.83543	9	19154

838.96518	1	19155

839.09493	10	19165

839.22468	3	19168

839.35443	6	19175

839.48418	2	19176

839.61393	4	19180

839.74368	3	19183

839.87343	3	19187

840.00318	5	19191

840.13293	3	19195

840.26268	2	19196

840.39243	3	19199

840.52218	1	19200

840.65193	13	19213

840.78168	2	19215

840.91143	5	19219

841.04118	14	19234

841.17093	2	19235

841.30068	1	19236

841.43043	2	19239

841.56018	2	19240

841.68993	2	19242

841.81968	5	19247

841.94943	1	19247

842.07919	2	19249

842.20894	1	19250

842.33869	5	19255

842.46844	1	19255

842.59819	2	19257

842.72794	3	19260

842.85769	1	19261

842.98744	1	19262

843.11719	5	19267

843.24694	2	19268

843.37669	6	19275

843.50644	17	19291

843.63619	15	19307

843.76594	12	19319

843.89569	14	19332

844.02544	3	19335

844.15519	18	19353

844.28494	1	19354

844.41469	2	19356

844.54444	9	19365

844.67419	1	19366

844.80394	6	19372

844.93369	2	19374

845.06344	2	19376

845.19319	1	19377

845.32294	1	19378

845.45269	1	19379

845.58244	16	19395

845.71219	2	19397

845.84194	34	19431

845.97169	1	19432

846.10144	22	19455

846.23119	6	19461

846.36094	6	19467

846.49069	8	19475

846.62044	1	19475

846.75019	2	19478

846.87994	2	19479

847.00969	5	19484

847.13945	2	19486

847.2692	2	19487

847.39895	1	19488

847.5287	1	19489

847.65845	2	19491

847.7882	6	19496

847.91795	2	19498

848.0477	4	19502

848.17745	6	19507

848.3072	6	19514

848.43695	10	19524

848.5667	2	19527

848.69645	1	19527

848.8262	5	19532

848.95595	5	19537

849.0857	2	19539

849.21545	1	19540

849.3452	2	19542

849.47495	1	19543

849.6047	6	19548

849.73445	1	19549

849.8642	2	19551

849.99395	2	19552

850.1237	15	19567

850.25345	10	19577

850.3832	1	19578

850.51295	2	19579

850.6427	1	19580

850.77245	1	19581

850.9022	22	19603

851.03195	1	19604

851.1617	2	19606

851.29145	1	19607

851.4212	14	19620

851.55095	2	19622

851.6807	4	19626

851.81045	2	19627

851.9402	1	19628

852.06996	2	19630

852.19971	1	19631

852.32946	2	19633

852.45921	1	19634

852.58896	10	19643

852.71871	1	19644

852.84846	14	19658

852.97821	22	19680

853.10796	2	19682

853.23771	3	19685

853.36746	4	19689

853.49721	7	19696

853.62696	1	19697

853.75671	1	19698

853.88646	1	19699

854.01621	4	19703

854.14596	14	19716

854.27571	2	19719

854.40546	4	19723

854.53521	1	19723

854.66496	4	19727

854.79471	2	19729

854.92446	1	19730

855.05421	1	19731

855.18396	1	19731

855.31371	1	19732

855.44346	2	19735

855.57321	9	19743

855.70296	1	19744

855.83271	2	19746

855.96246	1	19747

856.09221	1	19747

856.22196	5	19752

856.35171	8	19760

856.48146	2	19763

856.61121	1	19763

856.74096	1	19764

856.87071	2	19767

857.00046	1	19767

857.13022	3	19771

857.25997	17	19787

857.38972	27	19815

857.51947	1	19815

857.64922	2	19818

857.77897	18	19835

857.90872	8	19843

858.03847	5	19848

858.16822	2	19850

858.29797	18	19868

858.42772	1	19869

858.55747	1	19870

858.68722	27	19897

858.81697	12	19909

858.94672	1	19910

859.07647	1	19911

859.20622	1	19911

859.33597	1	19912

859.46572	2	19914

859.59547	1	19915

859.72522	1	19915

859.85497	6	19921

859.98472	2	19923

860.11447	1	19923

860.24422	7	19931

860.37397	2	19932

860.50372	1	19933

860.63347	1	19934

860.76322	6	19940

860.89297	1	19941

861.02272	3	19944

861.15247	2	19947

861.28222	2	19948

861.41197	3	19951

861.54172	2	19953

861.67147	2	19955

861.80122	1	19955

861.93097	1	19956

862.06073	1	19957

862.19048	3	19960

862.32023	9	19969

862.44998	1	19970

862.57973	2	19971

862.70948	3	19975

862.83923	3	19978

862.96898	5	19983

863.09873	2	19985

863.22848	1	19986

863.35823	2	19988

863.48798	6	19995

863.61773	2	19997

863.74748	2	19999

863.87723	2	20001

864.00698	1	20002

864.13673	2	20004

864.26648	1	20005

864.39623	3	20008

864.52598	1	20009

864.65573	1	20010

864.78548	1	20011

864.91523	2	20013

865.04498	1	20014

865.17473	6	20019

865.30448	2	20021

865.43423	2	20023

865.56398	2	20024

865.69373	1	20025

865.82348	6	20031

865.95323	8	20039

866.08298	1	20039

866.21273	7	20047

866.34248	2	20049

866.47223	5	20054

866.60198	2	20055

866.73173	5	20060

866.86148	1	20061

866.99123	28	20089

867.12099	4	20093

867.25074	6	20099

867.38049	1	20100

867.51024	2	20103

867.63999	1	20103

867.76974	3	20107

867.89949	1	20107

868.02924	1	20108

868.15899	1	20109

868.28874	1	20110

868.41849	8	20118

868.54824	4	20122

868.67799	25	20147

868.80774	1	20147

868.93749	2	20149

869.06724	2	20151

869.19699	1	20151

869.32674	2	20153

869.45649	1	20154

869.58624	1	20155

869.71599	10	20164

869.84574	2	20167

869.97549	1	20167

870.10524	4	20171

870.23499	2	20173

870.36474	6	20179

870.49449	6	20185

870.62424	1	20186

870.75399	2	20188

870.88374	1	20189

871.01349	2	20191

871.14324	1	20192

871.27299	2	20195

871.40274	1	20195

871.53249	1	20196

871.66224	1	20197

871.79199	1	20198

871.92174	1	20199

872.0515	1	20199

872.18125	3	20203

872.311	2	20204

872.44075	1	20205

872.5705	1	20206

872.70025	3	20209

872.83	3	20212

872.95975	2	20214

873.0895	1	20215

873.21925	1	20215

873.349	1	20216

873.47875	2	20218

873.6085	2	20219

873.73825	10	20230

873.868	1	20231

873.99775	2	20232

874.1275	2	20234

874.25725	1	20235

874.387	1	20235

874.51675	7	20243

874.6465	2	20245

874.77625	8	20253

874.906	1	20254

875.03575	2	20256

875.1655	2	20258

875.29525	8	20266

875.425	1	20267

875.55475	3	20270

875.6845	1	20271

875.81425	2	20272

875.944	1	20273

876.07375	1	20274

876.2035	10	20283

876.33325	1	20284

876.463	2	20286

876.59275	1	20287

876.7225	2	20289

876.85225	2	20291

876.982	2	20292

877.11176	1	20293

877.24151	1	20294

877.37126	4	20298

877.50101	1	20299

877.63076	2	20301

877.76051	2	20303

877.89026	2	20305

878.02001	3	20308

878.14976	1	20309

878.27951	1	20310

878.40926	2	20312

878.53901	2	20314

878.66876	1	20315

878.79851	2	20317

878.92826	1	20318

879.05801	3	20321

879.18776	7	20328

879.31751	14	20343

879.44726	2	20344

879.57701	1	20345

879.70676	2	20347

879.83651	1	20348

879.96626	5	20353

880.09601	2	20355

880.22576	1	20355

880.35551	2	20357

880.48526	6	20363

880.61501	5	20368

880.74476	1	20369

880.87451	14	20383

881.00426	1	20384

881.13401	1	20385

881.26376	1	20386

881.39351	1	20387

881.52326	2	20388

881.65301	1	20389

881.78276	1	20390

881.91251	1	20391

882.04227	1	20391

882.17202	1	20392

882.30177	17	20409

882.43152	3	20412

882.56127	1	20413

882.69102	1	20414

882.82077	2	20415

882.95052	1	20416

883.08027	6	20422

883.21002	1	20423

883.33977	1	20423

883.46952	2	20425

883.59927	1	20426

883.72902	1	20427

883.85877	1	20427

883.98852	1	20428

884.11827	3	20431

884.24802	1	20432

884.37777	1	20433

884.50752	8	20441

884.63727	4	20445

884.76702	14	20459

884.89677	2	20461

885.02652	1	20462

885.15627	1	20463

885.28602	2	20464

885.41577	2	20467

885.54552	1	20467

885.67527	1	20468

885.80502	5	20473

885.93477	1	20474

886.06452	1	20475

886.19427	1	20475

886.32402	2	20478

886.45377	1	20479

886.58352	1	20479

886.71327	1	20480

886.84302	1	20481

886.97277	2	20483

887.10253	1	20483

887.23228	10	20494

887.36203	1	20495

887.49178	10	20504

887.62153	2	20507

887.75128	1	20507

887.88103	2	20509

888.01078	6	20515

888.14053	2	20516

888.27028	1	20517

888.40003	2	20519

888.52978	1	20519

888.65953	2	20522

888.78928	5	20527

888.91903	5	20531

889.04878	5	20536

889.17853	2	20539

889.30828	2	20541

889.43803	2	20543

889.56778	7	20551

889.69753	5	20555

889.82728	2	20558

889.95703	7	20565

890.08678	2	20567

890.21653	5	20572

890.34628	19	20591

890.47603	2	20594

890.60578	2	20596

890.73553	12	20608

890.86528	5	20613

890.99503	2	20615

891.12478	2	20618

891.25453	5	20623

891.38428	2	20625

891.51403	2	20627

891.64378	17	20644

891.77353	2	20647

891.90328	10	20656

892.03304	7	20663

892.16279	10	20673

892.29254	2	20675

892.42229	2	20678

892.55204	2	20680

892.68179	5	20685

892.81154	2	20687

892.94129	2	20690

893.07104	36	20726

893.20079	2	20728

893.33054	7	20735

893.46029	2	20738

893.59004	2	20740

893.71979	2	20743

893.84954	2	20745

893.97929	22	20767

894.10904	2	20769

894.23879	2	20771

894.36854	5	20776

894.49829	2	20779

894.62804	5	20783

894.75779	10	20793

894.88754	5	20798

895.01729	7	20805

895.14704	34	20839

895.27679	2	20841

895.40654	5	20846

895.53629	2	20848

895.66604	2	20851

895.79579	2	20853

895.92554	12	20865

896.05529	7	20872

896.18504	7	20879

896.31479	5	20884

896.44454	2	20887

896.57429	7	20894

896.70404	2	20896

896.83379	2	20899

896.96354	2	20901

897.0933	5	20906

897.22305	2	20908

897.3528	2	20911

897.48255	12	20923

897.6123	36	20959

897.74205	10	20968

897.8718	2	20971

898.00155	2	20973

898.1313	5	20978

898.26105	26	21004

898.3908	5	21009

898.52055	2	21011

898.6503	7	21019

898.78005	5	21023

898.9098	5	21028

899.03955	2	21031

899.1693	5	21035

899.29905	2	21038

899.4288	7	21045

899.55855	2	21047

899.6883	2	21050

899.81805	10	21059

899.9478	2	21062

900.07755	2	21064

900.2073	2	21067

900.33705	5	21071

900.4668	5	21076

900.59655	2	21079

900.7263	10	21088

900.85605	41	21129

900.9858	2	21131

901.11555	2	21134

901.2453	2	21136

901.37505	5	21141

901.5048	2	21143

901.63455	2	21146

901.7643	2	21148

901.89405	7	21155

902.02381	2	21158

902.15356	2	21160

902.28331	7	21167

902.41306	2	21170

902.54281	2	21172

902.67256	22	21194

902.80231	5	21199

902.93206	5	21203

903.06181	2	21206

903.19156	2	21208

903.32131	14	21223

903.45106	5	21227

903.58081	7	21235

903.71056	12	21247

903.84031	5	21251

903.97006	2	21254

904.09981	2	21256

904.22956	2	21259

904.35931	5	21263

904.48906	12	21275

904.61881	7	21283

904.74856	2	21285

904.87831	2	21287

905.00806	2	21290

905.13781	14	21304

905.26756	2	21307

905.39731	12	21319

905.52706	5	21323

905.65681	10	21333

905.78656	2	21335

905.91631	2	21338

906.04606	2	21340

906.17581	2	21343

906.30556	10	21352

906.43531	2	21355

906.56506	2	21357

906.69481	5	21362

906.82456	7	21369

906.95431	2	21371

907.08407	2	21374

907.21382	5	21379

907.34357	2	21381

907.47332	2	21383

907.60307	5	21388

907.73282	2	21391

907.86257	2	21393

907.99232	2	21395

908.12207	2	21398

908.25182	10	21407

908.38157	2	21410

908.51132	2	21412

908.64107	2	21415

908.77082	2	21417

908.90057	10	21427

909.03032	2	21429

909.16007	5	21434

909.28982	22	21455

909.41957	2	21458

909.54932	5	21463

909.67907	2	21465

909.80882	2	21467

909.93857	2	21470

910.06832	2	21472

910.19807	2	21475

910.32782	5	21479

910.45757	5	21484

910.58732	10	21494

910.71707	7	21501

910.84682	10	21511

910.97657	2	21513

911.10632	2	21515

911.23607	2	21518

911.36582	2	21520

911.49557	5	21525

911.62532	2	21527

911.75507	5	21532

911.88482	5	21537

912.01458	5	21542

912.14433	7	21549

912.27408	5	21554

912.40383	2	21556

912.53358	5	21561

912.66333	2	21563

912.79308	2	21566

912.92283	7	21573

913.05258	2	21575

913.18233	2	21578

913.31208	2	21580

913.44183	2	21583

913.57158	2	21585

913.70133	5	21590

913.83108	5	21595

913.96083	2	21597

914.09058	7	21604

914.22033	2	21607

914.35008	2	21609

914.47983	2	21611

914.60958	2	21614

914.73933	7	21621

914.86908	2	21623

914.99883	5	21628

915.12858	7	21635

915.25833	24	21659

915.38808	5	21664

915.51783	2	21667

915.64758	12	21679

915.77733	2	21681

915.90708	2	21683

916.03683	2	21686

916.16658	5	21691

916.29633	10	21700

916.42608	17	21717

916.55583	2	21719

916.68558	2	21722

916.81533	7	21729

916.94508	2	21731

917.07484	7	21739

917.20459	2	21741

917.33434	5	21746

917.46409	2	21748

917.59384	5	21753

917.72359	2	21755

917.85334	2	21758

917.98309	10	21767

918.11284	17	21784

918.24259	2	21787

918.37234	2	21789

918.50209	2	21791

918.63184	2	21794

918.76159	5	21799

918.89134	7	21806

919.02109	5	21811

919.15084	2	21813

919.28059	5	21818

919.41034	5	21823

919.54009	2	21825

919.66984	10	21835

919.79959	2	21837

919.92934	2	21839

920.05909	2	21842

920.18884	5	21847

920.31859	14	21861

920.44834	2	21863

920.57809	12	21875

920.70784	7	21883

920.83759	34	21916

920.96734	5	21921

921.09709	2	21923

921.22684	2	21926

921.35659	2	21928

921.48634	2	21931

921.61609	2	21933

921.74584	7	21940

921.87559	7	21947

922.00535	7	21955

922.1351	2	21957

922.26485	5	21962

922.3946	2	21964

922.52435	2	21967

922.6541	2	21969

922.78385	5	21974

922.9136	2	21976

923.04335	2	21979

923.1731	10	21988

923.30285	2	21991

923.4326	12	22003

923.56235	5	22007

923.6921	7	22015

923.82185	19	22034

923.9516	2	22036

924.08135	2	22039

924.2111	2	22041

924.34085	2	22043

924.4706	2	22046

924.60035	12	22058

924.7301	19	22077

924.85985	2	22079

924.9896	2	22082

925.11935	7	22089

925.2491	2	22091

925.37885	5	22096

925.5086	7	22103

925.63835	5	22108

925.7681	5	22113

925.89785	2	22115

926.0276	2	22118

926.15735	2	22120

926.2871	7	22127

926.41685	2	22130

926.5466	2	22132

926.67635	7	22139

926.8061	2	22142

926.93585	2	22144

927.06561	5	22149

927.19536	5	22154

927.32511	2	22156

927.45486	5	22161

927.58461	10	22171

927.71436	2	22173

927.84411	2	22175

927.97386	7	22183

928.10361	2	22185

928.23336	2	22187

928.36311	5	22192

928.49286	14	22207

928.62261	5	22211

928.75236	2	22214

928.88211	2	22216

929.01186	7	22223

929.14161	7	22231

929.27136	2	22233

929.40111	5	22238

929.53086	29	22267

929.66061	2	22269

929.79036	5	22274

929.92011	5	22279

930.04986	2	22281

930.17961	2	22283

930.30936	2	22286

930.43911	2	22288

930.56886	2	22291

930.69861	2	22293

930.82836	5	22298

930.95811	10	22307

931.08786	5	22312

931.21761	5	22317

931.34736	2	22319

931.47711	12	22331

931.60686	14	22346

931.73661	2	22348

931.86636	2	22351

931.99612	2	22353

932.12587	5	22358

932.25562	2	22360

932.38537	2	22363

932.51512	2	22365

932.64487	7	22372

932.77462	2	22375

932.90437	5	22379

933.03412	2	22382

933.16387	2	22384

933.29362	2	22387

933.42337	5	22391

933.55312	2	22394

933.68287	5	22399

933.81262	5	22403

933.94237	5	22408

934.07212	2	22411

934.20187	2	22413

934.33162	5	22418

934.46137	7	22425

934.59112	2	22427

934.72087	5	22432

934.85062	5	22437

934.98037	2	22439

935.11012	12	22451

935.23987	2	22454

935.36962	5	22459

935.49937	10	22468

935.62912	2	22471

935.75887	7	22478

935.88862	5	22483

936.01837	2	22485

936.14812	2	22487

936.27787	2	22490

936.40762	10	22499

936.53737	2	22502

936.66712	2	22504

936.79687	5	22509

936.92662	5	22514

937.05638	2	22516

937.18613	7	22523

937.31588	2	22526

937.44563	10	22535

937.57538	5	22540

937.70513	2	22543

937.83488	5	22547

937.96463	2	22550

938.09438	2	22552

938.22413	5	22557

938.35388	2	22559

938.48363	5	22564

938.61338	5	22569

938.74313	5	22574

938.87288	2	22576

939.00263	2	22579

939.13238	2	22581

939.26213	2	22583

939.39188	2	22586

939.52163	2	22588

939.65138	2	22591

939.78113	2	22593

939.91088	2	22595

940.04063	2	22598

940.17038	2	22600

940.30013	2	22603

940.42988	2	22605

940.55963	17	22622

940.68938	7	22629

940.81913	2	22631

940.94888	2	22634

941.07863	12	22646

941.20838	2	22648

941.33813	5	22653

941.46788	5	22658

941.59763	7	22665

941.72738	2	22667

941.85713	2	22670

941.98689	2	22672

942.11664	5	22677

942.24639	2	22679

942.37614	5	22684

942.50589	2	22687

942.63564	5	22691

942.76539	2	22694

942.89514	2	22696

943.02489	2	22699

943.15464	2	22701

943.28439	2	22703

943.41414	2	22706

943.54389	5	22711

943.67364	2	22713

943.80339	2	22715

943.93314	2	22718

944.06289	2	22720

944.19264	2	22723

944.32239	5	22727

944.45214	22	22749

944.58189	2	22751

944.71164	2	22754

944.84139	2	22756

944.97114	17	22773

945.10089	7	22780

945.23064	2	22783

945.36039	2	22785

945.49014	5	22790

945.61989	7	22797

945.74964	2	22799

945.87939	2	22802

946.00914	17	22819

946.13889	12	22831

946.26864	2	22833

946.39839	2	22835

946.52814	89	22924

946.65789	2	22927

946.78764	2	22929

946.91739	2	22931

947.04715	5	22936

947.1769	5	22941

947.30665	2	22943

947.4364	2	22946

947.56615	5	22951

947.6959	12	22963

947.82565	24	22987

947.9554	2	22989

948.08515	17	23006

948.2149	2	23008

948.34465	2	23011

948.4744	2	23013

948.60415	5	23018

948.7339	2	23020

948.86365	2	23023

948.9934	12	23035

949.12315	5	23039

949.2529	2	23042

949.38265	5	23047

949.5124	2	23049

949.64215	10	23059

949.7719	2	23061

949.90165	2	23063

950.0314	10	23073

950.16115	2	23075

950.2909	10	23085

950.42065	2	23087

950.5504	10	23097

950.68015	2	23099

950.8099	2	23102

950.93965	2	23104

951.0694	10	23114

951.19915	5	23119

951.3289	14	23133

951.45865	14	23147

951.5884	2	23150

951.71815	2	23152

951.8479	5	23157

951.97766	5	23162

952.10741	2	23164

952.23716	2	23167

952.36691	5	23171

952.49666	2	23174

952.62641	2	23176

952.75616	5	23181

952.88591	2	23183

953.01566	5	23188

953.14541	7	23195

953.27516	5	23200

953.40491	17	23217

953.53466	7	23224

953.66441	7	23231

953.79416	10	23241

953.92391	14	23255

954.05366	19	23275

954.18341	2	23277

954.31316	2	23279

954.44291	7	23287

954.57266	2	23289

954.70241	2	23291

954.83216	2	23294

954.96191	2	23296

955.09166	2	23299

955.22141	5	23303

955.35116	5	23308

955.48091	14	23323

955.61066	2	23325

955.74041	10	23335

955.87016	2	23337

955.99991	2	23339

956.12966	7	23347

956.25941	2	23349

956.38916	2	23351

956.51891	2	23354

956.64866	7	23361

956.77841	2	23363

956.90816	5	23368

957.03792	2	23371

957.16767	2	23373

957.29742	5	23378

957.42717	5	23383

957.55692	5	23387

957.68667	5	23392

957.81642	5	23397

957.94617	2	23399

958.07592	10	23409

958.20567	17	23426

958.33542	2	23428

958.46517	5	23433

958.59492	7	23440

958.72467	2	23443

958.85442	2	23445

958.98417	2	23447

959.11392	5	23452

959.24367	2	23455

959.37342	12	23467

959.50317	2	23469

959.63292	2	23471

959.76267	12	23483

959.89242	5	23488

960.02217	2	23491

960.15192	2	23493

960.28167	5	23498

960.41142	2	23500

960.54117	2	23503

960.67092	2	23505

960.80067	5	23510

960.93042	2	23512

961.06017	2	23515

961.18992	2	23517

961.31967	2	23519

961.44942	5	23524

961.57917	12	23536

961.70892	14	23551

961.83867	14	23565

961.96843	29	23594

962.09818	2	23596

962.22793	2	23599

962.35768	2	23601

962.48743	2	23603

962.61718	2	23606

962.74693	2	23608

962.87668	5	23613

963.00643	5	23618

963.13618	5	23623

963.26593	2	23625

963.39568	2	23627

963.52543	2	23630

963.65518	5	23635

963.78493	5	23639

963.91468	2	23642

964.04443	12	23654

964.17418	10	23663

964.30393	7	23671

964.43368	2	23673

964.56343	2	23675

964.69318	2	23678

964.82293	2	23680

964.95268	5	23685

965.08243	2	23687

965.21218	5	23692

965.34193	7	23699

965.47168	2	23702

965.60143	2	23704

965.73118	2	23707

965.86093	2	23709

965.99068	10	23719

966.12043	24	23743

966.25018	2	23745

966.37993	5	23750

966.50968	2	23752

966.63943	2	23755

966.76918	5	23759

966.89893	2	23762

967.02869	2	23764

967.15844	2	23767

967.28819	12	23779

967.41794	7	23786

967.54769	2	23788

967.67744	2	23791

967.80719	2	23793

967.93694	2	23795

968.06669	2	23798

968.19644	2	23800

968.32619	2	23803

968.45594	2	23805

968.58569	2	23807

968.71544	7	23815

968.84519	5	23819

968.97494	2	23822

969.10469	5	23827

969.23444	5	23831

969.36419	2	23834

969.49394	2	23836

969.62369	2	23839

969.75344	2	23841

969.88319	7	23848

970.01294	5	23853

970.14269	14	23867

970.27244	5	23872

970.40219	2	23875

970.53194	2	23877

970.66169	2	23879

970.79144	2	23882

970.92119	7	23889

971.05094	2	23891

971.18069	2	23894

971.31044	5	23899

971.44019	2	23901

971.56994	2	23903

971.69969	2	23906

971.82944	2	23908

971.9592	2	23911

972.08895	2	23913

972.2187	5	23918

972.34845	2	23920

972.4782	2	23923

972.60795	2	23925

972.7377	14	23939

972.86745	5	23944

972.9972	5	23949

973.12695	2	23951

973.2567	2	23954

973.38645	5	23959

973.5162	2	23961

973.64595	2	23963

973.7757	2	23966

973.90545	2	23968

974.0352	7	23975

974.16495	2	23978

974.2947	2	23980

974.42445	10	23990

974.5542	2	23992

974.68395	10	24002

974.8137	2	24004

974.94345	10	24014

975.0732	5	24019

975.20295	7	24026

975.3327	2	24028

975.46245	2	24031

975.5922	2	24033

975.72195	2	24035

975.8517	5	24040

975.98145	2	24043

976.1112	5	24047

976.24095	5	24052

976.3707	2	24055

976.50045	2	24057

976.6302	2	24059

976.75995	10	24069

976.8897	10	24079

977.01946	2	24081

977.14921	2	24083

977.27896	2	24086

977.40871	2	24088

977.53846	2	24091

977.66821	5	24095

977.79796	10	24105

977.92771	2	24107

978.05746	2	24110

978.18721	12	24122

978.31696	2	24124

978.44671	2	24127

978.57646	5	24131

978.70621	2	24134

978.83596	2	24136

978.96571	2	24139

979.09546	2	24141

979.22521	2	24143

979.35496	7	24151

979.48471	2	24153

979.61446	2	24155

979.74421	19	24175

979.87396	5	24179

980.00371	5	24184

980.13346	5	24189

980.26321	2	24191

980.39296	5	24196

980.52271	10	24206

980.65246	2	24208

980.78221	7	24215

980.91196	17	24232

981.04171	7	24239

981.17146	2	24242

981.30121	2	24244

981.43096	2	24247

981.56071	2	24249

981.69046	10	24259

981.82021	7	24266

981.94997	10	24275

982.07972	2	24278

982.20947	5	24283

982.33922	2	24285

982.46897	5	24290

982.59872	5	24295

982.72847	2	24297

982.85822	10	24307

982.98797	2	24309

983.11772	2	24311

983.24747	2	24314

983.37722	7	24321

983.50697	2	24323

983.63672	7	24331

983.76647	2	24333

983.89622	2	24335

984.02597	2	24338

984.15572	2	24340

984.28547	2	24343

984.41522	2	24345

984.54497	5	24350

984.67472	5	24355

984.80447	7	24362

984.93422	2	24364

985.06397	5	24369

985.19372	2	24371

985.32347	2	24374

985.45322	2	24376

985.58297	5	24381

985.71272	2	24383

985.84247	7	24391

985.97222	14	24405

986.10197	2	24407

986.23172	2	24410

986.36147	5	24415

986.49122	7	24422

986.62097	2	24424

986.75072	5	24429

986.88047	2	24431

987.01023	5	24436

987.13998	2	24439

987.26973	2	24441

987.39948	2	24443

987.52923	2	24446

987.65898	14	24460

987.78873	7	24467

987.91848	5	24472

988.04823	2	24475

988.17798	10	24484

988.30773	5	24489

988.43748	7	24496

988.56723	2	24499

988.69698	5	24503

988.82673	5	24508

988.95648	2	24511

989.08623	2	24513

989.21598	2	24515

989.34573	2	24518

989.47548	5	24523

989.60523	2	24525

989.73498	7	24532

989.86473	2	24535

989.99448	2	24537

990.12423	10	24547

990.25398	26	24573

990.38373	5	24578

990.51348	7	24585

990.64323	2	24587

990.77298	2	24590

990.90273	12	24602

991.03248	2	24604

991.16223	5	24609

991.29198	2	24611

991.42173	5	24616

991.55148	12	24628

991.68123	7	24635

991.81098	7	24643

991.94074	7	24650

992.07049	2	24652

992.20024	2	24655

992.32999	2	24657

992.45974	2	24659

992.58949	2	24662

992.71924	5	24667

992.84899	2	24669

992.97874	2	24671

993.10849	2	24674

993.23824	5	24679

993.36799	5	24683

993.49774	2	24686

993.62749	2	24688

993.75724	2	24691

993.88699	5	24695

994.01674	2	24698

994.14649	5	24703

994.27624	5	24707

994.40599	10	24717

994.53574	5	24722

994.66549	2	24724

994.79524	7	24731

994.92499	5	24736

995.05474	7	24743

995.18449	2	24746

995.31424	2	24748

995.44399	12	24760

995.57374	12	24772

995.70349	2	24775

995.83324	2	24777

995.96299	10	24787

996.09274	2	24789

996.22249	2	24791

996.35224	2	24794

996.48199	7	24801

996.61174	17	24818

996.74149	5	24823

996.87124	2	24825

997.001	2	24827

997.13075	2	24830

997.2605	2	24832

997.39025	14	24847

997.52	5	24851

997.64975	10	24861

997.7795	7	24868

997.90925	19	24887

998.039	2	24890

998.16875	5	24895

998.2985	12	24907

998.42825	2	24909

998.558	7	24916

998.68775	2	24919

998.8175	2	24921

998.94725	2	24923

999.077	2	24926

999.20675	7	24933

999.3365	2	24935

999.46625	10	24945

999.596	5	24950

999.72575	7	24957

999.8555	5	24962

999.98525	2	24964

1000.115	2	24967

1000.24475	2	24969

1000.3745	26	24995

1000.50425	2	24998

1000.634	7	25005

1000.76375	2	25007

1000.8935	2	25010

1001.02325	2	25012

1001.153	7	25019

1001.28275	5	25024

1001.4125	14	25039

1001.54225	2	25041

1001.672	24	25065

1001.80176	10	25075

1001.93151	5	25079

1002.06126	5	25084

1002.19101	2	25087

1002.32076	2	25089

1002.45051	7	25096

1002.58026	2	25099

1002.71001	5	25103

1002.83976	2	25106

1002.96951	2	25108

1003.09926	2	25111

1003.22901	5	25115

1003.35876	2	25118

1003.48851	5	25123

1003.61826	5	25127

1003.74801	2	25130

1003.87776	5	25135

1004.00751	7	25142

1004.13726	2	25144

1004.26701	19	25163

1004.39676	2	25166

1004.52651	12	25178

1004.65626	2	25180

1004.78601	7	25187

1004.91576	7	25195

1005.04551	5	25199

1005.17526	10	25209

1005.30501	29	25238

1005.43476	5	25243

1005.56451	5	25247

1005.69426	2	25250

1005.82401	10	25259

1005.95376	2	25262

1006.08351	7	25269

1006.21326	5	25274

1006.34301	10	25283

1006.47276	2	25286

1006.60251	5	25291

1006.73227	2	25293

1006.86202	5	25298

1006.99177	5	25303

1007.12152	10	25312

1007.25127	10	25322

1007.38102	22	25343

1007.51077	2	25346

1007.64052	2	25348

1007.77027	2	25351

1007.90002	14	25365

1008.02977	2	25367

1008.15952	2	25370

1008.28927	2	25372

1008.41902	2	25375

1008.54877	2	25377

1008.67852	2	25379

1008.80827	2	25382

1008.93802	7	25389

1009.06777	5	25394

1009.19752	2	25396

1009.32727	5	25401

1009.45702	2	25403

1009.58677	5	25408

1009.71652	10	25418

1009.84627	2	25420

1009.97602	2	25423

1010.10577	2	25425

1010.23552	7	25432

1010.36527	2	25435

1010.49502	2	25437

1010.62477	5	25442

1010.75452	2	25444

1010.88427	10	25454

1011.01402	2	25456

1011.14377	5	25461

1011.27352	2	25463

1011.40327	2	25466

1011.53302	2	25468

1011.66277	2	25471

1011.79253	7	25478

1011.92228	2	25480

1012.05203	14	25495

1012.18178	2	25497

1012.31153	5	25502

1012.44128	2	25504

1012.57103	7	25511

1012.70078	2	25514

1012.83053	2	25516

1012.96028	2	25519

1013.09003	2	25521

1013.21978	5	25526

1013.34953	5	25531

1013.47928	10	25540

1013.60903	7	25547

1013.73878	2	25550

1013.86853	2	25552

1013.99828	2	25555

1014.12803	5	25559

1014.25778	2	25562

1014.38753	2	25564

1014.51728	2	25567

1014.64703	2	25569

1014.77678	2	25571

1014.90653	5	25576

1015.03628	2	25579

1015.16603	10	25588

1015.29578	7	25595

1015.42553	2	25598

1015.55528	5	25603

1015.68503	5	25607

1015.81478	2	25610

1015.94453	2	25612

1016.07428	2	25615

1016.20403	2	25617

1016.33378	2	25619

1016.46353	5	25624

1016.59328	5	25629

1016.72304	10	25639

1016.85279	10	25648

1016.98254	2	25651

1017.11229	5	25655

1017.24204	12	25667

1017.37179	2	25670

1017.50154	2	25672

1017.63129	2	25675

1017.76104	2	25677

1017.89079	2	25679

1018.02054	5	25684

1018.15029	2	25687

1018.28004	5	25691

1018.40979	2	25694

1018.53954	5	25699

1018.66929	2	25701

1018.79904	7	25708

1018.92879	2	25711

1019.05854	2	25713

1019.18829	7	25720

1019.31804	2	25723

1019.44779	5	25727

1019.57754	2	25730

1019.70729	2	25732

1019.83704	7	25739

1019.96679	10	25749

1020.09654	2	25751

1020.22629	2	25754

1020.35604	2	25756

1020.48579	5	25761

1020.61554	2	25763

1020.74529	5	25768

1020.87504	2	25771

1021.00479	5	25775

1021.13454	2	25778

1021.26429	5	25783

1021.39404	2	25785

1021.52379	2	25787

1021.65354	10	25797

1021.7833	19	25816

1021.91305	5	25821

1022.0428	2	25823

1022.17255	5	25828

1022.3023	2	25831

1022.43205	2	25833

1022.5618	2	25835

1022.69155	2	25838

1022.8213	2	25840

1022.95105	7	25847

1023.0808	2	25850

1023.21055	2	25852

1023.3403	10	25862

1023.47005	7	25869

1023.5998	2	25871

1023.72955	24	25895

1023.8593	7	25903

1023.98905	2	25905

1024.1188	5	25910

1024.24855	7	25917

1024.3783	2	25919

1024.50805	2	25922

1024.6378	2	25924

1024.76755	2	25927

1024.8973	7	25934

1025.02705	12	25946

1025.1568	2	25948

1025.28655	5	25953

1025.4163	5	25958

1025.54605	5	25963

1025.6758	5	25967

1025.80555	10	25977

1025.9353	7	25984

1026.06505	12	25996

1026.1948	2	25999

1026.32455	7	26006

1026.4543	10	26015

1026.58405	5	26020

1026.71381	2	26023

1026.84355	2	26025

1026.97331	2	26027

1027.10306	2	26030

1027.23281	2	26032

1027.36256	5	26037

1027.49231	5	26042

1027.62206	5	26047

1027.75181	2	26049

1027.88156	5	26054

1028.01131	12	26066

1028.14106	5	26071

1028.27081	2	26073

1028.40056	5	26078

1028.53031	2	26080

1028.66006	2	26083

1028.78981	2	26085

1028.91956	7	26092

1029.04931	2	26095

1029.17906	2	26097

1029.30881	10	26107

1029.43856	2	26109

1029.56831	7	26116

1029.69806	2	26119

1029.82781	2	26121

1029.95756	5	26126

1030.08731	26	26152

1030.21706	7	26159

1030.34681	2	26162

1030.47656	2	26164

1030.60631	2	26167

1030.73606	2	26169

1030.86581	2	26171

1030.99556	5	26176

1031.12531	2	26179

1031.25506	5	26183

1031.38481	2	26186

1031.51456	7	26193

1031.64431	7	26200

1031.77407	5	26205

1031.90381	2	26207

1032.03357	10	26217

1032.16332	5	26222

1032.29307	2	26224

1032.42282	10	26234

1032.55257	14	26248

1032.68232	5	26253

1032.81207	2	26255

1032.94182	5	26260

1033.07157	5	26265

1033.20132	2	26267

1033.33107	7	26275

1033.46082	10	26284

1033.59057	5	26289

1033.72032	2	26291

1033.85007	2	26294

1033.97982	7	26301

1034.10957	2	26303

1034.23932	2	26306

1034.36907	2	26308

1034.49882	10	26318

1034.62857	7	26325

1034.75832	2	26327

1034.88807	2	26330

1035.01782	7	26337

1035.14757	2	26339

1035.27732	5	26344

1035.40707	2	26347

1035.53682	5	26351

1035.66657	2	26354

1035.79632	5	26359

1035.92607	2	26361

1036.05582	2	26363

1036.18557	2	26366

1036.31532	2	26368

1036.44507	5	26373

1036.57482	5	26378

1036.70458	2	26380

1036.83433	5	26385

1036.96408	12	26397

1037.09382	2	26399

1037.22358	5	26404

1037.35333	2	26407

1037.48308	12	26419

1037.61283	2	26421

1037.74258	2	26423

1037.87233	7	26431

1038.00208	2	26433

1038.13183	10	26443

1038.26158	2	26445

1038.39133	2	26447

1038.52108	2	26450

1038.65083	2	26452

1038.78058	2	26455

1038.91033	5	26459

1039.04008	2	26462

1039.16983	2	26464

1039.29958	2	26467

1039.42933	2	26469

1039.55908	5	26474

1039.68883	2	26476

1039.81858	5	26481

1039.94833	7	26488

1040.07808	2	26491

1040.20783	2	26493

1040.33758	7	26500

1040.46733	2	26503

1040.59708	2	26505

1040.72683	2	26507

1040.85658	2	26510

1040.98633	2	26512

1041.11608	14	26527

1041.24583	2	26529

1041.37558	2	26531

1041.50533	5	26536

1041.63508	2	26539

1041.76483	5	26543

1041.89459	10	26553

1042.02434	2	26555

1042.15408	5	26560

1042.28384	2	26563

1042.41359	7	26570

1042.54334	10	26579

1042.67309	2	26582

1042.80284	2	26584

1042.93259	2	26587

1043.06234	5	26591

1043.19209	5	26596

1043.32184	2	26599

1043.45159	2	26601

1043.58134	17	26618

1043.71109	2	26620

1043.84084	26	26647

1043.97059	5	26651

1044.10034	2	26654

1044.23009	2	26656

1044.35984	10	26666

1044.48959	2	26668

1044.61934	5	26673

1044.74909	2	26675

1044.87884	5	26680

1045.00859	5	26685

1045.13834	5	26690

1045.26809	2	26692

1045.39784	2	26695

1045.52759	2	26697

1045.65734	2	26699

1045.78709	2	26702

1045.91684	2	26704

1046.04659	2	26707

1046.17634	5	26711

1046.30609	2	26714

1046.43584	10	26723

1046.56559	2	26726

1046.69535	5	26731

1046.82509	2	26733

1046.95485	10	26743

1047.0846	2	26745

1047.21435	10	26755

1047.3441	10	26764

1047.47385	2	26767

1047.6036	2	26769

1047.73335	2	26771

1047.8631	12	26783

1047.99285	19	26803

1048.1226	2	26805

1048.25235	2	26807

1048.3821	5	26812

1048.51185	5	26817

1048.6416	2	26819

1048.77135	5	26824

1048.9011	2	26827

1049.03085	14	26841

1049.1606	5	26846

1049.29035	2	26848

1049.4201	2	26851

1049.54985	5	26855

1049.6796	7	26863

1049.80935	2	26865

1049.9391	2	26867

1050.06885	5	26872

1050.1986	5	26877

1050.32835	2	26879

1050.4581	5	26884

1050.58785	2	26887

1050.7176	2	26889

1050.84735	2	26891

1050.9771	7	26899

1051.10685	2	26901

1051.2366	5	26906

1051.36635	2	26908

1051.4961	7	26915

1051.62585	2	26918

1051.75561	14	26932

1051.88535	2	26935

1052.0151	2	26937

1052.14486	5	26942

1052.27461	7	26949

1052.40436	2	26951

1052.53411	2	26954

1052.66386	2	26956

1052.79361	7	26963

1052.92336	5	26968

1053.05311	2	26971

1053.18286	2	26973

1053.31261	2	26975

1053.44236	2	26978

1053.57211	10	26987

1053.70186	5	26992

1053.83161	2	26995

1053.96136	5	26999

1054.09111	7	27007

1054.22086	2	27009

1054.35061	2	27011

1054.48036	7	27019

1054.61011	10	27028

1054.73986	9	27037

1054.86961	3	27040

1054.99936	3	27043

1055.12911	3	27046

1055.25886	3	27049

1055.38861	3	27052

1055.51836	3	27055

1055.64811	3	27058

1055.77786	6	27064

1055.90761	3	27067

1056.03736	3	27070

1056.16711	3	27073

1056.29686	3	27076

1056.42661	3	27079

1056.55636	36	27115

1056.68612	3	27118

1056.81587	3	27121

1056.94562	12	27133

1057.07536	3	27136

1057.20512	3	27139

1057.33487	6	27145

1057.46462	3	27148

1057.59437	30	27178

1057.72412	6	27184

1057.85387	3	27187

1057.98362	3	27190

1058.11337	3	27193

1058.24312	6	27199

1058.37287	3	27202

1058.50262	3	27205

1058.63237	3	27208

1058.76212	3	27211

1058.89187	6	27217

1059.02162	9	27226

1059.15137	3	27229

1059.28112	3	27232

1059.41087	6	27238

1059.54062	3	27241

1059.67037	9	27250

1059.80012	3	27253

1059.92987	9	27262

1060.05962	3	27265

1060.18937	15	27280

1060.31912	9	27289

1060.44887	3	27292

1060.57862	6	27298

1060.70837	3	27301

1060.83812	6	27307

1060.96787	9	27316

1061.09762	12	27328

1061.22737	6	27334

1061.35712	6	27340

1061.48687	18	27358

1061.61662	3	27361

1061.74637	6	27367

1061.87613	6	27373

1062.00588	3	27376

1062.13562	6	27382

1062.26538	3	27385

1062.39513	3	27388

1062.52488	3	27391

1062.65463	3	27394

1062.78438	3	27397

1062.91413	15	27412

1063.04388	6	27418

1063.17363	6	27424

1063.30338	3	27427

1063.43313	3	27430

1063.56288	3	27433

1063.69263	3	27436

1063.82238	9	27445

1063.95213	6	27451

1064.08188	18	27469

1064.21163	3	27472

1064.34138	3	27475

1064.47113	6	27481

1064.60088	6	27487

1064.73063	3	27490

1064.86038	3	27493

1064.99013	3	27496

1065.11988	3	27499

1065.24963	9	27508

1065.37938	3	27511

1065.50913	9	27520

1065.63888	6	27526

1065.76863	39	27565

1065.89838	3	27568

1066.02813	3	27571

1066.15788	6	27577

1066.28763	6	27583

1066.41738	9	27592

1066.54713	3	27595

1066.67689	3	27598

1066.80663	12	27610

1066.93639	3	27613

1067.06614	6	27619

1067.19589	3	27622

1067.32564	3	27625

1067.45539	6	27631

1067.58514	9	27640

1067.71489	9	27649

1067.84464	3	27652

1067.97439	3	27655

1068.10414	3	27658

1068.23389	6	27664

1068.36364	24	27688

1068.49339	3	27691

1068.62314	3	27694

1068.75289	3	27697

1068.88264	6	27703

1069.01239	3	27706

1069.14214	6	27712

1069.27189	3	27715

1069.40164	6	27721

1069.53139	3	27724

1069.66114	6	27730

1069.79089	3	27733

1069.92064	3	27736

1070.05039	6	27742

1070.18014	3	27745

1070.30989	9	27754

1070.43964	12	27766

1070.56939	3	27769

1070.69914	3	27772

1070.82889	6	27778

1070.95864	3	27781

1071.08839	3	27784

1071.21814	6	27790

1071.34789	3	27793

1071.47764	3	27796

1071.60739	3	27799

1071.73715	3	27802

1071.8669	3	27805

1071.99664	9	27814

1072.1264	3	27817

1072.25615	6	27823

1072.3859	3	27826

1072.51565	3	27829

1072.6454	9	27838

1072.77515	3	27841

1072.9049	3	27844

1073.03465	6	27850

1073.1644	3	27853

1073.29415	6	27859

1073.4239	3	27862

1073.55365	3	27865

1073.6834	3	27868

1073.81315	9	27877

1073.9429	6	27883

1074.07265	3	27886

1074.2024	9	27895

1074.33215	3	27898

1074.4619	15	27913

1074.59165	3	27916

1074.7214	6	27922

1074.85115	3	27925

1074.9809	3	27928

1075.11065	15	27943

1075.2404	3	27946

1075.37015	3	27949

1075.4999	6	27955

1075.62965	3	27958

1075.7594	6	27964

1075.88915	18	27982

1076.0189	3	27985

1076.14865	3	27988

1076.2784	3	27991

1076.40815	6	27997

1076.5379	6	28003

1076.66766	3	28006

1076.79741	6	28012

1076.92716	9	28021

1077.0569	3	28024

1077.18666	6	28030

1077.31641	3	28033

1077.44616	3	28036

1077.57591	3	28039

1077.70566	9	28048

1077.83541	9	28057

1077.96516	3	28060

1078.09491	3	28063

1078.22466	3	28066

1078.35441	6	28072

1078.48416	3	28075

1078.61391	3	28078

1078.74366	3	28081

1078.87341	39	28120

1079.00316	3	28123

1079.13291	3	28126

1079.26266	6	28132

1079.39241	9	28141

1079.52216	12	28153

1079.65191	6	28159

1079.78166	6	28165

1079.91141	3	28168

1080.04116	3	28171

1080.17091	3	28174

1080.30066	3	28177

1080.43041	3	28180

1080.56016	15	28195

1080.68991	3	28198

1080.81966	3	28201

1080.94941	12	28213

1081.07916	6	28219

1081.20891	3	28222

1081.33866	6	28228

1081.46841	3	28231

1081.59816	9	28240

1081.72791	3	28243

1081.85767	3	28246

1081.98742	3	28249

1082.11717	12	28261

1082.24692	3	28264

1082.37667	6	28270

1082.50642	3	28273

1082.63617	3	28276

1082.76592	3	28279

1082.89567	3	28282

1083.02542	3	28285

1083.15517	3	28288

1083.28492	3	28291

1083.41467	6	28297

1083.54442	3	28300

1083.67417	3	28303

1083.80392	3	28306

1083.93367	6	28312

1084.06342	3	28315

1084.19317	9	28324

1084.32292	6	28330

1084.45267	18	28348

1084.58242	18	28366

1084.71217	18	28384

1084.84192	3	28387

1084.97167	9	28396

1085.10142	6	28402

1085.23117	6	28408

1085.36092	9	28417

1085.49067	9	28426

1085.62042	6	28432

1085.75017	6	28438

1085.87992	3	28441

1086.00967	3	28444

1086.13942	9	28453

1086.26917	3	28456

1086.39892	3	28459

1086.52867	6	28465

1086.65843	3	28468

1086.78817	15	28483

1086.91793	6	28489

1087.04768	3	28492

1087.17743	6	28498

1087.30718	3	28501

1087.43693	3	28504

1087.56668	3	28507

1087.69643	3	28510

1087.82618	3	28513

1087.95593	3	28516

1088.08568	9	28525

1088.21543	3	28528

1088.34518	9	28537

1088.47493	3	28540

1088.60468	3	28543

1088.73443	3	28546

1088.86418	6	28552

1088.99393	18	28570

1089.12368	3	28573

1089.25343	3	28576

1089.38318	3	28579

1089.51293	9	28588

1089.64268	6	28594

1089.77243	6	28600

1089.90218	6	28606

1090.03193	6	28612

1090.16168	6	28618

1090.29143	6	28624

1090.42118	27	28651

1090.55093	6	28657

1090.68068	3	28660

1090.81043	6	28666

1090.94018	3	28669

1091.06993	39	28708

1091.19968	3	28711

1091.32943	3	28714

1091.45918	6	28720

1091.58893	6	28726

1091.71869	3	28729

1091.84844	9	28738

1091.97818	6	28744

1092.10794	12	28756

1092.23769	9	28765

1092.36744	6	28771

1092.49719	3	28774

1092.62694	3	28777

1092.75669	3	28780

1092.88644	9	28789

1093.01619	3	28792

1093.14594	3	28795

1093.27569	3	28798

1093.40544	3	28801

1093.53519	9	28810

1093.66494	3	28813

1093.79469	12	28825

1093.92444	9	28834

1094.05419	3	28837

1094.18394	3	28840

1094.31369	6	28846

1094.44344	6	28852

1094.57319	3	28855

1094.70294	9	28864

1094.83269	9	28873

1094.96244	9	28882

1095.09219	3	28885

1095.22194	6	28891

1095.35169	3	28894

1095.48144	3	28897

1095.61119	6	28903

1095.74094	3	28906

1095.87069	3	28909

1096.00044	3	28912

1096.13019	6	28918

1096.25994	6	28924

1096.38969	3	28927

1096.51944	3	28930

1096.6492	3	28933

1096.77895	30	28963

1096.9087	3	28966

1097.03844	3	28969

1097.1682	9	28978

1097.29795	3	28981

1097.4277	6	28987

1097.55745	6	28993

1097.6872	3	28996

1097.81695	9	29005

1097.9467	3	29008

1098.07645	3	29011

1098.2062	6	29017

1098.33595	6	29023

1098.4657	3	29026

1098.59545	9	29035

1098.7252	6	29041

1098.85495	3	29044

1098.9847	3	29047

1099.11445	12	29059

1099.2442	3	29062

1099.37395	9	29071

1099.5037	3	29074

1099.63345	3	29077

1099.7632	3	29080

1099.89295	6	29086

1100.0227	3	29089

1100.15245	3	29092

1100.2822	3	29095

1100.41195	6	29101

1100.5417	3	29104

1100.67145	3	29107

1100.8012	6	29113

1100.93095	3	29116

1101.0607	3	29119

1101.19045	3	29122

1101.3202	3	29125

1101.44995	3	29128

1101.5797	3	29131

1101.70945	3	29134

1101.83921	24	29158

1101.96896	3	29161

1102.09871	3	29164

1102.22846	3	29167

1102.35821	3	29170

1102.48796	6	29176

1102.61771	3	29179

1102.74746	6	29185

1102.87721	3	29188

1103.00696	3	29191

1103.13671	3	29194

1103.26646	6	29200

1103.39621	3	29203

1103.52596	6	29209

1103.65571	24	29233

1103.78546	12	29245

1103.91521	3	29248

1104.04496	3	29251

1104.17471	12	29263

1104.30446	3	29266

1104.43421	3	29269

1104.56396	6	29275

1104.69371	3	29278

1104.82346	3	29281

1104.95321	3	29284

1105.08296	9	29293

1105.21271	3	29296

1105.34246	21	29317

1105.47221	3	29320

1105.60196	3	29323

1105.73171	18	29341

1105.86146	6	29347

1105.99121	3	29350

1106.12096	6	29356

1106.25071	12	29368

1106.38046	6	29374

1106.51021	12	29386

1106.63997	6	29392

1106.76971	9	29401

1106.89947	6	29407

1107.02922	3	29410

1107.15897	3	29413

1107.28872	3	29416

1107.41847	6	29422

1107.54822	3	29425

1107.67797	3	29428

1107.80772	3	29431

1107.93747	3	29434

1108.06722	3	29437

1108.19697	6	29443

1108.32672	15	29458

1108.45647	6	29464

1108.58622	3	29467

1108.71597	3	29470

1108.84572	6	29476

1108.97547	3	29479

1109.10522	3	29482

1109.23497	6	29488

1109.36472	30	29518

1109.49447	3	29521

1109.62422	6	29527

1109.75397	3	29530

1109.88372	6	29536

1110.01347	3	29539

1110.14322	3	29542

1110.27297	3	29545

1110.40272	12	29557

1110.53247	6	29563

1110.66222	3	29566

1110.79197	6	29572

1110.92172	3	29575

1111.05147	3	29578

1111.18122	9	29587

1111.31097	3	29590

1111.44072	3	29593

1111.57047	18	29611

1111.70023	3	29614

1111.82998	3	29617

1111.95972	6	29623

1112.08948	3	29626

1112.21923	3	29629

1112.34898	9	29638

1112.47873	3	29641

1112.60848	6	29647

1112.73823	3	29650

1112.86798	1	29651

1112.99773	1	29652

1113.12748	1	29653

1113.25723	2	29655

1113.38698	2	29657

1113.51673	2	29659

1113.64648	1	29660

1113.77623	4	29664

1113.90598	6	29670

1114.03573	2	29672

1114.16548	2	29674

1114.29523	1	29675

1114.42498	1	29676

1114.55473	1	29677

1114.68448	2	29679

1114.81423	2	29681

1114.94398	1	29682

1115.07373	2	29684

1115.20348	6	29690

1115.33323	1	29691

1115.46298	3	29694

1115.59273	14	29708

1115.72248	1	29709

1115.85223	1	29710

1115.98198	1	29711

1116.11173	2	29713

1116.24148	17	29730

1116.37123	1	29731

1116.50098	2	29733

1116.63074	1	29734

1116.76049	2	29736

1116.89024	1	29737

1117.01998	1	29738

1117.14974	1	29739

1117.27949	2	29741

1117.40924	2	29743

1117.53899	1	29744

1117.66874	3	29747

1117.79849	4	29751

1117.92824	1	29752

1118.05799	5	29757

1118.18774	1	29758

1118.31749	1	29759

1118.44724	2	29761

1118.57699	1	29762

1118.70674	2	29764

1118.83649	1	29765

1118.96624	2	29767

1119.09599	1	29768

1119.22574	1	29769

1119.35549	1	29770

1119.48524	1	29771

1119.61499	1	29772

1119.74474	2	29774

1119.87449	1	29775

1120.00424	2	29777

1120.13399	1	29778

1120.26374	1	29779

1120.39349	2	29781

1120.52324	1	29782

1120.65299	4	29786

1120.78274	1	29787

1120.91249	1	29788

1121.04224	2	29790

1121.17199	1	29791

1121.30174	1	29792

1121.43149	7	29799

1121.56124	1	29800

1121.69099	1	29801

1121.82075	1	29802

1121.9505	1	29803

1122.08025	1	29804

1122.21	1	29805

1122.33975	1	29806

1122.4695	1	29807

1122.59925	32	29839

1122.729	1	29840

1122.85875	38	29878

1122.9885	2	29880

1123.11825	1	29881

1123.248	22	29903

1123.37775	1	29904

1123.5075	1	29905

1123.63725	5	29910

1123.767	1	29911

1123.89675	1	29912

1124.0265	3	29915

1124.15625	8	29923

1124.286	2	29925

1124.41575	4	29929

1124.5455	1	29930

1124.67525	1	29931

1124.805	2	29933

1124.93475	3	29936

1125.0645	1	29937

1125.19425	2	29939

1125.324	1	29940

1125.45375	1	29941

1125.5835	32	29973

1125.71325	1	29974

1125.843	38	30012

1125.97275	2	30014

1126.1025	1	30015

1126.23225	22	30037

1126.362	1	30038

1126.49175	1	30039

1126.62151	1	30040

1126.75125	1	30041

1126.88101	22	30063

1127.01076	1	30064

1127.14051	1	30065

1127.27026	32	30097

1127.40001	1	30098

1127.52976	38	30136

1127.65951	2	30138

1127.78926	1	30139

1127.91901	22	30161

1128.04876	1	30162

1128.17851	1	30163

1128.30826	32	30195

1128.43801	1	30196

1128.56776	38	30234

1128.69751	2	30236

1128.82726	1	30237

1128.95701	1	30238

1129.08676	5	30243

1129.21651	1	30244

1129.34626	1	30245

1129.47601	32	30277

1129.60576	1	30278

1129.73551	1	30279

1129.86526	2	30281

1129.99501	1	30282

1130.12476	1	30283

1130.25451	1	30284

1130.38426	1	30285

1130.51401	2	30287

1130.64376	27	30314

1130.77351	1	30315

1130.90326	1	30316

1131.03301	1	30317

1131.16276	1	30318

1131.29251	1	30319

1131.42226	1	30320

1131.55201	1	30321

1131.68177	2	30323

1131.81152	2	30325

1131.94126	1	30326

1132.07102	1	30327

1132.20077	37	30364

1132.33052	4	30368

1132.46027	1	30369

1132.59002	3	30372

1132.71977	1	30373

1132.84952	1	30374

1132.97927	44	30418

1133.10902	5	30423

1133.23877	1	30424

1133.36852	1	30425

1133.49827	6	30431

1133.62802	1	30432

1133.75777	1	30433

1133.88752	1	30434

1134.01727	6	30440

1134.14702	1	30441

1134.27677	1	30442

1134.40652	1	30443

1134.53627	33	30476

1134.66602	1	30477

1134.79577	3	30480

1134.92552	1	30481

1135.05527	1	30482

1135.18502	1	30483

1135.31477	2	30485

1135.44452	6	30491

1135.57427	1	30492

1135.70402	1	30493

1135.83377	67	30560

1135.96352	2	30562

1136.09327	1	30563

1136.22302	4	30567

1136.35277	1	30568

1136.48252	2	30570

1136.61227	1	30571

1136.74203	4	30575

1136.87178	2	30577

1137.00152	1	30578

1137.13128	75	30653

1137.26103	3	30656

1137.39078	1	30657

1137.52053	1	30658

1137.65028	1	30659

1137.78003	1	30660

1137.90978	88	30748

1138.03953	1	30749

1138.16928	1	30750

1138.29903	1	30751

1138.42878	1	30752

1138.55853	3	30755

1138.68828	4	30759

1138.81803	2	30761

1138.94778	68	30829

1139.07753	1	30830

1139.20728	2	30832

1139.33703	1	30833

1139.46678	7	30840

1139.59653	4	30844

1139.72628	3	30847

1139.85603	14	30861

1139.98578	5	30866

1140.11553	7	30873

1140.24528	2	30875

1140.37503	4	30879

1140.50478	1	30880

1140.63453	2	30882

1140.76428	77	30959

1140.89403	1	30960

1141.02378	2	30962

1141.15353	1	30963

1141.28328	1	30964

1141.41303	55	31019

1141.54278	2	31021

1141.67253	1	31022

1141.80229	1	31023

1141.93204	1	31024

1142.06179	1	31025

1142.19154	2	31027

1142.32129	3	31030

1142.45104	3	31033

1142.58079	17	31050

1142.71054	2	31052

1142.84029	1	31053

1142.97004	11	31064

1143.09979	1	31065

1143.22954	1	31066

1143.35929	22	31088

1143.48904	1	31089

1143.61879	2	31091

1143.74854	1	31092

1143.87829	28	31120

1144.00804	4	31124

1144.13779	1	31125

1144.26754	4	31129

1144.39729	7	31136

1144.52704	2	31138

1144.65679	2	31140

1144.78654	1	31141

1144.91629	40	31181

1145.04604	3	31184

1145.17579	1	31185

1145.30554	2	31187

1145.43529	25	31212

1145.56504	7	31219

1145.69479	1	31220

1145.82454	2	31222

1145.95429	1	31223

1146.08404	3	31226

1146.21379	1	31227

1146.34354	11	31238

1146.47329	1	31239

1146.60304	2	31241

1146.73279	1	31242

1146.86255	23	31265

1146.9923	1	31266

1147.12205	2	31268

1147.2518	24	31292

1147.38155	2	31294

1147.5113	6	31300

1147.64105	2	31302

1147.7708	27	31329

1147.90055	46	31375

1148.0303	27	31402

1148.16005	1	31403

1148.2898	38	31441

1148.41955	7	31448

1148.5493	26	31474

1148.67905	24	31498

1148.8088	2	31500

1148.93855	6	31506

1149.0683	2	31508

1149.19805	27	31535

1149.3278	1	31536

1149.45755	38	31574

1149.5873	75	31649

1149.71705	26	31675

1149.8468	24	31699

1149.97655	59	31758

1150.1063	2	31760

1150.23605	5	31765

1150.3658	120	31885

1150.49555	15	31900

1150.6253	5	31905

1150.75505	119	32024

1150.8848	2	32026

1151.01455	9	32035

1151.1443	155	32190

1151.27405	3	32193

1151.4038	3	32196

1151.53355	12677	44873

1151.66331	1	44874

1151.79306	3	44877

1151.9228	1	44878

1152.05256	1	44879

1152.18231	18	44897

1152.31206	21	44918

1152.44181	5	44923

1152.57156	1	44924

1152.70131	2	44926

1152.83106	14	44940

1152.96081	3	44943

1153.09056	3	44946

1153.22031	1	44947

1153.35006	1	44948

1153.47981	1	44949

1153.60956	1	44950

1153.73931	3	44953

1153.86906	2	44955

1153.99881	3	44958

**（*e*）Non-hydraulic-90°**

1	0.00211	0.3124	0	0

2	0.00911	0.3984	1	1

3	0.0255	0.498	1	2

4	0.03643	0.5976	1	3

5	0.05283	0.6972	2	5

6	0.07651	0.7968	1	7

7	0.10201	0.8964	0	7

8	0.12752	0.996	6	13

9	0.15484	1.0956	0	13

10	0.1767	1.1952	1	14

11	0.19674	1.2948	1	15

12	0.21496	1.3944	0	15

13	0.23135	1.494	0	16

14	0.24957	1.5936	1	17

15	0.27325	1.6932	3	19

16	0.29329	1.7928	3	22

17	0.30604	1.8924	0	22

18	0.32608	1.992	3	26

19	0.34612	2.0916	4	30

20	0.36251	2.1912	3	32

21	0.38073	2.2908	0	33

22	0.39712	2.3904	7	40

23	0.41716	2.49	1	41

24	0.43902	2.5896	2	43

25	0.4536	2.6892	0	43

26	0.46999	2.7888	4	47

27	0.49003	2.8884	0	47

28	0.51007	2.988	0	48

29	0.52646	3.0876	0	48

30	0.54104	3.1872	7	55

31	0.5629	3.2868	2	56

32	0.58111	3.3864	1	58

33	0.60115	3.486	3	60

34	0.61755	3.5856	4	64

35	0.63576	3.6852	17	81

36	0.65398	3.7848	3	84

37	0.67584	3.8844	2	87

38	0.69041	3.984	10	97

39	0.70681	4.0836	0	97

40	0.72502	4.1832	0	97

41	0.74688	4.2828	5	102

42	0.7651	4.3824	0	103

43	0.77785	4.482	0	103

44	0.79789	4.5816	4	108

45	0.81793	4.6812	2	110

46	0.83797	4.7808	1	111

47	0.85436	4.8804	8	119

48	0.86894	4.98	1	120

49	0.8908	5.0796	2	122

50	0.90719	5.1792	4	126

51	0.92541	5.2788	3	129

52	0.9418	5.3784	13	141

53	0.9582	5.478	0	142

54	0.98006	5.5776	1	142

55	0.99827	5.6772	1	144

56	1.01467	5.7768	2	146

57	1.03289	5.8764	1	146

58	1.0511	5.976	2	148

59	1.07114	6.0756	0	148

60	1.08936	6.1752	1	150

61	1.10029	6.2748	1	151

62	1.12215	6.3744	3	154

63	1.14036	6.474	2	156

64	1.1604	6.5736	4	160

65	1.17498	6.6732	0	160

66	1.18955	6.7728	0	161

67	1.21687	6.8724	2	163

68	1.23509	6.972	2	165

69	1.25149	7.0716	2	167

70	1.26242	7.1712	2	169

71	1.28428	7.2708	2	171

72	1.30796	7.3704	1	172

73	1.32435	7.47	2	173

74	1.33893	7.5696	5	178

75	1.35714	7.6692	6	185

76	1.379	7.7688	2	186

77	1.39904	7.8684	2	189

78	1.41361	7.968	1	190

79	1.43001	8.0676	1	191

80	1.45187	8.1672	1	192

81	1.46826	8.2668	0	192

82	1.49012	8.3664	0	192

83	1.50288	8.466	1	193

84	1.52109	8.5656	0	193

85	1.54477	8.6652	0	194

86	1.56299	8.7648	1	195

87	1.57574	8.8644	1	196

88	1.59578	8.964	2	197

89	1.61764	9.0636	2	199

90	1.63586	9.1632	4	202

91	1.65043	9.2628	6	208

92	1.66683	9.3624	0	209

93	1.68869	9.462	1	210

94	1.71055	9.5616	1	211

95	1.72512	9.6612	3	214

96	1.73969	9.7608	1	215

97	1.76155	9.8604	4	220

98	1.78341	9.96	6	226

99	1.80345	10.0596	1	227

100	1.81074	10.1592	1	227

101	1.8326	10.2588	1	228

102	1.85264	10.3584	2	230

103	1.8745	10.458	10	239

104	1.88907	10.5576	0	239

105	1.90364	10.6572	2	241

106	1.9255	10.7568	2	243

107	1.95101	10.8564	3	246

108	1.96558	10.956	1	246

109	1.97833	11.0556	2	248

110	1.99655	11.1552	1	249

111	2.01659	11.2548	1	250

112	2.03663	11.3544	0	250

113	2.05302	11.454	1	251

114	2.07124	11.5536	4	256

115	2.08945	11.6532	2	258

116	2.10949	11.7528	1	258

117	2.12771	11.8524	8	266

118	2.14228	11.952	11	277

119	2.16232	12.0516	0	278

120	2.18236	12.1512	5	283

121	2.2024	12.2508	1	284

122	2.21879	12.3504	2	286

123	2.23337	12.45	2	287

124	2.2534	12.5496	10	297

125	2.27526	12.6492	17	315

126	2.29348	12.7488	1	316

127	2.30441	12.8484	1	317

128	2.32445	12.948	0	317

129	2.34813	13.0476	3	320

130	2.36453	13.1472	1	321

131	2.37728	13.2468	17	338

132	2.39732	13.3464	18	356

133	2.41918	13.446	1	357

134	2.43921	13.5456	22	379

135	2.45561	13.6452	5	384

136	2.46654	13.7448	8	392

137	2.4884	13.8444	1	393

138	2.51572	13.944	0	393

139	2.5303	14.0436	0	394

140	2.54123	14.1432	1	395

141	2.56127	14.2428	0	395

142	2.58495	14.3424	0	395

143	2.59952	14.442	2	397

144	2.61774	14.5416	1	398

145	2.63231	14.6412	0	398

146	2.65417	14.7408	2	400

147	2.67421	14.8404	1	401

148	2.69425	14.94	4	405

149	2.70336	15.0396	0	405

150	2.72704	15.1392	0	405

151	2.74708	15.2388	1	407

152	2.76529	15.3384	5	412

153	2.77804	15.438	0	412

154	2.79808	15.5376	13	425

155	2.81812	15.6372	1	426

156	2.83634	15.7368	12	438

157	2.85455	15.8364	0	438

158	2.87095	15.936	2	441

159	2.88917	16.0356	2	443

160	2.9092	16.1352	1	444

161	2.92924	16.2348	1	446

162	2.94199	16.3344	1	446

163	2.96203	16.434	12	458

164	2.98207	16.5336	6	464

165	3.00211	16.6332	8	472

166	3.01668	16.7328	0	472

167	3.0349	16.8324	6	479

168	3.05858	16.932	2	480

169	3.07498	17.0316	0	481

170	3.09137	17.1312	2	482

171	3.10777	17.2308	2	485

172	3.12598	17.3304	0	485

173	3.14784	17.43	0	485

174	3.16424	17.5296	4	489

175	3.18063	17.6292	0	489

176	3.19703	17.7288	3	492

177	3.21889	17.8284	6	498

178	3.23893	17.928	3	501

179	3.24986	18.0276	5	506

180	3.26989	18.1272	1	507

181	3.28811	18.2268	1	508

182	3.31179	18.3264	2	510

183	3.32637	18.426	2	512

184	3.33912	18.5256	6	517

185	3.36098	18.6252	0	518

186	3.38284	18.7248	0	518

187	3.40105	18.8244	0	518

188	3.41563	18.924	2	520

189	3.43384	19.0236	1	521

190	3.45388	19.1232	2	523

191	3.47392	19.2228	1	524

192	3.48667	19.3224	1	525

193	3.50125	19.422	1	526

194	3.52311	19.5216	1	527

195	3.54314	19.6212	2	529

196	3.56318	19.7208	1	529

197	3.57411	19.8204	0	530

198	3.59415	19.92	14	544

199	3.61601	20.0196	0	544

200	3.63423	20.1192	2	546

201	3.6488	20.2188	0	547

202	3.66337	20.3184	1	548

203	3.68523	20.418	0	548

204	3.70527	20.5176	1	549

205	3.72167	20.6172	3	552

206	3.73624	20.7168	1	553

207	3.75628	20.8164	0	553

208	3.77632	20.916	0	554

209	3.79271	21.0156	0	554

210	3.80729	21.1152	1	555

211	3.8255	21.2148	0	556

212	3.84919	21.3144	0	556

213	3.8674	21.414	0	556

214	3.88015	21.5136	6	562

215	3.89837	21.6132	0	562

216	3.91659	21.7128	3	565

217	3.93845	21.8124	2	567

218	3.95302	21.912	4	571

219	3.96759	22.0116	7	578

220	3.98763	22.1112	1	579

221	4.01131	22.2108	4	583

222	4.03135	22.3104	1	584

223	4.04228	22.41	0	584

224	4.05686	22.5096	0	585

225	4.07872	22.6092	0	585

226	4.10058	22.7088	0	586

227	4.11333	22.8084	0	586

228	4.12972	22.908	6	592

229	4.15705	23.0076	0	592

230	4.17344	23.1072	0	593

231	4.19166	23.2068	1	594

232	4.20441	23.3064	7	601

233	4.22263	23.406	12	613

234	4.24631	23.5056	2	615

235	4.2627	23.6052	4	619

236	4.27728	23.7048	2	621

237	4.29185	23.8044	1	622

238	4.31735	23.904	2	624

239	4.33739	24.0036	0	624

240	4.35014	24.1032	0	625

241	4.36472	24.2028	4	628

242	4.38658	24.3024	0	628

243	4.40662	24.402	0	629

244	4.42301	24.5016	1	630

245	4.43758	24.6012	0	630

246	4.45398	24.7008	0	630

247	4.47766	24.8004	3	634

248	4.4977	24.9	0	634

249	4.51592	24.9996	0	634

250	4.53413	25.0992	0	634

251	4.55417	25.1988	0	635

252	4.57421	25.2984	2	637

253	4.58878	25.398	0	637

254	4.60518	25.4976	1	638

255	4.62522	25.5972	0	638

256	4.64343	25.6968	0	638

257	4.66347	25.7964	7	645

258	4.6744	25.896	1	646

259	4.69444	25.9956	0	646

260	4.71994	26.0952	3	649

261	4.73634	26.1948	6	655

262	4.75273	26.2944	1	657

263	4.76548	26.394	3	660

264	4.78552	26.4936	2	662

265	4.80738	26.5932	2	664

266	4.82378	26.6928	3	667

267	4.83653	26.7924	1	668

268	4.85292	26.892	2	670

269	4.87843	26.9916	1	671

270	4.90029	27.0912	6	677

271	4.91122	27.1908	1	678

272	4.92761	27.2904	1	679

273	4.94947	27.39	3	682

274	4.97315	27.4896	0	682

275	4.98591	27.5892	1	683

276	4.99866	27.6888	2	685

277	5.0187	27.7884	1	686

278	5.04238	27.888	0	686

279	5.05877	27.9876	1	688

280	5.07152	28.0872	0	688

281	5.09338	28.1868	0	688

282	5.11524	28.2864	2	690

283	5.13164	28.386	0	690

284	5.14621	28.4856	2	692

285	5.16261	28.5852	1	693

286	5.18447	28.6848	0	693

287	5.20633	28.7844	3	696

288	5.2209	28.884	1	697

289	5.2373	28.9836	2	699

290	5.25733	29.0832	0	699

291	5.27919	29.1828	0	700

292	5.29741	29.2824	0	700

293	5.31198	29.382	1	701

294	5.32838	29.4816	3	704

295	5.34842	29.5812	2	706

296	5.3721	29.6808	2	708

297	5.38303	29.7804	0	708

298	5.40125	29.88	2	710

299	5.42311	29.9796	1	711

300	5.44861	30.0792	5	716

301	5.45954	30.1788	1	717

302	5.47411	30.2784	9	727

303	5.49233	30.378	2	728

304	5.51783	30.4776	6	734

305	5.53423	30.5772	0	735

306	5.54698	30.6768	1	736

307	5.56884	30.7764	0	736

308	5.5907	30.876	2	739

309	5.61074	30.9756	1	739

310	5.62349	31.0752	0	740

311	5.63806	31.1748	1	740

312	5.66174	31.2744	1	742

313	5.68178	31.374	0	742

314	5.69453	31.4736	1	743

315	5.71093	31.5732	1	744

316	5.73097	31.6728	1	744

317	5.75283	31.7724	1	745

318	5.77287	31.872	3	748

319	5.78744	31.9716	1	750

320	5.80384	32.0712	1	751

321	5.82934	32.1708	0	751

322	5.84573	32.2704	0	751

323	5.85849	32.37	0	752

324	5.8767	32.4696	1	753

325	5.89674	32.5692	11	763

326	5.9186	32.6688	8	771

327	5.93317	32.7684	8	779

328	5.94957	32.868	1	780

329	5.97143	32.9676	5	785

330	5.99147	33.0672	0	785

331	6.00968	33.1668	0	785

332	6.02244	33.2664	1	786

333	6.04065	33.366	2	788

334	6.06251	33.4656	0	788

335	6.08255	33.5652	2	790

336	6.09712	33.6648	1	791

337	6.11352	33.7644	0	792

338	6.13902	33.864	0	792

339	6.15906	33.9636	2	794

340	6.17363	34.0632	2	796

341	6.19003	34.1628	0	796

342	6.20825	34.2624	0	797

343	6.22828	34.362	1	798

344	6.24832	34.4616	2	800

345	6.26107	34.5612	0	801

346	6.28111	34.6608	1	802

347	6.30297	34.7604	1	802

348	6.31937	34.86	1	804

349	6.33212	34.9596	3	806

350	6.35034	35.0592	0	806

351	6.37402	35.1588	3	810

352	6.39041	35.2584	2	812

353	6.41045	35.358	0	812

354	6.4232	35.4576	3	815

355	6.44506	35.5572	2	817

356	6.46692	35.6568	0	817

357	6.48332	35.7564	4	821

358	6.49971	35.856	1	822

359	6.51975	35.9556	8	830

360	6.54161	36.0552	6	837

361	6.55983	36.1548	7	844

362	6.56894	36.2544	15	859

363	6.58897	36.354	0	859

364	6.61266	36.4536	1	860

365	6.63269	36.5532	1	861

366	6.64909	36.6528	0	861

367	6.66366	36.7524	1	862

368	6.6837	36.852	2	864

369	6.70738	36.9516	0	864

370	6.7256	37.0512	0	865

371	6.73835	37.1508	7	872

372	6.75292	37.2504	0	872

373	6.77843	37.35	1	873

374	6.79482	37.4496	1	874

375	6.8094	37.5492	0	874

376	6.82761	37.6488	1	875

377	6.84583	37.7484	3	878

378	6.87133	37.848	19	897

379	6.88591	37.9476	4	901

380	6.90048	38.0472	1	902

381	6.9187	38.1468	0	902

382	6.94056	38.2464	1	903

383	6.95877	38.346	3	906

384	6.97517	38.4456	1	907

385	6.98974	38.5452	1	908

386	7.01524	38.6448	3	910

387	7.03528	38.7444	0	911

388	7.04803	38.844	1	912

389	7.06261	38.9436	2	914

390	7.08629	39.0432	1	915

391	7.10633	39.1428	1	916

392	7.12272	39.2424	1	917

393	7.1373	39.342	4	921

394	7.15916	39.4416	2	924

395	7.17919	39.5412	0	924

396	7.19741	39.6408	2	925

397	7.21198	39.7404	3	929

398	7.22656	39.84	1	930

399	7.25206	39.9396	1	931

400	7.2721	40.0392	2	933

401	7.28485	40.1388	0	933

402	7.30125	40.2384	0	934

403	7.32493	40.338	9	942

404	7.34314	40.4376	0	943

405	7.35772	40.5372	2	944

406	7.37776	40.6368	3	947

407	7.39415	40.7364	0	947

408	7.41965	40.836	0	948

409	7.43241	40.9356	2	950

410	7.44516	41.0352	0	950

411	7.4652	41.1348	1	951

412	7.48888	41.2344	2	952

413	7.50892	41.334	1	953

414	7.52167	41.4336	1	954

415	7.53806	41.5332	6	960

416	7.56357	41.6328	3	962

417	7.5836	41.7324	0	963

418	7.59818	41.832	1	964

419	7.61457	41.9316	9	972

420	7.63097	42.0312	5	977

421	7.65283	42.1308	3	980

422	7.67105	42.2304	2	982

423	7.6838	42.33	0	983

424	7.70201	42.4296	2	985

425	7.72752	42.5292	9	994

426	7.74391	42.6288	0	994

427	7.75849	42.7284	0	994

428	7.77852	42.828	0	995

429	7.79856	42.9276	1	995

430	7.82042	43.0272	1	996

431	7.83135	43.1268	3	999

432	7.84957	43.2264	3	1002

433	7.87143	43.326	0	1002

434	7.88965	43.4256	1	1003

435	7.90786	43.5252	3	1007

436	7.92426	43.6248	2	1009

437	7.9443	43.7244	2	1010

438	7.96616	43.824	3	1014

439	7.98437	43.9236	4	1018

440	7.9953	44.0232	6	1024

441	8.01352	44.1228	1	1026

442	8.03902	44.2224	2	1028

443	8.05542	44.322	2	1030

444	8.07181	44.4216	0	1030

445	8.09185	44.5212	1	1031

446	8.11007	44.6208	1	1032

447	8.13011	44.7204	0	1032

448	8.14832	44.82	3	1036

449	8.16107	44.9196	1	1036

450	8.17929	45.0192	8	1044

451	8.20479	45.1188	1	1045

452	8.21937	45.2184	3	1048

453	8.23576	45.318	6	1054

454	8.25034	45.4176	1	1056

455	8.27584	45.5172	0	1056

456	8.29588	45.6168	0	1056

457	8.30863	45.7164	0	1056

458	8.32502	45.816	2	1058

459	8.34688	45.9156	1	1059

460	8.36874	46.0152	4	1063

461	8.3815	46.1148	2	1065

462	8.39789	46.2144	1	1066

463	8.41793	46.314	1	1067

464	8.43979	46.4136	0	1068

465	8.45801	46.5132	0	1068

466	8.47258	46.6128	1	1068

467	8.49444	46.7124	0	1069

468	8.51266	46.812	0	1069

469	8.53269	46.9116	1	1070

470	8.54545	47.0112	3	1073

471	8.56548	47.1108	0	1073

472	8.5837	47.2104	1	1074

473	8.60556	47.31	3	1077

474	8.62013	47.4096	4	1081

475	8.63835	47.5092	12	1093

476	8.65657	47.6088	1	1094

477	8.67843	47.7084	7	1102

478	8.69664	47.808	0	1102

479	8.7094	47.9076	0	1102

480	8.72761	48.0072	0	1102

481	8.75312	48.1068	1	1103

482	8.77133	48.2064	0	1104

483	8.78226	48.306	10	1114

484	8.80048	48.4056	2	1115

485	8.82416	48.5052	9	1124

486	8.84602	48.6048	14	1138

487	8.85877	48.7044	3	1141

488	8.87335	48.804	4	1145

489	8.89521	48.9036	0	1145

490	8.91342	49.0032	1	1146

491	8.93164	49.1028	0	1146

492	8.94621	49.2024	0	1147

493	8.96807	49.302	4	1151

494	8.99175	49.4016	4	1155

495	9.00997	49.5012	1	1156

496	9.0209	49.6008	0	1156

497	9.04094	49.7004	6	1163

498	9.0628	49.8	0	1163

499	9.08284	49.8996	0	1163

500	9.09559	49.9992	2	1165

501	9.11198	50.0988	0	1165

502	9.13384	50.1984	0	1165

503	9.1557	50.298	1	1166

504	9.17392	50.3976	6	1172

505	9.18667	50.4972	1	1173

506	9.20489	50.5968	6	1180

507	9.22857	50.6964	1	1180

508	9.24679	50.796	2	1182

509	9.25954	50.8956	0	1183

510	9.27411	50.9952	1	1183

511	9.30326	51.0948	1	1185

512	9.31783	51.1944	2	1186

513	9.33058	51.294	6	1192

514	9.35244	51.3936	1	1194

515	9.37248	51.4932	2	1196

516	9.39434	51.5928	3	1199

517	9.40527	51.6924	0	1200

518	9.41985	51.792	0	1200

519	9.44535	51.8916	1	1200

520	9.46539	51.9912	4	1204

521	9.48178	52.0908	5	1209

522	9.49271	52.1904	3	1212

523	9.51457	52.29	6	1218

524	9.53643	52.3896	1	1220

525	9.55647	52.4892	1	1221

526	9.57105	52.5888	2	1222

527	9.58744	52.6884	2	1225

528	9.61112	52.788	1	1225

529	9.62934	52.8876	1	1227

530	9.64209	52.9872	0	1227

531	9.65849	53.0868	1	1228

532	9.68035	53.1864	5	1233

533	9.70221	53.286	1	1234

534	9.72042	53.3856	3	1237

535	9.73317	53.4852	1	1238

536	9.75503	53.5848	0	1238

537	9.77507	53.6844	8	1246

538	9.79147	53.784	3	1249

539	9.80422	53.8836	6	1255

540	9.82608	53.9832	1	1256

541	9.84976	54.0828	0	1256

542	9.86433	54.1824	0	1257

543	9.88073	54.282	0	1257

544	9.89895	54.3816	10	1267

545	9.91898	54.4812	7	1274

546	9.93902	54.5808	0	1274

547	9.9536	54.6804	0	1275

548	9.97181	54.78	1	1275

549	9.99003	54.8796	1	1276

550	10.01189	54.9792	0	1276

551	10.02828	55.0788	12	1288

552	10.04468	55.1784	0	1289

553	10.0629	55.278	1	1289

554	10.08293	55.3776	1	1290

555	10.10297	55.4772	4	1294

556	10.11572	55.5768	1	1295

557	10.13576	55.6764	0	1296

558	10.1558	55.776	1	1297

559	10.1722	55.8756	6	1303

560	10.18677	55.9752	2	1305

561	10.20681	56.0748	0	1305

562	10.22867	56.1744	2	1307

563	10.24871	56.274	0	1307

564	10.26328	56.3736	1	1308

565	10.27967	56.4732	0	1309

566	10.30153	56.5728	2	1310

567	10.32157	56.6724	1	1312

568	10.33797	56.772	24	1336

569	10.35072	56.8716	0	1336

570	10.36894	56.9712	0	1337

571	10.39626	57.0708	0	1337

572	10.40901	57.1704	1	1338

573	10.42359	57.27	0	1338

574	10.44727	57.3696	0	1339

575	10.46548	57.4692	1	1340

576	10.4837	57.5688	15	1355

577	10.5001	57.6684	0	1355

578	10.51649	57.768	1	1356

579	10.53653	57.8676	0	1356

580	10.55475	57.9672	2	1358

581	10.57114	58.0668	4	1362

582	10.58754	58.1664	1	1363

583	10.60757	58.266	2	1365

584	10.62943	58.3656	24	1388

585	10.64947	58.4652	1	1389

586	10.66405	58.5648	1	1390

587	10.68044	58.6644	1	1392

588	10.70048	58.764	2	1394

589	10.72052	58.8636	4	1398

590	10.73327	58.9632	4	1402

591	10.75149	59.0628	0	1402

592	10.77335	59.1624	1	1403

593	10.79156	59.262	0	1403

594	10.80978	59.3616	7	1410

595	10.82435	59.4612	1	1411

596	10.84439	59.5608	0	1411

597	10.86625	59.6604	0	1412

598	10.88265	59.76	3	1414

599	10.89722	59.8596	0	1414

600	10.91361	59.9592	1	1415

601	10.9373	60.0588	1	1416

602	10.95369	60.1584	1	1417

603	10.97191	60.258	0	1417

604	10.99012	60.3576	9	1426

605	11.01198	60.4572	8	1435

606	11.03202	60.5568	4	1439

607	11.04477	60.6564	1	1441

608	11.06117	60.756	2	1443

609	11.08303	60.8556	0	1443

610	11.10307	60.9552	3	1447

611	11.11582	61.0548	1	1448

612	11.13221	61.1544	0	1448

613	11.1559	61.254	0	1448

614	11.17411	61.3536	0	1449

615	11.19051	61.4532	0	1449

616	11.20508	61.5528	8	1457

617	11.2233	61.6524	7	1464

618	11.24516	61.752	4	1468

619	11.26337	61.8516	1	1469

620	11.27795	61.9512	2	1471

621	11.29616	62.0508	1	1472

622	11.31802	62.1504	0	1473

623	11.33806	62.25	1	1473

624	11.35081	62.3496	1	1474

625	11.36903	62.4492	3	1477

626	11.39089	62.5488	4	1481

627	11.40729	62.6484	6	1487

628	11.4255	62.748	0	1487

629	11.44008	62.8476	2	1489

630	11.46012	62.9472	0	1490

631	11.48198	63.0468	16	1505

632	11.50201	63.1464	0	1505

633	11.51294	63.246	1	1507

634	11.53116	63.3456	1	1508

635	11.55484	63.4452	10	1517

636	11.57124	63.5448	1	1518

637	11.58581	63.6444	0	1518

638	11.60221	63.744	22	1540

639	11.62407	63.8436	0	1541

640	11.6441	63.9432	0	1541

641	11.65686	64.0428	0	1541

642	11.67507	64.1424	1	1542

643	11.70058	64.242	2	1544

644	11.71879	64.3416	8	1552

645	11.73337	64.4412	1	1553

646	11.74794	64.5408	2	1555

647	11.76616	64.6404	1	1556

648	11.78802	64.74	0	1556

649	11.80623	64.8396	19	1575

650	11.82081	64.9392	4	1579

651	11.83902	65.0388	2	1581

652	11.8627	65.1384	0	1581

653	11.88092	65.238	0	1582

654	11.89367	65.3376	0	1582

655	11.91007	65.4372	1	1583

656	11.93375	65.5368	0	1583

657	11.95379	65.6364	3	1586

658	11.96472	65.736	3	1589

659	11.98293	65.8356	6	1595

660	12.00662	65.9352	0	1596

661	12.02665	66.0348	2	1597

662	12.04487	66.1344	0	1598

663	12.05762	66.234	0	1598

664	12.07584	66.3336	9	1607

665	12.0977	66.4332	3	1610

666	12.11227	66.5328	3	1614

667	12.13049	66.6324	1	1615

668	12.14688	66.732	6	1622

669	12.16692	66.8316	0	1622

670	12.18878	66.9312	0	1622

671	12.20336	67.0308	0	1622

672	12.22157	67.1304	1	1624

673	12.23979	67.23	13	1636

674	12.25983	67.3296	1	1637

675	12.27622	67.4292	1	1638

676	12.2908	67.5288	0	1638

677	12.30901	67.6284	2	1640

678	12.32905	67.728	0	1640

679	12.34909	67.8276	1	1641

680	12.36548	67.9272	5	1646

681	12.3837	68.0268	0	1646

682	12.40556	68.1264	7	1653

683	12.42378	68.226	4	1657

684	12.43653	68.3256	0	1658

685	12.4511	68.4252	1	1659

686	12.47478	68.5248	0	1659

687	12.49482	68.6244	4	1663

688	12.50757	68.724	6	1669

689	12.52761	68.8236	0	1669

690	12.54947	68.9232	1	1670

691	12.56769	69.0228	6	1676

692	12.58408	69.1224	0	1677

693	12.6023	69.222	2	1679

694	12.62052	69.3216	0	1679

695	12.64056	69.4212	32	1711

696	12.65513	69.5208	0	1712

697	12.67152	69.6204	1	1713

698	12.68974	69.72	0	1713

699	12.71342	69.8196	0	1713

700	12.728	69.9192	3	1716

701	12.74439	70.0188	8	1724

702	12.76261	70.1184	0	1724

703	12.78447	70.218	0	1725

704	12.80451	70.3176	2	1726

705	12.81544	70.4172	1	1728

706	12.83183	70.5168	0	1728

707	12.85369	70.6164	2	1730

708	12.87555	70.716	11	1741

709	12.89195	70.8156	2	1743

710	12.90834	70.9152	1	1744

711	12.92656	71.0148	7	1751

712	12.94842	71.1144	3	1754

713	12.96481	71.214	1	1754

714	12.97939	71.3136	6	1760

715	12.99578	71.4132	18	1778

716	13.01946	71.5128	1	1779

717	13.03586	71.6124	1	1780

718	13.05043	71.712	2	1782

719	13.07047	71.8116	4	1786

720	13.09415	71.9112	0	1787

721	13.11237	72.0108	1	1788

722	13.12512	72.1104	11	1799

723	13.14334	72.21	4	1803

724	13.16337	72.3096	1	1804

725	13.18341	72.4092	0	1804

726	13.19799	72.5088	0	1804

727	13.21438	72.6084	1	1805

728	13.23442	72.708	2	1807

729	13.2581	72.8076	1	1808

730	13.27267	72.9072	2	1811

731	13.28725	73.0068	2	1813

732	13.30729	73.1064	7	1820

733	13.33097	73.206	1	1820

734	13.34736	73.3056	0	1821

735	13.36012	73.4052	1	1822

736	13.37651	73.5048	1	1823

737	13.39837	73.6044	7	1830

738	13.41841	73.704	0	1830

739	13.43116	73.8036	0	1830

740	13.45302	73.9032	4	1834

741	13.47306	74.0028	0	1835

742	13.4931	74.1024	3	1838

743	13.50949	74.202	0	1838

744	13.52407	74.3016	0	1839

745	13.54228	74.4012	0	1839

746	13.56414	74.5008	0	1839

747	13.58054	74.6004	0	1840

748	13.59511	74.7	2	1842

749	13.61333	74.7996	5	1846

750	13.63701	74.8992	12	1859

751	13.65523	74.9988	0	1859

752	13.67162	75.0984	0	1859

753	13.68437	75.198	0	1860

754	13.70988	75.2976	2	1862

755	13.72809	75.3972	0	1862

756	13.74267	75.4968	0	1862

757	13.75724	75.5964	0	1863

758	13.7791	75.696	1	1863

759	13.79914	75.7956	2	1865

760	13.81553	75.8952	0	1866

761	13.83193	75.9948	0	1866

762	13.85379	76.0944	1	1867

763	13.872	76.194	4	1871

764	13.8884	76.2936	8	1878

765	13.90115	76.3932	6	1885

766	13.91937	76.4928	4	1889

767	13.94669	76.5924	0	1889

768	13.95944	76.692	0	1889

769	13.97402	76.7916	0	1890

770	13.99588	76.8912	0	1890

771	14.01774	76.9908	2	1892

772	14.03778	77.0904	1	1892

773	14.05053	77.19	1	1893

774	14.06874	77.2896	2	1895

775	14.08878	77.3892	1	1896

776	14.107	77.4888	3	1900

777	14.12157	77.5884	8	1907

778	14.13615	77.688	2	1910

779	14.16165	77.7876	0	1910

780	14.18169	77.8872	2	1912

781	14.19808	77.9868	4	1916

782	14.21266	78.0864	0	1916

783	14.23087	78.186	0	1916

784	14.25455	78.28561	0	1916

785	14.27095	78.38521	0	1917

786	14.28188	78.48481	1	1918

787	14.30192	78.58441	3	1921

788	14.32378	78.68401	1	1922

789	14.34564	78.78361	2	1924

790	14.35839	78.88321	3	1926

791	14.37661	78.98281	2	1928

792	14.39664	79.08241	1	1929

793	14.41668	79.18201	1	1931

794	14.43126	79.28161	0	1931

795	14.44947	79.38121	0	1931

796	14.46769	79.48081	0	1932

797	14.48773	79.58041	2	1934

798	14.50594	79.68001	9	1943

799	14.52052	79.77961	0	1943

800	14.54056	79.87921	0	1943

801	14.56242	79.97881	0	1944

802	14.57881	80.07841	1	1944

803	14.59338	80.17801	9	1954

804	14.61342	80.27761	6	1959

805	14.63528	80.37721	0	1960

806	14.6535	80.47681	2	1962

807	14.66625	80.57641	3	1965

808	14.68447	80.67601	6	1971

809	14.70815	80.77561	0	1971

810	14.72637	80.87521	1	1972

811	14.74276	80.97481	27	1999

812	14.75733	81.07441	0	1999

813	14.78102	81.17401	3	2002

814	14.79923	81.27361	3	2005

815	14.81381	81.37321	0	2006

816	14.8302	81.47281	1	2007

817	14.85024	81.57241	1	2008

818	14.8721	81.67201	1	2009

819	14.89032	81.77161	8	2017

820	14.90307	81.87121	2	2019

821	14.92675	81.97081	2	2021

822	14.94497	82.07041	1	2021

823	14.96136	82.17001	2	2024

824	14.97776	82.26961	1	2024

825	14.99415	82.36921	2	2026

826	15.01783	82.46881	4	2030

827	15.03423	82.56841	4	2034

828	15.0488	82.66801	0	2035

829	15.06884	82.76761	1	2036

830	15.08888	82.86721	0	2036

831	15.10892	82.96681	3	2040

832	15.12713	83.06641	1	2040

833	15.14353	83.16601	2	2042

834	15.15992	83.26561	2	2044

835	15.1836	83.36521	0	2044

836	15.19818	83.46481	2	2046

837	15.21275	83.56441	0	2046

838	15.23097	83.66401	1	2047

839	15.25465	83.76361	3	2049

840	15.27105	83.86321	9	2058

841	15.28744	83.96281	0	2058

842	15.30201	84.06241	4	2063

843	15.3257	84.16201	2	2065

844	15.34209	84.26161	11	2075

845	15.36031	84.36121	3	2078

846	15.37306	84.46081	0	2078

847	15.39674	84.56041	1	2079

848	15.41496	84.66001	13	2092

849	15.43135	84.75961	2	2095

850	15.44593	84.85921	0	2095

851	15.46779	84.95881	0	2095

852	15.49329	85.05841	0	2095

853	15.50786	85.15801	3	2098

854	15.51879	85.25761	8	2106

855	15.54247	85.35721	0	2106

856	15.56433	85.45681	1	2107

857	15.58073	85.55641	0	2107

858	15.59348	85.65601	0	2107

859	15.61352	85.75561	7	2114

860	15.63356	85.85521	6	2120

861	15.6536	85.95481	3	2123

862	15.66635	86.05441	0	2124

863	15.68456	86.15401	0	2124

864	15.70642	86.25361	4	2128

865	15.72646	86.35321	1	2129

866	15.73921	86.45281	6	2135

867	15.75561	86.55241	0	2135

868	15.77929	86.65201	1	2136

869	15.79751	86.75161	0	2136

870	15.8139	86.85121	1	2137

871	15.82848	86.95081	3	2140

872	15.84851	87.05041	1	2141

873	15.87402	87.15001	0	2141

874	15.88677	87.24961	0	2141

875	15.90316	87.34921	1	2142

876	15.92138	87.44881	0	2142

877	15.94506	87.54841	10	2152

878	15.95964	87.64801	1	2153

879	15.97421	87.74761	1	2154

880	15.99425	87.84721	2	2156

881	16.01611	87.94681	0	2156

882	16.03615	88.04641	0	2156

883	16.05072	88.14601	1	2157

884	16.06894	88.24561	0	2157

885	16.0908	88.34521	1	2158

886	16.10901	88.44481	4	2162

887	16.12359	88.54441	0	2162

888	16.1418	88.64401	2	2164

889	16.16002	88.74361	8	2172

890	16.17824	88.84321	4	2176

891	16.19645	88.94281	0	2177

892	16.21285	89.04241	3	2180

893	16.23471	89.14201	1	2181

894	16.25475	89.24161	2	2182

895	16.27114	89.34121	1	2184

896	16.28389	89.44081	1	2184

897	16.30575	89.54041	2	2186

898	16.32761	89.64001	1	2187

899	16.34401	89.73961	0	2188

900	16.35858	89.83921	0	2188

901	16.37862	89.93881	1	2189

902	16.39866	90.03841	0	2189

903	16.41687	90.13801	1	2190

904	16.43145	90.23761	0	2190

905	16.45149	90.33721	1	2192

906	16.47335	90.43681	1	2193

907	16.49338	90.53641	5	2198

908	16.50614	90.63601	2	2200

909	16.52253	90.73561	2	2203

910	16.54257	90.83521	1	2204

911	16.56443	90.93481	9	2213

912	16.579	91.03441	0	2213

913	16.5954	91.13401	0	2214

914	16.61544	91.23361	0	2214

915	16.63912	91.33321	0	2214

916	16.65551	91.43281	0	2214

917	16.66826	91.53241	0	2215

918	16.6883	91.63201	1	2216

919	16.71016	91.73161	24	2240

920	16.72656	91.83121	0	2240

921	16.74113	91.93081	0	2240

922	16.76117	92.03041	0	2240

923	16.78303	92.13001	2	2242

924	16.80307	92.22961	3	2245

925	16.814	92.32921	1	2246

926	16.83404	92.42881	1	2247

927	16.8559	92.52841	0	2248

928	16.87411	92.62801	2	2249

929	16.88504	92.72761	0	2249

930	16.90508	92.82721	0	2250

931	16.92512	92.92681	1	2250

932	16.94334	93.02641	1	2252

933	16.96337	93.12601	0	2252

934	16.97613	93.22561	0	2252

935	16.99616	93.32521	1	2253

936	17.01802	93.42481	4	2257

937	17.03624	93.52441	4	2261

938	17.05081	93.62401	4	2264

939	17.06903	93.72361	2	2266

940	17.09089	93.82321	4	2270

941	17.10729	93.92281	4	2274

942	17.12186	94.02241	0	2275

943	17.1419	94.12201	1	2276

944	17.16376	94.22161	2	2278

945	17.18198	94.32121	0	2278

946	17.19473	94.42081	0	2279

947	17.21294	94.52041	5	2284

948	17.23663	94.62001	0	2284

949	17.25302	94.71961	0	2284

950	17.26942	94.81921	4	2288

951	17.28581	94.91881	2	2291

952	17.30403	95.01841	0	2291

953	17.32771	95.11801	1	2292

954	17.3441	95.21761	1	2293

955	17.3605	95.31721	0	2293

956	17.37689	95.41681	2	2295

957	17.40058	95.51641	5	2301

958	17.41697	95.61601	12	2313

959	17.43154	95.71561	5	2318

960	17.44976	95.81521	1	2319

961	17.47162	95.91481	1	2320

962	17.48984	96.01441	1	2322

963	17.50441	96.11401	2	2323

964	17.52263	96.21361	2	2325

965	17.54631	96.31321	3	2328

966	17.56635	96.41281	0	2328

967	17.5791	96.51241	2	2329

968	17.59732	96.61201	3	2332

969	17.61918	96.71161	0	2333

970	17.63557	96.81121	0	2333

971	17.65197	96.91081	2	2335

972	17.66836	97.01041	4	2339

973	17.69204	97.11001	4	2343

974	17.7139	97.20961	0	2344

975	17.72665	97.30921	2	2346

976	17.74305	97.40881	6	2352

977	17.76309	97.50841	0	2353

978	17.78677	97.60801	2	2355

979	17.80134	97.70761	6	2361

980	17.81409	97.80721	2	2363

981	17.83231	97.90681	0	2363

982	17.85599	98.00641	1	2364

983	17.87239	98.10601	6	2370

984	17.88878	98.20561	2	2372

985	17.907	98.30521	2	2375

986	17.92704	98.40481	2	2377

987	17.94708	98.50441	1	2378

988	17.96347	98.60401	4	2383

989	17.97804	98.70361	0	2383

990	17.99808	98.80321	4	2387

991	18.01812	98.90281	1	2388

992	18.03452	99.00241	7	2395

993	18.05091	99.10201	1	2396

994	18.06913	99.20161	0	2396

995	18.09281	99.30121	2	2398

996	18.11103	99.40081	4	2402

997	18.1256	99.50041	2	2405

998	18.14382	99.60001	4	2408

999	18.16203	99.69961	4	2412

1000	18.18207	99.79921	2	2414

1001	18.193	99.89881	3	2418

1002	18.21668	99.99841	10	2427

1003	18.23854	100.09801	0	2428

1004	18.25858	100.19761	0	2428

1005	18.27133	100.29721	6	2433

1006	18.28773	100.39681	0	2434

1007	18.31141	100.49641	0	2434

1008	18.32963	100.59601	1	2435

1009	18.34238	100.69561	0	2436

1010	18.35877	100.79521	0	2436

1011	18.38245	100.89481	0	2436

1012	18.40067	100.99441	0	2436

1013	18.41524	101.09401	0	2437

1014	18.43164	101.19361	2	2439

1015	18.45168	101.29321	1	2440

1016	18.47718	101.39281	1	2441

1017	18.48993	101.49241	10	2451

1018	18.50815	101.59201	1	2452

1019	18.52637	101.69161	1	2453

1020	18.5464	101.79121	0	2453

1021	18.56462	101.89081	0	2454

1022	18.57919	101.99041	1	2455

1023	18.59923	102.09001	1	2456

1024	18.62109	102.18961	5	2460

1025	18.63931	102.28921	10	2471

1026	18.65206	102.38881	5	2476

1027	18.6721	102.48841	6	2481

1028	18.69396	102.58801	2	2483

1029	18.71035	102.68761	1	2484

1030	18.72493	102.78721	1	2485

1031	18.7395	102.88681	0	2485

1032	18.765	102.98641	1	2486

1033	18.7814	103.08601	2	2488

1034	18.79962	103.18561	1	2489

1035	18.81419	103.28521	0	2490

1036	18.83423	103.38481	0	2490

1037	18.85791	103.48441	1	2491

1038	18.87066	103.58401	4	2494

1039	18.88706	103.68361	3	2498

1040	18.90892	103.78321	4	2502

1041	18.93078	103.88281	0	2502

1042	18.94535	103.98241	1	2504

1043	18.96174	104.08201	1	2505

1044	18.98178	104.18161	7	2512

1045	19.00364	104.28121	8	2520

1046	19.01822	104.38081	0	2520

1047	19.03643	104.48041	0	2520

1048	19.05465	104.58001	5	2526

1049	19.07469	104.67961	1	2526

1050	19.09108	104.77921	1	2527

1051	19.10384	104.87881	0	2528

1052	19.1257	104.97841	0	2528

1053	19.14573	105.07801	6	2534

1054	19.16577	105.17761	0	2535

1055	19.17852	105.27721	2	2537

1056	19.19674	105.37681	1	2537

1057	19.22042	105.47641	6	2543

1058	19.23682	105.57601	1	2544

1059	19.25321	105.67561	0	2544

1060	19.27143	105.77521	8	2552

1061	19.28965	105.87481	1	2553

1062	19.31333	105.97441	1	2554

1063	19.32972	106.07401	12	2567

1064	19.34247	106.17361	0	2567

1065	19.36069	106.27321	0	2568

1066	19.38619	106.37281	8	2576

1067	19.40077	106.47241	0	2576

1068	19.41716	106.57201	0	2576

1069	19.43538	106.67161	1	2577

1070	19.45724	106.77121	1	2579

1071	19.47363	106.87081	0	2579

1072	19.48821	106.97041	0	2579

1073	19.50825	107.07001	3	2582

1074	19.52828	107.16961	0	2582

1075	19.54468	107.26921	1	2583

1076	19.55925	107.36881	0	2583

1077	19.57929	107.46841	2	2585

1078	19.60115	107.56801	2	2588

1079	19.62119	107.66761	0	2588

1080	19.63576	107.76721	0	2588

1081	19.65034	107.86681	2	2591

1082	19.67037	107.96641	3	2594

1083	19.69041	108.06601	0	2594

1084	19.70681	108.16561	11	2604

1085	19.7232	108.26521	1	2606

1086	19.74688	108.36481	0	2606

1087	19.7651	108.46441	1	2607

1088	19.7815	108.56401	10	2617

1089	19.79789	108.66361	4	2622

1090	19.81793	108.76321	6	2628

1091	19.83979	108.86281	1	2629

1092	19.85436	108.96241	0	2630

1093	19.87076	109.06201	5	2635

1094	19.88897	109.16161	0	2635

1095	19.91448	109.26121	2	2637

1096	19.93087	109.36081	0	2638

1097	19.94362	109.46041	2	2640

1098	19.96366	109.56001	1	2641

1099	19.98734	109.65961	1	2642

1100	20.00192	109.75921	0	2642

1101	20.01649	109.85881	4	2646

1102	20.03289	109.95841	4	2651

1103	20.05292	110.05801	0	2651

1104	20.07114	110.15761	1	2652

1105	20.08936	110.25721	1	2653

1106	20.10575	110.35681	2	2655

1107	20.12761	110.45641	0	2655

1108	20.14765	110.55601	12	2667

1109	20.16587	110.65561	0	2667

1110	20.17862	110.75521	1	2668

1111	20.19684	110.85481	4	2672

1112	20.21687	110.95441	1	2674

1113	20.23691	111.05401	8	2682

1114	20.24784	111.15361	4	2685

1115	20.27152	111.25321	2	2688

1116	20.29156	111.35281	2	2690

1117	20.3116	111.45241	11	2701

1118	20.32435	111.55201	0	2701

1119	20.34075	111.65161	3	2704

1120	20.36625	111.75121	0	2705

1121	20.38447	111.85081	0	2705

1122	20.39904	111.95041	5	2710

1123	20.41544	112.05001	14	2724

1124	20.43547	112.14961	0	2724

1125	20.45733	112.24921	6	2730

1126	20.47009	112.34881	2	2731

1127	20.49012	112.44841	0	2732

1128	20.51381	112.54801	0	2732

1129	20.53202	112.64761	3	2735

1130	20.53931	112.74721	1	2736

1131	20.55935	112.84681	8	2744

1132	20.58121	112.94641	1	2745

1133	20.60125	113.04601	3	2748

1134	20.614	113.14561	12	2761

1135	20.63221	113.24521	0	2761

1136	20.65407	113.34481	5	2766

1137	20.67593	113.44441	2	2767

1138	20.68869	113.54401	1	2769

1139	20.70508	113.64361	0	2769

1140	20.7233	113.74321	1	2770

1141	20.74516	113.84281	1	2770

1142	20.76155	113.94241	2	2773

1143	20.77613	114.04201	5	2778

1144	20.79252	114.14161	1	2779

1145	20.8162	114.24121	15	2794

1146	20.83442	114.34081	1	2795

1147	20.85081	114.44041	1	2796

1148	20.86721	114.54001	1	2796

1149	20.88907	114.63961	4	2800

1150	20.90729	114.73921	2	2802

1151	20.92004	114.83881	0	2802

1152	20.9419	114.93841	1	2803

1153	20.96012	115.03801	0	2803

1154	20.98015	115.13761	1	2804

1155	20.99291	115.23721	1	2805

1156	21.01294	115.33681	0	2805

1157	21.02934	115.43641	0	2805

1158	21.04938	115.53601	2	2807

1159	21.06577	115.63561	1	2808

1160	21.08035	115.73521	0	2808

1161	21.09674	115.83481	1	2809

1162	21.11314	115.93441	2	2811

1163	21.12224	116.03401	3	2814

1164	21.05484	116.13361	2	2816

1165	20.14401	116.23321	0	2816

1166	19.22224	116.33281	0	2817

1167	18.04545	116.43241	10	2826

1168	16.81946	116.53201	2	2828

1169	15.85034	116.63161	0	2829

1170	14.76826	116.73121	0	2829

1171	13.7372	116.83081	3	2832

1172	12.68792	116.93041	0	2832

1173	11.70968	117.03001	0	2833

1174	10.76424	117.12961	1	2834

1175	9.70585	117.22921	1	2835

1176	8.58188	117.32881	3	2838

1177	7.45973	117.42841	5	2843

1178	6.5325	117.52801	0	2843

1179	5.50326	117.62761	0	2844

1180	4.51227	117.72721	4	2848

1181	3.55954	117.82681	0	2849

1182	2.62685	117.92641	0	2849

118.02601	18	2867

118.12561	1	2867

118.22521	0	2868

118.32481	0	2868

118.42441	8	2876

118.52401	13	2889

118.62361	0	2889

118.72321	0	2889

118.82281	10	2899

118.92241	2	2902

119.02201	1	2903

119.12161	1	2904

119.22121	0	2904

119.32081	1	2905

119.42041	4	2908

119.52001	1	2910

119.61961	1	2910

119.71921	20	2930

119.81881	0	2930

119.91841	1	2932

120.01801	5	2936

120.11761	1	2938

120.21721	0	2938

120.31681	0	2938

120.41641	0	2939

120.51601	5	2944

120.61561	0	2944

120.71521	10	2954

120.81481	1	2955

120.91441	1	2955

121.01401	6	2961

121.11361	3	2964

121.21321	2	2966

121.31281	0	2966

121.41241	8	2974

121.51201	0	2974

121.61161	1	2975

121.71121	0	2976

121.81081	4	2980

121.91041	4	2984

122.01001	5	2989

122.10961	10	2999

122.20921	1	3000

122.30881	0	3000

122.40841	2	3003

122.50801	1	3003

122.60761	3	3006

122.70721	6	3012

122.80681	0	3013

122.90641	1	3014

123.00601	12	3026

123.10561	0	3026

123.20521	1	3027

123.30481	1	3028

123.40441	2	3029

123.50401	1	3030

123.60361	29	3060

123.70321	1	3061

123.80281	0	3061

123.90241	3	3064

124.00201	1	3065

124.10161	0	3066

124.20121	1	3067

124.30081	3	3070

124.40041	2	3072

124.50001	1	3073

124.59961	1	3074

124.69921	8	3082

124.79881	4	3086

124.89841	0	3086

124.99801	0	3086

125.09761	5	3092

125.19721	0	3092

125.29681	1	3093

125.39641	2	3096

125.49601	2	3097

125.59561	0	3098

125.69521	17	3115

125.79481	0	3115

125.89441	2	3117

125.99401	2	3119

126.09361	6	3125

126.19321	2	3126

126.29281	1	3127

126.39241	0	3128

126.49201	2	3129

126.59161	2	3131

126.69121	0	3131

126.79081	1	3132

126.89041	3	3135

126.99001	0	3135

127.08961	1	3136

127.18921	0	3137

127.28881	1	3138

127.38841	5	3143

127.48801	1	3144

127.58761	0	3144

127.68721	0	3144

127.78681	6	3150

127.88641	4	3154

127.98601	1	3155

128.08561	1	3156

128.18521	1	3156

128.28481	0	3157

128.38441	5	3162

128.48401	0	3162

128.58361	2	3164

128.68321	1	3164

128.78281	2	3167

128.88241	10	3177

128.98201	0	3177

129.08161	1	3178

129.18121	1	3179

129.28081	1	3180

129.38041	2	3182

129.48001	4	3186

129.57961	2	3187

129.67921	2	3189

129.77881	11	3199

129.87841	4	3203

129.97801	2	3205

130.07761	0	3205

130.17721	1	3206

130.27681	9	3215

130.37641	0	3215

130.47601	4	3220

130.57561	0	3220

130.67521	9	3229

130.77481	0	3229

130.87441	1	3230

130.97401	1	3231

131.07361	3	3233

131.17321	1	3234

131.27281	0	3235

131.37241	0	3235

131.47201	6	3241

131.57161	1	3242

131.67121	9	3251

131.77081	1	3252

131.87041	11	3263

131.97001	1	3264

132.06961	1	3265

132.16921	0	3265

132.26881	1	3267

132.36841	3	3269

132.46801	0	3270

132.56761	0	3270

132.66721	1	3271

132.76681	17	3288

132.86641	1	3288

132.96601	1	3289

133.06561	1	3290

133.16521	0	3290

133.26481	0	3291

133.36441	0	3291

133.46401	6	3297

133.56361	8	3305

133.66321	0	3305

133.76281	2	3308

133.86241	1	3308

133.96201	0	3308

134.06161	4	3312

134.16121	7	3319

134.26081	0	3319

134.36041	7	3327

134.46001	1	3328

134.55961	15	3343

134.65921	0	3343

134.75881	0	3344

134.85841	7	3351

134.95801	2	3353

135.05761	3	3356

135.15721	1	3357

135.25681	0	3357

135.35641	4	3361

135.45601	9	3370

135.55561	62	3432

135.65521	12	3444

135.75481	1	3445

135.85441	0	3445

135.95401	2	3448

136.05361	0	3448

136.15321	0	3448

136.25281	2	3450

136.35241	2	3452

136.45201	0	3452

136.55161	0	3453

136.65121	1	3454

136.75081	1	3454

136.85041	1	3455

136.95001	11	3466

137.04961	1	3467

137.14921	0	3467

137.24881	0	3467

137.34841	0	3468

137.44801	11	3478

137.54761	8	3487

137.64721	0	3487

137.74681	0	3487

137.84641	0	3488

137.94601	5	3492

138.04561	3	3495

138.14521	13	3508

138.24481	8	3517

138.34441	10	3526

138.44401	1	3528

138.54361	0	3528

138.64321	3	3531

138.74281	0	3531

138.84241	2	3533

138.94201	7	3540

139.04161	2	3542

139.14121	1	3542

139.24081	11	3553

139.34041	1	3554

139.44001	1	3556

139.53961	0	3556

139.63921	1	3557

139.73881	0	3557

139.83841	0	3557

139.93801	3	3560

140.03761	0	3561

140.13721	0	3561

140.23681	1	3562

140.33641	3	3564

140.43601	0	3564

140.53561	2	3566

140.63521	0	3567

140.73481	0	3567

140.83441	0	3567

140.93401	6	3574

141.03361	3	3576

141.13321	3	3579

141.23281	2	3581

141.33241	0	3582

141.43201	1	3583

141.53161	0	3583

141.63121	1	3584

141.73081	8	3592

141.83041	0	3592

141.93001	0	3593

142.02961	0	3593

142.12921	0	3594

142.22881	1	3594

142.32841	1	3595

142.42801	1	3596

142.52761	0	3596

142.62721	2	3598

142.72681	0	3598

142.82641	8	3606

142.92601	0	3606

143.02561	0	3607

143.12521	3	3609

143.22481	2	3612

143.32441	63	3675

143.42401	1	3676

143.52361	9	3685

143.62321	1	3686

143.72281	0	3687

143.82241	4	3691

143.92201	0	3691

144.02161	2	3693

144.12121	0	3694

144.22081	7	3701

144.32041	2	3703

144.42001	0	3703

144.51961	3	3706

144.61921	6	3711

144.71881	0	3712

144.81841	1	3713

144.91801	19	3732

145.01761	4	3737

145.11721	3	3740

145.21681	4	3744

145.31641	1	3745

145.41601	2	3747

145.51561	0	3748

145.61521	0	3748

145.71481	1	3749

145.81441	0	3749

145.91401	9	3758

146.01361	2	3760

146.11321	0	3760

146.21281	0	3760

146.31241	0	3760

146.41201	1	3761

146.51161	1	3762

146.61121	2	3765

146.71081	5	3770

146.81041	1	3770

146.91001	4	3774

147.00961	3	3778

147.10921	2	3780

147.20881	0	3780

147.30841	1	3780

147.40801	1	3781

147.50761	1	3782

147.60721	0	3782

147.70681	0	3783

147.80641	2	3785

147.90601	0	3785

148.00561	8	3793

148.10521	5	3798

148.20481	0	3798

148.30441	1	3800

148.40401	2	3802

148.50361	1	3803

148.60321	0	3803

148.70281	1	3804

148.80241	5	3808

148.90201	0	3809

149.00161	0	3809

149.10121	5	3814

149.20081	0	3814

149.30041	1	3815

149.40001	5	3820

149.49961	0	3821

149.59921	1	3822

149.69881	2	3823

149.79841	2	3825

149.89801	0	3826

149.99761	2	3827

150.09721	3	3830

150.19681	5	3835

150.29641	1	3836

150.39601	1	3837

150.49561	1	3838

150.59521	0	3838

150.69481	4	3842

150.79441	0	3843

150.89401	1	3844

150.99361	2	3846

151.09321	2	3848

151.19281	4	3852

151.29241	7	3859

151.39201	3	3862

151.49161	8	3870

151.59121	15	3884

151.69081	3	3887

151.79041	1	3888

151.89001	1	3889

151.98961	2	3891

152.08921	0	3891

152.18881	0	3891

152.28841	5	3896

152.38801	1	3897

152.48761	5	3902

152.58721	1	3903

152.68681	0	3903

152.78641	2	3905

152.88601	0	3906

152.98561	3	3909

153.08521	2	3911

153.18481	1	3912

153.28441	3	3915

153.38401	0	3915

153.48361	2	3917

153.58321	1	3918

153.68281	0	3918

153.78241	2	3920

153.88201	5	3925

153.98161	1	3926

154.08121	0	3926

154.18081	4	3931

154.28041	0	3931

154.38001	6	3937

154.47961	1	3938

154.57921	2	3940

154.67881	9	3949

154.77841	0	3949

154.87801	1	3950

154.97761	3	3954

155.07721	1	3955

155.17681	0	3955

155.27641	0	3956

155.37601	3	3958

155.47561	3	3962

155.57521	1	3962

155.67481	2	3964

155.77441	1	3965

155.87401	0	3965

155.97361	7	3972

156.07321	1	3973

156.17281	0	3973

156.27241	6	3980

156.37201	0	3980

156.47161	1	3981

156.57121	2	3983

156.67081	1	3984

156.77041	0	3985

156.87001	0	3985

156.96961	2	3987

157.06921	3	3990

157.16881	0	3990

157.26841	1	3991

157.36801	0	3991

157.46761	3	3994

157.56721	2	3996

157.66681	1	3997

157.76641	3	4000

157.86601	0	4000

157.96561	8	4008

158.06521	2	4011

158.16481	0	4011

158.26441	0	4011

158.36401	0	4012

158.46361	0	4012

158.56321	3	4015

158.66281	2	4017

158.76241	1	4018

158.86201	0	4018

158.96161	1	4019

159.06121	1	4020

159.16081	6	4026

159.26041	4	4030

159.36001	0	4031

159.45961	4	4035

159.55921	0	4035

159.65881	3	4038

159.75841	1	4039

159.85801	0	4039

159.95761	0	4040

160.05721	2	4042

160.15681	0	4042

160.25641	0	4042

160.35601	5	4047

160.45561	2	4049

160.55521	1	4050

160.65481	0	4051

160.75441	0	4051

160.85401	2	4053

160.95361	2	4054

161.05321	0	4055

161.15281	0	4055

161.25241	0	4055

161.35201	2	4057

161.45161	0	4057

161.55121	1	4058

161.65081	0	4058

161.75041	1	4060

161.85001	0	4060

161.94961	8	4068

162.04921	1	4069

162.14881	2	4071

162.24841	13	4084

162.34801	10	4094

162.44761	2	4096

162.54721	16	4112

162.64681	0	4112

162.74641	5	4117

162.84601	5	4121

162.94561	0	4122

163.04521	2	4124

163.14481	1	4124

163.24441	1	4125

163.34401	1	4126

163.44361	2	4128

163.54321	1	4129

163.64281	4	4133

163.74241	1	4134

163.84201	8	4142

163.94161	0	4142

164.04121	0	4142

164.14081	0	4142

164.24041	4	4146

164.34001	0	4147

164.43961	0	4147

164.53921	2	4149

164.63881	0	4150

164.73841	13	4162

164.83801	0	4162

164.93761	4	4166

165.03721	0	4166

165.13681	0	4167

165.23641	0	4167

165.33601	1	4168

165.43561	3	4170

165.53521	0	4171

165.63481	0	4171

165.73441	8	4179

165.83401	1	4179

165.93361	6	4185

166.03321	4	4189

166.13281	0	4190

166.23241	0	4190

166.33201	1	4191

166.43161	3	4194

166.53121	2	4196

166.63081	0	4196

166.73041	0	4196

166.83001	1	4197

166.92961	0	4198

167.02921	0	4198

167.12881	0	4198

167.22841	6	4204

167.32801	3	4207

167.42761	4	4210

167.52721	0	4211

167.62681	1	4212

167.72641	3	4215

167.82601	0	4215

167.92561	1	4216

168.02521	2	4218

168.12481	1	4219

168.22441	5	4224

168.32401	4	4228

168.42361	1	4229

168.52321	0	4229

168.62281	17	4246

168.72241	2	4248

168.82201	3	4250

168.92161	0	4251

169.02121	0	4251

169.12081	13	4264

169.22041	1	4265

169.32001	3	4268

169.41961	0	4268

169.51921	1	4269

169.61881	14	4283

169.71841	0	4284

169.81801	1	4284

169.91761	7	4291

170.01721	2	4294

170.11681	6	4299

170.21641	3	4302

170.31601	4	4306

170.41561	0	4306

170.51521	4	4310

170.61481	1	4311

170.71441	0	4311

170.81401	4	4315

170.91361	1	4316

171.01321	7	4323

171.11281	1	4324

171.21241	9	4333

171.31201	1	4334

171.41161	2	4336

171.51121	22	4358

171.61081	0	4359

171.71041	0	4359

171.81001	1	4360

171.90961	5	4365

172.00921	0	4365

172.10881	12	4377

172.20841	16	4393

172.30801	0	4393

172.40761	0	4393

172.50721	0	4394

172.60681	0	4394

172.70641	0	4394

172.80601	0	4395

172.90561	0	4395

173.00521	0	4395

173.10481	0	4395

173.20441	2	4398

173.30401	0	4398

173.40361	5	4403

173.50321	1	4404

173.60281	1	4405

173.70241	5	4410

173.80201	6	4416

173.90161	1	4417

174.00121	18	4435

174.10081	1	4436

174.20041	0	4436

174.30001	0	4436

174.39961	1	4437

174.49921	1	4438

174.59881	1	4439

174.69841	2	4440

174.79801	7	4447

174.89761	2	4448

174.99721	1	4450

175.09681	3	4452

175.19641	1	4454

175.29601	4	4458

175.39561	0	4458

175.49521	9	4467

175.59481	0	4467

175.69441	4	4471

175.79401	0	4472

175.89361	0	4472

175.99321	4	4476

176.09281	1	4477

176.19241	0	4477

176.29201	1	4478

176.39161	0	4478

176.49121	1	4479

176.59081	8	4487

176.69041	0	4487

176.79001	4	4491

176.88961	0	4492

176.98921	6	4498

177.08881	1	4499

177.18841	7	4506

177.28801	1	4507

177.38761	11	4518

177.48721	1	4519

177.58681	1	4520

177.68641	2	4522

177.78601	1	4522

177.88561	1	4523

177.98521	4	4528

178.08481	2	4530

178.18441	0	4530

178.28401	1	4531

178.38361	6	4536

178.48321	5	4542

178.58281	3	4544

178.68241	0	4545

178.78201	5	4549

178.88161	4	4553

178.98121	10	4562

179.08081	0	4563

179.18041	10	4573

179.28001	1	4574

179.37961	18	4592

179.47921	0	4592

179.57881	0	4592

179.67841	0	4593

179.77801	6	4599

179.87761	1	4599

179.97721	8	4607

180.07681	6	4614

180.17641	0	4614

180.27601	7	4621

180.37561	2	4623

180.47521	3	4626

180.57481	4	4630

180.67441	0	4630

180.77401	8	4639

180.87361	0	4639

180.97321	2	4641

181.07281	0	4641

181.17241	1	4642

181.27201	0	4642

181.37161	6	4649

181.47121	9	4658

181.57081	1	4659

181.67041	4	4663

181.77001	2	4665

181.86961	0	4665

181.96921	3	4668

182.06881	2	4670

182.16841	5	4675

182.26801	1	4675

182.36761	2	4677

182.46721	3	4680

182.56681	0	4680

182.66641	3	4683

182.76601	2	4684

182.86561	3	4687

182.96521	3	4690

183.06481	6	4696

183.16441	2	4698

183.26401	0	4698

183.36361	1	4700

183.46321	1	4700

183.56281	2	4702

183.66241	1	4704

183.76201	1	4704

183.86161	0	4704

183.96121	0	4705

184.06081	10	4715

184.16041	0	4716

184.26001	0	4716

184.35961	6	4722

184.45921	2	4724

184.55881	0	4725

184.65841	4	4729

184.75801	0	4730

184.85761	4	4734

184.95721	0	4734

185.05681	0	4735

185.15641	6	4740

185.25601	1	4741

185.35561	1	4742

185.45521	5	4747

185.55481	0	4747

185.65441	1	4748

185.75401	0	4749

185.85361	8	4756

185.95321	0	4756

186.05281	3	4759

186.15241	4	4764

186.25201	10	4773

186.35161	0	4774

186.45121	2	4775

186.55081	2	4777

186.65041	0	4778

186.75001	1	4778

186.84961	4	4783

186.94921	10	4793

187.04881	1	4794

187.14841	0	4795

187.24801	1	4796

187.34761	1	4796

187.44721	10	4806

187.54681	1	4807

187.64641	0	4807

187.74601	3	4810

187.84561	0	4810

187.94521	0	4810

188.04481	0	4811

188.14441	9	4819

188.24401	19	4838

188.34361	12	4850

188.44321	0	4850

188.54281	0	4851

188.64241	2	4852

188.74201	4	4856

188.84161	6	4863

188.94121	2	4865

189.04081	7	4872

189.14041	2	4874

189.24001	1	4876

189.33961	0	4876

189.43921	2	4878

189.53881	3	4881

189.63841	1	4883

189.73801	1	4884

189.83761	1	4885

189.93721	0	4886

190.03681	2	4887

190.13641	1	4888

190.23601	1	4889

190.33561	0	4889

190.43521	1	4890

190.53481	0	4890

190.63441	1	4891

190.73401	1	4893

190.83361	0	4893

190.93321	1	4894

191.03281	4	4898

191.13241	3	4901

191.23201	3	4904

191.33161	9	4913

191.43121	0	4913

191.53081	0	4913

191.63041	11	4924

191.73001	0	4924

191.82961	1	4925

191.92921	2	4927

192.02881	0	4927

192.12841	0	4928

192.22801	0	4928

192.32761	6	4934

192.42721	12	4946

192.52681	0	4947

192.62641	4	4951

192.72601	18	4969

192.82561	2	4971

192.92521	4	4976

193.02481	0	4976

193.12441	1	4977

193.22401	0	4977

193.32361	6	4983

193.42321	6	4988

193.52281	0	4989

193.62241	4	4993

193.72201	4	4996

193.82161	0	4996

193.92121	0	4997

194.02081	11	5008

194.12041	0	5008

194.22001	1	5009

194.31961	4	5013

194.41921	1	5014

194.51881	1	5014

194.61841	1	5015

194.71801	4	5019

194.81761	2	5020

194.91721	1	5022

195.01681	1	5023

195.11641	6	5028

195.21601	0	5029

195.31561	19	5048

195.41521	0	5048

195.51481	0	5049

195.61441	5	5054

195.71401	8	5061

195.81361	5	5066

195.91321	3	5069

196.01281	1	5070

196.11241	0	5070

196.21201	8	5078

196.31161	0	5078

196.41121	13	5091

196.51081	1	5093

196.61041	2	5095

196.71001	6	5101

196.80961	0	5101

196.90921	5	5106

197.00881	1	5108

197.10841	3	5110

197.20801	6	5116

197.30761	0	5116

197.40721	2	5118

197.50681	1	5120

197.60641	20	5139

197.70601	0	5140

197.80561	1	5140

197.90521	5	5145

198.00481	11	5156

198.10441	1	5157

198.20401	8	5166

198.30361	2	5167

198.40321	3	5170

198.50281	0	5170

198.60241	4	5174

198.70201	1	5175

198.80161	0	5175

198.90121	0	5176

199.00081	1	5177

199.10041	2	5179

199.20001	5	5184

199.29961	3	5187

199.39921	2	5188

199.49881	0	5189

199.59841	0	5189

199.69801	0	5189

199.79761	0	5190

199.89721	0	5190

199.99681	3	5193

200.09641	0	5193

200.19601	4	5198

200.29561	3	5200

200.39521	2	5202

200.49481	4	5206

200.59441	2	5207

200.69401	1	5208

200.79361	1	5209

200.89321	0	5209

200.99281	0	5209

201.09241	1	5210

201.19201	3	5213

201.29161	0	5214

201.39121	3	5216

201.49081	0	5217

201.59041	2	5219

201.69001	2	5221

201.78961	0	5221

201.88921	1	5222

201.98881	6	5228

202.08841	6	5234

202.18801	4	5238

202.28761	0	5238

202.38721	4	5242

202.48681	1	5243

202.58641	1	5244

202.68601	4	5248

202.78561	5	5252

202.88521	3	5255

202.98481	4	5259

203.08441	1	5260

203.18401	2	5263

203.28361	0	5263

203.38321	11	5274

203.48281	2	5276

203.58241	2	5278

203.68201	0	5278

203.78161	0	5279

203.88121	1	5280

203.98081	2	5282

204.08041	0	5282

204.18001	0	5283

204.27961	0	5283

204.37921	3	5286

204.47881	4	5290

204.57841	0	5290

204.67801	1	5291

204.77761	1	5292

204.87721	19	5311

204.97681	3	5313

205.07641	6	5319

205.17601	1	5320

205.27561	0	5321

205.37521	2	5322

205.47481	4	5326

205.57441	4	5330

205.67401	2	5332

205.77361	1	5333

205.87321	0	5333

205.97281	2	5335

206.07241	1	5336

206.17201	0	5336

206.27161	1	5337

206.37121	1	5338

206.47081	1	5339

206.57041	0	5340

206.67001	3	5343

206.76961	0	5343

206.86921	0	5344

206.96881	1	5344

207.06841	1	5345

207.16801	3	5348

207.26761	0	5349

207.36721	1	5350

207.46681	3	5353

207.56641	2	5355

207.66601	1	5356

207.76561	2	5358

207.86521	1	5358

207.96481	6	5364

208.06441	8	5372

208.16401	0	5373

208.26361	0	5373

208.36321	1	5374

208.46281	9	5383

208.56241	0	5383

208.66201	1	5384

208.76161	1	5385

208.86121	7	5392

208.96081	1	5393

209.06041	17	5410

209.16001	2	5412

209.25961	0	5412

209.35921	0	5412

209.45881	4	5416

209.55841	0	5416

209.65801	5	5421

209.75761	0	5422

209.85721	2	5424

209.95681	0	5425

210.05641	2	5427

210.15601	1	5428

210.25561	1	5429

210.35521	0	5430

210.45481	0	5430

210.55441	2	5432

210.65401	1	5433

210.75361	0	5433

210.85321	2	5435

210.95281	0	5436

211.05241	1	5436

211.15201	6	5442

211.25161	1	5444

211.35121	2	5446

211.45081	0	5446

211.55041	6	5452

211.65001	2	5454

211.74961	0	5454

211.84921	0	5454

211.94881	7	5461

212.04841	0	5462

212.14801	8	5469

212.24761	0	5470

212.34721	1	5470

212.44681	4	5474

212.54641	5	5479

212.64601	0	5479

212.74561	1	5480

212.84521	2	5482

212.94481	4	5486

213.04441	1	5488

213.14401	4	5491

213.24361	2	5493

213.34321	0	5494

213.44281	2	5496

213.54241	0	5496

213.64201	1	5496

213.74161	0	5497

213.84121	0	5497

213.94081	3	5500

214.04041	1	5501

214.14001	4	5504

214.23961	0	5505

214.33921	0	5505

214.43881	2	5507

214.53841	1	5509

214.63801	1	5509

214.73761	1	5510

214.83721	1	5511

214.93681	0	5511

215.03641	0	5512

215.13601	7	5519

215.23561	0	5519

215.33521	2	5521

215.43481	5	5526

215.53441	2	5528

215.63401	0	5528

215.73361	1	5529

215.83321	4	5533

215.93281	1	5534

216.03241	0	5535

216.13201	5	5539

216.23161	15	5554

216.33121	0	5555

216.43081	4	5559

216.53041	0	5559

216.63001	1	5560

216.72961	1	5561

216.82921	2	5563

216.92881	1	5564

217.02841	0	5564

217.12801	1	5565

217.22761	0	5565

217.32721	0	5565

217.42681	8	5573

217.52641	0	5573

217.62601	3	5576

217.72561	4	5580

217.82521	1	5581

217.92481	0	5582

218.02441	3	5584

218.12401	1	5585

218.22361	4	5590

218.32321	3	5592

218.42281	1	5593

218.52241	0	5593

218.62201	4	5598

218.72161	0	5598

218.82121	0	5599

218.92081	0	5599

219.02041	6	5605

219.12001	2	5608

219.21961	0	5608

219.31921	5	5612

219.41881	4	5617

219.51841	1	5617

219.61801	1	5618

219.71761	4	5622

219.81721	2	5624

219.91681	5	5629

220.01641	1	5630

220.11601	0	5631

220.21561	0	5631

220.31521	0	5631

220.41481	1	5632

220.51441	0	5632

220.61401	1	5633

220.71361	2	5635

220.81321	3	5638

220.91281	2	5640

221.01241	1	5640

221.11201	0	5641

221.21161	2	5643

221.31121	3	5646

221.41081	0	5646

221.51041	2	5648

221.61001	0	5648

221.70961	3	5651

221.80921	1	5651

221.90881	5	5656

222.00841	3	5660

222.10801	6	5666

222.20761	4	5670

222.30721	0	5670

222.40681	1	5671

222.50641	2	5673

222.60601	3	5676

222.70561	0	5676

222.80521	3	5679

222.90481	4	5683

223.00441	4	5687

223.10401	2	5688

223.20361	12	5701

223.30321	1	5702

223.40281	0	5702

223.50241	0	5703

223.60201	2	5704

223.70161	0	5705

223.80121	0	5705

223.90081	6	5712

224.00041	1	5713

224.10001	28	5740

224.19961	11	5752

224.29921	2	5753

224.39881	8	5761

224.49841	2	5764

224.59801	0	5764

224.69761	0	5764

224.79721	1	5765

224.89681	1	5767

224.99641	5	5772

225.09601	3	5774

225.19561	2	5776

225.29521	4	5781

225.39481	8	5788

225.49441	2	5790

225.59401	1	5792

225.69361	2	5793

225.79321	1	5794

225.89281	1	5796

225.99241	2	5797

226.09201	2	5799

226.19161	1	5800

226.29121	0	5800

226.39081	6	5806

226.49041	6	5812

226.59001	1	5814

226.68961	2	5816

226.78921	4	5820

226.88881	1	5821

226.98841	8	5828

227.08801	3	5832

227.18761	9	5840

227.28721	3	5843

227.38681	0	5844

227.48641	1	5845

227.58601	0	5845

227.68561	4	5849

227.78521	10	5859

227.88481	0	5860

227.98441	5	5864

228.08401	1	5865

228.18361	5	5870

228.28321	10	5880

228.38281	0	5881

228.48241	0	5881

228.58201	5	5886

228.68161	0	5887

228.78121	5	5892

228.88081	8	5900

228.98041	1	5901

229.08001	1	5902

229.17961	2	5904

229.27921	1	5906

229.37881	0	5906

229.47841	1	5907

229.57801	0	5908

229.67761	1	5908

229.77721	1	5909

229.87681	10	5920

229.97641	0	5920

230.07601	12	5932

230.17561	7	5939

230.27521	1	5940

230.37481	1	5942

230.47441	9	5951

230.57401	3	5953

230.67361	0	5954

230.77321	0	5954

230.87281	5	5960

230.97241	1	5960

231.07201	7	5968

231.17161	1	5969

231.27121	8	5977

231.37081	5	5983

231.47041	2	5984

231.57001	5	5989

231.66961	3	5992

231.76921	0	5993

231.86881	6	5999

231.96841	4	6004

232.06801	3	6006

232.16761	0	6007

232.26721	0	6007

232.36681	1	6008

232.46641	0	6009

232.56601	0	6009

232.66561	2	6012

232.76521	1	6012

232.86481	2	6014

232.96441	1	6015

233.06401	1	6016

233.16361	2	6019

233.26321	1	6020

233.36281	7	6027

233.46241	3	6030

233.56201	0	6030

233.66162	1	6032

233.76121	3	6035

233.86082	1	6036

233.96042	0	6036

234.06001	1	6038

234.15962	2	6040

234.25922	5	6045

234.35881	4	6049

234.45842	1	6050

234.55802	1	6051

234.65761	4	6055

234.75722	4	6060

234.85682	4	6064

234.95641	8	6072

235.05602	0	6072

235.15562	11	6084

235.25522	5	6089

235.35482	1	6090

235.45442	0	6091

235.55402	1	6092

235.65362	8	6100

235.75322	2	6101

235.85282	0	6102

235.95242	4	6106

236.05202	0	6107

236.15162	1	6108

236.25122	0	6108

236.35082	4	6112

236.45042	6	6119

236.55002	0	6119

236.64962	2	6121

236.74922	0	6122

236.84882	9	6131

236.94842	4	6135

237.04802	25	6160

237.14762	0	6160

237.24722	3	6163

237.34682	4	6167

237.44642	1	6168

237.54602	0	6169

237.64562	7	6176

237.74522	0	6176

237.84482	4	6180

237.94442	1	6181

238.04402	13	6194

238.14362	1	6195

238.24322	1	6196

238.34282	1	6197

238.44242	0	6198

238.54202	6	6204

238.64162	1	6205

238.74122	3	6208

238.84082	2	6210

238.94042	2	6212

239.04002	1	6212

239.13962	3	6216

239.23922	1	6216

239.33882	1	6218

239.43842	2	6220

239.53802	0	6220

239.63762	1	6221

239.73722	1	6222

239.83682	2	6224

239.93642	3	6227

240.03602	1	6228

240.13562	0	6228

240.23522	0	6229

240.33482	0	6229

240.43442	1	6230

240.53402	2	6232

240.63362	2	6234

240.73322	9	6243

240.83282	2	6245

240.93242	4	6250

241.03202	4	6254

241.13162	0	6255

241.23122	16	6271

241.33082	1	6272

241.43042	3	6275

241.53002	2	6277

241.62962	3	6280

241.72922	3	6283

241.82882	4	6287

241.92842	1	6288

242.02802	0	6288

242.12762	1	6289

242.22722	12	6301

242.32682	3	6304

242.42642	0	6304

242.52602	1	6305

242.62562	2	6307

242.72522	3	6310

242.82482	1	6311

242.92442	4	6315

243.02402	0	6315

243.12362	0	6316

243.22322	1	6317

243.32282	0	6317

243.42242	4	6321

243.52202	5	6326

243.62162	0	6327

243.72122	0	6327

243.82082	2	6329

243.92042	1	6330

244.02002	1	6331

244.11962	5	6336

244.21922	0	6336

244.31882	2	6339

244.41842	0	6339

244.51802	1	6340

244.61762	1	6341

244.71722	0	6341

244.81682	3	6344

244.91642	2	6347

245.01602	1	6348

245.11562	1	6349

245.21522	0	6350

245.31482	1	6351

245.41442	1	6352

245.51402	5	6356

245.61362	1	6357

245.71322	28	6385

245.81282	2	6388

245.91242	3	6390

246.01202	0	6391

246.11162	0	6391

246.21122	2	6393

246.31082	6	6399

246.41042	3	6401

246.51002	2	6404

246.60962	1	6404

246.70922	0	6405

246.80882	1	6406

246.90842	2	6408

247.00802	0	6409

247.10762	2	6411

247.20722	1	6412

247.30682	1	6413

247.40642	0	6413

247.50602	9	6423

247.60562	1	6424

247.70522	1	6424

247.80482	0	6425

247.90442	4	6428

248.00402	0	6429

248.10362	9	6438

248.20322	4	6443

248.30282	5	6448

248.40242	0	6448

248.50202	0	6449

248.60162	1	6450

248.70122	2	6452

248.80082	0	6452

248.90042	6	6458

249.00002	0	6458

249.09962	0	6459

249.19922	1	6460

249.29882	21	6481

249.39842	1	6482

249.49802	0	6482

249.59762	1	6484

249.69722	3	6486

249.79682	1	6487

249.89642	1	6488

249.99602	0	6489

250.09562	2	6491

250.19522	0	6492

250.29482	1	6492

250.39442	1	6494

250.49402	2	6496

250.59362	1	6497

250.69322	0	6497

250.79282	0	6498

250.89242	1	6499

250.99202	4	6502

251.09162	10	6512

251.19122	1	6513

251.29082	5	6518

251.39042	1	6519

251.49002	2	6521

251.58962	1	6522

251.68922	2	6524

251.78882	4	6528

251.88842	0	6528

251.98802	2	6530

252.08762	0	6530

252.18722	2	6532

252.28682	1	6533

252.38642	2	6535

252.48602	0	6536

252.58562	1	6537

252.68522	1	6538

252.78482	2	6540

252.88442	0	6540

252.98402	3	6543

253.08362	8	6551

253.18322	0	6552

253.28282	4	6556

253.38242	0	6556

253.48202	3	6560

253.58162	2	6562

253.68122	2	6564

253.78082	0	6564

253.88042	5	6569

253.98002	4	6573

254.07962	1	6574

254.17922	2	6576

254.27882	1	6578

254.37842	2	6580

254.47802	0	6580

254.57762	11	6591

254.67722	4	6594

254.77682	0	6595

254.87642	8	6602

254.97602	1	6604

255.07562	0	6604

255.17522	1	6605

255.27482	2	6608

255.37442	2	6610

255.47402	8	6618

255.57362	1	6619

255.67322	1	6620

255.77282	1	6621

255.87242	3	6624

255.97202	8	6632

256.07162	0	6632

256.17122	1	6633

256.27082	0	6633

256.37042	4	6638

256.47002	1	6639

256.56962	13	6652

256.66922	3	6655

256.76882	1	6656

256.86842	1	6656

256.96802	2	6659

257.06762	0	6659

257.16722	3	6662

257.26682	0	6662

257.36642	0	6663

257.46602	3	6666

257.56562	1	6667

257.66522	2	6669

257.76482	2	6671

257.86442	1	6672

257.96402	1	6673

258.06362	1	6675

258.16322	0	6675

258.26282	2	6677

258.36242	0	6677

258.46202	0	6678

258.56162	1	6679

258.66122	3	6681

258.76082	0	6682

258.86042	1	6683

258.96002	1	6684

259.05962	1	6685

259.15922	0	6685

259.25882	1	6686

259.35842	1	6687

259.45802	0	6688

259.55762	0	6688

259.65722	1	6689

259.75682	2	6691

259.85642	1	6692

259.95602	4	6696

260.05562	0	6696

260.15522	0	6697

260.25482	3	6700

260.35442	0	6700

260.45402	4	6704

260.55362	0	6704

260.65322	1	6706

260.75282	0	6706

260.85242	0	6707

260.95202	0	6707

261.05162	1	6708

261.15122	4	6712

261.25082	1	6712

261.35042	1	6713

261.45002	3	6716

261.54962	2	6718

261.64922	0	6719

261.74882	2	6721

261.84842	1	6722

261.94802	1	6723

262.04762	1	6724

262.14722	1	6725

262.24682	0	6725

262.34642	1	6726

262.44602	0	6727

262.54562	1	6728

262.64522	3	6731

262.74482	1	6732

262.84442	2	6733

262.94402	0	6734

263.04362	0	6734

263.14322	1	6736

263.24282	9	6744

263.34242	33	6777

263.44202	7	6784

263.54162	2	6786

263.64122	0	6787

263.74082	10	6796

263.84042	2	6799

263.94002	3	6802

264.03962	1	6803

264.13922	1	6804

264.23882	1	6805

264.33842	1	6806

264.43802	0	6807

264.53762	0	6807

264.63722	1	6808

264.73682	0	6808

264.83642	1	6810

264.93602	2	6812

265.03562	2	6814

265.13522	0	6814

265.23482	3	6817

265.33442	0	6817

265.43402	1	6818

265.53362	0	6819

265.63322	0	6819

265.73282	2	6821

265.83242	24	6845

265.93202	3	6848

266.03162	0	6848

266.13122	7	6855

266.23082	1	6856

266.33042	1	6857

266.43002	2	6859

266.52962	1	6860

266.62922	1	6861

266.72882	3	6864

266.82842	1	6865

266.92802	2	6867

267.02762	1	6868

267.12722	1	6869

267.22682	3	6872

267.32642	1	6873

267.42602	0	6873

267.52562	1	6874

267.62522	1	6876

267.72482	10	6885

267.82442	3	6888

267.92402	22	6911

268.02362	0	6911

268.12322	6	6917

268.22282	4	6922

268.32242	0	6922

268.42202	0	6923

268.52162	0	6923

268.62122	0	6924

268.72082	8	6932

268.82042	1	6933

268.92002	2	6935

269.01962	3	6938

269.11922	2	6940

269.21882	9	6949

269.31842	2	6952

269.41802	0	6952

269.51762	0	6952

269.61722	6	6958

269.71682	2	6960

269.81642	1	6961

269.91602	0	6961

270.01562	8	6969

270.11522	4	6973

270.21482	2	6975

270.31442	1	6976

270.41402	2	6978

270.51362	1	6979

270.61322	1	6980

270.71282	2	6981

270.81242	5	6986

270.91202	2	6988

271.01162	0	6989

271.11122	0	6989

271.21082	5	6995

271.31042	2	6996

271.41002	0	6997

271.50962	2	6999

271.60922	1	7000

271.70882	0	7000

271.80842	1	7001

271.90802	1	7002

272.00762	2	7004

272.10722	11	7015

272.20682	1	7016

272.30642	1	7016

272.40602	9	7026

272.50562	0	7026

272.60522	2	7028

272.70482	2	7031

272.80442	0	7031

272.90402	3	7034

273.00362	0	7034

273.10322	0	7035

273.20282	7	7042

273.30242	1	7043

273.40202	0	7043

273.50162	0	7044

273.60122	0	7044

273.70082	2	7046

273.80042	2	7048

273.90002	4	7051

273.99962	5	7056

274.09922	0	7057

274.19882	4	7060

274.29842	0	7061

274.39802	1	7062

274.49762	0	7063

274.59722	0	7063

274.69682	8	7071

274.79642	0	7071

274.89602	1	7072

274.99562	2	7075

275.09522	1	7076

275.19482	2	7077

275.29442	4	7081

275.39402	23	7104

275.49362	8	7112

275.59322	1	7112

275.69282	0	7113

275.79242	1	7114

275.89202	1	7115

275.99162	2	7117

276.09122	1	7118

276.19082	3	7121

276.29042	3	7124

276.39002	3	7126

276.48962	2	7128

276.58922	0	7129

276.68882	0	7129

276.78842	0	7130

276.88802	1	7131

276.98762	1	7132

277.08722	14	7146

277.18682	1	7147

277.28642	9	7156

277.38602	5	7161

277.48562	2	7164

277.58522	3	7166

277.68482	0	7167

277.78442	2	7169

277.88402	0	7169

277.98362	2	7172

278.08322	1	7173

278.18282	1	7174

278.28242	0	7175

278.38202	0	7175

278.48162	1	7176

278.58122	2	7178

278.68082	2	7180

278.78042	0	7180

278.88002	3	7184

278.97962	4	7188

279.07922	1	7189

279.17882	1	7190

279.27842	4	7194

279.37802	0	7195

279.47762	1	7196

279.57722	4	7200

279.67682	0	7201

279.77642	0	7201

279.87602	0	7202

279.97562	7	7208

280.07522	0	7209

280.17482	1	7210

280.27442	1	7211

280.37402	2	7213

280.47362	1	7214

280.57322	2	7216

280.67282	0	7216

280.77242	0	7217

280.87202	1	7218

280.97162	1	7219

281.07122	1	7220

281.17082	1	7221

281.27042	1	7222

281.37002	0	7223

281.46962	4	7227

281.56922	1	7228

281.66882	1	7229

281.76842	0	7230

281.86802	1	7231

281.96762	0	7231

282.06722	3	7234

282.16682	1	7236

282.26642	0	7236

282.36602	0	7236

282.46562	1	7237

282.56522	2	7239

282.66482	0	7240

282.76442	6	7246

282.86402	0	7246

282.96362	0	7247

283.06322	6	7253

283.16282	2	7255

283.26242	0	7255

283.36202	5	7260

283.46162	1	7261

283.56122	0	7262

283.66082	1	7263

283.76042	0	7263

283.86002	4	7267

283.95962	3	7270

284.05922	1	7272

284.15882	1	7273

284.25842	1	7274

284.35802	0	7274

284.45762	0	7275

284.55722	0	7275

284.65682	1	7276

284.75642	0	7276

284.85602	2	7278

284.95562	8	7287

285.05522	0	7287

285.15482	1	7288

285.25442	0	7288

285.35402	0	7289

285.45362	2	7291

285.55322	0	7291

285.65282	0	7292

285.75242	4	7295

285.85202	23	7318

285.95162	0	7318

286.05122	0	7319

286.15082	0	7319

286.25042	0	7320

286.35002	1	7320

286.44962	2	7322

286.54922	2	7324

286.64882	1	7326

286.74842	2	7328

286.84802	6	7334

286.94762	1	7335

287.04722	1	7336

287.14682	0	7336

287.24642	0	7336

287.34602	3	7340

287.44562	1	7340

287.54522	0	7341

287.64482	3	7344

287.74442	1	7345

287.84402	1	7346

287.94362	2	7348

288.04322	0	7348

288.14282	0	7349

288.24242	0	7349

288.34202	3	7352

288.44162	0	7353

288.54122	0	7353

288.64082	0	7354

288.74042	1	7355

288.84002	1	7356

288.93962	1	7357

289.03922	4	7361

289.13882	4	7365

289.23842	3	7368

289.33802	1	7369

289.43762	6	7375

289.53722	1	7376

289.63682	1	7376

289.73642	0	7377

289.83602	1	7378

289.93562	6	7384

290.03522	0	7384

290.13482	1	7386

290.23442	1	7387

290.33402	4	7390

290.43362	2	7392

290.53322	1	7394

290.63282	1	7395

290.73242	2	7396

290.83202	2	7399

290.93162	0	7399

291.03122	0	7400

291.13082	4	7404

291.23042	1	7404

291.33002	0	7405

291.42962	0	7405

291.52922	6	7411

291.62882	2	7413

291.72842	1	7414

291.82802	2	7416

291.92762	3	7419

292.02722	1	7420

292.12682	2	7423

292.22642	2	7424

292.32602	1	7425

292.42562	26	7451

292.52522	1	7452

292.62482	3	7455

292.72442	0	7455

292.82402	2	7457

292.92362	1	7458

293.02322	1	7460

293.12282	0	7460

293.22242	0	7460

293.32202	4	7464

293.42162	5	7470

293.52122	3	7473

293.62082	0	7473

293.72042	1	7474

293.82002	0	7475

293.91962	0	7475

294.01922	1	7476

294.11882	0	7476

294.21842	0	7477

294.31802	2	7479

294.41762	1	7480

294.51722	1	7481

294.61682	3	7484

294.71642	4	7488

294.81602	11	7499

294.91562	0	7500

295.01522	2	7502

295.11482	0	7502

295.21442	0	7503

295.31402	0	7503

295.41362	6	7509

295.51322	1	7510

295.61282	0	7511

295.71242	3	7514

295.81202	0	7514

295.91162	3	7517

296.01122	0	7518

296.11082	10	7528

296.21042	1	7529

296.31002	0	7529

296.40962	1	7530

296.50922	2	7532

296.60882	0	7533

296.70842	0	7533

296.80802	7	7540

296.90762	4	7544

297.00722	0	7544

297.10682	9	7553

297.20642	1	7555

297.30602	0	7555

297.40562	1	7556

297.50522	1	7558

297.60482	0	7558

297.70442	3	7561

297.80402	0	7562

297.90362	1	7563

298.00322	4	7567

298.10282	0	7568

298.20242	2	7570

298.30202	6	7576

298.40162	1	7577

298.50122	1	7578

298.60082	1	7579

298.70042	1	7580

298.80002	0	7580

298.89962	0	7581

298.99922	5	7586

299.09882	1	7587

299.19842	2	7589

299.29802	3	7592

299.39762	1	7593

299.49722	2	7595

299.59682	2	7597

299.69642	1	7598

299.79602	1	7599

299.89562	1	7600

299.99522	1	7601

300.09482	3	7604

300.19442	1	7605

300.29402	0	7605

300.39362	0	7606

300.49322	0	7606

300.59282	1	7608

300.69242	1	7608

300.79202	2	7610

300.89162	1	7611

300.99122	2	7613

301.09082	3	7616

301.19042	0	7616

301.29002	0	7617

301.38962	1	7618

301.48922	0	7619

301.58882	0	7619

301.68842	2	7621

301.78802	1	7623

301.88762	1	7624

301.98722	6	7629

302.08682	0	7630

302.18642	5	7635

302.28602	1	7636

302.38562	0	7636

302.48522	8	7644

302.58482	0	7645

302.68442	3	7648

302.78402	3	7651

302.88362	5	7656

302.98322	0	7656

303.08282	2	7659

303.18242	2	7660

303.28202	0	7661

303.38162	1	7662

303.48122	7	7669

303.58082	6	7675

303.68042	0	7675

303.78002	1	7676

303.87962	3	7680

303.97922	0	7680

304.07882	2	7682

304.17842	0	7682

304.27802	2	7684

304.37762	5	7689

304.47722	1	7691

304.57682	2	7692

304.67642	1	7693

304.77602	0	7694

304.87562	2	7696

304.97522	0	7696

305.07482	0	7696

305.17442	0	7697

305.27402	0	7697

305.37362	1	7698

305.47322	0	7699

305.57282	1	7700

305.67242	0	7700

305.77202	4	7704

305.87162	0	7704

305.97122	0	7705

306.07082	1	7706

306.17042	0	7707

306.27002	0	7707

306.36962	1	7708

306.46922	0	7709

306.56882	2	7711

306.66842	1	7712

306.76802	1	7712

306.86762	1	7714

306.96722	3	7717

307.06682	0	7717

307.16642	0	7718

307.26602	1	7719

307.36562	13	7732

307.46522	0	7732

307.56482	3	7735

307.66442	1	7736

307.76402	4	7739

307.86362	1	7740

307.96322	0	7741

308.06282	3	7744

308.16242	1	7745

308.26202	1	7746

308.36162	1	7747

308.46122	0	7748

308.56082	1	7749

308.66042	6	7755

308.76002	4	7760

308.85962	1	7760

308.95922	1	7761

309.05882	2	7764

309.15842	5	7768

309.25802	5	7774

309.35762	1	7775

309.45722	0	7775

309.55682	3	7778

309.65642	0	7779

309.75602	3	7781

309.85562	3	7784

309.95522	1	7785

310.05482	10	7795

310.15442	2	7797

310.25402	1	7798

310.35362	0	7799

310.45322	2	7800

310.55282	0	7801

310.65242	0	7801

310.75202	17	7819

310.85162	1	7820

310.95122	0	7820

311.05082	0	7821

311.15042	0	7821

311.25002	0	7822

311.34962	10	7832

311.44922	0	7832

311.54882	5	7837

311.64842	1	7838

311.74802	2	7840

311.84762	2	7843

311.94722	3	7846

312.04682	3	7849

312.14642	5	7854

312.24602	11	7865

312.34562	1	7866

312.44522	2	7868

312.54482	1	7869

312.64442	0	7869

312.74402	4	7873

312.84362	10	7884

312.94322	1	7885

313.04282	2	7887

313.14242	1	7888

313.24202	4	7893

313.34162	1	7894

313.44122	3	7897

313.54082	0	7897

313.64042	3	7900

313.74002	7	7907

313.83962	3	7910

313.93922	0	7910

314.03882	5	7915

314.13842	0	7916

314.23802	0	7916

314.33762	1	7917

314.43722	0	7918

314.53682	1	7919

314.63642	1	7920

314.73602	8	7928

314.83562	15	7942

314.93522	2	7944

315.03482	7	7951

315.13442	1	7952

315.23402	4	7955

315.33362	1	7956

315.43322	2	7959

315.53282	0	7959

315.63242	0	7960

315.73202	0	7960

315.83162	2	7962

315.93122	2	7964

316.03082	13	7977

316.13042	2	7979

316.23002	3	7982

316.32962	0	7983

316.42922	3	7985

316.52882	0	7986

316.62842	0	7986

316.72802	8	7994

316.82762	1	7995

316.92722	3	7998

317.02682	1	7999

317.12642	1	8000

317.22602	5	8006

317.32562	1	8007

317.42522	0	8007

317.52482	1	8008

317.62442	1	8009

317.72402	0	8010

317.82362	1	8011

317.92322	4	8015

318.02282	0	8015

318.12242	1	8016

318.22202	0	8017

318.32162	3	8020

318.42122	7	8027

318.52082	0	8027

318.62042	1	8028

318.72002	1	8029

318.81962	0	8030

318.91922	0	8030

319.01882	0	8031

319.11842	0	8031

319.21802	3	8034

319.31762	6	8040

319.41722	4	8044

319.51682	0	8044

319.61642	0	8044

319.71602	2	8047

319.81562	0	8047

319.91522	1	8048

320.01482	1	8049

320.11442	0	8049

320.21402	0	8050

320.31362	1	8051

320.41322	0	8051

320.51282	0	8052

320.61242	0	8052

320.71202	4	8056

320.81162	2	8058

320.91122	0	8058

321.01082	3	8061

321.11042	2	8063

321.21002	4	8067

321.30962	3	8069

321.40922	4	8073

321.50882	0	8074

321.60842	1	8075

321.70802	6	8080

321.80762	1	8081

321.90722	5	8087

322.00682	8	8095

322.10642	0	8096

322.20602	3	8098

322.30562	0	8099

322.40522	0	8099

322.50482	3	8102

322.60442	1	8103

322.70402	1	8104

322.80362	1	8105

322.90322	4	8109

323.00282	1	8110

323.10242	0	8111

323.20202	0	8111

323.30162	1	8112

323.40122	3	8115

323.50082	1	8116

323.60042	0	8116

323.70002	0	8116

323.79962	1	8117

323.89922	1	8118

323.99882	3	8121

324.09842	0	8121

324.19802	1	8123

324.29762	1	8124

324.39722	2	8126

324.49682	0	8127

324.59642	0	8127

324.69602	0	8128

324.79562	0	8128

324.89522	1	8129

324.99482	1	8130

325.09442	1	8131

325.19402	2	8133

325.29362	0	8133

325.39322	2	8136

325.49282	1	8136

325.59242	0	8137

325.69202	1	8138

325.79162	0	8139

325.89122	4	8143

325.99082	2	8145

326.09042	1	8146

326.19002	1	8148

326.28962	2	8149

326.38922	0	8150

326.48882	0	8150

326.58842	8	8159

326.68802	1	8160

326.78762	0	8160

326.88722	3	8163

326.98682	0	8164

327.08642	0	8164

327.18602	0	8164

327.28562	2	8166

327.38522	0	8167

327.48482	5	8172

327.58442	0	8172

327.68402	1	8173

327.78362	1	8175

327.88322	0	8175

327.98282	0	8176

328.08242	1	8176

328.18202	2	8178

328.28162	1	8179

328.38122	1	8180

328.48082	0	8180

328.58042	1	8181

328.68002	3	8184

328.77962	5	8189

328.87922	4	8193

328.97882	1	8194

329.07842	1	8195

329.17802	1	8196

329.27762	1	8197

329.37722	7	8204

329.47682	1	8204

329.57642	2	8206

329.67602	0	8207

329.77562	0	8207

329.87522	0	8208

329.97482	0	8208

330.07442	7	8215

330.17402	0	8216

330.27362	0	8216

330.37322	3	8219

330.47282	1	8220

330.57242	1	8221

330.67202	2	8223

330.77162	2	8225

330.87122	1	8226

330.97082	0	8226

331.07042	0	8227

331.17002	1	8228

331.26962	3	8231

331.36922	0	8231

331.46882	0	8232

331.56842	0	8232

331.66802	1	8233

331.76762	8	8241

331.86722	1	8242

331.96682	2	8244

332.06642	2	8246

332.16602	13	8259

332.26562	1	8260

332.36522	1	8260

332.46482	3	8263

332.56442	2	8265

332.66402	7	8272

332.76362	0	8272

332.86322	1	8273

332.96282	1	8274

333.06242	0	8275

333.16202	1	8276

333.26162	1	8277

333.36122	7	8284

333.46082	0	8285

333.56042	2	8287

333.66002	0	8288

333.75962	1	8288

333.85922	0	8289

333.95882	1	8290

334.05842	0	8291

334.15802	11	8302

334.25762	0	8302

334.35722	1	8303

334.45682	0	8304

334.55642	1	8304

334.65602	3	8307

334.75562	3	8310

334.85522	0	8310

334.95482	0	8311

335.05442	1	8312

335.15402	0	8312

335.25362	2	8314

335.35322	2	8316

335.45282	2	8319

335.55242	1	8320

335.65202	3	8323

335.75162	1	8324

335.85122	1	8324

335.95082	3	8328

336.05042	4	8332

336.15002	23	8355

336.24962	0	8355

336.34922	0	8356

336.44882	0	8356

336.54842	5	8361

336.64802	1	8362

336.74762	0	8363

336.84722	0	8363

336.94682	1	8364

337.04642	0	8364

337.14602	0	8365

337.24562	1	8366

337.34522	5	8371

337.44482	0	8371

337.54442	0	8372

337.64402	0	8372

337.74362	2	8374

337.84322	2	8376

337.94282	0	8376

338.04242	0	8376

338.14202	1	8378

338.24162	0	8378

338.34122	1	8379

338.44082	0	8380

338.54042	0	8380

338.64002	3	8383

338.73962	0	8383

338.83922	1	8384

338.93882	7	8391

339.03842	9	8400

339.13802	0	8400

339.23762	1	8402

339.33722	1	8403

339.43682	5	8408

339.53642	0	8409

339.63602	1	8410

339.73562	0	8410

339.83522	0	8411

339.93482	1	8412

340.03442	2	8413

340.13402	0	8414

340.23362	4	8418

340.33322	4	8423

340.43282	0	8423

340.53242	4	8427

340.63202	1	8428

340.73162	0	8428

340.83122	1	8429

340.93082	6	8436

341.03042	0	8436

341.13002	0	8436

341.22962	15	8452

341.32922	2	8453

341.42882	1	8454

341.52842	0	8455

341.62802	2	8456

341.72762	2	8459

341.82722	0	8459

341.92682	8	8468

342.02642	4	8472

342.12602	0	8472

342.22562	1	8474

342.32522	4	8477

342.42482	0	8478

342.52442	0	8478

342.62402	0	8479

342.72362	0	8479

342.82322	1	8480

342.92282	1	8481

343.02242	2	8483

343.12202	0	8483

343.22162	5	8488

343.32122	23	8511

343.42082	5	8516

343.52042	0	8516

343.62002	2	8518

343.71962	3	8521

343.81922	0	8521

343.91882	6	8527

344.01842	1	8528

344.11802	0	8529

344.21762	0	8529

344.31722	1	8531

344.41682	0	8531

344.51642	3	8534

344.61602	7	8540

344.71562	1	8541

344.81522	1	8542

344.91482	0	8543

345.01442	1	8544

345.11402	1	8545

345.21362	0	8546

345.31322	1	8547

345.41282	4	8551

345.51242	1	8552

345.61202	1	8553

345.71162	0	8553

345.81122	1	8554

345.91082	0	8555

346.01042	0	8555

346.11002	0	8556

346.20962	0	8556

346.30922	0	8556

346.40882	0	8557

346.50842	0	8557

346.60802	2	8560

346.70762	3	8562

346.80722	0	8563

346.90682	1	8564

347.00642	4	8568

347.10602	0	8568

347.20562	0	8568

347.30522	4	8572

347.40482	0	8573

347.50442	1	8574

347.60402	1	8575

347.70362	0	8576

347.80322	0	8576

347.90282	0	8576

348.00242	3	8580

348.10202	0	8580

348.20162	2	8582

348.30122	0	8582

348.40082	3	8585

348.50042	0	8585

348.60002	2	8588

348.69962	0	8588

348.79922	1	8589

348.89882	10	8599

348.99842	0	8599

349.09802	1	8600

349.19762	1	8601

349.29722	4	8604

349.39682	0	8605

349.49642	1	8606

349.59602	0	8606

349.69562	0	8607

349.79522	0	8607

349.89482	5	8612

349.99442	2	8614

350.09402	7	8621

350.19362	1	8622

350.29322	0	8623

350.39282	0	8623

350.49242	1	8624

350.59202	5	8629

350.69162	1	8631

350.79122	1	8632

350.89082	1	8633

350.99042	1	8634

351.09002	0	8635

351.18962	2	8636

351.28922	0	8637

351.38882	5	8642

351.48842	1	8644

351.58802	0	8644

351.68762	1	8645

351.78722	0	8645

351.88682	1	8646

351.98642	0	8647

352.08602	16	8663

352.18562	2	8664

352.28522	1	8665

352.38482	0	8666

352.48442	1	8667

352.58402	3	8670

352.68362	0	8670

352.78322	8	8679

352.88282	0	8679

352.98242	0	8680

353.08202	1	8681

353.18162	1	8682

353.28122	1	8683

353.38082	0	8683

353.48042	2	8685

353.58002	1	8686

353.67962	1	8687

353.77922	0	8688

353.87882	0	8688

353.97842	3	8691

354.07802	2	8692

354.17762	3	8695

354.27722	0	8696

354.37682	4	8699

354.47642	0	8700

354.57602	3	8702

354.67562	0	8703

354.77522	0	8703

354.87482	0	8704

354.97442	2	8705

355.07402	3	8708

355.17362	1	8709

355.27322	2	8711

355.37282	0	8711

355.47242	0	8712

355.57202	0	8712

355.67162	0	8712

355.77122	6	8719

355.87082	1	8720

355.97042	0	8720

356.07002	2	8723

356.16962	0	8723

356.26922	1	8724

356.36882	1	8725

356.46842	0	8726

356.56802	3	8728

356.66762	0	8729

356.76722	3	8732

356.86682	1	8733

356.96642	2	8735

357.06602	3	8738

357.16562	12	8750

357.26522	2	8752

357.36482	0	8753

357.46442	2	8755

357.56402	5	8760

357.66362	1	8761

357.76322	2	8764

357.86282	0	8764

357.96242	0	8764

358.06202	1	8766

358.16162	0	8766

358.26122	1	8768

358.36082	0	8768

358.46042	1	8769

358.56002	4	8773

358.65962	1	8774

358.75922	2	8776

358.85882	1	8777

358.95842	1	8778

359.05802	5	8783

359.15762	2	8785

359.25722	1	8786

359.35682	1	8787

359.45642	0	8787

359.55602	2	8789

359.65562	0	8789

359.75522	1	8790

359.85482	0	8791

359.95442	1	8792

360.05402	0	8792

360.15362	1	8793

360.25322	3	8796

360.35282	0	8796

360.45242	0	8797

360.55202	1	8798

360.65162	7	8805

360.75122	3	8808

360.85082	1	8808

360.95042	2	8810

361.05002	2	8812

361.14962	5	8817

361.24922	0	8817

361.34882	1	8819

361.44842	1	8820

361.54802	0	8820

361.64762	0	8821

361.74722	5	8826

361.84682	1	8827

361.94642	1	8828

362.04602	3	8831

362.14562	1	8832

362.24522	1	8833

362.34482	0	8833

362.44442	0	8834

362.54402	4	8838

362.64362	6	8844

362.74322	1	8845

362.84282	0	8845

362.94242	1	8846

363.04202	0	8847

363.14162	1	8848

363.24122	2	8849

363.34082	0	8850

363.44042	3	8853

363.54002	2	8855

363.63962	0	8855

363.73922	0	8856

363.83882	30	8885

363.93842	8	8893

364.03802	0	8894

364.13762	1	8895

364.23722	0	8896

364.33682	2	8898

364.43642	0	8898

364.53602	0	8899

364.63562	1	8900

364.73522	0	8900

364.83482	1	8901

364.93442	1	8902

365.03402	0	8902

365.13362	0	8903

365.23322	1	8904

365.33282	1	8905

365.43242	2	8907

365.53202	0	8908

365.63162	0	8908

365.73122	1	8909

365.83082	2	8912

365.93042	1	8912

366.03002	0	8913

366.12962	1	8914

366.22922	6	8920

366.32882	0	8921

366.42842	3	8924

366.52802	0	8924

366.62762	2	8926

366.72722	0	8927

366.82682	3	8929

366.92642	0	8930

367.02602	12	8941

367.12562	7	8948

367.22522	1	8949

367.32482	0	8949

367.42442	0	8950

367.52402	7	8956

367.62362	1	8958

367.72322	7	8964

367.82282	1	8965

367.92242	2	8967

368.02202	0	8968

368.12162	1	8968

368.22122	0	8969

368.32082	0	8969

368.42042	0	8970

368.52002	0	8970

368.61962	2	8972

368.71922	1	8973

368.81882	0	8974

368.91842	0	8974

369.01802	2	8976

369.11762	0	8976

369.21722	1	8978

369.31682	0	8978

369.41642	0	8979

369.51602	0	8979

369.61562	2	8981

369.71522	0	8981

369.81482	5	8987

369.91442	4	8990

370.01402	0	8991

370.11362	4	8994

370.21322	1	8996

370.31282	6	9001

370.41242	3	9004

370.51202	0	9004

370.61162	0	9005

370.71122	0	9005

370.81082	0	9006

370.91042	1	9007

371.01002	0	9008

371.10962	2	9009

371.20922	0	9010

371.30882	0	9010

371.40842	1	9012

371.50802	1	9012

371.60762	0	9013

371.70722	4	9017

371.80682	0	9017

371.90642	0	9018

372.00602	5	9023

372.10562	4	9027

372.20522	0	9027

372.30482	6	9033

372.40442	6	9039

372.50402	0	9039

372.60362	1	9040

372.70322	1	9041

372.80282	2	9043

372.90242	2	9045

373.00202	0	9046

373.10162	1	9047

373.20122	0	9047

373.30082	2	9049

373.40042	1	9050

373.50002	0	9051

373.59962	0	9051

373.69922	0	9052

373.79882	26	9077

373.89842	0	9078

373.99802	0	9078

374.09762	1	9080

374.19722	3	9082

374.29682	0	9083

374.39642	0	9083

374.49602	5	9088

374.59562	1	9089

374.69522	0	9090

374.79482	0	9090

374.89442	4	9094

374.99402	0	9095

375.09362	5	9100

375.19322	1	9101

375.29282	1	9102

375.39242	0	9102

375.49202	2	9104

375.59162	0	9105

375.69122	0	9105

375.79082	2	9108

375.89042	1	9108

375.99002	4	9113

376.08962	0	9113

376.18922	0	9114

376.28882	0	9114

376.38842	1	9116

376.48802	0	9116

376.58762	3	9119

376.68722	0	9119

376.78682	2	9121

376.88642	3	9124

376.98602	0	9124

377.08562	0	9125

377.18522	3	9128

377.28482	2	9130

377.38442	1	9131

377.48402	1	9132

377.58362	4	9135

377.68322	1	9136

377.78282	1	9137

377.88242	1	9138

377.98202	0	9139

378.08162	4	9143

378.18122	3	9146

378.28082	1	9147

378.38042	1	9148

378.48002	0	9148

378.57962	0	9149

378.67922	1	9150

378.77882	0	9151

378.87842	2	9152

378.97802	12	9164

379.07762	1	9165

379.17722	2	9167

379.27682	1	9168

379.37642	0	9169

379.47602	2	9171

379.57562	0	9171

379.67522	1	9172

379.77482	1	9173

379.87442	0	9174

379.97402	1	9175

380.07362	0	9175

380.17322	0	9176

380.27282	0	9176

380.37242	3	9179

380.47202	11	9190

380.57162	2	9192

380.67122	2	9194

380.77082	0	9194

380.87042	1	9195

380.97002	0	9196

381.06962	0	9196

381.16922	1	9197

381.26882	1	9198

381.36842	3	9201

381.46802	2	9203

381.56762	0	9203

381.66722	4	9207

381.76682	1	9208

381.86642	1	9208

381.96602	0	9209

382.06562	3	9212

382.16522	1	9213

382.26482	0	9213

382.36442	1	9214

382.46402	1	9215

382.56362	1	9216

382.66322	0	9216

382.76282	2	9218

382.86242	1	9219

382.96202	0	9220

383.06162	2	9221

383.16122	6	9228

383.26082	11	9238

383.36042	5	9244

383.46002	1	9245

383.55962	0	9245

383.65922	1	9247

383.75882	4	9251

383.85842	0	9252

383.95802	0	9252

384.05762	1	9253

384.15722	2	9255

384.25682	3	9258

384.35642	1	9260

384.45602	0	9260

384.55562	5	9265

384.65522	0	9265

384.75482	1	9266

384.85442	0	9267

384.95402	3	9270

385.05362	0	9270

385.15322	5	9276

385.25282	0	9276

385.35242	0	9276

385.45202	3	9280

385.55162	5	9284

385.65122	1	9285

385.75082	2	9288

385.85042	2	9289

385.95002	0	9290

386.04962	1	9291

386.14922	1	9292

386.24882	1	9293

386.34842	0	9293

386.44802	2	9295

386.54762	1	9296

386.64722	1	9297

386.74682	0	9298

386.84642	0	9298

386.94602	3	9301

387.04562	0	9302

387.14522	0	9302

387.24482	0	9303

387.34442	0	9303

387.44402	4	9308

387.54362	2	9309

387.64322	1	9310

387.74282	0	9311

387.84242	4	9314

387.94202	1	9316

388.04162	2	9318

388.14122	1	9319

388.24082	0	9319

388.34042	2	9321

388.44002	1	9323

388.53962	0	9323

388.63922	0	9324

388.73882	1	9325

388.83842	2	9327

388.93802	2	9329

389.03762	4	9333

389.13722	1	9334

389.23682	2	9336

389.33642	0	9337

389.43602	0	9337

389.53562	4	9341

389.63522	2	9344

389.73482	0	9344

389.83442	1	9345

389.93403	1	9346

390.03362	0	9347

390.13322	0	9347

390.23282	1	9348

390.33243	3	9352

390.43203	1	9352

390.53163	2	9354

390.63122	0	9355

390.73082	4	9359

390.83042	1	9360

390.93003	1	9361

391.02963	1	9363

391.12923	0	9363

391.22882	0	9364

391.32842	2	9365

391.42803	0	9366

391.52763	1	9367

391.62723	2	9369

391.72683	0	9370

391.82643	1	9371

391.92603	1	9372

392.02563	0	9372

392.12523	0	9373

392.22483	0	9373

392.32443	0	9374

392.42403	1	9375

392.52363	1	9376

392.62323	0	9376

392.72283	0	9377

392.82243	5	9382

392.92203	0	9382

393.02163	0	9383

393.12123	0	9383

393.22083	0	9384

393.32043	1	9384

393.42003	1	9385

393.51963	0	9386

393.61923	5	9391

393.71883	2	9393

393.81843	0	9393

393.91803	2	9395

394.01763	0	9396

394.11723	1	9396

394.21683	2	9398

394.31643	0	9399

394.41603	0	9399

394.51563	0	9400

394.61523	0	9400

394.71483	0	9400

394.81443	3	9403

394.91403	3	9406

395.01363	3	9408

395.11323	1	9409

395.21283	0	9410

395.31243	0	9410

395.41203	0	9411

395.51163	1	9412

395.61123	0	9412

395.71083	0	9412

395.81043	1	9414

395.91003	0	9414

396.00963	0	9415

396.10923	1	9416

396.20883	1	9417

396.30843	0	9417

396.40803	3	9420

396.50763	0	9420

396.60723	1	9422

396.70683	0	9422

396.80643	1	9423

396.90603	0	9424

397.00563	1	9425

397.10523	1	9426

397.20483	1	9427

397.30443	0	9427

397.40403	0	9428

397.50363	0	9428

397.60323	0	9428

397.70283	1	9429

397.80243	2	9432

397.90203	9	9441

398.00163	1	9442

398.10123	0	9443

398.20083	0	9443

398.30043	0	9444

398.40003	3	9446

398.49963	0	9447

398.59923	0	9447

398.69883	1	9448

398.79843	1	9449

398.89803	1	9450

398.99763	2	9452

399.09723	1	9453

399.19683	4	9457

399.29643	1	9459

399.39603	0	9459

399.49563	2	9461

399.59523	1	9463

399.69483	3	9465

399.79443	0	9466

399.89403	1	9467

399.99363	2	9469

400.09323	15	9484

400.19283	9	9493

400.29243	1	9494

400.39203	1	9496

400.49163	1	9497

400.59123	3	9500

400.69083	0	9500

400.79043	0	9501

400.89003	1	9502

400.98963	0	9502

401.08923	1	9503

401.18883	0	9504

401.28843	1	9505

401.38803	0	9505

401.48763	0	9506

401.58723	1	9507

401.68683	2	9509

401.78643	0	9509

401.88603	0	9510

401.98563	0	9510

402.08523	2	9512

402.18483	0	9512

402.28443	0	9513

402.38403	0	9513

402.48363	1	9515

402.58323	2	9516

402.68283	0	9517

402.78243	4	9521

402.88203	2	9523

402.98163	1	9524

403.08123	1	9525

403.18083	1	9526

403.28043	1	9527

403.38003	4	9530

403.47963	0	9531

403.57923	1	9532

403.67883	1	9533

403.77843	0	9534

403.87803	1	9535

403.97763	14	9549

404.07723	1	9550

404.17683	0	9551

404.27643	1	9552

404.37603	1	9553

404.47563	0	9554

404.57523	0	9554

404.67483	1	9555

404.77443	0	9556

404.87403	1	9556

404.97363	1	9557

405.07323	0	9558

405.17283	0	9558

405.27243	0	9559

405.37203	0	9559

405.47163	0	9560

405.57123	2	9561

405.67083	1	9562

405.77043	0	9563

405.87003	0	9563

405.96963	0	9564

406.06923	0	9564

406.16883	1	9565

406.26843	1	9567

406.36803	1	9568

406.46763	0	9568

406.56723	0	9568

406.66683	0	9569

406.76643	2	9571

406.86603	0	9572

406.96563	3	9574

407.06523	2	9576

407.16483	1	9577

407.26443	2	9579

407.36403	1	9580

407.46363	0	9580

407.56323	0	9581

407.66283	0	9581

407.76243	1	9582

407.86203	23	9605

407.96163	0	9606

408.06123	0	9606

408.16083	2	9608

408.26043	0	9609

408.36003	0	9609

408.45963	0	9610

408.55923	5	9615

408.65883	1	9616

408.75843	2	9617

408.85803	0	9618

408.95763	0	9618

409.05723	1	9620

409.15683	1	9620

409.25643	0	9621

409.35603	0	9621

409.45563	1	9623

409.55523	2	9625

409.65483	0	9625

409.75443	0	9626

409.85403	1	9627

409.95363	1	9628

410.05323	1	9628

410.15283	1	9629

410.25243	3	9632

410.35203	2	9634

410.45163	1	9635

410.55123	0	9635

410.65083	6	9641

410.75043	1	9642

410.85003	0	9643

410.94963	1	9644

411.04923	2	9646

411.14883	0	9646

411.24843	1	9647

411.34803	1	9648

411.44763	0	9648

411.54723	1	9649

411.64683	0	9650

411.74643	10	9660

411.84603	0	9660

411.94563	1	9661

412.04523	0	9662

412.14483	1	9663

412.24443	0	9663

412.34403	0	9664

412.44363	0	9664

412.54323	0	9664

412.64283	1	9665

412.74243	0	9666

412.84203	0	9666

412.94163	0	9667

413.04123	2	9668

413.14083	0	9669

413.24043	0	9669

413.34003	1	9671

413.43963	0	9671

413.53923	2	9673

413.63883	0	9673

413.73843	0	9674

413.83803	1	9675

413.93763	2	9676

414.03723	1	9678

414.13683	2	9680

414.23643	2	9681

414.33603	2	9684

414.43563	0	9684

414.53523	1	9685

414.63483	0	9685

414.73443	0	9686

414.83403	0	9686

414.93363	1	9688

415.03323	0	9688

415.13283	1	9689

415.23243	0	9689

415.33203	2	9691

415.43163	4	9695

415.53123	0	9696

415.63083	0	9696

415.73043	0	9696

415.83003	1	9697

415.92963	0	9698

416.02923	1	9699

416.12883	0	9699

416.22843	7	9706

416.32803	0	9707

416.42763	0	9707

416.52723	2	9709

416.62683	0	9710

416.72643	1	9711

416.82603	4	9714

416.92563	0	9715

417.02523	1	9716

417.12483	0	9716

417.22443	0	9716

417.32403	1	9717

417.42363	0	9718

417.52323	1	9719

417.62283	0	9720

417.72243	0	9720

417.82203	38	9758

417.92163	0	9759

418.02123	0	9759

418.12083	0	9760

418.22043	5	9764

418.32003	1	9765

418.41963	0	9766

418.51923	0	9766

418.61883	0	9767

418.71843	0	9767

418.81803	3	9770

418.91763	0	9771

419.01723	2	9773

419.11683	0	9773

419.21643	0	9774

419.31603	0	9774

419.41563	3	9777

419.51523	0	9777

419.61483	0	9778

419.71443	0	9778

419.81403	1	9779

419.91363	0	9780

420.01323	0	9780

420.11283	1	9781

420.21243	0	9781

420.31203	1	9782

420.41163	1	9783

420.51123	2	9785

420.61083	4	9790

420.71043	2	9792

420.81003	1	9793

420.90963	0	9793

421.00923	0	9794

421.10883	1	9795

421.20843	2	9797

421.30803	0	9797

421.40763	1	9798

421.50723	2	9800

421.60683	1	9801

421.70643	0	9801

421.80603	0	9802

421.90563	0	9802

422.00523	6	9808

422.10483	1	9809

422.20443	13	9823

422.30403	2	9824

422.40363	2	9826

422.50323	0	9827

422.60283	1	9828

422.70243	0	9828

422.80203	0	9828

422.90163	0	9829

423.00123	0	9829

423.10083	2	9832

423.20043	14	9845

423.30003	4	9849

423.39963	0	9850

423.49923	0	9850

423.59883	0	9851

423.69843	10	9860

423.79803	0	9861

423.89763	4	9864

423.99723	0	9865

424.09683	1	9866

424.19643	0	9866

424.29603	0	9867

424.39563	2	9868

424.49523	1	9869

424.59483	0	9870

424.69443	0	9870

424.79403	6	9876

424.89363	2	9878

424.99323	0	9879

425.09283	1	9880

425.19243	6	9885

425.29203	3	9888

425.39163	1	9889

425.49123	0	9889

425.59083	1	9890

425.69043	1	9891

425.79003	0	9892

425.88963	0	9892

425.98923	1	9893

426.08883	0	9893

426.18843	2	9895

426.28803	0	9896

426.38763	0	9896

426.48723	0	9896

426.58683	1	9897

426.68643	1	9898

426.78603	1	9899

426.88563	4	9903

426.98523	0	9903

427.08483	0	9904

427.18443	0	9904

427.28403	0	9904

427.38363	1	9905

427.48323	0	9906

427.58283	4	9909

427.68243	2	9911

427.78203	0	9912

427.88163	0	9912

427.98123	1	9913

428.08083	0	9913

428.18043	1	9915

428.28003	5	9920

428.37963	0	9920

428.47923	0	9920

428.57883	0	9921

428.67843	0	9921

428.77803	0	9922

428.87763	0	9922

428.97723	0	9923

429.07683	0	9923

429.17643	1	9924

429.27603	0	9924

429.37563	0	9925

429.47523	0	9925

429.57483	1	9926

429.67443	3	9929

429.77403	3	9932

429.87363	1	9933

429.97323	4	9937

430.07283	0	9938

430.17243	1	9939

430.27203	0	9940

430.37163	1	9940

430.47123	0	9941

430.57083	0	9941

430.67043	0	9942

430.77003	0	9942

430.86963	4	9947

430.96923	2	9948

431.06883	1	9950

431.16843	0	9950

431.26803	0	9951

431.36763	0	9951

431.46723	0	9952

431.56683	2	9954

431.66643	4	9958

431.76603	18	9976

431.86563	0	9976

431.96523	1	9978

432.06483	1	9979

432.16443	0	9980

432.26403	0	9980

432.36363	0	9980

432.46323	1	9981

432.56283	20	10001

432.66243	0	10001

432.76203	0	10002

432.86163	1	10003

432.96123	0	10003

433.06083	3	10006

433.16043	1	10007

433.26003	0	10007

433.35963	0	10008

433.45923	0	10008

433.55883	0	10008

433.65843	2	10010

433.75803	0	10011

433.85763	0	10011

433.95723	0	10012

434.05683	0	10012

434.15643	4	10016

434.25603	1	10016

434.35563	0	10017

434.45523	0	10017

434.55483	1	10019

434.65443	1	10020

434.75403	1	10020

434.85363	1	10022

434.95323	0	10022

435.05283	1	10023

435.15243	0	10024

435.25203	4	10028

435.35163	0	10028

435.45123	0	10029

435.55083	3	10032

435.65043	1	10033

435.75003	1	10034

435.84963	1	10035

435.94923	0	10035

436.04883	2	10037

436.14843	0	10038

436.24803	0	10038

436.34763	1	10039

436.44723	0	10040

436.54683	0	10040

436.64643	0	10040

436.74603	0	10041

436.84563	30	10071

436.94523	0	10071

437.04483	0	10072

437.14443	0	10072

437.24403	2	10074

437.34363	1	10075

437.44323	1	10076

437.54283	0	10076

437.64243	1	10077

437.74203	0	10078

437.84163	0	10078

437.94123	0	10079

438.04083	0	10079

438.14043	0	10080

438.24003	1	10080

438.33963	11	10092

438.43923	0	10092

438.53883	0	10092

438.63843	0	10093

438.73803	0	10093

438.83763	0	10094

438.93723	0	10094

439.03683	3	10097

439.13643	1	10098

439.23603	0	10099

439.33563	1	10100

439.43523	1	10100

439.53483	0	10101

439.63443	3	10104

439.73403	1	10104

439.83363	0	10105

439.93323	1	10106

440.03283	0	10106

440.13243	1	10107

440.23203	0	10108

440.33163	0	10108

440.43123	0	10108

440.53083	0	10109

440.63043	1	10110

440.73003	2	10112

440.82963	3	10115

440.92923	0	10115

441.02883	0	10116

441.12843	1	10117

441.22803	2	10119

441.32763	1	10120

441.42723	1	10122

441.52683	1	10123

441.62643	0	10124

441.72603	0	10124

441.82563	0	10124

441.92523	1	10126

442.02483	3	10129

442.12443	1	10130

442.22403	1	10131

442.32363	0	10132

442.42323	3	10135

442.52283	0	10135

442.62243	1	10136

442.72203	6	10142

442.82163	0	10142

442.92123	3	10145

443.02083	1	10146

443.12043	0	10146

443.22003	8	10154

443.31963	1	10155

443.41923	1	10156

443.51883	0	10156

443.61843	0	10157

443.71803	1	10158

443.81763	0	10158

443.91723	3	10161

444.01683	1	10162

444.11643	1	10163

444.21603	0	10164

444.31563	0	10164

444.41523	1	10165

444.51483	4	10169

444.61443	0	10170

444.71403	0	10170

444.81363	1	10171

444.91323	1	10172

445.01283	0	10173

445.11243	1	10174

445.21203	0	10174

445.31163	0	10175

445.41123	0	10175

445.51083	0	10176

445.61043	2	10177

445.71003	12	10190

445.80963	0	10190

445.90923	0	10191

446.00883	1	10192

446.10843	0	10192

446.20803	0	10193

446.30763	1	10194

446.40723	0	10194

446.50683	2	10196

446.60643	0	10197

446.70603	0	10197

446.80563	1	10199

446.90523	1	10200

447.00483	0	10200

447.10443	0	10201

447.20403	2	10203

447.30363	1	10204

447.40323	1	10205

447.50283	1	10206

447.60243	0	10207

447.70203	0	10207

447.80163	54	10261

447.90123	4	10266

448.00083	1	10267

448.10043	0	10267

448.20003	0	10268

448.29963	1	10269

448.39923	1	10270

448.49883	0	10270

448.59843	0	10271

448.69803	5	10276

448.79763	4	10280

448.89723	0	10280

448.99683	2	10282

449.09643	0	10283

449.19603	0	10283

449.29563	1	10284

449.39523	0	10285

449.49483	0	10285

449.59443	0	10286

449.69403	1	10287

449.79363	3	10290

449.89323	1	10291

449.99283	0	10291

450.09243	1	10292

450.19203	1	10293

450.29163	0	10293

450.39123	1	10294

450.49083	2	10296

450.59043	1	10297

450.69003	1	10298

450.78963	0	10298

450.88923	6	10304

450.98883	0	10304

451.08843	0	10305

451.18803	1	10306

451.28763	1	10307

451.38723	1	10308

451.48683	2	10310

451.58643	1	10311

451.68603	3	10314

451.78563	1	10315

451.88523	14	10329

451.98483	1	10330

452.08443	0	10331

452.18403	0	10331

452.28363	2	10333

452.38323	0	10333

452.48283	0	10334

452.58243	0	10334

452.68203	1	10335

452.78163	0	10336

452.88123	0	10336

452.98083	2	10338

453.08043	2	10340

453.18003	8	10349

453.27963	1	10350

453.37923	1	10351

453.47883	1	10352

453.57843	0	10352

453.67803	1	10353

453.77763	0	10354

453.87723	10	10364

453.97683	0	10364

454.07643	0	10364

454.17603	5	10369

454.27563	0	10370

454.37523	1	10371

454.47483	3	10373

454.57443	3	10376

454.67403	0	10377

454.77363	2	10379

454.87323	0	10380

454.97283	2	10381

455.07243	0	10382

455.17203	1	10383

455.27163	1	10384

455.37123	0	10384

455.47083	14	10398

455.57043	1	10399

455.67003	0	10400

455.76963	0	10400

455.86923	1	10401

455.96883	0	10401

456.06843	1	10402

456.16803	1	10404

456.26763	0	10404

456.36723	0	10404

456.46683	0	10405

456.56643	0	10405

456.66603	0	10406

456.76563	2	10408

456.86523	14	10421

456.96483	0	10422

457.06443	0	10422

457.16403	1	10423

457.26363	1	10424

457.36323	1	10425

457.46283	0	10425

457.56243	4	10429

457.66203	0	10430

457.76163	0	10430

457.86123	2	10432

457.96083	1	10433

458.06043	2	10435

458.16003	5	10440

458.25963	0	10440

458.35923	4	10444

458.45883	1	10445

458.55843	0	10445

458.65803	0	10446

458.75763	1	10447

458.85723	1	10448

458.95683	0	10448

459.05643	0	10448

459.15603	0	10449

459.25563	1	10450

459.35523	0	10450

459.45483	0	10451

459.55443	0	10451

459.65403	0	10452

459.75363	0	10452

459.85323	1	10453

459.95283	0	10453

460.05243	0	10454

460.15203	0	10454

460.25163	0	10455

460.35123	0	10455

460.45083	1	10456

460.55043	0	10456

460.65003	0	10457

460.74963	1	10458

460.84923	2	10460

460.94883	0	10460

461.04843	1	10461

461.14803	0	10462

461.24763	0	10462

461.34723	0	10463

461.44683	0	10463

461.54643	0	10464

461.64603	0	10464

461.74563	0	10464

461.84523	1	10465

461.94483	0	10466

462.04443	0	10466

462.14403	0	10467

462.24363	1	10468

462.34323	4	10472

462.44283	0	10472

462.54243	1	10473

462.64203	5	10478

462.74163	0	10479

462.84123	6	10484

462.94083	2	10486

463.04043	8	10494

463.14003	0	10494

463.23963	1	10496

463.33923	1	10496

463.43883	1	10497

463.53843	8	10505

463.63803	0	10506

463.73763	0	10506

463.83723	1	10508

463.93683	1	10509

464.03643	1	10510

464.13603	3	10512

464.23563	0	10513

464.33523	2	10515

464.43483	16	10531

464.53443	4	10535

464.63403	1	10536

464.73363	0	10536

464.83323	0	10537

464.93283	12	10549

465.03243	0	10549

465.13203	0	10550

465.23163	1	10551

465.33123	0	10551

465.43083	0	10552

465.53043	0	10552

465.63003	1	10553

465.72963	1	10555

465.82923	27	10582

465.92883	0	10582

466.02843	1	10583

466.12803	1	10584

466.22763	0	10585

466.32723	1	10586

466.42683	4	10591

466.52643	0	10591

466.62603	0	10592

466.72563	0	10592

466.82523	0	10592

466.92483	0	10593

467.02443	1	10594

467.12403	0	10594

467.22363	1	10595

467.32323	2	10597

467.42283	2	10599

467.52243	2	10601

467.62203	0	10601

467.72163	0	10602

467.82123	0	10602

467.92083	0	10603

468.02043	1	10604

468.12003	0	10604

468.21963	0	10604

468.31923	4	10608

468.41883	0	10609

468.51843	0	10609

468.61803	0	10610

468.71763	2	10612

468.81723	0	10612

468.91683	1	10613

469.01643	0	10613

469.11603	0	10614

469.21563	0	10614

469.31523	1	10616

469.41483	1	10616

469.51443	0	10617

469.61403	2	10619

469.71363	1	10620

469.81323	0	10620

469.91283	1	10621

470.01243	0	10622

470.11203	0	10622

470.21163	0	10623

470.31123	0	10623

470.41083	1	10624

470.51043	1	10625

470.61003	2	10627

470.70963	1	10628

470.80923	2	10630

470.90883	1	10631

471.00843	0	10632

471.10803	1	10632

471.20763	1	10633

471.30723	0	10634

471.40683	1	10635

471.50643	1	10636

471.60603	0	10636

471.70563	1	10637

471.80523	1	10638

471.90483	1	10640

472.00443	1	10641

472.10403	0	10641

472.20363	0	10642

472.30323	0	10642

472.40283	0	10643

472.50243	0	10643

472.60203	0	10644

472.70163	12	10656

472.80123	1	10656

472.90083	0	10657

473.00043	1	10658

473.10003	3	10661

473.19963	1	10662

473.29923	0	10662

473.39883	0	10663

473.49843	1	10664

473.59803	0	10664

473.69763	0	10664

473.79723	3	10667

473.89683	0	10668

473.99643	0	10668

474.09603	0	10668

474.19563	0	10669

474.29523	0	10669

474.39483	0	10670

474.49443	0	10670

474.59403	1	10671

474.69363	1	10672

474.79323	0	10672

474.89283	0	10673

474.99243	0	10673

475.09203	3	10676

475.19163	0	10676

475.29123	0	10677

475.39083	0	10677

475.49043	0	10678

475.59003	0	10678

475.68963	2	10680

475.78923	4	10684

475.88883	0	10684

475.98843	0	10685

476.08803	0	10685

476.18763	0	10686

476.28723	0	10686

476.38683	1	10687

476.48643	0	10688

476.58603	1	10688

476.68563	0	10689

476.78523	0	10689

476.88483	0	10690

476.98443	3	10693

477.08403	0	10693

477.18363	0	10694

477.28323	0	10694

477.38283	4	10698

477.48243	1	10699

477.58203	21	10720

477.68163	3	10723

477.78123	0	10723

477.88083	0	10724

477.98043	0	10724

478.08003	0	10724

478.17963	1	10725

478.27923	0	10726

478.37883	1	10727

478.47843	1	10728

478.57803	1	10728

478.67763	1	10729

478.77723	1	10731

478.87683	1	10732

478.97643	1	10733

479.07603	3	10736

479.17563	0	10736

479.27523	1	10737

479.37483	1	10738

479.47443	0	10739

479.57403	1	10740

479.67363	0	10740

479.77323	1	10741

479.87283	9	10750

479.97243	0	10751

480.07203	0	10751

480.17163	0	10752

480.27123	0	10752

480.37083	0	10752

480.47043	0	10753

480.57003	1	10754

480.66963	0	10754

480.76923	0	10755

480.86883	0	10755

480.96843	0	10756

481.06803	0	10756

481.16763	0	10756

481.26723	0	10757

481.36683	0	10757

481.46643	0	10758

481.56603	0	10758

481.66563	0	10759

481.76523	3	10761

481.86483	0	10762

481.96443	0	10762

482.06403	0	10763

482.16363	1	10764

482.26323	2	10766

482.36283	0	10767

482.46243	1	10768

482.56203	1	10769

482.66163	0	10770

482.76123	1	10771

482.86083	2	10772

482.96043	7	10780

483.06003	2	10782

483.15963	4	10785

483.25923	2	10788

483.35883	1	10788

483.45843	0	10789

483.55803	0	10789

483.65763	0	10790

483.75723	0	10790

483.85683	0	10791

483.95643	0	10791

484.05603	0	10792

484.15563	1	10792

484.25523	0	10793

484.35483	1	10794

484.45443	1	10795

484.55403	0	10795

484.65363	3	10798

484.75323	0	10799

484.85283	0	10799

484.95243	1	10800

485.05203	1	10801

485.15163	0	10802

485.25123	0	10802

485.35083	18	10820

485.45043	1	10821

485.55003	1	10822

485.64963	0	10823

485.74923	1	10824

485.84883	1	10825

485.94843	0	10825

486.04803	1	10827

486.14763	0	10827

486.24723	0	10828

486.34683	0	10828

486.44643	0	10828

486.54603	0	10829

486.64563	0	10829

486.74523	3	10832

486.84483	8	10840

486.94443	1	10840

487.04403	2	10842

487.14363	0	10843

487.24323	1	10844

487.34283	0	10844

487.44243	0	10845

487.54203	0	10845

487.64163	0	10846

487.74123	1	10847

487.84083	1	10848

487.94043	0	10848

488.04003	1	10849

488.13963	1	10851

488.23923	0	10851

488.33883	0	10852

488.43843	0	10852

488.53803	1	10853

488.63763	0	10853

488.73723	0	10854

488.83683	1	10855

488.93643	3	10858

489.03603	0	10859

489.13563	0	10859

489.23523	8	10867

489.33483	4	10872

489.43443	0	10872

489.53403	0	10872

489.63363	0	10873

489.73323	1	10874

489.83283	0	10875

489.93243	1	10876

490.03203	1	10876

490.13163	1	10877

490.23123	0	10878

490.33083	1	10879

490.43043	2	10881

490.53003	0	10881

490.62963	2	10883

490.72923	0	10884

490.82883	3	10887

490.92843	0	10887

491.02803	0	10888

491.12763	0	10888

491.22723	0	10888

491.32683	1	10890

491.42643	1	10891

491.52603	0	10892

491.62563	1	10893

491.72523	1	10894

491.82483	0	10895

491.92443	0	10895

492.02403	0	10896

492.12363	1	10896

492.22323	1	10898

492.32283	1	10899

492.42243	0	10899

492.52203	4	10903

492.62163	0	10904

492.72123	1	10904

492.82083	0	10905

492.92043	0	10905

493.02003	0	10906

493.11963	1	10907

493.21923	1	10908

493.31883	0	10909

493.41843	1	10910

493.51803	0	10910

493.61763	1	10911

493.71723	1	10912

493.81683	2	10915

493.91643	0	10915

494.01603	0	10916

494.11563	0	10916

494.21523	0	10916

494.31483	0	10917

494.41443	2	10919

494.51403	0	10919

494.61363	1	10920

494.71323	1	10921

494.81283	1	10922

494.91243	0	10923

495.01203	0	10923

495.11163	0	10924

495.21123	0	10924

495.31083	0	10924

495.41043	0	10925

495.51003	21	10946

495.60963	0	10946

495.70923	0	10947

495.80883	1	10948

495.90843	2	10949

496.00803	1	10950

496.10763	0	10951

496.20723	0	10951

496.30683	0	10952

496.40643	0	10952

496.50603	4	10956

496.60563	0	10957

496.70523	6	10963

496.80483	1	10964

496.90443	0	10964

497.00403	0	10965

497.10363	1	10966

497.20323	0	10966

497.30283	0	10967

497.40243	0	10967

497.50203	0	10968

497.60163	2	10969

497.70123	0	10970

497.80083	0	10970

497.90043	0	10971

498.00003	0	10971

498.09963	0	10972

498.19923	1	10972

498.29883	0	10973

498.39843	0	10973

498.49803	0	10974

498.59763	0	10974

498.69723	1	10975

498.79683	1	10976

498.89643	0	10977

498.99603	5	10982

499.09563	3	10985

499.19523	0	10985

499.29483	0	10986

499.39443	3	10988

499.49403	0	10989

499.59363	0	10989

499.69323	0	10990

499.79283	0	10990

499.89243	0	10991

499.99203	0	10991

500.09163	1	10992

500.19123	0	10992

500.29083	0	10993

500.39043	0	10993

500.49003	3	10996

500.58963	0	10996

500.68923	0	10997

500.78883	0	10997

500.88843	1	10999

500.98803	1	11000

501.08763	0	11000

501.18723	0	11001

501.28683	0	11001

501.38643	1	11002

501.48603	3	11005

501.58563	2	11007

501.68523	0	11007

501.78483	1	11008

501.88443	0	11008

501.98403	1	11009

502.08363	0	11010

502.18323	1	11011

502.28283	1	11012

502.38243	0	11012

502.48203	0	11012

502.58163	0	11013

502.68123	2	11015

502.78083	0	11016

502.88043	1	11017

502.98003	0	11017

503.07963	0	11018

503.17923	0	11018

503.27883	0	11019

503.37843	0	11019

503.47803	1	11020

503.57763	0	11020

503.67723	3	11023

503.77683	1	11024

503.87643	0	11024

503.97603	0	11025

504.07563	3	11028

504.17523	2	11029

504.27483	0	11030

504.37443	1	11031

504.47403	1	11032

504.57363	0	11032

504.67323	0	11033

504.77283	0	11033

504.87243	0	11034

504.97203	0	11034

505.07163	6	11040

505.17123	0	11040

505.27083	0	11041

505.37043	1	11042

505.47003	0	11042

505.56963	0	11043

505.66923	0	11043

505.76883	0	11044

505.86843	0	11044

505.96803	0	11044

506.06763	0	11045

506.16723	0	11045

506.26683	0	11046

506.36643	0	11046

506.46603	0	11047

506.56563	1	11048

506.66523	0	11048

506.76483	0	11048

506.86443	0	11049

506.96403	1	11050

507.06363	0	11051

507.16323	3	11054

507.26283	0	11054

507.36243	0	11055

507.46203	0	11055

507.56163	0	11056

507.66123	0	11056

507.76083	11	11067

507.86043	1	11068

507.96003	2	11070

508.05963	1	11071

508.15923	1	11072

508.25883	1	11073

508.35843	1	11073

508.45803	1	11074

508.55763	1	11075

508.65723	1	11075

508.75683	1	11076

508.85643	1	11077

508.95603	1	11078

509.05563	1	11079

509.15523	1	11079

509.25483	1	11081

509.35443	1	11081

509.45403	1	11082

509.55363	1	11083

509.65323	1	11084

509.75283	1	11085

509.85243	1	11086

509.95203	1	11087

510.05163	1	11088

510.15123	1	11089

510.25083	1	11089

510.35043	1	11091

510.45003	1	11091

510.54963	3	11094

510.64923	2	11096

510.74883	1	11097

510.84843	1	11098

510.94803	1	11099

511.04763	2	11101

511.14723	1	11102

511.24683	1	11103

511.34643	1	11103

511.44603	3	11107

511.54563	3	11109

511.64523	1	11111

511.74483	1	11111

511.84443	1	11113

511.94403	1	11113

512.04363	1	11114

512.14323	3	11117

512.24283	3	11121

512.34243	1	11122

512.44203	1	11123

512.54163	1	11123

512.64123	1	11124

512.74083	1	11125

512.84043	1	11127

512.94003	1	11127

513.03963	3	11130

513.13923	1	11131

513.23883	1	11132

513.33843	1	11133

513.43803	1	11133

513.53763	1	11134

513.63723	1	11135

513.73683	7	11142

513.83643	1	11143

513.93603	1	11145

514.03563	1	11145

514.13523	1	11146

514.23483	1	11147

514.33443	4	11151

514.43403	1	11152

514.53363	2	11154

514.63323	1	11155

514.73283	1	11155

514.83243	3	11158

514.93203	1	11159

515.03163	1	11159

515.13123	1	11161

515.23083	1	11161

515.33043	1	11163

515.43003	1	11164

515.52963	1	11165

515.62923	1	11165

515.72883	1	11166

515.82843	1	11167

515.92803	1	11167

516.02763	1	11169

516.12723	1	11169

516.22683	1	11170

516.32643	1	11171

516.42603	1	11171

516.52563	1	11172

516.62523	1	11173

516.72483	1	11173

516.82443	1	11174

516.92403	1	11175

517.02363	1	11175

517.12323	1	11176

517.22283	1	11177

517.32243	1	11179

517.42203	2	11181

517.52163	1	11181

517.62123	1	11182

517.72083	1	11183

517.82043	1	11184

517.92003	1	11185

518.01963	3	11187

518.11923	1	11189

518.21883	1	11190

518.31843	1	11191

518.41803	1	11192

518.51763	1	11193

518.61723	1	11193

518.71683	3	11196

518.81643	1	11197

518.91603	1	11197

519.01563	1	11199

519.11523	2	11201

519.21483	1	11202

519.31443	3	11205

519.41403	1	11205

519.51363	4	11209

519.61323	1	11210

519.71283	1	11211

519.81243	1	11211

519.91203	4	11215

520.01163	1	11216

520.11123	1	11217

520.21083	3	11220

520.31043	10	11230

520.41003	1	11231

520.50963	1	11231

520.60923	1	11232

520.70883	1	11233

520.80843	1	11234

520.90803	1	11235

521.00763	4	11239

521.10723	4	11243

521.20683	1	11243

521.30643	1	11244

521.40603	1	11245

521.50563	1	11246

521.60523	1	11247

521.70483	1	11247

521.80443	1	11248

521.90403	1	11249

522.00363	1	11250

522.10323	1	11251

522.20283	1	11251

522.30243	1	11252

522.40203	1	11253

522.50163	3	11257

522.60123	1	11257

522.70083	1	11258

522.80043	1	11259

522.90003	1	11260

522.99963	1	11261

523.09923	1	11263

523.19883	1	11264

523.29843	2	11266

523.39803	1	11267

523.49763	2	11269

523.59723	1	11269

523.69683	1	11270

523.79643	1	11271

523.89603	1	11272

523.99563	1	11273

524.09523	3	11275

524.19483	1	11276

524.29443	1	11277

524.39403	1	11278

524.49363	1	11279

524.59323	2	11281

524.69283	1	11282

524.79243	1	11283

524.89203	1	11284

524.99163	1	11285

525.09123	1	11286

525.19083	1	11287

525.29043	1	11287

525.39003	1	11288

525.48963	1	11289

525.58923	1	11290

525.68883	1	11291

525.78843	1	11291

525.88803	1	11292

525.98763	1	11293

526.08723	3	11296

526.18683	1	11297

526.28643	1	11297

526.38603	1	11298

526.48563	1	11299

526.58523	1	11299

526.68483	5	11304

526.78443	1	11305

526.88403	1	11306

526.98363	1	11307

527.08323	1	11307

527.18283	1	11308

527.28243	1	11309

527.38203	1	11310

527.48163	1	11311

527.58123	1	11312

527.68083	1	11313

527.78043	1	11314

527.88003	1	11315

527.97963	1	11315

528.07923	1	11317

528.17883	8	11325

528.27843	1	11326

528.37803	1	11327

528.47763	3	11330

528.57723	1	11331

528.67683	1	11332

528.77643	1	11333

528.87603	1	11333

528.97563	2	11335

529.07523	1	11336

529.17483	3	11339

529.27443	6	11345

529.37403	1	11346

529.47363	1	11347

529.57323	1	11347

529.67283	1	11349

529.77243	1	11350

529.87203	3	11353

529.97163	1	11353

530.07123	13	11367

530.17083	2	11369

530.27043	3	11371

530.37003	1	11372

530.46963	2	11374

530.56923	1	11375

530.66883	1	11375

530.76843	8	11383

530.86803	1	11384

530.96763	3	11387

531.06723	1	11387

531.16683	1	11389

531.26643	3	11392

531.36603	2	11394

531.46563	1	11395

531.56523	2	11397

531.66483	2	11399

531.76443	1	11400

531.86403	2	11402

531.96363	1	11403

532.06323	1	11404

532.16283	1	11405

532.26243	3	11407

532.36203	1	11408

532.46163	1	11409

532.56123	3	11411

532.66083	3	11414

532.76043	1	11415

532.86003	1	11415

532.95963	1	11416

533.05923	1	11417

533.15883	1	11418

533.25843	1	11419

533.35803	1	11421

533.45763	1	11421

533.55723	1	11423

533.65683	1	11423

533.75643	1	11424

533.85603	1	11425

533.95563	1	11426

534.05523	1	11427

534.15483	1	11428

534.25443	1	11429

534.35403	1	11429

534.45363	4	11433

534.55323	3	11436

534.65283	2	11438

534.75243	2	11440

534.85203	1	11441

534.95163	1	11442

535.05123	1	11443

535.15083	1	11443

535.25043	2	11445

535.35003	1	11446

535.44963	2	11448

535.54923	1	11449

535.64883	1	11449

535.74843	1	11450

535.84803	1	11451

535.94763	1	11451

536.04723	15	11467

536.14683	1	11468

536.24643	1	11469

536.34603	1	11469

536.44563	1	11470

536.54523	3	11473

536.64483	1	11473

536.74443	1	11474

536.84403	3	11477

536.94363	1	11477

537.04323	1	11478

537.14283	1	11479

537.24243	1	11479

537.34203	1	11481

537.44163	1	11481

537.54123	1	11483

537.64083	1	11483

537.74043	1	11484

537.84003	2	11486

537.93963	1	11487

538.03923	3	11489

538.13883	1	11490

538.23843	1	11491

538.33803	1	11491

538.43763	1	11493

538.53723	1	11493

538.63683	1	11494

538.73643	1	11495

538.83603	1	11495

538.93563	1	11496

539.03523	1	11497

539.13483	1	11498

539.23443	1	11499

539.33403	1	11499

539.43363	1	11500

539.53323	1	11501

539.63283	3	11503

539.73243	1	11504

539.83203	3	11507

539.93163	1	11508

540.03123	1	11509

540.13083	1	11510

540.23043	1	11511

540.33003	7	11518

540.42963	2	11520

540.52923	2	11522

540.62883	1	11523

540.72843	2	11525

540.82803	2	11527

540.92763	2	11529

541.02723	1	11529

541.12683	1	11531

541.22643	1	11531

541.32603	21	11552

541.42563	1	11553

541.52523	1	11553

541.62483	1	11554

541.72443	2	11556

541.82403	1	11557

541.92363	2	11559

542.02323	1	11561

542.12283	1	11561

542.22243	1	11562

542.32203	1	11563

542.42163	1	11563

542.52123	1	11564

542.62083	1	11565

542.72043	3	11567

542.82003	1	11568

542.91963	1	11569

543.01923	1	11570

543.11883	1	11571

543.21843	1	11571

543.31803	1	11572

543.41763	1	11573

543.51723	2	11575

543.61683	1	11576

543.71643	2	11578

543.81603	1	11579

543.91563	3	11581

544.01523	1	11583

544.11483	1	11583

544.21443	1	11584

544.31403	1	11585

544.41363	3	11587

544.51323	2	11589

544.61283	1	11590

544.71243	1	11591

544.81203	1	11591

544.91163	1	11592

545.01123	1	11593

545.11083	2	11595

545.21043	1	11597

545.31003	1	11597

545.40963	1	11598

545.50923	2	11600

545.60883	2	11602

545.70843	1	11603

545.80803	1	11603

545.90763	3	11606

546.00723	1	11607

546.10684	1	11609

546.20644	1	11609

546.30603	2	11611

546.40563	1	11612

546.50523	1	11613

546.60483	1	11613

546.70443	1	11615

546.80404	1	11615

546.90364	3	11618

547.00324	1	11619

547.10284	6	11625

547.20244	1	11626

547.30204	1	11627

547.40164	1	11627

547.50123	1	11628

547.60083	1	11629

547.70044	2	11631

547.80004	2	11633

547.89964	1	11634

547.99924	1	11635

548.09884	1	11636

548.19844	1	11637

548.29804	7	11645

548.39764	1	11645

548.49724	1	11647

548.59684	1	11647

548.69644	1	11648

548.79604	1	11649

548.89564	1	11650

548.99524	1	11651

549.09484	1	11651

549.19444	1	11653

549.29404	1	11653

549.39364	1	11654

549.49324	3	11657

549.59284	1	11658

549.69244	4	11662

549.79204	1	11663

549.89164	2	11665

549.99124	1	11665

550.09084	1	11667

550.19044	1	11667

550.29004	1	11669

550.38964	1	11669

550.48924	1	11670

550.58884	1	11671

550.68844	2	11673

550.78804	1	11673

550.88764	1	11674

550.98724	1	11675

551.08684	1	11675

551.18644	2	11677

551.28604	1	11678

551.38564	1	11679

551.48524	1	11679

551.58484	1	11680

551.68444	1	11681

551.78404	1	11681

551.88364	2	11683

551.98324	3	11687

552.08284	1	11687

552.18244	1	11689

552.28204	3	11691

552.38164	1	11692

552.48124	1	11693

552.58084	1	11694

552.68044	4	11698

552.78004	2	11700

552.87964	2	11702

552.97924	1	11703

553.07884	1	11704

553.17844	1	11705

553.27804	9	11714

553.37764	1	11715

553.47724	1	11715

553.57684	1	11716

553.67644	1	11717

553.77604	3	11720

553.87564	1	11721

553.97524	1	11721

554.07484	1	11722

554.17444	1	11723

554.27404	1	11723

554.37364	1	11724

554.47324	3	11727

554.57284	2	11729

554.67244	3	11733

554.77204	2	11735

554.87164	1	11736

554.97124	1	11737

555.07084	1	11738

555.17044	1	11739

555.27004	1	11739

555.36964	1	11740

555.46924	1	11741

555.56884	1	11742

555.66844	2	11744

555.76804	1	11745

555.86764	1	11746

555.96724	2	11748

556.06684	3	11751

556.16644	1	11751

556.26604	1	11752

556.36564	1	11753

556.46524	1	11753

556.56484	1	11755

556.66444	2	11757

556.76404	1	11757

556.86364	1	11758

556.96324	1	11759

557.06284	1	11759

557.16244	1	11761

557.26204	1	11761

557.36164	2	11763

557.46124	1	11764

557.56084	1	11765

557.66044	1	11765

557.76004	1	11766

557.85964	2	11768

557.95924	1	11769

558.05884	1	11770

558.15844	1	11771

558.25804	1	11773

558.35764	2	11775

558.45724	1	11775

558.55684	1	11777

558.65644	1	11778

558.75604	2	11780

558.85564	1	11781

558.95524	1	11782

559.05484	1	11783

559.15444	1	11784

559.25404	1	11785

559.35364	3	11787

559.45324	1	11789

559.55284	1	11790

559.65244	1	11791

559.75204	1	11791

559.85164	1	11793

559.95124	15	11807

560.05084	1	11808

560.15044	1	11809

560.25004	1	11809

560.34964	1	11810

560.44924	3	11813

560.54884	1	11814

560.64844	1	11815

560.74804	6	11821

560.84764	1	11822

560.94724	1	11823

561.04684	1	11823

561.14644	1	11824

561.24604	1	11825

561.34564	1	11825

561.44524	1	11826

561.54484	1	11827

561.64444	5	11831

561.74404	1	11833

561.84364	1	11833

561.94324	1	11835

562.04284	2	11837

562.14244	14	11851

562.24204	46	11897

562.34164	1	11897

562.44124	1	11898

562.54084	2	11900

562.64044	1	11901

562.74004	3	11903

562.83964	1	11904

562.93924	3	11907

563.03884	4	11911

563.13844	1	11911

563.23804	1	11912

563.33764	1	11913

563.43724	1	11914

563.53684	1	11915

563.63644	1	11915

563.73604	1	11916

563.83564	1	11917

563.93524	1	11917

564.03484	1	11919

564.13444	2	11921

564.23404	3	11924

564.33364	5	11929

564.43324	3	11932

564.53284	1	11933

564.63244	1	11934

564.73204	1	11935

564.83164	1	11936

564.93124	1	11937

565.03084	1	11937

565.13044	1	11938

565.23004	1	11939

565.32964	1	11939

565.42924	2	11941

565.52884	2	11943

565.62844	1	11945

565.72804	1	11946

565.82764	2	11948

565.92724	1	11949

566.02684	2	11951

566.12644	1	11951

566.22604	1	11952

566.32564	1	11953

566.42524	1	11953

566.52484	1	11955

566.62444	1	11955

566.72404	1	11956

566.82364	1	11957

566.92324	1	11957

567.02284	6	11963

567.12244	4	11967

567.22204	2	11969

567.32164	2	11971

567.42124	1	11972

567.52084	3	11975

567.62044	1	11976

567.72004	1	11977

567.81964	1	11977

567.91924	1	11978

568.01884	2	11980

568.11844	1	11981

568.21804	1	11982

568.31764	1	11983

568.41724	2	11985

568.51684	1	11985

568.61644	2	11987

568.71604	1	11988

568.81564	3	11991

568.91524	1	11992

569.01484	3	11995

569.11444	1	11995

569.21404	1	11996

569.31364	5	12001

569.41324	1	12002

569.51284	1	12003

569.61244	1	12004

569.71204	1	12005

569.81164	1	12006

569.91124	1	12007

570.01084	11	12017

570.11044	2	12019

570.21004	8	12027

570.30964	2	12029

570.40924	1	12030

570.50884	1	12031

570.60844	1	12031

570.70804	3	12034

570.80764	1	12035

570.90724	1	12035

571.00684	1	12036

571.10644	1	12037

571.20604	1	12038

571.30564	4	12042

571.40524	3	12045

571.50484	1	12046

571.60444	1	12047

571.70404	1	12047

571.80364	1	12049

571.90324	1	12049

572.00284	1	12050

572.10244	1	12051

572.20204	1	12052

572.30164	1	12053

572.40124	5	12058

572.50084	1	12059

572.60044	1	12059

572.70004	1	12060

572.79964	1	12061

572.89924	1	12061

572.99884	1	12063

573.09844	1	12064

573.19804	1	12065

573.29764	4	12069

573.39724	3	12072

573.49684	1	12073

573.59644	1	12075

573.69604	3	12078

573.79564	5	12083

573.89524	1	12084

573.99484	1	12085

574.09444	2	12087

574.19404	2	12089

574.29364	1	12090

574.39324	1	12091

574.49284	1	12092

574.59244	3	12095

574.69204	1	12096

574.79164	2	12098

574.89124	1	12099

574.99084	1	12100

575.09044	1	12101

575.19004	1	12101

575.28964	2	12103

575.38924	1	12104

575.48884	2	12106

575.58844	1	12107

575.68804	1	12107

575.78764	14	12121

575.88724	1	12123

575.98684	1	12123

576.08644	3	12126

576.18604	1	12127

576.28564	1	12127

576.38524	1	12128

576.48484	1	12129

576.58444	1	12130

576.68404	3	12133

576.78364	3	12135

576.88324	1	12136

576.98284	2	12138

577.08244	1	12139

577.18204	1	12140

577.28164	3	12143

577.38124	1	12144

577.48084	3	12147

577.58044	1	12147

577.68004	1	12148

577.77964	3	12151

577.87924	1	12151

577.97884	1	12152

578.07844	1	12153

578.17804	7	12161

578.27764	3	12163

578.37724	1	12164

578.47684	1	12165

578.57644	5	12170

578.67604	1	12171

578.77564	2	12173

578.87524	1	12174

578.97484	3	12177

579.07444	1	12177

579.17404	1	12179

579.27364	1	12179

579.37324	2	12181

579.47284	1	12182

579.57244	1	12183

579.67204	1	12183

579.77164	2	12185

579.87124	1	12186

579.97084	1	12187

580.07044	2	12189

580.17004	1	12189

580.26964	1	12190

580.36924	4	12194

580.46884	2	12196

580.56844	1	12197

580.66804	1	12197

580.76764	1	12198

580.86724	1	12199

580.96684	1	12201

581.06644	1	12201

581.16604	1	12202

581.26564	1	12203

581.36524	2	12205

581.46484	2	12207

581.56444	1	12207

581.66404	1	12208

581.76364	2	12210

581.86324	1	12211

581.96284	5	12215

582.06244	3	12218

582.16204	1	12219

582.26164	1	12219

582.36124	1	12221

582.46084	3	12224

582.56044	1	12225

582.66004	1	12226

582.75964	1	12227

582.85924	1	12227

582.95884	1	12228

583.05844	1	12229

583.15804	1	12229

583.25764	1	12230

583.35724	1	12231

583.45684	3	12234

583.55644	1	12235

583.65604	1	12235

583.75564	1	12237

583.85524	1	12238

583.95484	1	12239

584.05444	1	12240

584.15404	1	12241

584.25364	1	12242

584.35324	1	12243

584.45284	2	12245

584.55244	1	12246

584.65204	6	12252

584.75164	1	12253

584.85124	1	12253

584.95084	1	12255

585.05044	1	12255

585.15004	1	12256

585.24964	1	12257

585.34924	1	12258

585.44884	1	12259

585.54844	1	12260

585.64804	1	12261

585.74764	1	12261

585.84724	2	12263

585.94684	10	12273

586.04644	1	12275

586.14604	1	12275

586.24564	1	12276

586.34524	2	12278

586.44484	1	12279

586.54444	2	12281

586.64404	1	12282

586.74364	1	12283

586.84324	3	12286

586.94284	1	12287

587.04244	2	12289

587.14204	1	12289

587.24164	1	12291

587.34124	1	12291

587.44084	3	12294

587.54044	3	12297

587.64004	1	12298

587.73964	1	12299

587.83924	1	12300

587.93884	3	12303

588.03844	1	12303

588.13804	3	12306

588.23764	1	12307

588.33724	1	12308

588.43684	1	12309

588.53644	8	12317

588.63604	1	12318

588.73564	5	12323

588.83524	2	12325

588.93484	1	12327

589.03444	1	12327

589.13404	1	12328

589.23364	1	12329

589.33324	1	12330

589.43284	2	12332

589.53244	1	12333

589.63204	2	12335

589.73164	3	12338

589.83124	1	12339

589.93084	1	12340

590.03044	3	12343

590.13004	1	12343

590.22964	1	12344

590.32924	1	12345

590.42884	1	12346

590.52844	6	12352

590.62804	1	12353

590.72764	8	12361

590.82724	2	12363

590.92684	3	12367

591.02644	1	12367

591.12604	1	12368

591.22564	1	12369

591.32524	1	12371

591.42484	1	12371

591.52444	2	12373

591.62404	1	12374

591.72364	1	12375

591.82324	5	12379

591.92284	1	12380

592.02244	3	12383

592.12204	2	12385

592.22164	1	12385

592.32124	5	12390

592.42084	4	12394

592.52044	1	12395

592.62004	1	12396

592.71964	1	12397

592.81924	1	12397

592.91884	2	12399

593.01844	1	12400

593.11804	1	12401

593.21764	1	12402

593.31724	5	12407

593.41684	1	12407

593.51644	2	12409

593.61604	1	12410

593.71564	1	12411

593.81524	5	12416

593.91484	1	12417

594.01444	2	12419

594.11404	1	12419

594.21364	1	12421

594.31324	1	12421

594.41284	1	12423

594.51244	1	12424

594.61204	1	12425

594.71164	1	12425

594.81124	2	12427

594.91084	1	12428

595.01044	1	12429

595.11004	3	12432

595.20964	1	12433

595.30924	1	12434

595.40884	1	12435

595.50844	1	12436

595.60804	1	12437

595.70764	1	12437

595.80724	1	12438

595.90684	4	12442

596.00644	2	12444

596.10604	10	12454

596.20564	1	12455

596.30524	1	12456

596.40484	1	12457

596.50444	1	12459

596.60404	1	12459

596.70364	4	12463

596.80324	2	12465

596.90284	3	12469

597.00244	1	12469

597.10204	1	12470

597.20164	3	12473

597.30124	1	12474

597.40084	1	12475

597.50044	1	12476

597.60004	1	12477

597.69964	1	12477

597.79924	1	12478

597.89884	2	12480

597.99844	1	12481

598.09804	1	12483

598.19764	1	12484

598.29724	3	12487

598.39684	1	12487

598.49644	1	12488

598.59604	1	12489

598.69564	1	12490

598.79524	1	12491

598.89484	1	12492

598.99444	1	12493

599.09404	1	12494

599.19364	5	12499

599.29324	2	12501

599.39284	2	12503

599.49244	1	12504

599.59204	5	12509

599.69164	1	12509

599.79124	3	12513

599.89084	2	12515

599.99044	1	12516

600.09004	2	12518

600.18964	3	12521

600.28924	1	12521

600.38884	1	12522

600.48844	2	12524

600.58804	1	12525

600.68764	1	12527

600.78724	1	12527

600.88684	3	12530

600.98644	1	12531

601.08604	1	12531

601.18564	1	12533

601.28524	1	12533

601.38484	1	12534

601.48444	1	12535

601.58404	1	12536

601.68364	1	12537

601.78324	1	12537

601.88284	1	12538

601.98244	1	12539

602.08204	5	12544

602.18164	1	12545

602.28124	2	12547

602.38084	1	12547

602.48044	1	12548

602.58004	1	12549

602.67964	3	12551

602.77924	2	12553

602.87884	1	12554

602.97844	1	12555

603.07804	1	12556

603.17764	5	12561

603.27724	1	12561

603.37684	3	12564

603.47644	1	12565

603.57604	2	12567

603.67564	1	12567

603.77524	1	12568

603.87484	1	12569

603.97444	1	12570

604.07404	1	12571

604.17364	1	12571

604.27324	3	12575

604.37284	1	12575

604.47244	1	12576

604.57204	1	12577

604.67164	1	12577

604.77124	1	12578

604.87084	1	12579

604.97044	2	12581

605.07004	1	12581

605.16964	1	12583

605.26924	1	12584

605.36884	1	12585

605.46844	1	12586

605.56804	1	12587

605.66764	2	12589

605.76724	1	12590

605.86684	1	12591

605.96644	1	12591

606.06604	2	12593

606.16564	1	12595

606.26524	1	12595

606.36484	1	12597

606.46444	1	12597

606.56404	1	12598

606.66364	7	12605

606.76324	1	12605

606.86284	1	12607

606.96244	1	12608

607.06204	6	12614

607.16164	1	12615

607.26124	1	12615

607.36084	1	12616

607.46044	1	12617

607.56004	1	12618

607.65964	1	12619

607.75924	7	12627

607.85884	1	12627

607.95844	3	12630

608.05804	3	12633

608.15764	1	12634

608.25724	3	12637

608.35684	1	12637

608.45644	1	12638

608.55604	1	12639

608.65564	3	12641

608.75524	1	12642

608.85484	2	12644

608.95444	1	12645

609.05404	1	12646

609.15364	1	12647

609.25324	1	12647

609.35284	3	12651

609.45244	4	12655

609.55204	1	12655

609.65164	2	12657

609.75124	1	12658

609.85084	1	12659

609.95044	1	12659

610.05004	1	12661

610.14964	1	12661

610.24924	33	12694

610.34884	1	12695

610.44844	2	12697

610.54804	7	12704

610.64764	2	12706

610.74724	1	12707

610.84684	1	12707

610.94644	1	12708

611.04604	1	12709

611.14564	1	12710

611.24524	2	12712

611.34484	1	12713

611.44444	2	12715

611.54404	4	12719

611.64364	1	12720

611.74324	3	12723

611.84284	1	12725

611.94244	1	12726

612.04204	1	12727

612.14164	1	12727

612.24124	2	12729

612.34084	3	12733

612.44044	1	12733

612.54004	6	12739

612.63964	1	12740

612.73924	1	12741

612.83884	1	12742

612.93844	1	12743

613.03804	1	12743

613.13764	3	12747

613.23724	1	12747

613.33684	1	12748

613.43644	1	12749

613.53604	1	12749

613.63564	3	12753

613.73524	2	12755

613.83484	1	12755

613.93444	1	12756

614.03404	1	12757

614.13364	1	12758

614.23324	1	12759

614.33284	1	12759

614.43244	1	12760

614.53204	1	12761

614.63164	1	12761

614.73124	1	12763

614.83084	1	12763

614.93044	1	12765

615.03004	1	12766

615.12964	1	12767

615.22924	1	12769

615.32884	1	12770

615.42844	6	12776

615.52804	3	12779

615.62764	1	12779

615.72724	1	12780

615.82684	8	12788

615.92644	1	12789

616.02604	1	12790

616.12564	1	12791

616.22524	1	12793

616.32484	7	12800

616.42444	1	12801

616.52404	3	12804

616.62364	3	12807

616.72324	1	12808

616.82284	2	12810

616.92244	5	12815

617.02204	1	12815

617.12164	1	12817

617.22124	2	12819

617.32084	1	12819

617.42044	1	12820

617.52004	1	12821

617.61964	6	12827

617.71924	2	12829

617.81884	2	12831

617.91844	1	12832

618.01804	1	12833

618.11764	2	12835

618.21724	3	12837

618.31684	3	12841

618.41644	1	12841

618.51604	1	12842

618.61564	3	12845

618.71524	1	12845

618.81484	1	12846

618.91444	1	12847

619.01404	1	12848

619.11364	6	12854

619.21324	1	12855

619.31284	1	12855

619.41244	1	12856

619.51204	3	12859

619.61164	1	12860

619.71124	1	12861

619.81084	1	12861

619.91044	1	12862

620.01004	6	12868

620.10964	1	12869

620.20924	1	12870

620.30884	1	12871

620.40844	1	12872

620.50804	2	12874

620.60764	3	12877

620.70724	3	12881

620.80684	1	12881

620.90644	1	12883

621.00604	2	12885

621.10564	1	12885

621.20524	1	12886

621.30484	2	12888

621.40444	1	12889

621.50404	1	12889

621.60364	2	12891

621.70324	8	12899

621.80284	1	12901

621.90244	4	12905

622.00204	7	12911

622.10164	1	12912

622.20124	7	12919

622.30084	1	12921

622.40044	1	12922

622.50004	5	12927

622.59964	1	12927

622.69924	1	12928

622.79884	1	12929

622.89844	3	12931

622.99804	1	12933

623.09764	1	12934

623.19724	1	12935

623.29684	1	12936

623.39644	1	12937

623.49604	1	12937

623.59564	1	12938

623.69524	1	12939

623.79484	1	12939

623.89444	1	12940

623.99404	3	12943

624.09364	1	12944

624.19324	1	12945

624.29284	1	12946

624.39244	1	12947

624.49204	2	12949

624.59164	1	12949

624.69124	1	12950

624.79084	1	12951

624.89044	1	12951

624.99004	1	12953

625.08964	5	12957

625.18924	1	12959

625.28884	1	12959

625.38844	4	12963

625.48804	1	12964

625.58764	1	12965

625.68724	1	12966

625.78684	3	12969

625.88644	1	12970

625.98604	1	12971

626.08564	3	12974

626.18524	2	12976

626.28484	8	12984

626.38444	1	12985

626.48404	11	12995

626.58364	1	12996

626.68324	1	12997

626.78284	2	12999

626.88244	2	13001

626.98204	1	13003

627.08164	1	13003

627.18124	1	13004

627.28084	1	13005

627.38044	2	13007

627.48004	3	13009

627.57964	3	13013

627.67924	1	13014

627.77884	1	13015

627.87844	1	13016

627.97804	1	13017

628.07764	1	13018

628.17724	1	13019

628.27684	5	13023

628.37644	1	13024

628.47604	1	13025

628.57564	1	13026

628.67524	1	13027

628.77484	2	13029

628.87444	2	13031

628.97404	1	13031

629.07364	1	13033

629.17324	1	13033

629.27284	1	13034

629.37244	1	13035

629.47204	3	13039

629.57164	1	13039

629.67124	1	13040

629.77084	1	13041

629.87044	1	13041

629.97004	1	13042

630.06964	1	13043

630.16924	1	13044

630.26884	1	13045

630.36844	1	13045

630.46804	1	13046

630.56764	1	13047

630.66724	5	13052

630.76684	3	13055

630.86644	2	13057

630.96604	5	13063

631.06564	1	13063

631.16524	7	13071

631.26484	1	13071

631.36444	1	13073

631.46404	3	13076

631.56364	2	13078

631.66324	3	13081

631.76284	1	13081

631.86244	2	13083

631.96204	1	13085

632.06164	1	13085

632.16124	1	13087

632.26084	1	13087

632.36044	3	13090

632.46004	1	13091

632.55964	1	13091

632.65924	2	13093

632.75884	1	13094

632.85844	1	13095

632.95804	1	13095

633.05764	4	13099

633.15724	2	13101

633.25684	1	13102

633.35644	1	13103

633.45604	1	13104

633.55564	3	13107

633.65524	1	13107

633.75484	1	13108

633.85444	1	13109

633.95404	1	13109

634.05364	4	13113

634.15324	1	13114

634.25284	1	13115

634.35244	1	13116

634.45204	1	13117

634.55164	3	13121

634.65124	1	13121

634.75084	1	13122

634.85044	3	13125

634.95004	1	13125

635.04964	5	13130

635.14924	1	13131

635.24884	1	13131

635.34844	3	13134

635.44804	1	13135

635.54764	1	13135

635.64724	3	13139

635.74684	3	13141

635.84644	5	13146

635.94604	1	13147

636.04564	1	13149

636.14524	1	13150

636.24484	1	13151

636.34444	1	13151

636.44404	4	13155

636.54364	1	13156

636.64324	1	13157

636.74284	4	13161

636.84244	4	13165

636.94204	1	13165

637.04164	9	13174

637.14124	1	13175

637.24084	3	13178

637.34044	5	13183

637.44004	1	13183

637.53964	7	13191

637.63924	1	13191

637.73884	1	13192

637.83844	1	13193

637.93804	1	13193

638.03764	1	13194

638.13724	1	13195

638.23684	1	13195

638.33644	5	13201

638.43604	1	13201

638.53564	1	13202

638.63524	1	13203

638.73484	1	13203

638.83444	1	13204

638.93404	1	13205

639.03364	1	13205

639.13324	1	13206

639.23284	4	13210

639.33244	2	13212

639.43204	9	13221

639.53164	1	13221

639.63124	2	13223

639.73084	1	13224

639.83044	1	13225

639.93004	1	13225

640.02964	7	13233

640.12924	1	13234

640.22884	1	13235

640.32844	1	13235

640.42804	1	13237

640.52764	3	13240

640.62724	1	13241

640.72684	2	13243

640.82644	1	13244

640.92604	1	13245

641.02564	4	13249

641.12524	2	13251

641.22484	1	13252

641.32444	1	13253

641.42404	1	13253

641.52364	2	13255

641.62324	2	13257

641.72284	8	13265

641.82244	1	13266

641.92204	1	13267

642.02164	1	13267

642.12124	1	13269

642.22084	2	13271

642.32044	3	13273

642.42004	2	13275

642.51964	1	13276

642.61924	2	13278

642.71884	1	13279

642.81844	1	13279

642.91804	3	13283

643.01764	1	13284

643.11724	2	13286

643.21684	2	13288

643.31644	1	13289

643.41604	1	13290

643.51564	6	13296

643.61524	1	13297

643.71484	3	13299

643.81444	1	13300

643.91404	1	13301

644.01364	1	13302

644.11324	1	13303

644.21284	3	13305

644.31244	2	13307

644.41204	1	13308

644.51164	3	13311

644.61124	1	13312

644.71084	1	13313

644.81044	4	13317

644.91004	1	13317

645.00964	5	13323

645.10924	6	13329

645.20884	1	13330

645.30844	2	13332

645.40804	2	13334

645.50764	1	13335

645.60724	1	13335

645.70684	2	13337

645.80644	1	13339

645.90604	1	13339

646.00564	2	13341

646.10524	7	13348

646.20484	2	13350

646.30444	5	13355

646.40404	1	13356

646.50364	1	13357

646.60324	1	13358

646.70284	1	13359

646.80244	1	13360

646.90204	3	13363

647.00164	2	13365

647.10124	1	13365

647.20084	3	13369

647.30044	9	13377

647.40004	2	13379

647.49964	1	13380

647.59924	3	13383

647.69884	1	13384

647.79844	1	13385

647.89804	1	13385

647.99764	1	13386

648.09724	1	13387

648.19684	1	13388

648.29644	12	13400

648.39604	1	13401

648.49564	1	13401

648.59524	1	13403

648.69484	1	13403

648.79444	1	13404

648.89404	1	13405

648.99364	3	13407

649.09324	1	13408

649.19284	3	13411

649.29244	9	13420

649.39204	1	13421

649.49164	1	13421

649.59124	7	13429

649.69084	1	13430

649.79044	1	13431

649.89004	1	13432

649.98964	2	13434

650.08924	3	13437

650.18884	1	13439

650.28844	1	13440

650.38804	1	13441

650.48764	1	13443

650.58724	1	13443

650.68684	1	13444

650.78644	5	13449

650.88604	1	13449

650.98564	1	13450

651.08524	2	13452

651.18484	3	13455

651.28444	1	13455

651.38404	1	13457

651.48364	1	13457

651.58324	2	13459

651.68284	1	13460

651.78244	2	13462

651.88204	4	13466

651.98164	2	13468

652.08124	5	13473

652.18084	2	13475

652.28044	3	13478

652.38004	3	13481

652.47964	1	13483

652.57924	1	13483

652.67884	1	13484

652.77844	3	13487

652.87804	4	13491

652.97764	1	13492

653.07724	1	13493

653.17684	1	13493

653.27644	1	13494

653.37604	1	13495

653.47564	1	13496

653.57524	2	13498

653.67484	3	13501

653.77444	2	13503

653.87404	1	13504

653.97364	1	13505

654.07324	1	13506

654.17284	1	13507

654.27244	3	13510

654.37204	1	13511

654.47164	2	13513

654.57124	3	13515

654.67084	1	13516

654.77044	5	13521

654.87004	1	13521

654.96964	3	13525

655.06924	9	13533

655.16884	1	13534

655.26844	1	13535

655.36804	1	13537

655.46764	7	13543

655.56724	1	13545

655.66684	5	13549

655.76644	1	13550

655.86604	3	13553

655.96564	1	13555

656.06524	5	13559

656.16484	1	13561

656.26444	1	13562

656.36404	3	13565

656.46364	5	13569

656.56324	1	13570

656.66284	1	13571

656.76244	1	13573

656.86204	1	13573

656.96164	1	13575

657.06124	1	13575

657.16084	2	13577

657.26044	3	13580

657.36004	1	13581

657.45964	1	13582

657.55924	1	13583

657.65884	3	13585

657.75844	6	13591

657.85804	9	13600

657.95764	1	13601

658.05724	7	13607

658.15684	3	13611

658.25644	1	13611

658.35604	3	13614

658.45564	1	13615

658.55524	3	13619

658.65484	3	13621

658.75444	1	13622

658.85404	1	13623

658.95364	5	13627

659.05324	6	13633

659.15284	3	13636

659.25244	1	13637

659.35204	1	13637

659.45164	4	13641

659.55124	1	13642

659.65084	1	13643

659.75044	1	13644

659.85004	2	13646

659.94964	13	13659

660.04924	1	13660

660.14884	1	13661

660.24844	1	13661

660.34804	1	13663

660.44764	2	13665

660.54724	1	13666

660.64684	6	13672

660.74644	2	13674

660.84604	1	13675

660.94564	1	13675

661.04524	1	13676

661.14484	1	13677

661.24444	1	13678

661.34404	1	13679

661.44364	1	13679

661.54324	1	13680

661.64284	1	13681

661.74244	1	13682

661.84204	1	13683

661.94164	4	13687

662.04124	7	13694

662.14084	1	13695

662.24044	1	13697

662.34004	1	13697

662.43964	1	13698

662.53924	1	13699

662.63884	1	13700

662.73844	2	13702

662.83804	1	13703

662.93764	15	13717

663.03724	1	13719

663.13684	3	13722

663.23644	1	13723

663.33604	1	13724

663.43564	1	13725

663.53524	5	13730

663.63484	2	13732

663.73444	1	13733

663.83404	7	13739

663.93364	13	13753

664.03324	1	13753

664.13284	7	13760

664.23244	10	13770

664.33204	1	13771

664.43164	1	13771

664.53124	3	13775

664.63084	1	13775

664.73044	1	13776

664.83004	4	13780

664.92964	1	13781

665.02924	1	13781

665.12884	5	13786

665.22844	1	13787

665.32804	2	13789

665.42764	1	13789

665.52724	1	13790

665.62684	5	13795

665.72644	1	13795

665.82604	1	13796

665.92564	3	13799

666.02524	1	13800

666.12484	2	13802

666.22444	1	13803

666.32404	1	13803

666.42364	2	13805

666.52324	1	13806

666.62284	1	13807

666.72244	1	13809

666.82204	1	13809

666.92164	1	13810

667.02124	3	13813

667.12084	1	13815

667.22044	1	13815

667.32004	4	13819

667.41964	2	13821

667.51924	2	13823

667.61884	1	13824

667.71844	2	13826

667.81804	2	13828

667.91764	3	13831

668.01724	3	13833

668.11684	9	13843

668.21644	6	13849

668.31604	2	13851

668.41564	1	13851

668.51524	1	13852

668.61484	1	13853

668.71444	1	13854

668.81404	1	13855

668.91364	7	13861

669.01324	1	13862

669.11284	4	13866

669.21244	5	13871

669.31204	2	13873

669.41164	1	13874

669.51124	1	13875

669.61084	1	13876

669.71044	3	13879

669.81004	1	13881

669.90964	1	13881

670.00924	1	13882

670.10884	3	13885

670.20844	1	13887

670.30804	3	13889

670.40764	6	13895

670.50724	1	13896

670.60684	3	13899

670.70644	1	13900

670.80604	1	13901

670.90564	3	13903

671.00524	1	13904

671.10484	1	13905

671.20444	1	13905

671.30404	3	13908

671.40364	1	13909

671.50324	1	13911

671.60284	2	13913

671.70244	1	13914

671.80204	1	13915

671.90164	1	13916

672.00124	1	13917

672.10084	3	13921

672.20044	1	13921

672.30004	2	13923

672.39964	1	13925

672.49924	3	13928

672.59884	1	13929

672.69844	1	13930

672.79804	3	13933

672.89764	1	13933

672.99724	1	13935

673.09684	1	13935

673.19644	3	13938

673.29604	1	13939

673.39564	1	13940

673.49524	1	13941

673.59484	4	13945

673.69444	1	13945

673.79404	1	13946

673.89364	1	13947

673.99324	1	13947

674.09284	1	13948

674.19244	5	13953

674.29204	4	13957

674.39164	3	13959

674.49124	1	13960

674.59084	3	13963

674.69044	3	13967

674.79004	1	13967

674.88964	3	13971

674.98924	11	13981

675.08884	1	13983

675.18844	1	13983

675.28804	13	13997

675.38764	1	13997

675.48724	2	13999

675.58684	1	14000

675.68644	1	14001

675.78604	1	14001

675.88564	1	14002

675.98524	1	14003

676.08484	7	14009

676.18444	2	14011

676.28404	1	14012

676.38364	1	14013

676.48324	1	14014

676.58284	2	14016

676.68244	1	14017

676.78204	1	14018

676.88164	2	14020

676.98124	2	14022

677.08084	1	14023

677.18044	1	14024

677.28004	1	14025

677.37964	1	14026

677.47924	2	14028

677.57884	1	14029

677.67844	1	14029

677.77804	4	14033

677.87764	8	14041

677.97724	12	14053

678.07684	1	14054

678.17644	1	14055

678.27604	3	14057

678.37564	2	14059

678.47524	2	14061

678.57484	1	14062

678.67444	5	14067

678.77404	1	14068

678.87364	2	14070

678.97324	1	14071

679.07284	10	14081

679.17244	1	14081

679.27204	1	14082

679.37164	1	14083

679.47124	5	14088

679.57084	1	14089

679.67044	1	14091

679.77004	2	14093

679.86964	3	14095

679.96924	1	14097

680.06884	1	14097

680.16844	1	14098

680.26804	1	14099

680.36764	1	14099

680.46724	1	14100

680.56684	1	14101

680.66644	1	14102

680.76604	1	14103

680.86564	1	14104

680.96524	5	14109

681.06484	1	14110

681.16444	2	14112

681.26404	3	14115

681.36364	1	14115

681.46324	3	14119

681.56284	2	14121

681.66244	1	14121

681.76204	1	14123

681.86164	1	14124

681.96124	1	14125

682.06084	1	14125

682.16044	11	14136

682.26004	1	14137

682.35964	1	14137

682.45924	3	14140

682.55884	4	14144

682.65844	1	14145

682.75804	1	14147

682.85764	1	14147

682.95724	11	14158

683.05684	1	14159

683.15644	1	14159

683.25604	3	14163

683.35564	8	14171

683.45524	1	14171

683.55484	1	14172

683.65444	1	14173

683.75404	1	14173

683.85364	1	14174

683.95324	1	14175

684.05284	1	14176

684.15244	5	14181

684.25204	3	14183

684.35164	1	14185

684.45124	2	14187

684.55084	6	14193

684.65044	1	14194

684.75004	3	14197

684.84964	6	14203

684.94924	1	14203

685.04884	3	14206

685.14844	1	14207

685.24804	4	14211

685.34764	1	14212

685.44724	1	14213

685.54684	1	14213

685.64644	1	14214

685.74604	6	14220

685.84564	1	14221

685.94524	1	14223

686.04484	2	14225

686.14444	2	14227

686.24404	7	14234

686.34364	1	14235

686.44324	10	14245

686.54284	1	14245

686.64244	2	14247

686.74204	1	14249

686.84164	1	14249

686.94124	2	14251

687.04084	3	14255

687.14044	3	14258

687.24004	3	14261

687.33964	1	14261

687.43924	5	14266

687.53884	1	14267

687.63844	1	14268

687.73804	2	14270

687.83764	3	14273

687.93724	7	14280

688.03684	1	14281

688.13644	1	14281

688.23604	1	14282

688.33564	10	14292

688.43524	1	14293

688.53484	1	14294

688.63444	3	14297

688.73404	1	14297

688.83364	4	14301

688.93324	1	14303

689.03284	1	14303

689.13244	1	14304

689.23204	4	14308

689.33164	1	14309

689.43124	2	14311

689.53084	9	14320

689.63044	7	14327

689.73004	3	14329

689.82964	1	14330

689.92924	10	14340

690.02884	1	14341

690.12844	5	14347

690.22804	1	14347

690.32764	1	14348

690.42724	1	14349

690.52684	2	14351

690.62644	1	14352

690.72604	9	14361

690.82564	3	14363

690.92524	1	14364

691.02484	3	14367

691.12444	1	14368

691.22404	1	14369

691.32364	1	14370

691.42324	5	14375

691.52284	1	14376

691.62244	4	14380

691.72204	1	14381

691.82164	1	14382

691.92124	8	14390

692.02084	1	14391

692.12044	3	14394

692.22004	1	14395

692.31964	3	14398

692.41924	1	14399

692.51884	1	14400

692.61844	6	14406

692.71804	2	14408

692.81764	2	14410

692.91724	1	14411

693.01684	1	14412

693.11644	3	14415

693.21604	1	14416

693.31564	6	14422

693.41524	4	14426

693.51484	1	14427

693.61444	1	14427

693.71404	2	14429

693.81364	1	14431

693.91324	1	14432

694.01284	3	14435

694.11244	10	14445

694.21204	1	14446

694.31164	2	14448

694.41124	1	14449

694.51084	5	14454

694.61044	1	14455

694.71004	3	14457

694.80964	3	14460

694.90924	9	14469

695.00884	1	14470

695.10844	4	14474

695.20804	1	14475

695.30764	1	14476

695.40724	5	14481

695.50684	1	14482

695.60644	3	14485

695.70604	1	14487

695.80564	6	14493

695.90524	7	14500

696.00484	1	14501

696.10444	6	14507

696.20404	1	14509

696.30364	1	14509

696.40324	1	14511

696.50284	2	14513

696.60244	1	14514

696.70204	15	14529

696.80164	3	14532

696.90124	8	14540

697.00084	1	14541

697.10044	1	14542

697.20004	1	14543

697.29964	1	14545

697.39924	1	14545

697.49884	7	14553

697.59844	3	14556

697.69804	2	14558

697.79764	1	14559

697.89724	12	14571

697.99684	1	14571

698.09644	4	14575

698.19604	3	14578

698.29564	1	14579

698.39524	2	14581

698.49484	3	14585

698.59444	1	14585

698.69404	1	14586

698.79364	4	14590

698.89324	1	14591

698.99284	4	14595

699.09244	1	14595

699.19204	4	14599

699.29164	8	14607

699.39124	2	14609

699.49084	2	14611

699.59044	2	14613

699.69004	3	14616

699.78964	1	14617

699.88924	3	14620

699.98884	1	14621

700.08844	1	14621

700.18804	4	14625

700.28764	1	14627

700.38724	1	14627

700.48684	3	14631

700.58644	1	14632

700.68604	1	14633

700.78564	1	14633

700.88524	3	14636

700.98484	1	14637

701.08444	8	14645

701.18404	1	14645

701.28364	5	14650

701.38324	2	14652

701.48284	5	14657

701.58244	1	14659

701.68204	1	14659

701.78164	3	14663

701.88124	1	14663

701.98084	1	14665

702.08044	11	14676

702.18004	4	14680

702.27964	11	14691

702.37924	2	14693

702.47884	2	14695

702.57844	1	14696

702.67805	3	14699

702.77765	1	14699

702.87725	1	14700

702.97685	1	14701

703.07645	2	14703

703.17605	1	14705

703.27565	1	14706

703.37524	17	14723

703.47484	1	14723

703.57444	1	14724

703.67404	3	14727

703.77364	1	14728

703.87325	1	14729

703.97285	1	14730

704.07245	1	14731

704.17205	3	14733

704.27165	1	14735

704.37125	5	14740

704.47085	1	14741

704.57045	1	14741

704.67005	2	14743

704.76965	1	14745

704.86925	1	14746

704.96885	5	14751

705.06845	1	14753

705.16805	1	14753

705.26765	3	14757

705.36725	1	14758

705.46685	1	14759

705.56645	1	14759

705.66605	5	14764

705.76565	1	14765

705.86525	1	14767

705.96485	3	14770

706.06445	2	14772

706.16405	2	14774

706.26365	1	14775

706.36325	2	14777

706.46285	1	14777

706.56245	1	14779

706.66205	2	14781

706.76165	20	14801

706.86125	1	14801

706.96085	1	14803

707.06045	6	14809

707.16005	1	14809

707.25965	1	14810

707.35925	5	14815

707.45885	3	14818

707.55845	1	14819

707.65805	5	14823

707.75765	5	14829

707.85725	1	14829

707.95685	1	14830

708.05645	1	14831

708.15605	9	14840

708.25565	4	14844

708.35525	6	14850

708.45485	1	14851

708.55445	1	14853

708.65405	1	14854

708.75365	1	14855

708.85325	1	14855

708.95285	3	14859

709.05245	1	14859

709.15205	1	14860

709.25165	1	14861

709.35125	1	14861

709.45085	1	14863

709.55045	7	14869

709.65005	1	14871

709.74965	5	14876

709.84925	9	14885

709.94885	4	14889

710.04845	1	14890

710.14805	6	14896

710.24765	1	14897

710.34725	1	14898

710.44685	1	14899

710.54645	9	14908

710.64605	1	14909

710.74565	1	14910

710.84525	1	14911

710.94485	3	14915

711.04445	7	14921

711.14405	2	14923

711.24365	1	14924

711.34325	2	14926

711.44285	3	14929

711.54245	1	14929

711.64205	1	14930

711.74165	1	14931

711.84125	9	14939

711.94085	3	14943

712.04045	1	14943

712.14005	1	14944

712.23965	1	14945

712.33925	5	14949

712.43885	1	14950

712.53845	1	14951

712.63805	1	14952

712.73765	1	14953

712.83725	3	14955

712.93685	1	14956

713.03645	1	14957

713.13605	1	14958

713.23565	5	14963

713.33525	1	14963

713.43485	3	14967

713.53445	2	14969

713.63405	5	14973

713.73365	1	14975

713.83325	2	14977

713.93285	1	14977

714.03245	4	14981

714.13205	3	14985

714.23165	1	14985

714.33125	6	14991

714.43085	2	14993

714.53045	2	14995

714.63005	1	14996

714.72965	3	14999

714.82925	5	15004

714.92885	1	15005

715.02845	3	15007

715.12805	5	15012

715.22765	2	15014

715.32725	7	15021

715.42685	1	15021

715.52645	1	15023

715.62605	3	15026

715.72565	3	15029

715.82525	1	15030

715.92485	9	15039

716.02445	1	15040

716.12405	3	15043

716.22365	2	15045

716.32325	2	15047

716.42285	1	15047

716.52245	1	15048

716.62205	1	15049

716.72165	1	15050

716.82125	1	15051

716.92085	1	15052

717.02045	1	15053

717.12005	2	15055

717.21965	1	15057

717.31925	1	15057

717.41885	1	15058

717.51845	2	15060

717.61805	3	15063

717.71765	1	15064

717.81725	1	15065

717.91685	3	15067

718.01645	1	15068

718.11605	1	15069

718.21565	1	15069

718.31525	1	15070

718.41485	3	15073

718.51445	2	15075

718.61405	2	15077

718.71365	1	15078

718.81325	1	15079

718.91285	1	15080

719.01245	8	15088

719.11205	2	15090

719.21165	2	15092

719.31125	1	15093

719.41085	2	15095

719.51045	3	15098

719.61005	1	15099

719.70965	2	15101

719.80925	1	15102

719.90885	1	15103

720.00845	2	15105

720.10805	2	15107

720.20765	3	15111

720.30725	3	15114

720.40685	1	15115

720.50645	5	15119

720.60605	1	15121

720.70565	8	15129

720.80525	1	15130

720.90485	1	15131

721.00445	1	15132

721.10405	1	15133

721.20365	1	15134

721.30325	6	15140

721.40285	1	15141

721.50245	6	15147

721.60205	6	15153

721.70165	1	15153

721.80125	4	15157

721.90085	4	15161

722.00045	1	15162

722.10005	7	15169

722.19965	7	15176

722.29925	2	15178

722.39885	1	15179

722.49845	1	15179

722.59805	1	15180

722.69765	1	15181

722.79725	2	15183

722.89685	5	15189

722.99645	3	15191

723.09605	3	15194

723.19565	1	15195

723.29525	2	15197

723.39485	2	15199

723.49445	1	15199

723.59405	1	15200

723.69365	1	15201

723.79325	3	15203

723.89285	2	15205

723.99245	2	15207

724.09205	4	15211

724.19165	3	15215

724.29125	1	15215

724.39085	5	15220

724.49045	1	15221

724.59005	1	15221

724.68965	5	15226

724.78925	1	15227

724.88885	2	15229

724.98845	1	15231

725.08805	1	15232

725.18765	1	15233

725.28725	1	15234

725.38685	5	15239

725.48645	4	15243

725.58605	2	15245

725.68565	5	15251

725.78525	1	15251

725.88485	2	15253

725.98445	1	15254

726.08405	2	15256

726.18365	3	15259

726.28325	1	15260

726.38285	1	15261

726.48245	15	15275

726.58205	7	15283

726.68165	1	15284

726.78125	2	15286

726.88085	8	15294

726.98045	1	15295

727.08005	11	15305

727.17965	1	15306

727.27925	1	15307

727.37885	11	15318

727.47845	3	15321

727.57805	3	15323

727.67765	1	15325

727.77725	2	15327

727.87685	2	15329

727.97645	5	15333

728.07605	1	15335

728.17565	5	15339

728.27525	3	15343

728.37485	1	15343

728.47445	5	15349

728.57405	1	15349

728.67365	1	15351

728.77325	1	15351

728.87285	3	15354

728.97245	1	15355

729.07205	1	15355

729.17165	1	15356

729.27125	2	15358

729.37085	1	15359

729.47045	1	15360

729.57005	3	15363

729.66965	1	15363

729.76925	1	15364

729.86885	1	15365

729.96845	1	15367

730.06805	3	15369

730.16765	3	15372

730.26725	11	15383

730.36685	4	15387

730.46645	3	15389

730.56605	1	15390

730.66565	3	15393

730.76525	1	15395

730.86485	1	15395

730.96445	1	15396

731.06405	1	15397

731.16365	1	15398

731.26325	4	15402

731.36285	1	15403

731.46245	2	15405

731.56205	1	15406

731.66165	1	15407

731.76125	4	15411

731.86085	1	15411

731.96045	11	15423

732.06005	1	15424

732.15965	1	15425

732.25925	1	15426

732.35885	4	15430

732.45845	4	15434

732.55805	3	15437

732.65765	1	15439

732.75725	3	15441

732.85685	1	15442

732.95645	8	15450

733.05605	5	15455

733.15565	3	15458

733.25525	1	15459

733.35485	1	15460

733.45445	1	15461

733.55405	1	15462

733.65365	1	15463

733.75325	1	15465

733.85285	1	15465

733.95245	3	15468

734.05205	1	15469

734.15165	3	15471

734.25125	1	15473

734.35085	1	15473

734.45045	1	15475

734.55005	1	15475

734.64965	1	15477

734.74925	5	15481

734.84885	2	15483

734.94845	3	15486

735.04805	1	15487

735.14765	3	15489

735.24725	7	15497

735.34685	1	15497

735.44645	1	15498

735.54605	5	15503

735.64565	3	15506

735.74525	3	15509

735.84485	1	15510

735.94445	2	15512

736.04405	1	15513

736.14365	5	15518

736.24325	9	15527

736.34285	6	15533

736.44245	2	15535

736.54205	1	15536

736.64165	1	15537

736.74125	1	15538

736.84085	6	15544

736.94045	1	15545

737.04005	2	15547

737.13965	8	15555

737.23925	3	15559

737.33885	1	15559

737.43845	3	15562

737.53805	5	15567

737.63765	3	15569

737.73725	3	15572

737.83685	1	15573

737.93645	1	15574

738.03605	1	15575

738.13565	3	15579

738.23525	1	15580

738.33485	1	15581

738.43445	1	15581

738.53405	1	15582

738.63365	11	15593

738.73325	1	15594

738.83285	3	15597

738.93245	3	15599

739.03205	1	15600

739.13165	2	15602

739.23125	2	15604

739.33085	2	15606

739.43045	7	15613

739.53005	1	15614

739.62965	3	15617

739.72925	1	15618

739.82885	1	15619

739.92845	7	15627

740.02805	1	15627

740.12765	1	15628

740.22725	3	15631

740.32685	1	15632

740.42645	4	15636

740.52605	2	15638

740.62565	6	15644

740.72525	1	15645

740.82485	1	15646

740.92445	14	15660

741.02405	1	15661

741.12365	1	15662

741.22325	4	15666

741.32285	3	15669

741.42245	11	15679

741.52205	2	15681

741.62165	1	15683

741.72125	1	15683

741.82085	1	15684

741.92045	1	15685

742.02005	1	15686

742.11965	1	15687

742.21925	1	15689

742.31885	9	15697

742.41845	1	15698

742.51805	1	15699

742.61765	3	15703

742.71725	1	15703

742.81685	3	15706

742.91645	2	15708

743.01605	3	15711

743.11565	1	15713

743.21525	1	15713

743.31485	1	15714

743.41445	1	15715

743.51405	1	15715

743.61365	1	15716

743.71325	2	15718

743.81285	1	15719

743.91245	1	15720

744.01205	1	15721

744.11165	1	15722

744.21125	5	15727

744.31085	1	15727

744.41045	3	15730

744.51005	3	15733

744.60965	1	15733

744.70925	1	15734

744.80885	6	15740

744.90845	1	15741

745.00805	1	15742

745.10765	3	15745

745.20725	2	15747

745.30685	6	15753

745.40645	1	15753

745.50605	6	15759

745.60565	1	15760

745.70525	3	15763

745.80485	1	15765

745.90445	1	15765

746.00405	1	15766

746.10365	1	15767

746.20325	1	15768

746.30285	5	15773

746.40245	1	15773

746.50205	1	15775

746.60165	4	15779

746.70125	8	15787

746.80085	2	15789

746.90045	3	15792

747.00005	1	15793

747.09965	1	15794

747.19925	3	15797

747.29885	1	15797

747.39845	7	15804

747.49805	6	15810

747.59765	1	15811

747.69725	7	15819

747.79685	1	15819

747.89645	1	15820

747.99605	1	15821

748.09565	3	15825

748.19525	8	15833

748.29485	1	15833

748.39445	5	15839

748.49405	2	15841

748.59365	2	15843

748.69325	3	15846

748.79285	1	15847

748.89245	1	15848

748.99205	6	15854

749.09165	5	15859

749.19125	1	15859

749.29085	1	15860

749.39045	2	15862

749.49005	3	15865

749.58965	3	15868

749.68925	1	15869

749.78885	5	15874

749.88845	1	15875

749.98805	1	15875

750.08765	1	15876

750.18725	3	15879

750.28685	1	15881

750.38645	7	15887

750.48605	2	15889

750.58565	1	15891

750.68525	2	15893

750.78485	3	15895

750.88445	11	15906

750.98405	9	15915

751.08365	3	15918

751.18325	5	15923

751.28285	2	15925

751.38245	1	15927

751.48205	5	15931

751.58165	13	15944

751.68125	3	15947

751.78085	1	15948

751.88045	3	15951

751.98005	10	15961

752.07965	1	15962

752.17925	2	15964

752.27885	1	15965

752.37845	7	15972

752.47805	5	15977

752.57765	1	15978

752.67725	1	15979

752.77685	3	15981

752.87645	1	15982

752.97605	1	15983

753.07565	3	15987

753.17525	2	15989

753.27485	2	15991

753.37445	6	15997

753.47405	1	15998

753.57365	3	16001

753.67325	1	16001

753.77285	5	16006

753.87245	1	16007

753.97205	3	16009

754.07165	1	16010

754.17125	9	16019

754.27085	1	16019

754.37045	3	16023

754.47005	1	16023

754.56965	2	16025

754.66925	10	16035

754.76885	7	16042

754.86845	7	16049

754.96805	1	16049

755.06765	1	16050

755.16725	8	16058

755.26685	3	16061

755.36645	5	16067

755.46605	2	16069

755.56565	9	16078

755.66525	1	16079

755.76485	1	16081

755.86445	3	16083

755.96405	1	16084

756.06365	5	16089

756.16325	5	16094

756.26285	2	16096

756.36245	2	16098

756.46205	5	16103

756.56165	5	16109

756.66125	1	16109

756.76085	1	16110

756.86045	1	16111

756.96005	9	16121

757.05965	1	16121

757.15925	1	16122

757.25885	1	16123

757.35845	1	16124

757.45805	3	16127

757.55765	3	16130

757.65725	1	16131

757.75685	1	16132

757.85645	1	16133

757.95605	7	16140

758.05565	3	16143

758.15525	1	16145

758.25485	2	16147

758.35445	1	16148

758.45405	8	16156

758.55365	1	16157

758.65325	4	16161

758.75285	5	16166

758.85245	1	16167

758.95205	2	16169

759.05165	4	16173

759.15125	12	16185

759.25085	1	16185

759.35045	1	16186

759.45005	4	16190

759.54965	1	16191

759.64925	2	16193

759.74885	2	16195

759.84845	1	16196

759.94805	4	16200

760.04765	5	16205

760.14725	3	16207

760.24685	1	16208

760.34645	7	16215

760.44605	1	16215

760.54565	5	16220

760.64525	3	16223

760.74485	1	16224

760.84445	1	16225

760.94405	1	16226

761.04365	1	16227

761.14325	5	16231

761.24285	1	16233

761.34245	1	16234

761.44205	1	16235

761.54165	1	16236

761.64125	6	16242

761.74085	1	16243

761.84045	1	16244

761.94005	7	16251

762.03965	1	16251

762.13925	3	16254

762.23885	1	16255

762.33845	4	16259

762.43805	1	16261

762.53765	1	16261

762.63725	1	16262

762.73685	1	16263

762.83645	3	16265

762.93605	1	16266

763.03565	1	16267

763.13525	1	16268

763.23485	1	16269

763.33445	9	16278

763.43405	2	16280

763.53365	1	16281

763.63325	10	16291

763.73285	1	16292

763.83245	7	16299

763.93205	3	16303

764.03165	1	16303

764.13125	1	16305

764.23085	1	16305

764.33045	3	16309

764.43005	1	16310

764.52965	2	16312

764.62925	3	16315

764.72885	1	16316

764.82845	2	16318

764.92805	1	16319

765.02765	3	16323

765.12725	1	16323

765.22685	1	16324

765.32645	1	16325

765.42605	1	16325

765.52565	1	16327

765.62525	4	16331

765.72485	4	16335

765.82445	1	16336

765.92405	2	16338

766.02365	3	16341

766.12325	3	16344

766.22285	1	16345

766.32245	1	16345

766.42205	10	16355

766.52165	1	16357

766.62125	1	16358

766.72085	1	16359

766.82045	1	16361

766.92005	1	16362

767.01965	1	16363

767.11925	1	16365

767.21885	3	16368

767.31845	1	16369

767.41805	1	16370

767.51765	1	16371

767.61725	7	16378

767.71685	3	16381

767.81645	1	16382

767.91605	1	16383

768.01565	1	16384

768.11525	3	16387

768.21485	1	16389

768.31445	2	16391

768.41405	5	16396

768.51365	1	16397

768.61325	9	16405

768.71285	3	16409

768.81245	7	16416

768.91205	2	16418

769.01165	11	16429

769.11125	1	16430

769.21085	1	16431

769.31045	1	16431

769.41005	1	16433

769.50965	4	16437

769.60925	1	16437

769.70885	3	16441

769.80845	1	16441

769.90805	2	16443

770.00765	2	16445

770.10725	1	16446

770.20685	2	16448

770.30645	4	16452

770.40605	1	16453

770.50565	2	16455

770.60525	3	16458

770.70485	2	16460

770.80445	4	16464

770.90405	3	16467

771.00365	7	16474

771.10325	1	16475

771.20285	9	16485

771.30245	1	16485

771.40205	1	16486

771.50165	5	16491

771.60125	1	16491

771.70085	1	16492

771.80045	2	16494

771.90005	1	16495

771.99965	1	16496

772.09925	9	16505

772.19885	1	16505

772.29845	1	16506

772.39805	3	16509

772.49765	3	16512

772.59725	1	16513

772.69685	5	16518

772.79645	2	16520

772.89605	3	16523

772.99565	1	16523

773.09525	1	16525

773.19485	1	16525

773.29445	1	16527

773.39405	1	16527

773.49365	3	16530

773.59325	1	16531

773.69285	1	16531

773.79245	1	16532

773.89205	1	16533

773.99165	1	16534

774.09125	1	16535

774.19085	1	16535

774.29045	1	16537

774.39005	1	16538

774.48965	2	16540

774.58925	1	16541

774.68885	9	16550

774.78845	1	16551

774.88805	3	16555

774.98765	1	16555

775.08725	6	16561

775.18685	3	16564

775.28645	4	16568

775.38605	1	16569

775.48565	4	16573

775.58525	3	16576

775.68485	1	16577

775.78445	1	16577

775.88405	3	16580

775.98365	1	16581

776.08325	1	16581

776.18285	1	16582

776.28245	1	16583

776.38205	8	16591

776.48165	2	16593

776.58125	1	16594

776.68085	3	16597

776.78045	1	16597

776.88005	2	16599

776.97965	2	16601

777.07925	3	16604

777.17885	6	16610

777.27845	13	16623

777.37805	1	16625

777.47765	1	16626

777.57725	1	16627

777.67685	3	16630

777.77645	1	16631

777.87605	2	16633

777.97565	6	16639

778.07525	2	16641

778.17485	2	16643

778.27445	4	16647

778.37405	3	16649

778.47365	1	16650

778.57325	6	16656

778.67285	1	16657

778.77245	3	16660

778.87205	7	16667

778.97165	1	16667

779.07125	1	16669

779.17085	1	16669

779.27045	4	16673

779.37005	3	16677

779.46965	1	16677

779.56925	7	16685

779.66885	2	16687

779.76845	7	16693

779.86805	4	16697

779.96765	1	16699

780.06725	1	16700

780.16685	3	16703

780.26645	3	16706

780.36605	1	16707

780.46565	1	16708

780.56525	2	16710

780.66485	1	16711

780.76445	1	16712

780.86405	6	16718

780.96365	5	16723

781.06325	1	16724

781.16285	1	16725

781.26245	1	16725

781.36205	1	16726

781.46165	8	16734

781.56125	3	16737

781.66085	19	16756

781.76045	1	16757

781.86005	3	16760

781.95965	1	16761

782.05925	1	16761

782.15885	5	16766

782.25845	1	16767

782.35805	1	16768

782.45765	5	16773

782.55725	1	16774

782.65685	1	16775

782.75645	5	16781

782.85605	1	16781

782.95565	5	16786

783.05525	1	16787

783.15485	3	16789

783.25445	2	16791

783.35405	5	16797

783.45365	1	16797

783.55325	1	16798

783.65285	5	16803

783.75245	1	16804

783.85205	11	16815

783.95165	2	16817

784.05125	9	16825

784.15085	2	16827

784.25045	2	16829

784.35005	1	16831

784.44965	3	16833

784.54925	1	16834

784.64885	2	16836

784.74845	1	16837

784.84805	1	16838

784.94765	1	16839

785.04725	3	16842

785.14685	1	16843

785.24645	1	16843

785.34605	5	16849

785.44565	3	16852

785.54525	7	16859

785.64485	2	16861

785.74445	5	16867

785.84405	3	16870

785.94365	3	16873

786.04325	6	16879

786.14285	3	16882

786.24245	1	16883

786.34205	1	16883

786.44165	1	16885

786.54125	6	16891

786.64085	4	16895

786.74045	5	16900

786.84005	2	16902

786.93965	6	16908

787.03925	4	16912

787.13885	1	16913

787.23845	1	16914

787.33805	3	16917

787.43765	1	16917

787.53725	1	16919

787.63685	7	16926

787.73645	11	16937

787.83605	1	16939

787.93565	1	16939

788.03525	3	16942

788.13485	3	16945

788.23445	1	16947

788.33405	1	16948

788.43365	2	16950

788.53325	1	16951

788.63285	2	16953

788.73245	4	16957

788.83205	5	16962

788.93165	1	16963

789.03125	1	16963

789.13085	3	16966

789.23045	3	16969

789.33005	1	16970

789.42965	1	16971

789.52925	5	16975

789.62885	5	16981

789.72845	1	16981

789.82805	3	16985

789.92765	1	16986

790.02725	5	16991

790.12685	1	16991

790.22645	2	16993

790.32605	10	17003

790.42565	1	17005

790.52525	3	17007

790.62485	5	17012

790.72445	1	17013

790.82405	3	17016

790.92365	2	17018

791.02325	1	17019

791.12285	3	17023

791.22245	2	17025

791.32205	1	17025

791.42165	3	17029

791.52125	2	17031

791.62085	1	17031

791.72045	2	17033

791.82005	9	17043

791.91965	6	17049

792.01925	3	17051

792.11885	1	17052

792.21845	5	17057

792.31805	1	17058

792.41765	2	17060

792.51725	1	17061

792.61685	6	17067

792.71645	3	17070

792.81605	19	17089

792.91565	1	17089

793.01525	2	17091

793.11485	1	17093

793.21445	2	17095

793.31405	1	17095

793.41365	1	17096

793.51325	1	17097

793.61285	5	17102

793.71245	3	17105

793.81205	3	17109

793.91165	1	17109

794.01125	1	17111

794.11085	4	17115

794.21045	15	17130

794.31005	10	17140

794.40965	1	17141

794.50925	4	17145

794.60885	3	17148

794.70845	1	17149

794.80805	1	17149

794.90765	3	17152

795.00725	1	17153

795.10685	1	17153

795.20645	1	17154

795.30605	4	17158

795.40565	2	17160

795.50525	1	17161

795.60485	1	17162

795.70445	2	17164

795.80405	1	17165

795.90365	1	17165

796.00325	1	17166

796.10285	3	17169

796.20245	2	17171

796.30205	22	17193

796.40165	1	17195

796.50125	3	17197

796.60085	6	17203

796.70045	2	17205

796.80005	5	17210

796.89965	1	17211

796.99925	4	17215

797.09885	3	17218

797.19845	2	17220

797.29805	10	17230

797.39765	1	17231

797.49725	8	17239

797.59685	3	17243

797.69645	5	17248

797.79605	2	17250

797.89565	1	17251

797.99525	1	17251

798.09485	1	17252

798.19445	10	17262

798.29405	4	17266

798.39365	1	17267

798.49325	5	17273

798.59285	1	17273

798.69245	1	17274

798.79205	2	17276

798.89165	3	17279

798.99125	5	17285

799.09085	5	17289

799.19045	1	17290

799.29005	1	17291

799.38965	4	17295

799.48925	2	17297

799.58885	4	17301

799.68845	1	17302

799.78805	4	17306

799.88765	11	17317

799.98725	14	17331

800.08685	1	17331

800.18645	1	17333

800.28605	1	17333

800.38565	1	17334

800.48525	2	17336

800.58485	3	17339

800.68445	1	17339

800.78405	2	17341

800.88365	1	17342

800.98325	3	17345

801.08285	2	17347

801.18245	1	17347

801.28205	4	17351

801.38165	7	17358

801.48125	1	17359

801.58085	1	17359

801.68045	1	17361

801.78005	1	17361

801.87965	5	17367

801.97925	1	17368

802.07885	11	17379

802.17845	3	17381

802.27805	1	17382

802.37765	11	17393

802.47725	1	17393

802.57685	1	17394

802.67645	1	17395

802.77605	3	17399

802.87565	1	17399

802.97525	2	17401

803.07485	1	17402

803.17445	8	17410

803.27405	1	17411

803.37365	1	17412

803.47325	3	17415

803.57285	6	17421

803.67245	1	17421

803.77205	1	17422

803.87165	3	17425

803.97125	2	17427

804.07085	3	17430

804.17045	3	17433

804.27005	1	17433

804.36965	7	17441

804.46925	3	17444

804.56885	2	17446

804.66845	1	17447

804.76805	14	17461

804.86765	1	17462

804.96725	3	17465

805.06685	5	17470

805.16645	1	17471

805.26605	1	17472

805.36565	5	17477

805.46525	10	17487

805.56485	1	17488

805.66445	3	17491

805.76405	1	17493

805.86365	14	17507

805.96325	1	17508

806.06285	1	17509

806.16245	1	17510

806.26205	1	17511

806.36165	1	17512

806.46125	3	17515

806.56085	9	17524

806.66045	1	17525

806.76005	3	17528

806.85965	5	17533

806.95925	2	17535

807.05885	1	17535

807.15845	4	17539

807.25805	1	17541

807.35765	1	17541

807.45725	1	17542

807.55685	1	17543

807.65645	7	17549

807.75605	1	17550

807.85565	1	17551

807.95525	1	17552

808.05485	8	17560

808.15445	3	17563

808.25405	1	17563

808.35365	4	17567

808.45325	1	17569

808.55285	1	17569

808.65245	1	17570

808.75205	5	17575

808.85165	7	17582

808.95125	3	17585

809.05085	1	17585

809.15045	1	17586

809.25005	1	17587

809.34965	1	17589

809.44925	6	17595

809.54885	5	17599

809.64845	1	17600

809.74805	1	17601

809.84765	1	17602

809.94725	11	17613

810.04685	3	17616

810.14645	7	17623

810.24605	1	17624

810.34565	1	17625

810.44525	1	17625

810.54485	1	17626

810.64445	1	17627

810.74405	2	17629

810.84365	1	17631

810.94325	7	17637

811.04285	4	17641

811.14245	7	17649

811.24205	5	17654

811.34165	3	17657

811.44125	9	17665

811.54085	13	17679

811.64045	1	17679

811.74005	2	17681

811.83965	2	17683

811.93925	3	17686

812.03885	1	17687

812.13845	15	17702

812.23805	1	17703

812.33765	1	17705

812.43725	1	17705

812.53685	1	17706

812.63645	1	17707

812.73605	14	17721

812.83565	1	17722

812.93525	1	17723

813.03485	1	17725

813.13445	3	17727

813.23405	3	17730

813.33365	13	17743

813.43325	9	17753

813.53285	1	17753

813.63245	2	17755

813.73205	1	17756

813.83165	3	17759

813.93125	1	17759

814.03085	6	17765

814.13045	16	17781

814.23005	1	17782

814.32965	4	17786

814.42925	3	17789

814.52885	1	17790

814.62845	7	17797

814.72805	4	17801

814.82765	14	17815

814.92725	3	17817

815.02685	1	17819

815.12645	8	17827

815.22605	3	17829

815.32565	3	17832

815.42525	1	17833

815.52485	3	17836

815.62445	5	17841

815.72405	9	17850

815.82365	3	17853

815.92325	3	17855

816.02285	1	17857

816.12245	1	17857

816.22205	1	17858

816.32165	1	17859

816.42125	5	17863

816.52085	1	17864

816.62045	2	17866

816.72005	2	17868

816.81965	3	17871

816.91925	3	17874

817.01885	3	17877

817.11845	10	17887

817.21805	1	17889

817.31765	7	17896

817.41725	1	17897

817.51685	5	17903

817.61645	2	17905

817.71605	1	17906

817.81565	1	17907

817.91525	1	17908

818.01485	7	17915

818.11445	1	17916

818.21405	1	17917

818.31365	5	17921

818.41325	1	17923

818.51285	1	17923

818.61245	3	17926

818.71205	1	17927

818.81165	1	17929

818.91125	5	17933

819.01085	1	17935

819.11045	2	17937

819.21005	1	17937

819.30965	1	17938

819.40925	3	17941

819.50885	1	17942

819.60845	7	17949

819.70805	10	17959

819.80765	1	17960

819.90725	3	17963

820.00685	1	17963

820.10645	1	17964

820.20605	1	17965

820.30565	3	17968

820.40525	9	17977

820.50485	1	17977

820.60445	1	17979

820.70405	1	17979

820.80365	1	17980

820.90325	1	17981

821.00285	6	17987

821.10245	4	17991

821.20205	3	17994

821.30165	1	17995

821.40125	1	17995

821.50085	2	17997

821.60045	2	17999

821.70005	8	18007

821.79965	7	18015

821.89925	1	18015

821.99885	2	18017

822.09845	1	18018

822.19805	1	18019

822.29765	7	18027

822.39725	1	18027

822.49685	1	18029

822.59645	1	18029

822.69605	1	18031

822.79565	1	18031

822.89525	2	18033

822.99485	2	18035

823.09445	2	18037

823.19405	5	18042

823.29365	1	18043

823.39325	1	18044

823.49285	3	18047

823.59245	3	18050

823.69205	5	18055

823.79165	1	18055

823.89125	5	18061

823.99085	1	18062

824.09045	7	18069

824.19005	3	18072

824.28965	1	18073

824.38925	1	18073

824.48885	2	18075

824.58845	1	18077

824.68805	1	18077

824.78765	1	18078

824.88725	3	18081

824.98685	2	18083

825.08645	1	18083

825.18605	1	18084

825.28565	3	18087

825.38525	1	18088

825.48485	1	18089

825.58445	3	18092

825.68405	1	18093

825.78365	2	18095

825.88325	1	18096

825.98285	1	18097

826.08245	8	18105

826.18205	1	18106

826.28165	1	18107

826.38125	1	18108

826.48085	1	18109

826.58045	3	18111

826.68005	1	18112

826.77965	1	18113

826.87925	6	18119

826.97885	1	18119

827.07845	23	18142

827.17805	3	18145

827.27765	5	18151

827.37725	1	18151

827.47685	1	18152

827.57645	1	18153

827.67605	2	18155

827.77565	1	18155

827.87525	1	18157

827.97485	3	18159

828.07445	4	18163

828.17405	1	18164

828.27365	1	18165

828.37325	3	18169

828.47285	2	18171

828.57245	3	18173

828.67205	1	18174

828.77165	1	18175

828.87125	3	18177

828.97085	5	18183

829.07045	4	18187

829.17005	1	18187

829.26965	1	18188

829.36925	1	18189

829.46885	7	18195

829.56845	1	18196

829.66805	1	18197

829.76765	5	18201

829.86725	3	18204

829.96685	1	18205

830.06645	1	18205

830.16605	2	18207

830.26565	1	18209

830.36525	1	18209

830.46485	1	18211

830.56445	3	18213

830.66405	1	18214

830.76365	25	18239

830.86325	1	18241

830.96285	1	18241

831.06245	1	18242

831.16205	2	18244

831.26165	2	18246

831.36125	4	18250

831.46085	1	18251

831.56045	2	18253

831.66005	1	18253

831.75965	3	18257

831.85925	1	18257

831.95885	4	18261

832.05845	1	18262

832.15805	1	18263

832.25765	3	18267

832.35725	1	18267

832.45685	1	18268

832.55645	2	18270

832.65605	1	18271

832.75565	4	18275

832.85525	1	18276

832.95485	3	18279

833.05445	1	18280

833.15405	3	18283

833.25365	3	18286

833.35325	1	18287

833.45285	1	18288

833.55245	1	18289

833.65205	1	18290

833.75165	1	18291

833.85125	1	18292

833.95085	1	18293

834.05045	1	18294

834.15005	1	18295

834.24965	1	18295

834.34925	1	18297

834.44885	2	18299

834.54845	1	18299

834.64805	7	18307

834.74765	1	18307

834.84725	1	18308

834.94685	1	18309

835.04645	1	18311

835.14605	2	18313

835.24565	8	18321

835.34525	7	18328

835.44485	3	18331

835.54445	2	18333

835.64405	2	18335

835.74365	1	18336

835.84325	2	18338

835.94285	3	18341

836.04245	1	18341

836.14205	1	18342

836.24165	1	18343

836.34125	2	18345

836.44085	1	18345

836.54045	5	18351

836.64005	2	18353

836.73965	1	18353

836.83925	5	18358

836.93885	2	18360

837.03845	2	18362

837.13805	1	18363

837.23765	1	18365

837.33725	2	18367

837.43685	2	18369

837.53645	9	18377

837.63605	3	18381

837.73565	1	18382

837.83525	2	18384

837.93485	17	18401

838.03445	16	18417

838.13405	2	18419

838.23365	1	18419

838.33325	1	18420

838.43285	1	18421

838.53245	1	18422

838.63205	2	18424

838.73165	1	18425

838.83125	1	18425

838.93085	1	18427

839.03045	1	18427

839.13005	2	18429

839.22965	1	18431

839.32925	1	18432

839.42885	1	18433

839.52845	3	18436

839.62805	1	18437

839.72765	1	18438

839.82725	7	18445

839.92685	1	18445

840.02645	3	18449

840.12605	1	18449

840.22565	7	18457

840.32525	2	18459

840.42485	1	18460

840.52445	5	18465

840.62405	1	18467

840.72365	1	18467

840.82325	2	18469

840.92285	1	18470

841.02245	1	18471

841.12205	11	18481

841.22165	1	18482

841.32125	1	18483

841.42085	1	18485

841.52045	1	18485

841.62005	1	18486

841.71965	9	18495

841.81925	2	18497

841.91885	3	18500

842.01845	2	18502

842.11805	7	18509

842.21765	1	18509

842.31725	5	18514

842.41685	5	18519

842.51645	1	18519

842.61605	9	18528

842.71565	1	18529

842.81525	1	18530

842.91485	3	18533

843.01445	5	18537

843.11405	1	18538

843.21365	6	18544

843.31325	1	18545

843.41285	1	18545

843.51245	14	18559

843.61205	1	18560

843.71165	1	18561

843.81125	3	18563

843.91085	2	18565

844.01045	1	18566

844.11005	1	18567

844.20965	2	18569

844.30925	3	18571

844.40885	15	18587

844.50845	1	18587

844.60805	1	18589

844.70765	8	18597

844.80725	1	18597

844.90685	6	18603

845.00645	3	18606

845.10605	3	18609

845.20565	5	18615

845.30525	3	18617

845.40485	1	18619

845.50445	6	18625

845.60405	4	18629

845.70365	1	18630

845.80325	11	18641

845.90285	1	18642

846.00245	2	18644

846.10205	3	18647

846.20165	2	18649

846.30125	3	18652

846.40085	2	18654

846.50045	1	18655

846.60005	5	18660

846.69965	1	18661

846.79925	1	18661

846.89885	1	18662

846.99845	7	18669

847.09805	12	18681

847.19765	2	18683

847.29725	1	18683

847.39685	1	18685

847.49645	1	18685

847.59605	1	18686

847.69565	9	18695

847.79525	1	18696

847.89485	4	18700

847.99445	4	18704

848.09405	1	18705

848.19365	6	18711

848.29325	3	18713

848.39285	3	18716

848.49245	3	18719

848.59205	1	18719

848.69165	1	18720

848.79125	3	18723

848.89085	1	18724

848.99045	1	18725

849.09005	3	18727

849.18965	2	18729

849.28925	3	18732

849.38885	6	18738

849.48845	2	18740

849.58805	4	18744

849.68765	1	18745

849.78725	1	18745

849.88685	4	18749

849.98645	1	18750

850.08605	1	18751

850.18565	1	18752

850.28525	3	18755

850.38485	8	18763

850.48445	1	18764

850.58405	23	18787

850.68365	3	18790

850.78325	1	18791

850.88285	9	18800

850.98245	1	18801

851.08205	2	18803

851.18165	1	18803

851.28125	1	18804

851.38085	1	18805

851.48045	8	18813

851.58005	1	18814

851.67965	14	18828

851.77925	1	18829

851.87885	3	18831

851.97845	1	18832

852.07805	3	18835

852.17765	1	18836

852.27725	1	18837

852.37685	5	18842

852.47645	1	18843

852.57605	5	18847

852.67565	1	18848

852.77525	12	18860

852.87485	17	18877

852.97445	1	18877

853.07405	2	18879

853.17365	1	18880

853.27325	4	18884

853.37285	2	18886

853.47245	1	18887

853.57205	3	18890

853.67165	1	18891

853.77125	1	18891

853.87085	5	18897

853.97045	1	18898

854.07005	1	18899

854.16965	1	18899

854.26925	9	18908

854.36885	3	18911

854.46845	7	18918

854.56805	1	18919

854.66765	10	18929

854.76725	1	18930

854.86685	1	18931

854.96645	2	18933

855.06605	1	18934

855.16565	3	18937

855.26525	1	18938

855.36485	1	18939

855.46445	1	18940

855.56405	1	18941

855.66365	2	18943

855.76325	12	18955

855.86285	1	18955

855.96245	7	18963

856.06205	2	18965

856.16165	2	18967

856.26125	2	18969

856.36085	1	18970

856.46045	10	18980

856.56005	1	18981

856.65965	6	18987

856.75925	6	18993

856.85885	4	18997

856.95845	1	18999

857.05805	1	19000

857.15765	1	19001

857.25725	1	19002

857.35685	7	19009

857.45645	3	19012

857.55605	12	19024

857.65565	3	19027

857.75525	1	19028

857.85485	1	19029

857.95445	3	19031

858.05405	7	19038

858.15365	4	19042

858.25325	1	19043

858.35285	7	19050

858.45245	10	19060

858.55205	2	19062

858.65166	5	19067

858.75126	3	19070

858.85086	9	19079

858.95046	1	19079

859.05006	17	19097

859.14966	2	19099

859.24925	11	19109

859.34885	2	19111

859.44845	1	19113

859.54805	1	19114

859.64765	2	19116

859.74726	15	19131

859.84686	1	19132

859.94646	2	19134

860.04606	3	19137

860.14566	1	19137

860.24526	3	19140

860.34486	1	19141

860.44446	9	19151

860.54406	1	19151

860.64366	2	19153

860.74326	17	19171

860.84286	1	19171

860.94246	16	19187

861.04206	1	19188

861.14166	10	19198

861.24126	4	19202

861.34086	1	19203

861.44046	7	19209

861.54006	1	19210

861.63966	2	19212

861.73926	3	19215

861.83886	1	19215

861.93846	3	19218

862.03806	3	19221

862.13766	3	19223

862.23726	5	19229

862.33686	2	19231

862.43646	11	19242

862.53606	3	19245

862.63566	10	19255

862.73526	1	19255

862.83486	12	19267

862.93446	1	19268

863.03406	13	19281

863.13366	17	19299

863.23326	3	19301

863.33286	1	19302

863.43246	1	19303

863.53206	4	19307

863.63166	12	19319

863.73126	1	19320

863.83086	1	19321

863.93046	1	19321

864.03006	7	19329

864.12966	1	19329

864.22926	3	19332

864.32886	1	19333

864.42846	2	19335

864.52806	1	19335

864.62766	4	19339

864.72726	1	19340

864.82686	7	19347

864.92646	1	19348

865.02606	7	19355

865.12566	2	19357

865.22526	3	19360

865.32486	1	19361

865.42446	3	19364

865.52406	2	19366

865.62366	1	19367

865.72326	1	19367

865.82286	12	19379

865.92246	1	19381

866.02206	1	19382

866.12166	15	19397

866.22126	1	19398

866.32086	4	19402

866.42046	3	19405

866.52006	6	19411

866.61966	4	19415

866.71926	2	19417

866.81886	1	19419

866.91846	1	19419

867.01806	5	19425

867.11766	1	19425

867.21726	2	19427

867.31686	20	19447

867.41646	2	19449

867.51606	1	19451

867.61566	8	19459

867.71526	1	19460

867.81486	1	19461

867.91446	1	19461

868.01406	1	19463

868.11366	8	19471

868.21326	3	19474

868.31286	15	19489

868.41246	2	19491

868.51206	1	19493

868.61166	1	19494

868.71126	2	19496

868.81086	3	19499

868.91046	3	19502

869.01006	6	19508

869.10966	1	19509

869.20926	1	19509

869.30886	7	19516

869.40846	2	19518

869.50806	11	19529

869.60766	3	19532

869.70726	5	19537

869.80686	2	19539

869.90646	18	19557

870.00606	2	19559

870.10566	1	19560

870.20526	11	19571

870.30486	4	19575

870.40446	1	19577

870.50406	1	19577

870.60366	3	19581

870.70326	1	19581

870.80286	1	19583

870.90246	1	19584

871.00206	13	19597

871.10166	2	19599

871.20126	1	19599

871.30086	1	19601

871.40046	9	19610

871.50006	10	19620

871.59966	1	19621

871.69926	1	19622

871.79886	3	19625

871.89846	4	19629

871.99806	1	19631

872.09766	11	19642

872.19726	3	19645

872.29686	3	19649

872.39646	2	19651

872.49606	2	19653

872.59566	5	19657

872.69526	7	19665

872.79486	1	19665

872.89446	7	19673

872.99406	5	19678

873.09366	2	19680

873.19326	5	19685

873.29286	1	19686

873.39246	7	19693

873.49206	1	19693

873.59166	13	19707

873.69126	1	19707

873.79086	1	19708

873.89046	1	19709

873.99006	1	19709

874.08966	22	19731

874.18926	5	19737

874.28886	3	19740

874.38846	1	19741

874.48806	3	19744

874.58766	1	19745

874.68726	4	19749

874.78686	3	19752

874.88646	3	19755

874.98606	1	19756

875.08566	3	19759

875.18526	1	19759

875.28486	2	19761

875.38446	4	19765

875.48406	1	19766

875.58366	1	19767

875.68326	13	19779

875.78286	1	19780

875.88246	3	19783

875.98206	3	19786

876.08166	3	19789

876.18126	1	19789

876.28086	1	19790

876.38046	4	19794

876.48006	1	19795

876.57966	5	19800

876.67926	2	19802

876.77886	1	19803

876.87846	16	19819

876.97806	2	19821

877.07766	1	19822

877.17726	1	19823

877.27686	1	19825

877.37646	1	19826

877.47606	1	19827

877.57566	1	19828

877.67526	7	19835

877.77486	1	19835

877.87446	1	19836

877.97406	2	19838

878.07366	9	19847

878.17326	2	19849

878.27286	3	19851

878.37246	1	19852

878.47206	3	19855

878.57166	9	19863

878.67126	2	19865

878.77086	3	19869

878.87046	9	19877

878.97006	1	19878

879.06966	15	19893

879.16926	3	19896

879.26886	5	19901

879.36846	4	19905

879.46806	1	19907

879.56766	2	19909

879.66726	1	19909

879.76686	9	19918

879.86646	2	19920

879.96606	2	19922

880.06566	1	19923

880.16526	1	19924

880.26486	11	19935

880.36446	1	19936

880.46406	4	19940

880.56366	1	19941

880.66326	7	19948

880.76286	3	19951

880.86246	3	19954

880.96206	2	19956

881.06166	1	19957

881.16126	1	19958

881.26086	3	19961

881.36046	7	19968

881.46006	1	19969

881.55966	1	19969

881.65926	1	19971

881.75886	6	19977

881.85846	1	19977

881.95806	3	19981

882.05766	1	19982

882.15726	1	19983

882.25686	1	19985

882.35646	5	19989

882.45606	1	19990

882.55566	1	19991

882.65526	4	19995

882.75486	17	20012

882.85446	1	20013

882.95406	5	20018

883.05366	2	20020

883.15326	2	20022

883.25286	5	20027

883.35246	2	20029

883.45206	16	20045

883.55166	18	20063

883.65126	1	20065

883.75086	3	20067

883.85046	2	20069

883.95006	2	20071

884.04966	1	20072

884.14926	4	20076

884.24886	1	20077

884.34846	2	20079

884.44806	5	20084

884.54766	2	20086

884.64726	6	20092

884.74686	1	20093

884.84646	6	20099

884.94606	10	20109

885.04566	17	20125

885.14526	1	20126

885.24486	13	20139

885.34446	3	20141

885.44406	7	20148

885.54366	1	20149

885.64326	11	20160

885.74286	1	20161

885.84246	4	20165

885.94206	6	20171

886.04166	3	20174

886.14126	7	20181

886.24086	2	20183

886.34046	15	20198

886.44006	1	20199

886.53966	4	20203

886.63926	3	20206

886.73886	1	20207

886.83846	3	20209

886.93806	2	20211

887.03766	1	20212

887.13726	4	20216

887.23686	10	20226

887.33646	1	20227

887.43606	5	20231

887.53566	13	20244

887.63526	14	20258

887.73486	3	20261

887.83446	3	20264

887.93406	1	20265

888.03366	6	20271

888.13326	1	20272

888.23286	12	20284

888.33246	1	20285

888.43206	2	20287

888.53166	5	20292

888.63126	16	20308

888.73086	7	20315

888.83046	12	20327

888.93006	15	20341

889.02966	1	20342

889.12926	11	20353

889.22886	1	20354

889.32846	10	20364

889.42806	3	20367

889.52766	2	20369

889.62726	3	20372

889.72686	2	20374

889.82646	5	20379

889.92606	1	20379

890.02566	1	20380

890.12526	1	20381

890.22486	12	20393

890.32446	1	20393

890.42406	13	20407

890.52366	1	20407

890.62326	1	20408

890.72286	1	20409

890.82246	5	20414

890.92206	2	20416

891.02166	3	20419

891.12126	4	20423

891.22086	14	20437

891.32046	1	20439

891.42006	1	20439

891.51966	7	20447

891.61926	1	20447

891.71886	9	20457

891.81846	2	20459

891.91806	1	20459

892.01766	3	20462

892.11726	1	20463

892.21686	2	20465

892.31646	3	20467

892.41606	3	20471

892.51566	1	20472

892.61526	2	20474

892.71486	6	20480

892.81446	2	20482

892.91406	1	20483

893.01366	2	20485

893.11326	1	20487

893.21286	7	20494

893.31246	21	20515

893.41206	10	20525

893.51166	7	20531

893.61126	1	20533

893.71086	22	20555

893.81046	3	20557

893.91006	10	20567

894.00966	3	20571

894.10926	1	20571

894.20886	1	20572

894.30846	5	20577

894.40806	2	20579

894.50766	1	20581

894.60726	11	20592

894.70686	2	20594

894.80646	16	20610

894.90606	5	20615

895.00566	1	20616

895.10526	1	20617

895.20486	6	20623

895.30446	1	20623

895.40406	4	20627

895.50366	2	20629

895.60326	10	20639

895.70286	1	20640

895.80246	9	20649

895.90206	1	20651

896.00166	15	20665

896.10126	1	20666

896.20086	5	20671

896.30046	3	20675

896.40006	1	20676

896.49966	17	20693

896.59926	1	20694

896.69886	7	20701

896.79846	2	20703

896.89806	7	20710

896.99766	21	20731

897.09726	1	20731

897.19686	3	20734

897.29646	9	20743

897.39606	3	20747

897.49566	9	20756

897.59526	2	20758

897.69486	3	20761

897.79446	1	20762

897.89406	3	20765

897.99366	7	20772

898.09326	1	20773

898.19286	4	20777

898.29246	1	20778

898.39206	1	20779

898.49166	5	20784

898.59126	1	20785

898.69086	8	20793

898.79046	1	20793

898.89006	3	20796

898.98966	3	20799

899.08926	2	20801

899.18886	1	20802

899.28846	2	20804

899.38806	1	20805

899.48766	4	20809

899.58726	1	20809

899.68686	1	20810

899.78646	5	20815

899.88606	2	20817

899.98566	7	20825

900.08526	1	20825

900.18486	6	20831

900.28446	3	20835

900.38406	3	20837

900.48366	4	20841

900.58326	4	20845

900.68286	1	20847

900.78246	5	20852

900.88206	4	20856

900.98166	11	20867

901.08126	1	20867

901.18086	1	20869

901.28046	1	20869

901.38006	1	20871

901.47966	8	20879

901.57926	3	20882

901.67886	1	20883

901.77846	3	20886

901.87806	3	20889

901.97766	13	20902

902.07726	3	20905

902.17686	3	20908

902.27646	1	20909

902.37606	1	20910

902.47566	1	20911

902.57526	5	20916

902.67486	1	20917

902.77446	1	20918

902.87406	2	20920

902.97366	3	20923

903.07326	1	20925

903.17286	1	20925

903.27246	14	20939

903.37206	3	20943

903.47166	3	20946

903.57126	5	20951

903.67086	3	20954

903.77046	5	20959

903.87006	5	20963

903.96966	1	20964

904.06926	3	20967

904.16886	3	20970

904.26846	1	20971

904.36806	5	20976

904.46766	1	20977

904.56726	9	20986

904.66686	1	20987

904.76646	3	20991

904.86606	7	20997

904.96566	3	21000

905.06526	10	21010

905.16486	3	21013

905.26446	8	21021

905.36406	1	21021

905.46366	1	21022

905.56326	9	21031

905.66286	1	21032

905.76246	2	21034

905.86206	5	21039

905.96166	1	21039

906.06126	1	21041

906.16086	11	21051

906.26046	8	21059

906.36006	2	21061

906.45966	6	21067

906.55926	7	21074

906.65886	1	21075

906.75846	7	21081

906.85806	1	21083

906.95766	7	21090

907.05726	2	21092

907.15686	1	21093

907.25646	3	21096

907.35606	3	21099

907.45566	4	21103

907.55526	1	21104

907.65486	10	21114

907.75446	1	21115

907.85406	8	21123

907.95366	5	21128

908.05326	1	21129

908.15286	5	21134

908.25246	1	21135

908.35206	4	21139

908.45166	1	21140

908.55126	1	21141

908.65086	7	21148

908.75046	1	21149

908.85006	18	21167

908.94966	1	21168

909.04926	1	21169

909.14886	14	21183

909.24846	1	21183

909.34806	3	21186

909.44766	3	21189

909.54726	7	21196

909.64686	3	21199

909.74646	7	21207

909.84606	5	21212

909.94566	1	21213

910.04526	12	21225

910.14486	3	21228

910.24446	3	21231

910.34406	1	21232

910.44366	1	21233

910.54326	1	21234

910.64286	1	21235

910.74246	2	21237

910.84206	3	21240

910.94166	7	21247

911.04126	1	21247

911.14086	1	21248

911.24046	23	21271

911.34006	2	21273

911.43966	7	21280

911.53926	1	21281

911.63886	4	21285

911.73846	13	21297

911.83806	3	21301

911.93766	6	21307

912.03726	9	21315

912.13686	3	21318

912.23646	1	21319

912.33606	1	21320

912.43566	1	21321

912.53526	5	21325

912.63486	2	21327

912.73446	3	21330

912.83406	1	21331

912.93366	1	21331

913.03326	1	21332

913.13286	11	21343

913.23246	22	21365

913.33206	2	21367

913.43166	5	21371

913.53126	3	21374

913.63086	7	21381

913.73046	1	21382

913.83006	1	21383

913.92966	7	21389

914.02926	1	21390

914.12886	3	21393

914.22846	3	21395

914.32806	3	21398

914.42766	1	21399

914.52726	8	21407

914.62686	2	21409

914.72646	9	21417

914.82606	3	21420

914.92566	1	21421

915.02526	8	21429

915.12486	1	21430

915.22446	1	21431

915.32406	7	21438

915.42366	5	21443

915.52326	4	21447

915.62286	7	21455

915.72246	2	21457

915.82206	1	21457

915.92166	6	21463

916.02126	5	21469

916.12086	1	21469

916.22046	1	21471

916.32006	7	21477

916.41966	1	21479

916.51926	11	21489

916.61886	3	21492

916.71846	2	21494

916.81806	3	21497

916.91766	3	21499

917.01726	11	21510

917.11686	3	21513

917.21646	12	21525

917.31606	1	21526

917.41566	10	21536

917.51526	1	21537

917.61486	20	21557

917.71446	3	21559

917.81406	14	21573

917.91366	1	21575

918.01326	2	21577

918.11286	8	21585

918.21246	1	21586

918.31206	18	21604

918.41166	1	21605

918.51126	9	21614

918.61086	3	21617

918.71046	8	21625

918.81006	1	21625

918.90966	7	21633

919.00926	1	21633

919.10886	1	21634

919.20846	3	21637

919.30806	1	21639

919.40766	1	21639

919.50726	1	21640

919.60686	5	21645

919.70646	1	21646

919.80606	5	21651

919.90566	1	21651

920.00526	3	21654

920.10486	15	21669

920.20446	1	21669

920.30406	1	21671

920.40366	3	21674

920.50326	4	21678

920.60286	1	21679

920.70246	5	21683

920.80206	3	21686

920.90166	3	21689

921.00126	7	21695

921.10086	1	21697

921.20046	13	21710

921.30006	1	21711

921.39966	1	21711

921.49926	4	21715

921.59886	1	21717

921.69846	7	21723

921.79806	5	21729

921.89766	8	21737

921.99726	1	21737

922.09686	20	21757

922.19646	3	21760

922.29606	9	21769

922.39566	1	21769

922.49526	3	21772

922.59486	2	21774

922.69446	12	21786

922.79406	2	21788

922.89366	1	21789

922.99326	3	21793

923.09286	1	21793

923.19246	1	21794

923.29206	3	21797

923.39166	1	21798

923.49126	5	21803

923.59086	1	21804

923.69046	1	21805

923.79006	1	21805

923.88966	3	21808

923.98926	2	21810

924.08886	12	21822

924.18846	1	21823

924.28806	2	21825

924.38766	2	21827

924.48726	19	21846

924.58686	12	21858

924.68646	3	21861

924.78606	10	21871

924.88566	1	21873

924.98526	7	21879

925.08486	2	21881

925.18446	12	21893

925.28406	1	21895

925.38366	1	21896

925.48326	15	21911

925.58286	1	21913

925.68246	10	21923

925.78206	1	21923

925.88166	3	21927

925.98126	3	21929

926.08086	11	21941

926.18046	1	21941

926.28006	2	21943

926.37966	12	21955

926.47926	1	21956

926.57886	1	21957

926.67846	6	21963

926.77806	1	21963

926.87766	1	21965

926.97726	9	21974

927.07686	1	21975

927.17646	10	21985

927.27606	1	21985

927.37566	5	21991

927.47526	5	21995

927.57486	1	21996

927.67446	4	22000

927.77406	3	22003

927.87366	1	22004

927.97326	1	22005

928.07286	9	22014

928.17246	9	22023

928.27206	1	22024

928.37166	3	22027

928.47126	3	22030

928.57086	2	22032

928.67046	1	22033

928.77006	1	22034

928.86966	1	22035

928.96926	3	22039

929.06886	5	22043

929.16846	5	22049

929.26806	1	22049

929.36766	6	22055

929.46726	2	22057

929.56686	1	22059

929.66646	1	22059

929.76606	3	22063

929.86566	1	22063

929.96526	3	22066

930.06486	10	22076

930.16446	1	22077

930.26406	1	22078

930.36366	1	22079

930.46326	1	22081

930.56286	14	22095

930.66246	2	22097

930.76206	15	22111

930.86166	1	22112

930.96126	5	22117

931.06086	1	22117

931.16046	1	22118

931.26006	5	22123

931.35966	9	22131

931.45926	1	22132

931.55886	8	22140

931.65846	3	22143

931.75806	1	22144

931.85766	3	22147

931.95726	11	22158

932.05686	1	22159

932.15646	1	22159

932.25606	1	22161

932.35566	1	22161

932.45526	1	22162

932.55486	1	22163

932.65446	6	22169

932.75406	1	22169

932.85366	7	22177

932.95326	1	22178

933.05286	5	22183

933.15246	7	22189

933.25206	1	22190

933.35166	3	22193

933.45126	14	22207

933.55086	1	22208

933.65046	12	22220

933.75006	1	22221

933.84966	12	22233

933.94926	2	22235

934.04886	7	22242

934.14846	6	22248

934.24806	3	22251

934.34766	1	22253

934.44726	5	22257

934.54686	2	22259

934.64646	1	22261

934.74606	2	22263

934.84566	2	22265

934.94526	15	22279

935.04486	1	22281

935.14446	5	22285

935.24406	2	22287

935.34366	9	22296

935.44326	1	22297

935.54286	2	22299

935.64246	5	22303

935.74206	1	22305

935.84166	3	22308

935.94126	5	22313

936.04086	1	22313

936.14046	17	22331

936.24006	3	22333

936.33966	1	22334

936.43926	1	22335

936.53886	14	22349

936.63846	7	22356

936.73806	5	22361

936.83766	2	22363

936.93726	13	22377

937.03686	5	22381

937.13646	1	22383

937.23606	15	22398

937.33566	11	22409

937.43526	1	22410

937.53486	7	22417

937.63446	18	22435

937.73406	1	22435

937.83366	1	22436

937.93326	2	22438

938.03286	9	22447

938.13246	2	22449

938.23206	1	22450

938.33166	15	22465

938.43126	2	22467

938.53086	1	22469

938.63046	2	22471

938.73006	1	22471

938.82966	7	22479

938.92926	1	22479

939.02886	11	22490

939.12846	4	22494

939.22806	12	22506

939.32766	2	22508

939.42726	7	22515

939.52686	7	22521

939.62646	1	22522

939.72606	1	22523

939.82566	5	22529

939.92526	4	22533

940.02486	4	22537

940.12446	1	22537

940.22406	1	22538

940.32366	1	22539

940.42326	16	22555

940.52286	2	22557

940.62246	18	22575

940.72206	1	22576

940.82166	1	22577

940.92126	10	22587

941.02086	1	22587

941.12046	6	22593

941.22006	2	22595

941.31966	1	22596

941.41926	1	22597

941.51886	7	22604

941.61846	1	22605

941.71806	2	22607

941.81766	1	22607

941.91726	1	22608

942.01686	9	22617

942.11646	1	22617

942.21606	1	22619

942.31566	1	22619

942.41526	1	22621

942.51486	2	22623

942.61446	3	22625

942.71406	13	22639

942.81366	11	22650

942.91326	12	22662

943.01286	1	22663

943.11246	12	22675

943.21206	1	22675

943.31166	9	22684

943.41126	1	22685

943.51086	9	22695

943.61046	1	22695

943.71006	9	22704

943.80966	3	22707

943.90926	1	22707

944.00886	1	22708

944.10846	9	22717

944.20806	1	22718

944.30766	1	22719

944.40726	7	22726

944.50686	1	22727

944.60646	1	22727

944.70606	12	22739

944.80566	1	22741

944.90526	1	22741

945.00486	5	22746

945.10446	3	22749

945.20406	3	22751

945.30366	8	22759

945.40326	2	22761

945.50286	1	22762

945.60246	1	22763

945.70206	1	22763

945.80166	5	22769

945.90126	17	22786

946.00086	1	22787

946.10046	9	22795

946.20006	1	22796

946.29966	7	22803

946.39926	1	22805

946.49886	11	22816

946.59846	3	22819

946.69806	2	22821

946.79766	1	22821

946.89726	1	22822

946.99686	1	22823

947.09646	1	22823

947.19606	10	22833

947.29566	1	22834

947.39526	2	22836

947.49486	4	22840

947.59446	1	22841

947.69406	3	22843

947.79366	5	22848

947.89326	1	22849

947.99286	21	22870

948.09246	3	22873

948.19206	2	22875

948.29166	1	22876

948.39126	11	22887

948.49086	1	22888

948.59046	1	22889

948.69006	1	22889

948.78966	2	22891

948.88926	7	22899

948.98886	4	22903

949.08846	1	22904

949.18806	5	22909

949.28766	6	22915

949.38726	6	22921

949.48686	1	22922

949.58646	11	22933

949.68606	1	22933

949.78566	1	22934

949.88526	8	22942

949.98486	3	22945

950.08446	2	22947

950.18406	11	22958

950.28366	1	22959

950.38326	7	22965

950.48286	1	22967

950.58246	24	22991

950.68206	12	23003

950.78166	3	23005

950.88126	1	23006

950.98086	3	23009

951.08046	7	23016

951.18006	3	23019

951.27966	18	23037

951.37926	1	23038

951.47886	1	23039

951.57846	3	23041

951.67806	1	23042

951.77766	1	23043

951.87726	1	23043

951.97686	9	23052

952.07646	9	23061

952.17606	3	23065

952.27566	1	23065

952.37526	2	23067

952.47486	1	23068

952.57446	3	23071

952.67406	4	23075

952.77366	1	23075

952.87326	11	23087

952.97286	1	23087

953.07246	10	23097

953.17206	15	23113

953.27166	1	23114

953.37126	19	23133

953.47086	1	23133

953.57046	1	23134

953.67006	1	23135

953.76966	1	23136

953.86926	2	23138

953.96886	2	23140

954.06846	8	23148

954.16806	1	23149

954.26766	10	23159

954.36726	1	23160

954.46686	3	23163

954.56646	1	23164

954.66606	11	23175

954.76566	1	23175

954.86526	1	23176

954.96486	1	23177

955.06446	1	23178

955.16406	2	23180

955.26366	2	23182

955.36326	1	23183

955.46286	13	23196

955.56246	1	23197

955.66206	4	23201

955.76166	2	23203

955.86126	2	23205

955.96086	15	23220

956.06046	1	23221

956.16006	3	23224

956.25966	9	23233

956.35926	3	23236

956.45886	13	23249

956.55846	1	23250

956.65806	1	23251

956.75766	1	23253

956.85726	1	23253

956.95686	4	23257

957.05646	1	23258

957.15606	1	23259

957.25566	2	23261

957.35526	10	23271

957.45486	1	23272

957.55446	1	23273

957.65406	10	23283

957.75366	1	23284

957.85326	1	23285

957.95286	2	23287

958.05246	1	23289

958.15206	1	23290

958.25166	1	23291

958.35126	1	23292

958.45086	3	23295

958.55046	1	23295

958.65006	3	23298

958.74966	1	23299

958.84926	1	23300

958.94886	7	23307

959.04846	1	23309

959.14806	5	23313

959.24766	13	23327

959.34726	1	23327

959.44686	3	23331

959.54646	1	23332

959.64606	13	23345

959.74566	2	23347

959.84526	1	23348

959.94486	1	23349

960.04446	1	23350

960.14406	10	23360

960.24366	3	23363

960.34326	1	23364

960.44286	1	23365

960.54246	3	23368

960.64206	1	23369

960.74166	7	23377

960.84126	9	23386

960.94086	2	23388

961.04046	1	23389

961.14006	3	23392

961.23966	1	23393

961.33926	2	23395

961.43886	5	23401

961.53846	1	23402

961.63806	7	23409

961.73766	11	23421

961.83726	17	23437

961.93686	6	23443

962.03646	6	23449

962.13606	3	23452

962.23566	8	23460

962.33526	1	23461

962.43486	19	23480

962.53446	3	23483

962.63406	5	23488

962.73366	1	23489

962.83326	4	23493

962.93286	1	23494

963.03246	5	23499

963.13206	13	23511

963.23166	1	23512

963.33126	6	23518

963.43086	1	23519

963.53046	6	23525

963.63006	2	23527

963.72966	11	23537

963.82926	6	23543

963.92886	11	23555

964.02846	13	23567

964.12806	17	23584

964.22766	1	23585

964.32726	1	23586

964.42686	1	23587

964.52646	5	23592

964.62606	2	23594

964.72566	1	23595

964.82526	1	23596

964.92486	2	23598

965.02446	2	23600

965.12406	20	23620

965.22366	1	23621

965.32326	10	23631

965.42286	14	23645

965.52246	3	23647

965.62206	9	23656

965.72166	2	23658

965.82126	1	23659

965.92086	1	23659

966.02046	1	23660

966.12006	1	23661

966.21966	6	23667

966.31926	1	23668

966.41886	1	23669

966.51846	2	23671

966.61806	4	23675

966.71766	2	23677

966.81726	6	23683

966.91686	4	23687

967.01646	2	23689

967.11606	1	23691

967.21566	5	23695

967.31526	1	23696

967.41486	1	23697

967.51446	2	23699

967.61406	1	23699

967.71366	5	23705

967.81326	15	23719

967.91286	2	23721

968.01246	5	23727

968.11206	17	23744

968.21166	1	23745

968.31126	2	23747

968.41086	11	23758

968.51046	2	23760

968.61006	15	23775

968.70966	3	23778

968.80926	8	23786

968.90886	5	23791

969.00846	5	23797

969.10806	5	23801

969.20766	3	23804

969.30726	12	23816

969.40686	4	23820

969.50646	4	23824

969.60606	1	23825

969.70566	1	23826

969.80526	1	23827

969.90486	1	23827

970.00446	1	23828

970.10406	1	23829

970.20366	5	23834

970.30326	1	23835

970.40286	1	23836

970.50246	13	23849

970.60206	19	23869

970.70166	14	23883

970.80126	5	23888

970.90086	7	23895

971.00046	1	23895

971.10006	11	23907

971.19966	1	23908

971.29926	1	23909

971.39886	4	23913

971.49846	13	23925

971.59806	1	23926

971.69766	1	23927

971.79726	1	23928

971.89686	13	23941

971.99646	13	23955

972.09606	1	23955

972.19566	1	23957

972.29526	1	23958

972.39486	5	23963

972.49446	1	23964

972.59406	7	23971

972.69366	3	23975

972.79326	11	23986

972.89286	1	23987

972.99246	1	23987

973.09206	5	23992

973.19166	1	23993

973.29126	1	23994

973.39086	2	23996

973.49046	1	23997

973.59006	3	24000

973.68966	9	24009

973.78926	3	24011

973.88886	6	24017

973.98846	1	24019

974.08806	2	24021

974.18766	5	24025

974.28726	1	24026

974.38686	1	24027

974.48646	18	24045

974.58606	1	24045

974.68566	7	24052

974.78526	1	24053

974.88486	5	24057

974.98446	1	24059

975.08406	1	24059

975.18366	1	24060

975.28326	2	24062

975.38286	4	24066

975.48246	8	24074

975.58206	1	24075

975.68166	3	24078

975.78126	5	24083

975.88086	5	24088

975.98046	5	24093

976.08006	2	24095

976.17966	2	24097

976.27926	2	24099

976.37886	5	24103

976.47846	3	24106

976.57806	7	24113

976.67766	1	24115

976.77726	8	24123

976.87686	7	24129

976.97646	2	24131

977.07606	8	24139

977.17566	1	24140

977.27526	1	24141

977.37486	7	24149

977.47446	1	24149

977.57406	1	24150

977.67366	2	24152

977.77326	8	24160

977.87286	1	24161

977.97246	7	24167

978.07206	1	24168

978.17166	6	24174

978.27126	1	24175

978.37086	1	24175

978.47046	1	24176

978.57006	4	24180

978.66966	7	24187

978.76926	2	24189

978.86886	3	24193

978.96846	23	24215

979.06806	1	24216

979.16766	1	24217

979.26726	11	24228

979.36686	1	24229

979.46646	3	24231

979.56606	1	24232

979.66566	8	24240

979.76526	1	24241

979.86486	6	24247

979.96446	2	24249

980.06406	5	24254

980.16366	1	24255

980.26326	9	24263

980.36286	5	24269

980.46246	5	24273

980.56206	8	24281

980.66166	1	24282

980.76126	1	24283

980.86086	11	24293

980.96046	12	24305

981.06006	1	24306

981.15966	1	24307

981.25926	15	24322

981.35886	1	24323

981.45846	7	24330

981.55806	2	24332

981.65766	1	24333

981.75726	4	24337

981.85686	3	24340

981.95646	1	24341

982.05606	9	24350

982.15566	2	24352

982.25526	15	24367

982.35486	1	24368

982.45446	2	24370

982.55406	5	24375

982.65366	1	24377

982.75326	6	24383

982.85286	1	24384

982.95246	8	24392

983.05206	1	24393

983.15166	1	24394

983.25126	2	24396

983.35086	4	24400

983.45046	1	24401

983.55006	1	24402

983.64966	11	24413

983.74926	1	24414

983.84886	5	24419

983.94846	1	24420

984.04806	14	24434

984.14766	1	24435

984.24726	2	24437

984.34686	11	24447

984.44646	1	24449

984.54606	1	24449

984.64566	18	24467

984.74526	12	24479

984.84486	1	24480

984.94446	1	24481

985.04406	11	24492

985.14366	22	24514

985.24326	1	24515

985.34286	1	24515

985.44246	3	24518

985.54206	1	24519

985.64166	4	24523

985.74126	6	24529

985.84086	1	24530

985.94046	1	24531

986.04006	7	24539

986.13966	1	24539

986.23926	4	24543

986.33886	3	24546

986.43846	1	24547

986.53806	9	24556

986.63766	7	24563

986.73726	1	24563

986.83686	21	24584

986.93646	4	24588

987.03606	16	24604

987.13566	1	24605

987.23526	3	24608

987.33486	2	24610

987.43446	2	24612

987.53406	3	24615

987.63366	6	24621

987.73326	9	24630

987.83286	1	24631

987.93246	1	24632

988.03206	1	24633

988.13166	1	24634

988.23126	5	24639

988.33086	1	24640

988.43046	1	24641

988.53006	1	24641

988.62966	11	24653

988.72926	3	24655

988.82886	2	24657

988.92846	3	24660

989.02806	1	24661

989.12766	1	24661

989.22726	7	24669

989.32686	3	24671

989.42646	3	24674

989.52606	2	24676

989.62566	5	24681

989.72526	2	24683

989.82486	4	24687

989.92446	3	24690

990.02406	5	24695

990.12366	2	24697

990.22326	2	24699

990.32286	1	24700

990.42246	4	24704

990.52206	11	24715

990.62166	4	24719

990.72126	7	24725

990.82086	1	24727

990.92046	2	24729

991.02006	1	24729

991.11966	1	24731

991.21926	1	24732

991.31886	1	24733

991.41846	5	24738

991.51806	1	24739

991.61766	1	24740

991.71726	7	24747

991.81686	2	24749

991.91646	1	24750

992.01606	6	24756

992.11566	1	24757

992.21526	3	24760

992.31486	6	24766

992.41446	1	24767

992.51406	3	24770

992.61366	1	24771

992.71326	2	24773

992.81286	4	24777

992.91246	6	24783

993.01206	13	24796

993.11166	3	24799

993.21126	1	24800

993.31086	1	24801

993.41046	5	24805

993.51006	1	24807

993.60966	1	24808

993.70926	1	24809

993.80886	7	24816

993.90846	1	24817

994.00806	4	24821

994.10766	1	24823

994.20726	3	24826

994.30686	1	24827

994.40646	4	24831

994.50606	1	24832

994.60566	1	24833

994.70526	3	24835

994.80486	1	24837

994.90446	1	24837

995.00406	5	24843

995.10366	4	24847

995.20326	1	24847

995.30286	1	24848

995.40246	1	24849

995.50206	3	24852

995.60166	2	24854

995.70126	1	24855

995.80086	1	24855

995.90046	1	24857

996.00006	17	24873

996.09966	1	24874

996.19926	14	24888

996.29886	1	24889

996.39846	1	24891

996.49806	9	24899

996.59766	1	24900

996.69726	11	24911

996.79686	5	24915

996.89646	18	24933

996.99606	1	24935

997.09566	2	24937

997.19526	7	24943

997.29486	15	24958

997.39446	3	24961

997.49406	1	24963

997.59366	1	24964

997.69326	5	24969

997.79286	1	24970

997.89246	9	24979

997.99206	1	24979

998.09166	2	24981

998.19126	7	24989

998.29086	1	24990

998.39046	8	24998

998.49006	2	25000

998.58966	1	25001

998.68926	17	25018

998.78886	1	25019

998.88846	17	25036

998.98806	5	25041

999.08766	5	25047

999.18726	5	25051

999.28686	5	25057

999.38646	1	25058

999.48606	10	25068

999.58566	1	25069

999.68526	1	25070

999.78486	6	25076

999.88446	1	25077

999.98406	1	25077

1000.08366	1	25079

1000.18326	15	25093

1000.28286	3	25096

1000.38246	3	25099

1000.48206	1	25100

1000.58166	1	25101

1000.68126	1	25102

1000.78086	1	25103

1000.88046	1	25105

1000.98006	17	25122

1001.07966	1	25123

1001.17926	9	25131

1001.27886	1	25132

1001.37846	1	25133

1001.47806	14	25147

1001.57766	4	25151

1001.67726	1	25153

1001.77686	1	25154

1001.87646	3	25157

1001.97606	1	25158

1002.07566	2	25160

1002.17526	6	25166

1002.27486	1	25167

1002.37446	2	25169

1002.47406	1	25169

1002.57366	5	25175

1002.67326	7	25181

1002.77286	9	25190

1002.87246	1	25191

1002.97206	2	25193

1003.07166	1	25193

1003.17126	2	25195

1003.27086	3	25198

1003.37046	1	25199

1003.47006	10	25209

1003.56966	1	25210

1003.66926	3	25213

1003.76886	10	25223

1003.86846	6	25229

1003.96806	1	25230

1004.06766	10	25240

1004.16726	5	25245

1004.26686	10	25255

1004.36646	1	25257

1004.46606	3	25259

1004.56566	1	25260

1004.66526	11	25271

1004.76486	3	25275

1004.86446	1	25275

1004.96406	12	25287

1005.06366	2	25289

1005.16326	15	25305

1005.26286	1	25305

1005.36246	1	25306

1005.46206	1	25307

1005.56166	2	25309

1005.66126	7	25317

1005.76086	4	25321

1005.86046	5	25326

1005.96006	3	25329

1006.05966	6	25335

1006.15926	3	25338

1006.25886	7	25345

1006.35846	1	25347

1006.45806	13	25360

1006.55766	1	25361

1006.65726	1	25362

1006.75686	11	25373

1006.85646	7	25380

1006.95606	1	25381

1007.05566	1	25382

1007.15526	5	25387

1007.25486	2	25389

1007.35446	1	25389

1007.45406	5	25394

1007.55366	1	25395

1007.65326	17	25411

1007.75286	15	25427

1007.85247	1	25427

1007.95206	13	25441

1008.05167	1	25441

1008.15127	1	25443

1008.25087	3	25446

1008.35047	3	25449

1008.45007	5	25454

1008.54967	3	25457

1008.64927	13	25470

1008.74887	1	25471

1008.84847	6	25477

1008.94807	1	25478

1009.04767	25	25503

1009.14726	2	25505

1009.24687	5	25509

1009.34647	1	25510

1009.44607	9	25519

1009.54567	3	25522

1009.64527	17	25539

1009.74487	1	25541

1009.84447	1	25541

1009.94407	3	25544

1010.04367	1	25545

1010.14327	5	25550

1010.24287	1	25551

1010.34246	10	25561

1010.44207	1	25561

1010.54167	1	25562

1010.64127	11	25573

1010.74087	1	25574

1010.84047	1	25575

1010.94007	11	25586

1011.03967	2	25588

1011.13927	1	25589

1011.23887	1	25589

1011.33847	7	25596

1011.43807	1	25597

1011.53766	2	25599

1011.63727	5	25604

1011.73687	1	25605

1011.83647	1	25605

1011.93607	1	25606

1012.03567	1	25607

1012.13527	1	25608

1012.23487	1	25609

1012.33447	9	25618

1012.43407	3	25621

1012.53367	1	25622

1012.63327	10	25632

1012.73286	1	25633

1012.83247	3	25637

1012.93207	1	25637

1013.03167	15	25652

1013.13127	6	25658

1013.23087	2	25660

1013.33047	1	25661

1013.43007	11	25671

1013.52967	1	25672

1013.62927	5	25677

1013.72887	1	25677

1013.82847	4	25681

1013.92806	1	25682

1014.02767	2	25684

1014.12727	8	25692

1014.22687	1	25693

1014.32647	7	25699

1014.42607	7	25706

1014.52567	1	25707

1014.62527	7	25715

1014.72487	1	25715

1014.82447	1	25717

1014.92407	3	25719

1015.02367	4	25723

1015.12326	1	25724

1015.22287	1	25725

1015.32247	4	25729

1015.42207	11	25740

1015.52167	1	25741

1015.62127	1	25743

1015.72087	1	25744

1015.82047	6	25750

1015.92007	2	25752

1016.01967	4	25756

1016.11927	15	25771

1016.21887	1	25772

1016.31846	15	25787

1016.41807	8	25795

1016.51767	1	25795

1016.61727	1	25796

1016.71687	5	25801

1016.81647	1	25801

1016.91607	3	25804

1017.01567	3	25807

1017.11527	1	25808

1017.21487	19	25827

1017.31447	5	25832

1017.41407	1	25833

1017.51366	1	25833

1017.61327	1	25835

1017.71287	13	25848

1017.81247	1	25849

1017.91207	10	25859

1018.01167	3	25862

1018.11127	19	25881

1018.21087	12	25893

1018.31047	4	25897

1018.41007	3	25900

1018.50967	1	25901

1018.60927	1	25902

1018.70886	1	25903

1018.80847	1	25903

1018.90807	11	25915

1019.00767	14	25929

1019.10727	1	25930

1019.20687	1	25931

1019.30647	9	25940

1019.40607	1	25941

1019.50567	1	25941

1019.60527	5	25946

1019.70487	4	25950

1019.80447	4	25954

1019.90406	2	25956

1020.00367	1	25957

1020.10327	2	25959

1020.20287	1	25961

1020.30247	1	25962

1020.40207	3	25965

1020.50167	1	25965

1020.60127	1	25966

1020.70087	11	25977

1020.80047	1	25978

1020.90007	13	25991

1020.99967	1	25992

1021.09926	17	26009

1021.19887	1	26009

1021.29847	3	26013

1021.39807	7	26020

1021.49767	1	26021

1021.59727	1	26022

1021.69687	4	26026

1021.79647	4	26030

1021.89607	3	26033

1021.99567	6	26039

1022.09527	1	26039

1022.19487	18	26057

1022.29446	1	26058

1022.39407	4	26062

1022.49367	1	26063

1022.59327	1	26064

1022.69287	1	26065

1022.79247	1	26065

1022.89207	12	26077

1022.99167	13	26091

1023.09127	2	26093

1023.19087	5	26097

1023.29047	8	26105

1023.39007	1	26106

1023.48967	11	26117

1023.58927	1	26117

1023.68887	6	26123

1023.78847	1	26125

1023.88807	2	26127

1023.98767	6	26133

1024.08727	1	26134

1024.18687	13	26147

1024.28647	1	26148

1024.38607	7	26155

1024.48567	1	26155

1024.58527	7	26163

1024.68487	1	26163

1024.78447	3	26166

1024.88407	5	26171

1024.98367	1	26171

1025.08327	12	26183

1025.18287	3	26186

1025.28247	2	26188

1025.38207	3	26191

1025.48167	1	26191

1025.58127	1	26193

1025.68087	7	26200

1025.78047	1	26201

1025.88007	10	26211

1025.97967	6	26217

1026.07927	12	26229

1026.17887	1	26230

1026.27847	1	26231

1026.37807	1	26232

1026.47767	3	26235

1026.57727	3	26237

1026.67687	4	26241

1026.77647	1	26242

1026.87607	11	26253

1026.97567	2	26255

1027.07527	1	26256

1027.17487	15	26271

1027.27447	11	26282

1027.37407	1	26283

1027.47367	2	26285

1027.57327	7	26292

1027.67287	1	26293

1027.77247	11	26304

1027.87207	1	26305

1027.97167	2	26307

1028.07127	5	26312

1028.17087	3	26315

1028.27047	1	26317

1028.37007	3	26319

1028.46967	1	26321

1028.56927	1	26321

1028.66887	3	26324

1028.76847	1	26325

1028.86807	18	26343

1028.96767	1	26343

1029.06727	5	26348

1029.16687	1	26349

1029.26647	3	26352

1029.36607	9	26361

1029.46567	3	26363

1029.56527	1	26365

1029.66487	1	26365

1029.76447	1	26366

1029.86407	7	26373

1029.96367	5	26378

1030.06327	1	26379

1030.16287	8	26387

1030.26247	1	26388

1030.36207	1	26389

1030.46167	8	26397

1030.56127	2	26399

1030.66087	9	26408

1030.76047	1	26409

1030.86007	4	26413

1030.95967	12	26425

1031.05927	13	26437

1031.15887	1	26438

1031.25847	5	26443

1031.35807	1	26444

1031.45767	2	26446

1031.55727	3	26449

1031.65687	1	26451

1031.75647	1	26452

1031.85607	1	26453

1031.95567	6	26459

1032.05527	1	26460

1032.15487	3	26463

1032.25447	1	26463

1032.35407	2	26465

1032.45367	5	26470

1032.55327	1	26471

1032.65287	3	26474

1032.75247	13	26487

1032.85207	3	26489

1032.95167	18	26507

1033.05127	1	26508

1033.15087	1	26509

1033.25047	9	26518

1033.35007	17	26535

1033.44967	17	26553

1033.54927	11	26564

1033.64887	1	26565

1033.74847	2	26567

1033.84807	7	26574

1033.94767	1	26575

1034.04727	1	26576

1034.14687	1	26577

1034.24647	1	26577

1034.34607	1	26579

1034.44567	6	26585

1034.54527	2	26587

1034.64487	1	26588

1034.74447	1	26589

1034.84407	2	26591

1034.94367	11	26602

1035.04327	2	26604

1035.14287	7	26611

1035.24247	2	26613

1035.34207	5	26618

1035.44167	9	26627

1035.54127	3	26629

1035.64087	3	26632

1035.74047	1	26633

1035.84007	1	26633

1035.93967	1	26635

1036.03927	4	26639

1036.13887	3	26641

1036.23847	2	26643

1036.33807	2	26645

1036.43767	1	26646

1036.53727	5	26651

1036.63687	3	26654

1036.73647	1	26655

1036.83607	3	26657

1036.93567	3	26660

1037.03527	3	26663

1037.13487	1	26664

1037.23447	1	26665

1037.33407	1	26665

1037.43367	6	26671

1037.53327	1	26672

1037.63287	3	26675

1037.73247	7	26683

1037.83207	1	26683

1037.93167	1	26684

1038.03127	12	26696

1038.13087	3	26699

1038.23047	5	26704

1038.33007	4	26708

1038.42967	4	26712

1038.52927	1	26713

1038.62887	10	26723

1038.72847	2	26725

1038.82807	1	26726

1038.92767	19	26745

1039.02727	1	26745

1039.12687	1	26746

1039.22647	2	26748

1039.32607	4	26752

1039.42567	3	26755

1039.52527	3	26759

1039.62487	4	26763

1039.72447	5	26768

1039.82407	13	26781

1039.92367	20	26801

1040.02327	2	26803

1040.12287	5	26807

1040.22247	1	26809

1040.32207	1	26809

1040.42167	1	26810

1040.52127	4	26814

1040.62087	1	26815

1040.72047	7	26822

1040.82007	1	26823

1040.91967	1	26824

1041.01927	5	26829

1041.11887	5	26835

1041.21847	9	26843

1041.31807	1	26845

1041.41767	1	26845

1041.51727	3	26849

1041.61687	13	26862

1041.71647	1	26863

1041.81607	1	26864

1041.91567	5	26869

1042.01527	3	26873

1042.11487	1	26873

1042.21447	3	26877

1042.31407	1	26877

1042.41367	2	26879

1042.51327	1	26880

1042.61287	7	26887

1042.71247	3	26891

1042.81207	5	26895

1042.91167	5	26900

1043.01127	1	26901

1043.11087	13	26914

1043.21047	11	26925

1043.31007	5	26930

1043.40967	1	26931

1043.50927	1	26932

1043.60887	1	26933

1043.70847	1	26934

1043.80807	4	26938

1043.90767	1	26939

1044.00727	1	26940

1044.10687	5	26945

1044.20647	3	26948

1044.30607	16	26964

1044.40567	1	26965

1044.50527	2	26967

1044.60487	1	26968

1044.70447	1	26969

1044.80407	1	26970

1044.90367	1	26971

1045.00327	3	26973

1045.10287	1	26975

1045.20247	12	26987

1045.30207	1	26987

1045.40167	5	26993

1045.50127	3	26996

1045.60087	5	27001

1045.70047	8	27009

1045.80007	1	27011

1045.89967	5	27015

1045.99927	5	27021

1046.09887	8	27029

1046.19847	1	27030

1046.29807	10	27040

1046.39767	1	27041

1046.49727	5	27045

1046.59687	8	27053

1046.69647	1	27054

1046.79607	5	27059

1046.89567	6	27065

1046.99527	1	27066

1047.09487	2	27068

1047.19447	3	27071

1047.29407	1	27072

1047.39367	2	27074

1047.49327	6	27080

1047.59287	6	27086

1047.69247	1	27087

1047.79207	11	27097

1047.89167	1	27099

1047.99127	2	27101

1048.09087	11	27111

1048.19047	1	27112

1048.29007	2	27114

1048.38967	5	27119

1048.48927	1	27120

1048.58887	4	27124

1048.68847	3	27127

1048.78807	2	27129

1048.88767	7	27136

1048.98727	2	27138

1049.08687	1	27139

1049.18647	1	27140

1049.28607	13	27153

1049.38567	1	27153

1049.48527	1	27154

1049.58487	9	27163

1049.68447	1	27164

1049.78407	7	27171

1049.88367	1	27172

1049.98327	1	27173

1050.08287	5	27179

1050.18247	1	27179

1050.28207	5	27185

1050.38167	13	27197

1050.48127	1	27198

1050.58087	8	27206

1050.68047	3	27209

1050.78007	4	27213

1050.87967	8	27221

1050.97927	1	27222

1051.07887	4	27226

1051.17847	1	27227

1051.27807	7	27235

1051.37767	10	27245

1051.47727	2	27247

1051.57687	2	27249

1051.67647	6	27255

1051.77607	6	27261

1051.87567	5	27266

1051.97527	1	27267

1052.07487	11	27278

1052.17447	1	27279

1052.27407	1	27280

1052.37367	15	27295

1052.47327	1	27296

1052.57287	1	27297

1052.67247	3	27300

1052.77207	26	27326

1052.87167	1	27327

1052.97127	21	27348

1053.07087	1	27349

1053.17047	1	27350

1053.27007	7	27357

1053.36967	3	27360

1053.46927	1	27361

1053.56887	8	27369

1053.66847	1	27369

1053.76807	4	27373

1053.86767	4	27377

1053.96727	2	27379

1054.06687	1	27380

1054.16647	3	27383

1054.26607	3	27385

1054.36567	1	27387

1054.46527	17	27404

1054.56487	3	27407

1054.66447	1	27407

1054.76407	1	27409

1054.86367	12	27421

1054.96327	5	27426

1055.06287	3	27429

1055.16247	7	27435

1055.26207	1	27437

1055.36167	7	27444

1055.46127	4	27448

1055.56087	1	27449

1055.66047	1	27450

1055.76007	21	27471

1055.85967	1	27472

1055.95927	9	27481

1056.05887	1	27482

1056.15847	17	27499

1056.25807	1	27500

1056.35767	2	27502

1056.45727	1	27503

1056.55687	13	27516

1056.65647	1	27517

1056.75607	1	27517

1056.85567	10	27527

1056.95527	1	27528

1057.05487	2	27530

1057.15447	1	27531

1057.25407	2	27533

1057.35367	20	27553

1057.45327	2	27555

1057.55287	5	27559

1057.65247	3	27562

1057.75207	1	27563

1057.85167	3	27566

1057.95127	2	27568

1058.05087	19	27587

1058.15047	1	27588

1058.25007	8	27596

1058.34967	6	27602

1058.44927	5	27607

1058.54887	3	27609

1058.64847	17	27627

1058.74807	1	27627

1058.84767	1	27628

1058.94727	8	27636

1059.04687	1	27637

1059.14647	5	27642

1059.24607	1	27643

1059.34567	2	27645

1059.44527	7	27652

1059.54487	2	27654

1059.64447	1	27655

1059.74407	3	27658

1059.84367	7	27665

1059.94327	1	27666

1060.04287	3	27669

1060.14247	10	27679

1060.24207	1	27680

1060.34167	1	27681

1060.44127	11	27692

1060.54087	1	27693

1060.64047	1	27693

1060.74007	1	27694

1060.83967	4	27698

1060.93927	1	27699

1061.03887	1	27700

1061.13847	1	27701

1061.23807	6	27707

1061.33767	1	27709

1061.43727	6	27715

1061.53687	1	27716

1061.63647	9	27725

1061.73607	3	27727

1061.83567	8	27735

1061.93527	2	27737

1062.03487	3	27740

1062.13447	6	27746

1062.23407	1	27747

1062.33367	1	27747

1062.43327	15	27763

1062.53287	1	27764

1062.63247	6	27770

1062.73207	4	27774

1062.83167	7	27781

1062.93127	3	27783

1063.03087	8	27791

1063.13047	5	27796

1063.23007	1	27797

1063.32967	3	27800

1063.42927	1	27801

1063.52887	2	27803

1063.62847	1	27804

1063.72807	3	27807

1063.82767	5	27812

1063.92727	1	27813

1064.02687	1	27815

1064.12647	1	27815

1064.22607	1	27817

1064.32567	11	27827

1064.42527	1	27829

1064.52487	1	27829

1064.62447	5	27835

1064.72407	5	27840

1064.82367	1	27841

1064.92327	7	27847

1065.02287	1	27848

1065.12247	1	27849

1065.22207	3	27853

1065.32167	1	27853

1065.42127	2	27855

1065.52087	5	27861

1065.62047	6	27867

1065.72007	2	27869

1065.81967	5	27874

1065.91927	1	27875

1066.01887	1	27875

1066.11847	13	27889

1066.21807	2	27891

1066.31767	2	27893

1066.41727	7	27899

1066.51687	1	27900

1066.61647	7	27907

1066.71607	1	27907

1066.81567	1	27908

1066.91527	4	27912

1067.01487	12	27924

1067.11447	1	27925

1067.21407	19	27943

1067.31367	8	27951

1067.41327	8	27959

1067.51287	1	27960

1067.61247	2	27962

1067.71207	16	27978

1067.81167	1	27979

1067.91127	3	27981

1068.01087	11	27993

1068.11047	1	27993

1068.21007	1	27994

1068.30967	20	28014

1068.40927	5	28019

1068.50887	12	28031

1068.60847	4	28035

1068.70807	1	28036

1068.80767	1	28037

1068.90727	1	28037

1069.00687	1	28038

1069.10647	2	28040

1069.20607	13	28053

1069.30567	14	28067

1069.40527	5	28072

1069.50487	9	28081

1069.60447	1	28081

1069.70407	2	28083

1069.80367	1	28085

1069.90327	3	28087

1070.00287	13	28101

1070.10247	1	28101

1070.20207	3	28104

1070.30167	8	28112

1070.40127	4	28116

1070.50087	13	28129

1070.60047	1	28130

1070.70007	1	28131

1070.79967	7	28137

1070.89927	2	28139

1070.99887	1	28140

1071.09847	17	28157

1071.19807	5	28163

1071.29767	15	28178

1071.39727	1	28179

1071.49687	17	28196

1071.59647	1	28197

1071.69607	13	28209

1071.79567	1	28210

1071.89527	1	28211

1071.99487	16	28227

1072.09447	1	28227

1072.19407	2	28229

1072.29367	6	28235

1072.39327	3	28238

1072.49287	4	28242

1072.59247	3	28245

1072.69207	1	28246

1072.79167	1	28247

1072.89127	5	28251

1072.99087	1	28252

1073.09047	10	28262

1073.19007	6	28268

1073.28967	1	28269

1073.38927	8	28277

1073.48887	4	28281

1073.58847	2	28283

1073.68807	3	28286

1073.78767	4	28290

1073.88727	7	28297

1073.98687	18	28315

1074.08647	17	28332

1074.18607	6	28338

1074.28567	18	28356

1074.38527	1	28357

1074.48487	3	28359

1074.58447	1	28360

1074.68407	1	28361

1074.78367	11	28372

1074.88327	5	28377

1074.98287	3	28381

1075.08247	1	28381

1075.18207	1	28382

1075.28167	2	28384

1075.38127	1	28385

1075.48087	3	28388

1075.58047	1	28389

1075.68007	4	28393

1075.77967	8	28401

1075.87927	7	28408

1075.97887	5	28413

1076.07847	1	28415

1076.17807	23	28438

1076.27767	1	28439

1076.37727	25	28464

1076.47687	1	28465

1076.57647	7	28471

1076.67607	3	28474

1076.77567	1	28475

1076.87527	1	28476

1076.97487	4	28480

1077.07447	1	28481

1077.17407	1	28482

1077.27367	8	28490

1077.37327	1	28491

1077.47287	1	28491

1077.57247	1	28492

1077.67207	20	28512

1077.77167	1	28513

1077.87127	11	28524

1077.97087	1	28525

1078.07047	1	28526

1078.17007	11	28537

1078.26967	15	28553

1078.36927	1	28553

1078.46887	7	28560

1078.56847	1	28561

1078.66807	1	28563

1078.76767	6	28569

1078.86727	3	28572

1078.96687	2	28574

1079.06647	3	28577

1079.16607	2	28579

1079.26567	1	28580

1079.36527	3	28583

1079.46487	2	28585

1079.56447	2	28587

1079.66407	5	28592

1079.76367	3	28595

1079.86327	1	28596

1079.96287	1	28597

1080.06247	2	28599

1080.16207	1	28599

1080.26167	2	28601

1080.36127	4	28605

1080.46087	1	28606

1080.56047	2	28608

1080.66007	1	28609

1080.75967	1	28609

1080.85927	10	28619

1080.95887	1	28620

1081.05847	3	28623

1081.15807	6	28629

1081.25767	1	28630

1081.35727	1	28631

1081.45687	1	28633

1081.55647	21	28654

1081.65607	4	28658

1081.75567	1	28659

1081.85527	3	28661

1081.95487	9	28671

1082.05447	2	28673

1082.15407	1	28674

1082.25367	4	28678

1082.35327	1	28679

1082.45287	7	28687

1082.55247	7	28694

1082.65207	3	28697

1082.75167	3	28701

1082.85127	1	28701

1082.95087	7	28708

1083.05047	3	28711

1083.15007	3	28713

1083.24967	3	28717

1083.34927	22	28739

1083.44887	2	28741

1083.54847	6	28747

1083.64807	1	28748

1083.74767	1	28749

1083.84727	1	28749

1083.94687	1	28750

1084.04647	17	28767

1084.14607	1	28767

1084.24567	9	28776

1084.34527	17	28793

1084.44487	1	28795

1084.54447	2	28797

1084.64407	2	28799

1084.74367	9	28807

1084.84327	1	28808

1084.94287	3	28811

1085.04247	15	28825

1085.14207	1	28827

1085.24167	3	28829

1085.34127	3	28832

1085.44087	13	28845

1085.54047	1	28846

1085.64007	1	28847

1085.73967	1	28848

1085.83927	1	28849

1085.93887	1	28851

1086.03847	10	28861

1086.13807	1	28861

1086.23767	1	28862

1086.33727	1	28863

1086.43687	3	28865

1086.53647	1	28866

1086.63607	1	28867

1086.73567	3	28869

1086.83527	2	28871

1086.93487	1	28872

1087.03447	17	28889

1087.13407	2	28891

1087.23367	2	28893

1087.33327	7	28899

1087.43287	1	28901

1087.53247	23	28924

1087.63207	17	28941

1087.73167	13	28953

1087.83127	6	28959

1087.93087	2	28961

1088.03047	5	28967

1088.13007	8	28975

1088.22967	7	28982

1088.32927	1	28983

1088.42887	1	28984

1088.52847	7	28991

1088.62807	1	28992

1088.72767	3	28995

1088.82727	2	28997

1088.92687	11	29009

1089.02647	1	29010

1089.12607	1	29011

1089.22567	3	29014

1089.32527	1	29015

1089.42487	3	29018

1089.52447	24	29042

1089.62407	1	29043

1089.72367	1	29044

1089.82327	5	29049

1089.92287	1	29049

1090.02247	9	29059

1090.12207	1	29059

1090.22167	1	29061

1090.32127	23	29084

1090.42087	1	29085

1090.52047	1	29087

1090.62007	3	29089

1090.71967	5	29094

1090.81927	1	29095

1090.91887	1	29096

1091.01847	12	29108

1091.11807	1	29109

1091.21767	5	29114

1091.31727	1	29115

1091.41687	1	29117

1091.51647	13	29129

1091.61607	1	29131

1091.71567	1	29131

1091.81527	25	29157

1091.91487	3	29160

1092.01447	7	29167

1092.11407	1	29169

1092.21367	12	29181

1092.31327	1	29182

1092.41287	5	29187

1092.51247	4	29191

1092.61207	1	29191

1092.71167	10	29201

1092.81127	3	29204

1092.91087	15	29219

1093.01047	1	29220

1093.11007	1	29221

1093.20967	5	29226

1093.30927	21	29247

1093.40887	1	29248

1093.50847	15	29263

1093.60807	9	29271

1093.70767	1	29273

1093.80727	1	29274

1093.90687	1	29275

1094.00647	2	29277

1094.10607	14	29291

1094.20567	1	29291

1094.30527	15	29306

1094.40487	2	29308

1094.50447	11	29319

1094.60407	15	29333

1094.70367	1	29335

1094.80327	3	29337

1094.90287	3	29340

1095.00247	1	29341

1095.10207	1	29342

1095.20167	1	29343

1095.30127	2	29345

1095.40087	14	29359

1095.50047	4	29363

1095.60007	1	29364

1095.69967	17	29381

1095.79927	1	29381

1095.89887	1	29382

1095.99847	13	29395

1096.09807	2	29397

1096.19767	1	29397

1096.29727	1	29399

1096.39687	1	29399

1096.49647	1	29400

1096.59607	3	29403

1096.69567	6	29409

1096.79527	2	29411

1096.89487	5	29417

1096.99447	12	29429

1097.09407	1	29429

1097.19367	1	29430

1097.29327	4	29434

1097.39287	3	29437

1097.49247	5	29441

1097.59207	1	29442

1097.69167	1	29443

1097.79127	1	29443

1097.89087	27	29470

1097.99047	3	29473

1098.09007	9	29481

1098.18967	1	29482

1098.28927	1	29483

1098.38887	2	29485

1098.48847	4	29489

1098.58807	3	29493

1098.68767	13	29505

1098.78727	18	29523

1098.88687	4	29527

1098.98647	3	29530

1099.08607	1	29531

1099.18567	7	29537

1099.28527	2	29539

1099.38487	7	29547

1099.48447	5	29552

1099.58407	3	29555

1099.68367	1	29556

1099.78327	7	29563

1099.88287	13	29576

1099.98247	3	29579

1100.08207	1	29581

1100.18167	4	29585

1100.28127	11	29595

1100.38087	1	29596

1100.48047	5	29601

1100.58007	9	29610

1100.67967	1	29611

1100.77927	12	29623

1100.87887	4	29627

1100.97847	1	29627

1101.07807	3	29630

1101.17767	8	29638

1101.27727	5	29643

1101.37687	17	29659

1101.47647	4	29663

1101.57607	1	29664

1101.67567	9	29673

1101.77527	1	29675

1101.87487	1	29676

1101.97447	10	29686

1102.07407	18	29704

1102.17367	7	29711

1102.27327	1	29712

1102.37287	3	29715

1102.47247	15	29731

1102.57207	3	29733

1102.67167	1	29734

1102.77127	1	29735

1102.87087	9	29744

1102.97047	18	29762

1103.07007	1	29763

1103.16967	20	29783

1103.26927	3	29785

1103.36887	1	29787

1103.46847	14	29801

1103.56807	3	29803

1103.66767	9	29812

1103.76727	1	29813

1103.86687	5	29819

1103.96647	1	29819

1104.06607	1	29820

1104.16567	1	29821

1104.26527	2	29823

1104.36487	1	29824

1104.46447	5	29829

1104.56407	8	29837

1104.66367	1	29839

1104.76327	7	29846

1104.86287	5	29851

1104.96247	6	29857

1105.06207	1	29858

1105.16167	13	29871

1105.26127	4	29875

1105.36087	5	29881

1105.46047	1	29881

1105.56007	1	29882

1105.65967	21	29903

1105.75927	1	29905

1105.85887	1	29905

1105.95847	4	29909

1106.05807	2	29911

1106.15767	1	29912

1106.25727	8	29920

1106.35687	2	29922

1106.45647	11	29933

1106.55607	3	29936

1106.65567	3	29939

1106.75527	9	29948

1106.85487	1	29949

1106.95447	9	29958

1107.05407	11	29969

1107.15367	1	29969

1107.25327	3	29972

1107.35287	13	29985

1107.45247	5	29989

1107.55207	9	29999

1107.65167	3	30002

1107.75127	7	30009

1107.85087	1	30010

1107.95047	5	30015

1108.05007	3	30018

1108.14967	1	30019

1108.24927	3	30022

1108.34887	6	30028

1108.44847	3	30031

1108.54807	31	30063

1108.64767	1	30063

1108.74727	10	30073

1108.84687	3	30077

1108.94647	3	30080

1109.04607	1	30081

1109.14567	3	30083

1109.24527	2	30085

1109.34487	22	30107

1109.44447	9	30116

1109.54407	3	30119

1109.64367	3	30121

1109.74327	1	30123

1109.84287	23	30146

1109.94247	14	30160

1110.04207	1	30161

1110.14167	19	30180

1110.24127	1	30181

1110.34087	1	30182

1110.44047	14	30196

1110.54007	1	30197

1110.63967	1	30199

1110.73927	1	30199

1110.83887	3	30203

1110.93847	1	30203

1111.03807	9	30212

1111.13767	1	30213

1111.23727	13	30225

1111.33687	8	30233

1111.43647	1	30234

1111.53607	9	30243

1111.63567	1	30244

1111.73527	17	30261

1111.83487	3	30264

1111.93447	2	30266

1112.03407	5	30271

1112.13367	5	30275

1112.23327	15	30291

1112.33287	2	30293

1112.43247	5	30298

1112.53207	3	30301

1112.63167	2	30303

1112.73127	9	30313

1112.83087	5	30318

1112.93047	1	30319

1113.03007	1	30319

1113.12967	15	30335

1113.22927	1	30336

1113.32887	5	30341

1113.42847	2	30343

1113.52807	1	30344

1113.62767	3	30347

1113.72727	1	30348

1113.82687	1	30349

1113.92647	17	30366

1114.02607	2	30368

1114.12567	3	30371

1114.22527	11	30382

1114.32487	2	30384

1114.42447	10	30394

1114.52407	1	30395

1114.62367	3	30398

1114.72327	3	30401

1114.82287	1	30402

1114.92247	1	30403

1115.02207	1	30403

1115.12167	7	30410

1115.22127	1	30411

1115.32087	1	30411

1115.42047	7	30419

1115.52007	19	30438

1115.61967	2	30440

1115.71927	13	30453

1115.81887	2	30455

1115.91847	21	30476

1116.01807	11	30487

1116.11767	2	30489

1116.21727	3	30492

1116.31687	20	30512

1116.41647	13	30525

1116.51607	14	30539

1116.61567	1	30539

1116.71527	16	30555

1116.81487	1	30556

1116.91447	4	30560

1117.01407	1	30561

1117.11367	1	30562

1117.21327	3	30565

1117.31287	12	30577

1117.41247	3	30579

1117.51207	1	30581

1117.61167	9	30590

1117.71127	1	30591

1117.81087	1	30592

1117.91047	7	30599

1118.01007	3	30603

1118.10967	1	30603

1118.20927	16	30619

1118.30887	1	30620

1118.40847	3	30623

1118.50807	2	30625

1118.60767	1	30627

1118.70727	1	30627

1118.80687	1	30629

1118.90647	1	30630

1119.00607	4	30634

1119.10567	2	30636

1119.20527	3	30639

1119.30487	9	30647

1119.40447	1	30649

1119.50407	1	30649

1119.60367	5	30654

1119.70327	1	30655

1119.80287	1	30656

1119.90247	9	30665

1120.00207	2	30667

1120.10167	9	30676

1120.20127	1	30677

1120.30087	14	30691

1120.40047	1	30692

1120.50007	18	30710

1120.59967	1	30711

1120.69927	10	30721

1120.79887	1	30722

1120.89847	15	30737

1120.99807	7	30743

1121.09767	1	30744

1121.19727	2	30746

1121.29687	1	30747

1121.39647	3	30751

1121.49607	9	30759

1121.59567	2	30761

1121.69527	2	30763

1121.79487	2	30765

1121.89447	3	30768

1121.99407	3	30771

1122.09367	19	30789

1122.19327	19	30808

1122.29287	1	30809

1122.39247	24	30833

1122.49207	3	30835

1122.59167	3	30838

1122.69127	9	30847

1122.79087	2	30849

1122.89047	13	30861

1122.99007	1	30863

1123.08967	3	30866

1123.18927	6	30872

1123.28887	1	30873

1123.38847	9	30882

1123.48807	14	30896

1123.58767	1	30897

1123.68727	4	30901

1123.78687	5	30907

1123.88647	4	30911

1123.98607	7	30917

1124.08567	2	30919

1124.18527	1	30921

1124.28487	9	30929

1124.38447	3	30933

1124.48407	1	30933

1124.58367	4	30937

1124.68327	4	30941

1124.78287	1	30942

1124.88247	1	30943

1124.98207	1	30945

1125.08167	16	30961

1125.18127	7	30968

1125.28087	5	30973

1125.38047	1	30974

1125.48007	1	30975

1125.57967	4	30979

1125.67927	1	30980

1125.77887	1	30981

1125.87847	1	30982

1125.97807	1	30983

1126.07767	1	30985

1126.17727	7	30991

1126.27687	1	30993

1126.37647	2	30995

1126.47607	1	30995

1126.57567	1	30996

1126.67527	1	30997

1126.77487	2	30999

1126.87447	10	31009

1126.97407	1	31009

1127.07367	2	31011

1127.17327	15	31027

1127.27287	4	31031

1127.37247	1	31031

1127.47207	7	31038

1127.57167	1	31039

1127.67127	1	31039

1127.77087	9	31049

1127.87047	1	31050

1127.97007	11	31061

1128.06967	1	31061

1128.16927	3	31065

1128.26887	9	31074

1128.36847	2	31076

1128.46807	3	31079

1128.56767	2	31081

1128.66727	1	31082

1128.76687	11	31093

1128.86647	1	31094

1128.96607	6	31100

1129.06567	1	31101

1129.16527	1	31102

1129.26487	6	31108

1129.36447	11	31119

1129.46407	1	31121

1129.56367	1	31121

1129.66327	4	31125

1129.76287	1	31126

1129.86247	1	31127

1129.96207	14	31141

1130.06167	2	31143

1130.16127	6	31149

1130.26087	1	31150

1130.36047	11	31161

1130.46007	14	31175

1130.55967	2	31177

1130.65927	9	31187

1130.75887	1	31187

1130.85847	17	31205

1130.95807	1	31206

1131.05767	1	31207

1131.15727	3	31210

1131.25687	1	31211

1131.35647	1	31212

1131.45607	18	31230

1131.55567	2	31232

1131.65527	11	31243

1131.75487	1	31245

1131.85447	5	31250

1131.95407	1	31251

1132.05367	1	31252

1132.15327	5	31257

1132.25287	4	31261

1132.35247	2	31263

1132.45207	15	31278

1132.55167	1	31279

1132.65127	3	31281

1132.75087	5	31286

1132.85047	1	31287

1132.95007	3	31290

1133.04967	1	31291

1133.14927	3	31295

1133.24887	3	31298

1133.34847	2	31300

1133.44807	3	31303

1133.54767	11	31314

1133.64727	1	31315

1133.74687	2	31317

1133.84647	7	31323

1133.94607	2	31325

1134.04567	1	31327

1134.14527	4	31331

1134.24487	3	31333

1134.34447	3	31337

1134.44407	3	31340

1134.54367	1	31341

1134.64327	6	31347

1134.74287	3	31350

1134.84247	5	31355

1134.94207	1	31357

1135.04167	3	31360

1135.14127	3	31363

1135.24087	15	31379

1135.34047	6	31385

1135.44007	1	31385

1135.53967	3	31388

1135.63927	5	31393

1135.73887	1	31393

1135.83847	2	31395

1135.93807	14	31409

1136.03767	1	31410

1136.13727	2	31412

1136.23687	1	31413

1136.33647	7	31421

1136.43607	7	31428

1136.53567	1	31429

1136.63527	1	31429

1136.73487	1	31431

1136.83447	1	31431

1136.93407	7	31438

1137.03367	1	31439

1137.13327	9	31447

1137.23287	3	31450

1137.33247	4	31454

1137.43207	11	31465

1137.53167	3	31467

1137.63127	3	31471

1137.73087	1	31471

1137.83047	5	31476

1137.93007	6	31482

1138.02967	1	31483

1138.12927	1	31483

1138.22887	1	31485

1138.32847	5	31490

1138.42807	1	31491

1138.52767	4	31495

1138.62727	1	31496

1138.72687	1	31497

1138.82647	1	31498

1138.92607	3	31501

1139.02567	1	31501

1139.12527	1	31502

1139.22487	3	31505

1139.32447	1	31505

1139.42407	2	31507

1139.52367	1	31508

1139.62327	1	31509

1139.72287	1	31510

1139.82247	2	31512

1139.92207	17	31529

1140.02167	5	31534

1140.12127	3	31537

1140.22087	4	31541

1140.32047	4	31545

1140.42007	1	31545

1140.51967	1	31547

1140.61927	7	31554

1140.71887	7	31561

1140.81847	9	31571

1140.91807	5	31576

1141.01767	1	31577

1141.11727	2	31579

1141.21687	9	31588

1141.31647	1	31589

1141.41607	3	31592

1141.51567	2	31594

1141.61527	1	31595

1141.71487	1	31595

1141.81447	10	31605

1141.91407	1	31606

1142.01367	1	31607

1142.11327	1	31608

1142.21287	4	31612

1142.31247	5	31617

1142.41207	20	31637

1142.51167	13	31649

1142.61127	11	31661

1142.71087	1	31661

1142.81047	1	31663

1142.91007	6	31669

1143.00967	1	31669

1143.10927	6	31675

1143.20887	2	31677

1143.30847	1	31678

1143.40807	11	31689

1143.50767	1	31691

1143.60727	1	31691

1143.70687	7	31698

1143.80647	5	31703

1143.90607	1	31705

1144.00567	9	31714

1144.10527	1	31715

1144.20487	2	31717

1144.30447	3	31720

1144.40407	3	31723

1144.50367	21	31744

1144.60327	6	31750

1144.70287	5	31755

1144.80247	1	31756

1144.90207	17	31773

1145.00167	3	31777

1145.10127	1	31777

1145.20087	15	31793

1145.30047	1	31793

1145.40007	4	31797

1145.49967	1	31798

1145.59927	1	31799

1145.69887	1	31799

1145.79847	15	31814

1145.89807	12	31826

1145.99767	4	31830

1146.09727	1	31831

1146.19687	19	31850

1146.29647	1	31851

1146.39607	1	31851

1146.49567	10	31861

1146.59527	33	31894

1146.69487	1	31895

1146.79447	15	31909

1146.89407	7	31916

1146.99367	9	31925

1147.09327	1	31926

1147.19287	10	31936

1147.29247	1	31937

1147.39207	3	31940

1147.49167	1	31941

1147.59127	1	31941

1147.69087	1	31942

1147.79047	8	31950

1147.89007	2	31952

1147.98967	1	31953

1148.08927	21	31973

1148.18887	1	31975

1148.28847	2	31977

1148.38807	1	31977

1148.48767	3	31980

1148.58727	1	31981

1148.68687	1	31982

1148.78647	3	31985

1148.88607	1	31985

1148.98567	3	31988

1149.08527	1	31989

1149.18487	1	31990

1149.28447	2	31992

1149.38407	3	31995

1149.48367	1	31995

1149.58327	2	31997

1149.68287	2	31999

1149.78247	5	32004

1149.88207	1	32005

1149.98167	9	32013

1150.08127	1	32014

1150.18087	1	32015

1150.28047	3	32018

1150.38007	3	32021

1150.47967	1	32021

1150.57927	1	32023

1150.67887	10	32033

1150.77847	1	32033

1150.87807	1	32034

1150.97767	2	32036

1151.07727	4	32040

1151.17687	14	32054

1151.27647	1	32055

1151.37607	1	32055

1151.47567	1	32056

1151.57527	1	32057

1151.67487	1	32059

1151.77447	1	32060

1151.87407	7	32067

1151.97367	1	32069

1152.07327	1	32069

1152.17287	1	32070

1152.27247	10	32080

1152.37207	1	32081

1152.47167	3	32084

1152.57127	9	32093

1152.67087	1	32094

1152.77047	13	32107

1152.87007	1	32108

1152.96967	2	32110

1153.06927	1	32111

1153.16887	11	32123

1153.26847	5	32127

1153.36807	1	32128

1153.46767	1	32129

1153.56727	1	32129

1153.66687	16	32145

1153.76647	3	32149

1153.86607	1	32150

1153.96567	2	32152

1154.06527	1	32153

1154.16487	1	32153

1154.26447	5	32158

1154.36407	1	32159

1154.46367	1	32159

1154.56327	1	32160

1154.66287	1	32161

1154.76247	3	32163

1154.86207	1	32164

1154.96167	9	32173

1155.06127	2	32175

1155.16087	8	32183

1155.26047	1	32184

1155.36007	1	32185

1155.45967	1	32185

1155.55927	17	32203

1155.65887	1	32203

1155.75847	4	32207

1155.85807	1	32208

1155.95767	1	32209

1156.05727	1	32211

1156.15687	11	32222

1156.25647	7	32229

1156.35607	1	32230

1156.45567	19	32249

1156.55527	1	32249

1156.65487	13	32262

1156.75447	6	32268

1156.85407	4	32272

1156.95367	2	32274

1157.05327	22	32296

1157.15287	1	32297

1157.25247	4	32301

1157.35207	1	32302

1157.45167	11	32313

1157.55127	1	32313

1157.65087	3	32316

1157.75047	9	32325

1157.85007	1	32325

1157.94967	3	32329

1158.04927	15	32343

1158.14887	1	32345

1158.24847	2	32347

1158.34807	1	32348

1158.44767	1	32349

1158.54727	1	32350

1158.64687	1	32351

1158.74647	1	32351

1158.84607	1	32353

1158.94567	4	32357

1159.04527	19	32375

1159.14487	11	32387

1159.24447	3	32389

1159.34407	5	32395

1159.44367	6	32401

1159.54327	9	32410

1159.64287	2	32412

1159.74247	1	32413

1159.84207	1	32414

1159.94167	11	32425

1160.04127	5	32430

1160.14087	4	32434

1160.24047	5	32439

1160.34007	3	32441

1160.43967	7	32448

1160.53927	1	32449

1160.63887	20	32469

1160.73847	1	32469

1160.83807	4	32473

1160.93767	10	32483

1161.03727	1	32484

1161.13687	2	32486

1161.23647	4	32490

1161.33607	4	32494

1161.43567	1	32495

1161.53527	3	32498

1161.63487	8	32506

1161.73447	2	32508

1161.83407	2	32510

1161.93367	13	32523

1162.03327	3	32525

1162.13287	1	32527

1162.23247	1	32527

1162.33207	18	32545

1162.43167	11	32557

1162.53127	1	32557

1162.63087	3	32560

1162.73047	9	32569

1162.83007	3	32571

1162.92967	2	32573

1163.02927	1	32574

1163.12887	3	32577

1163.22847	5	32582

1163.32807	3	32585

1163.42767	1	32587

1163.52727	1	32587

1163.62687	1	32588

1163.72647	11	32599

1163.82607	4	32603

1163.92567	5	32609

1164.02527	4	32613

1164.12488	1	32614

1164.22448	11	32625

1164.32408	2	32627

1164.42368	1	32628

1164.52328	1	32629

1164.62288	18	32647

1164.72248	1	32648

1164.82208	9	32657

1164.92168	1	32658

1165.02128	1	32659

1165.12088	3	32661

1165.22048	11	32672

1165.32008	2	32674

1165.41968	15	32689

1165.51928	2	32691

1165.61887	3	32693

1165.71847	2	32695

1165.81807	1	32696

1165.91767	1	32697

1166.01727	4	32701

1166.11687	3	32704

1166.21647	11	32715

1166.31608	1	32716

1166.41568	2	32718

1166.51528	3	32721

1166.61488	8	32729

1166.71448	1	32729

1166.81408	7	32737

1166.91368	1	32738

1167.01328	1	32739

1167.11288	9	32747

1167.21248	5	32752

1167.31208	8	32760

1167.41168	1	32761

1167.51128	5	32765

1167.61088	7	32773

1167.71048	1	32773

1167.81008	1	32774

1167.90968	1	32775

1168.00927	13	32788

1168.10887	1	32789

1168.20847	2	32791

1168.30807	15	32805

1168.40767	1	32806

1168.50727	3	32809

1168.60687	3	32811

1168.70648	1	32812

1168.80608	17	32829

1168.90568	4	32833

1169.00528	13	32846

1169.10488	1	32847

1169.20448	8	32855

1169.30408	1	32856

1169.40368	8	32864

1169.50328	3	32867

1169.60288	1	32867

1169.70248	5	32873

1169.80208	11	32883

1169.90168	6	32889

1170.00128	9	32899

1170.10088	5	32903

1170.20048	1	32905

1170.30008	5	32909

1170.39967	9	32918

1170.49927	3	32921

1170.59887	6	32927

1170.69847	1	32928

1170.79807	19	32947

1170.89767	2	32949

1170.99727	4	32953

1171.09688	1	32953

1171.19648	1	32954

1171.29608	8	32962

1171.39568	1	32963

1171.49528	3	32966

1171.59488	6	32972

1171.69448	9	32981

1171.79408	1	32982

1171.89368	15	32997

1171.99328	2	32999

1172.09288	3	33002

1172.19248	3	33005

1172.29208	1	33007

1172.39168	1	33007

1172.49128	5	33013

1172.59088	3	33016

1172.69048	1	33017

1172.79007	7	33024

1172.88967	1	33025

1172.98927	1	33027

1173.08887	3	33029

1173.18847	14	33043

1173.28807	2	33045

1173.38767	7	33053

1173.48728	3	33056

1173.58688	1	33057

1173.68648	12	33069

1173.78608	1	33071

1173.88568	1	33071

1173.98528	29	33100

1174.08488	1	33101

1174.18448	15	33116

1174.28408	1	33117

1174.38368	1	33118

1174.48328	1	33119

1174.58288	8	33127

1174.68248	1	33127

1174.78208	7	33134

1174.88168	1	33135

1174.98128	15	33150

1175.08088	3	33153

1175.18047	1	33154

1175.28007	3	33157

1175.37967	1	33158

1175.47927	4	33162

1175.57887	1	33163

1175.67847	10	33173

1175.77807	1	33175

1175.87768	1	33175

1175.97728	13	33188

1176.07688	1	33189

1176.17648	8	33197

1176.27608	3	33200

1176.37568	1	33201

1176.47528	13	33213

1176.57488	4	33217

1176.67448	1	33218

1176.77408	6	33224

1176.87368	4	33228

1176.97328	7	33235

1177.07288	9	33245

1177.17248	1	33246

1177.27208	2	33248

1177.37168	10	33258

1177.47128	2	33260

1177.57087	2	33262

1177.67047	5	33267

1177.77007	1	33267

1177.86967	4	33271

1177.96927	5	33277

1178.06887	25	33301

1178.16847	1	33302

1178.26808	12	33314

1178.36768	1	33315

1178.46728	2	33317

1178.56688	6	33323

1178.66648	5	33328

1178.76608	5	33333

1178.86568	20	33353

1178.96528	3	33356

1179.06488	1	33357

1179.16448	20	33377

1179.26408	1	33378

1179.36368	1	33379

1179.46328	1	33380

1179.56288	44	33424

1179.66248	4	33428

1179.76208	3	33431

1179.86168	14	33445

1179.96128	4	33449

1180.06088	3	33452

1180.16048	1	33453

1180.26008	4	33457

1180.35968	46	33503

1180.45928	19	33522

1180.55888	21	33543

1180.65848	8	33551

1180.75808	2	33553

1180.85768	10	33563

1180.95728	5	33568

1181.05688	18	33586

1181.15648	1	33587

1181.25608	1	33587

1181.35568	13	33600

1181.45528	5	33605

1181.55488	18	33623

1181.65448	1	33623

1181.75408	11	33634

1181.85368	1	33635

1181.95328	12	33647

1182.05288	2	33649

1182.15248	4	33653

1182.25208	37	33689

1182.35168	1	33690

1182.45128	6	33696

1182.55088	5	33701

1182.65048	7	33707

1182.75008	1	33708

1182.84968	1	33709

1182.94928	1	33709

1183.04888	19	33729

1183.14848	2	33731

1183.24808	29	33760

1183.34768	4	33764

1183.44728	45	33809

1183.54688	24	33833

1183.64648	87	33920

1183.74608	11	33931

1183.84568	82	34013

1183.94528	118	34131

1184.04488	2	34133

1184.14448	67	34200

1184.24408	27	34227

1184.34368	87	34314

1184.44328	120	34434

1184.54288	50	34484

1184.64248	43	34526

1184.74208	1	34527

1184.84168	3	34530

1184.94128	11967	46497

1185.04088	57	46554

1185.14048	30	46584

1185.24008	20	46604

1185.33968	9	46613

1185.43928	11	46624

1185.53888	99	46723

1185.63848	41	46763

1185.73808	2	46765

1185.83768	3	46768

1185.93728	86	46854

1186.03688	494	47348

1186.13648	350	47698

1186.23608	44	47742

1186.33568	1	47743

1186.43528	1	47744

1186.53488	1	47745

**（*f*）Stabilized hydraulic-90°**

1	0.06358	0.55736	1	1

2	0.08935	0.6967	1	2

3	0.10654	0.83604	1	3

4	0.12372	0.97538	5	8

5	0.14091	1.11472	2	10

6	0.16324	1.25406	3	13

7	0.18558	1.3934	2	14

8	0.20277	1.53274	1	15

9	0.21479	1.67208	1	16

10	0.2337	1.81142	23	39

11	0.2526	1.95076	3	42

12	0.26806	2.0901	1	43

13	0.28525	2.22944	1	44

14	0.30071	2.36878	1	45

15	0.31961	2.50812	1	45

16	0.34023	2.64746	4	49

17	0.35742	2.7868	5	54

18	0.36773	2.92614	1	55

19	0.39178	3.06548	5	60

20	0.41241	3.20482	16	75

21	0.42615	3.34416	3	78

22	0.4399	3.4835	1	78

23	0.4588	3.62284	1	79

24	0.4777	3.76218	17	96

25	0.4966	3.90152	1	97

26	0.51207	4.04086	17	113

27	0.52582	4.1802	3	116

28	0.543	4.31954	3	119

29	0.56534	4.45889	2	120

30	0.58252	4.59823	6	126

31	0.59455	4.73757	1	127

32	0.6083	4.87691	2	129

33	0.63064	5.01625	1	129

34	0.64782	5.15559	3	132

35	0.66672	5.29493	1	133

36	0.67875	5.43427	5	138

37	0.69937	5.57361	3	141

38	0.71999	5.71295	1	141

39	0.73546	5.85229	9	150

40	0.7492	5.99163	1	151

41	0.76639	6.13097	1	152

42	0.78529	6.27031	2	153

43	0.80247	6.40965	1	154

44	0.82137	6.54899	1	154

45	0.83168	6.68833	1	155

46	0.85402	6.82767	12	167

47	0.87292	6.96701	1	167

48	0.88839	7.10635	5	172

49	0.90557	7.24569	3	174

50	0.91932	7.38503	1	175

51	0.93994	7.52437	1	175

52	0.95884	7.66371	1	176

53	0.97431	7.80305	2	177

54	0.98805	7.94239	6	183

55	1.00696	8.08173	1	184

56	1.02586	8.22107	1	184

57	1.04648	8.36041	5	189

58	1.06022	8.49975	2	191

59	1.07741	8.63909	3	194

60	1.09459	8.77843	1	194

61	1.11349	8.91777	44	238

62	1.13068	9.05711	1	239

63	1.14099	9.19645	7	246

64	1.15989	9.33579	3	248

65	1.18223	9.47513	3	251

66	1.20113	9.61447	8	258

67	1.21144	9.75381	5	263

68	1.22862	9.89315	1	263

69	1.24924	10.03249	1	264

70	1.26643	10.17183	1	265

71	1.28361	10.31117	1	265

72	1.29736	10.45051	1	266

73	1.31626	10.58985	3	269

74	1.3386	10.72919	3	272

75	1.35406	10.86853	3	275

76	1.36609	11.00787	8	283

77	1.38499	11.14721	1	283

78	1.40561	11.28655	1	284

79	1.4228	11.42589	4	288

80	1.43826	11.56523	1	289

81	1.45201	11.70457	2	290

82	1.47263	11.84391	3	293

83	1.49153	11.98325	1	294

84	1.507	12.12259	2	296

85	1.52074	12.26193	1	296

86	1.53793	12.40127	6	302

87	1.55855	12.54061	2	303

88	1.57573	12.67995	1	304

89	1.5912	12.81929	1	305

90	1.60494	12.95863	1	305

91	1.62556	13.09797	1	306

92	1.64618	13.23731	1	306

93	1.66165	13.37666	1	307

94	1.67368	13.516	2	309

95	1.69258	13.65534	1	309

96	1.71492	13.79468	1	310

97	1.73038	13.93402	1	311

98	1.74241	14.07336	1	312

99	1.75788	14.2127	1	313

100	1.78022	14.35204	2	315

101	1.79912	14.49138	1	316

102	1.8163	14.63072	1	316

103	1.82833	14.77006	5	321

104	1.84723	14.9094	1	321

105	1.86785	15.04874	2	323

106	1.88504	15.18808	1	324

107	1.89706	15.32742	1	325

108	1.91425	15.46676	1	326

109	1.93315	15.6061	1	326

110	1.95377	15.74544	1	327

111	1.96923	15.88478	3	329

112	1.98298	16.02412	1	330

113	2.00016	16.16346	5	334

114	2.0225	16.3028	2	336

115	2.03797	16.44214	7	343

116	2.05343	16.58148	1	344

117	2.05687	16.72082	2	346

118	2.0311	16.86016	4	349

119	2.03625	16.9995	1	350

120	2.06031	17.13884	9	358

121	2.08093	17.27818	3	361

122	2.09639	17.41752	9	369

123	2.10498	17.55686	3	372

124	2.12045	17.6962	1	373

125	2.14451	17.83554	1	373

126	2.16341	17.97488	1	374

127	2.17716	18.11422	1	375

128	2.1909	18.25356	1	375

129	2.2098	18.3929	1	376

130	2.23214	18.53224	29	404

131	2.24933	18.67158	5	409

132	2.26135	18.81092	1	410

133	2.27682	18.95026	2	412

134	2.30088	19.0896	1	413

135	2.31291	19.22894	1	414

136	2.32665	19.36828	1	415

137	2.34384	19.50762	1	415

138	2.36617	19.64696	1	416

139	2.38164	19.7863	2	417

140	2.40226	19.92564	1	418

141	2.41085	20.06498	1	418

142	2.42804	20.20432	1	419

143	2.45209	20.34366	1	420

144	2.46584	20.483	1	420

145	2.47959	20.62234	3	423

146	2.49677	20.76168	1	424

147	2.51911	20.90102	3	427

148	2.53285	21.04036	2	429

149	2.55176	21.1797	1	430

150	2.5655	21.31904	3	432

151	2.58612	21.45838	2	434

152	2.60331	21.59772	8	442

153	2.62049	21.73706	2	443

154	2.63424	21.8764	5	448

155	2.65486	22.01574	1	448

156	2.67204	22.15508	2	450

157	2.69094	22.29443	1	451

158	2.70469	22.43377	1	451

159	2.72359	22.57311	5	456

160	2.73906	22.71245	1	456

161	2.75796	22.85179	5	461

162	2.77342	22.99113	1	462

163	2.78717	23.13047	1	462

164	2.80779	23.26981	7	469

165	2.82841	23.40915	1	470

166	2.84216	23.54849	1	471

167	2.85934	23.68783	1	471

168	2.87481	23.82717	1	472

169	2.89715	23.96651	1	473

170	2.91777	24.10585	6	479

171	2.92808	24.24519	1	480

172	2.94354	24.38453	1	480

173	2.96244	24.52387	58	538

174	2.98135	24.66321	1	538

175	2.99853	24.80255	1	539

176	3.01399	24.94189	1	540

177	3.02946	25.08123	1	540

178	3.0518	25.22057	2	542

179	3.0707	25.35991	1	542

180	3.08101	25.49925	1	543

181	3.10163	25.63859	1	544

182	3.11881	25.77793	3	547

183	3.14115	25.91727	1	547

184	3.15146	26.05661	2	549

185	3.16865	26.19595	2	550

186	3.18755	26.33529	1	551

187	3.20989	26.47463	1	552

188	3.22363	26.61397	2	554

189	3.23738	26.75331	1	554

190	3.25456	26.89265	1	555

191	3.27518	27.03199	1	555

192	3.29237	27.17133	1	556

193	3.30268	27.31067	2	558

194	3.3233	27.45001	1	559

195	3.34564	27.58935	1	559

196	3.36282	27.72869	1	560

197	3.37829	27.86803	1	560

198	3.3886	28.00737	1	561

199	3.41265	28.14671	2	562

200	3.42812	28.28605	1	563

201	3.4453	28.42539	1	564

202	3.45733	28.56473	2	566

203	3.48139	28.70407	1	567

204	3.49857	28.84341	11	577

205	3.51575	28.98275	1	578

206	3.5295	29.12209	1	578

207	3.5484	29.26143	2	580

208	3.5673	29.40077	3	583

209	3.58792	29.54011	1	583

210	3.59823	29.67945	6	589

211	3.61026	29.81879	20	609

212	3.63432	29.95813	3	612

213	3.65666	30.09747	2	614

214	3.67041	30.23681	2	616

215	3.68415	30.37615	1	617

216	3.70305	30.51549	5	621

217	3.72367	30.65483	1	622

218	3.73914	30.79417	1	622

219	3.75117	30.93351	1	623

220	3.76663	31.07285	2	625

221	3.79241	31.2122	1	625

222	3.80959	31.35154	1	626

223	3.82334	31.49088	1	627

224	3.8388	31.63022	4	630

225	3.85771	31.76956	2	632

226	3.88005	31.9089	5	637

227	3.89379	32.04824	1	637

228	3.90754	32.18758	1	638

229	3.923	32.32692	1	638

230	3.94534	32.46626	2	640

231	3.96596	32.6056	1	641

232	3.97627	32.74494	2	642

233	3.99002	32.88428	29	671

234	4.01236	33.02362	4	675

235	4.03126	33.16296	4	678

236	4.04844	33.3023	2	680

237	4.06391	33.44164	1	681

238	4.08109	33.58098	8	689

239	4.09828	33.72032	1	689

240	4.12061	33.85966	1	690

241	4.12577	33.999	2	691

242	4.14639	34.13834	1	692

243	4.17217	34.27768	1	693

244	4.18591	34.41702	1	693

245	4.19622	34.55636	1	694

246	4.21341	34.6957	2	696

247	4.23403	34.83504	3	698

248	4.25465	34.97438	3	701

249	4.27011	35.11372	1	701

250	4.28386	35.25306	3	704

251	4.30276	35.3924	1	704

252	4.32338	35.53174	4	708

253	4.34056	35.67108	1	709

254	4.35603	35.81042	1	710

255	4.36978	35.94976	1	711

256	4.39383	36.0891	1	711

257	4.41274	36.22844	2	713

258	4.42133	36.36778	1	713

259	4.43679	36.50712	1	714

260	4.46085	36.64646	3	716

261	4.48319	36.7858	3	719

262	4.49178	36.92514	1	719

263	4.50724	37.06448	1	720

264	4.52958	37.20382	1	721

265	4.54849	37.34316	1	722

266	4.56395	37.4825	11	732

267	4.57254	37.62184	1	733

268	4.59144	37.76118	1	733

269	4.61206	37.90052	1	734

270	4.6344	38.03986	2	735

271	4.64643	38.1792	2	737

272	4.66018	38.31854	1	738

273	4.68424	38.45788	1	739

274	4.70486	38.59722	2	740

275	4.71688	38.73656	1	741

276	4.73063	38.8759	6	746

277	4.75125	39.01524	1	747

278	4.77187	39.15458	2	748

279	4.78562	39.29392	1	749

280	4.79593	39.43326	1	750

281	4.81827	39.5726	2	752

282	4.84061	39.71194	1	752

283	4.86123	39.85128	1	753

284	4.87154	39.99062	1	753

285	4.88356	40.12997	1	754

286	4.90762	40.26931	9	762

287	4.9248	40.40865	1	763

288	4.94027	40.54799	1	763

289	4.9523	40.68733	1	764

290	4.9712	40.82667	1	765

291	4.99526	40.96601	1	765

292	5.01244	41.10535	1	766

293	5.02619	41.24469	1	766

294	5.03993	41.38403	1	767

295	5.06399	41.52337	1	768

296	5.07946	41.66271	1	768

297	5.09149	41.80205	1	769

298	5.10867	41.94139	1	770

299	5.12757	42.08073	1	771

300	5.14647	42.22007	1	772

301	5.16194	42.35941	1	772

302	5.18084	42.49875	1	773

303	5.19974	42.63809	10	783

304	5.21693	42.77743	1	783

305	5.23239	42.91677	1	784

306	5.2427	43.05611	1	785

307	5.2616	43.19545	1	785

308	5.28566	43.33479	1	786

309	5.30112	43.47413	3	788

310	5.31659	43.61347	1	789

311	5.33377	43.75281	1	790

312	5.35268	43.89215	2	792

313	5.37158	44.03149	1	792

314	5.38189	44.17083	1	793

315	5.39563	44.31017	3	795

316	5.41969	44.44951	1	796

317	5.43859	44.58885	1	796

318	5.45406	44.72819	1	797

319	5.4678	44.86753	2	798

320	5.48671	45.00687	9	807

321	5.51248	45.14621	1	808

322	5.52967	45.28555	1	808

323	5.53826	45.42489	1	809

324	5.55716	45.56423	10	819

325	5.5795	45.70357	1	819

326	5.59668	45.84291	1	820

327	5.60699	45.98225	2	822

328	5.62418	46.12159	1	823

329	5.64823	46.26093	1	823

330	5.66713	46.40027	1	824

331	5.68088	46.53961	1	824

332	5.68947	46.67895	1	825

333	5.70837	46.81829	2	826

334	5.73243	46.95763	2	828

335	5.7479	47.09697	1	829

336	5.75821	47.23631	1	830

337	5.77883	47.37565	1	830

338	5.80117	47.51499	1	831

339	5.81663	47.65433	1	831

340	5.82866	47.79367	1	832

341	5.84756	47.93301	1	833

342	5.87162	48.07235	13	845

343	5.8888	48.21169	1	846

344	5.89911	48.35103	1	847

345	5.9163	48.49037	3	849

346	5.93692	48.62971	2	851

347	5.9541	48.76905	15	865

348	5.97472	48.90839	3	868

349	5.98675	49.04774	1	869

350	6.00221	49.18708	2	870

351	6.02455	49.32642	1	871

352	6.04002	49.46576	1	871

353	6.05376	49.6051	8	879

354	6.07267	49.74444	5	884

355	6.09329	49.88378	2	885

356	6.11047	50.02312	3	888

357	6.12593	50.16246	1	889

358	6.1414	50.3018	1	889

359	6.16202	50.44114	1	890

360	6.1792	50.58048	1	890

361	6.19639	50.71982	1	891

362	6.20498	50.85916	1	891

363	6.2256	50.9985	1	892

364	6.24794	51.13784	1	893

365	6.26512	51.27718	1	893

366	6.27715	51.41652	2	895

367	6.29605	51.55586	1	895

368	6.31839	51.6952	1	896

369	6.33729	51.83454	2	898

370	6.3476	51.97388	1	898

371	6.36307	52.11322	1	899

372	6.38197	52.25256	2	900

373	6.40603	52.3919	4	904

374	6.41977	52.53124	1	905

375	6.42837	52.67058	3	907

376	6.45242	52.80992	22	929

377	6.47132	52.94926	1	929

378	6.49023	53.0886	8	937

379	6.50054	53.22794	1	938

380	6.51944	53.36728	11	948

381	6.54178	53.50662	3	951

382	6.55724	53.64596	1	951

383	6.56755	53.7853	1	952

384	6.58817	53.92464	1	952

385	6.61395	54.06398	5	957

386	6.62769	54.20332	1	958

387	6.63972	54.34266	2	959

388	6.65691	54.482	1	960

389	6.67753	54.62134	7	966

390	6.69471	54.76068	2	968

391	6.70846	54.90002	2	970

392	6.7222	55.03936	13	983

393	6.74626	55.1787	1	983

394	6.76516	55.31804	4	987

395	6.78406	55.45738	1	988

396	6.79609	55.59672	1	989

397	6.81328	55.73606	1	989

398	6.83562	55.8754	1	990

399	6.85108	56.01474	3	993

400	6.86139	56.15408	1	993

401	6.88201	56.29342	1	994

402	6.90263	56.43276	3	996

403	6.91981	56.5721	9	1005

404	6.93184	56.71144	1	1006

405	6.94731	56.85078	1	1007

406	6.96965	56.99012	1	1008

407	6.99027	57.12946	2	1010

408	7.0023	57.2688	5	1014

409	7.01432	57.40814	1	1015

410	7.03494	57.54748	4	1018

411	7.05728	57.68682	3	1021

412	7.07447	57.82616	1	1021

413	7.0865	57.96551	2	1023

414	7.10368	58.10485	1	1024

415	7.1243	58.24419	1	1024

416	7.14492	58.38353	1	1025

417	7.15695	58.52287	2	1026

418	7.16898	58.66221	5	1031

419	7.19131	58.80155	1	1032

420	7.21194	58.94089	1	1032

421	7.22568	59.08023	2	1034

422	7.23599	59.21957	1	1034

423	7.26005	59.35891	1	1035

424	7.28239	59.49825	1	1035

425	7.29785	59.63759	1	1036

426	7.30816	59.77693	6	1042

427	7.32363	59.91627	4	1045

428	7.34769	60.05561	2	1047

429	7.36487	60.19495	1	1048

430	7.37518	60.33429	1	1049

431	7.39752	60.47363	7	1055

432	7.41814	60.61297	1	1056

433	7.43704	60.75231	1	1057

434	7.44735	60.89165	1	1058

435	7.46453	61.03099	2	1060

436	7.48515	61.17033	9	1068

437	7.50234	61.30967	2	1070

438	7.51608	61.44901	1	1071

439	7.52983	61.58835	1	1072

440	7.55045	61.72769	1	1072

441	7.57107	61.86703	1	1073

442	7.58825	62.00637	8	1081

443	7.59685	62.14571	3	1083

444	7.61919	62.28505	8	1091

445	7.64152	62.42439	1	1092

446	7.65527	62.56373	1	1093

447	7.6673	62.70307	4	1096

448	7.68448	62.84241	1	1097

449	7.70854	62.98175	1	1097

450	7.724	63.12109	1	1098

451	7.73947	63.26043	2	1100

452	7.75322	63.39977	8	1108

453	7.78243	63.53911	6	1114

454	7.79446	63.67845	1	1114

455	7.8082	63.81779	1	1115

456	7.82195	63.95713	5	1119

457	7.84429	64.09647	10	1129

458	7.86491	64.23581	1	1130

459	7.87694	64.37515	27	1156

460	7.88897	64.51449	4	1160

461	7.91302	64.65383	2	1162

462	7.93708	64.79317	4	1166

463	7.94911	64.93251	1	1166

464	7.96286	65.07185	2	1168

465	7.98004	65.21119	1	1168

466	8.00066	65.35053	1	1169

467	8.01956	65.48987	1	1170

468	8.03159	65.62921	1	1170

469	8.04877	65.76855	2	1172

470	8.06939	65.90789	1	1173

471	8.09001	66.04723	2	1175

472	8.10204	66.18657	1	1176

473	8.11407	66.32591	2	1178

474	8.13297	66.46525	1	1179

475	8.15703	66.60459	1	1179

476	8.17078	66.74393	3	1182

477	8.18281	66.88328	1	1182

478	8.19999	67.02262	3	1185

479	8.22233	67.16196	1	1185

480	8.23951	67.3013	1	1186

481	8.25154	67.44064	1	1186

482	8.27044	67.57998	1	1187

483	8.29278	67.71932	3	1189

484	8.30825	67.85866	1	1190

485	8.32199	67.998	16	1206

486	8.33918	68.13734	1	1207

487	8.35808	68.27668	1	1207

488	8.37698	68.41602	1	1208

489	8.39416	68.55536	5	1212

490	8.40963	68.6947	1	1213

491	8.42681	68.83404	3	1216

492	8.44743	68.97338	7	1223

493	8.45946	69.11272	6	1228

494	8.47149	69.25206	3	1231

495	8.49383	69.3914	8	1238

496	8.51788	69.53074	1	1239

497	8.53163	69.67008	6	1244

498	8.54538	69.80942	4	1248

499	8.56428	69.94876	1	1249

500	8.5849	70.0881	1	1249

501	8.60037	70.22744	13	1262

502	8.61068	70.36678	9	1271

503	8.62786	70.50612	1	1271

504	8.6502	70.64546	2	1273

505	8.66738	70.7848	1	1273

506	8.68113	70.92414	3	1276

507	8.69831	71.06348	1	1276

508	8.72065	71.20282	1	1277

509	8.74127	71.34216	6	1283

510	8.75502	71.4815	2	1284

511	8.76017	71.62084	1	1285

512	8.78423	71.76018	1	1285

513	8.80829	71.89952	2	1287

514	8.82375	72.03886	1	1287

515	8.83406	72.1782	10	1297

516	8.8564	72.31754	2	1298

517	8.87874	72.45688	4	1302

518	8.89592	72.59622	2	1304

519	8.90623	72.73556	1	1304

520	8.9217	72.8749	15	1319

521	8.94747	73.01424	4	1323

522	8.96122	73.15358	6	1328

523	8.97497	73.29292	2	1330

524	8.98871	73.43226	4	1334

525	9.01449	73.5716	9	1342

526	9.03511	73.71094	1	1343

527	9.04714	73.85028	3	1345

528	9.05745	73.98962	7	1352

529	9.07979	74.12896	1	1353

530	9.09697	74.2683	1	1354

531	9.11244	74.40764	3	1356

532	9.1279	74.54698	1	1357

533	9.1468	74.68632	1	1357

534	9.17258	74.82566	1	1358

535	9.18632	74.965	1	1359

536	9.19663	75.10434	1	1360

537	9.21726	75.24368	1	1360

538	9.23788	75.38302	1	1361

539	9.25334	75.52236	1	1361

540	9.26365	75.6617	6	1367

541	9.28427	75.80105	2	1369

542	9.30489	75.94039	1	1369

543	9.32036	76.07973	1	1370

544	9.33238	76.21907	1	1370

545	9.35129	76.35841	2	1372

546	9.37363	76.49775	2	1373

547	9.39253	76.63709	2	1375

548	9.40456	76.77643	1	1376

549	9.42002	76.91577	1	1376

550	9.44236	77.05511	1	1377

551	9.45954	77.19445	1	1378

552	9.47501	77.33379	1	1379

553	9.49047	77.47313	1	1379

554	9.50938	77.61247	1	1380

555	9.53343	77.75181	1	1381

556	9.54718	77.89115	1	1382

557	9.55921	78.03049	1	1383

558	9.57295	78.16983	1	1383

559	9.59873	78.30917	1	1384

560	9.61419	78.44851	1	1384

561	9.62794	78.58785	2	1386

562	9.64341	78.72719	7	1393

563	9.66575	78.86653	2	1395

564	9.6898	79.00587	1	1396

565	9.70183	79.14521	1	1396

566	9.71214	79.28455	1	1397

567	9.73104	79.42389	1	1398

568	9.74995	79.56323	1	1398

569	9.76541	79.70257	1	1399

570	9.77916	79.84191	2	1400

571	9.79634	79.98125	4	1404

572	9.82555	80.12059	1	1405

573	9.84102	80.25993	2	1406

574	9.85133	80.39927	2	1408

575	9.87023	80.53861	7	1415

576	9.89085	80.67795	9	1424

577	9.90803	80.81729	4	1427

578	9.91663	80.95663	1	1428

579	9.93553	81.09597	1	1429

580	9.95958	81.23531	1	1429

581	9.97677	81.37465	1	1430

582	9.99051	81.51399	2	1431

583	10.00598	81.65333	4	1435

584	10.02832	81.79267	1	1436

585	10.0455	81.93201	1	1436

586	10.05753	82.07135	2	1438

587	10.07128	82.21069	1	1439

588	10.09018	82.35003	1	1440

589	10.11252	82.48937	1	1440

590	10.1297	82.62871	2	1442

591	10.14173	82.76805	2	1444

592	10.16063	82.90739	12	1456

593	10.18469	83.04673	2	1457

594	10.195	83.18607	3	1460

595	10.20703	83.32541	1	1460

596	10.22421	83.46475	1	1461

597	10.24827	83.60409	1	1462

598	10.26717	83.74343	1	1462

599	10.2792	83.88277	10	1472

600	10.29638	84.02211	3	1475

601	10.32044	84.16145	1	1475

602	10.3359	84.30079	12	1487

603	10.34965	84.44013	1	1487

604	10.36168	84.57947	1	1488

605	10.38402	84.71882	10	1498

606	10.40636	84.85816	1	1498

607	10.41839	84.9975	4	1502

608	10.43385	85.13684	5	1507

609	10.45447	85.27618	1	1508

610	10.47509	85.41552	1	1509

611	10.48884	85.55486	1	1509

612	10.49915	85.6942	3	1512

613	10.52149	85.83354	4	1515

614	10.54382	85.97288	1	1516

615	10.55929	86.11222	5	1520

616	10.57132	86.25156	7	1527

617	10.5885	86.3909	1	1527

618	10.61256	86.53024	1	1528

619	10.63318	86.66958	4	1531

620	10.64177	86.80892	12	1543

621	10.65724	86.94826	1	1543

622	10.67786	87.0876	1	1544

623	10.69504	87.22694	1	1544

624	10.70879	87.36628	1	1545

625	10.72253	87.50562	2	1546

626	10.74659	87.64496	5	1551

627	10.76377	87.7843	1	1552

628	10.78096	87.92364	1	1552

629	10.79127	88.06298	1	1553

630	10.81532	88.20232	1	1554

631	10.83595	88.34166	1	1554

632	10.84626	88.481	1	1555

633	10.85828	88.62034	4	1558

634	10.88062	88.75968	1	1559

635	10.90468	88.89902	1	1559

636	10.91843	89.03836	1	1560

637	10.92874	89.1777	1	1560

638	10.94936	89.31704	18	1578

639	10.9717	89.45638	1	1578

640	10.98716	89.59572	19	1597

641	10.99575	89.73506	1	1598

642	11.01809	89.8744	7	1605

643	11.04215	90.01374	1	1606

644	11.05246	90.15308	1	1606

645	11.06792	90.29242	1	1607

646	11.08683	90.43176	1	1607

647	11.10745	90.5711	4	1611

648	11.12807	90.71044	1	1612

649	11.14181	90.84978	3	1614

650	11.15212	90.98912	14	1628

651	11.17618	91.12846	1	1629

652	11.19508	91.2678	1	1630

653	11.20883	91.40714	1	1630

654	11.22086	91.54648	1	1631

655	11.2432	91.68582	6	1636

656	11.26382	91.82516	5	1641

657	11.27928	91.9645	1	1642

658	11.29131	92.10384	13	1655

659	11.30849	92.24318	42	1697

660	11.33255	92.38252	2	1698

661	11.3463	92.52186	1	1699

662	11.35661	92.6612	2	1701

663	11.37551	92.80054	1	1701

664	11.39785	92.93988	3	1704

665	11.41847	93.07922	1	1704

666	11.4305	93.21856	1	1705

667	11.44768	93.3579	8	1712

668	11.4683	93.49725	1	1713

669	11.48376	93.63659	4	1716

670	11.50095	93.77593	1	1717

671	11.50954	93.91527	1	1717

672	11.53016	94.05461	15	1732

673	11.55594	94.19395	1	1733

674	11.56796	94.33329	2	1734

675	11.58171	94.47263	1	1735

676	11.60405	94.61197	2	1736

677	11.62639	94.75131	2	1738

678	11.63842	94.89065	1	1739

679	11.64701	95.02999	1	1740

680	11.66763	95.16933	1	1740

681	11.68997	95.30867	4	1744

682	11.70715	95.44801	1	1745

683	11.71746	95.58735	2	1746

684	11.7398	95.72669	3	1749

685	11.76042	95.86603	1	1750

686	11.7776	96.00537	15	1765

687	11.78963	96.14471	6	1771

688	11.8051	96.28405	1	1771

689	11.82744	96.42339	3	1774

690	11.8429	96.56273	1	1775

691	11.85837	96.70207	1	1775

692	11.87555	96.84141	1	1776

693	11.89617	96.98075	2	1777

694	11.91507	97.12009	3	1780

695	11.93054	97.25943	3	1783

696	11.93913	97.39877	1	1784

697	11.96147	97.53811	1	1785

698	11.98209	97.67745	3	1788

699	11.99755	97.81679	2	1789

700	12.01302	97.95613	1	1790

701	12.0302	98.09547	4	1793

702	12.05426	98.23481	1	1794

703	12.06629	98.37415	2	1796

704	12.08003	98.51349	2	1797

705	12.10065	98.65283	3	1800

706	12.12471	98.79217	3	1803

707	12.13674	98.93151	1	1804

708	12.14705	99.07085	21	1825

709	12.16767	99.21019	2	1827

710	12.19001	99.34953	8	1834

711	12.20547	99.48887	1	1835

712	12.21578	99.62821	1	1835

713	12.2364	99.76755	1	1836

714	12.25531	99.90689	1	1836

715	12.27421	100.04623	2	1838

716	12.28796	100.18557	7	1844

717	12.3017	100.32491	3	1847

718	12.32404	100.46425	1	1847

719	12.34122	100.60359	1	1848

720	12.35669	100.74293	1	1849

721	12.37215	100.88227	1	1850

722	12.39277	101.02161	1	1850

723	12.41511	101.16095	1	1851

724	12.42886	101.30029	7	1858

725	12.43745	101.43963	1	1858

726	12.45807	101.57897	1	1859

727	12.47697	101.71831	2	1861

728	12.49588	101.85765	16	1877

729	12.50962	101.99699	5	1881

730	12.52681	102.13633	1	1882

731	12.55258	102.27567	1	1882

732	12.56977	102.41502	1	1883

733	12.57836	102.55436	2	1885

734	12.59554	102.6937	1	1885

735	12.6196	102.83304	1	1886

736	12.63506	102.97238	3	1888

737	12.64365	103.11172	14	1902

738	12.66084	103.25106	21	1922

739	12.68833	103.3904	1	1923

740	12.70208	103.52974	13	1935

741	12.71754	103.66908	3	1938

742	12.73473	103.80842	1	1939

743	12.75707	103.94776	1	1939

744	12.77253	104.0871	1	1940

745	12.78456	104.22644	1	1941

746	12.80002	104.36578	3	1944

747	12.81893	104.50512	3	1947

748	12.83955	104.64446	10	1957

749	12.85329	104.7838	3	1959

750	12.87048	104.92314	2	1961

751	12.8911	105.06248	2	1962

752	12.91344	105.20182	1	1963

753	12.92375	105.34116	1	1963

754	12.93406	105.4805	1	1964

755	12.95983	105.61984	17	1981

756	12.97873	105.75918	1	1981

757	12.99248	105.89852	1	1982

758	13.00451	106.03786	30	2012

759	13.02685	106.1772	3	2015

760	13.0509	106.31654	1	2015

761	13.06121	106.45588	1	2016

762	13.07496	106.59522	1	2016

763	13.09386	106.73456	1	2017

764	13.11448	106.8739	1	2017

765	13.13167	107.01324	2	2019

766	13.14198	107.15258	1	2020

767	13.15744	107.29192	1	2020

768	13.18665	107.43126	1	2021

769	13.2004	107.5706	1	2021

770	13.21415	107.70994	1	2022

771	13.23477	107.84928	1	2023

772	13.24852	107.98862	1	2023

773	13.26914	108.12796	1	2024

774	13.28632	108.2673	4	2028

775	13.29319	108.40664	1	2028

776	13.31897	108.54598	1	2029

777	13.34302	108.68532	1	2029

778	13.35333	108.82466	1	2030

779	13.36708	108.964	4	2034

780	13.3877	109.10334	2	2036

781	13.4066	109.24268	1	2036

782	13.42207	109.38202	2	2038

783	13.43582	109.52136	1	2038

784	13.45128	109.6607	5	2043

785	13.47534	109.80004	15	2057

786	13.4908	109.93938	1	2058

787	13.50455	110.07872	2	2060

788	13.52517	110.21806	1	2060

789	13.54579	110.3574	2	2062

790	13.55954	110.49674	1	2063

791	13.575	110.63608	8	2071

792	13.59219	110.77542	1	2072

793	13.61281	110.91476	1	2073

794	13.63171	111.0541	10	2083

795	13.6403	111.19344	3	2086

796	13.6592	111.33279	12	2098

797	13.68498	111.47213	5	2102

798	13.69872	111.61147	1	2103

799	13.71075	111.75081	1	2103

800	13.7245	111.89015	3	2106

801	13.74684	112.02949	16	2122

802	13.76574	112.16883	1	2122

803	13.78121	112.30817	2	2124

804	13.79495	112.44751	1	2124

805	13.81729	112.58685	1	2125

806	13.83791	112.72619	16	2140

807	13.84994	112.86553	2	2142

808	13.86712	113.00487	1	2143

809	13.88602	113.14421	1	2144

810	13.90665	113.28355	6	2149

811	13.91867	113.42289	1	2150

812	13.93242	113.56223	5	2154

813	13.95304	113.70157	19	2173

814	13.97366	113.84091	2	2175

815	13.98741	113.98025	1	2176

816	13.99944	114.11959	3	2179

817	14.02006	114.25893	1	2179

818	14.04755	114.39827	22	2201

819	14.05786	114.53761	1	2201

820	14.06645	114.67695	12	2213

821	14.08707	114.81629	3	2216

822	14.11457	114.95563	5	2221

823	14.12831	115.09497	2	2222

824	14.1369	115.23431	16	2238

825	14.15753	115.37365	1	2239

826	14.1833	115.51299	1	2239

827	14.19877	115.65233	1	2240

828	14.20736	115.79167	5	2244

829	14.22626	115.93101	14	2258

830	14.2486	116.07035	1	2259

831	14.26578	116.20969	1	2259

832	14.27437	116.34903	21	2280

833	14.29671	116.48837	8	2288

834	14.31733	116.62771	6	2294

835	14.33795	116.76705	1	2295

836	14.34998	116.90639	1	2295

837	14.36545	117.04573	1	2296

838	14.38778	117.18507	1	2297

839	14.40153	117.32441	6	2303

840	14.41356	117.46375	1	2303

841	14.43246	117.60309	1	2304

842	14.45308	117.74243	2	2306

843	14.47198	117.88177	3	2309

844	14.48401	118.02111	2	2310

845	14.5012	118.16045	1	2311

846	14.52525	118.29979	1	2311

847	14.54244	118.43913	1	2312

848	14.55275	118.57847	1	2312

849	14.56821	118.71781	1	2313

850	14.58883	118.85715	1	2313

851	14.60773	118.99649	5	2318

852	14.6232	119.13583	4	2322

853	14.63695	119.27517	3	2324

854	14.65757	119.41451	1	2325

855	14.67819	119.55385	5	2330

856	14.69537	119.69319	1	2331

857	14.70396	119.83253	1	2332

858	14.7263	119.97187	33	2365

859	14.74864	120.11121	3	2367

860	14.7641	120.25056	1	2368

861	14.77098	120.3899	1	2369

862	14.7916	120.52924	17	2386

863	14.81565	120.66858	1	2386

864	14.83456	120.80792	1	2387

865	14.84487	120.94726	4	2390

866	14.86205	121.0866	9	2399

867	14.88783	121.22594	13	2411

868	14.89985	121.36528	14	2425

869	14.91016	121.50462	1	2425

870	14.93078	121.64396	10	2435

871	14.9514	121.7833	1	2436

872	14.97031	121.92264	6	2442

873	14.98234	122.06198	12	2454

874	14.99952	122.20132	3	2457

875	15.02186	122.34066	4	2460

876	15.03904	122.48	1	2461

877	15.05107	122.61934	2	2463

878	15.06482	122.75868	1	2463

879	15.08715	122.89802	1	2464

880	15.10606	123.03736	1	2464

881	15.12152	123.1767	11	2475

882	15.13699	123.31604	1	2475

883	15.15589	123.45538	1	2476

884	15.17823	123.59472	8	2483

885	15.19026	123.73406	2	2485

886	15.20057	123.8734	9	2494

887	15.22462	124.01274	4	2498

888	15.24696	124.15208	2	2499

889	15.25727	124.29142	1	2500

890	15.27102	124.43076	1	2500

891	15.29164	124.5701	1	2501

892	15.31398	124.70944	1	2502

893	15.33116	124.84878	21	2522

894	15.34319	124.98812	2	2524

895	15.36209	125.12746	1	2525

896	15.38099	125.2668	1	2525

897	15.3999	125.40614	27	2552

898	15.40677	125.54548	1	2553

899	15.43083	125.68482	7	2559

900	15.45145	125.82416	10	2569

901	15.47035	125.9635	1	2569

902	15.48066	126.10284	1	2570

903	15.49784	126.24218	1	2571

904	15.51846	126.38152	11	2581

905	15.53736	126.52086	51	2632

906	15.54767	126.6602	1	2632

907	15.56486	126.79954	1	2633

908	15.5872	126.93888	5	2637

909	15.6061	127.07822	5	2642

910	15.61813	127.21756	1	2643

911	15.63531	127.3569	3	2646

912	15.65765	127.49624	1	2646

913	15.67827	127.63558	1	2647

914	15.68514	127.77492	1	2647

915	15.70404	127.91426	7	2654

916	15.71951	128.0536	17	2671

917	15.74357	128.19294	18	2689

918	15.75388	128.33228	6	2695

919	15.7745	128.47162	1	2695

920	15.79512	128.61096	1	2696

921	15.81746	128.7503	5	2700

922	15.82948	128.88964	1	2701

923	15.83808	129.02898	7	2708

924	15.86213	129.16833	5	2713

925	15.88275	129.30767	2	2715

926	15.89134	129.44701	2	2716

927	15.90681	129.58635	1	2717

928	15.92743	129.72569	1	2717

929	15.95149	129.86503	1	2718

930	15.96695	130.00437	1	2719

931	15.97726	130.14371	2	2720

932	15.99788	130.28305	1	2721

933	16.0185	130.42239	1	2721

934	16.03397	130.56173	5	2726

935	16.04428	130.70107	1	2726

936	16.06318	130.84041	1	2727

937	16.08896	130.97975	1	2728

938	16.10442	131.11909	7	2734

939	16.11473	131.25843	2	2736

940	16.1302	131.39777	1	2736

941	16.15941	131.53711	6	2742

942	16.17316	131.67645	1	2743

943	16.18347	131.81579	3	2745

944	16.19721	131.95513	1	2746

945	16.22471	132.09447	2	2748

946	16.24017	132.23381	11	2759

947	16.25392	132.37315	4	2762

948	16.26938	132.51249	1	2763

949	16.2986	132.65183	6	2769

950	16.31406	132.79117	2	2771

951	16.32265	132.93051	3	2774

952	16.33812	133.06985	4	2777

953	16.35874	133.20919	1	2778

954	16.3742	133.34853	8	2786

955	16.38967	133.48787	2	2788

956	16.40685	133.62721	1	2789

957	16.42919	133.76655	1	2789

958	16.44981	133.90589	7	2796

959	16.46012	134.04523	3	2799

960	16.47559	134.18457	5	2804

961	16.49621	134.32391	1	2804

962	16.51339	134.46325	5	2809

963	16.52714	134.60259	27	2836

964	16.54432	134.74193	2	2837

965	16.5615	134.88127	20	2857

966	16.58556	135.02061	11	2868

967	16.59931	135.15995	8	2876

968	16.61305	135.29929	5	2880

969	16.63367	135.43863	1	2881

970	16.65601	135.57797	1	2881

971	16.67148	135.71731	1	2882

972	16.67835	135.85665	1	2883

973	16.69897	135.99599	22	2904

974	16.72303	136.13533	1	2905

975	16.73678	136.27467	2	2906

976	16.7488	136.41401	2	2908

977	16.76942	136.55335	2	2910

978	16.79348	136.69269	4	2914

979	16.80723	136.83203	1	2915

980	16.81754	136.97137	4	2918

981	16.83644	137.11071	1	2919

982	16.85878	137.25005	1	2919

983	16.87424	137.38939	6	2925

984	16.88627	137.52873	3	2928

985	16.90517	137.66807	23	2951

986	16.92751	137.80741	2	2952

987	16.94641	137.94675	5	2957

988	16.95672	138.0861	30	2987

989	16.97391	138.22544	1	2988

990	16.99625	138.36478	3	2990

991	17.00999	138.50412	1	2991

992	17.02202	138.64346	1	2991

993	17.04092	138.7828	7	2998

994	17.06326	138.92214	1	2999

995	17.08216	139.06148	1	2999

996	17.09591	139.20082	1	3000

997	17.1131	139.34016	3	3003

998	17.13543	139.4795	1	3003

999	17.15262	139.61884	1	3004

1000	17.16121	139.75818	2	3006

1001	17.17839	139.89752	1	3007

1002	17.20245	140.03686	3	3010

1003	17.2162	140.1762	3	3012

1004	17.22994	140.31554	1	3013

1005	17.24885	140.45488	2	3015

1006	17.27462	140.59422	3	3018

1007	17.29009	140.73356	1	3019

1008	17.3004	140.8729	1	3019

1009	17.31586	141.01224	5	3024

1010	17.33992	141.15158	1	3025

1011	17.3571	141.29092	2	3027

1012	17.37085	141.43026	10	3036

1013	17.38631	141.5696	1	3037

1014	17.41037	141.70894	10	3047

1015	17.43099	141.84828	6	3052

1016	17.4413	141.98762	1	3053

1017	17.45505	142.12696	1	3053

1018	17.47395	142.2663	8	3061

1019	17.49457	142.40564	4	3064

1020	17.51004	142.54498	1	3065

1021	17.52035	142.68432	2	3067

1022	17.54268	142.82366	1	3067

1023	17.56502	142.963	1	3068

1024	17.57877	143.10234	1	3068

1025	17.59252	143.24168	1	3069

1026	17.6097	143.38102	4	3072

1027	17.63376	143.52036	1	3073

1028	17.6475	143.6597	2	3074

1029	17.65953	143.79904	2	3076

1030	17.67843	143.93838	1	3076

1031	17.70249	144.07772	1	3077

1032	17.71796	144.21706	1	3077

1033	17.72998	144.3564	1	3078

1034	17.74889	144.49574	1	3078

1035	17.77294	144.63508	4	3082

1036	17.78841	144.77442	11	3093

1037	17.79528	144.91376	1	3093

1038	17.81418	145.0531	1	3094

1039	17.83996	145.19244	6	3099

1040	17.85714	145.33178	1	3100

1041	17.86745	145.47112	1	3100

1042	17.88292	145.61046	1	3101

1043	17.91385	145.7498	7	3107

1044	17.9276	145.88914	1	3108

1045	17.93619	146.02848	2	3109

1046	17.94993	146.16782	8	3117

1047	17.97571	146.30716	1	3117

1048	17.99461	146.4465	1	3118

1049	18.0032	146.58584	1	3118

1050	18.0221	146.72518	1	3119

1051	18.04788	146.86452	2	3121

1052	18.06678	147.00387	1	3122

1053	18.07709	147.14321	2	3124

1054	18.09256	147.28255	5	3128

1055	18.1149	147.42189	4	3132

1056	18.13036	147.56123	1	3132

1057	18.14067	147.70057	2	3134

1058	18.16129	147.83991	1	3134

1059	18.18191	147.97925	1	3135

1060	18.20081	148.11859	2	3137

1061	18.21456	148.25793	1	3137

1062	18.23003	148.39727	1	3138

1063	18.25236	148.53661	1	3139

1064	18.26955	148.67595	8	3147

1065	18.27814	148.81529	1	3147

1066	18.29532	148.95463	6	3153

1067	18.31938	149.09397	1	3154

1068	18.33485	149.23331	5	3158

1069	18.35203	149.37265	3	3161

1070	18.36406	149.51199	2	3163

1071	18.38983	149.65133	30	3193

1072	18.40873	149.79067	1	3193

1073	18.42076	149.93001	1	3194

1074	18.43451	150.06935	1	3194

1075	18.45685	150.20869	5	3199

1076	18.47403	150.34803	1	3200

1077	18.4895	150.48737	1	3200

1078	18.50153	150.62671	1	3201

1079	18.52215	150.76605	1	3201

1080	18.5462	150.90539	1	3202

1081	18.55995	151.04473	1	3202

1082	18.5737	151.18407	1	3203

1083	18.58916	151.32341	7	3210

1084	18.61322	151.46275	3	3213

1085	18.62525	151.60209	4	3217

1086	18.63556	151.74143	1	3218

1087	18.6579	151.88077	1	3219

1088	18.68023	152.02011	1	3219

1089	18.6957	152.15945	11	3230

1090	18.70945	152.29879	1	3231

1091	18.73007	152.43813	1	3231

1092	18.74897	152.57747	1	3232

1093	18.76443	152.71681	1	3233

1094	18.77474	152.85615	1	3234

1095	18.79193	152.99549	1	3234

1096	18.8177	153.13483	2	3236

1097	18.83317	153.27417	1	3236

1098	18.84692	153.41351	1	3237

1099	18.86582	153.55285	1	3237

1100	18.89159	153.69219	2	3239

1101	18.90534	153.83153	1	3239

1102	18.91221	153.97087	2	3241

1103	18.93111	154.11021	1	3242

1104	18.95173	154.24955	1	3242

1105	18.97236	154.38889	1	3243

1106	18.98267	154.52823	1	3244

1107	18.99985	154.66757	4	3248

1108	19.02391	154.80691	1	3249

1109	19.04624	154.94625	1	3250

1110	19.05484	155.08559	1	3251

1111	19.07202	155.22493	1	3251

1112	19.09264	155.36427	4	3255

1113	19.10982	155.50361	1	3256

1114	19.1167	155.64295	1	3256

1115	19.1356	155.78229	1	3257

1116	19.15794	155.92164	17	3273

1117	19.17684	156.06098	1	3274

1118	19.19059	156.20032	1	3275

1119	19.20949	156.33966	1	3276

1120	19.23011	156.479	1	3276

1121	19.24729	156.61834	1	3277

1122	19.25932	156.75768	1	3278

1123	19.27135	156.89702	3	3281

1124	19.29712	157.03636	1	3281

1125	19.31259	157.1757	1	3282

1126	19.32634	157.31504	1	3282

1127	19.34352	157.45438	13	3295

1128	19.36586	157.59372	2	3297

1129	19.38304	157.73306	1	3298

1130	19.39851	157.8724	1	3299

1131	19.41225	158.01174	7	3306

1132	19.43459	158.15108	1	3306

1133	19.44834	158.29042	1	3307

1134	19.46209	158.42976	1	3308

1135	19.47755	158.5691	10	3317

1136	19.49989	158.70844	1	3318

1137	19.52051	158.84778	1	3319

1138	19.53254	158.98712	1	3320

1139	19.55144	159.12646	1	3320

1140	19.56862	159.2658	1	3321

1141	19.58924	159.40514	1	3321

1142	19.60127	159.54448	1	3322

1143	19.6133	159.68382	1	3323

1144	19.63564	159.82316	1	3324

1145	19.65626	159.9625	5	3328

1146	19.67001	160.10184	1	3329

1147	19.68375	160.24118	1	3329

1148	19.70609	160.38052	1	3330

1149	19.72671	160.51986	1	3330

1150	19.74046	160.6592	1	3331

1151	19.74905	160.79854	1	3331

1152	19.77139	160.93788	2	3333

1153	19.79545	161.07722	1	3334

1154	19.80576	161.21656	7	3341

1155	19.8195	161.3559	20	3360

1156	19.84184	161.49524	1	3361

1157	19.86074	161.63458	2	3362

1158	19.87793	161.77392	1	3363

1159	19.88996	161.91326	1	3363

1160	19.91058	162.0526	1	3364

1161	19.93292	162.19194	1	3364

1162	19.94323	162.33128	1	3365

1163	19.95869	162.47062	1	3366

1164	19.97416	162.60996	1	3366

1165	20.00165	162.7493	10	3376

1166	20.01883	162.88864	1	3377

1167	20.02914	163.02798	3	3380

1168	20.04976	163.16732	1	3381

1169	20.0721	163.30666	1	3382

1170	20.08757	163.446	1	3383

1171	20.09444	163.58534	1	3383

1172	20.11506	163.72468	1	3384

1173	20.13568	163.86402	1	3384

1174	20.15115	164.00336	2	3386

1175	20.16317	164.1427	1	3387

1176	20.1838	164.28204	1	3387

1177	20.20785	164.42138	1	3388

1178	20.2216	164.56072	1	3388

1179	20.23363	164.70007	1	3389

1180	20.25253	164.83941	1	3390

1181	20.27659	164.97875	1	3390

1182	20.28861	165.11809	1	3391

1183	20.30064	165.25743	1	3391

1184	20.31783	165.39677	1	3392

1185	20.34188	165.53611	1	3392

1186	20.35735	165.67545	1	3393

1187	20.3711	165.81479	15	3407

1188	20.39172	165.95413	1	3408

1189	20.41234	166.09347	2	3410

1190	20.42608	166.23281	2	3411

1191	20.44155	166.37215	1	3412

1192	20.45701	166.51149	1	3412

1193	20.47935	166.65083	7	3419

1194	20.4931	166.79017	2	3421

1195	20.50856	166.92951	1	3421

1196	20.52747	167.06885	1	3422

1197	20.54809	167.20819	1	3423

1198	20.56527	167.34753	1	3423

1199	20.57558	167.48687	1	3424

1200	20.59276	167.62621	4	3428

1201	20.61338	167.76555	2	3430

1202	20.63229	167.90489	1	3430

1203	20.6426	168.04423	1	3431

1204	20.6615	168.18357	1	3431

1205	20.68212	168.32291	1	3432

1206	20.70102	168.46225	7	3439

1207	20.71305	168.60159	1	3439

1208	20.72336	168.74093	1	3440

1209	20.73882	168.88027	1	3440

1210	20.74054	169.01961	1	3441

1211	19.50232	169.15895	5	3445

1212	17.9421	169.29829	1	3446

1213	16.76435	169.43763	4	3449

1214	14.77286	169.57697	10	3459

1215	12.86041	169.71631	1	3459

1216	10.61976	169.85565	1	3460

1217	7.56813	169.99499	2	3462

1218	5.91507	170.13433	18	3479

170.27367	1	3480

170.41301	1	3481

170.55235	2	3482

170.69169	9	3491

170.83103	1	3491

170.97037	1	3492

171.10971	1	3493

171.24905	2	3495

171.38839	1	3495

171.52773	1	3496

171.66707	1	3496

171.80641	1	3497

171.94575	3	3499

172.08509	1	3500

172.22443	1	3500

172.36377	6	3506

172.50311	1	3506

172.64245	1	3507

172.78179	1	3507

172.92113	1	3508

173.06047	6	3514

173.19981	1	3515

173.33915	1	3516

173.47849	1	3516

173.61784	1	3517

173.75718	1	3517

173.89652	2	3519

174.03586	1	3520

174.1752	1	3520

174.31454	1	3521

174.45388	1	3521

174.59322	1	3522

174.73256	1	3522

174.8719	1	3523

175.01124	1	3523

175.15058	1	3524

175.28992	1	3524

175.42926	1	3525

175.5686	1	3525

175.70794	1	3526

175.84728	1	3526

175.98662	6	3532

176.12596	1	3533

176.2653	1	3534

176.40464	1	3535

176.54398	6	3540

176.68332	1	3541

176.82266	1	3541

176.962	1	3542

177.10134	4	3546

177.24068	1	3546

177.38002	3	3549

177.51936	1	3550

177.6587	2	3552

177.79804	1	3552

177.93738	1	3553

178.07672	1	3553

178.21606	2	3555

178.3554	1	3556

178.49474	1	3556

178.63408	1	3557

178.77342	1	3557

178.91276	1	3558

179.0521	1	3559

179.19144	4	3562

179.33078	1	3563

179.47012	1	3563

179.60946	1	3564

179.7488	1	3564

179.88814	9	3573

180.02748	1	3574

180.16682	1	3574

180.30616	1	3575

180.4455	1	3575

180.58484	1	3576

180.72418	1	3576

180.86352	1	3577

181.00286	1	3577

181.1422	1	3578

181.28154	1	3579

181.42088	1	3579

181.56022	2	3581

181.69956	1	3582

181.8389	1	3582

181.97824	1	3583

182.11758	3	3585

182.25692	1	3586

182.39626	1	3586

182.53561	1	3587

182.67495	1	3587

182.81429	1	3588

182.95363	4	3592

183.09297	7	3599

183.23231	2	3601

183.37165	1	3602

183.51099	5	3607

183.65033	2	3608

183.78967	1	3609

183.92901	1	3610

184.06835	2	3611

184.20769	1	3612

184.34703	2	3613

184.48637	2	3615

184.62571	3	3618

184.76505	1	3618

184.90439	1	3619

185.04373	1	3620

185.18307	1	3620

185.32241	1	3621

185.46175	1	3621

185.60109	1	3622

185.74043	1	3623

185.87977	1	3624

186.01911	1	3624

186.15845	1	3625

186.29779	1	3626

186.43713	2	3627

186.57647	1	3628

186.71581	1	3629

186.85515	8	3637

186.99449	1	3638

187.13383	1	3638

187.27317	1	3639

187.41251	1	3640

187.55185	1	3641

187.69119	1	3642

187.83053	1	3642

187.96987	1	3643

188.10921	3	3646

188.24855	3	3648

188.38789	1	3649

188.52723	1	3650

188.66657	1	3650

188.80591	1	3651

188.94525	1	3651

189.08459	1	3652

189.22393	1	3653

189.36327	1	3653

189.50261	1	3654

189.64195	2	3655

189.78129	8	3663

189.92063	1	3664

190.05997	1	3665

190.19931	3	3667

190.33865	1	3668

190.47799	1	3668

190.61733	1	3669

190.75667	1	3669

190.89601	1	3670

191.03535	1	3670

191.17469	1	3671

191.31403	1	3671

191.45337	1	3672

191.59272	2	3674

191.73206	1	3675

191.8714	1	3675

192.01074	1	3676

192.15008	1	3677

192.28942	1	3678

192.42876	1	3678

192.5681	1	3679

192.70744	1	3680

192.84678	1	3680

192.98612	1	3681

193.12546	1	3681

193.2648	1	3682

193.40414	1	3682

193.54348	1	3683

193.68282	1	3684

193.82216	6	3690

193.9615	1	3690

194.10084	3	3693

194.24018	1	3693

194.37952	1	3694

194.51886	1	3694

194.6582	2	3696

194.79754	1	3696

194.93688	1	3697

195.07622	1	3697

195.21556	2	3699

195.3549	2	3701

195.49424	1	3701

195.63358	1	3702

195.77292	1	3703

195.91226	1	3704

196.0516	7	3711

196.19094	1	3711

196.33028	2	3713

196.46962	1	3714

196.60896	3	3716

196.7483	1	3717

196.88764	1	3717

197.02698	1	3718

197.16632	2	3719

197.30566	1	3720

197.445	1	3720

197.58434	1	3721

197.72368	1	3722

197.86302	1	3722

198.00236	1	3723

198.1417	1	3724

198.28104	1	3725

198.42038	2	3726

198.55972	1	3727

198.69906	5	3731

198.8384	1	3732

198.97774	1	3733

199.11708	1	3734

199.25642	1	3734

199.39576	1	3735

199.5351	1	3735

199.67444	1	3736

199.81378	1	3737

199.95312	1	3738

200.09246	1	3738

200.2318	1	3739

200.37115	1	3739

200.51049	1	3740

200.64983	12	3751

200.78917	1	3752

200.92851	1	3753

201.06785	1	3754

201.20719	2	3755

201.34653	1	3756

201.48587	1	3757

201.62521	1	3758

201.76455	1	3758

201.90389	1	3759

202.04323	1	3759

202.18257	1	3760

202.32191	1	3760

202.46125	4	3764

202.60059	1	3765

202.73993	1	3765

202.87927	1	3766

203.01861	1	3766

203.15795	6	3772

203.29729	1	3772

203.43663	1	3773

203.57597	1	3773

203.71531	3	3776

203.85465	1	3776

203.99399	1	3777

204.13333	1	3778

204.27267	1	3778

204.41201	1	3779

204.55135	10	3788

204.69069	1	3789

204.83003	1	3789

204.96937	1	3790

205.10871	1	3791

205.24805	1	3791

205.38739	1	3792

205.52673	1	3792

205.66607	1	3793

205.80541	1	3793

205.94475	1	3794

206.08409	1	3795

206.22343	1	3795

206.36277	1	3796

206.50211	1	3796

206.64145	2	3798

206.78079	1	3798

206.92013	1	3799

207.05947	1	3800

207.19881	1	3800

207.33815	3	3803

207.47749	2	3805

207.61683	1	3805

207.75617	1	3806

207.89551	1	3806

208.03485	1	3807

208.17419	1	3807

208.31353	7	3814

208.45287	1	3815

208.59221	2	3817

208.73155	40	3856

208.87089	2	3858

209.01023	1	3859

209.14957	1	3860

209.28892	4	3864

209.42826	2	3866

209.5676	1	3867

209.70694	1	3868

209.84628	1	3869

209.98562	2	3871

210.12496	1	3872

210.2643	1	3873

210.40364	16	3889

210.54298	1	3890

210.68232	1	3891

210.82166	1	3892

210.961	1	3893

211.10034	1	3894

211.23968	2	3896

211.37902	1	3897

211.51836	8	3905

211.6577	1	3906

211.79704	1	3907

211.93638	2	3909

212.07572	1	3910

212.21506	2	3912

212.3544	2	3914

212.49374	1	3915

212.63308	1	3916

212.77242	1	3917

212.91176	1	3918

213.0511	1	3919

213.19044	1	3920

213.32978	1	3921

213.46912	1	3922

213.60846	2	3924

213.7478	2	3926

213.88714	1	3927

214.02648	1	3928

214.16582	1	3929

214.30516	1	3930

214.4445	1	3931

214.58384	9	3940

214.72318	1	3941

214.86252	1	3942

215.00186	12	3954

215.1412	1	3955

215.28054	1	3956

215.41988	1	3957

215.55922	1	3958

215.69856	2	3960

215.8379	1	3961

215.97724	2	3963

216.11658	1	3964

216.25592	1	3965

216.39526	1	3966

216.5346	2	3968

216.67394	1	3969

216.81328	1	3970

216.95262	1	3971

217.09196	2	3973

217.2313	1	3974

217.37064	1	3975

217.50998	1	3976

217.64932	1	3977

217.78866	1	3978

217.928	1	3979

218.06734	11	3990

218.20668	1	3991

218.34603	1	3992

218.48537	1	3993

218.62471	2	3995

218.76405	1	3996

218.90339	1	3997

219.04273	1	3998

219.18207	2	4000

219.32141	3	4003

219.46075	1	4004

219.60009	1	4005

219.73943	1	4006

219.87877	2	4008

220.01811	2	4010

220.15745	1	4011

220.29679	1	4012

220.43613	1	4013

220.57547	1	4014

220.71481	1	4015

220.85415	19	4034

220.99349	9	4043

221.13283	1	4044

221.27217	2	4046

221.41151	1	4047

221.55085	2	4049

221.69019	1	4050

221.82953	2	4052

221.96887	1	4053

222.10821	1	4054

222.24755	1	4055

222.38689	1	4056

222.52623	1	4057

222.66557	2	4059

222.80491	1	4060

222.94425	7	4067

223.08359	1	4068

223.22293	3	4071

223.36227	2	4073

223.50161	1	4074

223.64095	2	4076

223.78029	5	4081

223.91963	1	4082

224.05897	1	4083

224.19831	1	4084

224.33765	21	4105

224.47699	8	4113

224.61633	2	4115

224.75567	1	4116

224.89501	1	4117

225.03435	1	4118

225.17369	1	4119

225.31303	1	4120

225.45237	2	4122

225.59171	1	4123

225.73105	2	4125

225.87039	1	4126

226.00973	2	4128

226.14907	2	4130

226.28841	1	4131

226.42775	2	4133

226.56709	1	4134

226.70643	1	4135

226.84577	2	4137

226.98511	15	4152

227.12446	1	4153

227.2638	1	4154

227.40314	1	4155

227.54248	1	4156

227.68182	2	4158

227.82116	3	4161

227.9605	1	4162

228.09984	1	4163

228.23918	1	4164

228.37852	3	4167

228.51786	1	4168

228.6572	2	4170

228.79654	1	4171

228.93588	3	4174

229.07522	2	4176

229.21456	2	4178

229.3539	1	4179

229.49324	1	4180

229.63258	1	4181

229.77192	1	4182

229.91126	4	4186

230.0506	1	4187

230.18994	1	4188

230.32928	1	4189

230.46862	1	4190

230.60796	2	4192

230.7473	2	4194

230.88664	1	4195

231.02598	3	4198

231.16532	1	4199

231.30466	3	4202

231.444	7	4209

231.58334	1	4210

231.72268	1	4211

231.86202	2	4213

232.00136	8	4221

232.1407	3	4224

232.28004	1	4225

232.41938	2	4227

232.55872	11	4238

232.69806	2	4240

232.8374	1	4241

232.97674	1	4242

233.11608	1	4243

233.25542	1	4244

233.39476	1	4245

233.5341	6	4251

233.67344	2	4253

233.81278	1	4254

233.95212	2	4256

234.09146	1	4257

234.2308	3	4260

234.37014	1	4261

234.50948	2	4263

234.64882	1	4264

234.78816	1	4265

234.9275	14	4279

235.06684	1	4280

235.20618	1	4281

235.34552	3	4284

235.48486	1	4285

235.6242	3	4288

235.76354	2	4290

235.90288	2	4292

236.04223	1	4293

236.18157	1	4294

236.32091	1	4295

236.46025	1	4296

236.59959	1	4297

236.73893	1	4298

236.87827	1	4299

237.01761	39	4338

237.15695	1	4339

237.29629	1	4340

237.43563	1	4341

237.57497	3	4344

237.71431	2	4346

237.85365	4	4350

237.99299	1	4351

238.13233	2	4353

238.27167	2	4355

238.41101	7	4362

238.55035	2	4364

238.68969	2	4366

238.82903	2	4368

238.96837	1	4369

239.10771	2	4371

239.24705	1	4372

239.38639	2	4374

239.52573	1	4375

239.66507	3	4378

239.80441	2	4380

239.94375	2	4382

240.08309	2	4384

240.22243	1	4385

240.36177	3	4388

240.50111	1	4389

240.64045	7	4396

240.77979	3	4399

240.91913	1	4400

241.05847	1	4401

241.19781	47	4448

241.33715	1	4449

241.47649	2	4451

241.61583	3	4454

241.75517	1	4455

241.89451	2	4457

242.03385	1	4458

242.17319	2	4460

242.31253	1	4461

242.45187	1	4462

242.59121	1	4463

242.73055	1	4464

242.86989	3	4467

243.00923	3	4470

243.14857	1	4471

243.28791	1	4472

243.42725	1	4473

243.56659	2	4475

243.70593	1	4476

243.84527	1	4477

243.98461	1	4478

244.12395	1	4479

244.26329	2	4481

244.40263	1	4482

244.54197	1	4483

244.68131	3	4486

244.82065	4	4490

244.96	1	4491

245.09934	1	4492

245.23868	2	4494

245.37802	3	4497

245.51736	2	4499

245.6567	5	4504

245.79604	2	4506

245.93538	2	4508

246.07472	1	4509

246.21406	1	4510

246.3534	4	4514

246.49274	1	4515

246.63208	1	4516

246.77142	1	4517

246.91076	1	4518

247.0501	1	4519

247.18944	2	4521

247.32878	1	4522

247.46812	1	4523

247.60746	1	4524

247.7468	3	4527

247.88614	1	4528

248.02548	1	4529

248.16482	6	4535

248.30416	1	4536

248.4435	1	4537

248.58284	4	4541

248.72218	1	4542

248.86152	1	4543

249.00086	2	4545

249.1402	1	4546

249.27954	1	4547

249.41888	1	4548

249.55822	1	4549

249.69756	2	4551

249.8369	3	4554

249.97624	1	4555

250.11558	1	4556

250.25492	3	4559

250.39426	1	4560

250.5336	2	4562

250.67294	1	4563

250.81228	2	4565

250.95162	1	4566

251.09096	1	4567

251.2303	25	4592

251.36964	3	4595

251.50898	2	4597

251.64832	1	4598

251.78766	1	4599

251.927	1	4600

252.06634	2	4602

252.20568	5	4607

252.34502	13	4620

252.48436	1	4621

252.6237	1	4622

252.76304	3	4625

252.90238	2	4627

253.04172	2	4629

253.18106	1	4630

253.3204	1	4631

253.45974	1	4632

253.59908	1	4633

253.73842	1	4634

253.87777	2	4636

254.01711	1	4637

254.15645	1	4638

254.29579	2	4640

254.43513	1	4641

254.57447	24	4665

254.71381	13	4678

254.85315	1	4679

254.99249	2	4681

255.13183	1	4682

255.27117	1	4683

255.41051	1	4684

255.54985	1	4685

255.68919	1	4686

255.82853	1	4687

255.96787	4	4691

256.10721	5	4696

256.24655	2	4698

256.38589	3	4701

256.52523	3	4704

256.66457	1	4705

256.80391	2	4707

256.94325	1	4708

257.08259	1	4709

257.22193	3	4712

257.36127	1	4713

257.50061	16	4729

257.63995	1	4730

257.77929	2	4732

257.91863	1	4733

258.05797	2	4735

258.19731	1	4736

258.33665	1	4737

258.47599	1	4738

258.61533	1	4739

258.75467	1	4740

258.89401	1	4741

259.03335	1	4742

259.17269	2	4744

259.31203	5	4749

259.45137	1	4750

259.59071	2	4752

259.73005	1	4753

259.86939	1	4754

260.00873	4	4758

260.14807	1	4759

260.28741	1	4760

260.42675	1	4761

260.56609	13	4774

260.70543	1	4775

260.84477	1	4776

260.98411	1	4777

261.12345	1	4778

261.26279	1	4779

261.40213	2	4781

261.54147	1	4782

261.68081	2	4784

261.82015	2	4786

261.95949	1	4787

262.09883	1	4788

262.23817	1	4789

262.37751	1	4790

262.51685	2	4792

262.6562	1	4793

262.79554	1	4794

262.93488	1	4795

263.07422	1	4796

263.21356	1	4797

263.3529	2	4799

263.49224	1	4800

263.63158	1	4801

263.77092	3	4804

263.91026	1	4805

264.0496	2	4807

264.18894	1	4808

264.32828	4	4812

264.46762	2	4814

264.60696	3	4817

264.7463	2	4819

264.88564	2	4821

265.02498	1	4822

265.16432	1	4823

265.30366	2	4825

265.443	2	4827

265.58234	2	4829

265.72168	1	4830

265.86102	1	4831

266.00036	1	4832

266.1397	1	4833

266.27904	1	4834

266.41838	1	4835

266.55772	3	4838

266.69706	1	4839

266.8364	1	4840

266.97574	1	4841

267.11508	1	4842

267.25442	2	4844

267.39376	1	4845

267.5331	1	4846

267.67244	3	4849

267.81178	3	4852

267.95112	1	4853

268.09046	1	4854

268.2298	2	4856

268.36914	2	4858

268.50848	1	4859

268.64782	1	4860

268.78716	1	4861

268.9265	1	4862

269.06584	1	4863

269.20518	4	4867

269.34452	2	4869

269.48386	1	4870

269.6232	1	4871

269.76254	1	4872

269.90188	1	4873

270.04122	2	4875

270.18056	1	4876

270.3199	1	4877

270.45924	2	4879

270.59858	1	4880

270.73792	1	4881

270.87726	1	4882

271.0166	1	4883

271.15594	1	4884

271.29528	3	4887

271.43462	2	4889

271.57396	1	4890

271.71331	2	4892

271.85265	1	4893

271.99199	1	4894

272.13133	3	4897

272.27067	1	4898

272.41001	2	4900

272.54935	4	4904

272.68869	1	4905

272.82803	7	4912

272.96737	2	4914

273.10671	2	4916

273.24605	5	4921

273.38539	1	4922

273.52473	1	4923

273.66407	1	4924

273.80341	1	4925

273.94275	3	4928

274.08209	2	4930

274.22143	1	4931

274.36077	1	4932

274.50011	5	4937

274.63945	1	4938

274.77879	2	4940

274.91813	3	4943

275.05747	1	4944

275.19681	2	4946

275.33615	2	4948

275.47549	1	4949

275.61483	5	4954

275.75417	2	4956

275.89351	3	4959

276.03285	1	4960

276.17219	1	4961

276.31153	1	4962

276.45087	1	4963

276.59021	25	4988

276.72955	1	4989

276.86889	1	4990

277.00823	1	4991

277.14757	4	4995

277.28691	1	4996

277.42625	1	4997

277.56559	1	4998

277.70493	2	5000

277.84427	1	5001

277.98361	1	5002

278.12295	1	5003

278.26229	2	5005

278.40163	2	5007

278.54097	1	5008

278.68031	4	5012

278.81965	3	5015

278.95899	3	5018

279.09833	1	5019

279.23767	1	5020

279.37701	1	5021

279.51635	5	5026

279.65569	1	5027

279.79503	1	5028

279.93437	1	5029

280.07371	1	5030

280.21305	1	5031

280.35239	2	5033

280.49174	1	5034

280.63108	1	5035

280.77042	1	5036

280.90976	3	5039

281.0491	2	5041

281.18844	7	5048

281.32778	1	5049

281.46712	3	5052

281.60646	2	5054

281.7458	1	5055

281.88514	4	5059

282.02448	1	5060

282.16382	7	5067

282.30316	1	5068

282.4425	1	5069

282.58184	1	5070

282.72118	1	5071

282.86052	1	5072

282.99986	2	5074

283.1392	1	5075

283.27854	3	5078

283.41788	1	5079

283.55722	1	5080

283.69656	2	5082

283.8359	11	5093

283.97524	1	5094

284.11458	2	5096

284.25392	1	5097

284.39326	1	5098

284.5326	1	5099

284.67194	1	5100

284.81128	1	5101

284.95062	1	5102

285.08996	2	5104

285.2293	1	5105

285.36864	1	5106

285.50798	1	5107

285.64732	1	5108

285.78666	1	5109

285.926	1	5110

286.06534	2	5112

286.20468	1	5113

286.34402	1	5114

286.48336	1	5115

286.6227	1	5116

286.76204	2	5118

286.90138	1	5119

287.04072	4	5123

287.18006	1	5124

287.3194	4	5128

287.45874	3	5131

287.59808	2	5133

287.73742	3	5136

287.87676	3	5139

288.0161	1	5140

288.15544	1	5141

288.29478	1	5142

288.43412	1	5143

288.57346	1	5144

288.7128	2	5146

288.85214	5	5151

288.99148	1	5152

289.13082	2	5154

289.27016	3	5157

289.40951	2	5159

289.54885	1	5160

289.68819	1	5161

289.82753	2	5163

289.96687	1	5164

290.10621	1	5165

290.24555	2	5167

290.38489	2	5169

290.52423	2	5171

290.66357	1	5172

290.80291	1	5173

290.94225	1	5174

291.08159	1	5175

291.22093	3	5178

291.36027	1	5179

291.49961	3	5182

291.63895	1	5183

291.77829	1	5184

291.91763	3	5187

292.05697	1	5188

292.19631	2	5190

292.33565	9	5199

292.47499	1	5200

292.61433	1	5201

292.75367	2	5203

292.89301	2	5205

293.03235	1	5206

293.17169	23	5229

293.31103	2	5231

293.45037	6	5237

293.58971	2	5239

293.72905	2	5241

293.86839	1	5242

294.00773	1	5243

294.14707	2	5245

294.28641	5	5250

294.42575	1	5251

294.56509	2	5253

294.70443	1	5254

294.84377	1	5255

294.98311	1	5256

295.12245	1	5257

295.26179	2	5259

295.40113	5	5264

295.54047	2	5266

295.67981	19	5285

295.81915	2	5287

295.95849	1	5288

296.09783	1	5289

296.23717	3	5292

296.37651	1	5293

296.51585	1	5294

296.65519	4	5298

296.79453	1	5299

296.93387	1	5300

297.07321	1	5301

297.21255	4	5305

297.35189	1	5306

297.49123	2	5308

297.63057	9	5317

297.76991	2	5319

297.90925	3	5322

298.04859	1	5323

298.18793	1	5324

298.32727	1	5325

298.46662	2	5327

298.60596	1	5328

298.7453	1	5329

298.88464	8	5337

299.02398	1	5338

299.16332	2	5340

299.30266	1	5341

299.442	3	5344

299.58134	1	5345

299.72068	1	5346

299.86002	1	5347

299.99936	16	5363

300.1387	1	5364

300.27804	2	5366

300.41738	4	5370

300.55672	1	5371

300.69606	1	5372

300.8354	1	5373

300.97474	1	5374

301.11408	3	5377

301.25342	2	5379

301.39276	1	5380

301.5321	2	5382

301.67144	3	5385

301.81078	7	5392

301.95012	1	5393

302.08946	1	5394

302.2288	1	5395

302.36814	3	5398

302.50748	3	5401

302.64682	1	5402

302.78616	2	5404

302.9255	1	5405

303.06484	1	5406

303.20418	3	5409

303.34352	1	5410

303.48286	1	5411

303.6222	2	5413

303.76154	1	5414

303.90088	1	5415

304.04022	3	5418

304.17956	1	5419

304.3189	12	5431

304.45824	14	5445

304.59758	1	5446

304.73692	3	5449

304.87626	1	5450

305.0156	1	5451

305.15494	1	5452

305.29428	1	5453

305.43362	1	5454

305.57296	1	5455

305.7123	2	5457

305.85164	2	5459

305.99098	2	5461

306.13032	1	5462

306.26966	1	5463

306.409	2	5465

306.54834	2	5467

306.68768	3	5470

306.82702	1	5471

306.96636	2	5473

307.1057	3	5476

307.24505	1	5477

307.38439	6	5483

307.52373	1	5484

307.66307	2	5486

307.80241	2	5488

307.94175	1	5489

308.08109	1	5490

308.22043	1	5491

308.35977	1	5492

308.49911	6	5498

308.63845	2	5500

308.77779	1	5501

308.91713	3	5504

309.05647	4	5508

309.19581	1	5509

309.33515	4	5513

309.47449	2	5515

309.61383	3	5518

309.75317	1	5519

309.89251	1	5520

310.03185	5	5525

310.17119	1	5526

310.31053	2	5528

310.44987	2	5530

310.58921	2	5532

310.72855	1	5533

310.86789	1	5534

311.00723	1	5535

311.14657	2	5537

311.28591	1	5538

311.42525	1	5539

311.56459	11	5550

311.70393	1	5551

311.84327	2	5553

311.98261	3	5556

312.12195	1	5557

312.26129	2	5559

312.40063	2	5561

312.53997	3	5564

312.67931	1	5565

312.81865	1	5566

312.95799	4	5570

313.09733	1	5571

313.23667	4	5575

313.37601	3	5578

313.51535	1	5579

313.65469	4	5583

313.79403	1	5584

313.93337	5	5589

314.07271	5	5594

314.21205	1	5595

314.35139	1	5596

314.49073	1	5597

314.63007	3	5600

314.76941	1	5601

314.90875	1	5602

315.04809	2	5604

315.18743	1	5605

315.32677	1	5606

315.46611	1	5607

315.60545	1	5608

315.74479	14	5622

315.88413	2	5624

316.02347	4	5628

316.16282	1	5629

316.30216	1	5630

316.4415	1	5631

316.58084	4	5635

316.72018	3	5638

316.85952	2	5640

316.99886	1	5641

317.1382	11	5652

317.27754	1	5653

317.41688	3	5656

317.55622	1	5657

317.69556	3	5660

317.8349	1	5661

317.97424	5	5666

318.11358	2	5668

318.25292	3	5671

318.39226	5	5676

318.5316	3	5679

318.67094	1	5680

318.81028	1	5681

318.94962	2	5683

319.08896	2	5685

319.2283	1	5686

319.36764	2	5688

319.50698	6	5694

319.64632	2	5696

319.78566	1	5697

319.925	1	5698

320.06434	1	5699

320.20368	2	5701

320.34302	2	5703

320.48236	1	5704

320.6217	1	5705

320.76104	1	5706

320.90038	6	5712

321.03972	1	5713

321.17906	1	5714

321.3184	1	5715

321.45774	2	5717

321.59708	1	5718

321.73642	1	5719

321.87576	2	5721

322.0151	1	5722

322.15444	3	5725

322.29378	4	5729

322.43312	2	5731

322.57246	2	5733

322.7118	2	5735

322.85114	2	5737

322.99048	8	5745

323.12982	1	5746

323.26916	1	5747

323.4085	3	5750

323.54784	1	5751

323.68718	2	5753

323.82652	1	5754

323.96586	2	5756

324.1052	1	5757

324.24454	2	5759

324.38388	1	5760

324.52322	3	5763

324.66256	2	5765

324.8019	1	5766

324.94124	2	5768

325.08059	2	5770

325.21993	1	5771

325.35927	2	5773

325.49861	1	5774

325.63795	2	5776

325.77729	4	5780

325.91663	5	5785

326.05597	2	5787

326.19531	3	5790

326.33465	1	5791

326.47399	1	5792

326.61333	1	5793

326.75267	1	5794

326.89201	3	5797

327.03135	4	5801

327.17069	1	5802

327.31003	2	5804

327.44937	4	5808

327.58871	4	5812

327.72805	3	5815

327.86739	1	5816

328.00673	4	5820

328.14607	3	5823

328.28541	5	5828

328.42475	1	5829

328.56409	1	5830

328.70343	4	5834

328.84277	3	5837

328.98211	4	5841

329.12145	1	5842

329.26079	1	5843

329.40013	1	5844

329.53947	2	5846

329.67881	1	5847

329.81815	6	5853

329.95749	1	5854

330.09683	1	5855

330.23617	1	5856

330.37551	1	5857

330.51485	2	5859

330.65419	2	5861

330.79353	3	5864

330.93287	2	5866

331.07221	16	5882

331.21155	1	5883

331.35089	1	5884

331.49023	1	5885

331.62957	2	5887

331.76891	1	5888

331.90825	3	5891

332.04759	1	5892

332.18693	1	5893

332.32627	1	5894

332.46561	2	5896

332.60495	3	5899

332.74429	2	5901

332.88363	1	5902

333.02297	2	5904

333.16231	1	5905

333.30165	6	5911

333.44099	2	5913

333.58033	2	5915

333.71967	2	5917

333.85901	1	5918

333.99836	1	5919

334.1377	2	5921

334.27704	1	5922

334.41638	1	5923

334.55572	1	5924

334.69506	2	5926

334.8344	2	5928

334.97374	1	5929

335.11308	3	5932

335.25242	2	5934

335.39176	1	5935

335.5311	1	5936

335.67044	3	5939

335.80978	2	5941

335.94912	1	5942

336.08846	3	5945

336.2278	1	5946

336.36714	1	5947

336.50648	1	5948

336.64582	2	5950

336.78516	3	5953

336.9245	1	5954

337.06384	1	5955

337.20318	1	5956

337.34252	1	5957

337.48186	1	5958

337.6212	1	5959

337.76054	3	5962

337.89988	6	5968

338.03922	1	5969

338.17856	1	5970

338.3179	2	5972

338.45724	2	5974

338.59658	1	5975

338.73592	2	5977

338.87526	3	5980

339.0146	1	5981

339.15394	5	5986

339.29328	1	5987

339.43262	1	5988

339.57196	1	5989

339.7113	5	5994

339.85064	1	5995

339.98998	2	5997

340.12932	1	5998

340.26866	2	6000

340.408	2	6002

340.54734	2	6004

340.68668	1	6005

340.82602	2	6007

340.96536	3	6010

341.1047	1	6011

341.24404	3	6014

341.38338	1	6015

341.52272	4	6019

341.66206	2	6021

341.8014	2	6023

341.94074	1	6024

342.08008	1	6025

342.21942	2	6027

342.35876	2	6029

342.4981	2	6031

342.63744	3	6034

342.77678	1	6035

342.91613	2	6037

343.05547	5	6042

343.19481	1	6043

343.33415	1	6044

343.47349	3	6047

343.61283	2	6049

343.75217	1	6050

343.89151	2	6052

344.03085	2	6054

344.17019	1	6055

344.30953	1	6056

344.44887	1	6057

344.58821	1	6058

344.72755	1	6059

344.86689	1	6060

345.00623	2	6062

345.14557	1	6063

345.28491	3	6066

345.42425	3	6069

345.56359	2	6071

345.70293	4	6075

345.84227	4	6079

345.98161	1	6080

346.12095	3	6083

346.26029	1	6084

346.39963	1	6085

346.53897	1	6086

346.67831	1	6087

346.81765	2	6089

346.95699	1	6090

347.09633	2	6092

347.23567	1	6093

347.37501	1	6094

347.51435	1	6095

347.65369	1	6096

347.79303	4	6100

347.93237	2	6102

348.07171	4	6106

348.21105	2	6108

348.35039	1	6109

348.48973	2	6111

348.62907	1	6112

348.76841	3	6115

348.90775	6	6121

349.04709	3	6124

349.18643	2	6126

349.32577	1	6127

349.46511	1	6128

349.60445	1	6129

349.74379	2	6131

349.88313	1	6132

350.02247	2	6134

350.16181	2	6136

350.30115	1	6137

350.44049	4	6141

350.57983	1	6142

350.71917	2	6144

350.85851	1	6145

350.99785	1	6146

351.13719	7	6153

351.27653	1	6154

351.41587	1	6155

351.55521	2	6157

351.69455	1	6158

351.8339	2	6160

351.97324	1	6161

352.11258	2	6163

352.25192	2	6165

352.39126	2	6167

352.5306	1	6168

352.66994	7	6175

352.80928	5	6180

352.94862	1	6181

353.08796	1	6182

353.2273	2	6184

353.36664	1	6185

353.50598	1	6186

353.64532	2	6188

353.78466	2	6190

353.924	3	6193

354.06334	6	6199

354.20268	6	6205

354.34202	1	6206

354.48136	1	6207

354.6207	1	6208

354.76004	2	6210

354.89938	4	6214

355.03872	3	6217

355.17806	1	6218

355.3174	2	6220

355.45674	1	6221

355.59608	5	6226

355.73542	1	6227

355.87476	2	6229

356.0141	2	6231

356.15344	2	6233

356.29278	2	6235

356.43212	1	6236

356.57146	1	6237

356.7108	1	6238

356.85014	6	6244

356.98948	8	6252

357.12882	2	6254

357.26816	1	6255

357.4075	3	6258

357.54684	6	6264

357.68618	1	6265

357.82552	2	6267

357.96486	3	6270

358.1042	1	6271

358.24354	1	6272

358.38288	1	6273

358.52222	2	6275

358.66156	1	6276

358.8009	1	6277

358.94024	2	6279

359.07958	1	6280

359.21892	1	6281

359.35826	1	6282

359.4976	1	6283

359.63694	1	6284

359.77628	1	6285

359.91562	2	6287

360.05496	1	6288

360.1943	1	6289

360.33364	2	6291

360.47298	2	6293

360.61233	2	6295

360.75167	1	6296

360.89101	4	6300

361.03035	3	6303

361.16969	1	6304

361.30903	3	6307

361.44837	10	6317

361.58771	1	6318

361.72705	1	6319

361.86639	5	6324

362.00573	1	6325

362.14507	2	6327

362.28441	7	6334

362.42375	1	6335

362.56309	1	6336

362.70243	2	6338

362.84177	2	6340

362.98111	1	6341

363.12045	7	6348

363.25979	24	6372

363.39913	1	6373

363.53847	1	6374

363.67781	2	6376

363.81715	2	6378

363.95649	1	6379

364.09583	1	6380

364.23517	1	6381

364.37451	2	6383

364.51385	4	6387

364.65319	3	6390

364.79253	2	6392

364.93187	1	6393

365.07121	3	6396

365.21055	2	6398

365.34989	2	6400

365.48923	1	6401

365.62857	1	6402

365.76791	1	6403

365.90725	3	6406

366.04659	1	6407

366.18593	3	6410

366.32527	3	6413

366.46461	1	6414

366.60395	1	6415

366.74329	2	6417

366.88263	2	6419

367.02197	2	6421

367.16131	2	6423

367.30065	1	6424

367.43999	3	6427

367.57933	1	6428

367.71867	1	6429

367.85801	2	6431

367.99735	1	6432

368.13669	3	6435

368.27603	1	6436

368.41537	2	6438

368.55471	2	6440

368.69405	1	6441

368.83339	1	6442

368.97273	3	6445

369.11207	1	6446

369.25141	3	6449

369.39075	1	6450

369.5301	1	6451

369.66944	1	6452

369.80878	3	6455

369.94812	5	6460

370.08746	3	6463

370.2268	3	6466

370.36614	2	6468

370.50548	3	6471

370.64482	1	6472

370.78416	1	6473

370.9235	1	6474

371.06284	1	6475

371.20218	2	6477

371.34152	1	6478

371.48086	1	6479

371.6202	4	6483

371.75954	1	6484

371.89888	3	6487

372.03822	1	6488

372.17756	3	6491

372.3169	1	6492

372.45624	2	6494

372.59558	3	6497

372.73492	1	6498

372.87426	1	6499

373.0136	1	6500

373.15294	2	6502

373.29228	10	6512

373.43162	1	6513

373.57096	4	6517

373.7103	5	6522

373.84964	1	6523

373.98898	2	6525

374.12832	1	6526

374.26766	2	6528

374.407	1	6529

374.54634	3	6532

374.68568	2	6534

374.82502	1	6535

374.96436	1	6536

375.1037	3	6539

375.24304	1	6540

375.38238	2	6542

375.52172	2	6544

375.66106	1	6545

375.8004	2	6547

375.93974	2	6549

376.07908	3	6552

376.21842	2	6554

376.35776	4	6558

376.4971	4	6562

376.63644	2	6564

376.77578	4	6568

376.91512	2	6570

377.05446	1	6571

377.1938	1	6572

377.33314	1	6573

377.47248	1	6574

377.61182	1	6575

377.75116	3	6578

377.8905	2	6580

378.02984	4	6584

378.16918	2	6586

378.30852	5	6591

378.44786	2	6593

378.58721	3	6596

378.72655	4	6600

378.86589	4	6604

379.00523	1	6605

379.14457	1	6606

379.28391	2	6608

379.42325	1	6609

379.56259	1	6610

379.70193	1	6611

379.84127	1	6612

379.98061	1	6613

380.11995	1	6614

380.25929	1	6615

380.39863	3	6618

380.53797	3	6621

380.67731	6	6627

380.81665	1	6628

380.95599	1	6629

381.09533	5	6634

381.23467	1	6635

381.37401	1	6636

381.51335	1	6637

381.65269	1	6638

381.79203	3	6641

381.93137	1	6642

382.07071	1	6643

382.21005	1	6644

382.34939	1	6645

382.48873	2	6647

382.62807	2	6649

382.76741	1	6650

382.90675	2	6652

383.04609	1	6653

383.18543	1	6654

383.32477	1	6655

383.46411	1	6656

383.60345	2	6658

383.74279	1	6659

383.88213	5	6664

384.02147	1	6665

384.16081	3	6668

384.30015	3	6671

384.43949	6	6677

384.57883	4	6681

384.71817	1	6682

384.85751	1	6683

384.99685	4	6687

385.13619	3	6690

385.27553	1	6691

385.41487	3	6694

385.55421	1	6695

385.69355	1	6696

385.83289	3	6699

385.97223	1	6700

386.11157	4	6704

386.25091	1	6705

386.39025	1	6706

386.52959	4	6710

386.66893	1	6711

386.80827	3	6714

386.94761	1	6715

387.08695	7	6722

387.22629	2	6724

387.36564	2	6726

387.50498	1	6727

387.64432	2	6729

387.78366	2	6731

387.923	1	6732

388.06234	6	6738

388.20168	2	6740

388.34102	1	6741

388.48036	3	6744

388.6197	1	6745

388.75904	4	6749

388.89838	3	6752

389.03772	2	6754

389.17706	2	6756

389.3164	6	6762

389.45574	2	6764

389.59508	5	6769

389.73442	2	6771

389.87376	3	6774

390.0131	1	6775

390.15244	3	6778

390.29178	1	6779

390.43112	1	6780

390.57046	2	6782

390.7098	2	6784

390.84914	1	6785

390.98848	7	6792

391.12782	3	6795

391.26716	1	6796

391.4065	1	6797

391.54584	4	6801

391.68518	3	6804

391.82452	2	6806

391.96386	1	6807

392.1032	2	6809

392.24254	1	6810

392.38188	1	6811

392.52122	1	6812

392.66056	2	6814

392.7999	1	6815

392.93924	4	6819

393.07858	1	6820

393.21792	1	6821

393.35726	1	6822

393.4966	2	6824

393.63594	1	6825

393.77528	3	6828

393.91462	3	6831

394.05396	1	6832

394.1933	5	6837

394.33264	2	6839

394.47198	1	6840

394.61132	2	6842

394.75066	3	6845

394.89	1	6846

395.02934	2	6848

395.16868	1	6849

395.30802	1	6850

395.44736	3	6853

395.5867	1	6854

395.72604	5	6859

395.86538	3	6862

396.00472	1	6863

396.14406	5	6868

396.28341	1	6869

396.42275	3	6872

396.56209	4	6876

396.70143	2	6878

396.84077	1	6879

396.98011	1	6880

397.11945	1	6881

397.25879	3	6884

397.39813	1	6885

397.53747	8	6893

397.67681	1	6894

397.81615	1	6895

397.95549	5	6900

398.09483	3	6903

398.23417	1	6904

398.37351	1	6905

398.51285	5	6910

398.65219	4	6914

398.79153	2	6916

398.93087	1	6917

399.07021	2	6919

399.20955	3	6922

399.34889	1	6923

399.48823	1	6924

399.62757	2	6926

399.76691	3	6929

399.90625	1	6930

400.04559	2	6932

400.18493	1	6933

400.32427	16	6949

400.46361	2	6951

400.60295	3	6954

400.74229	2	6956

400.88163	2	6958

401.02097	4	6962

401.16031	1	6963

401.29965	1	6964

401.43899	3	6967

401.57833	1	6968

401.71767	2	6970

401.85701	22	6992

401.99635	1	6993

402.13569	1	6994

402.27503	1	6995

402.41437	3	6998

402.55371	1	6999

402.69305	7	7006

402.83239	1	7007

402.97173	1	7008

403.11107	1	7009

403.25041	6	7015

403.38975	1	7016

403.52909	1	7017

403.66843	3	7020

403.80777	1	7021

403.94711	3	7024

404.08645	1	7025

404.22579	1	7026

404.36513	5	7031

404.50447	2	7033

404.64381	2	7035

404.78315	2	7037

404.92249	1	7038

405.06183	1	7039

405.20117	1	7040

405.34052	2	7042

405.47986	1	7043

405.6192	2	7045

405.75854	1	7046

405.89788	7	7053

406.03722	3	7056

406.17656	2	7058

406.3159	1	7059

406.45524	3	7062

406.59458	2	7064

406.73392	3	7067

406.87326	1	7068

407.0126	2	7070

407.15194	4	7074

407.29128	2	7076

407.43062	6	7082

407.56996	1	7083

407.7093	1	7084

407.84864	5	7089

407.98798	1	7090

408.12732	1	7091

408.26666	1	7092

408.406	5	7097

408.54534	2	7099

408.68468	2	7101

408.82402	1	7102

408.96336	4	7106

409.1027	1	7107

409.24204	2	7109

409.38138	1	7110

409.52072	1	7111

409.66006	1	7112

409.7994	1	7113

409.93874	9	7122

410.07808	4	7126

410.21742	3	7129

410.35676	2	7131

410.4961	2	7133

410.63544	1	7134

410.77478	1	7135

410.91412	3	7138

411.05346	3	7141

411.1928	2	7143

411.33214	1	7144

411.47148	2	7146

411.61082	2	7148

411.75016	2	7150

411.8895	1	7151

412.02884	1	7152

412.16818	2	7154

412.30752	1	7155

412.44686	1	7156

412.5862	2	7158

412.72554	1	7159

412.86488	2	7161

413.00422	9	7170

413.14356	2	7172

413.2829	1	7173

413.42224	2	7175

413.56158	3	7178

413.70092	1	7179

413.84026	1	7180

413.9796	1	7181

414.11895	2	7183

414.25829	3	7186

414.39763	2	7188

414.53697	2	7190

414.67631	1	7191

414.81565	1	7192

414.95499	4	7196

415.09433	6	7202

415.23367	3	7205

415.37301	3	7208

415.51235	2	7210

415.65169	1	7211

415.79103	3	7214

415.93037	1	7215

416.06971	2	7217

416.20905	1	7218

416.34839	3	7221

416.48773	3	7224

416.62707	3	7227

416.76641	3	7230

416.90575	1	7231

417.04509	3	7234

417.18443	2	7236

417.32377	1	7237

417.46311	4	7241

417.60245	3	7244

417.74179	1	7245

417.88113	1	7246

418.02047	1	7247

418.15981	8	7255

418.29915	1	7256

418.43849	1	7257

418.57783	2	7259

418.71717	2	7261

418.85651	2	7263

418.99585	1	7264

419.13519	1	7265

419.27453	1	7266

419.41387	2	7268

419.55321	4	7272

419.69255	2	7274

419.83189	3	7277

419.97123	2	7279

420.11057	2	7281

420.24991	1	7282

420.38925	1	7283

420.52859	1	7284

420.66793	1	7285

420.80727	1	7286

420.94661	2	7288

421.08595	1	7289

421.22529	5	7294

421.36463	1	7295

421.50397	1	7296

421.64331	1	7297

421.78265	1	7298

421.92199	3	7301

422.06133	3	7304

422.20067	3	7307

422.34001	2	7309

422.47935	3	7312

422.61869	3	7315

422.75803	3	7318

422.89737	1	7319

423.03672	1	7320

423.17606	1	7321

423.3154	3	7324

423.45474	3	7327

423.59408	10	7337

423.73342	1	7338

423.87276	2	7340

424.0121	8	7348

424.15144	4	7352

424.29078	1	7353

424.43012	1	7354

424.56946	1	7355

424.7088	1	7356

424.84814	1	7357

424.98748	2	7359

425.12682	1	7360

425.26616	8	7368

425.4055	2	7370

425.54484	3	7373

425.68418	4	7377

425.82352	2	7379

425.96286	7	7386

426.1022	1	7387

426.24154	1	7388

426.38088	3	7391

426.52022	1	7392

426.65956	2	7394

426.7989	1	7395

426.93824	3	7398

427.07758	1	7399

427.21692	2	7401

427.35626	1	7402

427.4956	3	7405

427.63494	1	7406

427.77428	1	7407

427.91362	1	7408

428.05296	1	7409

428.1923	2	7411

428.33164	2	7413

428.47098	3	7416

428.61032	2	7418

428.74966	1	7419

428.889	1	7420

429.02834	5	7425

429.16768	2	7427

429.30702	1	7428

429.44636	1	7429

429.5857	4	7433

429.72504	2	7435

429.86438	6	7441

430.00372	2	7443

430.14306	4	7447

430.2824	1	7448

430.42174	5	7453

430.56108	4	7457

430.70042	1	7458

430.83976	1	7459

430.9791	2	7461

431.11844	3	7464

431.25778	8	7472

431.39712	3	7475

431.53646	3	7478

431.6758	1	7479

431.81514	1	7480

431.95449	1	7481

432.09383	6	7487

432.23317	2	7489

432.37251	3	7492

432.51185	3	7495

432.65119	3	7498

432.79053	1	7499

432.92987	7	7506

433.06921	3	7509

433.20855	4	7513

433.34789	1	7514

433.48723	3	7517

433.62657	3	7520

433.76591	3	7523

433.90525	1	7524

434.04459	1	7525

434.18393	1	7526

434.32327	1	7527

434.46261	1	7528

434.60195	1	7529

434.74129	1	7530

434.88063	1	7531

435.01997	4	7535

435.15931	2	7537

435.29865	4	7541

435.43799	3	7544

435.57733	2	7546

435.71667	1	7547

435.85601	2	7549

435.99535	1	7550

436.13469	6	7556

436.27403	1	7557

436.41337	1	7558

436.55271	1	7559

436.69205	3	7562

436.83139	7	7569

436.97073	3	7572

437.11007	2	7574

437.24941	1	7575

437.38875	2	7577

437.52809	2	7579

437.66743	3	7582

437.80677	1	7583

437.94611	1	7584

438.08545	1	7585

438.22479	2	7587

438.36413	1	7588

438.50347	6	7594

438.64281	1	7595

438.78215	1	7596

438.92149	1	7597

439.06083	5	7602

439.20017	1	7603

439.33951	6	7609

439.47885	3	7612

439.61819	1	7613

439.75753	2	7615

439.89687	1	7616

440.03621	1	7617

440.17555	2	7619

440.31489	9	7628

440.45423	1	7629

440.59357	2	7631

440.73291	5	7636

440.87226	2	7638

441.0116	2	7640

441.15094	8	7648

441.29028	3	7651

441.42962	1	7652

441.56896	2	7654

441.7083	2	7656

441.84764	1	7657

441.98698	1	7658

442.12632	2	7660

442.26566	1	7661

442.405	2	7663

442.54434	1	7664

442.68368	3	7667

442.82302	1	7668

442.96236	1	7669

443.1017	3	7672

443.24104	1	7673

443.38038	1	7674

443.51972	5	7679

443.65906	10	7689

443.7984	3	7692

443.93774	3	7695

444.07708	1	7696

444.21642	4	7700

444.35576	5	7705

444.4951	1	7706

444.63444	1	7707

444.77378	1	7708

444.91312	1	7709

445.05246	2	7711

445.1918	10	7721

445.33114	2	7723

445.47048	1	7724

445.60982	1	7725

445.74916	2	7727

445.8885	2	7729

446.02784	1	7730

446.16718	4	7734

446.30652	2	7736

446.44586	5	7741

446.5852	15	7756

446.72454	3	7759

446.86388	4	7763

447.00322	4	7767

447.14256	7	7774

447.2819	1	7775

447.42124	2	7777

447.56058	1	7778

447.69992	2	7780

447.83926	1	7781

447.9786	3	7784

448.11794	5	7789

448.25728	1	7790

448.39662	1	7791

448.53596	3	7794

448.6753	5	7799

448.81464	1	7800

448.95398	1	7801

449.09332	1	7802

449.23266	1	7803

449.372	1	7804

449.51134	1	7805

449.65068	1	7806

449.79003	3	7809

449.92937	2	7811

450.06871	2	7813

450.20805	2	7815

450.34739	1	7816

450.48673	6	7822

450.62607	1	7823

450.76541	2	7825

450.90475	4	7829

451.04409	2	7831

451.18343	5	7836

451.32277	3	7839

451.46211	3	7842

451.60145	2	7844

451.74079	1	7845

451.88013	1	7846

452.01947	1	7847

452.15881	1	7848

452.29815	3	7851

452.43749	4	7855

452.57683	1	7856

452.71617	3	7859

452.85551	1	7860

452.99485	1	7861

453.13419	3	7864

453.27353	2	7866

453.41287	6	7872

453.55221	2	7874

453.69155	3	7877

453.83089	4	7881

453.97023	1	7882

454.10957	2	7884

454.24891	1	7885

454.38825	1	7886

454.52759	1	7887

454.66693	1	7888

454.80627	1	7889

454.94561	3	7892

455.08495	1	7893

455.22429	1	7894

455.36363	4	7898

455.50297	4	7902

455.64231	1	7903

455.78165	3	7906

455.92099	3	7909

456.06033	1	7910

456.19967	1	7911

456.33901	1	7912

456.47835	1	7913

456.61769	4	7917

456.75703	4	7921

456.89637	2	7923

457.03571	5	7928

457.17505	1	7929

457.31439	2	7931

457.45373	2	7933

457.59307	1	7934

457.73241	2	7936

457.87175	6	7942

458.01109	8	7950

458.15043	6	7956

458.28977	1	7957

458.42911	3	7960

458.56845	3	7963

458.7078	1	7964

458.84714	1	7965

458.98648	1	7966

459.12582	3	7969

459.26516	2	7971

459.4045	1	7972

459.54384	1	7973

459.68318	2	7975

459.82252	2	7977

459.96186	8	7985

460.1012	1	7986

460.24054	1	7987

460.37988	1	7988

460.51922	3	7991

460.65856	1	7992

460.7979	11	8003

460.93724	7	8010

461.07658	6	8016

461.21592	1	8017

461.35526	1	8018

461.4946	1	8019

461.63394	11	8030

461.77328	1	8031

461.91262	1	8032

462.05196	2	8034

462.1913	2	8036

462.33064	4	8040

462.46998	9	8049

462.60932	2	8051

462.74866	4	8055

462.888	2	8057

463.02734	1	8058

463.16668	2	8060

463.30602	2	8062

463.44536	1	8063

463.5847	1	8064

463.72404	1	8065

463.86338	3	8068

464.00272	1	8069

464.14206	9	8078

464.2814	1	8079

464.42074	1	8080

464.56008	3	8083

464.69942	2	8085

464.83876	1	8086

464.9781	2	8088

465.11744	8	8096

465.25678	2	8098

465.39612	1	8099

465.53546	2	8101

465.6748	4	8105

465.81414	2	8107

465.95348	1	8108

466.09282	2	8110

466.23216	2	8112

466.3715	1	8113

466.51084	2	8115

466.65018	8	8123

466.78952	3	8126

466.92886	1	8127

467.0682	3	8130

467.20754	1	8131

467.34688	2	8133

467.48622	2	8135

467.62557	2	8137

467.76491	1	8138

467.90425	2	8140

468.04359	5	8145

468.18293	3	8148

468.32227	6	8154

468.46161	1	8155

468.60095	2	8157

468.74029	2	8159

468.87963	1	8160

469.01897	1	8161

469.15831	1	8162

469.29765	6	8168

469.43699	2	8170

469.57633	1	8171

469.71567	1	8172

469.85501	1	8173

469.99435	1	8174

470.13369	7	8181

470.27303	3	8184

470.41237	1	8185

470.55171	5	8190

470.69105	1	8191

470.83039	3	8194

470.96973	11	8205

471.10907	1	8206

471.24841	1	8207

471.38775	5	8212

471.52709	8	8220

471.66643	2	8222

471.80577	6	8228

471.94511	1	8229

472.08445	1	8230

472.22379	2	8232

472.36313	1	8233

472.50247	3	8236

472.64181	5	8241

472.78115	1	8242

472.92049	3	8245

473.05983	1	8246

473.19917	15	8261

473.33851	1	8262

473.47785	3	8265

473.61719	1	8266

473.75653	1	8267

473.89587	1	8268

474.03521	4	8272

474.17455	1	8273

474.31389	2	8275

474.45323	1	8276

474.59257	1	8277

474.73191	1	8278

474.87125	3	8281

475.01059	3	8284

475.14993	2	8286

475.28927	3	8289

475.42861	2	8291

475.56795	1	8292

475.70729	4	8296

475.84663	8	8304

475.98597	2	8306

476.12531	1	8307

476.26465	3	8310

476.404	1	8311

476.54334	1	8312

476.68268	4	8316

476.82202	13	8329

476.96136	7	8336

477.1007	1	8337

477.24004	7	8344

477.37938	3	8347

477.51872	1	8348

477.65806	3	8351

477.7974	2	8353

477.93674	1	8354

478.07608	1	8355

478.21542	6	8361

478.35476	3	8364

478.4941	4	8368

478.63344	3	8371

478.77278	1	8372

478.91212	5	8377

479.05146	3	8380

479.1908	10	8390

479.33014	1	8391

479.46948	4	8395

479.60882	2	8397

479.74816	2	8399

479.8875	1	8400

480.02684	10	8410

480.16618	3	8413

480.30552	3	8416

480.44486	2	8418

480.5842	4	8422

480.72354	1	8423

480.86288	1	8424

481.00222	1	8425

481.14156	2	8427

481.2809	1	8428

481.42024	1	8429

481.55958	1	8430

481.69892	1	8431

481.83826	1	8432

481.9776	1	8433

482.11694	1	8434

482.25628	3	8437

482.39562	2	8439

482.53496	2	8441

482.6743	3	8444

482.81364	2	8446

482.95298	1	8447

483.09232	1	8448

483.23166	1	8449

483.371	2	8451

483.51034	3	8454

483.64968	1	8455

483.78902	3	8458

483.92836	1	8459

484.0677	1	8460

484.20704	6	8466

484.34638	3	8469

484.48572	7	8476

484.62506	5	8481

484.7644	1	8482

484.90374	1	8483

485.04308	1	8484

485.18242	2	8486

485.32177	2	8488

485.46111	1	8489

485.60045	6	8495

485.73979	1	8496

485.87913	1	8497

486.01847	2	8499

486.15781	5	8504

486.29715	6	8510

486.43649	1	8511

486.57583	1	8512

486.71517	1	8513

486.85451	2	8515

486.99385	2	8517

487.13319	1	8518

487.27253	5	8523

487.41187	1	8524

487.55121	2	8526

487.69055	4	8530

487.82989	2	8532

487.96923	2	8534

488.10857	1	8535

488.24791	3	8538

488.38725	1	8539

488.52659	3	8542

488.66593	2	8544

488.80527	1	8545

488.94461	1	8546

489.08395	1	8547

489.22329	3	8550

489.36263	3	8553

489.50197	1	8554

489.64131	1	8555

489.78065	4	8559

489.91999	7	8566

490.05933	1	8567

490.19867	6	8573

490.33801	1	8574

490.47735	8	8582

490.61669	1	8583

490.75603	2	8585

490.89537	3	8588

491.03471	12	8600

491.17405	8	8608

491.31339	1	8609

491.45273	5	8614

491.59207	1	8615

491.73141	3	8618

491.87075	3	8621

492.01009	2	8623

492.14943	2	8625

492.28877	3	8628

492.42811	1	8629

492.56745	7	8636

492.70679	3	8639

492.84613	1	8640

492.98547	3	8643

493.12481	2	8645

493.26415	3	8648

493.40349	2	8650

493.54283	1	8651

493.68217	14	8665

493.82151	2	8667

493.96085	1	8668

494.10019	1	8669

494.23953	4	8673

494.37888	1	8674

494.51822	4	8678

494.65756	2	8680

494.7969	1	8681

494.93624	1	8682

495.07558	4	8686

495.21492	1	8687

495.35426	4	8691

495.4936	5	8696

495.63294	2	8698

495.77228	2	8700

495.91162	1	8701

496.05096	1	8702

496.1903	1	8703

496.32964	2	8705

496.46898	6	8711

496.60832	1	8712

496.74766	4	8716

496.887	6	8722

497.02634	1	8723

497.16568	2	8725

497.30502	1	8726

497.44436	13	8739

497.5837	5	8744

497.72304	2	8746

497.86238	1	8747

498.00172	2	8749

498.14106	1	8750

498.2804	2	8752

498.41974	2	8754

498.55908	3	8757

498.69842	1	8758

498.83776	5	8763

498.9771	3	8766

499.11644	11	8777

499.25578	3	8780

499.39512	1	8781

499.53446	3	8784

499.6738	3	8787

499.81314	1	8788

499.95248	3	8791

500.09182	1	8792

500.23116	1	8793

500.3705	1	8794

500.50984	1	8795

500.64918	3	8798

500.78852	1	8799

500.92786	1	8800

501.0672	4	8804

501.20654	1	8805

501.34588	2	8807

501.48522	1	8808

501.62456	3	8811

501.7639	7	8818

501.90324	2	8820

502.04258	1	8821

502.18192	1	8822

502.32126	3	8825

502.4606	2	8827

502.59994	1	8828

502.73928	3	8831

502.87862	1	8832

503.01796	1	8833

503.15731	1	8834

503.29665	4	8838

503.43599	1	8839

503.57533	4	8843

503.71467	1	8844

503.85401	4	8848

503.99335	1	8849

504.13269	2	8851

504.27203	1	8852

504.41137	1	8853

504.55071	1	8854

504.69005	1	8855

504.82939	4	8859

504.96873	2	8861

505.10807	1	8862

505.24741	4	8866

505.38675	4	8870

505.52609	4	8874

505.66543	4	8878

505.80477	2	8880

505.94411	2	8882

506.08345	2	8884

506.22279	3	8887

506.36213	2	8889

506.50147	1	8890

506.64081	1	8891

506.78015	4	8895

506.91949	1	8896

507.05883	2	8898

507.19817	2	8900

507.33751	1	8901

507.47685	1	8902

507.61619	1	8903

507.75553	3	8906

507.89487	2	8908

508.03421	1	8909

508.17355	2	8911

508.31289	7	8918

508.45223	5	8923

508.59157	1	8924

508.73091	1	8925

508.87025	4	8929

509.00959	1	8930

509.14893	2	8932

509.28827	6	8938

509.42761	3	8941

509.56695	2	8943

509.70629	1	8944

509.84563	2	8946

509.98497	2	8948

510.12431	1	8949

510.26365	2	8951

510.40299	1	8952

510.54233	2	8954

510.68167	2	8956

510.82101	5	8961

510.96035	1	8962

511.09969	3	8965

511.23903	1	8966

511.37837	2	8968

511.51771	2	8970

511.65705	2	8972

511.79639	1	8973

511.93573	10	8983

512.07507	1	8984

512.21442	5	8989

512.35376	1	8990

512.4931	3	8993

512.63244	1	8994

512.77178	1	8995

512.91112	4	8999

513.05046	1	9000

513.1898	1	9001

513.32914	1	9002

513.46848	1	9003

513.60782	3	9006

513.74716	2	9008

513.8865	1	9009

514.02584	6	9015

514.16518	1	9016

514.30452	9	9025

514.44386	5	9030

514.5832	1	9031

514.72254	1	9032

514.86188	9	9041

515.00122	7	9048

515.14056	5	9053

515.2799	6	9059

515.41924	2	9061

515.55858	1	9062

515.69792	3	9065

515.83726	3	9068

515.9766	1	9069

516.11594	1	9070

516.25528	7	9077

516.39462	1	9078

516.53396	8	9086

516.6733	3	9089

516.81264	12	9101

516.95198	4	9105

517.09132	9	9114

517.23066	1	9115

517.37	1	9116

517.50934	1	9117

517.64868	1	9118

517.78802	3	9121

517.92736	1	9122

518.0667	1	9123

518.20604	7	9130

518.34538	1	9131

518.48472	3	9134

518.62406	2	9136

518.7634	4	9140

518.90274	1	9141

519.04208	1	9142

519.18142	2	9144

519.32076	5	9149

519.4601	3	9152

519.59944	2	9154

519.73878	1	9155

519.87812	4	9159

520.01746	12	9171

520.1568	8	9179

520.29614	3	9182

520.43548	4	9186

520.57482	1	9187

520.71416	1	9188

520.8535	2	9190

520.99285	4	9194

521.13219	2	9196

521.27153	2	9198

521.41087	1	9199

521.55021	5	9204

521.68955	3	9207

521.82889	2	9209

521.96823	1	9210

522.10757	1	9211

522.24691	2	9213

522.38625	3	9216

522.52559	1	9217

522.66493	1	9218

522.80427	1	9219

522.94361	1	9220

523.08295	3	9223

523.22229	1	9224

523.36163	1	9225

523.50097	6	9231

523.64031	1	9232

523.77965	4	9236

523.91899	5	9241

524.05833	1	9242

524.19767	1	9243

524.33701	1	9244

524.47635	1	9245

524.61569	9	9254

524.75503	6	9260

524.89437	3	9263

525.03371	4	9267

525.17305	2	9269

525.31239	1	9270

525.45173	3	9273

525.59107	1	9274

525.73041	1	9275

525.86975	2	9277

526.00909	4	9281

526.14843	6	9287

526.28777	1	9288

526.42711	8	9296

526.56645	2	9298

526.70579	2	9300

526.84513	1	9301

526.98447	2	9303

527.12381	1	9304

527.26315	2	9306

527.40249	2	9308

527.54183	7	9315

527.68117	1	9316

527.82051	2	9318

527.95985	3	9321

528.09919	3	9324

528.23853	1	9325

528.37787	3	9328

528.51721	1	9329

528.65655	3	9332

528.79589	1	9333

528.93523	5	9338

529.07457	1	9339

529.21391	3	9342

529.35325	14	9356

529.49259	1	9357

529.63193	1	9358

529.77127	3	9361

529.91062	3	9364

530.04996	1	9365

530.1893	1	9366

530.32864	5	9371

530.46798	1	9372

530.60732	2	9374

530.74666	2	9376

530.886	20	9396

531.02534	4	9400

531.16468	1	9401

531.30402	2	9403

531.44336	1	9404

531.5827	1	9405

531.72204	1	9406

531.86138	7	9413

532.00072	1	9414

532.14006	4	9418

532.2794	1	9419

532.41874	1	9420

532.55808	1	9421

532.69742	5	9426

532.83676	2	9428

532.9761	1	9429

533.11544	1	9430

533.25478	3	9433

533.39412	3	9436

533.53346	1	9437

533.6728	2	9439

533.81214	5	9444

533.95148	1	9445

534.09082	3	9448

534.23016	1	9449

534.3695	2	9451

534.50884	1	9452

534.64818	2	9454

534.78752	3	9457

534.92686	1	9458

535.0662	1	9459

535.20554	2	9461

535.34488	1	9462

535.48422	1	9463

535.62356	2	9465

535.7629	5	9470

535.90224	1	9471

536.04158	6	9477

536.18092	49	9526

536.32026	1	9527

536.4596	12	9539

536.59894	4	9543

536.73828	1	9544

536.87762	1	9545

537.01696	2	9547

537.1563	1	9548

537.29564	1	9549

537.43498	1	9550

537.57432	4	9554

537.71366	5	9559

537.853	2	9561

537.99234	1	9562

538.13168	2	9564

538.27102	3	9567

538.41036	2	9569

538.5497	1	9570

538.68904	1	9571

538.82839	6	9577

538.96773	1	9578

539.10707	9	9587

539.24641	3	9590

539.38575	5	9595

539.52509	4	9599

539.66443	1	9600

539.80377	2	9602

539.94311	2	9604

540.08245	2	9606

540.22179	1	9607

540.36113	5	9612

540.50047	1	9613

540.63981	2	9615

540.77915	1	9616

540.91849	4	9620

541.05783	1	9621

541.19717	2	9623

541.33651	1	9624

541.47585	3	9627

541.61519	2	9629

541.75453	1	9630

541.89387	1	9631

542.03321	1	9632

542.17255	2	9634

542.31189	2	9636

542.45123	1	9637

542.59057	1	9638

542.72991	1	9639

542.86925	1	9640

543.00859	1	9641

543.14793	2	9643

543.28727	4	9647

543.42661	4	9651

543.56595	1	9652

543.70529	2	9654

543.84463	2	9656

543.98397	6	9662

544.12331	3	9665

544.26265	1	9666

544.40199	5	9671

544.54133	6	9677

544.68067	4	9681

544.82001	3	9684

544.95935	1	9685

545.09869	2	9687

545.23803	1	9688

545.37737	1	9689

545.51671	1	9690

545.65605	1	9691

545.79539	1	9692

545.93473	1	9693

546.07407	1	9694

546.21341	1	9695

546.35275	1	9696

546.49209	1	9697

546.63143	2	9699

546.77077	3	9702

546.91011	8	9710

547.04945	9	9719

547.18879	9	9728

547.32813	12	9740

547.46747	3	9743

547.60681	1	9744

547.74616	1	9745

547.8855	1	9746

548.02484	3	9749

548.16418	7	9756

548.30352	1	9757

548.44286	7	9764

548.5822	1	9765

548.72154	1	9766

548.86088	8	9774

549.00022	7	9781

549.13956	1	9782

549.2789	1	9783

549.41824	2	9785

549.55758	2	9787

549.69692	1	9788

549.83626	1	9789

549.9756	5	9794

550.11494	1	9795

550.25428	11	9806

550.39362	1	9807

550.53296	7	9814

550.6723	8	9822

550.81164	5	9827

550.95098	1	9828

551.09032	1	9829

551.22966	5	9834

551.369	2	9836

551.50834	4	9840

551.64768	1	9841

551.78702	1	9842

551.92636	2	9844

552.0657	4	9848

552.20504	4	9852

552.34438	12	9864

552.48372	4	9868

552.62306	2	9870

552.7624	3	9873

552.90174	1	9874

553.04108	5	9879

553.18042	1	9880

553.31976	4	9884

553.4591	6	9890

553.59844	1	9891

553.73778	4	9895

553.87712	1	9896

554.01646	11	9907

554.1558	3	9910

554.29514	10	9920

554.43448	5	9925

554.57382	2	9927

554.71316	1	9928

554.8525	14	9942

554.99184	1	9943

555.13118	3	9946

555.27052	5	9951

555.40986	1	9952

555.5492	1	9953

555.68854	1	9954

555.82788	3	9957

555.96722	1	9958

556.10656	1	9959

556.2459	1	9960

556.38524	1	9961

556.52459	4	9965

556.66393	3	9968

556.80327	6	9974

556.94261	4	9978

557.08195	5	9983

557.22129	1	9984

557.36063	2	9986

557.49997	2	9988

557.63931	6	9994

557.77865	4	9998

557.91799	1	9999

558.05733	6	10005

558.19667	1	10006

558.33601	1	10007

558.47535	2	10009

558.61469	5	10014

558.75403	1	10015

558.89337	2	10017

559.03271	2	10019

559.17205	2	10021

559.31139	2	10023

559.45073	3	10026

559.59007	2	10028

559.72941	1	10029

559.86875	2	10031

560.00809	1	10032

560.14743	1	10033

560.28677	1	10034

560.42611	6	10040

560.56545	1	10041

560.70479	1	10042

560.84413	3	10045

560.98347	6	10051

561.12281	2	10053

561.26215	2	10055

561.40149	1	10056

561.54083	10	10066

561.68017	8	10074

561.81951	1	10075

561.95885	1	10076

562.09819	5	10081

562.23753	6	10087

562.37687	1	10088

562.51621	2	10090

562.65555	5	10095

562.79489	2	10097

562.93423	1	10098

563.07357	5	10103

563.21291	1	10104

563.35225	1	10105

563.49159	1	10106

563.63093	2	10108

563.77027	1	10109

563.90961	2	10111

564.04895	3	10114

564.18829	1	10115

564.32763	1	10116

564.46697	1	10117

564.60631	2	10119

564.74565	1	10120

564.88499	2	10122

565.02433	1	10123

565.16367	1	10124

565.30301	1	10125

565.44236	3	10128

565.5817	3	10131

565.72104	1	10132

565.86038	1	10133

565.99972	4	10137

566.13906	1	10138

566.2784	6	10144

566.41774	2	10146

566.55708	6	10152

566.69642	2	10154

566.83576	3	10157

566.9751	4	10161

567.11444	1	10162

567.25378	1	10163

567.39312	1	10164

567.53246	2	10166

567.6718	4	10170

567.81114	2	10172

567.95048	2	10174

568.08982	3	10177

568.22916	2	10179

568.3685	1	10180

568.50784	4	10184

568.64718	2	10186

568.78652	5	10191

568.92586	5	10196

569.0652	2	10198

569.20454	3	10201

569.34388	6	10207

569.48322	3	10210

569.62256	1	10211

569.7619	11	10222

569.90124	8	10230

570.04058	1	10231

570.17992	1	10232

570.31926	3	10235

570.4586	4	10239

570.59794	4	10243

570.73728	1	10244

570.87662	12	10256

571.01596	1	10257

571.1553	3	10260

571.29464	5	10265

571.43398	2	10267

571.57332	1	10268

571.71266	3	10271

571.852	3	10274

571.99134	3	10277

572.13068	2	10279

572.27002	1	10280

572.40936	1	10281

572.5487	5	10286

572.68804	4	10290

572.82738	2	10292

572.96672	1	10293

573.10606	8	10301

573.2454	3	10304

573.38474	2	10306

573.52408	7	10313

573.66342	4	10317

573.80276	3	10320

573.9421	1	10321

574.08144	2	10323

574.22078	1	10324

574.36013	2	10326

574.49947	1	10327

574.63881	2	10329

574.77815	11	10340

574.91749	3	10343

575.05683	7	10350

575.19617	5	10355

575.33551	6	10361

575.47485	5	10366

575.61419	6	10372

575.75353	7	10379

575.89287	3	10382

576.03221	11	10393

576.17155	6	10399

576.31089	1	10400

576.45023	1	10401

576.58957	2	10403

576.72891	6	10409

576.86825	2	10411

577.00759	1	10412

577.14693	4	10416

577.28627	1	10417

577.42561	2	10419

577.56495	1	10420

577.70429	4	10424

577.84363	1	10425

577.98297	2	10427

578.12231	2	10429

578.26165	8	10437

578.40099	1	10438

578.54033	2	10440

578.67967	4	10444

578.81901	4	10448

578.95835	1	10449

579.09769	3	10452

579.23703	1	10453

579.37637	19	10472

579.51571	4	10476

579.65505	19	10495

579.79439	9	10504

579.93373	1	10505

580.07307	3	10508

580.21241	3	10511

580.35175	2	10513

580.49109	1	10514

580.63043	1	10515

580.76977	3	10518

580.90911	8	10526

581.04845	8	10534

581.18779	3	10537

581.32713	1	10538

581.46647	5	10543

581.60581	1	10544

581.74515	1	10545

581.88449	1	10546

582.02383	5	10551

582.16317	1	10552

582.30251	3	10555

582.44185	1	10556

582.58119	3	10559

582.72053	1	10560

582.85987	1	10561

582.99921	5	10566

583.13855	1	10567

583.27789	3	10570

583.41724	1	10571

583.55658	1	10572

583.69592	1	10573

583.83526	1	10574

583.9746	1	10575

584.11394	3	10578

584.25328	2	10580

584.39262	3	10583

584.53196	8	10591

584.6713	2	10593

584.81064	3	10596

584.94998	3	10599

585.08932	1	10600

585.22866	2	10602

585.368	1	10603

585.50734	1	10604

585.64668	1	10605

585.78602	1	10606

585.92536	1	10607

586.0647	2	10609

586.20404	4	10613

586.34338	4	10617

586.48272	2	10619

586.62206	9	10628

586.7614	1	10629

586.90074	1	10630

587.04008	3	10633

587.17942	8	10641

587.31876	1	10642

587.4581	2	10644

587.59744	5	10649

587.73678	2	10651

587.87612	9	10660

588.01546	2	10662

588.1548	1	10663

588.29414	2	10665

588.43348	2	10667

588.57282	5	10672

588.71216	1	10673

588.8515	5	10678

588.99084	1	10679

589.13018	2	10681

589.26952	6	10687

589.40886	2	10689

589.5482	4	10693

589.68754	1	10694

589.82688	2	10696

589.96622	14	10710

590.10556	3	10713

590.2449	17	10730

590.38424	1	10731

590.52358	1	10732

590.66292	5	10737

590.80226	1	10738

590.9416	3	10741

591.08094	9	10750

591.22028	7	10757

591.35962	1	10758

591.49896	1	10759

591.6383	3	10762

591.77764	7	10769

591.91698	3	10772

592.05632	5	10777

592.19566	2	10779

592.33501	3	10782

592.47435	4	10786

592.61369	4	10790

592.75303	5	10795

592.89237	1	10796

593.03171	5	10801

593.17105	3	10804

593.31039	6	10810

593.44973	2	10812

593.58907	2	10814

593.72841	4	10818

593.86775	4	10822

594.00709	1	10823

594.14643	2	10825

594.28577	2	10827

594.42511	3	10830

594.56445	7	10837

594.70379	2	10839

594.84313	1	10840

594.98247	1	10841

595.12181	1	10842

595.26115	1	10843

595.40049	2	10845

595.53983	1	10846

595.67917	12	10858

595.81851	2	10860

595.95785	1	10861

596.09719	1	10862

596.23653	2	10864

596.37587	8	10872

596.51521	1	10873

596.65455	3	10876

596.79389	2	10878

596.93323	1	10879

597.07257	2	10881

597.21191	3	10884

597.35125	7	10891

597.49059	3	10894

597.62993	2	10896

597.76927	1	10897

597.90861	2	10899

598.04795	1	10900

598.18729	2	10902

598.32663	1	10903

598.46597	4	10907

598.60531	1	10908

598.74465	1	10909

598.88399	2	10911

599.02333	1	10912

599.16267	3	10915

599.30201	11	10926

599.44135	1	10927

599.58069	4	10931

599.72003	6	10937

599.85937	1	10938

599.99871	1	10939

600.13805	2	10941

600.27739	3	10944

600.41673	1	10945

600.55607	1	10946

600.69541	2	10948

600.83475	8	10956

600.97409	3	10959

601.11343	1	10960

601.25278	1	10961

601.39212	3	10964

601.53146	1	10965

601.6708	3	10968

601.81014	12	10980

601.94948	2	10982

602.08882	3	10985

602.22816	1	10986

602.3675	3	10989

602.50684	3	10992

602.64618	6	10998

602.78552	1	10999

602.92486	1	11000

603.0642	1	11001

603.20354	2	11003

603.34288	1	11004

603.48222	5	11009

603.62156	19	11028

603.7609	7	11035

603.90024	6	11041

604.03958	1	11042

604.17892	2	11044

604.31826	1	11045

604.4576	4	11049

604.59694	2	11051

604.73628	2	11053

604.87562	6	11059

605.01496	1	11060

605.1543	1	11061

605.29364	1	11062

605.43298	1	11063

605.57232	1	11064

605.71166	1	11065

605.851	1	11066

605.99034	3	11069

606.12968	1	11070

606.26902	2	11072

606.40836	4	11076

606.5477	1	11077

606.68704	1	11078

606.82638	1	11079

606.96572	13	11092

607.10506	2	11094

607.2444	1	11095

607.38374	7	11102

607.52308	7	11109

607.66242	1	11110

607.80176	2	11112

607.9411	2	11114

608.08044	2	11116

608.21978	1	11117

608.35912	9	11126

608.49846	1	11127

608.6378	2	11129

608.77714	16	11145

608.91648	1	11146

609.05582	1	11147

609.19516	2	11149

609.3345	1	11150

609.47384	8	11158

609.61318	2	11160

609.75252	5	11165

609.89186	7	11172

610.03121	1	11173

610.17055	2	11175

610.30989	2	11177

610.44923	1	11178

610.58857	2	11180

610.72791	2	11182

610.86725	2	11184

611.00659	1	11185

611.14593	1	11186

611.28527	6	11192

611.42461	9	11201

611.56395	4	11205

611.70329	1	11206

611.84263	5	11211

611.98197	2	11213

612.12131	2	11215

612.26065	2	11217

612.39999	1	11218

612.53933	4	11222

612.67867	3	11225

612.81801	17	11242

612.95735	2	11244

613.09669	2	11246

613.23603	6	11252

613.37537	1	11253

613.51471	1	11254

613.65405	1	11255

613.79339	1	11256

613.93273	1	11257

614.07207	1	11258

614.21141	1	11259

614.35075	1	11260

614.49009	2	11262

614.62943	1	11263

614.76877	1	11264

614.90811	3	11267

615.04745	5	11272

615.18679	1	11273

615.32613	4	11277

615.46547	12	11289

615.60481	2	11291

615.74415	4	11295

615.88349	4	11299

616.02283	5	11304

616.16217	3	11307

616.30151	5	11312

616.44085	2	11314

616.58019	2	11316

616.71953	9	11325

616.85887	5	11330

616.99821	3	11333

617.13755	1	11334

617.27689	1	11335

617.41623	1	11336

617.55557	1	11337

617.69491	8	11345

617.83425	5	11350

617.97359	2	11352

618.11293	1	11353

618.25227	3	11356

618.39161	1	11357

618.53095	5	11362

618.67029	1	11363

618.80963	1	11364

618.94898	3	11367

619.08832	1	11368

619.22766	7	11375

619.367	1	11376

619.50634	1	11377

619.64568	1	11378

619.78502	4	11382

619.92436	1	11383

620.0637	3	11386

620.20304	3	11389

620.34238	4	11393

620.48172	1	11394

620.62106	2	11396

620.7604	1	11397

620.89974	5	11402

621.03908	3	11405

621.17842	4	11409

621.31776	1	11410

621.4571	1	11411

621.59644	1	11412

621.73578	11	11423

621.87512	1	11424

622.01446	4	11428

622.1538	2	11430

622.29314	3	11433

622.43248	2	11435

622.57182	3	11438

622.71116	3	11441

622.8505	3	11444

622.98984	1	11445

623.12918	1	11446

623.26852	4	11450

623.40786	1	11451

623.5472	2	11453

623.68654	4	11457

623.82588	3	11460

623.96522	2	11462

624.10456	4	11466

624.2439	5	11471

624.38324	1	11472

624.52258	4	11476

624.66192	7	11483

624.80126	2	11485

624.9406	1	11486

625.07994	8	11494

625.21928	5	11499

625.35862	3	11502

625.49796	4	11506

625.6373	1	11507

625.77664	3	11510

625.91598	1	11511

626.05532	2	11513

626.19466	2	11515

626.334	25	11540

626.47334	1	11541

626.61268	1	11542

626.75202	1	11543

626.89136	7	11550

627.0307	1	11551

627.17004	1	11552

627.30938	4	11556

627.44872	2	11558

627.58806	2	11560

627.7274	1	11561

627.86675	21	11582

628.00609	3	11585

628.14543	1	11586

628.28477	1	11587

628.42411	5	11592

628.56345	3	11595

628.70279	3	11598

628.84213	1	11599

628.98147	1	11600

629.12081	1	11601

629.26015	1	11602

629.39949	14	11616

629.53883	4	11620

629.67817	1	11621

629.81751	1	11622

629.95685	1	11623

630.09619	4	11627

630.23553	1	11628

630.37487	5	11633

630.51421	3	11636

630.65355	2	11638

630.79289	1	11639

630.93223	1	11640

631.07157	1	11641

631.21091	3	11644

631.35025	1	11645

631.48959	2	11647

631.62893	12	11659

631.76827	7	11666

631.90761	1	11667

632.04695	1	11668

632.18629	1	11669

632.32563	2	11671

632.46497	1	11672

632.60431	1	11673

632.74365	3	11676

632.88299	2	11678

633.02233	3	11681

633.16167	2	11683

633.30101	1	11684

633.44035	6	11690

633.57969	3	11693

633.71903	1	11694

633.85837	7	11701

633.99771	2	11703

634.13705	1	11704

634.27639	1	11705

634.41573	2	11707

634.55507	1	11708

634.69441	2	11710

634.83375	1	11711

634.97309	2	11713

635.11243	4	11717

635.25177	1	11718

635.39111	1	11719

635.53045	2	11721

635.66979	4	11725

635.80913	2	11727

635.94847	3	11730

636.08781	4	11734

636.22715	1	11735

636.36649	1	11736

636.50583	1	11737

636.64517	1	11738

636.78452	1	11739

636.92386	2	11741

637.0632	9	11750

637.20254	2	11752

637.34188	1	11753

637.48122	4	11757

637.62056	1	11758

637.7599	1	11759

637.89924	1	11760

638.03858	6	11766

638.17792	2	11768

638.31726	1	11769

638.4566	3	11772

638.59594	2	11774

638.73528	9	11783

638.87462	15	11798

639.01396	2	11800

639.1533	8	11808

639.29264	1	11809

639.43198	2	11811

639.57132	7	11818

639.71066	2	11820

639.85	3	11823

639.98934	4	11827

640.12868	2	11829

640.26802	6	11835

640.40736	1	11836

640.5467	1	11837

640.68604	1	11838

640.82538	3	11841

640.96472	2	11843

641.10406	1	11844

641.2434	1	11845

641.38274	14	11859

641.52208	1	11860

641.66142	1	11861

641.80076	8	11869

641.9401	2	11871

642.07944	1	11872

642.21878	8	11880

642.35812	1	11881

642.49746	1	11882

642.6368	1	11883

642.77614	2	11885

642.91548	3	11888

643.05482	2	11890

643.19416	6	11896

643.3335	1	11897

643.47284	2	11899

643.61218	4	11903

643.75152	3	11906

643.89086	3	11909

644.0302	1	11910

644.16954	1	11911

644.30888	11	11922

644.44822	1	11923

644.58756	10	11933

644.7269	1	11934

644.86624	2	11936

645.00558	4	11940

645.14492	10	11950

645.28426	1	11951

645.4236	2	11953

645.56295	2	11955

645.70229	3	11958

645.84163	1	11959

645.98097	2	11961

646.12031	3	11964

646.25965	1	11965

646.39899	1	11966

646.53833	5	11971

646.67767	1	11972

646.81701	3	11975

646.95635	4	11979

647.09569	1	11980

647.23503	1	11981

647.37437	1	11982

647.51371	1	11983

647.65305	4	11987

647.79239	1	11988

647.93173	6	11994

648.07107	13	12007

648.21041	3	12010

648.34975	7	12017

648.48909	1	12018

648.62843	1	12019

648.76777	5	12024

648.90711	6	12030

649.04645	2	12032

649.18579	1	12033

649.32513	3	12036

649.46447	2	12038

649.60381	2	12040

649.74315	4	12044

649.88249	24	12068

650.02183	2	12070

650.16117	1	12071

650.30051	2	12073

650.43985	11	12084

650.57919	3	12087

650.71853	6	12093

650.85787	4	12097

650.99721	1	12098

651.13655	1	12099

651.27589	1	12100

651.41523	6	12106

651.55457	1	12107

651.69391	2	12109

651.83325	4	12113

651.97259	1	12114

652.11193	2	12116

652.25127	4	12120

652.39061	5	12125

652.52995	1	12126

652.66929	3	12129

652.80863	1	12130

652.94797	22	12152

653.08731	2	12154

653.22665	3	12157

653.36599	4	12161

653.50533	2	12163

653.64467	5	12168

653.78401	2	12170

653.92335	14	12184

654.06269	1	12185

654.20203	4	12189

654.34137	1	12190

654.48072	4	12194

654.62006	3	12197

654.7594	2	12199

654.89874	1	12200

655.03808	2	12202

655.17742	4	12206

655.31676	8	12214

655.4561	5	12219

655.59544	2	12221

655.73478	1	12222

655.87412	1	12223

656.01346	1	12224

656.1528	1	12225

656.29214	1	12226

656.43148	6	12232

656.57082	3	12235

656.71016	1	12236

656.8495	4	12240

656.98884	1	12241

657.12818	3	12244

657.26752	2	12246

657.40686	1	12247

657.5462	2	12249

657.68554	12	12261

657.82488	3	12264

657.96422	1	12265

658.10356	6	12271

658.2429	1	12272

658.38224	3	12275

658.52158	1	12276

658.66092	5	12281

658.80026	1	12282

658.9396	5	12287

659.07894	1	12288

659.21828	1	12289

659.35762	2	12291

659.49696	4	12295

659.6363	1	12296

659.77564	4	12300

659.91498	9	12309

660.05432	1	12310

660.19366	9	12319

660.333	3	12322

660.47234	3	12325

660.61168	2	12327

660.75102	4	12331

660.89036	3	12334

661.0297	8	12342

661.16904	4	12346

661.30838	2	12348

661.44772	5	12353

661.58706	3	12356

661.7264	1	12357

661.86574	1	12358

662.00508	1	12359

662.14442	5	12364

662.28376	1	12365

662.4231	2	12367

662.56244	1	12368

662.70178	2	12370

662.84112	3	12373

662.98046	1	12374

663.1198	12	12386

663.25914	1	12387

663.39849	3	12390

663.53783	1	12391

663.67717	8	12399

663.81651	1	12400

663.95585	12	12412

664.09519	3	12415

664.23453	1	12416

664.37387	1	12417

664.51321	6	12423

664.65255	1	12424

664.79189	1	12425

664.93123	1	12426

665.07057	1	12427

665.20991	1	12428

665.34925	3	12431

665.48859	2	12433

665.62793	1	12434

665.76727	1	12435

665.90661	1	12436

666.04595	8	12444

666.18529	2	12446

666.32463	2	12448

666.46397	1	12449

666.60331	2	12451

666.74265	6	12457

666.88199	1	12458

667.02133	1	12459

667.16067	3	12462

667.30001	1	12463

667.43935	1	12464

667.57869	3	12467

667.71803	1	12468

667.85737	5	12473

667.99671	1	12474

668.13605	4	12478

668.27539	9	12487

668.41473	1	12488

668.55407	1	12489

668.69341	3	12492

668.83275	2	12494

668.97209	6	12500

669.11143	2	12502

669.25077	4	12506

669.39011	1	12507

669.52945	7	12514

669.66879	1	12515

669.80813	1	12516

669.94747	19	12535

670.08681	1	12536

670.22615	1	12537

670.36549	10	12547

670.50483	1	12548

670.64417	1	12549

670.78351	1	12550

670.92285	4	12554

671.06219	4	12558

671.20153	2	12560

671.34087	1	12561

671.48021	1	12562

671.61955	4	12566

671.75889	2	12568

671.89823	3	12571

672.03757	13	12584

672.17691	2	12586

672.31625	1	12587

672.4556	1	12588

672.59494	3	12591

672.73428	4	12595

672.87362	6	12601

673.01296	1	12602

673.1523	4	12606

673.29164	1	12607

673.43098	2	12609

673.57032	2	12611

673.70966	3	12614

673.849	6	12620

673.98834	3	12623

674.12768	1	12624

674.26702	1	12625

674.40636	2	12627

674.5457	3	12630

674.68504	4	12634

674.82438	2	12636

674.96372	1	12637

675.10306	2	12639

675.2424	2	12641

675.38174	1	12642

675.52108	8	12650

675.66042	1	12651

675.79976	1	12652

675.9391	5	12657

676.07844	4	12661

676.21778	1	12662

676.35712	5	12667

676.49646	2	12669

676.6358	2	12671

676.77514	1	12672

676.91448	1	12673

677.05382	2	12675

677.19316	1	12676

677.3325	3	12679

677.47184	5	12684

677.61118	1	12685

677.75052	5	12690

677.88986	2	12692

678.0292	11	12703

678.16854	4	12707

678.30788	1	12708

678.44722	1	12709

678.58656	1	12710

678.7259	2	12712

678.86524	3	12715

679.00458	1	12716

679.14392	1	12717

679.28326	2	12719

679.4226	1	12720

679.56194	6	12726

679.70128	4	12730

679.84062	2	12732

679.97996	2	12734

680.1193	3	12737

680.25864	5	12742

680.39798	5	12747

680.53732	1	12748

680.67666	1	12749

680.816	3	12752

680.95534	3	12755

681.09468	1	12756

681.23402	6	12762

681.37337	2	12764

681.51271	18	12782

681.65205	1	12783

681.79139	6	12789

681.93073	1	12790

682.07007	1	12791

682.20941	2	12793

682.34875	1	12794

682.48809	1	12795

682.62743	3	12798

682.76677	4	12802

682.90611	2	12804

683.04545	2	12806

683.18479	2	12808

683.32413	2	12810

683.46347	1	12811

683.60281	4	12815

683.74215	2	12817

683.88149	1	12818

684.02083	9	12827

684.16017	3	12830

684.29951	2	12832

684.43885	1	12833

684.57819	1	12834

684.71753	1	12835

684.85687	2	12837

684.99621	3	12840

685.13555	3	12843

685.27489	3	12846

685.41423	1	12847

685.55357	1	12848

685.69291	1	12849

685.83225	7	12856

685.97159	1	12857

686.11093	2	12859

686.25027	4	12863

686.38961	1	12864

686.52895	2	12866

686.66829	1	12867

686.80763	5	12872

686.94697	1	12873

687.08631	2	12875

687.22565	2	12877

687.36499	1	12878

687.50433	5	12883

687.64367	2	12885

687.78301	3	12888

687.92235	3	12891

688.06169	1	12892

688.20103	1	12893

688.34037	1	12894

688.47971	1	12895

688.61905	1	12896

688.75839	1	12897

688.89773	4	12901

689.03707	2	12903

689.17641	2	12905

689.31575	3	12908

689.45509	3	12911

689.59443	3	12914

689.73377	1	12915

689.87311	1	12916

690.01245	3	12919

690.15179	1	12920

690.29114	4	12924

690.43048	3	12927

690.56982	1	12928

690.70916	2	12930

690.8485	7	12937

690.98784	4	12941

691.12718	4	12945

691.26652	3	12948

691.40586	1	12949

691.5452	1	12950

691.68454	2	12952

691.82388	2	12954

691.96322	1	12955

692.10256	1	12956

692.2419	4	12960

692.38124	1	12961

692.52058	1	12962

692.65992	9	12971

692.79926	3	12974

692.9386	1	12975

693.07794	2	12977

693.21728	3	12980

693.35662	2	12982

693.49596	6	12988

693.6353	2	12990

693.77464	1	12991

693.91398	2	12993

694.05332	9	13002

694.19266	2	13004

694.332	3	13007

694.47134	1	13008

694.61068	1	13009

694.75002	20	13029

694.88936	4	13033

695.0287	1	13034

695.16804	3	13037

695.30738	1	13038

695.44672	1	13039

695.58606	1	13040

695.7254	2	13042

695.86474	5	13047

696.00408	1	13048

696.14342	1	13049

696.28276	6	13055

696.4221	7	13062

696.56144	1	13063

696.70078	1	13064

696.84012	5	13069

696.97946	11	13080

697.1188	3	13083

697.25814	1	13084

697.39748	2	13086

697.53682	4	13090

697.67616	1	13091

697.8155	1	13092

697.95484	14	13106

698.09418	5	13111

698.23352	5	13116

698.37286	2	13118

698.5122	1	13119

698.65154	4	13123

698.79088	1	13124

698.93022	1	13125

699.06957	4	13129

699.20891	1	13130

699.34825	9	13139

699.48759	5	13144

699.62693	4	13148

699.76627	1	13149

699.90561	5	13154

700.04495	2	13156

700.18429	1	13157

700.32363	3	13160

700.46297	2	13162

700.60231	1	13163

700.74165	1	13164

700.88099	3	13167

701.02033	2	13169

701.15967	5	13174

701.29901	5	13179

701.43835	4	13183

701.57769	1	13184

701.71703	2	13186

701.85637	1	13187

701.99571	2	13189

702.13505	3	13192

702.27439	11	13203

702.41373	1	13204

702.55307	6	13210

702.69241	2	13212

702.83175	5	13217

702.97109	2	13219

703.11043	2	13221

703.24977	3	13224

703.38911	2	13226

703.52845	2	13228

703.66779	2	13230

703.80713	1	13231

703.94647	1	13232

704.08581	2	13234

704.22515	2	13236

704.36449	1	13237

704.50383	2	13239

704.64317	2	13241

704.78251	2	13243

704.92185	1	13244

705.06119	1	13245

705.20053	2	13247

705.33987	7	13254

705.47921	4	13258

705.61855	2	13260

705.75789	8	13268

705.89723	1	13269

706.03657	1	13270

706.17591	1	13271

706.31525	7	13278

706.45459	1	13279

706.59393	1	13280

706.73327	2	13282

706.87261	2	13284

707.01195	3	13287

707.15129	1	13288

707.29063	2	13290

707.42997	1	13291

707.56931	4	13295

707.70865	4	13299

707.84799	1	13300

707.98734	2	13302

708.12668	9	13311

708.26602	2	13313

708.40536	4	13317

708.5447	2	13319

708.68404	1	13320

708.82338	1	13321

708.96272	1	13322

709.10206	2	13324

709.2414	3	13327

709.38074	1	13328

709.52008	3	13331

709.65942	3	13334

709.79876	3	13337

709.9381	6	13343

710.07744	6	13349

710.21678	8	13357

710.35612	1	13358

710.49546	1	13359

710.6348	1	13360

710.77414	1	13361

710.91348	1	13362

711.05282	1	13363

711.19216	3	13366

711.3315	4	13370

711.47084	1	13371

711.61018	5	13376

711.74952	8	13384

711.88886	2	13386

712.0282	2	13388

712.16754	2	13390

712.30688	1	13391

712.44622	1	13392

712.58556	1	13393

712.7249	6	13399

712.86424	2	13401

713.00358	4	13405

713.14292	1	13406

713.28226	9	13415

713.4216	1	13416

713.56094	6	13422

713.70028	2	13424

713.83962	2	13426

713.97896	5	13431

714.1183	14	13445

714.25764	1	13446

714.39698	1	13447

714.53632	3	13450

714.67566	1	13451

714.815	1	13452

714.95434	3	13455

715.09368	1	13456

715.23302	3	13459

715.37236	1	13460

715.5117	1	13461

715.65104	2	13463

715.79038	1	13464

715.92972	3	13467

716.06906	5	13472

716.2084	6	13478

716.34774	1	13479

716.48708	3	13482

716.62642	1	13483

716.76576	1	13484

716.90511	5	13489

717.04445	4	13493

717.18379	3	13496

717.32313	2	13498

717.46247	1	13499

717.60181	1	13500

717.74115	1	13501

717.88049	8	13509

718.01983	6	13515

718.15917	1	13516

718.29851	4	13520

718.43785	7	13527

718.57719	5	13532

718.71653	4	13536

718.85587	4	13540

718.99521	10	13550

719.13455	2	13552

719.27389	2	13554

719.41323	3	13557

719.55257	1	13558

719.69191	2	13560

719.83125	24	13584

719.97059	2	13586

720.10993	8	13594

720.24927	2	13596

720.38861	1	13597

720.52795	1	13598

720.66729	1	13599

720.80663	1	13600

720.94597	18	13618

721.08531	1	13619

721.22465	3	13622

721.36399	2	13624

721.50333	3	13627

721.64267	8	13635

721.78201	2	13637

721.92135	1	13638

722.06069	1	13639

722.20003	17	13656

722.33937	6	13662

722.47871	1	13663

722.61805	3	13666

722.75739	1	13667

722.89673	2	13669

723.03607	5	13674

723.17541	1	13675

723.31475	1	13676

723.45409	2	13678

723.59343	1	13679

723.73277	2	13681

723.87211	1	13682

724.01145	2	13684

724.15079	1	13685

724.29013	1	13686

724.42947	4	13690

724.56881	2	13692

724.70815	1	13693

724.84749	7	13700

724.98683	4	13704

725.12617	3	13707

725.26551	5	13712

725.40485	4	13716

725.54419	9	13725

725.68353	6	13731

725.82288	4	13735

725.96222	1	13736

726.10156	1	13737

726.2409	6	13743

726.38024	1	13744

726.51958	1	13745

726.65892	5	13750

726.79826	2	13752

726.9376	1	13753

727.07694	1	13754

727.21628	3	13757

727.35562	6	13763

727.49496	4	13767

727.6343	3	13770

727.77364	5	13775

727.91298	17	13792

728.05232	5	13797

728.19166	3	13800

728.331	5	13805

728.47034	1	13806

728.60968	5	13811

728.74902	2	13813

728.88836	1	13814

729.0277	2	13816

729.16704	6	13822

729.30638	4	13826

729.44572	5	13831

729.58506	3	13834

729.7244	4	13838

729.86374	11	13849

730.00308	5	13854

730.14242	2	13856

730.28176	4	13860

730.4211	3	13863

730.56044	6	13869

730.69978	4	13873

730.83912	1	13874

730.97846	5	13879

731.1178	1	13880

731.25714	2	13882

731.39648	2	13884

731.53582	13	13897

731.67516	1	13898

731.8145	13	13911

731.95384	1	13912

732.09318	1	13913

732.23252	1	13914

732.37186	4	13918

732.5112	1	13919

732.65054	2	13921

732.78988	1	13922

732.92922	8	13930

733.06856	5	13935

733.2079	2	13937

733.34724	9	13946

733.48658	3	13949

733.62592	1	13950

733.76526	1	13951

733.9046	3	13954

734.04394	4	13958

734.18328	1	13959

734.32262	1	13960

734.46196	1	13961

734.6013	2	13963

734.74065	1	13964

734.87999	2	13966

735.01933	1	13967

735.15867	2	13969

735.29801	3	13972

735.43735	2	13974

735.57669	8	13982

735.71603	2	13984

735.85537	1	13985

735.99471	11	13996

736.13405	2	13998

736.27339	1	13999

736.41273	1	14000

736.55207	11	14011

736.69141	1	14012

736.83075	1	14013

736.97009	1	14014

737.10943	1	14015

737.24877	2	14017

737.38811	2	14019

737.52745	1	14020

737.66679	1	14021

737.80613	1	14022

737.94547	3	14025

738.08481	12	14037

738.22415	2	14039

738.36349	1	14040

738.50283	2	14042

738.64217	2	14044

738.78151	2	14046

738.92085	2	14048

739.06019	2	14050

739.19953	1	14051

739.33887	7	14058

739.47821	3	14061

739.61755	2	14063

739.75689	1	14064

739.89623	4	14068

740.03557	3	14071

740.17491	2	14073

740.31425	1	14074

740.45359	1	14075

740.59293	1	14076

740.73227	1	14077

740.87161	2	14079

741.01095	4	14083

741.15029	2	14085

741.28963	1	14086

741.42897	3	14089

741.56831	1	14090

741.70765	6	14096

741.84699	3	14099

741.98633	1	14100

742.12567	1	14101

742.26501	9	14110

742.40435	1	14111

742.54369	2	14113

742.68303	2	14115

742.82237	1	14116

742.96171	3	14119

743.10105	5	14124

743.24039	2	14126

743.37973	1	14127

743.51908	1	14128

743.65842	1	14129

743.79776	1	14130

743.9371	2	14132

744.07644	13	14145

744.21578	1	14146

744.35512	2	14148

744.49446	2	14150

744.6338	2	14152

744.77314	3	14155

744.91248	1	14156

745.05182	7	14163

745.19116	1	14164

745.3305	3	14167

745.46984	1	14168

745.60918	2	14170

745.74852	8	14178

745.88786	2	14180

746.0272	2	14182

746.16654	13	14195

746.30588	1	14196

746.44522	2	14198

746.58456	1	14199

746.7239	5	14204

746.86324	2	14206

747.00258	4	14210

747.14192	6	14216

747.28126	3	14219

747.4206	1	14220

747.55994	2	14222

747.69928	4	14226

747.83862	1	14227

747.97796	3	14230

748.1173	4	14234

748.25664	1	14235

748.39598	1	14236

748.53532	7	14243

748.67466	3	14246

748.814	1	14247

748.95334	2	14249

749.09268	1	14250

749.23202	1	14251

749.37136	1	14252

749.5107	1	14253

749.65004	1	14254

749.78938	1	14255

749.92872	3	14258

750.06806	2	14260

750.2074	6	14266

750.34674	1	14267

750.48608	4	14271

750.62542	3	14274

750.76476	2	14276

750.9041	3	14279

751.04344	3	14282

751.18278	6	14288

751.32212	2	14290

751.46146	2	14292

751.6008	5	14297

751.74014	3	14300

751.87948	4	14304

752.01882	1	14305

752.15816	2	14307

752.2975	1	14308

752.43684	2	14310

752.57619	7	14317

752.71553	2	14319

752.85487	4	14323

752.99421	3	14326

753.13355	2	14328

753.27289	1	14329

753.41223	2	14331

753.55157	6	14337

753.69091	2	14339

753.83025	1	14340

753.96959	5	14345

754.10893	1	14346

754.24827	1	14347

754.38761	1	14348

754.52695	2	14350

754.66629	1	14351

754.80563	1	14352

754.94497	5	14357

755.08431	2	14359

755.22365	5	14364

755.36299	9	14373

755.50233	2	14375

755.64167	1	14376

755.78101	11	14387

755.92035	3	14390

756.05969	1	14391

756.19903	3	14394

756.33837	1	14395

756.47771	2	14397

756.61705	1	14398

756.75639	1	14399

756.89573	5	14404

757.03507	4	14408

757.17441	2	14410

757.31375	1	14411

757.45309	5	14416

757.59243	3	14419

757.73177	2	14421

757.87111	3	14424

758.01045	6	14430

758.14979	1	14431

758.28913	2	14433

758.42847	4	14437

758.56781	6	14443

758.70715	1	14444

758.84649	5	14449

758.98583	12	14461

759.12517	1	14462

759.26451	1	14463

759.40385	2	14465

759.54319	1	14466

759.68253	1	14467

759.82187	4	14471

759.96121	1	14472

760.10055	4	14476

760.23989	1	14477

760.37923	2	14479

760.51857	6	14485

760.65791	1	14486

760.79725	4	14490

760.93659	5	14495

761.07593	1	14496

761.21527	4	14500

761.35461	1	14501

761.49396	7	14508

761.6333	5	14513

761.77264	3	14516

761.91198	5	14521

762.05132	1	14522

762.19066	12	14534

762.33	11	14545

762.46934	2	14547

762.60868	3	14550

762.74802	2	14552

762.88736	4	14556

763.0267	2	14558

763.16604	1	14559

763.30538	2	14561

763.44472	4	14565

763.58406	3	14568

763.7234	3	14571

763.86274	1	14572

764.00208	1	14573

764.14142	3	14576

764.28076	6	14582

764.4201	2	14584

764.55944	2	14586

764.69878	8	14594

764.83812	1	14595

764.97746	5	14600

765.1168	1	14601

765.25614	6	14607

765.39548	2	14609

765.53482	5	14614

765.67416	2	14616

765.8135	3	14619

765.95284	6	14625

766.09218	7	14632

766.23152	14	14646

766.37086	1	14647

766.5102	3	14650

766.64954	1	14651

766.78888	6	14657

766.92822	1	14658

767.06756	1	14659

767.2069	2	14661

767.34624	2	14663

767.48558	3	14666

767.62492	2	14668

767.76426	7	14675

767.9036	6	14681

768.04294	1	14682

768.18228	1	14683

768.32162	10	14693

768.46096	1	14694

768.6003	1	14695

768.73964	1	14696

768.87898	7	14703

769.01832	1	14704

769.15766	3	14707

769.297	3	14710

769.43634	1	14711

769.57568	1	14712

769.71502	1	14713

769.85436	1	14714

769.9937	1	14715

770.13304	1	14716

770.27238	1	14717

770.41173	1	14718

770.55107	2	14720

770.69041	1	14721

770.82975	5	14726

770.96909	4	14730

771.10843	2	14732

771.24777	1	14733

771.38711	2	14735

771.52645	1	14736

771.66579	15	14751

771.80513	7	14758

771.94447	5	14763

772.08381	3	14766

772.22315	3	14769

772.36249	5	14774

772.50183	3	14777

772.64117	3	14780

772.78051	3	14783

772.91985	2	14785

773.05919	2	14787

773.19853	1	14788

773.33787	6	14794

773.47721	1	14795

773.61655	1	14796

773.75589	1	14797

773.89523	2	14799

774.03457	1	14800

774.17391	2	14802

774.31325	4	14806

774.45259	1	14807

774.59193	2	14809

774.73127	6	14815

774.87061	2	14817

775.00995	4	14821

775.14929	1	14822

775.28863	11	14833

775.42797	9	14842

775.56731	2	14844

775.70665	1	14845

775.84599	1	14846

775.98533	1	14847

776.12467	4	14851

776.26401	1	14852

776.40335	12	14864

776.54269	3	14867

776.68203	5	14872

776.82137	4	14876

776.96071	1	14877

777.10005	2	14879

777.23939	1	14880

777.37873	8	14888

777.51807	11	14899

777.65741	9	14908

777.79675	1	14909

777.93609	5	14914

778.07543	3	14917

778.21477	3	14920

778.35411	1	14921

778.49345	3	14924

778.63279	3	14927

778.77213	7	14934

778.91147	4	14938

779.05081	1	14939

779.19016	1	14940

779.3295	8	14948

779.46884	6	14954

779.60818	4	14958

779.74752	3	14961

779.88686	1	14962

780.0262	5	14967

780.16554	1	14968

780.30488	3	14971

780.44422	2	14973

780.58356	2	14975

780.7229	4	14979

780.86224	1	14980

781.00158	2	14982

781.14092	4	14986

781.28026	8	14994

781.4196	3	14997

781.55894	3	15000

781.69828	19	15019

781.83762	3	15022

781.97696	1	15023

782.1163	3	15026

782.25564	1	15027

782.39498	4	15031

782.53432	1	15032

782.67366	2	15034

782.813	3	15037

782.95234	1	15038

783.09168	1	15039

783.23102	3	15042

783.37036	2	15044

783.5097	2	15046

783.64904	1	15047

783.78838	3	15050

783.92772	2	15052

784.06706	20	15072

784.2064	3	15075

784.34574	1	15076

784.48508	5	15081

784.62442	8	15089

784.76376	2	15091

784.9031	3	15094

785.04244	6	15100

785.18178	1	15101

785.32112	10	15111

785.46046	1	15112

785.5998	4	15116

785.73914	12	15128

785.87848	1	15129

786.01782	1	15130

786.15716	11	15141

786.2965	5	15146

786.43584	1	15147

786.57518	2	15149

786.71452	1	15150

786.85386	4	15154

786.9932	1	15155

787.13254	8	15163

787.27188	1	15164

787.41122	13	15177

787.55056	2	15179

787.6899	4	15183

787.82924	3	15186

787.96858	1	15187

788.10793	3	15190

788.24727	4	15194

788.38661	5	15199

788.52595	3	15202

788.66529	2	15204

788.80463	10	15214

788.94397	2	15216

789.08331	5	15221

789.22265	1	15222

789.36199	2	15224

789.50133	3	15227

789.64067	2	15229

789.78001	4	15233

789.91935	3	15236

790.05869	2	15238

790.19803	2	15240

790.33737	1	15241

790.47671	3	15244

790.61605	1	15245

790.75539	4	15249

790.89473	2	15251

791.03407	1	15252

791.17341	5	15257

791.31275	2	15259

791.45209	1	15260

791.59143	4	15264

791.73077	3	15267

791.87011	2	15269

792.00945	2	15271

792.14879	1	15272

792.28813	1	15273

792.42747	2	15275

792.56681	1	15276

792.70615	2	15278

792.84549	4	15282

792.98483	1	15283

793.12417	3	15286

793.26351	1	15287

793.40285	4	15291

793.54219	3	15294

793.68153	3	15297

793.82087	2	15299

793.96021	4	15303

794.09955	1	15304

794.23889	2	15306

794.37823	1	15307

794.51757	3	15310

794.65691	1	15311

794.79625	4	15315

794.93559	2	15317

795.07493	14	15331

795.21427	3	15334

795.35361	2	15336

795.49295	2	15338

795.63229	3	15341

795.77163	2	15343

795.91097	2	15345

796.05031	1	15346

796.18965	5	15351

796.32899	2	15353

796.46833	1	15354

796.60767	1	15355

796.74701	1	15356

796.88635	2	15358

797.0257	1	15359

797.16504	1	15360

797.30438	2	15362

797.44372	3	15365

797.58306	3	15368

797.7224	1	15369

797.86174	4	15373

798.00108	1	15374

798.14042	1	15375

798.27976	2	15377

798.4191	1	15378

798.55844	1	15379

798.69778	2	15381

798.83712	10	15391

798.97646	5	15396

799.1158	2	15398

799.25514	2	15400

799.39448	2	15402

799.53382	5	15407

799.67316	1	15408

799.8125	1	15409

799.95184	2	15411

800.09118	4	15415

800.23052	1	15416

800.36986	2	15418

800.5092	6	15424

800.64854	2	15426

800.78788	2	15428

800.92722	1	15429

801.06656	1	15430

801.2059	1	15431

801.34524	2	15433

801.48458	2	15435

801.62392	5	15440

801.76326	7	15447

801.9026	1	15448

802.04194	6	15454

802.18128	3	15457

802.32062	8	15465

802.45996	2	15467

802.5993	11	15478

802.73864	8	15486

802.87798	1	15487

803.01732	1	15488

803.15666	1	15489

803.296	2	15491

803.43534	3	15494

803.57468	1	15495

803.71402	2	15497

803.85336	2	15499

803.9927	1	15500

804.13204	3	15503

804.27138	3	15506

804.41072	2	15508

804.55006	1	15509

804.6894	2	15511

804.82874	1	15512

804.96808	9	15521

805.10742	7	15528

805.24676	2	15530

805.3861	1	15531

805.52544	9	15540

805.66478	5	15545

805.80412	1	15546

805.94347	1	15547

806.08281	17	15564

806.22215	2	15566

806.36149	1	15567

806.50083	2	15569

806.64017	1	15570

806.77951	5	15575

806.91885	2	15577

807.05819	6	15583

807.19753	2	15585

807.33687	1	15586

807.47621	3	15589

807.61555	2	15591

807.75489	1	15592

807.89423	1	15593

808.03357	1	15594

808.17291	4	15598

808.31225	2	15600

808.45159	1	15601

808.59093	2	15603

808.73027	1	15604

808.86961	1	15605

809.00895	2	15607

809.14829	1	15608

809.28763	4	15612

809.42697	1	15613

809.56631	1	15614

809.70565	1	15615

809.84499	4	15619

809.98433	3	15622

810.12367	2	15624

810.26301	5	15629

810.40235	1	15630

810.54169	3	15633

810.68103	5	15638

810.82037	2	15640

810.95971	2	15642

811.09905	4	15646

811.23839	1	15647

811.37773	6	15653

811.51707	4	15657

811.65641	7	15664

811.79575	7	15671

811.93509	2	15673

812.07443	1	15674

812.21377	2	15676

812.35311	2	15678

812.49245	1	15679

812.63179	3	15682

812.77113	2	15684

812.91047	3	15687

813.04981	1	15688

813.18915	9	15697

813.32849	2	15699

813.46783	2	15701

813.60717	7	15708

813.74651	10	15718

813.88585	2	15720

814.02519	2	15722

814.16453	3	15725

814.30387	2	15727

814.44321	1	15728

814.58255	2	15730

814.72189	2	15732

814.86124	3	15735

815.00058	6	15741

815.13992	3	15744

815.27926	2	15746

815.4186	3	15749

815.55794	8	15757

815.69728	2	15759

815.83662	3	15762

815.97596	2	15764

816.1153	1	15765

816.25464	1	15766

816.39398	6	15772

816.53332	1	15773

816.67266	7	15780

816.812	1	15781

816.95134	1	15782

817.09068	1	15783

817.23002	2	15785

817.36936	1	15786

817.5087	3	15789

817.64804	4	15793

817.78738	4	15797

817.92672	1	15798

818.06606	1	15799

818.2054	1	15800

818.34474	10	15810

818.48408	1	15811

818.62342	1	15812

818.76276	1	15813

818.9021	10	15823

819.04144	4	15827

819.18078	2	15829

819.32012	3	15832

819.45946	1	15833

819.5988	2	15835

819.73814	1	15836

819.87748	1	15837

820.01682	1	15838

820.15616	1	15839

820.2955	1	15840

820.43484	2	15842

820.57418	1	15843

820.71352	11	15854

820.85286	1	15855

820.9922	1	15856

821.13154	1	15857

821.27088	1	15858

821.41022	6	15864

821.54956	1	15865

821.6889	1	15866

821.82824	1	15867

821.96758	3	15870

822.10692	1	15871

822.24626	10	15881

822.3856	4	15885

822.52494	1	15886

822.66428	2	15888

822.80362	10	15898

822.94296	1	15899

823.0823	2	15901

823.22164	4	15905

823.36098	4	15909

823.50032	6	15915

823.63966	3	15918

823.77901	1	15919

823.91835	1	15920

824.05769	3	15923

824.19703	1	15924

824.33637	1	15925

824.47571	3	15928

824.61505	3	15931

824.75439	1	15932

824.89373	3	15935

825.03307	2	15937

825.17241	1	15938

825.31175	2	15940

825.45109	1	15941

825.59043	1	15942

825.72977	1	15943

825.86911	7	15950

826.00845	1	15951

826.14779	2	15953

826.28713	14	15967

826.42647	1	15968

826.56581	2	15970

826.70515	1	15971

826.84449	2	15973

826.98383	12	15985

827.12317	1	15986

827.26251	1	15987

827.40185	5	15992

827.54119	2	15994

827.68053	1	15995

827.81987	2	15997

827.95921	8	16005

828.09855	1	16006

828.23789	1	16007

828.37723	7	16014

828.51657	2	16016

828.65591	2	16018

828.79525	4	16022

828.93459	1	16023

829.07393	1	16024

829.21327	6	16030

829.35261	1	16031

829.49195	1	16032

829.63129	1	16033

829.77063	1	16034

829.90997	3	16037

830.04931	9	16046

830.18865	4	16050

830.32799	1	16051

830.46733	1	16052

830.60667	13	16065

830.74601	1	16066

830.88535	2	16068

831.02469	2	16070

831.16403	1	16071

831.30337	7	16078

831.44271	11	16089

831.58205	2	16091

831.72139	8	16099

831.86073	2	16101

832.00007	3	16104

832.13941	6	16110

832.27875	3	16113

832.41809	1	16114

832.55743	4	16118

832.69678	2	16120

832.83612	1	16121

832.97546	2	16123

833.1148	1	16124

833.25414	4	16128

833.39348	3	16131

833.53282	3	16134

833.67216	2	16136

833.8115	7	16143

833.95084	1	16144

834.09018	2	16146

834.22952	3	16149

834.36886	15	16164

834.5082	1	16165

834.64754	1	16166

834.78688	6	16172

834.92622	1	16173

835.06556	2	16175

835.2049	1	16176

835.34424	1	16177

835.48358	3	16180

835.62292	1	16181

835.76226	2	16183

835.9016	2	16185

836.04094	3	16188

836.18028	7	16195

836.31962	1	16196

836.45896	1	16197

836.5983	5	16202

836.73764	1	16203

836.87698	3	16206

837.01632	3	16209

837.15566	4	16213

837.295	1	16214

837.43434	1	16215

837.57368	4	16219

837.71302	1	16220

837.85236	1	16221

837.9917	2	16223

838.13104	2	16225

838.27038	2	16227

838.40972	1	16228

838.54906	3	16231

838.6884	1	16232

838.82774	3	16235

838.96708	1	16236

839.10642	3	16239

839.24576	4	16243

839.3851	8	16251

839.52444	2	16253

839.66378	2	16255

839.80312	1	16256

839.94246	5	16261

840.0818	1	16262

840.22114	1	16263

840.36048	1	16264

840.49982	1	16265

840.63916	3	16268

840.7785	1	16269

840.91784	1	16270

841.05718	4	16274

841.19652	2	16276

841.33586	4	16280

841.4752	2	16282

841.61455	2	16284

841.75389	13	16297

841.89323	3	16300

842.03257	1	16301

842.17191	1	16302

842.31125	16	16318

842.45059	3	16321

842.58993	3	16324

842.72927	3	16327

842.86861	1	16328

843.00795	7	16335

843.14729	2	16337

843.28663	2	16339

843.42597	1	16340

843.56531	15	16355

843.70465	1	16356

843.84399	1	16357

843.98333	3	16360

844.12267	1	16361

844.26201	1	16362

844.40135	1	16363

844.54069	2	16365

844.68003	2	16367

844.81937	11	16378

844.95871	2	16380

845.09805	3	16383

845.23739	5	16388

845.37673	2	16390

845.51607	2	16392

845.65541	1	16393

845.79475	3	16396

845.93409	8	16404

846.07343	2	16406

846.21277	1	16407

846.35211	1	16408

846.49145	3	16411

846.63079	2	16413

846.77013	1	16414

846.90947	1	16415

847.04881	3	16418

847.18815	5	16423

847.32749	1	16424

847.46683	1	16425

847.60617	1	16426

847.74551	3	16429

847.88485	3	16432

848.02419	2	16434

848.16353	3	16437

848.30287	1	16438

848.44221	2	16440

848.58155	2	16442

848.72089	11	16453

848.86023	1	16454

848.99957	1	16455

849.13891	10	16465

849.27825	3	16468

849.41759	3	16471

849.55693	2	16473

849.69627	5	16478

849.83561	2	16480

849.97495	2	16482

850.11429	2	16484

850.25363	2	16486

850.39297	2	16488

850.53232	1	16489

850.67166	1	16490

850.811	6	16496

850.95034	1	16497

851.08968	2	16499

851.22902	6	16505

851.36836	1	16506

851.5077	6	16512

851.64704	2	16514

851.78638	1	16515

851.92572	2	16517

852.06506	10	16527

852.2044	3	16530

852.34374	1	16531

852.48308	1	16532

852.62242	2	16534

852.76176	4	16538

852.9011	11	16549

853.04044	6	16555

853.17978	3	16558

853.31912	17	16575

853.45846	1	16576

853.5978	1	16577

853.73714	4	16581

853.87648	2	16583

854.01582	1	16584

854.15516	8	16592

854.2945	2	16594

854.43384	2	16596

854.57318	2	16598

854.71252	2	16600

854.85186	9	16609

854.9912	1	16610

855.13054	2	16612

855.26988	3	16615

855.40922	4	16619

855.54856	1	16620

855.6879	9	16629

855.82724	2	16631

855.96658	11	16642

856.10592	3	16645

856.24526	1	16646

856.3846	1	16647

856.52394	1	16648

856.66328	2	16650

856.80262	3	16653

856.94196	9	16662

857.0813	3	16665

857.22064	1	16666

857.35998	2	16668

857.49932	8	16676

857.63866	6	16682

857.778	4	16686

857.91734	1	16687

858.05668	5	16692

858.19602	9	16701

858.33536	2	16703

858.4747	1	16704

858.61404	1	16705

858.75338	7	16712

858.89272	5	16717

859.03206	6	16723

859.1714	1	16724

859.31075	3	16727

859.45009	1	16728

859.58943	1	16729

859.72877	10	16739

859.86811	10	16749

860.00745	1	16750

860.14679	1	16751

860.28613	2	16753

860.42547	1	16754

860.56481	2	16756

860.70415	9	16765

860.84349	2	16767

860.98283	4	16771

861.12217	13	16784

861.26151	1	16785

861.40085	1	16786

861.54019	1	16787

861.67953	7	16794

861.81887	5	16799

861.95821	6	16805

862.09755	2	16807

862.23689	9	16816

862.37623	3	16819

862.51557	4	16823

862.65491	1	16824

862.79425	1	16825

862.93359	8	16833

863.07293	3	16836

863.21227	3	16839

863.35161	2	16841

863.49095	3	16844

863.63029	1	16845

863.76963	1	16846

863.90897	1	16847

864.04831	2	16849

864.18765	1	16850

864.32699	4	16854

864.46633	2	16856

864.60567	2	16858

864.74501	2	16860

864.88435	2	16862

865.02369	2	16864

865.16303	1	16865

865.30237	1	16866

865.44171	4	16870

865.58105	7	16877

865.72039	12	16889

865.85973	4	16893

865.99907	6	16899

866.13841	3	16902

866.27775	1	16903

866.41709	10	16913

866.55643	1	16914

866.69577	8	16922

866.83511	3	16925

866.97445	1	16926

867.11379	3	16929

867.25313	1	16930

867.39247	1	16931

867.53181	1	16932

867.67115	2	16934

867.81049	1	16935

867.94983	1	16936

868.08917	2	16938

868.22852	4	16942

868.36786	1	16943

868.5072	1	16944

868.64654	1	16945

868.78588	1	16946

868.92522	2	16948

869.06456	1	16949

869.2039	2	16951

869.34324	1	16952

869.48258	1	16953

869.62192	1	16954

869.76126	1	16955

869.9006	4	16959

870.03994	1	16960

870.17928	9	16969

870.31862	1	16970

870.45796	1	16971

870.5973	3	16974

870.73664	1	16975

870.87598	2	16977

871.01532	8	16985

871.15466	1	16986

871.294	1	16987

871.43334	3	16990

871.57268	4	16994

871.71202	6	17000

871.85136	3	17003

871.9907	13	17016

872.13004	4	17020

872.26938	3	17023

872.40872	1	17024

872.54806	1	17025

872.6874	1	17026

872.82674	1	17027

872.96608	5	17032

873.10542	1	17033

873.24476	1	17034

873.3841	3	17037

873.52344	1	17038

873.66278	2	17040

873.80212	13	17053

873.94146	1	17054

874.0808	1	17055

874.22014	1	17056

874.35948	1	17057

874.49882	1	17058

874.63816	1	17059

874.7775	7	17066

874.91684	1	17067

875.05618	2	17069

875.19552	1	17070

875.33486	1	17071

875.4742	10	17081

875.61354	8	17089

875.75288	2	17091

875.89222	1	17092

876.03156	1	17093

876.1709	12	17105

876.31024	3	17108

876.44958	11	17119

876.58892	1	17120

876.72826	1	17121

876.8676	2	17123

877.00694	5	17128

877.14629	1	17129

877.28563	2	17131

877.42497	1	17132

877.56431	1	17133

877.70365	1	17134

877.84299	2	17136

877.98233	4	17140

878.12167	1	17141

878.26101	1	17142

878.40035	7	17149

878.53969	1	17150

878.67903	4	17154

878.81837	4	17158

878.95771	1	17159

879.09705	2	17161

879.23639	1	17162

879.37573	6	17168

879.51507	4	17172

879.65441	1	17173

879.79375	5	17178

879.93309	1	17179

880.07243	5	17184

880.21177	3	17187

880.35111	2	17189

880.49045	1	17190

880.62979	4	17194

880.76913	1	17195

880.90847	1	17196

881.04781	2	17198

881.18715	7	17205

881.32649	7	17212

881.46583	6	17218

881.60517	14	17232

881.74451	1	17233

881.88385	1	17234

882.02319	1	17235

882.16253	1	17236

882.30187	5	17241

882.44121	5	17246

882.58055	1	17247

882.71989	1	17248

882.85923	3	17251

882.99857	6	17257

883.13791	8	17265

883.27725	3	17268

883.41659	4	17272

883.55593	3	17275

883.69527	1	17276

883.83461	1	17277

883.97395	2	17279

884.11329	2	17281

884.25263	7	17288

884.39197	5	17293

884.53131	1	17294

884.67065	3	17297

884.80999	4	17301

884.94933	2	17303

885.08867	2	17305

885.22801	3	17308

885.36735	3	17311

885.50669	2	17313

885.64603	2	17315

885.78537	1	17316

885.92471	2	17318

886.06406	2	17320

886.2034	5	17325

886.34274	3	17328

886.48208	1	17329

886.62142	3	17332

886.76076	3	17335

886.9001	3	17338

887.03944	1	17339

887.17878	1	17340

887.31812	7	17347

887.45746	3	17350

887.5968	1	17351

887.73614	4	17355

887.87548	1	17356

888.01482	2	17358

888.15416	1	17359

888.2935	5	17364

888.43284	6	17370

888.57218	1	17371

888.71152	1	17372

888.85086	2	17374

888.9902	1	17375

889.12954	1	17376

889.26888	1	17377

889.40822	1	17378

889.54756	1	17379

889.6869	1	17380

889.82624	1	17381

889.96558	1	17382

890.10492	1	17383

890.24426	2	17385

890.3836	3	17388

890.52294	4	17392

890.66228	2	17394

890.80162	1	17395

890.94096	5	17400

891.0803	1	17401

891.21964	1	17402

891.35898	1	17403

891.49832	2	17405

891.63766	1	17406

891.777	7	17413

891.91634	5	17418

892.05568	1	17419

892.19502	10	17429

892.33436	1	17430

892.4737	8	17438

892.61304	5	17443

892.75238	2	17445

892.89172	9	17454

893.03106	2	17456

893.1704	4	17460

893.30974	6	17466

893.44908	2	17468

893.58842	5	17473

893.72776	6	17479

893.8671	6	17485

894.00644	1	17486

894.14578	6	17492

894.28512	6	17498

894.42446	5	17503

894.5638	1	17504

894.70314	2	17506

894.84248	1	17507

894.98182	7	17514

895.12117	1	17515

895.26051	2	17517

895.39985	1	17518

895.53919	1	17519

895.67853	7	17526

895.81787	2	17528

895.95721	1	17529

896.09655	1	17530

896.23589	3	17533

896.37523	3	17536

896.51457	3	17539

896.65391	2	17541

896.79325	1	17542

896.93259	1	17543

897.07193	1	17544

897.21127	4	17548

897.35061	2	17550

897.48995	1	17551

897.62929	3	17554

897.76863	5	17559

897.90797	3	17562

898.04731	2	17564

898.18665	3	17567

898.32599	7	17574

898.46533	1	17575

898.60467	6	17581

898.74401	1	17582

898.88335	2	17584

899.02269	4	17588

899.16203	1	17589

899.30137	3	17592

899.44071	1	17593

899.58005	2	17595

899.71939	2	17597

899.85873	1	17598

899.99807	4	17602

900.13741	19	17621

900.27675	5	17626

900.41609	1	17627

900.55543	4	17631

900.69477	2	17633

900.83411	3	17636

900.97345	3	17639

901.11279	2	17641

901.25213	4	17645

901.39147	1	17646

901.53081	2	17648

901.67015	7	17655

901.80949	4	17659

901.94883	2	17661

902.08817	2	17663

902.22751	1	17664

902.36685	4	17668

902.50619	4	17672

902.64553	9	17681

902.78487	6	17687

902.92421	2	17689

903.06355	4	17693

903.20289	2	17695

903.34223	2	17697

903.48157	2	17699

903.62091	4	17703

903.76025	5	17708

903.8996	1	17709

904.03894	6	17715

904.17828	2	17717

904.31762	2	17719

904.45696	2	17721

904.5963	2	17723

904.73564	1	17724

904.87498	2	17726

905.01432	1	17727

905.15366	5	17732

905.293	3	17735

905.43234	1	17736

905.57168	1	17737

905.71102	2	17739

905.85036	4	17743

905.9897	1	17744

906.12904	2	17746

906.26838	3	17749

906.40772	3	17752

906.54706	4	17756

906.6864	1	17757

906.82574	1	17758

906.96508	9	17767

907.10442	5	17772

907.24376	2	17774

907.3831	2	17776

907.52244	1	17777

907.66178	1	17778

907.80112	6	17784

907.94046	5	17789

908.0798	1	17790

908.21914	5	17795

908.35848	4	17799

908.49782	1	17800

908.63716	1	17801

908.7765	2	17803

908.91584	2	17805

909.05518	2	17807

909.19452	1	17808

909.33386	3	17811

909.4732	2	17813

909.61254	1	17814

909.75188	1	17815

909.89122	3	17818

910.03056	1	17819

910.1699	1	17820

910.30924	1	17821

910.44858	3	17824

910.58792	8	17832

910.72726	1	17833

910.8666	1	17834

911.00594	1	17835

911.14528	1	17836

911.28462	1	17837

911.42396	1	17838

911.5633	1	17839

911.70264	4	17843

911.84198	3	17846

911.98132	1	17847

912.12066	1	17848

912.26	4	17852

912.39934	10	17862

912.53868	1	17863

912.67802	2	17865

912.81737	12	17877

912.95671	1	17878

913.09605	5	17883

913.23539	1	17884

913.37473	3	17887

913.51407	4	17891

913.65341	1	17892

913.79275	3	17895

913.93209	3	17898

914.07143	2	17900

914.21077	3	17903

914.35011	1	17904

914.48945	2	17906

914.62879	2	17908

914.76813	3	17911

914.90747	4	17915

915.04681	12	17927

915.18615	1	17928

915.32549	3	17931

915.46483	4	17935

915.60417	12	17947

915.74351	2	17949

915.88285	1	17950

916.02219	3	17953

916.16153	1	17954

916.30087	1	17955

916.44021	1	17956

916.57955	1	17957

916.71889	1	17958

916.85823	1	17959

916.99757	4	17963

917.13691	3	17966

917.27625	2	17968

917.41559	1	17969

917.55493	6	17975

917.69427	4	17979

917.83361	3	17982

917.97295	2	17984

918.11229	3	17987

918.25163	8	17995

918.39097	3	17998

918.53031	7	18005

918.66965	1	18006

918.80899	4	18010

918.94833	2	18012

919.08767	19	18031

919.22701	1	18032

919.36635	2	18034

919.50569	6	18040

919.64503	9	18049

919.78437	3	18052

919.92371	1	18053

920.06305	3	18056

920.20239	1	18057

920.34173	1	18058

920.48107	1	18059

920.62041	5	18064

920.75975	5	18069

920.89909	1	18070

921.03843	1	18071

921.17777	6	18077

921.31711	11	18088

921.45645	7	18095

921.59579	3	18098

921.73514	2	18100

921.87448	4	18104

922.01382	1	18105

922.15316	2	18107

922.2925	4	18111

922.43184	1	18112

922.57118	2	18114

922.71052	3	18117

922.84986	2	18119

922.9892	1	18120

923.12854	2	18122

923.26788	2	18124

923.40722	4	18128

923.54656	1	18129

923.6859	1	18130

923.82524	2	18132

923.96458	2	18134

924.10392	1	18135

924.24326	9	18144

924.3826	4	18148

924.52194	3	18151

924.66128	1	18152

924.80062	8	18160

924.93996	1	18161

925.0793	2	18163

925.21864	2	18165

925.35798	5	18170

925.49732	1	18171

925.63666	4	18175

925.776	4	18179

925.91534	7	18186

926.05468	19	18205

926.19402	1	18206

926.33336	1	18207

926.4727	12	18219

926.61204	1	18220

926.75138	1	18221

926.89072	1	18222

927.03006	1	18223

927.1694	1	18224

927.30874	1	18225

927.44808	3	18228

927.58742	2	18230

927.72676	2	18232

927.8661	1	18233

928.00544	1	18234

928.14478	2	18236

928.28412	1	18237

928.42346	1	18238

928.5628	4	18242

928.70214	2	18244

928.84148	2	18246

928.98082	2	18248

929.12016	1	18249

929.2595	1	18250

929.39884	1	18251

929.53818	4	18255

929.67752	2	18257

929.81686	1	18258

929.9562	2	18260

930.09554	3	18263

930.23488	2	18265

930.37422	1	18266

930.51356	3	18269

930.65291	2	18271

930.79225	2	18273

930.93159	7	18280

931.07093	2	18282

931.21027	4	18286

931.34961	6	18292

931.48895	4	18296

931.62829	1	18297

931.76763	5	18302

931.90697	5	18307

932.04631	2	18309

932.18565	3	18312

932.32499	1	18313

932.46433	1	18314

932.60367	7	18321

932.74301	2	18323

932.88235	2	18325

933.02169	3	18328

933.16103	3	18331

933.30037	9	18340

933.43971	1	18341

933.57905	3	18344

933.71839	1	18345

933.85773	2	18347

933.99707	5	18352

934.13641	1	18353

934.27575	1	18354

934.41509	6	18360

934.55443	5	18365

934.69377	7	18372

934.83311	10	18382

934.97245	1	18383

935.11179	2	18385

935.25113	3	18388

935.39047	2	18390

935.52981	1	18391

935.66915	2	18393

935.80849	3	18396

935.94783	4	18400

936.08717	32	18432

936.22651	2	18434

936.36585	1	18435

936.50519	2	18437

936.64453	2	18439

936.78387	11	18450

936.92321	2	18452

937.06255	4	18456

937.20189	6	18462

937.34123	1	18463

937.48057	1	18464

937.61991	2	18466

937.75925	1	18467

937.89859	1	18468

938.03793	6	18474

938.17727	2	18476

938.31661	2	18478

938.45595	1	18479

938.59529	10	18489

938.73463	1	18490

938.87397	19	18509

939.01331	2	18511

939.15265	4	18515

939.29199	1	18516

939.43133	6	18522

939.57068	2	18524

939.71002	4	18528

939.84936	9	18537

939.9887	1	18538

940.12804	2	18540

940.26738	1	18541

940.40672	2	18543

940.54606	1	18544

940.6854	4	18548

940.82474	1	18549

940.96408	2	18551

941.10342	2	18553

941.24276	2	18555

941.3821	1	18556

941.52144	6	18562

941.66078	7	18569

941.80012	1	18570

941.93946	3	18573

942.0788	2	18575

942.21814	1	18576

942.35748	9	18585

942.49682	4	18589

942.63616	10	18599

942.7755	4	18603

942.91484	1	18604

943.05418	1	18605

943.19352	1	18606

943.33286	2	18608

943.4722	6	18614

943.61154	1	18615

943.75088	3	18618

943.89022	5	18623

944.02956	2	18625

944.1689	4	18629

944.30824	2	18631

944.44758	1	18632

944.58692	1	18633

944.72626	3	18636

944.8656	3	18639

945.00494	1	18640

945.14428	3	18643

945.28362	3	18646

945.42296	1	18647

945.5623	2	18649

945.70164	5	18654

945.84098	11	18665

945.98032	1	18666

946.11966	2	18668

946.259	3	18671

946.39834	1	18672

946.53768	1	18673

946.67702	2	18675

946.81636	9	18684

946.9557	1	18685

947.09504	3	18688

947.23438	1	18689

947.37372	2	18691

947.51306	2	18693

947.6524	5	18698

947.79174	2	18700

947.93108	1	18701

948.07042	1	18702

948.20976	1	18703

948.34911	3	18706

948.48845	1	18707

948.62779	1	18708

948.76713	2	18710

948.90647	1	18711

949.04581	1	18712

949.18515	1	18713

949.32449	6	18719

949.46383	1	18720

949.60317	1	18721

949.74251	3	18724

949.88185	1	18725

950.02119	3	18728

950.16053	10	18738

950.29987	5	18743

950.43921	5	18748

950.57855	5	18753

950.71789	5	18758

950.85723	2	18760

950.99657	7	18767

951.13591	2	18769

951.27525	2	18771

951.41459	4	18775

951.55393	5	18780

951.69327	2	18782

951.83261	2	18784

951.97195	3	18787

952.11129	3	18790

952.25063	1	18791

952.38997	1	18792

952.52931	1	18793

952.66865	5	18798

952.80799	4	18802

952.94733	1	18803

953.08667	3	18806

953.22601	1	18807

953.36535	2	18809

953.50469	3	18812

953.64403	3	18815

953.78337	2	18817

953.92271	1	18818

954.06205	2	18820

954.20139	2	18822

954.34073	1	18823

954.48007	5	18828

954.61941	3	18831

954.75875	1	18832

954.89809	14	18846

955.03743	1	18847

955.17677	3	18850

955.31611	1	18851

955.45545	3	18854

955.59479	3	18857

955.73413	1	18858

955.87347	1	18859

956.01281	1	18860

956.15215	11	18871

956.29149	5	18876

956.43083	2	18878

956.57017	4	18882

956.70951	1	18883

956.84885	2	18885

956.98819	1	18886

957.12753	4	18890

957.26688	1	18891

957.40622	1	18892

957.54556	2	18894

957.6849	2	18896

957.82424	2	18898

957.96358	4	18902

958.10292	5	18907

958.24226	2	18909

958.3816	1	18910

958.52094	2	18912

958.66028	1	18913

958.79962	1	18914

958.93896	1	18915

959.0783	6	18921

959.21764	8	18929

959.35698	6	18935

959.49632	1	18936

959.63566	1	18937

959.775	2	18939

959.91434	2	18941

960.05368	2	18943

960.19302	2	18945

960.33236	10	18955

960.4717	1	18956

960.61104	1	18957

960.75038	1	18958

960.88972	1	18959

961.02906	4	18963

961.1684	1	18964

961.30774	2	18966

961.44708	1	18967

961.58642	4	18971

961.72576	1	18972

961.8651	1	18973

962.00444	5	18978

962.14378	1	18979

962.28312	2	18981

962.42246	1	18982

962.5618	1	18983

962.70114	3	18986

962.84048	2	18988

962.97982	1	18989

963.11916	3	18992

963.2585	1	18993

963.39784	3	18996

963.53718	5	19001

963.67652	1	19002

963.81586	2	19004

963.9552	1	19005

964.09454	4	19009

964.23388	7	19016

964.37322	3	19019

964.51256	2	19021

964.6519	2	19023

964.79124	3	19026

964.93058	5	19031

965.06992	3	19034

965.20926	4	19038

965.3486	1	19039

965.48794	5	19044

965.62728	4	19048

965.76662	2	19050

965.90596	1	19051

966.0453	6	19057

966.18465	6	19063

966.32399	11	19074

966.46333	6	19080

966.60267	1	19081

966.74201	11	19092

966.88135	1	19093

967.02069	9	19102

967.16003	3	19105

967.29937	2	19107

967.43871	1	19108

967.57805	5	19113

967.71739	3	19116

967.85673	2	19118

967.99607	2	19120

968.13541	5	19125

968.27475	1	19126

968.41409	9	19135

968.55343	1	19136

968.69277	1	19137

968.83211	3	19140

968.97145	2	19142

969.11079	1	19143

969.25013	1	19144

969.38947	1	19145

969.52881	3	19148

969.66815	4	19152

969.80749	2	19154

969.94683	1	19155

970.08617	1	19156

970.22551	1	19157

970.36485	1	19158

970.50419	1	19159

970.64353	2	19161

970.78287	1	19162

970.92221	3	19165

971.06155	2	19167

971.20089	1	19168

971.34023	3	19171

971.47957	2	19173

971.61891	2	19175

971.75825	2	19177

971.89759	2	19179

972.03693	1	19180

972.17627	12	19192

972.31561	10	19202

972.45495	4	19206

972.59429	3	19209

972.73363	3	19212

972.87297	2	19214

973.01231	3	19217

973.15165	9	19226

973.29099	1	19227

973.43033	1	19228

973.56967	4	19232

973.70901	1	19233

973.84835	3	19236

973.98769	3	19239

974.12703	2	19241

974.26637	2	19243

974.40571	4	19247

974.54505	2	19249

974.68439	4	19253

974.82373	1	19254

974.96307	2	19256

975.10241	2	19258

975.24176	2	19260

975.3811	21	19281

975.52044	1	19282

975.65978	7	19289

975.79912	2	19291

975.93846	1	19292

976.0778	2	19294

976.21714	4	19298

976.35648	2	19300

976.49582	1	19301

976.63516	5	19306

976.7745	1	19307

976.91384	2	19309

977.05318	1	19310

977.19252	3	19313

977.33186	3	19316

977.4712	2	19318

977.61054	2	19320

977.74988	5	19325

977.88922	4	19329

978.02856	1	19330

978.1679	2	19332

978.30724	5	19337

978.44658	1	19338

978.58592	3	19341

978.72526	1	19342

978.8646	2	19344

979.00394	1	19345

979.14328	9	19354

979.28262	1	19355

979.42196	6	19361

979.5613	1	19362

979.70064	3	19365

979.83998	1	19366

979.97932	1	19367

980.11866	1	19368

980.258	2	19370

980.39734	1	19371

980.53668	1	19372

980.67602	1	19373

980.81536	1	19374

980.9547	12	19386

981.09404	4	19390

981.23338	3	19393

981.37272	4	19397

981.51206	3	19400

981.6514	1	19401

981.79074	2	19403

981.93008	1	19404

982.06942	1	19405

982.20876	2	19407

982.3481	2	19409

982.48744	6	19415

982.62678	5	19420

982.76612	2	19422

982.90546	4	19426

983.0448	6	19432

983.18414	1	19433

983.32348	8	19441

983.46282	1	19442

983.60216	7	19449

983.7415	1	19450

983.88084	3	19453

984.02018	2	19455

984.15953	4	19459

984.29887	5	19464

984.43821	5	19469

984.57755	1	19470

984.71689	2	19472

984.85623	13	19485

984.99557	2	19487

985.13491	9	19496

985.27425	1	19497

985.41359	1	19498

985.55293	1	19499

985.69227	1	19500

985.83161	5	19505

985.97095	1	19506

986.11029	1	19507

986.24963	3	19510

986.38897	1	19511

986.52831	1	19512

986.66765	7	19519

986.80699	4	19523

986.94633	7	19530

987.08567	5	19535

987.22501	7	19542

987.36435	3	19545

987.50369	5	19550

987.64303	2	19552

987.78237	5	19557

987.92171	1	19558

988.06105	1	19559

988.20039	2	19561

988.33973	5	19566

988.47907	7	19573

988.61841	2	19575

988.75775	1	19576

988.89709	2	19578

989.03643	7	19585

989.17577	3	19588

989.31511	1	19589

989.45445	2	19591

989.59379	15	19606

989.73313	2	19608

989.87247	3	19611

990.01181	1	19612

990.15115	4	19616

990.29049	4	19620

990.42983	4	19624

990.56917	5	19629

990.70851	1	19630

990.84785	1	19631

990.98719	1	19632

991.12653	5	19637

991.26587	2	19639

991.40521	5	19644

991.54455	1	19645

991.68389	2	19647

991.82323	1	19648

991.96257	3	19651

992.10191	1	19652

992.24125	3	19655

992.38059	1	19656

992.51993	5	19661

992.65927	6	19667

992.79861	1	19668

992.93795	3	19671

993.0773	14	19685

993.21664	1	19686

993.35598	2	19688

993.49532	1	19689

993.63466	1	19690

993.774	1	19691

993.91334	4	19695

994.05268	4	19699

994.19202	1	19700

994.33136	4	19704

994.4707	1	19705

994.61004	5	19710

994.74938	3	19713

994.88872	7	19720

995.02806	10	19730

995.1674	2	19732

995.30674	1	19733

995.44608	2	19735

995.58542	5	19740

995.72476	1	19741

995.8641	2	19743

996.00344	2	19745

996.14278	2	19747

996.28212	5	19752

996.42146	2	19754

996.5608	2	19756

996.70014	1	19757

996.83948	2	19759

996.97882	3	19762

997.11816	1	19763

997.2575	2	19765

997.39684	1	19766

997.53618	2	19768

997.67552	2	19770

997.81486	3	19773

997.9542	2	19775

998.09354	2	19777

998.23288	1	19778

998.37222	2	19780

998.51156	2	19782

998.6509	5	19787

998.79024	6	19793

998.92958	1	19794

999.06892	1	19795

999.20826	5	19800

999.3476	7	19807

999.48694	3	19810

999.62628	1	19811

999.76562	4	19815

999.90496	1	19816

1000.0443	2	19818

1000.18364	1	19819

1000.32298	2	19821

1000.46232	4	19825

1000.60166	1	19826

1000.741	3	19829

1000.88034	2	19831

1001.01968	1	19832

1001.15902	4	19836

1001.29836	1	19837

1001.43771	3	19840

1001.57705	5	19845

1001.71638	3	19848

1001.85573	1	19849

1001.99507	1	19850

1002.13441	19	19869

1002.27375	2	19871

1002.41309	3	19874

1002.55243	17	19891

1002.69177	2	19893

1002.83111	4	19897

1002.97045	7	19904

1003.10979	3	19907

1003.24913	12	19919

1003.38847	4	19923

1003.52781	2	19925

1003.66715	3	19928

1003.80649	1	19929

1003.94583	5	19934

1004.08517	1	19935

1004.22451	1	19936

1004.36385	2	19938

1004.50319	1	19939

1004.64253	4	19943

1004.78187	1	19944

1004.92121	1	19945

1005.06055	3	19948

1005.19989	1	19949

1005.33923	3	19952

1005.47857	3	19955

1005.61791	2	19957

1005.75725	1	19958

1005.89659	3	19961

1006.03593	4	19965

1006.17527	4	19969

1006.31461	8	19977

1006.45395	12	19989

1006.59329	10	19999

1006.73263	1	20000

1006.87197	1	20001

1007.01131	6	20007

1007.15065	2	20009

1007.28999	5	20014

1007.42933	1	20015

1007.56867	11	20026

1007.70801	2	20028

1007.84735	4	20032

1007.98669	1	20033

1008.12603	1	20034

1008.26537	1	20035

1008.40471	6	20041

1008.54405	3	20044

1008.68339	1	20045

1008.82273	3	20048

1008.96207	1	20049

1009.10141	1	20050

1009.24075	2	20052

1009.38009	4	20056

1009.51943	6	20062

1009.65877	19	20081

1009.79811	4	20085

1009.93745	1	20086

1010.07679	9	20095

1010.21613	4	20099

1010.35548	1	20100

1010.49482	3	20103

1010.63416	2	20105

1010.7735	9	20114

1010.91284	15	20129

1011.05218	5	20134

1011.19152	5	20139

1011.33086	2	20141

1011.4702	1	20142

1011.60954	1	20143

1011.74888	1	20144

1011.88822	1	20145

1012.02756	1	20146

1012.1669	1	20147

1012.30624	1	20148

1012.44558	13	20161

1012.58492	1	20162

1012.72426	1	20163

1012.8636	2	20165

1013.00294	3	20168

1013.14228	1	20169

1013.28162	5	20174

1013.42096	2	20176

1013.5603	2	20178

1013.69964	14	20192

1013.83898	1	20193

1013.97832	2	20195

1014.11766	1	20196

1014.257	2	20198

1014.39634	1	20199

1014.53568	4	20203

1014.67502	1	20204

1014.81436	1	20205

1014.9537	1	20206

1015.09304	2	20208

1015.23238	1	20209

1015.37172	5	20214

1015.51106	3	20217

1015.6504	1	20218

1015.78974	5	20223

1015.92908	1	20224

1016.06842	3	20227

1016.20776	2	20229

1016.3471	2	20231

1016.48644	2	20233

1016.62578	2	20235

1016.76512	7	20242

1016.90446	6	20248

1017.0438	1	20249

1017.18314	11	20260

1017.32248	9	20269

1017.46182	1	20270

1017.60116	9	20279

1017.7405	10	20289

1017.87984	10	20299

1018.01918	2	20301

1018.15852	2	20303

1018.29786	1	20304

1018.4372	2	20306

1018.57654	1	20307

1018.71588	2	20309

1018.85522	1	20310

1018.99456	1	20311

1019.1339	2	20313

1019.27325	20	20333

1019.41259	2	20335

1019.55193	1	20336

1019.69127	2	20338

1019.83061	2	20340

1019.96995	2	20342

1020.10929	2	20344

1020.24863	4	20348

1020.38797	1	20349

1020.52731	1	20350

1020.66665	2	20352

1020.80599	4	20356

1020.94533	5	20361

1021.08467	1	20362

1021.22401	3	20365

1021.36335	1	20366

1021.50269	8	20374

1021.64203	6	20380

1021.78137	1	20381

1021.92071	3	20384

1022.06005	1	20385

1022.19939	2	20387

1022.33873	3	20390

1022.47807	1	20391

1022.61741	1	20392

1022.75675	2	20394

1022.89609	2	20396

1023.03543	4	20400

1023.17477	1	20401

1023.31411	8	20409

1023.45345	4	20413

1023.59279	3	20416

1023.73213	4	20420

1023.87147	6	20426

1024.01081	3	20429

1024.15015	2	20431

1024.28949	3	20434

1024.42883	4	20438

1024.56817	2	20440

1024.70751	3	20443

1024.84685	6	20449

1024.98619	2	20451

1025.12553	1	20452

1025.26487	1	20453

1025.40421	3	20456

1025.54355	3	20459

1025.68289	2	20461

1025.82223	1	20462

1025.96157	2	20464

1026.10091	1	20465

1026.24025	6	20471

1026.37959	1	20472

1026.51893	4	20476

1026.65827	1	20477

1026.79761	2	20479

1026.93695	5	20484

1027.07629	1	20485

1027.21563	1	20486

1027.35497	1	20487

1027.49431	3	20490

1027.63365	2	20492

1027.77299	3	20495

1027.91233	1	20496

1028.05167	2	20498

1028.19102	2	20500

1028.33036	8	20508

1028.4697	7	20515

1028.60903	1	20516

1028.74837	4	20520

1028.88772	1	20521

1029.02706	1	20522

1029.1664	1	20523

1029.30574	2	20525

1029.44508	4	20529

1029.58442	2	20531

1029.72376	7	20538

1029.8631	1	20539

1030.00244	1	20540

1030.14178	1	20541

1030.28112	6	20547

1030.42046	2	20549

1030.5598	2	20551

1030.69914	11	20562

1030.83848	1	20563

1030.97782	3	20566

1031.11716	4	20570

1031.2565	6	20576

1031.39584	1	20577

1031.53518	3	20580

1031.67452	2	20582

1031.81386	2	20584

1031.9532	1	20585

1032.09254	1	20586

1032.23188	1	20587

1032.37122	4	20591

1032.51056	15	20606

1032.6499	6	20612

1032.78924	2	20614

1032.92858	2	20616

1033.06792	3	20619

1033.20726	7	20626

1033.3466	4	20630

1033.48594	1	20631

1033.62528	3	20634

1033.76462	12	20646

1033.90396	6	20652

1034.0433	9	20661

1034.18264	3	20664

1034.32198	1	20665

1034.46132	1	20666

1034.60066	2	20668

1034.74	1	20669

1034.87934	3	20672

1035.01868	1	20673

1035.15802	4	20677

1035.29736	4	20681

1035.4367	1	20682

1035.57604	2	20684

1035.71538	2	20686

1035.85472	4	20690

1035.99406	1	20691

1036.1334	1	20692

1036.27274	2	20694

1036.41208	4	20698

1036.55142	1	20699

1036.69076	7	20706

1036.8301	3	20709

1036.96944	1	20710

1037.10878	5	20715

1037.24813	3	20718

1037.38747	1	20719

1037.52681	3	20722

1037.66614	2	20724

1037.80549	1	20725

1037.94483	5	20730

1038.08417	1	20731

1038.22351	2	20733

1038.36285	2	20735

1038.50219	1	20736

1038.64153	1	20737

1038.78087	1	20738

1038.92021	1	20739

1039.05955	3	20742

1039.19889	1	20743

1039.33823	5	20748

1039.47757	1	20749

1039.61691	5	20754

1039.75625	2	20756

1039.89559	1	20757

1040.03493	3	20760

1040.17427	17	20777

1040.31361	2	20779

1040.45295	17	20796

1040.59229	1	20797

1040.73163	7	20804

1040.87097	1	20805

1041.01031	1	20806

1041.14965	3	20809

1041.28899	2	20811

1041.42833	9	20820

1041.56767	15	20835

1041.70701	2	20837

1041.84635	3	20840

1041.98569	4	20844

1042.12503	7	20851

1042.26437	4	20855

1042.40371	2	20857

1042.54305	9	20866

1042.68239	1	20867

1042.82173	2	20869

1042.96107	1	20870

1043.10041	3	20873

1043.23975	2	20875

1043.37909	2	20877

1043.51843	1	20878

1043.65777	1	20879

1043.79711	4	20883

1043.93645	8	20891

1044.07579	8	20899

1044.21513	3	20902

1044.35447	1	20903

1044.49381	2	20905

1044.63315	2	20907

1044.77249	2	20909

1044.91183	15	20924

1045.05117	1	20925

1045.19051	1	20926

1045.32985	2	20928

1045.46919	7	20935

1045.60853	1	20936

1045.74787	2	20938

1045.88722	1	20939

1046.02655	4	20943

1046.1659	1	20944

1046.30524	1	20945

1046.44458	4	20949

1046.58391	1	20950

1046.72326	6	20956

1046.8626	5	20961

1047.00194	1	20962

1047.14128	3	20965

1047.28062	2	20967

1047.41996	2	20969

1047.5593	1	20970

1047.69864	4	20974

1047.83798	5	20979

1047.97732	1	20980

1048.11666	1	20981

1048.256	5	20986

1048.39534	4	20990

1048.53468	2	20992

1048.67402	4	20996

1048.81336	2	20998

1048.9527	1	20999

1049.09204	6	21005

1049.23138	2	21007

1049.37072	16	21023

1049.51006	4	21027

1049.6494	3	21030

1049.78874	2	21032

1049.92808	4	21036

1050.06742	1	21037

1050.20676	1	21038

1050.3461	5	21043

1050.48544	1	21044

1050.62478	1	21045

1050.76412	2	21047

1050.90346	4	21051

1051.0428	6	21057

1051.18214	3	21060

1051.32148	9	21069

1051.46082	2	21071

1051.60016	1	21072

1051.7395	4	21076

1051.87884	1	21077

1052.01818	1	21078

1052.15752	3	21081

1052.29686	15	21096

1052.4362	1	21097

1052.57554	4	21101

1052.71488	1	21102

1052.85422	2	21104

1052.99356	4	21108

1053.1329	11	21119

1053.27224	5	21124

1053.41158	6	21130

1053.55092	4	21134

1053.69026	7	21141

1053.8296	3	21144

1053.96894	2	21146

1054.10828	1	21147

1054.24762	3	21150

1054.38696	4	21154

1054.5263	5	21159

1054.66564	1	21160

1054.80499	1	21161

1054.94432	1	21162

1055.08367	2	21164

1055.22301	4	21168

1055.36235	1	21169

1055.50169	1	21170

1055.64102	4	21174

1055.78037	7	21181

1055.91971	2	21183

1056.05905	3	21186

1056.19839	4	21190

1056.33773	1	21191

1056.47707	1	21192

1056.61641	2	21194

1056.75575	1	21195

1056.89509	1	21196

1057.03443	6	21202

1057.17377	1	21203

1057.31311	3	21206

1057.45245	2	21208

1057.59179	1	21209

1057.73113	2	21211

1057.87047	1	21212

1058.00981	1	21213

1058.14915	4	21217

1058.28849	2	21219

1058.42783	1	21220

1058.56717	2	21222

1058.70651	1	21223

1058.84585	3	21226

1058.98519	1	21227

1059.12453	2	21229

1059.26387	5	21234

1059.40321	4	21238

1059.54255	2	21240

1059.68189	1	21241

1059.82123	1	21242

1059.96057	1	21243

1060.09991	1	21244

1060.23925	13	21257

1060.37859	1	21258

1060.51793	8	21266

1060.65727	1	21267

1060.79661	1	21268

1060.93595	4	21272

1061.07529	1	21273

1061.21463	1	21274

1061.35397	3	21277

1061.49331	1	21278

1061.63265	7	21285

1061.77199	2	21287

1061.91133	3	21290

1062.05067	5	21295

1062.19001	1	21296

1062.32935	5	21301

1062.46869	5	21306

1062.60803	1	21307

1062.74737	4	21311

1062.88671	1	21312

1063.02605	2	21314

1063.16539	1	21315

1063.30473	2	21317

1063.44407	3	21320

1063.58341	4	21324

1063.72276	1	21325

1063.8621	2	21327

1064.00143	4	21331

1064.14078	1	21332

1064.28012	1	21333

1064.41946	12	21345

1064.55879	1	21346

1064.69814	2	21348

1064.83748	6	21354

1064.97682	1	21355

1065.11616	1	21356

1065.2555	1	21357

1065.39484	11	21368

1065.53418	4	21372

1065.67352	1	21373

1065.81286	5	21378

1065.9522	2	21380

1066.09154	2	21382

1066.23088	3	21385

1066.37022	3	21388

1066.50956	1	21389

1066.6489	1	21390

1066.78824	1	21391

1066.92758	2	21393

1067.06692	1	21394

1067.20626	1	21395

1067.3456	2	21397

1067.48494	1	21398

1067.62428	1	21399

1067.76362	2	21401

1067.90296	3	21404

1068.0423	5	21409

1068.18164	2	21411

1068.32098	1	21412

1068.46032	1	21413

1068.59966	4	21417

1068.739	2	21419

1068.87834	4	21423

1069.01768	1	21424

1069.15702	1	21425

1069.29636	10	21435

1069.4357	4	21439

1069.57504	4	21443

1069.71438	1	21444

1069.85372	1	21445

1069.99306	3	21448

1070.1324	3	21451

1070.27174	3	21454

1070.41108	3	21457

1070.55042	4	21461

1070.68976	1	21462

1070.8291	1	21463

1070.96844	1	21464

1071.10778	1	21465

1071.24712	2	21467

1071.38646	1	21468

1071.5258	1	21469

1071.66514	5	21474

1071.80448	1	21475

1071.94382	7	21482

1072.08316	1	21483

1072.2225	1	21484

1072.36184	1	21485

1072.50118	1	21486

1072.64053	3	21489

1072.77987	2	21491

1072.9192	1	21492

1073.05855	1	21493

1073.19789	1	21494

1073.33723	4	21498

1073.47656	3	21501

1073.61591	1	21502

1073.75525	2	21504

1073.89459	10	21514

1074.03393	1	21515

1074.17327	7	21522

1074.31261	8	21530

1074.45195	1	21531

1074.59129	2	21533

1074.73063	4	21537

1074.86997	1	21538

1075.00931	1	21539

1075.14865	4	21543

1075.28799	10	21553

1075.42733	4	21557

1075.56667	1	21558

1075.70601	2	21560

1075.84535	6	21566

1075.98469	1	21567

1076.12403	6	21573

1076.26337	1	21574

1076.40271	4	21578

1076.54205	1	21579

1076.68139	2	21581

1076.82073	6	21587

1076.96007	1	21588

1077.09941	2	21590

1077.23875	5	21595

1077.37809	1	21596

1077.51743	2	21598

1077.65677	1	21599

1077.79611	1	21600

1077.93545	1	21601

1078.07479	3	21604

1078.21413	1	21605

1078.35347	1	21606

1078.49281	1	21607

1078.63215	1	21608

1078.77149	1	21609

1078.91083	1	21610

1079.05017	6	21616

1079.18951	1	21617

1079.32885	1	21618

1079.46819	2	21620

1079.60753	2	21622

1079.74687	1	21623

1079.88621	1	21624

1080.02555	1	21625

1080.16489	1	21626

1080.30423	4	21630

1080.44357	1	21631

1080.58291	2	21633

1080.72225	2	21635

1080.86159	3	21638

1081.00093	1	21639

1081.14027	1	21640

1081.27961	2	21642

1081.41895	6	21648

1081.5583	1	21649

1081.69764	4	21653

1081.83697	4	21657

1081.97632	3	21660

1082.11566	1	21661

1082.255	4	21665

1082.39434	4	21669

1082.53368	3	21672

1082.67302	3	21675

1082.81236	1	21676

1082.9517	2	21678

1083.09104	2	21680

1083.23038	1	21681

1083.36972	1	21682

1083.50906	1	21683

1083.6484	5	21688

1083.78774	1	21689

1083.92708	3	21692

1084.06642	2	21694

1084.20576	8	21702

1084.3451	1	21703

1084.48444	1	21704

1084.62378	3	21707

1084.76312	1	21708

1084.90246	2	21710

1085.0418	3	21713

1085.18114	1	21714

1085.32048	8	21722

1085.45982	4	21726

1085.59916	1	21727

1085.7385	1	21728

1085.87784	13	21741

1086.01718	1	21742

1086.15652	1	21743

1086.29586	1	21744

1086.4352	2	21746

1086.57454	2	21748

1086.71388	1	21749

1086.85322	2	21751

1086.99256	33	21784

1087.1319	5	21789

1087.27124	1	21790

1087.41058	4	21794

1087.54992	2	21796

1087.68926	1	21797

1087.8286	12	21809

1087.96794	1	21810

1088.10728	1	21811

1088.24662	7	21818

1088.38596	1	21819

1088.5253	1	21820

1088.66464	1	21821

1088.80398	1	21822

1088.94332	2	21824

1089.08266	3	21827

1089.222	2	21829

1089.36134	1	21830

1089.50068	2	21832

1089.64002	3	21835

1089.77936	1	21836

1089.9187	1	21837

1090.05804	11	21848

1090.19738	1	21849

1090.33672	1	21850

1090.47607	1	21851

1090.61541	2	21853

1090.75475	3	21856

1090.89408	3	21859

1091.03343	4	21863

1091.17277	2	21865

1091.31211	2	21867

1091.45145	2	21869

1091.59079	1	21870

1091.73013	1	21871

1091.86947	1	21872

1092.00881	16	21888

1092.14815	4	21892

1092.28749	2	21894

1092.42683	12	21906

1092.56617	2	21908

1092.70551	3	21911

1092.84485	6	21917

1092.98419	2	21919

1093.12353	2	21921

1093.26287	2	21923

1093.40221	1	21924

1093.54155	1	21925

1093.68089	1	21926

1093.82023	2	21928

1093.95957	44	21972

1094.09891	2	21974

1094.23825	9	21983

1094.37759	2	21985

1094.51693	1	21986

1094.65627	2	21988

1094.79561	1	21989

1094.93495	1	21990

1095.07429	1	21991

1095.21363	2	21993

1095.35297	9	22002

1095.49231	1	22003

1095.63165	7	22010

1095.77099	1	22011

1095.91033	5	22016

1096.04967	2	22018

1096.18901	2	22020

1096.32835	1	22021

1096.46769	4	22025

1096.60703	1	22026

1096.74637	1	22027

1096.88571	2	22029

1097.02505	1	22030

1097.16439	7	22037

1097.30373	2	22039

1097.44307	1	22040

1097.58241	3	22043

1097.72175	2	22045

1097.86109	2	22047

1098.00043	2	22049

1098.13977	10	22059

1098.27911	2	22061

1098.41845	7	22068

1098.55779	4	22072

1098.69713	1	22073

1098.83647	1	22074

1098.97581	1	22075

1099.11515	1	22076

1099.25449	2	22078

1099.39384	1	22079

1099.53318	3	22082

1099.67252	1	22083

1099.81185	1	22084

1099.9512	3	22087

1100.09054	2	22089

1100.22988	2	22091

1100.36922	1	22092

1100.50856	2	22094

1100.6479	2	22096

1100.78724	2	22098

1100.92658	1	22099

1101.06592	3	22102

1101.20526	1	22103

1101.3446	1	22104

1101.48394	1	22105

1101.62328	1	22106

1101.76262	1	22107

1101.90196	1	22108

1102.0413	4	22112

1102.18064	1	22113

1102.31998	1	22114

1102.45932	2	22116

1102.59866	3	22119

1102.738	1	22120

1102.87734	2	22122

1103.01668	3	22125

1103.15602	4	22129

1103.29536	3	22132

1103.4347	5	22137

1103.57404	5	22142

1103.71338	1	22143

1103.85272	1	22144

1103.99206	1	22145

1104.1314	3	22148

1104.27074	3	22151

1104.41008	2	22153

1104.54942	2	22155

1104.68876	2	22157

1104.8281	5	22162

1104.96744	1	22163

1105.10678	4	22167

1105.24612	1	22168

1105.38546	1	22169

1105.5248	4	22173

1105.66414	1	22174

1105.80348	7	22181

1105.94282	1	22182

1106.08216	5	22187

1106.2215	2	22189

1106.36084	5	22194

1106.50018	2	22196

1106.63952	4	22200

1106.77886	1	22201

1106.9182	5	22206

1107.05754	7	22213

1107.19688	1	22214

1107.33622	1	22215

1107.47556	9	22224

1107.6149	7	22231

1107.75424	3	22234

1107.89358	4	22238

1108.03292	7	22245

1108.17226	1	22246

1108.31161	1	22247

1108.45095	2	22249

1108.59029	1	22250

1108.72962	3	22253

1108.86896	3	22256

1109.00831	15	22271

1109.14765	13	22284

1109.28699	4	22288

1109.42633	16	22304

1109.56567	5	22309

1109.70501	1	22310

1109.84435	14	22324

1109.98369	1	22325

1110.12303	4	22329

1110.26237	1	22330

1110.40171	1	22331

1110.54105	5	22336

1110.68039	1	22337

1110.81973	2	22339

1110.95907	2	22341

1111.09841	2	22343

1111.23775	2	22345

1111.37709	2	22347

1111.51643	1	22348

1111.65577	1	22349

1111.79511	1	22350

1111.93445	5	22355

1112.07379	4	22359

1112.21313	2	22361

1112.35247	1	22362

1112.49181	1	22363

1112.63115	2	22365

1112.77049	1	22366

1112.90983	4	22370

1113.04917	8	22378

1113.18851	7	22385

1113.32785	1	22386

1113.46719	1	22387

1113.60653	11	22398

1113.74587	3	22401

1113.88521	1	22402

1114.02455	6	22408

1114.16389	3	22411

1114.30323	18	22429

1114.44257	2	22431

1114.58191	2	22433

1114.72125	7	22440

1114.86059	2	22442

1114.99993	1	22443

1115.13927	1	22444

1115.27861	2	22446

1115.41795	6	22452

1115.55729	2	22454

1115.69663	2	22456

1115.83597	4	22460

1115.97531	6	22466

1116.11465	2	22468

1116.25399	2	22470

1116.39333	7	22477

1116.53267	1	22478

1116.67201	1	22479

1116.81135	2	22481

1116.95069	3	22484

1117.09003	7	22491

1117.22937	3	22494

1117.36872	28	22522

1117.50806	1	22523

1117.6474	1	22524

1117.78673	15	22539

1117.92608	1	22540

1118.06542	1	22541

1118.20476	10	22551

1118.3441	1	22552

1118.48344	3	22555

1118.62278	8	22563

1118.76212	2	22565

1118.90146	1	22566

1119.0408	2	22568

1119.18014	1	22569

1119.31948	1	22570

1119.45882	1	22571

1119.59816	1	22572

1119.7375	2	22574

1119.87684	2	22576

1120.01618	1	22577

1120.15552	1	22578

1120.29486	2	22580

1120.4342	4	22584

1120.57354	4	22588

1120.71288	3	22591

1120.85222	1	22592

1120.99156	11	22603

1121.1309	18	22621

1121.27024	7	22628

1121.40958	2	22630

1121.54892	8	22638

1121.68826	1	22639

1121.8276	10	22649

1121.96694	3	22652

1122.10628	11	22663

1122.24562	2	22665

1122.38496	1	22666

1122.5243	2	22668

1122.66364	4	22672

1122.80298	3	22675

1122.94232	1	22676

1123.08166	1	22677

1123.221	2	22679

1123.36034	1	22680

1123.49968	2	22682

1123.63902	5	22687

1123.77836	7	22694

1123.9177	1	22695

1124.05704	8	22703

1124.19638	2	22705

1124.33572	2	22707

1124.47506	5	22712

1124.6144	1	22713

1124.75374	2	22715

1124.89308	1	22716

1125.03242	4	22720

1125.17176	3	22723

1125.3111	1	22724

1125.45044	1	22725

1125.58978	3	22728

1125.72912	9	22737

1125.86846	3	22740

1126.0078	1	22741

1126.14714	1	22742

1126.28649	1	22743

1126.42583	5	22748

1126.56517	5	22753

1126.7045	4	22757

1126.84385	2	22759

1126.98319	3	22762

1127.12253	3	22765

1127.26187	1	22766

1127.40121	3	22769

1127.54055	1	22770

1127.67989	2	22772

1127.81923	10	22782

1127.95857	3	22785

1128.09791	11	22796

1128.23725	5	22801

1128.37659	1	22802

1128.51593	4	22806

1128.65527	1	22807

1128.79461	5	22812

1128.93395	1	22813

1129.07329	1	22814

1129.21263	4	22818

1129.35197	1	22819

1129.49131	1	22820

1129.63065	4	22824

1129.76999	1	22825

1129.90933	1	22826

1130.04867	2	22828

1130.18801	1	22829

1130.32735	1	22830

1130.46669	1	22831

1130.60603	2	22833

1130.74537	1	22834

1130.88471	2	22836

1131.02405	4	22840

1131.16339	1	22841

1131.30273	1	22842

1131.44207	3	22845

1131.58141	1	22846

1131.72075	1	22847

1131.86009	4	22851

1131.99943	1	22852

1132.13877	1	22853

1132.27811	1	22854

1132.41745	1	22855

1132.55679	4	22859

1132.69613	1	22860

1132.83547	1	22861

1132.97481	1	22862

1133.11415	2	22864

1133.25349	7	22871

1133.39283	1	22872

1133.53217	2	22874

1133.67151	1	22875

1133.81085	8	22883

1133.95019	1	22884

1134.08953	2	22886

1134.22887	2	22888

1134.36821	5	22893

1134.50755	1	22894

1134.64689	3	22897

1134.78623	1	22898

1134.92557	1	22899

1135.06491	1	22900

1135.20426	1	22901

1135.3436	7	22908

1135.48294	2	22910

1135.62227	1	22911

1135.76161	1	22912

1135.90096	3	22915

1136.0403	3	22918

1136.17964	1	22919

1136.31898	1	22920

1136.45832	1	22921

1136.59766	4	22925

1136.737	2	22927

1136.87634	1	22928

1137.01568	1	22929

1137.15502	1	22930

1137.29436	2	22932

1137.4337	1	22933

1137.57304	1	22934

1137.71238	4	22938

1137.85172	8	22946

1137.99106	3	22949

1138.1304	3	22952

1138.26974	7	22959

1138.40908	3	22962

1138.54842	2	22964

1138.68776	1	22965

1138.8271	5	22970

1138.96644	8	22978

1139.10578	5	22983

1139.24512	1	22984

1139.38446	3	22987

1139.5238	1	22988

1139.66314	7	22995

1139.80248	2	22997

1139.94182	1	22998

1140.08116	1	22999

1140.2205	1	23000

1140.35984	1	23001

1140.49918	1	23002

1140.63852	1	23003

1140.77786	1	23004

1140.9172	1	23005

1141.05654	2	23007

1141.19588	3	23010

1141.33522	2	23012

1141.47456	1	23013

1141.6139	4	23017

1141.75324	1	23018

1141.89258	5	23023

1142.03192	1	23024

1142.17126	4	23028

1142.3106	1	23029

1142.44994	1	23030

1142.58928	2	23032

1142.72862	6	23038

1142.86796	1	23039

1143.0073	1	23040

1143.14664	3	23043

1143.28598	1	23044

1143.42532	1	23045

1143.56466	4	23049

1143.704	1	23050

1143.84335	3	23053

1143.98268	4	23057

1144.12202	1	23058

1144.26137	18	23076

1144.40071	1	23077

1144.54005	1	23078

1144.67938	3	23081

1144.81873	5	23086

1144.95807	3	23089

1145.09741	3	23092

1145.23675	1	23093

1145.37609	18	23111

1145.51543	3	23114

1145.65477	11	23125

1145.79411	19	23144

1145.93345	2	23146

1146.07279	1	23147

1146.21213	2	23149

1146.35147	4	23153

1146.49081	3	23156

1146.63015	2	23158

1146.76949	3	23161

1146.90883	1	23162

1147.04817	1	23163

1147.18751	1	23164

1147.32685	2	23166

1147.46619	4	23170

1147.60553	2	23172

1147.74487	1	23173

1147.88421	1	23174

1148.02355	1	23175

1148.16289	1	23176

1148.30223	3	23179

1148.44157	4	23183

1148.58091	1	23184

1148.72025	2	23186

1148.85959	14	23200

1148.99893	1	23201

1149.13827	4	23205

1149.27761	5	23210

1149.41695	1	23211

1149.55629	2	23213

1149.69563	1	23214

1149.83497	2	23216

1149.97431	1	23217

1150.11365	2	23219

1150.25299	1	23220

1150.39233	4	23224

1150.53167	1	23225

1150.67101	2	23227

1150.81035	1	23228

1150.94969	2	23230

1151.08903	1	23231

1151.22837	1	23232

1151.36771	1	23233

1151.50705	2	23235

1151.64639	1	23236

1151.78573	2	23238

1151.92507	3	23241

1152.06441	1	23242

1152.20375	2	23244

1152.34309	1	23245

1152.48243	22	23267

1152.62177	3	23270

1152.76112	2	23272

1152.90046	5	23277

1153.03979	1	23278

1153.17914	2	23280

1153.31848	3	23283

1153.45782	1	23284

1153.59715	1	23285

1153.7365	2	23287

1153.87584	4	23291

1154.01518	1	23292

1154.15452	1	23293

1154.29386	1	23294

1154.4332	1	23295

1154.57254	3	23298

1154.71188	1	23299

1154.85122	1	23300

1154.99056	1	23301

1155.1299	1	23302

1155.26924	2	23304

1155.40858	6	23310

1155.54792	3	23313

1155.68726	1	23314

1155.8266	1	23315

1155.96594	1	23316

1156.10528	1	23317

1156.24462	1	23318

1156.38396	2	23320

1156.5233	2	23322

1156.66264	1	23323

1156.80198	1	23324

1156.94132	1	23325

1157.08066	1	23326

1157.22	1	23327

1157.35934	4	23331

1157.49868	2	23333

1157.63802	1	23334

1157.77736	1	23335

1157.9167	1	23336

1158.05604	1	23337

1158.19538	1	23338

1158.33472	1	23339

1158.47406	9	23348

1158.6134	10	23358

1158.75274	1	23359

1158.89208	1	23360

1159.03142	4	23364

1159.17076	9	23373

1159.3101	4	23377

1159.44944	1	23378

1159.58878	1	23379

1159.72812	1	23380

1159.86746	2	23382

1160.0068	1	23383

1160.14614	2	23385

1160.28548	1	23386

1160.42482	4	23390

1160.56416	1	23391

1160.7035	2	23393

1160.84284	8	23401

1160.98218	1	23402

1161.12152	1	23403

1161.26086	3	23406

1161.4002	1	23407

1161.53954	3	23410

1161.67889	1	23411

1161.81823	3	23414

1161.95756	2	23416

1162.09691	8	23424

1162.23625	5	23429

1162.37559	4	23433

1162.51492	24	23457

1162.65427	2	23459

1162.79361	15	23474

1162.93295	6	23480

1163.07229	1	23481

1163.21163	1	23482

1163.35097	1	23483

1163.49031	3	23486

1163.62965	1	23487

1163.76899	1	23488

1163.90833	6	23494

1164.04767	1	23495

1164.18701	7	23502

1164.32635	2	23504

1164.46569	2	23506

1164.60503	3	23509

1164.74437	1	23510

1164.88371	2	23512

1165.02305	1	23513

1165.16239	6	23519

1165.30173	1	23520

1165.44107	1	23521

1165.58041	1	23522

1165.71975	2	23524

1165.85909	1	23525

1165.99843	6	23531

1166.13777	1	23532

1166.27711	1	23533

1166.41645	1	23534

1166.55579	1	23535

1166.69513	3	23538

1166.83447	1	23539

1166.97381	3	23542

1167.11315	26	23568

1167.25249	3	23571

1167.39183	17	23588

1167.53117	1	23589

1167.67051	5	23594

1167.80985	1	23595

1167.94919	1	23596

1168.08853	3	23599

1168.22787	4	23603

1168.36721	2	23605

1168.50655	2	23607

1168.64589	1	23608

1168.78523	1	23609

1168.92457	7	23616

1169.06391	5	23621

1169.20325	1	23622

1169.34259	3	23625

1169.48193	1	23626

1169.62127	2	23628

1169.76061	2	23630

1169.89995	1	23631

1170.03929	5	23636

1170.17863	1	23637

1170.31797	9	23646

1170.45731	1	23647

1170.59666	2	23649

1170.736	7	23656

1170.87534	1	23657

1171.01467	4	23661

1171.15402	2	23663

1171.29336	8	23671

1171.4327	1	23672

1171.57204	1	23673

1171.71138	5	23678

1171.85072	1	23679

1171.99006	1	23680

1172.1294	2	23682

1172.26874	5	23687

1172.40808	1	23688

1172.54742	6	23694

1172.68676	3	23697

1172.8261	1	23698

1172.96544	2	23700

1173.10478	7	23707

1173.24412	2	23709

1173.38346	1	23710

1173.5228	1	23711

1173.66214	2	23713

1173.80148	1	23714

1173.94082	2	23716

1174.08016	2	23718

1174.2195	1	23719

1174.35884	3	23722

1174.49818	10	23732

1174.63752	6	23738

1174.77686	6	23744

1174.9162	3	23747

1175.05554	2	23749

1175.19488	5	23754

1175.33422	1	23755

1175.47356	1	23756

1175.6129	2	23758

1175.75224	2	23760

1175.89158	4	23764

1176.03092	6	23770

1176.17026	2	23772

1176.3096	1	23773

1176.44894	1	23774

1176.58828	1	23775

1176.72762	1	23776

1176.86696	2	23778

1177.0063	2	23780

1177.14564	1	23781

1177.28498	1	23782

1177.42432	1	23783

1177.56366	2	23785

1177.703	3	23788

1177.84234	4	23792

1177.98168	2	23794

1178.12102	1	23795

1178.26036	1	23796

1178.3997	1	23797

1178.53904	3	23800

1178.67838	1	23801

1178.81772	2	23803

1178.95706	1	23804

1179.0964	3	23807

1179.23574	4	23811

1179.37508	1	23812

1179.51443	1	23813

1179.65377	3	23816

1179.79311	2	23818

1179.93244	1	23819

1180.07179	1	23820

1180.21113	1	23821

1180.35047	10	23831

1180.48981	1	23832

1180.62915	4	23836

1180.76849	52	23888

1180.90783	1	23889

1181.04717	1	23890

1181.18651	1	23891

1181.32585	2	23893

1181.46519	1	23894

1181.60453	2	23896

1181.74387	1	23897

1181.88321	1	23898

1182.02255	1	23899

1182.16189	3	23902

1182.30123	1	23903

1182.44057	1	23904

1182.57991	1	23905

1182.71925	1	23906

1182.85859	1	23907

1182.99793	4	23911

1183.13727	1	23912

1183.27661	1	23913

1183.41595	21	23934

1183.55529	2	23936

1183.69463	3	23939

1183.83397	1	23940

1183.97331	1	23941

1184.11265	10	23951

1184.25199	2	23953

1184.39133	1	23954

1184.53067	7	23961

1184.67001	1	23962

1184.80935	2	23964

1184.94869	5	23969

1185.08803	4	23973

1185.22737	4	23977

1185.36671	11	23988

1185.50605	1	23989

1185.64539	5	23994

1185.78473	28	24022

1185.92407	10	24032

1186.06341	1	24033

1186.20275	4	24037

1186.34209	2	24039

1186.48143	8	24047

1186.62077	9	24056

1186.76011	1	24057

1186.89945	2	24059

1187.03879	6	24065

1187.17813	2	24067

1187.31747	2	24069

1187.45681	3	24072

1187.59615	1	24073

1187.73549	1	24074

1187.87483	1	24075

1188.01417	3	24078

1188.15351	6	24084

1188.29285	2	24086

1188.4322	2	24088

1188.57154	1	24089

1188.71088	2	24091

1188.85021	1	24092

1188.98956	1	24093

1189.1289	4	24097

1189.26824	3	24100

1189.40758	4	24104

1189.54692	16	24120

1189.68626	4	24124

1189.8256	1	24125

1189.96494	1	24126

1190.10428	2	24128

1190.24362	8	24136

1190.38296	1	24137

1190.5223	1	24138

1190.66164	1	24139

1190.80098	1	24140

1190.94032	4	24144

1191.07966	2	24146

1191.219	4	24150

1191.35834	1	24151

1191.49768	6	24157

1191.63702	12	24169

1191.77636	3	24172

1191.9157	2	24174

1192.05504	5	24179

1192.19438	2	24181

1192.33372	6	24187

1192.47306	3	24190

1192.6124	1	24191

1192.75174	2	24193

1192.89108	1	24194

1193.03042	3	24197

1193.16976	2	24199

1193.3091	6	24205

1193.44844	1	24206

1193.58778	1	24207

1193.72712	1	24208

1193.86646	3	24211

1194.0058	3	24214

1194.14514	3	24217

1194.28448	5	24222

1194.42382	4	24226

1194.56316	1	24227

1194.7025	2	24229

1194.84184	6	24235

1194.98118	5	24240

1195.12052	10	24250

1195.25986	5	24255

1195.3992	1	24256

1195.53854	1	24257

1195.67788	5	24262

1195.81722	57	24319

1195.95656	7	24326

1196.0959	1	24327

1196.23524	3	24330

1196.37458	7	24337

1196.51392	3	24340

1196.65326	2	24342

1196.7926	1	24343

1196.93194	1	24344

1197.07128	1	24345

1197.21062	3	24348

1197.34997	1	24349

1197.48931	1	24350

1197.62865	5	24355

1197.76799	9	24364

1197.90732	1	24365

1198.04667	3	24368

1198.18601	2	24370

1198.32535	1	24371

1198.46469	2	24373

1198.60403	7	24380

1198.74337	4	24384

1198.88271	26	24410

1199.02205	61	24471

1199.16139	5	24476

1199.30073	8	24484

1199.44007	3	24487

1199.57941	4	24491

1199.71875	2	24493

1199.85809	5	24498

1199.99743	27	24525

1200.13677	2	24527

1200.27611	2	24529

1200.41545	4	24533

1200.55479	24	24557

1200.69413	1	24558

1200.83347	3	24561

1200.97281	1	24562

1201.11215	1	24563

1201.25149	1	24564

1201.39083	6	24570

1201.53017	8	24578

1201.66951	2	24580

1201.80885	3	24583

1201.94819	2	24585

1202.08753	3	24588

1202.22687	1	24589

1202.36621	3	24592

1202.50555	2	24594

1202.64489	2	24596

1202.78423	1	24597

1202.92357	2	24599

1203.06291	1	24600

1203.20225	2	24602

1203.34159	5	24607

1203.48093	11	24618

1203.62027	2	24620

1203.75961	4	24624

1203.89895	20	24644

1204.03829	1	24645

1204.17763	3	24648

1204.31697	3	24651

1204.45631	1	24652

1204.59565	1	24653

1204.73499	4	24657

1204.87433	1	24658

1205.01367	2	24660

1205.15301	1	24661

1205.29235	9	24670

1205.43169	11	24681

1205.57103	2	24683

1205.71037	2	24685

1205.84971	16	24701

1205.98905	5	24706

1206.12839	2	24708

1206.26773	2	24710

1206.40708	3	24713

1206.54642	1	24714

1206.68576	13	24727

1206.82509	4	24731

1206.96444	2	24733

1207.10378	1	24734

1207.24312	3	24737

1207.38246	4	24741

1207.5218	3	24744

1207.66114	1	24745

1207.80048	6	24751

1207.93982	1	24752

1208.07916	1	24753

1208.2185	1	24754

1208.35784	2	24756

1208.49718	3	24759

1208.63652	2	24761

1208.77586	1	24762

1208.9152	6	24768

1209.05454	6	24774

1209.19388	14	24788

1209.33322	1	24789

1209.47256	3	24792

1209.6119	3	24795

1209.75124	1	24796

1209.89058	4	24800

1210.02992	1	24801

1210.16926	18	24819

1210.3086	11	24830

1210.44794	10	24840

1210.58728	1	24841

1210.72662	1	24842

1210.86596	4	24846

1211.0053	4	24850

1211.14464	3	24853

1211.28398	10	24863

1211.42332	1	24864

1211.56266	3	24867

1211.702	2	24869

1211.84134	2	24871

1211.98068	1	24872

1212.12002	2	24874

1212.25936	1	24875

1212.3987	1	24876

1212.53804	2	24878

1212.67738	1	24879

1212.81672	4	24883

1212.95606	6	24889

1213.0954	1	24890

1213.23474	66	24956

1213.37408	2	24958

1213.51342	1	24959

1213.65276	9	24968

1213.7921	7	24975

1213.93144	1	24976

1214.07078	2	24978

1214.21012	6	24984

1214.34946	32	25016

1214.4888	4	25020

1214.62814	2	25022

1214.76748	2	25024

1214.90682	1	25025

1215.04616	2	25027

1215.1855	3	25030

1215.32485	1	25031

1215.46419	3	25034

1215.60353	1	25035

1215.74286	1	25036

1215.88221	3	25039

1216.02155	14	25053

1216.16089	1	25054

1216.30023	3	25057

1216.43957	2	25059

1216.57891	6	25065

1216.71825	6	25071

1216.85759	28	25099

1216.99693	5	25104

1217.13627	1	25105

1217.27561	1	25106

1217.41495	17	25123

1217.55429	12	25135

1217.69363	4	25139

1217.83297	1	25140

1217.97231	3	25143

1218.11165	6	25149

1218.25099	7	25156

1218.39033	4	25160

1218.52967	7	25167

1218.66901	12	25179

1218.80835	21	25200

1218.94769	11	25211

1219.08703	3	25214

1219.22637	17	25231

1219.36571	14	25245

1219.50505	2	25247

1219.64439	6	25253

1219.78373	9	25262

1219.92307	14	25276

1220.06241	23	25299

1220.20175	53	25352

1220.34109	56	25408

1220.48043	45	25453

1220.61977	1	25454

1220.75911	11438	36892

1220.89845	2	36894

1221.03779	1	36895

1221.17713	2	36897

1221.31647	1	36898

1221.45581	2	36900

1221.59515	4	36904

1221.73449	1	36905

1221.87383	2	36907

1222.01317	5	36912

1222.15251	2	36914

1222.29185	3	36917

1222.43119	2	36919

1222.57053	1	36920

**Fig 10. Acoustic emission peak frequency variation curve**

**（*a*）Non-hydraulic-0°**

1	0.15894	0.29035	164062

2	0.17339	0.38713	164062

3	0.18945	0.48391	0

4	0.20551	0.58069	164062

5	0.22477	0.67747	164062

6	0.23922	0.77425	0

7	0.25848	0.87104	234375

8	0.26973	0.96782	13183

9	0.28418	1.0646	2929

10	0.29862	1.16138	4394

11	0.3195	1.25816	0

12	0.33716	1.35495	5859

13	0.34679	1.45173	187500

14	0.36445	1.54851	46875

15	0.38212	1.64529	11718

16	0.39816	1.74207	0

17	0.41262	1.83885	140625

18	0.43188	1.93564	46875

19	0.44793	2.03242	46875

20	0.46078	2.1292	2929

21	0.47684	2.22598	162597

22	0.4945	2.32276	0

23	0.50894	2.41955	46875

24	0.5218	2.51633	8789

25	0.54106	2.61311	93750

26	0.56353	2.70989	93750

27	0.57317	2.80667	0

28	0.58761	2.90345	14648

29	0.61009	3.00024	0

30	0.62294	3.09702	187500

31	0.63578	3.1938	46875

32	0.65185	3.29058	0

33	0.67432	3.38736	46875

34	0.68395	3.48415	0

35	0.70161	3.58093	187500

36	0.71606	3.67771	5859

37	0.73532	3.77449	0

38	0.74977	3.87127	55664

39	0.76583	3.96805	93750

40	0.78028	4.06484	0

41	0.79954	4.16162	46875

42	0.81079	4.2584	46875

43	0.83005	4.35518	0

44	0.84771	4.45196	11718

45	0.86216	4.54875	46875

46	0.8734	4.64553	46875

47	0.88785	4.74231	0

48	0.90872	4.83909	23437

49	0.92317	4.93587	4394

50	0.93441	5.03265	13183

51	0.95849	5.12944	46875

52	0.97133	5.22622	93750

53	0.98418	5.323	46875

54	1.01628	5.41978	187500

55	1.03074	5.51656	46875

56	1.05001	5.61335	46875

57	1.05964	5.71013	0

58	1.0757	5.80691	0

59	1.09496	5.90369	0

60	1.10781	6.00047	0

61	1.11904	6.09725	93750

62	1.14473	6.19404	46875

63	1.15436	6.29082	11718

64	1.17203	6.3876	0

65	1.18647	6.48438	16113

66	1.20413	6.58116	0

67	1.2202	6.67794	13183

68	1.23464	6.77473	11718

69	1.2523	6.87151	0

70	1.26996	6.96829	6591

71	1.28441	7.06507	46875

72	1.29564	7.16185	169921

73	1.31652	7.25864	0

74	1.33258	7.35542	4394

75	1.34863	7.4522	14648

76	1.35988	7.54898	4394

77	1.38555	7.64576	8789

78	1.3984	7.74254	93750

79	1.40803	7.83933	0

80	1.42249	7.93611	46875

81	1.44497	8.03289	46875

82	1.46102	8.12967	8789

83	1.47226	8.22645	169921

84	1.49312	8.32324	46875

85	1.50757	8.42002	17578

86	1.52203	8.5168	46875

87	1.53808	8.61358	187500

88	1.55735	8.71036	4394

89	1.57019	8.80714	2929

90	1.58303	8.90393	0

91	1.6007	9.00071	5859

92	1.61996	9.09749	16113

93	1.63923	9.19427	5859

94	1.64725	9.29105	46875

95	1.66491	9.38784	187500

96	1.68579	9.48462	46875

97	1.69702	9.5814	93750

98	1.7163	9.67818	4394

99	1.73075	9.77496	0

100	1.75001	9.87174	23437

101	1.76446	9.96853	0

102	1.77569	10.06531	46875

103	1.79818	10.16209	11718

104	1.81263	10.25887	4394

105	1.82386	10.35565	46875

106	1.84152	10.45244	46875

107	1.86079	10.54922	46875

108	1.88165	10.646	35156

109	1.88647	10.74278	8789

110	1.90735	10.83956	0

111	1.92661	10.93634	0

112	1.93464	11.03313	0

113	1.94908	11.12991	17578

114	1.97157	11.22669	0

115	1.98762	11.32347	187500

116	2.00047	11.42025	0

117	2.01974	11.51704	0

118	2.039	11.61382	7324

119	2.05346	11.7106	46875

120	2.06629	11.80738	46875

121	2.07913	11.90416	187500

122	2.10322	12.00094	46875

123	2.11606	12.09773	0

124	2.12571	12.19451	0

125	2.14818	12.29129	0

126	2.16263	12.38807	46875

127	2.1835	12.48485	46875

128	2.19313	12.58164	46875

129	2.214	12.67842	0

130	2.22685	12.7752	187500

131	2.23969	12.87198	140625

132	2.25574	12.96876	17578

133	2.27983	13.06554	0

134	2.29106	13.16233	93750

135	2.30231	13.25911	46875

136	2.32157	13.35589	46875

137	2.34404	13.45267	11718

138	2.36011	13.54945	11718

139	2.36974	13.64624	38085

140	2.389	13.74302	17578

141	2.40827	13.8398	93750

142	2.4147	13.93658	58593

143	2.43396	14.03336	140625

144	2.45483	14.13014	6591

145	2.46928	14.22693	46875

146	2.48213	14.32371	8789

147	2.50299	14.42049	187500

148	2.52066	14.51727	23437

149	2.53189	14.61405	8789

150	2.54313	14.71084	23437

151	2.56401	14.80762	0

152	2.58167	14.9044	35156

153	2.58968	15.00118	0

154	2.61056	15.09796	46875

155	2.62982	15.19474	16113

156	2.64428	15.29153	2929

157	2.65711	15.38831	46875

158	2.67318	15.48509	46875

159	2.69726	15.58187	46875

160	2.7085	15.67865	187500

161	2.72135	15.77544	46875

162	2.74061	15.87222	46875

163	2.75666	15.969	46875

164	2.7695	16.06578	187500

165	2.78396	16.16256	16113

166	2.80965	16.25934	4394

167	2.81928	16.35613	8789

168	2.8273	16.45291	46875

169	2.853	16.54969	11718

170	2.87066	16.64647	4394

171	2.88189	16.74325	0

172	2.89634	16.84004	23437

173	2.91721	16.93682	46875

174	2.93327	17.0336	0

175	2.94772	17.13038	11718

176	2.96217	17.22716	3662

177	2.98625	17.32394	0

178	2.99589	17.42073	0

179	3.00713	17.51751	46875

180	3.02318	17.61429	46875

181	3.04566	17.71107	187500

182	3.06011	17.80785	46875

183	3.07615	17.90464	140625

184	3.09061	18.00142	46875

185	3.10827	18.0982	64453

186	3.11791	18.19498	46875

187	3.13717	18.29176	187500

188	3.15804	18.38854	46875

189	3.1757	18.48533	0

190	3.18694	18.58211	4394

191	3.20461	18.67889	140625

192	3.22226	18.77567	11718

193	3.23511	18.87245	5859

194	3.25116	18.96924	46875

195	3.26561	19.06602	187500

196	3.2913	19.1628	4394

197	3.30093	19.25958	13183

198	3.31538	19.35636	0

199	3.33143	19.45314	0

200	3.3507	19.54993	46875

201	3.36837	19.64671	14648

202	3.37639	19.74349	0

203	3.39887	19.84027	11718

204	3.41653	19.93705	0

205	3.42294	20.03383	140625

206	3.44222	20.13062	0

207	3.46309	20.2274	93750

208	3.47753	20.32418	46875

209	3.48878	20.42096	0

210	3.51286	20.51774	46875

211	3.53213	20.61453	0

212	3.53695	20.71131	0

213	3.55299	20.80809	93750

214	3.57066	20.90487	46875

215	3.59313	21.00165	0

216	3.59956	21.09843	187500

217	3.61883	21.19522	187500

218	3.63969	21.292	0

219	3.66057	21.38878	0

220	3.6702	21.48556	87890

221	3.68465	21.58234	0

222	3.70071	21.67913	0

223	3.71355	21.77591	117187

224	3.728	21.87269	140625

225	3.75047	21.96947	0

226	3.76814	22.06625	46875

227	3.78259	22.16303	0

228	3.79543	22.25982	0

229	3.81469	22.3566	46875

230	3.83235	22.45338	0

231	3.83717	22.55016	11718

232	3.85805	22.64694	0

233	3.88213	22.74373	187500

234	3.89176	22.84051	11718

235	3.903	22.93729	0

236	3.92869	23.03407	46875

237	3.94795	23.13085	0

238	3.95599	23.22763	46875

239	3.97364	23.32442	4394

240	3.99452	23.4212	0

241	4.00415	23.51798	46875

242	4.01699	23.61476	5859

243	4.03787	23.71154	11718

244	4.05713	23.80833	0

245	4.06837	23.90511	93750

246	4.08603	24.00189	8056

247	4.10368	24.09867	0

248	4.11653	24.19545	46875

249	4.12777	24.29223	46875

250	4.14383	24.38902	0

251	4.17112	24.4858	4394

252	4.18236	24.58258	187500

253	4.19359	24.67936	4394

254	4.21447	24.77614	0

255	4.23373	24.87293	0

256	4.24336	24.96971	46875

257	4.25782	25.06649	46875

258	4.2819	25.16327	46875

259	4.29474	25.26005	46875

260	4.30598	25.35683	46875

261	4.32524	25.45362	46875

262	4.34452	25.5504	0

263	4.36218	25.64718	0

264	4.37341	25.74396	46875

265	4.38466	25.84074	46875

266	4.41515	25.93753	0

267	4.41998	26.03431	11718

268	4.43282	26.13109	46875

269	4.45369	26.22787	46875

270	4.47136	26.32465	140625

271	4.4874	26.42143	46875

272	4.50025	26.51822	23437

273	4.52434	26.615	46875

274	4.53717	26.71178	46875

275	4.54681	26.80856	0

276	4.55965	26.90534	0

277	4.58534	27.00213	46875

278	4.59818	27.09891	46875

279	4.61263	27.19569	0

280	4.62708	27.29247	46875

281	4.64796	27.38925	0

282	4.6608	27.48603	187500

283	4.68006	27.58282	187500

284	4.68969	27.6796	0

285	4.70897	27.77638	166992

286	4.72181	27.87316	46875

287	4.73627	27.96994	152343

288	4.76035	28.06673	0

289	4.78443	28.16351	4394

290	4.78924	28.26029	140625

291	4.80208	28.35707	46875

292	4.82136	28.45385	175781

293	4.8358	28.55063	8789

294	4.84865	28.64742	0

295	4.86309	28.7442	46875

296	4.89199	28.84098	166992

297	4.90003	28.93776	17578

298	4.91929	29.03454	0

299	4.93695	29.13133	17578

300	4.95942	29.22811	0

301	4.96264	29.32489	140625

302	4.9803	29.42167	0

303	4.99475	29.51845	93750

304	5.01401	29.61523	16113

305	5.02364	29.71202	8789

306	5.04934	29.8088	46875

307	5.0686	29.90558	187500

308	5.08144	30.00236	187500

309	5.08947	30.09914	0

310	5.11836	30.19593	4394

311	5.13122	30.29271	0

312	5.14245	30.38949	0

313	5.15369	30.48627	11718

314	5.17616	30.58305	46875

315	5.19865	30.67983	0

316	5.20186	30.77662	0

317	5.22274	30.8734	82031

318	5.242	30.97018	13183

319	5.25003	31.06696	16113

320	5.26769	31.16374	46875

321	5.28374	31.26053	82031

322	5.30462	31.35731	46875

323	5.31264	31.45409	64453

324	5.33029	31.55087	11718

325	5.35438	31.64765	11718

326	5.37043	31.74443	16113

327	5.37846	31.84122	17578

328	5.39772	31.938	11718

329	5.42021	32.03478	8789

330	5.43305	32.13156	46875

331	5.44589	32.22834	0

332	5.46838	32.32512	0

333	5.48443	32.42191	140625

334	5.49567	32.51869	17578

335	5.51493	32.61547	35156

336	5.52618	32.71225	11718

337	5.54705	32.80903	11718

338	5.55026	32.90582	46875

339	5.56952	33.0026	93750

340	5.59199	33.09938	140625

341	5.61127	33.19616	4394

342	5.61608	33.29294	0

343	5.64498	33.38972	46875

344	5.66425	33.48651	0

345	5.66907	33.58329	93750

346	5.68512	33.68007	281250

347	5.70438	33.77685	93750

348	5.72204	33.87363	140625

349	5.72847	33.97042	20507

350	5.75255	34.0672	4394

351	5.7686	34.16398	0

352	5.79268	34.26076	0

353	5.7975	34.35754	0

354	5.81998	34.45432	164794

355	5.83604	34.55111	11718

356	5.84567	34.64789	0

357	5.85852	34.74467	0

358	5.88742	34.84145	4394

359	5.90186	34.93823	46875

360	5.90989	35.03502	187500

361	5.92915	35.1318	0

362	5.94681	35.22858	11718

363	5.95966	35.32536	169921

364	5.97572	35.42214	0

365	5.99177	35.51892	0

366	6.00943	35.61571	140625

367	6.02228	35.71249	0

368	6.03833	35.80927	35156

369	6.05599	35.90605	4394

370	6.07686	36.00283	187500

371	6.08649	36.09962	0

372	6.10255	36.1964	0

373	6.12182	36.29318	140625

374	6.14108	36.38996	11718

375	6.1459	36.48674	46875

376	6.16999	36.58352	140625

377	6.19727	36.68031	11718

378	6.20531	36.77709	0

379	6.21333	36.87387	46875

380	6.2326	36.97065	0

381	6.24865	37.06743	46875

382	6.25989	37.16422	46875

383	6.27915	37.261	11718

384	6.30003	37.35778	46875

385	6.3193	37.45456	5859

386	6.32411	37.55134	46875

387	6.34177	37.64812	46875

388	6.36585	37.74491	0

389	6.3803	37.84169	46875

390	6.38673	37.93847	187500

391	6.40599	38.03525	46875

392	6.43169	38.13203	46875

393	6.4381	38.22882	159667

394	6.45736	38.3256	187500

395	6.47342	38.42238	16113

396	6.4959	38.51916	46875

397	6.50393	38.61594	162597

398	6.51357	38.71272	46875

399	6.54086	38.80951	46875

400	6.55692	38.90629	11718

401	6.56173	39.00307	140625

402	6.58259	39.09985	12451

403	6.60668	39.19663	175781

404	6.62113	39.29342	17578

405	6.63237	39.3902	46875

406	6.65325	39.48698	46875

407	6.66769	39.58376	46875

408	6.67732	39.68054	0

409	6.69819	39.77732	16113

410	6.71906	39.87411	0

411	6.73512	39.97089	140625

412	6.74957	40.06767	0

413	6.76402	40.16445	0

414	6.78649	40.26123	11718

415	6.79774	40.35802	17578

416	6.80737	40.4548	234375

417	6.82664	40.55158	0

418	6.84911	40.64836	187500

419	6.86357	40.74514	140625

420	6.8732	40.84192	13183

421	6.89888	40.93871	46875

422	6.91494	41.03549	5859

423	6.91975	41.13227	41015

424	6.94705	41.22905	46875

425	6.9615	41.32583	93750

426	6.97113	41.42262	46875

427	6.98718	41.5194	46875

428	7.00325	41.61618	0

429	7.02251	41.71296	187500

430	7.04177	41.80974	140625

431	7.05784	41.90652	16113

432	7.06906	42.00331	16113

433	7.08673	42.10009	0

434	7.09797	42.19687	17578

435	7.11563	42.29365	11718

436	7.1381	42.39043	0

437	7.15094	42.48722	0

438	7.15898	42.584	16113

439	7.18627	42.68078	16113

440	7.20233	42.77756	0

441	7.21356	42.87434	152343

442	7.22801	42.97112	11718

443	7.25691	43.06791	46875

444	7.26495	43.16469	0

445	7.27618	43.26147	46875

446	7.28903	43.35825	0

447	7.31632	43.45503	0

448	7.32595	43.55182	46875

449	7.34361	43.6486	93750

450	7.35805	43.74538	46875

451	7.38054	43.84216	0

452	7.38696	43.93894	0

453	7.40141	44.03572	16113

454	7.42389	44.13251	0

455	7.43993	44.22929	46875

456	7.45279	44.32607	0

457	7.46723	44.42285	0

458	7.48651	44.51963	46875

459	7.50577	44.61641	46875

460	7.51701	44.7132	16113

461	7.52664	44.80998	0

462	7.54912	44.90676	166992

463	7.56839	45.00354	0

464	7.57641	45.10032	4394

465	7.6021	45.19711	0

466	7.62297	45.29389	46875

467	7.6326	45.39067	0

468	7.64063	45.48745	46875

469	7.66311	45.58423	0

470	7.6904	45.68101	17578

471	7.6888	45.7778	17578

472	7.71448	45.87458	46875

473	7.73054	45.97136	175781

474	7.7482	46.06814	93750

475	7.75462	46.16492	175781

476	7.7755	46.26171	0

477	7.79476	46.35849	0

478	7.80279	46.45527	99609

479	7.81724	46.55205	175781

480	7.83971	46.64883	0

481	7.85897	46.74561	16113

482	7.8654	46.8424	64453

483	7.88788	46.93918	0

484	7.91036	47.03596	46875

485	7.92159	47.13274	11718

486	7.93284	47.22952	16113

487	7.95371	47.32631	46875

488	7.96976	47.42309	187500

489	7.98743	47.51987	168457

490	7.99224	47.61665	140625

491	8.02435	47.71343	0

492	8.03559	47.81021	0

493	8.04201	47.907	166992

494	8.05807	48.00378	14648

495	8.08215	48.10056	16113

496	8.09499	48.19734	16113

497	8.11425	48.29412	46875

498	8.1287	48.39091	187500

499	8.14958	48.48769	0

500	8.15761	48.58447	0

501	8.17366	48.68125	187500

502	8.19775	48.77803	0

503	8.20576	48.87481	187500

504	8.21701	48.9716	140625

505	8.23146	49.06838	12451

506	8.25875	49.16516	166992

507	8.26679	49.26194	82031

508	8.27963	49.35872	187500

509	8.30371	49.45551	187500

510	8.32297	49.55229	16113

511	8.33742	49.64907	16113

512	8.34706	49.74585	17578

513	8.37114	49.84263	169921

514	8.38238	49.93941	17578

515	8.39522	50.0362	16845

516	8.41449	50.13298	187500

517	8.43215	50.22976	0

518	8.45302	50.32654	0

519	8.46104	50.42332	175781

520	8.47871	50.52011	16113

521	8.50279	50.61689	0

522	8.50761	50.71367	0

523	8.52527	50.81045	0

524	8.53971	50.90723	46875

525	8.57022	51.00401	0

526	8.57986	51.1008	105468

527	8.58949	51.19758	151611

528	8.61358	51.29436	11718

529	8.62802	51.39114	0

530	8.63605	51.48792	0

531	8.6521	51.58471	46875

532	8.67297	51.68149	234375

533	8.68742	51.77827	46875

534	8.69705	51.87505	0

535	8.71954	51.97183	17578

536	8.74201	52.06861	140625

537	8.75967	52.1654	0

538	8.75967	52.26218	11718

539	8.78215	52.35896	168457

540	8.7982	52.45574	4394

541	8.80623	52.55252	0

542	8.82389	52.64931	0

543	8.85279	52.74609	46875

544	8.87045	52.84287	46875

545	8.87687	52.93965	46875

546	8.89294	53.03643	11718

547	8.91702	53.13321	187500

548	8.92665	53.23	0

549	8.93949	53.32678	152343

550	8.96196	53.42356	234375

551	8.97803	53.52034	11718

552	8.98766	53.61712	93750

553	9.01013	53.71391	11718

554	9.0294	53.81069	3662

555	9.04384	53.90747	46875

556	9.05027	54.00425	23437

557	9.06793	54.10103	140625

558	9.09362	54.19781	0

559	9.10968	54.2946	17578

560	9.11609	54.39138	175781

561	9.14179	54.48816	6591

562	9.16586	54.58494	46875

563	9.17068	54.68172	46875

564	9.18193	54.77851	187500

565	9.20922	54.87529	0

566	9.22045	54.97207	0

567	9.23169	55.06885	17578

568	9.24936	55.16563	16113

569	9.27344	55.26241	16113

570	9.28628	55.3592	0

571	9.29591	55.45598	0

572	9.32161	55.55276	46875

573	9.33927	55.64954	0

574	9.33927	55.74632	0

575	9.36334	55.84311	46875

576	9.38582	55.93989	0

577	9.40027	56.03667	93750

578	9.4099	56.13345	23437

579	9.42757	56.23023	46875

580	9.45004	56.32701	0

581	9.45647	56.4238	155273

582	9.47413	56.52058	23437

583	9.48697	56.61736	187500

584	9.50623	56.71414	46875

585	9.51747	56.81092	0

586	9.53995	56.9077	82031

587	9.56082	57.00449	46875

588	9.57848	57.10127	23437

589	9.58652	57.19805	0

590	9.60256	57.29483	93750

591	9.62344	57.39161	8789

592	9.63789	57.4884	46875

593	9.64912	57.58518	93750

594	9.66518	57.68196	46875

595	9.68926	57.77874	46875

596	9.69729	57.87552	16113

597	9.71495	57.9723	0

598	9.73743	58.06909	0

599	9.75669	58.16587	46875

600	9.76312	58.26265	46875

601	9.77757	58.35943	0

602	9.80326	58.45621	0

603	9.81289	58.553	187500

604	9.82412	58.64978	0

605	9.84821	58.74656	17578

606	9.86908	58.84334	0

607	9.88192	58.94012	93750

608	9.88514	59.0369	16113

609	9.90922	59.13369	257812

610	9.93009	59.23047	0

611	9.93651	59.32725	23437

612	9.95096	59.42403	0

613	9.97665	59.52081	140625

614	9.99752	59.6176	17578

615	10.00876	59.71438	11718

616	10.02001	59.81116	16113

617	10.03927	59.90794	0

618	10.04568	60.00472	93750

619	10.06174	60.1015	5859

620	10.08422	60.19829	187500

621	10.1083	60.29507	46875

622	10.12115	60.39185	0

623	10.13078	60.48863	11718

624	10.15325	60.58541	6591

625	10.17734	60.6822	16113

626	10.17895	60.77898	35156

627	10.19179	60.87576	4394

628	10.21749	60.97254	46875

629	10.22712	61.06932	0

630	10.23996	61.1661	17578

631	10.25761	61.26289	187500

632	10.2833	61.35967	0

633	10.29293	61.45645	0

634	10.31221	61.55323	82031

635	10.32344	61.65001	58593

636	10.3411	61.7468	23437

637	10.34913	61.84358	11718

638	10.3668	61.94036	0

639	10.39409	62.03714	17578

640	10.40853	62.13392	0

641	10.41977	62.2307	93750

642	10.43743	62.32749	128906

643	10.46312	62.42427	0

644	10.47115	62.52105	93750

645	10.48239	62.61783	0

646	10.50486	62.71461	175781

647	10.51932	62.8114	0

648	10.53056	62.90818	0

649	10.54982	63.00496	16845

650	10.5755	63.10174	0

651	10.58514	63.19852	0

652	10.59157	63.2953	17578

653	10.60921	63.39209	0

654	10.6333	63.48887	0

655	10.64775	63.58565	46875

656	10.65579	63.68243	0

657	10.67665	63.77921	187500

658	10.69592	63.876	0

659	10.71037	63.97278	46875

660	10.72804	64.06956	93750

661	10.74889	64.16634	14648

662	10.75854	64.26312	0

663	10.76977	64.3599	0

664	10.78743	64.45669	5859

665	10.80991	64.55347	0

666	10.82114	64.65025	0

667	10.83881	64.74703	0

668	10.85327	64.84381	0

669	10.87735	64.9406	46875

670	10.89019	65.03738	93750

671	10.8934	65.13416	0

672	10.9207	65.23094	17578

673	10.93193	65.32772	0

674	10.94959	65.4245	0

675	10.96886	65.52129	0

676	10.99295	65.61807	93750

677	11.00258	65.71485	41015

678	11.01221	65.81163	187500

679	11.02505	65.90841	0

680	11.04913	66.0052	16845

681	11.05876	66.10198	46875

682	11.07321	66.19876	0

683	11.09248	66.29554	140625

684	11.11496	66.39232	17578

685	11.12459	66.4891	23437

686	11.13904	66.58589	0

687	11.16312	66.68267	46875

688	11.17436	66.77945	0

689	11.18079	66.87623	0

690	11.20326	66.97301	5859

691	11.22414	67.0698	16113

692	11.23377	67.16658	16113

693	11.24982	67.26336	187500

694	11.2739	67.36014	0

695	11.28995	67.45692	169921

696	11.3012	67.5537	0

697	11.31404	67.65049	187500

698	11.34133	67.74727	16113

699	11.34937	67.84405	0

700	11.3606	67.94083	46875

701	11.38468	68.03761	140625

702	11.40877	68.1344	0

703	11.4184	68.23118	0

704	11.42963	68.32796	17578

705	11.44409	68.42474	187500

706	11.46978	68.52152	165527

707	11.4778	68.6183	140625

708	11.49225	68.71509	152343

709	11.51472	68.81187	5859

710	11.53239	68.90865	16113

711	11.54202	69.00543	0

712	11.55648	69.10221	17578

713	11.57896	69.199	46875

714	11.59982	69.29578	23437

715	11.59822	69.39256	23437

716	11.62391	69.48934	46875

717	11.64318	69.58612	17578

718	11.65762	69.6829	0

719	11.67207	69.77969	0

720	11.69455	69.87647	0

721	11.71061	69.97325	16113

722	11.71864	70.07003	11718

723	11.72827	70.16681	17578

724	11.75716	70.26359	17578

725	11.77482	70.36038	0

726	11.78124	70.45716	187500

727	11.7989	70.55394	0

728	11.81978	70.65072	17578

729	11.83744	70.7475	0

730	11.84707	70.84429	0

731	11.86633	70.94107	0

732	11.8856	71.03785	187500

733	11.88721	71.13463	187500

734	11.91129	71.23141	0

735	11.93538	71.32819	0

736	11.94983	71.42498	0

737	11.95785	71.52176	11718

738	11.97712	71.61854	11718

739	11.9996	71.71532	16113

740	12.01564	71.8121	0

741	12.02689	71.90889	11718

742	12.03813	72.00567	0

743	12.059	72.10245	0

744	12.07344	72.19923	0

745	12.09272	72.29601	23437

746	12.10877	72.39279	46875

747	12.12803	72.48958	0

748	12.13767	72.58636	46875

749	12.15212	72.68314	5859

750	12.17299	72.77992	0

751	12.18904	72.8767	0

752	12.19065	72.97349	46875

753	12.21794	73.07027	17578

754	12.23882	73.16705	46875

755	12.25166	73.26383	46875

756	12.26771	73.36061	46875

757	12.28699	73.45739	269531

758	12.30143	73.55418	93750

759	12.30946	73.65096	0

760	12.32391	73.74774	46875

761	12.34799	73.84452	0

762	12.36725	73.9413	46875

763	12.3785	74.03809	46875

764	12.39455	74.13487	46875

765	12.42023	74.23165	46875

766	12.42827	74.32843	82031

767	12.43468	74.42521	17578

768	12.46198	74.52199	16113

769	12.47482	74.61878	165527

770	12.48607	74.71556	13183

771	12.50693	74.81234	0

772	12.5262	74.90912	0

773	12.54387	75.0059	46875

774	12.55189	75.10269	11718

775	12.56955	75.19947	16113

776	12.59042	75.29625	93750

777	12.60648	75.39303	64453

778	12.6145	75.48981	0

779	12.63698	75.58659	165527

780	12.64983	75.68338	9521

781	12.66428	75.78016	11718

782	12.68194	75.87694	46875

783	12.70281	75.97372	0

784	12.72047	76.0705	17578

785	12.72689	76.16729	13183

786	12.74776	76.26407	46875

787	12.76543	76.36085	46875

788	12.77506	76.45763	46875

789	12.7879	76.55441	0

790	12.81359	76.65119	46875

791	12.83606	76.74798	161865

792	12.84089	76.84476	16113

793	12.85694	76.94154	16113

794	12.8762	77.03832	0

795	12.88905	77.1351	0

796	12.90671	77.23189	0

797	12.91956	77.32867	140625

798	12.94524	77.42545	46875

799	12.95808	77.52223	140625

800	12.97414	77.61901	13183

801	12.99662	77.71579	187500

802	13.00465	77.81258	140625

803	13.02231	77.90936	16845

804	13.03354	78.00614	46875

805	13.05602	78.10292	187500

806	13.07368	78.1997	17578

807	13.0785	78.29649	46875

808	13.09937	78.39327	0

809	13.12024	78.49005	16113

810	13.1363	78.58683	5859

811	13.14593	78.68361	16113

812	13.17804	78.78039	16113

813	13.17965	78.87718	0

814	13.1941	78.97396	0

815	13.21818	79.07074	46875

816	13.23263	79.16752	187500

817	13.24868	79.2643	0

818	13.26474	79.36109	93750

819	13.27758	79.45787	46875

820	13.30006	79.55465	0

821	13.3113	79.65143	16113

822	13.31772	79.74821	0

823	13.34019	79.84499	46875

824	13.36107	79.94178	0

825	13.3707	80.03856	11718

826	13.38997	80.13534	17578

827	13.40603	80.23212	0

828	13.42207	80.3289	82031

829	13.43653	80.42569	187500

830	13.45579	80.52247	0

831	13.47667	80.61925	187500

832	13.48469	80.71603	11718

833	13.50075	80.81281	93750

834	13.5152	80.90959	0

835	13.53446	81.00638	0

836	13.54571	81.10316	140625

837	13.56818	81.19994	17578

838	13.58263	81.29672	140625

839	13.59547	81.3935	46875

840	13.60671	81.49029	0

841	13.62918	81.58707	0

842	13.64846	81.68385	16113

843	13.66451	81.78063	0

844	13.67254	81.87741	0

845	13.69823	81.97419	0

846	13.71107	82.07098	17578

847	13.72231	82.16776	46875

848	13.73034	82.26454	17578

849	13.76085	82.36132	16113

850	13.78332	82.4581	46875

851	13.78974	82.55488	187500

852	13.80258	82.65167	32958

853	13.82666	82.74845	187500

854	13.84754	82.84523	117187

855	13.84915	82.94201	0

856	13.86841	83.03879	187500

857	13.88607	83.13558	0

858	13.89891	83.23236	46875

859	13.91979	83.32914	17578

860	13.93584	83.42592	13183

861	13.95832	83.5227	17578

862	13.96796	83.61948	46875

863	13.98241	83.71627	165527

864	14.00327	83.81305	0

865	14.01933	83.90983	0

866	14.02255	84.00661	11718

867	14.05304	84.10339	93750

868	14.07231	84.20018	82031

869	14.08034	84.29696	46875

870	14.098	84.39374	46875

871	14.11245	84.49052	0

872	14.13011	84.5873	0

873	14.14135	84.68408	0

874	14.15098	84.78087	0

875	14.17667	84.87765	0

876	14.19112	84.97443	0

877	14.20075	85.07121	0

878	14.22644	85.16799	46875

879	14.24892	85.26478	152343

880	14.25695	85.36156	0

881	14.26658	85.45834	46875

882	14.28745	85.55512	16845

883	14.30832	85.6519	11718

884	14.31474	85.74868	41015

885	14.3324	85.84547	0

886	14.3597	85.94225	0

887	14.37093	86.03903	0

888	14.38379	86.13581	16113

889	14.39823	86.23259	0

890	14.4191	86.32938	46875

891	14.43034	86.42616	0

892	14.43997	86.52294	13183

893	14.46567	86.61972	46875

894	14.48332	86.7165	187500

895	14.49617	86.81328	58593

896	14.51061	86.91007	165527

897	14.5331	87.00685	0

898	14.54594	87.10363	17578

899	14.55718	87.20041	93750

900	14.57162	87.29719	16845

901	14.59891	87.39398	0

902	14.61176	87.49076	0

903	14.61979	87.58754	16113

904	14.64227	87.68432	46875

905	14.65833	87.7811	46875

906	14.67759	87.87788	17578

907	14.68722	87.97467	0

908	14.69686	88.07145	0

909	14.72095	88.16823	169921

910	14.73378	88.26501	0

911	14.74662	88.36179	16113

912	14.77071	88.45858	93750

913	14.79158	88.55536	0

914	14.80924	88.65214	0

915	14.81085	88.74892	0

916	14.84456	88.8457	0

917	14.85098	88.94248	0

918	14.85901	89.03927	0

919	14.87989	89.13605	46875

920	14.89755	89.23283	46875

921	14.91521	89.32961	0

922	14.93607	89.42639	17578

923	14.95855	89.52318	0

924	14.96979	89.61996	46875

925	14.96979	89.71674	5859

926	14.99387	89.81352	6591

927	15.01795	89.9103	82031

928	15.0308	90.00708	0

929	15.04043	90.10387	11718

930	15.0597	90.20065	0

931	15.08057	90.29743	46875

932	15.08539	90.39421	0

933	15.10626	90.49099	11718

934	15.12713	90.58778	0

935	15.13837	90.68456	46875

936	15.14962	90.78134	17578

937	15.16888	90.87812	140625

938	15.19135	90.9749	0

939	15.2042	91.07168	46875

940	15.21383	91.16847	169921

941	15.23309	91.26525	16113

942	15.25397	91.36203	155273

943	15.27163	91.45881	0

944	15.28126	91.55559	0

945	15.29731	91.65238	0

946	15.31819	91.74916	0

947	15.31979	91.84594	0

948	15.34388	91.94272	0

949	15.37118	92.0395	152343

950	15.3792	92.13628	0

951	15.39044	92.23307	152343

952	15.41291	92.32985	46875

953	15.42898	92.42663	16113

954	15.44342	92.52341	46875

955	15.44983	92.62019	46875

956	15.48034	92.71698	13183

957	15.49319	92.81376	46875

958	15.50924	92.91054	23437

959	15.52049	93.00732	0

960	15.54617	93.1041	93750

961	15.55901	93.20088	0

962	15.56384	93.29767	46875

963	15.58631	93.39445	0

964	15.60558	93.49123	16113

965	15.61842	93.58801	46875

966	15.63447	93.68479	46875

967	15.65695	93.78158	0

968	15.67139	93.87836	0

969	15.68104	93.97514	0

970	15.69548	94.07192	93750

971	15.71315	94.1687	105468

972	15.73081	94.26548	46875

973	15.74364	94.36227	0

974	15.76773	94.45905	16113

975	15.79021	94.55583	0

976	15.79663	94.65261	0

977	15.80144	94.74939	46875

978	15.82874	94.84617	35156

979	15.84961	94.94296	0

980	15.85603	95.03974	16113

981	15.86566	95.13652	0

982	15.89136	95.2333	11718

983	15.91062	95.33008	0

984	15.92026	95.42687	169921

985	15.93309	95.52365	11718

986	15.95557	95.62043	187500

987	15.97484	95.71721	0

988	15.98287	95.81399	0

989	16.00214	95.91077	17578

990	16.023	96.00756	11718

991	16.03264	96.10434	0

992	16.04227	96.20112	46875

993	16.07277	96.2979	0

994	16.08402	96.39468	16113

995	16.09205	96.49147	16113

996	16.11131	96.58825	0

997	16.13539	96.68503	0

998	16.14985	96.78181	46875

999	16.15786	96.87859	46875

1000	16.17714	96.97537	0

1001	16.19801	97.07216	16113

1002	16.21566	97.16894	0

1003	16.2253	97.26572	46875

1004	16.24296	97.3625	0

1005	16.26223	97.45928	0

1006	16.27347	97.55607	0

1007	16.2847	97.65285	0

1008	16.31682	97.74963	0

1009	16.32324	97.84641	152343

1010	16.3425	97.94319	0

1011	16.36498	98.03997	32958

1012	16.37622	98.13676	0

1013	16.38907	98.23354	166992

1014	16.40191	98.33032	16113

1015	16.42921	98.4271	0

1016	16.43722	98.52388	187500

1017	16.44044	98.62067	187500

1018	16.46773	98.71745	0

1019	16.49343	98.81423	164794

1020	16.50146	98.91101	46875

1021	16.51429	99.00779	93750

1022	16.53517	99.10457	0

1023	16.55443	99.20136	0

1024	16.56568	99.29814	0

1025	16.57852	99.39492	166992

1026	16.60099	99.4917	46875

1027	16.61866	99.58848	64453

1028	16.63149	99.68527	46875

1029	16.64114	99.78205	13183

1030	16.66361	99.87883	46875

1031	16.68127	99.97561	93750

1032	16.68608	100.07239	117187

1033	16.71017	100.16917	23437

1034	16.72301	100.26596	187500

1035	16.74067	100.36274	46875

1036	16.75351	100.45952	0

1037	16.78563	100.5563	140625

1038	16.79205	100.65308	140625

1039	16.80329	100.74987	0

1040	16.82095	100.84665	23437

1041	16.84182	100.94343	0

1042	16.85467	101.04021	17578

1043	16.86269	101.13699	41015

1044	16.88517	101.23377	0

1045	16.90765	101.33056	0

1046	16.92531	101.42734	64453

1047	16.93013	101.52412	0

1048	16.94778	101.6209	23437

1049	16.96865	101.71768	0

1050	16.97828	101.81447	175781

1051	16.99594	101.91125	187500

1052	17.01843	102.00803	23437

1053	17.03127	102.10481	46875

1054	17.0409	102.20159	46875

1055	17.06337	102.29837	117187

1056	17.07944	102.39516	0

1057	17.0971	102.49194	0

1058	17.10191	102.58872	46875

1059	17.1244	102.6855	35156

1060	17.14205	102.78228	0

1061	17.15971	102.87907	46875

1062	17.16934	102.97585	17578

1063	17.19503	103.07263	46875

1064	17.21109	103.16941	17578

1065	17.22072	103.26619	46875

1066	17.23678	103.36297	2929

1067	17.25926	103.45976	0

1068	17.2753	103.55654	140625

1069	17.28655	103.65332	0

1070	17.301	103.7501	16845

1071	17.31865	103.84688	0

1072	17.33792	103.94367	46875

1073	17.35237	104.04045	46875

1074	17.36682	104.13723	17578

1075	17.3893	104.23401	0

1076	17.39572	104.33079	93750

1077	17.41498	104.42757	46875

1078	17.42943	104.52436	0

1079	17.45031	104.62114	8789

1080	17.45673	104.71792	46875

1081	17.48082	104.8147	46875

1082	17.5049	104.91148	0

1083	17.51132	105.00827	187500

1084	17.52095	105.10505	152343

1085	17.54663	105.20183	0

1086	17.55788	105.29861	46875

1087	17.57715	105.39539	0

1088	17.58517	105.49217	7324

1089	17.60925	105.58896	46875

1090	17.63013	105.68574	0

1091	17.63976	105.78252	46875

1092	17.6526	105.8793	0

1093	17.67026	105.97608	16113

1094	17.68471	106.07287	187500

1095	17.69916	106.16965	0

1096	17.72004	106.26643	41015

1097	17.73769	106.36321	5859

1098	17.75214	106.45999	0

1099	17.76177	106.55677	46875

1100	17.78585	106.65356	17578

1101	17.80513	106.75034	16113

1102	17.80994	106.84712	16113

1103	17.83402	106.9439	0

1104	17.85008	107.04068	46875

1105	17.86293	107.13747	0

1106	17.87577	107.23425	0

1107	17.89504	107.33103	46875

1108	17.9159	107.42781	32226

1109	17.92875	107.52459	187500

1110	17.94159	107.62137	16845

1111	17.96247	107.71816	0

1112	17.97692	107.81494	46875

1113	17.98976	107.91172	7324

1114	18.00742	108.0085	0

1115	18.02829	108.10528	46875

1116	18.04113	108.20206	187500

1117	18.05558	108.29885	0

1118	18.06683	108.39563	46875

1119	18.09251	108.49241	166992

1120	18.10697	108.58919	169921

1121	18.1182	108.68597	17578

1122	18.13586	108.78276	46875

1123	18.15192	108.87954	16113

1124	18.16315	108.97632	46875

1125	18.18242	109.0731	17578

1126	18.20489	109.16988	187500

1127	18.22256	109.26666	0

1128	18.22738	109.36345	11718

1129	18.24022	109.46023	23437

1130	18.26591	109.55701	46875

1131	18.28036	109.65379	0

1132	18.29802	109.75057	17578

1133	18.31728	109.84736	16845

1134	18.32531	109.94414	0

1135	18.33816	110.04092	46875

1136	18.35903	110.1377	46875

1137	18.38151	110.23448	152343

1138	18.39434	110.33126	46875

1139	18.39916	110.42805	46875

1140	18.42486	110.52483	0

1141	18.44412	110.62161	0

1142	18.45536	110.71839	0

1143	18.46659	110.81517	46875

1144	18.48907	110.91196	11718

1145	18.50673	111.00874	187500

1146	18.54848	111.10552	0

1147	18.56775	111.2023	0

1148	18.57898	111.29908	0

1149	18.59664	111.39586	41015

1150	18.63839	111.49265	0

1151	18.65926	111.58943	46875

1152	18.67532	111.68621	23437

1153	18.85513	111.78299	140625

1154	18.94343	111.87977	187500

1155	19.11683	111.97656	171386

1156	18.92417	112.07334	0

1157	18.63518	112.17012	0

1158	18.24665	112.2669	46875

1159	17.19824	112.36368	166992

1160	16.18677	112.46046	0

1161	14.31956	112.55725	17578

1162	10.80188	112.65403	0

1163	7.50417	112.75081	46875

1164	5.75576	112.84759	0

1165	4.84865	112.94437	140625

1166	3.97525	113.04116	0

1167	3.14359	113.13794	0

1168	2.34083	113.23472	46875

113.3315	93750

113.42828	46875

113.52506	0

113.62185	17578

113.71863	11718

113.81541	0

113.91219	17578

114.00897	17578

114.10576	93750

114.20254	187500

114.29932	46875

114.3961	17578

114.49288	23437

114.58966	46875

114.68645	0

114.78323	187500

114.88001	33691

114.97679	46875

115.07357	17578

115.17036	140625

115.26714	17578

115.36392	17578

115.4607	46875

115.55748	46875

115.65426	17578

115.75105	46875

115.84783	46875

115.94461	93750

116.04139	140625

116.13817	0

116.23496	187500

116.33174	0

116.42852	46875

116.5253	0

116.62208	46875

116.71886	187500

116.81565	0

116.91243	46875

117.00921	46875

117.10599	140625

117.20277	93750

117.29956	166992

117.39634	140625

117.49312	187500

117.5899	46875

117.68668	46875

117.78346	46875

117.88025	0

117.97703	17578

118.07381	0

118.17059	187500

118.26737	11718

118.36416	166992

118.46094	0

118.55772	187500

118.6545	93750

118.75128	0

118.84806	46875

118.94485	0

119.04163	46875

119.13841	23437

119.23519	0

119.33197	0

119.42876	93750

119.52554	46875

119.62232	46875

119.7191	0

119.81588	0

119.91266	0

120.00945	0

120.10623	166992

120.20301	11718

120.29979	93750

120.39657	35156

120.49335	0

120.59014	169921

120.68692	23437

120.7837	0

120.88048	0

120.97726	11718

121.07405	187500

121.17083	5859

121.26761	93750

121.36439	11718

121.46117	11718

121.55795	0

121.65474	0

121.75152	93750

121.8483	46875

121.94508	16113

122.04186	187500

122.13865	0

122.23543	0

122.33221	46875

122.42899	0

122.52577	187500

122.62255	0

122.71934	166992

122.81612	93750

122.9129	46875

123.00968	0

123.10646	16113

123.20325	11718

123.30003	16845

123.39681	0

123.49359	0

123.59037	187500

123.68715	0

123.78394	0

123.88072	46875

123.9775	0

124.07428	152343

124.17106	0

124.26785	0

124.36463	11718

124.46141	0

124.55819	46875

124.65497	33691

124.75175	46875

124.84854	46875

124.94532	16113

125.0421	0

125.13888	46875

125.23566	17578

125.33245	82031

125.42923	187500

125.52601	16113

125.62279	0

125.71957	93750

125.81635	0

125.91314	46875

126.00992	0

126.1067	46875

126.20348	11718

126.30026	0

126.39705	165527

126.49383	46875

126.59061	140625

126.68739	46875

126.78417	4394

126.88095	175781

126.97774	93750

127.07452	187500

127.1713	0

127.26808	46875

127.36486	187500

127.46165	46875

127.55843	46875

127.65521	0

127.75199	16845

127.84877	46875

127.94555	35156

128.04234	140625

128.13912	0

128.2359	169921

128.33268	187500

128.42946	140625

128.52625	0

128.62303	187500

128.71981	11718

128.81659	152343

128.91337	17578

129.01015	46875

129.10694	166992

129.20372	152343

129.3005	0

129.39728	5859

129.49406	0

129.59085	58593

129.68763	46875

129.78441	166992

129.88119	5859

129.97797	0

130.07475	46875

130.17154	0

130.26832	0

130.3651	0

130.46188	0

130.55866	0

130.65545	46875

130.75223	0

130.84901	46875

130.94579	187500

131.04257	93750

131.13935	11718

131.23614	46875

131.33292	41015

131.4297	187500

131.52648	187500

131.62326	46875

131.72005	46875

131.81683	0

131.91361	46875

132.01039	0

132.10717	46875

132.20395	0

132.30074	140625

132.39752	0

132.4943	0

132.59108	0

132.68786	140625

132.78465	0

132.88143	0

132.97821	169921

133.07499	46875

133.17177	46875

133.26855	17578

133.36534	11718

133.46212	17578

133.5589	140625

133.65568	46875

133.75246	152343

133.84924	46875

133.94603	46875

134.04281	16113

134.13959	46875

134.23637	16845

134.33315	166992

134.42994	46875

134.52672	0

134.6235	46875

134.72028	11718

134.81706	46875

134.91384	46875

135.01063	0

135.10741	0

135.20419	175781

135.30097	46875

135.39775	0

135.49454	17578

135.59132	0

135.6881	169921

135.78488	23437

135.88166	5859

135.97844	46875

136.07523	0

136.17201	140625

136.26879	93750

136.36557	0

136.46235	0

136.55914	0

136.65592	0

136.7527	0

136.84948	11718

136.94626	23437

137.04304	0

137.13983	46875

137.23661	0

137.33339	46875

137.43017	82031

137.52695	46875

137.62374	140625

137.72052	187500

137.8173	140625

137.91408	93750

138.01086	0

138.10764	16845

138.20443	0

138.30121	0

138.39799	64453

138.49477	46875

138.59155	46875

138.68834	17578

138.78512	0

138.8819	187500

138.97868	0

139.07546	169921

139.17224	0

139.26903	46875

139.36581	17578

139.46259	17578

139.55937	0

139.65615	46875

139.75294	46875

139.84972	0

139.9465	0

140.04328	35156

140.14006	16113

140.23684	0

140.33363	93750

140.43041	16845

140.52719	17578

140.62397	0

140.72075	99609

140.81754	0

140.91432	46875

141.0111	0

141.10788	0

141.20466	166992

141.30144	46875

141.39823	46875

141.49501	0

141.59179	0

141.68857	0

141.78535	0

141.88214	46875

141.97892	0

142.0757	0

142.17248	0

142.26926	0

142.36604	93750

142.46283	0

142.55961	0

142.65639	0

142.75317	16845

142.84995	0

142.94674	46875

143.04352	93750

143.1403	46875

143.23708	7324

143.33386	0

143.43064	46875

143.52743	0

143.62421	0

143.72099	46875

143.81777	17578

143.91455	0

144.01134	0

144.10812	0

144.2049	175781

144.30168	140625

144.39846	0

144.49524	7324

144.59203	46875

144.68881	46875

144.78559	162597

144.88237	0

144.97915	46875

145.07593	33691

145.17272	140625

145.2695	46875

145.36628	0

145.46306	46875

145.55984	16113

145.65663	16845

145.75341	187500

145.85019	140625

145.94697	0

146.04375	17578

146.14053	0

146.23732	46875

146.3341	0

146.43088	0

146.52766	140625

146.62444	13183

146.72123	99609

146.81801	46875

146.91479	0

147.01157	140625

147.10835	0

147.20513	140625

147.30192	16113

147.3987	93750

147.49548	0

147.59226	35156

147.68904	46875

147.78583	17578

147.88261	11718

147.97939	0

148.07617	0

148.17295	0

148.26973	17578

148.36652	46875

148.4633	16113

148.56008	93750

148.65686	0

148.75364	0

148.85043	23437

148.94721	0

149.04399	0

149.14077	46875

149.23755	46875

149.33433	161865

149.43112	46875

149.5279	46875

149.62468	0

149.72146	5859

149.81824	5859

149.91503	0

150.01181	0

150.10859	0

150.20537	46875

150.30215	16113

150.39893	93750

150.49572	0

150.5925	0

150.68928	16113

150.78606	172851

150.88284	5859

150.97963	0

151.07641	0

151.17319	46875

151.26997	17578

151.36675	0

151.46353	187500

151.56032	152343

151.6571	0

151.75388	0

151.85066	0

151.94744	93750

152.04423	0

152.14101	0

152.23779	46875

152.33457	169921

152.43135	93750

152.52813	46875

152.62492	187500

152.7217	46875

152.81848	0

152.91526	0

153.01204	152343

153.10883	0

153.20561	0

153.30239	0

153.39917	46875

153.49595	0

153.59273	46875

153.68952	0

153.7863	0

153.88308	0

153.97986	46875

154.07664	0

154.17343	281250

154.27021	46875

154.36699	46875

154.46377	23437

154.56055	0

154.65733	0

154.75412	165527

154.8509	0

154.94768	0

155.04446	0

155.14124	93750

155.23803	175781

155.33481	46875

155.43159	0

155.52837	17578

155.62515	140625

155.72193	0

155.81872	46875

155.9155	140625

156.01228	11718

156.10906	162597

156.20584	281250

156.30263	0

156.39941	187500

156.49619	0

156.59297	169921

156.68975	0

156.78653	46875

156.88332	0

156.9801	0

157.07688	0

157.17366	0

157.27044	187500

157.36722	0

157.46401	46875

157.56079	23437

157.65757	46875

157.75435	46875

157.85113	35156

157.94792	46875

158.0447	38085

158.14148	11718

158.23826	46875

158.33504	11718

158.43183	162597

158.52861	46875

158.62539	0

158.72217	0

158.81895	0

158.91573	0

159.01252	16113

159.1093	0

159.20608	17578

159.30286	0

159.39964	0

159.49642	0

159.59321	46875

159.68999	0

159.78677	17578

159.88355	0

159.98033	46875

160.07712	0

160.1739	187500

160.27068	187500

160.36746	46875

160.46424	2929

160.56102	0

160.65781	93750

160.75459	152343

160.85137	0

160.94815	140625

161.04493	187500

161.14172	175781

161.2385	46875

161.33528	11718

161.43206	0

161.52884	82031

161.62562	23437

161.72241	11718

161.81919	16113

161.91597	0

162.01275	46875

162.10953	0

162.20632	46875

162.3031	46875

162.39988	46875

162.49666	46875

162.59344	0

162.69022	16113

162.78701	0

162.88379	0

162.98057	0

163.07735	0

163.17413	17578

163.27092	0

163.3677	93750

163.46448	169921

163.56126	187500

163.65804	46875

163.75482	169921

163.85161	140625

163.94839	0

164.04517	0

164.14195	152343

164.23873	23437

164.33552	46875

164.4323	17578

164.52908	0

164.62586	140625

164.72264	46875

164.81942	0

164.91621	0

165.01299	46875

165.10977	0

165.20655	46875

165.30333	0

165.40012	17578

165.4969	0

165.59368	187500

165.69046	0

165.78724	23437

165.88402	17578

165.98081	0

166.07759	17578

166.17437	0

166.27115	46875

166.36793	11718

166.46472	187500

166.5615	46875

166.65828	46875

166.75506	46875

166.85184	0

166.94862	0

167.04541	0

167.14219	93750

167.23897	46875

167.33575	0

167.43253	11718

167.52932	0

167.6261	0

167.72288	46875

167.81966	0

167.91644	46875

168.01322	46875

168.11001	0

168.20679	16845

168.30357	46875

168.40035	0

168.49713	140625

168.59392	0

168.6907	46875

168.78748	140625

168.88426	0

168.98104	0

169.07782	46875

169.17461	152343

169.27139	166992

169.36817	7324

169.46495	169921

169.56173	0

169.65852	0

169.7553	0

169.85208	17578

169.94886	0

170.04564	93750

170.14242	0

170.23921	0

170.33599	0

170.43277	93750

170.52955	0

170.62633	16113

170.72312	187500

170.8199	0

170.91668	152343

171.01346	0

171.11024	0

171.20702	0

171.30381	46875

171.40059	46875

171.49737	16113

171.59415	0

171.69093	0

171.78771	23437

171.8845	46875

171.98128	0

172.07806	169921

172.17484	46875

172.27162	0

172.36841	46875

172.46519	0

172.56197	0

172.65875	5859

172.75553	93750

172.85231	0

172.9491	152343

173.04588	46875

173.14266	152343

173.23944	0

173.33622	16113

173.43301	0

173.52979	0

173.62657	17578

173.72335	46875

173.82013	17578

173.91691	0

174.0137	0

174.11048	140625

174.20726	0

174.30404	0

174.40082	93750

174.49761	46875

174.59439	0

174.69117	0

174.78795	0

174.88473	0

174.98151	0

175.0783	0

175.17508	0

175.27186	140625

175.36864	0

175.46542	46875

175.56221	46875

175.65899	0

175.75577	17578

175.85255	0

175.94933	0

176.04611	0

176.1429	0

176.23968	46875

176.33646	17578

176.43324	93750

176.53002	0

176.62681	46875

176.72359	0

176.82037	0

176.91715	46875

177.01393	46875

177.11071	0

177.2075	0

177.30428	0

177.40106	93750

177.49784	0

177.59462	82031

177.69141	46875

177.78819	93750

177.88497	0

177.98175	16113

178.07853	16113

178.17531	187500

178.2721	0

178.36888	17578

178.46566	187500

178.56244	0

178.65922	0

178.75601	46875

178.85279	187500

178.94957	0

179.04635	46875

179.14313	143554

179.23991	46875

179.3367	16113

179.43348	0

179.53026	187500

179.62704	46875

179.72382	11718

179.82061	17578

179.91739	46875

180.01417	17578

180.11095	46875

180.20773	11718

180.30451	17578

180.4013	23437

180.49808	0

180.59486	46875

180.69164	0

180.78842	0

180.88521	166992

180.98199	17578

181.07877	0

181.17555	0

181.27233	0

181.36911	0

181.4659	169921

181.56268	152343

181.65946	0

181.75624	0

181.85302	0

181.94981	0

182.04659	58593

182.14337	46875

182.24015	0

182.33693	0

182.43371	0

182.5305	0

182.62728	0

182.72406	0

182.82084	17578

182.91762	33691

183.01441	0

183.11119	0

183.20797	0

183.30475	16113

183.40153	0

183.49831	5859

183.5951	0

183.69188	17578

183.78866	0

183.88544	93750

183.98222	46875

184.079	0

184.17579	0

184.27257	0

184.36935	0

184.46613	46875

184.56291	5859

184.6597	0

184.75648	0

184.85326	0

184.95004	17578

185.04682	0

185.1436	16113

185.24039	0

185.33717	0

185.43395	0

185.53073	0

185.62751	93750

185.7243	0

185.82108	0

185.91786	187500

186.01464	0

186.11142	166992

186.2082	0

186.30499	46875

186.40177	46875

186.49855	187500

186.59533	0

186.69211	17578

186.7889	17578

186.88568	0

186.98246	0

187.07924	46875

187.17602	0

187.2728	0

187.36959	46875

187.46637	0

187.56315	0

187.65993	0

187.75671	0

187.8535	46875

187.95028	0

188.04706	0

188.14384	0

188.24062	0

188.3374	0

188.43419	46875

188.53097	0

188.62775	0

188.72453	0

188.82131	187500

188.9181	0

189.01488	0

189.11166	11718

189.20844	140625

189.30522	0

189.402	16113

189.49879	0

189.59557	0

189.69235	16113

189.78913	0

189.88591	0

189.9827	5859

190.07948	0

190.17626	0

190.27304	17578

190.36982	0

190.4666	11718

190.56339	0

190.66017	46875

190.75695	46875

190.85373	46875

190.95051	0

191.0473	0

191.14408	0

191.24086	152343

191.33764	0

191.43442	0

191.5312	17578

191.62799	0

191.72477	0

191.82155	0

191.91833	187500

192.01511	0

192.1119	11718

192.20868	17578

192.30546	16113

192.40224	6591

192.49902	175781

192.5958	17578

192.69259	0

192.78937	0

192.88615	0

192.98293	0

193.07971	0

193.1765	82031

193.27328	0

193.37006	16113

193.46684	17578

193.56362	46875

193.6604	0

193.75719	0

193.85397	187500

193.95075	0

194.04753	93750

194.14431	0

194.2411	0

194.33788	46875

194.43466	0

194.53144	16113

194.62822	0

194.725	0

194.82179	17578

194.91857	46875

195.01535	0

195.11213	0

195.20891	0

195.30569	0

195.40248	0

195.49926	0

195.59604	0

195.69282	0

195.7896	0

195.88639	187500

195.98317	0

196.07995	0

196.17673	17578

196.27351	17578

196.3703	0

196.46708	0

196.56386	0

196.66064	0

196.75742	46875

196.8542	0

196.95099	0

197.04777	46875

197.14455	46875

197.24133	0

197.33811	0

197.43489	0

197.53168	0

197.62846	93750

197.72524	46875

197.82202	0

197.9188	17578

198.01559	0

198.11237	0

198.20915	0

198.30593	0

198.40271	0

198.49949	16113

198.59628	16113

198.69306	16113

198.78984	0

198.88662	93750

198.9834	0

199.08019	46875

199.17697	0

199.27375	0

199.37053	0

199.46731	0

199.56409	0

199.66088	0

199.75766	0

199.85444	16113

199.95122	93750

200.048	0

200.14479	17578

200.24157	16113

200.33835	0

200.43513	187500

200.53191	0

200.62869	17578

200.72548	0

200.82226	0

200.91904	0

201.01582	0

201.1126	0

201.20939	16113

201.30617	0

201.40295	0

201.49973	17578

201.59651	0

201.69329	0

201.79008	0

201.88686	0

201.98364	17578

202.08042	0

202.1772	0

202.27399	0

202.37077	0

202.46755	46875

202.56433	46875

202.66111	0

202.75789	0

202.85468	0

202.95146	17578

203.04824	16113

203.14502	0

203.2418	0

203.33859	0

203.43537	0

203.53215	0

203.62893	16113

203.72571	0

203.82249	17578

203.91928	169921

204.01606	5859

204.11284	0

204.20962	187500

204.3064	0

204.40319	0

204.49997	93750

204.59675	0

204.69353	0

204.79031	16113

204.88709	46875

204.98388	0

205.08066	187500

205.17744	0

205.27422	0

205.371	0

205.46779	0

205.56457	0

205.66135	0

205.75813	17578

205.85491	187500

205.95169	0

206.04848	16113

206.14526	46875

206.24204	0

206.33882	16113

206.4356	0

206.53239	0

206.62917	0

206.72595	0

206.82273	17578

206.91951	46875

207.01629	93750

207.11308	93750

207.20986	0

207.30664	0

207.40342	46875

207.5002	0

207.59699	187500

207.69377	0

207.79055	187500

207.88733	0

207.98411	23437

208.08089	0

208.17768	0

208.27446	187500

208.37124	140625

208.46802	17578

208.5648	0

208.66159	0

208.75837	0

208.85515	187500

208.95193	23437

209.04871	0

209.14549	0

209.24228	0

209.33906	93750

209.43584	4394

209.53262	0

209.6294	0

209.72618	0

209.82297	0

209.91975	16113

210.01653	0

210.11331	0

210.21009	16113

210.30688	0

210.40366	16113

210.50044	0

210.59722	11718

210.694	0

210.79078	17578

210.88757	0

210.98435	0

211.08113	17578

211.17791	0

211.27469	0

211.37148	0

211.46826	0

211.56504	0

211.66182	16113

211.7586	0

211.85538	0

211.95217	0

212.04895	0

212.14573	0

212.24251	0

212.33929	140625

212.43608	16845

212.53286	0

212.62964	0

212.72642	0

212.8232	16113

212.91998	0

213.01677	5859

213.11355	0

213.21033	0

213.30711	0

213.40389	0

213.50068	0

213.59746	0

213.69424	0

213.79102	46875

213.8878	187500

213.98458	0

214.08137	0

214.17815	16113

214.27493	17578

214.37171	17578

214.46849	93750

214.56528	0

214.66206	93750

214.75884	0

214.85562	2929

214.9524	17578

215.04918	0

215.14597	0

215.24275	0

215.33953	16113

215.43631	0

215.53309	0

215.62988	16113

215.72666	46875

215.82344	0

215.92022	0

216.017	0

216.11378	0

216.21057	16113

216.30735	0

216.40413	0

216.50091	0

216.59769	0

216.69448	93750

216.79126	169921

216.88804	0

216.98482	0

217.0816	0

217.17838	0

217.27517	93750

217.37195	0

217.46873	0

217.56551	23437

217.66229	0

217.75908	187500

217.85586	93750

217.95264	0

218.04942	17578

218.1462	0

218.24298	0

218.33977	0

218.43655	0

218.53333	46875

218.63011	0

218.72689	17578

218.82368	0

218.92046	0

219.01724	0

219.11402	0

219.2108	0

219.30758	0

219.40437	0

219.50115	0

219.59793	0

219.69471	93750

219.79149	0

219.88828	0

219.98506	0

220.08184	0

220.17862	17578

220.2754	46875

220.37218	23437

220.46897	0

220.56575	0

220.66253	0

220.75931	82031

220.85609	0

220.95288	0

221.04966	93750

221.14644	0

221.24322	187500

221.34	0

221.43678	16113

221.53357	17578

221.63035	0

221.72713	0

221.82391	0

221.92069	46875

222.01747	0

222.11426	16113

222.21104	0

222.30782	17578

222.4046	0

222.50138	93750

222.59817	16113

222.69495	0

222.79173	0

222.88851	0

222.98529	0

223.08207	0

223.17886	17578

223.27564	0

223.37242	17578

223.4692	0

223.56598	93750

223.66277	16113

223.75955	17578

223.85633	0

223.95311	0

224.04989	16113

224.14667	16113

224.24346	0

224.34024	0

224.43702	0

224.5338	0

224.63058	16113

224.72737	16113

224.82415	0

224.92093	0

225.01771	16113

225.11449	93750

225.21127	0

225.30806	0

225.40484	0

225.50162	0

225.5984	0

225.69518	187500

225.79197	93750

225.88875	93750

225.98553	93750

226.08231	0

226.17909	0

226.27587	23437

226.37266	0

226.46944	0

226.56622	0

226.663	93750

226.75978	16113

226.85657	0

226.95335	0

227.05013	0

227.14691	0

227.24369	0

227.34047	17578

227.43726	0

227.53404	0

227.63082	0

227.7276	0

227.82438	0

227.92117	0

228.01795	187500

228.11473	5859

228.21151	0

228.30829	0

228.40507	0

228.50186	23437

228.59864	0

228.69542	0

228.7922	0

228.88898	0

228.98577	0

229.08255	0

229.17933	17578

229.27611	5859

229.37289	0

229.46967	16113

229.56646	0

229.66324	0

229.76002	0

229.8568	16113

229.95358	16113

230.05037	46875

230.14715	0

230.24393	0

230.34071	0

230.43749	16113

230.53427	0

230.63106	0

230.72784	16113

230.82462	17578

230.9214	17578

231.01818	0

231.11497	0

231.21175	0

231.30853	0

231.40531	0

231.50209	0

231.59887	5859

231.69566	17578

231.79244	0

231.88922	23437

231.986	0

232.08278	5859

232.17957	0

232.27635	93750

232.37313	0

232.46991	0

232.56669	16113

232.66347	0

232.76026	0

232.85704	0

232.95382	0

233.0506	0

233.14738	17578

233.24417	0

233.34095	17578

233.43773	0

233.53451	93750

233.63129	0

233.72807	0

233.82486	93750

233.92164	46875

234.01842	0

234.1152	17578

234.21198	0

234.30876	93750

234.40555	0

234.50233	0

234.59911	0

234.69589	0

234.79267	0

234.88946	17578

234.98624	16845

235.08302	0

235.1798	16113

235.27658	17578

235.37336	0

235.47015	0

235.56693	0

235.66371	0

235.76049	0

235.85727	0

235.95406	0

236.05084	11718

236.14762	0

236.2444	93750

236.34118	0

236.43796	0

236.53475	23437

236.63153	0

236.72831	0

236.82509	0

236.92187	0

237.01866	16113

237.11544	0

237.21222	0

237.309	0

237.40578	93750

237.50256	17578

237.59935	0

237.69613	0

237.79291	0

237.88969	0

237.98647	0

238.08326	46875

238.18004	0

238.27682	187500

238.3736	0

238.47038	0

238.56716	0

238.66395	17578

238.76073	17578

238.85751	0

238.95429	0

239.05107	0

239.14786	5859

239.24464	0

239.34142	0

239.4382	6591

239.53498	46875

239.63176	46875

239.72855	0

239.82533	0

239.92211	0

240.01889	0

240.11567	0

240.21246	0

240.30924	93750

240.40602	0

240.5028	0

240.59958	0

240.69636	0

240.79315	11718

240.88993	16113

240.98671	0

241.08349	17578

241.18027	93750

241.27706	16113

241.37384	0

241.47062	0

241.5674	0

241.66418	0

241.76096	0

241.85775	18310

241.95453	16113

242.05131	16845

242.14809	0

242.24487	0

242.34166	0

242.43844	0

242.53522	17578

242.632	0

242.72878	93750

242.82556	23437

242.92235	23437

243.01913	46875

243.11591	41015

243.21269	0

243.30947	0

243.40626	0

243.50304	0

243.59982	0

243.6966	0

243.79338	0

243.89016	0

243.98695	6591

244.08373	17578

244.18051	0

244.27729	0

244.37407	0

244.47086	0

244.56764	23437

244.66442	0

244.7612	0

244.85798	0

244.95476	0

245.05155	0

245.14833	0

245.24511	0

245.34189	187500

245.43867	0

245.53546	0

245.63224	0

245.72902	0

245.8258	140625

245.92258	0

246.01936	2929

246.11615	16113

246.21293	16113

246.30971	0

246.40649	16113

246.50327	17578

246.60006	0

246.69684	0

246.79362	0

246.8904	0

246.98718	0

247.08396	93750

247.18075	16113

247.27753	0

247.37431	17578

247.47109	16113

247.56787	23437

247.66465	0

247.76144	0

247.85822	0

247.955	0

248.05178	16113

248.14856	0

248.24535	187500

248.34213	0

248.43891	0

248.53569	0

248.63247	0

248.72925	0

248.82604	0

248.92282	12451

249.0196	0

249.11638	140625

249.21316	0

249.30995	0

249.40673	17578

249.50351	17578

249.60029	23437

249.69707	0

249.79385	0

249.89064	16113

249.98742	0

250.0842	11718

250.18098	0

250.27776	0

250.37455	0

250.47133	0

250.56811	0

250.66489	0

250.76167	0

250.85845	0

250.95524	0

251.05202	0

251.1488	0

251.24558	17578

251.34236	0

251.43915	0

251.53593	17578

251.63271	17578

251.72949	5859

251.82627	0

251.92305	0

252.01984	17578

252.11662	0

252.2134	17578

252.31018	0

252.40696	17578

252.50375	0

252.60053	17578

252.69731	0

252.79409	0

252.89087	0

252.98765	17578

253.08444	0

253.18122	0

253.278	23437

253.37478	0

253.47156	93750

253.56835	0

253.66513	0

253.76191	0

253.85869	0

253.95547	0

254.05225	0

254.14904	0

254.24582	0

254.3426	0

254.43938	16113

254.53616	46875

254.63295	17578

254.72973	16113

254.82651	187500

254.92329	0

255.02007	16113

255.11685	0

255.21364	0

255.31042	16113

255.4072	0

255.50398	17578

255.60076	0

255.69755	23437

255.79433	0

255.89111	6591

255.98789	0

256.08467	16113

256.18145	0

256.27824	17578

256.37502	0

256.4718	0

256.56858	0

256.66536	46875

256.76215	0

256.85893	17578

256.95571	0

257.05249	0

257.14927	0

257.24605	0

257.34284	169921

257.43962	0

257.5364	6591

257.63318	0

257.72996	23437

257.82675	35156

257.92353	0

258.02031	17578

258.11709	0

258.21387	0

258.31065	0

258.40744	0

258.50422	0

258.601	0

258.69778	0

258.79456	0

258.89135	0

258.98813	0

259.08491	0

259.18169	0

259.27847	0

259.37525	0

259.47204	0

259.56882	46875

259.6656	0

259.76238	17578

259.85916	0

259.95594	0

260.05273	0

260.14951	0

260.24629	16113

260.34307	0

260.43985	16113

260.53664	0

260.63342	0

260.7302	0

260.82698	0

260.92376	17578

261.02054	0

261.11733	16113

261.21411	0

261.31089	0

261.40767	0

261.50445	93750

261.60124	0

261.69802	0

261.7948	46875

261.89158	16113

261.98836	187500

262.08514	11718

262.18193	0

262.27871	0

262.37549	16113

262.47227	46875

262.56905	17578

262.66584	16113

262.76262	93750

262.8594	0

262.95618	16845

263.05296	16113

263.14974	17578

263.24653	93750

263.34331	187500

263.44009	0

263.53687	16845

263.63365	0

263.73044	0

263.82722	0

263.924	93750

264.02078	23437

264.11756	0

264.21434	0

264.31113	46875

264.40791	0

264.50469	0

264.60147	0

264.69825	0

264.79504	17578

264.89182	0

264.9886	16113

265.08538	17578

265.18216	0

265.27894	93750

265.37573	0

265.47251	0

265.56929	0

265.66607	16113

265.76285	0

265.85964	0

265.95642	6591

266.0532	7324

266.14998	16113

266.24676	0

266.34354	93750

266.44033	0

266.53711	0

266.63389	0

266.73067	0

266.82745	16113

266.92424	23437

267.02102	0

267.1178	16113

267.21458	0

267.31136	0

267.40814	16113

267.50493	17578

267.60171	0

267.69849	23437

267.79527	0

267.89205	0

267.98884	0

268.08562	16113

268.1824	17578

268.27918	187500

268.37596	0

268.47274	17578

268.56953	5859

268.66631	0

268.76309	0

268.85987	16113

268.95665	0

269.05344	93750

269.15022	0

269.247	93750

269.34378	0

269.44056	17578

269.53734	0

269.63413	0

269.73091	17578

269.82769	16113

269.92447	5859

270.02125	17578

270.11804	0

270.21482	0

270.3116	23437

270.40838	8056

270.50516	0

270.60194	0

270.69873	0

270.79551	23437

270.89229	0

270.98907	187500

271.08585	17578

271.18264	0

271.27942	187500

271.3762	0

271.47298	0

271.56976	0

271.66654	13183

271.76333	93750

271.86011	0

271.95689	0

272.05367	0

272.15045	0

272.24723	0

272.34402	0

272.4408	16113

272.53758	0

272.63436	0

272.73114	0

272.82793	16113

272.92471	0

273.02149	0

273.11827	281250

273.21505	93750

273.31183	2929

273.40862	0

273.5054	93750

273.60218	0

273.69896	5859

273.79574	0

273.89253	0

273.98931	187500

274.08609	0

274.18287	17578

274.27965	16845

274.37643	0

274.47322	0

274.57	16113

274.66678	0

274.76356	0

274.86034	0

274.95713	93750

275.05391	5859

275.15069	5859

275.24747	0

275.34425	0

275.44103	17578

275.53782	11718

275.6346	0

275.73138	0

275.82816	0

275.92494	0

276.02173	0

276.11851	17578

276.21529	0

276.31207	0

276.40885	16845

276.50563	46875

276.60242	0

276.6992	0

276.79598	16113

276.89276	17578

276.98954	0

277.08633	0

277.18311	0

277.27989	17578

277.37667	0

277.47345	0

277.57023	16845

277.66702	17578

277.7638	0

277.86058	16113

277.95736	0

278.05414	17578

278.15093	0

278.24771	16113

278.34449	0

278.44127	0

278.53805	0

278.63483	16845

278.73162	0

278.8284	0

278.92518	16113

279.02196	0

279.11874	0

279.21553	16113

279.31231	93750

279.40909	0

279.50587	0

279.60265	0

279.69943	16113

279.79622	0

279.893	11718

279.98978	17578

280.08656	0

280.18334	0

280.28013	17578

280.37691	16845

280.47369	0

280.57047	17578

280.66725	0

280.76403	4394

280.86082	93750

280.9576	16113

281.05438	17578

281.15116	0

281.24794	93750

281.34473	17578

281.44151	0

281.53829	17578

281.63507	0

281.73185	16113

281.82863	17578

281.92542	16845

282.0222	0

282.11898	0

282.21576	0

282.31254	17578

282.40933	17578

282.50611	17578

282.60289	0

282.69967	6591

282.79645	7324

282.89323	0

282.99002	0

283.0868	0

283.18358	17578

283.28036	93750

283.37714	0

283.47393	0

283.57071	16113

283.66749	0

283.76427	93750

283.86105	17578

283.95783	93750

284.05462	0

284.1514	0

284.24818	17578

284.34496	0

284.44174	0

284.53852	16113

284.63531	0

284.73209	17578

284.82887	16845

284.92565	0

285.02243	0

285.11922	0

285.216	93750

285.31278	0

285.40956	0

285.50634	17578

285.60312	93750

285.69991	17578

285.79669	16845

285.89347	0

285.99025	0

286.08703	16845

286.18382	0

286.2806	0

286.37738	16845

286.47416	0

286.57094	187500

286.66772	6591

286.76451	0

286.86129	0

286.95807	0

287.05485	0

287.15163	16113

287.24842	0

287.3452	93750

287.44198	17578

287.53876	23437

287.63554	16113

287.73232	0

287.82911	0

287.92589	0

288.02267	0

288.11945	0

288.21623	0

288.31302	0

288.4098	0

288.50658	0

288.60336	17578

288.70014	0

288.79692	17578

288.89371	0

288.99049	0

289.08727	0

289.18405	0

289.28083	0

289.37762	0

289.4744	0

289.57118	11718

289.66796	0

289.76474	17578

289.86152	0

289.95831	187500

290.05509	17578

290.15187	0

290.24865	0

290.34543	17578

290.44222	17578

290.539	187500

290.63578	0

290.73256	0

290.82934	0

290.92612	0

291.02291	0

291.11969	93750

291.21647	5859

291.31325	46875

291.41003	0

291.50682	17578

291.6036	17578

291.70038	0

291.79716	0

291.89394	93750

291.99072	0

292.08751	0

292.18429	0

292.28107	0

292.37785	175781

292.47463	16113

292.57142	0

292.6682	0

292.76498	16113

292.86176	17578

292.95854	0

293.05532	0

293.15211	0

293.24889	16113

293.34567	0

293.44245	0

293.53923	17578

293.63602	17578

293.7328	0

293.82958	93750

293.92636	0

294.02314	93750

294.11992	93750

294.21671	0

294.31349	0

294.41027	0

294.50705	0

294.60383	0

294.70062	17578

294.7974	93750

294.89418	187500

294.99096	0

295.08774	0

295.18452	0

295.28131	0

295.37809	0

295.47487	0

295.57165	16113

295.66843	0

295.76522	0

295.862	0

295.95878	0

296.05556	17578

296.15234	0

296.24912	0

296.34591	0

296.44269	0

296.53947	187500

296.63625	17578

296.73303	0

296.82982	16113

296.9266	16113

297.02338	0

297.12016	0

297.21694	0

297.31372	16113

297.41051	0

297.50729	0

297.60407	0

297.70085	0

297.79763	0

297.89441	16113

297.9912	0

298.08798	0

298.18476	0

298.28154	0

298.37832	93750

298.47511	0

298.57189	16113

298.66867	0

298.76545	0

298.86223	16845

298.95901	0

299.0558	13183

299.15258	93750

299.24936	0

299.34614	0

299.44292	0

299.53971	0

299.63649	0

299.73327	0

299.83005	5859

299.92683	0

300.02361	0

300.1204	0

300.21718	0

300.31396	11718

300.41074	187500

300.50752	17578

300.60431	0

300.70109	0

300.79787	17578

300.89465	17578

300.99143	16113

301.08821	0

301.185	0

301.28178	0

301.37856	93750

301.47534	0

301.57212	0

301.66891	0

301.76569	0

301.86247	0

301.95925	0

302.05603	0

302.15281	93750

302.2496	16113

302.34638	0

302.44316	0

302.53994	93750

302.63672	0

302.73351	0

302.83029	5859

302.92707	0

303.02385	0

303.12063	0

303.21741	0

303.3142	187500

303.41098	16113

303.50776	0

303.60454	93750

303.70132	6591

303.79811	17578

303.89489	0

303.99167	0

304.08845	0

304.18523	17578

304.28201	93750

304.3788	11718

304.47558	5859

304.57236	0

304.66914	0

304.76592	17578

304.86271	0

304.95949	0

305.05627	187500

305.15305	17578

305.24983	11718

305.34661	0

305.4434	0

305.54018	17578

305.63696	187500

305.73374	0

305.83052	16113

305.92731	0

306.02409	163330

306.12087	0

306.21765	17578

306.31443	23437

306.41121	17578

306.508	0

306.60478	0

306.70156	16113

306.79834	0

306.89512	93750

306.99191	46875

307.08869	0

307.18547	0

307.28225	0

307.37903	0

307.47581	17578

307.5726	0

307.66938	93750

307.76616	0

307.86294	93750

307.95972	16845

308.05651	0

308.15329	0

308.25007	93750

308.34685	17578

308.44363	0

308.54041	17578

308.6372	0

308.73398	0

308.83076	0

308.92754	0

309.02432	0

309.12111	0

309.21789	0

309.31467	16845

309.41145	16113

309.50823	0

309.60501	0

309.7018	0

309.79858	0

309.89536	14648

309.99214	93750

310.08892	17578

310.1857	17578

310.28249	16113

310.37927	0

310.47605	11718

310.57283	93750

310.66961	16113

310.7664	0

310.86318	0

310.95996	0

311.05674	23437

311.15352	0

311.2503	17578

311.34709	0

311.44387	93750

311.54065	0

311.63743	0

311.73421	0

311.831	17578

311.92778	17578

312.02456	0

312.12134	0

312.21812	0

312.3149	0

312.41169	93750

312.50847	2929

312.60525	0

312.70203	17578

312.79881	0

312.8956	0

312.99238	17578

313.08916	0

313.18594	0

313.28272	0

313.3795	16845

313.47629	16113

313.57307	0

313.66985	0

313.76663	0

313.86341	0

313.9602	0

314.05698	187500

314.15376	0

314.25054	0

314.34732	0

314.4441	93750

314.54089	0

314.63767	0

314.73445	0

314.83123	0

314.92801	5859

315.0248	0

315.12158	117187

315.21836	5859

315.31514	6591

315.41192	16113

315.5087	0

315.60549	93750

315.70227	17578

315.79905	17578

315.89583	0

315.99261	93750

316.0894	0

316.18618	17578

316.28296	0

316.37974	0

316.47652	16113

316.5733	0

316.67009	0

316.76687	187500

316.86365	0

316.96043	17578

317.05721	7324

317.154	0

317.25078	0

317.34756	0

317.44434	16113

317.54112	93750

317.6379	17578

317.73469	0

317.83147	16845

317.92825	0

318.02503	93750

318.12181	0

318.2186	16113

318.31538	0

318.41216	0

318.50894	16113

318.60572	17578

318.7025	17578

318.79929	5859

318.89607	93750

318.99285	0

319.08963	93750

319.18641	0

319.2832	0

319.37998	16845

319.47676	16113

319.57354	0

319.67032	17578

319.7671	93750

319.86389	0

319.96067	16113

320.05745	0

320.15423	93750

320.25101	46875

320.3478	0

320.44458	0

320.54136	0

320.63814	0

320.73492	16113

320.8317	0

320.92849	0

321.02527	0

321.12205	17578

321.21883	17578

321.31561	0

321.4124	17578

321.50918	93750

321.60596	0

321.70274	17578

321.79952	0

321.8963	0

321.99309	0

322.08987	0

322.18665	2929

322.28343	93750

322.38021	0

322.47699	0

322.57378	2929

322.67056	0

322.76734	0

322.86412	0

322.9609	93750

323.05769	0

323.15447	0

323.25125	0

323.34803	17578

323.44481	0

323.54159	0

323.63838	5859

323.73516	0

323.83194	16845

323.92872	0

324.0255	0

324.12229	0

324.21907	0

324.31585	5859

324.41263	0

324.50941	16113

324.60619	0

324.70298	0

324.79976	0

324.89654	11718

324.99332	17578

325.0901	2929

325.18689	0

325.28367	16113

325.38045	0

325.47723	0

325.57401	0

325.67079	187500

325.76758	0

325.86436	93750

325.96114	0

326.05792	0

326.1547	93750

326.25149	0

326.34827	0

326.44505	0

326.54183	0

326.63861	16113

326.73539	0

326.83218	93750

326.92896	17578

327.02574	0

327.12252	0

327.2193	5859

327.31609	17578

327.41287	0

327.50965	17578

327.60643	0

327.70321	0

327.79999	0

327.89678	0

327.99356	6591

328.09034	0

328.18712	0

328.2839	0

328.38069	0

328.47747	17578

328.57425	0

328.67103	0

328.76781	0

328.86459	0

328.96138	17578

329.05816	0

329.15494	0

329.25172	17578

329.3485	0

329.44529	17578

329.54207	93750

329.63885	169921

329.73563	0

329.83241	2929

329.92919	93750

330.02598	0

330.12276	187500

330.21954	0

330.31632	23437

330.4131	17578

330.50989	0

330.60667	17578

330.70345	17578

330.80023	0

330.89701	0

330.99379	0

331.09058	0

331.18736	0

331.28414	0

331.38092	0

331.4777	0

331.57449	17578

331.67127	16845

331.76805	0

331.86483	0

331.96161	17578

332.05839	0

332.15518	0

332.25196	0

332.34874	93750

332.44552	0

332.5423	17578

332.63909	0

332.73587	0

332.83265	16845

332.92943	5859

333.02621	0

333.12299	0

333.21978	0

333.31656	0

333.41334	0

333.51012	0

333.6069	17578

333.70369	0

333.80047	0

333.89725	0

333.99403	0

334.09081	16113

334.18759	0

334.28438	16845

334.38116	0

334.47794	2929

334.57472	23437

334.6715	17578

334.76828	6591

334.86507	0

334.96185	0

335.05863	0

335.15541	0

335.25219	93750

335.34898	17578

335.44576	5859

335.54254	0

335.63932	16113

335.7361	0

335.83288	93750

335.92967	0

336.02645	0

336.12323	0

336.22001	17578

336.31679	0

336.41358	0

336.51036	17578

336.60714	0

336.70392	0

336.8007	0

336.89748	0

336.99427	0

337.09105	0

337.18783	93750

337.28461	0

337.38139	6591

337.47818	0

337.57496	93750

337.67174	0

337.76852	0

337.8653	0

337.96208	0

338.05887	0

338.15565	17578

338.25243	0

338.34921	0

338.44599	0

338.54278	0

338.63956	0

338.73634	17578

338.83312	0

338.9299	0

339.02668	17578

339.12347	0

339.22025	0

339.31703	17578

339.41381	0

339.51059	0

339.60738	17578

339.70416	187500

339.80094	0

339.89772	17578

339.9945	6591

340.09128	17578

340.18807	16113

340.28485	93750

340.38163	0

340.47841	93750

340.57519	17578

340.67198	0

340.76876	93750

340.86554	16113

340.96232	0

341.0591	17578

341.15588	0

341.25267	0

341.34945	0

341.44623	5859

341.54301	0

341.63979	0

341.73658	16113

341.83336	17578

341.93014	6591

342.02692	0

342.1237	93750

342.22048	0

342.31727	93750

342.41405	5859

342.51083	17578

342.60761	0

342.70439	0

342.80118	0

342.89796	17578

342.99474	0

343.09152	0

343.1883	17578

343.28508	17578

343.38187	0

343.47865	0

343.57543	0

343.67221	0

343.76899	0

343.86578	6591

343.96256	93750

344.05934	16113

344.15612	16113

344.2529	0

344.34968	0

344.44647	17578

344.54325	93750

344.64003	17578

344.73681	0

344.83359	2929

344.93038	0

345.02716	0

345.12394	0

345.22072	17578

345.3175	6591

345.41428	16113

345.51107	93750

345.60785	17578

345.70463	5859

345.80141	0

345.89819	0

345.99498	0

346.09176	6591

346.18854	17578

346.28532	0

346.3821	0

346.47888	17578

346.57567	93750

346.67245	17578

346.76923	16113

346.86601	0

346.96279	23437

347.05958	93750

347.15636	6591

347.25314	5859

347.34992	0

347.4467	0

347.54348	187500

347.64027	17578

347.73705	16113

347.83383	0

347.93061	0

348.02739	0

348.12417	17578

348.22096	0

348.31774	17578

348.41452	93750

348.5113	17578

348.60808	6591

348.70487	17578

348.80165	0

348.89843	16113

348.99521	0

349.09199	0

349.18877	0

349.28556	187500

349.38234	0

349.47912	0

349.5759	0

349.67268	0

349.76947	0

349.86625	0

349.96303	17578

350.05981	0

350.15659	0

350.25337	6591

350.35016	0

350.44694	0

350.54372	0

350.6405	17578

350.73728	93750

350.83407	0

350.93085	0

351.02763	82031

351.12441	0

351.22119	0

351.31797	152343

351.41476	0

351.51154	0

351.60832	93750

351.7051	0

351.80188	6591

351.89867	17578

351.99545	16845

352.09223	17578

352.18901	0

352.28579	0

352.38257	17578

352.47936	0

352.57614	2929

352.67292	0

352.7697	0

352.86648	2929

352.96327	0

353.06005	0

353.15683	93750

353.25361	6591

353.35039	0

353.44717	17578

353.54396	0

353.64074	0

353.73752	0

353.8343	17578

353.93108	0

354.02787	16113

354.12465	0

354.22143	0

354.31821	6591

354.41499	16113

354.51177	0

354.60856	17578

354.70534	17578

354.80212	0

354.8989	93750

354.99568	5859

355.09247	0

355.18925	17578

355.28603	0

355.38281	0

355.47959	0

355.57637	0

355.67316	0

355.76994	2929

355.86672	93750

355.9635	0

356.06028	17578

356.15707	0

356.25385	0

356.35063	17578

356.44741	0

356.54419	0

356.64097	0

356.73776	93750

356.83454	93750

356.93132	0

357.0281	0

357.12488	16113

357.22167	6591

357.31845	0

357.41523	2929

357.51201	0

357.60879	16845

357.70557	0

357.80236	13183

357.89914	0

357.99592	5859

358.0927	0

358.18948	0

358.28627	0

358.38305	0

358.47983	0

358.57661	0

358.67339	0

358.77017	0

358.86696	16113

358.96374	0

359.06052	0

359.1573	17578

359.25408	17578

359.35087	0

359.44765	0

359.54443	0

359.64121	187500

359.73799	16113

359.83477	6591

359.93156	0

360.02834	0

360.12512	0

360.2219	5859

360.31868	0

360.41546	16845

360.51225	0

360.60903	0

360.70581	0

360.80259	0

360.89937	0

360.99616	187500

361.09294	16113

361.18972	0

361.2865	17578

361.38328	16845

361.48006	17578

361.57685	0

361.67363	17578

361.77041	23437

361.86719	5859

361.96397	17578

362.06076	93750

362.15754	0

362.25432	0

362.3511	0

362.44788	0

362.54466	5859

362.64145	2929

362.73823	0

362.83501	7324

362.93179	0

363.02857	0

363.12536	0

363.22214	0

363.31892	0

363.4157	16113

363.51248	0

363.60926	0

363.70605	0

363.80283	0

363.89961	0

363.99639	0

364.09317	5859

364.18996	0

364.28674	0

364.38352	2929

364.4803	17578

364.57708	0

364.67386	0

364.77065	16113

364.86743	0

364.96421	0

365.06099	17578

365.15777	0

365.25456	6591

365.35134	0

365.44812	2929

365.5449	0

365.64168	0

365.73846	17578

365.83525	0

365.93203	16113

366.02881	0

366.12559	5859

366.22237	0

366.31916	0

366.41594	16113

366.51272	0

366.6095	17578

366.70628	93750

366.80306	6591

366.89985	11718

366.99663	0

367.09341	0

367.19019	0

367.28697	7324

367.38376	17578

367.48054	17578

367.57732	0

367.6741	0

367.77088	0

367.86766	0

367.96445	0

368.06123	0

368.15801	0

368.25479	0

368.35157	46875

368.44836	16113

368.54514	169921

368.64192	2929

368.7387	17578

368.83548	17578

368.93226	0

369.02905	0

369.12583	0

369.22261	17578

369.31939	0

369.41617	0

369.51296	0

369.60974	16845

369.70652	0

369.8033	0

369.90008	17578

369.99686	0

370.09365	0

370.19043	0

370.28721	0

370.38399	0

370.48077	0

370.57756	0

370.67434	17578

370.77112	0

370.8679	0

370.96468	16113

371.06146	93750

371.15825	0

371.25503	93750

371.35181	0

371.44859	0

371.54537	16113

371.64216	0

371.73894	0

371.83572	6591

371.9325	0

372.02928	0

372.12606	17578

372.22285	0

372.31963	17578

372.41641	93750

372.51319	0

372.60997	17578

372.70675	23437

372.80354	16113

372.90032	0

372.9971	187500

373.09388	17578

373.19066	17578

373.28745	0

373.38423	0

373.48101	0

373.57779	0

373.67457	17578

373.77135	0

373.86814	187500

373.96492	0

374.0617	93750

374.15848	0

374.25526	0

374.35205	17578

374.44883	0

374.54561	0

374.64239	0

374.73917	17578

374.83595	0

374.93274	17578

375.02952	0

375.1263	0

375.22308	0

375.31986	0

375.41665	6591

375.51343	0

375.61021	0

375.70699	5859

375.80377	0

375.90055	0

375.99734	93750

376.09412	17578

376.1909	6591

376.28768	5859

376.38446	0

376.48125	0

376.57803	0

376.67481	0

376.77159	17578

376.86837	16113

376.96515	0

377.06194	0

377.15872	0

377.2555	17578

377.35228	0

377.44906	5859

377.54585	6591

377.64263	187500

377.73941	93750

377.83619	0

377.93297	0

378.02975	6591

378.12654	17578

378.22332	0

378.3201	0

378.41688	0

378.51366	0

378.61045	17578

378.70723	187500

378.80401	0

378.90079	16113

378.99757	0

379.09435	6591

379.19114	46875

379.28792	11718

379.3847	0

379.48148	0

379.57826	17578

379.67505	0

379.77183	0

379.86861	17578

379.96539	0

380.06217	17578

380.15895	17578

380.25574	0

380.35252	0

380.4493	0

380.54608	0

380.64286	93750

380.73965	16113

380.83643	0

380.93321	0

381.02999	0

381.12677	6591

381.22355	0

381.32034	46875

381.41712	0

381.5139	0

381.61068	0

381.70746	0

381.80425	16113

381.90103	16845

381.99781	0

382.09459	0

382.19137	17578

382.28815	0

382.38494	0

382.48172	93750

382.5785	17578

382.67528	0

382.77206	93750

382.86885	0

382.96563	0

383.06241	5859

383.15919	17578

383.25597	17578

383.35275	0

383.44954	0

383.54632	0

383.6431	187500

383.73988	0

383.83666	0

383.93345	0

384.03023	5859

384.12701	0

384.22379	0

384.32057	17578

384.41735	93750

384.51414	0

384.61092	17578

384.7077	17578

384.80448	17578

384.90126	0

384.99804	0

385.09483	16845

385.19161	17578

385.28839	0

385.38517	17578

385.48195	0

385.57874	0

385.67552	17578

385.7723	0

385.86908	0

385.96586	46875

386.06264	0

386.15943	0

386.25621	140625

386.35299	0

386.44977	0

386.54655	0

386.64334	0

386.74012	0

386.8369	0

386.93368	17578

387.03046	16113

387.12724	2929

387.22403	23437

387.32081	17578

387.41759	0

387.51437	0

387.61115	0

387.70794	0

387.80472	0

387.9015	6591

387.99828	0

388.09506	17578

388.19184	0

388.28863	0

388.38541	5859

388.48219	6591

388.57897	0

388.67575	0

388.77254	6591

388.86932	0

388.9661	5859

389.06288	5859

389.15966	17578

389.25644	17578

389.35323	0

389.45001	0

389.54679	93750

389.64357	93750

389.74035	0

389.83714	7324

389.93392	17578

390.0307	16113

390.12748	187500

390.22426	187500

390.32104	93750

390.41783	0

390.51461	17578

390.61139	0

390.70817	17578

390.80495	17578

390.90174	17578

390.99852	0

391.0953	0

391.19208	46875

391.28886	17578

391.38564	0

391.48243	93750

391.57921	0

391.67599	0

391.77277	0

391.86955	17578

391.96634	0

392.06312	93750

392.1599	17578

392.25668	17578

392.35346	0

392.45024	0

392.54703	0

392.64381	0

392.74059	17578

392.83737	0

392.93415	2929

393.03094	0

393.12772	17578

393.2245	23437

393.32128	0

393.41806	5859

393.51484	17578

393.61163	0

393.70841	16113

393.80519	0

393.90197	0

393.99875	93750

394.09554	93750

394.19232	46875

394.2891	0

394.38588	0

394.48266	0

394.57944	17578

394.67623	0

394.77301	0

394.86979	6591

394.96657	0

395.06335	46875

395.16014	93750

395.25692	0

395.3537	0

395.45048	0

395.54726	17578

395.64404	0

395.74083	17578

395.83761	17578

395.93439	93750

396.03117	17578

396.12795	187500

396.22474	16845

396.32152	93750

396.4183	0

396.51508	17578

396.61186	17578

396.70864	0

396.80543	6591

396.90221	11718

396.99899	46875

397.09577	17578

397.19255	0

397.28933	0

397.38612	0

397.4829	0

397.57968	0

397.67646	16845

397.77324	0

397.87003	16113

397.96681	0

398.06359	0

398.16037	0

398.25715	0

398.35393	0

398.45072	0

398.5475	0

398.64428	0

398.74106	13183

398.83784	0

398.93463	23437

399.03141	0

399.12819	23437

399.22497	187500

399.32175	23437

399.41853	17578

399.51532	17578

399.6121	0

399.70888	0

399.80566	0

399.90244	0

399.99923	6591

400.09601	0

400.19279	0

400.28957	17578

400.38635	17578

400.48313	0

400.57992	0

400.6767	0

400.77348	6591

400.87026	93750

400.96704	0

401.06383	17578

401.16061	0

401.25739	0

401.35417	0

401.45095	0

401.54773	0

401.64452	0

401.7413	0

401.83808	0

401.93486	0

402.03164	0

402.12843	6591

402.22521	17578

402.32199	0

402.41877	0

402.51555	5859

402.61233	0

402.70912	17578

402.8059	0

402.90268	17578

402.99946	0

403.09624	46875

403.19303	0

403.28981	17578

403.38659	5859

403.48337	0

403.58015	0

403.67693	0

403.77372	17578

403.8705	0

403.96728	5859

404.06406	2929

404.16084	0

404.25763	5859

404.35441	16845

404.45119	0

404.54797	17578

404.64475	0

404.74153	6591

404.83832	0

404.9351	0

405.03188	17578

405.12866	0

405.22544	0

405.32223	0

405.41901	0

405.51579	17578

405.61257	0

405.70935	17578

405.80613	0

405.90292	0

405.9997	0

406.09648	17578

406.19326	17578

406.29004	0

406.38683	0

406.48361	0

406.58039	0

406.67717	93750

406.77395	0

406.87073	0

406.96752	0

407.0643	17578

407.16108	6591

407.25786	0

407.35464	93750

407.45143	0

407.54821	0

407.64499	0

407.74177	16845

407.83855	93750

407.93533	0

408.03212	0

408.1289	5859

408.22568	0

408.32246	17578

408.41924	0

408.51603	2929

408.61281	17578

408.70959	23437

408.80637	0

408.90315	0

408.99993	0

409.09672	0

409.1935	0

409.29028	5859

409.38706	17578

409.48384	17578

409.58063	0

409.67741	17578

409.77419	0

409.87097	0

409.96775	6591

410.06453	6591

410.16132	0

410.2581	0

410.35488	16113

410.45166	0

410.54844	6591

410.64522	0

410.74201	0

410.83879	0

410.93557	0

411.03235	0

411.12913	0

411.22592	16113

411.3227	23437

411.41948	169921

411.51626	0

411.61304	93750

411.70982	0

411.80661	17578

411.90339	0

412.00017	0

412.09695	0

412.19373	0

412.29052	93750

412.3873	0

412.48408	0

412.58086	17578

412.67764	93750

412.77442	17578

412.87121	5859

412.96799	0

413.06477	0

413.16155	17578

413.25833	0

413.35512	0

413.4519	0

413.54868	16845

413.64546	0

413.74224	0

413.83902	16113

413.93581	0

414.03259	17578

414.12937	6591

414.22615	0

414.32293	0

414.41972	0

414.5165	0

414.61328	187500

414.71006	0

414.80684	17578

414.90362	0

415.00041	0

415.09719	93750

415.19397	0

415.29075	0

415.38753	23437

415.48432	0

415.5811	16113

415.67788	5859

415.77466	0

415.87144	0

415.96822	0

416.06501	0

416.16179	0

416.25857	6591

416.35535	0

416.45213	0

416.54892	0

416.6457	0

416.74248	13183

416.83926	0

416.93604	0

417.03282	0

417.12961	17578

417.22639	93750

417.32317	0

417.41995	93750

417.51673	0

417.61352	0

417.7103	93750

417.80708	0

417.90386	0

418.00064	0

418.09742	0

418.19421	187500

418.29099	0

418.38777	93750

418.48455	17578

418.58133	0

418.67812	16113

418.7749	93750

418.87168	11718

418.96846	0

419.06524	17578

419.16202	93750

419.25881	0

419.35559	17578

419.45237	17578

419.54915	17578

419.64593	17578

419.74272	0

419.8395	17578

419.93628	17578

420.03306	0

420.12984	0

420.22662	17578

420.32341	17578

420.42019	17578

420.51697	17578

420.61375	0

420.71053	0

420.80732	46875

420.9041	0

421.00088	0

421.09766	17578

421.19444	6591

421.29122	16113

421.38801	0

421.48479	17578

421.58157	0

421.67835	0

421.77513	0

421.87192	6591

421.9687	0

422.06548	17578

422.16226	0

422.25904	23437

422.35582	17578

422.45261	0

422.54939	17578

422.64617	17578

422.74295	6591

422.83973	17578

422.93651	17578

423.0333	17578

423.13008	0

423.22686	17578

423.32364	0

423.42042	2929

423.51721	0

423.61399	0

423.71077	0

423.80755	0

423.90433	93750

424.00111	0

424.0979	0

424.19468	0

424.29146	17578

424.38824	5859

424.48502	5859

424.58181	0

424.67859	0

424.77537	0

424.87215	0

424.96893	17578

425.06571	5859

425.1625	17578

425.25928	0

425.35606	6591

425.45284	0

425.54962	0

425.64641	0

425.74319	0

425.83997	0

425.93675	93750

426.03353	93750

426.13031	0

426.2271	17578

426.32388	6591

426.42066	6591

426.51744	0

426.61422	0

426.71101	17578

426.80779	93750

426.90457	0

427.00135	0

427.09813	17578

427.19491	0

427.2917	6591

427.38848	0

427.48526	17578

427.58204	93750

427.67882	0

427.77561	0

427.87239	0

427.96917	2929

428.06595	17578

428.16273	16845

428.25951	0

428.3563	0

428.45308	0

428.54986	0

428.64664	17578

428.74342	0

428.84021	0

428.93699	0

429.03377	0

429.13055	17578

429.22733	93750

429.32411	0

429.4209	2929

429.51768	6591

429.61446	187500

429.71124	17578

429.80802	0

429.90481	6591

430.00159	0

430.09837	0

430.19515	0

430.29193	13183

430.38871	0

430.4855	0

430.58228	17578

430.67906	0

430.77584	0

430.87262	5859

430.96941	17578

431.06619	0

431.16297	0

431.25975	17578

431.35653	0

431.45331	0

431.5501	17578

431.64688	0

431.74366	16113

431.84044	16845

431.93722	0

432.03401	0

432.13079	0

432.22757	17578

432.32435	0

432.42113	16113

432.51791	6591

432.6147	0

432.71148	0

432.80826	13183

432.90504	0

433.00182	17578

433.09861	0

433.19539	6591

433.29217	0

433.38895	0

433.48573	0

433.58251	17578

433.6793	0

433.77608	93750

433.87286	13183

433.96964	0

434.06642	0

434.16321	0

434.25999	0

434.35677	0

434.45355	0

434.55033	5859

434.64711	0

434.7439	5859

434.84068	6591

434.93746	5859

435.03424	17578

435.13102	0

435.2278	6591

435.32459	6591

435.42137	0

435.51815	0

435.61493	187500

435.71171	0

435.8085	0

435.90528	6591

436.00206	93750

436.09884	93750

436.19562	0

436.2924	0

436.38919	0

436.48597	17578

436.58275	6591

436.67953	0

436.77631	0

436.8731	0

436.96988	0

437.06666	93750

437.16344	0

437.26022	0

437.357	5859

437.45379	0

437.55057	0

437.64735	0

437.74413	0

437.84091	0

437.9377	0

438.03448	0

438.13126	17578

438.22804	6591

438.32482	0

438.4216	0

438.51839	93750

438.61517	0

438.71195	5859

438.80873	17578

438.90551	0

439.0023	0

439.09908	0

439.19586	0

439.29264	6591

439.38942	0

439.4862	0

439.58299	0

439.67977	17578

439.77655	46875

439.87333	0

439.97011	0

440.0669	0

440.16368	6591

440.26046	17578

440.35724	6591

440.45402	2929

440.5508	0

440.64759	23437

440.74437	0

440.84115	0

440.93793	0

441.03471	0

441.1315	0

441.22828	17578

441.32506	5859

441.42184	0

441.51862	0

441.6154	17578

441.71219	0

441.80897	0

441.90575	0

442.00253	46875

442.09931	0

442.1961	5859

442.29288	17578

442.38966	23437

442.48644	0

442.58322	17578

442.68	17578

442.77679	17578

442.87357	2929

442.97035	17578

443.06713	0

443.16391	5859

443.2607	0

443.35748	17578

443.45426	5859

443.55104	0

443.64782	0

443.7446	0

443.84139	0

443.93817	0

444.03495	0

444.13173	17578

444.22851	5859

444.3253	0

444.42208	5859

444.51886	17578

444.61564	17578

444.71242	93750

444.8092	0

444.90599	0

445.00277	23437

445.09955	0

445.19633	0

445.29311	187500

445.3899	0

445.48668	6591

445.58346	17578

445.68024	17578

445.77702	93750

445.8738	93750

445.97059	0

446.06737	0

446.16415	0

446.26093	6591

446.35771	0

446.4545	0

446.55128	0

446.64806	0

446.74484	93750

446.84162	0

446.9384	0

447.03519	93750

447.13197	0

447.22875	0

447.32553	17578

447.42231	0

447.5191	17578

447.61588	17578

447.71266	0

447.80944	17578

447.90622	17578

448.003	0

448.09979	0

448.19657	0

448.29335	0

448.39013	0

448.48691	0

448.58369	11718

448.68048	0

448.77726	5859

448.87404	17578

448.97082	93750

449.0676	187500

449.16439	5859

449.26117	17578

449.35795	0

449.45473	0

449.55151	17578

449.64829	17578

449.74508	0

449.84186	0

449.93864	46875

450.03542	0

450.1322	6591

450.22899	93750

450.32577	17578

450.42255	0

450.51933	0

450.61611	6591

450.71289	93750

450.80968	0

450.90646	0

451.00324	17578

451.10002	0

451.1968	0

451.29359	0

451.39037	0

451.48715	0

451.58393	6591

451.68071	17578

451.77749	0

451.87428	187500

451.97106	93750

452.06784	0

452.16462	0

452.2614	187500

452.35819	0

452.45497	0

452.55175	0

452.64853	0

452.74531	0

452.84209	93750

452.93888	0

453.03566	0

453.13244	17578

453.22922	0

453.326	93750

453.42279	17578

453.51957	187500

453.61635	0

453.71313	0

453.80991	23437

453.90669	17578

454.00348	17578

454.10026	17578

454.19704	0

454.29382	0

454.3906	6591

454.48739	0

454.58417	17578

454.68095	0

454.77773	0

454.87451	0

454.97129	17578

455.06808	0

455.16486	5859

455.26164	93750

455.35842	0

455.4552	0

455.55199	17578

455.64877	17578

455.74555	0

455.84233	0

455.93911	0

456.03589	16113

456.13268	93750

456.22946	0

456.32624	0

456.42302	0

456.5198	17578

456.61659	93750

456.71337	0

456.81015	0

456.90693	93750

457.00371	17578

457.10049	46875

457.19728	0

457.29406	17578

457.39084	17578

457.48762	0

457.5844	187500

457.68119	5859

457.77797	0

457.87475	0

457.97153	0

458.06831	0

458.16509	0

458.26188	0

458.35866	0

458.45544	0

458.55222	0

458.649	0

458.74579	13183

458.84257	0

458.93935	187500

459.03613	0

459.13291	0

459.22969	187500

459.32648	0

459.42326	0

459.52004	0

459.61682	16113

459.7136	0

459.81039	0

459.90717	0

460.00395	0

460.10073	7324

460.19751	13183

460.29429	0

460.39108	93750

460.48786	0

460.58464	17578

460.68142	0

460.7782	93750

460.87498	0

460.97177	17578

461.06855	0

461.16533	0

461.26211	6591

461.35889	0

461.45568	6591

461.55246	0

461.64924	0

461.74602	0

461.8428	6591

461.93958	5859

462.03637	17578

462.13315	13183

462.22993	0

462.32671	0

462.42349	0

462.52028	0

462.61706	13183

462.71384	17578

462.81062	17578

462.9074	11718

463.00418	17578

463.10097	5859

463.19775	5859

463.29453	6591

463.39131	93750

463.48809	0

463.58488	0

463.68166	0

463.77844	0

463.87522	0

463.972	2929

464.06878	17578

464.16557	0

464.26235	0

464.35913	46875

464.45591	18310

464.55269	0

464.64948	0

464.74626	0

464.84304	23437

464.93982	0

465.0366	0

465.13338	0

465.23017	0

465.32695	0

465.42373	0

465.52051	0

465.61729	17578

465.71408	0

465.81086	0

465.90764	17578

466.00442	0

466.1012	16113

466.19798	16113

466.29477	5859

466.39155	0

466.48833	0

466.58511	17578

466.68189	0

466.77868	0

466.87546	0

466.97224	0

467.06902	5859

467.1658	0

467.26258	17578

467.35937	0

467.45615	0

467.55293	0

467.64971	0

467.74649	0

467.84328	0

467.94006	0

468.03684	0

468.13362	0

468.2304	0

468.32718	0

468.42397	2929

468.52075	0

468.61753	16113

468.71431	0

468.81109	0

468.90788	0

469.00466	0

469.10144	0

469.19822	17578

469.295	93750

469.39178	0

469.48857	0

469.58535	0

469.68213	187500

469.77891	0

469.87569	0

469.97248	0

470.06926	0

470.16604	0

470.26282	0

470.3596	0

470.45638	17578

470.55317	17578

470.64995	0

470.74673	0

470.84351	0

470.94029	0

471.03708	0

471.13386	0

471.23064	2929

471.32742	0

471.4242	93750

471.52098	0

471.61777	93750

471.71455	0

471.81133	17578

471.90811	0

472.00489	5859

472.10168	5859

472.19846	0

472.29524	0

472.39202	0

472.4888	0

472.58558	0

472.68237	0

472.77915	0

472.87593	0

472.97271	17578

473.06949	17578

473.16627	0

473.26306	0

473.35984	0

473.45662	0

473.5534	0

473.65018	0

473.74697	6591

473.84375	6591

473.94053	0

474.03731	0

474.13409	93750

474.23087	0

474.32766	0

474.42444	0

474.52122	17578

474.618	0

474.71478	16845

474.81157	17578

474.90835	0

475.00513	0

475.10191	0

475.19869	0

475.29547	5859

475.39226	0

475.48904	0

475.58582	5859

475.6826	5859

475.77938	0

475.87617	0

475.97295	0

476.06973	0

476.16651	6591

476.26329	0

476.36007	93750

476.45686	6591

476.55364	0

476.65042	0

476.7472	0

476.84398	2929

476.94077	5859

477.03755	0

477.13433	0

477.23111	0

477.32789	17578

477.42467	0

477.52146	0

477.61824	6591

477.71502	0

477.8118	0

477.90858	17578

478.00537	0

478.10215	0

478.19893	0

478.29571	0

478.39249	0

478.48927	0

478.58606	0

478.68284	93750

478.77962	0

478.8764	17578

478.97318	0

479.06997	0

479.16675	0

479.26353	93750

479.36031	5859

479.45709	23437

479.55387	0

479.65066	0

479.74744	17578

479.84422	0

479.941	0

480.03778	0

480.13457	0

480.23135	0

480.32813	0

480.42491	17578

480.52169	0

480.61847	17578

480.71526	0

480.81204	5859

480.90882	0

481.0056	17578

481.10238	0

481.19917	0

481.29595	0

481.39273	17578

481.48951	0

481.58629	0

481.68307	5859

481.77986	0

481.87664	0

481.97342	6591

482.0702	0

482.16698	0

482.26377	93750

482.36055	17578

482.45733	17578

482.55411	0

482.65089	0

482.74767	0

482.84446	6591

482.94124	0

483.03802	17578

483.1348	0

483.23158	0

483.32837	0

483.42515	0

483.52193	0

483.61871	5859

483.71549	0

483.81227	0

483.90906	0

484.00584	17578

484.10262	17578

484.1994	0

484.29618	0

484.39297	0

484.48975	17578

484.58653	0

484.68331	17578

484.78009	0

484.87687	0

484.97366	6591

485.07044	16113

485.16722	0

485.264	17578

485.36078	0

485.45756	0

485.55435	0

485.65113	6591

485.74791	187500

485.84469	6591

485.94147	0

486.03826	0

486.13504	6591

486.23182	0

486.3286	17578

486.42538	93750

486.52216	0

486.61895	0

486.71573	2929

486.81251	0

486.90929	2929

487.00607	0

487.10286	5859

487.19964	0

487.29642	0

487.3932	0

487.48998	23437

487.58676	0

487.68355	17578

487.78033	0

487.87711	0

487.97389	93750

488.07067	0

488.16746	17578

488.26424	5859

488.36102	0

488.4578	0

488.55458	0

488.65136	0

488.74815	5859

488.84493	17578

488.94171	0

489.03849	0

489.13527	0

489.23206	0

489.32884	0

489.42562	0

489.5224	5859

489.61918	0

489.71596	0

489.81275	17578

489.90953	93750

490.00631	17578

490.10309	0

490.19987	0

490.29666	0

490.39344	46875

490.49022	0

490.587	0

490.68378	93750

490.78056	0

490.87735	23437

490.97413	0

491.07091	0

491.16769	6591

491.26447	0

491.36126	0

491.45804	140625

491.55482	0

491.6516	93750

491.74838	0

491.84516	0

491.94195	93750

492.03873	0

492.13551	0

492.23229	17578

492.32907	17578

492.42586	166992

492.52264	0

492.61942	17578

492.7162	17578

492.81298	93750

492.90976	0

493.00655	6591

493.10333	0

493.20011	0

493.29689	2929

493.39367	0

493.49046	0

493.58724	23437

493.68402	23437

493.7808	0

493.87758	0

493.97436	0

494.07115	0

494.16793	17578

494.26471	6591

494.36149	17578

494.45827	16845

494.55506	0

494.65184	0

494.74862	16113

494.8454	17578

494.94218	0

495.03896	0

495.13575	16113

495.23253	0

495.32931	2929

495.42609	5859

495.52287	2929

495.61966	0

495.71644	93750

495.81322	0

495.91	0

496.00678	0

496.10356	5859

496.20035	93750

496.29713	5859

496.39391	0

496.49069	17578

496.58747	0

496.68426	17578

496.78104	0

496.87782	0

496.9746	0

497.07138	93750

497.16816	0

497.26495	0

497.36173	17578

497.45851	187500

497.55529	0

497.65207	0

497.74886	0

497.84564	0

497.94242	0

498.0392	17578

498.13598	0

498.23276	17578

498.32955	6591

498.42633	0

498.52311	0

498.61989	17578

498.71667	6591

498.81345	93750

498.91024	6591

499.00702	0

499.1038	16845

499.20058	17578

499.29736	93750

499.39415	93750

499.49093	2929

499.58771	0

499.68449	0

499.78127	6591

499.87805	0

499.97484	2929

500.07162	0

500.1684	0

500.26518	0

500.36196	0

500.45875	0

500.55553	93750

500.65231	17578

500.74909	17578

500.84587	0

500.94265	17578

501.03944	0

501.13622	6591

501.233	0

501.32978	17578

501.42656	93750

501.52335	0

501.62013	0

501.71691	0

501.81369	140625

501.91047	0

502.00725	0

502.10404	0

502.20082	0

502.2976	17578

502.39438	17578

502.49116	6591

502.58795	0

502.68473	0

502.78151	0

502.87829	0

502.97507	0

503.07185	0

503.16864	0

503.26542	0

503.3622	93750

503.45898	0

503.55576	93750

503.65255	5859

503.74933	2929

503.84611	0

503.94289	0

504.03967	0

504.13645	23437

504.23324	93750

504.33002	17578

504.4268	0

504.52358	17578

504.62036	0

504.71715	0

504.81393	5859

504.91071	6591

505.00749	6591

505.10427	5859

505.20105	0

505.29784	0

505.39462	93750

505.4914	0

505.58818	6591

505.68496	93750

505.78175	6591

505.87853	93750

505.97531	0

506.07209	0

506.16887	0

506.26565	0

506.36244	17578

506.45922	17578

506.556	0

506.65278	93750

506.74956	0

506.84635	0

506.94313	0

507.03991	0

507.13669	0

507.23347	2929

507.33025	6591

507.42704	93750

507.52382	187500

507.6206	0

507.71738	0

507.81416	17578

507.91095	0

508.00773	0

508.10451	5859

508.20129	17578

508.29807	0

508.39485	17578

508.49164	0

508.58842	2929

508.6852	0

508.78198	0

508.87876	11718

508.97555	0

509.07233	6591

509.16911	0

509.26589	0

509.36267	0

509.45945	17578

509.55624	0

509.65302	5859

509.7498	17578

509.84658	0

509.94336	0

510.04015	5859

510.13693	0

510.23371	6591

510.33049	0

510.42727	17578

510.52405	0

510.62084	0

510.71762	0

510.8144	0

510.91118	0

511.00796	6591

511.10474	2929

511.20153	0

511.29831	2929

511.39509	0

511.49187	0

511.58865	16113

511.68544	0

511.78222	0

511.879	0

511.97578	0

512.07256	6591

512.16934	17578

512.26613	5859

512.36291	0

512.45969	17578

512.55647	0

512.65325	0

512.75004	0

512.84682	0

512.9436	0

513.04038	0

513.13716	0

513.23394	5859

513.33073	93750

513.42751	0

513.52429	17578

513.62107	17578

513.71785	13183

513.81464	17578

513.91142	13183

514.0082	0

514.10498	0

514.20176	0

514.29854	0

514.39533	0

514.49211	2929

514.58889	0

514.68567	0

514.78245	0

514.87924	6591

514.97602	0

515.0728	187500

515.16958	0

515.26636	0

515.36314	93750

515.45993	17578

515.55671	93750

515.65349	0

515.75027	0

515.84705	0

515.94384	2929

516.04062	0

516.1374	0

516.23418	6591

516.33096	0

516.42774	0

516.52453	0

516.62131	0

516.71809	5859

516.81487	5859

516.91165	0

517.00844	93750

517.10522	6591

517.202	93750

517.29878	0

517.39556	93750

517.49234	6591

517.58913	6591

517.68591	0

517.78269	0

517.87947	17578

517.97625	2929

518.07304	0

518.16982	0

518.2666	0

518.36338	0

518.46016	17578

518.55694	0

518.65373	93750

518.75051	0

518.84729	0

518.94407	0

519.04085	93750

519.13764	0

519.23442	17578

519.3312	0

519.42798	93750

519.52476	0

519.62154	0

519.71833	0

519.81511	0

519.91189	0

520.00867	0

520.10545	0

520.20224	17578

520.29902	17578

520.3958	0

520.49258	6591

520.58936	0

520.68614	17578

520.78293	0

520.87971	17578

520.97649	17578

521.07327	0

521.17005	0

521.26684	16113

521.36362	17578

521.4604	0

521.55718	0

521.65396	6591

521.75074	0

521.84753	0

521.94431	0

522.04109	0

522.13787	17578

522.23465	0

522.33144	0

522.42822	0

522.525	0

522.62178	0

522.71856	93750

522.81534	0

522.91213	17578

523.00891	46875

523.10569	93750

523.20247	17578

523.29925	0

523.39603	17578

523.49282	17578

523.5896	0

523.68638	17578

523.78316	0

523.87994	93750

523.97673	0

524.07351	0

524.17029	0

524.26707	0

524.36385	0

524.46064	6591

524.55742	0

524.6542	17578

524.75098	0

524.84776	93750

524.94454	0

525.04133	187500

525.13811	0

525.23489	0

525.33167	46875

525.42845	17578

525.52523	0

525.62202	0

525.7188	46875

525.81558	7324

525.91236	2929

526.00914	0

526.10593	0

526.20271	17578

526.29949	17578

526.39627	6591

526.49305	17578

526.58983	0

526.68662	0

526.7834	6591

526.88018	0

526.97696	0

527.07374	0

527.17053	17578

527.26731	17578

527.36409	6591

527.46087	0

527.55765	93750

527.65443	5859

527.75122	93750

527.848	0

527.94478	2929

528.04156	0

528.13834	2929

528.23513	0

528.33191	17578

528.42869	0

528.52547	0

528.62225	0

528.71903	17578

528.81582	0

528.9126	17578

529.00938	17578

529.10616	0

529.20294	0

529.29973	0

529.39651	0

529.49329	0

529.59007	17578

529.68685	0

529.78363	0

529.88042	0

529.9772	0

530.07398	17578

530.17076	6591

530.26754	6591

530.36433	17578

530.46111	5859

530.55789	0

530.65467	0

530.75145	0

530.84823	0

530.94502	0

531.0418	0

531.13858	0

531.23536	17578

531.33214	13183

531.42893	0

531.52571	0

531.62249	0

531.71927	0

531.81605	0

531.91283	0

532.00962	5859

532.1064	187500

532.20318	5859

532.29996	0

532.39674	0

532.49353	0

532.59031	0

532.68709	0

532.78387	0

532.88065	0

532.97743	0

533.07422	0

533.171	0

533.26778	17578

533.36456	0

533.46134	0

533.55813	0

533.65491	5859

533.75169	17578

533.84847	17578

533.94525	93750

534.04203	0

534.13882	17578

534.2356	0

534.33238	5859

534.42916	17578

534.52594	0

534.62273	5859

534.71951	0

534.81629	0

534.91307	0

535.00985	93750

535.10663	0

535.20342	6591

535.3002	0

535.39698	0

535.49376	0

535.59054	5859

535.68733	5859

535.78411	0

535.88089	6591

535.97767	0

536.07445	0

536.17123	0

536.26802	0

536.3648	17578

536.46158	0

536.55836	0

536.65514	0

536.75193	23437

536.84871	93750

536.94549	17578

537.04227	0

537.13905	0

537.23583	0

537.33262	17578

537.4294	0

537.52618	0

537.62296	0

537.71974	0

537.81652	6591

537.91331	0

538.01009	0

538.10687	0

538.20365	0

538.30043	0

538.39722	93750

538.494	93750

538.59078	17578

538.68756	17578

538.78434	93750

538.88112	17578

538.97791	0

539.07469	0

539.17147	6591

539.26825	7324

539.36503	2929

539.46182	0

539.5586	17578

539.65538	6591

539.75216	2929

539.84894	23437

539.94572	93750

540.04251	0

540.13929	2929

540.23607	0

540.33285	93750

540.42963	17578

540.52642	17578

540.6232	0

540.71998	0

540.81676	0

540.91354	0

541.01032	0

541.10711	93750

541.20389	0

541.30067	0

541.39745	5859

541.49423	16845

541.59102	0

541.6878	0

541.78458	93750

541.88136	17578

541.97814	17578

542.07492	8056

542.17171	23437

542.26849	23437

542.36527	6591

542.46205	17578

542.55883	17578

542.65562	0

542.7524	93750

542.84918	5859

542.94596	13183
[truncated: 911,957 more chars]
