# Supplementary material for: Tunnel and underground engineering rock mass water inrush damage and acoustic emission characteristics
Source: PLoS One. 2024 Sep 20;19(9):e0307700. doi: 10.1371/journal.pone.0307700 (PMC11414891; doi:10.1371/journal.pone.0307700)
Supplement: S2 Dataset — (DOCX) [file pone.0307700.s002.docx]

**Fig 11. Damage variable evolution curve**

**（*a*）Non-hydraulic-0°**

1	0.03454	0.24544	0.0003702

2	0.07245	0.35487	0.00072

3	0.1125	0.4734	0.00094

4	0.15894	0.58069	0.001127

5	0.17339	0.67747	0.001137

6	0.18945	0.77425	0.001188

7	0.20551	0.87104	0.001192

8	0.22477	0.96782	0.001209

9	0.23922	1.0646	0.001217

10	0.25848	1.16138	0.00122

11	0.26973	1.25816	0.00129

12	0.28418	1.35495	0.00157

13	0.29862	1.45173	0.00158

14	0.3195	1.54851	0.00164

15	0.33716	1.64529	0.00175

16	0.34679	1.74207	0.00178

17	0.36445	1.83885	0.00193

18	0.38212	1.93564	0.00222

19	0.39816	2.03242	0.00224

20	0.41262	2.1292	0.00241

21	0.43188	2.22598	0.00247

22	0.44793	2.32276	0.00249

23	0.46078	2.41955	0.00252

24	0.47684	2.51633	0.00253

25	0.4945	2.61311	0.00263

26	0.50894	2.70989	0.00264

27	0.5218	2.80667	0.00266

28	0.54106	2.90345	0.00275

29	0.56353	3.00024	0.00276

30	0.57317	3.09702	0.00281

31	0.58761	3.1938	0.00282

32	0.61009	3.29058	0.00285

33	0.62294	3.38736	0.00301

34	0.63578	3.48415	0.00302

35	0.65185	3.58093	0.00319

36	0.67432	3.67771	0.00322

37	0.68395	3.77449	0.00323

38	0.70161	3.87127	0.00329

39	0.71606	3.96805	0.00334

40	0.73532	4.06484	0.00336

41	0.74977	4.16162	0.00343

42	0.76583	4.2584	0.00348

43	0.78028	4.35518	0.00359

44	0.79954	4.45196	0.0036

45	0.81079	4.54875	0.00362

46	0.83005	4.64553	0.00376

47	0.84771	4.74231	0.0041

48	0.86216	4.83909	0.00421

49	0.8734	4.93587	0.00423

50	0.88785	5.03265	0.0043

51	0.90872	5.12944	0.00432

52	0.92317	5.22622	0.00451

53	0.93441	5.323	0.00455

54	0.95849	5.41978	0.00456

55	0.97133	5.51656	0.00457

56	0.98418	5.61335	0.00458

57	1.01628	5.71013	0.00459

58	1.03074	5.80691	0.00461

59	1.05001	5.90369	0.00465

60	1.05964	6.00047	0.00487

61	1.0757	6.09725	0.00488

62	1.09496	6.19404	0.00525

63	1.10781	6.29082	0.00526

64	1.11904	6.3876	0.0055

65	1.14473	6.48438	0.00556

66	1.15436	6.58116	0.00557

67	1.17203	6.67794	0.00564

68	1.18647	6.77473	0.00573

69	1.20413	6.87151	0.0058

70	1.2202	6.96829	0.00581

71	1.23464	7.06507	0.006

72	1.2523	7.16185	0.00603

73	1.26996	7.25864	0.00611

74	1.28441	7.35542	0.00622

75	1.29564	7.4522	0.00624

76	1.31652	7.54898	0.00625

77	1.33258	7.64576	0.00627

78	1.34863	7.74254	0.00636

79	1.35988	7.83933	0.00644

80	1.38555	7.93611	0.00669

81	1.3984	8.03289	0.00673

82	1.40803	8.12967	0.00684

83	1.42249	8.22645	0.00695

84	1.44497	8.32324	0.00697

85	1.46102	8.42002	0.00741

86	1.47226	8.5168	0.00745

87	1.49312	8.61358	0.00746

88	1.50757	8.71036	0.00774

89	1.52203	8.80714	0.00802

90	1.53808	8.90393	0.00815

91	1.55735	9.00071	0.00827

92	1.57019	9.09749	0.00829

93	1.58303	9.19427	0.00845

94	1.6007	9.29105	0.00849

95	1.61996	9.38784	0.00883

96	1.63923	9.48462	0.00884

97	1.64725	9.5814	0.00889

98	1.66491	9.67818	0.00891

99	1.68579	9.77496	0.00897

100	1.69702	9.87174	0.00902

101	1.7163	9.96853	0.00931

102	1.73075	10.06531	0.01001

103	1.75001	10.16209	0.01009

104	1.76446	10.25887	0.0103

105	1.77569	10.35565	0.0104

106	1.79818	10.45244	0.01055

107	1.81263	10.54922	0.01056

108	1.82386	10.646	0.01057

109	1.84152	10.74278	0.01058

110	1.86079	10.83956	0.01071

111	1.88165	10.93634	0.01072

112	1.88647	11.03313	0.01076

113	1.90735	11.12991	0.01078

114	1.92661	11.22669	0.01079

115	1.93464	11.32347	0.01103

116	1.94908	11.42025	0.01105

117	1.97157	11.51704	0.01114

118	1.98762	11.61382	0.01116

119	2.00047	11.7106	0.01122

120	2.01974	11.80738	0.01123

121	2.039	11.90416	0.01124

122	2.05346	12.00094	0.01125

123	2.06629	12.09773	0.01132

124	2.07913	12.19451	0.0114

125	2.10322	12.29129	0.0115

126	2.11606	12.38807	0.01151

127	2.12571	12.48485	0.01157

128	2.14818	12.58164	0.01162

129	2.16263	12.67842	0.01186

130	2.1835	12.7752	0.01187

131	2.19313	12.87198	0.01189

132	2.214	12.96876	0.01204

133	2.22685	13.06554	0.01211

134	2.23969	13.16233	0.01224

135	2.25574	13.25911	0.01229

136	2.27983	13.35589	0.01246

137	2.29106	13.45267	0.01251

138	2.30231	13.54945	0.01253

139	2.32157	13.64624	0.01258

140	2.34404	13.74302	0.0126

141	2.36011	13.8398	0.01284

142	2.36974	13.93658	0.01291

143	2.389	14.03336	0.01299

144	2.40827	14.13014	0.01306

145	2.4147	14.22693	0.01313

146	2.43396	14.32371	0.01326

147	2.45483	14.42049	0.01329

148	2.46928	14.51727	0.01331

149	2.48213	14.61405	0.01336

150	2.50299	14.71084	0.01338

151	2.52066	14.80762	0.0134

152	2.53189	14.9044	0.01366

153	2.54313	15.00118	0.01374

154	2.56401	15.09796	0.01389

155	2.58167	15.19474	0.01399

156	2.58968	15.29153	0.01416

157	2.61056	15.38831	0.01418

158	2.62982	15.48509	0.01428

159	2.64428	15.58187	0.0143

160	2.65711	15.67865	0.01435

161	2.67318	15.77544	0.01438

162	2.69726	15.87222	0.01444

163	2.7085	15.969	0.01466

164	2.72135	16.06578	0.0147

165	2.74061	16.16256	0.01474

166	2.75666	16.25934	0.01485

167	2.7695	16.35613	0.01518

168	2.78396	16.45291	0.01519

169	2.80965	16.54969	0.01523

170	2.81928	16.64647	0.01531

171	2.8273	16.74325	0.01532

172	2.853	16.84004	0.01535

173	2.87066	16.93682	0.01558

174	2.88189	17.0336	0.01559

175	2.89634	17.13038	0.0156

176	2.91721	17.22716	0.0158

177	2.93327	17.32394	0.01583

178	2.94772	17.42073	0.01586

179	2.96217	17.51751	0.01595

180	2.98625	17.61429	0.01598

181	2.99589	17.71107	0.01601

182	3.00713	17.80785	0.01619

183	3.02318	17.90464	0.01638

184	3.04566	18.00142	0.0164

185	3.06011	18.0982	0.01651

186	3.07615	18.19498	0.01652

187	3.09061	18.29176	0.01692

188	3.10827	18.38854	0.01696

189	3.11791	18.48533	0.01707

190	3.13717	18.58211	0.01724

191	3.15804	18.67889	0.01732

192	3.1757	18.77567	0.01739

193	3.18694	18.87245	0.01759

194	3.20461	18.96924	0.0178

195	3.22226	19.06602	0.01782

196	3.23511	19.1628	0.01783

197	3.25116	19.25958	0.01787

198	3.26561	19.35636	0.01814

199	3.2913	19.45314	0.01815

200	3.30093	19.54993	0.01822

201	3.31538	19.64671	0.01823

202	3.33143	19.74349	0.01828

203	3.3507	19.84027	0.01829

204	3.36837	19.93705	0.01831

205	3.37639	20.03383	0.01874

206	3.39887	20.13062	0.01875

207	3.41653	20.2274	0.01893

208	3.42294	20.32418	0.01894

209	3.44222	20.42096	0.01895

210	3.46309	20.51774	0.01897

211	3.47753	20.61453	0.01903

212	3.48878	20.71131	0.01904

213	3.51286	20.80809	0.01907

214	3.53213	20.90487	0.0191

215	3.53695	21.00165	0.01911

216	3.55299	21.09843	0.01912

217	3.57066	21.19522	0.01928

218	3.59313	21.292	0.01929

219	3.59956	21.38878	0.0193

220	3.61883	21.48556	0.01935

221	3.63969	21.58234	0.01937

222	3.66057	21.67913	0.01938

223	3.6702	21.77591	0.01942

224	3.68465	21.87269	0.01943

225	3.70071	21.96947	0.01944

226	3.71355	22.06625	0.01946

227	3.728	22.16303	0.01948

228	3.75047	22.25982	0.01954

229	3.76814	22.3566	0.01955

230	3.78259	22.45338	0.01957

231	3.79543	22.55016	0.01972

232	3.81469	22.64694	0.01974

233	3.83235	22.74373	0.02012

234	3.83717	22.84051	0.02013

235	3.85805	22.93729	0.02025

236	3.88213	23.03407	0.02027

237	3.89176	23.13085	0.02028

238	3.903	23.22763	0.02048

239	3.92869	23.32442	0.02053

240	3.94795	23.4212	0.02065

241	3.95599	23.51798	0.02066

242	3.97364	23.61476	0.02071

243	3.99452	23.71154	0.02118

244	4.00415	23.80833	0.02119

245	4.01699	23.90511	0.02123

246	4.03787	24.00189	0.02128

247	4.05713	24.09867	0.02129

248	4.06837	24.19545	0.02136

249	4.08603	24.29223	0.0214

250	4.10368	24.38902	0.02148

251	4.11653	24.4858	0.02149

252	4.12777	24.58258	0.0215

253	4.14383	24.67936	0.02152

254	4.17112	24.77614	0.02158

255	4.18236	24.87293	0.02178

256	4.19359	24.96971	0.02193

257	4.21447	25.06649	0.022

258	4.23373	25.16327	0.02204

259	4.24336	25.26005	0.02206

260	4.25782	25.35683	0.02208

261	4.2819	25.45362	0.02226

262	4.29474	25.5504	0.02232

263	4.30598	25.64718	0.02233

264	4.32524	25.74396	0.02236

265	4.34452	25.84074	0.02241

266	4.36218	25.93753	0.02251

267	4.37341	26.03431	0.02254

268	4.38466	26.13109	0.0227

269	4.41515	26.22787	0.02274

270	4.41998	26.32465	0.02284

271	4.43282	26.42143	0.02288

272	4.45369	26.51822	0.02289

273	4.47136	26.615	0.0229

274	4.4874	26.71178	0.02295

275	4.50025	26.80856	0.02302

276	4.52434	26.90534	0.02303

277	4.53717	27.00213	0.0232

278	4.54681	27.09891	0.02321

279	4.55965	27.19569	0.02326

280	4.58534	27.29247	0.02328

281	4.59818	27.38925	0.02329

282	4.61263	27.48603	0.02343

283	4.62708	27.58282	0.02348

284	4.64796	27.6796	0.02356

285	4.6608	27.77638	0.02357

286	4.68006	27.87316	0.02365

287	4.68969	27.96994	0.0237

288	4.70897	28.06673	0.02373

289	4.72181	28.16351	0.02383

290	4.73627	28.26029	0.02408

291	4.76035	28.35707	0.0241

292	4.78443	28.45385	0.0243

293	4.78924	28.55063	0.02455

294	4.80208	28.64742	0.0246

295	4.82136	28.7442	0.02461

296	4.8358	28.84098	0.02465

297	4.84865	28.93776	0.02466

298	4.86309	29.03454	0.02471

299	4.89199	29.13133	0.02472

300	4.90003	29.22811	0.02474

301	4.91929	29.32489	0.02497

302	4.93695	29.42167	0.02499

303	4.95942	29.51845	0.02503

304	4.96264	29.61523	0.02505

305	4.9803	29.71202	0.02509

306	4.99475	29.8088	0.0251

307	5.01401	29.90558	0.02548

308	5.02364	30.00236	0.02549

309	5.04934	30.09914	0.0255

310	5.0686	30.19593	0.02553

311	5.08144	30.29271	0.02576

312	5.08947	30.38949	0.02577

313	5.11836	30.48627	0.02579

314	5.13122	30.58305	0.02588

315	5.14245	30.67983	0.02621

316	5.15369	30.77662	0.02633

317	5.17616	30.8734	0.02652

318	5.19865	30.97018	0.0267

319	5.20186	31.06696	0.02674

320	5.22274	31.16374	0.02708

321	5.242	31.26053	0.0271

322	5.25003	31.35731	0.02733

323	5.26769	31.45409	0.02743

324	5.28374	31.55087	0.02761

325	5.30462	31.64765	0.02765

326	5.31264	31.74443	0.02775

327	5.33029	31.84122	0.02784

328	5.35438	31.938	0.02785

329	5.37043	32.03478	0.02786

330	5.37846	32.13156	0.02789

331	5.39772	32.22834	0.028

332	5.42021	32.32512	0.02803

333	5.43305	32.42191	0.02809

334	5.44589	32.51869	0.0282

335	5.46838	32.61547	0.02832

336	5.48443	32.71225	0.02834

337	5.49567	32.80903	0.02842

338	5.51493	32.90582	0.02849

339	5.52618	33.0026	0.0285

340	5.54705	33.09938	0.02862

341	5.55026	33.19616	0.02863

342	5.56952	33.29294	0.02867

343	5.59199	33.38972	0.0287

344	5.61127	33.48651	0.02874

345	5.61608	33.58329	0.02876

346	5.64498	33.68007	0.02899

347	5.66425	33.77685	0.02901

348	5.66907	33.87363	0.02902

349	5.68512	33.97042	0.02903

350	5.70438	34.0672	0.02905

351	5.72204	34.16398	0.02959

352	5.72847	34.26076	0.02962

353	5.75255	34.35754	0.02963

354	5.7686	34.45432	0.02964

355	5.79268	34.55111	0.02966

356	5.7975	34.64789	0.02974

357	5.81998	34.74467	0.02976

358	5.83604	34.84145	0.02977

359	5.84567	34.93823	0.03012

360	5.85852	35.03502	0.03027

361	5.88742	35.1318	0.03028

362	5.90186	35.22858	0.03029

363	5.90989	35.32536	0.03033

364	5.92915	35.42214	0.03034

365	5.94681	35.51892	0.03043

366	5.95966	35.61571	0.03071

367	5.97572	35.71249	0.03074

368	5.99177	35.80927	0.03075

369	6.00943	35.90605	0.03076

370	6.02228	36.00283	0.03081

371	6.03833	36.09962	0.03098

372	6.05599	36.1964	0.03103

373	6.07686	36.29318	0.03107

374	6.08649	36.38996	0.03117

375	6.10255	36.48674	0.03118

376	6.12182	36.58352	0.03136

377	6.14108	36.68031	0.03137

378	6.1459	36.77709	0.03143

379	6.16999	36.87387	0.03151

380	6.19727	36.97065	0.03164

381	6.20531	37.06743	0.03166

382	6.21333	37.16422	0.03169

383	6.2326	37.261	0.03172

384	6.24865	37.35778	0.03188

385	6.25989	37.45456	0.03189

386	6.27915	37.55134	0.03199

387	6.30003	37.64812	0.03201

388	6.3193	37.74491	0.03205

389	6.32411	37.84169	0.03214

390	6.34177	37.93847	0.03244

391	6.36585	38.03525	0.03246

392	6.3803	38.13203	0.03278

393	6.38673	38.22882	0.03324

394	6.40599	38.3256	0.03369

395	6.43169	38.42238	0.03373

396	6.4381	38.51916	0.03384

397	6.45736	38.61594	0.03391

398	6.47342	38.71272	0.03395

399	6.4959	38.80951	0.03434

400	6.50393	38.90629	0.03444

401	6.51357	39.00307	0.03455

402	6.54086	39.09985	0.0346

403	6.55692	39.19663	0.03469

404	6.56173	39.29342	0.03474

405	6.58259	39.3902	0.03475

406	6.60668	39.48698	0.03481

407	6.62113	39.58376	0.03482

408	6.63237	39.68054	0.03488

409	6.65325	39.77732	0.0349

410	6.66769	39.87411	0.03491

411	6.67732	39.97089	0.03507

412	6.69819	40.06767	0.03512

413	6.71906	40.16445	0.03519

414	6.73512	40.26123	0.0352

415	6.74957	40.35802	0.03522

416	6.76402	40.4548	0.03525

417	6.78649	40.55158	0.0354

418	6.79774	40.64836	0.03566

419	6.80737	40.74514	0.03575

420	6.82664	40.84192	0.03587

421	6.84911	40.93871	0.03599

422	6.86357	41.03549	0.03602

423	6.8732	41.13227	0.03629

424	6.89888	41.22905	0.03633

425	6.91494	41.32583	0.03634

426	6.91975	41.42262	0.03636

427	6.94705	41.5194	0.03639

428	6.9615	41.61618	0.03672

429	6.97113	41.71296	0.03675

430	6.98718	41.80974	0.03677

431	7.00325	41.90652	0.03696

432	7.02251	42.00331	0.03706

433	7.04177	42.10009	0.03707

434	7.05784	42.19687	0.03708

435	7.06906	42.29365	0.03713

436	7.08673	42.39043	0.03772

437	7.09797	42.48722	0.03773

438	7.11563	42.584	0.03778

439	7.1381	42.68078	0.03784

440	7.15094	42.77756	0.03791

441	7.15898	42.87434	0.03792

442	7.18627	42.97112	0.03799

443	7.20233	43.06791	0.03801

444	7.21356	43.16469	0.03802

445	7.22801	43.26147	0.03817

446	7.25691	43.35825	0.03819

447	7.26495	43.45503	0.03825

448	7.27618	43.55182	0.03826

449	7.28903	43.6486	0.03827

450	7.31632	43.74538	0.0383

451	7.32595	43.84216	0.03831

452	7.34361	43.93894	0.03834

453	7.35805	44.03572	0.03835

454	7.38054	44.13251	0.03836

455	7.38696	44.22929	0.03841

456	7.40141	44.32607	0.03889

457	7.42389	44.42285	0.03895

458	7.43993	44.51963	0.03896

459	7.45279	44.61641	0.03914

460	7.46723	44.7132	0.03915

461	7.48651	44.80998	0.03919

462	7.50577	44.90676	0.0392

463	7.51701	45.00354	0.03926

464	7.52664	45.10032	0.03927

465	7.54912	45.19711	0.03929

466	7.56839	45.29389	0.0393

467	7.57641	45.39067	0.03937

468	7.6021	45.48745	0.03944

469	7.62297	45.58423	0.03947

470	7.6326	45.68101	0.03949

471	7.64063	45.7778	0.03953

472	7.66311	45.87458	0.03965

473	7.6904	45.97136	0.03967

474	7.6888	46.06814	0.03968

475	7.71448	46.16492	0.03986

476	7.73054	46.26171	0.03998

477	7.7482	46.35849	0.03999

478	7.75462	46.45527	0.04008

479	7.7755	46.55205	0.04019

480	7.79476	46.64883	0.0402

481	7.80279	46.74561	0.04039

482	7.81724	46.8424	0.04043

483	7.83971	46.93918	0.04062

484	7.85897	47.03596	0.04069

485	7.8654	47.13274	0.04075

486	7.88788	47.22952	0.04127

487	7.91036	47.32631	0.04131

488	7.92159	47.42309	0.04132

489	7.93284	47.51987	0.04133

490	7.95371	47.61665	0.04174

491	7.96976	47.71343	0.04176

492	7.98743	47.81021	0.0419

493	7.99224	47.907	0.04217

494	8.02435	48.00378	0.04227

495	8.03559	48.10056	0.04229

496	8.04201	48.19734	0.0423

497	8.05807	48.29412	0.04231

498	8.08215	48.39091	0.04234

499	8.09499	48.48769	0.04235

500	8.11425	48.58447	0.04239

501	8.1287	48.68125	0.04241

502	8.14958	48.77803	0.04289

503	8.15761	48.87481	0.04319

504	8.17366	48.9716	0.04325

505	8.19775	49.06838	0.04327

506	8.20576	49.16516	0.04331

507	8.21701	49.26194	0.04333

508	8.23146	49.35872	0.04357

509	8.25875	49.45551	0.04371

510	8.26679	49.55229	0.04411

511	8.27963	49.64907	0.04421

512	8.30371	49.74585	0.04494

513	8.32297	49.84263	0.04497

514	8.33742	49.93941	0.04498

515	8.34706	50.0362	0.04499

516	8.37114	50.13298	0.04509

517	8.38238	50.22976	0.04516

518	8.39522	50.32654	0.04517

519	8.41449	50.42332	0.04518

520	8.43215	50.52011	0.0452

521	8.45302	50.61689	0.04525

522	8.46104	50.71367	0.04526

523	8.47871	50.81045	0.04535

524	8.50279	50.90723	0.04588

525	8.50761	51.00401	0.04601

526	8.52527	51.1008	0.04602

527	8.53971	51.19758	0.04603

528	8.57022	51.29436	0.04617

529	8.57986	51.39114	0.04623

530	8.58949	51.48792	0.04635

531	8.61358	51.58471	0.04636

532	8.62802	51.68149	0.04644

533	8.63605	51.77827	0.04646

534	8.6521	51.87505	0.04647

535	8.67297	51.97183	0.04651

536	8.68742	52.06861	0.04685

537	8.69705	52.1654	0.04699

538	8.71954	52.26218	0.04701

539	8.74201	52.35896	0.04702

540	8.75967	52.45574	0.04707

541	8.75967	52.55252	0.04712

542	8.78215	52.64931	0.0472

543	8.7982	52.74609	0.04724

544	8.80623	52.84287	0.04726

545	8.82389	52.93965	0.04727

546	8.85279	53.03643	0.04745

547	8.87045	53.13321	0.0475

548	8.87687	53.23	0.0476

549	8.89294	53.32678	0.04762

550	8.91702	53.42356	0.0477

551	8.92665	53.52034	0.04782

552	8.93949	53.61712	0.04786

553	8.96196	53.71391	0.04788

554	8.97803	53.81069	0.04792

555	8.98766	53.90747	0.04793

556	9.01013	54.00425	0.04796

557	9.0294	54.10103	0.04807

558	9.04384	54.19781	0.04813

559	9.05027	54.2946	0.04817

560	9.06793	54.39138	0.04822

561	9.09362	54.48816	0.04824

562	9.10968	54.58494	0.04825

563	9.11609	54.68172	0.04826

564	9.14179	54.77851	0.04832

565	9.16586	54.87529	0.04873

566	9.17068	54.97207	0.04878

567	9.18193	55.06885	0.04879

568	9.20922	55.16563	0.0488

569	9.22045	55.26241	0.04891

570	9.23169	55.3592	0.04892

571	9.24936	55.45598	0.04893

572	9.27344	55.55276	0.04902

573	9.28628	55.64954	0.04904

574	9.29591	55.74632	0.04906

575	9.32161	55.84311	0.0491

576	9.33927	55.93989	0.04916

577	9.33927	56.03667	0.04918

578	9.36334	56.13345	0.04942

579	9.38582	56.23023	0.04948

580	9.40027	56.32701	0.04952

581	9.4099	56.4238	0.04964

582	9.42757	56.52058	0.04965

583	9.45004	56.61736	0.04977

584	9.45647	56.71414	0.04986

585	9.47413	56.81092	0.0499

586	9.48697	56.9077	0.04991

587	9.50623	57.00449	0.04993

588	9.51747	57.10127	0.05019

589	9.53995	57.19805	0.05032

590	9.56082	57.29483	0.05033

591	9.57848	57.39161	0.05041

592	9.58652	57.4884	0.05054

593	9.60256	57.58518	0.05059

594	9.62344	57.68196	0.0506

595	9.63789	57.77874	0.05061

596	9.64912	57.87552	0.05064

597	9.66518	57.9723	0.05067

598	9.68926	58.06909	0.05068

599	9.69729	58.16587	0.05069

600	9.71495	58.26265	0.05071

601	9.73743	58.35943	0.05072

602	9.75669	58.45621	0.05087

603	9.76312	58.553	0.05088

604	9.77757	58.64978	0.05091

605	9.80326	58.74656	0.05134

606	9.81289	58.84334	0.05145

607	9.82412	58.94012	0.05146

608	9.84821	59.0369	0.05151

609	9.86908	59.13369	0.05152

610	9.88192	59.23047	0.05155

611	9.88514	59.32725	0.05172

612	9.90922	59.42403	0.05179

613	9.93009	59.52081	0.05214

614	9.93651	59.6176	0.05215

615	9.95096	59.71438	0.05221

616	9.97665	59.81116	0.05229

617	9.99752	59.90794	0.05232

618	10.00876	60.00472	0.05241

619	10.02001	60.1015	0.05242

620	10.03927	60.19829	0.05245

621	10.04568	60.29507	0.05296

622	10.06174	60.39185	0.05326

623	10.08422	60.48863	0.05346

624	10.1083	60.58541	0.05354

625	10.12115	60.6822	0.05357

626	10.13078	60.77898	0.05358

627	10.15325	60.87576	0.0537

628	10.17734	60.97254	0.05372

629	10.17895	61.06932	0.05373

630	10.19179	61.1661	0.05374

631	10.21749	61.26289	0.05384

632	10.22712	61.35967	0.05387

633	10.23996	61.45645	0.05389

634	10.25761	61.55323	0.05397

635	10.2833	61.65001	0.05398

636	10.29293	61.7468	0.05404

637	10.31221	61.84358	0.05405

638	10.32344	61.94036	0.05407

639	10.3411	62.03714	0.05417

640	10.34913	62.13392	0.05419

641	10.3668	62.2307	0.05425

642	10.39409	62.32749	0.05426

643	10.40853	62.42427	0.05429

644	10.41977	62.52105	0.0543

645	10.43743	62.61783	0.05431

646	10.46312	62.71461	0.05438

647	10.47115	62.8114	0.05439

648	10.48239	62.90818	0.0544

649	10.50486	63.00496	0.05442

650	10.51932	63.10174	0.05443

651	10.53056	63.19852	0.05444

652	10.54982	63.2953	0.05447

653	10.5755	63.39209	0.05448

654	10.58514	63.48887	0.0545

655	10.59157	63.58565	0.05451

656	10.60921	63.68243	0.05464

657	10.6333	63.77921	0.0547

658	10.64775	63.876	0.05481

659	10.65579	63.97278	0.05482

660	10.67665	64.06956	0.05483

661	10.69592	64.16634	0.05494

662	10.71037	64.26312	0.05495

663	10.72804	64.3599	0.05496

664	10.74889	64.45669	0.05497

665	10.75854	64.55347	0.05499

666	10.76977	64.65025	0.05503

667	10.78743	64.74703	0.0551

668	10.80991	64.84381	0.05511

669	10.82114	64.9406	0.05513

670	10.83881	65.03738	0.05514

671	10.85327	65.13416	0.05515

672	10.87735	65.23094	0.05516

673	10.89019	65.32772	0.05518

674	10.8934	65.4245	0.0553

675	10.9207	65.52129	0.05531

676	10.93193	65.61807	0.05532

677	10.94959	65.71485	0.05535

678	10.96886	65.81163	0.05538

679	10.99295	65.90841	0.05539

680	11.00258	66.0052	0.05543

681	11.01221	66.10198	0.05545

682	11.02505	66.19876	0.05547

683	11.04913	66.29554	0.05548

684	11.05876	66.39232	0.05556

685	11.07321	66.4891	0.05557

686	11.09248	66.58589	0.05558

687	11.11496	66.68267	0.05567

688	11.12459	66.77945	0.05571

689	11.13904	66.87623	0.05578

690	11.16312	66.97301	0.0558

691	11.17436	67.0698	0.05581

692	11.18079	67.16658	0.05609

693	11.20326	67.26336	0.05611

694	11.22414	67.36014	0.05613

695	11.23377	67.45692	0.05625

696	11.24982	67.5537	0.05626

697	11.2739	67.65049	0.05629

698	11.28995	67.74727	0.05635

699	11.3012	67.84405	0.05637

700	11.31404	67.94083	0.05638

701	11.34133	68.03761	0.0564

702	11.34937	68.1344	0.05644

703	11.3606	68.23118	0.05685

704	11.38468	68.32796	0.05687

705	11.40877	68.42474	0.05691

706	11.4184	68.52152	0.05697

707	11.42963	68.6183	0.05705

708	11.44409	68.71509	0.05706

709	11.46978	68.81187	0.05714

710	11.4778	68.90865	0.05716

711	11.49225	69.00543	0.05718

712	11.51472	69.10221	0.0572

713	11.53239	69.199	0.05726

714	11.54202	69.29578	0.05761

715	11.55648	69.39256	0.05762

716	11.57896	69.48934	0.05763

717	11.59982	69.58612	0.05764

718	11.59822	69.6829	0.05772

719	11.62391	69.77969	0.05777

720	11.64318	69.87647	0.05779

721	11.65762	69.97325	0.05781

722	11.67207	70.07003	0.05782

723	11.69455	70.16681	0.05785

724	11.71061	70.26359	0.05786

725	11.71864	70.36038	0.05788

726	11.72827	70.45716	0.05789

727	11.75716	70.55394	0.05791

728	11.77482	70.65072	0.05792

729	11.78124	70.7475	0.05794

730	11.7989	70.84429	0.05797

731	11.81978	70.94107	0.05798

732	11.83744	71.03785	0.05799

733	11.84707	71.13463	0.058

734	11.86633	71.23141	0.05805

735	11.8856	71.32819	0.05808

736	11.88721	71.42498	0.0583

737	11.91129	71.52176	0.05831

738	11.93538	71.61854	0.05842

739	11.94983	71.71532	0.05843

740	11.95785	71.8121	0.05844

741	11.97712	71.90889	0.05845

742	11.9996	72.00567	0.05848

743	12.01564	72.10245	0.05851

744	12.02689	72.19923	0.05852

745	12.03813	72.29601	0.05879

746	12.059	72.39279	0.05881

747	12.07344	72.48958	0.05882

748	12.09272	72.58636	0.05883

749	12.10877	72.68314	0.0589

750	12.12803	72.77992	0.05892

751	12.13767	72.8767	0.05906

752	12.15212	72.97349	0.05932

753	12.17299	73.07027	0.05936

754	12.18904	73.16705	0.05943

755	12.19065	73.26383	0.05946

756	12.21794	73.36061	0.05947

757	12.23882	73.45739	0.05949

758	12.25166	73.55418	0.0595

759	12.26771	73.65096	0.05972

760	12.28699	73.74774	0.05983

761	12.30143	73.84452	0.05999

762	12.30946	73.9413	0.0604

763	12.32391	74.03809	0.06044

764	12.34799	74.13487	0.06046

765	12.36725	74.23165	0.06054

766	12.3785	74.32843	0.06093

767	12.39455	74.42521	0.06126

768	12.42023	74.52199	0.06127

769	12.42827	74.61878	0.06128

770	12.43468	74.71556	0.06135

771	12.46198	74.81234	0.06138

772	12.47482	74.90912	0.06146

773	12.48607	75.0059	0.06148

774	12.50693	75.10269	0.06166

775	12.5262	75.19947	0.06167

776	12.54387	75.29625	0.06214

777	12.55189	75.39303	0.06245

778	12.56955	75.48981	0.06248

779	12.59042	75.58659	0.06264

780	12.60648	75.68338	0.06265

781	12.6145	75.78016	0.06268

782	12.63698	75.87694	0.06303

783	12.64983	75.97372	0.06305

784	12.66428	76.0705	0.06307

785	12.68194	76.16729	0.06311

786	12.70281	76.26407	0.06312

787	12.72047	76.36085	0.06317

788	12.72689	76.45763	0.0638

789	12.74776	76.55441	0.06388

790	12.76543	76.65119	0.06396

791	12.77506	76.74798	0.06397

792	12.7879	76.84476	0.06398

793	12.81359	76.94154	0.06399

794	12.83606	77.03832	0.06403

795	12.84089	77.1351	0.06413

796	12.85694	77.23189	0.06421

797	12.8762	77.32867	0.06445

798	12.88905	77.42545	0.06447

799	12.90671	77.52223	0.06454

800	12.91956	77.61901	0.06469

801	12.94524	77.71579	0.06537

802	12.95808	77.81258	0.06539

803	12.97414	77.90936	0.06555

804	12.99662	78.00614	0.06569

805	13.00465	78.10292	0.0657

806	13.02231	78.1997	0.06599

807	13.03354	78.29649	0.06603

808	13.05602	78.39327	0.06607

809	13.07368	78.49005	0.06614

810	13.0785	78.58683	0.06616

811	13.09937	78.68361	0.06617

812	13.12024	78.78039	0.06641

813	13.1363	78.87718	0.06643

814	13.14593	78.97396	0.06644

815	13.17804	79.07074	0.06648

816	13.17965	79.16752	0.06661

817	13.1941	79.2643	0.06662

818	13.21818	79.36109	0.06674

819	13.23263	79.45787	0.06675

820	13.24868	79.55465	0.06698

821	13.26474	79.65143	0.06699

822	13.27758	79.74821	0.06702

823	13.30006	79.84499	0.06709

824	13.3113	79.94178	0.0671

825	13.31772	80.03856	0.06718

826	13.34019	80.13534	0.06722

827	13.36107	80.23212	0.06723

828	13.3707	80.3289	0.06725

829	13.38997	80.42569	0.06728

830	13.40603	80.52247	0.0673

831	13.42207	80.61925	0.06731

832	13.43653	80.71603	0.06732

833	13.45579	80.81281	0.06735

834	13.47667	80.90959	0.06746

835	13.48469	81.00638	0.0675

836	13.50075	81.10316	0.06768

837	13.5152	81.19994	0.06769

838	13.53446	81.29672	0.0677

839	13.54571	81.3935	0.06784

840	13.56818	81.49029	0.06786

841	13.58263	81.58707	0.06787

842	13.59547	81.68385	0.06788

843	13.60671	81.78063	0.0679

844	13.62918	81.87741	0.06796

845	13.64846	81.97419	0.06805

846	13.66451	82.07098	0.06808

847	13.67254	82.16776	0.0682

848	13.69823	82.26454	0.06826

849	13.71107	82.36132	0.0687

850	13.72231	82.4581	0.06873

851	13.73034	82.55488	0.06878

852	13.76085	82.65167	0.06879

853	13.78332	82.74845	0.06883

854	13.78974	82.84523	0.06884

855	13.80258	82.94201	0.06891

856	13.82666	83.03879	0.06893

857	13.84754	83.13558	0.0692

858	13.84915	83.23236	0.06923

859	13.86841	83.32914	0.06943

860	13.88607	83.42592	0.06979

861	13.89891	83.5227	0.0698

862	13.91979	83.61948	0.06981

863	13.93584	83.71627	0.06984

864	13.95832	83.81305	0.06986

865	13.96796	83.90983	0.06991

866	13.98241	84.00661	0.07017

867	14.00327	84.10339	0.07045

868	14.01933	84.20018	0.07046

869	14.02255	84.29696	0.07048

870	14.05304	84.39374	0.07049

871	14.07231	84.49052	0.0705

872	14.08034	84.5873	0.07051

873	14.098	84.68408	0.07052

874	14.11245	84.78087	0.07054

875	14.13011	84.87765	0.0706

876	14.14135	84.97443	0.07069

877	14.15098	85.07121	0.0707

878	14.17667	85.16799	0.07078

879	14.19112	85.26478	0.07102

880	14.20075	85.36156	0.07109

881	14.22644	85.45834	0.07123

882	14.24892	85.55512	0.07124

883	14.25695	85.6519	0.07125

884	14.26658	85.74868	0.07126

885	14.28745	85.84547	0.07137

886	14.30832	85.94225	0.07138

887	14.31474	86.03903	0.07149

888	14.3324	86.13581	0.0715

889	14.3597	86.23259	0.07164

890	14.37093	86.32938	0.07171

891	14.38379	86.42616	0.07173

892	14.39823	86.52294	0.07182

893	14.4191	86.61972	0.07195

894	14.43034	86.7165	0.07196

895	14.43997	86.81328	0.07198

896	14.46567	86.91007	0.07202

897	14.48332	87.00685	0.07207

898	14.49617	87.10363	0.07208

899	14.51061	87.20041	0.0721

900	14.5331	87.29719	0.0722

901	14.54594	87.39398	0.07224

902	14.55718	87.49076	0.0723

903	14.57162	87.58754	0.07238

904	14.59891	87.68432	0.07239

905	14.61176	87.7811	0.0724

906	14.61979	87.87788	0.07267

907	14.64227	87.97467	0.07268

908	14.65833	88.07145	0.07289

909	14.67759	88.16823	0.07293

910	14.68722	88.26501	0.07294

911	14.69686	88.36179	0.07295

912	14.72095	88.45858	0.07296

913	14.73378	88.55536	0.07297

914	14.74662	88.65214	0.07298

915	14.77071	88.74892	0.07299

916	14.79158	88.8457	0.07301

917	14.80924	88.94248	0.07309

918	14.81085	89.03927	0.0731

919	14.84456	89.13605	0.07312

920	14.85098	89.23283	0.07313

921	14.85901	89.32961	0.07325

922	14.87989	89.42639	0.0733

923	14.89755	89.52318	0.07359

924	14.91521	89.61996	0.07369

925	14.93607	89.71674	0.0737

926	14.95855	89.81352	0.07377

927	14.96979	89.9103	0.07378

928	14.96979	90.00708	0.07381

929	14.99387	90.10387	0.07382

930	15.01795	90.20065	0.07387

931	15.0308	90.29743	0.07389

932	15.04043	90.39421	0.07393

933	15.0597	90.49099	0.07395

934	15.08057	90.58778	0.07398

935	15.08539	90.68456	0.07399

936	15.10626	90.78134	0.07403

937	15.12713	90.87812	0.07414

938	15.13837	90.9749	0.07443

939	15.14962	91.07168	0.07462

940	15.16888	91.16847	0.07463

941	15.19135	91.26525	0.07464

942	15.2042	91.36203	0.07465

943	15.21383	91.45881	0.07466

944	15.23309	91.55559	0.07467

945	15.25397	91.65238	0.07468

946	15.27163	91.74916	0.07475

947	15.28126	91.84594	0.07476

948	15.29731	91.94272	0.07481

949	15.31819	92.0395	0.07484

950	15.31979	92.13628	0.07486

951	15.34388	92.23307	0.07488

952	15.37118	92.32985	0.07491

953	15.3792	92.42663	0.07532

954	15.39044	92.52341	0.07535

955	15.41291	92.62019	0.0754

956	15.42898	92.71698	0.07541

957	15.44342	92.81376	0.07545

958	15.44983	92.91054	0.07546

959	15.48034	93.00732	0.07567

960	15.49319	93.1041	0.07568

961	15.50924	93.20088	0.07572

962	15.52049	93.29767	0.07581

963	15.54617	93.39445	0.07614

964	15.55901	93.49123	0.07615

965	15.56384	93.58801	0.07616

966	15.58631	93.68479	0.07617

967	15.60558	93.78158	0.07618

968	15.61842	93.87836	0.07621

969	15.63447	93.97514	0.07634

970	15.65695	94.07192	0.07635

971	15.67139	94.1687	0.07637

972	15.68104	94.26548	0.07638

973	15.69548	94.36227	0.07639

974	15.71315	94.45905	0.07642

975	15.73081	94.55583	0.07645

976	15.74364	94.65261	0.07646

977	15.76773	94.74939	0.07687

978	15.79021	94.84617	0.07688

979	15.79663	94.94296	0.07692

980	15.80144	95.03974	0.07693

981	15.82874	95.13652	0.07722

982	15.84961	95.2333	0.07725

983	15.85603	95.33008	0.07727

984	15.86566	95.42687	0.07728

985	15.89136	95.52365	0.07729

986	15.91062	95.62043	0.07734

987	15.92026	95.71721	0.07737

988	15.93309	95.81399	0.07739

989	15.95557	95.91077	0.07745

990	15.97484	96.00756	0.07747

991	15.98287	96.10434	0.07767

992	16.00214	96.20112	0.07807

993	16.023	96.2979	0.07808

994	16.03264	96.39468	0.0781

995	16.04227	96.49147	0.07818

996	16.07277	96.58825	0.07836

997	16.08402	96.68503	0.07837

998	16.09205	96.78181	0.07858

999	16.11131	96.87859	0.07859

1000	16.13539	96.97537	0.07862

1001	16.14985	97.07216	0.07864

1002	16.15786	97.16894	0.07865

1003	16.17714	97.26572	0.07866

1004	16.19801	97.3625	0.07867

1005	16.21566	97.45928	0.07868

1006	16.2253	97.55607	0.07872

1007	16.24296	97.65285	0.07873

1008	16.26223	97.74963	0.07939

1009	16.27347	97.84641	0.0794

1010	16.2847	97.94319	0.07971

1011	16.31682	98.03997	0.07974

1012	16.32324	98.13676	0.07975

1013	16.3425	98.23354	0.07979

1014	16.36498	98.33032	0.07982

1015	16.37622	98.4271	0.07983

1016	16.38907	98.52388	0.08037

1017	16.40191	98.62067	0.0804

1018	16.42921	98.71745	0.08053

1019	16.43722	98.81423	0.08054

1020	16.44044	98.91101	0.08055

1021	16.46773	99.00779	0.08057

1022	16.49343	99.10457	0.08073

1023	16.50146	99.20136	0.08077

1024	16.51429	99.29814	0.08089

1025	16.53517	99.39492	0.08092

1026	16.55443	99.4917	0.08139

1027	16.56568	99.58848	0.08144

1028	16.57852	99.68527	0.08147

1029	16.60099	99.78205	0.08154

1030	16.61866	99.87883	0.08158

1031	16.63149	99.97561	0.08161

1032	16.64114	100.07239	0.08175

1033	16.66361	100.16917	0.08176

1034	16.68127	100.26596	0.08185

1035	16.68608	100.36274	0.0819

1036	16.71017	100.45952	0.08191

1037	16.72301	100.5563	0.08195

1038	16.74067	100.65308	0.08196

1039	16.75351	100.74987	0.08198

1040	16.78563	100.84665	0.08211

1041	16.79205	100.94343	0.08212

1042	16.80329	101.04021	0.08213

1043	16.82095	101.13699	0.08224

1044	16.84182	101.23377	0.08225

1045	16.85467	101.33056	0.08229

1046	16.86269	101.42734	0.0823

1047	16.88517	101.52412	0.08233

1048	16.90765	101.6209	0.08236

1049	16.92531	101.71768	0.08239

1050	16.93013	101.81447	0.08243

1051	16.94778	101.91125	0.0826

1052	16.96865	102.00803	0.08263

1053	16.97828	102.10481	0.08264

1054	16.99594	102.20159	0.08266

1055	17.01843	102.29837	0.08311

1056	17.03127	102.39516	0.08314

1057	17.0409	102.49194	0.08315

1058	17.06337	102.58872	0.08322

1059	17.07944	102.6855	0.08335

1060	17.0971	102.78228	0.08351

1061	17.10191	102.87907	0.08356

1062	17.1244	102.97585	0.08358

1063	17.14205	103.07263	0.08363

1064	17.15971	103.16941	0.08364

1065	17.16934	103.26619	0.08367

1066	17.19503	103.36297	0.08368

1067	17.21109	103.45976	0.08374

1068	17.22072	103.55654	0.08375

1069	17.23678	103.65332	0.0839

1070	17.25926	103.7501	0.08394

1071	17.2753	103.84688	0.08396

1072	17.28655	103.94367	0.08397

1073	17.301	104.04045	0.08399

1074	17.31865	104.13723	0.08403

1075	17.33792	104.23401	0.08404

1076	17.35237	104.33079	0.08415

1077	17.36682	104.42757	0.08422

1078	17.3893	104.52436	0.08424

1079	17.39572	104.62114	0.08425

1080	17.41498	104.71792	0.08427

1081	17.42943	104.8147	0.08448

1082	17.45031	104.91148	0.08449

1083	17.45673	105.00827	0.08454

1084	17.48082	105.10505	0.08455

1085	17.5049	105.20183	0.08477

1086	17.51132	105.29861	0.08485

1087	17.52095	105.39539	0.08486

1088	17.54663	105.49217	0.08488

1089	17.55788	105.58896	0.08489

1090	17.57715	105.68574	0.08497

1091	17.58517	105.78252	0.08499

1092	17.60925	105.8793	0.08501

1093	17.63013	105.97608	0.08514

1094	17.63976	106.07287	0.08519

1095	17.6526	106.16965	0.08522

1096	17.67026	106.26643	0.08525

1097	17.68471	106.36321	0.08528

1098	17.69916	106.45999	0.08544

1099	17.72004	106.55677	0.0867

1100	17.73769	106.65356	0.08671

1101	17.75214	106.75034	0.08678

1102	17.76177	106.84712	0.08679

1103	17.78585	106.9439	0.0868

1104	17.80513	107.04068	0.08686

1105	17.80994	107.13747	0.08707

1106	17.83402	107.23425	0.08711

1107	17.85008	107.33103	0.08727

1108	17.86293	107.42781	0.08728

1109	17.87577	107.52459	0.08736

1110	17.89504	107.62137	0.08811

1111	17.9159	107.71816	0.08812

1112	17.92875	107.81494	0.0882

1113	17.94159	107.91172	0.08823

1114	17.96247	108.0085	0.08824

1115	17.97692	108.10528	0.08859

1116	17.98976	108.20206	0.08881

1117	18.00742	108.29885	0.0889

1118	18.02829	108.39563	0.089

1119	18.04113	108.49241	0.0891

1120	18.05558	108.58919	0.08917

1121	18.06683	108.68597	0.08943

1122	18.09251	108.78276	0.08953

1123	18.10697	108.87954	0.08955

1124	18.1182	108.97632	0.08956

1125	18.13586	109.0731	0.08961

1126	18.15192	109.16988	0.08966

1127	18.16315	109.26666	0.08971

1128	18.18242	109.36345	0.08972

1129	18.20489	109.46023	0.08986

1130	18.22256	109.55701	0.08994

1131	18.22738	109.65379	0.08995

1132	18.24022	109.75057	0.09014

1133	18.26591	109.84736	0.09018

1134	18.28036	109.94414	0.09027

1135	18.29802	110.04092	0.09052

1136	18.31728	110.1377	0.09063

1137	18.32531	110.23448	0.09065

1138	18.33816	110.33126	0.09066

1139	18.35903	110.42805	0.09067

1140	18.38151	110.52483	0.0907

1141	18.39434	110.62161	0.0908

1142	18.39916	110.71839	0.09083

1143	18.42486	110.81517	0.09084

1144	18.44412	110.91196	0.09085

1145	18.45536	111.00874	0.09086

1146	18.46659	111.10552	0.09099

1147	18.48907	111.2023	0.09101

1148	18.50673	111.29908	0.09107

1149	18.54848	111.39586	0.09111

1150	18.56775	111.49265	0.09113

1151	18.57898	111.58943	0.09116

1152	18.59664	111.68621	0.09173

1153	18.63839	111.78299	0.09174

1154	18.65926	111.87977	0.09175

1155	18.67532	111.97656	0.09188

1156	18.85513	112.07334	0.09216

1157	18.94343	112.17012	0.09217

1158	19.11683	112.2669	0.09242

1159	18.92417	112.36368	0.09243

1160	18.63518	112.46046	0.0925

1161	18.24665	112.55725	0.09251

1162	17.19824	112.65403	0.09253

1163	16.18677	112.75081	0.09255

1164	14.31956	112.84759	0.09256

1165	10.80188	112.94437	0.0926

1166	7.50417	113.04116	0.09261

1167	5.75576	113.13794	0.09263

1168	4.84865	113.23472	0.09264

1169	3.97525	113.3315	0.09267

1170	3.14359	113.42828	0.09271

1171	2.34083	113.52506	0.09272

113.62185	0.09297

113.71863	0.09299

113.81541	0.09301

113.91219	0.09305

114.00897	0.09309

114.10576	0.09311

114.20254	0.09314

114.29932	0.09329

114.3961	0.0933

114.49288	0.09333

114.58966	0.09351

114.68645	0.09353

114.78323	0.09363

114.88001	0.09366

114.97679	0.09372

115.07357	0.09403

115.17036	0.0941

115.26714	0.09415

115.36392	0.09418

115.4607	0.09424

115.55748	0.09426

115.65426	0.09428

115.75105	0.09433

115.84783	0.09434

115.94461	0.09436

116.04139	0.09437

116.13817	0.09441

116.23496	0.09443

116.33174	0.09449

116.42852	0.09452

116.5253	0.09453

116.62208	0.09468

116.71886	0.09477

116.81565	0.09483

116.91243	0.09485

117.00921	0.09506

117.10599	0.09508

117.20277	0.0951

117.29956	0.09513

117.39634	0.0952

117.49312	0.09536

117.5899	0.09537

117.68668	0.09546

117.78346	0.09548

117.88025	0.0955

117.97703	0.09556

118.07381	0.09615

118.17059	0.09616

118.26737	0.09618

118.36416	0.09621

118.46094	0.09622

118.55772	0.09631

118.6545	0.09632

118.75128	0.09648

118.84806	0.09653

118.94485	0.09654

119.04163	0.09655

119.13841	0.09657

119.23519	0.09665

119.33197	0.09691

119.42876	0.09692

119.52554	0.09693

119.62232	0.09694

119.7191	0.09695

119.81588	0.09727

119.91266	0.09734

120.00945	0.09738

120.10623	0.09745

120.20301	0.09746

120.29979	0.09765

120.39657	0.09767

120.49335	0.09768

120.59014	0.09769

120.68692	0.09771

120.7837	0.09773

120.88048	0.09785

120.97726	0.09788

121.07405	0.09791

121.17083	0.09793

121.26761	0.09794

121.36439	0.09795

121.46117	0.09798

121.55795	0.098

121.65474	0.09811

121.75152	0.09814

121.8483	0.09815

121.94508	0.09816

122.04186	0.09819

122.13865	0.0982

122.23543	0.09822

122.33221	0.09823

122.42899	0.0986

122.52577	0.09864

122.62255	0.09868

122.71934	0.09869

122.81612	0.09871

122.9129	0.09874

123.00968	0.09898

123.10646	0.09899

123.20325	0.099

123.30003	0.09902

123.39681	0.09904

123.49359	0.09905

123.59037	0.09914

123.68715	0.09915

123.78394	0.09929

123.88072	0.0993

123.9775	0.09931

124.07428	0.0994

124.17106	0.09941

124.26785	0.09956

124.36463	0.09998

124.46141	0.10001

124.55819	0.10018

124.65497	0.1006

124.75175	0.10061

124.84854	0.10066

124.94532	0.10096

125.0421	0.101

125.13888	0.10102

125.23566	0.10131

125.33245	0.10132

125.42923	0.10134

125.52601	0.10136

125.62279	0.10138

125.71957	0.10139

125.81635	0.10148

125.91314	0.10151

126.00992	0.10152

126.1067	0.102

126.20348	0.10253

126.30026	0.10258

126.39705	0.10283

126.49383	0.10288

126.59061	0.10295

126.68739	0.10297

126.78417	0.10299

126.88095	0.103

126.97774	0.10306

127.07452	0.10309

127.1713	0.10312

127.26808	0.10318

127.36486	0.10319

127.46165	0.10337

127.55843	0.10357

127.65521	0.10363

127.75199	0.10368

127.84877	0.10369

127.94555	0.10389

128.04234	0.10392

128.13912	0.10396

128.2359	0.10397

128.33268	0.104

128.42946	0.10415

128.52625	0.10422

128.62303	0.1044

128.71981	0.10462

128.81659	0.10477

128.91337	0.10487

129.01015	0.10488

129.10694	0.10493

129.20372	0.10494

129.3005	0.10503

129.39728	0.1051

129.49406	0.10527

129.59085	0.10542

129.68763	0.10543

129.78441	0.10546

129.88119	0.10547

129.97797	0.10548

130.07475	0.1055

130.17154	0.10552

130.26832	0.10553

130.3651	0.10562

130.46188	0.10565

130.55866	0.1058

130.65545	0.10583

130.75223	0.10585

130.84901	0.10587

130.94579	0.10593

131.04257	0.10601

131.13935	0.10607

131.23614	0.10609

131.33292	0.10618

131.4297	0.10644

131.52648	0.10646

131.62326	0.1068

131.72005	0.10681

131.81683	0.10686

131.91361	0.10687

132.01039	0.1069

132.10717	0.10693

132.20395	0.10694

132.30074	0.10695

132.39752	0.10698

132.4943	0.10699

132.59108	0.10701

132.68786	0.1071

132.78465	0.10717

132.88143	0.10738

132.97821	0.1074

133.07499	0.10745

133.17177	0.10752

133.26855	0.10754

133.36534	0.10757

133.46212	0.10761

133.5589	0.10773

133.65568	0.10777

133.75246	0.10842

133.84924	0.1085

133.94603	0.10853

134.04281	0.10881

134.13959	0.10885

134.23637	0.10886

134.33315	0.10898

134.42994	0.109

134.52672	0.10907

134.6235	0.10911

134.72028	0.10912

134.81706	0.10913

134.91384	0.10917

135.01063	0.10921

135.10741	0.10922

135.20419	0.10925

135.30097	0.10926

135.39775	0.10939

135.49454	0.10941

135.59132	0.10948

135.6881	0.10959

135.78488	0.1096

135.88166	0.10963

135.97844	0.10968

136.07523	0.10969

136.17201	0.1097

136.26879	0.10971

136.36557	0.10972

136.46235	0.10973

136.55914	0.10981

136.65592	0.10985

136.7527	0.10986

136.84948	0.10993

136.94626	0.10994

137.04304	0.11015

137.13983	0.1102

137.23661	0.11023

137.33339	0.11026

137.43017	0.11028

137.52695	0.11031

137.62374	0.11043

137.72052	0.11044

137.8173	0.11048

137.91408	0.11049

138.01086	0.1105

138.10764	0.11058

138.20443	0.11063

138.30121	0.11085

138.39799	0.11101

138.49477	0.11102

138.59155	0.11108

138.68834	0.11109

138.78512	0.1112

138.8819	0.11122

138.97868	0.11137

139.07546	0.11144

139.17224	0.11152

139.26903	0.11153

139.36581	0.11156

139.46259	0.11158

139.55937	0.11159

139.65615	0.11161

139.75294	0.11168

139.84972	0.11172

139.9465	0.11173

140.04328	0.11175

140.14006	0.11179

140.23684	0.11188

140.33363	0.1119

140.43041	0.11196

140.52719	0.11197

140.62397	0.11212

140.72075	0.11213

140.81754	0.11215

140.91432	0.11245

141.0111	0.11249

141.10788	0.1127

141.20466	0.11271

141.30144	0.11272

141.39823	0.11273

141.49501	0.11274

141.59179	0.11277

141.68857	0.11278

141.78535	0.11279

141.88214	0.1128

141.97892	0.11281

142.0757	0.11283

142.17248	0.11284

142.26926	0.11285

142.36604	0.11286

142.46283	0.11311

142.55961	0.11312

142.65639	0.11317

142.75317	0.11318

142.84995	0.11324

142.94674	0.11354

143.04352	0.11355

143.1403	0.11373

143.23708	0.11375

143.33386	0.11377

143.43064	0.11388

143.52743	0.1139

143.62421	0.11391

143.72099	0.11392

143.81777	0.11394

143.91455	0.11398

144.01134	0.114

144.10812	0.11401

144.2049	0.11405

144.30168	0.11414

144.39846	0.11432

144.49524	0.11443

144.59203	0.11444

144.68881	0.11462

144.78559	0.11507

144.88237	0.1151

144.97915	0.11513

145.07593	0.11514

145.17272	0.11523

145.2695	0.11532

145.36628	0.11544

145.46306	0.11546

145.55984	0.11548

145.65663	0.11549

145.75341	0.11555

145.85019	0.11556

145.94697	0.11562

146.04375	0.11563

146.14053	0.11564

146.23732	0.11568

146.3341	0.11589

146.43088	0.11616

146.52766	0.11624

146.62444	0.11625

146.72123	0.11632

146.81801	0.11633

146.91479	0.11635

147.01157	0.11637

147.10835	0.11639

147.20513	0.11641

147.30192	0.1165

147.3987	0.11656

147.49548	0.11658

147.59226	0.11662

147.68904	0.11663

147.78583	0.11664

147.88261	0.11665

147.97939	0.11674

148.07617	0.11682

148.17295	0.11684

148.26973	0.11686

148.36652	0.11687

148.4633	0.11688

148.56008	0.11693

148.65686	0.11694

148.75364	0.11695

148.85043	0.11697

148.94721	0.11699

149.04399	0.11785

149.14077	0.11801

149.23755	0.11809

149.33433	0.1181

149.43112	0.11813

149.5279	0.11815

149.62468	0.11817

149.72146	0.11818

149.81824	0.11819

149.91503	0.11824

150.01181	0.11832

150.10859	0.11834

150.20537	0.11835

150.30215	0.11836

150.39893	0.1185

150.49572	0.11876

150.5925	0.11882

150.68928	0.11883

150.78606	0.11884

150.88284	0.11889

150.97963	0.11896

151.07641	0.11897

151.17319	0.119

151.26997	0.11906

151.36675	0.11908

151.46353	0.11909

151.56032	0.1191

151.6571	0.11912

151.75388	0.11913

151.85066	0.11914

151.94744	0.11918

152.04423	0.11927

152.14101	0.11929

152.23779	0.11938

152.33457	0.11941

152.43135	0.11945

152.52813	0.11947

152.62492	0.11948

152.7217	0.1195

152.81848	0.11951

152.91526	0.11953

153.01204	0.11954

153.10883	0.1196

153.20561	0.11961

153.30239	0.11963

153.39917	0.11964

153.49595	0.11965

153.59273	0.11966

153.68952	0.11971

153.7863	0.11972

153.88308	0.11975

153.97986	0.11979

154.07664	0.11984

154.17343	0.11986

154.27021	0.11987

154.36699	0.11988

154.46377	0.12019

154.56055	0.1202

154.65733	0.12021

154.75412	0.12022

154.8509	0.12024

154.94768	0.12029

155.04446	0.12035

155.14124	0.12036

155.23803	0.12039

155.33481	0.12044

155.43159	0.12045

155.52837	0.12052

155.62515	0.12055

155.72193	0.12058

155.81872	0.12097

155.9155	0.12099

156.01228	0.121

156.10906	0.12104

156.20584	0.12105

156.30263	0.12118

156.39941	0.12119

156.49619	0.12125

156.59297	0.12126

156.68975	0.12127

156.78653	0.12128

156.88332	0.12129

156.9801	0.12133

157.07688	0.12134

157.17366	0.12143

157.27044	0.12146

157.36722	0.12148

157.46401	0.1215

157.56079	0.12166

157.65757	0.12197

157.75435	0.12242

157.85113	0.12246

157.94792	0.12255

158.0447	0.12262

158.14148	0.1227

158.23826	0.12273

158.33504	0.12274

158.43183	0.12275

158.52861	0.12276

158.62539	0.12278

158.72217	0.12292

158.81895	0.12293

158.91573	0.12295

159.01252	0.12297

159.1093	0.12299

159.20608	0.123

159.30286	0.12303

159.39964	0.12305

159.49642	0.12308

159.59321	0.12309

159.68999	0.12344

159.78677	0.12345

159.88355	0.12347

159.98033	0.12349

160.07712	0.12398

160.1739	0.12411

160.27068	0.12412

160.36746	0.12413

160.46424	0.12426

160.56102	0.12427

160.65781	0.1243

160.75459	0.12433

160.85137	0.12442

160.94815	0.12447

161.04493	0.12454

161.14172	0.12455

161.2385	0.1246

161.33528	0.12462

161.43206	0.12465

161.52884	0.12468

161.62562	0.12469

161.72241	0.12499

161.81919	0.125

161.91597	0.12528

162.01275	0.12539

162.10953	0.12549

162.20632	0.12565

162.3031	0.12567

162.39988	0.12575

162.49666	0.12576

162.59344	0.12578

162.69022	0.12579

162.78701	0.12581

162.88379	0.12613

162.98057	0.12614

163.07735	0.12616

163.17413	0.12642

163.27092	0.12645

163.3677	0.12658

163.46448	0.12681

163.56126	0.1269

163.65804	0.12692

163.75482	0.12693

163.85161	0.12712

163.94839	0.12719

164.04517	0.1273

164.14195	0.12737

164.23873	0.12739

164.33552	0.12753

164.4323	0.12769

164.52908	0.12771

164.62586	0.12773

164.72264	0.12778

164.81942	0.1278

164.91621	0.12803

165.01299	0.12805

165.10977	0.1281

165.20655	0.12812

165.30333	0.12817

165.40012	0.12819

165.4969	0.12824

165.59368	0.12828

165.69046	0.12831

165.78724	0.12883

165.88402	0.12885

165.98081	0.12892

166.07759	0.12908

166.17437	0.12915

166.27115	0.1292

166.36793	0.12936

166.46472	0.1297

166.5615	0.12975

166.65828	0.12977

166.75506	0.12979

166.85184	0.12984

166.94862	0.12997

167.04541	0.13002

167.14219	0.13016

167.23897	0.13018

167.33575	0.1302

167.43253	0.13034

167.52932	0.13036

167.6261	0.13052

167.72288	0.13116

167.81966	0.13121

167.91644	0.13189

168.01322	0.13217

168.11001	0.13219

168.20679	0.13233

168.30357	0.13235

168.40035	0.13331

168.49713	0.13336

168.59392	0.13338

168.6907	0.13343

168.78748	0.13349

168.88426	0.13359

168.98104	0.13397

169.07782	0.13423

169.17461	0.13464

169.27139	0.13466

169.36817	0.13468

169.46495	0.13471

169.56173	0.13507

169.65852	0.13509

169.7553	0.13516

169.85208	0.13519

169.94886	0.13521

170.04564	0.13523

170.14242	0.13525

170.23921	0.13528

170.33599	0.13544

170.43277	0.13548

170.52955	0.13553

170.62633	0.1358

170.72312	0.13583

170.8199	0.13585

170.91668	0.13589

171.01346	0.13599

171.11024	0.13615

171.20702	0.13624

171.30381	0.13626

171.40059	0.13628

171.49737	0.13633

171.59415	0.1366

171.69093	0.13663

171.78771	0.13701

171.8845	0.1374

171.98128	0.13743

172.07806	0.13768

172.17484	0.1377

172.27162	0.13775

172.36841	0.13779

172.46519	0.13784

172.56197	0.13788

172.65875	0.13802

172.75553	0.13827

172.85231	0.13852

172.9491	0.13855

173.04588	0.13859

173.14266	0.13864

173.23944	0.13866

173.33622	0.13909

173.43301	0.1393

173.52979	0.13941

173.62657	0.13944

173.72335	0.13946

173.82013	0.13951

173.91691	0.13953

174.0137	0.13955

174.11048	0.13957

174.20726	0.13971

174.30404	0.13976

174.40082	0.13978

174.49761	0.1398

174.59439	0.13983

174.69117	0.13985

174.78795	0.13987

174.88473	0.13989

174.98151	0.13996

175.0783	0.14001

175.17508	0.1404

175.27186	0.14081

175.36864	0.14083

175.46542	0.14108

175.56221	0.14111

175.65899	0.14113

175.75577	0.14115

175.85255	0.14117

175.94933	0.14131

176.04611	0.14154

176.1429	0.14156

176.23968	0.14161

176.33646	0.14168

176.43324	0.1417

176.53002	0.14172

176.62681	0.14209

176.72359	0.14229

176.82037	0.14232

176.91715	0.14234

177.01393	0.14239

177.11071	0.14243

177.2075	0.14245

177.30428	0.14268

177.40106	0.14287

177.49784	0.14289

177.59462	0.14291

177.69141	0.14298

177.78819	0.14319

177.88497	0.14325

177.98175	0.14328

178.07853	0.14335

178.17531	0.14339

178.2721	0.14341

178.36888	0.14344

178.46566	0.14371

178.56244	0.14376

178.65922	0.14378

178.75601	0.14497

178.85279	0.1457

178.94957	0.14597

179.04635	0.14632

179.14313	0.14636

179.23991	0.14639

179.3367	0.14664

179.43348	0.14671

179.53026	0.14675

179.62704	0.1468

179.72382	0.14684

179.82061	0.14691

179.91739	0.14696

180.01417	0.147

180.11095	0.14709

180.20773	0.14712

180.30451	0.14719

180.4013	0.14721

180.49808	0.14723

180.59486	0.14757

180.69164	0.14764

180.78842	0.14767

180.88521	0.14769

180.98199	0.14771

181.07877	0.14773

181.17555	0.1481

181.27233	0.14815

181.36911	0.14817

181.4659	0.14819

181.56268	0.14821

181.65946	0.14826

181.75624	0.14831

181.85302	0.14842

181.94981	0.14844

182.04659	0.14847

182.14337	0.14849

182.24015	0.14851

182.33693	0.14853

182.43371	0.14856

182.5305	0.14867

182.62728	0.14927

182.72406	0.14929

182.82084	0.14931

182.91762	0.14933

183.01441	0.14938

183.11119	0.1494

183.20797	0.14959

183.30475	0.14961

183.40153	0.14965

183.49831	0.14968

183.5951	0.1497

183.69188	0.15016

183.78866	0.15018

183.88544	0.1502

183.98222	0.15023

184.079	0.15025

184.17579	0.15032

184.27257	0.15036

184.36935	0.15039

184.46613	0.15041

184.56291	0.15043

184.6597	0.15048

184.75648	0.1505

184.85326	0.15052

184.95004	0.15055

185.04682	0.15057

185.1436	0.15059

185.24039	0.15061

185.33717	0.15064

185.43395	0.15068

185.53073	0.15073

185.62751	0.1508

185.7243	0.15082

185.82108	0.15105

185.91786	0.15109

186.01464	0.15114

186.11142	0.15157

186.2082	0.15162

186.30499	0.15164

186.40177	0.15169

186.49855	0.15194

186.59533	0.15196

186.69211	0.15199

186.7889	0.15217

186.88568	0.15219

186.98246	0.15224

187.07924	0.15242

187.17602	0.15244

187.2728	0.15247

187.36959	0.15249

187.46637	0.15251

187.56315	0.15288

187.65993	0.1529

187.75671	0.15295

187.8535	0.15297

187.95028	0.15301

188.04706	0.15304

188.14384	0.15313

188.24062	0.15315

188.3374	0.15317

188.43419	0.15322

188.53097	0.15327

188.62775	0.15329

188.72453	0.15331

188.82131	0.15359

188.9181	0.15365

189.01488	0.15368

189.11166	0.15379

189.20844	0.15384

189.30522	0.15386

189.402	0.15397

189.49879	0.154

189.59557	0.15404

189.69235	0.15413

189.78913	0.15416

189.88591	0.15418

189.9827	0.15425

190.07948	0.15427

190.17626	0.15436

190.27304	0.15439

190.36982	0.15445

190.4666	0.15455

190.56339	0.15475

190.66017	0.15477

190.75695	0.1548

190.85373	0.15482

190.95051	0.15489

191.0473	0.15491

191.14408	0.15493

191.24086	0.155

191.33764	0.15503

191.43442	0.15505

191.5312	0.15507

191.62799	0.15519

191.72477	0.15521

191.82155	0.15537

191.91833	0.15544

192.01511	0.15548

192.1119	0.1556

192.20868	0.15567

192.30546	0.15571

192.40224	0.15573

192.49902	0.15576

192.5958	0.15578

192.69259	0.1558

192.78937	0.15583

192.88615	0.15599

192.98293	0.15601

193.07971	0.15606

193.1765	0.15608

193.27328	0.15631

193.37006	0.15633

193.46684	0.15635

193.56362	0.15638

193.6604	0.1564

193.75719	0.15642

193.85397	0.15644

193.95075	0.15647

194.04753	0.1569

194.14431	0.15692

194.2411	0.15704

194.33788	0.15706

194.43466	0.15708

194.53144	0.15713

194.62822	0.15779

194.725	0.15782

194.82179	0.15786

194.91857	0.15788

195.01535	0.15793

195.11213	0.15795

195.20891	0.158

195.30569	0.15802

195.40248	0.15804

195.49926	0.15807

195.59604	0.15811

195.69282	0.15814

195.7896	0.15818

195.88639	0.15823

195.98317	0.15827

196.07995	0.15832

196.17673	0.15834

196.27351	0.15836

196.3703	0.15839

196.46708	0.15857

196.56386	0.15862

196.66064	0.15864

196.75742	0.1588

196.8542	0.15891

196.95099	0.15894

197.04777	0.15898

197.14455	0.159

197.24133	0.15903

197.33811	0.15905

197.43489	0.15919

197.53168	0.15921

197.62846	0.15926

197.72524	0.15928

197.82202	0.1593

197.9188	0.15932

198.01559	0.15935

198.11237	0.15937

198.20915	0.15942

198.30593	0.15946

198.40271	0.15951

198.49949	0.15953

198.59628	0.15955

198.69306	0.15958

198.78984	0.15969

198.88662	0.15971

198.9834	0.15974

199.08019	0.15976

199.17697	0.15978

199.27375	0.1598

199.37053	0.15983

199.46731	0.15985

199.56409	0.15994

199.66088	0.15996

199.75766	0.15999

199.85444	0.16003

199.95122	0.16008

200.048	0.1601

200.14479	0.16012

200.24157	0.16015

200.33835	0.16019

200.43513	0.16022

200.53191	0.16024

200.62869	0.16026

200.72548	0.16028

200.82226	0.16031

200.91904	0.16076

201.01582	0.16079

201.1126	0.16081

201.20939	0.1609

201.30617	0.16092

201.40295	0.16095

201.49973	0.16099

201.59651	0.16102

201.69329	0.16108

201.79008	0.16111

201.88686	0.16113

201.98364	0.16115

202.08042	0.16118

202.1772	0.16129

202.27399	0.16134

202.37077	0.16136

202.46755	0.16138

202.56433	0.1614

202.66111	0.16145

202.75789	0.16152

202.85468	0.16154

202.95146	0.16156

203.04824	0.16159

203.14502	0.16161

203.2418	0.16163

203.33859	0.16168

203.43537	0.1617

203.53215	0.16177

203.62893	0.16198

203.72571	0.16202

203.82249	0.16204

203.91928	0.16209

204.01606	0.16211

204.11284	0.16214

204.20962	0.16216

204.3064	0.16218

204.40319	0.1622

204.49997	0.16232

204.59675	0.16239

204.69353	0.16241

204.79031	0.1625

204.88709	0.16252

204.98388	0.16257

205.08066	0.16262

205.17744	0.16264

205.27422	0.16266

205.371	0.16268

205.46779	0.16273

205.56457	0.16278

205.66135	0.1628

205.75813	0.16287

205.85491	0.16312

205.95169	0.16314

206.04848	0.16344

206.14526	0.16346

206.24204	0.16348

206.33882	0.16351

206.4356	0.16353

206.53239	0.1636

206.62917	0.16383

206.72595	0.16385

206.82273	0.16392

206.91951	0.16394

207.01629	0.16396

207.11308	0.16401

207.20986	0.16403

207.30664	0.16412

207.40342	0.16417

207.5002	0.16424

207.59699	0.16426

207.69377	0.16431

207.79055	0.16433

207.88733	0.16435

207.98411	0.16438

208.08089	0.16449

208.17768	0.1646

208.27446	0.16463

208.37124	0.16465

208.46802	0.16467

208.5648	0.16474

208.66159	0.16483

208.75837	0.16486

208.85515	0.1649

208.95193	0.16492

209.04871	0.16495

209.14549	0.16502

209.24228	0.16504

209.33906	0.16508

209.43584	0.16513

209.53262	0.16515

209.6294	0.16524

209.72618	0.16527

209.82297	0.16529

209.91975	0.16534

210.01653	0.16536

210.11331	0.16552

210.21009	0.16554

210.30688	0.16559

210.40366	0.16561

210.50044	0.16568

210.59722	0.1657

210.694	0.16572

210.79078	0.16579

210.88757	0.16584

210.98435	0.16586

211.08113	0.16588

211.17791	0.16591

211.27469	0.16593

211.37148	0.16602

211.46826	0.16604

211.56504	0.16607

211.66182	0.16609

211.7586	0.16611

211.85538	0.16614

211.95217	0.16616

212.04895	0.16625

212.14573	0.16634

212.24251	0.16636

212.33929	0.16641

212.43608	0.16646

212.53286	0.16652

212.62964	0.16655

212.72642	0.16703

212.8232	0.16705

212.91998	0.16707

213.01677	0.1671

213.11355	0.16712

213.21033	0.16716

213.30711	0.16719

213.40389	0.16721

213.50068	0.1673

213.59746	0.16737

213.69424	0.16742

213.79102	0.16744

213.8878	0.16771

213.98458	0.16799

214.08137	0.16824

214.17815	0.16826

214.27493	0.16828

214.37171	0.16831

214.46849	0.16833

214.56528	0.1684

214.66206	0.16844

214.75884	0.16847

214.85562	0.16849

214.9524	0.16851

215.04918	0.16874

215.14597	0.16876

215.24275	0.16879

215.33953	0.16886

215.43631	0.16904

215.53309	0.16906

215.62988	0.16908

215.72666	0.16913

215.82344	0.16915

215.92022	0.1692

216.017	0.16924

216.11378	0.16927

216.21057	0.16929

216.30735	0.16931

216.40413	0.16934

216.50091	0.16968

216.59769	0.1697

216.69448	0.16972

216.79126	0.16975

216.88804	0.16977

216.98482	0.16979

217.0816	0.16982

217.17838	0.16984

217.27517	0.16988

217.37195	0.16991

217.46873	0.16998

217.56551	0.17

217.66229	0.17002

217.75908	0.17007

217.85586	0.17011

217.95264	0.17014

218.04942	0.17018

218.1462	0.1702

218.24298	0.17046

218.33977	0.17048

218.43655	0.17052

218.53333	0.17055

218.63011	0.17057

218.72689	0.17059

218.82368	0.17062

218.92046	0.17066

219.01724	0.17068

219.11402	0.17071

219.2108	0.17073

219.30758	0.17075

219.40437	0.1708

219.50115	0.17082

219.59793	0.17084

219.69471	0.17087

219.79149	0.17089

219.88828	0.17105

219.98506	0.1711

220.08184	0.17114

220.17862	0.17116

220.2754	0.17121

220.37218	0.17123

220.46897	0.17144

220.56575	0.17146

220.66253	0.17148

220.75931	0.17153

220.85609	0.17155

220.95288	0.1716

221.04966	0.17162

221.14644	0.17169

221.24322	0.17178

221.34	0.1718

221.43678	0.17183

221.53357	0.17185

221.63035	0.17201

221.72713	0.17203

221.82391	0.17208

221.92069	0.1721

222.01747	0.17217

222.11426	0.17219

222.21104	0.17224

222.30782	0.17231

222.4046	0.17233

222.50138	0.17235

222.59817	0.17238

222.69495	0.17242

222.79173	0.17244

222.88851	0.17249

222.98529	0.17251

223.08207	0.17313

223.17886	0.17315

223.27564	0.17318

223.37242	0.17324

223.4692	0.17329

223.56598	0.17331

223.66277	0.17334

223.75955	0.17338

223.85633	0.17363

223.95311	0.17366

224.04989	0.17368

224.14667	0.1737

224.24346	0.17372

224.34024	0.17414

224.43702	0.17418

224.5338	0.1742

224.63058	0.17423

224.72737	0.17436

224.82415	0.17439

224.92093	0.17443

225.01771	0.17448

225.11449	0.1745

225.21127	0.17452

225.30806	0.17455

225.40484	0.17462

225.50162	0.17466

225.5984	0.17468

225.69518	0.17471

225.79197	0.17475

225.88875	0.1748

225.98553	0.17484

226.08231	0.17487

226.17909	0.17491

226.27587	0.17494

226.37266	0.17496

226.46944	0.17503

226.56622	0.17505

226.663	0.17507

226.75978	0.1751

226.85657	0.17512

226.95335	0.17514

227.05013	0.17519

227.14691	0.17521

227.24369	0.17523

227.34047	0.17526

227.43726	0.17528

227.53404	0.1753

227.63082	0.17532

227.7276	0.17542

227.82438	0.17548

227.92117	0.17551

228.01795	0.17553

228.11473	0.17555

228.21151	0.1756

228.30829	0.17562

228.40507	0.17564

228.50186	0.17569

228.59864	0.17571

228.69542	0.17574

228.7922	0.17576

228.88898	0.17583

228.98577	0.17587

229.08255	0.1759

229.17933	0.17596

229.27611	0.17599

229.37289	0.17601

229.46967	0.17603

229.56646	0.17608

229.66324	0.17617

229.76002	0.17628

229.8568	0.17631

229.95358	0.17633

230.05037	0.17635

230.14715	0.17644

230.24393	0.17647

230.34071	0.17649

230.43749	0.17654

230.53427	0.17663

230.63106	0.17695

230.72784	0.17697

230.82462	0.17699

230.9214	0.17702

231.01818	0.17704

231.11497	0.17706

231.21175	0.17708

231.30853	0.17713

231.40531	0.17718

231.50209	0.1772

231.59887	0.17724

231.69566	0.17727

231.79244	0.17736

231.88922	0.17738

231.986	0.1774

232.08278	0.17743

232.17957	0.17745

232.27635	0.17772

232.37313	0.17775

232.46991	0.17777

232.56669	0.17779

232.66347	0.17782

232.76026	0.17784

232.85704	0.17788

232.95382	0.17791

233.0506	0.178

233.14738	0.17802

233.24417	0.17804

233.34095	0.17807

233.43773	0.17809

233.53451	0.17814

233.63129	0.17887

233.72807	0.17889

233.82486	0.17896

233.92164	0.17898

234.01842	0.179

234.1152	0.17903

234.21198	0.17905

234.30876	0.17907

234.40555	0.1791

234.50233	0.17912

234.59911	0.17916

234.69589	0.1793

234.79267	0.17932

234.88946	0.17942

234.98624	0.17951

235.08302	0.17953

235.1798	0.17955

235.27658	0.17958

235.37336	0.1796

235.47015	0.17962

235.56693	0.17964

235.66371	0.17967

235.76049	0.17971

235.85727	0.17974

235.95406	0.17976

236.05084	0.17978

236.14762	0.1798

236.2444	0.17985

236.34118	0.17987

236.43796	0.1799

236.53475	0.17992

236.63153	0.17994

236.72831	0.17999

236.82509	0.18001

236.92187	0.18006

237.01866	0.18008

237.11544	0.18012

237.21222	0.18019

237.309	0.18022

237.40578	0.18024

237.50256	0.18026

237.59935	0.18028

237.69613	0.18031

237.79291	0.18047

237.88969	0.18049

237.98647	0.18051

238.08326	0.18054

238.18004	0.18056

238.27682	0.18058

238.3736	0.18063

238.47038	0.1807

238.56716	0.18072

238.66395	0.18074

238.76073	0.18076

238.85751	0.18081

238.95429	0.18083

239.05107	0.18086

239.14786	0.18104

239.24464	0.18118

239.34142	0.18127

239.4382	0.18129

239.53498	0.18131

239.63176	0.18134

239.72855	0.18136

239.82533	0.18138

239.92211	0.1814

240.01889	0.18143

240.11567	0.18145

240.21246	0.18147

240.30924	0.1815

240.40602	0.18152

240.5028	0.18159

240.59958	0.18163

240.69636	0.18166

240.79315	0.1817

240.88993	0.18172

240.98671	0.18179

241.08349	0.18182

241.18027	0.18184

241.27706	0.18188

241.37384	0.18191

241.47062	0.18193

241.5674	0.1825

241.66418	0.18259

241.76096	0.18273

241.85775	0.18275

241.95453	0.18278

242.05131	0.1828

242.14809	0.18282

242.24487	0.18287

242.34166	0.18289

242.43844	0.18291

242.53522	0.18298

242.632	0.18305

242.72878	0.18314

242.82556	0.18337

242.92235	0.18339

243.01913	0.18344

243.11591	0.18346

243.21269	0.18348

243.30947	0.18351

243.40626	0.18353

243.50304	0.18355

243.59982	0.18358

243.6966	0.18364

243.79338	0.18371

243.89016	0.18376

243.98695	0.18378

244.08373	0.1838

244.18051	0.18385

244.27729	0.1839

244.37407	0.18392

244.47086	0.18394

244.56764	0.18396

244.66442	0.18399

244.7612	0.18401

244.85798	0.18403

244.95476	0.18406

245.05155	0.18408

245.14833	0.1841

245.24511	0.18412

245.34189	0.18415

245.43867	0.18417

245.53546	0.18428

245.63224	0.18433

245.72902	0.1844

245.8258	0.18476

245.92258	0.1849

246.01936	0.18492

246.11615	0.18518

246.21293	0.18524

246.30971	0.18527

246.40649	0.18529

246.50327	0.18531

246.60006	0.18534

246.69684	0.18536

246.79362	0.18538

246.8904	0.18543

246.98718	0.18545

247.08396	0.18552

247.18075	0.18556

247.27753	0.18561

247.37431	0.18563

247.47109	0.18566

247.56787	0.18568

247.66465	0.1857

247.76144	0.18575

247.85822	0.18579

247.955	0.18588

248.05178	0.18591

248.14856	0.18593

248.24535	0.18598

248.34213	0.186

248.43891	0.18602

248.53569	0.18604

248.63247	0.18691

248.72925	0.18694

248.82604	0.18703

248.92282	0.18705

249.0196	0.18707

249.11638	0.18712

249.21316	0.18716

249.30995	0.18721

249.40673	0.18723

249.50351	0.18726

249.60029	0.1873

249.69707	0.18732

249.79385	0.18737

249.89064	0.18739

249.98742	0.18742

250.0842	0.18744

250.18098	0.18746

250.27776	0.18751

250.37455	0.18753

250.47133	0.18755

250.56811	0.18758

250.66489	0.1876

250.76167	0.18762

250.85845	0.18764

250.95524	0.18769

251.05202	0.18771

251.1488	0.18774

251.24558	0.18783

251.34236	0.18787

251.43915	0.18792

251.53593	0.18794

251.63271	0.18799

251.72949	0.18806

251.82627	0.18808

251.92305	0.18812

252.01984	0.18815

252.11662	0.18822

252.2134	0.18824

252.31018	0.18828

252.40696	0.18831

252.50375	0.18833

252.60053	0.18835

252.69731	0.1884

252.79409	0.18842

252.89087	0.18844

252.98765	0.18849

253.08444	0.18851

253.18122	0.18854

253.278	0.18858

253.37478	0.18863

253.47156	0.18865

253.56835	0.18867

253.66513	0.1887

253.76191	0.18874

253.85869	0.18876

253.95547	0.18879

254.05225	0.18881

254.14904	0.18886

254.24582	0.18892

254.3426	0.18897

254.43938	0.18904

254.53616	0.18906

254.63295	0.18911

254.72973	0.1892

254.82651	0.18922

254.92329	0.18925

255.02007	0.18934

255.11685	0.18936

255.21364	0.18943

255.31042	0.18945

255.4072	0.1895

255.50398	0.18952

255.60076	0.18961

255.69755	0.18963

255.79433	0.18975

255.89111	0.18977

255.98789	0.18984

256.08467	0.18986

256.18145	0.18991

256.27824	0.18993

256.37502	0.19002

256.4718	0.19007

256.56858	0.19011

256.66536	0.19016

256.76215	0.19018

256.85893	0.19021

256.95571	0.19023

257.05249	0.19059

257.14927	0.19062

257.24605	0.19071

257.34284	0.19073

257.43962	0.19085

257.5364	0.19101

257.63318	0.19103

257.72996	0.19107

257.82675	0.1911

257.92353	0.19112

258.02031	0.19114

258.11709	0.19117

258.21387	0.19119

258.31065	0.19121

258.40744	0.19126

258.50422	0.19128

258.601	0.1913

258.69778	0.19133

258.79456	0.19135

258.89135	0.19137

258.98813	0.19139

259.08491	0.19142

259.18169	0.19144

259.27847	0.19167

259.37525	0.19169

259.47204	0.19176

259.56882	0.19178

259.6656	0.19181

259.76238	0.19183

259.85916	0.19185

259.95594	0.19194

260.05273	0.19197

260.14951	0.19206

260.24629	0.19208

260.34307	0.1921

260.43985	0.19213

260.53664	0.19215

260.63342	0.19219

260.7302	0.19222

260.82698	0.19231

260.92376	0.19233

261.02054	0.19235

261.11733	0.19238

261.21411	0.1924

261.31089	0.19245

261.40767	0.19247

261.50445	0.19258

261.60124	0.19263

261.69802	0.19265

261.7948	0.19277

261.89158	0.19279

261.98836	0.19281

262.08514	0.19286

262.18193	0.19306

262.27871	0.19311

262.37549	0.19315

262.47227	0.19318

262.56905	0.1932

262.66584	0.19327

262.76262	0.19331

262.8594	0.19336

262.95618	0.19338

263.05296	0.19341

263.14974	0.19343

263.24653	0.19352

263.34331	0.19354

263.44009	0.19357

263.53687	0.19359

263.63365	0.19361

263.73044	0.19368

263.82722	0.1937

263.924	0.19375

264.02078	0.19389

264.11756	0.19391

264.21434	0.19393

264.31113	0.19395

264.40791	0.194

264.50469	0.19405

264.60147	0.19407

264.69825	0.19414

264.79504	0.19423

264.89182	0.19425

264.9886	0.19427

265.08538	0.1943

265.18216	0.19434

265.27894	0.19437

265.37573	0.19441

265.47251	0.19443

265.56929	0.19446

265.66607	0.19455

265.76285	0.19459

265.85964	0.19466

265.95642	0.19471

266.0532	0.19473

266.14998	0.19475

266.24676	0.19478

266.34354	0.1948

266.44033	0.19482

266.53711	0.19489

266.63389	0.19505

266.73067	0.19507

266.82745	0.19519

266.92424	0.19521

267.02102	0.19523

267.1178	0.19528

267.21458	0.19533

267.31136	0.19535

267.40814	0.19539

267.50493	0.19542

267.60171	0.19544

267.69849	0.19549

267.79527	0.19555

267.89205	0.19562

267.98884	0.19567

268.08562	0.19569

268.1824	0.19576

268.27918	0.1959

268.37596	0.19592

268.47274	0.19594

268.56953	0.19599

268.66631	0.19603

268.76309	0.19608

268.85987	0.1961

268.95665	0.19613

269.05344	0.19615

269.15022	0.19624

269.247	0.19629

269.34378	0.19631

269.44056	0.19638

269.53734	0.19688

269.63413	0.19693

269.73091	0.19699

269.82769	0.19704

269.92447	0.19706

270.02125	0.19711

270.11804	0.1972

270.21482	0.19722

270.3116	0.19727

270.40838	0.19729

270.50516	0.19734

270.60194	0.19736

270.69873	0.19741

270.79551	0.19747

270.89229	0.1975

270.98907	0.19757

271.08585	0.19759

271.18264	0.19761

271.27942	0.19763

271.3762	0.19768

271.47298	0.1977

271.56976	0.19773

271.66654	0.19775

271.76333	0.19777

271.86011	0.19779

271.95689	0.19782

272.05367	0.19784

272.15045	0.19789

272.24723	0.19791

272.34402	0.19793

272.4408	0.19795

272.53758	0.19805

272.63436	0.19807

272.73114	0.19809

272.82793	0.19814

272.92471	0.19816

273.02149	0.19821

273.11827	0.19823

273.21505	0.19825

273.31183	0.19827

273.40862	0.19832

273.5054	0.19834

273.60218	0.19837

273.69896	0.19839

273.79574	0.19841

273.89253	0.19848

273.98931	0.19855

274.08609	0.19857

274.18287	0.19859

274.27965	0.19866

274.37643	0.19869

274.47322	0.19871

274.57	0.19873

274.66678	0.19875

274.76356	0.19882

274.86034	0.19887

274.95713	0.19889

275.05391	0.19891

275.15069	0.19896

275.24747	0.19901

275.34425	0.19903

275.44103	0.19905

275.53782	0.19907

275.6346	0.1991

275.73138	0.19912

275.82816	0.19917

275.92494	0.19919

276.02173	0.19921

276.11851	0.19928

276.21529	0.19955

276.31207	0.19958

276.40885	0.1996

276.50563	0.19969

276.60242	0.19974

276.6992	0.19976

276.79598	0.19981

276.89276	0.19983

276.98954	0.19987

277.08633	0.1999

277.18311	0.19992

277.27989	0.19997

277.37667	0.20003

277.47345	0.20006

277.57023	0.20022

277.66702	0.20024

277.7638	0.20031

277.86058	0.20033

277.95736	0.20038

278.05414	0.2004

278.15093	0.20042

278.24771	0.20045

278.34449	0.20051

278.44127	0.20054

278.53805	0.20056

278.63483	0.20067

278.73162	0.2007

278.8284	0.20072

278.92518	0.20099

279.02196	0.20102

279.11874	0.20106

279.21553	0.20109

279.31231	0.20111

279.40909	0.20115

279.50587	0.20118

279.60265	0.20138

279.69943	0.20145

279.79622	0.20147

279.893	0.2015

279.98978	0.20163

280.08656	0.2017

280.18334	0.20173

280.28013	0.20191

280.37691	0.20193

280.47369	0.202

280.57047	0.20202

280.66725	0.20207

280.76403	0.20214

280.86082	0.20216

280.9576	0.20223

281.05438	0.20227

281.15116	0.2023

281.24794	0.20234

281.34473	0.20237

281.44151	0.20241

281.53829	0.20246

281.63507	0.20262

281.73185	0.20264

281.82863	0.20266

281.92542	0.20269

282.0222	0.20273

282.11898	0.20278

282.21576	0.20282

282.31254	0.20285

282.40933	0.20296

282.50611	0.20303

282.60289	0.20305

282.69967	0.20307

282.79645	0.2031

282.89323	0.20314

282.99002	0.20317

283.0868	0.20319

283.18358	0.20321

283.28036	0.20333

283.37714	0.20335

283.47393	0.20337

283.57071	0.20349

283.66749	0.20351

283.76427	0.20353

283.86105	0.20355

283.95783	0.20365

284.05462	0.20367

284.1514	0.20369

284.24818	0.20376

284.34496	0.20381

284.44174	0.20387

284.53852	0.20397

284.63531	0.20401

284.73209	0.20403

284.82887	0.20406

284.92565	0.20408

285.02243	0.2041

285.11922	0.20413

285.216	0.20417

285.31278	0.20419

285.40956	0.20426

285.50634	0.20435

285.60312	0.20438

285.69991	0.2044

285.79669	0.20449

285.89347	0.20451

285.99025	0.20454

286.08703	0.20472

286.18382	0.20474

286.2806	0.20477

286.37738	0.20481

286.47416	0.20483

286.57094	0.20486

286.66772	0.20488

286.76451	0.2049

286.86129	0.20499

286.95807	0.20502

287.05485	0.20504

287.15163	0.20513

287.24842	0.20518

287.3452	0.20529

287.44198	0.20531

287.53876	0.20534

287.63554	0.20536

287.73232	0.20538

287.82911	0.20541

287.92589	0.20543

288.02267	0.20545

288.11945	0.20547

288.21623	0.2055

288.31302	0.20554

288.4098	0.20559

288.50658	0.20563

288.60336	0.20566

288.70014	0.20568

288.79692	0.2057

288.89371	0.20573

288.99049	0.20577

289.08727	0.20579

289.18405	0.20582

289.28083	0.20586

289.37762	0.20589

289.4744	0.20593

289.57118	0.20595

289.66796	0.20598

289.76474	0.20605

289.86152	0.20607

289.95831	0.20609

290.05509	0.20616

290.15187	0.20621

290.24865	0.20623

290.34543	0.20625

290.44222	0.20627

290.539	0.2063

290.63578	0.20632

290.73256	0.20634

290.82934	0.20637

290.92612	0.20641

291.02291	0.20653

291.11969	0.20655

291.21647	0.20659

291.31325	0.20666

291.41003	0.20669

291.50682	0.20673

291.6036	0.2068

291.70038	0.20682

291.79716	0.20685

291.89394	0.20687

291.99072	0.20689

292.08751	0.20705

292.18429	0.2071

292.28107	0.20712

292.37785	0.20714

292.47463	0.20721

292.57142	0.20726

292.6682	0.20728

292.76498	0.2073

292.86176	0.20733

292.95854	0.20765

293.05532	0.20767

293.15211	0.20769

293.24889	0.20774

293.34567	0.20778

293.44245	0.20781

293.53923	0.20783

293.63602	0.20785

293.7328	0.20792

293.82958	0.20794

293.92636	0.20797

294.02314	0.20799

294.11992	0.20803

294.21671	0.20806

294.31349	0.20808

294.41027	0.20815

294.50705	0.20817

294.60383	0.20822

294.70062	0.20824

294.7974	0.20826

294.89418	0.20829

294.99096	0.20831

295.08774	0.20833

295.18452	0.20835

295.28131	0.20845

295.37809	0.20847

295.47487	0.20849

295.57165	0.20851

295.66843	0.20854

295.76522	0.20858

295.862	0.20863

295.95878	0.20865

296.05556	0.20867

296.15234	0.2087

296.24912	0.20874

296.34591	0.20881

296.44269	0.20886

296.53947	0.2089

296.63625	0.20902

296.73303	0.20904

296.82982	0.20906

296.9266	0.20909

297.02338	0.20915

297.12016	0.20918

297.21694	0.20922

297.31372	0.20925

297.41051	0.20927

297.50729	0.20931

297.60407	0.20941

297.70085	0.20945

297.79763	0.20947

297.89441	0.2095

297.9912	0.20952

298.08798	0.20954

298.18476	0.20957

298.28154	0.20961

298.37832	0.20963

298.47511	0.20966

298.57189	0.20975

298.66867	0.20977

298.76545	0.20982

298.86223	0.20984

298.95901	0.20986

299.0558	0.20989

299.15258	0.20991

299.24936	0.20995

299.34614	0.21

299.44292	0.21002

299.53971	0.21007

299.63649	0.21009

299.73327	0.21014

299.83005	0.21016

299.92683	0.21018

300.02361	0.21023

300.1204	0.21025

300.21718	0.21032

300.31396	0.21034

300.41074	0.21037

300.50752	0.21041

300.60431	0.21046

300.70109	0.21082

300.79787	0.21085

300.89465	0.21087

300.99143	0.21089

301.08821	0.21091

301.185	0.21094

301.28178	0.21096

301.37856	0.21098

301.47534	0.21101

301.57212	0.21103

301.66891	0.21105

301.76569	0.21107

301.86247	0.2111

301.95925	0.21117

302.05603	0.21119

302.15281	0.21121

302.2496	0.21123

302.34638	0.21126

302.44316	0.21128

302.53994	0.21133

302.63672	0.21135

302.73351	0.21137

302.83029	0.21139

302.92707	0.21142

303.02385	0.21146

303.12063	0.21155

303.21741	0.21158

303.3142	0.2116

303.41098	0.21165

303.50776	0.21169

303.60454	0.21171

303.70132	0.21174

303.79811	0.21176

303.89489	0.2119

303.99167	0.21194

304.08845	0.21199

304.18523	0.21206

304.28201	0.21208

304.3788	0.2121

304.47558	0.21215

304.57236	0.21217

304.66914	0.21219

304.76592	0.21222

304.86271	0.21229

304.95949	0.21233

305.05627	0.21235

305.15305	0.21238

305.24983	0.21245

305.34661	0.21251

305.4434	0.21254

305.54018	0.21272

305.63696	0.21274

305.73374	0.21359

305.83052	0.21361

305.92731	0.21368

306.02409	0.21391

306.12087	0.214

306.21765	0.21402

306.31443	0.21405

306.41121	0.21416

306.508	0.21421

306.60478	0.21423

306.70156	0.21437

306.79834	0.21439

306.89512	0.21441

306.99191	0.21443

307.08869	0.21446

307.18547	0.2145

307.28225	0.21453

307.37903	0.21455

307.47581	0.21457

307.5726	0.21459

307.66938	0.21473

307.76616	0.21475

307.86294	0.21478

307.95972	0.21482

308.05651	0.21491

308.15329	0.21494

308.25007	0.21498

308.34685	0.21501

308.44363	0.21503

308.54041	0.21505

308.6372	0.21507

308.73398	0.2151

308.83076	0.21512

308.92754	0.21514

309.02432	0.21519

309.12111	0.21526

309.21789	0.21528

309.31467	0.2153

309.41145	0.21533

309.50823	0.21535

309.60501	0.21539

309.7018	0.21542

309.79858	0.21549

309.89536	0.21553

309.99214	0.21558

310.08892	0.21562

310.1857	0.21578

310.28249	0.21581

310.37927	0.21587

310.47605	0.2159

310.57283	0.21592

310.66961	0.21594

310.7664	0.21599

310.86318	0.21601

310.95996	0.21606

311.05674	0.2161

311.15352	0.21613

311.2503	0.21615

311.34709	0.21617

311.44387	0.21619

311.54065	0.21626

311.63743	0.21631

311.73421	0.21633

311.831	0.21635

311.92778	0.21638

312.02456	0.2164

312.12134	0.21642

312.21812	0.21649

312.3149	0.21651

312.41169	0.21658

312.50847	0.21661

312.60525	0.21663

312.70203	0.21667

312.79881	0.2167

312.8956	0.21672

312.99238	0.21674

313.08916	0.21683

313.18594	0.2169

313.28272	0.21693

313.3795	0.21695

313.47629	0.21697

313.57307	0.21699

313.66985	0.21702

313.76663	0.21709

313.86341	0.21711

313.9602	0.21713

314.05698	0.21715

314.15376	0.21718

314.25054	0.21722

314.34732	0.21725

314.4441	0.21727

314.54089	0.21731

314.63767	0.21736

314.73445	0.21738

314.83123	0.21745

314.92801	0.2175

315.0248	0.21759

315.12158	0.21766

315.21836	0.21768

315.31514	0.2177

315.41192	0.21775

315.5087	0.21779

315.60549	0.21782

315.70227	0.21784

315.79905	0.21786

315.89583	0.21793

315.99261	0.21795

316.0894	0.21798

316.18618	0.21802

316.28296	0.21805

316.37974	0.21807

316.47652	0.21809

316.5733	0.21811

316.67009	0.21816

316.76687	0.21823

316.86365	0.21825

316.96043	0.21827

317.05721	0.2183

317.154	0.21834

317.25078	0.21837

317.34756	0.21843

317.44434	0.21846

317.54112	0.2185

317.6379	0.21853

317.73469	0.21855

317.83147	0.21857

317.92825	0.21862

318.02503	0.21864

318.12181	0.21866

318.2186	0.21871

318.31538	0.2188

318.41216	0.21885

318.50894	0.21889

318.60572	0.21891

318.7025	0.21894

318.79929	0.21896

318.89607	0.21898

318.99285	0.21901

319.08963	0.21907

319.18641	0.21912

319.2832	0.21914

319.37998	0.21923

319.47676	0.21926

319.57354	0.21928

319.67032	0.21946

319.7671	0.21949

319.86389	0.21951

319.96067	0.21962

320.05745	0.21967

320.15423	0.21969

320.25101	0.21971

320.3478	0.21974

320.44458	0.21981

320.54136	0.21983

320.63814	0.21985

320.73492	0.21987

320.8317	0.21992

320.92849	0.21997

321.02527	0.21999

321.12205	0.2201

321.21883	0.22013

321.31561	0.22015

321.4124	0.22022

321.50918	0.22024

321.60596	0.22026

321.70274	0.22029

321.79952	0.22031

321.8963	0.22038

321.99309	0.22042

322.08987	0.22045

322.18665	0.22047

322.28343	0.22051

322.38021	0.22054

322.47699	0.22056

322.57378	0.22058

322.67056	0.22061

322.76734	0.22063

322.86412	0.22065

322.9609	0.2207

323.05769	0.22077

323.15447	0.22079

323.25125	0.22081

323.34803	0.22086

323.44481	0.22088

323.54159	0.22097

323.63838	0.22099

323.73516	0.22102

323.83194	0.22104

323.92872	0.22106

324.0255	0.22113

324.12229	0.22118

324.21907	0.22143

324.31585	0.22145

324.41263	0.2215

324.50941	0.22152

324.60619	0.22168

324.70298	0.22173

324.79976	0.22177

324.89654	0.22182

324.99332	0.22195

325.0901	0.22198

325.18689	0.222

325.28367	0.22202

325.38045	0.22209

325.47723	0.22211

325.57401	0.22216

325.67079	0.22218

325.76758	0.22221

325.86436	0.22223

325.96114	0.22225

326.05792	0.22228

326.1547	0.2223

326.25149	0.22232

326.34827	0.22239

326.44505	0.22241

326.54183	0.22244

326.63861	0.22248

326.73539	0.2225

326.83218	0.22253

326.92896	0.22257

327.02574	0.22264

327.12252	0.22266

327.2193	0.22273

327.31609	0.22276

327.41287	0.22278

327.50965	0.2228

327.60643	0.22282

327.70321	0.22287

327.79999	0.22289

327.89678	0.22292

327.99356	0.22294

328.09034	0.22296

328.18712	0.22301

328.2839	0.22303

328.38069	0.22305

328.47747	0.22308

328.57425	0.2231

328.67103	0.22317

328.76781	0.22319

328.86459	0.22321

328.96138	0.22326

329.05816	0.22328

329.15494	0.22337

329.25172	0.2234

329.3485	0.22372

329.44529	0.22374

329.54207	0.22378

329.63885	0.22381

329.73563	0.22383

329.83241	0.2239

329.92919	0.22392

330.02598	0.22404

330.12276	0.22408

330.21954	0.2241

330.31632	0.22422

330.4131	0.22426

330.50989	0.22429

330.60667	0.22431

330.70345	0.22433

330.80023	0.22436

330.89701	0.22438

330.99379	0.2244

331.09058	0.22442

331.18736	0.22445

331.28414	0.22449

331.38092	0.22461

331.4777	0.22463

331.57449	0.22465

331.67127	0.2247

331.76805	0.22472

331.86483	0.22474

331.96161	0.22477

332.05839	0.22479

332.15518	0.22481

332.25196	0.2249

332.34874	0.22493

332.44552	0.22497

332.5423	0.22504

332.63909	0.22509

332.73587	0.22511

332.83265	0.22513

332.92943	0.22516

333.02621	0.22518

333.12299	0.2252

333.21978	0.22522

333.31656	0.22529

333.41334	0.22532

333.51012	0.22536

333.6069	0.22538

333.70369	0.22541

333.80047	0.22548

333.89725	0.2255

333.99403	0.22557

334.09081	0.22559

334.18759	0.22568

334.28438	0.22577

334.38116	0.22584

334.47794	0.22591

334.57472	0.22593

334.6715	0.22598

334.76828	0.226

334.86507	0.22602

334.96185	0.22605

335.05863	0.22609

335.15541	0.22614

335.25219	0.22616

335.34898	0.22628

335.44576	0.2263

335.54254	0.22632

335.63932	0.22634

335.7361	0.22637

335.83288	0.22639

335.92967	0.22646

336.02645	0.2265

336.12323	0.22653

336.22001	0.22662

336.31679	0.22664

336.41358	0.22669

336.51036	0.22671

336.60714	0.22673

336.70392	0.22678

336.8007	0.22682

336.89748	0.22685

336.99427	0.22687

337.09105	0.22698

337.18783	0.22701

337.28461	0.22703

337.38139	0.22705

337.47818	0.22708

337.57496	0.22712

337.67174	0.22714

337.76852	0.22717

337.8653	0.22721

337.96208	0.22724

338.05887	0.22726

338.15565	0.22728

338.25243	0.2273

338.34921	0.22733

338.44599	0.22737

338.54278	0.2274

338.63956	0.22744

338.73634	0.22751

338.83312	0.22753

338.9299	0.22756

339.02668	0.2276

339.12347	0.22762

339.22025	0.22767

339.31703	0.22772

339.41381	0.22776

339.51059	0.22778

339.60738	0.22792

339.70416	0.22799

339.80094	0.22806

339.89772	0.22815

339.9945	0.22817

340.09128	0.2282

340.18807	0.22822

340.28485	0.22831

340.38163	0.22833

340.47841	0.22838

340.57519	0.22845

340.67198	0.22847

340.76876	0.22852

340.86554	0.22854

340.96232	0.22856

341.0591	0.22858

341.15588	0.22865

341.25267	0.22868

341.34945	0.2287

341.44623	0.22886

341.54301	0.22893

341.63979	0.22906

341.73658	0.22909

341.83336	0.22913

341.93014	0.22916

342.02692	0.22918

342.1237	0.22922

342.22048	0.22932

342.31727	0.22936

342.41405	0.22938

342.51083	0.22941

342.60761	0.22945

342.70439	0.22948

342.80118	0.2295

342.89796	0.22957

342.99474	0.22959

343.09152	0.22961

343.1883	0.22964

343.28508	0.22966

343.38187	0.22968

343.47865	0.2297

343.57543	0.22984

343.67221	0.22989

343.76899	0.23012

343.86578	0.23016

343.96256	0.23018

344.05934	0.23021

344.15612	0.23028

344.2529	0.2303

344.34968	0.23041

344.44647	0.23044

344.54325	0.23048

344.64003	0.2305

344.73681	0.23053

344.83359	0.23055

344.93038	0.2306

345.02716	0.23064

345.12394	0.23073

345.22072	0.23076

345.3175	0.23085

345.41428	0.23092

345.51107	0.23096

345.60785	0.23098

345.70463	0.23101

345.80141	0.23192

345.89819	0.23206

345.99498	0.23208

346.09176	0.2321

346.18854	0.23217

346.28532	0.2322

346.3821	0.23224

346.47888	0.23229

346.57567	0.23231

346.67245	0.23236

346.76923	0.23238

346.86601	0.23249

346.96279	0.23254

347.05958	0.23256

347.15636	0.23258

347.25314	0.23263

347.34992	0.23274

347.4467	0.23318

347.54348	0.2332

347.64027	0.23322

347.73705	0.23325

347.83383	0.23329

347.93061	0.23332

348.02739	0.23336

348.12417	0.23338

348.22096	0.23348

348.31774	0.23354

348.41452	0.23359

348.5113	0.23361

348.60808	0.23366

348.70487	0.23368

348.80165	0.23373

348.89843	0.23377

348.99521	0.2338

349.09199	0.23382

349.18877	0.23384

349.28556	0.23386

349.38234	0.23389

349.47912	0.23391

349.5759	0.23393

349.67268	0.234

349.76947	0.23402

349.86625	0.23405

349.96303	0.23409

350.05981	0.23412

350.15659	0.23414

350.25337	0.23416

350.35016	0.23421

350.44694	0.23423

350.54372	0.23425

350.6405	0.23428

350.73728	0.23444

350.83407	0.23446

350.93085	0.23448

351.02763	0.23464

351.12441	0.23466

351.22119	0.23469

351.31797	0.23471

351.41476	0.23473

351.51154	0.2348

351.60832	0.23487

351.7051	0.23505

351.80188	0.2351

351.89867	0.23512

351.99545	0.23514

352.09223	0.23519

352.18901	0.23521

352.28579	0.2353

352.38257	0.23533

352.47936	0.23535

352.57614	0.23544

352.67292	0.23549

352.7697	0.23551

352.86648	0.23553

352.96327	0.23558

353.06005	0.2356

353.15683	0.23565

353.25361	0.23567

353.35039	0.23569

353.44717	0.23572

353.54396	0.23581

353.64074	0.23583

353.73752	0.23592

353.8343	0.23594

353.93108	0.23597

354.02787	0.23626

354.12465	0.23636

354.22143	0.23638

354.31821	0.23688

354.41499	0.23702

354.51177	0.23706

354.60856	0.23709

354.70534	0.23713

354.80212	0.23716

354.8989	0.2372

354.99568	0.23725

355.09247	0.23727

355.18925	0.23729

355.28603	0.23732

355.38281	0.23734

355.47959	0.23741

355.57637	0.23743

355.67316	0.23745

355.76994	0.23752

355.86672	0.23754

355.9635	0.23757

356.06028	0.23761

356.15707	0.23764

356.25385	0.23766

356.35063	0.23768

356.44741	0.2377

356.54419	0.23773

356.64097	0.23775

356.73776	0.23777

356.83454	0.23793

356.93132	0.23802

357.0281	0.23805

357.12488	0.23812

357.22167	0.23814

357.31845	0.23876

357.41523	0.23878

357.51201	0.23882

357.60879	0.23885

357.70557	0.23889

357.80236	0.23892

357.89914	0.23894

357.99592	0.23896

358.0927	0.23898

358.18948	0.23903

358.28627	0.23905

358.38305	0.23908

358.47983	0.2391

358.57661	0.23914

358.67339	0.23917

358.77017	0.23919

358.86696	0.23924

358.96374	0.23933

359.06052	0.23935

359.1573	0.23937

359.25408	0.2394

359.35087	0.23946

359.44765	0.23953

359.54443	0.23958

359.64121	0.2396

359.73799	0.23962

359.83477	0.23965

359.93156	0.23972

360.02834	0.23976

360.12512	0.23983

360.2219	0.23985

360.31868	0.23988

360.41546	0.23992

360.51225	0.23994

360.60903	0.23997

360.70581	0.24004

360.80259	0.24008

360.89937	0.2401

360.99616	0.24029

361.09294	0.24038

361.18972	0.24042

361.2865	0.24045

361.38328	0.24049

361.48006	0.24054

361.57685	0.24058

361.67363	0.24063

361.77041	0.24065

361.86719	0.24068

361.96397	0.2407

362.06076	0.24072

362.15754	0.24074

362.25432	0.24081

362.3511	0.2409

362.44788	0.24093

362.54466	0.24104

362.64145	0.24106

362.73823	0.24109

362.83501	0.24113

362.93179	0.24116

363.02857	0.24118

363.12536	0.24129

363.22214	0.24132

363.31892	0.24134

363.4157	0.24138

363.51248	0.24141

363.60926	0.24143

363.70605	0.24145

363.80283	0.2415

363.89961	0.24152

363.99639	0.24154

364.09317	0.24168

364.18996	0.24173

364.28674	0.24175

364.38352	0.24177

364.4803	0.24182

364.57708	0.24186

364.67386	0.24189

364.77065	0.24198

364.86743	0.242

364.96421	0.24207

365.06099	0.24209

365.15777	0.24214

365.25456	0.24218

365.35134	0.24223

365.44812	0.2423

365.5449	0.24232

365.64168	0.24241

365.73846	0.24244

365.83525	0.24253

365.93203	0.24255

366.02881	0.24257

366.12559	0.24262

366.22237	0.24264

366.31916	0.24271

366.41594	0.24273

366.51272	0.24285

366.6095	0.24289

366.70628	0.24292

366.80306	0.24294

366.89985	0.24296

366.99663	0.2431

367.09341	0.24314

367.19019	0.24319

367.28697	0.24321

367.38376	0.24324

367.48054	0.24326

367.57732	0.24328

367.6741	0.2433

367.77088	0.24333

367.86766	0.24335

367.96445	0.24337

368.06123	0.24346

368.15801	0.24381

368.25479	0.24424

368.35157	0.24431

368.44836	0.24438

368.54514	0.24442

368.64192	0.24445

368.7387	0.24447

368.83548	0.24449

368.93226	0.24458

369.02905	0.24461

369.12583	0.24463

369.22261	0.24465

369.31939	0.2447

369.41617	0.24472

369.51296	0.24474

369.60974	0.24479

369.70652	0.24481

369.8033	0.24486

369.90008	0.24488

369.99686	0.2449

370.09365	0.24493

370.19043	0.24495

370.28721	0.24497

370.38399	0.24506

370.48077	0.24509

370.57756	0.24511

370.67434	0.24522

370.77112	0.24525

370.8679	0.24527

370.96468	0.24529

371.06146	0.24532

371.15825	0.24534

371.25503	0.24538

371.35181	0.24541

371.44859	0.24543

371.54537	0.2455

371.64216	0.24552

371.73894	0.24554

371.83572	0.24559

371.9325	0.24561

372.02928	0.24568

372.12606	0.2457

372.22285	0.24573

372.31963	0.24577

372.41641	0.24582

372.51319	0.24589

372.60997	0.24591

372.70675	0.24598

372.80354	0.24602

372.90032	0.24609

372.9971	0.24612

373.09388	0.24614

373.19066	0.24616

373.28745	0.24618

373.38423	0.24628

373.48101	0.2463

373.57779	0.24632

373.67457	0.24634

373.77135	0.24637

373.86814	0.24639

373.96492	0.24641

374.0617	0.24646

374.15848	0.24648

374.25526	0.2465

374.35205	0.24653

374.44883	0.24662

374.54561	0.24664

374.64239	0.24673

374.73917	0.24676

374.83595	0.24678

374.93274	0.2468

375.02952	0.24682

375.1263	0.24694

375.22308	0.24698

375.31986	0.24701

375.41665	0.24705

375.51343	0.2471

375.61021	0.24712

375.70699	0.24714

375.80377	0.24721

375.90055	0.24733

375.99734	0.24737

376.09412	0.2474

376.1909	0.24742

376.28768	0.24744

376.38446	0.24746

376.48125	0.24788

376.57803	0.24792

376.67481	0.24794

376.77159	0.24797

376.86837	0.24799

376.96515	0.24804

377.06194	0.24806

377.15872	0.24813

377.2555	0.24822

377.35228	0.24829

377.44906	0.24831

377.54585	0.24833

377.64263	0.24836

377.73941	0.24845

377.83619	0.24847

377.93297	0.24849

378.02975	0.24852

378.12654	0.24854

378.22332	0.24856

378.3201	0.24868

378.41688	0.2487

378.51366	0.24874

378.61045	0.24895

378.70723	0.24897

378.80401	0.24902

378.90079	0.24918

378.99757	0.24922

379.09435	0.24925

379.19114	0.24927

379.28792	0.24943

379.3847	0.24945

379.48148	0.2495

379.57826	0.24966

379.67505	0.24968

379.77183	0.24977

379.86861	0.24982

379.96539	0.24984

380.06217	0.24986

380.15895	0.24989

380.25574	0.24991

380.35252	0.24993

380.4493	0.25

380.54608	0.25002

380.64286	0.25005

380.73965	0.25007

380.83643	0.25025

380.93321	0.25028

381.02999	0.25037

381.12677	0.25039

381.22355	0.25041

381.32034	0.25044

381.41712	0.25046

381.5139	0.25053

381.61068	0.25071

381.70746	0.25073

381.80425	0.25076

381.90103	0.2508

381.99781	0.25082

382.09459	0.25085

382.19137	0.25087

382.28815	0.25092

382.38494	0.25094

382.48172	0.25096

382.5785	0.25098

382.67528	0.25101

382.77206	0.25108

382.86885	0.25112

382.96563	0.25117

383.06241	0.25119

383.15919	0.25121

383.25597	0.25124

383.35275	0.25128

383.44954	0.2513

383.54632	0.25133

383.6431	0.25135

383.73988	0.25146

383.83666	0.25149

383.93345	0.25151

384.03023	0.2516

384.12701	0.25167

384.22379	0.25169

384.32057	0.25174

384.41735	0.25197

384.51414	0.25201

384.61092	0.25204

384.7077	0.25206

384.80448	0.25222

384.90126	0.25231

384.99804	0.25233

385.09483	0.25238

385.19161	0.2524

385.28839	0.25242

385.38517	0.25254

385.48195	0.25256

385.57874	0.25258

385.67552	0.25274

385.7723	0.25277

385.86908	0.25279

385.96586	0.25286

386.06264	0.25288

386.15943	0.2529

386.25621	0.25293

386.35299	0.25297

386.44977	0.253

386.54655	0.25302

386.64334	0.25309

386.74012	0.25313

386.8369	0.25318

386.93368	0.25325

387.03046	0.25332

387.12724	0.25334

387.22403	0.25336

387.32081	0.25338

387.41759	0.25343

387.51437	0.25345

387.61115	0.25354

387.70794	0.25357

387.80472	0.25361

387.9015	0.25364

387.99828	0.25366

388.09506	0.25375

388.19184	0.25386

388.28863	0.25389

388.38541	0.25393

388.48219	0.25398

388.57897	0.254

388.67575	0.25407

388.77254	0.25416

388.86932	0.25421

388.9661	0.25425

389.06288	0.25428

389.15966	0.2543

389.25644	0.25432

389.35323	0.25439

389.45001	0.25441

389.54679	0.25448

389.64357	0.25455

389.74035	0.2546

389.83714	0.25466

389.93392	0.25471

390.0307	0.25473

390.12748	0.25478

390.22426	0.25487

390.32104	0.25489

390.41783	0.25494

390.51461	0.25501

390.61139	0.25512

390.70817	0.25517

390.80495	0.25521

390.90174	0.25533

390.99852	0.25537

391.0953	0.2554

391.19208	0.25542

391.28886	0.25544

391.38564	0.25547

391.48243	0.25549

391.57921	0.25553

391.67599	0.25556

391.77277	0.25558

391.86955	0.25565

391.96634	0.25569

392.06312	0.25574

392.1599	0.25576

392.25668	0.25579

392.35346	0.25583

392.45024	0.25588

392.54703	0.2559

392.64381	0.25597

392.74059	0.25599

392.83737	0.25604

392.93415	0.25608

393.03094	0.25611

393.12772	0.25615

393.2245	0.25622

393.32128	0.25624

393.41806	0.25633

393.51484	0.25636

393.61163	0.25638

393.70841	0.2564

393.80519	0.25643

393.90197	0.25668

393.99875	0.2567

394.09554	0.25672

394.19232	0.25675

394.2891	0.25679

394.38588	0.25681

394.48266	0.25684

394.57944	0.25697

394.67623	0.257

394.77301	0.25718

394.86979	0.25725

394.96657	0.25727

395.06335	0.25729

395.16014	0.25732

395.25692	0.25741

395.3537	0.25745

395.45048	0.25752

395.54726	0.25757

395.64404	0.25759

395.74083	0.25766

395.83761	0.25771

395.93439	0.25775

396.03117	0.25777

396.12795	0.2578

396.22474	0.25784

396.32152	0.25791

396.4183	0.25793

396.51508	0.25805

396.61186	0.25825

396.70864	0.25832

396.80543	0.25837

396.90221	0.25839

396.99899	0.25844

397.09577	0.25846

397.19255	0.25848

397.28933	0.25851

397.38612	0.25864

397.4829	0.25867

397.57968	0.25885

397.67646	0.25887

397.77324	0.25892

397.87003	0.25894

397.96681	0.25896

398.06359	0.25899

398.16037	0.25901

398.25715	0.25903

398.35393	0.25905

398.45072	0.2591

398.5475	0.25912

398.64428	0.25917

398.74106	0.25919

398.83784	0.25924

398.93463	0.25926

399.03141	0.25935

399.12819	0.25942

399.22497	0.25947

399.32175	0.25949

399.41853	0.25953

399.51532	0.25956

399.6121	0.25958

399.70888	0.25976

399.80566	0.25979

399.90244	0.25981

399.99923	0.25985

400.09601	0.25992

400.19279	0.25995

400.28957	0.25999

400.38635	0.26001

400.48313	0.26049

400.57992	0.26054

400.6767	0.26056

400.77348	0.26061

400.87026	0.26063

400.96704	0.26065

401.06383	0.26068

401.16061	0.2607

401.25739	0.26072

401.35417	0.26075

401.45095	0.26077

401.54773	0.26079

401.64452	0.26084

401.7413	0.26086

401.83808	0.26102

401.93486	0.26109

402.03164	0.26111

402.12843	0.26113

402.22521	0.2612

402.32199	0.26123

402.41877	0.26127

402.51555	0.26129

402.61233	0.26139

402.70912	0.26141

402.8059	0.2615

402.90268	0.26152

402.99946	0.26157

403.09624	0.26161

403.19303	0.26164

403.28981	0.26166

403.38659	0.26168

403.48337	0.26177

403.58015	0.2618

403.67693	0.26184

403.77372	0.26189

403.8705	0.26191

403.96728	0.26196

404.06406	0.26205

404.16084	0.26207

404.25763	0.26221

404.35441	0.26225

404.45119	0.26235

404.54797	0.26237

404.64475	0.26239

404.74153	0.26244

404.83832	0.26248

404.9351	0.26251

405.03188	0.26253

405.12866	0.26255

405.22544	0.26262

405.32223	0.26264

405.41901	0.26269

405.51579	0.26271

405.61257	0.26273

405.70935	0.26276

405.80613	0.2628

405.90292	0.26285

405.9997	0.26287

406.09648	0.26289

406.19326	0.26292

406.29004	0.26294

406.38683	0.26296

406.48361	0.26299

406.58039	0.26301

406.67717	0.26305

406.77395	0.26317

406.87073	0.26324

406.96752	0.26326

407.0643	0.26328

407.16108	0.26331

407.25786	0.26333

407.35464	0.26337

407.45143	0.26349

407.54821	0.26351

407.64499	0.26353

407.74177	0.26356

407.83855	0.2636

407.93533	0.26363

408.03212	0.26367

408.1289	0.26369

408.22568	0.26376

408.32246	0.26381

408.41924	0.26385

408.51603	0.2639

408.61281	0.26392

408.70959	0.26395

408.80637	0.26397

408.90315	0.26399

408.99993	0.26406

409.09672	0.26413

409.1935	0.26417

409.29028	0.2642

409.38706	0.26424

409.48384	0.26427

409.58063	0.26429

409.67741	0.26433

409.77419	0.26443

409.87097	0.26445

409.96775	0.26447

410.06453	0.26465

410.16132	0.26468

410.2581	0.26472

410.35488	0.26475

410.45166	0.26477

410.54844	0.26479

410.64522	0.26481

410.74201	0.26484

410.83879	0.26486

410.93557	0.26491

411.03235	0.265

411.12913	0.26543

411.22592	0.26545

411.3227	0.2655

411.41948	0.26552

411.51626	0.26555

411.61304	0.26557

411.70982	0.26559

411.80661	0.26561

411.90339	0.26564

412.00017	0.26566

412.09695	0.26568

412.19373	0.26571

412.29052	0.26582

412.3873	0.26584

412.48408	0.26591

412.58086	0.26596

412.67764	0.26598

412.77442	0.266

412.87121	0.26605

412.96799	0.26607

413.06477	0.26609

413.16155	0.26612

413.25833	0.26619

413.35512	0.26621

413.4519	0.26623

413.54868	0.26632

413.64546	0.26635

413.74224	0.26641

413.83902	0.26657

413.93581	0.26662

414.03259	0.26664

414.12937	0.26667

414.22615	0.26669

414.32293	0.26676

414.41972	0.26678

414.5165	0.26696

414.61328	0.26699

414.71006	0.26701

414.80684	0.26703

414.90362	0.26705

415.00041	0.26708

415.09719	0.26712

415.19397	0.26715

415.29075	0.26721

415.38753	0.26728

415.48432	0.26731

415.5811	0.26733

415.67788	0.26735

415.77466	0.26737

415.87144	0.2674

415.96822	0.26751

416.06501	0.26753

416.16179	0.26756

416.25857	0.26758

416.35535	0.2676

416.45213	0.26776

416.54892	0.26779

416.6457	0.26783

416.74248	0.26785

416.83926	0.26792

416.93604	0.26795

417.03282	0.26797

417.12961	0.26799

417.22639	0.26801

417.32317	0.26804

417.41995	0.26806

417.51673	0.26808

417.61352	0.26811

417.7103	0.26813

417.80708	0.26815

417.90386	0.26817

418.00064	0.2682

418.09742	0.26822

418.19421	0.26827

418.29099	0.26829

418.38777	0.26833

418.48455	0.26836

418.58133	0.26843

418.67812	0.26845

418.7749	0.26849

418.87168	0.26852

418.96846	0.26856

419.06524	0.26861

419.16202	0.26865

419.25881	0.26877

419.35559	0.26893

419.45237	0.26895

419.54915	0.269

419.64593	0.26907

419.74272	0.26909

419.8395	0.26911

419.93628	0.26941

420.03306	0.2695

420.12984	0.26957

420.22662	0.26961

420.32341	0.26964

420.42019	0.26966

420.51697	0.26989

420.61375	0.26991

420.71053	0.26993

420.80732	0.26998

420.9041	0.27003

421.00088	0.27037

421.09766	0.27039

421.19444	0.27044

421.29122	0.27046

421.38801	0.27048

421.48479	0.27051

421.58157	0.27055

421.67835	0.27057

421.77513	0.27062

421.87192	0.27064

421.9687	0.27069

422.06548	0.27076

422.16226	0.27078

422.25904	0.27089

422.35582	0.27096

422.45261	0.27121

422.54939	0.27126

422.64617	0.27131

422.74295	0.2714

422.83973	0.27142

422.93651	0.27147

423.0333	0.27149

423.13008	0.27153

423.22686	0.27156

423.32364	0.27158

423.42042	0.2716

423.51721	0.27163

423.61399	0.27165

423.71077	0.27167

423.80755	0.27169

423.90433	0.27172

424.00111	0.27176

424.0979	0.27181

424.19468	0.27185

424.29146	0.2719

424.38824	0.27192

424.48502	0.27195

424.58181	0.27197

424.67859	0.27204

424.77537	0.27211

424.87215	0.27217

424.96893	0.2722

425.06571	0.27229

425.1625	0.27231

425.25928	0.27233

425.35606	0.27236

425.45284	0.27238

425.54962	0.2724

425.64641	0.27243

425.74319	0.27245

425.83997	0.27247

425.93675	0.27254

426.03353	0.27268

426.13031	0.27277

426.2271	0.27279

426.32388	0.27284

426.42066	0.27293

426.51744	0.27295

426.61422	0.27297

426.71101	0.273

426.80779	0.27329

426.90457	0.27332

427.00135	0.27345

427.09813	0.27348

427.19491	0.27355

427.2917	0.27357

427.38848	0.27359

427.48526	0.27361

427.58204	0.27366

427.67882	0.27373

427.77561	0.2738

427.87239	0.27389

427.96917	0.27391

428.06595	0.27393

428.16273	0.27398

428.25951	0.274

428.3563	0.27405

428.45308	0.27407

428.54986	0.27409

428.64664	0.27412

428.74342	0.27414

428.84021	0.27421

428.93699	0.27423

429.03377	0.27425

429.13055	0.2743

429.22733	0.27441

429.32411	0.27448

429.4209	0.27453

429.51768	0.27455

429.61446	0.27469

429.71124	0.27471

429.80802	0.27473

429.90481	0.27476

430.00159	0.2748

430.09837	0.27483

430.19515	0.27485

430.29193	0.27496

430.38871	0.27499

430.4855	0.27501

430.58228	0.27505

430.67906	0.2751

430.77584	0.27512

430.87262	0.27515

430.96941	0.27519

431.06619	0.27521

431.16297	0.27524

431.25975	0.27528

431.35653	0.27531

431.45331	0.27542

431.5501	0.27551

431.64688	0.27553

431.74366	0.27556

431.84044	0.27558

431.93722	0.27563

432.03401	0.27565

432.13079	0.27579

432.22757	0.27599

432.32435	0.27601

432.42113	0.27604

432.51791	0.27624

432.6147	0.27627

432.71148	0.27633

432.80826	0.27636

432.90504	0.27643

433.00182	0.27645

433.09861	0.27647

433.19539	0.27649

433.29217	0.27656

433.38895	0.27659

433.48573	0.27661

433.58251	0.27672

433.6793	0.27675

433.77608	0.27679

433.87286	0.27681

433.96964	0.27684

434.06642	0.27686

434.16321	0.27688

434.25999	0.277

434.35677	0.27702

434.45355	0.27711

434.55033	0.2772

434.64711	0.27725

434.7439	0.27729

434.84068	0.27732

434.93746	0.27743

435.03424	0.27752

435.13102	0.27757

435.2278	0.27759

435.32459	0.27761

435.42137	0.27766

435.51815	0.27768

435.61493	0.27773

435.71171	0.27775

435.8085	0.27777

435.90528	0.2778

436.00206	0.27782

436.09884	0.27787

436.19562	0.27796

436.2924	0.27803

436.38919	0.27805

436.48597	0.27807

436.58275	0.27809

436.67953	0.27812

436.77631	0.27814

436.8731	0.27816

436.96988	0.27819

437.06666	0.27823

437.16344	0.27825

437.26022	0.27828

437.357	0.2783

437.45379	0.27832

437.55057	0.27835

437.64735	0.27837

437.74413	0.27839

437.84091	0.27846

437.9377	0.27851

438.03448	0.27853

438.13126	0.27855

438.22804	0.27857

438.32482	0.2786

438.4216	0.27867

438.51839	0.27871

438.61517	0.27873

438.71195	0.27876

438.80873	0.27878

438.90551	0.2788

439.0023	0.27887

439.09908	0.27889

439.19586	0.27892

439.29264	0.27896

439.38942	0.27905

439.4862	0.27924

439.58299	0.27926

439.67977	0.27928

439.77655	0.27931

439.87333	0.27944

439.97011	0.27949

440.0669	0.27953

440.16368	0.27963

440.26046	0.27965

440.35724	0.27969

440.45402	0.27972

440.5508	0.27974

440.64759	0.27976

440.74437	0.27979

440.84115	0.27981

440.93793	0.27999

441.03471	0.28004

441.1315	0.28006

441.22828	0.28008

441.32506	0.28013

441.42184	0.28015

441.51862	0.28017

441.6154	0.28022

441.71219	0.28031

441.80897	0.28033

441.90575	0.28038

442.00253	0.28043

442.09931	0.28047

442.1961	0.28052

442.29288	0.28056

442.38966	0.28061

442.48644	0.28072

442.58322	0.28077

442.68	0.28081

442.77679	0.28084

442.87357	0.28088

442.97035	0.28091

443.06713	0.28095

443.16391	0.28104

443.2607	0.28107

443.35748	0.28109

443.45426	0.28111

443.55104	0.28113

443.64782	0.28116

443.7446	0.28118

443.84139	0.28123

443.93817	0.28132

444.03495	0.28134

444.13173	0.28139

444.22851	0.28145

444.3253	0.28152

444.42208	0.28157

444.51886	0.28161

444.61564	0.28164

444.71242	0.28168

444.8092	0.28171

444.90599	0.28173

445.00277	0.28175

445.09955	0.28177

445.19633	0.28184

445.29311	0.28189

445.3899	0.28193

445.48668	0.28198

445.58346	0.282

445.68024	0.28203

445.77702	0.28205

445.8738	0.28209

445.97059	0.28221

446.06737	0.28223

446.16415	0.28225

446.26093	0.28228

446.35771	0.2823

446.4545	0.28235

446.55128	0.28239

446.64806	0.28241

446.74484	0.28244

446.84162	0.28246

446.9384	0.28248

447.03519	0.28255

447.13197	0.28257

447.22875	0.28262

447.32553	0.28285

447.42231	0.28287

447.5191	0.28292

447.61588	0.28301

447.71266	0.28303

447.80944	0.28305

447.90622	0.28308

448.003	0.2831

448.09979	0.28312

448.19657	0.28315

448.29335	0.28321

448.39013	0.28324

448.48691	0.28331

448.58369	0.28335

448.68048	0.28337

448.77726	0.28342

448.87404	0.28347

448.97082	0.28353

449.0676	0.28356

449.16439	0.28358

449.26117	0.28365

449.35795	0.28369

449.45473	0.28372

449.55151	0.28374

449.64829	0.28392

449.74508	0.28395

449.84186	0.28408

449.93864	0.28411

450.03542	0.28415

450.1322	0.28417

450.22899	0.2842

450.32577	0.28433

450.42255	0.28436

450.51933	0.28438

450.61611	0.2844

450.71289	0.28449

450.80968	0.28452

450.90646	0.28454

451.00324	0.28456

451.10002	0.28459

451.1968	0.28461

451.29359	0.28475

451.39037	0.28488

451.48715	0.28491

451.58393	0.28493

451.68071	0.28495

451.77749	0.28497

451.87428	0.285

451.97106	0.28502

452.06784	0.28504

452.16462	0.28507

452.2614	0.28509

452.35819	0.28511

452.45497	0.28513

452.55175	0.28516

452.64853	0.28518

452.74531	0.2852

452.84209	0.28527

452.93888	0.28529

453.03566	0.28534

453.13244	0.28541

453.22922	0.28548

453.326	0.2855

453.42279	0.28552

453.51957	0.28557

453.61635	0.28561

453.71313	0.28566

453.80991	0.28575

453.90669	0.28577

454.00348	0.2858

454.10026	0.28589

454.19704	0.28591

454.29382	0.28598

454.3906	0.286

454.48739	0.28603

454.58417	0.28605

454.68095	0.28614

454.77773	0.28616

454.87451	0.28621

454.97129	0.28623

455.06808	0.28625

455.16486	0.28628

455.26164	0.28639

455.35842	0.28644

455.4552	0.28646

455.55199	0.28648

455.64877	0.28651

455.74555	0.28655

455.84233	0.28657

455.93911	0.2866

456.03589	0.28662

456.13268	0.28664

456.22946	0.28671

456.32624	0.28673

456.42302	0.28676

456.5198	0.28678

456.61659	0.2868

456.71337	0.28685

456.81015	0.28696

456.90693	0.28699

457.00371	0.28708

457.10049	0.28715

457.19728	0.28717

457.29406	0.28721

457.39084	0.28726

457.48762	0.28728

457.5844	0.28731

457.68119	0.28735

457.77797	0.28737

457.87475	0.2874

457.97153	0.28742

458.06831	0.28744

458.16509	0.28747

458.26188	0.28749

458.35866	0.28751

458.45544	0.28774

458.55222	0.28776

458.649	0.28781

458.74579	0.28783

458.84257	0.28788

458.93935	0.2879

459.03613	0.28792

459.13291	0.28795

459.22969	0.28797

459.32648	0.28801

459.42326	0.28804

459.52004	0.28806

459.61682	0.28808

459.7136	0.28811

459.81039	0.28817

459.90717	0.28831

460.00395	0.28833

460.10073	0.28836

460.19751	0.28838

460.29429	0.28845

460.39108	0.28847

460.48786	0.2885

460.58464	0.28852

460.68142	0.28859

460.7782	0.28861

460.87498	0.28866

460.97177	0.28877

461.06855	0.28879

461.16533	0.28884

461.26211	0.28886

461.35889	0.28888

461.45568	0.28891

461.55246	0.28898

461.64924	0.28907

461.74602	0.28914

461.8428	0.28918

461.93958	0.28923

462.03637	0.28925

462.13315	0.28927

462.22993	0.2893

462.32671	0.28936

462.42349	0.28948

462.52028	0.28952

462.61706	0.28957

462.71384	0.28968

462.81062	0.28973

462.9074	0.28978

463.00418	0.28991

463.10097	0.28994

463.19775	0.28996

463.29453	0.28998

463.39131	0.29

463.48809	0.29003

463.58488	0.29005

463.68166	0.29012

463.77844	0.29016

463.87522	0.29019

463.972	0.29021

464.06878	0.29039

464.16557	0.29046

464.26235	0.29048

464.35913	0.29051

464.45591	0.29053

464.55269	0.29058

464.64948	0.2906

464.74626	0.29062

464.84304	0.29064

464.93982	0.29067

465.0366	0.29069

465.13338	0.29071

465.23017	0.29074

465.32695	0.29078

465.42373	0.2908

465.52051	0.29083

465.61729	0.2909

465.71408	0.29092

465.81086	0.29096

465.90764	0.29101

466.00442	0.29106

466.1012	0.29108

466.19798	0.2911

466.29477	0.29115

466.39155	0.29117

466.48833	0.29119

466.58511	0.29122

466.68189	0.29124

466.77868	0.29128

466.87546	0.29131

466.97224	0.29142

467.06902	0.29144

467.1658	0.29147

467.26258	0.29149

467.35937	0.29151

467.45615	0.29154

467.55293	0.29156

467.64971	0.29158

467.74649	0.2916

467.84328	0.29163

467.94006	0.29165

468.03684	0.29167

468.13362	0.29174

468.2304	0.29176

468.32718	0.29181

468.42397	0.29183

468.52075	0.29186

468.61753	0.29188

468.71431	0.2919

468.81109	0.29192

468.90788	0.29199

469.00466	0.29202

469.10144	0.29204

469.19822	0.29206

469.295	0.29208

469.39178	0.29211

469.48857	0.29213

469.58535	0.29218

469.68213	0.2922

469.77891	0.29222

469.87569	0.29224

469.97248	0.29227

470.06926	0.29229

470.16604	0.29234

470.26282	0.29238

470.3596	0.2924

470.45638	0.29243

470.55317	0.29245

470.64995	0.29247

470.74673	0.2925

470.84351	0.29252

470.94029	0.29259

471.03708	0.29263

471.13386	0.29266

471.23064	0.29268

471.32742	0.29272

471.4242	0.29277

471.52098	0.29282

471.61777	0.29284

471.71455	0.29291

471.81133	0.29295

471.90811	0.29298

472.00489	0.29302

472.10168	0.29304

472.19846	0.29307

472.29524	0.29309

472.39202	0.29311

472.4888	0.29316

472.58558	0.2932

472.68237	0.29332

472.77915	0.29339

472.87593	0.29341

472.97271	0.29343

473.06949	0.29346

473.16627	0.2935

473.26306	0.29352

473.35984	0.29355

473.45662	0.29359

473.5534	0.29368

473.65018	0.29371

473.74697	0.29373

473.84375	0.29375

473.94053	0.29378

474.03731	0.2938

474.13409	0.29382

474.23087	0.29394

474.32766	0.29396

474.42444	0.29419

474.52122	0.29428

474.618	0.29432

474.71478	0.29435

474.81157	0.29437

474.90835	0.29439

475.00513	0.29444

475.10191	0.29446

475.19869	0.29448

475.29547	0.29453

475.39226	0.29458

475.48904	0.29462

475.58582	0.29467

475.6826	0.29469

475.77938	0.29471

475.87617	0.29478

475.97295	0.29483

476.06973	0.29485

476.16651	0.29499

476.26329	0.29503

476.36007	0.29506

476.45686	0.29508

476.55364	0.29517

476.65042	0.29522

476.7472	0.29524

476.84398	0.29526

476.94077	0.29528

477.03755	0.29535

477.13433	0.29538

477.23111	0.2954

477.32789	0.29551

477.42467	0.29554

477.52146	0.29556

477.61824	0.29563

477.71502	0.29565

477.8118	0.29567

477.90858	0.2957

478.00537	0.29572

478.10215	0.29574

478.19893	0.29576

478.29571	0.29579

478.39249	0.29581

478.48927	0.29583

478.58606	0.29588

478.68284	0.2959

478.77962	0.29592

478.8764	0.29595

478.97318	0.29597

479.06997	0.29606

479.16675	0.29611

479.26353	0.29613

479.36031	0.29615

479.45709	0.29622

479.55387	0.29624

479.65066	0.29627

479.74744	0.29629

479.84422	0.29631

479.941	0.29634

480.03778	0.29636

480.13457	0.2964

480.23135	0.29643

480.32813	0.29652

480.42491	0.29654

480.52169	0.29659

480.61847	0.29661

480.71526	0.29672

480.81204	0.29677

480.90882	0.29679

481.0056	0.29682

481.10238	0.29688

481.19917	0.29691

481.29595	0.29693

481.39273	0.29698

481.48951	0.297

481.58629	0.29702

481.68307	0.29709

481.77986	0.29711

481.87664	0.29714

481.97342	0.2972

482.0702	0.29732

482.16698	0.29739

482.26377	0.29741

482.36055	0.29743

482.45733	0.29746

482.55411	0.29759

482.65089	0.29762

482.74767	0.29766

482.84446	0.29768

482.94124	0.29771

483.03802	0.29773

483.1348	0.29775

483.23158	0.29778

483.32837	0.29784

483.42515	0.29787

483.52193	0.29789

483.61871	0.29791

483.71549	0.29796

483.81227	0.29803

483.90906	0.29805

484.00584	0.29807

484.10262	0.2981

484.1994	0.29814

484.29618	0.29816

484.39297	0.29823

484.48975	0.29828

484.58653	0.2983

484.68331	0.29837

484.78009	0.29846

484.87687	0.29848

484.97366	0.29855

485.07044	0.29858

485.16722	0.2986

485.264	0.29862

485.36078	0.29871

485.45756	0.29874

485.55435	0.29883

485.65113	0.29885

485.74791	0.29887

485.84469	0.29894

485.94147	0.29896

486.03826	0.29901

486.13504	0.29908

486.23182	0.2991

486.3286	0.29912

486.42538	0.29917

486.52216	0.29919

486.61895	0.29926

486.71573	0.29928

486.81251	0.29933

486.90929	0.29935

487.00607	0.29938

487.10286	0.2994

487.19964	0.29944

487.29642	0.29949

487.3932	0.29954

487.48998	0.29956

487.58676	0.29958

487.68355	0.2996

487.78033	0.29963

487.87711	0.2997

487.97389	0.29974

488.07067	0.29976

488.16746	0.29979

488.26424	0.29981

488.36102	0.29983

488.4578	0.2999

488.55458	0.29997

488.65136	0.29999

488.74815	0.30002

488.84493	0.30004

488.94171	0.30006

489.03849	0.30008

489.13527	0.30013

489.23206	0.30022

489.32884	0.30024

489.42562	0.30027

489.5224	0.30034

489.61918	0.30036

489.71596	0.3004

489.81275	0.30043

489.90953	0.30045

490.00631	0.30047

490.10309	0.3005

490.19987	0.30052

490.29666	0.30054

490.39344	0.30056

490.49022	0.30059

490.587	0.30063

490.68378	0.30066

490.78056	0.30068

490.87735	0.30072

490.97413	0.30075

491.07091	0.30077

491.16769	0.30084

491.26447	0.30086

491.36126	0.30088

491.45804	0.30091

491.55482	0.30093

491.6516	0.30095

491.74838	0.30098

491.84516	0.301

491.94195	0.30111

492.03873	0.30118

492.13551	0.30191

492.23229	0.30194

492.32907	0.30205

492.42586	0.30214

492.52264	0.30216

492.61942	0.30219

492.7162	0.30232

492.81298	0.30235

492.90976	0.30237

493.00655	0.30242

493.10333	0.30244

493.20011	0.30246

493.29689	0.30251

493.39367	0.30255

493.49046	0.30258

493.58724	0.3026

493.68402	0.30262

493.7808	0.30264

493.87758	0.30271

493.97436	0.30276

494.07115	0.3028

494.16793	0.30292

494.26471	0.30294

494.36149	0.30296

494.45827	0.30306

494.55506	0.3031

494.65184	0.30312

494.74862	0.30315

494.8454	0.3034

494.94218	0.30342

495.03896	0.30347

495.13575	0.30351

495.23253	0.30363

495.32931	0.30365

495.42609	0.30367

495.52287	0.3037

495.61966	0.30372

495.71644	0.30374

495.81322	0.30383

495.91	0.30386

496.00678	0.3039

496.10356	0.30392

496.20035	0.30397

496.29713	0.30399

496.39391	0.30411

496.49069	0.30413

496.58747	0.30415

496.68426	0.30418

496.78104	0.3042

496.87782	0.30422

496.9746	0.30424

497.07138	0.30436

497.16816	0.30438

497.26495	0.3044

497.36173	0.30443

497.45851	0.30445

497.55529	0.30447

497.65207	0.3045

497.74886	0.30454

497.84564	0.30456

497.94242	0.30468

498.0392	0.30482

498.13598	0.30484

498.23276	0.30486

498.32955	0.30493

498.42633	0.30498

498.52311	0.305

498.61989	0.30507

498.71667	0.30509

498.81345	0.30516

498.91024	0.3052

499.00702	0.30523

499.1038	0.30525

499.20058	0.30541

499.29736	0.30543

499.39415	0.30546

499.49093	0.3055

499.58771	0.30552

499.68449	0.30559

499.78127	0.30562

499.87805	0.30564

499.97484	0.30566

500.07162	0.30568

500.1684	0.30571

500.26518	0.30573

500.36196	0.30578

500.45875	0.30584

500.55553	0.30587

500.65231	0.30596

500.74909	0.30598

500.84587	0.30603

500.94265	0.30607

501.03944	0.30612

501.13622	0.30616

501.233	0.30619

501.32978	0.30621

501.42656	0.30626

501.52335	0.30635

501.62013	0.30637

501.71691	0.30639

501.81369	0.30644

501.91047	0.30646

502.00725	0.30651

502.10404	0.30658

502.20082	0.30662

502.2976	0.30664

502.39438	0.30667

502.49116	0.30669

502.58795	0.30674

502.68473	0.30676

502.78151	0.30678

502.87829	0.3068

502.97507	0.30683

503.07185	0.30685

503.16864	0.30687

503.26542	0.3069

503.3622	0.30694

503.45898	0.30701

503.55576	0.30703

503.65255	0.30706

503.74933	0.30708

503.84611	0.30712

503.94289	0.30715

504.03967	0.30719

504.13645	0.30724

504.23324	0.30731

504.33002	0.30735

504.4268	0.30738

504.52358	0.30742

504.62036	0.30749

504.71715	0.30758

504.81393	0.30763

504.91071	0.30765

505.00749	0.30767

505.10427	0.3077

505.20105	0.30772

505.29784	0.30783

505.39462	0.30786

505.4914	0.30792

505.58818	0.30795

505.68496	0.30797

505.78175	0.30799

505.87853	0.30802

505.97531	0.30804

506.07209	0.30808

506.16887	0.30818

506.26565	0.3082

506.36244	0.30824

506.45922	0.30827

506.556	0.30829

506.65278	0.30831

506.74956	0.30834

506.84635	0.30836

506.94313	0.30843

507.03991	0.3085

507.13669	0.30852

507.23347	0.30856

507.33025	0.30859

507.42704	0.30861

507.52382	0.30866

507.6206	0.30868

507.71738	0.3087

507.81416	0.30875

507.91095	0.30882

508.00773	0.30884

508.10451	0.30888

508.20129	0.30891

508.29807	0.30898

508.39485	0.309

508.49164	0.30902

508.58842	0.30909

508.6852	0.30911

508.78198	0.30916

508.87876	0.30918

508.97555	0.30923

509.07233	0.30925

509.16911	0.3093

509.26589	0.30934

509.36267	0.30939

509.45945	0.30946

509.55624	0.30948

509.65302	0.3095

509.7498	0.30955

509.84658	0.30957

509.94336	0.30964

510.04015	0.30966

510.13693	0.30971

510.23371	0.30973

510.33049	0.30975

510.42727	0.30978

510.52405	0.3098

510.62084	0.30982

510.71762	0.30989

510.8144	0.30998

510.91118	0.31

511.00796	0.31005

511.10474	0.31007

511.20153	0.3101

511.29831	0.31019

511.39509	0.31021

511.49187	0.31023

511.58865	0.31026

511.68544	0.31028

511.78222	0.31039

511.879	0.31053

511.97578	0.3106

512.07256	0.31062

512.16934	0.31067

512.26613	0.31069

512.36291	0.31071

512.45969	0.31074

512.55647	0.31076

512.65325	0.31078

512.75004	0.3108

512.84682	0.31083

512.9436	0.31087

513.04038	0.31092

513.13716	0.31094

513.23394	0.31101

513.33073	0.31106

513.42751	0.3111

513.52429	0.31117

513.62107	0.31124

513.71785	0.31126

513.81464	0.31128

513.91142	0.31131

514.0082	0.31133

514.10498	0.31138

514.20176	0.31142

514.29854	0.31144

514.39533	0.31147

514.49211	0.31149

514.58889	0.31154

514.68567	0.31156

514.78245	0.31158

514.87924	0.3116

514.97602	0.31163

515.0728	0.31167

515.16958	0.31181

515.26636	0.31183

515.36314	0.31188

515.45993	0.3119

515.55671	0.31192

515.65349	0.31197

515.75027	0.31199

515.84705	0.31202

515.94384	0.31211

516.04062	0.31213

516.1374	0.31215

516.23418	0.31218

516.33096	0.3122

516.42774	0.31224

516.52453	0.31229

516.62131	0.31231

516.71809	0.31234

516.81487	0.3124

516.91165	0.31243

517.00844	0.31245

517.10522	0.31247

517.202	0.31268

517.29878	0.31275

517.39556	0.31277

517.49234	0.31279

517.58913	0.31284

517.68591	0.31291

517.78269	0.31293

517.87947	0.31295

517.97625	0.31298

518.07304	0.313

518.16982	0.31307

518.2666	0.31309

518.36338	0.31314

518.46016	0.31316

518.55694	0.31318

518.65373	0.3132

518.75051	0.31323

518.84729	0.31325

518.94407	0.31332

519.04085	0.31334

519.13764	0.31336

519.23442	0.31339

519.3312	0.31341

519.42798	0.31343

519.52476	0.31346

519.62154	0.31348

519.71833	0.3135

519.81511	0.31355

519.91189	0.31364

520.00867	0.31371

520.10545	0.31373

520.20224	0.31391

520.29902	0.31394

520.3958	0.31403

520.49258	0.31407

520.58936	0.31412

520.68614	0.31426

520.78293	0.31428

520.87971	0.3143

520.97649	0.31435

521.07327	0.31442

521.17005	0.31444

521.26684	0.31446

521.36362	0.31453

521.4604	0.31455

521.55718	0.31458

521.65396	0.31462

521.75074	0.31464

521.84753	0.31469

521.94431	0.31474

522.04109	0.31476

522.13787	0.31478

522.23465	0.3148

522.33144	0.31483

522.42822	0.31485

522.525	0.31487

522.62178	0.31494

522.71856	0.31499

522.81534	0.31501

522.91213	0.31506

523.00891	0.31508

523.10569	0.31512

523.20247	0.31517

523.29925	0.31519

523.39603	0.31524

523.49282	0.31526

523.5896	0.31528

523.68638	0.31531

523.78316	0.31533

523.87994	0.31535

523.97673	0.31538

524.07351	0.3154

524.17029	0.31547

524.26707	0.31549

524.36385	0.31556

524.46064	0.31558

524.55742	0.3156

524.6542	0.31563

524.75098	0.31565

524.84776	0.31567

524.94454	0.3157

525.04133	0.31576

525.13811	0.3159

525.23489	0.31592

525.33167	0.31597

525.42845	0.31645

525.52523	0.31654

525.62202	0.31663

525.7188	0.31668

525.81558	0.3167

525.91236	0.31675

526.00914	0.31679

526.10593	0.31702

526.20271	0.31707

526.29949	0.31711

526.39627	0.31714

526.49305	0.31723

526.58983	0.31727

526.68662	0.3173

526.7834	0.31732

526.88018	0.31736

526.97696	0.31741

527.07374	0.31752

527.17053	0.31755

527.26731	0.31757

527.36409	0.31762

527.46087	0.31764

527.55765	0.31766

527.65443	0.31782

527.75122	0.31784

527.848	0.31791

527.94478	0.31794

528.04156	0.31805

528.13834	0.31807

528.23513	0.3181

528.33191	0.31812

528.42869	0.31816

528.52547	0.31819

528.62225	0.31826

528.71903	0.31835

528.81582	0.31837

528.9126	0.31839

529.00938	0.31844

529.10616	0.31846

529.20294	0.31848

529.29973	0.31853

529.39651	0.31855

529.49329	0.31858

529.59007	0.3186

529.68685	0.31862

529.78363	0.31867

529.88042	0.31885

529.9772	0.31894

530.07398	0.31903

530.17076	0.3191

530.26754	0.31912

530.36433	0.31915

530.46111	0.31917

530.55789	0.31922

530.65467	0.31924

530.75145	0.31926

530.84823	0.31928

530.94502	0.31933

531.0418	0.31942

531.13858	0.31944

531.23536	0.31947

531.33214	0.31949

531.42893	0.31951

531.52571	0.31956

531.62249	0.31958

531.71927	0.31963

531.81605	0.31965

531.91283	0.3197

532.00962	0.31972

532.1064	0.31976

532.20318	0.31979

532.29996	0.31981

532.39674	0.31983

532.49353	0.31986

532.59031	0.31988

532.68709	0.3199

532.78387	0.31992

532.88065	0.31995

532.97743	0.32002

533.07422	0.32004

533.171	0.32006

533.26778	0.32008

533.36456	0.32013

533.46134	0.32018

533.55813	0.32022

533.65491	0.32027

533.75169	0.32029

533.84847	0.32038

533.94525	0.32043

534.04203	0.32047

534.13882	0.32061

534.2356	0.32063

534.33238	0.32068

534.42916	0.3207

534.52594	0.32072

534.62273	0.32075

534.71951	0.32077

534.81629	0.32079

534.91307	0.32084

535.00985	0.32086

535.10663	0.32088

535.20342	0.32091

535.3002	0.32098

535.39698	0.32104

535.49376	0.32107

535.59054	0.32116

535.68733	0.3212

535.78411	0.32123

535.88089	0.32125

535.97767	0.32127

536.07445	0.32132

536.17123	0.32136

536.26802	0.32139

536.3648	0.32141

536.46158	0.32146

536.55836	0.3215

536.65514	0.32157

536.75193	0.32159

536.84871	0.32162

536.94549	0.32164

537.04227	0.32169

537.13905	0.32171

537.23583	0.32173

537.33262	0.32175

537.4294	0.32178

537.52618	0.32191

537.62296	0.32194

537.71974	0.32196

537.81652	0.32198

537.91331	0.32201

538.01009	0.32205

538.10687	0.32207

538.20365	0.3221

538.30043	0.32214

538.39722	0.32219

538.494	0.32221

538.59078	0.32226

538.68756	0.32228

538.78434	0.3223

538.88112	0.32267

538.97791	0.32271

539.07469	0.32276

539.17147	0.32278

539.26825	0.32283

539.36503	0.3229

539.46182	0.32294

539.5586	0.32299

539.65538	0.32301

539.75216	0.32303

539.84894	0.32308

539.94572	0.3231

540.04251	0.32313

540.13929	0.32319

540.23607	0.32329

540.33285	0.32331

540.42963	0.32333

540.52642	0.32335

540.6232	0.32338

540.71998	0.3234

540.81676	0.32345

540.91354	0.32347

541.01032	0.32349

541.10711	0.32354

541.20389	0.32361

541.30067	0.32363

541.39745	0.32365

541.49423	0.3237

541.59102	0.32374

541.6878	0.32388

541.78458	0.32393

541.88136	0.32397

541.97814	0.32402

542.07492	0.32406

542.17171	0.32411

542.26849	0.32415

542.36527	0.3242

542.46205	0.32422

542.55883	0.32427

542.65562	0.32429

542.7524	0.32431

542.84918	0.32434

542.94596	0.32445

543.04274	0.32457

543.13952	0.32461

543.23631	0.32463

543.33309	0.32466

543.42987	0.32473

543.52665	0.32479

543.62343	0.32484

543.72022	0.32486

543.817	0.32491

543.91378	0.32493

544.01056	0.32495

544.10734	0.32498

544.20412	0.32502

544.30091	0.32505

544.39769	0.32507

544.49447	0.32509

544.59125	0.32516

544.68803	0.32518

544.78482	0.32527

544.8816	0.3253

544.97838	0.32532

545.07516	0.32534

545.17194	0.32539

545.26872	0.32541

545.36551	0.32546

545.46229	0.32548

545.55907	0.32555

545.65585	0.32564

545.75263	0.32566

545.84942	0.32571

545.9462	0.32575

546.04298	0.32578

546.13976	0.32587

546.23654	0.32591

546.33332	0.32594

546.43011	0.32601

546.52689	0.32603

546.62367	0.32605

546.72045	0.32614

546.81723	0.32617

546.91402	0.32619

547.0108	0.32621

547.10758	0.32635

547.20436	0.32637

547.30114	0.32639

547.39792	0.32644

547.49471	0.32651

547.59149	0.32655

547.68827	0.32658

547.78505	0.3266

547.88183	0.32662

547.97862	0.32665

548.0754	0.32669

548.17218	0.32674

548.26896	0.32676

548.36574	0.32678

548.46252	0.32683

548.55931	0.3269

548.65609	0.32692

548.75287	0.32694

548.84965	0.32701

548.94643	0.32703

549.04322	0.32708

549.14	0.3271

549.23678	0.32715

549.33356	0.32717

549.43034	0.32719

549.52712	0.32722

549.62391	0.32724

549.72069	0.32726

549.81747	0.32735

549.91425	0.32738

550.01103	0.3274

550.10781	0.32745

550.2046	0.32747

550.30138	0.32751

550.39816	0.32754

550.49494	0.32756

550.59172	0.32758

550.68851	0.32765

550.78529	0.32767

550.88207	0.3277

550.97885	0.32772

551.07563	0.32774

551.17241	0.32783

551.2692	0.32802

551.36598	0.32804

551.46276	0.32811

551.55954	0.32813

551.65632	0.32815

551.75311	0.32818

551.84989	0.3282

551.94667	0.32825

552.04345	0.32827

552.14023	0.32829

552.23701	0.32831

552.3338	0.32834

552.43058	0.32836

552.52736	0.32838

552.62414	0.32843

552.72092	0.32845

552.81771	0.32847

552.91449	0.3285

553.01127	0.32854

553.10805	0.32857

553.20483	0.32859

553.30161	0.32863

553.3984	0.32868

553.49518	0.3287

553.59196	0.32877

553.68874	0.32886

553.78552	0.329

553.88231	0.32902

553.97909	0.32907

554.07587	0.32909

554.17265	0.32911

554.26943	0.32914

554.36621	0.32916

554.463	0.32918

554.55978	0.32921

554.65656	0.32923

554.75334	0.32925

554.85012	0.32939

554.94691	0.32946

555.04369	0.32948

555.14047	0.32953

555.23725	0.32955

555.33403	0.32957

555.43081	0.32962

555.5276	0.32964

555.62438	0.32966

555.72116	0.32969

555.81794	0.32971

555.91472	0.32975

556.01151	0.3298

556.10829	0.32982

556.20507	0.32985

556.30185	0.32987

556.39863	0.32989

556.49541	0.32991

556.5922	0.32994

556.68898	0.32996

556.78576	0.32998

556.88254	0.33001

556.97932	0.33005

557.07611	0.33007

557.17289	0.3301

557.26967	0.33012

557.36645	0.33014

557.46323	0.33017

557.56001	0.33021

557.6568	0.33023

557.75358	0.33026

557.85036	0.33028

557.94714	0.3303

558.04392	0.33037

558.14071	0.33039

558.23749	0.33042

558.33427	0.33049

558.43105	0.33053

558.52783	0.33055

558.62461	0.33062

558.7214	0.33065

558.81818	0.33071

558.91496	0.33081

559.01174	0.33085

559.10852	0.3309

559.20531	0.33099

559.30209	0.33101

559.39887	0.33103

559.49565	0.33106

559.59243	0.33108

559.68921	0.33115

559.786	0.33117

559.88278	0.33122

559.97956	0.33124

560.07634	0.33126

560.17312	0.33131

560.26991	0.33135

560.36669	0.33138

560.46347	0.33145

560.56025	0.33147

560.65703	0.33149

560.75381	0.33158

560.8506	0.33161

560.94738	0.33165

561.04416	0.33167

561.14094	0.3317

561.23772	0.33177

561.33451	0.33179

561.43129	0.33183

561.52807	0.33188

561.62485	0.33193

561.72163	0.33195

561.81841	0.33197

561.9152	0.33199

562.01198	0.33202

562.10876	0.33204

562.20554	0.33206

562.30232	0.33209

562.3991	0.33213

562.49589	0.33218

562.59267	0.3322

562.68945	0.33227

562.78623	0.33231

562.88301	0.33234

562.9798	0.33236

563.07658	0.33238

563.17336	0.33241

563.27014	0.33243

563.36692	0.33245

563.4637	0.3325

563.56049	0.33252

563.65727	0.33254

563.75405	0.33259

563.85083	0.33263

563.94761	0.33266

564.0444	0.33268

564.14118	0.3327

564.23796	0.33273

564.33474	0.33282

564.43152	0.33284

564.5283	0.33286

564.62509	0.33289

564.72187	0.33291

564.81865	0.33293

564.91543	0.33295

565.01221	0.333

565.109	0.33302

565.20578	0.33305

565.30256	0.33307

565.39934	0.33314

565.49612	0.33318

565.5929	0.33323

565.68969	0.3333

565.78647	0.33332

565.88325	0.33337

565.98003	0.33339

566.07681	0.33346

566.1736	0.33353

566.27038	0.33355

566.36716	0.33359

566.46394	0.33369

566.56072	0.33371

566.6575	0.3338

566.75429	0.33382

566.85107	0.33385

566.94785	0.33389

567.04463	0.33391

567.14141	0.33394

567.2382	0.33398

567.33498	0.33401

567.43176	0.33403

567.52854	0.33405

567.62532	0.33407

567.7221	0.33412

567.81889	0.33414

567.91567	0.33497

568.01245	0.33499

568.10923	0.33501

568.20601	0.33506

568.3028	0.33508

568.39958	0.3351

568.49636	0.33513

568.59314	0.33515

568.68992	0.33519

568.7867	0.33522

568.88349	0.33533

568.98027	0.33535

569.07705	0.33538

569.17383	0.33542

569.27061	0.33545

569.3674	0.33547

569.46418	0.33549

569.56096	0.33551

569.65774	0.33556

569.75452	0.33558

569.8513	0.33561

569.94809	0.33567

570.04487	0.3357

570.14165	0.33572

570.23843	0.33577

570.33521	0.33579

570.432	0.33581

570.52878	0.33583

570.62556	0.33586

570.72234	0.33588

570.81912	0.3359

570.9159	0.33595

571.01269	0.33597

571.10947	0.33602

571.20625	0.33604

571.30303	0.33609

571.39981	0.33615

571.4966	0.33618

571.59338	0.3362

571.69016	0.33622

571.78694	0.33625

571.88372	0.33627

571.9805	0.33629

572.07729	0.33631

572.17407	0.33636

572.27085	0.33638

572.36763	0.33643

572.46441	0.33645

572.5612	0.33647

572.65798	0.3365

572.75476	0.33652

572.85154	0.33659

572.94832	0.33661

573.0451	0.33663

573.14189	0.33668

573.23867	0.3367

573.33545	0.33675

573.43223	0.33677

573.52901	0.33684

573.6258	0.33691

573.72258	0.337

573.81936	0.33702

573.91614	0.33711

574.01292	0.33716

574.1097	0.33759

574.20649	0.33762

574.30327	0.33769

574.40005	0.33771

574.49683	0.33773

574.59361	0.33775

574.69039	0.3378

574.78718	0.33782

574.88396	0.33785

574.98074	0.33787

575.07752	0.33789

575.1743	0.33791

575.27109	0.33798

575.36787	0.33801

575.46465	0.33803

575.56143	0.33805

575.65821	0.33814

575.75499	0.33817

575.85178	0.33819

575.94856	0.33821

576.04534	0.33823

576.14212	0.33826

576.2389	0.33828

576.33569	0.33833

576.43247	0.33835

576.52925	0.33837

576.62603	0.33842

576.72281	0.33844

576.81959	0.33846

576.91638	0.33851

577.01316	0.33853

577.10994	0.33858

577.20672	0.3386

577.3035	0.33862

577.40029	0.33865

577.49707	0.33867

577.59385	0.33883

577.69063	0.33885

577.78741	0.33894

577.88419	0.33897

577.98098	0.33899

578.07776	0.33901

578.17454	0.33903

578.27132	0.3391

578.3681	0.33917

578.46489	0.33919

578.56167	0.33924

578.65845	0.33931

578.75523	0.33933

578.85201	0.33935

578.94879	0.33938

579.04558	0.3394

579.14236	0.33947

579.23914	0.33954

579.33592	0.33958

579.4327	0.33961

579.52949	0.33965

579.62627	0.33967

579.72305	0.33972

579.81983	0.33974

579.91661	0.33977

580.01339	0.33979

580.11018	0.33983

580.20696	0.33986

580.30374	0.33995

580.40052	0.33999

580.4973	0.34002

580.59409	0.34004

580.69087	0.34006

580.78765	0.34025

580.88443	0.34027

580.98121	0.34029

581.07799	0.34031

581.17478	0.34034

581.27156	0.34036

581.36834	0.34038

581.46512	0.34041

581.5619	0.34045

581.65869	0.34047

581.75547	0.3405

581.85225	0.34054

581.94903	0.34057

582.04581	0.34066

582.14259	0.34077

582.23938	0.34079

582.33616	0.34084

582.43294	0.34086

582.52972	0.34089

582.6265	0.34091

582.72329	0.34102

582.82007	0.34105

582.91685	0.34107

583.01363	0.34109

583.11041	0.34111

583.20719	0.34114

583.30398	0.34116

583.40076	0.34118

583.49754	0.34123

583.59432	0.34132

583.6911	0.34134

583.78789	0.34146

583.88467	0.34148

583.98145	0.3415

584.07823	0.34159

584.17501	0.34162

584.27179	0.34166

584.36858	0.34169

584.46536	0.34171

584.56214	0.34173

584.65892	0.34175

584.7557	0.3418

584.85249	0.34182

584.94927	0.34189

585.04605	0.34191

585.14283	0.34194

585.23961	0.34198

585.33639	0.34201

585.43318	0.34203

585.52996	0.34205

585.62674	0.34214

585.72352	0.34219

585.8203	0.34223

585.91709	0.34226

586.01387	0.34228

586.11065	0.3423

586.20743	0.34233

586.30421	0.34235

586.40099	0.34237

586.49778	0.34242

586.59456	0.34244

586.69134	0.34246

586.78812	0.34258

586.8849	0.3426

586.98168	0.34262

587.07847	0.34265

587.17525	0.34267

587.27203	0.34269

587.36881	0.34271

587.46559	0.34276

587.56238	0.34278

587.65916	0.34281

587.75594	0.34283

587.85272	0.34287

587.9495	0.34294

588.04628	0.34297

588.14307	0.34299

588.23985	0.34301

588.33663	0.34303

588.43341	0.34319

588.53019	0.34322

588.62698	0.34324

588.72376	0.34326

588.82054	0.34329

588.91732	0.34333

589.0141	0.34335

589.11088	0.34338

589.20767	0.34345

589.30445	0.34347

589.40123	0.34349

589.49801	0.34354

589.59479	0.34358

589.69158	0.34361

589.78836	0.34363

589.88514	0.34365

589.98192	0.34367

590.0787	0.3437

590.17548	0.34372

590.27227	0.34377

590.36905	0.34381

590.46583	0.34395

590.56261	0.34406

590.65939	0.34427

590.75618	0.34434

590.85296	0.34436

590.94974	0.34438

591.04652	0.34441

591.1433	0.3445

591.24008	0.34452

591.33687	0.34454

591.43365	0.34457

591.53043	0.34459

591.62721	0.34461

591.72399	0.34463

591.82078	0.34466

591.91756	0.34468

592.01434	0.3447

592.11112	0.34475

592.2079	0.34482

592.30468	0.34489

592.40147	0.34491

592.49825	0.34493

592.59503	0.34495

592.69181	0.34502

592.78859	0.34507

592.88538	0.34509

592.98216	0.34514

593.07894	0.34518

593.17572	0.34521

593.2725	0.34525

593.36928	0.34534

593.46607	0.34537

593.56285	0.34539

593.65963	0.34541

593.75641	0.34548

593.85319	0.34553

593.94998	0.34559

594.04676	0.34562

594.14354	0.34566

594.24032	0.34569

594.3371	0.34573

594.43388	0.34575

594.53067	0.34582

594.62745	0.34591

594.72423	0.34601

594.82101	0.34605

594.91779	0.3461

595.01458	0.34617

595.11136	0.34619

595.20814	0.34621

595.30492	0.3463

595.4017	0.34633

595.49848	0.34639

595.59527	0.34642

595.69205	0.34651

595.78883	0.34655

595.88561	0.34658

595.98239	0.34662

596.07918	0.34665

596.17596	0.34667

596.27274	0.34669

596.36952	0.34674

596.4663	0.34676

596.56308	0.34678

596.65987	0.34685

596.75665	0.3469

596.85343	0.34692

596.95021	0.34694

597.04699	0.34697

597.14378	0.34699

597.24056	0.34703

597.33734	0.34706

597.43412	0.34708

597.5309	0.34713

597.62768	0.34715

597.72447	0.34726

597.82125	0.34729

597.91803	0.34731

598.01481	0.34733

598.11159	0.34735

598.20838	0.34738

598.30516	0.34742

598.40194	0.34745

598.49872	0.34747

598.5955	0.34751

598.69228	0.34756

598.78907	0.34761

598.88585	0.34763

598.98263	0.34767

599.07941	0.3477

599.17619	0.34774

599.27297	0.34777

599.36976	0.34779

599.46654	0.34781

599.56332	0.34783

599.6601	0.34793

599.75688	0.34795

599.85367	0.34802

599.95045	0.34806

600.04723	0.34813

600.14401	0.34815

600.24079	0.34818

600.33758	0.3482

600.43436	0.34822

600.53114	0.34825

600.62792	0.34829

600.7247	0.34831

600.82148	0.34843

600.91827	0.34845

601.01505	0.34847

601.11183	0.3485

601.20861	0.34854

601.30539	0.34857

601.40217	0.34861

601.49896	0.34863

601.59574	0.34866

601.69252	0.34868

601.7893	0.3487

601.88608	0.34875

601.98287	0.34877

602.07965	0.34879

602.17643	0.34882

602.27321	0.34884

602.36999	0.34886

602.46677	0.34889

602.56356	0.34893

602.66034	0.34898

602.75712	0.34902

602.8539	0.34905

602.95068	0.34907

603.04747	0.34909

603.14425	0.34914

603.24103	0.34916

603.33781	0.34918

603.43459	0.34921

603.53137	0.34927

603.62816	0.34932

603.72494	0.34934

603.82172	0.34939

603.9185	0.34943

604.01528	0.34948

604.11207	0.34959

604.20885	0.34962

604.30563	0.34966

604.40241	0.34969

604.49919	0.34971

604.59597	0.34985

604.69276	0.34987

604.78954	0.34989

604.88632	0.34991

604.9831	0.34994

605.07988	0.34996

605.17667	0.35003

605.27345	0.35005

605.37023	0.35007

605.46701	0.3501

605.56379	0.35012

605.66057	0.35014

605.75736	0.35017

605.85414	0.35019

605.95092	0.35021

606.0477	0.35023

606.14448	0.35026

606.24127	0.3503

606.33805	0.35055

606.43483	0.35058

606.53161	0.3506

606.62839	0.35062

606.72517	0.35065

606.82196	0.35074

606.91874	0.35076

607.01552	0.35078

607.1123	0.35081

607.20908	0.35085

607.30587	0.3509

607.40265	0.35092

607.49943	0.35094

607.59621	0.35097

607.69299	0.35106

607.78977	0.35117

607.88656	0.35119

607.98334	0.35124

608.08012	0.35129

608.1769	0.35131

608.27368	0.35135

608.37047	0.35138

608.46725	0.35147

608.56403	0.35149

608.66081	0.35158

608.75759	0.35163

608.85437	0.35172

608.95116	0.35174

609.04794	0.35179

609.14472	0.35181

609.2415	0.35183

609.33828	0.35186

609.43507	0.3519

609.53185	0.35193

609.62863	0.35195

609.72541	0.35197

609.82219	0.35199

609.91897	0.35202

610.01576	0.35206

610.11254	0.35211

610.20932	0.35213

610.3061	0.35215

610.40288	0.35218

610.49967	0.35225

610.59645	0.35227

610.69323	0.35231

610.79001	0.35238

610.88679	0.35241

610.98357	0.35243

611.08036	0.35245

611.17714	0.3525

611.27392	0.35252

611.3707	0.35259

611.46748	0.35263

611.56427	0.3527

611.66105	0.35273

611.75783	0.35279

611.85461	0.35291

611.95139	0.35314

612.04817	0.35316

612.14496	0.35318

612.24174	0.35323

612.33852	0.35327

612.4353	0.35339

612.53208	0.35343

612.62887	0.35346

612.72565	0.35348

612.82243	0.35355

612.91921	0.35362

613.01599	0.35364

613.11277	0.35366

613.20956	0.35371

613.30634	0.35373

613.40312	0.35375

613.4999	0.35378

613.59668	0.3538

613.69346	0.35382

613.79025	0.35391

613.88703	0.35396

613.98381	0.35398

614.08059	0.35401

614.17737	0.35403

614.27416	0.35405

614.37094	0.35414

614.46772	0.35417

614.5645	0.35421

614.66128	0.35433

614.75806	0.35435

614.85485	0.35437

614.95163	0.35442

615.04841	0.35444

615.14519	0.35451

615.24197	0.35453

615.33876	0.35456

615.43554	0.3546

615.53232	0.35462

615.6291	0.35469

615.72588	0.35474

615.82266	0.35476

615.91945	0.35478

616.01623	0.35481

616.11301	0.35492

616.20979	0.35497

616.30657	0.35499

616.40336	0.3551

616.50014	0.35513

616.59692	0.35515

616.6937	0.35517

616.79048	0.35522

616.88726	0.35524

616.98405	0.35526

617.08083	0.35531

617.17761	0.35533

617.27439	0.35538

617.37117	0.35542

617.46796	0.35545

617.56474	0.35547

617.66152	0.35549

617.7583	0.35554

617.85508	0.35556

617.95186	0.35563

618.04865	0.3557

618.14543	0.35572

618.24221	0.35574

618.33899	0.35579

618.43577	0.35581

618.53256	0.35586

618.62934	0.35588

618.72612	0.3559

618.8229	0.35593

618.91968	0.35606

619.01646	0.35611

619.11325	0.35618

619.21003	0.35622

619.30681	0.35625

619.40359	0.35627

619.50037	0.35629

619.59716	0.35634

619.69394	0.35636

619.79072	0.35638

619.8875	0.35641

619.98428	0.35643

620.08106	0.35645

620.17785	0.3565

620.27463	0.35654

620.37141	0.35659

620.46819	0.35666

620.56497	0.35677

620.66176	0.3568

620.75854	0.35682

620.85532	0.35684

620.9521	0.35689

621.04888	0.35702

621.14566	0.35705

621.24245	0.35709

621.33923	0.35712

621.43601	0.35716

621.53279	0.35718

621.62957	0.35721

621.72636	0.35725

621.82314	0.35728

621.91992	0.35732

622.0167	0.35734

622.11348	0.35741

622.21026	0.35744

622.30705	0.35746

622.40383	0.35755

622.50061	0.35757

622.59739	0.3576

622.69417	0.35762

622.79096	0.35764

622.88774	0.35773

622.98452	0.35778

623.0813	0.3578

623.17808	0.35782

623.27486	0.35787

623.37165	0.35789

623.46843	0.35798

623.56521	0.35801

623.66199	0.35805

623.75877	0.35814

623.85556	0.35817

623.95234	0.35821

624.04912	0.35824

624.1459	0.35826

624.24268	0.35833

624.33946	0.35835

624.43625	0.35837

624.53303	0.35904

624.62981	0.35906

624.72659	0.35908

624.82337	0.35913

624.92016	0.35915

625.01694	0.3592

625.11372	0.35933

625.2105	0.35938

625.30728	0.35945

625.40406	0.35949

625.50085	0.35961

625.59763	0.35972

625.69441	0.35977

625.79119	0.35986

625.88797	0.3599

625.98475	0.35993

626.08154	0.35995

626.17832	0.35997

626.2751	0.36

626.37188	0.36002

626.46866	0.36006

626.56545	0.36009

626.66223	0.36011

626.75901	0.36013

626.85579	0.36016

626.95257	0.36022

627.04935	0.36025

627.14614	0.36032

627.24292	0.36036

627.3397	0.3605

627.43648	0.36052

627.53326	0.36054

627.63005	0.36057

627.72683	0.36059

627.82361	0.36061

627.92039	0.36064

628.01717	0.36068

628.11395	0.3607

628.21074	0.36075

628.30752	0.36077

628.4043	0.3608

628.50108	0.36082

628.59786	0.36086

628.69465	0.36093

628.79143	0.36096

628.88821	0.36098

628.98499	0.36102

629.08177	0.36107

629.17855	0.36109

629.27534	0.36112

629.37212	0.36114

629.4689	0.36123

629.56568	0.3613

629.66246	0.36137

629.75925	0.36141

629.85603	0.36144

629.95281	0.36146

630.04959	0.36148

630.14637	0.3615

630.24315	0.36153

630.33994	0.36155

630.43672	0.3616

630.5335	0.36169

630.63028	0.36178

630.72706	0.3618

630.82385	0.36182

630.92063	0.36185

631.01741	0.36187

631.11419	0.36189

631.21097	0.36203

631.30775	0.36205

631.40454	0.36212

631.50132	0.36217

631.5981	0.36221

631.69488	0.36226

631.79166	0.3623

631.88845	0.36235

631.98523	0.36237

632.08201	0.3624

632.17879	0.36242

632.27557	0.36244

632.37235	0.36249

632.46914	0.36251

632.56592	0.36256

632.6627	0.36265

632.75948	0.36267

632.85626	0.36278

632.95305	0.36281

633.04983	0.36288

633.14661	0.3629

633.24339	0.36294

633.34017	0.36297

633.43695	0.36299

633.53374	0.36301

633.63052	0.36304

633.7273	0.36308

633.82408	0.3631

633.92086	0.36315

634.01765	0.36322

634.11443	0.36324

634.21121	0.36326

634.30799	0.36329

634.40477	0.36331

634.50155	0.36333

634.59834	0.36336

634.69512	0.36338

634.7919	0.36342

634.88868	0.36345

634.98546	0.36349

635.08225	0.36354

635.17903	0.36361

635.27581	0.36372

635.37259	0.36374

635.46937	0.36377

635.56615	0.36381

635.66294	0.36384

635.75972	0.36386

635.8565	0.3639

635.95328	0.36393

636.05006	0.36395

636.14685	0.36402

636.24363	0.36409

636.34041	0.36411

636.43719	0.36413

636.53397	0.36416

636.63075	0.36418

636.72754	0.3642

636.82432	0.36422

636.9211	0.36427

637.01788	0.36429

637.11466	0.36432

637.21145	0.36434

637.30823	0.36436

637.40501	0.36438

637.50179	0.36445

637.59857	0.3645

637.69535	0.36459

637.79214	0.36468

637.88892	0.3647

637.9857	0.36473

638.08248	0.36475

638.17926	0.36477

638.27604	0.36486

638.37283	0.36493

638.46961	0.36496

638.56639	0.36498

638.66317	0.365

638.75995	0.36502

638.85674	0.36509

638.95352	0.36512

639.0503	0.36514

639.14708	0.36521

639.24386	0.36528

639.34064	0.36532

639.43743	0.36534

639.53421	0.36537

639.63099	0.36539

639.72777	0.36541

639.82455	0.36544

639.92134	0.36546

640.01812	0.3655

640.1149	0.36557

640.21168	0.36562

640.30846	0.36582

640.40524	0.36589

640.50203	0.36592

640.59881	0.36598

640.69559	0.36601

640.79237	0.36603

640.88915	0.36608

640.98594	0.3661

641.08272	0.36612

641.1795	0.36614

641.27628	0.36617

641.37306	0.36619

641.46984	0.36621

641.56663	0.36628

641.66341	0.36633

641.76019	0.36635

641.85697	0.36637

641.95375	0.3664

642.05054	0.36642

642.14732	0.36646

642.2441	0.36656

642.34088	0.3666

642.43766	0.36662

642.53444	0.36665

642.63123	0.36674

642.72801	0.36676

642.82479	0.36678

642.92157	0.36683

643.01835	0.36685

643.11514	0.36688

643.21192	0.3669

643.3087	0.36701

643.40548	0.36706

643.50226	0.3671

643.59904	0.36713

643.69583	0.36715

643.79261	0.36717

643.88939	0.36722

643.98617	0.36733

644.08295	0.36736

644.17974	0.36738

644.27652	0.36742

644.3733	0.36747

644.47008	0.36756

644.56686	0.36758

644.66364	0.36777

644.76043	0.36779

644.85721	0.36781

644.95399	0.36784

645.05077	0.36786

645.14755	0.36788

645.24434	0.36793

645.34112	0.36804

645.4379	0.36806

645.53468	0.36809

645.63146	0.36811

645.72824	0.36813

645.82503	0.36816

645.92181	0.36827

646.01859	0.36832

646.11537	0.36838

646.21215	0.36841

646.30894	0.36843

646.40572	0.36848

646.5025	0.3685

646.59928	0.36852

646.69606	0.36857

646.79284	0.36861

646.88963	0.36864

646.98641	0.3687

647.08319	0.36873

647.17997	0.36875

647.27675	0.3688

647.37354	0.36882

647.47032	0.36886

647.5671	0.36891

647.66388	0.36893

647.76066	0.36896

647.85744	0.36898

647.95423	0.36902

648.05101	0.36909

648.14779	0.36912

648.24457	0.36914

648.34135	0.36916

648.43814	0.36918

648.53492	0.36928

648.6317	0.36932

648.72848	0.36934

648.82526	0.36937

648.92204	0.36939

649.01883	0.36948

649.11561	0.36953

649.21239	0.36962

649.30917	0.36964

649.40595	0.36966

649.50274	0.36971

649.59952	0.36973

649.6963	0.36976

649.79308	0.36978

649.88986	0.3698

649.98664	0.36982

650.08343	0.36985

650.18021	0.36989

650.27699	0.36992

650.37377	0.36994

650.47055	0.37001

650.56733	0.37005

650.66412	0.37008

650.7609	0.37012

650.85768	0.37014

650.95446	0.37017

651.05124	0.37021

651.14803	0.37024

651.24481	0.37028

651.34159	0.3703

651.43837	0.37033

651.53515	0.37035

651.63193	0.37037

651.72872	0.37042

651.8255	0.37044

651.92228	0.37056

652.01906	0.37065

652.11584	0.37067

652.21263	0.37069

652.30941	0.37072

652.40619	0.37076

652.50297	0.37078

652.59975	0.37081

652.69653	0.37083

652.79332	0.3709

652.8901	0.37092

652.98688	0.37094

653.08366	0.37097

653.18044	0.37101

653.27723	0.37106

653.37401	0.37108

653.47079	0.37117

653.56757	0.3712

653.66435	0.37122

653.76113	0.37124

653.85792	0.37129

653.9547	0.37131

654.05148	0.37133

654.14826	0.37136

654.24504	0.37147

654.34183	0.37156

654.43861	0.37158

654.53539	0.37161

654.63217	0.3717

654.72895	0.37174

654.82573	0.37177

654.92252	0.37186

655.0193	0.37188

655.11608	0.3719

655.21286	0.37193

655.30964	0.37195

655.40643	0.37197

655.50321	0.37202

655.59999	0.37204

655.69677	0.37206

655.79355	0.37211

655.89033	0.37213

655.98712	0.37218

656.0839	0.37222

656.18068	0.37225

656.27746	0.37234

656.37424	0.37236

656.47103	0.37238

656.56781	0.37241

656.66459	0.37243

656.76137	0.37245

656.85815	0.37248

656.95493	0.3725

657.05172	0.37254

657.1485	0.37257

657.24528	0.37259

657.34206	0.37261

657.43884	0.37264

657.53563	0.37266

657.63241	0.3727

657.72919	0.37284

657.82597	0.37296

657.92275	0.37298

658.01953	0.37302

658.11632	0.37307

658.2131	0.37309

658.30988	0.37316

658.40666	0.37318

658.50344	0.37321

658.60023	0.37323

658.69701	0.37328

658.79379	0.37334

658.89057	0.37339

658.98735	0.37346

659.08413	0.37348

659.18092	0.3735

659.2777	0.37353

659.37448	0.37355

659.47126	0.37357

659.56804	0.3736

659.66483	0.37366

659.76161	0.37373

659.85839	0.37378

659.95517	0.3738

660.05195	0.37392

660.14873	0.37396

660.24552	0.37398

660.3423	0.37403

660.43908	0.3741

660.53586	0.37412

660.63264	0.37414

660.72943	0.37417

660.82621	0.37419

660.92299	0.37421

661.01977	0.37424

661.11655	0.37426

661.21333	0.37428

661.31012	0.3743

661.4069	0.37435

661.50368	0.37437

661.60046	0.3744

661.69724	0.37446

661.79403	0.37449

661.89081	0.37451

661.98759	0.37453

662.08437	0.37456

662.18115	0.37458

662.27793	0.3746

662.37472	0.37462

662.4715	0.37465

662.56828	0.37467

662.66506	0.37469

662.76184	0.37472

662.85862	0.37474

662.95541	0.37478

663.05219	0.37481

663.14897	0.37483

663.24575	0.37485

663.34253	0.37492

663.43932	0.37494

663.5361	0.37501

663.63288	0.37508

663.72966	0.3751

663.82644	0.37513

663.92322	0.37515

664.02001	0.37517

664.11679	0.37524

664.21357	0.37526

664.31035	0.37529

664.40713	0.37533

664.50392	0.37538

664.6007	0.3754

664.69748	0.37542

664.79426	0.37545

664.89104	0.37552

664.98782	0.37554

665.08461	0.37556

665.18139	0.37558

665.27817	0.37572

665.37495	0.37574

665.47173	0.37577

665.56852	0.37579

665.6653	0.37581

665.76208	0.37584

665.85886	0.37588

665.95564	0.37597

666.05242	0.376

666.14921	0.37602

666.24599	0.37604

666.34277	0.37606

666.43955	0.37609

666.53633	0.37611

666.63312	0.37618

666.7299	0.3762

666.82668	0.37625

666.92346	0.37627

667.02024	0.37636

667.11702	0.37641

667.21381	0.37643

667.31059	0.37645

667.40737	0.37648

667.50415	0.37652

667.60093	0.37661

667.69772	0.37675

667.7945	0.37682

667.89128	0.37689

667.98806	0.37691

668.08484	0.37693

668.18162	0.37698

668.27841	0.37702

668.37519	0.37705

668.47197	0.37707

668.56875	0.37709

668.66553	0.37714

668.76232	0.37716

668.8591	0.37723

668.95588	0.37725

669.05266	0.37728

669.14944	0.37737

669.24622	0.37739

669.34301	0.37744

669.43979	0.37746

669.53657	0.37748

669.63335	0.37753

669.73013	0.37755

669.82692	0.37757

669.9237	0.3776

670.02048	0.37764

670.11726	0.37766

670.21404	0.37769

670.31082	0.37773

670.40761	0.37776

670.50439	0.37778

670.60117	0.3778

670.69795	0.37782

670.79473	0.37787

670.89152	0.37789

670.9883	0.37796

671.08508	0.37798

671.18186	0.37808

671.27864	0.3781

671.37542	0.37812

671.47221	0.37814

671.56899	0.37817

671.66577	0.37819

671.76255	0.37821

671.85933	0.37824

671.95612	0.37826

672.0529	0.3783

672.14968	0.37833

672.24646	0.37835

672.34324	0.37842

672.44002	0.37844

672.53681	0.37846

672.63359	0.37849

672.73037	0.37851

672.82715	0.37853

672.92393	0.37856

673.02072	0.37858

673.1175	0.37862

673.21428	0.37865

673.31106	0.37872

673.40784	0.37883

673.50462	0.37897

673.60141	0.37901

673.69819	0.37904

673.79497	0.3791

673.89175	0.37913

673.98853	0.37929

674.08532	0.37931

674.1821	0.37933

674.27888	0.37942

674.37566	0.37949

674.47244	0.37958

674.56922	0.37963

674.66601	0.37968

674.76279	0.37972

674.85957	0.37974

674.95635	0.37977

675.05313	0.37986

675.14991	0.3799

675.2467	0.37993

675.34348	0.37995

675.44026	0.37997

675.53704	0.38002

675.63382	0.38004

675.73061	0.38006

675.82739	0.38016

675.92417	0.38032

676.02095	0.38034

676.11773	0.38036

676.21451	0.38045

676.3113	0.38052

676.40808	0.38054

676.50486	0.38057

676.60164	0.38061

676.69842	0.38064

676.79521	0.38066

676.89199	0.38068

676.98877	0.38073

677.08555	0.38077

677.18233	0.3808

677.27911	0.38082

677.3759	0.38086

677.47268	0.38089

677.56946	0.38098

677.66624	0.38105

677.76302	0.38107

677.85981	0.38112

677.95659	0.38114

678.05337	0.38116

678.15015	0.38118

678.24693	0.38121

678.34371	0.38123

678.4405	0.38125

678.53728	0.38134

678.63406	0.38141

678.73084	0.38144

678.82762	0.38146

678.92441	0.3815

679.02119	0.38153

679.11797	0.38155

679.21475	0.3816

679.31153	0.38162

679.40831	0.38164

679.5051	0.38166

679.60188	0.38182

679.69866	0.38185

679.79544	0.38187

679.89222	0.38189

679.98901	0.38192

680.08579	0.38198

680.18257	0.38203

680.27935	0.38205

680.37613	0.38208

680.47291	0.3821

680.5697	0.38214

680.66648	0.38219

680.76326	0.38221

680.86004	0.38226

680.95682	0.38228

681.05361	0.3823

681.15039	0.38235

681.24717	0.38246

681.34395	0.38249

681.44073	0.38251

681.53751	0.38262

681.6343	0.38265

681.73108	0.38281

681.82786	0.38283

681.92464	0.38285

682.02142	0.38288

682.11821	0.3829

682.21499	0.38294

682.31177	0.38301

682.40855	0.38304

682.50533	0.38306

682.60211	0.38308

682.6989	0.3831

682.79568	0.38313

682.89246	0.38315

682.98924	0.38317

683.08602	0.38347

683.18281	0.38352

683.27959	0.38354

683.37637	0.38358

683.47315	0.38363

683.56993	0.38365

683.66671	0.38368

683.7635	0.38372

683.86028	0.38374

683.95706	0.38379

684.05384	0.38381

684.15062	0.38384

684.24741	0.38388

684.34419	0.38393

684.44097	0.38395

684.53775	0.38397

684.63453	0.384

684.73131	0.38416

684.8281	0.3842

684.92488	0.38422

685.02166	0.38434

685.11844	0.38436

685.21522	0.38443

685.31201	0.38448

685.40879	0.3845

685.50557	0.38459

685.60235	0.38461

685.69913	0.38464

685.79591	0.38473

685.8927	0.38475

685.98948	0.38477

686.08626	0.3848

686.18304	0.38482

686.27982	0.38486

686.37661	0.38489

686.47339	0.38491

686.57017	0.38496

686.66695	0.38498

686.76373	0.38509

686.86051	0.38512

686.9573	0.38516

687.05408	0.38518

687.15086	0.38521

687.24764	0.38532

687.34442	0.38534

687.4412	0.38537

687.53799	0.38539

687.63477	0.38541

687.73155	0.38544

687.82833	0.38546

687.92511	0.38548

688.0219	0.38553

688.11868	0.38557

688.21546	0.3856

688.31224	0.38562

688.40902	0.38564

688.50581	0.38566

688.60259	0.38571

688.69937	0.38573

688.79615	0.3858

688.89293	0.38582

688.98971	0.38585

689.0865	0.38587

689.18328	0.38589

689.28006	0.38592

689.37684	0.38594

689.47362	0.38596

689.5704	0.38603

689.66719	0.38626

689.76397	0.38628

689.86075	0.3863

689.95753	0.38633

690.05431	0.38635

690.1511	0.38637

690.24788	0.3864

690.34466	0.38642

690.44144	0.38644

690.53822	0.38646

690.635	0.38649

690.73179	0.38653

690.82857	0.38656

690.92535	0.38658

691.02213	0.3866

691.11891	0.38662

691.2157	0.38672

691.31248	0.38681

691.40926	0.38683

691.50604	0.38685

691.60282	0.38688

691.6996	0.3869

691.79639	0.38692

691.89317	0.38694

691.98995	0.38697

692.08673	0.38699

692.18351	0.38704

692.2803	0.38708

692.37708	0.38713

692.47386	0.38715

692.57064	0.38717

692.66742	0.38724

692.7642	0.38726

692.86099	0.38729

692.95777	0.38733

693.05455	0.38738

693.15133	0.38742

693.24811	0.38749

693.3449	0.38752

693.44168	0.38756

693.53846	0.38761

693.63524	0.38763

693.73202	0.38765

693.8288	0.3877

693.92559	0.38772

694.02237	0.38775

694.11915	0.38779

694.21593	0.38781

694.31271	0.38784

694.4095	0.38788

694.50628	0.38791

694.60306	0.38793

694.69984	0.38795

694.79662	0.38797

694.8934	0.388

694.99019	0.38802

695.08697	0.38807

695.18375	0.3882

695.28053	0.38825

695.37731	0.38827

695.4741	0.38829

695.57088	0.38836

695.66766	0.38843

695.76444	0.3885

695.86122	0.38852

695.958	0.38857

696.05479	0.38859

696.15157	0.38861

696.24835	0.38864

696.34513	0.38866

696.44191	0.38868

696.5387	0.38871

696.63548	0.38877

696.73226	0.3888

696.82904	0.38882

696.92582	0.38893

697.0226	0.389

697.11939	0.38903

697.21617	0.38912

697.31295	0.38914

697.40973	0.38916

697.50651	0.38919

697.6033	0.38925

697.70008	0.38928

697.79686	0.3893

697.89364	0.38935

697.99042	0.38937

698.0872	0.38939

698.18399	0.38941

698.28077	0.38946

698.37755	0.38951

698.47433	0.38955

698.57111	0.38957

698.6679	0.3896

698.76468	0.38964

698.86146	0.38967

698.95824	0.38971

699.05502	0.38973

699.1518	0.38976

699.24859	0.38978

699.34537	0.3898

699.44215	0.38983

699.53893	0.38985

699.63571	0.38989

699.7325	0.38994

699.82928	0.38996

699.92606	0.38999

700.02284	0.39001

700.11962	0.39003

700.2164	0.39008

700.31319	0.3901

700.40997	0.39012

700.50675	0.39017

700.60353	0.39024

700.70031	0.39026

700.7971	0.39028

700.89388	0.39031

700.99066	0.39042

701.08744	0.39044

701.18422	0.39047

701.281	0.39049

701.37779	0.39053

701.47457	0.39056

701.57135	0.39058

701.66813	0.3906

701.76491	0.39065

701.86169	0.39069

701.95848	0.39072

702.05526	0.39076

702.15204	0.39081

702.24882	0.39083

702.3456	0.3909

702.44239	0.39095

702.53917	0.39097

702.63595	0.39101

702.73273	0.39104

702.82951	0.39106

702.92629	0.39108

703.02308	0.39111

703.11986	0.39113

703.21664	0.3912

703.31342	0.39124

703.4102	0.39127

703.50699	0.39133

703.60377	0.39136

703.70055	0.39138

703.79733	0.39143

703.89411	0.39152

703.99089	0.39156

704.08768	0.39163

704.18446	0.39165

704.28124	0.39168

704.37802	0.3917

704.4748	0.39172

704.57159	0.39175

704.66837	0.39179

704.76515	0.39181

704.86193	0.39184

704.95871	0.39186

705.05549	0.39188

705.15228	0.39191

705.24906	0.39193

705.34584	0.39195

705.44262	0.39197

705.5394	0.392

705.63619	0.39202

705.73297	0.39204

705.82975	0.39207

705.92653	0.39209

706.02331	0.39211

706.12009	0.3922

706.21688	0.39223

706.31366	0.39225

706.41044	0.39229

706.50722	0.39232

706.604	0.39234

706.70079	0.39239

706.79757	0.39259

706.89435	0.39266

706.99113	0.39287

707.08791	0.39289

707.18469	0.39293

707.28148	0.39296

707.37826	0.393

707.47504	0.39305

707.57182	0.39307

707.6686	0.39316

707.76539	0.39319

707.86217	0.39321

707.95895	0.39323

708.05573	0.39325

708.15251	0.39337

708.24929	0.39339

708.34608	0.39346

708.44286	0.39348

708.53964	0.39351

708.63642	0.39353

708.7332	0.39355

708.82999	0.3936

708.92677	0.39362

709.02355	0.39364

709.12033	0.39369

709.21711	0.39371

709.31389	0.39373

709.41068	0.39378

709.50746	0.39383

709.60424	0.39385

709.70102	0.39387

709.7978	0.39389

709.89459	0.39399

709.99137	0.39405

710.08815	0.39408

710.18493	0.3941

710.28171	0.39412

710.37849	0.39415

710.47528	0.39417

710.57206	0.39421

710.66884	0.39424

710.76562	0.39431

710.8624	0.39433

710.95919	0.39435

711.05597	0.39437

711.15275	0.39442

711.24953	0.39444

711.34631	0.39453

711.44309	0.39456

711.53988	0.39458

711.63666	0.3946

711.73344	0.39467

711.83022	0.39469

711.927	0.39474

712.02379	0.39476

712.12057	0.39481

712.21735	0.39483

712.31413	0.39485

712.41091	0.39488

712.50769	0.3949

712.60448	0.39492

712.70126	0.39499

712.79804	0.39504

712.89482	0.39506

712.9916	0.39513

713.08839	0.39515

713.18517	0.39517

713.28195	0.3952

713.37873	0.39527

713.47551	0.39529

713.57229	0.39533

713.66908	0.3954

713.76586	0.39543

713.86264	0.39547

713.95942	0.39549

714.0562	0.39552

714.15298	0.39556

714.24977	0.39559

714.34655	0.3957

714.44333	0.39572

714.54011	0.39577

714.63689	0.39579

714.73368	0.39581

714.83046	0.39584

714.92724	0.39586

715.02402	0.39588

715.1208	0.39602

715.21758	0.39604

715.31437	0.39607

715.41115	0.39613

715.50793	0.3962

715.60471	0.39623

715.70149	0.39625

715.79828	0.39627

715.89506	0.39629

715.99184	0.39632

716.08862	0.39634

716.1854	0.39636

716.28218	0.39639

716.37897	0.39641

716.47575	0.39645

716.57253	0.39652

716.66931	0.39657

716.76609	0.39659

716.86288	0.39661

716.95966	0.39668

717.05644	0.39671

717.15322	0.39673

717.25	0.39675

717.34678	0.3968

717.44357	0.39682

717.54035	0.39684

717.63713	0.39689

717.73391	0.39693

717.83069	0.39703

717.92748	0.39705

718.02426	0.39719

718.12104	0.39721

718.21782	0.39723

718.3146	0.39725

718.41138	0.39728

718.50817	0.39735

718.60495	0.39739

718.70173	0.39741

718.79851	0.39748

718.89529	0.39751

718.99208	0.39755

719.08886	0.39757

719.18564	0.3976

719.28242	0.39762

719.3792	0.39764

719.47598	0.39767

719.57277	0.39769

719.66955	0.39773

719.76633	0.39776

719.86311	0.39778

719.95989	0.3978

720.05668	0.39783

720.15346	0.39785

720.25024	0.39787

720.34702	0.39789

720.4438	0.39792

720.54058	0.39794

720.63737	0.39796

720.73415	0.39803

720.83093	0.39805

720.92771	0.3981

721.02449	0.39815

721.12128	0.39819

721.21806	0.39821

721.31484	0.39824

721.41162	0.39826

721.5084	0.39831

721.60518	0.39835

721.70197	0.39844

721.79875	0.39847

721.89553	0.39851

721.99231	0.39853

722.08909	0.39856

722.18588	0.39858

722.28266	0.39863

722.37944	0.39865

722.47622	0.39867

722.573	0.39872

722.66978	0.39874

722.76657	0.39876

722.86335	0.39879

722.96013	0.39881

723.05691	0.39883

723.15369	0.39888

723.25048	0.3989

723.34726	0.39895

723.44404	0.39897

723.54082	0.39899

723.6376	0.39901

723.73438	0.39904

723.83117	0.39911

723.92795	0.39913

724.02473	0.39915

724.12151	0.39922

724.21829	0.39924

724.31508	0.39927

724.41186	0.39929

724.50864	0.39931

724.60542	0.39936

724.7022	0.39938

724.79898	0.39945

724.89577	0.39947

724.99255	0.39949

725.08933	0.39952

725.18611	0.39956

725.28289	0.39959

725.37968	0.39961

725.47646	0.39963

725.57324	0.39965

725.67002	0.39968

725.7668	0.3997

725.86358	0.39975

725.96037	0.39977

726.05715	0.39981

726.15393	0.39984

726.25071	0.39988

726.34749	0.39993

726.44427	0.39997

726.54106	0.4

726.63784	0.40002

726.73462	0.40004

726.8314	0.40009

726.92818	0.40013

727.02497	0.40016

727.12175	0.40018

727.21853	0.4002

727.31531	0.40023

727.41209	0.40025

727.50887	0.40027

727.60566	0.40029

727.70244	0.40032

727.79922	0.40034

727.896	0.40039

727.99278	0.40041

728.08957	0.40043

728.18635	0.40045

728.28313	0.40048

728.37991	0.4005

728.47669	0.40052

728.57347	0.40055

728.67026	0.40057

728.76704	0.40059

728.86382	0.40061

728.9606	0.40064

729.05738	0.40066

729.15417	0.40068

729.25095	0.40071

729.34773	0.40075

729.44451	0.40077

729.54129	0.40089

729.63807	0.40091

729.73486	0.40096

729.83164	0.40098

729.92842	0.40103

730.0252	0.40105

730.12198	0.40107

730.21877	0.40109

730.31555	0.40112

730.41233	0.40114

730.50911	0.40119

730.60589	0.40121

730.70267	0.40123

730.79946	0.40125

730.89624	0.4013

730.99302	0.40132

731.0898	0.40137

731.18658	0.40139

731.28337	0.40141

731.38015	0.40148

731.47693	0.40151

731.57371	0.4016

731.67049	0.40169

731.76727	0.40171

731.86406	0.40176

731.96084	0.40178

732.05762	0.40187

732.1544	0.40189

732.25118	0.40192

732.34797	0.40194

732.44475	0.40196

732.54153	0.40199

732.63831	0.40203

732.73509	0.40208

732.83187	0.4021

732.92866	0.40212

733.02544	0.40215

733.12222	0.40217

733.219	0.40219

733.31578	0.40224

733.41257	0.40226

733.50935	0.40228

733.60613	0.40233

733.70291	0.40235

733.79969	0.40237

733.89647	0.4024

733.99326	0.40244

734.09004	0.40249

734.18682	0.40253

734.2836	0.40256

734.38038	0.40265

734.47717	0.40267

734.57395	0.40269

734.67073	0.40272

734.76751	0.40279

734.86429	0.40281

734.96107	0.40285

735.05786	0.40288

735.15464	0.4029

735.25142	0.40292

735.3482	0.40295

735.44498	0.40297

735.54177	0.40301

735.63855	0.40304

735.73533	0.40306

735.83211	0.40313

735.92889	0.40317

736.02567	0.4032

736.12246	0.40324

736.21924	0.40327

736.31602	0.40329

736.4128	0.40333

736.50958	0.40336

736.60637	0.40338

736.70315	0.40343

736.79993	0.40345

736.89671	0.40349

736.99349	0.40356

737.09027	0.40361

737.18706	0.4037

737.28384	0.40375

737.38062	0.40379

737.4774	0.40381

737.57418	0.40391

737.67097	0.40395

737.76775	0.40402

737.86453	0.40404

737.96131	0.40407

738.05809	0.40409

738.15487	0.40411

738.25166	0.40413

738.34844	0.40416

738.44522	0.40418

738.542	0.40423

738.63878	0.40427

738.73556	0.40432

738.83235	0.40434

738.92913	0.40436

739.02591	0.40439

739.12269	0.40441

739.21947	0.40443

739.31626	0.40445

739.41304	0.40448

739.50982	0.40452

739.6066	0.40459

739.70338	0.40461

739.80016	0.40468

739.89695	0.40471

739.99373	0.40473

740.09051	0.40475

740.18729	0.40477

740.28407	0.40482

740.38086	0.40487

740.47764	0.40493

740.57442	0.40496

740.6712	0.40498

740.76798	0.40505

740.86476	0.40514

740.96155	0.40516

741.05833	0.40519

741.15511	0.40521

741.25189	0.40525

741.34867	0.40528

741.44546	0.40532

741.54224	0.40537

741.63902	0.40541

741.7358	0.40544

741.83258	0.40546

741.92936	0.40548

742.02615	0.40551

742.12293	0.40553

742.21971	0.40557

742.31649	0.4056

742.41327	0.40562

742.51006	0.40567

742.60684	0.40571

742.70362	0.40576

742.8004	0.40583

742.89718	0.40585

742.99396	0.40587

743.09075	0.40589

743.18753	0.40592

743.28431	0.40594

743.38109	0.40596

743.47787	0.40599

743.57466	0.40603

743.67144	0.40608

743.76822	0.4061

743.865	0.40621

743.96178	0.40624

744.05856	0.40626

744.15535	0.40633

744.25213	0.40635

744.34891	0.40637

744.44569	0.4064

744.54247	0.40642

744.63926	0.40644

744.73604	0.40647

744.83282	0.40651

744.9296	0.40653

745.02638	0.40656

745.12316	0.4066

745.21995	0.40663

745.31673	0.40667

745.41351	0.40669

745.51029	0.40674

745.60707	0.40679

745.70386	0.40685

745.80064	0.4069

745.89742	0.40692

745.9942	0.40695

746.09098	0.40699

746.18776	0.40701

746.28455	0.40713

746.38133	0.40715

746.47811	0.40717

746.57489	0.4072

746.67167	0.40722

746.76846	0.40729

746.86524	0.40731

746.96202	0.4074

747.0588	0.40743

747.15558	0.40747

747.25236	0.40749

747.34915	0.40754

747.44593	0.40765

747.54271	0.40768

747.63949	0.4077

747.73627	0.40772

747.83306	0.40775

747.92984	0.40777

748.02662	0.40779

748.1234	0.40781

748.22018	0.40784

748.31696	0.40786

748.41375	0.40788

748.51053	0.40791

748.60731	0.40793

748.70409	0.40795

748.80087	0.40802

748.89766	0.40804

748.99444	0.40807

749.09122	0.40809

749.188	0.40811

749.28478	0.40818

749.38156	0.4082

749.47835	0.40823

749.57513	0.40825

749.67191	0.40827

749.76869	0.40829

749.86547	0.40832

749.96226	0.40834

750.05904	0.40836

750.15582	0.40839

750.2526	0.40841

750.34938	0.40843

750.44616	0.40845

750.54295	0.4085

750.63973	0.40852

750.73651	0.40855

750.83329	0.40859

750.93007	0.40861

751.02685	0.40868

751.12364	0.40873

751.22042	0.40875

751.3172	0.40877

751.41398	0.4088

751.51076	0.40882

751.60755	0.40889

751.70433	0.40891

751.80111	0.40893

751.89789	0.40896

751.99467	0.40898

752.09145	0.40905

752.18824	0.40909

752.28502	0.40914

752.3818	0.40916

752.47858	0.40919

752.57536	0.40921

752.67215	0.40932

752.76893	0.40935

752.86571	0.40937

752.96249	0.40939

753.05927	0.40941

753.15605	0.40944

753.25284	0.40946

753.34962	0.40953

753.4464	0.40955

753.54318	0.40957

753.63996	0.4096

753.73675	0.40964

753.83353	0.40967

753.93031	0.40971

754.02709	0.40973

754.12387	0.40978

754.22065	0.4098

754.31744	0.40985

754.41422	0.40989

754.511	0.40992

754.60778	0.40994

754.70456	0.41001

754.80135	0.41003

754.89813	0.41005

754.99491	0.41008

755.09169	0.4101

755.18847	0.41012

755.28525	0.41015

755.38204	0.41017

755.47882	0.41019

755.5756	0.41024

755.67238	0.41028

755.76916	0.41033

755.86595	0.41035

755.96273	0.41037

756.05951	0.41044

756.15629	0.41047

756.25307	0.41049

756.34985	0.41051

756.44664	0.41053

756.54342	0.41056

756.6402	0.41058

756.73698	0.4106

756.83376	0.41063

756.93055	0.41065

757.02733	0.41067

757.12411	0.41069

757.22089	0.41074

757.31767	0.41079

757.41445	0.41081

757.51124	0.41085

757.60802	0.41088

757.7048	0.41092

757.80158	0.41095

757.89836	0.41099

757.99515	0.41101

758.09193	0.41104

758.18871	0.41106

758.28549	0.41108

758.38227	0.41111

758.47905	0.41113

758.57584	0.41117

758.67262	0.4112

758.7694	0.41124

758.86618	0.41127

758.96296	0.41129

759.05975	0.41131

759.15653	0.41138

759.25331	0.4114

759.35009	0.41147

759.44687	0.41149

759.54365	0.41154

759.64044	0.41156

759.73722	0.41159

759.834	0.41161

759.93078	0.41163

760.02756	0.41165

760.12435	0.41168

760.22113	0.41172

760.31791	0.41177

760.41469	0.41179

760.51147	0.41181

760.60825	0.41184

760.70504	0.41186

760.80182	0.41188

760.8986	0.41191

760.99538	0.41193

761.09216	0.41195

761.18895	0.41202

761.28573	0.41204

761.38251	0.41213

761.47929	0.41216

761.57607	0.41218

761.67285	0.4122

761.76964	0.41223

761.86642	0.41225

761.9632	0.41229

762.05998	0.41232

762.15676	0.41234

762.25355	0.41239

762.35033	0.41241

762.44711	0.41248

762.54389	0.4125

762.64067	0.41252

762.73745	0.41255

762.83424	0.41257

762.93102	0.41259

763.0278	0.41261

763.12458	0.41264

763.22136	0.41266

763.31814	0.41268

763.41493	0.41271

763.51171	0.41273

763.60849	0.41275

763.70527	0.41277

763.80205	0.4128

763.89884	0.41291

763.99562	0.41293

764.0924	0.41296

764.18918	0.413

764.28596	0.41303

764.38275	0.41307

764.47953	0.41309

764.57631	0.41312

764.67309	0.41314

764.76987	0.41316

764.86665	0.41319

764.96344	0.41328

765.06022	0.4133

765.157	0.41332

765.25378	0.41335

765.35056	0.41339

765.44734	0.41344

765.54413	0.41346

765.64091	0.41348

765.73769	0.41351

765.83447	0.41355

765.93125	0.41357

766.02804	0.4136

766.12482	0.41362

766.2216	0.41364

766.31838	0.41373

766.41516	0.41376

766.51194	0.41378

766.60873	0.4138

766.70551	0.41383

766.80229	0.41385

766.89907	0.41387

766.99585	0.41389

767.09264	0.41399

767.18942	0.41403

767.2862	0.41405

767.38298	0.4141

767.47976	0.41412

767.57654	0.41417

767.67333	0.41421

767.77011	0.41428

767.86689	0.41431

767.96367	0.41433

768.06045	0.41444

768.15724	0.41447

768.25402	0.41449

768.3508	0.41451

768.44758	0.41453

768.54436	0.41458

768.64114	0.41463

768.73793	0.41465

768.83471	0.41467

768.93149	0.41472

769.02827	0.41481

769.12505	0.41485

769.22184	0.4149

769.31862	0.41492

769.4154	0.41497

769.51218	0.41499

769.60896	0.41501

769.70574	0.41506

769.80253	0.41508

769.89931	0.41511

769.99609	0.41515

770.09287	0.41517

770.18965	0.41522

770.28644	0.41524

770.38322	0.41527

770.48	0.41529

770.57678	0.41538

770.67356	0.4154

770.77034	0.41543

770.86713	0.41545

770.96391	0.41549

771.06069	0.41552

771.15747	0.41554

771.25425	0.41556

771.35104	0.41565

771.44782	0.4157

771.5446	0.41572

771.64138	0.41575

771.73816	0.41577

771.83494	0.41579

771.93173	0.41584

772.02851	0.41586

772.12529	0.41591

772.22207	0.41593

772.31885	0.416

772.41564	0.41602

772.51242	0.41604

772.6092	0.41607

772.70598	0.41611

772.80276	0.4162

772.89954	0.41625

772.99633	0.41629

773.09311	0.41632

773.18989	0.41639

773.28667	0.41641

773.38345	0.41643

773.48024	0.41645

773.57702	0.41648

773.6738	0.4165

773.77058	0.41655

773.86736	0.41659

773.96414	0.41661

774.06093	0.41666

774.15771	0.41675

774.25449	0.41677

774.35127	0.41682

774.44805	0.41684

774.54484	0.41687

774.64162	0.41689

774.7384	0.41691

774.83518	0.41693

774.93196	0.41707

775.02874	0.41709

775.12553	0.41712

775.22231	0.41719

775.31909	0.41721

775.41587	0.41723

775.51265	0.41725

775.60944	0.4173

775.70622	0.41732

775.803	0.41735

775.89978	0.41737

775.99656	0.41739

776.09334	0.41744

776.19013	0.41746

776.28691	0.41748

776.38369	0.41755

776.48047	0.41757

776.57725	0.41764

776.67404	0.41767

776.77082	0.41769

776.8676	0.41771

776.96438	0.41776

777.06116	0.41778

777.15794	0.4178

777.25473	0.41785

777.35151	0.41787

777.44829	0.41789

777.54507	0.41794

777.64185	0.41796

777.73863	0.41799

777.83542	0.41801

777.9322	0.41803

778.02898	0.41805

778.12576	0.41808

778.22254	0.4181

778.31933	0.41812

778.41611	0.41815

778.51289	0.41817

778.60967	0.41819

778.70645	0.41821

778.80323	0.41824

778.90002	0.41826

778.9968	0.41835

779.09358	0.41837

779.19036	0.41842

779.28714	0.41844

779.38393	0.41847

779.48071	0.41853

779.57749	0.41858

779.67427	0.4186

779.77105	0.41865

779.86783	0.41867

779.96462	0.41872

780.0614	0.41874

780.15818	0.41876

780.25496	0.41879

780.35174	0.41881

780.44853	0.41883

780.54531	0.41885

780.64209	0.41888

780.73887	0.41892

780.83565	0.41895

780.93243	0.41897

781.02922	0.41899

781.126	0.41906

781.22278	0.41908

781.31956	0.41911

781.41634	0.41915

781.51313	0.4192

781.60991	0.41924

781.70669	0.41927

781.80347	0.41931

781.90025	0.41936

781.99703	0.41938

782.09382	0.4194

782.1906	0.41947

782.28738	0.41949

782.38416	0.41959

782.48094	0.41961

782.57773	0.41963

782.67451	0.41965

782.77129	0.4197

782.86807	0.41972

782.96485	0.41977

783.06163	0.41979

783.15842	0.41981

783.2552	0.41984

783.35198	0.41986

783.44876	0.41988

783.54554	0.41991

783.64233	0.41993

783.73911	0.41997

783.83589	0.42

783.93267	0.42007

784.02945	0.42009

784.12623	0.42013

784.22302	0.42016

784.3198	0.4202

784.41658	0.42023

784.51336	0.42025

784.61014	0.42027

784.70693	0.42029

784.80371	0.42032

784.90049	0.42036

784.99727	0.42039

785.09405	0.42041

785.19083	0.42043

785.28762	0.42045

785.3844	0.42048

785.48118	0.4205

785.57796	0.42052

785.67474	0.42055

785.77153	0.42057

785.86831	0.42059

785.96509	0.42061

786.06187	0.42066

786.15865	0.42068

786.25543	0.42071

786.35222	0.42075

786.449	0.42078

786.54578	0.4208

786.64256	0.42084

786.73934	0.42094

786.83613	0.42098

786.93291	0.42103

787.02969	0.42105

787.12647	0.42107

787.22325	0.4211

787.32003	0.42132

787.41682	0.42135

787.5136	0.42142

787.61038	0.42144

787.70716	0.42146

787.80394	0.42148

787.90073	0.42153

787.99751	0.42155

788.09429	0.4216

788.19107	0.42167

788.28785	0.42169

788.38463	0.42174

788.48142	0.42176

788.5782	0.42178

788.67498	0.4218

788.77176	0.4219

788.86854	0.42192

788.96533	0.42199

789.06211	0.42201

789.15889	0.42203

789.25567	0.4221

789.35245	0.42212

789.44923	0.42219

789.54602	0.42222

789.6428	0.42224

789.73958	0.42226

789.83636	0.42228

789.93314	0.42233

790.02992	0.42235

790.12671	0.42238

790.22349	0.42242

790.32027	0.42244

790.41705	0.42251

790.51383	0.42254

790.61062	0.42256

790.7074	0.42258

790.80418	0.4226

790.90096	0.42267

790.99774	0.42272

791.09452	0.42276

791.19131	0.42279

791.28809	0.42281

791.38487	0.42283

791.48165	0.4229

791.57843	0.42292

791.67522	0.42295

791.772	0.42302

791.86878	0.42304

791.96556	0.42306

792.06234	0.42308

792.15912	0.42311

792.25591	0.42313

792.35269	0.42315

792.44947	0.42318

792.54625	0.4232

792.64303	0.42322

792.73982	0.42324

792.8366	0.42329

792.93338	0.42331

793.03016	0.42334

793.12694	0.42336

793.22372	0.4234

793.32051	0.42345

793.41729	0.42347

793.51407	0.42352

793.61085	0.42354

793.70763	0.42366

793.80442	0.42368

793.9012	0.4237

793.99798	0.42372

794.09476	0.42379

794.19154	0.42382

794.28832	0.42386

794.38511	0.42388

794.48189	0.42391

794.57867	0.42393

794.67545	0.42395

794.77223	0.42398

794.86902	0.424

794.9658	0.42404

795.06258	0.42409

795.15936	0.42411

795.25614	0.42418

795.35292	0.42423

795.44971	0.42425

795.54649	0.42427

795.64327	0.4243

795.74005	0.42434

795.83683	0.42436

795.93362	0.42439

796.0304	0.42443

796.12718	0.42446

796.22396	0.42457

796.32074	0.42462

796.41752	0.42471

796.51431	0.42478

796.61109	0.4248

796.70787	0.42482

796.80465	0.42484

796.90143	0.42487

796.99822	0.42489

797.095	0.42496

797.19178	0.425

797.28856	0.42503

797.38534	0.42505

797.48212	0.4251

797.57891	0.42514

797.67569	0.42516

797.77247	0.42519

797.86925	0.42521

797.96603	0.42526

798.06282	0.42528

798.1596	0.4253

798.25638	0.42532

798.35316	0.42535

798.44994	0.42537

798.54672	0.42542

798.64351	0.42544

798.74029	0.42546

798.83707	0.42548

798.93385	0.42553

799.03063	0.42558

799.12742	0.4256

799.2242	0.42564

799.32098	0.42571

799.41776	0.42576

799.51454	0.4258

799.61132	0.42585

799.70811	0.42587

799.80489	0.4259

799.90167	0.42592

799.99845	0.42594

800.09523	0.42596

800.19202	0.42599

800.2888	0.42601

800.38558	0.42603

800.48236	0.42606

800.57914	0.42612

800.67592	0.42615

800.77271	0.42617

800.86949	0.42619

800.96627	0.42622

801.06305	0.42624

801.15983	0.42626

801.25662	0.42628

801.3534	0.42631

801.45018	0.42635

801.54696	0.42638

801.64374	0.4264

801.74052	0.42642

801.83731	0.42644

801.93409	0.42647

802.03087	0.42649

802.12765	0.42654

802.22443	0.42658

802.32122	0.4266

802.418	0.42665

802.51478	0.42667

802.61156	0.42676

802.70834	0.42679

802.80512	0.42681

802.90191	0.42683

802.99869	0.42686

803.09547	0.42688

803.19225	0.42699

803.28903	0.42702

803.38581	0.42713

803.4826	0.42715

803.57938	0.42718

803.67616	0.42722

803.77294	0.42727

803.86972	0.42729

803.96651	0.42731

804.06329	0.42734

804.16007	0.42736

804.25685	0.42743

804.35363	0.42745

804.45041	0.42763

804.5472	0.42766

804.64398	0.42768

804.74076	0.4277

804.83754	0.42772

804.93432	0.42775

805.03111	0.42777

805.12789	0.42779

805.22467	0.42784

805.32145	0.42786

805.41823	0.42788

805.51501	0.42793

805.6118	0.42795

805.70858	0.42802

805.80536	0.42807

805.90214	0.42809

805.99892	0.42811

806.09571	0.42814

806.19249	0.42816

806.28927	0.42818

806.38605	0.4282

806.48283	0.42823

806.57961	0.42825

806.6764	0.42827

806.77318	0.42832

806.86996	0.42834

806.96674	0.42836

807.06352	0.42839

807.16031	0.42843

807.25709	0.42848

807.35387	0.4285

807.45065	0.42852

807.54743	0.42855

807.64421	0.42857

807.741	0.42859

807.83778	0.42862

807.93456	0.42864

808.03134	0.42866

808.12812	0.42871

808.22491	0.42878

808.32169	0.42882

808.41847	0.42889

808.51525	0.42891

808.61203	0.42894

808.70881	0.42896

808.8056	0.42898

808.90238	0.42905

808.99916	0.42907

809.09594	0.42912

809.19272	0.42914

809.28951	0.42937

809.38629	0.42951

809.48307	0.42955

809.57985	0.4296

809.67663	0.42962

809.77341	0.42964

809.8702	0.42967

809.96698	0.42974

810.06376	0.42978

810.16054	0.42985

810.25732	0.4299

810.35411	0.42992

810.45089	0.42994

810.54767	0.43001

810.64445	0.43003

810.74123	0.43008

810.83801	0.43012

810.9348	0.43015

811.03158	0.43017

811.12836	0.43038

811.22514	0.4304

811.32192	0.43044

811.41871	0.43047

811.51549	0.43049

811.61227	0.43051

811.70905	0.43058

811.80583	0.4306

811.90261	0.43063

811.9994	0.43067

812.09618	0.4307

812.19296	0.43072

812.28974	0.43074

812.38652	0.43079

812.48331	0.43088

812.58009	0.43095

812.67687	0.43097

812.77365	0.43106

812.87043	0.43111

812.96721	0.43113

813.064	0.43115

813.16078	0.4312

813.25756	0.43122

813.35434	0.43127

813.45112	0.43129

813.54791	0.43131

813.64469	0.43134

813.74147	0.43136

813.83825	0.43138

813.93503	0.43147

814.03181	0.4315

814.1286	0.43156

814.22538	0.43159

814.32216	0.43161

814.41894	0.43166

814.51572	0.4317

814.61251	0.43172

814.70929	0.43179

814.80607	0.43182

814.90285	0.43186

814.99963	0.43191

815.09641	0.43193

815.1932	0.43195

815.28998	0.432

815.38676	0.43209

815.48354	0.43211

815.58032	0.43214

815.6771	0.43216

815.77389	0.43218

815.87067	0.43225

815.96745	0.43227

816.06423	0.4323

816.16101	0.43232

816.2578	0.43234

816.35458	0.43236

816.45136	0.4325

816.54814	0.43255

816.64492	0.43257

816.7417	0.43259

816.83849	0.43262

816.93527	0.43264

817.03205	0.43266

817.12883	0.43268

817.22561	0.43273

817.3224	0.43278

817.41918	0.4328

817.51596	0.43282

817.61274	0.43287

817.70952	0.43289

817.8063	0.43291

817.90309	0.43294

817.99987	0.43296

818.09665	0.43298

818.19343	0.43305

818.29021	0.43307

818.387	0.43312

818.48378	0.43319

818.58056	0.43323

818.67734	0.43326

818.77412	0.4333

818.8709	0.43337

818.96769	0.43344

819.06447	0.43346

819.16125	0.43351

819.25803	0.43353

819.35481	0.43355

819.4516	0.43358

819.54838	0.4336

819.64516	0.43362

819.74194	0.43364

819.83872	0.43374

819.9355	0.43376

820.03229	0.4338

820.12907	0.43383

820.22585	0.43385

820.32263	0.43394

820.41941	0.43396

820.5162	0.43399

820.61298	0.43401

820.70976	0.43406

820.80654	0.43412

820.90332	0.43417

821.0001	0.43424

821.09689	0.43426

821.19367	0.43431

821.29045	0.43433

821.38723	0.43438

821.48401	0.4344

821.5808	0.43442

821.67758	0.43444

821.77436	0.43447

821.87114	0.43451

821.96792	0.43454

822.0647	0.4346

822.16149	0.43463

822.25827	0.43465

822.35505	0.43472

822.45183	0.43474

822.54861	0.43476

822.6454	0.43481

822.74218	0.43483

822.83896	0.43486

822.93574	0.4349

823.03252	0.43492

823.1293	0.43495

823.22609	0.43497

823.32287	0.43499

823.41965	0.43502

823.51643	0.43506

823.61321	0.43508

823.71	0.43511

823.80678	0.43513

823.90356	0.43515

824.00034	0.43518

824.09712	0.4352

824.1939	0.43524

824.29069	0.43527

824.38747	0.43531

824.48425	0.43534

824.58103	0.43536

824.67781	0.4354

824.7746	0.43545

824.87138	0.43547

824.96816	0.4355

825.06494	0.43552

825.16172	0.43556

825.2585	0.43563

825.35529	0.43572

825.45207	0.43575

825.54885	0.43577

825.64563	0.43579

825.74241	0.43586

825.8392	0.43588

825.93598	0.43591

826.03276	0.43593

826.12954	0.43598

826.22632	0.43604

826.3231	0.43614

826.41989	0.43616

826.51667	0.43618

826.61345	0.4362

826.71023	0.43623

826.80701	0.43627

826.90379	0.4363

827.00058	0.43632

827.09736	0.43634

827.19414	0.43636

827.29092	0.43641

827.3877	0.43643

827.48449	0.43646

827.58127	0.43648

827.67805	0.4365

827.77483	0.43655

827.87161	0.43657

827.96839	0.43659

828.06518	0.43662

828.16196	0.43666

828.25874	0.43668

828.35552	0.43671

828.4523	0.43673

828.54909	0.43678

828.64587	0.4368

828.74265	0.43682

828.83943	0.43687

828.93621	0.43694

829.03299	0.43696

829.12978	0.437

829.22656	0.43703

829.32334	0.43705

829.42012	0.4371

829.5169	0.43712

829.61369	0.43714

829.71047	0.43719

829.80725	0.43721

829.90403	0.43728

830.00081	0.4373

830.09759	0.43742

830.19438	0.43744

830.29116	0.43746

830.38794	0.43748

830.48472	0.43751

830.5815	0.43755

830.67829	0.43758

830.77507	0.43762

830.87185	0.43767

830.96863	0.43769

831.06541	0.43771

831.16219	0.43778

831.25898	0.43783

831.35576	0.43785

831.45254	0.43792

831.54932	0.43799

831.6461	0.43801

831.74289	0.43806

831.83967	0.43808

831.93645	0.4381

832.03323	0.43815

832.13001	0.43817

832.22679	0.43822

832.32358	0.43824

832.42036	0.43826

832.51714	0.43828

832.61392	0.43831

832.7107	0.43833

832.80749	0.43835

832.90427	0.43842

833.00105	0.43844

833.09783	0.43847

833.19461	0.43849

833.29139	0.43854

833.38818	0.43856

833.48496	0.43858

833.58174	0.4386

833.67852	0.43863

833.7753	0.43865

833.87209	0.4387

833.96887	0.43872

834.06565	0.43874

834.16243	0.43876

834.25921	0.43888

834.35599	0.4389

834.45278	0.43892

834.54956	0.43895

834.64634	0.43897

834.74312	0.43899

834.8399	0.43904

834.93669	0.43906

835.03347	0.43908

835.13025	0.43911

835.22703	0.43913

835.32381	0.43918

835.42059	0.43922

835.51738	0.43924

835.61416	0.43927

835.71094	0.43929

835.80772	0.43931

835.9045	0.43936

836.00129	0.43938

836.09807	0.4394

836.19485	0.43943

836.29163	0.43945

836.38841	0.43947

836.48519	0.43956

836.58198	0.43963

836.67876	0.43966

836.77554	0.43968

836.87232	0.43972

836.9691	0.43977

837.06589	0.43979

837.16267	0.43982

837.25945	0.43984

837.35623	0.43986

837.45301	0.43988

837.54979	0.43993

837.64658	0.43995

837.74336	0.44

837.84014	0.44002

837.93692	0.44004

838.0337	0.44007

838.13049	0.44009

838.22727	0.44011

838.32405	0.44014

838.42083	0.4402

838.51761	0.4403

838.61439	0.44032

838.71118	0.44034

838.80796	0.44036

838.90474	0.44039

839.00152	0.44041

839.0983	0.44043

839.19508	0.44048

839.29187	0.44052

839.38865	0.44055

839.48543	0.44059

839.58221	0.44066

839.67899	0.44071

839.77578	0.44073

839.87256	0.44075

839.96934	0.4408

840.06612	0.44082

840.1629	0.44084

840.25968	0.44087

840.35647	0.44089

840.45325	0.44094

840.55003	0.44096

840.64681	0.44098

840.74359	0.441

840.84038	0.44103

840.93716	0.44105

841.03394	0.44107

841.13072	0.4411

841.2275	0.44112

841.32428	0.44123

841.42107	0.44128

841.51785	0.4413

841.61463	0.44135

841.71141	0.44144

841.80819	0.44153

841.90498	0.44155

842.00176	0.44158

842.09854	0.4416

842.19532	0.44162

842.2921	0.44164

842.38888	0.44167

842.48567	0.44169

842.58245	0.44171

842.67923	0.44174

842.77601	0.44176

842.87279	0.44178

842.96958	0.4418

843.06636	0.44183

843.16314	0.44192

843.25992	0.44196

843.3567	0.44199

843.45348	0.44203

843.55027	0.44206

843.64705	0.44217

843.74383	0.44224

843.84061	0.44226

843.93739	0.44231

844.03418	0.44233

844.13096	0.44238

844.22774	0.4424

844.32452	0.44242

844.4213	0.44244

844.51808	0.44247

844.61487	0.44249

844.71165	0.44251

844.80843	0.44256

844.90521	0.44258

845.00199	0.4426

845.09878	0.44263

845.19556	0.44265

845.29234	0.44267

845.38912	0.44279

845.4859	0.44281

845.58268	0.44283

845.67947	0.44288

845.77625	0.4429

845.87303	0.44292

845.96981	0.44295

846.06659	0.44297

846.16338	0.44302

846.26016	0.44306

846.35694	0.44308

846.45372	0.44311

846.5505	0.44313

846.64728	0.44315

846.74407	0.44318

846.84085	0.4432

846.93763	0.44322

847.03441	0.44324

847.13119	0.44329

847.22798	0.44343

847.32476	0.44345

847.42154	0.44352

847.51832	0.44354

847.6151	0.44356

847.71188	0.44359

847.80867	0.44366

847.90545	0.44368

848.00223	0.4437

848.09901	0.44372

848.19579	0.44375

848.29258	0.44377

848.38936	0.44382

848.48614	0.44384

848.58292	0.44386

848.6797	0.44388

848.77648	0.44391

848.87327	0.44404

848.97005	0.44418

849.06683	0.4442

849.16361	0.44425

849.26039	0.44432

849.35718	0.44434

849.45396	0.44436

849.55074	0.44439

849.64752	0.44441

849.7443	0.44443

849.84108	0.44446

849.93787	0.44448

850.03465	0.44452

850.13143	0.44457

850.22821	0.44459

850.32499	0.44462

850.42178	0.44465

850.51856	0.44472

850.61534	0.44474

850.71212	0.4448

850.8089	0.44481

850.90568	0.44483

851.00247	0.44489

851.09925	0.44498

851.19603	0.44503

851.29281	0.44512

851.38959	0.44513

851.48638	0.4452

851.58316	0.44533

851.67994	0.44535

851.77672	0.44536

851.8735	0.44548

851.97028	0.44553

852.06707	0.44558

852.16385	0.44561

852.26063	0.44568

852.35741	0.4457

852.45419	0.44571

852.55097	0.44573

852.64776	0.44574

852.74454	0.44576

852.84132	0.4458

852.9381	0.44582

853.03488	0.44584

853.13167	0.44585

853.22845	0.44593

853.32523	0.44596

853.42201	0.44599

853.51879	0.446

853.61557	0.44603

853.71236	0.44609

853.80914	0.44625

853.90592	0.44626

854.0027	0.44629

854.09948	0.44634

854.19627	0.44635

854.29305	0.44637

854.38983	0.44638

854.48661	0.44644

854.58339	0.44646

854.68017	0.44648

854.77696	0.44649

854.87374	0.44657

854.97052	0.44658

855.0673	0.44673

855.16408	0.4468

855.26087	0.44681

855.35765	0.44686

855.45443	0.44687

855.55121	0.44692

855.64799	0.44695

855.74477	0.44699

855.84156	0.44701

855.93834	0.44705

856.03512	0.44708

856.1319	0.44727

856.22868	0.44728

856.32547	0.4473

856.42225	0.44736

856.51903	0.44737

856.61581	0.44751

856.71259	0.44753

856.80937	0.44754

856.90616	0.44757

857.00294	0.44759

857.09972	0.44774

857.1965	0.44776

857.29328	0.44777

857.39007	0.44779

857.48685	0.44789

857.58363	0.44791

857.68041	0.44798

857.77719	0.44801

857.87397	0.44803

857.97076	0.44806

858.06754	0.44808

858.16432	0.44811

858.2611	0.44812

858.35788	0.44815

858.45467	0.44817

858.55145	0.44818

858.64823	0.4482

858.74501	0.44821

858.84179	0.44823

858.93857	0.44826

859.03536	0.44827

859.13214	0.44829

859.22892	0.4483

859.3257	0.44833

859.42248	0.44835

859.51927	0.44843

859.61605	0.44844

859.71283	0.44849

859.80961	0.44852

859.90639	0.44856

860.00317	0.44859

860.09996	0.44865

860.19674	0.44867

860.29352	0.4487

860.3903	0.44872

860.48708	0.44873

860.58387	0.44876

860.68065	0.44882

860.77743	0.4489

860.87421	0.44893

860.97099	0.44896

861.06777	0.44897

861.16456	0.44902

861.26134	0.44905

861.35812	0.44911

861.4549	0.44914

861.55168	0.44916

861.64847	0.44917

861.74525	0.44937

861.84203	0.44939

861.93881	0.44949

862.03559	0.44952

862.13237	0.44954

862.22916	0.44963

862.32594	0.44966

862.42272	0.44968

862.5195	0.44969

862.61628	0.44972

862.71307	0.44975

862.80985	0.44981

862.90663	0.4499

863.00341	0.45

863.10019	0.45007

863.19697	0.45028

863.29376	0.45035

863.39054	0.45039

863.48732	0.4505

863.5841	0.45064

863.68088	0.45073

863.77767	0.45076

863.87445	0.45077

863.97123	0.45082

864.06801	0.45083

864.16479	0.45088

864.26157	0.45089

864.35836	0.45091

864.45514	0.45092

864.55192	0.45111

864.6487	0.4512

864.74548	0.45124

864.84227	0.45126

864.93905	0.45128

865.03583	0.45134

865.13261	0.45144

865.22939	0.45146

865.32617	0.4515

865.42296	0.45161

865.51974	0.45163

865.61652	0.4519

865.7133	0.45192

865.81008	0.45195

865.90686	0.45202

866.00365	0.45204

866.10043	0.45205

866.19721	0.45207

866.29399	0.4521

866.39077	0.45219

866.48756	0.4522

866.58434	0.45237

866.68112	0.45239

866.7779	0.45245

866.87468	0.4526

866.97146	0.45262

867.06825	0.45263

867.16503	0.45288

867.26181	0.45291

867.35859	0.45304

867.45537	0.45306

867.55216	0.45358

867.64894	0.45359

867.74572	0.45361

867.8425	0.45368

867.93928	0.45371

868.03606	0.45384

868.13285	0.45385

868.22963	0.45393

868.32641	0.45399

868.42319	0.454

868.51997	0.45402

868.61676	0.45406

868.71354	0.45408

868.81032	0.45411

868.9071	0.45417

869.00388	0.45419

869.10066	0.45422

869.19745	0.45425

869.29423	0.45432

869.39101	0.45434

869.48779	0.45443

869.58457	0.45455

869.68136	0.45463

869.77814	0.45469

869.87492	0.45475

869.9717	0.45477

870.06848	0.45478

870.16526	0.45489

870.26205	0.45492

870.35883	0.45496

870.45561	0.45509

870.55239	0.45519

870.64917	0.45521

870.74596	0.45522

870.84274	0.45524

870.93952	0.45527

871.0363	0.4553

871.13308	0.45533

871.22986	0.45536

871.32665	0.45541

871.42343	0.45547

871.52021	0.45562

871.61699	0.45565

871.71377	0.45568

871.81056	0.45569

871.90734	0.45574

872.00412	0.45576

872.1009	0.45585

872.19768	0.45586

872.29446	0.45589

872.39125	0.45591

872.48803	0.456

872.58481	0.45608

872.68159	0.45611

872.77837	0.45615

872.87516	0.45637

872.97194	0.45667

873.06872	0.4567

873.1655	0.45707

873.26228	0.45711

873.35906	0.45713

873.45585	0.45723

873.55263	0.45725

873.64941	0.45728

873.74619	0.45737

873.84297	0.45739

873.93976	0.45749

874.03654	0.45751

874.13332	0.45752

874.2301	0.45757

874.32688	0.45763

874.42366	0.45765

874.52045	0.45772

874.61723	0.45774

874.71401	0.45786

874.81079	0.45787

874.90757	0.45789

875.00436	0.45793

875.10114	0.45797

875.19792	0.45801

875.2947	0.45807

875.39148	0.4581

875.48826	0.45818

875.58505	0.45822

875.68183	0.45829

875.77861	0.45832

875.87539	0.45833

875.97217	0.45835

876.06896	0.45838

876.16574	0.45854

876.26252	0.45857

876.3593	0.45859

876.45608	0.45871

876.55286	0.45874

876.64965	0.45876

876.74643	0.45899

876.84321	0.45905

876.93999	0.45912

877.03677	0.45915

877.13356	0.45937

877.23034	0.4594

877.32712	0.4595

877.4239	0.45952

877.52068	0.45966

877.61746	0.4597

877.71425	0.45972

877.81103	0.45978

877.90781	0.45982

878.00459	0.4599

878.10137	0.45992

878.19816	0.46005

878.29494	0.46007

878.39172	0.46014

878.4885	0.46016

878.58528	0.46019

878.68206	0.46021

878.77885	0.46022

878.87563	0.46024

878.97241	0.46025

879.06919	0.4604

879.16597	0.46042

879.26275	0.46057

879.35954	0.46059

879.45632	0.4606

879.5531	0.46062

879.64988	0.46074

879.74666	0.46075

879.84345	0.46083

879.94023	0.46094

880.03701	0.46097

880.13379	0.46098

880.23057	0.46115

880.32735	0.46118

880.42414	0.46133

880.52092	0.46135

880.6177	0.46138

880.71448	0.46144

880.81126	0.46164

880.90805	0.46167

881.00483	0.4617

881.10161	0.46171

881.19839	0.46179

881.29517	0.46191

881.39195	0.46193

881.48874	0.46194

881.58552	0.462

881.6823	0.46205

881.77908	0.46208

881.87586	0.46211

881.97265	0.46213

882.06943	0.46214

882.16621	0.4622

882.26299	0.46223

882.35977	0.46225

882.45655	0.46226

882.55334	0.46228

882.65012	0.46238

882.7469	0.46243

882.84368	0.46245

882.94046	0.46246

883.03725	0.46252

883.13403	0.46261

883.23081	0.46278

883.32759	0.4628

883.42437	0.46281

883.52115	0.46322

883.61794	0.46324

883.71472	0.46327

883.8115	0.46331

883.90828	0.46336

884.00506	0.46337

884.10185	0.46347

884.19863	0.46351

884.29541	0.46353

884.39219	0.46362

884.48897	0.46363

884.58575	0.46379

884.68254	0.4638

884.77932	0.46398

884.8761	0.46412

884.97288	0.46418

885.06966	0.46427

885.16645	0.46429

885.26323	0.46437

885.36001	0.46455

885.45679	0.46464

885.55357	0.46469

885.65035	0.4647

885.74714	0.46472

885.84392	0.46473

885.9407	0.46475

886.03748	0.46479

886.13426	0.46484

886.23105	0.46485

886.32783	0.46487

886.42461	0.4649

886.52139	0.46494

886.61817	0.46496

886.71495	0.4651

886.81174	0.46511

886.90852	0.46514

887.0053	0.46516

887.10208	0.4652

887.19886	0.46522

887.29565	0.46525

887.39243	0.46533

887.48921	0.46536

887.58599	0.46551

887.68277	0.46555

887.77955	0.46557

887.87634	0.46571

887.97312	0.46578

888.0699	0.46598

888.16668	0.46601

888.26346	0.46603

888.36025	0.46606

888.45703	0.46607

888.55381	0.46622

888.65059	0.46624

888.74737	0.46626

888.84415	0.46629

888.94094	0.46632

889.03772	0.46635

889.1345	0.46644

889.23128	0.46645

889.32806	0.46647

889.42485	0.46653

889.52163	0.46673

889.61841	0.46683

889.71519	0.46685

889.81197	0.46688

889.90875	0.46705

890.00554	0.46706

890.10232	0.46709

890.1991	0.46711

890.29588	0.46717

890.39266	0.46718

890.48945	0.46722

890.58623	0.46725

890.68301	0.46729

890.77979	0.46732

890.87657	0.46734

890.97335	0.46741

891.07014	0.46746

891.16692	0.46758

891.2637	0.46761

891.36048	0.46778

891.45726	0.46781

891.55404	0.46796

891.65083	0.46798

891.74761	0.46799

891.84439	0.46805

891.94117	0.46807

892.03795	0.46808

892.13474	0.4681

892.23152	0.46811

892.3283	0.46821

892.42508	0.46822

892.52186	0.46827

892.61864	0.46833

892.71543	0.46836

892.81221	0.46839

892.90899	0.4684

893.00577	0.46853

893.10255	0.46856

893.19934	0.46857

893.29612	0.46859

893.3929	0.46862

893.48968	0.46863

893.58646	0.46865

893.68324	0.4688

893.78003	0.46894

893.87681	0.46897

893.97359	0.469

894.07037	0.46901

894.16715	0.46903

894.26394	0.46914

894.36072	0.46918

894.4575	0.46921

894.55428	0.46923

894.65106	0.46924

894.74784	0.4693

894.84463	0.46932

894.94141	0.46952

895.03819	0.46953

895.13497	0.4697

895.23175	0.46971

895.32854	0.46973

895.42532	0.46978

895.5221	0.46984

895.61888	0.46985

895.71566	0.46987

895.81244	0.46993

895.90923	0.46994

896.00601	0.46996

896.10279	0.46997

896.19957	0.47

896.29635	0.47002

896.39314	0.47006

896.48992	0.47008

896.5867	0.47017

896.68348	0.47022

896.78026	0.47023

896.87704	0.47025

896.97383	0.47038

897.07061	0.47042

897.16739	0.47045

897.26417	0.47046

897.36095	0.47048

897.45774	0.47058

897.55452	0.4706

897.6513	0.47072

897.74808	0.47074

897.84486	0.47075

897.94164	0.47077

898.03843	0.47102

898.13521	0.47104

898.23199	0.47113

898.32877	0.47115

898.42555	0.47118

898.52234	0.47124

898.61912	0.47125

898.7159	0.47142

898.81268	0.47144

898.90946	0.47145

899.00624	0.47157

899.10303	0.47165

899.19981	0.4717

899.29659	0.47171

899.39337	0.47173

899.49015	0.47176

899.58694	0.47182

899.68372	0.47183

899.7805	0.47188

899.87728	0.47212

899.97406	0.47214

900.07084	0.47215

900.16763	0.47218

900.26441	0.47221

900.36119	0.47224

900.45797	0.4723

900.55475	0.47232

900.65154	0.47234

900.74832	0.47238

900.8451	0.47256

900.94188	0.47258

901.03866	0.47269

901.13544	0.4727

901.23223	0.47284

901.32901	0.47287

901.42579	0.47288

901.52257	0.4729

901.61935	0.47293

901.71614	0.47304

901.81292	0.47305

901.9097	0.47307

902.00648	0.47308

902.10326	0.47313

902.20004	0.47314

902.29683	0.47316

902.39361	0.47317

902.49039	0.47339

902.58717	0.4734

902.68395	0.47342

902.78074	0.47343

902.87752	0.47345

902.9743	0.47354

903.07108	0.47355

903.16786	0.47357

903.26464	0.47366

903.36143	0.47368

903.45821	0.47381

903.55499	0.47383

903.65177	0.47387

903.74855	0.4739

903.84533	0.47397

903.94212	0.47409

904.0389	0.4741

904.13568	0.47412

904.23246	0.47413

904.32924	0.47415

904.42603	0.47441

904.52281	0.47445

904.61959	0.47453

904.71637	0.47454

904.81315	0.47459

904.90993	0.47471

905.00672	0.47476

905.1035	0.47486

905.20028	0.47488

905.29706	0.47502

905.39384	0.47511

905.49063	0.47512

905.58741	0.47514

905.68419	0.47517

905.78097	0.47518

905.87775	0.4752

905.97453	0.47537

906.07132	0.47541

906.1681	0.4755

906.26488	0.47552

906.36166	0.47554

906.45844	0.47567

906.55523	0.47569

906.65201	0.47581

906.74879	0.47584

906.84557	0.47593

906.94235	0.47596

907.03913	0.47598

907.13592	0.47601

907.2327	0.47607

907.32948	0.47633

907.42626	0.47634

907.52304	0.47637

907.61983	0.47653

907.71661	0.47654

907.81339	0.47663

907.91017	0.47666

908.00695	0.47668

908.10373	0.47674

908.20052	0.47675

908.2973	0.47677

908.39408	0.47691

908.49086	0.47692

908.58764	0.47703

908.68443	0.47704

908.78121	0.47706

908.87799	0.47707

908.97477	0.47739

909.07155	0.47741

909.16833	0.47744

909.26512	0.47756

909.3619	0.47758

909.45868	0.47764

909.55546	0.47765

909.65224	0.47767

909.74903	0.47768

909.84581	0.47785

909.94259	0.47787

910.03937	0.47788

910.13615	0.4779

910.23293	0.47794

910.32972	0.47799

910.4265	0.478

910.52328	0.47825

910.62006	0.47834

910.71684	0.47839

910.81363	0.47866

910.91041	0.47867

911.00719	0.47869

911.10397	0.47886

911.20075	0.47887

911.29753	0.47889

911.39432	0.47904

911.4911	0.47906

911.58788	0.47909

911.68466	0.4791

911.78144	0.47912

911.87823	0.47913

911.97501	0.47916

912.07179	0.47928

912.16857	0.4793

912.26535	0.47942

912.36213	0.47945

912.45892	0.47947

912.5557	0.47948

912.65248	0.47951

912.74926	0.47963

912.84604	0.47977

912.94283	0.4798

913.03961	0.47989

913.13639	0.47992

913.23317	0.47994

913.32995	0.48

913.42673	0.48029

913.52352	0.48037

913.6203	0.48041

913.71708	0.48052

913.81386	0.48053

913.91064	0.48072

914.00743	0.48073

914.10421	0.48075

914.20099	0.48078

914.29777	0.48084

914.39455	0.48085

914.49133	0.48093

914.58812	0.48101

914.6849	0.48102

914.78168	0.48122

914.87846	0.48123

914.97524	0.48125

915.07203	0.48128

915.16881	0.48133

915.26559	0.48134

915.36237	0.48181

915.45915	0.48183

915.55593	0.48184

915.65272	0.48192

915.7495	0.48197

915.84628	0.48204

915.94306	0.48206

916.03984	0.48216

916.13662	0.48218

916.23341	0.48229

916.33019	0.4823

916.42697	0.48247

916.52375	0.48248

916.62053	0.48251

916.71732	0.48253

916.8141	0.48255

916.91088	0.48256

917.00766	0.48271

917.10444	0.48273

917.20122	0.48274

917.29801	0.48277

917.39479	0.48279

917.49157	0.48282

917.58835	0.48283

917.68513	0.48317

917.78192	0.48329

917.8787	0.48331

917.97548	0.48343

918.07226	0.48369

918.16904	0.48389

918.26582	0.48404

918.36261	0.48405

918.45939	0.48407

918.55617	0.48415

918.65295	0.48419

918.74973	0.48421

918.84652	0.48422

918.9433	0.48424

919.04008	0.48425

919.13686	0.48428

919.23364	0.48439

919.33042	0.48445

919.42721	0.48447

919.52399	0.4845

919.62077	0.48456

919.71755	0.48463

919.81433	0.48466

919.91112	0.48477

920.0079	0.48479

920.10468	0.4848

920.20146	0.48501

920.29824	0.48503

920.39502	0.48517

920.49181	0.48518

920.58859	0.4852

920.68537	0.48533

920.78215	0.48552

920.87893	0.48553

920.97572	0.48561

921.0725	0.48564

921.16928	0.48576

921.26606	0.48579

921.36284	0.48582

921.45962	0.48605

921.55641	0.48607

921.65319	0.4861

921.74997	0.48611

921.84675	0.48625

921.94353	0.48645

922.04032	0.48649

922.1371	0.48651

922.23388	0.48675

922.33066	0.48683

922.42744	0.48684

922.52422	0.48719

922.62101	0.48722

922.71779	0.48728

922.81457	0.48739

922.91135	0.48742

923.00813	0.48744

923.10492	0.48751

923.2017	0.48754

923.29848	0.48771

923.39526	0.48779

923.49204	0.48788

923.58882	0.48789

923.68561	0.48791

923.78239	0.48794

923.87917	0.48803

923.97595	0.48809

924.07273	0.48814

924.16952	0.48815

924.2663	0.48846

924.36308	0.4887

924.45986	0.48872

924.55664	0.48892

924.65342	0.48893

924.75021	0.48898

924.84699	0.48905

924.94377	0.48908

925.04055	0.48911

925.13733	0.48913

925.23412	0.48917

925.3309	0.48924

925.42768	0.48927

925.52446	0.48931

925.62124	0.4896

925.71802	0.48975

925.81481	0.4898

925.91159	0.48981

926.00837	0.48994

926.10515	0.49

926.20193	0.49006

926.29872	0.49023

926.3955	0.49024

926.49228	0.49026

926.58906	0.49038

926.68584	0.49047

926.78262	0.49053

926.87941	0.4907

926.97619	0.49073

927.07297	0.49088

927.16975	0.4909

927.26653	0.49099

927.36331	0.49102

927.4601	0.49103

927.55688	0.4912

927.65366	0.49129

927.75044	0.49137

927.84722	0.49144

927.94401	0.49158

928.04079	0.4916

928.13757	0.4917

928.23435	0.49172

928.33113	0.49178

928.42791	0.49181

928.5247	0.49195

928.62148	0.49201

928.71826	0.49204

928.81504	0.49205

928.91182	0.49208

929.00861	0.49222

929.10539	0.49225

929.20217	0.49234

929.29895	0.49244

929.39573	0.49247

929.49251	0.4925

929.5893	0.49251

929.68608	0.49271

929.78286	0.49276

929.87964	0.49292

929.97642	0.49306

930.07321	0.49308

930.16999	0.49314

930.26677	0.49315

930.36355	0.4932

930.46033	0.49321

930.55711	0.49323

930.6539	0.4933

930.75068	0.49336

930.84746	0.49355

930.94424	0.49358

931.04102	0.49364

931.13781	0.49367

931.23459	0.49368

931.33137	0.4937

931.42815	0.49372

931.52493	0.49385

931.62171	0.49399

931.7185	0.49405

931.81528	0.49463

931.91206	0.49464

932.00884	0.49484

932.10562	0.49486

932.20241	0.49509

932.29919	0.4951

932.39597	0.49512

932.49275	0.49515

932.58953	0.49538

932.68631	0.49539

932.7831	0.49557

932.87988	0.49571

932.97666	0.49574

933.07344	0.49576

933.17022	0.49602

933.26701	0.4962

933.36379	0.49623

933.46057	0.49632

933.55735	0.49634

933.65413	0.49635

933.75091	0.49644

933.8477	0.49666

933.94448	0.4967

934.04126	0.49673

934.13804	0.49692

934.23482	0.49693

934.33161	0.49695

934.42839	0.49704

934.52517	0.49705

934.62195	0.49707

934.71873	0.49708

934.81551	0.49711

934.9123	0.49713

935.00908	0.49717

935.10586	0.49719

935.20264	0.49722

935.29942	0.49725

935.39621	0.49728

935.49299	0.49757

935.58977	0.49759

935.68655	0.49817

935.78333	0.49818

935.88011	0.4982

935.9769	0.49849

936.07368	0.49852

936.17046	0.49864

936.26724	0.49865

936.36402	0.49881

936.46081	0.49882

936.55759	0.49884

936.65437	0.4989

936.75115	0.49894

936.84793	0.49931

936.94471	0.49932

937.0415	0.49957

937.13828	0.49958

937.23506	0.49973

937.33184	0.49975

937.42862	0.49981

937.52541	0.49983

937.62219	0.49992

937.71897	0.49999

937.81575	0.50001

937.91253	0.50027

938.00931	0.50028

938.1061	0.50042

938.20288	0.50044

938.29966	0.5005

938.39644	0.50059

938.49322	0.50065

938.59001	0.5008

938.68679	0.50082

938.78357	0.50083

938.88035	0.50085

938.97713	0.50088

939.07391	0.50091

939.1707	0.50117

939.26748	0.5012

939.36426	0.50132

939.46104	0.50133

939.55782	0.50135

939.65461	0.50138

939.75139	0.50156

939.84817	0.50162

939.94495	0.50167

940.04173	0.50188

940.13851	0.5019

940.2353	0.50204

940.33208	0.50205

940.42886	0.50207

940.52564	0.5022

940.62242	0.50222

940.7192	0.50228

940.81599	0.50231

940.91277	0.50239

941.00955	0.50258

941.10633	0.50261

941.20311	0.50278

941.2999	0.50283

941.39668	0.50303

941.49346	0.50307

941.59024	0.5033

941.68702	0.50332

941.7838	0.50336

941.88059	0.50341

941.97737	0.50342

942.07415	0.50353

942.17093	0.50362

942.26771	0.50364

942.3645	0.50377

942.46128	0.50386

942.55806	0.50388

942.65484	0.50396

942.75162	0.50397

942.8484	0.50411

942.94519	0.50414

943.04197	0.50429

943.13875	0.50431

943.23553	0.50441

943.33231	0.50455

943.4291	0.50478

943.52588	0.50479

943.62266	0.50482

943.71944	0.50484

943.81622	0.50505

943.913	0.50525

944.00979	0.50542

944.10657	0.50545

944.20335	0.50546

944.30013	0.50548

944.39691	0.5056

944.4937	0.50563

944.59048	0.50565

944.68726	0.50572

944.78404	0.50575

944.88082	0.50577

944.9776	0.5058

945.07439	0.50604

945.17117	0.50606

945.26795	0.5062

945.36473	0.50621

945.46151	0.50623

945.5583	0.50624

945.65508	0.50632

945.75186	0.50635

945.84864	0.50636

945.94542	0.50647

946.0422	0.5065

946.13899	0.50653

946.23577	0.50656

946.33255	0.50667

946.42933	0.50668

946.52611	0.50694

946.6229	0.50696

946.71968	0.50697

946.81646	0.50703

946.91324	0.50706

947.01002	0.50709

947.1068	0.50734

947.20359	0.50737

947.30037	0.50745

947.39715	0.50746

947.49393	0.50752

947.59071	0.50764

947.6875	0.50766

947.78428	0.50767

947.88106	0.50773

947.97784	0.50775

948.07462	0.50777

948.1714	0.50781

948.26819	0.50784

948.36497	0.50824

948.46175	0.50825

948.55853	0.50842

948.65531	0.50844

948.7521	0.50845

948.84888	0.50859

948.94566	0.50865

949.04244	0.50869

949.13922	0.50885

949.236	0.50888

949.33279	0.50889

949.42957	0.50891

949.52635	0.50908

949.62313	0.50912

949.71991	0.50921

949.8167	0.50926

949.91348	0.50927

950.01026	0.50944

950.10704	0.50946

950.20382	0.50962

950.3006	0.50969

950.39739	0.50975

950.49417	0.50999

950.59095	0.51001

950.68773	0.51005

950.78451	0.51013

950.8813	0.51016

950.97808	0.51019

951.07486	0.51023

951.17164	0.51026

951.26842	0.51031

951.3652	0.51033

951.46199	0.51034

951.55877	0.51039

951.65555	0.51042

951.75233	0.51046

951.84911	0.51052

951.9459	0.51057

952.04268	0.5106

952.13946	0.51089

952.23624	0.51092

952.33302	0.51104

952.4298	0.51119

952.52659	0.51133

952.62337	0.51135

952.72015	0.51138

952.81693	0.51139

952.91371	0.51148

953.01049	0.5115

953.10728	0.51151

953.20406	0.51153

953.30084	0.51185

953.39762	0.51188

953.4944	0.51229

953.59119	0.51231

953.68797	0.51235

953.78475	0.51241

953.88153	0.51244

953.97831	0.51247

954.07509	0.51261

954.17188	0.51264

954.26866	0.51267

954.36544	0.51269

954.46222	0.51287

954.559	0.5129

954.65579	0.51292

954.75257	0.51296

954.84935	0.5131

954.94613	0.51311

955.04291	0.51313

955.13969	0.51321

955.23648	0.51324

955.33326	0.51325

955.43004	0.51328

955.52682	0.5133

955.6236	0.51334

955.72039	0.51351

955.81717	0.51356

955.91395	0.51357

956.01073	0.51359

956.10751	0.51372

956.20429	0.51374

956.30108	0.51375

956.39786	0.51409

956.49464	0.5141

956.59142	0.5143

956.6882	0.51432

956.78499	0.51458

956.88177	0.51467

956.97855	0.5147

957.07533	0.51471

957.17211	0.51488

957.26889	0.5149

957.36568	0.51506

957.46246	0.51513

957.55924	0.51519

957.65602	0.51543

957.7528	0.51545

957.84959	0.51549

957.94637	0.51557

958.04315	0.5156

958.13993	0.51563

958.23671	0.51567

958.33349	0.5157

958.43028	0.51575

958.52706	0.51577

958.62384	0.51578

958.72062	0.51583

958.8174	0.51586

958.91419	0.5159

959.01097	0.51596

959.10775	0.51601

959.20453	0.51604

959.30131	0.51633

959.39809	0.51636

959.49488	0.51648

959.59166	0.51663

959.68844	0.51677

959.78522	0.51679

959.882	0.51682

959.97879	0.51683

960.07557	0.51692

960.17235	0.51694

960.26913	0.51695

960.36591	0.51697

960.46269	0.51729

960.55948	0.51732

960.65626	0.51773

960.75304	0.51775

960.84982	0.51779

960.9466	0.51785

961.04339	0.51788

961.14017	0.51791

961.23695	0.51805

961.33373	0.51808

961.43051	0.51811

961.52729	0.51813

961.62408	0.51831

961.72086	0.51834

961.81764	0.51836

961.91442	0.5184

962.0112	0.51854

962.10799	0.51855

962.20477	0.51857

962.30155	0.51865

962.39833	0.51868

962.49511	0.51869

962.59189	0.51872

962.68868	0.51874

962.78546	0.51878

962.88224	0.51895

962.97902	0.519

963.0758	0.51901

963.17259	0.51903

963.26937	0.51916

963.36615	0.51918

963.46293	0.51919

963.55971	0.51953

963.65649	0.51954

963.75328	0.51974

963.85006	0.51976

963.94684	0.52002

964.04362	0.52005

964.1404	0.52008

964.23719	0.52014

964.33397	0.52015

964.43075	0.52023

964.52753	0.52028

964.62431	0.52075

964.72109	0.52076

964.81788	0.52113

964.91466	0.52134

965.01144	0.52139

965.10822	0.5214

965.205	0.52142

965.30179	0.52147

965.39857	0.52168

965.49535	0.52207

965.59213	0.52209

965.68891	0.52236

965.78569	0.52249

965.88248	0.5225

965.97926	0.52252

966.07604	0.52253

966.17282	0.52282

966.2696	0.52284

966.36639	0.52285

966.46317	0.523

966.55995	0.52305

966.65673	0.52307

966.75351	0.52313

966.85029	0.52317

966.94708	0.52319

967.04386	0.5232

967.14064	0.52322

967.23742	0.52325

967.3342	0.52349

967.43098	0.52351

967.52777	0.52352

967.62455	0.52355

967.72133	0.52393

967.81811	0.52395

967.91489	0.52415

968.01168	0.52416

968.10846	0.52435

968.20524	0.52438

968.30202	0.52439

968.3988	0.52441

968.49558	0.52471

968.59237	0.52474

968.68915	0.52479

968.78593	0.5248

968.88271	0.52482

968.97949	0.52483

969.07628	0.52534

969.17306	0.52535

969.26984	0.52549

969.36662	0.52572

969.4634	0.52584

969.56018	0.52587

969.65697	0.52588

969.75375	0.52611

969.85053	0.52617

969.94731	0.52619

970.04409	0.52652

970.14088	0.52654

970.23766	0.52672

970.33444	0.52706

970.43122	0.52761

970.528	0.52764

970.62478	0.52783

970.72157	0.52796

970.81835	0.52799

970.91513	0.528

971.01191	0.52837

971.10869	0.52878

971.20548	0.52881

971.30226	0.52924

971.39904	0.52925

971.49582	0.52939

971.5926	0.52951

971.68938	0.52953

971.78617	0.52994

971.88295	0.53

971.97973	0.53011

972.07651	0.53012

972.17329	0.53021

972.27008	0.53026

972.36686	0.53062

972.46364	0.53065

972.56042	0.53091

972.6572	0.53093

972.75398	0.53094

972.85077	0.53129

972.94755	0.53134

973.04433	0.53136

973.14111	0.53137

973.23789	0.5314

973.33468	0.53164

973.43146	0.53166

973.52824	0.53171

973.62502	0.53172

973.7218	0.53174

973.81858	0.53175

973.91537	0.53184

974.01215	0.53207

974.10893	0.53209

974.20571	0.5321

974.30249	0.53212

974.39928	0.53213

974.49606	0.53215

974.59284	0.53244

974.68962	0.53257

974.7864	0.53259

974.88318	0.53279

974.97997	0.53283

975.07675	0.53285

975.17353	0.53309

975.27031	0.53311

975.36709	0.53312

975.46388	0.5332

975.56066	0.53331

975.65744	0.53332

975.75422	0.53334

975.851	0.53356

975.94778	0.53361

976.04457	0.53399

976.14135	0.53401

976.23813	0.53402

976.33491	0.53407

976.43169	0.53425

976.52848	0.53431

976.62526	0.53466

976.72204	0.53468

976.81882	0.53469

976.9156	0.535

977.01238	0.53545

977.10917	0.53556

977.20595	0.53558

977.30273	0.53561

977.39951	0.53562

977.49629	0.53577

977.59308	0.53579

977.68986	0.5358

977.78664	0.53626

977.88342	0.53628

977.9802	0.53658

978.07698	0.5366

978.17377	0.53661

978.27055	0.5367

978.36733	0.53686

978.46411	0.53692

978.56089	0.53693

978.65768	0.53712

978.75446	0.53713

978.85124	0.53739

978.94802	0.53742

979.0448	0.53744

979.14158	0.53768

979.23837	0.53771

979.33515	0.53772

979.43193	0.53789

979.52871	0.53791

979.62549	0.53792

979.72227	0.53818

979.81906	0.53821

979.91584	0.53824

980.01262	0.53879

980.1094	0.53881

980.20618	0.53882

980.30297	0.53916

980.39975	0.53917

980.49653	0.53919

980.59331	0.53922

980.69009	0.53958

980.78687	0.53961

980.88366	0.53975

980.98044	0.53978

981.07722	0.5398

981.174	0.53981

981.27078	0.54007

981.36757	0.54009

981.46435	0.54012

981.56113	0.54032

981.65791	0.54039

981.75469	0.54042

981.85147	0.5408

981.94826	0.54082

982.04504	0.54102

982.14182	0.54105

982.2386	0.54111

982.33538	0.54112

982.43217	0.54117

982.52895	0.54123

982.62573	0.54124

982.72251	0.54134

982.81929	0.54138

982.91607	0.5415

983.01286	0.54153

983.10964	0.54155

983.20642	0.54158

983.3032	0.5416

983.39998	0.54166

983.49677	0.54169

983.59355	0.54172

983.69033	0.54208

983.78711	0.5421

983.88389	0.54224

983.98067	0.54225

984.07746	0.54227

984.17424	0.54233

984.27102	0.54239

984.3678	0.5424

984.46458	0.54243

984.56137	0.54249

984.65815	0.54265

984.75493	0.54266

984.85171	0.54269

984.94849	0.54278

985.04527	0.54318

985.14206	0.5432

985.23884	0.54364

985.33562	0.54365

985.4324	0.54367

985.52918	0.5437

985.62597	0.54384

985.72275	0.54385

985.81953	0.54387

985.91631	0.54388

986.01309	0.54391

986.10987	0.54414

986.20666	0.5442

986.30344	0.54422

986.40022	0.54483

986.497	0.54487

986.59378	0.54489

986.69057	0.54513

986.78735	0.54515

986.88413	0.54516

986.98091	0.54518

987.07769	0.54531

987.17447	0.54533

987.27126	0.54534

987.36804	0.54536

987.46482	0.54537

987.5616	0.54559

987.65838	0.54591

987.75517	0.54595

987.85195	0.54611

987.94873	0.54614

988.04551	0.54615

988.14229	0.54617

988.23907	0.54629

988.33586	0.54632

988.43264	0.54635

988.52942	0.54637

988.6262	0.54662

988.72298	0.54664

988.81977	0.54667

988.91655	0.54688

989.01333	0.5469

989.11011	0.54696

989.20689	0.54699

989.30367	0.54701

989.40046	0.54871

989.49724	0.54873

989.59402	0.54874

989.6908	0.54876

989.78758	0.549

989.88437	0.54935

989.98115	0.54941

990.07793	0.54943

990.17471	0.54966

990.27149	0.54969

990.36827	0.54975

990.46506	0.55007

990.56184	0.55008

990.65862	0.55025

990.7554	0.55027

990.85218	0.5503

990.94897	0.55046

991.04575	0.55051

991.14253	0.55078

991.23931	0.5508

991.33609	0.55081

991.43287	0.55121

991.52966	0.55126

991.62644	0.55141

991.72322	0.5515

991.82	0.55165

991.91678	0.55167

992.01356	0.55216

992.11035	0.55232

992.20713	0.55234

992.30391	0.5528

992.40069	0.55281

992.49747	0.55286

992.59426	0.55295

992.69104	0.55345

992.78782	0.55347

992.8846	0.55348

992.98138	0.55536

993.07816	0.55537

993.17495	0.55558

993.27173	0.55562

993.36851	0.5558

993.46529	0.55581

993.56207	0.55609

993.65886	0.5561

993.75564	0.55612

993.85242	0.55613

993.9492	0.55644

994.04598	0.55645

994.14276	0.55647

994.23955	0.55673

994.33633	0.55674

994.43311	0.55677

994.52989	0.55682

994.62667	0.55683

994.72346	0.55686

994.82024	0.55722

994.91702	0.55723

995.0138	0.55752

995.11058	0.55754

995.20736	0.55757

995.30415	0.55775

995.40093	0.55776

995.49771	0.55782

995.59449	0.55798

995.69127	0.55833

995.78806	0.55834

995.88484	0.55859

995.98162	0.55862

996.0784	0.55898

996.17518	0.55901

996.27196	0.55933

996.36875	0.55935

996.46553	0.55956

996.56231	0.55967

996.65909	0.5597

996.75587	0.56008

996.85266	0.56011

996.94944	0.56037

997.04622	0.5604

997.143	0.56042

997.23978	0.56081

997.33656	0.56084

997.43335	0.56102

997.53013	0.56104

997.62691	0.56116

997.72369	0.56119

997.82047	0.56229

997.91726	0.56235

998.01404	0.56237

998.11082	0.56247

998.2076	0.56258

998.30438	0.56264

998.40116	0.56278

998.49795	0.56281

998.59473	0.56285

998.69151	0.56287

998.78829	0.56293

998.88507	0.5632

998.98186	0.56342

999.07864	0.56349

999.17542	0.5636

999.2722	0.56368

999.36898	0.56389

999.46576	0.56407

999.56255	0.56409

999.65933	0.5641

999.75611	0.56429

999.85289	0.5643

999.94967	0.56432

1000.04646	0.56453

1000.14324	0.56455

1000.24002	0.56456

1000.3368	0.56458

1000.43358	0.56506

1000.53036	0.56508

1000.62715	0.56538

1000.72393	0.5654

1000.82071	0.56551

1000.91749	0.56558

1001.01427	0.5656

1001.11106	0.56563

1001.20784	0.56564

1001.30462	0.56579

1001.4014	0.56608

1001.49818	0.5661

1001.59496	0.56636

1001.69175	0.56637

1001.78853	0.56659

1001.88531	0.5666

1001.98209	0.56662

1002.07887	0.56665

1002.17566	0.56677

1002.27244	0.56685

1002.36922	0.56686

1002.466	0.56689

1002.56278	0.56692

1002.65956	0.56721

1002.75635	0.56729

1002.85313	0.56732

1002.94991	0.56733

1003.04669	0.56735

1003.14347	0.56738

1003.24026	0.56747

1003.33704	0.5675

1003.43382	0.56752

1003.5306	0.56753

1003.62738	0.56756

1003.72416	0.56767

1003.82095	0.5677

1003.91773	0.56778

1004.01451	0.56779

1004.11129	0.56781

1004.20807	0.56831

1004.30486	0.56843

1004.40164	0.56846

1004.49842	0.56877

1004.5952	0.56883

1004.69198	0.56884

1004.78876	0.56925

1004.88555	0.56927

1004.98233	0.56928

1005.07911	0.56967

1005.17589	0.57055

1005.27267	0.57056

1005.36946	0.57059

1005.46624	0.57069

1005.56302	0.57073

1005.6598	0.57101

1005.75658	0.57102

1005.85336	0.57136

1005.95015	0.5714

1006.04693	0.57142

1006.14371	0.57145

1006.24049	0.57151

1006.33727	0.57155

1006.43405	0.57183

1006.53084	0.57184

1006.62762	0.57186

1006.7244	0.57194

1006.82118	0.57201

1006.91796	0.57204

1007.01475	0.57215

1007.11153	0.57219

1007.20831	0.57223

1007.30509	0.57226

1007.40187	0.57256

1007.49866	0.57258

1007.59544	0.57265

1007.69222	0.57288

1007.789	0.57291

1007.88578	0.57293

1007.98256	0.57294

1008.07935	0.57341

1008.17613	0.57343

1008.27291	0.57351

1008.36969	0.57357

1008.46647	0.57358

1008.56326	0.57364

1008.66004	0.57383

1008.75682	0.57384

1008.8536	0.57425

1008.95038	0.57427

1009.04716	0.57437

1009.14395	0.57439

1009.24073	0.5744

1009.33751	0.5745

1009.43429	0.57463

1009.53107	0.57466

1009.62786	0.57469

1009.72464	0.57475

1009.82142	0.57492

1009.9182	0.57497

1010.01498	0.575

1010.11176	0.57504

1010.20855	0.57515

1010.30533	0.57517

1010.40211	0.57553

1010.49889	0.57555

1010.59567	0.57558

1010.69246	0.57572

1010.78924	0.57573

1010.88602	0.57576

1010.9828	0.576

1011.07958	0.5761

1011.17636	0.57611

1011.27315	0.57637

1011.36993	0.57645

1011.46671	0.57669

1011.56349	0.57672

1011.66027	0.57674

1011.75705	0.5773

1011.85384	0.57732

1011.95062	0.57733

1012.0474	0.57735

1012.14418	0.57765

1012.24096	0.57767

1012.33775	0.57783

1012.43453	0.57786

1012.53131	0.57821

1012.62809	0.57823

1012.72487	0.57824

1012.82165	0.57828

1012.91844	0.57867

1013.01522	0.5787

1013.112	0.57872

1013.20878	0.57873

1013.30556	0.57875

1013.40235	0.57884

1013.49913	0.57893

1013.59591	0.57896

1013.69269	0.57946

1013.78947	0.57949

1013.88625	0.57975

1013.98304	0.57977

1014.07982	0.57995

1014.1766	0.58001

1014.27338	0.58003

1014.37016	0.58033

1014.46695	0.58036

1014.56373	0.58038

1014.66051	0.58056

1014.75729	0.58061

1014.85407	0.58064

1014.95085	0.58084

1015.04764	0.58085

1015.14442	0.58088

1015.2412	0.58097

1015.33798	0.58108

1015.43476	0.58111

1015.53155	0.58116

1015.62833	0.58117

1015.72511	0.58141

1015.82189	0.58143

1015.91867	0.58151

1016.01545	0.58154

1016.11224	0.58155

1016.20902	0.58175

1016.3058	0.58176

1016.40258	0.58178

1016.49936	0.5818

1016.59615	0.58199

1016.69293	0.58201

1016.78971	0.58228

1016.88649	0.5823

1016.98327	0.58266

1017.08005	0.58269

1017.17684	0.58271

1017.27362	0.58274

1017.3704	0.58309

1017.46718	0.58317

1017.56396	0.58336

1017.66075	0.58365

1017.75753	0.58399

1017.85431	0.58407

1017.95109	0.58451

1018.04787	0.58452

1018.14465	0.58454

1018.24144	0.58455

1018.33822	0.58457

1018.435	0.58475

1018.53178	0.58483

1018.62856	0.58496

1018.72534	0.58521

1018.82213	0.58524

1018.91891	0.58547

1019.01569	0.58551

1019.11247	0.58565

1019.20925	0.58568

1019.30604	0.58571

1019.40282	0.58574

1019.4996	0.58576

1019.59638	0.5858

1019.69316	0.58586

1019.78995	0.58588

1019.88673	0.58609

1019.98351	0.58612

1020.08029	0.58637

1020.17707	0.5864

1020.27385	0.58652

1020.37064	0.58679

1020.46742	0.58682

1020.5642	0.58684

1020.66098	0.58685

1020.75776	0.58746

1020.85455	0.58748

1020.95133	0.58772

1021.04811	0.58774

1021.14489	0.58775

1021.24167	0.58777

1021.33845	0.58785

1021.43524	0.58791

1021.53202	0.58792

1021.6288	0.58798

1021.72558	0.58801

1021.82236	0.58833

1021.91915	0.58835

1022.01593	0.58838

1022.11271	0.58855

1022.20949	0.58868

1022.30627	0.58893

1022.40305	0.58894

1022.49984	0.58899

1022.59662	0.58908

1022.6934	0.58911

1022.79018	0.58913

1022.88696	0.58938

1022.98375	0.5894

1023.08053	0.58961

1023.17731	0.58964

1023.27409	0.58966

1023.37087	0.5898

1023.46765	0.58983

1023.56444	0.58984

1023.66122	0.59002

1023.758	0.59015

1023.85478	0.59042

1023.95156	0.59044

1024.04834	0.59066

1024.14513	0.59071

1024.24191	0.59073

1024.33869	0.59076

1024.43547	0.59079

1024.53225	0.59091

1024.62903	0.59092

1024.72582	0.59095

1024.8226	0.5913

1024.91938	0.59133

1025.01616	0.59135

1025.11294	0.59152

1025.20973	0.59167

1025.30651	0.59176

1025.40329	0.59178

1025.50007	0.5919

1025.59685	0.59193

1025.69364	0.59207

1025.79042	0.59226

1025.8872	0.59229

1025.98398	0.59233

1026.08076	0.59236

1026.17754	0.59265

1026.27433	0.59268

1026.37111	0.59281

1026.46789	0.59286

1026.56467	0.59287

1026.66145	0.59301

1026.75824	0.59312

1026.85502	0.59315

1026.9518	0.59322

1027.04858	0.59353

1027.14536	0.59354

1027.24214	0.59379

1027.33893	0.59397

1027.43571	0.59403

1027.53249	0.59414

1027.62927	0.59429

1027.72605	0.59431

1027.82283	0.59437

1027.91962	0.59446

1028.0164	0.59447

1028.11318	0.59458

1028.20996	0.59467

1028.30674	0.59478

1028.40353	0.59481

1028.50031	0.59516

1028.59709	0.59519

1028.69387	0.59522

1028.79065	0.59524

1028.88744	0.59525

1028.98422	0.59557

1029.081	0.59562

1029.17778	0.59563

1029.27456	0.59565

1029.37134	0.59585

1029.46813	0.59586

1029.56491	0.59588

1029.66169	0.59606

1029.75847	0.59607

1029.85525	0.5961

1029.95204	0.59629

1030.04882	0.59632

1030.1456	0.59662

1030.24238	0.59665

1030.33916	0.59667

1030.43594	0.59679

1030.53273	0.59681

1030.62951	0.59682

1030.72629	0.59684

1030.82307	0.59691

1030.91985	0.59694

1031.01664	0.59699

1031.11342	0.597

1031.2102	0.59723

1031.30698	0.59728

1031.40376	0.59731

1031.50054	0.59748

1031.59733	0.59789

1031.69411	0.59825

1031.79089	0.59828

1031.88767	0.5987

1031.98445	0.59882

1032.08124	0.59909

1032.17802	0.59934

1032.2748	0.59935

1032.37158	0.59938

1032.46836	0.59941

1032.56514	0.59949

1032.66193	0.59953

1032.75871	0.59955

1032.85549	0.5997

1032.95227	0.59973

1033.04905	0.59976

1033.14584	0.59978

1033.24262	0.60011

1033.3394	0.60014

1033.43618	0.60016

1033.53296	0.60022

1033.62974	0.60025

1033.72653	0.60028

1033.82331	0.60033

1033.92009	0.60034

1034.01687	0.60062

1034.11365	0.60066

1034.21044	0.60068

1034.30722	0.60075

1034.404	0.60078

1034.50078	0.60112

1034.59756	0.60113

1034.69434	0.60115

1034.79113	0.60116

1034.88791	0.60124

1034.98469	0.60133

1035.08147	0.60142

1035.17825	0.60168

1035.27503	0.60171

1035.37182	0.60174

1035.4686	0.60193

1035.56538	0.60196

1035.66216	0.60197

1035.75894	0.60232

1035.85572	0.6024

1035.95251	0.60257

1036.04929	0.60258

1036.14607	0.60267

1036.24285	0.60273

1036.33963	0.60289

1036.43642	0.6029

1036.5332	0.60337

1036.62998	0.6036

1036.72676	0.60362

1036.82354	0.60363

1036.92033	0.60397

1037.01711	0.60398

1037.11389	0.60421

1037.21067	0.60423

1037.30745	0.60439

1037.40423	0.6047

1037.50102	0.60474

1037.5978	0.60476

1037.69458	0.60478

1037.79136	0.60482

1037.88814	0.60496

1037.98493	0.60528

1038.08171	0.60529

1038.17849	0.60558

1038.27527	0.60561

1038.37205	0.60586

1038.46883	0.60589

1038.56562	0.60599

1038.6624	0.60606

1038.75918	0.60607

1038.85596	0.60625

1038.95274	0.60627

1039.04952	0.60648

1039.14631	0.6065

1039.24309	0.60651

1039.33987	0.60653

1039.43665	0.60654

1039.53343	0.60676

1039.63022	0.60677

1039.727	0.60679

1039.82378	0.60702

1039.92056	0.60709

1040.01734	0.60712

1040.11413	0.60729

1040.21091	0.60749

1040.30769	0.60761

1040.40447	0.60805

1040.50125	0.60807

1040.59803	0.60808

1040.69482	0.60811

1040.7916	0.60833

1040.88838	0.60837

1040.98516	0.60868

1041.08194	0.60869

1041.17873	0.60898

1041.27551	0.60903

1041.37229	0.60906

1041.46907	0.60907

1041.56585	0.60912

1041.66263	0.60932

1041.75942	0.60933

1041.8562	0.60956

1041.95298	0.60958

1042.04976	0.61005

1042.14654	0.61006

1042.24332	0.61037

1042.34011	0.61076

1042.43689	0.61078

1042.53367	0.61079

1042.63045	0.61081

1042.72723	0.61098

1042.82402	0.61147

1042.9208	0.6115

1043.01758	0.61177

1043.11436	0.61212

1043.21114	0.61218

1043.30793	0.6123

1043.40471	0.61233

1043.50149	0.61256

1043.59827	0.61258

1043.69505	0.61259

1043.79183	0.61262

1043.88862	0.61293

1043.9854	0.61294

1044.08218	0.61345

1044.17896	0.61346

1044.27574	0.61348

1044.37253	0.61351

1044.46931	0.61357

1044.56609	0.61363

1044.66287	0.61364

1044.75965	0.61369

1044.85643	0.61409

1044.95322	0.61415

1045.05	0.61416

1045.14678	0.61436

1045.24356	0.61438

1045.34034	0.6147

1045.43712	0.61471

1045.53391	0.61497

1045.63069	0.615

1045.72747	0.61502

1045.82425	0.61524

1045.92103	0.61559

1046.01782	0.61561

1046.1146	0.61564

1046.21138	0.61579

1046.30816	0.61631

1046.40494	0.61633

1046.50173	0.61651

1046.59851	0.6166

1046.69529	0.61747

1046.79207	0.61765

1046.88885	0.61783

1046.98563	0.61785

1047.08242	0.61791

1047.1792	0.618

1047.27598	0.61802

1047.37276	0.61803

1047.46954	0.6182

1047.56633	0.61844

1047.66311	0.61846

1047.75989	0.61883

1047.85667	0.61886

1047.95345	0.61887

1048.05023	0.61931

1048.14702	0.61933

1048.2438	0.61951

1048.34058	0.61953

1048.43736	0.61957

1048.53414	0.6196

1048.63092	0.61962

1048.72771	0.61986

1048.82449	0.61991

1048.92127	0.61994

1049.01805	0.62026

1049.11483	0.62033

1049.21161	0.62035

1049.3084	0.62068

1049.40518	0.6207

1049.50196	0.62105

1049.59874	0.62107

1049.69552	0.62139

1049.79231	0.6214

1049.88909	0.62142

1049.98587	0.62177

1050.08265	0.6218

1050.17943	0.62228

1050.27622	0.62232

1050.373	0.62233

1050.46978	0.62253

1050.56656	0.62254

1050.66334	0.62256

1050.76012	0.62288

1050.85691	0.62289

1050.95369	0.62305

1051.05047	0.62317

1051.14725	0.62318

1051.24403	0.62328

1051.34082	0.62331

1051.4376	0.62332

1051.53438	0.62334

1051.63116	0.62364

1051.72794	0.62392

1051.82472	0.62395

1051.92151	0.62398

1052.01829	0.62407

1052.11507	0.62414

1052.21185	0.62416

1052.30863	0.62424

1052.40541	0.62433

1052.5022	0.62434

1052.59898	0.62437

1052.69576	0.62446

1052.79254	0.62451

1052.88932	0.62454

1052.98611	0.62494

1053.08289	0.62523

1053.17967	0.62524

1053.27645	0.62526

1053.37323	0.6253

1053.47002	0.62548

1053.5668	0.6255

1053.66358	0.62552

1053.76036	0.62565

1053.85714	0.62623

1053.95392	0.62628

1054.05071	0.62667

1054.14749	0.62672

1054.24427	0.62705

1054.34105	0.62707

1054.43783	0.6271

1054.53462	0.62715

1054.6314	0.62747

1054.72818	0.6275

1054.82496	0.62774

1054.92174	0.62777

1055.01852	0.62779

1055.11531	0.62783

1055.21209	0.62791

1055.30887	0.62823

1055.40565	0.62827

1055.50243	0.62833

1055.59922	0.6284

1055.696	0.62841

1055.79278	0.62865

1055.88956	0.62867

1055.98634	0.62868

1056.08312	0.6287

1056.17991	0.62928

1056.27669	0.62929

1056.37347	0.62931

1056.47025	0.62936

1056.56703	0.62943

1056.66382	0.62945

1056.7606	0.62946

1056.85738	0.62948

1056.95416	0.62978

1057.05094	0.62984

1057.14772	0.63007

1057.24451	0.63009

1057.34129	0.63012

1057.43807	0.63036

1057.53485	0.63038

1057.63163	0.63039

1057.72842	0.63041

1057.8252	0.63053

1057.92198	0.63056

1058.01876	0.63059

1058.11554	0.63061

1058.21232	0.63079

1058.30911	0.63082

1058.40589	0.63097

1058.50267	0.63106

1058.59945	0.63108

1058.69623	0.63111

1058.79302	0.63112

1058.8898	0.63125

1058.98658	0.63128

1059.08336	0.63129

1059.18014	0.63144

1059.27692	0.63149

1059.37371	0.63153

1059.47049	0.63172

1059.56727	0.63187

1059.66405	0.63217

1059.76083	0.63219

1059.85761	0.63221

1059.9544	0.63248

1060.05118	0.63251

1060.14796	0.63253

1060.24474	0.63266

1060.34152	0.63274

1060.4383	0.63275

1060.53509	0.6328

1060.63187	0.63283

1060.72865	0.63286

1060.82543	0.63298

1060.92222	0.63303

1061.019	0.63312

1061.11578	0.63313

1061.21256	0.63317

1061.30934	0.63318

1061.40612	0.63361

1061.50291	0.63362

1061.59969	0.63364

1061.69647	0.63385

1061.79325	0.63387

1061.89003	0.63388

1061.98681	0.63413

1062.0836	0.63414

1062.18038	0.63419

1062.27716	0.63425

1062.37394	0.63428

1062.47072	0.63429

1062.56751	0.63448

1062.66429	0.63461

1062.76107	0.63501

1062.85785	0.63502

1062.95463	0.63505

1063.05141	0.63519

1063.1482	0.63551

1063.24498	0.63559

1063.34176	0.6356

1063.43854	0.63608

1063.53532	0.63612

1063.6321	0.63615

1063.72889	0.63618

1063.82567	0.6362

1063.92245	0.63621

1064.01923	0.6364

1064.11601	0.6365

1064.2128	0.63701

1064.30958	0.63702

1064.40636	0.63704

1064.50314	0.63737

1064.59992	0.63739

1064.69671	0.6374

1064.79349	0.63742

1064.89027	0.63749

1064.98705	0.63754

1065.08383	0.6379

1065.18061	0.63795

1065.2774	0.63798

1065.37418	0.63839

1065.47096	0.63888

1065.56774	0.63889

1065.66452	0.63893

1065.76131	0.63908

1065.85809	0.63911

1065.95487	0.63912

1066.05165	0.63944

1066.14843	0.63952

1066.24521	0.63953

1066.342	0.63978

1066.43878	0.63981

1066.53556	0.63982

1066.63234	0.63998

1066.72912	0.64004

1066.8259	0.64007

1066.92269	0.64008

1067.01947	0.6401

1067.11625	0.64046

1067.21303	0.64048

1067.30981	0.64088

1067.4066	0.64091

1067.50338	0.64092

1067.60016	0.64132

1067.69694	0.64133

1067.79372	0.64135

1067.89051	0.64146

1067.98729	0.6415

1068.08407	0.64152

1068.18085	0.64155

1068.27763	0.64181

1068.37441	0.64184

1068.4712	0.64187

1068.56798	0.6419

1068.66476	0.64203

1068.76154	0.64214

1068.85832	0.64219

1068.95511	0.64257

1069.05189	0.64258

1069.14867	0.64261

1069.24545	0.64283

1069.34223	0.64286

1069.43901	0.64298

1069.5358	0.64299

1069.63258	0.64301

1069.72936	0.64359

1069.82614	0.64391

1069.92292	0.64392

1070.0197	0.64394

1070.11649	0.64395

1070.21327	0.6442

1070.31005	0.64435

1070.40683	0.64444

1070.50361	0.6445

1070.6004	0.64455

1070.69718	0.64456

1070.79396	0.64458

1070.89074	0.64501

1070.98752	0.64505

1071.08431	0.64523

1071.18109	0.64536

1071.27787	0.64537

1071.37465	0.64539

1071.47143	0.64558

1071.56821	0.64566

1071.665	0.64571

1071.76178	0.64589

1071.85856	0.64597

1071.95534	0.64598

1072.05212	0.64639

1072.14891	0.64659

1072.24569	0.64662

1072.34247	0.64665

1072.43925	0.64697

1072.53603	0.647

1072.63281	0.64729

1072.7296	0.64731

1072.82638	0.64732

1072.92316	0.64735

1073.01994	0.64766

1073.11672	0.64767

1073.2135	0.64769

1073.31029	0.6477

1073.40707	0.64807

1073.50385	0.6481

1073.60063	0.64811

1073.69741	0.64836

1073.79419	0.64842

1073.89098	0.64843

1073.98776	0.64897

1074.08454	0.64932

1074.18132	0.64933

1074.2781	0.64953

1074.37489	0.64956

1074.47167	0.64993

1074.56845	0.65016

1074.66523	0.65019

1074.76201	0.6502

1074.8588	0.65035

1074.95558	0.65046

1075.05236	0.65048

1075.14914	0.65063

1075.24592	0.65064

1075.3427	0.65066

1075.43949	0.65078

1075.53627	0.6508

1075.63305	0.65081

1075.72983	0.65109

1075.82661	0.65115

1075.9234	0.65144

1076.02018	0.65147

1076.11696	0.65148

1076.21374	0.6515

1076.31052	0.6516

1076.4073	0.65177

1076.50409	0.65179

1076.60087	0.65195

1076.69765	0.65198

1076.79443	0.65214

1076.89121	0.65229

1076.988	0.65232

1077.08478	0.65234

1077.18156	0.6524

1077.27834	0.65241

1077.37512	0.65244

1077.4719	0.65249

1077.56869	0.6525

1077.66547	0.65258

1077.76225	0.65264

1077.85903	0.65266

1077.95581	0.6529

1078.0526	0.65291

1078.14938	0.65308

1078.24616	0.65311

1078.34294	0.65336

1078.43972	0.65339

1078.5365	0.6534

1078.63329	0.6536

1078.73007	0.65362

1078.82685	0.65363

1078.92363	0.65365

1079.02041	0.65386

1079.1172	0.65387

1079.21398	0.65391

1079.31076	0.65427

1079.40754	0.6543

1079.50432	0.65438

1079.6011	0.65464

1079.69789	0.6547

1079.79467	0.65473

1079.89145	0.65474

1079.98823	0.65494

1080.08501	0.65511

1080.1818	0.65514

1080.27858	0.65515

1080.37536	0.65547

1080.47214	0.65549

1080.56892	0.65551

1080.6657	0.65552

1080.76249	0.65555

1080.85927	0.65557

1080.95605	0.65558

1081.05283	0.65567

1081.14961	0.65581

1081.24639	0.65586

1081.34318	0.65587

1081.43996	0.65624

1081.53674	0.65625

1081.63352	0.65682

1081.7303	0.65691

1081.82709	0.65697

1081.92387	0.65701

1082.02065	0.65743

1082.11743	0.65744

1082.21421	0.65746

1082.311	0.65773

1082.40778	0.65775

1082.50456	0.65782

1082.60134	0.65793

1082.69812	0.65796

1082.7949	0.65802

1082.89169	0.65819

1082.98847	0.65828

1083.08525	0.65831

1083.18203	0.65832

1083.27881	0.65883

1083.3756	0.6591

1083.47238	0.65915

1083.56916	0.65919

1083.66594	0.65922

1083.76272	0.65928

1083.8595	0.65939

1083.95629	0.65941

1084.05307	0.65957

1084.14985	0.6596

1084.24663	0.65962

1084.34341	0.65965

1084.44019	0.65971

1084.53698	0.65974

1084.63376	0.65976

1084.73054	0.65979

1084.82732	0.66024

1084.9241	0.66066

1085.02089	0.66067

1085.11767	0.66122

1085.21445	0.66125

1085.31123	0.66139

1085.40801	0.66148

1085.5048	0.66149

1085.60158	0.66151

1085.69836	0.66174

1085.79514	0.6618

1085.89192	0.66184

1085.9887	0.66221

1086.08549	0.66227

1086.18227	0.66245

1086.27905	0.66247

1086.37583	0.66282

1086.47261	0.66285

1086.56939	0.66343

1086.66618	0.66346

1086.76296	0.66369

1086.85974	0.6637

1086.95652	0.66375

1087.0533	0.66386

1087.15009	0.66389

1087.24687	0.66421

1087.34365	0.66424

1087.44043	0.66474

1087.53721	0.66477

1087.63399	0.66479

1087.73078	0.665

1087.82756	0.66501

1087.92434	0.66523

1088.02112	0.66529

1088.1179	0.66532

1088.21468	0.66567

1088.31147	0.6657

1088.40825	0.66572

1088.50503	0.66578

1088.60181	0.66587

1088.69859	0.66588

1088.79538	0.6659

1088.89216	0.66591

1088.98894	0.66604

1089.08572	0.6662

1089.1825	0.66622

1089.27929	0.66623

1089.37607	0.66674

1089.47285	0.6668

1089.56963	0.66709

1089.66641	0.6671

1089.76319	0.66716

1089.85998	0.66719

1089.95676	0.66733

1090.05354	0.66751

1090.15032	0.66753

1090.2471	0.66789

1090.34389	0.66792

1090.44067	0.66832

1090.53745	0.66834

1090.63423	0.6686

1090.73101	0.66861

1090.82779	0.66904

1090.92458	0.66905

1091.02136	0.66907

1091.11814	0.66945

1091.21492	0.66949

1091.3117	0.66951

1091.40848	0.67001

1091.50527	0.67004

1091.60205	0.67029

1091.69883	0.67035

1091.79561	0.67058

1091.89239	0.67084

1091.98918	0.67085

1092.08596	0.67087

1092.18274	0.67135

1092.27952	0.67137

1092.3763	0.67143

1092.47309	0.67163

1092.56987	0.67172

1092.66665	0.67175

1092.76343	0.67176

1092.86021	0.67178

1092.95699	0.67183

1093.05378	0.67186

1093.15056	0.67187

1093.24734	0.67193

1093.34412	0.67195

1093.4409	0.6721

1093.53769	0.67216

1093.63447	0.67218

1093.73125	0.67221

1093.82803	0.6728

1093.92481	0.67282

1094.02159	0.67283

1094.11838	0.67291

1094.21516	0.67301

1094.31194	0.67303

1094.40872	0.67304

1094.5055	0.67318

1094.60228	0.67324

1094.69907	0.67326

1094.79585	0.67329

1094.89263	0.67336

1094.98941	0.67343

1095.08619	0.67344

1095.18298	0.67346

1095.27976	0.67365

1095.37654	0.6737

1095.47332	0.67372

1095.5701	0.67387

1095.66689	0.67394

1095.76367	0.67396

1095.86045	0.67399

1095.95723	0.6742

1096.05401	0.67426

1096.15079	0.6746

1096.24758	0.67461

1096.34436	0.67509

1096.44114	0.67513

1096.53792	0.67532

1096.6347	0.67544

1096.73149	0.67545

1096.82827	0.67548

1096.92505	0.67562

1097.02183	0.67565

1097.11861	0.6758

1097.21539	0.67582

1097.31218	0.67583

1097.40896	0.67629

1097.50574	0.67632

1097.60252	0.67634

1097.6993	0.67664

1097.79608	0.67669

1097.89287	0.6767

1097.98965	0.67702

1098.08643	0.67704

1098.18321	0.6771

1098.27999	0.67716

1098.37678	0.67717

1098.47356	0.67719

1098.57034	0.67774

1098.66712	0.6778

1098.7639	0.67803

1098.86069	0.67804

1098.95747	0.67806

1099.05425	0.67807

1099.15103	0.67835

1099.24781	0.67839

1099.34459	0.67847

1099.44138	0.6785

1099.53816	0.67871

1099.63494	0.67874

1099.73172	0.6789

1099.8285	0.67894

1099.92528	0.67896

1100.02207	0.67911

1100.11885	0.67913

1100.21563	0.67946

1100.31241	0.67949

1100.40919	0.67951

1100.50598	0.67957

1100.60276	0.67964

1100.69954	0.67996

1100.79632	0.67998

1100.8931	0.67999

1100.98988	0.6803

1101.08667	0.68037

1101.18345	0.68047

1101.28023	0.68065

1101.37701	0.68066

1101.47379	0.68068

1101.57058	0.68106

1101.66736	0.68126

1101.76414	0.68167

1101.86092	0.68169

1101.9577	0.68199

1102.05448	0.68216

1102.15127	0.68217

1102.24805	0.68219

1102.34483	0.68223

1102.44161	0.68249

1102.53839	0.68251

1102.63517	0.68274

1102.73196	0.68275

1102.82874	0.6828

1102.92552	0.68297

1103.0223	0.68298

1103.11908	0.68303

1103.21587	0.6833

1103.31265	0.68347

1103.40943	0.68357

1103.50621	0.68359

1103.60299	0.68386

1103.69978	0.68388

1103.79656	0.68389

1103.89334	0.68391

1103.99012	0.68412

1104.0869	0.68415

1104.18368	0.68417

1104.28047	0.68453

1104.37725	0.68464

1104.47403	0.68467

1104.57081	0.68499

1104.66759	0.68504

1104.76438	0.68505

1104.86116	0.6851

1104.95794	0.68534

1105.05472	0.68536

1105.1515	0.68568

1105.24828	0.68569

1105.34507	0.68572

1105.44185	0.68589

1105.53863	0.68595

1105.63541	0.68623

1105.73219	0.68624

1105.82897	0.68652

1105.92576	0.68659

1106.02254	0.68661

1106.11932	0.68664

1106.2161	0.68665

1106.31288	0.68668

1106.40967	0.6867

1106.50645	0.68678

1106.60323	0.68687

1106.70001	0.6869

1106.79679	0.68691

1106.89358	0.68732

1106.99036	0.68734

1107.08714	0.6876

1107.18392	0.68761

1107.2807	0.68766

1107.37748	0.68767

1107.47427	0.6881

1107.57105	0.68821

1107.66783	0.68827

1107.76461	0.68842

1107.86139	0.68844

1107.95818	0.68845

1108.05496	0.6886

1108.15174	0.68865

1108.24852	0.68866

1108.3453	0.68871

1108.44208	0.68873

1108.53887	0.68883

1108.63565	0.68903

1108.73243	0.68906

1108.82921	0.68908

1108.92599	0.68941

1109.02277	0.68947

1109.11956	0.68952

1109.21634	0.68953

1109.31312	0.68984

1109.4099	0.68985

1109.50668	0.68988

1109.60347	0.68994

1109.70025	0.69019

1109.79703	0.69054

1109.89381	0.69057

1109.99059	0.69087

1110.08738	0.6909

1110.18416	0.69106

1110.28094	0.69142

1110.37772	0.69144

1110.4745	0.69145

1110.57128	0.69183

1110.66807	0.69188

1110.76485	0.69203

1110.86163	0.69208

1110.95841	0.69214

1111.05519	0.69217

1111.15198	0.69218

1111.24876	0.6922

1111.34554	0.69223

1111.44232	0.69234

1111.5391	0.69255

1111.63588	0.69258

1111.73267	0.6926

1111.82945	0.69267

1111.92623	0.6927

1112.02301	0.69272

1112.11979	0.69289

1112.21657	0.6929

1112.31336	0.69293

1112.41014	0.69313

1112.50692	0.69314

1112.6037	0.69316

1112.70048	0.69318

1112.79726	0.69321

1112.89405	0.69325

1112.99083	0.69336

1113.08761	0.69397

1113.18439	0.69398

1113.28117	0.694

1113.37796	0.6942

1113.47474	0.69421

1113.57152	0.69423

1113.6683	0.69435

1113.76508	0.69438

1113.86187	0.69459

1113.95865	0.69465

1114.05543	0.69468

1114.15221	0.6947

1114.24899	0.69471

1114.34577	0.69473

1114.44256	0.6951

1114.53934	0.69516

1114.63612	0.69531

1114.7329	0.69555

1114.82968	0.69557

1114.92647	0.69558

1115.02325	0.6958

1115.12003	0.69583

1115.21681	0.6959

1115.31359	0.69592

1115.41037	0.69593

1115.50716	0.69613

1115.60394	0.69616

1115.70072	0.69618

1115.7975	0.69624

1115.89428	0.69633

1115.99106	0.69634

1116.08785	0.69656

1116.18463	0.6966

1116.28141	0.69665

1116.37819	0.69667

1116.47497	0.697

1116.57176	0.69709

1116.66854	0.69711

1116.76532	0.6974

1116.8621	0.69741

1116.95888	0.69743

1117.05567	0.69744

1117.15245	0.69746

1117.24923	0.69747

1117.34601	0.69767

1117.44279	0.69775

1117.53957	0.69782

1117.63636	0.69787

1117.73314	0.69804

1117.82992	0.69807

1117.9267	0.69808

1118.02348	0.6981

1118.12027	0.69831

1118.21705	0.69833

1118.31383	0.6984

1118.41061	0.69848

1118.50739	0.69851

1118.60417	0.69852

1118.70096	0.69857

1118.79774	0.69862

1118.89452	0.69863

1118.9913	0.69865

1119.08808	0.69916

1119.18486	0.69918

1119.28165	0.69933

1119.37843	0.69935

1119.47521	0.69938

1119.57199	0.69979

1119.66877	0.6998

1119.76556	0.69982

1119.86234	0.6999

1119.95912	0.69993

1120.0559	0.69997

1120.15268	0.70008

1120.24947	0.70009

1120.34625	0.70049

1120.44303	0.70051

1120.53981	0.70055

1120.63659	0.70064

1120.73337	0.70066

1120.83016	0.70067

1120.92694	0.70069

1121.02372	0.70078

1121.1205	0.70081

1121.21728	0.70084

1121.31407	0.70089

1121.41085	0.70093

1121.50763	0.70095

1121.60441	0.70096

1121.70119	0.70105

1121.79797	0.70108

1121.89476	0.70154

1121.99154	0.70182

1122.08832	0.70212

1122.1851	0.70241

1122.28188	0.70243

1122.37867	0.70285

1122.47545	0.70287

1122.57223	0.70305

1122.66901	0.7031

1122.76579	0.70311

1122.86257	0.70384

1122.95936	0.70413

1123.05614	0.70445

1123.15292	0.7045

1123.2497	0.70459

1123.34648	0.70474

1123.44327	0.70476

1123.54005	0.70509

1123.63683	0.70511

1123.73361	0.70549

1123.83039	0.7055

1123.92717	0.70559

1124.02395	0.70564

1124.12074	0.70567

1124.21752	0.7059

1124.3143	0.70613

1124.41108	0.70616

1124.50786	0.70642

1124.60465	0.70666

1124.70143	0.70668

1124.79821	0.70669

1124.89499	0.70674

1124.99177	0.70684

1125.08856	0.70687

1125.18534	0.70691

1125.28212	0.70732

1125.3789	0.70733

1125.47568	0.70736

1125.57246	0.70756

1125.66925	0.70788

1125.76603	0.7079

1125.86281	0.70808

1125.95959	0.70809

1126.05637	0.70831

1126.15316	0.70832

1126.24994	0.70837

1126.34672	0.7084

1126.4435	0.70844

1126.54028	0.70848

1126.63706	0.70887

1126.73385	0.70889

1126.83063	0.70915

1126.92741	0.70919

1127.02419	0.70939

1127.12097	0.70945

1127.21775	0.70985

1127.31454	0.70998

1127.41132	0.71047

1127.5081	0.71049

1127.60488	0.7105

1127.70166	0.71056

1127.79845	0.71061

1127.89523	0.7109

1127.99201	0.71111

1128.08879	0.71114

1128.18557	0.71116

1128.28236	0.71117

1128.37914	0.71134

1128.47592	0.71137

1128.5727	0.71154

1128.66948	0.71157

1128.76626	0.71158

1128.86305	0.71171

1128.95983	0.71174

1129.05661	0.71175

1129.15339	0.71177

1129.25017	0.71203

1129.34696	0.71204

1129.44374	0.71232

1129.54052	0.71233

1129.6373	0.71236

1129.73408	0.7127

1129.83086	0.71271

1129.92765	0.71302

1130.02443	0.71314

1130.12121	0.71318

1130.21799	0.71335

1130.31477	0.7136

1130.41155	0.71361

1130.50834	0.71363

1130.60512	0.71364

1130.7019	0.71375

1130.79868	0.71376

1130.89546	0.71379

1130.99225	0.71402

1131.08903	0.71411

1131.18581	0.71427

1131.28259	0.71431

1131.37937	0.71433

1131.47616	0.71465

1131.57294	0.71466

1131.66972	0.71489

1131.7665	0.71491

1131.86328	0.71501

1131.96006	0.71504

1132.05685	0.71545

1132.15363	0.71565

1132.25041	0.71571

1132.34719	0.71573

1132.44397	0.71574

1132.54076	0.7158

1132.63754	0.71597

1132.73432	0.71599

1132.8311	0.71616

1132.92788	0.71619

1133.02466	0.7164

1133.12145	0.71641

1133.21823	0.71644

1133.31501	0.71661

1133.41179	0.71684

1133.50857	0.71686

1133.60535	0.71687

1133.70214	0.71722

1133.79892	0.71724

1133.8957	0.71728

1133.99248	0.71747

1134.08926	0.71756

1134.18605	0.7176

1134.28283	0.71774

1134.37961	0.71776

1134.47639	0.71777

1134.57317	0.71783

1134.66996	0.71788

1134.76674	0.71789

1134.86352	0.71818

1134.9603	0.7182

1135.05708	0.71849

1135.15386	0.7185

1135.25065	0.71881

1135.34743	0.71882

1135.44421	0.71933

1135.54099	0.71934

1135.63777	0.71936

1135.73456	0.71945

1135.83134	0.71951

1135.92812	0.71952

1136.0249	0.71961

1136.12168	0.71968

1136.21846	0.71969

1136.31525	0.71978

1136.41203	0.7198

1136.50881	0.71983

1136.60559	0.71995

1136.70237	0.71997

1136.79915	0.7201

1136.89594	0.72018

1136.99272	0.72025

1137.0895	0.72027

1137.18628	0.72062

1137.28306	0.72074

1137.37984	0.72103

1137.47663	0.72105

1137.57341	0.72121

1137.67019	0.72128

1137.76697	0.72129

1137.86375	0.72149

1137.96054	0.7219

1138.05732	0.72192

1138.1541	0.72193

1138.25088	0.72195

1138.34766	0.72221

1138.44445	0.72248

1138.54123	0.72253

1138.63801	0.72259

1138.73479	0.72263

1138.83157	0.72295

1138.92835	0.72297

1139.02514	0.723

1139.12192	0.72317

1139.2187	0.72323

1139.31548	0.72327

1139.41226	0.72338

1139.50905	0.72341

1139.60583	0.72379

1139.70261	0.72381

1139.79939	0.72431

1139.89617	0.72434

1139.99295	0.7248

1140.08974	0.72481

1140.18652	0.72499

1140.2833	0.72513

1140.38008	0.72524

1140.47686	0.72527

1140.57364	0.72551

1140.67043	0.72554

1140.76721	0.72603

1140.86399	0.72608

1140.96077	0.72609

1141.05755	0.72623

1141.15434	0.72627

1141.25112	0.72633

1141.3479	0.72635

1141.44468	0.72656

1141.54146	0.72658

1141.63825	0.72684

1141.73503	0.72687

1141.83181	0.72731

1141.92859	0.7274

1142.02537	0.72775

1142.12215	0.72789

1142.21894	0.72795

1142.31572	0.72806

1142.4125	0.7281

1142.50928	0.72821

1142.60606	0.72825

1142.70285	0.72838

1142.79963	0.72844

1142.89641	0.7288

1142.99319	0.72886

1143.08997	0.72889

1143.18675	0.72896

1143.28354	0.72897

1143.38032	0.72915

1143.4771	0.72917

1143.57388	0.72949

1143.67066	0.7295

1143.76745	0.72952

1143.86423	0.72969

1143.96101	0.7299

1144.05779	0.73002

1144.15457	0.73004

1144.25135	0.73025

1144.34814	0.73027

1144.44492	0.73028

1144.5417	0.73054

1144.63848	0.73082

1144.73526	0.73085

1144.83205	0.73086

1144.92883	0.73123

1145.02561	0.73124

1145.12239	0.73152

1145.21917	0.73165

1145.31595	0.73168

1145.41274	0.7317

1145.50952	0.73171

1145.6063	0.73173

1145.70308	0.73197

1145.79986	0.73203

1145.89665	0.73206

1145.99343	0.73216

1146.09021	0.73223

1146.18699	0.73255

1146.28377	0.73269

1146.38055	0.73289

1146.47734	0.73296

1146.57412	0.73316

1146.6709	0.73318

1146.76768	0.73339

1146.86446	0.73342

1146.96125	0.73347

1147.05803	0.73348

1147.15481	0.73351

1147.25159	0.73353

1147.34837	0.73382

1147.44515	0.73405

1147.54194	0.73429

1147.63872	0.73435

1147.7355	0.73437

1147.83228	0.73456

1147.92906	0.73461

1148.02584	0.73462

1148.12263	0.73473

1148.21941	0.73478

1148.31619	0.73479

1148.41297	0.73487

1148.50975	0.7351

1148.60653	0.73511

1148.70332	0.73537

1148.8001	0.73539

1148.89688	0.7354

1148.99366	0.7359

1149.09045	0.73594

1149.18723	0.73609

1149.28401	0.73616

1149.38079	0.73618

1149.47757	0.73641

1149.57435	0.73642

1149.67114	0.73659

1149.76792	0.73661

1149.8647	0.73664

1149.96148	0.73679

1150.05826	0.7368

1150.15504	0.73697

1150.25183	0.73725

1150.34861	0.73729

1150.44539	0.73775

1150.54217	0.73776

1150.63895	0.73786

1150.73574	0.7379

1150.83252	0.73793

1150.9293	0.73795

1151.02608	0.73798

1151.12286	0.73799

1151.21964	0.73807

1151.31643	0.73813

1151.41321	0.73814

1151.50999	0.73833

1151.60677	0.73834

1151.70355	0.73869

1151.80033	0.73871

1151.89712	0.73871

1151.9939	0.73873

1152.09068	0.73882

1152.18746	0.73891

1152.28424	0.73894

1152.38103	0.73903

1152.47781	0.73905

1152.57459	0.73907

1152.67137	0.73912

1152.76815	0.73914

1152.86494	0.73933

1152.96172	0.73935

1153.0585	0.73939

1153.15528	0.73942

1153.25206	0.73944

1153.34884	0.73946

1153.44563	0.73949

1153.54241	0.73951

1153.63919	0.73953

1153.73597	0.73955

1153.83275	0.73965

1153.92954	0.73969

1154.02632	0.73971

1154.1231	0.73976

1154.21988	0.73987

1154.31666	0.7399

1154.41344	0.73992

1154.51023	0.73999

1154.60701	0.74001

1154.70379	0.74003

1154.80057	0.74006

1154.89735	0.74008

1154.99413	0.7401

1155.09092	0.74013

1155.1877	0.74015

1155.28448	0.74019

1155.38126	0.74022

1155.47804	0.74026

1155.57483	0.74029

1155.67161	0.74031

1155.76839	0.74035

1155.86517	0.74038

1155.96195	0.7404

1156.05874	0.74042

1156.15552	0.74061

1156.2523	0.74072

1156.34908	0.74074

1156.44586	0.74088

1156.54264	0.74093

1156.63943	0.74095

1156.73621	0.74097

1156.83299	0.74099

1156.92977	0.74104

1157.02655	0.74111

1157.12334	0.74113

1157.22012	0.74118

1157.3169	0.7412

1157.41368	0.74125

1157.51046	0.74131

1157.60724	0.74143

1157.70403	0.74145

1157.80081	0.74147

1157.89759	0.7415

1157.99437	0.74152

1158.09115	0.74166

1158.18793	0.74168

1158.28472	0.74175

1158.3815	0.74177

1158.47828	0.74179

1158.57506	0.74184

1158.67184	0.74186

1158.76863	0.74189

1158.86541	0.742

1158.96219	0.74211

1159.05897	0.74225

1159.15575	0.74227

1159.25254	0.7423

1159.34932	0.74232

1159.4461	0.74234

1159.54288	0.74239

1159.63966	0.7425

1159.73644	0.74253

1159.83323	0.74259

1159.93001	0.74269

1160.02679	0.74278

1160.12357	0.7428

1160.22035	0.74282

1160.31714	0.74285

1160.41392	0.74296

1160.5107	0.74303

1160.60748	0.74305

1160.70426	0.74312

1160.80104	0.74319

1160.89783	0.7465

1160.99461	0.74657

1161.09139	0.74659

1161.18817	0.74671

1161.28495	0.74673

1161.38173	0.74682

1161.47852	0.74685

1161.5753	0.74694

1161.67208	0.74698

1161.76886	0.74701

1161.86564	0.74705

1161.96242	0.74707

1162.05921	0.7471

1162.15599	0.74712

1162.25277	0.74714

1162.34955	0.74717

1162.44633	0.74719

1162.54312	0.74721

1162.6399	0.74723

1162.73668	0.74733

1162.83346	0.74739

1162.93024	0.74753

1163.02703	0.74767

1163.12381	0.74769

1163.22059	0.74774

1163.31737	0.74776

1163.41415	0.74785

1163.51093	0.74792

1163.60772	0.74794

1163.7045	0.74797

1163.80128	0.74799

1163.89806	0.74806

1163.99484	0.7481

1164.09163	0.74813

1164.18841	0.74819

1164.28519	0.74826

1164.38197	0.7521

1164.47875	0.75213

1164.57553	0.7562

1164.67232	0.75622

1164.7691	0.75626

1164.86588	0.75629

1164.96266	0.75649

1165.05944	0.75658

1165.15622	0.75661

1165.25301	0.75665

1165.34979	0.75668

1165.44657	0.7567

1165.54335	0.75672

1165.64013	0.75674

1165.73692	0.75677

1165.8337	0.75688

1165.93048	0.7569

1166.02726	0.75725

1166.12404	0.75727

1166.22083	0.75732

1166.31761	0.75738

1166.41439	0.75748

1166.51117	0.75752

1166.60795	0.75754

1166.70473	0.75761

1166.80152	0.75763

1166.8983	0.75769

1166.99508	0.75775

1167.09186	0.75778

1167.18864	0.75804

1167.28543	0.75818

1167.38221	0.75819

1167.47899	0.75821

1167.57577	0.75822

1167.67255	0.75824

1167.76933	0.75825

1167.86612	0.75833

1167.9629	0.75834

1168.05968	0.75837

1168.15646	0.75845

1168.25324	0.75856

1168.35003	0.75935

1168.44681	0.7594

1168.54359	0.75941

1168.64037	0.76029

1168.73715	0.76031

1168.83393	0.7604

1168.93072	0.7606

1169.0275	0.76061

1169.12428	0.76064

1169.22106	0.76095

1169.31784	0.76098

1169.41462	0.99281

1169.51141	0.99296

1169.60819	0.99378

1169.70497	0.9939

1169.80175	0.99628

1169.89853	0.99631

1169.99532	0.9964

1170.0921	0.99648

1170.18888	0.99736

1170.28566	0.99787

1170.38244	0.99808

1170.47923	0.99817

1170.57601	0.99927

1170.67279	0.99936

1170.76957	0.99957

1170.86635	0.99965

1170.96313	0.99966

1171.05992	0.99994

1171.1567	0.99998

1171.25348	1

**（*b*）Stabilized hydraulic-0°**

1	0.04596	2.41066	3.11219E-05

2	0.06524	2.57137	0.000103469

3	0.08303	2.73208	0.000158157

4	0.10676	2.89279	0.000211219

5	0.1201	3.0535	0.000234688

6	0.13048	3.21421	0.000258157

7	0.14827	3.37492	2.82E-04

8	0.16607	3.53563	3.29E-04

9	0.17645	3.69634	3.52E-04

10	0.19127	3.85706	3.76E-04

11	0.21203	4.01777	3.99E-04

12	0.22093	4.17848	4.46E-04

13	0.23575	4.33919	4.69E-04

14	0.25503	4.4999	4.93E-04

15	0.26986	4.66061	5.16E-04

16	0.27875	4.82132	5.40E-04

17	0.29951	4.98203	5.87E-04

18	0.31286	5.14274	6.34E-04

19	0.32472	5.30345	6.57E-04

20	0.34103	5.46416	9.39E-04

21	0.35882	5.62487	9.86E-04

22	0.37217	5.78558	0.00101

23	0.38551	5.94629	0.00103

24	0.40182	6.107	0.00108

25	0.41961	6.26772	0.00134

26	0.42999	6.42843	0.00136

27	0.4463	6.58914	0.00138

28	0.46261	6.74985	0.00143

29	0.47447	6.91056	0.00146

30	0.48634	7.07127	0.00148

31	0.50709	7.23198	0.00155

32	0.51896	7.39269	0.00157

33	0.5323	7.5534	0.0016

34	0.55009	7.71411	0.00162

35	0.5664	7.87482	0.00169

36	0.58271	8.03553	0.00174

37	0.59161	8.19624	0.00176

38	0.61237	8.35695	0.00183

39	0.61978	8.51766	0.00185

40	0.63609	8.67838	0.00188

41	0.65389	8.83909	0.00192

42	0.66871	8.9998	0.00207

43	0.67909	9.16051	0.00237

44	0.6954	9.32122	0.00244

45	0.71171	9.48193	0.00249

46	0.72357	9.64264	0.00256

47	0.74137	9.80335	0.00263

48	0.75619	9.96406	0.00265

49	0.76954	10.12477	0.00268

50	0.78437	10.28548	0.0027

51	0.80216	10.44619	0.00275

52	0.81847	10.6069	0.00277

53	0.82588	10.76761	0.003

54	0.84368	10.92832	0.00303

55	0.85999	11.08903	0.00305

56	0.87333	11.24975	0.00333

57	0.88816	11.41046	0.00338

58	0.90743	11.57117	0.00345

59	0.91929	11.73188	0.00347

60	0.93116	11.89259	0.0035

61	0.94895	12.0533	0.00354

62	0.96229	12.21401	0.00357

63	0.97564	12.37472	0.00359

64	0.99343	12.53543	0.00361

65	1.00974	12.69614	0.00364

66	1.02012	12.85685	0.00383

67	1.03643	13.01756	0.00385

68	1.05571	13.17827	0.00512

69	1.06757	13.33898	0.00514

70	1.07943	13.49969	0.00516

71	1.10019	13.66041	0.00521

72	1.11353	13.82112	0.00523

73	1.12243	13.98183	0.00526

74	1.1417	14.14254	0.00528

75	1.15801	14.30325	0.00537

76	1.16839	14.46396	0.0054

77	1.18322	14.62467	0.00554

78	1.20398	14.78538	0.00561

79	1.21732	14.94609	0.00563

80	1.22474	15.1068	0.00566

81	1.24401	15.26751	0.00568

82	1.26181	15.42822	0.0057

83	1.26922	15.58893	0.00573

84	1.28701	15.74964	0.00575

85	1.30629	15.91035	0.00577

86	1.31815	16.07107	0.0058

87	1.33001	16.23178	0.00582

88	1.34929	16.39249	0.00587

89	1.36411	16.5532	0.00591

90	1.37746	16.71391	0.00601

91	1.39525	16.87462	0.00603

92	1.4086	17.03533	0.0061

93	1.41749	17.19604	0.0062

94	1.4338	17.35675	0.00622

95	1.45456	17.51746	0.00667

96	1.46791	17.67817	0.00669

97	1.47977	17.83888	0.00683

98	1.50053	17.99959	0.00695

99	1.51239	18.1603	0.00697

100	1.52277	18.32101	0.00702

101	1.54056	18.48172	0.00704

102	1.55687	18.64244	0.00706

103	1.56577	18.80315	0.00709

104	1.58356	18.96386	0.00711

105	1.60432	19.12457	0.00713

106	1.6147	19.28528	0.00716

107	1.62656	19.44599	0.0072

108	1.64732	19.6067	0.00728

109	1.65918	19.76741	0.00735

110	1.66956	19.92812	0.00737

111	1.68883	20.08883	0.00739

112	1.70663	20.24954	0.00742

113	1.71552	20.41025	0.00744

114	1.73035	20.57096	0.00753

115	1.74963	20.73167	0.00756

116	1.75852	20.89238	0.0076

117	1.78225	21.0531	0.00763

118	1.75259	21.21381	0.00765

119	1.77187	21.37452	0.0077

120	1.78076	21.53523	0.00772

121	1.79559	21.69594	0.00774

122	1.8119	21.85665	0.00786

123	1.81931	22.01736	0.00789

124	1.83414	22.17807	0.00791

125	1.8549	22.33878	0.00805

126	1.86824	22.49949	0.00807

127	1.87862	22.6602	0.0081

128	1.89938	22.82091	0.00833

129	1.91273	22.98162	0.00835

130	1.92607	23.14233	0.0084

131	1.94238	23.30304	0.0085

132	1.96017	23.46375	0.00852

133	1.96907	23.62447	0.00857

134	1.98241	23.78518	0.00859

135	2.00317	23.94589	0.00864

136	2.01652	24.1066	0.00866

137	2.02541	24.26731	0.00868

138	2.04469	24.42802	0.00871

139	2.06248	24.58873	0.00873

140	2.07138	24.74944	0.00878

141	2.08917	24.91015	0.00892

142	2.10548	25.07086	0.00894

143	2.11734	25.23157	0.00897

144	2.12772	25.39228	0.00899

145	2.14996	25.55299	0.00906

146	2.16034	25.7137	0.00908

147	2.1722	25.87441	0.00911

148	2.19445	26.03513	0.00915

149	2.21076	26.19584	0.0092

150	2.22262	26.35655	0.00927

151	2.23596	26.51726	0.00929

152	2.25524	26.67797	0.00934

153	2.2671	26.83868	0.00936

154	2.276	26.99939	0.00943

155	2.29824	27.1601	0.00948

156	2.31306	27.32081	0.00967

157	2.32344	27.48152	0.00976

158	2.33975	27.64223	0.00981

159	2.35903	27.80294	0.00983

160	2.36644	27.96365	0.0099

161	2.38424	28.12436	0.00993

162	2.40203	28.28507	0.00997

163	2.41537	28.44579	0.01

164	2.42872	28.6065	0.0104

165	2.44651	28.76721	0.01049

166	2.46282	28.92792	0.01051

167	2.47468	29.08863	0.01058

168	2.48654	29.24934	0.01061

169	2.50878	29.41005	0.01065

170	2.51916	29.57076	0.01068

171	2.53399	29.73147	0.0107

172	2.5503	29.89218	0.01077

173	2.56513	30.05289	0.01091

174	2.57699	30.2136	0.01094

175	2.5933	30.37431	0.01155

176	2.61109	30.53502	0.01157

177	2.61999	30.69573	0.01162

178	2.6363	30.85644	0.01169

179	2.65558	31.01716	0.01176

180	2.66595	31.17787	0.0119

181	2.6793	31.33858	0.01199

182	2.69857	31.49929	0.01204

183	2.71192	31.66	0.01206

184	2.7223	31.82071	0.0122

185	2.74306	31.98142	0.01223

186	2.75937	32.14213	0.01227

187	2.76678	32.30284	0.0123

188	2.78457	32.46355	0.01232

189	2.80237	32.62426	0.01249

190	2.81275	32.78497	0.01253

191	2.82609	32.94568	0.01256

192	2.84537	33.10639	0.01258

193	2.86168	33.2671	0.0126

194	2.87057	33.42782	0.01263

195	2.89133	33.58853	0.01272

196	2.91061	33.74924	0.01277

197	2.9195	33.90995	0.01281

198	2.93136	34.07066	0.01286

199	2.95064	34.23137	0.01288

200	2.96102	34.39208	0.01291

201	2.97436	34.55279	0.01293

202	2.99512	34.7135	0.01298

203	3.01143	34.87421	0.013

204	3.02181	35.03492	0.01303

205	3.03812	35.19563	0.01314

206	3.0574	35.35634	0.01326

207	3.06778	35.51705	0.01352

208	3.08112	35.67776	0.01354

209	3.09891	35.83848	0.01364

210	3.11226	35.99919	0.01366

211	3.12708	36.1599	0.01368

212	3.14339	36.32061	0.01375

213	3.15674	36.48132	0.01382

214	3.16712	36.64203	0.01385

215	3.18491	36.80274	0.01394

216	3.2027	36.96345	0.01396

217	3.21605	37.12416	0.01399

218	3.22643	37.28487	0.01401

219	3.25015	37.44558	0.01403

220	3.26201	37.60629	0.01408

221	3.27239	37.767	0.01422

222	3.29315	37.92771	0.01429

223	3.3065	38.08842	0.01432

224	3.31391	38.24913	0.01434

225	3.33318	38.40985	0.01436

226	3.35246	38.57056	0.01439

227	3.36136	38.73127	0.01441

228	3.37915	38.89198	0.01446

229	3.39398	39.05269	0.01448

230	3.41029	39.2134	0.01511

231	3.42067	39.37411	0.01514

232	3.43846	39.53482	0.01516

233	3.45477	39.69553	0.01518

234	3.46515	39.85624	0.01521

235	3.48146	40.01695	0.01523

236	3.50073	40.17766	0.01525

237	3.5126	40.33837	0.0153

238	3.52594	40.49908	0.01533

239	3.54373	40.65979	0.01537

240	3.55559	40.82051	0.0154

241	3.56894	40.98122	0.01556

242	3.58821	41.14193	0.01558

243	3.60304	41.30264	0.01563

244	3.61342	41.46335	0.01565

245	3.62825	41.62406	0.01568

246	3.64752	41.78477	0.01575

247	3.66087	41.94548	0.01577

248	3.67273	42.10619	0.01582

249	3.69497	42.2669	0.01591

250	3.71128	42.42761	0.01598

251	3.71721	42.58832	0.01601

252	3.73204	42.74903	0.01612

253	3.75132	42.90974	0.01617

254	3.76318	43.07045	0.01619

255	3.77652	43.23116	0.01622

256	3.79728	43.39188	0.01626

257	3.81062	43.55259	0.01631

258	3.821	43.7133	0.01633

259	3.83731	43.87401	0.01751

260	3.85659	44.03472	0.01753

261	3.86549	44.19543	0.01755

262	3.88031	44.35614	0.01774

263	3.89959	44.51685	0.01777

264	3.91145	44.67756	0.01781

265	3.92776	44.83827	0.01786

266	3.94555	44.99898	0.01788

267	3.9589	45.15969	0.01791

268	3.97224	45.3204	0.01793

269	3.98855	45.48111	0.01847

270	4.00486	45.64182	0.01849

271	4.01524	45.80254	0.01863

272	4.03007	45.96325	0.01868

273	4.04934	46.12396	0.01873

274	4.06269	46.28467	0.01875

275	4.07307	46.44538	0.01882

276	4.09531	46.60609	0.01887

277	4.11162	46.7668	0.01899

278	4.11607	46.92751	0.01903

279	4.13979	47.08822	0.01906

280	4.1561	47.24893	0.01908

281	4.15907	47.40964	0.0191

282	4.17686	47.57035	0.01917

283	4.19762	47.73106	0.0192

284	4.20948	47.89177	0.01924

285	4.21986	48.05248	0.01927

286	4.24507	48.2132	0.01931

287	4.25693	48.37391	0.01943

288	4.26879	48.53462	0.01946

289	4.28362	48.69533	0.01962

290	4.30141	48.85604	0.01971

291	4.30882	49.01675	0.01976

292	4.32662	49.17746	0.01983

293	4.34737	49.33817	0.01985

294	4.35924	49.49888	0.02086

295	4.3711	49.65959	0.02089

296	4.38889	49.8203	0.02091

297	4.4052	49.98101	0.02117

298	4.41706	50.14172	0.02122

299	4.43189	50.30243	0.02124

300	4.4482	50.46314	0.02126

301	4.45858	50.62385	0.02131

302	4.47489	50.78457	0.02133

303	4.4912	50.94528	0.02147

304	4.50751	51.10599	0.02152

305	4.51789	51.2667	0.02154

306	4.53716	51.42741	0.02157

307	4.55347	51.58812	0.02159

308	4.56089	51.74883	0.02161

309	4.5772	51.90954	0.02164

310	4.59647	52.07025	0.02173

311	4.60389	52.23096	0.02178

312	4.62168	52.39167	0.0218

313	4.64096	52.55238	0.0219

314	4.65282	52.71309	0.02201

315	4.66468	52.8738	0.02204

316	4.68247	53.03451	0.02208

317	4.6973	53.19523	0.02213

318	4.71213	53.35594	0.02215

319	4.72844	53.51665	0.02225

320	4.74326	53.67736	0.02232

321	4.75809	53.83807	0.02234

322	4.76847	53.99878	0.02237

323	4.78775	54.15949	0.02239

324	4.80257	54.3202	0.02241

325	4.81444	54.48091	0.02248

326	4.83075	54.64162	0.02251

327	4.84854	54.80233	0.02253

328	4.85892	54.96304	0.02258

329	4.87226	55.12375	0.02265

330	4.89747	55.28446	0.02291

331	4.9034	55.44517	0.02293

332	4.9123	55.60589	0.02295

333	4.93602	55.7666	0.02305

334	4.95381	55.92731	0.02312

335	4.95826	56.08802	0.02316

336	4.9805	56.24873	0.02319

337	4.99533	56.40944	0.02321

338	5.00571	56.57015	0.02326

339	5.0235	56.73086	0.02328

340	5.04278	56.89157	0.02335

341	5.05612	57.05228	0.02337

342	5.0665	57.21299	0.02345

343	5.08726	57.3737	0.02361

344	5.10505	57.53441	0.02366

345	5.1095	57.69512	0.02377

346	5.12877	57.85583	0.02384

347	5.14657	58.01654	0.02387

348	5.15695	58.17726	0.02389

349	5.17029	58.33797	0.02391

350	5.18957	58.49868	0.02394

351	5.20439	58.65939	0.02396

352	5.21477	58.8201	0.02399

353	5.23405	58.98081	0.0241

354	5.24888	59.14152	0.02413

355	5.25926	59.30223	0.02441

356	5.27557	59.46294	0.02445

357	5.29336	59.62365	0.02448

358	5.3067	59.78436	0.02455

359	5.31708	59.94507	0.02457

360	5.34081	60.10578	0.02467

361	5.35563	60.26649	0.02469

362	5.36305	60.4272	0.02481

363	5.38232	60.58792	0.02483

364	5.40011	60.74863	0.0249

365	5.40605	60.90934	0.02492

366	5.4268	61.07005	0.02497

367	5.44608	61.23076	0.02499

368	5.45498	61.39147	0.02502

369	5.46832	61.55218	0.02506

370	5.48908	61.71289	0.02509

371	5.50242	61.8736	0.02511

372	5.51132	62.03431	0.02518

373	5.52911	62.19502	0.02523

374	5.54542	62.35573	0.0253

375	5.55432	62.51644	0.02537

376	5.57211	62.67715	0.02539

377	5.59139	62.83786	0.02542

378	5.60177	62.99858	0.02544

379	5.61659	63.15929	0.02551

380	5.63439	63.32	0.02553

381	5.64625	63.48071	0.02556

382	5.65811	63.64142	0.0256

383	5.67739	63.80213	0.0257

384	5.69666	63.96284	0.02575

385	5.70408	64.12355	0.02579

386	5.72187	64.28426	0.02589

387	5.73818	64.44497	0.02591

388	5.75301	64.60568	0.02596

389	5.7619	64.76639	0.02598

390	5.77969	64.9271	0.026

391	5.80045	65.08781	0.02605

392	5.8049	65.24852	0.02612

393	5.82418	65.40923	0.02617

394	5.84049	65.56995	0.02619

395	5.8568	65.73066	0.02621

396	5.86569	65.89137	0.02624

397	5.88497	66.05208	0.02629

398	5.90128	66.21279	0.02633

399	5.91018	66.3735	0.02636

400	5.92649	66.53421	0.02638

401	5.94873	66.69492	0.02647

402	5.95317	66.85563	0.0265

403	5.97097	67.01634	0.02654

404	5.99024	67.17705	0.02657

405	6.0021	67.33776	0.02659

406	6.01693	67.49847	0.02661

407	6.03621	67.65918	0.02713

408	6.04955	67.81989	0.02718

409	6.05993	67.98061	0.02744

410	6.08069	68.14132	0.02748

411	6.09552	68.30203	0.02769

412	6.10293	68.46274	0.02774

413	6.11776	68.62345	0.02776

414	6.14	68.78416	0.02788

415	6.15186	68.94487	0.0279

416	6.16224	69.10558	0.02793

417	6.18003	69.26629	0.028

418	6.20227	69.427	0.02802

419	6.20672	69.58771	0.02805

420	6.22303	69.74842	0.02809

421	6.24379	69.90913	0.02814

422	6.25417	70.06984	0.02816

423	6.269	70.23055	0.02819

424	6.28679	70.39126	0.02826

425	6.30013	70.55198	0.02828

426	6.30903	70.71269	0.0283

427	6.33127	70.8734	0.02833

428	6.34758	71.03411	0.02835

429	6.355	71.19482	0.02837

430	6.37131	71.35553	0.02842

431	6.39355	71.51624	0.02856

432	6.40244	71.67695	0.02882

433	6.4143	71.83766	0.02887

434	6.43655	71.99837	0.02898

435	6.44692	72.15908	0.02905

436	6.45879	72.31979	0.02912

437	6.47954	72.4805	0.02915

438	6.49585	72.64121	0.02917

439	6.50623	72.80192	0.02959

440	6.51958	72.96264	0.02962

441	6.53885	73.12335	0.02966

442	6.54923	73.28406	0.02969

443	6.56109	73.44477	0.02988

444	6.58037	73.60548	0.03002

445	6.5952	73.76619	0.03124

446	6.60706	73.9269	0.03128

447	6.62189	74.08761	0.03131

448	6.64413	74.24832	0.03135

449	6.65451	74.40903	0.0314

450	6.66489	74.56974	0.03145

451	6.68713	74.73045	0.03154

452	6.69602	74.89116	0.03161

453	6.71233	75.05187	0.03185

454	6.73161	75.21258	0.03189

455	6.74495	75.3733	0.03192

456	6.75237	75.53401	0.03194

457	6.77313	75.69472	0.03196

458	6.78795	75.85543	0.03201

459	6.8013	76.01614	0.03206

460	6.81613	76.17685	0.03211

461	6.83688	76.33756	0.03213

462	6.84578	76.49827	0.03222

463	6.85912	76.65898	0.03241

464	6.87988	76.81969	0.03243

465	6.89619	76.9804	0.03246

466	6.90509	77.14111	0.03253

467	6.91695	77.30182	0.03255

468	6.94067	77.46253	0.03257

469	6.94957	77.62324	0.03265

470	6.95995	77.78395	0.03272

471	6.98367	77.94467	0.03276

472	6.9985	78.10538	0.03304

473	7.00888	78.26609	0.03307

474	7.02519	78.4268	0.03309

475	7.04447	78.58751	0.03321

476	7.05485	78.74822	0.03333

477	7.06967	78.90893	0.03337

478	7.08598	79.06964	0.0334

479	7.09636	79.23035	0.03342

480	7.10822	79.39106	0.03344

481	7.1275	79.55177	0.03347

482	7.14233	79.71248	0.03354

483	7.15419	79.87319	0.03356

484	7.17346	80.0339	0.03358

485	7.19126	80.19461	0.03368

486	7.19867	80.35533	0.03372

487	7.21646	80.51604	0.03377

488	7.23574	80.67675	0.03384

489	7.24464	80.83746	0.03389

490	7.25798	80.99817	0.03394

491	7.2817	81.15888	0.03396

492	7.29505	81.31959	0.03398

493	7.30246	81.4803	0.03401

494	7.3247	81.64101	0.03403

495	7.34101	81.80172	0.03405

496	7.34694	81.96243	0.03422

497	7.36325	82.12314	0.03424

498	7.38253	82.28385	0.03426

499	7.39143	82.44456	0.03429

500	7.40774	82.60527	0.03431

501	7.42405	82.76599	0.03462

502	7.44184	82.9267	0.03464

503	7.45073	83.08741	0.03478

504	7.46705	83.24812	0.0348

505	7.4878	83.40883	0.03483

506	7.49522	83.56954	0.03487

507	7.51301	83.73025	0.0349

508	7.52784	83.89096	0.03495

509	7.54563	84.05167	0.0353

510	7.55304	84.21238	0.03534

511	7.57677	84.37309	0.03539

512	7.59011	84.5338	0.03541

513	7.60049	84.69451	0.03563

514	7.61532	84.85522	0.03577

515	7.63608	85.01593	0.03581

516	7.64794	85.17664	0.03584

517	7.6598	85.33736	0.03586

518	7.67908	85.49807	0.03595

519	7.69094	85.65878	0.03598

520	7.70428	85.81949	0.036

521	7.72504	85.9802	0.03602

522	7.73839	86.14091	0.03605

523	7.74728	86.30162	0.03607

524	7.76359	86.46233	0.03614

525	7.7799	86.62304	0.03617

526	7.79473	86.78375	0.03619

527	7.80659	86.94446	0.03626

528	7.82735	87.10517	0.03631

529	7.83921	87.26588	0.03633

530	7.85107	87.42659	0.03635

531	7.8659	87.5873	0.03645

532	7.88666	87.74802	0.03647

533	7.89555	87.90873	0.03649

534	7.91187	88.06944	0.03656

535	7.93262	88.23015	0.03663

536	7.94152	88.39086	0.03666

537	7.95338	88.55157	0.03678

538	7.97266	88.71228	0.03682

539	7.99045	88.87299	0.03685

540	8.00083	89.0337	0.03687

541	8.0201	89.19441	0.03699

542	8.03641	89.35512	0.03701

543	8.04828	89.51583	0.03706

544	8.05717	89.67654	0.03708

545	8.07941	89.83725	0.03715

546	8.09276	89.99796	0.03717

547	8.10462	90.15867	0.0372

548	8.12241	90.31939	0.03722

549	8.14021	90.4801	0.03724

550	8.1491	90.64081	0.03769

551	8.1669	90.80152	0.03774

552	8.18321	90.96223	0.03776

553	8.19358	91.12294	0.03778

554	8.20841	91.28365	0.03783

555	8.22769	91.44436	0.03786

556	8.23955	91.60507	0.03795

557	8.24993	91.76578	0.038

558	8.27069	91.92649	0.03809

559	8.28996	92.0872	0.03811

560	8.29589	92.24791	0.03821

561	8.3122	92.40862	0.03823

562	8.33148	92.56933	0.0383

563	8.34186	92.73005	0.03839

564	8.35669	92.89076	0.03847

565	8.373	93.05147	0.03875

566	8.38931	93.21218	0.03882

567	8.40413	93.37289	0.03886

568	8.41599	93.5336	0.03889

569	8.4412	93.69431	0.03901

570	8.44713	93.85502	0.0391

571	8.46048	94.01573	0.03915

572	8.4842	94.17644	0.03917

573	8.49161	94.33715	0.03926

574	8.50199	94.49786	0.03933

575	8.52572	94.65857	0.03936

576	8.54203	94.81928	0.03938

577	8.54944	94.97999	0.03947

578	8.56723	95.14071	0.03966

579	8.58354	95.30142	0.03985

580	8.59689	95.46213	0.03987

581	8.60727	95.62284	0.03994

582	8.62803	95.78355	0.03999

583	8.6384	95.94426	0.04001

584	8.64878	96.10497	0.04004

585	8.67251	96.26568	0.04016

586	8.68585	96.42639	0.04027

587	8.69475	96.5871	0.0403

588	8.71551	96.74781	0.04032

589	8.73033	96.90852	0.04039

590	8.7422	97.06923	0.04041

591	8.75702	97.22994	0.04044

592	8.7763	97.39065	0.04055

593	8.78816	97.55136	0.0406

594	8.79854	97.71208	0.04062

595	8.82078	97.87279	0.04069

596	8.83857	98.0335	0.04072

597	8.84747	98.19421	0.04086

598	8.86378	98.35492	0.04088

599	8.88306	98.51563	0.04133

600	8.89195	98.67634	0.04161

601	8.9053	98.83705	0.04163

602	8.92012	98.99776	0.04166

603	8.9394	99.15847	0.04173

604	8.94978	99.31918	0.04175

605	8.96757	99.47989	0.04189

606	8.98536	99.6406	0.04194

607	8.99426	99.80131	0.04196

608	9.0076	99.96202	0.04199

609	9.02836	100.12274	0.04201

610	9.03874	100.28345	0.04203

611	9.05209	100.44416	0.04206

612	9.07136	100.60487	0.0421

613	9.08767	100.76558	0.04222

614	9.09657	100.92629	0.04224

615	9.11881	101.087	0.04227

616	9.13215	101.24771	0.04236

617	9.14402	101.40842	0.04248

618	9.15588	101.56913	0.04253

619	9.18108	101.72984	0.04264

620	9.1885	101.89055	0.04267

621	9.19739	102.05126	0.04278

622	9.2226	102.21197	0.04283

623	9.23743	102.37268	0.04288

624	9.24633	102.5334	0.04302

625	9.2656	102.69411	0.04304

626	9.28339	102.85482	0.04311

627	9.29674	103.01553	0.04314

628	9.30119	103.17624	0.04316

629	9.32491	103.33695	0.04318

630	9.33677	103.49766	0.04321

631	9.35012	103.65837	0.04323

632	9.36939	103.81908	0.04325

633	9.3857	103.97979	0.04328

634	9.39756	104.1405	0.0433

635	9.41387	104.30121	0.04339

636	9.43018	104.46192	0.04342

637	9.44056	104.62263	0.04344

638	9.45242	104.78334	0.04346

639	9.47467	104.94405	0.04349

640	9.48356	105.10477	0.04353

641	9.49691	105.26548	0.04358

642	9.5147	105.42619	0.04363

643	9.53101	105.5869	0.04365

644	9.54139	105.74761	0.0437

645	9.55622	105.90832	0.04372

646	9.57994	106.06903	0.04375

647	9.58735	106.22974	0.04382

648	9.60366	106.39045	0.044

649	9.62294	106.55116	0.04403

650	9.63628	106.71187	0.04405

651	9.64963	106.87258	0.04407

652	9.66594	107.03329	0.04414

653	9.68225	107.194	0.04417

654	9.68966	107.35471	0.04424

655	9.70746	107.51543	0.04429

656	9.72821	107.67614	0.04497

657	9.73859	107.83685	0.04499

658	9.74897	107.99756	0.04504

659	9.7727	108.15827	0.04506

660	9.78752	108.31898	0.04513

661	9.79345	108.47969	0.04515

662	9.81273	108.6404	0.04518

663	9.83349	108.80111	0.04565

664	9.83942	108.96182	0.04567

665	9.85425	109.12253	0.04572

666	9.87352	109.28324	0.04574

667	9.88983	109.44395	0.04576

668	9.90169	109.60466	0.04623

669	9.918	109.76537	0.04696

670	9.93283	109.92609	0.04701

671	9.94173	110.0868	0.04708

672	9.95804	110.24751	0.04713

673	9.98028	110.40822	0.04717

674	9.98621	110.56893	0.04722

675	10.00104	110.72964	0.04729

676	10.02476	110.89035	0.04731

677	10.02921	111.05106	0.04738

678	10.04404	111.21177	0.04743

679	10.06628	111.37248	0.04745

680	10.0811	111.53319	0.04748

681	10.09	111.6939	0.04759

682	10.10928	111.85461	0.04762

683	10.13003	112.01532	0.04769

684	10.13597	112.17603	0.04776

685	10.14783	112.33674	0.04781

686	10.16859	112.49746	0.04783

687	10.1849	112.65817	0.04788

688	10.19379	112.81888	0.0479

689	10.21158	112.97959	0.04792

690	10.23086	113.1403	0.04795

691	10.23976	113.30101	0.04802

692	10.2531	113.46172	0.04804

693	10.27238	113.62243	0.04806

694	10.28276	113.78314	0.04809

695	10.2961	113.94385	0.04816

696	10.31686	114.10456	0.04818

697	10.33317	114.26527	0.0483

698	10.34355	114.42598	0.04832

699	10.35838	114.58669	0.04835

700	10.37469	114.7474	0.04846

701	10.3821	114.90812	0.04849

702	10.39841	115.06883	0.04851

703	10.42213	115.22954	0.04853

704	10.43399	115.39025	0.0486

705	10.44437	115.55096	0.04863

706	10.46365	115.71167	0.04867

707	10.48292	115.87238	0.0487

708	10.48737	116.03309	0.04874

709	10.50665	116.1938	0.04877

710	10.52592	116.35451	0.04879

711	10.53482	116.51522	0.04907

712	10.55261	116.67593	0.04917

713	10.57041	116.83664	0.04921

714	10.57634	116.99735	0.04924

715	10.59116	117.15806	0.04964

716	10.61785	117.31877	0.04966

717	10.62675	117.47949	0.04968

718	10.63416	117.6402	0.04982

719	10.6564	117.80091	0.04985

720	10.67271	117.96162	0.04992

721	10.68013	118.12233	0.04997

722	10.69792	118.28304	0.05004

723	10.72164	118.44375	0.05011

724	10.73351	118.60446	0.05046

725	10.74092	118.76517	0.0505

726	10.75871	118.92588	0.05055

727	10.77502	119.08659	0.05058

728	10.78689	119.2473	0.05097

729	10.80023	119.40801	0.051

730	10.81951	119.56872	0.05102

731	10.83137	119.72943	0.05112

732	10.84471	119.89015	0.05119

733	10.8714	120.05086	0.05121

734	10.88178	120.21157	0.05128

735	10.88771	120.37228	0.0513

736	10.91143	120.53299	0.05137

737	10.9233	120.6937	0.0514

738	10.93071	120.85441	0.05144

739	10.95147	121.01512	0.05161

740	10.96926	121.17583	0.05163

741	10.98112	121.33654	0.05168

742	10.99595	121.49725	0.0517

743	11.01523	121.65796	0.05182

744	11.02412	121.81867	0.05184

745	11.03598	121.97938	0.05187

746	11.05971	122.14009	0.05262

747	11.07454	122.30081	0.05264

748	11.08343	122.46152	0.05266

749	11.09974	122.62223	0.05271

750	11.11753	122.78294	0.05273

751	11.1294	122.94365	0.05285

752	11.13978	123.10436	0.05288

753	11.16202	123.26507	0.05292

754	11.17536	123.42578	0.05299

755	11.18574	123.58649	0.05304

756	11.20205	123.7472	0.05318

757	11.21984	123.90791	0.05323

758	11.22874	124.06862	0.05325

759	11.24505	124.22933	0.05327

760	11.26729	124.39004	0.0533

761	11.27619	124.55075	0.05332

762	11.28805	124.71146	0.05342

763	11.31029	124.87218	0.05344

764	11.32215	125.03289	0.05349

765	11.3355	125.1936	0.05358

766	11.35181	125.35431	0.0536

767	11.37405	125.51502	0.05363

768	11.38146	125.67573	0.05367

769	11.39184	125.83644	0.05372

770	11.41556	125.99715	0.05377

771	11.42743	126.15786	0.05384

772	11.43336	126.31857	0.05386

773	11.45856	126.47928	0.05391

774	11.47191	126.63999	0.05393

775	11.4808	126.8007	0.054

776	11.49415	126.96141	0.05403

777	11.51936	127.12212	0.05471

778	11.52825	127.28284	0.05475

779	11.54456	127.44355	0.05482

780	11.56384	127.60426	0.05489

781	11.57125	127.76497	0.05492

782	11.58608	127.92568	0.05494

783	11.60535	128.08639	0.05496

784	11.62166	128.2471	0.05499

785	11.62759	128.40781	0.05506

786	11.65132	128.56852	0.05508

787	11.66615	128.72923	0.05534

788	11.67801	128.88994	0.05536

789	11.68839	129.05065	0.05539

790	11.70766	129.21136	0.05541

791	11.71804	129.37207	0.05548

792	11.73435	129.53278	0.0555

793	11.7477	129.6935	0.05553

794	11.76697	129.85421	0.05557

795	11.77883	130.01492	0.05576

796	11.79366	130.17563	0.05581

797	11.82035	130.33634	0.05583

798	11.82628	130.49705	0.05595

799	11.83814	130.65776	0.05597

800	11.85594	130.81847	0.05602

801	11.86928	130.97918	0.05607

802	11.88114	131.13989	0.05609

803	11.90338	131.3006	0.0567

804	11.91673	131.46131	0.05672

805	11.93007	131.62202	0.05675

806	11.94045	131.78273	0.05679

807	11.95973	131.94344	0.05682

808	11.97011	132.10415	0.05694

809	11.98642	132.26487	0.0571

810	12.00569	132.42558	0.05715

811	12.01904	132.58629	0.05724

812	12.0309	132.747	0.05726

813	12.04721	132.90771	0.05729

814	12.065	133.06842	0.05731

815	12.07538	133.22913	0.05733

816	12.08576	133.38984	0.05738

817	12.11097	133.55055	0.05764

818	12.12431	133.71126	0.05766

819	12.13617	133.87197	0.05813

820	12.15248	134.03268	0.05816

821	12.16583	134.19339	0.0582

822	12.18065	134.3541	0.05823

823	12.19548	134.51481	0.05827

824	12.21327	134.67553	0.05839

825	12.2281	134.83624	0.05841

826	12.24589	134.99695	0.05844

827	12.25776	135.15766	0.05846

828	12.2711	135.31837	0.05848

829	12.28148	135.47908	0.05851

830	12.30224	135.63979	0.05853

831	12.323	135.8005	0.0586

832	12.32893	135.96121	0.05863

833	12.33931	136.12192	0.05867

834	12.366	136.28263	0.0587

835	12.37489	136.44334	0.05874

836	12.38527	136.60405	0.05895

837	12.409	136.76476	0.05898

838	12.41789	136.92547	0.05916

839	12.42827	137.08618	0.05919

840	12.45051	137.2469	0.05924

841	12.46386	137.40761	0.05949

842	12.48165	137.56832	0.05952

843	12.48906	137.72903	0.05985

844	12.51427	137.88974	0.05994

845	12.52317	138.05045	0.05999

846	12.53206	138.21116	0.06003

847	12.55134	138.37187	0.06006

848	12.57061	138.53258	0.06008

849	12.57951	138.69329	0.0601

850	12.60027	138.854	0.06015

851	12.6151	139.01471	0.06017

852	12.62399	139.17542	0.0602

853	12.64178	139.33613	0.06022

854	12.65661	139.49684	0.06024

855	12.66996	139.65756	0.06031

856	12.6833	139.81827	0.06036

857	12.70406	139.97898	0.06039

858	12.71444	140.13969	0.06048

859	12.72185	140.3004	0.06053

860	12.74113	140.46111	0.06055

861	12.76633	140.62182	0.06057

862	12.7693	140.78253	0.0606

863	12.79006	140.94324	0.06062

864	12.81082	141.10395	0.06107

865	12.81971	141.26466	0.06121

866	12.82713	141.42537	0.06128

867	12.8553	141.58608	0.0613

868	12.86271	141.74679	0.06135

869	12.87606	141.9075	0.06146

870	12.89681	142.06822	0.06161

871	12.91461	142.22893	0.06165

872	12.92499	142.38964	0.06168

873	12.93537	142.55035	0.06172

874	12.95909	142.71106	0.06175

875	12.97095	142.87177	0.06177

876	12.97836	143.03248	0.06179

877	13.00061	143.19319	0.06182

878	13.0184	143.3539	0.06189

879	13.02581	143.51461	0.06215

880	13.03916	143.67532	0.06217

881	13.06436	143.83603	0.06219

882	13.07029	143.99674	0.06229

883	13.08809	144.15745	0.06233

884	13.10143	144.31816	0.06236

885	13.11922	144.47887	0.0624

886	13.12812	144.63959	0.06261

887	13.15036	144.8003	0.06266

888	13.16519	144.96101	0.06269

889	13.17557	145.12172	0.06271

890	13.18891	145.28243	0.06273

891	13.20522	145.44314	0.06276

892	13.22005	145.60385	0.06278

893	13.23191	145.76456	0.0628

894	13.25415	145.92527	0.06292

895	13.26453	146.08598	0.06297

896	13.27788	146.24669	0.06344

897	13.29864	146.4074	0.06346

898	13.31198	146.56811	0.06351

899	13.31939	146.72882	0.06353

900	13.33422	146.88953	0.06355

901	13.35943	147.05025	0.06376

902	13.36388	147.21096	0.06381

903	13.3787	147.37167	0.06393

904	13.40094	147.53238	0.06405

905	13.41132	147.69309	0.06409

906	13.42318	147.8538	0.06412

907	13.44543	148.01451	0.06414

908	13.46025	148.17522	0.06428

909	13.46915	148.33593	0.06433

910	13.48843	148.49664	0.0644

911	13.5077	148.65735	0.06452

912	13.51956	148.81806	0.06454

913	13.52549	148.97877	0.06459

914	13.54477	149.13948	0.06463

915	13.56404	149.30019	0.06482

916	13.57442	149.46091	0.06484

917	13.59222	149.62162	0.06487

918	13.61001	149.78233	0.06489

919	13.62039	149.94304	0.06522

920	13.63522	150.10375	0.06527

921	13.64856	150.26446	0.06529

922	13.66635	150.42517	0.06548

923	13.6797	150.58588	0.0655

924	13.69601	150.74659	0.06552

925	13.70935	150.9073	0.06555

926	13.72121	151.06801	0.06557

927	13.74049	151.22872	0.0656

928	13.76125	151.38943	0.06564

929	13.76421	151.55014	0.06567

930	13.77904	151.71085	0.06569

931	13.80276	151.87156	0.06571

932	13.81759	152.03228	0.06574

933	13.82945	152.19299	0.06583

934	13.8428	152.3537	0.06602

935	13.86059	152.51441	0.06606

936	13.87245	152.67512	0.06611

937	13.88283	152.83583	0.06621

938	13.90804	152.99654	0.06623

939	13.9199	153.15725	0.0663

940	13.92583	153.31796	0.06632

941	13.94807	153.47867	0.06637

942	13.9629	153.63938	0.06639

943	13.97328	153.80009	0.06646

944	13.99107	153.9608	0.06649

945	14.00886	154.12151	0.06651

946	14.01776	154.28222	0.0666

947	14.03259	154.44294	0.0667

948	14.05483	154.60365	0.06677

949	14.06669	154.76436	0.06679

950	14.083	154.92507	0.06691

951	14.09486	155.08578	0.06696

952	14.11266	155.24649	0.06698

953	14.12304	155.4072	0.067

954	14.13638	155.56791	0.06703

955	14.15862	155.72862	0.06705

956	14.16455	155.88933	0.06721

957	14.18086	156.05004	0.06724

958	14.2031	156.21075	0.0675

959	14.21645	156.37146	0.06754

960	14.22238	156.53217	0.06757

961	14.24165	156.69288	0.06766

962	14.25796	156.85359	0.0678

963	14.26686	157.01431	0.06782

964	14.28465	157.17502	0.06785

965	14.30541	157.33573	0.06799

966	14.31282	157.49644	0.06801

967	14.32913	157.65715	0.06806

968	14.34989	157.81786	0.06818

969	14.36176	157.97857	0.06848

970	14.37213	158.13928	0.06858

971	14.38844	158.29999	0.0686

972	14.41069	158.4607	0.06862

973	14.41513	158.62141	0.06865

974	14.43293	158.78212	0.06876

975	14.45368	158.94283	0.06881

976	14.45665	159.10354	0.07003

977	14.47444	159.26425	0.07005

978	14.49817	159.42497	0.07012

979	14.51448	159.58568	0.07015

980	14.52189	159.74639	0.07022

981	14.53523	159.9071	0.07024

982	14.55451	160.06781	0.07027

983	14.56489	160.22852	0.07041

984	14.58268	160.38923	0.07043

985	14.59751	160.54994	0.07048

986	14.61085	160.71065	0.0705

987	14.62123	160.87136	0.07055

988	14.64792	161.03207	0.07059

989	14.6583	161.19278	0.07062

990	14.66868	161.35349	0.07064

991	14.68203	161.5142	0.07066

992	14.70427	161.67491	0.07076

993	14.70871	161.83563	0.07078

994	14.72354	161.99634	0.07088

995	14.74578	162.15705	0.0709

996	14.75616	162.31776	0.07095

997	14.77247	162.47847	0.07097

998	14.79175	162.63918	0.07102

999	14.80509	162.79989	0.07104

1000	14.81399	162.9606	0.07111

1001	14.83475	163.12131	0.07113

1002	14.84809	163.28202	0.07118

1003	14.86144	163.44273	0.0712

1004	14.87478	163.60344	0.07127

1005	14.89554	163.76415	0.0713

1006	14.9074	163.92486	0.07132

1007	14.91926	164.08557	0.07139

1008	14.93557	164.24628	0.07142

1009	14.9504	164.407	0.07144

1010	14.96374	164.56771	0.07146

1011	14.97857	164.72842	0.07158

1012	14.99785	164.88913	0.07165

1013	15.00823	165.04984	0.07167

1014	15.02305	165.21055	0.07172

1015	15.04381	165.37126	0.07174

1016	15.05716	165.53197	0.07177

1017	15.06012	165.69268	0.07179

1018	15.08829	165.85339	0.07181

1019	15.10312	166.0141	0.07184

1020	15.1135	166.17481	0.07186

1021	15.12981	166.33552	0.07193

1022	15.1476	166.49623	0.07198

1023	15.15947	166.65694	0.07203

1024	15.16984	166.81766	0.07205

1025	15.19357	166.97837	0.07207

1026	15.20691	167.13908	0.0721

1027	15.21729	167.29979	0.07214

1028	15.23509	167.4605	0.0724

1029	15.24991	167.62121	0.07245

1030	15.26326	167.78192	0.07247

1031	15.2766	167.94263	0.0725

1032	15.29143	168.10334	0.07257

1033	15.30626	168.26405	0.07264

1034	15.32108	168.42476	0.07322

1035	15.33739	168.58547	0.07327

1036	15.35519	168.74618	0.07332

1037	15.36557	168.90689	0.07454

1038	15.38039	169.0676	0.07458

1039	15.39819	169.22832	0.07461

1040	15.41005	169.38903	0.07463

1041	15.42784	169.54974	0.07465

1042	15.44415	169.71045	0.07468

1043	15.45305	169.87116	0.0747

1044	15.46787	170.03187	0.07475

1045	15.48715	170.19258	0.07477

1046	15.50939	170.35329	0.0748

1047	15.50939	170.514	0.07482

1048	15.53015	170.67471	0.07489

1049	15.54794	170.83542	0.07491

1050	15.55536	170.99613	0.07494

1051	15.5687	171.15684	0.07496

1052	15.59094	171.31755	0.07501

1053	15.60132	171.47826	0.07503

1054	15.61466	171.63897	0.07522

1055	15.63839	171.79969	0.07529

1056	15.65173	171.9604	0.07531

1057	15.6547	172.12111	0.07533

1058	15.67842	172.28182	0.0755

1059	15.69622	172.44253	0.07552

1060	15.70659	172.60324	0.07555

1061	15.72587	172.76395	0.07562

1062	15.7407	172.92466	0.07564

1063	15.75256	173.08537	0.07573

1064	15.76294	173.24608	0.0758

1065	15.78666	173.40679	0.07583

1066	15.79556	173.5675	0.07585

1067	15.80149	173.72821	0.07587

1068	15.82076	173.88892	0.07592

1069	15.84745	174.04963	0.07595

1070	15.85042	174.21035	0.07599

1071	15.87118	174.37106	0.07602

1072	15.88452	174.53177	0.07604

1073	15.90083	174.69248	0.07613

1074	15.91418	174.85319	0.0762

1075	15.929	175.0139	0.0763

1076	15.94383	175.17461	0.07632

1077	15.95569	175.33532	0.07634

1078	15.97349	175.49603	0.07639

1079	15.98831	175.65674	0.07646

1080	16.00314	175.81745	0.07653

1081	16.01204	175.97816	0.0766

1082	16.03576	176.13887	0.07663

1083	16.04762	176.29958	0.07667

1084	16.05652	176.46029	0.0767

1085	16.07431	176.621	0.07686

1086	16.09359	176.78172	0.07691

1087	16.09952	176.94243	0.07705

1088	16.11435	177.10314	0.07707

1089	16.144	177.26385	0.07712

1090	16.14845	177.42456	0.07721

1091	16.16328	177.58527	0.07728

1092	16.18255	177.74598	0.07735

1093	16.1959	177.90669	0.0774

1094	16.19886	178.0674	0.07742

1095	16.22852	178.22811	0.07745

1096	16.24038	178.38882	0.07747

1097	16.25224	178.54953	0.07761

1098	16.26707	178.71024	0.07763

1099	16.28486	178.87095	0.07768

1100	16.29672	179.03166	0.07773

1101	16.3071	179.19238	0.07775

1102	16.33082	179.35309	0.07778

1103	16.34269	179.5138	0.07785

1104	16.35455	179.67451	0.07787

1105	16.36938	179.83522	0.07789

1106	16.38717	179.99593	0.07792

1107	16.40051	180.15664	0.07796

1108	16.41831	180.31735	0.0781

1109	16.43165	180.47806	0.07815

1110	16.445	180.63877	0.07817

1111	16.45241	180.79948	0.07822

1112	16.4791	180.96019	0.07825

1113	16.49244	181.1209	0.07832

1114	16.50282	181.28161	0.07836

1115	16.51913	181.44232	0.07839

1116	16.53841	181.60304	0.07841

1117	16.54879	181.76375	0.07853

1118	16.56065	181.92446	0.07855

1119	16.58141	182.08517	0.0786

1120	16.59475	182.24588	0.07921

1121	16.60661	182.40659	0.07928

1122	16.62441	182.5673	0.0793

1123	16.63775	182.72801	0.07975

1124	16.64813	182.88872	0.07977

1125	16.66592	183.04943	0.07986

1126	16.68372	183.21014	0.07998

1127	16.69706	183.37085	0.08001

1128	16.70744	183.53156	0.08003

1129	16.72968	183.69227	0.08017

1130	16.74302	183.85298	0.08019

1131	16.74896	184.01369	0.08024

1132	16.76971	184.17441	0.08026

1133	16.78751	184.33512	0.08064

1134	16.79492	184.49583	0.08066

1135	16.80827	184.65654	0.08073

1136	16.82458	184.81725	0.08078

1137	16.8394	184.97796	0.08087

1138	16.84682	185.13867	0.0809

1139	16.86757	185.29938	0.08097

1140	16.87499	185.46009	0.08099

1141	16.12769	185.6208	0.08101

1142	15.22174	185.78151	0.08106

1143	14.39734	185.94222	0.08111

1144	13.49732	186.10293	0.08113

1145	12.52168	186.26364	0.08116

1146	11.63946	186.42435	0.08118

1147	10.69051	186.58507	0.0812

1148	9.94024	186.74578	0.08127

1149	9.02688	186.90649	0.08134

1150	8.23065	187.0672	0.08137

1151	7.42553	187.22791	0.08141

1152	6.60854	187.38862	0.08158

1153	5.78563	187.54933	0.0816

1154	5.01757	187.71004	0.08162

1155	4.19614	187.87075	0.08169

1156	3.41918	188.03146	0.08172

1157	2.64964	188.19217	0.08188

1158	2.64964	188.35288	0.08191

1159	2.08027	188.51359	0.08193

1160	1.39377	188.6743	0.08195

188.83501	0.082

188.99572	0.08209

189.15644	0.08214

189.31715	0.08216

189.47786	0.08226

189.63857	0.08228

189.79928	0.08238

189.95999	0.0824

190.1207	0.08249

190.28141	0.08261

190.44212	0.08263

190.60283	0.08266

190.76354	0.08306

190.92425	0.08313

191.08496	0.0832

191.24567	0.08327

191.40638	0.08331

191.5671	0.08341

191.72781	0.08357

191.88852	0.0836

192.04923	0.08364

192.20994	0.08367

192.37065	0.08374

192.53136	0.08381

192.69207	0.08385

192.85278	0.0839

193.01349	0.08416

193.1742	0.08418

193.33491	0.08421

193.49562	0.08423

193.65633	0.08425

193.81704	0.08428

193.97776	0.08435

194.13847	0.08439

194.29918	0.08442

194.45989	0.08444

194.6206	0.08446

194.78131	0.08453

194.94202	0.08461

195.10273	0.08463

195.26344	0.08465

195.42415	0.08468

195.58486	0.0847

195.74557	0.08475

195.90628	0.08477

196.06699	0.08482

196.2277	0.08491

196.38842	0.08496

196.54913	0.08505

196.70984	0.08512

196.87055	0.08514

197.03126	0.08517

197.19197	0.08519

197.35268	0.08526

197.51339	0.08529

197.6741	0.08531

197.83481	0.08533

197.99552	0.08552

198.15623	0.08559

198.31694	0.08561

198.47765	0.08564

198.63836	0.08571

198.79907	0.08576

198.95979	0.0858

199.1205	0.08587

199.28121	0.08599

199.44192	0.08601

199.60263	0.08611

199.76334	0.08613

199.92405	0.08615

200.08476	0.08618

200.24547	0.08625

200.40618	0.08634

200.56689	0.08637

200.7276	0.08676

200.88831	0.08679

201.04902	0.08681

201.20973	0.08683

201.37045	0.08688

201.53116	0.08693

201.69187	0.08698

201.85258	0.087

202.01329	0.08707

202.174	0.08712

202.33471	0.08714

202.49542	0.08723

202.65613	0.0873

202.81684	0.08773

202.97755	0.08787

203.13826	0.08791

203.29897	0.08794

203.45968	0.08796

203.62039	0.08888

203.7811	0.0889

203.94182	0.08892

204.10253	0.08899

204.26324	0.08902

204.42395	0.08904

204.58466	0.08916

204.74537	0.08986

204.90608	0.08989

205.06679	0.08998

205.2275	0.09014

205.38821	0.09017

205.54892	0.09026

205.70963	0.09031

205.87034	0.09033

206.03105	0.09035

206.19176	0.0904

206.35248	0.09043

206.51319	0.09047

206.6739	0.0905

206.83461	0.09052

206.99532	0.09054

207.15603	0.09073

207.31674	0.0908

207.47745	0.09082

207.63816	0.09085

207.79887	0.09087

207.95958	0.09089

208.12029	0.09099

208.281	0.09101

208.44171	0.09108

208.60242	0.09111

208.76314	0.09113

208.92385	0.09125

209.08456	0.09127

209.24527	0.0915

209.40598	0.09158

209.56669	0.09172

209.7274	0.09174

209.88811	0.09181

210.04882	0.09193

210.20953	0.09195

210.37024	0.092

210.53095	0.09202

210.69166	0.09209

210.85237	0.09214

211.01308	0.09216

211.17379	0.0923

211.33451	0.09235

211.49522	0.0924

211.65593	0.09242

211.81664	0.09247

211.97735	0.09249

212.13806	0.09258

212.29877	0.09268

212.45948	0.0927

212.62019	0.09273

212.7809	0.09289

212.94161	0.09298

213.10232	0.09301

213.26303	0.09303

213.42374	0.09308

213.58445	0.0931

213.74517	0.09312

213.90588	0.09315

214.06659	0.09317

214.2273	0.09319

214.38801	0.09324

214.54872	0.09329

214.70943	0.09376

214.87014	0.09385

215.03085	0.0939

215.19156	0.09392

215.35227	0.09402

215.51298	0.09404

215.67369	0.09406

215.8344	0.09413

215.99511	0.09432

216.15582	0.09446

216.31654	0.09451

216.47725	0.09467

216.63796	0.09474

216.79867	0.09477

216.95938	0.09479

217.12009	0.09484

217.2808	0.09495

217.44151	0.095

217.60222	0.09514

217.76293	0.09519

217.92364	0.09524

218.08435	0.09528

218.24506	0.09531

218.40577	0.09535

218.56648	0.09547

218.7272	0.09552

218.88791	0.09556

219.04862	0.09561

219.20933	0.09622

219.37004	0.09627

219.53075	0.09634

219.69146	0.0969

219.85217	0.09693

220.01288	0.09695

220.17359	0.09697

220.3343	0.09704

220.49501	0.09744

220.65572	0.09749

220.81643	0.09812

220.97714	0.09815

221.13786	0.09817

221.29857	0.09829

221.45928	0.09831

221.61999	0.09833

221.7807	0.09864

221.94141	0.0988

222.10212	0.09887

222.26283	0.0989

222.42354	0.09897

222.58425	0.09899

222.74496	0.09901

222.90567	0.09906

223.06638	0.09916

223.22709	0.0992

223.3878	0.09927

223.54851	0.0993

223.70923	0.09939

223.86994	0.09946

224.03065	0.09951

224.19136	0.09955

224.35207	0.09958

224.51278	0.0996

224.67349	0.09963

224.8342	0.09965

224.99491	0.09967

225.15562	0.0997

225.31633	0.09977

225.47704	0.09984

225.63775	0.09986

225.79846	0.09991

225.95917	0.09993

226.11989	0.09995

226.2806	0.10002

226.44131	0.10005

226.60202	0.10007

226.76273	0.10009

226.92344	0.10016

227.08415	0.10021

227.24486	0.10024

227.40557	0.10026

227.56628	0.10028

227.72699	0.10031

227.8877	0.10033

228.04841	0.10035

228.20912	0.1004

228.36983	0.10059

228.53055	0.10063

228.69126	0.10066

228.85197	0.10075

229.01268	0.1008

229.17339	0.10085

229.3341	0.10089

229.49481	0.10103

229.65552	0.10108

229.81623	0.1011

229.97694	0.10127

230.13765	0.10129

230.29836	0.10131

230.45907	0.10143

230.61978	0.10146

230.78049	0.10153

230.9412	0.10155

231.10192	0.10164

231.26263	0.10167

231.42334	0.10178

231.58405	0.10181

231.74476	0.10185

231.90547	0.10188

232.06618	0.1019

232.22689	0.10195

232.3876	0.10197

232.54831	0.10207

232.70902	0.10209

232.86973	0.10221

233.03044	0.10223

233.19115	0.10225

233.35186	0.10235

233.51258	0.10246

233.67329	0.10254

233.834	0.10258

233.99471	0.10261

234.15542	0.10263

234.31613	0.10272

234.47684	0.1031

234.63755	0.10312

234.79826	0.10319

234.95897	0.10324

235.11968	0.10326

235.28039	0.10331

235.4411	0.10333

235.60181	0.1034

235.76252	0.10343

235.92324	0.10345

236.08395	0.10354

236.24466	0.10357

236.40537	0.10359

236.56608	0.10361

236.72679	0.10364

236.8875	0.10366

237.04821	0.10369

237.20892	0.10383

237.36963	0.10385

237.53034	0.10387

237.69105	0.10397

237.85176	0.10399

238.01247	0.10401

238.17318	0.10406

238.33389	0.10408

238.49461	0.10418

238.65532	0.10427

238.81603	0.1043

238.97674	0.10432

239.13745	0.10434

239.29816	0.10437

239.45887	0.10439

239.61958	0.10441

239.78029	0.10448

239.941	0.10451

240.10171	0.1046

240.26242	0.10465

240.42313	0.10469

240.58384	0.10472

240.74455	0.10479

240.90527	0.10486

241.06598	0.10491

241.22669	0.10507

241.3874	0.10657

241.54811	0.10662

241.70882	0.10681

241.86953	0.10685

242.03024	0.1069

242.19095	0.10695

242.35166	0.10697

242.51237	0.10702

242.67308	0.10704

242.83379	0.10706

242.9945	0.10714

243.15521	0.10718

243.31592	0.10767

243.47664	0.1077

243.63735	0.10772

243.79806	0.10775

243.95877	0.10777

244.11948	0.10786

244.28019	0.10789

244.4409	0.10791

244.60161	0.10798

244.76232	0.108

244.92303	0.10803

245.08374	0.10805

245.24445	0.10807

245.40516	0.10812

245.56587	0.10814

245.72658	0.10817

245.8873	0.10821

246.04801	0.10826

246.20872	0.1084

246.36943	0.10847

246.53014	0.10852

246.69085	0.10854

246.85156	0.1088

247.01227	0.10887

247.17298	0.10908

247.33369	0.10913

247.4944	0.1092

247.65511	0.10934

247.81582	0.10936

247.97653	0.10939

248.13724	0.10951

248.29796	0.10953

248.45867	0.10955

248.61938	0.10962

248.78009	0.10965

248.9408	0.10967

249.10151	0.10972

249.26222	0.10983

249.42293	0.10986

249.58364	0.10995

249.74435	0.11002

249.90506	0.11005

250.06577	0.11009

250.22648	0.11016

250.38719	0.11023

250.5479	0.11026

250.70861	0.11028

250.86933	0.11033

251.03004	0.11035

251.19075	0.11044

251.35146	0.11051

251.51217	0.11054

251.67288	0.11059

251.83359	0.11068

251.9943	0.1107

252.15501	0.11075

252.31572	0.11082

252.47643	0.11084

252.63714	0.11091

252.79785	0.11094

252.95856	0.1111

253.11927	0.11122

253.27999	0.11124

253.4407	0.11127

253.60141	0.11129

253.76212	0.11145

253.92283	0.11164

254.08354	0.11166

254.24425	0.11178

254.40496	0.11183

254.56567	0.11188

254.72638	0.1119

254.88709	0.11195

255.0478	0.11197

255.20851	0.11199

255.36922	0.11204

255.52993	0.11206

255.69064	0.11211

255.85136	0.11213

256.01207	0.11216

256.17278	0.11218

256.33349	0.11223

256.4942	0.11225

256.65491	0.11227

256.81562	0.1123

256.97633	0.11237

257.13704	0.11239

257.29775	0.11242

257.45846	0.11244

257.61917	0.11246

257.77988	0.11293

257.94059	0.11298

258.1013	0.11303

258.26202	0.11307

258.42273	0.11312

258.58344	0.11317

258.74415	0.11321

258.90486	0.11324

259.06557	0.11338

259.22628	0.1134

259.38699	0.11342

259.5477	0.11385

259.70841	0.11387

259.86912	0.11389

260.02983	0.11392

260.19054	0.11394

260.35125	0.11399

260.51196	0.11403

260.67268	0.11408

260.83339	0.11418

260.9941	0.1142

261.15481	0.11422

261.31552	0.11432

261.47623	0.11434

261.63694	0.11441

261.79765	0.11443

261.95836	0.1145

262.11907	0.11455

262.27978	0.11457

262.44049	0.1146

262.6012	0.11469

262.76191	0.11474

262.92262	0.11483

263.08333	0.11486

263.24405	0.11493

263.40476	0.11495

263.56547	0.11509

263.72618	0.11511

263.88689	0.11514

264.0476	0.11516

264.20831	0.1153

264.36902	0.11533

264.52973	0.11558

264.69044	0.11561

264.85115	0.11563

265.01186	0.11568

265.17257	0.11572

265.33328	0.11575

265.49399	0.11615

265.65471	0.11617

265.81542	0.11619

265.97613	0.11641

266.13684	0.11643

266.29755	0.11655

266.45826	0.11664

266.61897	0.11678

266.77968	0.1168

266.94039	0.11683

267.1011	0.11685

267.26181	0.11692

267.42252	0.11695

267.58323	0.11699

267.74394	0.11702

267.90465	0.11706

268.06537	0.11709

268.22608	0.11711

268.38679	0.11718

268.5475	0.1172

268.70821	0.11725

268.86892	0.11727

269.02963	0.11737

269.19034	0.11741

269.35105	0.11781

269.51176	0.11784

269.67247	0.11786

269.83318	0.11791

269.99389	0.11795

270.1546	0.118

270.31531	0.11805

270.47602	0.11807

270.63674	0.11812

270.79745	0.11817

270.95816	0.11821

271.11887	0.11838

271.27958	0.1184

271.44029	0.11845

271.601	0.11849

271.76171	0.11854

271.92242	0.11856

272.08313	0.11861

272.24384	0.11875

272.40455	0.11929

272.56526	0.11939

272.72597	0.11941

272.88668	0.11948

273.0474	0.11955

273.20811	0.11957

273.36882	0.1196

273.52953	0.11962

273.69024	0.11964

273.85095	0.11971

274.01166	0.11974

274.17237	0.11976

274.33308	0.11978

274.49379	0.11988

274.6545	0.1199

274.81521	0.11995

274.97592	0.11997

275.13663	0.12002

275.29734	0.12004

275.45806	0.12007

275.61877	0.12011

275.77948	0.12014

275.94019	0.12016

276.1009	0.12072

276.26161	0.12075

276.42232	0.12077

276.58303	0.12089

276.74374	0.12091

276.90445	0.12096

277.06516	0.12098

277.22587	0.12101

277.38658	0.12108

277.54729	0.1211

277.708	0.12117

277.86871	0.12122

278.02943	0.12126

278.19014	0.12129

278.35085	0.12131

278.51156	0.12143

278.67227	0.12159

278.83298	0.12162

278.99369	0.12164

279.1544	0.12171

279.31511	0.12178

279.47582	0.12185

279.63653	0.12187

279.79724	0.12192

279.95795	0.12199

280.11866	0.12204

280.27937	0.12206

280.44009	0.12211

280.6008	0.12216

280.76151	0.12218

280.92222	0.1222

281.08293	0.12225

281.24364	0.12227

281.40435	0.12232

281.56506	0.12246

281.72577	0.12248

281.88648	0.12251

282.04719	0.12255

282.2079	0.12258

282.36861	0.1226

282.52932	0.12262

282.69003	0.12267

282.85074	0.12274

283.01146	0.12277

283.17217	0.12279

283.33288	0.12284

283.49359	0.12286

283.6543	0.12298

283.81501	0.123

283.97572	0.12302

284.13643	0.12335

284.29714	0.12347

284.45785	0.12349

284.61856	0.12352

284.77927	0.12354

284.93998	0.12368

285.10069	0.12373

285.2614	0.12382

285.42212	0.12384

285.58283	0.12389

285.74354	0.12396

285.90425	0.12399

286.06496	0.12403

286.22567	0.12406

286.38638	0.1241

286.54709	0.1242

286.7078	0.12422

286.86851	0.12424

287.02922	0.12429

287.18993	0.12431

287.35064	0.12438

287.51135	0.12448

287.67206	0.12453

287.83278	0.12483

287.99349	0.12488

288.1542	0.1249

288.31491	0.12492

288.47562	0.12495

288.63633	0.12497

288.79704	0.12504

288.95775	0.12507

289.11846	0.12509

289.27917	0.12511

289.43988	0.12514

289.60059	0.12516

289.7613	0.12518

289.92201	0.12523

290.08272	0.12525

290.24343	0.12528

290.40415	0.12535

290.56486	0.12544

290.72557	0.12546

290.88628	0.12549

291.04699	0.12563

291.2077	0.1257

291.36841	0.12572

291.52912	0.12579

291.68983	0.12582

291.85054	0.12584

292.01125	0.12586

292.17196	0.12589

292.33267	0.12591

292.49338	0.126

292.65409	0.12605

292.81481	0.12607

292.97552	0.12617

293.13623	0.12622

293.29694	0.12624

293.45765	0.12638

293.61836	0.12643

293.77907	0.12647

293.93978	0.1265

294.10049	0.12652

294.2612	0.12654

294.42191	0.12659

294.58262	0.12661

294.74333	0.12673

294.90404	0.1268

295.06475	0.12683

295.22546	0.12694

295.38618	0.12697

295.54689	0.12699

295.7076	0.12706

295.86831	0.12711

296.02902	0.1272

296.18973	0.12748

296.35044	0.12751

296.51115	0.12753

296.67186	0.12755

296.83257	0.12758

296.99328	0.1276

297.15399	0.12762

297.3147	0.12765

297.47541	0.12767

297.63612	0.12769

297.79684	0.12776

297.95755	0.12779

298.11826	0.12781

298.27897	0.12783

298.43968	0.12795

298.60039	0.12798

298.7611	0.12802

298.92181	0.12814

299.08252	0.12819

299.24323	0.12821

299.40394	0.12823

299.56465	0.12826

299.72536	0.12828

299.88607	0.1283

300.04678	0.12837

300.2075	0.12842

300.36821	0.12844

300.52892	0.12847

300.68963	0.12849

300.85034	0.12859

301.01105	0.12861

301.17176	0.12863

301.33247	0.12873

301.49318	0.12877

301.65389	0.1288

301.8146	0.12882

301.97531	0.12905

302.13602	0.1291

302.29673	0.12922

302.45744	0.12929

302.61816	0.12931

302.77887	0.12936

302.93958	0.12941

303.10029	0.12943

303.261	0.12945

303.42171	0.12948

303.58242	0.1295

303.74313	0.12952

303.90384	0.12955

304.06455	0.12969

304.22526	0.12974

304.38597	0.12976

304.54668	0.12978

304.70739	0.12981

304.8681	0.12983

305.02881	0.12992

305.18953	0.12995

305.35024	0.12999

305.51095	0.13002

305.67166	0.13004

305.83237	0.13009

305.99308	0.13011

306.15379	0.13013

306.3145	0.13016

306.47521	0.13018

306.63592	0.1302

306.79663	0.13025

306.95734	0.13028

307.11805	0.1303

307.27876	0.13039

307.43947	0.13049

307.60019	0.13051

307.7609	0.13053

307.92161	0.13056

308.08232	0.13063

308.24303	0.13067

308.40374	0.13091

308.56445	0.13096

308.72516	0.13103

308.88587	0.13107

309.04658	0.13126

309.20729	0.13128

309.368	0.13138

309.52871	0.13143

309.68942	0.13147

309.85013	0.13152

310.01084	0.13154

310.17156	0.13157

310.33227	0.13159

310.49298	0.13161

310.65369	0.13178

310.8144	0.13187

310.97511	0.13189

311.13582	0.13192

311.29653	0.13199

311.45724	0.13204

311.61795	0.13208

311.77866	0.13213

311.93937	0.13215

312.10008	0.13229

312.26079	0.13258

312.4215	0.1326

312.58222	0.13262

312.74293	0.13269

312.90364	0.13272

313.06435	0.13286

313.22506	0.13297

313.38577	0.13302

313.54648	0.13304

313.70719	0.13307

313.8679	0.13309

314.02861	0.13312

314.18932	0.13316

314.35003	0.13319

314.51074	0.13321

314.67145	0.13323

314.83216	0.13326

314.99288	0.1333

315.15359	0.13333

315.3143	0.13335

315.47501	0.13337

315.63572	0.13342

315.79643	0.13349

315.95714	0.13459

316.11785	0.13462

316.27856	0.13464

316.43927	0.13469

316.59998	0.13476

316.76069	0.13483

316.9214	0.13485

317.08211	0.13488

317.24282	0.1349

317.40353	0.13495

317.56425	0.13502

317.72496	0.13504

317.88567	0.13506

318.04638	0.13509

318.20709	0.13511

318.3678	0.13513

318.52851	0.13518

318.68922	0.13523

318.84993	0.1353

319.01064	0.13574

319.17135	0.13577

319.33206	0.13579

319.49277	0.13581

319.65348	0.13598

319.81419	0.13605

319.97491	0.13612

320.13562	0.13617

320.29633	0.13619

320.45704	0.13628

320.61775	0.13631

320.77846	0.13642

320.93917	0.1375

321.09988	0.13753

321.26059	0.13755

321.4213	0.13774

321.58201	0.13776

321.74272	0.13779

321.90343	0.13783

322.06414	0.13786

322.22485	0.13788

322.38556	0.1379

322.54628	0.13793

322.70699	0.13809

322.8677	0.13814

323.02841	0.13816

323.18912	0.13818

323.34983	0.13825

323.51054	0.1383

323.67125	0.1384

323.83196	0.13844

323.99267	0.13872

324.15338	0.13877

324.31409	0.13879

324.4748	0.13884

324.63551	0.13889

324.79622	0.13891

324.95694	0.13908

325.11765	0.13915

325.27836	0.13917

325.43907	0.13924

325.59978	0.13926

325.76049	0.13929

325.9212	0.13931

326.08191	0.13936

326.24262	0.13945

326.40333	0.13948

326.56404	0.13952

326.72475	0.13962

326.88546	0.13964

327.04617	0.13969

327.20688	0.13985

327.3676	0.13987

327.52831	0.1399

327.68902	0.13997

327.84973	0.14023

328.01044	0.14025

328.17115	0.1403

328.33186	0.14032

328.49257	0.14044

328.65328	0.14048

328.81399	0.14051

328.9747	0.14058

329.13541	0.1406

329.29612	0.14065

329.45683	0.14067

329.61754	0.1407

329.77825	0.14077

329.93897	0.14086

330.09968	0.14088

330.26039	0.14091

330.4211	0.14112

330.58181	0.14124

330.74252	0.14126

330.90323	0.14128

331.06394	0.14131

331.22465	0.14135

331.38536	0.1414

331.54607	0.14145

331.70678	0.14149

331.86749	0.14152

332.0282	0.14159

332.18891	0.14161

332.34963	0.14166

332.51034	0.1417

332.67105	0.14173

332.83176	0.14175

332.99247	0.1418

333.15318	0.14182

333.31389	0.14185

333.4746	0.14187

333.63531	0.14189

333.79602	0.14192

333.95673	0.14199

334.11744	0.14201

334.27815	0.14208

334.43886	0.14215

334.59957	0.14217

334.76029	0.1422

334.921	0.14222

335.08171	0.14224

335.24242	0.14227

335.40313	0.14243

335.56384	0.14246

335.72455	0.14262

335.88526	0.14264

336.04597	0.14267

336.20668	0.14269

336.36739	0.14281

336.5281	0.14283

336.68881	0.1429

336.84952	0.14293

337.01023	0.14307

337.17094	0.14309

337.33166	0.14314

337.49237	0.14332

337.65308	0.14339

337.81379	0.14342

337.9745	0.14354

338.13521	0.14356

338.29592	0.14358

338.45663	0.14361

338.61734	0.14363

338.77805	0.14377

338.93876	0.14389

339.09947	0.14391

339.26018	0.14393

339.42089	0.14403

339.5816	0.14405

339.74232	0.14419

339.90303	0.14422

340.06374	0.14429

340.22445	0.14431

340.38516	0.14436

340.54587	0.1444

340.70658	0.14443

340.86729	0.14459

341.028	0.14461

341.18871	0.14464

341.34942	0.14471

341.51013	0.1448

341.67084	0.14483

341.83155	0.14487

341.99226	0.1449

342.15298	0.14492

342.31369	0.14494

342.4744	0.14501

342.63511	0.14504

342.79582	0.14506

342.95653	0.14508

343.11724	0.14515

343.27795	0.14525

343.43866	0.1453

343.59937	0.14534

343.76008	0.14537

343.92079	0.14544

344.0815	0.14546

344.24221	0.14555

344.40292	0.14558

344.56363	0.14569

344.72435	0.14572

344.88506	0.14579

345.04577	0.14581

345.20648	0.14584

345.36719	0.14588

345.5279	0.14591

345.68861	0.14593

345.84932	0.14595

346.01003	0.14607

346.17074	0.14609

346.33145	0.14614

346.49216	0.14616

346.65287	0.14619

346.81358	0.14628

346.97429	0.1463

347.13501	0.14635

347.29572	0.1464

347.45643	0.14645

347.61714	0.14647

347.77785	0.14652

347.93856	0.14654

348.09927	0.14687

348.25998	0.14701

348.42069	0.14703

348.5814	0.1472

348.74211	0.14722

348.90282	0.14729

349.06353	0.14741

349.22424	0.1475

349.38495	0.14755

349.54566	0.14762

349.70638	0.14799

349.86709	0.14802

350.0278	0.14806

350.18851	0.14825

350.34922	0.1483

350.50993	0.14832

350.67064	0.14835

350.83135	0.14844

350.99206	0.14858

351.15277	0.14863

351.31348	0.14865

351.47419	0.1487

351.6349	0.14872

351.79561	0.14889

351.95632	0.149

352.11704	0.14907

352.27775	0.1491

352.43846	0.14936

352.59917	0.14938

352.75988	0.1494

352.92059	0.14943

353.0813	0.14947

353.24201	0.14952

353.40272	0.14959

353.56343	0.14961

353.72414	0.14978

353.88485	0.14982

354.04556	0.14994

354.20627	0.14997

354.36698	0.15001

354.5277	0.15036

354.68841	0.15039

354.84912	0.15055

355.00983	0.15067

355.17054	0.15072

355.33125	0.15076

355.49196	0.15079

355.65267	0.15081

355.81338	0.15086

355.97409	0.1509

356.1348	0.15095

356.29551	0.15119

356.45622	0.15151

356.61693	0.15177

356.77764	0.1518

356.93835	0.15182

357.09907	0.15184

357.25978	0.15187

357.42049	0.15189

357.5812	0.1521

357.74191	0.15217

357.90262	0.15222

358.06333	0.15224

358.22404	0.15229

358.38475	0.15231

358.54546	0.15241

358.70617	0.15243

358.86688	0.15245

359.02759	0.15248

359.1883	0.1525

359.34901	0.15257

359.50973	0.15259

359.67044	0.15264

359.83115	0.15266

359.99186	0.15269

360.15257	0.15273

360.31328	0.15285

360.47399	0.15288

360.6347	0.1529

360.79541	0.15292

360.95612	0.15295

361.11683	0.15313

361.27754	0.15316

361.43825	0.15318

361.59896	0.15323

361.75967	0.15325

361.92039	0.15353

362.0811	0.15356

362.24181	0.15367

362.40252	0.15372

362.56323	0.15381

362.72394	0.15384

362.88465	0.15388

363.04536	0.15393

363.20607	0.15396

363.36678	0.15405

363.52749	0.15417

363.6882	0.15419

363.84891	0.15421

364.00962	0.15431

364.17033	0.15433

364.33104	0.15438

364.49176	0.1544

364.65247	0.15442

364.81318	0.15445

364.97389	0.15447

365.1346	0.15452

365.29531	0.15454

365.45602	0.15457

365.61673	0.15468

365.77744	0.15473

365.93815	0.15482

366.09886	0.15494

366.25957	0.15499

366.42028	0.15501

366.58099	0.15508

366.7417	0.15515

366.90242	0.15518

367.06313	0.15522

367.22384	0.15525

367.38455	0.15529

367.54526	0.15534

367.70597	0.15539

367.86668	0.15541

368.02739	0.15555

368.1881	0.15565

368.34881	0.15567

368.50952	0.15569

368.67023	0.1563

368.83094	0.15687

368.99165	0.15689

369.15236	0.15698

369.31307	0.15703

369.47379	0.15705

369.6345	0.15708

369.79521	0.15715

369.95592	0.15717

370.11663	0.15726

370.27734	0.15729

370.43805	0.15738

370.59876	0.15743

370.75947	0.15745

370.92018	0.15748

371.08089	0.1575

371.2416	0.15757

371.40231	0.15759

371.56302	0.15766

371.72373	0.15771

371.88445	0.1579

372.04516	0.15792

372.20587	0.15795

372.36658	0.15804

372.52729	0.15809

372.688	0.15816

372.84871	0.1582

373.00942	0.15823

373.17013	0.15825

373.33084	0.15827

373.49155	0.1583

373.65226	0.15832

373.81297	0.15834

373.97368	0.15837

374.13439	0.15839

374.29511	0.15846

374.45582	0.15851

374.61653	0.15853

374.77724	0.15858

374.93795	0.15863

375.09866	0.15865

375.25937	0.1587

375.42008	0.15872

375.58079	0.15874

375.7415	0.15877

375.90221	0.15881

376.06292	0.15886

376.22363	0.15891

376.38434	0.15893

376.54505	0.1594

376.70576	0.15942

376.86648	0.15945

377.02719	0.15949

377.1879	0.15959

377.34861	0.15961

377.50932	0.15963

377.67003	0.1598

377.83074	0.15992

377.99145	0.15996

378.15216	0.16001

378.31287	0.16003

378.47358	0.16006

378.63429	0.16008

378.795	0.1601

378.95571	0.1602

379.11642	0.16022

379.27714	0.16025

379.43785	0.16029

379.59856	0.16032

379.75927	0.16034

379.91998	0.16039

380.08069	0.16043

380.2414	0.16046

380.40211	0.16067

380.56282	0.16083

380.72353	0.16086

380.88424	0.16088

381.04495	0.1609

381.20566	0.16097

381.36637	0.161

381.52708	0.16118

381.68779	0.16121

381.84851	0.16128

382.00922	0.16139

382.16993	0.16142

382.33064	0.16144

382.49135	0.16149

382.65206	0.16151

382.81277	0.16154

382.97348	0.16156

383.13419	0.16158

383.2949	0.16168

383.45561	0.1617

383.61632	0.16186

383.77703	0.16189

383.93774	0.16191

384.09845	0.16193

384.25917	0.16196

384.41988	0.16198

384.58059	0.16208

384.7413	0.1621

384.90201	0.16212

385.06272	0.16217

385.22343	0.16219

385.38414	0.1624

385.54485	0.16243

385.70556	0.16245

385.86627	0.1625

386.02698	0.16252

386.18769	0.16287

386.3484	0.16297

386.50911	0.16299

386.66983	0.16311

386.83054	0.1632

386.99125	0.16323

387.15196	0.16337

387.31267	0.16339

387.47338	0.16351

387.63409	0.16353

387.7948	0.16367

387.95551	0.16369

388.11622	0.16374

388.27693	0.16377

388.43764	0.16386

388.59835	0.16388

388.75906	0.16391

388.91977	0.16398

389.08048	0.164

389.2412	0.16402

389.40191	0.16405

389.56262	0.16412

389.72333	0.16414

389.88404	0.16416

390.04475	0.16428

390.20546	0.16435

390.36617	0.16442

390.52688	0.16445

390.68759	0.16447

390.8483	0.16449

391.00901	0.16459

391.16972	0.16461

391.33043	0.16466

391.49114	0.16473

391.65186	0.16475

391.81257	0.16496

391.97328	0.16499

392.13399	0.16503

392.2947	0.16506

392.45541	0.16513

392.61612	0.16515

392.77683	0.16546

392.93754	0.16548

393.09825	0.16555

393.25896	0.16557

393.41967	0.16562

393.58038	0.16567

393.74109	0.16569

393.9018	0.16571

394.06252	0.16574

394.22323	0.16576

394.38394	0.16595

394.54465	0.16597

394.70536	0.16602

394.86607	0.16604

395.02678	0.16607

395.18749	0.16609

395.3482	0.16611

395.50891	0.16614

395.66962	0.16616

395.83033	0.16618

395.99104	0.16621

396.15175	0.16628

396.31246	0.16632

396.47317	0.16639

396.63389	0.16642

396.7946	0.16644

396.95531	0.16646

397.11602	0.16653

397.27673	0.16658

397.43744	0.1667

397.59815	0.16679

397.75886	0.16682

397.91957	0.16693

398.08028	0.16696

398.24099	0.16714

398.4017	0.16717

398.56241	0.16719

398.72312	0.16722

398.88383	0.16724

399.04455	0.16738

399.20526	0.1674

399.36597	0.16743

399.52668	0.1682

399.68739	0.16822

399.8481	0.16827

400.00881	0.16832

400.16952	0.16834

400.33023	0.16837

400.49094	0.16846

400.65165	0.16851

400.81236	0.16853

400.97307	0.16862

401.13378	0.16869

401.29449	0.16879

401.4552	0.16881

401.61592	0.16893

401.77663	0.169

401.93734	0.16902

402.09805	0.16905

402.25876	0.16933

402.41947	0.16937

402.58018	0.1694

402.74089	0.16942

402.9016	0.16947

403.06231	0.16952

403.22302	0.16954

403.38373	0.16961

403.54444	0.1698

403.70515	0.16984

403.86586	0.16987

404.02658	0.16991

404.18729	0.16994

404.348	0.17015

404.50871	0.17017

404.66942	0.1702

404.83013	0.17027

404.99084	0.17029

405.15155	0.17031

405.31226	0.17034

405.47297	0.17038

405.63368	0.17045

405.79439	0.17048

405.9551	0.1705

406.11581	0.17085

406.27652	0.17095

406.43724	0.17097

406.59795	0.17113

406.75866	0.17118

406.91937	0.1712

407.08008	0.17123

407.24079	0.17125

407.4015	0.17128

407.56221	0.17135

407.72292	0.17142

407.88363	0.17144

408.04434	0.17146

408.20505	0.17153

408.36576	0.17163

408.52647	0.17165

408.68718	0.17167

408.84789	0.17182

409.00861	0.17184

409.16932	0.17186

409.33003	0.17191

409.49074	0.17193

409.65145	0.17196

409.81216	0.17203

409.97287	0.17207

410.13358	0.17217

410.29429	0.17238

410.455	0.1724

410.61571	0.17243

410.77642	0.17245

410.93713	0.17247

411.09784	0.17252

411.25855	0.17257

411.41927	0.17259

411.57998	0.17261

411.74069	0.17268

411.9014	0.17278

412.06211	0.1728

412.22282	0.17282

412.38353	0.17287

412.54424	0.17289

412.70495	0.17292

412.86566	0.17294

413.02637	0.17301

413.18708	0.17306

413.34779	0.1732

413.5085	0.17327

413.66921	0.17339

413.82993	0.17353

413.99064	0.1736

414.15135	0.17367

414.31206	0.17369

414.47277	0.17372

414.63348	0.17379

414.79419	0.1739

414.9549	0.17395

415.11561	0.17404

415.27632	0.17409

415.43703	0.17416

415.59774	0.17423

415.75845	0.17435

415.91916	0.17442

416.07987	0.17444

416.24058	0.17447

416.4013	0.17449

416.56201	0.17461

416.72272	0.17465

416.88343	0.17468

417.04414	0.17477

417.20485	0.1748

417.36556	0.17484

417.52627	0.17487

417.68698	0.17491

417.84769	0.17494

418.0084	0.17496

418.16911	0.17503

418.32982	0.17505

418.49053	0.17508

418.65124	0.17527

418.81196	0.17534

418.97267	0.17538

419.13338	0.17541

419.29409	0.17545

419.4548	0.17548

419.61551	0.17552

419.77622	0.17557

419.93693	0.17559

420.09764	0.17566

420.25835	0.17569

420.41906	0.17571

420.57977	0.17583

420.74048	0.17588

420.90119	0.17599

421.0619	0.17604

421.22262	0.17618

421.38333	0.1762

421.54404	0.17623

421.70475	0.17627

421.86546	0.1763

422.02617	0.17632

422.18688	0.17637

422.34759	0.17639

422.5083	0.17642

422.66901	0.17644

422.82972	0.17646

422.99043	0.17649

423.15114	0.17653

423.31185	0.17672

423.47256	0.17679

423.63327	0.17681

423.79399	0.17684

423.9547	0.17688

424.11541	0.17691

424.27612	0.177

424.43683	0.17707

424.59754	0.17712

424.75825	0.17714

424.91896	0.17745

425.07967	0.17747

425.24038	0.17752

425.40109	0.17756

425.5618	0.17764

425.72251	0.1778

425.88322	0.17787

426.04393	0.17789

426.20465	0.17792

426.36536	0.17794

426.52607	0.17796

426.68678	0.17803

426.84749	0.17808

427.0082	0.17832

427.16891	0.17834

427.32962	0.17836

427.49033	0.17839

427.65104	0.17843

427.81175	0.17846

427.97246	0.17848

428.13317	0.17855

428.29388	0.17857

428.45459	0.1786

428.6153	0.17862

428.77602	0.17867

428.93673	0.17869

429.09744	0.17876

429.25815	0.17879

429.41886	0.17883

429.57957	0.17888

429.74028	0.17893

429.90099	0.17902

430.0617	0.17907

430.22241	0.17916

430.38312	0.17921

430.54383	0.17923

430.70454	0.17933

430.86525	0.17935

431.02596	0.17949

431.18668	0.17954

431.34739	0.17956

431.5081	0.17958

431.66881	0.17963

431.82952	0.17965

431.99023	0.17968

432.15094	0.1797

432.31165	0.17972

432.47236	0.17986

432.63307	0.18001

432.79378	0.18003

432.95449	0.18031

433.1152	0.18033

433.27591	0.18036

433.43662	0.18038

433.59734	0.18048

433.75805	0.1805

433.91876	0.18064

434.07947	0.18076

434.24018	0.18078

434.40089	0.1808

434.5616	0.18094

434.72231	0.18097

434.88302	0.18127

435.04373	0.18141

435.20444	0.18165

435.36515	0.18167

435.52586	0.1817

435.68657	0.18172

435.84728	0.18174

436.00799	0.18177

436.16871	0.18186

436.32942	0.18188

436.49013	0.18191

436.65084	0.18212

436.81155	0.18216

436.97226	0.18219

437.13297	0.18221

437.29368	0.18224

437.45439	0.18226

437.6151	0.18228

437.77581	0.18231

437.93652	0.18233

438.09723	0.1824

438.25794	0.18242

438.41865	0.18266

438.57937	0.18268

438.74008	0.1827

438.90079	0.18273

439.0615	0.18275

439.22221	0.18289

439.38292	0.18292

439.54363	0.18296

439.70434	0.18306

439.86505	0.18308

440.02576	0.1831

440.18647	0.18315

440.34718	0.1832

440.50789	0.18329

440.6686	0.18334

440.82931	0.18341

440.99003	0.18346

441.15074	0.18348

441.31145	0.18355

441.47216	0.18362

441.63287	0.18371

441.79358	0.18374

441.95429	0.18376

442.115	0.18378

442.27571	0.18381

442.43642	0.18385

442.59713	0.18395

442.75784	0.18402

442.91855	0.18404

443.07926	0.18416

443.23997	0.18423

443.40068	0.1843

443.5614	0.18432

443.72211	0.18437

443.88282	0.18442

444.04353	0.18444

444.20424	0.18446

444.36495	0.18449

444.52566	0.18451

444.68637	0.18454

444.84708	0.18456

445.00779	0.18461

445.1685	0.1847

445.32921	0.18482

445.48992	0.18524

445.65063	0.18536

445.81134	0.18538

445.97206	0.18547

446.13277	0.1855

446.29348	0.18552

446.45419	0.18554

446.6149	0.18557

446.77561	0.18564

446.93632	0.18578

447.09703	0.18585

447.25774	0.18587

447.41845	0.1859

447.57916	0.18597

447.73987	0.18601

447.90058	0.18604

448.06129	0.18639

448.222	0.18641

448.38272	0.18653

448.54343	0.18658

448.70414	0.1866

448.86485	0.18662

449.02556	0.18669

449.18627	0.18672

449.34698	0.18674

449.50769	0.18679

449.6684	0.18693

449.82911	0.18698

449.98982	0.187

450.15053	0.18709

450.31124	0.18712

450.47195	0.18714

450.63266	0.18763

450.79337	0.18766

450.95409	0.18768

451.1148	0.1877

451.27551	0.18782

451.43622	0.18784

451.59693	0.18796

451.75764	0.18817

451.91835	0.18834

452.07906	0.18836

452.23977	0.18841

452.40048	0.18843

452.56119	0.1885

452.7219	0.18852

452.88261	0.18855

453.04332	0.18864

453.20403	0.18867

453.36475	0.18869

453.52546	0.18876

453.68617	0.18881

453.84688	0.18885

454.00759	0.18888

454.1683	0.1889

454.32901	0.18895

454.48972	0.18899

454.65043	0.18902

454.81114	0.18916

454.97185	0.18918

455.13256	0.18921

455.29327	0.18939

455.45398	0.18942

455.61469	0.18949

455.7754	0.18953

455.93612	0.18979

456.09683	0.18984

456.25754	0.18986

456.41825	0.18991

456.57896	0.18996

456.73967	0.19003

456.90038	0.19005

457.06109	0.19007

457.2218	0.1901

457.38251	0.19019

457.54322	0.19021

457.70393	0.19024

457.86464	0.19033

458.02535	0.19036

458.18606	0.19038

458.34678	0.1904

458.50749	0.19047

458.6682	0.19059

458.82891	0.19061

458.98962	0.19073

459.15033	0.19078

459.31104	0.19082

459.47175	0.1909

459.63246	0.19097

459.79317	0.19099

459.95388	0.19125

460.11459	0.19132

460.2753	0.19165

460.43601	0.19186

460.59672	0.19188

460.75744	0.19197

460.91815	0.192

461.07886	0.19216

461.23957	0.19242

461.40028	0.19244

461.56099	0.19275

461.7217	0.19277

461.88241	0.19282

462.04312	0.19284

462.20383	0.19303

462.36454	0.19308

462.52525	0.1932

462.68596	0.19324

462.84667	0.19327

463.00738	0.19338

463.16809	0.19341

463.32881	0.19343

463.48952	0.19352

463.65023	0.19355

463.81094	0.19357

463.97165	0.19359

464.13236	0.19364

464.29307	0.19366

464.45378	0.19376

464.61449	0.19378

464.7752	0.19381

464.93591	0.19392

465.09662	0.19395

465.25733	0.19397

465.41804	0.19404

465.57875	0.19409

465.73947	0.19411

465.90018	0.19432

466.06089	0.19456

466.2216	0.1946

466.38231	0.19463

466.54302	0.19467

466.70373	0.1947

466.86444	0.19472

467.02515	0.19474

467.18586	0.19477

467.34657	0.19491

467.50728	0.19496

467.66799	0.19498

467.8287	0.1951

467.98941	0.19514

468.15013	0.19517

468.31084	0.19519

468.47155	0.19524

468.63226	0.19526

468.79297	0.19533

468.95368	0.19538

469.11439	0.1954

469.2751	0.19552

469.43581	0.19561

469.59652	0.19564

469.75723	0.19571

469.91794	0.19573

470.07865	0.19575

470.23936	0.19578

470.40007	0.19596

470.56078	0.19601

470.7215	0.19603

470.88221	0.19606

471.04292	0.19608

471.20363	0.19613

471.36434	0.19615

471.52505	0.1962

471.68576	0.19751

471.84647	0.1978

472.00718	0.19782

472.16789	0.19784

472.3286	0.19787

472.48931	0.19789

472.65002	0.19791

472.81073	0.19798

472.97144	0.19803

473.13216	0.19805

473.29287	0.19808

473.45358	0.19812

473.61429	0.19817

473.775	0.19819

473.93571	0.19822

474.09642	0.19826

474.25713	0.19829

474.41784	0.19841

474.57855	0.19843

474.73926	0.19852

474.89997	0.19857

475.06068	0.19859

475.22139	0.19864

475.3821	0.19869

475.54281	0.19871

475.70353	0.19873

475.86424	0.19876

476.02495	0.1988

476.18566	0.19883

476.34637	0.19885

476.50708	0.19895

476.66779	0.19899

476.8285	0.19902

476.98921	0.19904

477.14992	0.19906

477.31063	0.19909

477.47134	0.19911

477.63205	0.19918

477.79276	0.1992

477.95347	0.19937

478.11419	0.1996

478.2749	0.1997

478.43561	0.19972

478.59632	0.19977

478.75703	0.19979

478.91774	0.19984

479.07845	0.19986

479.23916	0.19991

479.39987	0.19995

479.56058	0.20007

479.72129	0.20014

479.882	0.20038

480.04271	0.20045

480.20342	0.20047

480.36413	0.20054

480.52485	0.20068

480.68556	0.20075

480.84627	0.20078

481.00698	0.20089

481.16769	0.20094

481.3284	0.20129

481.48911	0.20139

481.64982	0.20143

481.81053	0.2016

481.97124	0.20186

482.13195	0.20188

482.29266	0.2019

482.45337	0.20193

482.61408	0.202

482.77479	0.20204

482.9355	0.20211

483.09622	0.20239

483.25693	0.20244

483.41764	0.20256

483.57835	0.20258

483.73906	0.20261

483.89977	0.20289

484.06048	0.20291

484.22119	0.20301

484.3819	0.20303

484.54261	0.20343

484.70332	0.20345

484.86403	0.20362

485.02474	0.20378

485.18545	0.2038

485.34616	0.20383

485.50688	0.20385

485.66759	0.20387

485.8283	0.2039

485.98901	0.20397

486.14972	0.20408

486.31043	0.20418

486.47114	0.20446

486.63185	0.20451

486.79256	0.20455

486.95327	0.20462

487.11398	0.20465

487.27469	0.20469

487.4354	0.20477

487.59611	0.20538

487.75682	0.20556

487.91753	0.20563

488.07825	0.20596

488.23896	0.2061

488.39967	0.20622

488.56038	0.20631

488.72109	0.20641

488.8818	0.20678

489.04251	0.20681

489.20322	0.20695

489.36393	0.20697

489.52464	0.20725

489.68535	0.20728

489.84606	0.2073

490.00677	0.20742

490.16748	0.20744

490.32819	0.20746

490.48891	0.20751

490.64962	0.20756

490.81033	0.20768

490.97104	0.2077

491.13175	0.20779

491.29246	0.20831

491.45317	0.20833

491.61388	0.20836

491.77459	0.20857

491.9353	0.20859

492.09601	0.20876

492.25672	0.20878

492.41743	0.20883

492.57814	0.20885

492.73885	0.20887

492.89957	0.20899

493.06028	0.20901

493.22099	0.20904

493.3817	0.20906

493.54241	0.20908

493.70312	0.20944

493.86383	0.20948

494.02454	0.20955

494.18525	0.20958

494.34596	0.20979

494.50667	0.20981

494.66738	0.20988

494.82809	0.20993

494.9888	0.20995

495.14951	0.20998

495.31023	0.21014

495.47094	0.21023

495.63165	0.21037

495.79236	0.21044

495.95307	0.21047

496.11378	0.21061

496.27449	0.21063

496.4352	0.21066

496.59591	0.21068

496.75662	0.21075

496.91733	0.2108

497.07804	0.21084

497.23875	0.21089

497.39946	0.21094

497.56017	0.21101

497.72088	0.21108

497.8816	0.21113

498.04231	0.21129

498.20302	0.21169

498.36373	0.21178

498.52444	0.21195

498.68515	0.21209

498.84586	0.21213

499.00657	0.21232

499.16728	0.21235

499.32799	0.21237

499.4887	0.21239

499.64941	0.21246

499.81012	0.2126

499.97083	0.21265

500.13154	0.21274

500.29226	0.21277

500.45297	0.21279

500.61368	0.21286

500.77439	0.21291

500.9351	0.21293

501.09581	0.21305

501.25652	0.2131

501.41723	0.21312

501.57794	0.21319

501.73865	0.2134

501.89936	0.21343

502.06007	0.21345

502.22078	0.21354

502.38149	0.21359

502.5422	0.21364

502.70291	0.21366

502.86363	0.21368

503.02434	0.21371

503.18505	0.21378

503.34576	0.21387

503.50647	0.21392

503.66718	0.21399

503.82789	0.21408

503.9886	0.21411

504.14931	0.2142

504.31002	0.21427

504.47073	0.21432

504.63144	0.21436

504.79215	0.21439

504.95286	0.21448

505.11357	0.21458

505.27429	0.21462

505.435	0.21476

505.59571	0.21479

505.75642	0.21481

505.91713	0.21497

506.07784	0.21507

506.23855	0.21509

506.39926	0.21528

506.55997	0.2154

506.72068	0.21549

506.88139	0.21551

507.0421	0.21554

507.20281	0.21561

507.36352	0.21596

507.52423	0.21617

507.68495	0.21629

507.84566	0.21648

508.00637	0.2165

508.16708	0.21652

508.32779	0.21657

508.4885	0.21664

508.64921	0.21671

508.80992	0.21706

508.97063	0.21709

509.13134	0.21734

509.29205	0.21737

509.45276	0.2176

509.61347	0.21763

509.77418	0.21779

509.93489	0.21786

510.0956	0.21788

510.25632	0.21793

510.41703	0.21805

510.57774	0.21807

510.73845	0.21812

510.89916	0.21814

511.05987	0.21849

511.22058	0.21852

511.38129	0.21861

511.542	0.21864

511.70271	0.21868

511.86342	0.21871

512.02413	0.21901

512.18484	0.21903

512.34555	0.21906

512.50626	0.21908

512.66698	0.21922

512.82769	0.21925

512.9884	0.21927

513.14911	0.21929

513.30982	0.21939

513.47053	0.21953

513.63124	0.21955

513.79195	0.21957

513.95266	0.21964

514.11337	0.21971

514.27408	0.21976

514.43479	0.21981

514.5955	0.21983

514.75621	0.22011

514.91692	0.22014

515.07763	0.22016

515.23835	0.22018

515.39906	0.2203

515.55977	0.22033

515.72048	0.22042

515.88119	0.22049

516.0419	0.22063

516.20261	0.22075

516.36332	0.22108

516.52403	0.22124

516.68474	0.22131

516.84545	0.22136

517.00616	0.22138

517.16687	0.22157

517.32758	0.22159

517.48829	0.22166

517.64901	0.22173

517.80972	0.22176

517.97043	0.22178

518.13114	0.2218

518.29185	0.22185

518.45256	0.22199

518.61327	0.22201

518.77398	0.22204

518.93469	0.22206

519.0954	0.22216

519.25611	0.22218

519.41682	0.2222

519.57753	0.22223

519.73824	0.22227

519.89895	0.2223

520.05967	0.22237

520.22038	0.22309

520.38109	0.22312

520.5418	0.22314

520.70251	0.22316

520.86322	0.22328

521.02393	0.22335

521.18464	0.2234

521.34535	0.22342

521.50606	0.22347

521.66677	0.22368

521.82748	0.22373

521.98819	0.22375

522.1489	0.2238

522.30961	0.22396

522.47032	0.22399

522.63104	0.22401

522.79175	0.22403

522.95246	0.22406

523.11317	0.22408

523.27388	0.2242

523.43459	0.22422

523.5953	0.22429

523.75601	0.22434

523.91672	0.22467

524.07743	0.22471

524.23814	0.22474

524.39885	0.22481

524.55956	0.22485

524.72027	0.22497

524.88098	0.225

525.0417	0.22521

525.20241	0.22525

525.36312	0.22528

525.52383	0.22535

525.68454	0.22539

525.84525	0.22544

526.00596	0.22565

526.16667	0.22584

526.32738	0.22586

526.48809	0.2261

526.6488	0.22617

526.80951	0.22619

526.97022	0.22629

527.13093	0.22631

527.29164	0.22633

527.45236	0.22636

527.61307	0.22645

527.77378	0.22647

527.93449	0.22673

528.0952	0.22676

528.25591	0.2268

528.41662	0.22683

528.57733	0.22685

528.73804	0.22687

528.89875	0.22701

529.05946	0.22704

529.22017	0.22708

529.38088	0.22739

529.54159	0.22758

529.7023	0.2276

529.86301	0.22772

530.02373	0.22774

530.18444	0.22814

530.34515	0.22816

530.50586	0.22819

530.66657	0.22823

530.82728	0.2283

530.98799	0.22835

531.1487	0.22837

531.30941	0.2284

531.47012	0.22842

531.63083	0.22845

531.79154	0.22854

531.95225	0.22856

532.11296	0.22859

532.27367	0.22868

532.43439	0.2287

532.5951	0.22873

532.75581	0.22875

532.91652	0.22877

533.07723	0.2288

533.23794	0.22889

533.39865	0.22899

533.55936	0.2291

533.72007	0.22913

533.88078	0.22924

534.04149	0.22927

534.2022	0.22936

534.36291	0.22938

534.52362	0.22945

534.68433	0.2295

534.84505	0.22981

535.00576	0.22983

535.16647	0.22985

535.32718	0.22992

535.48789	0.22995

535.6486	0.22997

535.80931	0.23002

535.97002	0.23011

536.13073	0.23014

536.29144	0.23016

536.45215	0.23028

536.61286	0.23032

536.77357	0.23051

536.93428	0.23053

537.09499	0.23063

537.2557	0.23082

537.41642	0.23084

537.57713	0.23086

537.73784	0.2311

537.89855	0.23112

538.05926	0.23114

538.21997	0.23121

538.38068	0.23126

538.54139	0.23129

538.7021	0.2314

538.86281	0.23143

539.02352	0.23145

539.18423	0.23147

539.34494	0.23154

539.50565	0.23157

539.66636	0.23164

539.82708	0.23168

539.98779	0.23171

540.1485	0.23173

540.30921	0.23178

540.46992	0.23182

540.63063	0.23187

540.79134	0.23192

540.95205	0.23194

541.11276	0.23201

541.27347	0.23204

541.43418	0.23215

541.59489	0.2322

541.7556	0.23222

541.91631	0.23225

542.07702	0.23251

542.23773	0.23253

542.39845	0.23258

542.55916	0.2326

542.71987	0.23262

542.88058	0.23267

543.04129	0.23269

543.202	0.23272

543.36271	0.23274

543.52342	0.23279

543.68413	0.23293

543.84484	0.23295

544.00555	0.23305

544.16626	0.23307

544.32697	0.23309

544.48768	0.23312

544.64839	0.23319

544.80911	0.23321

544.96982	0.23326

545.13053	0.23328

545.29124	0.23333

545.45195	0.23335

545.61266	0.23349

545.77337	0.23351

545.93408	0.23354

546.09479	0.23356

546.2555	0.23363

546.41621	0.23366

546.57692	0.23368

546.73763	0.23373

546.89834	0.23391

547.05905	0.23403

547.21977	0.23405

547.38048	0.23408

547.54119	0.2341

547.7019	0.23415

547.86261	0.2342

548.02332	0.23436

548.18403	0.23455

548.34474	0.23457

548.50545	0.23459

548.66616	0.23466

548.82687	0.23471

548.98758	0.23483

549.14829	0.23488

549.309	0.23502

549.46971	0.2352

549.63042	0.23523

549.79114	0.23527

549.95185	0.2353

550.11256	0.23535

550.27327	0.23537

550.43398	0.23542

550.59469	0.23544

550.7554	0.23549

550.91611	0.23558

551.07682	0.23563

551.23753	0.23586

551.39824	0.23588

551.55895	0.23603

551.71966	0.23605

551.88037	0.23607

552.04108	0.23612

552.2018	0.23614

552.36251	0.23617

552.52322	0.23619

552.68393	0.23621

552.84464	0.23631

553.00535	0.23633

553.16606	0.23642

553.32677	0.23647

553.48748	0.2365

553.64819	0.23654

553.8089	0.23657

553.96961	0.23659

554.13032	0.23664

554.29103	0.23666

554.45174	0.23668

554.61245	0.23673

554.77317	0.2372

554.93388	0.23725

555.09459	0.23727

555.2553	0.23729

555.41601	0.23743

555.57672	0.23746

555.73743	0.23781

555.89814	0.23786

556.05885	0.23793

556.21956	0.23804

556.38027	0.23807

556.54098	0.23809

556.70169	0.23811

556.8624	0.23814

557.02311	0.23816

557.18383	0.23823

557.34454	0.23826

557.50525	0.23842

557.66596	0.23851

557.82667	0.23875

557.98738	0.23884

558.14809	0.23898

558.3088	0.23901

558.46951	0.23915

558.63022	0.23917

558.79093	0.23926

558.95164	0.23929

559.11235	0.23938

559.27306	0.23957

559.43377	0.23959

559.59449	0.23997

559.7552	0.24004

559.91591	0.24006

560.07662	0.2403

560.23733	0.24034

560.39804	0.24044

560.55875	0.24051

560.71946	0.24053

560.88017	0.24056

561.04088	0.24063

561.20159	0.24065

561.3623	0.24084

561.52301	0.24086

561.68372	0.24088

561.84443	0.24093

562.00514	0.2411

562.16586	0.24112

562.32657	0.24168

562.48728	0.24171

562.64799	0.24178

562.8087	0.24185

562.96941	0.24257

563.13012	0.24262

563.29083	0.24264

563.45154	0.24269

563.61225	0.24271

563.77296	0.24274

563.93367	0.24278

564.09438	0.24293

564.25509	0.24304

564.4158	0.24318

564.57652	0.24325

564.73723	0.24349

564.89794	0.24354

565.05865	0.24358

565.21936	0.2437

565.38007	0.24379

565.54078	0.24386

565.70149	0.24403

565.8622	0.2441

566.02291	0.24417

566.18362	0.24438

566.34433	0.24447

566.50504	0.2445

566.66575	0.24452

566.82646	0.24462

566.98718	0.24494

567.14789	0.24518

567.3086	0.24525

567.46931	0.24527

567.63002	0.24532

567.79073	0.24537

567.95144	0.24544

568.11215	0.24546

568.27286	0.24555

568.43357	0.24562

568.59428	0.24574

568.75499	0.24577

568.9157	0.24581

569.07641	0.24598

569.23712	0.24609

569.39783	0.24614

569.55855	0.24633

569.71926	0.24647

569.87997	0.24654

570.04068	0.24656

570.20139	0.24677

570.3621	0.2468

570.52281	0.24689

570.68352	0.24696

570.84423	0.2471

571.00494	0.24717

571.16565	0.24722

571.32636	0.24731

571.48707	0.24746

571.64778	0.24748

571.80849	0.24753

571.96921	0.24755

572.12992	0.24771

572.29063	0.24799

572.45134	0.24807

572.61205	0.24809

572.77276	0.24835

572.93347	0.24837

573.09418	0.24842

573.25489	0.24856

573.4156	0.24865

573.57631	0.24879

573.73702	0.24886

573.89773	0.249

574.05844	0.24907

574.21915	0.24912

574.37987	0.24917

574.54058	0.24919

574.70129	0.24929

574.862	0.24933

575.02271	0.24947

575.18342	0.2495

575.34413	0.24952

575.50484	0.24957

575.66555	0.25053

575.82626	0.25055

575.98697	0.25058

576.14768	0.2506

576.30839	0.25065

576.4691	0.25069

576.62981	0.25112

576.79052	0.25123

576.95124	0.25126

577.11195	0.25128

577.27266	0.2513

577.43337	0.25133

577.59408	0.25147

577.75479	0.25159

577.9155	0.25177

578.07621	0.2518

578.23692	0.25182

578.39763	0.25229

578.55834	0.25236

578.71905	0.25245

578.87976	0.25248

579.04047	0.25274

579.20118	0.25276

579.3619	0.25292

579.52261	0.25295

579.68332	0.25302

579.84403	0.25309

580.00474	0.25372

580.16545	0.25374

580.32616	0.25389

580.48687	0.25391

580.64758	0.25393

580.80829	0.25396

580.969	0.254

581.12971	0.25405

581.29042	0.2541

581.45113	0.25412

581.61184	0.25414

581.77255	0.25421

581.93327	0.25424

582.09398	0.25426

582.25469	0.2544

582.4154	0.25443

582.57611	0.25459

582.73682	0.25461

582.89753	0.25468

583.05824	0.25473

583.21895	0.25485

583.37966	0.25487

583.54037	0.25489

583.70108	0.25492

583.86179	0.25497

584.0225	0.25501

584.18321	0.25504

584.34393	0.25515

584.50464	0.25518

584.66535	0.2552

584.82606	0.25536

584.98677	0.25539

585.14748	0.25543

585.30819	0.25553

585.4689	0.25555

585.62961	0.2556

585.79032	0.25567

585.95103	0.25572

586.11174	0.25581

586.27245	0.25583

586.43316	0.25597

586.59387	0.256

586.75459	0.25602

586.9153	0.25604

587.07601	0.25607

587.23672	0.25612

587.39743	0.25623

587.55814	0.25626

587.71885	0.2563

587.87956	0.25654

588.04027	0.25656

588.20098	0.25658

588.36169	0.25673

588.5224	0.25677

588.68311	0.2568

588.84382	0.25689

589.00453	0.25691

589.16524	0.25694

589.32596	0.25696

589.48667	0.25727

589.64738	0.25729

589.80809	0.25731

589.9688	0.25738

590.12951	0.25741

590.29022	0.2575

590.45093	0.25762

590.61164	0.25773

590.77235	0.25776

590.93306	0.2579

591.09377	0.25795

591.25448	0.25811

591.41519	0.25816

591.5759	0.25818

591.73662	0.25823

591.89733	0.25849

592.05804	0.25851

592.21875	0.25853

592.37946	0.2586

592.54017	0.25867

592.70088	0.2587

592.86159	0.25954

593.0223	0.25957

593.18301	0.25961

593.34372	0.25968

593.50443	0.25982

593.66514	0.25985

593.82585	0.25987

593.98656	0.25989

594.14728	0.25999

594.30799	0.26003

594.4687	0.26008

594.62941	0.26013

594.79012	0.26015

594.95083	0.26018

595.11154	0.26022

595.27225	0.26027

595.43296	0.26036

595.59367	0.26041

595.75438	0.2605

595.91509	0.26053

596.0758	0.26055

596.23651	0.26064

596.39722	0.26067

596.55793	0.26071

596.71865	0.26081

596.87936	0.26088

597.04007	0.2609

597.20078	0.26093

597.36149	0.261

597.5222	0.26107

597.68291	0.26111

597.84362	0.26116

598.00433	0.26118

598.16504	0.26121

598.32575	0.26125

598.48646	0.26135

598.64717	0.26137

598.80788	0.2614

598.96859	0.26149

599.12931	0.26156

599.29002	0.26161

599.45073	0.26163

599.61144	0.26165

599.77215	0.2617

599.93286	0.26177

600.09357	0.26179

600.25428	0.26182

600.41499	0.26186

600.5757	0.26208

600.73641	0.26215

600.89712	0.26217

601.05783	0.26219

601.21854	0.26222

601.37925	0.26245

601.53997	0.2625

601.70068	0.26257

601.86139	0.26259

602.0221	0.26264

602.18281	0.26266

602.34352	0.26269

602.50423	0.26276

602.66494	0.26283

602.82565	0.26292

602.98636	0.26299

603.14707	0.2632

603.30778	0.26332

603.46849	0.26341

603.6292	0.26344

603.78991	0.26355

603.95062	0.26358

604.11134	0.26365

604.27205	0.2637

604.43276	0.26372

604.59347	0.26377

604.75418	0.26379

604.91489	0.26391

605.0756	0.26405

605.23631	0.26407

605.39702	0.26426

605.55773	0.26428

605.71844	0.26435

605.87915	0.26438

606.03986	0.26452

606.20057	0.26461

606.36128	0.26463

606.522	0.2647

606.68271	0.26475

606.84342	0.26499

607.00413	0.26503

607.16484	0.26506

607.32555	0.26508

607.48626	0.26513

607.64697	0.26522

607.80768	0.26524

607.96839	0.26536

608.1291	0.26543

608.28981	0.2655

608.45052	0.26557

608.61123	0.26569

608.77194	0.26576

608.93265	0.26593

609.09337	0.26597

609.25408	0.26623

609.41479	0.2663

609.5755	0.26635

609.73621	0.26646

609.89692	0.26649

610.05763	0.26668

610.21834	0.26684

610.37905	0.26691

610.53976	0.26696

610.70047	0.26698

610.86118	0.267

611.02189	0.26719

611.1826	0.26745

611.34331	0.26747

611.50403	0.26773

611.66474	0.26778

611.82545	0.26783

611.98616	0.26785

612.14687	0.26794

612.30758	0.26797

612.46829	0.26822

612.629	0.26834

612.78971	0.26839

612.95042	0.26858

613.11113	0.2686

613.27184	0.26893

613.43255	0.26895

613.59326	0.26898

613.75397	0.26905

613.91469	0.26907

614.0754	0.26909

614.23611	0.26923

614.39682	0.26933

614.55753	0.26935

614.71824	0.26942

614.87895	0.26945

615.03966	0.26959

615.20037	0.26961

615.36108	0.26963

615.52179	0.26975

615.6825	0.2698

615.84321	0.26982

616.00392	0.26989

616.16463	0.26991

616.32534	0.2701

616.48606	0.27013

616.64677	0.27015

616.80748	0.2702

616.96819	0.27038

617.1289	0.27043

617.28961	0.2705

617.45032	0.27064

617.61103	0.27071

617.77174	0.27076

617.93245	0.27078

618.09316	0.27083

618.25387	0.27085

618.41458	0.27088

618.57529	0.2709

618.736	0.27092

618.89672	0.27109

619.05743	0.27111

619.21814	0.27114

619.37885	0.27121

619.53956	0.27123

619.70027	0.27125

619.86098	0.27163

620.02169	0.27165

620.1824	0.27172

620.34311	0.27182

620.50382	0.27184

620.66453	0.27203

620.82524	0.27221

620.98595	0.27224

621.14666	0.27238

621.30737	0.27245

621.46809	0.27247

621.6288	0.27252

621.78951	0.27261

621.95022	0.27268

622.11093	0.27271

622.27164	0.27292

622.43235	0.27297

622.59306	0.27299

622.75377	0.27306

622.91448	0.27308

623.07519	0.2732

623.2359	0.27365

623.39661	0.27367

623.55732	0.27386

623.71803	0.2739

623.87875	0.27402

624.03946	0.27407

624.20017	0.27416

624.36088	0.27419

624.52159	0.27421

624.6823	0.27423

624.84301	0.27428

625.00372	0.2743

625.16443	0.27433

625.32514	0.27442

625.48585	0.27444

625.64656	0.27447

625.80727	0.27459

625.96798	0.27491

626.12869	0.27517

626.28941	0.27522

626.45012	0.27531

626.61083	0.27541

626.77154	0.27559

626.93225	0.27562

627.09296	0.27564

627.25367	0.27566

627.41438	0.27574

627.57509	0.27576

627.7358	0.27583

627.89651	0.27602

628.05722	0.27604

628.21793	0.27644

628.37864	0.27649

628.53935	0.27653

628.70006	0.2766

628.86078	0.27665

629.02149	0.2767

629.1822	0.27674

629.34291	0.27684

629.50362	0.27712

629.66433	0.27717

629.82504	0.27733

629.98575	0.27735

630.14646	0.27738

630.30717	0.27764

630.46788	0.27775

630.62859	0.27778

630.7893	0.27782

630.95001	0.27806

631.11072	0.27815

631.27144	0.2782

631.43215	0.27822

631.59286	0.27827

631.75357	0.27839

631.91428	0.27853

632.07499	0.27855

632.2357	0.2786

632.39641	0.27883

632.55712	0.27907

632.71783	0.27909

632.87854	0.27921

633.03925	0.27923

633.19996	0.2793

633.36067	0.27937

633.52138	0.27961

633.6821	0.27963

633.84281	0.27965

634.00352	0.27975

634.16423	0.27977

634.32494	0.27998

634.48565	0.28001

634.64636	0.28029

634.80707	0.28036

634.96778	0.28038

635.12849	0.28045

635.2892	0.2805

635.44991	0.28055

635.61062	0.28059

635.77133	0.28069

635.93204	0.28071

636.09275	0.28073

636.25347	0.28087

636.41418	0.28092

636.57489	0.28104

636.7356	0.28111

636.89631	0.28123

637.05702	0.2813

637.21773	0.28134

637.37844	0.28137

637.53915	0.28141

637.69986	0.28144

637.86057	0.28148

638.02128	0.28151

638.18199	0.28153

638.3427	0.28156

638.50341	0.28158

638.66413	0.28184

638.82484	0.28186

638.98555	0.28195

639.14626	0.28198

639.30697	0.28202

639.46768	0.28207

639.62839	0.28233

639.7891	0.28256

639.94981	0.28278

640.11052	0.28325

640.27123	0.28329

640.43194	0.28332

640.59265	0.28343

640.75336	0.28367

640.91407	0.28369

641.07479	0.28371

641.2355	0.28378

641.39621	0.28388

641.55692	0.28404

641.71763	0.28461

641.87834	0.28463

642.03905	0.28475

642.19976	0.28479

642.36047	0.28484

642.52118	0.28486

642.68189	0.28489

642.8426	0.28503

643.00331	0.28512

643.16402	0.28515

643.32473	0.28517

643.48544	0.28526

643.64616	0.28529

643.80687	0.28559

643.96758	0.28578

644.12829	0.28583

644.289	0.28592

644.44971	0.28599

644.61042	0.28601

644.77113	0.28606

644.93184	0.28613

645.09255	0.28616

645.25326	0.2862

645.41397	0.28641

645.57468	0.28651

645.73539	0.28653

645.8961	0.28655

646.05682	0.28662

646.21753	0.28673

646.37824	0.28684

646.53895	0.28689

646.69966	0.28705

646.86037	0.2871

647.02108	0.28721

647.18179	0.28726

647.3425	0.28731

647.50321	0.28736

647.66392	0.28752

647.82463	0.28758

647.98534	0.28763

648.14605	0.28768

648.30676	0.28773

648.46747	0.28784

648.62819	0.28789

648.7889	0.28794

648.94961	0.288

649.11032	0.28816

649.27103	0.28821

649.43174	0.28842

649.59245	0.28847

649.75316	0.28858

649.91387	0.28863

650.07458	0.28874

650.23529	0.28879

650.396	0.28884

650.55671	0.2889

650.71742	0.28895

650.87813	0.28916

651.03885	0.28932

651.19956	0.28937

651.36027	0.28953

651.52098	0.28958

651.68169	0.28963

651.8424	0.28974

652.00311	0.28979

652.16382	0.28985

652.32453	0.2899

652.48524	0.28995

652.64595	0.29

652.80666	0.29006

652.96737	0.29016

653.12808	0.29022

653.28879	0.29027

653.44951	0.29032

653.61022	0.29037

653.77093	0.29043

653.93164	0.29048

654.09235	0.29053

654.25306	0.29058

654.41377	0.29064

654.57448	0.29069

654.73519	0.29074

654.8959	0.2908

655.05661	0.2909

655.21732	0.29095

655.37803	0.29101

655.53874	0.29106

655.69945	0.29111

655.86016	0.29122

656.02088	0.29132

656.18159	0.29138

656.3423	0.29143

656.50301	0.29154

656.66372	0.29159

656.82443	0.2918

656.98514	0.29191

657.14585	0.29196

657.30656	0.29212

657.46727	0.29222

657.62798	0.29233

657.78869	0.29238

657.9494	0.29243

658.11011	0.29275

658.27082	0.29291

658.43154	0.29301

658.59225	0.29307

658.75296	0.29333

658.91367	0.29344

659.07438	0.29349

659.23509	0.29354

659.3958	0.29359

659.55651	0.29375

659.71722	0.29381

659.87793	0.29391

660.03864	0.29407

660.19935	0.29418

660.36006	0.29428

660.52077	0.29433

660.68148	0.29439

660.84219	0.29455

661.00291	0.29465

661.16362	0.2947

661.32433	0.29476

661.48504	0.29481

661.64575	0.29486

661.80646	0.29497

661.96717	0.29502

662.12788	0.29513

662.28859	0.29518

662.4493	0.29528

662.61001	0.29534

662.77072	0.29539

662.93143	0.29544

663.09214	0.2955

663.25285	0.29555

663.41357	0.29565

663.57428	0.29571

663.73499	0.29576

663.8957	0.29581

664.05641	0.29587

664.21712	0.29592

664.37783	0.29597

664.53854	0.29602

664.69925	0.29608

664.85996	0.29613

665.02067	0.29618

665.18138	0.29624

665.34209	0.29639

665.5028	0.29645

665.66351	0.2965

665.82423	0.2966

665.98494	0.29666

666.14565	0.29671

666.30636	0.29676

666.46707	0.29682

666.62778	0.29703

666.78849	0.29708

666.9492	0.29719

667.10991	0.29724

667.27062	0.29729

667.43133	0.29734

667.59204	0.2974

667.75275	0.29766

667.91346	0.29771

668.07417	0.29777

668.23488	0.29787

668.3956	0.29814

668.55631	0.29819

668.71702	0.29829

668.87773	0.29835

669.03844	0.2984

669.19915	0.29845

669.35986	0.29861

669.52057	0.29866

669.68128	0.29888

669.84199	0.29893

670.0027	0.29898

670.16341	0.29924

670.32412	0.2993

670.48483	0.29935

670.64554	0.2994

670.80626	0.29946

670.96697	0.29951

671.12768	0.29956

671.28839	0.29961

671.4491	0.29967

671.60981	0.29972

671.77052	0.29977

671.93123	0.29988

672.09194	0.29993

672.25265	0.29998

672.41336	0.30004

672.57407	0.30009

672.73478	0.3002

672.89549	0.30025

673.0562	0.3003

673.21692	0.30035

673.37763	0.30041

673.53834	0.30051

673.69905	0.30067

673.85976	0.30072

674.02047	0.30083

674.18118	0.30093

674.34189	0.30099

674.5026	0.30104

674.66331	0.30109

674.82402	0.30115

674.98473	0.3012

675.14544	0.30125

675.30615	0.3013

675.46686	0.30136

675.62757	0.30141

675.78829	0.30146

675.949	0.30152

676.10971	0.30157

676.27042	0.30162

676.43113	0.30167

676.59184	0.30178

676.75255	0.30194

676.91326	0.3021

677.07397	0.30215

677.23468	0.30225

677.39539	0.30236

677.5561	0.30241

677.71681	0.30257

677.87752	0.30262

678.03823	0.30268

678.19895	0.30273

678.35966	0.30278

678.52037	0.30289

678.68108	0.30294

678.84179	0.30299

679.0025	0.30305

679.16321	0.3031

679.32392	0.30315

679.48463	0.30321

679.64534	0.30326

679.80605	0.30331

679.96676	0.30336

680.12747	0.30342

680.28818	0.30368

680.44889	0.30379

680.6096	0.30384

680.77032	0.30389

680.93103	0.30394

681.09174	0.304

681.25245	0.30405

681.41316	0.3041

681.57387	0.30416

681.73458	0.30421

681.89529	0.30426

682.056	0.30437

682.21671	0.30447

682.37742	0.30453

682.53813	0.30463

682.69884	0.30468

682.85955	0.30479

683.02026	0.30484

683.18098	0.3049

683.34169	0.30505

683.5024	0.30511

683.66311	0.30516

683.82382	0.30521

683.98453	0.30532

684.14524	0.30585

684.30595	0.30595

684.46666	0.30606

684.62737	0.30616

684.78808	0.30622

684.94879	0.30632

685.1095	0.30637

685.27021	0.30653

685.43092	0.30658

685.59164	0.30664

685.75235	0.30669

685.91306	0.30674

686.07377	0.3068

686.23448	0.30685

686.39519	0.3069

686.5559	0.30695

686.71661	0.30701

686.87732	0.30706

687.03803	0.30711

687.19874	0.30717

687.35945	0.30722

687.52016	0.30727

687.68087	0.30743

687.84158	0.30754

688.00229	0.30759

688.16301	0.30769

688.32372	0.30775

688.48443	0.3078

688.64514	0.30785

688.80585	0.3079

688.96656	0.30796

689.12727	0.30806

689.28798	0.30817

689.44869	0.30822

689.6094	0.30854

689.77011	0.3087

689.93082	0.3088

690.09153	0.30896

690.25224	0.30901

690.41295	0.30907

690.57367	0.30912

690.73438	0.30917

690.89509	0.30923

691.0558	0.30928

691.21651	0.30933

691.37722	0.30938

691.53793	0.30949

691.69864	0.30954

691.85935	0.30959

692.02006	0.3097

692.18077	0.30975

692.34148	0.30981

692.50219	0.30986

692.6629	0.31002

692.82361	0.31007

692.98433	0.31012

693.14504	0.31018

693.30575	0.31023

693.46646	0.31039

693.62717	0.31044

693.78788	0.31049

693.94859	0.31055

694.1093	0.3106

694.27001	0.31065

694.43072	0.31081

694.59143	0.31091

694.75214	0.31102

694.91285	0.31107

695.07356	0.31113

695.23427	0.31128

695.39498	0.31134

695.5557	0.3115

695.71641	0.31155

695.87712	0.3116

696.03783	0.31165

696.19854	0.31171

696.35925	0.31176

696.51996	0.31181

696.68067	0.31192

696.84138	0.31197

697.00209	0.31202

697.1628	0.31213

697.32351	0.31218

697.48422	0.31229

697.64493	0.31239

697.80564	0.31245

697.96636	0.31271

698.12707	0.31276

698.28778	0.31282

698.44849	0.31287

698.6092	0.31292

698.76991	0.31303

698.93062	0.31308

699.09133	0.31313

699.25204	0.31319

699.41275	0.31324

699.57346	0.31334

699.73417	0.3134

699.89488	0.31345

700.05559	0.3135

700.2163	0.31361

700.37702	0.31382

700.53773	0.31392

700.69844	0.31398

700.85915	0.31419

701.01986	0.31424

701.18057	0.31429

701.34128	0.31451

701.50199	0.31461

701.6627	0.31466

701.82341	0.31477

701.98412	0.31482

702.14483	0.31493

702.30554	0.31498

702.46625	0.31503

702.62696	0.31519

702.78767	0.31524

702.94839	0.3153

703.1091	0.3154

703.26981	0.31551

703.43052	0.31561

703.59123	0.31567

703.75194	0.31572

703.91265	0.31577

704.07336	0.31583

704.23407	0.31598

704.39478	0.31604

704.55549	0.31609

704.7162	0.31614

704.87691	0.3162

705.03762	0.31625

705.19833	0.3163

705.35905	0.31635

705.51976	0.31641

705.68047	0.31646

705.84118	0.31651

706.00189	0.31656

706.1626	0.31662

706.32331	0.31672

706.48402	0.31678

706.64473	0.31699

706.80544	0.31709

706.96615	0.31715

707.12686	0.3172

707.28757	0.31736

707.44828	0.31741

707.60899	0.31757

707.76971	0.31767

707.93042	0.31778

708.09113	0.31783

708.25184	0.31841

708.41255	0.31847

708.57326	0.31852

708.73397	0.31878

708.89468	0.31884

709.05539	0.31889

709.2161	0.31899

709.37681	0.31905

709.53752	0.3191

709.69823	0.31931

709.85894	0.31936

710.01965	0.31947

710.18036	0.31952

710.34108	0.31963

710.50179	0.31968

710.6625	0.31973

710.82321	0.31979

710.98392	0.31984

711.14463	0.31989

711.30534	0.31994

711.46605	0.32

711.62676	0.32005

711.78747	0.3201

711.94818	0.32016

712.10889	0.32026

712.2696	0.32058

712.43031	0.32084

712.59102	0.321

712.75174	0.32105

712.91245	0.32111

713.07316	0.32116

713.23387	0.32121

713.39458	0.32132

713.55529	0.32137

713.716	0.32148

713.87671	0.32153

714.03742	0.32174

714.19813	0.32195

714.35884	0.32206

714.51955	0.32216

714.68026	0.32221

714.84097	0.32248

715.00168	0.32269

715.16239	0.32274

715.32311	0.3228

715.48382	0.32295

715.64453	0.32301

715.80524	0.32311

715.96595	0.32322

716.12666	0.32327

716.28737	0.32332

716.44808	0.32338

716.60879	0.32343

716.7695	0.32348

716.93021	0.32354

717.09092	0.32359

717.25163	0.32369

717.41234	0.32375

717.57305	0.3238

717.73377	0.32385

717.89448	0.3239

718.05519	0.32396

718.2159	0.32406

718.37661	0.32433

718.53732	0.32438

718.69803	0.32443

718.85874	0.32449

719.01945	0.32454

719.18016	0.3247

719.34087	0.32475

719.50158	0.3248

719.66229	0.32486

719.823	0.32491

719.98371	0.32496

720.14443	0.32501

720.30514	0.32507

720.46585	0.32517

720.62656	0.32522

720.78727	0.32528

720.94798	0.32533

721.10869	0.32565

721.2694	0.32575

721.43011	0.32581

721.59082	0.32586

721.75153	0.32591

721.91224	0.32596

722.07295	0.32602

722.23366	0.32628

722.39437	0.32633

722.55508	0.32644

722.7158	0.32665

722.87651	0.3267

723.03722	0.32676

723.19793	0.32681

723.35864	0.32686

723.51935	0.32691

723.68006	0.32697

723.84077	0.32702

724.00148	0.32707

724.16219	0.32718

724.3229	0.32723

724.48361	0.32734

724.64432	0.32744

724.80503	0.3275

724.96574	0.32755

725.12646	0.3276

725.28717	0.32771

725.44788	0.32787

725.60859	0.32792

725.7693	0.32797

725.93001	0.32808

726.09072	0.32813

726.25143	0.32818

726.41214	0.32829

726.57285	0.32834

726.73356	0.3285

726.89427	0.3286

727.05498	0.32866

727.21569	0.32876

727.3764	0.32882

727.53711	0.32887

727.69783	0.32892

727.85854	0.32903

728.01925	0.32929

728.17996	0.3294

728.34067	0.32945

728.50138	0.3295

728.66209	0.32955

728.8228	0.32966

728.98351	0.32971

729.14422	0.32977

729.30493	0.32982

729.46564	0.32987

729.62635	0.32992

729.78706	0.33003

729.94777	0.33008

730.10849	0.33014

730.2692	0.33019

730.42991	0.33029

730.59062	0.33045

730.75133	0.33051

730.91204	0.33056

731.07275	0.33061

731.23346	0.33066

731.39417	0.33082

731.55488	0.33087

731.71559	0.33098

731.8763	0.33109

732.03701	0.33119

732.19772	0.33124

732.35843	0.3313

732.51915	0.33146

732.67986	0.33151

732.84057	0.33156

733.00128	0.33161

733.16199	0.33167

733.3227	0.33172

733.48341	0.33188

733.64412	0.33193

733.80483	0.33198

733.96554	0.33204

734.12625	0.33214

734.28696	0.33241

734.44767	0.33246

734.60838	0.33251

734.76909	0.33267

734.9298	0.33278

735.09052	0.33299

735.25123	0.33304

735.41194	0.33309

735.57265	0.33315

735.73336	0.33325

735.89407	0.3333

736.05478	0.33336

736.21549	0.33341

736.3762	0.33357

736.53691	0.33362

736.69762	0.33367

736.85833	0.33373

737.01904	0.33378

737.17975	0.33383

737.34046	0.33388

737.50118	0.33394

737.66189	0.33404

737.8226	0.3341

737.98331	0.3342

738.14402	0.33425

738.30473	0.33431

738.46544	0.33441

738.62615	0.33447

738.78686	0.33457

738.94757	0.33462

739.10828	0.33468

739.26899	0.33473

739.4297	0.33478

739.59041	0.33499

739.75112	0.33505

739.91184	0.3351

740.07255	0.33515

740.23326	0.3352

740.39397	0.33526

740.55468	0.33531

740.71539	0.33542

740.8761	0.33557

741.03681	0.33563

741.19752	0.33568

741.35823	0.33573

741.51894	0.33579

741.67965	0.33584

741.84036	0.33594

742.00107	0.33605

742.16178	0.3361

742.32249	0.33616

742.48321	0.33621

742.64392	0.33631

742.80463	0.33637

742.96534	0.33653

743.12605	0.33679

743.28676	0.33689

743.44747	0.33695

743.60818	0.337

743.76889	0.33737

743.9296	0.33742

744.09031	0.33748

744.25102	0.33769

744.41173	0.33774

744.57244	0.33779

744.73315	0.33795

744.89387	0.338

745.05458	0.33816

745.21529	0.33821

745.376	0.33827

745.53671	0.33832

745.69742	0.33837

745.85813	0.33843

746.01884	0.33848

746.17955	0.33853

746.34026	0.33864

746.50097	0.33869

746.66168	0.3389

746.82239	0.33895

746.9831	0.33911

747.14381	0.33922

747.30453	0.33927

747.46524	0.33932

747.62595	0.33943

747.78666	0.33953

747.94737	0.34017

748.10808	0.34022

748.26879	0.34027

748.4295	0.34033

748.59021	0.34043

748.75092	0.34049

748.91163	0.34059

749.07234	0.34064

749.23305	0.3407

749.39376	0.34075

749.55447	0.34086

749.71518	0.34091

749.8759	0.34101

750.03661	0.34107

750.19732	0.34128

750.35803	0.34138

750.51874	0.34144

750.67945	0.34154

750.84016	0.34165

751.00087	0.3417

751.16158	0.34175

751.32229	0.34181

751.483	0.34186

751.64371	0.34191

751.80442	0.34196

751.96513	0.34207

752.12584	0.34212

752.28656	0.34223

752.44727	0.34239

752.60798	0.34254

752.76869	0.34265

752.9294	0.34281

753.09011	0.34291

753.25082	0.34297

753.41153	0.34302

753.57224	0.34307

753.73295	0.34318

753.89366	0.34323

754.05437	0.34328

754.21508	0.34339

754.37579	0.34355

754.5365	0.34365

754.69721	0.34371

754.85793	0.34376

755.01864	0.34381

755.17935	0.34392

755.34006	0.34397

755.50077	0.34402

755.66148	0.34408

755.82219	0.34413

755.9829	0.34418

756.14361	0.34423

756.30432	0.34429

756.46503	0.34439

756.62574	0.3445

756.78645	0.34455

756.94716	0.3446

757.10787	0.34471

757.26859	0.34482

757.4293	0.34487

757.59001	0.34497

757.75072	0.34503

757.91143	0.34508

758.07214	0.34519

758.23285	0.34524

758.39356	0.34529

758.55427	0.34534

758.71498	0.3454

758.87569	0.34545

759.0364	0.3455

759.19711	0.34566

759.35782	0.34571

759.51853	0.34577

759.67925	0.34582

759.83996	0.34592

760.00067	0.34603

760.16138	0.34608

760.32209	0.34619

760.4828	0.34629

760.64351	0.3464

760.80422	0.34645

760.96493	0.34661

761.12564	0.34682

761.28635	0.34687

761.44706	0.34698

761.60777	0.34719

761.76848	0.3473

761.92919	0.34735

762.0899	0.34751

762.25062	0.34756

762.41133	0.34777

762.57204	0.34783

762.73275	0.34788

762.89346	0.34793

763.05417	0.34798

763.21488	0.34804

763.37559	0.34814

763.5363	0.34819

763.69701	0.34835

763.85772	0.34841

764.01843	0.34851

764.17914	0.34867

764.33985	0.34878

764.50056	0.34883

764.66128	0.34888

764.82199	0.34909

764.9827	0.34915

765.14341	0.34925

765.30412	0.34936

765.46483	0.34946

765.62554	0.34952

765.78625	0.34957

765.94696	0.34962

766.10767	0.34967

766.26838	0.34973

766.42909	0.34994

766.5898	0.35004

766.75051	0.3501

766.91122	0.3502

767.07194	0.35025

767.23265	0.35031

767.39336	0.35036

767.55407	0.35041

767.71478	0.35047

767.87549	0.35057

768.0362	0.35068

768.19691	0.35073

768.35762	0.35078

768.51833	0.35084

768.67904	0.35089

768.83975	0.35094

769.00046	0.35099

769.16117	0.35105

769.32188	0.3511

769.48259	0.3512

769.64331	0.35126

769.80402	0.35131

769.96473	0.35136

770.12544	0.35142

770.28615	0.35147

770.44686	0.35152

770.60757	0.35157

770.76828	0.35163

770.92899	0.35168

771.0897	0.35189

771.25041	0.35205

771.41112	0.35216

771.57183	0.35221

771.73254	0.35242

771.89325	0.35263

772.05397	0.35274

772.21468	0.35279

772.37539	0.35295

772.5361	0.353

772.69681	0.35305

772.85752	0.35311

773.01823	0.35326

773.17894	0.35332

773.33965	0.35337

773.50036	0.35342

773.66107	0.35348

773.82178	0.35353

773.98249	0.35358

774.1432	0.35369

774.30391	0.35385

774.46462	0.354

774.62534	0.35406

774.78605	0.35411

774.94676	0.35416

775.10747	0.35421

775.26818	0.35427

775.42889	0.35437

775.5896	0.35443

775.75031	0.35448

775.91102	0.35453

776.07173	0.35464

776.23244	0.35495

776.39315	0.35511

776.55386	0.35517

776.71457	0.35522

776.87528	0.35532

777.036	0.35538

777.19671	0.35553

777.35742	0.35564

777.51813	0.35575

777.67884	0.3558

777.83955	0.35585

778.00026	0.3559

778.16097	0.35596

778.32168	0.35601

778.48239	0.35612

778.6431	0.35617

778.80381	0.35622

778.96452	0.35643

779.12523	0.35649

779.28594	0.35659

779.44666	0.35664

779.60737	0.35675

779.76808	0.3568

779.92879	0.35685

780.0895	0.35696

780.25021	0.35701

780.41092	0.35707

780.57163	0.35712

780.73234	0.35722

780.89305	0.35744

781.05376	0.35749

781.21447	0.35754

781.37518	0.35759

781.53589	0.35765

781.6966	0.3577

781.85731	0.35775

782.01803	0.35781

782.17874	0.35812

782.33945	0.35818

782.50016	0.35828

782.66087	0.35849

782.82158	0.35854

782.98229	0.3586

783.143	0.35876

783.30371	0.35881

783.46442	0.35897

783.62513	0.35913

783.78584	0.35928

783.94655	0.3596

784.10726	0.35971

784.26797	0.35976

784.42869	0.35992

784.5894	0.36008

784.75011	0.36013

784.91082	0.36018

785.07153	0.36023

785.23224	0.36146

785.39295	0.36151

785.55366	0.36157

785.71437	0.36167

785.87508	0.36172

786.03579	0.36188

786.1965	0.36194

786.35721	0.36215

786.51792	0.3622

786.67863	0.36296

786.83934	0.36301

787.00006	0.36306

787.16077	0.36312

787.32148	0.36317

787.48219	0.36322

787.6429	0.36327

787.80361	0.36359

787.96432	0.36364

788.12503	0.3637

788.28574	0.36375

788.44645	0.3638

788.60716	0.36391

788.76787	0.36396

788.92858	0.36401

789.08929	0.36412

789.25	0.36422

789.41072	0.36428

789.57143	0.36433

789.73214	0.36438

789.89285	0.36444

790.05356	0.36459

790.21427	0.36475

790.37498	0.36481

790.53569	0.36486

790.6964	0.36491

790.85711	0.36507

791.01782	0.36512

791.17853	0.36533

791.33924	0.36539

791.49995	0.3656

791.66066	0.3657

791.82138	0.36576

791.98209	0.36581

792.1428	0.36586

792.30351	0.36591

792.46422	0.36602

792.62493	0.36613

792.78564	0.36623

792.94635	0.3666

793.10706	0.36671

793.26777	0.36681

793.42848	0.36686

793.58919	0.36692

793.7499	0.36796

793.91061	0.36801

794.07132	0.36807

794.23204	0.36812

794.39275	0.36833

794.55346	0.36844

794.71417	0.36896

794.87488	0.36912

795.03559	0.36918

795.1963	0.36933

795.35701	0.36939

795.51772	0.36944

795.67843	0.36965

795.83914	0.3697

795.99985	0.36976

796.16056	0.36981

796.32127	0.36992

796.48198	0.36997

796.64269	0.37002

796.80341	0.37013

796.96412	0.37018

797.12483	0.37023

797.28554	0.37034

797.44625	0.37039

797.60696	0.37044

797.76767	0.37071

797.92838	0.37076

798.08909	0.37081

798.2498	0.37087

798.41051	0.37092

798.57122	0.37097

798.73193	0.37102

798.89264	0.37108

799.05335	0.37118

799.21407	0.37124

799.37478	0.37145

799.53549	0.3715

799.6962	0.37155

799.85691	0.37166

800.01762	0.37171

800.17833	0.37182

800.33904	0.37187

800.49975	0.37192

800.66046	0.37203

800.82117	0.37208

800.98188	0.37219

801.14259	0.37224

801.3033	0.37229

801.46401	0.37375

801.62472	0.37381

801.78544	0.37386

801.94615	0.37391

802.10686	0.37396

802.26757	0.37402

802.42828	0.37407

802.58899	0.37412

802.7497	0.37423

802.91041	0.37433

803.07112	0.37444

803.23183	0.3746

803.39254	0.37465

803.55325	0.37476

803.71396	0.37481

803.87467	0.37486

804.03538	0.37491

804.1961	0.37497

804.35681	0.37502

804.51752	0.37528

804.67823	0.37534

804.83894	0.37539

804.99965	0.37544

805.16036	0.3755

805.32107	0.3756

805.48178	0.37565

805.64249	0.37576

805.8032	0.37581

805.96391	0.37586

806.12462	0.37592

806.28533	0.37602

806.44604	0.37613

806.60676	0.37618

806.76747	0.37623

806.92818	0.37629

807.08889	0.37634

807.2496	0.37639

807.41031	0.37645

807.57102	0.37655

807.73173	0.37671

807.89244	0.37676

808.05315	0.37692

808.21386	0.37703

808.37457	0.37708

808.53528	0.37713

808.69599	0.37724

808.8567	0.37729

809.01741	0.37734

809.17813	0.3774

809.33884	0.37755

809.49955	0.37761

809.66026	0.37766

809.82097	0.37777

809.98168	0.37798

810.14239	0.37808

810.3031	0.37814

810.46381	0.37819

810.62452	0.37835

810.78523	0.37845

810.94594	0.37866

811.10665	0.37872

811.26736	0.37893

811.42807	0.37898

811.58879	0.37903

811.7495	0.37909

811.91021	0.37919

812.07092	0.3793

812.23163	0.3794

812.39234	0.37946

812.55305	0.3803

812.71376	0.38041

812.87447	0.38046

813.03518	0.38051

813.19589	0.38067

813.3566	0.38072

813.51731	0.38078

813.67802	0.38088

813.83873	0.38093

813.99945	0.38099

814.16016	0.38104

814.32087	0.38115

814.48158	0.3812

814.64229	0.38125

814.803	0.38141

814.96371	0.38146

815.12442	0.38151

815.28513	0.38162

815.44584	0.38167

815.60655	0.38183

815.76726	0.38194

815.92797	0.38199

816.08868	0.3821

816.24939	0.38215

816.4101	0.38231

816.57082	0.38241

816.73153	0.38252

816.89224	0.38257

817.05295	0.38262

817.21366	0.38268

817.37437	0.38273

817.53508	0.38278

817.69579	0.38283

817.8565	0.38289

818.01721	0.38315

818.17792	0.38331

818.33863	0.38336

818.49934	0.38342

818.66005	0.38379

818.82076	0.38384

818.98148	0.38389

819.14219	0.38405

819.3029	0.3841

819.46361	0.38416

819.62432	0.38421

819.78503	0.38426

819.94574	0.38431

820.10645	0.38437

820.26716	0.38442

820.42787	0.38447

820.58858	0.38458

820.74929	0.38463

820.91	0.38474

821.07071	0.38479

821.23142	0.38484

821.39213	0.38495

821.55285	0.385

821.71356	0.38505

821.87427	0.38511

822.03498	0.38521

822.19569	0.38526

822.3564	0.38532

822.51711	0.38537

822.67782	0.38548

822.83853	0.38553

822.99924	0.38558

823.15995	0.38569

823.32066	0.38574

823.48137	0.38579

823.64208	0.38584

823.80279	0.3859

823.96351	0.38595

824.12422	0.386

824.28493	0.38606

824.44564	0.38611

824.60635	0.38637

824.76706	0.38648

824.92777	0.38653

825.08848	0.38669

825.24919	0.38674

825.4099	0.38685

825.57061	0.38695

825.73132	0.38701

825.89203	0.38706

826.05274	0.38711

826.21345	0.38716

826.37417	0.38732

826.53488	0.38738

826.69559	0.38743

826.8563	0.38753

827.01701	0.38775

827.17772	0.3878

827.33843	0.38785

827.49914	0.38806

827.65985	0.38822

827.82056	0.38833

827.98127	0.3887

828.14198	0.38875

828.30269	0.38885

828.4634	0.38891

828.62411	0.38896

828.78482	0.38907

828.94554	0.38912

829.10625	0.38922

829.26696	0.38965

829.42767	0.3897

829.58838	0.38986

829.74909	0.38991

829.9098	0.38996

830.07051	0.39002

830.23122	0.39017

830.39193	0.39023

830.55264	0.39028

830.71335	0.39039

830.87406	0.39044

831.03477	0.39049

831.19548	0.39054

831.3562	0.3906

831.51691	0.39076

831.67762	0.39081

831.83833	0.39086

831.99904	0.39097

832.15975	0.39277

832.32046	0.39324

832.48117	0.3933

832.64188	0.3934

832.80259	0.39351

832.9633	0.39356

833.12401	0.39361

833.28472	0.39377

833.44543	0.39388

833.60614	0.39398

833.76685	0.39404

833.92757	0.39409

834.08828	0.39414

834.24899	0.39419

834.4097	0.39425

834.57041	0.39456

834.73112	0.39462

834.89183	0.39467

835.05254	0.39477

835.21325	0.39488

835.37396	0.39493

835.53467	0.39499

835.69538	0.39514

835.85609	0.39525

836.0168	0.39541

836.17751	0.39546

836.33823	0.39551

836.49894	0.39557

836.65965	0.39562

836.82036	0.39567

836.98107	0.39573

837.14178	0.39578

837.30249	0.39599

837.4632	0.39604

837.62391	0.39609

837.78462	0.39615

837.94533	0.39646

838.10604	0.39652

838.26675	0.39657

838.42746	0.39662

838.58817	0.39668

838.74889	0.39678

838.9096	0.39741

839.07031	0.39747

839.23102	0.39752

839.39173	0.39757

839.55244	0.39763

839.71315	0.39778

839.87386	0.39789

840.03457	0.39794

840.19528	0.398

840.35599	0.39805

840.5167	0.3981

840.67741	0.39826

840.83812	0.39831

840.99883	0.39842

841.15954	0.39847

841.32026	0.39884

841.48097	0.399

841.64168	0.39905

841.80239	0.3991

841.9631	0.39916

842.12381	0.39921

842.28452	0.39926

842.44523	0.39932

842.60594	0.39953

842.76665	0.39958

842.92736	0.39963

843.08807	0.39974

843.24878	0.39979

843.40949	0.4

843.5702	0.40011

843.73092	0.40027

843.89163	0.40032

844.05234	0.40037

844.21305	0.40048

844.37376	0.40053

844.53447	0.40058

844.69518	0.40069

844.85589	0.40074

845.0166	0.40079

845.17731	0.40085

845.33802	0.40101

845.49873	0.40111

845.65944	0.40116

845.82015	0.40127

845.98086	0.40143

846.14158	0.40148

846.30229	0.40153

846.463	0.40159

846.62371	0.40164

846.78442	0.40174

846.94513	0.4018

847.10584	0.40185

847.26655	0.4019

847.42726	0.40196

847.58797	0.40201

847.74868	0.40233

847.90939	0.40248

848.0701	0.40264

848.23081	0.4027

848.39152	0.4028

848.55223	0.40285

848.71295	0.40307

848.87366	0.40317

849.03437	0.40333

849.19508	0.40343

849.35579	0.40354

849.5165	0.40365

849.67721	0.4037

849.83792	0.40375

849.99863	0.4038

850.15934	0.40391

850.32005	0.40407

850.48076	0.40412

850.64147	0.40423

850.80218	0.40428

850.96289	0.40433

851.12361	0.40444

851.28432	0.4046

851.44503	0.40465

851.60574	0.4047

851.76645	0.40475

851.92716	0.40481

852.08787	0.40486

852.24858	0.40491

852.40929	0.40497

852.57	0.40502

852.73071	0.40512

852.89142	0.40518

853.05213	0.40528

853.21284	0.40534

853.37355	0.40539

853.53427	0.40544

853.69498	0.40549

853.85569	0.40555

854.0164	0.4056

854.17711	0.40565

854.33782	0.40571

854.49853	0.40576

854.65924	0.40581

854.81995	0.40586

854.98066	0.40592

855.14137	0.40597

855.30208	0.40623

855.46279	0.40629

855.6235	0.40644

855.78421	0.40655

855.94492	0.40671

856.10564	0.40687

856.26635	0.40703

856.42706	0.40708

856.58777	0.40718

856.74848	0.40724

856.90919	0.4074

857.0699	0.40745

857.23061	0.4075

857.39132	0.40755

857.55203	0.40766

857.71274	0.40771

857.87345	0.40776

858.03416	0.40782

858.19487	0.40787

858.35558	0.40792

858.5163	0.40798

858.67701	0.40813

858.83772	0.40819

858.99843	0.40824

859.15914	0.40835

859.31985	0.4084

859.48056	0.40845

859.64127	0.40861

859.80198	0.40866

859.96269	0.40872

860.1234	0.40877

860.28411	0.40887

860.44482	0.40893

860.60553	0.40898

860.76624	0.40903

860.92695	0.40914

861.08767	0.40919

861.24838	0.40924

861.40909	0.4093

861.5698	0.40935

861.73051	0.40945

861.89122	0.40951

862.05193	0.40961

862.21264	0.40967

862.37335	0.40982

862.53406	0.40988

862.69477	0.40993

862.85548	0.40998

863.01619	0.41004

863.1769	0.41009

863.33761	0.41025

863.49833	0.4103

863.65904	0.4104

863.81975	0.41051

863.98046	0.41056

864.14117	0.41062

864.30188	0.41067

864.46259	0.41072

864.6233	0.41077

864.78401	0.41083

864.94472	0.41093

865.10543	0.41099

865.26614	0.41104

865.42685	0.41109

865.58756	0.41114

865.74827	0.41125

865.90899	0.4113

866.0697	0.41136

866.23041	0.41146

866.39112	0.41162

866.55183	0.41167

866.71254	0.41173

866.87325	0.41178

867.03396	0.41183

867.19467	0.41199

867.35538	0.41204

867.51609	0.41215

867.6768	0.41225

867.83751	0.41268

867.99822	0.41273

868.15893	0.41283

868.31964	0.41294

868.48036	0.41474

868.64107	0.41479

868.80178	0.41485

868.96249	0.4149

869.1232	0.41506

869.28391	0.41522

869.44462	0.41527

869.60533	0.41532

869.76604	0.41543

869.92675	0.41574

870.08746	0.41585

870.24817	0.4159

870.40888	0.41596

870.56959	0.41601

870.7303	0.41611

870.89102	0.41617

871.05173	0.41627

871.21244	0.41632

871.37315	0.41643

871.53386	0.41648

871.69457	0.41659

871.85528	0.41664

872.01599	0.41685

872.1767	0.41691

872.33741	0.41696

872.49812	0.41722

872.65883	0.41728

872.81954	0.41738

872.98025	0.41743

873.14096	0.41749

873.30168	0.41754

873.46239	0.4177

873.6231	0.4178

873.78381	0.41786

873.94452	0.41801

874.10523	0.41807

874.26594	0.41812

874.42665	0.41817

874.58736	0.41823

874.74807	0.41828

874.90878	0.41833

875.06949	0.41849

875.2302	0.4186

875.39091	0.41865

875.55162	0.41881

875.71233	0.41886

875.87305	0.41897

876.03376	0.41902

876.19447	0.41912

876.35518	0.41949

876.51589	0.41955

876.6766	0.41965

876.83731	0.4197

876.99802	0.41976

877.15873	0.41981

877.31944	0.41992

877.48015	0.42007

877.64086	0.42013

877.80157	0.42018

877.96228	0.42034

878.12299	0.4205

878.28371	0.42055

878.44442	0.42065

878.60513	0.42071

878.76584	0.42076

878.92655	0.42081

879.08726	0.42092

879.24797	0.42097

879.40868	0.42124

879.56939	0.42129

879.7301	0.42134

879.89081	0.42139

880.05152	0.42187

880.21223	0.42198

880.37294	0.42203

880.53365	0.42208

880.69436	0.4224

880.85508	0.42261

881.01579	0.42277

881.1765	0.42287

881.33721	0.42298

881.49792	0.42303

881.65863	0.42308

881.81934	0.42324

881.98005	0.42335

882.14076	0.42356

882.30147	0.42361

882.46218	0.42372

882.62289	0.42382

882.7836	0.42388

882.94431	0.42393

883.10502	0.42398

883.26574	0.42403

883.42645	0.42409

883.58716	0.42414

883.74787	0.42425

883.90858	0.42435

884.06929	0.42451

884.23	0.42456

884.39071	0.42462

884.55142	0.42472

884.71213	0.42477

884.87284	0.42483

885.03355	0.42504

885.19426	0.42514

885.35497	0.42551

885.51568	0.42557

885.6764	0.42562

885.83711	0.42583

885.99782	0.42588

886.15853	0.42599

886.31924	0.42615

886.47995	0.4262

886.64066	0.42625

886.80137	0.42636

886.96208	0.42652

887.12279	0.42657

887.2835	0.42662

887.44421	0.42667

887.60492	0.42673

887.76563	0.42683

887.92634	0.42689

888.08705	0.42699

888.24777	0.4272

888.40848	0.42731

888.56919	0.42741

888.7299	0.42747

888.89061	0.42763

889.05132	0.42768

889.21203	0.42773

889.37274	0.42778

889.53345	0.42789

889.69416	0.42794

889.85487	0.42805

890.01558	0.4281

890.17629	0.42815

890.337	0.42821

890.49771	0.42836

890.65843	0.42842

890.81914	0.42847

890.97985	0.42868

891.14056	0.42873

891.30127	0.42879

891.46198	0.42895

891.62269	0.42905

891.7834	0.4291

891.94411	0.42916

892.10482	0.42931

892.26553	0.42953

892.42624	0.42963

892.58695	0.42968

892.74766	0.42974

892.90837	0.42979

893.06908	0.43005

893.2298	0.43032

893.39051	0.43037

893.55122	0.43042

893.71193	0.43048

893.87264	0.43064

894.03335	0.43079

894.19406	0.43085

894.35477	0.4309

894.51548	0.431

894.67619	0.43106

894.8369	0.43111

894.99761	0.43143

895.15832	0.43148

895.31903	0.43153

895.47974	0.43159

895.64046	0.43164

895.80117	0.43169

895.96188	0.4319

896.12259	0.43201

896.2833	0.43206

896.44401	0.43217

896.60472	0.43222

896.76543	0.43227

896.92614	0.43232

897.08685	0.43238

897.24756	0.43243

897.40827	0.43248

897.56898	0.43264

897.72969	0.43269

897.8904	0.43285

898.05112	0.43291

898.21183	0.43312

898.37254	0.43322

898.53325	0.43333

898.69396	0.43354

898.85467	0.43391

899.01538	0.43396

899.17609	0.43407

899.3368	0.43417

899.49751	0.43423

899.65822	0.43428

899.81893	0.43438

899.97964	0.43449

900.14035	0.43454

900.30106	0.43465

900.46177	0.4347

900.62249	0.43502

900.7832	0.43533

900.94391	0.43549

901.10462	0.43555

901.26533	0.43565

901.42604	0.4357

901.58675	0.43576

901.74746	0.43581

901.90817	0.43586

902.06888	0.43592

902.22959	0.43602

902.3903	0.43607

902.55101	0.43613

902.71172	0.43623

902.87243	0.43634

903.03315	0.4365

903.19386	0.43655

903.35457	0.4366

903.51528	0.43676

903.67599	0.43692

903.8367	0.43697

903.99741	0.43708

904.15812	0.43713

904.31883	0.43724

904.47954	0.43729

904.64025	0.43734

904.80096	0.43745

904.96167	0.4375

905.12238	0.43755

905.28309	0.43761

905.44381	0.43766

905.60452	0.43776

905.76523	0.43782

905.92594	0.43787

906.08665	0.43792

906.24736	0.43797

906.40807	0.43813

906.56878	0.43829

906.72949	0.43834

906.8902	0.4384

907.05091	0.43861

907.21162	0.43877

907.37233	0.43893

907.53304	0.43903

907.69375	0.43914

907.85446	0.43919

908.01518	0.43924

908.17589	0.4394

908.3366	0.43951

908.49731	0.43956

908.65802	0.43972

908.81873	0.43977

908.97944	0.43982

909.14015	0.43988

909.30086	0.43998

909.46157	0.44009

909.62228	0.44025

909.78299	0.4403

909.9437	0.44035

910.10441	0.44046

910.26512	0.44056

910.42584	0.44062

910.58655	0.44067

910.74726	0.44072

910.90797	0.44077

911.06868	0.44083

911.22939	0.44093

911.3901	0.44098

911.55081	0.44104

911.71152	0.44114

911.87223	0.4413

912.03294	0.44146

912.19365	0.44151

912.35436	0.44162

912.51507	0.44167

912.67578	0.44172

912.8365	0.44178

912.99721	0.44188

913.15792	0.44194

913.31863	0.44204

913.47934	0.44209

913.64005	0.44236

913.80076	0.44241

913.96147	0.44252

914.12218	0.44257

914.28289	0.44262

914.4436	0.44273

914.60431	0.44278

914.76502	0.44283

914.92573	0.44315

915.08644	0.4432

915.24715	0.44326

915.40787	0.44331

915.56858	0.44336

915.72929	0.44341

915.89	0.44347

916.05071	0.44352

916.21142	0.44368

916.37213	0.44373

916.53284	0.44378

916.69355	0.44384

916.85426	0.44389

917.01497	0.44394

917.17568	0.44405

917.33639	0.4441

917.4971	0.44421

917.65781	0.44426

917.81853	0.44431

917.97924	0.44436

918.13995	0.44447

918.30066	0.44468

918.46137	0.44473

918.62208	0.44489

918.78279	0.44505

918.9435	0.44521

919.10421	0.44526

919.26492	0.44558

919.42563	0.44563

919.58634	0.44568

919.74705	0.44574

919.90776	0.44579

920.06847	0.44584

920.22919	0.4459

920.3899	0.44611

920.55061	0.44637

920.71132	0.44642

920.87203	0.44648

921.03274	0.44669

921.19345	0.44674

921.35416	0.44679

921.51487	0.44685

921.67558	0.4469

921.83629	0.44695

921.997	0.44727

922.15771	0.44732

922.31842	0.44743

922.47913	0.44753

922.63984	0.44764

922.80056	0.44785

922.96127	0.4479

923.12198	0.44795

923.28269	0.44801

923.4434	0.44822

923.60411	0.44827

923.76482	0.44838

923.92553	0.44843

924.08624	0.44864

924.24695	0.44869

924.40766	0.4488

924.56837	0.44885

924.72908	0.44901

924.88979	0.44906

925.0505	0.44912

925.21122	0.44917

925.37193	0.44933

925.53264	0.44943

925.69335	0.44954

925.85406	0.4497

926.01477	0.44986

926.17548	0.44991

926.33619	0.44996

926.4969	0.45017

926.65761	0.45028

926.81832	0.45033

926.97903	0.45038

927.13974	0.45054

927.30045	0.4506

927.46116	0.45065

927.62187	0.4507

927.78259	0.45075

927.9433	0.45081

928.10401	0.45096

928.26472	0.45102

928.42543	0.45112

928.58614	0.45118

928.74685	0.45123

928.90756	0.45133

929.06827	0.4516

929.22898	0.4517

929.38969	0.45202

929.5504	0.45207

929.71111	0.45218

929.87182	0.45244

930.03253	0.45255

930.19325	0.4526

930.35396	0.45265

930.51467	0.45276

930.67538	0.45281

930.83609	0.45292

930.9968	0.45297

931.15751	0.45308

931.31822	0.45313

931.47893	0.45329

931.63964	0.45334

931.80035	0.45345

931.96106	0.45355

932.12177	0.45376

932.28248	0.45382

932.44319	0.45387

932.60391	0.45392

932.76462	0.45403

932.92533	0.45408

933.08604	0.45413

933.24675	0.45429

933.40746	0.4544

933.56817	0.4545

933.72888	0.45456

933.88959	0.45466

934.0503	0.45477

934.21101	0.45493

934.37172	0.45503

934.53243	0.45524

934.69314	0.45551

934.85385	0.45561

935.01456	0.45625

935.17528	0.45635

935.33599	0.4564

935.4967	0.45646

935.65741	0.45651

935.81812	0.45667

935.97883	0.45672

936.13954	0.45677

936.30025	0.45683

936.46096	0.45688

936.62167	0.45693

936.78238	0.45698

936.94309	0.45714

937.1038	0.45725

937.26451	0.4573

937.42522	0.45741

937.58594	0.45746

937.74665	0.45783

937.90736	0.45788

938.06807	0.45794

938.22878	0.45815

938.38949	0.4582

938.5502	0.45825

938.71091	0.4583

938.87162	0.45836

939.03233	0.45841

939.19304	0.45846

939.35375	0.45857

939.51446	0.45862

939.67517	0.45867

939.83588	0.45878

939.99659	0.45883

940.15731	0.45894

940.31802	0.45904

940.47873	0.4592

940.63944	0.45926

940.80015	0.45931

940.96086	0.46006

941.12157	0.46022

941.28228	0.46043

941.44299	0.46054

941.6037	0.46059

941.76441	0.46075

941.92512	0.4608

942.08583	0.46091

942.24654	0.46096

942.40725	0.46102

942.56797	0.46117

942.72868	0.46123

942.88939	0.46128

943.0501	0.46133

943.21081	0.46139

943.37152	0.46144

943.53223	0.46149

943.69294	0.4616

943.85365	0.4617

944.01436	0.46212

944.17507	0.46218

944.33578	0.46223

944.49649	0.46249

944.6572	0.46255

944.81791	0.4626

944.97863	0.46265

945.13934	0.46276

945.30005	0.46292

945.46076	0.46302

945.62147	0.46307

945.78218	0.46313

945.94289	0.46323

946.1036	0.46329

946.26431	0.46428

946.42502	0.46433

946.58573	0.46438

946.74644	0.46454

946.90715	0.46459

947.06786	0.46465

947.22857	0.46475

947.38928	0.46486

947.55	0.46491

947.71071	0.46496

947.87142	0.46512

948.03213	0.46518

948.19284	0.46533

948.35355	0.46544

948.51426	0.46549

948.67497	0.46554

948.83568	0.46565

948.99639	0.4657

949.1571	0.46581

949.31781	0.46586

949.47852	0.46613

949.63923	0.46618

949.79994	0.46623

949.96066	0.46628

950.12137	0.46634

950.28208	0.46644

950.44279	0.4665

950.6035	0.46665

950.76421	0.46686

950.92492	0.46692

951.08563	0.46697

951.24634	0.46708

951.40705	0.46739

951.56776	0.46745

951.72847	0.4675

951.88918	0.46787

952.04989	0.46792

952.2106	0.46797

952.37132	0.46803

952.53203	0.46808

952.69274	0.46819

952.85345	0.46824

953.01416	0.46829

953.17487	0.46877

953.33558	0.46887

953.49629	0.46892

953.657	0.46898

953.81771	0.46903

953.97842	0.46908

954.13913	0.46919

954.29984	0.46929

954.46055	0.46935

954.62126	0.4694

954.78197	0.46951

954.94269	0.46956

955.1034	0.46987

955.26411	0.46993

955.42482	0.47009

955.58553	0.47014

955.74624	0.47019

955.90695	0.47035

956.06766	0.4704

956.22837	0.47056

956.38908	0.47083

956.54979	0.47088

956.7105	0.47093

956.87121	0.47109

957.03192	0.47114

957.19263	0.47119

957.35335	0.4713

957.51406	0.47141

957.67477	0.47151

957.83548	0.47162

957.99619	0.47178

958.1569	0.47209

958.31761	0.47215

958.47832	0.47236

958.63903	0.47241

958.79974	0.47252

958.96045	0.47262

959.12116	0.47273

959.28187	0.47278

959.44258	0.47283

959.60329	0.47288

959.76401	0.47299

959.92472	0.47304

960.08543	0.47325

960.24614	0.47336

960.40685	0.47347

960.56756	0.47352

960.72827	0.47373

960.88898	0.47378

961.04969	0.47389

961.2104	0.47394

961.37111	0.47405

961.53182	0.4741

961.69253	0.47415

961.85324	0.47426

962.01395	0.47431

962.17466	0.47447

962.33538	0.47452

962.49609	0.47463

962.6568	0.47468

962.81751	0.47473

962.97822	0.47479

963.13893	0.47484

963.29964	0.47494

963.46035	0.475

963.62106	0.47505

963.78177	0.47521

963.94248	0.47531

964.10319	0.47537

964.2639	0.47568

964.42461	0.47579

964.58532	0.47584

964.74604	0.47595

964.90675	0.47616

965.06746	0.47626

965.22817	0.47637

965.38888	0.47642

965.54959	0.47648

965.7103	0.47653

965.87101	0.47658

966.03172	0.47663

966.19243	0.47685

966.35314	0.47695

966.51385	0.477

966.67456	0.47706

966.83527	0.47716

966.99598	0.47721

967.15669	0.47732

967.31741	0.47743

967.47812	0.47748

967.63883	0.4778

967.79954	0.47785

967.96025	0.4779

968.12096	0.47795

968.28167	0.47801

968.44238	0.47811

968.60309	0.47817

968.7638	0.47832

968.92451	0.47853

969.08522	0.47859

969.24593	0.47864

969.40664	0.4788

969.56735	0.47896

969.72807	0.47901

969.88878	0.47964

970.04949	0.4797

970.2102	0.47985

970.37091	0.47991

970.53162	0.48033

970.69233	0.48038

970.85304	0.48059

971.01375	0.48065

971.17446	0.4807

971.33517	0.48075

971.49588	0.48081

971.65659	0.48086

971.8173	0.48096

971.97801	0.48107

972.13873	0.48118

972.29944	0.48128

972.46015	0.48133

972.62086	0.48165

972.78157	0.4817

972.94228	0.48176

973.10299	0.48191

973.2637	0.48228

973.42441	0.48234

973.58512	0.48239

973.74583	0.48244

973.90654	0.48255

974.06725	0.48265

974.22796	0.48271

974.38867	0.48276

974.54938	0.48286

974.7101	0.48297

974.87081	0.48323

975.03152	0.48339

975.19223	0.48345

975.35294	0.4835

975.51365	0.4836

975.67436	0.48366

975.83507	0.48371

975.99578	0.48376

976.15649	0.48413

976.3172	0.48418

976.47791	0.48429

976.63862	0.48434

976.79933	0.4844

976.96004	0.48445

977.12076	0.48455

977.28147	0.48466

977.44218	0.48471

977.60289	0.48477

977.7636	0.48503

977.92431	0.48519

978.08502	0.48535

978.24573	0.48545

978.40644	0.48561

978.56715	0.48572

978.72786	0.48577

978.88857	0.48587

979.04928	0.48593

979.20999	0.48609

979.3707	0.48614

979.53142	0.48619

979.69213	0.48624

979.85284	0.48635

980.01355	0.48646

980.17426	0.48651

980.33497	0.48656

980.49568	0.48667

980.65639	0.48672

980.8171	0.48677

980.97781	0.48683

981.13852	0.48688

981.29923	0.48714

981.45994	0.48725

981.62065	0.4873

981.78136	0.48735

981.94207	0.48741

982.10279	0.48746

982.2635	0.48751

982.42421	0.48756

982.58492	0.48762

982.74563	0.48767

982.90634	0.48778

983.06705	0.48793

983.22776	0.48799

983.38847	0.48804

983.54918	0.48809

983.70989	0.48815

983.8706	0.4882

984.03131	0.48825

984.19202	0.4883

984.35273	0.48836

984.51345	0.48841

984.67416	0.48851

984.83487	0.48873

984.99558	0.48878

985.15629	0.48883

985.317	0.48888

985.47771	0.48936

985.63842	0.48941

985.79913	0.48947

985.95984	0.48957

986.12055	0.4902

986.28126	0.49026

986.44197	0.49073

986.60268	0.49079

986.76339	0.49084

986.9241	0.49105

987.08482	0.4911

987.24553	0.49116

987.40624	0.49121

987.56695	0.49126

987.72766	0.49131

987.88837	0.49137

988.04908	0.49142

988.20979	0.49189

988.3705	0.49195

988.53121	0.492

988.69192	0.49216

988.85263	0.49221

989.01334	0.49232

989.17405	0.49237

989.33476	0.49242

989.49548	0.49248

989.65619	0.49263

989.8169	0.49274

989.97761	0.49284

990.13832	0.4929

990.29903	0.49353

990.45974	0.49369

990.62045	0.49374

990.78116	0.4956

990.94187	0.49565

991.10258	0.49586

991.26329	0.49597

991.424	0.49612

991.58471	0.49634

991.74542	0.49639

991.90614	0.49644

992.06685	0.49665

992.22756	0.49766

992.38827	0.49771

992.54898	0.49781

992.70969	0.49792

992.8704	0.49824

993.03111	0.49829

993.19182	0.49834

993.35253	0.49855

993.51324	0.49861

993.67395	0.49866

993.83466	0.49903

993.99537	0.49908

994.15608	0.49913

994.31679	0.49919

994.47751	0.49929

994.63822	0.49935

994.79893	0.4994

994.95964	0.4995

995.12035	0.49993

995.28106	0.50003

995.44177	0.50051

995.60248	0.50056

995.76319	0.50067

995.9239	0.50072

996.08461	0.50109

996.24532	0.50114

996.40603	0.50119

996.56674	0.50146

996.72745	0.50156

996.88817	0.50162

997.04888	0.50167

997.20959	0.50204

997.3703	0.50225

997.53101	0.5023

997.69172	0.50236

997.85243	0.50241

998.01314	0.50246

998.17385	0.50257

998.33456	0.50267

998.49527	0.50283

998.65598	0.50288

998.81669	0.50299

998.9774	0.50336

999.13811	0.50341

999.29882	0.50383

999.45954	0.50394

999.62025	0.50399

999.78096	0.50431

999.94167	0.50442

1000.10238	0.50447

1000.26309	0.50452

1000.4238	0.50468

1000.58451	0.50478

1000.74522	0.50484

1000.90593	0.50489

1001.06664	0.50494

1001.22735	0.50575

1001.38806	0.50581

1001.54877	0.50586

1001.70949	0.50591

1001.8702	0.50596

1002.03091	0.50607

1002.19162	0.50618

1002.35233	0.50623

1002.51304	0.50639

1002.67375	0.50644

1002.83446	0.50654

1002.99517	0.50665

1003.15588	0.5067

1003.31659	0.50686

1003.4773	0.50691

1003.63801	0.50697

1003.79872	0.50702

1003.95943	0.50744

1004.12015	0.5075

1004.28086	0.50755

1004.44157	0.50771

1004.60228	0.50776

1004.76299	0.50781

1004.9237	0.50786

1005.08441	0.50802

1005.24512	0.50818

1005.40583	0.50834

1005.56654	0.50839

1005.72725	0.50845

1005.88796	0.5085

1006.04867	0.50866

1006.20938	0.50871

1006.37009	0.50876

1006.5308	0.50882

1006.69152	0.50887

1006.85223	0.50897

1007.01294	0.50908

1007.17365	0.50919

1007.33436	0.50924

1007.49507	0.50929

1007.65578	0.50945

1007.81649	0.5095

1007.9772	0.50955

1008.13791	0.50961

1008.29862	0.50966

1008.45933	0.50971

1008.62004	0.50977

1008.78075	0.50987

1008.94146	0.51014

1009.10218	0.51019

1009.26289	0.51024

1009.4236	0.511

1009.58431	0.51121

1009.74502	0.51126

1009.90573	0.51131

1010.06644	0.51137

1010.22715	0.51158

1010.38786	0.51168

1010.54857	0.51174

1010.70928	0.51179

1010.86999	0.5119

1011.0307	0.512

1011.19141	0.51205

1011.35212	0.51211

1011.51283	0.51216

1011.67355	0.51221

1011.83426	0.51232

1011.99497	0.51242

1012.15568	0.51248

1012.31639	0.51253

1012.4771	0.51258

1012.63781	0.51264

1012.79852	0.51269

1012.95923	0.51274

1013.11994	0.51279

1013.28065	0.51322

1013.44136	0.51332

1013.60207	0.51337

1013.76278	0.51343

1013.92349	0.51448

1014.08421	0.51464

1014.24492	0.51475

1014.40563	0.5148

1014.56634	0.51485

1014.72705	0.51491

1014.88776	0.51496

1015.04847	0.51501

1015.20918	0.51506

1015.36989	0.51538

1015.5306	0.51549

1015.69131	0.51554

1015.85202	0.51617

1016.01273	0.51628

1016.17344	0.51633

1016.33415	0.51649

1016.49487	0.5166

1016.65558	0.51665

1016.81629	0.51675

1016.977	0.51691

1017.13771	0.51702

1017.29842	0.51712

1017.45913	0.51718

1017.61984	0.51723

1017.78055	0.51728

1017.94126	0.51733

1018.10197	0.51755

1018.26268	0.51765

1018.42339	0.5177

1018.5841	0.51776

1018.74481	0.51786

1018.90552	0.51855

1019.06624	0.51865

1019.22695	0.51871

1019.38766	0.51892

1019.54837	0.51897

1019.70908	0.51908

1019.86979	0.51918

1020.0305	0.51924

1020.19121	0.51934

1020.35192	0.51939

1020.51263	0.51945

1020.67334	0.5195

1020.83405	0.51971

1020.99476	0.5211

1021.15547	0.52154

1021.31618	0.52165

1021.4769	0.5217

1021.63761	0.52175

1021.79832	0.52181

1021.95903	0.52186

1022.11974	0.52191

1022.28045	0.52196

1022.44116	0.52202

1022.60187	0.52218

1022.76258	0.52223

1022.92329	0.52249

1023.084	0.52254

1023.24471	0.5227

1023.40542	0.52286

1023.56613	0.52297

1023.72684	0.52302

1023.88756	0.52307

1024.04826	0.52318

1024.20898	0.52334

1024.36969	0.52344

1024.5304	0.5236

1024.69111	0.52371

1024.85182	0.52376

1025.01253	0.52397

1025.17324	0.52408

1025.33395	0.52413

1025.49466	0.52418

1025.65537	0.52434

1025.81608	0.52445

1025.97679	0.52455

1026.1375	0.52476

1026.29821	0.52487

1026.45892	0.52503

1026.61964	0.52508

1026.78035	0.52518

1026.94106	0.52524

1027.10177	0.52534

1027.26248	0.5254

1027.42319	0.52561

1027.5839	0.52566

1027.74461	0.52571

1027.90532	0.52577

1028.06603	0.52598

1028.22674	0.52608

1028.38745	0.52635

1028.54816	0.52656

1028.70887	0.52666

1028.86958	0.52672

1029.0303	0.52698

1029.19101	0.52714

1029.35172	0.52724

1029.51243	0.5273

1029.67314	0.52746

1029.83385	0.52751

1029.99456	0.52761

1030.15527	0.52777

1030.31598	0.52783

1030.47669	0.52804

1030.6374	0.52809

1030.79811	0.52814

1030.95882	0.52819

1031.11953	0.5283

1031.28024	0.52867

1031.44096	0.52872

1031.60167	0.52915

1031.76238	0.5292

1031.92309	0.52925

1032.0838	0.52936

1032.24451	0.52951

1032.40522	0.52962

1032.56593	0.52973

1032.72664	0.52983

1032.88735	0.52988

1033.04806	0.52994

1033.20877	0.52999

1033.36948	0.53004

1033.53019	0.5301

1033.6909	0.53025

1033.85162	0.53052

1034.01233	0.53068

1034.17304	0.5312

1034.33375	0.53125

1034.49446	0.5313

1034.65517	0.53136

1034.81588	0.53146

1034.97659	0.53152

1035.1373	0.53173

1035.29801	0.53178

1035.45872	0.53183

1035.61943	0.53194

1035.78014	0.53199

1035.94085	0.53204

1036.10156	0.5321

1036.26228	0.53215

1036.42298	0.53231

1036.5837	0.53247

1036.74441	0.53257

1036.90512	0.53268

1037.06583	0.53278

1037.22654	0.53294

1037.38725	0.53315

1037.54796	0.53326

1037.70867	0.53331

1037.86938	0.53336

1038.03009	0.53358

1038.1908	0.53363

1038.35151	0.53368

1038.51222	0.53373

1038.67293	0.53394

1038.83364	0.534

1038.99436	0.53405

1039.15507	0.5341

1039.31578	0.53416

1039.47649	0.53437

1039.6372	0.53442

1039.79791	0.53447

1039.95862	0.53546

1040.11933	0.53552

1040.28004	0.53562

1040.44075	0.53583

1040.60146	0.53594

1040.76217	0.53599

1040.92288	0.53647

1041.08359	0.53652

1041.2443	0.53657

1041.40502	0.53663

1041.56573	0.53668

1041.72644	0.53673

1041.88715	0.53678

1042.04786	0.53684

1042.20857	0.53694

1042.36928	0.53721

1042.52999	0.53726

1042.6907	0.53737

1042.85141	0.53752

1043.01212	0.53758

1043.17283	0.53768

1043.33354	0.53773

1043.49425	0.53779

1043.65496	0.53795

1043.81568	0.538

1043.97639	0.53805

1044.1371	0.5381

1044.29781	0.53816

1044.45852	0.53821

1044.61923	0.53826

1044.77994	0.53832

1044.94065	0.53853

1045.10136	0.53863

1045.26207	0.53869

1045.42278	0.53874

1045.58349	0.53879

1045.7442	0.539

1045.90491	0.53906

1046.06562	0.53921

1046.22634	0.53927

1046.38705	0.53942

1046.54776	0.53948

1046.70847	0.53958

1046.86918	0.53979

1047.02989	0.54016

1047.1906	0.54048

1047.35131	0.54059

1047.51202	0.54064

1047.67273	0.54074

1047.83344	0.5408

1047.99415	0.54085

1048.15486	0.54096

1048.31557	0.54106

1048.47628	0.54127

1048.637	0.54133

1048.79771	0.54154

1048.95842	0.54159

1049.11913	0.54164

1049.27984	0.5417

1049.44055	0.5418

1049.60126	0.54185

1049.76197	0.54191

1049.92268	0.54201

1050.08339	0.54206

1050.2441	0.54212

1050.40481	0.54217

1050.56552	0.54222

1050.72623	0.54233

1050.88694	0.54238

1051.04766	0.54249

1051.20837	0.5427

1051.36908	0.54275

1051.52979	0.54291

1051.6905	0.54307

1051.85121	0.54323

1052.01192	0.54333

1052.17263	0.54349

1052.33334	0.5437

1052.49405	0.54375

1052.65476	0.54381

1052.81547	0.54386

1052.97618	0.54412

1053.13689	0.54465

1053.2976	0.54476

1053.45831	0.54481

1053.61903	0.54486

1053.77974	0.54502

1053.94045	0.54507

1054.10116	0.54513

1054.26187	0.54523

1054.42258	0.54529

1054.58329	0.54534

1054.744	0.5456

1054.90471	0.54597

1055.06542	0.54629

1055.22613	0.54639

1055.38684	0.54645

1055.54755	0.5465

1055.70826	0.54661

1055.86897	0.54666

1056.02969	0.54682

1056.1904	0.54687

1056.35111	0.54692

1056.51182	0.54713

1056.67253	0.54729

1056.83324	0.54735

1056.99395	0.54766

1057.15466	0.54905

1057.31537	0.5491

1057.47608	0.54915

1057.63679	0.54936

1057.7975	0.54942

1057.95821	0.54947

1058.11892	0.54952

1058.27963	0.54968

1058.44034	0.54973

1058.60106	0.54979

1058.76177	0.54984

1058.92248	0.54989

1059.08319	0.54994

1059.2439	0.55

1059.40461	0.5501

1059.56532	0.55021

1059.72603	0.55026

1059.88674	0.55037

1060.04745	0.55053

1060.20816	0.55058

1060.36887	0.55063

1060.52958	0.55074

1060.69029	0.55105

1060.851	0.55116

1061.01172	0.55121

1061.17243	0.55126

1061.33314	0.55132

1061.49385	0.55142

1061.65456	0.55153

1061.81527	0.55158

1061.97598	0.55169

1062.13669	0.55174

1062.2974	0.55179

1062.45811	0.55232

1062.61882	0.55237

1062.77953	0.55243

1062.94024	0.55248

1063.10095	0.55258

1063.26166	0.55264

1063.42238	0.5529

1063.58309	0.55295

1063.7438	0.55301

1063.90451	0.55306

1064.06522	0.55311

1064.22593	0.55317

1064.38664	0.55322

1064.54735	0.55327

1064.70806	0.55332

1064.86877	0.55338

1065.02948	0.55348

1065.19019	0.55359

1065.3509	0.5538

1065.51161	0.55385

1065.67232	0.55422

1065.83303	0.55438

1065.99375	0.55443

1066.15446	0.55449

1066.31517	0.55486

1066.47588	0.55491

1066.63659	0.55496

1066.7973	0.55512

1066.95801	0.55517

1067.11872	0.55544

1067.27943	0.55549

1067.44014	0.55554

1067.60085	0.55596

1067.76156	0.55618

1067.92227	0.55623

1068.08298	0.55628

1068.24369	0.55639

1068.40441	0.5567

1068.56512	0.55681

1068.72583	0.55691

1068.88654	0.55697

1069.04725	0.55713

1069.20796	0.55718

1069.36867	0.55723

1069.52938	0.55728

1069.69009	0.55744

1069.8508	0.5576

1070.01151	0.55776

1070.17222	0.55781

1070.33293	0.55792

1070.49364	0.55808

1070.65435	0.55818

1070.81507	0.55839

1070.97578	0.5585

1071.13649	0.55855

1071.2972	0.55882

1071.45791	0.55897

1071.61862	0.55919

1071.77933	0.55934

1071.94004	0.5595

1072.10075	0.55961

1072.26146	0.55966

1072.42217	0.55987

1072.58288	0.55992

1072.74359	0.56019

1072.9043	0.56024

1073.06501	0.56029

1073.22572	0.5604

1073.38643	0.56045

1073.54715	0.56051

1073.70786	0.56056

1073.86857	0.56061

1074.02928	0.56072

1074.18999	0.56082

1074.3507	0.56088

1074.51141	0.56093

1074.67212	0.56103

1074.83283	0.56109

1074.99354	0.56114

1075.15425	0.56124

1075.31496	0.56151

1075.47567	0.56156

1075.63638	0.56167

1075.79709	0.56177

1075.95781	0.56183

1076.11852	0.56188

1076.27923	0.56193

1076.43994	0.56198

1076.60065	0.5622

1076.76136	0.56225

1076.92207	0.5623

1077.08278	0.56235

1077.24349	0.56241

1077.4042	0.56246

1077.56491	0.56251

1077.72562	0.56262

1077.88633	0.56278

1078.04704	0.56283

1078.20775	0.56309

1078.36847	0.56325

1078.52918	0.5633

1078.68989	0.56336

1078.8506	0.56352

1079.01131	0.56357

1079.17202	0.56362

1079.33273	0.56378

1079.49344	0.56383

1079.65415	0.56415

1079.81486	0.56441

1079.97557	0.56462

1080.13628	0.56484

1080.29699	0.56489

1080.4577	0.56526

1080.61841	0.56536

1080.77913	0.56547

1080.93984	0.56589

1081.10055	0.56594

1081.26126	0.566

1081.42197	0.56605

1081.58268	0.5661

1081.74339	0.56621

1081.9041	0.56663

1082.06481	0.56668

1082.22552	0.56679

1082.38623	0.56684

1082.54694	0.56689

1082.70765	0.567

1082.86836	0.56705

1083.02907	0.56711

1083.18979	0.56721

1083.35049	0.56779

1083.51121	0.56785

1083.67192	0.5679

1083.83263	0.568

1083.99334	0.56806

1084.15405	0.56811

1084.31476	0.56822

1084.47547	0.56837

1084.63618	0.56843

1084.79689	0.56848

1084.9576	0.56853

1085.11831	0.56864

1085.27902	0.56869

1085.43973	0.5688

1085.60044	0.5689

1085.76115	0.56895

1085.92187	0.56911

1086.08258	0.56917

1086.24329	0.56922

1086.404	0.56948

1086.56471	0.56959

1086.72542	0.56985

1086.88613	0.57001

1087.04684	0.57012

1087.20755	0.57017

1087.36826	0.57022

1087.52897	0.57054

1087.68968	0.57059

1087.85039	0.57064

1088.0111	0.5707

1088.17181	0.57075

1088.33253	0.57096

1088.49324	0.57101

1088.65395	0.57117

1088.81466	0.57122

1088.97537	0.57128

1089.13608	0.57138

1089.29679	0.57154

1089.4575	0.57159

1089.61821	0.57165

1089.77892	0.57191

1089.93963	0.57202

1090.10034	0.57207

1090.26105	0.57212

1090.42176	0.57223

1090.58247	0.57233

1090.74319	0.57239

1090.9039	0.57249

1091.06461	0.5726

1091.22532	0.57291

1091.38603	0.57297

1091.54674	0.57318

1091.70745	0.57328

1091.86816	0.57355

1092.02887	0.5736

1092.18958	0.57365

1092.35029	0.57376

1092.511	0.57392

1092.67171	0.57397

1092.83242	0.57402

1092.99313	0.57408

1093.15385	0.57429

1093.31456	0.57434

1093.47527	0.57439

1093.63598	0.57455

1093.79669	0.5746

1093.9574	0.57476

1094.11811	0.57482

1094.27882	0.57497

1094.43953	0.57503

1094.60024	0.57508

1094.76095	0.57519

1094.92166	0.57534

1095.08237	0.5754

1095.24308	0.57566

1095.40379	0.57571

1095.56451	0.57587

1095.72521	0.57592

1095.88593	0.57635

1096.04664	0.5764

1096.20735	0.57661

1096.36806	0.57666

1096.52877	0.57677

1096.68948	0.57698

1096.85019	0.57703

1097.0109	0.57709

1097.17161	0.57724

1097.33232	0.5773

1097.49303	0.5774

1097.65374	0.57746

1097.81445	0.57751

1097.97516	0.57772

1098.13587	0.57777

1098.29659	0.57788

1098.4573	0.57798

1098.61801	0.57804

1098.77872	0.57814

1098.93943	0.5782

1099.10014	0.57825

1099.26085	0.57841

1099.42156	0.57856

1099.58227	0.57862

1099.74298	0.57867

1099.90369	0.57872

1100.0644	0.57878

1100.22511	0.57883

1100.38582	0.57888

1100.54653	0.5793

1100.70725	0.57941

1100.86796	0.57946

1101.02867	0.57952

1101.18938	0.57983

1101.35009	0.57994

1101.5108	0.57999

1101.67151	0.58004

1101.83222	0.5802

1101.99293	0.58031

1102.15364	0.58036

1102.31435	0.58041

1102.47506	0.58047

1102.63577	0.58147

1102.79648	0.58152

1102.9572	0.58157

1103.11791	0.58163

1103.27862	0.58173

1103.43933	0.58179

1103.60004	0.582

1103.76075	0.58205

1103.92146	0.5821

1104.08217	0.58216

1104.24288	0.58247

1104.40359	0.58258

1104.5643	0.58263

1104.72501	0.58284

1104.88572	0.58321

1105.04643	0.58342

1105.20714	0.58353

1105.36785	0.58363

1105.52857	0.58374

1105.68928	0.58379

1105.84999	0.58385

1106.0107	0.584

1106.17141	0.58406

1106.33212	0.58464

1106.49283	0.58469

1106.65354	0.58485

1106.81425	0.5849

1106.97496	0.58495

1107.13567	0.58501

1107.29638	0.58506

1107.45709	0.58517

1107.6178	0.58527

1107.77851	0.58532

1107.93923	0.58538

1108.09994	0.58575

1108.26065	0.58585

1108.42136	0.58596

1108.58207	0.58606

1108.74278	0.58612

1108.90349	0.58622

1109.0642	0.58627

1109.22491	0.58638

1109.38562	0.58643

1109.54633	0.58654

1109.70704	0.58664

1109.86775	0.5867

1110.02846	0.58675

1110.18917	0.58686

1110.34989	0.58691

1110.5106	0.58717

1110.67131	0.58728

1110.83202	0.58733

1110.99273	0.58738

1111.15344	0.58749

1111.31415	0.58765

1111.47486	0.58775

1111.63557	0.58791

1111.79628	0.58812

1111.95699	0.58818

1112.1177	0.58823

1112.27841	0.58828

1112.43912	0.58854

1112.59983	0.5886

1112.76054	0.58876

1112.92126	0.58886

1113.08197	0.58923

1113.24268	0.58955

1113.40339	0.5896

1113.5641	0.58976

1113.72481	0.58981

1113.88552	0.58986

1114.04623	0.58992

1114.20694	0.59002

1114.36765	0.59008

1114.52836	0.59018

1114.68907	0.59029

1114.84978	0.59034

1115.01049	0.5905

1115.1712	0.59066

1115.33192	0.59108

1115.49263	0.5914

1115.65334	0.59161

1115.81405	0.59166

1115.97476	0.59171

1116.13547	0.59182

1116.29618	0.59187

1116.45689	0.59198

1116.6176	0.59203

1116.77831	0.59214

1116.93902	0.59224

1117.09973	0.59229

1117.26044	0.59245

1117.42115	0.59256

1117.58186	0.59261

1117.74257	0.59266

1117.90329	0.59277

1118.064	0.59446

1118.22471	0.59457

1118.38542	0.59468

1118.54613	0.59473

1118.70684	0.59478

1118.86755	0.59483

1119.02826	0.59489

1119.18897	0.59494

1119.34968	0.59499

1119.51039	0.5951

1119.6711	0.59515

1119.83181	0.59526

1119.99252	0.59531

1120.15323	0.59568

1120.31395	0.59573

1120.47466	0.59579

1120.63537	0.59584

1120.79608	0.59594

1120.95679	0.59626

1121.1175	0.59647

1121.27821	0.59663

1121.43892	0.59668

1121.59963	0.59674

1121.76034	0.597

1121.92105	0.59705

1122.08176	0.59711

1122.24247	0.59721

1122.40318	0.59742

1122.56389	0.59758

1122.72461	0.59763

1122.88532	0.59811

1123.04603	0.59843

1123.20674	0.59848

1123.36745	0.59864

1123.52816	0.59874

1123.68887	0.59885

1123.84958	0.59895

1124.01029	0.59901

1124.171	0.59906

1124.33171	0.59916

1124.49242	0.59922

1124.65313	0.59927

1124.81384	0.59943

1124.97455	0.60053

1125.13526	0.60068

1125.29598	0.6009

1125.45669	0.601

1125.6174	0.60105

1125.77811	0.60111

1125.93882	0.60126

1126.09953	0.60137

1126.26024	0.60148

1126.42095	0.60153

1126.58166	0.60158

1126.74237	0.60163

1126.90308	0.60169

1127.06379	0.60195

1127.2245	0.60211

1127.38521	0.60216

1127.54592	0.60227

1127.70664	0.60232

1127.86734	0.60248

1128.02806	0.60253

1128.18877	0.60259

1128.34948	0.6029

1128.51019	0.60295

1128.6709	0.60301

1128.83161	0.60306

1128.99232	0.60311

1129.15303	0.60317

1129.31374	0.60327

1129.47445	0.60332

1129.63516	0.60338

1129.79587	0.60359

1129.95658	0.60375

1130.1173	0.60391

1130.278	0.60396

1130.43872	0.60406

1130.59943	0.60412

1130.76014	0.60422

1130.92085	0.60433

1131.08156	0.60443

1131.24227	0.60449

1131.40298	0.60454

1131.56369	0.60464

1131.7244	0.6047

1131.88511	0.60475

1132.04582	0.6048

1132.20653	0.60486

1132.36724	0.60491

1132.52795	0.60496

1132.68866	0.60528

1132.84938	0.60533

1133.01009	0.60549

1133.1708	0.60554

1133.33151	0.60565

1133.49222	0.60586

1133.65293	0.60596

1133.81364	0.60602

1133.97435	0.60607

1134.13506	0.60612

1134.29577	0.60618

1134.45648	0.60692

1134.61719	0.60728

1134.7779	0.60739

1134.93861	0.6075

1135.09932	0.60755

1135.26004	0.6076

1135.42075	0.60776

1135.58146	0.60781

1135.74217	0.60792

1135.90288	0.60797

1136.06359	0.60802

1136.2243	0.60824

1136.38501	0.60829

1136.54572	0.60834

1136.70643	0.60839

1136.86714	0.60845

1137.02785	0.60855

1137.18856	0.6086

1137.34927	0.60866

1137.50998	0.60892

1137.6707	0.60903

1137.83141	0.60908

1137.99212	0.60919

1138.15283	0.60934

1138.31354	0.60945

1138.47425	0.6095

1138.63496	0.60956

1138.79567	0.60961

1138.95638	0.60966

1139.11709	0.60971

1139.2778	0.60982

1139.43851	0.60992

1139.59922	0.61003

1139.75993	0.61008

1139.92064	0.61014

1140.08136	0.61019

1140.24207	0.61024

1140.40278	0.61029

1140.56349	0.61035

1140.7242	0.61051

1140.88491	0.61056

1141.04562	0.61061

1141.20633	0.61066

1141.36704	0.61072

1141.52775	0.61082

1141.68846	0.61093

1141.84917	0.61098

1142.00988	0.61109

1142.17059	0.61114

1142.3313	0.61119

1142.49202	0.6113

1142.65272	0.61135

1142.81344	0.61151

1142.97415	0.61172

1143.13486	0.61183

1143.29557	0.61188

1143.45628	0.61193

1143.61699	0.61204

1143.7777	0.61209

1143.93841	0.61214

1144.09912	0.61235

1144.25983	0.61246

1144.42054	0.61251

1144.58125	0.61257

1144.74196	0.61262

1144.90267	0.61267

1145.06338	0.61272

1145.2241	0.61278

1145.38481	0.61288

1145.54552	0.61309

1145.70623	0.61315

1145.86694	0.6133

1146.02765	0.61346

1146.18836	0.61362

1146.34907	0.61394

1146.50978	0.61399

1146.67049	0.61494

1146.8312	0.61521

1146.99191	0.6153

1147.15262	0.61544

1147.31333	0.61546

1147.47404	0.61549

1147.63476	0.61579

1147.79547	0.61584

1147.95618	0.61586

1148.11689	0.616

1148.2776	0.61631

1148.43831	0.61668

1148.59902	0.61671

1148.75973	0.61722

1148.92044	0.61725

1149.08115	0.61758

1149.24186	0.6176

1149.40257	0.61762

1149.56328	0.61795

1149.72399	0.61809

1149.8847	0.61812

1150.04542	0.61833

1150.20613	0.61835

1150.36684	0.61842

1150.52755	0.61844

1150.68826	0.61847

1150.84897	0.61863

1151.00968	0.61868

1151.17039	0.61898

1151.3311	0.61901

1151.49181	0.61903

1151.65252	0.61917

1151.81323	0.6192

1151.97394	0.61927

1152.13465	0.61931

1152.29536	0.6195

1152.45608	0.61955

1152.61679	0.61964

1152.7775	0.61981

1152.93821	0.61983

1153.09892	0.62025

1153.25963	0.62039

1153.42034	0.62093

1153.58105	0.621

1153.74176	0.62103

1153.90247	0.62119

1154.06318	0.62121

1154.22389	0.62124

1154.3846	0.62128

1154.54531	0.62138

1154.70602	0.62147

1154.86674	0.6215

1155.02744	0.62157

1155.18816	0.62161

1155.34887	0.62185

1155.50958	0.62187

1155.67029	0.62246

1155.831	0.62248

1155.99171	0.62283

1156.15242	0.62295

1156.31313	0.62344

1156.47384	0.62351

1156.63455	0.62372

1156.79526	0.62382

1156.95597	0.62384

1157.11668	0.62455

1157.2774	0.62462

1157.43811	0.62483

1157.59882	0.62487

1157.75953	0.63199

1157.92024	0.63612

1158.08095	0.63734

1158.24166	0.63738

1158.40237	0.63818

1158.56308	0.64203

1158.72379	0.65494

1158.8845	0.65578

1159.04521	0.99831

1159.20592	0.99843

1159.36663	0.99847

1159.52734	0.9985

1159.68805	0.99871

1159.84877	0.99878

1160.00948	0.99883

1160.17019	1

1160.3309	1

1160.49161	1

1160.65232	1

1160.81303	1

1160.97374	1

1161.13445	1

1161.29516	1

1161.45587	1

**（*c*）Non-hydraulic-45°**

1	0.04596	0.59398	1.68E-05

2	0.06524	0.69298	5.03E-05

3	0.08303	0.79198	0.000117464

4	0.05471	0.89097	1.68E-05

5	0.0724	0.98997	5.03E-05

6	0.0901	1.08897	1.17E-04

7	0.11263	1.18796	1.51E-04

8	0.13033	1.28696	3.36E-04

9	0.13998	1.38596	3.69E-04

10	0.15929	1.48496	4.36E-04

11	0.17699	1.58395	4.53E-04

12	0.19308	1.68295	4.70E-04

13	0.20917	1.78195	4.87E-04

14	0.22204	1.88094	7.38E-04

15	0.24296	1.97994	7.55E-04

16	0.25744	2.07894	7.89E-04

17	0.27031	2.17793	8.05E-04

18	0.28479	2.27693	8.22E-04

19	0.3041	2.37593	8.39E-04

20	0.32019	2.47493	8.73E-04

21	0.33145	2.57392	9.06E-04

22	0.34915	2.67292	9.23E-04

23	0.37168	2.77192	0.00106

24	0.38938	2.87091	0.00114

25	0.39742	2.96991	0.00131

26	0.41351	3.06891	0.00133

27	0.43765	3.1679	0.00154

28	0.4473	3.2669	0.00164

29	0.46178	3.3659	0.00183

30	0.4827	3.4649	0.00185

31	0.50362	3.56389	0.002

32	0.51488	3.66289	0.00203

33	0.52775	3.76189	0.00206

34	0.54706	3.86088	0.00225

35	0.55993	3.95988	0.00228

36	0.5728	4.05888	0.0023

37	0.5905	4.15788	0.0025

38	0.60981	4.25687	0.00252

39	0.6259	4.35587	0.00255

40	0.64038	4.45487	0.00263

41	0.65486	4.55386	0.00265

42	0.67417	4.65286	0.00284

43	0.68704	4.75186	0.00367

44	0.70152	4.85085	0.00388

45	0.716	4.94985	0.00391

46	0.73853	5.04885	0.00393

47	0.75462	5.14785	0.00396

48	0.77232	5.24684	0.00398

49	0.79002	5.34584	0.00401

50	0.81094	5.44484	0.00403

51	0.82542	5.54383	0.00404

52	0.83346	5.64283	0.00406

53	0.84955	5.74183	0.00425

54	0.87208	5.84082	0.00428

55	0.88173	5.93982	0.00433

56	0.89621	6.03882	0.00436

57	0.91713	6.13782	0.00441

58	0.93805	6.23681	0.00453

59	0.94931	6.33581	0.00456

60	0.96379	6.43481	0.00458

61	0.98471	6.5338	0.00467

62	0.99919	6.6328	0.00472

63	1.01045	6.7318	0.00498

64	1.02654	6.83079	0.00537

65	1.04746	6.92979	0.0054

66	1.06033	7.02879	0.00542

67	1.07642	7.12779	0.00545

68	1.09573	7.22678	0.00572

69	1.11343	7.32578	0.00576

70	1.12952	7.42478	0.00577

71	1.13756	7.52377	0.00579

72	1.15687	7.62277	0.00581

73	1.17457	7.72177	0.00584

74	1.18905	7.82077	0.00586

75	1.20353	7.91976	0.00589

76	1.22123	8.01876	0.00601

77	1.24215	8.11776	0.00602

78	1.25502	8.21675	0.00609

79	1.26789	8.31575	0.00616

80	1.2872	8.41475	0.00621

81	1.30651	8.51374	0.00623

82	1.31455	8.61274	0.00624

83	1.33064	8.71174	0.00626

84	1.35478	8.81074	0.00634

85	1.36926	8.90973	0.00638

86	1.38052	9.00873	0.00639

87	1.40305	9.10773	0.00641

88	1.41753	9.20672	0.00668

89	1.43201	9.30572	0.00675

90	1.44327	9.40472	0.00676

91	1.45936	9.50371	0.00678

92	1.48028	9.60271	0.0068

93	1.49798	9.70171	0.00701

94	1.50924	9.80071	0.00705

95	1.52694	9.8997	0.00713

96	1.54947	9.9987	0.00725

97	1.56556	10.0977	0.00737

98	1.5736	10.19669	0.00747

99	1.58969	10.29569	0.0075

100	1.61061	10.39469	0.00752

101	1.62187	10.49368	0.00755

102	1.63796	10.59268	0.00757

103	1.65727	10.69168	0.0076

104	1.67497	10.79068	0.00762

105	1.68784	10.88967	0.00764

106	1.70393	10.98867	0.00765

107	1.72485	11.08767	0.0077

108	1.73772	11.18666	0.00774

109	1.75059	11.28566	0.00805

110	1.76507	11.38466	0.00812

111	1.7876	11.48366	0.00814

112	1.80047	11.58265	0.00816

113	1.81656	11.68165	0.00819

114	1.83426	11.78065	0.00824

115	1.85518	11.87964	0.00826

116	1.86805	11.97864	0.00874

117	1.87931	12.07764	0.00876

118	1.8954	12.17663	0.00898

119	1.91471	12.27563	0.0092

120	1.93241	12.37463	0.00923

121	1.94206	12.47363	0.00926

122	1.95976	12.57262	0.00928

123	1.98229	12.67162	0.00931

124	1.99838	12.77062	0.00951

125	2.00964	12.86961	0.00982

126	2.02895	12.96861	0.00983

127	2.04665	13.06761	0.00987

128	2.0563	13.1666	0.00988

129	2.06917	13.2656	0.00997

130	2.08848	13.3646	0.01002

131	2.1094	13.4636	0.01113

132	2.12227	13.56259	0.01119

133	2.13836	13.66159	0.01121

134	2.15767	13.76059	0.01133

135	2.17215	13.85958	0.01139

136	2.18341	13.95858	0.01141

137	2.1995	14.05758	0.01143

138	2.21881	14.15658	0.0117

139	2.23651	14.25557	0.01186

140	2.24616	14.35457	0.01196

141	2.26386	14.45357	0.01198

142	2.288	14.55256	0.01205

143	2.30248	14.65156	0.01207

144	2.31213	14.75056	0.01215

145	2.32822	14.84955	0.01252

146	2.34753	14.94855	0.01257

147	2.36362	15.04755	0.01262

148	2.37488	15.14655	0.01265

149	2.39419	15.24554	0.01267

150	2.41189	15.34454	0.01269

151	2.42798	15.44354	0.01272

152	2.44085	15.54253	0.01312

153	2.46016	15.64153	0.01314

154	2.47625	15.74053	0.01316

155	2.48751	15.83952	0.01317

156	2.50199	15.93852	0.01339

157	2.52291	16.03752	0.01368

158	2.539	16.13652	0.01388

159	2.55026	16.23551	0.01391

160	2.56957	16.33451	0.01394

161	2.5921	16.43351	0.01405

162	2.60658	16.5325	0.01406

163	2.61623	16.6315	0.0141

164	2.63393	16.7305	0.01415

165	2.65324	16.82949	0.01416

166	2.66289	16.92849	0.01418

167	2.67898	17.02749	0.01433

168	2.69668	17.12649	0.01504

169	2.7176	17.22548	0.01509

170	2.73369	17.32448	0.0152

171	2.74656	17.42348	0.01535

172	2.76104	17.52247	0.01547

173	2.78035	17.62147	0.01551

174	2.79322	17.72047	0.01556

175	2.80609	17.81947	0.01557

176	2.82379	17.91846	0.01562

177	2.84149	18.01746	0.01564

178	2.85758	18.11646	0.01569

179	2.87367	18.21545	0.01572

180	2.89298	18.31445	0.01683

181	2.90907	18.41345	0.01686

182	2.92033	18.51244	0.01695

183	2.9316	18.61144	0.01697

184	2.95573	18.71044	0.01703

185	2.97182	18.80944	0.01712

186	2.98308	18.90843	0.01733

187	2.99917	19.00743	0.01735

188	3.0217	19.10643	0.0175

189	3.03779	19.20542	0.01767

190	3.05066	19.30442	0.01769

191	3.06514	19.40342	0.0177

192	3.08445	19.50241	0.01782

193	3.09732	19.60141	0.01784

194	3.10859	19.70041	0.01785

195	3.12789	19.79941	0.01787

196	3.14559	19.8984	0.01796

197	3.1649	19.9974	0.01799

198	3.17777	20.0964	0.01801

199	3.19547	20.19539	0.01804

200	3.21156	20.29439	0.01869

201	3.22443	20.39339	0.01873

202	3.23891	20.49238	0.01874

203	3.25661	20.59138	0.01879

204	3.27592	20.69038	0.01884

205	3.28558	20.78938	0.01886

206	3.30649	20.88837	0.01888

207	3.32741	20.98737	0.01926

208	3.3435	21.08637	0.01928

209	3.35315	21.18536	0.01938

210	3.36603	21.28436	0.0195

211	3.38694	21.38336	0.01953

212	3.40142	21.48236	0.01955

213	3.4143	21.58135	0.01958

214	3.43039	21.68035	0.01962

215	3.45452	21.77935	0.01963

216	3.469	21.87834	0.01975

217	3.47866	21.97734	0.01977

218	3.49796	22.07634	0.0198

219	3.51566	22.17533	0.01985

220	3.52853	22.27433	0.01989

221	3.5398	22.37333	0.01994

222	3.55428	22.47233	0.01995

223	3.57841	22.57132	0.01997

224	3.5945	22.67032	0.01999

225	3.60577	22.76932	0.02004

226	3.62668	22.86831	0.02009

227	3.64116	22.96731	0.0201

228	3.65565	23.06631	0.02012

229	3.6653	23.1653	0.02042

230	3.68622	23.2643	0.02044

231	3.70392	23.3633	0.02047

232	3.71518	23.4623	0.02054

233	3.73449	23.56129	0.02062

234	3.75862	23.66029	0.02064

235	3.7731	23.75929	0.02071

236	3.78597	23.85828	0.02072

237	3.79724	23.95728	0.02076

238	3.81654	24.05628	0.02079

239	3.83103	24.15527	0.02088

240	3.8439	24.25427	0.02089

241	3.8616	24.35327	0.02091

242	3.88412	24.45227	0.02093

243	3.89699	24.55126	0.02094

244	3.91308	24.65026	0.02099

245	3.92757	24.74926	0.02101

246	3.94848	24.84825	0.02104

247	3.95975	24.94725	0.02113

248	3.97262	25.04625	0.02114

249	3.98549	25.14525	0.02118

250	4.01123	25.24424	0.02119

251	4.02089	25.34324	0.02121

252	4.0402	25.44224	0.02123

253	4.06111	25.54123	0.02124

254	4.08042	25.64023	0.02126

255	4.08525	25.73923	0.02128

256	4.09973	25.83822	0.02129

257	4.12547	25.93722	0.02136

258	4.14156	26.03622	0.02151

259	4.14317	26.13522	0.02153

260	4.17213	26.23421	0.02155

261	4.19144	26.33321	0.02228

262	4.21236	26.43221	0.0223

263	4.2204	26.5312	0.02259

264	4.23649	26.6302	0.02262

265	4.25258	26.7292	0.02264

266	4.26546	26.82819	0.02267

267	4.27994	26.92719	0.02269

268	4.28959	27.02619	0.02272

269	4.31855	27.12519	0.02274

270	4.33303	27.22418	0.02277

271	4.34912	27.32318	0.02279

272	4.3636	27.42218	0.02282

273	4.3813	27.52117	0.02286

274	4.39096	27.62017	0.02289

275	4.40705	27.71917	0.02296

276	4.42475	27.81816	0.02356

277	4.44245	27.91716	0.02358

278	4.45854	28.01616	0.02359

279	4.47623	28.11516	0.02361

280	4.50037	28.21415	0.02363

281	4.51807	28.31315	0.02366

282	4.5245	28.41215	0.02368

283	4.5422	28.51114	0.02369

284	4.56473	28.61014	0.02371

285	4.57599	28.70914	0.02373

286	4.58886	28.80814	0.02374

287	4.60817	28.90713	0.02378

288	4.6307	29.00613	0.02383

289	4.64679	29.10513	0.02385

290	4.65644	29.20412	0.02386

291	4.66931	29.30312	0.0239

292	4.68058	29.40212	0.02391

293	4.69989	29.50111	0.02393

294	4.70954	29.60011	0.02395

295	4.72402	29.69911	0.02396

296	4.76264	29.79811	0.02411

297	4.77873	29.8971	0.02415

298	4.79321	29.9961	0.02418

299	4.80608	30.0951	0.0242

300	4.81734	30.19409	0.02423

301	4.83182	30.29309	0.02425

302	4.83504	30.39209	0.02426

303	4.85274	30.49108	0.02432

304	4.87366	30.59008	0.02433

305	4.88814	30.68908	0.02435

306	4.91549	30.78808	0.02437

307	4.94124	30.88707	0.02438

308	4.95572	30.98607	0.02443

309	4.95733	31.08507	0.02445

310	4.97181	31.18406	0.02447

311	4.98629	31.28306	0.02448

312	5.0072	31.38206	0.02455

313	5.01042	31.48105	0.02457

314	5.04421	31.58005	0.02458

315	5.06674	31.67905	0.0246

316	5.08765	31.77805	0.02462

317	5.08765	31.87704	0.02478

318	5.10053	31.97604	0.0248

319	5.12305	32.07504	0.02482

320	5.1311	32.17403	0.02499

321	5.14075	32.27303	0.025

322	5.15523	32.37203	0.02504

323	5.19063	32.47103	0.02505

324	5.2035	32.57002	0.02507

325	5.2212	32.66902	0.02509

326	5.2389	32.76802	0.0251

327	5.25177	32.86701	0.02527

328	5.25338	32.96601	0.02529

329	5.27108	33.06501	0.02531

330	5.30004	33.164	0.02532

331	5.3113	33.263	0.02593

332	5.32739	33.362	0.02598

333	5.35314	33.461	0.02604

334	5.36923	33.55999	0.02614

335	5.38049	33.65899	0.02616

336	5.39336	33.75799	0.02621

337	5.40945	33.85698	0.02631

338	5.42233	33.95598	0.02633

339	5.44163	34.05498	0.02635

340	5.44807	34.15397	0.0264

341	5.46255	34.25297	0.02645

342	5.4899	34.35197	0.02646

343	5.51243	34.45097	0.0265

344	5.53013	34.54996	0.02651

345	5.53978	34.64896	0.02653

346	5.55587	34.74796	0.02655

347	5.56874	34.84695	0.02656

348	5.57518	34.94595	0.02658

349	5.60253	35.04495	0.02668

350	5.62506	35.14395	0.0267

351	5.63793	35.24294	0.02671

352	5.65241	35.34194	0.02675

353	5.67816	35.44094	0.02677

354	5.68942	35.53993	0.02678

355	5.69264	35.63893	0.0268

356	5.70712	35.73793	0.02682

357	5.72643	35.83692	0.02693

358	5.7393	35.93592	0.02695

359	5.75378	36.03492	0.02697

360	5.78596	36.13392	0.02702

361	5.80366	36.23291	0.02703

362	5.82136	36.33191	0.02705

363	5.82297	36.43091	0.02708

364	5.8471	36.5299	0.0271

365	5.85676	36.6289	0.02713

366	5.87285	36.7279	0.02717

367	5.87767	36.82689	0.02718

368	5.90503	36.92589	0.0272

369	5.93238	37.02489	0.02722

370	5.93721	37.12389	0.02725

371	5.95651	37.22288	0.02729

372	5.97421	37.32188	0.0273

373	5.9903	37.42088	0.02732

374	5.99674	37.51987	0.02734

375	6.008	37.61887	0.02735

376	6.0257	37.71787	0.02742

377	6.05466	37.81686	0.02744

378	6.0611	37.91586	0.02745

379	6.08202	38.01486	0.0277

380	6.11259	38.11386	0.02776

381	6.11902	38.21285	0.02777

382	6.12868	38.31185	0.02806

383	6.13994	38.41085	0.02851

384	6.16247	38.50984	0.02853

385	6.17373	38.60884	0.02854

386	6.19143	38.70784	0.02861

387	6.21556	38.80684	0.02863

388	6.23809	38.90583	0.02864

389	6.25257	39.00483	0.02866

390	6.26544	39.10383	0.02873

391	6.27349	39.20282	0.02875

392	6.29601	39.30182	0.02876

393	6.30406	39.40082	0.02878

394	6.31371	39.49981	0.0288

395	6.34267	39.59881	0.02881

396	6.35876	39.69781	0.02885

397	6.37646	39.79681	0.02886

398	6.38612	39.8958	0.02891

399	6.41025	39.9948	0.02893

400	6.42634	40.0938	0.02895

401	6.43117	40.19279	0.02896

402	6.44404	40.29179	0.02957

403	6.46817	40.39079	0.02984

404	6.48587	40.48978	0.03034

405	6.49875	40.58878	0.03036

406	6.51805	40.68778	0.03039

407	6.5438	40.78678	0.03052

408	6.55506	40.88577	0.03054

409	6.55828	40.98477	0.03056

410	6.58241	41.08377	0.03057

411	6.59529	41.18276	0.03059

412	6.60655	41.28176	0.03061

413	6.62103	41.38076	0.03062

414	6.64195	41.47975	0.03064

415	6.66769	41.57875	0.03066

416	6.68217	41.67775	0.03068

417	6.69183	41.77675	0.03069

418	6.71596	41.87574	0.03071

419	6.73044	41.97474	0.03073

420	6.7417	42.07374	0.03074

421	6.75136	42.17273	0.03076

422	6.76584	42.27173	0.03081

423	6.79319	42.37073	0.03083

424	6.80606	42.46973	0.03084

425	6.82698	42.56872	0.03086

426	6.84468	42.66772	0.03089

427	6.85916	42.76672	0.03091

428	6.86077	42.86571	0.03093

429	6.87847	42.96471	0.03096

430	6.89617	43.06371	0.03098

431	6.91387	43.1627	0.03104

432	6.93318	43.2617	0.03111

433	6.94766	43.3607	0.03113

434	6.98145	43.4597	0.03116

435	6.98788	43.55869	0.03118

436	6.99754	43.65769	0.03123

437	7.01202	43.75669	0.03125

438	7.02972	43.85568	0.03126

439	7.04259	43.95468	0.0314

440	7.05546	44.05368	0.03141

441	7.08281	44.15267	0.03143

442	7.0989	44.25167	0.03145

443	7.11338	44.35067	0.03146

444	7.13108	44.44967	0.0315

445	7.15039	44.54866	0.03151

446	7.16648	44.64766	0.03153

447	7.17131	44.74666	0.03167

448	7.1874	44.84565	0.03172

449	7.20671	44.94465	0.03173

450	7.2244	45.04365	0.03175

451	7.23567	45.14264	0.03177

452	7.26141	45.24164	0.03178

453	7.27911	45.34064	0.03183

454	7.29359	45.43964	0.03187

455	7.30164	45.53863	0.03192

456	7.31612	45.63763	0.03193

457	7.34186	45.73663	0.03197

458	7.34991	45.83562	0.03198

459	7.35956	45.93462	0.032

460	7.38691	46.03362	0.03202

461	7.40622	46.13262	0.03203

462	7.41909	46.23161	0.03207

463	7.43679	46.33061	0.0321

464	7.45127	46.42961	0.03212

465	7.4738	46.5286	0.03213

466	7.48024	46.6276	0.03215

467	7.49633	46.7266	0.03217

468	7.5092	46.82559	0.03219

469	7.53172	46.92459	0.0322

470	7.54942	47.02359	0.03222

471	7.56229	47.12259	0.03224

472	7.57678	47.22158	0.03225

473	7.5993	47.32058	0.03229

474	7.60735	47.41958	0.0323

475	7.62344	47.51857	0.03234

476	7.64274	47.61757	0.03237

477	7.65883	47.71657	0.0324

478	7.67653	47.81556	0.03244

479	7.69101	47.91456	0.03249

480	7.71676	48.01356	0.03252

481	7.7248	48.11256	0.03254

482	7.73767	48.21155	0.03255

483	7.75698	48.31055	0.03257

484	7.77307	48.40955	0.03259

485	7.78594	48.50854	0.0326

486	7.79399	48.60754	0.03264

487	7.81812	48.70654	0.03266

488	7.84065	48.80553	0.03269

489	7.85352	48.90453	0.03271

490	7.87283	49.00353	0.03274

491	7.8857	49.10253	0.03282

492	7.90179	49.20152	0.03286

493	7.91627	49.30052	0.03289

494	7.92754	49.39952	0.03291

495	7.94845	49.49851	0.03292

496	7.96293	49.59751	0.03296

497	7.98224	49.69651	0.03299

498	7.99672	49.79551	0.03301

499	8.01764	49.8945	0.03302

500	8.0289	49.9935	0.03304

501	8.04017	50.0925	0.03306

502	8.05465	50.19149	0.03309

503	8.07717	50.29049	0.03312

504	8.09487	50.38949	0.03318

505	8.10453	50.48848	0.03319

506	8.12223	50.58748	0.03321

507	8.14636	50.68648	0.03343

508	8.16406	50.78548	0.03344

509	8.17532	50.88447	0.03346

510	8.1898	50.98347	0.03348

511	8.20911	51.08247	0.03349

512	8.22037	51.18146	0.03353

513	8.23003	51.28046	0.03354

514	8.25255	51.37946	0.03358

515	8.27186	51.47845	0.03359

516	8.28795	51.57745	0.03363

517	8.30565	51.67645	0.03366

518	8.32174	51.77545	0.03368

519	8.33944	51.87444	0.0337

520	8.34749	51.97344	0.03375

521	8.36679	52.07244	0.03376

522	8.38288	52.17143	0.0338

523	8.39736	52.27043	0.03385

524	8.40863	52.36943	0.03388

525	8.42954	52.46842	0.03406

526	8.45368	52.56742	0.03408

527	8.46816	52.66642	0.0341

528	8.47299	52.76542	0.03412

529	8.48908	52.86441	0.0342

530	8.5116	52.96341	0.03422

531	8.5293	53.06241	0.03427

532	8.53574	53.1614	0.03428

533	8.55826	53.2604	0.03435

534	8.57757	53.3594	0.03438

535	8.59366	53.4584	0.0344

536	8.60493	53.55739	0.03442

537	8.62745	53.65639	0.03443

538	8.64193	53.75539	0.03445

539	8.64998	53.85438	0.03448

540	8.66768	53.95338	0.03453

541	8.68698	54.05238	0.03458

542	8.70629	54.15137	0.0346

543	8.71916	54.25037	0.03462

544	8.74008	54.34937	0.0347

545	8.75295	54.44837	0.03475

546	8.76904	54.54736	0.0348

547	8.78192	54.64636	0.03482

548	8.79479	54.74536	0.03485

549	8.81892	54.84435	0.03489

550	8.83501	54.94335	0.0349

551	8.84306	55.04235	0.03492

552	8.86237	55.14134	0.03494

553	8.8865	55.24034	0.03499

554	8.90098	55.33934	0.03504

555	8.90903	55.43834	0.03505

556	8.92512	55.53733	0.03509

557	8.94442	55.63633	0.03511

558	8.96373	55.73533	0.03512

559	8.97499	55.83432	0.03516

560	8.9943	55.93332	0.03517

561	9.02005	56.03232	0.03519

562	9.03131	56.13132	0.03522

563	9.03935	56.23031	0.03526

564	9.06027	56.32931	0.03527

565	9.07636	56.42831	0.03536

566	9.09084	56.5273	0.03541

567	9.10371	56.6263	0.03546

568	9.12141	56.7253	0.03547

569	9.13911	56.82429	0.03549

570	9.15359	56.92329	0.03554

571	9.16968	57.02229	0.03556

572	9.1906	57.12129	0.03561

573	9.20991	57.22028	0.03563

574	9.22278	57.31928	0.03564

575	9.23404	57.41828	0.03568

576	9.25174	57.51727	0.03573

577	9.27427	57.61627	0.03574

578	9.28392	57.71527	0.03576

579	9.30323	57.81426	0.03578

580	9.32576	57.91326	0.03581

581	9.33702	58.01226	0.03584

582	9.34667	58.11126	0.03586

583	9.36598	58.21025	0.03599

584	9.38851	58.30925	0.03603

585	9.39494	58.40825	0.03604

586	9.40621	58.50724	0.03608

587	9.43195	58.60624	0.03611

588	9.44965	58.70524	0.03615

589	9.46252	58.80423	0.03616

590	9.477	58.90323	0.0362

591	9.49148	59.00223	0.03621

592	9.5124	59.10123	0.03623

593	9.52205	59.20022	0.03628

594	9.53493	59.29922	0.03638

595	9.56067	59.39822	0.03651

596	9.57998	59.49721	0.03655

597	9.58641	59.59621	0.03657

598	9.60894	59.69521	0.0366

599	9.62342	59.79421	0.03662

600	9.6379	59.8932	0.03663

601	9.65238	59.9922	0.03665

602	9.66847	60.0912	0.03668

603	9.68939	60.19019	0.0367

604	9.70548	60.28919	0.03672

605	9.71835	60.38819	0.03675

606	9.73766	60.48718	0.03677

607	9.75697	60.58618	0.03678

608	9.76823	60.68518	0.0368

609	9.78271	60.78418	0.03685

610	9.79558	60.88317	0.03688

611	9.81811	60.98217	0.03695

612	9.82776	61.08117	0.03697

613	9.84225	61.18016	0.037

614	9.86638	61.27916	0.03707

615	9.88247	61.37816	0.03709

616	9.89373	61.47715	0.0371

617	9.90661	61.57615	0.03714

618	9.93396	61.67515	0.03715

619	9.94522	61.77415	0.03719

620	9.96131	61.87314	0.0372

621	9.9774	61.97214	0.03727

622	9.99671	62.07114	0.03729

623	10.01441	62.17013	0.0373

624	10.02406	62.26913	0.03732

625	10.03854	62.36813	0.03749

626	10.06268	62.46712	0.03757

627	10.07233	62.56612	0.03761

628	10.09486	62.66512	0.03766

629	10.1029	62.76412	0.03771

630	10.12221	62.86311	0.03776

631	10.1383	62.96211	0.03782

632	10.15278	63.06111	0.03784

633	10.17209	63.1601	0.03791

634	10.18818	63.2591	0.03792

635	10.20749	63.3581	0.03796

636	10.21875	63.4571	0.03799

637	10.23484	63.55609	0.03802

638	10.24771	63.65509	0.03804

639	10.25737	63.75409	0.03811

640	10.27507	63.85308	0.03814

641	10.29759	63.95208	0.03816

642	10.3169	64.05108	0.03829

643	10.33782	64.15007	0.03831

644	10.34264	64.24907	0.03833

645	10.36678	64.34807	0.03834

646	10.37965	64.44707	0.03838

647	10.3893	64.54606	0.03844

648	10.407	64.64506	0.03849

649	10.42953	64.74406	0.03855

650	10.44723	64.84305	0.03858

651	10.46171	64.94205	0.0386

652	10.48102	65.04105	0.03861

653	10.50033	65.14004	0.03863

654	10.50837	65.23904	0.03865

655	10.52285	65.33804	0.03866

656	10.54216	65.43704	0.03868

657	10.56308	65.53603	0.03875

658	10.57273	65.63503	0.03876

659	10.58882	65.73403	0.03878

660	10.60974	65.83302	0.0388

661	10.62261	65.93202	0.03881

662	10.63387	66.03102	0.03888

663	10.65318	66.13001	0.03893

664	10.66927	66.22901	0.03895

665	10.68375	66.32801	0.03896

666	10.69341	66.42701	0.03898

667	10.71754	66.526	0.039

668	10.73846	66.625	0.03903

669	10.75294	66.724	0.03905

670	10.76903	66.82299	0.03907

671	10.77868	66.92199	0.03908

672	10.78995	67.02099	0.03915

673	10.82213	67.11999	0.0394

674	10.835	67.21898	0.03942

675	10.84465	67.31798	0.03943

676	10.86557	67.41698	0.03948

677	10.88327	67.51597	0.0395

678	10.89614	67.61497	0.03955

679	10.91223	67.71397	0.03962

680	10.9251	67.81296	0.03964

681	10.94763	67.91196	0.0397

682	10.9605	68.01096	0.03972

683	10.97498	68.10996	0.03975

684	10.99107	68.20895	0.03977

685	11.00394	68.30795	0.0398

686	11.01842	68.40695	0.03982

687	11.04578	68.50594	0.03984

688	11.06026	68.60494	0.0399

689	11.06669	68.70394	0.03992

690	11.07957	68.80293	0.03994

691	11.11175	68.90193	0.03997

692	11.11657	69.00093	0.04002

693	11.12462	69.09993	0.04011

694	11.15036	69.19892	0.04014

695	11.15841	69.29792	0.04022

696	11.18254	69.39692	0.04024

697	11.19863	69.49591	0.04034

698	11.20668	69.59491	0.04036

699	11.23242	69.69391	0.04037

700	11.24851	69.7929	0.04039

701	11.25012	69.8919	0.04044

702	11.27586	69.9909	0.04046

703	11.3	70.0899	0.04056

704	11.31609	70.18889	0.04059

705	11.32574	70.28789	0.04061

706	11.34022	70.38689	0.04066

707	11.35631	70.48588	0.04068

708	11.37562	70.58488	0.04081

709	11.38688	70.68388	0.04083

710	11.4078	70.78288	0.04093

711	11.42711	70.88187	0.04094

712	11.43194	70.98087	0.04098

713	11.44803	71.07987	0.04101

714	11.48181	71.17886	0.04103

715	11.48342	71.27786	0.04105

716	11.50112	71.37686	0.0411

717	11.51721	71.47585	0.04111

718	11.53813	71.57485	0.04123

719	11.55583	71.67385	0.04128

720	11.56226	71.77285	0.0413

721	11.58318	71.87184	0.04136

722	11.60893	71.97084	0.04153

723	11.6218	72.06984	0.04157

724	11.63145	72.16883	0.04162

725	11.64271	72.26783	0.04163

726	11.67007	72.36683	0.04165

727	11.67972	72.46582	0.04168

728	11.69742	72.56482	0.0417

729	11.71512	72.66382	0.04172

730	11.73604	72.76282	0.04173

731	11.74569	72.86181	0.04175

732	11.765	72.96081	0.04177

733	11.77787	73.05981	0.04178

734	11.80361	73.1588	0.0418

735	11.80683	73.2578	0.04187

736	11.82131	73.3568	0.04188

737	11.84545	73.45579	0.0419

738	11.85993	73.55479	0.04192

739	11.87602	73.65379	0.04195

740	11.88728	73.75279	0.04197

741	11.92429	73.85178	0.04202

742	11.91946	73.95078	0.04205

743	11.94038	74.04978	0.04207

744	11.94842	74.14877	0.04209

745	11.96934	74.24777	0.04214

746	11.98704	74.34677	0.04217

747	11.9983	74.44577	0.04219

748	12.02083	74.54476	0.0422

749	12.03531	74.64376	0.04222

750	12.05301	74.74276	0.04224

751	12.06105	74.84175	0.04225

752	12.08358	74.94075	0.04227

753	12.1045	75.03975	0.04229

754	12.10932	75.13874	0.04232

755	12.12541	75.23774	0.04235

756	12.14311	75.33674	0.04237

757	12.16725	75.43574	0.0424

758	12.17529	75.53473	0.04247

759	12.19943	75.63373	0.04249

760	12.21069	75.73273	0.04251

761	12.23483	75.83172	0.04261

762	12.24287	75.93072	0.04264

763	12.25896	76.02972	0.04267

764	12.28149	76.12871	0.04271

765	12.3008	76.22771	0.04272

766	12.30723	76.32671	0.04274

767	12.32171	76.42571	0.04276

768	12.34585	76.5247	0.04277

769	12.36355	76.6237	0.04279

770	12.36998	76.7227	0.04281

771	12.38768	76.82169	0.04284

772	12.41182	76.92069	0.04287

773	12.41986	77.01969	0.04289

774	12.43273	77.11868	0.04291

775	12.45687	77.21768	0.04292

776	12.47135	77.31668	0.04294

777	12.48583	77.41568	0.04301

778	12.50031	77.51467	0.04304

779	12.51962	77.61367	0.04306

780	12.5341	77.71267	0.04308

781	12.54697	77.81166	0.04309

782	12.56789	77.91066	0.04311

783	12.5872	78.00966	0.04313

784	12.60329	78.10866	0.04314

785	12.61455	78.20765	0.04316

786	12.62099	78.30665	0.04323

787	12.64673	78.40565	0.04328

788	12.66121	78.50464	0.04329

789	12.67247	78.60364	0.04331

790	12.695	78.70264	0.04336

791	12.71431	78.80163	0.04339

792	12.73362	78.90063	0.04341

793	12.73362	78.99963	0.04343

794	12.76419	79.09863	0.04345

795	12.77867	79.19762	0.04346

796	12.79154	79.29662	0.04348

797	12.80119	79.39562	0.0435

798	12.82372	79.49461	0.04351

799	12.84303	79.59361	0.04353

800	12.85429	79.69261	0.04355

801	12.87038	79.7916	0.04358

802	12.88647	79.8906	0.04368

803	12.909	79.9896	0.04371

804	12.91704	80.0886	0.04375

805	12.93635	80.18759	0.04376

806	12.95566	80.28659	0.04381

807	12.97175	80.38559	0.04383

808	12.98301	80.48458	0.04391

809	12.99427	80.58358	0.04395

810	13.02324	80.68258	0.04403

811	13.04093	80.78158	0.04405

812	13.0522	80.88057	0.04413

813	13.06185	80.97957	0.04415

814	13.0876	81.07857	0.04417

815	13.10047	81.17756	0.04418

816	13.10851	81.27656	0.0442

817	13.12943	81.37556	0.04422

818	13.14874	81.47455	0.04423

819	13.16805	81.57355	0.04427

820	13.17448	81.67255	0.04433

821	13.19701	81.77155	0.0444

822	13.21471	81.87054	0.04442

823	13.22275	81.96954	0.04445

824	13.24206	82.06854	0.04447

825	13.26137	82.16753	0.04449

826	13.27585	82.26653	0.0445

827	13.29033	82.36553	0.0446

828	13.30642	82.46452	0.04469

829	13.32734	82.56352	0.0447

830	13.34825	82.66252	0.04475

831	13.3563	82.76152	0.04477

832	13.36756	82.86051	0.04479

833	13.39652	82.95951	0.04482

834	13.40296	83.05851	0.04485

835	13.41744	83.1575	0.04489

836	13.43675	83.2565	0.0449

837	13.4641	83.3555	0.04492

838	13.47054	83.45449	0.04499

839	13.47858	83.55349	0.04501

840	13.50433	83.65249	0.04502

841	13.52203	83.75149	0.04504

842	13.53168	83.85048	0.04506

843	13.54294	83.94948	0.04511

844	13.56225	84.04848	0.04512

845	13.57995	84.14747	0.04514

846	13.60087	84.24647	0.04522

847	13.61374	84.34547	0.04524

848	13.63144	84.44447	0.04526

849	13.64431	84.54346	0.04527

850	13.6604	84.64246	0.04529

851	13.67166	84.74146	0.04536

852	13.68936	84.84045	0.04539

853	13.71028	84.93945	0.04548

854	13.72315	85.03845	0.04561

855	13.74568	85.13744	0.04563

856	13.76177	85.23644	0.04564

857	13.77303	85.33544	0.04566

858	13.78751	85.43444	0.04568

859	13.80682	85.53343	0.04579

860	13.82452	85.63243	0.04586

861	13.83739	85.73143	0.0459

862	13.84704	85.83042	0.04591

863	13.86957	85.92942	0.04593

864	13.89531	86.02842	0.04595

865	13.90014	86.12741	0.0462

866	13.91623	86.22641	0.04626

867	13.93393	86.32541	0.04628

868	13.95485	86.42441	0.0463

869	13.9645	86.5234	0.04631

870	13.97737	86.6224	0.04633

871	13.99829	86.7214	0.04635

872	14.02081	86.82039	0.04638

873	14.03369	86.91939	0.0464

874	14.04817	87.01839	0.04642

875	14.06104	87.11738	0.04643

876	14.08357	87.21638	0.04645

877	14.09	87.31538	0.0465

878	14.1077	87.41438	0.04658

879	14.13023	87.51337	0.04675

880	14.14793	87.61237	0.0468

881	14.15436	87.71137	0.04682

882	14.17689	87.81036	0.04685

883	14.1978	87.90936	0.04687

884	14.21068	88.00836	0.04705

885	14.22516	88.10736	0.04707

886	14.23481	88.20635	0.04712

887	14.25895	88.30535	0.04714

888	14.27021	88.40435	0.04722

889	14.28308	88.50334	0.04727

890	14.30722	88.60234	0.04729

891	14.32652	88.70134	0.0473

892	14.33296	88.80033	0.04732

893	14.35227	88.89933	0.04735

894	14.36836	88.99833	0.04739

895	14.38767	89.09733	0.04741

896	14.39893	89.19632	0.04744

897	14.41341	89.29532	0.04746

898	14.43433	89.39432	0.04749

899	14.45203	89.49331	0.04751

900	14.4649	89.59231	0.04752

901	14.47938	89.69131	0.04761

902	14.49386	89.7903	0.04811

903	14.51639	89.8893	0.04818

904	14.52604	89.9883	0.04819

905	14.5373	90.0873	0.04821

906	14.56144	90.18629	0.04836

907	14.58075	90.28529	0.04838

908	14.59201	90.38429	0.04905

909	14.61132	90.48328	0.04907

910	14.63223	90.58228	0.04913

911	14.64189	90.68128	0.04917

912	14.65154	90.78027	0.04918

913	14.67246	90.87927	0.0492

914	14.69338	90.97827	0.04922

915	14.70303	91.07727	0.04927

916	14.71912	91.17626	0.04928

917	14.74165	91.27526	0.0493

918	14.75935	91.37426	0.04932

919	14.769	91.47325	0.04934

920	14.78348	91.57225	0.04935

921	14.79957	91.67125	0.04939

922	14.81727	91.77025	0.0494

923	14.83175	91.86924	0.04942

924	14.84623	91.96824	0.04945

925	14.86554	92.06724	0.04947

926	14.88324	92.16623	0.04949

927	14.89933	92.26523	0.0495

928	14.91542	92.36423	0.04952

929	14.93312	92.46322	0.04955

930	14.94599	92.56222	0.0498

931	14.95725	92.66122	0.04986

932	14.97656	92.76022	0.04989

933	14.99587	92.85921	0.04991

934	15.01196	92.95821	0.04996

935	15.02483	93.05721	0.04997

936	15.04414	93.1562	0.04999

937	15.06506	93.2552	0.05001

938	15.07471	93.3542	0.05007

939	15.08758	93.45319	0.05009

940	15.10528	93.55219	0.05012

941	15.12459	93.65119	0.05017

942	15.13746	93.75019	0.05022

943	15.15516	93.84918	0.05029

944	15.17608	93.94818	0.05031

945	15.19217	94.04718	0.05038

946	15.20182	94.14617	0.05041

947	15.21791	94.24517	0.05043

948	15.23561	94.34417	0.05048

949	15.25331	94.44316	0.05049

950	15.26296	94.54216	0.05051

951	15.28227	94.64116	0.05054

952	15.29997	94.74016	0.05061

953	15.32089	94.83915	0.05078

954	15.33054	94.93815	0.05079

955	15.35307	95.03715	0.05081

956	15.36433	95.13614	0.05083

957	15.38042	95.23514	0.05085

958	15.38846	95.33414	0.05088

959	15.4126	95.43314	0.0509

960	15.4303	95.53213	0.05091

961	15.44156	95.63113	0.05093

962	15.46409	95.73013	0.05095

963	15.48179	95.82912	0.05096

964	15.49466	95.92812	0.05101

965	15.50592	96.02712	0.05106

966	15.52523	96.12611	0.05108

967	15.53971	96.22511	0.0511

968	15.55741	96.32411	0.05111

969	15.56867	96.42311	0.05113

970	15.58637	96.5221	0.05115

971	15.60729	96.6211	0.05116

972	15.62499	96.7201	0.0512

973	15.63947	96.81909	0.05125

974	15.65234	96.91809	0.05126

975	15.67165	97.01709	0.05133

976	15.68774	97.11608	0.05135

977	15.69739	97.21508	0.0514

978	15.71348	97.31408	0.05142

979	15.73762	97.41308	0.05143

980	15.7521	97.51207	0.05145

981	15.76658	97.61107	0.05147

982	15.78106	97.71007	0.05153

983	15.80198	97.80906	0.05155

984	15.81163	97.90806	0.05157

985	15.82611	98.00706	0.05158

986	15.84381	98.10605	0.0517

987	15.86151	98.20505	0.05172

988	15.87599	98.30405	0.05175

989	15.89369	98.40305	0.05185

990	15.91461	98.50204	0.05192

991	15.92909	98.60104	0.05194

992	15.93713	98.70004	0.052

993	15.95483	98.79903	0.05204

994	15.97253	98.89803	0.05214

995	15.99023	98.99703	0.05215

996	16.0031	99.09603	0.05217

997	16.01919	99.19502	0.05219

998	16.04333	99.29402	0.0522

999	16.05942	99.39302	0.05222

1000	16.07068	99.49201	0.05224

1001	16.08516	99.59101	0.05227

1002	16.10286	99.69001	0.05229

1003	16.11734	99.789	0.05231

1004	16.13021	99.888	0.05232

1005	16.14791	99.987	0.05234

1006	16.17044	100.086	0.05237

1007	16.18492	100.18499	0.05239

1008	16.19618	100.28399	0.05241

1009	16.2171	100.38299	0.05246

1010	16.2348	100.48198	0.05247

1011	16.24445	100.58098	0.05254

1012	16.25732	100.67998	0.05274

1013	16.27985	100.77897	0.05276

1014	16.29755	100.87797	0.05283

1015	16.3072	100.97697	0.05289

1016	16.3249	101.07597	0.05291

1017	16.34904	101.17496	0.05294

1018	16.36513	101.27396	0.05298

1019	16.37156	101.37296	0.05303

1020	16.39087	101.47195	0.05313

1021	16.40696	101.57095	0.05314

1022	16.42466	101.66995	0.05318

1023	16.43431	101.76895	0.05319

1024	16.45362	101.86794	0.05323

1025	16.47615	101.96694	0.05324

1026	16.49224	102.06594	0.05326

1027	16.50511	102.16493	0.05328

1028	16.51959	102.26393	0.0533

1029	16.5389	102.36293	0.05331

1030	16.55177	102.46192	0.05333

1031	16.56464	102.56092	0.05346

1032	16.58234	102.65992	0.05353

1033	16.60165	102.75892	0.05356

1034	16.61452	102.85791	0.05365

1035	16.63061	102.95691	0.05366

1036	16.65475	103.05591	0.05375

1037	16.66601	103.1549	0.05377

1038	16.67405	103.2539	0.05382

1039	16.69336	103.3529	0.0539

1040	16.71589	103.45189	0.05398

1041	16.72715	103.55089	0.05402

1042	16.74002	103.64989	0.05403

1043	16.75933	103.74889	0.05408

1044	16.78186	103.84788	0.05415

1045	16.79473	103.94688	0.05418

1046	16.80921	104.04588	0.0542

1047	16.82047	104.14487	0.05425

1048	16.843	104.24387	0.05427

1049	16.85587	104.34287	0.05432

1050	16.86874	104.44186	0.05435

1051	16.88483	104.54086	0.05442

1052	16.90897	104.63986	0.05444

1053	16.92184	104.73886	0.05445

1054	16.93632	104.83785	0.05449

1055	16.95402	104.93685	0.0545

1056	16.97333	105.03585	0.05452

1057	16.98459	105.13484	0.05454

1058	16.99907	105.23384	0.0546

1059	17.01838	105.33284	0.05462

1060	17.03608	105.43184	0.05467

1061	17.04734	105.53083	0.05469

1062	17.06343	105.62983	0.05472

1063	17.08596	105.72883	0.05476

1064	17.10044	105.82782	0.05484

1065	17.10687	105.92682	0.05486

1066	17.12779	106.02582	0.05487

1067	17.14871	106.12481	0.05489

1068	17.16158	106.22381	0.05491

1069	17.17445	106.32281	0.05494

1070	17.19537	106.42181	0.05502

1071	17.21468	106.5208	0.05504

1072	17.22916	106.6198	0.05514

1073	17.24042	106.7188	0.05519

1074	17.25812	106.81779	0.05529

1075	17.27421	106.91679	0.05534

1076	17.2903	107.01579	0.05539

1077	17.30478	107.11478	0.05541

1078	17.32087	107.21378	0.05546

1079	17.34018	107.31278	0.05549

1080	17.35466	107.41178	0.05551

1081	17.36914	107.51077	0.05553

1082	17.38845	107.60977	0.05554

1083	17.40776	107.70877	0.05558

1084	17.41741	107.80776	0.05559

1085	17.42867	107.90676	0.05561

1086	17.45281	108.00576	0.05563

1087	17.46729	108.10475	0.05571

1088	17.48016	108.20375	0.05575

1089	17.49464	108.30275	0.05578

1090	17.51556	108.40175	0.05583

1091	17.53165	108.50074	0.05585

1092	17.54291	108.59974	0.05586

1093	17.56383	108.69874	0.05588

1094	17.57992	108.79773	0.0559

1095	17.5944	108.89673	0.05591

1096	17.60406	108.99573	0.05593

1097	17.62497	109.09473	0.056

1098	17.64428	109.19372	0.05601

1099	17.66037	109.29272	0.05608

1100	17.67324	109.39172	0.05613

1101	17.68933	109.49071	0.05616

1102	17.70703	109.58971	0.05623

1103	17.72151	109.68871	0.05638

1104	17.73438	109.7877	0.05647

1105	17.75047	109.8867	0.05652

1106	17.77139	109.9857	0.05653

1107	17.78104	110.0847	0.05655

1108	17.79553	110.18369	0.05657

1109	17.81966	110.28269	0.0566

1110	17.83575	110.38169	0.05662

1111	17.8454	110.48068	0.05665

1112	17.86149	110.57968	0.0567

1113	17.88563	110.67868	0.0568

1114	17.8985	110.77767	0.05684

1115	17.91137	110.87667	0.0569

1116	17.92585	110.97567	0.057

1117	17.94677	111.07467	0.05702

1118	17.96286	111.17366	0.05704

1119	17.97895	111.27266	0.05709

1120	17.99343	111.37166	0.05712

1121	18.01274	111.47065	0.05714

1122	18.02561	111.56965	0.05721

1123	18.03848	111.66865	0.05724

1124	18.05618	111.76764	0.05727

1125	18.07227	111.86664	0.05754

1126	18.08675	111.96564	0.05759

1127	18.10767	112.06464	0.05762

1128	18.12859	112.16363	0.05769

1129	18.14146	112.26263	0.05781

1130	18.15272	112.36163	0.05786

1131	18.1656	112.46062	0.05794

1132	18.18651	112.55962	0.05796

1133	18.20099	112.65862	0.05798

1134	18.21387	112.75762	0.05804

1135	18.23156	112.85661	0.05806

1136	18.25409	112.95561	0.05808

1137	18.26857	113.05461	0.05813

1138	18.27983	113.1536	0.05816

1139	18.29753	113.2526	0.05818

1140	18.31684	113.3516	0.0582

1141	18.32971	113.45059	0.05825

1142	18.34098	113.54959	0.05826

1143	18.36028	113.64859	0.05831

1144	18.37959	113.74759	0.05846

1145	18.39246	113.84658	0.05848

1146	18.40695	113.94558	0.0585

1147	18.42625	114.04458	0.05858

1148	18.44234	114.14357	0.0586

1149	18.45843	114.24257	0.05861

1150	18.46809	114.34157	0.05868

1151	18.4874	114.44056	0.05878

1152	18.50509	114.53956	0.0588

1153	18.51797	114.63856	0.05883

1154	18.53406	114.73756	0.05885

1155	18.55176	114.83655	0.05895

1156	18.57106	114.93555	0.05897

1157	18.58554	115.03455	0.05905

1158	18.59842	115.13354	0.05913

1159	18.61612	115.23254	0.05915

1160	18.63221	115.33154	0.0592

1161	18.64508	115.43053	0.05922

1162	18.66278	115.52953	0.05924

1163	18.68369	115.62853	0.05925

1164	18.69817	115.72753	0.05927

1165	18.70622	115.82652	0.0593

1166	18.72392	115.92552	0.05934

1167	18.74323	116.02452	0.05935

1168	18.7561	116.12351	0.05939

1169	18.77058	116.22251	0.05942

1170	18.78667	116.32151	0.05944

1171	18.80437	116.42051	0.05945

1172	18.81402	116.5195	0.05955

1173	18.82207	116.6185	0.0596

1174	18.82529	116.7175	0.05962

1175	18.71909	116.81649	0.05964

1176	17.86954	116.91549	0.05966

1177	16.95241	117.01449	0.05967

1178	15.93231	117.11348	0.05971

1179	15.10206	117.21248	0.05972

1180	14.00151	117.31148	0.05974

1181	13.16483	117.41048	0.05977

1182	12.35872	117.50947	0.05979

1183	11.36114	117.60847	0.05981

1184	10.35873	117.70747	0.05982

1185	9.53493	117.80646	0.05987

1186	8.66768	117.90546	0.05989

1187	7.83743	118.00446	0.05991

118.10345	0.05994

118.20245	0.05997

118.30145	0.06007

118.40045	0.06011

118.49944	0.06014

118.59844	0.06019

118.69744	0.06026

118.79643	0.06028

118.89543	0.06029

118.99443	0.06033

119.09343	0.06034

119.19242	0.06036

119.29142	0.06038

119.39042	0.06039

119.48941	0.06048

119.58841	0.06049

119.68741	0.06059

119.7864	0.06061

119.8854	0.06076

119.9844	0.0608

120.0834	0.06086

120.18239	0.06091

120.28139	0.06093

120.38039	0.06106

120.47938	0.06108

120.57838	0.06115

120.67738	0.06122

120.77637	0.06123

120.87537	0.06125

120.97437	0.0613

121.07337	0.06132

121.17236	0.06133

121.27136	0.06148

121.37036	0.0615

121.46935	0.06157

121.56835	0.06162

121.66735	0.06165

121.76634	0.06169

121.86534	0.0618

121.96434	0.06184

122.06334	0.06185

122.16233	0.06187

122.26133	0.06189

122.36033	0.06199

122.45932	0.062

122.55832	0.06202

122.65732	0.06205

122.75632	0.06207

122.85531	0.06209

122.95431	0.06211

123.05331	0.06216

123.1523	0.06221

123.2513	0.06224

123.3503	0.06226

123.44929	0.06227

123.54829	0.06229

123.64729	0.06237

123.74629	0.06242

123.84528	0.06247

123.94428	0.06251

124.04328	0.06259

124.14227	0.06266

124.24127	0.06268

124.34027	0.06274

124.43926	0.06283

124.53826	0.06299

124.63726	0.06303

124.73626	0.06306

124.83525	0.0631

124.93425	0.0632

125.03325	0.06326

125.13224	0.06328

125.23124	0.06333

125.33024	0.06335

125.42923	0.06336

125.52823	0.06338

125.62723	0.0634

125.72623	0.06343

125.82522	0.06345

125.92422	0.06351

126.02322	0.06353

126.12221	0.06357

126.22121	0.06365

126.32021	0.06367

126.41921	0.06373

126.5182	0.06375

126.6172	0.0638

126.7162	0.06387

126.81519	0.06407

126.91419	0.0641

127.01319	0.06412

127.11218	0.06415

127.21118	0.0642

127.31018	0.0643

127.40918	0.06432

127.50817	0.06437

127.60717	0.06439

127.70617	0.0644

127.80516	0.06445

127.90416	0.06447

128.00316	0.0645

128.10215	0.06459

128.20115	0.06462

128.30015	0.06464

128.39915	0.06466

128.49814	0.06487

128.59714	0.06489

128.69614	0.06497

128.79513	0.06499

128.89413	0.06516

128.99313	0.06519

129.09212	0.06521

129.19112	0.06524

129.29012	0.06533

129.38912	0.06536

129.48811	0.06543

129.58711	0.06546

129.68611	0.06548

129.7851	0.06549

129.8841	0.06553

129.9831	0.06575

130.0821	0.06576

130.18109	0.0658

130.28009	0.06581

130.37909	0.06585

130.47808	0.0659

130.57708	0.06591

130.67608	0.06595

130.77507	0.06598

130.87407	0.066

130.97307	0.06605

131.07207	0.06608

131.17106	0.06617

131.27006	0.06618

131.36906	0.06622

131.46805	0.0663

131.56705	0.06638

131.66605	0.0664

131.76504	0.0665

131.86404	0.06654

131.96304	0.06655

132.06204	0.0666

132.16103	0.06662

132.26003	0.06665

132.35903	0.06672

132.45802	0.06674

132.55702	0.06675

132.65602	0.06707

132.75501	0.06719

132.85401	0.06724

132.95301	0.06726

133.05201	0.06737

133.151	0.06739

133.25	0.06741

133.349	0.06758

133.44799	0.06763

133.54699	0.06766

133.64599	0.06768

133.74499	0.06769

133.84398	0.06771

133.94298	0.06781

134.04198	0.06803

134.14097	0.06806

134.23997	0.06808

134.33897	0.0681

134.43796	0.06815

134.53696	0.0682

134.63596	0.06821

134.73496	0.06828

134.83395	0.0683

134.93295	0.06833

135.03195	0.06838

135.13094	0.06841

135.22994	0.0685

135.32894	0.06852

135.42793	0.06853

135.52693	0.06855

135.62593	0.06857

135.72493	0.0686

135.82392	0.06865

135.92292	0.06873

136.02192	0.06875

136.12091	0.0688

136.21991	0.06882

136.31891	0.06966

136.4179	0.06972

136.5169	0.06974

136.6159	0.06981

136.7149	0.06984

136.81389	0.06987

136.91289	0.06989

137.01189	0.06999

137.11088	0.07009

137.20988	0.07011

137.30888	0.07018

137.40788	0.07019

137.50687	0.07023

137.60587	0.07026

137.70487	0.07034

137.80386	0.07041

137.90286	0.07043

138.00186	0.07046

138.10085	0.07048

138.19985	0.07053

138.29885	0.07055

138.39785	0.07056

138.49684	0.07058

138.59584	0.0706

138.69484	0.07061

138.79383	0.07063

138.89283	0.07065

138.99183	0.07066

139.09082	0.07068

139.18982	0.07083

139.28882	0.0709

139.38782	0.07091

139.48681	0.07095

139.58581	0.07097

139.68481	0.07098

139.7838	0.071

139.8828	0.07102

139.9818	0.07105

140.08079	0.07112

140.17979	0.07113

140.27879	0.07115

140.37779	0.07118

140.47678	0.0712

140.57578	0.07122

140.67478	0.07123

140.77377	0.07125

140.87277	0.07127

140.97177	0.07128

141.07077	0.07133

141.16976	0.07135

141.26876	0.07138

141.36776	0.0714

141.46675	0.07142

141.56575	0.07145

141.66475	0.07149

141.76374	0.0715

141.86274	0.07152

141.96174	0.07155

142.06074	0.0716

142.15973	0.0717

142.25873	0.07174

142.35773	0.07177

142.45672	0.07187

142.55572	0.07197

142.65472	0.07202

142.75371	0.07207

142.85271	0.07209

142.95171	0.07216

143.05071	0.07222

143.1497	0.07224

143.2487	0.07226

143.3477	0.07234

143.44669	0.07236

143.54569	0.07243

143.64469	0.07244

143.74369	0.07246

143.84268	0.07249

143.94168	0.07251

144.04068	0.07253

144.13967	0.07258

144.23867	0.07259

144.33767	0.07261

144.43666	0.07263

144.53566	0.07266

144.63466	0.07268

144.73366	0.07271

144.83265	0.07288

144.93165	0.0729

145.03065	0.07291

145.12964	0.07303

145.22864	0.07306

145.32764	0.0731

145.42663	0.07311

145.52563	0.07313

145.62463	0.07326

145.72363	0.07331

145.82262	0.07335

145.92162	0.07336

146.02062	0.0734

146.11961	0.07345

146.21861	0.07352

146.31761	0.07355

146.4166	0.07357

146.5156	0.07377

146.6146	0.07394

146.7136	0.07395

146.81259	0.07397

146.91159	0.074

147.01059	0.07402

147.10958	0.07407

147.20858	0.0741

147.30758	0.07412

147.40658	0.07414

147.50557	0.07417

147.60457	0.0742

147.70357	0.07424

147.80256	0.0743

147.90156	0.07432

148.00056	0.07435

148.09955	0.07439

148.19855	0.07444

148.29755	0.07451

148.39655	0.07454

148.49554	0.07459

148.59454	0.07461

148.69354	0.07466

148.79253	0.07467

148.89153	0.07469

148.99053	0.07472

149.08952	0.07474

149.18852	0.07491

149.28752	0.07493

149.38652	0.07494

149.48551	0.07506

149.58451	0.07511

149.68351	0.07513

149.7825	0.07516

149.8815	0.07543

149.9805	0.07545

150.07949	0.07546

150.17849	0.07548

150.27749	0.0756

150.37649	0.07563

150.47548	0.07566

150.57448	0.07581

150.67348	0.07583

150.77247	0.07585

150.87147	0.07592

150.97047	0.07593

151.06947	0.07595

151.16846	0.07603

151.26746	0.07605

151.36646	0.07613

151.46545	0.07617

151.56445	0.0762

151.66345	0.07622

151.76244	0.07623

151.86144	0.07625

151.96044	0.07627

152.05944	0.07634

152.15843	0.07639

152.25743	0.07644

152.35643	0.07645

152.45542	0.07649

152.55442	0.0765

152.65342	0.07652

152.75241	0.07655

152.85141	0.07662

152.95041	0.0767

153.04941	0.07675

153.1484	0.07677

153.2474	0.07679

153.3464	0.0768

153.44539	0.07687

153.54439	0.07692

153.64339	0.07697

153.74238	0.07709

153.84138	0.07716

153.94038	0.07717

154.03938	0.07721

154.13837	0.07731

154.23737	0.07733

154.33637	0.07741

154.43536	0.07748

154.53436	0.07751

154.63336	0.07753

154.73236	0.07776

154.83135	0.0779

154.93035	0.07796

155.02935	0.07798

155.12834	0.07803

155.22734	0.07805

155.32634	0.07808

155.42533	0.0782

155.52433	0.07823

155.62333	0.07825

155.72233	0.0783

155.82132	0.07833

155.92032	0.07835

156.01932	0.07838

156.11831	0.0784

156.21731	0.07842

156.31631	0.0785

156.4153	0.07852

156.5143	0.07857

156.6133	0.0787

156.7123	0.07879

156.81129	0.07884

156.91029	0.07885

157.00929	0.07887

157.10828	0.0789

157.20728	0.07897

157.30628	0.079

157.40527	0.07902

157.50427	0.07904

157.60327	0.07905

157.70227	0.07912

157.80126	0.07917

157.90026	0.0792

157.99926	0.07922

158.09825	0.07924

158.19725	0.07925

158.29625	0.07927

158.39525	0.07937

158.49424	0.07941

158.59324	0.07944

158.69224	0.07946

158.79123	0.07947

158.89023	0.07966

158.98923	0.07999

159.08822	0.08001

159.18722	0.08004

159.28622	0.08009

159.38522	0.08013

159.48421	0.08014

159.58321	0.08016

159.68221	0.08018

159.7812	0.08019

159.8802	0.08023

159.9792	0.08026

160.07819	0.08028

160.17719	0.0803

160.27619	0.08031

160.37519	0.08035

160.47418	0.08036

160.57318	0.08043

160.67218	0.08045

160.77117	0.0805

160.87017	0.08056

160.96917	0.08058

161.06816	0.0806

161.16716	0.08061

161.26616	0.08063

161.36516	0.08065

161.46415	0.08066

161.56315	0.08068

161.66215	0.0807

161.76114	0.08071

161.86014	0.08075

161.95914	0.08077

162.05814	0.08078

162.15713	0.08087

162.25613	0.0809

162.35513	0.08092

162.45412	0.08093

162.55312	0.08102

162.65212	0.0811

162.75111	0.08117

162.85011	0.08124

162.94911	0.08125

163.04811	0.08127

163.1471	0.08132

163.2461	0.08134

163.3451	0.08152

163.44409	0.08157

163.54309	0.0816

163.64209	0.08164

163.74108	0.08174

163.84008	0.08176

163.93908	0.08177

164.03808	0.08182

164.13707	0.08184

164.23607	0.08186

164.33507	0.08191

164.43406	0.08212

164.53306	0.08221

164.63206	0.08223

164.73106	0.08224

164.83005	0.08228

164.92905	0.08229

165.02805	0.08231

165.12704	0.08238

165.22604	0.08258

165.32504	0.08259

165.42403	0.08275

165.52303	0.08276

165.62203	0.0828

165.72103	0.08281

165.82002	0.0829

165.91902	0.08291

166.01802	0.08298

166.11701	0.08306

166.21601	0.08311

166.31501	0.08315

166.414	0.08316

166.513	0.08323

166.612	0.08332

166.711	0.08335

166.80999	0.08338

166.90899	0.08342

167.00799	0.08471

167.10698	0.08483

167.20598	0.08484

167.30498	0.08486

167.40397	0.08488

167.50297	0.08489

167.60197	0.08493

167.70097	0.08494

167.79996	0.08503

167.89896	0.08506

167.99796	0.08511

168.09695	0.08513

168.19595	0.08531

168.29495	0.08533

168.39395	0.08548

168.49294	0.08551

168.59194	0.0856

168.69094	0.08563

168.78993	0.08567

168.88893	0.08572

168.98793	0.08575

169.08692	0.08577

169.18592	0.08578

169.28492	0.0858

169.38392	0.08582

169.48291	0.08583

169.58191	0.08585

169.68091	0.08587

169.7799	0.08612

169.8789	0.08632

169.9779	0.08635

170.07689	0.0864

170.17589	0.08642

170.27489	0.08655

170.37389	0.08659

170.47288	0.0866

170.57188	0.08662

170.67088	0.08664

170.76987	0.08679

170.86887	0.08691

170.96787	0.08697

171.06686	0.08699

171.16586	0.08706

171.26486	0.08713

171.36386	0.08714

171.46285	0.08719

171.56185	0.08721

171.66085	0.08723

171.75984	0.08731

171.85884	0.08733

171.95784	0.08738

172.05684	0.08746

172.15583	0.08751

172.25483	0.08754

172.35383	0.08758

172.45282	0.08775

172.55182	0.08785

172.65082	0.08786

172.74981	0.08788

172.84881	0.0879

172.94781	0.08798

173.04681	0.08812

173.1458	0.08813

173.2448	0.08817

173.3438	0.08835

173.44279	0.08837

173.54179	0.08845

173.64079	0.08847

173.73978	0.08853

173.83878	0.08862

173.93778	0.08869

174.03678	0.0887

174.13577	0.08872

174.23477	0.08874

174.33377	0.08887

174.43276	0.0889

174.53176	0.08892

174.63076	0.08894

174.72975	0.08897

174.82875	0.089

174.92775	0.08904

175.02675	0.08905

175.12574	0.08907

175.22474	0.08922

175.32374	0.08926

175.42273	0.08931

175.52173	0.08934

175.62073	0.08937

175.71973	0.08939

175.81872	0.08942

175.91772	0.08944

176.01672	0.08946

176.11571	0.08949

176.21471	0.08958

176.31371	0.08959

176.4127	0.08963

176.5117	0.08968

176.6107	0.08973

176.7097	0.08978

176.80869	0.08979

176.90769	0.08981

177.00669	0.08984

177.10568	0.08993

177.20468	0.08999

177.30368	0.09001

177.40267	0.09003

177.50167	0.09013

177.60067	0.09018

177.69967	0.09021

177.79866	0.09026

177.89766	0.09031

177.99666	0.09035

178.09565	0.09036

178.19465	0.09063

178.29365	0.09065

178.39264	0.0907

178.49164	0.09073

178.59064	0.09075

178.68964	0.09085

178.78863	0.09087

178.88763	0.0909

178.98663	0.09093

179.08562	0.09095

179.18462	0.09097

179.28362	0.091

179.38262	0.09103

179.48161	0.09109

179.58061	0.0911

179.67961	0.09115

179.7786	0.09119

179.8776	0.09122

179.9766	0.09132

180.07559	0.09135

180.17459	0.09137

180.27359	0.0914

180.37259	0.09144

180.47158	0.09145

180.57058	0.09157

180.66958	0.09162

180.76857	0.09164

180.86757	0.09166

180.96657	0.09172

181.06556	0.09176

181.16456	0.09186

181.26356	0.09189

181.36256	0.09192

181.46155	0.09203

181.56055	0.09206

181.65955	0.09209

181.75854	0.09224

181.85754	0.09226

181.95654	0.09228

182.05553	0.09231

182.15453	0.09239

182.25353	0.09241

182.35253	0.09249

182.45152	0.09251

182.55052	0.09263

182.64952	0.09275

182.74851	0.09276

182.84751	0.09283

182.94651	0.0929

183.04551	0.09291

183.1445	0.09293

183.2435	0.09295

183.3425	0.09296

183.44149	0.093

183.54049	0.09307

183.63949	0.09337

183.73848	0.0934

183.83748	0.09342

183.93648	0.09343

184.03548	0.09347

184.13447	0.09365

184.23347	0.09369

184.33247	0.09375

184.43146	0.09379

184.53046	0.0938

184.62946	0.09384

184.72845	0.09389

184.82745	0.0939

184.92645	0.09392

185.02545	0.09394

185.12444	0.09397

185.22344	0.09399

185.32244	0.09402

185.42143	0.09406

185.52043	0.09411

185.61943	0.09412

185.71843	0.09414

185.81742	0.09416

185.91642	0.09424

186.01542	0.09427

186.11441	0.09429

186.21341	0.09436

186.31241	0.09439

186.4114	0.09446

186.5104	0.09449

186.6094	0.09473

186.7084	0.09478

186.80739	0.09479

186.90639	0.09483

187.00539	0.09484

187.10438	0.09488

187.20338	0.09489

187.30238	0.09505

187.40137	0.09506

187.50037	0.09508

187.59937	0.09521

187.69837	0.09523

187.79736	0.09525

187.89636	0.09526

187.99536	0.09528

188.09435	0.09533

188.19335	0.09545

188.29235	0.09548

188.39134	0.0955

188.49034	0.09553

188.58934	0.0956

188.68834	0.09563

188.78733	0.09565

188.88633	0.09577

188.98533	0.09578

189.08432	0.0958

189.18332	0.09582

189.28232	0.09597

189.38132	0.09599

189.48031	0.09635

189.57931	0.09637

189.67831	0.09652

189.7773	0.09654

189.8763	0.09656

189.9753	0.09659

190.07429	0.09661

190.17329	0.09664

190.27229	0.09667

190.37129	0.09669

190.47028	0.09671

190.56928	0.09674

190.66828	0.09682

190.76727	0.09684

190.86627	0.09687

190.96527	0.09691

191.06426	0.09694

191.16326	0.09696

191.26226	0.09706

191.36126	0.09711

191.46025	0.09714

191.55925	0.09718

191.65825	0.09721

191.75724	0.09748

191.85624	0.0975

191.95524	0.09786

192.05423	0.09788

192.15323	0.09792

192.25223	0.09795

192.35123	0.09797

192.45022	0.09798

192.54922	0.098

192.64822	0.09803

192.74721	0.09812

192.84621	0.0984

192.94521	0.09844

193.04421	0.09852

193.1432	0.09889

193.2422	0.09892

193.3412	0.09897

193.44019	0.09911

193.53919	0.09914

193.63819	0.09919

193.73718	0.09921

193.83618	0.09924

193.93518	0.09926

194.03418	0.09927

194.13317	0.09931

194.23217	0.09934

194.33117	0.09936

194.43016	0.09954

194.52916	0.09956

194.62816	0.09961

194.72715	0.09963

194.82615	0.09966

194.92515	0.09973

195.02415	0.09976

195.12314	0.09979

195.22214	0.09981

195.32114	0.10001

195.42013	0.10003

195.51913	0.10005

195.61813	0.10006

195.71712	0.10013

195.81612	0.10015

195.91512	0.10021

196.01412	0.10042

196.11311	0.10043

196.21211	0.10048

196.31111	0.1005

196.4101	0.10053

196.5091	0.10063

196.6081	0.10065

196.7071	0.1007

196.80609	0.10072

196.90509	0.10075

197.00409	0.1008

197.10308	0.10082

197.20208	0.10083

197.30108	0.10085

197.40007	0.10087

197.49907	0.10089

197.59807	0.10092

197.69707	0.10105

197.79606	0.10107

197.89506	0.10109

197.99406	0.10119

198.09305	0.1013

198.19205	0.10137

198.29105	0.10152

198.39004	0.10157

198.48904	0.10161

198.58804	0.10162

198.68704	0.10166

198.78603	0.10176

198.88503	0.10177

198.98403	0.10179

199.08302	0.10184

199.18202	0.10193

199.28102	0.10206

199.38001	0.10221

199.47901	0.10223

199.57801	0.10224

199.67701	0.10226

199.776	0.10228

199.875	0.10229

199.974	0.10231

200.07299	0.10233

200.17199	0.10235

200.27099	0.10236

200.36999	0.10238

200.46898	0.1024

200.56798	0.10255

200.66698	0.10266

200.76597	0.10271

200.86497	0.10275

200.96397	0.1028

201.06296	0.1029

201.16196	0.10293

201.26096	0.10295

201.35996	0.10297

201.45895	0.10298

201.55795	0.103

201.65695	0.10303

201.75594	0.10318

201.85494	0.1032

201.95394	0.10323

202.05293	0.10325

202.15193	0.1033

202.25093	0.10334

202.34993	0.10339

202.44892	0.10342

202.54792	0.10345

202.64692	0.10347

202.74591	0.10354

202.84491	0.10357

202.94391	0.10365

203.0429	0.10367

203.1419	0.10369

203.2409	0.1037

203.3399	0.10374

203.43889	0.10387

203.53789	0.10396

203.63689	0.10397

203.73588	0.10402

203.83488	0.10404

203.93388	0.10406

204.03288	0.10412

204.13187	0.10438

204.23087	0.10441

204.32987	0.10444

204.42886	0.10451

204.52786	0.10453

204.62686	0.10474

204.72585	0.10476

204.82485	0.10478

204.92385	0.1048

205.02285	0.10483

205.12184	0.10486

205.22084	0.10491

205.31984	0.10493

205.41883	0.10495

205.51783	0.105

205.61683	0.10501

205.71582	0.10513

205.81482	0.10516

205.91382	0.10518

206.01282	0.1052

206.11181	0.10521

206.21081	0.10523

206.30981	0.10525

206.4088	0.10528

206.5078	0.10532

206.6068	0.10533

206.70579	0.10535

206.80479	0.10543

206.90379	0.10548

207.00279	0.10552

207.10178	0.10553

207.20078	0.10557

207.29978	0.10558

207.39877	0.1056

207.49777	0.10562

207.59677	0.10579

207.69577	0.10589

207.79476	0.10595

207.89376	0.10599

207.99276	0.106

208.09175	0.10607

208.19075	0.10614

208.28975	0.10617

208.38874	0.10622

208.48774	0.10624

208.58674	0.10631

208.68574	0.10632

208.78473	0.10646

208.88373	0.10649

208.98273	0.10651

209.08172	0.10656

209.18072	0.10661

209.27972	0.10662

209.37871	0.10666

209.47771	0.10669

209.57671	0.10671

209.67571	0.10672

209.7747	0.10674

209.8737	0.10676

209.9727	0.10725

210.07169	0.10731

210.17069	0.10735

210.26969	0.10745

210.36869	0.10748

210.46768	0.1075

210.56668	0.10751

210.66568	0.10756

210.76467	0.1076

210.86367	0.10785

210.96267	0.10787

211.06166	0.10803

211.16066	0.10808

211.25966	0.10812

211.35866	0.10813

211.45765	0.10815

211.55665	0.10817

211.65565	0.10818

211.75464	0.10825

211.85364	0.1084

211.95264	0.10849

212.05163	0.10852

212.15063	0.10854

212.24963	0.10855

212.34863	0.10855

212.44762	0.10856

212.54662	0.10857

212.64562	0.10863

212.74461	0.10867

212.84361	0.10868

212.94261	0.1087

213.0416	0.10876

213.1406	0.10877

213.2396	0.10878

213.3386	0.10885

213.43759	0.10887

213.53659	0.10889

213.63559	0.10893

213.73458	0.10897

213.83358	0.10907

213.93258	0.1091

214.03158	0.10919

214.13057	0.10922

214.22957	0.10923

214.32857	0.10924

214.42756	0.10928

214.52656	0.10928

214.62556	0.10929

214.72455	0.1093

214.82355	0.10932

214.92255	0.10936

215.02155	0.10939

215.12054	0.10941

215.21954	0.10946

215.31854	0.10947

215.41753	0.1095

215.51653	0.10957

215.61553	0.10958

215.71452	0.10959

215.81352	0.1096

215.91252	0.10964

216.01152	0.10972

216.11051	0.10973

216.20951	0.10976

216.30851	0.10978

216.4075	0.10979

216.5065	0.1098

216.6055	0.10994

216.70449	0.10996

216.80349	0.10997

216.90249	0.10998

217.00149	0.11003

217.10048	0.11004

217.19948	0.11007

217.29848	0.11009

217.39747	0.11011

217.49647	0.11012

217.59547	0.11013

217.69447	0.11016

217.79346	0.11017

217.89246	0.11019

217.99146	0.11021

218.09045	0.11022

218.18945	0.11022

218.28845	0.11025

218.38744	0.11032

218.48644	0.11033

218.58544	0.11034

218.68444	0.1104

218.78343	0.11043

218.88243	0.11044

218.98143	0.11045

219.08042	0.11046

219.17942	0.11047

219.27842	0.11048

219.37741	0.11048

219.47641	0.11053

219.57541	0.11054

219.67441	0.11056

219.7734	0.11058

219.8724	0.11059

219.9714	0.11063

220.07039	0.11068

220.16939	0.11069

220.26839	0.11081

220.36738	0.11083

220.46638	0.11085

220.56538	0.11086

220.66438	0.11087

220.76337	0.1109

220.86237	0.11096

220.96137	0.11097

221.06036	0.111

221.15936	0.11106

221.25836	0.1111

221.35736	0.11113

221.45635	0.11117

221.55535	0.11121

221.65435	0.11123

221.75334	0.11125

221.85234	0.11127

221.95134	0.11129

222.05033	0.1113

222.14933	0.11132

222.24833	0.11141

222.34733	0.11142

222.44632	0.11144

222.54532	0.11147

222.64432	0.11148

222.74331	0.11149

222.84231	0.11157

222.94131	0.11158

223.0403	0.11159

223.1393	0.1116

223.2383	0.11161

223.3373	0.11162

223.43629	0.11162

223.53529	0.11163

223.63429	0.11164

223.73328	0.11167

223.83228	0.11168

223.93128	0.11169

224.03027	0.11172

224.12927	0.11179

224.22827	0.1118

224.32727	0.11181

224.42626	0.11187

224.52526	0.11188

224.62426	0.11188

224.72325	0.1119

224.82225	0.11191

224.92125	0.11192

225.02025	0.11197

225.11924	0.11198

225.21824	0.11203

225.31724	0.11204

225.41623	0.11215

225.51523	0.11217

225.61423	0.1122

225.71322	0.11221

225.81222	0.11222

225.91122	0.11223

226.01022	0.11225

226.10921	0.11225

226.20821	0.11226

226.30721	0.11227

226.4062	0.11229

226.5052	0.1123

226.6042	0.1123

226.70319	0.11236

226.80219	0.11237

226.90119	0.11246

227.00019	0.11247

227.09918	0.11256

227.19818	0.11256

227.29718	0.1126

227.39617	0.11262

227.49517	0.11263

227.59417	0.11266

227.69316	0.11267

227.79216	0.11268

227.89116	0.11269

227.99016	0.1127

228.08915	0.11273

228.18815	0.11276

228.28715	0.11277

228.38614	0.11277

228.48514	0.11278

228.58414	0.11279

228.68314	0.1128

228.78213	0.11281

228.88113	0.11282

228.98013	0.11282

229.07912	0.11284

229.17812	0.11287

229.27712	0.11293

229.37611	0.11297

229.47511	0.11304

229.57411	0.11307

229.67311	0.11308

229.7721	0.11308

229.8711	0.11309

229.9701	0.1131

230.06909	0.11311

230.16809	0.11329

230.26709	0.11334

230.36608	0.11335

230.46508	0.11337

230.56408	0.11339

230.66308	0.1134

230.76207	0.1134

230.86107	0.11341

230.96007	0.11346

231.05906	0.11347

231.15806	0.11348

231.25706	0.11349

231.35606	0.1135

231.45505	0.11354

231.55405	0.11357

231.65305	0.11363

231.75204	0.11367

231.85104	0.11368

231.95004	0.11369

232.04903	0.1137

232.14803	0.11377

232.24703	0.1138

232.34603	0.11382

232.44502	0.11383

232.54402	0.11384

232.64302	0.11385

232.74201	0.11386

232.84101	0.1139

232.94001	0.11391

233.039	0.11407

233.138	0.11407

233.237	0.11413

233.336	0.11414

233.43499	0.11415

233.53399	0.11421

233.63299	0.11422

233.73198	0.11423

233.83098	0.11425

233.92998	0.11441

234.02897	0.11442

234.12797	0.11443

234.22697	0.11446

234.32597	0.11448

234.42496	0.11461

234.52396	0.11464

234.62296	0.11465

234.72195	0.11465

234.82095	0.11469

234.91995	0.1147

235.01895	0.11474

235.11794	0.11479

235.21694	0.11486

235.31594	0.11486

235.41493	0.11489

235.51393	0.11491

235.61293	0.11492

235.71192	0.11495

235.81092	0.11496

235.90992	0.11499

236.00892	0.11501

236.10791	0.11505

236.20691	0.11512

236.30591	0.11513

236.4049	0.11522

236.5039	0.11523

236.6029	0.11524

236.70189	0.11525

236.80089	0.1153

236.89989	0.11534

236.99889	0.11536

237.09788	0.11545

237.19688	0.11547

237.29588	0.11548

237.39487	0.11549

237.49387	0.11553

237.59287	0.11553

237.69186	0.1156

237.79086	0.11566

237.88986	0.11568

237.98886	0.11569

238.08785	0.11576

238.18685	0.11577

238.28585	0.11579

238.38484	0.1158

238.48384	0.11586

238.58284	0.11587

238.68184	0.11588

238.78083	0.11589

238.87983	0.11595

238.97883	0.11598

239.07782	0.11599

239.17682	0.116

239.27582	0.11601

239.37481	0.11603

239.47381	0.11604

239.57281	0.11617

239.67181	0.11618

239.7708	0.11621

239.8698	0.11622

239.9688	0.11626

240.06779	0.11631

240.16679	0.11631

240.26579	0.11633

240.36478	0.11634

240.46378	0.11636

240.56278	0.11637

240.66178	0.11638

240.76077	0.11647

240.85977	0.11649

240.95877	0.11658

241.05776	0.1166

241.15676	0.11668

241.25576	0.1167

241.35475	0.11673

241.45375	0.11674

241.55275	0.11678

241.65175	0.11678

241.75074	0.1168

241.84974	0.11681

241.94874	0.11682

242.04773	0.11683

242.14673	0.11684

242.24573	0.11689

242.34473	0.11691

242.44372	0.11699

242.54272	0.11701

242.64172	0.11703

242.74071	0.11716

242.83971	0.11717

242.93871	0.11718

243.0377	0.11725

243.1367	0.11728

243.2357	0.11737

243.3347	0.11739

243.43369	0.11741

243.53269	0.11742

243.63169	0.11746

243.73068	0.11747

243.82968	0.11748

243.92868	0.1175

244.02767	0.11753

244.12667	0.11757

244.22567	0.11758

244.32467	0.1176

244.42366	0.11765

244.52266	0.11766

244.62166	0.1177

244.72065	0.11772

244.81965	0.11783

244.91865	0.11783

245.01764	0.11784

245.11664	0.11785

245.21564	0.11788

245.31464	0.11788

245.41363	0.11796

245.51263	0.11797

245.61163	0.11798

245.71062	0.11803

245.80962	0.11805

245.90862	0.11806

246.00762	0.11823

246.10661	0.11825

246.20561	0.11832

246.30461	0.11834

246.4036	0.11835

246.5026	0.11839

246.6016	0.1184

246.70059	0.1184

246.79959	0.11841

246.89859	0.11842

246.99759	0.11844

247.09658	0.11848

247.19558	0.1185

247.29458	0.11853

247.39357	0.11855

247.49257	0.11856

247.59157	0.11857

247.69056	0.11859

247.78956	0.11871

247.88856	0.11871

247.98756	0.11872

248.08655	0.11876

248.18555	0.11879

248.28455	0.11881

248.38354	0.11882

248.48254	0.11892

248.58154	0.11893

248.68053	0.11896

248.77953	0.11897

248.87853	0.11898

248.97753	0.11899

249.07652	0.11903

249.17552	0.11903

249.27452	0.11908

249.37351	0.1191

249.47251	0.11915

249.57151	0.11916

249.67051	0.11917

249.7695	0.11918

249.8685	0.11919

249.9675	0.11921

250.06649	0.11922

250.16549	0.11931

250.26449	0.11933

250.36348	0.11944

250.46248	0.11949

250.56148	0.1195

250.66048	0.11963

250.75947	0.11966

250.85847	0.11971

250.95747	0.11972

251.05646	0.11976

251.15546	0.11979

251.25446	0.11981

251.35345	0.11981

251.45245	0.11982

251.55145	0.11985

251.65045	0.11986

251.74944	0.11986

251.84844	0.11991

251.94744	0.11996

252.04643	0.11998

252.14543	0.11999

252.24443	0.12

252.34343	0.12001

252.44242	0.12002

252.54142	0.12005

252.64042	0.12006

252.73941	0.12007

252.83841	0.1201

252.93741	0.12011

253.0364	0.12012

253.1354	0.12014

253.2344	0.12019

253.3334	0.12021

253.43239	0.12024

253.53139	0.12025

253.63039	0.12026

253.72938	0.1203

253.82838	0.12031

253.92738	0.12043

254.02637	0.12043

254.12537	0.12045

254.22437	0.12046

254.32337	0.12049

254.42236	0.12054

254.52136	0.12054

254.62036	0.12056

254.71935	0.12064

254.81835	0.12075

254.91735	0.12077

255.01634	0.1208

255.11534	0.12083

255.21434	0.12095

255.31334	0.12097

255.41233	0.12099

255.51133	0.12101

255.61033	0.12103

255.70932	0.12104

255.80832	0.12108

255.90732	0.12115

256.00631	0.12119

256.10531	0.1212

256.20431	0.12129

256.30331	0.12131

256.4023	0.12132

256.5013	0.12132

256.6003	0.12134

256.69929	0.12137

256.79829	0.12137

256.89729	0.1214

256.99629	0.12145

257.09528	0.12147

257.19428	0.12148

257.29328	0.12151

257.39227	0.12153

257.49127	0.12154

257.59027	0.12155

257.68926	0.12158

257.78826	0.12168

257.88726	0.12178

257.98626	0.12179

258.08525	0.12181

258.18425	0.12185

258.28325	0.12186

258.38224	0.12189

258.48124	0.1219

258.58024	0.12191

258.67923	0.12192

258.77823	0.12199

258.87723	0.122

258.97623	0.12202

259.07522	0.12203

259.17422	0.12204

259.27322	0.12205

259.37221	0.12212

259.47121	0.12213

259.57021	0.12214

259.66921	0.12215

259.7682	0.12217

259.8672	0.12219

259.9662	0.1222

260.06519	0.12222

260.16419	0.12223

260.26319	0.12233

260.36218	0.12234

260.46118	0.12236

260.56018	0.12236

260.65918	0.12237

260.75817	0.12238

260.85717	0.12247

260.95617	0.12247

261.05516	0.12249

261.15416	0.12251

261.25316	0.12255

261.35215	0.12256

261.45115	0.12258

261.55015	0.12267

261.64915	0.12269

261.74814	0.1227

261.84714	0.12283

261.94614	0.12284

262.04513	0.12285

262.14413	0.12286

262.24313	0.12287

262.34212	0.12295

262.44112	0.12296

262.54012	0.12299

262.63912	0.12301

262.73811	0.12302

262.83711	0.12303

262.93611	0.12307

263.0351	0.1231

263.1341	0.12311

263.2331	0.12313

263.3321	0.12314

263.43109	0.1232

263.53009	0.1232

263.62909	0.12324

263.72808	0.12325

263.82708	0.12325

263.92608	0.12326

264.02507	0.12328

264.12407	0.12337

264.22307	0.12339

264.32207	0.1234

264.42106	0.1234

264.52006	0.12341

264.61906	0.12351

264.71805	0.12353

264.81705	0.12354

264.91605	0.12355

265.01504	0.12357

265.11404	0.12361

265.21304	0.12366

265.31204	0.12369

265.41103	0.12371

265.51003	0.12372

265.60903	0.12376

265.70802	0.12381

265.80702	0.12386

265.90602	0.12391

266.00501	0.12392

266.10401	0.12393

266.20301	0.12395

266.30201	0.12405

266.401	0.12406

266.5	0.12408

266.599	0.12408

266.69799	0.12409

266.79699	0.12415

266.89599	0.12418

266.99499	0.12419

267.09398	0.1242

267.19298	0.12421

267.29198	0.12424

267.39097	0.1243

267.48997	0.12432

267.58897	0.12433

267.68796	0.12443

267.78696	0.12444

267.88596	0.12454

267.98496	0.12457

268.08395	0.12458

268.18295	0.12459

268.28195	0.12462

268.38094	0.12465

268.47994	0.12467

268.57894	0.1247

268.67793	0.12471

268.77693	0.12472

268.87593	0.12473

268.97493	0.12474

269.07392	0.12476

269.17292	0.12481

269.27192	0.12484

269.37091	0.12486

269.46991	0.12486

269.56891	0.12487

269.6679	0.12499

269.7669	0.125

269.8659	0.12504

269.9649	0.12507

270.06389	0.1251

270.16289	0.12511

270.26189	0.12512

270.36088	0.12526

270.45988	0.12527

270.55888	0.12532

270.65788	0.12533

270.75687	0.12533

270.85587	0.12542

270.95487	0.12544

271.05386	0.12545

271.15286	0.12553

271.25186	0.12559

271.35085	0.1256

271.44985	0.12565

271.54885	0.12591

271.64785	0.12596

271.74684	0.12598

271.84584	0.12604

271.94484	0.12606

272.04383	0.12606

272.14283	0.12624

272.24183	0.12629

272.34082	0.12635

272.43982	0.12637

272.53882	0.12638

272.63782	0.12657

272.73681	0.12662

272.83581	0.12667

272.93481	0.12668

273.0338	0.12669

273.1328	0.12672

273.2318	0.12675

273.33079	0.12676

273.42979	0.12681

273.52879	0.12682

273.62779	0.12687

273.72678	0.12698

273.82578	0.127

273.92478	0.12701

274.02377	0.12709

274.12277	0.12712

274.22177	0.12742

274.32077	0.12744

274.41976	0.12745

274.51876	0.12747

274.61776	0.12751

274.71675	0.12756

274.81575	0.12757

274.91475	0.1276

275.01374	0.12762

275.11274	0.12763

275.21174	0.12763

275.31074	0.12765

275.40973	0.12767

275.50873	0.12778

275.60773	0.12779

275.70672	0.12783

275.80572	0.12784

275.90472	0.12787

276.00371	0.1279

276.10271	0.12791

276.20171	0.12792

276.30071	0.12794

276.3997	0.12804

276.4987	0.12805

276.5977	0.12807

276.69669	0.12811

276.79569	0.12812

276.89469	0.12813

276.99369	0.12814

277.09268	0.12821

277.19168	0.12822

277.29068	0.12836

277.38967	0.12849

277.48867	0.1285

277.58767	0.12851

277.68666	0.12856

277.78566	0.1286

277.88466	0.12874

277.98366	0.12878

278.08265	0.12887

278.18165	0.12888

278.28065	0.12888

278.37964	0.12889

278.47864	0.12895

278.57764	0.12896

278.67663	0.12899

278.77563	0.12907

278.87463	0.12908

278.97363	0.12915

279.07262	0.12916

279.17162	0.12918

279.27062	0.12919

279.36961	0.12923

279.46861	0.12924

279.56761	0.12925

279.6666	0.12935

279.7656	0.12939

279.8646	0.1294

279.9636	0.12942

280.06259	0.12944

280.16159	0.12945

280.26059	0.12946

280.35958	0.12949

280.45858	0.12953

280.55758	0.12957

280.65657	0.12958

280.75557	0.12959

280.85457	0.12961

280.95357	0.12961

281.05256	0.12962

281.15156	0.12974

281.25056	0.12975

281.34955	0.12976

281.44855	0.12978

281.54755	0.12983

281.64655	0.1299

281.74554	0.12993

281.84454	0.12994

281.94354	0.12996

282.04253	0.12997

282.14153	0.12997

282.24053	0.13004

282.33952	0.13005

282.43852	0.13012

282.53752	0.13015

282.63652	0.13023

282.73551	0.13027

282.83451	0.13028

282.93351	0.1303

283.0325	0.13039

283.1315	0.13041

283.2305	0.13042

283.32949	0.13046

283.42849	0.13049

283.52749	0.13051

283.62649	0.13054

283.72548	0.13057

283.82448	0.13059

283.92348	0.1306

284.02247	0.13066

284.12147	0.13068

284.22047	0.1307

284.31947	0.13073

284.41846	0.13074

284.51746	0.1308

284.61646	0.13093

284.71545	0.13095

284.81445	0.13105

284.91345	0.13106

285.01244	0.13107

285.11144	0.13108

285.21044	0.13112

285.30944	0.13114

285.40843	0.13117

285.50743	0.13119

285.60643	0.13139

285.70542	0.13146

285.80442	0.13147

285.90342	0.13158

286.00241	0.1316

286.10141	0.13161

286.20041	0.13177

286.29941	0.13179

286.3984	0.1318

286.4974	0.1318

286.5964	0.1319

286.69539	0.13194

286.79439	0.13198

286.89339	0.132

286.99238	0.13202

287.09138	0.13203

287.19038	0.13211

287.28938	0.13212

287.38837	0.13215

287.48737	0.13223

287.58637	0.13224

287.68536	0.13234

287.78436	0.13237

287.88336	0.13238

287.98236	0.13248

288.08135	0.13249

288.18035	0.13272

288.27935	0.13284

288.37834	0.13292

288.47734	0.13293

288.57634	0.13299

288.67533	0.133

288.77433	0.13302

288.87333	0.13308

288.97233	0.13312

289.07132	0.13314

289.17032	0.13315

289.26932	0.13335

289.36831	0.13336

289.46731	0.13345

289.56631	0.13346

289.6653	0.13346

289.7643	0.1335

289.8633	0.13361

289.9623	0.13362

290.06129	0.13363

290.16029	0.13367

290.25929	0.13367

290.35828	0.13368

290.45728	0.13369

290.55628	0.13375

290.65527	0.1338

290.75427	0.13381

290.85327	0.13382

290.95227	0.13386

291.05126	0.1339

291.15026	0.13393

291.24926	0.13394

291.34825	0.13398

291.44725	0.13409

291.54625	0.1341

291.64525	0.13425

291.74424	0.13429

291.84324	0.13433

291.94224	0.13435

292.04123	0.13448

292.14023	0.13466

292.23923	0.13467

292.33822	0.13468

292.43722	0.13469

292.53622	0.13477

292.63522	0.13478

292.73421	0.13479

292.83321	0.1348

292.93221	0.13481

293.0312	0.13488

293.1302	0.13489

293.2292	0.1349

293.32819	0.13508

293.42719	0.13519

293.52619	0.13532

293.62519	0.13533

293.72418	0.13537

293.82318	0.13538

293.92218	0.13543

294.02117	0.13544

294.12017	0.13565

294.21917	0.13575

294.31816	0.13576

294.41716	0.13581

294.51616	0.13586

294.61516	0.13589

294.71415	0.13591

294.81315	0.13596

294.91215	0.13612

295.01114	0.13613

295.11014	0.13617

295.20914	0.13628

295.30814	0.13629

295.40713	0.1363

295.50613	0.13633

295.60513	0.13641

295.70412	0.13669

295.80312	0.13672

295.90212	0.13675

296.00111	0.13677

296.10011	0.1368

296.19911	0.13681

296.29811	0.13688

296.3971	0.13689

296.4961	0.13695

296.5951	0.13696

296.69409	0.13704

296.79309	0.13705

296.89209	0.13706

296.99108	0.13707

297.09008	0.13709

297.18908	0.13711

297.28808	0.13712

297.38707	0.13732

297.48607	0.13751

297.58507	0.13753

297.68406	0.13753

297.78306	0.13756

297.88206	0.13768

297.98106	0.13783

298.08005	0.13787

298.17905	0.13799

298.27805	0.13802

298.37704	0.13809

298.47604	0.13812

298.57504	0.13814

298.67403	0.13828

298.77303	0.13834

298.87203	0.13836

298.97103	0.13847

299.07002	0.13847

299.16902	0.13848

299.26802	0.13849

299.36701	0.13852

299.46601	0.1386

299.56501	0.13865

299.664	0.13866

299.763	0.13868

299.862	0.13871

299.961	0.13876

300.05999	0.13882

300.15899	0.13884

300.25799	0.13886

300.35698	0.13899

300.45598	0.139

300.55498	0.13908

300.65397	0.13909

300.75297	0.1391

300.85197	0.13928

300.95097	0.13929

301.04996	0.13939

301.14896	0.13941

301.24796	0.13942

301.34695	0.13943

301.44595	0.13948

301.54495	0.1395

301.64394	0.13951

301.74294	0.13952

301.84194	0.13954

301.94094	0.13961

302.03993	0.13975

302.13893	0.13984

302.23793	0.14002

302.33692	0.14004

302.43592	0.14005

302.53492	0.14031

302.63392	0.14034

302.73291	0.14036

302.83191	0.14042

302.93091	0.14043

303.0299	0.14051

303.1289	0.14052

303.2279	0.14053

303.32689	0.14057

303.42589	0.14073

303.52489	0.14078

303.62389	0.1408

303.72288	0.14081

303.82188	0.14082

303.92088	0.14083

304.01987	0.14087

304.11887	0.14087

304.21787	0.14088

304.31686	0.14095

304.41586	0.14096

304.51486	0.14097

304.61386	0.14098

304.71285	0.14112

304.81185	0.14116

304.91085	0.14117

305.00984	0.14124

305.10884	0.14132

305.20784	0.14133

305.30684	0.14133

305.40583	0.14159

305.50483	0.1416

305.60383	0.14186

305.70282	0.14186

305.80182	0.14189

305.90082	0.142

305.99981	0.14201

306.09881	0.14201

306.19781	0.14203

306.29681	0.14214

306.3958	0.14216

306.4948	0.14239

306.5938	0.1424

306.69279	0.14241

306.79179	0.14243

306.89079	0.14247

306.98978	0.14248

307.08878	0.1425

307.18778	0.14264

307.28678	0.14267

307.38577	0.14277

307.48477	0.14278

307.58377	0.14283

307.68276	0.1429

307.78176	0.14294

307.88076	0.14295

307.97975	0.14302

308.07875	0.14311

308.17775	0.14312

308.27675	0.14315

308.37574	0.14316

308.47474	0.14319

308.57374	0.14321

308.67273	0.14347

308.77173	0.1435

308.87073	0.14351

308.96973	0.14356

309.06872	0.14362

309.16772	0.14366

309.26672	0.14368

309.36571	0.14378

309.46471	0.14378

309.56371	0.14408

309.6627	0.1441

309.7617	0.14411

309.8607	0.14413

309.9597	0.1443

310.05869	0.14431

310.15769	0.14447

310.25669	0.14461

310.35568	0.14462

310.45468	0.14463

310.55368	0.14467

310.65267	0.14468

310.75167	0.14483

310.85067	0.14484

310.94967	0.14488

311.04866	0.14488

311.14766	0.14493

311.24666	0.145

311.34565	0.14504

311.44465	0.14514

311.54365	0.14515

311.64264	0.14516

311.74164	0.14521

311.84064	0.14524

311.93964	0.1455

312.03863	0.14551

312.13763	0.14561

312.23663	0.14564

312.33562	0.14576

312.43462	0.1458

312.53362	0.14594

312.63262	0.14603

312.73161	0.14613

312.83061	0.14617

312.92961	0.14619

313.0286	0.1462

313.1276	0.14627

313.2266	0.14639

313.32559	0.14639

313.42459	0.14646

313.52359	0.14648

313.62259	0.14649

313.72158	0.14677

313.82058	0.14678

313.91958	0.14679

314.01857	0.1468

314.11757	0.14683

314.21657	0.14686

314.31556	0.14694

314.41456	0.14695

314.51356	0.14717

314.61256	0.14719

314.71155	0.14729

314.81055	0.14731

314.90955	0.14732

315.00854	0.14737

315.10754	0.1474

315.20654	0.14741

315.30553	0.14742

315.40453	0.14746

315.50353	0.14752

315.60253	0.14763

315.70152	0.14769

315.80052	0.1477

315.89952	0.14774

315.99851	0.14785

316.09751	0.14785

316.19651	0.14789

316.29551	0.1479

316.3945	0.14791

316.4935	0.14792

316.5925	0.14809

316.69149	0.1481

316.79049	0.14811

316.88949	0.14812

316.98848	0.14817

317.08748	0.14824

317.18648	0.14825

317.28548	0.14827

317.38447	0.14835

317.48347	0.14837

317.58247	0.14837

317.68146	0.14847

317.78046	0.14862

317.87946	0.14864

317.97845	0.14865

318.07745	0.14869

318.17645	0.1487

318.27545	0.14874

318.37444	0.14876

318.47344	0.14877

318.57244	0.14879

318.67143	0.14881

318.77043	0.1491

318.86943	0.14911

318.96843	0.14912

319.06742	0.14923

319.16642	0.14926

319.26542	0.14927

319.36441	0.14939

319.46341	0.14943

319.56241	0.14965

319.6614	0.14966

319.7604	0.14967

319.8594	0.14973

319.9584	0.14977

320.05739	0.14978

320.15639	0.14993

320.25539	0.14995

320.35438	0.14996

320.45338	0.15002

320.55238	0.15011

320.65137	0.15013

320.75037	0.15052

320.84937	0.15072

320.94837	0.15074

321.04736	0.15075

321.14636	0.15078

321.24536	0.15079

321.34435	0.15102

321.44335	0.15103

321.54235	0.15127

321.64134	0.15129

321.74034	0.15131

321.83934	0.15137

321.93834	0.15139

322.03733	0.15142

322.13633	0.15144

322.23533	0.15157

322.33432	0.15163

322.43332	0.15174

322.53232	0.15175

322.63131	0.15183

322.73031	0.15186

322.82931	0.1519

322.92831	0.15202

323.0273	0.1521

323.1263	0.15212

323.2253	0.15215

323.32429	0.15226

323.42329	0.15227

323.52229	0.15228

323.62129	0.15245

323.72028	0.15246

323.81928	0.15247

323.91828	0.1525

324.01727	0.15263

324.11627	0.15264

324.21527	0.15271

324.31426	0.15275

324.41326	0.15276

324.51226	0.15287

324.61126	0.15296

324.71025	0.15301

324.80925	0.15304

324.90825	0.15305

325.00724	0.15313

325.10624	0.15317

325.20524	0.15335

325.30423	0.15337

325.40323	0.15338

325.50223	0.15348

325.60123	0.15349

325.70022	0.1535

325.79922	0.15351

325.89822	0.15354

325.99721	0.15359

326.09621	0.15364

326.19521	0.15371

326.29421	0.15374

326.3932	0.15377

326.4922	0.15382

326.5912	0.15383

326.69019	0.15405

326.78919	0.15405

326.88819	0.1543

326.98718	0.15431

327.08618	0.15437

327.18518	0.15438

327.28418	0.15439

327.38317	0.15447

327.48217	0.1545

327.58117	0.15451

327.68016	0.15478

327.77916	0.15483

327.87816	0.1549

327.97715	0.15491

328.07615	0.15498

328.17515	0.15499

328.27415	0.15499

328.37314	0.15513

328.47214	0.15517

328.57114	0.1552

328.67013	0.1552

328.76913	0.15524

328.86813	0.15538

328.96712	0.1554

329.06612	0.15577

329.16512	0.15584

329.26412	0.15588

329.36311	0.15588

329.46211	0.15589

329.56111	0.15598

329.6601	0.15599

329.7591	0.15603

329.8581	0.15613

329.9571	0.15614

330.05609	0.15615

330.15509	0.15616

330.25409	0.15619

330.35308	0.15626

330.45208	0.15627

330.55108	0.15632

330.65007	0.15653

330.74907	0.15655

330.84807	0.15657

330.94707	0.15663

331.04606	0.15664

331.14506	0.15666

331.24406	0.15679

331.34305	0.15682

331.44205	0.15684

331.54105	0.15685

331.64004	0.15702

331.73904	0.15704

331.83804	0.1571

331.93704	0.15712

332.03603	0.15713

332.13503	0.15719

332.23403	0.15722

332.33302	0.15739

332.43202	0.1574

332.53102	0.15741

332.63001	0.15757

332.72901	0.15759

332.82801	0.15765

332.92701	0.15796

333.026	0.15797

333.125	0.15816

333.224	0.15817

333.32299	0.15817

333.42199	0.15819

333.52099	0.15838

333.61999	0.15838

333.71898	0.1584

333.81798	0.15854

333.91698	0.15859

334.01597	0.15859

334.11497	0.15865

334.21397	0.1588

334.31296	0.15881

334.41196	0.15882

334.51096	0.15899

334.60996	0.159

334.70895	0.15902

334.80795	0.1591

334.90695	0.15916

335.00594	0.15917

335.10494	0.1592

335.20394	0.15932

335.30293	0.15935

335.40193	0.15936

335.50093	0.15943

335.59993	0.15946

335.69892	0.15947

335.79792	0.15949

335.89692	0.1595

335.99591	0.15976

336.09491	0.15987

336.19391	0.1599

336.2929	0.15994

336.3919	0.15995

336.4909	0.15999

336.5899	0.15999

336.68889	0.16002

336.78789	0.16024

336.88689	0.16031

336.98588	0.16048

337.08488	0.1605

337.18388	0.16052

337.28288	0.16057

337.38187	0.16059

337.48087	0.16062

337.57987	0.16068

337.67886	0.16072

337.77786	0.16075

337.87686	0.16083

337.97585	0.161

338.07485	0.16103

338.17385	0.16104

338.27285	0.16116

338.37184	0.16125

338.47084	0.16127

338.56984	0.16131

338.66883	0.16132

338.76783	0.16133

338.86683	0.16137

338.96582	0.16146

339.06482	0.16149

339.16382	0.1615

339.26282	0.16177

339.36181	0.16179

339.46081	0.16182

339.55981	0.16199

339.6588	0.162

339.7578	0.16222

339.8568	0.16223

339.95579	0.16224

340.05479	0.16224

340.15379	0.16241

340.25279	0.16264

340.35178	0.16268

340.45078	0.1627

340.54978	0.16271

340.64877	0.16278

340.74777	0.16284

340.84677	0.16285

340.94577	0.16312

341.04476	0.16313

341.14376	0.16315

341.24276	0.16318

341.34175	0.16326

341.44075	0.16328

341.53975	0.16351

341.63874	0.16354

341.73774	0.16378

341.83674	0.16379

341.93574	0.1638

342.03473	0.16385

342.13373	0.16385

342.23273	0.1639

342.33172	0.1639

342.43072	0.16398

342.52972	0.1641

342.62871	0.16411

342.72771	0.16411

342.82671	0.16416

342.92571	0.16416

343.0247	0.16422

343.1237	0.16434

343.2227	0.16435

343.32169	0.16436

343.42069	0.16443

343.51969	0.16445

343.61869	0.16446

343.71768	0.16447

343.81668	0.16489

343.91568	0.16492

344.01467	0.16495

344.11367	0.16509

344.21267	0.1651

344.31166	0.16529

344.41066	0.16532

344.50966	0.16534

344.60866	0.16535

344.70765	0.16544

344.80665	0.16547

344.90565	0.1656

345.00464	0.16561

345.10364	0.16562

345.20264	0.16584

345.30163	0.16587

345.40063	0.16588

345.49963	0.16588

345.59863	0.16609

345.69762	0.16611

345.79662	0.16625

345.89562	0.1663

345.99461	0.16632

346.09361	0.16634

346.19261	0.16646

346.2916	0.16647

346.3906	0.16676

346.4896	0.16677

346.5886	0.1668

346.68759	0.16681

346.78659	0.16687

346.88559	0.16688

346.98458	0.16688

347.08358	0.16701

347.18258	0.16703

347.28157	0.16703

347.38057	0.16732

347.47957	0.16735

347.57857	0.16742

347.67756	0.16753

347.77656	0.16754

347.87556	0.16755

347.97455	0.16757

348.07355	0.16759

348.17255	0.16763

348.27155	0.16768

348.37054	0.16787

348.46954	0.16788

348.56854	0.1679

348.66753	0.16814

348.76653	0.16815

348.86553	0.1682

348.96452	0.16833

349.06352	0.16838

349.16252	0.1684

349.26152	0.1686

349.36051	0.16863

349.45951	0.16869

349.55851	0.1687

349.6575	0.16886

349.7565	0.16899

349.8555	0.16901

349.95449	0.16901

350.05349	0.16903

350.15249	0.16906

350.25149	0.16906

350.35048	0.16909

350.44948	0.16914

350.54848	0.16917

350.64747	0.16917

350.74647	0.16919

350.84547	0.16948

350.94447	0.16949

351.04346	0.16954

351.14246	0.16971

351.24146	0.16972

351.34045	0.16974

351.43945	0.16997

351.53845	0.16999

351.63744	0.17003

351.73644	0.17023

351.83544	0.17025

351.93444	0.17029

352.03343	0.17052

352.13243	0.17052

352.23143	0.17057

352.33042	0.17071

352.42942	0.17073

352.52842	0.17074

352.62741	0.17076

352.72641	0.17084

352.82541	0.17089

352.92441	0.17129

353.0234	0.17131

353.1224	0.17133

353.2214	0.17144

353.32039	0.17146

353.41939	0.17181

353.51839	0.17183

353.61738	0.17183

353.71638	0.17184

353.81538	0.17195

353.91438	0.17197

354.01337	0.17198

354.11237	0.172

354.21137	0.17202

354.31036	0.17211

354.40936	0.17242

354.50836	0.17251

354.60736	0.17252

354.70635	0.17253

354.80535	0.17254

354.90435	0.17271

355.00334	0.17272

355.10234	0.17273

355.20134	0.17274

355.30033	0.17291

355.39933	0.17292

355.49833	0.17292

355.59733	0.17295

355.69632	0.17297

355.79532	0.17297

355.89432	0.17299

355.99331	0.17301

356.09231	0.17325

356.19131	0.17326

356.2903	0.17334

356.3893	0.17336

356.4883	0.17337

356.5873	0.17348

356.68629	0.17356

356.78529	0.17357

356.88429	0.17359

356.98328	0.17374

357.08228	0.17376

357.18128	0.17386

357.28027	0.17391

357.37927	0.17395

357.47827	0.17397

357.57727	0.17407

357.67626	0.17412

357.77526	0.1743

357.87426	0.17431

357.97325	0.17433

358.07225	0.1744

358.17125	0.17448

358.27025	0.17457

358.36924	0.17475

358.46824	0.17476

358.56724	0.17481

358.66623	0.1749

358.76523	0.17494

358.86423	0.17495

358.96322	0.17506

359.06222	0.17509

359.16122	0.17511

359.26022	0.17512

359.35921	0.17513

359.45821	0.17519

359.55721	0.17523

359.6562	0.17526

359.7552	0.17537

359.8542	0.17537

359.95319	0.17538

360.05219	0.17539

360.15119	0.17544

360.25019	0.17566

360.34918	0.1757

360.44818	0.17573

360.54718	0.17594

360.64617	0.17594

360.74517	0.17595

360.84417	0.17631

360.94316	0.17631

361.04216	0.17668

361.14116	0.1767

361.24016	0.17671

361.33915	0.1769

361.43815	0.17691

361.53715	0.17699

361.63614	0.1771

361.73514	0.17711

361.83414	0.1772

361.93314	0.17729

362.03213	0.17733

362.13113	0.17734

362.23013	0.17744

362.32912	0.17746

362.42812	0.17753

362.52712	0.17765

362.62611	0.17767

362.72511	0.17795

362.82411	0.17796

362.92311	0.17797

363.0221	0.17798

363.1211	0.17808

363.2201	0.1781

363.31909	0.17811

363.41809	0.17829

363.51709	0.1784

363.61608	0.17848

363.71508	0.17849

363.81408	0.17887

363.91308	0.17889

364.01207	0.17897

364.11107	0.17902

364.21007	0.17903

364.30906	0.17915

364.40806	0.17934

364.50706	0.17948

364.60606	0.17951

364.70505	0.17955

364.80405	0.17966

364.90305	0.17969

365.00204	0.17992

365.10104	0.17993

365.20004	0.17995

365.29903	0.17997

365.39803	0.18019

365.49703	0.18021

365.59603	0.18022

365.69502	0.18025

365.79402	0.18026

365.89302	0.18055

365.99201	0.18058

366.09101	0.18058

366.19001	0.1809

366.289	0.18091

366.388	0.18094

366.487	0.181

366.586	0.18112

366.68499	0.18113

366.78399	0.18115

366.88299	0.18133

366.98198	0.18135

367.08098	0.1814

367.17998	0.18142

367.27897	0.18143

367.37797	0.18145

367.47697	0.18154

367.57597	0.18155

367.67496	0.18157

367.77396	0.18157

367.87296	0.18173

367.97195	0.18174

368.07095	0.18175

368.16995	0.18183

368.26894	0.18184

368.36794	0.18193

368.46694	0.18209

368.56594	0.18212

368.66493	0.18243

368.76393	0.18245

368.86293	0.18246

368.96192	0.18262

369.06092	0.18262

369.15992	0.18264

369.25892	0.18288

369.35791	0.18292

369.45691	0.183

369.55591	0.18316

369.6549	0.18317

369.7539	0.18319

369.8529	0.18322

369.95189	0.18324

370.05089	0.18326

370.14989	0.18349

370.24889	0.1835

370.34788	0.18363

370.44688	0.18376

370.54588	0.18392

370.64487	0.18397

370.74387	0.18417

370.84287	0.18428

370.94186	0.18429

371.04086	0.18448

371.13986	0.18449

371.23886	0.18449

371.33785	0.18453

371.43685	0.18461

371.53585	0.18465

371.63484	0.18492

371.73384	0.18497

371.83284	0.18499

371.93184	0.18529

372.03083	0.18531

372.12983	0.18543

372.22883	0.18543

372.32782	0.18546

372.42682	0.18548

372.52582	0.18557

372.62481	0.1856

372.72381	0.18562

372.82281	0.1858

372.92181	0.18583

373.0208	0.18593

373.1198	0.18595

373.2188	0.18595

373.31779	0.18599

373.41679	0.18605

373.51579	0.18606

373.61478	0.18607

373.71378	0.18609

373.81278	0.18611

373.91178	0.18612

374.01077	0.18613

374.10977	0.18616

374.20877	0.18621

374.30776	0.18621

374.40676	0.18623

374.50576	0.18632

374.60475	0.18647

374.70375	0.18651

374.80275	0.1869

374.90175	0.18692

375.00074	0.18714

375.09974	0.18733

375.19874	0.18736

375.29773	0.18738

375.39673	0.18739

375.49573	0.18748

375.59473	0.18756

375.69372	0.18757

375.79272	0.1876

375.89172	0.18781

375.99071	0.18786

376.08971	0.18818

376.18871	0.18819

376.2877	0.18825

376.3867	0.18829

376.4857	0.1883

376.5847	0.18832

376.68369	0.18839

376.78269	0.18841

376.88169	0.18843

376.98068	0.18848

377.07968	0.18851

377.17868	0.18852

377.27767	0.18853

377.37667	0.18861

377.47567	0.18875

377.57467	0.18876

377.67366	0.18902

377.77266	0.18904

377.87166	0.18905

377.97065	0.18906

378.06965	0.18944

378.16865	0.18947

378.26764	0.18948

378.36664	0.18961

378.46564	0.18962

378.56464	0.18979

378.66363	0.1898

378.76263	0.18986

378.86163	0.18997

378.96062	0.18997

379.05962	0.19026

379.15862	0.19027

379.25762	0.19028

379.35661	0.19046

379.45561	0.19053

379.55461	0.19054

379.6536	0.19059

379.7526	0.19061

379.8516	0.19062

379.95059	0.19072

380.04959	0.19074

380.14859	0.19079

380.24759	0.19093

380.34658	0.19095

380.44558	0.19096

380.54458	0.19106

380.64357	0.1911

380.74257	0.19111

380.84157	0.19111

380.94056	0.19121

381.03956	0.19126

381.13856	0.19153

381.23756	0.19165

381.33655	0.19175

381.43555	0.19176

381.53455	0.19201

381.63354	0.19204

381.73254	0.19205

381.83154	0.19213

381.93053	0.1922

382.02953	0.19221

382.12853	0.19231

382.22753	0.19236

382.32652	0.19244

382.42552	0.19246

382.52452	0.19282

382.62351	0.19283

382.72251	0.19288

382.82151	0.19294

382.92051	0.19295

383.0195	0.19304

383.1185	0.19308

383.2175	0.19309

383.31649	0.19319

383.41549	0.19325

383.51449	0.19326

383.61348	0.19328

383.71248	0.19341

383.81148	0.19351

383.91048	0.19351

384.00947	0.19366

384.10847	0.19367

384.20747	0.19373

384.30646	0.19381

384.40546	0.19382

384.50446	0.19392

384.60345	0.19403

384.70245	0.19403

384.80145	0.19441

384.90045	0.19444

384.99944	0.19445

385.09844	0.19452

385.19744	0.19463

385.29643	0.19464

385.39543	0.19466

385.49443	0.19475

385.59343	0.19476

385.69242	0.19479

385.79142	0.19488

385.89042	0.19493

385.98941	0.195

386.08841	0.19501

386.18741	0.19502

386.2864	0.19504

386.3854	0.19532

386.4844	0.19533

386.5834	0.19537

386.68239	0.1954

386.78139	0.19566

386.88039	0.19568

386.97938	0.19571

387.07838	0.19574

387.17738	0.19575

387.27637	0.19577

387.37537	0.1958

387.47437	0.19583

387.57337	0.19586

387.67236	0.19586

387.77136	0.19591

387.87036	0.196

387.96935	0.19601

388.06835	0.19601

388.16735	0.19604

388.26634	0.19607

388.36534	0.19608

388.46434	0.19609

388.56334	0.1963

388.66233	0.19631

388.76133	0.19633

388.86033	0.19634

388.95932	0.19636

389.05832	0.19638

389.15732	0.19642

389.25631	0.19643

389.35531	0.19648

389.45431	0.19649

389.55331	0.19659

389.6523	0.19675

389.7513	0.19676

389.8503	0.19678

389.94929	0.19686

390.04829	0.19687

390.14729	0.19688

390.24629	0.19689

390.34528	0.19692

390.44428	0.19696

390.54328	0.19698

390.64227	0.19699

390.74127	0.19711

390.84027	0.19713

390.93926	0.19715

391.03826	0.19716

391.13726	0.19734

391.23626	0.19758

391.33525	0.19761

391.43425	0.19767

391.53325	0.19768

391.63224	0.19799

391.73124	0.19829

391.83024	0.1983

391.92923	0.19835

392.02823	0.19842

392.12723	0.19846

392.22623	0.19846

392.32522	0.19849

392.42422	0.19851

392.52322	0.19851

392.62221	0.19857

392.72121	0.19859

392.82021	0.19876

392.91921	0.19877

393.0182	0.1988

393.1172	0.19883

393.2162	0.19884

393.31519	0.19886

393.41419	0.19898

393.51319	0.19899

393.61218	0.19902

393.71118	0.19903

393.81018	0.1991

393.90918	0.19919

394.00817	0.19924

394.10717	0.19926

394.20617	0.19928

394.30516	0.19936

394.40416	0.19947

394.50316	0.1995

394.60215	0.19951

394.70115	0.19958

394.80015	0.19965

394.89915	0.19974

394.99814	0.19991

395.09714	0.20018

395.19614	0.20019

395.29513	0.2004

395.39413	0.20043

395.49313	0.20044

395.59212	0.20061

395.69112	0.20062

395.79012	0.20065

395.88912	0.20066

395.98811	0.20094

396.08711	0.20095

396.18611	0.20107

396.2851	0.20109

396.3841	0.20122

396.4831	0.20123

396.5821	0.20124

396.68109	0.20125

396.78009	0.20143

396.87909	0.20148

396.97808	0.2015

397.07708	0.20153

397.17608	0.20155

397.27507	0.20159

397.37407	0.20161

397.47307	0.20163

397.57207	0.20164

397.67106	0.20166

397.77006	0.20175

397.86906	0.20177

397.96805	0.20183

398.06705	0.20196

398.16605	0.20198

398.26504	0.20222

398.36404	0.20222

398.46304	0.20252

398.56204	0.20254

398.66103	0.2028

398.76003	0.20282

398.85903	0.203

398.95802	0.20312

399.05702	0.20326

399.15602	0.20336

399.25501	0.20358

399.35401	0.2036

399.45301	0.20361

399.55201	0.20364

399.651	0.20365

399.75	0.20367

399.849	0.20368

399.94799	0.20391

400.04699	0.20398

400.14599	0.20399

400.24499	0.20402

400.34398	0.20404

400.44298	0.20407

400.54198	0.20433

400.64097	0.20437

400.73997	0.20439

400.83897	0.20476

400.93796	0.20477

401.03696	0.20497

401.13596	0.20498

401.23496	0.205

401.33395	0.20519

401.43295	0.20523

401.53195	0.20528

401.63094	0.2053

401.72994	0.20555

401.82894	0.20555

401.92793	0.20566

402.02693	0.20566

402.12593	0.20569

402.22493	0.20573

402.32392	0.20588

402.42292	0.20594

402.52192	0.20615

402.62091	0.20616

402.71991	0.20619

402.81891	0.20621

402.9179	0.20652

403.0169	0.20653

403.1159	0.20654

403.2149	0.20655

403.31389	0.20657

403.41289	0.20672

403.51189	0.20673

403.61088	0.20675

403.70988	0.20687

403.80888	0.20689

403.90788	0.20716

404.00687	0.20717

404.10587	0.20719

404.20487	0.20723

404.30386	0.20727

404.40286	0.20728

404.50186	0.20731

404.60085	0.20741

404.69985	0.20743

404.79885	0.20746

404.89785	0.20747

404.99684	0.20759

405.09584	0.20762

405.19484	0.20764

405.29383	0.20791

405.39283	0.20792

405.49183	0.20795

405.59082	0.20797

405.68982	0.20837

405.78882	0.20868

405.88782	0.20869

405.98681	0.20872

406.08581	0.20873

406.18481	0.20879

406.2838	0.2089

406.3828	0.20891

406.4818	0.20896

406.58079	0.20902

406.67979	0.20912

406.77879	0.20913

406.87779	0.20927

406.97678	0.2093

407.07578	0.20937

407.17478	0.20964

407.27377	0.20965

407.37277	0.20968

407.47177	0.20991

407.57077	0.20992

407.66976	0.21004

407.76876	0.2101

407.86776	0.21044

407.96675	0.21045

408.06575	0.21058

408.16475	0.2106

408.26374	0.21083

408.36274	0.21084

408.46174	0.21093

408.56074	0.21115

408.65973	0.21118

408.75873	0.21124

408.85773	0.21155

408.95672	0.21159

409.05572	0.21172

409.15472	0.21173

409.25371	0.21181

409.35271	0.21186

409.45171	0.21189

409.55071	0.21197

409.6497	0.21199

409.7487	0.212

409.8477	0.21201

409.94669	0.21231

410.04569	0.21234

410.14469	0.21235

410.24369	0.21238

410.34268	0.2124

410.44168	0.21247

410.54068	0.21262

410.63967	0.21263

410.73867	0.21265

410.83767	0.21266

410.93666	0.21283

411.03566	0.21293

411.13466	0.21294

411.23366	0.21313

411.33265	0.21314

411.43165	0.21315

411.53065	0.21316

411.62964	0.21321

411.72864	0.21332

411.82764	0.21332

411.92663	0.21333

412.02563	0.21351

412.12463	0.21353

412.22363	0.21354

412.32262	0.21379

412.42162	0.21381

412.52062	0.21397

412.61961	0.21412

412.71861	0.21413

412.81761	0.21419

412.9166	0.2144

413.0156	0.21441

413.1146	0.21442

413.2136	0.21445

413.31259	0.21452

413.41159	0.21452

413.51059	0.21453

413.60958	0.21467

413.70858	0.21475

413.80758	0.21476

413.90657	0.21478

414.00557	0.21507

414.10457	0.21508

414.20357	0.21509

414.30256	0.21516

414.40156	0.21523

414.50056	0.21526

414.59955	0.21535

414.69855	0.21537

414.79755	0.2156

414.89655	0.21566

414.99554	0.21566

415.09454	0.21567

415.19354	0.21568

415.29253	0.21569

415.39153	0.21571

415.49053	0.21575

415.58952	0.21578

415.68852	0.21592

415.78752	0.21613

415.88652	0.21636

415.98551	0.21638

416.08451	0.21653

416.18351	0.21656

416.2825	0.21678

416.3815	0.21687

416.4805	0.21688

416.57949	0.21712

416.67849	0.21712

416.77749	0.21723

416.87649	0.21736

416.97548	0.21737

417.07448	0.21753

417.17348	0.21755

417.27247	0.21756

417.37147	0.21757

417.47047	0.21773

417.56947	0.21774

417.66846	0.21775

417.76746	0.21792

417.86646	0.21795

417.96545	0.21796

418.06445	0.21805

418.16345	0.21818

418.26244	0.21819

418.36144	0.21822

418.46044	0.21837

418.55944	0.21843

418.65843	0.21854

418.75743	0.21868

418.85643	0.21873

418.95542	0.21874

419.05442	0.21875

419.15342	0.21897

419.25241	0.21898

419.35141	0.21916

419.45041	0.21923

419.54941	0.21926

419.6484	0.21927

419.7474	0.21936

419.8464	0.21937

419.94539	0.21943

420.04439	0.21959

420.14339	0.21964

420.24238	0.21978

420.34138	0.21979

420.44038	0.2198

420.53938	0.21999

420.63837	0.22

420.73737	0.2202

420.83637	0.22021

420.93536	0.22024

421.03436	0.2204

421.13336	0.22041

421.23236	0.22041

421.33135	0.22056

421.43035	0.22057

421.52935	0.22063

421.62834	0.22066

421.72734	0.22069

421.82634	0.22071

421.92533	0.22072

422.02433	0.22113

422.12333	0.22115

422.22233	0.22122

422.32132	0.22123

422.42032	0.22126

422.51932	0.22128

422.61831	0.22141

422.71731	0.22143

422.81631	0.22156

422.9153	0.2216

423.0143	0.22161

423.1133	0.22163

423.2123	0.22201

423.31129	0.22202

423.41029	0.22224

423.50929	0.22225

423.60828	0.22228

423.70728	0.22245

423.80628	0.2227

423.90527	0.22271

424.00427	0.22272

424.10327	0.22273

424.20227	0.22301

424.30126	0.22303

424.40026	0.22326

424.49926	0.22327

424.59825	0.22331

424.69725	0.22353

424.79625	0.22354

424.89525	0.22375

424.99424	0.22379

425.09324	0.22391

425.19224	0.22392

425.29123	0.22395

425.39023	0.22396

425.48923	0.22403

425.58822	0.22404

425.68722	0.22419

425.78622	0.22423

425.88522	0.22427

425.98421	0.22428

426.08321	0.22429

426.18221	0.22456

426.2812	0.22458

426.3802	0.22459

426.4792	0.22494

426.57819	0.22515

426.67719	0.22517

426.77619	0.2252

426.87519	0.2253

426.97418	0.22531

427.07318	0.22536

427.17218	0.2255

427.27117	0.22551

427.37017	0.22552

427.46917	0.22563

427.56816	0.22565

427.66716	0.22567

427.76616	0.22568

427.86516	0.22598

427.96415	0.226

428.06315	0.22601

428.16215	0.22619

428.26114	0.22624

428.36014	0.22627

428.45914	0.22628

428.55814	0.22638

428.65713	0.22639

428.75613	0.22644

428.85513	0.22662

428.95412	0.22663

429.05312	0.22665

429.15212	0.22677

429.25111	0.22678

429.35011	0.22708

429.44911	0.22709

429.54811	0.22712

429.6471	0.22713

429.7461	0.22728

429.8451	0.22729

429.94409	0.22729

430.04309	0.2273

430.14209	0.22755

430.24108	0.22761

430.34008	0.22769

430.43908	0.22771

430.53808	0.22773

430.63707	0.22776

430.73607	0.22786

430.83507	0.22791

430.93406	0.22791

431.03306	0.2282

431.13206	0.22821

431.23106	0.22833

431.33005	0.2285

431.42905	0.22855

431.52805	0.22856

431.62704	0.22858

431.72604	0.2289

431.82504	0.22932

431.92403	0.22933

432.02303	0.22958

432.12203	0.22959

432.22103	0.22963

432.32002	0.22981

432.41902	0.22983

432.51802	0.22995

432.61701	0.22997

432.71601	0.22998

432.81501	0.22999

432.914	0.23

433.013	0.2302

433.112	0.23021

433.211	0.23042

433.30999	0.23049

433.40899	0.2305

433.50799	0.23051

433.60698	0.23083

433.70598	0.23084

433.80498	0.23105

433.90397	0.23107

434.00297	0.23139

434.10197	0.2314

434.20097	0.23158

434.29996	0.2316

434.39896	0.23192

434.49796	0.23193

434.59695	0.23218

434.69595	0.2322

434.79495	0.23249

434.89394	0.2325

434.99294	0.23255

435.09194	0.23265

435.19094	0.2327

435.28993	0.23305

435.38893	0.23306

435.48793	0.23307

435.58692	0.2333

435.68592	0.23333

435.78492	0.23335

435.88392	0.23336

435.98291	0.23365

436.08191	0.23368

436.18091	0.2337

436.2799	0.23371

436.3789	0.234

436.4779	0.23401

436.57689	0.23406

436.67589	0.23411

436.77489	0.23413

436.87389	0.23427

436.97288	0.23429

437.07188	0.23432

437.17088	0.23433

437.26987	0.23443

437.36887	0.23451

437.46787	0.23465

437.56686	0.23466

437.66586	0.23485

437.76486	0.23488

437.86386	0.23489

437.96285	0.23524

438.06185	0.23525

438.16085	0.23526

438.25984	0.23526

438.35884	0.23534

438.45784	0.23559

438.55684	0.23561

438.65583	0.23563

438.75483	0.23565

438.85383	0.23589

438.95282	0.2359

439.05182	0.23608

439.15082	0.2361

439.24981	0.2363

439.34881	0.23634

439.44781	0.23635

439.54681	0.23638

439.6458	0.23657

439.7448	0.23658

439.8438	0.23683

439.94279	0.23688

440.04179	0.23689

440.14079	0.2369

440.23978	0.23695

440.33878	0.23696

440.43778	0.23704

440.53678	0.23729

440.63577	0.23733

440.73477	0.23742

440.83377	0.2377

440.93276	0.23788

441.03176	0.23793

441.13076	0.23819

441.22975	0.2382

441.32875	0.23843

441.42775	0.23846

441.52675	0.23848

441.62574	0.23864

441.72474	0.23866

441.82374	0.23867

441.92273	0.23891

442.02173	0.23893

442.12073	0.23915

442.21973	0.23917

442.31872	0.23917

442.41772	0.23943

442.51672	0.23944

442.61571	0.23954

442.71471	0.23957

442.81371	0.23961

442.9127	0.23973

443.0117	0.23977

443.1107	0.2398

443.2097	0.24011

443.30869	0.24012

443.40769	0.2404

443.50669	0.24041

443.60568	0.2406

443.70468	0.24084

443.80368	0.24085

443.90267	0.24103

444.00167	0.24104

444.10067	0.24117

444.19967	0.24118

444.29866	0.2412

444.39766	0.2412

444.49666	0.24128

444.59565	0.24132

444.69465	0.24133

444.79365	0.24134

444.89264	0.24166

444.99164	0.24168

445.09064	0.2418

445.18964	0.24186

445.28863	0.24199

445.38763	0.24199

445.48663	0.242

445.58562	0.24222

445.68462	0.24223

445.78362	0.24235

445.88262	0.24237

445.98161	0.24239

446.08061	0.2424

446.17961	0.24263

446.2786	0.24266

446.3776	0.24267

446.4766	0.24282

446.57559	0.24283

446.67459	0.24289

446.77359	0.24305

446.87259	0.24306

446.97158	0.24308

447.07058	0.24317

447.16958	0.24319

447.26857	0.2432

447.36757	0.24321

447.46657	0.24326

447.56556	0.24357

447.66456	0.24359

447.76356	0.24388

447.86256	0.24389

447.96155	0.24419

448.06055	0.24422

448.15955	0.24453

448.25854	0.24459

448.35754	0.24502

448.45654	0.24533

448.55553	0.24535

448.65453	0.24536

448.75353	0.24569

448.85253	0.24597

448.95152	0.246

449.05052	0.24601

449.14952	0.24622

449.24851	0.2465

449.34751	0.24651

449.44651	0.24654

449.54551	0.2466

449.6445	0.24669

449.7435	0.24673

449.8425	0.24673

449.94149	0.24678

450.04049	0.24682

450.13949	0.24685

450.23848	0.24687

450.33748	0.24691

450.43648	0.2471

450.53548	0.24713

450.63447	0.24716

450.73347	0.24724

450.83247	0.24731

450.93146	0.24732

451.03046	0.24776

451.12946	0.24777

451.22845	0.24778

451.32745	0.24802

451.42645	0.24804

451.52545	0.24832

451.62444	0.24833

451.72344	0.24834

451.82244	0.24839

451.92143	0.24851

452.02043	0.24854

452.11943	0.24855

452.21843	0.24879

452.31742	0.24882

452.41642	0.24884

452.51542	0.24887

452.61441	0.24889

452.71341	0.24897

452.81241	0.24909

452.9114	0.2491

453.0104	0.24911

453.1094	0.24923

453.2084	0.24931

453.30739	0.24932

453.40639	0.24935

453.50539	0.24936

453.60438	0.24939

453.70338	0.2494

453.80238	0.24944

453.90137	0.24945

454.00037	0.2495

454.09937	0.24951

454.19837	0.24955

454.29736	0.24956

454.39636	0.24957

454.49536	0.24959

454.59435	0.24972

454.69335	0.24973

454.79235	0.24974

454.89134	0.24987

454.99034	0.24991

455.08934	0.24995

455.18834	0.24996

455.28733	0.25022

455.38633	0.25029

455.48533	0.25033

455.58432	0.25034

455.68332	0.2504

455.78232	0.25043

455.88131	0.25046

455.98031	0.25065

456.07931	0.25066

456.17831	0.25073

456.2773	0.25092

456.3763	0.25093

456.4753	0.25094

456.57429	0.2511

456.67329	0.25122

456.77229	0.25125

456.87129	0.25155

456.97028	0.25178

457.06928	0.25179

457.16828	0.25205

457.26727	0.25236

457.36627	0.25237

457.46527	0.2524

457.56426	0.25251

457.66326	0.25253

457.76226	0.25254

457.86126	0.2526

457.96025	0.25272

458.05925	0.25309

458.15825	0.2531

458.25724	0.25311

458.35624	0.25328

458.45524	0.25329

458.55423	0.25335

458.65323	0.25339

458.75223	0.2534

458.85123	0.25341

458.95022	0.25342

459.04922	0.25371

459.14822	0.25372

459.24721	0.25374

459.34621	0.2539

459.44521	0.25392

459.54421	0.25393

459.6432	0.25405

459.7422	0.25408

459.8412	0.25409

459.94019	0.25424

460.03919	0.25425

460.13819	0.25429

460.23718	0.25434

460.33618	0.25436

460.43518	0.25444

460.53418	0.25446

460.63317	0.25469

460.73217	0.2547

460.83117	0.25502

460.93016	0.25502

461.02916	0.25503

461.12816	0.2552

461.22715	0.25523

461.32615	0.25523

461.42515	0.25534

461.52415	0.25536

461.62314	0.25542

461.72214	0.25543

461.82114	0.25559

461.92013	0.25561

462.01913	0.25562

462.11813	0.25564

462.21712	0.25568

462.31612	0.25569

462.41512	0.25595

462.51412	0.25596

462.61311	0.25614

462.71211	0.25628

462.81111	0.25633

462.9101	0.25642

463.0091	0.25653

463.1081	0.25655

463.2071	0.25657

463.30609	0.25658

463.40509	0.25659

463.50409	0.25663

463.60308	0.25678

463.70208	0.25679

463.80108	0.25683

463.90007	0.25684

463.99907	0.25686

464.09807	0.25689

464.19707	0.2569

464.29606	0.25706

464.39506	0.25707

464.49406	0.25748

464.59305	0.25751

464.69205	0.25752

464.79105	0.25783

464.89004	0.25784

464.98904	0.25785

465.08804	0.25786

465.18704	0.25791

465.28603	0.25801

465.38503	0.25803

465.48403	0.25807

465.58302	0.25821

465.68202	0.25822

465.78102	0.25823

465.88001	0.25859

465.97901	0.2586

466.07801	0.25865

466.17701	0.25869

466.276	0.25904

466.375	0.25911

466.474	0.25917

466.57299	0.25918

466.67199	0.25923

466.77099	0.25925

466.86999	0.25929

466.96898	0.25929

467.06798	0.25935

467.16698	0.25938

467.26597	0.25939

467.36497	0.25942

467.46397	0.25964

467.56296	0.25965

467.66196	0.25966

467.76096	0.25972

467.85996	0.25976

467.95895	0.25976

468.05795	0.25981

468.15695	0.25992

468.25594	0.26016

468.35494	0.26019

468.45394	0.26032

468.55293	0.26035

468.65193	0.26043

468.75093	0.26047

468.84993	0.26049

468.94892	0.26049

469.04792	0.2605

469.14692	0.26071

469.24591	0.26076

469.34491	0.26087

469.44391	0.26096

469.5429	0.26106

469.6419	0.26107

469.7409	0.26115

469.8399	0.26116

469.93889	0.26138

470.03789	0.26139

470.13689	0.26175

470.23588	0.26176

470.33488	0.26199

470.43388	0.262

470.53288	0.26203

470.63187	0.26214

470.73087	0.26236

470.82987	0.26237

470.92886	0.26253

471.02786	0.26255

471.12686	0.26288

471.22585	0.26289

471.32485	0.2629

471.42385	0.26293

471.52285	0.26296

471.62184	0.26297

471.72084	0.26305

471.81984	0.26307

471.91883	0.26309

472.01783	0.2631

472.11683	0.26317

472.21582	0.26322

472.31482	0.26323

472.41382	0.26324

472.51282	0.26331

472.61181	0.26334

472.71081	0.26336

472.80981	0.26337

472.9088	0.26346

473.0078	0.26347

473.1068	0.26351

473.20579	0.26367

473.30479	0.26375

473.40379	0.26376

473.50279	0.26377

473.60178	0.26379

473.70078	0.26386

473.79978	0.26388

473.89877	0.26388

473.99777	0.26398

474.09677	0.26414

474.19577	0.26418

474.29476	0.26455

474.39376	0.26478

474.49276	0.26479

474.59175	0.26487

474.69075	0.26497

474.78975	0.26501

474.88874	0.26502

474.98774	0.26503

475.08674	0.26517

475.18574	0.26539

475.28473	0.2654

475.38373	0.26543

475.48273	0.26551

475.58172	0.26552

475.68072	0.2656

475.77972	0.26561

475.87871	0.26562

475.97771	0.26564

476.07671	0.26566

476.17571	0.26571

476.2747	0.26573

476.3737	0.26575

476.4727	0.26577

476.57169	0.26581

476.67069	0.26584

476.76969	0.26588

476.86869	0.26608

476.96768	0.26609

477.06668	0.26615

477.16568	0.2662

477.26467	0.26621

477.36367	0.26636

477.46267	0.2664

477.56166	0.26659

477.66066	0.2666

477.75966	0.26664

477.85866	0.26667

477.95765	0.26701

478.05665	0.26706

478.15565	0.26716

478.25464	0.26717

478.35364	0.26718

478.45264	0.26719

478.55163	0.26721

478.65063	0.26725

478.74963	0.26726

478.84863	0.26727

478.94762	0.26727

479.04662	0.2673

479.14562	0.26739

479.24461	0.26754

479.34361	0.26758

479.44261	0.26768

479.5416	0.26776

479.6406	0.26779

479.7396	0.26788

479.8386	0.26802

479.93759	0.26805

480.03659	0.26805

480.13559	0.26807

480.23458	0.26831

480.33358	0.26832

480.43258	0.26833

480.53157	0.26842

480.63057	0.26851

480.72957	0.26853

480.82857	0.26868

480.92756	0.26871

481.02656	0.26875

481.12556	0.26877

481.22455	0.26883

481.32355	0.26883

481.42255	0.26884

481.52155	0.26899

481.62054	0.26899

481.71954	0.26923

481.81854	0.26924

481.91753	0.26966

482.01653	0.2697

482.11553	0.26979

482.21452	0.2698

482.31352	0.26988

482.41252	0.26998

482.51152	0.26999

482.61051	0.27029

482.70951	0.27031

482.80851	0.27075

482.9075	0.27076

483.0065	0.27082

483.1055	0.27091

483.20449	0.27093

483.30349	0.27102

483.40249	0.27108

483.50149	0.27112

483.60048	0.27112

483.69948	0.27126

483.79848	0.27127

483.89747	0.27134

483.99647	0.27138

484.09547	0.27146

484.19447	0.2715

484.29346	0.27151

484.39246	0.27152

484.49146	0.27165

484.59045	0.27166

484.68945	0.27171

484.78845	0.27177

484.88744	0.27178

484.98644	0.27179

485.08544	0.27215

485.18444	0.27216

485.28343	0.27217

485.38243	0.27223

485.48143	0.27237

485.58042	0.27272

485.67942	0.27274

485.77842	0.27274

485.87741	0.27292

485.97641	0.27293

486.07541	0.27305

486.17441	0.27335

486.2734	0.2736

486.3724	0.27364

486.4714	0.27374

486.57039	0.27375

486.66939	0.27377

486.76839	0.27379

486.86738	0.27398

486.96638	0.27399

487.06538	0.27402

487.16438	0.2741

487.26337	0.2743

487.36237	0.27431

487.46137	0.27463

487.56036	0.27465

487.65936	0.27466

487.75836	0.27493

487.85736	0.27494

487.95635	0.27514

488.05535	0.27516

488.15435	0.27545

488.25334	0.27545

488.35234	0.27547

488.45134	0.27548

488.55033	0.27569

488.64933	0.27571

488.74833	0.27601

488.84733	0.27602

488.94632	0.27602

489.04532	0.27617

489.14432	0.27653

489.24331	0.27654

489.34231	0.27663

489.44131	0.27664

489.5403	0.27665

489.6393	0.27685

489.7383	0.27686

489.8373	0.27706

489.93629	0.27707

490.03529	0.27712

490.13429	0.27743

490.23328	0.27762

490.33228	0.27773

490.43128	0.27778

490.53027	0.2778

490.62927	0.27804

490.72827	0.27807

490.82727	0.27823

490.92626	0.27851

491.02526	0.27863

491.12426	0.2787

491.22325	0.27872

491.32225	0.27873

491.42125	0.27873

491.52025	0.27874

491.61924	0.27897

491.71824	0.2791

491.81724	0.27911

491.91623	0.27918

492.01523	0.27922

492.11423	0.27924

492.21322	0.27933

492.31222	0.27938

492.41122	0.27939

492.51022	0.2794

492.60921	0.27941

492.70821	0.27978

492.80721	0.27993

492.9062	0.28022

493.0052	0.28023

493.1042	0.28041

493.20319	0.28042

493.30219	0.28046

493.40119	0.28053

493.50019	0.28056

493.59918	0.28057

493.69818	0.28087

493.79718	0.28102

493.89617	0.28103

493.99517	0.28117

494.09417	0.2812

494.19316	0.28121

494.29216	0.28151

494.39116	0.28152

494.49016	0.28154

494.58915	0.28172

494.68815	0.28176

494.78715	0.28177

494.88614	0.28178

494.98514	0.28191

495.08414	0.28197

495.18314	0.28198

495.28213	0.282

495.38113	0.28225

495.48013	0.28228

495.57912	0.28243

495.67812	0.28254

495.77712	0.28257

495.87611	0.28258

495.97511	0.28259

496.07411	0.28276

496.17311	0.28278

496.2721	0.28307

496.3711	0.28311

496.4701	0.28334

496.56909	0.28336

496.66809	0.28346

496.76709	0.28349

496.86608	0.2835

496.96508	0.28371

497.06408	0.28411

497.16308	0.28415

497.26207	0.28419

497.36107	0.28441

497.46007	0.28442

497.55906	0.28451

497.65806	0.28455

497.75706	0.28456

497.85606	0.28458

497.95505	0.28463

498.05405	0.2848

498.15305	0.28482

498.25204	0.28514

498.35104	0.28516

498.45004	0.28525

498.54903	0.28526

498.64803	0.2853

498.74703	0.28546

498.84603	0.28546

498.94502	0.28547

499.04402	0.28567

499.14302	0.28567

499.24201	0.28574

499.34101	0.28577

499.44001	0.28585

499.539	0.28607

499.638	0.28608

499.737	0.28608

499.836	0.28617

499.93499	0.28619

500.03399	0.2862

500.13299	0.28642

500.23198	0.28663

500.33098	0.28666

500.42998	0.28679

500.52897	0.2868

500.62797	0.28682

500.72697	0.28683

500.82597	0.28685

500.92496	0.28686

501.02396	0.28687

501.12296	0.28691

501.22195	0.28702

501.32095	0.28723

501.41995	0.28723

501.51894	0.28746

501.61794	0.28747

501.71694	0.28753

501.81594	0.28762

501.91493	0.28766

502.01393	0.28767

502.11293	0.28768

502.21192	0.28791

502.31092	0.2881

502.40992	0.28812

502.50892	0.28813

502.60791	0.28833

502.70691	0.28835

502.80591	0.28837

502.9049	0.28838

503.0039	0.28838

503.1029	0.28839

503.20189	0.28851

503.30089	0.28853

503.39989	0.2886

503.49889	0.28863

503.59788	0.28869

503.69688	0.2887

503.79588	0.28908

503.89487	0.28909

503.99387	0.28911

504.09287	0.28916

504.19186	0.28955

504.29086	0.28956

504.38986	0.28957

504.48886	0.28963

504.58785	0.28968

504.68685	0.28968

504.78585	0.28973

504.88484	0.28978

504.98384	0.28981

505.08284	0.28982

505.18184	0.28986

505.28083	0.29003

505.37983	0.29017

505.47883	0.29025

505.57782	0.29026

505.67682	0.29027

505.77582	0.29037

505.87481	0.29043

505.97381	0.29044

506.07281	0.29046

506.17181	0.29064

506.2708	0.29087

506.3698	0.29088

506.4688	0.29088

506.56779	0.29092

506.66679	0.29103

506.76579	0.29106

506.86478	0.29107

506.96378	0.29108

507.06278	0.29109

507.16178	0.29109

507.26077	0.29122

507.35977	0.29123

507.45877	0.29124

507.55776	0.29126

507.65676	0.2914

507.75576	0.29141

507.85475	0.2915

507.95375	0.29153

508.05275	0.29155

508.15175	0.29156

508.25074	0.29158

508.34974	0.29172

508.44874	0.29176

508.54773	0.29177

508.64673	0.29179

508.74573	0.29197

508.84473	0.29197

508.94372	0.29217

509.04272	0.29218

509.14172	0.29219

509.24071	0.29234

509.33971	0.29244

509.43871	0.29244

509.5377	0.29254

509.6367	0.2926

509.7357	0.29261

509.8347	0.29276

509.93369	0.29277

510.03269	0.29278

510.13169	0.29291

510.23068	0.29291

510.32968	0.29294

510.42868	0.29315

510.52767	0.29331

510.62667	0.29333

510.72567	0.29335

510.82467	0.2934

510.92366	0.29344

511.02266	0.29356

511.12166	0.29357

511.22065	0.29381

511.31965	0.29383

511.41865	0.29384

511.51764	0.29391

511.61664	0.294

511.71564	0.29406

511.81464	0.29412

511.91363	0.29413

512.01263	0.29414

512.11163	0.29421

512.21062	0.29422

512.30962	0.29438

512.40862	0.29453

512.50762	0.29454

512.60661	0.29458

512.70561	0.2946

512.80461	0.29461

512.9036	0.29483

513.0026	0.29484

513.1016	0.29485

513.20059	0.29495

513.29959	0.29499

513.39859	0.29505

513.49759	0.29507

513.59658	0.29508

513.69558	0.29509

513.79458	0.29527

513.89357	0.29535

513.99257	0.29536

514.09157	0.29564

514.19056	0.29566

514.28956	0.29567

514.38856	0.29579

514.48756	0.29581

514.58655	0.29607

514.68555	0.29608

514.78455	0.29609

514.88354	0.29622

514.98254	0.29625

515.08154	0.29625

515.18053	0.29651

515.27953	0.29679

515.37853	0.2968

515.47753	0.29683

515.57652	0.29684

515.67552	0.29709

515.77452	0.29717

515.87351	0.29719

515.97251	0.29724

516.07151	0.29748

516.17051	0.29749

516.2695	0.29781

516.3685	0.29782

516.4675	0.29785

516.56649	0.29802

516.66549	0.29803

516.76449	0.29804

516.86348	0.29806

516.96248	0.29812

517.06148	0.29813

517.16048	0.29821

517.25947	0.29828

517.35847	0.29831

517.45747	0.29839

517.55646	0.2984

517.65546	0.29852

517.75446	0.29855

517.85345	0.29858

517.95245	0.29867

518.05145	0.2988

518.15045	0.29881

518.24944	0.29899

518.34844	0.29901

518.44744	0.29905

518.54643	0.29919

518.64543	0.29943

518.74443	0.29945

518.84343	0.29977

518.94242	0.29984

519.04142	0.29985

519.14042	0.30006

519.23941	0.30016

519.33841	0.30021

519.43741	0.30022

519.5364	0.30042

519.6354	0.30043

519.7344	0.30044

519.8334	0.30045

519.93239	0.30052

520.03139	0.30054

520.13039	0.30075

520.22938	0.30076

520.32838	0.30091

520.42738	0.30092

520.52637	0.30094

520.62537	0.30111

520.72437	0.30112

520.82337	0.30113

520.92236	0.30126

521.02136	0.30127

521.12036	0.30128

521.21935	0.30138

521.31835	0.30142

521.41735	0.30179

521.51634	0.3018

521.61534	0.30213

521.71434	0.30237

521.81334	0.30238

521.91233	0.30239

522.01133	0.30245

522.11033	0.30247

522.20932	0.30251

522.30832	0.30257

522.40732	0.30258

522.50631	0.30269

522.60531	0.3027

522.70431	0.30306

522.80331	0.30307

522.9023	0.30316

523.0013	0.30317

523.1003	0.30318

523.19929	0.30338

523.29829	0.30339

523.39729	0.30362

523.49629	0.30365

523.59528	0.30379

523.69428	0.3038

523.79328	0.3039

523.89227	0.30391

523.99127	0.30396

524.09027	0.30408

524.18926	0.3041

524.28826	0.30426

524.38726	0.30427

524.48626	0.30432

524.58525	0.30442

524.68425	0.30443

524.78325	0.30446

524.88224	0.30455

524.98124	0.30456

525.08024	0.30485

525.17923	0.30511

525.27823	0.30517

525.37723	0.30518

525.47623	0.30519

525.57522	0.30521

525.67422	0.30526

525.77322	0.30537

525.87221	0.30539

525.97121	0.30542

526.07021	0.30547

526.16921	0.30547

526.2682	0.30548

526.3672	0.3057

526.4662	0.30574

526.56519	0.30575

526.66419	0.30578

526.76319	0.30589

526.86218	0.30594

526.96118	0.30596

527.06018	0.30597

527.15918	0.30598

527.25817	0.30618

527.35717	0.30621

527.45617	0.30623

527.55516	0.30625

527.65416	0.30626

527.75316	0.30628

527.85215	0.3064

527.95115	0.30648

528.05015	0.30683

528.14915	0.30684

528.24814	0.30685

528.34714	0.3069

528.44614	0.30706

528.54513	0.30707

528.64413	0.30708

528.74313	0.3071

528.84212	0.30711

528.94112	0.30714

529.04012	0.30721

529.13912	0.30722

529.23811	0.30745

529.33711	0.30746

529.43611	0.30765

529.5351	0.30766

529.6341	0.30775

529.7331	0.30779

529.8321	0.3079

529.93109	0.30791

530.03009	0.30805

530.12909	0.30808

530.22808	0.30828

530.32708	0.3083

530.42608	0.30835

530.52507	0.30836

530.62407	0.30838

530.72307	0.30839

530.82207	0.3084

530.92106	0.30844

531.02006	0.30846

531.11906	0.30848

531.21805	0.30858

531.31705	0.30859

531.41605	0.3086

531.51504	0.3086

531.61404	0.30864

531.71304	0.30865

531.81204	0.30865

531.91103	0.30874

532.01003	0.30876

532.10903	0.30876

532.20802	0.30902

532.30702	0.30902

532.40602	0.30903

532.50501	0.30919

532.60401	0.30926

532.70301	0.30927

532.80201	0.30929

532.901	0.3093

533	0.3094

533.099	0.30943

533.19799	0.30964

533.29699	0.3097

533.39599	0.30972

533.49499	0.30973

533.59398	0.30974

533.69298	0.30975

533.79198	0.30976

533.89097	0.30978

533.98997	0.3098

534.08897	0.30981

534.18796	0.30982

534.28696	0.30994

534.38596	0.30997

534.48496	0.31034

534.58395	0.31041

534.68295	0.31042

534.78195	0.31045

534.88094	0.31054

534.97994	0.31058

535.07894	0.31062

535.17793	0.31077

535.27693	0.31078

535.37593	0.31099

535.47493	0.31101

535.57392	0.31103

535.67292	0.3111

535.77192	0.31111

535.87091	0.31114

535.96991	0.31128

536.06891	0.31131

536.1679	0.31136

536.2669	0.31154

536.3659	0.31184

536.4649	0.31185

536.56389	0.31194

536.66289	0.31201

536.76189	0.31202

536.86088	0.31222

536.95988	0.31224

537.05888	0.31243

537.15788	0.31246

537.25687	0.31252

537.35587	0.31262

537.45487	0.31263

537.55386	0.31296

537.65286	0.31304

537.75186	0.31308

537.85085	0.3131

537.94985	0.31315

538.04885	0.31317

538.14785	0.3132

538.24684	0.31335

538.34584	0.31336

538.44484	0.31351

538.54383	0.31352

538.64283	0.31353

538.74183	0.31355

538.84082	0.31371

538.93982	0.31372

539.03882	0.31374

539.13782	0.31402

539.23681	0.31402

539.33581	0.31406

539.43481	0.31409

539.5338	0.3141

539.6328	0.31413

539.7318	0.31422

539.83079	0.31423

539.92979	0.31425

540.02879	0.31426

540.12779	0.31432

540.22678	0.31434

540.32578	0.31437

540.42478	0.31439

540.52377	0.3144

540.62277	0.3146

540.72177	0.31462

540.82077	0.31463

540.91976	0.31486

541.01876	0.31496

541.11776	0.31503

541.21675	0.31504

541.31575	0.31509

541.41475	0.31516

541.51374	0.31517

541.61274	0.31525

541.71174	0.31527

541.81074	0.31528

541.90973	0.31536

542.00873	0.31538

542.10773	0.31539

542.20672	0.31565

542.30572	0.31566

542.40472	0.31567

542.50371	0.31588

542.60271	0.31589

542.70171	0.3159

542.80071	0.31594

542.8997	0.316

542.9987	0.31613

543.0977	0.31614

543.19669	0.31624

543.29569	0.31631

543.39469	0.31631

543.49369	0.31656

543.59268	0.31657

543.69168	0.31658

543.79068	0.31673

543.88967	0.31674

543.98867	0.31675

544.08767	0.31677

544.18666	0.31693

544.28566	0.31694

544.38466	0.31717

544.48366	0.31718

544.58265	0.31735

544.68165	0.31736

544.78065	0.31736

544.87964	0.31751

544.97864	0.31751

545.07764	0.31771

545.17663	0.31774

545.27563	0.31788

545.37463	0.3179

545.47363	0.31798

545.57262	0.318

545.67162	0.31801

545.77062	0.31802

545.86961	0.31803

545.96861	0.31819

546.06761	0.3182

546.1666	0.31822

546.2656	0.31827

546.3646	0.31829

546.4636	0.31835

546.56259	0.31837

546.66159	0.3184

546.76059	0.31845

546.85958	0.31845

546.95858	0.31879

547.05758	0.3188

547.15657	0.31892

547.25557	0.31894

547.35457	0.31896

547.45357	0.31899

547.55256	0.31908

547.65156	0.31908

547.75056	0.31931

547.84955	0.31933

547.94855	0.31949

548.04755	0.31951

548.14655	0.31952

548.24554	0.31953

548.34454	0.31993

548.44354	0.31995

548.54253	0.32001

548.64153	0.32007

548.74053	0.32007

548.83952	0.3201

548.93852	0.32021

549.03752	0.32022

549.13652	0.32024

549.23551	0.32027

549.33451	0.32028

549.43351	0.32068

549.5325	0.32069

549.6315	0.32075

549.7305	0.32082

549.82949	0.32083

549.92849	0.32084

550.02749	0.3209

550.12649	0.32101

550.22548	0.32127

550.32448	0.32141

550.42348	0.32142

550.52247	0.32143

550.62147	0.32144

550.72047	0.3216

550.81947	0.32162

550.91846	0.32174

551.01746	0.32179

551.11646	0.32181

551.21545	0.32188

551.31445	0.32189

551.41345	0.3219

551.51244	0.32193

551.61144	0.322

551.71044	0.3222

551.80944	0.32222

551.90843	0.32238

552.00743	0.32246

552.10643	0.32247

552.20542	0.32256

552.30442	0.32262

552.40342	0.32275

552.50241	0.32277

552.60141	0.32285

552.70041	0.32301

552.79941	0.32302

552.8984	0.3232

552.9974	0.32325

553.0964	0.3235

553.19539	0.32352

553.29439	0.32367

553.39339	0.32371

553.49238	0.32372

553.59138	0.32399

553.69038	0.324

553.78938	0.32427

553.88837	0.32429

553.98737	0.32438

554.08637	0.3244

554.18536	0.32445

554.28436	0.32446

554.38336	0.32453

554.48236	0.32477

554.58135	0.32478

554.68035	0.32496

554.77935	0.32497

554.87834	0.32498

554.97734	0.32507

555.07634	0.32511

555.17533	0.32512

555.27433	0.32512

555.37333	0.32528

555.47233	0.32529

555.57132	0.3253

555.67032	0.32538

555.76932	0.32538

555.86831	0.32539

555.96731	0.32554

556.06631	0.32558

556.1653	0.32559

556.2643	0.32583

556.3633	0.32586

556.4623	0.32613

556.56129	0.32617

556.66029	0.32633

556.75929	0.32634

556.85828	0.32636

556.95728	0.32652

557.05628	0.32653

557.15527	0.32661

557.25427	0.32666

557.35327	0.32695

557.45227	0.32695

557.55126	0.32698

557.65026	0.32712

557.74926	0.32731

557.84825	0.32731

557.94725	0.32734

558.04625	0.32738

558.14525	0.3274

558.24424	0.32742

558.34324	0.32748

558.44224	0.32749

558.54123	0.32752

558.64023	0.32756

558.73923	0.32775

558.83822	0.32776

558.93722	0.32777

559.03622	0.32787

559.13522	0.32788

559.23421	0.32791

559.33321	0.32792

559.43221	0.32793

559.5312	0.32801

559.6302	0.32806

559.7292	0.32809

559.82819	0.32831

559.92719	0.32832

560.02619	0.32833

560.12519	0.32846

560.22418	0.32848

560.32318	0.32858

560.42218	0.32867

560.52117	0.32874

560.62017	0.3288

560.71917	0.32881

560.81816	0.32882

560.91716	0.32898

561.01616	0.329

561.11516	0.32901

561.21415	0.32903

561.31315	0.32914

561.41215	0.32933

561.51114	0.32935

561.61014	0.32936

561.70914	0.32937

561.80814	0.32938

561.90713	0.32958

562.00613	0.32959

562.10513	0.32961

562.20412	0.32966

562.30312	0.32979

562.40212	0.32992

562.50111	0.32993

562.60011	0.3303

562.69911	0.3306

562.79811	0.3307

562.8971	0.33072

562.9961	0.33073

563.0951	0.33074

563.19409	0.33088

563.29309	0.33089

563.39209	0.33105

563.49108	0.33111

563.59008	0.33112

563.68908	0.33117

563.78808	0.33124

563.88707	0.33125

563.98607	0.33133

564.08507	0.3314

564.18406	0.33141

564.28306	0.33142

564.38206	0.33154

564.48105	0.33159

564.58005	0.33162

564.67905	0.33178

564.77805	0.33179

564.87704	0.3318

564.97604	0.33184

565.07504	0.33186

565.17403	0.33187

565.27303	0.33188

565.37203	0.33197

565.47103	0.33198

565.57002	0.332

565.66902	0.33201

565.76802	0.33218

565.86701	0.3322

565.96601	0.33221

566.06501	0.33225

566.164	0.33236

566.263	0.33238

566.362	0.3324

566.461	0.33255

566.55999	0.33256

566.65899	0.33283

566.75799	0.33303

566.85698	0.33305

566.95598	0.33305

567.05498	0.33308

567.15397	0.33317

567.25297	0.33324

567.35197	0.33357

567.45097	0.33372

567.54996	0.33373

567.64896	0.33374

567.74796	0.33392

567.84695	0.33393

567.94595	0.33394

568.04495	0.33397

568.14395	0.33399

568.24294	0.334

568.34194	0.33401

568.44094	0.33403

568.53993	0.33407

568.63893	0.3341

568.73793	0.33411

568.83692	0.33418

568.93592	0.33426

569.03492	0.33427

569.13392	0.33428

569.23291	0.33462

569.33191	0.33463

569.43091	0.33464

569.5299	0.3347

569.6289	0.33471

569.7279	0.33474

569.82689	0.33485

569.92589	0.33487

570.02489	0.33487

570.12389	0.33492

570.22288	0.335

570.32188	0.33503

570.42088	0.33503

570.51987	0.33513

570.61887	0.33514

570.71787	0.33515

570.81686	0.33557

570.91586	0.33558

571.01486	0.3356

571.11386	0.33582

571.21285	0.33586

571.31185	0.33592

571.41085	0.33606

571.50984	0.33607

571.60884	0.33609

571.70784	0.33635

571.80684	0.33636

571.90583	0.33637

572.00483	0.33661

572.10383	0.33664

572.20282	0.33671

572.30182	0.33674

572.40082	0.33675

572.49981	0.33694

572.59881	0.337

572.69781	0.33706

572.79681	0.33707

572.8958	0.33709

572.9948	0.33721

573.0938	0.33723

573.19279	0.33724

573.29179	0.33726

573.39079	0.33727

573.48978	0.33768

573.58878	0.33798

573.68778	0.338

573.78678	0.33809

573.88577	0.33816

573.98477	0.33846

574.08377	0.33847

574.18276	0.33861

574.28176	0.33865

574.38076	0.33867

574.47975	0.33879

574.57875	0.3388

574.67775	0.3391

574.77675	0.33912

574.87574	0.33915

574.97474	0.33932

575.07374	0.3394

575.17273	0.33941

575.27173	0.33963

575.37073	0.33966

575.46973	0.33975

575.56872	0.33982

575.66772	0.33983

575.76672	0.33995

575.86571	0.33998

575.96471	0.34019

576.06371	0.34019

576.1627	0.34023

576.2617	0.34033

576.3607	0.34034

576.4597	0.34045

576.55869	0.34048

576.65769	0.3405

576.75669	0.34065

576.85568	0.34066

576.95468	0.34069

577.05368	0.34071

577.15267	0.34075

577.25167	0.34077

577.35067	0.34084

577.44967	0.34086

577.54866	0.34087

577.64766	0.34104

577.74666	0.34107

577.84565	0.34108

577.94465	0.34109

578.04365	0.34118

578.14264	0.34152

578.24164	0.34153

578.34064	0.3418

578.43964	0.34181

578.53863	0.34182

578.63763	0.34183

578.73663	0.34185

578.83562	0.34212

578.93462	0.34216

579.03362	0.34217

579.13262	0.34234

579.23161	0.34235

579.33061	0.34236

579.42961	0.34237

579.5286	0.3424

579.6276	0.34247

579.7266	0.3425

579.82559	0.34255

579.92459	0.34262

580.02359	0.34274

580.12259	0.34303

580.22158	0.34305

580.32058	0.3431

580.41958	0.34313

580.51857	0.34315

580.61757	0.34337

580.71657	0.34344

580.81556	0.34345

580.91456	0.34355

581.01356	0.34359

581.11256	0.34361

581.21155	0.34363

581.31055	0.34379

581.40955	0.34383

581.50854	0.34384

581.60754	0.34388

581.70654	0.34392

581.80553	0.34396

581.90453	0.34408

582.00353	0.34417

582.10253	0.34418

582.20152	0.34419

582.30052	0.34424

582.39952	0.34428

582.49851	0.34429

582.59751	0.3443

582.69651	0.34433

582.79551	0.34434

582.8945	0.34436

582.9935	0.34439

583.0925	0.34446

583.19149	0.34446

583.29049	0.34447

583.38949	0.34467

583.48848	0.34484

583.58748	0.34519

583.68648	0.34529

583.78548	0.34535

583.88447	0.34566

583.98347	0.34569

584.08247	0.34588

584.18146	0.34589

584.28046	0.34592

584.37946	0.34613

584.47845	0.34613

584.57745	0.34614

584.67645	0.34644

584.77545	0.34648

584.87444	0.34655

584.97344	0.34656

585.07244	0.3466

585.17143	0.3466

585.27043	0.34674

585.36943	0.34676

585.46843	0.34679

585.56742	0.3468

585.66642	0.34696

585.76542	0.34697

585.86441	0.34706

585.96341	0.34719

586.06241	0.3472

586.1614	0.34722

586.2604	0.34725

586.3594	0.34726

586.4584	0.34727

586.55739	0.34728

586.65639	0.34735

586.75539	0.34736

586.85438	0.3475

586.95338	0.34751

587.05238	0.34753

587.15137	0.34754

587.25037	0.34766

587.34937	0.34767

587.44837	0.34788

587.54736	0.34789

587.64636	0.3479

587.74536	0.34794

587.84435	0.34797

587.94335	0.34798

588.04235	0.34819

588.14134	0.3482

588.24034	0.34849

588.33934	0.3485

588.43834	0.34851

588.53733	0.34852

588.63633	0.34855

588.73533	0.34858

588.83432	0.34865

588.93332	0.34868

589.03232	0.3487

589.13131	0.34874

589.23031	0.34886

589.32931	0.34887

589.42831	0.34891

589.5273	0.34898

589.6263	0.34905

589.7253	0.34911

589.82429	0.34914

589.92329	0.34915

590.02229	0.34915

590.12129	0.34916

590.22028	0.34934

590.31928	0.34935

590.41828	0.34941

590.51727	0.34953

590.61627	0.34957

590.71527	0.34957

590.81426	0.34967

590.91326	0.34973

591.01226	0.34999

591.11126	0.34999

591.21025	0.35

591.30925	0.3502

591.40825	0.35025

591.50724	0.35044

591.60624	0.35047

591.70524	0.35049

591.80423	0.3505

591.90323	0.35075

592.00223	0.35076

592.10123	0.35093

592.20022	0.35094

592.29922	0.35119

592.39822	0.35135

592.49721	0.3514

592.59621	0.35142

592.69521	0.35157

592.79421	0.35162

592.8932	0.35169

592.9922	0.3517

593.0912	0.35171

593.19019	0.35202

593.28919	0.35203

593.38819	0.35207

593.48718	0.3523

593.58618	0.35232

593.68518	0.35234

593.78418	0.35236

593.88317	0.35237

593.98217	0.35238

594.08117	0.35239

594.18016	0.3524

594.27916	0.35245

594.37816	0.35249

594.47715	0.3525

594.57615	0.35261

594.67515	0.3527

594.77415	0.35277

594.87314	0.35291

594.97214	0.35313

595.07114	0.35315

595.17013	0.35332

595.26913	0.35334

595.36813	0.35358

595.46712	0.3536

595.56612	0.35361

595.66512	0.35374

595.76412	0.35376

595.86311	0.35378

595.96211	0.35381

596.06111	0.35382

596.1601	0.35383

596.2591	0.35394

596.3581	0.35395

596.4571	0.35404

596.55609	0.35409

596.65509	0.3541

596.75409	0.35414

596.85308	0.35425

596.95208	0.35426

597.05108	0.35444

597.15007	0.35446

597.24907	0.35454

597.34807	0.35455

597.44707	0.3546

597.54606	0.35478

597.64506	0.3548

597.74406	0.35487

597.84305	0.35488

597.94205	0.35489

598.04105	0.35489

598.14004	0.35492

598.23904	0.35505

598.33804	0.35506

598.43704	0.35516

598.53603	0.35517

598.63503	0.35518

598.73403	0.3553

598.83302	0.35536

598.93202	0.35537

599.03102	0.35539

599.13001	0.35566

599.22901	0.35567

599.32801	0.35591

599.42701	0.35592

599.526	0.35593

599.625	0.35606

599.724	0.35607

599.82299	0.35635

599.92199	0.35639

600.02099	0.35643

600.11999	0.35644

600.21898	0.35666

600.31798	0.35671

600.41698	0.35692

600.51597	0.35692

600.61497	0.35697

600.71397	0.35703

600.81296	0.35705

600.91196	0.35707

601.01096	0.35708

601.10996	0.35727

601.20895	0.35728

601.30795	0.35728

601.40695	0.35729

601.50594	0.35759

601.60494	0.35797

601.70394	0.35798

601.80293	0.35823

601.90193	0.35824

602.00093	0.35825

602.09993	0.35831

602.19892	0.35833

602.29792	0.35834

602.39692	0.35836

602.49591	0.35848

602.59491	0.35849

602.69391	0.35855

602.7929	0.35888

602.8919	0.3589

602.9909	0.35896

603.0899	0.35897

603.18889	0.35898

603.28789	0.35923

603.38689	0.35944

603.48588	0.35945

603.58488	0.35946

603.68388	0.35947

603.78288	0.35951

603.88187	0.35954

603.98087	0.35957

604.07987	0.3597

604.17886	0.35973

604.27786	0.35974

604.37686	0.35975

604.47585	0.35983

604.57485	0.35996

604.67385	0.35999

604.77285	0.36003

604.87184	0.36004

604.97084	0.36011

605.06984	0.36012

605.16883	0.36013

605.26783	0.36021

605.36683	0.36041

605.46582	0.36049

605.56482	0.36052

605.66382	0.36054

605.76282	0.3606

605.86181	0.36078

605.96081	0.36078

606.05981	0.36089

606.1588	0.3609

606.2578	0.36094

606.3568	0.36117

606.45579	0.36118

606.55479	0.36127

606.65379	0.36129

606.75279	0.36146

606.85178	0.36147

606.95078	0.36164

607.04978	0.36165

607.14877	0.36166

607.24777	0.36184

607.34677	0.36185

607.44577	0.36187

607.54476	0.36192

607.64376	0.36197

607.74276	0.36199

607.84175	0.36221

607.94075	0.36222

608.03975	0.3623

608.13874	0.36239

608.23774	0.36239

608.33674	0.3624

608.43574	0.36267

608.53473	0.36283

608.63373	0.36284

608.73273	0.36317

608.83172	0.36317

608.93072	0.36341

609.02972	0.36343

609.12871	0.36346

609.22771	0.36348

609.32671	0.36359

609.42571	0.36359

609.5247	0.36362

609.6237	0.36367

609.7227	0.36376

609.82169	0.36416

609.92069	0.36419

610.01969	0.36421

610.11869	0.36423

610.21768	0.36444

610.31668	0.36445

610.41568	0.36446

610.51467	0.36453

610.61367	0.36458

610.71267	0.36461

610.81166	0.36464

610.91066	0.36469

611.00966	0.36472

611.10866	0.36478

611.20765	0.36479

611.30665	0.36485

611.40565	0.36494

611.50464	0.36495

611.60364	0.36496

611.70264	0.3651

611.80163	0.36511

611.90063	0.36514

611.99963	0.36526

612.09863	0.36526

612.19762	0.36547

612.29662	0.36551

612.39562	0.36556

612.49461	0.36559

612.59361	0.36561

612.69261	0.36565

612.7916	0.36576

612.8906	0.36577

612.9896	0.36578

613.0886	0.36593

613.18759	0.36594

613.28659	0.36595

613.38559	0.36615

613.48458	0.36615

613.58358	0.36617

613.68258	0.36628

613.78157	0.3663

613.88057	0.36631

613.97957	0.36649

614.07857	0.36651

614.17756	0.36652

614.27656	0.36653

614.37556	0.36655

614.47455	0.36656

614.57355	0.36657

614.67255	0.36658

614.77155	0.36684

614.87054	0.36686

614.96954	0.36687

615.06854	0.36706

615.16753	0.36707

615.26653	0.36708

615.36553	0.36728

615.46452	0.36729

615.56352	0.36731

615.66252	0.36732

615.76152	0.36736

615.86051	0.36742

615.95951	0.36743

616.05851	0.36745

616.1575	0.36752

616.2565	0.36753

616.3555	0.36755

616.45449	0.36781

616.55349	0.36807

616.65249	0.36807

616.75149	0.36813

616.85048	0.36826

616.94948	0.36828

617.04848	0.36828

617.14747	0.36844

617.24647	0.36844

617.34547	0.36845

617.44447	0.36846

617.54346	0.36862

617.64246	0.36863

617.74146	0.36864

617.84045	0.36877

617.93945	0.36884

618.03845	0.36885

618.13744	0.36886

618.23644	0.36888

618.33544	0.36899

618.43444	0.369

618.53343	0.36901

618.63243	0.36908

618.73143	0.3691

618.83042	0.36911

618.92942	0.36912

619.02842	0.36933

619.12741	0.36948

619.22641	0.3695

619.32541	0.36981

619.42441	0.36982

619.5234	0.36995

619.6224	0.37

619.7214	0.37

619.82039	0.37019

619.91939	0.3702

620.01839	0.37039

620.11738	0.3704

620.21638	0.37042

620.31538	0.37048

620.41438	0.37065

620.51337	0.37068

620.61237	0.37072

620.71137	0.37084

620.81036	0.37089

620.90936	0.37096

621.00836	0.37097

621.10736	0.37102

621.20635	0.37108

621.30535	0.37115

621.40435	0.37131

621.50334	0.37132

621.60234	0.37133

621.70134	0.37157

621.80033	0.37157

621.89933	0.37158

621.99833	0.37161

622.09733	0.3717

622.19632	0.37171

622.29532	0.37175

622.39432	0.37177

622.49331	0.37178

622.59231	0.37187

622.69131	0.37196

622.7903	0.37198

622.8893	0.37199

622.9883	0.3721

623.0873	0.37211

623.18629	0.37212

623.28529	0.37213

623.38429	0.37214

623.48328	0.37246

623.58228	0.37248

623.68128	0.37249

623.78027	0.37256

623.87927	0.37258

623.97827	0.3726

624.07727	0.37265

624.17626	0.37271

624.27526	0.37272

624.37426	0.37273

624.47325	0.37275

624.57225	0.37289

624.67125	0.37292

624.77025	0.37292

624.86924	0.37326

624.96824	0.37327

625.06724	0.37342

625.16623	0.37347

625.26523	0.37349

625.36423	0.37351

625.46322	0.37359

625.56222	0.37366

625.66122	0.37396

625.76022	0.37422

625.85921	0.37425

625.95821	0.37427

626.05721	0.37443

626.1562	0.37444

626.2552	0.37455

626.3542	0.37456

626.45319	0.37457

626.55219	0.37471

626.65119	0.37475

626.75019	0.37475

626.84918	0.37503

626.94818	0.37504

627.04718	0.37505

627.14617	0.37506

627.24517	0.37527

627.34417	0.37529

627.44316	0.37554

627.54216	0.37555

627.64116	0.37556

627.74016	0.37557

627.83915	0.37562

627.93815	0.37563

628.03715	0.37563

628.13614	0.37605

628.23514	0.37607

628.33414	0.3763

628.43314	0.37661

628.53213	0.37665

628.63113	0.37678

628.73013	0.37679

628.82912	0.37707

628.92812	0.37709

629.02712	0.37738

629.12611	0.37739

629.22511	0.37766

629.32411	0.37768

629.42311	0.37773

629.5221	0.37782

629.6211	0.37782

629.7201	0.37791

629.81909	0.37796

629.91809	0.37797

630.01709	0.37815

630.11608	0.37819

630.21508	0.3782

630.31408	0.37821

630.41308	0.37836

630.51207	0.37839

630.61107	0.3784

630.71007	0.3786

630.80906	0.37864

630.90806	0.37865

631.00706	0.37887

631.10605	0.37888

631.20505	0.37925

631.30405	0.37927

631.40305	0.37928

631.50204	0.37934

631.60104	0.37935

631.70004	0.37936

631.79903	0.37969

631.89803	0.3797

631.99703	0.37987

632.09603	0.37992

632.19502	0.37995

632.29402	0.37998

632.39302	0.38001

632.49201	0.38001

632.59101	0.38005

632.69001	0.38006

632.789	0.38006

632.888	0.38016

632.987	0.38018

633.086	0.38019

633.18499	0.38021

633.28399	0.38026

633.38299	0.38033

633.48198	0.38034

633.58098	0.3805

633.67998	0.38051

633.77897	0.38073

633.87797	0.38079

633.97697	0.38079

634.07597	0.38091

634.17496	0.38093

634.27396	0.38094

634.37296	0.38095

634.47195	0.38109

634.57095	0.3811

634.66995	0.38111

634.76895	0.38112

634.86794	0.38113

634.96694	0.38116

635.06594	0.38132

635.16493	0.38133

635.26393	0.38137

635.36293	0.38153

635.46192	0.38155

635.56092	0.38161

635.65992	0.38163

635.75892	0.38165

635.85791	0.38166

635.95691	0.38167

636.05591	0.38168

636.1549	0.3817

636.2539	0.38187

636.3529	0.38188

636.45189	0.38189

636.55089	0.38191

636.64989	0.38192

636.74889	0.382

636.84788	0.38201

636.94688	0.38202

637.04588	0.38207

637.14487	0.38211

637.24387	0.38212

637.34287	0.38213

637.44186	0.38245

637.54086	0.38247

637.63986	0.38257

637.73886	0.38259

637.83785	0.3826

637.93685	0.38262

638.03585	0.38276

638.13484	0.38277

638.23384	0.38278

638.33284	0.3828

638.43184	0.38292

638.53083	0.38293

638.62983	0.38295

638.72883	0.38306

638.82782	0.38308

638.92682	0.38309

639.02582	0.3833

639.12481	0.38331

639.22381	0.38355

639.32281	0.38356

639.42181	0.38357

639.5208	0.38383

639.6198	0.38384

639.7188	0.38419

639.81779	0.3842

639.91679	0.38421

640.01579	0.38439

640.11478	0.3844

640.21378	0.38455

640.31278	0.38456

640.41178	0.38457

640.51077	0.38478

640.60977	0.3848

640.70877	0.38481

640.80776	0.38498

640.90676	0.38501

641.00576	0.38518

641.10475	0.38526

641.20375	0.38527

641.30275	0.38543

641.40175	0.38545

641.50074	0.38564

641.59974	0.38565

641.69874	0.38566

641.79773	0.38573

641.89673	0.38579

641.99573	0.38602

642.09473	0.38603

642.19372	0.38626

642.29272	0.38627

642.39172	0.38653

642.49071	0.38656

642.58971	0.38672

642.68871	0.38673

642.7877	0.38673

642.8867	0.38701

642.9857	0.38702

643.0847	0.38707

643.18369	0.3871

643.28269	0.38718

643.38169	0.38719

643.48068	0.3872

643.57968	0.38727

643.67868	0.38728

643.77767	0.38729

643.87667	0.38732

643.97567	0.38736

644.07467	0.38753

644.17366	0.38754

644.27266	0.38755

644.37166	0.38757

644.47065	0.38787

644.56965	0.38788

644.66865	0.38788

644.76764	0.38789

644.86664	0.38804

644.96564	0.38806

645.06464	0.3883

645.16363	0.38831

645.26263	0.38876

645.36163	0.38877

645.46062	0.38877

645.55962	0.38898

645.65862	0.38898

645.75762	0.389

645.85661	0.38924

645.95561	0.38926

646.05461	0.38927

646.1536	0.38939

646.2526	0.3894

646.3516	0.38942

646.45059	0.38948

646.54959	0.38953

646.64859	0.38975

646.74759	0.38981

646.84658	0.38987

646.94558	0.38989

647.04458	0.38992

647.14357	0.39006

647.24257	0.39008

647.34157	0.39022

647.44056	0.39029

647.53956	0.3903

647.63856	0.39038

647.73756	0.39038

647.83655	0.39039

647.93555	0.39053

648.03455	0.39059

648.13354	0.3906

648.23254	0.39061

648.33154	0.39062

648.43053	0.3907

648.52953	0.39078

648.62853	0.39079

648.72753	0.3908

648.82652	0.39109

648.92552	0.39111

649.02452	0.39111

649.12351	0.39112

649.22251	0.39113

649.32151	0.39119

649.42051	0.39122

649.5195	0.39123

649.6185	0.39125

649.7175	0.39133

649.81649	0.39136

649.91549	0.39137

650.01449	0.39143

650.11348	0.39149

650.21248	0.3915

650.31148	0.39174

650.41048	0.39175

650.50947	0.39207

650.60847	0.39208

650.70747	0.39222

650.80646	0.39223

650.90546	0.39253

651.00446	0.39254

651.10345	0.39272

651.20245	0.39273

651.30145	0.39276

651.40045	0.39278

651.49944	0.39278

651.59844	0.39293

651.69744	0.39294

651.79643	0.39319

651.89543	0.3932

651.99443	0.39327

652.09343	0.39341

652.19242	0.39343

652.29142	0.39353

652.39042	0.39355

652.48941	0.39382

652.58841	0.39388

652.68741	0.3941

652.7864	0.39411

652.8854	0.39419

652.9844	0.39437

653.0834	0.39438

653.18239	0.3944

653.28139	0.39442

653.38039	0.39449

653.47938	0.39458

653.57838	0.3946

653.67738	0.39464

653.77637	0.39468

653.87537	0.39469

653.97437	0.39487

654.07337	0.39488

654.17236	0.39517

654.27136	0.39518

654.37036	0.39523

654.46935	0.39525

654.56835	0.39544

654.66735	0.39545

654.76634	0.39549

654.86534	0.39549

654.96434	0.39573

655.06334	0.39574

655.16233	0.39595

655.26133	0.39596

655.36033	0.39597

655.45932	0.39599

655.55832	0.3962

655.65732	0.39622

655.75631	0.39635

655.85531	0.39636

655.95431	0.39652

656.05331	0.39653

656.1523	0.39654

656.2513	0.39681

656.3503	0.39682

656.44929	0.39689

656.54829	0.39707

656.64729	0.39708

656.74629	0.39709

656.84528	0.39719

656.94428	0.3972

657.04328	0.39721

657.14227	0.39721

657.24127	0.39733

657.34027	0.39735

657.43926	0.39737

657.53826	0.39741

657.63726	0.39742

657.73626	0.39742

657.83525	0.39782

657.93425	0.39786

658.03325	0.39806

658.13224	0.39807

658.23124	0.39809

658.33024	0.39811

658.42923	0.39828

658.52823	0.3983

658.62723	0.39831

658.72623	0.39851

658.82522	0.39872

658.92422	0.39872

659.02322	0.39876

659.12221	0.39877

659.22121	0.39888

659.32021	0.39888

659.41921	0.39896

659.5182	0.39897

659.6172	0.39924

659.7162	0.39925

659.81519	0.39927

659.91419	0.39936

660.01319	0.39937

660.11218	0.39938

660.21118	0.39959

660.31018	0.3996

660.40918	0.39962

660.50817	0.39967

660.60717	0.39974

660.70617	0.39978

660.80516	0.3998

660.90416	0.39997

661.00316	0.39997

661.10215	0.39998

661.20115	0.39999

661.30015	0.4002

661.39915	0.40021

661.49814	0.40035

661.59714	0.40037

661.69614	0.4006

661.79513	0.40061

661.89413	0.40065

661.99313	0.40071

662.09212	0.40072

662.19112	0.40091

662.29012	0.40091

662.38912	0.40092

662.48811	0.40093

662.58711	0.40122

662.68611	0.40126

662.7851	0.40127

662.8841	0.40146

662.9831	0.40151

663.0821	0.40164

663.18109	0.40165

663.28009	0.40166

663.37909	0.4019

663.47808	0.402

663.57708	0.40211

663.67608	0.4023

663.77507	0.40231

663.87407	0.40233

663.97307	0.40259

664.07207	0.4026

664.17106	0.40287

664.27006	0.40289

664.36906	0.403

664.46805	0.40312

664.56705	0.40313

664.66605	0.40314

664.76504	0.40329

664.86404	0.4033

664.96304	0.40344

665.06204	0.40346

665.16103	0.40348

665.26003	0.40379

665.35903	0.40381

665.45802	0.40408

665.55702	0.40409

665.65602	0.40415

665.75501	0.40422

665.85401	0.40426

665.95301	0.40448

666.05201	0.4045

666.151	0.40451

666.25	0.40487

666.349	0.40488

666.44799	0.40493

666.54699	0.40499

666.64599	0.40512

666.74499	0.40513

666.84398	0.40516

666.94298	0.40518

667.04198	0.4054

667.14097	0.40541

667.23997	0.40555

667.33897	0.40557

667.43796	0.40558

667.53696	0.40585

667.63596	0.40586

667.73496	0.40588

667.83395	0.40602

667.93295	0.40606

668.03195	0.40607

668.13094	0.40641

668.22994	0.40644

668.32894	0.40648

668.42793	0.40652

668.52693	0.40653

668.62593	0.40654

668.72493	0.40662

668.82392	0.40665

668.92292	0.40667

669.02192	0.40681

669.12091	0.40684

669.21991	0.40685

669.31891	0.40688

669.4179	0.40716

669.5169	0.40718

669.6159	0.40724

669.7149	0.40747

669.81389	0.4077

669.91289	0.40772

670.01189	0.40773

670.11088	0.4078

670.20988	0.40784

670.30888	0.40785

670.40788	0.40802

670.50687	0.40803

670.60587	0.40818

670.70487	0.4082

670.80386	0.40843

670.90286	0.40847

671.00186	0.40847

671.10085	0.40848

671.19985	0.40864

671.29885	0.40865

671.39785	0.40866

671.49684	0.40895

671.59584	0.40896

671.69484	0.40912

671.79383	0.40915

671.89283	0.40916

671.99183	0.40931

672.09082	0.40936

672.18982	0.40936

672.28882	0.40959

672.38782	0.40988

672.48681	0.41008

672.58581	0.41009

672.68481	0.41033

672.7838	0.41035

672.8828	0.41037

672.9818	0.4106

673.08079	0.41061

673.17979	0.41063

673.27879	0.41064

673.37779	0.41068

673.47678	0.41072

673.57578	0.41073

673.67478	0.41076

673.77377	0.411

673.87277	0.41102

673.97177	0.41102

674.07077	0.41108

674.16976	0.41109

674.26876	0.41111

674.36776	0.41112

674.46675	0.41113

674.56575	0.41114

674.66475	0.41119

674.76374	0.4112

674.86274	0.41121

674.96174	0.41147

675.06074	0.41149

675.15973	0.41163

675.25873	0.41164

675.35773	0.41172

675.45672	0.41186

675.55572	0.41187

675.65472	0.41188

675.75371	0.41189

675.85271	0.41215

675.95171	0.41216

676.05071	0.41217

676.1497	0.41217

676.2487	0.41219

676.3477	0.4123

676.44669	0.41251

676.54569	0.41254

676.64469	0.41255

676.74369	0.41272

676.84268	0.41274

676.94168	0.41275

677.04068	0.41276

677.13967	0.41298

677.23867	0.41306

677.33767	0.41307

677.43666	0.41325

677.53566	0.41334

677.63466	0.41335

677.73366	0.41337

677.83265	0.41337

677.93165	0.41346

678.03065	0.41366

678.12964	0.41367

678.22864	0.414

678.32764	0.41404

678.42663	0.4142

678.52563	0.41427

678.62463	0.41428

678.72363	0.41471

678.82262	0.41472

678.92162	0.41473

679.02062	0.41503

679.11961	0.41504

679.21861	0.41534

679.31761	0.41535

679.4166	0.41561

679.5156	0.41562

679.6146	0.41586

679.7136	0.41587

679.81259	0.41621

679.91159	0.41626

680.01059	0.41627

680.10958	0.41645

680.20858	0.4165

680.30758	0.41651

680.40657	0.41676

680.50557	0.41676

680.60457	0.41682

680.70357	0.41686

680.80256	0.41687

680.90156	0.41699

681.00056	0.41702

681.09955	0.41705

681.19855	0.41707

681.29755	0.4172

681.39655	0.41721

681.49554	0.41722

681.59454	0.41748

681.69354	0.41749

681.79253	0.41761

681.89153	0.41762

681.99053	0.41764

682.08952	0.41776

682.18852	0.41778

682.28752	0.41783

682.38652	0.41796

682.48551	0.41796

682.58451	0.41797

682.68351	0.41821

682.7825	0.41822

682.8815	0.41843

682.9805	0.41844

683.07949	0.41845

683.17849	0.41846

683.27749	0.41847

683.37649	0.41848

683.47548	0.41859

683.57448	0.4186

683.67348	0.41875

683.77247	0.41876

683.87147	0.41877

683.97047	0.41907

684.06947	0.41911

684.16846	0.41916

684.26746	0.41918

684.36646	0.4194

684.46545	0.41941

684.56445	0.41942

684.66345	0.41962

684.76244	0.41962

684.86144	0.41964

684.96044	0.41988

685.05944	0.41989

685.15843	0.41989

685.25743	0.42013

685.35643	0.42017

685.45542	0.42018

685.55442	0.42031

685.65342	0.42033

685.75241	0.42034

685.85141	0.42037

685.95041	0.42043

686.04941	0.42044

686.1484	0.42046

686.2474	0.42047

686.3464	0.42049

686.44539	0.42055

686.54439	0.42062

686.64339	0.42065

686.74238	0.42107

686.84138	0.42108

686.94038	0.42115

687.03938	0.42122

687.13837	0.42123

687.23737	0.42167

687.33637	0.42169

687.43536	0.42193

687.53436	0.42194

687.63336	0.42195

687.73236	0.42196

687.83135	0.42212

687.93035	0.42221

688.02935	0.42222

688.12834	0.42244

688.22734	0.42255

688.32634	0.42257

688.42533	0.42276

688.52433	0.42283

688.62333	0.42284

688.72233	0.42315

688.82132	0.42316

688.92032	0.42317

689.01932	0.42318

689.11831	0.42326

689.21731	0.4233

689.31631	0.42348

689.4153	0.42349

689.5143	0.4235

689.6133	0.42352

689.7123	0.42353

689.81129	0.42395

689.91029	0.42396

690.00929	0.42416

690.10828	0.42417

690.20728	0.42452

690.30628	0.42452

690.40527	0.42458

690.50427	0.42476

690.60327	0.42477

690.70227	0.42478

690.80126	0.42495

690.90026	0.42497

690.99926	0.42498

691.09825	0.42499

691.19725	0.42516

691.29625	0.42518

691.39525	0.42521

691.49424	0.42531

691.59324	0.42536

691.69224	0.42554

691.79123	0.42555

691.89023	0.42563

691.98923	0.42569

692.08822	0.42577

692.18722	0.42579

692.28622	0.4258

692.38522	0.42582

692.48421	0.42583

692.58321	0.42606

692.68221	0.42608

692.7812	0.42613

692.8802	0.42615

692.9792	0.42617

693.07819	0.42619

693.17719	0.42642

693.27619	0.42643

693.37519	0.42645

693.47418	0.42647

693.57318	0.42652

693.67218	0.42658

693.77117	0.42659

693.87017	0.42661

693.96917	0.42681

694.06816	0.42682

694.16716	0.42685

694.26616	0.42689

694.36516	0.427

694.46415	0.42701

694.56315	0.42725

694.66215	0.42726

694.76114	0.42759

694.86014	0.42762

694.95914	0.42772

695.05814	0.42774

695.15713	0.42776

695.25613	0.42777

695.35513	0.428

695.45412	0.42802

695.55312	0.42802

695.65212	0.42803

695.75111	0.42804

695.85011	0.42835

695.94911	0.42838

696.04811	0.42838

696.1471	0.42868

696.2461	0.42872

696.3451	0.4288

696.44409	0.4288

696.54309	0.42881

696.64209	0.42882

696.74108	0.42885

696.84008	0.42897

696.93908	0.42898

697.03808	0.42901

697.13707	0.42917

697.23607	0.42925

697.33507	0.42927

697.43406	0.42942

697.53306	0.42946

697.63206	0.42949

697.73105	0.42982

697.83005	0.42995

697.92905	0.43

698.02805	0.43

698.12704	0.43015

698.22604	0.43021

698.32504	0.43021

698.42403	0.43023

698.52303	0.43027

698.62203	0.43041

698.72103	0.43043

698.82002	0.43044

698.91902	0.43066

699.01802	0.43069

699.11701	0.43073

699.21601	0.43077

699.31501	0.43078

699.414	0.43119

699.513	0.4312

699.612	0.4312

699.711	0.43155

699.80999	0.43158

699.90899	0.43166

700.00799	0.43167

700.10698	0.43169

700.20598	0.4317

700.30498	0.43171

700.40397	0.43181

700.50297	0.43187

700.60197	0.43188

700.70097	0.43203

700.79996	0.43204

700.89896	0.43205

700.99796	0.43208

701.09695	0.43222

701.19595	0.43223

701.29495	0.43224

701.39395	0.43234

701.49294	0.43244

701.59194	0.43248

701.69094	0.43269

701.78993	0.43276

701.88893	0.43277

701.98793	0.43281

702.08692	0.43286

702.18592	0.43286

702.28492	0.43287

702.38392	0.43295

702.48291	0.43296

702.58191	0.43297

702.68091	0.433

702.7799	0.43313

702.8789	0.43342

702.9779	0.43343

703.07689	0.43368

703.17589	0.43394

703.27489	0.43395

703.37389	0.43396

703.47288	0.4343

703.57188	0.43431

703.67088	0.43459

703.76987	0.43462

703.86887	0.43468

703.96787	0.43471

704.06686	0.43472

704.16586	0.43491

704.26486	0.43492

704.36386	0.43516

704.46285	0.43517

704.56185	0.43519

704.66085	0.43537

704.75984	0.43538

704.85884	0.43564

704.95784	0.43568

705.05684	0.43573

705.15583	0.43573

705.25483	0.43584

705.35383	0.43588

705.45282	0.43595

705.55182	0.43617

705.65082	0.4362

705.74981	0.43642

705.84881	0.43643

705.94781	0.43676

706.04681	0.43677

706.1458	0.43681

706.2448	0.43693

706.3438	0.43693

706.44279	0.43698

706.54179	0.43709

706.64079	0.43709

706.73978	0.4371

706.83878	0.43724

706.93778	0.43726

707.03678	0.43727

707.13577	0.43755

707.23477	0.43756

707.33377	0.43757

707.43276	0.43772

707.53176	0.43774

707.63076	0.43775

707.72975	0.43776

707.82875	0.43778

707.92775	0.43779

708.02675	0.43781

708.12574	0.43813

708.22474	0.43815

708.32374	0.43818

708.42273	0.43835

708.52173	0.43836

708.62073	0.43846

708.71973	0.43847

708.81872	0.43848

708.91772	0.43849

709.01672	0.4385

709.11571	0.43858

709.21471	0.43867

709.31371	0.43872

709.4127	0.43875

709.5117	0.43877

709.6107	0.43907

709.7097	0.4392

709.80869	0.43921

709.90769	0.43923

710.00669	0.43924

710.10568	0.43926

710.20468	0.43928

710.30368	0.43935

710.40267	0.4394

710.50167	0.43968

710.60067	0.43969

710.69967	0.4397

710.79866	0.43998

710.89766	0.44004

710.99666	0.44005

711.09565	0.44019

711.19465	0.44021

711.29365	0.44022

711.39264	0.44028

711.49164	0.44042

711.59064	0.44044

711.68964	0.44045

711.78863	0.44077

711.88763	0.44084

711.98663	0.44089

712.08562	0.4409

712.18462	0.44091

712.28362	0.44103

712.38262	0.44105

712.48161	0.44106

712.58061	0.44126

712.67961	0.44158

712.7786	0.44159

712.8776	0.44161

712.9766	0.4418

713.07559	0.44182

713.17459	0.44185

713.27359	0.44191

713.37259	0.44192

713.47158	0.44193

713.57058	0.44193

713.66958	0.44194

713.76857	0.44195

713.86757	0.44196

713.96657	0.4421

714.06556	0.44211

714.16456	0.44212

714.26356	0.44213

714.36256	0.44214

714.46155	0.44223

714.56055	0.44237

714.65955	0.4424

714.75854	0.44279

714.85754	0.44306

714.95654	0.44308

715.05553	0.44308

715.15453	0.44335

715.25353	0.44339

715.35253	0.4434

715.45152	0.44355

715.55052	0.44358

715.64952	0.44361

715.74851	0.44362

715.84751	0.44363

715.94651	0.44381

716.04551	0.44381

716.1445	0.44388

716.2435	0.44391

716.3425	0.44392

716.44149	0.44393

716.54049	0.44415

716.63949	0.44416

716.73848	0.44454

716.83748	0.44469

716.93648	0.44474

717.03548	0.44476

717.13447	0.44503

717.23347	0.44505

717.33247	0.44506

717.43146	0.44527

717.53046	0.44528

717.62946	0.44529

717.72845	0.44531

717.82745	0.44563

717.92645	0.44573

718.02545	0.44579

718.12444	0.4458

718.22344	0.4462

718.32244	0.44621

718.42143	0.44622

718.52043	0.44642

718.61943	0.44643

718.71843	0.44665

718.81742	0.44666

718.91642	0.44668

719.01542	0.44669

719.11441	0.44688

719.21341	0.44689

719.31241	0.44689

719.4114	0.44691

719.5104	0.44709

719.6094	0.44724

719.7084	0.44725

719.80739	0.44727

719.90639	0.4475

720.00539	0.44751

720.10438	0.44753

720.20338	0.44768

720.30238	0.44769

720.40137	0.4477

720.50037	0.44809

720.59937	0.44811

720.69837	0.44826

720.79736	0.44827

720.89636	0.44828

720.99536	0.44829

721.09435	0.44872

721.19335	0.44873

721.29235	0.44876

721.39134	0.44892

721.49034	0.44892

721.58934	0.44897

721.68834	0.44906

721.78733	0.44911

721.88633	0.44912

721.98533	0.44944

722.08432	0.44949

722.18332	0.44949

722.28232	0.44953

722.38131	0.44965

722.48031	0.44965

722.57931	0.4497

722.67831	0.44973

722.7773	0.44975

722.8763	0.44976

722.9753	0.44978

723.07429	0.44991

723.17329	0.44998

723.27229	0.45

723.37129	0.45001

723.47028	0.45045

723.56928	0.45046

723.66828	0.45075

723.76727	0.45076

723.86627	0.45078

723.96527	0.45079

724.06426	0.45094

724.16326	0.45097

724.26226	0.45098

724.36126	0.45135

724.46025	0.45137

724.55925	0.45138

724.65825	0.45139

724.75724	0.45158

724.85624	0.4516

724.95524	0.45177

725.05423	0.45185

725.15323	0.45202

725.25223	0.45205

725.35123	0.45205

725.45022	0.45222

725.54922	0.45225

725.64822	0.45248

725.74721	0.4525

725.84621	0.45251

725.94521	0.45276

726.04421	0.45277

726.1432	0.45296

726.2422	0.45297

726.3412	0.45305

726.44019	0.45306

726.53919	0.45325

726.63819	0.45327

726.73718	0.45328

726.83618	0.45329

726.93518	0.45356

727.03418	0.45358

727.13317	0.4536

727.23217	0.45388

727.33117	0.45389

727.43016	0.45408

727.52916	0.45408

727.62816	0.45409

727.72715	0.45413

727.82615	0.45415

727.92515	0.45439

728.02415	0.45441

728.12314	0.45443

728.22214	0.4545

728.32114	0.45452

728.42013	0.45481

728.51913	0.45482

728.61813	0.45485

728.71712	0.45511

728.81612	0.45512

728.91512	0.45527

729.01412	0.4553

729.11311	0.45532

729.21211	0.45533

729.31111	0.45556

729.4101	0.45557

729.5091	0.45558

729.6081	0.45581

729.7071	0.45582

729.80609	0.45616

729.90509	0.45616

730.00409	0.45621

730.10308	0.4563

730.20208	0.45632

730.30108	0.45632

730.40007	0.45657

730.49907	0.45659

730.59807	0.45689

730.69707	0.45693

730.79606	0.45705

730.89506	0.45707

730.99406	0.45731

731.09305	0.45733

731.19205	0.45735

731.29105	0.45736

731.39004	0.45752

731.48904	0.45752

731.58804	0.45754

731.68704	0.45778

731.78603	0.45778

731.88503	0.45781

731.98403	0.458

732.08302	0.45803

732.18202	0.45804

732.28102	0.45806

732.38001	0.45835

732.47901	0.45836

732.57801	0.45837

732.67701	0.45853

732.776	0.45854

732.875	0.45855

732.974	0.45856

733.07299	0.45857

733.17199	0.45882

733.27099	0.45886

733.36999	0.45903

733.46898	0.45903

733.56798	0.45906

733.66698	0.45945

733.76597	0.45945

733.86497	0.45946

733.96397	0.45947

734.06296	0.45959

734.16196	0.4596

734.26096	0.45964

734.35996	0.45978

734.45895	0.45981

734.55795	0.45981

734.65695	0.46012

734.75594	0.46013

734.85494	0.46015

734.95394	0.46017

735.05293	0.46029

735.15193	0.4603

735.25093	0.46059

735.34993	0.46061

735.44892	0.46065

735.54792	0.4608

735.64692	0.46086

735.74591	0.46123

735.84491	0.46127

735.94391	0.46147

736.0429	0.46148

736.1419	0.46148

736.2409	0.46149

736.3399	0.4615

736.43889	0.46151

736.53789	0.46161

736.63689	0.46163

736.73588	0.46185

736.83488	0.46189

736.93388	0.4619

737.03288	0.46206

737.13187	0.4621

737.23087	0.46211

737.32987	0.46212

737.42886	0.46235

737.52786	0.46236

737.62686	0.46237

737.72585	0.46256

737.82485	0.46268

737.92385	0.46268

738.02285	0.46276

738.12184	0.46311

738.22084	0.46312

738.31984	0.46313

738.41883	0.46332

738.51783	0.46343

738.61683	0.46344

738.71582	0.46346

738.81482	0.46348

738.91382	0.46349

739.01282	0.46363

739.11181	0.46364

739.21081	0.46366

739.30981	0.46367

739.4088	0.46386

739.5078	0.46387

739.6068	0.46388

739.70579	0.46388

739.80479	0.46414

739.90379	0.46415

740.00279	0.46416

740.10178	0.46417

740.20078	0.46419

740.29978	0.4643

740.39877	0.4643

740.49777	0.46459

740.59677	0.46486

740.69577	0.46488

740.79476	0.46508

740.89376	0.46514

740.99276	0.46516

741.09175	0.46518

741.19075	0.46535

741.28975	0.46537

741.38874	0.46538

741.48774	0.46565

741.58674	0.46565

741.68574	0.46566

741.78473	0.46581

741.88373	0.46584

741.98273	0.46586

742.08172	0.46596

742.18072	0.46601

742.27972	0.46603

742.37871	0.46604

742.47771	0.46642

742.57671	0.46643

742.67571	0.46647

742.7747	0.46651

742.8737	0.46656

742.9727	0.46673

743.07169	0.46674

743.17069	0.46675

743.26969	0.46692

743.36869	0.46701

743.46768	0.46732

743.56668	0.46733

743.66568	0.46734

743.76467	0.46765

743.86367	0.46766

743.96267	0.46767

744.06166	0.46805

744.16066	0.46806

744.25966	0.46807

744.35866	0.46809

744.45765	0.46821

744.55665	0.46822

744.65565	0.46823

744.75464	0.46824

744.85364	0.46844

744.95264	0.46846

745.05163	0.46847

745.15063	0.46848

745.24963	0.46876

745.34863	0.46878

745.44762	0.4688

745.54662	0.46904

745.64562	0.46904

745.74461	0.46907

745.84361	0.46908

745.94261	0.46937

746.0416	0.4694

746.1406	0.4694

746.2396	0.4697

746.3386	0.46971

746.43759	0.46974

746.53659	0.46976

746.63559	0.46982

746.73458	0.46983

746.83358	0.46985

746.93258	0.46987

747.03157	0.47011

747.13057	0.47012

747.22957	0.47013

747.32857	0.4704

747.42756	0.47043

747.52656	0.47044

747.62556	0.47045

747.72455	0.47058

747.82355	0.47062

747.92255	0.47093

748.02155	0.47095

748.12054	0.47097

748.21954	0.47113

748.31854	0.47116

748.41753	0.47118

748.51653	0.47118

748.61553	0.47119

748.71452	0.4712

748.81352	0.47138

748.91252	0.47143

749.01152	0.47165

749.11051	0.47166

749.20951	0.47167

749.30851	0.47178

749.4075	0.47179

749.5065	0.4718

749.6055	0.4719

749.70449	0.47196

749.80349	0.47197

749.90249	0.47198

750.00149	0.47226

750.10048	0.47227

750.19948	0.47255

750.29848	0.47275

750.39747	0.47276

750.49647	0.47277

750.59547	0.47279

750.69447	0.47296

750.79346	0.47298

750.89246	0.47299

750.99146	0.47329

751.09045	0.47336

751.18945	0.47337

751.28845	0.47358

751.38744	0.47364

751.48644	0.47378

751.58544	0.47379

751.68444	0.47408

751.78343	0.47409

751.88243	0.47409

751.98143	0.47425

752.08042	0.47428

752.17942	0.47429

752.27842	0.47462

752.37741	0.47472

752.47641	0.47473

752.57541	0.47485

752.67441	0.47487

752.7734	0.47488

752.8724	0.47491

752.9714	0.47506

753.07039	0.47508

753.16939	0.47509

753.26839	0.47533

753.36738	0.47535

753.46638	0.47535

753.56538	0.47548

753.66438	0.47549

753.76337	0.4755

753.86237	0.47551

753.96137	0.47582

754.06036	0.47584

754.15936	0.47587

754.25836	0.47605

754.35736	0.47606

754.45635	0.47608

754.55535	0.47641

754.65435	0.47643

754.75334	0.47644

754.85234	0.47657

754.95134	0.47659

755.05033	0.4766

755.14933	0.47663

755.24833	0.4768

755.34733	0.47685

755.44632	0.47686

755.54532	0.47686

755.64432	0.47709

755.74331	0.4771

755.84231	0.47712

755.94131	0.47724

756.0403	0.47729

756.1393	0.47756

756.2383	0.47762

756.3373	0.47777

756.43629	0.47779

756.53529	0.4778

756.63429	0.47794

756.73328	0.47795

756.83228	0.47795

756.93128	0.47796

757.03027	0.47806

757.12927	0.47813

757.22827	0.47816

757.32727	0.47818

757.42626	0.47827

757.52526	0.4786

757.62426	0.47861

757.72325	0.47862

757.82225	0.47885

757.92125	0.47886

758.02025	0.47887

758.11924	0.47918

758.21824	0.4792

758.31724	0.47924

758.41623	0.47928

758.51523	0.47946

758.61423	0.47948

758.71322	0.47949

758.81222	0.47957

758.91122	0.47965

759.01022	0.47967

759.10921	0.47967

759.20821	0.47976

759.30721	0.47979

759.4062	0.47982

759.5052	0.47983

759.6042	0.47983

759.70319	0.47984

759.80219	0.48005

759.90119	0.48006

760.00019	0.48019

760.09918	0.4802

760.19818	0.4803

760.29718	0.4804

760.39617	0.4804

760.49517	0.48041

760.59417	0.4806

760.69316	0.48062

760.79216	0.48063

760.89116	0.48085

760.99016	0.48087

761.08915	0.48107

761.18815	0.48108

761.28715	0.48108

761.38614	0.48109

761.48514	0.48141

761.58414	0.48142

761.68314	0.48145

761.78213	0.48168

761.88113	0.4817

761.98013	0.48175

762.07912	0.48176

762.17812	0.48181

762.27712	0.48184

762.37611	0.48211

762.47511	0.48212

762.57411	0.48215

762.67311	0.48238

762.7721	0.48239

762.8711	0.48261

762.9701	0.48262

763.06909	0.48264

763.16809	0.48265

763.26709	0.48286

763.36608	0.48287

763.46508	0.48299

763.56408	0.48301

763.66308	0.48311

763.76207	0.48314

763.86107	0.48316

763.96007	0.48329

764.05906	0.4835

764.15806	0.48352

764.25706	0.48353

764.35605	0.48355

764.45505	0.48359

764.55405	0.48361

764.65305	0.48362

764.75204	0.48366

764.85104	0.48375

764.95004	0.48379

765.04903	0.4838

765.14803	0.48418

765.24703	0.48419

765.34603	0.48441

765.44502	0.48443

765.54402	0.48456

765.64302	0.48457

765.74201	0.48458

765.84101	0.48489

765.94001	0.48494

766.039	0.48495

766.138	0.48496

766.237	0.48519

766.336	0.4852

766.43499	0.48523

766.53399	0.48538

766.63299	0.4854

766.73198	0.48579

766.83098	0.4858

766.92998	0.48582

767.02897	0.48608

767.12797	0.4861

767.22697	0.48618

767.32597	0.48621

767.42496	0.48622

767.52396	0.48624

767.62296	0.48642

767.72195	0.48644

767.82095	0.48669

767.91995	0.48671

768.01895	0.48672

768.11794	0.48683

768.21694	0.48691

768.31594	0.48692

768.41493	0.48693

768.51393	0.48694

768.61293	0.48695

768.71192	0.48713

768.81092	0.48714

768.90992	0.48735

769.00892	0.48765

769.10791	0.4877

769.20691	0.48789

769.30591	0.48792

769.4049	0.48793

769.5039	0.48812

769.6029	0.48813

769.70189	0.48816

769.80089	0.48819

769.89989	0.48834

769.99889	0.48838

770.09788	0.48861

770.19688	0.48869

770.29588	0.48869

770.39487	0.4887

770.49387	0.48875

770.59287	0.48877

770.69186	0.48879

770.79086	0.48897

770.88986	0.48899

770.98886	0.4892

771.08785	0.48921

771.18685	0.48926

771.28585	0.48943

771.38484	0.48969

771.48384	0.48971

771.58284	0.48993

771.68184	0.48994

771.78083	0.49004

771.87983	0.49008

771.97883	0.4901

772.07782	0.49039

772.17682	0.49041

772.27582	0.49066

772.37481	0.49067

772.47381	0.49078

772.57281	0.49089

772.67181	0.49092

772.7708	0.49093

772.8698	0.49118

772.9688	0.49133

773.06779	0.49134

773.16679	0.49135

773.26579	0.4915

773.36478	0.49152

773.46378	0.49156

773.56278	0.49182

773.66178	0.4919

773.76077	0.49191

773.85977	0.49192

773.95877	0.49203

774.05776	0.4921

774.15676	0.49214

774.25576	0.49242

774.35475	0.49243

774.45375	0.49244

774.55275	0.49252

774.65175	0.49255

774.75074	0.49259

774.84974	0.49272

774.94874	0.49276

775.04773	0.49277

775.14673	0.49278

775.24573	0.49281

775.34473	0.49292

775.44372	0.49294

775.54272	0.49295

775.64172	0.49329

775.74071	0.4933

775.83971	0.49331

775.93871	0.49369

776.0377	0.49372

776.1367	0.49398

776.2357	0.49399

776.3347	0.49405

776.43369	0.49435

776.53269	0.49436

776.63169	0.49437

776.73068	0.49438

776.82968	0.49443

776.92868	0.4945

777.02767	0.49452

777.12667	0.49453

777.22567	0.49453

777.32467	0.4947

777.42366	0.49475

777.52266	0.49477

777.62166	0.49505

777.72065	0.49507

777.81965	0.49531

777.91865	0.49532

778.01764	0.49533

778.11664	0.49534

778.21564	0.49536

778.31464	0.49546

778.41363	0.49547

778.51263	0.49565

778.61163	0.49566

778.71062	0.49567

778.80962	0.49568

778.90862	0.49571

779.00762	0.4959

779.10661	0.49592

779.20561	0.4963

779.30461	0.49631

779.4036	0.49672

779.5026	0.49672

779.6016	0.49673

779.70059	0.49679

779.79959	0.4969

779.89859	0.49691

779.99759	0.49717

780.09658	0.49719

780.19558	0.49745

780.29458	0.49748

780.39357	0.49749

780.49257	0.49751

780.59157	0.49772

780.69056	0.49774

780.78956	0.49776

780.88856	0.49801

780.98756	0.49802

781.08655	0.49802

781.18555	0.49803

781.28455	0.49821

781.38354	0.49823

781.48254	0.49823

781.58154	0.49824

781.68053	0.49847

781.77953	0.49849

781.87853	0.4985

781.97753	0.49851

782.07652	0.49857

782.17552	0.49868

782.27452	0.49871

782.37351	0.49901

782.47251	0.49901

782.57151	0.49926

782.67051	0.49927

782.7695	0.49928

782.8685	0.49934

782.9675	0.49937

783.06649	0.4994

783.16549	0.49941

783.26449	0.49942

783.36348	0.49956

783.46248	0.49959

783.56148	0.4996

783.66048	0.49981

783.75947	0.49989

783.85847	0.4999

783.95747	0.4999

784.05646	0.50016

784.15546	0.50017

784.25446	0.50046

784.35345	0.50055

784.45245	0.50063

784.55145	0.50067

784.65045	0.50073

784.74944	0.50075

784.84844	0.50102

784.94744	0.50104

785.04643	0.50122

785.14543	0.50124

785.24443	0.50128

785.34343	0.50145

785.44242	0.5015

785.54142	0.50151

785.64042	0.50154

785.73941	0.50163

785.83841	0.50164

785.93741	0.50165

786.0364	0.50205

786.1354	0.50208

786.2344	0.50209

786.3334	0.50243

786.43239	0.50245

786.53139	0.50246

786.63039	0.5025

786.72938	0.50251

786.82838	0.50257

786.92738	0.50259

787.02637	0.50276

787.12537	0.50284

787.22437	0.50288

787.32337	0.50313

787.42236	0.50314

787.52136	0.50333

787.62036	0.50334

787.71935	0.50347

787.81835	0.50352

787.91735	0.50354

788.01634	0.50358

788.11534	0.5037

788.21434	0.50386

788.31334	0.50386

788.41233	0.50412

788.51133	0.50412

788.61033	0.50428

788.70932	0.50435

788.80832	0.50436

788.90732	0.50438

789.00631	0.50457

789.10531	0.50458

789.20431	0.50459

789.30331	0.50493

789.4023	0.50494

789.5013	0.50495

789.6003	0.50495

789.69929	0.50521

789.79829	0.50526

789.89729	0.5053

789.99629	0.50531

790.09528	0.50589

790.19428	0.50591

790.29328	0.50597

790.39227	0.50601

790.49127	0.50604

790.59027	0.50606

790.68926	0.50615

790.78826	0.50615

790.88726	0.50617

790.98626	0.50618

791.08525	0.50667

791.18425	0.50667

791.28325	0.50678

791.38224	0.50686

791.48124	0.50687

791.58024	0.50688

791.67923	0.50733

791.77823	0.50734

791.87723	0.50757

791.97623	0.50759

792.07522	0.5076

792.17422	0.50761

792.27322	0.50783

792.37221	0.50787

792.47121	0.50788

792.57021	0.5079

792.66921	0.50804

792.7682	0.50807

792.8672	0.50835

792.9662	0.50844

793.06519	0.50845

793.16419	0.50859

793.26319	0.50862

793.36218	0.50863

793.46118	0.50874

793.56018	0.50877

793.65918	0.50882

793.75817	0.50883

793.85717	0.50884

793.95617	0.50887

794.05516	0.50914

794.15416	0.50915

794.25316	0.50916

794.35215	0.5095

794.45115	0.50957

794.55015	0.50962

794.64915	0.50963

794.74814	0.50968

794.84714	0.50969

794.94614	0.50972

795.04513	0.50993

795.14413	0.50994

795.24313	0.50996

795.34212	0.51016

795.44112	0.51016

795.54012	0.51051

795.63912	0.51052

795.73811	0.51053

795.83711	0.51053

795.93611	0.5108

796.0351	0.51083

796.1341	0.51084

796.2331	0.5111

796.3321	0.51111

796.43109	0.51126

796.53009	0.51137

796.62909	0.51139

796.72808	0.5114

796.82708	0.51174

796.92608	0.51175

797.02507	0.51188

797.12407	0.51196

797.22307	0.51197

797.32207	0.51235

797.42106	0.51235

797.52006	0.51236

797.61906	0.51237

797.71805	0.51258

797.81705	0.5126

797.91605	0.51261

798.01504	0.51261

798.11404	0.51262

798.21304	0.51263

798.31204	0.51267

798.41103	0.51268

798.51003	0.5127

798.60903	0.51282

798.70802	0.51287

798.80702	0.51293

798.90602	0.513

799.00501	0.5131

799.10401	0.51313

799.20301	0.51328

799.30201	0.51333

799.401	0.51334

799.5	0.51356

799.599	0.5136

799.69799	0.5136

799.79699	0.51361

799.89599	0.51362

799.99499	0.51364

800.09398	0.5137

800.19298	0.51373

800.29198	0.51374

800.39097	0.51379

800.48997	0.51381

800.58897	0.51388

800.68796	0.51397

800.78696	0.51398

800.88596	0.51399

800.98496	0.51401

801.08395	0.51418

801.18295	0.51419

801.28195	0.5142

801.38094	0.51421

801.47994	0.51454

801.57894	0.51456

801.67793	0.51459

801.77693	0.51482

801.87593	0.51486

801.97493	0.51486

802.07392	0.51503

802.17292	0.51505

802.27192	0.51506

802.37091	0.5153

802.46991	0.51531

802.56891	0.51535

802.6679	0.51536

802.7669	0.51554

802.8659	0.51592

802.9649	0.51594

803.06389	0.51595

803.16289	0.51595

803.26189	0.51622

803.36088	0.51637

803.45988	0.51642

803.55888	0.51646

803.65788	0.5166

803.75687	0.51661

803.85587	0.51662

803.95487	0.51663

804.05386	0.51666

804.15286	0.51673

804.25186	0.51673

804.35085	0.51675

804.44985	0.51699

804.54885	0.517

804.64785	0.51702

804.74684	0.51721

804.84584	0.51725

804.94484	0.51751

805.04383	0.51751

805.14283	0.51754

805.24183	0.51781

805.34082	0.51782

805.43982	0.51783

805.53882	0.51783

805.63782	0.5181

805.73681	0.51811

805.83581	0.51813

805.93481	0.5183

806.0338	0.51831

806.1328	0.51853

806.2318	0.51854

806.33079	0.51855

806.42979	0.51871

806.52879	0.51872

806.62779	0.51876

806.72678	0.51902

806.82578	0.51903

806.92478	0.51908

807.02377	0.51912

807.12277	0.51913

807.22177	0.51914

807.32077	0.51915

807.41976	0.51949

807.51876	0.5195

807.61776	0.51955

807.71675	0.51955

807.81575	0.51963

807.91475	0.51964

808.01374	0.51986

808.11274	0.51986

808.21174	0.5199

808.31074	0.52012

808.40973	0.52016

808.50873	0.52029

808.60773	0.52031

808.70672	0.52033

808.80572	0.52042

808.90472	0.52044

809.00371	0.52046

809.10271	0.52061

809.20171	0.52062

809.30071	0.52066

809.3997	0.52067

809.4987	0.52072

809.5977	0.5208

809.69669	0.52081

809.79569	0.52082

809.89469	0.52083

809.99369	0.5211

810.09268	0.52112

810.19168	0.52127

810.29068	0.52132

810.38967	0.52133

810.48867	0.52136

810.58767	0.52152

810.68666	0.52153

810.78566	0.52154

810.88466	0.52184

810.98366	0.52188

811.08265	0.52219

811.18165	0.5222

811.28065	0.5222

811.37964	0.52254

811.47864	0.52255

811.57764	0.52256

811.67663	0.52257

811.77563	0.52275

811.87463	0.52281

811.97363	0.52282

812.07262	0.52302

812.17162	0.52303

812.27062	0.52304

812.36961	0.52304

812.46861	0.52305

812.56761	0.5233

812.6666	0.52332

812.7656	0.52333

812.8646	0.52369

812.9636	0.52371

813.06259	0.52372

813.16159	0.52402

813.26059	0.52404

813.35958	0.52405

813.45858	0.52406

813.55758	0.52424

813.65657	0.52427

813.75557	0.52428

813.85457	0.52462

813.95357	0.52465

814.05256	0.52466

814.15156	0.52475

814.25056	0.52476

814.34955	0.52478

814.44855	0.52479

814.54755	0.52493

814.64655	0.52494

814.74554	0.52496

814.84454	0.52497

814.94354	0.52499

815.04253	0.52516

815.14153	0.52518

815.24053	0.52537

815.33952	0.52538

815.43852	0.52541

815.53752	0.52544

815.63652	0.52544

815.73551	0.52545

815.83451	0.52546

815.93351	0.52549

816.0325	0.52568

816.1315	0.52572

816.2305	0.52573

816.32949	0.52574

816.42849	0.52601

816.52749	0.52605

816.62649	0.52606

816.72548	0.52609

816.82448	0.52615

816.92348	0.52624

817.02247	0.52629

817.12147	0.5263

817.22047	0.52657

817.31947	0.52671

817.41846	0.52674

817.51746	0.52678

817.61646	0.52685

817.71545	0.527

817.81445	0.52701

817.91345	0.52702

818.01244	0.52731

818.11144	0.52733

818.21044	0.52757

818.30944	0.52764

818.40843	0.52765

818.50743	0.52795

818.60643	0.52797

818.70542	0.52798

818.80442	0.52799

818.90342	0.52815

819.00241	0.52816

819.10141	0.52819

819.20041	0.52821

819.29941	0.52841

819.3984	0.52841

819.4974	0.5286

819.5964	0.52866

819.69539	0.52867

819.79439	0.52888

819.89339	0.52888

819.99238	0.52889

820.09138	0.52893

820.19038	0.52914

820.28938	0.52918

820.38837	0.52919

820.48737	0.52919

820.58637	0.52941

820.68536	0.52942

820.78436	0.52943

820.88336	0.52944

820.98236	0.52961

821.08135	0.52962

821.18035	0.52987

821.27935	0.52989

821.37834	0.53013

821.47734	0.53015

821.57634	0.53023

821.67533	0.53031

821.77433	0.53032

821.87333	0.53045

821.97233	0.5305

822.07132	0.53051

822.17032	0.53072

822.26932	0.53075

822.36831	0.53076

822.46731	0.53077

822.56631	0.53082

822.6653	0.53089

822.7643	0.53095

822.8633	0.53096

822.9623	0.53112

823.06129	0.53113

823.16029	0.53115

823.25929	0.53116

823.35828	0.53117

823.45728	0.53117

823.55628	0.53133

823.65527	0.53133

823.75427	0.53134

823.85327	0.5315

823.95227	0.53153

824.05126	0.53156

824.15026	0.53159

824.24926	0.53159

824.34825	0.53172

824.44725	0.53173

824.54625	0.53174

824.64525	0.53176

824.74424	0.53204

824.84324	0.53206

824.94224	0.5321

825.04123	0.53228

825.14023	0.53234

825.23923	0.53235

825.33822	0.53251

825.43722	0.53255

825.53622	0.53294

825.63522	0.53294

825.73421	0.53296

825.83321	0.53333

825.93221	0.53334

826.0312	0.53336

826.1302	0.53364

826.2292	0.53365

826.32819	0.53366

826.42719	0.53368

826.52619	0.53396

826.62519	0.53398

826.72418	0.53399

826.82318	0.534

826.92218	0.53414

827.02117	0.53419

827.12017	0.53423

827.21917	0.53424

827.31816	0.53425

827.41716	0.53456

827.51616	0.53456

827.61516	0.53491

827.71415	0.53497

827.81315	0.53538

827.91215	0.53539

828.01114	0.53539

828.11014	0.5354

828.20914	0.53541

828.30814	0.53548

828.40713	0.53593

828.50613	0.53595

828.60513	0.53596

828.70412	0.53633

828.80312	0.53658

828.90212	0.53661

829.00111	0.53664

829.10011	0.53677

829.19911	0.53679

829.29811	0.5368

829.3971	0.53707

829.4961	0.53708

829.5951	0.53744

829.69409	0.53745

829.79309	0.53774

829.89209	0.53776

829.99108	0.53777

830.09008	0.53804

830.18908	0.53806

830.28808	0.53826

830.38707	0.53826

830.48607	0.53829

830.58507	0.53883

830.68406	0.53884

830.78306	0.53916

830.88206	0.5392

830.98105	0.5392

831.08005	0.53922

831.17905	0.5396

831.27805	0.53965

831.37704	0.53967

831.47604	0.53995

831.57504	0.54002

831.67403	0.54003

831.77303	0.54004

831.87203	0.54015

831.97103	0.54018

832.07002	0.54019

832.16902	0.54053

832.26802	0.54054

832.36701	0.54064

832.46601	0.54086

832.56501	0.54087

832.664	0.54088

832.763	0.54113

832.862	0.54114

832.961	0.54116

833.05999	0.54149

833.15899	0.54151

833.25799	0.54191

833.35698	0.54192

833.45598	0.54194

833.55498	0.54212

833.65397	0.54215

833.75297	0.54216

833.85197	0.54243

833.95097	0.54246

834.04996	0.54247

834.14896	0.54248

834.24796	0.54249

834.34695	0.54255

834.44595	0.54264

834.54495	0.54265

834.64395	0.54266

834.74294	0.54285

834.84194	0.54287

834.94094	0.543

835.03993	0.54303

835.13893	0.54304

835.23793	0.54308

835.33692	0.54312

835.43592	0.54343

835.53492	0.54344

835.63392	0.5437

835.73291	0.54371

835.83191	0.54372

835.93091	0.54401

836.0299	0.54435

836.1289	0.54436

836.2279	0.54437

836.32689	0.54439

836.42589	0.54452

836.52489	0.54456

836.62389	0.54474

836.72288	0.54475

836.82188	0.54476

836.92088	0.54477

837.01987	0.54481

837.11887	0.54498

837.21787	0.54501

837.31686	0.54527

837.41586	0.54528

837.51486	0.54529

837.61386	0.5453

837.71285	0.54565

837.81185	0.54593

837.91085	0.54597

838.00984	0.54601

838.10884	0.54623

838.20784	0.54624

838.30684	0.54626

838.40583	0.54633

838.50483	0.54634

838.60383	0.54641

838.70282	0.54642

838.80182	0.54644

838.90082	0.54663

838.99981	0.54664

839.09881	0.5467

839.19781	0.54711

839.29681	0.54712

839.3958	0.54728

839.4948	0.54729

839.5938	0.5473

839.69279	0.54731

839.79179	0.54732

839.89079	0.54764

839.98978	0.54774

840.08878	0.54795

840.18778	0.54797

840.28678	0.54811

840.38577	0.54811

840.48477	0.54812

840.58377	0.54815

840.68276	0.54842

840.78176	0.5485

840.88076	0.54851

840.97975	0.54856

841.07875	0.54862

841.17775	0.54863

841.27675	0.54864

841.37574	0.54869

841.47474	0.54877

841.57374	0.54905

841.67273	0.54906

841.77173	0.5491

841.87073	0.54911

841.96973	0.54912

842.06872	0.54949

842.16772	0.5495

842.26672	0.54952

842.36571	0.54995

842.46471	0.54996

842.56371	0.54998

842.6627	0.54999

842.7617	0.55022

842.8607	0.55023

842.9597	0.55051

843.05869	0.55052

843.15769	0.55054

843.25669	0.55069

843.35568	0.55072

843.45468	0.55086

843.55368	0.5509

843.65267	0.55092

843.75167	0.55093

843.85067	0.55093

843.94967	0.55123

844.04866	0.55124

844.14766	0.5514

844.24666	0.55141

844.34565	0.55142

844.44465	0.55143

844.54365	0.55144

844.64264	0.55163

844.74164	0.55165

844.84064	0.55165

844.93964	0.55167

845.03863	0.55194

845.13763	0.55195

845.23663	0.55197

845.33562	0.55227

845.43462	0.55228

845.53362	0.55228

845.63262	0.55245

845.73161	0.55248

845.83061	0.55249

845.92961	0.55254

846.0286	0.55257

846.1276	0.55258

846.2266	0.55259

846.32559	0.55261

846.42459	0.55262

846.52359	0.55294

846.62259	0.55295

846.72158	0.55306

846.82058	0.55313

846.91958	0.55317

847.01857	0.55317

847.11757	0.55319

847.21657	0.55338

847.31556	0.5534

847.41456	0.55341

847.51356	0.55343

847.61256	0.55348

847.71155	0.5535

847.81055	0.55351

847.90955	0.55352

848.00854	0.55354

848.10754	0.55374

848.20654	0.55374

848.30553	0.55375

848.40453	0.55381

848.50353	0.554

848.60253	0.554

848.70152	0.55437

848.80052	0.5544

848.89952	0.55448

848.99851	0.55457

849.09751	0.55457

849.19651	0.55458

849.29551	0.55469

849.3945	0.5547

849.4935	0.55474

849.5925	0.55482

849.69149	0.55492

849.79049	0.55494

849.88949	0.55497

849.98848	0.55498

850.08748	0.55504

850.18648	0.55518

850.28548	0.55519

850.38447	0.55523

850.48347	0.55524

850.58247	0.5553

850.68146	0.55538

850.78046	0.55546

850.87946	0.55557

850.97845	0.55558

851.07745	0.55592

851.17645	0.55594

851.27545	0.55595

851.37444	0.55599

851.47344	0.55611

851.57244	0.55612

851.67143	0.55624

851.77043	0.55627

851.86943	0.55628

851.96843	0.55629

852.06742	0.55629

852.16642	0.55659

852.26542	0.55664

852.36441	0.55666

852.46341	0.55668

852.56241	0.55669

852.6614	0.5567

852.7604	0.55671

852.8594	0.55672

852.9584	0.55696

853.05739	0.55702

853.15639	0.55702

853.25539	0.55734

853.35438	0.55739

853.45338	0.55739

853.55238	0.55775

853.65137	0.55777

853.75037	0.55787

853.84937	0.55789

853.94837	0.55791

854.04736	0.55803

854.14636	0.55812

854.24536	0.55813

854.34435	0.55837

854.44335	0.55838

854.54235	0.55838

854.64134	0.55875

854.74034	0.55879

854.83934	0.5588

854.93834	0.55885

855.03733	0.55901

855.13633	0.55904

855.23533	0.5594

855.33432	0.55942

855.43332	0.55945

855.53232	0.55958

855.63131	0.55966

855.73031	0.55967

855.82931	0.56009

855.92831	0.56043

856.0273	0.56045

856.1263	0.56046

856.2253	0.56046

856.32429	0.56069

856.42329	0.56072

856.52229	0.56098

856.62129	0.56099

856.72028	0.561

856.81928	0.56128

856.91828	0.56129

857.01727	0.5613

857.11627	0.56131

857.21527	0.56146

857.31426	0.56149

857.41326	0.5615

857.51226	0.56151

857.61126	0.56156

857.71025	0.56162

857.80925	0.56164

857.90825	0.56165

858.00724	0.56197

858.10624	0.56198

858.20524	0.56239

858.30423	0.5626

858.40323	0.56261

858.50223	0.56263

858.60123	0.56266

858.70022	0.56286

858.79922	0.56288

858.89822	0.56306

858.99721	0.56316

859.09621	0.56319

859.19521	0.5634

859.29421	0.56341

859.3932	0.56343

859.4922	0.56366

859.5912	0.56367

859.69019	0.56368

859.78919	0.5638

859.88819	0.56381

859.98718	0.56382

860.08618	0.56385

860.18518	0.56413

860.28418	0.56421

860.38317	0.56422

860.48217	0.56424

860.58117	0.56425

860.68016	0.56443

860.77916	0.56448

860.87816	0.5645

860.97715	0.56452

861.07615	0.56453

861.17515	0.56454

861.27415	0.56466

861.37314	0.5647

861.47214	0.56471

861.57114	0.56495

861.67013	0.56495

861.76913	0.56496

861.86813	0.56533

861.96712	0.56538

862.06612	0.56541

862.16512	0.56542

862.26412	0.56544

862.36311	0.56557

862.46211	0.56557

862.56111	0.56567

862.6601	0.5658

862.7591	0.56581

862.8581	0.56582

862.9571	0.56598

863.05609	0.56607

863.15509	0.56609

863.25409	0.5661

863.35308	0.56613

863.45208	0.56635

863.55108	0.5664

863.65007	0.56641

863.74907	0.56641

863.84807	0.56642

863.94707	0.56672

864.04606	0.56674

864.14506	0.56679

864.24406	0.56708

864.34305	0.56711

864.44205	0.56713

864.54105	0.56729

864.64004	0.56729

864.73904	0.5673

864.83804	0.56745

864.93704	0.56746

865.03603	0.56747

865.13503	0.56748

865.23403	0.5675

865.33302	0.56776

865.43202	0.56777

865.53102	0.56779

865.63001	0.56781

865.72901	0.56782

865.82801	0.56823

865.92701	0.56827

866.026	0.5685

866.125	0.56851

866.224	0.56854

866.32299	0.56857

866.42199	0.56875

866.52099	0.56878

866.61999	0.56903

866.71898	0.56904

866.81798	0.56905

866.91698	0.56924

867.01597	0.56926

867.11497	0.56927

867.21397	0.5693

867.31296	0.56952

867.41196	0.56955

867.51096	0.56979

867.60996	0.56981

867.70895	0.57001

867.80795	0.57004

867.90695	0.57005

868.00594	0.57031

868.10494	0.57032

868.20394	0.57036

868.30293	0.57046

868.40193	0.57048

868.50093	0.57052

868.59993	0.57076

868.69892	0.57079

868.79792	0.57081

868.89692	0.57084

868.99591	0.57095

869.09491	0.57097

869.19391	0.57099

869.2929	0.57101

869.3919	0.57131

869.4909	0.57134

869.5899	0.57135

869.68889	0.57156

869.78789	0.57158

869.88689	0.5719

869.98588	0.57217

870.08488	0.57219

870.18388	0.5722

870.28288	0.57222

870.38187	0.57228

870.48087	0.5723

870.57987	0.57234

870.67886	0.57235

870.77786	0.57235

870.87686	0.5725

870.97585	0.57251

871.07485	0.57252

871.17385	0.57253

871.27285	0.57266

871.37184	0.57269

871.47084	0.57271

871.56984	0.57282

871.66883	0.57289

871.76783	0.57296

871.86683	0.57297

871.96582	0.57314

872.06482	0.57315

872.16382	0.57318

872.26282	0.57337

872.36181	0.57339

872.46081	0.57339

872.55981	0.57341

872.6588	0.57342

872.7578	0.57351

872.8568	0.57357

872.95579	0.57358

873.05479	0.57359

873.15379	0.57365

873.25279	0.57376

873.35178	0.57376

873.45078	0.57377

873.54978	0.57379

873.64877	0.57381

873.74777	0.57411

873.84677	0.57412

873.94577	0.57414

874.04476	0.57442

874.14376	0.57443

874.24276	0.57471

874.34175	0.57473

874.44075	0.57474

874.53975	0.57475

874.63874	0.57477

874.73774	0.57508

874.83674	0.5751

874.93574	0.57511

875.03473	0.57537

875.13373	0.57538

875.23273	0.57543

875.33172	0.57551

875.43072	0.57554

875.52972	0.57578

875.62871	0.57579

875.72771	0.5758

875.82671	0.57583

875.92571	0.57584

876.0247	0.57589

876.1237	0.57589

876.2227	0.57615

876.32169	0.57615

876.42069	0.57618

876.51969	0.57622

876.61869	0.57649

876.71768	0.57651

876.81668	0.57652

876.91568	0.57678

877.01467	0.57679

877.11367	0.5768

877.21267	0.57695

877.31166	0.57709

877.41066	0.5771

877.50966	0.57737

877.60866	0.57738

877.70765	0.5774

877.80665	0.5774

877.90565	0.57772

878.00464	0.57773

878.10364	0.57775

878.20264	0.57776

878.30163	0.57807

878.40063	0.57832

878.49963	0.57834

878.59863	0.57846

878.69762	0.57849

878.79662	0.5785

878.89562	0.5785

878.99461	0.5786

879.09361	0.57861

879.19261	0.57863

879.2916	0.57864

879.3906	0.57889

879.4896	0.5789

879.5886	0.57891

879.68759	0.57921

879.78659	0.57923

879.88559	0.57924

879.98458	0.57926

880.08358	0.57945

880.18258	0.57946

880.28157	0.57947

880.38057	0.57948

880.47957	0.57976

880.57857	0.5798

880.67756	0.57981

880.77656	0.58014

880.87556	0.58015

880.97455	0.58045

881.07355	0.58046

881.17255	0.58047

881.27155	0.58052

881.37054	0.5807

881.46954	0.58072

881.56854	0.58073

881.66753	0.58078

881.76653	0.58096

881.86553	0.58097

881.96452	0.58099

882.06352	0.58104

882.16252	0.58121

882.26152	0.58122

882.36051	0.58146

882.45951	0.58147

882.55851	0.58148

882.6575	0.58173

882.7565	0.58174

882.8555	0.58175

882.95449	0.58176

883.05349	0.58181

883.15249	0.58188

883.25149	0.58219

883.35048	0.58221

883.44948	0.58223

883.54848	0.58225

883.64747	0.58242

883.74647	0.58245

883.84547	0.58246

883.94447	0.58246

884.04346	0.58259

884.14246	0.58265

884.24146	0.58267

884.34045	0.58298

884.43945	0.583

884.53845	0.58331

884.63744	0.58337

884.73644	0.58338

884.83544	0.58361

884.93444	0.58361

885.03343	0.58363

885.13243	0.58365

885.23143	0.5838

885.33042	0.58382

885.42942	0.58384

885.52842	0.58409

885.62741	0.5841

885.72641	0.58426

885.82541	0.58427

885.92441	0.58428

886.0234	0.58454

886.1224	0.58455

886.2214	0.58482

886.32039	0.58483

886.41939	0.58522

886.51839	0.58526

886.61738	0.58528

886.71638	0.58559

886.81538	0.58563

886.91438	0.58564

887.01337	0.58568

887.11237	0.5858

887.21137	0.58581

887.31036	0.58583

887.40936	0.58586

887.50836	0.58588

887.60736	0.58589

887.70635	0.5859

887.80535	0.58614

887.90435	0.58616

888.00334	0.58621

888.10234	0.58622

888.20134	0.58658

888.30033	0.58658

888.39933	0.58659

888.49833	0.58687

888.59733	0.58691

888.69632	0.58692

888.79532	0.58719

888.89432	0.5872

888.99331	0.58721

889.09231	0.58722

889.19131	0.58746

889.2903	0.58746

889.3893	0.5878

889.4883	0.58783

889.5873	0.58783

889.68629	0.58785

889.78529	0.58823

889.88429	0.58825

889.98328	0.58828

890.08228	0.58852

890.18128	0.58854

890.28027	0.58872

890.37927	0.58876

890.47827	0.58877

890.57727	0.58898

890.67626	0.589

890.77526	0.58944

890.87426	0.58947

890.97325	0.58977

891.07225	0.58979

891.17125	0.59011

891.27025	0.59012

891.36924	0.59013

891.46824	0.59014

891.56724	0.59039

891.66623	0.5904

891.76523	0.59042

891.86423	0.59068

891.96322	0.59069

892.06222	0.5907

892.16122	0.59073

892.26022	0.59109

892.35921	0.5911

892.45821	0.59111

892.55721	0.59112

892.6562	0.59123

892.7552	0.59125

892.8542	0.59165

892.95319	0.59168

893.05219	0.59169

893.15119	0.59169

893.25019	0.59188

893.34918	0.59189

893.44818	0.5919

893.54718	0.59192

893.64617	0.59194

893.74517	0.592

893.84417	0.59212

893.94316	0.59216

894.04216	0.59217

894.14116	0.59221

894.24016	0.59222

894.33915	0.59224

894.43815	0.59227

894.53715	0.59229

894.63614	0.59232

894.73514	0.59236

894.83414	0.59244

894.93314	0.59246

895.03213	0.59251

895.13113	0.59257

895.23013	0.59261

895.32912	0.59262

895.42812	0.59264

895.52712	0.59266

895.62611	0.59271

895.72511	0.59273

895.82411	0.59274

895.92311	0.59278

896.0221	0.59279

896.1211	0.59281

896.2201	0.59284

896.31909	0.59286

896.41809	0.59288

896.51709	0.59293

896.61608	0.59294

896.71508	0.59301

896.81408	0.59304

896.91308	0.59311

897.01207	0.59313

897.11107	0.59316

897.21007	0.59318

897.30906	0.5932

897.40806	0.59321

897.50706	0.59328

897.60605	0.59331

897.70505	0.59333

897.80405	0.5934

897.90305	0.59356

898.00204	0.5936

898.10104	0.59363

898.20004	0.59367

898.29903	0.59368

898.39803	0.59373

898.49703	0.59382

898.59603	0.59383

898.69502	0.59397

898.79402	0.59398

898.89302	0.59403

898.99201	0.59412

899.09101	0.59415

899.19001	0.59417

899.289	0.59442

899.388	0.59445

899.487	0.59454

899.586	0.59457

899.68499	0.59459

899.78399	0.59461

899.88299	0.59466

899.98198	0.59467

900.08098	0.59471

900.17998	0.59472

900.27897	0.59474

900.37797	0.59476

900.47697	0.59479

900.57597	0.59481

900.67496	0.59491

900.77396	0.59492

900.87296	0.59499

900.97195	0.59501

901.07095	0.59502

901.16995	0.59509

901.26895	0.59511

901.36794	0.59524

901.46694	0.59526

901.56594	0.59528

901.66493	0.59531

901.76393	0.59536

901.86293	0.59538

901.96192	0.59563

902.06092	0.59565

902.15992	0.5957

902.25892	0.59576

902.35791	0.59578

902.45691	0.59583

902.55591	0.5959

902.6549	0.59593

902.7539	0.596

902.8529	0.59603

902.95189	0.5961

903.05089	0.59612

903.14989	0.59615

903.24889	0.59617

903.34788	0.59618

903.44688	0.5962

903.54588	0.59622

903.64487	0.59623

903.74387	0.59627

903.84287	0.59628

903.94186	0.5963

904.04086	0.59633

904.13986	0.59635

904.23886	0.5964

904.33785	0.59642

904.43685	0.59643

904.53585	0.59645

904.63484	0.59647

904.73384	0.59652

904.83284	0.59653

904.93184	0.59655

905.03083	0.5966

905.12983	0.59662

905.22883	0.59664

905.32782	0.59665

905.42682	0.59667

905.52582	0.5967

905.62481	0.59672

905.72381	0.59674

905.82281	0.59675

905.92181	0.59677

906.0208	0.59679

906.1198	0.59712

906.2188	0.59717

906.31779	0.59719

906.41679	0.59726

906.51579	0.59727

906.61478	0.59739

906.71378	0.59741

906.81278	0.59744

906.91178	0.59746

907.01077	0.59756

907.10977	0.59759

907.20877	0.59761

907.30776	0.59768

907.40676	0.59774

907.50576	0.59776

907.60475	0.59778

907.70375	0.59783

907.80275	0.59784

907.90175	0.59786

908.00074	0.59788

908.09974	0.59791

908.19874	0.59826

908.29773	0.59845

908.39673	0.59848

908.49573	0.59853

908.59473	0.59858

908.69372	0.59863

908.79272	0.59865

908.89172	0.5987

908.99071	0.59872

909.08971	0.59873

909.18871	0.5988

909.2877	0.59882

909.3867	0.59883

909.4857	0.59888

909.5847	0.5989

909.68369	0.59898

909.78269	0.599

909.88169	0.59902

909.98068	0.59904

910.07968	0.59905

910.17868	0.59909

910.27767	0.5991

910.37667	0.59912

910.47567	0.59927

910.57467	0.5993

910.67366	0.59932

910.77266	0.59935

910.87166	0.5994

910.97065	0.59942

911.06965	0.59944

911.16865	0.59947

911.26764	0.59956

911.36664	0.59957

911.46564	0.59962

911.56464	0.59966

911.66363	0.59969

911.76263	0.59971

911.86163	0.59992

911.96062	0.60003

912.05962	0.60004

912.15862	0.60006

912.25762	0.60009

912.35661	0.60013

912.45561	0.60014

912.55461	0.60026

912.6536	0.60028

912.7526	0.60029

912.8516	0.6005

912.95059	0.60053

913.04959	0.60055

913.14859	0.60056

913.24759	0.6006

913.34658	0.60061

913.44558	0.60068

913.54458	0.60071

913.64357	0.60073

913.74257	0.60076

913.84157	0.6008

913.94056	0.60081

914.03956	0.60083

914.13856	0.60085

914.23756	0.60088

914.33655	0.60091

914.43555	0.60098

914.53455	0.601

914.63354	0.60102

914.73254	0.60108

914.83154	0.60123

914.93053	0.60125

915.02953	0.60127

915.12853	0.60135

915.22753	0.60143

915.32652	0.60145

915.42552	0.60149

915.52452	0.6015

915.62351	0.60152

915.72251	0.6018

915.82151	0.60184

915.92051	0.60185

916.0195	0.60187

916.1185	0.6019

916.2175	0.60195

916.31649	0.60199

916.41549	0.60204

916.51449	0.60207

916.61348	0.60209

916.71248	0.60212

916.81148	0.60214

916.91048	0.60216

917.00947	0.60217

917.10847	0.60219

917.20747	0.60224

917.30646	0.60226

917.40546	0.60227

917.50446	0.60229

917.60345	0.60231

917.70245	0.60236

917.80145	0.60237

917.90045	0.60242

917.99944	0.60246

918.09844	0.60248

918.19744	0.60253

918.29643	0.60254

918.39543	0.60256

918.49443	0.60261

918.59343	0.60264

918.69242	0.60271

918.79142	0.60278

918.89042	0.60279

918.98941	0.60281

919.08841	0.60283

919.18741	0.60284

919.2864	0.60288

919.3854	0.60289

919.4844	0.60296

919.5834	0.60301

919.68239	0.60303

919.78139	0.60305

919.88039	0.60308

919.97938	0.60315

920.07838	0.60316

920.17738	0.60318

920.27637	0.60326

920.37537	0.60328

920.47437	0.60333

920.57337	0.60338

920.67236	0.60345

920.77136	0.60347

920.87036	0.60355

920.96935	0.60358

921.06835	0.60362

921.16735	0.60363

921.26634	0.60365

921.36534	0.60368

921.46434	0.6037

921.56334	0.60372

921.66233	0.60377

921.76133	0.60378

921.86033	0.6038

921.95932	0.60383

922.05832	0.60394

922.15732	0.60395

922.25631	0.60397

922.35531	0.60399

922.45431	0.604

922.55331	0.60402

922.6523	0.60404

922.7513	0.60407

922.8503	0.60409

922.94929	0.6041

923.04829	0.60419

923.14729	0.6042

923.24629	0.60422

923.34528	0.60424

923.44428	0.60425

923.54328	0.60429

923.64227	0.60432

923.74127	0.60434

923.84027	0.60435

923.93926	0.6044

924.03826	0.60442

924.13726	0.60444

924.23626	0.60447

924.33525	0.60451

924.43425	0.60452

924.53325	0.60454

924.63224	0.60461

924.73124	0.60462

924.83024	0.60464

924.92923	0.60471

925.02823	0.60474

925.12723	0.60476

925.22623	0.60477

925.32522	0.60479

925.42422	0.60481

925.52322	0.60482

925.62221	0.60484

925.72121	0.60486

925.82021	0.60504

925.91921	0.60506

926.0182	0.60508

926.1172	0.60509

926.2162	0.60513

926.31519	0.60514

926.41419	0.60519

926.51319	0.60523

926.61218	0.60524

926.71118	0.60526

926.81018	0.60528

926.90918	0.60529

927.00817	0.60533

927.10717	0.60538

927.20617	0.60539

927.30516	0.60543

927.40416	0.60545

927.50316	0.60546

927.60215	0.60548

927.70115	0.60551

927.80015	0.60553

927.89915	0.60555

927.99814	0.60563

928.09714	0.60568

928.19614	0.60571

928.29513	0.60575

928.39413	0.60576

928.49313	0.60585

928.59212	0.60586

928.69112	0.60588

928.79012	0.6059

928.88912	0.60592

928.98811	0.60593

929.08711	0.60597

929.18611	0.60607

929.2851	0.60618

929.3841	0.6062

929.4831	0.60622

929.5821	0.60623

929.68109	0.60625

929.78009	0.60627

929.87909	0.60628

929.97808	0.6063

930.07708	0.60632

930.17608	0.60633

930.27507	0.60642

930.37407	0.60645

930.47307	0.60647

930.57207	0.60649

930.67106	0.6065

930.77006	0.6066

930.86906	0.60664

930.96805	0.60665

931.06705	0.60669

931.16605	0.6067

931.26504	0.60682

931.36404	0.60687

931.46304	0.60689

931.56204	0.60692

931.66103	0.60694

931.76003	0.60696

931.85903	0.60697

931.95802	0.60699

932.05702	0.60701

932.15602	0.60704

932.25501	0.60706

932.35401	0.60709

932.45301	0.60711

932.55201	0.60712

932.651	0.60719

932.75	0.60721

932.849	0.60722

932.94799	0.60724

933.04699	0.60726

933.14599	0.60727

933.24499	0.60732

933.34398	0.60734

933.44298	0.60738

933.54198	0.60739

933.64097	0.60741

933.73997	0.60744

933.83897	0.60746

933.93796	0.60748

934.03696	0.60749

934.13596	0.60751

934.23496	0.60753

934.33395	0.60754

934.43295	0.60763

934.53195	0.60766

934.63094	0.60768

934.72994	0.60773

934.82894	0.60774

934.92793	0.60776

935.02693	0.60778

935.12593	0.60779

935.22493	0.60781

935.32392	0.60783

935.42292	0.60784

935.52192	0.60786

935.62091	0.60788

935.71991	0.60811

935.81891	0.60813

935.9179	0.60818

936.0169	0.60828

936.1159	0.6083

936.2149	0.60831

936.31389	0.60833

936.41289	0.60835

936.51189	0.60837

936.61088	0.60838

936.70988	0.6084

936.80888	0.60842

936.90788	0.60843

937.00687	0.60845

937.10587	0.60847

937.20487	0.60853

937.30386	0.60855

937.40286	0.60868

937.50186	0.60872

937.60085	0.60873

937.69985	0.60875

937.79885	0.60877

937.89785	0.60878

937.99684	0.60884

938.09584	0.60885

938.19484	0.60894

938.29383	0.60899

938.39283	0.60902

938.49183	0.60904

938.59082	0.60905

938.68982	0.60909

938.78882	0.6091

938.88782	0.60912

938.98681	0.60914

939.08581	0.60919

939.18481	0.6092

939.2838	0.60922

939.3828	0.60924

939.4818	0.60927

939.58079	0.60929

939.67979	0.60932

939.77879	0.60934

939.87779	0.60936

939.97678	0.60937

940.07578	0.60941

940.17478	0.60944

940.27377	0.60947

940.37277	0.60949

940.47177	0.60951

940.57077	0.60952

940.66976	0.60956

940.76876	0.60957

940.86776	0.60981

940.96675	0.60983

941.06575	0.60984

941.16475	0.60988

941.26374	0.60989

941.36274	0.60993

941.46174	0.60994

941.56074	0.60996

941.65973	0.61001

941.75873	0.61006

941.85773	0.61009

941.95672	0.61014

942.05572	0.61016

942.15472	0.61018

942.25371	0.61019

942.35271	0.61026

942.45171	0.61033

942.55071	0.61035

942.6497	0.61043

942.7487	0.61046

942.8477	0.6105

942.94669	0.61051

943.04569	0.61053

943.14469	0.61056

943.24369	0.61058

943.34268	0.61093

943.44168	0.61097

943.54068	0.61098

943.63967	0.61103

943.73867	0.6111

943.83767	0.61112

943.93666	0.61115

944.03566	0.61117

944.13466	0.61118

944.23366	0.61122

944.33265	0.61125

944.43165	0.61127

944.53065	0.61128

944.62964	0.6113

944.72864	0.61137

944.82764	0.61139

944.92663	0.61142

945.02563	0.61145

945.12463	0.61152

945.22363	0.61154

945.32262	0.61155

945.42162	0.61157

945.52062	0.6116

945.61961	0.61172

945.71861	0.61181

945.81761	0.61182

945.9166	0.61187

946.0156	0.61194

946.1146	0.61197

946.2136	0.61199

946.31259	0.61201

946.41159	0.61206

946.51059	0.61207

946.60958	0.61209

946.70858	0.61211

946.80758	0.61212

946.90657	0.61214

947.00557	0.61216

947.10457	0.61217

947.20357	0.61224

947.30256	0.61229

947.40156	0.61231

947.50056	0.61243

947.59955	0.61244

947.69855	0.61246

947.79755	0.61248

947.89655	0.61249

947.99554	0.61266

948.09454	0.61269

948.19354	0.61271

948.29253	0.61273

948.39153	0.6128

948.49053	0.61285

948.58952	0.61288

948.68852	0.6129

948.78752	0.61291

948.88652	0.61293

948.98551	0.61316

949.08451	0.6132

949.18351	0.61321

949.2825	0.61327

949.3815	0.61332

949.4805	0.61333

949.57949	0.61335

949.67849	0.61337

949.77749	0.61338

949.87649	0.61342

949.97548	0.61345

950.07448	0.61347

950.17348	0.61348

950.27247	0.6135

950.37147	0.61352

950.47047	0.61353

950.56947	0.61355

950.66846	0.61358

950.76746	0.6136

950.86646	0.61363

950.96545	0.61367

951.06445	0.61368

951.16345	0.6137

951.26244	0.61372

951.36144	0.61373

951.46044	0.61375

951.55944	0.6138

951.65843	0.61385

951.75743	0.61387

951.85643	0.6139

951.95542	0.61392

952.05442	0.61394

952.15342	0.61395

952.25241	0.61397

952.35141	0.614

952.45041	0.61405

952.54941	0.61439

952.6484	0.61442

952.7474	0.61447

952.8464	0.61449

952.94539	0.61452

953.04439	0.61456

953.14339	0.61457

953.24238	0.61462

953.34138	0.61469

953.44038	0.61473

953.53938	0.61476

953.63837	0.61478

953.73737	0.61483

953.83637	0.61488

953.93536	0.61489

954.03436	0.6154

954.13336	0.61541

954.23236	0.61543

954.33135	0.61545

954.43035	0.6155

954.52935	0.61551

954.62834	0.61553

954.72734	0.6156

954.82634	0.61582

954.92533	0.61583

955.02433	0.61585

955.12333	0.61587

955.22233	0.61595

955.32132	0.61597

955.42032	0.61598

955.51932	0.61602

955.61831	0.61612

955.71731	0.61613

955.81631	0.61615

955.9153	0.61618

956.0143	0.61622

956.1133	0.61624

956.2123	0.61625

956.31129	0.61629

956.41029	0.61635

956.50929	0.61637

956.60828	0.61639

956.70728	0.6164

956.80628	0.61649

956.90527	0.6165

957.00427	0.61655

957.10327	0.61852

957.20227	0.61855

957.30126	0.61857

957.40026	0.61858

957.49926	0.6186

957.59825	0.61862

957.69725	0.61863

957.79625	0.61865

957.89525	0.61867

957.99424	0.6187

958.09324	0.61872

958.19224	0.61874

958.29123	0.61934

958.39023	0.61937

958.48923	0.61939

958.58822	0.61942

958.68722	0.61944

958.78622	0.61946

958.88522	0.61947

958.98421	0.61949

959.08321	0.61951

959.18221	0.61956

959.2812	0.61957

959.3802	0.61959

959.4792	0.61961

959.57819	0.61962

959.67719	0.61968

959.77619	0.61973

959.87519	0.61974

959.97418	0.61976

960.07318	0.61984

960.17218	0.62008

960.27117	0.62009

960.37017	0.62016

960.46917	0.62018

960.56816	0.6202

960.66716	0.62021

960.76616	0.62025

960.86516	0.62031

960.96415	0.62035

961.06315	0.62036

961.16215	0.6204

961.26114	0.6205

961.36014	0.62051

961.45914	0.62055

961.55814	0.6206

961.65713	0.62062

961.75613	0.62063

961.85513	0.62065

961.95412	0.62067

962.05312	0.62072

962.15212	0.62073

962.25111	0.62075

962.35011	0.62077

962.44911	0.62092

962.54811	0.62097

962.6471	0.62098

962.7461	0.621

962.8451	0.62102

962.94409	0.62112

963.04309	0.62115

963.14209	0.62117

963.24108	0.62119

963.34008	0.6212

963.43908	0.62134

963.53808	0.62137

963.63707	0.62139

963.73607	0.6214

963.83507	0.62142

963.93406	0.62144

964.03306	0.62145

964.13206	0.62159

964.23105	0.62161

964.33005	0.62164

964.42905	0.62167

964.52805	0.62169

964.62704	0.62171

964.72604	0.62174

964.82504	0.62177

964.92403	0.62179

965.02303	0.62182

965.12203	0.62186

965.22103	0.62187

965.32002	0.62189

965.41902	0.62191

965.51802	0.62192

965.61701	0.62197

965.71601	0.62199

965.81501	0.62201

965.914	0.62206

966.013	0.62207

966.112	0.62209

966.211	0.62211

966.30999	0.62213

966.40899	0.62214

966.50799	0.62218

966.60698	0.62221

966.70598	0.62223

966.80498	0.62226

966.90397	0.62228

967.00297	0.62231

967.10197	0.62233

967.20097	0.62234

967.29996	0.62236

967.39896	0.62238

967.49796	0.62239

967.59695	0.62243

967.69595	0.62251

967.79495	0.62253

967.89395	0.62258

967.99294	0.6226

968.09194	0.62263

968.19094	0.62265

968.28993	0.6227

968.38893	0.62271

968.48793	0.62273

968.58692	0.62275

968.68592	0.62276

968.78492	0.62278

968.88392	0.62288

968.98291	0.6229

969.08191	0.62291

969.18091	0.62296

969.2799	0.62305

969.3789	0.62306

969.4779	0.6231

969.57689	0.62312

969.67589	0.62318

969.77489	0.6232

969.87389	0.62322

969.97288	0.62337

970.07188	0.62338

970.17088	0.6234

970.26987	0.62397

970.36887	0.62404

970.46787	0.62406

970.56686	0.62409

970.66586	0.62412

970.76486	0.62416

970.86386	0.62417

970.96285	0.62419

971.06185	0.62421

971.16085	0.62422

971.25984	0.62429

971.35884	0.62437

971.45784	0.62439

971.55684	0.62446

971.65583	0.62447

971.75483	0.62449

971.85383	0.62451

971.95282	0.62454

972.05182	0.62456

972.15082	0.62469

972.24981	0.62471

972.34881	0.62474

972.44781	0.62476

972.54681	0.62478

972.6458	0.62481

972.7448	0.62493

972.8438	0.62498

972.94279	0.62499

973.04179	0.62501

973.14079	0.62505

973.23978	0.6251

973.33878	0.62511

973.43778	0.62515

973.53678	0.62516

973.63577	0.6252

973.73477	0.62523

973.83377	0.62526

973.93276	0.62528

974.03176	0.6253

974.13076	0.62533

974.22975	0.62535

974.32875	0.6255

974.42775	0.62553

974.52675	0.62557

974.62574	0.62568

974.72474	0.62573

974.82374	0.62587

974.92273	0.62588

975.02173	0.62595

975.12073	0.62598

975.21973	0.62602

975.31872	0.62604

975.41772	0.62605

975.51672	0.62609

975.61571	0.6262

975.71471	0.62622

975.81371	0.62624

975.9127	0.62625

976.0117	0.62627

976.1107	0.6263

976.2097	0.62639

976.30869	0.62642

976.40769	0.62644

976.50669	0.62654

976.60568	0.62659

976.70468	0.62664

976.80368	0.62667

976.90267	0.62669

977.00167	0.62671

977.10067	0.62674

977.19967	0.62676

977.29866	0.62677

977.39766	0.62684

977.49666	0.62709

977.59565	0.62711

977.69465	0.62718

977.79365	0.62723

977.89264	0.62724

977.99164	0.62726

978.09064	0.62728

978.18964	0.62729

978.28863	0.62733

978.38763	0.62734

978.48663	0.62736

978.58562	0.62738

978.68462	0.62744

978.78362	0.62753

978.88262	0.62755

978.98161	0.62756

979.08061	0.6276

979.17961	0.62763

979.2786	0.62765

979.3776	0.62766

979.4766	0.62793

979.57559	0.62795

979.67459	0.62796

979.77359	0.62798

979.87259	0.628

979.97158	0.62803

980.07058	0.62805

980.16958	0.62807

980.26857	0.62808

980.36757	0.62812

980.46657	0.62813

980.56556	0.62818

980.66456	0.62825

980.76356	0.62827

980.86256	0.62828

980.96155	0.6283

981.06055	0.62832

981.15955	0.62854

981.25854	0.62855

981.35754	0.62857

981.45654	0.6286

981.55553	0.62862

981.65453	0.62864

981.75353	0.62882

981.85253	0.62885

981.95152	0.62899

982.05052	0.62902

982.14952	0.62904

982.24851	0.62906

982.34751	0.62907

982.44651	0.62909

982.54551	0.62926

982.6445	0.62927

982.7435	0.62929

982.8425	0.62931

982.94149	0.62932

983.04049	0.62934

983.13949	0.62936

983.23848	0.62942

983.33748	0.62944

983.43648	0.62949

983.53548	0.62951

983.63447	0.62953

983.73347	0.62954

983.83247	0.62961

983.93146	0.62963

984.03046	0.6302

984.12946	0.63023

984.22845	0.63025

984.32745	0.63026

984.42645	0.6303

984.52545	0.63038

984.62444	0.6304

984.72344	0.63041

984.82244	0.63052

984.92143	0.63053

985.02043	0.63057

985.11943	0.6306

985.21843	0.63062

985.31742	0.63067

985.41642	0.63068

985.51542	0.63072

985.61441	0.63075

985.71341	0.6309

985.81241	0.63094

985.9114	0.63095

986.0104	0.63097

986.1094	0.63099

986.2084	0.631

986.30739	0.63102

986.40639	0.63104

986.50539	0.63105

986.60438	0.6311

986.70338	0.63114

986.80238	0.63115

986.90137	0.6312

987.00037	0.63122

987.09937	0.63127

987.19837	0.63135

987.29736	0.63137

987.39636	0.63149

987.49536	0.63166

987.59435	0.63167

987.69335	0.63169

987.79235	0.63172

987.89134	0.63176

987.99034	0.63177

988.08934	0.63179

988.18834	0.63181

988.28733	0.63186

988.38633	0.63191

988.48533	0.63194

988.58432	0.63198

988.68332	0.63199

988.78232	0.63201

988.88131	0.63203

988.98031	0.63204

989.07931	0.63206

989.17831	0.63208

989.2773	0.63209

989.3763	0.63214

989.4753	0.6326

989.57429	0.63263

989.67329	0.63265

989.77229	0.63266

989.87129	0.63268

989.97028	0.6327

990.06928	0.63273

990.16828	0.63275

990.26727	0.63285

990.36627	0.63286

990.46527	0.63288

990.56426	0.6329

990.66326	0.63308

990.76226	0.63328

990.86126	0.6333

990.96025	0.63335

991.05925	0.63339

991.15825	0.6334

991.25724	0.63342

991.35624	0.63344

991.45524	0.63345

991.55423	0.63347

991.65323	0.63354

991.75223	0.63355

991.85123	0.63357

991.95022	0.63362

992.04922	0.63379

992.14822	0.6338

992.24721	0.63419

992.34621	0.63421

992.44521	0.63429

992.54421	0.63431

992.6432	0.63432

992.7422	0.63439

992.8412	0.63441

992.94019	0.63443

993.03919	0.63444

993.13819	0.63449

993.23718	0.63453

993.33618	0.63454

993.43518	0.63481

993.53418	0.63484

993.63317	0.63486

993.73217	0.63488

993.83117	0.6349

993.93016	0.63491

994.02916	0.63496

994.12816	0.63498

994.22715	0.635

994.32615	0.63501

994.42515	0.63506

994.52415	0.6351

994.62314	0.63518

994.72214	0.6352

994.82114	0.63528

994.92013	0.6353

995.01913	0.63531

995.11813	0.63533

995.21712	0.63537

995.31612	0.63538

995.41512	0.63543

995.51412	0.63545

995.61311	0.63547

995.71211	0.63548

995.81111	0.6355

995.9101	0.63553

996.0091	0.63555

996.1081	0.63557

996.2071	0.63595

996.30609	0.63597

996.40509	0.63617

996.50409	0.63619

996.60308	0.6362

996.70208	0.63636

996.80108	0.63637

996.90007	0.63641

996.99907	0.63644

997.09807	0.63646

997.19707	0.63647

997.29606	0.63649

997.39506	0.63652

997.49406	0.63654

997.59305	0.63701

997.69205	0.63704

997.79105	0.63709

997.89004	0.63711

997.98904	0.63716

998.08804	0.63718

998.18704	0.63721

998.28603	0.63724

998.38503	0.63728

998.48403	0.63729

998.58302	0.63731

998.68202	0.63733

998.78102	0.63735

998.88001	0.63736

998.97901	0.63738

999.07801	0.63741

999.17701	0.63743

999.276	0.63745

999.375	0.63746

999.474	0.63748

999.57299	0.63753

999.67199	0.63756

999.77099	0.6376

999.86999	0.63761

999.96898	0.63763

1000.06798	0.6377

1000.16698	0.63775

1000.26597	0.63776

1000.36497	0.63785

1000.46397	0.63787

1000.56296	0.63792

1000.66196	0.63793

1000.76096	0.63802

1000.85996	0.63808

1000.95895	0.6381

1001.05795	0.63813

1001.15695	0.63818

1001.25594	0.6382

1001.35494	0.63823

1001.45394	0.6383

1001.55294	0.63834

1001.65193	0.63842

1001.75093	0.63847

1001.84993	0.6385

1001.94892	0.63857

1002.04792	0.63859

1002.14692	0.6386

1002.24591	0.63862

1002.34491	0.63864

1002.44391	0.63872

1002.54291	0.63875

1002.6419	0.63889

1002.7409	0.63894

1002.8399	0.63897

1002.93889	0.63901

1003.03789	0.63904

1003.13689	0.63906

1003.23588	0.63907

1003.33488	0.63911

1003.43388	0.63916

1003.53288	0.63921

1003.63187	0.63922

1003.73087	0.63926

1003.82987	0.63929

1003.92886	0.63986

1004.02786	0.63988

1004.12686	0.6399

1004.22585	0.63991

1004.32485	0.63993

1004.42385	0.64033

1004.52285	0.64038

1004.62184	0.6404

1004.72084	0.64053

1004.81984	0.64055

1004.91883	0.64057

1005.01783	0.64062

1005.11683	0.64073

1005.21583	0.64089

1005.31482	0.64092

1005.41382	0.64099

1005.51282	0.641

1005.61181	0.64102

1005.71081	0.64104

1005.80981	0.64211

1005.9088	0.64214

1006.0078	0.64216

1006.1068	0.64218

1006.2058	0.64219

1006.30479	0.64221

1006.40379	0.64223

1006.50279	0.64228

1006.60178	0.64241

1006.70078	0.64243

1006.79978	0.64245

1006.89877	0.64251

1006.99777	0.64253

1007.09677	0.64255

1007.19577	0.64272

1007.29476	0.64344

1007.39376	0.64347

1007.49276	0.64349

1007.59175	0.6435

1007.69075	0.64352

1007.78975	0.64362

1007.88874	0.64364

1007.98774	0.64384

1008.08674	0.64386

1008.18574	0.64387

1008.28473	0.64389

1008.38373	0.64392

1008.48273	0.64394

1008.58172	0.64396

1008.68072	0.64416

1008.77972	0.64418

1008.87872	0.64433

1008.97771	0.64434

1009.07671	0.64436

1009.17571	0.64439

1009.2747	0.64451

1009.3737	0.64454

1009.4727	0.64456

1009.57169	0.64458

1009.67069	0.64459

1009.76969	0.64461

1009.86869	0.64466

1009.96768	0.64471

1010.06668	0.6448

1010.16568	0.6449

1010.26467	0.6454

1010.36367	0.64542

1010.46267	0.64577

1010.56166	0.64579

1010.66066	0.64582

1010.75966	0.64585

1010.85866	0.64587

1010.95765	0.6459

1011.05665	0.64594

1011.15565	0.64599

1011.25464	0.64609

1011.35364	0.64616

1011.45264	0.64617

1011.55163	0.64619

1011.65063	0.64621

1011.74963	0.64647

1011.84863	0.64651

1011.94762	0.64657

1012.04662	0.64659

1012.14562	0.64674

1012.24461	0.64676

1012.34361	0.64678

1012.44261	0.64679

1012.54161	0.64681

1012.6406	0.64683

1012.7396	0.64684

1012.8386	0.64691

1012.93759	0.64693

1013.03659	0.64696

1013.13559	0.64701

1013.23458	0.64703

1013.33358	0.64704

1013.43258	0.64706

1013.53158	0.64708

1013.63057	0.64715

1013.72957	0.64718

1013.82857	0.6472

1013.92756	0.64721

1014.02656	0.64733

1014.12556	0.64735

1014.22455	0.64736

1014.32355	0.64738

1014.42255	0.6474

1014.52154	0.64741

1014.62054	0.64743

1014.71954	0.64745

1014.81854	0.64746

1014.91753	0.64751

1015.01653	0.64753

1015.11553	0.64762

1015.21453	0.6477

1015.31352	0.64772

1015.41252	0.64773

1015.51152	0.64775

1015.61051	0.64777

1015.70951	0.64783

1015.80851	0.64787

1015.9075	0.6479

1016.0065	0.64792

1016.1055	0.64793

1016.2045	0.64798

1016.30349	0.64803

1016.40249	0.64805

1016.50149	0.64807

1016.60048	0.64808

1016.69948	0.64824

1016.79848	0.64827

1016.89747	0.64832

1016.99647	0.64839

1017.09547	0.64844

1017.19447	0.64852

1017.29346	0.64854

1017.39246	0.64855

1017.49146	0.64857

1017.59045	0.64859

1017.68945	0.64861

1017.78845	0.64862

1017.88744	0.64867

1017.98644	0.64872

1018.08544	0.64876

1018.18444	0.64877

1018.28343	0.64879

1018.38243	0.64881

1018.48143	0.64882

1018.58042	0.64936

1018.67942	0.64938

1018.77842	0.64978

1018.87742	0.6498

1018.97641	0.64981

1019.07541	0.64983

1019.17441	0.64993

1019.2734	0.65003

1019.3724	0.65005

1019.4714	0.65008

1019.57039	0.6501

1019.66939	0.65018

1019.76839	0.65025

1019.86739	0.65027

1019.96638	0.65032

1020.06538	0.65033

1020.16438	0.65035

1020.26337	0.65037

1020.36237	0.65042

1020.46137	0.65043

1020.56036	0.65048

1020.65936	0.6505

1020.75836	0.65052

1020.85736	0.65053

1020.95635	0.65055

1021.05535	0.65057

1021.15435	0.6506

1021.25334	0.65102

1021.35234	0.65106

1021.45134	0.65238

1021.55033	0.65241

1021.64933	0.65287

1021.74833	0.6529

1021.84733	0.65295

1021.94632	0.653

1022.04532	0.65302

1022.14432	0.65337

1022.24331	0.65339

1022.34231	0.65344

1022.44131	0.65347

1022.54031	0.65351

1022.6393	0.65352

1022.7383	0.65354

1022.8373	0.65357

1022.93629	0.65367

1023.03529	0.65369

1023.13429	0.65371

1023.23328	0.65372

1023.33228	0.65376

1023.43128	0.65391

1023.53028	0.65392

1023.62927	0.65394

1023.72827	0.65396

1023.82727	0.65401

1023.92626	0.65403

1024.02526	0.65406

1024.12426	0.65429

1024.22325	0.65436

1024.32225	0.65441

1024.42125	0.65443

1024.52024	0.65448

1024.61924	0.6545

1024.71824	0.65451

1024.81724	0.65453

1024.91623	0.65458

1025.01523	0.65463

1025.11423	0.65465

1025.21322	0.65475

1025.31222	0.65476

1025.41122	0.65478

1025.51022	0.65481

1025.60921	0.65485

1025.70821	0.65488

1025.80721	0.6549

1025.9062	0.65507

1026.0052	0.65508

1026.1042	0.65512

1026.2032	0.65513

1026.30219	0.65515

1026.40119	0.65517

1026.50019	0.65518

1026.59918	0.6552

1026.69818	0.65522

1026.79718	0.65532

1026.89617	0.65557

1026.99517	0.6556

1027.09417	0.65562

1027.19316	0.65565

1027.29216	0.65567

1027.39116	0.65569

1027.49016	0.6557

1027.58915	0.65572

1027.68815	0.65574

1027.78715	0.65575

1027.88614	0.65577

1027.98514	0.65585

1028.08414	0.65589

1028.18314	0.65632

1028.28213	0.65648

1028.38113	0.65649

1028.48013	0.65651

1028.57912	0.65654

1028.67812	0.65661

1028.77712	0.65663

1028.87611	0.65668

1028.97511	0.65671

1029.07411	0.65693

1029.17311	0.65696

1029.2721	0.65698

1029.3711	0.657

1029.4701	0.65701

1029.56909	0.65703

1029.66809	0.65705

1029.76709	0.65706

1029.86609	0.65708

1029.96508	0.65711

1030.06408	0.65715

1030.16308	0.65716

1030.26207	0.65718

1030.36107	0.6572

1030.46007	0.65721

1030.55906	0.65723

1030.65806	0.65738

1030.75706	0.6574

1030.85606	0.65741

1030.95505	0.65743

1031.05405	0.65745

1031.15305	0.65748

1031.25204	0.65752

1031.35104	0.65777

1031.45004	0.65782

1031.54903	0.65785

1031.64803	0.65787

1031.74703	0.65794

1031.84603	0.65799

1031.94502	0.658

1032.04402	0.65802

1032.14302	0.65804

1032.24201	0.6581

1032.34101	0.65812

1032.44001	0.65815

1032.539	0.65817

1032.638	0.65819

1032.737	0.6582

1032.836	0.65822

1032.93499	0.65825

1033.03399	0.65827

1033.13299	0.65829

1033.23198	0.6583

1033.33098	0.65832

1033.42998	0.65837

1033.52897	0.65841

1033.62797	0.65842

1033.72697	0.65847

1033.82597	0.65849

1033.92496	0.65852

1034.02396	0.65857

1034.12296	0.65887

1034.22195	0.65889

1034.32095	0.65893

1034.41995	0.65896

1034.51895	0.65898

1034.61794	0.65899

1034.71694	0.65901

1034.81594	0.65903

1034.91493	0.65909

1035.01393	0.65911

1035.11293	0.65913

1035.21192	0.65914

1035.31092	0.65916

1035.40992	0.65918

1035.50891	0.65919

1035.60791	0.65921

1035.70691	0.65923

1035.80591	0.65926

1035.9049	0.65929

1036.0039	0.65931

1036.1029	0.65933

1036.20189	0.65936

1036.30089	0.6594

1036.39989	0.65941

1036.49889	0.65945

1036.59788	0.65946

1036.69688	0.65953

1036.79588	0.65955

1036.89487	0.65965

1036.99387	0.65966

1037.09287	0.6597

1037.19187	0.65983

1037.29086	0.65986

1037.38986	0.65988

1037.48886	0.6599

1037.58785	0.65992

1037.68685	0.66003

1037.78585	0.66027

1037.88484	0.66028

1037.98384	0.6603

1038.08284	0.66033

1038.18184	0.66035

1038.28083	0.66037

1038.37983	0.66069

1038.47883	0.6607

1038.57782	0.66072

1038.67682	0.66074

1038.77582	0.66077

1038.87481	0.66084

1038.97381	0.66085

1039.07281	0.66087

1039.1718	0.66107

1039.2708	0.66109

1039.3698	0.66111

1039.4688	0.66122

1039.56779	0.66127

1039.66679	0.66129

1039.76579	0.66136

1039.86479	0.66139

1039.96378	0.66141

1040.06278	0.66143

1040.16178	0.66166

1040.26077	0.66171

1040.35977	0.66174

1040.45877	0.66178

1040.55776	0.66179

1040.65676	0.66183

1040.75576	0.66188

1040.85476	0.6619

1040.95375	0.66195

1041.05275	0.66201

1041.15175	0.66203

1041.25074	0.66205

1041.34974	0.66206

1041.44874	0.6621

1041.54773	0.66213

1041.64673	0.66216

1041.74573	0.66228

1041.84472	0.6623

1041.94372	0.66233

1042.04272	0.66235

1042.14172	0.66237

1042.24071	0.66272

1042.33971	0.66273

1042.43871	0.66275

1042.5377	0.66277

1042.6367	0.66278

1042.7357	0.66294

1042.8347	0.66295

1042.93369	0.66297

1043.03269	0.66299

1043.13169	0.663

1043.23068	0.66304

1043.32968	0.66305

1043.42868	0.66309

1043.52768	0.6631

1043.62667	0.66312

1043.72567	0.66314

1043.82467	0.66315

1043.92366	0.66317

1044.02266	0.66332

1044.12166	0.66334

1044.22065	0.66336

1044.31965	0.66337

1044.41865	0.66339

1044.51764	0.66341

1044.61664	0.66342

1044.71564	0.66344

1044.81464	0.66346

1044.91363	0.66347

1045.01263	0.66356

1045.11163	0.66372

1045.21062	0.66389

1045.30962	0.66391

1045.40862	0.66393

1045.50762	0.66396

1045.60661	0.66399

1045.70561	0.66401

1045.80461	0.66403

1045.9036	0.66404

1046.0026	0.66406

1046.1016	0.66409

1046.20059	0.66433

1046.29959	0.66436

1046.39859	0.66438

1046.49759	0.66446

1046.59658	0.66513

1046.69558	0.66547

1046.79458	0.66549

1046.89357	0.66552

1046.99257	0.66565

1047.09157	0.66592

1047.19057	0.66596

1047.28956	0.66597

1047.38856	0.66601

1047.48756	0.66606

1047.58655	0.66611

1047.68555	0.66626

1047.78455	0.66631

1047.88354	0.66633

1047.98254	0.66634

1048.08154	0.66639

1048.18054	0.66641

1048.27953	0.66703

1048.37853	0.66706

1048.47753	0.6671

1048.57652	0.66732

1048.67552	0.66733

1048.77452	0.66738

1048.87351	0.6674

1048.97251	0.66743

1049.07151	0.66748

1049.17051	0.6675

1049.2695	0.66767

1049.3685	0.66768

1049.4675	0.66772

1049.56649	0.66774

1049.66549	0.66785

1049.76449	0.66787

1049.86348	0.66814

1049.96248	0.66815

1050.06148	0.66817

1050.16047	0.66869

1050.25947	0.66871

1050.35847	0.66878

1050.45747	0.66879

1050.55646	0.66881

1050.65546	0.66883

1050.75446	0.66884

1050.85345	0.66889

1050.95245	0.66956

1051.05145	0.66958

1051.15045	0.66963

1051.24944	0.66972

1051.34844	0.66978

1051.44744	0.6698

1051.54643	0.66982

1051.64543	0.66983

1051.74443	0.66985

1051.84343	0.66987

1051.94242	0.6699

1052.04142	0.66992

1052.14042	0.66993

1052.23941	0.67003

1052.33841	0.67007

1052.43741	0.67008

1052.5364	0.67012

1052.6354	0.67013

1052.7344	0.67022

1052.8334	0.67025

1052.93239	0.67027

1053.03139	0.67029

1053.13039	0.67035

1053.22938	0.67037

1053.32838	0.67039

1053.42738	0.6704

1053.52637	0.67042

1053.62537	0.67059

1053.72437	0.6706

1053.82337	0.67064

1053.92236	0.67067

1054.02136	0.67069

1054.12036	0.67071

1054.21935	0.67074

1054.31835	0.67077

1054.41735	0.67081

1054.51635	0.67084

1054.61534	0.67107

1054.71434	0.67109

1054.81334	0.67111

1054.91233	0.67114

1055.01133	0.67116

1055.11033	0.67118

1055.20932	0.67119

1055.30832	0.67148

1055.40732	0.67149

1055.50632	0.67153

1055.60531	0.67156

1055.70431	0.67159

1055.80331	0.67161

1055.9023	0.67163

1056.0013	0.67164

1056.1003	0.67166

1056.19929	0.6717

1056.29829	0.67193

1056.39729	0.67203

1056.49628	0.67247

1056.59528	0.6725

1056.69428	0.67252

1056.79328	0.67253

1056.89227	0.67255

1056.99127	0.67258

1057.09027	0.67262

1057.18926	0.67265

1057.28826	0.67267

1057.38726	0.67279

1057.48626	0.6728

1057.58525	0.6729

1057.68425	0.67294

1057.78325	0.67297

1057.88224	0.67299

1057.98124	0.673

1058.08024	0.67302

1058.17924	0.67304

1058.27823	0.67305

1058.37723	0.67307

1058.47623	0.67317

1058.57522	0.67322

1058.67422	0.67329

1058.77322	0.67331

1058.87221	0.67332

1058.97121	0.67336

1059.07021	0.67339

1059.1692	0.67341

1059.2682	0.67342

1059.3672	0.67344

1059.4662	0.67346

1059.56519	0.67354

1059.66419	0.67356

1059.76319	0.67357

1059.86218	0.67366

1059.96118	0.67368

1060.06018	0.67369

1060.15918	0.67423

1060.25817	0.67425

1060.35717	0.67426

1060.45617	0.67428

1060.55516	0.67436

1060.65416	0.67438

1060.75316	0.6744

1060.85216	0.6745

1060.95115	0.67455

1061.05015	0.67456

1061.14915	0.67458

1061.24814	0.6746

1061.34714	0.67463

1061.44614	0.67465

1061.54513	0.67467

1061.64413	0.67468

1061.74313	0.6747

1061.84212	0.67472

1061.94112	0.67473

1062.04012	0.67475

1062.13912	0.67477

1062.23811	0.67478

1062.33711	0.67482

1062.43611	0.67483

1062.5351	0.67485

1062.6341	0.67487

1062.7331	0.67488

1062.8321	0.6749

1062.93109	0.67492

1063.03009	0.67493

1063.12909	0.67495

1063.22808	0.67497

1063.32708	0.675

1063.42608	0.67503

1063.52507	0.67517

1063.62407	0.67519

1063.72307	0.6752

1063.82207	0.67524

1063.92106	0.67527

1064.02006	0.6753

1064.11906	0.67534

1064.21805	0.67535

1064.31705	0.67581

1064.41605	0.67582

1064.51505	0.67584

1064.61404	0.67587

1064.71304	0.67589

1064.81204	0.67592

1064.91103	0.67594

1065.01003	0.67599

1065.10903	0.67601

1065.20802	0.67604

1065.30702	0.67606

1065.40602	0.67609

1065.50502	0.67611

1065.60401	0.67613

1065.70301	0.67614

1065.80201	0.67616

1065.901	0.67618

1066	0.67619

1066.099	0.67623

1066.19799	0.67626

1066.29699	0.67628

1066.39599	0.67629

1066.49499	0.67631

1066.59398	0.67633

1066.69298	0.67636

1066.79198	0.67639

1066.89097	0.67643

1066.98997	0.67658

1067.08897	0.67661

1067.18796	0.67666

1067.28696	0.67671

1067.38596	0.67673

1067.48495	0.67676

1067.58395	0.67678

1067.68295	0.6768

1067.78195	0.67681

1067.88094	0.67683

1067.97994	0.67685

1068.07894	0.67722

1068.17793	0.67723

1068.27693	0.67725

1068.37593	0.67727

1068.47493	0.67728

1068.57392	0.6773

1068.67292	0.67732

1068.77192	0.67735

1068.87091	0.6774

1068.96991	0.67742

1069.06891	0.67743

1069.16791	0.67752

1069.2669	0.67755

1069.3659	0.67757

1069.4649	0.6776

1069.56389	0.67762

1069.66289	0.67767

1069.76189	0.67769

1069.86088	0.6777

1069.95988	0.67772

1070.05888	0.67774

1070.15788	0.67779

1070.25687	0.6778

1070.35587	0.67787

1070.45487	0.67789

1070.55386	0.6779

1070.65286	0.67792

1070.75186	0.67794

1070.85085	0.67795

1070.94985	0.67797

1071.04885	0.678

1071.14785	0.67802

1071.24684	0.67822

1071.34584	0.67826

1071.44484	0.67827

1071.54383	0.67829

1071.64283	0.67841

1071.74183	0.67847

1071.84083	0.67849

1071.93982	0.67851

1072.03882	0.67852

1072.13782	0.67859

1072.23681	0.67863

1072.33581	0.67866

1072.43481	0.67868

1072.5338	0.67869

1072.6328	0.67915

1072.7318	0.67916

1072.8308	0.6792

1072.92979	0.67921

1073.02879	0.67923

1073.12779	0.67926

1073.22678	0.67928

1073.32578	0.6794

1073.42478	0.67958

1073.52377	0.6796

1073.62277	0.67962

1073.72177	0.67963

1073.82076	0.67965

1073.91976	0.67968

1074.01876	0.6797

1074.11776	0.67972

1074.21675	0.67973

1074.31575	0.67975

1074.41475	0.67977

1074.51374	0.68019

1074.61274	0.68022

1074.71174	0.68024

1074.81074	0.6803

1074.90973	0.6804

1075.00873	0.68042

1075.10773	0.68044

1075.20672	0.68045

1075.30572	0.68047

1075.40472	0.68051

1075.50372	0.68074

1075.60271	0.68076

1075.70171	0.68077

1075.80071	0.68079

1075.8997	0.68081

1075.9987	0.68082

1076.0977	0.68134

1076.19669	0.68136

1076.29569	0.68138

1076.39469	0.68141

1076.49368	0.68143

1076.59268	0.68144

1076.69168	0.68153

1076.79068	0.68155

1076.88967	0.68156

1076.98867	0.68165

1077.08767	0.68166

1077.18666	0.68168

1077.28566	0.6817

1077.38466	0.68171

1077.48366	0.68173

1077.58265	0.68178

1077.68165	0.6818

1077.78065	0.68181

1077.87964	0.68183

1077.97864	0.68191

1078.07764	0.68195

1078.17663	0.682

1078.27563	0.68202

1078.37463	0.6822

1078.47363	0.68222

1078.57262	0.68238

1078.67162	0.6824

1078.77062	0.68247

1078.86961	0.68277

1078.96861	0.68279

1079.06761	0.6828

1079.1666	0.68284

1079.2656	0.68285

1079.3646	0.68294

1079.4636	0.68296

1079.56259	0.68297

1079.66159	0.68299

1079.76059	0.68319

1079.85958	0.68321

1079.95858	0.68326

1080.05758	0.68327

1080.15658	0.68336

1080.25557	0.68337

1080.35457	0.68339

1080.45357	0.68341

1080.55256	0.68342

1080.65156	0.68358

1080.75056	0.68359

1080.84955	0.68361

1080.94855	0.68366

1081.04755	0.68368

1081.14655	0.68415

1081.24554	0.68416

1081.34454	0.6842

1081.44354	0.68436

1081.54253	0.68438

1081.64153	0.68441

1081.74053	0.68458

1081.83953	0.68504

1081.93852	0.68505

1082.03752	0.68509

1082.13652	0.68537

1082.23551	0.68539

1082.33451	0.68542

1082.43351	0.68544

1082.5325	0.68546

1082.6315	0.68551

1082.7305	0.68552

1082.8295	0.68559

1082.92849	0.68561

1083.02749	0.68562

1083.12649	0.68564

1083.22548	0.68566

1083.32448	0.68567

1083.42348	0.68569

1083.52247	0.68571

1083.62147	0.68577

1083.72047	0.68579

1083.81947	0.68581

1083.91846	0.68589

1084.01746	0.68599

1084.11646	0.68613

1084.21545	0.68631

1084.31445	0.68636

1084.41345	0.68651

1084.51244	0.68653

1084.61144	0.68655

1084.71044	0.68656

1084.80943	0.68661

1084.90843	0.68663

1085.00743	0.68665

1085.10643	0.68668

1085.20542	0.68671

1085.30442	0.68673

1085.40342	0.68675

1085.50241	0.68676

1085.60141	0.68678

1085.70041	0.6868

1085.79941	0.68681

1085.8984	0.68755

1085.9974	0.68757

1086.0964	0.6876

1086.19539	0.68762

1086.29439	0.68764

1086.39339	0.68765

1086.49239	0.68767

1086.59138	0.68769

1086.69038	0.6877

1086.78938	0.68774

1086.88837	0.68775

1086.98737	0.68777

1087.08637	0.68779

1087.18536	0.6878

1087.28436	0.68782

1087.38336	0.68784

1087.48236	0.68831

1087.58135	0.68834

1087.68035	0.68836

1087.77935	0.68838

1087.87834	0.68841

1087.97734	0.68844

1088.07634	0.68861

1088.17533	0.68864

1088.27433	0.68866

1088.37333	0.68868

1088.47233	0.68869

1088.57132	0.68871

1088.67032	0.68874

1088.76932	0.68876

1088.86831	0.68879

1088.96731	0.68881

1089.06631	0.68885

1089.16531	0.68886

1089.2643	0.6889

1089.3633	0.68921

1089.4623	0.68923

1089.56129	0.68925

1089.66029	0.68926

1089.75929	0.68928

1089.85828	0.6893

1089.95728	0.68931

1090.05628	0.68935

1090.15528	0.68937

1090.25427	0.68945

1090.35327	0.68948

1090.45227	0.68952

1090.55126	0.68953

1090.65026	0.68955

1090.74926	0.69

1090.84825	0.69002

1090.94725	0.69004

1091.04625	0.69005

1091.14524	0.69007

1091.24424	0.69009

1091.34324	0.6901

1091.44224	0.69014

1091.54123	0.69015

1091.64023	0.69017

1091.73923	0.69022

1091.83822	0.69032

1091.93722	0.69034

1092.03622	0.69118

1092.13522	0.69119

1092.23421	0.69121

1092.33321	0.69124

1092.43221	0.69126

1092.5312	0.69141

1092.6302	0.69143

1092.7292	0.69165

1092.8282	0.69245

1092.92719	0.69249

1093.02619	0.69257

1093.12519	0.69259

1093.22418	0.6926

1093.32318	0.69262

1093.42218	0.69264

1093.52117	0.69265

1093.62017	0.69267

1093.71917	0.6927

1093.81816	0.69274

1093.91716	0.69275

1094.01616	0.69277

1094.11516	0.69279

1094.21415	0.69281

1094.31315	0.69282

1094.41215	0.69287

1094.51114	0.69289

1094.61014	0.69291

1094.70914	0.69294

1094.80814	0.69296

1094.90713	0.69299

1095.00613	0.69301

1095.10513	0.69304

1095.20412	0.69309

1095.30312	0.69312

1095.40212	0.69314

1095.50111	0.69317

1095.60011	0.69319

1095.69911	0.69321

1095.79811	0.69322

1095.8971	0.69324

1095.9961	0.69328

1096.0951	0.69329

1096.19409	0.69331

1096.29309	0.69334

1096.39209	0.69336

1096.49109	0.69338

1096.59008	0.69339

1096.68908	0.69341

1096.78808	0.69343

1096.88707	0.69348

1096.98607	0.69356

1097.08507	0.69358

1097.18406	0.69361

1097.28306	0.69364

1097.38206	0.69366

1097.48106	0.69368

1097.58005	0.69376

1097.67905	0.69378

1097.77805	0.6938

1097.87704	0.69381

1097.97604	0.69385

1098.07504	0.69391

1098.17403	0.69393

1098.27303	0.69395

1098.37203	0.69396

1098.47103	0.69398

1098.57002	0.69401

1098.66902	0.69405

1098.76802	0.69406

1098.86701	0.6941

1098.96601	0.69411

1099.06501	0.69413

1099.164	0.69415

1099.263	0.69423

1099.362	0.69427

1099.461	0.69428

1099.55999	0.6943

1099.65899	0.69432

1099.75799	0.69539

1099.85698	0.69557

1099.95598	0.69559

1100.05498	0.69561

1100.15397	0.69564

1100.25297	0.69566

1100.35197	0.69578

1100.45097	0.69598

1100.54996	0.69599

1100.64896	0.69601

1100.74796	0.69604

1100.84695	0.69606

1100.94595	0.69628

1101.04495	0.69635

1101.14395	0.69651

1101.24294	0.69661

1101.34194	0.69673

1101.44094	0.69675

1101.53993	0.69677

1101.63893	0.69678

1101.73793	0.69685

1101.83692	0.69687

1101.93592	0.69688

1102.03492	0.69695

1102.13391	0.69697

1102.23291	0.697

1102.33191	0.69703

1102.43091	0.69744

1102.5299	0.69745

1102.6289	0.69747

1102.7279	0.69749

1102.82689	0.69796

1102.92589	0.69814

1103.02489	0.69818

1103.12389	0.69849

1103.22288	0.69868

1103.32188	0.69883

1103.42088	0.69885

1103.51987	0.69886

1103.61887	0.69888

1103.71787	0.69891

1103.81687	0.69893

1103.91586	0.69913

1104.01486	0.69923

1104.11386	0.69925

1104.21285	0.6993

1104.31185	0.69932

1104.41085	0.69933

1104.50984	0.69943

1104.60884	0.69945

1104.70784	0.69947

1104.80684	0.69948

1104.90583	0.69977

1105.00483	0.69979

1105.10383	0.69982

1105.20282	0.69987

1105.30182	0.69989

1105.40082	0.6999

1105.49981	0.69995

1105.59881	0.70007

1105.69781	0.70009

1105.7968	0.70104

1105.8958	0.70109

1105.9948	0.70111

1106.0938	0.70123

1106.19279	0.70131

1106.29179	0.70133

1106.39079	0.70136

1106.48979	0.70138

1106.58878	0.7014

1106.68778	0.7016

1106.78678	0.70162

1106.88577	0.70165

1106.98477	0.70168

1107.08377	0.7017

1107.18276	0.70172

1107.28176	0.7018

1107.38076	0.70187

1107.47976	0.70188

1107.57875	0.7019

1107.67775	0.70192

1107.77675	0.70217

1107.87574	0.7023

1107.97474	0.70237

1108.07374	0.70239

1108.17273	0.7024

1108.27173	0.70242

1108.37073	0.70267

1108.46972	0.70269

1108.56872	0.70272

1108.66772	0.70274

1108.76672	0.70297

1108.86571	0.70301

1108.96471	0.70311

1109.06371	0.70316

1109.1627	0.70319

1109.2617	0.70323

1109.3607	0.70328

1109.4597	0.70329

1109.55869	0.70333

1109.65769	0.70344

1109.75669	0.70346

1109.85568	0.70349

1109.95468	0.70376

1110.05368	0.704

1110.15268	0.70401

1110.25167	0.70403

1110.35067	0.70407

1110.44967	0.70408

1110.54866	0.70417

1110.64766	0.7042

1110.74666	0.70422

1110.84565	0.70423

1110.94465	0.70425

1111.04365	0.70428

1111.14264	0.7043

1111.24164	0.70432

1111.34064	0.70435

1111.43964	0.70438

1111.53863	0.70442

1111.63763	0.70443

1111.73663	0.70445

1111.83562	0.70462

1111.93462	0.70464

1112.03362	0.70465

1112.13262	0.70467

1112.23161	0.70487

1112.33061	0.70492

1112.42961	0.70497

1112.5286	0.70499

1112.6276	0.70504

1112.7266	0.70506

1112.82559	0.70588

1112.92459	0.70589

1113.02359	0.7061

1113.12259	0.70635

1113.22158	0.70636

1113.32058	0.70638

1113.41958	0.70641

1113.51857	0.70645

1113.61757	0.70646

1113.71657	0.70648

1113.81557	0.70652

1113.91456	0.70653

1114.01356	0.70662

1114.11256	0.70663

1114.21155	0.70665

1114.31055	0.70668

1114.40955	0.7071

1114.50854	0.70712

1114.60754	0.70714

1114.70654	0.70776

1114.80554	0.70791

1114.90453	0.70796

1115.00353	0.70797

1115.10253	0.70803

1115.20152	0.70804

1115.30052	0.70806

1115.39952	0.70809

1115.49851	0.70811

1115.59751	0.70828

1115.69651	0.70855

1115.79551	0.70856

1115.8945	0.70868

1115.9935	0.7087

1116.0925	0.70873

1116.19149	0.70937

1116.29049	0.70955

1116.38949	0.70957

1116.48848	0.7096

1116.58748	0.70962

1116.68648	0.70964

1116.78547	0.70965

1116.88447	0.70967

1116.98347	0.71009

1117.08247	0.71014

1117.18146	0.71016

1117.28046	0.71017

1117.37946	0.71019

1117.47845	0.71027

1117.57745	0.71056

1117.67645	0.71058

1117.77545	0.71061

1117.87444	0.71064

1117.97344	0.71066

1118.07244	0.71068

1118.17143	0.71069

1118.27043	0.71101

1118.36943	0.71103

1118.46843	0.71105

1118.56742	0.71106

1118.66642	0.7111

1118.76542	0.71111

1118.86441	0.71113

1118.96341	0.71118

1119.06241	0.71121

1119.1614	0.71123

1119.2604	0.71125

1119.3594	0.71126

1119.4584	0.71128

1119.55739	0.71131

1119.65639	0.71133

1119.75539	0.71135

1119.85438	0.71136

1119.95338	0.71138

1120.05238	0.71142

1120.15137	0.71143

1120.25037	0.71147

1120.34937	0.7115

1120.44837	0.71152

1120.54736	0.71153

1120.64636	0.71158

1120.74536	0.71162

1120.84435	0.71167

1120.94335	0.71168

1121.04235	0.7117

1121.14135	0.71172

1121.24034	0.71178

1121.33934	0.7118

1121.43834	0.71182

1121.53733	0.71183

1121.63633	0.71185

1121.73533	0.71187

1121.83432	0.71188

1121.93332	0.7119

1122.03232	0.71192

1122.13132	0.71194

1122.23031	0.71197

1122.32931	0.71199

1122.42831	0.71205

1122.5273	0.71209

1122.6263	0.7121

1122.7253	0.71214

1122.82429	0.71215

1122.92329	0.71217

1123.02229	0.71219

1123.12128	0.7122

1123.22028	0.71222

1123.31928	0.71229

1123.41828	0.7123

1123.51727	0.71241

1123.61627	0.71244

1123.71527	0.71262

1123.81426	0.71264

1123.91326	0.71267

1124.01226	0.71269

1124.11126	0.71271

1124.21025	0.71274

1124.30925	0.71277

1124.40825	0.71281

1124.50724	0.71282

1124.60624	0.71286

1124.70524	0.71287

1124.80424	0.71289

1124.90323	0.71291

1125.00223	0.71299

1125.10123	0.71304

1125.20022	0.71309

1125.29922	0.71314

1125.39822	0.71316

1125.49721	0.71318

1125.59621	0.71319

1125.69521	0.71323

1125.7942	0.71326

1125.8932	0.71328

1125.9922	0.71329

1126.0912	0.71331

1126.19019	0.71333

1126.28919	0.7136

1126.38819	0.71363

1126.48718	0.71365

1126.58618	0.71366

1126.68518	0.71368

1126.78418	0.71383

1126.88317	0.71385

1126.98217	0.71387

1127.08117	0.71388

1127.18016	0.7139

1127.27916	0.71392

1127.37816	0.71393

1127.47716	0.71395

1127.57615	0.71398

1127.67515	0.71405

1127.77415	0.7141

1127.87314	0.71412

1127.97214	0.71413

1128.07114	0.71415

1128.17013	0.7142

1128.26913	0.7143

1128.36813	0.71447

1128.46712	0.71449

1128.56612	0.71452

1128.66512	0.71454

1128.76412	0.71457

1128.86311	0.71459

1128.96211	0.71464

1129.06111	0.71465

1129.1601	0.71521

1129.2591	0.71522

1129.3581	0.71526

1129.4571	0.71527

1129.55609	0.71529

1129.65509	0.71531

1129.75409	0.71532

1129.85308	0.71534

1129.95208	0.71563

1130.05108	0.71581

1130.15007	0.71583

1130.24907	0.71585

1130.34807	0.71611

1130.44707	0.71626

1130.54606	0.71628

1130.64506	0.71687

1130.74406	0.71689

1130.84305	0.71692

1130.94205	0.71694

1131.04105	0.71699

1131.14005	0.71725

1131.23904	0.71731

1131.33804	0.71732

1131.43704	0.71736

1131.53603	0.71756

1131.63503	0.71761

1131.73403	0.71764

1131.83302	0.71818

1131.93202	0.71819

1132.03102	0.71823

1132.13002	0.71824

1132.22901	0.71826

1132.32801	0.71841

1132.42701	0.71843

1132.526	0.71845

1132.625	0.71846

1132.724	0.71848

1132.82299	0.71866

1132.92199	0.71868

1133.02099	0.71871

1133.11999	0.71875

1133.21898	0.71898

1133.31798	0.71913

1133.41698	0.71915

1133.51597	0.71918

1133.61497	0.7192

1133.71397	0.71922

1133.81296	0.71923

1133.91196	0.71935

1134.01096	0.71937

1134.10995	0.71939

1134.20895	0.7194

1134.30795	0.71949

1134.40695	0.7195

1134.50594	0.71952

1134.60494	0.71954

1134.70394	0.7197

1134.80293	0.71979

1134.90193	0.71981

1135.00093	0.71994

1135.09993	0.71999

1135.19892	0.72009

1135.29792	0.72011

1135.39692	0.72016

1135.49591	0.72017

1135.59491	0.72019

1135.69391	0.72021

1135.79291	0.72022

1135.8919	0.72061

1135.9909	0.72063

1136.0899	0.72064

1136.18889	0.72076

1136.28789	0.72187

1136.38689	0.72189

1136.48588	0.7219

1136.58488	0.72194

1136.68388	0.72197

1136.78288	0.72254

1136.88187	0.72266

1136.98087	0.72267

1137.07987	0.72273

1137.17886	0.72274

1137.27786	0.72276

1137.37686	0.72281

1137.47585	0.72283

1137.57485	0.72284

1137.67385	0.72286

1137.77285	0.72288

1137.87184	0.72294

1137.97084	0.72296

1138.06984	0.72308

1138.16883	0.72309

1138.26783	0.72311

1138.36683	0.72313

1138.46583	0.72314

1138.56482	0.72316

1138.66382	0.72323

1138.76282	0.72328

1138.86181	0.72351

1138.96081	0.72353

1139.05981	0.72355

1139.1588	0.72372

1139.2578	0.72377

1139.3568	0.7238

1139.4558	0.72382

1139.55479	0.72383

1139.65379	0.72385

1139.75279	0.72387

1139.85178	0.72388

1139.95078	0.72397

1140.04978	0.72398

1140.14877	0.72402

1140.24777	0.72403

1140.34677	0.72471

1140.44576	0.72474

1140.54476	0.72477

1140.64376	0.72482

1140.74276	0.72496

1140.84175	0.72504

1140.94075	0.72509

1141.03975	0.72511

1141.13874	0.72512

1141.23774	0.72514

1141.33674	0.72536

1141.43574	0.72538

1141.53473	0.72541

1141.63373	0.72546

1141.73273	0.72551

1141.83172	0.72553

1141.93072	0.72563

1142.02972	0.72565

1142.12872	0.72566

1142.22771	0.7257

1142.32671	0.72571

1142.42571	0.72585

1142.5247	0.72586

1142.6237	0.72588

1142.7227	0.7259

1142.82169	0.72591

1142.92069	0.72593

1143.01969	0.72595

1143.11868	0.72632

1143.21768	0.7267

1143.31668	0.72672

1143.41568	0.72674

1143.51467	0.72675

1143.61367	0.72717

1143.71267	0.72719

1143.81166	0.72721

1143.91066	0.72722

1144.00966	0.72724

1144.10866	0.72726

1144.20765	0.72763

1144.30665	0.72764

1144.40565	0.72766

1144.50464	0.72768

1144.60364	0.72778

1144.70264	0.72779

1144.80163	0.72781

1144.90063	0.72783

1144.99963	0.72784

1145.09863	0.72815

1145.19762	0.72816

1145.29662	0.72818

1145.39562	0.7282

1145.49461	0.72821

1145.59361	0.7284

1145.69261	0.72865

1145.7916	0.72867

1145.8906	0.72885

1145.9896	0.72893

1146.0886	0.729

1146.18759	0.72905

1146.28659	0.7293

1146.38559	0.72934

1146.48458	0.72984

1146.58358	0.72987

1146.68258	0.72996

1146.78158	0.73002

1146.88057	0.73004

1146.97957	0.73006

1147.07857	0.73009

1147.17756	0.73011

1147.27656	0.73013

1147.37556	0.73014

1147.47455	0.73016

1147.57355	0.73019

1147.67255	0.73023

1147.77155	0.73024

1147.87054	0.73028

1147.96954	0.73029

1148.06854	0.73036

1148.16753	0.73038

1148.26653	0.73039

1148.36553	0.73041

1148.46453	0.73043

1148.56352	0.73044

1148.66252	0.73046

1148.76152	0.73048

1148.86051	0.73049

1148.95951	0.73051

1149.05851	0.73122

1149.1575	0.73123

1149.2565	0.73125

1149.3555	0.73127

1149.4545	0.73128

1149.55349	0.7313

1149.65249	0.73133

1149.75149	0.7319

1149.85048	0.73192

1149.94948	0.73194

1150.04848	0.73197

1150.14747	0.73199

1150.24647	0.732

1150.34547	0.73202

1150.44447	0.73204

1150.54346	0.73206

1150.64246	0.73224

1150.74146	0.73237

1150.84045	0.73239

1150.93945	0.73241

1151.03845	0.73244

1151.13744	0.73246

1151.23644	0.73247

1151.33544	0.73251

1151.43443	0.73271

1151.53343	0.73274

1151.63243	0.73279

1151.73143	0.73281

1151.83042	0.73283

1151.92942	0.73284

1152.02842	0.73286

1152.12741	0.73288

1152.22641	0.73289

1152.32541	0.73293

1152.42441	0.73294

1152.5234	0.73296

1152.6224	0.73308

1152.7214	0.7331

1152.82039	0.73323

1152.91939	0.73325

1153.01839	0.73326

1153.11739	0.73328

1153.21638	0.7334

1153.31538	0.73341

1153.41438	0.73343

1153.51337	0.73345

1153.61237	0.73346

1153.71137	0.73348

1153.81036	0.73352

1153.90936	0.73353

1154.00836	0.73355

1154.10736	0.73358

1154.20635	0.73362

1154.30535	0.73372

1154.40435	0.73373

1154.50334	0.73375

1154.60234	0.73377

1154.70134	0.73378

1154.80033	0.7338

1154.89933	0.73385

1154.99833	0.73398

1155.09733	0.734

1155.19632	0.73402

1155.29532	0.73404

1155.39432	0.73407

1155.49331	0.73409

1155.59231	0.73414

1155.69131	0.73415

1155.79031	0.73429

1155.8893	0.7343

1155.9883	0.73432

1156.0873	0.73434

1156.18629	0.73435

1156.28529	0.73437

1156.38429	0.73439

1156.48328	0.7344

1156.58228	0.73442

1156.68128	0.73444

1156.78028	0.73445

1156.87927	0.73459

1156.97827	0.73461

1157.07727	0.73466

1157.17626	0.73467

1157.27526	0.73477

1157.37426	0.73479

1157.47325	0.73482

1157.57225	0.73484

1157.67125	0.73486

1157.77024	0.73487

1157.86924	0.73489

1157.96824	0.73492

1158.06724	0.73496

1158.16623	0.73501

1158.26523	0.73503

1158.36423	0.73504

1158.46322	0.73511

1158.56222	0.73513

1158.66122	0.73558

1158.76022	0.73571

1158.85921	0.73575

1158.95821	0.73576

1159.05721	0.73578

1159.1562	0.7358

1159.2552	0.73581

1159.3542	0.73583

1159.4532	0.73585

1159.55219	0.73591

1159.65119	0.73598

1159.75019	0.736

1159.84918	0.73602

1159.94818	0.73603

1160.04718	0.73607

1160.14617	0.73623

1160.24517	0.73655

1160.34417	0.73657

1160.44316	0.73659

1160.54216	0.7366

1160.64116	0.73662

1160.74016	0.73665

1160.83915	0.73667

1160.93815	0.73674

1161.03715	0.73677

1161.13614	0.73677

1161.23514	0.73679

1161.33414	0.73681

1161.43314	0.73682

1161.53213	0.73686

1161.63113	0.73688

1161.73013	0.73689

1161.82912	0.73691

1161.92812	0.73692

1162.02712	0.73696

1162.12611	0.737

1162.22511	0.73704

1162.32411	0.73705

1162.42311	0.73708

1162.5221	0.7372

1162.6211	0.73721

1162.7201	0.73725

1162.81909	0.73728

1162.91809	0.73743

1163.01709	0.73744

1163.11609	0.73746

1163.21508	0.73753

1163.31408	0.73766

1163.41308	0.7377

1163.51207	0.73771

1163.61107	0.73773

1163.71007	0.73776

1163.80906	0.73779

1163.90806	0.7378

1164.00706	0.73782

1164.10606	0.73783

1164.20505	0.73784

1164.30405	0.73793

1164.40305	0.73795

1164.50204	0.73796

1164.60104	0.738

1164.70004	0.73803

1164.79903	0.73804

1164.89803	0.73805

1164.99703	0.73807

1165.09603	0.7381

1165.19502	0.73814

1165.29402	0.73821

1165.39302	0.73821

1165.49201	0.73826

1165.59101	0.73836

1165.69001	0.73841

1165.789	0.73861

1165.888	0.73862

1165.987	0.73862

1166.086	0.73868

1166.18499	0.73869

1166.28399	0.73872

1166.38299	0.73887

1166.48198	0.73889

1166.58098	0.73899

1166.67998	0.73901

1166.77897	0.73902

1166.87797	0.73904

1166.97697	0.73905

1167.07597	0.73907

1167.17496	0.73908

1167.27396	0.73909

1167.37296	0.7391

1167.47195	0.73913

1167.57095	0.73919

1167.66995	0.73922

1167.76895	0.73928

1167.86794	0.73929

1167.96694	0.73948

1168.06594	0.73949

1168.16493	0.73961

1168.26393	0.73969

1168.36293	0.73972

1168.46192	0.73974

1168.56092	0.73975

1168.65992	0.73988

1168.75891	0.73989

1168.85791	0.7399

1168.95691	0.73997

1169.05591	0.73998

1169.1549	0.74032

1169.2539	0.74038

1169.3529	0.74039

1169.45189	0.74057

1169.55089	0.74058

1169.64989	0.74059

1169.74889	0.74062

1169.84788	0.74066

1169.94688	0.74068

1170.04588	0.74069

1170.14487	0.7407

1170.24387	0.74072

1170.34287	0.74074

1170.44187	0.74075

1170.54086	0.74076

1170.63986	0.74077

1170.73886	0.74078

1170.83785	0.74086

1170.93685	0.74089

1171.03585	0.74118

1171.13484	0.74123

1171.23384	0.74123

1171.33284	0.74124

1171.43184	0.74125

1171.53083	0.74127

1171.62983	0.74136

1171.72883	0.74139

1171.82782	0.74139

1171.92682	0.74144

1172.02582	0.74144

1172.12481	0.74158

1172.22381	0.74159

1172.32281	0.74165

1172.4218	0.7417

1172.5208	0.74171

1172.6198	0.74172

1172.7188	0.74174

1172.81779	0.74179

1172.91679	0.7418

1173.01579	0.7418

1173.11479	0.74199

1173.21378	0.74202

1173.31278	0.74203

1173.41178	0.74206

1173.51077	0.74208

1173.60977	0.74212

1173.70877	0.74214

1173.80776	0.74215

1173.90676	0.74216

1174.00576	0.74217

1174.10476	0.74225

1174.20375	0.74227

1174.30275	0.74228

1174.40175	0.74242

1174.50074	0.74245

1174.59974	0.74249

1174.69874	0.74253

1174.79773	0.74258

1174.89673	0.74264

1174.99573	0.74268

1175.09472	0.7427

1175.19372	0.74271

1175.29272	0.74277

1175.39172	0.74279

1175.49071	0.74279

1175.58971	0.7428

1175.68871	0.74285

1175.7877	0.74295

1175.8867	0.74297

1175.9857	0.74317

1176.0847	0.74318

1176.18369	0.74322

1176.28269	0.74328

1176.38169	0.74329

1176.48068	0.7433

1176.57968	0.74334

1176.67868	0.74337

1176.77768	0.74338

1176.87667	0.74341

1176.97567	0.74342

1177.07467	0.74344

1177.17366	0.74345

1177.27266	0.74347

1177.37166	0.74404

1177.47065	0.74405

1177.56965	0.74406

1177.66865	0.74408

1177.76764	0.74419

1177.86664	0.7442

1177.96564	0.7442

1178.06464	0.74421

1178.16363	0.74422

1178.26263	0.74493

1178.36163	0.74494

1178.46062	0.74495

1178.55962	0.74497

1178.65862	0.74498

1178.75762	0.745

1178.85661	0.74501

1178.95561	0.74502

1179.05461	0.74503

1179.1536	0.74504

1179.2526	0.74529

1179.3516	0.7453

1179.45059	0.74532

1179.54959	0.74534

1179.64859	0.74535

1179.74759	0.74535

1179.84658	0.74545

1179.94558	0.74545

1180.04458	0.7455

1180.14357	0.74591

1180.24257	0.74593

1180.34157	0.74597

1180.44057	0.74597

1180.53956	0.746

1180.63856	0.74603

1180.73756	0.74608

1180.83655	0.74659

1180.93555	0.7466

1181.03455	0.74663

1181.13354	0.74664

1181.23254	0.74665

1181.33154	0.74705

1181.43054	0.74708

1181.52953	0.74709

1181.62853	0.74796

1181.72753	0.74797

1181.82652	0.748

1181.92552	0.74804

1182.02452	0.74806

1182.12351	0.74807

1182.22251	0.74873

1182.32151	0.74881

1182.42051	0.74882

1182.5195	0.74887

1182.6185	0.74889

1182.7175	0.75004

1182.81649	0.75005

1182.91549	0.75006

1183.01449	0.75007

1183.11348	0.75009

1183.21248	0.75009

1183.31148	0.75011

1183.41047	0.75013

1183.50947	0.75014

1183.60847	0.7502

1183.70747	0.75025

1183.80646	0.75027

1183.90546	0.75028

1184.00446	0.75034

1184.10345	0.75035

1184.20245	0.75036

1184.30145	0.75115

1184.40045	0.75123

1184.49944	0.75124

1184.59844	0.75127

1184.69744	0.75139

1184.79643	0.7514

1184.89543	0.75141

1184.99443	0.75143

1185.09343	0.75145

1185.19242	0.7515

1185.29142	0.75151

1185.39042	0.75152

1185.48941	0.75211

1185.58841	0.75212

1185.68741	0.75214

1185.7864	0.75215

1185.8854	0.75216

1185.9844	0.75217

1186.0834	0.75218

1186.18239	0.75219

1186.28139	0.75221

1186.38039	0.75222

1186.47938	0.75296

1186.57838	0.75298

1186.67738	0.75301

1186.77637	0.75301

1186.87537	0.75304

1186.97437	0.75306

1187.07337	0.75307

1187.17236	0.75326

1187.27136	0.75327

1187.37036	0.7533

1187.46935	0.75331

1187.56835	0.75336

1187.66735	0.75338

1187.76635	0.75344

1187.86534	0.75348

1187.96434	0.75348

1188.06334	0.75432

1188.16233	0.75437

1188.26133	0.75438

1188.36033	0.75439

1188.45932	0.75441

1188.55832	0.75442

1188.65732	0.7545

1188.75632	0.75451

1188.85531	0.75452

1188.95431	0.7546

1189.05331	0.75468

1189.1523	0.7547

1189.2513	0.75474

1189.3503	0.75477

1189.44929	0.7548

1189.54829	0.75514

1189.64729	0.75515

1189.74628	0.75525

1189.84528	0.75538

1189.94428	0.75541

1190.04328	0.75542

1190.14227	0.75543

1190.24127	0.75546

1190.34027	0.75547

1190.43926	0.75648

1190.53826	0.75657

1190.63726	0.75661

1190.73626	0.75661

1190.83525	0.75745

1190.93425	0.75749

1191.03325	0.75754

1191.13224	0.75754

1191.23124	0.75756

1191.33024	0.75757

1191.42924	0.7577

1191.52823	0.75782

1191.62723	0.75816

1191.72623	0.7582

1191.82522	0.75824

1191.92422	0.75827

1192.02322	0.75901

1192.12221	0.75903

1192.22121	0.75908

1192.32021	0.75921

1192.4192	0.75923

1192.5182	0.75925

1192.6172	0.75931

1192.7162	0.75937

1192.81519	0.7594

1192.91419	0.7595

1193.01319	0.75962

1193.11218	0.75973

1193.21118	0.76088

1193.31018	0.76094

1193.40918	0.76099

1193.50817	0.76104

1193.60717	0.76138

1193.70617	0.76146

1193.80516	0.76151

1193.90416	0.7623

1194.00316	0.76244

1194.10216	0.76336

1194.20115	0.76346

1194.30015	0.76373

1194.39915	0.7638

1194.49814	0.76383

1194.59714	0.76396

1194.69614	0.76494

1194.79513	0.76593

1194.89413	0.76611

1194.99313	0.76945

1195.09212	0.98896

1195.19112	0.99059

1195.29012	0.99492

1195.38912	0.99517

1195.48811	0.99522

1195.58711	0.99528

1195.68611	0.9953

1195.7851	0.9954

1195.8841	0.99555

1195.9831	0.99564

1196.0821	0.99569

1196.18109	0.9957

1196.28009	0.99626

1196.37909	0.99661

1196.47808	0.99671

1196.57708	0.99674

1196.67608	0.99705

1196.77507	0.99733

1196.87407	0.99737

1196.97307	0.99752

1197.07207	0.99762

1197.17106	0.99765

1197.27006	0.99784

1197.36906	0.99785

1197.46805	0.9979

1197.56705	0.99797

1197.66605	0.99812

1197.76505	0.99814

1197.86404	0.99857

1197.96304	0.99883

1198.06204	0.99884

1198.16103	0.99886

1198.26003	0.99903

1198.35903	0.99904

1198.45802	0.99918

1198.55702	0.99921

1198.65602	0.99924

1198.75502	0.99933

1198.85401	0.99953

1198.95301	0.99961

1199.05201	0.99968

1199.151	0.99973

1199.25	0.9998

1199.33	0.99987

1199.45	0.99988

1199.57	1

**（*d*）Stabilized hydraulic-45°**

1	0.024452	0.4772	0.000152702

2	0.05754	1.0347	0.00027427

3	0.08102	1.454	0.000457057

4	0.10349	1.94625	0.000511586

5	0.12048	2.076	0.000533829

6	0.13593	2.20575	0.000622801

7	0.1452	2.3355	0.000511586

8	0.16064	2.46525	0.000533829

9	0.17918	2.59501	0.000622801

10	0.18999	2.72476	6.45E-04

11	0.20544	2.85451	6.90E-04

12	0.22397	2.98426	7.56E-04

13	0.24251	3.11401	7.79E-04

14	0.25795	3.24376	8.01E-04

15	0.27186	3.37351	8.23E-04

16	0.29039	3.50326	8.45E-04

17	0.30584	3.63301	1.05E-03

18	0.31511	3.76276	1.11E-03

19	0.3321	3.89251	1.13E-03

20	0.35372	4.02226	1.16E-03

21	0.36608	4.15201	1.18E-03

22	0.37998	4.28176	1.49E-03

23	0.39852	4.41151	1.51E-03

24	0.41551	4.54126	1.53E-03

25	0.42787	4.67101	1.58E-03

26	0.44486	4.80076	1.60E-03

27	0.46339	4.93051	1.65E-03

28	0.47729	5.06026	1.80E-03

29	0.48811	5.19001	0.00189

30	0.50664	5.31976	0.00191

31	0.52363	5.44951	0.002

32	0.53753	5.57926	0.00205

33	0.55144	5.70901	0.00209

34	0.56997	5.83876	0.00216

35	0.58851	5.96851	0.00238

36	0.59932	6.09826	0.0024

37	0.61013	6.22801	0.00245

38	0.62867	6.35776	0.00247

39	0.64411	6.48751	0.00249

40	0.65647	6.61726	0.00251

41	0.67501	6.74701	0.00256

42	0.69045	6.87676	0.00287

43	0.7059	7.00651	0.00289

44	0.71826	7.13626	0.00291

45	0.73525	7.26601	0.00294

46	0.75224	7.39576	0.00296

47	0.7646	7.52552	0.00298

48	0.78159	7.65527	0.00307

49	0.80012	7.78502	0.0032

50	0.81557	7.91477	0.00323

51	0.82638	8.04452	0.00325

52	0.84183	8.17427	0.00327

53	0.86345	8.30402	0.00329

54	0.87118	8.43377	0.00331

55	0.88508	8.56352	0.00336

56	0.9067	8.69327	0.00338

57	0.92524	8.82302	0.0034

58	0.93451	8.95277	0.00347

59	0.94995	9.08252	0.00349

60	0.97003	9.21227	0.00351

61	0.98548	9.34202	0.00356

62	0.9932	9.47177	0.00365

63	1.01174	9.60152	0.00367

64	1.03027	9.73127	0.00371

65	1.04109	9.86102	0.00376

66	1.05653	9.99077	0.00378

67	1.07816	10.12052	0.00383

68	1.09206	10.25027	0.00385

69	1.09978	10.38002	0.00387

70	1.11986	10.50977	0.00391

71	1.15539	10.63952	0.00409

72	1.15694	10.76927	0.00438

73	1.1662	10.89902	0.00445

74	1.18937	11.02877	0.00449

75	1.20636	11.15852	0.00467

76	1.21563	11.28827	0.00469

77	1.23108	11.41802	0.00474

78	1.24961	11.54777	0.00476

79	1.2666	11.67752	0.0048

80	1.27587	11.80727	0.00498

81	1.28977	11.93702	0.005

82	1.31294	12.06677	0.00565

83	1.3253	12.19652	0.00567

84	1.3392	12.32627	0.00572

85	1.35774	12.45602	0.00594

86	1.37319	12.58578	0.00596

87	1.38709	12.71553	0.00598

88	1.40099	12.84528	0.00601

89	1.41798	12.97503	0.00649

90	1.43188	13.10478	0.00663

91	1.44578	13.23453	0.00665

92	1.46432	13.36428	0.00667

93	1.48285	13.49403	0.00678

94	1.49676	13.62378	0.00685

95	1.50602	13.75353	0.00687

96	1.52456	13.88328	0.00712

97	1.54464	14.01303	0.00723

98	1.55236	14.14278	0.00725

99	1.5709	14.27253	0.00732

100	1.59252	14.40228	0.00734

101	1.60488	14.53203	0.00736

102	1.61724	14.66178	0.00741

103	1.63268	14.79153	0.00743

104	1.64968	14.92128	0.00745

105	1.66512	15.05103	0.00754

106	1.67902	15.18078	0.00756

107	1.69447	15.31053	0.00767

108	1.70992	15.44028	0.00832

109	1.72227	15.57003	0.00834

110	1.74235	15.69978	0.00836

111	1.76089	15.82953	0.00839

112	1.77479	15.95928	0.00841

113	1.78406	16.08903	0.00843

114	1.80105	16.21878	0.00845

115	1.81959	16.34853	0.0085

116	1.83349	16.47828	0.00852

117	1.85048	16.60803	0.00856

118	1.82576	16.73778	0.00863

119	1.83812	16.86753	0.00865

120	1.85202	16.99728	0.00867

121	1.86747	17.12703	0.0087

122	1.89064	17.25678	0.00872

123	1.90145	17.38653	0.00874

124	1.91535	17.51629	0.00883

125	1.93543	17.64604	0.0089

126	1.94625	17.77579	0.00892

127	1.95706	17.90554	0.00908

128	1.97405	18.03529	0.00921

129	1.99722	18.16504	0.00956

130	2.00803	18.29479	0.00959

131	2.02502	18.42454	0.00968

132	2.04356	18.55429	0.00974

133	2.05901	18.68404	0.00976

134	2.07136	18.81379	0.00981

135	2.07754	18.94354	0.00983

136	2.10226	19.07329	0.01008

137	2.11925	19.20304	0.0105

138	2.12697	19.33279	0.01052

139	2.14705	19.46254	0.01054

140	2.16713	19.59229	0.01068

141	2.17949	19.72204	0.0107

142	2.18876	19.85179	0.01077

143	2.20884	19.98154	0.01079

144	2.22583	20.11129	0.01081

145	2.23509	20.24104	0.01083

146	2.25054	20.37079	0.01092

147	2.27525	20.50054	0.01094

148	2.28916	20.63029	0.01099

149	2.30151	20.76004	0.01103

150	2.31542	20.88979	0.01105

151	2.3355	21.01954	0.01114

152	2.34631	21.14929	0.01117

153	2.35712	21.27904	0.01121

154	2.38029	21.40879	0.01123

155	2.39883	21.53854	0.01125

156	2.40964	21.66829	0.01128

157	2.42508	21.79804	0.0113

158	2.44362	21.92779	0.01132

159	2.46061	22.05754	0.01134

160	2.46833	22.18729	0.01137

161	2.48224	22.31704	0.01139

162	2.50077	22.44679	0.01148

163	2.51313	22.57655	0.0115

164	2.52703	22.7063	0.01152

165	2.54866	22.83605	0.01161

166	2.56565	22.9658	0.01163

167	2.578	23.09555	0.01174

168	2.595	23.2253	0.01177

169	2.61044	23.35505	0.01179

170	2.62434	23.4848	0.01181

171	2.63825	23.61455	0.01183

172	2.65678	23.7443	0.01186

173	2.67223	23.87405	0.01188

174	2.68767	24.0038	0.0119

175	2.70158	24.13355	0.01194

176	2.72011	24.2633	0.01197

177	2.7371	24.39305	0.01199

178	2.74174	24.5228	0.01226

179	2.76182	24.65255	0.01228

180	2.7819	24.7823	0.01232

181	2.79734	24.91205	0.01246

182	2.80507	25.0418	0.01257

183	2.83133	25.17155	0.01268

184	2.84368	25.3013	0.01277

185	2.85913	25.43105	0.01281

186	2.87149	25.5608	0.01283

187	2.88848	25.69055	0.01286

188	2.89929	25.8203	0.01288

189	2.91474	25.95005	0.01295

190	2.93327	26.0798	0.01297

191	2.95335	26.20955	0.01299

192	2.96571	26.3393	0.01301

193	2.98116	26.46905	0.01303

194	2.9966	26.5988	0.01306

195	3.02286	26.72855	0.01308

196	3.02286	26.8583	0.01312

197	3.04294	26.98805	0.01315

198	3.05839	27.1178	0.01317

199	3.07229	27.24755	0.01319

200	3.08774	27.3773	0.01321

201	3.10782	27.50706	0.01326

202	3.12635	27.63681	0.0133

203	3.13871	27.76656	0.01335

204	3.14798	27.89631	0.01341

205	3.16651	28.02606	0.01343

206	3.1835	28.15581	0.01348

207	3.19586	28.28556	0.01352

208	3.20822	28.41531	0.01355

209	3.23602	28.54506	0.01361

210	3.24992	28.67481	0.01366

211	3.25919	28.80456	0.0139

212	3.27927	28.93431	0.01392

213	3.29626	29.06406	0.01395

214	3.30707	29.19381	0.01404

215	3.31943	29.32356	0.01406

216	3.33797	29.45331	0.01417

217	3.3565	29.58306	0.01419

218	3.36732	29.71281	0.01421

219	3.38431	29.84256	0.01426

220	3.40593	29.97231	0.0145

221	3.41829	30.10206	0.01459

222	3.42756	30.23181	0.01461

223	3.44455	30.36156	0.01464

224	3.46463	30.49131	0.01466

225	3.47853	30.62106	0.01468

226	3.4878	30.75081	0.0147

227	3.50942	30.88056	0.01475

228	3.52641	31.01031	0.01481

229	3.5434	31.14006	0.01484

230	3.55422	31.26981	0.01486

231	3.56812	31.39956	0.0149

232	3.58357	31.52931	0.01492

233	3.59438	31.65906	0.01499

234	3.61291	31.78881	0.01501

235	3.63608	31.91856	0.01506

236	3.6469	32.04831	0.01508

237	3.66389	32.17806	0.0151

238	3.67933	32.30781	0.01513

239	3.70405	32.43756	0.01515

240	3.70868	32.56732	0.01526

241	3.71949	32.69707	0.01528

242	3.74112	32.82682	0.0153

243	3.75811	32.95657	0.01533

244	3.76892	33.08632	0.01535

245	3.78746	33.21607	0.01537

246	3.80754	33.34582	0.01541

247	3.82298	33.47557	0.01546

248	3.82762	33.60532	0.01548

249	3.85079	33.73507	0.01564

250	3.86778	33.86482	0.01566

251	3.87241	33.99457	0.0157

252	3.8894	34.12432	0.01573

253	3.91103	34.25407	0.01575

254	3.92493	34.38382	0.01577

255	3.93729	34.51357	0.01581

256	3.95582	34.64332	0.01584

257	3.9759	34.77307	0.01586

258	3.98826	34.90282	0.01588

259	3.99907	35.03257	0.01593

260	4.01915	35.16232	0.01597

261	4.03151	35.29207	0.01608

262	4.04387	35.42182	0.0161

263	4.06549	35.55157	0.01613

264	4.08712	35.68132	0.01615

265	4.09793	35.81107	0.01619

266	4.10256	35.94082	0.01624

267	4.12419	36.07057	0.01635

268	4.1489	36.20032	0.01637

269	4.15045	36.33007	0.01639

270	4.17053	36.45982	0.01673

271	4.18906	36.58957	0.01675

272	4.20605	36.71932	0.01677

273	4.21841	36.84907	0.01715

274	4.23231	36.97882	0.01719

275	4.25239	37.10857	0.01724

276	4.26166	37.23832	0.01728

277	4.27247	37.36807	0.01735

278	4.29564	37.49783	0.01755

279	4.31109	37.62758	0.01759

280	4.3219	37.75733	0.01766

281	4.33889	37.88708	0.01775

282	4.36361	38.01683	0.01777

283	4.37751	38.14658	0.01782

284	4.38987	38.27633	0.01788

285	4.40222	38.40608	0.01802

286	4.42539	38.53583	0.01804

287	4.43775	38.66558	0.01806

288	4.44702	38.79533	0.01808

289	4.46555	38.92508	0.01815

290	4.48718	39.05483	0.01819

291	4.49645	39.18458	0.01831

292	4.51498	39.31433	0.01833

293	4.53352	39.44408	0.01844

294	4.54742	39.57383	0.01846

295	4.5536	39.70358	0.01848

296	4.57522	39.83333	0.01851

297	4.59067	39.96308	0.01853

298	4.60612	40.09283	0.01855

299	4.61693	40.22258	0.01857

300	4.63855	40.35233	0.0186

301	4.65709	40.48208	0.01862

302	4.66636	40.61183	0.01864

303	4.68026	40.74158	0.01866

304	4.69571	40.87133	0.01871

305	4.70806	41.00108	0.01873

306	4.72196	41.13083	0.01948

307	4.74359	41.26058	0.01951

308	4.76058	41.39033	0.01953

309	4.77757	41.52008	0.01955

310	4.7853	41.64983	0.0196

311	4.80692	41.77958	0.01968

312	4.82082	41.90933	0.01973

313	4.83009	42.03908	0.0198

314	4.84863	42.16883	0.01986

315	4.86716	42.29858	0.01991

316	4.88261	42.42833	0.01993

317	4.90114	42.55809	0.01995

318	4.91504	42.68784	0.02

319	4.93204	42.81759	0.02015

320	4.9413	42.94734	0.02017

321	4.95675	43.07709	0.0202

322	4.97374	43.20684	0.02022

323	4.99073	43.33659	0.02024

324	5	43.46634	0.02026

325	5.02162	43.59609	0.02033

326	5.04325	43.72584	0.02046

327	5.05715	43.85559	0.02053

328	5.06333	43.98534	0.02057

329	5.07569	44.11509	0.0206

330	5.09731	44.24484	0.02062

331	5.10812	44.37459	0.02064

332	5.12512	44.50434	0.02066

333	5.14829	44.63409	0.02069

334	5.16528	44.76384	0.02073

335	5.17146	44.89359	0.02077

336	5.18999	45.02334	0.02082

337	5.20853	45.15309	0.02089

338	5.22397	45.28284	0.02093

339	5.22861	45.41259	0.02098

340	5.25332	45.54234	0.021

341	5.26877	45.67209	0.02102

342	5.2873	45.80184	0.02113

343	5.29657	45.93159	0.02118

344	5.3182	46.06134	0.02122

345	5.3321	46.19109	0.02126

346	5.34137	46.32084	0.02129

347	5.36145	46.45059	0.02131

348	5.37535	46.58034	0.0214

349	5.39388	46.71009	0.02144

350	5.4047	46.83984	0.02146

351	5.42169	46.96959	0.02153

352	5.44486	47.09934	0.02155

353	5.45258	47.22909	0.02162

354	5.46648	47.35884	0.02164

355	5.48038	47.4886	0.02166

356	5.49892	47.61835	0.02169

357	5.51128	47.7481	0.02171

358	5.52518	47.87785	0.0218

359	5.5468	48.0076	0.02198

360	5.55762	48.13735	0.02202

361	5.57461	48.2671	0.02206

362	5.59932	48.39685	0.02209

363	5.61168	48.5266	0.02211

364	5.61631	48.65635	0.02213

365	5.63021	48.7861	0.02215

366	5.65802	48.91585	0.02218

367	5.67037	49.0456	0.0222

368	5.67655	49.17535	0.02224

369	5.69818	49.3051	0.02227

370	5.72135	49.43485	0.02229

371	5.73216	49.5646	0.02233

372	5.74143	49.69435	0.02235

373	5.76305	49.8241	0.02238

374	5.78004	49.95385	0.02244

375	5.79086	50.0836	0.02247

376	5.80167	50.21335	0.02249

377	5.82793	50.3431	0.02251

378	5.84028	50.47285	0.02253

379	5.85728	50.6026	0.02255

380	5.86963	50.73235	0.02264

381	5.89435	50.8621	0.02267

382	5.90053	50.99185	0.02278

383	5.90979	51.1216	0.02311

384	5.93142	51.25135	0.02313

385	5.94995	51.3811	0.02315

386	5.96231	51.51085	0.02318

387	5.97621	51.6406	0.0232

388	5.99784	51.77035	0.02322

389	6.01174	51.9001	0.02327

390	6.01946	52.02985	0.02329

391	6.04263	52.1596	0.02333

392	6.05808	52.28935	0.02347

393	6.06889	52.4191	0.02349

394	6.08588	52.54886	0.02351

395	6.10287	52.67861	0.02436

396	6.11677	52.80836	0.02438

397	6.13068	52.93811	0.02447

398	6.14767	53.06786	0.02451

399	6.16775	53.19761	0.02453

400	6.17702	53.32736	0.02489

401	6.18474	53.45711	0.02491

402	6.20636	53.58686	0.02493

403	6.22335	53.71661	0.02496

404	6.23262	53.84636	0.02502

405	6.25425	53.97611	0.02505

406	6.27587	54.10586	0.02538

407	6.29286	54.23561	0.0254

408	6.29904	54.36536	0.02549

409	6.31758	54.49511	0.02551

410	6.33766	54.62486	0.02553

411	6.34847	54.75461	0.02558

412	6.35774	54.88436	0.02562

413	6.37936	55.01411	0.02567

414	6.39944	55.14386	0.02569

415	6.40871	55.27361	0.02571

416	6.4257	55.40336	0.02574

417	6.44733	55.53311	0.02576

418	6.45968	55.66286	0.02578

419	6.46586	55.79261	0.02585

420	6.4844	55.92236	0.02587

421	6.50139	56.05211	0.02591

422	6.51993	56.18186	0.026

423	6.53228	56.31161	0.02605

424	6.557	56.44136	0.02607

425	6.56472	56.57111	0.02614

426	6.57862	56.70086	0.02616

427	6.5987	56.83061	0.0262

428	6.6126	56.96036	0.02622

429	6.62342	57.09011	0.02627

430	6.63268	57.21986	0.02629

431	6.66049	57.34961	0.02654

432	6.67748	57.47937	0.02656

433	6.69447	57.60912	0.02658

434	6.70374	57.73887	0.02662

435	6.72227	57.86862	0.02669

436	6.73618	57.99837	0.02783

437	6.74081	58.12812	0.02789

438	6.76089	58.25787	0.02794

439	6.77943	58.38762	0.02796

440	6.80105	58.51737	0.028

441	6.80723	58.64712	0.02803

442	6.82731	58.77687	0.02805

443	6.84584	58.90662	0.02807

444	6.86129	59.03637	0.02814

445	6.87365	59.16612	0.02816

446	6.89064	59.29587	0.02834

447	6.90609	59.42562	0.02836

448	6.91226	59.55537	0.0284

449	6.93234	59.68512	0.02843

450	6.9586	59.81487	0.02845

451	6.97251	59.94462	0.02856

452	6.97868	60.07437	0.02858

453	6.99413	60.20412	0.0286

454	7.0173	60.33387	0.02863

455	7.02039	60.46362	0.02865

456	7.03275	60.59337	0.02874

457	7.06364	60.72312	0.02876

458	7.08063	60.85287	0.02883

459	7.08835	60.98262	0.02887

460	7.10998	61.11237	0.02892

461	7.12234	61.24212	0.02894

462	7.13933	61.37187	0.02896

463	7.14396	61.50162	0.02898

464	7.16559	61.63137	0.029

465	7.18721	61.76112	0.02903

466	7.19339	61.89087	0.02905

467	7.21501	62.02062	0.02907

468	7.23664	62.15037	0.02909

469	7.25054	62.28012	0.02916

470	7.25826	62.40987	0.02918

471	7.27371	62.53963	0.0292

472	7.29534	62.66938	0.02923

473	7.29997	62.79913	0.02927

474	7.31387	62.92888	0.02932

475	7.3355	63.05863	0.02934

476	7.35867	63.18838	0.02936

477	7.36948	63.31813	0.02938

478	7.38492	63.44788	0.02943

479	7.405	63.57763	0.02945

480	7.422	63.70738	0.02952

481	7.42817	63.83713	0.02956

482	7.44208	63.96688	0.02961

483	7.46061	64.09663	0.02965

484	7.47451	64.22638	0.02967

485	7.49305	64.35613	0.02969

486	7.51158	64.48588	0.02972

487	7.52858	64.61563	0.02974

488	7.53321	64.74538	0.02976

489	7.5502	64.87513	0.02978

490	7.57492	65.00488	0.03005

491	7.58109	65.13463	0.03007

492	7.59036	65.26438	0.03012

493	7.61353	65.39413	0.03014

494	7.63052	65.52388	0.03021

495	7.64751	65.65363	0.03025

496	7.65678	65.78338	0.03027

497	7.67841	65.91313	0.03032

498	7.69849	66.04288	0.03043

499	7.70466	66.17263	0.03045

500	7.72629	66.30238	0.03047

501	7.74174	66.43213	0.03049

502	7.74946	66.56188	0.03056

503	7.768	66.69163	0.03058

504	7.78499	66.82138	0.03061

505	7.80661	66.95113	0.0307

506	7.82051	67.08088	0.03078

507	7.82515	67.21063	0.03083

508	7.84677	67.34038	0.03085

509	7.86531	67.47014	0.03094

510	7.87303	67.59989	0.03096

511	7.89157	67.72964	0.03101

512	7.91628	67.85939	0.03103

513	7.924	67.98914	0.03105

514	7.93636	68.11889	0.03112

515	7.9549	68.24864	0.03114

516	7.97652	68.37839	0.03116

517	7.9827	68.50814	0.03123

518	7.99815	68.63789	0.03125

519	8.01977	68.76764	0.03127

520	8.03213	68.89739	0.0313

521	8.05066	69.02714	0.03134

522	8.05993	69.15689	0.03136

523	8.08156	69.28664	0.03138

524	8.09082	69.41639	0.03141

525	8.10318	69.54614	0.03143

526	8.12326	69.67589	0.03145

527	8.14025	69.80564	0.0315

528	8.14798	69.93539	0.03152

529	8.17269	70.06514	0.03158

530	8.19432	70.19489	0.03167

531	8.20667	70.32464	0.0317

532	8.21903	70.45439	0.03172

533	8.23448	70.58414	0.03179

534	8.24838	70.71389	0.03194

535	8.26537	70.84364	0.03196

536	8.27464	70.97339	0.03199

537	8.29317	71.10314	0.03203

538	8.31634	71.23289	0.03205

539	8.32252	71.36264	0.03207

540	8.34106	71.49239	0.0321

541	8.36268	71.62214	0.03227

542	8.37504	71.75189	0.03234

543	8.37658	71.88164	0.03239

544	8.40284	72.01139	0.03241

545	8.41983	72.14114	0.03243

546	8.4291	72.27089	0.03247

547	8.44455	72.40064	0.03256

548	8.46463	72.5304	0.03259

549	8.48471	72.66015	0.03263

550	8.49089	72.7899	0.03265

551	8.50942	72.91965	0.0327

552	8.52487	73.0494	0.03274

553	8.54186	73.17915	0.03276

554	8.55113	73.3089	0.03285

555	8.56812	73.43865	0.03292

556	8.5882	73.5684	0.03294

557	8.59747	73.69815	0.03305

558	8.62373	73.8279	0.03316

559	8.64072	73.95765	0.03321

560	8.65153	74.0874	0.03323

561	8.65925	74.21715	0.03334

562	8.6747	74.3469	0.03412

563	8.69787	74.47665	0.03419

564	8.70714	74.6064	0.03421

565	8.72722	74.73615	0.03423

566	8.74421	74.8659	0.03432

567	8.7612	74.99565	0.03434

568	8.77201	75.1254	0.03437

569	8.78128	75.25515	0.03439

570	8.80599	75.3849	0.03443

571	8.81681	75.51465	0.03445

572	8.83225	75.6444	0.03452

573	8.8477	75.77415	0.03454

574	8.86778	75.9039	0.03459

575	8.88168	76.03365	0.03461

576	8.89713	76.1634	0.03479

577	8.91103	76.29315	0.03485

578	8.92956	76.4229	0.03501

579	8.94038	76.55265	0.03503

580	8.95582	76.6824	0.03505

581	8.9759	76.81215	0.0351

582	8.98517	76.9419	0.03519

583	8.99907	77.07165	0.03528

584	9.0207	77.2014	0.0353

585	9.04232	77.33115	0.03532

586	9.05314	77.46091	0.03534

587	9.06395	77.59066	0.03546

588	9.08712	77.72041	0.03552

589	9.09947	77.85016	0.03554

590	9.1072	77.97991	0.03561

591	9.12264	78.10966	0.03572

592	9.15045	78.23941	0.03574

593	9.15972	78.36916	0.03579

594	9.17207	78.49891	0.03581

595	9.19061	78.62866	0.03583

596	9.2076	78.75841	0.03586

597	9.21687	78.88816	0.03588

598	9.23386	79.01791	0.0359

599	9.25085	79.14766	0.03592

600	9.26784	79.27741	0.03597

601	9.2802	79.40716	0.03601

602	9.29719	79.53691	0.03603

603	9.32499	79.66666	0.03628

604	9.32963	79.79641	0.0363

605	9.33735	79.92616	0.03632

606	9.35434	80.05591	0.03634

607	9.37288	80.18566	0.0365

608	9.38678	80.31541	0.03652

609	9.39759	80.44516	0.03657

610	9.42076	80.57491	0.03659

611	9.44238	80.70466	0.03661

612	9.45474	80.83441	0.03663

613	9.46092	80.96416	0.03668

614	9.48872	81.09391	0.03672

615	9.4949	81.22366	0.03679

616	9.50417	81.35341	0.03683

617	9.52888	81.48316	0.0369

618	9.54897	81.61291	0.03692

619	9.55205	81.74266	0.03701

620	9.57059	81.87241	0.03703

621	9.59376	82.00216	0.0371

622	9.61075	82.13191	0.03712

623	9.61538	82.26166	0.03715

624	9.63392	82.39141	0.03719

625	9.65246	82.52117	0.03721

626	9.66327	82.65092	0.03726

627	9.67563	82.78067	0.03728

628	9.70497	82.91042	0.0373

629	9.72042	83.04017	0.03732

630	9.73432	83.16992	0.03735

631	9.74513	83.29967	0.03737

632	9.76985	83.42942	0.03739

633	9.77294	83.55917	0.03741

634	9.78838	83.68892	0.03748

635	9.80846	83.81867	0.0375

636	9.82237	83.94842	0.03752

637	9.84245	84.07817	0.03755

638	9.85171	84.20792	0.03757

639	9.87488	84.33767	0.03759

640	9.88724	84.46742	0.03763

641	9.89805	84.59717	0.03766

642	9.9135	84.72692	0.03768

643	9.93821	84.85667	0.03772

644	9.93821	84.98642	0.03795

645	9.95521	85.11617	0.03801

646	9.97838	85.24592	0.03806

647	9.99846	85.37567	0.03815

648	10.0139	85.50542	0.03819

649	10.02626	85.63517	0.03821

650	10.04788	85.76492	0.03824

651	10.05561	85.89467	0.03826

652	10.06333	86.02442	0.0383

653	10.08032	86.15417	0.03832

654	10.1004	86.28392	0.03837

655	10.12048	86.41367	0.03839

656	10.13284	86.54342	0.03859

657	10.14674	86.67317	0.03861

658	10.16837	86.80292	0.03864

659	10.17609	86.93267	0.03866

660	10.18381	87.06242	0.03868

661	10.21007	87.19217	0.0387

662	10.22861	87.32192	0.03917

663	10.23479	87.45167	0.03919

664	10.25641	87.58143	0.03941

665	10.28112	87.71118	0.03953

666	10.29039	87.84093	0.03968

667	10.29966	87.97068	0.0403

668	10.32129	88.10043	0.04033

669	10.33519	88.23018	0.04048

670	10.34445	88.35993	0.0405

671	10.35372	88.48968	0.04053

672	10.38153	88.61943	0.04064

673	10.40315	88.74918	0.04066

674	10.40624	88.87893	0.04068

675	10.42632	89.00868	0.04075

676	10.44486	89.13843	0.04077

677	10.45567	89.26818	0.04079

678	10.46185	89.39793	0.04082

679	10.4912	89.52768	0.04084

680	10.50201	89.65743	0.04086

681	10.51128	89.78718	0.0409

682	10.53599	89.91693	0.04093

683	10.55298	90.04668	0.04095

684	10.56225	90.17643	0.04102

685	10.5777	90.30618	0.04104

686	10.59469	90.43593	0.04106

687	10.61631	90.56568	0.04113

688	10.6194	90.69543	0.04115

689	10.6333	90.82518	0.04124

690	10.65493	90.95493	0.04126

691	10.67501	91.08468	0.04131

692	10.68891	91.21443	0.04135

693	10.70436	91.34418	0.04139

694	10.72753	91.47393	0.04142

695	10.73679	91.60368	0.04144

696	10.74143	91.73343	0.04146

697	10.75842	91.86318	0.04151

698	10.7785	91.99293	0.04164

699	10.7924	92.12268	0.04168

700	10.80476	92.25243	0.04171

701	10.83102	92.38218	0.04177

702	10.84337	92.51194	0.04179

703	10.85419	92.64169	0.04182

704	10.86963	92.77144	0.04197

705	10.88971	92.90119	0.04199

706	10.89898	93.03094	0.04202

707	10.91288	93.16069	0.04211

708	10.92987	93.29044	0.04213

709	10.95304	93.42019	0.04215

710	10.9654	93.54994	0.04219

711	10.97776	93.67969	0.04244

712	11.00093	93.80944	0.04246

713	11.01792	93.93919	0.04253

714	11.02101	94.06894	0.04255

715	11.038	94.19869	0.04257

716	11.05344	94.32844	0.0426

717	11.07044	94.45819	0.04266

718	11.08897	94.58794	0.04268

719	11.11214	94.71769	0.04306

720	11.12913	94.84744	0.04328

721	11.13686	94.97719	0.04337

722	11.14767	95.10694	0.04353

723	11.16775	95.23669	0.0436

724	11.17856	95.36644	0.04391

725	11.19246	95.49619	0.04393

726	11.21254	95.62594	0.04395

727	11.23262	95.75569	0.044

728	11.2388	95.88544	0.04413

729	11.25579	96.01519	0.04424

730	11.27896	96.14494	0.04426

731	11.29286	96.27469	0.04429

732	11.29904	96.40444	0.04446

733	11.31603	96.53419	0.04451

734	11.33766	96.66394	0.04457

735	11.34847	96.79369	0.04462

736	11.36237	96.92344	0.04498

737	11.38245	97.05319	0.04502

738	11.39944	97.18294	0.04569

739	11.41489	97.31269	0.04573

740	11.42261	97.44244	0.04578

741	11.44115	97.5722	0.0458

742	11.45814	97.70195	0.04595

743	11.46586	97.8317	0.04598

744	11.48594	97.96145	0.046

745	11.51529	98.0912	0.04602

746	11.52765	98.22095	0.04609

747	11.53383	98.3507	0.04611

748	11.55236	98.48045	0.04624

749	11.5709	98.6102	0.04627

750	11.58171	98.73995	0.04629

751	11.59098	98.8697	0.04631

752	11.61569	98.99945	0.04633

753	11.62651	99.1292	0.04638

754	11.63577	99.25895	0.04644

755	11.66049	99.3887	0.0466

756	11.67902	99.51845	0.04695

757	11.68984	99.6482	0.04698

758	11.6991	99.77795	0.047

759	11.72691	99.9077	0.04702

760	11.73463	100.03745	0.04711

761	11.74544	100.1672	0.04713

762	11.75935	100.29695	0.04724

763	11.78869	100.4267	0.04729

764	11.80105	100.55645	0.04731

765	11.80877	100.6862	0.0474

766	11.8304	100.81595	0.04742

767	11.8443	100.9457	0.04744

768	11.8582	101.07545	0.04747

769	11.86747	101.2052	0.04753

770	11.88601	101.33495	0.04758

771	11.90609	101.4647	0.04762

772	11.9169	101.59445	0.04767

773	11.93852	101.7242	0.04771

774	11.9586	101.85395	0.04776

775	11.97096	101.9837	0.04778

776	11.97405	102.11345	0.0478

777	11.99104	102.2432	0.04782

778	12.0173	102.37295	0.04784

779	12.02811	102.50271	0.04791

780	12.0451	102.63246	0.04796

781	12.06827	102.76221	0.04798

782	12.08372	102.89196	0.04804

783	12.09762	103.02171	0.04807

784	12.1038	103.15146	0.04809

785	12.13006	103.28121	0.0482

786	12.14242	103.41096	0.04822

787	12.15323	103.54071	0.04827

788	12.16559	103.67046	0.04829

789	12.18876	103.80021	0.04833

790	12.20729	103.92996	0.04838

791	12.21965	104.05971	0.0484

792	12.23664	104.18946	0.04842

793	12.24745	104.31921	0.04845

794	12.25054	104.44896	0.04847

795	12.27217	104.57871	0.04849

796	12.29534	104.70846	0.04851

797	12.30306	104.83821	0.0486

798	12.31542	104.96796	0.04869

799	12.34476	105.09771	0.04871

800	12.35712	105.22746	0.04873

801	12.37257	105.35721	0.04889

802	12.38801	105.48696	0.04891

803	12.40192	105.61671	0.04893

804	12.41736	105.74646	0.049

805	12.41891	105.87621	0.04913

806	12.44053	106.00596	0.04916

807	12.46679	106.13571	0.04922

808	12.4776	106.26546	0.04925

809	12.49305	106.39521	0.04927

810	12.51467	106.52496	0.04929

811	12.52858	106.65471	0.04931

812	12.53475	106.78446	0.04942

813	12.54402	106.91421	0.04945

814	12.57028	107.04396	0.04951

815	12.58418	107.17371	0.04958

816	12.60117	107.30346	0.0496

817	12.61662	107.43322	0.04962

818	12.63825	107.56297	0.04967

819	12.65369	107.69272	0.04987

820	12.65369	107.82247	0.04991

821	12.67841	107.95222	0.05002

822	12.68767	108.08197	0.05005

823	12.70312	108.21172	0.05009

824	12.71548	108.34147	0.05011

825	12.7371	108.47122	0.05025

826	12.75873	108.60097	0.05027

827	12.76954	108.73072	0.05029

828	12.78499	108.86047	0.05034

829	12.8097	108.99022	0.05038

830	12.81433	109.11997	0.05042

831	12.82978	109.24972	0.05045

832	12.85604	109.37947	0.05047

833	12.86222	109.50922	0.05051

834	12.87303	109.63897	0.05054

835	12.89774	109.76872	0.05056

836	12.91783	109.89847	0.05062

837	12.92864	110.02822	0.0508

838	12.94099	110.15797	0.05082

839	12.95026	110.28772	0.05098

840	12.96571	110.41747	0.05105

841	12.97343	110.54722	0.05107

842	12.99197	110.67697	0.05114

843	13.01977	110.80672	0.05118

844	13.03522	110.93647	0.0512

845	13.04603	111.06622	0.05123

846	13.0692	111.19597	0.05127

847	13.08156	111.32572	0.05131

848	13.09391	111.45547	0.05134

849	13.10473	111.58522	0.05136

850	13.12481	111.71497	0.05149

851	13.14489	111.84472	0.05151

852	13.1557	111.97447	0.05158

853	13.17732	112.10422	0.0516

854	13.18968	112.23397	0.05163

855	13.20822	112.36372	0.05171

856	13.21594	112.49348	0.05174

857	13.22521	112.62323	0.05178

858	13.24374	112.75298	0.0518

859	13.26228	112.88273	0.05185

860	13.27618	113.01248	0.05187

861	13.30399	113.14223	0.05189

862	13.31943	113.27198	0.052

863	13.32407	113.40173	0.05203

864	13.33797	113.53148	0.05209

865	13.35959	113.66123	0.05212

866	13.36886	113.79098	0.05214

867	13.38431	113.92073	0.05216

868	13.39975	114.05048	0.05218

869	13.41829	114.18023	0.0522

870	13.43837	114.30998	0.05225

871	13.45536	114.43973	0.05229

872	13.4739	114.56948	0.05232

873	13.48625	114.69923	0.05234

874	13.49089	114.82898	0.05238

875	13.50633	114.95873	0.05243

876	13.53105	115.08848	0.05256

877	13.53723	115.21823	0.05258

878	13.55422	115.34798	0.05263

879	13.57584	115.47773	0.05267

880	13.59283	115.60748	0.05269

881	13.60365	115.73723	0.05276

882	13.62218	115.86698	0.05278

883	13.63454	115.99673	0.05292

884	13.6469	116.12648	0.05294

885	13.6608	116.25623	0.05303

886	13.67779	116.38598	0.05307

887	13.69632	116.51573	0.05312

888	13.7164	116.64548	0.05314

889	13.73031	116.77523	0.05323

890	13.7473	116.90498	0.05327

891	13.75811	117.03473	0.05329

892	13.76738	117.16448	0.05332

893	13.78437	117.29423	0.05334

894	13.80445	117.42399	0.05336

895	13.81835	117.55374	0.05338

896	13.83225	117.68349	0.05341

897	13.85233	117.81324	0.05343

898	13.87396	117.94299	0.05347

899	13.88323	118.07274	0.05349

900	13.89558	118.20249	0.05352

901	13.90948	118.33224	0.05363

902	13.92802	118.46199	0.05365

903	13.94192	118.59174	0.05372

904	13.95737	118.72149	0.05374

905	13.98363	118.85124	0.05376

906	13.99907	118.98099	0.05378

907	14.00834	119.11074	0.05381

908	14.02688	119.24049	0.05392

909	14.03923	119.37024	0.05394

910	14.04696	119.49999	0.05452

911	14.05777	119.62974	0.05454

912	14.08403	119.75949	0.05467

913	14.10102	119.88924	0.05474

914	14.11338	120.01899	0.05478

915	14.13809	120.14874	0.05485

916	14.1489	120.27849	0.05494

917	14.16126	120.40824	0.05498

918	14.16898	120.53799	0.05503

919	14.1937	120.66774	0.05507

920	14.20605	120.79749	0.05514

921	14.21996	120.92724	0.05558

922	14.23849	121.05699	0.05623

923	14.26166	121.18674	0.05728

924	14.27711	121.31649	0.05752

925	14.28638	121.44624	0.05754

926	14.29873	121.57599	0.05772

927	14.31572	121.70574	0.05779

928	14.32345	121.83549	0.05781

929	14.34044	121.96524	0.05783

930	14.36515	122.09499	0.05785

931	14.38214	122.22474	0.05788

932	14.39296	122.35449	0.0579

933	14.40068	122.48425	0.05794

934	14.42694	122.614	0.05801

935	14.4393	122.74375	0.05808

936	14.44547	122.8735	0.05819

937	14.46401	123.00325	0.05848

938	14.48255	123.133	0.05863

939	14.49954	123.26275	0.05868

940	14.51653	123.3925	0.0587

941	14.5397	123.52225	0.05872

942	14.5536	123.652	0.05877

943	14.55823	123.78175	0.05879

944	14.57522	123.9115	0.05881

945	14.59067	124.04125	0.05883

946	14.60457	124.171	0.05885

947	14.61538	124.30075	0.0589

948	14.64319	124.4305	0.05892

949	14.66481	124.56025	0.05897

950	14.66945	124.69	0.05899

951	14.68026	124.81975	0.05921

952	14.6988	124.9495	0.05923

953	14.71424	125.07925	0.05926

954	14.72351	125.209	0.0593

955	14.74359	125.33875	0.05932

956	14.7683	125.4685	0.05937

957	14.78221	125.59825	0.05939

958	14.78993	125.728	0.05941

959	14.81001	125.85775	0.05943

960	14.82546	125.9875	0.05946

961	14.82854	126.11725	0.05986

962	14.84554	126.247	0.05988

963	14.87025	126.37675	0.0599

964	14.88724	126.5065	0.05992

965	14.8996	126.63625	0.05997

966	14.91659	126.766	0.06003

967	14.94285	126.89575	0.06006

968	14.94439	127.0255	0.06012

969	14.95984	127.15525	0.06023

970	14.9722	127.285	0.06028

971	14.99382	127.41476	0.0603

972	14.99537	127.54451	0.06034

973	15.02317	127.67426	0.06037

974	15.04634	127.80401	0.06039

975	15.06024	127.93376	0.06041

976	15.06951	128.06351	0.06043

977	15.08496	128.19326	0.06055

978	15.10504	128.32301	0.0607

979	15.11121	128.45276	0.06072

980	15.12357	128.58251	0.06075

981	15.14829	128.71226	0.06083

982	15.16219	128.84201	0.06086

983	15.17918	128.97176	0.06088

984	15.19771	129.10151	0.06092

985	15.21934	129.23126	0.06095

986	15.23015	129.36101	0.06097

987	15.2317	129.49076	0.06101

988	15.25487	129.62051	0.06103

989	15.27186	129.75026	0.06106

990	15.28421	129.88001	0.06123

991	15.29657	130.00976	0.06128

992	15.32592	130.13951	0.0613

993	15.33519	130.26926	0.06132

994	15.34291	130.39901	0.06141

995	15.36608	130.52876	0.06146

996	15.38307	130.65851	0.06148

997	15.38925	130.78826	0.0615

998	15.39852	130.91801	0.06152

999	15.42941	131.04776	0.06155

1000	15.45103	131.17751	0.06159

1001	15.45721	131.30726	0.06166

1002	15.4742	131.43701	0.06168

1003	15.49737	131.56676	0.0617

1004	15.5051	131.69651	0.06237

1005	15.51128	131.82626	0.06244

1006	15.52672	131.95601	0.06248

1007	15.54989	132.08576	0.0625

1008	15.5607	132.21551	0.0627

1009	15.58233	132.34526	0.06275

1010	15.60241	132.47502	0.06279

1011	15.6194	132.60477	0.06281

1012	15.62712	132.73452	0.06288

1013	15.63794	132.86427	0.0629

1014	15.65802	132.99402	0.06293

1015	15.6642	133.12377	0.06295

1016	15.67346	133.25352	0.06297

1017	15.70281	133.38327	0.06299

1018	15.72444	133.51302	0.06306

1019	15.73679	133.64277	0.06308

1020	15.75378	133.77252	0.0631

1021	15.76923	133.90227	0.06313

1022	15.78622	134.03202	0.06317

1023	15.78622	134.16177	0.06319

1024	15.80476	134.29152	0.06328

1025	15.82793	134.42127	0.0633

1026	15.84337	134.55102	0.06335

1027	15.85728	134.68077	0.06341

1028	15.87272	134.81052	0.06353

1029	15.89898	134.94027	0.06357

1030	15.90207	135.07002	0.06361

1031	15.91443	135.19977	0.06368

1032	15.93296	135.32952	0.0637

1033	15.94995	135.45927	0.06375

1034	15.95304	135.58902	0.06377

1035	15.9793	135.71877	0.06379

1036	16.00556	135.84852	0.06381

1037	16.01637	135.97827	0.06384

1038	16.03027	136.10802	0.06386

1039	16.04263	136.23777	0.06393

1040	16.06117	136.36752	0.06395

1041	16.05499	136.49727	0.06413

1042	16.08897	136.62702	0.06433

1043	16.10751	136.75677	0.06437

1044	16.10905	136.88652	0.06442

1045	16.12759	137.01627	0.06455

1046	16.15539	137.14602	0.06457

1047	16.16929	137.27577	0.06464

1048	16.17856	137.40553	0.06466

1049	16.20173	137.53528	0.06477

1050	16.21872	137.66503	0.06495

1051	16.22181	137.79478	0.06499

1052	16.23262	137.92453	0.06506

1053	16.26043	138.05428	0.06508

1054	16.27587	138.18403	0.06515

1055	16.28977	138.31378	0.06539

1056	16.3114	138.44353	0.06551

1057	16.33302	138.57328	0.06555

1058	16.34229	138.70303	0.06557

1059	16.3531	138.83278	0.06566

1060	16.36701	138.96253	0.06577

1061	16.37473	139.09228	0.06579

1062	16.39172	139.22203	0.06631

1063	16.40871	139.35178	0.06633

1064	16.43034	139.48153	0.06635

1065	16.45042	139.61128	0.06648

1066	16.45968	139.74103	0.06651

1067	16.48131	139.87078	0.06655

1068	16.49521	140.00053	0.06657

1069	16.50139	140.13028	0.06662

1070	16.5122	140.26003	0.06668

1071	16.52919	140.38978	0.06671

1072	16.55082	140.51953	0.06684

1073	16.5709	140.64928	0.06693

1074	16.5848	140.77903	0.06695

1075	16.60797	140.90878	0.067

1076	16.61724	141.03853	0.06702

1077	16.62651	141.16828	0.06704

1078	16.6435	141.29803	0.06706

1079	16.65585	141.42778	0.06708

1080	16.66667	141.55753	0.06717

1081	16.68984	141.68728	0.0672

1082	16.71301	141.81703	0.06726

1083	16.72382	141.94678	0.06733

1084	16.73309	142.07653	0.06735

1085	16.75471	142.20628	0.0674

1086	16.77325	142.33603	0.06742

1087	16.77943	142.46579	0.06744

1088	16.78715	142.59554	0.06746

1089	16.80877	142.72529	0.06751

1090	16.83194	142.85504	0.06771

1091	16.84584	142.98479	0.06777

1092	16.86129	143.11454	0.06784

1093	16.87983	143.24429	0.06786

1094	16.89373	143.37404	0.06791

1095	16.90454	143.50379	0.06795

1096	16.91381	143.63354	0.06797

1097	16.93543	143.76329	0.06802

1098	16.94625	143.89304	0.06813

1099	16.96478	144.02279	0.06815

1100	16.98795	144.15254	0.06817

1101	17.00803	144.28229	0.0682

1102	17.01884	144.41204	0.06849

1103	17.02966	144.54179	0.06851

1104	17.04665	144.67154	0.0688

1105	17.06055	144.80129	0.06882

1106	17.06055	144.93104	0.06891

1107	17.08217	145.06079	0.06895

1108	17.10998	145.19054	0.069

1109	17.12388	145.32029	0.06902

1110	17.14087	145.45004	0.06904

1111	17.15323	145.57979	0.06909

1112	17.16867	145.70954	0.06913

1113	17.1764	145.83929	0.06915

1114	17.19339	145.96904	0.06918

1115	17.21656	146.09879	0.06924

1116	17.22583	146.22854	0.06926

1117	17.23664	146.35829	0.06929

1118	17.26444	146.48804	0.06931

1119	17.28452	146.61779	0.06938

1120	17.28761	146.74754	0.0694

1121	17.30615	146.87729	0.06942

1122	17.32468	147.00704	0.06949

1123	17.33086	147.13679	0.06951

1124	17.34013	147.26654	0.06953

1125	17.35867	147.3963	0.06958

1126	17.3772	147.52605	0.0696

1127	17.39574	147.6558	0.06971

1128	17.41118	147.78555	0.06975

1129	17.42663	147.9153	0.06995

1130	17.44053	148.04505	0.06998

1131	17.4498	148.1748	0.07002

1132	17.46216	148.30455	0.07007

1133	17.47451	148.4343	0.07013

1134	17.47142	148.56405	0.07015

1135	16.68675	148.6938	0.07018

1136	15.90053	148.82355	0.07022

1137	15.08496	148.9533	0.07024

1138	14.1798	149.08305	0.07027

1139	13.29317	149.2128	0.07029

1140	12.45907	149.34255	0.07033

1141	11.49985	149.4723	0.0704

1142	10.57461	149.60205	0.07044

1143	9.73741	149.7318	0.07051

149.86155	0.07053

149.9913	0.07064

150.12105	0.07071

150.2508	0.0708

150.38055	0.07082

150.5103	0.07084

150.64005	0.07091

150.7698	0.07093

150.89955	0.07098

151.0293	0.071

151.15905	0.0712

151.2888	0.07124

151.41855	0.07127

151.5483	0.07138

151.67805	0.07142

151.8078	0.07153

151.93755	0.0716

152.0673	0.07164

152.19705	0.07167

152.3268	0.07171

152.45656	0.07173

152.58631	0.07182

152.71606	0.07189

152.84581	0.07196

152.97556	0.07198

153.10531	0.072

153.23506	0.07209

153.36481	0.07216

153.49456	0.07218

153.62431	0.07262

153.75406	0.07267

153.88381	0.07271

154.01356	0.07273

154.14331	0.07276

154.27306	0.07278

154.40281	0.07289

154.53256	0.07298

154.66231	0.073

154.79206	0.07305

154.92181	0.07307

155.05156	0.07316

155.18131	0.0732

155.31106	0.07322

155.44081	0.07327

155.57056	0.07329

155.70031	0.07331

155.83006	0.07333

155.95981	0.07336

156.08956	0.0734

156.21931	0.07345

156.34906	0.07351

156.47881	0.07353

156.60856	0.0736

156.73831	0.07362

156.86806	0.07365

156.99781	0.07367

157.12756	0.07369

157.25731	0.0738

157.38707	0.07394

157.51682	0.074

157.64657	0.07402

157.77632	0.07407

157.90607	0.07409

158.03582	0.07411

158.16557	0.07438

158.29532	0.0744

158.42507	0.07442

158.55482	0.07445

158.68457	0.0746

158.81432	0.07462

158.94407	0.07467

159.07382	0.07474

159.20357	0.07476

159.33332	0.07478

159.46307	0.0748

159.59282	0.07483

159.72257	0.07485

159.85232	0.07487

159.98207	0.07489

160.11182	0.07491

160.24157	0.07494

160.37132	0.075

160.50107	0.07505

160.63082	0.07507

160.76057	0.07509

160.89032	0.07511

161.02007	0.07523

161.14982	0.07531

161.27957	0.07536

161.40932	0.07538

161.53907	0.07545

161.66882	0.07547

161.79857	0.07549

161.92832	0.07551

162.05807	0.07554

162.18782	0.07558

162.31757	0.0756

162.44733	0.07563

162.57708	0.07565

162.70683	0.07567

162.83658	0.07569

162.96633	0.07574

163.09608	0.0758

163.22583	0.07589

163.35558	0.07594

163.48533	0.07596

163.61508	0.07598

163.74483	0.076

163.87458	0.07603

164.00433	0.07609

164.13408	0.07612

164.26383	0.07614

164.39358	0.0762

164.52333	0.07623

164.65308	0.07625

164.78283	0.07627

164.91258	0.07709

165.04233	0.07714

165.17208	0.07716

165.30183	0.07729

165.43158	0.07732

165.56133	0.07741

165.69108	0.07743

165.82083	0.07745

165.95058	0.07747

166.08033	0.07749

166.21008	0.07756

166.33983	0.07769

166.46958	0.07781

166.59933	0.07783

166.72908	0.07789

166.85883	0.07792

166.98858	0.07803

167.11833	0.07805

167.24808	0.07807

167.37783	0.07818

167.50759	0.07823

167.63734	0.07825

167.76709	0.07827

167.89684	0.07832

168.02659	0.07834

168.15634	0.07836

168.28609	0.07852

168.41584	0.07856

168.54559	0.07858

168.67534	0.07865

168.80509	0.07867

168.93484	0.07896

169.06459	0.07898

169.19434	0.07901

169.32409	0.07907

169.45384	0.07914

169.58359	0.07916

169.71334	0.07923

169.84309	0.07925

169.97284	0.0793

170.10259	0.07932

170.23234	0.07936

170.36209	0.07938

170.49184	0.07941

170.62159	0.07943

170.75134	0.07976

170.88109	0.07979

171.01084	0.07983

171.14059	0.0799

171.27034	0.07992

171.40009	0.07994

171.52984	0.07996

171.65959	0.07999

171.78934	0.08003

171.91909	0.08007

172.04884	0.0801

172.17859	0.08014

172.30834	0.08016

172.4381	0.08021

172.56785	0.08023

172.6976	0.08025

172.82735	0.08032

172.9571	0.08036

173.08685	0.08047

173.2166	0.0805

173.34635	0.08052

173.4761	0.08065

173.60585	0.08067

173.7356	0.0807

173.86535	0.08072

173.9951	0.08079

174.12485	0.08081

174.2546	0.08083

174.38435	0.0809

174.5141	0.08094

174.64385	0.08136

174.7736	0.0815

174.90335	0.08154

175.0331	0.08156

175.16285	0.08163

175.2926	0.08165

175.42235	0.08168

175.5521	0.08181

175.68185	0.08185

175.8116	0.0819

175.94135	0.08203

176.0711	0.08205

176.20085	0.0821

176.3306	0.08212

176.46035	0.08243

176.5901	0.08299

176.71985	0.08301

176.8496	0.08303

176.97935	0.08305

177.1091	0.0831

177.23885	0.08312

177.3686	0.08328

177.49836	0.0833

177.62811	0.08332

177.75786	0.0835

177.88761	0.08354

178.01736	0.08363

178.14711	0.08366

178.27686	0.08374

178.40661	0.08377

178.53636	0.08381

178.66611	0.08383

178.79586	0.08386

178.92561	0.08394

179.05536	0.08397

179.18511	0.08399

179.31486	0.08401

179.44461	0.08406

179.57436	0.08412

179.70411	0.08439

179.83386	0.08446

179.96361	0.08448

180.09336	0.0845

180.22311	0.08455

180.35286	0.08466

180.48261	0.08475

180.61236	0.08477

180.74211	0.08479

180.87186	0.08481

181.00161	0.08486

181.13136	0.08488

181.26111	0.0849

181.39086	0.08492

181.52061	0.08495

181.65036	0.08497

181.78011	0.08499

181.90986	0.08503

182.03961	0.08508

182.16936	0.08515

182.29911	0.08535

182.42887	0.08539

182.55862	0.08541

182.68837	0.08546

182.81812	0.0855

182.94787	0.08557

183.07762	0.08559

183.20737	0.0857

183.33712	0.08581

183.46687	0.08595

183.59662	0.08599

183.72637	0.08601

183.85612	0.08606

183.98587	0.08608

184.11562	0.08612

184.24537	0.08615

184.37512	0.08624

184.50487	0.08626

184.63462	0.08641

184.76437	0.08644

184.89412	0.08648

185.02387	0.08655

185.15362	0.08664

185.28337	0.08668

185.41312	0.08677

185.54287	0.08681

185.67262	0.08684

185.80237	0.08693

185.93212	0.08695

186.06187	0.08697

186.19162	0.08699

186.32137	0.08701

186.45112	0.08704

186.58087	0.08717

186.71062	0.08719

186.84037	0.08721

186.97012	0.08728

187.09987	0.0873

187.22962	0.08733

187.35937	0.08735

187.48913	0.08737

187.61888	0.08741

187.74863	0.08744

187.87838	0.08746

188.00813	0.08748

188.13788	0.0875

188.26763	0.08753

188.39738	0.08755

188.52713	0.08759

188.65688	0.08761

188.78663	0.08764

188.91638	0.08766

189.04613	0.08768

189.17588	0.08773

189.30563	0.08775

189.43538	0.08779

189.56513	0.08786

189.69488	0.0879

189.82463	0.08795

189.95438	0.08797

190.08413	0.08799

190.21388	0.08802

190.34363	0.08804

190.47338	0.08806

190.60313	0.08808

190.73288	0.08817

190.86263	0.08822

190.99238	0.08824

191.12213	0.08828

191.25188	0.08842

191.38163	0.08844

191.51138	0.08853

191.64113	0.08857

191.77088	0.08859

191.90063	0.08862

192.03038	0.08864

192.16013	0.08866

192.28988	0.0887

192.41964	0.08877

192.54939	0.08882

192.67914	0.08886

192.80889	0.08888

192.93864	0.0889

193.06839	0.08893

193.19814	0.08897

193.32789	0.08899

193.45764	0.08904

193.58739	0.08913

193.71714	0.08915

193.84689	0.08924

193.97664	0.08926

194.10639	0.08928

194.23614	0.08935

194.36589	0.08937

194.49564	0.08942

194.62539	0.08946

194.75514	0.08951

194.88489	0.08953

195.01464	0.08955

195.14439	0.08957

195.27414	0.08959

195.40389	0.08966

195.53364	0.08982

195.66339	0.08984

195.79314	0.08986

195.92289	0.08988

196.05264	0.08997

196.18239	0.09002

196.31214	0.09004

196.44189	0.09006

196.57164	0.09011

196.70139	0.09013

196.83114	0.09017

196.96089	0.09019

197.09064	0.09028

197.22039	0.09031

197.35014	0.09042

197.4799	0.09046

197.60965	0.09048

197.7394	0.09051

197.86915	0.09057

197.9989	0.09064

198.12865	0.09068

198.2584	0.09075

198.38815	0.09077

198.5179	0.091

198.64765	0.09104

198.7774	0.09108

198.90715	0.09113

199.0369	0.09115

199.16665	0.09117

199.2964	0.0912

199.42615	0.09124

199.5559	0.09131

199.68565	0.09135

199.8154	0.09153

199.94515	0.0916

200.0749	0.09162

200.20465	0.09171

200.3344	0.09173

200.46415	0.09177

200.5939	0.0918

200.72365	0.09184

200.8534	0.09186

200.98315	0.09191

201.1129	0.09206

201.24265	0.09211

201.3724	0.09213

201.50215	0.09215

201.6319	0.09217

201.76165	0.0922

201.8914	0.09222

202.02115	0.09224

202.1509	0.09226

202.28065	0.09229

202.41041	0.09233

202.54016	0.09235

202.66991	0.09237

202.79966	0.0924

202.92941	0.09251

203.05916	0.09253

203.18891	0.09255

203.31866	0.09262

203.44841	0.09264

203.57816	0.09278

203.70791	0.09284

203.83766	0.09286

203.96741	0.09291

204.09716	0.09293

204.22691	0.09295

204.35666	0.09302

204.48641	0.09309

204.61616	0.09311

204.74591	0.09313

204.87566	0.09318

205.00541	0.09322

205.13516	0.09326

205.26491	0.09329

205.39466	0.09331

205.52441	0.09333

205.65416	0.09342

205.78391	0.09346

205.91366	0.09349

206.04341	0.09351

206.17316	0.09353

206.30291	0.09358

206.43266	0.09362

206.56241	0.09366

206.69216	0.09371

206.82191	0.09373

206.95166	0.09375

207.08141	0.09378

207.21116	0.09384

207.34091	0.09386

207.47067	0.09389

207.60042	0.09398

207.73017	0.09407

207.85992	0.09411

207.98967	0.09418

208.11942	0.09478

208.24917	0.09487

208.37892	0.09491

208.50867	0.09509

208.63842	0.09513

208.76817	0.09516

208.89792	0.09527

209.02767	0.09529

209.15742	0.09536

209.28717	0.09538

209.41692	0.09551

209.54667	0.09558

209.67642	0.09571

209.80617	0.09573

209.93592	0.09576

210.06567	0.09578

210.19542	0.09602

210.32517	0.09604

210.45492	0.09607

210.58467	0.09609

210.71442	0.09613

210.84417	0.09616

210.97392	0.09624

211.10367	0.09627

211.23342	0.09631

211.36317	0.09633

211.49292	0.09636

211.62267	0.0964

211.75242	0.09642

211.88217	0.09645

212.01192	0.09647

212.14167	0.09649

212.27142	0.09651

212.40117	0.09653

212.53093	0.0966

212.66068	0.09662

212.79043	0.09669

212.92018	0.09671

213.04993	0.09673

213.17968	0.09676

213.30943	0.09685

213.43918	0.09693

213.56893	0.09698

213.69868	0.09707

213.82843	0.09713

213.95818	0.09718

214.08793	0.09722

214.21768	0.09727

214.34743	0.09731

214.47718	0.09742

214.60693	0.09745

214.73668	0.09749

214.86643	0.09754

214.99618	0.09756

215.12593	0.09762

215.25568	0.09767

215.38543	0.09769

215.51518	0.09776

215.64493	0.0978

215.77468	0.09782

215.90443	0.09831

216.03418	0.09834

216.16393	0.09838

216.29368	0.09842

216.42343	0.09845

216.55318	0.09847

216.68293	0.09851

216.81268	0.09856

216.94243	0.09858

217.07218	0.09862

217.20193	0.09865

217.33168	0.09871

217.46144	0.09874

217.59119	0.09878

217.72094	0.0988

217.85069	0.09883

217.98044	0.09885

218.11019	0.09889

218.23994	0.09891

218.36969	0.09894

218.49944	0.099

218.62919	0.09909

218.75894	0.0992

218.88869	0.09923

219.01844	0.09925

219.14819	0.09945

219.27794	0.09947

219.40769	0.09954

219.53744	0.09956

219.66719	0.09958

219.79694	0.0996

219.92669	0.09965

220.05644	0.09978

220.18619	0.09983

220.31594	0.09985

220.44569	0.09995

220.57544	0.10007

220.70519	0.10008

220.83494	0.10013

220.96469	0.10015

221.09444	0.10017

221.22419	0.10047

221.35394	0.10053

221.48369	0.10089

221.61344	0.1009

221.74319	0.10092

221.87294	0.10119

222.00269	0.10139

222.13244	0.10141

222.26219	0.10177

222.39194	0.10179

222.5217	0.10181

222.65145	0.10193

222.7812	0.10196

222.91095	0.10199

223.0407	0.10201

223.17045	0.10204

223.3002	0.10211

223.42995	0.10216

223.5597	0.10226

223.68945	0.10227

223.8192	0.10228

223.94895	0.10248

224.0787	0.10251

224.20845	0.10252

224.3382	0.10295

224.46795	0.10296

224.5977	0.10297

224.72745	0.10298

224.8572	0.103

224.98695	0.10315

225.1167	0.10322

225.24645	0.10324

225.3762	0.10327

225.50595	0.10345

225.6357	0.10354

225.76545	0.10355

225.8952	0.10371

226.02495	0.10372

226.1547	0.10373

226.28445	0.10376

226.4142	0.1039

226.54395	0.10391

226.6737	0.10392

226.80345	0.10431

226.9332	0.10432

227.06295	0.10434

227.1927	0.10436

227.32245	0.10456

227.45221	0.10461

227.58196	0.10462

227.71171	0.10464

227.84146	0.10471

227.97121	0.10473

228.10096	0.10521

228.23071	0.10523

228.36046	0.10528

228.49021	0.10552

228.61996	0.10554

228.74971	0.10555

228.87946	0.10556

229.00921	0.10594

229.13896	0.10599

229.26871	0.10621

229.39846	0.10622

229.52821	0.10623

229.65796	0.10625

229.78771	0.10651

229.91746	0.10654

230.04721	0.10658

230.17696	0.10693

230.30671	0.10694

230.43646	0.10698

230.56621	0.10729

230.69596	0.1073

230.82571	0.10732

230.95546	0.10741

231.08521	0.10743

231.21496	0.10748

231.34471	0.10754

231.47446	0.10763

231.60421	0.10778

231.73396	0.10779

231.86371	0.10782

231.99346	0.10818

232.12321	0.10819

232.25296	0.10846

232.38271	0.10847

232.51247	0.10862

232.64222	0.10871

232.77197	0.10873

232.90172	0.10875

233.03147	0.10877

233.16122	0.10901

233.29097	0.10903

233.42072	0.10905

233.55047	0.10956

233.68022	0.10958

233.80997	0.10959

233.93972	0.1096

234.06947	0.10962

234.19922	0.10964

234.32897	0.10965

234.45872	0.10966

234.58847	0.10971

234.71822	0.10986

234.84797	0.10987

234.97772	0.10988

235.10747	0.11026

235.23722	0.1103

235.36697	0.11031

235.49672	0.11074

235.62647	0.11076

235.75622	0.11083

235.88597	0.11113

236.01572	0.11115

236.14547	0.11143

236.27522	0.11146

236.40497	0.11147

236.53472	0.11149

236.66447	0.11151

236.79422	0.11154

236.92397	0.11155

237.05372	0.11209

237.18347	0.11213

237.31322	0.11215

237.44298	0.11248

237.57273	0.11253

237.70248	0.11254

237.83223	0.11297

237.96198	0.11299

238.09173	0.11307

238.22148	0.11309

238.35123	0.11334

238.48098	0.11337

238.61073	0.11338

238.74048	0.11341

238.87023	0.11343

238.99998	0.11371

239.12973	0.11372

239.25948	0.11373

239.38923	0.11375

239.51898	0.11378

239.64873	0.11379

239.77848	0.11381

239.90823	0.11383

240.03798	0.11405

240.16773	0.11413

240.29748	0.11416

240.42723	0.11445

240.55698	0.11446

240.68673	0.11447

240.81648	0.11448

240.94623	0.1145

241.07598	0.11471

241.20573	0.11475

241.33548	0.11477

241.46523	0.11478

241.59498	0.11504

241.72473	0.11505

241.85448	0.11506

241.98423	0.11507

242.11398	0.1151

242.24373	0.11524

242.37348	0.11532

242.50324	0.11545

242.63299	0.11555

242.76274	0.11559

242.89249	0.1156

243.02224	0.1158

243.15199	0.11582

243.28174	0.11585

243.41149	0.11587

243.54124	0.11596

243.67099	0.11597

243.80074	0.11601

243.93049	0.11603

244.06024	0.11605

244.18999	0.11607

244.31974	0.11609

244.44949	0.11611

244.57924	0.11612

244.70899	0.11664

244.83874	0.11666

244.96849	0.11668

245.09824	0.11672

245.22799	0.11705

245.35774	0.11706

245.48749	0.11709

245.61724	0.11712

245.74699	0.11721

245.87674	0.11735

246.00649	0.11736

246.13624	0.11765

246.26599	0.11774

246.39574	0.11775

246.52549	0.11778

246.65524	0.11779

246.78499	0.11781

246.91474	0.11799

247.04449	0.118

247.17424	0.11801

247.30399	0.11802

247.43375	0.11822

247.5635	0.11837

247.69325	0.1184

247.823	0.11877

247.95275	0.11878

248.0825	0.11879

248.21225	0.11881

248.342	0.11882

248.47175	0.11884

248.6015	0.11887

248.73125	0.1189

248.861	0.11891

248.99075	0.11893

249.1205	0.11909

249.25025	0.11913

249.38	0.11914

249.50975	0.11929

249.6395	0.11958

249.76925	0.11959

249.899	0.1196

250.02875	0.11993

250.1585	0.11994

250.28825	0.12001

250.418	0.12003

250.54775	0.12045

250.6775	0.12046

250.80725	0.12048

250.937	0.1205

251.06675	0.12051

251.1965	0.12062

251.32625	0.12091

251.456	0.12096

251.58575	0.12125

251.7155	0.12126

251.84525	0.12128

251.975	0.1213

252.10475	0.12131

252.2345	0.12132

252.36426	0.12133

252.49401	0.12159

252.62376	0.1216

252.75351	0.12162

252.88326	0.12165

253.01301	0.1218

253.14276	0.12182

253.27251	0.12194

253.40226	0.12196

253.53201	0.12231

253.66176	0.12235

253.79151	0.12236

253.92126	0.12237

254.05101	0.12273

254.18076	0.12276

254.31051	0.12277

254.44026	0.12279

254.57001	0.12281

254.69976	0.12284

254.82951	0.12286

254.95926	0.12288

255.08901	0.12307

255.21876	0.12333

255.34851	0.12336

255.47826	0.12337

255.60801	0.12338

255.73776	0.12359

255.86751	0.1236

255.99726	0.12363

256.12701	0.12366

256.25676	0.12378

256.38651	0.12382

256.51626	0.12384

256.64601	0.12385

256.77576	0.12388

256.90551	0.1239

257.03526	0.12392

257.16501	0.12395

257.29476	0.12396

257.42452	0.12422

257.55427	0.12427

257.68402	0.1243

257.81377	0.12433

257.94352	0.12434

258.07327	0.12437

258.20302	0.1247

258.33277	0.12506

258.46252	0.12513

258.59227	0.12514

258.72202	0.12516

258.85177	0.12534

258.98152	0.12535

259.11127	0.12541

259.24102	0.12565

259.37077	0.12566

259.50052	0.12567

259.63027	0.12571

259.76002	0.12573

259.88977	0.12588

260.01952	0.12593

260.14927	0.12613

260.27902	0.12615

260.40877	0.12616

260.53852	0.12617

260.66827	0.12656

260.79802	0.12657

260.92777	0.1266

261.05752	0.12664

261.18727	0.12665

261.31702	0.12667

261.44677	0.12694

261.57652	0.12696

261.70627	0.12703

261.83602	0.12705

261.96577	0.12708

262.09552	0.12742

262.22527	0.12746

262.35503	0.12747

262.48478	0.1275

262.61453	0.12753

262.74428	0.12756

262.87403	0.12759

263.00378	0.12761

263.13353	0.12784

263.26328	0.12787

263.39303	0.12789

263.52278	0.12844

263.65253	0.12846

263.78228	0.12854

263.91203	0.12855

264.04178	0.12857

264.17153	0.1286

264.30128	0.12904

264.43103	0.12906

264.56078	0.12908

264.69053	0.12938

264.82028	0.1294

264.95003	0.12942

265.07978	0.12948

265.20953	0.1295

265.33928	0.12952

265.46903	0.12955

265.59878	0.12981

265.72853	0.12982

265.85828	0.12984

265.98803	0.12985

266.11778	0.1299

266.24753	0.13012

266.37728	0.13013

266.50703	0.13014

266.63678	0.13015

266.76653	0.13033

266.89628	0.13037

267.02603	0.13039

267.15578	0.1304

267.28553	0.13042

267.41529	0.13043

267.54504	0.13044

267.67479	0.13049

267.80454	0.13053

267.93429	0.13079

268.06404	0.13084

268.19379	0.13087

268.32354	0.13089

268.45329	0.13092

268.58304	0.13095

268.71279	0.13099

268.84254	0.13101

268.97229	0.13121

269.10204	0.13124

269.23179	0.13127

269.36154	0.13129

269.49129	0.1313

269.62104	0.13133

269.75079	0.13189

269.88054	0.13194

270.01029	0.13232

270.14004	0.13237

270.26979	0.13238

270.39954	0.13269

270.52929	0.1327

270.65904	0.13273

270.78879	0.13275

270.91854	0.13277

271.04829	0.13287

271.17804	0.13289

271.30779	0.1329

271.43754	0.13296

271.56729	0.13313

271.69704	0.13316

271.82679	0.13325

271.95654	0.13326

272.08629	0.13328

272.21604	0.13331

272.3458	0.13332

272.47555	0.13361

272.6053	0.13365

272.73505	0.13368

272.8648	0.1337

272.99455	0.13371

273.1243	0.13379

273.25405	0.1339

273.3838	0.13392

273.51355	0.13395

273.6433	0.13396

273.77305	0.13412

273.9028	0.13414

274.03255	0.13415

274.1623	0.13451

274.29205	0.13452

274.4218	0.13458

274.55155	0.1346

274.6813	0.13461

274.81105	0.13464

274.9408	0.13471

275.07055	0.13474

275.2003	0.13476

275.33005	0.13478

275.4598	0.1348

275.58955	0.13481

275.7193	0.13499

275.84905	0.13503

275.9788	0.13505

276.10855	0.1351

276.2383	0.13511

276.36805	0.13514

276.4978	0.13515

276.62755	0.1355

276.7573	0.13557

276.88705	0.13604

277.0168	0.13607

277.14655	0.13608

277.2763	0.13609

277.40606	0.13612

277.53581	0.13615

277.66556	0.13616

277.79531	0.13618

277.92506	0.13623

278.05481	0.13633

278.18456	0.13635

278.31431	0.13637

278.44406	0.13638

278.57381	0.13689

278.70356	0.13692

278.83331	0.13693

278.96306	0.13699

279.09281	0.13712

279.22256	0.13714

279.35231	0.13717

279.48206	0.13758

279.61181	0.13761

279.74156	0.13762

279.87131	0.13763

280.00106	0.13792

280.13081	0.13795

280.26056	0.13797

280.39031	0.13802

280.52006	0.13804

280.64981	0.13815

280.77956	0.13817

280.90931	0.1382

281.03906	0.13843

281.16881	0.13881

281.29856	0.13882

281.42831	0.13883

281.55806	0.13885

281.68781	0.13913

281.81756	0.13915

281.94731	0.13937

282.07706	0.13941

282.20681	0.13942

282.33657	0.13964

282.46632	0.13965

282.59607	0.13966

282.72582	0.13967

282.85557	0.13995

282.98532	0.14

283.11507	0.14027

283.24482	0.14029

283.37457	0.14033

283.50432	0.14034

283.63407	0.14036

283.76382	0.14037

283.89357	0.1404

284.02332	0.14041

284.15307	0.14043

284.28282	0.14074

284.41257	0.14075

284.54232	0.14082

284.67207	0.14118

284.80182	0.14125

284.93157	0.14128

285.06132	0.14129

285.19107	0.14131

285.32082	0.14133

285.45057	0.14134

285.58032	0.14136

285.71007	0.14143

285.83982	0.14144

285.96957	0.14146

286.09932	0.14151

286.22907	0.14162

286.35882	0.14164

286.48857	0.14168

286.61832	0.1417

286.74807	0.14172

286.87782	0.14204

287.00757	0.14208

287.13732	0.14211

287.26707	0.14224

287.39683	0.14257

287.52658	0.14261

287.65633	0.14263

287.78608	0.14265

287.91583	0.14267

288.04558	0.14268

288.17533	0.14272

288.30508	0.14289

288.43483	0.14291

288.56458	0.14293

288.69433	0.14313

288.82408	0.14317

288.95383	0.14321

289.08358	0.14323

289.21333	0.14326

289.34308	0.14328

289.47283	0.14343

289.60258	0.14346

289.73233	0.14349

289.86208	0.14352

289.99183	0.14354

290.12158	0.14379

290.25133	0.1438

290.38108	0.14381

290.51083	0.14382

290.64058	0.14383

290.77033	0.14394

290.90008	0.14396

291.02983	0.14429

291.15958	0.1443

291.28933	0.14433

291.41908	0.14436

291.54883	0.14438

291.67858	0.14472

291.80833	0.14475

291.93808	0.14477

292.06783	0.14479

292.19758	0.1448

292.32734	0.14481

292.45709	0.14483

292.58684	0.14486

292.71659	0.14501

292.84634	0.14508

292.97609	0.14528

293.10584	0.14529

293.23559	0.14536

293.36534	0.14538

293.49509	0.1454

293.62484	0.14544

293.75459	0.14547

293.88434	0.14549

294.01409	0.14551

294.14384	0.14552

294.27359	0.14557

294.40334	0.1456

294.53309	0.14564

294.66284	0.14587

294.79259	0.14592

294.92234	0.14597

295.05209	0.14599

295.18184	0.14612

295.31159	0.14614

295.44134	0.14617

295.57109	0.14618

295.70084	0.1464

295.83059	0.14642

295.96034	0.14646

296.09009	0.14648

296.21984	0.14681

296.34959	0.14684

296.47934	0.14685

296.60909	0.14686

296.73884	0.14688

296.86859	0.14689

296.99834	0.1469

297.12809	0.14691

297.25784	0.14726

297.3876	0.14727

297.51735	0.14728

297.6471	0.14731

297.77685	0.14754

297.9066	0.14755

298.03635	0.14756

298.1661	0.14757

298.29585	0.14766

298.4256	0.14786

298.55535	0.14788

298.6851	0.14789

298.81485	0.1479

298.9446	0.14793

299.07435	0.14796

299.2041	0.14797

299.33385	0.14806

299.4636	0.14808

299.59335	0.14837

299.7231	0.14838

299.85285	0.14839

299.9826	0.14866

300.11235	0.14867

300.2421	0.14868

300.37185	0.1487

300.5016	0.14873

300.63135	0.14874

300.7611	0.14876

300.89085	0.14905

301.0206	0.14911

301.15035	0.14913

301.2801	0.14936

301.40985	0.14938

301.5396	0.14939

301.66935	0.14942

301.7991	0.14944

301.92885	0.14946

302.0586	0.14957

302.18835	0.14963

302.31811	0.14964

302.44786	0.14991

302.57761	0.14993

302.70736	0.14996

302.83711	0.14998

302.96686	0.15006

303.09661	0.15008

303.22636	0.15017

303.35611	0.15018

303.48586	0.1502

303.61561	0.15043

303.74536	0.15044

303.87511	0.15048

304.00486	0.1505

304.13461	0.15051

304.26436	0.15084

304.39411	0.15086

304.52386	0.15092

304.65361	0.15096

304.78336	0.15125

304.91311	0.15126

305.04286	0.15127

305.17261	0.15177

305.30236	0.15179

305.43211	0.15181

305.56186	0.15183

305.69161	0.15185

305.82136	0.15186

305.95111	0.15189

306.08086	0.1519

306.21061	0.15191

306.34036	0.15237

306.47011	0.1524

306.59986	0.15242

306.72961	0.15244

306.85936	0.1525

306.98911	0.15253

307.11886	0.15254

307.24861	0.1526

307.37837	0.15286

307.50812	0.15288

307.63787	0.1529

307.76762	0.15294

307.89737	0.15295

308.02712	0.15299

308.15687	0.153

308.28662	0.15302

308.41637	0.15304

308.54612	0.15305

308.67587	0.1531

308.80562	0.1535

308.93537	0.15354

309.06512	0.15358

309.19487	0.15359

309.32462	0.15361

309.45437	0.15363

309.58412	0.15364

309.71387	0.15371

309.84362	0.15381

309.97337	0.15383

310.10312	0.15388

310.23287	0.15389

310.36262	0.1539

310.49237	0.15402

310.62212	0.15403

310.75187	0.15405

310.88162	0.15407

311.01137	0.15412

311.14112	0.15415

311.27087	0.15422

311.40062	0.15423

311.53037	0.15425

311.66012	0.15427

311.78987	0.15458

311.91962	0.15464

312.04937	0.15465

312.17912	0.15485

312.30888	0.15487

312.43863	0.15488

312.56838	0.15489

312.69813	0.15508

312.82788	0.15512

312.95763	0.15519

313.08738	0.15523

313.21713	0.15563

313.34688	0.15564

313.47663	0.15566

313.60638	0.15568

313.73613	0.15591

313.86588	0.15592

313.99563	0.15594

314.12538	0.15597

314.25513	0.15617

314.38488	0.15618

314.51463	0.1565

314.64438	0.15651

314.77413	0.15655

314.90388	0.15659

315.03363	0.15677

315.16338	0.15678

315.29313	0.1568

315.42288	0.15689

315.55263	0.15693

315.68238	0.15696

315.81213	0.15697

315.94188	0.15698

316.07163	0.157

316.20138	0.15701

316.33113	0.15703

316.46088	0.15734

316.59063	0.15742

316.72038	0.15771

316.85013	0.15774

316.97988	0.15806

317.10963	0.15808

317.23938	0.1581

317.36914	0.1583

317.49889	0.15832

317.62864	0.15837

317.75839	0.15838

317.88814	0.1584

318.01789	0.1585

318.14764	0.15885

318.27739	0.15886

318.40714	0.15888

318.53689	0.1589

318.66664	0.15893

318.79639	0.15895

318.92614	0.15897

319.05589	0.15898

319.18564	0.15899

319.31539	0.15904

319.44514	0.15909

319.57489	0.15913

319.70464	0.15914

319.83439	0.15915

319.96414	0.15945

320.09389	0.15946

320.22364	0.15968

320.35339	0.15969

320.48314	0.1597

320.61289	0.15973

320.74264	0.15977

320.87239	0.15979

321.00214	0.1598

321.13189	0.15982

321.26164	0.16024

321.39139	0.1603

321.52114	0.16032

321.65089	0.16033

321.78064	0.16068

321.91039	0.1607

322.04014	0.16074

322.16989	0.16076

322.29965	0.16078

322.4294	0.16079

322.55915	0.1608

322.6889	0.16083

322.81865	0.16088

322.9484	0.16106

323.07815	0.16108

323.2079	0.16109

323.33765	0.16112

323.4674	0.16114

323.59715	0.16155

323.7269	0.16156

323.85665	0.16157

323.9864	0.16159

324.11615	0.16162

324.2459	0.16163

324.37565	0.16164

324.5054	0.16185

324.63515	0.16186

324.7649	0.16187

324.89465	0.1621

325.0244	0.16214

325.15415	0.16215

325.2839	0.16217

325.41365	0.16225

325.5434	0.16233

325.67315	0.16271

325.8029	0.16272

325.93265	0.16274

326.0624	0.16276

326.19215	0.16277

326.3219	0.16278

326.45165	0.16283

326.5814	0.16285

326.71115	0.16286

326.8409	0.16288

326.97065	0.1629

327.1004	0.16291

327.23015	0.16293

327.35991	0.16294

327.48966	0.16296

327.61941	0.16335

327.74916	0.16336

327.87891	0.16339

328.00866	0.1634

328.13841	0.16342

328.26816	0.16344

328.39791	0.16349

328.52766	0.1635

328.65741	0.16352

328.78716	0.16353

328.91691	0.16355

329.04666	0.16356

329.17641	0.16363

329.30616	0.16369

329.43591	0.16395

329.56566	0.16396

329.69541	0.16399

329.82516	0.164

329.95491	0.16411

330.08466	0.16414

330.21441	0.16422

330.34416	0.16424

330.47391	0.16425

330.60366	0.16426

330.73341	0.1643

330.86316	0.16468

330.99291	0.16469

331.12266	0.16472

331.25241	0.16474

331.38216	0.16476

331.51191	0.16481

331.64166	0.16483

331.77141	0.16484

331.90116	0.16526

332.03091	0.1653

332.16066	0.16531

332.29042	0.16551

332.42017	0.16552

332.54992	0.16554

332.67967	0.16555

332.80942	0.16559

332.93917	0.16561

333.06892	0.16563

333.19867	0.1658

333.32842	0.16581

333.45817	0.16583

333.58792	0.16589

333.71767	0.1659

333.84742	0.16619

333.97717	0.1662

334.10692	0.16621

334.23667	0.16623

334.36642	0.16625

334.49617	0.16627

334.62592	0.1664

334.75567	0.16643

334.88542	0.16645

335.01517	0.1666

335.14492	0.16663

335.27467	0.16669

335.40442	0.16704

335.53417	0.16706

335.66392	0.16708

335.79367	0.16747

335.92342	0.16752

336.05317	0.16756

336.18292	0.16757

336.31267	0.16777

336.44242	0.16779

336.57217	0.16783

336.70192	0.16786

336.83167	0.16789

336.96142	0.1679

337.09117	0.16797

337.22092	0.16823

337.35068	0.16825

337.48043	0.16826

337.61018	0.16828

337.73993	0.1683

337.86968	0.16846

337.99943	0.1685

338.12918	0.16851

338.25893	0.16882

338.38868	0.16886

338.51843	0.16892

338.64818	0.16908

338.77793	0.16909

338.90768	0.16925

339.03743	0.1693

339.16718	0.16937

339.29693	0.16939

339.42668	0.1694

339.55643	0.16961

339.68618	0.16962

339.81593	0.16968

339.94568	0.1698

340.07543	0.16982

340.20518	0.16985

340.33493	0.16987

340.46468	0.17027

340.59443	0.17029

340.72418	0.1704

340.85393	0.17044

340.98368	0.17045

341.11343	0.17047

341.24318	0.17048

341.37293	0.17049

341.50268	0.1705

341.63243	0.17088

341.76218	0.17089

341.89193	0.1709

342.02168	0.17091

342.15143	0.17094

342.28118	0.17096

342.41094	0.17104

342.54069	0.17105

342.67044	0.17125

342.80019	0.17126

342.92994	0.17128

343.05969	0.17167

343.18944	0.1717

343.31919	0.17172

343.44894	0.17183

343.57869	0.17184

343.70844	0.17187

343.83819	0.1722

343.96794	0.17223

344.09769	0.17224

344.22744	0.17225

344.35719	0.17262

344.48694	0.17266

344.61669	0.17267

344.74644	0.17272

344.87619	0.17278

345.00594	0.17282

345.13569	0.17283

345.26544	0.17298

345.39519	0.17302

345.52494	0.17305

345.65469	0.17307

345.78444	0.17311

345.91419	0.17313

346.04394	0.17314

346.17369	0.17332

346.30344	0.17337

346.43319	0.17341

346.56294	0.17343

346.69269	0.17367

346.82244	0.17371

346.95219	0.17372

347.08194	0.17373

347.21169	0.17375

347.34145	0.17377

347.4712	0.17378

347.60095	0.17379

347.7307	0.17381

347.86045	0.17383

347.9902	0.17384

348.11995	0.17388

348.2497	0.17389

348.37945	0.17391

348.5092	0.17392

348.63895	0.17394

348.7687	0.17395

348.89845	0.17396

349.0282	0.17397

349.15795	0.17399

349.2877	0.17411

349.41745	0.17413

349.5472	0.17416

349.67695	0.17418

349.8067	0.17421

349.93645	0.17426

350.0662	0.17428

350.19595	0.17457

350.3257	0.1746

350.45545	0.17461

350.5852	0.17463

350.71495	0.17467

350.8447	0.1749

350.97445	0.17491

351.1042	0.17494

351.23395	0.17498

351.3637	0.175

351.49345	0.17501

351.6232	0.1751

351.75295	0.17516

351.8827	0.17517

352.01245	0.17518

352.1422	0.17521

352.27195	0.17523

352.40171	0.17524

352.53146	0.17526

352.66121	0.17529

352.79096	0.1753

352.92071	0.17531

353.05046	0.17532

353.18021	0.17534

353.30996	0.17539

353.43971	0.1754

353.56946	0.17546

353.69921	0.1755

353.82896	0.17555

353.95871	0.17556

354.08846	0.17585

354.21821	0.17586

354.34796	0.17587

354.47771	0.17589

354.60746	0.1762

354.73721	0.17623

354.86696	0.17625

354.99671	0.17645

355.12646	0.17659

355.25621	0.17661

355.38596	0.17664

355.51571	0.17666

355.64546	0.17713

355.77521	0.17719

355.90496	0.1772

356.03471	0.17726

356.16446	0.17729

356.29421	0.1773

356.42396	0.17731

356.55371	0.17733

356.68346	0.17751

356.81321	0.17752

356.94296	0.17754

357.07271	0.17755

357.20246	0.17787

357.33222	0.17792

357.46197	0.17795

357.59172	0.17798

357.72147	0.17802

357.85122	0.17804

357.98097	0.17805

358.11072	0.17808

358.24047	0.17824

358.37022	0.17825

358.49997	0.17827

358.62972	0.17828

358.75947	0.1783

358.88922	0.17834

359.01897	0.17837

359.14872	0.17838

359.27847	0.17849

359.40822	0.17851

359.53797	0.17852

359.66772	0.17853

359.79747	0.17858

359.92722	0.17875

360.05697	0.17877

360.18672	0.17878

360.31647	0.17881

360.44622	0.17882

360.57597	0.17902

360.70572	0.17904

360.83547	0.17907

360.96522	0.17909

361.09497	0.17911

361.22472	0.17917

361.35447	0.17918

361.48422	0.1792

361.61397	0.17953

361.74372	0.17957

361.87347	0.17959

362.00322	0.17961

362.13297	0.17963

362.26272	0.17966

362.39248	0.17967

362.52223	0.17994

362.65198	0.17996

362.78173	0.17999

362.91148	0.18

363.04123	0.18058

363.17098	0.1806

363.30073	0.18096

363.43048	0.18098

363.56023	0.18109

363.68998	0.18134

363.81973	0.18135

363.94948	0.18136

364.07923	0.18138

364.20898	0.1814

364.33873	0.18141

364.46848	0.18142

364.59823	0.18144

364.72798	0.18145

364.85773	0.1816

364.98748	0.18169

365.11723	0.18171

365.24698	0.18172

365.37673	0.18174

365.50648	0.18179

365.63623	0.18202

365.76598	0.18205

365.89573	0.18207

366.02548	0.1821

366.15523	0.18211

366.28498	0.18218

366.41473	0.1822

366.54448	0.18222

366.67423	0.18224

366.80398	0.18226

366.93373	0.1823

367.06348	0.18232

367.19323	0.18277

367.32299	0.18278

367.45274	0.1828

367.58249	0.18323

367.71224	0.18324

367.84199	0.18356

367.97174	0.18357

368.10149	0.18358

368.23124	0.1836

368.36099	0.18374

368.49074	0.18387

368.62049	0.18388

368.75024	0.18408

368.87999	0.18409

369.00974	0.1841

369.13949	0.18412

369.26924	0.18432

369.39899	0.18434

369.52874	0.18436

369.65849	0.18438

369.78824	0.1844

369.91799	0.18442

370.04774	0.18444

370.17749	0.18446

370.30724	0.1845

370.43699	0.18453

370.56674	0.18455

370.69649	0.18457

370.82624	0.1846

370.95599	0.18463

371.08574	0.18466

371.21549	0.18467

371.34524	0.18468

371.47499	0.1847

371.60474	0.18472

371.73449	0.18474

371.86424	0.18476

371.99399	0.18479

372.12374	0.1848

372.25349	0.18487

372.38325	0.18489

372.513	0.18498

372.64275	0.18554

372.7725	0.18558

372.90225	0.18562

373.032	0.18594

373.16175	0.18601

373.2915	0.18602

373.42125	0.18604

373.551	0.18605

373.68075	0.18606

373.8105	0.18607

373.94025	0.18612

374.07	0.18614

374.19975	0.18652

374.3295	0.18657

374.45925	0.18658

374.589	0.18661

374.71875	0.18662

374.8485	0.18664

374.97825	0.18668

375.108	0.18677

375.23775	0.18678

375.3675	0.1868

375.49725	0.18708

375.627	0.18711

375.75675	0.18712

375.8865	0.18713

376.01625	0.18744

376.146	0.18745

376.27575	0.18746

376.4055	0.18753

376.53525	0.18773

376.665	0.18774

376.79475	0.18823

376.9245	0.18825

377.05425	0.18826

377.184	0.18837

377.31376	0.1884

377.44351	0.18844

377.57326	0.18845

377.70301	0.18848

377.83276	0.1885

377.96251	0.18851

378.09226	0.18853

378.22201	0.18855

378.35176	0.18858

378.48151	0.18859

378.61126	0.18886

378.74101	0.1889

378.87076	0.18898

379.00051	0.18899

379.13026	0.18901

379.26001	0.18908

379.38976	0.18911

379.51951	0.18913

379.64926	0.18915

379.77901	0.18919

379.90876	0.18921

380.03851	0.18923

380.16826	0.18924

380.29801	0.18926

380.42776	0.18928

380.55751	0.18944

380.68726	0.18945

380.81701	0.18952

380.94676	0.18954

381.07651	0.18955

381.20626	0.18958

381.33601	0.18962

381.46576	0.1897

381.59551	0.18971

381.72526	0.18972

381.85501	0.18973

381.98476	0.19017

382.11451	0.19018

382.24426	0.1902

382.37402	0.19022

382.50377	0.19024

382.63352	0.19028

382.76327	0.19029

382.89302	0.1903

383.02277	0.19031

383.15252	0.19045

383.28227	0.1905

383.41202	0.19052

383.54177	0.19053

383.67152	0.19055

383.80127	0.19057

383.93102	0.19058

384.06077	0.19065

384.19052	0.19072

384.32027	0.19073

384.45002	0.19077

384.57977	0.1909

384.70952	0.19106

384.83927	0.19107

384.96902	0.19109

385.09877	0.19111

385.22852	0.19113

385.35827	0.19114

385.48802	0.19132

385.61777	0.19137

385.74752	0.19138

385.87727	0.1914

386.00702	0.19189

386.13677	0.1919

386.26652	0.19228

386.39627	0.19229

386.52602	0.1923

386.65577	0.19232

386.78552	0.19241

386.91527	0.19242

387.04502	0.19247

387.17477	0.19249

387.30453	0.1925

387.43428	0.19255

387.56403	0.19257

387.69378	0.19259

387.82353	0.19266

387.95328	0.19267

388.08303	0.19268

388.21278	0.19269

388.34253	0.19298

388.47228	0.19301

388.60203	0.19302

388.73178	0.19307

388.86153	0.19308

388.99128	0.19309

389.12103	0.1931

389.25078	0.19316

389.38053	0.19327

389.51028	0.19334

389.64003	0.19335

389.76978	0.19336

389.89953	0.19338

390.02928	0.1934

390.15903	0.19345

390.28878	0.19378

390.41853	0.19379

390.54828	0.1938

390.67803	0.19381

390.80778	0.19382

390.93753	0.19388

391.06728	0.19401

391.19703	0.19402

391.32678	0.19409

391.45653	0.19415

391.58628	0.19437

391.71603	0.19439

391.84578	0.1944

391.97553	0.19441

392.10528	0.19443

392.23503	0.19445

392.36479	0.19446

392.49454	0.19447

392.62429	0.19481

392.75404	0.19488

392.88379	0.1949

393.01354	0.19495

393.14329	0.19496

393.27304	0.19531

393.40279	0.19533

393.53254	0.19544

393.66229	0.19546

393.79204	0.19547

393.92179	0.19549

394.05154	0.1955

394.18129	0.19558

394.31104	0.1956

394.44079	0.19563

394.57054	0.19564

394.70029	0.19567

394.83004	0.19568

394.95979	0.1957

395.08954	0.19572

395.21929	0.19592

395.34904	0.19593

395.47879	0.19595

395.60854	0.19599

395.73829	0.19602

395.86804	0.19606

395.99779	0.19607

396.12754	0.19609

396.25729	0.19613

396.38704	0.19615

396.51679	0.19616

396.64654	0.19618

396.77629	0.19623

396.90604	0.19657

397.03579	0.1966

397.16554	0.19662

397.2953	0.19713

397.42505	0.19714

397.5548	0.19718

397.68455	0.19724

397.8143	0.19725

397.94405	0.19727

398.0738	0.19748

398.20355	0.19749

398.3333	0.19756

398.46305	0.19758

398.5928	0.19763

398.72255	0.19767

398.8523	0.19768

398.98205	0.1982

399.1118	0.19823

399.24155	0.19825

399.3713	0.19827

399.50105	0.1983

399.6308	0.19831

399.76055	0.19833

399.8903	0.19835

400.02005	0.19838

400.1498	0.19841

400.27955	0.19842

400.4093	0.19897

400.53905	0.199

400.6688	0.19901

400.79855	0.19902

400.9283	0.19904

401.05805	0.19914

401.1878	0.19915

401.31755	0.19921

401.4473	0.19934

401.57705	0.19939

401.7068	0.19941

401.83655	0.19942

401.9663	0.19944

402.09605	0.19946

402.2258	0.19951

402.35556	0.19953

402.48531	0.19989

402.61506	0.1999

402.74481	0.19992

402.87456	0.20036

403.00431	0.20038

403.13406	0.20039

403.26381	0.20044

403.39356	0.20045

403.52331	0.20059

403.65306	0.20062

403.78281	0.20063

403.91256	0.20103

404.04231	0.20105

404.17206	0.20106

404.30181	0.20108

404.43156	0.20145

404.56131	0.20149

404.69106	0.20153

404.82081	0.20184

404.95056	0.20187

405.08031	0.20188

405.21006	0.20189

405.33981	0.20191

405.46956	0.20195

405.59931	0.20207

405.72906	0.20209

405.85881	0.20214

405.98856	0.20219

406.11831	0.20222

406.24806	0.20225

406.37781	0.20228

406.50756	0.20234

406.63731	0.20237

406.76706	0.20269

406.89681	0.20276

407.02656	0.20283

407.15631	0.20284

407.28607	0.20286

407.41582	0.20323

407.54557	0.20327

407.67532	0.20328

407.80507	0.20332

407.93482	0.20334

408.06457	0.20336

408.19432	0.20337

408.32407	0.20338

408.45382	0.2034

408.58357	0.20342

408.71332	0.20347

408.84307	0.20364

408.97282	0.20366

409.10257	0.20368

409.23232	0.20372

409.36207	0.20373

409.49182	0.20377

409.62157	0.20399

409.75132	0.20409

409.88107	0.20411

410.01082	0.20418

410.14057	0.20423

410.27032	0.20425

410.40007	0.20429

410.52982	0.20436

410.65957	0.20439

410.78932	0.20442

410.91907	0.20455

411.04882	0.20479

411.17857	0.2048

411.30832	0.20485

411.43807	0.20493

411.56782	0.20496

411.69757	0.20497

411.82732	0.20502

411.95707	0.20511

412.08682	0.20513

412.21657	0.20516

412.34633	0.20538

412.47608	0.20539

412.60583	0.20541

412.73558	0.20549

412.86533	0.20551

412.99508	0.20562

413.12483	0.20574

413.25458	0.20581

413.38433	0.20585

413.51408	0.20586

413.64383	0.20621

413.77358	0.20627

413.90333	0.20628

414.03308	0.20639

414.16283	0.20644

414.29258	0.20651

414.42233	0.20655

414.55208	0.20659

414.68183	0.20661

414.81158	0.20663

414.94133	0.20687

415.07108	0.20694

415.20083	0.20696

415.33058	0.20734

415.46033	0.20737

415.59008	0.20744

415.71983	0.20746

415.84958	0.20747

415.97933	0.20751

416.10908	0.20783

416.23883	0.20784

416.36858	0.20786

416.49833	0.20788

416.62808	0.20789

416.75783	0.2079

416.88758	0.20792

417.01733	0.20807

417.14708	0.20816

417.27684	0.20823

417.40659	0.20824

417.53634	0.20825

417.66609	0.20827

417.79584	0.20829

417.92559	0.2083

418.05534	0.20833

418.18509	0.20836

418.31484	0.20837

418.44459	0.20839

418.57434	0.20845

418.70409	0.20854

418.83384	0.20855

418.96359	0.20896

419.09334	0.20897

419.22309	0.20899

419.35284	0.20901

419.48259	0.20927

419.61234	0.20929

419.74209	0.20934

419.87184	0.20936

420.00159	0.20938

420.13134	0.20941

420.26109	0.20943

420.39084	0.20958

420.52059	0.20959

420.65034	0.20962

420.78009	0.20963

420.90984	0.20965

421.03959	0.2101

421.16934	0.21015

421.29909	0.21046

421.42884	0.21048

421.55859	0.2105

421.68834	0.21051

421.81809	0.21052

421.94784	0.21105

422.07759	0.21107

422.20734	0.2111

422.3371	0.21114

422.46685	0.21123

422.5966	0.21132

422.72635	0.21134

422.8561	0.21142

422.98585	0.21143

423.1156	0.21146

423.24535	0.21147

423.3751	0.21174

423.50485	0.21176

423.6346	0.21177

423.76435	0.2118

423.8941	0.21181

424.02385	0.21183

424.1536	0.21185

424.28335	0.21186

424.4131	0.21187

424.54285	0.21189

424.6726	0.21202

424.80235	0.21203

424.9321	0.21207

425.06185	0.21212

425.1916	0.21213

425.32135	0.21216

425.4511	0.21231

425.58085	0.21238

425.7106	0.21242

425.84035	0.21244

425.9701	0.21249

426.09985	0.21251

426.2296	0.21252

426.35935	0.21258

426.4891	0.21266

426.61885	0.21269

426.7486	0.2127

426.87835	0.21272

427.0081	0.21295

427.13785	0.213

427.26761	0.21301

427.39736	0.21303

427.52711	0.21305

427.65686	0.21306

427.78661	0.21314

427.91636	0.21351

428.04611	0.21358

428.17586	0.21362

428.30561	0.21364

428.43536	0.21382

428.56511	0.21389

428.69486	0.21391

428.82461	0.21397

428.95436	0.21399

429.08411	0.214

429.21386	0.21402

429.34361	0.21435

429.47336	0.21437

429.60311	0.21438

429.73286	0.2144

429.86261	0.21442

429.99236	0.21445

430.12211	0.21472

430.25186	0.21476

430.38161	0.21478

430.51136	0.2148

430.64111	0.21481

430.77086	0.21482

430.90061	0.21513

431.03036	0.21518

431.16011	0.21519

431.28986	0.21524

431.41961	0.21534

431.54936	0.21537

431.67911	0.21538

431.80886	0.21539

431.93861	0.2154

432.06836	0.21541

432.19811	0.21543

432.32787	0.21544

432.45762	0.21547

432.58737	0.21548

432.71712	0.2159

432.84687	0.21591

432.97662	0.21622

433.10637	0.21623

433.23612	0.21626

433.36587	0.21636

433.49562	0.21638

433.62537	0.21678

433.75512	0.21679

433.88487	0.21682

434.01462	0.21691

434.14437	0.21693

434.27412	0.21695

434.40387	0.21696

434.53362	0.21697

434.66337	0.21725

434.79312	0.21728

434.92287	0.21729

435.05262	0.21731

435.18237	0.21735

435.31212	0.21736

435.44187	0.21776

435.57162	0.21777

435.70137	0.21779

435.83112	0.2181

435.96087	0.21812

436.09062	0.21816

436.22037	0.21817

436.35012	0.21818

436.47987	0.21819

436.60962	0.21821

436.73937	0.21824

436.86912	0.21828

436.99887	0.21829

437.12862	0.21836

437.25838	0.2184

437.38813	0.21841

437.51788	0.21869

437.64763	0.21871

437.77738	0.21873

437.90713	0.21874

438.03688	0.21875

438.16663	0.21886

438.29638	0.21903

438.42613	0.21904

438.55588	0.21919

438.68563	0.21937

438.81538	0.21938

438.94513	0.21939

439.07488	0.21949

439.20463	0.21963

439.33438	0.21964

439.46413	0.21995

439.59388	0.21996

439.72363	0.21997

439.85338	0.22019

439.98313	0.2202

440.11288	0.22022

440.24263	0.22023

440.37238	0.22047

440.50213	0.22049

440.63188	0.22052

440.76163	0.22056

440.89138	0.22057

441.02113	0.22058

441.15088	0.2206

441.28063	0.22067

441.41038	0.22069

441.54013	0.22073

441.66988	0.22074

441.79963	0.22076

441.92938	0.22085

442.05913	0.22088

442.18888	0.22102

442.31864	0.22105

442.44839	0.22106

442.57814	0.22108

442.70789	0.22111

442.83764	0.22112

442.96739	0.22113

443.09714	0.22114

443.22689	0.22118

443.35664	0.22119

443.48639	0.22121

443.61614	0.22125

443.74589	0.22149

443.87564	0.22154

444.00539	0.22155

444.13514	0.22158

444.26489	0.22172

444.39464	0.22174

444.52439	0.22175

444.65414	0.22179

444.78389	0.22184

444.91364	0.22185

445.04339	0.22186

445.17314	0.22187

445.30289	0.22188

445.43264	0.22236

445.56239	0.22238

445.69214	0.2224

445.82189	0.22286

445.95164	0.22293

446.08139	0.22295

446.21114	0.223

446.34089	0.22304

446.47064	0.22307

446.60039	0.22314

446.73014	0.22315

446.85989	0.22355

446.98964	0.22377

447.11939	0.22391

447.24915	0.22393

447.3789	0.22397

447.50865	0.22401

447.6384	0.22402

447.76815	0.22404

447.8979	0.22406

448.02765	0.2242

448.1574	0.22425

448.28715	0.22427

448.4169	0.22429

448.54665	0.22466

448.6764	0.22472

448.80615	0.22474

448.9359	0.22479

449.06565	0.22484

449.1954	0.22505

449.32515	0.2251

449.4549	0.22539

449.58465	0.22541

449.7144	0.22543

449.84415	0.22546

449.9739	0.22551

450.10365	0.22558

450.2334	0.22562

450.36315	0.22567

450.4929	0.22568

450.62265	0.22569

450.7524	0.2257

450.88215	0.22571

451.0119	0.22573

451.14165	0.22608

451.2714	0.22609

451.40115	0.2261

451.5309	0.22612

451.66065	0.22613

451.7904	0.22614

451.92015	0.22618

452.0499	0.22647

452.17965	0.22648

452.30941	0.22649

452.43916	0.22651

452.56891	0.22652

452.69866	0.22654

452.82841	0.22655

452.95816	0.22658

453.08791	0.2266

453.21766	0.22664

453.34741	0.22668

453.47716	0.22693

453.60691	0.22694

453.73666	0.22696

453.86641	0.227

453.99616	0.22701

454.12591	0.22703

454.25566	0.22704

454.38541	0.22706

454.51516	0.2273

454.64491	0.22731

454.77466	0.22732

454.90441	0.22763

455.03416	0.22764

455.16391	0.22769

455.29366	0.22805

455.42341	0.22807

455.55316	0.22808

455.68291	0.2284

455.81266	0.22845

455.94241	0.22847

456.07216	0.22869

456.20191	0.22871

456.33166	0.22873

456.46141	0.22875

456.59116	0.22876

456.72091	0.2288

456.85066	0.22885

456.98041	0.22902

457.11016	0.2292

457.23992	0.22921

457.36967	0.22922

457.49942	0.22925

457.62917	0.22954

457.75892	0.22956

457.88867	0.22958

458.01842	0.22959

458.14817	0.2296

458.27792	0.22964

458.40767	0.22971

458.53742	0.22972

458.66717	0.23001

458.79692	0.23004

458.92667	0.23005

459.05642	0.23014

459.18617	0.23022

459.31592	0.23029

459.44567	0.2303

459.57542	0.23049

459.70517	0.2305

459.83492	0.23051

459.96467	0.23053

460.09442	0.23097

460.22417	0.23101

460.35392	0.23103

460.48367	0.23107

460.61342	0.23111

460.74317	0.23113

460.87292	0.2313

461.00267	0.23155

461.13242	0.23156

461.26217	0.23158

461.39192	0.23159

461.52167	0.2318

461.65142	0.23182

461.78117	0.23183

461.91092	0.23184

462.04067	0.23242

462.17042	0.23243

462.30018	0.23244

462.42993	0.23248

462.55968	0.23252

462.68943	0.23254

462.81918	0.23256

462.94893	0.23257

463.07868	0.23259

463.20843	0.23262

463.33818	0.23263

463.46793	0.23264

463.59768	0.23265

463.72743	0.23266

463.85718	0.23271

463.98693	0.23275

464.11668	0.23276

464.24643	0.23279

464.37618	0.23282

464.50593	0.23306

464.63568	0.23307

464.76543	0.23308

464.89518	0.23313

465.02493	0.23315

465.15468	0.23318

465.28443	0.23321

465.41418	0.23355

465.54393	0.23356

465.67368	0.23361

465.80343	0.23366

465.93318	0.23368

466.06293	0.23374

466.19268	0.23381

466.32243	0.23383

466.45218	0.23384

466.58193	0.23387

466.71168	0.23402

466.84143	0.23406

466.97118	0.23407

467.10093	0.23445

467.23068	0.23446

467.36044	0.23447

467.49019	0.2345

467.61994	0.23451

467.74969	0.23452

467.87944	0.23481

468.00919	0.23482

468.13894	0.23483

468.26869	0.23492

468.39844	0.23497

468.52819	0.235

468.65794	0.23501

468.78769	0.23507

468.91744	0.23519

469.04719	0.2352

469.17694	0.23522

469.30669	0.23524

469.43644	0.23525

469.56619	0.23527

469.69594	0.23555

469.82569	0.23559

469.95544	0.2356

470.08519	0.23561

470.21494	0.23565

470.34469	0.23566

470.47444	0.23572

470.60419	0.23574

470.73394	0.23576

470.86369	0.23577

470.99344	0.23579

471.12319	0.2358

471.25294	0.23582

471.38269	0.23583

471.51244	0.23586

471.64219	0.23611

471.77194	0.2362

471.90169	0.23621

472.03144	0.23622

472.16119	0.23628

472.29095	0.23632

472.4207	0.23638

472.55045	0.23639

472.6802	0.2364

472.80995	0.23651

472.9397	0.23652

473.06945	0.23654

473.1992	0.2366

473.32895	0.23673

473.4587	0.23674

473.58845	0.23675

473.7182	0.23676

473.84795	0.23678

473.9777	0.23706

474.10745	0.23718

474.2372	0.23721

474.36695	0.23725

474.4967	0.23732

474.62645	0.2374

474.7562	0.23742

474.88595	0.23745

475.0157	0.23781

475.14545	0.23782

475.2752	0.23783

475.40495	0.23785

475.5347	0.23791

475.66445	0.23795

475.7942	0.23804

475.92395	0.23805

476.0537	0.23808

476.18345	0.23809

476.3132	0.2381

476.44295	0.23811

476.5727	0.23815

476.70245	0.23818

476.8322	0.23821

476.96195	0.23822

477.0917	0.2384

477.22145	0.23843

477.35121	0.23845

477.48096	0.23848

477.61071	0.23852

477.74046	0.23853

477.87021	0.23855

477.99996	0.23867

478.12971	0.23869

478.25946	0.2387

478.38921	0.23873

478.51896	0.23899

478.64871	0.239

478.77846	0.23901

478.90821	0.2391

479.03796	0.23923

479.16771	0.23924

479.29746	0.23933

479.42721	0.23939

479.55696	0.23941

479.68671	0.23942

479.81646	0.2395

479.94621	0.23954

480.07596	0.23959

480.20571	0.2396

480.33546	0.23971

480.46521	0.23974

480.59496	0.23979

480.72471	0.2398

480.85446	0.24013

480.98421	0.24018

481.11396	0.24026

481.24371	0.24038

481.37346	0.2404

481.50321	0.24041

481.63296	0.2406

481.76271	0.24072

481.89246	0.24073

482.02221	0.24076

482.15196	0.24077

482.28172	0.2411

482.41147	0.24111

482.54122	0.24112

482.67097	0.24117

482.80072	0.24152

482.93047	0.24154

483.06022	0.24158

483.18997	0.2416

483.31972	0.24185

483.44947	0.24189

483.57922	0.2419

483.70897	0.24197

483.83872	0.24205

483.96847	0.24206

484.09822	0.24209

484.22797	0.24223

484.35772	0.24224

484.48747	0.24253

484.61722	0.24264

484.74697	0.24265

484.87672	0.24269

485.00647	0.2427

485.13622	0.24271

485.26597	0.24273

485.39572	0.24295

485.52547	0.24297

485.65522	0.24298

485.78497	0.24306

485.91472	0.24326

486.04447	0.24329

486.17422	0.24368

486.30397	0.24372

486.43372	0.24374

486.56347	0.24375

486.69322	0.24377

486.82297	0.24378

486.95272	0.24379

487.08247	0.24413

487.21222	0.24416

487.34198	0.24422

487.47173	0.24428

487.60148	0.24429

487.73123	0.2443

487.86098	0.24433

487.99073	0.24453

488.12048	0.24455

488.25023	0.24465

488.37998	0.24469

488.50973	0.24471

488.63948	0.24472

488.76923	0.24476

488.89898	0.24477

489.02873	0.24478

489.15848	0.24479

489.28823	0.24508

489.41798	0.24509

489.54773	0.24511

489.67748	0.24512

489.80723	0.24518

489.93698	0.24529

490.06673	0.24531

490.19648	0.24554

490.32623	0.24555

490.45598	0.24556

490.58573	0.24559

490.71548	0.24578

490.84523	0.24579

490.97498	0.24581

491.10473	0.24583

491.23448	0.24598

491.36423	0.24623

491.49398	0.24624

491.62373	0.24657

491.75348	0.2466

491.88323	0.24662

492.01298	0.24665

492.14273	0.24666

492.27249	0.24667

492.40224	0.24675

492.53199	0.24676

492.66174	0.24704

492.79149	0.24711

492.92124	0.24712

493.05099	0.24714

493.18074	0.24725

493.31049	0.24727

493.44024	0.2473

493.56999	0.24754

493.69974	0.24755

493.82949	0.2476

493.95924	0.24765

494.08899	0.24766

494.21874	0.24773

494.34849	0.24774

494.47824	0.24775

494.60799	0.24789

494.73774	0.24792

494.86749	0.24794

494.99724	0.24795

495.12699	0.24815

495.25674	0.24822

495.38649	0.24825

495.51624	0.24836

495.64599	0.24843

495.77574	0.24844

495.90549	0.24874

496.03524	0.2488

496.16499	0.24886

496.29474	0.24891

496.42449	0.24892

496.55424	0.24894

496.68399	0.24908

496.81374	0.24911

496.94349	0.24912

497.07324	0.24919

497.20299	0.24921

497.33275	0.24926

497.4625	0.24933

497.59225	0.24934

497.722	0.24935

497.85175	0.24939

497.9815	0.24961

498.11125	0.24962

498.241	0.24967

498.37075	0.24984

498.5005	0.24991

498.63025	0.2503

498.76	0.25031

498.88975	0.25032

499.0195	0.25034

499.14925	0.25035

499.279	0.25038

499.40875	0.25039

499.5385	0.25042

499.66825	0.2506

499.798	0.25061

499.92775	0.25063

500.0575	0.25066

500.18725	0.25067

500.317	0.25104

500.44675	0.25106

500.5765	0.25108

500.70625	0.2511

500.836	0.25137

500.96575	0.25143

501.0955	0.25144

501.22525	0.2515

501.355	0.25152

501.48475	0.25157

501.6145	0.25186

501.74425	0.25188

501.874	0.25189

502.00375	0.25191

502.1335	0.25198

502.26326	0.252

502.39301	0.25211

502.52276	0.25219

502.65251	0.25221

502.78226	0.25222

502.91201	0.25225

503.04176	0.25227

503.17151	0.25245

503.30126	0.25246

503.43101	0.25277

503.56076	0.25278

503.69051	0.25287

503.82026	0.2529

503.95001	0.25291

504.07976	0.25296

504.20951	0.25301

504.33926	0.25302

504.46901	0.2531

504.59876	0.25312

504.72851	0.25315

504.85826	0.25317

504.98801	0.25318

505.11776	0.25327

505.24751	0.2533

505.37726	0.25334

505.50701	0.25335

505.63676	0.25367

505.76651	0.25368

505.89626	0.25384

506.02601	0.25387

506.15576	0.25389

506.28551	0.25396

506.41526	0.25397

506.54501	0.25398

506.67476	0.25435

506.80451	0.25437

506.93426	0.25444

507.06401	0.25445

507.19376	0.25449

507.32352	0.2545

507.45327	0.25453

507.58302	0.25461

507.71277	0.25463

507.84252	0.25464

507.97227	0.25493

508.10202	0.25495

508.23177	0.25496

508.36152	0.25511

508.49127	0.25514

508.62102	0.25515

508.75077	0.25516

508.88052	0.25518

509.01027	0.2552

509.14002	0.25525

509.26977	0.25527

509.39952	0.25528

509.52927	0.25534

509.65902	0.25535

509.78877	0.2554

509.91852	0.25542

510.04827	0.25543

510.17802	0.25544

510.30777	0.25546

510.43752	0.25547

510.56727	0.2555

510.69702	0.25565

510.82677	0.25567

510.95652	0.25568

511.08627	0.2557

511.21602	0.25602

511.34577	0.25605

511.47552	0.25606

511.60527	0.25612

511.73502	0.25614

511.86477	0.25615

511.99452	0.25619

512.12427	0.25629

512.25403	0.2563

512.38378	0.25643

512.51353	0.25646

512.64328	0.25649

512.77303	0.25671

512.90278	0.25673

513.03253	0.25674

513.16228	0.25682

513.29203	0.25684

513.42178	0.25687

513.55153	0.25691

513.68128	0.25693

513.81103	0.25698

513.94078	0.25699

514.07053	0.25724

514.20028	0.25727

514.33003	0.25732

514.45978	0.25733

514.58953	0.25734

514.71928	0.25738

514.84903	0.25739

514.97878	0.25742

515.10853	0.25743

515.23828	0.25747

515.36803	0.25749

515.49778	0.25751

515.62753	0.25752

515.75728	0.25754

515.88703	0.25759

516.01678	0.25769

516.14653	0.25771

516.27628	0.25773

516.40603	0.25783

516.53578	0.25787

516.66553	0.25793

516.79528	0.25811

516.92503	0.25813

517.05478	0.25816

517.18453	0.25817

517.31429	0.25818

517.44404	0.25822

517.57379	0.25825

517.70354	0.25826

517.83329	0.25827

517.96304	0.2583

518.09279	0.25834

518.22254	0.25836

518.35229	0.25837

518.48204	0.25841

518.61179	0.25843

518.74154	0.25845

518.87129	0.25848

519.00104	0.25885

519.13079	0.25888

519.26054	0.2589

519.39029	0.25891

519.52004	0.25909

519.64979	0.25911

519.77954	0.25915

519.90929	0.25917

520.03904	0.25919

520.16879	0.2592

520.29854	0.2594

520.42829	0.25941

520.55804	0.25942

520.68779	0.25943

520.81754	0.25961

520.94729	0.25963

521.07704	0.25965

521.20679	0.26007

521.33654	0.26009

521.46629	0.26038

521.59604	0.26039

521.72579	0.26048

521.85554	0.26055

521.98529	0.26058

522.11504	0.26059

522.2448	0.26062

522.37455	0.26066

522.5043	0.26069

522.63405	0.26073

522.7638	0.26076

522.89355	0.26079

523.0233	0.26081

523.15305	0.26115

523.2828	0.26119

523.41255	0.26122

523.5423	0.26123

523.67205	0.26124

523.8018	0.26126

523.93155	0.26134

524.0613	0.26135

524.19105	0.26137

524.3208	0.26142

524.45055	0.26144

524.5803	0.26147

524.71005	0.26162

524.8398	0.26167

524.96955	0.26172

525.0993	0.26174

525.22905	0.26179

525.3588	0.26208

525.48855	0.26209

525.6183	0.26223

525.74805	0.26227

525.8778	0.26229

526.00755	0.2623

526.1373	0.26231

526.26705	0.26245

526.3968	0.26247

526.52655	0.26251

526.6563	0.26258

526.78605	0.26259

526.9158	0.26261

527.04555	0.26262

527.1753	0.26264

527.30506	0.26268

527.43481	0.26271

527.56456	0.2628

527.69431	0.26281

527.82406	0.26284

527.95381	0.26287

528.08356	0.2629

528.21331	0.26291

528.34306	0.26307

528.47281	0.26308

528.60256	0.2631

528.73231	0.26348

528.86206	0.26349

528.99181	0.2635

529.12156	0.26372

529.25131	0.26374

529.38106	0.26377

529.51081	0.26395

529.64056	0.26396

529.77031	0.26397

529.90006	0.26403

530.02981	0.26419

530.15956	0.26425

530.28931	0.26427

530.41906	0.26435

530.54881	0.26436

530.67856	0.26437

530.80831	0.26475

530.93806	0.26477

531.06781	0.26479

531.19756	0.26485

531.32731	0.26486

531.45706	0.26487

531.58681	0.26492

531.71656	0.26497

531.84631	0.26499

531.97606	0.265

532.10581	0.26534

532.23557	0.26536

532.36532	0.26538

532.49507	0.2654

532.62482	0.26544

532.75457	0.26545

532.88432	0.26546

533.01407	0.26557

533.14382	0.26564

533.27357	0.26567

533.40332	0.2657

533.53307	0.26572

533.66282	0.26608

533.79257	0.26609

533.92232	0.26611

534.05207	0.26614

534.18182	0.26616

534.31157	0.26618

534.44132	0.2662

534.57107	0.26623

534.70082	0.26625

534.83057	0.26626

534.96032	0.26634

535.09007	0.26638

535.21982	0.2664

535.34957	0.26649

535.47932	0.26654

535.60907	0.26657

535.73882	0.26659

535.86857	0.2666

535.99832	0.26678

536.12807	0.26704

536.25782	0.26705

536.38757	0.26714

536.51732	0.26717

536.64707	0.26719

536.77682	0.26721

536.90657	0.26727

537.03632	0.26731

537.16607	0.26734

537.29583	0.26747

537.42558	0.26754

537.55533	0.26773

537.68508	0.26775

537.81483	0.26777

537.94458	0.2678

538.07433	0.26784

538.20408	0.26785

538.33383	0.26786

538.46358	0.26788

538.59333	0.26789

538.72308	0.26793

538.85283	0.26794

538.98258	0.26795

539.11233	0.26796

539.24208	0.26805

539.37183	0.26812

539.50158	0.26813

539.63133	0.26815

539.76108	0.26824

539.89083	0.26825

540.02058	0.26838

540.15033	0.26839

540.28008	0.26843

540.40983	0.2685

540.53958	0.26852

540.66933	0.26853

540.79908	0.26855

540.92883	0.26856

541.05858	0.26857

541.18833	0.26879

541.31808	0.26881

541.44783	0.26882

541.57758	0.26896

541.70733	0.26897

541.83708	0.26899

541.96683	0.26903

542.09658	0.26904

542.22634	0.26905

542.35609	0.26906

542.48584	0.26921

542.61559	0.26922

542.74534	0.26923

542.87509	0.26925

543.00484	0.26929

543.13459	0.26932

543.26434	0.26933

543.39409	0.26935

543.52384	0.26938

543.65359	0.26941

543.78334	0.26942

543.91309	0.26952

544.04284	0.26966

544.17259	0.26967

544.30234	0.26981

544.43209	0.26991

544.56184	0.27004

544.69159	0.27015

544.82134	0.27016

544.95109	0.27017

545.08084	0.2702

545.21059	0.27024

545.34034	0.27026

545.47009	0.27042

545.59984	0.27044

545.72959	0.27046

545.85934	0.27052

545.98909	0.27054

546.11884	0.27056

546.24859	0.27058

546.37834	0.2706

546.50809	0.27063

546.63784	0.27083

546.76759	0.27085

546.89734	0.27086

547.02709	0.27104

547.15684	0.27106

547.2866	0.2711

547.41635	0.27114

547.5461	0.2712

547.67585	0.27122

547.8056	0.27124

547.93535	0.27125

548.0651	0.27133

548.19485	0.2714

548.3246	0.27142

548.45435	0.27143

548.5841	0.27145

548.71385	0.27146

548.8436	0.27147

548.97335	0.27149

549.1031	0.27152

549.23285	0.27153

549.3626	0.27155

549.49235	0.27157

549.6221	0.27179

549.75185	0.27199

549.8816	0.27201

550.01135	0.27204

550.1411	0.27224

550.27085	0.27228

550.4006	0.27229

550.53035	0.27231

550.6601	0.27236

550.78985	0.27245

550.9196	0.27246

551.04935	0.27249

551.1791	0.2725

551.30885	0.27274

551.4386	0.27275

551.56835	0.27278

551.6981	0.27279

551.82785	0.2728

551.9576	0.27281

552.08735	0.27282

552.21711	0.27284

552.34686	0.27285

552.47661	0.27298

552.60636	0.273

552.73611	0.27301

552.86586	0.27305

552.99561	0.27308

553.12536	0.27311

553.25511	0.27316

553.38486	0.27318

553.51461	0.27319

553.64436	0.2732

553.77411	0.27321

553.90386	0.27325

554.03361	0.27329

554.16336	0.27335

554.29311	0.27337

554.42286	0.27338

554.55261	0.27339

554.68236	0.27348

554.81211	0.27349

554.94186	0.2735

555.07161	0.27354

555.20136	0.27367

555.33111	0.27368

555.46086	0.27387

555.59061	0.27388

555.72036	0.27389

555.85011	0.2739

555.97986	0.27393

556.10961	0.27399

556.23936	0.274

556.36911	0.27401

556.49886	0.27403

556.62861	0.27404

556.75836	0.27415

556.88811	0.27417

557.01786	0.27419

557.14761	0.27427

557.27737	0.27431

557.40712	0.27432

557.53687	0.27437

557.66662	0.27448

557.79637	0.2745

557.92612	0.27452

558.05587	0.27463

558.18562	0.27466

558.31537	0.27469

558.44512	0.27472

558.57487	0.27479

558.70462	0.27481

558.83437	0.27482

558.96412	0.27484

559.09387	0.275

559.22362	0.27501

559.35337	0.27502

559.48312	0.27503

559.61287	0.27504

559.74262	0.27508

559.87237	0.27532

560.00212	0.27534

560.13187	0.27543

560.26162	0.27544

560.39137	0.27549

560.52112	0.27552

560.65087	0.27553

560.78062	0.27554

560.91037	0.27568

561.04012	0.27573

561.16987	0.27577

561.29962	0.27578

561.42937	0.27579

561.55912	0.2758

561.68887	0.27581

561.81862	0.27595

561.94837	0.27596

562.07812	0.27598

562.20787	0.27616

562.33763	0.27621

562.46738	0.27622

562.59713	0.27629

562.72688	0.27632

562.85663	0.27637

562.98638	0.2764

563.11613	0.27648

563.24588	0.27649

563.37563	0.27657

563.50538	0.27659

563.63513	0.27661

563.76488	0.27662

563.89463	0.27663

564.02438	0.27668

564.15413	0.27669

564.28388	0.27675

564.41363	0.27676

564.54338	0.27696

564.67313	0.27697

564.80288	0.27699

564.93263	0.27702

565.06238	0.27715

565.19213	0.27725

565.32188	0.27727

565.45163	0.27729

565.58138	0.2775

565.71113	0.27765

565.84088	0.27767

565.97063	0.27769

566.10038	0.27771

566.23013	0.27779

566.35988	0.2778

566.48963	0.27781

566.61938	0.27791

566.74913	0.27794

566.87888	0.27795

567.00863	0.27804

567.13838	0.27806

567.26814	0.27807

567.39789	0.27808

567.52764	0.2781

567.65739	0.27814

567.78714	0.27815

567.91689	0.27817

568.04664	0.2782

568.17639	0.27821

568.30614	0.27827

568.43589	0.27833

568.56564	0.27834

568.69539	0.27844

568.82514	0.27846

568.95489	0.27848

569.08464	0.27875

569.21439	0.27876

569.34414	0.27879

569.47389	0.27887

569.60364	0.27889

569.73339	0.2789

569.86314	0.27896

569.99289	0.27898

570.12264	0.27905

570.25239	0.27908

570.38214	0.2791

570.51189	0.27926

570.64164	0.2793

570.77139	0.27945

570.90114	0.27955

571.03089	0.27958

571.16064	0.27962

571.29039	0.27982

571.42014	0.27993

571.54989	0.27995

571.67964	0.27997

571.80939	0.27999

571.93914	0.28002

572.06889	0.28004

572.19865	0.28006

572.3284	0.28008

572.45815	0.2801

572.5879	0.28013

572.71765	0.28015

572.8474	0.28037

572.97715	0.28039

573.1069	0.28057

573.23665	0.28062

573.3664	0.28068

573.49615	0.28071

573.6259	0.28077

573.75565	0.28082

573.8854	0.28084

574.01515	0.28086

574.1449	0.28088

574.27465	0.28091

574.4044	0.28099

574.53415	0.28106

574.6639	0.28108

574.79365	0.28113

574.9234	0.28117

575.05315	0.28119

575.1829	0.28122

575.31265	0.28126

575.4424	0.28133

575.57215	0.28135

575.7019	0.28148

575.83165	0.28151

575.9614	0.28153

576.09115	0.28186

576.2209	0.28206

576.35065	0.28208

576.4804	0.28215

576.61015	0.28222

576.7399	0.28235

576.86965	0.28237

576.9994	0.28242

577.12915	0.28248

577.25891	0.28251

577.38866	0.28255

577.51841	0.28257

577.64816	0.28268

577.77791	0.28271

577.90766	0.28337

578.03741	0.2834

578.16716	0.28342

578.29691	0.28353

578.42666	0.28357

578.55641	0.2836

578.68616	0.28366

578.81591	0.28369

578.94566	0.28371

579.07541	0.28373

579.20516	0.28375

579.33491	0.28382

579.46466	0.28384

579.59441	0.28393

579.72416	0.28395

579.85391	0.28397

579.98366	0.284

580.11341	0.28413

580.24316	0.28415

580.37291	0.28418

580.50266	0.2842

580.63241	0.28422

580.76216	0.28429

580.89191	0.28431

581.02166	0.28433

581.15141	0.2844

581.28116	0.28451

581.41091	0.28455

581.54066	0.28458

581.67041	0.2846

581.80016	0.28462

581.92991	0.28464

582.05966	0.2848

582.18942	0.28482

582.31917	0.28484

582.44892	0.28486

582.57867	0.28491

582.70842	0.28493

582.83817	0.28495

582.96792	0.28498

583.09767	0.28504

583.22742	0.28506

583.35717	0.28518

583.48692	0.2852

583.61667	0.28524

583.74642	0.28526

583.87617	0.28529

584.00592	0.28535

584.13567	0.2854

584.26542	0.28542

584.39517	0.28558

584.52492	0.2856

584.65467	0.28567

584.78442	0.28569

584.91417	0.28573

585.04392	0.28575

585.17367	0.28578

585.30342	0.28582

585.43317	0.28587

585.56292	0.28589

585.69267	0.28593

585.82242	0.28595

585.95217	0.28598

586.08192	0.286

586.21167	0.28607

586.34142	0.28611

586.47117	0.28613

586.60092	0.28615

586.73067	0.28624

586.86042	0.28631

586.99017	0.28635

587.11992	0.28638

587.24968	0.2864

587.37943	0.28642

587.50918	0.28647

587.63893	0.28671

587.76868	0.2868

587.89843	0.28682

588.02818	0.28684

588.15793	0.28689

588.28768	0.28698

588.41743	0.287

588.54718	0.28704

588.67693	0.2872

588.80668	0.28724

588.93643	0.28727

589.06618	0.28729

589.19593	0.28731

589.32568	0.28733

589.45543	0.28736

589.58518	0.28738

589.71493	0.28742

589.84468	0.28744

589.97443	0.28751

590.10418	0.28756

590.23393	0.28758

590.36368	0.2876

590.49343	0.28762

590.62318	0.28764

590.75293	0.28767

590.88268	0.28769

591.01243	0.28771

591.14218	0.28773

591.27193	0.28778

591.40168	0.2878

591.53143	0.28782

591.66118	0.288

591.79093	0.28805

591.92068	0.28807

592.05043	0.28809

592.18019	0.28831

592.30994	0.28833

592.43969	0.28836

592.56944	0.28867

592.69919	0.28869

592.82894	0.28873

592.95869	0.28911

593.08844	0.28914

593.21819	0.28916

593.34794	0.28918

593.47769	0.2892

593.60744	0.28922

593.73719	0.28938

593.86694	0.2894

593.99669	0.28942

594.12644	0.28949

594.25619	0.28976

594.38594	0.28991

594.51569	0.29051

594.64544	0.29054

594.77519	0.29058

594.90494	0.2906

595.03469	0.29063

595.16444	0.29076

595.29419	0.29078

595.42394	0.29087

595.55369	0.29091

595.68344	0.29094

595.81319	0.29096

595.94294	0.29098

596.07269	0.29109

596.20244	0.29111

596.33219	0.29118

596.46194	0.2912

596.59169	0.29125

596.72144	0.29127

596.85119	0.29129

596.98094	0.29132

597.11069	0.29143

597.24045	0.29169

597.3702	0.29178

597.49995	0.2918

597.6297	0.29187

597.75945	0.29189

597.8892	0.29192

598.01895	0.29198

598.1487	0.292

598.27845	0.29205

598.4082	0.29207

598.53795	0.29209

598.6677	0.29212

598.79745	0.2922

598.9272	0.29223

599.05695	0.29227

599.1867	0.29232

599.31645	0.29243

599.4462	0.29247

599.57595	0.29249

599.7057	0.29252

599.83545	0.29267

599.9652	0.29281

600.09495	0.29283

600.2247	0.29285

600.35445	0.29289

600.4842	0.29292

600.61395	0.29294

600.7437	0.29296

600.87345	0.29301

601.0032	0.29307

601.13295	0.29309

601.2627	0.29312

601.39245	0.29316

601.5222	0.29318

601.65195	0.29323

601.7817	0.29325

601.91145	0.29327

602.0412	0.29329

602.17096	0.29334

602.30071	0.29336

602.43046	0.29478

602.56021	0.29485

602.68996	0.29503

602.81971	0.29507

602.94946	0.2951

603.07921	0.29514

603.20896	0.29545

603.33871	0.29547

603.46846	0.2955

603.59821	0.29554

603.72796	0.29556

603.85771	0.29559

603.98746	0.29561

604.11721	0.29563

604.24696	0.29565

604.37671	0.29567

604.50646	0.2957

604.63621	0.29572

604.76596	0.29574

604.89571	0.29576

605.02546	0.29579

605.15521	0.29581

605.28496	0.29585

605.41471	0.29587

605.54446	0.2959

605.67421	0.29599

605.80396	0.29601

605.93371	0.29603

606.06346	0.29605

606.19321	0.29608

606.32296	0.2961

606.45271	0.29612

606.58246	0.29614

606.71221	0.29619

606.84196	0.29621

606.97171	0.29625

607.10146	0.29628

607.23122	0.29659

607.36097	0.2975

607.49072	0.29752

607.62047	0.29754

607.75022	0.29757

607.87997	0.29765

608.00972	0.29768

608.13947	0.2977

608.26922	0.29772

608.39897	0.29774

608.52872	0.29777

608.65847	0.29779

608.78822	0.29799

608.91797	0.29803

609.04772	0.2981

609.17747	0.29812

609.30722	0.29819

609.43697	0.29834

609.56672	0.29839

609.69647	0.29843

609.82622	0.2985

609.95597	0.29852

610.08572	0.29854

610.21547	0.29857

610.34522	0.29859

610.47497	0.29861

610.60472	0.29863

610.73447	0.29868

610.86422	0.2987

610.99397	0.29872

611.12372	0.29874

611.25347	0.29877

611.38322	0.29879

611.51297	0.29886

611.64272	0.29888

611.77247	0.29892

611.90222	0.29897

612.03197	0.29899

612.16173	0.29908

612.29148	0.29986

612.42123	0.29995

612.55098	0.29997

612.68073	0.29999

612.81048	0.30001

612.94023	0.30003

613.06998	0.30006

613.19973	0.30008

613.32948	0.30015

613.45923	0.30017

613.58898	0.30019

613.71873	0.30021

613.84848	0.30023

613.97823	0.30026

614.10798	0.30028

614.23773	0.3003

614.36748	0.30032

614.49723	0.30035

614.62698	0.30037

614.75673	0.30039

614.88648	0.30052

615.01623	0.30055

615.14598	0.30057

615.27573	0.30059

615.40548	0.30066

615.53523	0.30068

615.66498	0.30072

615.79473	0.30075

615.92448	0.30077

616.05423	0.30079

616.18398	0.30086

616.31373	0.30088

616.44348	0.30092

616.57323	0.30108

616.70298	0.3011

616.83273	0.30112

616.96248	0.30121

617.09223	0.30124

617.22199	0.3013

617.35174	0.30135

617.48149	0.30137

617.61124	0.30139

617.74099	0.30141

617.87074	0.30144

618.00049	0.30152

618.13024	0.30159

618.25999	0.30161

618.38974	0.30164

618.51949	0.30168

618.64924	0.30172

618.77899	0.30175

618.90874	0.30181

619.03849	0.30186

619.16824	0.30192

619.29799	0.30195

619.42774	0.30197

619.55749	0.3021

619.68724	0.30215

619.81699	0.30217

619.94674	0.30219

620.07649	0.30221

620.20624	0.30224

620.33599	0.30226

620.46574	0.30228

620.59549	0.30237

620.72524	0.30239

620.85499	0.30244

620.98474	0.30246

621.11449	0.30248

621.24424	0.3025

621.37399	0.30253

621.50374	0.30255

621.63349	0.30257

621.76324	0.30259

621.89299	0.30264

622.02274	0.30266

622.1525	0.30268

622.28225	0.30273

622.412	0.30279

622.54175	0.30284

622.6715	0.3029

622.80125	0.30293

622.931	0.30297

623.06075	0.30382

623.1905	0.3039

623.32025	0.30397

623.45	0.30399

623.57975	0.30402

623.7095	0.30404

623.83925	0.30406

623.969	0.3041

624.09875	0.30428

624.2285	0.30437

624.35825	0.30442

624.488	0.30444

624.61775	0.30446

624.7475	0.30448

624.87725	0.30451

625.007	0.30453

625.13675	0.30459

625.2665	0.30464

625.39625	0.30466

625.526	0.30468

625.65575	0.30471

625.7855	0.30475

625.91525	0.30477

626.045	0.30484

626.17475	0.30488

626.3045	0.30491

626.43425	0.30495

626.564	0.30497

626.69375	0.30504

626.8235	0.30508

626.95325	0.30519

627.083	0.30522

627.21276	0.30524

627.34251	0.30528

627.47226	0.30531

627.60201	0.30533

627.73176	0.30535

627.86151	0.30537

627.99126	0.30577

628.12101	0.30582

628.25076	0.30584

628.38051	0.30588

628.51026	0.30593

628.64001	0.30595

628.76976	0.30602

628.89951	0.30611

629.02926	0.30631

629.15901	0.30635

629.28876	0.30637

629.41851	0.3064

629.54826	0.30642

629.67801	0.30644

629.80776	0.3066

629.93751	0.30662

630.06726	0.30664

630.19701	0.30671

630.32676	0.30673

630.45651	0.30675

630.58626	0.30677

630.71601	0.30686

630.84576	0.30702

630.97551	0.30706

631.10526	0.30709

631.23501	0.30713

631.36476	0.3072

631.49451	0.30749

631.62426	0.30751

631.75401	0.3076

631.88376	0.30771

632.01351	0.30775

632.14327	0.3078

632.27302	0.30791

632.40277	0.30795

632.53252	0.30804

632.66227	0.30806

632.79202	0.30811

632.92177	0.30813

633.05152	0.30818

633.18127	0.30822

633.31102	0.30824

633.44077	0.30838

633.57052	0.30851

633.70027	0.30853

633.83002	0.30855

633.95977	0.30862

634.08952	0.30864

634.21927	0.30866

634.34902	0.30869

634.47877	0.30871

634.60852	0.30873

634.73827	0.30875

634.86802	0.3088

634.99777	0.30886

635.12752	0.30889

635.25727	0.30893

635.38702	0.30904

635.51677	0.30913

635.64652	0.30915

635.77627	0.3092

635.90602	0.30929

636.03577	0.30931

636.16552	0.30933

636.29527	0.30935

636.42502	0.30964

636.55477	0.30967

636.68452	0.30969

636.81427	0.30971

636.94402	0.30975

637.07377	0.30984

637.20353	0.30987

637.33328	0.30991

637.46303	0.30993

637.59278	0.30995

637.72253	0.31

637.85228	0.31007

637.98203	0.31009

638.11178	0.31013

638.24153	0.31015

638.37128	0.31018

638.50103	0.3102

638.63078	0.31024

638.76053	0.31035

638.89028	0.31038

639.02003	0.3104

639.14978	0.31042

639.27953	0.31044

639.40928	0.31058

639.53903	0.3106

639.66878	0.31067

639.79853	0.31076

639.92828	0.3108

640.05803	0.31082

640.18778	0.31116

640.31753	0.3112

640.44728	0.31122

640.57703	0.31124

640.70678	0.31127

640.83653	0.3114

640.96628	0.31142

641.09603	0.31147

641.22578	0.31149

641.35553	0.3116

641.48528	0.31162

641.61503	0.31165

641.74478	0.31169

641.87453	0.31171

642.00428	0.3118

642.13404	0.31182

642.26379	0.31196

642.39354	0.31205

642.52329	0.31209

642.65304	0.3122

642.78279	0.31227

642.91254	0.31229

643.04229	0.31233

643.17204	0.31236

643.30179	0.31247

643.43154	0.31253

643.56129	0.3126

643.69104	0.31267

643.82079	0.31269

643.95054	0.31273

644.08029	0.31276

644.21004	0.31278

644.33979	0.31287

644.46954	0.31291

644.59929	0.31296

644.72904	0.31298

644.85879	0.313

644.98854	0.31314

645.11829	0.31318

645.24804	0.3132

645.37779	0.31326

645.50754	0.31331

645.63729	0.31333

645.76704	0.31334

645.89679	0.31336

646.02654	0.31338

646.15629	0.31349

646.28604	0.3135

646.41579	0.31352

646.54554	0.31354

646.67529	0.31358

646.80504	0.31359

646.93479	0.31361

647.06454	0.31365

647.1943	0.31366

647.32405	0.31377

647.4538	0.31381

647.58355	0.31388

647.7133	0.31411

647.84305	0.31415

647.9728	0.31418

648.10255	0.31429

648.2323	0.31432

648.36205	0.31434

648.4918	0.31436

648.62155	0.31438

648.7513	0.31445

648.88105	0.31447

649.0108	0.31448

649.14055	0.3145

649.2703	0.31455

649.40005	0.31463

649.5298	0.31466

649.65955	0.31468

649.7893	0.31471

649.91905	0.31575

650.0488	0.31576

650.17855	0.3158

650.3083	0.31582

650.43805	0.31584

650.5678	0.31585

650.69755	0.31587

650.8273	0.31589

650.95705	0.31592

651.0868	0.31596

651.21655	0.31598

651.3463	0.31605

651.47605	0.3161

651.6058	0.31612

651.73555	0.31621

651.8653	0.31623

651.99505	0.3163

652.12481	0.31632

652.25456	0.31633

652.38431	0.31635

652.51406	0.31637

652.64381	0.31639

652.77356	0.31641

652.90331	0.31642

653.03306	0.31665

653.16281	0.31669

653.29256	0.31671

653.42231	0.31673

653.55206	0.31674

653.68181	0.31678

653.81156	0.31683

653.94131	0.31685

654.07106	0.31694

654.20081	0.31699

654.33056	0.31701

654.46031	0.31703

654.59006	0.31705

654.71981	0.31706

654.84956	0.31715

654.97931	0.31717

655.10906	0.31719

655.23881	0.31721

655.36856	0.31722

655.49831	0.31724

655.62806	0.31726

655.75781	0.31729

655.88756	0.31742

656.01731	0.31744

656.14706	0.31745

656.27681	0.31747

656.40656	0.31765

656.53631	0.31769

656.66606	0.3177

656.79581	0.31776

656.92556	0.31779

657.05531	0.31781

657.18507	0.31783

657.31482	0.31786

657.44457	0.31788

657.57432	0.31794

657.70407	0.31795

657.83382	0.31797

657.96357	0.31799

658.09332	0.31801

658.22307	0.31802

658.35282	0.31808

658.48257	0.31813

658.61232	0.31815

658.74207	0.31827

658.87182	0.31829

659.00157	0.31831

659.13132	0.31836

659.26107	0.31838

659.39082	0.3184

659.52057	0.31843

659.65032	0.31845

659.78007	0.31874

659.90982	0.31877

660.03957	0.31881

660.16932	0.31884

660.29907	0.31893

660.42882	0.31897

660.55857	0.31899

660.68832	0.31904

660.81807	0.31906

660.94782	0.31907

661.07757	0.31909

661.20732	0.31913

661.33707	0.31915

661.46682	0.31916

661.59657	0.31918

661.72632	0.31922

661.85607	0.31923

661.98582	0.31948

662.11558	0.3195

662.24533	0.31952

662.37508	0.31954

662.50483	0.31955

662.63458	0.31957

662.76433	0.31959

662.89408	0.31961

663.02383	0.31963

663.15358	0.31966

663.28333	0.31968

663.41308	0.3197

663.54283	0.31973

663.67258	0.31975

663.80233	0.31977

663.93208	0.31989

664.06183	0.31991

664.19158	0.31995

664.32133	0.31996

664.45108	0.31998

664.58083	0.32

664.71058	0.32002

664.84033	0.32004

664.97008	0.32009

665.09983	0.32011

665.22958	0.32012

665.35933	0.32016

665.48908	0.32046

665.61883	0.32048

665.74858	0.3205

665.87833	0.32052

666.00808	0.32053

666.13783	0.32055

666.26758	0.32064

666.39733	0.32066

666.52708	0.32068

666.65683	0.32069

666.78658	0.32073

666.91633	0.32075

667.04608	0.32076

667.17584	0.32078

667.30559	0.32084

667.43534	0.32091

667.56509	0.32092

667.69484	0.32094

667.82459	0.32096

667.95434	0.32098

668.08409	0.321

668.21384	0.32103

668.34359	0.32112

668.47334	0.32114

668.60309	0.32135

668.73284	0.32144

668.86259	0.32146

668.99234	0.32148

669.12209	0.32153

669.25184	0.32155

669.38159	0.32157

669.51134	0.32165

669.64109	0.32167

669.77084	0.32169

669.90059	0.32174

670.03034	0.32176

670.16009	0.32178

670.28984	0.32181

670.41959	0.32183

670.54934	0.32185

670.67909	0.32189

670.80884	0.32194

670.93859	0.32199

671.06834	0.32201

671.19809	0.32205

671.32784	0.32213

671.45759	0.32221

671.58734	0.32222

671.71709	0.32226

671.84684	0.32244

671.97659	0.32249

672.10635	0.3229

672.2361	0.32292

672.36585	0.32294

672.4956	0.32297

672.62535	0.32301

672.7551	0.32302

672.88485	0.32306

673.0146	0.32311

673.14435	0.32313

673.2741	0.32315

673.40385	0.32322

673.5336	0.32326

673.66335	0.32349

673.7931	0.3235

673.92285	0.32352

674.0526	0.32354

674.18235	0.32358

674.3121	0.32359

674.44185	0.32361

674.5716	0.32367

674.70135	0.32372

674.8311	0.32374

674.96085	0.32381

675.0906	0.32406

675.22035	0.32436

675.3501	0.32439

675.47985	0.32441

675.6096	0.32445

675.73935	0.32447

675.8691	0.32448

675.99885	0.3245

676.1286	0.32452

676.25835	0.32454

676.3881	0.32457

676.51785	0.32463

676.6476	0.32464

676.77735	0.32468

676.9071	0.3247

677.03685	0.32496

677.16661	0.32498

677.29636	0.325

677.42611	0.32502

677.55586	0.32512

677.68561	0.32536

677.81536	0.32543

677.94511	0.32544

678.07486	0.32582

678.20461	0.32584

678.33436	0.32585

678.46411	0.32589

678.59386	0.32592

678.72361	0.32601

678.85336	0.32607

678.98311	0.3261

679.11286	0.32617

679.24261	0.32619

679.37236	0.32632

679.50211	0.32635

679.63186	0.32637

679.76161	0.32639

679.89136	0.32641

680.02111	0.32642

680.15086	0.32662

680.28061	0.32664

680.41036	0.32665

680.54011	0.32667

680.66986	0.32669

680.79961	0.32671

680.92936	0.32674

681.05911	0.32683

681.18886	0.32692

681.31861	0.3273

681.44836	0.32731

681.57811	0.32733

681.70786	0.32738

681.83761	0.32746

681.96736	0.32747

682.09712	0.32751

682.22687	0.32753

682.35662	0.32754

682.48637	0.32756

682.61612	0.32762

682.74587	0.32767

682.87562	0.32769

683.00537	0.32772

683.13512	0.32781

683.26487	0.32785

683.39462	0.32786

683.52437	0.32788

683.65412	0.3279

683.78387	0.32795

683.91362	0.32797

684.04337	0.32815

684.17312	0.32817

684.30287	0.32818

684.43262	0.32822

684.56237	0.32826

684.69212	0.32827

684.82187	0.32829

684.95162	0.32833

685.08137	0.32836

685.21112	0.32843

685.34087	0.32847

685.47062	0.32849

685.60037	0.32863

685.73012	0.32865

685.85987	0.32868

685.98962	0.32874

686.11937	0.32884

686.24912	0.32886

686.37887	0.3289

686.50862	0.32893

686.63837	0.32909

686.76812	0.32918

686.89787	0.32925

687.02762	0.32927

687.15738	0.3295

687.28713	0.32964

687.41688	0.32966

687.54663	0.32968

687.67638	0.3297

687.80613	0.32979

687.93588	0.32995

688.06563	0.32996

688.19538	0.32998

688.32513	0.33002

688.45488	0.33004

688.58463	0.33007

688.71438	0.33009

688.84413	0.33011

688.97388	0.33012

689.10363	0.33018

689.23338	0.33039

689.36313	0.33041

689.49288	0.33048

689.62263	0.3305

689.75238	0.33055

689.88213	0.33059

690.01188	0.3306

690.14163	0.33062

690.27138	0.33085

690.40113	0.33171

690.53088	0.33173

690.66063	0.33178

690.79038	0.33185

690.92013	0.33187

691.04988	0.3319

691.17963	0.33205

691.30938	0.33215

691.43913	0.33217

691.56888	0.33224

691.69863	0.33226

691.82838	0.33228

691.95813	0.3323

692.08788	0.33235

692.21764	0.3324

692.34739	0.33242

692.47714	0.33246

692.60689	0.33254

692.73664	0.3326

692.86639	0.33262

692.99614	0.33263

693.12589	0.33267

693.25564	0.33269

693.38539	0.3327

693.51514	0.33278

693.64489	0.33279

693.77464	0.33281

693.90439	0.33283

694.03414	0.33285

694.16389	0.33288

694.29364	0.3329

694.42339	0.33295

694.55314	0.33297

694.68289	0.33299

694.81264	0.33301

694.94239	0.33304

695.07214	0.33306

695.20189	0.33308

695.33164	0.33315

695.46139	0.33319

695.59114	0.33326

695.72089	0.33333

695.85064	0.33335

695.98039	0.33336

696.11014	0.33342

696.23989	0.33345

696.36964	0.33347

696.49939	0.33351

696.62914	0.33354

696.75889	0.33356

696.88864	0.33358

697.01839	0.33359

697.14815	0.33361

697.2779	0.3337

697.40765	0.33372

697.5374	0.33377

697.66715	0.33379

697.7969	0.33381

697.92665	0.33388

698.0564	0.3339

698.18615	0.33397

698.3159	0.33402

698.44565	0.33406

698.5754	0.33409

698.70515	0.33411

698.8349	0.33413

698.96465	0.33416

699.0944	0.33418

699.22415	0.33422

699.3539	0.33427

699.48365	0.33429

699.6134	0.33438

699.74315	0.33441

699.8729	0.33443

700.00265	0.33445

700.1324	0.33448

700.26215	0.33454

700.3919	0.33457

700.52165	0.33477

700.6514	0.33479

700.78115	0.3348

700.9109	0.33482

701.04065	0.33484

701.1704	0.33486

701.30015	0.33489

701.4299	0.33491

701.55965	0.33493

701.6894	0.33496

701.81915	0.33502

701.9489	0.33504

702.07865	0.33512

702.20841	0.33514

702.33816	0.33518

702.46791	0.33521

702.59766	0.33539

702.72741	0.33541

702.85716	0.33543

702.98691	0.33546

703.11666	0.33548

703.24641	0.3355

703.37616	0.33553

703.50591	0.33561

703.63566	0.33562

703.76541	0.33564

703.89516	0.33566

704.02491	0.33578

704.15466	0.33582

704.28441	0.33585

704.41416	0.33616

704.54391	0.33617

704.67366	0.33619

704.80341	0.33621

704.93316	0.33626

705.06291	0.33628

705.19266	0.3363

705.32241	0.33635

705.45216	0.33637

705.58191	0.33639

705.71166	0.33641

705.84141	0.33646

705.97116	0.33648

706.10091	0.33655

706.23066	0.33657

706.36041	0.33662

706.49016	0.33669

706.61991	0.33673

706.74966	0.33682

706.87941	0.3369

707.00916	0.33692

707.13892	0.33698

707.26867	0.33701

707.39842	0.33703

707.52817	0.33705

707.65792	0.33706

707.78767	0.33708

707.91742	0.33724

708.04717	0.33726

708.17692	0.3373

708.30667	0.33733

708.43642	0.33738

708.56617	0.33744

708.69592	0.33763

708.82567	0.33785

708.95542	0.33788

709.08517	0.33792

709.21492	0.33795

709.34467	0.33797

709.47442	0.33799

709.60417	0.33803

709.73392	0.33806

709.86367	0.33829

709.99342	0.33833

710.12317	0.33835

710.25292	0.33836

710.38267	0.33838

710.51242	0.33842

710.64217	0.33858

710.77192	0.33859

710.90167	0.33861

711.03142	0.33867

711.16117	0.33868

711.29092	0.3387

711.42067	0.33874

711.55042	0.33875

711.68017	0.33877

711.80992	0.33884

711.93967	0.3389

712.06942	0.33891

712.19918	0.33895

712.32893	0.33899

712.45868	0.33904

712.58843	0.33911

712.71818	0.33913

712.84793	0.33915

712.97768	0.33916

713.10743	0.33918

713.23718	0.33922

713.36693	0.33927

713.49668	0.33931

713.62643	0.33932

713.75618	0.33934

713.88593	0.33936

714.01568	0.33938

714.14543	0.33945

714.27518	0.33947

714.40493	0.33954

714.53468	0.33961

714.66443	0.33963

714.79418	0.33966

714.92393	0.33968

715.05368	0.3397

715.18343	0.33973

715.31318	0.33977

715.44293	0.33979

715.57268	0.3398

715.70243	0.33984

715.83218	0.33986

715.96193	0.33989

716.09168	0.33991

716.22143	0.33993

716.35118	0.34002

716.48093	0.34016

716.61068	0.34018

716.74043	0.3402

716.87018	0.34023

716.99993	0.34025

717.12969	0.34028

717.25944	0.34036

717.38919	0.34037

717.51894	0.34039

717.64869	0.34041

717.77844	0.34048

717.90819	0.34052

718.03794	0.34053

718.16769	0.34055

718.29744	0.34057

718.42719	0.34059

718.55694	0.34061

718.68669	0.34064

718.81644	0.34069

718.94619	0.34071

719.07594	0.34073

719.20569	0.34084

719.33544	0.34085

719.46519	0.34087

719.59494	0.34089

719.72469	0.34096

719.85444	0.341

719.98419	0.34105

720.11394	0.34114

720.24369	0.34119

720.37344	0.34121

720.50319	0.34132

720.63294	0.34133

720.76269	0.34142

720.89244	0.34144

721.02219	0.34148

721.15194	0.34149

721.28169	0.34151

721.41144	0.34153

721.54119	0.3416

721.67094	0.34174

721.80069	0.34178

721.93044	0.34182

722.06019	0.34183

722.18995	0.34185

722.3197	0.34187

722.44945	0.34189

722.5792	0.3419

722.70895	0.34203

722.8387	0.34205

722.96845	0.34219

723.0982	0.34228

723.22795	0.34233

723.3577	0.3424

723.48745	0.34247

723.6172	0.34256

723.74695	0.34258

723.8767	0.34262

724.00645	0.34263

724.1362	0.34269

724.26595	0.34285

724.3957	0.3429

724.52545	0.34292

724.6552	0.34294

724.78495	0.34295

724.9147	0.34299

725.04445	0.34303

725.1742	0.3434

725.30395	0.34343

725.4337	0.34349

725.56345	0.34361

725.6932	0.34368

725.82295	0.3437

725.9527	0.34372

726.08245	0.34374

726.2122	0.34375

726.34195	0.34379

726.4717	0.34383

726.60145	0.34388

726.7312	0.3439

726.86095	0.34393

726.9907	0.34395

727.12046	0.34397

727.25021	0.34399

727.37996	0.344

727.50971	0.34404

727.63946	0.34406

727.76921	0.34409

727.89896	0.34415

728.02871	0.34431

728.15846	0.34432

728.28821	0.34445

728.41796	0.34452

728.54771	0.34464

728.67746	0.34472

728.80721	0.34484

728.93696	0.34495

729.06671	0.34504

729.19646	0.34516

729.32621	0.34532

729.45596	0.34534

729.58571	0.34536

729.71546	0.34585

729.84521	0.34591

729.97496	0.34593

730.10471	0.34598

730.23446	0.34603

730.36421	0.3461

730.49396	0.34614

730.62371	0.34623

730.75346	0.34644

730.88321	0.34646

731.01296	0.3465

731.14271	0.34655

731.27246	0.34657

731.40221	0.34658

731.53196	0.34694

731.66171	0.34701

731.79146	0.34703

731.92121	0.34708

732.05096	0.3471

732.18072	0.34714

732.31047	0.34715

732.44022	0.34717

732.56997	0.34722

732.69972	0.34724

732.82947	0.3473

732.95922	0.34738

733.08897	0.34742

733.21872	0.34746

733.34847	0.34749

733.47822	0.34755

733.60797	0.34758

733.73772	0.34763

733.86747	0.34767

733.99722	0.34769

734.12697	0.34776

734.25672	0.34779

734.38647	0.34785

734.51622	0.34788

734.64597	0.34794

734.77572	0.34795

734.90547	0.34799

735.03522	0.3481

735.16497	0.34815

735.29472	0.34819

735.42447	0.34822

735.55422	0.34829

735.68397	0.34833

735.81372	0.34835

735.94347	0.34836

736.07322	0.34851

736.20297	0.34852

736.33272	0.34854

736.46247	0.34859

736.59222	0.34867

736.72197	0.34881

736.85172	0.34883

736.98147	0.34884

737.11122	0.34886

737.24098	0.34897

737.37073	0.34911

737.50048	0.34924

737.63023	0.34927

737.75998	0.34931

737.88973	0.34961

738.01948	0.34963

738.14923	0.34966

738.27898	0.3497

738.40873	0.34973

738.53848	0.34979

738.66823	0.3498

738.79798	0.34982

738.92773	0.34984

739.05748	0.34986

739.18723	0.34991

739.31698	0.35007

739.44673	0.3502

739.57648	0.35027

739.70623	0.35064

739.83598	0.35119

739.96573	0.35121

740.09548	0.35123

740.22523	0.35125

740.35498	0.35205

740.48473	0.35219

740.61448	0.35222

740.74423	0.35224

740.87398	0.35226

741.00373	0.35233

741.13348	0.35239

741.26323	0.3524

741.39298	0.35283

741.52273	0.35292

741.65248	0.35295

741.78223	0.35313

741.91198	0.35333

742.04173	0.35336

742.17149	0.35342

742.30124	0.35347

742.43099	0.35356

742.56074	0.35358

742.69049	0.3537

742.82024	0.35374

742.94999	0.35376

743.07974	0.3539

743.20949	0.35392

743.33924	0.35393

743.46899	0.35395

743.59874	0.35422

743.72849	0.35425

743.85824	0.35441

743.98799	0.35448

744.11774	0.3545

744.24749	0.35454

744.37724	0.35456

744.50699	0.35457

744.63674	0.35463

744.76649	0.35466

744.89624	0.35473

745.02599	0.35475

745.15574	0.35488

745.28549	0.35489

745.41524	0.35493

745.54499	0.35513

745.67474	0.35516

745.80449	0.35518

745.93424	0.35532

746.06399	0.35536

746.19374	0.35569

746.32349	0.35577

746.45324	0.35593

746.58299	0.35596

746.71274	0.35598

746.84249	0.356

746.97224	0.35612

747.10199	0.35625

747.23175	0.3565

747.3615	0.35655

747.49125	0.35657

747.621	0.35699

747.75075	0.35701

747.8805	0.35715

748.01025	0.35719

748.14	0.35733

748.26975	0.35763

748.3995	0.35785

748.52925	0.35792

748.659	0.35794

748.78875	0.35806

748.9185	0.35808

749.04825	0.3581

749.178	0.35847

749.30775	0.3586

749.4375	0.35863

749.56725	0.35865

749.697	0.35877

749.82675	0.35879

749.9565	0.35881

750.08625	0.35884

750.216	0.35886

750.34575	0.35893

750.4755	0.35897

750.60525	0.359

750.735	0.35902

750.86475	0.35904

750.9945	0.35906

751.12425	0.35908

751.254	0.35909

751.38375	0.35911

751.5135	0.35924

751.64325	0.35931

751.773	0.35941

751.90275	0.35943

752.0325	0.35947

752.16226	0.3595

752.29201	0.35957

752.42176	0.35961

752.55151	0.35963

752.68126	0.35965

752.81101	0.35966

752.94076	0.35968

753.07051	0.35973

753.20026	0.35979

753.33001	0.35984

753.45976	0.35986

753.58951	0.35991

753.71926	0.35993

753.84901	0.35997

753.97876	0.36

754.10851	0.36021

754.23826	0.36023

754.36801	0.36025

754.49776	0.36027

754.62751	0.3605

754.75726	0.36052

754.88701	0.36053

755.01676	0.36096

755.14651	0.3611

755.27626	0.36116

755.40601	0.36139

755.53576	0.36141

755.66551	0.36144

755.79526	0.36148

755.92501	0.3615

756.05476	0.36151

756.18451	0.36153

756.31426	0.36157

756.44401	0.36158

756.57376	0.3616

756.70351	0.36167

756.83326	0.36171

756.96301	0.36192

757.09276	0.36199

757.22252	0.36214

757.35227	0.36217

757.48202	0.36221

757.61177	0.36223

757.74152	0.36224

757.87127	0.36231

758.00102	0.36233

758.13077	0.36235

758.26052	0.36242

758.39027	0.36246

758.52002	0.36247

758.64977	0.36255

758.77952	0.36256

758.90927	0.36262

759.03902	0.36263

759.16877	0.36265

759.29852	0.36269

759.42827	0.36271

759.55802	0.36274

759.68777	0.36276

759.81752	0.36279

759.94727	0.36281

760.07702	0.36285

760.20677	0.36288

760.33652	0.36297

760.46627	0.36299

760.59602	0.36308

760.72577	0.3632

760.85552	0.36333

760.98527	0.36335

761.11502	0.36336

761.24477	0.36349

761.37452	0.36361

761.50427	0.36363

761.63402	0.36365

761.76377	0.36372

761.89352	0.36376

762.02327	0.36383

762.15303	0.36397

762.28278	0.36399

762.41253	0.36404

762.54228	0.36406

762.67203	0.36408

762.80178	0.36409

762.93153	0.36411

763.06128	0.36413

763.19103	0.36424

763.32078	0.36429

763.45053	0.36433

763.58028	0.3644

763.71003	0.36441

763.83978	0.36452

763.96953	0.36456

764.09928	0.36463

764.22903	0.36479

764.35878	0.36481

764.48853	0.36488

764.61828	0.36534

764.74803	0.36537

764.87778	0.36539

765.00753	0.36545

765.13728	0.36546

765.26703	0.3655

765.39678	0.36554

765.52653	0.36577

765.65628	0.36582

765.78603	0.36584

765.91578	0.36586

766.04553	0.36587

766.17528	0.36589

766.30503	0.36603

766.43478	0.3661

766.56453	0.36621

766.69428	0.36626

766.82403	0.36648

766.95378	0.3665

767.08353	0.36655

767.21329	0.36657

767.34304	0.3666

767.47279	0.36664

767.60254	0.36666

767.73229	0.36667

767.86204	0.36678

767.99179	0.3668

768.12154	0.36751

768.25129	0.36753

768.38104	0.36756

768.51079	0.36765

768.64054	0.3679

768.77029	0.36792

768.90004	0.36797

769.02979	0.36804

769.15954	0.36815

769.28929	0.36826

769.41904	0.36831

769.54879	0.36833

769.67854	0.36835

769.80829	0.36842

769.93804	0.36845

770.06779	0.36874

770.19754	0.36876

770.32729	0.36895

770.45704	0.36902

770.58679	0.36909

770.71654	0.36922

770.84629	0.36924

770.97604	0.36927

771.10579	0.36929

771.23554	0.36938

771.36529	0.36941

771.49504	0.36961

771.62479	0.36965

771.75454	0.36982

771.88429	0.36984

772.01404	0.36986

772.1438	0.36989

772.27355	0.36993

772.4033	0.36995

772.53305	0.36997

772.6628	0.37002

772.79255	0.37014

772.9223	0.37016

773.05205	0.37022

773.1818	0.37023

773.31155	0.37027

773.4413	0.37032

773.57105	0.37034

773.7008	0.37038

773.83055	0.37071

773.9603	0.37075

774.09005	0.37078

774.2198	0.37082

774.34955	0.37093

774.4793	0.37094

774.60905	0.37096

774.7388	0.37114

774.86855	0.37123

774.9983	0.37125

775.12805	0.37128

775.2578	0.37134

775.38755	0.37135

775.5173	0.37141

775.64705	0.37143

775.7768	0.37144

775.90655	0.37146

776.0363	0.37148

776.16605	0.37153

776.2958	0.37157

776.42555	0.37159

776.5553	0.37166

776.68505	0.37167

776.8148	0.37169

776.94455	0.37173

777.0743	0.37176

777.20406	0.37182

777.33381	0.37183

777.46356	0.37199

777.59331	0.37207

777.72306	0.37219

777.85281	0.37233

777.98256	0.37235

778.11231	0.37237

778.24206	0.37239

778.37181	0.37247

778.50156	0.37253

778.63131	0.37255

778.76106	0.37258

778.89081	0.37264

779.02056	0.37267

779.15031	0.37269

779.28006	0.37271

779.40981	0.37274

779.53956	0.37288

779.66931	0.37297

779.79906	0.37299

779.92881	0.37306

780.05856	0.37312

780.18831	0.37313

780.31806	0.37336

780.44781	0.3734

780.57756	0.37345

780.70731	0.37347

780.83706	0.3737

780.96681	0.37372

781.09656	0.37376

781.22631	0.37379

781.35606	0.37385

781.48581	0.37386

781.61556	0.37393

781.74531	0.37399

781.87506	0.37415

782.00481	0.37417

782.13457	0.37422

782.26432	0.37424

782.39407	0.37425

782.52382	0.37427

782.65357	0.37429

782.78332	0.37433

782.91307	0.37443

783.04282	0.37449

783.17257	0.37463

783.30232	0.37493

783.43207	0.37495

783.56182	0.37502

783.69157	0.37506

783.82132	0.3752

783.95107	0.37522

784.08082	0.37525

784.21057	0.3753

784.34032	0.37536

784.47007	0.37539

784.59982	0.3755

784.72957	0.37552

784.85932	0.37566

784.98907	0.3757

785.11882	0.37571

785.24857	0.376

785.37832	0.37619

785.50807	0.37627

785.63782	0.37628

785.76757	0.37644

785.89732	0.37648

786.02707	0.3765

786.15682	0.37655

786.28657	0.37657

786.41632	0.37719

786.54607	0.37735

786.67582	0.37764

786.80557	0.37771

786.93532	0.37772

787.06507	0.37774

787.19483	0.37783

787.32458	0.37785

787.45433	0.37796

787.58408	0.37806

787.71383	0.37813

787.84358	0.37817

787.97333	0.3782

788.10308	0.37828

788.23283	0.37831

788.36258	0.37833

788.49233	0.37835

788.62208	0.37844

788.75183	0.37845

788.88158	0.37847

789.01133	0.37854

789.14108	0.37861

789.27083	0.37863

789.40058	0.3787

789.53033	0.37874

789.66008	0.37885

789.78983	0.37886

789.91958	0.3789

790.04933	0.37892

790.17908	0.37899

790.30883	0.37901

790.43858	0.37908

790.56833	0.37922

790.69808	0.37927

790.82783	0.37934

790.95758	0.37954

791.08733	0.3799

791.21708	0.37991

791.34683	0.37993

791.47658	0.37997

791.60633	0.38023

791.73608	0.38025

791.86583	0.38029

791.99558	0.38034

792.12534	0.38055

792.25509	0.38112

792.38484	0.38121

792.51459	0.38127

792.64434	0.38134

792.77409	0.38139

792.90384	0.38141

793.03359	0.38143

793.16334	0.38144

793.29309	0.38151

793.42284	0.38162

793.55259	0.3823

793.68234	0.38232

793.81209	0.38233

793.94184	0.38235

794.07159	0.38237

794.20134	0.38249

794.33109	0.38253

794.46084	0.38256

794.59059	0.38262

794.72034	0.38264

794.85009	0.38271

794.97984	0.38272

795.10959	0.38283

795.23934	0.38312

795.36909	0.38322

795.49884	0.38324

795.62859	0.38328

795.75834	0.38329

795.88809	0.38331

796.01784	0.38333

796.14759	0.38335

796.27734	0.38336

796.40709	0.38353

796.53684	0.38354

796.66659	0.3836

796.79634	0.38367

796.92609	0.3837

797.05584	0.38395

797.1856	0.38399

797.31535	0.38401

797.4451	0.38408

797.57485	0.3847

797.7046	0.38472

797.83435	0.38475

797.9641	0.38481

798.09385	0.38482

798.2236	0.38484

798.35335	0.38486

798.4831	0.38495

798.61285	0.38497

798.7426	0.38509

798.87235	0.38518

799.0021	0.3852

799.13185	0.38523

799.2616	0.38529

799.39135	0.38539

799.5211	0.38546

799.65085	0.38554

799.7806	0.38555

799.91035	0.38564

800.0401	0.3857

800.16985	0.38579

800.2996	0.3858

800.42935	0.38582

800.5591	0.38591

800.68885	0.386

800.8186	0.38603

800.94835	0.38609

801.0781	0.38614

801.20785	0.38616

801.3376	0.38619

801.46735	0.38627

801.5971	0.38648

801.72685	0.38691

801.8566	0.38694

801.98635	0.38728

802.11611	0.38735

802.24586	0.38737

802.37561	0.38739

802.50536	0.38746

802.63511	0.38748

802.76486	0.38751

802.89461	0.38753

803.02436	0.3878

803.15411	0.38781

803.28386	0.38783

803.41361	0.38813

803.54336	0.38837

803.67311	0.38838

803.80286	0.38845

803.93261	0.38847

804.06236	0.38849

804.19211	0.38851

804.32186	0.38856

804.45161	0.38858

804.58136	0.38865

804.71111	0.3887

804.84086	0.38897

804.97061	0.38899

805.10036	0.38904

805.23011	0.3892

805.35986	0.38931

805.48961	0.38933

805.61936	0.38949

805.74911	0.39057

805.87886	0.39059

806.00861	0.39068

806.13836	0.39071

806.26811	0.39084

806.39786	0.39086

806.52761	0.391

806.65736	0.39107

806.78711	0.39109

806.91686	0.39111

807.04661	0.39118

807.17637	0.39132

807.30612	0.39159

807.43587	0.3916

807.56562	0.39164

807.69537	0.39182

807.82512	0.39192

807.95487	0.39196

808.08462	0.39201

808.21437	0.39208

808.34412	0.39216

808.47387	0.39219

808.60362	0.39224

808.73337	0.39228

808.86312	0.39232

808.99287	0.39246

809.12262	0.39258

809.25237	0.39262

809.38212	0.3928

809.51187	0.39308

809.64162	0.39313

809.77137	0.39324

809.90112	0.39328

810.03087	0.39333

810.16062	0.39345

810.29037	0.39356

810.42012	0.39363

810.54987	0.3937

810.67962	0.39379

810.80937	0.39381

810.93912	0.39385

811.06887	0.3939

811.19862	0.39392

811.32837	0.39401

811.45812	0.39442

811.58787	0.39498

811.71762	0.39525

811.84737	0.39529

811.97712	0.39548

812.10688	0.39557

812.23663	0.39561

812.36638	0.39563

812.49613	0.39566

812.62588	0.39573

812.75563	0.39614

812.88538	0.39646

813.01513	0.39648

813.14488	0.39666

813.27463	0.39669

813.40438	0.39673

813.53413	0.39676

813.66388	0.39678

813.79363	0.39691

813.92338	0.39692

814.05313	0.39723

814.18288	0.39726

814.31263	0.3973

814.44238	0.39737

814.57213	0.39842

814.70188	0.39876

814.83163	0.39877

814.96138	0.39902

815.09113	0.39906

815.22088	0.3991

815.35063	0.39922

815.48038	0.39931

815.61013	0.39933

815.73988	0.39965

815.86963	0.39966

815.99938	0.39975

816.12913	0.39998

816.25888	0.40011

816.38863	0.40015

816.51838	0.40018

816.64813	0.4002

816.77788	0.40023

816.90763	0.40027

817.03738	0.40029

817.16714	0.40039

817.29689	0.40045

817.42664	0.40048

817.55639	0.40084

817.68614	0.40089

817.81589	0.40137

817.94564	0.40139

818.07539	0.40144

818.20514	0.40162

818.33489	0.40171

818.46464	0.40221

818.59439	0.4023

818.72414	0.40239

818.85389	0.4024

818.98364	0.40242

819.11339	0.40262

819.24314	0.40273

819.37289	0.40278

819.50264	0.40287

819.63239	0.40289

819.76214	0.40308

819.89189	0.40315

820.02164	0.40326

820.15139	0.4036

820.28114	0.40413

820.41089	0.40417

820.54064	0.40447

820.67039	0.40461

820.80014	0.40465

820.92989	0.40491

821.05964	0.40499

821.18939	0.405

821.31914	0.40509

821.44889	0.40513

821.57864	0.40515

821.70839	0.40525

821.83814	0.40538

821.96789	0.40539

822.09765	0.40548

822.2274	0.40552

822.35715	0.40557

822.4869	0.40568

822.61665	0.40573

822.7464	0.40575

822.87615	0.40577

823.0059	0.4058

823.13565	0.40584

823.2654	0.40616

823.39515	0.40618

823.5249	0.40643

823.65465	0.40662

823.7844	0.40664

823.91415	0.40671

824.0439	0.40726

824.17365	0.40728

824.3034	0.40733

824.43315	0.40748

824.5629	0.40762

824.69265	0.40776

824.8224	0.40789

824.95215	0.40792

825.0819	0.40794

825.21165	0.40813

825.3414	0.40945

825.47115	0.40954

825.6009	0.40956

825.73065	0.40979

825.8604	0.40981

825.99015	0.40983

826.1199	0.40984

826.24965	0.41007

826.3794	0.41011

826.50915	0.41013

826.6389	0.41016

826.76865	0.41022

826.8984	0.41048

827.02815	0.41054

827.15791	0.41055

827.28766	0.41061

827.41741	0.41064

827.54716	0.4107

827.67691	0.41075

827.80666	0.41077

827.93641	0.41086

828.06616	0.41087

828.19591	0.41089

828.32566	0.411

828.45541	0.41102

828.58516	0.41107

828.71491	0.41112

828.84466	0.41118

828.97441	0.4112

829.10416	0.41153

829.23391	0.41155

829.36366	0.41209

829.49341	0.41212

829.62316	0.4123

829.75291	0.41248

829.88266	0.41294

830.01241	0.41312

830.14216	0.41317

830.27191	0.4133

830.40166	0.41351

830.53141	0.41406

830.66116	0.41408

830.79091	0.41431

830.92066	0.4147

831.05041	0.4149

831.18016	0.41499

831.30991	0.41509

831.43966	0.41555

831.56941	0.41563

831.69916	0.41588

831.82891	0.416

831.95866	0.41604

832.08842	0.41636

832.21817	0.41652

832.34792	0.41657

832.47767	0.41675

832.60742	0.41717

832.73717	0.41726

832.86692	0.41728

832.99667	0.41733

833.12642	0.41753

833.25617	0.41792

833.38592	0.41794

833.51567	0.41797

833.64542	0.41805

833.77517	0.41806

833.90492	0.41808

834.03467	0.41833

834.16442	0.4187

834.29417	0.41883

834.42392	0.4189

834.55367	0.41894

834.68342	0.41906

834.81317	0.41911

834.94292	0.41922

835.07267	0.41924

835.20242	0.41926

835.33217	0.41938

835.46192	0.41942

835.59167	0.41943

835.72142	0.41952

835.85117	0.42098

835.98092	0.42105

836.11067	0.42111

836.24042	0.42132

836.37017	0.42144

836.49992	0.42173

836.62967	0.42216

836.75942	0.42244

836.88917	0.4226

837.01892	0.42283

837.14868	0.42365

837.27843	0.42379

837.40818	0.42411

837.53793	0.42413

837.66768	0.42442

837.79743	0.42445

837.92718	0.42461

838.05693	0.42532

838.18668	0.42538

838.31643	0.42572

838.44618	0.42579

838.57593	0.4258

838.70568	0.42584

838.83543	0.42604

838.96518	0.42605

839.09493	0.42628

839.22468	0.42636

839.35443	0.4265

839.48418	0.42653

839.61393	0.42662

839.74368	0.42669

839.87343	0.42677

840.00318	0.42687

840.13293	0.42694

840.26268	0.42698

840.39243	0.42705

840.52218	0.42707

840.65193	0.42735

840.78168	0.42739

840.91143	0.42749

841.04118	0.42782

841.17093	0.42785

841.30068	0.42787

841.43043	0.42792

841.56018	0.42796

841.68993	0.42799

841.81968	0.4281

841.94943	0.42812

842.07919	0.42815

842.20894	0.42817

842.33869	0.42828

842.46844	0.4283

842.59819	0.42833

842.72794	0.4284

842.85769	0.42842

842.98744	0.42844

843.11719	0.42854

843.24694	0.42858

843.37669	0.42872

843.50644	0.4291

843.63619	0.42943

843.76594	0.4297

843.89569	0.43

844.02544	0.43008

844.15519	0.43047

844.28494	0.43048

844.41469	0.43054

844.54444	0.43073

844.67419	0.43075

844.80394	0.43089

844.93369	0.43093

845.06344	0.43098

845.19319	0.431

845.32294	0.43102

845.45269	0.43104

845.58244	0.43139

845.71219	0.43145

845.84194	0.43221

845.97169	0.43223

846.10144	0.43273

846.23119	0.43287

846.36094	0.43299

846.49069	0.43317

846.62044	0.43319

846.75019	0.43324

846.87994	0.43328

847.00969	0.43338

847.13945	0.43342

847.2692	0.43346

847.39895	0.43347

847.5287	0.43349

847.65845	0.43353

847.7882	0.43365

847.91795	0.43369

848.0477	0.43378

848.17745	0.4339

848.3072	0.43404

848.43695	0.43427

848.5667	0.43433

848.69645	0.43435

848.8262	0.43445

848.95595	0.43456

849.0857	0.43461

849.21545	0.43463

849.3452	0.43467

849.47495	0.43468

849.6047	0.43481

849.73445	0.43483

849.8642	0.43486

849.99395	0.4349

850.1237	0.43524

850.25345	0.43545

850.3832	0.43547

850.51295	0.4355

850.6427	0.43552

850.77245	0.43554

850.9022	0.43604

851.03195	0.43605

851.1617	0.43609

851.29145	0.43611

851.4212	0.43641

851.55095	0.43645

851.6807	0.43653

851.81045	0.43657

851.9402	0.43659

852.06996	0.43662

852.19971	0.43664

852.32946	0.43669

852.45921	0.43671

852.58896	0.43693

852.71871	0.43694

852.84846	0.43725

852.97821	0.43774

853.10796	0.43778

853.23771	0.43785

853.36746	0.43794

853.49721	0.4381

853.62696	0.43812

853.75671	0.43814

853.88646	0.43815

854.01621	0.43824

854.14596	0.43855

854.27571	0.4386

854.40546	0.43869

854.53521	0.43871

854.66496	0.43879

854.79471	0.43883

854.92446	0.43885

855.05421	0.43887

855.18396	0.43888

855.31371	0.4389

855.44346	0.43895

855.57321	0.43915

855.70296	0.43917

855.83271	0.4392

855.96246	0.43922

856.09221	0.43924

856.22196	0.43935

856.35171	0.43952

856.48146	0.43958

856.61121	0.4396

856.74096	0.43961

856.87071	0.43967

857.00046	0.43968

857.13022	0.43976

857.25997	0.44013

857.38972	0.44073

857.51947	0.44075

857.64922	0.44081

857.77897	0.4412

857.90872	0.44137

858.03847	0.44148

858.16822	0.44152

858.29797	0.44193

858.42772	0.44194

858.55747	0.44196

858.68722	0.44257

858.81697	0.44283

858.94672	0.44285

859.07647	0.44287

859.20622	0.44289

859.33597	0.4429

859.46572	0.44294

859.59547	0.44296

859.72522	0.44298

859.85497	0.4431

859.98472	0.44314

860.11447	0.44315

860.24422	0.44331

860.37397	0.44335

860.50372	0.44337

860.63347	0.44339

860.76322	0.44353

860.89297	0.44355

861.02272	0.44362

861.15247	0.44367

861.28222	0.44371

861.41197	0.44378

861.54172	0.44381

861.67147	0.44385

861.80122	0.44387

861.93097	0.44388

862.06073	0.4439

862.19048	0.44397

862.32023	0.44417

862.44998	0.44419

862.57973	0.44422

862.70948	0.44429

862.83923	0.44436

862.96898	0.44447

863.09873	0.44452

863.22848	0.44454

863.35823	0.4446

863.48798	0.44474

863.61773	0.44479

863.74748	0.44483

863.87723	0.44488

864.00698	0.4449

864.13673	0.44495

864.26648	0.44497

864.39623	0.44504

864.52598	0.44506

864.65573	0.44508

864.78548	0.44509

864.91523	0.44515

865.04498	0.44516

865.17473	0.44529

865.30448	0.44532

865.43423	0.44536

865.56398	0.4454

865.69373	0.44541

865.82348	0.44554

865.95323	0.44572

866.08298	0.44573

866.21273	0.44589

866.34248	0.44595

866.47223	0.44605

866.60198	0.44609

866.73173	0.4462

866.86148	0.44621

866.99123	0.44684

867.12099	0.44693

867.25074	0.44707

867.38049	0.44709

867.51024	0.44714

867.63999	0.44716

867.76974	0.44723

867.89949	0.44725

868.02924	0.44726

868.15899	0.44728

868.28874	0.4473

868.41849	0.44748

868.54824	0.44757

868.67799	0.44812

868.80774	0.44814

868.93749	0.44817

869.06724	0.44821

869.19699	0.44823

869.32674	0.44826

869.45649	0.44828

869.58624	0.4483

869.71599	0.44851

869.84574	0.44856

869.97549	0.44858

870.10524	0.44867

870.23499	0.44871

870.36474	0.44883

870.49449	0.44897

870.62424	0.44899

870.75399	0.44904

870.88374	0.44906

871.01349	0.44911

871.14324	0.44913

871.27299	0.44919

871.40274	0.4492

871.53249	0.44922

871.66224	0.44924

871.79199	0.44926

871.92174	0.44928

872.0515	0.44929

872.18125	0.44936

872.311	0.4494

872.44075	0.44942

872.5705	0.44944

872.70025	0.44951

872.83	0.44958

872.95975	0.44961

873.0895	0.44963

873.21925	0.44965

873.349	0.44967

873.47875	0.4497

873.6085	0.44974

873.73825	0.44997

873.868	0.44999

873.99775	0.45002

874.1275	0.45006

874.25725	0.45008

874.387	0.45009

874.51675	0.45025

874.6465	0.45031

874.77625	0.45049

874.906	0.4505

875.03575	0.45056

875.1655	0.45059

875.29525	0.45077

875.425	0.45079

875.55475	0.45086

875.6845	0.45088

875.81425	0.45091

875.944	0.45093

876.07375	0.45095

876.2035	0.45116

876.33325	0.45118

876.463	0.45121

876.59275	0.45123

876.7225	0.45129

876.85225	0.45132

876.982	0.45136

877.11176	0.45137

877.24151	0.45139

877.37126	0.45148

877.50101	0.4515

877.63076	0.45155

877.76051	0.45159

877.89026	0.45164

878.02001	0.45171

878.14976	0.45173

878.27951	0.45175

878.40926	0.4518

878.53901	0.45184

878.66876	0.45186

878.79851	0.45191

878.92826	0.45193

879.05801	0.452

879.18776	0.45216

879.31751	0.45248

879.44726	0.45251

879.57701	0.45253

879.70676	0.45258

879.83651	0.4526

879.96626	0.45271

880.09601	0.45274

880.22576	0.45276

880.35551	0.4528

880.48526	0.45294

880.61501	0.45305

880.74476	0.45307

880.87451	0.45339

881.00426	0.4534

881.13401	0.45342

881.26376	0.45344

881.39351	0.45346

881.52326	0.45349

881.65301	0.45351

881.78276	0.45353

881.91251	0.45355

882.04227	0.45356

882.17202	0.45358

882.30177	0.45396

882.43152	0.45403

882.56127	0.45404

882.69102	0.45406

882.82077	0.4541

882.95052	0.45412

883.08027	0.45424

883.21002	0.45426

883.33977	0.45428

883.46952	0.45431

883.59927	0.45433

883.72902	0.45435

883.85877	0.45436

883.98852	0.45438

884.11827	0.45445

884.24802	0.45447

884.37777	0.45449

884.50752	0.45467

884.63727	0.45476

884.76702	0.45508

884.89677	0.45511

885.02652	0.45513

885.15627	0.45515

885.28602	0.45518

885.41577	0.45524

885.54552	0.45525

885.67527	0.45527

885.80502	0.45538

885.93477	0.4554

886.06452	0.45541

886.19427	0.45543

886.32402	0.45549

886.45377	0.4555

886.58352	0.45552

886.71327	0.45554

886.84302	0.45556

886.97277	0.45559

887.10253	0.45561

887.23228	0.45584

887.36203	0.45586

887.49178	0.45607

887.62153	0.45613

887.75128	0.45614

887.88103	0.45618

888.01078	0.4563

888.14053	0.45634

888.27028	0.45636

888.40003	0.45639

888.52978	0.45641

888.65953	0.45646

888.78928	0.45657

888.91903	0.45668

889.04878	0.45678

889.17853	0.45684

889.30828	0.45689

889.43803	0.45694

889.56778	0.4571

889.69753	0.45721

889.82728	0.45726

889.95703	0.45742

890.08678	0.45748

890.21653	0.45759

890.34628	0.45801

890.47603	0.45807

890.60578	0.45812

890.73553	0.45839

890.86528	0.45849

890.99503	0.45855

891.12478	0.4586

891.25453	0.45871

891.38428	0.45876

891.51403	0.45881

891.64378	0.45919

891.77353	0.45924

891.90328	0.45945

892.03304	0.45961

892.16279	0.45983

892.29254	0.45988

892.42229	0.45993

892.55204	0.45999

892.68179	0.46009

892.81154	0.46015

892.94129	0.4602

893.07104	0.461

893.20079	0.46105

893.33054	0.46122

893.46029	0.46127

893.59004	0.46132

893.71979	0.46138

893.84954	0.46143

893.97929	0.46191

894.10904	0.46196

894.23879	0.46202

894.36854	0.46212

894.49829	0.46218

894.62804	0.46228

894.75779	0.4625

894.88754	0.4626

895.01729	0.46276

895.14704	0.46351

895.27679	0.46356

895.40654	0.46367

895.53629	0.46372

895.66604	0.46378

895.79579	0.46383

895.92554	0.4641

896.05529	0.46426

896.18504	0.46442

896.31479	0.46452

896.44454	0.46458

896.57429	0.46474

896.70404	0.46479

896.83379	0.46485

896.96354	0.4649

897.0933	0.46501

897.22305	0.46506

897.3528	0.46511

897.48255	0.46538

897.6123	0.46618

897.74205	0.46639

897.8718	0.46645

898.00155	0.4665

898.1313	0.46661

898.26105	0.46719

898.3908	0.4673

898.52055	0.46735

898.6503	0.46751

898.78005	0.46762

898.9098	0.46773

899.03955	0.46778

899.1693	0.46789

899.29905	0.46794

899.4288	0.4681

899.55855	0.46815

899.6883	0.46821

899.81805	0.46842

899.9478	0.46848

900.07755	0.46853

900.2073	0.46858

900.33705	0.46869

900.4668	0.4688

900.59655	0.46885

900.7263	0.46906

900.85605	0.46997

900.9858	0.47002

901.11555	0.47008

901.2453	0.47013

901.37505	0.47024

901.5048	0.47029

901.63455	0.47034

901.7643	0.4704

901.89405	0.47056

902.02381	0.47061

902.15356	0.47066

902.28331	0.47082

902.41306	0.47088

902.54281	0.47093

902.67256	0.47141

902.80231	0.47152

902.93206	0.47162

903.06181	0.47168

903.19156	0.47173

903.32131	0.47205

903.45106	0.47216

903.58081	0.47232

903.71056	0.47259

903.84031	0.47269

903.97006	0.47275

904.09981	0.4728

904.22956	0.47285

904.35931	0.47296

904.48906	0.47323

904.61881	0.47339

904.74856	0.47344

904.87831	0.47349

905.00806	0.47355

905.13781	0.47387

905.26756	0.47392

905.39731	0.47419

905.52706	0.47429

905.65681	0.47451

905.78656	0.47456

905.91631	0.47461

906.04606	0.47467

906.17581	0.47472

906.30556	0.47493

906.43531	0.47499

906.56506	0.47504

906.69481	0.47515

906.82456	0.47531

906.95431	0.47536

907.08407	0.47541

907.21382	0.47552

907.34357	0.47558

907.47332	0.47563

907.60307	0.47574

907.73282	0.47579

907.86257	0.47584

907.99232	0.4759

908.12207	0.47595

908.25182	0.47616

908.38157	0.47622

908.51132	0.47627

908.64107	0.47632

908.77082	0.47638

908.90057	0.47659

909.03032	0.47664

909.16007	0.47675

909.28982	0.47723

909.41957	0.47728

909.54932	0.47739

909.67907	0.47744

909.80882	0.4775

909.93857	0.47755

910.06832	0.4776

910.19807	0.47766

910.32782	0.47776

910.45757	0.47787

910.58732	0.47808

910.71707	0.47824

910.84682	0.47846

910.97657	0.47851

911.10632	0.47856

911.23607	0.47862

911.36582	0.47867

911.49557	0.47878

911.62532	0.47883

911.75507	0.47894

911.88482	0.47904

912.01458	0.47915

912.14433	0.47931

912.27408	0.47942

912.40383	0.47947

912.53358	0.47958

912.66333	0.47963

912.79308	0.47969

912.92283	0.47985

913.05258	0.4799

913.18233	0.47995

913.31208	0.48001

913.44183	0.48006

913.57158	0.48011

913.70133	0.48022

913.83108	0.48033

913.96083	0.48038

914.09058	0.48054

914.22033	0.48059

914.35008	0.48065

914.47983	0.4807

914.60958	0.48075

914.73933	0.48091

914.86908	0.48097

914.99883	0.48107

915.12858	0.48123

915.25833	0.48177

915.38808	0.48187

915.51783	0.48193

915.64758	0.48219

915.77733	0.48225

915.90708	0.4823

916.03683	0.48235

916.16658	0.48246

916.29633	0.48268

916.42608	0.48305

916.55583	0.4831

916.68558	0.48316

916.81533	0.48332

916.94508	0.48337

917.07484	0.48353

917.20459	0.48358

917.33434	0.48369

917.46409	0.48374

917.59384	0.48385

917.72359	0.4839

917.85334	0.48396

917.98309	0.48417

918.11284	0.48454

918.24259	0.4846

918.37234	0.48465

918.50209	0.4847

918.63184	0.48476

918.76159	0.48486

918.89134	0.48502

919.02109	0.48513

919.15084	0.48518

919.28059	0.48529

919.41034	0.4854

919.54009	0.48545

919.66984	0.48566

919.79959	0.48572

919.92934	0.48577

920.05909	0.48582

920.18884	0.48593

920.31859	0.48625

920.44834	0.48631

920.57809	0.48657

920.70784	0.48673

920.83759	0.48748

920.96734	0.48759

921.09709	0.48764

921.22684	0.48769

921.35659	0.48775

921.48634	0.4878

921.61609	0.48785

921.74584	0.48801

921.87559	0.48817

922.00535	0.48833

922.1351	0.48839

922.26485	0.48849

922.3946	0.48855

922.52435	0.4886

922.6541	0.48865

922.78385	0.48876

922.9136	0.48881

923.04335	0.48887

923.1731	0.48908

923.30285	0.48913

923.4326	0.4894

923.56235	0.48951

923.6921	0.48967

923.82185	0.4901

923.9516	0.49015

924.08135	0.4902

924.2111	0.49026

924.34085	0.49031

924.4706	0.49036

924.60035	0.49063

924.7301	0.49106

924.85985	0.49111

924.9896	0.49116

925.11935	0.49132

925.2491	0.49138

925.37885	0.49148

925.5086	0.49164

925.63835	0.49175

925.7681	0.49186

925.89785	0.49191

926.0276	0.49196

926.15735	0.49202

926.2871	0.49218

926.41685	0.49223

926.5466	0.49228

926.67635	0.49244

926.8061	0.4925

926.93585	0.49255

927.06561	0.49266

927.19536	0.49276

927.32511	0.49282

927.45486	0.49292

927.58461	0.49314

927.71436	0.49319

927.84411	0.49324

927.97386	0.4934

928.10361	0.49346

928.23336	0.49351

928.36311	0.49362

928.49286	0.49394

928.62261	0.49405

928.75236	0.4941

928.88211	0.49415

929.01186	0.49431

929.14161	0.49447

929.27136	0.49453

929.40111	0.49463

929.53086	0.49527

929.66061	0.49533

929.79036	0.49543

929.92011	0.49554

930.04986	0.49559

930.17961	0.49565

930.30936	0.4957

930.43911	0.49575

930.56886	0.49581

930.69861	0.49586

930.82836	0.49597

930.95811	0.49618

931.08786	0.49629

931.21761	0.49639

931.34736	0.49645

931.47711	0.49671

931.60686	0.49704

931.73661	0.49709

931.86636	0.49714

931.99612	0.4972

932.12587	0.4973

932.25562	0.49736

932.38537	0.49741

932.51512	0.49746

932.64487	0.49762

932.77462	0.49768

932.90437	0.49778

933.03412	0.49784

933.16387	0.49789

933.29362	0.49794

933.42337	0.49805

933.55312	0.4981

933.68287	0.49821

933.81262	0.49832

933.94237	0.49842

934.07212	0.49848

934.20187	0.49853

934.33162	0.49864

934.46137	0.4988

934.59112	0.49885

934.72087	0.49896

934.85062	0.49906

934.98037	0.49912

935.11012	0.49938

935.23987	0.49944

935.36962	0.49954

935.49937	0.49976

935.62912	0.49981

935.75887	0.49997

935.88862	0.50008

936.01837	0.50013

936.14812	0.50018

936.27787	0.50024

936.40762	0.50045

936.53737	0.5005

936.66712	0.50056

936.79687	0.50067

936.92662	0.50077

937.05638	0.50083

937.18613	0.50099

937.31588	0.50104

937.44563	0.50125

937.57538	0.50136

937.70513	0.50141

937.83488	0.50152

937.96463	0.50157

938.09438	0.50163

938.22413	0.50173

938.35388	0.50179

938.48363	0.50189

938.61338	0.502

938.74313	0.50211

938.87288	0.50216

939.00263	0.50221

939.13238	0.50227

939.26213	0.50232

939.39188	0.50237

939.52163	0.50243

939.65138	0.50248

939.78113	0.50253

939.91088	0.50259

940.04063	0.50264

940.17038	0.50269

940.30013	0.50275

940.42988	0.5028

940.55963	0.50317

940.68938	0.50333

940.81913	0.50339

940.94888	0.50344

941.07863	0.50371

941.20838	0.50376

941.33813	0.50387

941.46788	0.50397

941.59763	0.50413

941.72738	0.50419

941.85713	0.50424

941.98689	0.5043

942.11664	0.5044

942.24639	0.50446

942.37614	0.50456

942.50589	0.50462

942.63564	0.50472

942.76539	0.50478

942.89514	0.50483

943.02489	0.50488

943.15464	0.50494

943.28439	0.50499

943.41414	0.50504

943.54389	0.50515

943.67364	0.5052

943.80339	0.50526

943.93314	0.50531

944.06289	0.50536

944.19264	0.50542

944.32239	0.50552

944.45214	0.506

944.58189	0.50606

944.71164	0.50611

944.84139	0.50616

944.97114	0.50654

945.10089	0.5067

945.23064	0.50675

945.36039	0.5068

945.49014	0.50691

945.61989	0.50707

945.74964	0.50712

945.87939	0.50718

946.00914	0.50755

946.13889	0.50782

946.26864	0.50787

946.39839	0.50793

946.52814	0.5099

946.65789	0.50995

946.78764	0.51001

946.91739	0.51006

947.04715	0.51017

947.1769	0.51027

947.30665	0.51033

947.4364	0.51038

947.56615	0.51049

947.6959	0.51075

947.82565	0.51129

947.9554	0.51134

948.08515	0.51172

948.2149	0.51177

948.34465	0.51182

948.4744	0.51188

948.60415	0.51198

948.7339	0.51204

948.86365	0.51209

948.9934	0.51236

949.12315	0.51246

949.2529	0.51252

949.38265	0.51262

949.5124	0.51268

949.64215	0.51289

949.7719	0.51294

949.90165	0.513

950.0314	0.51321

950.16115	0.51326

950.2909	0.51348

950.42065	0.51353

950.5504	0.51374

950.68015	0.5138

950.8099	0.51385

950.93965	0.5139

951.0694	0.51412

951.19915	0.51422

951.3289	0.51454

951.45865	0.51486

951.5884	0.51492

951.71815	0.51497

951.8479	0.51508

951.97766	0.51519

952.10741	0.51524

952.23716	0.51529

952.36691	0.5154

952.49666	0.51545

952.62641	0.51551

952.75616	0.51561

952.88591	0.51567

953.01566	0.51577

953.14541	0.51593

953.27516	0.51604

953.40491	0.51641

953.53466	0.51657

953.66441	0.51673

953.79416	0.51695

953.92391	0.51727

954.05366	0.51769

954.18341	0.51775

954.31316	0.5178

954.44291	0.51796

954.57266	0.51801

954.70241	0.51807

954.83216	0.51812

954.96191	0.51817

955.09166	0.51823

955.22141	0.51833

955.35116	0.51844

955.48091	0.51876

955.61066	0.51882

955.74041	0.51903

955.87016	0.51908

955.99991	0.51914

956.12966	0.5193

956.25941	0.51935

956.38916	0.5194

956.51891	0.51946

956.64866	0.51962

956.77841	0.51967

956.90816	0.51978

957.03792	0.51983

957.16767	0.51988

957.29742	0.51999

957.42717	0.5201

957.55692	0.5202

957.68667	0.52031

957.81642	0.52042

957.94617	0.52047

958.07592	0.52068

958.20567	0.52106

958.33542	0.52111

958.46517	0.52122

958.59492	0.52138

958.72467	0.52143

958.85442	0.52148

958.98417	0.52154

959.11392	0.52164

959.24367	0.5217

959.37342	0.52196

959.50317	0.52202

959.63292	0.52207

959.76267	0.52234

959.89242	0.52245

960.02217	0.5225

960.15192	0.52255

960.28167	0.52266

960.41142	0.52271

960.54117	0.52277

960.67092	0.52282

960.80067	0.52293

960.93042	0.52298

961.06017	0.52303

961.18992	0.52309

961.31967	0.52314

961.44942	0.52325

961.57917	0.52351

961.70892	0.52383

961.83867	0.52415

961.96843	0.52479

962.09818	0.52485

962.22793	0.5249

962.35768	0.52495

962.48743	0.52501

962.61718	0.52506

962.74693	0.52511

962.87668	0.52522

963.00643	0.52533

963.13618	0.52543

963.26593	0.52549

963.39568	0.52554

963.52543	0.52559

963.65518	0.5257

963.78493	0.52581

963.91468	0.52586

964.04443	0.52613

964.17418	0.52634

964.30393	0.5265

964.43368	0.52656

964.56343	0.52661

964.69318	0.52666

964.82293	0.52672

964.95268	0.52682

965.08243	0.52688

965.21218	0.52698

965.34193	0.52714

965.47168	0.5272

965.60143	0.52725

965.73118	0.5273

965.86093	0.52736

965.99068	0.52757

966.12043	0.5281

966.25018	0.52816

966.37993	0.52826

966.50968	0.52832

966.63943	0.52837

966.76918	0.52848

966.89893	0.52853

967.02869	0.52858

967.15844	0.52864

967.28819	0.5289

967.41794	0.52906

967.54769	0.52912

967.67744	0.52917

967.80719	0.52922

967.93694	0.52928

968.06669	0.52933

968.19644	0.52939

968.32619	0.52944

968.45594	0.52949

968.58569	0.52955

968.71544	0.52971

968.84519	0.52981

968.97494	0.52987

969.10469	0.52997

969.23444	0.53008

969.36419	0.53013

969.49394	0.53019

969.62369	0.53024

969.75344	0.53029

969.88319	0.53045

970.01294	0.53056

970.14269	0.53088

970.27244	0.53099

970.40219	0.53104

970.53194	0.53109

970.66169	0.53115

970.79144	0.5312

970.92119	0.53136

971.05094	0.53141

971.18069	0.53147

971.31044	0.53157

971.44019	0.53163

971.56994	0.53168

971.69969	0.53173

971.82944	0.53179

971.9592	0.53184

972.08895	0.53189

972.2187	0.532

972.34845	0.53205

972.4782	0.53211

972.60795	0.53216

972.7377	0.53248

972.86745	0.53259

972.9972	0.53269

973.12695	0.53275

973.2567	0.5328

973.38645	0.53291

973.5162	0.53296

973.64595	0.53302

973.7757	0.53307

973.90545	0.53312

974.0352	0.53328

974.16495	0.53334

974.2947	0.53339

974.42445	0.5336

974.5542	0.53366

974.68395	0.53387

974.8137	0.53392

974.94345	0.53414

975.0732	0.53424

975.20295	0.5344

975.3327	0.53446

975.46245	0.53451

975.5922	0.53456

975.72195	0.53462

975.8517	0.53472

975.98145	0.53478

976.1112	0.53488

976.24095	0.53499

976.3707	0.53504

976.50045	0.5351

976.6302	0.53515

976.75995	0.53536

976.8897	0.53558

977.01946	0.53563

977.14921	0.53568

977.27896	0.53574

977.40871	0.53579

977.53846	0.53584

977.66821	0.53595

977.79796	0.53616

977.92771	0.53622

978.05746	0.53627

978.18721	0.53654

978.31696	0.53659

978.44671	0.53665

978.57646	0.53675

978.70621	0.53681

978.83596	0.53686

978.96571	0.53691

979.09546	0.53697

979.22521	0.53702

979.35496	0.53718

979.48471	0.53723

979.61446	0.53729

979.74421	0.53771

979.87396	0.53782

980.00371	0.53793

980.13346	0.53803

980.26321	0.53809

980.39296	0.53819

980.52271	0.53841

980.65246	0.53846

980.78221	0.53862

980.91196	0.53899

981.04171	0.53915

981.17146	0.53921

981.30121	0.53926

981.43096	0.53931

981.56071	0.53937

981.69046	0.53958

981.82021	0.53974

981.94997	0.53995

982.07972	0.54001

982.20947	0.54012

982.33922	0.54017

982.46897	0.54028

982.59872	0.54038

982.72847	0.54044

982.85822	0.54065

982.98797	0.5407

983.11772	0.54076

983.24747	0.54081

983.37722	0.54097

983.50697	0.54102

983.63672	0.54118

983.76647	0.54124

983.89622	0.54129

984.02597	0.54134

984.15572	0.5414

984.28547	0.54145

984.41522	0.5415

984.54497	0.54161

984.67472	0.54172

984.80447	0.54188

984.93422	0.54193

985.06397	0.54204

985.19372	0.54209

985.32347	0.54214

985.45322	0.5422

985.58297	0.5423

985.71272	0.54236

985.84247	0.54252

985.97222	0.54284

986.10197	0.54289

986.23172	0.54294

986.36147	0.54305

986.49122	0.54321

986.62097	0.54326

986.75072	0.54337

986.88047	0.54342

987.01023	0.54353

987.13998	0.54358

987.26973	0.54364

987.39948	0.54369

987.52923	0.54375

987.65898	0.54407

987.78873	0.54423

987.91848	0.54433

988.04823	0.54439

988.17798	0.5446

988.30773	0.54471

988.43748	0.54487

988.56723	0.54492

988.69698	0.54503

988.82673	0.54513

988.95648	0.54519

989.08623	0.54524

989.21598	0.54529

989.34573	0.54535

989.47548	0.54545

989.60523	0.54551

989.73498	0.54567

989.86473	0.54572

989.99448	0.54577

990.12423	0.54599

990.25398	0.54657

990.38373	0.54668

990.51348	0.54684

990.64323	0.54689

990.77298	0.54695

990.90273	0.54721

991.03248	0.54727

991.16223	0.54738

991.29198	0.54743

991.42173	0.54754

991.55148	0.5478

991.68123	0.54796

991.81098	0.54812

991.94074	0.54828

992.07049	0.54834

992.20024	0.54839

992.32999	0.54844

992.45974	0.5485

992.58949	0.54855

992.71924	0.54866

992.84899	0.54871

992.97874	0.54876

993.10849	0.54882

993.23824	0.54892

993.36799	0.54903

993.49774	0.54908

993.62749	0.54914

993.75724	0.54919

993.88699	0.5493

994.01674	0.54935

994.14649	0.54946

994.27624	0.54956

994.40599	0.54978

994.53574	0.54988

994.66549	0.54994

994.79524	0.5501

994.92499	0.5502

995.05474	0.55036

995.18449	0.55042

995.31424	0.55047

995.44399	0.55074

995.57374	0.55101

995.70349	0.55106

995.83324	0.55111

995.96299	0.55133

996.09274	0.55138

996.22249	0.55143

996.35224	0.55149

996.48199	0.55165

996.61174	0.55202

996.74149	0.55213

996.87124	0.55218

997.001	0.55223

997.13075	0.55229

997.2605	0.55234

997.39025	0.55266

997.52	0.55277

997.64975	0.55298

997.7795	0.55314

997.90925	0.55357

998.039	0.55362

998.16875	0.55373

998.2985	0.55399

998.42825	0.55405

998.558	0.55421

998.68775	0.55426

998.8175	0.55431

998.94725	0.55437

999.077	0.55442

999.20675	0.55458

999.3365	0.55464

999.46625	0.55485

999.596	0.55496

999.72575	0.55512

999.8555	0.55522

999.98525	0.55528

1000.115	0.55533

1000.24475	0.55538

1000.3745	0.55597

1000.50425	0.55602

1000.634	0.55618

1000.76375	0.55624

1000.8935	0.55629

1001.02325	0.55634

1001.153	0.5565

1001.28275	0.55661

1001.4125	0.55693

1001.54225	0.55698

1001.672	0.55752

1001.80176	0.55773

1001.93151	0.55784

1002.06126	0.55794

1002.19101	0.558

1002.32076	0.55805

1002.45051	0.55821

1002.58026	0.55827

1002.71001	0.55837

1002.83976	0.55843

1002.96951	0.55848

1003.09926	0.55853

1003.22901	0.55864

1003.35876	0.55869

1003.48851	0.5588

1003.61826	0.55891

1003.74801	0.55896

1003.87776	0.55907

1004.00751	0.55923

1004.13726	0.55928

1004.26701	0.55971

1004.39676	0.55976

1004.52651	0.56003

1004.65626	0.56008

1004.78601	0.56024

1004.91576	0.5604

1005.04551	0.56051

1005.17526	0.56072

1005.30501	0.56136

1005.43476	0.56147

1005.56451	0.56157

1005.69426	0.56163

1005.82401	0.56184

1005.95376	0.5619

1006.08351	0.56206

1006.21326	0.56216

1006.34301	0.56238

1006.47276	0.56243

1006.60251	0.56254

1006.73227	0.56259

1006.86202	0.5627

1006.99177	0.5628

1007.12152	0.56302

1007.25127	0.56323

1007.38102	0.56371

1007.51077	0.56376

1007.64052	0.56382

1007.77027	0.56387

1007.90002	0.56419

1008.02977	0.56424

1008.15952	0.5643

1008.28927	0.56435

1008.41902	0.5644

1008.54877	0.56446

1008.67852	0.56451

1008.80827	0.56456

1008.93802	0.56472

1009.06777	0.56483

1009.19752	0.56488

1009.32727	0.56499

1009.45702	0.56504

1009.58677	0.56515

1009.71652	0.56537

1009.84627	0.56542

1009.97602	0.56547

1010.10577	0.56553

1010.23552	0.56569

1010.36527	0.56574

1010.49502	0.56579

1010.62477	0.5659

1010.75452	0.56595

1010.88427	0.56617

1011.01402	0.56622

1011.14377	0.56633

1011.27352	0.56638

1011.40327	0.56643

1011.53302	0.56649

1011.66277	0.56654

1011.79253	0.5667

1011.92228	0.56675

1012.05203	0.56707

1012.18178	0.56713

1012.31153	0.56723

1012.44128	0.56729

1012.57103	0.56745

1012.70078	0.5675

1012.83053	0.56755

1012.96028	0.56761

1013.09003	0.56766

1013.21978	0.56777

1013.34953	0.56787

1013.47928	0.56809

1013.60903	0.56825

1013.73878	0.5683

1013.86853	0.56835

1013.99828	0.56841

1014.12803	0.56851

1014.25778	0.56857

1014.38753	0.56862

1014.51728	0.56867

1014.64703	0.56873

1014.77678	0.56878

1014.90653	0.56889

1015.03628	0.56894

1015.16603	0.56916

1015.29578	0.56932

1015.42553	0.56937

1015.55528	0.56948

1015.68503	0.56958

1015.81478	0.56964

1015.94453	0.56969

1016.07428	0.56974

1016.20403	0.5698

1016.33378	0.56985

1016.46353	0.56996

1016.59328	0.57006

1016.72304	0.57028

1016.85279	0.57049

1016.98254	0.57054

1017.11229	0.57065

1017.24204	0.57092

1017.37179	0.57097

1017.50154	0.57102

1017.63129	0.57108

1017.76104	0.57113

1017.89079	0.57118

1018.02054	0.57129

1018.15029	0.57134

1018.28004	0.57145

1018.40979	0.5715

1018.53954	0.57161

1018.66929	0.57166

1018.79904	0.57182

1018.92879	0.57188

1019.05854	0.57193

1019.18829	0.57209

1019.31804	0.57214

1019.44779	0.57225

1019.57754	0.5723

1019.70729	0.57236

1019.83704	0.57252

1019.96679	0.57273

1020.09654	0.57279

1020.22629	0.57284

1020.35604	0.57289

1020.48579	0.573

1020.61554	0.57305

1020.74529	0.57316

1020.87504	0.57321

1021.00479	0.57332

1021.13454	0.57337

1021.26429	0.57348

1021.39404	0.57353

1021.52379	0.57359

1021.65354	0.5738

1021.7833	0.57423

1021.91305	0.57433

1022.0428	0.57439

1022.17255	0.57449

1022.3023	0.57455

1022.43205	0.5746

1022.5618	0.57465

1022.69155	0.57471

1022.8213	0.57476

1022.95105	0.57492

1023.0808	0.57497

1023.21055	0.57503

1023.3403	0.57524

1023.47005	0.5754

1023.5998	0.57545

1023.72955	0.57599

1023.8593	0.57615

1023.98905	0.5762

1024.1188	0.57631

1024.24855	0.57647

1024.3783	0.57652

1024.50805	0.57658

1024.6378	0.57663

1024.76755	0.57668

1024.8973	0.57684

1025.02705	0.57711

1025.1568	0.57716

1025.28655	0.57727

1025.4163	0.57738

1025.54605	0.57748

1025.6758	0.57759

1025.80555	0.5778

1025.9353	0.57796

1026.06505	0.57823

1026.1948	0.57828

1026.32455	0.57844

1026.4543	0.57866

1026.58405	0.57876

1026.71381	0.57882

1026.84355	0.57887

1026.97331	0.57892

1027.10306	0.57898

1027.23281	0.57903

1027.36256	0.57914

1027.49231	0.57924

1027.62206	0.57935

1027.75181	0.5794

1027.88156	0.57951

1028.01131	0.57978

1028.14106	0.57989

1028.27081	0.57994

1028.40056	0.58005

1028.53031	0.5801

1028.66006	0.58015

1028.78981	0.58021

1028.91956	0.58037

1029.04931	0.58042

1029.17906	0.58047

1029.30881	0.58069

1029.43856	0.58074

1029.56831	0.5809

1029.69806	0.58095

1029.82781	0.58101

1029.95756	0.58111

1030.08731	0.5817

1030.21706	0.58186

1030.34681	0.58191

1030.47656	0.58197

1030.60631	0.58202

1030.73606	0.58207

1030.86581	0.58213

1030.99556	0.58223

1031.12531	0.58229

1031.25506	0.58239

1031.38481	0.58245

1031.51456	0.58261

1031.64431	0.58277

1031.77407	0.58287

1031.90381	0.58293

1032.03357	0.58314

1032.16332	0.58325

1032.29307	0.5833

1032.42282	0.58352

1032.55257	0.58384

1032.68232	0.58394

1032.81207	0.584

1032.94182	0.5841

1033.07157	0.58421

1033.20132	0.58426

1033.33107	0.58442

1033.46082	0.58464

1033.59057	0.58474

1033.72032	0.5848

1033.85007	0.58485

1033.97982	0.58501

1034.10957	0.58506

1034.23932	0.58512

1034.36907	0.58517

1034.49882	0.58538

1034.62857	0.58554

1034.75832	0.5856

1034.88807	0.58565

1035.01782	0.58581

1035.14757	0.58586

1035.27732	0.58597

1035.40707	0.58602

1035.53682	0.58613

1035.66657	0.58618

1035.79632	0.58629

1035.92607	0.58634

1036.05582	0.5864

1036.18557	0.58645

1036.31532	0.5865

1036.44507	0.58661

1036.57482	0.58672

1036.70458	0.58677

1036.83433	0.58688

1036.96408	0.58715

1037.09382	0.5872

1037.22358	0.58731

1037.35333	0.58736

1037.48308	0.58763

1037.61283	0.58768

1037.74258	0.58773

1037.87233	0.58789

1038.00208	0.58795

1038.13183	0.58816

1038.26158	0.58821

1038.39133	0.58827

1038.52108	0.58832

1038.65083	0.58837

1038.78058	0.58843

1038.91033	0.58853

1039.04008	0.58859

1039.16983	0.58864

1039.29958	0.58869

1039.42933	0.58875

1039.55908	0.58885

1039.68883	0.58891

1039.81858	0.58901

1039.94833	0.58917

1040.07808	0.58923

1040.20783	0.58928

1040.33758	0.58944

1040.46733	0.58949

1040.59708	0.58955

1040.72683	0.5896

1040.85658	0.58965

1040.98633	0.58971

1041.11608	0.59003

1041.24583	0.59008

1041.37558	0.59013

1041.50533	0.59024

1041.63508	0.59029

1041.76483	0.5904

1041.89459	0.59062

1042.02434	0.59067

1042.15408	0.59078

1042.28384	0.59083

1042.41359	0.59099

1042.54334	0.5912

1042.67309	0.59126

1042.80284	0.59131

1042.93259	0.59136

1043.06234	0.59147

1043.19209	0.59158

1043.32184	0.59163

1043.45159	0.59168

1043.58134	0.59206

1043.71109	0.59211

1043.84084	0.5927

1043.97059	0.5928

1044.10034	0.59286

1044.23009	0.59291

1044.35984	0.59312

1044.48959	0.59318

1044.61934	0.59328

1044.74909	0.59334

1044.87884	0.59344

1045.00859	0.59355

1045.13834	0.59366

1045.26809	0.59371

1045.39784	0.59376

1045.52759	0.59382

1045.65734	0.59387

1045.78709	0.59393

1045.91684	0.59398

1046.04659	0.59403

1046.17634	0.59414

1046.30609	0.59419

1046.43584	0.59441

1046.56559	0.59446

1046.69535	0.59457

1046.82509	0.59462

1046.95485	0.59483

1047.0846	0.59489

1047.21435	0.5951

1047.3441	0.59531

1047.47385	0.59537

1047.6036	0.59542

1047.73335	0.59547

1047.8631	0.59574

1047.99285	0.59617

1048.1226	0.59622

1048.25235	0.59627

1048.3821	0.59638

1048.51185	0.59649

1048.6416	0.59654

1048.77135	0.59665

1048.9011	0.5967

1049.03085	0.59702

1049.1606	0.59713

1049.29035	0.59718

1049.4201	0.59723

1049.54985	0.59734

1049.6796	0.5975

1049.80935	0.59756

1049.9391	0.59761

1050.06885	0.59772

1050.1986	0.59782

1050.32835	0.59788

1050.4581	0.59798

1050.58785	0.59804

1050.7176	0.59809

1050.84735	0.59814

1050.9771	0.5983

1051.10685	0.59836

1051.2366	0.59846

1051.36635	0.59852

1051.4961	0.59868

1051.62585	0.59873

1051.75561	0.59905

1051.88535	0.5991

1052.0151	0.59916

1052.14486	0.59926

1052.27461	0.59942

1052.40436	0.59948

1052.53411	0.59953

1052.66386	0.59958

1052.79361	0.59974

1052.92336	0.59985

1053.05311	0.5999

1053.18286	0.59996

1053.31261	0.60001

1053.44236	0.60006

1053.57211	0.60028

1053.70186	0.60038

1053.83161	0.60044

1053.96136	0.60054

1054.09111	0.6007

1054.22086	0.60076

1054.35061	0.60081

1054.48036	0.60097

1054.61011	0.60119

1054.73986	0.60139

1054.86961	0.60145

1054.99936	0.60152

1055.12911	0.60159

1055.25886	0.60165

1055.38861	0.60172

1055.51836	0.60179

1055.64811	0.60185

1055.77786	0.60199

1055.90761	0.60205

1056.03736	0.60212

1056.16711	0.60219

1056.29686	0.60225

1056.42661	0.60232

1056.55636	0.60312

1056.68612	0.60319

1056.81587	0.60325

1056.94562	0.60352

1057.07536	0.60359

1057.20512	0.60365

1057.33487	0.60379

1057.46462	0.60385

1057.59437	0.60452

1057.72412	0.60465

1057.85387	0.60472

1057.98362	0.60479

1058.11337	0.60486

1058.24312	0.60499

1058.37287	0.60506

1058.50262	0.60512

1058.63237	0.60519

1058.76212	0.60526

1058.89187	0.60539

1059.02162	0.60559

1059.15137	0.60566

1059.28112	0.60572

1059.41087	0.60586

1059.54062	0.60592

1059.67037	0.60612

1059.80012	0.60619

1059.92987	0.60639

1060.05962	0.60646

1060.18937	0.60679

1060.31912	0.60699

1060.44887	0.60706

1060.57862	0.60719

1060.70837	0.60726

1060.83812	0.60739

1060.96787	0.60759

1061.09762	0.60786

1061.22737	0.60799

1061.35712	0.60812

1061.48687	0.60853

1061.61662	0.60859

1061.74637	0.60873

1061.87613	0.60886

1062.00588	0.60893

1062.13562	0.60906

1062.26538	0.60913

1062.39513	0.60919

1062.52488	0.60926

1062.65463	0.60933

1062.78438	0.60939

1062.91413	0.60973

1063.04388	0.60986

1063.17363	0.60999

1063.30338	0.61006

1063.43313	0.61013

1063.56288	0.61019

1063.69263	0.61026

1063.82238	0.61046

1063.95213	0.61059

1064.08188	0.61099

1064.21163	0.61106

1064.34138	0.61113

1064.47113	0.61126

1064.60088	0.61139

1064.73063	0.61146

1064.86038	0.61153

1064.99013	0.61159

1065.11988	0.61166

1065.24963	0.61186

1065.37938	0.61193

1065.50913	0.61213

1065.63888	0.61226

1065.76863	0.61313

1065.89838	0.6132

1066.02813	0.61326

1066.15788	0.6134

1066.28763	0.61353

1066.41738	0.61373

1066.54713	0.6138

1066.67689	0.61386

1066.80663	0.61413

1066.93639	0.6142

1067.06614	0.61433

1067.19589	0.6144

1067.32564	0.61446

1067.45539	0.6146

1067.58514	0.6148

1067.71489	0.615

1067.84464	0.61506

1067.97439	0.61513

1068.10414	0.6152

1068.23389	0.61533

1068.36364	0.61587

1068.49339	0.61593

1068.62314	0.616

1068.75289	0.61607

1068.88264	0.6162

1069.01239	0.61627

1069.14214	0.6164

1069.27189	0.61647

1069.40164	0.6166

1069.53139	0.61667

1069.66114	0.6168

1069.79089	0.61687

1069.92064	0.61693

1070.05039	0.61707

1070.18014	0.61713

1070.30989	0.61733

1070.43964	0.6176

1070.56939	0.61767

1070.69914	0.61773

1070.82889	0.61787

1070.95864	0.61793

1071.08839	0.618

1071.21814	0.61813

1071.34789	0.6182

1071.47764	0.61827

1071.60739	0.61833

1071.73715	0.6184

1071.8669	0.61847

1071.99664	0.61867

1072.1264	0.61873

1072.25615	0.61887

1072.3859	0.61893

1072.51565	0.619

1072.6454	0.6192

1072.77515	0.61927

1072.9049	0.61934

1073.03465	0.61947

1073.1644	0.61954

1073.29415	0.61967

1073.4239	0.61974

1073.55365	0.6198

1073.6834	0.61987

1073.81315	0.62007

1073.9429	0.6202

1074.07265	0.62027

1074.2024	0.62047

1074.33215	0.62054

1074.4619	0.62087

1074.59165	0.62094

1074.7214	0.62107

1074.85115	0.62114

1074.9809	0.6212

1075.11065	0.62154

1075.2404	0.6216

1075.37015	0.62167

1075.4999	0.6218

1075.62965	0.62187

1075.7594	0.622

1075.88915	0.6224

1076.0189	0.62247

1076.14865	0.62254

1076.2784	0.6226

1076.40815	0.62274

1076.5379	0.62287

1076.66766	0.62294

1076.79741	0.62307

1076.92716	0.62327

1077.0569	0.62334

1077.18666	0.62347

1077.31641	0.62354

1077.44616	0.62361

1077.57591	0.62367

1077.70566	0.62387

1077.83541	0.62407

1077.96516	0.62414

1078.09491	0.62421

1078.22466	0.62427

1078.35441	0.62441

1078.48416	0.62447

1078.61391	0.62454

1078.74366	0.62461

1078.87341	0.62547

1079.00316	0.62554

1079.13291	0.62561

1079.26266	0.62574

1079.39241	0.62594

1079.52216	0.62621

1079.65191	0.62634

1079.78166	0.62648

1079.91141	0.62654

1080.04116	0.62661

1080.17091	0.62668

1080.30066	0.62674

1080.43041	0.62681

1080.56016	0.62714

1080.68991	0.62721

1080.81966	0.62728

1080.94941	0.62754

1081.07916	0.62768

1081.20891	0.62774

1081.33866	0.62788

1081.46841	0.62794

1081.59816	0.62814

1081.72791	0.62821

1081.85767	0.62828

1081.98742	0.62834

1082.11717	0.62861

1082.24692	0.62868

1082.37667	0.62881

1082.50642	0.62888

1082.63617	0.62894

1082.76592	0.62901

1082.89567	0.62908

1083.02542	0.62914

1083.15517	0.62921

1083.28492	0.62928

1083.41467	0.62941

1083.54442	0.62948

1083.67417	0.62954

1083.80392	0.62961

1083.93367	0.62974

1084.06342	0.62981

1084.19317	0.63001

1084.32292	0.63015

1084.45267	0.63055

1084.58242	0.63095

1084.71217	0.63135

1084.84192	0.63141

1084.97167	0.63161

1085.10142	0.63175

1085.23117	0.63188

1085.36092	0.63208

1085.49067	0.63228

1085.62042	0.63241

1085.75017	0.63255

1085.87992	0.63261

1086.00967	0.63268

1086.13942	0.63288

1086.26917	0.63295

1086.39892	0.63301

1086.52867	0.63315

1086.65843	0.63321

1086.78817	0.63355

1086.91793	0.63368

1087.04768	0.63375

1087.17743	0.63388

1087.30718	0.63395

1087.43693	0.63402

1087.56668	0.63408

1087.69643	0.63415

1087.82618	0.63422

1087.95593	0.63428

1088.08568	0.63448

1088.21543	0.63455

1088.34518	0.63475

1088.47493	0.63482

1088.60468	0.63488

1088.73443	0.63495

1088.86418	0.63508

1088.99393	0.63548

1089.12368	0.63555

1089.25343	0.63562

1089.38318	0.63568

1089.51293	0.63588

1089.64268	0.63602

1089.77243	0.63615

1089.90218	0.63628

1090.03193	0.63642

1090.16168	0.63655

1090.29143	0.63668

1090.42118	0.63729

1090.55093	0.63742

1090.68068	0.63749

1090.81043	0.63762

1090.94018	0.63769

1091.06993	0.63855

1091.19968	0.63862

1091.32943	0.63869

1091.45918	0.63882

1091.58893	0.63895

1091.71869	0.63902

1091.84844	0.63922

1091.97818	0.63935

1092.10794	0.63962

1092.23769	0.63982

1092.36744	0.63995

1092.49719	0.64002

1092.62694	0.64009

1092.75669	0.64015

1092.88644	0.64035

1093.01619	0.64042

1093.14594	0.64049

1093.27569	0.64056

1093.40544	0.64062

1093.53519	0.64082

1093.66494	0.64089

1093.79469	0.64116

1093.92444	0.64136

1094.05419	0.64142

1094.18394	0.64149

1094.31369	0.64162

1094.44344	0.64176

1094.57319	0.64182

1094.70294	0.64202

1094.83269	0.64222

1094.96244	0.64242

1095.09219	0.64249

1095.22194	0.64262

1095.35169	0.64269

1095.48144	0.64276

1095.61119	0.64289

1095.74094	0.64296

1095.87069	0.64302

1096.00044	0.64309

1096.13019	0.64322

1096.25994	0.64336

1096.38969	0.64342

1096.51944	0.64349

1096.6492	0.64356

1096.77895	0.64423

1096.9087	0.64429

1097.03844	0.64436

1097.1682	0.64456

1097.29795	0.64463

1097.4277	0.64476

1097.55745	0.64489

1097.6872	0.64496

1097.81695	0.64516

1097.9467	0.64523

1098.07645	0.64529

1098.2062	0.64543

1098.33595	0.64556

1098.4657	0.64563

1098.59545	0.64583

1098.7252	0.64596

1098.85495	0.64603

1098.9847	0.64609

1099.11445	0.64636

1099.2442	0.64643

1099.37395	0.64663

1099.5037	0.64669

1099.63345	0.64676

1099.7632	0.64683

1099.89295	0.64696

1100.0227	0.64703

1100.15245	0.64709

1100.2822	0.64716

1100.41195	0.64729

1100.5417	0.64736

1100.67145	0.64743

1100.8012	0.64756

1100.93095	0.64763

1101.0607	0.64769

1101.19045	0.64776

1101.3202	0.64783

1101.44995	0.6479

1101.5797	0.64796

1101.70945	0.64803

1101.83921	0.64856

1101.96896	0.64863

1102.09871	0.6487

1102.22846	0.64876

1102.35821	0.64883

1102.48796	0.64896

1102.61771	0.64903

1102.74746	0.64916

1102.87721	0.64923

1103.00696	0.6493

1103.13671	0.64936

1103.26646	0.6495

1103.39621	0.64956

1103.52596	0.6497

1103.65571	0.65023

1103.78546	0.6505

1103.91521	0.65056

1104.04496	0.65063

1104.17471	0.6509

1104.30446	0.65096

1104.43421	0.65103

1104.56396	0.65116

1104.69371	0.65123

1104.82346	0.6513

1104.95321	0.65137

1105.08296	0.65157

1105.21271	0.65163

1105.34246	0.6521

1105.47221	0.65217

1105.60196	0.65223

1105.73171	0.65263

1105.86146	0.65277

1105.99121	0.65283

1106.12096	0.65297

1106.25071	0.65323

1106.38046	0.65337

1106.51021	0.65363

1106.63997	0.65377

1106.76971	0.65397

1106.89947	0.6541

1107.02922	0.65417

1107.15897	0.65423

1107.28872	0.6543

1107.41847	0.65443

1107.54822	0.6545

1107.67797	0.65457

1107.80772	0.65463

1107.93747	0.6547

1108.06722	0.65477

1108.19697	0.6549

1108.32672	0.65524

1108.45647	0.65537

1108.58622	0.65544

1108.71597	0.6555

1108.84572	0.65564

1108.97547	0.6557

1109.10522	0.65577

1109.23497	0.6559

1109.36472	0.65657

1109.49447	0.65664

1109.62422	0.65677

1109.75397	0.65684

1109.88372	0.65697

1110.01347	0.65704

1110.14322	0.6571

1110.27297	0.65717

1110.40272	0.65744

1110.53247	0.65757

1110.66222	0.65764

1110.79197	0.65777

1110.92172	0.65784

1111.05147	0.6579

1111.18122	0.6581

1111.31097	0.65817

1111.44072	0.65824

1111.57047	0.65864

1111.70023	0.65871

1111.82998	0.65877

1111.95972	0.65891

1112.08948	0.65897

1112.21923	0.65904

1112.34898	0.65924

1112.47873	0.65931

1112.60848	0.65944

1112.73823	0.65951

1112.86798	0.65953

1112.99773	0.65955

1113.12748	0.65957

1113.25723	0.65962

1113.38698	0.65966

1113.51673	0.65971

1113.64648	0.65973

1113.77623	0.65982

1113.90598	0.65995

1114.03573	0.66

1114.16548	0.66004

1114.29523	0.66006

1114.42498	0.66008

1114.55473	0.66011

1114.68448	0.66015

1114.81423	0.6602

1114.94398	0.66022

1115.07373	0.66026

1115.20348	0.6604

1115.33323	0.66042

1115.46298	0.66048

1115.59273	0.6608

1115.72248	0.66082

1115.85223	0.66084

1115.98198	0.66086

1116.11173	0.66091

1116.24148	0.66129

1116.37123	0.66131

1116.50098	0.66135

1116.63074	0.66137

1116.76049	0.66142

1116.89024	0.66144

1117.01998	0.66146

1117.14974	0.66149

1117.27949	0.66153

1117.40924	0.66157

1117.53899	0.6616

1117.66874	0.66166

1117.79849	0.66175

1117.92824	0.66177

1118.05799	0.66189

1118.18774	0.66191

1118.31749	0.66193

1118.44724	0.66197

1118.57699	0.662

1118.70674	0.66204

1118.83649	0.66206

1118.96624	0.66211

1119.09599	0.66213

1119.22574	0.66215

1119.35549	0.66218

1119.48524	0.6622

1119.61499	0.66222

1119.74474	0.66226

1119.87449	0.66229

1120.00424	0.66233

1120.13399	0.66235

1120.26374	0.66238

1120.39349	0.66242

1120.52324	0.66244

1120.65299	0.66253

1120.78274	0.66255

1120.91249	0.66258

1121.04224	0.66262

1121.17199	0.66264

1121.30174	0.66266

1121.43149	0.66282

1121.56124	0.66284

1121.69099	0.66286

1121.82075	0.66289

1121.9505	0.66291

1122.08025	0.66293

1122.21	0.66295

1122.33975	0.66298

1122.4695	0.663

1122.59925	0.66371

1122.729	0.66373

1122.85875	0.66458

1122.9885	0.66462

1123.11825	0.66464

1123.248	0.66513

1123.37775	0.66516

1123.5075	0.66518

1123.63725	0.66529

1123.767	0.66531

1123.89675	0.66533

1124.0265	0.6654

1124.15625	0.66558

1124.286	0.66562

1124.41575	0.66571

1124.5455	0.66573

1124.67525	0.66576

1124.805	0.6658

1124.93475	0.66587

1125.0645	0.66589

1125.19425	0.66593

1125.324	0.66596

1125.45375	0.66598

1125.5835	0.66669

1125.71325	0.66671

1125.843	0.66756

1125.97275	0.6676

1126.1025	0.66762

1126.23225	0.66811

1126.362	0.66814

1126.49175	0.66816

1126.62151	0.66818

1126.75125	0.6682

1126.88101	0.66869

1127.01076	0.66871

1127.14051	0.66874

1127.27026	0.66945

1127.40001	0.66947

1127.52976	0.67032

1127.65951	0.67036

1127.78926	0.67038

1127.91901	0.67087

1128.04876	0.67089

1128.17851	0.67092

1128.30826	0.67163

1128.43801	0.67165

1128.56776	0.6725

1128.69751	0.67254

1128.82726	0.67256

1128.95701	0.67258

1129.08676	0.6727

1129.21651	0.67272

1129.34626	0.67274

1129.47601	0.67345

1129.60576	0.67347

1129.73551	0.6735

1129.86526	0.67354

1129.99501	0.67356

1130.12476	0.67359

1130.25451	0.67361

1130.38426	0.67363

1130.51401	0.67367

1130.64376	0.67428

1130.77351	0.6743

1130.90326	0.67432

1131.03301	0.67434

1131.16276	0.67436

1131.29251	0.67439

1131.42226	0.67441

1131.55201	0.67443

1131.68177	0.67448

1131.81152	0.67452

1131.94126	0.67454

1132.07102	0.67456

1132.20077	0.67539

1132.33052	0.67548

1132.46027	0.6755

1132.59002	0.67557

1132.71977	0.67559

1132.84952	0.67561

1132.97927	0.67659

1133.10902	0.6767

1133.23877	0.67672

1133.36852	0.67674

1133.49827	0.67688

1133.62802	0.6769

1133.75777	0.67692

1133.88752	0.67694

1134.01727	0.67708

1134.14702	0.6771

1134.27677	0.67712

1134.40652	0.67714

1134.53627	0.67788

1134.66602	0.6779

1134.79577	0.67797

1134.92552	0.67799

1135.05527	0.67801

1135.18502	0.67803

1135.31477	0.67808

1135.44452	0.67821

1135.57427	0.67823

1135.70402	0.67826

1135.83377	0.67975

1135.96352	0.67979

1136.09327	0.67981

1136.22302	0.6799

1136.35277	0.67992

1136.48252	0.67997

1136.61227	0.67999

1136.74203	0.68008

1136.87178	0.68013

1137.00152	0.68015

1137.13128	0.68182

1137.26103	0.68188

1137.39078	0.6819

1137.52053	0.68193

1137.65028	0.68195

1137.78003	0.68197

1137.90978	0.68393

1138.03953	0.68395

1138.16928	0.68397

1138.29903	0.684

1138.42878	0.68402

1138.55853	0.68408

1138.68828	0.68417

1138.81803	0.68422

1138.94778	0.68573

1139.07753	0.68575

1139.20728	0.6858

1139.33703	0.68582

1139.46678	0.68597

1139.59653	0.68606

1139.72628	0.68613

1139.85603	0.68644

1139.98578	0.68655

1140.11553	0.68671

1140.24528	0.68675

1140.37503	0.68684

1140.50478	0.68686

1140.63453	0.68691

1140.76428	0.68862

1140.89403	0.68864

1141.02378	0.68869

1141.15353	0.68871

1141.28328	0.68873

1141.41303	0.68996

1141.54278	0.69

1141.67253	0.69002

1141.80229	0.69005

1141.93204	0.69007

1142.06179	0.69009

1142.19154	0.69013

1142.32129	0.6902

1142.45104	0.69027

1142.58079	0.69065

1142.71054	0.69069

1142.84029	0.69071

1142.97004	0.69096

1143.09979	0.69098

1143.22954	0.691

1143.35929	0.69149

1143.48904	0.69151

1143.61879	0.69156

1143.74854	0.69158

1143.87829	0.6922

1144.00804	0.69229

1144.13779	0.69231

1144.26754	0.6924

1144.39729	0.69256

1144.52704	0.6926

1144.65679	0.69265

1144.78654	0.69267

1144.91629	0.69356

1145.04604	0.69363

1145.17579	0.69365

1145.30554	0.69369

1145.43529	0.69425

1145.56504	0.69441

1145.69479	0.69443

1145.82454	0.69447

1145.95429	0.69449

1146.08404	0.69456

1146.21379	0.69458

1146.34354	0.69483

1146.47329	0.69485

1146.60304	0.69489

1146.73279	0.69492

1146.86255	0.69543

1146.9923	0.69545

1147.12205	0.69549

1147.2518	0.69603

1147.38155	0.69607

1147.5113	0.69621

1147.64105	0.69625

1147.7708	0.69685

1147.90055	0.69787

1148.0303	0.69848

1148.16005	0.6985

1148.2898	0.69934

1148.41955	0.6995

1148.5493	0.70008

1148.67905	0.70061

1148.8088	0.70066

1148.93855	0.70079

1149.0683	0.70083

1149.19805	0.70143

1149.3278	0.70146

1149.45755	0.7023

1149.5873	0.70397

1149.71705	0.70455

1149.8468	0.70508

1149.97655	0.70639

1150.1063	0.70644

1150.23605	0.70655

1150.3658	0.70922

1150.49555	0.70955

1150.6253	0.70966

1150.75505	0.71231

1150.8848	0.71236

1151.01455	0.71256

1151.1443	0.716

1151.27405	0.71607

1151.4038	0.71614

1151.53355	0.99811

1151.66331	0.99813

1151.79306	0.9982

1151.9228	0.99822

1152.05256	0.99824

1152.18231	0.99864

1152.31206	0.99911

1152.44181	0.99922

1152.57156	0.99924

1152.70131	0.99929

1152.83106	0.9996

1152.96081	0.99967

1153.09056	0.99973

1153.22031	0.99976

1153.35006	0.99978

1153.47981	0.9998

1153.60956	0.99982

1153.73931	0.99989

1153.86906	0.99993

1153.99881	1

**（*e*）Non-hydraulic-90°**

1	0	0.1988	0.00E+00

2	0.00911	0.2384	0.000024702

3	0.0255	0.498	0.0000437

4	0.03643	0.5976	6.52E-05

5	0.05283	0.6972	0.000111704

6	0.07651	0.7968	0.00013963

7	0.10201	0.8964	1.44E-04

8	0.12752	0.996	2.70E-04

9	0.15484	1.0956	2.75E-04

10	0.1767	1.1952	2.98E-04

11	0.19674	1.2948	3.16E-04

12	0.21496	1.3944	3.21E-04

13	0.23135	1.494	3.26E-04

14	0.24957	1.5936	3.49E-04

15	0.27325	1.6932	4.05E-04

16	0.29329	1.7928	4.65E-04

17	0.30604	1.8924	4.70E-04

18	0.32608	1.992	5.35E-04

19	0.34612	2.0916	6.24E-04

20	0.36251	2.1912	6.80E-04

21	0.38073	2.2908	6.84E-04

22	0.39712	2.3904	8.38E-04

23	0.41716	2.49	8.56E-04

24	0.43902	2.5896	8.94E-04

25	0.4536	2.6892	8.98E-04

26	0.46999	2.7888	9.82E-04

27	0.49003	2.8884	9.91E-04

28	0.51007	2.988	9.96E-04

29	0.52646	3.0876	0.001

30	0.54104	3.1872	0.00114

31	0.5629	3.2868	0.00118

32	0.58111	3.3864	0.00121

33	0.60115	3.486	0.00127

34	0.61755	3.5856	0.00135

35	0.63576	3.6852	0.0017

36	0.65398	3.7848	0.00176

37	0.67584	3.8844	0.00182

38	0.69041	3.984	0.00202

39	0.70681	4.0836	0.00203

40	0.72502	4.1832	0.00203

41	0.74688	4.2828	0.00215

42	0.7651	4.3824	0.00215

43	0.77785	4.482	0.00216

44	0.79789	4.5816	0.00225

45	0.81793	4.6812	0.0023

46	0.83797	4.7808	0.00232

47	0.85436	4.8804	0.00249

48	0.86894	4.98	0.0025

49	0.8908	5.0796	0.00255

50	0.90719	5.1792	0.00263

51	0.92541	5.2788	0.00269

52	0.9418	5.3784	0.00296

53	0.9582	5.478	0.00296

54	0.98006	5.5776	0.00298

55	0.99827	5.6772	0.00301

56	1.01467	5.7768	0.00305

57	1.03289	5.8764	0.00307

58	1.0511	5.976	0.0031

59	1.07114	6.0756	0.00311

60	1.08936	6.1752	0.00314

61	1.10029	6.2748	0.00316

62	1.12215	6.3744	0.00323

63	1.14036	6.474	0.00326

64	1.1604	6.5736	0.00336

65	1.17498	6.6732	0.00336

66	1.18955	6.7728	0.00337

67	1.21687	6.8724	0.00341

68	1.23509	6.972	0.00346

69	1.25149	7.0716	0.00349

70	1.26242	7.1712	0.00354

71	1.28428	7.2708	0.00358

72	1.30796	7.3704	0.0036

73	1.32435	7.47	0.00363

74	1.33893	7.5696	0.00374

75	1.35714	7.6692	0.00387

76	1.379	7.7688	0.0039

77	1.39904	7.8684	0.00396

78	1.41361	7.968	0.00397

79	1.43001	8.0676	0.004

80	1.45187	8.1672	0.00402

81	1.46826	8.2668	0.00402

82	1.49012	8.3664	0.00403

83	1.50288	8.466	0.00404

84	1.52109	8.5656	0.00405

85	1.54477	8.6652	0.00405

86	1.56299	8.7648	0.00408

87	1.57574	8.8644	0.0041

88	1.59578	8.964	0.00413

89	1.61764	9.0636	0.00416

90	1.63586	9.1632	0.00424

91	1.65043	9.2628	0.00437

92	1.66683	9.3624	0.00437

93	1.68869	9.462	0.00439

94	1.71055	9.5616	0.00442

95	1.72512	9.6612	0.00448

96	1.73969	9.7608	0.00451

97	1.76155	9.8604	0.0046

98	1.78341	9.96	0.00473

99	1.80345	10.0596	0.00475

100	1.81074	10.1592	0.00476

101	1.8326	10.2588	0.00478

102	1.85264	10.3584	0.00481

103	1.8745	10.458	0.00501

104	1.88907	10.5576	0.00501

105	1.90364	10.6572	0.00505

106	1.9255	10.7568	0.00508

107	1.95101	10.8564	0.00514

108	1.96558	10.956	0.00516

109	1.97833	11.0556	0.0052

110	1.99655	11.1552	0.00522

111	2.01659	11.2548	0.00524

112	2.03663	11.3544	0.00524

113	2.05302	11.454	0.00526

114	2.07124	11.5536	0.00536

115	2.08945	11.6532	0.00539

116	2.10949	11.7528	0.00541

117	2.12771	11.8524	0.00558

118	2.14228	11.952	0.00581

119	2.16232	12.0516	0.00582

120	2.18236	12.1512	0.00592

121	2.2024	12.2508	0.00595

122	2.21879	12.3504	0.00598

123	2.23337	12.45	0.00601

124	2.2534	12.5496	0.00623

125	2.27526	12.6492	0.00659

126	2.29348	12.7488	0.00661

127	2.30441	12.8484	0.00663

128	2.32445	12.948	0.00664

129	2.34813	13.0476	0.0067

130	2.36453	13.1472	0.00673

131	2.37728	13.2468	0.00708

132	2.39732	13.3464	0.00746

133	2.41918	13.446	0.00748

134	2.43921	13.5456	0.00794

135	2.45561	13.6452	0.00805

136	2.46654	13.7448	0.00821

137	2.4884	13.8444	0.00823

138	2.51572	13.944	0.00823

139	2.5303	14.0436	0.00824

140	2.54123	14.1432	0.00827

141	2.56127	14.2428	0.00828

142	2.58495	14.3424	0.00828

143	2.59952	14.442	0.00831

144	2.61774	14.5416	0.00833

145	2.63231	14.6412	0.00833

146	2.65417	14.7408	0.00838

147	2.67421	14.8404	0.0084

148	2.69425	14.94	0.00848

149	2.70336	15.0396	0.00848

150	2.72704	15.1392	0.00849

151	2.74708	15.2388	0.00852

152	2.76529	15.3384	0.00862

153	2.77804	15.438	0.00863

154	2.79808	15.5376	0.00889

155	2.81812	15.6372	0.00892

156	2.83634	15.7368	0.00917

157	2.85455	15.8364	0.00918

158	2.87095	15.936	0.00923

159	2.88917	16.0356	0.00928

160	2.9092	16.1352	0.0093

161	2.92924	16.2348	0.00933

162	2.94199	16.3344	0.00935

163	2.96203	16.434	0.0096

164	2.98207	16.5336	0.00973

165	3.00211	16.6332	0.00989

166	3.01668	16.7328	0.00989

167	3.0349	16.8324	0.01003

168	3.05858	16.932	0.01006

169	3.07498	17.0316	0.01007

170	3.09137	17.1312	0.0101

171	3.10777	17.2308	0.01016

172	3.12598	17.3304	0.01016

173	3.14784	17.43	0.01017

174	3.16424	17.5296	0.01024

175	3.18063	17.6292	0.01024

176	3.19703	17.7288	0.0103

177	3.21889	17.8284	0.01043

178	3.23893	17.928	0.0105

179	3.24986	18.0276	0.0106

180	3.26989	18.1272	0.01063

181	3.28811	18.2268	0.01064

182	3.31179	18.3264	0.01068

183	3.32637	18.426	0.01071

184	3.33912	18.5256	0.01083

185	3.36098	18.6252	0.01084

186	3.38284	18.7248	0.01085

187	3.40105	18.8244	0.01085

188	3.41563	18.924	0.0109

189	3.43384	19.0236	0.01092

190	3.45388	19.1232	0.01096

191	3.47392	19.2228	0.01097

192	3.48667	19.3224	0.011

193	3.50125	19.422	0.01101

194	3.52311	19.5216	0.01104

195	3.54314	19.6212	0.01107

196	3.56318	19.7208	0.01109

197	3.57411	19.8204	0.01109

198	3.59415	19.92	0.01139

199	3.61601	20.0196	0.01139

200	3.63423	20.1192	0.01144

201	3.6488	20.2188	0.01145

202	3.66337	20.3184	0.01148

203	3.68523	20.418	0.01149

204	3.70527	20.5176	0.0115

205	3.72167	20.6172	0.01156

206	3.73624	20.7168	0.01158

207	3.75628	20.8164	0.01159

208	3.77632	20.916	0.01159

209	3.79271	21.0156	0.0116

210	3.80729	21.1152	0.01163

211	3.8255	21.2148	0.01164

212	3.84919	21.3144	0.01164

213	3.8674	21.414	0.01165

214	3.88015	21.5136	0.01176

215	3.89837	21.6132	0.01177

216	3.91659	21.7128	0.01184

217	3.93845	21.8124	0.01187

218	3.95302	21.912	0.01197

219	3.96759	22.0116	0.01212

220	3.98763	22.1112	0.01213

221	4.01131	22.2108	0.01221

222	4.03135	22.3104	0.01223

223	4.04228	22.41	0.01224

224	4.05686	22.5096	0.01225

225	4.07872	22.6092	0.01226

226	4.10058	22.7088	0.01226

227	4.11333	22.8084	0.01227

228	4.12972	22.908	0.01239

229	4.15705	23.0076	0.0124

230	4.17344	23.1072	0.01241

231	4.19166	23.2068	0.01244

232	4.20441	23.3064	0.01259

233	4.22263	23.406	0.01283

234	4.24631	23.5056	0.01288

235	4.2627	23.6052	0.01296

236	4.27728	23.7048	0.013

237	4.29185	23.8044	0.01303

238	4.31735	23.904	0.01307

239	4.33739	24.0036	0.01308

240	4.35014	24.1032	0.01308

241	4.36472	24.2028	0.01316

242	4.38658	24.3024	0.01316

243	4.40662	24.402	0.01317

244	4.42301	24.5016	0.0132

245	4.43758	24.6012	0.0132

246	4.45398	24.7008	0.0132

247	4.47766	24.8004	0.01327

248	4.4977	24.9	0.01328

249	4.51592	24.9996	0.01328

250	4.53413	25.0992	0.01329

251	4.55417	25.1988	0.01329

252	4.57421	25.2984	0.01333

253	4.58878	25.398	0.01334

254	4.60518	25.4976	0.01336

255	4.62522	25.5972	0.01336

256	4.64343	25.6968	0.01337

257	4.66347	25.7964	0.01352

258	4.6744	25.896	0.01353

259	4.69444	25.9956	0.01354

260	4.71994	26.0952	0.0136

261	4.73634	26.1948	0.01373

262	4.75273	26.2944	0.01375

263	4.76548	26.394	0.01381

264	4.78552	26.4936	0.01386

265	4.80738	26.5932	0.0139

266	4.82378	26.6928	0.01396

267	4.83653	26.7924	0.01399

268	4.85292	26.892	0.01404

269	4.87843	26.9916	0.01405

270	4.90029	27.0912	0.01418

271	4.91122	27.1908	0.01419

272	4.92761	27.2904	0.01421

273	4.94947	27.39	0.01428

274	4.97315	27.4896	0.01429

275	4.98591	27.5892	0.01431

276	4.99866	27.6888	0.01435

277	5.0187	27.7884	0.01437

278	5.04238	27.888	0.01437

279	5.05877	27.9876	0.0144

280	5.07152	28.0872	0.01441

281	5.09338	28.1868	0.01442

282	5.11524	28.2864	0.01445

283	5.13164	28.386	0.01446

284	5.14621	28.4856	0.01449

285	5.16261	28.5852	0.01451

286	5.18447	28.6848	0.01451

287	5.20633	28.7844	0.01458

288	5.2209	28.884	0.01461

289	5.2373	28.9836	0.01464

290	5.25733	29.0832	0.01465

291	5.27919	29.1828	0.01465

292	5.29741	29.2824	0.01466

293	5.31198	29.382	0.01468

294	5.32838	29.4816	0.01475

295	5.34842	29.5812	0.01479

296	5.3721	29.6808	0.01483

297	5.38303	29.7804	0.01484

298	5.40125	29.88	0.01487

299	5.42311	29.9796	0.01489

300	5.44861	30.0792	0.015

301	5.45954	30.1788	0.01502

302	5.47411	30.2784	0.01522

303	5.49233	30.378	0.01525

304	5.51783	30.4776	0.01538

305	5.53423	30.5772	0.01539

306	5.54698	30.6768	0.01541

307	5.56884	30.7764	0.01542

308	5.5907	30.876	0.01547

309	5.61074	30.9756	0.01548

310	5.62349	31.0752	0.01549

311	5.63806	31.1748	0.0155

312	5.66174	31.2744	0.01553

313	5.68178	31.374	0.01554

314	5.69453	31.4736	0.01556

315	5.71093	31.5732	0.01557

316	5.73097	31.6728	0.01559

317	5.75283	31.7724	0.0156

318	5.77287	31.872	0.01567

319	5.78744	31.9716	0.0157

320	5.80384	32.0712	0.01572

321	5.82934	32.1708	0.01573

322	5.84573	32.2704	0.01574

323	5.85849	32.37	0.01575

324	5.8767	32.4696	0.01576

325	5.89674	32.5692	0.01599

326	5.9186	32.6688	0.01615

327	5.93317	32.7684	0.01631

328	5.94957	32.868	0.01633

329	5.97143	32.9676	0.01643

330	5.99147	33.0672	0.01644

331	6.00968	33.1668	0.01645

332	6.02244	33.2664	0.01646

333	6.04065	33.366	0.0165

334	6.06251	33.4656	0.0165

335	6.08255	33.5652	0.01655

336	6.09712	33.6648	0.01657

337	6.11352	33.7644	0.01658

338	6.13902	33.864	0.01658

339	6.15906	33.9636	0.01663

340	6.17363	34.0632	0.01667

341	6.19003	34.1628	0.01668

342	6.20825	34.2624	0.01669

343	6.22828	34.362	0.01671

344	6.24832	34.4616	0.01676

345	6.26107	34.5612	0.01677

346	6.28111	34.6608	0.01679

347	6.30297	34.7604	0.01681

348	6.31937	34.86	0.01683

349	6.33212	34.9596	0.01689

350	6.35034	35.0592	0.01689

351	6.37402	35.1588	0.01696

352	6.39041	35.2584	0.017

353	6.41045	35.358	0.01701

354	6.4232	35.4576	0.01707

355	6.44506	35.5572	0.01711

356	6.46692	35.6568	0.01712

357	6.48332	35.7564	0.0172

358	6.49971	35.856	0.01722

359	6.51975	35.9556	0.01739

360	6.54161	36.0552	0.01752

361	6.55983	36.1548	0.01767

362	6.56894	36.2544	0.01799

363	6.58897	36.354	0.01799

364	6.61266	36.4536	0.01801

365	6.63269	36.5532	0.01803

366	6.64909	36.6528	0.01804

367	6.66366	36.7524	0.01806

368	6.6837	36.852	0.0181

369	6.70738	36.9516	0.01811

370	6.7256	37.0512	0.01811

371	6.73835	37.1508	0.01826

372	6.75292	37.2504	0.01827

373	6.77843	37.35	0.01829

374	6.79482	37.4496	0.01831

375	6.8094	37.5492	0.01831

376	6.82761	37.6488	0.01833

377	6.84583	37.7484	0.0184

378	6.87133	37.848	0.01879

379	6.88591	37.9476	0.01886

380	6.90048	38.0472	0.01889

381	6.9187	38.1468	0.0189

382	6.94056	38.2464	0.01891

383	6.95877	38.346	0.01897

384	6.97517	38.4456	0.01899

385	6.98974	38.5452	0.01901

386	7.01524	38.6448	0.01907

387	7.03528	38.7444	0.01907

388	7.04803	38.844	0.01909

389	7.06261	38.9436	0.01914

390	7.08629	39.0432	0.01916

391	7.10633	39.1428	0.01919

392	7.12272	39.2424	0.01921

393	7.1373	39.342	0.01929

394	7.15916	39.4416	0.01934

395	7.17919	39.5412	0.01935

396	7.19741	39.6408	0.01938

397	7.21198	39.7404	0.01945

398	7.22656	39.84	0.01948

399	7.25206	39.9396	0.0195

400	7.2721	40.0392	0.01954

401	7.28485	40.1388	0.01954

402	7.30125	40.2384	0.01955

403	7.32493	40.338	0.01974

404	7.34314	40.4376	0.01974

405	7.35772	40.5372	0.01978

406	7.37776	40.6368	0.01984

407	7.39415	40.7364	0.01984

408	7.41965	40.836	0.01985

409	7.43241	40.9356	0.01989

410	7.44516	41.0352	0.0199

411	7.4652	41.1348	0.01991

412	7.48888	41.2344	0.01995

413	7.50892	41.334	0.01996

414	7.52167	41.4336	0.01998

415	7.53806	41.5332	0.0201

416	7.56357	41.6328	0.02015

417	7.5836	41.7324	0.02016

418	7.59818	41.832	0.02018

419	7.61457	41.9316	0.02037

420	7.63097	42.0312	0.02047

421	7.65283	42.1308	0.02053

422	7.67105	42.2304	0.02057

423	7.6838	42.33	0.02058

424	7.70201	42.4296	0.02063

425	7.72752	42.5292	0.02081

426	7.74391	42.6288	0.02082

427	7.75849	42.7284	0.02082

428	7.77852	42.828	0.02083

429	7.79856	42.9276	0.02085

430	7.82042	43.0272	0.02086

431	7.83135	43.1268	0.02093

432	7.84957	43.2264	0.02099

433	7.87143	43.326	0.021

434	7.88965	43.4256	0.02101

435	7.90786	43.5252	0.02108

436	7.92426	43.6248	0.02113

437	7.9443	43.7244	0.02116

438	7.96616	43.824	0.02123

439	7.98437	43.9236	0.02132

440	7.9953	44.0232	0.02145

441	8.01352	44.1228	0.02148

442	8.03902	44.2224	0.02152

443	8.05542	44.322	0.02156

444	8.07181	44.4216	0.02157

445	8.09185	44.5212	0.0216

446	8.11007	44.6208	0.02161

447	8.13011	44.7204	0.02162

448	8.14832	44.82	0.02169

449	8.16107	44.9196	0.02171

450	8.17929	45.0192	0.02187

451	8.20479	45.1188	0.02188

452	8.21937	45.2184	0.02195

453	8.23576	45.318	0.02208

454	8.25034	45.4176	0.02211

455	8.27584	45.5172	0.02211

456	8.29588	45.6168	0.02212

457	8.30863	45.7164	0.02213

458	8.32502	45.816	0.02216

459	8.34688	45.9156	0.02218

460	8.36874	46.0152	0.02227

461	8.3815	46.1148	0.02231

462	8.39789	46.2144	0.02234

463	8.41793	46.314	0.02235

464	8.43979	46.4136	0.02236

465	8.45801	46.5132	0.02236

466	8.47258	46.6128	0.02238

467	8.49444	46.7124	0.02238

468	8.51266	46.812	0.02239

469	8.53269	46.9116	0.02241

470	8.54545	47.0112	0.02247

471	8.56548	47.1108	0.02247

472	8.5837	47.2104	0.02249

473	8.60556	47.31	0.02255

474	8.62013	47.4096	0.02264

475	8.63835	47.5092	0.0229

476	8.65657	47.6088	0.02292

477	8.67843	47.7084	0.02307

478	8.69664	47.808	0.02308

479	8.7094	47.9076	0.02308

480	8.72761	48.0072	0.02309

481	8.75312	48.1068	0.02311

482	8.77133	48.2064	0.02311

483	8.78226	48.306	0.02332

484	8.80048	48.4056	0.02336

485	8.82416	48.5052	0.02355

486	8.84602	48.6048	0.02383

487	8.85877	48.7044	0.02389

488	8.87335	48.804	0.02398

489	8.89521	48.9036	0.02399

490	8.91342	49.0032	0.024

491	8.93164	49.1028	0.02401

492	8.94621	49.2024	0.02402

493	8.96807	49.302	0.0241

494	8.99175	49.4016	0.02419

495	9.00997	49.5012	0.02421

496	9.0209	49.6008	0.02422

497	9.04094	49.7004	0.02435

498	9.0628	49.8	0.02436

499	9.08284	49.8996	0.02436

500	9.09559	49.9992	0.02439

501	9.11198	50.0988	0.0244

502	9.13384	50.1984	0.02441

503	9.1557	50.298	0.02442

504	9.17392	50.3976	0.02454

505	9.18667	50.4972	0.02457

506	9.20489	50.5968	0.02471

507	9.22857	50.6964	0.02472

508	9.24679	50.796	0.02476

509	9.25954	50.8956	0.02477

510	9.27411	50.9952	0.02478

511	9.30326	51.0948	0.02481

512	9.31783	51.1944	0.02485

513	9.33058	51.294	0.02498

514	9.35244	51.3936	0.025

515	9.37248	51.4932	0.02505

516	9.39434	51.5928	0.02512

517	9.40527	51.6924	0.02512

518	9.41985	51.792	0.02513

519	9.44535	51.8916	0.02514

520	9.46539	51.9912	0.02522

521	9.48178	52.0908	0.02532

522	9.49271	52.1904	0.02539

523	9.51457	52.29	0.02552

524	9.53643	52.3896	0.02555

525	9.55647	52.4892	0.02557

526	9.57105	52.5888	0.0256

527	9.58744	52.6884	0.02565

528	9.61112	52.788	0.02566

529	9.62934	52.8876	0.02569

530	9.64209	52.9872	0.0257

531	9.65849	53.0868	0.02571

532	9.68035	53.1864	0.02582

533	9.70221	53.286	0.02585

534	9.72042	53.3856	0.02591

535	9.73317	53.4852	0.02593

536	9.75503	53.5848	0.02593

537	9.77507	53.6844	0.02609

538	9.79147	53.784	0.02616

539	9.80422	53.8836	0.02629

540	9.82608	53.9832	0.02631

541	9.84976	54.0828	0.02631

542	9.86433	54.1824	0.02632

543	9.88073	54.282	0.02632

544	9.89895	54.3816	0.02654

545	9.91898	54.4812	0.02668

546	9.93902	54.5808	0.02669

547	9.9536	54.6804	0.0267

548	9.97181	54.78	0.02671

549	9.99003	54.8796	0.02673

550	10.01189	54.9792	0.02673

551	10.02828	55.0788	0.02698

552	10.04468	55.1784	0.02699

553	10.0629	55.278	0.027

554	10.08293	55.3776	0.02703

555	10.10297	55.4772	0.02711

556	10.11572	55.5768	0.02713

557	10.13576	55.6764	0.02714

558	10.1558	55.776	0.02716

559	10.1722	55.8756	0.02729

560	10.18677	55.9752	0.02733

561	10.20681	56.0748	0.02733

562	10.22867	56.1744	0.02738

563	10.24871	56.274	0.02738

564	10.26328	56.3736	0.0274

565	10.27967	56.4732	0.02741

566	10.30153	56.5728	0.02745

567	10.32157	56.6724	0.02747

568	10.33797	56.772	0.02798

569	10.35072	56.8716	0.02799

570	10.36894	56.9712	0.028

571	10.39626	57.0708	0.028

572	10.40901	57.1704	0.02802

573	10.42359	57.27	0.02803

574	10.44727	57.3696	0.02804

575	10.46548	57.4692	0.02806

576	10.4837	57.5688	0.02837

577	10.5001	57.6684	0.02838

578	10.51649	57.768	0.02839

579	10.53653	57.8676	0.0284

580	10.55475	57.9672	0.02844

581	10.57114	58.0668	0.02853

582	10.58754	58.1664	0.02855

583	10.60757	58.266	0.02858

584	10.62943	58.3656	0.02908

585	10.64947	58.4652	0.02909

586	10.66405	58.5648	0.02912

587	10.68044	58.6644	0.02915

588	10.70048	58.764	0.02919

589	10.72052	58.8636	0.02928

590	10.73327	58.9632	0.02935

591	10.75149	59.0628	0.02936

592	10.77335	59.1624	0.02938

593	10.79156	59.262	0.02939

594	10.80978	59.3616	0.02953

595	10.82435	59.4612	0.02955

596	10.84439	59.5608	0.02955

597	10.86625	59.6604	0.02956

598	10.88265	59.76	0.02962

599	10.89722	59.8596	0.02962

600	10.91361	59.9592	0.02964

601	10.9373	60.0588	0.02966

602	10.95369	60.1584	0.02968

603	10.97191	60.258	0.02968

604	10.99012	60.3576	0.02988

605	11.01198	60.4572	0.03005

606	11.03202	60.5568	0.03015

607	11.04477	60.6564	0.03017

608	11.06117	60.756	0.03023

609	11.08303	60.8556	0.03023

610	11.10307	60.9552	0.0303

611	11.11582	61.0548	0.03032

612	11.13221	61.1544	0.03033

613	11.1559	61.254	0.03033

614	11.17411	61.3536	0.03034

615	11.19051	61.4532	0.03035

616	11.20508	61.5528	0.03051

617	11.2233	61.6524	0.03066

618	11.24516	61.752	0.03075

619	11.26337	61.8516	0.03077

620	11.27795	61.9512	0.03082

621	11.29616	62.0508	0.03083

622	11.31802	62.1504	0.03084

623	11.33806	62.25	0.03086

624	11.35081	62.3496	0.03088

625	11.36903	62.4492	0.03094

626	11.39089	62.5488	0.03102

627	11.40729	62.6484	0.03114

628	11.4255	62.748	0.03115

629	11.44008	62.8476	0.03119

630	11.46012	62.9472	0.0312

631	11.48198	63.0468	0.03152

632	11.50201	63.1464	0.03153

633	11.51294	63.246	0.03156

634	11.53116	63.3456	0.03157

635	11.55484	63.4452	0.03178

636	11.57124	63.5448	0.03179

637	11.58581	63.6444	0.03179

638	11.60221	63.744	0.03226

639	11.62407	63.8436	0.03227

640	11.6441	63.9432	0.03227

641	11.65686	64.0428	0.03228

642	11.67507	64.1424	0.0323

643	11.70058	64.242	0.03233

644	11.71879	64.3416	0.03251

645	11.73337	64.4412	0.03252

646	11.74794	64.5408	0.03256

647	11.76616	64.6404	0.03259

648	11.78802	64.74	0.03259

649	11.80623	64.8396	0.03299

650	11.82081	64.9392	0.03308

651	11.83902	65.0388	0.03311

652	11.8627	65.1384	0.03312

653	11.88092	65.238	0.03313

654	11.89367	65.3376	0.03313

655	11.91007	65.4372	0.03315

656	11.93375	65.5368	0.03316

657	11.95379	65.6364	0.03322

658	11.96472	65.736	0.03328

659	11.98293	65.8356	0.03341

660	12.00662	65.9352	0.03342

661	12.02665	66.0348	0.03345

662	12.04487	66.1344	0.03346

663	12.05762	66.234	0.03346

664	12.07584	66.3336	0.03366

665	12.0977	66.4332	0.03373

666	12.11227	66.5328	0.0338

667	12.13049	66.6324	0.03383

668	12.14688	66.732	0.03396

669	12.16692	66.8316	0.03397

670	12.18878	66.9312	0.03398

671	12.20336	67.0308	0.03398

672	12.22157	67.1304	0.034

673	12.23979	67.23	0.03427

674	12.25983	67.3296	0.03428

675	12.27622	67.4292	0.0343

676	12.2908	67.5288	0.0343

677	12.30901	67.6284	0.03435

678	12.32905	67.728	0.03436

679	12.34909	67.8276	0.03437

680	12.36548	67.9272	0.03448

681	12.3837	68.0268	0.03448

682	12.40556	68.1264	0.03463

683	12.42378	68.226	0.03471

684	12.43653	68.3256	0.03472

685	12.4511	68.4252	0.03474

686	12.47478	68.5248	0.03475

687	12.49482	68.6244	0.03482

688	12.50757	68.724	0.03495

689	12.52761	68.8236	0.03496

690	12.54947	68.9232	0.03498

691	12.56769	69.0228	0.03511

692	12.58408	69.1224	0.03512

693	12.6023	69.222	0.03517

694	12.62052	69.3216	0.03517

695	12.64056	69.4212	0.03584

696	12.65513	69.5208	0.03585

697	12.67152	69.6204	0.03587

698	12.68974	69.72	0.03588

699	12.71342	69.8196	0.03588

700	12.728	69.9192	0.03595

701	12.74439	70.0188	0.03611

702	12.76261	70.1184	0.03611

703	12.78447	70.218	0.03612

704	12.80451	70.3176	0.03615

705	12.81544	70.4172	0.03618

706	12.83183	70.5168	0.03619

707	12.85369	70.6164	0.03623

708	12.87555	70.716	0.03647

709	12.89195	70.8156	0.03651

710	12.90834	70.9152	0.03653

711	12.92656	71.0148	0.03668

712	12.94842	71.1144	0.03673

713	12.96481	71.214	0.03675

714	12.97939	71.3136	0.03687

715	12.99578	71.4132	0.03725

716	13.01946	71.5128	0.03726

717	13.03586	71.6124	0.03728

718	13.05043	71.712	0.03732

719	13.07047	71.8116	0.03741

720	13.09415	71.9112	0.03742

721	13.11237	72.0108	0.03745

722	13.12512	72.1104	0.03767

723	13.14334	72.21	0.03776

724	13.16337	72.3096	0.03778

725	13.18341	72.4092	0.03778

726	13.19799	72.5088	0.03779

727	13.21438	72.6084	0.03781

728	13.23442	72.708	0.03785

729	13.2581	72.8076	0.03787

730	13.27267	72.9072	0.03792

731	13.28725	73.0068	0.03797

732	13.30729	73.1064	0.03811

733	13.33097	73.206	0.03813

734	13.34736	73.3056	0.03813

735	13.36012	73.4052	0.03816

736	13.37651	73.5048	0.03818

737	13.39837	73.6044	0.03832

738	13.41841	73.704	0.03832

739	13.43116	73.8036	0.03833

740	13.45302	73.9032	0.03842

741	13.47306	74.0028	0.03843

742	13.4931	74.1024	0.0385

743	13.50949	74.202	0.0385

744	13.52407	74.3016	0.03851

745	13.54228	74.4012	0.03852

746	13.56414	74.5008	0.03852

747	13.58054	74.6004	0.03853

748	13.59511	74.7	0.03858

749	13.61333	74.7996	0.03867

750	13.63701	74.8992	0.03893

751	13.65523	74.9988	0.03893

752	13.67162	75.0984	0.03894

753	13.68437	75.198	0.03895

754	13.70988	75.2976	0.03899

755	13.72809	75.3972	0.039

756	13.74267	75.4968	0.03901

757	13.75724	75.5964	0.03901

758	13.7791	75.696	0.03903

759	13.79914	75.7956	0.03907

760	13.81553	75.8952	0.03907

761	13.83193	75.9948	0.03908

762	13.85379	76.0944	0.03911

763	13.872	76.194	0.03918

764	13.8884	76.2936	0.03934

765	13.90115	76.3932	0.03947

766	13.91937	76.4928	0.03956

767	13.94669	76.5924	0.03957

768	13.95944	76.692	0.03957

769	13.97402	76.7916	0.03958

770	13.99588	76.8912	0.03958

771	14.01774	76.9908	0.03962

772	14.03778	77.0904	0.03964

773	14.05053	77.19	0.03965

774	14.06874	77.2896	0.03969

775	14.08878	77.3892	0.03972

776	14.107	77.4888	0.03979

777	14.12157	77.5884	0.03995

778	14.13615	77.688	0.03999

779	14.16165	77.7876	0.04

780	14.18169	77.8872	0.04004

781	14.19808	77.9868	0.04012

782	14.21266	78.0864	0.04013

783	14.23087	78.186	0.04013

784	14.25455	78.28561	0.04014

785	14.27095	78.38521	0.04015

786	14.28188	78.48481	0.04017

787	14.30192	78.58441	0.04023

788	14.32378	78.68401	0.04026

789	14.34564	78.78361	0.04029

790	14.35839	78.88321	0.04034

791	14.37661	78.98281	0.04039

792	14.39664	79.08241	0.04041

793	14.41668	79.18201	0.04044

794	14.43126	79.28161	0.04044

795	14.44947	79.38121	0.04045

796	14.46769	79.48081	0.04046

797	14.48773	79.58041	0.04051

798	14.50594	79.68001	0.04069

799	14.52052	79.77961	0.04069

800	14.54056	79.87921	0.0407

801	14.56242	79.97881	0.04071

802	14.57881	80.07841	0.04073

803	14.59338	80.17801	0.04092

804	14.61342	80.27761	0.04104

805	14.63528	80.37721	0.04104

806	14.6535	80.47681	0.04109

807	14.66625	80.57641	0.04115

808	14.68447	80.67601	0.04127

809	14.70815	80.77561	0.04128

810	14.72637	80.87521	0.0413

811	14.74276	80.97481	0.04187

812	14.75733	81.07441	0.04187

813	14.78102	81.17401	0.04194

814	14.79923	81.27361	0.042

815	14.81381	81.37321	0.04201

816	14.8302	81.47281	0.04203

817	14.85024	81.57241	0.04205

818	14.8721	81.67201	0.04207

819	14.89032	81.77161	0.04224

820	14.90307	81.87121	0.04229

821	14.92675	81.97081	0.04232

822	14.94497	82.07041	0.04234

823	14.96136	82.17001	0.04238

824	14.97776	82.26961	0.0424

825	14.99415	82.36921	0.04244

826	15.01783	82.46881	0.04253

827	15.03423	82.56841	0.04261

828	15.0488	82.66801	0.04262

829	15.06884	82.76761	0.04265

830	15.08888	82.86721	0.04265

831	15.10892	82.96681	0.04272

832	15.12713	83.06641	0.04274

833	15.14353	83.16601	0.04277

834	15.15992	83.26561	0.04281

835	15.1836	83.36521	0.04282

836	15.19818	83.46481	0.04285

837	15.21275	83.56441	0.04285

838	15.23097	83.66401	0.04287

839	15.25465	83.76361	0.04292

840	15.27105	83.86321	0.04311

841	15.28744	83.96281	0.04311

842	15.30201	84.06241	0.0432

843	15.3257	84.16201	0.04324

844	15.34209	84.26161	0.04347

845	15.36031	84.36121	0.04352

846	15.37306	84.46081	0.04353

847	15.39674	84.56041	0.04355

848	15.41496	84.66001	0.04382

849	15.43135	84.75961	0.04387

850	15.44593	84.85921	0.04388

851	15.46779	84.95881	0.04388

852	15.49329	85.05841	0.04389

853	15.50786	85.15801	0.04394

854	15.51879	85.25761	0.0441

855	15.54247	85.35721	0.04411

856	15.56433	85.45681	0.04412

857	15.58073	85.55641	0.04413

858	15.59348	85.65601	0.04413

859	15.61352	85.75561	0.04428

860	15.63356	85.85521	0.04441

861	15.6536	85.95481	0.04447

862	15.66635	86.05441	0.04448

863	15.68456	86.15401	0.04449

864	15.70642	86.25361	0.04457

865	15.72646	86.35321	0.04459

866	15.73921	86.45281	0.04471

867	15.75561	86.55241	0.04472

868	15.77929	86.65201	0.04474

869	15.79751	86.75161	0.04475

870	15.8139	86.85121	0.04477

871	15.82848	86.95081	0.04482

872	15.84851	87.05041	0.04484

873	15.87402	87.15001	0.04484

874	15.88677	87.24961	0.04485

875	15.90316	87.34921	0.04486

876	15.92138	87.44881	0.04487

877	15.94506	87.54841	0.04508

878	15.95964	87.64801	0.0451

879	15.97421	87.74761	0.04511

880	15.99425	87.84721	0.04516

881	16.01611	87.94681	0.04516

882	16.03615	88.04641	0.04517

883	16.05072	88.14601	0.04518

884	16.06894	88.24561	0.04518

885	16.0908	88.34521	0.04521

886	16.10901	88.44481	0.04529

887	16.12359	88.54441	0.04529

888	16.1418	88.64401	0.04533

889	16.16002	88.74361	0.0455

890	16.17824	88.84321	0.04558

891	16.19645	88.94281	0.04559

892	16.21285	89.04241	0.04565

893	16.23471	89.14201	0.04567

894	16.25475	89.24161	0.04571

895	16.27114	89.34121	0.04573

896	16.28389	89.44081	0.04575

897	16.30575	89.54041	0.04578

898	16.32761	89.64001	0.04581

899	16.34401	89.73961	0.04582

900	16.35858	89.83921	0.04582

901	16.37862	89.93881	0.04584

902	16.39866	90.03841	0.04585

903	16.41687	90.13801	0.04587

904	16.43145	90.23761	0.04588

905	16.45149	90.33721	0.04591

906	16.47335	90.43681	0.04593

907	16.49338	90.53641	0.04605

908	16.50614	90.63601	0.04609

909	16.52253	90.73561	0.04614

910	16.54257	90.83521	0.04616

911	16.56443	90.93481	0.04635

912	16.579	91.03441	0.04636

913	16.5954	91.13401	0.04636

914	16.61544	91.23361	0.04637

915	16.63912	91.33321	0.04637

916	16.65551	91.43281	0.04638

917	16.66826	91.53241	0.04638

918	16.6883	91.63201	0.04641

919	16.71016	91.73161	0.04691

920	16.72656	91.83121	0.04691

921	16.74113	91.93081	0.04692

922	16.76117	92.03041	0.04692

923	16.78303	92.13001	0.04696

924	16.80307	92.22961	0.04703

925	16.814	92.32921	0.04705

926	16.83404	92.42881	0.04707

927	16.8559	92.52841	0.04707

928	16.87411	92.62801	0.04711

929	16.88504	92.72761	0.04711

930	16.90508	92.82721	0.04712

931	16.92512	92.92681	0.04713

932	16.94334	93.02641	0.04716

933	16.96337	93.12601	0.04716

934	16.97613	93.22561	0.04717

935	16.99616	93.32521	0.04719

936	17.01802	93.42481	0.04726

937	17.03624	93.52441	0.04735

938	17.05081	93.62401	0.04743

939	17.06903	93.72361	0.04746

940	17.09089	93.82321	0.04754

941	17.10729	93.92281	0.04764

942	17.12186	94.02241	0.04764

943	17.1419	94.12201	0.04766

944	17.16376	94.22161	0.04771

945	17.18198	94.32121	0.04772

946	17.19473	94.42081	0.04773

947	17.21294	94.52041	0.04784

948	17.23663	94.62001	0.04784

949	17.25302	94.71961	0.04785

950	17.26942	94.81921	0.04793

951	17.28581	94.91881	0.04798

952	17.30403	95.01841	0.04798

953	17.32771	95.11801	0.048

954	17.3441	95.21761	0.04802

955	17.3605	95.31721	0.04802

956	17.37689	95.41681	0.04807

957	17.40058	95.51641	0.04819

958	17.41697	95.61601	0.04845

959	17.43154	95.71561	0.04854

960	17.44976	95.81521	0.04857

961	17.47162	95.91481	0.0486

962	17.48984	96.01441	0.04862

963	17.50441	96.11401	0.04866

964	17.52263	96.21361	0.04869

965	17.54631	96.31321	0.04875

966	17.56635	96.41281	0.04875

967	17.5791	96.51241	0.04879

968	17.59732	96.61201	0.04885

969	17.61918	96.71161	0.04886

970	17.63557	96.81121	0.04887

971	17.65197	96.91081	0.04891

972	17.66836	97.01041	0.049

973	17.69204	97.11001	0.04908

974	17.7139	97.20961	0.04909

975	17.72665	97.30921	0.04913

976	17.74305	97.40881	0.04927

977	17.76309	97.50841	0.04928

978	17.78677	97.60801	0.04932

979	17.80134	97.70761	0.04945

980	17.81409	97.80721	0.04949

981	17.83231	97.90681	0.04949

982	17.85599	98.00641	0.04952

983	17.87239	98.10601	0.04964

984	17.88878	98.20561	0.04968

985	17.907	98.30521	0.04974

986	17.92704	98.40481	0.04979

987	17.94708	98.50441	0.04982

988	17.96347	98.60401	0.04991

989	17.97804	98.70361	0.04991

990	17.99808	98.80321	0.05

991	18.01812	98.90281	0.05002

992	18.03452	99.00241	0.05016

993	18.05091	99.10201	0.05019

994	18.06913	99.20161	0.05019

995	18.09281	99.30121	0.05023

996	18.11103	99.40081	0.05032

997	18.1256	99.50041	0.05037

998	18.14382	99.60001	0.05044

999	18.16203	99.69961	0.05052

1000	18.18207	99.79921	0.05057

1001	18.193	99.89881	0.05063

1002	18.21668	99.99841	0.05084

1003	18.23854	100.09801	0.05084

1004	18.25858	100.19761	0.05085

1005	18.27133	100.29721	0.05096

1006	18.28773	100.39681	0.05097

1007	18.31141	100.49641	0.05098

1008	18.32963	100.59601	0.051

1009	18.34238	100.69561	0.05101

1010	18.35877	100.79521	0.05102

1011	18.38245	100.89481	0.05103

1012	18.40067	100.99441	0.05103

1013	18.41524	101.09401	0.05103

1014	18.43164	101.19361	0.05108

1015	18.45168	101.29321	0.0511

1016	18.47718	101.39281	0.05113

1017	18.48993	101.49241	0.05134

1018	18.50815	101.59201	0.05136

1019	18.52637	101.69161	0.05137

1020	18.5464	101.79121	0.05138

1021	18.56462	101.89081	0.05139

1022	18.57919	101.99041	0.05141

1023	18.59923	102.09001	0.05143

1024	18.62109	102.18961	0.05153

1025	18.63931	102.28921	0.05175

1026	18.65206	102.38881	0.05185

1027	18.6721	102.48841	0.05197

1028	18.69396	102.58801	0.052

1029	18.71035	102.68761	0.05203

1030	18.72493	102.78721	0.05204

1031	18.7395	102.88681	0.05205

1032	18.765	102.98641	0.05208

1033	18.7814	103.08601	0.05211

1034	18.79962	103.18561	0.05213

1035	18.81419	103.28521	0.05214

1036	18.83423	103.38481	0.05215

1037	18.85791	103.48441	0.05217

1038	18.87066	103.58401	0.05224

1039	18.88706	103.68361	0.05231

1040	18.90892	103.78321	0.0524

1041	18.93078	103.88281	0.05241

1042	18.94535	103.98241	0.05244

1043	18.96174	104.08201	0.05246

1044	18.98178	104.18161	0.05261

1045	19.00364	104.28121	0.05278

1046	19.01822	104.38081	0.05278

1047	19.03643	104.48041	0.05279

1048	19.05465	104.58001	0.0529

1049	19.07469	104.67961	0.05291

1050	19.09108	104.77921	0.05293

1051	19.10384	104.87881	0.05294

1052	19.1257	104.97841	0.05295

1053	19.14573	105.07801	0.05308

1054	19.16577	105.17761	0.05309

1055	19.17852	105.27721	0.05313

1056	19.19674	105.37681	0.05314

1057	19.22042	105.47641	0.05326

1058	19.23682	105.57601	0.05328

1059	19.25321	105.67561	0.05328

1060	19.27143	105.77521	0.05346

1061	19.28965	105.87481	0.05348

1062	19.31333	105.97441	0.0535

1063	19.32972	106.07401	0.05376

1064	19.34247	106.17361	0.05377

1065	19.36069	106.27321	0.05378

1066	19.38619	106.37281	0.05395

1067	19.40077	106.47241	0.05395

1068	19.41716	106.57201	0.05396

1069	19.43538	106.67161	0.05398

1070	19.45724	106.77121	0.05401

1071	19.47363	106.87081	0.05402

1072	19.48821	106.97041	0.05402

1073	19.50825	107.07001	0.05408

1074	19.52828	107.16961	0.05408

1075	19.54468	107.26921	0.0541

1076	19.55925	107.36881	0.05411

1077	19.57929	107.46841	0.05415

1078	19.60115	107.56801	0.0542

1079	19.62119	107.66761	0.0542

1080	19.63576	107.76721	0.05421

1081	19.65034	107.86681	0.05426

1082	19.67037	107.96641	0.05432

1083	19.69041	108.06601	0.05433

1084	19.70681	108.16561	0.05455

1085	19.7232	108.26521	0.05458

1086	19.74688	108.36481	0.05459

1087	19.7651	108.46441	0.0546

1088	19.7815	108.56401	0.05481

1089	19.79789	108.66361	0.05491

1090	19.81793	108.76321	0.05504

1091	19.83979	108.86281	0.05507

1092	19.85436	108.96241	0.05507

1093	19.87076	109.06201	0.05518

1094	19.88897	109.16161	0.05519

1095	19.91448	109.26121	0.05524

1096	19.93087	109.36081	0.05524

1097	19.94362	109.46041	0.05529

1098	19.96366	109.56001	0.05531

1099	19.98734	109.65961	0.05533

1100	20.00192	109.75921	0.05534

1101	20.01649	109.85881	0.05543

1102	20.03289	109.95841	0.05552

1103	20.05292	110.05801	0.05552

1104	20.07114	110.15761	0.05554

1105	20.08936	110.25721	0.05556

1106	20.10575	110.35681	0.0556

1107	20.12761	110.45641	0.05561

1108	20.14765	110.55601	0.05585

1109	20.16587	110.65561	0.05586

1110	20.17862	110.75521	0.05589

1111	20.19684	110.85481	0.05597

1112	20.21687	110.95441	0.056

1113	20.23691	111.05401	0.05617

1114	20.24784	111.15361	0.05624

1115	20.27152	111.25321	0.05629

1116	20.29156	111.35281	0.05633

1117	20.3116	111.45241	0.05656

1118	20.32435	111.55201	0.05657

1119	20.34075	111.65161	0.05664

1120	20.36625	111.75121	0.05665

1121	20.38447	111.85081	0.05665

1122	20.39904	111.95041	0.05675

1123	20.41544	112.05001	0.05705

1124	20.43547	112.14961	0.05705

1125	20.45733	112.24921	0.05717

1126	20.47009	112.34881	0.0572

1127	20.49012	112.44841	0.05721

1128	20.51381	112.54801	0.05722

1129	20.53202	112.64761	0.05729

1130	20.53931	112.74721	0.05731

1131	20.55935	112.84681	0.05748

1132	20.58121	112.94641	0.0575

1133	20.60125	113.04601	0.05756

1134	20.614	113.14561	0.05782

1135	20.63221	113.24521	0.05783

1136	20.65407	113.34481	0.05792

1137	20.67593	113.44441	0.05796

1138	20.68869	113.54401	0.05799

1139	20.70508	113.64361	0.05799

1140	20.7233	113.74321	0.05801

1141	20.74516	113.84281	0.05802

1142	20.76155	113.94241	0.05807

1143	20.77613	114.04201	0.05817

1144	20.79252	114.14161	0.0582

1145	20.8162	114.24121	0.05852

1146	20.83442	114.34081	0.05854

1147	20.85081	114.44041	0.05856

1148	20.86721	114.54001	0.05857

1149	20.88907	114.63961	0.05864

1150	20.90729	114.73921	0.05868

1151	20.92004	114.83881	0.05869

1152	20.9419	114.93841	0.0587

1153	20.96012	115.03801	0.05871

1154	20.98015	115.13761	0.05872

1155	20.99291	115.23721	0.05874

1156	21.01294	115.33681	0.05875

1157	21.02934	115.43641	0.05875

1158	21.04938	115.53601	0.05879

1159	21.06577	115.63561	0.0588

1160	21.08035	115.73521	0.05881

1161	21.09674	115.83481	0.05883

1162	21.11314	115.93441	0.05887

1163	21.12224	116.03401	0.05893

1164	21.05484	116.13361	0.05898

1165	20.14401	116.23321	0.05899

1166	19.22224	116.33281	0.05899

1167	18.04545	116.43241	0.05919

1168	16.81946	116.53201	0.05924

1169	15.85034	116.63161	0.05924

1170	14.76826	116.73121	0.05925

1171	13.7372	116.83081	0.05932

1172	12.68792	116.93041	0.05932

1173	11.70968	117.03001	0.05933

1174	10.76424	117.12961	0.05936

1175	9.70585	117.22921	0.05938

1176	8.58188	117.32881	0.05945

1177	7.45973	117.42841	0.05955

1178	6.5325	117.52801	0.05955

1179	5.50326	117.62761	0.05956

1180	4.51227	117.72721	0.05965

1181	3.55954	117.82681	0.05966

1182	2.62685	117.92641	0.05967

118.02601	0.06004

118.12561	0.06005

118.22521	0.06006

118.32481	0.06006

118.42441	0.06023

118.52401	0.0605

118.62361	0.06051

118.72321	0.06051

118.82281	0.06072

118.92241	0.06078

119.02201	0.06079

119.12161	0.06082

119.22121	0.06083

119.32081	0.06084

119.42041	0.06092

119.52001	0.06094

119.61961	0.06095

119.71921	0.06137

119.81881	0.06137

119.91841	0.0614

120.01801	0.0615

120.11761	0.06153

120.21721	0.06153

120.31681	0.06154

120.41641	0.06155

120.51601	0.06165

120.61561	0.06166

120.71521	0.06187

120.81481	0.06188

120.91441	0.0619

121.01401	0.06202

121.11361	0.06208

121.21321	0.06212

121.31281	0.06213

121.41241	0.06229

121.51201	0.0623

121.61161	0.06231

121.71121	0.06232

121.81081	0.06241

121.91041	0.0625

122.01001	0.06261

122.10961	0.06281

122.20921	0.06283

122.30881	0.06284

122.40841	0.06289

122.50801	0.0629

122.60761	0.06296

122.70721	0.06309

122.80681	0.0631

122.90641	0.06312

123.00601	0.06337

123.10561	0.06338

123.20521	0.06339

123.30481	0.06341

123.40441	0.06344

123.50401	0.06347

123.60361	0.06408

123.70321	0.06411

123.80281	0.06411

123.90241	0.06417

124.00201	0.0642

124.10161	0.06421

124.20121	0.06423

124.30081	0.0643

124.40041	0.06434

124.50001	0.06436

124.59961	0.06438

124.69921	0.06455

124.79881	0.06463

124.89841	0.06464

124.99801	0.06464

125.09761	0.06476

125.19721	0.06476

125.29681	0.06478

125.39641	0.06483

125.49601	0.06487

125.59561	0.06488

125.69521	0.06523

125.79481	0.06524

125.89441	0.06528

125.99401	0.06533

126.09361	0.06544

126.19321	0.06548

126.29281	0.0655

126.39241	0.0655

126.49201	0.06554

126.59161	0.06557

126.69121	0.06557

126.79081	0.06559

126.89041	0.06565

126.99001	0.06566

127.08961	0.06569

127.18921	0.0657

127.28881	0.06572

127.38841	0.06582

127.48801	0.06584

127.58761	0.06584

127.68721	0.06585

127.78681	0.06597

127.88641	0.06605

127.98601	0.06607

128.08561	0.06609

128.18521	0.06611

128.28481	0.06611

128.38441	0.06622

128.48401	0.06623

128.58361	0.06626

128.68321	0.06628

128.78281	0.06632

128.88241	0.06654

128.98201	0.06654

129.08161	0.06656

129.18121	0.06658

129.28081	0.06659

129.38041	0.06664

129.48001	0.06672

129.57961	0.06675

129.67921	0.06678

129.77881	0.06701

129.87841	0.06709

129.97801	0.06712

130.07761	0.06712

130.17721	0.06714

130.27681	0.06734

130.37641	0.06734

130.47601	0.06744

130.57561	0.06744

130.67521	0.06762

130.77481	0.06763

130.87441	0.06765

130.97401	0.06766

131.07361	0.06772

131.17321	0.06774

131.27281	0.06775

131.37241	0.06775

131.47201	0.06788

131.57161	0.0679

131.67121	0.06808

131.77081	0.06811

131.87041	0.06834

131.97001	0.06836

132.06961	0.06838

132.16921	0.06839

132.26881	0.06842

132.36841	0.06847

132.46801	0.06848

132.56761	0.06849

132.66721	0.06851

132.76681	0.06886

132.86641	0.06887

132.96601	0.06889

133.06561	0.06891

133.16521	0.06892

133.26481	0.06892

133.36441	0.06893

133.46401	0.06906

133.56361	0.06922

133.66321	0.06923

133.76281	0.06927

133.86241	0.06929

133.96201	0.06929

134.06161	0.06937

134.16121	0.06951

134.26081	0.06952

134.36041	0.06968

134.46001	0.06969

134.55961	0.07001

134.65921	0.07002

134.75881	0.07003

134.85841	0.07018

134.95801	0.07023

135.05761	0.07029

135.15721	0.07031

135.25681	0.07031

135.35641	0.0704

135.45601	0.07059

135.55561	0.07189

135.65521	0.07214

135.75481	0.07216

135.85441	0.07216

135.95401	0.07221

136.05361	0.07222

136.15321	0.07222

136.25281	0.07226

136.35241	0.0723

136.45201	0.07231

136.55161	0.07231

136.65121	0.07233

136.75081	0.07235

136.85041	0.07236

136.95001	0.07259

137.04961	0.07261

137.14921	0.07262

137.24881	0.07262

137.34841	0.07263

137.44801	0.07285

137.54761	0.07303

137.64721	0.07303

137.74681	0.07304

137.84641	0.07304

137.94601	0.07314

138.04561	0.0732

138.14521	0.07348

138.24481	0.07365

138.34441	0.07386

138.44401	0.07389

138.54361	0.07389

138.64321	0.07395

138.74281	0.07396

138.84241	0.07399

138.94201	0.07413

139.04161	0.07418

139.14121	0.07419

139.24081	0.07442

139.34041	0.07445

139.44001	0.07447

139.53961	0.07448

139.63921	0.07449

139.73881	0.0745

139.83841	0.07451

139.93801	0.07457

140.03761	0.07458

140.13721	0.07458

140.23681	0.07459

140.33641	0.07465

140.43601	0.07466

140.53561	0.0747

140.63521	0.07471

140.73481	0.07471

140.83441	0.07472

140.93401	0.07485

141.03361	0.07491

141.13321	0.07496

141.23281	0.07501

141.33241	0.07502

141.43201	0.07505

141.53161	0.07505

141.63121	0.07507

141.73081	0.07523

141.83041	0.07524

141.93001	0.07525

142.02961	0.07526

142.12921	0.07527

142.22881	0.07528

142.32841	0.0753

142.42801	0.07531

142.52761	0.07532

142.62721	0.07536

142.72681	0.07537

142.82641	0.07553

142.92601	0.07554

143.02561	0.07554

143.12521	0.0756

143.22481	0.07564

143.32441	0.07697

143.42401	0.077

143.52361	0.07719

143.62321	0.07721

143.72281	0.07722

143.82241	0.07731

143.92201	0.07731

144.02161	0.07735

144.12121	0.07736

144.22081	0.07751

144.32041	0.07755

144.42001	0.07755

144.51961	0.07761

144.61921	0.07773

144.71881	0.07774

144.81841	0.07776

144.91801	0.07817

145.01761	0.07826

145.11721	0.07833

145.21681	0.07841

145.31641	0.07843

145.41601	0.07849

145.51561	0.07849

145.61521	0.0785

145.71481	0.07851

145.81441	0.07852

145.91401	0.0787

146.01361	0.07874

146.11321	0.07875

146.21281	0.07875

146.31241	0.07876

146.41201	0.07877

146.51161	0.0788

146.61121	0.07885

146.71081	0.07896

146.81041	0.07897

146.91001	0.07905

147.00961	0.07912

147.10921	0.07916

147.20881	0.07917

147.30841	0.07918

147.40801	0.07919

147.50761	0.07921

147.60721	0.07922

147.70681	0.07923

147.80641	0.07927

147.90601	0.07927

148.00561	0.07944

148.10521	0.07955

148.20481	0.07956

148.30441	0.07958

148.40401	0.07963

148.50361	0.07965

148.60321	0.07965

148.70281	0.07967

148.80241	0.07977

148.90201	0.07977

149.00161	0.07978

149.10121	0.07987

149.20081	0.07988

149.30041	0.07991

149.40001	0.08002

149.49961	0.08003

149.59921	0.08004

149.69881	0.08008

149.79841	0.08012

149.89801	0.08012

149.99761	0.08016

150.09721	0.08022

150.19681	0.08032

150.29641	0.08035

150.39601	0.08037

150.49561	0.08038

150.59521	0.08039

150.69481	0.08048

150.79441	0.08048

150.89401	0.08051

150.99361	0.08056

151.09321	0.08059

151.19281	0.08067

151.29241	0.08082

151.39201	0.08089

151.49161	0.08105

151.59121	0.08136

151.69081	0.08142

151.79041	0.08143

151.89001	0.08146

151.98961	0.08149

152.08921	0.0815

152.18881	0.0815

152.28841	0.0816

152.38801	0.08162

152.48761	0.08173

152.58721	0.08174

152.68681	0.08174

152.78641	0.0818

152.88601	0.0818

152.98561	0.08187

153.08521	0.08191

153.18481	0.08193

153.28441	0.08199

153.38401	0.082

153.48361	0.08204

153.58321	0.08206

153.68281	0.08207

153.78241	0.08211

153.88201	0.0822

153.98161	0.08223

154.08121	0.08224

154.18081	0.08233

154.28041	0.08233

154.38001	0.08247

154.47961	0.08248

154.57921	0.08252

154.67881	0.08271

154.77841	0.08272

154.87801	0.08274

154.97761	0.08281

155.07721	0.08283

155.17681	0.08284

155.27641	0.08285

155.37601	0.08291

155.47561	0.08297

155.57521	0.08299

155.67481	0.08302

155.77441	0.08304

155.87401	0.08305

155.97361	0.08319

156.07321	0.08321

156.17281	0.08321

156.27241	0.08335

156.37201	0.08336

156.47161	0.08338

156.57121	0.08343

156.67081	0.08345

156.77041	0.08346

156.87001	0.08347

156.96961	0.0835

157.06921	0.08357

157.16881	0.08357

157.26841	0.08359

157.36801	0.0836

157.46761	0.08365

157.56721	0.0837

157.66681	0.08372

157.76641	0.08378

157.86601	0.08378

157.96561	0.08395

158.06521	0.084

158.16481	0.08401

158.26441	0.08402

158.36401	0.08402

158.46361	0.08403

158.56321	0.08409

158.66281	0.08414

158.76241	0.08415

158.86201	0.08415

158.96161	0.08417

159.06121	0.0842

159.16081	0.08433

159.26041	0.08441

159.36001	0.08442

159.45961	0.0845

159.55921	0.08451

159.65881	0.08457

159.75841	0.08459

159.85801	0.0846

159.95761	0.08461

160.05721	0.08465

160.15681	0.08466

160.25641	0.08466

160.35601	0.08476

160.45561	0.08481

160.55521	0.08483

160.65481	0.08484

160.75441	0.08484

160.85401	0.08488

160.95361	0.08492

161.05321	0.08492

161.15281	0.08493

161.25241	0.08493

161.35201	0.08496

161.45161	0.08497

161.55121	0.085

161.65081	0.085

161.75041	0.08503

161.85001	0.08503

161.94961	0.08521

162.04921	0.08523

162.14881	0.08526

162.24841	0.08553

162.34801	0.08574

162.44761	0.08578

162.54721	0.08611

162.64681	0.08612

162.74641	0.08622

162.84601	0.08632

162.94561	0.08632

163.04521	0.08637

163.14481	0.08638

163.24441	0.0864

163.34401	0.08642

163.44361	0.08646

163.54321	0.08648

163.64281	0.08656

163.74241	0.08658

163.84201	0.08674

163.94161	0.08675

164.04121	0.08676

164.14081	0.08676

164.24041	0.08685

164.34001	0.08685

164.43961	0.08686

164.53921	0.08691

164.63881	0.08691

164.73841	0.08718

164.83801	0.08718

164.93761	0.08726

165.03721	0.08726

165.13681	0.08727

165.23641	0.08727

165.33601	0.08729

165.43561	0.08734

165.53521	0.08735

165.63481	0.08736

165.73441	0.08752

165.83401	0.08753

165.93361	0.08765

166.03321	0.08774

166.13281	0.08775

166.23241	0.08775

166.33201	0.08777

166.43161	0.08783

166.53121	0.08788

166.63081	0.08789

166.73041	0.08789

166.83001	0.08791

166.92961	0.08792

167.02921	0.08792

167.12881	0.08793

167.22841	0.08805

167.32801	0.08811

167.42761	0.08819

167.52721	0.08819

167.62681	0.08821

167.72641	0.08827

167.82601	0.08828

167.92561	0.0883

168.02521	0.08835

168.12481	0.08836

168.22441	0.08847

168.32401	0.08855

168.42361	0.08857

168.52321	0.08858

168.62281	0.08893

168.72241	0.08897

168.82201	0.08902

168.92161	0.08903

169.02121	0.08904

169.12081	0.08932

169.22041	0.08933

169.32001	0.08939

169.41961	0.08939

169.51921	0.0894

169.61881	0.08971

169.71841	0.08972

169.81801	0.08973

169.91761	0.08988

170.01721	0.08993

170.11681	0.09004

170.21641	0.09011

170.31601	0.09019

170.41561	0.09019

170.51521	0.09028

170.61481	0.09029

170.71441	0.0903

170.81401	0.09038

170.91361	0.09041

171.01321	0.09055

171.11281	0.09057

171.21241	0.09075

171.31201	0.09077

171.41161	0.09082

171.51121	0.09128

171.61081	0.09129

171.71041	0.09129

171.81001	0.09132

171.90961	0.09142

172.00921	0.09142

172.10881	0.09167

172.20841	0.092

172.30801	0.09201

172.40761	0.09202

172.50721	0.09203

172.60681	0.09203

172.70641	0.09204

172.80601	0.09204

172.90561	0.09205

173.00521	0.09205

173.10481	0.09206

173.20441	0.09211

173.30401	0.09212

173.40361	0.09222

173.50321	0.09224

173.60281	0.09225

173.70241	0.09237

173.80201	0.09249

173.90161	0.09251

174.00121	0.09288

174.10081	0.0929

174.20041	0.09291

174.30001	0.09292

174.39961	0.09293

174.49921	0.09295

174.59881	0.09297

174.69841	0.093

174.79801	0.09314

174.89761	0.09317

174.99721	0.09319

175.09681	0.09325

175.19641	0.09328

175.29601	0.09336

175.39561	0.09337

175.49521	0.09356

175.59481	0.09357

175.69441	0.09365

175.79401	0.09366

175.89361	0.09366

175.99321	0.09374

176.09281	0.09376

176.19241	0.09377

176.29201	0.09378

176.39161	0.09379

176.49121	0.09381

176.59081	0.09398

176.69041	0.09398

176.79001	0.09407

176.88961	0.09407

176.98921	0.09421

177.08881	0.09423

177.18841	0.09439

177.28801	0.0944

177.38761	0.09462

177.48721	0.09464

177.58681	0.09466

177.68641	0.0947

177.78601	0.09472

177.88561	0.09474

177.98521	0.09483

178.08481	0.09487

178.18441	0.09488

178.28401	0.09489

178.38361	0.09501

178.48321	0.09512

178.58281	0.09518

178.68241	0.09519

178.78201	0.09528

178.88161	0.09536

178.98121	0.09556

179.08081	0.09557

179.18041	0.09577

179.28001	0.0958

179.37961	0.09618

179.47921	0.09618

179.57881	0.09619

179.67841	0.09619

179.77801	0.09632

179.87761	0.09633

179.97721	0.0965

180.07681	0.09663

180.17641	0.09663

180.27601	0.09678

180.37561	0.09683

180.47521	0.09689

180.57481	0.09697

180.67441	0.09698

180.77401	0.09715

180.87361	0.09716

180.97321	0.0972

181.07281	0.0972

181.17241	0.09722

181.27201	0.09723

181.37161	0.09736

181.47121	0.09756

181.57081	0.09757

181.67041	0.09766

181.77001	0.0977

181.86961	0.09771

181.96921	0.09777

182.06881	0.0978

182.16841	0.09791

182.26801	0.09792

182.36761	0.09796

182.46721	0.09802

182.56681	0.09802

182.66641	0.09808

182.76601	0.09811

182.86561	0.09817

182.96521	0.09823

183.06481	0.09836

183.16441	0.0984

183.26401	0.09841

183.36361	0.09843

183.46321	0.09844

183.56281	0.09849

183.66241	0.09851

183.76201	0.09853

183.86161	0.09853

183.96121	0.09854

184.06081	0.09876

184.16041	0.09877

184.26001	0.09877

184.35961	0.0989

184.45921	0.09895

184.55881	0.09896

184.65841	0.09905

184.75801	0.09906

184.85761	0.09916

184.95721	0.09916

185.05681	0.09917

185.15641	0.09929

185.25601	0.0993

185.35561	0.09932

185.45521	0.09943

185.55481	0.09943

185.65441	0.09945

185.75401	0.09946

185.85361	0.09962

185.95321	0.09962

186.05281	0.09968

186.15241	0.09977

186.25201	0.09997

186.35161	0.09998

186.45121	0.10002

186.55081	0.10006

186.65041	0.10007

186.75001	0.10008

186.84961	0.10018

186.94921	0.10039

187.04881	0.10042

187.14841	0.10043

187.24801	0.10044

187.34761	0.10045

187.44721	0.10066

187.54681	0.10067

187.64641	0.10068

187.74601	0.10074

187.84561	0.10075

187.94521	0.10075

188.04481	0.10076

188.14441	0.10094

188.24401	0.10134

188.34361	0.10159

188.44321	0.10159

188.54281	0.10159

188.64241	0.10163

188.74201	0.10171

188.84161	0.10185

188.94121	0.1019

189.04081	0.10205

189.14041	0.10209

189.24001	0.10212

189.33961	0.10213

189.43921	0.10218

189.53881	0.10224

189.63841	0.10226

189.73801	0.10229

189.83761	0.10232

189.93721	0.10233

190.03681	0.10236

190.13641	0.10237

190.23601	0.1024

190.33561	0.1024

190.43521	0.10241

190.53481	0.10242

190.63441	0.10245

190.73401	0.10247

190.83361	0.10248

190.93321	0.1025

191.03281	0.10258

191.13241	0.10265

191.23201	0.10271

191.33161	0.1029

191.43121	0.1029

191.53081	0.10291

191.63041	0.10313

191.73001	0.10314

191.82961	0.10315

191.92921	0.1032

192.02881	0.1032

192.12841	0.1032

192.22801	0.10321

192.32761	0.10335

192.42721	0.1036

192.52681	0.10361

192.62641	0.10369

192.72601	0.10408

192.82561	0.10412

192.92521	0.10421

193.02481	0.10422

193.12441	0.10423

193.22401	0.10424

193.32361	0.10436

193.42321	0.10448

193.52281	0.10448

193.62241	0.10457

193.72201	0.10464

193.82161	0.10465

193.92121	0.10465

194.02081	0.10489

194.12041	0.1049

194.22001	0.10491

194.31961	0.10499

194.41921	0.10501

194.51881	0.10502

194.61841	0.10504

194.71801	0.10512

194.81761	0.10515

194.91721	0.10518

195.01681	0.1052

195.11641	0.10532

195.21601	0.10532

195.31561	0.10573

195.41521	0.10574

195.51481	0.10574

195.61441	0.10584

195.71401	0.106

195.81361	0.1061

195.91321	0.10617

196.01281	0.10619

196.11241	0.1062

196.21201	0.10636

196.31161	0.10636

196.41121	0.10664

196.51081	0.10666

196.61041	0.10671

196.71001	0.10684

196.80961	0.10684

196.90921	0.10695

197.00881	0.10698

197.10841	0.10704

197.20801	0.10715

197.30761	0.10716

197.40721	0.1072

197.50681	0.10723

197.60641	0.10764

197.70601	0.10765

197.80561	0.10766

197.90521	0.10776

198.00481	0.108

198.10441	0.10801

198.20401	0.10819

198.30361	0.10822

198.40321	0.10828

198.50281	0.10828

198.60241	0.10836

198.70201	0.10838

198.80161	0.10839

198.90121	0.1084

199.00081	0.10843

199.10041	0.10848

199.20001	0.10858

199.29961	0.10864

199.39921	0.10867

199.49881	0.10867

199.59841	0.10868

199.69801	0.10868

199.79761	0.10869

199.89721	0.1087

199.99681	0.10877

200.09641	0.10877

200.19601	0.10886

200.29561	0.10892

200.39521	0.10895

200.49481	0.10903

200.59441	0.10906

200.69401	0.10908

200.79361	0.1091

200.89321	0.1091

200.99281	0.10911

201.09241	0.10912

201.19201	0.10919

201.29161	0.1092

201.39121	0.10926

201.49081	0.10926

201.59041	0.1093

201.69001	0.10935

201.78961	0.10936

201.88921	0.10937

201.98881	0.1095

202.08841	0.10962

202.18801	0.1097

202.28761	0.10971

202.38721	0.10979

202.48681	0.10981

202.58641	0.10983

202.68601	0.10991

202.78561	0.11001

202.88521	0.11007

202.98481	0.11015

203.08441	0.11018

203.18401	0.11022

203.28361	0.11023

203.38321	0.11047

203.48281	0.11051

203.58241	0.11054

203.68201	0.11055

203.78161	0.11056

203.88121	0.11059

203.98081	0.11063

204.08041	0.11063

204.18001	0.11064

204.27961	0.11065

204.37921	0.11071

204.47881	0.11079

204.57841	0.1108

204.67801	0.11082

204.77761	0.11084

204.87721	0.11123

204.97681	0.11128

205.07641	0.11141

205.17601	0.11143

205.27561	0.11144

205.37521	0.11147

205.47481	0.11155

205.57441	0.11163

205.67401	0.11168

205.77361	0.1117

205.87321	0.1117

205.97281	0.11174

206.07241	0.11176

206.17201	0.11177

206.27161	0.11178

206.37121	0.11181

206.47081	0.11183

206.57041	0.11183

206.67001	0.1119

206.76961	0.11191

206.86921	0.11192

206.96881	0.11194

207.06841	0.11196

207.16801	0.11202

207.26761	0.11203

207.36721	0.11205

207.46681	0.11211

207.56641	0.11216

207.66601	0.11218

207.76561	0.11222

207.86521	0.11223

207.96481	0.11235

208.06441	0.11252

208.16401	0.11253

208.26361	0.11253

208.36321	0.11256

208.46281	0.11274

208.56241	0.11275

208.66201	0.11277

208.76161	0.11279

208.86121	0.11293

208.96081	0.11295

209.06041	0.1133

209.16001	0.11335

209.25961	0.11336

209.35921	0.11336

209.45881	0.11344

209.55841	0.11344

209.65801	0.11355

209.75761	0.11356

209.85721	0.11361

209.95681	0.11362

210.05641	0.11367

210.15601	0.11368

210.25561	0.11371

210.35521	0.11372

210.45481	0.11372

210.55441	0.11377

210.65401	0.11379

210.75361	0.11379

210.85321	0.11384

210.95281	0.11384

211.05241	0.11386

211.15201	0.11399

211.25161	0.11401

211.35121	0.11405

211.45081	0.11406

211.55041	0.11418

211.65001	0.11423

211.74961	0.11423

211.84921	0.11424

211.94881	0.11438

212.04841	0.11439

212.14801	0.11455

212.24761	0.11456

212.34721	0.11458

212.44681	0.11465

212.54641	0.11475

212.64601	0.11476

212.74561	0.11477

212.84521	0.11481

212.94481	0.11491

213.04441	0.11493

213.14401	0.11501

213.24361	0.11505

213.34321	0.11506

213.44281	0.1151

213.54241	0.11511

213.64201	0.11512

213.74161	0.11512

213.84121	0.11513

213.94081	0.11519

214.04041	0.11521

214.14001	0.11529

214.23961	0.1153

214.33921	0.1153

214.43881	0.11535

214.53841	0.11538

214.63801	0.11539

214.73761	0.11541

214.83721	0.11542

214.93681	0.11543

215.03641	0.11544

215.13601	0.11559

215.23561	0.11559

215.33521	0.11563

215.43481	0.11573

215.53441	0.11577

215.63401	0.11578

215.73361	0.1158

215.83321	0.11589

215.93281	0.11591

216.03241	0.11592

216.13201	0.11602

216.23161	0.11633

216.33121	0.11634

216.43081	0.11643

216.53041	0.11644

216.63001	0.11645

216.72961	0.11647

216.82921	0.11651

216.92881	0.11653

217.02841	0.11653

217.12801	0.11655

217.22761	0.11655

217.32721	0.11656

217.42681	0.11672

217.52641	0.11673

217.62601	0.1168

217.72561	0.11688

217.82521	0.11689

217.92481	0.1169

218.02441	0.11696

218.12401	0.11698

218.22361	0.11707

218.32321	0.11713

218.42281	0.11714

218.52241	0.11715

218.62201	0.11724

218.72161	0.11725

218.82121	0.11726

218.92081	0.11727

219.02041	0.1174

219.12001	0.11745

219.21961	0.11745

219.31921	0.11755

219.41881	0.11764

219.51841	0.11765

219.61801	0.11767

219.71761	0.11775

219.81721	0.1178

219.91681	0.1179

220.01641	0.11792

220.11601	0.11793

220.21561	0.11794

220.31521	0.11794

220.41481	0.11796

220.51441	0.11797

220.61401	0.11799

220.71361	0.11802

220.81321	0.11808

220.91281	0.11812

221.01241	0.11814

221.11201	0.11815

221.21161	0.11819

221.31121	0.11824

221.41081	0.11825

221.51041	0.11829

221.61001	0.11829

221.70961	0.11835

221.80921	0.11836

221.90881	0.11847

222.00841	0.11854

222.10801	0.11867

222.20761	0.11875

222.30721	0.11876

222.40681	0.11878

222.50641	0.11882

222.60601	0.11887

222.70561	0.11888

222.80521	0.11894

222.90481	0.11902

223.00441	0.1191

223.10401	0.11914

223.20361	0.1194

223.30321	0.11942

223.40281	0.11943

223.50241	0.11944

223.60201	0.11948

223.70161	0.11949

223.80121	0.1195

223.90081	0.11963

224.00041	0.11965

224.10001	0.12023

224.19961	0.12046

224.29921	0.1205

224.39881	0.12067

224.49841	0.12071

224.59801	0.12072

224.69761	0.12073

224.79721	0.12075

224.89681	0.12078

224.99641	0.12088

225.09601	0.12094

225.19561	0.12098

225.29521	0.12108

225.39481	0.12124

225.49441	0.12127

225.59401	0.1213

225.69361	0.12134

225.79321	0.12136

225.89281	0.12138

225.99241	0.12142

226.09201	0.12146

226.19161	0.12148

226.29121	0.12149

226.39081	0.12161

226.49041	0.12174

226.59001	0.12177

226.68961	0.12181

226.78921	0.1219

226.88881	0.12192

226.98841	0.12207

227.08801	0.12214

227.18761	0.12232

227.28721	0.12238

227.38681	0.12239

227.48641	0.12242

227.58601	0.12243

227.68561	0.1225

227.78521	0.12272

227.88481	0.12273

227.98441	0.12283

228.08401	0.12285

228.18361	0.12295

228.28321	0.12316

228.38281	0.12317

228.48241	0.12318

228.58201	0.12328

228.68161	0.12329

228.78121	0.1234

228.88081	0.12357

228.98041	0.12359

229.08001	0.12362

229.17961	0.12367

229.27921	0.12369

229.37881	0.1237

229.47841	0.12372

229.57801	0.12373

229.67761	0.12375

229.77721	0.12377

229.87681	0.12398

229.97641	0.12399

230.07601	0.12424

230.17561	0.12439

230.27521	0.12442

230.37481	0.12445

230.47441	0.12463

230.57401	0.12469

230.67361	0.1247

230.77321	0.12471

230.87281	0.12482

230.97241	0.12484

231.07201	0.12499

231.17161	0.12502

231.27121	0.12519

231.37081	0.1253

231.47041	0.12534

231.57001	0.12544

231.66961	0.12551

231.76921	0.12552

231.86881	0.12565

231.96841	0.12574

232.06801	0.1258

232.16761	0.12581

232.26721	0.12582

232.36681	0.12584

232.46641	0.12585

232.56601	0.12586

232.66561	0.12591

232.76521	0.12593

232.86481	0.12596

232.96441	0.12598

233.06401	0.12601

233.16361	0.12606

233.26321	0.12609

233.36281	0.12623

233.46241	0.12629

233.56201	0.1263

233.66162	0.12633

233.76121	0.12639

233.86082	0.12642

233.96042	0.12643

234.06001	0.12646

234.15962	0.1265

234.25922	0.12661

234.35881	0.12669

234.45842	0.12672

234.55802	0.12674

234.65761	0.12682

234.75722	0.12691

234.85682	0.12701

234.95641	0.12717

235.05602	0.12718

235.15562	0.12742

235.25522	0.12753

235.35482	0.12756

235.45442	0.12757

235.55402	0.12759

235.65362	0.12775

235.75322	0.12779

235.85282	0.1278

235.95242	0.12789

236.05202	0.1279

236.15162	0.12792

236.25122	0.12793

236.35082	0.12802

236.45042	0.12815

236.55002	0.12816

236.64962	0.12821

236.74922	0.12822

236.84882	0.12841

236.94842	0.12849

237.04802	0.12901

237.14762	0.12902

237.24722	0.12908

237.34682	0.12917

237.44642	0.12919

237.54602	0.1292

237.64562	0.12934

237.74522	0.12935

237.84482	0.12944

237.94442	0.12946

238.04402	0.12973

238.14362	0.12974

238.24322	0.12977

238.34282	0.1298

238.44242	0.12981

238.54202	0.12993

238.64162	0.12996

238.74122	0.13002

238.84082	0.13006

238.94042	0.1301

239.04002	0.13012

239.13962	0.13018

239.23922	0.1302

239.33882	0.13023

239.43842	0.13027

239.53802	0.13028

239.63762	0.1303

239.73722	0.13032

239.83682	0.13036

239.93642	0.13041

240.03602	0.13044

240.13562	0.13045

240.23522	0.13046

240.33482	0.13047

240.43442	0.13049

240.53402	0.13053

240.63362	0.13056

240.73322	0.13076

240.83282	0.13081

240.93242	0.1309

241.03202	0.13099

241.13162	0.131

241.23122	0.13135

241.33082	0.13136

241.43042	0.13143

241.53002	0.13148

241.62962	0.13153

241.72922	0.1316

241.82882	0.13167

241.92842	0.13169

242.02802	0.1317

242.12762	0.13172

242.22722	0.13198

242.32682	0.13203

242.42642	0.13204

242.52602	0.13206

242.62562	0.1321

242.72522	0.13216

242.82482	0.13218

242.92442	0.13226

243.02402	0.13227

243.12362	0.13228

243.22322	0.1323

243.32282	0.13231

243.42242	0.13239

243.52202	0.1325

243.62162	0.13251

243.72122	0.13252

243.82082	0.13256

243.92042	0.13258

244.02002	0.1326

244.11962	0.1327

244.21922	0.13271

244.31882	0.13276

244.41842	0.13277

244.51802	0.13279

244.61762	0.13281

244.71722	0.13282

244.81682	0.13288

244.91642	0.13293

245.01602	0.13296

245.11562	0.13298

245.21522	0.13299

245.31482	0.13301

245.41442	0.13303

245.51402	0.13313

245.61362	0.13315

245.71322	0.13374

245.81282	0.13378

245.91242	0.13384

246.01202	0.13385

246.11162	0.13386

246.21122	0.1339

246.31082	0.13402

246.41042	0.13407

246.51002	0.13412

246.60962	0.13414

246.70922	0.13415

246.80882	0.13417

246.90842	0.13422

247.00802	0.13423

247.10762	0.13428

247.20722	0.1343

247.30682	0.13431

247.40642	0.13432

247.50602	0.13452

247.60562	0.13454

247.70522	0.13456

247.80482	0.13457

247.90442	0.13464

248.00402	0.13465

248.10362	0.13485

248.20322	0.13494

248.30282	0.13505

248.40242	0.13506

248.50202	0.13507

248.60162	0.13509

248.70122	0.13512

248.80082	0.13513

248.90042	0.13525

249.00002	0.13526

249.09962	0.13527

249.19922	0.1353

249.29882	0.13574

249.39842	0.13576

249.49802	0.13577

249.59762	0.13579

249.69722	0.13585

249.79682	0.13587

249.89642	0.1359

249.99602	0.13591

250.09562	0.13595

250.19522	0.13596

250.29482	0.13598

250.39442	0.13601

250.49402	0.13606

250.59362	0.13607

250.69322	0.13608

250.79282	0.13609

250.89242	0.13611

250.99202	0.13619

251.09162	0.1364

251.19122	0.13642

251.29082	0.13652

251.39042	0.13654

251.49002	0.13658

251.58962	0.1366

251.68922	0.13663

251.78882	0.13672

251.88842	0.13673

251.98802	0.13676

252.08762	0.13677

252.18722	0.13681

252.28682	0.13683

252.38642	0.13687

252.48602	0.13688

252.58562	0.13691

252.68522	0.13693

252.78482	0.13697

252.88442	0.13698

252.98402	0.13703

253.08362	0.13721

253.18322	0.13722

253.28282	0.13731

253.38242	0.13732

253.48202	0.13739

253.58162	0.13743

253.68122	0.13748

253.78082	0.13749

253.88042	0.13759

253.98002	0.13767

254.07962	0.13769

254.17922	0.13774

254.27882	0.13777

254.37842	0.13781

254.47802	0.13781

254.57762	0.13804

254.67722	0.13811

254.77682	0.13812

254.87642	0.13828

254.97602	0.13831

255.07562	0.13832

255.17522	0.13835

255.27482	0.13839

255.37442	0.13844

255.47402	0.13862

255.57362	0.13863

255.67322	0.13865

255.77282	0.13867

255.87242	0.13873

255.97202	0.13889

256.07162	0.1389

256.17122	0.13892

256.27082	0.13893

256.37042	0.13902

256.47002	0.13904

256.56962	0.13931

256.66922	0.13938

256.76882	0.1394

256.86842	0.13942

256.96802	0.13946

257.06762	0.13947

257.16722	0.13953

257.26682	0.13954

257.36642	0.13955

257.46602	0.13961

257.56562	0.13964

257.66522	0.13968

257.76482	0.13972

257.86442	0.13975

257.96402	0.13977

258.06362	0.1398

258.16322	0.13981

258.26282	0.13984

258.36242	0.13985

258.46202	0.13986

258.56162	0.13988

258.66122	0.13994

258.76082	0.13995

258.86042	0.13997

258.96002	0.13999

259.05962	0.14001

259.15922	0.14002

259.25882	0.14004

259.35842	0.14006

259.45802	0.14007

259.55762	0.14008

259.65722	0.1401

259.75682	0.14014

259.85642	0.14017

259.95602	0.14024

260.05562	0.14025

260.15522	0.14026

260.25482	0.14032

260.35442	0.14033

260.45402	0.14041

260.55362	0.14042

260.65322	0.14045

260.75282	0.14046

260.85242	0.14047

260.95202	0.14048

261.05162	0.1405

261.15122	0.14057

261.25082	0.14059

261.35042	0.14061

261.45002	0.14066

261.54962	0.14071

261.64922	0.14072

261.74882	0.14077

261.84842	0.14078

261.94802	0.1408

262.04762	0.14082

262.14722	0.14085

262.24682	0.14086

262.34642	0.14088

262.44602	0.14089

262.54562	0.1409

262.64522	0.14097

262.74482	0.14099

262.84442	0.14103

262.94402	0.14104

263.04362	0.14104

263.14322	0.14107

263.24282	0.14126

263.34242	0.14195

263.44202	0.14209

263.54162	0.14213

263.64122	0.14214

263.74082	0.14235

263.84042	0.14239

263.94002	0.14246

264.03962	0.14249

264.13922	0.14251

264.23882	0.14253

264.33842	0.14255

264.43802	0.14256

264.53762	0.14257

264.63722	0.14259

264.73682	0.1426

264.83642	0.14263

264.93602	0.14267

265.03562	0.14271

265.13522	0.14272

265.23482	0.14278

265.33442	0.14279

265.43402	0.1428

265.53362	0.14281

265.63322	0.14282

265.73282	0.14287

265.83242	0.14336

265.93202	0.14343

266.03162	0.14344

266.13122	0.14358

266.23082	0.1436

266.33042	0.14362

266.43002	0.14366

266.52962	0.14368

266.62922	0.14371

266.72882	0.14376

266.82842	0.14378

266.92802	0.14382

267.02762	0.14384

267.12722	0.14387

267.22682	0.14392

267.32642	0.14395

267.42602	0.14396

267.52562	0.14398

267.62522	0.144

267.72482	0.14421

267.82442	0.14427

267.92402	0.14474

268.02362	0.14475

268.12322	0.14488

268.22282	0.14497

268.32242	0.14498

268.42202	0.14499

268.52162	0.145

268.62122	0.14501

268.72082	0.14519

268.82042	0.14521

268.92002	0.14525

269.01962	0.14531

269.11922	0.14535

269.21882	0.14555

269.31842	0.1456

269.41802	0.14561

269.51762	0.14562

269.61722	0.14574

269.71682	0.14577

269.81642	0.14579

269.91602	0.1458

270.01562	0.14596

270.11522	0.14604

270.21482	0.14608

270.31442	0.14611

270.41402	0.14615

270.51362	0.14616

270.61322	0.14618

270.71282	0.14622

270.81242	0.14632

270.91202	0.14637

271.01162	0.14638

271.11122	0.14639

271.21082	0.1465

271.31042	0.14654

271.41002	0.14655

271.50962	0.14658

271.60922	0.1466

271.70882	0.14661

271.80842	0.14663

271.90802	0.14666

272.00762	0.14669

272.10722	0.14692

272.20682	0.14694

272.30642	0.14696

272.40602	0.14715

272.50562	0.14716

272.60522	0.14721

272.70482	0.14725

272.80442	0.14726

272.90402	0.14732

273.00362	0.14733

273.10322	0.14734

273.20282	0.14749

273.30242	0.1475

273.40202	0.14751

273.50162	0.14752

273.60122	0.14753

273.70082	0.14757

273.80042	0.14761

273.90002	0.14768

273.99962	0.14779

274.09922	0.1478

274.19882	0.14788

274.29842	0.14789

274.39802	0.14791

274.49762	0.14792

274.59722	0.14793

274.69682	0.14809

274.79642	0.1481

274.89602	0.14813

274.99562	0.14818

275.09522	0.14819

275.19482	0.14823

275.29442	0.14831

275.39402	0.14879

275.49362	0.14895

275.59322	0.14897

275.69282	0.14898

275.79242	0.14899

275.89202	0.14902

275.99162	0.14906

276.09122	0.14908

276.19082	0.14914

276.29042	0.1492

276.39002	0.14925

276.48962	0.1493

276.58922	0.14931

276.68882	0.14932

276.78842	0.14933

276.88802	0.14935

276.98762	0.14938

277.08722	0.14966

277.18682	0.14969

277.28642	0.14988

277.38602	0.14999

277.48562	0.15004

277.58522	0.15009

277.68482	0.1501

277.78442	0.15015

277.88402	0.15016

277.98362	0.1502

278.08322	0.15023

278.18282	0.15026

278.28242	0.15027

278.38202	0.15028

278.48162	0.1503

278.58122	0.15034

278.68082	0.15038

278.78042	0.15039

278.88002	0.15046

278.97962	0.15055

279.07922	0.15057

279.17882	0.15059

279.27842	0.15068

279.37802	0.15069

279.47762	0.15072

279.57722	0.15081

279.67682	0.15082

279.77642	0.15083

279.87602	0.15084

279.97562	0.15098

280.07522	0.15099

280.17482	0.151

280.27442	0.15103

280.37402	0.15108

280.47362	0.1511

280.57322	0.15114

280.67282	0.15114

280.77242	0.15115

280.87202	0.15117

280.97162	0.1512

281.07122	0.15122

281.17082	0.15124

281.27042	0.15127

281.37002	0.15127

281.46962	0.15136

281.56922	0.15139

281.66882	0.15141

281.76842	0.15142

281.86802	0.15144

281.96762	0.15145

282.06722	0.15152

282.16682	0.15154

282.26642	0.15155

282.36602	0.15156

282.46562	0.15158

282.56522	0.15162

282.66482	0.15163

282.76442	0.15176

282.86402	0.15177

282.96362	0.15178

283.06322	0.15191

283.16282	0.15195

283.26242	0.15195

283.36202	0.15206

283.46162	0.15208

283.56122	0.15209

283.66082	0.15211

283.76042	0.15212

283.86002	0.15221

283.95962	0.15227

284.05922	0.1523

284.15882	0.15233

284.25842	0.15235

284.35802	0.15235

284.45762	0.15236

284.55722	0.15237

284.65682	0.15239

284.75642	0.1524

284.85602	0.15244

284.95562	0.15262

285.05522	0.15262

285.15482	0.15264

285.25442	0.15265

285.35402	0.15266

285.45362	0.1527

285.55322	0.15271

285.65282	0.15272

285.75242	0.15279

285.85202	0.15327

285.95162	0.15328

286.05122	0.15329

286.15082	0.15329

286.25042	0.1533

286.35002	0.15332

286.44962	0.15336

286.54922	0.15341

286.64882	0.15343

286.74842	0.15347

286.84802	0.1536

286.94762	0.15362

287.04722	0.15364

287.14682	0.15365

287.24642	0.15366

287.34602	0.15372

287.44562	0.15374

287.54522	0.15375

287.64482	0.15382

287.74442	0.15383

287.84402	0.15386

287.94362	0.1539

288.04322	0.15391

288.14282	0.15392

288.24242	0.15393

288.34202	0.15399

288.44162	0.154

288.54122	0.15401

288.64082	0.15402

288.74042	0.15405

288.84002	0.15408

288.93962	0.1541

289.03922	0.15418

289.13882	0.15426

289.23842	0.15432

289.33802	0.15434

289.43762	0.15446

289.53722	0.15448

289.63682	0.1545

289.73642	0.1545

289.83602	0.15452

289.93562	0.15465

290.03522	0.15466

290.13482	0.15469

290.23442	0.15471

290.33402	0.15478

290.43362	0.15483

290.53322	0.15486

290.63282	0.15488

290.73242	0.15491

290.83202	0.15496

290.93162	0.15497

291.03122	0.15498

291.13082	0.15506

291.23042	0.15508

291.33002	0.15509

291.42962	0.1551

291.52922	0.15522

291.62882	0.15526

291.72842	0.15528

291.82802	0.15532

291.92762	0.15539

292.02722	0.15542

292.12682	0.15546

292.22642	0.1555

292.32602	0.15552

292.42562	0.15606

292.52522	0.15608

292.62482	0.15613

292.72442	0.15614

292.82402	0.15618

292.92362	0.15621

293.02322	0.15624

293.12282	0.15625

293.22242	0.15625

293.32202	0.15634

293.42162	0.15645

293.52122	0.15652

293.62082	0.15652

293.72042	0.15654

293.82002	0.15655

293.91962	0.15656

294.01922	0.15658

294.11882	0.15659

294.21842	0.1566

294.31802	0.15665

294.41762	0.15666

294.51722	0.15669

294.61682	0.15675

294.71642	0.15683

294.81602	0.15706

294.91562	0.15707

295.01522	0.15712

295.11482	0.15713

295.21442	0.15714

295.31402	0.15715

295.41362	0.15727

295.51322	0.1573

295.61282	0.15731

295.71242	0.15737

295.81202	0.15738

295.91162	0.15745

296.01122	0.15746

296.11082	0.15767

296.21042	0.15769

296.31002	0.1577

296.40962	0.15772

296.50922	0.15776

296.60882	0.15777

296.70842	0.15778

296.80802	0.15792

296.90762	0.158

297.00722	0.15801

297.10682	0.1582

297.20642	0.15823

297.30602	0.15824

297.40562	0.15827

297.50522	0.15829

297.60482	0.1583

297.70442	0.15837

297.80402	0.15838

297.90362	0.15841

298.00322	0.15849

298.10282	0.1585

298.20242	0.15854

298.30202	0.15867

298.40162	0.15869

298.50122	0.15871

298.60082	0.15873

298.70042	0.15876

298.80002	0.15877

298.89962	0.15878

298.99922	0.15888

299.09882	0.1589

299.19842	0.15895

299.29802	0.159

299.39762	0.15903

299.49722	0.15908

299.59682	0.15911

299.69642	0.15913

299.79602	0.15916

299.89562	0.15918

299.99522	0.1592

300.09482	0.15925

300.19442	0.15928

300.29402	0.15929

300.39362	0.1593

300.49322	0.15931

300.59282	0.15934

300.69242	0.15935

300.79202	0.15939

300.89162	0.15941

300.99122	0.15945

301.09082	0.15951

301.19042	0.15952

301.29002	0.15953

301.38962	0.15956

301.48922	0.15957

301.58882	0.15958

301.68842	0.15962

301.78802	0.15965

301.88762	0.15967

301.98722	0.15979

302.08682	0.1598

302.18642	0.15991

302.28602	0.15993

302.38562	0.15994

302.48522	0.16011

302.58482	0.16012

302.68442	0.16018

302.78402	0.16025

302.88362	0.16035

302.98322	0.16036

303.08282	0.16041

303.18242	0.16044

303.28202	0.16045

303.38162	0.16048

303.48122	0.16062

303.58082	0.16074

303.68042	0.16075

303.78002	0.16078

303.87962	0.16084

303.97922	0.16085

304.07882	0.16089

304.17842	0.1609

304.27802	0.16095

304.37762	0.16105

304.47722	0.16108

304.57682	0.16111

304.67642	0.16113

304.77602	0.16114

304.87562	0.16118

304.97522	0.16119

305.07482	0.1612

305.17442	0.16121

305.27402	0.16122

305.37362	0.16124

305.47322	0.16124

305.57282	0.16126

305.67242	0.16127

305.77202	0.16136

305.87162	0.16137

305.97122	0.16137

306.07082	0.1614

306.17042	0.16141

306.27002	0.16142

306.36962	0.16145

306.46922	0.16146

306.56882	0.1615

306.66842	0.16151

306.76802	0.16153

306.86762	0.16156

306.96722	0.16163

307.06682	0.16164

307.16642	0.16164

307.26602	0.16166

307.36562	0.16193

307.46522	0.16194

307.56482	0.162

307.66442	0.16202

307.76402	0.16209

307.86362	0.16212

307.96322	0.16213

308.06282	0.16219

308.16242	0.16221

308.26202	0.16224

308.36162	0.16226

308.46122	0.16227

308.56082	0.1623

308.66042	0.16243

308.76002	0.16252

308.85962	0.16254

308.95922	0.16256

309.05882	0.1626

309.15842	0.16271

309.25802	0.16282

309.35762	0.16284

309.45722	0.16285

309.55682	0.16291

309.65642	0.16292

309.75602	0.16298

309.85562	0.16303

309.95522	0.16305

310.05482	0.16326

310.15442	0.16331

310.25402	0.16333

310.35362	0.16334

310.45322	0.16338

310.55282	0.16339

310.65242	0.16339

310.75202	0.16376

310.85162	0.16379

310.95122	0.16379

311.05082	0.1638

311.15042	0.16381

311.25002	0.16382

311.34962	0.16403

311.44922	0.16404

311.54882	0.16414

311.64842	0.16417

311.74802	0.16421

311.84762	0.16426

311.94722	0.16433

312.04682	0.16439

312.14642	0.1645

312.24602	0.16473

312.34562	0.16474

312.44522	0.16479

312.54482	0.16481

312.64442	0.16482

312.74402	0.1649

312.84362	0.16512

312.94322	0.16514

313.04282	0.16519

313.14242	0.16522

313.24202	0.16531

313.34162	0.16533

313.44122	0.1654

313.54082	0.16541

313.64042	0.16546

313.74002	0.1656

313.83962	0.16567

313.93922	0.16568

314.03882	0.16578

314.13842	0.16579

314.23802	0.1658

314.33762	0.16582

314.43722	0.16583

314.53682	0.16586

314.63642	0.16588

314.73602	0.16604

314.83562	0.16635

314.93522	0.16638

315.03482	0.16652

315.13442	0.16654

315.23402	0.16662

315.33362	0.16664

315.43322	0.16669

315.53282	0.1667

315.63242	0.16671

315.73202	0.16672

315.83162	0.16676

315.93122	0.1668

316.03082	0.16707

316.13042	0.16712

316.23002	0.16718

316.32962	0.16719

316.42922	0.16725

316.52882	0.16726

316.62842	0.16727

316.72802	0.16743

316.82762	0.16745

316.92722	0.16751

317.02682	0.16754

317.12642	0.16756

317.22602	0.16768

317.32562	0.1677

317.42522	0.1677

317.52482	0.16773

317.62442	0.16775

317.72402	0.16776

317.82362	0.16778

317.92322	0.16786

318.02282	0.16787

318.12242	0.1679

318.22202	0.16791

318.32162	0.16797

318.42122	0.16811

318.52082	0.16812

318.62042	0.16814

318.72002	0.16817

318.81962	0.16818

318.91922	0.16819

319.01882	0.1682

319.11842	0.16821

319.21802	0.16827

319.31762	0.16839

319.41722	0.16847

319.51682	0.16848

319.61642	0.16849

319.71602	0.16853

319.81562	0.16854

319.91522	0.16856

320.01482	0.16858

320.11442	0.16859

320.21402	0.1686

320.31362	0.16862

320.41322	0.16863

320.51282	0.16864

320.61242	0.16864

320.71202	0.16873

320.81162	0.16877

320.91122	0.16878

321.01082	0.16884

321.11042	0.16888

321.21002	0.16895

321.30962	0.16901

321.40922	0.16909

321.50882	0.1691

321.60842	0.16912

321.70802	0.16924

321.80762	0.16926

321.90722	0.16937

322.00682	0.16955

322.10642	0.16956

322.20602	0.16961

322.30562	0.16962

322.40522	0.16963

322.50482	0.16969

322.60442	0.16972

322.70402	0.16973

322.80362	0.16976

322.90322	0.16985

323.00282	0.16986

323.10242	0.16987

323.20202	0.16988

323.30162	0.1699

323.40122	0.16996

323.50082	0.16998

323.60042	0.16999

323.70002	0.16999

323.79962	0.17001

323.89922	0.17003

323.99882	0.17009

324.09842	0.1701

324.19802	0.17012

324.29762	0.17015

324.39722	0.1702

324.49682	0.17021

324.59642	0.17022

324.69602	0.17023

324.79562	0.17024

324.89522	0.17026

324.99482	0.17028

325.09442	0.1703

325.19402	0.17034

325.29362	0.17035

325.39322	0.17039

325.49282	0.17041

325.59242	0.17042

325.69202	0.17045

325.79162	0.17046

325.89122	0.17055

325.99082	0.1706

326.09042	0.17062

326.19002	0.17065

326.28962	0.17068

326.38922	0.17069

326.48882	0.1707

326.58842	0.17088

326.68802	0.17091

326.78762	0.17092

326.88722	0.17097

326.98682	0.17098

327.08642	0.17099

327.18602	0.171

327.28562	0.17104

327.38522	0.17105

327.48482	0.17116

327.58442	0.17117

327.68402	0.17119

327.78362	0.17121

327.88322	0.17122

327.98282	0.17123

328.08242	0.17125

328.18202	0.17129

328.28162	0.17131

328.38122	0.17133

328.48082	0.17133

328.58042	0.17135

328.68002	0.17142

328.77962	0.17152

328.87922	0.1716

328.97882	0.17161

329.07842	0.17163

329.17802	0.17165

329.27762	0.17168

329.37722	0.17182

329.47682	0.17184

329.57642	0.17187

329.67602	0.17188

329.77562	0.17189

329.87522	0.1719

329.97482	0.17191

330.07442	0.17206

330.17402	0.17207

330.27362	0.17208

330.37322	0.17214

330.47282	0.17216

330.57242	0.17218

330.67202	0.17222

330.77162	0.17227

330.87122	0.17228

330.97082	0.17229

331.07042	0.1723

331.17002	0.17232

331.26962	0.17239

331.36922	0.1724

331.46882	0.17241

331.56842	0.17241

331.66802	0.17244

331.76762	0.17261

331.86722	0.17263

331.96682	0.17267

332.06642	0.1727

332.16602	0.17297

332.26562	0.17299

332.36522	0.17301

332.46482	0.17307

332.56442	0.17311

332.66402	0.17325

332.76362	0.17326

332.86322	0.17328

332.96282	0.1733

333.06242	0.17331

333.16202	0.17334

333.26162	0.17336

333.36122	0.17351

333.46082	0.17352

333.56042	0.17357

333.66002	0.17358

333.75962	0.1736

333.85922	0.17361

333.95882	0.17363

334.05842	0.17364

334.15802	0.17388

334.25762	0.17389

334.35722	0.1739

334.45682	0.17391

334.55642	0.17393

334.65602	0.17399

334.75562	0.17404

334.85522	0.17405

334.95482	0.17406

335.05442	0.17408

335.15402	0.17409

335.25362	0.17414

335.35322	0.17418

335.45282	0.17423

335.55242	0.17426

335.65202	0.17431

335.75162	0.17433

335.85122	0.17435

335.95082	0.17442

336.05042	0.17451

336.15002	0.17498

336.24962	0.17499

336.34922	0.175

336.44882	0.17501

336.54842	0.17512

336.64802	0.17514

336.74762	0.17515

336.84722	0.17516

336.94682	0.17518

337.04642	0.17519

337.14602	0.1752

337.24562	0.17522

337.34522	0.17532

337.44482	0.17533

337.54442	0.17534

337.64402	0.17535

337.74362	0.17538

337.84322	0.17542

337.94282	0.17543

338.04242	0.17544

338.14202	0.17547

338.24162	0.17548

338.34122	0.1755

338.44082	0.17551

338.54042	0.17551

338.64002	0.17557

338.73962	0.17558

338.83922	0.1756

338.93882	0.17575

339.03842	0.17593

339.13802	0.17594

339.23762	0.17597

339.33722	0.176

339.43682	0.17611

339.53642	0.17612

339.63602	0.17614

339.73562	0.17615

339.83522	0.17616

339.93482	0.17618

340.03442	0.17621

340.13402	0.17622

340.23362	0.17632

340.33322	0.17641

340.43282	0.17642

340.53242	0.1765

340.63202	0.17652

340.73162	0.17653

340.83122	0.17655

340.93082	0.17668

341.03042	0.17669

341.13002	0.1767

341.22962	0.17701

341.32922	0.17705

341.42882	0.17707

341.52842	0.17708

341.62802	0.17712

341.72762	0.17716

341.82722	0.17717

341.92682	0.17735

342.02642	0.17744

342.12602	0.17745

342.22562	0.17748

342.32522	0.17755

342.42482	0.17756

342.52442	0.17757

342.62402	0.17758

342.72362	0.17759

342.82322	0.17761

342.92282	0.17763

343.02242	0.17766

343.12202	0.17767

343.22162	0.17779

343.32122	0.17826

343.42082	0.17836

343.52042	0.17837

343.62002	0.17841

343.71962	0.17847

343.81922	0.17847

343.91882	0.1786

344.01842	0.17862

344.11802	0.17863

344.21762	0.17864

344.31722	0.17867

344.41682	0.17868

344.51642	0.17874

344.61602	0.17887

344.71562	0.17889

344.81522	0.17891

344.91482	0.17892

345.01442	0.17895

345.11402	0.17898

345.21362	0.17899

345.31322	0.17901

345.41282	0.1791

345.51242	0.17912

345.61202	0.17914

345.71162	0.17914

345.81122	0.17916

345.91082	0.17917

346.01042	0.17918

346.11002	0.17919

346.20962	0.1792

346.30922	0.17921

346.40882	0.17922

346.50842	0.17923

346.60802	0.17928

346.70762	0.17933

346.80722	0.17934

346.90682	0.17937

347.00642	0.17944

347.10602	0.17945

347.20562	0.17946

347.30522	0.17955

347.40482	0.17955

347.50442	0.17958

347.60402	0.1796

347.70362	0.17961

347.80322	0.17962

347.90282	0.17963

348.00242	0.17969

348.10202	0.1797

348.20162	0.17974

348.30122	0.17975

348.40082	0.17981

348.50042	0.17982

348.60002	0.17986

348.69962	0.17987

348.79922	0.17989

348.89882	0.18009

348.99842	0.1801

349.09802	0.18012

349.19762	0.18014

349.29722	0.18022

349.39682	0.18022

349.49642	0.18024

349.59602	0.18025

349.69562	0.18026

349.79522	0.18027

349.89482	0.18037

349.99442	0.18042

350.09402	0.18057

350.19362	0.18059

350.29322	0.1806

350.39282	0.18061

350.49242	0.18063

350.59202	0.18074

350.69162	0.18076

350.79122	0.18079

350.89082	0.18082

350.99042	0.18084

351.09002	0.18085

351.18962	0.18089

351.28922	0.18089

351.38882	0.18101

351.48842	0.18103

351.58802	0.18104

351.68762	0.18106

351.78722	0.18107

351.88682	0.18109

351.98642	0.1811

352.08602	0.18143

352.18562	0.18147

352.28522	0.18149

352.38482	0.1815

352.48442	0.18153

352.58402	0.18158

352.68362	0.18159

352.78322	0.18177

352.88282	0.18178

352.98242	0.18179

353.08202	0.18182

353.18162	0.18184

353.28122	0.18185

353.38082	0.18186

353.48042	0.18191

353.58002	0.18193

353.67962	0.18195

353.77922	0.18196

353.87882	0.18197

353.97842	0.18202

354.07802	0.18206

354.17762	0.18211

354.27722	0.18212

354.37682	0.1822

354.47642	0.18221

354.57602	0.18226

354.67562	0.18227

354.77522	0.18228

354.87482	0.18229

354.97442	0.18233

355.07402	0.18238

355.17362	0.1824

355.27322	0.18244

355.37282	0.18245

355.47242	0.18246

355.57202	0.18247

355.67162	0.18248

355.77122	0.18261

355.87082	0.18264

355.97042	0.18264

356.07002	0.18269

356.16962	0.1827

356.26922	0.18273

356.36882	0.18275

356.46842	0.18276

356.56802	0.18281

356.66762	0.18282

356.76722	0.18288

356.86682	0.18291

356.96642	0.18294

357.06602	0.18301

357.16562	0.18327

357.26522	0.18332

357.36482	0.18332

357.46442	0.18337

357.56402	0.18347

357.66362	0.1835

357.76322	0.18355

357.86282	0.18356

357.96242	0.18357

358.06202	0.18359

358.16162	0.1836

358.26122	0.18363

358.36082	0.18364

358.46042	0.18367

358.56002	0.18374

358.65962	0.18377

358.75922	0.18382

358.85882	0.18384

358.95842	0.18386

359.05802	0.18396

359.15762	0.18399

359.25722	0.18401

359.35682	0.18403

359.45642	0.18404

359.55602	0.18408

359.65562	0.18409

359.75522	0.18411

359.85482	0.18412

359.95442	0.18413

360.05402	0.18414

360.15362	0.18416

360.25322	0.18423

360.35282	0.18424

360.45242	0.18425

360.55202	0.18427

360.65162	0.18441

360.75122	0.18447

360.85082	0.18449

360.95042	0.18453

361.05002	0.18456

361.14962	0.18466

361.24922	0.18467

361.34882	0.1847

361.44842	0.18473

361.54802	0.18474

361.64762	0.18475

361.74722	0.18486

361.84682	0.18488

361.94642	0.18491

362.04602	0.18496

362.14562	0.18498

362.24522	0.185

362.34482	0.18501

362.44442	0.18502

362.54402	0.1851

362.64362	0.18523

362.74322	0.18525

362.84282	0.18526

362.94242	0.18528

363.04202	0.18529

363.14162	0.18531

363.24122	0.18534

363.34082	0.18535

363.44042	0.18542

363.54002	0.18546

363.63962	0.18547

363.73922	0.18547

363.83882	0.1861

363.93842	0.18627

364.03802	0.18628

364.13762	0.1863

364.23722	0.18631

364.33682	0.18636

364.43642	0.18637

364.53602	0.18638

364.63562	0.1864

364.73522	0.18641

364.83482	0.18642

364.93442	0.18644

365.03402	0.18645

365.13362	0.18646

365.23322	0.18649

365.33282	0.18651

365.43242	0.18655

365.53202	0.18656

365.63162	0.18657

365.73122	0.1866

365.83082	0.18665

365.93042	0.18667

366.03002	0.18668

366.12962	0.1867

366.22922	0.18683

366.32882	0.18684

366.42842	0.1869

366.52802	0.18691

366.62762	0.18695

366.72722	0.18696

366.82682	0.18702

366.92642	0.18703

367.02602	0.18727

367.12562	0.18741

367.22522	0.18743

367.32482	0.18744

367.42442	0.18745

367.52402	0.18759

367.62362	0.18762

367.72322	0.18776

367.82282	0.18777

367.92242	0.18781

368.02202	0.18782

368.12162	0.18784

368.22122	0.18785

368.32082	0.18786

368.42042	0.18787

368.52002	0.18788

368.61962	0.18792

368.71922	0.18794

368.81882	0.18795

368.91842	0.18796

369.01802	0.188

369.11762	0.18801

369.21722	0.18803

369.31682	0.18804

369.41642	0.18805

369.51602	0.18806

369.61562	0.1881

369.71522	0.18811

369.81482	0.18822

369.91442	0.1883

370.01402	0.1883

370.11362	0.18838

370.21322	0.18841

370.31282	0.18853

370.41242	0.18858

370.51202	0.18859

370.61162	0.1886

370.71122	0.18861

370.81082	0.18862

370.91042	0.18865

371.01002	0.18866

371.10962	0.1887

371.20922	0.1887

371.30882	0.18871

371.40842	0.18874

371.50802	0.18876

371.60762	0.18877

371.70722	0.18885

371.80682	0.18886

371.90642	0.18887

372.00602	0.18897

372.10562	0.18906

372.20522	0.18907

372.30482	0.18919

372.40442	0.18931

372.50402	0.18932

372.60362	0.18934

372.70322	0.18937

372.80282	0.1894

372.90242	0.18945

373.00202	0.18946

373.10162	0.18948

373.20122	0.18949

373.30082	0.18952

373.40042	0.18955

373.50002	0.18956

373.59962	0.18957

373.69922	0.18958

373.79882	0.19012

373.89842	0.19013

373.99802	0.19014

374.09762	0.19017

374.19722	0.19022

374.29682	0.19023

374.39642	0.19024

374.49602	0.19034

374.59562	0.19037

374.69522	0.19038

374.79482	0.19039

374.89442	0.19047

374.99402	0.19048

375.09362	0.19059

375.19322	0.19061

375.29282	0.19063

375.39242	0.19064

375.49202	0.19069

375.59162	0.1907

375.69122	0.19071

375.79082	0.19075

375.89042	0.19077

375.99002	0.19086

376.08962	0.19087

376.18922	0.19088

376.28882	0.19089

376.38842	0.19092

376.48802	0.19093

376.58762	0.19099

376.68722	0.19099

376.78682	0.19104

376.88642	0.1911

376.98602	0.19111

377.08562	0.19112

377.18522	0.19118

377.28482	0.19122

377.38442	0.19124

377.48402	0.19126

377.58362	0.19133

377.68322	0.19135

377.78282	0.19138

377.88242	0.1914

377.98202	0.1914

378.08162	0.1915

378.18122	0.19155

378.28082	0.19157

378.38042	0.1916

378.48002	0.19161

378.57962	0.19162

378.67922	0.19165

378.77882	0.19166

378.87842	0.19169

378.97802	0.19194

379.07762	0.19196

379.17722	0.192

379.27682	0.19203

379.37642	0.19204

379.47602	0.19207

379.57562	0.19208

379.67522	0.1921

379.77482	0.19213

379.87442	0.19214

379.97402	0.19216

380.07362	0.19217

380.17322	0.19218

380.27282	0.19219

380.37242	0.19224

380.47202	0.19247

380.57162	0.19251

380.67122	0.19256

380.77082	0.19257

380.87042	0.19259

380.97002	0.1926

381.06962	0.19261

381.16922	0.19263

381.26882	0.19265

381.36842	0.19271

381.46802	0.19274

381.56762	0.19275

381.66722	0.19283

381.76682	0.19285

381.86642	0.19287

381.96602	0.19288

382.06562	0.19294

382.16522	0.19296

382.26482	0.19297

382.36442	0.19299

382.46402	0.19301

382.56362	0.19302

382.66322	0.19303

382.76282	0.19307

382.86242	0.19309

382.96202	0.1931

383.06162	0.19314

383.16122	0.19327

383.26082	0.19349

383.36042	0.1936

383.46002	0.19363

383.55962	0.19364

383.65922	0.19367

383.75882	0.19376

383.85842	0.19377

383.95802	0.19378

384.05762	0.1938

384.15722	0.19384

384.25682	0.19391

384.35642	0.19394

384.45602	0.19395

384.55562	0.19405

384.65522	0.19406

384.75482	0.19408

384.85442	0.19409

384.95402	0.19415

385.05362	0.19416

385.15322	0.19427

385.25282	0.19428

385.35242	0.19429

385.45202	0.19436

385.55162	0.19446

385.65122	0.19448

385.75082	0.19452

385.85042	0.19456

385.95002	0.19457

386.04962	0.1946

386.14922	0.19462

386.24882	0.19463

386.34842	0.19464

386.44802	0.19468

386.54762	0.19471

386.64722	0.19473

386.74682	0.19474

386.84642	0.19475

386.94602	0.19481

387.04562	0.19482

387.14522	0.19483

387.24482	0.19484

387.34442	0.19485

387.44402	0.19494

387.54362	0.19498

387.64322	0.195

387.74282	0.19501

387.84242	0.19508

387.94202	0.19511

388.04162	0.19516

388.14122	0.19517

388.24082	0.19518

388.34042	0.19523

388.44002	0.19526

388.53962	0.19527

388.63922	0.19528

388.73882	0.1953

388.83842	0.19535

388.93802	0.19539

389.03762	0.19548

389.13722	0.1955

389.23682	0.19555

389.33642	0.19556

389.43602	0.19557

389.53562	0.19565

389.63522	0.1957

389.73482	0.1957

389.83442	0.19572

389.93403	0.19575

390.03362	0.19576

390.13322	0.19577

390.23282	0.1958

390.33243	0.19586

390.43203	0.19588

390.53163	0.19592

390.63122	0.19593

390.73082	0.19601

390.83042	0.19604

390.93003	0.19607

391.02963	0.1961

391.12923	0.19611

391.22882	0.19611

391.32842	0.19615

391.42803	0.19616

391.52763	0.19619

391.62723	0.19624

391.72683	0.19624

391.82643	0.19627

391.92603	0.19629

392.02563	0.1963

392.12523	0.19631

392.22483	0.19632

392.32443	0.19633

392.42403	0.19635

392.52363	0.19638

392.62323	0.19638

392.72283	0.19639

392.82243	0.1965

392.92203	0.19651

393.02163	0.19651

393.12123	0.19652

393.22083	0.19653

393.32043	0.19655

393.42003	0.19657

393.51963	0.19658

393.61923	0.19668

393.71883	0.19673

393.81843	0.19674

393.91803	0.19678

394.01763	0.19678

394.11723	0.1968

394.21683	0.19684

394.31643	0.19685

394.41603	0.19686

394.51563	0.19687

394.61523	0.19688

394.71483	0.19689

394.81443	0.19694

394.91403	0.197

395.01363	0.19705

395.11323	0.19707

395.21283	0.19708

395.31243	0.19709

395.41203	0.1971

395.51163	0.19712

395.61123	0.19713

395.71083	0.19714

395.81043	0.19717

395.91003	0.19718

396.00963	0.19719

396.10923	0.19721

396.20883	0.19723

396.30843	0.19724

396.40803	0.1973

396.50763	0.19731

396.60723	0.19733

396.70683	0.19734

396.80643	0.19736

396.90603	0.19737

397.00563	0.1974

397.10523	0.19742

397.20483	0.19744

397.30443	0.19745

397.40403	0.19746

397.50363	0.19746

397.60323	0.19747

397.70283	0.19749

397.80243	0.19754

397.90203	0.19773

398.00163	0.19776

398.10123	0.19777

398.20083	0.19778

398.30043	0.19779

398.40003	0.19785

398.49963	0.19786

398.59923	0.19786

398.69883	0.19788

398.79843	0.1979

398.89803	0.19792

398.99763	0.19797

399.09723	0.19799

399.19683	0.19808

399.29643	0.19811

399.39603	0.19812

399.49563	0.19816

399.59523	0.19819

399.69483	0.19825

399.79443	0.19826

399.89403	0.19828

399.99363	0.19833

400.09323	0.19864

400.19283	0.19882

400.29243	0.19885

400.39203	0.19888

400.49163	0.19891

400.59123	0.19897

400.69083	0.19898

400.79043	0.19899

400.89003	0.19901

400.98963	0.19902

401.08923	0.19904

401.18883	0.19905

401.28843	0.19907

401.38803	0.19908

401.48763	0.19909

401.58723	0.19912

401.68683	0.19916

401.78643	0.19917

401.88603	0.19918

401.98563	0.19919

402.08523	0.19922

402.18483	0.19923

402.28443	0.19924

402.38403	0.19925

402.48363	0.19928

402.58323	0.19932

402.68283	0.19933

402.78243	0.19942

402.88203	0.19946

402.98163	0.19947

403.08123	0.19949

403.18083	0.19951

403.28043	0.19953

403.38003	0.19961

403.47963	0.19961

403.57923	0.19964

403.67883	0.19967

403.77843	0.19968

403.87803	0.1997

403.97763	0.2

404.07723	0.20002

404.17683	0.20003

404.27643	0.20006

404.37603	0.20009

404.47563	0.2001

404.57523	0.20011

404.67483	0.20013

404.77443	0.20014

404.87403	0.20015

404.97363	0.20017

405.07323	0.20018

405.17283	0.20019

405.27243	0.2002

405.37203	0.20021

405.47163	0.20022

405.57123	0.20026

405.67083	0.20028

405.77043	0.20028

405.87003	0.20029

405.96963	0.2003

406.06923	0.20031

406.16883	0.20034

406.26843	0.20037

406.36803	0.20039

406.46763	0.2004

406.56723	0.20041

406.66683	0.20042

406.76643	0.20046

406.86603	0.20047

406.96563	0.20053

407.06523	0.20056

407.16483	0.20059

407.26443	0.20063

407.36403	0.20065

407.46363	0.20066

407.56323	0.20067

407.66283	0.20068

407.76243	0.20069

407.86203	0.20118

407.96163	0.20119

408.06123	0.2012

408.16083	0.20124

408.26043	0.20125

408.36003	0.20126

408.45963	0.20127

408.55923	0.20137

408.65883	0.20139

408.75843	0.20143

408.85803	0.20144

408.95763	0.20145

409.05723	0.20148

409.15683	0.20149

409.25643	0.2015

409.35603	0.20151

409.45563	0.20154

409.55523	0.20159

409.65483	0.2016

409.75443	0.20161

409.85403	0.20163

409.95363	0.20164

410.05323	0.20166

410.15283	0.20168

410.25243	0.20174

410.35203	0.20177

410.45163	0.20179

410.55123	0.2018

410.65083	0.20192

410.75043	0.20195

410.85003	0.20196

410.94963	0.20198

411.04923	0.20203

411.14883	0.20203

411.24843	0.20205

411.34803	0.20207

411.44763	0.20208

411.54723	0.2021

411.64683	0.20211

411.74643	0.20232

411.84603	0.20233

411.94563	0.20235

412.04523	0.20236

412.14483	0.20238

412.24443	0.20239

412.34403	0.2024

412.44363	0.20241

412.54323	0.20242

412.64283	0.20244

412.74243	0.20244

412.84203	0.20245

412.94163	0.20246

413.04123	0.2025

413.14083	0.20251

413.24043	0.20252

413.34003	0.20255

413.43963	0.20256

413.53923	0.20259

413.63883	0.2026

413.73843	0.20261

413.83803	0.20263

413.93763	0.20267

414.03723	0.2027

414.13683	0.20273

414.23643	0.20277

414.33603	0.20282

414.43563	0.20283

414.53523	0.20284

414.63483	0.20285

414.73443	0.20286

414.83403	0.20287

414.93363	0.2029

415.03323	0.20291

415.13283	0.20293

415.23243	0.20294

415.33203	0.20298

415.43163	0.20306

415.53123	0.20307

415.63083	0.20308

415.73043	0.20309

415.83003	0.20311

415.92963	0.20311

416.02923	0.20313

416.12883	0.20314

416.22843	0.20329

416.32803	0.2033

416.42763	0.20331

416.52723	0.20336

416.62683	0.20337

416.72643	0.20338

416.82603	0.20346

416.92563	0.20347

417.02523	0.20349

417.12483	0.2035

417.22443	0.20351

417.32403	0.20352

417.42363	0.20353

417.52323	0.20356

417.62283	0.20357

417.72243	0.20358

417.82203	0.20438

417.92163	0.20439

418.02123	0.2044

418.12083	0.20441

418.22043	0.20451

418.32003	0.20453

418.41963	0.20454

418.51923	0.20455

418.61883	0.20456

418.71843	0.20457

418.81803	0.20463

418.91763	0.20464

419.01723	0.20469

419.11683	0.2047

419.21643	0.20471

419.31603	0.20472

419.41563	0.20477

419.51523	0.20478

419.61483	0.20479

419.71443	0.2048

419.81403	0.20482

419.91363	0.20483

420.01323	0.20484

420.11283	0.20486

420.21243	0.20486

420.31203	0.20488

420.41163	0.2049

420.51123	0.20495

420.61083	0.20504

420.71043	0.20508

420.81003	0.20511

420.90963	0.20512

421.00923	0.20513

421.10883	0.20514

421.20843	0.20519

421.30803	0.2052

421.40763	0.20522

421.50723	0.20526

421.60683	0.20527

421.70643	0.20528

421.80603	0.20529

421.90563	0.2053

422.00523	0.20543

422.10483	0.20545

422.20443	0.20573

422.30403	0.20577

422.40363	0.2058

422.50323	0.20581

422.60283	0.20583

422.70243	0.20584

422.80203	0.20585

422.90163	0.20586

423.00123	0.20587

423.10083	0.20592

423.20043	0.20621

423.30003	0.20629

423.39963	0.2063

423.49923	0.20631

423.59883	0.20632

423.69843	0.20652

423.79803	0.20653

423.89763	0.20661

423.99723	0.20661

424.09683	0.20663

424.19643	0.20664

424.29603	0.20665

424.39563	0.20669

424.49523	0.20671

424.59483	0.20672

424.69443	0.20673

424.79403	0.20685

424.89363	0.20689

424.99323	0.2069

425.09283	0.20692

425.19243	0.20704

425.29203	0.2071

425.39163	0.20712

425.49123	0.20713

425.59083	0.20715

425.69043	0.20716

425.79003	0.20717

425.88963	0.20718

425.98923	0.2072

426.08883	0.20721

426.18843	0.20725

426.28803	0.20726

426.38763	0.20727

426.48723	0.20728

426.58683	0.20729

426.68643	0.20731

426.78603	0.20733

426.88563	0.20741

426.98523	0.20742

427.08483	0.20742

427.18443	0.20743

427.28403	0.20744

427.38363	0.20746

427.48323	0.20747

427.58283	0.20755

427.68243	0.20758

427.78203	0.20759

427.88163	0.2076

427.98123	0.20762

428.08083	0.20763

428.18043	0.20766

428.28003	0.20776

428.37963	0.20777

428.47923	0.20778

428.57883	0.20779

428.67843	0.2078

428.77803	0.20781

428.87763	0.20782

428.97723	0.20782

429.07683	0.20783

429.17643	0.20785

429.27603	0.20786

429.37563	0.20787

429.47523	0.20788

429.57483	0.2079

429.67443	0.20796

429.77403	0.20802

429.87363	0.20805

429.97323	0.20813

430.07283	0.20814

430.17243	0.20817

430.27203	0.20818

430.37163	0.2082

430.47123	0.20821

430.57083	0.20822

430.67043	0.20823

430.77003	0.20823

430.86963	0.20833

430.96923	0.20836

431.06883	0.20839

431.16843	0.2084

431.26803	0.20841

431.36763	0.20842

431.46723	0.20843

431.56683	0.20848

431.66643	0.20856

431.76603	0.20894

431.86563	0.20895

431.96523	0.20898

432.06483	0.20901

432.16443	0.20902

432.26403	0.20903

432.36363	0.20903

432.46323	0.20905

432.56283	0.20946

432.66243	0.20947

432.76203	0.20948

432.86163	0.2095

432.96123	0.20951

433.06083	0.20957

433.16043	0.20958

433.26003	0.20959

433.35963	0.2096

433.45923	0.20961

433.55883	0.20962

433.65843	0.20966

433.75803	0.20967

433.85763	0.20968

433.95723	0.20969

434.05683	0.2097

434.15643	0.20977

434.25603	0.20979

434.35563	0.2098

434.45523	0.20981

434.55483	0.20984

434.65443	0.20985

434.75403	0.20987

434.85363	0.2099

434.95323	0.20991

435.05283	0.20993

435.15243	0.20994

435.25203	0.21003

435.35163	0.21004

435.45123	0.21005

435.55083	0.21011

435.65043	0.21013

435.75003	0.21015

435.84963	0.21017

435.94923	0.21018

436.04883	0.21023

436.14843	0.21024

436.24803	0.21025

436.34763	0.21026

436.44723	0.21027

436.54683	0.21028

436.64643	0.21029

436.74603	0.2103

436.84563	0.21092

436.94523	0.21093

437.04483	0.21094

437.14443	0.21095

437.24403	0.21099

437.34363	0.21102

437.44323	0.21104

437.54283	0.21105

437.64243	0.21106

437.74203	0.21107

437.84163	0.21108

437.94123	0.21109

438.04083	0.2111

438.14043	0.21111

438.24003	0.21113

438.33963	0.21136

438.43923	0.21137

438.53883	0.21138

438.63843	0.21139

438.73803	0.2114

438.83763	0.21141

438.93723	0.21142

439.03683	0.21148

439.13643	0.2115

439.23603	0.21151

439.33563	0.21153

439.43523	0.21155

439.53483	0.21156

439.63443	0.21161

439.73403	0.21163

439.83363	0.21164

439.93323	0.21166

440.03283	0.21167

440.13243	0.21169

440.23203	0.2117

440.33163	0.21171

440.43123	0.21172

440.53083	0.21173

440.63043	0.21174

440.73003	0.21178

440.82963	0.21185

440.92923	0.21186

441.02883	0.21186

441.12843	0.21189

441.22803	0.21194

441.32763	0.21197

441.42723	0.212

441.52683	0.21202

441.62643	0.21203

441.72603	0.21204

441.82563	0.21205

441.92523	0.21208

442.02483	0.21214

442.12443	0.21217

442.22403	0.21219

442.32363	0.2122

442.42323	0.21227

442.52283	0.21227

442.62243	0.21229

442.72203	0.21241

442.82163	0.21242

442.92123	0.21248

443.02083	0.2125

443.12043	0.21251

443.22003	0.21267

443.31963	0.21269

443.41923	0.21271

443.51883	0.21272

443.61843	0.21273

443.71803	0.21275

443.81763	0.21276

443.91723	0.21282

444.01683	0.21284

444.11643	0.21286

444.21603	0.21287

444.31563	0.21288

444.41523	0.2129

444.51483	0.21299

444.61443	0.213

444.71403	0.21301

444.81363	0.21303

444.91323	0.21306

445.01283	0.21307

445.11243	0.21308

445.21203	0.21309

445.31163	0.2131

445.41123	0.21311

445.51083	0.21312

445.61043	0.21316

445.71003	0.21342

445.80963	0.21343

445.90923	0.21344

446.00883	0.21347

446.10843	0.21348

446.20803	0.21348

446.30763	0.2135

446.40723	0.21351

446.50683	0.21356

446.60643	0.21357

446.70603	0.21358

446.80563	0.21361

446.90523	0.21363

447.00483	0.21364

447.10443	0.21365

447.20403	0.21369

447.30363	0.21372

447.40323	0.21374

447.50283	0.21376

447.60243	0.21377

447.70203	0.21378

447.80163	0.21492

447.90123	0.21501

448.00083	0.21503

448.10043	0.21504

448.20003	0.21505

448.29963	0.21508

448.39923	0.21509

448.49883	0.2151

448.59843	0.21511

448.69803	0.21522

448.79763	0.2153

448.89723	0.21531

448.99683	0.21536

449.09643	0.21536

449.19603	0.21537

449.29563	0.2154

449.39523	0.21541

449.49483	0.21542

449.59443	0.21543

449.69403	0.21546

449.79363	0.21551

449.89323	0.21553

449.99283	0.21554

450.09243	0.21556

450.19203	0.21558

450.29163	0.21559

450.39123	0.21561

450.49083	0.21564

450.59043	0.21566

450.69003	0.21568

450.78963	0.21569

450.88923	0.21581

450.98883	0.21582

451.08843	0.21583

451.18803	0.21585

451.28763	0.21587

451.38723	0.21589

451.48683	0.21593

451.58643	0.21596

451.68603	0.21603

451.78563	0.21604

451.88523	0.21634

451.98483	0.21636

452.08443	0.21637

452.18403	0.21638

452.28363	0.21642

452.38323	0.21643

452.48283	0.21644

452.58243	0.21644

452.68203	0.21646

452.78163	0.21647

452.88123	0.21648

452.98083	0.21653

453.08043	0.21657

453.18003	0.21675

453.27963	0.21677

453.37923	0.21679

453.47883	0.21681

453.57843	0.21682

453.67803	0.21684

453.77763	0.21685

453.87723	0.21706

453.97683	0.21707

454.07643	0.21708

454.17603	0.21718

454.27563	0.21719

454.37523	0.21721

454.47483	0.21726

454.57443	0.21733

454.67403	0.21734

454.77363	0.21738

454.87323	0.21739

454.97283	0.21743

455.07243	0.21744

455.17203	0.21746

455.27163	0.21748

455.37123	0.21749

455.47083	0.21779

455.57043	0.2178

455.67003	0.21781

455.76963	0.21782

455.86923	0.21784

455.96883	0.21785

456.06843	0.21787

456.16803	0.2179

456.26763	0.21791

456.36723	0.21792

456.46683	0.21792

456.56643	0.21793

456.66603	0.21794

456.76563	0.21798

456.86523	0.21827

456.96483	0.21828

457.06443	0.21829

457.16403	0.21831

457.26363	0.21833

457.36323	0.21834

457.46283	0.21835

457.56243	0.21844

457.66203	0.21845

457.76163	0.21846

457.86123	0.21849

457.96083	0.21851

458.06043	0.21855

458.16003	0.21865

458.25963	0.21866

458.35923	0.21874

458.45883	0.21876

458.55843	0.21877

458.65803	0.21878

458.75763	0.2188

458.85723	0.21882

458.95683	0.21883

459.05643	0.21884

459.15603	0.21885

459.25563	0.21886

459.35523	0.21887

459.45483	0.21888

459.55443	0.21889

459.65403	0.2189

459.75363	0.21891

459.85323	0.21893

459.95283	0.21894

460.05243	0.21895

460.15203	0.21896

460.25163	0.21897

460.35123	0.21898

460.45083	0.219

460.55043	0.219

460.65003	0.21901

460.74963	0.21904

460.84923	0.21908

460.94883	0.21909

461.04843	0.21911

461.14803	0.21912

461.24763	0.21913

461.34723	0.21913

461.44683	0.21914

461.54643	0.21915

461.64603	0.21916

461.74563	0.21917

461.84523	0.21919

461.94483	0.2192

462.04443	0.21921

462.14403	0.21922

462.24363	0.21924

462.34323	0.21932

462.44283	0.21933

462.54243	0.21935

462.64203	0.21946

462.74163	0.21947

462.84123	0.21959

462.94083	0.21963

463.04043	0.21979

463.14003	0.2198

463.23963	0.21982

463.33923	0.21984

463.43883	0.21986

463.53843	0.22003

463.63803	0.22004

463.73763	0.22005

463.83723	0.22008

463.93683	0.2201

464.03643	0.22012

464.13603	0.22018

464.23563	0.22019

464.33523	0.22022

464.43483	0.22056

464.53443	0.22065

464.63403	0.22067

464.73363	0.22068

464.83323	0.22069

464.93283	0.22094

465.03243	0.22095

465.13203	0.22096

465.23163	0.22098

465.33123	0.22099

465.43083	0.221

465.53043	0.22101

465.63003	0.22103

465.72963	0.22106

465.82923	0.22163

465.92883	0.22164

466.02843	0.22166

466.12803	0.22169

466.22763	0.22169

466.32723	0.22172

466.42683	0.22182

466.52643	0.22183

466.62603	0.22183

466.72563	0.22184

466.82523	0.22185

466.92483	0.22186

467.02443	0.22188

467.12403	0.22189

467.22363	0.22191

467.32323	0.22195

467.42283	0.22198

467.52243	0.22203

467.62203	0.22204

467.72163	0.22205

467.82123	0.22206

467.92083	0.22207

468.02043	0.22209

468.12003	0.2221

468.21963	0.2221

468.31923	0.22219

468.41883	0.2222

468.51843	0.22221

468.61803	0.22222

468.71763	0.22225

468.81723	0.22226

468.91683	0.22228

469.01643	0.22229

469.11603	0.2223

469.21563	0.22231

469.31523	0.22234

469.41483	0.22236

469.51443	0.22236

469.61403	0.22241

469.71363	0.22243

469.81323	0.22244

469.91283	0.22246

470.01243	0.22247

470.11203	0.22248

470.21163	0.22249

470.31123	0.2225

470.41083	0.22252

470.51043	0.22254

470.61003	0.22258

470.70963	0.2226

470.80923	0.22263

470.90883	0.22266

471.00843	0.22267

471.10803	0.22269

471.20763	0.22271

471.30723	0.22272

471.40683	0.22274

471.50643	0.22276

471.60603	0.22277

471.70563	0.22278

471.80523	0.22281

471.90483	0.22284

472.00443	0.22287

472.10403	0.22288

472.20363	0.22289

472.30323	0.2229

472.40283	0.2229

472.50243	0.22291

472.60203	0.22292

472.70163	0.22317

472.80123	0.22319

472.90083	0.2232

473.00043	0.22323

473.10003	0.22329

473.19963	0.22331

473.29923	0.22331

473.39883	0.22332

473.49843	0.22334

473.59803	0.22335

473.69763	0.22336

473.79723	0.22342

473.89683	0.22343

473.99643	0.22344

474.09603	0.22344

474.19563	0.22345

474.29523	0.22346

474.39483	0.22347

474.49443	0.22348

474.59403	0.2235

474.69363	0.22352

474.79323	0.22353

474.89283	0.22354

474.99243	0.22355

475.09203	0.2236

475.19163	0.22361

475.29123	0.22362

475.39083	0.22363

475.49043	0.22364

475.59003	0.22365

475.68963	0.22369

475.78923	0.22377

475.88883	0.22378

475.98843	0.22379

476.08803	0.2238

476.18763	0.22381

476.28723	0.22382

476.38683	0.22384

476.48643	0.22385

476.58603	0.22386

476.68563	0.22387

476.78523	0.22388

476.88483	0.22389

476.98443	0.22396

477.08403	0.22397

477.18363	0.22398

477.28323	0.22398

477.38283	0.22407

477.48243	0.22409

477.58203	0.22452

477.68163	0.22458

477.78123	0.22459

477.88083	0.2246

477.98043	0.22461

478.08003	0.22462

478.17963	0.22464

478.27923	0.22465

478.37883	0.22466

478.47843	0.22468

478.57803	0.2247

478.67763	0.22472

478.77723	0.22475

478.87683	0.22478

478.97643	0.22479

479.07603	0.22486

479.17563	0.22487

479.27523	0.22489

479.37483	0.22491

479.47443	0.22492

479.57403	0.22493

479.67363	0.22494

479.77323	0.22496

479.87283	0.22516

479.97243	0.22517

480.07203	0.22518

480.17163	0.22519

480.27123	0.22519

480.37083	0.2252

480.47043	0.22521

480.57003	0.22523

480.66963	0.22524

480.76923	0.22525

480.86883	0.22526

480.96843	0.22527

481.06803	0.22528

481.16763	0.22529

481.26723	0.2253

481.36683	0.22531

481.46643	0.22532

481.56603	0.22533

481.66563	0.22533

481.76523	0.22539

481.86483	0.2254

481.96443	0.22541

482.06403	0.22542

482.16363	0.22545

482.26323	0.22549

482.36283	0.2255

482.46243	0.22553

482.56203	0.22556

482.66163	0.22557

482.76123	0.22559

482.86083	0.22562

482.96043	0.22577

483.06003	0.22582

483.15963	0.22589

483.25923	0.22594

483.35883	0.22596

483.45843	0.22597

483.55803	0.22598

483.65763	0.22599

483.75723	0.226

483.85683	0.226

483.95643	0.22601

484.05603	0.22602

484.15563	0.22604

484.25523	0.22605

484.35483	0.22607

484.45443	0.22609

484.55403	0.2261

484.65363	0.22616

484.75323	0.22617

484.85283	0.22618

484.95243	0.2262

485.05203	0.22623

485.15163	0.22624

485.25123	0.22625

485.35083	0.22663

485.45043	0.22665

485.55003	0.22667

485.64963	0.22667

485.74923	0.2267

485.84883	0.22672

485.94843	0.22673

486.04803	0.22676

486.14763	0.22677

486.24723	0.22678

486.34683	0.22679

486.44643	0.2268

486.54603	0.22681

486.64563	0.22681

486.74523	0.22687

486.84483	0.22703

486.94443	0.22705

487.04403	0.22708

487.14363	0.22709

487.24323	0.22712

487.34283	0.22713

487.44243	0.22714

487.54203	0.22715

487.64163	0.22716

487.74123	0.22718

487.84083	0.2272

487.94043	0.22721

488.04003	0.22723

488.13963	0.22726

488.23923	0.22727

488.33883	0.22728

488.43843	0.22729

488.53803	0.22731

488.63763	0.22732

488.73723	0.22733

488.83683	0.22735

488.93643	0.22742

489.03603	0.22743

489.13563	0.22744

489.23523	0.22761

489.33483	0.2277

489.43443	0.22771

489.53403	0.22772

489.63363	0.22773

489.73323	0.22775

489.83283	0.22776

489.93243	0.22778

490.03203	0.2278

490.13163	0.22782

490.23123	0.22783

490.33083	0.22786

490.43043	0.22789

490.53003	0.2279

490.62963	0.22794

490.72923	0.22795

490.82883	0.22802

490.92843	0.22802

491.02803	0.22803

491.12763	0.22804

491.22723	0.22805

491.32683	0.22808

491.42643	0.22811

491.52603	0.22812

491.62563	0.22815

491.72523	0.22817

491.82483	0.22818

491.92443	0.22819

492.02403	0.2282

492.12363	0.22822

492.22323	0.22825

492.32283	0.22827

492.42243	0.22828

492.52203	0.22836

492.62163	0.22837

492.72123	0.22839

492.82083	0.2284

492.92043	0.22841

493.02003	0.22842

493.11963	0.22844

493.21923	0.22847

493.31883	0.22848

493.41843	0.2285

493.51803	0.22851

493.61763	0.22853

493.71723	0.22856

493.81683	0.2286

493.91643	0.22861

494.01603	0.22862

494.11563	0.22863

494.21523	0.22864

494.31483	0.22865

494.41443	0.22869

494.51403	0.22869

494.61363	0.22871

494.71323	0.22873

494.81283	0.22876

494.91243	0.22877

495.01203	0.22878

495.11163	0.22879

495.21123	0.2288

495.31083	0.22881

495.41043	0.22882

495.51003	0.22925

495.60963	0.22926

495.70923	0.22927

495.80883	0.22929

495.90843	0.22933

496.00803	0.22935

496.10763	0.22936

496.20723	0.22937

496.30683	0.22937

496.40643	0.22938

496.50603	0.22948

496.60563	0.22949

496.70523	0.22962

496.80483	0.22964

496.90443	0.22964

497.00403	0.22965

497.10363	0.22967

497.20323	0.22968

497.30283	0.22969

497.40243	0.2297

497.50203	0.22971

497.60163	0.22975

497.70123	0.22976

497.80083	0.22977

497.90043	0.22977

498.00003	0.22978

498.09963	0.22979

498.19923	0.22981

498.29883	0.22982

498.39843	0.22983

498.49803	0.22984

498.59763	0.22985

498.69723	0.22987

498.79683	0.2299

498.89643	0.2299

498.99603	0.23002

499.09563	0.23007

499.19523	0.23008

499.29483	0.23009

499.39443	0.23015

499.49403	0.23016

499.59363	0.23017

499.69323	0.23017

499.79283	0.23018

499.89243	0.23019

499.99203	0.2302

500.09163	0.23022

500.19123	0.23023

500.29083	0.23024

500.39043	0.23025

500.49003	0.23031

500.58963	0.23031

500.68923	0.23032

500.78883	0.23033

500.88843	0.23036

500.98803	0.23039

501.08763	0.2304

501.18723	0.23041

501.28683	0.23042

501.38643	0.23044

501.48603	0.23049

501.58563	0.23053

501.68523	0.23054

501.78483	0.23056

501.88443	0.23057

501.98403	0.23058

502.08363	0.23059

502.18323	0.23061

502.28283	0.23063

502.38243	0.23064

502.48203	0.23065

502.58163	0.23066

502.68123	0.23071

502.78083	0.23071

502.88043	0.23074

502.98003	0.23075

503.07963	0.23076

503.17923	0.23077

503.27883	0.23078

503.37843	0.23079

503.47803	0.23081

503.57763	0.23082

503.67723	0.23087

503.77683	0.23089

503.87643	0.2309

503.97603	0.23091

504.07563	0.23097

504.17523	0.231

504.27483	0.23101

504.37443	0.23104

504.47403	0.23106

504.57363	0.23107

504.67323	0.23108

504.77283	0.23109

504.87243	0.2311

504.97203	0.23111

505.07163	0.23123

505.17123	0.23124

505.27083	0.23125

505.37043	0.23126

505.47003	0.23127

505.56963	0.23128

505.66923	0.23129

505.76883	0.2313

505.86843	0.23131

505.96803	0.23132

506.06763	0.23133

506.16723	0.23134

506.26683	0.23135

506.36643	0.23136

506.46603	0.23137

506.56563	0.23139

506.66523	0.23139

506.76483	0.2314

506.86443	0.23141

506.96403	0.23144

507.06363	0.23145

507.16323	0.23152

507.26283	0.23152

507.36243	0.23153

507.46203	0.23154

507.56163	0.23155

507.66123	0.23156

507.76083	0.23179

507.86043	0.23181

507.96003	0.23186

508.05963	0.23188

508.15923	0.2319

508.25883	0.23191

508.35843	0.23192

508.45803	0.23194

508.55763	0.23195

508.65723	0.23197

508.75683	0.23198

508.85643	0.23199

508.95603	0.23202

509.05563	0.23204

509.15523	0.23205

509.25483	0.23208

509.35443	0.23209

509.45403	0.23211

509.55363	0.23213

509.65323	0.23215

509.75283	0.23216

509.85243	0.23219

509.95203	0.23222

510.05163	0.23223

510.15123	0.23225

510.25083	0.23226

510.35043	0.23229

510.45003	0.2323

510.54963	0.23236

510.64923	0.2324

510.74883	0.23241

510.84843	0.23244

510.94803	0.23246

511.04763	0.2325

511.14723	0.23253

511.24683	0.23254

511.34643	0.23255

511.44603	0.23262

511.54563	0.23268

511.64523	0.23271

511.74483	0.23272

511.84443	0.23275

511.94403	0.23276

512.04363	0.23278

512.14323	0.23285

512.24283	0.23292

512.34243	0.23294

512.44203	0.23296

512.54163	0.23297

512.64123	0.23299

512.74083	0.23301

512.84043	0.23304

512.94003	0.23306

513.03963	0.23311

513.13923	0.23313

513.23883	0.23315

513.33843	0.23317

513.43803	0.23318

513.53763	0.2332

513.63723	0.23321

513.73683	0.23336

513.83643	0.23339

513.93603	0.23342

514.03563	0.23343

514.13523	0.23345

514.23483	0.23347

514.33443	0.23356

514.43403	0.23357

514.53363	0.23361

514.63323	0.23363

514.73283	0.23364

514.83243	0.2337

514.93203	0.23371

515.03163	0.23373

515.13123	0.23375

515.23083	0.23377

515.33043	0.2338

515.43003	0.23382

515.52963	0.23384

515.62923	0.23385

515.72883	0.23387

515.82843	0.23388

515.92803	0.23389

516.02763	0.23392

516.12723	0.23394

516.22683	0.23395

516.32643	0.23396

516.42603	0.23398

516.52563	0.23399

516.62523	0.23401

516.72483	0.23402

516.82443	0.23403

516.92403	0.23405

517.02363	0.23406

517.12323	0.23408

517.22283	0.2341

517.32243	0.23413

517.42203	0.23417

517.52163	0.23419

517.62123	0.2342

517.72083	0.23423

517.82043	0.23424

517.92003	0.23426

518.01963	0.23431

518.11923	0.23434

518.21883	0.23437

518.31843	0.23438

518.41803	0.23441

518.51763	0.23442

518.61723	0.23444

518.71683	0.23449

518.81643	0.23451

518.91603	0.23452

519.01563	0.23455

519.11523	0.23459

519.21483	0.23462

519.31443	0.23468

519.41403	0.23469

519.51363	0.23477

519.61323	0.23479

519.71283	0.2348

519.81243	0.23482

519.91203	0.2349

520.01163	0.23491

520.11123	0.23494

520.21083	0.235

520.31043	0.23521

520.41003	0.23522

520.50963	0.23523

520.60923	0.23525

520.70883	0.23526

520.80843	0.23529

520.90803	0.2353

521.00763	0.23539

521.10723	0.23547

521.20683	0.23549

521.30643	0.2355

521.40603	0.23551

521.50563	0.23554

521.60523	0.23556

521.70483	0.23557

521.80443	0.23558

521.90403	0.23561

522.00363	0.23563

522.10323	0.23564

522.20283	0.23565

522.30243	0.23567

522.40203	0.23569

522.50163	0.23576

522.60123	0.23578

522.70083	0.23579

522.80043	0.23581

522.90003	0.23583

522.99963	0.23586

523.09923	0.23589

523.19883	0.23592

523.29843	0.23596

523.39803	0.23597

523.49763	0.23602

523.59723	0.23603

523.69683	0.23604

523.79643	0.23607

523.89603	0.23609

523.99563	0.2361

524.09523	0.23616

524.19483	0.23617

524.29443	0.2362

524.39403	0.23621

524.49363	0.23624

524.59323	0.23628

524.69283	0.2363

524.79243	0.23632

524.89203	0.23634

524.99163	0.23637

525.09123	0.23638

525.19083	0.23639

525.29043	0.23641

525.39003	0.23642

525.48963	0.23643

525.58923	0.23646

525.68883	0.23648

525.78843	0.23649

525.88803	0.2365

525.98763	0.23653

526.08723	0.23659

526.18683	0.2366

526.28643	0.23662

526.38603	0.23663

526.48563	0.23664

526.58523	0.23666

526.68483	0.23676

526.78443	0.23677

526.88403	0.2368

526.98363	0.23681

527.08323	0.23683

527.18283	0.23684

527.28243	0.23685

527.38203	0.23688

527.48163	0.2369

527.58123	0.23692

527.68083	0.23694

527.78043	0.23697

527.88003	0.23698

527.97963	0.23699

528.07923	0.23702

528.17883	0.23719

528.27843	0.23722

528.37803	0.23724

528.47763	0.2373

528.57723	0.23733

528.67683	0.23734

528.77643	0.23736

528.87603	0.23737

528.97563	0.23741

529.07523	0.23743

529.17483	0.2375

529.27443	0.23762

529.37403	0.23764

529.47363	0.23765

529.57323	0.23766

529.67283	0.23769

529.77243	0.23772

529.87203	0.23778

529.97163	0.23779

530.07123	0.23807

530.17083	0.23811

530.27043	0.23817

530.37003	0.23818

530.46963	0.23822

530.56923	0.23824

530.66883	0.23825

530.76843	0.23842

530.86803	0.23843

530.96763	0.23849

531.06723	0.2385

531.16683	0.23853

531.26643	0.2386

531.36603	0.23864

531.46563	0.23866

531.56523	0.2387

531.66483	0.23874

531.76443	0.23877

531.86403	0.23881

531.96363	0.23882

532.06323	0.23885

532.16283	0.23886

532.26243	0.23892

532.36203	0.23893

532.46163	0.23895

532.56123	0.239

532.66083	0.23906

532.76043	0.23907

532.86003	0.23909

532.95963	0.2391

533.05923	0.23912

533.15883	0.23914

533.25843	0.23917

533.35803	0.2392

533.45763	0.23921

533.55723	0.23924

533.65683	0.23926

533.75643	0.23927

533.85603	0.23928

533.95563	0.23931

534.05523	0.23934

534.15483	0.23935

534.25443	0.23937

534.35403	0.23938

534.45363	0.23946

534.55323	0.23952

534.65283	0.23956

534.75243	0.2396

534.85203	0.23963

534.95163	0.23965

535.05123	0.23966

535.15083	0.23967

535.25043	0.23972

535.35003	0.23973

535.44963	0.23977

535.54923	0.23979

535.64883	0.2398

535.74843	0.23981

535.84803	0.23983

535.94763	0.23984

536.04723	0.24016

536.14683	0.24019

536.24643	0.2402

536.34603	0.24022

536.44563	0.24023

536.54523	0.24029

536.64483	0.2403

536.74443	0.24032

536.84403	0.24037

536.94363	0.24039

537.04323	0.2404

537.14283	0.24041

537.24243	0.24043

537.34203	0.24046

537.44163	0.24047

537.54123	0.2405

537.64083	0.24051

537.74043	0.24053

537.84003	0.24057

537.93963	0.24058

538.03923	0.24064

538.13883	0.24065

538.23843	0.24067

538.33803	0.24068

538.43763	0.24071

538.53723	0.24072

538.63683	0.24074

538.73643	0.24075

538.83603	0.24076

538.93563	0.24078

539.03523	0.24079

539.13483	0.24082

539.23443	0.24083

539.33403	0.24085

539.43363	0.24086

539.53323	0.24088

539.63283	0.24093

539.73243	0.24095

539.83203	0.24101

539.93163	0.24103

540.03123	0.24106

540.13083	0.24107

540.23043	0.24108

540.33003	0.24124

540.42963	0.24128

540.52923	0.24132

540.62883	0.24134

540.72843	0.24138

540.82803	0.24142

540.92763	0.24146

541.02723	0.24148

541.12683	0.2415

541.22643	0.24152

541.32603	0.24195

541.42563	0.24196

541.52523	0.24198

541.62483	0.24199

541.72443	0.24203

541.82403	0.24206

541.92363	0.2421

542.02323	0.24213

542.12283	0.24215

542.22243	0.24216

542.32203	0.24217

542.42163	0.24219

542.52123	0.2422

542.62083	0.24222

542.72043	0.24227

542.82003	0.24229

542.91963	0.24231

543.01923	0.24233

543.11883	0.24234

543.21843	0.24236

543.31803	0.24237

543.41763	0.24238

543.51723	0.24243

543.61683	0.24245

543.71643	0.24249

543.81603	0.24251

543.91563	0.24256

544.01523	0.24259

544.11483	0.24261

544.21443	0.24262

544.31403	0.24263

544.41363	0.24269

544.51323	0.24273

544.61283	0.24275

544.71243	0.24276

544.81203	0.24277

544.91163	0.24279

545.01123	0.24282

545.11083	0.24286

545.21043	0.24289

545.31003	0.2429

545.40963	0.24291

545.50923	0.24296

545.60883	0.243

545.70843	0.24301

545.80803	0.24303

545.90763	0.24308

546.00723	0.24311

546.10684	0.24314

546.20644	0.24315

546.30603	0.24319

546.40563	0.24321

546.50523	0.24322

546.60483	0.24323

546.70443	0.24326

546.80404	0.24328

546.90364	0.24333

547.00324	0.24335

547.10284	0.24347

547.20244	0.2435

547.30204	0.24351

547.40164	0.24353

547.50123	0.24354

547.60083	0.24356

547.70044	0.2436

547.80004	0.24364

547.89964	0.24367

547.99924	0.2437

548.09884	0.24371

548.19844	0.24374

548.29804	0.24389

548.39764	0.24391

548.49724	0.24393

548.59684	0.24395

548.69644	0.24396

548.79604	0.24399

548.89564	0.244

548.99524	0.24402

549.09484	0.24403

549.19444	0.24406

549.29404	0.24407

549.39364	0.24409

549.49324	0.24414

549.59284	0.24417

549.69244	0.24425

549.79204	0.24427

549.89164	0.24431

549.99124	0.24432

550.09084	0.24435

550.19044	0.24437

550.29004	0.24439

550.38964	0.24441

550.48924	0.24442

550.58884	0.24444

550.68844	0.24448

550.78804	0.24449

550.88764	0.24451

550.98724	0.24452

551.08684	0.24453

551.18644	0.24458

551.28604	0.24459

551.38564	0.2446

551.48524	0.24462

551.58484	0.24463

551.68444	0.24465

551.78404	0.24466

551.88364	0.2447

551.98324	0.24477

552.08284	0.24478

552.18244	0.24481

552.28204	0.24487

552.38164	0.24488

552.48124	0.24491

552.58084	0.24492

552.68044	0.24501

552.78004	0.24505

552.87964	0.24509

552.97924	0.24512

553.07884	0.24513

553.17844	0.24515

553.27804	0.24534

553.37764	0.24536

553.47724	0.24537

553.57684	0.24539

553.67644	0.2454

553.77604	0.24547

553.87564	0.24548

553.97524	0.2455

554.07484	0.24551

554.17444	0.24552

554.27404	0.24554

554.37364	0.24555

554.47324	0.24562

554.57284	0.24566

554.67244	0.24573

554.77204	0.24578

554.87164	0.2458

554.97124	0.24583

555.07084	0.24585

555.17044	0.24586

555.27004	0.24587

555.36964	0.24589

555.46924	0.24592

555.56884	0.24593

555.66844	0.24597

555.76804	0.24599

555.86764	0.24601

555.96724	0.24606

556.06684	0.24611

556.16644	0.24613

556.26604	0.24614

556.36564	0.24615

556.46524	0.24617

556.56484	0.2462

556.66444	0.24624

556.76404	0.24625

556.86364	0.24626

556.96324	0.24628

557.06284	0.24629

557.16244	0.24632

557.26204	0.24633

557.36164	0.24638

557.46124	0.24639

557.56084	0.2464

557.66044	0.24642

557.76004	0.24643

557.85964	0.24647

557.95924	0.2465

558.05884	0.24652

558.15844	0.24654

558.25804	0.24657

558.35764	0.24661

558.45724	0.24663

558.55684	0.24666

558.65644	0.24668

558.75604	0.24673

558.85564	0.24675

558.95524	0.24677

559.05484	0.2468

559.15444	0.24681

559.25404	0.24682

559.35364	0.24688

559.45324	0.24691

559.55284	0.24694

559.65244	0.24695

559.75204	0.24696

559.85164	0.24699

559.95124	0.2473

560.05084	0.24731

560.15044	0.24733

560.25004	0.24734

560.34964	0.24735

560.44924	0.24742

560.54884	0.24744

560.64844	0.24747

560.74804	0.24759

560.84764	0.24761

560.94724	0.24762

561.04684	0.24763

561.14644	0.24765

561.24604	0.24766

561.34564	0.24768

561.44524	0.24769

561.54484	0.2477

561.64444	0.2478

561.74404	0.24783

561.84364	0.24784

561.94324	0.24787

562.04284	0.24791

562.14244	0.24821

562.24204	0.24917

562.34164	0.24918

562.44124	0.2492

562.54084	0.24924

562.64044	0.24925

562.74004	0.24931

562.83964	0.24932

562.93924	0.24938

563.03884	0.24946

563.13844	0.24948

563.23804	0.24949

563.33764	0.24952

563.43724	0.24953

563.53684	0.24955

563.63644	0.24956

563.73604	0.24957

563.83564	0.24959

563.93524	0.2496

564.03484	0.24963

564.13444	0.24967

564.23404	0.24974

564.33364	0.24984

564.43324	0.24991

564.53284	0.24994

564.63244	0.24995

564.73204	0.24998

564.83164	0.24999

564.93124	0.25001

565.03084	0.25002

565.13044	0.25003

565.23004	0.25005

565.32964	0.25006

565.42924	0.2501

565.52884	0.25015

565.62844	0.25017

565.72804	0.2502

565.82764	0.25024

565.92724	0.25026

566.02684	0.2503

566.12644	0.25031

566.22604	0.25033

566.32564	0.25034

566.42524	0.25036

566.52484	0.25038

566.62444	0.2504

566.72404	0.25041

566.82364	0.25043

566.92324	0.25044

567.02284	0.25057

567.12244	0.25065

567.22204	0.25069

567.32164	0.25073

567.42124	0.25075

567.52084	0.25082

567.62044	0.25083

567.72004	0.25084

567.81964	0.25086

567.91924	0.25087

568.01884	0.25091

568.11844	0.25093

568.21804	0.25096

568.31764	0.25097

568.41724	0.25101

568.51684	0.25103

568.61644	0.25107

568.71604	0.25108

568.81564	0.25114

568.91524	0.25117

569.01484	0.25122

569.11444	0.25124

569.21404	0.25125

569.31364	0.25136

569.41324	0.25138

569.51284	0.25139

569.61244	0.25142

569.71204	0.25145

569.81164	0.25146

569.91124	0.25147

570.01084	0.2517

570.11044	0.25174

570.21004	0.25191

570.30964	0.25195

570.40924	0.25196

570.50884	0.25198

570.60844	0.25199

570.70804	0.25205

570.80764	0.25206

570.90724	0.25207

571.00684	0.25209

571.10644	0.25212

571.20604	0.25213

571.30564	0.25221

571.40524	0.25228

571.50484	0.2523

571.60444	0.25231

571.70404	0.25232

571.80364	0.25235

571.90324	0.25237

572.00284	0.25238

572.10244	0.25241

572.20204	0.25242

572.30164	0.25244

572.40124	0.25255

572.50084	0.25256

572.60044	0.25258

572.70004	0.25259

572.79964	0.2526

572.89924	0.25262

572.99884	0.25265

573.09844	0.25267

573.19804	0.25269

573.29764	0.25277

573.39724	0.25284

573.49684	0.25287

573.59644	0.2529

573.69604	0.25297

573.79564	0.25308

573.89524	0.25309

573.99484	0.25312

574.09444	0.25316

574.19404	0.2532

574.29364	0.25322

574.39324	0.25325

574.49284	0.25326

574.59244	0.25333

574.69204	0.25334

574.79164	0.25339

574.89124	0.25341

574.99084	0.25343

575.09044	0.25344

575.19004	0.25346

575.28964	0.2535

575.38924	0.25351

575.48884	0.25355

575.58844	0.25357

575.68804	0.25358

575.78764	0.25387

575.88724	0.2539

575.98684	0.25392

576.08644	0.25397

576.18604	0.25399

576.28564	0.254

576.38524	0.25401

576.48484	0.25404

576.58444	0.25406

576.68404	0.25411

576.78364	0.25417

576.88324	0.25418

576.98284	0.25422

577.08244	0.25425

577.18204	0.25427

577.28164	0.25434

577.38124	0.25435

577.48084	0.25441

577.58044	0.25442

577.68004	0.25443

577.77964	0.25449

577.87924	0.2545

577.97884	0.25452

578.07844	0.25454

578.17804	0.2547

578.27764	0.25475

578.37724	0.25477

578.47684	0.25478

578.57644	0.25489

578.67604	0.25492

578.77564	0.25496

578.87524	0.25498

578.97484	0.25503

579.07444	0.25505

579.17404	0.25508

579.27364	0.25509

579.37324	0.25513

579.47284	0.25515

579.57244	0.25516

579.67204	0.25517

579.77164	0.25522

579.87124	0.25523

579.97084	0.25524

580.07044	0.25528

580.17004	0.2553

580.26964	0.25531

580.36924	0.2554

580.46884	0.25544

580.56844	0.25545

580.66804	0.25547

580.76764	0.25548

580.86724	0.25551

580.96684	0.25554

581.06644	0.25555

581.16604	0.25556

581.26564	0.25558

581.36524	0.25562

581.46484	0.25566

581.56444	0.25568

581.66404	0.25569

581.76364	0.25573

581.86324	0.25575

581.96284	0.25584

582.06244	0.2559

582.16204	0.25591

582.26164	0.25593

582.36124	0.25596

582.46084	0.25603

582.56044	0.25604

582.66004	0.25607

582.75964	0.25608

582.85924	0.25609

582.95884	0.25611

583.05844	0.25612

583.15804	0.25614

583.25764	0.25615

583.35724	0.25616

583.45684	0.25623

583.55644	0.25625

583.65604	0.25626

583.75564	0.25629

583.85524	0.25632

583.95484	0.25633

584.05444	0.25636

584.15404	0.25637

584.25364	0.2564

584.35324	0.25642

584.45284	0.25646

584.55244	0.25649

584.65204	0.25661

584.75164	0.25663

584.85124	0.25664

584.95084	0.25667

585.05044	0.25668

585.15004	0.2567

585.24964	0.25671

585.34924	0.25674

585.44884	0.25675

585.54844	0.25678

585.64804	0.25679

585.74764	0.25681

585.84724	0.25685

585.94684	0.25706

586.04644	0.25709

586.14604	0.2571

586.24564	0.25711

586.34524	0.25716

586.44484	0.25717

586.54444	0.25721

586.64404	0.25724

586.74364	0.25727

586.84324	0.25732

586.94284	0.25734

587.04244	0.25738

587.14204	0.25739

587.24164	0.25742

587.34124	0.25744

587.44084	0.25749

587.54044	0.25755

587.64004	0.25757

587.73964	0.25759

587.83924	0.25762

587.93884	0.25767

588.03844	0.25769

588.13804	0.25774

588.23764	0.25777

588.33724	0.25778

588.43684	0.25781

588.53644	0.25798

588.63604	0.25799

588.73564	0.25811

588.83524	0.25815

588.93484	0.25818

589.03444	0.25819

589.13404	0.2582

589.23364	0.25823

589.33324	0.25825

589.43284	0.25829

589.53244	0.2583

589.63204	0.25834

589.73164	0.25841

589.83124	0.25843

589.93084	0.25845

590.03044	0.25851

590.13004	0.25852

590.22964	0.25854

590.32924	0.25857

590.42884	0.25858

590.52844	0.25871

590.62804	0.25873

590.72764	0.2589

590.82724	0.25894

590.92684	0.25901

591.02644	0.25903

591.12604	0.25904

591.22564	0.25907

591.32524	0.2591

591.42484	0.25911

591.52444	0.25915

591.62404	0.25917

591.72364	0.25918

591.82324	0.25928

591.92284	0.25929

592.02244	0.25935

592.12204	0.25939

592.22164	0.2594

592.32124	0.2595

592.42084	0.25959

592.52044	0.25961

592.62004	0.25963

592.71964	0.25964

592.81924	0.25966

592.91884	0.2597

593.01844	0.25971

593.11804	0.25973

593.21764	0.25975

593.31724	0.25985

593.41684	0.25986

593.51644	0.25991

593.61604	0.25992

593.71564	0.25993

593.81524	0.26005

593.91484	0.26006

594.01444	0.2601

594.11404	0.26012

594.21364	0.26014

594.31324	0.26016

594.41284	0.26019

594.51244	0.26021

594.61204	0.26023

594.71164	0.26024

594.81124	0.26028

594.91084	0.2603

595.01044	0.26033

595.11004	0.26038

595.20964	0.2604

595.30924	0.26042

595.40884	0.26045

595.50844	0.26047

595.60804	0.26048

595.70764	0.26049

595.80724	0.26051

595.90684	0.26059

596.00644	0.26063

596.10604	0.26084

596.20564	0.26087

596.30524	0.26088

596.40484	0.26091

596.50444	0.26094

596.60404	0.26095

596.70364	0.26104

596.80324	0.26108

596.90284	0.26115

597.00244	0.26116

597.10204	0.26118

597.20164	0.26123

597.30124	0.26126

597.40084	0.26128

597.50044	0.2613

597.60004	0.26132

597.69964	0.26133

597.79924	0.26134

597.89884	0.26139

597.99844	0.26141

598.09804	0.26144

598.19764	0.26147

598.29724	0.26153

598.39684	0.26154

598.49644	0.26155

598.59604	0.26157

598.69564	0.2616

598.79524	0.26161

598.89484	0.26164

598.99444	0.26167

599.09404	0.26168

599.19364	0.26179

599.29324	0.26183

599.39284	0.26188

599.49244	0.26189

599.59204	0.26199

599.69164	0.262

599.79124	0.26207

599.89084	0.26211

599.99044	0.26214

600.09004	0.26218

600.18964	0.26224

600.28924	0.26225

600.38884	0.26227

600.48844	0.26231

600.58804	0.26234

600.68764	0.26236

600.78724	0.26238

600.88684	0.26243

600.98644	0.26245

601.08604	0.26246

601.18564	0.26249

601.28524	0.2625

601.38484	0.26252

601.48444	0.26255

601.58404	0.26256

601.68364	0.26257

601.78324	0.26259

601.88284	0.2626

601.98244	0.26263

602.08204	0.26273

602.18164	0.26274

602.28124	0.26278

602.38084	0.2628

602.48044	0.26281

602.58004	0.26282

602.67964	0.26288

602.77924	0.26292

602.87884	0.26294

602.97844	0.26296

603.07804	0.26298

603.17764	0.26308

603.27724	0.26309

603.37684	0.26315

603.47644	0.26316

603.57604	0.2632

603.67564	0.26322

603.77524	0.26323

603.87484	0.26324

603.97444	0.26327

604.07404	0.26329

604.17364	0.2633

604.27324	0.26337

604.37284	0.26338

604.47244	0.2634

604.57204	0.26341

604.67164	0.26343

604.77124	0.26344

604.87084	0.26345

604.97044	0.2635

605.07004	0.26351

605.16964	0.26354

605.26924	0.26357

605.36884	0.26358

605.46844	0.26361

605.56804	0.26362

605.66764	0.26366

605.76724	0.26369

605.86684	0.2637

605.96644	0.26372

606.06604	0.26376

606.16564	0.26379

606.26524	0.2638

606.36484	0.26383

606.46444	0.26384

606.56404	0.26386

606.66364	0.264

606.76324	0.26401

606.86284	0.26404

606.96244	0.26407

607.06204	0.26419

607.16164	0.26421

607.26124	0.26422

607.36084	0.26424

607.46044	0.26426

607.56004	0.26428

607.65964	0.26431

607.75924	0.26446

607.85884	0.26447

607.95844	0.26453

608.05804	0.2646

608.15764	0.26461

608.25724	0.26467

608.35684	0.26468

608.45644	0.2647

608.55604	0.26471

608.65564	0.26477

608.75524	0.26478

608.85484	0.26482

608.95444	0.26485

609.05404	0.26486

609.15364	0.26488

609.25324	0.26489

609.35284	0.26496

609.45244	0.26505

609.55204	0.26506

609.65164	0.2651

609.75124	0.26511

609.85084	0.26513

609.95044	0.26514

610.05004	0.26517

610.14964	0.26518

610.24924	0.26587

610.34884	0.26588

610.44844	0.26592

610.54804	0.26608

610.64764	0.26612

610.74724	0.26613

610.84684	0.26615

610.94644	0.26616

611.04604	0.26619

611.14564	0.2662

611.24524	0.26625

611.34484	0.26627

611.44444	0.26632

611.54404	0.2664

611.64364	0.26641

611.74324	0.26648

611.84284	0.26651

611.94244	0.26654

612.04204	0.26655

612.14164	0.26657

612.24124	0.26661

612.34084	0.26668

612.44044	0.26669

612.54004	0.26682

612.63964	0.26683

612.73924	0.26686

612.83884	0.26687

612.93844	0.26689

613.03804	0.2669

613.13764	0.26697

613.23724	0.26699

613.33684	0.267

613.43644	0.26701

613.53604	0.26703

613.63564	0.2671

613.73524	0.26714

613.83484	0.26715

613.93444	0.26717

614.03404	0.26718

614.13364	0.26721

614.23324	0.26722

614.33284	0.26724

614.43244	0.26725

614.53204	0.26727

614.63164	0.26728

614.73124	0.26731

614.83084	0.26732

614.93044	0.26735

615.03004	0.26738

615.12964	0.2674

615.22924	0.26743

615.32884	0.26746

615.42844	0.26759

615.52804	0.26764

615.62764	0.26766

615.72724	0.26767

615.82684	0.26784

615.92644	0.26787

616.02604	0.26788

616.12564	0.26791

616.22524	0.26794

616.32484	0.26809

616.42444	0.2681

616.52404	0.26817

616.62364	0.26823

616.72324	0.26826

616.82284	0.2683

616.92244	0.2684

617.02204	0.26841

617.12164	0.26844

617.22124	0.26848

617.32084	0.26849

617.42044	0.26851

617.52004	0.26852

617.61964	0.26865

617.71924	0.26869

617.81884	0.26873

617.91844	0.26876

618.01804	0.26877

618.11764	0.26882

618.21724	0.26887

618.31684	0.26894

618.41644	0.26895

618.51604	0.26897

618.61564	0.26902

618.71524	0.26904

618.81484	0.26905

618.91444	0.26907

619.01404	0.26909

619.11364	0.26922

619.21324	0.26923

619.31284	0.26925

619.41244	0.26926

619.51204	0.26932

619.61164	0.26935

619.71124	0.26936

619.81084	0.26937

619.91044	0.26939

620.01004	0.26951

620.10964	0.26953

620.20924	0.26956

620.30884	0.26957

620.40844	0.2696

620.50804	0.26964

620.60764	0.26971

620.70724	0.26978

620.80684	0.26979

620.90644	0.26982

621.00604	0.26986

621.10564	0.26988

621.20524	0.26989

621.30484	0.26993

621.40444	0.26995

621.50404	0.26996

621.60364	0.27

621.70324	0.27017

621.80284	0.2702

621.90244	0.27028

622.00204	0.27042

622.10164	0.27043

622.20124	0.27059

622.30084	0.27062

622.40044	0.27064

622.50004	0.27074

622.59964	0.27076

622.69924	0.27077

622.79884	0.27078

622.89844	0.27084

622.99804	0.27087

623.09764	0.2709

623.19724	0.27092

623.29684	0.27094

623.39644	0.27095

623.49604	0.27097

623.59564	0.27098

623.69524	0.27099

623.79484	0.27101

623.89444	0.27102

623.99404	0.27109

624.09364	0.27111

624.19324	0.27113

624.29284	0.27115

624.39244	0.27116

624.49204	0.2712

624.59164	0.27122

624.69124	0.27123

624.79084	0.27124

624.89044	0.27126

624.99004	0.27129

625.08964	0.27138

625.18924	0.27141

625.28884	0.27143

625.38844	0.27151

625.48804	0.27152

625.58764	0.27154

625.68724	0.27157

625.78684	0.27164

625.88644	0.27165

625.98604	0.27166

626.08564	0.27173

626.18524	0.27178

626.28484	0.27194

626.38444	0.27196

626.48404	0.27218

626.58364	0.27219

626.68324	0.27222

626.78284	0.27226

626.88244	0.27231

626.98204	0.27233

627.08164	0.27235

627.18124	0.27236

627.28084	0.27238

627.38044	0.27242

627.48004	0.27247

627.57964	0.27254

627.67924	0.27257

627.77884	0.2726

627.87844	0.27261

627.97804	0.27264

628.07764	0.27265

628.17724	0.27267

628.27684	0.27277

628.37644	0.27278

628.47604	0.27281

628.57564	0.27282

628.67524	0.27284

628.77484	0.27288

628.87444	0.27292

628.97404	0.27293

629.07364	0.27296

629.17324	0.27298

629.27284	0.27299

629.37244	0.27302

629.47204	0.27309

629.57164	0.2731

629.67124	0.27312

629.77084	0.27313

629.87044	0.27314

629.97004	0.27316

630.06964	0.27317

630.16924	0.2732

630.26884	0.27321

630.36844	0.27323

630.46804	0.27324

630.56764	0.27326

630.66724	0.27337

630.76684	0.27344

630.86644	0.27348

630.96604	0.27359

631.06564	0.2736

631.16524	0.27376

631.26484	0.27377

631.36444	0.2738

631.46404	0.27387

631.56364	0.27391

631.66324	0.27397

631.76284	0.27398

631.86244	0.27402

631.96204	0.27405

632.06164	0.27407

632.16124	0.27409

632.26084	0.27411

632.36044	0.27416

632.46004	0.27418

632.55964	0.27419

632.65924	0.27423

632.75884	0.27425

632.85844	0.27426

632.95804	0.27427

633.05764	0.27436

633.15724	0.2744

633.25684	0.27441

633.35644	0.27443

633.45604	0.27446

633.55564	0.27451

633.65524	0.27453

633.75484	0.27454

633.85444	0.27455

633.95404	0.27457

634.05364	0.27465

634.15324	0.27467

634.25284	0.27468

634.35244	0.27471

634.45204	0.27474

634.55164	0.27481

634.65124	0.27482

634.75084	0.27483

634.85044	0.27489

634.95004	0.2749

635.04964	0.275

635.14924	0.27501

635.24884	0.27503

635.34844	0.27508

635.44804	0.2751

635.54764	0.27511

635.64724	0.27518

635.74684	0.27524

635.84644	0.27534

635.94604	0.27536

636.04564	0.27539

636.14524	0.27542

636.24484	0.27543

636.34444	0.27545

636.44404	0.27553

636.54364	0.27555

636.64324	0.27556

636.74284	0.27564

636.84244	0.27573

636.94204	0.27574

637.04164	0.27592

637.14124	0.27594

637.24084	0.27601

637.34044	0.2761

637.44004	0.27612

637.53964	0.27627

637.63924	0.27629

637.73884	0.2763

637.83844	0.27631

637.93804	0.27633

638.03764	0.27634

638.13724	0.27636

638.23684	0.27637

638.33644	0.27648

638.43604	0.27649

638.53564	0.27651

638.63524	0.27652

638.73484	0.27654

638.83444	0.27655

638.93404	0.27656

639.03364	0.27658

639.13324	0.27659

639.23284	0.27668

639.33244	0.27672

639.43204	0.2769

639.53164	0.27691

639.63124	0.27696

639.73084	0.27697

639.83044	0.27698

639.93004	0.277

640.02964	0.27715

640.12924	0.27718

640.22884	0.27719

640.32844	0.27721

640.42804	0.27723

640.52764	0.2773

640.62724	0.27732

640.72684	0.27736

640.82644	0.27739

640.92604	0.2774

641.02564	0.27749

641.12524	0.27753

641.22484	0.27756

641.32444	0.27757

641.42404	0.27758

641.52364	0.27763

641.62324	0.27767

641.72284	0.27784

641.82244	0.27785

641.92204	0.27786

642.02164	0.27788

642.12124	0.2779

642.22084	0.27795

642.32044	0.278

642.42004	0.27804

642.51964	0.27806

642.61924	0.2781

642.71884	0.27811

642.81844	0.27813

642.91804	0.2782

643.01764	0.27823

643.11724	0.27827

643.21684	0.27831

643.31644	0.27834

643.41604	0.27835

643.51564	0.27848

643.61524	0.27849

643.71484	0.27855

643.81444	0.27856

643.91404	0.27858

644.01364	0.2786

644.11324	0.27862

644.21284	0.27867

644.31244	0.27871

644.41204	0.27873

644.51164	0.2788

644.61124	0.27881

644.71084	0.27883

644.81044	0.27891

644.91004	0.27892

645.00964	0.27904

645.10924	0.27916

645.20884	0.27919

645.30844	0.27923

645.40804	0.27927

645.50764	0.27929

645.60724	0.2793

645.70684	0.27934

645.80644	0.27937

645.90604	0.27939

646.00564	0.27943

646.10524	0.27957

646.20484	0.27961

646.30444	0.27971

646.40404	0.27973

646.50364	0.27975

646.60324	0.27978

646.70284	0.27979

646.80244	0.27982

646.90204	0.27987

647.00164	0.27992

647.10124	0.27993

647.20084	0.28

647.30044	0.28018

647.40004	0.28022

647.49964	0.28024

647.59924	0.28031

647.69884	0.28032

647.79844	0.28033

647.89804	0.28035

647.99764	0.28036

648.09724	0.28039

648.19684	0.2804

648.29644	0.28066

648.39604	0.28067

648.49564	0.28068

648.59524	0.28071

648.69484	0.28073

648.79444	0.28074

648.89404	0.28075

648.99364	0.28081

649.09324	0.28082

649.19284	0.28088

649.29244	0.28107

649.39204	0.28109

649.49164	0.2811

649.59124	0.28126

649.69084	0.28128

649.79044	0.28131

649.89004	0.28133

649.98964	0.28137

650.08924	0.28144

650.18884	0.28147

650.28844	0.28149

650.38804	0.28152

650.48764	0.28155

650.58724	0.28156

650.68684	0.28158

650.78644	0.28167

650.88604	0.28169

650.98564	0.2817

651.08524	0.28174

651.18484	0.2818

651.28444	0.28181

651.38404	0.28184

651.48364	0.28186

651.58324	0.2819

651.68284	0.28191

651.78244	0.28195

651.88204	0.28204

651.98164	0.28208

652.08124	0.28218

652.18084	0.28222

652.28044	0.28229

652.38004	0.28236

652.47964	0.28239

652.57924	0.2824

652.67884	0.28242

652.77844	0.28247

652.87804	0.28255

652.97764	0.28258

653.07724	0.2826

653.17684	0.28261

653.27644	0.28262

653.37604	0.28264

653.47564	0.28267

653.57524	0.28271

653.67484	0.28278

653.77444	0.28282

653.87404	0.28283

653.97364	0.28286

654.07324	0.28288

654.17284	0.2829

654.27244	0.28296

654.37204	0.28297

654.47164	0.28302

654.57124	0.28307

654.67084	0.28309

654.77044	0.28318

654.87004	0.2832

654.96964	0.28327

655.06924	0.28345

655.16884	0.28346

655.26844	0.28349

655.36804	0.28352

655.46764	0.28366

655.56724	0.28369

655.66684	0.28378

655.76644	0.2838

655.86604	0.28387

655.96564	0.2839

656.06524	0.28399

656.16484	0.28402

656.26444	0.28405

656.36404	0.2841

656.46364	0.2842

656.56324	0.28422

656.66284	0.28424

656.76244	0.28427

656.86204	0.28429

656.96164	0.28431

657.06124	0.28433

657.16084	0.28437

657.26044	0.28443

657.36004	0.28444

657.45964	0.28447

657.55924	0.28448

657.65884	0.28454

657.75844	0.28466

657.85804	0.28484

657.95764	0.28486

658.05724	0.285

658.15684	0.28507

658.25644	0.28508

658.35604	0.28514

658.45564	0.28517

658.55524	0.28524

658.65484	0.28529

658.75444	0.28531

658.85404	0.28532

658.95364	0.28542

659.05324	0.28554

659.15284	0.2856

659.25244	0.28561

659.35204	0.28563

659.45164	0.28571

659.55124	0.28572

659.65084	0.28575

659.75044	0.28577

659.85004	0.28581

659.94964	0.28607

660.04924	0.2861

660.14884	0.28612

660.24844	0.28613

660.34804	0.28616

660.44764	0.2862

660.54724	0.28623

660.64684	0.28635

660.74644	0.28639

660.84604	0.28641

660.94564	0.28642

661.04524	0.28644

661.14484	0.28645

661.24444	0.28648

661.34404	0.28649

661.44364	0.28651

661.54324	0.28652

661.64284	0.28655

661.74244	0.28656

661.84204	0.28659

661.94164	0.28667

662.04124	0.28681

662.14084	0.28684

662.24044	0.28687

662.34004	0.28688

662.43964	0.2869

662.53924	0.28691

662.63884	0.28694

662.73844	0.28698

662.83804	0.28699

662.93764	0.2873

663.03724	0.28733

663.13684	0.2874

663.23644	0.28743

663.33604	0.28744

663.43564	0.28746

663.53524	0.28757

663.63484	0.28761

663.73444	0.28762

663.83404	0.28776

663.93364	0.28804

664.03324	0.28806

664.13284	0.2882

664.23244	0.28841

664.33204	0.28842

664.43164	0.28843

664.53124	0.2885

664.63084	0.28852

664.73044	0.28853

664.83004	0.28861

664.92964	0.28863

665.02924	0.28864

665.12884	0.28874

665.22844	0.28875

665.32804	0.2888

665.42764	0.28881

665.52724	0.28882

665.62684	0.28892

665.72644	0.28894

665.82604	0.28895

665.92564	0.28901

666.02524	0.28903

666.12484	0.28908

666.22444	0.28909

666.32404	0.2891

666.42364	0.28915

666.52324	0.28916

666.62284	0.28919

666.72244	0.28922

666.82204	0.28923

666.92164	0.28924

667.02124	0.28931

667.12084	0.28934

667.22044	0.28935

667.32004	0.28944

667.41964	0.28948

667.51924	0.28952

667.61884	0.28954

667.71844	0.28958

667.81804	0.28962

667.91764	0.28968

668.01724	0.28973

668.11684	0.28993

668.21644	0.29005

668.31604	0.29009

668.41564	0.29011

668.51524	0.29012

668.61484	0.29014

668.71444	0.29016

668.81404	0.29018

668.91364	0.29032

669.01324	0.29033

669.11284	0.29042

669.21244	0.29053

669.31204	0.29057

669.41164	0.29058

669.51124	0.2906

669.61084	0.29063

669.71044	0.2907

669.81004	0.29072

669.90964	0.29074

670.00924	0.29075

670.10884	0.29082

670.20844	0.29085

670.30804	0.2909

670.40764	0.29103

670.50724	0.29104

670.60684	0.29111

670.70644	0.29113

670.80604	0.29114

670.90564	0.2912

671.00524	0.29121

671.10484	0.29123

671.20444	0.29124

671.30404	0.2913

671.40364	0.29132

671.50324	0.29135

671.60284	0.29139

671.70244	0.29142

671.80204	0.29145

671.90164	0.29146

672.00124	0.29149

672.10084	0.29156

672.20044	0.29157

672.30004	0.29162

672.39964	0.29164

672.49924	0.29171

672.59884	0.29174

672.69844	0.29176

672.79804	0.29181

672.89764	0.29183

672.99724	0.29185

673.09684	0.29187

673.19644	0.29192

673.29604	0.29194

673.39564	0.29197

673.49524	0.29198

673.59484	0.29206

673.69444	0.29208

673.79404	0.29209

673.89364	0.29211

673.99324	0.29212

674.09284	0.29213

674.19244	0.29223

674.29204	0.29231

674.39164	0.29237

674.49124	0.29238

674.59084	0.29245

674.69044	0.29252

674.79004	0.29254

674.88964	0.29261

674.98924	0.29283

675.08884	0.29286

675.18844	0.29287

675.28804	0.29315

675.38764	0.29317

675.48724	0.29321

675.58684	0.29322

675.68644	0.29324

675.78604	0.29325

675.88564	0.29326

675.98524	0.29328

676.08484	0.29342

676.18444	0.29346

676.28404	0.29347

676.38364	0.29349

676.48324	0.29352

676.58284	0.29356

676.68244	0.29357

676.78204	0.2936

676.88164	0.29364

676.98124	0.29368

677.08084	0.2937

677.18044	0.29373

677.28004	0.29375

677.37964	0.29377

677.47924	0.29381

677.57884	0.29382

677.67844	0.29384

677.77804	0.29392

677.87764	0.29409

677.97724	0.29434

678.07684	0.29435

678.17644	0.29437

678.27604	0.29442

678.37564	0.29447

678.47524	0.29451

678.57484	0.29452

678.67444	0.29462

678.77404	0.29465

678.87364	0.29469

678.97324	0.2947

679.07284	0.29491

679.17244	0.29493

679.27204	0.29494

679.37164	0.29497

679.47124	0.29507

679.57084	0.29509

679.67044	0.29512

679.77004	0.29516

679.86964	0.29522

679.96924	0.29525

680.06884	0.29526

680.16844	0.29527

680.26804	0.29529

680.36764	0.2953

680.46724	0.29532

680.56684	0.29533

680.66644	0.29536

680.76604	0.29537

680.86564	0.2954

680.96524	0.2955

681.06484	0.29553

681.16444	0.29557

681.26404	0.29562

681.36364	0.29564

681.46324	0.29571

681.56284	0.29575

681.66244	0.29576

681.76204	0.29579

681.86164	0.29582

681.96124	0.29583

682.06084	0.29585

682.16044	0.29607

682.26004	0.29608

682.35964	0.2961

682.45924	0.29615

682.55884	0.29624

682.65844	0.29627

682.75804	0.29629

682.85764	0.29631

682.95724	0.29653

683.05684	0.29655

683.15644	0.29656

683.25604	0.29663

683.35564	0.2968

683.45524	0.29681

683.55484	0.29682

683.65444	0.29684

683.75404	0.29685

683.85364	0.29687

683.95324	0.29689

684.05284	0.29691

684.15244	0.29701

684.25204	0.29706

684.35164	0.29709

684.45124	0.29713

684.55084	0.29726

684.65044	0.29729

684.75004	0.29734

684.84964	0.29747

684.94924	0.29748

685.04884	0.29754

685.14844	0.29755

685.24804	0.29763

685.34764	0.29766

685.44724	0.29768

685.54684	0.29769

685.64644	0.2977

685.74604	0.29783

685.84564	0.29786

685.94524	0.29789

686.04484	0.29793

686.14444	0.29797

686.24404	0.29812

686.34364	0.29814

686.44324	0.29835

686.54284	0.29836

686.64244	0.2984

686.74204	0.29843

686.84164	0.29844

686.94124	0.29849

687.04084	0.29856

687.14044	0.29863

687.24004	0.29868

687.33964	0.2987

687.43924	0.29879

687.53884	0.29882

687.63844	0.29884

687.73804	0.29888

687.83764	0.29895

687.93724	0.29909

688.03684	0.2991

688.13644	0.29911

688.23604	0.29913

688.33564	0.29934

688.43524	0.29937

688.53484	0.29938

688.63444	0.29944

688.73404	0.29945

688.83364	0.29953

688.93324	0.29956

689.03284	0.29958

689.13244	0.29959

689.23204	0.29967

689.33164	0.2997

689.43124	0.29974

689.53084	0.29992

689.63044	0.30006

689.73004	0.30012

689.82964	0.30013

689.92924	0.30034

690.02884	0.30037

690.12844	0.30048

690.22804	0.3005

690.32764	0.30051

690.42724	0.30054

690.52684	0.30058

690.62644	0.30059

690.72604	0.30078

690.82564	0.30083

690.92524	0.30085

691.02484	0.3009

691.12444	0.30093

691.22404	0.30096

691.32364	0.30097

691.42324	0.30107

691.52284	0.3011

691.62244	0.30118

691.72204	0.30121

691.82164	0.30122

691.92124	0.30139

692.02084	0.3014

692.12044	0.30147

692.22004	0.3015

692.31964	0.30156

692.41924	0.30159

692.51884	0.3016

692.61844	0.30173

692.71804	0.30177

692.81764	0.30181

692.91724	0.30184

693.01684	0.30185

693.11644	0.30191

693.21604	0.30194

693.31564	0.30206

693.41524	0.30214

693.51484	0.30216

693.61444	0.30217

693.71404	0.30221

693.81364	0.30224

693.91324	0.30227

694.01284	0.30234

694.11244	0.30255

694.21204	0.30256

694.31164	0.30261

694.41124	0.30263

694.51084	0.30273

694.61044	0.30275

694.71004	0.3028

694.80964	0.30286

694.90924	0.30304

695.00884	0.30307

695.10844	0.30315

695.20804	0.30318

695.30764	0.30319

695.40724	0.3033

695.50684	0.30332

695.60644	0.30339

695.70604	0.30342

695.80564	0.30354

695.90524	0.30369

696.00484	0.30372

696.10444	0.30385

696.20404	0.30388

696.30364	0.30389

696.40324	0.30392

696.50284	0.30396

696.60244	0.30399

696.70204	0.3043

696.80164	0.30436

696.90124	0.30453

697.00084	0.30456

697.10044	0.30457

697.20004	0.3046

697.29964	0.30463

697.39924	0.30464

697.49884	0.3048

697.59844	0.30487

697.69804	0.30491

697.79764	0.30492

697.89724	0.30517

697.99684	0.30519

698.09644	0.30527

698.19604	0.30533

698.29564	0.30536

698.39524	0.3054

698.49484	0.30547

698.59444	0.30548

698.69404	0.3055

698.79364	0.30558

698.89324	0.30559

698.99284	0.30568

699.09244	0.30569

699.19204	0.30578

699.29164	0.30594

699.39124	0.30598

699.49084	0.30603

699.59044	0.30607

699.69004	0.30612

699.78964	0.30614

699.88924	0.30621

699.98884	0.30622

700.08844	0.30624

700.18804	0.30632

700.28764	0.30635

700.38724	0.30636

700.48684	0.30643

700.58644	0.30646

700.68604	0.30647

700.78564	0.30649

700.88524	0.30654

700.98484	0.30656

701.08444	0.30672

701.18404	0.30674

701.28364	0.30684

701.38324	0.30688

701.48284	0.30699

701.58244	0.30702

701.68204	0.30703

701.78164	0.3071

701.88124	0.30712

701.98084	0.30714

702.08044	0.30738

702.18004	0.30746

702.27964	0.3077

702.37924	0.30774

702.47884	0.30779

702.57844	0.3078

702.67805	0.30786

702.77765	0.30787

702.87725	0.30788

702.97685	0.30791

703.07645	0.30795

703.17605	0.30798

703.27565	0.30801

703.37524	0.30836

703.47484	0.30837

703.57444	0.30839

703.67404	0.30846

703.77364	0.30847

703.87325	0.3085

703.97285	0.30851

704.07245	0.30853

704.17205	0.30858

704.27165	0.30861

704.37125	0.30872

704.47085	0.30874

704.57045	0.30875

704.67005	0.30879

704.76965	0.30882

704.86925	0.30885

704.96885	0.30896

705.06845	0.30899

705.16805	0.309

705.26765	0.30907

705.36725	0.3091

705.46685	0.30911

705.56645	0.30913

705.66605	0.30922

705.76565	0.30925

705.86525	0.30928

705.96485	0.30935

706.06445	0.30939

706.16405	0.30943

706.26365	0.30945

706.36325	0.30949

706.46285	0.3095

706.56245	0.30953

706.66205	0.30957

706.76165	0.30999

706.86125	0.31001

706.96085	0.31003

707.06045	0.31016

707.16005	0.31017

707.25965	0.31019

707.35925	0.31029

707.45885	0.31035

707.55845	0.31037

707.65805	0.31047

707.75765	0.31058

707.85725	0.31059

707.95685	0.31061

708.05645	0.31062

708.15605	0.31082

708.25565	0.3109

708.35525	0.31103

708.45485	0.31105

708.55445	0.31108

708.65405	0.31111

708.75365	0.31112

708.85325	0.31114

708.95285	0.31121

709.05245	0.31122

709.15205	0.31123

709.25165	0.31125

709.35125	0.31126

709.45085	0.31129

709.55045	0.31143

709.65005	0.31146

709.74965	0.31157

709.84925	0.31177

709.94885	0.31185

710.04845	0.31186

710.14805	0.31199

710.24765	0.31202

710.34725	0.31203

710.44685	0.31206

710.54645	0.31224

710.64605	0.31225

710.74565	0.31228

710.84525	0.31231

710.94485	0.31238

711.04445	0.31252

711.14405	0.31256

711.24365	0.31258

711.34325	0.31262

711.44285	0.31267

711.54245	0.31269

711.64205	0.3127

711.74165	0.31271

711.84125	0.3129

711.94085	0.31297

712.04045	0.31298

712.14005	0.31299

712.23965	0.31301

712.33925	0.31311

712.43885	0.31312

712.53845	0.31315

712.63805	0.31316

712.73765	0.31318

712.83725	0.31323

712.93685	0.31325

713.03645	0.31327

713.13605	0.31329

713.23565	0.31338

713.33525	0.3134

713.43485	0.31347

713.53445	0.31351

713.63405	0.31361

713.73365	0.31364

713.83325	0.31368

713.93285	0.31369

714.03245	0.31378

714.13205	0.31385

714.23165	0.31386

714.33125	0.31399

714.43085	0.31403

714.53045	0.31407

714.63005	0.31408

714.72965	0.31414

714.82925	0.31425

714.92885	0.31426

715.02845	0.31432

715.12805	0.31442

715.22765	0.31446

715.32725	0.3146

715.42685	0.31461

715.52645	0.31464

715.62605	0.31471

715.72565	0.31478

715.82525	0.3148

715.92485	0.31498

716.02445	0.315

716.12405	0.31506

716.22365	0.3151

716.32325	0.31514

716.42285	0.31516

716.52245	0.31517

716.62205	0.3152

716.72165	0.31521

716.82125	0.31524

716.92085	0.31526

717.02045	0.31528

717.12005	0.31533

717.21965	0.31535

717.31925	0.31537

717.41885	0.31538

717.51845	0.31542

717.61805	0.31548

717.71765	0.31551

717.81725	0.31552

717.91685	0.31558

718.01645	0.31559

718.11605	0.31561

718.21565	0.31562

718.31525	0.31563

718.41485	0.31569

718.51445	0.31573

718.61405	0.31577

718.71365	0.3158

718.81325	0.31581

718.91285	0.31584

719.01245	0.31601

719.11205	0.31605

719.21165	0.31609

719.31125	0.31611

719.41085	0.31615

719.51045	0.31622

719.61005	0.31625

719.70965	0.31629

719.80925	0.3163

719.90885	0.31633

720.00845	0.31637

720.10805	0.31641

720.20765	0.31648

720.30725	0.31655

720.40685	0.31657

720.50645	0.31667

720.60605	0.31669

720.70565	0.31686

720.80525	0.31689

720.90485	0.3169

721.00445	0.31693

721.10405	0.31695

721.20365	0.31697

721.30325	0.3171

721.40285	0.31711

721.50245	0.31724

721.60205	0.31736

721.70165	0.31738

721.80125	0.31746

721.90085	0.31755

722.00045	0.31756

722.10005	0.31771

722.19965	0.31785

722.29925	0.31789

722.39885	0.31791

722.49845	0.31792

722.59805	0.31794

722.69765	0.31796

722.79725	0.31801

722.89685	0.31812

722.99645	0.31817

723.09605	0.31823

723.19565	0.31824

723.29525	0.31829

723.39485	0.31833

723.49445	0.31834

723.59405	0.31836

723.69365	0.31837

723.79325	0.31843

723.89285	0.31847

723.99245	0.31851

724.09205	0.31859

724.19165	0.31866

724.29125	0.31868

724.39085	0.31877

724.49045	0.31879

724.59005	0.3188

724.68965	0.3189

724.78925	0.31893

724.88885	0.31897

724.98845	0.319

725.08805	0.31903

725.18765	0.31905

725.28725	0.31907

725.38685	0.31918

725.48645	0.31926

725.58605	0.31931

725.68565	0.31942

725.78525	0.31943

725.88485	0.31947

725.98445	0.31949

726.08405	0.31953

726.18365	0.3196

726.28325	0.31961

726.38285	0.31963

726.48245	0.31993

726.58205	0.32009

726.68165	0.32012

726.78125	0.32016

726.88085	0.32032

726.98045	0.32034

727.08005	0.32056

727.17965	0.32058

727.27925	0.3206

727.37885	0.32083

727.47845	0.32088

727.57805	0.32094

727.67765	0.32097

727.77725	0.32101

727.87685	0.32105

727.97645	0.32115

728.07605	0.32118

728.17565	0.32127

728.27525	0.32134

728.37485	0.32136

728.47445	0.32147

728.57405	0.32148

728.67365	0.32151

728.77325	0.32153

728.87285	0.32158

728.97245	0.3216

729.07205	0.32161

729.17165	0.32162

729.27125	0.32166

729.37085	0.32168

729.47045	0.32171

729.57005	0.32176

729.66965	0.32178

729.76925	0.32179

729.86885	0.32182

729.96845	0.32185

730.06805	0.3219

730.16765	0.32196

730.26725	0.32218

730.36685	0.32227

730.46645	0.32232

730.56605	0.32234

730.66565	0.3224

730.76525	0.32243

730.86485	0.32245

730.96445	0.32246

731.06405	0.32247

731.16365	0.3225

731.26325	0.32259

731.36285	0.3226

731.46245	0.32264

731.56205	0.32267

731.66165	0.32268

731.76125	0.32277

731.86085	0.32278

731.96045	0.32302

732.06005	0.32305

732.15965	0.32308

732.25925	0.32309

732.35885	0.32317

732.45845	0.32326

732.55805	0.32333

732.65765	0.32335

732.75725	0.32341

732.85685	0.32342

732.95645	0.32359

733.05605	0.3237

733.15565	0.32376

733.25525	0.32379

733.35485	0.3238

733.45445	0.32382

733.55405	0.32384

733.65365	0.32387

733.75325	0.3239

733.85285	0.32391

733.95245	0.32397

734.05205	0.32398

734.15165	0.32404

734.25125	0.32407

734.35085	0.32408

734.45045	0.32411

734.55005	0.32412

734.64965	0.32415

734.74925	0.32425

734.84885	0.32429

734.94845	0.32435

735.04805	0.32436

735.14765	0.32442

735.24725	0.32457

735.34685	0.32458

735.44645	0.3246

735.54605	0.32469

735.64565	0.32476

735.74525	0.32482

735.84485	0.32485

735.94445	0.32489

736.04405	0.3249

736.14365	0.32502

736.24325	0.3252

736.34285	0.32532

736.44245	0.32537

736.54205	0.32539

736.64165	0.32541

736.74125	0.32543

736.84085	0.32556

736.94045	0.32559

737.04005	0.32563

737.13965	0.3258

737.23925	0.32587

737.33885	0.32588

737.43845	0.32594

737.53805	0.32604

737.63765	0.32609

737.73725	0.32615

737.83685	0.32617

737.93645	0.32619

738.03605	0.32622

738.13565	0.32629

738.23525	0.32631

738.33485	0.32633

738.43445	0.32634

738.53405	0.32636

738.63365	0.32659

738.73325	0.32661

738.83285	0.32666

738.93245	0.32672

739.03205	0.32673

739.13165	0.32678

739.23125	0.32682

739.33085	0.32686

739.43045	0.327

739.53005	0.32703

739.62965	0.32708

739.72925	0.32711

739.82885	0.32714

739.92845	0.32729

740.02805	0.32731

740.12765	0.32732

740.22725	0.32738

740.32685	0.3274

740.42645	0.32749

740.52605	0.32753

740.62565	0.32766

740.72525	0.32768

740.82485	0.3277

740.92445	0.32799

741.02405	0.328

741.12365	0.32803

741.22325	0.32812

741.32285	0.32817

741.42245	0.3284

741.52205	0.32844

741.62165	0.32846

741.72125	0.32848

741.82085	0.32849

741.92045	0.32852

742.02005	0.32853

742.11965	0.32856

742.21925	0.32859

742.31885	0.32877

742.41845	0.32879

742.51805	0.32881

742.61765	0.32888

742.71725	0.3289

742.81685	0.32895

742.91645	0.329

743.01605	0.32907

743.11565	0.32909

743.21525	0.32911

743.31485	0.32912

743.41445	0.32914

743.51405	0.32915

743.61365	0.32916

743.71325	0.3292

743.81285	0.32923

743.91245	0.32925

744.01205	0.32926

744.11165	0.32929

744.21125	0.32939

744.31085	0.3294

744.41045	0.32946

744.51005	0.32951

744.60965	0.32953

744.70925	0.32954

744.80885	0.32967

744.90845	0.32968

745.00805	0.32971

745.10765	0.32976

745.20725	0.32981

745.30685	0.32993

745.40645	0.32994

745.50605	0.33007

745.60565	0.33008

745.70525	0.33015

745.80485	0.33018

745.90445	0.3302

746.00405	0.33021

746.10365	0.33022

746.20325	0.33025

746.30285	0.33035

746.40245	0.33036

746.50205	0.33039

746.60165	0.33048

746.70125	0.33064

746.80085	0.33069

746.90045	0.33075

747.00005	0.33078

747.09965	0.3308

747.19925	0.33085

747.29885	0.33087

747.39845	0.33101

747.49805	0.33113

747.59765	0.33116

747.69725	0.33131

747.79685	0.33133

747.89645	0.33134

747.99605	0.33137

748.09565	0.33144

748.19525	0.33161

748.29485	0.33162

748.39445	0.33173

748.49405	0.33177

748.59365	0.33182

748.69325	0.33189

748.79285	0.3319

748.89245	0.33193

748.99205	0.33205

749.09165	0.33215

749.19125	0.33217

749.29085	0.33218

749.39045	0.33222

749.49005	0.33228

749.58965	0.33235

749.68925	0.33237

749.78885	0.33247

749.88845	0.33249

749.98805	0.3325

750.08765	0.33251

750.18725	0.33258

750.28685	0.33261

750.38645	0.33275

750.48605	0.33279

750.58565	0.33282

750.68525	0.33286

750.78485	0.33292

750.88445	0.33314

750.98405	0.33332

751.08365	0.33339

751.18325	0.33351

751.28285	0.33355

751.38245	0.33358

751.48205	0.33367

751.58165	0.33394

751.68125	0.33401

751.78085	0.33402

751.88045	0.33409

751.98005	0.3343

752.07965	0.33432

752.17925	0.33436

752.27885	0.33437

752.37845	0.33452

752.47805	0.33464

752.57765	0.33465

752.67725	0.33466

752.77685	0.33472

752.87645	0.33473

752.97605	0.33476

753.07565	0.33483

753.17525	0.33487

753.27485	0.33492

753.37445	0.33504

753.47405	0.33507

753.57365	0.33513

753.67325	0.33514

753.77285	0.33524

753.87245	0.33525

753.97205	0.33531

754.07165	0.33532

754.17125	0.3355

754.27085	0.33552

754.37045	0.33559

754.47005	0.3356

754.56965	0.33564

754.66925	0.33585

754.76885	0.33599

754.86845	0.33613

754.96805	0.33614

755.06765	0.33616

755.16725	0.33633

755.26685	0.3364

755.36645	0.33651

755.46605	0.33655

755.56565	0.33674

755.66525	0.33677

755.76485	0.3368

755.86445	0.33686

755.96405	0.33687

756.06365	0.33697

756.16325	0.33708

756.26285	0.33712

756.36245	0.33716

756.46205	0.33728

756.56165	0.33739

756.66125	0.3374

756.76085	0.33742

756.86045	0.33744

756.96005	0.33764

757.05965	0.33765

757.15925	0.33767

757.25885	0.33769

757.35845	0.33771

757.45805	0.33776

757.55765	0.33783

757.65725	0.33785

757.75685	0.33788

757.85645	0.33789

757.95605	0.33804

758.05565	0.33811

758.15525	0.33814

758.25485	0.33818

758.35445	0.33821

758.45405	0.33838

758.55365	0.33839

758.65325	0.33848

758.75285	0.33859

758.85245	0.3386

758.95205	0.33864

759.05165	0.33873

759.15125	0.33898

759.25085	0.33899

759.35045	0.33901

759.45005	0.33909

759.54965	0.3391

759.64925	0.33915

759.74885	0.33919

759.84845	0.33922

759.94805	0.3393

760.04765	0.3394

760.14725	0.33945

760.24685	0.33947

760.34645	0.33961

760.44605	0.33962

760.54565	0.33972

760.64525	0.33979

760.74485	0.3398

760.84445	0.33983

760.94405	0.33984

761.04365	0.33986

761.14325	0.33996

761.24285	0.33998

761.34245	0.34001

761.44205	0.34004

761.54165	0.34005

761.64125	0.34018

761.74085	0.34019

761.84045	0.34022

761.94005	0.34036

762.03965	0.34038

762.13925	0.34043

762.23885	0.34046

762.33845	0.34054

762.43805	0.34057

762.53765	0.34058

762.63725	0.3406

762.73685	0.34061

762.83645	0.34067

762.93605	0.34068

763.03565	0.34071

763.13525	0.34072

763.23485	0.34074

763.33445	0.34093

763.43405	0.34098

763.53365	0.341

763.63325	0.34121

763.73285	0.34123

763.83245	0.34138

763.93205	0.34145

764.03165	0.34146

764.13125	0.34149

764.23085	0.34151

764.33045	0.34158

764.43005	0.3416

764.52965	0.34165

764.62925	0.3417

764.72885	0.34173

764.82845	0.34177

764.92805	0.3418

765.02765	0.34187

765.12725	0.34188

765.22685	0.3419

765.32645	0.34191

765.42605	0.34193

765.52565	0.34195

765.62525	0.34204

765.72485	0.34212

765.82445	0.34215

765.92405	0.34219

766.02365	0.34226

766.12325	0.34232

766.22285	0.34233

766.32245	0.34234

766.42205	0.34255

766.52165	0.34258

766.62125	0.34261

766.72085	0.34264

766.82045	0.34267

766.92005	0.34269

767.01965	0.34272

767.11925	0.34275

767.21885	0.34282

767.31845	0.34283

767.41805	0.34286

767.51765	0.34287

767.61725	0.34303

767.71685	0.3431

767.81645	0.34311

767.91605	0.34313

768.01565	0.34315

768.11525	0.34322

768.21485	0.34325

768.31445	0.34329

768.41405	0.34341

768.51365	0.34342

768.61325	0.3436

768.71285	0.34367

768.81245	0.34382

768.91205	0.34387

769.01165	0.3441

769.11125	0.34412

769.21085	0.34413

769.31045	0.34415

769.41005	0.34417

769.50965	0.34426

769.60925	0.34427

769.70885	0.34434

769.80845	0.34435

769.90805	0.3444

770.00765	0.34444

770.10725	0.34445

770.20685	0.34449

770.30645	0.34458

770.40605	0.34459

770.50565	0.34463

770.60525	0.3447

770.70485	0.34475

770.80445	0.34483

770.90405	0.3449

771.00365	0.34504

771.10325	0.34507

771.20285	0.34526

771.30245	0.34528

771.40205	0.34529

771.50165	0.34539

771.60125	0.3454

771.70085	0.34542

771.80045	0.34546

771.90005	0.34549

771.99965	0.3455

772.09925	0.34568

772.19885	0.3457

772.29845	0.34571

772.39805	0.34578

772.49765	0.34583

772.59725	0.34585

772.69685	0.34596

772.79645	0.346

772.89605	0.34606

772.99565	0.34607

773.09525	0.3461

773.19485	0.34611

773.29445	0.34614

773.39405	0.34616

773.49365	0.34621

773.59325	0.34623

773.69285	0.34624

773.79245	0.34625

773.89205	0.34628

773.99165	0.3463

774.09125	0.34631

774.19085	0.34632

774.29045	0.34635

774.39005	0.34638

774.48965	0.34642

774.58925	0.34645

774.68885	0.34663

774.78845	0.34666

774.88805	0.34673

774.98765	0.34674

775.08725	0.34687

775.18685	0.34692

775.28645	0.34701

775.38605	0.34704

775.48565	0.34712

775.58525	0.34718

775.68485	0.34719

775.78445	0.3472

775.88405	0.34726

775.98365	0.34727

776.08325	0.34729

776.18285	0.3473

776.28245	0.34731

776.38205	0.34748

776.48165	0.34752

776.58125	0.34755

776.68085	0.34761

776.78045	0.34762

776.88005	0.34766

776.97965	0.34771

777.07925	0.34776

777.17885	0.34789

777.27845	0.34817

777.37805	0.34819

777.47765	0.34822

777.57725	0.34824

777.67685	0.34831

777.77645	0.34832

777.87605	0.34836

777.97565	0.34849

778.07525	0.34853

778.17485	0.34857

778.27445	0.34866

778.37405	0.34871

778.47365	0.34873

778.57325	0.34885

778.67285	0.34888

778.77245	0.34893

778.87205	0.34907

778.97165	0.34909

779.07125	0.34912

779.17085	0.34913

779.27045	0.34921

779.37005	0.34928

779.46965	0.3493

779.56925	0.34945

779.66885	0.34949

779.76845	0.34963

779.86805	0.34972

779.96765	0.34974

780.06725	0.34977

780.16685	0.34984

780.26645	0.3499

780.36605	0.34991

780.46565	0.34994

780.56525	0.34998

780.66485	0.35

780.76445	0.35002

780.86405	0.35015

780.96365	0.35025

781.06325	0.35028

781.16285	0.35029

781.26245	0.3503

781.36205	0.35032

781.46165	0.35048

781.56125	0.35055

781.66085	0.35095

781.76045	0.35096

781.86005	0.35103

781.95965	0.35104

782.05925	0.35106

782.15885	0.35115

782.25845	0.35117

782.35805	0.3512

782.45765	0.35129

782.55725	0.35132

782.65685	0.35135

782.75645	0.35146

782.85605	0.35148

782.95565	0.35157

783.05525	0.35159

783.15485	0.35164

783.25445	0.35169

783.35405	0.3518

783.45365	0.35181

783.55325	0.35182

783.65285	0.35194

783.75245	0.35195

783.85205	0.35217

783.95165	0.35222

784.05125	0.3524

784.15085	0.35244

784.25045	0.35248

784.35005	0.35251

784.44965	0.35256

784.54925	0.35258

784.64885	0.35262

784.74845	0.35265

784.84805	0.35266

784.94765	0.35268

785.04725	0.35275

785.14685	0.35276

785.24645	0.35277

785.34605	0.35289

785.44565	0.35296

785.54525	0.35311

785.64485	0.35315

785.74445	0.35326

785.84405	0.35333

785.94365	0.35339

786.04325	0.35351

786.14285	0.35358

786.24245	0.3536

786.34205	0.35361

786.44165	0.35364

786.54125	0.35377

786.64085	0.35385

786.74045	0.35396

786.84005	0.354

786.93965	0.35413

787.03925	0.35421

787.13885	0.35423

787.23845	0.35425

787.33805	0.35431

787.43765	0.35432

787.53725	0.35435

787.63685	0.35451

787.73645	0.35474

787.83605	0.35477

787.93565	0.35479

788.03525	0.35484

788.13485	0.35491

788.23445	0.35494

788.33405	0.35497

788.43365	0.35501

788.53325	0.35504

788.63285	0.35508

788.73245	0.35516

788.83205	0.35526

788.93165	0.35527

789.03125	0.35529

789.13085	0.35534

789.23045	0.35541

789.33005	0.35543

789.42965	0.35544

789.52925	0.35554

789.62885	0.35565

789.72845	0.35566

789.82805	0.35573

789.92765	0.35576

790.02725	0.35586

790.12685	0.35587

790.22645	0.35592

790.32605	0.35613

790.42565	0.35615

790.52525	0.35621

790.62485	0.35631

790.72445	0.35632

790.82405	0.35639

790.92365	0.35643

791.02325	0.35646

791.12285	0.35653

791.22245	0.35657

791.32205	0.35659

791.42165	0.35666

791.52125	0.3567

791.62085	0.35671

791.72045	0.35675

791.82005	0.35695

791.91965	0.35708

792.01925	0.35713

792.11885	0.35714

792.21845	0.35726

792.31805	0.35727

792.41765	0.35731

792.51725	0.35733

792.61685	0.35745

792.71645	0.35752

792.81605	0.35791

792.91565	0.35793

793.01525	0.35797

793.11485	0.358

793.21445	0.35804

793.31405	0.35805

793.41365	0.35807

793.51325	0.35808

793.61285	0.35819

793.71245	0.35826

793.81205	0.35833

793.91165	0.35835

794.01125	0.35837

794.11085	0.35846

794.21045	0.35878

794.31005	0.35899

794.40965	0.359

794.50925	0.35909

794.60885	0.35916

794.70845	0.35917

794.80805	0.35918

794.90765	0.35924

795.00725	0.35925

795.10685	0.35927

795.20645	0.35928

795.30605	0.35936

795.40565	0.35941

795.50525	0.35942

795.60485	0.35945

795.70445	0.35949

795.80405	0.3595

795.90365	0.35952

796.00325	0.35953

796.10285	0.3596

796.20245	0.35964

796.30205	0.36011

796.40165	0.36013

796.50125	0.36019

796.60085	0.36031

796.70045	0.36036

796.80005	0.36045

796.89965	0.36047

796.99925	0.36055

797.09885	0.36062

797.19845	0.36066

797.29805	0.36087

797.39765	0.3609

797.49725	0.36107

797.59685	0.36114

797.69645	0.36125

797.79605	0.36129

797.89565	0.36131

797.99525	0.36132

798.09485	0.36133

798.19445	0.36154

798.29405	0.36163

798.39365	0.36165

798.49325	0.36177

798.59285	0.36178

798.69245	0.36179

798.79205	0.36184

798.89165	0.36191

798.99125	0.36202

799.09085	0.36212

799.19045	0.36213

799.29005	0.36216

799.38965	0.36224

799.48925	0.36228

799.58885	0.36237

799.68845	0.36238

799.78805	0.36246

799.88765	0.36269

799.98725	0.36298

800.08685	0.363

800.18645	0.36302

800.28605	0.36304

800.38565	0.36305

800.48525	0.36309

800.58485	0.36315

800.68445	0.36316

800.78405	0.3632

800.88365	0.36322

800.98325	0.36327

801.08285	0.36332

801.18245	0.36333

801.28205	0.36341

801.38165	0.36355

801.48125	0.36357

801.58085	0.36358

801.68045	0.36361

801.78005	0.36362

801.87965	0.36374

801.97925	0.36376

802.07885	0.36399

802.17845	0.36404

802.27805	0.36406

802.37765	0.36428

802.47725	0.36429

802.57685	0.36431

802.67645	0.36434

802.77605	0.36441

802.87565	0.36442

802.97525	0.36446

803.07485	0.36448

803.17445	0.36464

803.27405	0.36467

803.37365	0.36468

803.47325	0.36474

803.57285	0.36487

803.67245	0.36488

803.77205	0.36489

803.87165	0.36496

803.97125	0.36501

804.07085	0.36506

804.17045	0.36512

804.27005	0.36513

804.36965	0.36529

804.46925	0.36536

804.56885	0.3654

804.66845	0.36541

804.76805	0.3657

804.86765	0.36573

804.96725	0.36579

805.06685	0.3659

805.16645	0.36591

805.26605	0.36594

805.36565	0.36605

805.46525	0.36626

805.56485	0.36628

805.66445	0.36635

805.76405	0.36637

805.86365	0.36667

805.96325	0.3667

806.06285	0.36671

806.16245	0.36674

806.26205	0.36677

806.36165	0.36678

806.46125	0.36685

806.56085	0.36703

806.66045	0.36704

806.76005	0.36711

806.85965	0.36721

806.95925	0.36725

807.05885	0.36727

807.15845	0.36735

807.25805	0.36738

807.35765	0.36739

807.45725	0.36741

807.55685	0.36742

807.65645	0.36756

807.75605	0.36758

807.85565	0.36759

807.95525	0.36762

808.05485	0.36778

808.15445	0.36784

808.25405	0.36785

808.35365	0.36794

808.45325	0.36797

808.55285	0.36798

808.65245	0.36799

808.75205	0.36811

808.85165	0.36825

808.95125	0.3683

809.05085	0.36832

809.15045	0.36833

809.25005	0.36836

809.34965	0.36839

809.44925	0.36851

809.54885	0.36861

809.64845	0.36862

809.74805	0.36864

809.84765	0.36866

809.94725	0.3689

810.04685	0.36896

810.14645	0.36911

810.24605	0.36913

810.34565	0.36914

810.44525	0.36915

810.54485	0.36917

810.64445	0.36919

810.74405	0.36924

810.84365	0.36926

810.94325	0.3694

811.04285	0.36949

811.14245	0.36964

811.24205	0.36975

811.34165	0.36981

811.44125	0.36999

811.54085	0.37027

811.64045	0.37028

811.74005	0.37033

811.83965	0.37037

811.93925	0.37042

812.03885	0.37045

812.13845	0.37076

812.23805	0.37079

812.33765	0.37081

812.43725	0.37083

812.53685	0.37084

812.63645	0.37086

812.73605	0.37115

812.83565	0.37118

812.93525	0.37121

813.03485	0.37123

813.13445	0.37129

813.23405	0.37135

813.33365	0.37162

813.43325	0.37182

813.53285	0.37183

813.63245	0.37188

813.73205	0.37189

813.83165	0.37195

813.93125	0.37196

814.03085	0.37209

814.13045	0.37242

814.23005	0.37243

814.32965	0.37252

814.42925	0.37259

814.52885	0.3726

814.62845	0.37274

814.72805	0.37283

814.82765	0.37312

814.92725	0.37317

815.02685	0.3732

815.12645	0.37337

815.22605	0.37343

815.32565	0.37348

815.42525	0.3735

815.52485	0.37357

815.62445	0.37368

815.72405	0.37386

815.82365	0.37391

815.92325	0.37397

816.02285	0.374

816.12245	0.37401

816.22205	0.37403

816.32165	0.37404

816.42125	0.37414

816.52085	0.37415

816.62045	0.37419

816.72005	0.37424

816.81965	0.37429

816.91925	0.37436

817.01885	0.37443

817.11845	0.37464

817.21805	0.37467

817.31765	0.37482

817.41725	0.37485

817.51685	0.37496

817.61645	0.375

817.71605	0.37503

817.81565	0.37505

817.91525	0.37507

818.01485	0.37521

818.11445	0.37524

818.21405	0.37525

818.31365	0.37535

818.41325	0.37538

818.51285	0.37539

818.61245	0.37545

818.71205	0.37548

818.81165	0.37551

818.91125	0.3756

819.01085	0.37563

819.11045	0.37567

819.21005	0.37569

819.30965	0.3757

819.40925	0.37577

819.50885	0.37579

819.60845	0.37594

819.70805	0.37615

819.80765	0.37616

819.90725	0.37622

820.00685	0.37623

820.10645	0.37625

820.20605	0.37627

820.30565	0.37633

820.40525	0.37651

820.50485	0.37653

820.60445	0.37655

820.70405	0.37657

820.80365	0.37658

820.90325	0.3766

821.00285	0.37672

821.10245	0.3768

821.20205	0.37687

821.30165	0.37689

821.40125	0.3769

821.50085	0.37694

821.60045	0.37699

821.70005	0.37715

821.79965	0.37731

821.89925	0.37732

821.99885	0.37736

822.09845	0.37738

822.19805	0.37741

822.29765	0.37756

822.39725	0.37757

822.49685	0.3776

822.59645	0.37761

822.69605	0.37764

822.79565	0.37766

822.89525	0.3777

822.99485	0.37774

823.09445	0.37778

823.19405	0.37788

823.29365	0.37791

823.39325	0.37792

823.49285	0.37799

823.59245	0.37805

823.69205	0.37815

823.79165	0.37816

823.89125	0.37827

823.99085	0.3783

824.09045	0.37844

824.19005	0.37851

824.28965	0.37852

824.38925	0.37854

824.48885	0.37858

824.58845	0.37861

824.68805	0.37862

824.78765	0.37863

824.88725	0.37869

824.98685	0.37873

825.08645	0.37875

825.18605	0.37876

825.28565	0.37883

825.38525	0.37884

825.48485	0.37886

825.58445	0.37893

825.68405	0.37896

825.78365	0.379

825.88325	0.37901

825.98285	0.37904

826.08245	0.37921

826.18205	0.37922

826.28165	0.37925

826.38125	0.37926

826.48085	0.37928

826.58045	0.37933

826.68005	0.37935

826.77965	0.37936

826.87925	0.37949

826.97885	0.3795

827.07845	0.37997

827.17805	0.38004

827.27765	0.38016

827.37725	0.38017

827.47685	0.38018

827.57645	0.3802

827.67605	0.38024

827.77565	0.38025

827.87525	0.38028

827.97485	0.38034

828.07445	0.38042

828.17405	0.38044

828.27365	0.38046

828.37325	0.38053

828.47285	0.38057

828.57245	0.38063

828.67205	0.38064

828.77165	0.38066

828.87125	0.38071

828.97085	0.38083

829.07045	0.38091

829.17005	0.38092

829.26965	0.38094

829.36925	0.38095

829.46885	0.38109

829.56845	0.38111

829.66805	0.38112

829.76765	0.38122

829.86725	0.38127

829.96685	0.38129

830.06645	0.3813

830.16605	0.38134

830.26565	0.38137

830.36525	0.38138

830.46485	0.38141

830.56445	0.38147

830.66405	0.38148

830.76365	0.38201

830.86325	0.38204

830.96285	0.38205

831.06245	0.38207

831.16205	0.38211

831.26165	0.38215

831.36125	0.38224

831.46085	0.38225

831.56045	0.38229

831.66005	0.38231

831.75965	0.38238

831.85925	0.38239

831.95885	0.38247

832.05845	0.38249

832.15805	0.38252

832.25765	0.38259

832.35725	0.3826

832.45685	0.38261

832.55645	0.38266

832.65605	0.38268

832.75565	0.38277

832.85525	0.38278

832.95485	0.38285

833.05445	0.38286

833.15405	0.38292

833.25365	0.38299

833.35325	0.383

833.45285	0.38303

833.55245	0.38306

833.65205	0.38307

833.75165	0.3831

833.85125	0.38312

833.95085	0.38313

834.05045	0.38316

834.15005	0.38317

834.24965	0.38319

834.34925	0.38321

834.44885	0.38326

834.54845	0.38327

834.64805	0.38342

834.74765	0.38344

834.84725	0.38345

834.94685	0.38348

835.04645	0.38351

835.14605	0.38355

835.24565	0.38372

835.34525	0.38387

835.44485	0.38394

835.54445	0.38398

835.64405	0.38402

835.74365	0.38404

835.84325	0.38408

835.94285	0.38414

836.04245	0.38415

836.14205	0.38416

836.24165	0.38418

836.34125	0.38422

836.44085	0.38423

836.54045	0.38434

836.64005	0.38439

836.73965	0.3844

836.83925	0.3845

836.93885	0.38454

837.03845	0.38458

837.13805	0.38461

837.23765	0.38464

837.33725	0.38468

837.43685	0.38472

837.53645	0.3849

837.63605	0.38497

837.73565	0.385

837.83525	0.38504

837.93485	0.38539

838.03445	0.38573

838.13405	0.38577

838.23365	0.38578

838.33325	0.3858

838.43285	0.38581

838.53245	0.38584

838.63205	0.38588

838.73165	0.38589

838.83125	0.38591

838.93085	0.38594

839.03045	0.38595

839.13005	0.38599

839.22965	0.38602

839.32925	0.38605

839.42885	0.38606

839.52845	0.38613

839.62805	0.38615

839.72765	0.38617

839.82725	0.38631

839.92685	0.38633

840.02645	0.3864

840.12605	0.38641

840.22565	0.38656

840.32525	0.38661

840.42485	0.38663

840.52445	0.38675

840.62405	0.38677

840.72365	0.38679

840.82325	0.38683

840.92285	0.38684

841.02245	0.38686

841.12205	0.38708

841.22165	0.3871

841.32125	0.38712

841.42085	0.38715

841.52045	0.38717

841.62005	0.38718

841.71965	0.38736

841.81925	0.3874

841.91885	0.38747

842.01845	0.38751

842.11805	0.38765

842.21765	0.38767

842.31725	0.38777

842.41685	0.38786

842.51645	0.38788

842.61605	0.38806

842.71565	0.38807

842.81525	0.3881

842.91485	0.38816

843.01445	0.38825

843.11405	0.38827

843.21365	0.38839

843.31325	0.38841

843.41285	0.38842

843.51245	0.38872

843.61205	0.38873

843.71165	0.38874

843.81125	0.3888

843.91085	0.38884

844.01045	0.38885

844.11005	0.38887

844.20965	0.38891

844.30925	0.38897

844.40885	0.38929

844.50845	0.3893

844.60805	0.38933

844.70765	0.3895

844.80725	0.38951

844.90685	0.38964

845.00645	0.38969

845.10605	0.38976

845.20565	0.38987

845.30525	0.38993

845.40485	0.38996

845.50445	0.39008

845.60405	0.39017

845.70365	0.3902

845.80325	0.39043

845.90285	0.39045

846.00245	0.39049

846.10205	0.39056

846.20165	0.3906

846.30125	0.39066

846.40085	0.3907

846.50045	0.39073

846.60005	0.39082

846.69965	0.39084

846.79925	0.39085

846.89885	0.39087

846.99845	0.39101

847.09805	0.39126

847.19765	0.3913

847.29725	0.39131

847.39685	0.39134

847.49645	0.39135

847.59605	0.39137

847.69565	0.39156

847.79525	0.39158

847.89485	0.39166

847.99445	0.39175

848.09405	0.39176

848.19365	0.39188

848.29325	0.39194

848.39285	0.392

848.49245	0.39205

848.59205	0.39207

848.69165	0.39208

848.79125	0.39214

848.89085	0.39216

848.99045	0.39218

849.09005	0.39223

849.18965	0.39228

849.28925	0.39233

849.38885	0.39246

849.48845	0.3925

849.58805	0.39258

849.68765	0.3926

849.78725	0.39261

849.88685	0.39269

849.98645	0.39271

850.08605	0.39272

850.18565	0.39275

850.28525	0.39282

850.38485	0.39299

850.48445	0.393

850.58405	0.39348

850.68365	0.39355

850.78325	0.39356

850.88285	0.39376

850.98245	0.39377

851.08205	0.39381

851.18165	0.39383

851.28125	0.39384

851.38085	0.39385

851.48045	0.39402

851.58005	0.39405

851.67965	0.39434

851.77925	0.39436

851.87885	0.39441

851.97845	0.39443

852.07805	0.3945

852.17765	0.39451

852.27725	0.39452

852.37685	0.39464

852.47645	0.39465

852.57605	0.39475

852.67565	0.39476

852.77525	0.39501

852.87485	0.39536

852.97445	0.39538

853.07405	0.39542

853.17365	0.39543

853.27325	0.39552

853.37285	0.39556

853.47245	0.39558

853.57205	0.39564

853.67165	0.39565

853.77125	0.39567

853.87085	0.39578

853.97045	0.39581

854.07005	0.39582

854.16965	0.39584

854.26925	0.39602

854.36885	0.39609

854.46845	0.39623

854.56805	0.39626

854.66765	0.39646

854.76725	0.39648

854.86685	0.39649

854.96645	0.39653

855.06605	0.39656

855.16565	0.39662

855.26525	0.39665

855.36485	0.39667

855.46445	0.39669

855.56405	0.3967

855.66365	0.39674

855.76325	0.397

855.86285	0.39701

855.96245	0.39716

856.06205	0.3972

856.16165	0.39725

856.26125	0.39729

856.36085	0.39732

856.46045	0.39753

856.56005	0.39755

856.65965	0.39768

856.75925	0.39781

856.85885	0.39789

856.95845	0.39792

857.05805	0.39794

857.15765	0.39797

857.25725	0.39799

857.35685	0.39814

857.45645	0.3982

857.55605	0.39845

857.65565	0.3985

857.75525	0.39853

857.85485	0.39855

857.95445	0.3986

858.05405	0.39874

858.15365	0.39882

858.25325	0.39885

858.35285	0.39899

858.45245	0.3992

858.55205	0.39924

858.65166	0.39935

858.75126	0.39941

858.85086	0.39959

858.95046	0.39961

859.05006	0.39997

859.14966	0.40001

859.24925	0.40023

859.34885	0.40028

859.44845	0.4003

859.54805	0.40033

859.64765	0.40037

859.74726	0.40068

859.84686	0.40071

859.94646	0.40075

860.04606	0.40081

860.14566	0.40082

860.24526	0.40088

860.34486	0.4009

860.44446	0.4011

860.54406	0.40111

860.64366	0.40116

860.74326	0.40152

860.84286	0.40153

860.94246	0.40187

861.04206	0.40188

861.14166	0.40209

861.24126	0.40218

861.34086	0.40219

861.44046	0.40233

861.54006	0.40234

861.63966	0.40238

861.73926	0.40244

861.83886	0.40245

861.93846	0.40251

862.03806	0.40257

862.13766	0.40262

862.23726	0.40273

862.33686	0.40278

862.43646	0.40301

862.53606	0.40307

862.63566	0.40328

862.73526	0.40329

862.83486	0.40354

862.93446	0.40356

863.03406	0.40384

863.13366	0.4042

863.23326	0.40426

863.33286	0.40427

863.43246	0.4043

863.53206	0.40438

863.63166	0.40463

863.73126	0.40465

863.83086	0.40466

863.93046	0.40467

864.03006	0.40483

864.12966	0.40484

864.22926	0.4049

864.32886	0.40491

864.42846	0.40495

864.52806	0.40497

864.62766	0.40505

864.72726	0.40507

864.82686	0.40521

864.92646	0.40523

865.02606	0.40537

865.12566	0.40541

865.22526	0.40548

865.32486	0.40551

865.42446	0.40557

865.52406	0.40561

865.62366	0.40562

865.72326	0.40564

865.82286	0.40589

865.92246	0.40592

866.02206	0.40595

866.12166	0.40627

866.22126	0.40628

866.32086	0.40636

866.42046	0.40643

866.52006	0.40656

866.61966	0.40664

866.71926	0.40669

866.81886	0.40671

866.91846	0.40673

867.01806	0.40684

867.11766	0.40685

867.21726	0.40689

867.31686	0.40731

867.41646	0.40736

867.51606	0.40738

867.61566	0.40755

867.71526	0.40758

867.81486	0.40759

867.91446	0.40761

868.01406	0.40763

868.11366	0.4078

868.21326	0.40787

868.31286	0.40819

868.41246	0.40824

868.51206	0.40826

868.61166	0.40829

868.71126	0.40833

868.81086	0.40839

868.91046	0.40846

869.01006	0.40858

869.10966	0.4086

869.20926	0.40861

869.30886	0.40875

869.40846	0.40879

869.50806	0.40902

869.60766	0.40909

869.70726	0.40918

869.80686	0.40923

869.90646	0.4096

870.00606	0.40965

870.10566	0.40967

870.20526	0.40991

870.30486	0.40999

870.40446	0.41002

870.50406	0.41004

870.60366	0.41011

870.70326	0.41012

870.80286	0.41015

870.90246	0.41018

871.00206	0.41044

871.10166	0.41048

871.20126	0.4105

871.30086	0.41053

871.40046	0.41072

871.50006	0.41093

871.59966	0.41094

871.69926	0.41097

871.79886	0.41104

871.89846	0.41113

871.99806	0.41115

872.09766	0.41139

872.19726	0.41146

872.29686	0.41153

872.39646	0.41157

872.49606	0.41161

872.59566	0.41171

872.69526	0.41187

872.79486	0.41188

872.89446	0.41203

872.99406	0.41214

873.09366	0.41219

873.19326	0.41228

873.29286	0.41231

873.39246	0.41245

873.49206	0.41247

873.59166	0.41275

873.69126	0.41276

873.79086	0.41277

873.89046	0.41279

873.99006	0.4128

874.08966	0.41326

874.18926	0.41337

874.28886	0.41344

874.38846	0.41346

874.48806	0.41353

874.58766	0.41356

874.68726	0.41364

874.78686	0.41369

874.88646	0.41376

874.98606	0.41378

875.08566	0.41383

875.18526	0.41385

875.28486	0.41389

875.38446	0.41397

875.48406	0.41399

875.58366	0.414

875.68326	0.41427

875.78286	0.41428

875.88246	0.41435

875.98206	0.41441

876.08166	0.41446

876.18126	0.41448

876.28086	0.41449

876.38046	0.41457

876.48006	0.4146

876.57966	0.4147

876.67926	0.41474

876.77886	0.41477

876.87846	0.41511

876.97806	0.41515

877.07766	0.41516

877.17726	0.41519

877.27686	0.41522

877.37646	0.41524

877.47606	0.41527

877.57566	0.41529

877.67526	0.41543

877.77486	0.41544

877.87446	0.41545

877.97406	0.4155

878.07366	0.41568

878.17326	0.41572

878.27286	0.41578

878.37246	0.41579

878.47206	0.41585

878.57166	0.41603

878.67126	0.41607

878.77086	0.41614

878.87046	0.41632

878.97006	0.41633

879.06966	0.41666

879.16926	0.41671

879.26886	0.41682

879.36846	0.41691

879.46806	0.41693

879.56766	0.41698

879.66726	0.41699

879.76686	0.41717

879.86646	0.41721

879.96606	0.41726

880.06566	0.41727

880.16526	0.4173

880.26486	0.41753

880.36446	0.41755

880.46406	0.41763

880.56366	0.41765

880.66326	0.4178

880.76286	0.41786

880.86246	0.41793

880.96206	0.41797

881.06166	0.41798

881.16126	0.41801

881.26086	0.41808

881.36046	0.41822

881.46006	0.41823

881.55966	0.41825

881.65926	0.41827

881.75886	0.4184

881.85846	0.41841

881.95806	0.41848

882.05766	0.41851

882.15726	0.41854

882.25686	0.41857

882.35646	0.41867

882.45606	0.41868

882.55566	0.41871

882.65526	0.41879

882.75486	0.41914

882.85446	0.41915

882.95406	0.41927

883.05366	0.41931

883.15326	0.41935

883.25286	0.41946

883.35246	0.4195

883.45206	0.41984

883.55166	0.42022

883.65126	0.42024

883.75086	0.4203

883.85046	0.42034

883.95006	0.42038

884.04966	0.4204

884.14926	0.42048

884.24886	0.42049

884.34846	0.42054

884.44806	0.42065

884.54766	0.42069

884.64726	0.42082

884.74686	0.42083

884.84646	0.42096

884.94606	0.42117

885.04566	0.42151

885.14526	0.42153

885.24486	0.42179

885.34446	0.42185

885.44406	0.42199

885.54366	0.422

885.64326	0.42224

885.74286	0.42225

885.84246	0.42234

885.94206	0.42246

886.04166	0.42253

886.14126	0.42269

886.24086	0.42273

886.34046	0.42304

886.44006	0.42305

886.53966	0.42313

886.63926	0.4232

886.73886	0.42322

886.83846	0.42327

886.93806	0.42332

887.03766	0.42333

887.13726	0.42341

887.23686	0.42362

887.33646	0.42364

887.43606	0.42373

887.53566	0.424

887.63526	0.42429

887.73486	0.42435

887.83446	0.42442

887.93406	0.42445

888.03366	0.42457

888.13326	0.42459

888.23286	0.42484

888.33246	0.42485

888.43206	0.42489

888.53166	0.425

888.63126	0.42534

888.73086	0.42548

888.83046	0.42573

888.93006	0.42604

889.02966	0.42605

889.12926	0.42629

889.22886	0.4263

889.32846	0.42651

889.42806	0.42658

889.52766	0.42662

889.62726	0.42668

889.72686	0.42672

889.82646	0.42682

889.92606	0.42683

890.02566	0.42685

890.12526	0.42686

890.22486	0.42711

890.32446	0.42713

890.42406	0.42741

890.52366	0.42742

890.62326	0.42743

890.72286	0.42746

890.82246	0.42756

890.92206	0.4276

891.02166	0.42767

891.12126	0.42776

891.22086	0.42805

891.32046	0.42808

891.42006	0.42809

891.51966	0.42824

891.61926	0.42826

891.71886	0.42845

891.81846	0.4285

891.91806	0.42851

892.01766	0.42857

892.11726	0.42858

892.21686	0.42862

892.31646	0.42868

892.41606	0.42875

892.51566	0.42877

892.61526	0.42882

892.71486	0.42894

892.81446	0.42898

892.91406	0.42901

893.01366	0.42905

893.11326	0.42908

893.21286	0.42924

893.31246	0.42967

893.41206	0.42988

893.51166	0.43002

893.61126	0.43005

893.71086	0.43051

893.81046	0.43056

893.91006	0.43077

894.00966	0.43084

894.10926	0.43086

894.20886	0.43087

894.30846	0.43098

894.40806	0.43102

894.50766	0.43105

894.60726	0.43129

894.70686	0.43133

894.80646	0.43167

894.90606	0.43176

895.00566	0.43179

895.10526	0.4318

895.20486	0.43193

895.30446	0.43194

895.40406	0.43203

895.50366	0.43207

895.60326	0.43228

895.70286	0.43229

895.80246	0.43249

895.90206	0.43252

896.00166	0.43282

896.10126	0.43284

896.20086	0.43295

896.30046	0.43302

896.40006	0.43305

896.49966	0.43341

896.59926	0.43342

896.69886	0.43358

896.79846	0.43362

896.89806	0.43376

896.99766	0.43419

897.09726	0.43421

897.19686	0.43426

897.29646	0.43446

897.39606	0.43453

897.49566	0.43472

897.59526	0.43477

897.69486	0.43483

897.79446	0.43485

897.89406	0.4349

897.99366	0.43506

898.09326	0.43507

898.19286	0.43516

898.29246	0.43518

898.39206	0.43521

898.49166	0.43531

898.59126	0.43532

898.69086	0.43549

898.79046	0.43551

898.89006	0.43556

898.98966	0.43562

899.08926	0.43566

899.18886	0.43569

899.28846	0.43573

899.38806	0.43574

899.48766	0.43583

899.58726	0.43584

899.68686	0.43585

899.78646	0.43597

899.88606	0.43601

899.98566	0.43616

900.08526	0.43618

900.18486	0.4363

900.28446	0.43637

900.38406	0.43643

900.48366	0.43651

900.58326	0.43659

900.68286	0.43662

900.78246	0.43673

900.88206	0.43682

900.98166	0.43704

901.08126	0.43705

901.18086	0.43708

901.28046	0.4371

901.38006	0.43712

901.47966	0.43729

901.57926	0.43736

901.67886	0.43739

901.77846	0.43745

901.87806	0.4375

901.97766	0.43778

902.07726	0.43784

902.17686	0.43791

902.27646	0.43793

902.37606	0.43795

902.47566	0.43796

902.57526	0.43807

902.67486	0.43809

902.77446	0.43812

902.87406	0.43816

902.97366	0.43823

903.07326	0.43826

903.17286	0.43827

903.27246	0.43856

903.37206	0.43863

903.47166	0.4387

903.57126	0.4388

903.67086	0.43887

903.77046	0.43897

903.87006	0.43907

903.96966	0.43908

904.06926	0.43914

904.16886	0.43921

904.26846	0.43922

904.36806	0.43933

904.46766	0.43936

904.56726	0.43954

904.66686	0.43957

904.76646	0.43964

904.86606	0.43978

904.96566	0.43983

905.06526	0.44004

905.16486	0.4401

905.26446	0.44027

905.36406	0.44028

905.46366	0.44029

905.56326	0.44049

905.66286	0.4405

905.76246	0.44055

905.86206	0.44064

905.96166	0.44066

906.06126	0.44069

906.16086	0.44091

906.26046	0.44108

906.36006	0.44112

906.45966	0.44124

906.55926	0.44138

906.65886	0.4414

906.75846	0.44154

906.85806	0.44156

906.95766	0.44172

907.05726	0.44176

907.15686	0.44177

907.25646	0.44184

907.35606	0.44191

907.45566	0.442

907.55526	0.44201

907.65486	0.44222

907.75446	0.44224

907.85406	0.4424

907.95366	0.44251

908.05326	0.44254

908.15286	0.44264

908.25246	0.44267

908.35206	0.44275

908.45166	0.44277

908.55126	0.44278

908.65086	0.44293

908.75046	0.44296

908.85006	0.44334

908.94966	0.44335

909.04926	0.44337

909.14886	0.44366

909.24846	0.44367

909.34806	0.44373

909.44766	0.4438

909.54726	0.44394

909.64686	0.44401

909.74646	0.44416

909.84606	0.44427

909.94566	0.44429

910.04526	0.44454

910.14486	0.44461

910.24446	0.44468

910.34406	0.44469

910.44366	0.44472

910.54326	0.44473

910.64286	0.44475

910.74246	0.44479

910.84206	0.44486

910.94166	0.445

911.04126	0.44501

911.14086	0.44503

911.24046	0.4455

911.34006	0.44554

911.43966	0.4457

911.53926	0.44571

911.63886	0.4458

911.73846	0.44606

911.83806	0.44613

911.93766	0.44626

912.03726	0.44644

912.13686	0.44649

912.23646	0.44652

912.33606	0.44654

912.43566	0.44655

912.53526	0.44665

912.63486	0.44669

912.73446	0.44675

912.83406	0.44676

912.93366	0.44677

913.03326	0.44679

913.13286	0.44701

913.23246	0.44747

913.33206	0.44751

913.43166	0.44761

913.53126	0.44767

913.63086	0.44782

913.73046	0.44783

913.83006	0.44785

913.92966	0.44799

914.02926	0.448

914.12886	0.44806

914.22846	0.44811

914.32806	0.44817

914.42766	0.44818

914.52726	0.44835

914.62686	0.44839

914.72646	0.44857

914.82606	0.44863

914.92566	0.44864

915.02526	0.44881

915.12486	0.44884

915.22446	0.44885

915.32406	0.44901

915.42366	0.44912

915.52326	0.4492

915.62286	0.44936

915.72246	0.4494

915.82206	0.44941

915.92166	0.44954

916.02126	0.44965

916.12086	0.44966

916.22046	0.44969

916.32006	0.44983

916.41966	0.44986

916.51926	0.45008

916.61886	0.45014

916.71846	0.45018

916.81806	0.45024

916.91766	0.45029

917.01726	0.45052

917.11686	0.45057

917.21646	0.45082

917.31606	0.45085

917.41566	0.45106

917.51526	0.45107

917.61486	0.45149

917.71446	0.45155

917.81406	0.45184

917.91366	0.45187

918.01326	0.45191

918.11286	0.45208

918.21246	0.45211

918.31206	0.45248

918.41166	0.45251

918.51126	0.45269

918.61086	0.45275

918.71046	0.45292

918.81006	0.45293

918.90966	0.45308

919.00926	0.4531

919.10886	0.45311

919.20846	0.45318

919.30806	0.45321

919.40766	0.45322

919.50726	0.45324

919.60686	0.45335

919.70646	0.45336

919.80606	0.45346

919.90566	0.45348

920.00526	0.45353

920.10486	0.45384

920.20446	0.45385

920.30406	0.45388

920.40366	0.45395

920.50326	0.45403

920.60286	0.45405

920.70246	0.45415

920.80206	0.4542

920.90166	0.45426

921.00126	0.4544

921.10086	0.45442

921.20046	0.4547

921.30006	0.45472

921.39966	0.45473

921.49926	0.45482

921.59886	0.45484

921.69846	0.45498

921.79806	0.4551

921.89766	0.45526

921.99726	0.45528

922.09686	0.4557

922.19646	0.45575

922.29606	0.45593

922.39566	0.45595

922.49526	0.456

922.59486	0.45604

922.69446	0.4563

922.79406	0.45634

922.89366	0.45637

922.99326	0.45644

923.09286	0.45645

923.19246	0.45646

923.29206	0.45652

923.39166	0.45655

923.49126	0.45666

923.59086	0.45667

923.69046	0.45669

923.79006	0.4567

923.88966	0.45676

923.98926	0.4568

924.08886	0.45705

924.18846	0.45706

924.28806	0.45711

924.38766	0.45715

924.48726	0.45755

924.58686	0.4578

924.68646	0.45787

924.78606	0.45808

924.88566	0.45811

924.98526	0.45825

925.08486	0.45829

925.18446	0.45854

925.28406	0.45857

925.38366	0.4586

925.48326	0.45892

925.58286	0.45895

925.68246	0.45916

925.78206	0.45917

925.88166	0.45924

925.98126	0.4593

926.08086	0.45954

926.18046	0.45955

926.28006	0.45959

926.37966	0.45984

926.47926	0.45986

926.57886	0.45987

926.67846	0.46

926.77806	0.46001

926.87766	0.46004

926.97726	0.46023

927.07686	0.46025

927.17646	0.46046

927.27606	0.46047

927.37566	0.46058

927.47526	0.46068

927.57486	0.46069

927.67446	0.46078

927.77406	0.46085

927.87366	0.46086

927.97326	0.46088

928.07286	0.46107

928.17246	0.46127

928.27206	0.46128

928.37166	0.46135

928.47126	0.46141

928.57086	0.46145

928.67046	0.46146

928.77006	0.46149

928.86966	0.46152

928.96926	0.46159

929.06886	0.46169

929.16846	0.4618

929.26806	0.46181

929.36766	0.46194

929.46726	0.46198

929.56686	0.46201

929.66646	0.46202

929.76606	0.46209

929.86566	0.4621

929.96526	0.46216

930.06486	0.46237

930.16446	0.46238

930.26406	0.46241

930.36366	0.46244

930.46326	0.46247

930.56286	0.46276

930.66246	0.4628

930.76206	0.46311

930.86166	0.46312

930.96126	0.46322

931.06086	0.46324

931.16046	0.46325

931.26006	0.46335

931.35966	0.46353

931.45926	0.46354

931.55886	0.46371

931.65846	0.46378

931.75806	0.46379

931.85766	0.46385

931.95726	0.46409

932.05686	0.4641

932.15646	0.46412

932.25606	0.46414

932.35566	0.46416

932.45526	0.46417

932.55486	0.46418

932.65446	0.46431

932.75406	0.46432

932.85366	0.46448

932.95326	0.46451

933.05286	0.4646

933.15246	0.46474

933.25206	0.46476

933.35166	0.46481

933.45126	0.46511

933.55086	0.46513

933.65046	0.46539

933.75006	0.4654

933.84966	0.46565

933.94926	0.46569

934.04886	0.46585

934.14846	0.46597

934.24806	0.46604

934.34766	0.46607

934.44726	0.46617

934.54686	0.46621

934.64646	0.46624

934.74606	0.46628

934.84566	0.46632

934.94526	0.46663

935.04486	0.46666

935.14446	0.46675

935.24406	0.4668

935.34366	0.46698

935.44326	0.46699

935.54286	0.46703

935.64246	0.46713

935.74206	0.46716

935.84166	0.46723

935.94126	0.46733

936.04086	0.46734

936.14046	0.4677

936.24006	0.46776

936.33966	0.46777

936.43926	0.4678

936.53886	0.46809

936.63846	0.46823

936.73806	0.46835

936.83766	0.46839

936.93726	0.46867

937.03686	0.46876

937.13646	0.46879

937.23606	0.46911

937.33566	0.46935

937.43526	0.46937

937.53486	0.4695

937.63446	0.46988

937.73406	0.4699

937.83366	0.46991

937.93326	0.46995

938.03286	0.47013

938.13246	0.47018

938.23206	0.4702

938.33166	0.47052

938.43126	0.47057

938.53086	0.47059

938.63046	0.47064

938.73006	0.47065

938.82966	0.4708

938.92926	0.47082

939.02886	0.47104

939.12846	0.47112

939.22806	0.47138

939.32766	0.47142

939.42726	0.47156

939.52686	0.4717

939.62646	0.47171

939.72606	0.47174

939.82566	0.47185

939.92526	0.47193

940.02486	0.47202

940.12446	0.47203

940.22406	0.47205

940.32366	0.47207

940.42326	0.47241

940.52286	0.47245

940.62246	0.47283

940.72206	0.47284

940.82166	0.47286

940.92126	0.47307

941.02086	0.47308

941.12046	0.47321

941.22006	0.47325

941.31966	0.47326

941.41926	0.47327

941.51886	0.47343

941.61846	0.47344

941.71806	0.47348

941.81766	0.4735

941.91726	0.47351

942.01686	0.47369

942.11646	0.47371

942.21606	0.47374

942.31566	0.47375

942.41526	0.47378

942.51486	0.47382

942.61446	0.47388

942.71406	0.47415

942.81366	0.47439

942.91326	0.47464

943.01286	0.47466

943.11246	0.47491

943.21206	0.47492

943.31166	0.4751

943.41126	0.47513

943.51086	0.47533

943.61046	0.47534

943.71006	0.47552

943.80966	0.47558

943.90926	0.47559

944.00886	0.47561

944.10846	0.4758

944.20806	0.47582

944.30766	0.47584

944.40726	0.47598

944.50686	0.476

944.60646	0.47601

944.70606	0.47626

944.80566	0.47629

944.90526	0.4763

945.00486	0.4764

945.10446	0.47646

945.20406	0.47651

945.30366	0.47668

945.40326	0.47672

945.50286	0.47674

945.60246	0.47675

945.70206	0.47677

945.80166	0.47688

945.90126	0.47724

946.00086	0.47725

946.10046	0.47744

946.20006	0.47745

946.29966	0.4776

946.39926	0.47763

946.49886	0.47787

946.59846	0.47792

946.69806	0.47797

946.79766	0.47798

946.89726	0.47799

946.99686	0.47801

947.09646	0.47802

947.19606	0.47823

947.29566	0.47825

947.39526	0.47829

947.49486	0.47837

947.59446	0.47839

947.69406	0.47844

947.79366	0.47854

947.89326	0.47855

947.99286	0.479

948.09246	0.47906

948.19206	0.4791

948.29166	0.47913

948.39126	0.47936

948.49086	0.47938

948.59046	0.47939

948.69006	0.4794

948.78966	0.47945

948.88926	0.4796

948.98886	0.47968

949.08846	0.47971

949.18806	0.47981

949.28766	0.47994

949.38726	0.48006

949.48686	0.48009

949.58646	0.48031

949.68606	0.48033

949.78566	0.48034

949.88526	0.48051

949.98486	0.48056

950.08446	0.48061

950.18406	0.48084

950.28366	0.48086

950.38326	0.481

950.48286	0.48102

950.58246	0.48153

950.68206	0.48178

950.78166	0.48183

950.88126	0.48185

950.98086	0.48192

951.08046	0.48206

951.18006	0.48213

951.27966	0.4825

951.37926	0.48252

951.47886	0.48253

951.57846	0.48259

951.67806	0.4826

951.77766	0.48262

951.87726	0.48263

951.97686	0.48281

952.07646	0.48301

952.17606	0.48308

952.27566	0.48309

952.37526	0.48313

952.47486	0.48315

952.57446	0.4832

952.67406	0.48329

952.77366	0.4833

952.87326	0.48354

952.97286	0.48355

953.07246	0.48376

953.17206	0.48408

953.27166	0.48411

953.37126	0.4845

953.47086	0.48452

953.57046	0.48453

953.67006	0.48454

953.76966	0.48457

953.86926	0.48461

953.96886	0.48465

954.06846	0.48482

954.16806	0.48484

954.26766	0.48505

954.36726	0.48507

954.46686	0.48514

954.56646	0.48516

954.66606	0.48538

954.76566	0.48539

954.86526	0.48541

954.96486	0.48544

955.06446	0.48545

955.16406	0.48549

955.26366	0.48553

955.36326	0.48555

955.46286	0.48583

955.56246	0.48584

955.66206	0.48593

955.76166	0.48597

955.86126	0.48601

955.96086	0.48633

956.06046	0.48636

956.16006	0.48641

956.25966	0.4866

956.35926	0.48667

956.45886	0.48694

956.55846	0.48696

956.65806	0.48699

956.75766	0.48701

956.85726	0.48703

956.95686	0.48711

957.05646	0.48713

957.15606	0.48715

957.25566	0.4872

957.35526	0.48741

957.45486	0.48742

957.55446	0.48745

957.65406	0.48766

957.75366	0.48767

957.85326	0.4877

957.95286	0.48774

958.05246	0.48777

958.15206	0.4878

958.25166	0.48781

958.35126	0.48784

958.45086	0.48789

958.55046	0.48791

958.65006	0.48796

958.74966	0.48799

958.84926	0.48801

958.94886	0.48816

959.04846	0.48819

959.14806	0.48829

959.24766	0.48856

959.34726	0.48858

959.44686	0.48865

959.54646	0.48868

959.64606	0.48894

959.74566	0.48898

959.84526	0.48901

959.94486	0.48903

960.04446	0.48905

960.14406	0.48926

960.24366	0.48933

960.34326	0.48935

960.44286	0.48936

960.54246	0.48943

960.64206	0.48946

960.74166	0.48961

960.84126	0.48981

960.94086	0.48985

961.04046	0.48986

961.14006	0.48993

961.23966	0.48996

961.33926	0.49

961.43886	0.49011

961.53846	0.49014

961.63806	0.4903

961.73766	0.49053

961.83726	0.49088

961.93686	0.49101

962.03646	0.49113

962.13606	0.49119

962.23566	0.49136

962.33526	0.49137

962.43486	0.49178

962.53446	0.49183

962.63406	0.49194

962.73366	0.49197

962.83326	0.49206

962.93286	0.49207

963.03246	0.49217

963.13206	0.49243

963.23166	0.49245

963.33126	0.49257

963.43086	0.49259

963.53046	0.49271

963.63006	0.49275

963.72966	0.49298

963.82926	0.4931

963.92886	0.49334

964.02846	0.4936

964.12806	0.49395

964.22766	0.49397

964.32726	0.494

964.42686	0.49402

964.52646	0.49412

964.62606	0.49416

964.72566	0.49419

964.82526	0.49421

964.92486	0.49425

965.02446	0.49429

965.12406	0.49471

965.22366	0.49472

965.32326	0.49493

965.42286	0.49522

965.52246	0.49528

965.62206	0.49546

965.72166	0.4955

965.82126	0.49552

965.92086	0.49553

966.02046	0.49555

966.12006	0.49557

966.21966	0.4957

966.31926	0.49571

966.41886	0.49574

966.51846	0.49578

966.61806	0.49587

966.71766	0.49591

966.81726	0.49603

966.91686	0.49612

967.01646	0.49616

967.11606	0.49619

967.21566	0.49629

967.31526	0.4963

967.41486	0.49631

967.51446	0.49636

967.61406	0.49637

967.71366	0.49648

967.81326	0.49679

967.91286	0.49683

968.01246	0.49694

968.11206	0.49731

968.21166	0.49733

968.31126	0.49737

968.41086	0.4976

968.51046	0.49764

968.61006	0.49796

968.70966	0.49802

968.80926	0.49818

968.90886	0.4983

969.00846	0.49841

969.10806	0.49851

969.20766	0.49856

969.30726	0.49881

969.40686	0.4989

969.50646	0.49898

969.60606	0.49901

969.70566	0.49902

969.80526	0.49904

969.90486	0.49905

970.00446	0.49906

970.10406	0.49908

970.20366	0.49919

970.30326	0.49922

970.40286	0.49923

970.50246	0.49951

970.60206	0.49992

970.70166	0.50021

970.80126	0.50032

970.90086	0.50046

971.00046	0.50047

971.10006	0.50071

971.19966	0.50074

971.29926	0.50075

971.39886	0.50084

971.49846	0.5011

971.59806	0.50112

971.69766	0.50113

971.79726	0.50116

971.89686	0.50144

971.99646	0.50172

972.09606	0.50173

972.19566	0.50176

972.29526	0.50179

972.39486	0.50189

972.49446	0.50191

972.59406	0.50207

972.69366	0.50214

972.79326	0.50237

972.89286	0.50239

972.99246	0.5024

973.09206	0.5025

973.19166	0.50251

973.29126	0.50254

973.39086	0.50258

973.49046	0.5026

973.59006	0.50267

973.68966	0.50285

973.78926	0.5029

973.88886	0.50303

973.98846	0.50306

974.08806	0.5031

974.18766	0.5032

974.28726	0.50321

974.38686	0.50323

974.48646	0.5036

974.58606	0.50362

974.68566	0.50376

974.78526	0.50377

974.88486	0.50387

974.98446	0.5039

975.08406	0.50391

975.18366	0.50392

975.28326	0.50397

975.38286	0.50405

975.48246	0.50422

975.58206	0.50423

975.68166	0.5043

975.78126	0.50441

975.88086	0.50451

975.98046	0.50461

976.08006	0.50465

976.17966	0.50469

976.27926	0.50473

976.37886	0.50483

976.47846	0.50489

976.57806	0.50504

976.67766	0.50507

976.77726	0.50524

976.87686	0.50538

976.97646	0.50542

977.07606	0.50559

977.17566	0.5056

977.27526	0.50563

977.37486	0.50578

977.47446	0.50579

977.57406	0.50581

977.67366	0.50585

977.77326	0.50602

977.87286	0.50603

977.97246	0.50617

978.07206	0.50619

978.17166	0.50631

978.27126	0.50633

978.37086	0.50634

978.47046	0.50635

978.57006	0.50644

978.66966	0.50659

978.76926	0.50663

978.86886	0.5067

978.96846	0.50718

979.06806	0.50719

979.16766	0.50722

979.26726	0.50744

979.36686	0.50746

979.46646	0.50751

979.56606	0.50753

979.66566	0.50769

979.76526	0.50771

979.86486	0.50783

979.96446	0.50788

980.06406	0.50799

980.16366	0.508

980.26326	0.50818

980.36286	0.50829

980.46246	0.50839

980.56206	0.50856

980.66166	0.50857

980.76126	0.50859

980.86086	0.50881

980.96046	0.50906

981.06006	0.50908

981.15966	0.5091

981.25926	0.50941

981.35886	0.50943

981.45846	0.50958

981.55806	0.50962

981.65766	0.50963

981.75726	0.50972

981.85686	0.50979

981.95646	0.50982

982.05606	0.51

982.15566	0.51004

982.25526	0.51035

982.35486	0.51037

982.45446	0.51042

982.55406	0.51053

982.65366	0.51056

982.75326	0.51068

982.85286	0.51071

982.95246	0.51088

983.05206	0.51089

983.15166	0.51092

983.25126	0.51096

983.35086	0.51104

983.45046	0.51107

983.55006	0.51109

983.64966	0.51132

983.74926	0.51134

983.84886	0.51144

983.94846	0.51146

984.04806	0.51176

984.14766	0.51177

984.24726	0.51181

984.34686	0.51204

984.44646	0.51206

984.54606	0.51208

984.64566	0.51245

984.74526	0.51271

984.84486	0.51272

984.94446	0.51275

985.04406	0.51297

985.14366	0.51343

985.24326	0.51345

985.34286	0.51346

985.44246	0.51352

985.54206	0.51354

985.64166	0.51363

985.74126	0.51375

985.84086	0.51377

985.94046	0.5138

986.04006	0.51395

986.13966	0.51396

986.23926	0.51405

986.33886	0.5141

986.43846	0.51412

986.53806	0.51431

986.63766	0.51445

986.73726	0.51447

986.83686	0.5149

986.93646	0.51498

987.03606	0.51532

987.13566	0.51533

987.23526	0.5154

987.33486	0.51544

987.43446	0.51548

987.53406	0.51555

987.63366	0.51568

987.73326	0.51586

987.83286	0.51589

987.93246	0.5159

988.03206	0.51592

988.13166	0.51595

988.23126	0.51606

988.33086	0.51607

988.43046	0.51609

988.53006	0.5161

988.62966	0.51634

988.72926	0.51639

988.82886	0.51643

988.92846	0.51649

989.02806	0.5165

989.12766	0.51652

989.22726	0.51667

989.32686	0.51673

989.42646	0.51678

989.52606	0.51683

989.62566	0.51692

989.72526	0.51697

989.82486	0.51705

989.92446	0.51712

990.02406	0.51723

990.12366	0.51727

990.22326	0.51731

990.32286	0.51733

990.42246	0.51741

990.52206	0.51764

990.62166	0.51772

990.72126	0.51786

990.82086	0.51789

990.92046	0.51793

991.02006	0.51794

991.11966	0.51797

991.21926	0.518

991.31886	0.51803

991.41846	0.51812

991.51806	0.51814

991.61766	0.51817

991.71726	0.51832

991.81686	0.51836

991.91646	0.51838

992.01606	0.5185

992.11566	0.51853

992.21526	0.51858

992.31486	0.51871

992.41446	0.51872

992.51406	0.51879

992.61366	0.51882

992.71326	0.51886

992.81286	0.51895

992.91246	0.51907

993.01206	0.51934

993.11166	0.51941

993.21126	0.51942

993.31086	0.51944

993.41046	0.51953

993.51006	0.51956

993.60966	0.51959

993.70926	0.51962

993.80886	0.51976

993.90846	0.51979

994.00806	0.51987

994.10766	0.5199

994.20726	0.51997

994.30686	0.51998

994.40646	0.52006

994.50606	0.52009

994.60566	0.52011

994.70526	0.52016

994.80486	0.52019

994.90446	0.5202

995.00406	0.52032

995.10366	0.5204

995.20326	0.52041

995.30286	0.52043

995.40246	0.52046

995.50206	0.52051

995.60166	0.52055

995.70126	0.52057

995.80086	0.52058

995.90046	0.52061

996.00006	0.52096

996.09966	0.52097

996.19926	0.52127

996.29886	0.52129

996.39846	0.52132

996.49806	0.5215

996.59766	0.52152

996.69726	0.52174

996.79686	0.52184

996.89646	0.52222

996.99606	0.52224

997.09566	0.52228

997.19526	0.52242

997.29486	0.52273

997.39446	0.5228

997.49406	0.52283

997.59366	0.52286

997.69326	0.52296

997.79286	0.52298

997.89246	0.52316

997.99206	0.52318

998.09166	0.52322

998.19126	0.52337

998.29086	0.5234

998.39046	0.52357

998.49006	0.52361

998.58966	0.52364

998.68926	0.52399

998.78886	0.52402

998.88846	0.52437

998.98806	0.52448

999.08766	0.52459

999.18726	0.52469

999.28686	0.5248

999.38646	0.52483

999.48606	0.52504

999.58566	0.52505

999.68526	0.52508

999.78486	0.5252

999.88446	0.52522

999.98406	0.52523

1000.08366	0.52526

1000.18326	0.52557

1000.28286	0.52562

1000.38246	0.52569

1000.48206	0.52571

1000.58166	0.52572

1000.68126	0.52575

1000.78086	0.52578

1000.88046	0.5258

1000.98006	0.52617

1001.07966	0.52618

1001.17926	0.52636

1001.27886	0.52638

1001.37846	0.5264

1001.47806	0.5267

1001.57766	0.52678

1001.67726	0.52681

1001.77686	0.52684

1001.87646	0.52689

1001.97606	0.52692

1002.07566	0.52696

1002.17526	0.52709

1002.27486	0.5271

1002.37446	0.52714

1002.47406	0.52716

1002.57366	0.52727

1002.67326	0.52741

1002.77286	0.52759

1002.87246	0.5276

1002.97206	0.52765

1003.07166	0.52766

1003.17126	0.5277

1003.27086	0.52776

1003.37046	0.52779

1003.47006	0.528

1003.56966	0.52801

1003.66926	0.52808

1003.76886	0.52829

1003.86846	0.52841

1003.96806	0.52843

1004.06766	0.52864

1004.16726	0.52875

1004.26686	0.52896

1004.36646	0.52899

1004.46606	0.52904

1004.56566	0.52906

1004.66526	0.52929

1004.76486	0.52936

1004.86446	0.52938

1004.96406	0.52963

1005.06366	0.52967

1005.16326	0.52999

1005.26286	0.53001

1005.36246	0.53002

1005.46206	0.53005

1005.56166	0.53009

1005.66126	0.53024

1005.76086	0.53033

1005.86046	0.53044

1005.96006	0.53051

1006.05966	0.53063

1006.15926	0.53069

1006.25886	0.53084

1006.35846	0.53087

1006.45806	0.53115

1006.55766	0.53117

1006.65726	0.53119

1006.75686	0.53143

1006.85646	0.53157

1006.95606	0.5316

1007.05566	0.53161

1007.15526	0.53171

1007.25486	0.53175

1007.35446	0.53177

1007.45406	0.53186

1007.55366	0.53188

1007.65326	0.53223

1007.75286	0.53255

1007.85247	0.53256

1007.95206	0.53284

1008.05167	0.53285

1008.15127	0.53288

1008.25087	0.53295

1008.35047	0.53301

1008.45007	0.53312

1008.54967	0.53319

1008.64927	0.53346

1008.74887	0.53348

1008.84847	0.53361

1008.94807	0.53362

1009.04767	0.53414

1009.14726	0.53418

1009.24687	0.53428

1009.34647	0.53429

1009.44607	0.53449

1009.54567	0.53454

1009.64527	0.53491

1009.74487	0.53494

1009.84447	0.53495

1009.94407	0.53501

1010.04367	0.53503

1010.14327	0.53513

1010.24287	0.53514

1010.34246	0.53535

1010.44207	0.53537

1010.54167	0.53538

1010.64127	0.53561

1010.74087	0.53563

1010.84047	0.53565

1010.94007	0.53588

1011.03967	0.53593

1011.13927	0.53594

1011.23887	0.53595

1011.33847	0.53609

1011.43807	0.53611

1011.53766	0.53615

1011.63727	0.53626

1011.73687	0.53628

1011.83647	0.53629

1011.93607	0.5363

1012.03567	0.53632

1012.13527	0.53635

1012.23487	0.53636

1012.33447	0.53656

1012.43407	0.53662

1012.53367	0.53664

1012.63327	0.53685

1012.73286	0.53688

1012.83247	0.53695

1012.93207	0.53696

1013.03167	0.53727

1013.13127	0.53739

1013.23087	0.53743

1013.33047	0.53745

1013.43007	0.53767

1013.52967	0.53769

1013.62927	0.53778

1013.72887	0.5378

1013.82847	0.53788

1013.92806	0.5379

1014.02767	0.53794

1014.12727	0.5381

1014.22687	0.53812

1014.32647	0.53826

1014.42607	0.5384

1014.52567	0.53843

1014.62527	0.53858

1014.72487	0.53859

1014.82447	0.53862

1014.92407	0.53868

1015.02367	0.53876

1015.12326	0.53878

1015.22287	0.53879

1015.32247	0.53887

1015.42207	0.53911

1015.52167	0.53914

1015.62127	0.53917

1015.72087	0.53919

1015.82047	0.53932

1015.92007	0.53936

1016.01967	0.53945

1016.11927	0.53975

1016.21887	0.53978

1016.31846	0.54009

1016.41807	0.54026

1016.51767	0.54027

1016.61727	0.54028

1016.71687	0.54038

1016.81647	0.54039

1016.91607	0.54045

1017.01567	0.54052

1017.11527	0.54053

1017.21487	0.54093

1017.31447	0.54104

1017.41407	0.54105

1017.51366	0.54107

1017.61327	0.54109

1017.71287	0.54137

1017.81247	0.54139

1017.91207	0.5416

1018.01167	0.54167

1018.11127	0.54206

1018.21087	0.54231

1018.31047	0.54239

1018.41007	0.54246

1018.50967	0.54248

1018.60927	0.5425

1018.70886	0.54252

1018.80847	0.54253

1018.90807	0.54277

1019.00767	0.54306

1019.10727	0.54309

1019.20687	0.54312

1019.30647	0.5433

1019.40607	0.54331

1019.50567	0.54333

1019.60527	0.54342

1019.70487	0.54351

1019.80447	0.54359

1019.90406	0.54363

1020.00367	0.54366

1020.10327	0.5437

1020.20287	0.54373

1020.30247	0.54376

1020.40207	0.54382

1020.50167	0.54383

1020.60127	0.54384

1020.70087	0.54408

1020.80047	0.5441

1020.90007	0.54437

1020.99967	0.54439

1021.09926	0.54474

1021.19887	0.54475

1021.29847	0.54482

1021.39807	0.54497

1021.49767	0.545

1021.59727	0.54502

1021.69687	0.5451

1021.79647	0.54518

1021.89607	0.54524

1021.99567	0.54537

1022.09527	0.54538

1022.19487	0.54576

1022.29446	0.54577

1022.39407	0.54585

1022.49367	0.54587

1022.59327	0.5459

1022.69287	0.54591

1022.79247	0.54592

1022.89207	0.54618

1022.99167	0.54645

1023.09127	0.5465

1023.19087	0.54659

1023.29047	0.54676

1023.39007	0.54678

1023.48967	0.547

1023.58927	0.54701

1023.68887	0.54714

1023.78847	0.54717

1023.88807	0.54721

1023.98767	0.54733

1024.08727	0.54736

1024.18687	0.54763

1024.28647	0.54766

1024.38607	0.5478

1024.48567	0.54781

1024.58527	0.54796

1024.68487	0.54798

1024.78447	0.54803

1024.88407	0.54813

1024.98367	0.54814

1025.08327	0.5484

1025.18287	0.54845

1025.28247	0.54849

1025.38207	0.54855

1025.48167	0.54856

1025.58127	0.54859

1025.68087	0.54874

1025.78047	0.54876

1025.88007	0.54897

1025.97967	0.54909

1026.07927	0.54935

1026.17887	0.54937

1026.27847	0.54939

1026.37807	0.54941

1026.47767	0.54947

1026.57727	0.54953

1026.67687	0.54961

1026.77647	0.54962

1026.87607	0.54985

1026.97567	0.54989

1027.07527	0.54992

1027.17487	0.55022

1027.27447	0.55046

1027.37407	0.55049

1027.47367	0.55053

1027.57327	0.55067

1027.67287	0.55069

1027.77247	0.55092

1027.87207	0.55095

1027.97167	0.55099

1028.07127	0.55109

1028.17087	0.55116

1028.27047	0.55119

1028.37007	0.55124

1028.46967	0.55127

1028.56927	0.55129

1028.66887	0.55134

1028.76847	0.55136

1028.86807	0.55173

1028.96767	0.55175

1029.06727	0.55184

1029.16687	0.55186

1029.26647	0.55193

1029.36607	0.55211

1029.46567	0.55217

1029.56527	0.55219

1029.66487	0.55221

1029.76447	0.55222

1029.86407	0.55236

1029.96367	0.55247

1030.06327	0.55249

1030.16287	0.55265

1030.26247	0.55268

1030.36207	0.55271

1030.46167	0.55288

1030.56127	0.55292

1030.66087	0.5531

1030.76047	0.55312

1030.86007	0.5532

1030.95967	0.55345

1031.05927	0.55372

1031.15887	0.55373

1031.25847	0.55384

1031.35807	0.55386

1031.45767	0.5539

1031.55727	0.55397

1031.65687	0.55399

1031.75647	0.55402

1031.85607	0.55404

1031.95567	0.55416

1032.05527	0.55419

1032.15487	0.55425

1032.25447	0.55426

1032.35407	0.5543

1032.45367	0.5544

1032.55327	0.55441

1032.65287	0.55448

1032.75247	0.55475

1032.85207	0.5548

1032.95167	0.55518

1033.05127	0.5552

1033.15087	0.55522

1033.25047	0.55541

1033.35007	0.55577

1033.44967	0.55613

1033.54927	0.55637

1033.64887	0.55638

1033.74847	0.55642

1033.84807	0.55658

1033.94767	0.55659

1034.04727	0.55662

1034.14687	0.55663

1034.24647	0.55665

1034.34607	0.55668

1034.44567	0.5568

1034.54527	0.55684

1034.64487	0.55687

1034.74447	0.5569

1034.84407	0.55694

1034.94367	0.55716

1035.04327	0.55721

1035.14287	0.55735

1035.24247	0.55739

1035.34207	0.5575

1035.44167	0.55768

1035.54127	0.55774

1035.64087	0.55779

1035.74047	0.55781

1035.84007	0.55782

1035.93967	0.55785

1036.03927	0.55793

1036.13887	0.55799

1036.23847	0.55803

1036.33807	0.55807

1036.43767	0.55809

1036.53727	0.5582

1036.63687	0.55825

1036.73647	0.55827

1036.83607	0.55832

1036.93567	0.55838

1037.03527	0.55844

1037.13487	0.55846

1037.23447	0.55848

1037.33407	0.55849

1037.43367	0.55862

1037.53327	0.55863

1037.63287	0.5587

1037.73247	0.55885

1037.83207	0.55887

1037.93167	0.55888

1038.03127	0.55913

1038.13087	0.55919

1038.23047	0.5593

1038.33007	0.55938

1038.42967	0.55947

1038.52927	0.55948

1038.62887	0.55969

1038.72847	0.55973

1038.82807	0.55976

1038.92767	0.56015

1039.02727	0.56017

1039.12687	0.56018

1039.22647	0.56022

1039.32607	0.56031

1039.42567	0.56038

1039.52527	0.56045

1039.62487	0.56053

1039.72447	0.56064

1039.82407	0.56091

1039.92367	0.56133

1040.02327	0.56137

1040.12287	0.56146

1040.22247	0.56149

1040.32207	0.56151

1040.42167	0.56152

1040.52127	0.5616

1040.62087	0.56163

1040.72047	0.56177

1040.82007	0.56179

1040.91967	0.56181

1041.01927	0.56193

1041.11887	0.56204

1041.21847	0.56222

1041.31807	0.56225

1041.41767	0.56226

1041.51727	0.56233

1041.61687	0.56261

1041.71647	0.56262

1041.81607	0.56265

1041.91567	0.56276

1042.01527	0.56283

1042.11487	0.56285

1042.21447	0.56292

1042.31407	0.56293

1042.41367	0.56297

1042.51327	0.56299

1042.61287	0.56314

1042.71247	0.56321

1042.81207	0.56331

1042.91167	0.56341

1043.01127	0.56342

1043.11087	0.5637

1043.21047	0.56394

1043.31007	0.56403

1043.40967	0.56406

1043.50927	0.56408

1043.60887	0.5641

1043.70847	0.56412

1043.80807	0.5642

1043.90767	0.56422

1044.00727	0.56424

1044.10687	0.56434

1044.20647	0.56441

1044.30607	0.56475

1044.40567	0.56477

1044.50527	0.56482

1044.60487	0.56483

1044.70447	0.56486

1044.80407	0.56487

1044.90367	0.56489

1045.00327	0.56494

1045.10287	0.56497

1045.20247	0.56522

1045.30207	0.56523

1045.40167	0.56535

1045.50127	0.56542

1045.60087	0.56553

1045.70047	0.5657

1045.80007	0.56572

1045.89967	0.56582

1045.99927	0.56593

1046.09887	0.5661

1046.19847	0.56613

1046.29807	0.56634

1046.39767	0.56635

1046.49727	0.56645

1046.59687	0.56662

1046.69647	0.56663

1046.79607	0.56674

1046.89567	0.56687

1046.99527	0.56688

1047.09487	0.56692

1047.19447	0.56699

1047.29407	0.56701

1047.39367	0.56705

1047.49327	0.56718

1047.59287	0.5673

1047.69247	0.56732

1047.79207	0.56754

1047.89167	0.56757

1047.99127	0.56761

1048.09087	0.56783

1048.19047	0.56785

1048.29007	0.56789

1048.38967	0.56799

1048.48927	0.56801

1048.58887	0.5681

1048.68847	0.56817

1048.78807	0.56821

1048.88767	0.56835

1048.98727	0.56839

1049.08687	0.5684

1049.18647	0.56843

1049.28607	0.5687

1049.38567	0.56871

1049.48527	0.56873

1049.58487	0.56892

1049.68447	0.56894

1049.78407	0.56907

1049.88367	0.5691

1049.98327	0.56913

1050.08287	0.56924

1050.18247	0.56926

1050.28207	0.56937

1050.38167	0.56963

1050.48127	0.56965

1050.58087	0.56981

1050.68047	0.56988

1050.78007	0.56997

1050.87967	0.57014

1050.97927	0.57015

1051.07887	0.57023

1051.17847	0.57026

1051.27807	0.57042

1051.37767	0.57062

1051.47727	0.57067

1051.57687	0.57071

1051.67647	0.57083

1051.77607	0.57096

1051.87567	0.57107

1051.97527	0.57109

1052.07487	0.57132

1052.17447	0.57134

1052.27407	0.57136

1052.37367	0.57167

1052.47327	0.5717

1052.57287	0.57173

1052.67247	0.57178

1052.77207	0.57233

1052.87167	0.57236

1052.97127	0.57279

1053.07087	0.5728

1053.17047	0.57283

1053.27007	0.57298

1053.36967	0.57304

1053.46927	0.57305

1053.56887	0.57322

1053.66847	0.57324

1053.76807	0.57332

1053.86767	0.5734

1053.96727	0.57345

1054.06687	0.57346

1054.16647	0.57352

1054.26607	0.57357

1054.36567	0.5736

1054.46527	0.57396

1054.56487	0.57402

1054.66447	0.57403

1054.76407	0.57406

1054.86367	0.57431

1054.96327	0.57442

1055.06287	0.57448

1055.16247	0.57462

1055.26207	0.57465

1055.36167	0.5748

1055.46127	0.57488

1055.56087	0.5749

1055.66047	0.57493

1055.76007	0.57537

1055.85967	0.57539

1055.95927	0.57557

1056.05887	0.5756

1056.15847	0.57596

1056.25807	0.57597

1056.35767	0.57601

1056.45727	0.57603

1056.55687	0.57631

1056.65647	0.57632

1056.75607	0.57634

1056.85567	0.57655

1056.95527	0.57656

1057.05487	0.5766

1057.15447	0.57661

1057.25407	0.57666

1057.35367	0.57708

1057.45327	0.57712

1057.55287	0.57722

1057.65247	0.57727

1057.75207	0.5773

1057.85167	0.57735

1057.95127	0.5774

1058.05087	0.57779

1058.15047	0.57782

1058.25007	0.57798

1058.34967	0.57811

1058.44927	0.57821

1058.54887	0.57826

1058.64847	0.57863

1058.74807	0.57864

1058.84767	0.57865

1058.94727	0.57882

1059.04687	0.57883

1059.14647	0.57895

1059.24607	0.57896

1059.34567	0.579

1059.44527	0.57916

1059.54487	0.5792

1059.64447	0.57921

1059.74407	0.57928

1059.84367	0.57942

1059.94327	0.57945

1060.04287	0.57951

1060.14247	0.57971

1060.24207	0.57974

1060.34167	0.57976

1060.44127	0.57999

1060.54087	0.58001

1060.64047	0.58002

1060.74007	0.58004

1060.83967	0.58012

1060.93927	0.58013

1061.03887	0.58016

1061.13847	0.58019

1061.23807	0.58032

1061.33767	0.58034

1061.43727	0.58047

1061.53687	0.5805

1061.63647	0.58068

1061.73607	0.58073

1061.83567	0.5809

1061.93527	0.58094

1062.03487	0.581

1062.13447	0.58112

1062.23407	0.58114

1062.33367	0.58115

1062.43327	0.58147

1062.53287	0.5815

1062.63247	0.58163

1062.73207	0.58171

1062.83167	0.58185

1062.93127	0.58191

1063.03087	0.58207

1063.13047	0.58217

1063.23007	0.58219

1063.32967	0.58226

1063.42927	0.58228

1063.52887	0.58233

1063.62847	0.58234

1063.72807	0.5824

1063.82767	0.58251

1063.92727	0.58254

1064.02687	0.58256

1064.12647	0.58258

1064.22607	0.5826

1064.32567	0.58283

1064.42527	0.58286

1064.52487	0.58287

1064.62447	0.58298

1064.72407	0.58309

1064.82367	0.58311

1064.92327	0.58325

1065.02287	0.58326

1065.12247	0.58329

1065.22207	0.58336

1065.32167	0.58337

1065.42127	0.58341

1065.52087	0.58353

1065.62047	0.58365

1065.72007	0.58369

1065.81967	0.58381

1065.91927	0.58382

1066.01887	0.58383

1066.11847	0.58411

1066.21807	0.58415

1066.31767	0.5842

1066.41727	0.58434

1066.51687	0.58435

1066.61647	0.58449

1066.71607	0.5845

1066.81567	0.58452

1066.91527	0.5846

1067.01487	0.58485

1067.11447	0.58487

1067.21407	0.58526

1067.31367	0.58543

1067.41327	0.58559

1067.51287	0.58561

1067.61247	0.58565

1067.71207	0.58598

1067.81167	0.586

1067.91127	0.58605

1068.01087	0.58629

1068.11047	0.58631

1068.21007	0.58632

1068.30967	0.58674

1068.40927	0.58684

1068.50887	0.58709

1068.60847	0.58717

1068.70807	0.5872

1068.80767	0.58721

1068.90727	0.58723

1069.00687	0.58724

1069.10647	0.58728

1069.20607	0.58755

1069.30567	0.58784

1069.40527	0.58795

1069.50487	0.58813

1069.60447	0.58815

1069.70407	0.58819

1069.80367	0.58822

1069.90327	0.58827

1070.00287	0.58855

1070.10247	0.58857

1070.20207	0.58862

1070.30167	0.58879

1070.40127	0.58887

1070.50087	0.58915

1070.60047	0.58917

1070.70007	0.58918

1070.79967	0.58932

1070.89927	0.58936

1070.99887	0.58938

1071.09847	0.58974

1071.19807	0.58985

1071.29767	0.59017

1071.39727	0.59019

1071.49687	0.59055

1071.59647	0.59056

1071.69607	0.59083

1071.79567	0.59084

1071.89527	0.59086

1071.99487	0.59119

1072.09447	0.59121

1072.19407	0.59125

1072.29367	0.59137

1072.39327	0.59143

1072.49287	0.59151

1072.59247	0.59158

1072.69207	0.5916

1072.79167	0.59161

1072.89127	0.59171

1072.99087	0.59172

1073.09047	0.59193

1073.19007	0.59206

1073.28967	0.59207

1073.38927	0.59224

1073.48887	0.59232

1073.58847	0.59237

1073.68807	0.59243

1073.78767	0.59252

1073.88727	0.59267

1073.98687	0.59305

1074.08647	0.5934

1074.18607	0.59352

1074.28567	0.5939

1074.38527	0.59391

1074.48487	0.59397

1074.58447	0.59398

1074.68407	0.594

1074.78367	0.59424

1074.88327	0.59435

1074.98287	0.59442

1075.08247	0.59443

1075.18207	0.59445

1075.28167	0.59449

1075.38127	0.59452

1075.48087	0.59457

1075.58047	0.59459

1075.68007	0.59467

1075.77967	0.59484

1075.87927	0.59499

1075.97887	0.5951

1076.07847	0.59513

1076.17807	0.59562

1076.27767	0.59563

1076.37727	0.59616

1076.47687	0.59618

1076.57647	0.59632

1076.67607	0.59637

1076.77567	0.5964

1076.87527	0.59641

1076.97487	0.5965

1077.07447	0.59651

1077.17407	0.59654

1077.27367	0.59671

1077.37327	0.59672

1077.47287	0.59674

1077.57247	0.59675

1077.67207	0.59717

1077.77167	0.5972

1077.87127	0.59742

1077.97087	0.59743

1078.07047	0.59746

1078.17007	0.5977

1078.26967	0.59802

1078.36927	0.59803

1078.46887	0.59817

1078.56847	0.5982

1078.66807	0.59823

1078.76767	0.59836

1078.86727	0.59842

1078.96687	0.59847

1079.06647	0.59854

1079.16607	0.59858

1079.26567	0.59859

1079.36527	0.59866

1079.46487	0.5987

1079.56447	0.59875

1079.66407	0.59884

1079.76367	0.5989

1079.86327	0.59893

1079.96287	0.59894

1080.06247	0.59898

1080.16207	0.599

1080.26167	0.59904

1080.36127	0.59912

1080.46087	0.59914

1080.56047	0.59918

1080.66007	0.59919

1080.75967	0.59921

1080.85927	0.59942

1080.95887	0.59943

1081.05847	0.5995

1081.15807	0.59963

1081.25767	0.59964

1081.35727	0.59967

1081.45687	0.5997

1081.55647	0.60014

1081.65607	0.60023

1081.75567	0.60024

1081.85527	0.6003

1081.95487	0.60049

1082.05447	0.60053

1082.15407	0.60056

1082.25367	0.60065

1082.35327	0.60067

1082.45287	0.60083

1082.55247	0.60098

1082.65207	0.60105

1082.75167	0.60112

1082.85127	0.60113

1082.95087	0.60127

1083.05047	0.60133

1083.15007	0.60139

1083.24967	0.60145

1083.34927	0.60192

1083.44887	0.60196

1083.54847	0.60208

1083.64807	0.60211

1083.74767	0.60213

1083.84727	0.60214

1083.94687	0.60215

1084.04647	0.6025

1084.14607	0.60252

1084.24567	0.6027

1084.34527	0.60306

1084.44487	0.60309

1084.54447	0.60313

1084.64407	0.60317

1084.74367	0.60335

1084.84327	0.60337

1084.94287	0.60342

1085.04247	0.60373

1085.14207	0.60376

1085.24167	0.60381

1085.34127	0.60387

1085.44087	0.60415

1085.54047	0.60416

1085.64007	0.60419

1085.73967	0.60421

1085.83927	0.60423

1085.93887	0.60426

1086.03847	0.60447

1086.13807	0.60448

1086.23767	0.6045

1086.33727	0.60451

1086.43687	0.60457

1086.53647	0.60458

1086.63607	0.6046

1086.73567	0.60465

1086.83527	0.60469

1086.93487	0.60471

1087.03447	0.60506

1087.13407	0.6051

1087.23367	0.60514

1087.33327	0.60528

1087.43287	0.60531

1087.53247	0.6058

1087.63207	0.60615

1087.73167	0.60641

1087.83127	0.60654

1087.93087	0.60658

1088.03047	0.60669

1088.13007	0.60686

1088.22967	0.60701

1088.32927	0.60704

1088.42887	0.60705

1088.52847	0.60721

1088.62807	0.60722

1088.72767	0.60729

1088.82727	0.60733

1088.92687	0.60757

1089.02647	0.6076

1089.12607	0.60761

1089.22567	0.60768

1089.32527	0.60771

1089.42487	0.60777

1089.52447	0.60827

1089.62407	0.60828

1089.72367	0.60831

1089.82327	0.60841

1089.92287	0.60842

1090.02247	0.60862

1090.12207	0.60863

1090.22167	0.60866

1090.32127	0.60915

1090.42087	0.60918

1090.52047	0.6092

1090.62007	0.60926

1090.71967	0.60936

1090.81927	0.60937

1090.91887	0.6094

1091.01847	0.60965

1091.11807	0.60967

1091.21767	0.60978

1091.31727	0.6098

1091.41687	0.60983

1091.51647	0.6101

1091.61607	0.61013

1091.71567	0.61014

1091.81527	0.61067

1091.91487	0.61074

1092.01447	0.61089

1092.11407	0.61092

1092.21367	0.61117

1092.31327	0.6112

1092.41287	0.6113

1092.51247	0.61138

1092.61207	0.6114

1092.71167	0.61161

1092.81127	0.61166

1092.91087	0.61198

1093.01047	0.612

1093.11007	0.61201

1093.20967	0.61212

1093.30927	0.61257

1093.40887	0.61258

1093.50847	0.61289

1093.60807	0.61307

1093.70767	0.6131

1093.80727	0.61313

1093.90687	0.61314

1094.00647	0.61318

1094.10607	0.61348

1094.20567	0.61349

1094.30527	0.6138

1094.40487	0.61384

1094.50447	0.61406

1094.60407	0.61437

1094.70367	0.6144

1094.80327	0.61445

1094.90287	0.61451

1095.00247	0.61454

1095.10207	0.61455

1095.20167	0.61457

1095.30127	0.61461

1095.40087	0.6149

1095.50047	0.61499

1095.60007	0.61501

1095.69967	0.61536

1095.79927	0.61538

1095.89887	0.61539

1095.99847	0.61566

1096.09807	0.6157

1096.19767	0.61571

1096.29727	0.61574

1096.39687	0.61575

1096.49647	0.61577

1096.59607	0.61584

1096.69567	0.61596

1096.79527	0.616

1096.89487	0.61612

1096.99447	0.61637

1097.09407	0.61638

1097.19367	0.6164

1097.29327	0.61648

1097.39287	0.61653

1097.49247	0.61663

1097.59207	0.61665

1097.69167	0.61666

1097.79127	0.61667

1097.89087	0.61723

1097.99047	0.61729

1098.09007	0.61747

1098.18967	0.61748

1098.28927	0.61751

1098.38887	0.61755

1098.48847	0.61764

1098.58807	0.61771

1098.68767	0.61797

1098.78727	0.61835

1098.88687	0.61843

1098.98647	0.61849

1099.08607	0.6185

1099.18567	0.61864

1099.28527	0.61869

1099.38487	0.61884

1099.48447	0.61895

1099.58407	0.61901

1099.68367	0.61903

1099.78327	0.61919

1099.88287	0.61945

1099.98247	0.61952

1100.08207	0.61955

1100.18167	0.61963

1100.28127	0.61986

1100.38087	0.61987

1100.48047	0.61998

1100.58007	0.62017

1100.67967	0.62018

1100.77927	0.62043

1100.87887	0.62051

1100.97847	0.62053

1101.07807	0.62058

1101.17767	0.62075

1101.27727	0.62085

1101.37687	0.6212

1101.47647	0.62128

1101.57607	0.6213

1101.67567	0.62149

1101.77527	0.62152

1101.87487	0.62155

1101.97447	0.62176

1102.07407	0.62213

1102.17367	0.62229

1102.27327	0.6223

1102.37287	0.62237

1102.47247	0.62269

1102.57207	0.62275

1102.67167	0.62276

1102.77127	0.62278

1102.87087	0.62297

1102.97047	0.62335

1103.07007	0.62336

1103.16967	0.62378

1103.26927	0.62384

1103.36887	0.62387

1103.46847	0.62416

1103.56807	0.62421

1103.66767	0.6244

1103.76727	0.62442

1103.86687	0.62454

1103.96647	0.62455

1104.06607	0.62456

1104.16567	0.62459

1104.26527	0.62463

1104.36487	0.62465

1104.46447	0.62476

1104.56407	0.62493

1104.66367	0.62495

1104.76327	0.62511

1104.86287	0.62521

1104.96247	0.62533

1105.06207	0.62536

1105.16167	0.62564

1105.26127	0.62572

1105.36087	0.62583

1105.46047	0.62585

1105.56007	0.62586

1105.65967	0.62631

1105.75927	0.62634

1105.85887	0.62635

1105.95847	0.62643

1106.05807	0.62648

1106.15767	0.62649

1106.25727	0.62666

1106.35687	0.6267

1106.45647	0.62694

1106.55607	0.62699

1106.65567	0.62705

1106.75527	0.62724

1106.85487	0.62726

1106.95447	0.62745

1107.05407	0.62768

1107.15367	0.62769

1107.25327	0.62775

1107.35287	0.62801

1107.45247	0.62811

1107.55207	0.62831

1107.65167	0.62838

1107.75127	0.62853

1107.85087	0.62854

1107.95047	0.62864

1108.05007	0.62871

1108.14967	0.62874

1108.24927	0.62879

1108.34887	0.62892

1108.44847	0.62899

1108.54807	0.62965

1108.64767	0.62966

1108.74727	0.62987

1108.84687	0.62994

1108.94647	0.63001

1109.04607	0.63002

1109.14567	0.63008

1109.24527	0.63012

1109.34487	0.63058

1109.44447	0.63076

1109.54407	0.63082

1109.64367	0.63087

1109.74327	0.6309

1109.84287	0.63139

1109.94247	0.63168

1110.04207	0.6317

1110.14167	0.6321

1110.24127	0.63212

1110.34087	0.63215

1110.44047	0.63244

1110.54007	0.63247

1110.63967	0.63249

1110.73927	0.63251

1110.83887	0.63258

1110.93847	0.63259

1111.03807	0.63277

1111.13767	0.63279

1111.23727	0.63305

1111.33687	0.63322

1111.43647	0.63323

1111.53607	0.63343

1111.63567	0.63344

1111.73527	0.63381

1111.83487	0.63386

1111.93447	0.6339

1112.03407	0.634

1112.13367	0.6341

1112.23327	0.63442

1112.33287	0.63446

1112.43247	0.63458

1112.53207	0.63464

1112.63167	0.63469

1112.73127	0.63488

1112.83087	0.63499

1112.93047	0.63501

1113.03007	0.63502

1113.12967	0.63534

1113.22927	0.63537

1113.32887	0.63547

1113.42847	0.63551

1113.52807	0.63554

1113.62767	0.63559

1113.72727	0.63562

1113.82687	0.63564

1113.92647	0.636

1114.02607	0.63604

1114.12567	0.63611

1114.22527	0.63633

1114.32487	0.63638

1114.42447	0.63659

1114.52407	0.63661

1114.62367	0.63667

1114.72327	0.63674

1114.82287	0.63675

1114.92247	0.63677

1115.02207	0.63678

1115.12167	0.63692

1115.22127	0.63693

1115.32087	0.63695

1115.42047	0.6371

1115.52007	0.63751

1115.61967	0.63755

1115.71927	0.63783

1115.81887	0.63787

1115.91847	0.6383

1116.01807	0.63854

1116.11767	0.63858

1116.21727	0.63864

1116.31687	0.63906

1116.41647	0.63932

1116.51607	0.63962

1116.61567	0.63963

1116.71527	0.63996

1116.81487	0.63998

1116.91447	0.64006

1117.01407	0.64008

1117.11367	0.6401

1117.21327	0.64016

1117.31287	0.64041

1117.41247	0.64047

1117.51207	0.6405

1117.61167	0.64069

1117.71127	0.64072

1117.81087	0.64073

1117.91047	0.64089

1118.01007	0.64096

1118.10967	0.64097

1118.20927	0.64131

1118.30887	0.64132

1118.40847	0.64139

1118.50807	0.64143

1118.60767	0.64146

1118.70727	0.64147

1118.80687	0.6415

1118.90647	0.64153

1119.00607	0.64161

1119.10567	0.64165

1119.20527	0.64171

1119.30487	0.64189

1119.40447	0.64192

1119.50407	0.64193

1119.60367	0.64203

1119.70327	0.64206

1119.80287	0.64207

1119.90247	0.64225

1120.00207	0.6423

1120.10167	0.64249

1120.20127	0.64251

1120.30087	0.6428

1120.40047	0.64283

1120.50007	0.6432

1120.59967	0.64323

1120.69927	0.64344

1120.79887	0.64346

1120.89847	0.64376

1120.99807	0.6439

1121.09767	0.64392

1121.19727	0.64396

1121.29687	0.64399

1121.39647	0.64406

1121.49607	0.64424

1121.59567	0.64428

1121.69527	0.64432

1121.79487	0.64436

1121.89447	0.64442

1121.99407	0.64447

1122.09367	0.64487

1122.19327	0.64526

1122.29287	0.64527

1122.39247	0.64577

1122.49207	0.64583

1122.59167	0.64589

1122.69127	0.64607

1122.79087	0.64611

1122.89047	0.64637

1122.99007	0.6464

1123.08967	0.64647

1123.18927	0.6466

1123.28887	0.64661

1123.38847	0.64681

1123.48807	0.6471

1123.58767	0.64713

1123.68727	0.64721

1123.78687	0.64732

1123.88647	0.64741

1123.98607	0.64755

1124.08567	0.64759

1124.18527	0.64762

1124.28487	0.6478

1124.38447	0.64787

1124.48407	0.64788

1124.58367	0.64797

1124.68327	0.64805

1124.78287	0.64806

1124.88247	0.64809

1124.98207	0.64812

1125.08167	0.64845

1125.18127	0.64861

1125.28087	0.64871

1125.38047	0.64873

1125.48007	0.64875

1125.57967	0.64883

1125.67927	0.64886

1125.77887	0.64887

1125.87847	0.6489

1125.97807	0.64893

1126.07767	0.64896

1126.17727	0.6491

1126.27687	0.64912

1126.37647	0.64917

1126.47607	0.64918

1126.57567	0.64919

1126.67527	0.64921

1126.77487	0.64925

1126.87447	0.64946

1126.97407	0.64947

1127.07367	0.64952

1127.17327	0.64984

1127.27287	0.64992

1127.37247	0.64993

1127.47207	0.65007

1127.57167	0.65009

1127.67127	0.6501

1127.77087	0.6503

1127.87047	0.65033

1127.97007	0.65055

1128.06967	0.65056

1128.16927	0.65063

1128.26887	0.65083

1128.36847	0.65087

1128.46807	0.65093

1128.56767	0.65097

1128.66727	0.651

1128.76687	0.65123

1128.86647	0.65125

1128.96607	0.65137

1129.06567	0.65139

1129.16527	0.65141

1129.26487	0.65154

1129.36447	0.65178

1129.46407	0.65181

1129.56367	0.65182

1129.66327	0.6519

1129.76287	0.65192

1129.86247	0.65193

1129.96207	0.65222

1130.06167	0.65227

1130.16127	0.65239

1130.26087	0.65242

1130.36047	0.65266

1130.46007	0.65295

1130.55967	0.65299

1130.65927	0.65319

1130.75887	0.6532

1130.85847	0.65356

1130.95807	0.65359

1131.05767	0.65361

1131.15727	0.65368

1131.25687	0.65369

1131.35647	0.65372

1131.45607	0.6541

1131.55567	0.65414

1131.65527	0.65437

1131.75487	0.6544

1131.85447	0.65451

1131.95407	0.65453

1132.05367	0.65456

1132.15327	0.65467

1132.25287	0.65475

1132.35247	0.65479

1132.45207	0.6551

1132.55167	0.65511

1132.65127	0.65517

1132.75087	0.65527

1132.85047	0.65528

1132.95007	0.65535

1133.04967	0.65538

1133.14927	0.65545

1133.24887	0.65552

1133.34847	0.65556

1133.44807	0.65562

1133.54767	0.65585

1133.64727	0.65587

1133.74687	0.65591

1133.84647	0.65605

1133.94607	0.65609

1134.04567	0.65612

1134.14527	0.6562

1134.24487	0.65626

1134.34447	0.65633

1134.44407	0.6564

1134.54367	0.65641

1134.64327	0.65654

1134.74287	0.65661

1134.84247	0.65672

1134.94207	0.65675

1135.04167	0.65682

1135.14127	0.65689

1135.24087	0.65721

1135.34047	0.65733

1135.44007	0.65735

1135.53967	0.6574

1135.63927	0.6575

1135.73887	0.65752

1135.83847	0.65756

1135.93807	0.65785

1136.03767	0.65787

1136.13727	0.65791

1136.23687	0.65794

1136.33647	0.65809

1136.43607	0.65824

1136.53567	0.65826

1136.63527	0.65827

1136.73487	0.6583

1136.83447	0.65831

1136.93407	0.65845

1137.03367	0.65847

1137.13327	0.65865

1137.23287	0.6587

1137.33247	0.65879

1137.43207	0.65901

1137.53167	0.65907

1137.63127	0.65914

1137.73087	0.65915

1137.83047	0.65925

1137.93007	0.65937

1138.02967	0.65939

1138.12927	0.6594

1138.22887	0.65943

1138.32847	0.65954

1138.42807	0.65955

1138.52767	0.65964

1138.62727	0.65967

1138.72687	0.65968

1138.82647	0.65971

1138.92607	0.65976

1139.02567	0.65978

1139.12527	0.65979

1139.22487	0.65985

1139.32447	0.65986

1139.42407	0.6599

1139.52367	0.65992

1139.62327	0.65995

1139.72287	0.65996

1139.82247	0.66

1139.92207	0.66035

1140.02167	0.66046

1140.12127	0.66052

1140.22087	0.6606

1140.32047	0.66069

1140.42007	0.6607

1140.51967	0.66073

1140.61927	0.66088

1140.71887	0.66103

1140.81847	0.66123

1140.91807	0.66134

1141.01767	0.66136

1141.11727	0.6614

1141.21687	0.66159

1141.31647	0.66161

1141.41607	0.66168

1141.51567	0.66172

1141.61527	0.66173

1141.71487	0.66175

1141.81447	0.66196

1141.91407	0.66197

1142.01367	0.662

1142.11327	0.66201

1142.21287	0.6621

1142.31247	0.66219

1142.41207	0.66261

1142.51167	0.66288

1142.61127	0.66312

1142.71087	0.66313

1142.81047	0.66316

1142.91007	0.66328

1143.00967	0.6633

1143.10927	0.66342

1143.20887	0.66346

1143.30847	0.66348

1143.40807	0.66372

1143.50767	0.66374

1143.60727	0.66376

1143.70687	0.6639

1143.80647	0.66401

1143.90607	0.66404

1144.00567	0.66423

1144.10527	0.66425

1144.20487	0.66429

1144.30447	0.66436

1144.40407	0.66441

1144.50367	0.66486

1144.60327	0.66499

1144.70287	0.6651

1144.80247	0.66511

1144.90207	0.66548

1145.00167	0.66554

1145.10127	0.66556

1145.20087	0.66588

1145.30047	0.66589

1145.40007	0.66598

1145.49967	0.66599

1145.59927	0.66601

1145.69887	0.66602

1145.79847	0.66633

1145.89807	0.66658

1145.99767	0.66666

1146.09727	0.66668

1146.19687	0.66708

1146.29647	0.66709

1146.39607	0.66711

1146.49567	0.66732

1146.59527	0.668

1146.69487	0.66802

1146.79447	0.66832

1146.89407	0.66846

1146.99367	0.66866

1147.09327	0.66867

1147.19287	0.66888

1147.29247	0.66891

1147.39207	0.66897

1147.49167	0.66898

1147.59127	0.66899

1147.69087	0.66901

1147.79047	0.66918

1147.89007	0.66922

1147.98967	0.66923

1148.08927	0.66966

1148.18887	0.66969

1148.28847	0.66973

1148.38807	0.66975

1148.48767	0.6698

1148.58727	0.66983

1148.68687	0.66985

1148.78647	0.6699

1148.88607	0.66992

1148.98567	0.66997

1149.08527	0.67

1149.18487	0.67001

1149.28447	0.67006

1149.38407	0.67011

1149.48367	0.67012

1149.58327	0.67017

1149.68287	0.67021

1149.78247	0.67031

1149.88207	0.67032

1149.98167	0.6705

1150.08127	0.67052

1150.18087	0.67053

1150.28047	0.6706

1150.38007	0.67066

1150.47967	0.67067

1150.57927	0.6707

1150.67887	0.67091

1150.77847	0.67092

1150.87807	0.67093

1150.97767	0.67098

1151.07727	0.67106

1151.17687	0.67135

1151.27647	0.67137

1151.37607	0.67138

1151.47567	0.6714

1151.57527	0.67142

1151.67487	0.67145

1151.77447	0.67148

1151.87407	0.67163

1151.97367	0.67166

1152.07327	0.67167

1152.17287	0.67169

1152.27247	0.6719

1152.37207	0.67191

1152.47167	0.67198

1152.57127	0.67216

1152.67087	0.67219

1152.77047	0.67247

1152.87007	0.67248

1152.96967	0.67253

1153.06927	0.67255

1153.16887	0.67279

1153.26847	0.67289

1153.36807	0.6729

1153.46767	0.67292

1153.56727	0.67293

1153.66687	0.67327

1153.76647	0.67334

1153.86607	0.67336

1153.96567	0.67341

1154.06527	0.67342

1154.16487	0.67343

1154.26447	0.67353

1154.36407	0.67355

1154.46367	0.67356

1154.56327	0.67357

1154.66287	0.67359

1154.76247	0.67364

1154.86207	0.67366

1154.96167	0.67385

1155.06127	0.67389

1155.16087	0.67406

1155.26047	0.67408

1155.36007	0.67409

1155.45967	0.6741

1155.55927	0.67447

1155.65887	0.67448

1155.75847	0.67457

1155.85807	0.67458

1155.95767	0.67461

1156.05727	0.67463

1156.15687	0.67487

1156.25647	0.67503

1156.35607	0.67504

1156.45567	0.67543

1156.55527	0.67544

1156.65487	0.67571

1156.75447	0.67584

1156.85407	0.67592

1156.95367	0.67596

1157.05327	0.67642

1157.15287	0.67645

1157.25247	0.67653

1157.35207	0.67655

1157.45167	0.67677

1157.55127	0.67679

1157.65087	0.67684

1157.75047	0.67702

1157.85007	0.67704

1157.94967	0.67711

1158.04927	0.67741

1158.14887	0.67744

1158.24847	0.67748

1158.34807	0.67751

1158.44767	0.67754

1158.54727	0.67755

1158.64687	0.67757

1158.74647	0.67758

1158.84607	0.67761

1158.94567	0.67769

1159.04527	0.67808

1159.14487	0.67832

1159.24447	0.67838

1159.34407	0.67849

1159.44367	0.67861

1159.54327	0.67881

1159.64287	0.67885

1159.74247	0.67888

1159.84207	0.67889

1159.94167	0.67913

1160.04127	0.67923

1160.14087	0.67931

1160.24047	0.67941

1160.34007	0.67947

1160.43967	0.67961

1160.53927	0.67962

1160.63887	0.68004

1160.73847	0.68005

1160.83807	0.68014

1160.93767	0.68035

1161.03727	0.68036

1161.13687	0.6804

1161.23647	0.68049

1161.33607	0.68057

1161.43567	0.6806

1161.53527	0.68065

1161.63487	0.68082

1161.73447	0.68086

1161.83407	0.6809

1161.93367	0.68117

1162.03327	0.68123

1162.13287	0.68125

1162.23247	0.68127

1162.33207	0.68164

1162.43167	0.68188

1162.53127	0.6819

1162.63087	0.68195

1162.73047	0.68213

1162.83007	0.68219

1162.92967	0.68223

1163.02927	0.68224

1163.12887	0.68231

1163.22847	0.68241

1163.32807	0.68248

1163.42767	0.68251

1163.52727	0.68252

1163.62687	0.68254

1163.72647	0.68278

1163.82607	0.68286

1163.92567	0.68297

1164.02527	0.68305

1164.12488	0.68308

1164.22448	0.68332

1164.32408	0.68336

1164.42368	0.68338

1164.52328	0.68339

1164.62288	0.68377

1164.72248	0.68379

1164.82208	0.68398

1164.92168	0.684

1165.02128	0.68402

1165.12088	0.68407

1165.22048	0.6843

1165.32008	0.68434

1165.41968	0.68465

1165.51928	0.68469

1165.61887	0.68474

1165.71847	0.68479

1165.81807	0.6848

1165.91767	0.68481

1166.01727	0.6849

1166.11687	0.68497

1166.21647	0.68519

1166.31608	0.68522

1166.41568	0.68526

1166.51528	0.68532

1166.61488	0.68548

1166.71448	0.6855

1166.81408	0.68565

1166.91368	0.68568

1167.01328	0.68569

1167.11288	0.68588

1167.21248	0.68597

1167.31208	0.68614

1167.41168	0.68615

1167.51128	0.68625

1167.61088	0.68641

1167.71048	0.68642

1167.81008	0.68643

1167.90968	0.68645

1168.00927	0.68673

1168.10887	0.68674

1168.20847	0.68678

1168.30807	0.68709

1168.40767	0.6871

1168.50727	0.68716

1168.60687	0.68722

1168.70648	0.68723

1168.80608	0.68758

1168.90568	0.68766

1169.00528	0.68794

1169.10488	0.68796

1169.20448	0.68812

1169.30408	0.68815

1169.40368	0.68832

1169.50328	0.68837

1169.60288	0.68839

1169.70248	0.6885

1169.80208	0.68872

1169.90168	0.68885

1170.00128	0.68904

1170.10088	0.68914

1170.20048	0.68917

1170.30008	0.68927

1170.39967	0.68945

1170.49927	0.68952

1170.59887	0.68965

1170.69847	0.68966

1170.79807	0.69005

1170.89767	0.69009

1170.99727	0.69018

1171.09688	0.69019

1171.19648	0.6902

1171.29608	0.69037

1171.39568	0.69039

1171.49528	0.69045

1171.59488	0.69058

1171.69448	0.69078

1171.79408	0.69079

1171.89368	0.6911

1171.99328	0.69114

1172.09288	0.69121

1172.19248	0.69128

1172.29208	0.69131

1172.39168	0.69132

1172.49128	0.69143

1172.59088	0.6915

1172.69048	0.69152

1172.79007	0.69167

1172.88967	0.6917

1172.98927	0.69173

1173.08887	0.69178

1173.18847	0.69207

1173.28807	0.69212

1173.38767	0.69227

1173.48728	0.69234

1173.58688	0.69237

1173.68648	0.69262

1173.78608	0.69265

1173.88568	0.69266

1173.98528	0.69326

1174.08488	0.69328

1174.18448	0.6936

1174.28408	0.69361

1174.38368	0.69364

1174.48328	0.69365

1174.58288	0.69382

1174.68248	0.69383

1174.78208	0.69397

1174.88168	0.69399

1174.98128	0.69431

1175.08088	0.69438

1175.18047	0.69439

1175.28007	0.69445

1175.37967	0.69448

1175.47927	0.69456

1175.57887	0.69459

1175.67847	0.6948

1175.77807	0.69483

1175.87768	0.69484

1175.97728	0.6951

1176.07688	0.69512

1176.17648	0.69529

1176.27608	0.69536

1176.37568	0.69537

1176.47528	0.69564

1176.57488	0.69572

1176.67448	0.69573

1176.77408	0.69586

1176.87368	0.69594

1176.97328	0.6961

1177.07288	0.69629

1177.17248	0.69632

1177.27208	0.69636

1177.37168	0.69657

1177.47128	0.69661

1177.57087	0.69665

1177.67047	0.69675

1177.77007	0.69677

1177.86967	0.69685

1177.96927	0.69696

1178.06887	0.69748

1178.16847	0.69749

1178.26808	0.69774

1178.36768	0.69776

1178.46728	0.6978

1178.56688	0.69793

1178.66648	0.69804

1178.76608	0.69813

1178.86568	0.69855

1178.96528	0.69862

1179.06488	0.69865

1179.16448	0.69907

1179.26408	0.69908

1179.36368	0.6991

1179.46328	0.69913

1179.56288	0.70005

1179.66248	0.70013

1179.76208	0.70019

1179.86168	0.70048

1179.96128	0.70056

1180.06088	0.70063

1180.16048	0.70066

1180.26008	0.70075

1180.35968	0.70171

1180.45928	0.7021

1180.55888	0.70255

1180.65848	0.70271

1180.75808	0.70276

1180.85768	0.70297

1180.95728	0.70306

1181.05688	0.70344

1181.15648	0.70345

1181.25608	0.70347

1181.35568	0.70373

1181.45528	0.70383

1181.55488	0.70421

1181.65448	0.70422

1181.75408	0.70445

1181.85368	0.70446

1181.95328	0.70471

1182.05288	0.70475

1182.15248	0.70484

1182.25208	0.7056

1182.35168	0.70562

1182.45128	0.70574

1182.55088	0.70584

1182.65048	0.70598

1182.75008	0.706

1182.84968	0.70601

1182.94928	0.70602

1183.04888	0.70643

1183.14848	0.70647

1183.24808	0.70708

1183.34768	0.70717

1183.44728	0.7081

1183.54688	0.70861

1183.64648	0.71043

1183.74608	0.71066

1183.84568	0.71238

1183.94528	0.71485

1184.04488	0.71489

1184.14448	0.71629

1184.24408	0.71686

1184.34368	0.71868

1184.44328	0.72119

1184.54288	0.72224

1184.64248	0.72314

1184.74208	0.72315

1184.84168	0.72321

1184.94128	0.97385

1185.04088	0.97504

1185.14048	0.97567

1185.24008	0.97609

1185.33968	0.97628

1185.43928	0.97651

1185.53888	0.97858

1185.63848	0.97943

1185.73808	0.97947

1185.83768	0.97953

1185.93728	0.98133

1186.03688	0.99168

1186.13648	0.99901

1186.23608	0.99993

1186.33568	0.99996

1186.43528	0.99997

1186.53488	1

**（*f*）Stabilized hydraulic-90°**

1	0.01952	0.21802	1.20E-05

2	0.04358	0.55736	0.0000247

3	0.08935	0.6967	0.000047275

4	0.10654	0.83604	6.77E-05

5	0.12372	0.97538	0.000203142

6	0.14091	1.11472	0.000257313

7	0.16324	1.25406	3.39E-04

8	0.18558	1.3934	3.79E-04

9	0.20277	1.53274	4.06E-04

10	0.21479	1.67208	4.33E-04

11	0.2337	1.81142	0.00106

12	0.2526	1.95076	0.00114

13	0.26806	2.0901	0.00115

14	0.28525	2.22944	0.00118

15	0.30071	2.36878	0.00121

16	0.31961	2.50812	0.00122

17	0.34023	2.64746	0.00133

18	0.35742	2.7868	0.00146

19	0.36773	2.92614	0.00149

20	0.39178	3.06548	0.00161

21	0.41241	3.20482	0.00203

22	0.42615	3.34416	0.0021

23	0.4399	3.4835	0.00211

24	0.4588	3.62284	0.00213

25	0.4777	3.76218	0.00259

26	0.4966	3.90152	0.00261

27	0.51207	4.04086	0.00306

28	0.52582	4.1802	0.00313

29	0.543	4.31954	0.00321

30	0.56534	4.45889	0.00325

31	0.58252	4.59823	0.00341

32	0.59455	4.73757	0.00343

33	0.6083	4.87691	0.00348

34	0.63064	5.01625	0.00349

35	0.64782	5.15559	0.00358

36	0.66672	5.29493	0.00359

37	0.67875	5.43427	0.00372

38	0.69937	5.57361	0.00381

39	0.71999	5.71295	0.00382

40	0.73546	5.85229	0.00406

41	0.7492	5.99163	0.00408

42	0.76639	6.13097	0.0041

43	0.78529	6.27031	0.00414

44	0.80247	6.40965	0.00416

45	0.82137	6.54899	0.00417

46	0.83168	6.68833	0.0042

47	0.85402	6.82767	0.00451

48	0.87292	6.96701	0.00452

49	0.88839	7.10635	0.00465

50	0.90557	7.24569	0.00471

51	0.91932	7.38503	0.00473

52	0.93994	7.52437	0.00474

53	0.95884	7.66371	0.00475

54	0.97431	7.80305	0.00479

55	0.98805	7.94239	0.00494

56	1.00696	8.08173	0.00497

57	1.02586	8.22107	0.00498

58	1.04648	8.36041	0.00511

59	1.06022	8.49975	0.00516

60	1.07741	8.63909	0.00524

61	1.09459	8.77843	0.00525

62	1.11349	8.91777	0.00645

63	1.13068	9.05711	0.00646

64	1.14099	9.19645	0.00665

65	1.15989	9.33579	0.00672

66	1.18223	9.47513	0.00678

67	1.20113	9.61447	0.00699

68	1.21144	9.75381	0.00711

69	1.22862	9.89315	0.00712

70	1.24924	10.03249	0.00714

71	1.26643	10.17183	0.00716

72	1.28361	10.31117	0.00718

73	1.29736	10.45051	0.0072

74	1.31626	10.58985	0.00729

75	1.3386	10.72919	0.00737

76	1.35406	10.86853	0.00743

77	1.36609	11.00787	0.00765

78	1.38499	11.14721	0.00767

79	1.40561	11.28655	0.00769

80	1.4228	11.42589	0.0078

81	1.43826	11.56523	0.00781

82	1.45201	11.70457	0.00785

83	1.47263	11.84391	0.00792

84	1.49153	11.98325	0.00795

85	1.507	12.12259	0.008

86	1.52074	12.26193	0.00802

87	1.53793	12.40127	0.00817

88	1.55855	12.54061	0.00821

89	1.57573	12.67995	0.00823

90	1.5912	12.81929	0.00825

91	1.60494	12.95863	0.00826

92	1.62556	13.09797	0.00827

93	1.64618	13.23731	0.00829

94	1.66165	13.37666	0.0083

95	1.67368	13.516	0.00836

96	1.69258	13.65534	0.00837

97	1.71492	13.79468	0.0084

98	1.73038	13.93402	0.00841

99	1.74241	14.07336	0.00844

100	1.75788	14.2127	0.00846

101	1.78022	14.35204	0.00852

102	1.79912	14.49138	0.00855

103	1.8163	14.63072	0.00856

104	1.82833	14.77006	0.00868

105	1.84723	14.9094	0.00869

106	1.86785	15.04874	0.00874

107	1.88504	15.18808	0.00876

108	1.89706	15.32742	0.00879

109	1.91425	15.46676	0.00882

110	1.93315	15.6061	0.00883

111	1.95377	15.74544	0.00884

112	1.96923	15.88478	0.00891

113	1.98298	16.02412	0.00892

114	2.00016	16.16346	0.00905

115	2.0225	16.3028	0.0091

116	2.03797	16.44214	0.00928

117	2.05343	16.58148	0.0093

118	2.05687	16.72082	0.00936

119	2.0311	16.86016	0.00945

120	2.03625	16.9995	0.00947

121	2.06031	17.13884	0.0097

122	2.08093	17.27818	0.00976

123	2.09639	17.41752	0.00999

124	2.10498	17.55686	0.01008

125	2.12045	17.6962	0.01009

126	2.14451	17.83554	0.0101

127	2.16341	17.97488	0.01013

128	2.17716	18.11422	0.01014

129	2.1909	18.25356	0.01016

130	2.2098	18.3929	0.01017

131	2.23214	18.53224	0.01094

132	2.24933	18.67158	0.01108

133	2.26135	18.81092	0.01109

134	2.27682	18.95026	0.01115

135	2.30088	19.0896	0.01117

136	2.31291	19.22894	0.0112

137	2.32665	19.36828	0.01123

138	2.34384	19.50762	0.01124

139	2.36617	19.64696	0.01125

140	2.38164	19.7863	0.01129

141	2.40226	19.92564	0.01131

142	2.41085	20.06498	0.01132

143	2.42804	20.20432	0.01134

144	2.45209	20.34366	0.01136

145	2.46584	20.483	0.01138

146	2.47959	20.62234	0.01146

147	2.49677	20.76168	0.01147

148	2.51911	20.90102	0.01155

149	2.53285	21.04036	0.01161

150	2.55176	21.1797	0.01163

151	2.5655	21.31904	0.0117

152	2.58612	21.45838	0.01174

153	2.60331	21.59772	0.01196

154	2.62049	21.73706	0.012

155	2.63424	21.8764	0.01212

156	2.65486	22.01574	0.01213

157	2.67204	22.15508	0.01217

158	2.69094	22.29443	0.0122

159	2.70469	22.43377	0.01222

160	2.72359	22.57311	0.01234

161	2.73906	22.71245	0.01235

162	2.75796	22.85179	0.01249

163	2.77342	22.99113	0.0125

164	2.78717	23.13047	0.01251

165	2.80779	23.26981	0.0127

166	2.82841	23.40915	0.01272

167	2.84216	23.54849	0.01274

168	2.85934	23.68783	0.01276

169	2.87481	23.82717	0.01278

170	2.89715	23.96651	0.0128

171	2.91777	24.10585	0.01296

172	2.92808	24.24519	0.01299

173	2.94354	24.38453	0.013

174	2.96244	24.52387	0.01456

175	2.98135	24.66321	0.01457

176	2.99853	24.80255	0.0146

177	3.01399	24.94189	0.01461

178	3.02946	25.08123	0.01463

179	3.0518	25.22057	0.01467

180	3.0707	25.35991	0.01468

181	3.08101	25.49925	0.01471

182	3.10163	25.63859	0.01472

183	3.11881	25.77793	0.0148

184	3.14115	25.91727	0.01482

185	3.15146	26.05661	0.01486

186	3.16865	26.19595	0.0149

187	3.18755	26.33529	0.01491

188	3.20989	26.47463	0.01494

189	3.22363	26.61397	0.01499

190	3.23738	26.75331	0.01501

191	3.25456	26.89265	0.01502

192	3.27518	27.03199	0.01503

193	3.29237	27.17133	0.01506

194	3.30268	27.31067	0.01511

195	3.3233	27.45001	0.01513

196	3.34564	27.58935	0.01514

197	3.36282	27.72869	0.01515

198	3.37829	27.86803	0.01517

199	3.3886	28.00737	0.01518

200	3.41265	28.14671	0.01522

201	3.42812	28.28605	0.01525

202	3.4453	28.42539	0.01526

203	3.45733	28.56473	0.01532

204	3.48139	28.70407	0.01534

205	3.49857	28.84341	0.01563

206	3.51575	28.98275	0.01564

207	3.5295	29.12209	0.01566

208	3.5484	29.26143	0.01571

209	3.5673	29.40077	0.01578

210	3.58792	29.54011	0.01579

211	3.59823	29.67945	0.01595

212	3.61026	29.81879	0.0165

213	3.63432	29.95813	0.01658

214	3.65666	30.09747	0.01663

215	3.67041	30.23681	0.01668

216	3.68415	30.37615	0.0167

217	3.70305	30.51549	0.01682

218	3.72367	30.65483	0.01683

219	3.73914	30.79417	0.01685

220	3.75117	30.93351	0.01687

221	3.76663	31.07285	0.01691

222	3.79241	31.2122	0.01693

223	3.80959	31.35154	0.01694

224	3.82334	31.49088	0.01697

225	3.8388	31.63022	0.01706

226	3.85771	31.76956	0.0171

227	3.88005	31.9089	0.01724

228	3.89379	32.04824	0.01725

229	3.90754	32.18758	0.01727

230	3.923	32.32692	0.01728

231	3.94534	32.46626	0.01733

232	3.96596	32.6056	0.01735

233	3.97627	32.74494	0.01739

234	3.99002	32.88428	0.01816

235	4.01236	33.02362	0.01827

236	4.03126	33.16296	0.01836

237	4.04844	33.3023	0.01842

238	4.06391	33.44164	0.01843

239	4.08109	33.58098	0.01865

240	4.09828	33.72032	0.01866

241	4.12061	33.85966	0.01868

242	4.12577	33.999	0.01872

243	4.14639	34.13834	0.01873

244	4.17217	34.27768	0.01876

245	4.18591	34.41702	0.01877

246	4.19622	34.55636	0.01878

247	4.21341	34.6957	0.01884

248	4.23403	34.83504	0.01891

249	4.25465	34.97438	0.01897

250	4.27011	35.11372	0.01899

251	4.28386	35.25306	0.01905

252	4.30276	35.3924	0.01907

253	4.32338	35.53174	0.01918

254	4.34056	35.67108	0.0192

255	4.35603	35.81042	0.01923

256	4.36978	35.94976	0.01924

257	4.39383	36.0891	0.01926

258	4.41274	36.22844	0.0193

259	4.42133	36.36778	0.01931

260	4.43679	36.50712	0.01933

261	4.46085	36.64646	0.01939

262	4.48319	36.7858	0.01946

263	4.49178	36.92514	0.01947

264	4.50724	37.06448	0.01949

265	4.52958	37.20382	0.01952

266	4.54849	37.34316	0.01954

267	4.56395	37.4825	0.01983

268	4.57254	37.62184	0.01984

269	4.59144	37.76118	0.01985

270	4.61206	37.90052	0.01987

271	4.6344	38.03986	0.01991

272	4.64643	38.1792	0.01996

273	4.66018	38.31854	0.01999

274	4.68424	38.45788	0.02

275	4.70486	38.59722	0.02004

276	4.71688	38.73656	0.02006

277	4.73063	38.8759	0.02021

278	4.75125	39.01524	0.02022

279	4.77187	39.15458	0.02026

280	4.78562	39.29392	0.02027

281	4.79593	39.43326	0.0203

282	4.81827	39.5726	0.02035

283	4.84061	39.71194	0.02037

284	4.86123	39.85128	0.02038

285	4.87154	39.99062	0.0204

286	4.88356	40.12997	0.02041

287	4.90762	40.26931	0.02064

288	4.9248	40.40865	0.02065

289	4.94027	40.54799	0.02067

290	4.9523	40.68733	0.02068

291	4.9712	40.82667	0.02071

292	4.99526	40.96601	0.02072

293	5.01244	41.10535	0.02073

294	5.02619	41.24469	0.02075

295	5.03993	41.38403	0.02077

296	5.06399	41.52337	0.02079

297	5.07946	41.66271	0.0208

298	5.09149	41.80205	0.02083

299	5.10867	41.94139	0.02084

300	5.12757	42.08073	0.02087

301	5.14647	42.22007	0.0209

302	5.16194	42.35941	0.02091

303	5.18084	42.49875	0.02094

304	5.19974	42.63809	0.02119

305	5.21693	42.77743	0.02121

306	5.23239	42.91677	0.02124

307	5.2427	43.05611	0.02125

308	5.2616	43.19545	0.02126

309	5.28566	43.33479	0.02128

310	5.30112	43.47413	0.02134

311	5.31659	43.61347	0.02136

312	5.33377	43.75281	0.02138

313	5.35268	43.89215	0.02144

314	5.37158	44.03149	0.02145

315	5.38189	44.17083	0.02147

316	5.39563	44.31017	0.02153

317	5.41969	44.44951	0.02155

318	5.43859	44.58885	0.02156

319	5.45406	44.72819	0.02157

320	5.4678	44.86753	0.02161

321	5.48671	45.00687	0.02186

322	5.51248	45.14621	0.02187

323	5.52967	45.28555	0.02189

324	5.53826	45.42489	0.0219

325	5.55716	45.56423	0.02217

326	5.5795	45.70357	0.02218

327	5.59668	45.84291	0.0222

328	5.60699	45.98225	0.02225

329	5.62418	46.12159	0.02228

330	5.64823	46.26093	0.02229

331	5.66713	46.40027	0.0223

332	5.68088	46.53961	0.02232

333	5.68947	46.67895	0.02233

334	5.70837	46.81829	0.02237

335	5.73243	46.95763	0.02243

336	5.7479	47.09697	0.02245

337	5.75821	47.23631	0.02247

338	5.77883	47.37565	0.02248

339	5.80117	47.51499	0.02249

340	5.81663	47.65433	0.02251

341	5.82866	47.79367	0.02252

342	5.84756	47.93301	0.02255

343	5.87162	48.07235	0.02289

344	5.8888	48.21169	0.0229

345	5.89911	48.35103	0.02293

346	5.9163	48.49037	0.023

347	5.93692	48.62971	0.02304

348	5.9541	48.76905	0.02343

349	5.97472	48.90839	0.02351

350	5.98675	49.04774	0.02352

351	6.00221	49.18708	0.02356

352	6.02455	49.32642	0.02358

353	6.04002	49.46576	0.02359

354	6.05376	49.6051	0.02379

355	6.07267	49.74444	0.02393

356	6.09329	49.88378	0.02397

357	6.11047	50.02312	0.02405

358	6.12593	50.16246	0.02407

359	6.1414	50.3018	0.02408

360	6.16202	50.44114	0.02409

361	6.1792	50.58048	0.02411

362	6.19639	50.71982	0.02412

363	6.20498	50.85916	0.02413

364	6.2256	50.9985	0.02415

365	6.24794	51.13784	0.02417

366	6.26512	51.27718	0.02419

367	6.27715	51.41652	0.02423

368	6.29605	51.55586	0.02424

369	6.31839	51.6952	0.02426

370	6.33729	51.83454	0.02431

371	6.3476	51.97388	0.02432

372	6.36307	52.11322	0.02434

373	6.38197	52.25256	0.02438

374	6.40603	52.3919	0.02449

375	6.41977	52.53124	0.0245

376	6.42837	52.67058	0.02457

377	6.45242	52.80992	0.02515

378	6.47132	52.94926	0.02516

379	6.49023	53.0886	0.02537

380	6.50054	53.22794	0.02539

381	6.51944	53.36728	0.02568

382	6.54178	53.50662	0.02574

383	6.55724	53.64596	0.02576

384	6.56755	53.7853	0.02577

385	6.58817	53.92464	0.02579

386	6.61395	54.06398	0.02592

387	6.62769	54.20332	0.02593

388	6.63972	54.34266	0.02598

389	6.65691	54.482	0.02599

390	6.67753	54.62134	0.02616

391	6.69471	54.76068	0.02622

392	6.70846	54.90002	0.02627

393	6.7222	55.03936	0.02661

394	6.74626	55.1787	0.02663

395	6.76516	55.31804	0.02673

396	6.78406	55.45738	0.02675

397	6.79609	55.59672	0.02677

398	6.81328	55.73606	0.02679

399	6.83562	55.8754	0.0268

400	6.85108	56.01474	0.02688

401	6.86139	56.15408	0.0269

402	6.88201	56.29342	0.02691

403	6.90263	56.43276	0.02698

404	6.91981	56.5721	0.02721

405	6.93184	56.71144	0.02723

406	6.94731	56.85078	0.02726

407	6.96965	56.99012	0.02729

408	6.99027	57.12946	0.02734

409	7.0023	57.2688	0.02746

410	7.01432	57.40814	0.02748

411	7.03494	57.54748	0.02757

412	7.05728	57.68682	0.02764

413	7.07447	57.82616	0.02765

414	7.0865	57.96551	0.02771

415	7.10368	58.10485	0.02772

416	7.1243	58.24419	0.02774

417	7.14492	58.38353	0.02775

418	7.15695	58.52287	0.02779

419	7.16898	58.66221	0.02793

420	7.19131	58.80155	0.02794

421	7.21194	58.94089	0.02795

422	7.22568	59.08023	0.02799

423	7.23599	59.21957	0.02801

424	7.26005	59.35891	0.02802

425	7.28239	59.49825	0.02803

426	7.29785	59.63759	0.02806

427	7.30816	59.77693	0.02821

428	7.32363	59.91627	0.0283

429	7.34769	60.05561	0.02836

430	7.36487	60.19495	0.02839

431	7.37518	60.33429	0.0284

432	7.39752	60.47363	0.02858

433	7.41814	60.61297	0.0286

434	7.43704	60.75231	0.02863

435	7.44735	60.89165	0.02864

436	7.46453	61.03099	0.0287

437	7.48515	61.17033	0.02893

438	7.50234	61.30967	0.02898

439	7.51608	61.44901	0.02901

440	7.52983	61.58835	0.02902

441	7.55045	61.72769	0.02904

442	7.57107	61.86703	0.02905

443	7.58825	62.00637	0.02927

444	7.59685	62.14571	0.02933

445	7.61919	62.28505	0.02955

446	7.64152	62.42439	0.02956

447	7.65527	62.56373	0.02959

448	7.6673	62.70307	0.02969

449	7.68448	62.84241	0.0297

450	7.70854	62.98175	0.02971

451	7.724	63.12109	0.02973

452	7.73947	63.26043	0.02978

453	7.75322	63.39977	0.03

454	7.78243	63.53911	0.03016

455	7.79446	63.67845	0.03017

456	7.8082	63.81779	0.03019

457	7.82195	63.95713	0.03031

458	7.84429	64.09647	0.03057

459	7.86491	64.23581	0.03059

460	7.87694	64.37515	0.03131

461	7.88897	64.51449	0.03141

462	7.91302	64.65383	0.03146

463	7.93708	64.79317	0.03157

464	7.94911	64.93251	0.03158

465	7.96286	65.07185	0.03162

466	7.98004	65.21119	0.03164

467	8.00066	65.35053	0.03166

468	8.01956	65.48987	0.03168

469	8.03159	65.62921	0.03169

470	8.04877	65.76855	0.03174

471	8.06939	65.90789	0.03176

472	8.09001	66.04723	0.03181

473	8.10204	66.18657	0.03184

474	8.11407	66.32591	0.03189

475	8.13297	66.46525	0.03192

476	8.15703	66.60459	0.03193

477	8.17078	66.74393	0.032

478	8.18281	66.88328	0.03202

479	8.19999	67.02262	0.03208

480	8.22233	67.16196	0.0321

481	8.23951	67.3013	0.03211

482	8.25154	67.44064	0.03212

483	8.27044	67.57998	0.03214

484	8.29278	67.71932	0.0322

485	8.30825	67.85866	0.03222

486	8.32199	67.998	0.03265

487	8.33918	68.13734	0.03268

488	8.35808	68.27668	0.03269

489	8.37698	68.41602	0.03271

490	8.39416	68.55536	0.03283

491	8.40963	68.6947	0.03285

492	8.42681	68.83404	0.03292

493	8.44743	68.97338	0.03311

494	8.45946	69.11272	0.03326

495	8.47149	69.25206	0.03333

496	8.49383	69.3914	0.03353

497	8.51788	69.53074	0.03355

498	8.53163	69.67008	0.03369

499	8.54538	69.80942	0.0338

500	8.56428	69.94876	0.03382

501	8.5849	70.0881	0.03383

502	8.60037	70.22744	0.03418

503	8.61068	70.36678	0.03441

504	8.62786	70.50612	0.03443

505	8.6502	70.64546	0.03447

506	8.66738	70.7848	0.03448

507	8.68113	70.92414	0.03455

508	8.69831	71.06348	0.03456

509	8.72065	71.20282	0.03457

510	8.74127	71.34216	0.03474

511	8.75502	71.4815	0.03478

512	8.76017	71.62084	0.03479

513	8.78423	71.76018	0.0348

514	8.80829	71.89952	0.03485

515	8.82375	72.03886	0.03486

516	8.83406	72.1782	0.03512

517	8.8564	72.31754	0.03516

518	8.87874	72.45688	0.03527

519	8.89592	72.59622	0.03531

520	8.90623	72.73556	0.03532

521	8.9217	72.8749	0.03571

522	8.94747	73.01424	0.03582

523	8.96122	73.15358	0.03597

524	8.97497	73.29292	0.03601

525	8.98871	73.43226	0.03612

526	9.01449	73.5716	0.03635

527	9.03511	73.71094	0.03636

528	9.04714	73.85028	0.03643

529	9.05745	73.98962	0.03661

530	9.07979	74.12896	0.03663

531	9.09697	74.2683	0.03666

532	9.11244	74.40764	0.03673

533	9.1279	74.54698	0.03674

534	9.1468	74.68632	0.03676

535	9.17258	74.82566	0.03677

536	9.18632	74.965	0.0368

537	9.19663	75.10434	0.03682

538	9.21726	75.24368	0.03684

539	9.23788	75.38302	0.03685

540	9.25334	75.52236	0.03686

541	9.26365	75.6617	0.03701

542	9.28427	75.80105	0.03707

543	9.30489	75.94039	0.03708

544	9.32036	76.07973	0.03709

545	9.33238	76.21907	0.03711

546	9.35129	76.35841	0.03715

547	9.37363	76.49775	0.03719

548	9.39253	76.63709	0.03724

549	9.40456	76.77643	0.03726

550	9.42002	76.91577	0.03727

551	9.44236	77.05511	0.03728

552	9.45954	77.19445	0.03731

553	9.47501	77.33379	0.03734

554	9.49047	77.47313	0.03735

555	9.50938	77.61247	0.03736

556	9.53343	77.75181	0.03739

557	9.54718	77.89115	0.03742

558	9.55921	78.03049	0.03745

559	9.57295	78.16983	0.03746

560	9.59873	78.30917	0.03747

561	9.61419	78.44851	0.03749

562	9.62794	78.58785	0.03754

563	9.64341	78.72719	0.03772

564	9.66575	78.86653	0.03777

565	9.6898	79.00587	0.0378

566	9.70183	79.14521	0.03781

567	9.71214	79.28455	0.03784

568	9.73104	79.42389	0.03785

569	9.74995	79.56323	0.03787

570	9.76541	79.70257	0.03788

571	9.77916	79.84191	0.03792

572	9.79634	79.98125	0.03803

573	9.82555	80.12059	0.03804

574	9.84102	80.25993	0.03808

575	9.85133	80.39927	0.03814

576	9.87023	80.53861	0.03833

577	9.89085	80.67795	0.03856

578	9.90803	80.81729	0.03865

579	9.91663	80.95663	0.03866

580	9.93553	81.09597	0.03869

581	9.95958	81.23531	0.03871

582	9.97677	81.37465	0.03872

583	9.99051	81.51399	0.03876

584	10.00598	81.65333	0.03885

585	10.02832	81.79267	0.03888

586	10.0455	81.93201	0.03889

587	10.05753	82.07135	0.03895

588	10.07128	82.21069	0.03896

589	10.09018	82.35003	0.03899

590	10.11252	82.48937	0.039

591	10.1297	82.62871	0.03904

592	10.14173	82.76805	0.0391

593	10.16063	82.90739	0.03942

594	10.18469	83.04673	0.03946

595	10.195	83.18607	0.03953

596	10.20703	83.32541	0.03954

597	10.22421	83.46475	0.03957

598	10.24827	83.60409	0.03959

599	10.26717	83.74343	0.0396

600	10.2792	83.88277	0.03987

601	10.29638	84.02211	0.03994

602	10.32044	84.16145	0.03995

603	10.3359	84.30079	0.04026

604	10.34965	84.44013	0.04028

605	10.36168	84.57947	0.04029

606	10.38402	84.71882	0.04056

607	10.40636	84.85816	0.04057

608	10.41839	84.9975	0.04068

609	10.43385	85.13684	0.04082

610	10.45447	85.27618	0.04085

611	10.47509	85.41552	0.04086

612	10.48884	85.55486	0.04087

613	10.49915	85.6942	0.04094

614	10.52149	85.83354	0.04103

615	10.54382	85.97288	0.04105

616	10.55929	86.11222	0.04117

617	10.57132	86.25156	0.04135

618	10.5885	86.3909	0.04136

619	10.61256	86.53024	0.04137

620	10.63318	86.66958	0.04147

621	10.64177	86.80892	0.04178

622	10.65724	86.94826	0.04179

623	10.67786	87.0876	0.04181

624	10.69504	87.22694	0.04182

625	10.70879	87.36628	0.04183

626	10.72253	87.50562	0.04187

627	10.74659	87.64496	0.04201

628	10.76377	87.7843	0.04202

629	10.78096	87.92364	0.04204

630	10.79127	88.06298	0.04206

631	10.81532	88.20232	0.04208

632	10.83595	88.34166	0.04209

633	10.84626	88.481	0.0421

634	10.85828	88.62034	0.0422

635	10.88062	88.75968	0.04221

636	10.90468	88.89902	0.04223

637	10.91843	89.03836	0.04224

638	10.92874	89.1777	0.04225

639	10.94936	89.31704	0.04273

640	10.9717	89.45638	0.04274

641	10.98716	89.59572	0.04326

642	10.99575	89.73506	0.04327

643	11.01809	89.8744	0.04346

644	11.04215	90.01374	0.04349

645	11.05246	90.15308	0.0435

646	11.06792	90.29242	0.04351

647	11.08683	90.43176	0.04353

648	11.10745	90.5711	0.04363

649	11.12807	90.71044	0.04365

650	11.14181	90.84978	0.04372

651	11.15212	90.98912	0.0441

652	11.17618	91.12846	0.04411

653	11.19508	91.2678	0.04414

654	11.20883	91.40714	0.04415

655	11.22086	91.54648	0.04416

656	11.2432	91.68582	0.04431

657	11.26382	91.82516	0.04445

658	11.27928	91.9645	0.04447

659	11.29131	92.10384	0.04481

660	11.30849	92.24318	0.04595

661	11.33255	92.38252	0.04599

662	11.3463	92.52186	0.046

663	11.35661	92.6612	0.04606

664	11.37551	92.80054	0.04607

665	11.39785	92.93988	0.04614

666	11.41847	93.07922	0.04615

667	11.4305	93.21856	0.04617

668	11.44768	93.3579	0.04637

669	11.4683	93.49725	0.04638

670	11.48376	93.63659	0.04648

671	11.50095	93.77593	0.04649

672	11.50954	93.91527	0.04651

673	11.53016	94.05461	0.04691

674	11.55594	94.19395	0.04693

675	11.56796	94.33329	0.04697

676	11.58171	94.47263	0.04698

677	11.60405	94.61197	0.04702

678	11.62639	94.75131	0.04707

679	11.63842	94.89065	0.04709

680	11.64701	95.02999	0.04712

681	11.66763	95.16933	0.04713

682	11.68997	95.30867	0.04724

683	11.70715	95.44801	0.04725

684	11.71746	95.58735	0.04729

685	11.7398	95.72669	0.04737

686	11.76042	95.86603	0.0474

687	11.7776	96.00537	0.04779

688	11.78963	96.14471	0.04796

689	11.8051	96.28405	0.04797

690	11.82744	96.42339	0.04805

691	11.8429	96.56273	0.04806

692	11.85837	96.70207	0.04808

693	11.87555	96.84141	0.04809

694	11.89617	96.98075	0.04813

695	11.91507	97.12009	0.04821

696	11.93054	97.25943	0.04829

697	11.93913	97.39877	0.04831

698	11.96147	97.53811	0.04833

699	11.98209	97.67745	0.04842

700	11.99755	97.81679	0.04846

701	12.01302	97.95613	0.04847

702	12.0302	98.09547	0.04856

703	12.05426	98.23481	0.04859

704	12.06629	98.37415	0.04863

705	12.08003	98.51349	0.04867

706	12.10065	98.65283	0.04875

707	12.12471	98.79217	0.04884

708	12.13674	98.93151	0.04886

709	12.14705	99.07085	0.04943

710	12.16767	99.21019	0.04947

711	12.19001	99.34953	0.04967

712	12.20547	99.48887	0.04969

713	12.21578	99.62821	0.0497

714	12.2364	99.76755	0.04972

715	12.25531	99.90689	0.04973

716	12.27421	100.04623	0.04977

717	12.28796	100.18557	0.04995

718	12.3017	100.32491	0.05001

719	12.32404	100.46425	0.05003

720	12.34122	100.60359	0.05004

721	12.35669	100.74293	0.05007

722	12.37215	100.88227	0.05009

723	12.39277	101.02161	0.05011

724	12.41511	101.16095	0.05012

725	12.42886	101.30029	0.05031

726	12.43745	101.43963	0.05033

727	12.45807	101.57897	0.05035

728	12.47697	101.71831	0.05039

729	12.49588	101.85765	0.05083

730	12.50962	101.99699	0.05095

731	12.52681	102.13633	0.05096

732	12.55258	102.27567	0.05098

733	12.56977	102.41502	0.05099

734	12.57836	102.55436	0.05104

735	12.59554	102.6937	0.05106

736	12.6196	102.83304	0.05107

737	12.63506	102.97238	0.05114

738	12.64365	103.11172	0.0515

739	12.66084	103.25106	0.05206

740	12.68833	103.3904	0.05207

741	12.70208	103.52974	0.05241

742	12.71754	103.66908	0.05249

743	12.73473	103.80842	0.05251

744	12.75707	103.94776	0.05252

745	12.77253	104.0871	0.05255

746	12.78456	104.22644	0.05257

747	12.80002	104.36578	0.05264

748	12.81893	104.50512	0.05272

749	12.83955	104.64446	0.05299

750	12.85329	104.7838	0.05306

751	12.87048	104.92314	0.0531

752	12.8911	105.06248	0.05314

753	12.91344	105.20182	0.05316

754	12.92375	105.34116	0.05317

755	12.93406	105.4805	0.0532

756	12.95983	105.61984	0.05364

757	12.97873	105.75918	0.05366

758	12.99248	105.89852	0.05368

759	13.00451	106.03786	0.05448

760	13.02685	106.1772	0.05456

761	13.0509	106.31654	0.05458

762	13.06121	106.45588	0.05459

763	13.07496	106.59522	0.0546

764	13.09386	106.73456	0.05462

765	13.11448	106.8739	0.05463

766	13.13167	107.01324	0.05467

767	13.14198	107.15258	0.0547

768	13.15744	107.29192	0.05471

769	13.18665	107.43126	0.05473

770	13.2004	107.5706	0.05474

771	13.21415	107.70994	0.05477

772	13.23477	107.84928	0.05478

773	13.24852	107.98862	0.05479

774	13.26914	108.12796	0.05481

775	13.28632	108.2673	0.05492

776	13.29319	108.40664	0.05493

777	13.31897	108.54598	0.05494

778	13.34302	108.68532	0.05496

779	13.35333	108.82466	0.05497

780	13.36708	108.964	0.05508

781	13.3877	109.10334	0.05513

782	13.4066	109.24268	0.05515

783	13.42207	109.38202	0.05519

784	13.43582	109.52136	0.0552

785	13.45128	109.6607	0.05532

786	13.47534	109.80004	0.05572

787	13.4908	109.93938	0.05574

788	13.50455	110.07872	0.05578

789	13.52517	110.21806	0.0558

790	13.54579	110.3574	0.05585

791	13.55954	110.49674	0.05586

792	13.575	110.63608	0.05608

793	13.59219	110.77542	0.05611

794	13.61281	110.91476	0.05613

795	13.63171	111.0541	0.05641

796	13.6403	111.19344	0.05649

797	13.6592	111.33279	0.05681

798	13.68498	111.47213	0.05693

799	13.69872	111.61147	0.05695

800	13.71075	111.75081	0.05696

801	13.7245	111.89015	0.05704

802	13.74684	112.02949	0.05746

803	13.76574	112.16883	0.05748

804	13.78121	112.30817	0.05752

805	13.79495	112.44751	0.05753

806	13.81729	112.58685	0.05754

807	13.83791	112.72619	0.05796

808	13.84994	112.86553	0.05802

809	13.86712	113.00487	0.05803

810	13.88602	113.14421	0.05806

811	13.90665	113.28355	0.05821

812	13.91867	113.42289	0.05822

813	13.93242	113.56223	0.05834

814	13.95304	113.70157	0.05886

815	13.97366	113.84091	0.05891

816	13.98741	113.98025	0.05892

817	13.99944	114.11959	0.05901

818	14.02006	114.25893	0.05902

819	14.04755	114.39827	0.0596

820	14.05786	114.53761	0.05962

821	14.06645	114.67695	0.05994

822	14.08707	114.81629	0.06001

823	14.11457	114.95563	0.06014

824	14.12831	115.09497	0.06018

825	14.1369	115.23431	0.0606

826	14.15753	115.37365	0.06063

827	14.1833	115.51299	0.06064

828	14.19877	115.65233	0.06066

829	14.20736	115.79167	0.06078

830	14.22626	115.93101	0.06116

831	14.2486	116.07035	0.06117

832	14.26578	116.20969	0.06119

833	14.27437	116.34903	0.06176

834	14.29671	116.48837	0.06197

835	14.31733	116.62771	0.06213

836	14.33795	116.76705	0.06215

837	14.34998	116.90639	0.06216

838	14.36545	117.04573	0.06217

839	14.38778	117.18507	0.0622

840	14.40153	117.32441	0.06236

841	14.41356	117.46375	0.06238

842	14.43246	117.60309	0.06241

843	14.45308	117.74243	0.06245

844	14.47198	117.88177	0.06253

845	14.48401	118.02111	0.06257

846	14.5012	118.16045	0.06258

847	14.52525	118.29979	0.06259

848	14.54244	118.43913	0.06261

849	14.55275	118.57847	0.06262

850	14.56821	118.71781	0.06264

851	14.58883	118.85715	0.06265

852	14.60773	118.99649	0.06278

853	14.6232	119.13583	0.06288

854	14.63695	119.27517	0.06295

855	14.65757	119.41451	0.06296

856	14.67819	119.55385	0.0631

857	14.69537	119.69319	0.06312

858	14.70396	119.83253	0.06315

859	14.7263	119.97187	0.06404

860	14.74864	120.11121	0.06411

861	14.7641	120.25056	0.06414

862	14.77098	120.3899	0.06415

863	14.7916	120.52924	0.06461

864	14.81565	120.66858	0.06463

865	14.83456	120.80792	0.06464

866	14.84487	120.94726	0.06473

867	14.86205	121.0866	0.06496

868	14.88783	121.22594	0.0653

869	14.89985	121.36528	0.06567

870	14.91016	121.50462	0.06568

871	14.93078	121.64396	0.06595

872	14.9514	121.7833	0.06598

873	14.97031	121.92264	0.06614

874	14.98234	122.06198	0.06645

875	14.99952	122.20132	0.06654

876	15.02186	122.34066	0.06663

877	15.03904	122.48	0.06664

878	15.05107	122.61934	0.0667

879	15.06482	122.75868	0.06671

880	15.08715	122.89802	0.06673

881	15.10606	123.03736	0.06674

882	15.12152	123.1767	0.06702

883	15.13699	123.31604	0.06704

884	15.15589	123.45538	0.06705

885	15.17823	123.59472	0.06725

886	15.19026	123.73406	0.06731

887	15.20057	123.8734	0.06755

888	15.22462	124.01274	0.06765

889	15.24696	124.15208	0.06769

890	15.25727	124.29142	0.0677

891	15.27102	124.43076	0.06771

892	15.29164	124.5701	0.06774

893	15.31398	124.70944	0.06775

894	15.33116	124.84878	0.06831

895	15.34319	124.98812	0.06836

896	15.36209	125.12746	0.06838

897	15.38099	125.2668	0.06839

898	15.3999	125.40614	0.06912

899	15.40677	125.54548	0.06914

900	15.43083	125.68482	0.06931

901	15.45145	125.82416	0.06957

902	15.47035	125.9635	0.06958

903	15.48066	126.10284	0.06961

904	15.49784	126.24218	0.06962

905	15.51846	126.38152	0.06991

906	15.53736	126.52086	0.07128

907	15.54767	126.6602	0.07129

908	15.56486	126.79954	0.0713

909	15.5872	126.93888	0.07142

910	15.6061	127.07822	0.07156

911	15.61813	127.21756	0.07157

912	15.63531	127.3569	0.07165

913	15.65765	127.49624	0.07167

914	15.67827	127.63558	0.07168

915	15.68514	127.77492	0.0717

916	15.70404	127.91426	0.07189

917	15.71951	128.0536	0.07235

918	15.74357	128.19294	0.07282

919	15.75388	128.33228	0.07298

920	15.7745	128.47162	0.073

921	15.79512	128.61096	0.07301

922	15.81746	128.7503	0.07313

923	15.82948	128.88964	0.07316

924	15.83808	129.02898	0.07333

925	15.86213	129.16833	0.07347

926	15.88275	129.30767	0.07352

927	15.89134	129.44701	0.07356

928	15.90681	129.58635	0.07358

929	15.92743	129.72569	0.07359

930	15.95149	129.86503	0.07361

931	15.96695	130.00437	0.07363

932	15.97726	130.14371	0.07367

933	15.99788	130.28305	0.07369

934	16.0185	130.42239	0.0737

935	16.03397	130.56173	0.07382

936	16.04428	130.70107	0.07384

937	16.06318	130.84041	0.07386

938	16.08896	130.97975	0.07388

939	16.10442	131.11909	0.07405

940	16.11473	131.25843	0.07409

941	16.1302	131.39777	0.07411

942	16.15941	131.53711	0.07427

943	16.17316	131.67645	0.07428

944	16.18347	131.81579	0.07435

945	16.19721	131.95513	0.07438

946	16.22471	132.09447	0.07443

947	16.24017	132.23381	0.07472

948	16.25392	132.37315	0.07481

949	16.26938	132.51249	0.07482

950	16.2986	132.65183	0.07499

951	16.31406	132.79117	0.07504

952	16.32265	132.93051	0.07512

953	16.33812	133.06985	0.07522

954	16.35874	133.20919	0.07524

955	16.3742	133.34853	0.07546

956	16.38967	133.48787	0.07551

957	16.40685	133.62721	0.07553

958	16.42919	133.76655	0.07554

959	16.44981	133.90589	0.07573

960	16.46012	134.04523	0.07581

961	16.47559	134.18457	0.07593

962	16.49621	134.32391	0.07595

963	16.51339	134.46325	0.07608

964	16.52714	134.60259	0.0768

965	16.54432	134.74193	0.07684

966	16.5615	134.88127	0.07737

967	16.58556	135.02061	0.07767

968	16.59931	135.15995	0.07788

969	16.61305	135.29929	0.07801

970	16.63367	135.43863	0.07802

971	16.65601	135.57797	0.07803

972	16.67148	135.71731	0.07805

973	16.67835	135.85665	0.07807

974	16.69897	135.99599	0.07866

975	16.72303	136.13533	0.07867

976	16.73678	136.27467	0.07871

977	16.7488	136.41401	0.07876

978	16.76942	136.55335	0.07881

979	16.79348	136.69269	0.07891

980	16.80723	136.83203	0.07894

981	16.81754	136.97137	0.07904

982	16.83644	137.11071	0.07905

983	16.85878	137.25005	0.07906

984	16.87424	137.38939	0.07923

985	16.88627	137.52873	0.07931

986	16.90517	137.66807	0.07992

987	16.92751	137.80741	0.07996

988	16.94641	137.94675	0.08009

989	16.95672	138.0861	0.08089

990	16.97391	138.22544	0.08092

991	16.99625	138.36478	0.08099

992	17.00999	138.50412	0.081

993	17.02202	138.64346	0.08101

994	17.04092	138.7828	0.0812

995	17.06326	138.92214	0.08122

996	17.08216	139.06148	0.08123

997	17.09591	139.20082	0.08124

998	17.1131	139.34016	0.08132

999	17.13543	139.4795	0.08134

1000	17.15262	139.61884	0.08137

1001	17.16121	139.75818	0.08142

1002	17.17839	139.89752	0.08143

1003	17.20245	140.03686	0.08151

1004	17.2162	140.1762	0.08158

1005	17.22994	140.31554	0.0816

1006	17.24885	140.45488	0.08165

1007	17.27462	140.59422	0.08173

1008	17.29009	140.73356	0.08176

1009	17.3004	140.8729	0.08177

1010	17.31586	141.01224	0.08191

1011	17.33992	141.15158	0.08193

1012	17.3571	141.29092	0.08197

1013	17.37085	141.43026	0.08223

1014	17.38631	141.5696	0.08225

1015	17.41037	141.70894	0.08252

1016	17.43099	141.84828	0.08267

1017	17.4413	141.98762	0.08268

1018	17.45505	142.12696	0.08269

1019	17.47395	142.2663	0.0829

1020	17.49457	142.40564	0.08299

1021	17.51004	142.54498	0.083

1022	17.52035	142.68432	0.08306

1023	17.54268	142.82366	0.08307

1024	17.56502	142.963	0.08309

1025	17.57877	143.10234	0.0831

1026	17.59252	143.24168	0.08311

1027	17.6097	143.38102	0.08321

1028	17.63376	143.52036	0.08322

1029	17.6475	143.6597	0.08326

1030	17.65953	143.79904	0.0833

1031	17.67843	143.93838	0.08332

1032	17.70249	144.07772	0.08333

1033	17.71796	144.21706	0.08334

1034	17.72998	144.3564	0.08336

1035	17.74889	144.49574	0.08337

1036	17.77294	144.63508	0.08348

1037	17.78841	144.77442	0.08376

1038	17.79528	144.91376	0.08378

1039	17.81418	145.0531	0.08379

1040	17.83996	145.19244	0.08394

1041	17.85714	145.33178	0.08395

1042	17.86745	145.47112	0.08397

1043	17.88292	145.61046	0.08398

1044	17.91385	145.7498	0.08415

1045	17.9276	145.88914	0.08417

1046	17.93619	146.02848	0.08421

1047	17.94993	146.16782	0.08441

1048	17.97571	146.30716	0.08443

1049	17.99461	146.4465	0.08444

1050	18.0032	146.58584	0.08445

1051	18.0221	146.72518	0.08448

1052	18.04788	146.86452	0.08452

1053	18.06678	147.00387	0.08455

1054	18.07709	147.14321	0.0846

1055	18.09256	147.28255	0.08472

1056	18.1149	147.42189	0.08482

1057	18.13036	147.56123	0.08483

1058	18.14067	147.70057	0.08487

1059	18.16129	147.83991	0.08489

1060	18.18191	147.97925	0.0849

1061	18.20081	148.11859	0.08495

1062	18.21456	148.25793	0.08497

1063	18.23003	148.39727	0.08498

1064	18.25236	148.53661	0.08501

1065	18.26955	148.67595	0.08522

1066	18.27814	148.81529	0.08524

1067	18.29532	148.95463	0.0854

1068	18.31938	149.09397	0.08541

1069	18.33485	149.23331	0.08554

1070	18.35203	149.37265	0.0856

1071	18.36406	149.51199	0.08566

1072	18.38983	149.65133	0.08647

1073	18.40873	149.79067	0.08648

1074	18.42076	149.93001	0.0865

1075	18.43451	150.06935	0.08651

1076	18.45685	150.20869	0.08665

1077	18.47403	150.34803	0.08666

1078	18.4895	150.48737	0.08667

1079	18.50153	150.62671	0.08669

1080	18.52215	150.76605	0.0867

1081	18.5462	150.90539	0.08671

1082	18.55995	151.04473	0.08673

1083	18.5737	151.18407	0.08676

1084	18.58916	151.32341	0.08694

1085	18.61322	151.46275	0.08703

1086	18.62525	151.60209	0.08712

1087	18.63556	151.74143	0.08715

1088	18.6579	151.88077	0.08717

1089	18.68023	152.02011	0.08719

1090	18.6957	152.15945	0.08749

1091	18.70945	152.29879	0.0875

1092	18.73007	152.43813	0.08751

1093	18.74897	152.57747	0.08754

1094	18.76443	152.71681	0.08757

1095	18.77474	152.85615	0.08758

1096	18.79193	152.99549	0.08759

1097	18.8177	153.13483	0.08764

1098	18.83317	153.27417	0.08765

1099	18.84692	153.41351	0.08766

1100	18.86582	153.55285	0.08768

1101	18.89159	153.69219	0.08772

1102	18.90534	153.83153	0.08773

1103	18.91221	153.97087	0.08777

1104	18.93111	154.11021	0.0878

1105	18.95173	154.24955	0.08781

1106	18.97236	154.38889	0.08784

1107	18.98267	154.52823	0.08785

1108	18.99985	154.66757	0.08796

1109	19.02391	154.80691	0.08799

1110	19.04624	154.94625	0.08801

1111	19.05484	155.08559	0.08804

1112	19.07202	155.22493	0.08806

1113	19.09264	155.36427	0.08815

1114	19.10982	155.50361	0.08818

1115	19.1167	155.64295	0.08819

1116	19.1356	155.78229	0.0882

1117	19.15794	155.92164	0.08865

1118	19.17684	156.06098	0.08866

1119	19.19059	156.20032	0.08869

1120	19.20949	156.33966	0.08872

1121	19.23011	156.479	0.08873

1122	19.24729	156.61834	0.08875

1123	19.25932	156.75768	0.08877

1124	19.27135	156.89702	0.08885

1125	19.29712	157.03636	0.08887

1126	19.31259	157.1757	0.08888

1127	19.32634	157.31504	0.08889

1128	19.34352	157.45438	0.08925

1129	19.36586	157.59372	0.0893

1130	19.38304	157.73306	0.08933

1131	19.39851	157.8724	0.08934

1132	19.41225	158.01174	0.08953

1133	19.43459	158.15108	0.08954

1134	19.44834	158.29042	0.08956

1135	19.46209	158.42976	0.08959

1136	19.47755	158.5691	0.08984

1137	19.49989	158.70844	0.08986

1138	19.52051	158.84778	0.08988

1139	19.53254	158.98712	0.08991

1140	19.55144	159.12646	0.08992

1141	19.56862	159.2658	0.08994

1142	19.58924	159.40514	0.08995

1143	19.60127	159.54448	0.08996

1144	19.6133	159.68382	0.08999

1145	19.63564	159.82316	0.09002

1146	19.65626	159.9625	0.09014

1147	19.67001	160.10184	0.09015

1148	19.68375	160.24118	0.09017

1149	19.70609	160.38052	0.09018

1150	19.72671	160.51986	0.0902

1151	19.74046	160.6592	0.09021

1152	19.74905	160.79854	0.09022

1153	19.77139	160.93788	0.09028

1154	19.79545	161.07722	0.09029

1155	19.80576	161.21656	0.09048

1156	19.8195	161.3559	0.09101

1157	19.84184	161.49524	0.09102

1158	19.86074	161.63458	0.09106

1159	19.87793	161.77392	0.09108

1160	19.88996	161.91326	0.09109

1161	19.91058	162.0526	0.0911

1162	19.93292	162.19194	0.09112

1163	19.94323	162.33128	0.09114

1164	19.95869	162.47062	0.09116

1165	19.97416	162.60996	0.09117

1166	20.00165	162.7493	0.09144

1167	20.01883	162.88864	0.09145

1168	20.02914	163.02798	0.09154

1169	20.04976	163.16732	0.09156

1170	20.0721	163.30666	0.09159

1171	20.08757	163.446	0.09162

1172	20.09444	163.58534	0.09163

1173	20.11506	163.72468	0.09164

1174	20.13568	163.86402	0.09166

1175	20.15115	164.00336	0.09171

1176	20.16317	164.1427	0.09173

1177	20.1838	164.28204	0.09174

1178	20.20785	164.42138	0.09175

1179	20.2216	164.56072	0.09177

1180	20.23363	164.70007	0.09179

1181	20.25253	164.83941	0.09181

1182	20.27659	164.97875	0.09182

1183	20.28861	165.11809	0.09183

1184	20.30064	165.25743	0.09185

1185	20.31783	165.39677	0.09186

1186	20.34188	165.53611	0.09187

1187	20.35735	165.67545	0.09189

1188	20.3711	165.81479	0.09228

1189	20.39172	165.95413	0.09231

1190	20.41234	166.09347	0.09235

1191	20.42608	166.23281	0.09239

1192	20.44155	166.37215	0.0924

1193	20.45701	166.51149	0.09242

1194	20.47935	166.65083	0.09261

1195	20.4931	166.79017	0.09265

1196	20.50856	166.92951	0.09266

1197	20.52747	167.06885	0.09269

1198	20.54809	167.20819	0.0927

1199	20.56527	167.34753	0.09271

1200	20.57558	167.48687	0.09273

1201	20.59276	167.62621	0.09284

1202	20.61338	167.76555	0.09289

1203	20.63229	167.90489	0.0929

1204	20.6426	168.04423	0.09292

1205	20.6615	168.18357	0.09293

1206	20.68212	168.32291	0.09294

1207	20.70102	168.46225	0.09313

1208	20.71305	168.60159	0.09315

1209	20.72336	168.74093	0.09316

1210	20.73882	168.88027	0.09317

1211	20.74054	169.01961	0.09319

1212	19.50232	169.15895	0.09331

1213	17.9421	169.29829	0.09332

1214	16.76435	169.43763	0.09342

1215	14.77286	169.57697	0.09368

1216	12.86041	169.71631	0.09369

1217	10.61976	169.85565	0.0937

1218	7.56813	169.99499	0.09376

1219	5.91507	170.13433	0.09423

170.27367	0.09426

170.41301	0.09427

170.55235	0.09431

170.69169	0.09454

170.83103	0.09456

170.97037	0.09458

171.10971	0.09461

171.24905	0.09465

171.38839	0.09466

171.52773	0.09468

171.66707	0.09469

171.80641	0.0947

171.94575	0.09477

172.08509	0.09479

172.22443	0.0948

172.36377	0.09495

172.50311	0.09496

172.64245	0.09498

172.78179	0.09499

172.92113	0.09502

173.06047	0.09518

173.19981	0.09521

173.33915	0.09522

173.47849	0.09523

173.61784	0.09525

173.75718	0.09526

173.89652	0.09531

174.03586	0.09533

174.1752	0.09534

174.31454	0.09535

174.45388	0.09537

174.59322	0.09538

174.73256	0.0954

174.8719	0.09541

175.01124	0.09542

175.15058	0.09544

175.28992	0.09545

175.42926	0.09546

175.5686	0.09548

175.70794	0.09549

175.84728	0.0955

175.98662	0.09567

176.12596	0.09568

176.2653	0.09571

176.40464	0.09573

176.54398	0.09588

176.68332	0.0959

176.82266	0.09591

176.962	0.09594

177.10134	0.09603

177.24068	0.09605

177.38002	0.09611

177.51936	0.09614

177.6587	0.09619

177.79804	0.09621

177.93738	0.09622

178.07672	0.09624

178.21606	0.09628

178.3554	0.0963

178.49474	0.09632

178.63408	0.09633

178.77342	0.09634

178.91276	0.09636

179.0521	0.09638

179.19144	0.09648

179.33078	0.09649

179.47012	0.09651

179.60946	0.09652

179.7488	0.09653

179.88814	0.09678

180.02748	0.09679

180.16682	0.0968

180.30616	0.09682

180.4455	0.09683

180.58484	0.09684

180.72418	0.09686

180.86352	0.09687

181.00286	0.09689

181.1422	0.09691

181.28154	0.09693

181.42088	0.09694

181.56022	0.09699

181.69956	0.09701

181.8389	0.09702

181.97824	0.09703

182.11758	0.0971

182.25692	0.09712

182.39626	0.09713

182.53561	0.09714

182.67495	0.09716

182.81429	0.09718

182.95363	0.09729

183.09297	0.09748

183.23231	0.09752

183.37165	0.09755

183.51099	0.09768

183.65033	0.09772

183.78967	0.09775

183.92901	0.09777

184.06835	0.09781

184.20769	0.09782

184.34703	0.09786

184.48637	0.0979

184.62571	0.09798

184.76505	0.098

184.90439	0.09801

185.04373	0.09804

185.18307	0.09805

185.32241	0.09806

185.46175	0.09808

185.60109	0.0981

185.74043	0.09813

185.87977	0.09814

186.01911	0.09816

186.15845	0.09817

186.29779	0.0982

186.43713	0.09824

186.57647	0.09827

186.71581	0.09829

186.85515	0.0985

186.99449	0.09852

187.13383	0.09854

187.27317	0.09856

187.41251	0.09859

187.55185	0.09861

187.69119	0.09863

187.83053	0.09865

187.96987	0.09866

188.10921	0.09874

188.24855	0.09881

188.38789	0.09882

188.52723	0.09885

188.66657	0.09886

188.80591	0.09888

188.94525	0.09889

189.08459	0.0989

189.22393	0.09893

189.36327	0.09894

189.50261	0.09896

189.64195	0.099

189.78129	0.09921

189.92063	0.09924

190.05997	0.09926

190.19931	0.09932

190.33865	0.09934

190.47799	0.09935

190.61733	0.09936

190.75667	0.09938

190.89601	0.09939

191.03535	0.0994

191.17469	0.09942

191.31403	0.09943

191.45337	0.09944

191.59272	0.0995

191.73206	0.09953

191.8714	0.09954

192.01074	0.09955

192.15008	0.09958

192.28942	0.09961

192.42876	0.09962

192.5681	0.09963

192.70744	0.09966

192.84678	0.09967

192.98612	0.09969

193.12546	0.0997

193.2648	0.09972

193.40414	0.09973

193.54348	0.09974

193.68282	0.09977

193.82216	0.09993

193.9615	0.09995

194.10084	0.10001

194.24018	0.10003

194.37952	0.10004

194.51886	0.10005

194.6582	0.10009

194.79754	0.10011

194.93688	0.10012

195.07622	0.10014

195.21556	0.10018

195.3549	0.10023

195.49424	0.10024

195.63358	0.10027

195.77292	0.1003

195.91226	0.10031

196.0516	0.1005

196.19094	0.10051

196.33028	0.10056

196.46962	0.10058

196.60896	0.10065

196.7483	0.10066

196.88764	0.10068

197.02698	0.10069

197.16632	0.10073

197.30566	0.10074

197.445	0.10076

197.58434	0.10077

197.72368	0.1008

197.86302	0.10081

198.00236	0.10084

198.1417	0.10085

198.28104	0.10088

198.42038	0.10092

198.55972	0.10093

198.69906	0.10106

198.8384	0.10108

198.97774	0.10111

199.11708	0.10112

199.25642	0.10114

199.39576	0.10115

199.5351	0.10116

199.67444	0.10119

199.81378	0.10121

199.95312	0.10123

200.09246	0.10125

200.2318	0.10126

200.37115	0.10127

200.51049	0.10129

200.64983	0.1016

200.78917	0.10163

200.92851	0.10164

201.06785	0.10167

201.20719	0.10171

201.34653	0.10173

201.48587	0.10175

201.62521	0.10177

201.76455	0.10179

201.90389	0.1018

202.04323	0.10181

202.18257	0.10183

202.32191	0.10184

202.46125	0.10195

202.60059	0.10196

202.73993	0.10198

202.87927	0.10199

203.01861	0.102

203.15795	0.10215

203.29729	0.10217

203.43663	0.10218

203.57597	0.10219

203.71531	0.10226

203.85465	0.10228

203.99399	0.10229

204.13333	0.10232

204.27267	0.10233

204.41201	0.10234

204.55135	0.1026

204.69069	0.10261

204.83003	0.10263

204.96937	0.10265

205.10871	0.10267

205.24805	0.10268

205.38739	0.1027

205.52673	0.10271

205.66607	0.10272

205.80541	0.10274

205.94475	0.10276

206.08409	0.10278

206.22343	0.10279

206.36277	0.1028

206.50211	0.10282

206.64145	0.10286

206.78079	0.10287

206.92013	0.10288

207.05947	0.10291

207.19881	0.10293

207.33815	0.10301

207.47749	0.10305

207.61683	0.10306

207.75617	0.10307

207.89551	0.10309

208.03485	0.1031

208.17419	0.10311

208.31353	0.1033

208.45287	0.10332

208.59221	0.10337

208.73155	0.10444

208.87089	0.1045

209.01023	0.10452

209.14957	0.10455

209.28892	0.10466

209.42826	0.10471

209.5676	0.10474

209.70694	0.10477

209.84628	0.10479

209.98562	0.10485

210.12496	0.10488

210.2643	0.1049

210.40364	0.10534

210.54298	0.10536

210.68232	0.10539

210.82166	0.10542

210.961	0.10544

211.10034	0.10547

211.23968	0.10553

211.37902	0.10555

211.51836	0.10577

211.6577	0.1058

211.79704	0.10582

211.93638	0.10588

212.07572	0.1059

212.21506	0.10596

212.3544	0.10601

212.49374	0.10604

212.63308	0.10607

212.77242	0.10609

212.91176	0.10612

213.0511	0.10615

213.19044	0.10618

213.32978	0.1062

213.46912	0.10623

213.60846	0.10628

213.7478	0.10634

213.88714	0.10637

214.02648	0.10639

214.16582	0.10642

214.30516	0.10645

214.4445	0.10647

214.58384	0.10672

214.72318	0.10674

214.86252	0.10677

215.00186	0.1071

215.1412	0.10712

215.28054	0.10715

215.41988	0.10718

215.55922	0.1072

215.69856	0.10726

215.8379	0.10729

215.97724	0.10734

216.11658	0.10737

216.25592	0.10739

216.39526	0.10742

216.5346	0.10748

216.67394	0.1075

216.81328	0.10753

216.95262	0.10756

217.09196	0.10761

217.2313	0.10764

217.37064	0.10767

217.50998	0.10769

217.64932	0.10772

217.78866	0.10775

217.928	0.10777

218.06734	0.10807

218.20668	0.1081

218.34603	0.10813

218.48537	0.10815

218.62471	0.10821

218.76405	0.10823

218.90339	0.10826

219.04273	0.10829

219.18207	0.10834

219.32141	0.10842

219.46075	0.10845

219.60009	0.10848

219.73943	0.1085

219.87877	0.10856

220.01811	0.10861

220.15745	0.10864

220.29679	0.10867

220.43613	0.10869

220.57547	0.10872

220.71481	0.10875

220.85415	0.10926

220.99349	0.10951

221.13283	0.10953

221.27217	0.10959

221.41151	0.10962

221.55085	0.10967

221.69019	0.1097

221.82953	0.10975

221.96887	0.10978

222.10821	0.1098

222.24755	0.10983

222.38689	0.10986

222.52623	0.10989

222.66557	0.10994

222.80491	0.10997

222.94425	0.11016

223.08359	0.11018

223.22293	0.11027

223.36227	0.11032

223.50161	0.11035

223.64095	0.1104

223.78029	0.11054

223.91963	0.11056

224.05897	0.11059

224.19831	0.11062

224.33765	0.11119

224.47699	0.1114

224.61633	0.11146

224.75567	0.11148

224.89501	0.11151

225.03435	0.11154

225.17369	0.11157

225.31303	0.11159

225.45237	0.11165

225.59171	0.11167

225.73105	0.11173

225.87039	0.11176

226.00973	0.11181

226.14907	0.11186

226.28841	0.11189

226.42775	0.11194

226.56709	0.11197

226.70643	0.112

226.84577	0.11205

226.98511	0.11246

227.12446	0.11249

227.2638	0.11251

227.40314	0.11254

227.54248	0.11257

227.68182	0.11262

227.82116	0.1127

227.9605	0.11273

228.09984	0.11276

228.23918	0.11278

228.37852	0.11287

228.51786	0.11289

228.6572	0.11295

228.79654	0.11297

228.93588	0.11306

229.07522	0.11311

229.21456	0.11316

229.3539	0.11319

229.49324	0.11322

229.63258	0.11324

229.77192	0.11327

229.91126	0.11338

230.0506	0.11341

230.18994	0.11343

230.32928	0.11346

230.46862	0.11349

230.60796	0.11354

230.7473	0.1136

230.88664	0.11362

231.02598	0.11371

231.16532	0.11373

231.30466	0.11381

231.444	0.114

231.58334	0.11403

231.72268	0.11406

231.86202	0.11411

232.00136	0.11433

232.1407	0.11441

232.28004	0.11444

232.41938	0.11449

232.55872	0.11479

232.69806	0.11484

232.8374	0.11487

232.97674	0.1149

233.11608	0.11492

233.25542	0.11495

233.39476	0.11498

233.5341	0.11514

233.67344	0.1152

233.81278	0.11522

233.95212	0.11528

234.09146	0.1153

234.2308	0.11538

234.37014	0.11541

234.50948	0.11547

234.64882	0.11549

234.78816	0.11552

234.9275	0.1159

235.06684	0.11593

235.20618	0.11595

235.34552	0.11603

235.48486	0.11606

235.6242	0.11614

235.76354	0.1162

235.90288	0.11625

236.04223	0.11628

236.18157	0.11631

236.32091	0.11633

236.46025	0.11636

236.59959	0.11639

236.73893	0.11641

236.87827	0.11644

237.01761	0.1175

237.15695	0.11752

237.29629	0.11755

237.43563	0.11758

237.57497	0.11766

237.71431	0.11771

237.85365	0.11782

237.99299	0.11785

238.13233	0.1179

238.27167	0.11796

238.41101	0.11815

238.55035	0.1182

238.68969	0.11826

238.82903	0.11831

238.96837	0.11834

239.10771	0.11839

239.24705	0.11842

239.38639	0.11847

239.52573	0.1185

239.66507	0.11858

239.80441	0.11863

239.94375	0.11869

240.08309	0.11874

240.22243	0.11877

240.36177	0.11885

240.50111	0.11888

240.64045	0.11907

240.77979	0.11915

240.91913	0.11918

241.05847	0.1192

241.19781	0.12048

241.33715	0.1205

241.47649	0.12056

241.61583	0.12064

241.75517	0.12067

241.89451	0.12072

242.03385	0.12075

242.17319	0.1208

242.31253	0.12083

242.45187	0.12086

242.59121	0.12088

242.73055	0.12091

242.86989	0.12099

243.00923	0.12107

243.14857	0.1211

243.28791	0.12113

243.42725	0.12115

243.56659	0.12121

243.70593	0.12124

243.84527	0.12126

243.98461	0.12129

244.12395	0.12132

244.26329	0.12137

244.40263	0.1214

244.54197	0.12142

244.68131	0.12151

244.82065	0.12161

244.96	0.12164

245.09934	0.12167

245.23868	0.12172

245.37802	0.1218

245.51736	0.12186

245.6567	0.12199

245.79604	0.12205

245.93538	0.1221

246.07472	0.12213

246.21406	0.12216

246.3534	0.12226

246.49274	0.12229

246.63208	0.12232

246.77142	0.12235

246.91076	0.12237

247.0501	0.1224

247.18944	0.12245

247.32878	0.12248

247.46812	0.12251

247.60746	0.12254

247.7468	0.12262

247.88614	0.12264

248.02548	0.12267

248.16482	0.12283

248.30416	0.12286

248.4435	0.12289

248.58284	0.123

248.72218	0.12302

248.86152	0.12305

249.00086	0.1231

249.1402	0.12313

249.27954	0.12316

249.41888	0.12319

249.55822	0.12321

249.69756	0.12327

249.8369	0.12335

249.97624	0.12337

250.11558	0.1234

250.25492	0.12348

250.39426	0.12351

250.5336	0.12356

250.67294	0.12359

250.81228	0.12365

250.95162	0.12367

251.09096	0.1237

251.2303	0.12438

251.36964	0.12446

251.50898	0.12451

251.64832	0.12454

251.78766	0.12457

251.927	0.12459

252.06634	0.12465

252.20568	0.12478

252.34502	0.12514

252.48436	0.12516

252.6237	0.12519

252.76304	0.12527

252.90238	0.12533

253.04172	0.12538

253.18106	0.12541

253.3204	0.12543

253.45974	0.12546

253.59908	0.12549

253.73842	0.12551

253.87777	0.12557

254.01711	0.1256

254.15645	0.12562

254.29579	0.12568

254.43513	0.1257

254.57447	0.12635

254.71381	0.12671

254.85315	0.12673

254.99249	0.12679

255.13183	0.12681

255.27117	0.12684

255.41051	0.12687

255.54985	0.1269

255.68919	0.12692

255.82853	0.12695

255.96787	0.12706

256.10721	0.12719

256.24655	0.12725

256.38589	0.12733

256.52523	0.12741

256.66457	0.12744

256.80391	0.12749

256.94325	0.12752

257.08259	0.12755

257.22193	0.12763

257.36127	0.12765

257.50061	0.12809

257.63995	0.12811

257.77929	0.12817

257.91863	0.1282

258.05797	0.12825

258.19731	0.12828

258.33665	0.1283

258.47599	0.12833

258.61533	0.12836

258.75467	0.12839

258.89401	0.12841

259.03335	0.12844

259.17269	0.12849

259.31203	0.12863

259.45137	0.12866

259.59071	0.12871

259.73005	0.12874

259.86939	0.12876

260.00873	0.12887

260.14807	0.1289

260.28741	0.12893

260.42675	0.12895

260.56609	0.12931

260.70543	0.12933

260.84477	0.12936

260.98411	0.12939

261.12345	0.12941

261.26279	0.12944

261.40213	0.1295

261.54147	0.12952

261.68081	0.12958

261.82015	0.12963

261.95949	0.12966

262.09883	0.12969

262.23817	0.12971

262.37751	0.12974

262.51685	0.12979

262.6562	0.12982

262.79554	0.12985

262.93488	0.12988

263.07422	0.1299

263.21356	0.12993

263.3529	0.12998

263.49224	0.13001

263.63158	0.13004

263.77092	0.13012

263.91026	0.13015

264.0496	0.1302

264.18894	0.13023

264.32828	0.13034

264.46762	0.13039

264.60696	0.13047

264.7463	0.13053

264.88564	0.13058

265.02498	0.13061

265.16432	0.13063

265.30366	0.13069

265.443	0.13074

265.58234	0.1308

265.72168	0.13082

265.86102	0.13085

266.00036	0.13088

266.1397	0.1309

266.27904	0.13093

266.41838	0.13096

266.55772	0.13104

266.69706	0.13107

266.8364	0.13109

266.97574	0.13112

267.11508	0.13115

267.25442	0.1312

267.39376	0.13123

267.5331	0.13126

267.67244	0.13134

267.81178	0.13142

267.95112	0.13145

268.09046	0.13147

268.2298	0.13153

268.36914	0.13158

268.50848	0.13161

268.64782	0.13164

268.78716	0.13166

268.9265	0.13169

269.06584	0.13172

269.20518	0.13183

269.34452	0.13188

269.48386	0.13191

269.6232	0.13193

269.76254	0.13196

269.90188	0.13199

270.04122	0.13204

270.18056	0.13207

270.3199	0.1321

270.45924	0.13215

270.59858	0.13218

270.73792	0.1322

270.87726	0.13223

271.0166	0.13226

271.15594	0.13229

271.29528	0.13237

271.43462	0.13242

271.57396	0.13245

271.71331	0.1325

271.85265	0.13253

271.99199	0.13256

272.13133	0.13264

272.27067	0.13267

272.41001	0.13272

272.54935	0.13283

272.68869	0.13285

272.82803	0.13304

272.96737	0.1331

273.10671	0.13315

273.24605	0.13329

273.38539	0.13332

273.52473	0.13334

273.66407	0.13337

273.80341	0.1334

273.94275	0.13348

274.08209	0.13353

274.22143	0.13356

274.36077	0.13359

274.50011	0.13372

274.63945	0.13375

274.77879	0.1338

274.91813	0.13388

275.05747	0.13391

275.19681	0.13397

275.33615	0.13402

275.47549	0.13405

275.61483	0.13418

275.75417	0.13424

275.89351	0.13432

276.03285	0.13434

276.17219	0.13437

276.31153	0.1344

276.45087	0.13443

276.59021	0.1351

276.72955	0.13513

276.86889	0.13516

277.00823	0.13518

277.14757	0.13529

277.28691	0.13532

277.42625	0.13535

277.56559	0.13537

277.70493	0.13543

277.84427	0.13546

277.98361	0.13548

278.12295	0.13551

278.26229	0.13556

278.40163	0.13562

278.54097	0.13564

278.68031	0.13575

278.81965	0.13583

278.95899	0.13592

279.09833	0.13594

279.23767	0.13597

279.37701	0.136

279.51635	0.13613

279.65569	0.13616

279.79503	0.13619

279.93437	0.13621

280.07371	0.13624

280.21305	0.13627

280.35239	0.13632

280.49174	0.13635

280.63108	0.13638

280.77042	0.1364

280.90976	0.13648

281.0491	0.13654

281.18844	0.13673

281.32778	0.13676

281.46712	0.13684

281.60646	0.13689

281.7458	0.13692

281.88514	0.13703

282.02448	0.13705

282.16382	0.13724

282.30316	0.13727

282.4425	0.1373

282.58184	0.13732

282.72118	0.13735

282.86052	0.13738

282.99986	0.13743

283.1392	0.13746

283.27854	0.13754

283.41788	0.13757

283.55722	0.13759

283.69656	0.13765

283.8359	0.13795

283.97524	0.13797

284.11458	0.13803

284.25392	0.13806

284.39326	0.13808

284.5326	0.13811

284.67194	0.13814

284.81128	0.13816

284.95062	0.13819

285.08996	0.13824

285.2293	0.13827

285.36864	0.1383

285.50798	0.13833

285.64732	0.13835

285.78666	0.13838

285.926	0.13841

286.06534	0.13846

286.20468	0.13849

286.34402	0.13852

286.48336	0.13854

286.6227	0.13857

286.76204	0.13862

286.90138	0.13865

287.04072	0.13876

287.18006	0.13879

287.3194	0.13889

287.45874	0.13898

287.59808	0.13903

287.73742	0.13911

287.87676	0.13919

288.0161	0.13922

288.15544	0.13925

288.29478	0.13927

288.43412	0.1393

288.57346	0.13933

288.7128	0.13938

288.85214	0.13952

288.99148	0.13954

289.13082	0.1396

289.27016	0.13968

289.40951	0.13973

289.54885	0.13976

289.68819	0.13979

289.82753	0.13984

289.96687	0.13987

290.10621	0.1399

290.24555	0.13995

290.38489	0.14001

290.52423	0.14006

290.66357	0.14009

290.80291	0.14011

290.94225	0.14014

291.08159	0.14017

291.22093	0.14025

291.36027	0.14028

291.49961	0.14036

291.63895	0.14038

291.77829	0.14041

291.91763	0.14049

292.05697	0.14052

292.19631	0.14057

292.33565	0.14082

292.47499	0.14085

292.61433	0.14087

292.75367	0.14093

292.89301	0.14098

293.03235	0.14101

293.17169	0.14163

293.31103	0.14168

293.45037	0.14185

293.58971	0.1419

293.72905	0.14196

293.86839	0.14198

294.00773	0.14201

294.14707	0.14206

294.28641	0.1422

294.42575	0.14223

294.56509	0.14228

294.70443	0.14231

294.84377	0.14233

294.98311	0.14236

295.12245	0.14239

295.26179	0.14244

295.40113	0.14258

295.54047	0.14263

295.67981	0.14315

295.81915	0.1432

295.95849	0.14323

296.09783	0.14326

296.23717	0.14334

296.37651	0.14336

296.51585	0.14339

296.65519	0.1435

296.79453	0.14353

296.93387	0.14355

297.07321	0.14358

297.21255	0.14369

297.35189	0.14372

297.49123	0.14377

297.63057	0.14401

297.76991	0.14407

297.90925	0.14415

298.04859	0.14418

298.18793	0.1442

298.32727	0.14423

298.46662	0.14428

298.60596	0.14431

298.7453	0.14434

298.88464	0.14456

299.02398	0.14458

299.16332	0.14464

299.30266	0.14466

299.442	0.14475

299.58134	0.14477

299.72068	0.1448

299.86002	0.14483

299.99936	0.14526

300.1387	0.14529

300.27804	0.14534

300.41738	0.14545

300.55672	0.14548

300.69606	0.1455

300.8354	0.14553

300.97474	0.14556

301.11408	0.14564

301.25342	0.14569

301.39276	0.14572

301.5321	0.14577

301.67144	0.14586

301.81078	0.14605

301.95012	0.14607

302.08946	0.1461

302.2288	0.14613

302.36814	0.14621

302.50748	0.14629

302.64682	0.14632

302.78616	0.14637

302.9255	0.1464

303.06484	0.14642

303.20418	0.14651

303.34352	0.14653

303.48286	0.14656

303.6222	0.14661

303.76154	0.14664

303.90088	0.14667

304.04022	0.14675

304.17956	0.14678

304.3189	0.1471

304.45824	0.14748

304.59758	0.14751

304.73692	0.14759

304.87626	0.14762

305.0156	0.14764

305.15494	0.14767

305.29428	0.1477

305.43362	0.14772

305.57296	0.14775

305.7123	0.14781

305.85164	0.14786

305.99098	0.14791

306.13032	0.14794

306.26966	0.14797

306.409	0.14802

306.54834	0.14808

306.68768	0.14816

306.82702	0.14819

306.96636	0.14824

307.1057	0.14832

307.24505	0.14835

307.38439	0.14851

307.52373	0.14854

307.66307	0.14859

307.80241	0.14865

307.94175	0.14867

308.08109	0.1487

308.22043	0.14873

308.35977	0.14875

308.49911	0.14892

308.63845	0.14897

308.77779	0.149

308.91713	0.14908

309.05647	0.14919

309.19581	0.14921

309.33515	0.14932

309.47449	0.14938

309.61383	0.14946

309.75317	0.14949

309.89251	0.14951

310.03185	0.14965

310.17119	0.14967

310.31053	0.14973

310.44987	0.14978

310.58921	0.14984

310.72855	0.14986

310.86789	0.14989

311.00723	0.14992

311.14657	0.14997

311.28591	0.15

311.42525	0.15003

311.56459	0.15033

311.70393	0.15035

311.84327	0.15041

311.98261	0.15049

312.12195	0.15051

312.26129	0.15057

312.40063	0.15062

312.53997	0.1507

312.67931	0.15073

312.81865	0.15076

312.95799	0.15087

313.09733	0.15089

313.23667	0.151

313.37601	0.15108

313.51535	0.15111

313.65469	0.15122

313.79403	0.15125

313.93337	0.15138

314.07271	0.15152

314.21205	0.15154

314.35139	0.15157

314.49073	0.1516

314.63007	0.15168

314.76941	0.15171

314.90875	0.15173

315.04809	0.15179

315.18743	0.15181

315.32677	0.15184

315.46611	0.15187

315.60545	0.1519

315.74479	0.15228

315.88413	0.15233

316.02347	0.15244

316.16282	0.15246

316.30216	0.15249

316.4415	0.15252

316.58084	0.15263

316.72018	0.15271

316.85952	0.15276

316.99886	0.15279

317.1382	0.15309

317.27754	0.15311

317.41688	0.1532

317.55622	0.15322

317.69556	0.1533

317.8349	0.15333

317.97424	0.15347

318.11358	0.15352

318.25292	0.1536

318.39226	0.15374

318.5316	0.15382

318.67094	0.15385

318.81028	0.15387

318.94962	0.15393

319.08896	0.15398

319.2283	0.15401

319.36764	0.15406

319.50698	0.15423

319.64632	0.15428

319.78566	0.15431

319.925	0.15433

320.06434	0.15436

320.20368	0.15441

320.34302	0.15447

320.48236	0.1545

320.6217	0.15452

320.76104	0.15455

320.90038	0.15471

321.03972	0.15474

321.17906	0.15477

321.3184	0.15479

321.45774	0.15485

321.59708	0.15488

321.73642	0.1549

321.87576	0.15496

322.0151	0.15498

322.15444	0.15507

322.29378	0.15517

322.43312	0.15523

322.57246	0.15528

322.7118	0.15534

322.85114	0.15539

322.99048	0.15561

323.12982	0.15563

323.26916	0.15566

323.4085	0.15574

323.54784	0.15577

323.68718	0.15582

323.82652	0.15585

323.96586	0.1559

324.1052	0.15593

324.24454	0.15599

324.38388	0.15601

324.52322	0.15609

324.66256	0.15615

324.8019	0.15618

324.94124	0.15623

325.08059	0.15628

325.21993	0.15631

325.35927	0.15637

325.49861	0.15639

325.63795	0.15645

325.77729	0.15655

325.91663	0.15669

326.05597	0.15674

326.19531	0.15683

326.33465	0.15685

326.47399	0.15688

326.61333	0.15691

326.75267	0.15693

326.89201	0.15702

327.03135	0.15712

327.17069	0.15715

327.31003	0.1572

327.44937	0.15731

327.58871	0.15742

327.72805	0.1575

327.86739	0.15753

328.00673	0.15764

328.14607	0.15772

328.28541	0.15785

328.42475	0.15788

328.56409	0.15791

328.70343	0.15802

328.84277	0.1581

328.98211	0.15821

329.12145	0.15823

329.26079	0.15826

329.40013	0.15829

329.53947	0.15834

329.67881	0.15837

329.81815	0.15853

329.95749	0.15856

330.09683	0.15859

330.23617	0.15861

330.37551	0.15864

330.51485	0.15869

330.65419	0.15875

330.79353	0.15883

330.93287	0.15888

331.07221	0.15932

331.21155	0.15934

331.35089	0.15937

331.49023	0.1594

331.62957	0.15945

331.76891	0.15948

331.90825	0.15956

332.04759	0.15959

332.18693	0.15962

332.32627	0.15964

332.46561	0.1597

332.60495	0.15978

332.74429	0.15983

332.88363	0.15986

333.02297	0.15991

333.16231	0.15994

333.30165	0.1601

333.44099	0.16016

333.58033	0.16021

333.71967	0.16027

333.85901	0.16029

333.99836	0.16032

334.1377	0.16037

334.27704	0.1604

334.41638	0.16043

334.55572	0.16046

334.69506	0.16051

334.8344	0.16056

334.97374	0.16059

335.11308	0.16067

335.25242	0.16073

335.39176	0.16075

335.5311	0.16078

335.67044	0.16086

335.80978	0.16092

335.94912	0.16094

336.08846	0.16102

336.2278	0.16105

336.36714	0.16108

336.50648	0.16111

336.64582	0.16116

336.78516	0.16124

336.9245	0.16127

337.06384	0.16129

337.20318	0.16132

337.34252	0.16135

337.48186	0.16138

337.6212	0.1614

337.76054	0.16148

337.89988	0.16165

338.03922	0.16167

338.17856	0.1617

338.3179	0.16176

338.45724	0.16181

338.59658	0.16184

338.73592	0.16189

338.87526	0.16197

339.0146	0.162

339.15394	0.16213

339.29328	0.16216

339.43262	0.16219

339.57196	0.16222

339.7113	0.16235

339.85064	0.16238

339.98998	0.16243

340.12932	0.16246

340.26866	0.16251

340.408	0.16257

340.54734	0.16262

340.68668	0.16265

340.82602	0.1627

340.96536	0.16278

341.1047	0.16281

341.24404	0.16289

341.38338	0.16292

341.52272	0.16303

341.66206	0.16308

341.8014	0.16314

341.94074	0.16316

342.08008	0.16319

342.21942	0.16324

342.35876	0.1633

342.4981	0.16335

342.63744	0.16343

342.77678	0.16346

342.91613	0.16352

343.05547	0.16365

343.19481	0.16368

343.33415	0.16371

343.47349	0.16379

343.61283	0.16384

343.75217	0.16387

343.89151	0.16392

344.03085	0.16398

344.17019	0.164

344.30953	0.16403

344.44887	0.16406

344.58821	0.16408

344.72755	0.16411

344.86689	0.16414

345.00623	0.16419

345.14557	0.16422

345.28491	0.1643

345.42425	0.16438

345.56359	0.16444

345.70293	0.16454

345.84227	0.16465

345.98161	0.16468

346.12095	0.16476

346.26029	0.16479

346.39963	0.16482

346.53897	0.16484

346.67831	0.16487

346.81765	0.16492

346.95699	0.16495

347.09633	0.16501

347.23567	0.16503

347.37501	0.16506

347.51435	0.16509

347.65369	0.16511

347.79303	0.16522

347.93237	0.16528

348.07171	0.16538

348.21105	0.16544

348.35039	0.16547

348.48973	0.16552

348.62907	0.16555

348.76841	0.16563

348.90775	0.16579

349.04709	0.16587

349.18643	0.16593

349.32577	0.16595

349.46511	0.16598

349.60445	0.16601

349.74379	0.16606

349.88313	0.16609

350.02247	0.16614

350.16181	0.1662

350.30115	0.16622

350.44049	0.16633

350.57983	0.16636

350.71917	0.16641

350.85851	0.16644

350.99785	0.16647

351.13719	0.16666

351.27653	0.16668

351.41587	0.16671

351.55521	0.16677

351.69455	0.16679

351.8339	0.16685

351.97324	0.16687

352.11258	0.16693

352.25192	0.16698

352.39126	0.16704

352.5306	0.16706

352.66994	0.16725

352.80928	0.16739

352.94862	0.16742

353.08796	0.16744

353.2273	0.1675

353.36664	0.16752

353.50598	0.16755

353.64532	0.16761

353.78466	0.16766

353.924	0.16774

354.06334	0.1679

354.20268	0.16807

354.34202	0.16809

354.48136	0.16812

354.6207	0.16815

354.76004	0.1682

354.89938	0.16831

355.03872	0.16839

355.17806	0.16842

355.3174	0.16847

355.45674	0.1685

355.59608	0.16863

355.73542	0.16866

355.87476	0.16872

356.0141	0.16877

356.15344	0.16882

356.29278	0.16888

356.43212	0.16891

356.57146	0.16893

356.7108	0.16896

356.85014	0.16912

356.98948	0.16934

357.12882	0.16939

357.26816	0.16942

357.4075	0.1695

357.54684	0.16966

357.68618	0.16969

357.82552	0.16975

357.96486	0.16983

358.1042	0.16985

358.24354	0.16988

358.38288	0.16991

358.52222	0.16996

358.66156	0.16999

358.8009	0.17002

358.94024	0.17007

359.07958	0.1701

359.21892	0.17012

359.35826	0.17015

359.4976	0.17018

359.63694	0.17021

359.77628	0.17023

359.91562	0.17029

360.05496	0.17031

360.1943	0.17034

360.33364	0.1704

360.47298	0.17045

360.61233	0.1705

360.75167	0.17053

360.89101	0.17064

361.03035	0.17072

361.16969	0.17075

361.30903	0.17083

361.44837	0.1711

361.58771	0.17113

361.72705	0.17115

361.86639	0.17129

362.00573	0.17132

362.14507	0.17137

362.28441	0.17156

362.42375	0.17159

362.56309	0.17161

362.70243	0.17167

362.84177	0.17172

362.98111	0.17175

363.12045	0.17194

363.25979	0.17259

363.39913	0.17262

363.53847	0.17264

363.67781	0.1727

363.81715	0.17275

363.95649	0.17278

364.09583	0.17281

364.23517	0.17283

364.37451	0.17289

364.51385	0.173

364.65319	0.17308

364.79253	0.17313

364.93187	0.17316

365.07121	0.17324

365.21055	0.17329

365.34989	0.17335

365.48923	0.17337

365.62857	0.1734

365.76791	0.17343

365.90725	0.17351

366.04659	0.17354

366.18593	0.17362

366.32527	0.1737

366.46461	0.17373

366.60395	0.17375

366.74329	0.17381

366.88263	0.17386

367.02197	0.17392

367.16131	0.17397

367.30065	0.174

367.43999	0.17408

367.57933	0.17411

367.71867	0.17413

367.85801	0.17419

367.99735	0.17421

368.13669	0.1743

368.27603	0.17432

368.41537	0.17438

368.55471	0.17443

368.69405	0.17446

368.83339	0.17449

368.97273	0.17457

369.11207	0.17459

369.25141	0.17467

369.39075	0.1747

369.5301	0.17473

369.66944	0.17476

369.80878	0.17484

369.94812	0.17497

370.08746	0.17505

370.2268	0.17514

370.36614	0.17519

370.50548	0.17527

370.64482	0.1753

370.78416	0.17533

370.9235	0.17535

371.06284	0.17538

371.20218	0.17543

371.34152	0.17546

371.48086	0.17549

371.6202	0.1756

371.75954	0.17562

371.89888	0.1757

372.03822	0.17573

372.17756	0.17581

372.3169	0.17584

372.45624	0.17589

372.59558	0.17598

372.73492	0.176

372.87426	0.17603

373.0136	0.17606

373.15294	0.17611

373.29228	0.17638

373.43162	0.17641

373.57096	0.17652

373.7103	0.17665

373.84964	0.17668

373.98898	0.17673

374.12832	0.17676

374.26766	0.17681

374.407	0.17684

374.54634	0.17692

374.68568	0.17698

374.82502	0.177

374.96436	0.17703

375.1037	0.17711

375.24304	0.17714

375.38238	0.17719

375.52172	0.17725

375.66106	0.17728

375.8004	0.17733

375.93974	0.17738

376.07908	0.17746

376.21842	0.17752

376.35776	0.17763

376.4971	0.17774

376.63644	0.17779

376.77578	0.1779

376.91512	0.17795

377.05446	0.17798

377.1938	0.17801

377.33314	0.17803

377.47248	0.17806

377.61182	0.17809

377.75116	0.17817

377.8905	0.17822

378.02984	0.17833

378.16918	0.17839

378.30852	0.17852

378.44786	0.17858

378.58721	0.17866

378.72655	0.17876

378.86589	0.17887

379.00523	0.1789

379.14457	0.17893

379.28391	0.17898

379.42325	0.17901

379.56259	0.17904

379.70193	0.17906

379.84127	0.17909

379.98061	0.17912

380.11995	0.17914

380.25929	0.17917

380.39863	0.17925

380.53797	0.17933

380.67731	0.1795

380.81665	0.17952

380.95599	0.17955

381.09533	0.17969

381.23467	0.17971

381.37401	0.17974

381.51335	0.17977

381.65269	0.17979

381.79203	0.17988

381.93137	0.1799

382.07071	0.17993

382.21005	0.17996

382.34939	0.17998

382.48873	0.18004

382.62807	0.18009

382.76741	0.18012

382.90675	0.18017

383.04609	0.1802

383.18543	0.18023

383.32477	0.18025

383.46411	0.18028

383.60345	0.18034

383.74279	0.18036

383.88213	0.1805

384.02147	0.18053

384.16081	0.18061

384.30015	0.18069

384.43949	0.18085

384.57883	0.18096

384.71817	0.18099

384.85751	0.18101

384.99685	0.18112

385.13619	0.1812

385.27553	0.18123

385.41487	0.18131

385.55421	0.18134

385.69355	0.18137

385.83289	0.18145

385.97223	0.18147

386.11157	0.18158

386.25091	0.18161

386.39025	0.18164

386.52959	0.18174

386.66893	0.18177

386.80827	0.18185

386.94761	0.18188

387.08695	0.18207

387.22629	0.18212

387.36564	0.18218

387.50498	0.1822

387.64432	0.18226

387.78366	0.18231

387.923	0.18234

388.06234	0.1825

388.20168	0.18256

388.34102	0.18258

388.48036	0.18267

388.6197	0.18269

388.75904	0.1828

388.89838	0.18288

389.03772	0.18294

389.17706	0.18299

389.3164	0.18315

389.45574	0.18321

389.59508	0.18334

389.73442	0.1834

389.87376	0.18348

390.0131	0.1835

390.15244	0.18359

390.29178	0.18361

390.43112	0.18364

390.57046	0.18369

390.7098	0.18375

390.84914	0.18378

390.98848	0.18397

391.12782	0.18405

391.26716	0.18407

391.4065	0.1841

391.54584	0.18421

391.68518	0.18429

391.82452	0.18434

391.96386	0.18437

392.1032	0.18443

392.24254	0.18445

392.38188	0.18448

392.52122	0.18451

392.66056	0.18456

392.7999	0.18459

392.93924	0.1847

393.07858	0.18472

393.21792	0.18475

393.35726	0.18478

393.4966	0.18483

393.63594	0.18486

393.77528	0.18494

393.91462	0.18502

394.05396	0.18505

394.1933	0.18518

394.33264	0.18524

394.47198	0.18527

394.61132	0.18532

394.75066	0.1854

394.89	0.18543

395.02934	0.18548

395.16868	0.18551

395.30802	0.18554

395.44736	0.18562

395.5867	0.18564

395.72604	0.18578

395.86538	0.18586

396.00472	0.18589

396.14406	0.18602

396.28341	0.18605

396.42275	0.18613

396.56209	0.18624

396.70143	0.18629

396.84077	0.18632

396.98011	0.18635

397.11945	0.18638

397.25879	0.18646

397.39813	0.18648

397.53747	0.1867

397.67681	0.18673

397.81615	0.18676

397.95549	0.18689

398.09483	0.18697

398.23417	0.187

398.37351	0.18703

398.51285	0.18716

398.65219	0.18727

398.79153	0.18732

398.93087	0.18735

399.07021	0.18741

399.20955	0.18749

399.34889	0.18751

399.48823	0.18754

399.62757	0.18759

399.76691	0.18768

399.90625	0.1877

400.04559	0.18776

400.18493	0.18778

400.32427	0.18822

400.46361	0.18827

400.60295	0.18835

400.74229	0.18841

400.88163	0.18846

401.02097	0.18857

401.16031	0.1886

401.29965	0.18862

401.43899	0.18871

401.57833	0.18873

401.71767	0.18879

401.85701	0.18938

401.99635	0.18941

402.13569	0.18944

402.27503	0.18946

402.41437	0.18954

402.55371	0.18957

402.69305	0.18976

402.83239	0.18979

402.97173	0.18982

403.11107	0.18984

403.25041	0.19001

403.38975	0.19003

403.52909	0.19006

403.66843	0.19014

403.80777	0.19017

403.94711	0.19025

404.08645	0.19028

404.22579	0.1903

404.36513	0.19044

404.50447	0.19049

404.64381	0.19055

404.78315	0.1906

404.92249	0.19063

405.06183	0.19066

405.20117	0.19068

405.34052	0.19074

405.47986	0.19076

405.6192	0.19082

405.75854	0.19085

405.89788	0.19103

406.03722	0.19112

406.17656	0.19117

406.3159	0.1912

406.45524	0.19128

406.59458	0.19133

406.73392	0.19141

406.87326	0.19144

407.0126	0.1915

407.15194	0.1916

407.29128	0.19166

407.43062	0.19182

407.56996	0.19185

407.7093	0.19187

407.84864	0.19201

407.98798	0.19204

408.12732	0.19206

408.26666	0.19209

408.406	0.19223

408.54534	0.19228

408.68468	0.19233

408.82402	0.19236

408.96336	0.19247

409.1027	0.1925

409.24204	0.19255

409.38138	0.19258

409.52072	0.19261

409.66006	0.19263

409.7994	0.19266

409.93874	0.1929

410.07808	0.19301

410.21742	0.19309

410.35676	0.19315

410.4961	0.1932

410.63544	0.19323

410.77478	0.19326

410.91412	0.19334

411.05346	0.19342

411.1928	0.19347

411.33214	0.1935

411.47148	0.19355

411.61082	0.19361

411.75016	0.19366

411.8895	0.19369

412.02884	0.19372

412.16818	0.19377

412.30752	0.1938

412.44686	0.19382

412.5862	0.19388

412.72554	0.19391

412.86488	0.19396

413.00422	0.1942

413.14356	0.19426

413.2829	0.19428

413.42224	0.19434

413.56158	0.19442

413.70092	0.19445

413.84026	0.19447

413.9796	0.1945

414.11895	0.19456

414.25829	0.19464

414.39763	0.19469

414.53697	0.19475

414.67631	0.19477

414.81565	0.1948

414.95499	0.19491

415.09433	0.19507

415.23367	0.19515

415.37301	0.19523

415.51235	0.19529

415.65169	0.19531

415.79103	0.1954

415.93037	0.19542

416.06971	0.19548

416.20905	0.1955

416.34839	0.19559

416.48773	0.19567

416.62707	0.19575

416.76641	0.19583

416.90575	0.19586

417.04509	0.19594

417.18443	0.19599

417.32377	0.19602

417.46311	0.19613

417.60245	0.19621

417.74179	0.19624

417.88113	0.19626

418.02047	0.19629

418.15981	0.19651

418.29915	0.19653

418.43849	0.19656

418.57783	0.19661

418.71717	0.19667

418.85651	0.19672

418.99585	0.19675

419.13519	0.19678

419.27453	0.1968

419.41387	0.19686

419.55321	0.19697

419.69255	0.19702

419.83189	0.1971

419.97123	0.19716

420.11057	0.19721

420.24991	0.19724

420.38925	0.19726

420.52859	0.19729

420.66793	0.19732

420.80727	0.19735

420.94661	0.1974

421.08595	0.19743

421.22529	0.19756

421.36463	0.19759

421.50397	0.19762

421.64331	0.19764

421.78265	0.19767

421.92199	0.19775

422.06133	0.19783

422.20067	0.19791

422.34001	0.19797

422.47935	0.19805

422.61869	0.19813

422.75803	0.19821

422.89737	0.19824

423.03672	0.19827

423.17606	0.19829

423.3154	0.19837

423.45474	0.19846

423.59408	0.19873

423.73342	0.19875

423.87276	0.19881

424.0121	0.19902

424.15144	0.19913

424.29078	0.19916

424.43012	0.19919

424.56946	0.19921

424.7088	0.19924

424.84814	0.19927

424.98748	0.19932

425.12682	0.19935

425.26616	0.19957

425.4055	0.19962

425.54484	0.1997

425.68418	0.19981

425.82352	0.19986

425.96286	0.20005

426.1022	0.20008

426.24154	0.20011

426.38088	0.20019

426.52022	0.20022

426.65956	0.20027

426.7989	0.2003

426.93824	0.20038

427.07758	0.20041

427.21692	0.20046

427.35626	0.20049

427.4956	0.20057

427.63494	0.2006

427.77428	0.20062

427.91362	0.20065

428.05296	0.20068

428.1923	0.20073

428.33164	0.20079

428.47098	0.20087

428.61032	0.20092

428.74966	0.20095

428.889	0.20098

429.02834	0.20111

429.16768	0.20116

429.30702	0.20119

429.44636	0.20122

429.5857	0.20133

429.72504	0.20138

429.86438	0.20154

430.00372	0.2016

430.14306	0.20171

430.2824	0.20173

430.42174	0.20187

430.56108	0.20198

430.70042	0.202

430.83976	0.20203

430.9791	0.20209

431.11844	0.20217

431.25778	0.20238

431.39712	0.20246

431.53646	0.20255

431.6758	0.20257

431.81514	0.2026

431.95449	0.20263

432.09383	0.20279

432.23317	0.20284

432.37251	0.20293

432.51185	0.20301

432.65119	0.20309

432.79053	0.20311

432.92987	0.2033

433.06921	0.20339

433.20855	0.20349

433.34789	0.20352

433.48723	0.2036

433.62657	0.20368

433.76591	0.20376

433.90525	0.20379

434.04459	0.20382

434.18393	0.20385

434.32327	0.20387

434.46261	0.2039

434.60195	0.20393

434.74129	0.20395

434.88063	0.20398

435.01997	0.20409

435.15931	0.20414

435.29865	0.20425

435.43799	0.20433

435.57733	0.20439

435.71667	0.20441

435.85601	0.20447

435.99535	0.2045

436.13469	0.20466

436.27403	0.20469

436.41337	0.20471

436.55271	0.20474

436.69205	0.20482

436.83139	0.20501

436.97073	0.20509

437.11007	0.20515

437.24941	0.20517

437.38875	0.20523

437.52809	0.20528

437.66743	0.20536

437.80677	0.20539

437.94611	0.20542

438.08545	0.20544

438.22479	0.2055

438.36413	0.20553

438.50347	0.20569

438.64281	0.20572

438.78215	0.20574

438.92149	0.20577

439.06083	0.2059

439.20017	0.20593

439.33951	0.20609

439.47885	0.20618

439.61819	0.2062

439.75753	0.20626

439.89687	0.20628

440.03621	0.20631

440.17555	0.20637

440.31489	0.20661

440.45423	0.20664

440.59357	0.20669

440.73291	0.20683

440.87226	0.20688

441.0116	0.20693

441.15094	0.20715

441.29028	0.20723

441.42962	0.20726

441.56896	0.20731

441.7083	0.20737

441.84764	0.20739

441.98698	0.20742

442.12632	0.20748

442.26566	0.2075

442.405	0.20756

442.54434	0.20758

442.68368	0.20767

442.82302	0.20769

442.96236	0.20772

443.1017	0.2078

443.24104	0.20783

443.38038	0.20785

443.51972	0.20799

443.65906	0.20826

443.7984	0.20834

443.93774	0.20842

444.07708	0.20845

444.21642	0.20856

444.35576	0.20869

444.4951	0.20872

444.63444	0.20875

444.77378	0.20878

444.91312	0.2088

445.05246	0.20886

445.1918	0.20913

445.33114	0.20918

445.47048	0.20921

445.60982	0.20924

445.74916	0.20929

445.8885	0.20934

446.02784	0.20937

446.16718	0.20948

446.30652	0.20953

446.44586	0.20967

446.5852	0.21008

446.72454	0.21016

446.86388	0.21027

447.00322	0.21037

447.14256	0.21056

447.2819	0.21059

447.42124	0.21064

447.56058	0.21067

447.69992	0.21073

447.83926	0.21075

447.9786	0.21083

448.11794	0.21097

448.25728	0.211

448.39662	0.21102

448.53596	0.21111

448.6753	0.21124

448.81464	0.21127

448.95398	0.21129

449.09332	0.21132

449.23266	0.21135

449.372	0.21138

449.51134	0.2114

449.65068	0.21143

449.79003	0.21151

449.92937	0.21157

450.06871	0.21162

450.20805	0.21167

450.34739	0.2117

450.48673	0.21186

450.62607	0.21189

450.76541	0.21194

450.90475	0.21205

451.04409	0.21211

451.18343	0.21224

451.32277	0.21232

451.46211	0.21241

451.60145	0.21246

451.74079	0.21249

451.88013	0.21251

452.01947	0.21254

452.15881	0.21257

452.29815	0.21265

452.43749	0.21276

452.57683	0.21278

452.71617	0.21287

452.85551	0.21289

452.99485	0.21292

453.13419	0.213

453.27353	0.21306

453.41287	0.21322

453.55221	0.21327

453.69155	0.21335

453.83089	0.21346

453.97023	0.21349

454.10957	0.21354

454.24891	0.21357

454.38825	0.2136

454.52759	0.21362

454.66693	0.21365

454.80627	0.21368

454.94561	0.21376

455.08495	0.21379

455.22429	0.21381

455.36363	0.21392

455.50297	0.21403

455.64231	0.21406

455.78165	0.21414

455.92099	0.21422

456.06033	0.21425

456.19967	0.21427

456.33901	0.2143

456.47835	0.21433

456.61769	0.21444

456.75703	0.21454

456.89637	0.2146

457.03571	0.21473

457.17505	0.21476

457.31439	0.21482

457.45373	0.21487

457.59307	0.2149

457.73241	0.21495

457.87175	0.21511

458.01109	0.21533

458.15043	0.21549

458.28977	0.21552

458.42911	0.2156

458.56845	0.21568

458.7078	0.21571

458.84714	0.21574

458.98648	0.21576

459.12582	0.21585

459.26516	0.2159

459.4045	0.21593

459.54384	0.21595

459.68318	0.21601

459.82252	0.21606

459.96186	0.21628

460.1012	0.21631

460.24054	0.21633

460.37988	0.21636

460.51922	0.21644

460.65856	0.21647

460.7979	0.21677

460.93724	0.21696

461.07658	0.21712

461.21592	0.21715

461.35526	0.21717

461.4946	0.2172

461.63394	0.2175

461.77328	0.21752

461.91262	0.21755

462.05196	0.21761

462.1913	0.21766

462.33064	0.21777

462.46998	0.21801

462.60932	0.21807

462.74866	0.21817

462.888	0.21823

463.02734	0.21826

463.16668	0.21831

463.30602	0.21836

463.44536	0.21839

463.5847	0.21842

463.72404	0.21845

463.86338	0.21853

464.00272	0.21855

464.14206	0.2188

464.2814	0.21882

464.42074	0.21885

464.56008	0.21893

464.69942	0.21899

464.83876	0.21901

464.9781	0.21907

465.11744	0.21928

465.25678	0.21934

465.39612	0.21937

465.53546	0.21942

465.6748	0.21953

465.81414	0.21958

465.95348	0.21961

466.09282	0.21966

466.23216	0.21972

466.3715	0.21975

466.51084	0.2198

466.65018	0.22002

466.78952	0.2201

466.92886	0.22012

467.0682	0.22021

467.20754	0.22023

467.34688	0.22029

467.48622	0.22034

467.62557	0.2204

467.76491	0.22042

467.90425	0.22048

468.04359	0.22061

468.18293	0.22069

468.32227	0.22086

468.46161	0.22088

468.60095	0.22094

468.74029	0.22099

468.87963	0.22102

469.01897	0.22105

469.15831	0.22107

469.29765	0.22124

469.43699	0.22129

469.57633	0.22132

469.71567	0.22134

469.85501	0.22137

469.99435	0.2214

470.13369	0.22159

470.27303	0.22167

470.41237	0.2217

470.55171	0.22183

470.69105	0.22186

470.83039	0.22194

470.96973	0.22224

471.10907	0.22226

471.24841	0.22229

471.38775	0.22243

471.52709	0.22264

471.66643	0.2227

471.80577	0.22286

471.94511	0.22289

472.08445	0.22291

472.22379	0.22297

472.36313	0.223

472.50247	0.22308

472.64181	0.22321

472.78115	0.22324

472.92049	0.22332

473.05983	0.22335

473.19917	0.22375

473.33851	0.22378

473.47785	0.22386

473.61719	0.22389

473.75653	0.22392

473.89587	0.22394

474.03521	0.22405

474.17455	0.22408

474.31389	0.22413

474.45323	0.22416

474.59257	0.22419

474.73191	0.22421

474.87125	0.2243

475.01059	0.22438

475.14993	0.22443

475.28927	0.22451

475.42861	0.22457

475.56795	0.22459

475.70729	0.2247

475.84663	0.22492

475.98597	0.22497

476.12531	0.225

476.26465	0.22508

476.404	0.22511

476.54334	0.22514

476.68268	0.22524

476.82202	0.2256

476.96136	0.22579

477.1007	0.22581

477.24004	0.226

477.37938	0.22608

477.51872	0.22611

477.65806	0.22619

477.7974	0.22625

477.93674	0.22627

478.07608	0.2263

478.21542	0.22646

478.35476	0.22654

478.4941	0.22665

478.63344	0.22673

478.77278	0.22676

478.91212	0.2269

479.05146	0.22698

479.1908	0.22725

479.33014	0.22728

479.46948	0.22738

479.60882	0.22744

479.74816	0.22749

479.8875	0.22752

480.02684	0.22779

480.16618	0.22787

480.30552	0.22795

480.44486	0.22801

480.5842	0.22811

480.72354	0.22814

480.86288	0.22817

481.00222	0.2282

481.14156	0.22825

481.2809	0.22828

481.42024	0.2283

481.55958	0.22833

481.69892	0.22836

481.83826	0.22839

481.9776	0.22841

482.11694	0.22844

482.25628	0.22852

482.39562	0.22858

482.53496	0.22863

482.6743	0.22871

482.81364	0.22876

482.95298	0.22879

483.09232	0.22882

483.23166	0.22885

483.371	0.2289

483.51034	0.22898

483.64968	0.22901

483.78902	0.22909

483.92836	0.22912

484.0677	0.22914

484.20704	0.22931

484.34638	0.22939

484.48572	0.22958

484.62506	0.22971

484.7644	0.22974

484.90374	0.22977

485.04308	0.22979

485.18242	0.22985

485.32177	0.2299

485.46111	0.22993

485.60045	0.23009

485.73979	0.23012

485.87913	0.23015

486.01847	0.2302

486.15781	0.23034

486.29715	0.2305

486.43649	0.23053

486.57583	0.23055

486.71517	0.23058

486.85451	0.23063

486.99385	0.23069

487.13319	0.23072

487.27253	0.23085

487.41187	0.23088

487.55121	0.23093

487.69055	0.23104

487.82989	0.23109

487.96923	0.23115

488.10857	0.23118

488.24791	0.23126

488.38725	0.23128

488.52659	0.23137

488.66593	0.23142

488.80527	0.23145

488.94461	0.23147

489.08395	0.2315

489.22329	0.23158

489.36263	0.23166

489.50197	0.23169

489.64131	0.23172

489.78065	0.23183

489.91999	0.23202

490.05933	0.23204

490.19867	0.2322

490.33801	0.23223

490.47735	0.23245

490.61669	0.23248

490.75603	0.23253

490.89537	0.23261

491.03471	0.23294

491.17405	0.23315

491.31339	0.23318

491.45273	0.23332

491.59207	0.23334

491.73141	0.23342

491.87075	0.2335

492.01009	0.23356

492.14943	0.23361

492.28877	0.23369

492.42811	0.23372

492.56745	0.23391

492.70679	0.23399

492.84613	0.23402

492.98547	0.2341

493.12481	0.23415

493.26415	0.23424

493.40349	0.23429

493.54283	0.23432

493.68217	0.2347

493.82151	0.23475

493.96085	0.23478

494.10019	0.2348

494.23953	0.23491

494.37888	0.23494

494.51822	0.23505

494.65756	0.2351

494.7969	0.23513

494.93624	0.23516

495.07558	0.23527

495.21492	0.23529

495.35426	0.2354

495.4936	0.23554

495.63294	0.23559

495.77228	0.23564

495.91162	0.23567

496.05096	0.2357

496.1903	0.23573

496.32964	0.23578

496.46898	0.23594

496.60832	0.23597

496.74766	0.23608

496.887	0.23624

497.02634	0.23627

497.16568	0.23632

497.30502	0.23635

497.44436	0.2367

497.5837	0.23684

497.72304	0.23689

497.86238	0.23692

498.00172	0.23697

498.14106	0.237

498.2804	0.23705

498.41974	0.23711

498.55908	0.23719

498.69842	0.23722

498.83776	0.23735

498.9771	0.23743

499.11644	0.23773

499.25578	0.23781

499.39512	0.23784

499.53446	0.23792

499.6738	0.238

499.81314	0.23803

499.95248	0.23811

500.09182	0.23814

500.23116	0.23816

500.3705	0.23819

500.50984	0.23822

500.64918	0.2383

500.78852	0.23833

500.92786	0.23835

501.0672	0.23846

501.20654	0.23849

501.34588	0.23854

501.48522	0.23857

501.62456	0.23865

501.7639	0.23884

501.90324	0.23889

502.04258	0.23892

502.18192	0.23895

502.32126	0.23903

502.4606	0.23908

502.59994	0.23911

502.73928	0.23919

502.87862	0.23922

503.01796	0.23925

503.15731	0.23927

503.29665	0.23938

503.43599	0.23941

503.57533	0.23952

503.71467	0.23954

503.85401	0.23965

503.99335	0.23968

504.13269	0.23973

504.27203	0.23976

504.41137	0.23979

504.55071	0.23982

504.69005	0.23984

504.82939	0.23995

504.96873	0.24001

505.10807	0.24003

505.24741	0.24014

505.38675	0.24025

505.52609	0.24036

505.66543	0.24047

505.80477	0.24052

505.94411	0.24057

506.08345	0.24063

506.22279	0.24071

506.36213	0.24076

506.50147	0.24079

506.64081	0.24082

506.78015	0.24093

506.91949	0.24095

507.05883	0.24101

507.19817	0.24106

507.33751	0.24109

507.47685	0.24112

507.61619	0.24114

507.75553	0.24122

507.89487	0.24128

508.03421	0.24131

508.17355	0.24136

508.31289	0.24155

508.45223	0.24168

508.59157	0.24171

508.73091	0.24174

508.87025	0.24185

509.00959	0.24187

509.14893	0.24193

509.28827	0.24209

509.42761	0.24217

509.56695	0.24223

509.70629	0.24225

509.84563	0.24231

509.98497	0.24236

510.12431	0.24239

510.26365	0.24244

510.40299	0.24247

510.54233	0.24252

510.68167	0.24258

510.82101	0.24271

510.96035	0.24274

511.09969	0.24282

511.23903	0.24285

511.37837	0.2429

511.51771	0.24296

511.65705	0.24301

511.79639	0.24304

511.93573	0.24331

512.07507	0.24334

512.21442	0.24347

512.35376	0.2435

512.4931	0.24358

512.63244	0.24361

512.77178	0.24363

512.91112	0.24374

513.05046	0.24377

513.1898	0.2438

513.32914	0.24382

513.46848	0.24385

513.60782	0.24393

513.74716	0.24399

513.8865	0.24401

514.02584	0.24418

514.16518	0.2442

514.30452	0.24445

514.44386	0.24458

514.5832	0.24461

514.72254	0.24464

514.86188	0.24488

515.00122	0.24507

515.14056	0.24521

515.2799	0.24537

515.41924	0.24542

515.55858	0.24545

515.69792	0.24553

515.83726	0.24561

515.9766	0.24564

516.11594	0.24567

516.25528	0.24586

516.39462	0.24588

516.53396	0.2461

516.6733	0.24618

516.81264	0.24651

516.95198	0.24661

517.09132	0.24686

517.23066	0.24689

517.37	0.24691

517.50934	0.24694

517.64868	0.24697

517.78802	0.24705

517.92736	0.24707

518.0667	0.2471

518.20604	0.24729

518.34538	0.24732

518.48472	0.2474

518.62406	0.24745

518.7634	0.24756

518.90274	0.24759

519.04208	0.24762

519.18142	0.24767

519.32076	0.24781

519.4601	0.24789

519.59944	0.24794

519.73878	0.24797

519.87812	0.24808

520.01746	0.2484

520.1568	0.24862

520.29614	0.2487

520.43548	0.24881

520.57482	0.24884

520.71416	0.24886

520.8535	0.24892

520.99285	0.24902

521.13219	0.24908

521.27153	0.24913

521.41087	0.24916

521.55021	0.2493

521.68955	0.24938

521.82889	0.24943

521.96823	0.24946

522.10757	0.24949

522.24691	0.24954

522.38625	0.24962

522.52559	0.24965

522.66493	0.24967

522.80427	0.2497

522.94361	0.24973

523.08295	0.24981

523.22229	0.24984

523.36163	0.24986

523.50097	0.25003

523.64031	0.25005

523.77965	0.25016

523.91899	0.2503

524.05833	0.25033

524.19767	0.25035

524.33701	0.25038

524.47635	0.25041

524.61569	0.25065

524.75503	0.25081

524.89437	0.25089

525.03371	0.251

525.17305	0.25106

525.31239	0.25108

525.45173	0.25116

525.59107	0.25119

525.73041	0.25122

525.86975	0.25127

526.00909	0.25138

526.14843	0.25154

526.28777	0.25157

526.42711	0.25179

526.56645	0.25184

526.70579	0.2519

526.84513	0.25192

526.98447	0.25198

527.12381	0.252

527.26315	0.25206

527.40249	0.25211

527.54183	0.2523

527.68117	0.25233

527.82051	0.25238

527.95985	0.25246

528.09919	0.25255

528.23853	0.25257

528.37787	0.25265

528.51721	0.25268

528.65655	0.25276

528.79589	0.25279

528.93523	0.25293

529.07457	0.25295

529.21391	0.25303

529.35325	0.25341

529.49259	0.25344

529.63193	0.25347

529.77127	0.25355

529.91062	0.25363

530.04996	0.25366

530.1893	0.25368

530.32864	0.25382

530.46798	0.25385

530.60732	0.2539

530.74666	0.25395

530.886	0.2545

531.02534	0.2546

531.16468	0.25463

531.30402	0.25469

531.44336	0.25471

531.5827	0.25474

531.72204	0.25477

531.86138	0.25496

532.00072	0.25498

532.14006	0.25509

532.2794	0.25512

532.41874	0.25515

532.55808	0.25517

532.69742	0.25531

532.83676	0.25536

532.9761	0.25539

533.11544	0.25542

533.25478	0.2555

533.39412	0.25558

533.53346	0.25561

533.6728	0.25566

533.81214	0.2558

533.95148	0.25582

534.09082	0.2559

534.23016	0.25593

534.3695	0.25599

534.50884	0.25601

534.64818	0.25607

534.78752	0.25615

534.92686	0.25618

535.0662	0.2562

535.20554	0.25626

535.34488	0.25628

535.48422	0.25631

535.62356	0.25637

535.7629	0.2565

535.90224	0.25653

536.04158	0.25669

536.18092	0.25802

536.32026	0.25804

536.4596	0.25837

536.59894	0.25848

536.73828	0.2585

536.87762	0.25853

537.01696	0.25859

537.1563	0.25861

537.29564	0.25864

537.43498	0.25867

537.57432	0.25878

537.71366	0.25891

537.853	0.25897

537.99234	0.25899

538.13168	0.25905

538.27102	0.25913

538.41036	0.25918

538.5497	0.25921

538.68904	0.25924

538.82839	0.2594

538.96773	0.25943

539.10707	0.25967

539.24641	0.25975

539.38575	0.25989

539.52509	0.25999

539.66443	0.26002

539.80377	0.26008

539.94311	0.26013

540.08245	0.26018

540.22179	0.26021

540.36113	0.26035

540.50047	0.26037

540.63981	0.26043

540.77915	0.26046

540.91849	0.26056

541.05783	0.26059

541.19717	0.26064

541.33651	0.26067

541.47585	0.26075

541.61519	0.26081

541.75453	0.26083

541.89387	0.26086

542.03321	0.26089

542.17255	0.26094

542.31189	0.261

542.45123	0.26102

542.59057	0.26105

542.72991	0.26108

542.86925	0.26111

543.00859	0.26113

543.14793	0.26119

543.28727	0.26129

543.42661	0.2614

543.56595	0.26143

543.70529	0.26148

543.84463	0.26154

543.98397	0.2617

544.12331	0.26178

544.26265	0.26181

544.40199	0.26194

544.54133	0.26211

544.68067	0.26222

544.82001	0.2623

544.95935	0.26232

545.09869	0.26238

545.23803	0.26241

545.37737	0.26243

545.51671	0.26246

545.65605	0.26249

545.79539	0.26251

545.93473	0.26254

546.07407	0.26257

546.21341	0.26259

546.35275	0.26262

546.49209	0.26265

546.63143	0.2627

546.77077	0.26278

546.91011	0.263

547.04945	0.26324

547.18879	0.26349

547.32813	0.26381

547.46747	0.26389

547.60681	0.26392

547.74616	0.26395

547.8855	0.26398

548.02484	0.26406

548.16418	0.26425

548.30352	0.26427

548.44286	0.26446

548.5822	0.26449

548.72154	0.26452

548.86088	0.26473

549.00022	0.26492

549.13956	0.26495

549.2789	0.26498

549.41824	0.26503

549.55758	0.26509

549.69692	0.26511

549.83626	0.26514

549.9756	0.26528

550.11494	0.2653

550.25428	0.2656

550.39362	0.26563

550.53296	0.26582

550.6723	0.26603

550.81164	0.26617

550.95098	0.2662

551.09032	0.26622

551.22966	0.26636

551.369	0.26641

551.50834	0.26652

551.64768	0.26655

551.78702	0.26658

551.92636	0.26663

552.0657	0.26674

552.20504	0.26685

552.34438	0.26717

552.48372	0.26728

552.62306	0.26733

552.7624	0.26742

552.90174	0.26744

553.04108	0.26758

553.18042	0.26761

553.31976	0.26771

553.4591	0.26788

553.59844	0.2679

553.73778	0.26801

553.87712	0.26804

554.01646	0.26834

554.1558	0.26842

554.29514	0.26869

554.43448	0.26882

554.57382	0.26888

554.71316	0.26891

554.8525	0.26928

554.99184	0.26931

555.13118	0.26939

555.27052	0.26953

555.40986	0.26956

555.5492	0.26958

555.68854	0.26961

555.82788	0.26969

555.96722	0.26972

556.10656	0.26975

556.2459	0.26977

556.38524	0.2698

556.52459	0.26991

556.66393	0.26999

556.80327	0.27015

556.94261	0.27026

557.08195	0.2704

557.22129	0.27042

557.36063	0.27048

557.49997	0.27053

557.63931	0.27069

557.77865	0.2708

557.91799	0.27083

558.05733	0.27099

558.19667	0.27102

558.33601	0.27105

558.47535	0.2711

558.61469	0.27124

558.75403	0.27126

558.89337	0.27132

559.03271	0.27137

559.17205	0.27142

559.31139	0.27148

559.45073	0.27156

559.59007	0.27161

559.72941	0.27164

559.86875	0.2717

560.00809	0.27172

560.14743	0.27175

560.28677	0.27178

560.42611	0.27194

560.56545	0.27197

560.70479	0.27199

560.84413	0.27207

560.98347	0.27224

561.12281	0.27229

561.26215	0.27235

561.40149	0.27237

561.54083	0.27264

561.68017	0.27286

561.81951	0.27289

561.95885	0.27291

562.09819	0.27305

562.23753	0.27321

562.37687	0.27324

562.51621	0.27329

562.65555	0.27343

562.79489	0.27348

562.93423	0.27351

563.07357	0.27365

563.21291	0.27367

563.35225	0.2737

563.49159	0.27373

563.63093	0.27378

563.77027	0.27381

563.90961	0.27386

564.04895	0.27394

564.18829	0.27397

564.32763	0.274

564.46697	0.27402

564.60631	0.27408

564.74565	0.27411

564.88499	0.27416

565.02433	0.27419

565.16367	0.27421

565.30301	0.27424

565.44236	0.27432

565.5817	0.2744

565.72104	0.27443

565.86038	0.27446

565.99972	0.27457

566.13906	0.27459

566.2784	0.27476

566.41774	0.27481

566.55708	0.27497

566.69642	0.27503

566.83576	0.27511

566.9751	0.27522

567.11444	0.27524

567.25378	0.27527

567.39312	0.2753

567.53246	0.27535

567.6718	0.27546

567.81114	0.27551

567.95048	0.27557

568.08982	0.27565

568.22916	0.2757

568.3685	0.27573

568.50784	0.27584

568.64718	0.27589

568.78652	0.27603

568.92586	0.27616

569.0652	0.27622

569.20454	0.2763

569.34388	0.27646

569.48322	0.27654

569.62256	0.27657

569.7619	0.27687

569.90124	0.27709

570.04058	0.27711

570.17992	0.27714

570.31926	0.27722

570.4586	0.27733

570.59794	0.27744

570.73728	0.27746

570.87662	0.27779

571.01596	0.27782

571.1553	0.2779

571.29464	0.27803

571.43398	0.27809

571.57332	0.27811

571.71266	0.2782

571.852	0.27828

571.99134	0.27836

572.13068	0.27841

572.27002	0.27844

572.40936	0.27847

572.5487	0.2786

572.68804	0.27871

572.82738	0.27876

572.96672	0.27879

573.10606	0.27901

573.2454	0.27909

573.38474	0.27914

573.52408	0.27933

573.66342	0.27944

573.80276	0.27952

573.9421	0.27955

574.08144	0.2796

574.22078	0.27963

574.36013	0.27969

574.49947	0.27971

574.63881	0.27977

574.77815	0.28007

574.91749	0.28015

575.05683	0.28034

575.19617	0.28047

575.33551	0.28063

575.47485	0.28077

575.61419	0.28093

575.75353	0.28112

575.89287	0.2812

576.03221	0.2815

576.17155	0.28166

576.31089	0.28169

576.45023	0.28172

576.58957	0.28177

576.72891	0.28193

576.86825	0.28199

577.00759	0.28202

577.14693	0.28212

577.28627	0.28215

577.42561	0.2822

577.56495	0.28223

577.70429	0.28234

577.84363	0.28237

577.98297	0.28242

578.12231	0.28248

578.26165	0.28269

578.40099	0.28272

578.54033	0.28277

578.67967	0.28288

578.81901	0.28299

578.95835	0.28302

579.09769	0.2831

579.23703	0.28313

579.37637	0.28364

579.51571	0.28375

579.65505	0.28426

579.79439	0.28451

579.93373	0.28453

580.07307	0.28462

580.21241	0.2847

580.35175	0.28475

580.49109	0.28478

580.63043	0.2848

580.76977	0.28489

580.90911	0.2851

581.04845	0.28532

581.18779	0.2854

581.32713	0.28543

581.46647	0.28556

581.60581	0.28559

581.74515	0.28562

581.88449	0.28564

582.02383	0.28578

582.16317	0.28581

582.30251	0.28589

582.44185	0.28592

582.58119	0.286

582.72053	0.28602

582.85987	0.28605

582.99921	0.28619

583.13855	0.28621

583.27789	0.28629

583.41724	0.28632

583.55658	0.28635

583.69592	0.28638

583.83526	0.2864

583.9746	0.28643

584.11394	0.28651

584.25328	0.28657

584.39262	0.28665

584.53196	0.28686

584.6713	0.28692

584.81064	0.287

584.94998	0.28708

585.08932	0.28711

585.22866	0.28716

585.368	0.28719

585.50734	0.28722

585.64668	0.28724

585.78602	0.28727

585.92536	0.2873

586.0647	0.28735

586.20404	0.28746

586.34338	0.28757

586.48272	0.28762

586.62206	0.28787

586.7614	0.28789

586.90074	0.28792

587.04008	0.288

587.17942	0.28822

587.31876	0.28824

587.4581	0.2883

587.59744	0.28843

587.73678	0.28849

587.87612	0.28873

588.01546	0.28879

588.1548	0.28881

588.29414	0.28887

588.43348	0.28892

588.57282	0.28906

588.71216	0.28908

588.8515	0.28922

588.99084	0.28925

589.13018	0.2893

589.26952	0.28946

589.40886	0.28952

589.5482	0.28963

589.68754	0.28965

589.82688	0.28971

589.96622	0.29009

590.10556	0.29017

590.2449	0.29063

590.38424	0.29066

590.52358	0.29068

590.66292	0.29082

590.80226	0.29085

590.9416	0.29093

591.08094	0.29117

591.22028	0.29136

591.35962	0.29139

591.49896	0.29141

591.6383	0.2915

591.77764	0.29168

591.91698	0.29177

592.05632	0.2919

592.19566	0.29196

592.33501	0.29204

592.47435	0.29215

592.61369	0.29225

592.75303	0.29239

592.89237	0.29242

593.03171	0.29255

593.17105	0.29263

593.31039	0.2928

593.44973	0.29285

593.58907	0.2929

593.72841	0.29301

593.86775	0.29312

594.00709	0.29315

594.14643	0.2932

594.28577	0.29326

594.42511	0.29334

594.56445	0.29353

594.70379	0.29358

594.84313	0.29361

594.98247	0.29363

595.12181	0.29366

595.26115	0.29369

595.40049	0.29374

595.53983	0.29377

595.67917	0.2941

595.81851	0.29415

595.95785	0.29418

596.09719	0.2942

596.23653	0.29426

596.37587	0.29447

596.51521	0.2945

596.65455	0.29458

596.79389	0.29464

596.93323	0.29466

597.07257	0.29472

597.21191	0.2948

597.35125	0.29499

597.49059	0.29507

597.62993	0.29512

597.76927	0.29515

597.90861	0.29521

598.04795	0.29523

598.18729	0.29529

598.32663	0.29531

598.46597	0.29542

598.60531	0.29545

598.74465	0.29548

598.88399	0.29553

599.02333	0.29556

599.16267	0.29564

599.30201	0.29594

599.44135	0.29596

599.58069	0.29607

599.72003	0.29624

599.85937	0.29626

599.99871	0.29629

600.13805	0.29634

600.27739	0.29642

600.41673	0.29645

600.55607	0.29648

600.69541	0.29653

600.83475	0.29675

600.97409	0.29683

601.11343	0.29686

601.25278	0.29689

601.39212	0.29697

601.53146	0.29699

601.6708	0.29707

601.81014	0.2974

601.94948	0.29745

602.08882	0.29754

602.22816	0.29756

602.3675	0.29764

602.50684	0.29772

602.64618	0.29789

602.78552	0.29791

602.92486	0.29794

603.0642	0.29797

603.20354	0.29802

603.34288	0.29805

603.48222	0.29819

603.62156	0.2987

603.7609	0.29889

603.90024	0.29905

604.03958	0.29908

604.17892	0.29913

604.31826	0.29916

604.4576	0.29927

604.59694	0.29932

604.73628	0.29938

604.87562	0.29954

605.01496	0.29957

605.1543	0.29959

605.29364	0.29962

605.43298	0.29965

605.57232	0.29967

605.71166	0.2997

605.851	0.29973

605.99034	0.29981

606.12968	0.29984

606.26902	0.29989

606.40836	0.3

606.5477	0.30003

606.68704	0.30005

606.82638	0.30008

606.96572	0.30043

607.10506	0.30049

607.2444	0.30051

607.38374	0.3007

607.52308	0.30089

607.66242	0.30092

607.80176	0.30098

607.9411	0.30103

608.08044	0.30108

608.21978	0.30111

608.35912	0.30135

608.49846	0.30138

608.6378	0.30144

608.77714	0.30187

608.91648	0.3019

609.05582	0.30192

609.19516	0.30198

609.3345	0.302

609.47384	0.30222

609.61318	0.30228

609.75252	0.30241

609.89186	0.3026

610.03121	0.30263

610.17055	0.30268

610.30989	0.30274

610.44923	0.30276

610.58857	0.30282

610.72791	0.30287

610.86725	0.30293

611.00659	0.30295

611.14593	0.30298

611.28527	0.30314

611.42461	0.30339

611.56395	0.30349

611.70329	0.30352

611.84263	0.30366

611.98197	0.30371

612.12131	0.30376

612.26065	0.30382

612.39999	0.30385

612.53933	0.30395

612.67867	0.30404

612.81801	0.3045

612.95735	0.30455

613.09669	0.3046

613.23603	0.30477

613.37537	0.30479

613.51471	0.30482

613.65405	0.30485

613.79339	0.30488

613.93273	0.3049

614.07207	0.30493

614.21141	0.30496

614.35075	0.30498

614.49009	0.30504

614.62943	0.30507

614.76877	0.30509

614.90811	0.30517

615.04745	0.30531

615.18679	0.30534

615.32613	0.30544

615.46547	0.30577

615.60481	0.30582

615.74415	0.30593

615.88349	0.30604

616.02283	0.30618

616.16217	0.30626

616.30151	0.30639

616.44085	0.30645

616.58019	0.3065

616.71953	0.30674

616.85887	0.30688

616.99821	0.30696

617.13755	0.30699

617.27689	0.30702

617.41623	0.30704

617.55557	0.30707

617.69491	0.30729

617.83425	0.30742

617.97359	0.30748

618.11293	0.3075

618.25227	0.30758

618.39161	0.30761

618.53095	0.30775

618.67029	0.30777

618.80963	0.3078

618.94898	0.30788

619.08832	0.30791

619.22766	0.3081

619.367	0.30813

619.50634	0.30815

619.64568	0.30818

619.78502	0.30829

619.92436	0.30832

620.0637	0.3084

620.20304	0.30848

620.34238	0.30859

620.48172	0.30861

620.62106	0.30867

620.7604	0.30869

620.89974	0.30883

621.03908	0.30891

621.17842	0.30902

621.31776	0.30905

621.4571	0.30907

621.59644	0.3091

621.73578	0.3094

621.87512	0.30943

622.01446	0.30953

622.1538	0.30959

622.29314	0.30967

622.43248	0.30972

622.57182	0.3098

622.71116	0.30989

622.8505	0.30997

622.98984	0.30999

623.12918	0.31002

623.26852	0.31013

623.40786	0.31016

623.5472	0.31021

623.68654	0.31032

623.82588	0.3104

623.96522	0.31046

624.10456	0.31056

624.2439	0.3107

624.38324	0.31073

624.52258	0.31083

624.66192	0.31102

624.80126	0.31108

624.9406	0.31111

625.07994	0.31132

625.21928	0.31146

625.35862	0.31154

625.49796	0.31165

625.6373	0.31167

625.77664	0.31176

625.91598	0.31178

626.05532	0.31184

626.19466	0.31189

626.334	0.31257

626.47334	0.31259

626.61268	0.31262

626.75202	0.31265

626.89136	0.31284

627.0307	0.31287

627.17004	0.31289

627.30938	0.313

627.44872	0.31306

627.58806	0.31311

627.7274	0.31314

627.86675	0.31371

628.00609	0.31379

628.14543	0.31381

628.28477	0.31384

628.42411	0.31398

628.56345	0.31406

628.70279	0.31414

628.84213	0.31417

628.98147	0.31419

629.12081	0.31422

629.26015	0.31425

629.39949	0.31463

629.53883	0.31473

629.67817	0.31476

629.81751	0.31479

629.95685	0.31482

630.09619	0.31492

630.23553	0.31495

630.37487	0.31509

630.51421	0.31517

630.65355	0.31522

630.79289	0.31525

630.93223	0.31528

631.07157	0.3153

631.21091	0.31538

631.35025	0.31541

631.48959	0.31547

631.62893	0.31579

631.76827	0.31598

631.90761	0.31601

632.04695	0.31603

632.18629	0.31606

632.32563	0.31612

632.46497	0.31614

632.60431	0.31617

632.74365	0.31625

632.88299	0.31631

633.02233	0.31639

633.16167	0.31644

633.30101	0.31647

633.44035	0.31663

633.57969	0.31671

633.71903	0.31674

633.85837	0.31693

633.99771	0.31698

634.13705	0.31701

634.27639	0.31704

634.41573	0.31709

634.55507	0.31712

634.69441	0.31717

634.83375	0.3172

634.97309	0.31725

635.11243	0.31736

635.25177	0.31739

635.39111	0.31742

635.53045	0.31747

635.66979	0.31758

635.80913	0.31763

635.94847	0.31771

636.08781	0.31782

636.22715	0.31785

636.36649	0.31788

636.50583	0.3179

636.64517	0.31793

636.78452	0.31796

636.92386	0.31801

637.0632	0.31826

637.20254	0.31831

637.34188	0.31834

637.48122	0.31845

637.62056	0.31847

637.7599	0.3185

637.89924	0.31853

638.03858	0.31869

638.17792	0.31874

638.31726	0.31877

638.4566	0.31885

638.59594	0.31891

638.73528	0.31915

638.87462	0.31956

639.01396	0.31961

639.1533	0.31983

639.29264	0.31985

639.43198	0.31991

639.57132	0.3201

639.71066	0.32015

639.85	0.32023

639.98934	0.32034

640.12868	0.3204

640.26802	0.32056

640.40736	0.32059

640.5467	0.32061

640.68604	0.32064

640.82538	0.32072

640.96472	0.32077

641.10406	0.3208

641.2434	0.32083

641.38274	0.32121

641.52208	0.32124

641.66142	0.32126

641.80076	0.32148

641.9401	0.32153

642.07944	0.32156

642.21878	0.32178

642.35812	0.3218

642.49746	0.32183

642.6368	0.32186

642.77614	0.32191

642.91548	0.32199

643.05482	0.32205

643.19416	0.32221

643.3335	0.32224

643.47284	0.32229

643.61218	0.3224

643.75152	0.32248

643.89086	0.32256

644.0302	0.32259

644.16954	0.32262

644.30888	0.32291

644.44822	0.32294

644.58756	0.32321

644.7269	0.32324

644.86624	0.32329

645.00558	0.3234

645.14492	0.32367

645.28426	0.3237

645.4236	0.32375

645.56295	0.32381

645.70229	0.32389

645.84163	0.32392

645.98097	0.32397

646.12031	0.32405

646.25965	0.32408

646.39899	0.32411

646.53833	0.32424

646.67767	0.32427

646.81701	0.32435

646.95635	0.32446

647.09569	0.32449

647.23503	0.32451

647.37437	0.32454

647.51371	0.32457

647.65305	0.32467

647.79239	0.3247

647.93173	0.32486

648.07107	0.32522

648.21041	0.3253

648.34975	0.32549

648.48909	0.32551

648.62843	0.32554

648.76777	0.32568

648.90711	0.32584

649.04645	0.32589

649.18579	0.32592

649.32513	0.326

649.46447	0.32606

649.60381	0.32611

649.74315	0.32622

649.88249	0.32687

650.02183	0.32692

650.16117	0.32695

650.30051	0.327

650.43985	0.3273

650.57919	0.32738

650.71853	0.32755

650.85787	0.32765

650.99721	0.32768

651.13655	0.32771

651.27589	0.32774

651.41523	0.3279

651.55457	0.32793

651.69391	0.32798

651.83325	0.32809

651.97259	0.32811

652.11193	0.32817

652.25127	0.32828

652.39061	0.32841

652.52995	0.32844

652.66929	0.32852

652.80863	0.32855

652.94797	0.32914

653.08731	0.3292

653.22665	0.32928

653.36599	0.32939

653.50533	0.32944

653.64467	0.32958

653.78401	0.32963

653.92335	0.33001

654.06269	0.33004

654.20203	0.33015

654.34137	0.33017

654.48072	0.33028

654.62006	0.33036

654.7594	0.33042

654.89874	0.33044

655.03808	0.3305

655.17742	0.33061

655.31676	0.33082

655.4561	0.33096

655.59544	0.33101

655.73478	0.33104

655.87412	0.33107

656.01346	0.33109

656.1528	0.33112

656.29214	0.33115

656.43148	0.33131

656.57082	0.33139

656.71016	0.33142

656.8495	0.33153

656.98884	0.33155

657.12818	0.33164

657.26752	0.33169

657.40686	0.33172

657.5462	0.33177

657.68554	0.3321

657.82488	0.33218

657.96422	0.3322

658.10356	0.33237

658.2429	0.33239

658.38224	0.33248

658.52158	0.3325

658.66092	0.33264

658.80026	0.33267

658.9396	0.3328

659.07894	0.33283

659.21828	0.33285

659.35762	0.33291

659.49696	0.33302

659.6363	0.33304

659.77564	0.33315

659.91498	0.3334

660.05432	0.33342

660.19366	0.33367

660.333	0.33375

660.47234	0.33383

660.61168	0.33388

660.75102	0.33399

660.89036	0.33407

661.0297	0.33429

661.16904	0.3344

661.30838	0.33445

661.44772	0.33459

661.58706	0.33467

661.7264	0.3347

661.86574	0.33472

662.00508	0.33475

662.14442	0.33489

662.28376	0.33491

662.4231	0.33497

662.56244	0.33499

662.70178	0.33505

662.84112	0.33513

662.98046	0.33516

663.1198	0.33548

663.25914	0.33551

663.39849	0.33559

663.53783	0.33562

663.67717	0.33583

663.81651	0.33586

663.95585	0.33619

664.09519	0.33627

664.23453	0.33629

664.37387	0.33632

664.51321	0.33648

664.65255	0.33651

664.79189	0.33654

664.93123	0.33657

665.07057	0.33659

665.20991	0.33662

665.34925	0.3367

665.48859	0.33676

665.62793	0.33678

665.76727	0.33681

665.90661	0.33684

666.04595	0.33705

666.18529	0.33711

666.32463	0.33716

666.46397	0.33719

666.60331	0.33724

666.74265	0.33741

666.88199	0.33743

667.02133	0.33746

667.16067	0.33754

667.30001	0.33757

667.43935	0.33759

667.57869	0.33768

667.71803	0.3377

667.85737	0.33784

667.99671	0.33787

668.13605	0.33797

668.27539	0.33822

668.41473	0.33824

668.55407	0.33827

668.69341	0.33835

668.83275	0.33841

668.97209	0.33857

669.11143	0.33862

669.25077	0.33873

669.39011	0.33876

669.52945	0.33895

669.66879	0.33898

669.80813	0.339

669.94747	0.33952

670.08681	0.33954

670.22615	0.33957

670.36549	0.33984

670.50483	0.33987

670.64417	0.3399

670.78351	0.33992

670.92285	0.34003

671.06219	0.34014

671.20153	0.3402

671.34087	0.34022

671.48021	0.34025

671.61955	0.34036

671.75889	0.34041

671.89823	0.34049

672.03757	0.34085

672.17691	0.3409

672.31625	0.34093

672.4556	0.34095

672.59494	0.34103

672.73428	0.34114

672.87362	0.34131

673.01296	0.34133

673.1523	0.34144

673.29164	0.34147

673.43098	0.34152

673.57032	0.34158

673.70966	0.34166

673.849	0.34182

673.98834	0.3419

674.12768	0.34193

674.26702	0.34196

674.40636	0.34201

674.5457	0.34209

674.68504	0.3422

674.82438	0.34225

674.96372	0.34228

675.10306	0.34233

675.2424	0.34239

675.38174	0.34242

675.52108	0.34263

675.66042	0.34266

675.79976	0.34269

675.9391	0.34282

676.07844	0.34293

676.21778	0.34296

676.35712	0.34309

676.49646	0.34315

676.6358	0.3432

676.77514	0.34323

676.91448	0.34326

677.05382	0.34331

677.19316	0.34334

677.3325	0.34342

677.47184	0.34355

677.61118	0.34358

677.75052	0.34372

677.88986	0.34377

678.0292	0.34407

678.16854	0.34418

678.30788	0.3442

678.44722	0.34423

678.58656	0.34426

678.7259	0.34431

678.86524	0.34439

679.00458	0.34442

679.14392	0.34445

679.28326	0.3445

679.4226	0.34453

679.56194	0.34469

679.70128	0.3448

679.84062	0.34485

679.97996	0.34491

680.1193	0.34499

680.25864	0.34512

680.39798	0.34526

680.53732	0.34529

680.67666	0.34531

680.816	0.3454

680.95534	0.34548

681.09468	0.3455

681.23402	0.34567

681.37337	0.34572

681.51271	0.34621

681.65205	0.34624

681.79139	0.3464

681.93073	0.34642

682.07007	0.34645

682.20941	0.34651

682.34875	0.34653

682.48809	0.34656

682.62743	0.34664

682.76677	0.34675

682.90611	0.3468

683.04545	0.34686

683.18479	0.34691

683.32413	0.34697

683.46347	0.34699

683.60281	0.3471

683.74215	0.34716

683.88149	0.34718

684.02083	0.34743

684.16017	0.34751

684.29951	0.34756

684.43885	0.34759

684.57819	0.34762

684.71753	0.34764

684.85687	0.3477

684.99621	0.34778

685.13555	0.34786

685.27489	0.34794

685.41423	0.34797

685.55357	0.348

685.69291	0.34802

685.83225	0.34821

685.97159	0.34824

686.11093	0.34829

686.25027	0.3484

686.38961	0.34843

686.52895	0.34848

686.66829	0.34851

686.80763	0.34865

686.94697	0.34867

687.08631	0.34873

687.22565	0.34878

687.36499	0.34881

687.50433	0.34894

687.64367	0.349

687.78301	0.34908

687.92235	0.34916

688.06169	0.34919

688.20103	0.34921

688.34037	0.34924

688.47971	0.34927

688.61905	0.3493

688.75839	0.34932

688.89773	0.34943

689.03707	0.34949

689.17641	0.34954

689.31575	0.34962

689.45509	0.3497

689.59443	0.34978

689.73377	0.34981

689.87311	0.34984

690.01245	0.34992

690.15179	0.34995

690.29114	0.35005

690.43048	0.35014

690.56982	0.35016

690.70916	0.35022

690.8485	0.35041

690.98784	0.35051

691.12718	0.35062

691.26652	0.3507

691.40586	0.35073

691.5452	0.35076

691.68454	0.35081

691.82388	0.35087

691.96322	0.35089

692.10256	0.35092

692.2419	0.35103

692.38124	0.35106

692.52058	0.35108

692.65992	0.35133

692.79926	0.35141

692.9386	0.35144

693.07794	0.35149

693.21728	0.35157

693.35662	0.35163

693.49596	0.35179

693.6353	0.35184

693.77464	0.35187

693.91398	0.35192

694.05332	0.35217

694.19266	0.35222

694.332	0.3523

694.47134	0.35233

694.61068	0.35236

694.75002	0.3529

694.88936	0.35301

695.0287	0.35303

695.16804	0.35311

695.30738	0.35314

695.44672	0.35317

695.58606	0.3532

695.7254	0.35325

695.86474	0.35339

696.00408	0.35341

696.14342	0.35344

696.28276	0.3536

696.4221	0.35379

696.56144	0.35382

696.70078	0.35385

696.84012	0.35398

696.97946	0.35428

697.1188	0.35436

697.25814	0.35439

697.39748	0.35444

697.53682	0.35455

697.67616	0.35458

697.8155	0.3546

697.95484	0.35498

698.09418	0.35512

698.23352	0.35525

698.37286	0.35531

698.5122	0.35534

698.65154	0.35544

698.79088	0.35547

698.93022	0.3555

699.06957	0.35561

699.20891	0.35563

699.34825	0.35588

699.48759	0.35601

699.62693	0.35612

699.76627	0.35615

699.90561	0.35628

700.04495	0.35634

700.18429	0.35637

700.32363	0.35645

700.46297	0.3565

700.60231	0.35653

700.74165	0.35655

700.88099	0.35664

701.02033	0.35669

701.15967	0.35683

701.29901	0.35696

701.43835	0.35707

701.57769	0.3571

701.71703	0.35715

701.85637	0.35718

701.99571	0.35723

702.13505	0.35731

702.27439	0.35761

702.41373	0.35764

702.55307	0.3578

702.69241	0.35785

702.83175	0.35799

702.97109	0.35804

703.11043	0.3581

703.24977	0.35818

703.38911	0.35823

703.52845	0.35829

703.66779	0.35834

703.80713	0.35837

703.94647	0.3584

704.08581	0.35845

704.22515	0.3585

704.36449	0.35853

704.50383	0.35859

704.64317	0.35864

704.78251	0.35869

704.92185	0.35872

705.06119	0.35875

705.20053	0.3588

705.33987	0.35899

705.47921	0.3591

705.61855	0.35915

705.75789	0.35937

705.89723	0.3594

706.03657	0.35943

706.17591	0.35945

706.31525	0.35964

706.45459	0.35967

706.59393	0.3597

706.73327	0.35975

706.87261	0.3598

707.01195	0.35989

707.15129	0.35991

707.29063	0.35997

707.42997	0.35999

707.56931	0.3601

707.70865	0.36021

707.84799	0.36024

707.98734	0.36029

708.12668	0.36054

708.26602	0.36059

708.40536	0.3607

708.5447	0.36075

708.68404	0.36078

708.82338	0.36081

708.96272	0.36083

709.10206	0.36089

709.2414	0.36097

709.38074	0.361

709.52008	0.36108

709.65942	0.36116

709.79876	0.36124

709.9381	0.3614

710.07744	0.36157

710.21678	0.36178

710.35612	0.36181

710.49546	0.36184

710.6348	0.36186

710.77414	0.36189

710.91348	0.36192

711.05282	0.36194

711.19216	0.36203

711.3315	0.36213

711.47084	0.36216

711.61018	0.3623

711.74952	0.36251

711.88886	0.36257

712.0282	0.36262

712.16754	0.36268

712.30688	0.3627

712.44622	0.36273

712.58556	0.36276

712.7249	0.36292

712.86424	0.36297

713.00358	0.36308

713.14292	0.36311

713.28226	0.36335

713.4216	0.36338

713.56094	0.36354

713.70028	0.3636

713.83962	0.36365

713.97896	0.36379

714.1183	0.36417

714.25764	0.36419

714.39698	0.36422

714.53632	0.3643

714.67566	0.36433

714.815	0.36436

714.95434	0.36444

715.09368	0.36446

715.23302	0.36454

715.37236	0.36457

715.5117	0.3646

715.65104	0.36465

715.79038	0.36468

715.92972	0.36476

716.06906	0.3649

716.2084	0.36506

716.34774	0.36509

716.48708	0.36517

716.62642	0.3652

716.76576	0.36522

716.90511	0.36536

717.04445	0.36547

717.18379	0.36555

717.32313	0.3656

717.46247	0.36563

717.60181	0.36566

717.74115	0.36568

717.88049	0.3659

718.01983	0.36606

718.15917	0.36609

718.29851	0.3662

718.43785	0.36639

718.57719	0.36652

718.71653	0.36663

718.85587	0.36674

718.99521	0.36701

719.13455	0.36706

719.27389	0.36712

719.41323	0.3672

719.55257	0.36723

719.69191	0.36728

719.83125	0.36793

719.97059	0.36798

720.10993	0.3682

720.24927	0.36826

720.38861	0.36828

720.52795	0.36831

720.66729	0.36834

720.80663	0.36836

720.94597	0.36885

721.08531	0.36888

721.22465	0.36896

721.36399	0.36901

721.50333	0.3691

721.64267	0.36931

721.78201	0.36937

721.92135	0.36939

722.06069	0.36942

722.20003	0.36988

722.33937	0.37004

722.47871	0.37007

722.61805	0.37015

722.75739	0.37018

722.89673	0.37023

723.03607	0.37037

723.17541	0.3704

723.31475	0.37042

723.45409	0.37048

723.59343	0.3705

723.73277	0.37056

723.87211	0.37059

724.01145	0.37064

724.15079	0.37067

724.29013	0.37069

724.42947	0.3708

724.56881	0.37086

724.70815	0.37088

724.84749	0.37107

724.98683	0.37118

725.12617	0.37126

725.26551	0.3714

725.40485	0.37151

725.54419	0.37175

725.68353	0.37191

725.82288	0.37202

725.96222	0.37205

726.10156	0.37207

726.2409	0.37224

726.38024	0.37226

726.51958	0.37229

726.65892	0.37243

726.79826	0.37248

726.9376	0.37251

727.07694	0.37254

727.21628	0.37262

727.35562	0.37278

727.49496	0.37289

727.6343	0.37297

727.77364	0.3731

727.91298	0.37356

728.05232	0.3737

728.19166	0.37378

728.331	0.37392

728.47034	0.37394

728.60968	0.37408

728.74902	0.37413

728.88836	0.37416

729.0277	0.37421

729.16704	0.37438

729.30638	0.37449

729.44572	0.37462

729.58506	0.3747

729.7244	0.37481

729.86374	0.37511

730.00308	0.37524

730.14242	0.3753

730.28176	0.37541

730.4211	0.37549

730.56044	0.37565

730.69978	0.37576

730.83912	0.37579

730.97846	0.37592

731.1178	0.37595

731.25714	0.376

731.39648	0.37606

731.53582	0.37641

731.67516	0.37644

731.8145	0.37679

731.95384	0.37681

732.09318	0.37684

732.23252	0.37687

732.37186	0.37698

732.5112	0.377

732.65054	0.37706

732.78988	0.37709

732.92922	0.3773

733.06856	0.37744

733.2079	0.37749

733.34724	0.37774

733.48658	0.37782

733.62592	0.37784

733.76526	0.37787

733.9046	0.37795

734.04394	0.37806

734.18328	0.37809

734.32262	0.37811

734.46196	0.37814

734.6013	0.3782

734.74065	0.37822

734.87999	0.37828

735.01933	0.3783

735.15867	0.37836

735.29801	0.37844

735.43735	0.37849

735.57669	0.37871

735.71603	0.37876

735.85537	0.37879

735.99471	0.37909

736.13405	0.37914

736.27339	0.37917

736.41273	0.3792

736.55207	0.3795

736.69141	0.37952

736.83075	0.37955

736.97009	0.37958

737.10943	0.3796

737.24877	0.37966

737.38811	0.37971

737.52745	0.37974

737.66679	0.37977

737.80613	0.37979

737.94547	0.37988

738.08481	0.3802

738.22415	0.38025

738.36349	0.38028

738.50283	0.38034

738.64217	0.38039

738.78151	0.38044

738.92085	0.3805

739.06019	0.38055

739.19953	0.38058

739.33887	0.38077

739.47821	0.38085

739.61755	0.3809

739.75689	0.38093

739.89623	0.38104

740.03557	0.38112

740.17491	0.38118

740.31425	0.3812

740.45359	0.38123

740.59293	0.38126

740.73227	0.38128

740.87161	0.38134

741.01095	0.38145

741.15029	0.3815

741.28963	0.38153

741.42897	0.38161

741.56831	0.38164

741.70765	0.3818

741.84699	0.38188

741.98633	0.38191

742.12567	0.38193

742.26501	0.38218

742.40435	0.3822

742.54369	0.38226

742.68303	0.38231

742.82237	0.38234

742.96171	0.38242

743.10105	0.38256

743.24039	0.38261

743.37973	0.38264

743.51908	0.38267

743.65842	0.38269

743.79776	0.38272

743.9371	0.38277

744.07644	0.38313

744.21578	0.38315

744.35512	0.38321

744.49446	0.38326

744.6338	0.38332

744.77314	0.3834

744.91248	0.38342

745.05182	0.38361

745.19116	0.38364

745.3305	0.38372

745.46984	0.38375

745.60918	0.3838

745.74852	0.38402

745.88786	0.38407

746.0272	0.38413

746.16654	0.38448

746.30588	0.38451

746.44522	0.38456

746.58456	0.38459

746.7239	0.38472

746.86324	0.38478

747.00258	0.38489

747.14192	0.38505

747.28126	0.38513

747.4206	0.38516

747.55994	0.38521

747.69928	0.38532

747.83862	0.38535

747.97796	0.38543

748.1173	0.38554

748.25664	0.38556

748.39598	0.38559

748.53532	0.38578

748.67466	0.38586

748.814	0.38589

748.95334	0.38594

749.09268	0.38597

749.23202	0.386

749.37136	0.38602

749.5107	0.38605

749.65004	0.38608

749.78938	0.38611

749.92872	0.38619

750.06806	0.38624

750.2074	0.3864

750.34674	0.38643

750.48608	0.38654

750.62542	0.38662

750.76476	0.38667

750.9041	0.38676

751.04344	0.38684

751.18278	0.387

751.32212	0.38705

751.46146	0.38711

751.6008	0.38724

751.74014	0.38732

751.87948	0.38743

752.01882	0.38746

752.15816	0.38751

752.2975	0.38754

752.43684	0.38759

752.57619	0.38778

752.71553	0.38784

752.85487	0.38795

752.99421	0.38803

753.13355	0.38808

753.27289	0.38811

753.41223	0.38816

753.55157	0.38833

753.69091	0.38838

753.83025	0.38841

753.96959	0.38854

754.10893	0.38857

754.24827	0.3886

754.38761	0.38862

754.52695	0.38868

754.66629	0.38871

754.80563	0.38873

754.94497	0.38887

755.08431	0.38892

755.22365	0.38906

755.36299	0.3893

755.50233	0.38936

755.64167	0.38938

755.78101	0.38968

755.92035	0.38976

756.05969	0.38979

756.19903	0.38987

756.33837	0.3899

756.47771	0.38995

756.61705	0.38998

756.75639	0.39001

756.89573	0.39014

757.03507	0.39025

757.17441	0.3903

757.31375	0.39033

757.45309	0.39047

757.59243	0.39055

757.73177	0.3906

757.87111	0.39068

758.01045	0.39085

758.14979	0.39087

758.28913	0.39093

758.42847	0.39103

758.56781	0.3912

758.70715	0.39122

758.84649	0.39136

758.98583	0.39168

759.12517	0.39171

759.26451	0.39174

759.40385	0.39179

759.54319	0.39182

759.68253	0.39185

759.82187	0.39196

759.96121	0.39198

760.10055	0.39209

760.23989	0.39212

760.37923	0.39217

760.51857	0.39233

760.65791	0.39236

760.79725	0.39247

760.93659	0.39261

761.07593	0.39263

761.21527	0.39274

761.35461	0.39277

761.49396	0.39296

761.6333	0.39309

761.77264	0.39317

761.91198	0.39331

762.05132	0.39334

762.19066	0.39366

762.33	0.39396

762.46934	0.39401

762.60868	0.3941

762.74802	0.39415

762.88736	0.39426

763.0267	0.39431

763.16604	0.39434

763.30538	0.39439

763.44472	0.3945

763.58406	0.39458

763.7234	0.39466

763.86274	0.39469

764.00208	0.39472

764.14142	0.3948

764.28076	0.39496

764.4201	0.39502

764.55944	0.39507

764.69878	0.39529

764.83812	0.39531

764.97746	0.39545

765.1168	0.39548

765.25614	0.39564

765.39548	0.39569

765.53482	0.39583

765.67416	0.39588

765.8135	0.39596

765.95284	0.39613

766.09218	0.39632

766.23152	0.3967

766.37086	0.39672

766.5102	0.3968

766.64954	0.39683

766.78888	0.39699

766.92822	0.39702

767.06756	0.39705

767.2069	0.3971

767.34624	0.39716

767.48558	0.39724

767.62492	0.39729

767.76426	0.39748

767.9036	0.39764

768.04294	0.39767

768.18228	0.3977

768.32162	0.39797

768.46096	0.398

768.6003	0.39802

768.73964	0.39805

768.87898	0.39824

769.01832	0.39827

769.15766	0.39835

769.297	0.39843

769.43634	0.39846

769.57568	0.39848

769.71502	0.39851

769.85436	0.39854

769.9937	0.39856

770.13304	0.39859

770.27238	0.39862

770.41173	0.39865

770.55107	0.3987

770.69041	0.39873

770.82975	0.39886

770.96909	0.39897

771.10843	0.39902

771.24777	0.39905

771.38711	0.39911

771.52645	0.39913

771.66579	0.39954

771.80513	0.39973

771.94447	0.39986

772.08381	0.39995

772.22315	0.40003

772.36249	0.40016

772.50183	0.40024

772.64117	0.40033

772.78051	0.40041

772.91985	0.40046

773.05919	0.40051

773.19853	0.40054

773.33787	0.4007

773.47721	0.40073

773.61655	0.40076

773.75589	0.40079

773.89523	0.40084

774.03457	0.40087

774.17391	0.40092

774.31325	0.40103

774.45259	0.40106

774.59193	0.40111

774.73127	0.40127

774.87061	0.40133

775.00995	0.40144

775.14929	0.40146

775.28863	0.40176

775.42797	0.402

775.56731	0.40206

775.70665	0.40209

775.84599	0.40211

775.98533	0.40214

776.12467	0.40225

776.26401	0.40228

776.40335	0.4026

776.54269	0.40268

776.68203	0.40282

776.82137	0.40293

776.96071	0.40295

777.10005	0.40301

777.23939	0.40303

777.37873	0.40325

777.51807	0.40355

777.65741	0.40379

777.79675	0.40382

777.93609	0.40395

778.07543	0.40404

778.21477	0.40412

778.35411	0.40414

778.49345	0.40423

778.63279	0.40431

778.77213	0.4045

778.91147	0.4046

779.05081	0.40463

779.19016	0.40466

779.3295	0.40488

779.46884	0.40504

779.60818	0.40515

779.74752	0.40523

779.88686	0.40525

780.0262	0.40539

780.16554	0.40542

780.30488	0.4055

780.44422	0.40555

780.58356	0.40561

780.7229	0.40572

780.86224	0.40574

781.00158	0.4058

781.14092	0.4059

781.28026	0.40612

781.4196	0.4062

781.55894	0.40628

781.69828	0.4068

781.83762	0.40688

781.97696	0.40691

782.1163	0.40699

782.25564	0.40702

782.39498	0.40712

782.53432	0.40715

782.67366	0.4072

782.813	0.40729

782.95234	0.40731

783.09168	0.40734

783.23102	0.40742

783.37036	0.40748

783.5097	0.40753

783.64904	0.40756

783.78838	0.40764

783.92772	0.40769

784.06706	0.40823

784.2064	0.40832

784.34574	0.40834

784.48508	0.40848

784.62442	0.40869

784.76376	0.40875

784.9031	0.40883

785.04244	0.40899

785.18178	0.40902

785.32112	0.40929

785.46046	0.40932

785.5998	0.40943

785.73914	0.40975

785.87848	0.40978

786.01782	0.4098

786.15716	0.4101

786.2965	0.41024

786.43584	0.41027

786.57518	0.41032

786.71452	0.41035

786.85386	0.41046

786.9932	0.41048

787.13254	0.4107

787.27188	0.41073

787.41122	0.41108

787.55056	0.41113

787.6899	0.41124

787.82924	0.41132

787.96858	0.41135

788.10793	0.41143

788.24727	0.41154

788.38661	0.41167

788.52595	0.41176

788.66529	0.41181

788.80463	0.41208

788.94397	0.41213

789.08331	0.41227

789.22265	0.4123

789.36199	0.41235

789.50133	0.41243

789.64067	0.41249

789.78001	0.41259

789.91935	0.41268

790.05869	0.41273

790.19803	0.41278

790.33737	0.41281

790.47671	0.41289

790.61605	0.41292

790.75539	0.41303

790.89473	0.41308

791.03407	0.41311

791.17341	0.41324

791.31275	0.4133

791.45209	0.41333

791.59143	0.41343

791.73077	0.41352

791.87011	0.41357

792.00945	0.41362

792.14879	0.41365

792.28813	0.41368

792.42747	0.41373

792.56681	0.41376

792.70615	0.41381

792.84549	0.41392

792.98483	0.41395

793.12417	0.41403

793.26351	0.41406

793.40285	0.41417

793.54219	0.41425

793.68153	0.41433

793.82087	0.41438

793.96021	0.41449

794.09955	0.41452

794.23889	0.41457

794.37823	0.4146

794.51757	0.41468

794.65691	0.41471

794.79625	0.41482

794.93559	0.41487

795.07493	0.41525

795.21427	0.41533

795.35361	0.41538

795.49295	0.41544

795.63229	0.41552

795.77163	0.41557

795.91097	0.41563

796.05031	0.41566

796.18965	0.41579

796.32899	0.41585

796.46833	0.41587

796.60767	0.4159

796.74701	0.41593

796.88635	0.41598

797.0257	0.41601

797.16504	0.41603

797.30438	0.41609

797.44372	0.41617

797.58306	0.41625

797.7224	0.41628

797.86174	0.41639

798.00108	0.41641

798.14042	0.41644

798.27976	0.4165

798.4191	0.41652

798.55844	0.41655

798.69778	0.4166

798.83712	0.41687

798.97646	0.41701

799.1158	0.41706

799.25514	0.41712

799.39448	0.41717

799.53382	0.41731

799.67316	0.41733

799.8125	0.41736

799.95184	0.41742

800.09118	0.41752

800.23052	0.41755

800.36986	0.41761

800.5092	0.41777

800.64854	0.41782

800.78788	0.41788

800.92722	0.4179

801.06656	0.41793

801.2059	0.41796

801.34524	0.41801

801.48458	0.41807

801.62392	0.4182

801.76326	0.41839

801.9026	0.41842

802.04194	0.41858

802.18128	0.41866

802.32062	0.41888

802.45996	0.41893

802.5993	0.41923

802.73864	0.41945

802.87798	0.41947

803.01732	0.4195

803.15666	0.41953

803.296	0.41958

803.43534	0.41966

803.57468	0.41969

803.71402	0.41975

803.85336	0.4198

803.9927	0.41983

804.13204	0.41991

804.27138	0.41999

804.41072	0.42004

804.55006	0.42007

804.6894	0.42012

804.82874	0.42015

804.96808	0.4204

805.10742	0.42059

805.24676	0.42064

805.3861	0.42067

805.52544	0.42091

805.66478	0.42105

805.80412	0.42107

805.94347	0.4211

806.08281	0.42156

806.22215	0.42161

806.36149	0.42164

806.50083	0.4217

806.64017	0.42172

806.77951	0.42186

806.91885	0.42191

807.05819	0.42207

807.19753	0.42213

807.33687	0.42216

807.47621	0.42224

807.61555	0.42229

807.75489	0.42232

807.89423	0.42235

808.03357	0.42237

808.17291	0.42248

808.31225	0.42254

808.45159	0.42256

808.59093	0.42262

808.73027	0.42264

808.86961	0.42267

809.00895	0.42272

809.14829	0.42275

809.28763	0.42286

809.42697	0.42289

809.56631	0.42291

809.70565	0.42294

809.84499	0.42305

809.98433	0.42313

810.12367	0.42319

810.26301	0.42332

810.40235	0.42335

810.54169	0.42343

810.68103	0.42356

810.82037	0.42362

810.95971	0.42367

811.09905	0.42378

811.23839	0.42381

811.37773	0.42397

811.51707	0.42408

811.65641	0.42427

811.79575	0.42446

811.93509	0.42451

812.07443	0.42454

812.21377	0.42459

812.35311	0.42465

812.49245	0.42467

812.63179	0.42476

812.77113	0.42481

812.91047	0.42489

813.04981	0.42492

813.18915	0.42516

813.32849	0.42522

813.46783	0.42527

813.60717	0.42546

813.74651	0.42573

813.88585	0.42579

814.02519	0.42584

814.16453	0.42592

814.30387	0.42598

814.44321	0.426

814.58255	0.42606

814.72189	0.42611

814.86124	0.42619

815.00058	0.42635

815.13992	0.42644

815.27926	0.42649

815.4186	0.42657

815.55794	0.42679

815.69728	0.42684

815.83662	0.42692

815.97596	0.42698

816.1153	0.427

816.25464	0.42703

816.39398	0.42719

816.53332	0.42722

816.67266	0.42741

816.812	0.42744

816.95134	0.42746

817.09068	0.42749

817.23002	0.42755

817.36936	0.42757

817.5087	0.42765

817.64804	0.42776

817.78738	0.42787

817.92672	0.4279

818.06606	0.42793

818.2054	0.42795

818.34474	0.42822

818.48408	0.42825

818.62342	0.42828

818.76276	0.4283

818.9021	0.42858

819.04144	0.42868

819.18078	0.42874

819.32012	0.42882

819.45946	0.42885

819.5988	0.4289

819.73814	0.42893

819.87748	0.42895

820.01682	0.42898

820.15616	0.42901

820.2955	0.42904

820.43484	0.42909

820.57418	0.42912

820.71352	0.42941

820.85286	0.42944

820.9922	0.42947

821.13154	0.4295

821.27088	0.42952

821.41022	0.42969

821.54956	0.42971

821.6889	0.42974

821.82824	0.42977

821.96758	0.42985

822.10692	0.42988

822.24626	0.43015

822.3856	0.43025

822.52494	0.43028

822.66428	0.43034

822.80362	0.43061

822.94296	0.43063

823.0823	0.43069

823.22164	0.4308

823.36098	0.4309

823.50032	0.43107

823.63966	0.43115

823.77901	0.43118

823.91835	0.4312

824.05769	0.43128

824.19703	0.43131

824.33637	0.43134

824.47571	0.43142

824.61505	0.4315

824.75439	0.43153

824.89373	0.43161

825.03307	0.43166

825.17241	0.43169

825.31175	0.43174

825.45109	0.43177

825.59043	0.4318

825.72977	0.43183

825.86911	0.43202

826.00845	0.43204

826.14779	0.4321

826.28713	0.43248

826.42647	0.4325

826.56581	0.43256

826.70515	0.43258

826.84449	0.43264

826.98383	0.43296

827.12317	0.43299

827.26251	0.43302

827.40185	0.43315

827.54119	0.43321

827.68053	0.43323

827.81987	0.43329

827.95921	0.4335

828.09855	0.43353

828.23789	0.43356

828.37723	0.43375

828.51657	0.4338

828.65591	0.43386

828.79525	0.43397

828.93459	0.43399

829.07393	0.43402

829.21327	0.43418

829.35261	0.43421

829.49195	0.43424

829.63129	0.43426

829.77063	0.43429

829.90997	0.43437

830.04931	0.43462

830.18865	0.43472

830.32799	0.43475

830.46733	0.43478

830.60667	0.43513

830.74601	0.43516

830.88535	0.43521

831.02469	0.43527

831.16403	0.43529

831.30337	0.43548

831.44271	0.43578

831.58205	0.43583

831.72139	0.43605

831.86073	0.43611

832.00007	0.43619

832.13941	0.43635

832.27875	0.43643

832.41809	0.43646

832.55743	0.43657

832.69678	0.43662

832.83612	0.43665

832.97546	0.4367

833.1148	0.43673

833.25414	0.43684

833.39348	0.43692

833.53282	0.437

833.67216	0.43705

833.8115	0.43724

833.95084	0.43727

834.09018	0.43732

834.22952	0.43741

834.36886	0.43781

834.5082	0.43784

834.64754	0.43787

834.78688	0.43803

834.92622	0.43806

835.06556	0.43811

835.2049	0.43814

835.34424	0.43816

835.48358	0.43824

835.62292	0.43827

835.76226	0.43833

835.9016	0.43838

836.04094	0.43846

836.18028	0.43865

836.31962	0.43868

836.45896	0.43871

836.5983	0.43884

836.73764	0.43887

836.87698	0.43895

837.01632	0.43903

837.15566	0.43914

837.295	0.43917

837.43434	0.43919

837.57368	0.4393

837.71302	0.43933

837.85236	0.43936

837.9917	0.43941

838.13104	0.43946

838.27038	0.43952

838.40972	0.43954

838.54906	0.43963

838.6884	0.43965

838.82774	0.43973

838.96708	0.43976

839.10642	0.43984

839.24576	0.43995

839.3851	0.44017

839.52444	0.44022

839.66378	0.44028

839.80312	0.4403

839.94246	0.44044

840.0818	0.44047

840.22114	0.44049

840.36048	0.44052

840.49982	0.44055

840.63916	0.44063

840.7785	0.44066

840.91784	0.44068

841.05718	0.44079

841.19652	0.44085

841.33586	0.44095

841.4752	0.44101

841.61455	0.44106

841.75389	0.44141

841.89323	0.4415

842.03257	0.44152

842.17191	0.44155

842.31125	0.44198

842.45059	0.44206

842.58993	0.44215

842.72927	0.44223

842.86861	0.44225

843.00795	0.44244

843.14729	0.4425

843.28663	0.44255

843.42597	0.44258

843.56531	0.44298

843.70465	0.44301

843.84399	0.44304

843.98333	0.44312

844.12267	0.44315

844.26201	0.44317

844.40135	0.4432

844.54069	0.44326

844.68003	0.44331

844.81937	0.44361

844.95871	0.44366

845.09805	0.44374

845.23739	0.44388

845.37673	0.44393

845.51607	0.44399

845.65541	0.44401

845.79475	0.4441

845.93409	0.44431

846.07343	0.44437

846.21277	0.44439

846.35211	0.44442

846.49145	0.4445

846.63079	0.44456

846.77013	0.44458

846.90947	0.44461

847.04881	0.44469

847.18815	0.44483

847.32749	0.44485

847.46683	0.44488

847.60617	0.44491

847.74551	0.44499

847.88485	0.44507

848.02419	0.44512

848.16353	0.44521

848.30287	0.44523

848.44221	0.44529

848.58155	0.44534

848.72089	0.44564

848.86023	0.44567

848.99957	0.44569

849.13891	0.44596

849.27825	0.44605

849.41759	0.44613

849.55693	0.44618

849.69627	0.44632

849.83561	0.44637

849.97495	0.44642

850.11429	0.44648

850.25363	0.44653

850.39297	0.44659

850.53232	0.44661

850.67166	0.44664

850.811	0.4468

850.95034	0.44683

851.08968	0.44689

851.22902	0.44705

851.36836	0.44707

851.5077	0.44724

851.64704	0.44729

851.78638	0.44732

851.92572	0.44737

852.06506	0.44764

852.2044	0.44772

852.34374	0.44775

852.48308	0.44778

852.62242	0.44783

852.76176	0.44794

852.9011	0.44824

853.04044	0.4484

853.17978	0.44848

853.31912	0.44894

853.45846	0.44897

853.5978	0.449

853.73714	0.44911

853.87648	0.44916

854.01582	0.44919

854.15516	0.4494

854.2945	0.44946

854.43384	0.44951

854.57318	0.44957

854.71252	0.44962

854.85186	0.44986

854.9912	0.44989

855.13054	0.44995

855.26988	0.45003

855.40922	0.45014

855.54856	0.45016

855.6879	0.45041

855.82724	0.45046

855.96658	0.45076

856.10592	0.45084

856.24526	0.45087

856.3846	0.45089

856.52394	0.45092

856.66328	0.45098

856.80262	0.45106

856.94196	0.4513

857.0813	0.45138

857.22064	0.45141

857.35998	0.45146

857.49932	0.45168

857.63866	0.45184

857.778	0.45195

857.91734	0.45198

858.05668	0.45211

858.19602	0.45236

858.33536	0.45241

858.4747	0.45244

858.61404	0.45246

858.75338	0.45265

858.89272	0.45279

859.03206	0.45295

859.1714	0.45298

859.31075	0.45306

859.45009	0.45309

859.58943	0.45311

859.72877	0.45339

859.86811	0.45366

860.00745	0.45368

860.14679	0.45371

860.28613	0.45376

860.42547	0.45379

860.56481	0.45385

860.70415	0.45409

860.84349	0.45414

860.98283	0.45425

861.12217	0.4546

861.26151	0.45463

861.40085	0.45466

861.54019	0.45469

861.67953	0.45488

861.81887	0.45501

861.95821	0.45517

862.09755	0.45523

862.23689	0.45547

862.37623	0.45555

862.51557	0.45566

862.65491	0.45569

862.79425	0.45572

862.93359	0.45593

863.07293	0.45601

863.21227	0.45609

863.35161	0.45615

863.49095	0.45623

863.63029	0.45626

863.76963	0.45628

863.90897	0.45631

864.04831	0.45637

864.18765	0.45639

864.32699	0.4565

864.46633	0.45655

864.60567	0.45661

864.74501	0.45666

864.88435	0.45672

865.02369	0.45677

865.16303	0.4568

865.30237	0.45683

865.44171	0.45693

865.58105	0.45712

865.72039	0.45745

865.85973	0.45756

865.99907	0.45772

866.13841	0.4578

866.27775	0.45783

866.41709	0.4581

866.55643	0.45813

866.69577	0.45834

866.83511	0.45842

866.97445	0.45845

867.11379	0.45853

867.25313	0.45856

867.39247	0.45859

867.53181	0.45861

867.67115	0.45867

867.81049	0.45869

867.94983	0.45872

868.08917	0.45878

868.22852	0.45888

868.36786	0.45891

868.5072	0.45894

868.64654	0.45897

868.78588	0.45899

868.92522	0.45905

869.06456	0.45907

869.2039	0.45913

869.34324	0.45915

869.48258	0.45918

869.62192	0.45921

869.76126	0.45924

869.9006	0.45934

870.03994	0.45937

870.17928	0.45962

870.31862	0.45964

870.45796	0.45967

870.5973	0.45975

870.73664	0.45978

870.87598	0.45983

871.01532	0.46005

871.15466	0.46008

871.294	0.4601

871.43334	0.46018

871.57268	0.46029

871.71202	0.46046

871.85136	0.46054

871.9907	0.46089

872.13004	0.461

872.26938	0.46108

872.40872	0.46111

872.54806	0.46113

872.6874	0.46116

872.82674	0.46119

872.96608	0.46132

873.10542	0.46135

873.24476	0.46138

873.3841	0.46146

873.52344	0.46148

873.66278	0.46154

873.80212	0.46189

873.94146	0.46192

874.0808	0.46194

874.22014	0.46197

874.35948	0.462

874.49882	0.46203

874.63816	0.46205

874.7775	0.46224

874.91684	0.46227

875.05618	0.46232

875.19552	0.46235

875.33486	0.46238

875.4742	0.46265

875.61354	0.46287

875.75288	0.46292

875.89222	0.46295

876.03156	0.46297

876.1709	0.4633

876.31024	0.46338

876.44958	0.46368

876.58892	0.46371

876.72826	0.46373

876.8676	0.46379

877.00694	0.46392

877.14629	0.46395

877.28563	0.464

877.42497	0.46403

877.56431	0.46406

877.70365	0.46408

877.84299	0.46414

877.98233	0.46425

878.12167	0.46427

878.26101	0.4643

878.40035	0.46449

878.53969	0.46452

878.67903	0.46463

878.81837	0.46473

878.95771	0.46476

879.09705	0.46482

879.23639	0.46484

879.37573	0.46501

879.51507	0.46511

879.65441	0.46514

879.79375	0.46528

879.93309	0.4653

880.07243	0.46544

880.21177	0.46552

880.35111	0.46557

880.49045	0.4656

880.62979	0.46571

880.76913	0.46574

880.90847	0.46576

881.04781	0.46582

881.18715	0.46601

881.32649	0.4662

881.46583	0.46636

881.60517	0.46674

881.74451	0.46677

881.88385	0.46679

882.02319	0.46682

882.16253	0.46685

882.30187	0.46698

882.44121	0.46712

882.58055	0.46715

882.71989	0.46717

882.85923	0.46725

882.99857	0.46742

883.13791	0.46763

883.27725	0.46771

883.41659	0.46782

883.55593	0.4679

883.69527	0.46793

883.83461	0.46796

883.97395	0.46801

884.11329	0.46807

884.25263	0.46826

884.39197	0.46839

884.53131	0.46842

884.67065	0.4685

884.80999	0.46861

884.94933	0.46866

885.08867	0.46872

885.22801	0.4688

885.36735	0.46888

885.50669	0.46893

885.64603	0.46899

885.78537	0.46901

885.92471	0.46907

886.06406	0.46912

886.2034	0.46926

886.34274	0.46934

886.48208	0.46937

886.62142	0.46945

886.76076	0.46953

886.9001	0.46961

887.03944	0.46964

887.17878	0.46966

887.31812	0.46985

887.45746	0.46993

887.5968	0.46996

887.73614	0.47007

887.87548	0.4701

888.01482	0.47015

888.15416	0.47018

888.2935	0.47031

888.43284	0.47048

888.57218	0.4705

888.71152	0.47053

888.85086	0.47059

888.9902	0.47061

889.12954	0.47064

889.26888	0.47067

889.40822	0.47069

889.54756	0.47072

889.6869	0.47075

889.82624	0.47077

889.96558	0.4708

890.10492	0.47083

890.24426	0.47088

890.3836	0.47096

890.52294	0.47107

890.66228	0.47113

890.80162	0.47115

890.94096	0.47129

891.0803	0.47132

891.21964	0.47134

891.35898	0.47137

891.49832	0.47142

891.63766	0.47145

891.777	0.47164

891.91634	0.47178

892.05568	0.4718

892.19502	0.47207

892.33436	0.4721

892.4737	0.47232

892.61304	0.47245

892.75238	0.47251

892.89172	0.47275

893.03106	0.47281

893.1704	0.47291

893.30974	0.47308

893.44908	0.47313

893.58842	0.47327

893.72776	0.47343

893.8671	0.47359

894.00644	0.47362

894.14578	0.47378

894.28512	0.47394

894.42446	0.47408

894.5638	0.47411

894.70314	0.47416

894.84248	0.47419

894.98182	0.47438

895.12117	0.4744

895.26051	0.47446

895.39985	0.47449

895.53919	0.47451

895.67853	0.4747

895.81787	0.47476

895.95721	0.47478

896.09655	0.47481

896.23589	0.47489

896.37523	0.47497

896.51457	0.47505

896.65391	0.47511

896.79325	0.47514

896.93259	0.47516

897.07193	0.47519

897.21127	0.4753

897.35061	0.47535

897.48995	0.47538

897.62929	0.47546

897.76863	0.4756

897.90797	0.47568

898.04731	0.47573

898.18665	0.47581

898.32599	0.476

898.46533	0.47603

898.60467	0.47619

898.74401	0.47622

898.88335	0.47627

899.02269	0.47638

899.16203	0.47641

899.30137	0.47649

899.44071	0.47652

899.58005	0.47657

899.71939	0.47663

899.85873	0.47665

899.99807	0.47676

900.13741	0.47728

900.27675	0.47741

900.41609	0.47744

900.55543	0.47755

900.69477	0.4776

900.83411	0.47768

900.97345	0.47776

901.11279	0.47782

901.25213	0.47793

901.39147	0.47795

901.53081	0.47801

901.67015	0.4782

901.80949	0.4783

901.94883	0.47836

902.08817	0.47841

902.22751	0.47844

902.36685	0.47855

902.50619	0.47866

902.64553	0.4789

902.78487	0.47906

902.92421	0.47912

903.06355	0.47923

903.20289	0.47928

903.34223	0.47933

903.48157	0.47939

903.62091	0.4795

903.76025	0.47963

903.8996	0.47966

904.03894	0.47982

904.17828	0.47988

904.31762	0.47993

904.45696	0.47998

904.5963	0.48004

904.73564	0.48007

904.87498	0.48012

905.01432	0.48015

905.15366	0.48028

905.293	0.48036

905.43234	0.48039

905.57168	0.48042

905.71102	0.48047

905.85036	0.48058

905.9897	0.48061

906.12904	0.48066

906.26838	0.48074

906.40772	0.48082

906.54706	0.48093

906.6864	0.48096

906.82574	0.48099

906.96508	0.48123

907.10442	0.48137

907.24376	0.48142

907.3831	0.48147

907.52244	0.4815

907.66178	0.48153

907.80112	0.48169

907.94046	0.48183

908.0798	0.48185

908.21914	0.48199

908.35848	0.4821

908.49782	0.48212

908.63716	0.48215

908.7765	0.4822

908.91584	0.48226

909.05518	0.48231

909.19452	0.48234

909.33386	0.48242

909.4732	0.48248

909.61254	0.4825

909.75188	0.48253

909.89122	0.48261

910.03056	0.48264

910.1699	0.48267

910.30924	0.48269

910.44858	0.48277

910.58792	0.48299

910.72726	0.48302

910.8666	0.48304

911.00594	0.48307

911.14528	0.4831

911.28462	0.48313

911.42396	0.48315

911.5633	0.48318

911.70264	0.48329

911.84198	0.48337

911.98132	0.4834

912.12066	0.48342

912.26	0.48353

912.39934	0.4838

912.53868	0.48383

912.67802	0.48388

912.81737	0.48421

912.95671	0.48424

913.09605	0.48437

913.23539	0.4844

913.37473	0.48448

913.51407	0.48459

913.65341	0.48462

913.79275	0.4847

913.93209	0.48478

914.07143	0.48483

914.21077	0.48491

914.35011	0.48494

914.48945	0.48499

914.62879	0.48505

914.76813	0.48513

914.90747	0.48524

915.04681	0.48556

915.18615	0.48559

915.32549	0.48567

915.46483	0.48578

915.60417	0.48611

915.74351	0.48616

915.88285	0.48619

916.02219	0.48627

916.16153	0.48629

916.30087	0.48632

916.44021	0.48635

916.57955	0.48638

916.71889	0.4864

916.85823	0.48643

916.99757	0.48654

917.13691	0.48662

917.27625	0.48667

917.41559	0.4867

917.55493	0.48686

917.69427	0.48697

917.83361	0.48705

917.97295	0.48711

918.11229	0.48719

918.25163	0.48741

918.39097	0.48749

918.53031	0.48768

918.66965	0.4877

918.80899	0.48781

918.94833	0.48787

919.08767	0.48838

919.22701	0.48841

919.36635	0.48846

919.50569	0.48862

919.64503	0.48887

919.78437	0.48895

919.92371	0.48898

920.06305	0.48906

920.20239	0.48908

920.34173	0.48911

920.48107	0.48914

920.62041	0.48927

920.75975	0.48941

920.89909	0.48944

921.03843	0.48946

921.17777	0.48963

921.31711	0.48992

921.45645	0.49011

921.59579	0.4902

921.73514	0.49025

921.87448	0.49036

922.01382	0.49038

922.15316	0.49044

922.2925	0.49055

922.43184	0.49057

922.57118	0.49063

922.71052	0.49071

922.84986	0.49076

922.9892	0.49079

923.12854	0.49085

923.26788	0.4909

923.40722	0.49101

923.54656	0.49103

923.6859	0.49106

923.82524	0.49112

923.96458	0.49117

924.10392	0.4912

924.24326	0.49144

924.3826	0.49155

924.52194	0.49163

924.66128	0.49166

924.80062	0.49187

924.93996	0.4919

925.0793	0.49196

925.21864	0.49201

925.35798	0.49215

925.49732	0.49217

925.63666	0.49228

925.776	0.49239

925.91534	0.49258

926.05468	0.49309

926.19402	0.49312

926.33336	0.49315

926.4727	0.49347

926.61204	0.4935

926.75138	0.49353

926.89072	0.49355

927.03006	0.49358

927.1694	0.49361

927.30874	0.49363

927.44808	0.49372

927.58742	0.49377

927.72676	0.49382

927.8661	0.49385

928.00544	0.49388

928.14478	0.49393

928.28412	0.49396

928.42346	0.49399

928.5628	0.4941

928.70214	0.49415

928.84148	0.4942

928.98082	0.49426

929.12016	0.49428

929.2595	0.49431

929.39884	0.49434

929.53818	0.49445

929.67752	0.4945

929.81686	0.49453

929.9562	0.49458

930.09554	0.49466

930.23488	0.49472

930.37422	0.49475

930.51356	0.49483

930.65291	0.49488

930.79225	0.49493

930.93159	0.49512

931.07093	0.49518

931.21027	0.49529

931.34961	0.49545

931.48895	0.49556

931.62829	0.49559

931.76763	0.49572

931.90697	0.49586

932.04631	0.49591

932.18565	0.49599

932.32499	0.49602

932.46433	0.49605

932.60367	0.49624

932.74301	0.49629

932.88235	0.49634

933.02169	0.49642

933.16103	0.49651

933.30037	0.49675

933.43971	0.49678

933.57905	0.49686

933.71839	0.49689

933.85773	0.49694

933.99707	0.49707

934.13641	0.4971

934.27575	0.49713

934.41509	0.49729

934.55443	0.49743

934.69377	0.49762

934.83311	0.49789

934.97245	0.49791

935.11179	0.49797

935.25113	0.49805

935.39047	0.4981

935.52981	0.49813

935.66915	0.49819

935.80849	0.49827

935.94783	0.49837

936.08717	0.49924

936.22651	0.4993

936.36585	0.49932

936.50519	0.49938

936.64453	0.49943

936.78387	0.49973

936.92321	0.49978

937.06255	0.49989

937.20189	0.50005

937.34123	0.50008

937.48057	0.50011

937.61991	0.50016

937.75925	0.50019

937.89859	0.50022

938.03793	0.50038

938.17727	0.50043

938.31661	0.50049

938.45595	0.50051

938.59529	0.50079

938.73463	0.50081

938.87397	0.50133

939.01331	0.50138

939.15265	0.50149

939.29199	0.50152

939.43133	0.50168

939.57068	0.50173

939.71002	0.50184

939.84936	0.50209

939.9887	0.50211

940.12804	0.50217

940.26738	0.50219

940.40672	0.50225

940.54606	0.50228

940.6854	0.50238

940.82474	0.50241

940.96408	0.50246

941.10342	0.50252

941.24276	0.50257

941.3821	0.5026

941.52144	0.50276

941.66078	0.50295

941.80012	0.50298

941.93946	0.50306

942.0788	0.50311

942.21814	0.50314

942.35748	0.50339

942.49682	0.50349

942.63616	0.50376

942.7755	0.50387

942.91484	0.5039

943.05418	0.50393

943.19352	0.50395

943.33286	0.50401

943.4722	0.50417

943.61154	0.5042

943.75088	0.50428

943.89022	0.50441

944.02956	0.50447

944.1689	0.50458

944.30824	0.50463

944.44758	0.50466

944.58692	0.50469

944.72626	0.50477

944.8656	0.50485

945.00494	0.50488

945.14428	0.50496

945.28362	0.50504

945.42296	0.50507

945.5623	0.50512

945.70164	0.50525

945.84098	0.50555

945.98032	0.50558

946.11966	0.50563

946.259	0.50572

946.39834	0.50574

946.53768	0.50577

946.67702	0.50582

946.81636	0.50607

946.9557	0.50609

947.09504	0.50618

947.23438	0.5062

947.37372	0.50626

947.51306	0.50631

947.6524	0.50645

947.79174	0.5065

947.93108	0.50653

948.07042	0.50655

948.20976	0.50658

948.34911	0.50666

948.48845	0.50669

948.62779	0.50672

948.76713	0.50677

948.90647	0.5068

949.04581	0.50683

949.18515	0.50685

949.32449	0.50702

949.46383	0.50704

949.60317	0.50707

949.74251	0.50715

949.88185	0.50718

950.02119	0.50726

950.16053	0.50753

950.29987	0.50767

950.43921	0.5078

950.57855	0.50794

950.71789	0.50807

950.85723	0.50813

950.99657	0.50832

951.13591	0.50837

951.27525	0.50842

951.41459	0.50853

951.55393	0.50867

951.69327	0.50872

951.83261	0.50878

951.97195	0.50886

952.11129	0.50894

952.25063	0.50897

952.38997	0.50899

952.52931	0.50902

952.66865	0.50915

952.80799	0.50926

952.94733	0.50929

953.08667	0.50937

953.22601	0.5094

953.36535	0.50945

953.50469	0.50953

953.64403	0.50962

953.78337	0.50967

953.92271	0.5097

954.06205	0.50975

954.20139	0.5098

954.34073	0.50983

954.48007	0.50997

954.61941	0.51005

954.75875	0.51008

954.89809	0.51046

955.03743	0.51048

955.17677	0.51056

955.31611	0.51059

955.45545	0.51067

955.59479	0.51075

955.73413	0.51078

955.87347	0.51081

956.01281	0.51083

956.15215	0.51113

956.29149	0.51127

956.43083	0.51132

956.57017	0.51143

956.70951	0.51146

956.84885	0.51151

956.98819	0.51154

957.12753	0.51165

957.26688	0.51167

957.40622	0.5117

957.54556	0.51176

957.6849	0.51181

957.82424	0.51186

957.96358	0.51197

958.10292	0.51211

958.24226	0.51216

958.3816	0.51219

958.52094	0.51224

958.66028	0.51227

958.79962	0.5123

958.93896	0.51232

959.0783	0.51249

959.21764	0.5127

959.35698	0.51287

959.49632	0.51289

959.63566	0.51292

959.775	0.51297

959.91434	0.51303

960.05368	0.51308

960.19302	0.51314

960.33236	0.51341

960.4717	0.51343

960.61104	0.51346

960.75038	0.51349

960.88972	0.51352

961.02906	0.51362

961.1684	0.51365

961.30774	0.51371

961.44708	0.51373

961.58642	0.51384

961.72576	0.51387

961.8651	0.51389

962.00444	0.51403

962.14378	0.51406

962.28312	0.51411

962.42246	0.51414

962.5618	0.51417

962.70114	0.51425

962.84048	0.5143

962.97982	0.51433

963.11916	0.51441

963.2585	0.51444

963.39784	0.51452

963.53718	0.51465

963.67652	0.51468

963.81586	0.51473

963.9552	0.51476

964.09454	0.51487

964.23388	0.51506

964.37322	0.51514

964.51256	0.5152

964.6519	0.51525

964.79124	0.51533

964.93058	0.51547

965.06992	0.51555

965.20926	0.51566

965.3486	0.51568

965.48794	0.51582

965.62728	0.51593

965.76662	0.51598

965.90596	0.51601

966.0453	0.51617

966.18465	0.51633

966.32399	0.51663

966.46333	0.51679

966.60267	0.51682

966.74201	0.51712

966.88135	0.51715

967.02069	0.51739

967.16003	0.51747

967.29937	0.51752

967.43871	0.51755

967.57805	0.51769

967.71739	0.51777

967.85673	0.51782

967.99607	0.51788

968.13541	0.51801

968.27475	0.51804

968.41409	0.51828

968.55343	0.51831

968.69277	0.51834

968.83211	0.51842

968.97145	0.51847

969.11079	0.5185

969.25013	0.51853

969.38947	0.51855

969.52881	0.51863

969.66815	0.51874

969.80749	0.5188

969.94683	0.51882

970.08617	0.51885

970.22551	0.51888

970.36485	0.51891

970.50419	0.51893

970.64353	0.51899

970.78287	0.51901

970.92221	0.5191

971.06155	0.51915

971.20089	0.51918

971.34023	0.51926

971.47957	0.51931

971.61891	0.51937

971.75825	0.51942

971.89759	0.51947

972.03693	0.5195

972.17627	0.51983

972.31561	0.5201

972.45495	0.52021

972.59429	0.52029

972.73363	0.52037

972.87297	0.52042

973.01231	0.5205

973.15165	0.52075

973.29099	0.52077

973.43033	0.5208

973.56967	0.52091

973.70901	0.52094

973.84835	0.52102

973.98769	0.5211

974.12703	0.52115

974.26637	0.52121

974.40571	0.52132

974.54505	0.52137

974.68439	0.52148

974.82373	0.52151

974.96307	0.52156

975.10241	0.52161

975.24176	0.52167

975.3811	0.52224

975.52044	0.52226

975.65978	0.52245

975.79912	0.52251

975.93846	0.52254

976.0778	0.52259

976.21714	0.5227

976.35648	0.52275

976.49582	0.52278

976.63516	0.52291

976.7745	0.52294

976.91384	0.523

977.05318	0.52302

977.19252	0.5231

977.33186	0.52319

977.4712	0.52324

977.61054	0.52329

977.74988	0.52343

977.88922	0.52354

978.02856	0.52356

978.1679	0.52362

978.30724	0.52375

978.44658	0.52378

978.58592	0.52386

978.72526	0.52389

978.8646	0.52394

979.00394	0.52397

979.14328	0.52421

979.28262	0.52424

979.42196	0.5244

979.5613	0.52443

979.70064	0.52451

979.83998	0.52454

979.97932	0.52457

980.11866	0.52459

980.258	0.52465

980.39734	0.52467

980.53668	0.5247

980.67602	0.52473

980.81536	0.52476

980.9547	0.52508

981.09404	0.52519

981.23338	0.52527

981.37272	0.52538

981.51206	0.52546

981.6514	0.52549

981.79074	0.52554

981.93008	0.52557

982.06942	0.5256

982.20876	0.52565

982.3481	0.5257

982.48744	0.52587

982.62678	0.526

982.76612	0.52606

982.90546	0.52616

983.0448	0.52633

983.18414	0.52635

983.32348	0.52657

983.46282	0.5266

983.60216	0.52679

983.7415	0.52681

983.88084	0.5269

984.02018	0.52695

984.15953	0.52706

984.29887	0.52719

984.43821	0.52733

984.57755	0.52736

984.71689	0.52741

984.85623	0.52776

984.99557	0.52782

985.13491	0.52806

985.27425	0.52809

985.41359	0.52811

985.55293	0.52814

985.69227	0.52817

985.83161	0.5283

985.97095	0.52833

986.11029	0.52836

986.24963	0.52844

986.38897	0.52847

986.52831	0.52849

986.66765	0.52868

986.80699	0.52879

986.94633	0.52898

987.08567	0.52912

987.22501	0.52931

987.36435	0.52939

987.50369	0.52952

987.64303	0.52958

987.78237	0.52971

987.92171	0.52974

988.06105	0.52977

988.20039	0.52982

988.33973	0.52996

988.47907	0.53015

988.61841	0.5302

988.75775	0.53023

988.89709	0.53028

989.03643	0.53047

989.17577	0.53055

989.31511	0.53058

989.45445	0.53063

989.59379	0.53104

989.73313	0.53109

989.87247	0.53118

990.01181	0.5312

990.15115	0.53131

990.29049	0.53142

990.42983	0.53153

990.56917	0.53166

990.70851	0.53169

990.84785	0.53172

990.98719	0.53174

991.12653	0.53188

991.26587	0.53193

991.40521	0.53207

991.54455	0.5321

991.68389	0.53215

991.82323	0.53218

991.96257	0.53226

992.10191	0.53229

992.24125	0.53237

992.38059	0.53239

992.51993	0.53253

992.65927	0.53269

992.79861	0.53272

992.93795	0.5328

993.0773	0.53318

993.21664	0.53321

993.35598	0.53326

993.49532	0.53329

993.63466	0.53332

993.774	0.53334

993.91334	0.53345

994.05268	0.53356

994.19202	0.53359

994.33136	0.53369

994.4707	0.53372

994.61004	0.53386

994.74938	0.53394

994.88872	0.53413

995.02806	0.5344

995.1674	0.53445

995.30674	0.53448

995.44608	0.53453

995.58542	0.53467

995.72476	0.5347

995.8641	0.53475

996.00344	0.5348

996.14278	0.53486

996.28212	0.53499

996.42146	0.53505

996.5608	0.5351

996.70014	0.53513

996.83948	0.53518

996.97882	0.53527

997.11816	0.53529

997.2575	0.53535

997.39684	0.53537

997.53618	0.53543

997.67552	0.53548

997.81486	0.53556

997.9542	0.53562

998.09354	0.53567

998.23288	0.5357

998.37222	0.53575

998.51156	0.53581

998.6509	0.53594

998.79024	0.53611

998.92958	0.53613

999.06892	0.53616

999.20826	0.53629

999.3476	0.53648

999.48694	0.53657

999.62628	0.53659

999.76562	0.5367

999.90496	0.53673

1000.0443	0.53678

1000.18364	0.53681

1000.32298	0.53686

1000.46232	0.53697

1000.60166	0.537

1000.741	0.53708

1000.88034	0.53713

1001.01968	0.53716

1001.15902	0.53727

1001.29836	0.5373

1001.43771	0.53738

1001.57705	0.53751

1001.71638	0.53759

1001.85573	0.53762

1001.99507	0.53765

1002.13441	0.53816

1002.27375	0.53822

1002.41309	0.5383

1002.55243	0.53876

1002.69177	0.53881

1002.83111	0.53892

1002.97045	0.53911

1003.10979	0.53919

1003.24913	0.53952

1003.38847	0.53963

1003.52781	0.53968

1003.66715	0.53976

1003.80649	0.53979

1003.94583	0.53992

1004.08517	0.53995

1004.22451	0.53998

1004.36385	0.54003

1004.50319	0.54006

1004.64253	0.54017

1004.78187	0.5402

1004.92121	0.54022

1005.06055	0.5403

1005.19989	0.54033

1005.33923	0.54041

1005.47857	0.54049

1005.61791	0.54055

1005.75725	0.54057

1005.89659	0.54066

1006.03593	0.54076

1006.17527	0.54087

1006.31461	0.54109

1006.45395	0.54141

1006.59329	0.54168

1006.73263	0.54171

1006.87197	0.54174

1007.01131	0.5419

1007.15065	0.54196

1007.28999	0.54209

1007.42933	0.54212

1007.56867	0.54242

1007.70801	0.54247

1007.84735	0.54258

1007.98669	0.54261

1008.12603	0.54263

1008.26537	0.54266

1008.40471	0.54282

1008.54405	0.5429

1008.68339	0.54293

1008.82273	0.54301

1008.96207	0.54304

1009.10141	0.54307

1009.24075	0.54312

1009.38009	0.54323

1009.51943	0.54339

1009.65877	0.54391

1009.79811	0.54401

1009.93745	0.54404

1010.07679	0.54428

1010.21613	0.54439

1010.35548	0.54442

1010.49482	0.5445

1010.63416	0.54456

1010.7735	0.5448

1010.91284	0.54521

1011.05218	0.54534

1011.19152	0.54548

1011.33086	0.54553

1011.4702	0.54556

1011.60954	0.54559

1011.74888	0.54561

1011.88822	0.54564

1012.02756	0.54567

1012.1669	0.54569

1012.30624	0.54572

1012.44558	0.54607

1012.58492	0.5461

1012.72426	0.54613

1012.8636	0.54618

1013.00294	0.54626

1013.14228	0.54629

1013.28162	0.54642

1013.42096	0.54648

1013.5603	0.54653

1013.69964	0.54691

1013.83898	0.54694

1013.97832	0.54699

1014.11766	0.54702

1014.257	0.54707

1014.39634	0.5471

1014.53568	0.54721

1014.67502	0.54724

1014.81436	0.54726

1014.9537	0.54729

1015.09304	0.54735

1015.23238	0.54737

1015.37172	0.54751

1015.51106	0.54759

1015.6504	0.54762

1015.78974	0.54775

1015.92908	0.54778

1016.06842	0.54786

1016.20776	0.54791

1016.3471	0.54797

1016.48644	0.54802

1016.62578	0.54808

1016.76512	0.54827

1016.90446	0.54843

1017.0438	0.54846

1017.18314	0.54875

1017.32248	0.549

1017.46182	0.54902

1017.60116	0.54927

1017.7405	0.54954

1017.87984	0.54981

1018.01918	0.54986

1018.15852	0.54992

1018.29786	0.54995

1018.4372	0.55

1018.57654	0.55003

1018.71588	0.55008

1018.85522	0.55011

1018.99456	0.55014

1019.1339	0.55019

1019.27325	0.55073

1019.41259	0.55079

1019.55193	0.55081

1019.69127	0.55087

1019.83061	0.55092

1019.96995	0.55098

1020.10929	0.55103

1020.24863	0.55114

1020.38797	0.55116

1020.52731	0.55119

1020.66665	0.55125

1020.80599	0.55135

1020.94533	0.55149

1021.08467	0.55152

1021.22401	0.5516

1021.36335	0.55163

1021.50269	0.55184

1021.64203	0.552

1021.78137	0.55203

1021.92071	0.55211

1022.06005	0.55214

1022.19939	0.55219

1022.33873	0.55228

1022.47807	0.5523

1022.61741	0.55233

1022.75675	0.55238

1022.89609	0.55244

1023.03543	0.55255

1023.17477	0.55257

1023.31411	0.55279

1023.45345	0.5529

1023.59279	0.55298

1023.73213	0.55309

1023.87147	0.55325

1024.01081	0.55333

1024.15015	0.55339

1024.28949	0.55347

1024.42883	0.55358

1024.56817	0.55363

1024.70751	0.55371

1024.84685	0.55387

1024.98619	0.55393

1025.12553	0.55395

1025.26487	0.55398

1025.40421	0.55406

1025.54355	0.55414

1025.68289	0.5542

1025.82223	0.55423

1025.96157	0.55428

1026.10091	0.55431

1026.24025	0.55447

1026.37959	0.5545

1026.51893	0.5546

1026.65827	0.55463

1026.79761	0.55469

1026.93695	0.55482

1027.07629	0.55485

1027.21563	0.55488

1027.35497	0.5549

1027.49431	0.55498

1027.63365	0.55504

1027.77299	0.55512

1027.91233	0.55515

1028.05167	0.5552

1028.19102	0.55525

1028.33036	0.55547

1028.4697	0.55566

1028.60903	0.55569

1028.74837	0.5558

1028.88772	0.55582

1029.02706	0.55585

1029.1664	0.55588

1029.30574	0.55593

1029.44508	0.55604

1029.58442	0.55609

1029.72376	0.55628

1029.8631	0.55631

1030.00244	0.55634

1030.14178	0.55637

1030.28112	0.55653

1030.42046	0.55658

1030.5598	0.55664

1030.69914	0.55693

1030.83848	0.55696

1030.97782	0.55704

1031.11716	0.55715

1031.2565	0.55731

1031.39584	0.55734

1031.53518	0.55742

1031.67452	0.55748

1031.81386	0.55753

1031.9532	0.55756

1032.09254	0.55758

1032.23188	0.55761

1032.37122	0.55772

1032.51056	0.55813

1032.6499	0.55829

1032.78924	0.55834

1032.92858	0.5584

1033.06792	0.55848

1033.20726	0.55867

1033.3466	0.55878

1033.48594	0.5588

1033.62528	0.55888

1033.76462	0.55921

1033.90396	0.55937

1034.0433	0.55962

1034.18264	0.5597

1034.32198	0.55972

1034.46132	0.55975

1034.60066	0.5598

1034.74	0.55983

1034.87934	0.55991

1035.01868	0.55994

1035.15802	0.56005

1035.29736	0.56016

1035.4367	0.56018

1035.57604	0.56024

1035.71538	0.56029

1035.85472	0.5604

1035.99406	0.56043

1036.1334	0.56046

1036.27274	0.56051

1036.41208	0.56062

1036.55142	0.56064

1036.69076	0.56083

1036.8301	0.56092

1036.96944	0.56094

1037.10878	0.56108

1037.24813	0.56116

1037.38747	0.56119

1037.52681	0.56127

1037.66614	0.56132

1037.80549	0.56135

1037.94483	0.56148

1038.08417	0.56151

1038.22351	0.56157

1038.36285	0.56162

1038.50219	0.56165

1038.64153	0.56167

1038.78087	0.5617

1038.92021	0.56173

1039.05955	0.56181

1039.19889	0.56184

1039.33823	0.56197

1039.47757	0.562

1039.61691	0.56213

1039.75625	0.56219

1039.89559	0.56222

1040.03493	0.5623

1040.17427	0.56276

1040.31361	0.56281

1040.45295	0.56327

1040.59229	0.5633

1040.73163	0.56349

1040.87097	0.56352

1041.01031	0.56354

1041.14965	0.56362

1041.28899	0.56368

1041.42833	0.56392

1041.56767	0.56433

1041.70701	0.56438

1041.84635	0.56446

1041.98569	0.56457

1042.12503	0.56476

1042.26437	0.56487

1042.40371	0.56492

1042.54305	0.56517

1042.68239	0.5652

1042.82173	0.56525

1042.96107	0.56528

1043.10041	0.56536

1043.23975	0.56541

1043.37909	0.56547

1043.51843	0.56549

1043.65777	0.56552

1043.79711	0.56563

1043.93645	0.56585

1044.07579	0.56606

1044.21513	0.56614

1044.35447	0.56617

1044.49381	0.56622

1044.63315	0.56628

1044.77249	0.56633

1044.91183	0.56674

1045.05117	0.56677

1045.19051	0.56679

1045.32985	0.56685

1045.46919	0.56704

1045.60853	0.56706

1045.74787	0.56712

1045.88722	0.56715

1046.02655	0.56725

1046.1659	0.56728

1046.30524	0.56731

1046.44458	0.56742

1046.58391	0.56744

1046.72326	0.56761

1046.8626	0.56774

1047.00194	0.56777

1047.14128	0.56785

1047.28062	0.5679

1047.41996	0.56796

1047.5593	0.56798

1047.69864	0.56809

1047.83798	0.56823

1047.97732	0.56826

1048.11666	0.56828

1048.256	0.56842

1048.39534	0.56853

1048.53468	0.56858

1048.67402	0.56869

1048.81336	0.56874

1048.9527	0.56877

1049.09204	0.56893

1049.23138	0.56899

1049.37072	0.56942

1049.51006	0.56953

1049.6494	0.56961

1049.78874	0.56966

1049.92808	0.56977

1050.06742	0.5698

1050.20676	0.56983

1050.3461	0.56996

1050.48544	0.56999

1050.62478	0.57002

1050.76412	0.57007

1050.90346	0.57018

1051.0428	0.57034

1051.18214	0.57042

1051.32148	0.57067

1051.46082	0.57072

1051.60016	0.57075

1051.7395	0.57086

1051.87884	0.57088

1052.01818	0.57091

1052.15752	0.57099

1052.29686	0.5714

1052.4362	0.57142

1052.57554	0.57153

1052.71488	0.57156

1052.85422	0.57161

1052.99356	0.57172

1053.1329	0.57202

1053.27224	0.57216

1053.41158	0.57232

1053.55092	0.57243

1053.69026	0.57262

1053.8296	0.5727

1053.96894	0.57275

1054.10828	0.57278

1054.24762	0.57286

1054.38696	0.57297

1054.5263	0.5731

1054.66564	0.57313

1054.80499	0.57316

1054.94432	0.57319

1055.08367	0.57324

1055.22301	0.57335

1055.36235	0.57337

1055.50169	0.5734

1055.64102	0.57351

1055.78037	0.5737

1055.91971	0.57375

1056.05905	0.57384

1056.19839	0.57394

1056.33773	0.57397

1056.47707	0.574

1056.61641	0.57405

1056.75575	0.57408

1056.89509	0.57411

1057.03443	0.57427

1057.17377	0.5743

1057.31311	0.57438

1057.45245	0.57443

1057.59179	0.57446

1057.73113	0.57451

1057.87047	0.57454

1058.00981	0.57457

1058.14915	0.57467

1058.28849	0.57473

1058.42783	0.57476

1058.56717	0.57481

1058.70651	0.57484

1058.84585	0.57492

1058.98519	0.57495

1059.12453	0.575

1059.26387	0.57514

1059.40321	0.57524

1059.54255	0.5753

1059.68189	0.57533

1059.82123	0.57535

1059.96057	0.57538

1060.09991	0.57541

1060.23925	0.57576

1060.37859	0.57579

1060.51793	0.576

1060.65727	0.57603

1060.79661	0.57606

1060.93595	0.57616

1061.07529	0.57619

1061.21463	0.57622

1061.35397	0.5763

1061.49331	0.57633

1061.63265	0.57652

1061.77199	0.57657

1061.91133	0.57665

1062.05067	0.57679

1062.19001	0.57681

1062.32935	0.57695

1062.46869	0.57709

1062.60803	0.57711

1062.74737	0.57722

1062.88671	0.57725

1063.02605	0.5773

1063.16539	0.57733

1063.30473	0.57738

1063.44407	0.57746

1063.58341	0.57757

1063.72276	0.5776

1063.8621	0.57765

1064.00143	0.57776

1064.14078	0.57779

1064.28012	0.57782

1064.41946	0.57814

1064.55879	0.57817

1064.69814	0.57822

1064.83748	0.57839

1064.97682	0.57841

1065.11616	0.57844

1065.2555	0.57847

1065.39484	0.57876

1065.53418	0.57887

1065.67352	0.5789

1065.81286	0.57904

1065.9522	0.57909

1066.09154	0.57914

1066.23088	0.57923

1066.37022	0.57931

1066.50956	0.57933

1066.6489	0.57936

1066.78824	0.57939

1066.92758	0.57944

1067.06692	0.57947

1067.20626	0.5795

1067.3456	0.57955

1067.48494	0.57958

1067.62428	0.5796

1067.76362	0.57966

1067.90296	0.57974

1068.0423	0.57988

1068.18164	0.57993

1068.32098	0.57996

1068.46032	0.57998

1068.59966	0.58009

1068.739	0.58015

1068.87834	0.58025

1069.01768	0.58028

1069.15702	0.58031

1069.29636	0.58058

1069.4357	0.58069

1069.57504	0.5808

1069.71438	0.58082

1069.85372	0.58085

1069.99306	0.58093

1070.1324	0.58101

1070.27174	0.58109

1070.41108	0.58118

1070.55042	0.58128

1070.68976	0.58131

1070.8291	0.58134

1070.96844	0.58137

1071.10778	0.58139

1071.24712	0.58145

1071.38646	0.58147

1071.5258	0.5815

1071.66514	0.58164

1071.80448	0.58166

1071.94382	0.58185

1072.08316	0.58188

1072.2225	0.58191

1072.36184	0.58193

1072.50118	0.58196

1072.64053	0.58204

1072.77987	0.5821

1072.9192	0.58212

1073.05855	0.58215

1073.19789	0.58218

1073.33723	0.58229

1073.47656	0.58237

1073.61591	0.58239

1073.75525	0.58245

1073.89459	0.58272

1074.03393	0.58275

1074.17327	0.58294

1074.31261	0.58315

1074.45195	0.58318

1074.59129	0.58323

1074.73063	0.58334

1074.86997	0.58337

1075.00931	0.5834

1075.14865	0.5835

1075.28799	0.58378

1075.42733	0.58388

1075.56667	0.58391

1075.70601	0.58397

1075.84535	0.58413

1075.98469	0.58415

1076.12403	0.58432

1076.26337	0.58434

1076.40271	0.58445

1076.54205	0.58448

1076.68139	0.58453

1076.82073	0.5847

1076.96007	0.58472

1077.09941	0.58478

1077.23875	0.58491

1077.37809	0.58494

1077.51743	0.58499

1077.65677	0.58502

1077.79611	0.58505

1077.93545	0.58508

1078.07479	0.58516

1078.21413	0.58518

1078.35347	0.58521

1078.49281	0.58524

1078.63215	0.58527

1078.77149	0.58529

1078.91083	0.58532

1079.05017	0.58548

1079.18951	0.58551

1079.32885	0.58554

1079.46819	0.58559

1079.60753	0.58564

1079.74687	0.58567

1079.88621	0.5857

1080.02555	0.58573

1080.16489	0.58575

1080.30423	0.58586

1080.44357	0.58589

1080.58291	0.58594

1080.72225	0.586

1080.86159	0.58608

1081.00093	0.58611

1081.14027	0.58613

1081.27961	0.58619

1081.41895	0.58635

1081.5583	0.58638

1081.69764	0.58648

1081.83697	0.58659

1081.97632	0.58667

1082.11566	0.5867

1082.255	0.58681

1082.39434	0.58692

1082.53368	0.587

1082.67302	0.58708

1082.81236	0.58711

1082.9517	0.58716

1083.09104	0.58722

1083.23038	0.58724

1083.36972	0.58727

1083.50906	0.5873

1083.6484	0.58743

1083.78774	0.58746

1083.92708	0.58754

1084.06642	0.58759

1084.20576	0.58781

1084.3451	0.58784

1084.48444	0.58787

1084.62378	0.58795

1084.76312	0.58797

1084.90246	0.58803

1085.0418	0.58811

1085.18114	0.58814

1085.32048	0.58835

1085.45982	0.58846

1085.59916	0.58849

1085.7385	0.58852

1085.87784	0.58887

1086.01718	0.58889

1086.15652	0.58892

1086.29586	0.58895

1086.4352	0.589

1086.57454	0.58906

1086.71388	0.58908

1086.85322	0.58914

1086.99256	0.59003

1087.1319	0.59017

1087.27124	0.5902

1087.41058	0.5903

1087.54992	0.59036

1087.68926	0.59038

1087.8286	0.59071

1087.96794	0.59074

1088.10728	0.59076

1088.24662	0.59095

1088.38596	0.59098

1088.5253	0.59101

1088.66464	0.59103

1088.80398	0.59106

1088.94332	0.59112

1089.08266	0.5912

1089.222	0.59125

1089.36134	0.59128

1089.50068	0.59133

1089.64002	0.59141

1089.77936	0.59144

1089.9187	0.59147

1090.05804	0.59177

1090.19738	0.59179

1090.33672	0.59182

1090.47607	0.59185

1090.61541	0.5919

1090.75475	0.59198

1090.89408	0.59206

1091.03343	0.59217

1091.17277	0.59223

1091.31211	0.59228

1091.45145	0.59233

1091.59079	0.59236

1091.73013	0.59239

1091.86947	0.59242

1092.00881	0.59285

1092.14815	0.59296

1092.28749	0.59301

1092.42683	0.59334

1092.56617	0.59339

1092.70551	0.59347

1092.84485	0.59363

1092.98419	0.59369

1093.12353	0.59374

1093.26287	0.5938

1093.40221	0.59382

1093.54155	0.59385

1093.68089	0.59388

1093.82023	0.59393

1093.95957	0.59512

1094.09891	0.59518

1094.23825	0.59542

1094.37759	0.59548

1094.51693	0.5955

1094.65627	0.59556

1094.79561	0.59559

1094.93495	0.59561

1095.07429	0.59564

1095.21363	0.59569

1095.35297	0.59594

1095.49231	0.59596

1095.63165	0.59615

1095.77099	0.59618

1095.91033	0.59632

1096.04967	0.59637

1096.18901	0.59642

1096.32835	0.59645

1096.46769	0.59656

1096.60703	0.59659

1096.74637	0.59661

1096.88571	0.59667

1097.02505	0.5967

1097.16439	0.59689

1097.30373	0.59694

1097.44307	0.59697

1097.58241	0.59705

1097.72175	0.5971

1097.86109	0.59716

1098.00043	0.59721

1098.13977	0.59748

1098.27911	0.59754

1098.41845	0.59772

1098.55779	0.59783

1098.69713	0.59786

1098.83647	0.59789

1098.97581	0.59791

1099.11515	0.59794

1099.25449	0.598

1099.39384	0.59802

1099.53318	0.5981

1099.67252	0.59813

1099.81185	0.59816

1099.9512	0.59824

1100.09054	0.59829

1100.22988	0.59835

1100.36922	0.59837

1100.50856	0.59843

1100.6479	0.59848

1100.78724	0.59854

1100.92658	0.59856

1101.06592	0.59865

1101.20526	0.59867

1101.3446	0.5987

1101.48394	0.59873

1101.62328	0.59875

1101.76262	0.59878

1101.90196	0.59881

1102.0413	0.59892

1102.18064	0.59894

1102.31998	0.59897

1102.45932	0.59902

1102.59866	0.59911

1102.738	0.59913

1102.87734	0.59919

1103.01668	0.59927

1103.15602	0.59938

1103.29536	0.59946

1103.4347	0.59959

1103.57404	0.59973

1103.71338	0.59976

1103.85272	0.59978

1103.99206	0.59981

1104.1314	0.59989

1104.27074	0.59997

1104.41008	0.60003

1104.54942	0.60008

1104.68876	0.60014

1104.8281	0.60027

1104.96744	0.6003

1105.10678	0.60041

1105.24612	0.60043

1105.38546	0.60046

1105.5248	0.60057

1105.66414	0.6006

1105.80348	0.60079

1105.94282	0.60081

1106.08216	0.60095

1106.2215	0.601

1106.36084	0.60114

1106.50018	0.60119

1106.63952	0.6013

1106.77886	0.60133

1106.9182	0.60146

1107.05754	0.60165

1107.19688	0.60168

1107.33622	0.60171

1107.47556	0.60195

1107.6149	0.60214

1107.75424	0.60222

1107.89358	0.60233

1108.03292	0.60252

1108.17226	0.60255

1108.31161	0.60257

1108.45095	0.60263

1108.59029	0.60265

1108.72962	0.60274

1108.86896	0.60282

1109.00831	0.60322

1109.14765	0.60358

1109.28699	0.60368

1109.42633	0.60412

1109.56567	0.60425

1109.70501	0.60428

1109.84435	0.60466

1109.98369	0.60469

1110.12303	0.60479

1110.26237	0.60482

1110.40171	0.60485

1110.54105	0.60498

1110.68039	0.60501

1110.81973	0.60507

1110.95907	0.60512

1111.09841	0.60517

1111.23775	0.60523

1111.37709	0.60528

1111.51643	0.60531

1111.65577	0.60534

1111.79511	0.60536

1111.93445	0.6055

1112.07379	0.60561

1112.21313	0.60566

1112.35247	0.60569

1112.49181	0.60572

1112.63115	0.60577

1112.77049	0.6058

1112.90983	0.6059

1113.04917	0.60612

1113.18851	0.60631

1113.32785	0.60634

1113.46719	0.60637

1113.60653	0.60666

1113.74587	0.60674

1113.88521	0.60677

1114.02455	0.60693

1114.16389	0.60702

1114.30323	0.6075

1114.44257	0.60756

1114.58191	0.60761

1114.72125	0.6078

1114.86059	0.60785

1114.99993	0.60788

1115.13927	0.60791

1115.27861	0.60796

1115.41795	0.60813

1115.55729	0.60818

1115.69663	0.60823

1115.83597	0.60834

1115.97531	0.6085

1116.11465	0.60856

1116.25399	0.60861

1116.39333	0.6088

1116.53267	0.60883

1116.67201	0.60886

1116.81135	0.60891

1116.95069	0.60899

1117.09003	0.60918

1117.22937	0.60926

1117.36872	0.61002

1117.50806	0.61005

1117.6474	0.61008

1117.78673	0.61048

1117.92608	0.61051

1118.06542	0.61054

1118.20476	0.61081

1118.3441	0.61083

1118.48344	0.61092

1118.62278	0.61113

1118.76212	0.61119

1118.90146	0.61121

1119.0408	0.61127

1119.18014	0.61129

1119.31948	0.61132

1119.45882	0.61135

1119.59816	0.61138

1119.7375	0.61143

1119.87684	0.61148

1120.01618	0.61151

1120.15552	0.61154

1120.29486	0.61159

1120.4342	0.6117

1120.57354	0.61181

1120.71288	0.61189

1120.85222	0.61192

1120.99156	0.61222

1121.1309	0.6127

1121.27024	0.61289

1121.40958	0.61295

1121.54892	0.61316

1121.68826	0.61319

1121.8276	0.61346

1121.96694	0.61354

1122.10628	0.61384

1122.24562	0.61389

1122.38496	0.61392

1122.5243	0.61398

1122.66364	0.61408

1122.80298	0.61417

1122.94232	0.61419

1123.08166	0.61422

1123.221	0.61427

1123.36034	0.6143

1123.49968	0.61436

1123.63902	0.61449

1123.77836	0.61468

1123.9177	0.61471

1124.05704	0.61492

1124.19638	0.61498

1124.33572	0.61503

1124.47506	0.61517

1124.6144	0.6152

1124.75374	0.61525

1124.89308	0.61528

1125.03242	0.61538

1125.17176	0.61547

1125.3111	0.61549

1125.45044	0.61552

1125.58978	0.6156

1125.72912	0.61585

1125.86846	0.61593

1126.0078	0.61595

1126.14714	0.61598

1126.28649	0.61601

1126.42583	0.61614

1126.56517	0.61628

1126.7045	0.61639

1126.84385	0.61644

1126.98319	0.61652

1127.12253	0.6166

1127.26187	0.61663

1127.40121	0.61671

1127.54055	0.61674

1127.67989	0.61679

1127.81923	0.61706

1127.95857	0.61715

1128.09791	0.61744

1128.23725	0.61758

1128.37659	0.61761

1128.51593	0.61771

1128.65527	0.61774

1128.79461	0.61788

1128.93395	0.6179

1129.07329	0.61793

1129.21263	0.61804

1129.35197	0.61807

1129.49131	0.61809

1129.63065	0.6182

1129.76999	0.61823

1129.90933	0.61826

1130.04867	0.61831

1130.18801	0.61834

1130.32735	0.61836

1130.46669	0.61839

1130.60603	0.61845

1130.74537	0.61847

1130.88471	0.61853

1131.02405	0.61863

1131.16339	0.61866

1131.30273	0.61869

1131.44207	0.61877

1131.58141	0.6188

1131.72075	0.61882

1131.86009	0.61893

1131.99943	0.61896

1132.13877	0.61899

1132.27811	0.61901

1132.41745	0.61904

1132.55679	0.61915

1132.69613	0.61918

1132.83547	0.6192

1132.97481	0.61923

1133.11415	0.61928

1133.25349	0.61947

1133.39283	0.6195

1133.53217	0.61956

1133.67151	0.61958

1133.81085	0.6198

1133.95019	0.61983

1134.08953	0.61988

1134.22887	0.61993

1134.36821	0.62007

1134.50755	0.6201

1134.64689	0.62018

1134.78623	0.62021

1134.92557	0.62023

1135.06491	0.62026

1135.20426	0.62029

1135.3436	0.62048

1135.48294	0.62053

1135.62227	0.62056

1135.76161	0.62059

1135.90096	0.62067

1136.0403	0.62075

1136.17964	0.62077

1136.31898	0.6208

1136.45832	0.62083

1136.59766	0.62094

1136.737	0.62099

1136.87634	0.62102

1137.01568	0.62105

1137.15502	0.62107

1137.29436	0.62113

1137.4337	0.62115

1137.57304	0.62118

1137.71238	0.62129

1137.85172	0.62151

1137.99106	0.62159

1138.1304	0.62167

1138.26974	0.62186

1138.40908	0.62194

1138.54842	0.62199

1138.68776	0.62202

1138.8271	0.62216

1138.96644	0.62237

1139.10578	0.62251

1139.24512	0.62254

1139.38446	0.62262

1139.5238	0.62264

1139.66314	0.62283

1139.80248	0.62289

1139.94182	0.62291

1140.08116	0.62294

1140.2205	0.62297

1140.35984	0.623

1140.49918	0.62302

1140.63852	0.62305

1140.77786	0.62308

1140.9172	0.6231

1141.05654	0.62316

1141.19588	0.62324

1141.33522	0.62329

1141.47456	0.62332

1141.6139	0.62343

1141.75324	0.62346

1141.89258	0.62359

1142.03192	0.62362

1142.17126	0.62373

1142.3106	0.62375

1142.44994	0.62378

1142.58928	0.62384

1142.72862	0.624

1142.86796	0.62402

1143.0073	0.62405

1143.14664	0.62413

1143.28598	0.62416

1143.42532	0.62419

1143.56466	0.6243

1143.704	0.62432

1143.84335	0.6244

1143.98268	0.62451

1144.12202	0.62454

1144.26137	0.62503

1144.40071	0.62505

1144.54005	0.62508

1144.67938	0.62516

1144.81873	0.6253

1144.95807	0.62538

1145.09741	0.62546

1145.23675	0.62549

1145.37609	0.62598

1145.51543	0.62606

1145.65477	0.62635

1145.79411	0.62687

1145.93345	0.62692

1146.07279	0.62695

1146.21213	0.627

1146.35147	0.62711

1146.49081	0.62719

1146.63015	0.62725

1146.76949	0.62733

1146.90883	0.62736

1147.04817	0.62738

1147.18751	0.62741

1147.32685	0.62746

1147.46619	0.62757

1147.60553	0.62763

1147.74487	0.62765

1147.88421	0.62768

1148.02355	0.62771

1148.16289	0.62774

1148.30223	0.62782

1148.44157	0.62793

1148.58091	0.62795

1148.72025	0.62801

1148.85959	0.62839

1148.99893	0.62841

1149.13827	0.62852

1149.27761	0.62866

1149.41695	0.62868

1149.55629	0.62874

1149.69563	0.62876

1149.83497	0.62882

1149.97431	0.62885

1150.11365	0.6289

1150.25299	0.62893

1150.39233	0.62904

1150.53167	0.62906

1150.67101	0.62912

1150.81035	0.62914

1150.94969	0.6292

1151.08903	0.62923

1151.22837	0.62925

1151.36771	0.62928

1151.50705	0.62933

1151.64639	0.62936

1151.78573	0.62941

1151.92507	0.6295

1152.06441	0.62952

1152.20375	0.62958

1152.34309	0.6296

1152.48243	0.6302

1152.62177	0.63028

1152.76112	0.63034

1152.90046	0.63047

1153.03979	0.6305

1153.17914	0.63055

1153.31848	0.63063

1153.45782	0.63066

1153.59715	0.63069

1153.7365	0.63074

1153.87584	0.63085

1154.01518	0.63088

1154.15452	0.6309

1154.29386	0.63093

1154.4332	0.63096

1154.57254	0.63104

1154.71188	0.63107

1154.85122	0.63109

1154.99056	0.63112

1155.1299	0.63115

1155.26924	0.6312

1155.40858	0.63137

1155.54792	0.63145

1155.68726	0.63147

1155.8266	0.6315

1155.96594	0.63153

1156.10528	0.63155

1156.24462	0.63158

1156.38396	0.63164

1156.5233	0.63169

1156.66264	0.63172

1156.80198	0.63174

1156.94132	0.63177

1157.08066	0.6318

1157.22	0.63183

1157.35934	0.63193

1157.49868	0.63199

1157.63802	0.63202

1157.77736	0.63204

1157.9167	0.63207

1158.05604	0.6321

1158.19538	0.63212

1158.33472	0.63215

1158.47406	0.63239

1158.6134	0.63267

1158.75274	0.63269

1158.89208	0.63272

1159.03142	0.63283

1159.17076	0.63307

1159.3101	0.63318

1159.44944	0.63321

1159.58878	0.63323

1159.72812	0.63326

1159.86746	0.63332

1160.0068	0.63334

1160.14614	0.6334

1160.28548	0.63342

1160.42482	0.63353

1160.56416	0.63356

1160.7035	0.63361

1160.84284	0.63383

1160.98218	0.63386

1161.12152	0.63388

1161.26086	0.63397

1161.4002	0.63399

1161.53954	0.63407

1161.67889	0.6341

1161.81823	0.63418

1161.95756	0.63424

1162.09691	0.63445

1162.23625	0.63459

1162.37559	0.6347

1162.51492	0.63535

1162.65427	0.6354

1162.79361	0.63581

1162.93295	0.63597

1163.07229	0.636

1163.21163	0.63602

1163.35097	0.63605

1163.49031	0.63613

1163.62965	0.63616

1163.76899	0.63619

1163.90833	0.63635

1164.04767	0.63638

1164.18701	0.63657

1164.32635	0.63662

1164.46569	0.63667

1164.60503	0.63676

1164.74437	0.63678

1164.88371	0.63684

1165.02305	0.63686

1165.16239	0.63703

1165.30173	0.63705

1165.44107	0.63708

1165.58041	0.63711

1165.71975	0.63716

1165.85909	0.63719

1165.99843	0.63735

1166.13777	0.63738

1166.27711	0.63741

1166.41645	0.63743

1166.55579	0.63746

1166.69513	0.63754

1166.83447	0.63757

1166.97381	0.63765

1167.11315	0.63835

1167.25249	0.63843

1167.39183	0.63889

1167.53117	0.63892

1167.67051	0.63906

1167.80985	0.63908

1167.94919	0.63911

1168.08853	0.63919

1168.22787	0.6393

1168.36721	0.63936

1168.50655	0.63941

1168.64589	0.63944

1168.78523	0.63946

1168.92457	0.63965

1169.06391	0.63979

1169.20325	0.63982

1169.34259	0.6399

1169.48193	0.63992

1169.62127	0.63998

1169.76061	0.64003

1169.89995	0.64006

1170.03929	0.6402

1170.17863	0.64022

1170.31797	0.64047

1170.45731	0.64049

1170.59666	0.64055

1170.736	0.64074

1170.87534	0.64076

1171.01467	0.64087

1171.15402	0.64093

1171.29336	0.64114

1171.4327	0.64117

1171.57204	0.6412

1171.71138	0.64133

1171.85072	0.64136

1171.99006	0.64139

1172.1294	0.64144

1172.26874	0.64158

1172.40808	0.6416

1172.54742	0.64177

1172.68676	0.64185

1172.8261	0.64187

1172.96544	0.64193

1173.10478	0.64212

1173.24412	0.64217

1173.38346	0.6422

1173.5228	0.64223

1173.66214	0.64228

1173.80148	0.64231

1173.94082	0.64236

1174.08016	0.64242

1174.2195	0.64244

1174.35884	0.64252

1174.49818	0.6428

1174.63752	0.64296

1174.77686	0.64312

1174.9162	0.6432

1175.05554	0.64326

1175.19488	0.64339

1175.33422	0.64342

1175.47356	0.64345

1175.6129	0.6435

1175.75224	0.64355

1175.89158	0.64366

1176.03092	0.64382

1176.17026	0.64388

1176.3096	0.64391

1176.44894	0.64393

1176.58828	0.64396

1176.72762	0.64399

1176.86696	0.64404

1177.0063	0.6441

1177.14564	0.64412

1177.28498	0.64415

1177.42432	0.64418

1177.56366	0.64423

1177.703	0.64431

1177.84234	0.64442

1177.98168	0.64447

1178.12102	0.6445

1178.26036	0.64453

1178.3997	0.64456

1178.53904	0.64464

1178.67838	0.64466

1178.81772	0.64472

1178.95706	0.64475

1179.0964	0.64483

1179.23574	0.64493

1179.37508	0.64496

1179.51443	0.64499

1179.65377	0.64507

1179.79311	0.64512

1179.93244	0.64515

1180.07179	0.64518

1180.21113	0.64521

1180.35047	0.64548

1180.48981	0.6455

1180.62915	0.64561

1180.76849	0.64702

1180.90783	0.64705

1181.04717	0.64707

1181.18651	0.6471

1181.32585	0.64716

1181.46519	0.64718

1181.60453	0.64724

1181.74387	0.64726

1181.88321	0.64729

1182.02255	0.64732

1182.16189	0.6474

1182.30123	0.64743

1182.44057	0.64745

1182.57991	0.64748

1182.71925	0.64751

1182.85859	0.64754

1182.99793	0.64764

1183.13727	0.64767

1183.27661	0.6477

1183.41595	0.64827

1183.55529	0.64832

1183.69463	0.6484

1183.83397	0.64843

1183.97331	0.64846

1184.11265	0.64873

1184.25199	0.64878

1184.39133	0.64881

1184.53067	0.649

1184.67001	0.64902

1184.80935	0.64908

1184.94869	0.64921

1185.08803	0.64932

1185.22737	0.64943

1185.36671	0.64973

1185.50605	0.64976

1185.64539	0.64989

1185.78473	0.65065

1185.92407	0.65092

1186.06341	0.65095

1186.20275	0.65106

1186.34209	0.65111

1186.48143	0.65133

1186.62077	0.65157

1186.76011	0.6516

1186.89945	0.65165

1187.03879	0.65181

1187.17813	0.65187

1187.31747	0.65192

1187.45681	0.652

1187.59615	0.65203

1187.73549	0.65206

1187.87483	0.65209

1188.01417	0.65217

1188.15351	0.65233

1188.29285	0.65238

1188.4322	0.65244

1188.57154	0.65246

1188.71088	0.65252

1188.85021	0.65255

1188.98956	0.65257

1189.1289	0.65268

1189.26824	0.65276

1189.40758	0.65287

1189.54692	0.6533

1189.68626	0.65341

1189.8256	0.65344

1189.96494	0.65347

1190.10428	0.65352

1190.24362	0.65374

1190.38296	0.65376

1190.5223	0.65379

1190.66164	0.65382

1190.80098	0.65385

1190.94032	0.65395

1191.07966	0.65401

1191.219	0.65412

1191.35834	0.65414

1191.49768	0.65431

1191.63702	0.65463

1191.77636	0.65471

1191.9157	0.65477

1192.05504	0.6549

1192.19438	0.65496

1192.33372	0.65512

1192.47306	0.6552

1192.6124	0.65523

1192.75174	0.65528

1192.89108	0.65531

1193.03042	0.65539

1193.16976	0.65544

1193.3091	0.65561

1193.44844	0.65563

1193.58778	0.65566

1193.72712	0.65569

1193.86646	0.65577

1194.0058	0.65585

1194.14514	0.65593

1194.28448	0.65607

1194.42382	0.65618

1194.56316	0.6562

1194.7025	0.65626

1194.84184	0.65642

1194.98118	0.65655

1195.12052	0.65683

1195.25986	0.65696

1195.3992	0.65699

1195.53854	0.65702

1195.67788	0.65715

1195.81722	0.65869

1195.95656	0.65888

1196.0959	0.65891

1196.23524	0.65899

1196.37458	0.65918

1196.51392	0.65926

1196.65326	0.65932

1196.7926	0.65934

1196.93194	0.65937

1197.07128	0.6594

1197.21062	0.65948

1197.34997	0.65951

1197.48931	0.65953

1197.62865	0.65967

1197.76799	0.65991

1197.90732	0.65994

1198.04667	0.66002

1198.18601	0.66008

1198.32535	0.6601

1198.46469	0.66016

1198.60403	0.66035

1198.74337	0.66046

1198.88271	0.66116

1199.02205	0.66281

1199.16139	0.66295

1199.30073	0.66316

1199.44007	0.66324

1199.57941	0.66335

1199.71875	0.66341

1199.85809	0.66354

1199.99743	0.66427

1200.13677	0.66433

1200.27611	0.66438

1200.41545	0.66449

1200.55479	0.66514

1200.69413	0.66517

1200.83347	0.66525

1200.97281	0.66528

1201.11215	0.6653

1201.25149	0.66533

1201.39083	0.66549

1201.53017	0.66571

1201.66951	0.66576

1201.80885	0.66585

1201.94819	0.6659

1202.08753	0.66598

1202.22687	0.66601

1202.36621	0.66609

1202.50555	0.66614

1202.64489	0.6662

1202.78423	0.66622

1202.92357	0.66628

1203.06291	0.66631

1203.20225	0.66636

1203.34159	0.6665

1203.48093	0.66679

1203.62027	0.66685

1203.75961	0.66696

1203.89895	0.6675

1204.03829	0.66752

1204.17763	0.66761

1204.31697	0.66769

1204.45631	0.66771

1204.59565	0.66774

1204.73499	0.66785

1204.87433	0.66788

1205.01367	0.66793

1205.15301	0.66796

1205.29235	0.6682

1205.43169	0.6685

1205.57103	0.66855

1205.71037	0.66861

1205.84971	0.66904

1205.98905	0.66918

1206.12839	0.66923

1206.26773	0.66928

1206.40708	0.66937

1206.54642	0.66939

1206.68576	0.66975

1206.82509	0.66985

1206.96444	0.66991

1207.10378	0.66993

1207.24312	0.67002

1207.38246	0.67012

1207.5218	0.67021

1207.66114	0.67023

1207.80048	0.6704

1207.93982	0.67042

1208.07916	0.67045

1208.2185	0.67048

1208.35784	0.67053

1208.49718	0.67061

1208.63652	0.67067

1208.77586	0.67069

1208.9152	0.67086

1209.05454	0.67102

1209.19388	0.6714

1209.33322	0.67142

1209.47256	0.67151

1209.6119	0.67159

1209.75124	0.67161

1209.89058	0.67172

1210.02992	0.67175

1210.16926	0.67224

1210.3086	0.67254

1210.44794	0.67281

1210.58728	0.67283

1210.72662	0.67286

1210.86596	0.67297

1211.0053	0.67308

1211.14464	0.67316

1211.28398	0.67343

1211.42332	0.67346

1211.56266	0.67354

1211.702	0.67359

1211.84134	0.67365

1211.98068	0.67367

1212.12002	0.67373

1212.25936	0.67375

1212.3987	0.67378

1212.53804	0.67384

1212.67738	0.67386

1212.81672	0.67397

1212.95606	0.67413

1213.0954	0.67416

1213.23474	0.67595

1213.37408	0.676

1213.51342	0.67603

1213.65276	0.67627

1213.7921	0.67646

1213.93144	0.67649

1214.07078	0.67654

1214.21012	0.67671

1214.34946	0.67757

1214.4888	0.67768

1214.62814	0.67774

1214.76748	0.67779

1214.90682	0.67782

1215.04616	0.67787

1215.1855	0.67795

1215.32485	0.67798

1215.46419	0.67806

1215.60353	0.67809

1215.74286	0.67811

1215.88221	0.6782

1216.02155	0.67858

1216.16089	0.6786

1216.30023	0.67868

1216.43957	0.67874

1216.57891	0.6789

1216.71825	0.67906

1216.85759	0.67982

1216.99693	0.67996

1217.13627	0.67998

1217.27561	0.68001

1217.41495	0.68047

1217.55429	0.6808

1217.69363	0.6809

1217.83297	0.68093

1217.97231	0.68101

1218.11165	0.68118

1218.25099	0.68137

1218.39033	0.68147

1218.52967	0.68166

1218.66901	0.68199

1218.80835	0.68256

1218.94769	0.68285

1219.08703	0.68294

1219.22637	0.6834

1219.36571	0.68378

1219.50505	0.68383

1219.64439	0.68399

1219.78373	0.68424

1219.92307	0.68462

1220.06241	0.68524

1220.20175	0.68667

1220.34109	0.68819

1220.48043	0.68941

1220.61977	0.68944

1220.75911	0.99924

1220.89845	0.9993

1221.03779	0.99932

1221.17713	0.99938

1221.31647	0.9994

1221.45581	0.99946

1221.59515	0.99957

1221.73449	0.99959

1221.87383	0.99965

1222.01317	0.99978

1222.15251	0.99984

1222.29185	0.99992

1222.43119	0.99997

1222.57053	1

**Fig 12. Characteristic curve of damage variable stage**

1	0.03454	0.24544	0.0003702

2	0.07245	0.35487	0.00072

3	0.1125	0.4734	0.00094

4	0.15894	0.58069	0.001127

5	0.17339	0.67747	0.001137

6	0.18945	0.77425	0.001188

7	0.20551	0.87104	0.001192

8	0.22477	0.96782	0.001209

9	0.23922	1.0646	0.001217

10	0.25848	1.16138	0.00122

11	0.26973	1.25816	0.00129

12	0.28418	1.35495	0.00157

13	0.29862	1.45173	0.00158

14	0.3195	1.54851	0.00164

15	0.33716	1.64529	0.00175

16	0.34679	1.74207	0.00178

17	0.36445	1.83885	0.00193

18	0.38212	1.93564	0.00222

19	0.39816	2.03242	0.00224

20	0.41262	2.1292	0.00241

21	0.43188	2.22598	0.00247

22	0.44793	2.32276	0.00249

23	0.46078	2.41955	0.00252

24	0.47684	2.51633	0.00253

25	0.4945	2.61311	0.00263

26	0.50894	2.70989	0.00264

27	0.5218	2.80667	0.00266

28	0.54106	2.90345	0.00275

29	0.56353	3.00024	0.00276

30	0.57317	3.09702	0.00281

31	0.58761	3.1938	0.00282

32	0.61009	3.29058	0.00285

33	0.62294	3.38736	0.00301

34	0.63578	3.48415	0.00302

35	0.65185	3.58093	0.00319

36	0.67432	3.67771	0.00322

37	0.68395	3.77449	0.00323

38	0.70161	3.87127	0.00329

39	0.71606	3.96805	0.00334

40	0.73532	4.06484	0.00336

41	0.74977	4.16162	0.00343

42	0.76583	4.2584	0.00348

43	0.78028	4.35518	0.00359

44	0.79954	4.45196	0.0036

45	0.81079	4.54875	0.00362

46	0.83005	4.64553	0.00376

47	0.84771	4.74231	0.0041

48	0.86216	4.83909	0.00421

49	0.8734	4.93587	0.00423

50	0.88785	5.03265	0.0043

51	0.90872	5.12944	0.00432

52	0.92317	5.22622	0.00451

53	0.93441	5.323	0.00455

54	0.95849	5.41978	0.00456

55	0.97133	5.51656	0.00457

56	0.98418	5.61335	0.00458

57	1.01628	5.71013	0.00459

58	1.03074	5.80691	0.00461

59	1.05001	5.90369	0.00465

60	1.05964	6.00047	0.00487

61	1.0757	6.09725	0.00488

62	1.09496	6.19404	0.00525

63	1.10781	6.29082	0.00526

64	1.11904	6.3876	0.0055

65	1.14473	6.48438	0.00556

66	1.15436	6.58116	0.00557

67	1.17203	6.67794	0.00564

68	1.18647	6.77473	0.00573

69	1.20413	6.87151	0.0058

70	1.2202	6.96829	0.00581

71	1.23464	7.06507	0.006

72	1.2523	7.16185	0.00603

73	1.26996	7.25864	0.00611

74	1.28441	7.35542	0.00622

75	1.29564	7.4522	0.00624

76	1.31652	7.54898	0.00625

77	1.33258	7.64576	0.00627

78	1.34863	7.74254	0.00636

79	1.35988	7.83933	0.00644

80	1.38555	7.93611	0.00669

81	1.3984	8.03289	0.00673

82	1.40803	8.12967	0.00684

83	1.42249	8.22645	0.00695

84	1.44497	8.32324	0.00697

85	1.46102	8.42002	0.00741

86	1.47226	8.5168	0.00745

87	1.49312	8.61358	0.00746

88	1.50757	8.71036	0.00774

89	1.52203	8.80714	0.00802

90	1.53808	8.90393	0.00815

91	1.55735	9.00071	0.00827

92	1.57019	9.09749	0.00829

93	1.58303	9.19427	0.00845

94	1.6007	9.29105	0.00849

95	1.61996	9.38784	0.00883

96	1.63923	9.48462	0.00884

97	1.64725	9.5814	0.00889

98	1.66491	9.67818	0.00891

99	1.68579	9.77496	0.00897

100	1.69702	9.87174	0.00902

101	1.7163	9.96853	0.00931

102	1.73075	10.06531	0.01001

103	1.75001	10.16209	0.01009

104	1.76446	10.25887	0.0103

105	1.77569	10.35565	0.0104

106	1.79818	10.45244	0.01055

107	1.81263	10.54922	0.01056

108	1.82386	10.646	0.01057

109	1.84152	10.74278	0.01058

110	1.86079	10.83956	0.01071

111	1.88165	10.93634	0.01072

112	1.88647	11.03313	0.01076

113	1.90735	11.12991	0.01078

114	1.92661	11.22669	0.01079

115	1.93464	11.32347	0.01103

116	1.94908	11.42025	0.01105

117	1.97157	11.51704	0.01114

118	1.98762	11.61382	0.01116

119	2.00047	11.7106	0.01122

120	2.01974	11.80738	0.01123

121	2.039	11.90416	0.01124

122	2.05346	12.00094	0.01125

123	2.06629	12.09773	0.01132

124	2.07913	12.19451	0.0114

125	2.10322	12.29129	0.0115

126	2.11606	12.38807	0.01151

127	2.12571	12.48485	0.01157

128	2.14818	12.58164	0.01162

129	2.16263	12.67842	0.01186

130	2.1835	12.7752	0.01187

131	2.19313	12.87198	0.01189

132	2.214	12.96876	0.01204

133	2.22685	13.06554	0.01211

134	2.23969	13.16233	0.01224

135	2.25574	13.25911	0.01229

136	2.27983	13.35589	0.01246

137	2.29106	13.45267	0.01251

138	2.30231	13.54945	0.01253

139	2.32157	13.64624	0.01258

140	2.34404	13.74302	0.0126

141	2.36011	13.8398	0.01284

142	2.36974	13.93658	0.01291

143	2.389	14.03336	0.01299

144	2.40827	14.13014	0.01306

145	2.4147	14.22693	0.01313

146	2.43396	14.32371	0.01326

147	2.45483	14.42049	0.01329

148	2.46928	14.51727	0.01331

149	2.48213	14.61405	0.01336

150	2.50299	14.71084	0.01338

151	2.52066	14.80762	0.0134

152	2.53189	14.9044	0.01366

153	2.54313	15.00118	0.01374

154	2.56401	15.09796	0.01389

155	2.58167	15.19474	0.01399

156	2.58968	15.29153	0.01416

157	2.61056	15.38831	0.01418

158	2.62982	15.48509	0.01428

159	2.64428	15.58187	0.0143

160	2.65711	15.67865	0.01435

161	2.67318	15.77544	0.01438

162	2.69726	15.87222	0.01444

163	2.7085	15.969	0.01466

164	2.72135	16.06578	0.0147

165	2.74061	16.16256	0.01474

166	2.75666	16.25934	0.01485

167	2.7695	16.35613	0.01518

168	2.78396	16.45291	0.01519

169	2.80965	16.54969	0.01523

170	2.81928	16.64647	0.01531

171	2.8273	16.74325	0.01532

172	2.853	16.84004	0.01535

173	2.87066	16.93682	0.01558

174	2.88189	17.0336	0.01559

175	2.89634	17.13038	0.0156

176	2.91721	17.22716	0.0158

177	2.93327	17.32394	0.01583

178	2.94772	17.42073	0.01586

179	2.96217	17.51751	0.01595

180	2.98625	17.61429	0.01598

181	2.99589	17.71107	0.01601

182	3.00713	17.80785	0.01619

183	3.02318	17.90464	0.01638

184	3.04566	18.00142	0.0164

185	3.06011	18.0982	0.01651

186	3.07615	18.19498	0.01652

187	3.09061	18.29176	0.01692

188	3.10827	18.38854	0.01696

189	3.11791	18.48533	0.01707

190	3.13717	18.58211	0.01724

191	3.15804	18.67889	0.01732

192	3.1757	18.77567	0.01739

193	3.18694	18.87245	0.01759

194	3.20461	18.96924	0.0178

195	3.22226	19.06602	0.01782

196	3.23511	19.1628	0.01783

197	3.25116	19.25958	0.01787

198	3.26561	19.35636	0.01814

199	3.2913	19.45314	0.01815

200	3.30093	19.54993	0.01822

201	3.31538	19.64671	0.01823

202	3.33143	19.74349	0.01828

203	3.3507	19.84027	0.01829

204	3.36837	19.93705	0.01831

205	3.37639	20.03383	0.01874

206	3.39887	20.13062	0.01875

207	3.41653	20.2274	0.01893

208	3.42294	20.32418	0.01894

209	3.44222	20.42096	0.01895

210	3.46309	20.51774	0.01897

211	3.47753	20.61453	0.01903

212	3.48878	20.71131	0.01904

213	3.51286	20.80809	0.01907

214	3.53213	20.90487	0.0191

215	3.53695	21.00165	0.01911

216	3.55299	21.09843	0.01912

217	3.57066	21.19522	0.01928

218	3.59313	21.292	0.01929

219	3.59956	21.38878	0.0193

220	3.61883	21.48556	0.01935

221	3.63969	21.58234	0.01937

222	3.66057	21.67913	0.01938

223	3.6702	21.77591	0.01942

224	3.68465	21.87269	0.01943

225	3.70071	21.96947	0.01944

226	3.71355	22.06625	0.01946

227	3.728	22.16303	0.01948

228	3.75047	22.25982	0.01954

229	3.76814	22.3566	0.01955

230	3.78259	22.45338	0.01957

231	3.79543	22.55016	0.01972

232	3.81469	22.64694	0.01974

233	3.83235	22.74373	0.02012

234	3.83717	22.84051	0.02013

235	3.85805	22.93729	0.02025

236	3.88213	23.03407	0.02027

237	3.89176	23.13085	0.02028

238	3.903	23.22763	0.02048

239	3.92869	23.32442	0.02053

240	3.94795	23.4212	0.02065

241	3.95599	23.51798	0.02066

242	3.97364	23.61476	0.02071

243	3.99452	23.71154	0.02118

244	4.00415	23.80833	0.02119

245	4.01699	23.90511	0.02123

246	4.03787	24.00189	0.02128

247	4.05713	24.09867	0.02129

248	4.06837	24.19545	0.02136

249	4.08603	24.29223	0.0214

250	4.10368	24.38902	0.02148

251	4.11653	24.4858	0.02149

252	4.12777	24.58258	0.0215

253	4.14383	24.67936	0.02152

254	4.17112	24.77614	0.02158

255	4.18236	24.87293	0.02178

256	4.19359	24.96971	0.02193

257	4.21447	25.06649	0.022

258	4.23373	25.16327	0.02204

259	4.24336	25.26005	0.02206

260	4.25782	25.35683	0.02208

261	4.2819	25.45362	0.02226

262	4.29474	25.5504	0.02232

263	4.30598	25.64718	0.02233

264	4.32524	25.74396	0.02236

265	4.34452	25.84074	0.02241

266	4.36218	25.93753	0.02251

267	4.37341	26.03431	0.02254

268	4.38466	26.13109	0.0227

269	4.41515	26.22787	0.02274

270	4.41998	26.32465	0.02284

271	4.43282	26.42143	0.02288

272	4.45369	26.51822	0.02289

273	4.47136	26.615	0.0229

274	4.4874	26.71178	0.02295

275	4.50025	26.80856	0.02302

276	4.52434	26.90534	0.02303

277	4.53717	27.00213	0.0232

278	4.54681	27.09891	0.02321

279	4.55965	27.19569	0.02326

280	4.58534	27.29247	0.02328

281	4.59818	27.38925	0.02329

282	4.61263	27.48603	0.02343

283	4.62708	27.58282	0.02348

284	4.64796	27.6796	0.02356

285	4.6608	27.77638	0.02357

286	4.68006	27.87316	0.02365

287	4.68969	27.96994	0.0237

288	4.70897	28.06673	0.02373

289	4.72181	28.16351	0.02383

290	4.73627	28.26029	0.02408

291	4.76035	28.35707	0.0241

292	4.78443	28.45385	0.0243

293	4.78924	28.55063	0.02455

294	4.80208	28.64742	0.0246

295	4.82136	28.7442	0.02461

296	4.8358	28.84098	0.02465

297	4.84865	28.93776	0.02466

298	4.86309	29.03454	0.02471

299	4.89199	29.13133	0.02472

300	4.90003	29.22811	0.02474

301	4.91929	29.32489	0.02497

302	4.93695	29.42167	0.02499

303	4.95942	29.51845	0.02503

304	4.96264	29.61523	0.02505

305	4.9803	29.71202	0.02509

306	4.99475	29.8088	0.0251

307	5.01401	29.90558	0.02548

308	5.02364	30.00236	0.02549

309	5.04934	30.09914	0.0255

310	5.0686	30.19593	0.02553

311	5.08144	30.29271	0.02576

312	5.08947	30.38949	0.02577

313	5.11836	30.48627	0.02579

314	5.13122	30.58305	0.02588

315	5.14245	30.67983	0.02621

316	5.15369	30.77662	0.02633

317	5.17616	30.8734	0.02652

318	5.19865	30.97018	0.0267

319	5.20186	31.06696	0.02674

320	5.22274	31.16374	0.02708

321	5.242	31.26053	0.0271

322	5.25003	31.35731	0.02733

323	5.26769	31.45409	0.02743

324	5.28374	31.55087	0.02761

325	5.30462	31.64765	0.02765

326	5.31264	31.74443	0.02775

327	5.33029	31.84122	0.02784

328	5.35438	31.938	0.02785

329	5.37043	32.03478	0.02786

330	5.37846	32.13156	0.02789

331	5.39772	32.22834	0.028

332	5.42021	32.32512	0.02803

333	5.43305	32.42191	0.02809

334	5.44589	32.51869	0.0282

335	5.46838	32.61547	0.02832

336	5.48443	32.71225	0.02834

337	5.49567	32.80903	0.02842

338	5.51493	32.90582	0.02849

339	5.52618	33.0026	0.0285

340	5.54705	33.09938	0.02862

341	5.55026	33.19616	0.02863

342	5.56952	33.29294	0.02867

343	5.59199	33.38972	0.0287

344	5.61127	33.48651	0.02874

345	5.61608	33.58329	0.02876

346	5.64498	33.68007	0.02899

347	5.66425	33.77685	0.02901

348	5.66907	33.87363	0.02902

349	5.68512	33.97042	0.02903

350	5.70438	34.0672	0.02905

351	5.72204	34.16398	0.02959

352	5.72847	34.26076	0.02962

353	5.75255	34.35754	0.02963

354	5.7686	34.45432	0.02964

355	5.79268	34.55111	0.02966

356	5.7975	34.64789	0.02974

357	5.81998	34.74467	0.02976

358	5.83604	34.84145	0.02977

359	5.84567	34.93823	0.03012

360	5.85852	35.03502	0.03027

361	5.88742	35.1318	0.03028

362	5.90186	35.22858	0.03029

363	5.90989	35.32536	0.03033

364	5.92915	35.42214	0.03034

365	5.94681	35.51892	0.03043

366	5.95966	35.61571	0.03071

367	5.97572	35.71249	0.03074

368	5.99177	35.80927	0.03075

369	6.00943	35.90605	0.03076

370	6.02228	36.00283	0.03081

371	6.03833	36.09962	0.03098

372	6.05599	36.1964	0.03103

373	6.07686	36.29318	0.03107

374	6.08649	36.38996	0.03117

375	6.10255	36.48674	0.03118

376	6.12182	36.58352	0.03136

377	6.14108	36.68031	0.03137

378	6.1459	36.77709	0.03143

379	6.16999	36.87387	0.03151

380	6.19727	36.97065	0.03164

381	6.20531	37.06743	0.03166

382	6.21333	37.16422	0.03169

383	6.2326	37.261	0.03172

384	6.24865	37.35778	0.03188

385	6.25989	37.45456	0.03189

386	6.27915	37.55134	0.03199

387	6.30003	37.64812	0.03201

388	6.3193	37.74491	0.03205

389	6.32411	37.84169	0.03214

390	6.34177	37.93847	0.03244

391	6.36585	38.03525	0.03246

392	6.3803	38.13203	0.03278

393	6.38673	38.22882	0.03324

394	6.40599	38.3256	0.03369

395	6.43169	38.42238	0.03373

396	6.4381	38.51916	0.03384

397	6.45736	38.61594	0.03391

398	6.47342	38.71272	0.03395

399	6.4959	38.80951	0.03434

400	6.50393	38.90629	0.03444

401	6.51357	39.00307	0.03455

402	6.54086	39.09985	0.0346

403	6.55692	39.19663	0.03469

404	6.56173	39.29342	0.03474

405	6.58259	39.3902	0.03475

406	6.60668	39.48698	0.03481

407	6.62113	39.58376	0.03482

408	6.63237	39.68054	0.03488

409	6.65325	39.77732	0.0349

410	6.66769	39.87411	0.03491

411	6.67732	39.97089	0.03507

412	6.69819	40.06767	0.03512

413	6.71906	40.16445	0.03519

414	6.73512	40.26123	0.0352

415	6.74957	40.35802	0.03522

416	6.76402	40.4548	0.03525

417	6.78649	40.55158	0.0354

418	6.79774	40.64836	0.03566

419	6.80737	40.74514	0.03575

420	6.82664	40.84192	0.03587

421	6.84911	40.93871	0.03599

422	6.86357	41.03549	0.03602

423	6.8732	41.13227	0.03629

424	6.89888	41.22905	0.03633

425	6.91494	41.32583	0.03634

426	6.91975	41.42262	0.03636

427	6.94705	41.5194	0.03639

428	6.9615	41.61618	0.03672

429	6.97113	41.71296	0.03675

430	6.98718	41.80974	0.03677

431	7.00325	41.90652	0.03696

432	7.02251	42.00331	0.03706

433	7.04177	42.10009	0.03707

434	7.05784	42.19687	0.03708

435	7.06906	42.29365	0.03713

436	7.08673	42.39043	0.03772

437	7.09797	42.48722	0.03773

438	7.11563	42.584	0.03778

439	7.1381	42.68078	0.03784

440	7.15094	42.77756	0.03791

441	7.15898	42.87434	0.03792

442	7.18627	42.97112	0.03799

443	7.20233	43.06791	0.03801

444	7.21356	43.16469	0.03802

445	7.22801	43.26147	0.03817

446	7.25691	43.35825	0.03819

447	7.26495	43.45503	0.03825

448	7.27618	43.55182	0.03826

449	7.28903	43.6486	0.03827

450	7.31632	43.74538	0.0383

451	7.32595	43.84216	0.03831

452	7.34361	43.93894	0.03834

453	7.35805	44.03572	0.03835

454	7.38054	44.13251	0.03836

455	7.38696	44.22929	0.03841

456	7.40141	44.32607	0.03889

457	7.42389	44.42285	0.03895

458	7.43993	44.51963	0.03896

459	7.45279	44.61641	0.03914

460	7.46723	44.7132	0.03915

461	7.48651	44.80998	0.03919

462	7.50577	44.90676	0.0392

463	7.51701	45.00354	0.03926

464	7.52664	45.10032	0.03927

465	7.54912	45.19711	0.03929

466	7.56839	45.29389	0.0393

467	7.57641	45.39067	0.03937

468	7.6021	45.48745	0.03944

469	7.62297	45.58423	0.03947

470	7.6326	45.68101	0.03949

471	7.64063	45.7778	0.03953

472	7.66311	45.87458	0.03965

473	7.6904	45.97136	0.03967

474	7.6888	46.06814	0.03968

475	7.71448	46.16492	0.03986

476	7.73054	46.26171	0.03998

477	7.7482	46.35849	0.03999

478	7.75462	46.45527	0.04008

479	7.7755	46.55205	0.04019

480	7.79476	46.64883	0.0402

481	7.80279	46.74561	0.04039

482	7.81724	46.8424	0.04043

483	7.83971	46.93918	0.04062

484	7.85897	47.03596	0.04069

485	7.8654	47.13274	0.04075

486	7.88788	47.22952	0.04127

487	7.91036	47.32631	0.04131

488	7.92159	47.42309	0.04132

489	7.93284	47.51987	0.04133

490	7.95371	47.61665	0.04174

491	7.96976	47.71343	0.04176

492	7.98743	47.81021	0.0419

493	7.99224	47.907	0.04217

494	8.02435	48.00378	0.04227

495	8.03559	48.10056	0.04229

496	8.04201	48.19734	0.0423

497	8.05807	48.29412	0.04231

498	8.08215	48.39091	0.04234

499	8.09499	48.48769	0.04235

500	8.11425	48.58447	0.04239

501	8.1287	48.68125	0.04241

502	8.14958	48.77803	0.04289

503	8.15761	48.87481	0.04319

504	8.17366	48.9716	0.04325

505	8.19775	49.06838	0.04327

506	8.20576	49.16516	0.04331

507	8.21701	49.26194	0.04333

508	8.23146	49.35872	0.04357

509	8.25875	49.45551	0.04371

510	8.26679	49.55229	0.04411

511	8.27963	49.64907	0.04421

512	8.30371	49.74585	0.04494

513	8.32297	49.84263	0.04497

514	8.33742	49.93941	0.04498

515	8.34706	50.0362	0.04499

516	8.37114	50.13298	0.04509

517	8.38238	50.22976	0.04516

518	8.39522	50.32654	0.04517

519	8.41449	50.42332	0.04518

520	8.43215	50.52011	0.0452

521	8.45302	50.61689	0.04525

522	8.46104	50.71367	0.04526

523	8.47871	50.81045	0.04535

524	8.50279	50.90723	0.04588

525	8.50761	51.00401	0.04601

526	8.52527	51.1008	0.04602

527	8.53971	51.19758	0.04603

528	8.57022	51.29436	0.04617

529	8.57986	51.39114	0.04623

530	8.58949	51.48792	0.04635

531	8.61358	51.58471	0.04636

532	8.62802	51.68149	0.04644

533	8.63605	51.77827	0.04646

534	8.6521	51.87505	0.04647

535	8.67297	51.97183	0.04651

536	8.68742	52.06861	0.04685

537	8.69705	52.1654	0.04699

538	8.71954	52.26218	0.04701

539	8.74201	52.35896	0.04702

540	8.75967	52.45574	0.04707

541	8.75967	52.55252	0.04712

542	8.78215	52.64931	0.0472

543	8.7982	52.74609	0.04724

544	8.80623	52.84287	0.04726

545	8.82389	52.93965	0.04727

546	8.85279	53.03643	0.04745

547	8.87045	53.13321	0.0475

548	8.87687	53.23	0.0476

549	8.89294	53.32678	0.04762

550	8.91702	53.42356	0.0477

551	8.92665	53.52034	0.04782

552	8.93949	53.61712	0.04786

553	8.96196	53.71391	0.04788

554	8.97803	53.81069	0.04792

555	8.98766	53.90747	0.04793

556	9.01013	54.00425	0.04796

557	9.0294	54.10103	0.04807

558	9.04384	54.19781	0.04813

559	9.05027	54.2946	0.04817

560	9.06793	54.39138	0.04822

561	9.09362	54.48816	0.04824

562	9.10968	54.58494	0.04825

563	9.11609	54.68172	0.04826

564	9.14179	54.77851	0.04832

565	9.16586	54.87529	0.04873

566	9.17068	54.97207	0.04878

567	9.18193	55.06885	0.04879

568	9.20922	55.16563	0.0488

569	9.22045	55.26241	0.04891

570	9.23169	55.3592	0.04892

571	9.24936	55.45598	0.04893

572	9.27344	55.55276	0.04902

573	9.28628	55.64954	0.04904

574	9.29591	55.74632	0.04906

575	9.32161	55.84311	0.0491

576	9.33927	55.93989	0.04916

577	9.33927	56.03667	0.04918

578	9.36334	56.13345	0.04942

579	9.38582	56.23023	0.04948

580	9.40027	56.32701	0.04952

581	9.4099	56.4238	0.04964

582	9.42757	56.52058	0.04965

583	9.45004	56.61736	0.04977

584	9.45647	56.71414	0.04986

585	9.47413	56.81092	0.0499

586	9.48697	56.9077	0.04991

587	9.50623	57.00449	0.04993

588	9.51747	57.10127	0.05019

589	9.53995	57.19805	0.05032

590	9.56082	57.29483	0.05033

591	9.57848	57.39161	0.05041

592	9.58652	57.4884	0.05054

593	9.60256	57.58518	0.05059

594	9.62344	57.68196	0.0506

595	9.63789	57.77874	0.05061

596	9.64912	57.87552	0.05064

597	9.66518	57.9723	0.05067

598	9.68926	58.06909	0.05068

599	9.69729	58.16587	0.05069

600	9.71495	58.26265	0.05071

601	9.73743	58.35943	0.05072

602	9.75669	58.45621	0.05087

603	9.76312	58.553	0.05088

604	9.77757	58.64978	0.05091

605	9.80326	58.74656	0.05134

606	9.81289	58.84334	0.05145

607	9.82412	58.94012	0.05146

608	9.84821	59.0369	0.05151

609	9.86908	59.13369	0.05152

610	9.88192	59.23047	0.05155

611	9.88514	59.32725	0.05172

612	9.90922	59.42403	0.05179

613	9.93009	59.52081	0.05214

614	9.93651	59.6176	0.05215

615	9.95096	59.71438	0.05221

616	9.97665	59.81116	0.05229

617	9.99752	59.90794	0.05232

618	10.00876	60.00472	0.05241

619	10.02001	60.1015	0.05242

620	10.03927	60.19829	0.05245

621	10.04568	60.29507	0.05296

622	10.06174	60.39185	0.05326

623	10.08422	60.48863	0.05346

624	10.1083	60.58541	0.05354

625	10.12115	60.6822	0.05357

626	10.13078	60.77898	0.05358

627	10.15325	60.87576	0.0537

628	10.17734	60.97254	0.05372

629	10.17895	61.06932	0.05373

630	10.19179	61.1661	0.05374

631	10.21749	61.26289	0.05384

632	10.22712	61.35967	0.05387

633	10.23996	61.45645	0.05389

634	10.25761	61.55323	0.05397

635	10.2833	61.65001	0.05398

636	10.29293	61.7468	0.05404

637	10.31221	61.84358	0.05405

638	10.32344	61.94036	0.05407

639	10.3411	62.03714	0.05417

640	10.34913	62.13392	0.05419

641	10.3668	62.2307	0.05425

642	10.39409	62.32749	0.05426

643	10.40853	62.42427	0.05429

644	10.41977	62.52105	0.0543

645	10.43743	62.61783	0.05431

646	10.46312	62.71461	0.05438

647	10.47115	62.8114	0.05439

648	10.48239	62.90818	0.0544

649	10.50486	63.00496	0.05442

650	10.51932	63.10174	0.05443

651	10.53056	63.19852	0.05444

652	10.54982	63.2953	0.05447

653	10.5755	63.39209	0.05448

654	10.58514	63.48887	0.0545

655	10.59157	63.58565	0.05451

656	10.60921	63.68243	0.05464

657	10.6333	63.77921	0.0547

658	10.64775	63.876	0.05481

659	10.65579	63.97278	0.05482

660	10.67665	64.06956	0.05483

661	10.69592	64.16634	0.05494

662	10.71037	64.26312	0.05495

663	10.72804	64.3599	0.05496

664	10.74889	64.45669	0.05497

665	10.75854	64.55347	0.05499

666	10.76977	64.65025	0.05503

667	10.78743	64.74703	0.0551

668	10.80991	64.84381	0.05511

669	10.82114	64.9406	0.05513

670	10.83881	65.03738	0.05514

671	10.85327	65.13416	0.05515

672	10.87735	65.23094	0.05516

673	10.89019	65.32772	0.05518

674	10.8934	65.4245	0.0553

675	10.9207	65.52129	0.05531

676	10.93193	65.61807	0.05532

677	10.94959	65.71485	0.05535

678	10.96886	65.81163	0.05538

679	10.99295	65.90841	0.05539

680	11.00258	66.0052	0.05543

681	11.01221	66.10198	0.05545

682	11.02505	66.19876	0.05547

683	11.04913	66.29554	0.05548

684	11.05876	66.39232	0.05556

685	11.07321	66.4891	0.05557

686	11.09248	66.58589	0.05558

687	11.11496	66.68267	0.05567

688	11.12459	66.77945	0.05571

689	11.13904	66.87623	0.05578

690	11.16312	66.97301	0.0558

691	11.17436	67.0698	0.05581

692	11.18079	67.16658	0.05609

693	11.20326	67.26336	0.05611

694	11.22414	67.36014	0.05613

695	11.23377	67.45692	0.05625

696	11.24982	67.5537	0.05626

697	11.2739	67.65049	0.05629

698	11.28995	67.74727	0.05635

699	11.3012	67.84405	0.05637

700	11.31404	67.94083	0.05638

701	11.34133	68.03761	0.0564

702	11.34937	68.1344	0.05644

703	11.3606	68.23118	0.05685

704	11.38468	68.32796	0.05687

705	11.40877	68.42474	0.05691

706	11.4184	68.52152	0.05697

707	11.42963	68.6183	0.05705

708	11.44409	68.71509	0.05706

709	11.46978	68.81187	0.05714

710	11.4778	68.90865	0.05716

711	11.49225	69.00543	0.05718

712	11.51472	69.10221	0.0572

713	11.53239	69.199	0.05726

714	11.54202	69.29578	0.05761

715	11.55648	69.39256	0.05762

716	11.57896	69.48934	0.05763

717	11.59982	69.58612	0.05764

718	11.59822	69.6829	0.05772

719	11.62391	69.77969	0.05777

720	11.64318	69.87647	0.05779

721	11.65762	69.97325	0.05781

722	11.67207	70.07003	0.05782

723	11.69455	70.16681	0.05785

724	11.71061	70.26359	0.05786

725	11.71864	70.36038	0.05788

726	11.72827	70.45716	0.05789

727	11.75716	70.55394	0.05791

728	11.77482	70.65072	0.05792

729	11.78124	70.7475	0.05794

730	11.7989	70.84429	0.05797

731	11.81978	70.94107	0.05798

732	11.83744	71.03785	0.05799

733	11.84707	71.13463	0.058

734	11.86633	71.23141	0.05805

735	11.8856	71.32819	0.05808

736	11.88721	71.42498	0.0583

737	11.91129	71.52176	0.05831

738	11.93538	71.61854	0.05842

739	11.94983	71.71532	0.05843

740	11.95785	71.8121	0.05844

741	11.97712	71.90889	0.05845

742	11.9996	72.00567	0.05848

743	12.01564	72.10245	0.05851

744	12.02689	72.19923	0.05852

745	12.03813	72.29601	0.05879

746	12.059	72.39279	0.05881

747	12.07344	72.48958	0.05882

748	12.09272	72.58636	0.05883

749	12.10877	72.68314	0.0589

750	12.12803	72.77992	0.05892

751	12.13767	72.8767	0.05906

752	12.15212	72.97349	0.05932

753	12.17299	73.07027	0.05936

754	12.18904	73.16705	0.05943

755	12.19065	73.26383	0.05946

756	12.21794	73.36061	0.05947

757	12.23882	73.45739	0.05949

758	12.25166	73.55418	0.0595

759	12.26771	73.65096	0.05972

760	12.28699	73.74774	0.05983

761	12.30143	73.84452	0.05999

762	12.30946	73.9413	0.0604

763	12.32391	74.03809	0.06044

764	12.34799	74.13487	0.06046

765	12.36725	74.23165	0.06054

766	12.3785	74.32843	0.06093

767	12.39455	74.42521	0.06126

768	12.42023	74.52199	0.06127

769	12.42827	74.61878	0.06128

770	12.43468	74.71556	0.06135

771	12.46198	74.81234	0.06138

772	12.47482	74.90912	0.06146

773	12.48607	75.0059	0.06148

774	12.50693	75.10269	0.06166

775	12.5262	75.19947	0.06167

776	12.54387	75.29625	0.06214

777	12.55189	75.39303	0.06245

778	12.56955	75.48981	0.06248

779	12.59042	75.58659	0.06264

780	12.60648	75.68338	0.06265

781	12.6145	75.78016	0.06268

782	12.63698	75.87694	0.06303

783	12.64983	75.97372	0.06305

784	12.66428	76.0705	0.06307

785	12.68194	76.16729	0.06311

786	12.70281	76.26407	0.06312

787	12.72047	76.36085	0.06317

788	12.72689	76.45763	0.0638

789	12.74776	76.55441	0.06388

790	12.76543	76.65119	0.06396

791	12.77506	76.74798	0.06397

792	12.7879	76.84476	0.06398

793	12.81359	76.94154	0.06399

794	12.83606	77.03832	0.06403

795	12.84089	77.1351	0.06413

796	12.85694	77.23189	0.06421

797	12.8762	77.32867	0.06445

798	12.88905	77.42545	0.06447

799	12.90671	77.52223	0.06454

800	12.91956	77.61901	0.06469

801	12.94524	77.71579	0.06537

802	12.95808	77.81258	0.06539

803	12.97414	77.90936	0.06555

804	12.99662	78.00614	0.06569

805	13.00465	78.10292	0.0657

806	13.02231	78.1997	0.06599

807	13.03354	78.29649	0.06603

808	13.05602	78.39327	0.06607

809	13.07368	78.49005	0.06614

810	13.0785	78.58683	0.06616

811	13.09937	78.68361	0.06617

812	13.12024	78.78039	0.06641

813	13.1363	78.87718	0.06643

814	13.14593	78.97396	0.06644

815	13.17804	79.07074	0.06648

816	13.17965	79.16752	0.06661

817	13.1941	79.2643	0.06662

818	13.21818	79.36109	0.06674

819	13.23263	79.45787	0.06675

820	13.24868	79.55465	0.06698

821	13.26474	79.65143	0.06699

822	13.27758	79.74821	0.06702

823	13.30006	79.84499	0.06709

824	13.3113	79.94178	0.0671

825	13.31772	80.03856	0.06718

826	13.34019	80.13534	0.06722

827	13.36107	80.23212	0.06723

828	13.3707	80.3289	0.06725

829	13.38997	80.42569	0.06728

830	13.40603	80.52247	0.0673

831	13.42207	80.61925	0.06731

832	13.43653	80.71603	0.06732

833	13.45579	80.81281	0.06735

834	13.47667	80.90959	0.06746

835	13.48469	81.00638	0.0675

836	13.50075	81.10316	0.06768

837	13.5152	81.19994	0.06769

838	13.53446	81.29672	0.0677

839	13.54571	81.3935	0.06784

840	13.56818	81.49029	0.06786

841	13.58263	81.58707	0.06787

842	13.59547	81.68385	0.06788

843	13.60671	81.78063	0.0679

844	13.62918	81.87741	0.06796

845	13.64846	81.97419	0.06805

846	13.66451	82.07098	0.06808

847	13.67254	82.16776	0.0682

848	13.69823	82.26454	0.06826

849	13.71107	82.36132	0.0687

850	13.72231	82.4581	0.06873

851	13.73034	82.55488	0.06878

852	13.76085	82.65167	0.06879

853	13.78332	82.74845	0.06883

854	13.78974	82.84523	0.06884

855	13.80258	82.94201	0.06891

856	13.82666	83.03879	0.06893

857	13.84754	83.13558	0.0692

858	13.84915	83.23236	0.06923

859	13.86841	83.32914	0.06943

860	13.88607	83.42592	0.06979

861	13.89891	83.5227	0.0698

862	13.91979	83.61948	0.06981

863	13.93584	83.71627	0.06984

864	13.95832	83.81305	0.06986

865	13.96796	83.90983	0.06991

866	13.98241	84.00661	0.07017

867	14.00327	84.10339	0.07045

868	14.01933	84.20018	0.07046

869	14.02255	84.29696	0.07048

870	14.05304	84.39374	0.07049

871	14.07231	84.49052	0.0705

872	14.08034	84.5873	0.07051

873	14.098	84.68408	0.07052

874	14.11245	84.78087	0.07054

875	14.13011	84.87765	0.0706

876	14.14135	84.97443	0.07069

877	14.15098	85.07121	0.0707

878	14.17667	85.16799	0.07078

879	14.19112	85.26478	0.07102

880	14.20075	85.36156	0.07109

881	14.22644	85.45834	0.07123

882	14.24892	85.55512	0.07124

883	14.25695	85.6519	0.07125

884	14.26658	85.74868	0.07126

885	14.28745	85.84547	0.07137

886	14.30832	85.94225	0.07138

887	14.31474	86.03903	0.07149

888	14.3324	86.13581	0.0715

889	14.3597	86.23259	0.07164

890	14.37093	86.32938	0.07171

891	14.38379	86.42616	0.07173

892	14.39823	86.52294	0.07182

893	14.4191	86.61972	0.07195

894	14.43034	86.7165	0.07196

895	14.43997	86.81328	0.07198

896	14.46567	86.91007	0.07202

897	14.48332	87.00685	0.07207

898	14.49617	87.10363	0.07208

899	14.51061	87.20041	0.0721

900	14.5331	87.29719	0.0722

901	14.54594	87.39398	0.07224

902	14.55718	87.49076	0.0723

903	14.57162	87.58754	0.07238

904	14.59891	87.68432	0.07239

905	14.61176	87.7811	0.0724

906	14.61979	87.87788	0.07267

907	14.64227	87.97467	0.07268

908	14.65833	88.07145	0.07289

909	14.67759	88.16823	0.07293

910	14.68722	88.26501	0.07294

911	14.69686	88.36179	0.07295

912	14.72095	88.45858	0.07296

913	14.73378	88.55536	0.07297

914	14.74662	88.65214	0.07298

915	14.77071	88.74892	0.07299

916	14.79158	88.8457	0.07301

917	14.80924	88.94248	0.07309

918	14.81085	89.03927	0.0731

919	14.84456	89.13605	0.07312

920	14.85098	89.23283	0.07313

921	14.85901	89.32961	0.07325

922	14.87989	89.42639	0.0733

923	14.89755	89.52318	0.07359

924	14.91521	89.61996	0.07369

925	14.93607	89.71674	0.0737

926	14.95855	89.81352	0.07377

927	14.96979	89.9103	0.07378

928	14.96979	90.00708	0.07381

929	14.99387	90.10387	0.07382

930	15.01795	90.20065	0.07387

931	15.0308	90.29743	0.07389

932	15.04043	90.39421	0.07393

933	15.0597	90.49099	0.07395

934	15.08057	90.58778	0.07398

935	15.08539	90.68456	0.07399

936	15.10626	90.78134	0.07403

937	15.12713	90.87812	0.07414

938	15.13837	90.9749	0.07443

939	15.14962	91.07168	0.07462

940	15.16888	91.16847	0.07463

941	15.19135	91.26525	0.07464

942	15.2042	91.36203	0.07465

943	15.21383	91.45881	0.07466

944	15.23309	91.55559	0.07467

945	15.25397	91.65238	0.07468

946	15.27163	91.74916	0.07475

947	15.28126	91.84594	0.07476

948	15.29731	91.94272	0.07481

949	15.31819	92.0395	0.07484

950	15.31979	92.13628	0.07486

951	15.34388	92.23307	0.07488

952	15.37118	92.32985	0.07491

953	15.3792	92.42663	0.07532

954	15.39044	92.52341	0.07535

955	15.41291	92.62019	0.0754

956	15.42898	92.71698	0.07541

957	15.44342	92.81376	0.07545

958	15.44983	92.91054	0.07546

959	15.48034	93.00732	0.07567

960	15.49319	93.1041	0.07568

961	15.50924	93.20088	0.07572

962	15.52049	93.29767	0.07581

963	15.54617	93.39445	0.07614

964	15.55901	93.49123	0.07615

965	15.56384	93.58801	0.07616

966	15.58631	93.68479	0.07617

967	15.60558	93.78158	0.07618

968	15.61842	93.87836	0.07621

969	15.63447	93.97514	0.07634

970	15.65695	94.07192	0.07635

971	15.67139	94.1687	0.07637

972	15.68104	94.26548	0.07638

973	15.69548	94.36227	0.07639

974	15.71315	94.45905	0.07642

975	15.73081	94.55583	0.07645

976	15.74364	94.65261	0.07646

977	15.76773	94.74939	0.07687

978	15.79021	94.84617	0.07688

979	15.79663	94.94296	0.07692

980	15.80144	95.03974	0.07693

981	15.82874	95.13652	0.07722

982	15.84961	95.2333	0.07725

983	15.85603	95.33008	0.07727

984	15.86566	95.42687	0.07728

985	15.89136	95.52365	0.07729

986	15.91062	95.62043	0.07734

987	15.92026	95.71721	0.07737

988	15.93309	95.81399	0.07739

989	15.95557	95.91077	0.07745

990	15.97484	96.00756	0.07747

991	15.98287	96.10434	0.07767

992	16.00214	96.20112	0.07807

993	16.023	96.2979	0.07808

994	16.03264	96.39468	0.0781

995	16.04227	96.49147	0.07818

996	16.07277	96.58825	0.07836

997	16.08402	96.68503	0.07837

998	16.09205	96.78181	0.07858

999	16.11131	96.87859	0.07859

1000	16.13539	96.97537	0.07862

1001	16.14985	97.07216	0.07864

1002	16.15786	97.16894	0.07865

1003	16.17714	97.26572	0.07866

1004	16.19801	97.3625	0.07867

1005	16.21566	97.45928	0.07868

1006	16.2253	97.55607	0.07872

1007	16.24296	97.65285	0.07873

1008	16.26223	97.74963	0.07939

1009	16.27347	97.84641	0.0794

1010	16.2847	97.94319	0.07971

1011	16.31682	98.03997	0.07974

1012	16.32324	98.13676	0.07975

1013	16.3425	98.23354	0.07979

1014	16.36498	98.33032	0.07982

1015	16.37622	98.4271	0.07983

1016	16.38907	98.52388	0.08037

1017	16.40191	98.62067	0.0804

1018	16.42921	98.71745	0.08053

1019	16.43722	98.81423	0.08054

1020	16.44044	98.91101	0.08055

1021	16.46773	99.00779	0.08057

1022	16.49343	99.10457	0.08073

1023	16.50146	99.20136	0.08077

1024	16.51429	99.29814	0.08089

1025	16.53517	99.39492	0.08092

1026	16.55443	99.4917	0.08139

1027	16.56568	99.58848	0.08144

1028	16.57852	99.68527	0.08147

1029	16.60099	99.78205	0.08154

1030	16.61866	99.87883	0.08158

1031	16.63149	99.97561	0.08161

1032	16.64114	100.07239	0.08175

1033	16.66361	100.16917	0.08176

1034	16.68127	100.26596	0.08185

1035	16.68608	100.36274	0.0819

1036	16.71017	100.45952	0.08191

1037	16.72301	100.5563	0.08195

1038	16.74067	100.65308	0.08196

1039	16.75351	100.74987	0.08198

1040	16.78563	100.84665	0.08211

1041	16.79205	100.94343	0.08212

1042	16.80329	101.04021	0.08213

1043	16.82095	101.13699	0.08224

1044	16.84182	101.23377	0.08225

1045	16.85467	101.33056	0.08229

1046	16.86269	101.42734	0.0823

1047	16.88517	101.52412	0.08233

1048	16.90765	101.6209	0.08236

1049	16.92531	101.71768	0.08239

1050	16.93013	101.81447	0.08243

1051	16.94778	101.91125	0.0826

1052	16.96865	102.00803	0.08263

1053	16.97828	102.10481	0.08264

1054	16.99594	102.20159	0.08266

1055	17.01843	102.29837	0.08311

1056	17.03127	102.39516	0.08314

1057	17.0409	102.49194	0.08315

1058	17.06337	102.58872	0.08322

1059	17.07944	102.6855	0.08335

1060	17.0971	102.78228	0.08351

1061	17.10191	102.87907	0.08356

1062	17.1244	102.97585	0.08358

1063	17.14205	103.07263	0.08363

1064	17.15971	103.16941	0.08364

1065	17.16934	103.26619	0.08367

1066	17.19503	103.36297	0.08368

1067	17.21109	103.45976	0.08374

1068	17.22072	103.55654	0.08375

1069	17.23678	103.65332	0.0839

1070	17.25926	103.7501	0.08394

1071	17.2753	103.84688	0.08396

1072	17.28655	103.94367	0.08397

1073	17.301	104.04045	0.08399

1074	17.31865	104.13723	0.08403

1075	17.33792	104.23401	0.08404

1076	17.35237	104.33079	0.08415

1077	17.36682	104.42757	0.08422

1078	17.3893	104.52436	0.08424

1079	17.39572	104.62114	0.08425

1080	17.41498	104.71792	0.08427

1081	17.42943	104.8147	0.08448

1082	17.45031	104.91148	0.08449

1083	17.45673	105.00827	0.08454

1084	17.48082	105.10505	0.08455

1085	17.5049	105.20183	0.08477

1086	17.51132	105.29861	0.08485

1087	17.52095	105.39539	0.08486

1088	17.54663	105.49217	0.08488

1089	17.55788	105.58896	0.08489

1090	17.57715	105.68574	0.08497

1091	17.58517	105.78252	0.08499

1092	17.60925	105.8793	0.08501

1093	17.63013	105.97608	0.08514

1094	17.63976	106.07287	0.08519

1095	17.6526	106.16965	0.08522

1096	17.67026	106.26643	0.08525

1097	17.68471	106.36321	0.08528

1098	17.69916	106.45999	0.08544

1099	17.72004	106.55677	0.0867

1100	17.73769	106.65356	0.08671

1101	17.75214	106.75034	0.08678

1102	17.76177	106.84712	0.08679

1103	17.78585	106.9439	0.0868

1104	17.80513	107.04068	0.08686

1105	17.80994	107.13747	0.08707

1106	17.83402	107.23425	0.08711

1107	17.85008	107.33103	0.08727

1108	17.86293	107.42781	0.08728

1109	17.87577	107.52459	0.08736

1110	17.89504	107.62137	0.08811

1111	17.9159	107.71816	0.08812

1112	17.92875	107.81494	0.0882

1113	17.94159	107.91172	0.08823

1114	17.96247	108.0085	0.08824

1115	17.97692	108.10528	0.08859

1116	17.98976	108.20206	0.08881

1117	18.00742	108.29885	0.0889

1118	18.02829	108.39563	0.089

1119	18.04113	108.49241	0.0891

1120	18.05558	108.58919	0.08917

1121	18.06683	108.68597	0.08943

1122	18.09251	108.78276	0.08953

1123	18.10697	108.87954	0.08955

1124	18.1182	108.97632	0.08956

1125	18.13586	109.0731	0.08961

1126	18.15192	109.16988	0.08966

1127	18.16315	109.26666	0.08971

1128	18.18242	109.36345	0.08972

1129	18.20489	109.46023	0.08986

1130	18.22256	109.55701	0.08994

1131	18.22738	109.65379	0.08995

1132	18.24022	109.75057	0.09014

1133	18.26591	109.84736	0.09018

1134	18.28036	109.94414	0.09027

1135	18.29802	110.04092	0.09052

1136	18.31728	110.1377	0.09063

1137	18.32531	110.23448	0.09065

1138	18.33816	110.33126	0.09066

1139	18.35903	110.42805	0.09067

1140	18.38151	110.52483	0.0907

1141	18.39434	110.62161	0.0908

1142	18.39916	110.71839	0.09083

1143	18.42486	110.81517	0.09084

1144	18.44412	110.91196	0.09085

1145	18.45536	111.00874	0.09086

1146	18.46659	111.10552	0.09099

1147	18.48907	111.2023	0.09101

1148	18.50673	111.29908	0.09107

1149	18.54848	111.39586	0.09111

1150	18.56775	111.49265	0.09113

1151	18.57898	111.58943	0.09116

1152	18.59664	111.68621	0.09173

1153	18.63839	111.78299	0.09174

1154	18.65926	111.87977	0.09175

1155	18.67532	111.97656	0.09188

1156	18.85513	112.07334	0.09216

1157	18.94343	112.17012	0.09217

1158	19.11683	112.2669	0.09242

1159	18.92417	112.36368	0.09243

1160	18.63518	112.46046	0.0925

1161	18.24665	112.55725	0.09251

1162	17.19824	112.65403	0.09253

1163	16.18677	112.75081	0.09255

1164	14.31956	112.84759	0.09256

1165	10.80188	112.94437	0.0926

1166	7.50417	113.04116	0.09261

1167	5.75576	113.13794	0.09263

1168	4.84865	113.23472	0.09264

1169	3.97525	113.3315	0.09267

1170	3.14359	113.42828	0.09271

1171	2.34083	113.52506	0.09272

113.62185	0.09297

113.71863	0.09299

113.81541	0.09301

113.91219	0.09305

114.00897	0.09309

114.10576	0.09311

114.20254	0.09314

114.29932	0.09329

114.3961	0.0933

114.49288	0.09333

114.58966	0.09351

114.68645	0.09353

114.78323	0.09363

114.88001	0.09366

114.97679	0.09372

115.07357	0.09403

115.17036	0.0941

115.26714	0.09415

115.36392	0.09418

115.4607	0.09424

115.55748	0.09426

115.65426	0.09428

115.75105	0.09433

115.84783	0.09434

115.94461	0.09436

116.04139	0.09437

116.13817	0.09441

116.23496	0.09443

116.33174	0.09449

116.42852	0.09452

116.5253	0.09453

116.62208	0.09468

116.71886	0.09477

116.81565	0.09483

116.91243	0.09485

117.00921	0.09506

117.10599	0.09508

117.20277	0.0951

117.29956	0.09513

117.39634	0.0952

117.49312	0.09536

117.5899	0.09537

117.68668	0.09546

117.78346	0.09548

117.88025	0.0955

117.97703	0.09556

118.07381	0.09615

118.17059	0.09616

118.26737	0.09618

118.36416	0.09621

118.46094	0.09622

118.55772	0.09631

118.6545	0.09632

118.75128	0.09648

118.84806	0.09653

118.94485	0.09654

119.04163	0.09655

119.13841	0.09657

119.23519	0.09665

119.33197	0.09691

119.42876	0.09692

119.52554	0.09693

119.62232	0.09694

119.7191	0.09695

119.81588	0.09727

119.91266	0.09734

120.00945	0.09738

120.10623	0.09745

120.20301	0.09746

120.29979	0.09765

120.39657	0.09767

120.49335	0.09768

120.59014	0.09769

120.68692	0.09771

120.7837	0.09773

120.88048	0.09785

120.97726	0.09788

121.07405	0.09791

121.17083	0.09793

121.26761	0.09794

121.36439	0.09795

121.46117	0.09798

121.55795	0.098

121.65474	0.09811

121.75152	0.09814

121.8483	0.09815

121.94508	0.09816

122.04186	0.09819

122.13865	0.0982

122.23543	0.09822

122.33221	0.09823

122.42899	0.0986

122.52577	0.09864

122.62255	0.09868

122.71934	0.09869

122.81612	0.09871

122.9129	0.09874

123.00968	0.09898

123.10646	0.09899

123.20325	0.099

123.30003	0.09902

123.39681	0.09904

123.49359	0.09905

123.59037	0.09914

123.68715	0.09915

123.78394	0.09929

123.88072	0.0993

123.9775	0.09931

124.07428	0.0994

124.17106	0.09941

124.26785	0.09956

124.36463	0.09998

124.46141	0.10001

124.55819	0.10018

124.65497	0.1006

124.75175	0.10061

124.84854	0.10066

124.94532	0.10096

125.0421	0.101

125.13888	0.10102

125.23566	0.10131

125.33245	0.10132

125.42923	0.10134

125.52601	0.10136

125.62279	0.10138

125.71957	0.10139

125.81635	0.10148

125.91314	0.10151

126.00992	0.10152

126.1067	0.102

126.20348	0.10253

126.30026	0.10258

126.39705	0.10283

126.49383	0.10288

126.59061	0.10295

126.68739	0.10297

126.78417	0.10299

126.88095	0.103

126.97774	0.10306

127.07452	0.10309

127.1713	0.10312

127.26808	0.10318

127.36486	0.10319

127.46165	0.10337

127.55843	0.10357

127.65521	0.10363

127.75199	0.10368

127.84877	0.10369

127.94555	0.10389

128.04234	0.10392

128.13912	0.10396

128.2359	0.10397

128.33268	0.104

128.42946	0.10415

128.52625	0.10422

128.62303	0.1044

128.71981	0.10462

128.81659	0.10477

128.91337	0.10487

129.01015	0.10488

129.10694	0.10493

129.20372	0.10494

129.3005	0.10503

129.39728	0.1051

129.49406	0.10527

129.59085	0.10542

129.68763	0.10543

129.78441	0.10546

129.88119	0.10547

129.97797	0.10548

130.07475	0.1055

130.17154	0.10552

130.26832	0.10553

130.3651	0.10562

130.46188	0.10565

130.55866	0.1058

130.65545	0.10583

130.75223	0.10585

130.84901	0.10587

130.94579	0.10593

131.04257	0.10601

131.13935	0.10607

131.23614	0.10609

131.33292	0.10618

131.4297	0.10644

131.52648	0.10646

131.62326	0.1068

131.72005	0.10681

131.81683	0.10686

131.91361	0.10687

132.01039	0.1069

132.10717	0.10693

132.20395	0.10694

132.30074	0.10695

132.39752	0.10698

132.4943	0.10699

132.59108	0.10701

132.68786	0.1071

132.78465	0.10717

132.88143	0.10738

132.97821	0.1074

133.07499	0.10745

133.17177	0.10752

133.26855	0.10754

133.36534	0.10757

133.46212	0.10761

133.5589	0.10773

133.65568	0.10777

133.75246	0.10842

133.84924	0.1085

133.94603	0.10853

134.04281	0.10881

134.13959	0.10885

134.23637	0.10886

134.33315	0.10898

134.42994	0.109

134.52672	0.10907

134.6235	0.10911

134.72028	0.10912

134.81706	0.10913

134.91384	0.10917

135.01063	0.10921

135.10741	0.10922

135.20419	0.10925

135.30097	0.10926

135.39775	0.10939

135.49454	0.10941

135.59132	0.10948

135.6881	0.10959

135.78488	0.1096

135.88166	0.10963

135.97844	0.10968

136.07523	0.10969

136.17201	0.1097

136.26879	0.10971

136.36557	0.10972

136.46235	0.10973

136.55914	0.10981

136.65592	0.10985

136.7527	0.10986

136.84948	0.10993

136.94626	0.10994

137.04304	0.11015

137.13983	0.1102

137.23661	0.11023

137.33339	0.11026

137.43017	0.11028

137.52695	0.11031

137.62374	0.11043

137.72052	0.11044

137.8173	0.11048

137.91408	0.11049

138.01086	0.1105

138.10764	0.11058

138.20443	0.11063

138.30121	0.11085

138.39799	0.11101

138.49477	0.11102

138.59155	0.11108

138.68834	0.11109

138.78512	0.1112

138.8819	0.11122

138.97868	0.11137

139.07546	0.11144

139.17224	0.11152

139.26903	0.11153

139.36581	0.11156

139.46259	0.11158

139.55937	0.11159

139.65615	0.11161

139.75294	0.11168

139.84972	0.11172

139.9465	0.11173

140.04328	0.11175

140.14006	0.11179

140.23684	0.11188

140.33363	0.1119

140.43041	0.11196

140.52719	0.11197

140.62397	0.11212

140.72075	0.11213

140.81754	0.11215

140.91432	0.11245

141.0111	0.11249

141.10788	0.1127

141.20466	0.11271

141.30144	0.11272

141.39823	0.11273

141.49501	0.11274

141.59179	0.11277

141.68857	0.11278

141.78535	0.11279

141.88214	0.1128

141.97892	0.11281

142.0757	0.11283

142.17248	0.11284

142.26926	0.11285

142.36604	0.11286

142.46283	0.11311

142.55961	0.11312

142.65639	0.11317

142.75317	0.11318

142.84995	0.11324

142.94674	0.11354

143.04352	0.11355

143.1403	0.11373

143.23708	0.11375

143.33386	0.11377

143.43064	0.11388

143.52743	0.1139

143.62421	0.11391

143.72099	0.11392

143.81777	0.11394

143.91455	0.11398

144.01134	0.114

144.10812	0.11401

144.2049	0.11405

144.30168	0.11414

144.39846	0.11432

144.49524	0.11443

144.59203	0.11444

144.68881	0.11462

144.78559	0.11507

144.88237	0.1151

144.97915	0.11513

145.07593	0.11514

145.17272	0.11523

145.2695	0.11532

145.36628	0.11544

145.46306	0.11546

145.55984	0.11548

145.65663	0.11549

145.75341	0.11555

145.85019	0.11556

145.94697	0.11562

146.04375	0.11563

146.14053	0.11564

146.23732	0.11568

146.3341	0.11589

146.43088	0.11616

146.52766	0.11624

146.62444	0.11625

146.72123	0.11632

146.81801	0.11633

146.91479	0.11635

147.01157	0.11637

147.10835	0.11639

147.20513	0.11641

147.30192	0.1165

147.3987	0.11656

147.49548	0.11658

147.59226	0.11662

147.68904	0.11663

147.78583	0.11664

147.88261	0.11665

147.97939	0.11674

148.07617	0.11682

148.17295	0.11684

148.26973	0.11686

148.36652	0.11687

148.4633	0.11688

148.56008	0.11693

148.65686	0.11694

148.75364	0.11695

148.85043	0.11697

148.94721	0.11699

149.04399	0.11785

149.14077	0.11801

149.23755	0.11809

149.33433	0.1181

149.43112	0.11813

149.5279	0.11815

149.62468	0.11817

149.72146	0.11818

149.81824	0.11819

149.91503	0.11824

150.01181	0.11832

150.10859	0.11834

150.20537	0.11835

150.30215	0.11836

150.39893	0.1185

150.49572	0.11876

150.5925	0.11882

150.68928	0.11883

150.78606	0.11884

150.88284	0.11889

150.97963	0.11896

151.07641	0.11897

151.17319	0.119

151.26997	0.11906

151.36675	0.11908

151.46353	0.11909

151.56032	0.1191

151.6571	0.11912

151.75388	0.11913

151.85066	0.11914

151.94744	0.11918

152.04423	0.11927

152.14101	0.11929

152.23779	0.11938

152.33457	0.11941

152.43135	0.11945

152.52813	0.11947

152.62492	0.11948

152.7217	0.1195

152.81848	0.11951

152.91526	0.11953

153.01204	0.11954

153.10883	0.1196

153.20561	0.11961

153.30239	0.11963

153.39917	0.11964

153.49595	0.11965

153.59273	0.11966

153.68952	0.11971

153.7863	0.11972

153.88308	0.11975

153.97986	0.11979

154.07664	0.11984

154.17343	0.11986

154.27021	0.11987

154.36699	0.11988

154.46377	0.12019

154.56055	0.1202

154.65733	0.12021

154.75412	0.12022

154.8509	0.12024

154.94768	0.12029

155.04446	0.12035

155.14124	0.12036

155.23803	0.12039

155.33481	0.12044

155.43159	0.12045

155.52837	0.12052

155.62515	0.12055

155.72193	0.12058

155.81872	0.12097

155.9155	0.12099

156.01228	0.121

156.10906	0.12104

156.20584	0.12105

156.30263	0.12118

156.39941	0.12119

156.49619	0.12125

156.59297	0.12126

156.68975	0.12127

156.78653	0.12128

156.88332	0.12129

156.9801	0.12133

157.07688	0.12134

157.17366	0.12143

157.27044	0.12146

157.36722	0.12148

157.46401	0.1215

157.56079	0.12166

157.65757	0.12197

157.75435	0.12242

157.85113	0.12246

157.94792	0.12255

158.0447	0.12262

158.14148	0.1227

158.23826	0.12273

158.33504	0.12274

158.43183	0.12275

158.52861	0.12276

158.62539	0.12278

158.72217	0.12292

158.81895	0.12293

158.91573	0.12295

159.01252	0.12297

159.1093	0.12299

159.20608	0.123

159.30286	0.12303

159.39964	0.12305

159.49642	0.12308

159.59321	0.12309

159.68999	0.12344

159.78677	0.12345

159.88355	0.12347

159.98033	0.12349

160.07712	0.12398

160.1739	0.12411

160.27068	0.12412

160.36746	0.12413

160.46424	0.12426

160.56102	0.12427

160.65781	0.1243

160.75459	0.12433

160.85137	0.12442

160.94815	0.12447

161.04493	0.12454

161.14172	0.12455

161.2385	0.1246

161.33528	0.12462

161.43206	0.12465

161.52884	0.12468

161.62562	0.12469

161.72241	0.12499

161.81919	0.125

161.91597	0.12528

162.01275	0.12539

162.10953	0.12549

162.20632	0.12565

162.3031	0.12567

162.39988	0.12575

162.49666	0.12576

162.59344	0.12578

162.69022	0.12579

162.78701	0.12581

162.88379	0.12613

162.98057	0.12614

163.07735	0.12616

163.17413	0.12642

163.27092	0.12645

163.3677	0.12658

163.46448	0.12681

163.56126	0.1269

163.65804	0.12692

163.75482	0.12693

163.85161	0.12712

163.94839	0.12719

164.04517	0.1273

164.14195	0.12737

164.23873	0.12739

164.33552	0.12753

164.4323	0.12769

164.52908	0.12771

164.62586	0.12773

164.72264	0.12778

164.81942	0.1278

164.91621	0.12803

165.01299	0.12805

165.10977	0.1281

165.20655	0.12812

165.30333	0.12817

165.40012	0.12819

165.4969	0.12824

165.59368	0.12828

165.69046	0.12831

165.78724	0.12883

165.88402	0.12885

165.98081	0.12892

166.07759	0.12908

166.17437	0.12915

166.27115	0.1292

166.36793	0.12936

166.46472	0.1297

166.5615	0.12975

166.65828	0.12977

166.75506	0.12979

166.85184	0.12984

166.94862	0.12997

167.04541	0.13002

167.14219	0.13016

167.23897	0.13018

167.33575	0.1302

167.43253	0.13034

167.52932	0.13036

167.6261	0.13052

167.72288	0.13116

167.81966	0.13121

167.91644	0.13189

168.01322	0.13217

168.11001	0.13219

168.20679	0.13233

168.30357	0.13235

168.40035	0.13331

168.49713	0.13336

168.59392	0.13338

168.6907	0.13343

168.78748	0.13349

168.88426	0.13359

168.98104	0.13397

169.07782	0.13423

169.17461	0.13464

169.27139	0.13466

169.36817	0.13468

169.46495	0.13471

169.56173	0.13507

169.65852	0.13509

169.7553	0.13516

169.85208	0.13519

169.94886	0.13521

170.04564	0.13523

170.14242	0.13525

170.23921	0.13528

170.33599	0.13544

170.43277	0.13548

170.52955	0.13553

170.62633	0.1358

170.72312	0.13583

170.8199	0.13585

170.91668	0.13589

171.01346	0.13599

171.11024	0.13615

171.20702	0.13624

171.30381	0.13626

171.40059	0.13628

171.49737	0.13633

171.59415	0.1366

171.69093	0.13663

171.78771	0.13701

171.8845	0.1374

171.98128	0.13743

172.07806	0.13768

172.17484	0.1377

172.27162	0.13775

172.36841	0.13779

172.46519	0.13784

172.56197	0.13788

172.65875	0.13802

172.75553	0.13827

172.85231	0.13852

172.9491	0.13855

173.04588	0.13859

173.14266	0.13864

173.23944	0.13866

173.33622	0.13909

173.43301	0.1393

173.52979	0.13941

173.62657	0.13944

173.72335	0.13946

173.82013	0.13951

173.91691	0.13953

174.0137	0.13955

174.11048	0.13957

174.20726	0.13971

174.30404	0.13976

174.40082	0.13978

174.49761	0.1398

174.59439	0.13983

174.69117	0.13985

174.78795	0.13987

174.88473	0.13989

174.98151	0.13996

175.0783	0.14001

175.17508	0.1404

175.27186	0.14081

175.36864	0.14083

175.46542	0.14108

175.56221	0.14111

175.65899	0.14113

175.75577	0.14115

175.85255	0.14117

175.94933	0.14131

176.04611	0.14154

176.1429	0.14156

176.23968	0.14161

176.33646	0.14168

176.43324	0.1417

176.53002	0.14172

176.62681	0.14209

176.72359	0.14229

176.82037	0.14232

176.91715	0.14234

177.01393	0.14239

177.11071	0.14243

177.2075	0.14245

177.30428	0.14268

177.40106	0.14287

177.49784	0.14289

177.59462	0.14291

177.69141	0.14298

177.78819	0.14319

177.88497	0.14325

177.98175	0.14328

178.07853	0.14335

178.17531	0.14339

178.2721	0.14341

178.36888	0.14344

178.46566	0.14371

178.56244	0.14376

178.65922	0.14378

178.75601	0.14497

178.85279	0.1457

178.94957	0.14597

179.04635	0.14632

179.14313	0.14636

179.23991	0.14639

179.3367	0.14664

179.43348	0.14671

179.53026	0.14675

179.62704	0.1468

179.72382	0.14684

179.82061	0.14691

179.91739	0.14696

180.01417	0.147

180.11095	0.14709

180.20773	0.14712

180.30451	0.14719

180.4013	0.14721

180.49808	0.14723

180.59486	0.14757

180.69164	0.14764

180.78842	0.14767

180.88521	0.14769

180.98199	0.14771

181.07877	0.14773

181.17555	0.1481

181.27233	0.14815

181.36911	0.14817

181.4659	0.14819

181.56268	0.14821

181.65946	0.14826

181.75624	0.14831

181.85302	0.14842

181.94981	0.14844

182.04659	0.14847

182.14337	0.14849

182.24015	0.14851

182.33693	0.14853

182.43371	0.14856

182.5305	0.14867

182.62728	0.14927

182.72406	0.14929

182.82084	0.14931

182.91762	0.14933

183.01441	0.14938

183.11119	0.1494

183.20797	0.14959

183.30475	0.14961

183.40153	0.14965

183.49831	0.14968

183.5951	0.1497

183.69188	0.15016

183.78866	0.15018

183.88544	0.1502

183.98222	0.15023

184.079	0.15025

184.17579	0.15032

184.27257	0.15036

184.36935	0.15039

184.46613	0.15041

184.56291	0.15043

184.6597	0.15048

184.75648	0.1505

184.85326	0.15052

184.95004	0.15055

185.04682	0.15057

185.1436	0.15059

185.24039	0.15061

185.33717	0.15064

185.43395	0.15068

185.53073	0.15073

185.62751	0.1508

185.7243	0.15082

185.82108	0.15105

185.91786	0.15109

186.01464	0.15114

186.11142	0.15157

186.2082	0.15162

186.30499	0.15164

186.40177	0.15169

186.49855	0.15194

186.59533	0.15196

186.69211	0.15199

186.7889	0.15217

186.88568	0.15219

186.98246	0.15224

187.07924	0.15242

187.17602	0.15244

187.2728	0.15247

187.36959	0.15249

187.46637	0.15251

187.56315	0.15288

187.65993	0.1529

187.75671	0.15295

187.8535	0.15297

187.95028	0.15301

188.04706	0.15304

188.14384	0.15313

188.24062	0.15315

188.3374	0.15317

188.43419	0.15322

188.53097	0.15327

188.62775	0.15329

188.72453	0.15331

188.82131	0.15359

188.9181	0.15365

189.01488	0.15368

189.11166	0.15379

189.20844	0.15384

189.30522	0.15386

189.402	0.15397

189.49879	0.154

189.59557	0.15404

189.69235	0.15413

189.78913	0.15416

189.88591	0.15418

189.9827	0.15425

190.07948	0.15427

190.17626	0.15436

190.27304	0.15439

190.36982	0.15445

190.4666	0.15455

190.56339	0.15475

190.66017	0.15477

190.75695	0.1548

190.85373	0.15482

190.95051	0.15489

191.0473	0.15491

191.14408	0.15493

191.24086	0.155

191.33764	0.15503

191.43442	0.15505

191.5312	0.15507

191.62799	0.15519

191.72477	0.15521

191.82155	0.15537

191.91833	0.15544

192.01511	0.15548

192.1119	0.1556

192.20868	0.15567

192.30546	0.15571

192.40224	0.15573

192.49902	0.15576

192.5958	0.15578

192.69259	0.1558

192.78937	0.15583

192.88615	0.15599

192.98293	0.15601

193.07971	0.15606

193.1765	0.15608

193.27328	0.15631

193.37006	0.15633

193.46684	0.15635

193.56362	0.15638

193.6604	0.1564

193.75719	0.15642

193.85397	0.15644

193.95075	0.15647

194.04753	0.1569

194.14431	0.15692

194.2411	0.15704

194.33788	0.15706

194.43466	0.15708

194.53144	0.15713

194.62822	0.15779

194.725	0.15782

194.82179	0.15786

194.91857	0.15788

195.01535	0.15793

195.11213	0.15795

195.20891	0.158

195.30569	0.15802

195.40248	0.15804

195.49926	0.15807

195.59604	0.15811

195.69282	0.15814

195.7896	0.15818

195.88639	0.15823

195.98317	0.15827

196.07995	0.15832

196.17673	0.15834

196.27351	0.15836

196.3703	0.15839

196.46708	0.15857

196.56386	0.15862

196.66064	0.15864

196.75742	0.1588

196.8542	0.15891

196.95099	0.15894

197.04777	0.15898

197.14455	0.159

197.24133	0.15903

197.33811	0.15905

197.43489	0.15919

197.53168	0.15921

197.62846	0.15926

197.72524	0.15928

197.82202	0.1593

197.9188	0.15932

198.01559	0.15935

198.11237	0.15937

198.20915	0.15942

198.30593	0.15946

198.40271	0.15951

198.49949	0.15953

198.59628	0.15955

198.69306	0.15958

198.78984	0.15969

198.88662	0.15971

198.9834	0.15974

199.08019	0.15976

199.17697	0.15978

199.27375	0.1598

199.37053	0.15983

199.46731	0.15985

199.56409	0.15994

199.66088	0.15996

199.75766	0.15999

199.85444	0.16003

199.95122	0.16008

200.048	0.1601

200.14479	0.16012

200.24157	0.16015

200.33835	0.16019

200.43513	0.16022

200.53191	0.16024

200.62869	0.16026

200.72548	0.16028

200.82226	0.16031

200.91904	0.16076

201.01582	0.16079

201.1126	0.16081

201.20939	0.1609

201.30617	0.16092

201.40295	0.16095

201.49973	0.16099

201.59651	0.16102

201.69329	0.16108

201.79008	0.16111

201.88686	0.16113

201.98364	0.16115

202.08042	0.16118

202.1772	0.16129

202.27399	0.16134

202.37077	0.16136

202.46755	0.16138

202.56433	0.1614

202.66111	0.16145

202.75789	0.16152

202.85468	0.16154

202.95146	0.16156

203.04824	0.16159

203.14502	0.16161

203.2418	0.16163

203.33859	0.16168

203.43537	0.1617

203.53215	0.16177

203.62893	0.16198

203.72571	0.16202

203.82249	0.16204

203.91928	0.16209

204.01606	0.16211

204.11284	0.16214

204.20962	0.16216

204.3064	0.16218

204.40319	0.1622

204.49997	0.16232

204.59675	0.16239

204.69353	0.16241

204.79031	0.1625

204.88709	0.16252

204.98388	0.16257

205.08066	0.16262

205.17744	0.16264

205.27422	0.16266

205.371	0.16268

205.46779	0.16273

205.56457	0.16278

205.66135	0.1628

205.75813	0.16287

205.85491	0.16312

205.95169	0.16314

206.04848	0.16344

206.14526	0.16346

206.24204	0.16348

206.33882	0.16351

206.4356	0.16353

206.53239	0.1636

206.62917	0.16383

206.72595	0.16385

206.82273	0.16392

206.91951	0.16394

207.01629	0.16396

207.11308	0.16401

207.20986	0.16403

207.30664	0.16412

207.40342	0.16417

207.5002	0.16424

207.59699	0.16426

207.69377	0.16431

207.79055	0.16433

207.88733	0.16435

207.98411	0.16438

208.08089	0.16449

208.17768	0.1646

208.27446	0.16463

208.37124	0.16465

208.46802	0.16467

208.5648	0.16474

208.66159	0.16483

208.75837	0.16486

208.85515	0.1649

208.95193	0.16492

209.04871	0.16495

209.14549	0.16502

209.24228	0.16504

209.33906	0.16508

209.43584	0.16513

209.53262	0.16515

209.6294	0.16524

209.72618	0.16527

209.82297	0.16529

209.91975	0.16534

210.01653	0.16536

210.11331	0.16552

210.21009	0.16554

210.30688	0.16559

210.40366	0.16561

210.50044	0.16568

210.59722	0.1657

210.694	0.16572

210.79078	0.16579

210.88757	0.16584

210.98435	0.16586

211.08113	0.16588

211.17791	0.16591

211.27469	0.16593

211.37148	0.16602

211.46826	0.16604

211.56504	0.16607

211.66182	0.16609

211.7586	0.16611

211.85538	0.16614

211.95217	0.16616

212.04895	0.16625

212.14573	0.16634

212.24251	0.16636

212.33929	0.16641

212.43608	0.16646

212.53286	0.16652

212.62964	0.16655

212.72642	0.16703

212.8232	0.16705

212.91998	0.16707

213.01677	0.1671

213.11355	0.16712

213.21033	0.16716

213.30711	0.16719

213.40389	0.16721

213.50068	0.1673

213.59746	0.16737

213.69424	0.16742

213.79102	0.16744

213.8878	0.16771

213.98458	0.16799

214.08137	0.16824

214.17815	0.16826

214.27493	0.16828

214.37171	0.16831

214.46849	0.16833

214.56528	0.1684

214.66206	0.16844

214.75884	0.16847

214.85562	0.16849

214.9524	0.16851

215.04918	0.16874

215.14597	0.16876

215.24275	0.16879

215.33953	0.16886

215.43631	0.16904

215.53309	0.16906

215.62988	0.16908

215.72666	0.16913

215.82344	0.16915

215.92022	0.1692

216.017	0.16924

216.11378	0.16927

216.21057	0.16929

216.30735	0.16931

216.40413	0.16934

216.50091	0.16968

216.59769	0.1697

216.69448	0.16972

216.79126	0.16975

216.88804	0.16977

216.98482	0.16979

217.0816	0.16982

217.17838	0.16984

217.27517	0.16988

217.37195	0.16991

217.46873	0.16998

217.56551	0.17

217.66229	0.17002

217.75908	0.17007

217.85586	0.17011

217.95264	0.17014

218.04942	0.17018

218.1462	0.1702

218.24298	0.17046

218.33977	0.17048

218.43655	0.17052

218.53333	0.17055

218.63011	0.17057

218.72689	0.17059

218.82368	0.17062

218.92046	0.17066

219.01724	0.17068

219.11402	0.17071

219.2108	0.17073

219.30758	0.17075

219.40437	0.1708

219.50115	0.17082

219.59793	0.17084

219.69471	0.17087

219.79149	0.17089

219.88828	0.17105

219.98506	0.1711

220.08184	0.17114

220.17862	0.17116

220.2754	0.17121

220.37218	0.17123

220.46897	0.17144

220.56575	0.17146

220.66253	0.17148

220.75931	0.17153

220.85609	0.17155

220.95288	0.1716

221.04966	0.17162

221.14644	0.17169

221.24322	0.17178

221.34	0.1718

221.43678	0.17183

221.53357	0.17185

221.63035	0.17201

221.72713	0.17203

221.82391	0.17208

221.92069	0.1721

222.01747	0.17217

222.11426	0.17219

222.21104	0.17224

222.30782	0.17231

222.4046	0.17233

222.50138	0.17235

222.59817	0.17238

222.69495	0.17242

222.79173	0.17244

222.88851	0.17249

222.98529	0.17251

223.08207	0.17313

223.17886	0.17315

223.27564	0.17318

223.37242	0.17324

223.4692	0.17329

223.56598	0.17331

223.66277	0.17334

223.75955	0.17338

223.85633	0.17363

223.95311	0.17366

224.04989	0.17368

224.14667	0.1737

224.24346	0.17372

224.34024	0.17414

224.43702	0.17418

224.5338	0.1742

224.63058	0.17423

224.72737	0.17436

224.82415	0.17439

224.92093	0.17443

225.01771	0.17448

225.11449	0.1745

225.21127	0.17452

225.30806	0.17455

225.40484	0.17462

225.50162	0.17466

225.5984	0.17468

225.69518	0.17471

225.79197	0.17475

225.88875	0.1748

225.98553	0.17484

226.08231	0.17487

226.17909	0.17491

226.27587	0.17494

226.37266	0.17496

226.46944	0.17503

226.56622	0.17505

226.663	0.17507

226.75978	0.1751

226.85657	0.17512

226.95335	0.17514

227.05013	0.17519

227.14691	0.17521

227.24369	0.17523

227.34047	0.17526

227.43726	0.17528

227.53404	0.1753

227.63082	0.17532

227.7276	0.17542

227.82438	0.17548

227.92117	0.17551

228.01795	0.17553

228.11473	0.17555

228.21151	0.1756

228.30829	0.17562

228.40507	0.17564

228.50186	0.17569

228.59864	0.17571

228.69542	0.17574

228.7922	0.17576

228.88898	0.17583

228.98577	0.17587

229.08255	0.1759

229.17933	0.17596

229.27611	0.17599

229.37289	0.17601

229.46967	0.17603

229.56646	0.17608

229.66324	0.17617

229.76002	0.17628

229.8568	0.17631

229.95358	0.17633

230.05037	0.17635

230.14715	0.17644

230.24393	0.17647

230.34071	0.17649

230.43749	0.17654

230.53427	0.17663

230.63106	0.17695

230.72784	0.17697

230.82462	0.17699

230.9214	0.17702

231.01818	0.17704

231.11497	0.17706

231.21175	0.17708

231.30853	0.17713

231.40531	0.17718

231.50209	0.1772

231.59887	0.17724

231.69566	0.17727

231.79244	0.17736

231.88922	0.17738

231.986	0.1774

232.08278	0.17743

232.17957	0.17745

232.27635	0.17772

232.37313	0.17775

232.46991	0.17777

232.56669	0.17779

232.66347	0.17782

232.76026	0.17784

232.85704	0.17788

232.95382	0.17791

233.0506	0.178

233.14738	0.17802

233.24417	0.17804

233.34095	0.17807

233.43773	0.17809

233.53451	0.17814

233.63129	0.17887

233.72807	0.17889

233.82486	0.17896

233.92164	0.17898

234.01842	0.179

234.1152	0.17903

234.21198	0.17905

234.30876	0.17907

234.40555	0.1791

234.50233	0.17912

234.59911	0.17916

234.69589	0.1793

234.79267	0.17932

234.88946	0.17942

234.98624	0.17951

235.08302	0.17953

235.1798	0.17955

235.27658	0.17958

235.37336	0.1796

235.47015	0.17962

235.56693	0.17964

235.66371	0.17967

235.76049	0.17971

235.85727	0.17974

235.95406	0.17976

236.05084	0.17978

236.14762	0.1798

236.2444	0.17985

236.34118	0.17987

236.43796	0.1799

236.53475	0.17992

236.63153	0.17994

236.72831	0.17999

236.82509	0.18001

236.92187	0.18006

237.01866	0.18008

237.11544	0.18012

237.21222	0.18019

237.309	0.18022

237.40578	0.18024

237.50256	0.18026

237.59935	0.18028

237.69613	0.18031

237.79291	0.18047

237.88969	0.18049

237.98647	0.18051

238.08326	0.18054

238.18004	0.18056

238.27682	0.18058

238.3736	0.18063

238.47038	0.1807

238.56716	0.18072

238.66395	0.18074

238.76073	0.18076

238.85751	0.18081

238.95429	0.18083

239.05107	0.18086

239.14786	0.18104

239.24464	0.18118

239.34142	0.18127

239.4382	0.18129

239.53498	0.18131

239.63176	0.18134

239.72855	0.18136

239.82533	0.18138

239.92211	0.1814

240.01889	0.18143

240.11567	0.18145

240.21246	0.18147

240.30924	0.1815

240.40602	0.18152

240.5028	0.18159

240.59958	0.18163

240.69636	0.18166

240.79315	0.1817

240.88993	0.18172

240.98671	0.18179

241.08349	0.18182

241.18027	0.18184

241.27706	0.18188

241.37384	0.18191

241.47062	0.18193

241.5674	0.1825

241.66418	0.18259

241.76096	0.18273

241.85775	0.18275

241.95453	0.18278

242.05131	0.1828

242.14809	0.18282

242.24487	0.18287

242.34166	0.18289

242.43844	0.18291

242.53522	0.18298

242.632	0.18305

242.72878	0.18314

242.82556	0.18337

242.92235	0.18339

243.01913	0.18344

243.11591	0.18346

243.21269	0.18348

243.30947	0.18351

243.40626	0.18353

243.50304	0.18355

243.59982	0.18358

243.6966	0.18364

243.79338	0.18371

243.89016	0.18376

243.98695	0.18378

244.08373	0.1838

244.18051	0.18385

244.27729	0.1839

244.37407	0.18392

244.47086	0.18394

244.56764	0.18396

244.66442	0.18399

244.7612	0.18401

244.85798	0.18403

244.95476	0.18406

245.05155	0.18408

245.14833	0.1841

245.24511	0.18412

245.34189	0.18415

245.43867	0.18417

245.53546	0.18428

245.63224	0.18433

245.72902	0.1844

245.8258	0.18476

245.92258	0.1849

246.01936	0.18492

246.11615	0.18518

246.21293	0.18524

246.30971	0.18527

246.40649	0.18529

246.50327	0.18531

246.60006	0.18534

246.69684	0.18536

246.79362	0.18538

246.8904	0.18543

246.98718	0.18545

247.08396	0.18552

247.18075	0.18556

247.27753	0.18561

247.37431	0.18563

247.47109	0.18566

247.56787	0.18568

247.66465	0.1857

247.76144	0.18575

247.85822	0.18579

247.955	0.18588

248.05178	0.18591

248.14856	0.18593

248.24535	0.18598

248.34213	0.186

248.43891	0.18602

248.53569	0.18604

248.63247	0.18691

248.72925	0.18694

248.82604	0.18703

248.92282	0.18705

249.0196	0.18707

249.11638	0.18712

249.21316	0.18716

249.30995	0.18721

249.40673	0.18723

249.50351	0.18726

249.60029	0.1873

249.69707	0.18732

249.79385	0.18737

249.89064	0.18739

249.98742	0.18742

250.0842	0.18744

250.18098	0.18746

250.27776	0.18751

250.37455	0.18753

250.47133	0.18755

250.56811	0.18758

250.66489	0.1876

250.76167	0.18762

250.85845	0.18764

250.95524	0.18769

251.05202	0.18771

251.1488	0.18774

251.24558	0.18783

251.34236	0.18787

251.43915	0.18792

251.53593	0.18794

251.63271	0.18799

251.72949	0.18806

251.82627	0.18808

251.92305	0.18812

252.01984	0.18815

252.11662	0.18822

252.2134	0.18824

252.31018	0.18828

252.40696	0.18831

252.50375	0.18833

252.60053	0.18835

252.69731	0.1884

252.79409	0.18842

252.89087	0.18844

252.98765	0.18849

253.08444	0.18851

253.18122	0.18854

253.278	0.18858

253.37478	0.18863

253.47156	0.18865

253.56835	0.18867

253.66513	0.1887

253.76191	0.18874

253.85869	0.18876

253.95547	0.18879

254.05225	0.18881

254.14904	0.18886

254.24582	0.18892

254.3426	0.18897

254.43938	0.18904

254.53616	0.18906

254.63295	0.18911

254.72973	0.1892

254.82651	0.18922

254.92329	0.18925

255.02007	0.18934

255.11685	0.18936

255.21364	0.18943

255.31042	0.18945

255.4072	0.1895

255.50398	0.18952

255.60076	0.18961

255.69755	0.18963

255.79433	0.18975

255.89111	0.18977

255.98789	0.18984

256.08467	0.18986

256.18145	0.18991

256.27824	0.18993

256.37502	0.19002

256.4718	0.19007

256.56858	0.19011

256.66536	0.19016

256.76215	0.19018

256.85893	0.19021

256.95571	0.19023

257.05249	0.19059

257.14927	0.19062

257.24605	0.19071

257.34284	0.19073

257.43962	0.19085

257.5364	0.19101

257.63318	0.19103

257.72996	0.19107

257.82675	0.1911

257.92353	0.19112

258.02031	0.19114

258.11709	0.19117

258.21387	0.19119

258.31065	0.19121

258.40744	0.19126

258.50422	0.19128

258.601	0.1913

258.69778	0.19133

258.79456	0.19135

258.89135	0.19137

258.98813	0.19139

259.08491	0.19142

259.18169	0.19144

259.27847	0.19167

259.37525	0.19169

259.47204	0.19176

259.56882	0.19178

259.6656	0.19181

259.76238	0.19183

259.85916	0.19185

259.95594	0.19194

260.05273	0.19197

260.14951	0.19206

260.24629	0.19208

260.34307	0.1921

260.43985	0.19213

260.53664	0.19215

260.63342	0.19219

260.7302	0.19222

260.82698	0.19231

260.92376	0.19233

261.02054	0.19235

261.11733	0.19238

261.21411	0.1924

261.31089	0.19245

261.40767	0.19247

261.50445	0.19258

261.60124	0.19263

261.69802	0.19265

261.7948	0.19277

261.89158	0.19279

261.98836	0.19281

262.08514	0.19286

262.18193	0.19306

262.27871	0.19311

262.37549	0.19315

262.47227	0.19318

262.56905	0.1932

262.66584	0.19327

262.76262	0.19331

262.8594	0.19336

262.95618	0.19338

263.05296	0.19341

263.14974	0.19343

263.24653	0.19352

263.34331	0.19354

263.44009	0.19357

263.53687	0.19359

263.63365	0.19361

263.73044	0.19368

263.82722	0.1937

263.924	0.19375

264.02078	0.19389

264.11756	0.19391

264.21434	0.19393

264.31113	0.19395

264.40791	0.194

264.50469	0.19405

264.60147	0.19407

264.69825	0.19414

264.79504	0.19423

264.89182	0.19425

264.9886	0.19427

265.08538	0.1943

265.18216	0.19434

265.27894	0.19437

265.37573	0.19441

265.47251	0.19443

265.56929	0.19446

265.66607	0.19455

265.76285	0.19459

265.85964	0.19466

265.95642	0.19471

266.0532	0.19473

266.14998	0.19475

266.24676	0.19478

266.34354	0.1948

266.44033	0.19482

266.53711	0.19489

266.63389	0.19505

266.73067	0.19507

266.82745	0.19519

266.92424	0.19521

267.02102	0.19523

267.1178	0.19528

267.21458	0.19533

267.31136	0.19535

267.40814	0.19539

267.50493	0.19542

267.60171	0.19544

267.69849	0.19549

267.79527	0.19555

267.89205	0.19562

267.98884	0.19567

268.08562	0.19569

268.1824	0.19576

268.27918	0.1959

268.37596	0.19592

268.47274	0.19594

268.56953	0.19599

268.66631	0.19603

268.76309	0.19608

268.85987	0.1961

268.95665	0.19613

269.05344	0.19615

269.15022	0.19624

269.247	0.19629

269.34378	0.19631

269.44056	0.19638

269.53734	0.19688

269.63413	0.19693

269.73091	0.19699

269.82769	0.19704

269.92447	0.19706

270.02125	0.19711

270.11804	0.1972

270.21482	0.19722

270.3116	0.19727

270.40838	0.19729

270.50516	0.19734

270.60194	0.19736

270.69873	0.19741

270.79551	0.19747

270.89229	0.1975

270.98907	0.19757

271.08585	0.19759

271.18264	0.19761

271.27942	0.19763

271.3762	0.19768

271.47298	0.1977

271.56976	0.19773

271.66654	0.19775

271.76333	0.19777

271.86011	0.19779

271.95689	0.19782

272.05367	0.19784

272.15045	0.19789

272.24723	0.19791

272.34402	0.19793

272.4408	0.19795

272.53758	0.19805

272.63436	0.19807

272.73114	0.19809

272.82793	0.19814

272.92471	0.19816

273.02149	0.19821

273.11827	0.19823

273.21505	0.19825

273.31183	0.19827

273.40862	0.19832

273.5054	0.19834

273.60218	0.19837

273.69896	0.19839

273.79574	0.19841

273.89253	0.19848

273.98931	0.19855

274.08609	0.19857

274.18287	0.19859

274.27965	0.19866

274.37643	0.19869

274.47322	0.19871

274.57	0.19873

274.66678	0.19875

274.76356	0.19882

274.86034	0.19887

274.95713	0.19889

275.05391	0.19891

275.15069	0.19896

275.24747	0.19901

275.34425	0.19903

275.44103	0.19905

275.53782	0.19907

275.6346	0.1991

275.73138	0.19912

275.82816	0.19917

275.92494	0.19919

276.02173	0.19921

276.11851	0.19928

276.21529	0.19955

276.31207	0.19958

276.40885	0.1996

276.50563	0.19969

276.60242	0.19974

276.6992	0.19976

276.79598	0.19981

276.89276	0.19983

276.98954	0.19987

277.08633	0.1999

277.18311	0.19992

277.27989	0.19997

277.37667	0.20003

277.47345	0.20006

277.57023	0.20022

277.66702	0.20024

277.7638	0.20031

277.86058	0.20033

277.95736	0.20038

278.05414	0.2004

278.15093	0.20042

278.24771	0.20045

278.34449	0.20051

278.44127	0.20054

278.53805	0.20056

278.63483	0.20067

278.73162	0.2007

278.8284	0.20072

278.92518	0.20099

279.02196	0.20102

279.11874	0.20106

279.21553	0.20109

279.31231	0.20111

279.40909	0.20115

279.50587	0.20118

279.60265	0.20138

279.69943	0.20145

279.79622	0.20147

279.893	0.2015

279.98978	0.20163

280.08656	0.2017

280.18334	0.20173

280.28013	0.20191

280.37691	0.20193

280.47369	0.202

280.57047	0.20202

280.66725	0.20207

280.76403	0.20214

280.86082	0.20216

280.9576	0.20223

281.05438	0.20227

281.15116	0.2023

281.24794	0.20234

281.34473	0.20237

281.44151	0.20241

281.53829	0.20246

281.63507	0.20262

281.73185	0.20264

281.82863	0.20266

281.92542	0.20269

282.0222	0.20273

282.11898	0.20278

282.21576	0.20282

282.31254	0.20285

282.40933	0.20296

282.50611	0.20303

282.60289	0.20305

282.69967	0.20307

282.79645	0.2031

282.89323	0.20314

282.99002	0.20317

283.0868	0.20319

283.18358	0.20321

283.28036	0.20333

283.37714	0.20335

283.47393	0.20337

283.57071	0.20349

283.66749	0.20351

283.76427	0.20353

283.86105	0.20355

283.95783	0.20365

284.05462	0.20367

284.1514	0.20369

284.24818	0.20376

284.34496	0.20381

284.44174	0.20387

284.53852	0.20397

284.63531	0.20401

284.73209	0.20403

284.82887	0.20406

284.92565	0.20408

285.02243	0.2041

285.11922	0.20413

285.216	0.20417

285.31278	0.20419

285.40956	0.20426

285.50634	0.20435

285.60312	0.20438

285.69991	0.2044

285.79669	0.20449

285.89347	0.20451

285.99025	0.20454

286.08703	0.20472

286.18382	0.20474

286.2806	0.20477

286.37738	0.20481

286.47416	0.20483

286.57094	0.20486

286.66772	0.20488

286.76451	0.2049

286.86129	0.20499

286.95807	0.20502

287.05485	0.20504

287.15163	0.20513

287.24842	0.20518

287.3452	0.20529

287.44198	0.20531

287.53876	0.20534

287.63554	0.20536

287.73232	0.20538

287.82911	0.20541

287.92589	0.20543

288.02267	0.20545

288.11945	0.20547

288.21623	0.2055

288.31302	0.20554

288.4098	0.20559

288.50658	0.20563

288.60336	0.20566

288.70014	0.20568

288.79692	0.2057

288.89371	0.20573

288.99049	0.20577

289.08727	0.20579

289.18405	0.20582

289.28083	0.20586

289.37762	0.20589

289.4744	0.20593

289.57118	0.20595

289.66796	0.20598

289.76474	0.20605

289.86152	0.20607

289.95831	0.20609

290.05509	0.20616

290.15187	0.20621

290.24865	0.20623

290.34543	0.20625

290.44222	0.20627

290.539	0.2063

290.63578	0.20632

290.73256	0.20634

290.82934	0.20637

290.92612	0.20641

291.02291	0.20653

291.11969	0.20655

291.21647	0.20659

291.31325	0.20666

291.41003	0.20669

291.50682	0.20673

291.6036	0.2068

291.70038	0.20682

291.79716	0.20685

291.89394	0.20687

291.99072	0.20689

292.08751	0.20705

292.18429	0.2071

292.28107	0.20712

292.37785	0.20714

292.47463	0.20721

292.57142	0.20726

292.6682	0.20728

292.76498	0.2073

292.86176	0.20733

292.95854	0.20765

293.05532	0.20767

293.15211	0.20769

293.24889	0.20774

293.34567	0.20778

293.44245	0.20781

293.53923	0.20783

293.63602	0.20785

293.7328	0.20792

293.82958	0.20794

293.92636	0.20797

294.02314	0.20799

294.11992	0.20803

294.21671	0.20806

294.31349	0.20808

294.41027	0.20815

294.50705	0.20817

294.60383	0.20822

294.70062	0.20824

294.7974	0.20826

294.89418	0.20829

294.99096	0.20831

295.08774	0.20833

295.18452	0.20835

295.28131	0.20845

295.37809	0.20847

295.47487	0.20849

295.57165	0.20851

295.66843	0.20854

295.76522	0.20858

295.862	0.20863

295.95878	0.20865

296.05556	0.20867

296.15234	0.2087

296.24912	0.20874

296.34591	0.20881

296.44269	0.20886

296.53947	0.2089

296.63625	0.20902

296.73303	0.20904

296.82982	0.20906

296.9266	0.20909

297.02338	0.20915

297.12016	0.20918

297.21694	0.20922

297.31372	0.20925

297.41051	0.20927

297.50729	0.20931

297.60407	0.20941

297.70085	0.20945

297.79763	0.20947

297.89441	0.2095

297.9912	0.20952

298.08798	0.20954

298.18476	0.20957

298.28154	0.20961

298.37832	0.20963

298.47511	0.20966

298.57189	0.20975

298.66867	0.20977

298.76545	0.20982

298.86223	0.20984

298.95901	0.20986

299.0558	0.20989

299.15258	0.20991

299.24936	0.20995

299.34614	0.21

299.44292	0.21002

299.53971	0.21007

299.63649	0.21009

299.73327	0.21014

299.83005	0.21016

299.92683	0.21018

300.02361	0.21023

300.1204	0.21025

300.21718	0.21032

300.31396	0.21034

300.41074	0.21037

300.50752	0.21041

300.60431	0.21046

300.70109	0.21082

300.79787	0.21085

300.89465	0.21087

300.99143	0.21089

301.08821	0.21091

301.185	0.21094

301.28178	0.21096

301.37856	0.21098

301.47534	0.21101

301.57212	0.21103

301.66891	0.21105

301.76569	0.21107

301.86247	0.2111

301.95925	0.21117

302.05603	0.21119

302.15281	0.21121

302.2496	0.21123

302.34638	0.21126

302.44316	0.21128

302.53994	0.21133

302.63672	0.21135

302.73351	0.21137

302.83029	0.21139

302.92707	0.21142

303.02385	0.21146

303.12063	0.21155

303.21741	0.21158

303.3142	0.2116

303.41098	0.21165

303.50776	0.21169

303.60454	0.21171

303.70132	0.21174

303.79811	0.21176

303.89489	0.2119

303.99167	0.21194

304.08845	0.21199

304.18523	0.21206

304.28201	0.21208

304.3788	0.2121

304.47558	0.21215

304.57236	0.21217

304.66914	0.21219

304.76592	0.21222

304.86271	0.21229

304.95949	0.21233

305.05627	0.21235

305.15305	0.21238

305.24983	0.21245

305.34661	0.21251

305.4434	0.21254

305.54018	0.21272

305.63696	0.21274

305.73374	0.21359

305.83052	0.21361

305.92731	0.21368

306.02409	0.21391

306.12087	0.214

306.21765	0.21402

306.31443	0.21405

306.41121	0.21416

306.508	0.21421

306.60478	0.21423

306.70156	0.21437

306.79834	0.21439

306.89512	0.21441

306.99191	0.21443

307.08869	0.21446

307.18547	0.2145

307.28225	0.21453

307.37903	0.21455

307.47581	0.21457

307.5726	0.21459

307.66938	0.21473

307.76616	0.21475

307.86294	0.21478

307.95972	0.21482

308.05651	0.21491

308.15329	0.21494

308.25007	0.21498

308.34685	0.21501

308.44363	0.21503

308.54041	0.21505

308.6372	0.21507

308.73398	0.2151

308.83076	0.21512

308.92754	0.21514

309.02432	0.21519

309.12111	0.21526

309.21789	0.21528

309.31467	0.2153

309.41145	0.21533

309.50823	0.21535

309.60501	0.21539

309.7018	0.21542

309.79858	0.21549

309.89536	0.21553

309.99214	0.21558

310.08892	0.21562

310.1857	0.21578

310.28249	0.21581

310.37927	0.21587

310.47605	0.2159

310.57283	0.21592

310.66961	0.21594

310.7664	0.21599

310.86318	0.21601

310.95996	0.21606

311.05674	0.2161

311.15352	0.21613

311.2503	0.21615

311.34709	0.21617

311.44387	0.21619

311.54065	0.21626

311.63743	0.21631

311.73421	0.21633

311.831	0.21635

311.92778	0.21638

312.02456	0.2164

312.12134	0.21642

312.21812	0.21649

312.3149	0.21651

312.41169	0.21658

312.50847	0.21661

312.60525	0.21663

312.70203	0.21667

312.79881	0.2167

312.8956	0.21672

312.99238	0.21674

313.08916	0.21683

313.18594	0.2169

313.28272	0.21693

313.3795	0.21695

313.47629	0.21697

313.57307	0.21699

313.66985	0.21702

313.76663	0.21709

313.86341	0.21711

313.9602	0.21713

314.05698	0.21715

314.15376	0.21718

314.25054	0.21722

314.34732	0.21725

314.4441	0.21727

314.54089	0.21731

314.63767	0.21736

314.73445	0.21738

314.83123	0.21745

314.92801	0.2175

315.0248	0.21759

315.12158	0.21766

315.21836	0.21768

315.31514	0.2177

315.41192	0.21775

315.5087	0.21779

315.60549	0.21782

315.70227	0.21784

315.79905	0.21786

315.89583	0.21793

315.99261	0.21795

316.0894	0.21798

316.18618	0.21802

316.28296	0.21805

316.37974	0.21807

316.47652	0.21809

316.5733	0.21811

316.67009	0.21816

316.76687	0.21823

316.86365	0.21825

316.96043	0.21827

317.05721	0.2183

317.154	0.21834

317.25078	0.21837

317.34756	0.21843

317.44434	0.21846

317.54112	0.2185

317.6379	0.21853

317.73469	0.21855

317.83147	0.21857

317.92825	0.21862

318.02503	0.21864

318.12181	0.21866

318.2186	0.21871

318.31538	0.2188

318.41216	0.21885

318.50894	0.21889

318.60572	0.21891

318.7025	0.21894

318.79929	0.21896

318.89607	0.21898

318.99285	0.21901

319.08963	0.21907

319.18641	0.21912

319.2832	0.21914

319.37998	0.21923

319.47676	0.21926

319.57354	0.21928

319.67032	0.21946

319.7671	0.21949

319.86389	0.21951

319.96067	0.21962

320.05745	0.21967

320.15423	0.21969

320.25101	0.21971

320.3478	0.21974

320.44458	0.21981

320.54136	0.21983

320.63814	0.21985

320.73492	0.21987

320.8317	0.21992

320.92849	0.21997

321.02527	0.21999

321.12205	0.2201

321.21883	0.22013

321.31561	0.22015

321.4124	0.22022

321.50918	0.22024

321.60596	0.22026

321.70274	0.22029

321.79952	0.22031

321.8963	0.22038

321.99309	0.22042

322.08987	0.22045

322.18665	0.22047

322.28343	0.22051

322.38021	0.22054

322.47699	0.22056

322.57378	0.22058

322.67056	0.22061

322.76734	0.22063

322.86412	0.22065

322.9609	0.2207

323.05769	0.22077

323.15447	0.22079

323.25125	0.22081

323.34803	0.22086

323.44481	0.22088

323.54159	0.22097

323.63838	0.22099

323.73516	0.22102

323.83194	0.22104

323.92872	0.22106

324.0255	0.22113

324.12229	0.22118

324.21907	0.22143

324.31585	0.22145

324.41263	0.2215

324.50941	0.22152

324.60619	0.22168

324.70298	0.22173

324.79976	0.22177

324.89654	0.22182

324.99332	0.22195

325.0901	0.22198

325.18689	0.222

325.28367	0.22202

325.38045	0.22209

325.47723	0.22211

325.57401	0.22216

325.67079	0.22218

325.76758	0.22221

325.86436	0.22223

325.96114	0.22225

326.05792	0.22228

326.1547	0.2223

326.25149	0.22232

326.34827	0.22239

326.44505	0.22241

326.54183	0.22244

326.63861	0.22248

326.73539	0.2225

326.83218	0.22253

326.92896	0.22257

327.02574	0.22264

327.12252	0.22266

327.2193	0.22273

327.31609	0.22276

327.41287	0.22278

327.50965	0.2228

327.60643	0.22282

327.70321	0.22287

327.79999	0.22289

327.89678	0.22292

327.99356	0.22294

328.09034	0.22296

328.18712	0.22301

328.2839	0.22303

328.38069	0.22305

328.47747	0.22308

328.57425	0.2231

328.67103	0.22317

328.76781	0.22319

328.86459	0.22321

328.96138	0.22326

329.05816	0.22328

329.15494	0.22337

329.25172	0.2234

329.3485	0.22372

329.44529	0.22374

329.54207	0.22378

329.63885	0.22381

329.73563	0.22383

329.83241	0.2239

329.92919	0.22392

330.02598	0.22404

330.12276	0.22408

330.21954	0.2241

330.31632	0.22422

330.4131	0.22426

330.50989	0.22429

330.60667	0.22431

330.70345	0.22433

330.80023	0.22436

330.89701	0.22438

330.99379	0.2244

331.09058	0.22442

331.18736	0.22445

331.28414	0.22449

331.38092	0.22461

331.4777	0.22463

331.57449	0.22465

331.67127	0.2247

331.76805	0.22472

331.86483	0.22474

331.96161	0.22477

332.05839	0.22479

332.15518	0.22481

332.25196	0.2249

332.34874	0.22493

332.44552	0.22497

332.5423	0.22504

332.63909	0.22509

332.73587	0.22511

332.83265	0.22513

332.92943	0.22516

333.02621	0.22518

333.12299	0.2252

333.21978	0.22522

333.31656	0.22529

333.41334	0.22532

333.51012	0.22536

333.6069	0.22538

333.70369	0.22541

333.80047	0.22548

333.89725	0.2255

333.99403	0.22557

334.09081	0.22559

334.18759	0.22568

334.28438	0.22577

334.38116	0.22584

334.47794	0.22591

334.57472	0.22593

334.6715	0.22598

334.76828	0.226

334.86507	0.22602

334.96185	0.22605

335.05863	0.22609

335.15541	0.22614

335.25219	0.22616

335.34898	0.22628

335.44576	0.2263

335.54254	0.22632

335.63932	0.22634

335.7361	0.22637

335.83288	0.22639

335.92967	0.22646

336.02645	0.2265

336.12323	0.22653

336.22001	0.22662

336.31679	0.22664

336.41358	0.22669

336.51036	0.22671

336.60714	0.22673

336.70392	0.22678

336.8007	0.22682

336.89748	0.22685

336.99427	0.22687

337.09105	0.22698

337.18783	0.22701

337.28461	0.22703

337.38139	0.22705

337.47818	0.22708

337.57496	0.22712

337.67174	0.22714

337.76852	0.22717

337.8653	0.22721

337.96208	0.22724

338.05887	0.22726

338.15565	0.22728

338.25243	0.2273

338.34921	0.22733

338.44599	0.22737

338.54278	0.2274

338.63956	0.22744

338.73634	0.22751

338.83312	0.22753

338.9299	0.22756

339.02668	0.2276

339.12347	0.22762

339.22025	0.22767

339.31703	0.22772

339.41381	0.22776

339.51059	0.22778

339.60738	0.22792

339.70416	0.22799

339.80094	0.22806

339.89772	0.22815

339.9945	0.22817

340.09128	0.2282

340.18807	0.22822

340.28485	0.22831

340.38163	0.22833

340.47841	0.22838

340.57519	0.22845

340.67198	0.22847

340.76876	0.22852

340.86554	0.22854

340.96232	0.22856

341.0591	0.22858

341.15588	0.22865

341.25267	0.22868

341.34945	0.2287

341.44623	0.22886

341.54301	0.22893

341.63979	0.22906

341.73658	0.22909

341.83336	0.22913

341.93014	0.22916

342.02692	0.22918

342.1237	0.22922

342.22048	0.22932

342.31727	0.22936

342.41405	0.22938

342.51083	0.22941

342.60761	0.22945

342.70439	0.22948

342.80118	0.2295

342.89796	0.22957

342.99474	0.22959

343.09152	0.22961

343.1883	0.22964

343.28508	0.22966

343.38187	0.22968

343.47865	0.2297

343.57543	0.22984

343.67221	0.22989

343.76899	0.23012

343.86578	0.23016

343.96256	0.23018

344.05934	0.23021

344.15612	0.23028

344.2529	0.2303

344.34968	0.23041

344.44647	0.23044

344.54325	0.23048

344.64003	0.2305

344.73681	0.23053

344.83359	0.23055

344.93038	0.2306

345.02716	0.23064

345.12394	0.23073

345.22072	0.23076

345.3175	0.23085

345.41428	0.23092

345.51107	0.23096

345.60785	0.23098

345.70463	0.23101

345.80141	0.23192

345.89819	0.23206

345.99498	0.23208

346.09176	0.2321

346.18854	0.23217

346.28532	0.2322

346.3821	0.23224

346.47888	0.23229

346.57567	0.23231

346.67245	0.23236

346.76923	0.23238

346.86601	0.23249

346.96279	0.23254

347.05958	0.23256

347.15636	0.23258

347.25314	0.23263

347.34992	0.23274

347.4467	0.23318

347.54348	0.2332

347.64027	0.23322

347.73705	0.23325

347.83383	0.23329

347.93061	0.23332

348.02739	0.23336

348.12417	0.23338

348.22096	0.23348

348.31774	0.23354

348.41452	0.23359

348.5113	0.23361

348.60808	0.23366

348.70487	0.23368

348.80165	0.23373

348.89843	0.23377

348.99521	0.2338

349.09199	0.23382

349.18877	0.23384

349.28556	0.23386

349.38234	0.23389

349.47912	0.23391

349.5759	0.23393

349.67268	0.234

349.76947	0.23402

349.86625	0.23405

349.96303	0.23409

350.05981	0.23412

350.15659	0.23414

350.25337	0.23416

350.35016	0.23421

350.44694	0.23423

350.54372	0.23425

350.6405	0.23428

350.73728	0.23444

350.83407	0.23446

350.93085	0.23448

351.02763	0.23464

351.12441	0.23466

351.22119	0.23469

351.31797	0.23471

351.41476	0.23473

351.51154	0.2348

351.60832	0.23487

351.7051	0.23505

351.80188	0.2351

351.89867	0.23512

351.99545	0.23514

352.09223	0.23519

352.18901	0.23521

352.28579	0.2353

352.38257	0.23533

352.47936	0.23535

352.57614	0.23544

352.67292	0.23549

352.7697	0.23551

352.86648	0.23553

352.96327	0.23558

353.06005	0.2356

353.15683	0.23565

353.25361	0.23567

353.35039	0.23569

353.44717	0.23572

353.54396	0.23581

353.64074	0.23583

353.73752	0.23592

353.8343	0.23594

353.93108	0.23597

354.02787	0.23626

354.12465	0.23636

354.22143	0.23638

354.31821	0.23688

354.41499	0.23702

354.51177	0.23706

354.60856	0.23709

354.70534	0.23713

354.80212	0.23716

354.8989	0.2372

354.99568	0.23725

355.09247	0.23727

355.18925	0.23729

355.28603	0.23732

355.38281	0.23734

355.47959	0.23741

355.57637	0.23743

355.67316	0.23745

355.76994	0.23752

355.86672	0.23754

355.9635	0.23757

356.06028	0.23761

356.15707	0.23764

356.25385	0.23766

356.35063	0.23768

356.44741	0.2377

356.54419	0.23773

356.64097	0.23775

356.73776	0.23777

356.83454	0.23793

356.93132	0.23802

357.0281	0.23805

357.12488	0.23812

357.22167	0.23814

357.31845	0.23876

357.41523	0.23878

357.51201	0.23882

357.60879	0.23885

357.70557	0.23889

357.80236	0.23892

357.89914	0.23894

357.99592	0.23896

358.0927	0.23898

358.18948	0.23903

358.28627	0.23905

358.38305	0.23908

358.47983	0.2391

358.57661	0.23914

358.67339	0.23917

358.77017	0.23919

358.86696	0.23924

358.96374	0.23933

359.06052	0.23935

359.1573	0.23937

359.25408	0.2394

359.35087	0.23946

359.44765	0.23953

359.54443	0.23958

359.64121	0.2396

359.73799	0.23962

359.83477	0.23965

359.93156	0.23972

360.02834	0.23976

360.12512	0.23983

360.2219	0.23985

360.31868	0.23988

360.41546	0.23992

360.51225	0.23994

360.60903	0.23997

360.70581	0.24004

360.80259	0.24008

360.89937	0.2401

360.99616	0.24029

361.09294	0.24038

361.18972	0.24042

361.2865	0.24045

361.38328	0.24049

361.48006	0.24054

361.57685	0.24058

361.67363	0.24063

361.77041	0.24065

361.86719	0.24068

361.96397	0.2407

362.06076	0.24072

362.15754	0.24074

362.25432	0.24081

362.3511	0.2409

362.44788	0.24093

362.54466	0.24104

362.64145	0.24106

362.73823	0.24109

362.83501	0.24113

362.93179	0.24116

363.02857	0.24118

363.12536	0.24129

363.22214	0.24132

363.31892	0.24134

363.4157	0.24138

363.51248	0.24141

363.60926	0.24143

363.70605	0.24145

363.80283	0.2415

363.89961	0.24152

363.99639	0.24154

364.09317	0.24168

364.18996	0.24173

364.28674	0.24175

364.38352	0.24177

364.4803	0.24182

364.57708	0.24186

364.67386	0.24189

364.77065	0.24198

364.86743	0.242

364.96421	0.24207

365.06099	0.24209

365.15777	0.24214

365.25456	0.24218

365.35134	0.24223

365.44812	0.2423

365.5449	0.24232

365.64168	0.24241

365.73846	0.24244

365.83525	0.24253

365.93203	0.24255

366.02881	0.24257

366.12559	0.24262

366.22237	0.24264

366.31916	0.24271

366.41594	0.24273

366.51272	0.24285

366.6095	0.24289

366.70628	0.24292

366.80306	0.24294

366.89985	0.24296

366.99663	0.2431

367.09341	0.24314

367.19019	0.24319

367.28697	0.24321

367.38376	0.24324

367.48054	0.24326

367.57732	0.24328

367.6741	0.2433

367.77088	0.24333

367.86766	0.24335

367.96445	0.24337

368.06123	0.24346

368.15801	0.24381

368.25479	0.24424

368.35157	0.24431

368.44836	0.24438

368.54514	0.24442

368.64192	0.24445

368.7387	0.24447

368.83548	0.24449

368.93226	0.24458

369.02905	0.24461

369.12583	0.24463

369.22261	0.24465

369.31939	0.2447

369.41617	0.24472

369.51296	0.24474

369.60974	0.24479

369.70652	0.24481

369.8033	0.24486

369.90008	0.24488

369.99686	0.2449

370.09365	0.24493

370.19043	0.24495

370.28721	0.24497

370.38399	0.24506

370.48077	0.24509

370.57756	0.24511

370.67434	0.24522

370.77112	0.24525

370.8679	0.24527

370.96468	0.24529

371.06146	0.24532

371.15825	0.24534

371.25503	0.24538

371.35181	0.24541

371.44859	0.24543

371.54537	0.2455

371.64216	0.24552

371.73894	0.24554

371.83572	0.24559

371.9325	0.24561

372.02928	0.24568

372.12606	0.2457

372.22285	0.24573

372.31963	0.24577

372.41641	0.24582

372.51319	0.24589

372.60997	0.24591

372.70675	0.24598

372.80354	0.24602

372.90032	0.24609

372.9971	0.24612

373.09388	0.24614

373.19066	0.24616

373.28745	0.24618

373.38423	0.24628

373.48101	0.2463

373.57779	0.24632

373.67457	0.24634

373.77135	0.24637

373.86814	0.24639

373.96492	0.24641

374.0617	0.24646

374.15848	0.24648

374.25526	0.2465

374.35205	0.24653

374.44883	0.24662

374.54561	0.24664

374.64239	0.24673

374.73917	0.24676

374.83595	0.24678

374.93274	0.2468

375.02952	0.24682

375.1263	0.24694

375.22308	0.24698

375.31986	0.24701

375.41665	0.24705

375.51343	0.2471

375.61021	0.24712

375.70699	0.24714

375.80377	0.24721

375.90055	0.24733

375.99734	0.24737

376.09412	0.2474

376.1909	0.24742

376.28768	0.24744

376.38446	0.24746

376.48125	0.24788

376.57803	0.24792

376.67481	0.24794

376.77159	0.24797

376.86837	0.24799

376.96515	0.24804

377.06194	0.24806

377.15872	0.24813

377.2555	0.24822

377.35228	0.24829

377.44906	0.24831

377.54585	0.24833

377.64263	0.24836

377.73941	0.24845

377.83619	0.24847

377.93297	0.24849

378.02975	0.24852

378.12654	0.24854

378.22332	0.24856

378.3201	0.24868

378.41688	0.2487

378.51366	0.24874

378.61045	0.24895

378.70723	0.24897

378.80401	0.24902

378.90079	0.24918

378.99757	0.24922

379.09435	0.24925

379.19114	0.24927

379.28792	0.24943

379.3847	0.24945

379.48148	0.2495

379.57826	0.24966

379.67505	0.24968

379.77183	0.24977

379.86861	0.24982

379.96539	0.24984

380.06217	0.24986

380.15895	0.24989

380.25574	0.24991

380.35252	0.24993

380.4493	0.25

380.54608	0.25002

380.64286	0.25005

380.73965	0.25007

380.83643	0.25025

380.93321	0.25028

381.02999	0.25037

381.12677	0.25039

381.22355	0.25041

381.32034	0.25044

381.41712	0.25046

381.5139	0.25053

381.61068	0.25071

381.70746	0.25073

381.80425	0.25076

381.90103	0.2508

381.99781	0.25082

382.09459	0.25085

382.19137	0.25087

382.28815	0.25092

382.38494	0.25094

382.48172	0.25096

382.5785	0.25098

382.67528	0.25101

382.77206	0.25108

382.86885	0.25112

382.96563	0.25117

383.06241	0.25119

383.15919	0.25121

383.25597	0.25124

383.35275	0.25128

383.44954	0.2513

383.54632	0.25133

383.6431	0.25135

383.73988	0.25146

383.83666	0.25149

383.93345	0.25151

384.03023	0.2516

384.12701	0.25167

384.22379	0.25169

384.32057	0.25174

384.41735	0.25197

384.51414	0.25201

384.61092	0.25204

384.7077	0.25206

384.80448	0.25222

384.90126	0.25231

384.99804	0.25233

385.09483	0.25238

385.19161	0.2524

385.28839	0.25242

385.38517	0.25254

385.48195	0.25256

385.57874	0.25258

385.67552	0.25274

385.7723	0.25277

385.86908	0.25279

385.96586	0.25286

386.06264	0.25288

386.15943	0.2529

386.25621	0.25293

386.35299	0.25297

386.44977	0.253

386.54655	0.25302

386.64334	0.25309

386.74012	0.25313

386.8369	0.25318

386.93368	0.25325

387.03046	0.25332

387.12724	0.25334

387.22403	0.25336

387.32081	0.25338

387.41759	0.25343

387.51437	0.25345

387.61115	0.25354

387.70794	0.25357

387.80472	0.25361

387.9015	0.25364

387.99828	0.25366

388.09506	0.25375

388.19184	0.25386

388.28863	0.25389

388.38541	0.25393

388.48219	0.25398

388.57897	0.254

388.67575	0.25407

388.77254	0.25416

388.86932	0.25421

388.9661	0.25425

389.06288	0.25428

389.15966	0.2543

389.25644	0.25432

389.35323	0.25439

389.45001	0.25441

389.54679	0.25448

389.64357	0.25455

389.74035	0.2546

389.83714	0.25466

389.93392	0.25471

390.0307	0.25473

390.12748	0.25478

390.22426	0.25487

390.32104	0.25489

390.41783	0.25494

390.51461	0.25501

390.61139	0.25512

390.70817	0.25517

390.80495	0.25521

390.90174	0.25533

390.99852	0.25537

391.0953	0.2554

391.19208	0.25542

391.28886	0.25544

391.38564	0.25547

391.48243	0.25549

391.57921	0.25553

391.67599	0.25556

391.77277	0.25558

391.86955	0.25565

391.96634	0.25569

392.06312	0.25574

392.1599	0.25576

392.25668	0.25579

392.35346	0.25583

392.45024	0.25588

392.54703	0.2559

392.64381	0.25597

392.74059	0.25599

392.83737	0.25604

392.93415	0.25608

393.03094	0.25611

393.12772	0.25615

393.2245	0.25622

393.32128	0.25624

393.41806	0.25633

393.51484	0.25636

393.61163	0.25638

393.70841	0.2564

393.80519	0.25643

393.90197	0.25668

393.99875	0.2567

394.09554	0.25672

394.19232	0.25675

394.2891	0.25679

394.38588	0.25681

394.48266	0.25684

394.57944	0.25697

394.67623	0.257

394.77301	0.25718

394.86979	0.25725

394.96657	0.25727

395.06335	0.25729

395.16014	0.25732

395.25692	0.25741

395.3537	0.25745

395.45048	0.25752

395.54726	0.25757

395.64404	0.25759

395.74083	0.25766

395.83761	0.25771

395.93439	0.25775

396.03117	0.25777

396.12795	0.2578

396.22474	0.25784

396.32152	0.25791

396.4183	0.25793

396.51508	0.25805

396.61186	0.25825

396.70864	0.25832

396.80543	0.25837

396.90221	0.25839

396.99899	0.25844

397.09577	0.25846

397.19255	0.25848

397.28933	0.25851

397.38612	0.25864

397.4829	0.25867

397.57968	0.25885

397.67646	0.25887

397.77324	0.25892

397.87003	0.25894

397.96681	0.25896

398.06359	0.25899

398.16037	0.25901

398.25715	0.25903

398.35393	0.25905

398.45072	0.2591

398.5475	0.25912

398.64428	0.25917

398.74106	0.25919

398.83784	0.25924

398.93463	0.25926

399.03141	0.25935

399.12819	0.25942

399.22497	0.25947

399.32175	0.25949

399.41853	0.25953

399.51532	0.25956

399.6121	0.25958

399.70888	0.25976

399.80566	0.25979

399.90244	0.25981

399.99923	0.25985

400.09601	0.25992

400.19279	0.25995

400.28957	0.25999

400.38635	0.26001

400.48313	0.26049

400.57992	0.26054

400.6767	0.26056

400.77348	0.26061

400.87026	0.26063

400.96704	0.26065

401.06383	0.26068

401.16061	0.2607

401.25739	0.26072

401.35417	0.26075

401.45095	0.26077

401.54773	0.26079

401.64452	0.26084

401.7413	0.26086

401.83808	0.26102

401.93486	0.26109

402.03164	0.26111

402.12843	0.26113

402.22521	0.2612

402.32199	0.26123

402.41877	0.26127

402.51555	0.26129

402.61233	0.26139

402.70912	0.26141

402.8059	0.2615

402.90268	0.26152

402.99946	0.26157

403.09624	0.26161

403.19303	0.26164

403.28981	0.26166

403.38659	0.26168

403.48337	0.26177

403.58015	0.2618

403.67693	0.26184

403.77372	0.26189

403.8705	0.26191

403.96728	0.26196

404.06406	0.26205

404.16084	0.26207

404.25763	0.26221

404.35441	0.26225

404.45119	0.26235

404.54797	0.26237

404.64475	0.26239

404.74153	0.26244

404.83832	0.26248

404.9351	0.26251

405.03188	0.26253

405.12866	0.26255

405.22544	0.26262

405.32223	0.26264

405.41901	0.26269

405.51579	0.26271

405.61257	0.26273

405.70935	0.26276

405.80613	0.2628

405.90292	0.26285

405.9997	0.26287

406.09648	0.26289

406.19326	0.26292

406.29004	0.26294

406.38683	0.26296

406.48361	0.26299

406.58039	0.26301

406.67717	0.26305

406.77395	0.26317

406.87073	0.26324

406.96752	0.26326

407.0643	0.26328

407.16108	0.26331

407.25786	0.26333

407.35464	0.26337

407.45143	0.26349

407.54821	0.26351

407.64499	0.26353

407.74177	0.26356

407.83855	0.2636

407.93533	0.26363

408.03212	0.26367

408.1289	0.26369

408.22568	0.26376

408.32246	0.26381

408.41924	0.26385

408.51603	0.2639

408.61281	0.26392

408.70959	0.26395

408.80637	0.26397

408.90315	0.26399

408.99993	0.26406

409.09672	0.26413

409.1935	0.26417

409.29028	0.2642

409.38706	0.26424

409.48384	0.26427

409.58063	0.26429

409.67741	0.26433

409.77419	0.26443

409.87097	0.26445

409.96775	0.26447

410.06453	0.26465

410.16132	0.26468

410.2581	0.26472

410.35488	0.26475

410.45166	0.26477

410.54844	0.26479

410.64522	0.26481

410.74201	0.26484

410.83879	0.26486

410.93557	0.26491

411.03235	0.265

411.12913	0.26543

411.22592	0.26545

411.3227	0.2655

411.41948	0.26552

411.51626	0.26555

411.61304	0.26557

411.70982	0.26559

411.80661	0.26561

411.90339	0.26564

412.00017	0.26566

412.09695	0.26568

412.19373	0.26571

412.29052	0.26582

412.3873	0.26584

412.48408	0.26591

412.58086	0.26596

412.67764	0.26598

412.77442	0.266

412.87121	0.26605

412.96799	0.26607

413.06477	0.26609

413.16155	0.26612

413.25833	0.26619

413.35512	0.26621

413.4519	0.26623

413.54868	0.26632

413.64546	0.26635

413.74224	0.26641

413.83902	0.26657

413.93581	0.26662

414.03259	0.26664

414.12937	0.26667

414.22615	0.26669

414.32293	0.26676

414.41972	0.26678

414.5165	0.26696

414.61328	0.26699

414.71006	0.26701

414.80684	0.26703

414.90362	0.26705

415.00041	0.26708

415.09719	0.26712

415.19397	0.26715

415.29075	0.26721

415.38753	0.26728

415.48432	0.26731

415.5811	0.26733

415.67788	0.26735

415.77466	0.26737

415.87144	0.2674

415.96822	0.26751

416.06501	0.26753

416.16179	0.26756

416.25857	0.26758

416.35535	0.2676

416.45213	0.26776

416.54892	0.26779

416.6457	0.26783

416.74248	0.26785

416.83926	0.26792

416.93604	0.26795

417.03282	0.26797

417.12961	0.26799

417.22639	0.26801

417.32317	0.26804

417.41995	0.26806

417.51673	0.26808

417.61352	0.26811

417.7103	0.26813

417.80708	0.26815

417.90386	0.26817

418.00064	0.2682

418.09742	0.26822

418.19421	0.26827

418.29099	0.26829

418.38777	0.26833

418.48455	0.26836

418.58133	0.26843

418.67812	0.26845

418.7749	0.26849

418.87168	0.26852

418.96846	0.26856

419.06524	0.26861

419.16202	0.26865

419.25881	0.26877

419.35559	0.26893

419.45237	0.26895

419.54915	0.269

419.64593	0.26907

419.74272	0.26909

419.8395	0.26911

419.93628	0.26941

420.03306	0.2695

420.12984	0.26957

420.22662	0.26961

420.32341	0.26964

420.42019	0.26966

420.51697	0.26989

420.61375	0.26991

420.71053	0.26993

420.80732	0.26998

420.9041	0.27003

421.00088	0.27037

421.09766	0.27039

421.19444	0.27044

421.29122	0.27046

421.38801	0.27048

421.48479	0.27051

421.58157	0.27055

421.67835	0.27057

421.77513	0.27062

421.87192	0.27064

421.9687	0.27069

422.06548	0.27076

422.16226	0.27078

422.25904	0.27089

422.35582	0.27096

422.45261	0.27121

422.54939	0.27126

422.64617	0.27131

422.74295	0.2714

422.83973	0.27142

422.93651	0.27147

423.0333	0.27149

423.13008	0.27153

423.22686	0.27156

423.32364	0.27158

423.42042	0.2716

423.51721	0.27163

423.61399	0.27165

423.71077	0.27167

423.80755	0.27169

423.90433	0.27172

424.00111	0.27176

424.0979	0.27181

424.19468	0.27185

424.29146	0.2719

424.38824	0.27192

424.48502	0.27195

424.58181	0.27197

424.67859	0.27204

424.77537	0.27211

424.87215	0.27217

424.96893	0.2722

425.06571	0.27229

425.1625	0.27231

425.25928	0.27233

425.35606	0.27236

425.45284	0.27238

425.54962	0.2724

425.64641	0.27243

425.74319	0.27245

425.83997	0.27247

425.93675	0.27254

426.03353	0.27268

426.13031	0.27277

426.2271	0.27279

426.32388	0.27284

426.42066	0.27293

426.51744	0.27295

426.61422	0.27297

426.71101	0.273

426.80779	0.27329

426.90457	0.27332

427.00135	0.27345

427.09813	0.27348

427.19491	0.27355

427.2917	0.27357

427.38848	0.27359

427.48526	0.27361

427.58204	0.27366

427.67882	0.27373

427.77561	0.2738

427.87239	0.27389

427.96917	0.27391

428.06595	0.27393

428.16273	0.27398

428.25951	0.274

428.3563	0.27405

428.45308	0.27407

428.54986	0.27409

428.64664	0.27412

428.74342	0.27414

428.84021	0.27421

428.93699	0.27423

429.03377	0.27425

429.13055	0.2743

429.22733	0.27441

429.32411	0.27448

429.4209	0.27453

429.51768	0.27455

429.61446	0.27469

429.71124	0.27471

429.80802	0.27473

429.90481	0.27476

430.00159	0.2748

430.09837	0.27483

430.19515	0.27485

430.29193	0.27496

430.38871	0.27499

430.4855	0.27501

430.58228	0.27505

430.67906	0.2751

430.77584	0.27512

430.87262	0.27515

430.96941	0.27519

431.06619	0.27521

431.16297	0.27524

431.25975	0.27528

431.35653	0.27531

431.45331	0.27542

431.5501	0.27551

431.64688	0.27553

431.74366	0.27556

431.84044	0.27558

431.93722	0.27563

432.03401	0.27565

432.13079	0.27579

432.22757	0.27599

432.32435	0.27601

432.42113	0.27604

432.51791	0.27624

432.6147	0.27627

432.71148	0.27633

432.80826	0.27636

432.90504	0.27643

433.00182	0.27645

433.09861	0.27647

433.19539	0.27649

433.29217	0.27656

433.38895	0.27659

433.48573	0.27661

433.58251	0.27672

433.6793	0.27675

433.77608	0.27679

433.87286	0.27681

433.96964	0.27684

434.06642	0.27686

434.16321	0.27688

434.25999	0.277

434.35677	0.27702

434.45355	0.27711

434.55033	0.2772

434.64711	0.27725

434.7439	0.27729

434.84068	0.27732

434.93746	0.27743

435.03424	0.27752

435.13102	0.27757

435.2278	0.27759

435.32459	0.27761

435.42137	0.27766

435.51815	0.27768

435.61493	0.27773

435.71171	0.27775

435.8085	0.27777

435.90528	0.2778

436.00206	0.27782

436.09884	0.27787

436.19562	0.27796

436.2924	0.27803

436.38919	0.27805

436.48597	0.27807

436.58275	0.27809

436.67953	0.27812

436.77631	0.27814

436.8731	0.27816

436.96988	0.27819

437.06666	0.27823

437.16344	0.27825

437.26022	0.27828

437.357	0.2783

437.45379	0.27832

437.55057	0.27835

437.64735	0.27837

437.74413	0.27839

437.84091	0.27846

437.9377	0.27851

438.03448	0.27853

438.13126	0.27855

438.22804	0.27857

438.32482	0.2786

438.4216	0.27867

438.51839	0.27871

438.61517	0.27873

438.71195	0.27876

438.80873	0.27878

438.90551	0.2788

439.0023	0.27887

439.09908	0.27889

439.19586	0.27892

439.29264	0.27896

439.38942	0.27905

439.4862	0.27924

439.58299	0.27926

439.67977	0.27928

439.77655	0.27931

439.87333	0.27944

439.97011	0.27949

440.0669	0.27953

440.16368	0.27963

440.26046	0.27965

440.35724	0.27969

440.45402	0.27972

440.5508	0.27974

440.64759	0.27976

440.74437	0.27979

440.84115	0.27981

440.93793	0.27999

441.03471	0.28004

441.1315	0.28006

441.22828	0.28008

441.32506	0.28013

441.42184	0.28015

441.51862	0.28017

441.6154	0.28022

441.71219	0.28031

441.80897	0.28033

441.90575	0.28038

442.00253	0.28043

442.09931	0.28047

442.1961	0.28052

442.29288	0.28056

442.38966	0.28061

442.48644	0.28072

442.58322	0.28077

442.68	0.28081

442.77679	0.28084

442.87357	0.28088

442.97035	0.28091

443.06713	0.28095

443.16391	0.28104

443.2607	0.28107

443.35748	0.28109

443.45426	0.28111

443.55104	0.28113

443.64782	0.28116

443.7446	0.28118

443.84139	0.28123

443.93817	0.28132

444.03495	0.28134

444.13173	0.28139

444.22851	0.28145

444.3253	0.28152

444.42208	0.28157

444.51886	0.28161

444.61564	0.28164

444.71242	0.28168

444.8092	0.28171

444.90599	0.28173

445.00277	0.28175

445.09955	0.28177

445.19633	0.28184

445.29311	0.28189

445.3899	0.28193

445.48668	0.28198

445.58346	0.282

445.68024	0.28203

445.77702	0.28205

445.8738	0.28209

445.97059	0.28221

446.06737	0.28223

446.16415	0.28225

446.26093	0.28228

446.35771	0.2823

446.4545	0.28235

446.55128	0.28239

446.64806	0.28241

446.74484	0.28244

446.84162	0.28246

446.9384	0.28248

447.03519	0.28255

447.13197	0.28257

447.22875	0.28262

447.32553	0.28285

447.42231	0.28287

447.5191	0.28292

447.61588	0.28301

447.71266	0.28303

447.80944	0.28305

447.90622	0.28308

448.003	0.2831

448.09979	0.28312

448.19657	0.28315

448.29335	0.28321

448.39013	0.28324

448.48691	0.28331

448.58369	0.28335

448.68048	0.28337

448.77726	0.28342

448.87404	0.28347

448.97082	0.28353

449.0676	0.28356

449.16439	0.28358

449.26117	0.28365

449.35795	0.28369

449.45473	0.28372

449.55151	0.28374

449.64829	0.28392

449.74508	0.28395

449.84186	0.28408

449.93864	0.28411

450.03542	0.28415

450.1322	0.28417

450.22899	0.2842

450.32577	0.28433

450.42255	0.28436

450.51933	0.28438

450.61611	0.2844

450.71289	0.28449

450.80968	0.28452

450.90646	0.28454

451.00324	0.28456

451.10002	0.28459

451.1968	0.28461

451.29359	0.28475

451.39037	0.28488

451.48715	0.28491

451.58393	0.28493

451.68071	0.28495

451.77749	0.28497

451.87428	0.285

451.97106	0.28502

452.06784	0.28504

452.16462	0.28507

452.2614	0.28509

452.35819	0.28511

452.45497	0.28513

452.55175	0.28516

452.64853	0.28518

452.74531	0.2852

452.84209	0.28527

452.93888	0.28529

453.03566	0.28534

453.13244	0.28541

453.22922	0.28548

453.326	0.2855

453.42279	0.28552

453.51957	0.28557

453.61635	0.28561

453.71313	0.28566

453.80991	0.28575

453.90669	0.28577

454.00348	0.2858

454.10026	0.28589

454.19704	0.28591

454.29382	0.28598

454.3906	0.286

454.48739	0.28603

454.58417	0.28605

454.68095	0.28614

454.77773	0.28616

454.87451	0.28621

454.97129	0.28623

455.06808	0.28625

455.16486	0.28628

455.26164	0.28639

455.35842	0.28644

455.4552	0.28646

455.55199	0.28648

455.64877	0.28651

455.74555	0.28655

455.84233	0.28657

455.93911	0.2866

456.03589	0.28662

456.13268	0.28664

456.22946	0.28671

456.32624	0.28673

456.42302	0.28676

456.5198	0.28678

456.61659	0.2868

456.71337	0.28685

456.81015	0.28696

456.90693	0.28699

457.00371	0.28708

457.10049	0.28715

457.19728	0.28717

457.29406	0.28721

457.39084	0.28726

457.48762	0.28728

457.5844	0.28731

457.68119	0.28735

457.77797	0.28737

457.87475	0.2874

457.97153	0.28742

458.06831	0.28744

458.16509	0.28747

458.26188	0.28749

458.35866	0.28751

458.45544	0.28774

458.55222	0.28776

458.649	0.28781

458.74579	0.28783

458.84257	0.28788

458.93935	0.2879

459.03613	0.28792

459.13291	0.28795

459.22969	0.28797

459.32648	0.28801

459.42326	0.28804

459.52004	0.28806

459.61682	0.28808

459.7136	0.28811

459.81039	0.28817

459.90717	0.28831

460.00395	0.28833

460.10073	0.28836

460.19751	0.28838

460.29429	0.28845

460.39108	0.28847

460.48786	0.2885

460.58464	0.28852

460.68142	0.28859

460.7782	0.28861

460.87498	0.28866

460.97177	0.28877

461.06855	0.28879

461.16533	0.28884

461.26211	0.28886

461.35889	0.28888

461.45568	0.28891

461.55246	0.28898

461.64924	0.28907

461.74602	0.28914

461.8428	0.28918

461.93958	0.28923

462.03637	0.28925

462.13315	0.28927

462.22993	0.2893

462.32671	0.28936

462.42349	0.28948

462.52028	0.28952

462.61706	0.28957

462.71384	0.28968

462.81062	0.28973

462.9074	0.28978

463.00418	0.28991

463.10097	0.28994

463.19775	0.28996

463.29453	0.28998

463.39131	0.29

463.48809	0.29003

463.58488	0.29005

463.68166	0.29012

463.77844	0.29016

463.87522	0.29019

463.972	0.29021

464.06878	0.29039

464.16557	0.29046

464.26235	0.29048

464.35913	0.29051

464.45591	0.29053

464.55269	0.29058

464.64948	0.2906

464.74626	0.29062

464.84304	0.29064

464.93982	0.29067

465.0366	0.29069

465.13338	0.29071

465.23017	0.29074

465.32695	0.29078

465.42373	0.2908

465.52051	0.29083

465.61729	0.2909

465.71408	0.29092

465.81086	0.29096

465.90764	0.29101

466.00442	0.29106

466.1012	0.29108

466.19798	0.2911

466.29477	0.29115

466.39155	0.29117

466.48833	0.29119

466.58511	0.29122

466.68189	0.29124

466.77868	0.29128

466.87546	0.29131

466.97224	0.29142

467.06902	0.29144

467.1658	0.29147

467.26258	0.29149

467.35937	0.29151

467.45615	0.29154

467.55293	0.29156

467.64971	0.29158

467.74649	0.2916

467.84328	0.29163

467.94006	0.29165

468.03684	0.29167

468.13362	0.29174

468.2304	0.29176

468.32718	0.29181

468.42397	0.29183

468.52075	0.29186

468.61753	0.29188

468.71431	0.2919

468.81109	0.29192

468.90788	0.29199

469.00466	0.29202

469.10144	0.29204

469.19822	0.29206

469.295	0.29208

469.39178	0.29211

469.48857	0.29213

469.58535	0.29218

469.68213	0.2922

469.77891	0.29222

469.87569	0.29224

469.97248	0.29227

470.06926	0.29229

470.16604	0.29234

470.26282	0.29238

470.3596	0.2924

470.45638	0.29243

470.55317	0.29245

470.64995	0.29247

470.74673	0.2925

470.84351	0.29252

470.94029	0.29259

471.03708	0.29263

471.13386	0.29266

471.23064	0.29268

471.32742	0.29272

471.4242	0.29277

471.52098	0.29282

471.61777	0.29284

471.71455	0.29291

471.81133	0.29295

471.90811	0.29298

472.00489	0.29302

472.10168	0.29304

472.19846	0.29307

472.29524	0.29309

472.39202	0.29311

472.4888	0.29316

472.58558	0.2932

472.68237	0.29332

472.77915	0.29339

472.87593	0.29341

472.97271	0.29343

473.06949	0.29346

473.16627	0.2935

473.26306	0.29352

473.35984	0.29355

473.45662	0.29359

473.5534	0.29368

473.65018	0.29371

473.74697	0.29373

473.84375	0.29375

473.94053	0.29378

474.03731	0.2938

474.13409	0.29382

474.23087	0.29394

474.32766	0.29396

474.42444	0.29419

474.52122	0.29428

474.618	0.29432

474.71478	0.29435

474.81157	0.29437

474.90835	0.29439

475.00513	0.29444

475.10191	0.29446

475.19869	0.29448

475.29547	0.29453

475.39226	0.29458

475.48904	0.29462

475.58582	0.29467

475.6826	0.29469

475.77938	0.29471

475.87617	0.29478

475.97295	0.29483

476.06973	0.29485

476.16651	0.29499

476.26329	0.29503

476.36007	0.29506

476.45686	0.29508

476.55364	0.29517

476.65042	0.29522

476.7472	0.29524

476.84398	0.29526

476.94077	0.29528

477.03755	0.29535

477.13433	0.29538

477.23111	0.2954

477.32789	0.29551

477.42467	0.29554

477.52146	0.29556

477.61824	0.29563

477.71502	0.29565

477.8118	0.29567

477.90858	0.2957

478.00537	0.29572

478.10215	0.29574

478.19893	0.29576

478.29571	0.29579

478.39249	0.29581

478.48927	0.29583

478.58606	0.29588

478.68284	0.2959

478.77962	0.29592

478.8764	0.29595

478.97318	0.29597

479.06997	0.29606

479.16675	0.29611

479.26353	0.29613

479.36031	0.29615

479.45709	0.29622

479.55387	0.29624

479.65066	0.29627

479.74744	0.29629

479.84422	0.29631

479.941	0.29634

480.03778	0.29636

480.13457	0.2964

480.23135	0.29643

480.32813	0.29652

480.42491	0.29654

480.52169	0.29659

480.61847	0.29661

480.71526	0.29672

480.81204	0.29677

480.90882	0.29679

481.0056	0.29682

481.10238	0.29688

481.19917	0.29691

481.29595	0.29693

481.39273	0.29698

481.48951	0.297

481.58629	0.29702

481.68307	0.29709

481.77986	0.29711

481.87664	0.29714

481.97342	0.2972

482.0702	0.29732

482.16698	0.29739

482.26377	0.29741

482.36055	0.29743

482.45733	0.29746

482.55411	0.29759

482.65089	0.29762

482.74767	0.29766

482.84446	0.29768

482.94124	0.29771

483.03802	0.29773

483.1348	0.29775

483.23158	0.29778

483.32837	0.29784

483.42515	0.29787

483.52193	0.29789

483.61871	0.29791

483.71549	0.29796

483.81227	0.29803

483.90906	0.29805

484.00584	0.29807

484.10262	0.2981

484.1994	0.29814

484.29618	0.29816

484.39297	0.29823

484.48975	0.29828

484.58653	0.2983

484.68331	0.29837

484.78009	0.29846

484.87687	0.29848

484.97366	0.29855

485.07044	0.29858

485.16722	0.2986

485.264	0.29862

485.36078	0.29871

485.45756	0.29874

485.55435	0.29883

485.65113	0.29885

485.74791	0.29887

485.84469	0.29894

485.94147	0.29896

486.03826	0.29901

486.13504	0.29908

486.23182	0.2991

486.3286	0.29912

486.42538	0.29917

486.52216	0.29919

486.61895	0.29926

486.71573	0.29928

486.81251	0.29933

486.90929	0.29935

487.00607	0.29938

487.10286	0.2994

487.19964	0.29944

487.29642	0.29949

487.3932	0.29954

487.48998	0.29956

487.58676	0.29958

487.68355	0.2996

487.78033	0.29963

487.87711	0.2997

487.97389	0.29974

488.07067	0.29976

488.16746	0.29979

488.26424	0.29981

488.36102	0.29983

488.4578	0.2999

488.55458	0.29997

488.65136	0.29999

488.74815	0.30002

488.84493	0.30004

488.94171	0.30006

489.03849	0.30008

489.13527	0.30013

489.23206	0.30022

489.32884	0.30024

489.42562	0.30027

489.5224	0.30034

489.61918	0.30036

489.71596	0.3004

489.81275	0.30043

489.90953	0.30045

490.00631	0.30047

490.10309	0.3005

490.19987	0.30052

490.29666	0.30054

490.39344	0.30056

490.49022	0.30059

490.587	0.30063

490.68378	0.30066

490.78056	0.30068

490.87735	0.30072

490.97413	0.30075

491.07091	0.30077

491.16769	0.30084

491.26447	0.30086

491.36126	0.30088

491.45804	0.30091

491.55482	0.30093

491.6516	0.30095

491.74838	0.30098

491.84516	0.301

491.94195	0.30111

492.03873	0.30118

492.13551	0.30191

492.23229	0.30194

492.32907	0.30205

492.42586	0.30214

492.52264	0.30216

492.61942	0.30219

492.7162	0.30232

492.81298	0.30235

492.90976	0.30237

493.00655	0.30242

493.10333	0.30244

493.20011	0.30246

493.29689	0.30251

493.39367	0.30255

493.49046	0.30258

493.58724	0.3026

493.68402	0.30262

493.7808	0.30264

493.87758	0.30271

493.97436	0.30276

494.07115	0.3028

494.16793	0.30292

494.26471	0.30294

494.36149	0.30296

494.45827	0.30306

494.55506	0.3031

494.65184	0.30312

494.74862	0.30315

494.8454	0.3034

494.94218	0.30342

495.03896	0.30347

495.13575	0.30351

495.23253	0.30363

495.32931	0.30365

495.42609	0.30367

495.52287	0.3037

495.61966	0.30372

495.71644	0.30374

495.81322	0.30383

495.91	0.30386

496.00678	0.3039

496.10356	0.30392

496.20035	0.30397

496.29713	0.30399

496.39391	0.30411

496.49069	0.30413

496.58747	0.30415

496.68426	0.30418

496.78104	0.3042

496.87782	0.30422

496.9746	0.30424

497.07138	0.30436

497.16816	0.30438

497.26495	0.3044

497.36173	0.30443

497.45851	0.30445

497.55529	0.30447

497.65207	0.3045

497.74886	0.30454

497.84564	0.30456

497.94242	0.30468

498.0392	0.30482

498.13598	0.30484

498.23276	0.30486

498.32955	0.30493

498.42633	0.30498

498.52311	0.305

498.61989	0.30507

498.71667	0.30509

498.81345	0.30516

498.91024	0.3052

499.00702	0.30523

499.1038	0.30525

499.20058	0.30541

499.29736	0.30543

499.39415	0.30546

499.49093	0.3055

499.58771	0.30552

499.68449	0.30559

499.78127	0.30562

499.87805	0.30564

499.97484	0.30566

500.07162	0.30568

500.1684	0.30571

500.26518	0.30573

500.36196	0.30578

500.45875	0.30584

500.55553	0.30587

500.65231	0.30596

500.74909	0.30598

500.84587	0.30603

500.94265	0.30607

501.03944	0.30612

501.13622	0.30616

501.233	0.30619

501.32978	0.30621

501.42656	0.30626

501.52335	0.30635

501.62013	0.30637

501.71691	0.30639

501.81369	0.30644

501.91047	0.30646

502.00725	0.30651

502.10404	0.30658

502.20082	0.30662

502.2976	0.30664

502.39438	0.30667

502.49116	0.30669

502.58795	0.30674

502.68473	0.30676

502.78151	0.30678

502.87829	0.3068

502.97507	0.30683

503.07185	0.30685

503.16864	0.30687

503.26542	0.3069

503.3622	0.30694

503.45898	0.30701

503.55576	0.30703

503.65255	0.30706

503.74933	0.30708

503.84611	0.30712

503.94289	0.30715

504.03967	0.30719

504.13645	0.30724

504.23324	0.30731

504.33002	0.30735

504.4268	0.30738

504.52358	0.30742

504.62036	0.30749

504.71715	0.30758

504.81393	0.30763

504.91071	0.30765

505.00749	0.30767

505.10427	0.3077

505.20105	0.30772

505.29784	0.30783

505.39462	0.30786

505.4914	0.30792

505.58818	0.30795

505.68496	0.30797

505.78175	0.30799

505.87853	0.30802

505.97531	0.30804

506.07209	0.30808

506.16887	0.30818

506.26565	0.3082

506.36244	0.30824

506.45922	0.30827

506.556	0.30829

506.65278	0.30831

506.74956	0.30834

506.84635	0.30836

506.94313	0.30843

507.03991	0.3085

507.13669	0.30852

507.23347	0.30856

507.33025	0.30859

507.42704	0.30861

507.52382	0.30866

507.6206	0.30868

507.71738	0.3087

507.81416	0.30875

507.91095	0.30882

508.00773	0.30884

508.10451	0.30888

508.20129	0.30891

508.29807	0.30898

508.39485	0.309

508.49164	0.30902

508.58842	0.30909

508.6852	0.30911

508.78198	0.30916

508.87876	0.30918

508.97555	0.30923

509.07233	0.30925

509.16911	0.3093

509.26589	0.30934

509.36267	0.30939

509.45945	0.30946

509.55624	0.30948

509.65302	0.3095

509.7498	0.30955

509.84658	0.30957

509.94336	0.30964

510.04015	0.30966

510.13693	0.30971

510.23371	0.30973

510.33049	0.30975

510.42727	0.30978

510.52405	0.3098

510.62084	0.30982

510.71762	0.30989

510.8144	0.30998

510.91118	0.31

511.00796	0.31005

511.10474	0.31007

511.20153	0.3101

511.29831	0.31019

511.39509	0.31021

511.49187	0.31023

511.58865	0.31026

511.68544	0.31028

511.78222	0.31039

511.879	0.31053

511.97578	0.3106

512.07256	0.31062

512.16934	0.31067

512.26613	0.31069

512.36291	0.31071

512.45969	0.31074

512.55647	0.31076

512.65325	0.31078

512.75004	0.3108

512.84682	0.31083

512.9436	0.31087

513.04038	0.31092

513.13716	0.31094

513.23394	0.31101

513.33073	0.31106

513.42751	0.3111

513.52429	0.31117

513.62107	0.31124

513.71785	0.31126

513.81464	0.31128

513.91142	0.31131

514.0082	0.31133

514.10498	0.31138

514.20176	0.31142

514.29854	0.31144

514.39533	0.31147

514.49211	0.31149

514.58889	0.31154

514.68567	0.31156

514.78245	0.31158

514.87924	0.3116

514.97602	0.31163

515.0728	0.31167

515.16958	0.31181

515.26636	0.31183

515.36314	0.31188

515.45993	0.3119

515.55671	0.31192

515.65349	0.31197

515.75027	0.31199

515.84705	0.31202

515.94384	0.31211

516.04062	0.31213

516.1374	0.31215

516.23418	0.31218

516.33096	0.3122

516.42774	0.31224

516.52453	0.31229

516.62131	0.31231

516.71809	0.31234

516.81487	0.3124

516.91165	0.31243

517.00844	0.31245

517.10522	0.31247

517.202	0.31268

517.29878	0.31275

517.39556	0.31277

517.49234	0.31279

517.58913	0.31284

517.68591	0.31291

517.78269	0.31293

517.87947	0.31295

517.97625	0.31298

518.07304	0.313

518.16982	0.31307

518.2666	0.31309

518.36338	0.31314

518.46016	0.31316

518.55694	0.31318

518.65373	0.3132

518.75051	0.31323

518.84729	0.31325

518.94407	0.31332

519.04085	0.31334

519.13764	0.31336

519.23442	0.31339

519.3312	0.31341

519.42798	0.31343

519.52476	0.31346

519.62154	0.31348

519.71833	0.3135

519.81511	0.31355

519.91189	0.31364

520.00867	0.31371

520.10545	0.31373

520.20224	0.31391

520.29902	0.31394

520.3958	0.31403

520.49258	0.31407

520.58936	0.31412

520.68614	0.31426

520.78293	0.31428

520.87971	0.3143

520.97649	0.31435

521.07327	0.31442

521.17005	0.31444

521.26684	0.31446

521.36362	0.31453

521.4604	0.31455

521.55718	0.31458

521.65396	0.31462

521.75074	0.31464

521.84753	0.31469

521.94431	0.31474

522.04109	0.31476

522.13787	0.31478

522.23465	0.3148

522.33144	0.31483

522.42822	0.31485

522.525	0.31487

522.62178	0.31494

522.71856	0.31499

522.81534	0.31501

522.91213	0.31506

523.00891	0.31508

523.10569	0.31512

523.20247	0.31517

523.29925	0.31519

523.39603	0.31524

523.49282	0.31526

523.5896	0.31528

523.68638	0.31531

523.78316	0.31533

523.87994	0.31535

523.97673	0.31538

524.07351	0.3154

524.17029	0.31547

524.26707	0.31549

524.36385	0.31556

524.46064	0.31558

524.55742	0.3156

524.6542	0.31563

524.75098	0.31565

524.84776	0.31567

524.94454	0.3157

525.04133	0.31576

525.13811	0.3159

525.23489	0.31592

525.33167	0.31597

525.42845	0.31645

525.52523	0.31654

525.62202	0.31663

525.7188	0.31668

525.81558	0.3167

525.91236	0.31675

526.00914	0.31679

526.10593	0.31702

526.20271	0.31707

526.29949	0.31711

526.39627	0.31714

526.49305	0.31723

526.58983	0.31727

526.68662	0.3173

526.7834	0.31732

526.88018	0.31736

526.97696	0.31741

527.07374	0.31752

527.17053	0.31755

527.26731	0.31757

527.36409	0.31762

527.46087	0.31764

527.55765	0.31766

527.65443	0.31782

527.75122	0.31784

527.848	0.31791

527.94478	0.31794

528.04156	0.31805

528.13834	0.31807

528.23513	0.3181

528.33191	0.31812

528.42869	0.31816

528.52547	0.31819

528.62225	0.31826

528.71903	0.31835

528.81582	0.31837

528.9126	0.31839

529.00938	0.31844

529.10616	0.31846

529.20294	0.31848

529.29973	0.31853

529.39651	0.31855

529.49329	0.31858

529.59007	0.3186

529.68685	0.31862

529.78363	0.31867

529.88042	0.31885

529.9772	0.31894

530.07398	0.31903

530.17076	0.3191

530.26754	0.31912

530.36433	0.31915

530.46111	0.31917

530.55789	0.31922

530.65467	0.31924

530.75145	0.31926

530.84823	0.31928

530.94502	0.31933

531.0418	0.31942

531.13858	0.31944

531.23536	0.31947

531.33214	0.31949

531.42893	0.31951

531.52571	0.31956

531.62249	0.31958

531.71927	0.31963

531.81605	0.31965

531.91283	0.3197

532.00962	0.31972

532.1064	0.31976

532.20318	0.31979

532.29996	0.31981

532.39674	0.31983

532.49353	0.31986

532.59031	0.31988

532.68709	0.3199

532.78387	0.31992

532.88065	0.31995

532.97743	0.32002

533.07422	0.32004

533.171	0.32006

533.26778	0.32008

533.36456	0.32013

533.46134	0.32018

533.55813	0.32022

533.65491	0.32027

533.75169	0.32029

533.84847	0.32038

533.94525	0.32043

534.04203	0.32047

534.13882	0.32061

534.2356	0.32063

534.33238	0.32068

534.42916	0.3207

534.52594	0.32072

534.62273	0.32075

534.71951	0.32077

534.81629	0.32079

534.91307	0.32084

535.00985	0.32086

535.10663	0.32088

535.20342	0.32091

535.3002	0.32098

535.39698	0.32104

535.49376	0.32107

535.59054	0.32116

535.68733	0.3212

535.78411	0.32123

535.88089	0.32125

535.97767	0.32127

536.07445	0.32132

536.17123	0.32136

536.26802	0.32139

536.3648	0.32141

536.46158	0.32146

536.55836	0.3215

536.65514	0.32157

536.75193	0.32159

536.84871	0.32162

536.94549	0.32164

537.04227	0.32169

537.13905	0.32171

537.23583	0.32173

537.33262	0.32175

537.4294	0.32178

537.52618	0.32191

537.62296	0.32194

537.71974	0.32196

537.81652	0.32198

537.91331	0.32201

538.01009	0.32205

538.10687	0.32207

538.20365	0.3221

538.30043	0.32214

538.39722	0.32219

538.494	0.32221

538.59078	0.32226

538.68756	0.32228

538.78434	0.3223

538.88112	0.32267

538.97791	0.32271

539.07469	0.32276

539.17147	0.32278

539.26825	0.32283

539.36503	0.3229

539.46182	0.32294

539.5586	0.32299

539.65538	0.32301

539.75216	0.32303

539.84894	0.32308

539.94572	0.3231

540.04251	0.32313

540.13929	0.32319

540.23607	0.32329

540.33285	0.32331

540.42963	0.32333

540.52642	0.32335

540.6232	0.32338

540.71998	0.3234

540.81676	0.32345

540.91354	0.32347

541.01032	0.32349

541.10711	0.32354

541.20389	0.32361

541.30067	0.32363

541.39745	0.32365

541.49423	0.3237

541.59102	0.32374

541.6878	0.32388

541.78458	0.32393

541.88136	0.32397

541.97814	0.32402

542.07492	0.32406

542.17171	0.32411

542.26849	0.32415

542.36527	0.3242

542.46205	0.32422

542.55883	0.32427

542.65562	0.32429

542.7524	0.32431

542.84918	0.32434

542.94596	0.32445

543.04274	0.32457

543.13952	0.32461

543.23631	0.32463

543.33309	0.32466

543.42987	0.32473

543.52665	0.32479

543.62343	0.32484

543.72022	0.32486

543.817	0.32491

543.91378	0.32493

544.01056	0.32495

544.10734	0.32498

544.20412	0.32502

544.30091	0.32505

544.39769	0.32507

544.49447	0.32509

544.59125	0.32516

544.68803	0.32518

544.78482	0.32527

544.8816	0.3253

544.97838	0.32532

545.07516	0.32534

545.17194	0.32539

545.26872	0.32541

545.36551	0.32546

545.46229	0.32548

545.55907	0.32555

545.65585	0.32564

545.75263	0.32566

545.84942	0.32571

545.9462	0.32575

546.04298	0.32578

546.13976	0.32587

546.23654	0.32591

546.33332	0.32594

546.43011	0.32601

546.52689	0.32603

546.62367	0.32605

546.72045	0.32614

546.81723	0.32617

546.91402	0.32619

547.0108	0.32621

547.10758	0.32635

547.20436	0.32637

547.30114	0.32639

547.39792	0.32644

547.49471	0.32651

547.59149	0.32655

547.68827	0.32658

547.78505	0.3266

547.88183	0.32662

547.97862	0.32665

548.0754	0.32669

548.17218	0.32674

548.26896	0.32676

548.36574	0.32678

548.46252	0.32683

548.55931	0.3269

548.65609	0.32692

548.75287	0.32694

548.84965	0.32701

548.94643	0.32703

549.04322	0.32708

549.14	0.3271

549.23678	0.32715

549.33356	0.32717

549.43034	0.32719

549.52712	0.32722

549.62391	0.32724

549.72069	0.32726

549.81747	0.32735

549.91425	0.32738

550.01103	0.3274

550.10781	0.32745

550.2046	0.32747

550.30138	0.32751

550.39816	0.32754

550.49494	0.32756

550.59172	0.32758

550.68851	0.32765

550.78529	0.32767

550.88207	0.3277

550.97885	0.32772

551.07563	0.32774

551.17241	0.32783

551.2692	0.32802

551.36598	0.32804

551.46276	0.32811

551.55954	0.32813

551.65632	0.32815

551.75311	0.32818

551.84989	0.3282

551.94667	0.32825

552.04345	0.32827

552.14023	0.32829

552.23701	0.32831

552.3338	0.32834

552.43058	0.32836

552.52736	0.32838

552.62414	0.32843

552.72092	0.32845

552.81771	0.32847

552.91449	0.3285

553.01127	0.32854

553.10805	0.32857

553.20483	0.32859

553.30161	0.32863

553.3984	0.32868

553.49518	0.3287

553.59196	0.32877

553.68874	0.32886

553.78552	0.329

553.88231	0.32902

553.97909	0.32907

554.07587	0.32909

554.17265	0.32911

554.26943	0.32914

554.36621	0.32916

554.463	0.32918

554.55978	0.32921

554.65656	0.32923

554.75334	0.32925

554.85012	0.32939

554.94691	0.32946

555.04369	0.32948

555.14047	0.32953

555.23725	0.32955

555.33403	0.32957

555.43081	0.32962

555.5276	0.32964

555.62438	0.32966

555.72116	0.32969

555.81794	0.32971

555.91472	0.32975

556.01151	0.3298

556.10829	0.32982

556.20507	0.32985

556.30185	0.32987

556.39863	0.32989

556.49541	0.32991

556.5922	0.32994

556.68898	0.32996

556.78576	0.32998

556.88254	0.33001

556.97932	0.33005

557.07611	0.33007

557.17289	0.3301

557.26967	0.33012

557.36645	0.33014

557.46323	0.33017

557.56001	0.33021

557.6568	0.33023

557.75358	0.33026

557.85036	0.33028

557.94714	0.3303

558.04392	0.33037

558.14071	0.33039

558.23749	0.33042

558.33427	0.33049

558.43105	0.33053

558.52783	0.33055

558.62461	0.33062

558.7214	0.33065

558.81818	0.33071

558.91496	0.33081

559.01174	0.33085

559.10852	0.3309

559.20531	0.33099

559.30209	0.33101

559.39887	0.33103

559.49565	0.33106

559.59243	0.33108

559.68921	0.33115

559.786	0.33117

559.88278	0.33122

559.97956	0.33124

560.07634	0.33126

560.17312	0.33131

560.26991	0.33135

560.36669	0.33138

560.46347	0.33145

560.56025	0.33147

560.65703	0.33149

560.75381	0.33158

560.8506	0.33161

560.94738	0.33165

561.04416	0.33167

561.14094	0.3317

561.23772	0.33177

561.33451	0.33179

561.43129	0.33183

561.52807	0.33188

561.62485	0.33193

561.72163	0.33195

561.81841	0.33197

561.9152	0.33199

562.01198	0.33202

562.10876	0.33204

562.20554	0.33206

562.30232	0.33209

562.3991	0.33213

562.49589	0.33218

562.59267	0.3322

562.68945	0.33227

562.78623	0.33231

562.88301	0.33234

562.9798	0.33236

563.07658	0.33238

563.17336	0.33241

563.27014	0.33243

563.36692	0.33245

563.4637	0.3325

563.56049	0.33252

563.65727	0.33254

563.75405	0.33259

563.85083	0.33263

563.94761	0.33266

564.0444	0.33268

564.14118	0.3327

564.23796	0.33273

564.33474	0.33282

564.43152	0.33284

564.5283	0.33286

564.62509	0.33289

564.72187	0.33291

564.81865	0.33293

564.91543	0.33295

565.01221	0.333

565.109	0.33302

565.20578	0.33305

565.30256	0.33307

565.39934	0.33314

565.49612	0.33318

565.5929	0.33323

565.68969	0.3333

565.78647	0.33332

565.88325	0.33337

565.98003	0.33339

566.07681	0.33346

566.1736	0.33353

566.27038	0.33355

566.36716	0.33359

566.46394	0.33369

566.56072	0.33371

566.6575	0.3338

566.75429	0.33382

566.85107	0.33385

566.94785	0.33389

567.04463	0.33391

567.14141	0.33394

567.2382	0.33398

567.33498	0.33401

567.43176	0.33403

567.52854	0.33405

567.62532	0.33407

567.7221	0.33412

567.81889	0.33414

567.91567	0.33497

568.01245	0.33499

568.10923	0.33501

568.20601	0.33506

568.3028	0.33508

568.39958	0.3351

568.49636	0.33513

568.59314	0.33515

568.68992	0.33519

568.7867	0.33522

568.88349	0.33533

568.98027	0.33535

569.07705	0.33538

569.17383	0.33542

569.27061	0.33545

569.3674	0.33547

569.46418	0.33549

569.56096	0.33551

569.65774	0.33556

569.75452	0.33558

569.8513	0.33561

569.94809	0.33567

570.04487	0.3357

570.14165	0.33572

570.23843	0.33577

570.33521	0.33579

570.432	0.33581

570.52878	0.33583

570.62556	0.33586

570.72234	0.33588

570.81912	0.3359

570.9159	0.33595

571.01269	0.33597

571.10947	0.33602

571.20625	0.33604

571.30303	0.33609

571.39981	0.33615

571.4966	0.33618

571.59338	0.3362

571.69016	0.33622

571.78694	0.33625

571.88372	0.33627

571.9805	0.33629

572.07729	0.33631

572.17407	0.33636

572.27085	0.33638

572.36763	0.33643

572.46441	0.33645

572.5612	0.33647

572.65798	0.3365

572.75476	0.33652

572.85154	0.33659

572.94832	0.33661

573.0451	0.33663

573.14189	0.33668

573.23867	0.3367

573.33545	0.33675

573.43223	0.33677

573.52901	0.33684

573.6258	0.33691

573.72258	0.337

573.81936	0.33702

573.91614	0.33711

574.01292	0.33716

574.1097	0.33759

574.20649	0.33762

574.30327	0.33769

574.40005	0.33771

574.49683	0.33773

574.59361	0.33775

574.69039	0.3378

574.78718	0.33782

574.88396	0.33785

574.98074	0.33787

575.07752	0.33789

575.1743	0.33791

575.27109	0.33798

575.36787	0.33801

575.46465	0.33803

575.56143	0.33805

575.65821	0.33814

575.75499	0.33817

575.85178	0.33819

575.94856	0.33821

576.04534	0.33823

576.14212	0.33826

576.2389	0.33828

576.33569	0.33833

576.43247	0.33835

576.52925	0.33837

576.62603	0.33842

576.72281	0.33844

576.81959	0.33846

576.91638	0.33851

577.01316	0.33853

577.10994	0.33858

577.20672	0.3386

577.3035	0.33862

577.40029	0.33865

577.49707	0.33867

577.59385	0.33883

577.69063	0.33885

577.78741	0.33894

577.88419	0.33897

577.98098	0.33899

578.07776	0.33901

578.17454	0.33903

578.27132	0.3391

578.3681	0.33917

578.46489	0.33919

578.56167	0.33924

578.65845	0.33931

578.75523	0.33933

578.85201	0.33935

578.94879	0.33938

579.04558	0.3394

579.14236	0.33947

579.23914	0.33954

579.33592	0.33958

579.4327	0.33961

579.52949	0.33965

579.62627	0.33967

579.72305	0.33972

579.81983	0.33974

579.91661	0.33977

580.01339	0.33979

580.11018	0.33983

580.20696	0.33986

580.30374	0.33995

580.40052	0.33999

580.4973	0.34002

580.59409	0.34004

580.69087	0.34006

580.78765	0.34025

580.88443	0.34027

580.98121	0.34029

581.07799	0.34031

581.17478	0.34034

581.27156	0.34036

581.36834	0.34038

581.46512	0.34041

581.5619	0.34045

581.65869	0.34047

581.75547	0.3405

581.85225	0.34054

581.94903	0.34057

582.04581	0.34066

582.14259	0.34077

582.23938	0.34079

582.33616	0.34084

582.43294	0.34086

582.52972	0.34089

582.6265	0.34091

582.72329	0.34102

582.82007	0.34105

582.91685	0.34107

583.01363	0.34109

583.11041	0.34111

583.20719	0.34114

583.30398	0.34116

583.40076	0.34118

583.49754	0.34123

583.59432	0.34132

583.6911	0.34134

583.78789	0.34146

583.88467	0.34148

583.98145	0.3415

584.07823	0.34159

584.17501	0.34162

584.27179	0.34166

584.36858	0.34169

584.46536	0.34171

584.56214	0.34173

584.65892	0.34175

584.7557	0.3418

584.85249	0.34182

584.94927	0.34189

585.04605	0.34191

585.14283	0.34194

585.23961	0.34198

585.33639	0.34201

585.43318	0.34203

585.52996	0.34205

585.62674	0.34214

585.72352	0.34219

585.8203	0.34223

585.91709	0.34226

586.01387	0.34228

586.11065	0.3423

586.20743	0.34233

586.30421	0.34235

586.40099	0.34237

586.49778	0.34242

586.59456	0.34244

586.69134	0.34246

586.78812	0.34258

586.8849	0.3426

586.98168	0.34262

587.07847	0.34265

587.17525	0.34267

587.27203	0.34269

587.36881	0.34271

587.46559	0.34276

587.56238	0.34278

587.65916	0.34281

587.75594	0.34283

587.85272	0.34287

587.9495	0.34294

588.04628	0.34297

588.14307	0.34299

588.23985	0.34301

588.33663	0.34303

588.43341	0.34319

588.53019	0.34322

588.62698	0.34324

588.72376	0.34326

588.82054	0.34329

588.91732	0.34333

589.0141	0.34335

589.11088	0.34338

589.20767	0.34345

589.30445	0.34347

589.40123	0.34349

589.49801	0.34354

589.59479	0.34358

589.69158	0.34361

589.78836	0.34363

589.88514	0.34365

589.98192	0.34367

590.0787	0.3437

590.17548	0.34372

590.27227	0.34377

590.36905	0.34381

590.46583	0.34395

590.56261	0.34406

590.65939	0.34427

590.75618	0.34434

590.85296	0.34436

590.94974	0.34438

591.04652	0.34441

591.1433	0.3445

591.24008	0.34452

591.33687	0.34454

591.43365	0.34457

591.53043	0.34459

591.62721	0.34461

591.72399	0.34463

591.82078	0.34466

591.91756	0.34468

592.01434	0.3447

592.11112	0.34475

592.2079	0.34482

592.30468	0.34489

592.40147	0.34491

592.49825	0.34493

592.59503	0.34495

592.69181	0.34502

592.78859	0.34507

592.88538	0.34509

592.98216	0.34514

593.07894	0.34518

593.17572	0.34521

593.2725	0.34525

593.36928	0.34534

593.46607	0.34537

593.56285	0.34539

593.65963	0.34541

593.75641	0.34548

593.85319	0.34553

593.94998	0.34559

594.04676	0.34562

594.14354	0.34566

594.24032	0.34569

594.3371	0.34573

594.43388	0.34575

594.53067	0.34582

594.62745	0.34591

594.72423	0.34601

594.82101	0.34605

594.91779	0.3461

595.01458	0.34617

595.11136	0.34619

595.20814	0.34621

595.30492	0.3463

595.4017	0.34633

595.49848	0.34639

595.59527	0.34642

595.69205	0.34651

595.78883	0.34655

595.88561	0.34658

595.98239	0.34662

596.07918	0.34665

596.17596	0.34667

596.27274	0.34669

596.36952	0.34674

596.4663	0.34676

596.56308	0.34678

596.65987	0.34685

596.75665	0.3469

596.85343	0.34692

596.95021	0.34694

597.04699	0.34697

597.14378	0.34699

597.24056	0.34703

597.33734	0.34706

597.43412	0.34708

597.5309	0.34713

597.62768	0.34715

597.72447	0.34726

597.82125	0.34729

597.91803	0.34731

598.01481	0.34733

598.11159	0.34735

598.20838	0.34738

598.30516	0.34742

598.40194	0.34745

598.49872	0.34747

598.5955	0.34751

598.69228	0.34756

598.78907	0.34761

598.88585	0.34763

598.98263	0.34767

599.07941	0.3477

599.17619	0.34774

599.27297	0.34777

599.36976	0.34779

599.46654	0.34781

599.56332	0.34783

599.6601	0.34793

599.75688	0.34795

599.85367	0.34802

599.95045	0.34806

600.04723	0.34813

600.14401	0.34815

600.24079	0.34818

600.33758	0.3482

600.43436	0.34822

600.53114	0.34825

600.62792	0.34829

600.7247	0.34831

600.82148	0.34843

600.91827	0.34845

601.01505	0.34847

601.11183	0.3485

601.20861	0.34854

601.30539	0.34857

601.40217	0.34861

601.49896	0.34863

601.59574	0.34866

601.69252	0.34868

601.7893	0.3487

601.88608	0.34875

601.98287	0.34877

602.07965	0.34879

602.17643	0.34882

602.27321	0.34884

602.36999	0.34886

602.46677	0.34889

602.56356	0.34893

602.66034	0.34898

602.75712	0.34902

602.8539	0.34905

602.95068	0.34907

603.04747	0.34909

603.14425	0.34914

603.24103	0.34916

603.33781	0.34918

603.43459	0.34921

603.53137	0.34927

603.62816	0.34932

603.72494	0.34934

603.82172	0.34939

603.9185	0.34943

604.01528	0.34948

604.11207	0.34959

604.20885	0.34962

604.30563	0.34966

604.40241	0.34969

604.49919	0.34971

604.59597	0.34985

604.69276	0.34987

604.78954	0.34989

604.88632	0.34991

604.9831	0.34994

605.07988	0.34996

605.17667	0.35003

605.27345	0.35005

605.37023	0.35007

605.46701	0.3501

605.56379	0.35012

605.66057	0.35014

605.75736	0.35017

605.85414	0.35019

605.95092	0.35021

606.0477	0.35023

606.14448	0.35026

606.24127	0.3503

606.33805	0.35055

606.43483	0.35058

606.53161	0.3506

606.62839	0.35062

606.72517	0.35065

606.82196	0.35074

606.91874	0.35076

607.01552	0.35078

607.1123	0.35081

607.20908	0.35085

607.30587	0.3509

607.40265	0.35092

607.49943	0.35094

607.59621	0.35097

607.69299	0.35106

607.78977	0.35117

607.88656	0.35119

607.98334	0.35124

608.08012	0.35129

608.1769	0.35131

608.27368	0.35135

608.37047	0.35138

608.46725	0.35147

608.56403	0.35149

608.66081	0.35158

608.75759	0.35163

608.85437	0.35172

608.95116	0.35174

609.04794	0.35179

609.14472	0.35181

609.2415	0.35183

609.33828	0.35186

609.43507	0.3519

609.53185	0.35193

609.62863	0.35195

609.72541	0.35197

609.82219	0.35199

609.91897	0.35202

610.01576	0.35206

610.11254	0.35211

610.20932	0.35213

610.3061	0.35215

610.40288	0.35218

610.49967	0.35225

610.59645	0.35227

610.69323	0.35231

610.79001	0.35238

610.88679	0.35241

610.98357	0.35243

611.08036	0.35245

611.17714	0.3525

611.27392	0.35252

611.3707	0.35259

611.46748	0.35263

611.56427	0.3527

611.66105	0.35273

611.75783	0.35279

611.85461	0.35291

611.95139	0.35314

612.04817	0.35316

612.14496	0.35318

612.24174	0.35323

612.33852	0.35327

612.4353	0.35339

612.53208	0.35343

612.62887	0.35346

612.72565	0.35348

612.82243	0.35355

612.91921	0.35362

613.01599	0.35364

613.11277	0.35366

613.20956	0.35371

613.30634	0.35373

613.40312	0.35375

613.4999	0.35378

613.59668	0.3538

613.69346	0.35382

613.79025	0.35391

613.88703	0.35396

613.98381	0.35398

614.08059	0.35401

614.17737	0.35403

614.27416	0.35405

614.37094	0.35414

614.46772	0.35417

614.5645	0.35421

614.66128	0.35433

614.75806	0.35435

614.85485	0.35437

614.95163	0.35442

615.04841	0.35444

615.14519	0.35451

615.24197	0.35453

615.33876	0.35456

615.43554	0.3546

615.53232	0.35462

615.6291	0.35469

615.72588	0.35474

615.82266	0.35476

615.91945	0.35478

616.01623	0.35481

616.11301	0.35492

616.20979	0.35497

616.30657	0.35499

616.40336	0.3551

616.50014	0.35513

616.59692	0.35515

616.6937	0.35517

616.79048	0.35522

616.88726	0.35524

616.98405	0.35526

617.08083	0.35531

617.17761	0.35533

617.27439	0.35538

617.37117	0.35542

617.46796	0.35545

617.56474	0.35547

617.66152	0.35549

617.7583	0.35554

617.85508	0.35556

617.95186	0.35563

618.04865	0.3557

618.14543	0.35572

618.24221	0.35574

618.33899	0.35579

618.43577	0.35581

618.53256	0.35586

618.62934	0.35588

618.72612	0.3559

618.8229	0.35593

618.91968	0.35606

619.01646	0.35611

619.11325	0.35618

619.21003	0.35622

619.30681	0.35625

619.40359	0.35627

619.50037	0.35629

619.59716	0.35634

619.69394	0.35636

619.79072	0.35638

619.8875	0.35641

619.98428	0.35643

620.08106	0.35645

620.17785	0.3565

620.27463	0.35654

620.37141	0.35659

620.46819	0.35666

620.56497	0.35677

620.66176	0.3568

620.75854	0.35682

620.85532	0.35684

620.9521	0.35689

621.04888	0.35702

621.14566	0.35705

621.24245	0.35709

621.33923	0.35712

621.43601	0.35716

621.53279	0.35718

621.62957	0.35721

621.72636	0.35725

621.82314	0.35728

621.91992	0.35732

622.0167	0.35734

622.11348	0.35741

622.21026	0.35744

622.30705	0.35746

622.40383	0.35755

622.50061	0.35757

622.59739	0.3576

622.69417	0.35762

622.79096	0.35764

622.88774	0.35773

622.98452	0.35778

623.0813	0.3578

623.17808	0.35782

623.27486	0.35787

623.37165	0.35789

623.46843	0.35798

623.56521	0.35801

623.66199	0.35805

623.75877	0.35814

623.85556	0.35817

623.95234	0.35821

624.04912	0.35824

624.1459	0.35826

624.24268	0.35833

624.33946	0.35835

624.43625	0.35837

624.53303	0.35904

624.62981	0.35906

624.72659	0.35908

624.82337	0.35913

624.92016	0.35915

625.01694	0.3592

625.11372	0.35933

625.2105	0.35938

625.30728	0.35945

625.40406	0.35949

625.50085	0.35961

625.59763	0.35972

625.69441	0.35977

625.79119	0.35986

625.88797	0.3599

625.98475	0.35993

626.08154	0.35995

626.17832	0.35997

626.2751	0.36

626.37188	0.36002

626.46866	0.36006

626.56545	0.36009

626.66223	0.36011

626.75901	0.36013

626.85579	0.36016

626.95257	0.36022

627.04935	0.36025

627.14614	0.36032

627.24292	0.36036

627.3397	0.3605

627.43648	0.36052

627.53326	0.36054

627.63005	0.36057

627.72683	0.36059

627.82361	0.36061

627.92039	0.36064

628.01717	0.36068

628.11395	0.3607

628.21074	0.36075

628.30752	0.36077

628.4043	0.3608

628.50108	0.36082

628.59786	0.36086

628.69465	0.36093

628.79143	0.36096

628.88821	0.36098

628.98499	0.36102

629.08177	0.36107

629.17855	0.36109

629.27534	0.36112

629.37212	0.36114

629.4689	0.36123

629.56568	0.3613

629.66246	0.36137

629.75925	0.36141

629.85603	0.36144

629.95281	0.36146

630.04959	0.36148

630.14637	0.3615

630.24315	0.36153

630.33994	0.36155

630.43672	0.3616

630.5335	0.36169

630.63028	0.36178

630.72706	0.3618

630.82385	0.36182

630.92063	0.36185

631.01741	0.36187

631.11419	0.36189

631.21097	0.36203

631.30775	0.36205

631.40454	0.36212

631.50132	0.36217

631.5981	0.36221

631.69488	0.36226

631.79166	0.3623

631.88845	0.36235

631.98523	0.36237

632.08201	0.3624

632.17879	0.36242

632.27557	0.36244

632.37235	0.36249

632.46914	0.36251

632.56592	0.36256

632.6627	0.36265

632.75948	0.36267

632.85626	0.36278

632.95305	0.36281

633.04983	0.36288

633.14661	0.3629

633.24339	0.36294

633.34017	0.36297

633.43695	0.36299

633.53374	0.36301

633.63052	0.36304

633.7273	0.36308

633.82408	0.3631

633.92086	0.36315

634.01765	0.36322

634.11443	0.36324

634.21121	0.36326

634.30799	0.36329

634.40477	0.36331

634.50155	0.36333

634.59834	0.36336

634.69512	0.36338

634.7919	0.36342

634.88868	0.36345

634.98546	0.36349

635.08225	0.36354

635.17903	0.36361

635.27581	0.36372

635.37259	0.36374

635.46937	0.36377

635.56615	0.36381

635.66294	0.36384

635.75972	0.36386

635.8565	0.3639

635.95328	0.36393

636.05006	0.36395

636.14685	0.36402

636.24363	0.36409

636.34041	0.36411

636.43719	0.36413

636.53397	0.36416

636.63075	0.36418

636.72754	0.3642

636.82432	0.36422

636.9211	0.36427

637.01788	0.36429

637.11466	0.36432

637.21145	0.36434

637.30823	0.36436

637.40501	0.36438

637.50179	0.36445

637.59857	0.3645

637.69535	0.36459

637.79214	0.36468

637.88892	0.3647

637.9857	0.36473

638.08248	0.36475

638.17926	0.36477

638.27604	0.36486

638.37283	0.36493

638.46961	0.36496

638.56639	0.36498

638.66317	0.365

638.75995	0.36502

638.85674	0.36509

638.95352	0.36512

639.0503	0.36514

639.14708	0.36521

639.24386	0.36528

639.34064	0.36532

639.43743	0.36534

639.53421	0.36537

639.63099	0.36539

639.72777	0.36541

639.82455	0.36544

639.92134	0.36546

640.01812	0.3655

640.1149	0.36557

640.21168	0.36562

640.30846	0.36582

640.40524	0.36589

640.50203	0.36592

640.59881	0.36598

640.69559	0.36601

640.79237	0.36603

640.88915	0.36608

640.98594	0.3661

641.08272	0.36612

641.1795	0.36614

641.27628	0.36617

641.37306	0.36619

641.46984	0.36621

641.56663	0.36628

641.66341	0.36633

641.76019	0.36635

641.85697	0.36637

641.95375	0.3664

642.05054	0.36642

642.14732	0.36646

642.2441	0.36656

642.34088	0.3666

642.43766	0.36662

642.53444	0.36665

642.63123	0.36674

642.72801	0.36676

642.82479	0.36678

642.92157	0.36683

643.01835	0.36685

643.11514	0.36688

643.21192	0.3669

643.3087	0.36701

643.40548	0.36706

643.50226	0.3671

643.59904	0.36713

643.69583	0.36715

643.79261	0.36717

643.88939	0.36722

643.98617	0.36733

644.08295	0.36736

644.17974	0.36738

644.27652	0.36742

644.3733	0.36747

644.47008	0.36756

644.56686	0.36758

644.66364	0.36777

644.76043	0.36779

644.85721	0.36781

644.95399	0.36784

645.05077	0.36786

645.14755	0.36788

645.24434	0.36793

645.34112	0.36804

645.4379	0.36806

645.53468	0.36809

645.63146	0.36811

645.72824	0.36813

645.82503	0.36816

645.92181	0.36827

646.01859	0.36832

646.11537	0.36838

646.21215	0.36841

646.30894	0.36843

646.40572	0.36848

646.5025	0.3685

646.59928	0.36852

646.69606	0.36857

646.79284	0.36861

646.88963	0.36864

646.98641	0.3687

647.08319	0.36873

647.17997	0.36875

647.27675	0.3688

647.37354	0.36882

647.47032	0.36886

647.5671	0.36891

647.66388	0.36893

647.76066	0.36896

647.85744	0.36898

647.95423	0.36902

648.05101	0.36909

648.14779	0.36912

648.24457	0.36914

648.34135	0.36916

648.43814	0.36918

648.53492	0.36928

648.6317	0.36932

648.72848	0.36934

648.82526	0.36937

648.92204	0.36939

649.01883	0.36948

649.11561	0.36953

649.21239	0.36962

649.30917	0.36964

649.40595	0.36966

649.50274	0.36971

649.59952	0.36973

649.6963	0.36976

649.79308	0.36978

649.88986	0.3698

649.98664	0.36982

650.08343	0.36985

650.18021	0.36989

650.27699	0.36992

650.37377	0.36994

650.47055	0.37001

650.56733	0.37005

650.66412	0.37008

650.7609	0.37012

650.85768	0.37014

650.95446	0.37017

651.05124	0.37021

651.14803	0.37024

651.24481	0.37028

651.34159	0.3703

651.43837	0.37033

651.53515	0.37035

651.63193	0.37037

651.72872	0.37042

651.8255	0.37044

651.92228	0.37056

652.01906	0.37065

652.11584	0.37067

652.21263	0.37069

652.30941	0.37072

652.40619	0.37076

652.50297	0.37078

652.59975	0.37081

652.69653	0.37083

652.79332	0.3709

652.8901	0.37092

652.98688	0.37094

653.08366	0.37097

653.18044	0.37101

653.27723	0.37106

653.37401	0.37108

653.47079	0.37117

653.56757	0.3712

653.66435	0.37122

653.76113	0.37124

653.85792	0.37129

653.9547	0.37131

654.05148	0.37133

654.14826	0.37136

654.24504	0.37147

654.34183	0.37156

654.43861	0.37158

654.53539	0.37161

654.63217	0.3717

654.72895	0.37174

654.82573	0.37177

654.92252	0.37186

655.0193	0.37188

655.11608	0.3719

655.21286	0.37193

655.30964	0.37195

655.40643	0.37197

655.50321	0.37202

655.59999	0.37204

655.69677	0.37206

655.79355	0.37211

655.89033	0.37213

655.98712	0.37218

656.0839	0.37222

656.18068	0.37225

656.27746	0.37234

656.37424	0.37236

656.47103	0.37238

656.56781	0.37241

656.66459	0.37243

656.76137	0.37245

656.85815	0.37248

656.95493	0.3725

657.05172	0.37254

657.1485	0.37257

657.24528	0.37259

657.34206	0.37261

657.43884	0.37264

657.53563	0.37266

657.63241	0.3727

657.72919	0.37284

657.82597	0.37296

657.92275	0.37298

658.01953	0.37302

658.11632	0.37307

658.2131	0.37309

658.30988	0.37316

658.40666	0.37318

658.50344	0.37321

658.60023	0.37323

658.69701	0.37328

658.79379	0.37334

658.89057	0.37339

658.98735	0.37346

659.08413	0.37348

659.18092	0.3735

659.2777	0.37353

659.37448	0.37355

659.47126	0.37357

659.56804	0.3736

659.66483	0.37366

659.76161	0.37373

659.85839	0.37378

659.95517	0.3738

660.05195	0.37392

660.14873	0.37396

660.24552	0.37398

660.3423	0.37403

660.43908	0.3741

660.53586	0.37412

660.63264	0.37414

660.72943	0.37417

660.82621	0.37419

660.92299	0.37421

661.01977	0.37424

661.11655	0.37426

661.21333	0.37428

661.31012	0.3743

661.4069	0.37435

661.50368	0.37437

661.60046	0.3744

661.69724	0.37446

661.79403	0.37449

661.89081	0.37451

661.98759	0.37453

662.08437	0.37456

662.18115	0.37458

662.27793	0.3746

662.37472	0.37462

662.4715	0.37465

662.56828	0.37467

662.66506	0.37469

662.76184	0.37472

662.85862	0.37474

662.95541	0.37478

663.05219	0.37481

663.14897	0.37483

663.24575	0.37485

663.34253	0.37492

663.43932	0.37494

663.5361	0.37501

663.63288	0.37508

663.72966	0.3751

663.82644	0.37513

663.92322	0.37515

664.02001	0.37517

664.11679	0.37524

664.21357	0.37526

664.31035	0.37529

664.40713	0.37533

664.50392	0.37538

664.6007	0.3754

664.69748	0.37542

664.79426	0.37545

664.89104	0.37552

664.98782	0.37554

665.08461	0.37556

665.18139	0.37558

665.27817	0.37572

665.37495	0.37574

665.47173	0.37577

665.56852	0.37579

665.6653	0.37581

665.76208	0.37584

665.85886	0.37588

665.95564	0.37597

666.05242	0.376

666.14921	0.37602

666.24599	0.37604

666.34277	0.37606

666.43955	0.37609

666.53633	0.37611

666.63312	0.37618

666.7299	0.3762

666.82668	0.37625

666.92346	0.37627

667.02024	0.37636

667.11702	0.37641

667.21381	0.37643

667.31059	0.37645

667.40737	0.37648

667.50415	0.37652

667.60093	0.37661

667.69772	0.37675

667.7945	0.37682

667.89128	0.37689

667.98806	0.37691

668.08484	0.37693

668.18162	0.37698

668.27841	0.37702

668.37519	0.37705

668.47197	0.37707

668.56875	0.37709

668.66553	0.37714

668.76232	0.37716

668.8591	0.37723

668.95588	0.37725

669.05266	0.37728

669.14944	0.37737

669.24622	0.37739

669.34301	0.37744

669.43979	0.37746

669.53657	0.37748

669.63335	0.37753

669.73013	0.37755

669.82692	0.37757

669.9237	0.3776

670.02048	0.37764

670.11726	0.37766

670.21404	0.37769

670.31082	0.37773

670.40761	0.37776

670.50439	0.37778

670.60117	0.3778

670.69795	0.37782

670.79473	0.37787

670.89152	0.37789

670.9883	0.37796

671.08508	0.37798

671.18186	0.37808

671.27864	0.3781

671.37542	0.37812

671.47221	0.37814

671.56899	0.37817

671.66577	0.37819

671.76255	0.37821

671.85933	0.37824

671.95612	0.37826

672.0529	0.3783

672.14968	0.37833

672.24646	0.37835

672.34324	0.37842

672.44002	0.37844

672.53681	0.37846

672.63359	0.37849

672.73037	0.37851

672.82715	0.37853

672.92393	0.37856

673.02072	0.37858

673.1175	0.37862

673.21428	0.37865

673.31106	0.37872

673.40784	0.37883

673.50462	0.37897

673.60141	0.37901

673.69819	0.37904

673.79497	0.3791

673.89175	0.37913

673.98853	0.37929

674.08532	0.37931

674.1821	0.37933

674.27888	0.37942

674.37566	0.37949

674.47244	0.37958

674.56922	0.37963

674.66601	0.37968

674.76279	0.37972

674.85957	0.37974

674.95635	0.37977

675.05313	0.37986

675.14991	0.3799

675.2467	0.37993

675.34348	0.37995

675.44026	0.37997

675.53704	0.38002

675.63382	0.38004

675.73061	0.38006

675.82739	0.38016

675.92417	0.38032

676.02095	0.38034

676.11773	0.38036

676.21451	0.38045

676.3113	0.38052

676.40808	0.38054

676.50486	0.38057

676.60164	0.38061

676.69842	0.38064

676.79521	0.38066

676.89199	0.38068

676.98877	0.38073

677.08555	0.38077

677.18233	0.3808

677.27911	0.38082

677.3759	0.38086

677.47268	0.38089

677.56946	0.38098

677.66624	0.38105

677.76302	0.38107

677.85981	0.38112

677.95659	0.38114

678.05337	0.38116

678.15015	0.38118

678.24693	0.38121

678.34371	0.38123

678.4405	0.38125

678.53728	0.38134

678.63406	0.38141

678.73084	0.38144

678.82762	0.38146

678.92441	0.3815

679.02119	0.38153

679.11797	0.38155

679.21475	0.3816

679.31153	0.38162

679.40831	0.38164

679.5051	0.38166

679.60188	0.38182

679.69866	0.38185

679.79544	0.38187

679.89222	0.38189

679.98901	0.38192

680.08579	0.38198

680.18257	0.38203

680.27935	0.38205

680.37613	0.38208

680.47291	0.3821

680.5697	0.38214

680.66648	0.38219

680.76326	0.38221

680.86004	0.38226

680.95682	0.38228

681.05361	0.3823

681.15039	0.38235

681.24717	0.38246

681.34395	0.38249

681.44073	0.38251

681.53751	0.38262

681.6343	0.38265

681.73108	0.38281

681.82786	0.38283

681.92464	0.38285

682.02142	0.38288

682.11821	0.3829

682.21499	0.38294

682.31177	0.38301

682.40855	0.38304

682.50533	0.38306

682.60211	0.38308

682.6989	0.3831

682.79568	0.38313

682.89246	0.38315

682.98924	0.38317

683.08602	0.38347

683.18281	0.38352

683.27959	0.38354

683.37637	0.38358

683.47315	0.38363

683.56993	0.38365

683.66671	0.38368

683.7635	0.38372

683.86028	0.38374

683.95706	0.38379

684.05384	0.38381

684.15062	0.38384

684.24741	0.38388

684.34419	0.38393

684.44097	0.38395

684.53775	0.38397

684.63453	0.384

684.73131	0.38416

684.8281	0.3842

684.92488	0.38422

685.02166	0.38434

685.11844	0.38436

685.21522	0.38443

685.31201	0.38448

685.40879	0.3845

685.50557	0.38459

685.60235	0.38461

685.69913	0.38464

685.79591	0.38473

685.8927	0.38475

685.98948	0.38477

686.08626	0.3848

686.18304	0.38482

686.27982	0.38486

686.37661	0.38489

686.47339	0.38491

686.57017	0.38496

686.66695	0.38498

686.76373	0.38509

686.86051	0.38512

686.9573	0.38516

687.05408	0.38518

687.15086	0.38521

687.24764	0.38532

687.34442	0.38534

687.4412	0.38537

687.53799	0.38539

687.63477	0.38541

687.73155	0.38544

687.82833	0.38546

687.92511	0.38548

688.0219	0.38553

688.11868	0.38557

688.21546	0.3856

688.31224	0.38562

688.40902	0.38564

688.50581	0.38566

688.60259	0.38571

688.69937	0.38573

688.79615	0.3858

688.89293	0.38582

688.98971	0.38585

689.0865	0.38587

689.18328	0.38589

689.28006	0.38592

689.37684	0.38594

689.47362	0.38596

689.5704	0.38603

689.66719	0.38626

689.76397	0.38628

689.86075	0.3863

689.95753	0.38633

690.05431	0.38635

690.1511	0.38637

690.24788	0.3864

690.34466	0.38642

690.44144	0.38644

690.53822	0.38646

690.635	0.38649

690.73179	0.38653

690.82857	0.38656

690.92535	0.38658

691.02213	0.3866

691.11891	0.38662

691.2157	0.38672

691.31248	0.38681

691.40926	0.38683

691.50604	0.38685

691.60282	0.38688

691.6996	0.3869

691.79639	0.38692

691.89317	0.38694

691.98995	0.38697

692.08673	0.38699

692.18351	0.38704

692.2803	0.38708

692.37708	0.38713

692.47386	0.38715

692.57064	0.38717

692.66742	0.38724

692.7642	0.38726

692.86099	0.38729

692.95777	0.38733

693.05455	0.38738

693.15133	0.38742

693.24811	0.38749

693.3449	0.38752

693.44168	0.38756

693.53846	0.38761

693.63524	0.38763

693.73202	0.38765

693.8288	0.3877

693.92559	0.38772

694.02237	0.38775

694.11915	0.38779

694.21593	0.38781

694.31271	0.38784

694.4095	0.38788

694.50628	0.38791

694.60306	0.38793

694.69984	0.38795

694.79662	0.38797

694.8934	0.388

694.99019	0.38802

695.08697	0.38807

695.18375	0.3882

695.28053	0.38825

695.37731	0.38827

695.4741	0.38829

695.57088	0.38836

695.66766	0.38843

695.76444	0.3885

695.86122	0.38852

695.958	0.38857

696.05479	0.38859

696.15157	0.38861

696.24835	0.38864

696.34513	0.38866

696.44191	0.38868

696.5387	0.38871

696.63548	0.38877

696.73226	0.3888

696.82904	0.38882

696.92582	0.38893

697.0226	0.389

697.11939	0.38903

697.21617	0.38912

697.31295	0.38914

697.40973	0.38916

697.50651	0.38919

697.6033	0.38925

697.70008	0.38928

697.79686	0.3893

697.89364	0.38935

697.99042	0.38937

698.0872	0.38939

698.18399	0.38941

698.28077	0.38946

698.37755	0.38951

698.47433	0.38955

698.57111	0.38957

698.6679	0.3896

698.76468	0.38964

698.86146	0.38967

698.95824	0.38971

699.05502	0.38973

699.1518	0.38976

699.24859	0.38978

699.34537	0.3898

699.44215	0.38983

699.53893	0.38985

699.63571	0.38989

699.7325	0.38994

699.82928	0.38996

699.92606	0.38999

700.02284	0.39001

700.11962	0.39003

700.2164	0.39008

700.31319	0.3901

700.40997	0.39012

700.50675	0.39017

700.60353	0.39024

700.70031	0.39026

700.7971	0.39028

700.89388	0.39031

700.99066	0.39042

701.08744	0.39044

701.18422	0.39047

701.281	0.39049

701.37779	0.39053

701.47457	0.39056

701.57135	0.39058

701.66813	0.3906

701.76491	0.39065

701.86169	0.39069

701.95848	0.39072

702.05526	0.39076

702.15204	0.39081

702.24882	0.39083

702.3456	0.3909

702.44239	0.39095

702.53917	0.39097

702.63595	0.39101

702.73273	0.39104

702.82951	0.39106

702.92629	0.39108

703.02308	0.39111

703.11986	0.39113

703.21664	0.3912

703.31342	0.39124

703.4102	0.39127

703.50699	0.39133

703.60377	0.39136

703.70055	0.39138

703.79733	0.39143

703.89411	0.39152

703.99089	0.39156

704.08768	0.39163

704.18446	0.39165

704.28124	0.39168

704.37802	0.3917

704.4748	0.39172

704.57159	0.39175

704.66837	0.39179

704.76515	0.39181

704.86193	0.39184

704.95871	0.39186

705.05549	0.39188

705.15228	0.39191

705.24906	0.39193

705.34584	0.39195

705.44262	0.39197

705.5394	0.392

705.63619	0.39202

705.73297	0.39204

705.82975	0.39207

705.92653	0.39209

706.02331	0.39211

706.12009	0.3922

706.21688	0.39223

706.31366	0.39225

706.41044	0.39229

706.50722	0.39232

706.604	0.39234

706.70079	0.39239

706.79757	0.39259

706.89435	0.39266

706.99113	0.39287

707.08791	0.39289

707.18469	0.39293

707.28148	0.39296

707.37826	0.393

707.47504	0.39305

707.57182	0.39307

707.6686	0.39316

707.76539	0.39319

707.86217	0.39321

707.95895	0.39323

708.05573	0.39325

708.15251	0.39337

708.24929	0.39339

708.34608	0.39346

708.44286	0.39348

708.53964	0.39351

708.63642	0.39353

708.7332	0.39355

708.82999	0.3936

708.92677	0.39362

709.02355	0.39364

709.12033	0.39369

709.21711	0.39371

709.31389	0.39373

709.41068	0.39378

709.50746	0.39383

709.60424	0.39385

709.70102	0.39387

709.7978	0.39389

709.89459	0.39399

709.99137	0.39405

710.08815	0.39408

710.18493	0.3941

710.28171	0.39412

710.37849	0.39415

710.47528	0.39417

710.57206	0.39421

710.66884	0.39424

710.76562	0.39431

710.8624	0.39433

710.95919	0.39435

711.05597	0.39437

711.15275	0.39442

711.24953	0.39444

711.34631	0.39453

711.44309	0.39456

711.53988	0.39458

711.63666	0.3946

711.73344	0.39467

711.83022	0.39469

711.927	0.39474

712.02379	0.39476

712.12057	0.39481

712.21735	0.39483

712.31413	0.39485

712.41091	0.39488

712.50769	0.3949

712.60448	0.39492

712.70126	0.39499

712.79804	0.39504

712.89482	0.39506

712.9916	0.39513

713.08839	0.39515

713.18517	0.39517

713.28195	0.3952

713.37873	0.39527

713.47551	0.39529

713.57229	0.39533

713.66908	0.3954

713.76586	0.39543

713.86264	0.39547

713.95942	0.39549

714.0562	0.39552

714.15298	0.39556

714.24977	0.39559

714.34655	0.3957

714.44333	0.39572

714.54011	0.39577

714.63689	0.39579

714.73368	0.39581

714.83046	0.39584

714.92724	0.39586

715.02402	0.39588

715.1208	0.39602

715.21758	0.39604

715.31437	0.39607

715.41115	0.39613

715.50793	0.3962

715.60471	0.39623

715.70149	0.39625

715.79828	0.39627

715.89506	0.39629

715.99184	0.39632

716.08862	0.39634

716.1854	0.39636

716.28218	0.39639

716.37897	0.39641

716.47575	0.39645

716.57253	0.39652

716.66931	0.39657

716.76609	0.39659

716.86288	0.39661

716.95966	0.39668

717.05644	0.39671

717.15322	0.39673

717.25	0.39675

717.34678	0.3968

717.44357	0.39682

717.54035	0.39684

717.63713	0.39689

717.73391	0.39693

717.83069	0.39703

717.92748	0.39705

718.02426	0.39719

718.12104	0.39721

718.21782	0.39723

718.3146	0.39725

718.41138	0.39728

718.50817	0.39735

718.60495	0.39739

718.70173	0.39741

718.79851	0.39748

718.89529	0.39751

718.99208	0.39755

719.08886	0.39757

719.18564	0.3976

719.28242	0.39762

719.3792	0.39764

719.47598	0.39767

719.57277	0.39769

719.66955	0.39773

719.76633	0.39776

719.86311	0.39778

719.95989	0.3978

720.05668	0.39783

720.15346	0.39785

720.25024	0.39787

720.34702	0.39789

720.4438	0.39792

720.54058	0.39794

720.63737	0.39796

720.73415	0.39803

720.83093	0.39805

720.92771	0.3981

721.02449	0.39815

721.12128	0.39819

721.21806	0.39821

721.31484	0.39824

721.41162	0.39826

721.5084	0.39831

721.60518	0.39835

721.70197	0.39844

721.79875	0.39847

721.89553	0.39851

721.99231	0.39853

722.08909	0.39856

722.18588	0.39858

722.28266	0.39863

722.37944	0.39865

722.47622	0.39867

722.573	0.39872

722.66978	0.39874

722.76657	0.39876

722.86335	0.39879

722.96013	0.39881

723.05691	0.39883

723.15369	0.39888

723.25048	0.3989

723.34726	0.39895

723.44404	0.39897

723.54082	0.39899

723.6376	0.39901

723.73438	0.39904

723.83117	0.39911

723.92795	0.39913

724.02473	0.39915

724.12151	0.39922

724.21829	0.39924

724.31508	0.39927

724.41186	0.39929

724.50864	0.39931

724.60542	0.39936

724.7022	0.39938

724.79898	0.39945

724.89577	0.39947

724.99255	0.39949

725.08933	0.39952

725.18611	0.39956

725.28289	0.39959

725.37968	0.39961

725.47646	0.39963

725.57324	0.39965

725.67002	0.39968

725.7668	0.3997

725.86358	0.39975

725.96037	0.39977

726.05715	0.39981

726.15393	0.39984

726.25071	0.39988

726.34749	0.39993

726.44427	0.39997

726.54106	0.4

726.63784	0.40002

726.73462	0.40004

726.8314	0.40009

726.92818	0.40013

727.02497	0.40016

727.12175	0.40018

727.21853	0.4002

727.31531	0.40023

727.41209	0.40025

727.50887	0.40027

727.60566	0.40029

727.70244	0.40032

727.79922	0.40034

727.896	0.40039

727.99278	0.40041

728.08957	0.40043

728.18635	0.40045

728.28313	0.40048

728.37991	0.4005

728.47669	0.40052

728.57347	0.40055

728.67026	0.40057

728.76704	0.40059

728.86382	0.40061

728.9606	0.40064

729.05738	0.40066

729.15417	0.40068

729.25095	0.40071

729.34773	0.40075

729.44451	0.40077

729.54129	0.40089

729.63807	0.40091

729.73486	0.40096

729.83164	0.40098

729.92842	0.40103

730.0252	0.40105

730.12198	0.40107

730.21877	0.40109

730.31555	0.40112

730.41233	0.40114

730.50911	0.40119

730.60589	0.40121

730.70267	0.40123

730.79946	0.40125

730.89624	0.4013

730.99302	0.40132

731.0898	0.40137

731.18658	0.40139

731.28337	0.40141

731.38015	0.40148

731.47693	0.40151

731.57371	0.4016

731.67049	0.40169

731.76727	0.40171

731.86406	0.40176

731.96084	0.40178

732.05762	0.40187

732.1544	0.40189

732.25118	0.40192

732.34797	0.40194

732.44475	0.40196

732.54153	0.40199

732.63831	0.40203

732.73509	0.40208

732.83187	0.4021

732.92866	0.40212

733.02544	0.40215

733.12222	0.40217

733.219	0.40219

733.31578	0.40224

733.41257	0.40226

733.50935	0.40228

733.60613	0.40233

733.70291	0.40235

733.79969	0.40237

733.89647	0.4024

733.99326	0.40244

734.09004	0.40249

734.18682	0.40253

734.2836	0.40256

734.38038	0.40265

734.47717	0.40267

734.57395	0.40269

734.67073	0.40272

734.76751	0.40279

734.86429	0.40281

734.96107	0.40285

735.05786	0.40288

735.15464	0.4029

735.25142	0.40292

735.3482	0.40295

735.44498	0.40297

735.54177	0.40301

735.63855	0.40304

735.73533	0.40306

735.83211	0.40313

735.92889	0.40317

736.02567	0.4032

736.12246	0.40324

736.21924	0.40327

736.31602	0.40329

736.4128	0.40333

736.50958	0.40336

736.60637	0.40338

736.70315	0.40343

736.79993	0.40345

736.89671	0.40349

736.99349	0.40356

737.09027	0.40361

737.18706	0.4037

737.28384	0.40375

737.38062	0.40379

737.4774	0.40381

737.57418	0.40391

737.67097	0.40395

737.76775	0.40402

737.86453	0.40404

737.96131	0.40407

738.05809	0.40409

738.15487	0.40411

738.25166	0.40413

738.34844	0.40416

738.44522	0.40418

738.542	0.40423

738.63878	0.40427

738.73556	0.40432

738.83235	0.40434

738.92913	0.40436

739.02591	0.40439

739.12269	0.40441

739.21947	0.40443

739.31626	0.40445

739.41304	0.40448

739.50982	0.40452

739.6066	0.40459

739.70338	0.40461

739.80016	0.40468

739.89695	0.40471

739.99373	0.40473

740.09051	0.40475

740.18729	0.40477

740.28407	0.40482

740.38086	0.40487

740.47764	0.40493

740.57442	0.40496

740.6712	0.40498

740.76798	0.40505

740.86476	0.40514

740.96155	0.40516

741.05833	0.40519

741.15511	0.40521

741.25189	0.40525

741.34867	0.40528

741.44546	0.40532

741.54224	0.40537

741.63902	0.40541

741.7358	0.40544

741.83258	0.40546

741.92936	0.40548

742.02615	0.40551

742.12293	0.40553

742.21971	0.40557

742.31649	0.4056

742.41327	0.40562

742.51006	0.40567

742.60684	0.40571

742.70362	0.40576

742.8004	0.40583

742.89718	0.40585

742.99396	0.40587

743.09075	0.40589

743.18753	0.40592

743.28431	0.40594

743.38109	0.40596

743.47787	0.40599

743.57466	0.40603

743.67144	0.40608

743.76822	0.4061

743.865	0.40621

743.96178	0.40624

744.05856	0.40626

744.15535	0.40633

744.25213	0.40635

744.34891	0.40637

744.44569	0.4064

744.54247	0.40642

744.63926	0.40644

744.73604	0.40647

744.83282	0.40651

744.9296	0.40653

745.02638	0.40656

745.12316	0.4066

745.21995	0.40663

745.31673	0.40667

745.41351	0.40669

745.51029	0.40674

745.60707	0.40679

745.70386	0.40685

745.80064	0.4069

745.89742	0.40692

745.9942	0.40695

746.09098	0.40699

746.18776	0.40701

746.28455	0.40713

746.38133	0.40715

746.47811	0.40717

746.57489	0.4072

746.67167	0.40722

746.76846	0.40729

746.86524	0.40731

746.96202	0.4074

747.0588	0.40743

747.15558	0.40747

747.25236	0.40749

747.34915	0.40754

747.44593	0.40765

747.54271	0.40768

747.63949	0.4077

747.73627	0.40772

747.83306	0.40775

747.92984	0.40777

748.02662	0.40779

748.1234	0.40781

748.22018	0.40784

748.31696	0.40786

748.41375	0.40788

748.51053	0.40791

748.60731	0.40793

748.70409	0.40795

748.80087	0.40802

748.89766	0.40804

748.99444	0.40807

749.09122	0.40809

749.188	0.40811

749.28478	0.40818

749.38156	0.4082

749.47835	0.40823

749.57513	0.40825

749.67191	0.40827

749.76869	0.40829

749.86547	0.40832

749.96226	0.40834

750.05904	0.40836

750.15582	0.40839

750.2526	0.40841

750.34938	0.40843

750.44616	0.40845

750.54295	0.4085

750.63973	0.40852

750.73651	0.40855

750.83329	0.40859

750.93007	0.40861

751.02685	0.40868

751.12364	0.40873

751.22042	0.40875

751.3172	0.40877

751.41398	0.4088

751.51076	0.40882

751.60755	0.40889

751.70433	0.40891

751.80111	0.40893

751.89789	0.40896

751.99467	0.40898

752.09145	0.40905

752.18824	0.40909

752.28502	0.40914

752.3818	0.40916

752.47858	0.40919

752.57536	0.40921

752.67215	0.40932

752.76893	0.40935

752.86571	0.40937

752.96249	0.40939

753.05927	0.40941

753.15605	0.40944

753.25284	0.40946

753.34962	0.40953

753.4464	0.40955

753.54318	0.40957

753.63996	0.4096

753.73675	0.40964

753.83353	0.40967

753.93031	0.40971

754.02709	0.40973

754.12387	0.40978

754.22065	0.4098

754.31744	0.40985

754.41422	0.40989

754.511	0.40992

754.60778	0.40994

754.70456	0.41001

754.80135	0.41003

754.89813	0.41005

754.99491	0.41008

755.09169	0.4101

755.18847	0.41012

755.28525	0.41015

755.38204	0.41017

755.47882	0.41019

755.5756	0.41024

755.67238	0.41028

755.76916	0.41033

755.86595	0.41035

755.96273	0.41037

756.05951	0.41044

756.15629	0.41047

756.25307	0.41049

756.34985	0.41051

756.44664	0.41053

756.54342	0.41056

756.6402	0.41058

756.73698	0.4106

756.83376	0.41063

756.93055	0.41065

757.02733	0.41067

757.12411	0.41069

757.22089	0.41074

757.31767	0.41079

757.41445	0.41081

757.51124	0.41085

757.60802	0.41088

757.7048	0.41092

757.80158	0.41095

757.89836	0.41099

757.99515	0.41101

758.09193	0.41104

758.18871	0.41106

758.28549	0.41108

758.38227	0.41111

758.47905	0.41113

758.57584	0.41117

758.67262	0.4112

758.7694	0.41124

758.86618	0.41127

758.96296	0.41129

759.05975	0.41131

759.15653	0.41138

759.25331	0.4114

759.35009	0.41147

759.44687	0.41149

759.54365	0.41154

759.64044	0.41156

759.73722	0.41159

759.834	0.41161

759.93078	0.41163

760.02756	0.41165

760.12435	0.41168

760.22113	0.41172

760.31791	0.41177

760.41469	0.41179

760.51147	0.41181

760.60825	0.41184

760.70504	0.41186

760.80182	0.41188

760.8986	0.41191

760.99538	0.41193

761.09216	0.41195

761.18895	0.41202

761.28573	0.41204

761.38251	0.41213

761.47929	0.41216

761.57607	0.41218

761.67285	0.4122

761.76964	0.41223

761.86642	0.41225

761.9632	0.41229

762.05998	0.41232

762.15676	0.41234

762.25355	0.41239

762.35033	0.41241

762.44711	0.41248

762.54389	0.4125

762.64067	0.41252

762.73745	0.41255

762.83424	0.41257

762.93102	0.41259

763.0278	0.41261

763.12458	0.41264

763.22136	0.41266

763.31814	0.41268

763.41493	0.41271

763.51171	0.41273

763.60849	0.41275

763.70527	0.41277

763.80205	0.4128

763.89884	0.41291

763.99562	0.41293

764.0924	0.41296

764.18918	0.413

764.28596	0.41303

764.38275	0.41307

764.47953	0.41309

764.57631	0.41312

764.67309	0.41314

764.76987	0.41316

764.86665	0.41319

764.96344	0.41328

765.06022	0.4133

765.157	0.41332

765.25378	0.41335

765.35056	0.41339

765.44734	0.41344

765.54413	0.41346

765.64091	0.41348

765.73769	0.41351

765.83447	0.41355

765.93125	0.41357

766.02804	0.4136

766.12482	0.41362

766.2216	0.41364

766.31838	0.41373

766.41516	0.41376

766.51194	0.41378

766.60873	0.4138

766.70551	0.41383

766.80229	0.41385

766.89907	0.41387

766.99585	0.41389

767.09264	0.41399

767.18942	0.41403

767.2862	0.41405

767.38298	0.4141

767.47976	0.41412

767.57654	0.41417

767.67333	0.41421

767.77011	0.41428

767.86689	0.41431

767.96367	0.41433

768.06045	0.41444

768.15724	0.41447

768.25402	0.41449

768.3508	0.41451

768.44758	0.41453

768.54436	0.41458

768.64114	0.41463

768.73793	0.41465

768.83471	0.41467

768.93149	0.41472

769.02827	0.41481

769.12505	0.41485

769.22184	0.4149

769.31862	0.41492

769.4154	0.41497

769.51218	0.41499

769.60896	0.41501

769.70574	0.41506

769.80253	0.41508

769.89931	0.41511

769.99609	0.41515

770.09287	0.41517

770.18965	0.41522

770.28644	0.41524

770.38322	0.41527

770.48	0.41529

770.57678	0.41538

770.67356	0.4154

770.77034	0.41543

770.86713	0.41545

770.96391	0.41549

771.06069	0.41552

771.15747	0.41554

771.25425	0.41556

771.35104	0.41565

771.44782	0.4157

771.5446	0.41572

771.64138	0.41575

771.73816	0.41577

771.83494	0.41579

771.93173	0.41584

772.02851	0.41586

772.12529	0.41591

772.22207	0.41593

772.31885	0.416

772.41564	0.41602

772.51242	0.41604

772.6092	0.41607

772.70598	0.41611

772.80276	0.4162

772.89954	0.41625

772.99633	0.41629

773.09311	0.41632

773.18989	0.41639

773.28667	0.41641

773.38345	0.41643

773.48024	0.41645

773.57702	0.41648

773.6738	0.4165

773.77058	0.41655

773.86736	0.41659

773.96414	0.41661

774.06093	0.41666

774.15771	0.41675

774.25449	0.41677

774.35127	0.41682

774.44805	0.41684

774.54484	0.41687

774.64162	0.41689

774.7384	0.41691

774.83518	0.41693

774.93196	0.41707

775.02874	0.41709

775.12553	0.41712

775.22231	0.41719

775.31909	0.41721

775.41587	0.41723

775.51265	0.41725

775.60944	0.4173

775.70622	0.41732

775.803	0.41735

775.89978	0.41737

775.99656	0.41739

776.09334	0.41744

776.19013	0.41746

776.28691	0.41748

776.38369	0.41755

776.48047	0.41757

776.57725	0.41764

776.67404	0.41767

776.77082	0.41769

776.8676	0.41771

776.96438	0.41776

777.06116	0.41778

777.15794	0.4178

777.25473	0.41785

777.35151	0.41787

777.44829	0.41789

777.54507	0.41794

777.64185	0.41796

777.73863	0.41799

777.83542	0.41801

777.9322	0.41803

778.02898	0.41805

778.12576	0.41808

778.22254	0.4181

778.31933	0.41812

778.41611	0.41815

778.51289	0.41817

778.60967	0.41819

778.70645	0.41821

778.80323	0.41824

778.90002	0.41826

778.9968	0.41835

779.09358	0.41837

779.19036	0.41842

779.28714	0.41844

779.38393	0.41847

779.48071	0.41853

779.57749	0.41858

779.67427	0.4186

779.77105	0.41865

779.86783	0.41867

779.96462	0.41872

780.0614	0.41874

780.15818	0.41876

780.25496	0.41879

780.35174	0.41881

780.44853	0.41883

780.54531	0.41885

780.64209	0.41888

780.73887	0.41892

780.83565	0.41895

780.93243	0.41897

781.02922	0.41899

781.126	0.41906

781.22278	0.41908

781.31956	0.41911

781.41634	0.41915

781.51313	0.4192

781.60991	0.41924

781.70669	0.41927

781.80347	0.41931

781.90025	0.41936

781.99703	0.41938

782.09382	0.4194

782.1906	0.41947

782.28738	0.41949

782.38416	0.41959

782.48094	0.41961

782.57773	0.41963

782.67451	0.41965

782.77129	0.4197

782.86807	0.41972

782.96485	0.41977

783.06163	0.41979

783.15842	0.41981

783.2552	0.41984

783.35198	0.41986

783.44876	0.41988

783.54554	0.41991

783.64233	0.41993

783.73911	0.41997

783.83589	0.42

783.93267	0.42007

784.02945	0.42009

784.12623	0.42013

784.22302	0.42016

784.3198	0.4202

784.41658	0.42023

784.51336	0.42025

784.61014	0.42027

784.70693	0.42029

784.80371	0.42032

784.90049	0.42036

784.99727	0.42039

785.09405	0.42041

785.19083	0.42043

785.28762	0.42045

785.3844	0.42048

785.48118	0.4205

785.57796	0.42052

785.67474	0.42055

785.77153	0.42057

785.86831	0.42059

785.96509	0.42061

786.06187	0.42066

786.15865	0.42068

786.25543	0.42071

786.35222	0.42075

786.449	0.42078

786.54578	0.4208

786.64256	0.42084

786.73934	0.42094

786.83613	0.42098

786.93291	0.42103

787.02969	0.42105

787.12647	0.42107

787.22325	0.4211

787.32003	0.42132

787.41682	0.42135

787.5136	0.42142

787.61038	0.42144

787.70716	0.42146

787.80394	0.42148

787.90073	0.42153

787.99751	0.42155

788.09429	0.4216

788.19107	0.42167

788.28785	0.42169

788.38463	0.42174

788.48142	0.42176

788.5782	0.42178

788.67498	0.4218

788.77176	0.4219

788.86854	0.42192

788.96533	0.42199

789.06211	0.42201

789.15889	0.42203

789.25567	0.4221

789.35245	0.42212

789.44923	0.42219

789.54602	0.42222

789.6428	0.42224

789.73958	0.42226

789.83636	0.42228

789.93314	0.42233

790.02992	0.42235

790.12671	0.42238

790.22349	0.42242

790.32027	0.42244

790.41705	0.42251

790.51383	0.42254

790.61062	0.42256

790.7074	0.42258

790.80418	0.4226

790.90096	0.42267

790.99774	0.42272

791.09452	0.42276

791.19131	0.42279

791.28809	0.42281

791.38487	0.42283

791.48165	0.4229

791.57843	0.42292

791.67522	0.42295

791.772	0.42302

791.86878	0.42304

791.96556	0.42306

792.06234	0.42308

792.15912	0.42311

792.25591	0.42313

792.35269	0.42315

792.44947	0.42318

792.54625	0.4232

792.64303	0.42322

792.73982	0.42324

792.8366	0.42329

792.93338	0.42331

793.03016	0.42334

793.12694	0.42336

793.22372	0.4234

793.32051	0.42345

793.41729	0.42347

793.51407	0.42352

793.61085	0.42354

793.70763	0.42366

793.80442	0.42368

793.9012	0.4237

793.99798	0.42372

794.09476	0.42379

794.19154	0.42382

794.28832	0.42386

794.38511	0.42388

794.48189	0.42391

794.57867	0.42393

794.67545	0.42395

794.77223	0.42398

794.86902	0.424

794.9658	0.42404

795.06258	0.42409

795.15936	0.42411

795.25614	0.42418

795.35292	0.42423

795.44971	0.42425

795.54649	0.42427

795.64327	0.4243

795.74005	0.42434

795.83683	0.42436

795.93362	0.42439

796.0304	0.42443

796.12718	0.42446

796.22396	0.42457

796.32074	0.42462

796.41752	0.42471

796.51431	0.42478

796.61109	0.4248

796.70787	0.42482

796.80465	0.42484

796.90143	0.42487

796.99822	0.42489

797.095	0.42496

797.19178	0.425

797.28856	0.42503

797.38534	0.42505

797.48212	0.4251

797.57891	0.42514

797.67569	0.42516

797.77247	0.42519

797.86925	0.42521

797.96603	0.42526

798.06282	0.42528

798.1596	0.4253

798.25638	0.42532

798.35316	0.42535

798.44994	0.42537

798.54672	0.42542

798.64351	0.42544

798.74029	0.42546

798.83707	0.42548

798.93385	0.42553

799.03063	0.42558

799.12742	0.4256

799.2242	0.42564

799.32098	0.42571

799.41776	0.42576

799.51454	0.4258

799.61132	0.42585

799.70811	0.42587

799.80489	0.4259

799.90167	0.42592

799.99845	0.42594

800.09523	0.42596

800.19202	0.42599

800.2888	0.42601

800.38558	0.42603

800.48236	0.42606

800.57914	0.42612

800.67592	0.42615

800.77271	0.42617

800.86949	0.42619

800.96627	0.42622

801.06305	0.42624

801.15983	0.42626

801.25662	0.42628

801.3534	0.42631

801.45018	0.42635

801.54696	0.42638

801.64374	0.4264

801.74052	0.42642

801.83731	0.42644

801.93409	0.42647

802.03087	0.42649

802.12765	0.42654

802.22443	0.42658

802.32122	0.4266

802.418	0.42665

802.51478	0.42667

802.61156	0.42676

802.70834	0.42679

802.80512	0.42681

802.90191	0.42683

802.99869	0.42686

803.09547	0.42688

803.19225	0.42699

803.28903	0.42702

803.38581	0.42713

803.4826	0.42715

803.57938	0.42718

803.67616	0.42722

803.77294	0.42727

803.86972	0.42729

803.96651	0.42731

804.06329	0.42734

804.16007	0.42736

804.25685	0.42743

804.35363	0.42745

804.45041	0.42763

804.5472	0.42766

804.64398	0.42768

804.74076	0.4277

804.83754	0.42772

804.93432	0.42775

805.03111	0.42777

805.12789	0.42779

805.22467	0.42784

805.32145	0.42786

805.41823	0.42788

805.51501	0.42793

805.6118	0.42795

805.70858	0.42802

805.80536	0.42807

805.90214	0.42809

805.99892	0.42811

806.09571	0.42814

806.19249	0.42816

806.28927	0.42818

806.38605	0.4282

806.48283	0.42823

806.57961	0.42825

806.6764	0.42827

806.77318	0.42832

806.86996	0.42834

806.96674	0.42836

807.06352	0.42839

807.16031	0.42843

807.25709	0.42848

807.35387	0.4285

807.45065	0.42852

807.54743	0.42855

807.64421	0.42857

807.741	0.42859

807.83778	0.42862

807.93456	0.42864

808.03134	0.42866

808.12812	0.42871

808.22491	0.42878

808.32169	0.42882

808.41847	0.42889

808.51525	0.42891

808.61203	0.42894

808.70881	0.42896

808.8056	0.42898

808.90238	0.42905

808.99916	0.42907

809.09594	0.42912

809.19272	0.42914

809.28951	0.42937

809.38629	0.42951

809.48307	0.42955

809.57985	0.4296

809.67663	0.42962

809.77341	0.42964

809.8702	0.42967

809.96698	0.42974

810.06376	0.42978

810.16054	0.42985

810.25732	0.4299

810.35411	0.42992

810.45089	0.42994

810.54767	0.43001

810.64445	0.43003

810.74123	0.43008

810.83801	0.43012

810.9348	0.43015

811.03158	0.43017

811.12836	0.43038

811.22514	0.4304

811.32192	0.43044

811.41871	0.43047

811.51549	0.43049

811.61227	0.43051

811.70905	0.43058

811.80583	0.4306

811.90261	0.43063

811.9994	0.43067

812.09618	0.4307

812.19296	0.43072

812.28974	0.43074

812.38652	0.43079

812.48331	0.43088

812.58009	0.43095

812.67687	0.43097

812.77365	0.43106

812.87043	0.43111

812.96721	0.43113

813.064	0.43115

813.16078	0.4312

813.25756	0.43122

813.35434	0.43127

813.45112	0.43129

813.54791	0.43131

813.64469	0.43134

813.74147	0.43136

813.83825	0.43138

813.93503	0.43147

814.03181	0.4315

814.1286	0.43156

814.22538	0.43159

814.32216	0.43161

814.41894	0.43166

814.51572	0.4317

814.61251	0.43172

814.70929	0.43179

814.80607	0.43182

814.90285	0.43186

814.99963	0.43191

815.09641	0.43193

815.1932	0.43195

815.28998	0.432

815.38676	0.43209

815.48354	0.43211

815.58032	0.43214

815.6771	0.43216

815.77389	0.43218

815.87067	0.43225

815.96745	0.43227

816.06423	0.4323

816.16101	0.43232

816.2578	0.43234

816.35458	0.43236

816.45136	0.4325

816.54814	0.43255

816.64492	0.43257

816.7417	0.43259

816.83849	0.43262

816.93527	0.43264

817.03205	0.43266

817.12883	0.43268

817.22561	0.43273

817.3224	0.43278

817.41918	0.4328

817.51596	0.43282

817.61274	0.43287

817.70952	0.43289

817.8063	0.43291

817.90309	0.43294

817.99987	0.43296

818.09665	0.43298

818.19343	0.43305

818.29021	0.43307

818.387	0.43312

818.48378	0.43319

818.58056	0.43323

818.67734	0.43326

818.77412	0.4333

818.8709	0.43337

818.96769	0.43344

819.06447	0.43346

819.16125	0.43351

819.25803	0.43353

819.35481	0.43355

819.4516	0.43358

819.54838	0.4336

819.64516	0.43362

819.74194	0.43364

819.83872	0.43374

819.9355	0.43376

820.03229	0.4338

820.12907	0.43383

820.22585	0.43385

820.32263	0.43394

820.41941	0.43396

820.5162	0.43399

820.61298	0.43401

820.70976	0.43406

820.80654	0.43412

820.90332	0.43417

821.0001	0.43424

821.09689	0.43426

821.19367	0.43431

821.29045	0.43433

821.38723	0.43438

821.48401	0.4344

821.5808	0.43442

821.67758	0.43444

821.77436	0.43447

821.87114	0.43451

821.96792	0.43454

822.0647	0.4346

822.16149	0.43463

822.25827	0.43465

822.35505	0.43472

822.45183	0.43474

822.54861	0.43476

822.6454	0.43481

822.74218	0.43483

822.83896	0.43486

822.93574	0.4349

823.03252	0.43492

823.1293	0.43495

823.22609	0.43497

823.32287	0.43499

823.41965	0.43502

823.51643	0.43506

823.61321	0.43508

823.71	0.43511

823.80678	0.43513

823.90356	0.43515

824.00034	0.43518

824.09712	0.4352

824.1939	0.43524

824.29069	0.43527

824.38747	0.43531

824.48425	0.43534

824.58103	0.43536

824.67781	0.4354

824.7746	0.43545

824.87138	0.43547

824.96816	0.4355

825.06494	0.43552

825.16172	0.43556

825.2585	0.43563

825.35529	0.43572

825.45207	0.43575

825.54885	0.43577

825.64563	0.43579

825.74241	0.43586

825.8392	0.43588

825.93598	0.43591

826.03276	0.43593

826.12954	0.43598

826.22632	0.43604

826.3231	0.43614

826.41989	0.43616

826.51667	0.43618

826.61345	0.4362

826.71023	0.43623

826.80701	0.43627

826.90379	0.4363

827.00058	0.43632

827.09736	0.43634

827.19414	0.43636

827.29092	0.43641

827.3877	0.43643

827.48449	0.43646

827.58127	0.43648

827.67805	0.4365

827.77483	0.43655

827.87161	0.43657

827.96839	0.43659

828.06518	0.43662

828.16196	0.43666

828.25874	0.43668

828.35552	0.43671

828.4523	0.43673

828.54909	0.43678

828.64587	0.4368

828.74265	0.43682

828.83943	0.43687

828.93621	0.43694

829.03299	0.43696

829.12978	0.437

829.22656	0.43703

829.32334	0.43705

829.42012	0.4371

829.5169	0.43712

829.61369	0.43714

829.71047	0.43719

829.80725	0.43721

829.90403	0.43728

830.00081	0.4373

830.09759	0.43742

830.19438	0.43744

830.29116	0.43746

830.38794	0.43748

830.48472	0.43751

830.5815	0.43755

830.67829	0.43758

830.77507	0.43762

830.87185	0.43767

830.96863	0.43769

831.06541	0.43771

831.16219	0.43778

831.25898	0.43783

831.35576	0.43785

831.45254	0.43792

831.54932	0.43799

831.6461	0.43801

831.74289	0.43806

831.83967	0.43808

831.93645	0.4381

832.03323	0.43815

832.13001	0.43817

832.22679	0.43822

832.32358	0.43824

832.42036	0.43826

832.51714	0.43828

832.61392	0.43831

832.7107	0.43833

832.80749	0.43835

832.90427	0.43842

833.00105	0.43844

833.09783	0.43847

833.19461	0.43849

833.29139	0.43854

833.38818	0.43856

833.48496	0.43858

833.58174	0.4386

833.67852	0.43863

833.7753	0.43865

833.87209	0.4387

833.96887	0.43872

834.06565	0.43874

834.16243	0.43876

834.25921	0.43888

834.35599	0.4389

834.45278	0.43892

834.54956	0.43895

834.64634	0.43897

834.74312	0.43899

834.8399	0.43904

834.93669	0.43906

835.03347	0.43908

835.13025	0.43911

835.22703	0.43913

835.32381	0.43918

835.42059	0.43922

835.51738	0.43924

835.61416	0.43927

835.71094	0.43929

835.80772	0.43931

835.9045	0.43936

836.00129	0.43938

836.09807	0.4394

836.19485	0.43943

836.29163	0.43945

836.38841	0.43947

836.48519	0.43956

836.58198	0.43963

836.67876	0.43966

836.77554	0.43968

836.87232	0.43972

836.9691	0.43977

837.06589	0.43979

837.16267	0.43982

837.25945	0.43984

837.35623	0.43986

837.45301	0.43988

837.54979	0.43993

837.64658	0.43995

837.74336	0.44

837.84014	0.44002

837.93692	0.44004

838.0337	0.44007

838.13049	0.44009

838.22727	0.44011

838.32405	0.44014

838.42083	0.4402

838.51761	0.4403

838.61439	0.44032

838.71118	0.44034

838.80796	0.44036

838.90474	0.44039

839.00152	0.44041

839.0983	0.44043

839.19508	0.44048

839.29187	0.44052

839.38865	0.44055

839.48543	0.44059

839.58221	0.44066

839.67899	0.44071

839.77578	0.44073

839.87256	0.44075

839.96934	0.4408

840.06612	0.44082

840.1629	0.44084

840.25968	0.44087

840.35647	0.44089

840.45325	0.44094

840.55003	0.44096

840.64681	0.44098

840.74359	0.441

840.84038	0.44103

840.93716	0.44105

841.03394	0.44107

841.13072	0.4411

841.2275	0.44112

841.32428	0.44123

841.42107	0.44128

841.51785	0.4413

841.61463	0.44135

841.71141	0.44144

841.80819	0.44153

841.90498	0.44155

842.00176	0.44158

842.09854	0.4416

842.19532	0.44162

842.2921	0.44164

842.38888	0.44167

842.48567	0.44169

842.58245	0.44171

842.67923	0.44174

842.77601	0.44176

842.87279	0.44178

842.96958	0.4418

843.06636	0.44183

843.16314	0.44192

843.25992	0.44196

843.3567	0.44199

843.45348	0.44203

843.55027	0.44206

843.64705	0.44217

843.74383	0.44224

843.84061	0.44226

843.93739	0.44231

844.03418	0.44233

844.13096	0.44238

844.22774	0.4424

844.32452	0.44242

844.4213	0.44244

844.51808	0.44247

844.61487	0.44249

844.71165	0.44251

844.80843	0.44256

844.90521	0.44258

845.00199	0.4426

845.09878	0.44263

845.19556	0.44265

845.29234	0.44267

845.38912	0.44279

845.4859	0.44281

845.58268	0.44283

845.67947	0.44288

845.77625	0.4429

845.87303	0.44292

845.96981	0.44295

846.06659	0.44297

846.16338	0.44302

846.26016	0.44306

846.35694	0.44308

846.45372	0.44311

846.5505	0.44313

846.64728	0.44315

846.74407	0.44318

846.84085	0.4432

846.93763	0.44322

847.03441	0.44324

847.13119	0.44329

847.22798	0.44343

847.32476	0.44345

847.42154	0.44352

847.51832	0.44354

847.6151	0.44356

847.71188	0.44359

847.80867	0.44366

847.90545	0.44368

848.00223	0.4437

848.09901	0.44372

848.19579	0.44375

848.29258	0.44377

848.38936	0.44382

848.48614	0.44384

848.58292	0.44386

848.6797	0.44388

848.77648	0.44391

848.87327	0.44404

848.97005	0.44418

849.06683	0.4442

849.16361	0.44425

849.26039	0.44432

849.35718	0.44434

849.45396	0.44436

849.55074	0.44439

849.64752	0.44441

849.7443	0.44443

849.84108	0.44446

849.93787	0.44448

850.03465	0.44452

850.13143	0.44457

850.22821	0.44459

850.32499	0.44462

850.42178	0.44465

850.51856	0.44472

850.61534	0.44474

850.71212	0.4448

850.8089	0.44481

850.90568	0.44483

851.00247	0.44489

851.09925	0.44498

851.19603	0.44503

851.29281	0.44512

851.38959	0.44513

851.48638	0.4452

851.58316	0.44533

851.67994	0.44535

851.77672	0.44536

851.8735	0.44548

851.97028	0.44553

852.06707	0.44558

852.16385	0.44561

852.26063	0.44568

852.35741	0.4457

852.45419	0.44571

852.55097	0.44573

852.64776	0.44574

852.74454	0.44576

852.84132	0.4458

852.9381	0.44582

853.03488	0.44584

853.13167	0.44585

853.22845	0.44593

853.32523	0.44596

853.42201	0.44599

853.51879	0.446

853.61557	0.44603

853.71236	0.44609

853.80914	0.44625

853.90592	0.44626

854.0027	0.44629

854.09948	0.44634

854.19627	0.44635

854.29305	0.44637

854.38983	0.44638

854.48661	0.44644

854.58339	0.44646

854.68017	0.44648

854.77696	0.44649

854.87374	0.44657

854.97052	0.44658

855.0673	0.44673

855.16408	0.4468

855.26087	0.44681

855.35765	0.44686

855.45443	0.44687

855.55121	0.44692

855.64799	0.44695

855.74477	0.44699

855.84156	0.44701

855.93834	0.44705

856.03512	0.44708

856.1319	0.44727

856.22868	0.44728

856.32547	0.4473

856.42225	0.44736

856.51903	0.44737

856.61581	0.44751

856.71259	0.44753

856.80937	0.44754

856.90616	0.44757

857.00294	0.44759

857.09972	0.44774

857.1965	0.44776

857.29328	0.44777

857.39007	0.44779

857.48685	0.44789

857.58363	0.44791

857.68041	0.44798

857.77719	0.44801

857.87397	0.44803

857.97076	0.44806

858.06754	0.44808

858.16432	0.44811

858.2611	0.44812

858.35788	0.44815

858.45467	0.44817

858.55145	0.44818

858.64823	0.4482

858.74501	0.44821

858.84179	0.44823

858.93857	0.44826

859.03536	0.44827

859.13214	0.44829

859.22892	0.4483

859.3257	0.44833

859.42248	0.44835

859.51927	0.44843

859.61605	0.44844

859.71283	0.44849

859.80961	0.44852

859.90639	0.44856

860.00317	0.44859

860.09996	0.44865

860.19674	0.44867

860.29352	0.4487

860.3903	0.44872

860.48708	0.44873

860.58387	0.44876

860.68065	0.44882

860.77743	0.4489

860.87421	0.44893

860.97099	0.44896

861.06777	0.44897

861.16456	0.44902

861.26134	0.44905

861.35812	0.44911

861.4549	0.44914

861.55168	0.44916

861.64847	0.44917

861.74525	0.44937

861.84203	0.44939

861.93881	0.44949

862.03559	0.44952

862.13237	0.44954

862.22916	0.44963

862.32594	0.44966

862.42272	0.44968

862.5195	0.44969

862.61628	0.44972

862.71307	0.44975

862.80985	0.44981

862.90663	0.4499

863.00341	0.45

863.10019	0.45007

863.19697	0.45028

863.29376	0.45035

863.39054	0.45039

863.48732	0.4505

863.5841	0.45064

863.68088	0.45073

863.77767	0.45076

863.87445	0.45077

863.97123	0.45082

864.06801	0.45083

864.16479	0.45088

864.26157	0.45089

864.35836	0.45091

864.45514	0.45092

864.55192	0.45111

864.6487	0.4512

864.74548	0.45124

864.84227	0.45126

864.93905	0.45128

865.03583	0.45134

865.13261	0.45144

865.22939	0.45146

865.32617	0.4515

865.42296	0.45161

865.51974	0.45163

865.61652	0.4519

865.7133	0.45192

865.81008	0.45195

865.90686	0.45202

866.00365	0.45204

866.10043	0.45205

866.19721	0.45207

866.29399	0.4521

866.39077	0.45219

866.48756	0.4522

866.58434	0.45237

866.68112	0.45239

866.7779	0.45245

866.87468	0.4526

866.97146	0.45262

867.06825	0.45263

867.16503	0.45288

867.26181	0.45291

867.35859	0.45304

867.45537	0.45306

867.55216	0.45358

867.64894	0.45359

867.74572	0.45361

867.8425	0.45368

867.93928	0.45371

868.03606	0.45384

868.13285	0.45385

868.22963	0.45393

868.32641	0.45399

868.42319	0.454

868.51997	0.45402

868.61676	0.45406

868.71354	0.45408

868.81032	0.45411

868.9071	0.45417

869.00388	0.45419

869.10066	0.45422

869.19745	0.45425

869.29423	0.45432

869.39101	0.45434

869.48779	0.45443

869.58457	0.45455

869.68136	0.45463

869.77814	0.45469

869.87492	0.45475

869.9717	0.45477

870.06848	0.45478

870.16526	0.45489

870.26205	0.45492

870.35883	0.45496

870.45561	0.45509

870.55239	0.45519

870.64917	0.45521

870.74596	0.45522

870.84274	0.45524

870.93952	0.45527

871.0363	0.4553

871.13308	0.45533

871.22986	0.45536

871.32665	0.45541

871.42343	0.45547

871.52021	0.45562

871.61699	0.45565

871.71377	0.45568

871.81056	0.45569

871.90734	0.45574

872.00412	0.45576

872.1009	0.45585

872.19768	0.45586

872.29446	0.45589

872.39125	0.45591

872.48803	0.456

872.58481	0.45608

872.68159	0.45611

872.77837	0.45615

872.87516	0.45637

872.97194	0.45667

873.06872	0.4567

873.1655	0.45707

873.26228	0.45711

873.35906	0.45713

873.45585	0.45723

873.55263	0.45725

873.64941	0.45728

873.74619	0.45737

873.84297	0.45739

873.93976	0.45749

874.03654	0.45751

874.13332	0.45752

874.2301	0.45757

874.32688	0.45763

874.42366	0.45765

874.52045	0.45772

874.61723	0.45774

874.71401	0.45786

874.81079	0.45787

874.90757	0.45789

875.00436	0.45793

875.10114	0.45797

875.19792	0.45801

875.2947	0.45807

875.39148	0.4581

875.48826	0.45818

875.58505	0.45822

875.68183	0.45829

875.77861	0.45832

875.87539	0.45833

875.97217	0.45835

876.06896	0.45838

876.16574	0.45854

876.26252	0.45857

876.3593	0.45859

876.45608	0.45871

876.55286	0.45874

876.64965	0.45876

876.74643	0.45899

876.84321	0.45905

876.93999	0.45912

877.03677	0.45915

877.13356	0.45937

877.23034	0.4594

877.32712	0.4595

877.4239	0.45952

877.52068	0.45966

877.61746	0.4597

877.71425	0.45972

877.81103	0.45978

877.90781	0.45982

878.00459	0.4599

878.10137	0.45992

878.19816	0.46005

878.29494	0.46007

878.39172	0.46014

878.4885	0.46016

878.58528	0.46019

878.68206	0.46021

878.77885	0.46022

878.87563	0.46024

878.97241	0.46025

879.06919	0.4604

879.16597	0.46042

879.26275	0.46057

879.35954	0.46059

879.45632	0.4606

879.5531	0.46062

879.64988	0.46074

879.74666	0.46075

879.84345	0.46083

879.94023	0.46094

880.03701	0.46097

880.13379	0.46098

880.23057	0.46115

880.32735	0.46118

880.42414	0.46133

880.52092	0.46135

880.6177	0.46138

880.71448	0.46144

880.81126	0.46164

880.90805	0.46167

881.00483	0.4617

881.10161	0.46171

881.19839	0.46179

881.29517	0.46191

881.39195	0.46193

881.48874	0.46194

881.58552	0.462

881.6823	0.46205

881.77908	0.46208

881.87586	0.46211

881.97265	0.46213

882.06943	0.46214

882.16621	0.4622

882.26299	0.46223

882.35977	0.46225

882.45655	0.46226

882.55334	0.46228

882.65012	0.46238

882.7469	0.46243

882.84368	0.46245

882.94046	0.46246

883.03725	0.46252

883.13403	0.46261

883.23081	0.46278

883.32759	0.4628

883.42437	0.46281

883.52115	0.46322

883.61794	0.46324

883.71472	0.46327

883.8115	0.46331

883.90828	0.46336

884.00506	0.46337

884.10185	0.46347

884.19863	0.46351

884.29541	0.46353

884.39219	0.46362

884.48897	0.46363

884.58575	0.46379

884.68254	0.4638

884.77932	0.46398

884.8761	0.46412

884.97288	0.46418

885.06966	0.46427

885.16645	0.46429

885.26323	0.46437

885.36001	0.46455

885.45679	0.46464

885.55357	0.46469

885.65035	0.4647

885.74714	0.46472

885.84392	0.46473

885.9407	0.46475

886.03748	0.46479

886.13426	0.46484

886.23105	0.46485

886.32783	0.46487

886.42461	0.4649

886.52139	0.46494

886.61817	0.46496

886.71495	0.4651

886.81174	0.46511

886.90852	0.46514

887.0053	0.46516

887.10208	0.4652

887.19886	0.46522

887.29565	0.46525

887.39243	0.46533

887.48921	0.46536

887.58599	0.46551

887.68277	0.46555

887.77955	0.46557

887.87634	0.46571

887.97312	0.46578

888.0699	0.46598

888.16668	0.46601

888.26346	0.46603

888.36025	0.46606

888.45703	0.46607

888.55381	0.46622

888.65059	0.46624

888.74737	0.46626

888.84415	0.46629

888.94094	0.46632

889.03772	0.46635

889.1345	0.46644

889.23128	0.46645

889.32806	0.46647

889.42485	0.46653

889.52163	0.46673

889.61841	0.46683

889.71519	0.46685

889.81197	0.46688

889.90875	0.46705

890.00554	0.46706

890.10232	0.46709

890.1991	0.46711

890.29588	0.46717

890.39266	0.46718

890.48945	0.46722

890.58623	0.46725

890.68301	0.46729

890.77979	0.46732

890.87657	0.46734

890.97335	0.46741

891.07014	0.46746

891.16692	0.46758

891.2637	0.46761

891.36048	0.46778

891.45726	0.46781

891.55404	0.46796

891.65083	0.46798

891.74761	0.46799

891.84439	0.46805

891.94117	0.46807

892.03795	0.46808

892.13474	0.4681

892.23152	0.46811

892.3283	0.46821

892.42508	0.46822

892.52186	0.46827

892.61864	0.46833

892.71543	0.46836

892.81221	0.46839

892.90899	0.4684

893.00577	0.46853

893.10255	0.46856

893.19934	0.46857

893.29612	0.46859

893.3929	0.46862

893.48968	0.46863

893.58646	0.46865

893.68324	0.4688

893.78003	0.46894

893.87681	0.46897

893.97359	0.469

894.07037	0.46901

894.16715	0.46903

894.26394	0.46914

894.36072	0.46918

894.4575	0.46921

894.55428	0.46923

894.65106	0.46924

894.74784	0.4693

894.84463	0.46932

894.94141	0.46952

895.03819	0.46953

895.13497	0.4697

895.23175	0.46971

895.32854	0.46973

895.42532	0.46978

895.5221	0.46984

895.61888	0.46985

895.71566	0.46987

895.81244	0.46993

895.90923	0.46994

896.00601	0.46996

896.10279	0.46997

896.19957	0.47

896.29635	0.47002

896.39314	0.47006

896.48992	0.47008

896.5867	0.47017

896.68348	0.47022

896.78026	0.47023

896.87704	0.47025

896.97383	0.47038

897.07061	0.47042

897.16739	0.47045

897.26417	0.47046

897.36095	0.47048

897.45774	0.47058

897.55452	0.4706

897.6513	0.47072

897.74808	0.47074

897.84486	0.47075

897.94164	0.47077

898.03843	0.47102

898.13521	0.47104

898.23199	0.47113

898.32877	0.47115

898.42555	0.47118

898.52234	0.47124

898.61912	0.47125

898.7159	0.47142

898.81268	0.47144

898.90946	0.47145

899.00624	0.47157

899.10303	0.47165

899.19981	0.4717

899.29659	0.47171

899.39337	0.47173

899.49015	0.47176

899.58694	0.47182

899.68372	0.47183

899.7805	0.47188

899.87728	0.47212

899.97406	0.47214

900.07084	0.47215

900.16763	0.47218

900.26441	0.47221

900.36119	0.47224

900.45797	0.4723

900.55475	0.47232

900.65154	0.47234

900.74832	0.47238

900.8451	0.47256

900.94188	0.47258

901.03866	0.47269

901.13544	0.4727

901.23223	0.47284

901.32901	0.47287

901.42579	0.47288

901.52257	0.4729

901.61935	0.47293

901.71614	0.47304

901.81292	0.47305

901.9097	0.47307

902.00648	0.47308

902.10326	0.47313

902.20004	0.47314

902.29683	0.47316

902.39361	0.47317

902.49039	0.47339

902.58717	0.4734

902.68395	0.47342

902.78074	0.47343

902.87752	0.47345

902.9743	0.47354

903.07108	0.47355

903.16786	0.47357

903.26464	0.47366

903.36143	0.47368

903.45821	0.47381

903.55499	0.47383

903.65177	0.47387

903.74855	0.4739

903.84533	0.47397

903.94212	0.47409

904.0389	0.4741

904.13568	0.47412

904.23246	0.47413

904.32924	0.47415

904.42603	0.47441

904.52281	0.47445

904.61959	0.47453

904.71637	0.47454

904.81315	0.47459

904.90993	0.47471

905.00672	0.47476

905.1035	0.47486

905.20028	0.47488

905.29706	0.47502

905.39384	0.47511

905.49063	0.47512

905.58741	0.47514

905.68419	0.47517

905.78097	0.47518

905.87775	0.4752

905.97453	0.47537

906.07132	0.47541

906.1681	0.4755

906.26488	0.47552

906.36166	0.47554

906.45844	0.47567

906.55523	0.47569

906.65201	0.47581

906.74879	0.47584

906.84557	0.47593

906.94235	0.47596

907.03913	0.47598

907.13592	0.47601

907.2327	0.47607

907.32948	0.47633

907.42626	0.47634

907.52304	0.47637

907.61983	0.47653

907.71661	0.47654

907.81339	0.47663

907.91017	0.47666

908.00695	0.47668

908.10373	0.47674

908.20052	0.47675

908.2973	0.47677

908.39408	0.47691

908.49086	0.47692

908.58764	0.47703

908.68443	0.47704

908.78121	0.47706

908.87799	0.47707

908.97477	0.47739

909.07155	0.47741

909.16833	0.47744

909.26512	0.47756

909.3619	0.47758

909.45868	0.47764

909.55546	0.47765

909.65224	0.47767

909.74903	0.47768

909.84581	0.47785

909.94259	0.47787

910.03937	0.47788

910.13615	0.4779

910.23293	0.47794

910.32972	0.47799

910.4265	0.478

910.52328	0.47825

910.62006	0.47834

910.71684	0.47839

910.81363	0.47866

910.91041	0.47867

911.00719	0.47869

911.10397	0.47886

911.20075	0.47887

911.29753	0.47889

911.39432	0.47904

911.4911	0.47906

911.58788	0.47909

911.68466	0.4791

911.78144	0.47912

911.87823	0.47913

911.97501	0.47916

912.07179	0.47928

912.16857	0.4793

912.26535	0.47942

912.36213	0.47945

912.45892	0.47947

912.5557	0.47948

912.65248	0.47951

912.74926	0.47963

912.84604	0.47977

912.94283	0.4798

913.03961	0.47989

913.13639	0.47992

913.23317	0.47994

913.32995	0.48

913.42673	0.48029

913.52352	0.48037

913.6203	0.48041

913.71708	0.48052

913.81386	0.48053

913.91064	0.48072

914.00743	0.48073

914.10421	0.48075

914.20099	0.48078

914.29777	0.48084

914.39455	0.48085

914.49133	0.48093

914.58812	0.48101

914.6849	0.48102

914.78168	0.48122

914.87846	0.48123

914.97524	0.48125

915.07203	0.48128

915.16881	0.48133

915.26559	0.48134

915.36237	0.48181

915.45915	0.48183

915.55593	0.48184

915.65272	0.48192

915.7495	0.48197

915.84628	0.48204

915.94306	0.48206

916.03984	0.48216

916.13662	0.48218

916.23341	0.48229

916.33019	0.4823

916.42697	0.48247

916.52375	0.48248

916.62053	0.48251

916.71732	0.48253

916.8141	0.48255

916.91088	0.48256

917.00766	0.48271

917.10444	0.48273

917.20122	0.48274

917.29801	0.48277

917.39479	0.48279

917.49157	0.48282

917.58835	0.48283

917.68513	0.48317

917.78192	0.48329

917.8787	0.48331

917.97548	0.48343

918.07226	0.48369

918.16904	0.48389

918.26582	0.48404

918.36261	0.48405

918.45939	0.48407

918.55617	0.48415

918.65295	0.48419

918.74973	0.48421

918.84652	0.48422

918.9433	0.48424

919.04008	0.48425

919.13686	0.48428

919.23364	0.48439

919.33042	0.48445

919.42721	0.48447

919.52399	0.4845

919.62077	0.48456

919.71755	0.48463

919.81433	0.48466

919.91112	0.48477

920.0079	0.48479

920.10468	0.4848

920.20146	0.48501

920.29824	0.48503

920.39502	0.48517

920.49181	0.48518

920.58859	0.4852

920.68537	0.48533

920.78215	0.48552

920.87893	0.48553

920.97572	0.48561

921.0725	0.48564

921.16928	0.48576

921.26606	0.48579

921.36284	0.48582

921.45962	0.48605

921.55641	0.48607

921.65319	0.4861

921.74997	0.48611

921.84675	0.48625

921.94353	0.48645

922.04032	0.48649

922.1371	0.48651

922.23388	0.48675

922.33066	0.48683

922.42744	0.48684

922.52422	0.48719

922.62101	0.48722

922.71779	0.48728

922.81457	0.48739

922.91135	0.48742

923.00813	0.48744

923.10492	0.48751

923.2017	0.48754

923.29848	0.48771

923.39526	0.48779

923.49204	0.48788

923.58882	0.48789

923.68561	0.48791

923.78239	0.48794

923.87917	0.48803

923.97595	0.48809

924.07273	0.48814

924.16952	0.48815

924.2663	0.48846

924.36308	0.4887

924.45986	0.48872

924.55664	0.48892

924.65342	0.48893

924.75021	0.48898

924.84699	0.48905

924.94377	0.48908

925.04055	0.48911

925.13733	0.48913

925.23412	0.48917

925.3309	0.48924

925.42768	0.48927

925.52446	0.48931

925.62124	0.4896

925.71802	0.48975

925.81481	0.4898

925.91159	0.48981

926.00837	0.48994

926.10515	0.49

926.20193	0.49006

926.29872	0.49023

926.3955	0.49024

926.49228	0.49026

926.58906	0.49038

926.68584	0.49047

926.78262	0.49053

926.87941	0.4907

926.97619	0.49073

927.07297	0.49088

927.16975	0.4909

927.26653	0.49099

927.36331	0.49102

927.4601	0.49103

927.55688	0.4912

927.65366	0.49129

927.75044	0.49137

927.84722	0.49144

927.94401	0.49158

928.04079	0.4916

928.13757	0.4917

928.23435	0.49172

928.33113	0.49178

928.42791	0.49181

928.5247	0.49195

928.62148	0.49201

928.71826	0.49204

928.81504	0.49205

928.91182	0.49208

929.00861	0.49222

929.10539	0.49225

929.20217	0.49234

929.29895	0.49244

929.39573	0.49247

929.49251	0.4925

929.5893	0.49251

929.68608	0.49271

929.78286	0.49276

929.87964	0.49292

929.97642	0.49306

930.07321	0.49308

930.16999	0.49314

930.26677	0.49315

930.36355	0.4932

930.46033	0.49321

930.55711	0.49323

930.6539	0.4933

930.75068	0.49336

930.84746	0.49355

930.94424	0.49358

931.04102	0.49364

931.13781	0.49367

931.23459	0.49368

931.33137	0.4937

931.42815	0.49372

931.52493	0.49385

931.62171	0.49399

931.7185	0.49405

931.81528	0.49463

931.91206	0.49464

932.00884	0.49484

932.10562	0.49486

932.20241	0.49509

932.29919	0.4951

932.39597	0.49512

932.49275	0.49515

932.58953	0.49538

932.68631	0.49539

932.7831	0.49557

932.87988	0.49571

932.97666	0.49574

933.07344	0.49576

933.17022	0.49602

933.26701	0.4962

933.36379	0.49623

933.46057	0.49632

933.55735	0.49634

933.65413	0.49635

933.75091	0.49644

933.8477	0.49666

933.94448	0.4967

934.04126	0.49673

934.13804	0.49692

934.23482	0.49693

934.33161	0.49695

934.42839	0.49704

934.52517	0.49705

934.62195	0.49707

934.71873	0.49708

934.81551	0.49711

934.9123	0.49713

935.00908	0.49717

935.10586	0.49719

935.20264	0.49722

935.29942	0.49725

935.39621	0.49728

935.49299	0.49757

935.58977	0.49759

935.68655	0.49817

935.78333	0.49818

935.88011	0.4982

935.9769	0.49849

936.07368	0.49852

936.17046	0.49864

936.26724	0.49865

936.36402	0.49881

936.46081	0.49882

936.55759	0.49884

936.65437	0.4989

936.75115	0.49894

936.84793	0.49931

936.94471	0.49932

937.0415	0.49957

937.13828	0.49958

937.23506	0.49973

937.33184	0.49975

937.42862	0.49981

937.52541	0.49983

937.62219	0.49992

937.71897	0.49999

937.81575	0.50001

937.91253	0.50027

938.00931	0.50028

938.1061	0.50042

938.20288	0.50044

938.29966	0.5005

938.39644	0.50059

938.49322	0.50065

938.59001	0.5008

938.68679	0.50082

938.78357	0.50083

938.88035	0.50085

938.97713	0.50088

939.07391	0.50091

939.1707	0.50117

939.26748	0.5012

939.36426	0.50132

939.46104	0.50133

939.55782	0.50135

939.65461	0.50138

939.75139	0.50156

939.84817	0.50162

939.94495	0.50167

940.04173	0.50188

940.13851	0.5019

940.2353	0.50204

940.33208	0.50205

940.42886	0.50207

940.52564	0.5022

940.62242	0.50222

940.7192	0.50228

940.81599	0.50231

940.91277	0.50239

941.00955	0.50258

941.10633	0.50261

941.20311	0.50278

941.2999	0.50283

941.39668	0.50303

941.49346	0.50307

941.59024	0.5033

941.68702	0.50332

941.7838	0.50336

941.88059	0.50341

941.97737	0.50342

942.07415	0.50353

942.17093	0.50362

942.26771	0.50364

942.3645	0.50377

942.46128	0.50386

942.55806	0.50388

942.65484	0.50396

942.75162	0.50397

942.8484	0.50411

942.94519	0.50414

943.04197	0.50429

943.13875	0.50431

943.23553	0.50441

943.33231	0.50455

943.4291	0.50478

943.52588	0.50479

943.62266	0.50482

943.71944	0.50484

943.81622	0.50505

943.913	0.50525

944.00979	0.50542

944.10657	0.50545

944.20335	0.50546

944.30013	0.50548

944.39691	0.5056

944.4937	0.50563

944.59048	0.50565

944.68726	0.50572

944.78404	0.50575

944.88082	0.50577

944.9776	0.5058

945.07439	0.50604

945.17117	0.50606

945.26795	0.5062

945.36473	0.50621

945.46151	0.50623

945.5583	0.50624

945.65508	0.50632

945.75186	0.50635

945.84864	0.50636

945.94542	0.50647

946.0422	0.5065

946.13899	0.50653

946.23577	0.50656

946.33255	0.50667

946.42933	0.50668

946.52611	0.50694

946.6229	0.50696

946.71968	0.50697

946.81646	0.50703

946.91324	0.50706

947.01002	0.50709

947.1068	0.50734

947.20359	0.50737

947.30037	0.50745

947.39715	0.50746

947.49393	0.50752

947.59071	0.50764

947.6875	0.50766

947.78428	0.50767

947.88106	0.50773

947.97784	0.50775

948.07462	0.50777

948.1714	0.50781

948.26819	0.50784

948.36497	0.50824

948.46175	0.50825

948.55853	0.50842

948.65531	0.50844

948.7521	0.50845

948.84888	0.50859

948.94566	0.50865

949.04244	0.50869

949.13922	0.50885

949.236	0.50888

949.33279	0.50889

949.42957	0.50891

949.52635	0.50908

949.62313	0.50912

949.71991	0.50921

949.8167	0.50926

949.91348	0.50927

950.01026	0.50944

950.10704	0.50946

950.20382	0.50962

950.3006	0.50969

950.39739	0.50975

950.49417	0.50999

950.59095	0.51001

950.68773	0.51005

950.78451	0.51013

950.8813	0.51016

950.97808	0.51019

951.07486	0.51023

951.17164	0.51026

951.26842	0.51031

951.3652	0.51033

951.46199	0.51034

951.55877	0.51039

951.65555	0.51042

951.75233	0.51046

951.84911	0.51052

951.9459	0.51057

952.04268	0.5106

952.13946	0.51089

952.23624	0.51092

952.33302	0.51104

952.4298	0.51119

952.52659	0.51133

952.62337	0.51135

952.72015	0.51138

952.81693	0.51139

952.91371	0.51148

953.01049	0.5115

953.10728	0.51151

953.20406	0.51153

953.30084	0.51185

953.39762	0.51188

953.4944	0.51229

953.59119	0.51231

953.68797	0.51235

953.78475	0.51241

953.88153	0.51244

953.97831	0.51247

954.07509	0.51261

954.17188	0.51264

954.26866	0.51267

954.36544	0.51269

954.46222	0.51287

954.559	0.5129

954.65579	0.51292

954.75257	0.51296

954.84935	0.5131

954.94613	0.51311

955.04291	0.51313

955.13969	0.51321

955.23648	0.51324

955.33326	0.51325

955.43004	0.51328

955.52682	0.5133

955.6236	0.51334

955.72039	0.51351

955.81717	0.51356

955.91395	0.51357

956.01073	0.51359

956.10751	0.51372

956.20429	0.51374

956.30108	0.51375

956.39786	0.51409

956.49464	0.5141

956.59142	0.5143

956.6882	0.51432

956.78499	0.51458

956.88177	0.51467

956.97855	0.5147

957.07533	0.51471

957.17211	0.51488

957.26889	0.5149

957.36568	0.51506

957.46246	0.51513

957.55924	0.51519

957.65602	0.51543

957.7528	0.51545

957.84959	0.51549

957.94637	0.51557

958.04315	0.5156

958.13993	0.51563

958.23671	0.51567

958.33349	0.5157

958.43028	0.51575

958.52706	0.51577

958.62384	0.51578

958.72062	0.51583

958.8174	0.51586

958.91419	0.5159

959.01097	0.51596

959.10775	0.51601

959.20453	0.51604

959.30131	0.51633

959.39809	0.51636

959.49488	0.51648

959.59166	0.51663

959.68844	0.51677

959.78522	0.51679

959.882	0.51682

959.97879	0.51683

960.07557	0.51692

960.17235	0.51694

960.26913	0.51695

960.36591	0.51697

960.46269	0.51729

960.55948	0.51732

960.65626	0.51773

960.75304	0.51775

960.84982	0.51779

960.9466	0.51785

961.04339	0.51788

961.14017	0.51791

961.23695	0.51805

961.33373	0.51808

961.43051	0.51811

961.52729	0.51813

961.62408	0.51831

961.72086	0.51834

961.81764	0.51836

961.91442	0.5184

962.0112	0.51854

962.10799	0.51855

962.20477	0.51857

962.30155	0.51865

962.39833	0.51868

962.49511	0.51869

962.59189	0.51872

962.68868	0.51874

962.78546	0.51878

962.88224	0.51895

962.97902	0.519

963.0758	0.51901

963.17259	0.51903

963.26937	0.51916

963.36615	0.51918

963.46293	0.51919

963.55971	0.51953

963.65649	0.51954

963.75328	0.51974

963.85006	0.51976

963.94684	0.52002

964.04362	0.52005

964.1404	0.52008

964.23719	0.52014

964.33397	0.52015

964.43075	0.52023

964.52753	0.52028

964.62431	0.52075

964.72109	0.52076

964.81788	0.52113

964.91466	0.52134

965.01144	0.52139

965.10822	0.5214

965.205	0.52142

965.30179	0.52147

965.39857	0.52168

965.49535	0.52207

965.59213	0.52209

965.68891	0.52236

965.78569	0.52249

965.88248	0.5225

965.97926	0.52252

966.07604	0.52253

966.17282	0.52282

966.2696	0.52284

966.36639	0.52285

966.46317	0.523

966.55995	0.52305

966.65673	0.52307

966.75351	0.52313

966.85029	0.52317

966.94708	0.52319

967.04386	0.5232

967.14064	0.52322

967.23742	0.52325

967.3342	0.52349

967.43098	0.52351

967.52777	0.52352

967.62455	0.52355

967.72133	0.52393

967.81811	0.52395

967.91489	0.52415

968.01168	0.52416

968.10846	0.52435

968.20524	0.52438

968.30202	0.52439

968.3988	0.52441

968.49558	0.52471

968.59237	0.52474

968.68915	0.52479

968.78593	0.5248

968.88271	0.52482

968.97949	0.52483

969.07628	0.52534

969.17306	0.52535

969.26984	0.52549

969.36662	0.52572

969.4634	0.52584

969.56018	0.52587

969.65697	0.52588

969.75375	0.52611

969.85053	0.52617

969.94731	0.52619

970.04409	0.52652

970.14088	0.52654

970.23766	0.52672

970.33444	0.52706

970.43122	0.52761

970.528	0.52764

970.62478	0.52783

970.72157	0.52796

970.81835	0.52799

970.91513	0.528

971.01191	0.52837

971.10869	0.52878

971.20548	0.52881

971.30226	0.52924

971.39904	0.52925

971.49582	0.52939

971.5926	0.52951

971.68938	0.52953

971.78617	0.52994

971.88295	0.53

971.97973	0.53011

972.07651	0.53012

972.17329	0.53021

972.27008	0.53026

972.36686	0.53062

972.46364	0.53065

972.56042	0.53091

972.6572	0.53093

972.75398	0.53094

972.85077	0.53129

972.94755	0.53134

973.04433	0.53136

973.14111	0.53137

973.23789	0.5314

973.33468	0.53164

973.43146	0.53166

973.52824	0.53171

973.62502	0.53172

973.7218	0.53174

973.81858	0.53175

973.91537	0.53184

974.01215	0.53207

974.10893	0.53209

974.20571	0.5321

974.30249	0.53212

974.39928	0.53213

974.49606	0.53215

974.59284	0.53244

974.68962	0.53257

974.7864	0.53259

974.88318	0.53279

974.97997	0.53283

975.07675	0.53285

975.17353	0.53309

975.27031	0.53311

975.36709	0.53312

975.46388	0.5332

975.56066	0.53331

975.65744	0.53332

975.75422	0.53334

975.851	0.53356

975.94778	0.53361

976.04457	0.53399

976.14135	0.53401

976.23813	0.53402

976.33491	0.53407

976.43169	0.53425

976.52848	0.53431

976.62526	0.53466

976.72204	0.53468

976.81882	0.53469

976.9156	0.535

977.01238	0.53545

977.10917	0.53556

977.20595	0.53558

977.30273	0.53561

977.39951	0.53562

977.49629	0.53577

977.59308	0.53579

977.68986	0.5358

977.78664	0.53626

977.88342	0.53628

977.9802	0.53658

978.07698	0.5366

978.17377	0.53661

978.27055	0.5367

978.36733	0.53686

978.46411	0.53692

978.56089	0.53693

978.65768	0.53712

978.75446	0.53713

978.85124	0.53739

978.94802	0.53742

979.0448	0.53744

979.14158	0.53768

979.23837	0.53771

979.33515	0.53772

979.43193	0.53789

979.52871	0.53791

979.62549	0.53792

979.72227	0.53818

979.81906	0.53821

979.91584	0.53824

980.01262	0.53879

980.1094	0.53881

980.20618	0.53882

980.30297	0.53916

980.39975	0.53917

980.49653	0.53919

980.59331	0.53922

980.69009	0.53958

980.78687	0.53961

980.88366	0.53975

980.98044	0.53978

981.07722	0.5398

981.174	0.53981

981.27078	0.54007

981.36757	0.54009

981.46435	0.54012

981.56113	0.54032

981.65791	0.54039

981.75469	0.54042

981.85147	0.5408

981.94826	0.54082

982.04504	0.54102

982.14182	0.54105

982.2386	0.54111

982.33538	0.54112

982.43217	0.54117

982.52895	0.54123

982.62573	0.54124

982.72251	0.54134

982.81929	0.54138

982.91607	0.5415

983.01286	0.54153

983.10964	0.54155

983.20642	0.54158

983.3032	0.5416

983.39998	0.54166

983.49677	0.54169

983.59355	0.54172

983.69033	0.54208

983.78711	0.5421

983.88389	0.54224

983.98067	0.54225

984.07746	0.54227

984.17424	0.54233

984.27102	0.54239

984.3678	0.5424

984.46458	0.54243

984.56137	0.54249

984.65815	0.54265

984.75493	0.54266

984.85171	0.54269

984.94849	0.54278

985.04527	0.54318

985.14206	0.5432

985.23884	0.54364

985.33562	0.54365

985.4324	0.54367

985.52918	0.5437

985.62597	0.54384

985.72275	0.54385

985.81953	0.54387

985.91631	0.54388

986.01309	0.54391

986.10987	0.54414

986.20666	0.5442

986.30344	0.54422

986.40022	0.54483

986.497	0.54487

986.59378	0.54489

986.69057	0.54513

986.78735	0.54515

986.88413	0.54516

986.98091	0.54518

987.07769	0.54531

987.17447	0.54533

987.27126	0.54534

987.36804	0.54536

987.46482	0.54537

987.5616	0.54559

987.65838	0.54591

987.75517	0.54595

987.85195	0.54611

987.94873	0.54614

988.04551	0.54615

988.14229	0.54617

988.23907	0.54629

988.33586	0.54632

988.43264	0.54635

988.52942	0.54637

988.6262	0.54662

988.72298	0.54664

988.81977	0.54667

988.91655	0.54688

989.01333	0.5469

989.11011	0.54696

989.20689	0.54699

989.30367	0.54701

989.40046	0.54871

989.49724	0.54873

989.59402	0.54874

989.6908	0.54876

989.78758	0.549

989.88437	0.54935

989.98115	0.54941

990.07793	0.54943

990.17471	0.54966

990.27149	0.54969

990.36827	0.54975

990.46506	0.55007

990.56184	0.55008

990.65862	0.55025

990.7554	0.55027

990.85218	0.5503

990.94897	0.55046

991.04575	0.55051

991.14253	0.55078

991.23931	0.5508

991.33609	0.55081

991.43287	0.55121

991.52966	0.55126

991.62644	0.55141

991.72322	0.5515

991.82	0.55165

991.91678	0.55167

992.01356	0.55216

992.11035	0.55232

992.20713	0.55234

992.30391	0.5528

992.40069	0.55281

992.49747	0.55286

992.59426	0.55295

992.69104	0.55345

992.78782	0.55347

992.8846	0.55348

992.98138	0.55536

993.07816	0.55537

993.17495	0.55558

993.27173	0.55562

993.36851	0.5558

993.46529	0.55581

993.56207	0.55609

993.65886	0.5561

993.75564	0.55612

993.85242	0.55613

993.9492	0.55644

994.04598	0.55645

994.14276	0.55647

994.23955	0.55673

994.33633	0.55674

994.43311	0.55677

994.52989	0.55682

994.62667	0.55683

994.72346	0.55686

994.82024	0.55722

994.91702	0.55723

995.0138	0.55752

995.11058	0.55754

995.20736	0.55757

995.30415	0.55775

995.40093	0.55776

995.49771	0.55782

995.59449	0.55798

995.69127	0.55833

995.78806	0.55834

995.88484	0.55859

995.98162	0.55862

996.0784	0.55898

996.17518	0.55901

996.27196	0.55933

996.36875	0.55935

996.46553	0.55956

996.56231	0.55967

996.65909	0.5597

996.75587	0.56008

996.85266	0.56011

996.94944	0.56037

997.04622	0.5604

997.143	0.56042

997.23978	0.56081

997.33656	0.56084

997.43335	0.56102

997.53013	0.56104

997.62691	0.56116

997.72369	0.56119

997.82047	0.56229

997.91726	0.56235

998.01404	0.56237

998.11082	0.56247

998.2076	0.56258

998.30438	0.56264

998.40116	0.56278

998.49795	0.56281

998.59473	0.56285

998.69151	0.56287

998.78829	0.56293

998.88507	0.5632

998.98186	0.56342

999.07864	0.56349

999.17542	0.5636

999.2722	0.56368

999.36898	0.56389

999.46576	0.56407

999.56255	0.56409

999.65933	0.5641

999.75611	0.56429

999.85289	0.5643

999.94967	0.56432

1000.04646	0.56453

1000.14324	0.56455

1000.24002	0.56456

1000.3368	0.56458

1000.43358	0.56506

1000.53036	0.56508

1000.62715	0.56538

1000.72393	0.5654

1000.82071	0.56551

1000.91749	0.56558

1001.01427	0.5656

1001.11106	0.56563

1001.20784	0.56564

1001.30462	0.56579

1001.4014	0.56608

1001.49818	0.5661

1001.59496	0.56636

1001.69175	0.56637

1001.78853	0.56659

1001.88531	0.5666

1001.98209	0.56662

1002.07887	0.56665

1002.17566	0.56677

1002.27244	0.56685

1002.36922	0.56686

1002.466	0.56689

1002.56278	0.56692

1002.65956	0.56721

1002.75635	0.56729

1002.85313	0.56732

1002.94991	0.56733

1003.04669	0.56735

1003.14347	0.56738

1003.24026	0.56747

1003.33704	0.5675

1003.43382	0.56752

1003.5306	0.56753

1003.62738	0.56756

1003.72416	0.56767

1003.82095	0.5677

1003.91773	0.56778

1004.01451	0.56779

1004.11129	0.56781

1004.20807	0.56831

1004.30486	0.56843

1004.40164	0.56846

1004.49842	0.56877

1004.5952	0.56883

1004.69198	0.56884

1004.78876	0.56925

1004.88555	0.56927

1004.98233	0.56928

1005.07911	0.56967

1005.17589	0.57055

1005.27267	0.57056

1005.36946	0.57059

1005.46624	0.57069

1005.56302	0.57073

1005.6598	0.57101

1005.75658	0.57102

1005.85336	0.57136

1005.95015	0.5714

1006.04693	0.57142

1006.14371	0.57145

1006.24049	0.57151

1006.33727	0.57155

1006.43405	0.57183

1006.53084	0.57184

1006.62762	0.57186

1006.7244	0.57194

1006.82118	0.57201

1006.91796	0.57204

1007.01475	0.57215

1007.11153	0.57219

1007.20831	0.57223

1007.30509	0.57226

1007.40187	0.57256

1007.49866	0.57258

1007.59544	0.57265

1007.69222	0.57288

1007.789	0.57291

1007.88578	0.57293

1007.98256	0.57294

1008.07935	0.57341

1008.17613	0.57343

1008.27291	0.57351

1008.36969	0.57357

1008.46647	0.57358

1008.56326	0.57364

1008.66004	0.57383

1008.75682	0.57384

1008.8536	0.57425

1008.95038	0.57427

1009.04716	0.57437

1009.14395	0.57439

1009.24073	0.5744

1009.33751	0.5745

1009.43429	0.57463

1009.53107	0.57466

1009.62786	0.57469

1009.72464	0.57475

1009.82142	0.57492

1009.9182	0.57497

1010.01498	0.575

1010.11176	0.57504

1010.20855	0.57515

1010.30533	0.57517

1010.40211	0.57553

1010.49889	0.57555

1010.59567	0.57558

1010.69246	0.57572

1010.78924	0.57573

1010.88602	0.57576

1010.9828	0.576

1011.07958	0.5761

1011.17636	0.57611

1011.27315	0.57637

1011.36993	0.57645

1011.46671	0.57669

1011.56349	0.57672

1011.66027	0.57674

1011.75705	0.5773

1011.85384	0.57732

1011.95062	0.57733

1012.0474	0.57735

1012.14418	0.57765

1012.24096	0.57767

1012.33775	0.57783

1012.43453	0.57786

1012.53131	0.57821

1012.62809	0.57823

1012.72487	0.57824

1012.82165	0.57828

1012.91844	0.57867

1013.01522	0.5787

1013.112	0.57872

1013.20878	0.57873

1013.30556	0.57875

1013.40235	0.57884

1013.49913	0.57893

1013.59591	0.57896

1013.69269	0.57946

1013.78947	0.57949

1013.88625	0.57975

1013.98304	0.57977

1014.07982	0.57995

1014.1766	0.58001

1014.27338	0.58003

1014.37016	0.58033

1014.46695	0.58036

1014.56373	0.58038

1014.66051	0.58056

1014.75729	0.58061

1014.85407	0.58064

1014.95085	0.58084

1015.04764	0.58085

1015.14442	0.58088

1015.2412	0.58097

1015.33798	0.58108

1015.43476	0.58111

1015.53155	0.58116

1015.62833	0.58117

1015.72511	0.58141

1015.82189	0.58143

1015.91867	0.58151

1016.01545	0.58154

1016.11224	0.58155

1016.20902	0.58175

1016.3058	0.58176

1016.40258	0.58178

1016.49936	0.5818

1016.59615	0.58199

1016.69293	0.58201

1016.78971	0.58228

1016.88649	0.5823

1016.98327	0.58266

1017.08005	0.58269

1017.17684	0.58271

1017.27362	0.58274

1017.3704	0.58309

1017.46718	0.58317

1017.56396	0.58336

1017.66075	0.58365

1017.75753	0.58399

1017.85431	0.58407

1017.95109	0.58451

1018.04787	0.58452

1018.14465	0.58454

1018.24144	0.58455

1018.33822	0.58457

1018.435	0.58475

1018.53178	0.58483

1018.62856	0.58496

1018.72534	0.58521

1018.82213	0.58524

1018.91891	0.58547

1019.01569	0.58551

1019.11247	0.58565

1019.20925	0.58568

1019.30604	0.58571

1019.40282	0.58574

1019.4996	0.58576

1019.59638	0.5858

1019.69316	0.58586

1019.78995	0.58588

1019.88673	0.58609

1019.98351	0.58612

1020.08029	0.58637

1020.17707	0.5864

1020.27385	0.58652

1020.37064	0.58679

1020.46742	0.58682

1020.5642	0.58684

1020.66098	0.58685

1020.75776	0.58746

1020.85455	0.58748

1020.95133	0.58772

1021.04811	0.58774

1021.14489	0.58775

1021.24167	0.58777

1021.33845	0.58785

1021.43524	0.58791

1021.53202	0.58792

1021.6288	0.58798

1021.72558	0.58801

1021.82236	0.58833

1021.91915	0.58835

1022.01593	0.58838

1022.11271	0.58855

1022.20949	0.58868

1022.30627	0.58893

1022.40305	0.58894

1022.49984	0.58899

1022.59662	0.58908

1022.6934	0.58911

1022.79018	0.58913

1022.88696	0.58938

1022.98375	0.5894

1023.08053	0.58961

1023.17731	0.58964

1023.27409	0.58966

1023.37087	0.5898

1023.46765	0.58983

1023.56444	0.58984

1023.66122	0.59002

1023.758	0.59015

1023.85478	0.59042

1023.95156	0.59044

1024.04834	0.59066

1024.14513	0.59071

1024.24191	0.59073

1024.33869	0.59076

1024.43547	0.59079

1024.53225	0.59091

1024.62903	0.59092

1024.72582	0.59095

1024.8226	0.5913

1024.91938	0.59133

1025.01616	0.59135

1025.11294	0.59152

1025.20973	0.59167

1025.30651	0.59176

1025.40329	0.59178

1025.50007	0.5919

1025.59685	0.59193

1025.69364	0.59207

1025.79042	0.59226

1025.8872	0.59229

1025.98398	0.59233

1026.08076	0.59236

1026.17754	0.59265

1026.27433	0.59268

1026.37111	0.59281

1026.46789	0.59286

1026.56467	0.59287

1026.66145	0.59301

1026.75824	0.59312

1026.85502	0.59315

1026.9518	0.59322

1027.04858	0.59353

1027.14536	0.59354

1027.24214	0.59379

1027.33893	0.59397

1027.43571	0.59403

1027.53249	0.59414

1027.62927	0.59429

1027.72605	0.59431

1027.82283	0.59437

1027.91962	0.59446

1028.0164	0.59447

1028.11318	0.59458

1028.20996	0.59467

1028.30674	0.59478

1028.40353	0.59481

1028.50031	0.59516

1028.59709	0.59519

1028.69387	0.59522

1028.79065	0.59524

1028.88744	0.59525

1028.98422	0.59557

1029.081	0.59562

1029.17778	0.59563

1029.27456	0.59565

1029.37134	0.59585

1029.46813	0.59586

1029.56491	0.59588

1029.66169	0.59606

1029.75847	0.59607

1029.85525	0.5961

1029.95204	0.59629

1030.04882	0.59632

1030.1456	0.59662

1030.24238	0.59665

1030.33916	0.59667

1030.43594	0.59679

1030.53273	0.59681

1030.62951	0.59682

1030.72629	0.59684

1030.82307	0.59691

1030.91985	0.59694

1031.01664	0.59699

1031.11342	0.597

1031.2102	0.59723

1031.30698	0.59728

1031.40376	0.59731

1031.50054	0.59748

1031.59733	0.59789

1031.69411	0.59825

1031.79089	0.59828

1031.88767	0.5987

1031.98445	0.59882

1032.08124	0.59909

1032.17802	0.59934

1032.2748	0.59935

1032.37158	0.59938

1032.46836	0.59941

1032.56514	0.59949

1032.66193	0.59953

1032.75871	0.59955

1032.85549	0.5997

1032.95227	0.59973

1033.04905	0.59976

1033.14584	0.59978

1033.24262	0.60011

1033.3394	0.60014

1033.43618	0.60016

1033.53296	0.60022

1033.62974	0.60025

1033.72653	0.60028

1033.82331	0.60033

1033.92009	0.60034

1034.01687	0.60062

1034.11365	0.60066

1034.21044	0.60068

1034.30722	0.60075

1034.404	0.60078

1034.50078	0.60112

1034.59756	0.60113

1034.69434	0.60115

1034.79113	0.60116

1034.88791	0.60124

1034.98469	0.60133

1035.08147	0.60142

1035.17825	0.60168

1035.27503	0.60171

1035.37182	0.60174

1035.4686	0.60193

1035.56538	0.60196

1035.66216	0.60197

1035.75894	0.60232

1035.85572	0.6024

1035.95251	0.60257

1036.04929	0.60258

1036.14607	0.60267

1036.24285	0.60273

1036.33963	0.60289

1036.43642	0.6029

1036.5332	0.60337

1036.62998	0.6036

1036.72676	0.60362

1036.82354	0.60363

1036.92033	0.60397

1037.01711	0.60398

1037.11389	0.60421

1037.21067	0.60423

1037.30745	0.60439

1037.40423	0.6047

1037.50102	0.60474

1037.5978	0.60476

1037.69458	0.60478

1037.79136	0.60482

1037.88814	0.60496

1037.98493	0.60528

1038.08171	0.60529

1038.17849	0.60558

1038.27527	0.60561

1038.37205	0.60586

1038.46883	0.60589

1038.56562	0.60599

1038.6624	0.60606

1038.75918	0.60607

1038.85596	0.60625

1038.95274	0.60627

1039.04952	0.60648

1039.14631	0.6065

1039.24309	0.60651

1039.33987	0.60653

1039.43665	0.60654

1039.53343	0.60676

1039.63022	0.60677

1039.727	0.60679

1039.82378	0.60702

1039.92056	0.60709

1040.01734	0.60712

1040.11413	0.60729

1040.21091	0.60749

1040.30769	0.60761

1040.40447	0.60805

1040.50125	0.60807

1040.59803	0.60808

1040.69482	0.60811

1040.7916	0.60833

1040.88838	0.60837

1040.98516	0.60868

1041.08194	0.60869

1041.17873	0.60898

1041.27551	0.60903

1041.37229	0.60906

1041.46907	0.60907

1041.56585	0.60912

1041.66263	0.60932

1041.75942	0.60933

1041.8562	0.60956

1041.95298	0.60958

1042.04976	0.61005

1042.14654	0.61006

1042.24332	0.61037

1042.34011	0.61076

1042.43689	0.61078

1042.53367	0.61079

1042.63045	0.61081

1042.72723	0.61098

1042.82402	0.61147

1042.9208	0.6115

1043.01758	0.61177

1043.11436	0.61212

1043.21114	0.61218

1043.30793	0.6123

1043.40471	0.61233

1043.50149	0.61256

1043.59827	0.61258

1043.69505	0.61259

1043.79183	0.61262

1043.88862	0.61293

1043.9854	0.61294

1044.08218	0.61345

1044.17896	0.61346

1044.27574	0.61348

1044.37253	0.61351

1044.46931	0.61357

1044.56609	0.61363

1044.66287	0.61364

1044.75965	0.61369

1044.85643	0.61409

1044.95322	0.61415

1045.05	0.61416

1045.14678	0.61436

1045.24356	0.61438

1045.34034	0.6147

1045.43712	0.61471

1045.53391	0.61497

1045.63069	0.615

1045.72747	0.61502

1045.82425	0.61524

1045.92103	0.61559

1046.01782	0.61561

1046.1146	0.61564

1046.21138	0.61579

1046.30816	0.61631

1046.40494	0.61633

1046.50173	0.61651

1046.59851	0.6166

1046.69529	0.61747

1046.79207	0.61765

1046.88885	0.61783

1046.98563	0.61785

1047.08242	0.61791

1047.1792	0.618

1047.27598	0.61802

1047.37276	0.61803

1047.46954	0.6182

1047.56633	0.61844

1047.66311	0.61846

1047.75989	0.61883

1047.85667	0.61886

1047.95345	0.61887

1048.05023	0.61931

1048.14702	0.61933

1048.2438	0.61951

1048.34058	0.61953

1048.43736	0.61957

1048.53414	0.6196

1048.63092	0.61962

1048.72771	0.61986

1048.82449	0.61991

1048.92127	0.61994

1049.01805	0.62026

1049.11483	0.62033

1049.21161	0.62035

1049.3084	0.62068

1049.40518	0.6207

1049.50196	0.62105

1049.59874	0.62107

1049.69552	0.62139

1049.79231	0.6214

1049.88909	0.62142

1049.98587	0.62177

1050.08265	0.6218

1050.17943	0.62228

1050.27622	0.62232

1050.373	0.62233

1050.46978	0.62253

1050.56656	0.62254

1050.66334	0.62256

1050.76012	0.62288

1050.85691	0.62289

1050.95369	0.62305

1051.05047	0.62317

1051.14725	0.62318

1051.24403	0.62328

1051.34082	0.62331

1051.4376	0.62332

1051.53438	0.62334

1051.63116	0.62364

1051.72794	0.62392

1051.82472	0.62395

1051.92151	0.62398

1052.01829	0.62407

1052.11507	0.62414

1052.21185	0.62416

1052.30863	0.62424

1052.40541	0.62433

1052.5022	0.62434

1052.59898	0.62437

1052.69576	0.62446

1052.79254	0.62451

1052.88932	0.62454

1052.98611	0.62494

1053.08289	0.62523

1053.17967	0.62524

1053.27645	0.62526

1053.37323	0.6253

1053.47002	0.62548

1053.5668	0.6255

1053.66358	0.62552

1053.76036	0.62565

1053.85714	0.62623

1053.95392	0.62628

1054.05071	0.62667

1054.14749	0.62672

1054.24427	0.62705

1054.34105	0.62707

1054.43783	0.6271

1054.53462	0.62715

1054.6314	0.62747

1054.72818	0.6275

1054.82496	0.62774

1054.92174	0.62777

1055.01852	0.62779

1055.11531	0.62783

1055.21209	0.62791

1055.30887	0.62823

1055.40565	0.62827

1055.50243	0.62833

1055.59922	0.6284

1055.696	0.62841

1055.79278	0.62865

1055.88956	0.62867

1055.98634	0.62868

1056.08312	0.6287

1056.17991	0.62928

1056.27669	0.62929

1056.37347	0.62931

1056.47025	0.62936

1056.56703	0.62943

1056.66382	0.62945

1056.7606	0.62946

1056.85738	0.62948

1056.95416	0.62978

1057.05094	0.62984

1057.14772	0.63007

1057.24451	0.63009

1057.34129	0.63012

1057.43807	0.63036

1057.53485	0.63038

1057.63163	0.63039

1057.72842	0.63041

1057.8252	0.63053

1057.92198	0.63056

1058.01876	0.63059

1058.11554	0.63061

1058.21232	0.63079

1058.30911	0.63082

1058.40589	0.63097

1058.50267	0.63106

1058.59945	0.63108

1058.69623	0.63111

1058.79302	0.63112

1058.8898	0.63125

1058.98658	0.63128

1059.08336	0.63129

1059.18014	0.63144

1059.27692	0.63149

1059.37371	0.63153

1059.47049	0.63172

1059.56727	0.63187

1059.66405	0.63217

1059.76083	0.63219

1059.85761	0.63221

1059.9544	0.63248

1060.05118	0.63251

1060.14796	0.63253

1060.24474	0.63266

1060.34152	0.63274

1060.4383	0.63275

1060.53509	0.6328

1060.63187	0.63283

1060.72865	0.63286

1060.82543	0.63298

1060.92222	0.63303

1061.019	0.63312

1061.11578	0.63313

1061.21256	0.63317

1061.30934	0.63318

1061.40612	0.63361

1061.50291	0.63362

1061.59969	0.63364

1061.69647	0.63385

1061.79325	0.63387

1061.89003	0.63388

1061.98681	0.63413

1062.0836	0.63414

1062.18038	0.63419

1062.27716	0.63425

1062.37394	0.63428

1062.47072	0.63429

1062.56751	0.63448

1062.66429	0.63461

1062.76107	0.63501

1062.85785	0.63502

1062.95463	0.63505

1063.05141	0.63519

1063.1482	0.63551

1063.24498	0.63559

1063.34176	0.6356

1063.43854	0.63608

1063.53532	0.63612

1063.6321	0.63615

1063.72889	0.63618

1063.82567	0.6362

1063.92245	0.63621

1064.01923	0.6364

1064.11601	0.6365

1064.2128	0.63701

1064.30958	0.63702

1064.40636	0.63704

1064.50314	0.63737

1064.59992	0.63739

1064.69671	0.6374

1064.79349	0.63742

1064.89027	0.63749

1064.98705	0.63754

1065.08383	0.6379

1065.18061	0.63795

1065.2774	0.63798

1065.37418	0.63839

1065.47096	0.63888

1065.56774	0.63889

1065.66452	0.63893

1065.76131	0.63908

1065.85809	0.63911

1065.95487	0.63912

1066.05165	0.63944

1066.14843	0.63952

1066.24521	0.63953

1066.342	0.63978

1066.43878	0.63981

1066.53556	0.63982

1066.63234	0.63998

1066.72912	0.64004

1066.8259	0.64007

1066.92269	0.64008

1067.01947	0.6401

1067.11625	0.64046

1067.21303	0.64048

1067.30981	0.64088

1067.4066	0.64091

1067.50338	0.64092

1067.60016	0.64132

1067.69694	0.64133

1067.79372	0.64135

1067.89051	0.64146

1067.98729	0.6415

1068.08407	0.64152

1068.18085	0.64155

1068.27763	0.64181

1068.37441	0.64184

1068.4712	0.64187

1068.56798	0.6419

1068.66476	0.64203

1068.76154	0.64214

1068.85832	0.64219

1068.95511	0.64257

1069.05189	0.64258

1069.14867	0.64261

1069.24545	0.64283

1069.34223	0.64286

1069.43901	0.64298

1069.5358	0.64299

1069.63258	0.64301

1069.72936	0.64359

1069.82614	0.64391

1069.92292	0.64392

1070.0197	0.64394

1070.11649	0.64395

1070.21327	0.6442

1070.31005	0.64435

1070.40683	0.64444

1070.50361	0.6445

1070.6004	0.64455

1070.69718	0.64456

1070.79396	0.64458

1070.89074	0.64501

1070.98752	0.64505

1071.08431	0.64523

1071.18109	0.64536

1071.27787	0.64537

1071.37465	0.64539

1071.47143	0.64558

1071.56821	0.64566

1071.665	0.64571

1071.76178	0.64589

1071.85856	0.64597

1071.95534	0.64598

1072.05212	0.64639

1072.14891	0.64659

1072.24569	0.64662

1072.34247	0.64665

1072.43925	0.64697

1072.53603	0.647

1072.63281	0.64729

1072.7296	0.64731

1072.82638	0.64732

1072.92316	0.64735

1073.01994	0.64766

1073.11672	0.64767

1073.2135	0.64769

1073.31029	0.6477

1073.40707	0.64807

1073.50385	0.6481

1073.60063	0.64811

1073.69741	0.64836

1073.79419	0.64842

1073.89098	0.64843

1073.98776	0.64897

1074.08454	0.64932

1074.18132	0.64933

1074.2781	0.64953

1074.37489	0.64956

1074.47167	0.64993

1074.56845	0.65016

1074.66523	0.65019

1074.76201	0.6502

1074.8588	0.65035

1074.95558	0.65046

1075.05236	0.65048

1075.14914	0.65063

1075.24592	0.65064

1075.3427	0.65066

1075.43949	0.65078

1075.53627	0.6508

1075.63305	0.65081

1075.72983	0.65109

1075.82661	0.65115

1075.9234	0.65144

1076.02018	0.65147

1076.11696	0.65148

1076.21374	0.6515

1076.31052	0.6516

1076.4073	0.65177

1076.50409	0.65179

1076.60087	0.65195

1076.69765	0.65198

1076.79443	0.65214

1076.89121	0.65229

1076.988	0.65232

1077.08478	0.65234

1077.18156	0.6524

1077.27834	0.65241

1077.37512	0.65244

1077.4719	0.65249

1077.56869	0.6525

1077.66547	0.65258

1077.76225	0.65264

1077.85903	0.65266

1077.95581	0.6529

1078.0526	0.65291

1078.14938	0.65308

1078.24616	0.65311

1078.34294	0.65336

1078.43972	0.65339

1078.5365	0.6534

1078.63329	0.6536

1078.73007	0.65362

1078.82685	0.65363

1078.92363	0.65365

1079.02041	0.65386

1079.1172	0.65387

1079.21398	0.65391

1079.31076	0.65427

1079.40754	0.6543

1079.50432	0.65438

1079.6011	0.65464

1079.69789	0.6547

1079.79467	0.65473

1079.89145	0.65474

1079.98823	0.65494

1080.08501	0.65511

1080.1818	0.65514

1080.27858	0.65515

1080.37536	0.65547

1080.47214	0.65549

1080.56892	0.65551

1080.6657	0.65552

1080.76249	0.65555

1080.85927	0.65557

1080.95605	0.65558

1081.05283	0.65567

1081.14961	0.65581

1081.24639	0.65586

1081.34318	0.65587

1081.43996	0.65624

1081.53674	0.65625

1081.63352	0.65682

1081.7303	0.65691

1081.82709	0.65697

1081.92387	0.65701

1082.02065	0.65743

1082.11743	0.65744

1082.21421	0.65746

1082.311	0.65773

1082.40778	0.65775

1082.50456	0.65782

1082.60134	0.65793

1082.69812	0.65796

1082.7949	0.65802

1082.89169	0.65819

1082.98847	0.65828

1083.08525	0.65831

1083.18203	0.65832

1083.27881	0.65883

1083.3756	0.6591

1083.47238	0.65915

1083.56916	0.65919

1083.66594	0.65922

1083.76272	0.65928

1083.8595	0.65939

1083.95629	0.65941

1084.05307	0.65957

1084.14985	0.6596

1084.24663	0.65962

1084.34341	0.65965

1084.44019	0.65971

1084.53698	0.65974

1084.63376	0.65976

1084.73054	0.65979

1084.82732	0.66024

1084.9241	0.66066

1085.02089	0.66067

1085.11767	0.66122

1085.21445	0.66125

1085.31123	0.66139

1085.40801	0.66148

1085.5048	0.66149

1085.60158	0.66151

1085.69836	0.66174

1085.79514	0.6618

1085.89192	0.66184

1085.9887	0.66221

1086.08549	0.66227

1086.18227	0.66245

1086.27905	0.66247

1086.37583	0.66282

1086.47261	0.66285

1086.56939	0.66343

1086.66618	0.66346

1086.76296	0.66369

1086.85974	0.6637

1086.95652	0.66375

1087.0533	0.66386

1087.15009	0.66389

1087.24687	0.66421

1087.34365	0.66424

1087.44043	0.66474

1087.53721	0.66477

1087.63399	0.66479

1087.73078	0.665

1087.82756	0.66501

1087.92434	0.66523

1088.02112	0.66529

1088.1179	0.66532

1088.21468	0.66567

1088.31147	0.6657

1088.40825	0.66572

1088.50503	0.66578

1088.60181	0.66587

1088.69859	0.66588

1088.79538	0.6659

1088.89216	0.66591

1088.98894	0.66604

1089.08572	0.6662

1089.1825	0.66622

1089.27929	0.66623

1089.37607	0.66674

1089.47285	0.6668

1089.56963	0.66709

1089.66641	0.6671

1089.76319	0.66716

1089.85998	0.66719

1089.95676	0.66733

1090.05354	0.66751

1090.15032	0.66753

1090.2471	0.66789

1090.34389	0.66792

1090.44067	0.66832

1090.53745	0.66834

1090.63423	0.6686

1090.73101	0.66861

1090.82779	0.66904

1090.92458	0.66905

1091.02136	0.66907

1091.11814	0.66945

1091.21492	0.66949

1091.3117	0.66951

1091.40848	0.67001

1091.50527	0.67004

1091.60205	0.67029

1091.69883	0.67035

1091.79561	0.67058

1091.89239	0.67084

1091.98918	0.67085

1092.08596	0.67087

1092.18274	0.67135

1092.27952	0.67137

1092.3763	0.67143

1092.47309	0.67163

1092.56987	0.67172

1092.66665	0.67175

1092.76343	0.67176

1092.86021	0.67178

1092.95699	0.67183

1093.05378	0.67186

1093.15056	0.67187

1093.24734	0.67193

1093.34412	0.67195

1093.4409	0.6721

1093.53769	0.67216

1093.63447	0.67218

1093.73125	0.67221

1093.82803	0.6728

1093.92481	0.67282

1094.02159	0.67283

1094.11838	0.67291

1094.21516	0.67301

1094.31194	0.67303

1094.40872	0.67304

1094.5055	0.67318

1094.60228	0.67324

1094.69907	0.67326

1094.79585	0.67329

1094.89263	0.67336

1094.98941	0.67343

1095.08619	0.67344

1095.18298	0.67346

1095.27976	0.67365

1095.37654	0.6737

1095.47332	0.67372

1095.5701	0.67387

1095.66689	0.67394

1095.76367	0.67396

1095.86045	0.67399

1095.95723	0.6742

1096.05401	0.67426

1096.15079	0.6746

1096.24758	0.67461

1096.34436	0.67509

1096.44114	0.67513

1096.53792	0.67532

1096.6347	0.67544

1096.73149	0.67545

1096.82827	0.67548

1096.92505	0.67562

1097.02183	0.67565

1097.11861	0.6758

1097.21539	0.67582

1097.31218	0.67583

1097.40896	0.67629

1097.50574	0.67632

1097.60252	0.67634

1097.6993	0.67664

1097.79608	0.67669

1097.89287	0.6767

1097.98965	0.67702

1098.08643	0.67704

1098.18321	0.6771

1098.27999	0.67716

1098.37678	0.67717

1098.47356	0.67719

1098.57034	0.67774

1098.66712	0.6778

1098.7639	0.67803

1098.86069	0.67804

1098.95747	0.67806

1099.05425	0.67807

1099.15103	0.67835

1099.24781	0.67839

1099.34459	0.67847

1099.44138	0.6785

1099.53816	0.67871

1099.63494	0.67874

1099.73172	0.6789

1099.8285	0.67894

1099.92528	0.67896

1100.02207	0.67911

1100.11885	0.67913

1100.21563	0.67946

1100.31241	0.67949

1100.40919	0.67951

1100.50598	0.67957

1100.60276	0.67964

1100.69954	0.67996

1100.79632	0.67998

1100.8931	0.67999

1100.98988	0.6803

1101.08667	0.68037

1101.18345	0.68047

1101.28023	0.68065

1101.37701	0.68066

1101.47379	0.68068

1101.57058	0.68106

1101.66736	0.68126

1101.76414	0.68167

1101.86092	0.68169

1101.9577	0.68199

1102.05448	0.68216

1102.15127	0.68217

1102.24805	0.68219

1102.34483	0.68223

1102.44161	0.68249

1102.53839	0.68251

1102.63517	0.68274

1102.73196	0.68275

1102.82874	0.6828

1102.92552	0.68297

1103.0223	0.68298

1103.11908	0.68303

1103.21587	0.6833

1103.31265	0.68347

1103.40943	0.68357

1103.50621	0.68359

1103.60299	0.68386

1103.69978	0.68388

1103.79656	0.68389

1103.89334	0.68391

1103.99012	0.68412

1104.0869	0.68415

1104.18368	0.68417

1104.28047	0.68453

1104.37725	0.68464

1104.47403	0.68467

1104.57081	0.68499

1104.66759	0.68504

1104.76438	0.68505

1104.86116	0.6851

1104.95794	0.68534

1105.05472	0.68536

1105.1515	0.68568

1105.24828	0.68569

1105.34507	0.68572

1105.44185	0.68589

1105.53863	0.68595

1105.63541	0.68623

1105.73219	0.68624

1105.82897	0.68652

1105.92576	0.68659

1106.02254	0.68661

1106.11932	0.68664

1106.2161	0.68665

1106.31288	0.68668

1106.40967	0.6867

1106.50645	0.68678

1106.60323	0.68687

1106.70001	0.6869

1106.79679	0.68691

1106.89358	0.68732

1106.99036	0.68734

1107.08714	0.6876

1107.18392	0.68761

1107.2807	0.68766

1107.37748	0.68767

1107.47427	0.6881

1107.57105	0.68821

1107.66783	0.68827

1107.76461	0.68842

1107.86139	0.68844

1107.95818	0.68845

1108.05496	0.6886

1108.15174	0.68865

1108.24852	0.68866

1108.3453	0.68871

1108.44208	0.68873

1108.53887	0.68883

1108.63565	0.68903

1108.73243	0.68906

1108.82921	0.68908

1108.92599	0.68941

1109.02277	0.68947

1109.11956	0.68952

1109.21634	0.68953

1109.31312	0.68984

1109.4099	0.68985

1109.50668	0.68988

1109.60347	0.68994

1109.70025	0.69019

1109.79703	0.69054

1109.89381	0.69057

1109.99059	0.69087

1110.08738	0.6909

1110.18416	0.69106

1110.28094	0.69142

1110.37772	0.69144

1110.4745	0.69145

1110.57128	0.69183

1110.66807	0.69188

1110.76485	0.69203

1110.86163	0.69208

1110.95841	0.69214

1111.05519	0.69217

1111.15198	0.69218

1111.24876	0.6922

1111.34554	0.69223

1111.44232	0.69234

1111.5391	0.69255

1111.63588	0.69258

1111.73267	0.6926

1111.82945	0.69267

1111.92623	0.6927

1112.02301	0.69272

1112.11979	0.69289

1112.21657	0.6929

1112.31336	0.69293

1112.41014	0.69313

1112.50692	0.69314

1112.6037	0.69316

1112.70048	0.69318

1112.79726	0.69321

1112.89405	0.69325

1112.99083	0.69336

1113.08761	0.69397

1113.18439	0.69398

1113.28117	0.694

1113.37796	0.6942

1113.47474	0.69421

1113.57152	0.69423

1113.6683	0.69435

1113.76508	0.69438

1113.86187	0.69459

1113.95865	0.69465

1114.05543	0.69468

1114.15221	0.6947

1114.24899	0.69471

1114.34577	0.69473

1114.44256	0.6951

1114.53934	0.69516

1114.63612	0.69531

1114.7329	0.69555

1114.82968	0.69557

1114.92647	0.69558

1115.02325	0.6958

1115.12003	0.69583

1115.21681	0.6959

1115.31359	0.69592

1115.41037	0.69593

1115.50716	0.69613

1115.60394	0.69616

1115.70072	0.69618

1115.7975	0.69624

1115.89428	0.69633

1115.99106	0.69634

1116.08785	0.69656

1116.18463	0.6966

1116.28141	0.69665

1116.37819	0.69667

1116.47497	0.697

1116.57176	0.69709

1116.66854	0.69711

1116.76532	0.6974

1116.8621	0.69741

1116.95888	0.69743

1117.05567	0.69744

1117.15245	0.69746

1117.24923	0.69747

1117.34601	0.69767

1117.44279	0.69775

1117.53957	0.69782

1117.63636	0.69787

1117.73314	0.69804

1117.82992	0.69807

1117.9267	0.69808

1118.02348	0.6981

1118.12027	0.69831

1118.21705	0.69833

1118.31383	0.6984

1118.41061	0.69848

1118.50739	0.69851

1118.60417	0.69852

1118.70096	0.69857

1118.79774	0.69862

1118.89452	0.69863

1118.9913	0.69865

1119.08808	0.69916

1119.18486	0.69918

1119.28165	0.69933

1119.37843	0.69935

1119.47521	0.69938

1119.57199	0.69979

1119.66877	0.6998

1119.76556	0.69982

1119.86234	0.6999

1119.95912	0.69993

1120.0559	0.69997

1120.15268	0.70008

1120.24947	0.70009

1120.34625	0.70049

1120.44303	0.70051

1120.53981	0.70055

1120.63659	0.70064

1120.73337	0.70066

1120.83016	0.70067

1120.92694	0.70069

1121.02372	0.70078

1121.1205	0.70081

1121.21728	0.70084

1121.31407	0.70089

1121.41085	0.70093

1121.50763	0.70095

1121.60441	0.70096

1121.70119	0.70105

1121.79797	0.70108

1121.89476	0.70154

1121.99154	0.70182

1122.08832	0.70212

1122.1851	0.70241

1122.28188	0.70243

1122.37867	0.70285

1122.47545	0.70287

1122.57223	0.70305

1122.66901	0.7031

1122.76579	0.70311

1122.86257	0.70384

1122.95936	0.70413

1123.05614	0.70445

1123.15292	0.7045

1123.2497	0.70459

1123.34648	0.70474

1123.44327	0.70476

1123.54005	0.70509

1123.63683	0.70511

1123.73361	0.70549

1123.83039	0.7055

1123.92717	0.70559

1124.02395	0.70564

1124.12074	0.70567

1124.21752	0.7059

1124.3143	0.70613

1124.41108	0.70616

1124.50786	0.70642

1124.60465	0.70666

1124.70143	0.70668

1124.79821	0.70669

1124.89499	0.70674

1124.99177	0.70684

1125.08856	0.70687

1125.18534	0.70691

1125.28212	0.70732

1125.3789	0.70733

1125.47568	0.70736

1125.57246	0.70756

1125.66925	0.70788

1125.76603	0.7079

1125.86281	0.70808

1125.95959	0.70809

1126.05637	0.70831

1126.15316	0.70832

1126.24994	0.70837

1126.34672	0.7084

1126.4435	0.70844

1126.54028	0.70848

1126.63706	0.70887

1126.73385	0.70889

1126.83063	0.70915

1126.92741	0.70919

1127.02419	0.70939

1127.12097	0.70945

1127.21775	0.70985

1127.31454	0.70998

1127.41132	0.71047

1127.5081	0.71049

1127.60488	0.7105

1127.70166	0.71056

1127.79845	0.71061

1127.89523	0.7109

1127.99201	0.71111

1128.08879	0.71114

1128.18557	0.71116

1128.28236	0.71117

1128.37914	0.71134

1128.47592	0.71137

1128.5727	0.71154

1128.66948	0.71157

1128.76626	0.71158

1128.86305	0.71171

1128.95983	0.71174

1129.05661	0.71175

1129.15339	0.71177

1129.25017	0.71203

1129.34696	0.71204

1129.44374	0.71232

1129.54052	0.71233

1129.6373	0.71236

1129.73408	0.7127

1129.83086	0.71271

1129.92765	0.71302

1130.02443	0.71314

1130.12121	0.71318

1130.21799	0.71335

1130.31477	0.7136

1130.41155	0.71361

1130.50834	0.71363

1130.60512	0.71364

1130.7019	0.71375

1130.79868	0.71376

1130.89546	0.71379

1130.99225	0.71402

1131.08903	0.71411

1131.18581	0.71427

1131.28259	0.71431

1131.37937	0.71433

1131.47616	0.71465

1131.57294	0.71466

1131.66972	0.71489

1131.7665	0.71491

1131.86328	0.71501

1131.96006	0.71504

1132.05685	0.71545

1132.15363	0.71565

1132.25041	0.71571

1132.34719	0.71573

1132.44397	0.71574

1132.54076	0.7158

1132.63754	0.71597

1132.73432	0.71599

1132.8311	0.71616

1132.92788	0.71619

1133.02466	0.7164

1133.12145	0.71641

1133.21823	0.71644

1133.31501	0.71661

1133.41179	0.71684

1133.50857	0.71686

1133.60535	0.71687

1133.70214	0.71722

1133.79892	0.71724

1133.8957	0.71728

1133.99248	0.71747

1134.08926	0.71756

1134.18605	0.7176

1134.28283	0.71774

1134.37961	0.71776

1134.47639	0.71777

1134.57317	0.71783

1134.66996	0.71788

1134.76674	0.71789

1134.86352	0.71818

1134.9603	0.7182

1135.05708	0.71849

1135.15386	0.7185

1135.25065	0.71881

1135.34743	0.71882

1135.44421	0.71933

1135.54099	0.71934

1135.63777	0.71936

1135.73456	0.71945

1135.83134	0.71951

1135.92812	0.71952

1136.0249	0.71961

1136.12168	0.71968

1136.21846	0.71969

1136.31525	0.71978

1136.41203	0.7198

1136.50881	0.71983

1136.60559	0.71995

1136.70237	0.71997

1136.79915	0.7201

1136.89594	0.72018

1136.99272	0.72025

1137.0895	0.72027

1137.18628	0.72062

1137.28306	0.72074

1137.37984	0.72103

1137.47663	0.72105

1137.57341	0.72121

1137.67019	0.72128

1137.76697	0.72129

1137.86375	0.72149

1137.96054	0.7219

1138.05732	0.72192

1138.1541	0.72193

1138.25088	0.72195

1138.34766	0.72221

1138.44445	0.72248

1138.54123	0.72253

1138.63801	0.72259

1138.73479	0.72263

1138.83157	0.72295

1138.92835	0.72297

1139.02514	0.723

1139.12192	0.72317

1139.2187	0.72323

1139.31548	0.72327

1139.41226	0.72338

1139.50905	0.72341

1139.60583	0.72379

1139.70261	0.72381

1139.79939	0.72431

1139.89617	0.72434

1139.99295	0.7248

1140.08974	0.72481

1140.18652	0.72499

1140.2833	0.72513

1140.38008	0.72524

1140.47686	0.72527

1140.57364	0.72551

1140.67043	0.72554

1140.76721	0.72603

1140.86399	0.72608

1140.96077	0.72609

1141.05755	0.72623

1141.15434	0.72627

1141.25112	0.72633

1141.3479	0.72635

1141.44468	0.72656

1141.54146	0.72658

1141.63825	0.72684

1141.73503	0.72687

1141.83181	0.72731

1141.92859	0.7274

1142.02537	0.72775

1142.12215	0.72789

1142.21894	0.72795

1142.31572	0.72806

1142.4125	0.7281

1142.50928	0.72821

1142.60606	0.72825

1142.70285	0.72838

1142.79963	0.72844

1142.89641	0.7288

1142.99319	0.72886

1143.08997	0.72889

1143.18675	0.72896

1143.28354	0.72897

1143.38032	0.72915

1143.4771	0.72917

1143.57388	0.72949

1143.67066	0.7295

1143.76745	0.72952

1143.86423	0.72969

1143.96101	0.7299

1144.05779	0.73002

1144.15457	0.73004

1144.25135	0.73025

1144.34814	0.73027

1144.44492	0.73028

1144.5417	0.73054

1144.63848	0.73082

1144.73526	0.73085

1144.83205	0.73086

1144.92883	0.73123

1145.02561	0.73124

1145.12239	0.73152

1145.21917	0.73165

1145.31595	0.73168

1145.41274	0.7317

1145.50952	0.73171

1145.6063	0.73173

1145.70308	0.73197

1145.79986	0.73203

1145.89665	0.73206

1145.99343	0.73216

1146.09021	0.73223

1146.18699	0.73255

1146.28377	0.73269

1146.38055	0.73289

1146.47734	0.73296

1146.57412	0.73316

1146.6709	0.73318

1146.76768	0.73339

1146.86446	0.73342

1146.96125	0.73347

1147.05803	0.73348

1147.15481	0.73351

1147.25159	0.73353

1147.34837	0.73382

1147.44515	0.73405

1147.54194	0.73429

1147.63872	0.73435

1147.7355	0.73437

1147.83228	0.73456

1147.92906	0.73461

1148.02584	0.73462

1148.12263	0.73473

1148.21941	0.73478

1148.31619	0.73479

1148.41297	0.73487

1148.50975	0.7351

1148.60653	0.73511

1148.70332	0.73537

1148.8001	0.73539

1148.89688	0.7354

1148.99366	0.7359

1149.09045	0.73594

1149.18723	0.73609

1149.28401	0.73616

1149.38079	0.73618

1149.47757	0.73641

1149.57435	0.73642

1149.67114	0.73659

1149.76792	0.73661

1149.8647	0.73664

1149.96148	0.73679

1150.05826	0.7368

1150.15504	0.73697

1150.25183	0.73725

1150.34861	0.73729

1150.44539	0.73775

1150.54217	0.73776

1150.63895	0.73786

1150.73574	0.7379

1150.83252	0.73793

1150.9293	0.73795

1151.02608	0.73798

1151.12286	0.73799

1151.21964	0.73807

1151.31643	0.73813

1151.41321	0.73814

1151.50999	0.73833

1151.60677	0.73834

1151.70355	0.73869

1151.80033	0.73871

1151.89712	0.73871

1151.9939	0.73873

1152.09068	0.73882

1152.18746	0.73891

1152.28424	0.73894

1152.38103	0.73903

1152.47781	0.73905

1152.57459	0.73907

1152.67137	0.73912

1152.76815	0.73914

1152.86494	0.73933

1152.96172	0.73935

1153.0585	0.73939

1153.15528	0.73942

1153.25206	0.73944

1153.34884	0.73946

1153.44563	0.73949

1153.54241	0.73951

1153.63919	0.73953

1153.73597	0.73955

1153.83275	0.73965

1153.92954	0.73969

1154.02632	0.73971

1154.1231	0.73976

1154.21988	0.73987

1154.31666	0.7399

1154.41344	0.73992

1154.51023	0.73999

1154.60701	0.74001

1154.70379	0.74003

1154.80057	0.74006

1154.89735	0.74008

1154.99413	0.7401

1155.09092	0.74013

1155.1877	0.74015

1155.28448	0.74019

1155.38126	0.74022

1155.47804	0.74026

1155.57483	0.74029

1155.67161	0.74031

1155.76839	0.74035

1155.86517	0.74038

1155.96195	0.7404

1156.05874	0.74042

1156.15552	0.74061

1156.2523	0.74072

1156.34908	0.74074

1156.44586	0.74088

1156.54264	0.74093

1156.63943	0.74095

1156.73621	0.74097

1156.83299	0.74099

1156.92977	0.74104

1157.02655	0.74111

1157.12334	0.74113

1157.22012	0.74118

1157.3169	0.7412

1157.41368	0.74125

1157.51046	0.74131

1157.60724	0.74143

1157.70403	0.74145

1157.80081	0.74147

1157.89759	0.7415

1157.99437	0.74152

1158.09115	0.74166

1158.18793	0.74168

1158.28472	0.74175

1158.3815	0.74177

1158.47828	0.74179

1158.57506	0.74184

1158.67184	0.74186

1158.76863	0.74189

1158.86541	0.742

1158.96219	0.74211

1159.05897	0.74225

1159.15575	0.74227

1159.25254	0.7423

1159.34932	0.74232

1159.4461	0.74234

1159.54288	0.74239

1159.63966	0.7425

1159.73644	0.74253

1159.83323	0.74259

1159.93001	0.74269

1160.02679	0.74278

1160.12357	0.7428

1160.22035	0.74282

1160.31714	0.74285

1160.41392	0.74296

1160.5107	0.74303

1160.60748	0.74305

1160.70426	0.74312

1160.80104	0.74319

1160.89783	0.7465

1160.99461	0.74657

1161.09139	0.74659

1161.18817	0.74671

1161.28495	0.74673

1161.38173	0.74682

1161.47852	0.74685

1161.5753	0.74694

1161.67208	0.74698

1161.76886	0.74701

1161.86564	0.74705

1161.96242	0.74707

1162.05921	0.7471

1162.15599	0.74712

1162.25277	0.74714

1162.34955	0.74717

1162.44633	0.74719

1162.54312	0.74721

1162.6399	0.74723

1162.73668	0.74733

1162.83346	0.74739

1162.93024	0.74753

1163.02703	0.74767

1163.12381	0.74769

1163.22059	0.74774

1163.31737	0.74776

1163.41415	0.74785

1163.51093	0.74792

1163.60772	0.74794

1163.7045	0.74797

1163.80128	0.74799

1163.89806	0.74806

1163.99484	0.7481

1164.09163	0.74813

1164.18841	0.74819

1164.28519	0.74826

1164.38197	0.7521

1164.47875	0.75213

1164.57553	0.7562

1164.67232	0.75622

1164.7691	0.75626

1164.86588	0.75629

1164.96266	0.75649

1165.05944	0.75658

1165.15622	0.75661

1165.25301	0.75665

1165.34979	0.75668

1165.44657	0.7567

1165.54335	0.75672

1165.64013	0.75674

1165.73692	0.75677

1165.8337	0.75688

1165.93048	0.7569

1166.02726	0.75725

1166.12404	0.75727

1166.22083	0.75732

1166.31761	0.75738

1166.41439	0.75748

1166.51117	0.75752

1166.60795	0.75754

1166.70473	0.75761

1166.80152	0.75763

1166.8983	0.75769

1166.99508	0.75775

1167.09186	0.75778

1167.18864	0.75804

1167.28543	0.75818

1167.38221	0.75819

1167.47899	0.75821

1167.57577	0.75822

1167.67255	0.75824

1167.76933	0.75825

1167.86612	0.75833

1167.9629	0.75834

1168.05968	0.75837

1168.15646	0.75845

1168.25324	0.75856

1168.35003	0.75935

1168.44681	0.7594

1168.54359	0.75941

1168.64037	0.76029

1168.73715	0.76031

1168.83393	0.7604

1168.93072	0.7606

1169.0275	0.76061

1169.12428	0.76064

1169.22106	0.76095

1169.31784	0.76098

1169.41462	0.99281

1169.51141	0.99296

1169.60819	0.99378

1169.70497	0.9939

1169.80175	0.99628

1169.89853	0.99631

1169.99532	0.9964

1170.0921	0.99648

1170.18888	0.99736

1170.28566	0.99787

1170.38244	0.99808

1170.47923	0.99817

1170.57601	0.99927

1170.67279	0.99936

1170.76957	0.99957

1170.86635	0.99965

1170.96313	0.99966

1171.05992	0.99994

1171.1567	0.99998

1171.25348	1
